# Supplementary material for: A Handle on Mass Coincidence Errors in De Novo Sequencing of Antibodies by Bottom-up Proteomics
Source: J Proteome Res. 2024 Jun 27;23(8):3552–9. doi: 10.1021/acs.jproteome.4c00188 (PMC11301774; doi:10.1021/acs.jproteome.4c00188)
Supplement: Supplementary file 1 — pr4c00188_si_001.zip [file pr4c00188_si_001.zip › supplementary data/xln-disambiguation/2023-12-13@14-36-36 f59/report/reads/Combined_005.html]

Details Combined\_005 | Stitch OverviewUndefined

# Read Combined\_005

## Sequence (length=9)

TFDDYAMHW

## Spectrum 7317? Spectrum 7317 The raw spectrum of this peptide as annotated by Hecklib. The fragments are coloured according to ion type (see legend). Any peaks with a star '\*' as text can be hovered over to see the full details, first the ion type second the mass shift type. By hovering over the amino acids in the peptide or ions in the legend the corresponding peaks are highlighted. By toggling the 'Unassigned' label you can turn the background (unassigned) peaks on or off in the plot. By updating the slider in the Ion legend you can update the spectrum to only show the top X% of the peaks with labels. The top X% means any peak that is within X% of the highest intensity. By dragging in the spectrum you can zoom in to a specific part of the spectrum and use 'Zoom Out' to get back to the original zoom level. The annotation of the spectrum is based on the given sequence in the peptides file and is done with different software so inconsistencies are likely. The peaks are annotated based on the given sequence, with 20 ppm tolerance.

Copy Data

### Spectrum 7317 (TSV)

#### Preview

```
Loading example...
```

*Click on the button to copy the data to your clipboard.*

Mz MinMz MaxIntensity Max

WidthHeightPeptide font sizePeptide stroke widthSpectrum font sizeSpectrum stroke widthCompact peptide

Ion legend

wxyz

abcd

OtherUnassignedIonChargePositionShow for top:%

TFDDYAMHW

07.44e+61.49e+72.23e+72.98e+7

Zoom Out

b+23a+12a+12y+11a+12b+24b+12y+23b+12d+13a+13a+13y+12b+13b+26y+25b+13b+14y+27y+27b+14y+13b+28y+28y+28y+14\*\*b+15b+15b+16b+16y+15y+16y+16b+17b+17y+17y+17b+18b+18b+18y+18y+18

0616123318492466

Fragment Matches Table

Show background peaks

| Position | Ion type | Intensity | mz Theoretical | mz Error (Th) | mz Error (ppm) | Charge | Series Number |
| --- | --- | --- | --- | --- | --- | --- | --- |
| - | - | 7.092E+06 | 120.1 | - | - | 0 | - |
| - | - | 2.212E+04 | 121 | - | - | 0 | - |
| - | - | 2.139E+04 | 121.1 | - | - | 0 | - |
| - | - | 5.55E+05 | 121.1 | - | - | 0 | - |
| - | - | 3.172E+04 | 122.1 | - | - | 0 | - |
| - | - | 2.007E+04 | 122.1 | - | - | 0 | - |
| - | - | 1.502E+04 | 125.1 | - | - | 0 | - |
| - | - | 3.206E+04 | 127.1 | - | - | 0 | - |
| - | - | 1.358E+04 | 129.1 | - | - | 0 | - |
| - | - | 1.994E+05 | 130.1 | - | - | 0 | - |
| - | - | 1.681E+04 | 131.1 | - | - | 0 | - |
| - | - | 3.675E+05 | 132.1 | - | - | 0 | - |
| - | - | 4.066E+04 | 133.1 | - | - | 0 | - |
| - | - | 4.033E+04 | 134.1 | - | - | 0 | - |
| - | - | 1.395E+04 | 135.1 | - | - | 0 | - |
| - | - | 2.585E+06 | 136.1 | - | - | 0 | - |
| - | - | 7.759E+04 | 136.1 | - | - | 0 | - |
| - | - | 1.827E+05 | 137.1 | - | - | 0 | - |
| - | - | 1.259E+04 | 137.1 | - | - | 0 | - |
| - | - | 1.942E+05 | 138.1 | - | - | 0 | - |
| - | - | 3.076E+05 | 138.1 | - | - | 0 | - |
| - | - | 1.509E+04 | 139.1 | - | - | 0 | - |
| - | - | 1.679E+04 | 139.1 | - | - | 0 | - |
| - | - | 1.01E+04 | 141.6 | - | - | 0 | - |
| - | - | 4.82E+04 | 143.1 | - | - | 0 | - |
| - | - | 1.208E+04 | 143.1 | - | - | 0 | - |
| - | - | 4.587E+04 | 143.1 | - | - | 0 | - |
| - | - | 5.166E+04 | 144.1 | - | - | 0 | - |
| - | - | 9.894E+04 | 146.1 | - | - | 0 | - |
| - | - | 5.26E+04 | 146.1 | - | - | 0 | - |
| - | - | 1.122E+04 | 147 | - | - | 0 | - |
| - | - | 1.846E+04 | 147.1 | - | - | 0 | - |
| - | - | 3.351E+04 | 148.1 | - | - | 0 | - |
| - | - | 9.316E+04 | 148.1 | - | - | 0 | - |
| - | - | 1.315E+04 | 148.1 | - | - | 0 | - |
| - | - | 1.219E+04 | 150.1 | - | - | 0 | - |
| - | - | 2.26E+04 | 153.1 | - | - | 0 | - |
| - | - | 4.355E+04 | 155.1 | - | - | 0 | - |
| - | - | 6.302E+04 | 155.1 | - | - | 0 | - |
| - | - | 6.427E+05 | 156.1 | - | - | 0 | - |
| - | - | 4.064E+04 | 157.1 | - | - | 0 | - |
| - | - | 1.079E+06 | 158.1 | - | - | 0 | - |
| - | - | 1.471E+04 | 159.1 | - | - | 0 | - |
| - | - | 5.712E+05 | 159.1 | - | - | 0 | - |
| - | - | 1.182E+05 | 159.1 | - | - | 0 | - |
| - | - | 2.066E+05 | 160.1 | - | - | 0 | - |
| - | - | 2.173E+04 | 160.1 | - | - | 0 | - |
| - | - | 4.241E+04 | 160.1 | - | - | 0 | - |
| - | - | 4.865E+05 | 160.1 | - | - | 0 | - |
| - | - | 5.282E+04 | 161.1 | - | - | 0 | - |
| - | - | 1.968E+04 | 162.1 | - | - | 0 | - |
| - | - | 1.821E+04 | 164.1 | - | - | 0 | - |
| - | - | 2.865E+04 | 165.1 | - | - | 0 | - |
| - | - | 1.31E+05 | 165.1 | - | - | 0 | - |
| - | - | 1.376E+04 | 165.1 | - | - | 0 | - |
| - | - | 4.225E+04 | 166.1 | - | - | 0 | - |
| - | - | 9.407E+04 | 166.1 | - | - | 0 | - |
| - | - | 1.359E+04 | 166.1 | - | - | 0 | - |
| - | - | 1.393E+04 | 167 | - | - | 0 | - |
| - | - | 1.165E+05 | 167.1 | - | - | 0 | - |
| - | - | 3.248E+04 | 169.1 | - | - | 0 | - |
| - | - | 1.672E+04 | 170.1 | - | - | 0 | - |
| - | - | 8.393E+04 | 171.1 | - | - | 0 | - |
| - | - | 1.76E+04 | 172.1 | - | - | 0 | - |
| - | - | 1.727E+04 | 172.1 | - | - | 0 | - |
| - | - | 1.398E+04 | 173.1 | - | - | 0 | - |
| - | - | 2.036E+04 | 173.4 | - | - | 0 | - |
| 3 | b | 7.996E+04 | 174.1 | 0.001177 | 6.759 | +2 | 3 |
| - | - | 1.345E+04 | 174.1 | - | - | 0 | - |
| - | - | 1.164E+04 | 175.1 | - | - | 0 | - |
| - | - | 3.813E+04 | 175.1 | - | - | 0 | - |
| - | - | 1.432E+04 | 175.1 | - | - | 0 | - |
| - | - | 2.83E+04 | 175.1 | - | - | 0 | - |
| - | - | 1.78E+05 | 176.1 | - | - | 0 | - |
| - | - | 2.176E+06 | 176.1 | - | - | 0 | - |
| - | - | 1.492E+06 | 177.1 | - | - | 0 | - |
| - | - | 2.127E+05 | 177.1 | - | - | 0 | - |
| - | - | 2.102E+04 | 178.1 | - | - | 0 | - |
| - | - | 1.242E+04 | 178.1 | - | - | 0 | - |
| - | - | 1.605E+05 | 178.1 | - | - | 0 | - |
| - | - | 1.121E+05 | 180.1 | - | - | 0 | - |
| - | - | 1.805E+05 | 185.1 | - | - | 0 | - |
| - | - | 1.852E+05 | 186.1 | - | - | 0 | - |
| - | - | 2.875E+04 | 187.1 | - | - | 0 | - |
| - | - | 7.801E+05 | 188.1 | - | - | 0 | - |
| - | - | 1.059E+05 | 189.1 | - | - | 0 | - |
| - | - | 1.18E+04 | 189.1 | - | - | 0 | - |
| - | - | 2.943E+04 | 190.1 | - | - | 0 | - |
| - | - | 7.766E+04 | 191.1 | - | - | 0 | - |
| - | - | 1.339E+05 | 191.1 | - | - | 0 | - |
| - | - | 2.19E+04 | 192.1 | - | - | 0 | - |
| - | - | 3.934E+04 | 193.1 | - | - | 0 | - |
| - | - | 6.274E+05 | 193.1 | - | - | 0 | - |
| - | - | 2.437E+04 | 194.1 | - | - | 0 | - |
| - | - | 1.129E+04 | 194.1 | - | - | 0 | - |
| - | - | 5.268E+04 | 194.1 | - | - | 0 | - |
| - | - | 1.686E+05 | 195.1 | - | - | 0 | - |
| - | - | 1.523E+04 | 197.1 | - | - | 0 | - |
| - | - | 7.945E+04 | 202.1 | - | - | 0 | - |
| - | - | 1.805E+05 | 203.1 | - | - | 0 | - |
| - | - | 6.063E+04 | 203.1 | - | - | 0 | - |
| 2 | a | 7.366E+05 | 203.1 | 0.0003813 | 1.877 | +1 | 2 |
| - | - | 3.337E+05 | 204.1 | - | - | 0 | - |
| 2 | a | 5.33E+04 | 204.1 | 0.0001762 | 0.8632 | +1 | 2 |
| - | - | 1.045E+05 | 204.1 | - | - | 0 | - |
| - | - | 2.748E+04 | 205.1 | - | - | 0 | - |
| 9 | y | 5.802E+05 | 205.1 | 0.0004716 | 2.3 | +1 | 1 |
| - | - | 4.376E+04 | 205.1 | - | - | 0 | - |
| - | - | 5.878E+04 | 206.1 | - | - | 0 | - |
| - | - | 1.516E+05 | 207.1 | - | - | 0 | - |
| - | - | 9.289E+04 | 208.1 | - | - | 0 | - |
| - | - | 2.695E+04 | 208.1 | - | - | 0 | - |
| - | - | 3.891E+04 | 212.1 | - | - | 0 | - |
| - | - | 1.594E+04 | 213.1 | - | - | 0 | - |
| - | - | 1.425E+04 | 213.1 | - | - | 0 | - |
| - | - | 1.264E+04 | 214 | - | - | 0 | - |
| - | - | 3.747E+04 | 215.1 | - | - | 0 | - |
| - | - | 4.05E+05 | 217.1 | - | - | 0 | - |
| - | - | 2.258E+04 | 218.1 | - | - | 0 | - |
| - | - | 5.618E+04 | 218.1 | - | - | 0 | - |
| - | - | 1.216E+05 | 219.1 | - | - | 0 | - |
| - | - | 1.8E+04 | 219.1 | - | - | 0 | - |
| - | - | 1.889E+04 | 220.1 | - | - | 0 | - |
| - | - | 1.702E+06 | 221.1 | - | - | 0 | - |
| 2 | a | 2.947E+07 | 221.1 | 0.0006199 | 2.803 | +1 | 2 |
| - | - | 1.618E+05 | 222.1 | - | - | 0 | - |
| - | - | 3.583E+06 | 222.1 | - | - | 0 | - |
| - | - | 4.136E+04 | 223.1 | - | - | 0 | - |
| - | - | 2.203E+05 | 223.1 | - | - | 0 | - |
| - | - | 2.111E+04 | 224.1 | - | - | 0 | - |
| - | - | 1.647E+04 | 225.1 | - | - | 0 | - |
| - | - | 8.469E+04 | 228.1 | - | - | 0 | - |
| - | - | 6.141E+04 | 229.1 | - | - | 0 | - |
| - | - | 4.534E+05 | 231.1 | - | - | 0 | - |
| 4 | b | 1.493E+04 | 231.1 | 0.001319 | 5.709 | +2 | 4 |
| 2 | b | 3.008E+05 | 231.1 | 0.0004771 | 2.064 | +1 | 2 |
| - | - | 1.981E+04 | 231.1 | - | - | 0 | - |
| - | - | 3.57E+04 | 232.1 | - | - | 0 | - |
| - | - | 3.004E+04 | 232.1 | - | - | 0 | - |
| - | - | 3.643E+04 | 233.1 | - | - | 0 | - |
| - | - | 8.076E+05 | 235.1 | - | - | 0 | - |
| - | - | 9.08E+04 | 236.1 | - | - | 0 | - |
| - | - | 2.61E+04 | 237.1 | - | - | 0 | - |
| - | - | 2.192E+04 | 237.1 | - | - | 0 | - |
| - | - | 2.848E+04 | 238.1 | - | - | 0 | - |
| - | - | 1.664E+05 | 239.1 | - | - | 0 | - |
| - | - | 1.806E+04 | 240.1 | - | - | 0 | - |
| - | - | 1.779E+04 | 240.1 | - | - | 0 | - |
| 7 | y | 3.482E+04 | 245.1 | 0.004094 | 16.7 | +2 | 3 |
| - | - | 2.855E+04 | 245.1 | - | - | 0 | - |
| - | - | 1.542E+04 | 246.1 | - | - | 0 | - |
| - | - | 5.131E+04 | 246.1 | - | - | 0 | - |
| - | - | 3.4E+04 | 247.1 | - | - | 0 | - |
| - | - | 3.905E+04 | 247.1 | - | - | 0 | - |
| - | - | 1.851E+04 | 247.1 | - | - | 0 | - |
| - | - | 2.053E+04 | 248.1 | - | - | 0 | - |
| - | - | 7.327E+04 | 248.2 | - | - | 0 | - |
| 2 | b | 5.132E+06 | 249.1 | 0.0005936 | 2.383 | +1 | 2 |
| - | - | 6.919E+05 | 250.1 | - | - | 0 | - |
| - | - | 5.155E+05 | 251.1 | - | - | 0 | - |
| - | - | 8.308E+04 | 251.1 | - | - | 0 | - |
| - | - | 5.55E+04 | 252.1 | - | - | 0 | - |
| - | - | 2.388E+04 | 253.1 | - | - | 0 | - |
| - | - | 1.495E+04 | 255.1 | - | - | 0 | - |
| - | - | 3.327E+04 | 256.1 | - | - | 0 | - |
| - | - | 2.774E+04 | 257.1 | - | - | 0 | - |
| - | - | 3.441E+05 | 257.1 | - | - | 0 | - |
| - | - | 3.34E+04 | 258.1 | - | - | 0 | - |
| - | - | 1.99E+04 | 258.1 | - | - | 0 | - |
| - | - | 9.03E+04 | 261.1 | - | - | 0 | - |
| - | - | 4.817E+04 | 263.1 | - | - | 0 | - |
| - | - | 1.039E+06 | 263.1 | - | - | 0 | - |
| - | - | 9.945E+04 | 263.1 | - | - | 0 | - |
| - | - | 1.675E+05 | 264.1 | - | - | 0 | - |
| - | - | 2.169E+04 | 264.1 | - | - | 0 | - |
| - | - | 1.052E+05 | 264.1 | - | - | 0 | - |
| - | - | 4.273E+04 | 265.1 | - | - | 0 | - |
| - | - | 2.31E+04 | 266.1 | - | - | 0 | - |
| - | - | 6.202E+04 | 267.1 | - | - | 0 | - |
| - | - | 5.77E+04 | 268.1 | - | - | 0 | - |
| - | - | 1.899E+04 | 268.1 | - | - | 0 | - |
| - | - | 3.667E+04 | 268.1 | - | - | 0 | - |
| - | - | 4.996E+04 | 269.1 | - | - | 0 | - |
| - | - | 1.399E+04 | 270.1 | - | - | 0 | - |
| - | - | 2.534E+04 | 270.1 | - | - | 0 | - |
| - | - | 4.612E+04 | 271.1 | - | - | 0 | - |
| - | - | 2.447E+04 | 272.1 | - | - | 0 | - |
| - | - | 1.651E+04 | 273.1 | - | - | 0 | - |
| - | - | 2.277E+04 | 273.1 | - | - | 0 | - |
| - | - | 2.828E+05 | 274.1 | - | - | 0 | - |
| - | - | 2.51E+04 | 274.2 | - | - | 0 | - |
| - | - | 4.325E+04 | 275.1 | - | - | 0 | - |
| - | - | 3.553E+04 | 275.1 | - | - | 0 | - |
| - | - | 1.738E+04 | 277.6 | - | - | 0 | - |
| - | - | 1.428E+04 | 277.6 | - | - | 0 | - |
| - | - | 4.893E+05 | 279.1 | - | - | 0 | - |
| - | - | 4.284E+04 | 279.1 | - | - | 0 | - |
| - | - | 4.353E+04 | 280.1 | - | - | 0 | - |
| - | - | 1.881E+04 | 283.1 | - | - | 0 | - |
| - | - | 2E+04 | 283.2 | - | - | 0 | - |
| - | - | 6.77E+05 | 285.1 | - | - | 0 | - |
| - | - | 7.442E+04 | 286.1 | - | - | 0 | - |
| - | - | 1.439E+04 | 286.1 | - | - | 0 | - |
| - | - | 3.454E+04 | 287.1 | - | - | 0 | - |
| - | - | 3.383E+04 | 289.1 | - | - | 0 | - |
| - | - | 1.591E+04 | 289.1 | - | - | 0 | - |
| - | - | 2.72E+04 | 290.1 | - | - | 0 | - |
| - | - | 4.082E+05 | 292.1 | - | - | 0 | - |
| 3 | d | 9.963E+04 | 292.2 | 0.0006612 | 2.263 | +1 | 3 |
| - | - | 4.242E+04 | 293.1 | - | - | 0 | - |
| - | - | 2.002E+04 | 294.2 | - | - | 0 | - |
| - | - | 1.352E+04 | 295.1 | - | - | 0 | - |
| - | - | 5.391E+05 | 296.2 | - | - | 0 | - |
| - | - | 2.216E+04 | 297.1 | - | - | 0 | - |
| - | - | 8.926E+04 | 297.2 | - | - | 0 | - |
| - | - | 3.438E+04 | 299.2 | - | - | 0 | - |
| - | - | 6.348E+04 | 300.1 | - | - | 0 | - |
| - | - | 3.336E+04 | 301.1 | - | - | 0 | - |
| - | - | 4.723E+04 | 302.1 | - | - | 0 | - |
| - | - | 9.16E+04 | 303.1 | - | - | 0 | - |
| - | - | 1.723E+04 | 304.1 | - | - | 0 | - |
| - | - | 1.57E+04 | 305.5 | - | - | 0 | - |
| - | - | 2.102E+04 | 306.1 | - | - | 0 | - |
| - | - | 1.584E+04 | 306.1 | - | - | 0 | - |
| - | - | 7.32E+04 | 310.2 | - | - | 0 | - |
| - | - | 4.283E+04 | 313.1 | - | - | 0 | - |
| - | - | 7.011E+04 | 314.1 | - | - | 0 | - |
| - | - | 2.502E+04 | 315.1 | - | - | 0 | - |
| 3 | a | 1.661E+05 | 318.1 | 0.0006752 | 2.122 | +1 | 3 |
| - | - | 3.497E+04 | 319.1 | - | - | 0 | - |
| - | - | 2.64E+04 | 320.1 | - | - | 0 | - |
| - | - | 1.977E+05 | 323.1 | - | - | 0 | - |
| - | - | 1.485E+04 | 323.1 | - | - | 0 | - |
| - | - | 1.181E+06 | 324.1 | - | - | 0 | - |
| - | - | 2.016E+05 | 325.1 | - | - | 0 | - |
| - | - | 1.857E+04 | 326.2 | - | - | 0 | - |
| - | - | 5.518E+04 | 327.1 | - | - | 0 | - |
| - | - | 1.14E+05 | 328.1 | - | - | 0 | - |
| - | - | 4.926E+04 | 328.1 | - | - | 0 | - |
| - | - | 1.846E+04 | 329.1 | - | - | 0 | - |
| - | - | 6.51E+04 | 330.1 | - | - | 0 | - |
| - | - | 2.631E+04 | 331.1 | - | - | 0 | - |
| - | - | 1.334E+04 | 331.1 | - | - | 0 | - |
| - | - | 8.633E+04 | 332.1 | - | - | 0 | - |
| - | - | 3.808E+04 | 333.1 | - | - | 0 | - |
| - | - | 2.211E+04 | 334.1 | - | - | 0 | - |
| - | - | 2.245E+04 | 334.1 | - | - | 0 | - |
| - | - | 2.385E+04 | 335.2 | - | - | 0 | - |
| 3 | a | 2.595E+04 | 336.2 | 0.0007002 | 2.083 | +1 | 3 |
| - | - | 7.542E+04 | 338.1 | - | - | 0 | - |
| - | - | 1.095E+05 | 339.2 | - | - | 0 | - |
| - | - | 1.705E+04 | 340.2 | - | - | 0 | - |
| 8 | y | 6.117E+06 | 342.2 | 0.0007334 | 2.143 | +1 | 2 |
| - | - | 1.121E+06 | 343.2 | - | - | 0 | - |
| - | - | 8.662E+04 | 344.2 | - | - | 0 | - |
| 3 | b | 3.654E+05 | 346.1 | 0.0006032 | 1.743 | +1 | 3 |
| - | - | 6.295E+04 | 347.1 | - | - | 0 | - |
| - | - | 1.655E+05 | 348.1 | - | - | 0 | - |
| - | - | 1.797E+04 | 349.1 | - | - | 0 | - |
| - | - | 1.668E+04 | 349.1 | - | - | 0 | - |
| - | - | 3.538E+05 | 350.1 | - | - | 0 | - |
| - | - | 5.513E+04 | 351.1 | - | - | 0 | - |
| - | - | 1.085E+05 | 352.1 | - | - | 0 | - |
| - | - | 2.38E+04 | 353.1 | - | - | 0 | - |
| - | - | 1.513E+04 | 354.2 | - | - | 0 | - |
| - | - | 3.383E+05 | 356.1 | - | - | 0 | - |
| 6 | b | 4.834E+04 | 357.1 | 0.0002749 | 0.7698 | +2 | 6 |
| - | - | 2.037E+04 | 358.1 | - | - | 0 | - |
| - | - | 2.094E+04 | 359.1 | - | - | 0 | - |
| - | - | 9.46E+04 | 360.1 | - | - | 0 | - |
| - | - | 2.147E+04 | 360.1 | - | - | 0 | - |
| - | - | 1.94E+04 | 361.1 | - | - | 0 | - |
| 5 | y | 5.52E+04 | 362.1 | 0.002981 | 8.232 | +2 | 5 |
| 3 | b | 1.533E+06 | 364.2 | 0.0005365 | 1.473 | +1 | 3 |
| - | - | 2.6E+05 | 365.2 | - | - | 0 | - |
| - | - | 1.356E+05 | 366.1 | - | - | 0 | - |
| - | - | 4.048E+04 | 366.2 | - | - | 0 | - |
| - | - | 3.769E+04 | 367.1 | - | - | 0 | - |
| - | - | 7.089E+04 | 367.2 | - | - | 0 | - |
| - | - | 1.789E+04 | 368.1 | - | - | 0 | - |
| - | - | 4.528E+04 | 371.1 | - | - | 0 | - |
| - | - | 6.732E+04 | 374.1 | - | - | 0 | - |
| - | - | 1.589E+05 | 376.1 | - | - | 0 | - |
| - | - | 1.499E+04 | 377.1 | - | - | 0 | - |
| - | - | 2.22E+04 | 377.1 | - | - | 0 | - |
| - | - | 5.557E+04 | 378.1 | - | - | 0 | - |
| - | - | 8.416E+05 | 378.1 | - | - | 0 | - |
| - | - | 1.674E+05 | 379.1 | - | - | 0 | - |
| - | - | 5.396E+04 | 380.1 | - | - | 0 | - |
| - | - | 1.678E+05 | 382.1 | - | - | 0 | - |
| - | - | 1.961E+04 | 383.1 | - | - | 0 | - |
| - | - | 4.301E+04 | 384.1 | - | - | 0 | - |
| - | - | 5.008E+04 | 385.1 | - | - | 0 | - |
| - | - | 1.765E+04 | 386.1 | - | - | 0 | - |
| - | - | 2.527E+04 | 388.1 | - | - | 0 | - |
| - | - | 3.338E+04 | 388.1 | - | - | 0 | - |
| - | - | 1.235E+05 | 390.2 | - | - | 0 | - |
| - | - | 2.105E+04 | 391.2 | - | - | 0 | - |
| - | - | 1.093E+06 | 394.1 | - | - | 0 | - |
| - | - | 2.052E+05 | 395.1 | - | - | 0 | - |
| - | - | 2.468E+04 | 395.2 | - | - | 0 | - |
| - | - | 2.619E+04 | 396.1 | - | - | 0 | - |
| - | - | 4.089E+04 | 397.2 | - | - | 0 | - |
| - | - | 2.386E+04 | 398.2 | - | - | 0 | - |
| - | - | 6.006E+04 | 399.2 | - | - | 0 | - |
| - | - | 1.8E+04 | 401.2 | - | - | 0 | - |
| - | - | 3.083E+04 | 404.1 | - | - | 0 | - |
| - | - | 2.902E+04 | 404.1 | - | - | 0 | - |
| - | - | 1.586E+04 | 405.1 | - | - | 0 | - |
| - | - | 4.157E+04 | 406.2 | - | - | 0 | - |
| - | - | 1.139E+05 | 407.2 | - | - | 0 | - |
| - | - | 2.417E+05 | 408.2 | - | - | 0 | - |
| - | - | 5.339E+04 | 409.2 | - | - | 0 | - |
| - | - | 2.086E+04 | 410.1 | - | - | 0 | - |
| - | - | 1.758E+04 | 410.2 | - | - | 0 | - |
| - | - | 7.861E+04 | 415.2 | - | - | 0 | - |
| - | - | 1.625E+04 | 416.1 | - | - | 0 | - |
| - | - | 1.587E+04 | 417.1 | - | - | 0 | - |
| - | - | 8.144E+04 | 419.2 | - | - | 0 | - |
| - | - | 1.63E+04 | 423.2 | - | - | 0 | - |
| - | - | 9.406E+05 | 425.2 | - | - | 0 | - |
| - | - | 1.993E+05 | 426.2 | - | - | 0 | - |
| - | - | 1.834E+05 | 427.2 | - | - | 0 | - |
| - | - | 3.113E+04 | 428.2 | - | - | 0 | - |
| - | - | 1.86E+04 | 429.1 | - | - | 0 | - |
| - | - | 1.451E+04 | 429.2 | - | - | 0 | - |
| - | - | 2.208E+04 | 430.1 | - | - | 0 | - |
| - | - | 1.919E+04 | 431.2 | - | - | 0 | - |
| - | - | 4.005E+04 | 432.1 | - | - | 0 | - |
| - | - | 1.276E+05 | 433.2 | - | - | 0 | - |
| - | - | 3.345E+04 | 434.2 | - | - | 0 | - |
| - | - | 5.625E+04 | 435.2 | - | - | 0 | - |
| - | - | 1.809E+04 | 436.1 | - | - | 0 | - |
| - | - | 2.252E+04 | 436.2 | - | - | 0 | - |
| - | - | 3.364E+04 | 437.2 | - | - | 0 | - |
| - | - | 2.536E+04 | 438.2 | - | - | 0 | - |
| - | - | 4.036E+04 | 442.2 | - | - | 0 | - |
| - | - | 4.566E+04 | 443.2 | - | - | 0 | - |
| - | - | 2.614E+04 | 443.2 | - | - | 0 | - |
| - | - | 1.217E+05 | 447.2 | - | - | 0 | - |
| - | - | 4.753E+04 | 449.1 | - | - | 0 | - |
| - | - | 3.203E+04 | 449.2 | - | - | 0 | - |
| - | - | 1.774E+04 | 452.7 | - | - | 0 | - |
| - | - | 7.414E+04 | 454.2 | - | - | 0 | - |
| - | - | 1.883E+05 | 455.2 | - | - | 0 | - |
| - | - | 3.899E+04 | 456.2 | - | - | 0 | - |
| - | - | 4.331E+04 | 458.2 | - | - | 0 | - |
| - | - | 3.582E+04 | 459.2 | - | - | 0 | - |
| 4 | b | 2.671E+05 | 461.2 | 0.0003935 | 0.8533 | +1 | 4 |
| - | - | 9.997E+04 | 461.2 | - | - | 0 | - |
| - | - | 5.612E+04 | 462.2 | - | - | 0 | - |
| - | - | 3.915E+04 | 462.2 | - | - | 0 | - |
| - | - | 8.267E+05 | 465.2 | - | - | 0 | - |
| - | - | 1.699E+05 | 466.2 | - | - | 0 | - |
| - | - | 2.192E+04 | 467.2 | - | - | 0 | - |
| 3 | y | 3.754E+04 | 468.2 | 0.00485 | 10.36 | +2 | 7 |
| - | - | 2.796E+04 | 469.2 | - | - | 0 | - |
| - | - | 1.894E+04 | 470.2 | - | - | 0 | - |
| - | - | 1.397E+04 | 470.2 | - | - | 0 | - |
| - | - | 1.095E+05 | 471.2 | - | - | 0 | - |
| - | - | 3.591E+04 | 472.2 | - | - | 0 | - |
| - | - | 6.613E+04 | 473.2 | - | - | 0 | - |
| - | - | 1.996E+04 | 475.1 | - | - | 0 | - |
| 3 | y | 1.476E+05 | 477.2 | 0.004095 | 8.581 | +2 | 7 |
| - | - | 4.074E+04 | 477.7 | - | - | 0 | - |
| - | - | 3.939E+04 | 478.2 | - | - | 0 | - |
| - | - | 5.092E+04 | 478.2 | - | - | 0 | - |
| 4 | b | 8.281E+05 | 479.2 | 0.0008762 | 1.829 | +1 | 4 |
| - | - | 1.143E+04 | 480.1 | - | - | 0 | - |
| - | - | 2.054E+05 | 480.2 | - | - | 0 | - |
| - | - | 4.133E+04 | 481.2 | - | - | 0 | - |
| - | - | 5.482E+04 | 482.2 | - | - | 0 | - |
| - | - | 2.416E+04 | 483.2 | - | - | 0 | - |
| - | - | 1.135E+05 | 486.2 | - | - | 0 | - |
| - | - | 3.548E+04 | 487.2 | - | - | 0 | - |
| 7 | y | 1.403E+06 | 489.2 | 0.005893 | 12.05 | +1 | 3 |
| - | - | 3.981E+05 | 490.2 | - | - | 0 | - |
| - | - | 2.377E+04 | 490.7 | - | - | 0 | - |
| - | - | 1.94E+05 | 491.2 | - | - | 0 | - |
| - | - | 4.192E+04 | 492.2 | - | - | 0 | - |
| - | - | 1.792E+04 | 493.2 | - | - | 0 | - |
| - | - | 1.137E+05 | 495.2 | - | - | 0 | - |
| - | - | 1.481E+04 | 495.7 | - | - | 0 | - |
| - | - | 3.726E+04 | 496.2 | - | - | 0 | - |
| - | - | 3.955E+04 | 496.2 | - | - | 0 | - |
| - | - | 7.567E+05 | 496.2 | - | - | 0 | - |
| - | - | 1.322E+05 | 497.2 | - | - | 0 | - |
| - | - | 1.926E+05 | 497.2 | - | - | 0 | - |
| - | - | 2.299E+04 | 498.2 | - | - | 0 | - |
| - | - | 3.492E+04 | 498.2 | - | - | 0 | - |
| 8 | b | 6.528E+04 | 499.2 | 0.0002344 | 0.4696 | +2 | 8 |
| - | - | 2.089E+04 | 500.2 | - | - | 0 | - |
| - | - | 2.664E+04 | 503.2 | - | - | 0 | - |
| - | - | 2.94E+04 | 504.2 | - | - | 0 | - |
| - | - | 2.858E+04 | 505.2 | - | - | 0 | - |
| - | - | 2.154E+04 | 506.2 | - | - | 0 | - |
| - | - | 2.922E+04 | 508.2 | - | - | 0 | - |
| - | - | 2.442E+04 | 511.2 | - | - | 0 | - |
| - | - | 1.603E+04 | 513.2 | - | - | 0 | - |
| - | - | 1.015E+05 | 513.2 | - | - | 0 | - |
| - | - | 2.234E+04 | 514.2 | - | - | 0 | - |
| - | - | 2.131E+04 | 514.2 | - | - | 0 | - |
| - | - | 3.847E+04 | 516.2 | - | - | 0 | - |
| - | - | 1.665E+04 | 517.2 | - | - | 0 | - |
| - | - | 4.493E+04 | 518.7 | - | - | 0 | - |
| - | - | 2.14E+05 | 519.2 | - | - | 0 | - |
| - | - | 1.191E+05 | 520.2 | - | - | 0 | - |
| - | - | 3.548E+04 | 521.2 | - | - | 0 | - |
| - | - | 2.967E+04 | 523.2 | - | - | 0 | - |
| - | - | 2.831E+04 | 524.2 | - | - | 0 | - |
| - | - | 2.161E+04 | 525.2 | - | - | 0 | - |
| - | - | 5.251E+04 | 530.2 | - | - | 0 | - |
| - | - | 2.855E+04 | 531.2 | - | - | 0 | - |
| - | - | 2.324E+04 | 532.7 | - | - | 0 | - |
| - | - | 6.267E+04 | 537.2 | - | - | 0 | - |
| - | - | 2.535E+04 | 538.2 | - | - | 0 | - |
| - | - | 5.843E+04 | 541.2 | - | - | 0 | - |
| 2 | y | 6.441E+04 | 541.7 | 0.003235 | 5.973 | +2 | 8 |
| - | - | 1.114E+05 | 542.2 | - | - | 0 | - |
| - | - | 2.786E+04 | 542.7 | - | - | 0 | - |
| - | - | 2.599E+04 | 543.2 | - | - | 0 | - |
| - | - | 2.082E+04 | 544.2 | - | - | 0 | - |
| - | - | 2.358E+04 | 547.2 | - | - | 0 | - |
| - | - | 2.501E+05 | 548.2 | - | - | 0 | - |
| - | - | 6.937E+04 | 549.2 | - | - | 0 | - |
| - | - | 1.694E+04 | 550.2 | - | - | 0 | - |
| 2 | y | 7.237E+05 | 550.7 | 0.003019 | 5.482 | +2 | 8 |
| - | - | 5.213E+05 | 551.2 | - | - | 0 | - |
| - | - | 2.326E+05 | 551.7 | - | - | 0 | - |
| - | - | 5.052E+04 | 552.2 | - | - | 0 | - |
| - | - | 2.155E+04 | 553.2 | - | - | 0 | - |
| - | - | 1.925E+04 | 556.2 | - | - | 0 | - |
| - | - | 1.255E+05 | 557.2 | - | - | 0 | - |
| - | - | 4.191E+04 | 558.2 | - | - | 0 | - |
| 6 | y | 2.332E+06 | 560.2 | 0.00534 | 9.531 | +1 | 4 |
| - | - | 7.284E+04 | 560.7 | - | - | 0 | - |
| - | - | 7.474E+05 | 561.2 | - | - | 0 | - |
| - | - | 1.769E+05 | 562.2 | - | - | 0 | - |
| - | - | 1.506E+05 | 569.2 | - | - | 0 | - |
| - | - | 1.024E+05 | 569.7 | - | - | 0 | - |
| - | - | 2.426E+05 | 570.2 | - | - | 0 | - |
| - | - | 3.708E+04 | 570.7 | - | - | 0 | - |
| - | - | 5.908E+04 | 571.2 | - | - | 0 | - |
| - | - | 1.813E+04 | 574.7 | - | - | 0 | - |
| - | - | 8.928E+04 | 578.2 | - | - | 0 | - |
| - | - | 4.611E+04 | 578.7 | - | - | 0 | - |
| - | - | 1.877E+04 | 579.2 | - | - | 0 | - |
| - | - | 7.152E+04 | 583.2 | - | - | 0 | - |
| - | - | 6.132E+04 | 583.7 | - | - | 0 | - |
| - | - | 3.915E+04 | 584.2 | - | - | 0 | - |
| - | - | 3.852E+04 | 588.2 | - | - | 0 | - |
| 0 | Precursor | 4.085E+05 | 592.2 | 0.003139 | 5.3 | +2 | -1 |
| - | - | 2.967E+05 | 592.7 | - | - | 0 | - |
| - | - | 1.13E+05 | 593.2 | - | - | 0 | - |
| - | - | 2.883E+04 | 593.7 | - | - | 0 | - |
| - | - | 7.999E+04 | 594.2 | - | - | 0 | - |
| - | - | 3.373E+04 | 595.2 | - | - | 0 | - |
| - | - | 1.357E+05 | 596.2 | - | - | 0 | - |
| - | - | 1.044E+05 | 597.2 | - | - | 0 | - |
| - | - | 5.224E+04 | 598.2 | - | - | 0 | - |
| 0 | Precursor | 1.071E+06 | 601.2 | 0.003411 | 5.673 | +2 | -1 |
| - | - | 7.931E+05 | 601.7 | - | - | 0 | - |
| - | - | 3.461E+05 | 602.2 | - | - | 0 | - |
| - | - | 4.7E+04 | 602.7 | - | - | 0 | - |
| - | - | 2.309E+05 | 606.2 | - | - | 0 | - |
| - | - | 6.022E+04 | 607.2 | - | - | 0 | - |
| - | - | 2.704E+04 | 608.2 | - | - | 0 | - |
| - | - | 3.648E+05 | 612.2 | - | - | 0 | - |
| - | - | 1.057E+05 | 613.2 | - | - | 0 | - |
| - | - | 2.277E+04 | 613.3 | - | - | 0 | - |
| - | - | 1.613E+04 | 614.2 | - | - | 0 | - |
| - | - | 2.876E+05 | 614.2 | - | - | 0 | - |
| - | - | 1.093E+05 | 615.2 | - | - | 0 | - |
| - | - | 1.92E+04 | 622.2 | - | - | 0 | - |
| 5 | b | 2.023E+05 | 624.2 | 0.00106 | 1.699 | +1 | 5 |
| - | - | 5.032E+04 | 625.2 | - | - | 0 | - |
| - | - | 1.696E+04 | 626.2 | - | - | 0 | - |
| - | - | 1.03E+05 | 631.2 | - | - | 0 | - |
| - | - | 4.347E+04 | 632.3 | - | - | 0 | - |
| - | - | 2.651E+05 | 634.2 | - | - | 0 | - |
| - | - | 5.802E+04 | 635.2 | - | - | 0 | - |
| - | - | 1.855E+04 | 636.2 | - | - | 0 | - |
| - | - | 6.004E+04 | 639.3 | - | - | 0 | - |
| - | - | 1.167E+05 | 640.2 | - | - | 0 | - |
| - | - | 4.97E+04 | 641.3 | - | - | 0 | - |
| 5 | b | 1.716E+05 | 642.2 | 0.0006885 | 1.072 | +1 | 5 |
| - | - | 6.447E+04 | 643.2 | - | - | 0 | - |
| - | - | 1.97E+04 | 644.2 | - | - | 0 | - |
| - | - | 1.906E+04 | 650.2 | - | - | 0 | - |
| - | - | 6.632E+04 | 652.2 | - | - | 0 | - |
| - | - | 1.582E+04 | 653.2 | - | - | 0 | - |
| - | - | 5.362E+05 | 657.3 | - | - | 0 | - |
| - | - | 1.618E+05 | 658.3 | - | - | 0 | - |
| - | - | 8.746E+05 | 659.3 | - | - | 0 | - |
| - | - | 3.251E+05 | 660.3 | - | - | 0 | - |
| - | - | 5.664E+04 | 661.3 | - | - | 0 | - |
| - | - | 9.632E+04 | 667.3 | - | - | 0 | - |
| - | - | 4.595E+04 | 668.3 | - | - | 0 | - |
| - | - | 1.328E+04 | 669.3 | - | - | 0 | - |
| - | - | 1.422E+05 | 676.3 | - | - | 0 | - |
| - | - | 7.123E+04 | 677.3 | - | - | 0 | - |
| - | - | 2.169E+04 | 678.3 | - | - | 0 | - |
| - | - | 7.51E+05 | 685.3 | - | - | 0 | - |
| - | - | 2.57E+05 | 686.3 | - | - | 0 | - |
| - | - | 4.771E+04 | 687.3 | - | - | 0 | - |
| 6 | b | 1.385E+05 | 695.3 | 0.0008362 | 1.203 | +1 | 6 |
| - | - | 4.502E+04 | 696.3 | - | - | 0 | - |
| - | - | 2.257E+04 | 702.3 | - | - | 0 | - |
| - | - | 2.12E+05 | 703.3 | - | - | 0 | - |
| - | - | 4.014E+04 | 704.2 | - | - | 0 | - |
| - | - | 4.193E+04 | 704.3 | - | - | 0 | - |
| - | - | 1.002E+05 | 705.3 | - | - | 0 | - |
| - | - | 6.066E+04 | 706.3 | - | - | 0 | - |
| - | - | 1.47E+04 | 708.3 | - | - | 0 | - |
| 6 | b | 9.379E+04 | 713.3 | 0.0001958 | 0.2746 | +1 | 6 |
| - | - | 2.682E+04 | 714.3 | - | - | 0 | - |
| - | - | 7.167E+05 | 721.3 | - | - | 0 | - |
| - | - | 2.844E+05 | 722.3 | - | - | 0 | - |
| 5 | y | 2.251E+06 | 723.3 | 0.005182 | 7.165 | +1 | 5 |
| - | - | 8.954E+05 | 724.3 | - | - | 0 | - |
| - | - | 3.052E+05 | 725.3 | - | - | 0 | - |
| - | - | 3.125E+04 | 728.3 | - | - | 0 | - |
| - | - | 8.181E+04 | 731.2 | - | - | 0 | - |
| - | - | 3.635E+04 | 732.2 | - | - | 0 | - |
| - | - | 2.874E+04 | 733.3 | - | - | 0 | - |
| - | - | 2.123E+04 | 747.3 | - | - | 0 | - |
| - | - | 1.72E+04 | 748.3 | - | - | 0 | - |
| - | - | 1.032E+06 | 749.3 | - | - | 0 | - |
| - | - | 4.035E+05 | 750.3 | - | - | 0 | - |
| - | - | 1.261E+05 | 751.3 | - | - | 0 | - |
| - | - | 2.281E+04 | 752.3 | - | - | 0 | - |
| - | - | 7.121E+04 | 756.3 | - | - | 0 | - |
| - | - | 4.273E+04 | 757.3 | - | - | 0 | - |
| - | - | 2.544E+04 | 759.3 | - | - | 0 | - |
| - | - | 2.618E+05 | 767.3 | - | - | 0 | - |
| - | - | 9.797E+04 | 768.3 | - | - | 0 | - |
| - | - | 4.099E+04 | 769.3 | - | - | 0 | - |
| - | - | 8.773E+05 | 774.3 | - | - | 0 | - |
| - | - | 3.821E+05 | 775.3 | - | - | 0 | - |
| - | - | 1.017E+05 | 776.3 | - | - | 0 | - |
| - | - | 3.291E+04 | 778.3 | - | - | 0 | - |
| - | - | 2.295E+04 | 779.3 | - | - | 0 | - |
| - | - | 1.664E+04 | 787.3 | - | - | 0 | - |
| - | - | 5.595E+04 | 792.3 | - | - | 0 | - |
| - | - | 1.981E+04 | 793.3 | - | - | 0 | - |
| - | - | 2.736E+04 | 796.3 | - | - | 0 | - |
| - | - | 2.257E+04 | 803.3 | - | - | 0 | - |
| - | - | 4.01E+04 | 804.3 | - | - | 0 | - |
| - | - | 1.68E+04 | 805.3 | - | - | 0 | - |
| - | - | 2.656E+04 | 814.3 | - | - | 0 | - |
| - | - | 2.153E+04 | 817.3 | - | - | 0 | - |
| - | - | 2.32E+04 | 818.3 | - | - | 0 | - |
| - | - | 1.75E+04 | 819.3 | - | - | 0 | - |
| 4 | y | 1.423E+05 | 820.3 | 0.006077 | 7.408 | +1 | 6 |
| - | - | 7.973E+04 | 821.3 | - | - | 0 | - |
| - | - | 2.994E+04 | 822.3 | - | - | 0 | - |
| - | - | 2.172E+04 | 823.3 | - | - | 0 | - |
| - | - | 1.675E+04 | 825.3 | - | - | 0 | - |
| - | - | 1.848E+04 | 827.3 | - | - | 0 | - |
| - | - | 8.053E+04 | 832.3 | - | - | 0 | - |
| - | - | 3.477E+04 | 833.3 | - | - | 0 | - |
| 4 | y | 2.833E+06 | 838.3 | 0.005278 | 6.296 | +1 | 6 |
| - | - | 1.291E+06 | 839.3 | - | - | 0 | - |
| - | - | 4.27E+05 | 840.3 | - | - | 0 | - |
| - | - | 4.093E+04 | 841.3 | - | - | 0 | - |
| 7 | b | 6.276E+04 | 842.3 | 0.00408 | 4.843 | +1 | 7 |
| - | - | 1.1E+05 | 843.3 | - | - | 0 | - |
| - | - | 5.416E+04 | 844.3 | - | - | 0 | - |
| - | - | 1.746E+04 | 845.4 | - | - | 0 | - |
| - | - | 1.85E+04 | 846.4 | - | - | 0 | - |
| - | - | 2.48E+04 | 848.3 | - | - | 0 | - |
| - | - | 4.082E+04 | 850.2 | - | - | 0 | - |
| - | - | 2.694E+04 | 850.3 | - | - | 0 | - |
| - | - | 2.214E+04 | 853.3 | - | - | 0 | - |
| - | - | 2.714E+04 | 854.3 | - | - | 0 | - |
| 7 | b | 6.683E+04 | 860.3 | 0.009323 | 10.84 | +1 | 7 |
| - | - | 3.282E+04 | 861.3 | - | - | 0 | - |
| - | - | 8.008E+04 | 862.3 | - | - | 0 | - |
| - | - | 4.335E+04 | 863.3 | - | - | 0 | - |
| - | - | 6.28E+04 | 868.3 | - | - | 0 | - |
| - | - | 4.239E+05 | 871.3 | - | - | 0 | - |
| - | - | 1.931E+05 | 872.3 | - | - | 0 | - |
| - | - | 7.782E+04 | 873.3 | - | - | 0 | - |
| - | - | 3.969E+06 | 889.3 | - | - | 0 | - |
| - | - | 1.996E+06 | 890.4 | - | - | 0 | - |
| - | - | 3.105E+04 | 890.4 | - | - | 0 | - |
| - | - | 5.132E+05 | 891.4 | - | - | 0 | - |
| - | - | 4.751E+04 | 892.3 | - | - | 0 | - |
| - | - | 8.08E+04 | 896.3 | - | - | 0 | - |
| - | - | 3.911E+04 | 897.3 | - | - | 0 | - |
| - | - | 2.225E+04 | 898.3 | - | - | 0 | - |
| - | - | 2.341E+04 | 899.3 | - | - | 0 | - |
| - | - | 1.49E+05 | 907.3 | - | - | 0 | - |
| - | - | 8.263E+04 | 908.3 | - | - | 0 | - |
| - | - | 2.834E+04 | 909.3 | - | - | 0 | - |
| - | - | 1.98E+04 | 914.3 | - | - | 0 | - |
| - | - | 2.392E+04 | 917.3 | - | - | 0 | - |
| - | - | 3.898E+04 | 918.3 | - | - | 0 | - |
| - | - | 2.767E+04 | 919.3 | - | - | 0 | - |
| - | - | 3.676E+04 | 933.4 | - | - | 0 | - |
| 3 | y | 7.442E+05 | 935.3 | 0.005196 | 5.555 | +1 | 7 |
| - | - | 4.882E+05 | 936.3 | - | - | 0 | - |
| - | - | 1.706E+05 | 937.3 | - | - | 0 | - |
| - | - | 1.222E+05 | 938.3 | - | - | 0 | - |
| - | - | 3.497E+04 | 939.3 | - | - | 0 | - |
| - | - | 2.076E+04 | 951.4 | - | - | 0 | - |
| 3 | y | 1.21E+07 | 953.3 | 0.00519 | 5.444 | +1 | 7 |
| - | - | 6.461E+06 | 954.3 | - | - | 0 | - |
| - | - | 2.286E+06 | 955.3 | - | - | 0 | - |
| - | - | 2.312E+05 | 956.3 | - | - | 0 | - |
| - | - | 9.231E+04 | 963.3 | - | - | 0 | - |
| - | - | 3.356E+04 | 964.3 | - | - | 0 | - |
| - | - | 5.118E+04 | 969.4 | - | - | 0 | - |
| - | - | 3.517E+04 | 970.4 | - | - | 0 | - |
| 8 | b | 2.374E+04 | 979.4 | 0.001991 | 2.033 | +1 | 8 |
| 8 | b | 1.897E+04 | 980.3 | 0.0195 | 19.89 | +1 | 8 |
| 8 | b | 1.296E+05 | 997.4 | 0.004366 | 4.378 | +1 | 8 |
| - | - | 7.063E+04 | 998.4 | - | - | 0 | - |
| - | - | 2.095E+04 | 999.4 | - | - | 0 | - |
| - | - | 4.72E+04 | 1015 | - | - | 0 | - |
| - | - | 2.586E+04 | 1016 | - | - | 0 | - |
| - | - | 6.461E+04 | 1018 | - | - | 0 | - |
| - | - | 5.421E+04 | 1019 | - | - | 0 | - |
| - | - | 2.625E+05 | 1036 | - | - | 0 | - |
| - | - | 1.689E+05 | 1037 | - | - | 0 | - |
| - | - | 3.802E+04 | 1038 | - | - | 0 | - |
| - | - | 1.787E+04 | 1046 | - | - | 0 | - |
| - | - | 1.761E+04 | 1056 | - | - | 0 | - |
| 2 | y | 9.476E+04 | 1082 | 0.005447 | 5.032 | +1 | 8 |
| - | - | 5.339E+04 | 1083 | - | - | 0 | - |
| 2 | y | 7.1E+05 | 1100 | 0.004648 | 4.223 | +1 | 8 |
| - | - | 4.524E+05 | 1101 | - | - | 0 | - |
| - | - | 1.852E+05 | 1102 | - | - | 0 | - |
| - | - | 2.792E+04 | 1103 | - | - | 0 | - |
| - | - | 1.209E+05 | 1110 | - | - | 0 | - |
| - | - | 8.447E+04 | 1111 | - | - | 0 | - |
| - | - | 4.446E+04 | 1112 | - | - | 0 | - |
| - | - | 1.49E+04 | 2401 | - | - | 0 | - |
| - | - | 1.719E+04 | 2441 | - | - | 0 | - |

m/z Charge Intensity FragmentType MassShift Position
120.08125305175781 0 7091824
121.04000091552734 0 22120.4
121.07906341552734 0 21391.3
121.08455657958984 0 554972.5
122.07174682617188 0 31723.475
122.0879898071289 0 20072.482
125.07132720947266 0 15021.966
127.0870361328125 0 32059.105
129.07052612304688 0 13581.879
130.06556701660156 0 199376.14
131.0684814453125 0 16810.377
132.08120727539062 0 367484.75
133.0846405029297 0 40655.637
134.09693908691406 0 40326.64
135.0999755859375 0 13953.187
136.07614135742188 0 2584963.5
136.08746337890625 0 77592.38
137.0794677734375 0 182685.56
137.09120178222656 0 12589.119
138.0553741455078 0 194238.75
138.06661987304688 0 307647.88
139.0592498779297 0 15089.203
139.0699462890625 0 16790.79
141.5588836669922 0 10099.392
143.07350158691406 0 48200.76
143.080078125 0 12080.138
143.08595275878906 0 45874.727
144.08120727539062 0 51663.48
146.06051635742188 0 98938.21
146.0968475341797 0 52597.51
147.04437255859375 0 11223.638
147.09190368652344 0 18455.066
148.0763702392578 0 33506.887
148.0873565673828 0 93157.78
148.1126708984375 0 13146.571
150.06591796875 0 12190.28
153.06643676757812 0 22596.104
155.08197021484375 0 43545.14
155.09312438964844 0 63021.57
156.0771942138672 0 642724.06
157.0805206298828 0 40636.016
158.0968475341797 0 1079209.1
159.05596923828125 0 14712.73
159.09213256835938 0 571159.9
159.10009765625 0 118211.93
160.07614135742188 0 206598.5
160.08779907226562 0 21734.955
160.0957489013672 0 42408.652
160.11248779296875 0 486542.5
161.11590576171875 0 52815.125
162.09225463867188 0 19684.922
164.0823516845703 0 18208.635
165.070068359375 0 28652.992
165.07757568359375 0 131011.414
165.10256958007812 0 13757.527
166.05368041992188 0 42249.56
166.06158447265625 0 94070.18
166.0863037109375 0 13592.713
167.045166015625 0 13930.623
167.0932159423828 0 116456.34
169.0765380859375 0 32475.537
170.0606689453125 0 16724.562
171.07687377929688 0 83934.62
172.07650756835938 0 17595.84
172.10855102539062 0 17268.973
173.0921173095703 0 13975.182
173.43838500976562 0 20356.822
174.0666961669922 0 79959.51 b Ammonia loss 2
174.09226989746094 0 13449.933
175.0697479248047 0 11640.266
175.0870361328125 0 38126.297
175.0979766845703 0 14319.765
175.12355041503906 0 28299.873
176.0824432373047 0 177990.14
176.10748291015625 0 2176386.8
177.10272216796875 0 1491951.9
177.11134338378906 0 212685.4
178.0616455078125 0 21021.857
178.09829711914062 0 12423.441
178.10606384277344 0 160506.55
180.0772705078125 0 112085.43
185.05618286132812 0 180524.08
186.0919189453125 0 185211.06
187.0631561279297 0 28753.375
188.07106018066406 0 780071.6
189.07443237304688 0 105914.8
189.10354614257812 0 11797.865
190.08688354492188 0 29427.6
191.0931854248047 0 77657.49
191.1183624267578 0 133873.02
192.1022186279297 0 21900.92
193.0727996826172 0 39338.87
193.10877990722656 0 627374.44
194.08038330078125 0 24371.896
194.0914306640625 0 11294.174
194.11231994628906 0 52683.793
195.08807373046875 0 168648.25
197.10464477539062 0 15232.787
202.05380249023438 0 79446.984
203.0665740966797 0 180502.05
203.0934295654297 0 60632.39
203.11827087402344 0 736588.94 a Water loss 1
204.0771484375 0 333707.66
204.10208129882812 0 53299.23 a Ammonia loss 1
204.1217498779297 0 104456.35
205.08135986328125 0 27482.154
205.09762573242188 0 580222.1 y 8
205.10818481445312 0 43762.242
206.10128784179688 0 58784.086
207.11334228515625 0 151594.2
208.07217407226562 0 92894.48
208.1168212890625 0 26954.988
212.11720275878906 0 38909.61
213.0502166748047 0 15942.259
213.09934997558594 0 14254.291
214.03570556640625 0 12637.427
215.08201599121094 0 37469.164
217.09765625 0 405020.78
218.08216857910156 0 22576.346
218.10110473632812 0 56179.3
219.08041381835938 0 121560.33
219.11318969726562 0 18001.064
220.12094116210938 0 18886.168
221.1041259765625 0 1701863
221.1290740966797 0 29472186 a 1
222.1071014404297 0 161774.6
222.1322479248047 0 3583316.8
223.11935424804688 0 41356.246
223.1347198486328 0 220344.48
224.11778259277344 0 21107.822
225.0990753173828 0 16474.602
228.11337280273438 0 84686.37
229.10865783691406 0 61412.816
231.0616912841797 0 453406.94
231.0883026123047 0 14929.592 b Water loss 3
231.11328125 0 300844.5 b Water loss 1
231.12620544433594 0 19814.086
232.06497192382812 0 35698.066
232.11688232421875 0 30037.113
233.0927734375 0 36433.324
235.10826110839844 0 807556.9
236.1109619140625 0 90795.734
237.08973693847656 0 26099.092
237.1241912841797 0 21918.414
238.1313018798828 0 28482.307
239.11448669433594 0 166438.62
240.08131408691406 0 18056.799
240.11700439453125 0 17790.857
245.0928192138672 0 34820.273 y 6
245.1290740966797 0 28546.729
246.07447814941406 0 15415.251
246.13531494140625 0 51312.098
247.10736083984375 0 33999.992
247.1199493408203 0 39053.004
247.14503479003906 0 18505.752
248.11526489257812 0 20531.105
248.15106201171875 0 73272.016
249.12396240234375 0 5132272.5 b 1
250.12730407714844 0 691860.06
251.1031494140625 0 515539.6
251.1292724609375 0 83075.69
252.10678100585938 0 55501.97
253.09353637695312 0 23877.201
255.11231994628906 0 14946.366
256.1084289550781 0 33272.297
257.0752868652344 0 27740.34
257.1068115234375 0 344114.3
258.11016845703125 0 33404.67
258.12432861328125 0 19896.186
261.0875244140625 0 90300.875
263.07012939453125 0 48171.45
263.10302734375 0 1039388.9
263.12933349609375 0 99452.945
264.1066589355469 0 167543.64
264.13177490234375 0 21689.162
264.1461181640625 0 105177.13
265.1173095703125 0 42726.055
266.1235046386719 0 23095.809
267.0914306640625 0 62016.17
268.0751953125 0 57698.758
268.09197998046875 0 18991.299
268.14013671875 0 36671.152
269.0773010253906 0 49964.836
270.0788269042969 0 13992.194
270.109375 0 25339.457
271.1087341308594 0 46116.293
272.1133728027344 0 24471.035
273.0876159667969 0 16507.945
273.1239013671875 0 22773.203
274.13018798828125 0 282823.7
274.1558837890625 0 25103.445
275.11444091796875 0 43252.918
275.1336364746094 0 35526.137
277.59674072265625 0 17379.633
277.6205749511719 0 14277.675
279.09808349609375 0 489292.44
279.125244140625 0 42838.312
280.10137939453125 0 43526.074
283.1064453125 0 18806.129
283.15252685546875 0 19998.6
285.1021423339844 0 676977.2
286.1048889160156 0 74415.36
286.12017822265625 0 14392.861
287.100830078125 0 34535.527
289.0824890136719 0 33828.72
289.1190185546875 0 15912.827
290.1260986328125 0 27203.725
292.1409912109375 0 408179.62
292.1662292480469 0 99628.516 d 2
293.1435852050781 0 42419.7
294.1546936035156 0 20023.74
295.14385986328125 0 13518.481
296.1512145996094 0 539114.7
297.13525390625 0 22160.12
297.15478515625 0 89264.84
299.1508483886719 0 34379.918
300.13482666015625 0 63476.777
301.1190490722656 0 33364.152
302.114501953125 0 47228.824
303.1136474609375 0 91601.23
304.1147155761719 0 17232.934
305.4649353027344 0 15703.436
306.1333923339844 0 21017.219
306.1468200683594 0 15839.155
310.15167236328125 0 73200.15
313.0967712402344 0 42826.98
314.0987854003906 0 70105.1
315.0997009277344 0 25023.266
318.1455078125 0 166064.39 a Water loss 2
319.1489562988281 0 34968.24
320.1355285644531 0 26396.791
323.09942626953125 0 197730.75
323.14013671875 0 14853.643
324.1462707519531 0 1181274.5
325.14910888671875 0 201630.5
326.154296875 0 18567.598
327.14593505859375 0 55179.363
328.1294860839844 0 113971.17
328.1466064453125 0 49255.79
329.13189697265625 0 18456.887
330.1100158691406 0 65099.27
331.0929260253906 0 26305.56
331.1131286621094 0 13343.931
332.12457275390625 0 86330.23
333.10882568359375 0 38082.9
334.1083984375 0 22110.426
334.1302490234375 0 22453.457
335.17132568359375 0 23854.68
336.1560974121094 0 25949.467 a 2
338.12884521484375 0 75424.28
339.171142578125 0 109545.414
340.1752014160156 0 17046.537
342.15679931640625 0 6117012 y 7
343.1598815917969 0 1121017.2
344.162353515625 0 86624.555
346.1403503417969 0 365387.1 b Water loss 2
347.14337158203125 0 62950.97
348.1194152832031 0 165547.98
349.10198974609375 0 17972.873
349.1243591308594 0 16675.88
350.13525390625 0 353847.25
351.1380310058594 0 55128.45
352.1407470703125 0 108494.516
353.1465759277344 0 23797.516
354.1554870605469 0 15125.087
356.1390686035156 0 338278.34
357.1422119140625 0 48344.164 b 5
358.1046447753906 0 20365.488
359.0865173339844 0 20942.352
360.1195373535156 0 94603.32
360.14306640625 0 21470.03
361.123779296875 0 19404.912
362.1501159667969 0 55202.16 y 4
364.1508483886719 0 1533393.8 b 2
365.15411376953125 0 259976.33
366.1294860839844 0 135582.11
366.15631103515625 0 40478.703
367.13250732421875 0 37693.54
367.16571044921875 0 70892.51
368.1190490722656 0 17890.582
371.1353759765625 0 45279.965
374.14923095703125 0 67324.73
376.11456298828125 0 158904.62
377.0954895019531 0 14987.46
377.11981201171875 0 22201.352
378.09979248046875 0 55572.652
378.1301574707031 0 841624.2
379.1331787109375 0 167445.56
380.1393127441406 0 53956.055
382.14373779296875 0 167816.05
383.14617919921875 0 19612.463
384.13519287109375 0 43006.934
385.1361389160156 0 50082.742
386.13726806640625 0 17651.154
388.11602783203125 0 25273.578
388.149658203125 0 33380.54
390.1570129394531 0 123475.86
391.15966796875 0 21049.473
394.12518310546875 0 1092798.9
395.12823486328125 0 205218.47
395.1567687988281 0 24677.285
396.13177490234375 0 26186.62
397.16314697265625 0 40892.2
398.1690368652344 0 23864.39
399.17681884765625 0 60064.41
401.1961975097656 0 18002.166
404.1130065917969 0 30831.734
404.145263671875 0 29022.496
405.1146545410156 0 15861.225
406.1518249511719 0 41573.016
407.1836242675781 0 113943.336
408.1673278808594 0 241731
409.1696472167969 0 53388.66
410.1404113769531 0 20862.219
410.18212890625 0 17581.076
415.1618347167969 0 78611.77
416.14508056640625 0 16252.024
417.144775390625 0 15870.451
419.1568603515625 0 81439.83
423.17840576171875 0 16295.685
425.1940612792969 0 940629
426.1971130371094 0 199303.53
427.2091064453125 0 183421.38
428.2106628417969 0 31130.363
429.143310546875 0 18602.459
429.19097900390625 0 14505.265
430.1235656738281 0 22080.316
431.1575927734375 0 19186.416
432.1413269042969 0 40046.9
433.1729736328125 0 127627.29
434.1763916015625 0 33446.184
435.1800842285156 0 56252.543
436.14752197265625 0 18093.51
436.181640625 0 22522.129
437.1673889160156 0 33639.03
438.1706848144531 0 25360.713
442.17303466796875 0 40355.234
443.1556701660156 0 45661.68
443.1866455078125 0 26143.566
447.15142822265625 0 121650.49
449.1344909667969 0 47526.46
449.1679382324219 0 32030.584
452.6690368652344 0 17740.193
454.1968688964844 0 74139.195
455.2041931152344 0 188332.23
456.2077331542969 0 38989.684
458.1688232421875 0 43310.41
459.15362548828125 0 35817.305
461.1670837402344 0 267069.72 b Water loss 3
461.1960144042969 0 99971.234
462.1698303222656 0 56123.58
462.20025634765625 0 39146.586
465.1625061035156 0 826656.2
466.16571044921875 0 169914.06
467.16748046875 0 21922.553
468.17364501953125 0 37536.184 y Water loss 2
469.20965576171875 0 27963.074
470.1679992675781 0 18938.62
470.21240234375 0 13965.2705
471.1808776855469 0 109504.43
472.1824645996094 0 35905.223
473.2148742675781 0 66127.01
475.1444091796875 0 19962.004
477.16998291015625 0 147606.14 y 2
477.6781005859375 0 40736.805
478.16595458984375 0 39389.117
478.22113037109375 0 50917.164
479.1781311035156 0 828099.2 b 3
480.1445617675781 0 11430.541
480.1812438964844 0 205365.92
481.18206787109375 0 41329.89
482.19342041015625 0 54823.266
483.19647216796875 0 24159.973
486.1631164550781 0 113471.32
487.16668701171875 0 35479.88
489.19244384765625 0 1402828.8 y 6
490.19464111328125 0 398135.12
490.68585205078125 0 23773.553
491.2055358886719 0 193974.7
492.20977783203125 0 41917.445
493.20306396484375 0 17922.95
495.1868896484375 0 113702.586
495.69329833984375 0 14810.388
496.1729431152344 0 37258.367
496.1885070800781 0 39546.875
496.2311096191406 0 756706.56
497.1711730957031 0 132197.81
497.2343444824219 0 192576.52
498.1737976074219 0 22990.46
498.2389221191406 0 34921.066
499.18695068359375 0 65282.605 b 7
500.18804931640625 0 20894.54
503.1781311035156 0 26642.234
504.1814880371094 0 29397.338
505.19317626953125 0 28582.809
506.21563720703125 0 21542.047
508.19476318359375 0 29215.78
511.19354248046875 0 24422.688
513.1630249023438 0 16025.253
513.1991577148438 0 101534.78
514.1925659179688 0 22339.537
514.22900390625 0 21307.936
516.2232055664062 0 38473.617
517.2260131835938 0 16646.162
518.7092895507812 0 44931.723
519.2041015625 0 214008.55
520.2052612304688 0 119136.49
521.20556640625 0 35481.543
523.1859741210938 0 29667.137
524.223388671875 0 28305.275
525.1992797851562 0 21614.746
530.1896362304688 0 52507.496
531.18994140625 0 28549.568
532.70068359375 0 23238.268
537.2135620117188 0 62672.75
538.2155151367188 0 25351.807
541.1942138671875 0 58426.08
541.7062377929688 0 64413.434 y Water loss 1
542.234375 0 111427.586
542.710205078125 0 27860.082
543.2239990234375 0 25986.758
544.2119750976562 0 20820.752
547.195068359375 0 23580.62
548.1995849609375 0 250130.2
549.2020874023438 0 69374.15
550.2078247070312 0 16941.42
550.7113037109375 0 723724.7 y 1
551.2130737304688 0 521344.88
551.7138671875 0 232558.61
552.215576171875 0 50518.207
553.2003173828125 0 21550.092
556.210693359375 0 19246.875
557.19970703125 0 125481.59
558.2023315429688 0 41912
560.22900390625 0 2331863.5 y 5
560.7299194335938 0 72842.92
561.2321166992188 0 747350.2
562.2308959960938 0 176872.72
569.2340698242188 0 150616.28
569.7352294921875 0 102422.25
570.2291870117188 0 242603.75
570.720458984375 0 37080.816
571.2305297851562 0 59076.535
574.7166137695312 0 18129.379
578.2305297851562 0 89280.84
578.7325439453125 0 46113.383
579.2156982421875 0 18765.475
583.2258911132812 0 71517.17
583.7255249023438 0 61316.992
584.2031860351562 0 39154.95
588.240234375 0 38517.82
592.22998046875 0 408459.25 Precursor Water loss
592.7318115234375 0 296696.03
593.2322387695312 0 113009.51
593.7312622070312 0 28834.582
594.186279296875 0 79985.91
595.1869506835938 0 33729.34
596.2355346679688 0 135728.6
597.2254638671875 0 104437.01
598.2254638671875 0 52235.19
601.2355346679688 0 1071055 Precursor
601.7366943359375 0 793070.94
602.2374267578125 0 346144.7
602.737060546875 0 47001.742
606.233154296875 0 230944.55
607.23681640625 0 60216.496
608.2337646484375 0 27038.58
612.1978149414062 0 364796
613.2012329101562 0 105690.48
613.29052734375 0 22766.865
614.2008666992188 0 16126.004
614.246337890625 0 287555
615.2483520507812 0 109298.76
622.2219848632812 0 19202.357
624.2310791015625 0 202283.47 b Water loss 4
625.2332153320312 0 50317.55
626.2349243164062 0 16957.771
631.2467041015625 0 103007.23
632.2583618164062 0 43473.555
634.2295532226562 0 265102.2
635.2333374023438 0 58021.3
636.2343139648438 0 18546.445
639.2531127929688 0 60044.01
640.2382202148438 0 116724.33
641.2804565429688 0 49695.445
642.2412719726562 0 171637.66 b 4
643.2451171875 0 64466.04
644.24658203125 0 19699.273
650.2454833984375 0 19056.543
652.2403564453125 0 66319.29
653.2449340820312 0 15817.383
657.263427734375 0 536225.7
658.2657470703125 0 161762.14
659.294189453125 0 874613.5
660.297119140625 0 325069
661.29931640625 0 56643.938
667.2515258789062 0 96323.93
668.251220703125 0 45951.023
669.27783203125 0 13282.995
676.2696533203125 0 142213.83
677.2758178710938 0 71227.414
678.2951049804688 0 21691.428
685.2583618164062 0 750989.3
686.2612915039062 0 257029.88
687.2650146484375 0 47711.516
695.2662963867188 0 138460.03 b Water loss 5
696.2725830078125 0 45015.1
702.28662109375 0 22572.508
703.2653198242188 0 212025.97
704.2440185546875 0 40143.445
704.2664794921875 0 41927.61
705.2815551757812 0 100177.26
706.2854614257812 0 60657.54
708.2720947265625 0 14695.937
713.2778930664062 0 93788.875 b 5
714.2777099609375 0 26820.889
721.2614135742188 0 716726.25
722.2645874023438 0 284427.78
723.2921752929688 0 2251368.8 y 4
724.2952880859375 0 895409.75
725.295166015625 0 305183.9
728.3198852539062 0 31251.404
731.2474975585938 0 81812.29
732.2432250976562 0 36352.227
733.2720336914062 0 28742.07
747.2918090820312 0 21234.957
748.2979125976562 0 17199.387
749.2561645507812 0 1032349.4
750.2588500976562 0 403495.75
751.259521484375 0 126069.5
752.25341796875 0 22811.729
756.310546875 0 71207.36
757.30517578125 0 42727.7
759.259521484375 0 25444.688
767.2667236328125 0 261791.95
768.2706298828125 0 97966.02
769.2672729492188 0 40989.246
774.320556640625 0 877278.3
775.323486328125 0 382132.34
776.32666015625 0 101700.03
778.298095703125 0 32909.516
779.31103515625 0 22947.367
787.3148803710938 0 16640.75
792.3156127929688 0 55948.45
793.3134765625 0 19811.488
796.3153686523438 0 27357.713
803.284912109375 0 22574.84
804.32763671875 0 40102.793
805.3328247070312 0 16797.027
814.3160400390625 0 26555.697
817.3228759765625 0 21534.867
818.33447265625 0 23195.697
819.3413696289062 0 17500.926
820.3094482421875 0 142270 y Water loss 3
821.3070068359375 0 79731.14
822.299560546875 0 29935.748
823.2855224609375 0 21721.725
825.3106079101562 0 16753.254
827.3470458984375 0 18483.537
832.3264770507812 0 80530.99
833.3284301757812 0 34774.934
838.3192138671875 0 2832887.5 y 3
839.3218383789062 0 1290682.8
840.321533203125 0 427000.9
841.3250732421875 0 40934.477
842.3016967773438 0 62756.58 b Water loss 6
843.3391723632812 0 109953.484
844.34228515625 0 54159.844
845.3539428710938 0 17456.76
846.3591918945312 0 18496.676
848.30224609375 0 24801.084
850.1714477539062 0 40816.23
850.3236083984375 0 26940.258
853.3203735351562 0 22138.746
854.3191528320312 0 27143.225
860.3175048828125 0 66830.13 b 6
861.314697265625 0 32816.91
862.3238525390625 0 80082.89
863.3256225585938 0 43352.4
868.3311767578125 0 62796.49
871.3368530273438 0 423891.62
872.3364868164062 0 193145.03
873.3384399414062 0 77824.11
889.3473510742188 0 3969402.8
890.3502197265625 0 1995913.8
890.4302978515625 0 31046.857
891.35302734375 0 513234.4
892.3488159179688 0 47507.44
896.3245239257812 0 80802.3
897.3297729492188 0 39106.176
898.3217163085938 0 22253.932
899.3314819335938 0 23411.113
907.3394775390625 0 149010.17
908.342041015625 0 82629.92
909.3403930664062 0 28336.707
914.3306274414062 0 19799.23
917.3245849609375 0 23919.973
918.3199462890625 0 38980.496
919.318603515625 0 27668.904
933.3786010742188 0 36758.055
935.3355102539062 0 744199.2 y Water loss 2
936.3350219726562 0 488167.2
937.3347778320312 0 170649.77
938.3265991210938 0 122225.1
939.3259887695312 0 34972.613
951.3657836914062 0 20760.234
953.3460693359375 0 12102720 y 2
954.3486328125 0 6460993.5
955.348876953125 0 2285785.2
956.3493041992188 0 231213.11
963.3313598632812 0 92309.11
964.3329467773438 0 33563.17
969.376953125 0 51178.1
970.3757934570312 0 35174.973
979.3585205078125 0 23744.436 b Water loss 7
980.3600463867188 0 18966.85 b Ammonia loss 7
997.3714599609375 0 129582.09 b 7
998.375732421875 0 70625.555
999.3756713867188 0 20945.385
1015.3811645507812 0 47200.61
1016.39111328125 0 25858.322
1018.3936157226562 0 64606.22
1019.4008178710938 0 54206.047
1036.413818359375 0 262479.56
1037.419189453125 0 168900.1
1038.4210205078125 0 38022.637
1046.3929443359375 0 17869.68
1055.9176025390625 0 17607.543
1082.4041748046875 0 94762.484 y Water loss 1
1083.4022216796875 0 53388.46
1100.4139404296875 0 709954.5 y 1
1101.4166259765625 0 452356.28
1102.417236328125 0 185164.64
1103.4202880859375 0 27918.594
1110.3992919921875 0 120917.45
1111.4007568359375 0 84474.38
1112.4019775390625 0 44457.62
2401.114013671875 0 14902.679
2441.31640625 0 17188.86

Spectrum Details

|  |  |
| --- | --- |
| Matched peaks? Matched peaksThe total absolute number of peaks matched. Additionally in brackets the total fraction of peaks matched and the total number of peaks is shown. | 44 (6.89% of 639) |
| FDR? FDRThe false discovery rate estimated for this peptide. It is calculated by matching all theoretical fragments with a non-integer shift with the raw peaks for this spectrum. This is done with 40 different shifts. The resulting percentage is the average number of annotated peaks over the number of annotated peaks with the correct spectrum. | 0.00% |
| Satellite FDR? Satellite FDRSee the FDR for details on its calculation. This satellite ion specific FDR only contains the satellite ions (d/w) for I/L/J positions. | - |
| PSM Score? PSM ScoreThe PSM Score as given by Hecklib to this annotated spectrum. It is shown with three significant figures. | 536 |

## Spectrum 7379? Spectrum 7379 The raw spectrum of this peptide as annotated by Hecklib. The fragments are coloured according to ion type (see legend). Any peaks with a star '\*' as text can be hovered over to see the full details, first the ion type second the mass shift type. By hovering over the amino acids in the peptide or ions in the legend the corresponding peaks are highlighted. By toggling the 'Unassigned' label you can turn the background (unassigned) peaks on or off in the plot. By updating the slider in the Ion legend you can update the spectrum to only show the top X% of the peaks with labels. The top X% means any peak that is within X% of the highest intensity. By dragging in the spectrum you can zoom in to a specific part of the spectrum and use 'Zoom Out' to get back to the original zoom level. The annotation of the spectrum is based on the given sequence in the peptides file and is done with different software so inconsistencies are likely. The peaks are annotated based on the given sequence, with 20 ppm tolerance.

Copy Data

### Spectrum 7379 (TSV)

#### Preview

```
Loading example...
```

*Click on the button to copy the data to your clipboard.*

Mz MinMz MaxIntensity Max

WidthHeightPeptide font sizePeptide stroke widthSpectrum font sizeSpectrum stroke widthCompact peptide

Ion legend

wxyz

abcd

OtherUnassignedIonChargePositionShow for top:%

TFDDYAMHW

02.23e+64.47e+66.70e+68.94e+6

Zoom Out

b+23a+12a+12y+11a+12b+24b+12y+23b+12d+13a+13y+12b+13b+26b+26y+25b+13b+14y+27y+27b+14y+13b+28y+28y+28y+14\*\*b+15b+15b+16b+16y+15y+16y+16b+17b+17y+17y+17b+18b+18y+18y+18

0818163624543272

Fragment Matches Table

Show background peaks

| Position | Ion type | Intensity | mz Theoretical | mz Error (Th) | mz Error (ppm) | Charge | Series Number |
| --- | --- | --- | --- | --- | --- | --- | --- |
| - | - | 2.182E+06 | 120.1 | - | - | 0 | - |
| - | - | 5911 | 121 | - | - | 0 | - |
| - | - | 1.627E+05 | 121.1 | - | - | 0 | - |
| - | - | 1.277E+04 | 122.1 | - | - | 0 | - |
| - | - | 6464 | 122.1 | - | - | 0 | - |
| - | - | 6640 | 127.1 | - | - | 0 | - |
| - | - | 9324 | 129.1 | - | - | 0 | - |
| - | - | 3154 | 129.2 | - | - | 0 | - |
| - | - | 4.657E+04 | 130.1 | - | - | 0 | - |
| - | - | 5443 | 131.1 | - | - | 0 | - |
| - | - | 1.12E+05 | 132.1 | - | - | 0 | - |
| - | - | 6168 | 133.1 | - | - | 0 | - |
| - | - | 8821 | 133.1 | - | - | 0 | - |
| - | - | 9515 | 134.1 | - | - | 0 | - |
| - | - | 7.861E+05 | 136.1 | - | - | 0 | - |
| - | - | 2.627E+04 | 136.1 | - | - | 0 | - |
| - | - | 5548 | 137.1 | - | - | 0 | - |
| - | - | 6.285E+04 | 137.1 | - | - | 0 | - |
| - | - | 5369 | 137.1 | - | - | 0 | - |
| - | - | 5.913E+04 | 138.1 | - | - | 0 | - |
| - | - | 8.726E+04 | 138.1 | - | - | 0 | - |
| - | - | 4916 | 139.1 | - | - | 0 | - |
| - | - | 6577 | 141.1 | - | - | 0 | - |
| - | - | 5459 | 143 | - | - | 0 | - |
| - | - | 1.395E+04 | 143.1 | - | - | 0 | - |
| - | - | 1.259E+04 | 143.1 | - | - | 0 | - |
| - | - | 3006 | 144.1 | - | - | 0 | - |
| - | - | 1.091E+04 | 144.1 | - | - | 0 | - |
| - | - | 3.001E+04 | 146.1 | - | - | 0 | - |
| - | - | 4785 | 146.1 | - | - | 0 | - |
| - | - | 1.386E+04 | 146.1 | - | - | 0 | - |
| - | - | 7203 | 148.1 | - | - | 0 | - |
| - | - | 2.491E+04 | 148.1 | - | - | 0 | - |
| - | - | 6613 | 150.1 | - | - | 0 | - |
| - | - | 1.391E+04 | 155.1 | - | - | 0 | - |
| - | - | 2.172E+04 | 155.1 | - | - | 0 | - |
| - | - | 2.044E+05 | 156.1 | - | - | 0 | - |
| - | - | 1.233E+04 | 157.1 | - | - | 0 | - |
| - | - | 3.26E+05 | 158.1 | - | - | 0 | - |
| - | - | 3697 | 159.1 | - | - | 0 | - |
| - | - | 1.649E+05 | 159.1 | - | - | 0 | - |
| - | - | 3.406E+04 | 159.1 | - | - | 0 | - |
| - | - | 5.868E+04 | 160.1 | - | - | 0 | - |
| - | - | 6060 | 160.1 | - | - | 0 | - |
| - | - | 1.54E+04 | 160.1 | - | - | 0 | - |
| - | - | 1.486E+05 | 160.1 | - | - | 0 | - |
| - | - | 3859 | 161.1 | - | - | 0 | - |
| - | - | 1.65E+04 | 161.1 | - | - | 0 | - |
| - | - | 5179 | 162.1 | - | - | 0 | - |
| - | - | 4468 | 164.1 | - | - | 0 | - |
| - | - | 9347 | 165.1 | - | - | 0 | - |
| - | - | 3.29E+04 | 165.1 | - | - | 0 | - |
| - | - | 1.337E+04 | 166.1 | - | - | 0 | - |
| - | - | 2.806E+04 | 166.1 | - | - | 0 | - |
| - | - | 5245 | 166.1 | - | - | 0 | - |
| - | - | 2.515E+04 | 167.1 | - | - | 0 | - |
| - | - | 1.202E+04 | 169.1 | - | - | 0 | - |
| - | - | 2.719E+04 | 171.1 | - | - | 0 | - |
| - | - | 5293 | 171.1 | - | - | 0 | - |
| - | - | 5993 | 172.1 | - | - | 0 | - |
| - | - | 5835 | 172.1 | - | - | 0 | - |
| - | - | 8810 | 173.1 | - | - | 0 | - |
| 3 | b | 2.021E+04 | 174.1 | 0.001222 | 7.022 | +2 | 3 |
| - | - | 4125 | 174.1 | - | - | 0 | - |
| - | - | 1.27E+04 | 175.1 | - | - | 0 | - |
| - | - | 5952 | 175.1 | - | - | 0 | - |
| - | - | 8749 | 175.1 | - | - | 0 | - |
| - | - | 5.372E+04 | 176.1 | - | - | 0 | - |
| - | - | 6.578E+05 | 176.1 | - | - | 0 | - |
| - | - | 4.675E+05 | 177.1 | - | - | 0 | - |
| - | - | 7.02E+04 | 177.1 | - | - | 0 | - |
| - | - | 4.455E+04 | 178.1 | - | - | 0 | - |
| - | - | 4998 | 178.1 | - | - | 0 | - |
| - | - | 2.927E+04 | 180.1 | - | - | 0 | - |
| - | - | 5605 | 182 | - | - | 0 | - |
| - | - | 5.017E+04 | 185.1 | - | - | 0 | - |
| - | - | 5.44E+04 | 186.1 | - | - | 0 | - |
| - | - | 1.201E+04 | 187.1 | - | - | 0 | - |
| - | - | 2.233E+05 | 188.1 | - | - | 0 | - |
| - | - | 2.768E+04 | 189.1 | - | - | 0 | - |
| - | - | 8221 | 190.1 | - | - | 0 | - |
| - | - | 5370 | 191.1 | - | - | 0 | - |
| - | - | 2.356E+04 | 191.1 | - | - | 0 | - |
| - | - | 4.673E+04 | 191.1 | - | - | 0 | - |
| - | - | 1.12E+04 | 192.1 | - | - | 0 | - |
| - | - | 4599 | 192.1 | - | - | 0 | - |
| - | - | 1.346E+04 | 193.1 | - | - | 0 | - |
| - | - | 1.859E+05 | 193.1 | - | - | 0 | - |
| - | - | 9673 | 194.1 | - | - | 0 | - |
| - | - | 1.411E+04 | 194.1 | - | - | 0 | - |
| - | - | 4155 | 194.3 | - | - | 0 | - |
| - | - | 4.846E+04 | 195.1 | - | - | 0 | - |
| - | - | 3839 | 196.7 | - | - | 0 | - |
| - | - | 1.592E+04 | 202.1 | - | - | 0 | - |
| - | - | 5.248E+04 | 203.1 | - | - | 0 | - |
| - | - | 1.184E+04 | 203.1 | - | - | 0 | - |
| 2 | a | 2.237E+05 | 203.1 | 0.0004729 | 2.328 | +1 | 2 |
| - | - | 1.023E+05 | 204.1 | - | - | 0 | - |
| 2 | a | 1.362E+04 | 204.1 | 0.0003898 | 1.91 | +1 | 2 |
| - | - | 3.078E+04 | 204.1 | - | - | 0 | - |
| - | - | 8510 | 205.1 | - | - | 0 | - |
| 9 | y | 1.731E+05 | 205.1 | 0.0005327 | 2.597 | +1 | 1 |
| - | - | 1.346E+04 | 205.1 | - | - | 0 | - |
| - | - | 2.532E+04 | 206.1 | - | - | 0 | - |
| - | - | 4.775E+04 | 207.1 | - | - | 0 | - |
| - | - | 2.809E+04 | 208.1 | - | - | 0 | - |
| - | - | 4858 | 209.1 | - | - | 0 | - |
| - | - | 5479 | 211.1 | - | - | 0 | - |
| - | - | 1.209E+04 | 212.1 | - | - | 0 | - |
| - | - | 1.131E+04 | 215.1 | - | - | 0 | - |
| - | - | 7610 | 216.1 | - | - | 0 | - |
| - | - | 1.171E+05 | 217.1 | - | - | 0 | - |
| - | - | 1.368E+04 | 218.1 | - | - | 0 | - |
| - | - | 3.737E+04 | 219.1 | - | - | 0 | - |
| - | - | 7069 | 219.1 | - | - | 0 | - |
| - | - | 4828 | 220.1 | - | - | 0 | - |
| - | - | 5.394E+05 | 221.1 | - | - | 0 | - |
| 2 | a | 8.848E+06 | 221.1 | 0.0007267 | 3.286 | +1 | 2 |
| - | - | 5.126E+04 | 222.1 | - | - | 0 | - |
| - | - | 1.075E+06 | 222.1 | - | - | 0 | - |
| - | - | 8257 | 223.1 | - | - | 0 | - |
| - | - | 6.325E+04 | 223.1 | - | - | 0 | - |
| - | - | 8979 | 224.1 | - | - | 0 | - |
| - | - | 6291 | 225.1 | - | - | 0 | - |
| - | - | 2.429E+04 | 228.1 | - | - | 0 | - |
| - | - | 1.875E+04 | 229.1 | - | - | 0 | - |
| - | - | 5973 | 230.1 | - | - | 0 | - |
| - | - | 1.493E+05 | 231.1 | - | - | 0 | - |
| 4 | b | 5860 | 231.1 | 0.001899 | 8.218 | +2 | 4 |
| 2 | b | 8.454E+04 | 231.1 | 0.0005839 | 2.526 | +1 | 2 |
| - | - | 4046 | 231.1 | - | - | 0 | - |
| - | - | 1.373E+04 | 232.1 | - | - | 0 | - |
| - | - | 1.832E+04 | 232.1 | - | - | 0 | - |
| - | - | 4682 | 233.1 | - | - | 0 | - |
| - | - | 5954 | 234.1 | - | - | 0 | - |
| - | - | 2.085E+05 | 235.1 | - | - | 0 | - |
| - | - | 2.329E+04 | 236.1 | - | - | 0 | - |
| - | - | 8882 | 237.1 | - | - | 0 | - |
| - | - | 6521 | 237.1 | - | - | 0 | - |
| - | - | 1.373E+04 | 238.1 | - | - | 0 | - |
| - | - | 5.744E+04 | 239.1 | - | - | 0 | - |
| - | - | 8397 | 240.1 | - | - | 0 | - |
| - | - | 4632 | 241.1 | - | - | 0 | - |
| - | - | 5882 | 241.1 | - | - | 0 | - |
| 7 | y | 8183 | 245.1 | 0.003514 | 14.34 | +2 | 3 |
| - | - | 8516 | 245.1 | - | - | 0 | - |
| - | - | 6426 | 246.1 | - | - | 0 | - |
| - | - | 1.646E+04 | 246.1 | - | - | 0 | - |
| - | - | 7315 | 247.1 | - | - | 0 | - |
| - | - | 7701 | 247.1 | - | - | 0 | - |
| - | - | 5871 | 248.1 | - | - | 0 | - |
| - | - | 1.699E+04 | 248.2 | - | - | 0 | - |
| 2 | b | 1.626E+06 | 249.1 | 0.0007156 | 2.873 | +1 | 2 |
| - | - | 2.247E+05 | 250.1 | - | - | 0 | - |
| - | - | 1.538E+05 | 251.1 | - | - | 0 | - |
| - | - | 2.944E+04 | 251.1 | - | - | 0 | - |
| - | - | 2.557E+04 | 252.1 | - | - | 0 | - |
| - | - | 1.233E+04 | 253.1 | - | - | 0 | - |
| - | - | 5021 | 255.1 | - | - | 0 | - |
| - | - | 7279 | 256.1 | - | - | 0 | - |
| - | - | 4329 | 257.1 | - | - | 0 | - |
| - | - | 8.671E+04 | 257.1 | - | - | 0 | - |
| - | - | 1.677E+04 | 261.1 | - | - | 0 | - |
| - | - | 1.117E+04 | 263.1 | - | - | 0 | - |
| - | - | 3.091E+05 | 263.1 | - | - | 0 | - |
| - | - | 3.163E+04 | 263.1 | - | - | 0 | - |
| - | - | 4.37E+04 | 264.1 | - | - | 0 | - |
| - | - | 6830 | 264.1 | - | - | 0 | - |
| - | - | 2.738E+04 | 264.1 | - | - | 0 | - |
| - | - | 1.321E+04 | 265.1 | - | - | 0 | - |
| - | - | 4818 | 265.1 | - | - | 0 | - |
| - | - | 2.037E+04 | 267.1 | - | - | 0 | - |
| - | - | 1.207E+04 | 268.1 | - | - | 0 | - |
| - | - | 4823 | 268.1 | - | - | 0 | - |
| - | - | 9172 | 268.1 | - | - | 0 | - |
| - | - | 1.544E+04 | 269.1 | - | - | 0 | - |
| - | - | 6127 | 270.1 | - | - | 0 | - |
| - | - | 5975 | 270.1 | - | - | 0 | - |
| - | - | 7538 | 271.1 | - | - | 0 | - |
| - | - | 6759 | 273.1 | - | - | 0 | - |
| - | - | 7786 | 273.1 | - | - | 0 | - |
| - | - | 7.739E+04 | 274.1 | - | - | 0 | - |
| - | - | 5396 | 277.7 | - | - | 0 | - |
| - | - | 1.318E+05 | 279.1 | - | - | 0 | - |
| - | - | 1.455E+04 | 279.1 | - | - | 0 | - |
| - | - | 1.184E+04 | 280.1 | - | - | 0 | - |
| - | - | 6148 | 283.1 | - | - | 0 | - |
| - | - | 6417 | 283.2 | - | - | 0 | - |
| - | - | 1.92E+05 | 285.1 | - | - | 0 | - |
| - | - | 2.465E+04 | 286.1 | - | - | 0 | - |
| - | - | 6761 | 287.1 | - | - | 0 | - |
| - | - | 1.731E+04 | 289.1 | - | - | 0 | - |
| - | - | 5360 | 289.1 | - | - | 0 | - |
| - | - | 1.001E+04 | 290.1 | - | - | 0 | - |
| - | - | 1.382E+05 | 292.1 | - | - | 0 | - |
| 3 | d | 3.028E+04 | 292.2 | 0.000295 | 1.01 | +1 | 3 |
| - | - | 1.682E+04 | 293.1 | - | - | 0 | - |
| - | - | 1.567E+05 | 296.2 | - | - | 0 | - |
| - | - | 2.645E+04 | 297.2 | - | - | 0 | - |
| - | - | 6630 | 299.2 | - | - | 0 | - |
| - | - | 1.264E+04 | 300.1 | - | - | 0 | - |
| - | - | 7678 | 301.1 | - | - | 0 | - |
| - | - | 8491 | 302.1 | - | - | 0 | - |
| - | - | 1.486E+04 | 302.1 | - | - | 0 | - |
| - | - | 4904 | 303.1 | - | - | 0 | - |
| - | - | 2.627E+04 | 303.1 | - | - | 0 | - |
| - | - | 1.773E+04 | 306.1 | - | - | 0 | - |
| - | - | 7465 | 307.1 | - | - | 0 | - |
| - | - | 2.044E+04 | 310.2 | - | - | 0 | - |
| - | - | 5165 | 311.1 | - | - | 0 | - |
| - | - | 3980 | 311.2 | - | - | 0 | - |
| - | - | 8922 | 313.1 | - | - | 0 | - |
| - | - | 1.893E+04 | 314.1 | - | - | 0 | - |
| - | - | 1.066E+04 | 315.1 | - | - | 0 | - |
| - | - | 4818 | 316.1 | - | - | 0 | - |
| 3 | a | 5.111E+04 | 318.1 | 0.0008889 | 2.794 | +1 | 3 |
| - | - | 1.03E+04 | 319.1 | - | - | 0 | - |
| - | - | 9409 | 320.1 | - | - | 0 | - |
| - | - | 6.266E+04 | 323.1 | - | - | 0 | - |
| - | - | 6259 | 323.1 | - | - | 0 | - |
| - | - | 5492 | 323.1 | - | - | 0 | - |
| - | - | 3.813E+05 | 324.1 | - | - | 0 | - |
| - | - | 5.56E+04 | 325.1 | - | - | 0 | - |
| - | - | 1.271E+04 | 327.1 | - | - | 0 | - |
| - | - | 2.977E+04 | 328.1 | - | - | 0 | - |
| - | - | 1.415E+04 | 328.1 | - | - | 0 | - |
| - | - | 1.755E+04 | 330.1 | - | - | 0 | - |
| - | - | 2.406E+04 | 332.1 | - | - | 0 | - |
| - | - | 1.385E+04 | 333.1 | - | - | 0 | - |
| - | - | 4755 | 333.2 | - | - | 0 | - |
| - | - | 1.603E+04 | 334.1 | - | - | 0 | - |
| - | - | 8096 | 334.1 | - | - | 0 | - |
| - | - | 7293 | 335.2 | - | - | 0 | - |
| - | - | 2.355E+04 | 338.1 | - | - | 0 | - |
| - | - | 7153 | 339.1 | - | - | 0 | - |
| - | - | 2.704E+04 | 339.2 | - | - | 0 | - |
| 8 | y | 1.827E+06 | 342.2 | 0.000947 | 2.768 | +1 | 2 |
| - | - | 3.588E+05 | 343.2 | - | - | 0 | - |
| - | - | 2.856E+04 | 344.2 | - | - | 0 | - |
| 3 | b | 9.36E+04 | 346.1 | 0.0008778 | 2.536 | +1 | 3 |
| - | - | 7926 | 346.2 | - | - | 0 | - |
| - | - | 1.607E+04 | 347.1 | - | - | 0 | - |
| - | - | 5.845E+04 | 348.1 | - | - | 0 | - |
| 6 | b | 4545 | 348.1 | 0.006747 | 19.38 | +2 | 6 |
| - | - | 1.006E+05 | 350.1 | - | - | 0 | - |
| - | - | 2.365E+04 | 351.1 | - | - | 0 | - |
| - | - | 4.29E+04 | 352.1 | - | - | 0 | - |
| - | - | 8560 | 353.1 | - | - | 0 | - |
| - | - | 9569 | 354.2 | - | - | 0 | - |
| - | - | 9.969E+04 | 356.1 | - | - | 0 | - |
| 6 | b | 1.456E+04 | 357.1 | 9.128E-05 | 0.2556 | +2 | 6 |
| - | - | 5464 | 359.1 | - | - | 0 | - |
| - | - | 2.419E+04 | 360.1 | - | - | 0 | - |
| - | - | 6571 | 361.1 | - | - | 0 | - |
| 5 | y | 1.5E+04 | 362.1 | 0.004049 | 11.18 | +2 | 5 |
| - | - | 5483 | 362.7 | - | - | 0 | - |
| 3 | b | 4.671E+05 | 364.2 | 0.0007807 | 2.144 | +1 | 3 |
| - | - | 7.811E+04 | 365.2 | - | - | 0 | - |
| - | - | 4.784E+04 | 366.1 | - | - | 0 | - |
| - | - | 1.13E+04 | 366.2 | - | - | 0 | - |
| - | - | 7633 | 367.1 | - | - | 0 | - |
| - | - | 1.805E+04 | 367.2 | - | - | 0 | - |
| - | - | 5990 | 368.2 | - | - | 0 | - |
| - | - | 1.536E+04 | 371.1 | - | - | 0 | - |
| - | - | 2.138E+04 | 374.1 | - | - | 0 | - |
| - | - | 5521 | 375.2 | - | - | 0 | - |
| - | - | 4.046E+04 | 376.1 | - | - | 0 | - |
| - | - | 1.396E+04 | 378.1 | - | - | 0 | - |
| - | - | 2.521E+05 | 378.1 | - | - | 0 | - |
| - | - | 4.303E+04 | 379.1 | - | - | 0 | - |
| - | - | 1.891E+04 | 380.1 | - | - | 0 | - |
| - | - | 5.375E+04 | 382.1 | - | - | 0 | - |
| - | - | 1.33E+04 | 383.1 | - | - | 0 | - |
| - | - | 1.519E+04 | 384.1 | - | - | 0 | - |
| - | - | 1.582E+04 | 385.1 | - | - | 0 | - |
| - | - | 8243 | 388.1 | - | - | 0 | - |
| - | - | 9694 | 388.2 | - | - | 0 | - |
| - | - | 3.445E+04 | 390.2 | - | - | 0 | - |
| - | - | 3.366E+05 | 394.1 | - | - | 0 | - |
| - | - | 5.749E+04 | 395.1 | - | - | 0 | - |
| - | - | 7100 | 396.1 | - | - | 0 | - |
| - | - | 8875 | 397.2 | - | - | 0 | - |
| - | - | 5902 | 398.2 | - | - | 0 | - |
| - | - | 1.251E+04 | 399.2 | - | - | 0 | - |
| - | - | 1.013E+04 | 401.2 | - | - | 0 | - |
| - | - | 6794 | 404.1 | - | - | 0 | - |
| - | - | 7389 | 404.1 | - | - | 0 | - |
| - | - | 1.022E+04 | 406.1 | - | - | 0 | - |
| - | - | 1.396E+04 | 406.2 | - | - | 0 | - |
| - | - | 2.945E+04 | 407.2 | - | - | 0 | - |
| - | - | 8.365E+04 | 408.2 | - | - | 0 | - |
| - | - | 2.411E+04 | 409.2 | - | - | 0 | - |
| - | - | 6922 | 409.2 | - | - | 0 | - |
| - | - | 9429 | 410.1 | - | - | 0 | - |
| - | - | 5756 | 410.2 | - | - | 0 | - |
| - | - | 4947 | 414.7 | - | - | 0 | - |
| - | - | 1.565E+04 | 415.2 | - | - | 0 | - |
| - | - | 1.953E+04 | 419.2 | - | - | 0 | - |
| - | - | 7481 | 423.2 | - | - | 0 | - |
| - | - | 2.871E+05 | 425.2 | - | - | 0 | - |
| - | - | 6.62E+04 | 426.2 | - | - | 0 | - |
| - | - | 5.123E+04 | 427.2 | - | - | 0 | - |
| - | - | 9265 | 428.2 | - | - | 0 | - |
| - | - | 6007 | 429.2 | - | - | 0 | - |
| - | - | 4599 | 431.2 | - | - | 0 | - |
| - | - | 6746 | 432.1 | - | - | 0 | - |
| - | - | 4.036E+04 | 433.2 | - | - | 0 | - |
| - | - | 1.175E+04 | 434.2 | - | - | 0 | - |
| - | - | 1.11E+04 | 435.2 | - | - | 0 | - |
| - | - | 8973 | 437.2 | - | - | 0 | - |
| - | - | 1.14E+04 | 442.2 | - | - | 0 | - |
| - | - | 9807 | 443.2 | - | - | 0 | - |
| - | - | 5243 | 443.2 | - | - | 0 | - |
| - | - | 7072 | 446.2 | - | - | 0 | - |
| - | - | 4.13E+04 | 447.2 | - | - | 0 | - |
| - | - | 1.449E+04 | 449.1 | - | - | 0 | - |
| - | - | 1.572E+04 | 449.2 | - | - | 0 | - |
| - | - | 8409 | 450.2 | - | - | 0 | - |
| - | - | 5890 | 451.2 | - | - | 0 | - |
| - | - | 6856 | 452.7 | - | - | 0 | - |
| - | - | 5417 | 453.2 | - | - | 0 | - |
| - | - | 8726 | 454.2 | - | - | 0 | - |
| - | - | 2.236E+04 | 454.2 | - | - | 0 | - |
| - | - | 6.239E+04 | 455.2 | - | - | 0 | - |
| - | - | 1.536E+04 | 456.2 | - | - | 0 | - |
| - | - | 7958 | 458.2 | - | - | 0 | - |
| - | - | 9358 | 459.1 | - | - | 0 | - |
| 4 | b | 6.972E+04 | 461.2 | 0.0004546 | 0.9857 | +1 | 4 |
| - | - | 3.024E+04 | 461.2 | - | - | 0 | - |
| - | - | 2.187E+04 | 462.2 | - | - | 0 | - |
| - | - | 5442 | 464.2 | - | - | 0 | - |
| - | - | 2.29E+05 | 465.2 | - | - | 0 | - |
| - | - | 5.377E+04 | 466.2 | - | - | 0 | - |
| - | - | 9939 | 467.2 | - | - | 0 | - |
| 3 | y | 8171 | 468.2 | 0.004178 | 8.925 | +2 | 7 |
| - | - | 7272 | 469.2 | - | - | 0 | - |
| - | - | 1.082E+04 | 469.2 | - | - | 0 | - |
| - | - | 8448 | 470.2 | - | - | 0 | - |
| - | - | 9669 | 470.2 | - | - | 0 | - |
| - | - | 3.734E+04 | 471.2 | - | - | 0 | - |
| - | - | 8905 | 472.2 | - | - | 0 | - |
| - | - | 1.911E+04 | 473.2 | - | - | 0 | - |
| - | - | 4548 | 473.6 | - | - | 0 | - |
| - | - | 7060 | 475.1 | - | - | 0 | - |
| 3 | y | 4.41E+04 | 477.2 | 0.006231 | 13.06 | +2 | 7 |
| - | - | 1.808E+04 | 477.7 | - | - | 0 | - |
| - | - | 1.651E+04 | 478.2 | - | - | 0 | - |
| - | - | 1.431E+04 | 478.2 | - | - | 0 | - |
| 4 | b | 2.495E+05 | 479.2 | 0.001181 | 2.465 | +1 | 4 |
| - | - | 4.857E+04 | 480.2 | - | - | 0 | - |
| - | - | 9983 | 481.2 | - | - | 0 | - |
| - | - | 1.439E+04 | 482.2 | - | - | 0 | - |
| - | - | 8179 | 483.2 | - | - | 0 | - |
| - | - | 3.468E+04 | 486.2 | - | - | 0 | - |
| - | - | 8830 | 487.2 | - | - | 0 | - |
| 7 | y | 4.556E+05 | 489.2 | 0.006259 | 12.8 | +1 | 3 |
| - | - | 1.115E+05 | 490.2 | - | - | 0 | - |
| - | - | 5.633E+04 | 491.2 | - | - | 0 | - |
| - | - | 1.757E+04 | 492.2 | - | - | 0 | - |
| - | - | 3.459E+04 | 495.2 | - | - | 0 | - |
| - | - | 8806 | 496.2 | - | - | 0 | - |
| - | - | 1.222E+04 | 496.2 | - | - | 0 | - |
| - | - | 2.324E+05 | 496.2 | - | - | 0 | - |
| - | - | 4.232E+04 | 497.2 | - | - | 0 | - |
| - | - | 6.379E+04 | 497.2 | - | - | 0 | - |
| - | - | 8224 | 498.2 | - | - | 0 | - |
| 8 | b | 1.873E+04 | 499.2 | 0.0007727 | 1.548 | +2 | 8 |
| - | - | 1.241E+04 | 499.7 | - | - | 0 | - |
| - | - | 8652 | 502.2 | - | - | 0 | - |
| - | - | 5070 | 504.2 | - | - | 0 | - |
| - | - | 5862 | 507.2 | - | - | 0 | - |
| - | - | 8343 | 508.2 | - | - | 0 | - |
| - | - | 7708 | 511.2 | - | - | 0 | - |
| - | - | 2.85E+04 | 513.2 | - | - | 0 | - |
| - | - | 1.276E+04 | 514.2 | - | - | 0 | - |
| - | - | 9907 | 516.2 | - | - | 0 | - |
| - | - | 1.845E+04 | 518.7 | - | - | 0 | - |
| - | - | 6.22E+04 | 519.2 | - | - | 0 | - |
| - | - | 3.636E+04 | 520.2 | - | - | 0 | - |
| - | - | 1.461E+04 | 521.2 | - | - | 0 | - |
| - | - | 1.015E+04 | 523.2 | - | - | 0 | - |
| - | - | 5838 | 525.2 | - | - | 0 | - |
| - | - | 6058 | 525.2 | - | - | 0 | - |
| - | - | 5501 | 528.2 | - | - | 0 | - |
| - | - | 2.24E+04 | 530.2 | - | - | 0 | - |
| - | - | 6908 | 531.2 | - | - | 0 | - |
| - | - | 8045 | 532.2 | - | - | 0 | - |
| - | - | 8174 | 533.2 | - | - | 0 | - |
| - | - | 2.26E+04 | 537.2 | - | - | 0 | - |
| - | - | 7732 | 538.2 | - | - | 0 | - |
| - | - | 1.476E+04 | 541.2 | - | - | 0 | - |
| 2 | y | 3.855E+04 | 541.7 | 0.003174 | 5.86 | +2 | 8 |
| - | - | 3.343E+04 | 542.2 | - | - | 0 | - |
| - | - | 1.31E+04 | 542.7 | - | - | 0 | - |
| - | - | 9713 | 543.2 | - | - | 0 | - |
| - | - | 5650 | 547.2 | - | - | 0 | - |
| - | - | 7.836E+04 | 548.2 | - | - | 0 | - |
| - | - | 1.983E+04 | 549.2 | - | - | 0 | - |
| - | - | 9859 | 550.2 | - | - | 0 | - |
| 2 | y | 2.159E+05 | 550.7 | 0.003629 | 6.59 | +2 | 8 |
| - | - | 1.633E+05 | 551.2 | - | - | 0 | - |
| - | - | 6E+04 | 551.7 | - | - | 0 | - |
| - | - | 1.543E+04 | 552.2 | - | - | 0 | - |
| - | - | 7353 | 555.7 | - | - | 0 | - |
| - | - | 4.859E+04 | 557.2 | - | - | 0 | - |
| - | - | 1.196E+04 | 558.2 | - | - | 0 | - |
| 6 | y | 7.084E+05 | 560.2 | 0.005889 | 10.51 | +1 | 4 |
| - | - | 1.665E+04 | 560.7 | - | - | 0 | - |
| - | - | 2.326E+05 | 561.2 | - | - | 0 | - |
| - | - | 5.797E+04 | 562.2 | - | - | 0 | - |
| - | - | 8525 | 566.2 | - | - | 0 | - |
| - | - | 4.321E+04 | 569.2 | - | - | 0 | - |
| - | - | 3.177E+04 | 569.7 | - | - | 0 | - |
| - | - | 7.277E+04 | 570.2 | - | - | 0 | - |
| - | - | 1.432E+04 | 571.2 | - | - | 0 | - |
| - | - | 1.206E+04 | 574.7 | - | - | 0 | - |
| - | - | 2.953E+04 | 578.2 | - | - | 0 | - |
| - | - | 1.57E+04 | 578.7 | - | - | 0 | - |
| - | - | 7582 | 579.2 | - | - | 0 | - |
| - | - | 5964 | 580.2 | - | - | 0 | - |
| - | - | 3.034E+04 | 583.2 | - | - | 0 | - |
| - | - | 1.553E+04 | 583.7 | - | - | 0 | - |
| - | - | 8031 | 584.2 | - | - | 0 | - |
| - | - | 1.723E+04 | 588.2 | - | - | 0 | - |
| - | - | 6184 | 589.2 | - | - | 0 | - |
| 0 | Precursor | 1.125E+05 | 592.2 | 0.003505 | 5.919 | +2 | -1 |
| - | - | 1.005E+05 | 592.7 | - | - | 0 | - |
| - | - | 3.886E+04 | 593.2 | - | - | 0 | - |
| - | - | 7093 | 593.7 | - | - | 0 | - |
| - | - | 2.049E+04 | 594.2 | - | - | 0 | - |
| - | - | 9600 | 595.2 | - | - | 0 | - |
| - | - | 5.067E+04 | 596.2 | - | - | 0 | - |
| - | - | 3.03E+04 | 597.2 | - | - | 0 | - |
| - | - | 1.494E+04 | 598.2 | - | - | 0 | - |
| 0 | Precursor | 3.377E+05 | 601.2 | 0.003838 | 6.384 | +2 | -1 |
| - | - | 2.666E+05 | 601.7 | - | - | 0 | - |
| - | - | 1.009E+05 | 602.2 | - | - | 0 | - |
| - | - | 1.212E+04 | 602.7 | - | - | 0 | - |
| - | - | 7.198E+04 | 606.2 | - | - | 0 | - |
| - | - | 1.832E+04 | 607.2 | - | - | 0 | - |
| - | - | 8878 | 608.2 | - | - | 0 | - |
| - | - | 1.147E+05 | 612.2 | - | - | 0 | - |
| - | - | 3.383E+04 | 613.2 | - | - | 0 | - |
| - | - | 9533 | 613.3 | - | - | 0 | - |
| - | - | 7.626E+04 | 614.2 | - | - | 0 | - |
| - | - | 2.18E+04 | 615.2 | - | - | 0 | - |
| - | - | 6415 | 616.3 | - | - | 0 | - |
| - | - | 5554 | 622.2 | - | - | 0 | - |
| 5 | b | 5.808E+04 | 624.2 | 0.001854 | 2.97 | +1 | 5 |
| - | - | 1.992E+04 | 625.2 | - | - | 0 | - |
| - | - | 2.318E+04 | 631.2 | - | - | 0 | - |
| - | - | 1.197E+04 | 632.3 | - | - | 0 | - |
| - | - | 7.041E+04 | 634.2 | - | - | 0 | - |
| - | - | 1.974E+04 | 635.2 | - | - | 0 | - |
| - | - | 1.676E+04 | 639.3 | - | - | 0 | - |
| - | - | 3.276E+04 | 640.2 | - | - | 0 | - |
| - | - | 1.458E+04 | 641.3 | - | - | 0 | - |
| 5 | b | 5.193E+04 | 642.2 | 0.001848 | 2.878 | +1 | 5 |
| - | - | 2.062E+04 | 643.2 | - | - | 0 | - |
| - | - | 2.488E+04 | 652.2 | - | - | 0 | - |
| - | - | 7899 | 653.2 | - | - | 0 | - |
| - | - | 1.667E+05 | 657.3 | - | - | 0 | - |
| - | - | 5.107E+04 | 658.3 | - | - | 0 | - |
| - | - | 2.62E+05 | 659.3 | - | - | 0 | - |
| - | - | 6420 | 660.2 | - | - | 0 | - |
| - | - | 1.051E+05 | 660.3 | - | - | 0 | - |
| - | - | 1.467E+04 | 661.3 | - | - | 0 | - |
| - | - | 1.992E+04 | 667.3 | - | - | 0 | - |
| - | - | 1.518E+04 | 668.2 | - | - | 0 | - |
| - | - | 7123 | 669.3 | - | - | 0 | - |
| - | - | 3.371E+04 | 676.3 | - | - | 0 | - |
| - | - | 2.143E+04 | 677.3 | - | - | 0 | - |
| - | - | 6506 | 678.3 | - | - | 0 | - |
| - | - | 2.119E+05 | 685.3 | - | - | 0 | - |
| - | - | 7.631E+04 | 686.3 | - | - | 0 | - |
| - | - | 1.852E+04 | 687.3 | - | - | 0 | - |
| 6 | b | 4.35E+04 | 695.3 | 0.0001037 | 0.1492 | +1 | 6 |
| - | - | 1.183E+04 | 696.3 | - | - | 0 | - |
| - | - | 6.582E+04 | 703.3 | - | - | 0 | - |
| - | - | 1.884E+04 | 704.3 | - | - | 0 | - |
| - | - | 2.502E+04 | 705.3 | - | - | 0 | - |
| - | - | 9097 | 706.3 | - | - | 0 | - |
| 6 | b | 2.368E+04 | 713.3 | 0.002088 | 2.927 | +1 | 6 |
| - | - | 5567 | 714.3 | - | - | 0 | - |
| - | - | 9375 | 721.2 | - | - | 0 | - |
| - | - | 2.119E+05 | 721.3 | - | - | 0 | - |
| - | - | 6.895E+04 | 722.3 | - | - | 0 | - |
| 5 | y | 6.873E+05 | 723.3 | 0.005854 | 8.093 | +1 | 5 |
| - | - | 2.711E+05 | 724.3 | - | - | 0 | - |
| - | - | 7.824E+04 | 725.3 | - | - | 0 | - |
| - | - | 9781 | 726.3 | - | - | 0 | - |
| - | - | 9625 | 728.3 | - | - | 0 | - |
| - | - | 2.115E+04 | 731.2 | - | - | 0 | - |
| - | - | 1.312E+04 | 732.2 | - | - | 0 | - |
| - | - | 1.262E+04 | 733.3 | - | - | 0 | - |
| - | - | 6316 | 734.3 | - | - | 0 | - |
| - | - | 6185 | 740.3 | - | - | 0 | - |
| - | - | 1.183E+04 | 746.3 | - | - | 0 | - |
| - | - | 5763 | 747.3 | - | - | 0 | - |
| - | - | 7229 | 748.3 | - | - | 0 | - |
| - | - | 2.899E+05 | 749.3 | - | - | 0 | - |
| - | - | 1.166E+05 | 750.3 | - | - | 0 | - |
| - | - | 3.809E+04 | 751.3 | - | - | 0 | - |
| - | - | 1.859E+04 | 756.3 | - | - | 0 | - |
| - | - | 1.425E+04 | 757.3 | - | - | 0 | - |
| - | - | 1.223E+04 | 759.3 | - | - | 0 | - |
| - | - | 7.267E+04 | 767.3 | - | - | 0 | - |
| - | - | 2.929E+04 | 768.3 | - | - | 0 | - |
| - | - | 7544 | 769.3 | - | - | 0 | - |
| - | - | 2.825E+05 | 774.3 | - | - | 0 | - |
| - | - | 1.158E+05 | 775.3 | - | - | 0 | - |
| - | - | 2.259E+04 | 776.3 | - | - | 0 | - |
| - | - | 1.433E+04 | 778.3 | - | - | 0 | - |
| - | - | 6974 | 779.3 | - | - | 0 | - |
| - | - | 7479 | 784.3 | - | - | 0 | - |
| - | - | 9612 | 792.3 | - | - | 0 | - |
| - | - | 5774 | 793.3 | - | - | 0 | - |
| - | - | 7951 | 796.3 | - | - | 0 | - |
| - | - | 8156 | 803.3 | - | - | 0 | - |
| - | - | 9148 | 804.3 | - | - | 0 | - |
| - | - | 8710 | 805.3 | - | - | 0 | - |
| - | - | 8866 | 818.3 | - | - | 0 | - |
| 4 | y | 4.236E+04 | 820.3 | 0.005894 | 7.185 | +1 | 6 |
| - | - | 2.216E+04 | 821.3 | - | - | 0 | - |
| - | - | 1.089E+04 | 822.3 | - | - | 0 | - |
| - | - | 6426 | 823.3 | - | - | 0 | - |
| - | - | 7256 | 824.3 | - | - | 0 | - |
| - | - | 9211 | 828.4 | - | - | 0 | - |
| - | - | 2.446E+04 | 832.3 | - | - | 0 | - |
| - | - | 1.677E+04 | 833.3 | - | - | 0 | - |
| 4 | y | 8.721E+05 | 838.3 | 0.006193 | 7.388 | +1 | 6 |
| - | - | 3.874E+05 | 839.3 | - | - | 0 | - |
| - | - | 1.309E+05 | 840.3 | - | - | 0 | - |
| - | - | 9753 | 841.3 | - | - | 0 | - |
| 7 | b | 1.44E+04 | 842.3 | 0.01049 | 12.45 | +1 | 7 |
| - | - | 3.66E+04 | 843.3 | - | - | 0 | - |
| - | - | 1.578E+04 | 844.3 | - | - | 0 | - |
| - | - | 1.336E+04 | 848.3 | - | - | 0 | - |
| - | - | 6242 | 850.3 | - | - | 0 | - |
| - | - | 5755 | 851.3 | - | - | 0 | - |
| - | - | 5538 | 853.3 | - | - | 0 | - |
| - | - | 1.026E+04 | 854.3 | - | - | 0 | - |
| 7 | b | 1.436E+04 | 860.3 | 0.003891 | 4.523 | +1 | 7 |
| - | - | 9270 | 861.3 | - | - | 0 | - |
| - | - | 2.743E+04 | 862.3 | - | - | 0 | - |
| - | - | 1.377E+04 | 863.3 | - | - | 0 | - |
| - | - | 1.58E+04 | 868.3 | - | - | 0 | - |
| - | - | 9692 | 869.3 | - | - | 0 | - |
| - | - | 1.168E+05 | 871.3 | - | - | 0 | - |
| - | - | 6.629E+04 | 872.3 | - | - | 0 | - |
| - | - | 2.3E+04 | 873.3 | - | - | 0 | - |
| - | - | 1.203E+06 | 889.3 | - | - | 0 | - |
| - | - | 5.57E+05 | 890.4 | - | - | 0 | - |
| - | - | 9090 | 890.5 | - | - | 0 | - |
| - | - | 1.548E+05 | 891.4 | - | - | 0 | - |
| - | - | 1.574E+04 | 892.4 | - | - | 0 | - |
| - | - | 2.32E+04 | 896.3 | - | - | 0 | - |
| - | - | 1.297E+04 | 897.3 | - | - | 0 | - |
| - | - | 7818 | 899.3 | - | - | 0 | - |
| - | - | 4.579E+04 | 907.3 | - | - | 0 | - |
| - | - | 2.637E+04 | 908.3 | - | - | 0 | - |
| - | - | 1.115E+04 | 909.3 | - | - | 0 | - |
| - | - | 7221 | 914.3 | - | - | 0 | - |
| - | - | 1.26E+04 | 917.3 | - | - | 0 | - |
| - | - | 1.291E+04 | 918.3 | - | - | 0 | - |
| - | - | 1.241E+04 | 933.4 | - | - | 0 | - |
| 3 | y | 2.38E+05 | 935.3 | 0.005989 | 6.404 | +1 | 7 |
| - | - | 1.365E+05 | 936.3 | - | - | 0 | - |
| - | - | 5.475E+04 | 937.3 | - | - | 0 | - |
| - | - | 2.544E+04 | 938.3 | - | - | 0 | - |
| - | - | 8975 | 939.3 | - | - | 0 | - |
| 3 | y | 3.722E+06 | 953.3 | 0.006289 | 6.597 | +1 | 7 |
| - | - | 1.945E+06 | 954.3 | - | - | 0 | - |
| - | - | 6.993E+05 | 955.4 | - | - | 0 | - |
| - | - | 6.848E+04 | 956.4 | - | - | 0 | - |
| - | - | 2.345E+04 | 963.3 | - | - | 0 | - |
| - | - | 1.18E+04 | 964.3 | - | - | 0 | - |
| - | - | 1.423E+04 | 969.4 | - | - | 0 | - |
| - | - | 5420 | 970.4 | - | - | 0 | - |
| 8 | b | 1.028E+04 | 979.4 | 0.003639 | 3.716 | +1 | 8 |
| - | - | 5809 | 990.1 | - | - | 0 | - |
| 8 | b | 4.149E+04 | 997.4 | 0.005587 | 5.602 | +1 | 8 |
| - | - | 2.141E+04 | 998.4 | - | - | 0 | - |
| - | - | 1.088E+04 | 999.4 | - | - | 0 | - |
| - | - | 1.248E+04 | 1015 | - | - | 0 | - |
| - | - | 5830 | 1016 | - | - | 0 | - |
| - | - | 1.879E+04 | 1018 | - | - | 0 | - |
| - | - | 9061 | 1019 | - | - | 0 | - |
| - | - | 5689 | 1020 | - | - | 0 | - |
| - | - | 8.097E+04 | 1036 | - | - | 0 | - |
| - | - | 5.15E+04 | 1037 | - | - | 0 | - |
| - | - | 1.791E+04 | 1038 | - | - | 0 | - |
| - | - | 6202 | 1046 | - | - | 0 | - |
| 2 | y | 2.351E+04 | 1082 | 0.006545 | 6.047 | +1 | 8 |
| - | - | 1.369E+04 | 1083 | - | - | 0 | - |
| 2 | y | 2.073E+05 | 1100 | 0.006479 | 5.887 | +1 | 8 |
| - | - | 1.385E+05 | 1101 | - | - | 0 | - |
| - | - | 5.836E+04 | 1102 | - | - | 0 | - |
| - | - | 9629 | 1103 | - | - | 0 | - |
| - | - | 3.557E+04 | 1110 | - | - | 0 | - |
| - | - | 2.573E+04 | 1111 | - | - | 0 | - |
| - | - | 9870 | 1112 | - | - | 0 | - |
| - | - | 5642 | 1956 | - | - | 0 | - |
| - | - | 6472 | 3240 | - | - | 0 | - |

m/z Charge Intensity FragmentType MassShift Position
120.08128356933594 0 2181933.5
121.04022216796875 0 5911.368
121.08458709716797 0 162669.42
122.0720443725586 0 12771.19
122.08824920654297 0 6464.0454
127.08721923828125 0 6639.904
129.07052612304688 0 9323.835
129.16705322265625 0 3154.443
130.0655517578125 0 46569.223
131.0685272216797 0 5443.433
132.08123779296875 0 111954.1
133.06103515625 0 6168.472
133.0843963623047 0 8820.505
134.09689331054688 0 9514.965
136.076171875 0 786052.4
136.08749389648438 0 26270.564
137.07366943359375 0 5548.151
137.0795440673828 0 62848.367
137.0844268798828 0 5368.931
138.05543518066406 0 59126.77
138.06666564941406 0 87262.48
139.07008361816406 0 4916.3735
141.0663299560547 0 6576.707
143.0456085205078 0 5458.708
143.073486328125 0 13951.242
143.08607482910156 0 12589.368
144.0749969482422 0 3006.4016
144.08119201660156 0 10914.035
146.06056213378906 0 30014.475
146.0717315673828 0 4784.646
146.09686279296875 0 13855.525
148.07640075683594 0 7202.824
148.08741760253906 0 24912.656
150.06700134277344 0 6612.655
155.0819854736328 0 13912.131
155.0931854248047 0 21720.398
156.0772247314453 0 204384.56
157.08058166503906 0 12332.426
158.09690856933594 0 325995.34
159.054931640625 0 3697.449
159.0922088623047 0 164924.53
159.10018920898438 0 34061.094
160.07615661621094 0 58682.53
160.08753967285156 0 6059.764
160.09539794921875 0 15395.32
160.112548828125 0 148562.6
161.0790557861328 0 3859.1362
161.1159210205078 0 16495.67
162.09239196777344 0 5179.444
164.08201599121094 0 4468.3647
165.0701141357422 0 9346.841
165.07760620117188 0 32898.152
166.05369567871094 0 13367.219
166.0616455078125 0 28061.467
166.0860137939453 0 5245.241
167.09320068359375 0 25148.924
169.07664489746094 0 12021.771
171.076904296875 0 27188.268
171.0919647216797 0 5292.718
172.07723999023438 0 5993.219
172.10867309570312 0 5835.1836
173.09262084960938 0 8810.467
174.06674194335938 0 20212.145 b Ammonia loss 2
174.09173583984375 0 4125.1265
175.0872802734375 0 12704.858
175.09779357910156 0 5951.9893
175.12351989746094 0 8748.858
176.0824432373047 0 53717.37
176.1075439453125 0 657830.3
177.102783203125 0 467468.94
177.11135864257812 0 70200.25
178.10614013671875 0 44548.84
178.11419677734375 0 4997.9736
180.0771942138672 0 29272.6
182.0456085205078 0 5604.774
185.05624389648438 0 50171.668
186.09185791015625 0 54400.4
187.06356811523438 0 12011.196
188.0711212158203 0 223348.25
189.0745849609375 0 27677.74
190.0868682861328 0 8221.209
191.08358764648438 0 5370.167
191.0935821533203 0 23561.326
191.11842346191406 0 46733.445
192.1027069091797 0 11195.881
192.1204376220703 0 4598.6733
193.07254028320312 0 13456.464
193.10888671875 0 185870
194.0802459716797 0 9672.893
194.11248779296875 0 14105.567
194.33055114746094 0 4155.0615
195.0881805419922 0 48464.77
196.6621856689453 0 3839.406
202.05381774902344 0 15919.494
203.0667266845703 0 52484.848
203.0931854248047 0 11838.531
203.1183624267578 0 223670.89 a Water loss 1
204.07725524902344 0 102332.87
204.102294921875 0 13616.174 a Ammonia loss 1
204.12156677246094 0 30775.748
205.08135986328125 0 8510.439
205.09768676757812 0 173131.36 y 8
205.10829162597656 0 13461.29
206.1012420654297 0 25323.28
207.11334228515625 0 47748.52
208.0721893310547 0 28086.008
209.07562255859375 0 4857.9556
211.08340454101562 0 5478.818
212.11679077148438 0 12089.758
215.0823211669922 0 11311.131
216.0655517578125 0 7609.9453
217.0977325439453 0 117130.99
218.10150146484375 0 13684.508
219.08035278320312 0 37374.92
219.09072875976562 0 7069.462
220.12049865722656 0 4827.6895
221.10406494140625 0 539384.9
221.12918090820312 0 8847761 a 1
222.10714721679688 0 51256.75
222.13238525390625 0 1074879.2
223.1199951171875 0 8257.199
223.1348419189453 0 63254.65
224.118408203125 0 8979.486
225.09967041015625 0 6291.1206
228.11351013183594 0 24287.598
229.1089324951172 0 18746.22
230.12936401367188 0 5973.041
231.06179809570312 0 149280.97
231.08888244628906 0 5860.252 b Water loss 3
231.11338806152344 0 84543.94 b Water loss 1
231.1240997314453 0 4046.3032
232.0647735595703 0 13725.806
232.1166534423828 0 18324.592
233.0930938720703 0 4681.994
234.12408447265625 0 5954.1973
235.10838317871094 0 208485.81
236.11109924316406 0 23285.402
237.09042358398438 0 8882.131
237.1244354248047 0 6521.125
238.1304473876953 0 13730.005
239.11444091796875 0 57436.402
240.1169891357422 0 8396.95
241.08460998535156 0 4632.0483
241.09864807128906 0 5881.937
245.09339904785156 0 8182.7495 y 6
245.1292724609375 0 8516.108
246.09893798828125 0 6426.07
246.1354522705078 0 16460.455
247.10707092285156 0 7315.119
247.12034606933594 0 7700.661
248.08914184570312 0 5871.0977
248.15103149414062 0 16991.596
249.12408447265625 0 1625605.9 b 1
250.1273956298828 0 224715.83
251.10324096679688 0 153772.47
251.12974548339844 0 29440.498
252.10691833496094 0 25574.51
253.09397888183594 0 12325.319
255.11279296875 0 5021.4565
256.1087951660156 0 7279.006
257.07879638671875 0 4329.331
257.1069641113281 0 86709.24
261.0870361328125 0 16772.875
263.0703430175781 0 11170.293
263.10321044921875 0 309139.16
263.1295471191406 0 31627.879
264.106689453125 0 43696.414
264.1312561035156 0 6829.831
264.14605712890625 0 27382.709
265.1171875 0 13212.423
265.14349365234375 0 4817.633
267.0916748046875 0 20366.047
268.0757751464844 0 12071.028
268.0950622558594 0 4822.7007
268.140380859375 0 9172.347
269.077392578125 0 15436.0205
270.07244873046875 0 6126.639
270.1083679199219 0 5975.0366
271.1079406738281 0 7537.863
273.088134765625 0 6759.008
273.12298583984375 0 7786.3438
274.1304626464844 0 77390.11
277.6948547363281 0 5395.6963
279.0982666015625 0 131750.97
279.12542724609375 0 14545.06
280.1016845703125 0 11844.262
283.10784912109375 0 6148.2227
283.15167236328125 0 6416.617
285.1023254394531 0 192045.72
286.1045227050781 0 24645.846
287.100830078125 0 6760.728
289.08270263671875 0 17313.047
289.1191101074219 0 5359.882
290.1257019042969 0 10007.213
292.1412353515625 0 138245.48
292.1658630371094 0 30276.068 d 2
293.144287109375 0 16823.74
296.1513671875 0 156707.73
297.154052734375 0 26454.1
299.1521301269531 0 6630.267
300.1346130371094 0 12635.563
301.11859130859375 0 7677.919
302.09783935546875 0 8491.267
302.1150207519531 0 14855.417
303.09619140625 0 4903.748
303.113525390625 0 26272.299
306.14520263671875 0 17727.746
307.12115478515625 0 7464.934
310.15185546875 0 20438.406
311.11895751953125 0 5164.9688
311.155029296875 0 3979.7737
313.0979309082031 0 8922.309
314.0994567871094 0 18933.344
315.09881591796875 0 10661.101
316.1296081542969 0 4818.3384
318.1457214355469 0 51108.703 a Water loss 2
319.1494445800781 0 10298.334
320.1371154785156 0 9408.925
323.0995178222656 0 62656.844
323.11871337890625 0 6258.8984
323.1402587890625 0 5492.2476
324.1464538574219 0 381259.78
325.1490478515625 0 55595.285
327.14642333984375 0 12713.654
328.1296081542969 0 29765.594
328.1465148925781 0 14154.25
330.10955810546875 0 17545.176
332.12530517578125 0 24060.355
333.1093444824219 0 13852.653
333.21673583984375 0 4754.676
334.1075439453125 0 16033.018
334.1295471191406 0 8095.9546
335.1717224121094 0 7292.821
338.1288757324219 0 23553.598
339.133056640625 0 7153.0366
339.17108154296875 0 27036.098
342.1570129394531 0 1826543.5 y 7
343.1600646972656 0 358795.5
344.1622314453125 0 28558.496
346.140625 0 93602.09 b Water loss 2
346.1625061035156 0 7926.03
347.14337158203125 0 16065.131
348.119873046875 0 58449.57
348.1439514160156 0 4544.755 b Water loss 5
350.1353759765625 0 100575.73
351.137939453125 0 23653.082
352.1408996582031 0 42897.49
353.1481628417969 0 8559.761
354.1556396484375 0 9569.318
356.1396484375 0 99694.03
357.142578125 0 14563.707 b 5
359.0858154296875 0 5464.26
360.1199951171875 0 24191.043
361.1219787597656 0 6570.986
362.15118408203125 0 15000.353 y 4
362.6501770019531 0 5483.0293
364.1510925292969 0 467080.4 b 2
365.1545104980469 0 78110.125
366.1298522949219 0 47838.87
366.1559753417969 0 11298.902
367.1334533691406 0 7633.1934
367.1657409667969 0 18054.309
368.16650390625 0 5989.5073
371.13525390625 0 15358.471
374.1499938964844 0 21378.459
375.1528625488281 0 5520.721
376.11468505859375 0 40462.17
378.1008605957031 0 13960.353
378.13043212890625 0 252102.14
379.13348388671875 0 43030.25
380.1390075683594 0 18908.71
382.1435546875 0 53745.918
383.1467590332031 0 13303.781
384.1355895996094 0 15185.886
385.1370849609375 0 15824.381
388.11572265625 0 8243.153
388.15032958984375 0 9694.376
390.15667724609375 0 34450.492
394.12554931640625 0 336600
395.1290588378906 0 57494.51
396.1305236816406 0 7100.391
397.1635437011719 0 8875.369
398.1702880859375 0 5901.903
399.1772155761719 0 12514.386
401.193603515625 0 10126.145
404.11346435546875 0 6794.243
404.1451110839844 0 7388.6323
406.1238708496094 0 10217.56
406.1522216796875 0 13962.809
407.1835021972656 0 29445.678
408.16754150390625 0 83649.59
409.1707458496094 0 24114.117
409.1991271972656 0 6921.582
410.1392822265625 0 9429.155
410.1814270019531 0 5755.977
414.72283935546875 0 4947.156
415.1619873046875 0 15654.642
419.1566162109375 0 19527.266
423.1805114746094 0 7480.9185
425.1943664550781 0 287123.34
426.1971740722656 0 66202.86
427.2097473144531 0 51234.81
428.2128601074219 0 9265.369
429.1883239746094 0 6006.9043
431.1598205566406 0 4598.7935
432.1419982910156 0 6745.7275
433.1728820800781 0 40360.484
434.1766357421875 0 11753.126
435.1807861328125 0 11100.952
437.1676025390625 0 8973.459
442.1737976074219 0 11400.148
443.1556091308594 0 9806.575
443.18743896484375 0 5242.531
446.21630859375 0 7072.4116
447.1521301269531 0 41297.05
449.1341552734375 0 14489.806
449.1668395996094 0 15722.004
450.22686767578125 0 8408.951
451.1778564453125 0 5889.5913
452.6684875488281 0 6855.554
453.1895751953125 0 5417.3354
454.158935546875 0 8725.863
454.1971740722656 0 22361.383
455.2043151855469 0 62394.336
456.2062072753906 0 15362.8125
458.1673583984375 0 7957.529
459.1479797363281 0 9357.519
461.1671447753906 0 69722.35 b Water loss 3
461.1961975097656 0 30241.57
462.1709899902344 0 21872.447
464.1863098144531 0 5441.8887
465.162841796875 0 228991.92
466.1658630371094 0 53768.285
467.1670837402344 0 9938.92
468.1729736328125 0 8171.347 y Water loss 2
469.1731872558594 0 7272.144
469.2090759277344 0 10817.193
470.16693115234375 0 8447.771
470.2126770019531 0 9669.2295
471.18182373046875 0 37344.355
472.1853332519531 0 8904.687
473.2153625488281 0 19110.312
473.5928039550781 0 4547.584
475.14483642578125 0 7059.5293
477.1678466796875 0 44098.54 y 2
477.6787109375 0 18076.326
478.1681213378906 0 16512.145
478.22021484375 0 14312.334
479.1784362792969 0 249481.45 b 3
480.1817932128906 0 48570.465
481.1827087402344 0 9982.827
482.19232177734375 0 14385.575
483.1963806152344 0 8179.4663
486.1636657714844 0 34680.895
487.1650695800781 0 8830.274
489.19281005859375 0 455575.84 y 6
490.19512939453125 0 111502.94
491.205078125 0 56330.855
492.20989990234375 0 17569.576
495.1868896484375 0 34591.03
496.1698913574219 0 8806.172
496.1889343261719 0 12222.2
496.2315979003906 0 232371.8
497.1713562011719 0 42315.387
497.23455810546875 0 63789.766
498.23785400390625 0 8224.265
499.1879577636719 0 18729.596 b 7
499.6936950683594 0 12409.62
502.1936340332031 0 8651.736
504.1790771484375 0 5069.562
507.1520080566406 0 5861.7856
508.196533203125 0 8342.51
511.1936950683594 0 7707.979
513.1993408203125 0 28504.137
514.224365234375 0 12756.905
516.2210083007812 0 9907.169
518.7117919921875 0 18454.428
519.20458984375 0 62202.71
520.2054443359375 0 36355.47
521.20654296875 0 14613.535
523.189697265625 0 10149.605
525.1622314453125 0 5837.811
525.2067260742188 0 6057.9106
528.2100830078125 0 5501.368
530.189697265625 0 22400.621
531.1885375976562 0 6907.521
532.2052001953125 0 8044.824
533.2040405273438 0 8174.3096
537.212646484375 0 22604.74
538.21337890625 0 7731.9727
541.1947021484375 0 14759.629
541.7061767578125 0 38553.758 y Water loss 1
542.2341918945312 0 33432.74
542.7105102539062 0 13098.348
543.2423095703125 0 9712.734
547.19677734375 0 5650.0557
548.199951171875 0 78361.055
549.2035522460938 0 19827.977
550.2059936523438 0 9859.31
550.7119140625 0 215872.4 y 1
551.2137451171875 0 163313.36
551.714111328125 0 59995.45
552.2168579101562 0 15428.408
555.7022705078125 0 7352.665
557.2001953125 0 48587.24
558.2012329101562 0 11958.626
560.2295532226562 0 708430.7 y 5
560.730712890625 0 16649.041
561.2324829101562 0 232633.45
562.230712890625 0 57973.145
566.1953125 0 8524.865
569.2343139648438 0 43209.883
569.7352905273438 0 31766.398
570.229736328125 0 72771.39
571.2306518554688 0 14321.262
574.7137451171875 0 12060.506
578.2310791015625 0 29527.422
578.7354125976562 0 15696.626
579.2308349609375 0 7581.79
580.2115478515625 0 5964.396
583.2246704101562 0 30344.54
583.72607421875 0 15527.608
584.1993408203125 0 8031.3174
588.241943359375 0 17231.535
589.2054443359375 0 6183.9473
592.2303466796875 0 112470.54 Precursor Water loss
592.73193359375 0 100496.05
593.2328491210938 0 38861.484
593.728515625 0 7092.8706
594.1886596679688 0 20487.16
595.1889038085938 0 9600.0625
596.236328125 0 50667.094
597.2257080078125 0 30298.082
598.2246704101562 0 14941.212
601.2359619140625 0 337733.66 Precursor
601.7373657226562 0 266579.88
602.2379150390625 0 100882.13
602.7376098632812 0 12118.864
606.2332153320312 0 71978.78
607.2353515625 0 18319.09
608.2390747070312 0 8877.875
612.1982421875 0 114740.48
613.200927734375 0 33832.63
613.2879028320312 0 9532.743
614.2467651367188 0 76263.96
615.2488403320312 0 21799.9
616.2564086914062 0 6414.5103
622.2164916992188 0 5553.948
624.2318725585938 0 58077.707 b Water loss 4
625.2364501953125 0 19916.186
631.2484130859375 0 23184.887
632.2551879882812 0 11965.216
634.23046875 0 70413.61
635.2330322265625 0 19742.078
639.2538452148438 0 16762.365
640.2393798828125 0 32756.66
641.2820434570312 0 14575.529
642.242431640625 0 51928.777 b 4
643.244140625 0 20617.04
652.2413330078125 0 24881.35
653.245361328125 0 7898.9634
657.2639770507812 0 166738.8
658.2658081054688 0 51069.844
659.2947998046875 0 262025.52
660.2374877929688 0 6420.137
660.2978515625 0 105090.125
661.3015747070312 0 14666.085
667.2503662109375 0 19922.95
668.2452392578125 0 15183.845
669.2774658203125 0 7122.9614
676.2698364257812 0 33711.523
677.27685546875 0 21433.71
678.27880859375 0 6506.113
685.2589721679688 0 211878.38
686.2616577148438 0 76306.805
687.2659301757812 0 18517.84
695.2670288085938 0 43497.242 b Water loss 5
696.2720336914062 0 11833.97
703.2664794921875 0 65822.734
704.2676391601562 0 18837.438
705.2794189453125 0 25022.406
706.2910766601562 0 9097.447
713.27978515625 0 23675.873 b 5
714.280029296875 0 5566.757
721.1887817382812 0 9374.528
721.2622680664062 0 211906.77
722.2652587890625 0 68950.97
723.2928466796875 0 687285.06 y 4
724.2958984375 0 271128.66
725.2962036132812 0 78237.64
726.2960815429688 0 9780.581
728.3192749023438 0 9625.308
731.2472534179688 0 21148.012
732.2402954101562 0 13118.889
733.2714233398438 0 12615.959
734.2821044921875 0 6316.319
740.297607421875 0 6184.7153
746.2752685546875 0 11829.718
747.295166015625 0 5763.4155
748.30712890625 0 7229.3857
749.2572021484375 0 289933.2
750.259765625 0 116633.945
751.2608032226562 0 38087.63
756.310302734375 0 18586.658
757.3065185546875 0 14253.49
759.2623291015625 0 12226.231
767.2681884765625 0 72669.625
768.2711181640625 0 29294.533
769.2700805664062 0 7544.035
774.3216552734375 0 282454.1
775.3247680664062 0 115798.516
776.3257446289062 0 22587.059
778.3037719726562 0 14330.035
779.3119506835938 0 6973.727
784.3017578125 0 7478.7085
792.322021484375 0 9612.122
793.31640625 0 5773.977
796.3175048828125 0 7951.3936
803.2791748046875 0 8155.8433
804.3356323242188 0 9148.472
805.333740234375 0 8709.848
818.3333740234375 0 8866.148
820.3092651367188 0 42355.02 y Water loss 3
821.3036499023438 0 22160.145
822.305908203125 0 10893.616
823.2994384765625 0 6426.346
824.3014526367188 0 7256.0527
828.4381103515625 0 9210.656
832.32666015625 0 24464.412
833.32470703125 0 16772.865
838.3201293945312 0 872098.5 y 3
839.3228759765625 0 387434.5
840.323974609375 0 130885.41
841.3272094726562 0 9753.028
842.30810546875 0 14399.812 b Water loss 6
843.3414916992188 0 36602.555
844.34228515625 0 15778.613
848.3036499023438 0 13359.339
850.3196411132812 0 6242.312
851.3267822265625 0 5755.324
853.3162841796875 0 5538.022
854.3164672851562 0 10263.047
860.3120727539062 0 14361.596 b 6
861.3201293945312 0 9269.537
862.3258056640625 0 27431.871
863.3323974609375 0 13768.885
868.3306884765625 0 15801.212
869.3309936523438 0 9691.689
871.3385620117188 0 116843.28
872.3367919921875 0 66285.31
873.33837890625 0 23002.104
889.3484497070312 0 1202962
890.3511352539062 0 556980.1
890.4560546875 0 9089.946
891.3541259765625 0 154758.25
892.3532104492188 0 15740.692
896.3237915039062 0 23196.402
897.3292236328125 0 12974.866
899.3331298828125 0 7817.9595
907.3412475585938 0 45789.594
908.3453369140625 0 26367.908
909.3482055664062 0 11150.911
914.3378295898438 0 7221.38
917.3282470703125 0 12598.105
918.3123779296875 0 12909.952
933.375 0 12414.952
935.3363037109375 0 237980.16 y Water loss 2
936.3361206054688 0 136542.23
937.33544921875 0 54746.68
938.3240356445312 0 25444.453
939.3244018554688 0 8975.313
953.34716796875 0 3721935.5 y 2
954.3497314453125 0 1945402.8
955.3500366210938 0 699255.94
956.3515014648438 0 68475.93
963.3317260742188 0 23447.814
964.3347778320312 0 11802.063
969.37841796875 0 14226.93
970.3892211914062 0 5419.886
979.3601684570312 0 10277.703 b Water loss 7
990.0547485351562 0 5808.9736
997.3726806640625 0 41487.027 b 7
998.37353515625 0 21413.25
999.3815307617188 0 10884.492
1015.3798217773438 0 12477.065
1016.3836059570312 0 5829.796
1018.3960571289062 0 18790.969
1019.4061279296875 0 9060.508
1020.3939208984375 0 5689.033
1036.4158935546875 0 80972.93
1037.418212890625 0 51501.32
1038.42333984375 0 17914.045
1046.398681640625 0 6202.001
1082.4052734375 0 23512.863 y Water loss 1
1083.40771484375 0 13691.651
1100.415771484375 0 207322.73 y 1
1101.41796875 0 138519.31
1102.4185791015625 0 58362.56
1103.4189453125 0 9629.02
1110.399658203125 0 35571.395
1111.4024658203125 0 25730.951
1112.4095458984375 0 9870.391
1955.6422119140625 0 5642.3633
3239.639404296875 0 6471.89

Spectrum Details

|  |  |
| --- | --- |
| Matched peaks? Matched peaksThe total absolute number of peaks matched. Additionally in brackets the total fraction of peaks matched and the total number of peaks is shown. | 43 (7.12% of 604) |
| FDR? FDRThe false discovery rate estimated for this peptide. It is calculated by matching all theoretical fragments with a non-integer shift with the raw peaks for this spectrum. This is done with 40 different shifts. The resulting percentage is the average number of annotated peaks over the number of annotated peaks with the correct spectrum. | 0.06% |
| Satellite FDR? Satellite FDRSee the FDR for details on its calculation. This satellite ion specific FDR only contains the satellite ions (d/w) for I/L/J positions. | - |
| PSM Score? PSM ScoreThe PSM Score as given by Hecklib to this annotated spectrum. It is shown with three significant figures. | 487 |

## Spectrum 7537? Spectrum 7537 The raw spectrum of this peptide as annotated by Hecklib. The fragments are coloured according to ion type (see legend). Any peaks with a star '\*' as text can be hovered over to see the full details, first the ion type second the mass shift type. By hovering over the amino acids in the peptide or ions in the legend the corresponding peaks are highlighted. By toggling the 'Unassigned' label you can turn the background (unassigned) peaks on or off in the plot. By updating the slider in the Ion legend you can update the spectrum to only show the top X% of the peaks with labels. The top X% means any peak that is within X% of the highest intensity. By dragging in the spectrum you can zoom in to a specific part of the spectrum and use 'Zoom Out' to get back to the original zoom level. The annotation of the spectrum is based on the given sequence in the peptides file and is done with different software so inconsistencies are likely. The peaks are annotated based on the given sequence, with 20 ppm tolerance.

Copy Data

### Spectrum 7537 (TSV)

#### Preview

```
Loading example...
```

*Click on the button to copy the data to your clipboard.*

Mz MinMz MaxIntensity Max

WidthHeightPeptide font sizePeptide stroke widthSpectrum font sizeSpectrum stroke widthCompact peptide

Ion legend

wxyz

abcd

OtherUnassignedIonChargePositionShow for top:%

TFDDYAMHW

06.64e+51.33e+61.99e+62.65e+6

Zoom Out

b+23a+12a+12y+11a+12b+12y+23b+12d+13a+13a+13y+12b+13b+26y+25b+13y+26b+14y+27y+27b+14y+13b+28y+28y+28y+14\*\*b+15b+15b+16b+16y+15y+16y+16b+17b+17y+17y+17b+18b+18y+18y+18

02815628431124

Fragment Matches Table

Show background peaks

| Position | Ion type | Intensity | mz Theoretical | mz Error (Th) | mz Error (ppm) | Charge | Series Number |
| --- | --- | --- | --- | --- | --- | --- | --- |
| - | - | 6.55E+05 | 120.1 | - | - | 0 | - |
| - | - | 1944 | 121 | - | - | 0 | - |
| - | - | 4.978E+04 | 121.1 | - | - | 0 | - |
| - | - | 2414 | 122.1 | - | - | 0 | - |
| - | - | 910 | 122.1 | - | - | 0 | - |
| - | - | 1979 | 127.1 | - | - | 0 | - |
| - | - | 1676 | 129.1 | - | - | 0 | - |
| - | - | 3201 | 129.1 | - | - | 0 | - |
| - | - | 894.4 | 130.1 | - | - | 0 | - |
| - | - | 1196 | 130.1 | - | - | 0 | - |
| - | - | 2.041E+04 | 130.1 | - | - | 0 | - |
| - | - | 920.2 | 131 | - | - | 0 | - |
| - | - | 1375 | 131.1 | - | - | 0 | - |
| - | - | 883.7 | 131.1 | - | - | 0 | - |
| - | - | 3.287E+04 | 132.1 | - | - | 0 | - |
| - | - | 3510 | 133.1 | - | - | 0 | - |
| - | - | 2417 | 134.1 | - | - | 0 | - |
| - | - | 2.231E+05 | 136.1 | - | - | 0 | - |
| - | - | 7133 | 136.1 | - | - | 0 | - |
| - | - | 1.57E+04 | 137.1 | - | - | 0 | - |
| - | - | 1.636E+04 | 138.1 | - | - | 0 | - |
| - | - | 2.571E+04 | 138.1 | - | - | 0 | - |
| - | - | 863.8 | 138.4 | - | - | 0 | - |
| - | - | 1418 | 139.1 | - | - | 0 | - |
| - | - | 1374 | 141.1 | - | - | 0 | - |
| - | - | 1988 | 142.1 | - | - | 0 | - |
| - | - | 1429 | 143 | - | - | 0 | - |
| - | - | 4598 | 143.1 | - | - | 0 | - |
| - | - | 4643 | 143.1 | - | - | 0 | - |
| - | - | 3032 | 144.1 | - | - | 0 | - |
| - | - | 8304 | 146.1 | - | - | 0 | - |
| - | - | 1313 | 146.1 | - | - | 0 | - |
| - | - | 3289 | 146.1 | - | - | 0 | - |
| - | - | 1020 | 147 | - | - | 0 | - |
| - | - | 1023 | 147.1 | - | - | 0 | - |
| - | - | 1112 | 147.1 | - | - | 0 | - |
| - | - | 1775 | 148.1 | - | - | 0 | - |
| - | - | 4720 | 148.1 | - | - | 0 | - |
| - | - | 1512 | 150.1 | - | - | 0 | - |
| - | - | 4895 | 155.1 | - | - | 0 | - |
| - | - | 4759 | 155.1 | - | - | 0 | - |
| - | - | 5.819E+04 | 156.1 | - | - | 0 | - |
| - | - | 3457 | 157.1 | - | - | 0 | - |
| - | - | 2822 | 157.1 | - | - | 0 | - |
| - | - | 9.55E+04 | 158.1 | - | - | 0 | - |
| - | - | 1132 | 159.1 | - | - | 0 | - |
| - | - | 4.987E+04 | 159.1 | - | - | 0 | - |
| - | - | 8698 | 159.1 | - | - | 0 | - |
| - | - | 1.871E+04 | 160.1 | - | - | 0 | - |
| - | - | 2453 | 160.1 | - | - | 0 | - |
| - | - | 4462 | 160.1 | - | - | 0 | - |
| - | - | 4.322E+04 | 160.1 | - | - | 0 | - |
| - | - | 5381 | 161.1 | - | - | 0 | - |
| - | - | 2025 | 165.1 | - | - | 0 | - |
| - | - | 1.285E+04 | 165.1 | - | - | 0 | - |
| - | - | 1260 | 165.1 | - | - | 0 | - |
| - | - | 3096 | 166.1 | - | - | 0 | - |
| - | - | 8882 | 166.1 | - | - | 0 | - |
| - | - | 2558 | 166.1 | - | - | 0 | - |
| - | - | 1416 | 167.1 | - | - | 0 | - |
| - | - | 8704 | 167.1 | - | - | 0 | - |
| - | - | 4054 | 169.1 | - | - | 0 | - |
| - | - | 1259 | 169.1 | - | - | 0 | - |
| - | - | 8600 | 171.1 | - | - | 0 | - |
| - | - | 1567 | 171.1 | - | - | 0 | - |
| - | - | 2313 | 172.1 | - | - | 0 | - |
| - | - | 1199 | 172.1 | - | - | 0 | - |
| - | - | 2844 | 173.1 | - | - | 0 | - |
| - | - | 1137 | 173.1 | - | - | 0 | - |
| - | - | 2225 | 173.4 | - | - | 0 | - |
| 3 | b | 4974 | 174.1 | 0.0009782 | 5.62 | +2 | 3 |
| - | - | 1138 | 174.1 | - | - | 0 | - |
| - | - | 3101 | 175.1 | - | - | 0 | - |
| - | - | 2131 | 175.1 | - | - | 0 | - |
| - | - | 2967 | 175.1 | - | - | 0 | - |
| - | - | 1.713E+04 | 176.1 | - | - | 0 | - |
| - | - | 1.873E+05 | 176.1 | - | - | 0 | - |
| - | - | 1.357E+05 | 177.1 | - | - | 0 | - |
| - | - | 1.94E+04 | 177.1 | - | - | 0 | - |
| - | - | 1224 | 178.1 | - | - | 0 | - |
| - | - | 1734 | 178.1 | - | - | 0 | - |
| - | - | 1.352E+04 | 178.1 | - | - | 0 | - |
| - | - | 9863 | 180.1 | - | - | 0 | - |
| - | - | 1.794E+04 | 185.1 | - | - | 0 | - |
| - | - | 1.432E+04 | 186.1 | - | - | 0 | - |
| - | - | 2544 | 187.1 | - | - | 0 | - |
| - | - | 1055 | 187.1 | - | - | 0 | - |
| - | - | 6.426E+04 | 188.1 | - | - | 0 | - |
| - | - | 7657 | 189.1 | - | - | 0 | - |
| - | - | 1353 | 189.1 | - | - | 0 | - |
| - | - | 1613 | 190.1 | - | - | 0 | - |
| - | - | 2040 | 191.1 | - | - | 0 | - |
| - | - | 6178 | 191.1 | - | - | 0 | - |
| - | - | 1.389E+04 | 191.1 | - | - | 0 | - |
| - | - | 2709 | 192.1 | - | - | 0 | - |
| - | - | 1277 | 192.1 | - | - | 0 | - |
| - | - | 3328 | 193.1 | - | - | 0 | - |
| - | - | 6.075E+04 | 193.1 | - | - | 0 | - |
| - | - | 3312 | 194.1 | - | - | 0 | - |
| - | - | 1100 | 194.1 | - | - | 0 | - |
| - | - | 3393 | 194.1 | - | - | 0 | - |
| - | - | 1.37E+04 | 195.1 | - | - | 0 | - |
| - | - | 1497 | 196.1 | - | - | 0 | - |
| - | - | 1208 | 199.1 | - | - | 0 | - |
| - | - | 1120 | 201.1 | - | - | 0 | - |
| - | - | 5986 | 202.1 | - | - | 0 | - |
| - | - | 1.536E+04 | 203.1 | - | - | 0 | - |
| - | - | 4591 | 203.1 | - | - | 0 | - |
| 2 | a | 6.406E+04 | 203.1 | 0.0003661 | 1.802 | +1 | 2 |
| - | - | 1992 | 204.1 | - | - | 0 | - |
| - | - | 2.468E+04 | 204.1 | - | - | 0 | - |
| 2 | a | 4105 | 204.1 | 0.0001304 | 0.6389 | +1 | 2 |
| - | - | 9034 | 204.1 | - | - | 0 | - |
| - | - | 2442 | 205.1 | - | - | 0 | - |
| 9 | y | 4.798E+04 | 205.1 | 0.0003648 | 1.779 | +1 | 1 |
| - | - | 2655 | 205.1 | - | - | 0 | - |
| - | - | 6278 | 206.1 | - | - | 0 | - |
| - | - | 1.366E+04 | 207.1 | - | - | 0 | - |
| - | - | 8575 | 208.1 | - | - | 0 | - |
| - | - | 1527 | 208.1 | - | - | 0 | - |
| - | - | 1175 | 208.9 | - | - | 0 | - |
| - | - | 1326 | 211.1 | - | - | 0 | - |
| - | - | 4132 | 212.1 | - | - | 0 | - |
| - | - | 1362 | 213.1 | - | - | 0 | - |
| - | - | 2010 | 213.1 | - | - | 0 | - |
| - | - | 3970 | 215.1 | - | - | 0 | - |
| - | - | 1325 | 216.1 | - | - | 0 | - |
| - | - | 3.176E+04 | 217.1 | - | - | 0 | - |
| - | - | 1642 | 218.1 | - | - | 0 | - |
| - | - | 3419 | 218.1 | - | - | 0 | - |
| - | - | 7784 | 219.1 | - | - | 0 | - |
| - | - | 1840 | 219.1 | - | - | 0 | - |
| - | - | 1091 | 219.1 | - | - | 0 | - |
| - | - | 1997 | 220.1 | - | - | 0 | - |
| - | - | 1.588E+05 | 221.1 | - | - | 0 | - |
| 2 | a | 2.628E+06 | 221.1 | 0.0005588 | 2.527 | +1 | 2 |
| - | - | 1.327E+04 | 222.1 | - | - | 0 | - |
| - | - | 3.172E+05 | 222.1 | - | - | 0 | - |
| - | - | 1230 | 223.1 | - | - | 0 | - |
| - | - | 2452 | 223.1 | - | - | 0 | - |
| - | - | 1.788E+04 | 223.1 | - | - | 0 | - |
| - | - | 1015 | 223.2 | - | - | 0 | - |
| - | - | 2042 | 224.1 | - | - | 0 | - |
| - | - | 8330 | 228.1 | - | - | 0 | - |
| - | - | 5112 | 229.1 | - | - | 0 | - |
| - | - | 1122 | 230.1 | - | - | 0 | - |
| - | - | 4.22E+04 | 231.1 | - | - | 0 | - |
| 2 | b | 2.794E+04 | 231.1 | 0.0004618 | 1.998 | +1 | 2 |
| - | - | 3191 | 232.1 | - | - | 0 | - |
| - | - | 3905 | 232.1 | - | - | 0 | - |
| - | - | 2660 | 233.1 | - | - | 0 | - |
| - | - | 6.408E+04 | 235.1 | - | - | 0 | - |
| - | - | 8763 | 236.1 | - | - | 0 | - |
| - | - | 4179 | 238.1 | - | - | 0 | - |
| - | - | 1062 | 239.1 | - | - | 0 | - |
| - | - | 1.41E+04 | 239.1 | - | - | 0 | - |
| - | - | 1342 | 240.1 | - | - | 0 | - |
| - | - | 1346 | 240.1 | - | - | 0 | - |
| - | - | 1396 | 241.1 | - | - | 0 | - |
| 7 | y | 3247 | 245.1 | 0.004842 | 19.76 | +2 | 3 |
| - | - | 1397 | 245.1 | - | - | 0 | - |
| - | - | 1395 | 246.1 | - | - | 0 | - |
| - | - | 4958 | 246.1 | - | - | 0 | - |
| - | - | 1236 | 247.1 | - | - | 0 | - |
| - | - | 1867 | 247.1 | - | - | 0 | - |
| - | - | 2580 | 248.1 | - | - | 0 | - |
| - | - | 4227 | 248.2 | - | - | 0 | - |
| 2 | b | 4.648E+05 | 249.1 | 0.0005478 | 2.199 | +1 | 2 |
| - | - | 2467 | 250.1 | - | - | 0 | - |
| - | - | 5.837E+04 | 250.1 | - | - | 0 | - |
| - | - | 4.963E+04 | 251.1 | - | - | 0 | - |
| - | - | 7028 | 251.1 | - | - | 0 | - |
| - | - | 5055 | 252.1 | - | - | 0 | - |
| - | - | 2347 | 253.1 | - | - | 0 | - |
| - | - | 2342 | 255.1 | - | - | 0 | - |
| - | - | 3702 | 256.1 | - | - | 0 | - |
| - | - | 2.781E+04 | 257.1 | - | - | 0 | - |
| - | - | 2964 | 258.1 | - | - | 0 | - |
| - | - | 1381 | 260.7 | - | - | 0 | - |
| - | - | 7077 | 261.1 | - | - | 0 | - |
| - | - | 1348 | 262.1 | - | - | 0 | - |
| - | - | 1675 | 262.1 | - | - | 0 | - |
| - | - | 2826 | 263.1 | - | - | 0 | - |
| - | - | 9.19E+04 | 263.1 | - | - | 0 | - |
| - | - | 9810 | 263.1 | - | - | 0 | - |
| - | - | 1.171E+04 | 264.1 | - | - | 0 | - |
| - | - | 9057 | 264.1 | - | - | 0 | - |
| - | - | 3167 | 265.1 | - | - | 0 | - |
| - | - | 6861 | 267.1 | - | - | 0 | - |
| - | - | 3701 | 268.1 | - | - | 0 | - |
| - | - | 2343 | 268.1 | - | - | 0 | - |
| - | - | 3139 | 268.1 | - | - | 0 | - |
| - | - | 2029 | 269.1 | - | - | 0 | - |
| - | - | 1399 | 270.1 | - | - | 0 | - |
| - | - | 1797 | 270.1 | - | - | 0 | - |
| - | - | 4815 | 271.1 | - | - | 0 | - |
| - | - | 1551 | 272.1 | - | - | 0 | - |
| - | - | 2254 | 273.1 | - | - | 0 | - |
| - | - | 2.325E+04 | 274.1 | - | - | 0 | - |
| - | - | 1695 | 274.2 | - | - | 0 | - |
| - | - | 4435 | 275.1 | - | - | 0 | - |
| - | - | 3682 | 275.1 | - | - | 0 | - |
| - | - | 1598 | 277.7 | - | - | 0 | - |
| - | - | 1128 | 277.8 | - | - | 0 | - |
| - | - | 1012 | 277.8 | - | - | 0 | - |
| - | - | 1482 | 278.1 | - | - | 0 | - |
| - | - | 3.822E+04 | 279.1 | - | - | 0 | - |
| - | - | 3821 | 279.1 | - | - | 0 | - |
| - | - | 6055 | 280.1 | - | - | 0 | - |
| - | - | 1645 | 282.1 | - | - | 0 | - |
| - | - | 1355 | 283.2 | - | - | 0 | - |
| - | - | 5.931E+04 | 285.1 | - | - | 0 | - |
| - | - | 8055 | 286.1 | - | - | 0 | - |
| - | - | 2215 | 287.1 | - | - | 0 | - |
| - | - | 1569 | 288.1 | - | - | 0 | - |
| - | - | 4265 | 289.1 | - | - | 0 | - |
| - | - | 1639 | 290.1 | - | - | 0 | - |
| - | - | 3.869E+04 | 292.1 | - | - | 0 | - |
| 3 | d | 9110 | 292.2 | 0.0005392 | 1.845 | +1 | 3 |
| - | - | 4635 | 293.1 | - | - | 0 | - |
| - | - | 1863 | 296.1 | - | - | 0 | - |
| - | - | 4.329E+04 | 296.2 | - | - | 0 | - |
| - | - | 1574 | 297.1 | - | - | 0 | - |
| - | - | 7805 | 297.2 | - | - | 0 | - |
| - | - | 4326 | 300.1 | - | - | 0 | - |
| - | - | 3466 | 301.1 | - | - | 0 | - |
| - | - | 4370 | 302.1 | - | - | 0 | - |
| - | - | 6029 | 303.1 | - | - | 0 | - |
| - | - | 1698 | 306.1 | - | - | 0 | - |
| - | - | 1134 | 306.1 | - | - | 0 | - |
| - | - | 1879 | 307.1 | - | - | 0 | - |
| - | - | 6705 | 310.2 | - | - | 0 | - |
| - | - | 2776 | 313.1 | - | - | 0 | - |
| - | - | 4091 | 314.1 | - | - | 0 | - |
| - | - | 3427 | 315.1 | - | - | 0 | - |
| - | - | 1762 | 316.1 | - | - | 0 | - |
| 3 | a | 1.516E+04 | 318.1 | 0.0006447 | 2.027 | +1 | 3 |
| - | - | 1537 | 319.1 | - | - | 0 | - |
| - | - | 3902 | 320.1 | - | - | 0 | - |
| - | - | 1.761E+04 | 323.1 | - | - | 0 | - |
| - | - | 1.031E+05 | 324.1 | - | - | 0 | - |
| - | - | 1.56E+04 | 325.1 | - | - | 0 | - |
| - | - | 1233 | 327.1 | - | - | 0 | - |
| - | - | 2513 | 327.1 | - | - | 0 | - |
| - | - | 7216 | 328.1 | - | - | 0 | - |
| - | - | 4932 | 328.1 | - | - | 0 | - |
| - | - | 5824 | 330.1 | - | - | 0 | - |
| - | - | 2568 | 331.1 | - | - | 0 | - |
| - | - | 5319 | 332.1 | - | - | 0 | - |
| - | - | 3791 | 333.1 | - | - | 0 | - |
| - | - | 1675 | 333.1 | - | - | 0 | - |
| - | - | 2844 | 334.1 | - | - | 0 | - |
| - | - | 3270 | 334.1 | - | - | 0 | - |
| - | - | 2638 | 335.2 | - | - | 0 | - |
| 3 | a | 1325 | 336.2 | 0.0001814 | 0.5395 | +1 | 3 |
| - | - | 7243 | 338.1 | - | - | 0 | - |
| - | - | 9064 | 339.2 | - | - | 0 | - |
| - | - | 1649 | 340.2 | - | - | 0 | - |
| 8 | y | 5.377E+05 | 342.2 | 0.0007944 | 2.322 | +1 | 2 |
| - | - | 9.701E+04 | 343.2 | - | - | 0 | - |
| - | - | 9883 | 344.2 | - | - | 0 | - |
| 3 | b | 3.078E+04 | 346.1 | 0.0004811 | 1.39 | +1 | 3 |
| - | - | 4527 | 347.1 | - | - | 0 | - |
| - | - | 1.408E+04 | 348.1 | - | - | 0 | - |
| - | - | 1574 | 349.1 | - | - | 0 | - |
| - | - | 3093 | 349.1 | - | - | 0 | - |
| - | - | 3.146E+04 | 350.1 | - | - | 0 | - |
| - | - | 4398 | 351.1 | - | - | 0 | - |
| - | - | 9164 | 352.1 | - | - | 0 | - |
| - | - | 2013 | 353.1 | - | - | 0 | - |
| - | - | 2629 | 354.2 | - | - | 0 | - |
| - | - | 3.236E+04 | 356.1 | - | - | 0 | - |
| 6 | b | 4701 | 357.1 | 0.000458 | 1.282 | +2 | 6 |
| - | - | 2559 | 358.1 | - | - | 0 | - |
| - | - | 2605 | 359.1 | - | - | 0 | - |
| - | - | 6722 | 360.1 | - | - | 0 | - |
| 5 | y | 4639 | 362.1 | 0.003531 | 9.749 | +2 | 5 |
| - | - | 3286 | 362.7 | - | - | 0 | - |
| 3 | b | 1.298E+05 | 364.2 | 0.0006281 | 1.725 | +1 | 3 |
| - | - | 2.64E+04 | 365.2 | - | - | 0 | - |
| - | - | 1.422E+04 | 366.1 | - | - | 0 | - |
| - | - | 2124 | 366.2 | - | - | 0 | - |
| - | - | 2159 | 367.1 | - | - | 0 | - |
| - | - | 6502 | 367.2 | - | - | 0 | - |
| - | - | 1870 | 368.2 | - | - | 0 | - |
| - | - | 3382 | 371.1 | - | - | 0 | - |
| - | - | 6205 | 374.1 | - | - | 0 | - |
| - | - | 1.36E+04 | 376.1 | - | - | 0 | - |
| - | - | 2851 | 377.1 | - | - | 0 | - |
| - | - | 3634 | 378.1 | - | - | 0 | - |
| - | - | 7.319E+04 | 378.1 | - | - | 0 | - |
| - | - | 1.244E+04 | 379.1 | - | - | 0 | - |
| - | - | 5090 | 380.1 | - | - | 0 | - |
| - | - | 2640 | 381.1 | - | - | 0 | - |
| - | - | 1.564E+04 | 382.1 | - | - | 0 | - |
| - | - | 3144 | 383.1 | - | - | 0 | - |
| - | - | 4377 | 384.1 | - | - | 0 | - |
| - | - | 3858 | 385.1 | - | - | 0 | - |
| - | - | 1882 | 386.1 | - | - | 0 | - |
| - | - | 1893 | 388.1 | - | - | 0 | - |
| - | - | 1988 | 388.1 | - | - | 0 | - |
| - | - | 1720 | 389.1 | - | - | 0 | - |
| - | - | 9620 | 390.2 | - | - | 0 | - |
| - | - | 1813 | 391.2 | - | - | 0 | - |
| - | - | 9.138E+04 | 394.1 | - | - | 0 | - |
| - | - | 1.792E+04 | 395.1 | - | - | 0 | - |
| - | - | 1789 | 395.2 | - | - | 0 | - |
| - | - | 1773 | 396.1 | - | - | 0 | - |
| - | - | 1707 | 397.2 | - | - | 0 | - |
| - | - | 2599 | 398.2 | - | - | 0 | - |
| - | - | 4252 | 399.2 | - | - | 0 | - |
| - | - | 2578 | 404.1 | - | - | 0 | - |
| - | - | 2373 | 404.1 | - | - | 0 | - |
| - | - | 2146 | 406.1 | - | - | 0 | - |
| - | - | 2772 | 406.2 | - | - | 0 | - |
| - | - | 9792 | 407.2 | - | - | 0 | - |
| - | - | 2.123E+04 | 408.2 | - | - | 0 | - |
| - | - | 6368 | 409.2 | - | - | 0 | - |
| - | - | 2926 | 410.1 | - | - | 0 | - |
| - | - | 2098 | 410.2 | - | - | 0 | - |
| - | - | 4603 | 415.2 | - | - | 0 | - |
| - | - | 2253 | 416.1 | - | - | 0 | - |
| - | - | 1530 | 417.2 | - | - | 0 | - |
| - | - | 3928 | 419.2 | - | - | 0 | - |
| 4 | y | 1427 | 419.7 | 0.00129 | 3.073 | +2 | 6 |
| - | - | 1495 | 420.2 | - | - | 0 | - |
| - | - | 2198 | 423.2 | - | - | 0 | - |
| - | - | 7.709E+04 | 425.2 | - | - | 0 | - |
| - | - | 1.676E+04 | 426.2 | - | - | 0 | - |
| - | - | 1.604E+04 | 427.2 | - | - | 0 | - |
| - | - | 3659 | 428.2 | - | - | 0 | - |
| - | - | 2145 | 430.1 | - | - | 0 | - |
| - | - | 1654 | 431.1 | - | - | 0 | - |
| - | - | 1529 | 431.2 | - | - | 0 | - |
| - | - | 2240 | 432.1 | - | - | 0 | - |
| - | - | 1567 | 433.1 | - | - | 0 | - |
| - | - | 1.47E+04 | 433.2 | - | - | 0 | - |
| - | - | 2187 | 434.2 | - | - | 0 | - |
| - | - | 4260 | 435.2 | - | - | 0 | - |
| - | - | 3920 | 437.2 | - | - | 0 | - |
| - | - | 1942 | 438.2 | - | - | 0 | - |
| - | - | 2966 | 442.2 | - | - | 0 | - |
| - | - | 3066 | 443.2 | - | - | 0 | - |
| - | - | 1423 | 445.2 | - | - | 0 | - |
| - | - | 1449 | 446.2 | - | - | 0 | - |
| - | - | 1.155E+04 | 447.2 | - | - | 0 | - |
| - | - | 2343 | 448.2 | - | - | 0 | - |
| - | - | 2895 | 449.1 | - | - | 0 | - |
| - | - | 3223 | 449.2 | - | - | 0 | - |
| - | - | 2548 | 450.2 | - | - | 0 | - |
| - | - | 1671 | 451.2 | - | - | 0 | - |
| - | - | 2608 | 454.2 | - | - | 0 | - |
| - | - | 5796 | 454.2 | - | - | 0 | - |
| - | - | 1.448E+04 | 455.2 | - | - | 0 | - |
| - | - | 3441 | 456.2 | - | - | 0 | - |
| - | - | 3602 | 458.2 | - | - | 0 | - |
| - | - | 3522 | 459.2 | - | - | 0 | - |
| 4 | b | 2.036E+04 | 461.2 | 0.0006377 | 1.383 | +1 | 4 |
| - | - | 7611 | 461.2 | - | - | 0 | - |
| - | - | 6412 | 462.2 | - | - | 0 | - |
| - | - | 2194 | 462.2 | - | - | 0 | - |
| - | - | 7.285E+04 | 465.2 | - | - | 0 | - |
| - | - | 1.681E+04 | 466.2 | - | - | 0 | - |
| - | - | 1570 | 467.2 | - | - | 0 | - |
| 3 | y | 3130 | 468.2 | 0.002805 | 5.991 | +2 | 7 |
| - | - | 3204 | 469.2 | - | - | 0 | - |
| - | - | 1593 | 470.2 | - | - | 0 | - |
| - | - | 1485 | 470.2 | - | - | 0 | - |
| - | - | 1.176E+04 | 471.2 | - | - | 0 | - |
| - | - | 3264 | 472.2 | - | - | 0 | - |
| - | - | 1270 | 472.2 | - | - | 0 | - |
| - | - | 6696 | 473.2 | - | - | 0 | - |
| - | - | 1544 | 475.1 | - | - | 0 | - |
| - | - | 1516 | 476.2 | - | - | 0 | - |
| 3 | y | 1.204E+04 | 477.2 | 0.006933 | 14.53 | +2 | 7 |
| - | - | 3512 | 477.7 | - | - | 0 | - |
| - | - | 3535 | 478.2 | - | - | 0 | - |
| - | - | 2294 | 478.2 | - | - | 0 | - |
| 4 | b | 6.895E+04 | 479.2 | 0.0008762 | 1.829 | +1 | 4 |
| - | - | 1.559E+04 | 480.2 | - | - | 0 | - |
| - | - | 1777 | 481.2 | - | - | 0 | - |
| - | - | 3813 | 482.2 | - | - | 0 | - |
| - | - | 1704 | 483.2 | - | - | 0 | - |
| - | - | 1513 | 485.7 | - | - | 0 | - |
| - | - | 1.199E+04 | 486.2 | - | - | 0 | - |
| - | - | 2175 | 487.2 | - | - | 0 | - |
| 7 | y | 1.283E+05 | 489.2 | 0.005954 | 12.17 | +1 | 3 |
| - | - | 3.609E+04 | 490.2 | - | - | 0 | - |
| - | - | 2378 | 490.7 | - | - | 0 | - |
| - | - | 1.684E+04 | 491.2 | - | - | 0 | - |
| - | - | 4277 | 492.2 | - | - | 0 | - |
| - | - | 1284 | 493.2 | - | - | 0 | - |
| - | - | 9251 | 495.2 | - | - | 0 | - |
| - | - | 2297 | 496.2 | - | - | 0 | - |
| - | - | 3675 | 496.2 | - | - | 0 | - |
| - | - | 6.561E+04 | 496.2 | - | - | 0 | - |
| - | - | 1.201E+04 | 497.2 | - | - | 0 | - |
| - | - | 1.965E+04 | 497.2 | - | - | 0 | - |
| - | - | 2571 | 498.2 | - | - | 0 | - |
| - | - | 2010 | 498.2 | - | - | 0 | - |
| 8 | b | 5624 | 499.2 | 0.001719 | 3.443 | +2 | 8 |
| - | - | 1745 | 502.2 | - | - | 0 | - |
| - | - | 3198 | 503.2 | - | - | 0 | - |
| - | - | 1653 | 506.2 | - | - | 0 | - |
| - | - | 1687 | 509.7 | - | - | 0 | - |
| - | - | 1611 | 511.2 | - | - | 0 | - |
| - | - | 1.103E+04 | 513.2 | - | - | 0 | - |
| - | - | 2730 | 514.2 | - | - | 0 | - |
| - | - | 3745 | 516.2 | - | - | 0 | - |
| - | - | 4520 | 518.7 | - | - | 0 | - |
| - | - | 1.938E+04 | 519.2 | - | - | 0 | - |
| - | - | 1483 | 519.7 | - | - | 0 | - |
| - | - | 8478 | 520.2 | - | - | 0 | - |
| - | - | 2053 | 521.2 | - | - | 0 | - |
| - | - | 3271 | 523.2 | - | - | 0 | - |
| - | - | 1393 | 525.2 | - | - | 0 | - |
| - | - | 4007 | 525.2 | - | - | 0 | - |
| - | - | 1613 | 526.2 | - | - | 0 | - |
| - | - | 1604 | 527.2 | - | - | 0 | - |
| - | - | 2068 | 527.7 | - | - | 0 | - |
| - | - | 3920 | 530.2 | - | - | 0 | - |
| - | - | 1533 | 532.2 | - | - | 0 | - |
| - | - | 1710 | 533.2 | - | - | 0 | - |
| - | - | 6359 | 537.2 | - | - | 0 | - |
| - | - | 5089 | 541.2 | - | - | 0 | - |
| 2 | y | 9249 | 541.7 | 0.002686 | 4.959 | +2 | 8 |
| - | - | 1.191E+04 | 542.2 | - | - | 0 | - |
| - | - | 5138 | 542.7 | - | - | 0 | - |
| - | - | 3207 | 543.2 | - | - | 0 | - |
| - | - | 2025 | 545.2 | - | - | 0 | - |
| - | - | 2.392E+04 | 548.2 | - | - | 0 | - |
| - | - | 8286 | 549.2 | - | - | 0 | - |
| - | - | 2009 | 550.2 | - | - | 0 | - |
| 2 | y | 6.756E+04 | 550.7 | 0.003141 | 5.704 | +2 | 8 |
| - | - | 4.973E+04 | 551.2 | - | - | 0 | - |
| - | - | 1.997E+04 | 551.7 | - | - | 0 | - |
| - | - | 4202 | 552.2 | - | - | 0 | - |
| - | - | 2317 | 553.2 | - | - | 0 | - |
| - | - | 3081 | 555.7 | - | - | 0 | - |
| - | - | 1.17E+04 | 557.2 | - | - | 0 | - |
| - | - | 1615 | 558.2 | - | - | 0 | - |
| 6 | y | 2.042E+05 | 560.2 | 0.005462 | 9.749 | +1 | 4 |
| - | - | 5372 | 560.7 | - | - | 0 | - |
| - | - | 6.736E+04 | 561.2 | - | - | 0 | - |
| - | - | 1.413E+04 | 562.2 | - | - | 0 | - |
| - | - | 1558 | 563.2 | - | - | 0 | - |
| - | - | 1.388E+04 | 569.2 | - | - | 0 | - |
| - | - | 1.131E+04 | 569.7 | - | - | 0 | - |
| - | - | 2.025E+04 | 570.2 | - | - | 0 | - |
| - | - | 1356 | 570.7 | - | - | 0 | - |
| - | - | 3570 | 571.2 | - | - | 0 | - |
| - | - | 2606 | 574.7 | - | - | 0 | - |
| - | - | 1.048E+04 | 578.2 | - | - | 0 | - |
| - | - | 5561 | 578.7 | - | - | 0 | - |
| - | - | 1760 | 579.2 | - | - | 0 | - |
| - | - | 6215 | 583.2 | - | - | 0 | - |
| - | - | 3611 | 583.7 | - | - | 0 | - |
| - | - | 4942 | 588.2 | - | - | 0 | - |
| 0 | Precursor | 4.421E+04 | 592.2 | 0.003444 | 5.815 | +2 | -1 |
| - | - | 2.84E+04 | 592.7 | - | - | 0 | - |
| - | - | 1.416E+04 | 593.2 | - | - | 0 | - |
| - | - | 2856 | 593.7 | - | - | 0 | - |
| - | - | 5815 | 594.2 | - | - | 0 | - |
| - | - | 1.41E+04 | 596.2 | - | - | 0 | - |
| - | - | 9118 | 597.2 | - | - | 0 | - |
| - | - | 3522 | 598.2 | - | - | 0 | - |
| 0 | Precursor | 1.054E+05 | 601.2 | 0.003411 | 5.673 | +2 | -1 |
| - | - | 7.35E+04 | 601.7 | - | - | 0 | - |
| - | - | 1814 | 601.8 | - | - | 0 | - |
| - | - | 3.26E+04 | 602.2 | - | - | 0 | - |
| - | - | 5006 | 602.7 | - | - | 0 | - |
| - | - | 2.057E+04 | 606.2 | - | - | 0 | - |
| - | - | 6713 | 607.2 | - | - | 0 | - |
| - | - | 2552 | 608.2 | - | - | 0 | - |
| - | - | 3.572E+04 | 612.2 | - | - | 0 | - |
| - | - | 1.12E+04 | 613.2 | - | - | 0 | - |
| - | - | 2301 | 613.3 | - | - | 0 | - |
| - | - | 2079 | 614.2 | - | - | 0 | - |
| - | - | 2.479E+04 | 614.2 | - | - | 0 | - |
| - | - | 1319 | 614.3 | - | - | 0 | - |
| - | - | 6558 | 615.2 | - | - | 0 | - |
| - | - | 1661 | 622.2 | - | - | 0 | - |
| 5 | b | 1.603E+04 | 624.2 | 0.001243 | 1.992 | +1 | 5 |
| - | - | 5388 | 625.2 | - | - | 0 | - |
| - | - | 9085 | 631.2 | - | - | 0 | - |
| - | - | 4627 | 632.3 | - | - | 0 | - |
| - | - | 1370 | 633.3 | - | - | 0 | - |
| - | - | 1.864E+04 | 634.2 | - | - | 0 | - |
| - | - | 7531 | 635.2 | - | - | 0 | - |
| - | - | 1572 | 636.2 | - | - | 0 | - |
| - | - | 6264 | 639.3 | - | - | 0 | - |
| - | - | 8924 | 640.2 | - | - | 0 | - |
| - | - | 2170 | 641.2 | - | - | 0 | - |
| - | - | 2126 | 641.3 | - | - | 0 | - |
| 5 | b | 1.523E+04 | 642.2 | 0.001299 | 2.022 | +1 | 5 |
| - | - | 3374 | 643.2 | - | - | 0 | - |
| - | - | 5391 | 652.2 | - | - | 0 | - |
| - | - | 1383 | 653.2 | - | - | 0 | - |
| - | - | 4.736E+04 | 657.3 | - | - | 0 | - |
| - | - | 1.353E+04 | 658.3 | - | - | 0 | - |
| - | - | 7.355E+04 | 659.3 | - | - | 0 | - |
| - | - | 2.692E+04 | 660.3 | - | - | 0 | - |
| - | - | 6523 | 661.3 | - | - | 0 | - |
| - | - | 5791 | 667.3 | - | - | 0 | - |
| - | - | 4322 | 668.3 | - | - | 0 | - |
| - | - | 9196 | 676.3 | - | - | 0 | - |
| - | - | 5837 | 677.3 | - | - | 0 | - |
| - | - | 2333 | 678.3 | - | - | 0 | - |
| - | - | 6.358E+04 | 685.3 | - | - | 0 | - |
| - | - | 2.12E+04 | 686.3 | - | - | 0 | - |
| - | - | 5234 | 687.3 | - | - | 0 | - |
| 6 | b | 1.241E+04 | 695.3 | 0.0002258 | 0.3248 | +1 | 6 |
| - | - | 4211 | 696.3 | - | - | 0 | - |
| - | - | 2680 | 702.3 | - | - | 0 | - |
| - | - | 1.678E+04 | 703.3 | - | - | 0 | - |
| - | - | 3969 | 704.2 | - | - | 0 | - |
| - | - | 4331 | 704.3 | - | - | 0 | - |
| - | - | 8059 | 705.3 | - | - | 0 | - |
| - | - | 3007 | 706.3 | - | - | 0 | - |
| - | - | 1516 | 708.3 | - | - | 0 | - |
| 6 | b | 6364 | 713.3 | 0.001818 | 2.549 | +1 | 6 |
| - | - | 1825 | 714.3 | - | - | 0 | - |
| - | - | 5.745E+04 | 721.3 | - | - | 0 | - |
| - | - | 2.343E+04 | 722.3 | - | - | 0 | - |
| 5 | y | 1.891E+05 | 723.3 | 0.005366 | 7.418 | +1 | 5 |
| - | - | 7.931E+04 | 724.3 | - | - | 0 | - |
| - | - | 2.496E+04 | 725.3 | - | - | 0 | - |
| - | - | 1610 | 728.3 | - | - | 0 | - |
| - | - | 6410 | 731.2 | - | - | 0 | - |
| - | - | 3536 | 732.2 | - | - | 0 | - |
| - | - | 4372 | 733.3 | - | - | 0 | - |
| - | - | 1845 | 739.3 | - | - | 0 | - |
| - | - | 3377 | 746.3 | - | - | 0 | - |
| - | - | 1816 | 747.3 | - | - | 0 | - |
| - | - | 2006 | 748.3 | - | - | 0 | - |
| - | - | 8.518E+04 | 749.3 | - | - | 0 | - |
| - | - | 2.974E+04 | 750.3 | - | - | 0 | - |
| - | - | 8775 | 751.3 | - | - | 0 | - |
| - | - | 5940 | 756.3 | - | - | 0 | - |
| - | - | 3725 | 757.3 | - | - | 0 | - |
| - | - | 2607 | 759.3 | - | - | 0 | - |
| - | - | 2.077E+04 | 767.3 | - | - | 0 | - |
| - | - | 8136 | 768.3 | - | - | 0 | - |
| - | - | 2013 | 769.3 | - | - | 0 | - |
| - | - | 7.819E+04 | 774.3 | - | - | 0 | - |
| - | - | 3.359E+04 | 775.3 | - | - | 0 | - |
| - | - | 8560 | 776.3 | - | - | 0 | - |
| - | - | 3662 | 778.3 | - | - | 0 | - |
| - | - | 4651 | 792.3 | - | - | 0 | - |
| - | - | 3281 | 793.3 | - | - | 0 | - |
| - | - | 2225 | 796.3 | - | - | 0 | - |
| - | - | 2442 | 803.3 | - | - | 0 | - |
| - | - | 1930 | 804.3 | - | - | 0 | - |
| - | - | 2114 | 814.3 | - | - | 0 | - |
| - | - | 1564 | 817.3 | - | - | 0 | - |
| - | - | 2959 | 818.3 | - | - | 0 | - |
| - | - | 1825 | 819.3 | - | - | 0 | - |
| 4 | y | 1.077E+04 | 820.3 | 0.005711 | 6.962 | +1 | 6 |
| - | - | 7344 | 821.3 | - | - | 0 | - |
| - | - | 2508 | 822.3 | - | - | 0 | - |
| - | - | 1475 | 823.3 | - | - | 0 | - |
| - | - | 5917 | 832.3 | - | - | 0 | - |
| - | - | 2501 | 833.3 | - | - | 0 | - |
| - | - | 1791 | 834.3 | - | - | 0 | - |
| 4 | y | 2.356E+05 | 838.3 | 0.005522 | 6.587 | +1 | 6 |
| - | - | 1.093E+05 | 839.3 | - | - | 0 | - |
| - | - | 3.664E+04 | 840.3 | - | - | 0 | - |
| - | - | 4770 | 841.3 | - | - | 0 | - |
| 7 | b | 6596 | 842.3 | 0.006582 | 7.814 | +1 | 7 |
| - | - | 7051 | 843.3 | - | - | 0 | - |
| - | - | 4077 | 844.3 | - | - | 0 | - |
| - | - | 2320 | 845.4 | - | - | 0 | - |
| - | - | 2442 | 848.3 | - | - | 0 | - |
| - | - | 2490 | 850.6 | - | - | 0 | - |
| - | - | 2675 | 853.3 | - | - | 0 | - |
| - | - | 3938 | 854.3 | - | - | 0 | - |
| 7 | b | 4325 | 860.3 | 0.005356 | 6.225 | +1 | 7 |
| - | - | 5395 | 862.3 | - | - | 0 | - |
| - | - | 2968 | 863.3 | - | - | 0 | - |
| - | - | 4006 | 868.3 | - | - | 0 | - |
| - | - | 1676 | 869.3 | - | - | 0 | - |
| - | - | 3.236E+04 | 871.3 | - | - | 0 | - |
| - | - | 1.933E+04 | 872.3 | - | - | 0 | - |
| - | - | 7252 | 873.3 | - | - | 0 | - |
| - | - | 2044 | 874.3 | - | - | 0 | - |
| - | - | 1901 | 888.3 | - | - | 0 | - |
| - | - | 3.297E+05 | 889.3 | - | - | 0 | - |
| - | - | 1.685E+05 | 890.4 | - | - | 0 | - |
| - | - | 2206 | 890.5 | - | - | 0 | - |
| - | - | 4.351E+04 | 891.4 | - | - | 0 | - |
| - | - | 4450 | 892.4 | - | - | 0 | - |
| - | - | 7324 | 896.3 | - | - | 0 | - |
| - | - | 3911 | 897.3 | - | - | 0 | - |
| - | - | 1600 | 899.3 | - | - | 0 | - |
| - | - | 1.535E+04 | 907.3 | - | - | 0 | - |
| - | - | 9560 | 908.3 | - | - | 0 | - |
| - | - | 3097 | 909.3 | - | - | 0 | - |
| - | - | 2909 | 915.4 | - | - | 0 | - |
| - | - | 2028 | 917.3 | - | - | 0 | - |
| - | - | 3593 | 918.3 | - | - | 0 | - |
| - | - | 3012 | 933.4 | - | - | 0 | - |
| 3 | y | 5.99E+04 | 935.3 | 0.005562 | 5.947 | +1 | 7 |
| - | - | 3.975E+04 | 936.3 | - | - | 0 | - |
| - | - | 1.533E+04 | 937.3 | - | - | 0 | - |
| - | - | 8958 | 938.3 | - | - | 0 | - |
| - | - | 3917 | 939.3 | - | - | 0 | - |
| 3 | y | 1.085E+06 | 953.3 | 0.005679 | 5.957 | +1 | 7 |
| - | - | 5.653E+05 | 954.3 | - | - | 0 | - |
| - | - | 1.926E+05 | 955.3 | - | - | 0 | - |
| - | - | 2.591E+04 | 956.4 | - | - | 0 | - |
| - | - | 8431 | 963.3 | - | - | 0 | - |
| - | - | 3868 | 964.3 | - | - | 0 | - |
| - | - | 4569 | 969.4 | - | - | 0 | - |
| - | - | 2930 | 970.4 | - | - | 0 | - |
| 8 | b | 3178 | 979.4 | 0.001686 | 1.722 | +1 | 8 |
| - | - | 1576 | 980.4 | - | - | 0 | - |
| - | - | 1594 | 981.4 | - | - | 0 | - |
| 8 | b | 1.297E+04 | 997.4 | 0.005587 | 5.602 | +1 | 8 |
| - | - | 6537 | 998.4 | - | - | 0 | - |
| - | - | 2532 | 999.4 | - | - | 0 | - |
| - | - | 4391 | 1015 | - | - | 0 | - |
| - | - | 5215 | 1018 | - | - | 0 | - |
| - | - | 1805 | 1019 | - | - | 0 | - |
| - | - | 2.415E+04 | 1036 | - | - | 0 | - |
| - | - | 1.513E+04 | 1037 | - | - | 0 | - |
| - | - | 4800 | 1038 | - | - | 0 | - |
| - | - | 1720 | 1046 | - | - | 0 | - |
| 2 | y | 7427 | 1082 | 0.005202 | 4.806 | +1 | 8 |
| - | - | 4194 | 1083 | - | - | 0 | - |
| 2 | y | 6.038E+04 | 1100 | 0.005136 | 4.667 | +1 | 8 |
| - | - | 3.974E+04 | 1101 | - | - | 0 | - |
| - | - | 1.643E+04 | 1102 | - | - | 0 | - |
| - | - | 1836 | 1103 | - | - | 0 | - |
| - | - | 9855 | 1110 | - | - | 0 | - |
| - | - | 4929 | 1111 | - | - | 0 | - |
| - | - | 2976 | 1112 | - | - | 0 | - |

m/z Charge Intensity FragmentType MassShift Position
120.08120727539062 0 655006.7
121.03990173339844 0 1943.9277
121.08448791503906 0 49775.227
122.07177734375 0 2414.2556
122.08861541748047 0 910.02686
127.08715057373047 0 1979.1365
129.07061767578125 0 1675.9445
129.10269165039062 0 3201.2234
130.050537109375 0 894.4042
130.05996704101562 0 1196.4757
130.06552124023438 0 20409.764
131.0491180419922 0 920.2438
131.0688934326172 0 1374.8514
131.08584594726562 0 883.71545
132.0811309814453 0 32865.227
133.084716796875 0 3510.1946
134.09674072265625 0 2416.5183
136.0760955810547 0 223087.67
136.08729553222656 0 7132.913
137.0794677734375 0 15697.696
138.0552978515625 0 16358.1455
138.0666046142578 0 25712.2
138.36717224121094 0 863.8476
139.06997680664062 0 1417.796
141.10238647460938 0 1373.5
142.06533813476562 0 1988.4291
143.0454864501953 0 1429.1755
143.0734100341797 0 4597.922
143.08592224121094 0 4643.113
144.0811767578125 0 3032.2705
146.06044006347656 0 8304.412
146.0718994140625 0 1312.8082
146.09693908691406 0 3289.0571
147.04412841796875 0 1020.25903
147.0635223388672 0 1023.02454
147.09231567382812 0 1111.8215
148.07606506347656 0 1775.0371
148.08721923828125 0 4720.4697
150.06663513183594 0 1512.2742
155.08193969726562 0 4894.689
155.09315490722656 0 4758.6963
156.07713317871094 0 58194.367
157.08053588867188 0 3457.0464
157.1342315673828 0 2821.9756
158.09678649902344 0 95504.77
159.05563354492188 0 1131.5476
159.09207153320312 0 49865.984
159.0999755859375 0 8698.0205
160.07606506347656 0 18714.943
160.08749389648438 0 2453.4377
160.09555053710938 0 4462.1016
160.11241149902344 0 43224.086
161.11570739746094 0 5381.053
165.070068359375 0 2024.6824
165.07742309570312 0 12845.015
165.10313415527344 0 1259.9515
166.0536346435547 0 3096.393
166.06153869628906 0 8881.914
166.08627319335938 0 2557.6475
167.0567626953125 0 1416.1543
167.0932159423828 0 8704.479
169.07643127441406 0 4053.958
169.09738159179688 0 1258.6539
171.07691955566406 0 8599.688
171.09185791015625 0 1566.6725
172.0762481689453 0 2312.754
172.10833740234375 0 1199.0736
173.0924072265625 0 2843.531
173.12904357910156 0 1137.3196
173.4388427734375 0 2224.8162
174.06649780273438 0 4973.751 b Ammonia loss 2
174.09214782714844 0 1137.8798
175.08743286132812 0 3100.5913
175.09837341308594 0 2131.1687
175.1234893798828 0 2967.1675
176.08236694335938 0 17134.12
176.10743713378906 0 187296.58
177.10264587402344 0 135737.72
177.11109924316406 0 19404.389
178.06134033203125 0 1224.4556
178.09814453125 0 1733.5239
178.10610961914062 0 13517.441
180.0772247314453 0 9862.59
185.05615234375 0 17935.03
186.09173583984375 0 14324.095
187.0633087158203 0 2544.0771
187.0952911376953 0 1055.0028
188.0709991455078 0 64259.207
189.07432556152344 0 7656.75
189.10198974609375 0 1352.9362
190.08663940429688 0 1613.4431
191.08285522460938 0 2040.1997
191.09329223632812 0 6177.99
191.11837768554688 0 13888.793
192.10272216796875 0 2709.0974
192.12205505371094 0 1276.7059
193.07276916503906 0 3328.0964
193.10877990722656 0 60748.617
194.080078125 0 3311.878
194.0930938720703 0 1099.8557
194.1122589111328 0 3392.9294
195.0880584716797 0 13703.3955
196.09194946289062 0 1496.7706
199.07142639160156 0 1207.5057
201.06582641601562 0 1119.8401
202.05355834960938 0 5986.0903
203.06666564941406 0 15357.032
203.0931396484375 0 4591.217
203.11825561523438 0 64058.535 a Water loss 1
204.06809997558594 0 1991.7843
204.0771484375 0 24677.11
204.10203552246094 0 4104.7876 a Ammonia loss 1
204.12164306640625 0 9033.745
205.08132934570312 0 2441.938
205.09751892089844 0 47983.45 y 8
205.10781860351562 0 2655.1733
206.10101318359375 0 6278.4917
207.11331176757812 0 13659.186
208.0720672607422 0 8574.618
208.11724853515625 0 1527.1578
208.926513671875 0 1174.64
211.05856323242188 0 1326.2236
212.11610412597656 0 4131.636
213.05136108398438 0 1362.4452
213.10006713867188 0 2010.371
215.08216857910156 0 3970.1853
216.06680297851562 0 1324.7069
217.09759521484375 0 31759.615
218.08206176757812 0 1641.8201
218.10177612304688 0 3419.3108
219.0801239013672 0 7784.0356
219.09005737304688 0 1839.91
219.11367797851562 0 1090.6678
220.12022399902344 0 1996.6882
221.10406494140625 0 158804.08
221.12901306152344 0 2628171.2 a 1
222.10694885253906 0 13270.981
222.13217163085938 0 317154.7
223.10699462890625 0 1230.0045
223.1195068359375 0 2451.7454
223.13462829589844 0 17880.385
223.18272399902344 0 1015.42053
224.11795043945312 0 2042.1597
228.1134033203125 0 8330.455
229.10882568359375 0 5111.579
230.1139678955078 0 1121.8392
231.0615997314453 0 42202.7
231.11326599121094 0 27938.076 b Water loss 1
232.0651092529297 0 3190.6362
232.11660766601562 0 3904.7039
233.0914764404297 0 2659.8142
235.10821533203125 0 64083.48
236.1111602783203 0 8762.927
238.13038635253906 0 4178.581
239.0952911376953 0 1062.4667
239.11428833007812 0 14097.047
240.08151245117188 0 1342.1515
240.117431640625 0 1346.0073
241.08407592773438 0 1395.9797
245.09207153320312 0 3246.8574 y 6
245.1301727294922 0 1396.6715
246.09873962402344 0 1395.065
246.13519287109375 0 4957.724
247.10675048828125 0 1236.2273
247.12025451660156 0 1866.5511
248.11569213867188 0 2579.8066
248.1510009765625 0 4227.208
249.12391662597656 0 464768.44 b 1
250.10537719726562 0 2466.8389
250.12721252441406 0 58368.453
251.10311889648438 0 49630.31
251.1297149658203 0 7027.679
252.10684204101562 0 5055.468
253.09371948242188 0 2347.303
255.11199951171875 0 2342.2397
256.1084289550781 0 3701.948
257.1068420410156 0 27806.127
258.1099548339844 0 2963.686
260.7489013671875 0 1380.918
261.087646484375 0 7077.4253
262.070068359375 0 1347.5609
262.1191101074219 0 1674.8418
263.0707092285156 0 2825.8135
263.10302734375 0 91900.73
263.1294250488281 0 9809.604
264.10662841796875 0 11706.672
264.14593505859375 0 9056.768
265.11785888671875 0 3167.3584
267.0914611816406 0 6860.9126
268.07568359375 0 3701.3262
268.0929870605469 0 2342.748
268.141845703125 0 3139.3254
269.07708740234375 0 2028.8745
270.07342529296875 0 1399.3889
270.10821533203125 0 1797.0527
271.108642578125 0 4815.0146
272.1141662597656 0 1551.4655
273.1228332519531 0 2254.2485
274.13031005859375 0 23247.102
274.1563415527344 0 1694.586
275.1146240234375 0 4435.3286
275.1341857910156 0 3681.8403
277.7330017089844 0 1598.2203
277.7564697265625 0 1128.4482
277.7767639160156 0 1011.8174
278.1391296386719 0 1482.3912
279.0981140136719 0 38224.727
279.125 0 3820.945
280.1014099121094 0 6055.007
282.1217346191406 0 1645.3188
283.1540222167969 0 1354.6404
285.1021423339844 0 59312.684
286.1051330566406 0 8055.236
287.0997009277344 0 2214.7285
288.13421630859375 0 1568.8612
289.0825500488281 0 4265.169
290.1258239746094 0 1639.1357
292.1409912109375 0 38686.08
292.1661071777344 0 9109.908 d 2
293.1432800292969 0 4634.535
296.0887145996094 0 1862.8988
296.151123046875 0 43289.906
297.1373291015625 0 1573.6985
297.15496826171875 0 7804.9487
300.13525390625 0 4325.6377
301.1189270019531 0 3466.4255
302.1147155761719 0 4370.415
303.1133728027344 0 6028.7393
306.1334533691406 0 1697.8877
306.1471862792969 0 1133.5023
307.1199645996094 0 1878.5795
310.151611328125 0 6705.1694
313.09783935546875 0 2775.9673
314.0992736816406 0 4090.8848
315.0984191894531 0 3427.1433
316.1307373046875 0 1762.3309
318.1454772949219 0 15163.056 a Water loss 2
319.14837646484375 0 1536.9923
320.13507080078125 0 3902.01
323.09942626953125 0 17606.857
324.1462707519531 0 103113.125
325.14898681640625 0 15601.766
327.09722900390625 0 1232.9373
327.145751953125 0 2513.2034
328.12921142578125 0 7216.108
328.1454772949219 0 4931.6323
330.110107421875 0 5823.602
331.09295654296875 0 2568.4587
332.12506103515625 0 5318.584
333.10882568359375 0 3791.35
333.129638671875 0 1675.2139
334.1072692871094 0 2843.7715
334.132568359375 0 3269.737
335.1714782714844 0 2637.89
336.15557861328125 0 1324.6097 a 2
338.1288757324219 0 7243.4263
339.1712646484375 0 9064.027
340.1754455566406 0 1648.5146
342.1568603515625 0 537688.75 y 7
343.1598815917969 0 97014.96
344.16192626953125 0 9882.805
346.1402282714844 0 30784.844 b Water loss 2
347.1429138183594 0 4526.8184
348.1197814941406 0 14077.768
349.10113525390625 0 1573.5166
349.12322998046875 0 3092.6528
350.1351318359375 0 31457.81
351.1382751464844 0 4397.6357
352.1407470703125 0 9164.232
353.1473083496094 0 2012.7504
354.1562194824219 0 2628.785
356.13916015625 0 32357.812
357.14202880859375 0 4701.4863 b 5
358.1041259765625 0 2558.8076
359.08807373046875 0 2605.2234
360.11981201171875 0 6722.1733
362.1506652832031 0 4639.373 y 4
362.6512451171875 0 3285.5906
364.15093994140625 0 129821.72 b 2
365.1541442871094 0 26402.525
366.12969970703125 0 14222.144
366.1544189453125 0 2124.0303
367.13250732421875 0 2159.3225
367.165771484375 0 6501.922
368.1688232421875 0 1869.5585
371.1365966796875 0 3382.0254
374.1493835449219 0 6204.742
376.1145935058594 0 13599.233
377.12005615234375 0 2850.7893
378.1006164550781 0 3634.4292
378.13018798828125 0 73193.71
379.1331787109375 0 12444.761
380.13916015625 0 5090.3335
381.14508056640625 0 2640.296
382.1438903808594 0 15643.522
383.14715576171875 0 3144.0332
384.1354064941406 0 4376.6367
385.1358642578125 0 3857.9119
386.1371765136719 0 1881.9977
388.1155700683594 0 1893.4998
388.1489562988281 0 1987.8489
389.1481628417969 0 1720.214
390.1567687988281 0 9620.155
391.15997314453125 0 1812.7428
394.1252746582031 0 91379.18
395.128173828125 0 17918.506
395.1558837890625 0 1788.8707
396.12908935546875 0 1772.8109
397.1629943847656 0 1706.7963
398.170166015625 0 2599.4084
399.1752624511719 0 4252.1816
404.110595703125 0 2577.737
404.1455078125 0 2372.5444
406.12567138671875 0 2146.3435
406.15289306640625 0 2771.889
407.18359375 0 9791.642
408.1675109863281 0 21227.877
409.1713562011719 0 6368.0522
410.1392822265625 0 2926.4688
410.18267822265625 0 2097.652
415.1620178222656 0 4602.6855
416.1457214355469 0 2252.651
417.1748352050781 0 1530.1332
419.1566162109375 0 3927.7764
419.6618957519531 0 1426.5105 y 3
420.1636047363281 0 1494.8773
423.17791748046875 0 2198.1235
425.194091796875 0 77093.33
426.196533203125 0 16760.396
427.20880126953125 0 16044.458
428.214599609375 0 3659.0024
430.12548828125 0 2144.6853
431.1253356933594 0 1653.9756
431.1571044921875 0 1529.4131
432.13909912109375 0 2239.7896
433.1419677734375 0 1566.7491
433.17266845703125 0 14703.074
434.1787414550781 0 2187.4734
435.17864990234375 0 4260.065
437.16748046875 0 3920.4036
438.1724548339844 0 1942.2416
442.17413330078125 0 2966.106
443.156982421875 0 3066.0146
445.1757507324219 0 1422.5725
446.2176818847656 0 1448.9598
447.15167236328125 0 11545.06
448.1573791503906 0 2342.854
449.1327209472656 0 2894.6155
449.1666259765625 0 3223.3372
450.2287902832031 0 2548.3936
451.1802673339844 0 1671.1417
454.1561584472656 0 2607.8362
454.1972961425781 0 5796.191
455.2039489746094 0 14480.469
456.2068786621094 0 3440.8374
458.16729736328125 0 3602.0552
459.1531066894531 0 3522.2688
461.1673278808594 0 20361.676 b Water loss 3
461.19586181640625 0 7610.704
462.1693420410156 0 6412.4155
462.2013244628906 0 2194.3726
465.16253662109375 0 72848.61
466.1656188964844 0 16808.992
467.1675109863281 0 1569.6323
468.1716003417969 0 3129.6274 y Water loss 2
469.2063903808594 0 3203.7458
470.1700744628906 0 1592.5618
470.208984375 0 1484.776
471.181884765625 0 11756.759
472.18560791015625 0 3263.8557
472.2305603027344 0 1269.6356
473.2149963378906 0 6696.3965
475.1459045410156 0 1543.9972
476.1869201660156 0 1516.2251
477.1671447753906 0 12035.732 y 2
477.678955078125 0 3512.0713
478.169677734375 0 3534.6606
478.2201843261719 0 2293.587
479.1781311035156 0 68951.01 b 3
480.1817321777344 0 15586.481
481.18243408203125 0 1776.5442
482.1914978027344 0 3813.0486
483.1986389160156 0 1704.2844
485.6944274902344 0 1513.2848
486.1632080078125 0 11992.325
487.1629943847656 0 2175.3762
489.1925048828125 0 128345.06 y 6
490.19482421875 0 36088.45
490.68499755859375 0 2377.7703
491.2050476074219 0 16837.896
492.20904541015625 0 4277.1616
493.156494140625 0 1283.8553
495.18499755859375 0 9251.376
496.1690368652344 0 2296.5547
496.1887512207031 0 3675.4946
496.231201171875 0 65613.85
497.1714782714844 0 12005.437
497.2343444824219 0 19647.162
498.1733093261719 0 2570.7952
498.2394104003906 0 2009.8298
499.18890380859375 0 5624.365 b 7
502.19696044921875 0 1745.2897
503.1819763183594 0 3198.4514
506.214111328125 0 1652.6471
509.7073059082031 0 1687.0619
511.1950378417969 0 1610.9193
513.198486328125 0 11031.513
514.196533203125 0 2730.1594
516.2207641601562 0 3745.3845
518.7103881835938 0 4519.645
519.2041015625 0 19376.031
519.7161254882812 0 1483.3477
520.2056884765625 0 8478.418
521.2060546875 0 2053.3203
523.1860961914062 0 3271.4307
525.1605834960938 0 1393.0721
525.2042846679688 0 4007.4792
526.206298828125 0 1613.4353
527.2122802734375 0 1604.4269
527.7095947265625 0 2067.975
530.1912231445312 0 3919.9192
532.203369140625 0 1532.8994
533.2030029296875 0 1710.099
537.2139892578125 0 6359.2275
541.1947631835938 0 5089.152
541.7056884765625 0 9249.052 y Water loss 1
542.2332153320312 0 11913.791
542.7101440429688 0 5137.8726
543.2404174804688 0 3206.689
545.209716796875 0 2024.921
548.1997680664062 0 23922.46
549.2026977539062 0 8285.62
550.2078247070312 0 2009.0723
550.71142578125 0 67558.28 y 1
551.2130737304688 0 49729.35
551.7137451171875 0 19965.602
552.2146606445312 0 4201.944
553.1980590820312 0 2317.0164
555.7045288085938 0 3080.674
557.19970703125 0 11698.4
558.2073364257812 0 1615.367
560.2291259765625 0 204233.61 y 5
560.73095703125 0 5372.221
561.2323608398438 0 67360.266
562.230712890625 0 14126.073
563.2342529296875 0 1558.4706
569.2338256835938 0 13877.834
569.7357788085938 0 11305.058
570.22998046875 0 20245.309
570.7149658203125 0 1355.5648
571.233154296875 0 3569.8162
574.7113037109375 0 2606.3413
578.2305908203125 0 10482.999
578.7332153320312 0 5560.869
579.2340698242188 0 1760.1838
583.2241821289062 0 6214.603
583.72607421875 0 3611.2769
588.24072265625 0 4941.6636
592.2302856445312 0 44207.453 Precursor Water loss
592.7319946289062 0 28396.787
593.232421875 0 14160.639
593.7296142578125 0 2856.4438
594.1875610351562 0 5814.7773
596.2363891601562 0 14097.564
597.2255249023438 0 9117.759
598.2293701171875 0 3521.769
601.2355346679688 0 105427.33 Precursor
601.7369384765625 0 73497.78
601.8181762695312 0 1813.9718
602.2374267578125 0 32600.924
602.73876953125 0 5006.261
606.233642578125 0 20569.645
607.235595703125 0 6713.252
608.2372436523438 0 2551.7012
612.1978759765625 0 35722.22
613.2008056640625 0 11200.814
613.2879028320312 0 2301.4966
614.2005004882812 0 2078.974
614.2465209960938 0 24793.73
614.29296875 0 1318.7683
615.24951171875 0 6558.428
622.2181396484375 0 1661.1803
624.2312622070312 0 16030.322 b Water loss 4
625.235595703125 0 5388.152
631.2476196289062 0 9084.823
632.2593383789062 0 4627.26
633.2838134765625 0 1370.0878
634.2299194335938 0 18639.95
635.2338256835938 0 7530.553
636.2266845703125 0 1571.9669
639.251220703125 0 6264.0513
640.2380981445312 0 8924.194
641.2344970703125 0 2169.5774
641.2880859375 0 2126.2847
642.2418823242188 0 15233.086 b 4
643.2450561523438 0 3374.1445
652.2405395507812 0 5390.76
653.2381591796875 0 1382.7831
657.263427734375 0 47360.008
658.265380859375 0 13532.226
659.294189453125 0 73548.19
660.2971801757812 0 26923.234
661.2997436523438 0 6523.4214
667.2503051757812 0 5790.733
668.2513427734375 0 4321.5464
676.2689208984375 0 9195.509
677.2723999023438 0 5836.85
678.2920532226562 0 2333.2332
685.2586059570312 0 63575.582
686.2617797851562 0 21203.24
687.2622680664062 0 5233.762
695.2669067382812 0 12405.139 b Water loss 5
696.27099609375 0 4210.7373
702.2882080078125 0 2679.7637
703.2667846679688 0 16775.934
704.2403564453125 0 3968.647
704.2681884765625 0 4330.847
705.27783203125 0 8059.436
706.2854614257812 0 3006.7356
708.270263671875 0 1516.3378
713.27587890625 0 6363.864 b 5
714.2827758789062 0 1824.9425
721.2615356445312 0 57452.547
722.2646484375 0 23430.174
723.2923583984375 0 189148.81 y 4
724.2955932617188 0 79311.45
725.2946166992188 0 24959.102
728.3179321289062 0 1610.1587
731.2471313476562 0 6410.461
732.238037109375 0 3535.897
733.2744750976562 0 4372.475
739.2919921875 0 1845.1597
746.2737426757812 0 3377.276
747.2976684570312 0 1815.8197
748.302978515625 0 2006.1334
749.2564086914062 0 85176.51
750.2590942382812 0 29740.576
751.2589111328125 0 8775.1
756.3106689453125 0 5939.6123
757.3074340820312 0 3725.1243
759.2630004882812 0 2606.8542
767.2672729492188 0 20765.27
768.2706909179688 0 8136.4985
769.2611083984375 0 2012.7195
774.3212280273438 0 78194.26
775.3237915039062 0 33592.58
776.326416015625 0 8559.693
778.302001953125 0 3661.6836
792.315673828125 0 4650.9434
793.3177490234375 0 3280.5852
796.3170776367188 0 2224.9224
803.2808837890625 0 2441.9912
804.3418579101562 0 1929.7942
814.3135986328125 0 2113.7737
817.32080078125 0 1563.6152
818.3331298828125 0 2958.5852
819.3389282226562 0 1825.1858
820.30908203125 0 10771.703 y Water loss 3
821.30224609375 0 7343.6265
822.3143920898438 0 2508.3843
823.2891845703125 0 1474.9518
832.3289794921875 0 5916.859
833.3307495117188 0 2500.9763
834.3251953125 0 1790.6102
838.3194580078125 0 235587.19 y 3
839.3220825195312 0 109252.94
840.3231201171875 0 36638.305
841.32275390625 0 4769.812
842.30419921875 0 6595.5825 b Water loss 6
843.3377685546875 0 7051.218
844.3375854492188 0 4077.3523
845.3507080078125 0 2319.513
848.3008422851562 0 2441.8115
850.5880126953125 0 2489.7927
853.3255615234375 0 2675.1775
854.321533203125 0 3937.7664
860.3135375976562 0 4325.3833 b 6
862.3243408203125 0 5395.102
863.3316650390625 0 2967.7612
868.3290405273438 0 4005.861
869.3340454101562 0 1675.8552
871.337158203125 0 32358.209
872.3369750976562 0 19332.559
873.3384399414062 0 7252.373
874.3203125 0 2044.0474
888.3489990234375 0 1901.258
889.3477783203125 0 329666.22
890.3504638671875 0 168475.12
890.4552612304688 0 2205.647
891.353759765625 0 43508.2
892.3524780273438 0 4449.9253
896.3252563476562 0 7323.5894
897.3292846679688 0 3911.262
899.3383178710938 0 1599.8584
907.3411865234375 0 15351.666
908.3438110351562 0 9560.227
909.3446044921875 0 3097.1619
915.354248046875 0 2909.054
917.3283081054688 0 2027.724
918.3145751953125 0 3592.6091
933.3751831054688 0 3012.0293
935.3358764648438 0 59900.863 y Water loss 2
936.3358764648438 0 39751.543
937.3339233398438 0 15325.871
938.3248291015625 0 8957.561
939.3258666992188 0 3916.6414
953.3465576171875 0 1085217.2 y 2
954.3491821289062 0 565254.56
955.3494873046875 0 192562.45
956.3505249023438 0 25907.246
963.3322143554688 0 8430.684
964.33056640625 0 3868.374
969.381103515625 0 4569.3354
970.381591796875 0 2930.327
979.3582153320312 0 3177.5261 b Water loss 7
980.3811645507812 0 1576.1697
981.3692016601562 0 1594.4335
997.3726806640625 0 12968.312 b 7
998.3759765625 0 6536.64
999.3818969726562 0 2531.641
1015.3814086914062 0 4390.8877
1018.3994140625 0 5214.942
1019.4024658203125 0 1804.873
1036.4156494140625 0 24147.96
1037.418212890625 0 15128.36
1038.4237060546875 0 4800.4204
1046.39501953125 0 1720.4977
1082.4039306640625 0 7427.4375 y Water loss 1
1083.4061279296875 0 4194.392
1100.4144287109375 0 60379.797 y 1
1101.41748046875 0 39741.734
1102.417724609375 0 16431.43
1103.4149169921875 0 1836.0184
1110.3994140625 0 9854.918
1111.40380859375 0 4928.8857
1112.4013671875 0 2975.5261

Spectrum Details

|  |  |
| --- | --- |
| Matched peaks? Matched peaksThe total absolute number of peaks matched. Additionally in brackets the total fraction of peaks matched and the total number of peaks is shown. | 43 (6.76% of 636) |
| FDR? FDRThe false discovery rate estimated for this peptide. It is calculated by matching all theoretical fragments with a non-integer shift with the raw peaks for this spectrum. This is done with 40 different shifts. The resulting percentage is the average number of annotated peaks over the number of annotated peaks with the correct spectrum. | 0.00% |
| Satellite FDR? Satellite FDRSee the FDR for details on its calculation. This satellite ion specific FDR only contains the satellite ions (d/w) for I/L/J positions. | - |
| PSM Score? PSM ScoreThe PSM Score as given by Hecklib to this annotated spectrum. It is shown with three significant figures. | 511 |

## Spectrum 7636? Spectrum 7636 The raw spectrum of this peptide as annotated by Hecklib. The fragments are coloured according to ion type (see legend). Any peaks with a star '\*' as text can be hovered over to see the full details, first the ion type second the mass shift type. By hovering over the amino acids in the peptide or ions in the legend the corresponding peaks are highlighted. By toggling the 'Unassigned' label you can turn the background (unassigned) peaks on or off in the plot. By updating the slider in the Ion legend you can update the spectrum to only show the top X% of the peaks with labels. The top X% means any peak that is within X% of the highest intensity. By dragging in the spectrum you can zoom in to a specific part of the spectrum and use 'Zoom Out' to get back to the original zoom level. The annotation of the spectrum is based on the given sequence in the peptides file and is done with different software so inconsistencies are likely. The peaks are annotated based on the given sequence, with 20 ppm tolerance.

Copy Data

### Spectrum 7636 (TSV)

#### Preview

```
Loading example...
```

*Click on the button to copy the data to your clipboard.*

Mz MinMz MaxIntensity Max

WidthHeightPeptide font sizePeptide stroke widthSpectrum font sizeSpectrum stroke widthCompact peptide

Ion legend

wxyz

abcd

OtherUnassignedIonChargePositionShow for top:%

TFDDYAMHW

03.87e+57.73e+51.16e+61.55e+6

Zoom Out

b+23a+12a+12y+11a+12b+24b+12y+23b+12d+13a+13a+13y+12b+13b+26y+25b+13b+14y+27b+14y+13b+28y+28y+28y+14\*\*b+15b+15b+16b+16y+15y+16y+16b+17b+17y+17y+17b+18y+18y+18

0830166024903320

Fragment Matches Table

Show background peaks

| Position | Ion type | Intensity | mz Theoretical | mz Error (Th) | mz Error (ppm) | Charge | Series Number |
| --- | --- | --- | --- | --- | --- | --- | --- |
| - | - | 3.695E+05 | 120.1 | - | - | 0 | - |
| - | - | 1208 | 121 | - | - | 0 | - |
| - | - | 1307 | 121.1 | - | - | 0 | - |
| - | - | 2.755E+04 | 121.1 | - | - | 0 | - |
| - | - | 489.4 | 121.6 | - | - | 0 | - |
| - | - | 1817 | 122.1 | - | - | 0 | - |
| - | - | 1008 | 122.1 | - | - | 0 | - |
| - | - | 511 | 124.8 | - | - | 0 | - |
| - | - | 1513 | 127.1 | - | - | 0 | - |
| - | - | 1445 | 129.1 | - | - | 0 | - |
| - | - | 3941 | 129.1 | - | - | 0 | - |
| - | - | 1.162E+04 | 130.1 | - | - | 0 | - |
| - | - | 789.1 | 131.1 | - | - | 0 | - |
| - | - | 2.035E+04 | 132.1 | - | - | 0 | - |
| - | - | 539.5 | 132.1 | - | - | 0 | - |
| - | - | 645.7 | 133.1 | - | - | 0 | - |
| - | - | 1658 | 133.1 | - | - | 0 | - |
| - | - | 1629 | 134.1 | - | - | 0 | - |
| - | - | 1.339E+05 | 136.1 | - | - | 0 | - |
| - | - | 4454 | 136.1 | - | - | 0 | - |
| - | - | 9722 | 137.1 | - | - | 0 | - |
| - | - | 667.1 | 137.1 | - | - | 0 | - |
| - | - | 1.066E+04 | 138.1 | - | - | 0 | - |
| - | - | 1.381E+04 | 138.1 | - | - | 0 | - |
| - | - | 736.1 | 139.1 | - | - | 0 | - |
| - | - | 1008 | 140.1 | - | - | 0 | - |
| - | - | 699.9 | 141.1 | - | - | 0 | - |
| - | - | 883.4 | 141.1 | - | - | 0 | - |
| - | - | 660.9 | 143 | - | - | 0 | - |
| - | - | 2011 | 143.1 | - | - | 0 | - |
| - | - | 2261 | 143.1 | - | - | 0 | - |
| - | - | 2276 | 144.1 | - | - | 0 | - |
| - | - | 4361 | 146.1 | - | - | 0 | - |
| - | - | 2561 | 146.1 | - | - | 0 | - |
| - | - | 701.7 | 147.1 | - | - | 0 | - |
| - | - | 1456 | 148.1 | - | - | 0 | - |
| - | - | 4070 | 148.1 | - | - | 0 | - |
| - | - | 552.5 | 148.1 | - | - | 0 | - |
| - | - | 693.3 | 148.3 | - | - | 0 | - |
| - | - | 694.8 | 152.1 | - | - | 0 | - |
| - | - | 1965 | 155.1 | - | - | 0 | - |
| - | - | 3944 | 155.1 | - | - | 0 | - |
| - | - | 3.29E+04 | 156.1 | - | - | 0 | - |
| - | - | 2000 | 157.1 | - | - | 0 | - |
| - | - | 824.7 | 157.1 | - | - | 0 | - |
| - | - | 5.631E+04 | 158.1 | - | - | 0 | - |
| - | - | 1150 | 159.1 | - | - | 0 | - |
| - | - | 2.757E+04 | 159.1 | - | - | 0 | - |
| - | - | 7101 | 159.1 | - | - | 0 | - |
| - | - | 9129 | 160.1 | - | - | 0 | - |
| - | - | 1156 | 160.1 | - | - | 0 | - |
| - | - | 2227 | 160.1 | - | - | 0 | - |
| - | - | 2.67E+04 | 160.1 | - | - | 0 | - |
| - | - | 1487 | 161.1 | - | - | 0 | - |
| - | - | 2637 | 161.1 | - | - | 0 | - |
| - | - | 606 | 161.7 | - | - | 0 | - |
| - | - | 1040 | 162.1 | - | - | 0 | - |
| - | - | 1580 | 165.1 | - | - | 0 | - |
| - | - | 6783 | 165.1 | - | - | 0 | - |
| - | - | 2634 | 166.1 | - | - | 0 | - |
| - | - | 6618 | 166.1 | - | - | 0 | - |
| - | - | 2793 | 166.1 | - | - | 0 | - |
| - | - | 776 | 167 | - | - | 0 | - |
| - | - | 663.8 | 167.1 | - | - | 0 | - |
| - | - | 4959 | 167.1 | - | - | 0 | - |
| - | - | 1900 | 169.1 | - | - | 0 | - |
| - | - | 1172 | 170.1 | - | - | 0 | - |
| - | - | 4927 | 171.1 | - | - | 0 | - |
| - | - | 862.4 | 172.1 | - | - | 0 | - |
| - | - | 1531 | 173.1 | - | - | 0 | - |
| - | - | 885.4 | 173.1 | - | - | 0 | - |
| 3 | b | 4072 | 174.1 | 0.001283 | 7.373 | +2 | 3 |
| - | - | 884.5 | 174.1 | - | - | 0 | - |
| - | - | 1880 | 175.1 | - | - | 0 | - |
| - | - | 1640 | 175.1 | - | - | 0 | - |
| - | - | 1808 | 175.1 | - | - | 0 | - |
| - | - | 9822 | 176.1 | - | - | 0 | - |
| - | - | 1.107E+05 | 176.1 | - | - | 0 | - |
| - | - | 7.463E+04 | 177.1 | - | - | 0 | - |
| - | - | 1.32E+04 | 177.1 | - | - | 0 | - |
| - | - | 1443 | 178.1 | - | - | 0 | - |
| - | - | 1218 | 178.1 | - | - | 0 | - |
| - | - | 6980 | 178.1 | - | - | 0 | - |
| - | - | 6032 | 180.1 | - | - | 0 | - |
| - | - | 1883 | 183.1 | - | - | 0 | - |
| - | - | 9426 | 185.1 | - | - | 0 | - |
| - | - | 887.4 | 185.1 | - | - | 0 | - |
| - | - | 9670 | 186.1 | - | - | 0 | - |
| - | - | 1405 | 187.1 | - | - | 0 | - |
| - | - | 3.707E+04 | 188.1 | - | - | 0 | - |
| - | - | 4291 | 189.1 | - | - | 0 | - |
| - | - | 999.5 | 189.1 | - | - | 0 | - |
| - | - | 1639 | 190.1 | - | - | 0 | - |
| - | - | 1085 | 191.1 | - | - | 0 | - |
| - | - | 3587 | 191.1 | - | - | 0 | - |
| - | - | 6999 | 191.1 | - | - | 0 | - |
| - | - | 2095 | 192.1 | - | - | 0 | - |
| - | - | 621 | 192.1 | - | - | 0 | - |
| - | - | 1324 | 193.1 | - | - | 0 | - |
| - | - | 3.298E+04 | 193.1 | - | - | 0 | - |
| - | - | 2916 | 194.1 | - | - | 0 | - |
| - | - | 2691 | 194.1 | - | - | 0 | - |
| - | - | 8696 | 195.1 | - | - | 0 | - |
| - | - | 958.8 | 195.1 | - | - | 0 | - |
| - | - | 710.7 | 198.1 | - | - | 0 | - |
| - | - | 772.8 | 199.1 | - | - | 0 | - |
| - | - | 1023 | 201.1 | - | - | 0 | - |
| - | - | 3500 | 202.1 | - | - | 0 | - |
| - | - | 8499 | 203.1 | - | - | 0 | - |
| - | - | 3310 | 203.1 | - | - | 0 | - |
| 2 | a | 3.726E+04 | 203.1 | 0.0004424 | 2.178 | +1 | 2 |
| - | - | 1.856E+04 | 204.1 | - | - | 0 | - |
| 2 | a | 2109 | 204.1 | 0.0001137 | 0.5573 | +1 | 2 |
| - | - | 4949 | 204.1 | - | - | 0 | - |
| - | - | 660.6 | 204.5 | - | - | 0 | - |
| - | - | 1687 | 205.1 | - | - | 0 | - |
| 9 | y | 3.026E+04 | 205.1 | 0.0005327 | 2.597 | +1 | 1 |
| - | - | 1157 | 205.1 | - | - | 0 | - |
| - | - | 3787 | 206.1 | - | - | 0 | - |
| - | - | 8167 | 207.1 | - | - | 0 | - |
| - | - | 4753 | 208.1 | - | - | 0 | - |
| - | - | 1505 | 212.1 | - | - | 0 | - |
| - | - | 811.7 | 213.1 | - | - | 0 | - |
| - | - | 758.6 | 213.1 | - | - | 0 | - |
| - | - | 1433 | 213.1 | - | - | 0 | - |
| - | - | 2272 | 215.1 | - | - | 0 | - |
| - | - | 887.9 | 216.1 | - | - | 0 | - |
| - | - | 983.3 | 216.1 | - | - | 0 | - |
| - | - | 1.938E+04 | 217.1 | - | - | 0 | - |
| - | - | 852.3 | 218.1 | - | - | 0 | - |
| - | - | 2325 | 218.1 | - | - | 0 | - |
| - | - | 5949 | 219.1 | - | - | 0 | - |
| - | - | 1131 | 219.1 | - | - | 0 | - |
| - | - | 675.5 | 220.1 | - | - | 0 | - |
| - | - | 1883 | 220.1 | - | - | 0 | - |
| - | - | 8.811E+04 | 221.1 | - | - | 0 | - |
| 2 | a | 1.531E+06 | 221.1 | 0.0006962 | 3.148 | +1 | 2 |
| - | - | 7442 | 222.1 | - | - | 0 | - |
| - | - | 1.858E+05 | 222.1 | - | - | 0 | - |
| - | - | 9969 | 223.1 | - | - | 0 | - |
| - | - | 1163 | 224.1 | - | - | 0 | - |
| - | - | 1031 | 225.1 | - | - | 0 | - |
| - | - | 920.2 | 226.2 | - | - | 0 | - |
| - | - | 6017 | 228.1 | - | - | 0 | - |
| - | - | 3038 | 229.1 | - | - | 0 | - |
| - | - | 2.067E+04 | 229.2 | - | - | 0 | - |
| - | - | 1276 | 229.2 | - | - | 0 | - |
| - | - | 3329 | 230.2 | - | - | 0 | - |
| - | - | 2.336E+04 | 231.1 | - | - | 0 | - |
| 4 | b | 806.5 | 231.1 | 0.0004495 | 1.945 | +2 | 4 |
| 2 | b | 1.482E+04 | 231.1 | 0.0005686 | 2.46 | +1 | 2 |
| - | - | 1953 | 232.1 | - | - | 0 | - |
| - | - | 1685 | 232.1 | - | - | 0 | - |
| - | - | 1782 | 233.1 | - | - | 0 | - |
| - | - | 837 | 233.1 | - | - | 0 | - |
| - | - | 4.054E+04 | 235.1 | - | - | 0 | - |
| - | - | 5356 | 236.1 | - | - | 0 | - |
| - | - | 713.1 | 237.1 | - | - | 0 | - |
| - | - | 1746 | 238.1 | - | - | 0 | - |
| - | - | 1115 | 239.1 | - | - | 0 | - |
| - | - | 1.031E+04 | 239.1 | - | - | 0 | - |
| - | - | 786.7 | 240.1 | - | - | 0 | - |
| - | - | 1277 | 240.1 | - | - | 0 | - |
| 7 | y | 1302 | 245.1 | 0.004537 | 18.51 | +2 | 3 |
| - | - | 1931 | 245.1 | - | - | 0 | - |
| - | - | 2953 | 246.1 | - | - | 0 | - |
| - | - | 1637 | 247.1 | - | - | 0 | - |
| - | - | 949.3 | 247.1 | - | - | 0 | - |
| - | - | 1016 | 247.1 | - | - | 0 | - |
| - | - | 959 | 248.1 | - | - | 0 | - |
| - | - | 3423 | 248.2 | - | - | 0 | - |
| 2 | b | 2.682E+05 | 249.1 | 0.0006699 | 2.689 | +1 | 2 |
| - | - | 3.373E+04 | 250.1 | - | - | 0 | - |
| - | - | 2.895E+04 | 251.1 | - | - | 0 | - |
| - | - | 4737 | 251.1 | - | - | 0 | - |
| - | - | 4439 | 252.1 | - | - | 0 | - |
| - | - | 874.8 | 252.1 | - | - | 0 | - |
| - | - | 1236 | 253.1 | - | - | 0 | - |
| - | - | 1093 | 253.1 | - | - | 0 | - |
| - | - | 1805 | 256.1 | - | - | 0 | - |
| - | - | 1.666E+04 | 257.1 | - | - | 0 | - |
| - | - | 2214 | 258.1 | - | - | 0 | - |
| - | - | 1053 | 258.1 | - | - | 0 | - |
| - | - | 3149 | 261.1 | - | - | 0 | - |
| - | - | 1021 | 261.1 | - | - | 0 | - |
| - | - | 1436 | 262.1 | - | - | 0 | - |
| - | - | 5.39E+04 | 263.1 | - | - | 0 | - |
| - | - | 5990 | 263.1 | - | - | 0 | - |
| - | - | 5898 | 264.1 | - | - | 0 | - |
| - | - | 4897 | 264.1 | - | - | 0 | - |
| - | - | 2700 | 265.1 | - | - | 0 | - |
| - | - | 765.8 | 265.1 | - | - | 0 | - |
| - | - | 1123 | 266.1 | - | - | 0 | - |
| - | - | 923 | 266.2 | - | - | 0 | - |
| - | - | 3883 | 267.1 | - | - | 0 | - |
| - | - | 1445 | 268.1 | - | - | 0 | - |
| - | - | 915.7 | 268.1 | - | - | 0 | - |
| - | - | 2450 | 268.1 | - | - | 0 | - |
| - | - | 1599 | 269.1 | - | - | 0 | - |
| - | - | 1150 | 271.1 | - | - | 0 | - |
| - | - | 875.2 | 273.1 | - | - | 0 | - |
| - | - | 1552 | 273.1 | - | - | 0 | - |
| - | - | 1.466E+04 | 274.1 | - | - | 0 | - |
| - | - | 1259 | 274.2 | - | - | 0 | - |
| - | - | 1807 | 275.1 | - | - | 0 | - |
| - | - | 1067 | 278 | - | - | 0 | - |
| - | - | 1064 | 278 | - | - | 0 | - |
| - | - | 2.131E+04 | 279.1 | - | - | 0 | - |
| - | - | 1952 | 279.1 | - | - | 0 | - |
| - | - | 3030 | 280.1 | - | - | 0 | - |
| - | - | 870.9 | 283.1 | - | - | 0 | - |
| - | - | 1223 | 283.2 | - | - | 0 | - |
| - | - | 1696 | 285.1 | - | - | 0 | - |
| - | - | 3.478E+04 | 285.1 | - | - | 0 | - |
| - | - | 4216 | 286.1 | - | - | 0 | - |
| - | - | 1156 | 287.1 | - | - | 0 | - |
| - | - | 2037 | 289.1 | - | - | 0 | - |
| - | - | 1133 | 290.1 | - | - | 0 | - |
| - | - | 2.086E+04 | 292.1 | - | - | 0 | - |
| 3 | d | 5014 | 292.2 | 0.0008749 | 2.994 | +1 | 3 |
| - | - | 2410 | 293.1 | - | - | 0 | - |
| - | - | 947.9 | 293.2 | - | - | 0 | - |
| - | - | 876.1 | 296.1 | - | - | 0 | - |
| - | - | 2.767E+04 | 296.2 | - | - | 0 | - |
| - | - | 891.3 | 297.1 | - | - | 0 | - |
| - | - | 4534 | 297.2 | - | - | 0 | - |
| - | - | 940.8 | 299.2 | - | - | 0 | - |
| - | - | 2094 | 300.1 | - | - | 0 | - |
| - | - | 1478 | 301.1 | - | - | 0 | - |
| - | - | 1134 | 302.1 | - | - | 0 | - |
| - | - | 2541 | 302.1 | - | - | 0 | - |
| - | - | 837.1 | 302.2 | - | - | 0 | - |
| - | - | 5272 | 303.1 | - | - | 0 | - |
| - | - | 914.5 | 306.1 | - | - | 0 | - |
| - | - | 719.9 | 307.1 | - | - | 0 | - |
| - | - | 1513 | 307.1 | - | - | 0 | - |
| - | - | 3125 | 310.2 | - | - | 0 | - |
| - | - | 2129 | 313.1 | - | - | 0 | - |
| - | - | 2669 | 314.1 | - | - | 0 | - |
| - | - | 1982 | 315.1 | - | - | 0 | - |
| 3 | a | 7854 | 318.1 | 0.0008278 | 2.602 | +1 | 3 |
| - | - | 1689 | 319.1 | - | - | 0 | - |
| - | - | 3065 | 320.1 | - | - | 0 | - |
| - | - | 1.102E+04 | 323.1 | - | - | 0 | - |
| - | - | 2066 | 324.1 | - | - | 0 | - |
| - | - | 6.16E+04 | 324.1 | - | - | 0 | - |
| - | - | 9130 | 325.1 | - | - | 0 | - |
| - | - | 2402 | 327.1 | - | - | 0 | - |
| - | - | 4849 | 328.1 | - | - | 0 | - |
| - | - | 1983 | 328.1 | - | - | 0 | - |
| - | - | 1611 | 329.1 | - | - | 0 | - |
| - | - | 2553 | 330.1 | - | - | 0 | - |
| - | - | 1373 | 331.1 | - | - | 0 | - |
| - | - | 4138 | 332.1 | - | - | 0 | - |
| - | - | 2002 | 333.1 | - | - | 0 | - |
| - | - | 1879 | 334.1 | - | - | 0 | - |
| - | - | 2220 | 334.1 | - | - | 0 | - |
| - | - | 1477 | 335.2 | - | - | 0 | - |
| 3 | a | 1255 | 336.2 | 0.0005781 | 1.72 | +1 | 3 |
| - | - | 4200 | 338.1 | - | - | 0 | - |
| - | - | 1347 | 339.1 | - | - | 0 | - |
| - | - | 6747 | 339.2 | - | - | 0 | - |
| 8 | y | 3.164E+05 | 342.2 | 0.0009165 | 2.679 | +1 | 2 |
| - | - | 5.928E+04 | 343.2 | - | - | 0 | - |
| - | - | 5625 | 344.2 | - | - | 0 | - |
| 3 | b | 1.616E+04 | 346.1 | 0.0008168 | 2.36 | +1 | 3 |
| - | - | 2660 | 347.1 | - | - | 0 | - |
| - | - | 7438 | 348.1 | - | - | 0 | - |
| - | - | 1.786E+04 | 350.1 | - | - | 0 | - |
| - | - | 4759 | 351.1 | - | - | 0 | - |
| - | - | 6267 | 352.1 | - | - | 0 | - |
| - | - | 1214 | 353.1 | - | - | 0 | - |
| - | - | 1154 | 354.2 | - | - | 0 | - |
| - | - | 1.687E+04 | 356.1 | - | - | 0 | - |
| 6 | b | 2611 | 357.1 | 0.0002134 | 0.5974 | +2 | 6 |
| - | - | 1446 | 358.1 | - | - | 0 | - |
| - | - | 1615 | 359.1 | - | - | 0 | - |
| - | - | 5005 | 360.1 | - | - | 0 | - |
| - | - | 914 | 360.1 | - | - | 0 | - |
| - | - | 962.3 | 361.1 | - | - | 0 | - |
| 5 | y | 2732 | 362.1 | 0.004721 | 13.04 | +2 | 5 |
| - | - | 1283 | 362.7 | - | - | 0 | - |
| 3 | b | 7.65E+04 | 364.2 | 0.0008112 | 2.228 | +1 | 3 |
| - | - | 1.455E+04 | 365.2 | - | - | 0 | - |
| - | - | 8593 | 366.1 | - | - | 0 | - |
| - | - | 1083 | 366.2 | - | - | 0 | - |
| - | - | 1028 | 367.1 | - | - | 0 | - |
| - | - | 3859 | 367.2 | - | - | 0 | - |
| - | - | 2587 | 371.1 | - | - | 0 | - |
| - | - | 5006 | 374.2 | - | - | 0 | - |
| - | - | 9233 | 376.1 | - | - | 0 | - |
| - | - | 2403 | 378.1 | - | - | 0 | - |
| - | - | 4.191E+04 | 378.1 | - | - | 0 | - |
| - | - | 7957 | 379.1 | - | - | 0 | - |
| - | - | 2785 | 380.1 | - | - | 0 | - |
| - | - | 947.1 | 381.1 | - | - | 0 | - |
| - | - | 7597 | 382.1 | - | - | 0 | - |
| - | - | 2036 | 383.1 | - | - | 0 | - |
| - | - | 1897 | 384.1 | - | - | 0 | - |
| - | - | 2403 | 385.1 | - | - | 0 | - |
| - | - | 1589 | 388.2 | - | - | 0 | - |
| - | - | 759.3 | 389.1 | - | - | 0 | - |
| - | - | 5691 | 390.2 | - | - | 0 | - |
| - | - | 1352 | 391.2 | - | - | 0 | - |
| - | - | 1049 | 392.2 | - | - | 0 | - |
| - | - | 5.327E+04 | 394.1 | - | - | 0 | - |
| - | - | 1.098E+04 | 395.1 | - | - | 0 | - |
| - | - | 1376 | 396.1 | - | - | 0 | - |
| - | - | 2721 | 397.2 | - | - | 0 | - |
| - | - | 897.9 | 398.1 | - | - | 0 | - |
| - | - | 1187 | 398.2 | - | - | 0 | - |
| - | - | 1929 | 399.2 | - | - | 0 | - |
| - | - | 1212 | 401.2 | - | - | 0 | - |
| - | - | 1502 | 404.1 | - | - | 0 | - |
| - | - | 1829 | 404.1 | - | - | 0 | - |
| - | - | 1570 | 406.1 | - | - | 0 | - |
| - | - | 2104 | 406.2 | - | - | 0 | - |
| - | - | 4915 | 407.2 | - | - | 0 | - |
| - | - | 1.302E+04 | 408.2 | - | - | 0 | - |
| - | - | 3536 | 409.2 | - | - | 0 | - |
| - | - | 838 | 409.2 | - | - | 0 | - |
| - | - | 1813 | 410.1 | - | - | 0 | - |
| - | - | 1315 | 410.2 | - | - | 0 | - |
| - | - | 2637 | 415.2 | - | - | 0 | - |
| - | - | 853 | 417.2 | - | - | 0 | - |
| - | - | 2158 | 419.2 | - | - | 0 | - |
| - | - | 1282 | 420.2 | - | - | 0 | - |
| - | - | 5.024E+04 | 425.2 | - | - | 0 | - |
| - | - | 1.101E+04 | 426.2 | - | - | 0 | - |
| - | - | 8383 | 427.2 | - | - | 0 | - |
| - | - | 1774 | 428.2 | - | - | 0 | - |
| - | - | 1068 | 429.2 | - | - | 0 | - |
| - | - | 1167 | 430.1 | - | - | 0 | - |
| - | - | 938 | 431.1 | - | - | 0 | - |
| - | - | 931.3 | 431.2 | - | - | 0 | - |
| - | - | 1809 | 432.1 | - | - | 0 | - |
| - | - | 8106 | 433.2 | - | - | 0 | - |
| - | - | 973.7 | 434.2 | - | - | 0 | - |
| - | - | 1504 | 435.2 | - | - | 0 | - |
| - | - | 2363 | 437.2 | - | - | 0 | - |
| - | - | 910.1 | 438.2 | - | - | 0 | - |
| - | - | 2481 | 442.2 | - | - | 0 | - |
| - | - | 1843 | 443.2 | - | - | 0 | - |
| - | - | 1765 | 443.2 | - | - | 0 | - |
| - | - | 871.6 | 446.2 | - | - | 0 | - |
| - | - | 6363 | 447.2 | - | - | 0 | - |
| - | - | 2050 | 449.1 | - | - | 0 | - |
| - | - | 2741 | 449.2 | - | - | 0 | - |
| - | - | 1152 | 450.2 | - | - | 0 | - |
| - | - | 3278 | 454.2 | - | - | 0 | - |
| - | - | 1.081E+04 | 455.2 | - | - | 0 | - |
| - | - | 1770 | 456.2 | - | - | 0 | - |
| - | - | 1881 | 458.2 | - | - | 0 | - |
| - | - | 1130 | 459.1 | - | - | 0 | - |
| 4 | b | 1.197E+04 | 461.2 | 0.0006682 | 1.449 | +1 | 4 |
| - | - | 4093 | 461.2 | - | - | 0 | - |
| - | - | 3766 | 462.2 | - | - | 0 | - |
| - | - | 1639 | 462.2 | - | - | 0 | - |
| - | - | 4.128E+04 | 465.2 | - | - | 0 | - |
| - | - | 1482 | 466.1 | - | - | 0 | - |
| - | - | 9517 | 466.2 | - | - | 0 | - |
| - | - | 946.5 | 467.2 | - | - | 0 | - |
| - | - | 1776 | 469.2 | - | - | 0 | - |
| - | - | 888.5 | 470.2 | - | - | 0 | - |
| - | - | 6042 | 471.2 | - | - | 0 | - |
| - | - | 1233 | 472.2 | - | - | 0 | - |
| - | - | 3577 | 473.2 | - | - | 0 | - |
| - | - | 1237 | 475.1 | - | - | 0 | - |
| - | - | 1102 | 476.2 | - | - | 0 | - |
| 3 | y | 7626 | 477.2 | 0.006689 | 14.02 | +2 | 7 |
| - | - | 2134 | 477.7 | - | - | 0 | - |
| - | - | 2217 | 478.2 | - | - | 0 | - |
| - | - | 2039 | 478.2 | - | - | 0 | - |
| 4 | b | 4.211E+04 | 479.2 | 0.001273 | 2.656 | +1 | 4 |
| - | - | 1.008E+04 | 480.2 | - | - | 0 | - |
| - | - | 1179 | 481.2 | - | - | 0 | - |
| - | - | 2739 | 482.2 | - | - | 0 | - |
| - | - | 958.2 | 483.2 | - | - | 0 | - |
| - | - | 6749 | 486.2 | - | - | 0 | - |
| - | - | 1199 | 487.2 | - | - | 0 | - |
| 7 | y | 7.481E+04 | 489.2 | 0.006259 | 12.8 | +1 | 3 |
| - | - | 2.084E+04 | 490.2 | - | - | 0 | - |
| - | - | 1.053E+04 | 491.2 | - | - | 0 | - |
| - | - | 2938 | 492.2 | - | - | 0 | - |
| - | - | 971.8 | 493.2 | - | - | 0 | - |
| - | - | 5530 | 495.2 | - | - | 0 | - |
| - | - | 2309 | 496.2 | - | - | 0 | - |
| - | - | 3.618E+04 | 496.2 | - | - | 0 | - |
| - | - | 6676 | 497.2 | - | - | 0 | - |
| - | - | 9366 | 497.2 | - | - | 0 | - |
| - | - | 2003 | 498.2 | - | - | 0 | - |
| - | - | 1214 | 498.2 | - | - | 0 | - |
| 8 | b | 3376 | 499.2 | 0.002726 | 5.46 | +2 | 8 |
| - | - | 1022 | 499.7 | - | - | 0 | - |
| - | - | 1286 | 502.2 | - | - | 0 | - |
| - | - | 1899 | 506.2 | - | - | 0 | - |
| - | - | 1628 | 511.2 | - | - | 0 | - |
| - | - | 6676 | 513.2 | - | - | 0 | - |
| - | - | 1931 | 514.2 | - | - | 0 | - |
| - | - | 1446 | 516.2 | - | - | 0 | - |
| - | - | 2632 | 518.7 | - | - | 0 | - |
| - | - | 1.16E+04 | 519.2 | - | - | 0 | - |
| - | - | 6822 | 520.2 | - | - | 0 | - |
| - | - | 2406 | 521.2 | - | - | 0 | - |
| - | - | 1424 | 522.2 | - | - | 0 | - |
| - | - | 1971 | 523.2 | - | - | 0 | - |
| - | - | 865.6 | 525.2 | - | - | 0 | - |
| - | - | 1176 | 525.2 | - | - | 0 | - |
| - | - | 1556 | 529.2 | - | - | 0 | - |
| - | - | 3209 | 530.2 | - | - | 0 | - |
| - | - | 1358 | 531.2 | - | - | 0 | - |
| - | - | 854.8 | 532.2 | - | - | 0 | - |
| - | - | 994.9 | 533.2 | - | - | 0 | - |
| - | - | 1014 | 535.3 | - | - | 0 | - |
| - | - | 1016 | 536.2 | - | - | 0 | - |
| - | - | 3250 | 537.2 | - | - | 0 | - |
| - | - | 2329 | 538.2 | - | - | 0 | - |
| - | - | 2384 | 541.2 | - | - | 0 | - |
| 2 | y | 5630 | 541.7 | 0.003174 | 5.86 | +2 | 8 |
| - | - | 6189 | 542.2 | - | - | 0 | - |
| - | - | 2030 | 542.7 | - | - | 0 | - |
| - | - | 2125 | 543.2 | - | - | 0 | - |
| - | - | 1073 | 544.2 | - | - | 0 | - |
| - | - | 1.249E+04 | 548.2 | - | - | 0 | - |
| - | - | 4172 | 549.2 | - | - | 0 | - |
| 2 | y | 3.93E+04 | 550.7 | 0.003568 | 6.48 | +2 | 8 |
| - | - | 2.756E+04 | 551.2 | - | - | 0 | - |
| - | - | 1.052E+04 | 551.7 | - | - | 0 | - |
| - | - | 3398 | 552.2 | - | - | 0 | - |
| - | - | 824.2 | 553.2 | - | - | 0 | - |
| - | - | 1193 | 555.7 | - | - | 0 | - |
| - | - | 6603 | 557.2 | - | - | 0 | - |
| - | - | 2580 | 558.2 | - | - | 0 | - |
| 6 | y | 1.287E+05 | 560.2 | 0.00595 | 10.62 | +1 | 4 |
| - | - | 2456 | 560.7 | - | - | 0 | - |
| - | - | 3.694E+04 | 561.2 | - | - | 0 | - |
| - | - | 8376 | 562.2 | - | - | 0 | - |
| - | - | 811 | 563.2 | - | - | 0 | - |
| - | - | 7590 | 569.2 | - | - | 0 | - |
| - | - | 7508 | 569.7 | - | - | 0 | - |
| - | - | 1.076E+04 | 570.2 | - | - | 0 | - |
| - | - | 1073 | 570.7 | - | - | 0 | - |
| - | - | 3311 | 571.2 | - | - | 0 | - |
| - | - | 982.2 | 574.7 | - | - | 0 | - |
| - | - | 6543 | 578.2 | - | - | 0 | - |
| - | - | 2676 | 578.7 | - | - | 0 | - |
| - | - | 1816 | 579.2 | - | - | 0 | - |
| - | - | 3450 | 583.2 | - | - | 0 | - |
| - | - | 2543 | 583.7 | - | - | 0 | - |
| - | - | 1991 | 584.2 | - | - | 0 | - |
| - | - | 2155 | 588.2 | - | - | 0 | - |
| 0 | Precursor | 2.049E+04 | 592.2 | 0.003688 | 6.228 | +2 | -1 |
| - | - | 1.566E+04 | 592.7 | - | - | 0 | - |
| - | - | 7889 | 593.2 | - | - | 0 | - |
| - | - | 2025 | 593.7 | - | - | 0 | - |
| - | - | 3013 | 594.2 | - | - | 0 | - |
| - | - | 1233 | 596.2 | - | - | 0 | - |
| - | - | 7803 | 596.2 | - | - | 0 | - |
| - | - | 3462 | 597.2 | - | - | 0 | - |
| - | - | 1864 | 598.2 | - | - | 0 | - |
| 0 | Precursor | 5.926E+04 | 601.2 | 0.003777 | 6.282 | +2 | -1 |
| - | - | 3.875E+04 | 601.7 | - | - | 0 | - |
| - | - | 1.883E+04 | 602.2 | - | - | 0 | - |
| - | - | 1237 | 602.3 | - | - | 0 | - |
| - | - | 2594 | 602.7 | - | - | 0 | - |
| - | - | 1106 | 605.2 | - | - | 0 | - |
| - | - | 1.045E+04 | 606.2 | - | - | 0 | - |
| - | - | 3691 | 607.2 | - | - | 0 | - |
| - | - | 2.14E+04 | 612.2 | - | - | 0 | - |
| - | - | 959.5 | 612.4 | - | - | 0 | - |
| - | - | 6548 | 613.2 | - | - | 0 | - |
| - | - | 1800 | 613.3 | - | - | 0 | - |
| - | - | 1.194E+04 | 614.2 | - | - | 0 | - |
| - | - | 4013 | 615.3 | - | - | 0 | - |
| - | - | 956.8 | 623.2 | - | - | 0 | - |
| 5 | b | 8819 | 624.2 | 0.002037 | 3.263 | +1 | 5 |
| - | - | 3843 | 625.2 | - | - | 0 | - |
| - | - | 5014 | 631.2 | - | - | 0 | - |
| - | - | 1244 | 632.3 | - | - | 0 | - |
| - | - | 1.509E+04 | 634.2 | - | - | 0 | - |
| - | - | 3841 | 635.2 | - | - | 0 | - |
| - | - | 3210 | 639.3 | - | - | 0 | - |
| - | - | 5461 | 640.2 | - | - | 0 | - |
| - | - | 2172 | 641.2 | - | - | 0 | - |
| - | - | 1543 | 641.3 | - | - | 0 | - |
| 5 | b | 1.035E+04 | 642.2 | 0.001421 | 2.212 | +1 | 5 |
| - | - | 2494 | 643.2 | - | - | 0 | - |
| - | - | 1023 | 649.3 | - | - | 0 | - |
| - | - | 3368 | 652.2 | - | - | 0 | - |
| - | - | 951.3 | 653.2 | - | - | 0 | - |
| - | - | 903.8 | 654.7 | - | - | 0 | - |
| - | - | 2.903E+04 | 657.3 | - | - | 0 | - |
| - | - | 8206 | 658.3 | - | - | 0 | - |
| - | - | 4.596E+04 | 659.3 | - | - | 0 | - |
| - | - | 1.683E+04 | 660.3 | - | - | 0 | - |
| - | - | 4315 | 661.3 | - | - | 0 | - |
| - | - | 888.7 | 662.2 | - | - | 0 | - |
| - | - | 1060 | 663.3 | - | - | 0 | - |
| - | - | 960.9 | 663.8 | - | - | 0 | - |
| - | - | 4282 | 667.3 | - | - | 0 | - |
| - | - | 2574 | 668.3 | - | - | 0 | - |
| - | - | 1939 | 669.3 | - | - | 0 | - |
| - | - | 6093 | 676.3 | - | - | 0 | - |
| - | - | 3853 | 677.3 | - | - | 0 | - |
| - | - | 1182 | 678.3 | - | - | 0 | - |
| - | - | 3.768E+04 | 685.3 | - | - | 0 | - |
| - | - | 1.427E+04 | 686.3 | - | - | 0 | - |
| - | - | 1643 | 687.3 | - | - | 0 | - |
| 6 | b | 9091 | 695.3 | 0.001239 | 1.782 | +1 | 6 |
| - | - | 3661 | 696.3 | - | - | 0 | - |
| - | - | 1052 | 700.8 | - | - | 0 | - |
| - | - | 1522 | 702.3 | - | - | 0 | - |
| - | - | 1.133E+04 | 703.3 | - | - | 0 | - |
| - | - | 3365 | 704.3 | - | - | 0 | - |
| - | - | 4484 | 705.3 | - | - | 0 | - |
| - | - | 1597 | 706.3 | - | - | 0 | - |
| - | - | 1449 | 712.3 | - | - | 0 | - |
| 6 | b | 4822 | 713.3 | 0.00105 | 1.473 | +1 | 6 |
| - | - | 1680 | 714.3 | - | - | 0 | - |
| - | - | 1678 | 719.4 | - | - | 0 | - |
| - | - | 3.465E+04 | 721.3 | - | - | 0 | - |
| - | - | 809.6 | 721.8 | - | - | 0 | - |
| - | - | 1.286E+04 | 722.3 | - | - | 0 | - |
| 5 | y | 1.179E+05 | 723.3 | 0.005854 | 8.093 | +1 | 5 |
| - | - | 4.906E+04 | 724.3 | - | - | 0 | - |
| - | - | 1.507E+04 | 725.3 | - | - | 0 | - |
| - | - | 1464 | 725.8 | - | - | 0 | - |
| - | - | 3752 | 726.3 | - | - | 0 | - |
| - | - | 848.2 | 727.8 | - | - | 0 | - |
| - | - | 1455 | 728.3 | - | - | 0 | - |
| - | - | 877.6 | 729.3 | - | - | 0 | - |
| - | - | 3899 | 731.2 | - | - | 0 | - |
| - | - | 2286 | 732.2 | - | - | 0 | - |
| - | - | 1924 | 733.3 | - | - | 0 | - |
| - | - | 924.2 | 734.8 | - | - | 0 | - |
| - | - | 1052 | 735.8 | - | - | 0 | - |
| - | - | 1610 | 739.3 | - | - | 0 | - |
| - | - | 888.4 | 742.3 | - | - | 0 | - |
| - | - | 1731 | 746.3 | - | - | 0 | - |
| - | - | 1285 | 747.3 | - | - | 0 | - |
| - | - | 1086 | 747.8 | - | - | 0 | - |
| - | - | 2272 | 748.3 | - | - | 0 | - |
| - | - | 5.429E+04 | 749.3 | - | - | 0 | - |
| - | - | 1.972E+04 | 750.3 | - | - | 0 | - |
| - | - | 5816 | 751.3 | - | - | 0 | - |
| - | - | 5139 | 756.3 | - | - | 0 | - |
| - | - | 2259 | 756.8 | - | - | 0 | - |
| - | - | 4747 | 757.3 | - | - | 0 | - |
| - | - | 1033 | 758.3 | - | - | 0 | - |
| - | - | 1294 | 759.3 | - | - | 0 | - |
| - | - | 1028 | 760.3 | - | - | 0 | - |
| - | - | 1.28E+04 | 767.3 | - | - | 0 | - |
| - | - | 4197 | 768.3 | - | - | 0 | - |
| - | - | 1379 | 769.3 | - | - | 0 | - |
| - | - | 4.646E+04 | 774.3 | - | - | 0 | - |
| - | - | 2.013E+04 | 775.3 | - | - | 0 | - |
| - | - | 5502 | 776.3 | - | - | 0 | - |
| - | - | 959.7 | 777.3 | - | - | 0 | - |
| - | - | 1868 | 778.3 | - | - | 0 | - |
| - | - | 2529 | 792.3 | - | - | 0 | - |
| - | - | 1524 | 796.3 | - | - | 0 | - |
| - | - | 1398 | 803.3 | - | - | 0 | - |
| - | - | 2173 | 804.3 | - | - | 0 | - |
| - | - | 1312 | 804.4 | - | - | 0 | - |
| - | - | 1492 | 805.3 | - | - | 0 | - |
| - | - | 1628 | 810.3 | - | - | 0 | - |
| - | - | 1164 | 814.3 | - | - | 0 | - |
| - | - | 2633 | 818.3 | - | - | 0 | - |
| 4 | y | 6215 | 820.3 | 0.007054 | 8.599 | +1 | 6 |
| - | - | 3480 | 821.3 | - | - | 0 | - |
| - | - | 986.8 | 822.3 | - | - | 0 | - |
| - | - | 1081 | 827.3 | - | - | 0 | - |
| - | - | 3755 | 832.3 | - | - | 0 | - |
| - | - | 2085 | 833.3 | - | - | 0 | - |
| 4 | y | 1.4E+05 | 838.3 | 0.006193 | 7.388 | +1 | 6 |
| - | - | 6.677E+04 | 839.3 | - | - | 0 | - |
| - | - | 2.288E+04 | 840.3 | - | - | 0 | - |
| - | - | 2323 | 841.3 | - | - | 0 | - |
| 7 | b | 2692 | 842.3 | 0.004446 | 5.278 | +1 | 7 |
| - | - | 4919 | 843.3 | - | - | 0 | - |
| - | - | 2719 | 844.3 | - | - | 0 | - |
| - | - | 1596 | 850.3 | - | - | 0 | - |
| - | - | 959.4 | 851.4 | - | - | 0 | - |
| - | - | 1912 | 853.3 | - | - | 0 | - |
| - | - | 1118 | 854.3 | - | - | 0 | - |
| 7 | b | 2917 | 860.3 | 0.005478 | 6.367 | +1 | 7 |
| - | - | 3511 | 862.3 | - | - | 0 | - |
| - | - | 1037 | 863.3 | - | - | 0 | - |
| - | - | 3153 | 868.3 | - | - | 0 | - |
| - | - | 978.6 | 869.3 | - | - | 0 | - |
| - | - | 2.249E+04 | 871.3 | - | - | 0 | - |
| - | - | 1.093E+04 | 872.3 | - | - | 0 | - |
| - | - | 3102 | 873.3 | - | - | 0 | - |
| - | - | 2.004E+05 | 889.3 | - | - | 0 | - |
| - | - | 9.568E+04 | 890.4 | - | - | 0 | - |
| - | - | 2.642E+04 | 891.4 | - | - | 0 | - |
| - | - | 2954 | 892.4 | - | - | 0 | - |
| - | - | 4769 | 896.3 | - | - | 0 | - |
| - | - | 1576 | 897.3 | - | - | 0 | - |
| - | - | 1309 | 898.3 | - | - | 0 | - |
| - | - | 1331 | 899.3 | - | - | 0 | - |
| - | - | 9516 | 907.3 | - | - | 0 | - |
| - | - | 4059 | 908.3 | - | - | 0 | - |
| - | - | 2404 | 909.3 | - | - | 0 | - |
| - | - | 1375 | 914.3 | - | - | 0 | - |
| - | - | 1273 | 915.3 | - | - | 0 | - |
| - | - | 1323 | 917.3 | - | - | 0 | - |
| - | - | 3350 | 918.3 | - | - | 0 | - |
| - | - | 1301 | 919.3 | - | - | 0 | - |
| - | - | 822.2 | 924.3 | - | - | 0 | - |
| - | - | 1764 | 933.4 | - | - | 0 | - |
| 3 | y | 3.812E+04 | 935.3 | 0.006234 | 6.665 | +1 | 7 |
| - | - | 2.54E+04 | 936.3 | - | - | 0 | - |
| - | - | 8626 | 937.3 | - | - | 0 | - |
| - | - | 5909 | 938.3 | - | - | 0 | - |
| - | - | 1301 | 939.3 | - | - | 0 | - |
| - | - | 1120 | 940.3 | - | - | 0 | - |
| 3 | y | 6.363E+05 | 953.3 | 0.006289 | 6.597 | +1 | 7 |
| - | - | 3.334E+05 | 954.3 | - | - | 0 | - |
| - | - | 1.212E+05 | 955.4 | - | - | 0 | - |
| - | - | 1.425E+04 | 956.4 | - | - | 0 | - |
| - | - | 4602 | 963.3 | - | - | 0 | - |
| - | - | 2018 | 964.3 | - | - | 0 | - |
| - | - | 1806 | 969.4 | - | - | 0 | - |
| - | - | 1773 | 970.4 | - | - | 0 | - |
| - | - | 1752 | 980.4 | - | - | 0 | - |
| 8 | b | 7970 | 997.4 | 0.005587 | 5.602 | +1 | 8 |
| - | - | 4168 | 998.4 | - | - | 0 | - |
| - | - | 1109 | 999.4 | - | - | 0 | - |
| - | - | 948.7 | 1009 | - | - | 0 | - |
| - | - | 2494 | 1015 | - | - | 0 | - |
| - | - | 1341 | 1016 | - | - | 0 | - |
| - | - | 3240 | 1018 | - | - | 0 | - |
| - | - | 1405 | 1019 | - | - | 0 | - |
| - | - | 1.468E+04 | 1036 | - | - | 0 | - |
| - | - | 8789 | 1037 | - | - | 0 | - |
| - | - | 2054 | 1038 | - | - | 0 | - |
| - | - | 1252 | 1046 | - | - | 0 | - |
| - | - | 903 | 1047 | - | - | 0 | - |
| 2 | y | 3918 | 1082 | 0.003249 | 3.002 | +1 | 8 |
| - | - | 2442 | 1083 | - | - | 0 | - |
| 2 | y | 3.58E+04 | 1100 | 0.005868 | 5.333 | +1 | 8 |
| - | - | 2.288E+04 | 1101 | - | - | 0 | - |
| - | - | 9240 | 1102 | - | - | 0 | - |
| - | - | 1295 | 1103 | - | - | 0 | - |
| - | - | 4962 | 1110 | - | - | 0 | - |
| - | - | 2754 | 1111 | - | - | 0 | - |
| - | - | 1391 | 1112 | - | - | 0 | - |
| - | - | 779.4 | 1241 | - | - | 0 | - |
| - | - | 975.3 | 2080 | - | - | 0 | - |
| - | - | 1035 | 3081 | - | - | 0 | - |
| - | - | 1093 | 3287 | - | - | 0 | - |

m/z Charge Intensity FragmentType MassShift Position
120.08126831054688 0 369533.38
121.03996276855469 0 1207.5842
121.07914733886719 0 1307.13
121.0845718383789 0 27553.94
121.58333587646484 0 489.36572
122.07172393798828 0 1816.6228
122.0877456665039 0 1008.4808
124.76318359375 0 511.0499
127.08699798583984 0 1512.5953
129.0704803466797 0 1445.2957
129.10267639160156 0 3941.0527
130.0655517578125 0 11624.403
131.08583068847656 0 789.0686
132.08123779296875 0 20348.256
132.10214233398438 0 539.47314
133.0612335205078 0 645.7221
133.0846405029297 0 1657.7229
134.096923828125 0 1628.8036
136.07615661621094 0 133914.56
136.08743286132812 0 4453.6353
137.0795135498047 0 9721.661
137.09075927734375 0 667.1475
138.055419921875 0 10661.192
138.06663513183594 0 13808.415
139.05889892578125 0 736.14294
140.08250427246094 0 1007.7129
141.0662384033203 0 699.9486
141.10267639160156 0 883.359
143.0457305908203 0 660.8744
143.07350158691406 0 2010.8842
143.0859832763672 0 2261.086
144.08126831054688 0 2275.7314
146.0605010986328 0 4360.7217
146.096923828125 0 2561.2817
147.09213256835938 0 701.6676
148.07635498046875 0 1456.346
148.08741760253906 0 4069.7698
148.09335327148438 0 552.5485
148.30393981933594 0 693.29504
152.08248901367188 0 694.8452
155.08175659179688 0 1964.6946
155.09326171875 0 3943.6726
156.07723999023438 0 32895.633
157.08053588867188 0 1999.7927
157.1342010498047 0 824.65845
158.09689331054688 0 56312.305
159.0561981201172 0 1150.2598
159.09217834472656 0 27573.992
159.1001739501953 0 7101.0522
160.07620239257812 0 9128.996
160.08778381347656 0 1156.4255
160.0957489013672 0 2227.4683
160.11253356933594 0 26700.236
161.07945251464844 0 1486.6498
161.1157684326172 0 2637.4126
161.71182250976562 0 605.98486
162.0917205810547 0 1040.3629
165.07012939453125 0 1580.3054
165.07762145996094 0 6783.0425
166.0537109375 0 2633.8076
166.06155395507812 0 6618.1636
166.0865020751953 0 2792.7449
167.0464324951172 0 775.9618
167.08160400390625 0 663.8125
167.09304809570312 0 4959.469
169.07640075683594 0 1899.6855
170.0605926513672 0 1172.2335
171.07687377929688 0 4927.483
172.10845947265625 0 862.3955
173.09259033203125 0 1531.2952
173.12953186035156 0 885.43854
174.06680297851562 0 4071.7725 b Ammonia loss 2
174.09210205078125 0 884.48395
175.0873565673828 0 1880.0366
175.09823608398438 0 1640.1676
175.1236114501953 0 1808.3518
176.08238220214844 0 9822.318
176.10751342773438 0 110690.33
177.1027374267578 0 74629.82
177.11129760742188 0 13197.494
178.06163024902344 0 1443.2832
178.09793090820312 0 1218.2572
178.10609436035156 0 6980.274
180.07725524902344 0 6031.501
183.14955139160156 0 1883.2098
185.0562744140625 0 9425.522
185.0924835205078 0 887.3963
186.09185791015625 0 9669.72
187.06353759765625 0 1404.5299
188.07115173339844 0 37074.05
189.07435607910156 0 4290.7837
189.0865478515625 0 999.5287
190.08741760253906 0 1639.286
191.0828857421875 0 1084.9984
191.09323120117188 0 3586.5825
191.118408203125 0 6999.119
192.10276794433594 0 2095.4119
192.1211700439453 0 620.9766
193.07276916503906 0 1324.1307
193.10882568359375 0 32978.082
194.0802001953125 0 2916.1338
194.1125030517578 0 2691.2256
195.08815002441406 0 8695.608
195.1239776611328 0 958.7778
198.08831787109375 0 710.66974
199.0720977783203 0 772.81177
201.12393188476562 0 1023.177
202.05340576171875 0 3499.8098
203.0668182373047 0 8499.061
203.09336853027344 0 3310.113
203.1183319091797 0 37262.375 a Water loss 1
204.07725524902344 0 18559.23
204.10179138183594 0 2109.0461 a Ammonia loss 1
204.12158203125 0 4948.6943
204.48049926757812 0 660.60724
205.08106994628906 0 1687.0348
205.09768676757812 0 30262.715 y 8
205.10733032226562 0 1156.7955
206.10122680664062 0 3787.3303
207.1133270263672 0 8167.493
208.07223510742188 0 4752.7104
212.1177520751953 0 1504.5682
213.05177307128906 0 811.6828
213.0876922607422 0 758.60657
213.09881591796875 0 1433.1163
215.08187866210938 0 2271.8845
216.06610107421875 0 887.8647
216.09754943847656 0 983.276
217.09768676757812 0 19376.842
218.0824432373047 0 852.27936
218.1015625 0 2325.0254
219.08033752441406 0 5949.175
219.1140594482422 0 1131.0269
220.095458984375 0 675.50903
220.1198272705078 0 1882.5734
221.10421752929688 0 88107.99
221.129150390625 0 1530951.4 a 1
222.10691833496094 0 7442.231
222.13235473632812 0 185797.19
223.1346893310547 0 9968.911
224.11740112304688 0 1163.0834
225.09957885742188 0 1030.9445
226.1554718017578 0 920.17944
228.1136932373047 0 6017.3306
229.10902404785156 0 3038.138
229.15524291992188 0 20674.908
229.16775512695312 0 1276.4375
230.1586456298828 0 3329.2817
231.06179809570312 0 23359.87
231.08743286132812 0 806.5371 b Water loss 3
231.11337280273438 0 14821.493 b Water loss 1
232.0648651123047 0 1952.6062
232.11656188964844 0 1685.351
233.0924530029297 0 1781.8008
233.10464477539062 0 836.97797
235.1083526611328 0 40535.35
236.1116943359375 0 5356.4795
237.0895233154297 0 713.07947
238.13145446777344 0 1745.8398
239.10044860839844 0 1115.4666
239.11441040039062 0 10313.442
240.0814666748047 0 786.6572
240.11676025390625 0 1277.3099
245.09237670898438 0 1302.2407 y 6
245.129638671875 0 1930.8486
246.13571166992188 0 2952.579
247.10647583007812 0 1636.8016
247.1199493408203 0 949.3331
247.1453094482422 0 1016.357
248.08912658691406 0 959.03503
248.15127563476562 0 3423.3188
249.12403869628906 0 268155.62 b 1
250.1273193359375 0 33727.574
251.1032257080078 0 28947.514
251.12966918945312 0 4736.941
252.10678100585938 0 4438.872
252.13409423828125 0 874.75214
253.0929718017578 0 1236.4319
253.1184844970703 0 1092.7721
256.10797119140625 0 1804.8723
257.1069641113281 0 16655.578
258.1100158691406 0 2213.5793
258.1242370605469 0 1052.5659
261.0874328613281 0 3149.185
261.112060546875 0 1021.34625
262.11883544921875 0 1435.9072
263.1031494140625 0 53903.137
263.1294250488281 0 5990.3394
264.1065979003906 0 5898.2134
264.1459045410156 0 4897.021
265.1182556152344 0 2700.2832
265.14788818359375 0 765.82745
266.1229248046875 0 1123.4323
266.15118408203125 0 923.0448
267.0918273925781 0 3882.6865
268.0762634277344 0 1444.7598
268.09332275390625 0 915.6614
268.1407165527344 0 2450.2798
269.07757568359375 0 1598.798
271.1065368652344 0 1149.6643
273.0872497558594 0 875.21796
273.123779296875 0 1552.0111
274.13037109375 0 14663.498
274.156005859375 0 1258.5438
275.1146240234375 0 1806.707
278.0209045410156 0 1067.2247
278.0382995605469 0 1064.3228
279.0982666015625 0 21312.654
279.125244140625 0 1951.6755
280.1018371582031 0 3029.918
283.1063537597656 0 870.8762
283.1531066894531 0 1223.4875
285.0841064453125 0 1696.1274
285.102294921875 0 34776.36
286.1051330566406 0 4216.1016
287.0995788574219 0 1155.8438
289.082275390625 0 2037.4564
290.1264343261719 0 1133.0947
292.14129638671875 0 20863.588
292.16644287109375 0 5013.9966 d 2
293.14404296875 0 2409.54
293.1700744628906 0 947.91785
296.0880126953125 0 876.11975
296.15142822265625 0 27672.64
297.1358337402344 0 891.31415
297.1543884277344 0 4533.5146
299.15228271484375 0 940.8059
300.1352844238281 0 2093.5005
301.11822509765625 0 1477.5521
302.09783935546875 0 1133.7694
302.1151123046875 0 2540.864
302.1516418457031 0 837.1236
303.1136474609375 0 5271.7695
306.14306640625 0 914.52106
307.0963439941406 0 719.8869
307.1200256347656 0 1513.0942
310.1520080566406 0 3124.6387
313.09759521484375 0 2129.0994
314.09954833984375 0 2668.8567
315.09832763671875 0 1981.8035
318.1456604003906 0 7854.228 a Water loss 2
319.1490783691406 0 1688.895
320.13543701171875 0 3065.0176
323.099609375 0 11018.823
324.1020812988281 0 2065.5024
324.1464538574219 0 61604.68
325.1490173339844 0 9129.726
327.1454772949219 0 2402.0051
328.12969970703125 0 4849.2134
328.1479797363281 0 1982.6252
329.13348388671875 0 1610.8302
330.1103820800781 0 2553.0107
331.0937194824219 0 1373.278
332.12518310546875 0 4137.6934
333.10894775390625 0 2001.7806
334.1077575683594 0 1878.8301
334.1322021484375 0 2219.5613
335.1708679199219 0 1477.1292
336.1559753417969 0 1254.5404 a 2
338.1290283203125 0 4199.815
339.1321716308594 0 1346.6335
339.1714782714844 0 6746.824
342.156982421875 0 316370.22 y 7
343.1600646972656 0 59281.637
344.1623229980469 0 5625.2837
346.14056396484375 0 16157.04 b Water loss 2
347.1429748535156 0 2660.439
348.11968994140625 0 7437.813
350.13543701171875 0 17856.102
351.1390075683594 0 4758.9644
352.1410827636719 0 6267.3076
353.1467590332031 0 1213.8318
354.1556091308594 0 1153.541
356.13946533203125 0 16870.473
357.1427001953125 0 2610.7542 b 5
358.10333251953125 0 1445.6514
359.089111328125 0 1615.2916
360.11968994140625 0 5005.278
360.1441345214844 0 913.9657
361.1220397949219 0 962.3293
362.15185546875 0 2731.8896 y 4
362.65325927734375 0 1283.485
364.151123046875 0 76503.54 b 2
365.1541748046875 0 14549.737
366.1302795410156 0 8593.221
366.1549987792969 0 1083.0033
367.1327209472656 0 1028.1401
367.1661376953125 0 3858.5066
371.13690185546875 0 2586.782
374.15008544921875 0 5006.3804
376.1148681640625 0 9233.423
378.1004638671875 0 2402.787
378.13043212890625 0 41906.812
379.1329040527344 0 7956.971
380.1398010253906 0 2784.6562
381.14642333984375 0 947.1171
382.14398193359375 0 7596.799
383.1474304199219 0 2035.9601
384.13580322265625 0 1897.1499
385.13751220703125 0 2402.5125
388.1507263183594 0 1589.0054
389.11767578125 0 759.3144
390.1572265625 0 5690.772
391.15838623046875 0 1352.0978
392.17352294921875 0 1049.3707
394.1255187988281 0 53269.188
395.1285400390625 0 10979.697
396.13116455078125 0 1375.9882
397.1633605957031 0 2721.1553
398.1398620605469 0 897.91064
398.17071533203125 0 1186.6538
399.1749267578125 0 1929.0332
401.1942443847656 0 1211.9545
404.112060546875 0 1501.6896
404.14599609375 0 1828.7499
406.1242980957031 0 1569.6888
406.1534729003906 0 2104.084
407.1837463378906 0 4915.261
408.16766357421875 0 13019.779
409.1710510253906 0 3535.6943
409.1977844238281 0 837.9531
410.1404113769531 0 1812.8235
410.1813659667969 0 1315.3694
415.1625061035156 0 2636.865
417.1740417480469 0 852.9834
419.15643310546875 0 2157.5671
420.16363525390625 0 1282.3754
425.1943054199219 0 50237.66
426.1969299316406 0 11010.228
427.208984375 0 8382.899
428.213623046875 0 1774.469
429.1896667480469 0 1068.0969
430.1252136230469 0 1166.7334
431.1240234375 0 937.98706
431.15643310546875 0 931.3225
432.1409606933594 0 1808.9692
433.1726379394531 0 8106.2856
434.17864990234375 0 973.6549
435.1795349121094 0 1504.2036
437.16851806640625 0 2362.804
438.1750793457031 0 910.119
442.1737365722656 0 2481.106
443.15625 0 1843.137
443.1873779296875 0 1764.5148
446.2175598144531 0 871.6047
447.15216064453125 0 6363.314
449.13446044921875 0 2050.3755
449.16802978515625 0 2741.459
450.2285461425781 0 1152.1323
454.1971740722656 0 3278.291
455.2046203613281 0 10811.344
456.2062683105469 0 1769.8401
458.168212890625 0 1880.6782
459.14984130859375 0 1129.992
461.1673583984375 0 11967.915 b Water loss 3
461.1963195800781 0 4093.338
462.169921875 0 3765.7307
462.20166015625 0 1639.2656
465.1628112792969 0 41278.234
466.1289367675781 0 1481.5679
466.16607666015625 0 9517.224
467.1696472167969 0 946.54297
469.20831298828125 0 1775.8745
470.21221923828125 0 888.49384
471.18231201171875 0 6041.95
472.1827087402344 0 1233.26
473.2152404785156 0 3576.5654
475.1448974609375 0 1236.7594
476.18682861328125 0 1102.4692
477.1673889160156 0 7625.798 y 2
477.679443359375 0 2134.4534
478.1668701171875 0 2216.8718
478.221435546875 0 2039.3588
479.17852783203125 0 42109.875 b 3
480.1812744140625 0 10079.487
481.1817321777344 0 1178.7336
482.1903076171875 0 2738.9888
483.19970703125 0 958.2207
486.1637878417969 0 6749.3257
487.16259765625 0 1199.3423
489.19281005859375 0 74809.32 y 6
490.1950378417969 0 20842.783
491.2054443359375 0 10532.051
492.2101135253906 0 2937.9282
493.2091979980469 0 971.8331
495.1867980957031 0 5530.059
496.1894226074219 0 2309.069
496.2315673828125 0 36176.816
497.17144775390625 0 6676.275
497.2343444824219 0 9366.469
498.17352294921875 0 2003.0073
498.23870849609375 0 1213.9717
499.1899108886719 0 3376.3655 b 7
499.6943664550781 0 1022.3287
502.19677734375 0 1286.1273
506.21728515625 0 1898.6476
511.19232177734375 0 1628.1487
513.19873046875 0 6675.7793
514.2283935546875 0 1930.519
516.2210693359375 0 1446.2681
518.7106323242188 0 2632.191
519.2039794921875 0 11599.8
520.2058715820312 0 6822.062
521.205322265625 0 2405.828
522.1897583007812 0 1424.2976
523.1834716796875 0 1971.0592
525.1611328125 0 865.58105
525.2023315429688 0 1176.2136
529.2060546875 0 1556.417
530.1907348632812 0 3208.763
531.19091796875 0 1357.9058
532.2051391601562 0 854.78754
533.2044677734375 0 994.91003
535.3050537109375 0 1014.20654
536.2176513671875 0 1015.9198
537.214111328125 0 3250.3013
538.2188110351562 0 2329.1519
541.1937255859375 0 2383.6445
541.7061767578125 0 5630.32 y Water loss 1
542.23388671875 0 6189.2827
542.70849609375 0 2030.2551
543.236328125 0 2124.8123
544.2117309570312 0 1072.816
548.2002563476562 0 12491.029
549.2032470703125 0 4172.1953
550.7118530273438 0 39302.68 y 1
551.2133178710938 0 27559.225
551.713623046875 0 10524.197
552.2169799804688 0 3398.0234
553.202392578125 0 824.1542
555.7022094726562 0 1192.6975
557.200927734375 0 6603.143
558.2044067382812 0 2580.2576
560.2296142578125 0 128711.914 y 5
560.7275390625 0 2455.7017
561.2327880859375 0 36939.15
562.2308959960938 0 8375.878
563.2348022460938 0 811.01294
569.2343139648438 0 7590.2856
569.736328125 0 7507.6626
570.2300415039062 0 10755.997
570.7168579101562 0 1073.3827
571.231689453125 0 3310.9805
574.7139892578125 0 982.2282
578.2308959960938 0 6543.3623
578.7346801757812 0 2675.6045
579.2105102539062 0 1816.3082
583.2252807617188 0 3449.6099
583.7277221679688 0 2543.187
584.237060546875 0 1991.4823
588.2396850585938 0 2154.5925
592.2305297851562 0 20486.133 Precursor Water loss
592.7318725585938 0 15655.008
593.23291015625 0 7888.6533
593.7310180664062 0 2025.2734
594.1871337890625 0 3012.7734
596.1884765625 0 1233.18
596.236572265625 0 7803.232
597.231201171875 0 3462.2043
598.2265625 0 1864.394
601.2359008789062 0 59257.78 Precursor
601.7373046875 0 38745.81
602.2379760742188 0 18832.604
602.3209228515625 0 1236.6936
602.7395629882812 0 2593.6545
605.244384765625 0 1105.5488
606.2343139648438 0 10445.888
607.2372436523438 0 3691.0815
612.1981201171875 0 21397.86
612.3595581054688 0 959.4559
613.2005004882812 0 6548.3833
613.289794921875 0 1800.4471
614.2462158203125 0 11935.501
615.2507934570312 0 4013.2712
623.2496337890625 0 956.814
624.2320556640625 0 8819.381 b Water loss 4
625.2352294921875 0 3843.3455
631.2471923828125 0 5013.7607
632.2630004882812 0 1244.1031
634.2301635742188 0 15089.239
635.2333374023438 0 3840.8965
639.2542724609375 0 3209.8357
640.2406005859375 0 5461.028
641.2369384765625 0 2172.3188
641.289306640625 0 1542.7241
642.2420043945312 0 10354.506 b 4
643.245361328125 0 2493.9797
649.2626342773438 0 1022.5062
652.2424926757812 0 3368.285
653.2479248046875 0 951.3083
654.7432250976562 0 903.814
657.2640991210938 0 29034.69
658.2659301757812 0 8205.683
659.2947387695312 0 45962.508
660.2977294921875 0 16825.96
661.3001098632812 0 4314.89
662.2227783203125 0 888.6825
663.2703247070312 0 1059.9816
663.7672729492188 0 960.94336
667.2503662109375 0 4281.8354
668.2520141601562 0 2573.588
669.2732543945312 0 1939.2961
676.27001953125 0 6093.258
677.2738647460938 0 3852.7227
678.2713012695312 0 1181.7362
685.259033203125 0 37680.383
686.2620239257812 0 14270.177
687.2640991210938 0 1643.0481
695.2683715820312 0 9090.67 b Water loss 5
696.2720336914062 0 3661.4866
700.7830200195312 0 1051.925
702.2838745117188 0 1521.8458
703.2669677734375 0 11329.895
704.2689208984375 0 3364.8455
705.279296875 0 4483.6533
706.292236328125 0 1597.1151
712.305419921875 0 1448.8716
713.2787475585938 0 4821.6553 b 5
714.2803955078125 0 1680.313
719.3951416015625 0 1678.0222
721.2621459960938 0 34652.168
721.8115844726562 0 809.61865
722.2652587890625 0 12858.927
723.2928466796875 0 117859.73 y 4
724.2960815429688 0 49055.336
725.2963256835938 0 15066.804
725.8079223632812 0 1464.296
726.2982788085938 0 3751.536
727.8041381835938 0 848.18317
728.3137817382812 0 1454.5986
729.3131103515625 0 877.56964
731.2482299804688 0 3899.429
732.238525390625 0 2286.066
733.2740478515625 0 1924.1624
734.8175048828125 0 924.1877
735.816650390625 0 1051.7399
739.2874755859375 0 1609.9445
742.3193969726562 0 888.37103
746.2747802734375 0 1731.3643
747.292724609375 0 1285.4813
747.8209838867188 0 1085.9565
748.3140258789062 0 2271.9734
749.257080078125 0 54289.83
750.2601928710938 0 19720.3
751.2606201171875 0 5816.048
756.3131103515625 0 5138.903
756.825927734375 0 2258.8167
757.31640625 0 4747.445
758.3151245117188 0 1032.8044
759.2647705078125 0 1294.3291
760.2976684570312 0 1028.1599
767.267578125 0 12796.494
768.2699584960938 0 4196.7026
769.2705078125 0 1379.4155
774.3215942382812 0 46459.81
775.324462890625 0 20125.848
776.3280029296875 0 5501.7573
777.32421875 0 959.6713
778.3043823242188 0 1867.6146
792.3145141601562 0 2529.065
796.3184204101562 0 1523.8451
803.2844848632812 0 1397.7631
804.3298950195312 0 2173.0798
804.4264526367188 0 1312.0624
805.3302001953125 0 1492.4342
810.3225708007812 0 1627.7957
814.3148803710938 0 1163.7596
818.3355712890625 0 2633.1123
820.3104248046875 0 6215.3037 y Water loss 3
821.3055419921875 0 3480.0525
822.303466796875 0 986.82733
827.3479614257812 0 1080.79
832.3258666992188 0 3755.109
833.3271484375 0 2085.02
838.3201293945312 0 139990.25 y 3
839.3231201171875 0 66769.11
840.3230590820312 0 22884.54
841.3257446289062 0 2322.8323
842.3020629882812 0 2691.6553 b Water loss 6
843.33984375 0 4918.6504
844.3394775390625 0 2719.1736
850.3287353515625 0 1595.6416
851.4258422851562 0 959.3688
853.3267211914062 0 1911.5938
854.31201171875 0 1117.5721
860.3136596679688 0 2917.1064 b 6
862.3233032226562 0 3511.4058
863.3312377929688 0 1036.9823
868.3313598632812 0 3152.7627
869.328857421875 0 978.5715
871.3381958007812 0 22486.387
872.3373413085938 0 10930.817
873.3395385742188 0 3102.1072
889.3484497070312 0 200363.47
890.3511352539062 0 95679.19
891.3543090820312 0 26418.492
892.354248046875 0 2953.562
896.3260498046875 0 4768.5537
897.3248901367188 0 1575.6599
898.3250122070312 0 1308.507
899.3304443359375 0 1330.6941
907.3412475585938 0 9515.653
908.3432006835938 0 4059.1858
909.3422241210938 0 2404.067
914.3370971679688 0 1374.9932
915.3482055664062 0 1273.2108
917.3256225585938 0 1322.5947
918.3145751953125 0 3349.876
919.3212890625 0 1300.5671
924.3329467773438 0 822.2163
933.3740844726562 0 1764.0918
935.3365478515625 0 38116.48 y Water loss 2
936.3357543945312 0 25396.135
937.3359985351562 0 8625.703
938.325439453125 0 5909.4966
939.3244018554688 0 1300.7046
940.3269653320312 0 1120.2905
953.34716796875 0 636301.56 y 2
954.3497314453125 0 333384.22
955.350341796875 0 121194.43
956.3521118164062 0 14254.829
963.3311767578125 0 4601.5195
964.3370361328125 0 2017.5824
969.3761596679688 0 1806.0498
970.3794555664062 0 1773.4933
980.3678588867188 0 1752.0121
997.3726806640625 0 7969.669 b 7
998.3761596679688 0 4168.1143
999.3746948242188 0 1108.7039
1009.3821411132812 0 948.6528
1015.384521484375 0 2494.2285
1016.3828125 0 1340.649
1018.3985595703125 0 3239.5188
1019.400146484375 0 1405.0002
1036.4154052734375 0 14678.127
1037.41845703125 0 8788.928
1038.4276123046875 0 2054.348
1046.4007568359375 0 1251.6556
1047.40625 0 903.0405
1082.4019775390625 0 3918.0427 y Water loss 1
1083.403564453125 0 2441.8914
1100.4151611328125 0 35804.97 y 1
1101.4185791015625 0 22878.395
1102.4185791015625 0 9240.098
1103.4200439453125 0 1295.085
1110.3988037109375 0 4962.005
1111.403076171875 0 2753.9102
1112.40283203125 0 1391.1752
1240.9527587890625 0 779.44104
2079.87109375 0 975.27826
3080.990966796875 0 1035.0864
3287.3818359375 0 1093.2383

Spectrum Details

|  |  |
| --- | --- |
| Matched peaks? Matched peaksThe total absolute number of peaks matched. Additionally in brackets the total fraction of peaks matched and the total number of peaks is shown. | 41 (6.29% of 652) |
| FDR? FDRThe false discovery rate estimated for this peptide. It is calculated by matching all theoretical fragments with a non-integer shift with the raw peaks for this spectrum. This is done with 40 different shifts. The resulting percentage is the average number of annotated peaks over the number of annotated peaks with the correct spectrum. | 0.17% |
| Satellite FDR? Satellite FDRSee the FDR for details on its calculation. This satellite ion specific FDR only contains the satellite ions (d/w) for I/L/J positions. | - |
| PSM Score? PSM ScoreThe PSM Score as given by Hecklib to this annotated spectrum. It is shown with three significant figures. | 487 |

## Spectrum 9198? Spectrum 9198 The raw spectrum of this peptide as annotated by Hecklib. The fragments are coloured according to ion type (see legend). Any peaks with a star '\*' as text can be hovered over to see the full details, first the ion type second the mass shift type. By hovering over the amino acids in the peptide or ions in the legend the corresponding peaks are highlighted. By toggling the 'Unassigned' label you can turn the background (unassigned) peaks on or off in the plot. By updating the slider in the Ion legend you can update the spectrum to only show the top X% of the peaks with labels. The top X% means any peak that is within X% of the highest intensity. By dragging in the spectrum you can zoom in to a specific part of the spectrum and use 'Zoom Out' to get back to the original zoom level. The annotation of the spectrum is based on the given sequence in the peptides file and is done with different software so inconsistencies are likely. The peaks are annotated based on the given sequence, with 20 ppm tolerance.

Copy Data

### Spectrum 9198 (TSV)

#### Preview

```
Loading example...
```

*Click on the button to copy the data to your clipboard.*

Mz MinMz MaxIntensity Max

WidthHeightPeptide font sizePeptide stroke widthSpectrum font sizeSpectrum stroke widthCompact peptide

Ion legend

wxyz

abcd

OtherUnassignedIonChargePositionShow for top:%

TFDDYAMHW

05.48e+41.10e+51.64e+52.19e+5

Zoom Out

a+12y+11a+12b+12b+24b+12a+13y+12b+13b+13b+14y+13b+14b+28b+28y+28y+14\*\*b+15b+15b+16y+15b+16y+16y+16y+17y+17b+18y+18

0675135020252700

Fragment Matches Table

Show background peaks

| Position | Ion type | Intensity | mz Theoretical | mz Error (Th) | mz Error (ppm) | Charge | Series Number |
| --- | --- | --- | --- | --- | --- | --- | --- |
| - | - | 6.346E+04 | 120.1 | - | - | 0 | - |
| - | - | 4943 | 121.1 | - | - | 0 | - |
| - | - | 608.7 | 127.1 | - | - | 0 | - |
| - | - | 352 | 129 | - | - | 0 | - |
| - | - | 2873 | 129.1 | - | - | 0 | - |
| - | - | 2027 | 130.1 | - | - | 0 | - |
| - | - | 2811 | 132.1 | - | - | 0 | - |
| - | - | 1692 | 132.1 | - | - | 0 | - |
| - | - | 3877 | 133.1 | - | - | 0 | - |
| - | - | 539.5 | 134 | - | - | 0 | - |
| - | - | 418.4 | 134.1 | - | - | 0 | - |
| - | - | 411.7 | 134.2 | - | - | 0 | - |
| - | - | 2.992E+04 | 136.1 | - | - | 0 | - |
| - | - | 2423 | 137.1 | - | - | 0 | - |
| - | - | 2507 | 138.1 | - | - | 0 | - |
| - | - | 427.1 | 139.1 | - | - | 0 | - |
| - | - | 465.2 | 141.1 | - | - | 0 | - |
| - | - | 553.2 | 142.4 | - | - | 0 | - |
| - | - | 916.5 | 146.1 | - | - | 0 | - |
| - | - | 775.1 | 146.1 | - | - | 0 | - |
| - | - | 482.1 | 149 | - | - | 0 | - |
| - | - | 3175 | 149 | - | - | 0 | - |
| - | - | 6671 | 156.1 | - | - | 0 | - |
| - | - | 8258 | 158.1 | - | - | 0 | - |
| - | - | 4077 | 159.1 | - | - | 0 | - |
| - | - | 905.7 | 159.1 | - | - | 0 | - |
| - | - | 2023 | 160.1 | - | - | 0 | - |
| - | - | 3481 | 160.1 | - | - | 0 | - |
| - | - | 417.8 | 161.1 | - | - | 0 | - |
| - | - | 565.5 | 165.1 | - | - | 0 | - |
| - | - | 698.5 | 165.1 | - | - | 0 | - |
| - | - | 2724 | 166.1 | - | - | 0 | - |
| - | - | 3949 | 166.1 | - | - | 0 | - |
| - | - | 4600 | 167.1 | - | - | 0 | - |
| - | - | 573.4 | 167.1 | - | - | 0 | - |
| - | - | 1389 | 167.1 | - | - | 0 | - |
| - | - | 1093 | 168.1 | - | - | 0 | - |
| - | - | 620.5 | 169.1 | - | - | 0 | - |
| - | - | 904.7 | 171.1 | - | - | 0 | - |
| - | - | 437 | 171.1 | - | - | 0 | - |
| - | - | 1032 | 173.1 | - | - | 0 | - |
| - | - | 540 | 173.4 | - | - | 0 | - |
| - | - | 616 | 175.1 | - | - | 0 | - |
| - | - | 579.9 | 175.1 | - | - | 0 | - |
| - | - | 1.616E+04 | 176.1 | - | - | 0 | - |
| - | - | 1.234E+04 | 177.1 | - | - | 0 | - |
| - | - | 2977 | 177.1 | - | - | 0 | - |
| - | - | 905.8 | 178.1 | - | - | 0 | - |
| - | - | 834.3 | 180.1 | - | - | 0 | - |
| - | - | 496.6 | 181.1 | - | - | 0 | - |
| - | - | 874.6 | 182.1 | - | - | 0 | - |
| - | - | 452.4 | 182.5 | - | - | 0 | - |
| - | - | 462.1 | 183.1 | - | - | 0 | - |
| - | - | 1656 | 185.1 | - | - | 0 | - |
| - | - | 1129 | 186.1 | - | - | 0 | - |
| - | - | 528.7 | 187.1 | - | - | 0 | - |
| - | - | 421.2 | 187.1 | - | - | 0 | - |
| - | - | 777.1 | 188.1 | - | - | 0 | - |
| - | - | 7627 | 188.1 | - | - | 0 | - |
| - | - | 1096 | 189.1 | - | - | 0 | - |
| - | - | 736.5 | 189.1 | - | - | 0 | - |
| - | - | 1160 | 191.1 | - | - | 0 | - |
| - | - | 464.9 | 193.1 | - | - | 0 | - |
| - | - | 1188 | 195.1 | - | - | 0 | - |
| - | - | 1217 | 196.1 | - | - | 0 | - |
| - | - | 583.6 | 197.1 | - | - | 0 | - |
| - | - | 651.3 | 199.2 | - | - | 0 | - |
| - | - | 1004 | 201.1 | - | - | 0 | - |
| - | - | 1421 | 203.1 | - | - | 0 | - |
| 2 | a | 4906 | 203.1 | 0.0002135 | 1.051 | +1 | 2 |
| - | - | 1161 | 204.1 | - | - | 0 | - |
| 9 | y | 4672 | 205.1 | 0.0001665 | 0.8116 | +1 | 1 |
| - | - | 1929 | 207.1 | - | - | 0 | - |
| - | - | 651.5 | 208.1 | - | - | 0 | - |
| - | - | 477.2 | 211.5 | - | - | 0 | - |
| - | - | 617.8 | 212.1 | - | - | 0 | - |
| - | - | 698 | 215.1 | - | - | 0 | - |
| - | - | 2526 | 217.1 | - | - | 0 | - |
| - | - | 4749 | 221.1 | - | - | 0 | - |
| 2 | a | 2.168E+05 | 221.1 | 0.00033 | 1.492 | +1 | 2 |
| - | - | 538.8 | 221.6 | - | - | 0 | - |
| - | - | 940.7 | 222.1 | - | - | 0 | - |
| - | - | 2.81E+04 | 222.1 | - | - | 0 | - |
| - | - | 839.3 | 223.1 | - | - | 0 | - |
| - | - | 1644 | 223.1 | - | - | 0 | - |
| - | - | 688 | 224.1 | - | - | 0 | - |
| - | - | 1830 | 225 | - | - | 0 | - |
| - | - | 1202 | 226.2 | - | - | 0 | - |
| - | - | 3768 | 231.1 | - | - | 0 | - |
| 2 | b | 2111 | 231.1 | 0.0004771 | 2.064 | +1 | 2 |
| - | - | 576.1 | 231.2 | - | - | 0 | - |
| - | - | 1077 | 231.2 | - | - | 0 | - |
| - | - | 648.2 | 233.1 | - | - | 0 | - |
| - | - | 1522 | 233.2 | - | - | 0 | - |
| - | - | 6465 | 235.1 | - | - | 0 | - |
| - | - | 608.2 | 236.1 | - | - | 0 | - |
| - | - | 582.8 | 238.1 | - | - | 0 | - |
| - | - | 1.155E+04 | 239.1 | - | - | 0 | - |
| 4 | b | 2229 | 240.1 | 0.003102 | 12.92 | +2 | 4 |
| - | - | 4150 | 241.1 | - | - | 0 | - |
| - | - | 625.8 | 243.1 | - | - | 0 | - |
| - | - | 529 | 244.1 | - | - | 0 | - |
| 2 | b | 3.617E+04 | 249.1 | 0.0002731 | 1.096 | +1 | 2 |
| - | - | 3992 | 250.1 | - | - | 0 | - |
| - | - | 5188 | 251.1 | - | - | 0 | - |
| - | - | 3314 | 252.1 | - | - | 0 | - |
| - | - | 1623 | 253.1 | - | - | 0 | - |
| - | - | 791.3 | 261.1 | - | - | 0 | - |
| - | - | 7855 | 263.1 | - | - | 0 | - |
| - | - | 700.7 | 263.1 | - | - | 0 | - |
| - | - | 559.5 | 265.1 | - | - | 0 | - |
| - | - | 513.1 | 265.3 | - | - | 0 | - |
| - | - | 732.8 | 267 | - | - | 0 | - |
| - | - | 1590 | 269 | - | - | 0 | - |
| - | - | 1.629E+04 | 269.1 | - | - | 0 | - |
| - | - | 1872 | 270.1 | - | - | 0 | - |
| - | - | 3523 | 279.1 | - | - | 0 | - |
| - | - | 1667 | 281.1 | - | - | 0 | - |
| - | - | 949.9 | 283 | - | - | 0 | - |
| - | - | 2.275E+04 | 285 | - | - | 0 | - |
| - | - | 3602 | 286 | - | - | 0 | - |
| - | - | 1567 | 287.1 | - | - | 0 | - |
| - | - | 4763 | 296.2 | - | - | 0 | - |
| - | - | 641.2 | 297.2 | - | - | 0 | - |
| - | - | 1.483E+04 | 299.1 | - | - | 0 | - |
| - | - | 641.9 | 299.2 | - | - | 0 | - |
| - | - | 2556 | 300.1 | - | - | 0 | - |
| - | - | 706.9 | 301.1 | - | - | 0 | - |
| - | - | 2906 | 303 | - | - | 0 | - |
| - | - | 673.9 | 304 | - | - | 0 | - |
| - | - | 1004 | 315.2 | - | - | 0 | - |
| 3 | a | 708.1 | 318.1 | 0.0006065 | 1.906 | +1 | 3 |
| - | - | 1645 | 322.1 | - | - | 0 | - |
| - | - | 1335 | 323.1 | - | - | 0 | - |
| - | - | 632.4 | 323.2 | - | - | 0 | - |
| - | - | 1.135E+04 | 324.1 | - | - | 0 | - |
| - | - | 2259 | 325.1 | - | - | 0 | - |
| - | - | 726.1 | 328.1 | - | - | 0 | - |
| - | - | 642.3 | 332.1 | - | - | 0 | - |
| - | - | 710 | 339.2 | - | - | 0 | - |
| - | - | 5241 | 340.1 | - | - | 0 | - |
| - | - | 1492 | 341.1 | - | - | 0 | - |
| 8 | y | 5.677E+04 | 342.2 | 0.0003977 | 1.162 | +1 | 2 |
| - | - | 1.151E+04 | 343.2 | - | - | 0 | - |
| - | - | 619.7 | 344.2 | - | - | 0 | - |
| - | - | 2814 | 345 | - | - | 0 | - |
| - | - | 927.2 | 346 | - | - | 0 | - |
| 3 | b | 2065 | 346.1 | 0.0002675 | 0.7727 | +1 | 3 |
| - | - | 1774 | 348.1 | - | - | 0 | - |
| - | - | 4231 | 350.1 | - | - | 0 | - |
| - | - | 1201 | 352.1 | - | - | 0 | - |
| - | - | 5.824E+04 | 355.1 | - | - | 0 | - |
| - | - | 1.514E+04 | 356.1 | - | - | 0 | - |
| - | - | 589.1 | 357.6 | - | - | 0 | - |
| - | - | 770.1 | 358.2 | - | - | 0 | - |
| 3 | b | 8930 | 364.2 | 0.0003839 | 1.054 | +1 | 3 |
| - | - | 1581 | 365.2 | - | - | 0 | - |
| - | - | 1063 | 366.1 | - | - | 0 | - |
| - | - | 1403 | 371.1 | - | - | 0 | - |
| - | - | 5452 | 373.1 | - | - | 0 | - |
| - | - | 2008 | 374.1 | - | - | 0 | - |
| - | - | 755.9 | 376.1 | - | - | 0 | - |
| - | - | 6225 | 378.1 | - | - | 0 | - |
| - | - | 1.205E+04 | 394.1 | - | - | 0 | - |
| - | - | 2471 | 395.1 | - | - | 0 | - |
| - | - | 1636 | 401 | - | - | 0 | - |
| - | - | 2899 | 415 | - | - | 0 | - |
| - | - | 582.3 | 415.2 | - | - | 0 | - |
| - | - | 783.9 | 416 | - | - | 0 | - |
| - | - | 4754 | 419 | - | - | 0 | - |
| - | - | 1667 | 420 | - | - | 0 | - |
| - | - | 968.3 | 432.1 | - | - | 0 | - |
| - | - | 989.3 | 433.2 | - | - | 0 | - |
| - | - | 825.8 | 438.2 | - | - | 0 | - |
| - | - | 537.1 | 449.2 | - | - | 0 | - |
| - | - | 1156 | 455.2 | - | - | 0 | - |
| 4 | b | 1014 | 461.2 | 0.001065 | 2.309 | +1 | 4 |
| - | - | 3163 | 462.3 | - | - | 0 | - |
| - | - | 1267 | 462.8 | - | - | 0 | - |
| - | - | 959.4 | 463.3 | - | - | 0 | - |
| - | - | 1.189E+04 | 465.2 | - | - | 0 | - |
| - | - | 2821 | 466.2 | - | - | 0 | - |
| 7 | y | 1.229E+04 | 473.2 | 0.0001352 | 0.2858 | +1 | 3 |
| - | - | 3181 | 474.2 | - | - | 0 | - |
| - | - | 1681 | 475.2 | - | - | 0 | - |
| - | - | 783.7 | 477.2 | - | - | 0 | - |
| - | - | 706.8 | 478.8 | - | - | 0 | - |
| 4 | b | 5486 | 479.2 | 0.0006626 | 1.383 | +1 | 4 |
| - | - | 1006 | 480.2 | - | - | 0 | - |
| 8 | b | 772.5 | 482.2 | 0.002062 | 4.277 | +2 | 8 |
| - | - | 695.5 | 483.2 | - | - | 0 | - |
| - | - | 1740 | 486.2 | - | - | 0 | - |
| 8 | b | 787.7 | 491.2 | 0.001327 | 2.701 | +2 | 8 |
| - | - | 2371 | 503.2 | - | - | 0 | - |
| - | - | 888.4 | 513.2 | - | - | 0 | - |
| - | - | 789.3 | 521.2 | - | - | 0 | - |
| - | - | 1029 | 540.2 | - | - | 0 | - |
| 2 | y | 4115 | 542.7 | 0.0006436 | 1.186 | +2 | 8 |
| - | - | 3237 | 543.2 | - | - | 0 | - |
| - | - | 1046 | 543.7 | - | - | 0 | - |
| 6 | y | 1.334E+04 | 544.2 | 3.926E-05 | 0.07214 | +1 | 4 |
| - | - | 3484 | 545.2 | - | - | 0 | - |
| - | - | 938.2 | 546.2 | - | - | 0 | - |
| - | - | 741.9 | 548.2 | - | - | 0 | - |
| - | - | 2166 | 557.2 | - | - | 0 | - |
| - | - | 2208 | 575.4 | - | - | 0 | - |
| 0 | Precursor | 2923 | 584.2 | 0.0005803 | 0.9933 | +2 | -1 |
| - | - | 1381 | 584.7 | - | - | 0 | - |
| - | - | 855.4 | 585.2 | - | - | 0 | - |
| - | - | 1595 | 590.2 | - | - | 0 | - |
| - | - | 1052 | 591.2 | - | - | 0 | - |
| - | - | 771.8 | 592.3 | - | - | 0 | - |
| - | - | 910.6 | 592.3 | - | - | 0 | - |
| - | - | 796.2 | 593.1 | - | - | 0 | - |
| 0 | Precursor | 2693 | 593.2 | 0.0003685 | 0.6212 | +2 | -1 |
| - | - | 826 | 593.3 | - | - | 0 | - |
| - | - | 1856 | 593.7 | - | - | 0 | - |
| - | - | 1433 | 594.2 | - | - | 0 | - |
| - | - | 2064 | 596.2 | - | - | 0 | - |
| - | - | 1549 | 614.2 | - | - | 0 | - |
| - | - | 3635 | 618.2 | - | - | 0 | - |
| - | - | 803.6 | 619.2 | - | - | 0 | - |
| 5 | b | 1582 | 624.2 | 0.001686 | 2.701 | +1 | 5 |
| - | - | 660.7 | 636.2 | - | - | 0 | - |
| 5 | b | 824.6 | 642.2 | 0.0009594 | 1.494 | +1 | 5 |
| - | - | 667.1 | 649.3 | - | - | 0 | - |
| - | - | 917.3 | 656.3 | - | - | 0 | - |
| - | - | 1309 | 688.2 | - | - | 0 | - |
| - | - | 747.6 | 689.2 | - | - | 0 | - |
| 6 | b | 1224 | 695.3 | 0.003033 | 4.363 | +1 | 6 |
| - | - | 953.6 | 699.4 | - | - | 0 | - |
| - | - | 7835 | 705.3 | - | - | 0 | - |
| - | - | 2238 | 706.3 | - | - | 0 | - |
| 5 | y | 1.451E+04 | 707.3 | 0.0007282 | 1.03 | +1 | 5 |
| - | - | 6918 | 708.3 | - | - | 0 | - |
| - | - | 1083 | 709.3 | - | - | 0 | - |
| 6 | b | 838.1 | 713.3 | 0.001086 | 1.522 | +1 | 6 |
| - | - | 1.426E+04 | 733.3 | - | - | 0 | - |
| - | - | 5974 | 734.3 | - | - | 0 | - |
| - | - | 943 | 735.3 | - | - | 0 | - |
| - | - | 2819 | 751.3 | - | - | 0 | - |
| - | - | 1071 | 752.3 | - | - | 0 | - |
| - | - | 1252 | 788.4 | - | - | 0 | - |
| 4 | y | 888.5 | 804.3 | 0.002458 | 3.056 | +1 | 6 |
| 4 | y | 1.758E+04 | 822.3 | 8.338E-05 | 0.1014 | +1 | 6 |
| - | - | 8637 | 823.3 | - | - | 0 | - |
| - | - | 2328 | 824.3 | - | - | 0 | - |
| - | - | 1315 | 880.3 | - | - | 0 | - |
| - | - | 963.1 | 891.3 | - | - | 0 | - |
| 3 | y | 5696 | 919.3 | 0.001996 | 2.172 | +1 | 7 |
| - | - | 3422 | 920.3 | - | - | 0 | - |
| - | - | 1411 | 921.3 | - | - | 0 | - |
| - | - | 1019 | 923.5 | - | - | 0 | - |
| - | - | 735.8 | 924.5 | - | - | 0 | - |
| 3 | y | 9.121E+04 | 937.4 | 0.0007203 | 0.7684 | +1 | 7 |
| - | - | 4.925E+04 | 938.4 | - | - | 0 | - |
| - | - | 1.478E+04 | 939.4 | - | - | 0 | - |
| - | - | 1972 | 940.4 | - | - | 0 | - |
| - | - | 668.9 | 942 | - | - | 0 | - |
| - | - | 3484 | 956.4 | - | - | 0 | - |
| - | - | 1228 | 957.5 | - | - | 0 | - |
| 8 | b | 1359 | 981.4 | 0.001789 | 1.822 | +1 | 8 |
| - | - | 793.1 | 1056 | - | - | 0 | - |
| 2 | y | 6851 | 1084 | 0.00169 | 1.559 | +1 | 8 |
| - | - | 4589 | 1085 | - | - | 0 | - |
| - | - | 1529 | 1086 | - | - | 0 | - |
| - | - | 683.7 | 1394 | - | - | 0 | - |
| - | - | 743.1 | 2033 | - | - | 0 | - |
| - | - | 601.6 | 2304 | - | - | 0 | - |
| - | - | 651.1 | 2674 | - | - | 0 | - |

m/z Charge Intensity FragmentType MassShift Position
120.08106994628906 0 63463.07
121.0844497680664 0 4943.2837
127.08684539794922 0 608.72955
129.0480499267578 0 351.9599
129.10250854492188 0 2872.5771
130.06546020507812 0 2027.4053
132.08103942871094 0 2811.0896
132.1022491455078 0 1691.9514
133.08612060546875 0 3876.8486
134.02732849121094 0 539.45844
134.08946228027344 0 418.37253
134.2315673828125 0 411.71304
136.0759735107422 0 29924.674
137.07931518554688 0 2422.7576
138.06637573242188 0 2506.8855
139.11697387695312 0 427.0782
141.10264587402344 0 465.15707
142.43756103515625 0 553.2468
146.060302734375 0 916.5293
146.09640502929688 0 775.13605
148.95509338378906 0 482.08994
149.0452117919922 0 3174.7915
156.07701110839844 0 6670.889
158.09664916992188 0 8257.678
159.09201049804688 0 4076.647
159.09933471679688 0 905.6695
160.07601928710938 0 2023.3156
160.11236572265625 0 3481.4277
161.09228515625 0 417.78287
165.05467224121094 0 565.4593
165.07720947265625 0 698.54944
166.06134033203125 0 2723.8357
166.0865020751953 0 3948.7222
167.05569458007812 0 4600.2383
167.08206176757812 0 573.3935
167.08985900878906 0 1388.6356
168.07701110839844 0 1092.6461
169.09764099121094 0 620.5149
171.0771026611328 0 904.69434
171.11512756347656 0 436.99844
173.1285400390625 0 1032.042
173.43765258789062 0 540.00024
175.08937072753906 0 616.0417
175.0977020263672 0 579.88074
176.10726928710938 0 16156.024
177.10243225097656 0 12342.484
177.11134338378906 0 2976.642
178.1061553955078 0 905.77905
180.0771942138672 0 834.323
181.09780883789062 0 496.6479
182.08145141601562 0 874.6306
182.45204162597656 0 452.39743
183.06336975097656 0 462.0876
185.0559844970703 0 1655.6514
186.0914306640625 0 1129.24
187.13427734375 0 528.706
187.14480590820312 0 421.1652
188.06227111816406 0 777.0703
188.07086181640625 0 7626.7744
189.07437133789062 0 1096.1381
189.0865936279297 0 736.50525
191.1183319091797 0 1160.4044
193.1083984375 0 464.90628
195.0879669189453 0 1188.4327
196.09059143066406 0 1217.4354
197.1280975341797 0 583.5654
199.17019653320312 0 651.28864
201.1236572265625 0 1003.7478
203.0663299560547 0 1420.6113
203.11810302734375 0 4905.576 a Water loss 1
204.1216583251953 0 1161.4464
205.09732055664062 0 4672.1895 y 8
207.11289978027344 0 1928.6068
208.07208251953125 0 651.4979
211.45167541503906 0 477.1709
212.1179656982422 0 617.8308
215.13818359375 0 697.9901
217.09759521484375 0 2525.92
221.08480834960938 0 4748.5376
221.1287841796875 0 216849.97 a 1
221.55271911621094 0 538.78064
222.08445739746094 0 940.6876
222.1320037841797 0 28102.133
223.1081085205078 0 839.2829
223.1342315673828 0 1643.8467
224.10289001464844 0 688.0253
225.0430908203125 0 1830.4349
226.1554412841797 0 1202.3064
231.0614471435547 0 3767.5603
231.11328125 0 2110.6055 b Water loss 1
231.15846252441406 0 576.0734
231.17051696777344 0 1076.9641
233.09072875976562 0 648.17334
233.16531372070312 0 1522.2173
235.1080780029297 0 6464.576
236.1117401123047 0 608.1571
238.1208953857422 0 582.79474
239.09530639648438 0 11554.261
240.09536743164062 0 2229.1902 b 3
241.11212158203125 0 4149.667
243.1343231201172 0 625.81744
244.0926513671875 0 529.0495
249.12364196777344 0 36172.62 b 1
250.126953125 0 3992.4045
251.10276794433594 0 5188.27
252.0802764892578 0 3313.9465
253.1182403564453 0 1623.4304
261.0870056152344 0 791.3241
263.1028747558594 0 7854.6074
263.12744140625 0 700.6518
265.11883544921875 0 559.52765
265.3362731933594 0 513.10034
266.9991760253906 0 732.81335
268.9784851074219 0 1589.8026
269.1069030761719 0 16290.364
270.1093444824219 0 1872.3152
279.0976257324219 0 3522.6038
281.05169677734375 0 1666.7754
283.0304870605469 0 949.8768
285.0100402832031 0 22752.56
286.0101013183594 0 3601.6416
287.11712646484375 0 1566.9789
296.15106201171875 0 4763.4004
297.15411376953125 0 641.2104
299.0619201660156 0 14827.998
299.15118408203125 0 641.9195
300.06317138671875 0 2555.8398
301.13873291015625 0 706.9432
303.0205078125 0 2905.7004
304.0209045410156 0 673.85565
315.1669921875 0 1004.1676
318.14422607421875 0 708.101 a Water loss 2
322.1342468261719 0 1645.1953
323.0985107421875 0 1334.8047
323.17138671875 0 632.4083
324.14581298828125 0 11349.111
325.14898681640625 0 2258.9412
328.13006591796875 0 726.12476
332.123779296875 0 642.3066
339.17034912109375 0 710.04065
340.14410400390625 0 5241.2554
341.14544677734375 0 1491.9161
342.1564636230469 0 56771.38 y 7
343.15960693359375 0 11513.827
344.1634521484375 0 619.72595
344.9764709472656 0 2813.6372
345.9755554199219 0 927.2224
346.1400146484375 0 2064.596 b Water loss 2
348.1185607910156 0 1773.586
350.13494873046875 0 4230.8325
352.1404113769531 0 1201.3813
355.07000732421875 0 58242.668
356.0707092285156 0 15135.749
357.6465759277344 0 589.0754
358.155517578125 0 770.14795
364.15069580078125 0 8930.311 b 2
365.15277099609375 0 1580.6095
366.13037109375 0 1063.0286
371.1005554199219 0 1403.0896
373.0807189941406 0 5451.9146
374.0818176269531 0 2008.4823
376.1155700683594 0 755.89496
378.1297607421875 0 6225.06
394.1248779296875 0 12047.196
395.12841796875 0 2471.0498
400.98565673828125 0 1635.9397
415.0373840332031 0 2899.445
415.15325927734375 0 582.2796
416.0365295410156 0 783.8743
418.99566650390625 0 4754.246
419.99530029296875 0 1667.429
432.1413269042969 0 968.265
433.1722412109375 0 989.3294
438.1603698730469 0 825.7883
449.16668701171875 0 537.0791
455.1861572265625 0 1155.6952
461.1677551269531 0 1014.16907 b Water loss 3
462.26416015625 0 3163.4797
462.7658996582031 0 1266.7484
463.2648010253906 0 959.4008
465.1621398925781 0 11885.224
466.1655578613281 0 2821.2695
473.1966857910156 0 12285.362 y 6
474.1999206542969 0 3180.8892
475.2105712890625 0 1680.7576
477.1611328125 0 783.6565
478.8359375 0 706.81885
479.17791748046875 0 5485.563 b 3
480.1810302734375 0 1006.1308
482.18896484375 0 772.545 b Water loss 7
483.1791687011719 0 695.54004
486.1610412597656 0 1740.234
491.1935119628906 0 787.653 b 7
503.20782470703125 0 2370.6223
513.19775390625 0 888.4142
521.2176513671875 0 789.2529
540.24267578125 0 1029.2953
542.7139282226562 0 4115.433 y 1
543.2135620117188 0 3236.8538
543.7171020507812 0 1045.558
544.2337036132812 0 13343.203 y 5
545.2364501953125 0 3483.6943
546.232666015625 0 938.16583
548.1981811523438 0 741.8588
557.1986083984375 0 2165.679
575.363525390625 0 2207.8738
584.232421875 0 2922.7405 Precursor Water loss
584.7340698242188 0 1381.0369
585.2349243164062 0 855.44965
590.2384033203125 0 1594.6599
591.23974609375 0 1051.869
592.2561645507812 0 771.786
592.33984375 0 910.61237
593.1439208984375 0 796.1597
593.2367553710938 0 2692.8782 Precursor
593.33984375 0 825.98883
593.7389526367188 0 1856.157
594.24072265625 0 1432.7947
596.20263671875 0 2064.4514
614.2450561523438 0 1549.4419
618.2338256835938 0 3634.9995
619.2378540039062 0 803.6471
624.2283325195312 0 1581.6228 b Water loss 4
636.2454833984375 0 660.6835
642.2396240234375 0 824.6229 b 4
649.3399047851562 0 667.14014
656.3020629882812 0 917.26965
688.2399291992188 0 1308.6904
689.2371215820312 0 747.64136
695.2640991210938 0 1223.6624 b Water loss 5
699.4032592773438 0 953.61896
705.2656860351562 0 7834.577
706.2687377929688 0 2237.7646
707.2962646484375 0 14508.633 y 4
708.2996826171875 0 6917.6025
709.297119140625 0 1083.1698
713.276611328125 0 838.12177 b 5
733.2605590820312 0 14260.914
734.2639770507812 0 5973.7974
735.264404296875 0 942.97534
751.27099609375 0 2818.8066
752.2725830078125 0 1071.257
788.3558959960938 0 1252.1725
804.3109130859375 0 888.4528 y Water loss 3
822.3238525390625 0 17582.615 y 3
823.326171875 0 8637.4375
824.3276977539062 0 2327.5647
880.330078125 0 1314.8188
891.3427124023438 0 963.0831
919.3383178710938 0 5695.8975 y Water loss 2
920.3377685546875 0 3422.2842
921.3384399414062 0 1410.7673
923.5155639648438 0 1019.2341
924.5242919921875 0 735.79034
937.3501586914062 0 91207.125 y 2
938.3529663085938 0 49246.152
939.3532104492188 0 14775.649
940.3558959960938 0 1971.8398
941.9912109375 0 668.9095
956.4464111328125 0 3484.233
957.4514770507812 0 1228.4435
981.3753051757812 0 1358.687 b 7
1055.515625 0 793.11145
1084.4176025390625 0 6850.86 y 1
1085.4207763671875 0 4588.953
1086.425537109375 0 1528.6871
1394.168212890625 0 683.69366
2033.496826171875 0 743.0636
2303.85595703125 0 601.57996
2673.571044921875 0 651.0887

Spectrum Details

|  |  |
| --- | --- |
| Matched peaks? Matched peaksThe total absolute number of peaks matched. Additionally in brackets the total fraction of peaks matched and the total number of peaks is shown. | 30 (11.11% of 270) |
| FDR? FDRThe false discovery rate estimated for this peptide. It is calculated by matching all theoretical fragments with a non-integer shift with the raw peaks for this spectrum. This is done with 40 different shifts. The resulting percentage is the average number of annotated peaks over the number of annotated peaks with the correct spectrum. | 0.71% |
| Satellite FDR? Satellite FDRSee the FDR for details on its calculation. This satellite ion specific FDR only contains the satellite ions (d/w) for I/L/J positions. | - |
| PSM Score? PSM ScoreThe PSM Score as given by Hecklib to this annotated spectrum. It is shown with three significant figures. | 373 |

## Spectrum 8296? Spectrum 8296 The raw spectrum of this peptide as annotated by Hecklib. The fragments are coloured according to ion type (see legend). Any peaks with a star '\*' as text can be hovered over to see the full details, first the ion type second the mass shift type. By hovering over the amino acids in the peptide or ions in the legend the corresponding peaks are highlighted. By toggling the 'Unassigned' label you can turn the background (unassigned) peaks on or off in the plot. By updating the slider in the Ion legend you can update the spectrum to only show the top X% of the peaks with labels. The top X% means any peak that is within X% of the highest intensity. By dragging in the spectrum you can zoom in to a specific part of the spectrum and use 'Zoom Out' to get back to the original zoom level. The annotation of the spectrum is based on the given sequence in the peptides file and is done with different software so inconsistencies are likely. The peaks are annotated based on the given sequence, with 20 ppm tolerance.

Copy Data

### Spectrum 8296 (TSV)

#### Preview

```
Loading example...
```

*Click on the button to copy the data to your clipboard.*

Mz MinMz MaxIntensity Max

WidthHeightPeptide font sizePeptide stroke widthSpectrum font sizeSpectrum stroke widthCompact peptide

Ion legend

wxyz

abcd

OtherUnassignedIonChargePositionShow for top:%

TFDDYAMHW

09.90e+41.98e+52.97e+53.96e+5

Zoom Out

b+23a+12a+12y+11a+12b+12b+12d+13a+13a+13y+12b+13b+13b+14y+27b+14y+13y+28y+28y+14\*\*b+15b+15b+16b+16y+15y+16y+16b+17y+17y+17b+18y+18

0840167925193358

Fragment Matches Table

Show background peaks

| Position | Ion type | Intensity | mz Theoretical | mz Error (Th) | mz Error (ppm) | Charge | Series Number |
| --- | --- | --- | --- | --- | --- | --- | --- |
| - | - | 1.068E+05 | 120.1 | - | - | 0 | - |
| - | - | 7907 | 121.1 | - | - | 0 | - |
| - | - | 798.4 | 122.1 | - | - | 0 | - |
| - | - | 1048 | 127.1 | - | - | 0 | - |
| - | - | 388.6 | 128.1 | - | - | 0 | - |
| - | - | 1343 | 128.1 | - | - | 0 | - |
| - | - | 567.5 | 129.1 | - | - | 0 | - |
| - | - | 5974 | 129.1 | - | - | 0 | - |
| - | - | 584.6 | 129.1 | - | - | 0 | - |
| - | - | 4779 | 130.1 | - | - | 0 | - |
| - | - | 696.4 | 131.1 | - | - | 0 | - |
| - | - | 438 | 131.1 | - | - | 0 | - |
| - | - | 6124 | 132.1 | - | - | 0 | - |
| - | - | 673.5 | 133.1 | - | - | 0 | - |
| - | - | 3.762E+04 | 136.1 | - | - | 0 | - |
| - | - | 651.5 | 137 | - | - | 0 | - |
| - | - | 3156 | 137.1 | - | - | 0 | - |
| - | - | 3073 | 138.1 | - | - | 0 | - |
| - | - | 3554 | 138.1 | - | - | 0 | - |
| - | - | 475.1 | 139.1 | - | - | 0 | - |
| - | - | 382.3 | 139.1 | - | - | 0 | - |
| - | - | 344.7 | 140.6 | - | - | 0 | - |
| - | - | 444.3 | 141.1 | - | - | 0 | - |
| - | - | 1036 | 141.1 | - | - | 0 | - |
| - | - | 428.3 | 142.3 | - | - | 0 | - |
| - | - | 873.4 | 143.1 | - | - | 0 | - |
| - | - | 501.7 | 143.1 | - | - | 0 | - |
| - | - | 1683 | 143.1 | - | - | 0 | - |
| - | - | 547.1 | 144.1 | - | - | 0 | - |
| - | - | 448.1 | 145.1 | - | - | 0 | - |
| - | - | 2461 | 146.1 | - | - | 0 | - |
| - | - | 432.4 | 146.5 | - | - | 0 | - |
| - | - | 1151 | 148.1 | - | - | 0 | - |
| - | - | 384.4 | 150.1 | - | - | 0 | - |
| - | - | 397.9 | 154.2 | - | - | 0 | - |
| - | - | 1622 | 155.1 | - | - | 0 | - |
| - | - | 651.8 | 155.1 | - | - | 0 | - |
| - | - | 482.2 | 155.1 | - | - | 0 | - |
| - | - | 1.142E+04 | 156.1 | - | - | 0 | - |
| - | - | 587.1 | 157.1 | - | - | 0 | - |
| - | - | 2275 | 157.1 | - | - | 0 | - |
| - | - | 1.356E+04 | 158.1 | - | - | 0 | - |
| - | - | 543 | 159.1 | - | - | 0 | - |
| - | - | 9096 | 159.1 | - | - | 0 | - |
| - | - | 784 | 159.1 | - | - | 0 | - |
| - | - | 2230 | 160.1 | - | - | 0 | - |
| - | - | 758.2 | 160.1 | - | - | 0 | - |
| - | - | 1089 | 160.1 | - | - | 0 | - |
| - | - | 5823 | 160.1 | - | - | 0 | - |
| - | - | 410.1 | 160.4 | - | - | 0 | - |
| - | - | 1232 | 161.1 | - | - | 0 | - |
| - | - | 468.4 | 165.1 | - | - | 0 | - |
| - | - | 1144 | 165.1 | - | - | 0 | - |
| - | - | 795.8 | 165.1 | - | - | 0 | - |
| - | - | 793 | 166.1 | - | - | 0 | - |
| - | - | 1629 | 166.1 | - | - | 0 | - |
| - | - | 3554 | 166.1 | - | - | 0 | - |
| - | - | 832.9 | 167.1 | - | - | 0 | - |
| - | - | 2501 | 167.1 | - | - | 0 | - |
| - | - | 885.2 | 167.1 | - | - | 0 | - |
| - | - | 869.3 | 169.1 | - | - | 0 | - |
| - | - | 616.4 | 169.1 | - | - | 0 | - |
| - | - | 1001 | 169.1 | - | - | 0 | - |
| - | - | 936.6 | 169.1 | - | - | 0 | - |
| - | - | 1449 | 171.1 | - | - | 0 | - |
| - | - | 938.3 | 171.1 | - | - | 0 | - |
| - | - | 428.5 | 172.1 | - | - | 0 | - |
| - | - | 3725 | 173.1 | - | - | 0 | - |
| - | - | 699.8 | 173.4 | - | - | 0 | - |
| 3 | b | 1337 | 174.1 | 9.318E-05 | 0.5353 | +2 | 3 |
| - | - | 1358 | 175.1 | - | - | 0 | - |
| - | - | 2273 | 176.1 | - | - | 0 | - |
| - | - | 2.683E+04 | 176.1 | - | - | 0 | - |
| - | - | 2.204E+04 | 177.1 | - | - | 0 | - |
| - | - | 3302 | 177.1 | - | - | 0 | - |
| - | - | 2199 | 178.1 | - | - | 0 | - |
| - | - | 1583 | 180.1 | - | - | 0 | - |
| - | - | 1254 | 182.1 | - | - | 0 | - |
| - | - | 535.3 | 183.1 | - | - | 0 | - |
| - | - | 2666 | 185.1 | - | - | 0 | - |
| - | - | 992.1 | 185.1 | - | - | 0 | - |
| - | - | 1049 | 186.1 | - | - | 0 | - |
| - | - | 699 | 187.1 | - | - | 0 | - |
| - | - | 6590 | 187.1 | - | - | 0 | - |
| - | - | 1.532E+04 | 188.1 | - | - | 0 | - |
| - | - | 1582 | 189.1 | - | - | 0 | - |
| - | - | 1711 | 191.1 | - | - | 0 | - |
| - | - | 755.7 | 192.1 | - | - | 0 | - |
| - | - | 472.5 | 193 | - | - | 0 | - |
| - | - | 523.3 | 193.1 | - | - | 0 | - |
| - | - | 8679 | 193.1 | - | - | 0 | - |
| - | - | 1472 | 195.1 | - | - | 0 | - |
| - | - | 557.8 | 197.1 | - | - | 0 | - |
| - | - | 1998 | 199.1 | - | - | 0 | - |
| - | - | 512.6 | 201.1 | - | - | 0 | - |
| - | - | 1662 | 201.1 | - | - | 0 | - |
| - | - | 1085 | 202.1 | - | - | 0 | - |
| - | - | 2878 | 203.1 | - | - | 0 | - |
| - | - | 621.8 | 203.1 | - | - | 0 | - |
| - | - | 981.2 | 203.1 | - | - | 0 | - |
| - | - | 1616 | 203.1 | - | - | 0 | - |
| 2 | a | 1.085E+04 | 203.1 | 9.168E-05 | 0.4514 | +1 | 2 |
| - | - | 485.6 | 204.1 | - | - | 0 | - |
| - | - | 3508 | 204.1 | - | - | 0 | - |
| 2 | a | 647.1 | 204.1 | 0.000222 | 1.087 | +1 | 2 |
| - | - | 1046 | 204.1 | - | - | 0 | - |
| - | - | 559.6 | 205.1 | - | - | 0 | - |
| 9 | y | 8562 | 205.1 | 0.0001082 | 0.5275 | +1 | 1 |
| - | - | 848.5 | 205.1 | - | - | 0 | - |
| - | - | 1033 | 206.1 | - | - | 0 | - |
| - | - | 2722 | 207.1 | - | - | 0 | - |
| - | - | 1068 | 208.1 | - | - | 0 | - |
| - | - | 603.6 | 211.1 | - | - | 0 | - |
| - | - | 638.9 | 212.1 | - | - | 0 | - |
| - | - | 646.4 | 213.2 | - | - | 0 | - |
| - | - | 831.3 | 215.1 | - | - | 0 | - |
| - | - | 7291 | 215.1 | - | - | 0 | - |
| - | - | 585.9 | 217.1 | - | - | 0 | - |
| - | - | 5322 | 217.1 | - | - | 0 | - |
| - | - | 2222 | 219.1 | - | - | 0 | - |
| - | - | 519.8 | 220.1 | - | - | 0 | - |
| - | - | 2.162E+04 | 221.1 | - | - | 0 | - |
| 2 | a | 3.922E+05 | 221.1 | 0.0001011 | 0.4571 | +1 | 2 |
| - | - | 2049 | 222.1 | - | - | 0 | - |
| - | - | 4.885E+04 | 222.1 | - | - | 0 | - |
| - | - | 603.6 | 223.1 | - | - | 0 | - |
| - | - | 528.4 | 223.1 | - | - | 0 | - |
| - | - | 2929 | 223.1 | - | - | 0 | - |
| - | - | 3085 | 226.2 | - | - | 0 | - |
| - | - | 3344 | 227.1 | - | - | 0 | - |
| - | - | 728.9 | 227.2 | - | - | 0 | - |
| - | - | 1529 | 228.1 | - | - | 0 | - |
| - | - | 769.7 | 229.1 | - | - | 0 | - |
| - | - | 2228 | 229.2 | - | - | 0 | - |
| - | - | 5983 | 231.1 | - | - | 0 | - |
| 2 | b | 3246 | 231.1 | 0.0002024 | 0.8759 | +1 | 2 |
| - | - | 1316 | 231.2 | - | - | 0 | - |
| - | - | 880.6 | 232.1 | - | - | 0 | - |
| - | - | 1453 | 233.2 | - | - | 0 | - |
| - | - | 8716 | 235.1 | - | - | 0 | - |
| - | - | 577.3 | 235.1 | - | - | 0 | - |
| - | - | 1351 | 236.1 | - | - | 0 | - |
| - | - | 584.2 | 238.1 | - | - | 0 | - |
| - | - | 904.2 | 239.1 | - | - | 0 | - |
| - | - | 2745 | 239.1 | - | - | 0 | - |
| - | - | 571.6 | 240.1 | - | - | 0 | - |
| - | - | 567 | 242.2 | - | - | 0 | - |
| - | - | 716.5 | 244.1 | - | - | 0 | - |
| - | - | 563.4 | 245.1 | - | - | 0 | - |
| - | - | 649.4 | 246.1 | - | - | 0 | - |
| - | - | 514.7 | 247.1 | - | - | 0 | - |
| - | - | 2562 | 248.1 | - | - | 0 | - |
| - | - | 871.2 | 248.2 | - | - | 0 | - |
| 2 | b | 6.796E+04 | 249.1 | 5.95E-05 | 0.2388 | +1 | 2 |
| - | - | 9360 | 250.1 | - | - | 0 | - |
| - | - | 6503 | 251.1 | - | - | 0 | - |
| - | - | 911 | 251.1 | - | - | 0 | - |
| - | - | 862.1 | 254.1 | - | - | 0 | - |
| - | - | 949.4 | 254.2 | - | - | 0 | - |
| - | - | 843.7 | 255.1 | - | - | 0 | - |
| - | - | 926.8 | 256.1 | - | - | 0 | - |
| - | - | 4609 | 257.1 | - | - | 0 | - |
| - | - | 519.3 | 257.2 | - | - | 0 | - |
| - | - | 957.4 | 261.1 | - | - | 0 | - |
| - | - | 700.1 | 261.1 | - | - | 0 | - |
| - | - | 1.406E+04 | 263.1 | - | - | 0 | - |
| - | - | 1719 | 263.1 | - | - | 0 | - |
| - | - | 1666 | 264.1 | - | - | 0 | - |
| - | - | 760.7 | 264.1 | - | - | 0 | - |
| - | - | 871.3 | 264.1 | - | - | 0 | - |
| - | - | 638.2 | 265.2 | - | - | 0 | - |
| - | - | 776.5 | 266.1 | - | - | 0 | - |
| - | - | 984.5 | 267.1 | - | - | 0 | - |
| - | - | 1312 | 270.2 | - | - | 0 | - |
| - | - | 548.5 | 271.1 | - | - | 0 | - |
| - | - | 2494 | 273.1 | - | - | 0 | - |
| - | - | 3525 | 274.1 | - | - | 0 | - |
| - | - | 678.5 | 275.2 | - | - | 0 | - |
| - | - | 6598 | 279.1 | - | - | 0 | - |
| - | - | 689.8 | 280.1 | - | - | 0 | - |
| - | - | 641.2 | 284.2 | - | - | 0 | - |
| - | - | 8046 | 285.1 | - | - | 0 | - |
| - | - | 914.6 | 286.1 | - | - | 0 | - |
| - | - | 6190 | 292.1 | - | - | 0 | - |
| 3 | d | 978.8 | 292.2 | 0.0001424 | 0.4875 | +1 | 3 |
| - | - | 749.9 | 293.1 | - | - | 0 | - |
| - | - | 775.8 | 293.1 | - | - | 0 | - |
| - | - | 6940 | 296.2 | - | - | 0 | - |
| - | - | 506.1 | 297 | - | - | 0 | - |
| - | - | 1101 | 297.2 | - | - | 0 | - |
| - | - | 719.9 | 302.1 | - | - | 0 | - |
| - | - | 6181 | 303.1 | - | - | 0 | - |
| - | - | 1115 | 310.2 | - | - | 0 | - |
| - | - | 1043 | 314.1 | - | - | 0 | - |
| - | - | 2706 | 314.2 | - | - | 0 | - |
| - | - | 928.3 | 315.1 | - | - | 0 | - |
| - | - | 1242 | 316.1 | - | - | 0 | - |
| 3 | a | 2412 | 318.1 | 0.0003013 | 0.9471 | +1 | 3 |
| - | - | 756.4 | 321.1 | - | - | 0 | - |
| - | - | 2695 | 323.1 | - | - | 0 | - |
| - | - | 596.5 | 323.2 | - | - | 0 | - |
| - | - | 996.3 | 323.2 | - | - | 0 | - |
| - | - | 1.611E+04 | 324.1 | - | - | 0 | - |
| - | - | 1818 | 325.1 | - | - | 0 | - |
| - | - | 882.2 | 328.1 | - | - | 0 | - |
| - | - | 1066 | 328.1 | - | - | 0 | - |
| - | - | 750.4 | 330.1 | - | - | 0 | - |
| - | - | 806.3 | 332.1 | - | - | 0 | - |
| - | - | 772.8 | 333.2 | - | - | 0 | - |
| 3 | a | 783.1 | 336.2 | 0.0001849 | 0.5499 | +1 | 3 |
| - | - | 1365 | 339.2 | - | - | 0 | - |
| - | - | 658.8 | 339.3 | - | - | 0 | - |
| 8 | y | 8.034E+04 | 342.2 | 6.01E-05 | 0.1756 | +1 | 2 |
| - | - | 1.387E+04 | 343.2 | - | - | 0 | - |
| - | - | 1029 | 344.2 | - | - | 0 | - |
| 3 | b | 4850 | 346.1 | 7.199E-06 | 0.0208 | +1 | 3 |
| - | - | 644.1 | 347.1 | - | - | 0 | - |
| - | - | 1798 | 348.1 | - | - | 0 | - |
| - | - | 4687 | 350.1 | - | - | 0 | - |
| - | - | 902.3 | 351.1 | - | - | 0 | - |
| - | - | 1885 | 352.1 | - | - | 0 | - |
| - | - | 1306 | 353.2 | - | - | 0 | - |
| - | - | 1706 | 355.2 | - | - | 0 | - |
| - | - | 3847 | 356.1 | - | - | 0 | - |
| - | - | 986.9 | 358.2 | - | - | 0 | - |
| - | - | 688.9 | 359.1 | - | - | 0 | - |
| - | - | 1375 | 360.1 | - | - | 0 | - |
| 3 | b | 2.006E+04 | 364.2 | 0.0002264 | 0.6218 | +1 | 3 |
| - | - | 3561 | 365.2 | - | - | 0 | - |
| - | - | 2199 | 366.1 | - | - | 0 | - |
| - | - | 987 | 367.2 | - | - | 0 | - |
| - | - | 744.5 | 367.2 | - | - | 0 | - |
| - | - | 849.1 | 368.2 | - | - | 0 | - |
| - | - | 3462 | 371.2 | - | - | 0 | - |
| - | - | 616.2 | 374.1 | - | - | 0 | - |
| - | - | 2164 | 376.1 | - | - | 0 | - |
| - | - | 8227 | 378.1 | - | - | 0 | - |
| - | - | 1874 | 379.1 | - | - | 0 | - |
| - | - | 2256 | 382.1 | - | - | 0 | - |
| - | - | 984.7 | 385.2 | - | - | 0 | - |
| - | - | 1660 | 387.3 | - | - | 0 | - |
| - | - | 1394 | 390.2 | - | - | 0 | - |
| - | - | 1.435E+04 | 394.1 | - | - | 0 | - |
| - | - | 2090 | 395.1 | - | - | 0 | - |
| - | - | 4270 | 402.2 | - | - | 0 | - |
| - | - | 1172 | 407.2 | - | - | 0 | - |
| - | - | 3249 | 408.2 | - | - | 0 | - |
| - | - | 855.2 | 409.2 | - | - | 0 | - |
| - | - | 566.1 | 413.8 | - | - | 0 | - |
| - | - | 1190 | 420.2 | - | - | 0 | - |
| - | - | 636.2 | 421.2 | - | - | 0 | - |
| - | - | 1.167E+04 | 425.2 | - | - | 0 | - |
| - | - | 2159 | 426.2 | - | - | 0 | - |
| - | - | 2253 | 427.2 | - | - | 0 | - |
| - | - | 771.2 | 428.2 | - | - | 0 | - |
| - | - | 2331 | 433.2 | - | - | 0 | - |
| - | - | 1962 | 447.2 | - | - | 0 | - |
| - | - | 1352 | 448.8 | - | - | 0 | - |
| - | - | 873.9 | 449.1 | - | - | 0 | - |
| - | - | 652.8 | 449.3 | - | - | 0 | - |
| - | - | 1043 | 454.2 | - | - | 0 | - |
| - | - | 2166 | 455.2 | - | - | 0 | - |
| - | - | 2344 | 456.3 | - | - | 0 | - |
| - | - | 864.8 | 457.3 | - | - | 0 | - |
| - | - | 728 | 460.8 | - | - | 0 | - |
| 4 | b | 3413 | 461.2 | 0.0003694 | 0.8011 | +1 | 4 |
| - | - | 1043 | 461.2 | - | - | 0 | - |
| - | - | 887.2 | 462.2 | - | - | 0 | - |
| - | - | 703.7 | 462.3 | - | - | 0 | - |
| - | - | 1.006E+04 | 465.2 | - | - | 0 | - |
| - | - | 1989 | 466.2 | - | - | 0 | - |
| - | - | 1601 | 466.3 | - | - | 0 | - |
| - | - | 1470 | 471.2 | - | - | 0 | - |
| 3 | y | 1648 | 477.2 | 0.006719 | 14.08 | +2 | 7 |
| - | - | 680 | 477.7 | - | - | 0 | - |
| 4 | b | 1.122E+04 | 479.2 | 0.0001309 | 0.2731 | +1 | 4 |
| - | - | 2685 | 480.2 | - | - | 0 | - |
| - | - | 966.9 | 482.2 | - | - | 0 | - |
| - | - | 5594 | 484.3 | - | - | 0 | - |
| - | - | 1461 | 485.3 | - | - | 0 | - |
| - | - | 2051 | 486.2 | - | - | 0 | - |
| 7 | y | 2.061E+04 | 489.2 | 0.004825 | 9.864 | +1 | 3 |
| - | - | 5550 | 490.2 | - | - | 0 | - |
| - | - | 2079 | 491.2 | - | - | 0 | - |
| - | - | 1699 | 495.2 | - | - | 0 | - |
| - | - | 8064 | 496.2 | - | - | 0 | - |
| - | - | 1605 | 497.2 | - | - | 0 | - |
| - | - | 2838 | 497.2 | - | - | 0 | - |
| - | - | 1816 | 513.2 | - | - | 0 | - |
| - | - | 912.9 | 513.3 | - | - | 0 | - |
| - | - | 630.7 | 518.7 | - | - | 0 | - |
| - | - | 3569 | 519.2 | - | - | 0 | - |
| - | - | 1981 | 520.2 | - | - | 0 | - |
| - | - | 638.3 | 527.7 | - | - | 0 | - |
| - | - | 978.9 | 534.3 | - | - | 0 | - |
| - | - | 934.9 | 537.2 | - | - | 0 | - |
| 2 | y | 1095 | 541.7 | 0.004212 | 7.776 | +2 | 8 |
| - | - | 2043 | 542.2 | - | - | 0 | - |
| - | - | 3426 | 548.2 | - | - | 0 | - |
| - | - | 722.2 | 549.2 | - | - | 0 | - |
| 2 | y | 1.129E+04 | 550.7 | 0.002042 | 3.709 | +2 | 8 |
| - | - | 6500 | 551.2 | - | - | 0 | - |
| - | - | 2273 | 551.7 | - | - | 0 | - |
| - | - | 1250 | 553.3 | - | - | 0 | - |
| - | - | 1421 | 557.2 | - | - | 0 | - |
| - | - | 1590 | 559.3 | - | - | 0 | - |
| 6 | y | 3.067E+04 | 560.2 | 0.004485 | 8.006 | +1 | 4 |
| - | - | 9028 | 561.2 | - | - | 0 | - |
| - | - | 2067 | 562.2 | - | - | 0 | - |
| - | - | 2410 | 569.2 | - | - | 0 | - |
| - | - | 1471 | 569.7 | - | - | 0 | - |
| - | - | 3464 | 570.2 | - | - | 0 | - |
| - | - | 1107 | 574.3 | - | - | 0 | - |
| - | - | 1348 | 578.2 | - | - | 0 | - |
| - | - | 1103 | 579.2 | - | - | 0 | - |
| - | - | 1055 | 590.8 | - | - | 0 | - |
| - | - | 764.2 | 591.3 | - | - | 0 | - |
| 0 | Precursor | 5903 | 592.2 | 0.002468 | 4.167 | +2 | -1 |
| - | - | 4704 | 592.7 | - | - | 0 | - |
| - | - | 1860 | 593.2 | - | - | 0 | - |
| - | - | 1693 | 596.2 | - | - | 0 | - |
| - | - | 795.1 | 599.8 | - | - | 0 | - |
| - | - | 754.3 | 600.3 | - | - | 0 | - |
| - | - | 744.9 | 601 | - | - | 0 | - |
| 0 | Precursor | 1.496E+04 | 601.2 | 0.001946 | 3.237 | +2 | -1 |
| - | - | 854.2 | 601.3 | - | - | 0 | - |
| - | - | 3141 | 601.4 | - | - | 0 | - |
| - | - | 9649 | 601.7 | - | - | 0 | - |
| - | - | 4875 | 602.2 | - | - | 0 | - |
| - | - | 3507 | 606.2 | - | - | 0 | - |
| - | - | 5060 | 612.2 | - | - | 0 | - |
| - | - | 1385 | 613.2 | - | - | 0 | - |
| - | - | 2866 | 614.2 | - | - | 0 | - |
| - | - | 1118 | 615.2 | - | - | 0 | - |
| 5 | b | 2535 | 624.2 | 0.001793 | 2.872 | +1 | 5 |
| - | - | 801.2 | 631.2 | - | - | 0 | - |
| - | - | 3021 | 634.2 | - | - | 0 | - |
| - | - | 789 | 635.2 | - | - | 0 | - |
| - | - | 1589 | 640.2 | - | - | 0 | - |
| - | - | 1106 | 641.3 | - | - | 0 | - |
| 5 | b | 2091 | 642.2 | 0.0008984 | 1.399 | +1 | 5 |
| - | - | 780.9 | 655.3 | - | - | 0 | - |
| - | - | 5450 | 657.3 | - | - | 0 | - |
| - | - | 1829 | 658.3 | - | - | 0 | - |
| - | - | 790.4 | 659.2 | - | - | 0 | - |
| - | - | 9749 | 659.3 | - | - | 0 | - |
| - | - | 3940 | 660.3 | - | - | 0 | - |
| - | - | 645.5 | 672.3 | - | - | 0 | - |
| - | - | 1206 | 676.3 | - | - | 0 | - |
| - | - | 685 | 677.3 | - | - | 0 | - |
| - | - | 8208 | 685.3 | - | - | 0 | - |
| - | - | 2918 | 686.3 | - | - | 0 | - |
| - | - | 931.5 | 691.3 | - | - | 0 | - |
| 6 | b | 1310 | 695.3 | 0.0008972 | 1.29 | +1 | 6 |
| - | - | 982 | 696.3 | - | - | 0 | - |
| - | - | 673.6 | 696.8 | - | - | 0 | - |
| - | - | 2945 | 703.3 | - | - | 0 | - |
| - | - | 591.8 | 704.2 | - | - | 0 | - |
| - | - | 1872 | 705.3 | - | - | 0 | - |
| 6 | b | 1371 | 713.3 | 0.002062 | 2.892 | +1 | 6 |
| - | - | 9393 | 721.3 | - | - | 0 | - |
| - | - | 2801 | 722.3 | - | - | 0 | - |
| 5 | y | 2.955E+04 | 723.3 | 0.003718 | 5.14 | +1 | 5 |
| - | - | 1.119E+04 | 724.3 | - | - | 0 | - |
| - | - | 3052 | 725.3 | - | - | 0 | - |
| - | - | 1337 | 731.2 | - | - | 0 | - |
| - | - | 803.7 | 733.3 | - | - | 0 | - |
| - | - | 1.254E+04 | 749.3 | - | - | 0 | - |
| - | - | 3753 | 750.3 | - | - | 0 | - |
| - | - | 1394 | 751.3 | - | - | 0 | - |
| - | - | 709.5 | 754.4 | - | - | 0 | - |
| - | - | 1042 | 756.3 | - | - | 0 | - |
| - | - | 3433 | 767.3 | - | - | 0 | - |
| - | - | 1.092E+04 | 774.3 | - | - | 0 | - |
| - | - | 5146 | 775.3 | - | - | 0 | - |
| - | - | 900.8 | 776.3 | - | - | 0 | - |
| - | - | 649.4 | 796.3 | - | - | 0 | - |
| - | - | 768.2 | 804.3 | - | - | 0 | - |
| - | - | 1646 | 810 | - | - | 0 | - |
| 4 | y | 1649 | 820.3 | 0.004856 | 5.92 | +1 | 6 |
| - | - | 1133 | 821.3 | - | - | 0 | - |
| - | - | 994.5 | 832.3 | - | - | 0 | - |
| 4 | y | 3.342E+04 | 838.3 | 0.003752 | 4.476 | +1 | 6 |
| - | - | 1.755E+04 | 839.3 | - | - | 0 | - |
| - | - | 5476 | 840.3 | - | - | 0 | - |
| 7 | b | 869.2 | 842.3 | 0.007498 | 8.901 | +1 | 7 |
| - | - | 1041 | 862.3 | - | - | 0 | - |
| - | - | 727 | 865.7 | - | - | 0 | - |
| - | - | 4133 | 871.3 | - | - | 0 | - |
| - | - | 3015 | 872.3 | - | - | 0 | - |
| - | - | 902.2 | 872.4 | - | - | 0 | - |
| - | - | 5.008E+04 | 889.3 | - | - | 0 | - |
| - | - | 2.257E+04 | 890.3 | - | - | 0 | - |
| - | - | 5787 | 891.4 | - | - | 0 | - |
| - | - | 776.2 | 892.3 | - | - | 0 | - |
| - | - | 1577 | 895.5 | - | - | 0 | - |
| - | - | 2957 | 896.5 | - | - | 0 | - |
| - | - | 1042 | 897.5 | - | - | 0 | - |
| - | - | 1784 | 899.4 | - | - | 0 | - |
| - | - | 1466 | 900.4 | - | - | 0 | - |
| - | - | 2043 | 907.3 | - | - | 0 | - |
| - | - | 1210 | 908.3 | - | - | 0 | - |
| - | - | 856.1 | 918.3 | - | - | 0 | - |
| - | - | 750.2 | 933.4 | - | - | 0 | - |
| 3 | y | 9800 | 935.3 | 0.003121 | 3.337 | +1 | 7 |
| - | - | 7265 | 936.3 | - | - | 0 | - |
| - | - | 1944 | 937.3 | - | - | 0 | - |
| - | - | 1233 | 938.3 | - | - | 0 | - |
| 3 | y | 1.558E+05 | 953.3 | 0.003726 | 3.908 | +1 | 7 |
| - | - | 8.248E+04 | 954.3 | - | - | 0 | - |
| - | - | 3.161E+04 | 955.3 | - | - | 0 | - |
| - | - | 724.5 | 955.5 | - | - | 0 | - |
| - | - | 3411 | 956.3 | - | - | 0 | - |
| - | - | 1051 | 969.4 | - | - | 0 | - |
| 8 | b | 2419 | 997.4 | 0.00162 | 1.624 | +1 | 8 |
| - | - | 1184 | 998.4 | - | - | 0 | - |
| - | - | 979.5 | 998.6 | - | - | 0 | - |
| - | - | 765.3 | 1018 | - | - | 0 | - |
| - | - | 2713 | 1036 | - | - | 0 | - |
| - | - | 2455 | 1037 | - | - | 0 | - |
| 2 | y | 8248 | 1100 | 0.00184 | 1.672 | +1 | 8 |
| - | - | 5654 | 1101 | - | - | 0 | - |
| - | - | 2818 | 1102 | - | - | 0 | - |
| - | - | 820.9 | 1110 | - | - | 0 | - |
| - | - | 1181 | 1111 | - | - | 0 | - |
| - | - | 600.6 | 2147 | - | - | 0 | - |
| - | - | 670.2 | 2426 | - | - | 0 | - |
| - | - | 903.8 | 3080 | - | - | 0 | - |
| - | - | 1005 | 3080 | - | - | 0 | - |
| - | - | 667.2 | 3325 | - | - | 0 | - |

m/z Charge Intensity FragmentType MassShift Position
120.0809555053711 0 106798.125
121.08427429199219 0 7907.037
122.07159423828125 0 798.3794
127.08661651611328 0 1047.9135
128.0900115966797 0 388.5552
128.1071319580078 0 1343.0938
129.06581115722656 0 567.5327
129.10238647460938 0 5974.3735
129.1105194091797 0 584.62994
130.0652313232422 0 4779.4883
131.0684356689453 0 696.4083
131.11795043945312 0 437.97498
132.08094787597656 0 6124.4326
133.0845489501953 0 673.5101
136.0758056640625 0 37618.215
137.03475952148438 0 651.54333
137.0791473388672 0 3156.461
138.0549774169922 0 3072.54
138.0662841796875 0 3553.585
139.06988525390625 0 475.0821
139.08644104003906 0 382.2755
140.5623321533203 0 344.67453
141.0657958984375 0 444.293
141.10215759277344 0 1035.8916
142.28428649902344 0 428.31143
143.07275390625 0 873.36145
143.08580017089844 0 501.7298
143.11805725097656 0 1682.9429
144.08096313476562 0 547.1384
145.06141662597656 0 448.12396
146.0601348876953 0 2460.9429
146.47555541992188 0 432.37518
148.0869598388672 0 1151.4911
150.0662384033203 0 384.39108
154.20001220703125 0 397.94513
155.08160400390625 0 1621.7831
155.09317016601562 0 651.79205
155.11773681640625 0 482.2
156.0768280029297 0 11422.032
157.08035278320312 0 587.1345
157.09716796875 0 2274.8584
158.09645080566406 0 13560.104
159.07635498046875 0 543.0043
159.0917205810547 0 9095.888
159.09921264648438 0 784.0109
160.0758056640625 0 2229.6206
160.08726501464844 0 758.1922
160.09506225585938 0 1088.7548
160.11207580566406 0 5822.62
160.3658905029297 0 410.06024
161.1158447265625 0 1232.2871
165.05516052246094 0 468.4261
165.0771484375 0 1143.668
165.10238647460938 0 795.8435
166.05360412597656 0 792.97516
166.06117248535156 0 1629.354
166.08631896972656 0 3553.8992
167.0552520751953 0 832.88916
167.0814666748047 0 2501.1953
167.0926055908203 0 885.1744
169.06063842773438 0 869.3293
169.07638549804688 0 616.42957
169.09718322753906 0 1000.86365
169.13348388671875 0 936.63367
171.07667541503906 0 1449.1265
171.11276245117188 0 938.30396
172.10821533203125 0 428.49704
173.12850952148438 0 3725.2449
173.4374237060547 0 699.81055
174.06561279296875 0 1336.8674 b Ammonia loss 2
175.0865936279297 0 1358.4573
176.0819854736328 0 2273.4126
176.1070556640625 0 26827.625
177.10223388671875 0 22042.564
177.11099243164062 0 3301.6367
178.10572814941406 0 2198.8784
180.0768585205078 0 1582.5162
182.0806884765625 0 1254.3962
183.1133575439453 0 535.2904
185.05581665039062 0 2665.7734
185.09207153320312 0 992.0648
186.0902099609375 0 1049.0244
187.0629119873047 0 698.99536
187.14413452148438 0 6589.6265
188.0706787109375 0 15317.38
189.0739288330078 0 1582.2743
191.11810302734375 0 1711.3572
192.10218811035156 0 755.7131
193.02513122558594 0 472.50452
193.07249450683594 0 523.2602
193.10848999023438 0 8679.317
195.08731079101562 0 1471.7247
197.12850952148438 0 557.83417
199.1079559326172 0 1997.9836
201.0657958984375 0 512.6197
201.12353515625 0 1662.1328
202.053466796875 0 1084.7106
203.0662384033203 0 2878.0747
203.08241271972656 0 621.8125
203.09239196777344 0 981.2484
203.10226440429688 0 1616.1309
203.1177978515625 0 10850.271 a Water loss 1
204.0677032470703 0 485.64728
204.07675170898438 0 3507.5642
204.1021270751953 0 647.1122 a Ammonia loss 1
204.12156677246094 0 1046.3539
205.0806884765625 0 559.5864
205.0970458984375 0 8561.772 y 8
205.10775756835938 0 848.46814
206.10113525390625 0 1032.6315
207.1127471923828 0 2721.6196
208.0716552734375 0 1067.526
211.1444091796875 0 603.5995
212.1160888671875 0 638.90857
213.1599578857422 0 646.4364
215.08143615722656 0 831.2855
215.1389923095703 0 7291.468
217.08456420898438 0 585.9422
217.09707641601562 0 5321.521
219.07972717285156 0 2221.5332
220.1200408935547 0 519.7583
221.10333251953125 0 21615.977
221.12855529785156 0 392150.8 a 1
222.10650634765625 0 2048.5159
222.13182067871094 0 48848.6
223.0623779296875 0 603.6405
223.1194305419922 0 528.42053
223.13409423828125 0 2928.8975
226.15501403808594 0 3085.1448
227.1022491455078 0 3344.0151
227.15826416015625 0 728.9442
228.11285400390625 0 1529.2017
229.10751342773438 0 769.68585
229.15484619140625 0 2227.9058
231.06121826171875 0 5982.9087
231.11300659179688 0 3245.5784 b Water loss 1
231.169921875 0 1315.6846
232.1165771484375 0 880.556
233.1654815673828 0 1453.3765
235.1077423095703 0 8715.595
235.14303588867188 0 577.31976
236.11155700683594 0 1350.6312
238.13026428222656 0 584.2482
239.0955047607422 0 904.23486
239.1136932373047 0 2745.357
240.1328582763672 0 571.56854
242.1865997314453 0 566.96594
244.12994384765625 0 716.529
245.1290283203125 0 563.4058
246.13482666015625 0 649.3727
247.1070098876953 0 514.70844
248.10714721679688 0 2561.645
248.15003967285156 0 871.19025
249.12342834472656 0 67958.22 b 1
250.12660217285156 0 9360.445
251.10260009765625 0 6503.3867
251.12738037109375 0 910.97894
254.11370849609375 0 862.10565
254.15025329589844 0 949.3522
255.14501953125 0 843.706
256.10870361328125 0 926.75116
257.106201171875 0 4609.4844
257.1631164550781 0 519.31537
261.08697509765625 0 957.37494
261.1255187988281 0 700.1145
263.1025390625 0 14064.565
263.1288146972656 0 1718.8212
264.10595703125 0 1666.0881
264.1312561035156 0 760.66266
264.14556884765625 0 871.2748
265.1538391113281 0 638.2119
266.1490478515625 0 776.4583
267.0907287597656 0 984.4569
270.1815185546875 0 1311.5237
271.10760498046875 0 548.5063
273.134521484375 0 2493.6023
274.1297302246094 0 3524.9482
275.1746826171875 0 678.54364
279.0975036621094 0 6598.422
280.12677001953125 0 689.822
284.1605224609375 0 641.23816
285.1016540527344 0 8045.669
286.1040954589844 0 914.59656
292.1403503417969 0 6190.029
292.16571044921875 0 978.76764 d 2
293.12347412109375 0 749.89935
293.1430358886719 0 775.7666
296.1506042480469 0 6939.9614
297.0419006347656 0 506.06216
297.1539001464844 0 1101.3086
302.1139221191406 0 719.91766
303.10986328125 0 6181.2495
310.1507873535156 0 1114.6819
314.0989990234375 0 1043.0653
314.2077941894531 0 2706.1172
315.1462707519531 0 928.2921
316.12890625 0 1241.8998
318.14453125 0 2412.3315 a Water loss 2
321.11883544921875 0 756.3512
323.098388671875 0 2695.265
323.1731262207031 0 596.5278
323.208251953125 0 996.32904
324.1455078125 0 16107.247
325.1485900878906 0 1818.3453
328.12646484375 0 882.2406
328.14703369140625 0 1065.941
330.11004638671875 0 750.4062
332.124267578125 0 806.2669
333.1574401855469 0 772.82074
336.15521240234375 0 783.0653 a 2
339.1710205078125 0 1364.535
339.26507568359375 0 658.8195
342.156005859375 0 80335.18 y 7
343.1591796875 0 13873.534
344.1614685058594 0 1029.3921
346.1397399902344 0 4850.243 b Water loss 2
347.1414794921875 0 644.0719
348.1189880371094 0 1797.7812
350.1344909667969 0 4687.2666
351.1360778808594 0 902.3134
352.1401062011719 0 1884.7158
353.2185974121094 0 1305.8281
355.1610412597656 0 1705.9282
356.13848876953125 0 3846.6428
358.15032958984375 0 986.9089
359.08892822265625 0 688.89484
360.1199645996094 0 1375.3713
364.15008544921875 0 20064.127 b 2
365.1529235839844 0 3560.6316
366.1287536621094 0 2199.0137
367.1640319824219 0 987.0173
367.23480224609375 0 744.51746
368.22955322265625 0 849.05817
371.22882080078125 0 3461.5208
374.1497802734375 0 616.20447
376.11395263671875 0 2163.6155
378.12945556640625 0 8227.12
379.13226318359375 0 1873.7908
382.1429748535156 0 2256.0588
385.2430114746094 0 984.66187
387.2594299316406 0 1660.2007
390.15545654296875 0 1394.3318
394.1242980957031 0 14349.946
395.1276550292969 0 2089.5925
402.1767883300781 0 4270.1533
407.1838073730469 0 1172.2126
408.16680908203125 0 3248.9326
409.168701171875 0 855.24585
413.84857177734375 0 566.1343
420.1870422363281 0 1189.8822
421.2234191894531 0 636.2462
425.1929931640625 0 11668.6045
426.1958923339844 0 2159.391
427.20806884765625 0 2252.5232
428.21051025390625 0 771.1779
433.1723327636719 0 2330.671
447.1510314941406 0 1961.799
448.7696533203125 0 1352.0507
449.1335144042969 0 873.888
449.2691650390625 0 652.8371
454.1957702636719 0 1043.0098
455.2050476074219 0 2166.4033
456.3185119628906 0 2343.8958
457.32025146484375 0 864.7553
460.76593017578125 0 727.99756
461.16632080078125 0 3413.1406 b Water loss 3
461.1976318359375 0 1042.962
462.1693420410156 0 887.22205
462.2654724121094 0 703.66705
465.1617736816406 0 10061.072
466.16363525390625 0 1988.8601
466.30316162109375 0 1601.2472
471.1806640625 0 1470.0734
477.1673583984375 0 1647.5569 y 2
477.6753845214844 0 679.99023
479.1771240234375 0 11218.919 b 3
480.1800537109375 0 2684.5432
482.19146728515625 0 966.91626
484.3128662109375 0 5594.161
485.3162536621094 0 1461.3495
486.16107177734375 0 2050.5159
489.1913757324219 0 20612.762 y 6
490.1935729980469 0 5549.6357
491.20208740234375 0 2079.196
495.18499755859375 0 1698.5656
496.2303466796875 0 8063.629
497.1705017089844 0 1604.7181
497.2333068847656 0 2838.2986
513.2000122070312 0 1815.6592
513.3134155273438 0 912.8737
518.7142333984375 0 630.6696
519.2032470703125 0 3569.3074
520.2039794921875 0 1980.5526
527.7089233398438 0 638.34595
534.3006591796875 0 978.894
537.2144165039062 0 934.8719
541.7072143554688 0 1095.1273 y Water loss 1
542.22900390625 0 2042.6029
548.1991577148438 0 3426.424
549.1998901367188 0 722.24286
550.7103271484375 0 11285.784 y 1
551.2119140625 0 6499.652
551.7120971679688 0 2273.2437
553.3102416992188 0 1249.7638
557.1978149414062 0 1420.8402
559.2501831054688 0 1590.0543
560.2281494140625 0 30672.615 y 5
561.23095703125 0 9028.359
562.2313842773438 0 2067.2012
569.2329711914062 0 2410.043
569.7352294921875 0 1470.5496
570.2276611328125 0 3464.2346
574.3278198242188 0 1106.6741
578.2301025390625 0 1348.0715
579.232666015625 0 1102.7257
590.8449096679688 0 1054.7443
591.3427734375 0 764.169
592.2293090820312 0 5903.066 Precursor Water loss
592.7311401367188 0 4704.3877
593.2296752929688 0 1860.423
596.2340087890625 0 1693.0798
599.8474731445312 0 795.06085
600.347412109375 0 754.2893
601.046630859375 0 744.9211
601.2340698242188 0 14955.613 Precursor
601.316162109375 0 854.217
601.3919067382812 0 3140.6465
601.7355346679688 0 9649.133
602.2360229492188 0 4875.017
606.2330322265625 0 3507.1619
612.1964111328125 0 5060.417
613.1980590820312 0 1385.3534
614.245361328125 0 2866.2852
615.248046875 0 1117.5612
624.2318115234375 0 2535.0344 b Water loss 4
631.2437133789062 0 801.222
634.2276000976562 0 3021.0513
635.2360229492188 0 788.9862
640.2359008789062 0 1588.5123
641.2886962890625 0 1105.9518
642.2396850585938 0 2090.7974 b 4
655.3031005859375 0 780.91486
657.2623901367188 0 5450.128
658.2647094726562 0 1829.0197
659.2359008789062 0 790.396
659.2926025390625 0 9748.505
660.2957763671875 0 3940.1294
672.3223876953125 0 645.4686
676.2640991210938 0 1205.8364
677.2772216796875 0 684.98926
685.25732421875 0 8207.969
686.2610473632812 0 2917.8591
691.33837890625 0 931.4651
695.2662353515625 0 1310.0208 b Water loss 5
696.2726440429688 0 982.00073
696.7733764648438 0 673.5925
703.2628173828125 0 2944.9424
704.2459716796875 0 591.837
705.2770385742188 0 1871.7349
713.275634765625 0 1370.9537 b 5
721.2601318359375 0 9392.79
722.2640380859375 0 2800.8389
723.2907104492188 0 29548.973 y 4
724.2935180664062 0 11186.157
725.293212890625 0 3052.0513
731.2449340820312 0 1337.0775
733.2622680664062 0 803.749
749.2546997070312 0 12535.747
750.2576293945312 0 3753.1423
751.25732421875 0 1394.0764
754.3675537109375 0 709.5104
756.3111572265625 0 1041.9985
767.2667236328125 0 3433.0637
774.3196411132812 0 10922.18
775.3225708007812 0 5145.764
776.3246459960938 0 900.76044
796.305419921875 0 649.3784
804.3320922851562 0 768.22723
810.0420532226562 0 1645.8474
820.3082275390625 0 1649.4116 y Water loss 3
821.3095703125 0 1132.5508
832.3189697265625 0 994.5229
838.3176879882812 0 33416.992 y 3
839.3207397460938 0 17552.451
840.3198852539062 0 5476.0605
842.3051147460938 0 869.23975 b Water loss 6
862.3184204101562 0 1041.0304
865.7373046875 0 727.017
871.3367309570312 0 4133.103
872.3362426757812 0 3014.8445
872.4375610351562 0 902.2493
889.3460693359375 0 50080.74
890.3488159179688 0 22569.238
891.35107421875 0 5787.34
892.3444213867188 0 776.23254
895.5223999023438 0 1576.5991
896.52392578125 0 2956.6208
897.5274047851562 0 1041.9243
899.4246826171875 0 1783.887
900.4262084960938 0 1465.7043
907.3358154296875 0 2042.5051
908.340087890625 0 1210.252
918.31201171875 0 856.0934
933.3724975585938 0 750.22675
935.3334350585938 0 9799.988 y Water loss 2
936.3350830078125 0 7265.265
937.32763671875 0 1944.0225
938.3173828125 0 1232.7847
953.3446044921875 0 155779.64 y 2
954.3472290039062 0 82478.914
955.3474731445312 0 31608.64
955.4636840820312 0 724.50244
956.3473510742188 0 3410.9895
969.3678588867188 0 1050.834
997.3687133789062 0 2419.1653 b 7
998.3734741210938 0 1183.9127
998.5703735351562 0 979.48804
1018.4050903320312 0 765.2556
1036.413330078125 0 2712.9668
1037.4161376953125 0 2454.9006
1100.4111328125 0 8247.889 y 1
1101.416259765625 0 5654.3867
1102.4173583984375 0 2817.632
1110.3946533203125 0 820.9077
1111.3974609375 0 1181.3713
2146.81005859375 0 600.5825
2425.674560546875 0 670.19977
3079.611328125 0 903.78436
3080.35498046875 0 1004.76794
3325.0634765625 0 667.1938

Spectrum Details

|  |  |
| --- | --- |
| Matched peaks? Matched peaksThe total absolute number of peaks matched. Additionally in brackets the total fraction of peaks matched and the total number of peaks is shown. | 34 (7.91% of 430) |
| FDR? FDRThe false discovery rate estimated for this peptide. It is calculated by matching all theoretical fragments with a non-integer shift with the raw peaks for this spectrum. This is done with 40 different shifts. The resulting percentage is the average number of annotated peaks over the number of annotated peaks with the correct spectrum. | 0.77% |
| Satellite FDR? Satellite FDRSee the FDR for details on its calculation. This satellite ion specific FDR only contains the satellite ions (d/w) for I/L/J positions. | - |
| PSM Score? PSM ScoreThe PSM Score as given by Hecklib to this annotated spectrum. It is shown with three significant figures. | 440 |

## Spectrum 10292? Spectrum 10292 The raw spectrum of this peptide as annotated by Hecklib. The fragments are coloured according to ion type (see legend). Any peaks with a star '\*' as text can be hovered over to see the full details, first the ion type second the mass shift type. By hovering over the amino acids in the peptide or ions in the legend the corresponding peaks are highlighted. By toggling the 'Unassigned' label you can turn the background (unassigned) peaks on or off in the plot. By updating the slider in the Ion legend you can update the spectrum to only show the top X% of the peaks with labels. The top X% means any peak that is within X% of the highest intensity. By dragging in the spectrum you can zoom in to a specific part of the spectrum and use 'Zoom Out' to get back to the original zoom level. The annotation of the spectrum is based on the given sequence in the peptides file and is done with different software so inconsistencies are likely. The peaks are annotated based on the given sequence, with 20 ppm tolerance.

Copy Data

### Spectrum 10292 (TSV)

#### Preview

```
Loading example...
```

*Click on the button to copy the data to your clipboard.*

Mz MinMz MaxIntensity Max

WidthHeightPeptide font sizePeptide stroke widthSpectrum font sizeSpectrum stroke widthCompact peptide

Ion legend

wxyz

abcd

OtherUnassignedIonChargePositionShow for top:%

TFDDYAMHW

02.11e+44.23e+46.34e+48.45e+4

Zoom Out

a+12y+11a+12b+12b+12a+13y+12b+13b+13b+14y+13y+28y+14\*\*b+15y+15y+16y+17y+17y+18

0779155723363115

Fragment Matches Table

Show background peaks

| Position | Ion type | Intensity | mz Theoretical | mz Error (Th) | mz Error (ppm) | Charge | Series Number |
| --- | --- | --- | --- | --- | --- | --- | --- |
| - | - | 7.483E+04 | 120.1 | - | - | 0 | - |
| - | - | 760.6 | 121.1 | - | - | 0 | - |
| - | - | 6623 | 121.1 | - | - | 0 | - |
| - | - | 524.7 | 122.1 | - | - | 0 | - |
| - | - | 355.3 | 122.5 | - | - | 0 | - |
| - | - | 937.8 | 123 | - | - | 0 | - |
| - | - | 464.9 | 127.1 | - | - | 0 | - |
| - | - | 689.6 | 129.1 | - | - | 0 | - |
| - | - | 7613 | 129.1 | - | - | 0 | - |
| - | - | 2188 | 130.1 | - | - | 0 | - |
| - | - | 592 | 130.1 | - | - | 0 | - |
| - | - | 2820 | 132.1 | - | - | 0 | - |
| - | - | 867.5 | 132.1 | - | - | 0 | - |
| - | - | 1193 | 133.1 | - | - | 0 | - |
| - | - | 3.091E+04 | 136.1 | - | - | 0 | - |
| - | - | 2776 | 137.1 | - | - | 0 | - |
| - | - | 685.1 | 138.1 | - | - | 0 | - |
| - | - | 1118 | 138.1 | - | - | 0 | - |
| - | - | 474.7 | 141.9 | - | - | 0 | - |
| - | - | 448.5 | 143 | - | - | 0 | - |
| - | - | 876.8 | 144.1 | - | - | 0 | - |
| - | - | 5361 | 146.1 | - | - | 0 | - |
| - | - | 1138 | 147 | - | - | 0 | - |
| - | - | 1042 | 147.1 | - | - | 0 | - |
| - | - | 645.8 | 148.9 | - | - | 0 | - |
| - | - | 491.9 | 148.9 | - | - | 0 | - |
| - | - | 643.4 | 148.9 | - | - | 0 | - |
| - | - | 611.9 | 148.9 | - | - | 0 | - |
| - | - | 963.5 | 148.9 | - | - | 0 | - |
| - | - | 1191 | 148.9 | - | - | 0 | - |
| - | - | 1329 | 148.9 | - | - | 0 | - |
| - | - | 3216 | 148.9 | - | - | 0 | - |
| - | - | 5391 | 148.9 | - | - | 0 | - |
| - | - | 3706 | 149 | - | - | 0 | - |
| - | - | 1854 | 149 | - | - | 0 | - |
| - | - | 1121 | 149 | - | - | 0 | - |
| - | - | 979.6 | 149 | - | - | 0 | - |
| - | - | 870.9 | 149 | - | - | 0 | - |
| - | - | 660.8 | 149 | - | - | 0 | - |
| - | - | 554 | 149 | - | - | 0 | - |
| - | - | 511.5 | 149 | - | - | 0 | - |
| - | - | 529.6 | 149 | - | - | 0 | - |
| - | - | 425.3 | 149 | - | - | 0 | - |
| - | - | 1159 | 149 | - | - | 0 | - |
| - | - | 427.8 | 155.1 | - | - | 0 | - |
| - | - | 2041 | 156.1 | - | - | 0 | - |
| - | - | 1340 | 157.1 | - | - | 0 | - |
| - | - | 2966 | 158.1 | - | - | 0 | - |
| - | - | 8511 | 159.1 | - | - | 0 | - |
| - | - | 775.6 | 160.1 | - | - | 0 | - |
| - | - | 1163 | 160.1 | - | - | 0 | - |
| - | - | 1506 | 160.1 | - | - | 0 | - |
| - | - | 8466 | 165.1 | - | - | 0 | - |
| - | - | 665.4 | 165.1 | - | - | 0 | - |
| - | - | 530.5 | 165.1 | - | - | 0 | - |
| - | - | 1577 | 166.1 | - | - | 0 | - |
| - | - | 1767 | 167.1 | - | - | 0 | - |
| - | - | 703.3 | 169.1 | - | - | 0 | - |
| - | - | 768.3 | 170.1 | - | - | 0 | - |
| - | - | 3968 | 173.1 | - | - | 0 | - |
| - | - | 735.8 | 174.1 | - | - | 0 | - |
| - | - | 694.3 | 175.1 | - | - | 0 | - |
| - | - | 593.4 | 176.1 | - | - | 0 | - |
| - | - | 7048 | 176.1 | - | - | 0 | - |
| - | - | 4356 | 177.1 | - | - | 0 | - |
| - | - | 916.5 | 177.1 | - | - | 0 | - |
| - | - | 844.3 | 178.1 | - | - | 0 | - |
| - | - | 569.3 | 181.1 | - | - | 0 | - |
| - | - | 1.642E+04 | 182.1 | - | - | 0 | - |
| - | - | 1306 | 183.1 | - | - | 0 | - |
| - | - | 1004 | 185.1 | - | - | 0 | - |
| - | - | 1715 | 185.1 | - | - | 0 | - |
| - | - | 697.6 | 186.1 | - | - | 0 | - |
| - | - | 652.4 | 187.1 | - | - | 0 | - |
| - | - | 763.9 | 187.1 | - | - | 0 | - |
| - | - | 2.84E+04 | 188.1 | - | - | 0 | - |
| - | - | 3589 | 189.1 | - | - | 0 | - |
| - | - | 480.3 | 189.1 | - | - | 0 | - |
| - | - | 460 | 189.1 | - | - | 0 | - |
| - | - | 1692 | 191.1 | - | - | 0 | - |
| - | - | 1671 | 193.1 | - | - | 0 | - |
| - | - | 772.5 | 195.1 | - | - | 0 | - |
| - | - | 497.2 | 199.1 | - | - | 0 | - |
| - | - | 941.8 | 200.1 | - | - | 0 | - |
| - | - | 2961 | 201.1 | - | - | 0 | - |
| - | - | 1168 | 203.1 | - | - | 0 | - |
| 2 | a | 3032 | 203.1 | 1.539E-05 | 0.07577 | +1 | 2 |
| - | - | 1183 | 204.1 | - | - | 0 | - |
| 9 | y | 1.078E+04 | 205.1 | 0.0001665 | 0.8116 | +1 | 1 |
| - | - | 1691 | 206.1 | - | - | 0 | - |
| - | - | 749 | 214.1 | - | - | 0 | - |
| - | - | 1441 | 217.1 | - | - | 0 | - |
| - | - | 774.9 | 219.1 | - | - | 0 | - |
| - | - | 5434 | 221.1 | - | - | 0 | - |
| 2 | a | 8.37E+04 | 221.1 | 0.0002079 | 0.9401 | +1 | 2 |
| - | - | 941 | 222.1 | - | - | 0 | - |
| - | - | 751.1 | 222.1 | - | - | 0 | - |
| - | - | 9170 | 222.1 | - | - | 0 | - |
| - | - | 1494 | 223.1 | - | - | 0 | - |
| - | - | 681.7 | 223.1 | - | - | 0 | - |
| - | - | 503.7 | 224.6 | - | - | 0 | - |
| - | - | 478 | 225.9 | - | - | 0 | - |
| - | - | 765.9 | 226.2 | - | - | 0 | - |
| - | - | 712.9 | 229.1 | - | - | 0 | - |
| - | - | 500.6 | 229.2 | - | - | 0 | - |
| - | - | 1941 | 231.1 | - | - | 0 | - |
| - | - | 637.3 | 231.1 | - | - | 0 | - |
| 2 | b | 1469 | 231.1 | 3.459E-05 | 0.1497 | +1 | 2 |
| - | - | 2241 | 233.1 | - | - | 0 | - |
| - | - | 1553 | 233.1 | - | - | 0 | - |
| - | - | 827.3 | 233.2 | - | - | 0 | - |
| - | - | 538.9 | 233.2 | - | - | 0 | - |
| - | - | 2433 | 235.1 | - | - | 0 | - |
| - | - | 640.9 | 237.1 | - | - | 0 | - |
| - | - | 927.1 | 239.1 | - | - | 0 | - |
| - | - | 815.7 | 239.1 | - | - | 0 | - |
| - | - | 1284 | 239.2 | - | - | 0 | - |
| - | - | 981.1 | 241.1 | - | - | 0 | - |
| 2 | b | 1.368E+04 | 249.1 | 0.0002579 | 1.035 | +1 | 2 |
| - | - | 1981 | 250.1 | - | - | 0 | - |
| - | - | 2138 | 251.1 | - | - | 0 | - |
| - | - | 898.6 | 251.2 | - | - | 0 | - |
| - | - | 771.7 | 253.1 | - | - | 0 | - |
| - | - | 1058 | 253.2 | - | - | 0 | - |
| - | - | 942 | 257.1 | - | - | 0 | - |
| - | - | 559.3 | 257.2 | - | - | 0 | - |
| - | - | 1399 | 261.1 | - | - | 0 | - |
| - | - | 2791 | 263.1 | - | - | 0 | - |
| - | - | 632.8 | 274.1 | - | - | 0 | - |
| - | - | 2063 | 279.1 | - | - | 0 | - |
| - | - | 581.4 | 282.1 | - | - | 0 | - |
| - | - | 598.9 | 284.2 | - | - | 0 | - |
| - | - | 958.7 | 285 | - | - | 0 | - |
| - | - | 1604 | 285.1 | - | - | 0 | - |
| - | - | 1051 | 292.1 | - | - | 0 | - |
| - | - | 1590 | 296.2 | - | - | 0 | - |
| 3 | a | 738.4 | 318.1 | 0.0008278 | 2.602 | +1 | 3 |
| - | - | 1203 | 319.1 | - | - | 0 | - |
| - | - | 682 | 321.2 | - | - | 0 | - |
| - | - | 3610 | 324.1 | - | - | 0 | - |
| - | - | 717.2 | 325.1 | - | - | 0 | - |
| - | - | 1.07E+04 | 329.1 | - | - | 0 | - |
| - | - | 2242 | 330.2 | - | - | 0 | - |
| - | - | 1813 | 340.2 | - | - | 0 | - |
| 8 | y | 1.815E+04 | 342.2 | 0.0002451 | 0.7163 | +1 | 2 |
| - | - | 1131 | 342.2 | - | - | 0 | - |
| - | - | 3447 | 343.2 | - | - | 0 | - |
| 3 | b | 1294 | 346.1 | 0.00135 | 3.9 | +1 | 3 |
| - | - | 554.2 | 349.4 | - | - | 0 | - |
| - | - | 1092 | 350.1 | - | - | 0 | - |
| - | - | 986.7 | 352.2 | - | - | 0 | - |
| - | - | 1284 | 355.1 | - | - | 0 | - |
| - | - | 673.1 | 356.1 | - | - | 0 | - |
| - | - | 1163 | 356.1 | - | - | 0 | - |
| - | - | 2458 | 357.2 | - | - | 0 | - |
| 3 | b | 3299 | 364.2 | 7.384E-05 | 0.2028 | +1 | 3 |
| - | - | 527.7 | 365.2 | - | - | 0 | - |
| - | - | 630.1 | 366.1 | - | - | 0 | - |
| - | - | 608.3 | 366.2 | - | - | 0 | - |
| - | - | 842.7 | 368.2 | - | - | 0 | - |
| - | - | 2795 | 378.1 | - | - | 0 | - |
| - | - | 2905 | 394.1 | - | - | 0 | - |
| - | - | 1012 | 398.2 | - | - | 0 | - |
| - | - | 728.5 | 401.7 | - | - | 0 | - |
| - | - | 709.7 | 408.2 | - | - | 0 | - |
| - | - | 1102 | 413.3 | - | - | 0 | - |
| - | - | 661.5 | 414.3 | - | - | 0 | - |
| - | - | 794.8 | 422.7 | - | - | 0 | - |
| - | - | 612.6 | 423.2 | - | - | 0 | - |
| - | - | 2175 | 425.2 | - | - | 0 | - |
| - | - | 1050 | 427.7 | - | - | 0 | - |
| - | - | 1082 | 433.3 | - | - | 0 | - |
| - | - | 629 | 436.7 | - | - | 0 | - |
| - | - | 954.4 | 437.2 | - | - | 0 | - |
| - | - | 820.6 | 455.2 | - | - | 0 | - |
| - | - | 2181 | 465.2 | - | - | 0 | - |
| 4 | b | 2218 | 479.2 | 0.0007846 | 1.637 | +1 | 4 |
| - | - | 923.7 | 480.2 | - | - | 0 | - |
| - | - | 592.7 | 480.8 | - | - | 0 | - |
| - | - | 729.1 | 483.2 | - | - | 0 | - |
| 7 | y | 5124 | 489.2 | 0.005313 | 10.86 | +1 | 3 |
| - | - | 1371 | 490.2 | - | - | 0 | - |
| - | - | 1136 | 496.2 | - | - | 0 | - |
| - | - | 693.8 | 497.2 | - | - | 0 | - |
| - | - | 625.3 | 499.7 | - | - | 0 | - |
| - | - | 692.4 | 501.3 | - | - | 0 | - |
| - | - | 742.6 | 513.4 | - | - | 0 | - |
| - | - | 624.6 | 540.2 | - | - | 0 | - |
| - | - | 640 | 547.1 | - | - | 0 | - |
| - | - | 1236 | 547.3 | - | - | 0 | - |
| 2 | y | 1748 | 550.7 | 0.003141 | 5.704 | +2 | 8 |
| - | - | 1694 | 551.2 | - | - | 0 | - |
| - | - | 1020 | 551.7 | - | - | 0 | - |
| - | - | 655.1 | 557.2 | - | - | 0 | - |
| 6 | y | 6552 | 560.2 | 0.004607 | 8.224 | +1 | 4 |
| - | - | 2376 | 561.2 | - | - | 0 | - |
| - | - | 735 | 562.2 | - | - | 0 | - |
| - | - | 691 | 569.2 | - | - | 0 | - |
| - | - | 577.2 | 570.2 | - | - | 0 | - |
| - | - | 4210 | 585.3 | - | - | 0 | - |
| - | - | 1443 | 586.3 | - | - | 0 | - |
| 0 | Precursor | 1025 | 592.2 | 0.001674 | 2.827 | +2 | -1 |
| - | - | 707.3 | 592.7 | - | - | 0 | - |
| 0 | Precursor | 2982 | 601.2 | 0.002984 | 4.962 | +2 | -1 |
| - | - | 629.1 | 601.3 | - | - | 0 | - |
| - | - | 1892 | 601.7 | - | - | 0 | - |
| - | - | 732.3 | 601.8 | - | - | 0 | - |
| - | - | 1388 | 612.2 | - | - | 0 | - |
| - | - | 771.6 | 614.2 | - | - | 0 | - |
| - | - | 628.3 | 614.8 | - | - | 0 | - |
| 5 | b | 1149 | 624.2 | 0.0004045 | 0.648 | +1 | 5 |
| - | - | 1021 | 628.4 | - | - | 0 | - |
| - | - | 605.7 | 640.2 | - | - | 0 | - |
| - | - | 1074 | 657.3 | - | - | 0 | - |
| - | - | 644 | 658.3 | - | - | 0 | - |
| - | - | 2634 | 659.3 | - | - | 0 | - |
| - | - | 5850 | 670.8 | - | - | 0 | - |
| - | - | 6108 | 671.3 | - | - | 0 | - |
| - | - | 3015 | 671.8 | - | - | 0 | - |
| - | - | 663.5 | 672.3 | - | - | 0 | - |
| - | - | 656.4 | 682.3 | - | - | 0 | - |
| - | - | 1524 | 685.3 | - | - | 0 | - |
| - | - | 1390 | 693.8 | - | - | 0 | - |
| - | - | 1201 | 694.3 | - | - | 0 | - |
| - | - | 683.8 | 695.3 | - | - | 0 | - |
| - | - | 9676 | 700.4 | - | - | 0 | - |
| - | - | 4312 | 701.4 | - | - | 0 | - |
| - | - | 1419 | 721.3 | - | - | 0 | - |
| 5 | y | 5580 | 723.3 | 0.003351 | 4.633 | +1 | 5 |
| - | - | 1889 | 724.3 | - | - | 0 | - |
| - | - | 826.8 | 725.3 | - | - | 0 | - |
| - | - | 1509 | 730.4 | - | - | 0 | - |
| - | - | 1832 | 730.9 | - | - | 0 | - |
| - | - | 3412 | 744.4 | - | - | 0 | - |
| - | - | 3664 | 744.9 | - | - | 0 | - |
| - | - | 1371 | 745.4 | - | - | 0 | - |
| - | - | 2395 | 749.3 | - | - | 0 | - |
| - | - | 883.2 | 750.3 | - | - | 0 | - |
| - | - | 1016 | 757.4 | - | - | 0 | - |
| - | - | 638.9 | 767.3 | - | - | 0 | - |
| - | - | 2449 | 774.3 | - | - | 0 | - |
| - | - | 1270 | 775.3 | - | - | 0 | - |
| - | - | 2833 | 783.4 | - | - | 0 | - |
| - | - | 1387 | 784.4 | - | - | 0 | - |
| - | - | 2816 | 794.4 | - | - | 0 | - |
| - | - | 1345 | 795.4 | - | - | 0 | - |
| - | - | 1387 | 800.4 | - | - | 0 | - |
| - | - | 4901 | 801.4 | - | - | 0 | - |
| - | - | 2529 | 802.4 | - | - | 0 | - |
| - | - | 2811 | 828.4 | - | - | 0 | - |
| - | - | 792.2 | 829.4 | - | - | 0 | - |
| 4 | y | 7332 | 838.3 | 0.003752 | 4.476 | +1 | 6 |
| - | - | 3518 | 839.3 | - | - | 0 | - |
| - | - | 1201 | 840.3 | - | - | 0 | - |
| - | - | 6471 | 844.5 | - | - | 0 | - |
| - | - | 3356 | 845.5 | - | - | 0 | - |
| - | - | 664.6 | 846.5 | - | - | 0 | - |
| - | - | 926.9 | 854.4 | - | - | 0 | - |
| - | - | 714.7 | 855.4 | - | - | 0 | - |
| - | - | 616.5 | 862.3 | - | - | 0 | - |
| - | - | 837.2 | 871.3 | - | - | 0 | - |
| - | - | 983.5 | 871.4 | - | - | 0 | - |
| - | - | 2.642E+04 | 872.4 | - | - | 0 | - |
| - | - | 1.363E+04 | 873.4 | - | - | 0 | - |
| - | - | 3138 | 874.5 | - | - | 0 | - |
| - | - | 8885 | 889.3 | - | - | 0 | - |
| - | - | 6132 | 890.3 | - | - | 0 | - |
| - | - | 1067 | 891.4 | - | - | 0 | - |
| - | - | 814.2 | 896.3 | - | - | 0 | - |
| - | - | 2245 | 909.4 | - | - | 0 | - |
| - | - | 1001 | 910.4 | - | - | 0 | - |
| 3 | y | 1996 | 935.3 | 0.005013 | 5.359 | +1 | 7 |
| - | - | 1452 | 936.3 | - | - | 0 | - |
| 3 | y | 3.244E+04 | 953.3 | 0.003481 | 3.652 | +1 | 7 |
| - | - | 1.776E+04 | 954.3 | - | - | 0 | - |
| - | - | 6996 | 955.3 | - | - | 0 | - |
| - | - | 5203 | 991.5 | - | - | 0 | - |
| - | - | 1936 | 992.5 | - | - | 0 | - |
| - | - | 1.172E+04 | 1020 | - | - | 0 | - |
| - | - | 7033 | 1021 | - | - | 0 | - |
| - | - | 2884 | 1022 | - | - | 0 | - |
| - | - | 750.1 | 1036 | - | - | 0 | - |
| - | - | 809.2 | 1038 | - | - | 0 | - |
| - | - | 655.8 | 1039 | - | - | 0 | - |
| - | - | 768.1 | 1056 | - | - | 0 | - |
| 2 | y | 1785 | 1100 | 0.001718 | 1.561 | +1 | 8 |
| - | - | 1644 | 1101 | - | - | 0 | - |
| - | - | 805 | 1178 | - | - | 0 | - |
| - | - | 640 | 1220 | - | - | 0 | - |
| - | - | 739.4 | 1325 | - | - | 0 | - |
| - | - | 1351 | 1341 | - | - | 0 | - |
| - | - | 727.2 | 1342 | - | - | 0 | - |
| - | - | 667.2 | 1343 | - | - | 0 | - |
| - | - | 793.5 | 1387 | - | - | 0 | - |
| - | - | 640.8 | 2239 | - | - | 0 | - |
| - | - | 621.3 | 3007 | - | - | 0 | - |
| - | - | 706.5 | 3083 | - | - | 0 | - |
| - | - | 736.6 | 3084 | - | - | 0 | - |

m/z Charge Intensity FragmentType MassShift Position
120.0810775756836 0 74825.69
121.07963562011719 0 760.59607
121.0843734741211 0 6622.862
122.08763885498047 0 524.7402
122.54342651367188 0 355.2934
123.04446411132812 0 937.8296
127.08678436279297 0 464.88043
129.06622314453125 0 689.5675
129.1024932861328 0 7612.9683
130.06544494628906 0 2187.7705
130.1060791015625 0 591.957
132.0810089111328 0 2819.6099
132.10191345214844 0 867.5407
133.0860595703125 0 1193.0459
136.0759735107422 0 30910.54
137.07937622070312 0 2776.1365
138.05543518066406 0 685.0808
138.06640625 0 1118.3257
141.92776489257812 0 474.72717
143.04627990722656 0 448.52484
144.08108520507812 0 876.7502
146.06028747558594 0 5361.3223
147.0443115234375 0 1137.6934
147.06393432617188 0 1042.0813
148.88705444335938 0 645.76886
148.89427185058594 0 491.92996
148.90174865722656 0 643.44
148.90902709960938 0 611.87714
148.91615295410156 0 963.5283
148.92298889160156 0 1190.9082
148.93060302734375 0 1329.0435
148.9380645751953 0 3216.1963
148.94590759277344 0 5391.2007
148.9626007080078 0 3706.0193
148.97032165527344 0 1854.2363
148.97735595703125 0 1121.4265
148.98477172851562 0 979.57355
148.9920196533203 0 870.8611
148.99908447265625 0 660.7506
149.00631713867188 0 554.0174
149.0138397216797 0 511.4777
149.02175903320312 0 529.6324
149.03555297851562 0 425.28384
149.04469299316406 0 1159.2622
155.1179962158203 0 427.8363
156.07696533203125 0 2040.6235
157.1342010498047 0 1340.2266
158.09669494628906 0 2965.566
159.0919189453125 0 8510.533
160.07569885253906 0 775.5916
160.09539794921875 0 1163.3433
160.1122589111328 0 1505.7382
165.05484008789062 0 8465.788
165.07716369628906 0 665.42255
165.1027374267578 0 530.53723
166.08636474609375 0 1577.4423
167.05567932128906 0 1767.2898
169.09759521484375 0 703.3233
170.0601806640625 0 768.2877
173.12869262695312 0 3968.0977
174.0552520751953 0 735.7778
175.08738708496094 0 694.34564
176.08270263671875 0 593.35864
176.1072235107422 0 7047.6416
177.10256958007812 0 4356.3384
177.1110076904297 0 916.468
178.1062774658203 0 844.27704
181.09707641601562 0 569.3438
182.0813751220703 0 16423.047
183.0848388671875 0 1306.2047
185.05593872070312 0 1003.5048
185.128662109375 0 1715.4496
186.09144592285156 0 697.56824
187.08726501464844 0 652.3694
187.14486694335938 0 763.931
188.07081604003906 0 28400.797
189.07432556152344 0 3588.8406
189.083251953125 0 480.27866
189.08689880371094 0 459.99008
191.1179962158203 0 1691.8612
193.10899353027344 0 1670.6501
195.0878448486328 0 772.4851
199.14369201660156 0 497.22894
200.13986206054688 0 941.7689
201.12347412109375 0 2961.0789
203.0663299560547 0 1168.0022
203.1178741455078 0 3031.9849 a Water loss 1
204.07701110839844 0 1183.024
205.09732055664062 0 10779.662 y 8
206.10086059570312 0 1691.1487
214.1192169189453 0 749.0025
217.0974578857422 0 1440.784
219.113037109375 0 774.94556
221.10348510742188 0 5433.93
221.128662109375 0 83697.1 a 1
222.1064453125 0 941.0348
222.12191772460938 0 751.05615
222.13201904296875 0 9169.681
223.06394958496094 0 1493.6649
223.13414001464844 0 681.68536
224.63760375976562 0 503.66226
225.85354614257812 0 477.96643
226.1544952392578 0 765.889
229.11915588378906 0 712.93756
229.1718292236328 0 500.56186
231.06143188476562 0 1941.256
231.09791564941406 0 637.337
231.1128387451172 0 1469.3253 b Water loss 1
233.09207153320312 0 2241.3352
233.12884521484375 0 1552.7098
233.15052795410156 0 827.29083
233.22940063476562 0 538.9385
235.1080780029297 0 2433.3772
237.13531494140625 0 640.8737
239.09564208984375 0 927.1436
239.1146697998047 0 815.6695
239.1500244140625 0 1283.7881
241.0742950439453 0 981.1117
249.12362670898438 0 13681.495 b 1
250.12677001953125 0 1980.6306
251.1028594970703 0 2137.5713
251.15045166015625 0 898.6455
253.11892700195312 0 771.69116
253.16542053222656 0 1057.6295
257.1064147949219 0 941.9957
257.1636047363281 0 559.2632
261.12408447265625 0 1398.5833
263.1026916503906 0 2791.0024
274.12921142578125 0 632.78656
279.097900390625 0 2062.9368
282.0733642578125 0 581.42426
284.19696044921875 0 598.9029
285.0096435546875 0 958.7087
285.102294921875 0 1603.6631
292.14013671875 0 1050.6718
296.15057373046875 0 1589.5886
318.1456604003906 0 738.38727 a Water loss 2
319.1406555175781 0 1202.5692
321.15643310546875 0 682.01855
324.1455078125 0 3609.6624
325.1466979980469 0 717.17334
329.1498718261719 0 10697.475
330.1529846191406 0 2242.4946
340.1976013183594 0 1812.782
342.15631103515625 0 18146.129 y 7
342.17938232421875 0 1130.6898
343.1593933105469 0 3446.9165
346.1383972167969 0 1294.2783 b Water loss 2
349.4438781738281 0 554.20886
350.1356506347656 0 1092.267
352.16522216796875 0 986.74945
355.0696105957031 0 1284.3989
356.0703430175781 0 673.05475
356.13836669921875 0 1163.2494
357.22430419921875 0 2457.7888
364.1502380371094 0 3298.979 b 2
365.1522521972656 0 527.68
366.1318664550781 0 630.059
366.1788635253906 0 608.3415
368.19207763671875 0 842.69257
378.1294860839844 0 2795.4949
394.12469482421875 0 2904.7056
398.2403869628906 0 1012.112
401.7113952636719 0 728.5161
408.1669921875 0 709.70416
413.2662353515625 0 1101.8892
414.2691650390625 0 661.485
422.7315979003906 0 794.81476
423.2313232421875 0 612.64325
425.19342041015625 0 2174.502
427.7229309082031 0 1050.3813
433.2784423828125 0 1082.2023
436.72760009765625 0 628.99384
437.2298889160156 0 954.4045
455.1998596191406 0 820.5557
465.16162109375 0 2181.1929
479.17803955078125 0 2218.1455 b 3
480.1819763183594 0 923.6663
480.7956848144531 0 592.6813
483.2198791503906 0 729.08966
489.1918640136719 0 5124.308 y 6
490.1963806152344 0 1371.2264
496.231201171875 0 1136.0952
497.2349853515625 0 693.8341
499.67803955078125 0 625.3261
501.2513122558594 0 692.42896
513.3526611328125 0 742.5673
540.2152099609375 0 624.597
547.10205078125 0 639.99725
547.2503051757812 0 1236.1854
550.71142578125 0 1748.3896 y 1
551.21142578125 0 1693.643
551.7146606445312 0 1020.2867
557.1973876953125 0 655.0757
560.228271484375 0 6551.6577 y 5
561.2307739257812 0 2376.007
562.2269287109375 0 735.0182
569.2363891601562 0 691.0455
570.224365234375 0 577.17126
585.3350830078125 0 4209.502
586.3395385742188 0 1442.9493
592.228515625 0 1025.3196 Precursor Water loss
592.7317504882812 0 707.33093
601.235107421875 0 2982.3735 Precursor
601.3348999023438 0 629.0687
601.7377319335938 0 1891.8937
601.8200073242188 0 732.2514
612.1963500976562 0 1387.7468
614.24267578125 0 771.609
614.7567138671875 0 628.27606
624.2296142578125 0 1148.6498 b Water loss 4
628.3759765625 0 1020.86426
640.24072265625 0 605.66595
657.2596435546875 0 1074.2959
658.263671875 0 644.01294
659.29248046875 0 2634.085
670.8169555664062 0 5849.8486
671.318359375 0 6107.933
671.818359375 0 3015.292
672.320068359375 0 663.53125
682.3495483398438 0 656.44965
685.2571411132812 0 1523.9951
693.8124389648438 0 1390.0758
694.3053588867188 0 1200.5438
695.316162109375 0 683.7854
700.3618774414062 0 9676.276
701.3641967773438 0 4311.9946
721.2625732421875 0 1418.9855
723.2903442382812 0 5580.415 y 4
724.2950439453125 0 1888.9052
725.3018798828125 0 826.75714
730.3539428710938 0 1509.2874
730.8550415039062 0 1831.6211
744.350830078125 0 3412.2964
744.852294921875 0 3664.3735
745.3519897460938 0 1371.0907
749.2554931640625 0 2395.3867
750.2579956054688 0 883.1646
757.3824462890625 0 1015.9397
767.2647705078125 0 638.89655
774.3203735351562 0 2448.7825
775.3240356445312 0 1269.5634
783.3988037109375 0 2833.4917
784.4013061523438 0 1386.6251
794.3824462890625 0 2815.9001
795.385986328125 0 1344.6469
800.4276733398438 0 1387.0732
801.4111938476562 0 4901.196
802.4136352539062 0 2528.6985
828.4185791015625 0 2811.206
829.419921875 0 792.17456
838.3176879882812 0 7332.0864 y 3
839.3213500976562 0 3518.31
840.3238525390625 0 1201.4911
844.4503784179688 0 6470.7227
845.4526977539062 0 3356.4448
846.4612426757812 0 664.6456
854.4331665039062 0 926.9094
855.4400024414062 0 714.722
862.3141479492188 0 616.46576
871.3304443359375 0 837.1832
871.414306640625 0 983.4592
872.4462280273438 0 26415.834
873.44873046875 0 13626.743
874.451416015625 0 3138.4548
889.3466796875 0 8884.795
890.349609375 0 6131.971
891.3570556640625 0 1067.3441
896.32763671875 0 814.19745
909.4090576171875 0 2245.028
910.4092407226562 0 1000.9867
935.3353271484375 0 1995.5905 y Water loss 2
936.3323974609375 0 1451.911
953.3443603515625 0 32441.254 y 2
954.3469848632812 0 17762.115
955.347412109375 0 6996.055
991.5183715820312 0 5202.6807
992.519775390625 0 1935.9857
1019.5133056640625 0 11717.781
1020.515869140625 0 7032.5596
1021.5178833007812 0 2884.462
1036.4190673828125 0 750.0618
1037.5079345703125 0 809.18756
1038.513427734375 0 655.7966
1056.4842529296875 0 768.11694
1100.4110107421875 0 1784.5399 y 1
1101.41650390625 0 1643.7509
1177.568359375 0 805.03235
1220.1669921875 0 639.9993
1324.6290283203125 0 739.3899
1340.6229248046875 0 1350.9779
1341.6287841796875 0 727.211
1342.593017578125 0 667.2108
1386.580810546875 0 793.5208
2238.658447265625 0 640.8371
3007.208740234375 0 621.2717
3083.005615234375 0 706.52844
3083.75537109375 0 736.63104

Spectrum Details

|  |  |
| --- | --- |
| Matched peaks? Matched peaksThe total absolute number of peaks matched. Additionally in brackets the total fraction of peaks matched and the total number of peaks is shown. | 21 (7.05% of 298) |
| FDR? FDRThe false discovery rate estimated for this peptide. It is calculated by matching all theoretical fragments with a non-integer shift with the raw peaks for this spectrum. This is done with 40 different shifts. The resulting percentage is the average number of annotated peaks over the number of annotated peaks with the correct spectrum. | 1.47% |
| Satellite FDR? Satellite FDRSee the FDR for details on its calculation. This satellite ion specific FDR only contains the satellite ions (d/w) for I/L/J positions. | - |
| PSM Score? PSM ScoreThe PSM Score as given by Hecklib to this annotated spectrum. It is shown with three significant figures. | 250 |

## Spectrum 10402? Spectrum 10402 The raw spectrum of this peptide as annotated by Hecklib. The fragments are coloured according to ion type (see legend). Any peaks with a star '\*' as text can be hovered over to see the full details, first the ion type second the mass shift type. By hovering over the amino acids in the peptide or ions in the legend the corresponding peaks are highlighted. By toggling the 'Unassigned' label you can turn the background (unassigned) peaks on or off in the plot. By updating the slider in the Ion legend you can update the spectrum to only show the top X% of the peaks with labels. The top X% means any peak that is within X% of the highest intensity. By dragging in the spectrum you can zoom in to a specific part of the spectrum and use 'Zoom Out' to get back to the original zoom level. The annotation of the spectrum is based on the given sequence in the peptides file and is done with different software so inconsistencies are likely. The peaks are annotated based on the given sequence, with 20 ppm tolerance.

Copy Data

### Spectrum 10402 (TSV)

#### Preview

```
Loading example...
```

*Click on the button to copy the data to your clipboard.*

Mz MinMz MaxIntensity Max

WidthHeightPeptide font sizePeptide stroke widthSpectrum font sizeSpectrum stroke widthCompact peptide

Ion legend

wxyz

abcd

OtherUnassignedIonChargePositionShow for top:%

TFDDYAMHW

02.10e+44.20e+46.30e+48.40e+4

Zoom Out

a+12y+11a+12b+12b+12a+13y+12b+13b+13b+14b+14y+13y+28y+14\*\*y+15y+16y+17y+17y+18

0698139520932790

Fragment Matches Table

Show background peaks

| Position | Ion type | Intensity | mz Theoretical | mz Error (Th) | mz Error (ppm) | Charge | Series Number |
| --- | --- | --- | --- | --- | --- | --- | --- |
| - | - | 528.4 | 120.1 | - | - | 0 | - |
| - | - | 7.228E+04 | 120.1 | - | - | 0 | - |
| - | - | 5420 | 121.1 | - | - | 0 | - |
| - | - | 694.8 | 123 | - | - | 0 | - |
| - | - | 8080 | 129.1 | - | - | 0 | - |
| - | - | 2503 | 130.1 | - | - | 0 | - |
| - | - | 397.7 | 130.1 | - | - | 0 | - |
| - | - | 396.9 | 130.1 | - | - | 0 | - |
| - | - | 2595 | 132.1 | - | - | 0 | - |
| - | - | 1083 | 132.1 | - | - | 0 | - |
| - | - | 1009 | 133.1 | - | - | 0 | - |
| - | - | 2.942E+04 | 136.1 | - | - | 0 | - |
| - | - | 2448 | 137.1 | - | - | 0 | - |
| - | - | 662.6 | 138.1 | - | - | 0 | - |
| - | - | 950.2 | 138.1 | - | - | 0 | - |
| - | - | 394.4 | 140.1 | - | - | 0 | - |
| - | - | 478.1 | 141.6 | - | - | 0 | - |
| - | - | 388.6 | 141.7 | - | - | 0 | - |
| - | - | 557.2 | 142.1 | - | - | 0 | - |
| - | - | 485.3 | 144.1 | - | - | 0 | - |
| - | - | 6072 | 146.1 | - | - | 0 | - |
| - | - | 1249 | 147 | - | - | 0 | - |
| - | - | 988.7 | 148.1 | - | - | 0 | - |
| - | - | 832.8 | 148.9 | - | - | 0 | - |
| - | - | 1362 | 149 | - | - | 0 | - |
| - | - | 397.9 | 149.7 | - | - | 0 | - |
| - | - | 451 | 154.2 | - | - | 0 | - |
| - | - | 496.2 | 155.1 | - | - | 0 | - |
| - | - | 1303 | 156.1 | - | - | 0 | - |
| - | - | 1435 | 157.1 | - | - | 0 | - |
| - | - | 2427 | 158.1 | - | - | 0 | - |
| - | - | 8936 | 159.1 | - | - | 0 | - |
| - | - | 1024 | 159.1 | - | - | 0 | - |
| - | - | 657.9 | 160.1 | - | - | 0 | - |
| - | - | 1177 | 160.1 | - | - | 0 | - |
| - | - | 1272 | 160.1 | - | - | 0 | - |
| - | - | 8156 | 165.1 | - | - | 0 | - |
| - | - | 442.3 | 165.1 | - | - | 0 | - |
| - | - | 839.1 | 166.1 | - | - | 0 | - |
| - | - | 1727 | 166.1 | - | - | 0 | - |
| - | - | 1360 | 167.1 | - | - | 0 | - |
| - | - | 539.9 | 167.1 | - | - | 0 | - |
| - | - | 550.9 | 169.1 | - | - | 0 | - |
| - | - | 649.4 | 170.1 | - | - | 0 | - |
| - | - | 520.5 | 173.1 | - | - | 0 | - |
| - | - | 5419 | 173.1 | - | - | 0 | - |
| - | - | 912.1 | 173.5 | - | - | 0 | - |
| - | - | 1421 | 175.1 | - | - | 0 | - |
| - | - | 744.7 | 175.1 | - | - | 0 | - |
| - | - | 501.8 | 176.1 | - | - | 0 | - |
| - | - | 5825 | 176.1 | - | - | 0 | - |
| - | - | 4576 | 177.1 | - | - | 0 | - |
| - | - | 791 | 177.1 | - | - | 0 | - |
| - | - | 1.552E+04 | 182.1 | - | - | 0 | - |
| - | - | 813.3 | 182.1 | - | - | 0 | - |
| - | - | 1101 | 183.1 | - | - | 0 | - |
| - | - | 533.2 | 185.1 | - | - | 0 | - |
| - | - | 1758 | 185.1 | - | - | 0 | - |
| - | - | 432.1 | 186.1 | - | - | 0 | - |
| - | - | 2.779E+04 | 188.1 | - | - | 0 | - |
| - | - | 3451 | 189.1 | - | - | 0 | - |
| - | - | 484.9 | 189.8 | - | - | 0 | - |
| - | - | 1519 | 191.1 | - | - | 0 | - |
| - | - | 1492 | 193.1 | - | - | 0 | - |
| - | - | 539.3 | 195.1 | - | - | 0 | - |
| - | - | 557 | 195.1 | - | - | 0 | - |
| - | - | 907.7 | 200.1 | - | - | 0 | - |
| - | - | 2484 | 201.1 | - | - | 0 | - |
| - | - | 595.1 | 202.1 | - | - | 0 | - |
| - | - | 493.5 | 203.1 | - | - | 0 | - |
| 2 | a | 2394 | 203.1 | 0.0002745 | 1.352 | +1 | 2 |
| - | - | 943.6 | 204.1 | - | - | 0 | - |
| 9 | y | 1.143E+04 | 205.1 | 0.0003648 | 1.779 | +1 | 1 |
| - | - | 1210 | 206.1 | - | - | 0 | - |
| - | - | 911.3 | 207.1 | - | - | 0 | - |
| - | - | 669.8 | 211.1 | - | - | 0 | - |
| - | - | 730.5 | 214.1 | - | - | 0 | - |
| - | - | 531.9 | 214.9 | - | - | 0 | - |
| - | - | 696.8 | 215.1 | - | - | 0 | - |
| - | - | 828.3 | 216.1 | - | - | 0 | - |
| - | - | 1165 | 217.1 | - | - | 0 | - |
| - | - | 500.7 | 218.7 | - | - | 0 | - |
| - | - | 1029 | 219.1 | - | - | 0 | - |
| - | - | 4260 | 221.1 | - | - | 0 | - |
| 2 | a | 8.316E+04 | 221.1 | 0.0003605 | 1.63 | +1 | 2 |
| - | - | 595.1 | 222.1 | - | - | 0 | - |
| - | - | 8928 | 222.1 | - | - | 0 | - |
| - | - | 1513 | 223.1 | - | - | 0 | - |
| - | - | 539.1 | 223.1 | - | - | 0 | - |
| - | - | 485.2 | 224.1 | - | - | 0 | - |
| - | - | 604.7 | 225.1 | - | - | 0 | - |
| - | - | 1174 | 225.2 | - | - | 0 | - |
| - | - | 558.9 | 226.1 | - | - | 0 | - |
| - | - | 796.7 | 227.1 | - | - | 0 | - |
| - | - | 545 | 228.1 | - | - | 0 | - |
| - | - | 1321 | 231.1 | - | - | 0 | - |
| 2 | b | 1103 | 231.1 | 0.0002706 | 1.171 | +1 | 2 |
| - | - | 1092 | 233.1 | - | - | 0 | - |
| - | - | 1583 | 233.1 | - | - | 0 | - |
| - | - | 605.4 | 233.1 | - | - | 0 | - |
| - | - | 3151 | 235.1 | - | - | 0 | - |
| - | - | 545.6 | 239.1 | - | - | 0 | - |
| - | - | 1825 | 239.2 | - | - | 0 | - |
| - | - | 641.7 | 241.6 | - | - | 0 | - |
| - | - | 484 | 243.1 | - | - | 0 | - |
| - | - | 817.7 | 249.1 | - | - | 0 | - |
| 2 | b | 1.525E+04 | 249.1 | 0.0004105 | 1.648 | +1 | 2 |
| - | - | 2221 | 250.1 | - | - | 0 | - |
| - | - | 1442 | 251.1 | - | - | 0 | - |
| - | - | 497 | 251.1 | - | - | 0 | - |
| - | - | 743.6 | 252.1 | - | - | 0 | - |
| - | - | 671.8 | 253.1 | - | - | 0 | - |
| - | - | 1515 | 253.2 | - | - | 0 | - |
| - | - | 800.2 | 256.2 | - | - | 0 | - |
| - | - | 1040 | 257.1 | - | - | 0 | - |
| - | - | 1458 | 261.1 | - | - | 0 | - |
| - | - | 3714 | 263.1 | - | - | 0 | - |
| - | - | 505.4 | 268 | - | - | 0 | - |
| - | - | 2262 | 279.1 | - | - | 0 | - |
| - | - | 514.9 | 280.2 | - | - | 0 | - |
| - | - | 1362 | 285 | - | - | 0 | - |
| - | - | 594.4 | 285.1 | - | - | 0 | - |
| - | - | 1404 | 285.1 | - | - | 0 | - |
| - | - | 805.3 | 292.1 | - | - | 0 | - |
| - | - | 515.3 | 294.9 | - | - | 0 | - |
| - | - | 1884 | 296.2 | - | - | 0 | - |
| - | - | 809.4 | 302.1 | - | - | 0 | - |
| - | - | 1229 | 304.1 | - | - | 0 | - |
| - | - | 628 | 315.2 | - | - | 0 | - |
| 3 | a | 652.8 | 318.1 | 0.001072 | 3.369 | +1 | 3 |
| - | - | 1300 | 319.1 | - | - | 0 | - |
| - | - | 731.4 | 323.1 | - | - | 0 | - |
| - | - | 2614 | 324.1 | - | - | 0 | - |
| - | - | 1019 | 325.1 | - | - | 0 | - |
| - | - | 9764 | 329.2 | - | - | 0 | - |
| - | - | 2147 | 330.2 | - | - | 0 | - |
| - | - | 758.2 | 335.2 | - | - | 0 | - |
| - | - | 636.5 | 336.2 | - | - | 0 | - |
| - | - | 689 | 337.2 | - | - | 0 | - |
| - | - | 2320 | 340.2 | - | - | 0 | - |
| 8 | y | 1.642E+04 | 342.2 | 0.0005808 | 1.697 | +1 | 2 |
| - | - | 1107 | 342.2 | - | - | 0 | - |
| - | - | 2873 | 343.2 | - | - | 0 | - |
| 3 | b | 685.8 | 346.1 | 0.0009694 | 2.8 | +1 | 3 |
| - | - | 905.1 | 350.1 | - | - | 0 | - |
| - | - | 1030 | 352.2 | - | - | 0 | - |
| - | - | 1295 | 355.1 | - | - | 0 | - |
| - | - | 663.4 | 356.1 | - | - | 0 | - |
| - | - | 1949 | 357.2 | - | - | 0 | - |
| 3 | b | 3490 | 364.2 | 0.0006281 | 1.725 | +1 | 3 |
| - | - | 839.3 | 365.2 | - | - | 0 | - |
| - | - | 1082 | 368.2 | - | - | 0 | - |
| - | - | 625.7 | 374.2 | - | - | 0 | - |
| - | - | 2142 | 378.1 | - | - | 0 | - |
| - | - | 627.3 | 390.2 | - | - | 0 | - |
| - | - | 1110 | 392.2 | - | - | 0 | - |
| - | - | 2469 | 394.1 | - | - | 0 | - |
| - | - | 554.5 | 401.2 | - | - | 0 | - |
| - | - | 680.4 | 401.7 | - | - | 0 | - |
| - | - | 626.7 | 410.8 | - | - | 0 | - |
| - | - | 1197 | 413.3 | - | - | 0 | - |
| - | - | 2511 | 425.2 | - | - | 0 | - |
| - | - | 967.5 | 427.7 | - | - | 0 | - |
| - | - | 634.2 | 428.2 | - | - | 0 | - |
| - | - | 549 | 436.7 | - | - | 0 | - |
| - | - | 729 | 437.2 | - | - | 0 | - |
| 4 | b | 759.8 | 461.2 | 0.0007597 | 1.647 | +1 | 4 |
| - | - | 1764 | 465.2 | - | - | 0 | - |
| 4 | b | 2160 | 479.2 | 0.0008152 | 1.701 | +1 | 4 |
| - | - | 826.5 | 480.2 | - | - | 0 | - |
| - | - | 839 | 483.2 | - | - | 0 | - |
| - | - | 636 | 487.1 | - | - | 0 | - |
| - | - | 681.6 | 487.3 | - | - | 0 | - |
| 7 | y | 3419 | 489.2 | 0.006076 | 12.42 | +1 | 3 |
| - | - | 1060 | 490.2 | - | - | 0 | - |
| - | - | 1754 | 496.2 | - | - | 0 | - |
| - | - | 704.5 | 510.3 | - | - | 0 | - |
| - | - | 881.6 | 519.2 | - | - | 0 | - |
| - | - | 1153 | 539.2 | - | - | 0 | - |
| - | - | 902.7 | 547.3 | - | - | 0 | - |
| - | - | 963.9 | 548.2 | - | - | 0 | - |
| 2 | y | 2251 | 550.7 | 0.00369 | 6.701 | +2 | 8 |
| - | - | 1651 | 551.2 | - | - | 0 | - |
| - | - | 665.3 | 559.3 | - | - | 0 | - |
| 6 | y | 6158 | 560.2 | 0.00534 | 9.531 | +1 | 4 |
| - | - | 2002 | 561.2 | - | - | 0 | - |
| - | - | 576.7 | 579.8 | - | - | 0 | - |
| - | - | 2376 | 585.3 | - | - | 0 | - |
| - | - | 1070 | 586.3 | - | - | 0 | - |
| - | - | 741.3 | 589.3 | - | - | 0 | - |
| 0 | Precursor | 1350 | 592.2 | 0.003627 | 6.125 | +2 | -1 |
| 0 | Precursor | 3811 | 601.2 | 0.003472 | 5.774 | +2 | -1 |
| - | - | 798.4 | 601.3 | - | - | 0 | - |
| - | - | 1646 | 601.7 | - | - | 0 | - |
| - | - | 1301 | 612.2 | - | - | 0 | - |
| - | - | 675.4 | 614.2 | - | - | 0 | - |
| - | - | 947.4 | 628.4 | - | - | 0 | - |
| - | - | 864.1 | 651.4 | - | - | 0 | - |
| - | - | 1461 | 657.3 | - | - | 0 | - |
| - | - | 1961 | 659.3 | - | - | 0 | - |
| - | - | 1073 | 660.3 | - | - | 0 | - |
| - | - | 7254 | 670.8 | - | - | 0 | - |
| - | - | 3637 | 671.3 | - | - | 0 | - |
| - | - | 702.7 | 671.4 | - | - | 0 | - |
| - | - | 2367 | 671.8 | - | - | 0 | - |
| - | - | 897.1 | 672.3 | - | - | 0 | - |
| - | - | 2344 | 685.3 | - | - | 0 | - |
| - | - | 1148 | 686.3 | - | - | 0 | - |
| - | - | 1553 | 693.8 | - | - | 0 | - |
| - | - | 1249 | 694.3 | - | - | 0 | - |
| - | - | 646.8 | 696.3 | - | - | 0 | - |
| - | - | 9650 | 700.4 | - | - | 0 | - |
| - | - | 3366 | 701.4 | - | - | 0 | - |
| - | - | 1202 | 702.4 | - | - | 0 | - |
| - | - | 1524 | 721.3 | - | - | 0 | - |
| - | - | 1012 | 722.4 | - | - | 0 | - |
| 5 | y | 6350 | 723.3 | 0.004755 | 6.574 | +1 | 5 |
| - | - | 2091 | 724.3 | - | - | 0 | - |
| - | - | 1654 | 730.4 | - | - | 0 | - |
| - | - | 1476 | 730.9 | - | - | 0 | - |
| - | - | 1167 | 731.4 | - | - | 0 | - |
| - | - | 4143 | 744.4 | - | - | 0 | - |
| - | - | 2368 | 744.9 | - | - | 0 | - |
| - | - | 1599 | 745.4 | - | - | 0 | - |
| - | - | 2821 | 749.3 | - | - | 0 | - |
| - | - | 705.7 | 754.9 | - | - | 0 | - |
| - | - | 846.8 | 757.4 | - | - | 0 | - |
| - | - | 706.6 | 768.3 | - | - | 0 | - |
| - | - | 2300 | 774.3 | - | - | 0 | - |
| - | - | 1063 | 775.3 | - | - | 0 | - |
| - | - | 3082 | 783.4 | - | - | 0 | - |
| - | - | 1322 | 784.4 | - | - | 0 | - |
| - | - | 3670 | 794.4 | - | - | 0 | - |
| - | - | 1748 | 795.4 | - | - | 0 | - |
| - | - | 1822 | 800.4 | - | - | 0 | - |
| - | - | 5687 | 801.4 | - | - | 0 | - |
| - | - | 2161 | 802.4 | - | - | 0 | - |
| - | - | 1814 | 828.4 | - | - | 0 | - |
| - | - | 889.4 | 829.4 | - | - | 0 | - |
| 4 | y | 7480 | 838.3 | 0.004973 | 5.932 | +1 | 6 |
| - | - | 3211 | 839.3 | - | - | 0 | - |
| - | - | 1151 | 840.3 | - | - | 0 | - |
| - | - | 8452 | 844.5 | - | - | 0 | - |
| - | - | 2167 | 845.5 | - | - | 0 | - |
| - | - | 834.6 | 846.5 | - | - | 0 | - |
| - | - | 1187 | 854.4 | - | - | 0 | - |
| - | - | 824.9 | 855.4 | - | - | 0 | - |
| - | - | 1446 | 871.3 | - | - | 0 | - |
| - | - | 2.648E+04 | 872.4 | - | - | 0 | - |
| - | - | 1.258E+04 | 873.5 | - | - | 0 | - |
| - | - | 4349 | 874.5 | - | - | 0 | - |
| - | - | 9290 | 889.3 | - | - | 0 | - |
| - | - | 4976 | 890.4 | - | - | 0 | - |
| - | - | 1106 | 891.4 | - | - | 0 | - |
| - | - | 788.6 | 896.5 | - | - | 0 | - |
| - | - | 2530 | 909.4 | - | - | 0 | - |
| - | - | 1626 | 910.4 | - | - | 0 | - |
| 3 | y | 1289 | 935.3 | 0.003487 | 3.728 | +1 | 7 |
| - | - | 723.3 | 936.3 | - | - | 0 | - |
| 3 | y | 3.01E+04 | 953.3 | 0.004885 | 5.124 | +1 | 7 |
| - | - | 1.588E+04 | 954.3 | - | - | 0 | - |
| - | - | 5985 | 955.3 | - | - | 0 | - |
| - | - | 4260 | 991.5 | - | - | 0 | - |
| - | - | 2378 | 992.5 | - | - | 0 | - |
| - | - | 644.1 | 1006 | - | - | 0 | - |
| - | - | 1.172E+04 | 1020 | - | - | 0 | - |
| - | - | 6955 | 1021 | - | - | 0 | - |
| - | - | 2403 | 1022 | - | - | 0 | - |
| - | - | 1016 | 1038 | - | - | 0 | - |
| 2 | y | 2195 | 1100 | 0.005014 | 4.556 | +1 | 8 |
| - | - | 789.2 | 1101 | - | - | 0 | - |
| - | - | 691.3 | 1178 | - | - | 0 | - |
| - | - | 891 | 1179 | - | - | 0 | - |
| - | - | 796 | 1325 | - | - | 0 | - |
| - | - | 935.3 | 1326 | - | - | 0 | - |
| - | - | 977.5 | 1341 | - | - | 0 | - |
| - | - | 761.7 | 1388 | - | - | 0 | - |
| - | - | 725 | 1691 | - | - | 0 | - |
| - | - | 599.4 | 1743 | - | - | 0 | - |
| - | - | 663.6 | 2454 | - | - | 0 | - |
| - | - | 668.1 | 2763 | - | - | 0 | - |

m/z Charge Intensity FragmentType MassShift Position
120.05274963378906 0 528.3845
120.08113098144531 0 72283.88
121.08444213867188 0 5420.418
123.04449462890625 0 694.8314
129.10255432128906 0 8079.9556
130.06546020507812 0 2502.8594
130.08653259277344 0 397.6913
130.10586547851562 0 396.9001
132.0811004638672 0 2595.2246
132.10231018066406 0 1082.6255
133.0862274169922 0 1008.84467
136.07601928710938 0 29422.805
137.07936096191406 0 2447.597
138.05517578125 0 662.6209
138.06666564941406 0 950.1739
140.08758544921875 0 394.40488
141.6322479248047 0 478.147
141.73451232910156 0 388.62628
142.0651092529297 0 557.1789
144.08099365234375 0 485.2975
146.06036376953125 0 6072.176
147.04452514648438 0 1248.9517
148.07606506347656 0 988.68085
148.947998046875 0 832.76105
149.04525756835938 0 1362.4324
149.69105529785156 0 397.88406
154.24655151367188 0 450.9836
155.0819091796875 0 496.1757
156.07687377929688 0 1302.9392
157.1336212158203 0 1435.4164
158.09664916992188 0 2427.0198
159.09202575683594 0 8936.187
159.09890747070312 0 1024.0952
160.0762481689453 0 657.9106
160.0948944091797 0 1177.3562
160.1123046875 0 1271.7322
165.05491638183594 0 8156.28
165.10301208496094 0 442.3448
166.0581512451172 0 839.14166
166.08644104003906 0 1726.8243
167.05564880371094 0 1360.4402
167.09288024902344 0 539.87634
169.07603454589844 0 550.8842
170.0604705810547 0 649.37164
173.09291076660156 0 520.5413
173.1288299560547 0 5418.854
173.4529571533203 0 912.0618
175.08685302734375 0 1421.4373
175.1190948486328 0 744.69147
176.08245849609375 0 501.8116
176.10736083984375 0 5824.5522
177.10247802734375 0 4575.506
177.1109619140625 0 791.033
182.08157348632812 0 15517.532
182.0906982421875 0 813.3195
183.08505249023438 0 1101.259
185.05615234375 0 533.2248
185.12864685058594 0 1758.3844
186.09120178222656 0 432.06287
188.0709228515625 0 27792.283
189.07440185546875 0 3450.7537
189.8454132080078 0 484.87366
191.1185760498047 0 1518.7468
193.10879516601562 0 1492.2798
195.0874481201172 0 539.3215
195.11257934570312 0 556.99
200.13954162597656 0 907.6705
201.1234130859375 0 2484.2866
202.05197143554688 0 595.08496
203.06784057617188 0 493.50003
203.1181640625 0 2394.265 a Water loss 1
204.07745361328125 0 943.59393
205.09751892089844 0 11434.23 y 8
206.10116577148438 0 1210.4684
207.1129913330078 0 911.27277
211.06040954589844 0 669.77386
214.11880493164062 0 730.528
214.88795471191406 0 531.8751
215.13987731933594 0 696.8246
216.06533813476562 0 828.3242
217.0972900390625 0 1164.5037
218.7314910888672 0 500.73312
219.1132354736328 0 1029.3645
221.10365295410156 0 4259.7983
221.12881469726562 0 83159.5 a 1
222.1060791015625 0 595.061
222.13217163085938 0 8927.736
223.06358337402344 0 1513.0472
223.13572692871094 0 539.11383
224.13980102539062 0 485.23343
225.1227264404297 0 604.712
225.1717529296875 0 1173.8484
226.11996459960938 0 558.90546
227.1028289794922 0 796.657
228.1125030517578 0 545.018
231.0616455078125 0 1320.6053
231.11253356933594 0 1103.0365 b Water loss 1
233.0924072265625 0 1091.7612
233.129150390625 0 1583.0413
233.14979553222656 0 605.41144
235.1080322265625 0 3151.181
239.0836639404297 0 545.58124
239.15052795410156 0 1825.4329
241.58250427246094 0 641.678
243.11087036132812 0 483.9956
249.10897827148438 0 817.7079
249.123779296875 0 15247.668 b 1
250.1268310546875 0 2221.3718
251.10305786132812 0 1442.2742
251.14939880371094 0 496.98138
252.13380432128906 0 743.5798
253.11874389648438 0 671.7779
253.1660614013672 0 1514.731
256.17706298828125 0 800.20776
257.1069030761719 0 1040.4934
261.1233825683594 0 1458.0654
263.1028747558594 0 3713.7124
267.95928955078125 0 505.4244
279.09771728515625 0 2261.883
280.16400146484375 0 514.8922
285.0101013183594 0 1361.8192
285.08544921875 0 594.3824
285.1020812988281 0 1403.9703
292.1411437988281 0 805.3313
294.921142578125 0 515.2848
296.1507873535156 0 1884.0399
302.11407470703125 0 809.4395
304.1287841796875 0 1229.244
315.167236328125 0 628.04663
318.1459045410156 0 652.75806 a Water loss 2
319.1400451660156 0 1300.4761
323.09832763671875 0 731.44885
324.1459045410156 0 2614.4216
325.1480712890625 0 1019.0516
329.1501159667969 0 9764.449
330.1533508300781 0 2147.1729
335.1808776855469 0 758.1664
336.18145751953125 0 636.5052
337.1866149902344 0 689.022
340.1982421875 0 2319.8186
342.1566467285156 0 16416.9 y 7
342.17962646484375 0 1107.1902
343.1593322753906 0 2872.6091
346.1407165527344 0 685.75616 b Water loss 2
350.13433837890625 0 905.10504
352.1661071777344 0 1030.4681
355.07025146484375 0 1294.9414
356.1387023925781 0 663.3715
357.2247619628906 0 1949.4021
364.15093994140625 0 3490.025 b 2
365.1542663574219 0 839.34576
368.1927795410156 0 1082.0171
374.15081787109375 0 625.6587
378.1298828125 0 2142.2017
390.15673828125 0 627.2715
392.20428466796875 0 1109.9601
394.12457275390625 0 2468.5225
401.20745849609375 0 554.49805
401.71282958984375 0 680.402
410.8466491699219 0 626.7346
413.2677307128906 0 1196.8252
425.1930236816406 0 2511.1375
427.7223815917969 0 967.4936
428.223876953125 0 634.2296
436.727294921875 0 549.02264
437.2280578613281 0 729.0428
461.1674499511719 0 759.8383 b Water loss 3
465.162841796875 0 1763.8226
479.1780700683594 0 2160.253 b 3
480.1837463378906 0 826.4528
483.2218017578125 0 838.9502
487.1086120605469 0 636.0113
487.33331298828125 0 681.57
489.192626953125 0 3418.6392 y 6
490.1949157714844 0 1059.9303
496.2297668457031 0 1754.1301
510.2618713378906 0 704.46735
519.2019653320312 0 881.6235
539.217041015625 0 1152.7689
547.25244140625 0 902.6691
548.1970825195312 0 963.91846
550.7119750976562 0 2251.4963 y 1
551.21240234375 0 1651.2352
559.2646484375 0 665.2907
560.22900390625 0 6157.582 y 5
561.2323608398438 0 2002.11
579.794921875 0 576.72424
585.3348999023438 0 2376.4978
586.338623046875 0 1070.0841
589.2849731445312 0 741.25653
592.23046875 0 1349.8389 Precursor Water loss
601.235595703125 0 3811.3972 Precursor
601.3343505859375 0 798.39105
601.738037109375 0 1645.5261
612.1961669921875 0 1300.6646
614.244384765625 0 675.41174
628.3781127929688 0 947.407
651.3648681640625 0 864.133
657.2647094726562 0 1460.7903
659.2937622070312 0 1960.5596
660.2958374023438 0 1072.5854
670.8173217773438 0 7253.5312
671.3195190429688 0 3637.4927
671.4031982421875 0 702.6588
671.819580078125 0 2366.8293
672.319580078125 0 897.11774
685.2590942382812 0 2343.6028
686.2600708007812 0 1147.5306
693.8112182617188 0 1552.8441
694.315185546875 0 1249.0684
696.3206787109375 0 646.77325
700.3623657226562 0 9649.598
701.3651123046875 0 3366.1868
702.3687744140625 0 1202.4656
721.26513671875 0 1524.0164
722.40087890625 0 1012.3358
723.291748046875 0 6350.138 y 4
724.2950439453125 0 2091.479
730.353759765625 0 1654.1277
730.8547973632812 0 1476.0222
731.3551635742188 0 1166.9066
744.351318359375 0 4142.8105
744.8533325195312 0 2368.1338
745.3521728515625 0 1599.4075
749.2551879882812 0 2821.3032
754.8512573242188 0 705.72156
757.3843994140625 0 846.8036
768.336669921875 0 706.55444
774.31982421875 0 2299.9895
775.3220825195312 0 1063.4358
783.399658203125 0 3082.0486
784.3994750976562 0 1321.6467
794.3839111328125 0 3670.3308
795.3856811523438 0 1747.9928
800.4281616210938 0 1822.284
801.41259765625 0 5686.5625
802.4133911132812 0 2161.1755
828.4200439453125 0 1813.7668
829.4193725585938 0 889.39844
838.3189086914062 0 7480.1587 y 3
839.3217163085938 0 3210.8767
840.3141479492188 0 1150.6272
844.45263671875 0 8451.682
845.4541625976562 0 2166.8442
846.4559326171875 0 834.59564
854.4364013671875 0 1186.7605
855.4330444335938 0 824.8736
871.3399047851562 0 1445.5566
872.4476318359375 0 26481.18
873.4501342773438 0 12581.717
874.45361328125 0 4349.2827
889.3473510742188 0 9289.597
890.3502197265625 0 4976.3765
891.3535766601562 0 1106.0988
896.52490234375 0 788.5963
909.4109497070312 0 2530.1116
910.4146728515625 0 1625.7242
935.3338012695312 0 1288.8478 y Water loss 2
936.3380126953125 0 723.33575
953.3457641601562 0 30104.13 y 2
954.3482666015625 0 15883.617
955.3494873046875 0 5984.744
991.5211791992188 0 4260.4575
992.5239868164062 0 2377.7476
1006.4817504882812 0 644.06494
1019.5150146484375 0 11715.347
1020.5181274414062 0 6954.882
1021.5191040039062 0 2403.1746
1037.50927734375 0 1016.1239
1100.414306640625 0 2195.4097 y 1
1101.4200439453125 0 789.1866
1177.5618896484375 0 691.29736
1178.5701904296875 0 891.00305
1324.6229248046875 0 796.02985
1325.6304931640625 0 935.32733
1340.61962890625 0 977.45764
1387.629638671875 0 761.7111
1691.113037109375 0 725.02747
1743.4324951171875 0 599.4033
2454.062744140625 0 663.6124
2762.576416015625 0 668.1058

Spectrum Details

|  |  |
| --- | --- |
| Matched peaks? Matched peaksThe total absolute number of peaks matched. Additionally in brackets the total fraction of peaks matched and the total number of peaks is shown. | 21 (7.47% of 281) |
| FDR? FDRThe false discovery rate estimated for this peptide. It is calculated by matching all theoretical fragments with a non-integer shift with the raw peaks for this spectrum. This is done with 40 different shifts. The resulting percentage is the average number of annotated peaks over the number of annotated peaks with the correct spectrum. | 1.36% |
| Satellite FDR? Satellite FDRSee the FDR for details on its calculation. This satellite ion specific FDR only contains the satellite ions (d/w) for I/L/J positions. | - |
| PSM Score? PSM ScoreThe PSM Score as given by Hecklib to this annotated spectrum. It is shown with three significant figures. | 250 |

## Spectrum 10187? Spectrum 10187 The raw spectrum of this peptide as annotated by Hecklib. The fragments are coloured according to ion type (see legend). Any peaks with a star '\*' as text can be hovered over to see the full details, first the ion type second the mass shift type. By hovering over the amino acids in the peptide or ions in the legend the corresponding peaks are highlighted. By toggling the 'Unassigned' label you can turn the background (unassigned) peaks on or off in the plot. By updating the slider in the Ion legend you can update the spectrum to only show the top X% of the peaks with labels. The top X% means any peak that is within X% of the highest intensity. By dragging in the spectrum you can zoom in to a specific part of the spectrum and use 'Zoom Out' to get back to the original zoom level. The annotation of the spectrum is based on the given sequence in the peptides file and is done with different software so inconsistencies are likely. The peaks are annotated based on the given sequence, with 20 ppm tolerance.

Copy Data

### Spectrum 10187 (TSV)

#### Preview

```
Loading example...
```

*Click on the button to copy the data to your clipboard.*

Mz MinMz MaxIntensity Max

WidthHeightPeptide font sizePeptide stroke widthSpectrum font sizeSpectrum stroke widthCompact peptide

Ion legend

wxyz

abcd

OtherUnassignedIonChargePositionShow for top:%

TFDDYAMHW

02.20e+44.40e+46.60e+48.80e+4

Zoom Out

a+12y+11a+12b+12b+12y+12b+13b+14y+13y+28y+14\*\*y+15y+16y+17y+17y+18

0837167325103347

Fragment Matches Table

Show background peaks

| Position | Ion type | Intensity | mz Theoretical | mz Error (Th) | mz Error (ppm) | Charge | Series Number |
| --- | --- | --- | --- | --- | --- | --- | --- |
| - | - | 7.869E+04 | 120.1 | - | - | 0 | - |
| - | - | 570.1 | 121.1 | - | - | 0 | - |
| - | - | 5937 | 121.1 | - | - | 0 | - |
| - | - | 341.5 | 121.2 | - | - | 0 | - |
| - | - | 1364 | 123 | - | - | 0 | - |
| - | - | 430.3 | 127.1 | - | - | 0 | - |
| - | - | 606.7 | 129.1 | - | - | 0 | - |
| - | - | 5627 | 129.1 | - | - | 0 | - |
| - | - | 1614 | 130.1 | - | - | 0 | - |
| - | - | 1819 | 132.1 | - | - | 0 | - |
| - | - | 1171 | 132.1 | - | - | 0 | - |
| - | - | 1127 | 133.1 | - | - | 0 | - |
| - | - | 2.689E+04 | 136.1 | - | - | 0 | - |
| - | - | 2874 | 137.1 | - | - | 0 | - |
| - | - | 567.7 | 138.1 | - | - | 0 | - |
| - | - | 1109 | 138.1 | - | - | 0 | - |
| - | - | 602.7 | 141.1 | - | - | 0 | - |
| - | - | 898.4 | 143.1 | - | - | 0 | - |
| - | - | 2294 | 146.1 | - | - | 0 | - |
| - | - | 813.2 | 147 | - | - | 0 | - |
| - | - | 401 | 148.3 | - | - | 0 | - |
| - | - | 415.6 | 148.9 | - | - | 0 | - |
| - | - | 751.2 | 149 | - | - | 0 | - |
| - | - | 462.9 | 155.1 | - | - | 0 | - |
| - | - | 2291 | 156.1 | - | - | 0 | - |
| - | - | 2024 | 157.1 | - | - | 0 | - |
| - | - | 3361 | 158.1 | - | - | 0 | - |
| - | - | 5102 | 159.1 | - | - | 0 | - |
| - | - | 812.8 | 159.1 | - | - | 0 | - |
| - | - | 640.6 | 160.1 | - | - | 0 | - |
| - | - | 1048 | 160.1 | - | - | 0 | - |
| - | - | 1614 | 160.1 | - | - | 0 | - |
| - | - | 531.9 | 161 | - | - | 0 | - |
| - | - | 1.024E+04 | 165.1 | - | - | 0 | - |
| - | - | 420.8 | 165.3 | - | - | 0 | - |
| - | - | 848.1 | 166.1 | - | - | 0 | - |
| - | - | 1292 | 166.1 | - | - | 0 | - |
| - | - | 1787 | 167.1 | - | - | 0 | - |
| - | - | 520.7 | 169.1 | - | - | 0 | - |
| - | - | 698.6 | 169.1 | - | - | 0 | - |
| - | - | 657.4 | 170.1 | - | - | 0 | - |
| - | - | 583.9 | 171.1 | - | - | 0 | - |
| - | - | 669.6 | 171.1 | - | - | 0 | - |
| - | - | 4873 | 173.1 | - | - | 0 | - |
| - | - | 2286 | 173.5 | - | - | 0 | - |
| - | - | 811.8 | 174.1 | - | - | 0 | - |
| - | - | 588.6 | 174.1 | - | - | 0 | - |
| - | - | 755.9 | 175.1 | - | - | 0 | - |
| - | - | 6179 | 176.1 | - | - | 0 | - |
| - | - | 5512 | 177.1 | - | - | 0 | - |
| - | - | 1345 | 177.1 | - | - | 0 | - |
| - | - | 536.5 | 181.1 | - | - | 0 | - |
| - | - | 1.754E+04 | 182.1 | - | - | 0 | - |
| - | - | 433.2 | 182.1 | - | - | 0 | - |
| - | - | 2337 | 183.1 | - | - | 0 | - |
| - | - | 500.4 | 183.2 | - | - | 0 | - |
| - | - | 638.1 | 184.1 | - | - | 0 | - |
| - | - | 556.7 | 185.1 | - | - | 0 | - |
| - | - | 540.4 | 185.1 | - | - | 0 | - |
| - | - | 2152 | 185.1 | - | - | 0 | - |
| - | - | 818.3 | 186.1 | - | - | 0 | - |
| - | - | 1.199E+04 | 188.1 | - | - | 0 | - |
| - | - | 1414 | 189.1 | - | - | 0 | - |
| - | - | 679.5 | 191.1 | - | - | 0 | - |
| - | - | 1677 | 191.1 | - | - | 0 | - |
| - | - | 1406 | 192.1 | - | - | 0 | - |
| - | - | 2029 | 193.1 | - | - | 0 | - |
| - | - | 723.5 | 197.1 | - | - | 0 | - |
| - | - | 709.9 | 199.1 | - | - | 0 | - |
| - | - | 3472 | 201.1 | - | - | 0 | - |
| - | - | 532.3 | 202.1 | - | - | 0 | - |
| - | - | 804.7 | 203.1 | - | - | 0 | - |
| 2 | a | 3111 | 203.1 | 7.616E-05 | 0.375 | +1 | 2 |
| - | - | 1061 | 204.1 | - | - | 0 | - |
| 9 | y | 5882 | 205.1 | 0.0001054 | 0.514 | +1 | 1 |
| - | - | 951.2 | 205.1 | - | - | 0 | - |
| - | - | 603.8 | 206.1 | - | - | 0 | - |
| - | - | 1051 | 207.1 | - | - | 0 | - |
| - | - | 2290 | 209.1 | - | - | 0 | - |
| - | - | 712.6 | 209.1 | - | - | 0 | - |
| - | - | 839.1 | 214.1 | - | - | 0 | - |
| - | - | 701.5 | 215.1 | - | - | 0 | - |
| - | - | 571.8 | 217.1 | - | - | 0 | - |
| - | - | 1555 | 217.1 | - | - | 0 | - |
| - | - | 1605 | 219.1 | - | - | 0 | - |
| - | - | 5238 | 221.1 | - | - | 0 | - |
| 2 | a | 8.717E+04 | 221.1 | 0.0002842 | 1.285 | +1 | 2 |
| - | - | 838.6 | 222.1 | - | - | 0 | - |
| - | - | 1.129E+04 | 222.1 | - | - | 0 | - |
| - | - | 1093 | 223.1 | - | - | 0 | - |
| - | - | 671.9 | 224.1 | - | - | 0 | - |
| - | - | 720.3 | 225.1 | - | - | 0 | - |
| - | - | 604.1 | 225.2 | - | - | 0 | - |
| - | - | 1213 | 229.1 | - | - | 0 | - |
| - | - | 1311 | 231.1 | - | - | 0 | - |
| 2 | b | 850.2 | 231.1 | 5.696E-05 | 0.2465 | +1 | 2 |
| - | - | 1197 | 233.1 | - | - | 0 | - |
| - | - | 3096 | 235.1 | - | - | 0 | - |
| - | - | 501.6 | 239.1 | - | - | 0 | - |
| - | - | 792.9 | 239.1 | - | - | 0 | - |
| - | - | 1160 | 239.2 | - | - | 0 | - |
| - | - | 913.9 | 241.1 | - | - | 0 | - |
| - | - | 468.3 | 242.1 | - | - | 0 | - |
| 2 | b | 1.57E+04 | 249.1 | 0.0003189 | 1.28 | +1 | 2 |
| - | - | 2079 | 250.1 | - | - | 0 | - |
| - | - | 2123 | 251.1 | - | - | 0 | - |
| - | - | 1314 | 253.2 | - | - | 0 | - |
| - | - | 542.9 | 257.1 | - | - | 0 | - |
| - | - | 2920 | 263.1 | - | - | 0 | - |
| - | - | 694 | 270.1 | - | - | 0 | - |
| - | - | 1824 | 279.1 | - | - | 0 | - |
| - | - | 718.1 | 284.2 | - | - | 0 | - |
| - | - | 2132 | 285.1 | - | - | 0 | - |
| - | - | 534.9 | 285.2 | - | - | 0 | - |
| - | - | 612.1 | 287.1 | - | - | 0 | - |
| - | - | 1080 | 292.1 | - | - | 0 | - |
| - | - | 1409 | 296.2 | - | - | 0 | - |
| - | - | 881.5 | 300.1 | - | - | 0 | - |
| - | - | 656.9 | 300.2 | - | - | 0 | - |
| - | - | 995.2 | 302.1 | - | - | 0 | - |
| - | - | 614.6 | 309.2 | - | - | 0 | - |
| - | - | 1307 | 319.1 | - | - | 0 | - |
| - | - | 865.4 | 320.1 | - | - | 0 | - |
| - | - | 4120 | 324.1 | - | - | 0 | - |
| - | - | 722.5 | 325.1 | - | - | 0 | - |
| - | - | 665.4 | 329.1 | - | - | 0 | - |
| - | - | 1.262E+04 | 329.2 | - | - | 0 | - |
| - | - | 2248 | 330.2 | - | - | 0 | - |
| - | - | 663.2 | 333.1 | - | - | 0 | - |
| - | - | 520.7 | 333.2 | - | - | 0 | - |
| - | - | 724.3 | 338.1 | - | - | 0 | - |
| - | - | 554.7 | 339.2 | - | - | 0 | - |
| - | - | 2425 | 340.2 | - | - | 0 | - |
| 8 | y | 1.684E+04 | 342.2 | 0.0004892 | 1.43 | +1 | 2 |
| - | - | 3021 | 343.2 | - | - | 0 | - |
| - | - | 793.4 | 350.1 | - | - | 0 | - |
| - | - | 708.7 | 355.1 | - | - | 0 | - |
| - | - | 1094 | 356.1 | - | - | 0 | - |
| - | - | 3504 | 357.2 | - | - | 0 | - |
| 3 | b | 4451 | 364.2 | 4.823E-05 | 0.1325 | +1 | 3 |
| - | - | 912.4 | 365.2 | - | - | 0 | - |
| - | - | 759.7 | 366.1 | - | - | 0 | - |
| - | - | 695.8 | 368.2 | - | - | 0 | - |
| - | - | 828.1 | 376.1 | - | - | 0 | - |
| - | - | 2377 | 378.1 | - | - | 0 | - |
| - | - | 637.5 | 392.2 | - | - | 0 | - |
| - | - | 2542 | 394.1 | - | - | 0 | - |
| - | - | 589.6 | 409.2 | - | - | 0 | - |
| - | - | 681.8 | 418.7 | - | - | 0 | - |
| - | - | 694.6 | 422.7 | - | - | 0 | - |
| - | - | 2565 | 425.2 | - | - | 0 | - |
| - | - | 1763 | 427.7 | - | - | 0 | - |
| - | - | 1106 | 428.2 | - | - | 0 | - |
| - | - | 980.6 | 432.2 | - | - | 0 | - |
| - | - | 1548 | 436.7 | - | - | 0 | - |
| - | - | 1193 | 437.2 | - | - | 0 | - |
| - | - | 710.5 | 441.3 | - | - | 0 | - |
| - | - | 572.3 | 447.2 | - | - | 0 | - |
| - | - | 2146 | 465.2 | - | - | 0 | - |
| 4 | b | 2229 | 479.2 | 0.0006321 | 1.319 | +1 | 4 |
| - | - | 1028 | 483.2 | - | - | 0 | - |
| 7 | y | 3884 | 489.2 | 0.00571 | 11.67 | +1 | 3 |
| - | - | 1563 | 490.2 | - | - | 0 | - |
| - | - | 1713 | 496.2 | - | - | 0 | - |
| - | - | 680.7 | 500.2 | - | - | 0 | - |
| - | - | 669.4 | 501.3 | - | - | 0 | - |
| - | - | 1339 | 519.2 | - | - | 0 | - |
| - | - | 902 | 524.3 | - | - | 0 | - |
| - | - | 971.7 | 539.2 | - | - | 0 | - |
| - | - | 615.1 | 547.3 | - | - | 0 | - |
| - | - | 743.3 | 548.2 | - | - | 0 | - |
| 2 | y | 2301 | 550.7 | 0.002897 | 5.26 | +2 | 8 |
| - | - | 1198 | 551.2 | - | - | 0 | - |
| - | - | 706.3 | 551.7 | - | - | 0 | - |
| 6 | y | 6772 | 560.2 | 0.005523 | 9.858 | +1 | 4 |
| - | - | 1690 | 561.2 | - | - | 0 | - |
| - | - | 4166 | 585.3 | - | - | 0 | - |
| - | - | 1133 | 586.3 | - | - | 0 | - |
| 0 | Precursor | 824.9 | 592.2 | 0.003078 | 5.197 | +2 | -1 |
| - | - | 1089 | 592.7 | - | - | 0 | - |
| - | - | 866.9 | 595.3 | - | - | 0 | - |
| - | - | 781.8 | 600.3 | - | - | 0 | - |
| - | - | 895.5 | 601 | - | - | 0 | - |
| 0 | Precursor | 2598 | 601.2 | 0.001946 | 3.237 | +2 | -1 |
| - | - | 892.7 | 601.3 | - | - | 0 | - |
| - | - | 1597 | 601.7 | - | - | 0 | - |
| - | - | 748.9 | 602.2 | - | - | 0 | - |
| - | - | 808.5 | 606.9 | - | - | 0 | - |
| - | - | 1143 | 612.2 | - | - | 0 | - |
| - | - | 813 | 614.2 | - | - | 0 | - |
| - | - | 853.3 | 628.4 | - | - | 0 | - |
| - | - | 1539 | 657.3 | - | - | 0 | - |
| - | - | 1614 | 659.3 | - | - | 0 | - |
| - | - | 1422 | 660.3 | - | - | 0 | - |
| - | - | 2579 | 670.8 | - | - | 0 | - |
| - | - | 875.4 | 671.3 | - | - | 0 | - |
| - | - | 1014 | 671.8 | - | - | 0 | - |
| - | - | 642.7 | 672.4 | - | - | 0 | - |
| - | - | 717.7 | 682.4 | - | - | 0 | - |
| - | - | 2131 | 685.3 | - | - | 0 | - |
| - | - | 819 | 686.3 | - | - | 0 | - |
| - | - | 1.144E+04 | 700.4 | - | - | 0 | - |
| - | - | 3866 | 701.4 | - | - | 0 | - |
| - | - | 906.8 | 702.4 | - | - | 0 | - |
| - | - | 1715 | 721.3 | - | - | 0 | - |
| - | - | 950.2 | 722.3 | - | - | 0 | - |
| - | - | 634.1 | 722.4 | - | - | 0 | - |
| 5 | y | 4872 | 723.3 | 0.005366 | 7.418 | +1 | 5 |
| - | - | 2594 | 724.3 | - | - | 0 | - |
| - | - | 713.1 | 725.3 | - | - | 0 | - |
| - | - | 1463 | 744.3 | - | - | 0 | - |
| - | - | 2670 | 749.3 | - | - | 0 | - |
| - | - | 1542 | 750.3 | - | - | 0 | - |
| - | - | 928.2 | 757.4 | - | - | 0 | - |
| - | - | 753.9 | 773.4 | - | - | 0 | - |
| - | - | 3437 | 774.3 | - | - | 0 | - |
| - | - | 2734 | 783.4 | - | - | 0 | - |
| - | - | 1186 | 784.4 | - | - | 0 | - |
| - | - | 1254 | 794.4 | - | - | 0 | - |
| - | - | 1367 | 800.4 | - | - | 0 | - |
| - | - | 6096 | 801.4 | - | - | 0 | - |
| - | - | 2937 | 802.4 | - | - | 0 | - |
| - | - | 656.7 | 803.2 | - | - | 0 | - |
| - | - | 807.5 | 803.4 | - | - | 0 | - |
| - | - | 2357 | 828.4 | - | - | 0 | - |
| - | - | 901.7 | 829.4 | - | - | 0 | - |
| 4 | y | 6770 | 838.3 | 0.005827 | 6.951 | +1 | 6 |
| - | - | 3053 | 839.3 | - | - | 0 | - |
| - | - | 707.7 | 840.3 | - | - | 0 | - |
| - | - | 8928 | 844.5 | - | - | 0 | - |
| - | - | 2823 | 845.5 | - | - | 0 | - |
| - | - | 1206 | 846.5 | - | - | 0 | - |
| - | - | 1783 | 854.4 | - | - | 0 | - |
| - | - | 3.28E+04 | 872.4 | - | - | 0 | - |
| - | - | 1.368E+04 | 873.4 | - | - | 0 | - |
| - | - | 3211 | 874.5 | - | - | 0 | - |
| - | - | 9799 | 889.3 | - | - | 0 | - |
| - | - | 4965 | 890.4 | - | - | 0 | - |
| - | - | 1468 | 891.4 | - | - | 0 | - |
| - | - | 658.8 | 907.3 | - | - | 0 | - |
| - | - | 843.9 | 909.4 | - | - | 0 | - |
| 3 | y | 2271 | 935.3 | 0.003487 | 3.728 | +1 | 7 |
| - | - | 1176 | 936.3 | - | - | 0 | - |
| 3 | y | 3.337E+04 | 953.3 | 0.004763 | 4.996 | +1 | 7 |
| - | - | 1.865E+04 | 954.3 | - | - | 0 | - |
| - | - | 521 | 954.4 | - | - | 0 | - |
| - | - | 6277 | 955.3 | - | - | 0 | - |
| - | - | 906.7 | 956.3 | - | - | 0 | - |
| - | - | 5555 | 991.5 | - | - | 0 | - |
| - | - | 2919 | 992.5 | - | - | 0 | - |
| - | - | 1096 | 993.5 | - | - | 0 | - |
| - | - | 924.9 | 1003 | - | - | 0 | - |
| - | - | 723.2 | 1004 | - | - | 0 | - |
| - | - | 1.181E+04 | 1020 | - | - | 0 | - |
| - | - | 8219 | 1021 | - | - | 0 | - |
| - | - | 3371 | 1022 | - | - | 0 | - |
| 2 | y | 1870 | 1100 | 0.006357 | 5.777 | +1 | 8 |
| - | - | 1003 | 1101 | - | - | 0 | - |
| - | - | 646.6 | 1810 | - | - | 0 | - |
| - | - | 684.2 | 2603 | - | - | 0 | - |
| - | - | 759.5 | 2744 | - | - | 0 | - |
| - | - | 726.4 | 3314 | - | - | 0 | - |

m/z Charge Intensity FragmentType MassShift Position
120.0810775756836 0 78690.08
121.0796127319336 0 570.1216
121.08438110351562 0 5936.549
121.24666595458984 0 341.5262
123.04437255859375 0 1364.3822
127.0870132446289 0 430.3237
129.06597900390625 0 606.6612
129.10247802734375 0 5627.443
130.0653076171875 0 1613.845
132.08106994628906 0 1818.9316
132.10206604003906 0 1171.1364
133.08627319335938 0 1126.7461
136.0759735107422 0 26892.732
137.07919311523438 0 2874.2207
138.05477905273438 0 567.6762
138.06639099121094 0 1108.9232
141.10243225097656 0 602.7204
143.1180419921875 0 898.37695
146.0603485107422 0 2294.2375
147.04412841796875 0 813.2065
148.3268585205078 0 401.04553
148.89614868164062 0 415.6424
149.04530334472656 0 751.2287
155.09286499023438 0 462.9214
156.07708740234375 0 2291.2764
157.13385009765625 0 2023.5339
158.0967559814453 0 3360.6655
159.0919952392578 0 5102.24
159.0991973876953 0 812.7911
160.07601928710938 0 640.6468
160.09532165527344 0 1047.9307
160.1121368408203 0 1614.3533
161.04547119140625 0 531.866
165.0548858642578 0 10237.037
165.3275604248047 0 420.7727
166.058837890625 0 848.0728
166.08644104003906 0 1292.0577
167.0557861328125 0 1787.2887
169.09725952148438 0 520.6956
169.1336212158203 0 698.6039
170.05984497070312 0 657.4058
171.07598876953125 0 583.92944
171.1129608154297 0 669.58997
173.12867736816406 0 4873.456
173.4512176513672 0 2285.898
174.05511474609375 0 811.8099
174.13253784179688 0 588.62305
175.08656311035156 0 755.9475
176.1072540283203 0 6179.049
177.10250854492188 0 5512.4385
177.111572265625 0 1345.1095
181.0974578857422 0 536.55
182.0814666748047 0 17540.992
182.1283721923828 0 433.1541
183.084716796875 0 2337.0542
183.15020751953125 0 500.42737
184.07203674316406 0 638.1284
185.05528259277344 0 556.7222
185.0916290283203 0 540.4078
185.128662109375 0 2152.0977
186.09158325195312 0 818.329
188.07086181640625 0 11993.623
189.07421875 0 1414.0117
191.08175659179688 0 679.5059
191.1178741455078 0 1676.8235
192.06570434570312 0 1406.2291
193.10861206054688 0 2029.1111
197.12828063964844 0 723.4927
199.07119750976562 0 709.94507
201.1236114501953 0 3472.134
202.05113220214844 0 532.3108
203.066162109375 0 804.69666
203.1179656982422 0 3110.9902 a Water loss 1
204.07708740234375 0 1060.64
205.09725952148438 0 5882.139 y 8
205.10763549804688 0 951.2351
206.1006622314453 0 603.7553
207.11279296875 0 1050.553
209.09217834472656 0 2290.264
209.1028594970703 0 712.59283
214.11915588378906 0 839.08887
215.13909912109375 0 701.522
217.08303833007812 0 571.7717
217.09751892089844 0 1555.207
219.11309814453125 0 1605.1743
221.103515625 0 5237.607
221.1287384033203 0 87165.14 a 1
222.10638427734375 0 838.64185
222.13211059570312 0 11292.048
223.06382751464844 0 1092.5588
224.14012145996094 0 671.9308
225.10963439941406 0 720.29333
225.1722869873047 0 604.1222
229.11862182617188 0 1212.7778
231.06182861328125 0 1311.1582
231.1127471923828 0 850.16327 b Water loss 1
233.12872314453125 0 1197.2301
235.1080322265625 0 3095.9243
239.0952606201172 0 501.5524
239.11410522460938 0 792.9403
239.15048217773438 0 1160.3221
241.07444763183594 0 913.933
242.11427307128906 0 468.3336
249.12368774414062 0 15697.872 b 1
250.12696838378906 0 2079.0918
251.1028594970703 0 2122.9773
253.16636657714844 0 1313.5834
257.105224609375 0 542.94446
263.10272216796875 0 2920.3162
270.1068115234375 0 693.9526
279.09814453125 0 1823.9956
284.19696044921875 0 718.1012
285.1020812988281 0 2132.3987
285.1540222167969 0 534.8652
287.1007995605469 0 612.1033
292.14068603515625 0 1080.0632
296.15142822265625 0 1408.5326
300.11767578125 0 881.51904
300.15631103515625 0 656.89484
302.11395263671875 0 995.2231
309.203369140625 0 614.6229
319.1399230957031 0 1306.7936
320.1241760253906 0 865.42737
324.1459045410156 0 4120.1733
325.1491394042969 0 722.54987
329.127685546875 0 665.43866
329.15008544921875 0 12620.763
330.1534423828125 0 2247.9902
333.12054443359375 0 663.2038
333.19293212890625 0 520.6717
338.1355895996094 0 724.34717
339.1690979003906 0 554.66095
340.19854736328125 0 2424.7886
342.15655517578125 0 16843.191 y 7
343.15960693359375 0 3020.8516
350.1343688964844 0 793.3789
355.0683898925781 0 708.6547
356.1380615234375 0 1093.8615
357.2247009277344 0 3504.449
364.1503601074219 0 4451.24 b 2
365.1554260253906 0 912.44214
366.13006591796875 0 759.6565
368.19134521484375 0 695.8249
376.114990234375 0 828.0991
378.1288757324219 0 2376.748
392.20635986328125 0 637.5134
394.12481689453125 0 2541.795
409.1697692871094 0 589.5602
418.7174377441406 0 681.78894
422.7294006347656 0 694.5545
425.19378662109375 0 2565.2214
427.7220764160156 0 1762.855
428.22369384765625 0 1105.5438
432.1868591308594 0 980.6327
436.7281188964844 0 1548.3325
437.22845458984375 0 1192.6357
441.2966003417969 0 710.5404
447.1556091308594 0 572.2836
465.16253662109375 0 2145.6528
479.1778869628906 0 2229.3816 b 3
483.21923828125 0 1027.8107
489.1922607421875 0 3883.888 y 6
490.1950378417969 0 1563.1538
496.229736328125 0 1712.7854
500.2446594238281 0 680.7254
501.2556457519531 0 669.3983
519.2006225585938 0 1338.5464
524.251220703125 0 901.9906
539.2145385742188 0 971.72424
547.252685546875 0 615.08765
548.1990966796875 0 743.30066
550.711181640625 0 2301.1035 y 1
551.21337890625 0 1198.2449
551.713623046875 0 706.2917
560.2291870117188 0 6772.2935 y 5
561.230712890625 0 1690.0574
585.3355712890625 0 4165.8843
586.340576171875 0 1133.0725
592.2299194335938 0 824.87085 Precursor Water loss
592.7260131835938 0 1088.9312
595.3255004882812 0 866.89215
600.3356323242188 0 781.809
600.9881591796875 0 895.5088
601.2340698242188 0 2597.7944 Precursor
601.3220825195312 0 892.7103
601.7366333007812 0 1597.3378
602.236328125 0 748.9384
606.9269409179688 0 808.49603
612.197509765625 0 1143.132
614.2463989257812 0 812.9945
628.3775024414062 0 853.2888
657.26123046875 0 1538.764
659.2939453125 0 1613.9319
660.2996826171875 0 1421.8481
670.8168334960938 0 2578.762
671.3197631835938 0 875.3535
671.8202514648438 0 1013.8709
672.392333984375 0 642.7142
682.3529663085938 0 717.7321
685.2567138671875 0 2130.5425
686.2603759765625 0 818.9505
700.3623657226562 0 11441.81
701.36572265625 0 3865.6538
702.3703002929688 0 906.77594
721.2598266601562 0 1715.3807
722.263427734375 0 950.2233
722.4005126953125 0 634.1129
723.2923583984375 0 4871.7 y 4
724.293212890625 0 2593.922
725.2947998046875 0 713.13336
744.346435546875 0 1462.5355
749.2566528320312 0 2669.5156
750.2601318359375 0 1541.9106
757.3831787109375 0 928.20575
773.4103393554688 0 753.9234
774.318359375 0 3436.627
783.3984375 0 2733.8423
784.4073486328125 0 1186.3274
794.3832397460938 0 1253.7246
800.4257202148438 0 1367.3721
801.4131469726562 0 6095.712
802.413330078125 0 2937.092
803.1714477539062 0 656.65173
803.41650390625 0 807.532
828.419189453125 0 2356.8916
829.427001953125 0 901.7207
838.3197631835938 0 6769.509 y 3
839.32177734375 0 3052.6948
840.3307495117188 0 707.6905
844.4519653320312 0 8927.713
845.4529418945312 0 2823.262
846.4580078125 0 1206.1481
854.4329223632812 0 1783.331
872.4470825195312 0 32802.242
873.4498291015625 0 13678.922
874.454345703125 0 3210.5642
889.3472290039062 0 9798.565
890.3507690429688 0 4965.0034
891.3523559570312 0 1468.2529
907.3327026367188 0 658.83746
909.408447265625 0 843.8872
935.3338012695312 0 2271.268 y Water loss 2
936.3345336914062 0 1175.8663
953.3456420898438 0 33368.1 y 2
954.3486328125 0 18651.074
954.4338989257812 0 520.9684
955.3496704101562 0 6276.839
956.3479614257812 0 906.70667
991.5186157226562 0 5555.4976
992.523681640625 0 2919.2688
993.5260620117188 0 1095.9982
1003.3854370117188 0 924.9484
1004.3820190429688 0 723.1774
1019.5140991210938 0 11814.348
1020.5172729492188 0 8218.838
1021.5198364257812 0 3371.0964
1100.4156494140625 0 1870.2688 y 1
1101.4136962890625 0 1002.5679
1810.0751953125 0 646.63135
2603.2861328125 0 684.22723
2744.458740234375 0 759.4819
3313.851806640625 0 726.4017

Spectrum Details

|  |  |
| --- | --- |
| Matched peaks? Matched peaksThe total absolute number of peaks matched. Additionally in brackets the total fraction of peaks matched and the total number of peaks is shown. | 18 (6.87% of 262) |
| FDR? FDRThe false discovery rate estimated for this peptide. It is calculated by matching all theoretical fragments with a non-integer shift with the raw peaks for this spectrum. This is done with 40 different shifts. The resulting percentage is the average number of annotated peaks over the number of annotated peaks with the correct spectrum. | 1.46% |
| Satellite FDR? Satellite FDRSee the FDR for details on its calculation. This satellite ion specific FDR only contains the satellite ions (d/w) for I/L/J positions. | - |
| PSM Score? PSM ScoreThe PSM Score as given by Hecklib to this annotated spectrum. It is shown with three significant figures. | 195 |

## Spectrum 10126? Spectrum 10126 The raw spectrum of this peptide as annotated by Hecklib. The fragments are coloured according to ion type (see legend). Any peaks with a star '\*' as text can be hovered over to see the full details, first the ion type second the mass shift type. By hovering over the amino acids in the peptide or ions in the legend the corresponding peaks are highlighted. By toggling the 'Unassigned' label you can turn the background (unassigned) peaks on or off in the plot. By updating the slider in the Ion legend you can update the spectrum to only show the top X% of the peaks with labels. The top X% means any peak that is within X% of the highest intensity. By dragging in the spectrum you can zoom in to a specific part of the spectrum and use 'Zoom Out' to get back to the original zoom level. The annotation of the spectrum is based on the given sequence in the peptides file and is done with different software so inconsistencies are likely. The peaks are annotated based on the given sequence, with 20 ppm tolerance.

Copy Data

### Spectrum 10126 (TSV)

#### Preview

```
Loading example...
```

*Click on the button to copy the data to your clipboard.*

Mz MinMz MaxIntensity Max

WidthHeightPeptide font sizePeptide stroke widthSpectrum font sizeSpectrum stroke widthCompact peptide

Ion legend

wxyz

abcd

OtherUnassignedIonChargePositionShow for top:%

TFDDYAMHW

02.32e+44.64e+46.96e+49.29e+4

Zoom Out

a+12y+11a+12b+12b+12a+13y+12b+13b+13y+27b+14y+13y+28y+14\*\*b+15y+15y+16y+17y+17y+18

0835167025043339

Fragment Matches Table

Show background peaks

| Position | Ion type | Intensity | mz Theoretical | mz Error (Th) | mz Error (ppm) | Charge | Series Number |
| --- | --- | --- | --- | --- | --- | --- | --- |
| - | - | 7.531E+04 | 120.1 | - | - | 0 | - |
| - | - | 837.2 | 121.1 | - | - | 0 | - |
| - | - | 7596 | 121.1 | - | - | 0 | - |
| - | - | 1149 | 123 | - | - | 0 | - |
| - | - | 577.5 | 127.1 | - | - | 0 | - |
| - | - | 4718 | 129.1 | - | - | 0 | - |
| - | - | 426.8 | 129.3 | - | - | 0 | - |
| - | - | 368.1 | 130.1 | - | - | 0 | - |
| - | - | 1740 | 130.1 | - | - | 0 | - |
| - | - | 929.9 | 132.1 | - | - | 0 | - |
| - | - | 864.9 | 132.1 | - | - | 0 | - |
| - | - | 824.1 | 133.1 | - | - | 0 | - |
| - | - | 490.3 | 133.4 | - | - | 0 | - |
| - | - | 385.1 | 134.5 | - | - | 0 | - |
| - | - | 2.549E+04 | 136.1 | - | - | 0 | - |
| - | - | 2440 | 137.1 | - | - | 0 | - |
| - | - | 893.2 | 138.1 | - | - | 0 | - |
| - | - | 1182 | 138.1 | - | - | 0 | - |
| - | - | 424.5 | 141.1 | - | - | 0 | - |
| - | - | 448 | 141.1 | - | - | 0 | - |
| - | - | 1358 | 146.1 | - | - | 0 | - |
| - | - | 1232 | 147 | - | - | 0 | - |
| - | - | 577.6 | 147.1 | - | - | 0 | - |
| - | - | 467.5 | 149 | - | - | 0 | - |
| - | - | 441 | 149 | - | - | 0 | - |
| - | - | 1036 | 155.1 | - | - | 0 | - |
| - | - | 2821 | 156.1 | - | - | 0 | - |
| - | - | 2073 | 157.1 | - | - | 0 | - |
| - | - | 656.4 | 158.1 | - | - | 0 | - |
| - | - | 3860 | 158.1 | - | - | 0 | - |
| - | - | 3280 | 159.1 | - | - | 0 | - |
| - | - | 456.2 | 159.1 | - | - | 0 | - |
| - | - | 1027 | 160.1 | - | - | 0 | - |
| - | - | 1346 | 160.1 | - | - | 0 | - |
| - | - | 407.4 | 163.5 | - | - | 0 | - |
| - | - | 1.194E+04 | 165.1 | - | - | 0 | - |
| - | - | 662.5 | 166.1 | - | - | 0 | - |
| - | - | 3014 | 166.1 | - | - | 0 | - |
| - | - | 518.4 | 167 | - | - | 0 | - |
| - | - | 1472 | 167.1 | - | - | 0 | - |
| - | - | 513.8 | 167.1 | - | - | 0 | - |
| - | - | 863.6 | 171.1 | - | - | 0 | - |
| - | - | 547.6 | 173.1 | - | - | 0 | - |
| - | - | 5826 | 173.1 | - | - | 0 | - |
| - | - | 2628 | 173.5 | - | - | 0 | - |
| - | - | 1423 | 175.1 | - | - | 0 | - |
| - | - | 459.5 | 175.6 | - | - | 0 | - |
| - | - | 653.3 | 176.1 | - | - | 0 | - |
| - | - | 7376 | 176.1 | - | - | 0 | - |
| - | - | 3778 | 177.1 | - | - | 0 | - |
| - | - | 637 | 178.1 | - | - | 0 | - |
| - | - | 438.2 | 181.1 | - | - | 0 | - |
| - | - | 1.992E+04 | 182.1 | - | - | 0 | - |
| - | - | 1364 | 183.1 | - | - | 0 | - |
| - | - | 736.4 | 183.1 | - | - | 0 | - |
| - | - | 677.6 | 183.1 | - | - | 0 | - |
| - | - | 1839 | 185.1 | - | - | 0 | - |
| - | - | 604.6 | 186.1 | - | - | 0 | - |
| - | - | 519.7 | 187.1 | - | - | 0 | - |
| - | - | 4678 | 188.1 | - | - | 0 | - |
| - | - | 655.1 | 189.1 | - | - | 0 | - |
| - | - | 2568 | 191.1 | - | - | 0 | - |
| - | - | 1929 | 193.1 | - | - | 0 | - |
| - | - | 773.3 | 195.1 | - | - | 0 | - |
| - | - | 648.4 | 199.1 | - | - | 0 | - |
| - | - | 456.4 | 200.6 | - | - | 0 | - |
| - | - | 3609 | 201.1 | - | - | 0 | - |
| - | - | 895.6 | 203.1 | - | - | 0 | - |
| 2 | a | 2430 | 203.1 | 0.0002135 | 1.051 | +1 | 2 |
| - | - | 724.2 | 204.1 | - | - | 0 | - |
| 9 | y | 3383 | 205.1 | 0.0004411 | 2.151 | +1 | 1 |
| - | - | 564.2 | 207.1 | - | - | 0 | - |
| - | - | 1049 | 207.1 | - | - | 0 | - |
| - | - | 1103 | 211.1 | - | - | 0 | - |
| - | - | 909 | 214.1 | - | - | 0 | - |
| - | - | 634 | 215.1 | - | - | 0 | - |
| - | - | 1108 | 217.1 | - | - | 0 | - |
| - | - | 1364 | 219.1 | - | - | 0 | - |
| - | - | 5088 | 221.1 | - | - | 0 | - |
| 2 | a | 9.194E+04 | 221.1 | 0.00033 | 1.492 | +1 | 2 |
| - | - | 528.4 | 222.1 | - | - | 0 | - |
| - | - | 645.6 | 222.1 | - | - | 0 | - |
| - | - | 1.184E+04 | 222.1 | - | - | 0 | - |
| - | - | 1401 | 223.1 | - | - | 0 | - |
| - | - | 688.5 | 225.2 | - | - | 0 | - |
| - | - | 546.3 | 226.2 | - | - | 0 | - |
| - | - | 816.6 | 229.2 | - | - | 0 | - |
| - | - | 1574 | 231.1 | - | - | 0 | - |
| 2 | b | 804.8 | 231.1 | 0.001026 | 4.441 | +1 | 2 |
| - | - | 1198 | 233.1 | - | - | 0 | - |
| - | - | 515.2 | 234.8 | - | - | 0 | - |
| - | - | 2090 | 235.1 | - | - | 0 | - |
| - | - | 656 | 238.1 | - | - | 0 | - |
| - | - | 919.8 | 239.1 | - | - | 0 | - |
| - | - | 878.8 | 239.1 | - | - | 0 | - |
| - | - | 967.4 | 241.1 | - | - | 0 | - |
| - | - | 562.1 | 241.5 | - | - | 0 | - |
| - | - | 594.9 | 246.9 | - | - | 0 | - |
| 2 | b | 1.583E+04 | 249.1 | 0.0003036 | 1.219 | +1 | 2 |
| - | - | 2425 | 250.1 | - | - | 0 | - |
| - | - | 2035 | 251.1 | - | - | 0 | - |
| - | - | 1397 | 253.1 | - | - | 0 | - |
| - | - | 660.4 | 253.2 | - | - | 0 | - |
| - | - | 880 | 257.1 | - | - | 0 | - |
| - | - | 3294 | 263.1 | - | - | 0 | - |
| - | - | 690.4 | 264.1 | - | - | 0 | - |
| - | - | 1465 | 279.1 | - | - | 0 | - |
| - | - | 689.2 | 283.1 | - | - | 0 | - |
| - | - | 1238 | 285 | - | - | 0 | - |
| - | - | 2285 | 285.1 | - | - | 0 | - |
| - | - | 612.2 | 286.1 | - | - | 0 | - |
| - | - | 1545 | 292.1 | - | - | 0 | - |
| - | - | 1354 | 296.2 | - | - | 0 | - |
| - | - | 732.3 | 302.1 | - | - | 0 | - |
| - | - | 823.1 | 314.1 | - | - | 0 | - |
| 3 | a | 903.6 | 318.1 | 0.0005532 | 1.739 | +1 | 3 |
| - | - | 1066 | 319.1 | - | - | 0 | - |
| - | - | 767.2 | 323.1 | - | - | 0 | - |
| - | - | 2928 | 324.1 | - | - | 0 | - |
| - | - | 788.2 | 325.1 | - | - | 0 | - |
| - | - | 1.294E+04 | 329.1 | - | - | 0 | - |
| - | - | 2529 | 330.2 | - | - | 0 | - |
| - | - | 798.1 | 333.1 | - | - | 0 | - |
| - | - | 2857 | 340.2 | - | - | 0 | - |
| - | - | 1069 | 342.1 | - | - | 0 | - |
| 8 | y | 1.986E+04 | 342.2 | 0.0005197 | 1.519 | +1 | 2 |
| - | - | 3349 | 343.2 | - | - | 0 | - |
| 3 | b | 1450 | 346.1 | 2.332E-05 | 0.06737 | +1 | 3 |
| - | - | 994.5 | 350.1 | - | - | 0 | - |
| - | - | 928.2 | 355.1 | - | - | 0 | - |
| - | - | 957.4 | 356.1 | - | - | 0 | - |
| - | - | 2731 | 357.2 | - | - | 0 | - |
| 3 | b | 4849 | 364.2 | 0.0002569 | 0.7056 | +1 | 3 |
| - | - | 825.4 | 368.2 | - | - | 0 | - |
| - | - | 2045 | 378.1 | - | - | 0 | - |
| - | - | 760.9 | 379.1 | - | - | 0 | - |
| - | - | 3939 | 394.1 | - | - | 0 | - |
| - | - | 602.5 | 395.1 | - | - | 0 | - |
| - | - | 889.2 | 401.2 | - | - | 0 | - |
| - | - | 679.9 | 401.7 | - | - | 0 | - |
| - | - | 560.4 | 402.2 | - | - | 0 | - |
| - | - | 619.3 | 408.2 | - | - | 0 | - |
| - | - | 705.3 | 413.3 | - | - | 0 | - |
| - | - | 2708 | 425.2 | - | - | 0 | - |
| - | - | 832.7 | 426.2 | - | - | 0 | - |
| - | - | 878.6 | 427.7 | - | - | 0 | - |
| - | - | 1042 | 428.2 | - | - | 0 | - |
| - | - | 991.7 | 432.2 | - | - | 0 | - |
| - | - | 2198 | 436.7 | - | - | 0 | - |
| - | - | 786.8 | 455.2 | - | - | 0 | - |
| - | - | 2628 | 465.2 | - | - | 0 | - |
| - | - | 692.4 | 470.3 | - | - | 0 | - |
| - | - | 804.8 | 471.3 | - | - | 0 | - |
| 3 | y | 646.1 | 477.2 | 0.004553 | 9.541 | +2 | 7 |
| - | - | 624.9 | 479 | - | - | 0 | - |
| 4 | b | 1665 | 479.2 | 0.002066 | 4.312 | +1 | 4 |
| - | - | 1253 | 483.2 | - | - | 0 | - |
| - | - | 734.7 | 487.3 | - | - | 0 | - |
| 7 | y | 4047 | 489.2 | 0.005893 | 12.05 | +1 | 3 |
| - | - | 1326 | 490.2 | - | - | 0 | - |
| - | - | 2057 | 496.2 | - | - | 0 | - |
| - | - | 721.4 | 497.2 | - | - | 0 | - |
| - | - | 805.3 | 497.2 | - | - | 0 | - |
| - | - | 838.4 | 500.2 | - | - | 0 | - |
| - | - | 939.5 | 501.3 | - | - | 0 | - |
| - | - | 885 | 510.3 | - | - | 0 | - |
| - | - | 703.5 | 513.4 | - | - | 0 | - |
| - | - | 671.1 | 519.2 | - | - | 0 | - |
| - | - | 568.1 | 525.5 | - | - | 0 | - |
| - | - | 675 | 527.7 | - | - | 0 | - |
| - | - | 773 | 533.2 | - | - | 0 | - |
| - | - | 938.5 | 548.2 | - | - | 0 | - |
| 2 | y | 2511 | 550.7 | 0.002531 | 4.595 | +2 | 8 |
| - | - | 1513 | 551.2 | - | - | 0 | - |
| 6 | y | 5597 | 560.2 | 0.005279 | 9.422 | +1 | 4 |
| - | - | 1502 | 561.2 | - | - | 0 | - |
| - | - | 835.5 | 570.2 | - | - | 0 | - |
| - | - | 658.6 | 580.3 | - | - | 0 | - |
| - | - | 736.1 | 583.3 | - | - | 0 | - |
| - | - | 4948 | 585.3 | - | - | 0 | - |
| - | - | 1583 | 586.3 | - | - | 0 | - |
| 0 | Precursor | 1343 | 592.2 | 0.0009416 | 1.59 | +2 | -1 |
| - | - | 1009 | 593.3 | - | - | 0 | - |
| - | - | 718.2 | 600.3 | - | - | 0 | - |
| 0 | Precursor | 1626 | 601.2 | 0.001702 | 2.83 | +2 | -1 |
| - | - | 737 | 601.7 | - | - | 0 | - |
| - | - | 1851 | 601.7 | - | - | 0 | - |
| - | - | 1073 | 612.2 | - | - | 0 | - |
| - | - | 885.3 | 613.2 | - | - | 0 | - |
| - | - | 1107 | 614.2 | - | - | 0 | - |
| - | - | 938.5 | 616.3 | - | - | 0 | - |
| 5 | b | 845.1 | 624.2 | 0.002647 | 4.241 | +1 | 5 |
| - | - | 1522 | 628.4 | - | - | 0 | - |
| - | - | 1333 | 657.3 | - | - | 0 | - |
| - | - | 2433 | 659.3 | - | - | 0 | - |
| - | - | 902.6 | 682.3 | - | - | 0 | - |
| - | - | 1934 | 685.3 | - | - | 0 | - |
| - | - | 776.4 | 699.4 | - | - | 0 | - |
| - | - | 1.372E+04 | 700.4 | - | - | 0 | - |
| - | - | 3564 | 701.4 | - | - | 0 | - |
| - | - | 828.1 | 702.4 | - | - | 0 | - |
| - | - | 823.4 | 703.3 | - | - | 0 | - |
| - | - | 668.6 | 715.4 | - | - | 0 | - |
| - | - | 1796 | 721.3 | - | - | 0 | - |
| 5 | y | 6159 | 723.3 | 0.004755 | 6.574 | +1 | 5 |
| - | - | 2921 | 724.3 | - | - | 0 | - |
| - | - | 2097 | 749.3 | - | - | 0 | - |
| - | - | 997.7 | 750.3 | - | - | 0 | - |
| - | - | 1638 | 757.4 | - | - | 0 | - |
| - | - | 2827 | 774.3 | - | - | 0 | - |
| - | - | 1367 | 775.3 | - | - | 0 | - |
| - | - | 2691 | 783.4 | - | - | 0 | - |
| - | - | 1407 | 784.4 | - | - | 0 | - |
| - | - | 1822 | 800.4 | - | - | 0 | - |
| - | - | 6413 | 801.4 | - | - | 0 | - |
| - | - | 2057 | 802.4 | - | - | 0 | - |
| - | - | 878.1 | 803.4 | - | - | 0 | - |
| - | - | 2795 | 828.4 | - | - | 0 | - |
| 4 | y | 7659 | 838.3 | 0.004973 | 5.932 | +1 | 6 |
| - | - | 3405 | 839.3 | - | - | 0 | - |
| - | - | 875.9 | 840.3 | - | - | 0 | - |
| - | - | 8475 | 844.5 | - | - | 0 | - |
| - | - | 4523 | 845.5 | - | - | 0 | - |
| - | - | 1433 | 846.5 | - | - | 0 | - |
| - | - | 1226 | 854.4 | - | - | 0 | - |
| - | - | 911.9 | 871.3 | - | - | 0 | - |
| - | - | 3.383E+04 | 872.4 | - | - | 0 | - |
| - | - | 1.489E+04 | 873.4 | - | - | 0 | - |
| - | - | 5080 | 874.5 | - | - | 0 | - |
| - | - | 862.3 | 875.5 | - | - | 0 | - |
| - | - | 1.058E+04 | 889.3 | - | - | 0 | - |
| - | - | 5781 | 890.3 | - | - | 0 | - |
| - | - | 1202 | 891.4 | - | - | 0 | - |
| 3 | y | 1998 | 935.3 | 0.003914 | 4.185 | +1 | 7 |
| - | - | 1346 | 936.3 | - | - | 0 | - |
| 3 | y | 3.642E+04 | 953.3 | 0.004336 | 4.548 | +1 | 7 |
| - | - | 1.762E+04 | 954.3 | - | - | 0 | - |
| - | - | 5814 | 955.3 | - | - | 0 | - |
| - | - | 981.3 | 956.3 | - | - | 0 | - |
| - | - | 5348 | 991.5 | - | - | 0 | - |
| - | - | 3000 | 992.5 | - | - | 0 | - |
| - | - | 1094 | 993.5 | - | - | 0 | - |
| - | - | 942.7 | 1002 | - | - | 0 | - |
| - | - | 1.604E+04 | 1020 | - | - | 0 | - |
| - | - | 8426 | 1021 | - | - | 0 | - |
| - | - | 3202 | 1022 | - | - | 0 | - |
| - | - | 655.9 | 1023 | - | - | 0 | - |
| 2 | y | 2080 | 1100 | 0.004037 | 3.669 | +1 | 8 |
| - | - | 1685 | 1101 | - | - | 0 | - |
| - | - | 725.1 | 1981 | - | - | 0 | - |
| - | - | 647 | 3306 | - | - | 0 | - |

m/z Charge Intensity FragmentType MassShift Position
120.08108520507812 0 75313.11
121.07976531982422 0 837.21857
121.08442687988281 0 7595.673
123.04444885253906 0 1149.4572
127.0868911743164 0 577.4934
129.10252380371094 0 4718.2197
129.33848571777344 0 426.78607
130.05010986328125 0 368.10193
130.06539916992188 0 1740.4673
132.08108520507812 0 929.94666
132.10220336914062 0 864.90906
133.08599853515625 0 824.11664
133.42918395996094 0 490.32425
134.47633361816406 0 385.08597
136.0760040283203 0 25494.3
137.0794219970703 0 2440.355
138.05502319335938 0 893.19275
138.06643676757812 0 1182.4369
141.06655883789062 0 424.4572
141.10264587402344 0 448.0341
146.0603485107422 0 1357.7173
147.0443115234375 0 1231.6442
147.11294555664062 0 577.6242
149.0234832763672 0 467.47702
149.04510498046875 0 441.03702
155.08145141601562 0 1036.4138
156.0769805908203 0 2821.449
157.1337432861328 0 2073.303
158.06048583984375 0 656.42865
158.0966796875 0 3859.863
159.09190368652344 0 3280.147
159.09854125976562 0 456.1568
160.07579040527344 0 1027.09
160.1123504638672 0 1345.5756
163.53880310058594 0 407.44986
165.05490112304688 0 11935.008
166.0605926513672 0 662.48395
166.08663940429688 0 3014.46
167.04444885253906 0 518.38873
167.05569458007812 0 1472.06
167.08197021484375 0 513.77594
171.07691955566406 0 863.616
173.09217834472656 0 547.5696
173.12879943847656 0 5825.8994
173.45138549804688 0 2628.3096
175.0868377685547 0 1422.6965
175.55300903320312 0 459.52643
176.0820770263672 0 653.2757
176.1072540283203 0 7376.197
177.1026153564453 0 3777.718
178.10610961914062 0 637.04504
181.0970001220703 0 438.2238
182.0814666748047 0 19922.693
183.0849151611328 0 1363.787
183.11300659179688 0 736.3883
183.1492919921875 0 677.59973
185.12881469726562 0 1839.3284
186.09201049804688 0 604.5733
187.14466857910156 0 519.7314
188.07093811035156 0 4678.211
189.08729553222656 0 655.10175
191.11834716796875 0 2567.9182
193.10858154296875 0 1928.8748
195.08773803710938 0 773.31915
199.07147216796875 0 648.40216
200.64329528808594 0 456.39062
201.12355041503906 0 3609.0654
203.06631469726562 0 895.57587
203.11810302734375 0 2430.1292 a Water loss 1
204.07730102539062 0 724.2379
205.09759521484375 0 3383.2754 y 8
207.07684326171875 0 564.1747
207.1131591796875 0 1048.6593
211.14385986328125 0 1102.5006
214.11924743652344 0 908.9845
215.1389617919922 0 633.9618
217.0970001220703 0 1107.5686
219.11302185058594 0 1363.551
221.10354614257812 0 5088.451
221.1287841796875 0 91939.45 a 1
222.0859375 0 528.3535
222.10691833496094 0 645.6466
222.13214111328125 0 11838.674
223.06398010253906 0 1400.7198
225.17141723632812 0 688.5134
226.15469360351562 0 546.2718
229.1710205078125 0 816.6411
231.06175231933594 0 1574.2847
231.11383056640625 0 804.81604 b Water loss 1
233.14993286132812 0 1197.5289
234.7512664794922 0 515.1508
235.10784912109375 0 2090.2585
238.11915588378906 0 656.041
239.09535217285156 0 919.7835
239.11416625976562 0 878.8365
241.0741729736328 0 967.41943
241.4734344482422 0 562.09717
246.91885375976562 0 594.88544
249.12367248535156 0 15827.041 b 1
250.12725830078125 0 2425.1958
251.102783203125 0 2034.9456
253.11866760253906 0 1397.4177
253.1654815673828 0 660.4244
257.1065673828125 0 880.0471
263.10272216796875 0 3294.147
264.14666748046875 0 690.3915
279.09808349609375 0 1465.3009
283.1440734863281 0 689.2331
285.0103759765625 0 1238.1849
285.10205078125 0 2285.3196
286.10321044921875 0 612.21
292.1413269042969 0 1545.1833
296.15032958984375 0 1354.423
302.1145935058594 0 732.3015
314.0978088378906 0 823.12897
318.1453857421875 0 903.63586 a Water loss 2
319.14129638671875 0 1065.7781
323.0990905761719 0 767.1947
324.1459655761719 0 2928.2283
325.14990234375 0 788.2388
329.1499938964844 0 12936.524
330.15350341796875 0 2529.2366
333.120849609375 0 798.1457
340.19830322265625 0 2857.0867
342.13311767578125 0 1069.4796
342.1565856933594 0 19856.361 y 7
343.159423828125 0 3349.1792
346.1397705078125 0 1449.9137 b Water loss 2
350.1351013183594 0 994.5183
355.0696105957031 0 928.22644
356.1395263671875 0 957.3887
357.22442626953125 0 2731.333
364.1500549316406 0 4849.104 b 2
368.1927795410156 0 825.38855
378.12939453125 0 2045.2319
379.1338806152344 0 760.92883
394.125 0 3938.9583
395.128173828125 0 602.48145
401.20928955078125 0 889.2479
401.7116394042969 0 679.928
402.215087890625 0 560.35284
408.1673583984375 0 619.32697
413.2671813964844 0 705.27216
425.19384765625 0 2707.5747
426.1971130371094 0 832.7487
427.72308349609375 0 878.6435
428.22314453125 0 1041.8668
432.1887512207031 0 991.674
436.7279357910156 0 2198.057
455.20330810546875 0 786.75214
465.1629333496094 0 2627.5413
470.3082580566406 0 692.4232
471.2555847167969 0 804.8368
477.1695251464844 0 646.06146 y 2
479.0285949707031 0 624.91486
479.1793212890625 0 1664.7766 b 3
483.21990966796875 0 1253.4845
487.3341064453125 0 734.6992
489.19244384765625 0 4047.4382 y 6
490.1951904296875 0 1326.0809
496.2319641113281 0 2057.4912
497.1672668457031 0 721.4251
497.23199462890625 0 805.26874
500.24615478515625 0 838.44543
501.2557373046875 0 939.5417
510.26220703125 0 885.02026
513.3521118164062 0 703.49426
519.207763671875 0 671.14197
525.5235595703125 0 568.09985
527.66943359375 0 674.9945
533.2044067382812 0 772.9859
548.1991577148438 0 938.51874
550.7108154296875 0 2511.055 y 1
551.2138061523438 0 1512.8279
560.2289428710938 0 5597.2495 y 5
561.2322998046875 0 1501.6659
570.2266845703125 0 835.51587
580.2970581054688 0 658.5911
583.28173828125 0 736.0546
585.3357543945312 0 4948.1997
586.3386840820312 0 1582.5524
592.227783203125 0 1343.161 Precursor Water loss
593.3148193359375 0 1008.6783
600.2871704101562 0 718.23486
601.2338256835938 0 1625.7046 Precursor
601.6558227539062 0 736.9764
601.7380981445312 0 1850.7911
612.198486328125 0 1073.254
613.1975708007812 0 885.3042
614.2483520507812 0 1106.5028
616.3095092773438 0 938.51245
624.232666015625 0 845.07227 b Water loss 4
628.3784790039062 0 1521.8993
657.2609252929688 0 1332.6609
659.2958374023438 0 2432.685
682.3488159179688 0 902.55676
685.2589721679688 0 1934.3044
699.4033203125 0 776.4241
700.3629760742188 0 13715.5625
701.364990234375 0 3563.889
702.3705444335938 0 828.13367
703.2666015625 0 823.4165
715.3754272460938 0 668.56683
721.2595825195312 0 1795.7366
723.291748046875 0 6158.6953 y 4
724.2938842773438 0 2921.4302
749.2564086914062 0 2096.7004
750.2608642578125 0 997.7303
757.3872680664062 0 1637.9763
774.3208618164062 0 2826.5117
775.3209228515625 0 1367.2075
783.3997802734375 0 2691.378
784.39990234375 0 1407.4503
800.4234008789062 0 1821.8845
801.4130859375 0 6413.291
802.4126586914062 0 2056.5269
803.4159545898438 0 878.058
828.4219360351562 0 2794.8774
838.3189086914062 0 7658.6465 y 3
839.321533203125 0 3405.1143
840.3233642578125 0 875.9065
844.451904296875 0 8474.893
845.4556884765625 0 4522.677
846.4552612304688 0 1432.6406
854.4349365234375 0 1225.779
871.3381958007812 0 911.9389
872.4473876953125 0 33826.96
873.4498291015625 0 14887.167
874.451171875 0 5079.5034
875.4544677734375 0 862.2736
889.3475341796875 0 10578.498
890.3499755859375 0 5780.8477
891.3543090820312 0 1201.7155
935.334228515625 0 1997.8395 y Water loss 2
936.3299560546875 0 1346.3616
953.34521484375 0 36415.29 y 2
954.347900390625 0 17624.684
955.3485717773438 0 5814.4946
956.3483276367188 0 981.2815
991.5196533203125 0 5347.761
992.5234985351562 0 3000.0815
993.5233154296875 0 1094.038
1001.5028076171875 0 942.6851
1019.5145874023438 0 16041.866
1020.5187377929688 0 8426.085
1021.5185546875 0 3201.665
1022.5311889648438 0 655.93243
1100.413330078125 0 2079.8105 y 1
1101.4190673828125 0 1684.9229
1980.8382568359375 0 725.11957
3306.2109375 0 646.9964

Spectrum Details

|  |  |
| --- | --- |
| Matched peaks? Matched peaksThe total absolute number of peaks matched. Additionally in brackets the total fraction of peaks matched and the total number of peaks is shown. | 22 (8.76% of 251) |
| FDR? FDRThe false discovery rate estimated for this peptide. It is calculated by matching all theoretical fragments with a non-integer shift with the raw peaks for this spectrum. This is done with 40 different shifts. The resulting percentage is the average number of annotated peaks over the number of annotated peaks with the correct spectrum. | 1.08% |
| Satellite FDR? Satellite FDRSee the FDR for details on its calculation. This satellite ion specific FDR only contains the satellite ions (d/w) for I/L/J positions. | - |
| PSM Score? PSM ScoreThe PSM Score as given by Hecklib to this annotated spectrum. It is shown with three significant figures. | 250 |

## Spectrum 7474? Spectrum 7474 The raw spectrum of this peptide as annotated by Hecklib. The fragments are coloured according to ion type (see legend). Any peaks with a star '\*' as text can be hovered over to see the full details, first the ion type second the mass shift type. By hovering over the amino acids in the peptide or ions in the legend the corresponding peaks are highlighted. By toggling the 'Unassigned' label you can turn the background (unassigned) peaks on or off in the plot. By updating the slider in the Ion legend you can update the spectrum to only show the top X% of the peaks with labels. The top X% means any peak that is within X% of the highest intensity. By dragging in the spectrum you can zoom in to a specific part of the spectrum and use 'Zoom Out' to get back to the original zoom level. The annotation of the spectrum is based on the given sequence in the peptides file and is done with different software so inconsistencies are likely. The peaks are annotated based on the given sequence, with 20 ppm tolerance.

Copy Data

### Spectrum 7474 (TSV)

#### Preview

```
Loading example...
```

*Click on the button to copy the data to your clipboard.*

Mz MinMz MaxIntensity Max

WidthHeightPeptide font sizePeptide stroke widthSpectrum font sizeSpectrum stroke widthCompact peptide

Ion legend

wxyz

abcd

OtherUnassignedIonChargePositionShow for top:%

TFDDYAMHW

04.03e+58.05e+51.21e+61.61e+6

Zoom Out

y+11c+12y+12c+13z+13y+27c+14y+13c+28y+28z+28z+14y+28y+14c+15z+15c+16y+15c+16w+16y+16z+16y+16c+17c+17c+17w+17y+17z+17y+17c+18c+18c+18z+18y+18

0801160224043205

Fragment Matches Table

Show background peaks

| Position | Ion type | Intensity | mz Theoretical | mz Error (Th) | mz Error (ppm) | Charge | Series Number |
| --- | --- | --- | --- | --- | --- | --- | --- |
| - | - | 1597 | 120.1 | - | - | 0 | - |
| - | - | 1.762E+04 | 120.1 | - | - | 0 | - |
| - | - | 1405 | 121.1 | - | - | 0 | - |
| - | - | 953.6 | 124.6 | - | - | 0 | - |
| - | - | 944.9 | 126.9 | - | - | 0 | - |
| - | - | 3660 | 136.1 | - | - | 0 | - |
| - | - | 1169 | 143.1 | - | - | 0 | - |
| - | - | 2183 | 148.9 | - | - | 0 | - |
| - | - | 1167 | 152.5 | - | - | 0 | - |
| - | - | 6063 | 159.1 | - | - | 0 | - |
| - | - | 1414 | 160.1 | - | - | 0 | - |
| - | - | 1387 | 171.1 | - | - | 0 | - |
| - | - | 1107 | 171.3 | - | - | 0 | - |
| - | - | 1360 | 173.4 | - | - | 0 | - |
| - | - | 1399 | 173.4 | - | - | 0 | - |
| - | - | 1965 | 173.5 | - | - | 0 | - |
| - | - | 3460 | 176.1 | - | - | 0 | - |
| - | - | 6950 | 177.1 | - | - | 0 | - |
| - | - | 4618 | 188.1 | - | - | 0 | - |
| - | - | 4979 | 203.1 | - | - | 0 | - |
| 9 | y | 1.111E+04 | 205.1 | 0.0002122 | 1.035 | +1 | 1 |
| - | - | 5.573E+05 | 221.1 | - | - | 0 | - |
| - | - | 6.481E+04 | 222.1 | - | - | 0 | - |
| - | - | 3097 | 223.1 | - | - | 0 | - |
| - | - | 3890 | 231.1 | - | - | 0 | - |
| - | - | 6074 | 231.1 | - | - | 0 | - |
| - | - | 3334 | 235.1 | - | - | 0 | - |
| 2 | c | 2.167E+05 | 249.1 | 0.0002731 | 1.096 | +1 | 2 |
| - | - | 2.516E+04 | 250.1 | - | - | 0 | - |
| - | - | 1825 | 251.1 | - | - | 0 | - |
| - | - | 2254 | 251.1 | - | - | 0 | - |
| - | - | 1726 | 262 | - | - | 0 | - |
| - | - | 1.364E+04 | 263.1 | - | - | 0 | - |
| - | - | 2251 | 264.1 | - | - | 0 | - |
| - | - | 3.208E+04 | 264.1 | - | - | 0 | - |
| - | - | 7258 | 265.1 | - | - | 0 | - |
| - | - | 1.197E+04 | 282.1 | - | - | 0 | - |
| - | - | 1.622E+04 | 283.2 | - | - | 0 | - |
| - | - | 1330 | 283.7 | - | - | 0 | - |
| - | - | 2085 | 284.2 | - | - | 0 | - |
| - | - | 1397 | 287 | - | - | 0 | - |
| - | - | 2177 | 292.2 | - | - | 0 | - |
| - | - | 2110 | 324.1 | - | - | 0 | - |
| - | - | 1.343E+04 | 327.1 | - | - | 0 | - |
| - | - | 2367 | 328.1 | - | - | 0 | - |
| 8 | y | 5.771E+04 | 342.2 | 0.0003977 | 1.162 | +1 | 2 |
| - | - | 1.147E+04 | 343.2 | - | - | 0 | - |
| - | - | 5073 | 346.1 | - | - | 0 | - |
| - | - | 2814 | 356.1 | - | - | 0 | - |
| - | - | 4179 | 358.2 | - | - | 0 | - |
| 3 | c | 5.133E+04 | 364.2 | 0.0001093 | 0.3001 | +1 | 3 |
| - | - | 8093 | 365.2 | - | - | 0 | - |
| - | - | 8951 | 378.1 | - | - | 0 | - |
| - | - | 2240 | 379.1 | - | - | 0 | - |
| - | - | 5356 | 411.2 | - | - | 0 | - |
| - | - | 2545 | 412.2 | - | - | 0 | - |
| - | - | 5178 | 415.2 | - | - | 0 | - |
| - | - | 4173 | 425.2 | - | - | 0 | - |
| - | - | 9013 | 429.2 | - | - | 0 | - |
| - | - | 3927 | 430.2 | - | - | 0 | - |
| - | - | 1760 | 433.2 | - | - | 0 | - |
| - | - | 1676 | 454.2 | - | - | 0 | - |
| - | - | 1631 | 454.5 | - | - | 0 | - |
| - | - | 1781 | 455.2 | - | - | 0 | - |
| - | - | 7121 | 461.2 | - | - | 0 | - |
| - | - | 2215 | 465.2 | - | - | 0 | - |
| - | - | 1784 | 467.2 | - | - | 0 | - |
| - | - | 2884 | 471.2 | - | - | 0 | - |
| 7 | z | 1.965E+04 | 473.2 | 0.005513 | 11.65 | +1 | 3 |
| - | - | 1.037E+05 | 474.2 | - | - | 0 | - |
| - | - | 2.744E+04 | 475.2 | - | - | 0 | - |
| - | - | 4031 | 476.2 | - | - | 0 | - |
| 3 | y | 4593 | 477.2 | 0.001074 | 2.25 | +2 | 7 |
| - | - | 2175 | 477.7 | - | - | 0 | - |
| 4 | c | 3.069E+04 | 479.2 | 0.00051 | 1.064 | +1 | 4 |
| - | - | 6058 | 480.2 | - | - | 0 | - |
| - | - | 4385 | 481.2 | - | - | 0 | - |
| - | - | 4569 | 488.2 | - | - | 0 | - |
| 7 | y | 5.572E+04 | 489.2 | 0.005222 | 10.67 | +1 | 3 |
| - | - | 1.579E+04 | 490.2 | - | - | 0 | - |
| - | - | 2778 | 491.2 | - | - | 0 | - |
| - | - | 7223 | 496.2 | - | - | 0 | - |
| 8 | c | 3442 | 499.2 | 0.003275 | 6.561 | +2 | 8 |
| - | - | 2219 | 499.7 | - | - | 0 | - |
| - | - | 1.427E+04 | 500.2 | - | - | 0 | - |
| - | - | 5421 | 501.2 | - | - | 0 | - |
| - | - | 3280 | 513.2 | - | - | 0 | - |
| - | - | 4277 | 516.2 | - | - | 0 | - |
| - | - | 5666 | 519.2 | - | - | 0 | - |
| - | - | 1974 | 520.2 | - | - | 0 | - |
| - | - | 1.203E+04 | 521.2 | - | - | 0 | - |
| - | - | 3198 | 522.2 | - | - | 0 | - |
| 2 | y | 4614 | 541.7 | 0.002747 | 5.071 | +2 | 8 |
| - | - | 4490 | 542.2 | - | - | 0 | - |
| 2 | z | 2422 | 542.7 | 0.007193 | 13.25 | +2 | 8 |
| 6 | z | 3.448E+05 | 544.2 | 0.005326 | 9.786 | +1 | 4 |
| - | - | 1.638E+05 | 545.2 | - | - | 0 | - |
| - | - | 3.637E+04 | 546.2 | - | - | 0 | - |
| - | - | 6422 | 547.2 | - | - | 0 | - |
| 2 | y | 3.82E+04 | 550.7 | 0.002775 | 5.039 | +2 | 8 |
| - | - | 2.355E+04 | 551.2 | - | - | 0 | - |
| - | - | 8282 | 551.7 | - | - | 0 | - |
| - | - | 1537 | 552.2 | - | - | 0 | - |
| - | - | 1693 | 559.2 | - | - | 0 | - |
| 6 | y | 9.274E+04 | 560.2 | 0.004973 | 8.877 | +1 | 4 |
| - | - | 2.497E+04 | 561.2 | - | - | 0 | - |
| - | - | 6961 | 562.2 | - | - | 0 | - |
| - | - | 8226 | 569.2 | - | - | 0 | - |
| - | - | 3827 | 569.7 | - | - | 0 | - |
| - | - | 7979 | 578.2 | - | - | 0 | - |
| - | - | 2840 | 578.7 | - | - | 0 | - |
| - | - | 2542 | 579.2 | - | - | 0 | - |
| - | - | 2936 | 583.2 | - | - | 0 | - |
| - | - | 1.298E+04 | 587.2 | - | - | 0 | - |
| - | - | 4284 | 588.2 | - | - | 0 | - |
| - | - | 2117 | 591.2 | - | - | 0 | - |
| - | - | 2.335E+04 | 592.2 | - | - | 0 | - |
| - | - | 1.459E+04 | 592.7 | - | - | 0 | - |
| - | - | 7148 | 593.2 | - | - | 0 | - |
| - | - | 4062 | 596.2 | - | - | 0 | - |
| - | - | 1577 | 597.2 | - | - | 0 | - |
| - | - | 7.403E+04 | 601.2 | - | - | 0 | - |
| - | - | 3071 | 601.3 | - | - | 0 | - |
| - | - | 4.208E+04 | 601.7 | - | - | 0 | - |
| - | - | 1.692E+04 | 602.2 | - | - | 0 | - |
| - | - | 4590 | 602.7 | - | - | 0 | - |
| - | - | 1579 | 606.2 | - | - | 0 | - |
| - | - | 2458 | 612.2 | - | - | 0 | - |
| - | - | 1.03E+04 | 614.2 | - | - | 0 | - |
| - | - | 4855 | 615.2 | - | - | 0 | - |
| - | - | 2121 | 618.3 | - | - | 0 | - |
| - | - | 1560 | 619.3 | - | - | 0 | - |
| - | - | 5975 | 624.2 | - | - | 0 | - |
| - | - | 4205 | 634.2 | - | - | 0 | - |
| - | - | 1.325E+04 | 636.2 | - | - | 0 | - |
| - | - | 4040 | 637.2 | - | - | 0 | - |
| 5 | c | 8901 | 642.2 | 0.001482 | 2.308 | +1 | 5 |
| - | - | 1825 | 643.2 | - | - | 0 | - |
| - | - | 3285 | 649.3 | - | - | 0 | - |
| - | - | 9779 | 659.3 | - | - | 0 | - |
| - | - | 2685 | 660.3 | - | - | 0 | - |
| - | - | 4681 | 662.3 | - | - | 0 | - |
| - | - | 1.364E+04 | 663.3 | - | - | 0 | - |
| - | - | 3716 | 664.3 | - | - | 0 | - |
| - | - | 2174 | 665.3 | - | - | 0 | - |
| - | - | 4529 | 685.3 | - | - | 0 | - |
| - | - | 3983 | 688.3 | - | - | 0 | - |
| - | - | 3580 | 693.2 | - | - | 0 | - |
| - | - | 2106 | 695.3 | - | - | 0 | - |
| - | - | 2370 | 696.3 | - | - | 0 | - |
| - | - | 5141 | 706.3 | - | - | 0 | - |
| 5 | z | 1.916E+05 | 707.3 | 0.005352 | 7.567 | +1 | 5 |
| - | - | 9.103E+04 | 708.3 | - | - | 0 | - |
| - | - | 2204 | 708.4 | - | - | 0 | - |
| - | - | 2.721E+04 | 709.3 | - | - | 0 | - |
| - | - | 4263 | 710.3 | - | - | 0 | - |
| 6 | c | 3791 | 713.3 | 0.003187 | 4.467 | +1 | 6 |
| - | - | 1763 | 714.3 | - | - | 0 | - |
| - | - | 1.1E+04 | 715.3 | - | - | 0 | - |
| - | - | 6051 | 716.3 | - | - | 0 | - |
| - | - | 8310 | 721.3 | - | - | 0 | - |
| - | - | 7641 | 722.3 | - | - | 0 | - |
| 5 | y | 9.911E+04 | 723.3 | 0.004755 | 6.574 | +1 | 5 |
| - | - | 3.806E+04 | 724.3 | - | - | 0 | - |
| - | - | 1.136E+04 | 725.3 | - | - | 0 | - |
| - | - | 3844 | 729.3 | - | - | 0 | - |
| 6 | c | 5403 | 730.3 | 0.001512 | 2.07 | +1 | 6 |
| - | - | 2197 | 733.3 | - | - | 0 | - |
| - | - | 5378 | 734.3 | - | - | 0 | - |
| - | - | 3483 | 735.3 | - | - | 0 | - |
| - | - | 2.694E+04 | 749.3 | - | - | 0 | - |
| - | - | 2.203E+04 | 750.3 | - | - | 0 | - |
| - | - | 1.505E+04 | 751.3 | - | - | 0 | - |
| - | - | 3104 | 752.3 | - | - | 0 | - |
| - | - | 7859 | 759.3 | - | - | 0 | - |
| - | - | 5807 | 760.3 | - | - | 0 | - |
| - | - | 1841 | 761.3 | - | - | 0 | - |
| - | - | 3247 | 763.3 | - | - | 0 | - |
| - | - | 5416 | 764.3 | - | - | 0 | - |
| - | - | 1878 | 765.3 | - | - | 0 | - |
| - | - | 3834 | 767.3 | - | - | 0 | - |
| - | - | 2642 | 771.3 | - | - | 0 | - |
| - | - | 2318 | 772.3 | - | - | 0 | - |
| - | - | 1.007E+04 | 774.3 | - | - | 0 | - |
| - | - | 5489 | 775.3 | - | - | 0 | - |
| 4 | w | 4865 | 777.3 | 0.0006215 | 0.7996 | +1 | 6 |
| - | - | 1.067E+05 | 778.3 | - | - | 0 | - |
| - | - | 4.7E+04 | 779.3 | - | - | 0 | - |
| - | - | 1.317E+04 | 780.3 | - | - | 0 | - |
| - | - | 2247 | 781.3 | - | - | 0 | - |
| - | - | 3611 | 787.4 | - | - | 0 | - |
| - | - | 1723 | 793.3 | - | - | 0 | - |
| - | - | 2405 | 805.4 | - | - | 0 | - |
| - | - | 4762 | 808.3 | - | - | 0 | - |
| - | - | 2502 | 815.3 | - | - | 0 | - |
| - | - | 2997 | 816.3 | - | - | 0 | - |
| 4 | y | 3693 | 820.3 | 0.004368 | 5.325 | +1 | 6 |
| - | - | 2202 | 821.3 | - | - | 0 | - |
| 4 | z | 1.32E+05 | 822.3 | 0.004715 | 5.734 | +1 | 6 |
| - | - | 1.328E+05 | 823.3 | - | - | 0 | - |
| - | - | 5.717E+04 | 824.3 | - | - | 0 | - |
| - | - | 1.237E+04 | 825.3 | - | - | 0 | - |
| - | - | 2035 | 829.3 | - | - | 0 | - |
| - | - | 2.479E+04 | 830.3 | - | - | 0 | - |
| - | - | 1.543E+04 | 831.3 | - | - | 0 | - |
| - | - | 4591 | 832.4 | - | - | 0 | - |
| - | - | 5.641E+04 | 833.3 | - | - | 0 | - |
| - | - | 3.89E+04 | 834.3 | - | - | 0 | - |
| - | - | 1.423E+04 | 835.3 | - | - | 0 | - |
| - | - | 2525 | 836.3 | - | - | 0 | - |
| 4 | y | 1.044E+05 | 838.3 | 0.004729 | 5.641 | +1 | 6 |
| - | - | 4.823E+04 | 839.3 | - | - | 0 | - |
| - | - | 1.567E+04 | 840.3 | - | - | 0 | - |
| - | - | 7.329E+04 | 849.3 | - | - | 0 | - |
| - | - | 3.518E+04 | 850.3 | - | - | 0 | - |
| - | - | 1.1E+04 | 851.4 | - | - | 0 | - |
| - | - | 9916 | 858.3 | - | - | 0 | - |
| 7 | c | 7109 | 859.3 | 0.007028 | 8.178 | +1 | 7 |
| 7 | c | 8271 | 860.3 | 0.006699 | 7.786 | +1 | 7 |
| - | - | 3147 | 861.3 | - | - | 0 | - |
| - | - | 2073 | 862.3 | - | - | 0 | - |
| - | - | 4907 | 866.3 | - | - | 0 | - |
| - | - | 4190 | 871.3 | - | - | 0 | - |
| - | - | 2136 | 872.3 | - | - | 0 | - |
| - | - | 1.021E+04 | 874.3 | - | - | 0 | - |
| - | - | 7585 | 875.3 | - | - | 0 | - |
| - | - | 6.708E+04 | 876.3 | - | - | 0 | - |
| 7 | c | 2.877E+05 | 877.3 | 0.004136 | 4.715 | +1 | 7 |
| - | - | 1.305E+05 | 878.3 | - | - | 0 | - |
| - | - | 4.212E+04 | 879.3 | - | - | 0 | - |
| - | - | 4919 | 880.3 | - | - | 0 | - |
| - | - | 6.529E+04 | 889.3 | - | - | 0 | - |
| - | - | 3.484E+04 | 890.3 | - | - | 0 | - |
| - | - | 9392 | 891.4 | - | - | 0 | - |
| 3 | w | 4743 | 892.3 | 0.01034 | 11.59 | +1 | 7 |
| - | - | 1.66E+05 | 893.3 | - | - | 0 | - |
| - | - | 8.978E+04 | 894.3 | - | - | 0 | - |
| - | - | 2.695E+04 | 895.3 | - | - | 0 | - |
| - | - | 5284 | 896.3 | - | - | 0 | - |
| - | - | 4520 | 898.3 | - | - | 0 | - |
| - | - | 1990 | 907.3 | - | - | 0 | - |
| - | - | 2628 | 907.4 | - | - | 0 | - |
| - | - | 2261 | 908.4 | - | - | 0 | - |
| - | - | 2209 | 925.3 | - | - | 0 | - |
| - | - | 6387 | 926.4 | - | - | 0 | - |
| - | - | 3728 | 927.4 | - | - | 0 | - |
| 3 | y | 2.135E+04 | 935.3 | 0.004158 | 4.446 | +1 | 7 |
| - | - | 1.717E+04 | 936.3 | - | - | 0 | - |
| 3 | z | 3.81E+04 | 937.3 | 0.005604 | 5.979 | +1 | 7 |
| - | - | 2.348E+04 | 938.3 | - | - | 0 | - |
| - | - | 8793 | 939.3 | - | - | 0 | - |
| - | - | 2136 | 940.3 | - | - | 0 | - |
| - | - | 1.113E+04 | 952.4 | - | - | 0 | - |
| 3 | y | 5.05E+05 | 953.3 | 0.005129 | 5.38 | +1 | 7 |
| - | - | 2.567E+05 | 954.3 | - | - | 0 | - |
| - | - | 1.01E+05 | 955.3 | - | - | 0 | - |
| - | - | 1.156E+04 | 956.3 | - | - | 0 | - |
| - | - | 2287 | 963.3 | - | - | 0 | - |
| - | - | 6.007E+04 | 970.4 | - | - | 0 | - |
| - | - | 3.168E+04 | 971.4 | - | - | 0 | - |
| - | - | 1.174E+04 | 972.4 | - | - | 0 | - |
| - | - | 1658 | 981.4 | - | - | 0 | - |
| 8 | c | 2085 | 996.4 | 0.01866 | 18.72 | +1 | 8 |
| 8 | c | 8350 | 997.4 | 0.01151 | 11.54 | +1 | 8 |
| - | - | 5648 | 998.4 | - | - | 0 | - |
| - | - | 9167 | 999.4 | - | - | 0 | - |
| - | - | 3494 | 1000 | - | - | 0 | - |
| 8 | c | 5.235E+05 | 1014 | 0.004612 | 4.546 | +1 | 8 |
| - | - | 2.997E+05 | 1015 | - | - | 0 | - |
| - | - | 1.007E+05 | 1016 | - | - | 0 | - |
| - | - | 1.455E+04 | 1017 | - | - | 0 | - |
| - | - | 2015 | 1026 | - | - | 0 | - |
| - | - | 4976 | 1036 | - | - | 0 | - |
| - | - | 4221 | 1037 | - | - | 0 | - |
| - | - | 1831 | 1038 | - | - | 0 | - |
| - | - | 2.401E+04 | 1040 | - | - | 0 | - |
| - | - | 1.546E+04 | 1041 | - | - | 0 | - |
| - | - | 7035 | 1042 | - | - | 0 | - |
| - | - | 2400 | 1056 | - | - | 0 | - |
| - | - | 3140 | 1069 | - | - | 0 | - |
| - | - | 6563 | 1071 | - | - | 0 | - |
| - | - | 3622 | 1072 | - | - | 0 | - |
| - | - | 2045 | 1079 | - | - | 0 | - |
| - | - | 2749 | 1080 | - | - | 0 | - |
| 2 | z | 5.528E+04 | 1084 | 0.00384 | 3.542 | +1 | 8 |
| - | - | 3.55E+04 | 1085 | - | - | 0 | - |
| - | - | 1.367E+04 | 1086 | - | - | 0 | - |
| - | - | 7629 | 1094 | - | - | 0 | - |
| - | - | 3807 | 1095 | - | - | 0 | - |
| - | - | 3408 | 1096 | - | - | 0 | - |
| - | - | 4148 | 1097 | - | - | 0 | - |
| - | - | 2840 | 1098 | - | - | 0 | - |
| 2 | y | 2.606E+04 | 1100 | 0.004648 | 4.223 | +1 | 8 |
| - | - | 1.532E+04 | 1101 | - | - | 0 | - |
| - | - | 5510 | 1102 | - | - | 0 | - |
| - | - | 1.995E+04 | 1103 | - | - | 0 | - |
| - | - | 1.245E+04 | 1104 | - | - | 0 | - |
| - | - | 4096 | 1105 | - | - | 0 | - |
| - | - | 9414 | 1110 | - | - | 0 | - |
| - | - | 8167 | 1111 | - | - | 0 | - |
| - | - | 1910 | 1112 | - | - | 0 | - |
| - | - | 1971 | 1113 | - | - | 0 | - |
| - | - | 1.202E+04 | 1122 | - | - | 0 | - |
| - | - | 1.083E+04 | 1123 | - | - | 0 | - |
| - | - | 8797 | 1124 | - | - | 0 | - |
| - | - | 2217 | 1128 | - | - | 0 | - |
| - | - | 1.513E+04 | 1138 | - | - | 0 | - |
| - | - | 9183 | 1139 | - | - | 0 | - |
| - | - | 1.093E+05 | 1140 | - | - | 0 | - |
| - | - | 1.699E+05 | 1141 | - | - | 0 | - |
| - | - | 1.928E+05 | 1142 | - | - | 0 | - |
| - | - | 1.045E+05 | 1143 | - | - | 0 | - |
| - | - | 3.782E+04 | 1144 | - | - | 0 | - |
| - | - | 6187 | 1145 | - | - | 0 | - |
| - | - | 7394 | 1156 | - | - | 0 | - |
| - | - | 4223 | 1157 | - | - | 0 | - |
| - | - | 1.168E+04 | 1158 | - | - | 0 | - |
| - | - | 5509 | 1159 | - | - | 0 | - |
| - | - | 2931 | 1160 | - | - | 0 | - |
| - | - | 3928 | 1166 | - | - | 0 | - |
| - | - | 2950 | 1167 | - | - | 0 | - |
| - | - | 3118 | 1168 | - | - | 0 | - |
| - | - | 2414 | 1169 | - | - | 0 | - |
| - | - | 6672 | 1170 | - | - | 0 | - |
| - | - | 5212 | 1171 | - | - | 0 | - |
| - | - | 2076 | 1172 | - | - | 0 | - |
| - | - | 1.418E+04 | 1174 | - | - | 0 | - |
| - | - | 9386 | 1175 | - | - | 0 | - |
| - | - | 3883 | 1176 | - | - | 0 | - |
| - | - | 8.446E+04 | 1184 | - | - | 0 | - |
| - | - | 7.281E+05 | 1185 | - | - | 0 | - |
| - | - | 5.004E+05 | 1186 | - | - | 0 | - |
| - | - | 2.239E+05 | 1187 | - | - | 0 | - |
| - | - | 4.364E+04 | 1188 | - | - | 0 | - |
| - | - | 4706 | 1189 | - | - | 0 | - |
| - | - | 4.917E+05 | 1201 | - | - | 0 | - |
| - | - | 1.595E+06 | 1202 | - | - | 0 | - |
| - | - | 1.028E+06 | 1203 | - | - | 0 | - |
| - | - | 4.076E+05 | 1204 | - | - | 0 | - |
| - | - | 5.561E+04 | 1205 | - | - | 0 | - |
| - | - | 2235 | 3083 | - | - | 0 | - |
| - | - | 1883 | 3173 | - | - | 0 | - |

m/z Charge Intensity FragmentType MassShift Position
120.0763931274414 0 1597.0543
120.0810317993164 0 17615.98
121.08451843261719 0 1404.7292
124.58456420898438 0 953.57666
126.87700653076172 0 944.8678
136.07603454589844 0 3659.5237
143.0876922607422 0 1168.69
148.9464569091797 0 2182.7212
152.51382446289062 0 1167.3524
159.091796875 0 6062.777
160.11241149902344 0 1413.7346
171.0773162841797 0 1387.3049
171.34498596191406 0 1107.3647
173.4191131591797 0 1359.5571
173.4320831298828 0 1398.6136
173.45870971679688 0 1964.608
176.1072540283203 0 3460.2454
177.10243225097656 0 6949.8306
188.07095336914062 0 4618.0757
203.11810302734375 0 4978.7285
205.0973663330078 0 11108.559 y 8
221.1287384033203 0 557328.44
222.1320343017578 0 64813.535
223.13406372070312 0 3097.472
231.06126403808594 0 3890.468
231.11306762695312 0 6074.2295
235.1077117919922 0 3334.0747
249.12364196777344 0 216681.56 c Ammonia loss 1
250.12680053710938 0 25158.428
251.1029510498047 0 1824.9471
251.12948608398438 0 2254.347
262.03521728515625 0 1725.7743
263.10272216796875 0 13639.7
264.1064453125 0 2251.4421
264.1369934082031 0 32078.24
265.1413879394531 0 7257.9087
282.1478576660156 0 11965.072
283.1552734375 0 16222.265
283.7259216308594 0 1330.1963
284.1591491699219 0 2085.0156
287.0005187988281 0 1397.1726
292.16595458984375 0 2177.206
324.14654541015625 0 2110.3877
327.14556884765625 0 13426.602
328.14837646484375 0 2366.9255
342.1564636230469 0 57711.605 y 7
343.1595153808594 0 11465.715
346.1398010253906 0 5073.472
356.138916015625 0 2814.2498
358.1552734375 0 4178.832
364.1504211425781 0 51327.16 c Ammonia loss 2
365.15313720703125 0 8092.882
378.12969970703125 0 8951.372
379.1343078613281 0 2239.6248
411.1907043457031 0 5356.2207
412.1964111328125 0 2545.2217
415.1522216796875 0 5177.705
425.19317626953125 0 4172.9526
429.1831970214844 0 9013.047
430.18829345703125 0 3927.4434
433.1720886230469 0 1759.7737
454.197021484375 0 1676.2046
454.48876953125 0 1630.9086
455.20367431640625 0 1780.7893
461.16632080078125 0 7121.136
465.1632385253906 0 2214.968
467.2041015625 0 1784.1581
471.1825256347656 0 2884.402
473.17333984375 0 19650.596 z 6
474.1807556152344 0 103713.55
475.18359375 0 27435.912
476.18438720703125 0 4031.4663
477.1730041503906 0 4592.758 y 2
477.6753845214844 0 2175.0872
479.1777648925781 0 30694.164 c Ammonia loss 3
480.1806335449219 0 6058.0024
481.2210388183594 0 4384.7837
488.1833190917969 0 4568.978
489.1917724609375 0 55717.38 y 6
490.19384765625 0 15793.053
491.1948547363281 0 2778.3582
496.23040771484375 0 7222.529
499.1904602050781 0 3442.2793 c Ammonia loss 7
499.68914794921875 0 2218.8774
500.2200927734375 0 14270.626
501.2238464355469 0 5421.379
513.199462890625 0 3279.5068
516.178466796875 0 4276.696
519.2054443359375 0 5665.834
520.2051391601562 0 1973.9126
521.217529296875 0 12025.353
522.2206420898438 0 3198.1738
541.7057495117188 0 4613.9746 y Water loss 1
542.2055053710938 0 4490.375
542.7061157226562 0 2421.9854 z 1
544.2102661132812 0 344751.7 z 5
545.21484375 0 163803.11
546.21630859375 0 36367.37
547.224609375 0 6421.6987
550.7110595703125 0 38201.516 y 1
551.212646484375 0 23546.27
551.7138061523438 0 8281.598
552.2130737304688 0 1536.5061
559.21875 0 1692.6699
560.2286376953125 0 92735.39 y 5
561.23095703125 0 24971.309
562.2316284179688 0 6961.0674
569.2324829101562 0 8225.671
569.7335205078125 0 3826.651
578.22216796875 0 7978.8623
578.7315063476562 0 2840.4521
579.224853515625 0 2542.0461
583.2222290039062 0 2936.0356
587.216064453125 0 12983.528
588.222412109375 0 4283.953
591.2242431640625 0 2117.0596
592.2308349609375 0 23350.852
592.731201171875 0 14591.516
593.233154296875 0 7148.121
596.2354125976562 0 4061.8787
597.2260131835938 0 1577.225
601.2352905273438 0 74027.125
601.2835693359375 0 3070.556
601.736572265625 0 42082.266
602.2372436523438 0 16918.95
602.7361450195312 0 4589.638
606.2230834960938 0 1579.2554
612.196533203125 0 2458.268
614.2454223632812 0 10297.064
615.2467651367188 0 4855.448
618.271240234375 0 2120.9849
619.2765502929688 0 1559.7692
624.229736328125 0 5974.616
634.229248046875 0 4205.4146
636.2446899414062 0 13250.695
637.2457275390625 0 4040.0508
642.2420654296875 0 8901.32 c Ammonia loss 4
643.2413330078125 0 1824.7704
649.251220703125 0 3284.5652
659.2946166992188 0 9779.032
660.2965698242188 0 2685.4949
662.2590942382812 0 4680.7495
663.281494140625 0 13639.992
664.2841186523438 0 3715.7314
665.28857421875 0 2174.4072
685.2592163085938 0 4528.993
688.2975463867188 0 3982.9556
693.2442626953125 0 3579.5244
695.2677001953125 0 2105.8315
696.2699584960938 0 2370.4941
706.2610473632812 0 5141.3374
707.2736206054688 0 191584.39 z 4
708.2772827148438 0 91031.37
708.381103515625 0 2204.0024
709.2769165039062 0 27212.291
710.2749633789062 0 4262.874
713.2808837890625 0 3790.659 c Ammonia loss 5
714.3076171875 0 1762.5967
715.3191528320312 0 11000.93
716.3233642578125 0 6050.7954
721.2646484375 0 8309.877
722.2774047851562 0 7641.135
723.291748046875 0 99109.336 y 4
724.2948608398438 0 38055.344
725.294921875 0 11356.094
729.2942504882812 0 3843.6746
730.302734375 0 5402.939 c 5
733.29345703125 0 2196.6577
734.3177490234375 0 5378.452
735.323486328125 0 3483.4216
749.2562255859375 0 26937.627
750.2725219726562 0 22028.885
751.2739868164062 0 15046.661
752.2736206054688 0 3103.999
759.3095092773438 0 7858.878
760.313720703125 0 5807.1367
761.3201293945312 0 1840.9949
763.2886962890625 0 3247.2373
764.2849731445312 0 5416.136
765.2915649414062 0 1878.4666
767.2691650390625 0 3834.0593
771.3499145507812 0 2642.4888
772.347900390625 0 2317.8865
774.3197631835938 0 10073.877
775.3224487304688 0 5489.1045
777.2969360351562 0 4864.698 w 3
778.3099365234375 0 106737.71
779.3131103515625 0 46995.07
780.3137817382812 0 13169.536
781.3087158203125 0 2247.3118
787.3616943359375 0 3611.454
793.3167724609375 0 1723.1306
805.3583984375 0 2404.6553
808.2723388671875 0 4761.777
815.308349609375 0 2502.107
816.3087768554688 0 2997.4678
820.3077392578125 0 3693.0762 y Water loss 3
821.2907104492188 0 2201.553
822.2999267578125 0 131984.4 z 3
823.3057250976562 0 132803.73
824.3082275390625 0 57170.227
825.310791015625 0 12372.384
829.33740234375 0 2035.3494
830.3468627929688 0 24786.312
831.349853515625 0 15432.03
832.3513793945312 0 4591.1353
833.3257446289062 0 56410.293
834.3298950195312 0 38902.76
835.3318481445312 0 14227.51
836.3388671875 0 2524.6023
838.3186645507812 0 104423.09 y 3
839.3214111328125 0 48232.805
840.3222045898438 0 15674.408
849.34716796875 0 73294.58
850.3485717773438 0 35180.69
851.3502807617188 0 11001.07
858.3218383789062 0 9915.586
859.317138671875 0 7108.556 c Water loss 6
860.3148803710938 0 8270.832 c Ammonia loss 6
861.320068359375 0 3146.5435
862.3247680664062 0 2072.6572
866.313720703125 0 4907.376
871.3350219726562 0 4190.0537
872.3369750976562 0 2136.0176
874.3389892578125 0 10209.176
875.3345336914062 0 7584.87
876.3321533203125 0 67084.586
877.3388671875 0 287736.38 c 6
878.3416748046875 0 130465.79
879.3411865234375 0 42122.504
880.33935546875 0 4919.1855
889.346923828125 0 65286.207
890.3499755859375 0 34838.31
891.352783203125 0 9392.203
892.3348388671875 0 4743.4097 w 2
893.3369140625 0 165971.12
894.3400268554688 0 89777.7
895.3400268554688 0 26948.412
896.3323974609375 0 5284.4775
898.3367919921875 0 4519.5977
907.3273315429688 0 1989.6703
907.4049072265625 0 2627.7842
908.4090576171875 0 2261.0588
925.322998046875 0 2209.2256
926.396484375 0 6386.915
927.39599609375 0 3727.9612
935.33447265625 0 21353.395 y Water loss 2
936.3356323242188 0 17169.137
937.3277587890625 0 38096.42 z 2
938.329345703125 0 23477.918
939.3325805664062 0 8792.837
940.3386840820312 0 2135.9404
952.3734741210938 0 11131.198
953.3460083007812 0 504993 y 2
954.348876953125 0 256701.5
955.3491821289062 0 100988.016
956.3491821289062 0 11558.796
963.3301391601562 0 2286.812
970.38427734375 0 60065.094
971.38671875 0 31679.46
972.3889770507812 0 11738.292
981.3685302734375 0 1657.6431
996.4017333984375 0 2085.241 c Water loss 7
997.3786010742188 0 8350.193 c Ammonia loss 7
998.3869018554688 0 5647.6187
999.3867797851562 0 9167.1045
1000.393310546875 0 3493.549
1014.3982543945312 0 523461.03 c 7
1015.4010009765625 0 299738.38
1016.4011840820312 0 100694.23
1017.4039916992188 0 14552.26
1026.404541015625 0 2014.9081
1036.414306640625 0 4975.741
1037.4326171875 0 4221.003
1038.437255859375 0 1831.331
1040.405029296875 0 24005.008
1041.408203125 0 15463.059
1042.411376953125 0 7035.342
1056.3870849609375 0 2400.2166
1069.39013671875 0 3139.6824
1071.3927001953125 0 6562.8604
1072.400634765625 0 3621.5208
1079.466796875 0 2044.6904
1080.4573974609375 0 2749.2424
1084.3944091796875 0 55282.957 z 1
1085.398681640625 0 35500.156
1086.3988037109375 0 13671.765
1094.4775390625 0 7629.344
1095.4859619140625 0 3807.4016
1096.482177734375 0 3408.2117
1097.46142578125 0 4148.365
1098.4625244140625 0 2839.8127
1100.4139404296875 0 26058.34 y 1
1101.415771484375 0 15318.007
1102.4195556640625 0 5509.8823
1103.413330078125 0 19950.416
1104.4156494140625 0 12445.658
1105.420166015625 0 4096.07
1110.42236328125 0 9413.883
1111.42919921875 0 8166.6343
1112.4608154296875 0 1910.3658
1113.4951171875 0 1971.0558
1122.453857421875 0 12021.51
1123.4521484375 0 10832.114
1124.4522705078125 0 8796.666
1128.435302734375 0 2217.3965
1138.47265625 0 15125.29
1139.46826171875 0 9183.48
1140.467529296875 0 109341.3
1141.4598388671875 0 169922.12
1142.453369140625 0 192751.39
1143.452392578125 0 104523.12
1144.45263671875 0 37817.246
1145.453125 0 6187.409
1156.4620361328125 0 7393.529
1157.4688720703125 0 4222.756
1158.4853515625 0 11676.415
1159.49365234375 0 5508.6426
1160.4832763671875 0 2930.585
1166.44287109375 0 3928.3643
1167.452880859375 0 2949.732
1168.4361572265625 0 3118.3965
1169.4443359375 0 2413.972
1170.426025390625 0 6671.845
1171.426513671875 0 5212.219
1172.42822265625 0 2076.184
1174.4736328125 0 14184.857
1175.4749755859375 0 9385.6455
1176.4739990234375 0 3883.0718
1184.457763671875 0 84460.04
1185.446533203125 0 728107.06
1186.449462890625 0 500403.78
1187.4503173828125 0 223928.34
1188.45068359375 0 43638.516
1189.448974609375 0 4705.818
1201.4608154296875 0 491710.34
1202.4677734375 0 1594982.4
1203.47119140625 0 1028292.7
1204.4720458984375 0 407550.5
1205.473876953125 0 55607.14
3083.405517578125 0 2234.9524
3173.217041015625 0 1883.3218

Spectrum Details

|  |  |
| --- | --- |
| Matched peaks? Matched peaksThe total absolute number of peaks matched. Additionally in brackets the total fraction of peaks matched and the total number of peaks is shown. | 35 (10.23% of 342) |
| FDR? FDRThe false discovery rate estimated for this peptide. It is calculated by matching all theoretical fragments with a non-integer shift with the raw peaks for this spectrum. This is done with 40 different shifts. The resulting percentage is the average number of annotated peaks over the number of annotated peaks with the correct spectrum. | 0.48% |
| Satellite FDR? Satellite FDRSee the FDR for details on its calculation. This satellite ion specific FDR only contains the satellite ions (d/w) for I/L/J positions. | - |
| PSM Score? PSM ScoreThe PSM Score as given by Hecklib to this annotated spectrum. It is shown with three significant figures. | 449 |

## Spectrum 10919? Spectrum 10919 The raw spectrum of this peptide as annotated by Hecklib. The fragments are coloured according to ion type (see legend). Any peaks with a star '\*' as text can be hovered over to see the full details, first the ion type second the mass shift type. By hovering over the amino acids in the peptide or ions in the legend the corresponding peaks are highlighted. By toggling the 'Unassigned' label you can turn the background (unassigned) peaks on or off in the plot. By updating the slider in the Ion legend you can update the spectrum to only show the top X% of the peaks with labels. The top X% means any peak that is within X% of the highest intensity. By dragging in the spectrum you can zoom in to a specific part of the spectrum and use 'Zoom Out' to get back to the original zoom level. The annotation of the spectrum is based on the given sequence in the peptides file and is done with different software so inconsistencies are likely. The peaks are annotated based on the given sequence, with 20 ppm tolerance.

Copy Data

### Spectrum 10919 (TSV)

#### Preview

```
Loading example...
```

*Click on the button to copy the data to your clipboard.*

Mz MinMz MaxIntensity Max

WidthHeightPeptide font sizePeptide stroke widthSpectrum font sizeSpectrum stroke widthCompact peptide

Ion legend

wxyz

abcd

OtherUnassignedIonChargePositionShow for top:%

TFDDYAMHW

01.63e+43.26e+44.89e+46.52e+4

Zoom Out

a+12y+11a+12b+12b+12a+13y+12b+13b+14y+13y+28y+14\*y+15y+16y+17y+17y+18

0778155623343112

Fragment Matches Table

Show background peaks

| Position | Ion type | Intensity | mz Theoretical | mz Error (Th) | mz Error (ppm) | Charge | Series Number |
| --- | --- | --- | --- | --- | --- | --- | --- |
| - | - | 5.054E+04 | 120.1 | - | - | 0 | - |
| - | - | 435.3 | 120.7 | - | - | 0 | - |
| - | - | 345 | 120.9 | - | - | 0 | - |
| - | - | 4087 | 121.1 | - | - | 0 | - |
| - | - | 418.9 | 122.1 | - | - | 0 | - |
| - | - | 776.2 | 123 | - | - | 0 | - |
| - | - | 414.9 | 127.1 | - | - | 0 | - |
| - | - | 560.8 | 129.1 | - | - | 0 | - |
| - | - | 7488 | 129.1 | - | - | 0 | - |
| - | - | 1568 | 130.1 | - | - | 0 | - |
| - | - | 717.6 | 130.1 | - | - | 0 | - |
| - | - | 816.6 | 132.1 | - | - | 0 | - |
| - | - | 466 | 133.1 | - | - | 0 | - |
| - | - | 1534 | 133.1 | - | - | 0 | - |
| - | - | 1.635E+04 | 136.1 | - | - | 0 | - |
| - | - | 1556 | 137.1 | - | - | 0 | - |
| - | - | 765.6 | 138.1 | - | - | 0 | - |
| - | - | 916.4 | 138.1 | - | - | 0 | - |
| - | - | 499.3 | 146.1 | - | - | 0 | - |
| - | - | 564.5 | 147 | - | - | 0 | - |
| - | - | 943.5 | 147.1 | - | - | 0 | - |
| - | - | 858.2 | 149 | - | - | 0 | - |
| - | - | 569.9 | 155.1 | - | - | 0 | - |
| - | - | 1853 | 156.1 | - | - | 0 | - |
| - | - | 1331 | 157.1 | - | - | 0 | - |
| - | - | 2479 | 158.1 | - | - | 0 | - |
| - | - | 1873 | 159.1 | - | - | 0 | - |
| - | - | 504.3 | 160.1 | - | - | 0 | - |
| - | - | 1002 | 160.1 | - | - | 0 | - |
| - | - | 654.1 | 162.1 | - | - | 0 | - |
| - | - | 5031 | 165.1 | - | - | 0 | - |
| - | - | 494.9 | 165.1 | - | - | 0 | - |
| - | - | 1485 | 166.1 | - | - | 0 | - |
| - | - | 1169 | 167.1 | - | - | 0 | - |
| - | - | 531.8 | 171.1 | - | - | 0 | - |
| - | - | 2984 | 173.1 | - | - | 0 | - |
| - | - | 551 | 173.5 | - | - | 0 | - |
| - | - | 5543 | 176.1 | - | - | 0 | - |
| - | - | 3586 | 177.1 | - | - | 0 | - |
| - | - | 1483 | 177.1 | - | - | 0 | - |
| - | - | 1.169E+04 | 182.1 | - | - | 0 | - |
| - | - | 994.4 | 183.1 | - | - | 0 | - |
| - | - | 3745 | 183.1 | - | - | 0 | - |
| - | - | 1006 | 185.1 | - | - | 0 | - |
| - | - | 3567 | 188.1 | - | - | 0 | - |
| - | - | 927.1 | 191.1 | - | - | 0 | - |
| - | - | 1745 | 193.1 | - | - | 0 | - |
| - | - | 797.5 | 197.1 | - | - | 0 | - |
| - | - | 890.5 | 200.1 | - | - | 0 | - |
| - | - | 3674 | 201.1 | - | - | 0 | - |
| - | - | 1060 | 203.1 | - | - | 0 | - |
| 2 | a | 923.4 | 203.1 | 0.0003203 | 1.577 | +1 | 2 |
| - | - | 606.7 | 204.1 | - | - | 0 | - |
| 9 | y | 2349 | 205.1 | 0.000197 | 0.9604 | +1 | 1 |
| - | - | 898.4 | 211.1 | - | - | 0 | - |
| - | - | 866.4 | 211.1 | - | - | 0 | - |
| - | - | 680.3 | 213.1 | - | - | 0 | - |
| - | - | 688.2 | 213.1 | - | - | 0 | - |
| - | - | 617.3 | 213.1 | - | - | 0 | - |
| - | - | 4348 | 221.1 | - | - | 0 | - |
| 2 | a | 6.459E+04 | 221.1 | 0.0001926 | 0.8711 | +1 | 2 |
| - | - | 8727 | 222.1 | - | - | 0 | - |
| - | - | 620.2 | 222.1 | - | - | 0 | - |
| - | - | 1207 | 223.1 | - | - | 0 | - |
| - | - | 541.1 | 223.1 | - | - | 0 | - |
| - | - | 606.7 | 227.1 | - | - | 0 | - |
| - | - | 917.2 | 231.1 | - | - | 0 | - |
| - | - | 548.2 | 231.1 | - | - | 0 | - |
| 2 | b | 683.2 | 231.1 | 0.0002024 | 0.8759 | +1 | 2 |
| - | - | 881.6 | 233.1 | - | - | 0 | - |
| - | - | 1523 | 235.1 | - | - | 0 | - |
| - | - | 1116 | 239.1 | - | - | 0 | - |
| - | - | 626.1 | 239.1 | - | - | 0 | - |
| - | - | 816 | 241.1 | - | - | 0 | - |
| 2 | b | 1.061E+04 | 249.1 | 0.0001968 | 0.7901 | +1 | 2 |
| - | - | 1408 | 250.1 | - | - | 0 | - |
| - | - | 1112 | 251.1 | - | - | 0 | - |
| - | - | 949.4 | 253.1 | - | - | 0 | - |
| - | - | 828.7 | 253.2 | - | - | 0 | - |
| - | - | 600.3 | 257.2 | - | - | 0 | - |
| - | - | 2300 | 263.1 | - | - | 0 | - |
| - | - | 1204 | 279.1 | - | - | 0 | - |
| - | - | 1441 | 285.1 | - | - | 0 | - |
| - | - | 894 | 296.2 | - | - | 0 | - |
| - | - | 701.9 | 302.1 | - | - | 0 | - |
| 3 | a | 601 | 318.1 | 0.002537 | 7.974 | +1 | 3 |
| - | - | 2313 | 324.1 | - | - | 0 | - |
| - | - | 5988 | 329.1 | - | - | 0 | - |
| - | - | 1514 | 330.2 | - | - | 0 | - |
| - | - | 1481 | 340.2 | - | - | 0 | - |
| 8 | y | 1.196E+04 | 342.2 | 0.0003672 | 1.073 | +1 | 2 |
| - | - | 2525 | 343.2 | - | - | 0 | - |
| - | - | 705.8 | 350.1 | - | - | 0 | - |
| - | - | 1526 | 355.1 | - | - | 0 | - |
| - | - | 711.6 | 356.1 | - | - | 0 | - |
| - | - | 1464 | 357.2 | - | - | 0 | - |
| 3 | b | 2932 | 364.2 | 0.000379 | 1.041 | +1 | 3 |
| - | - | 872.8 | 368.2 | - | - | 0 | - |
| - | - | 1271 | 378.1 | - | - | 0 | - |
| - | - | 670.4 | 392.2 | - | - | 0 | - |
| - | - | 2161 | 394.1 | - | - | 0 | - |
| - | - | 746.3 | 413.3 | - | - | 0 | - |
| - | - | 632.7 | 422.7 | - | - | 0 | - |
| - | - | 2310 | 425.2 | - | - | 0 | - |
| - | - | 1053 | 427.7 | - | - | 0 | - |
| - | - | 688.3 | 436.7 | - | - | 0 | - |
| - | - | 638.2 | 458.8 | - | - | 0 | - |
| - | - | 1546 | 465.2 | - | - | 0 | - |
| - | - | 677.5 | 472 | - | - | 0 | - |
| 4 | b | 2197 | 479.2 | 0.000571 | 1.192 | +1 | 4 |
| - | - | 757.8 | 483.2 | - | - | 0 | - |
| - | - | 780.6 | 487.3 | - | - | 0 | - |
| 7 | y | 2717 | 489.2 | 0.005374 | 10.99 | +1 | 3 |
| - | - | 754.8 | 490.2 | - | - | 0 | - |
| - | - | 1399 | 496.2 | - | - | 0 | - |
| - | - | 788.5 | 511.8 | - | - | 0 | - |
| 2 | y | 1499 | 550.7 | 0.002836 | 5.15 | +2 | 8 |
| - | - | 705.8 | 551.2 | - | - | 0 | - |
| 6 | y | 5290 | 560.2 | 0.004607 | 8.224 | +1 | 4 |
| - | - | 1398 | 561.2 | - | - | 0 | - |
| - | - | 2264 | 585.3 | - | - | 0 | - |
| - | - | 880.1 | 586.3 | - | - | 0 | - |
| - | - | 616.7 | 588 | - | - | 0 | - |
| - | - | 695.2 | 590.9 | - | - | 0 | - |
| - | - | 733.9 | 600.3 | - | - | 0 | - |
| 0 | Precursor | 2033 | 601.2 | 0.004143 | 6.891 | +2 | -1 |
| - | - | 636 | 602.2 | - | - | 0 | - |
| - | - | 1411 | 612.2 | - | - | 0 | - |
| - | - | 705 | 639.7 | - | - | 0 | - |
| - | - | 1068 | 643 | - | - | 0 | - |
| - | - | 641.3 | 649.3 | - | - | 0 | - |
| - | - | 967.3 | 657.3 | - | - | 0 | - |
| - | - | 972.2 | 659.3 | - | - | 0 | - |
| - | - | 727.3 | 660.3 | - | - | 0 | - |
| - | - | 1028 | 663.4 | - | - | 0 | - |
| - | - | 906.9 | 682.3 | - | - | 0 | - |
| - | - | 1913 | 685.3 | - | - | 0 | - |
| - | - | 5982 | 700.4 | - | - | 0 | - |
| - | - | 3165 | 701.4 | - | - | 0 | - |
| - | - | 966.6 | 702.4 | - | - | 0 | - |
| - | - | 776 | 705.1 | - | - | 0 | - |
| - | - | 1309 | 721.3 | - | - | 0 | - |
| 5 | y | 3837 | 723.3 | 0.004572 | 6.321 | +1 | 5 |
| - | - | 1744 | 724.3 | - | - | 0 | - |
| - | - | 2198 | 749.3 | - | - | 0 | - |
| - | - | 763 | 750.4 | - | - | 0 | - |
| - | - | 706.3 | 753.5 | - | - | 0 | - |
| - | - | 822.8 | 757.4 | - | - | 0 | - |
| - | - | 1930 | 774.3 | - | - | 0 | - |
| - | - | 1916 | 783.4 | - | - | 0 | - |
| - | - | 1006 | 785.4 | - | - | 0 | - |
| - | - | 3273 | 801.4 | - | - | 0 | - |
| - | - | 1084 | 802.4 | - | - | 0 | - |
| - | - | 1327 | 828.4 | - | - | 0 | - |
| 4 | y | 5596 | 838.3 | 0.003569 | 4.257 | +1 | 6 |
| - | - | 3052 | 839.3 | - | - | 0 | - |
| - | - | 1126 | 840.3 | - | - | 0 | - |
| - | - | 4929 | 844.5 | - | - | 0 | - |
| - | - | 3103 | 845.5 | - | - | 0 | - |
| - | - | 1070 | 854.4 | - | - | 0 | - |
| - | - | 952.8 | 871.3 | - | - | 0 | - |
| - | - | 1.802E+04 | 872.4 | - | - | 0 | - |
| - | - | 8073 | 873.4 | - | - | 0 | - |
| - | - | 1793 | 874.5 | - | - | 0 | - |
| - | - | 8877 | 889.3 | - | - | 0 | - |
| - | - | 3383 | 890.3 | - | - | 0 | - |
| - | - | 791.4 | 891.3 | - | - | 0 | - |
| 3 | y | 845.7 | 935.3 | 0.007515 | 8.035 | +1 | 7 |
| - | - | 639.2 | 936.3 | - | - | 0 | - |
| 3 | y | 2.446E+04 | 953.3 | 0.004336 | 4.548 | +1 | 7 |
| - | - | 1.321E+04 | 954.3 | - | - | 0 | - |
| - | - | 3556 | 955.3 | - | - | 0 | - |
| - | - | 2852 | 991.5 | - | - | 0 | - |
| - | - | 1760 | 992.5 | - | - | 0 | - |
| - | - | 680 | 995.6 | - | - | 0 | - |
| - | - | 7993 | 1020 | - | - | 0 | - |
| - | - | 4054 | 1021 | - | - | 0 | - |
| - | - | 1657 | 1022 | - | - | 0 | - |
| 2 | y | 1808 | 1100 | 0.005258 | 4.778 | +1 | 8 |
| - | - | 817.3 | 1101 | - | - | 0 | - |
| - | - | 963.6 | 3081 | - | - | 0 | - |

m/z Charge Intensity FragmentType MassShift Position
120.08102416992188 0 50540.28
120.68478393554688 0 435.31186
120.9048843383789 0 344.9729
121.08440399169922 0 4086.7212
122.0712890625 0 418.92365
123.04439544677734 0 776.1738
127.0868148803711 0 414.93036
129.06593322753906 0 560.80524
129.1024627685547 0 7487.6235
130.06521606445312 0 1568.4825
130.08657836914062 0 717.6031
132.0808563232422 0 816.61993
133.06092834472656 0 465.99066
133.08627319335938 0 1534.1483
136.07591247558594 0 16348.97
137.079345703125 0 1555.5374
138.06651306152344 0 765.636
138.0917510986328 0 916.4373
146.0603790283203 0 499.3232
147.04443359375 0 564.51135
147.11317443847656 0 943.5045
149.04519653320312 0 858.1831
155.08126831054688 0 569.8621
156.0769500732422 0 1852.9752
157.13365173339844 0 1330.8273
158.09664916992188 0 2478.6638
159.09194946289062 0 1873.1482
160.07586669921875 0 504.28635
160.11224365234375 0 1001.827
162.09173583984375 0 654.1201
165.05484008789062 0 5031.024
165.07684326171875 0 494.90668
166.08645629882812 0 1484.9368
167.05604553222656 0 1169.0874
171.0763397216797 0 531.7902
173.12876892089844 0 2984.0554
173.45396423339844 0 550.9648
176.1072235107422 0 5542.8228
177.10232543945312 0 3586.0159
177.1114959716797 0 1483.0769
182.08139038085938 0 11688.185
183.0849151611328 0 994.3702
183.1129608154297 0 3744.7197
185.12875366210938 0 1005.69464
188.07061767578125 0 3566.7585
191.11766052246094 0 927.11273
193.108642578125 0 1744.8597
197.1287078857422 0 797.5241
200.13914489746094 0 890.5057
201.12342834472656 0 3673.7085
203.06642150878906 0 1059.9158
203.1182098388672 0 923.4112 a Water loss 1
204.0770263671875 0 606.6942
205.09735107421875 0 2348.9526 y 8
211.10748291015625 0 898.4252
211.14480590820312 0 866.39746
213.08782958984375 0 680.2529
213.09959411621094 0 688.192
213.11138916015625 0 617.2801
221.1034698486328 0 4347.877
221.12864685058594 0 64589.9 a 1
222.1320037841797 0 8726.68
222.14422607421875 0 620.2389
223.0635223388672 0 1206.6619
223.13502502441406 0 541.0863
227.10328674316406 0 606.73755
231.06109619140625 0 917.21
231.0987091064453 0 548.16156
231.11300659179688 0 683.15454 b Water loss 1
233.09197998046875 0 881.58136
235.1082305908203 0 1523.2767
239.0950469970703 0 1116.334
239.1151123046875 0 626.0793
241.07382202148438 0 816.0028
249.12356567382812 0 10614.167 b 1
250.12684631347656 0 1408.0583
251.10317993164062 0 1112.2915
253.1184844970703 0 949.4325
253.16648864746094 0 828.71375
257.16357421875 0 600.26776
263.1028747558594 0 2299.5806
279.0977478027344 0 1203.876
285.1022644042969 0 1440.8046
296.150634765625 0 893.9806
302.1145935058594 0 701.87756
318.1473693847656 0 600.9904 a Water loss 2
324.1459045410156 0 2313.028
329.14971923828125 0 5988.3125
330.153076171875 0 1513.7043
340.1980895996094 0 1480.9598
342.15643310546875 0 11959.498 y 7
343.1598205566406 0 2524.564
350.1357421875 0 705.7987
355.070068359375 0 1525.6775
356.13916015625 0 711.6282
357.2242431640625 0 1463.7773
364.1499328613281 0 2931.907 b 2
368.19281005859375 0 872.80664
378.12939453125 0 1271.241
392.2042236328125 0 670.36066
394.1248779296875 0 2161.1377
413.266845703125 0 746.309
422.7298889160156 0 632.6772
425.1930236816406 0 2309.826
427.7208251953125 0 1053.0953
436.7270202636719 0 688.2705
458.7972717285156 0 638.15704
465.1615905761719 0 1545.6748
471.9722900390625 0 677.4635
479.1778259277344 0 2196.9753 b 3
483.2227783203125 0 757.8341
487.3342590332031 0 780.60284
489.1919250488281 0 2716.6753 y 6
490.19342041015625 0 754.81433
496.2298278808594 0 1399.2815
511.76568603515625 0 788.506
550.7111206054688 0 1498.575 y 1
551.2097778320312 0 705.8169
560.228271484375 0 5289.619 y 5
561.2304077148438 0 1398.2249
585.3350830078125 0 2264.1042
586.338134765625 0 880.1071
587.9902954101562 0 616.7396
590.85400390625 0 695.2135
600.310791015625 0 733.9289
601.2362670898438 0 2033.1282 Precursor
602.2351684570312 0 635.98236
612.1967163085938 0 1411.1196
639.6577758789062 0 705.03424
642.9663696289062 0 1068.4215
649.3262939453125 0 641.2659
657.2603759765625 0 967.28754
659.2932739257812 0 972.2236
660.2963256835938 0 727.3058
663.36376953125 0 1027.7887
682.3455200195312 0 906.87213
685.2564697265625 0 1912.824
700.3617553710938 0 5981.5635
701.3648681640625 0 3164.6738
702.3656005859375 0 966.6085
705.091796875 0 776.02405
721.2635498046875 0 1308.644
723.2915649414062 0 3837.2505 y 4
724.29296875 0 1744.2095
749.255615234375 0 2197.6213
750.4104614257812 0 763.0373
753.5126342773438 0 706.3173
757.3824462890625 0 822.7959
774.3187255859375 0 1930.1649
783.3973999023438 0 1916.4873
785.4105834960938 0 1005.7189
801.40869140625 0 3272.9
802.4091796875 0 1084.0396
828.4214477539062 0 1326.6582
838.3175048828125 0 5596.2104 y 3
839.3206787109375 0 3052.0068
840.3231811523438 0 1126.2335
844.4522094726562 0 4929.4014
845.4537353515625 0 3103.1523
854.4343872070312 0 1069.6958
871.3311157226562 0 952.7786
872.447021484375 0 18021.78
873.4497680664062 0 8073.206
874.452880859375 0 1793.049
889.345947265625 0 8877.343
890.34912109375 0 3383.4092
891.3487548828125 0 791.39417
935.3378295898438 0 845.6565 y Water loss 2
936.3406372070312 0 639.19055
953.34521484375 0 24456.56 y 2
954.34814453125 0 13208.013
955.3465576171875 0 3555.9307
991.5183715820312 0 2852.0442
992.5261840820312 0 1759.953
995.56689453125 0 679.9838
1019.5144653320312 0 7992.6353
1020.518310546875 0 4054.4326
1021.5200805664062 0 1656.7563
1100.41455078125 0 1807.6875 y 1
1101.4144287109375 0 817.34894
3081.4970703125 0 963.6072

Spectrum Details

|  |  |
| --- | --- |
| Matched peaks? Matched peaksThe total absolute number of peaks matched. Additionally in brackets the total fraction of peaks matched and the total number of peaks is shown. | 18 (9.94% of 181) |
| FDR? FDRThe false discovery rate estimated for this peptide. It is calculated by matching all theoretical fragments with a non-integer shift with the raw peaks for this spectrum. This is done with 40 different shifts. The resulting percentage is the average number of annotated peaks over the number of annotated peaks with the correct spectrum. | 1.46% |
| Satellite FDR? Satellite FDRSee the FDR for details on its calculation. This satellite ion specific FDR only contains the satellite ions (d/w) for I/L/J positions. | - |
| PSM Score? PSM ScoreThe PSM Score as given by Hecklib to this annotated spectrum. It is shown with three significant figures. | 213 |

## Spectrum 9794? Spectrum 9794 The raw spectrum of this peptide as annotated by Hecklib. The fragments are coloured according to ion type (see legend). Any peaks with a star '\*' as text can be hovered over to see the full details, first the ion type second the mass shift type. By hovering over the amino acids in the peptide or ions in the legend the corresponding peaks are highlighted. By toggling the 'Unassigned' label you can turn the background (unassigned) peaks on or off in the plot. By updating the slider in the Ion legend you can update the spectrum to only show the top X% of the peaks with labels. The top X% means any peak that is within X% of the highest intensity. By dragging in the spectrum you can zoom in to a specific part of the spectrum and use 'Zoom Out' to get back to the original zoom level. The annotation of the spectrum is based on the given sequence in the peptides file and is done with different software so inconsistencies are likely. The peaks are annotated based on the given sequence, with 20 ppm tolerance.

Copy Data

### Spectrum 9794 (TSV)

#### Preview

```
Loading example...
```

*Click on the button to copy the data to your clipboard.*

Mz MinMz MaxIntensity Max

WidthHeightPeptide font sizePeptide stroke widthSpectrum font sizeSpectrum stroke widthCompact peptide

Ion legend

wxyz

abcd

OtherUnassignedIonChargePositionShow for top:%

TFDDYAMHW

02.83e+45.67e+48.50e+41.13e+5

Zoom Out

a+12y+11a+12b+12b+12y+12b+13b+13b+14b+14y+13y+28y+14\*\*y+15y+16y+17y+17b+18y+18

036272310851447

Fragment Matches Table

Show background peaks

| Position | Ion type | Intensity | mz Theoretical | mz Error (Th) | mz Error (ppm) | Charge | Series Number |
| --- | --- | --- | --- | --- | --- | --- | --- |
| - | - | 1.041E+05 | 120.1 | - | - | 0 | - |
| - | - | 9603 | 121.1 | - | - | 0 | - |
| - | - | 1334 | 123 | - | - | 0 | - |
| - | - | 464 | 128.6 | - | - | 0 | - |
| - | - | 510.7 | 129.1 | - | - | 0 | - |
| - | - | 5479 | 129.1 | - | - | 0 | - |
| - | - | 649.7 | 130.1 | - | - | 0 | - |
| - | - | 2924 | 130.1 | - | - | 0 | - |
| - | - | 461.2 | 132 | - | - | 0 | - |
| - | - | 1651 | 132.1 | - | - | 0 | - |
| - | - | 1062 | 132.1 | - | - | 0 | - |
| - | - | 592.7 | 133.1 | - | - | 0 | - |
| - | - | 3.281E+04 | 136.1 | - | - | 0 | - |
| - | - | 3393 | 137.1 | - | - | 0 | - |
| - | - | 1290 | 138.1 | - | - | 0 | - |
| - | - | 422.1 | 139.9 | - | - | 0 | - |
| - | - | 448.1 | 144 | - | - | 0 | - |
| - | - | 2132 | 146.1 | - | - | 0 | - |
| - | - | 939.8 | 147 | - | - | 0 | - |
| - | - | 622.1 | 148.1 | - | - | 0 | - |
| - | - | 1989 | 149 | - | - | 0 | - |
| - | - | 456 | 151.1 | - | - | 0 | - |
| - | - | 401.2 | 151.5 | - | - | 0 | - |
| - | - | 522.2 | 153.1 | - | - | 0 | - |
| - | - | 511 | 155.1 | - | - | 0 | - |
| - | - | 552 | 155.1 | - | - | 0 | - |
| - | - | 2853 | 156.1 | - | - | 0 | - |
| - | - | 403.4 | 156.1 | - | - | 0 | - |
| - | - | 567.1 | 157.1 | - | - | 0 | - |
| - | - | 669 | 157.1 | - | - | 0 | - |
| - | - | 1669 | 157.1 | - | - | 0 | - |
| - | - | 664.9 | 158.1 | - | - | 0 | - |
| - | - | 3979 | 158.1 | - | - | 0 | - |
| - | - | 5139 | 159.1 | - | - | 0 | - |
| - | - | 1124 | 160.1 | - | - | 0 | - |
| - | - | 651.4 | 160.1 | - | - | 0 | - |
| - | - | 1898 | 160.1 | - | - | 0 | - |
| - | - | 1.3E+04 | 165.1 | - | - | 0 | - |
| - | - | 1061 | 166.1 | - | - | 0 | - |
| - | - | 3231 | 166.1 | - | - | 0 | - |
| - | - | 2046 | 167.1 | - | - | 0 | - |
| - | - | 641.6 | 167.1 | - | - | 0 | - |
| - | - | 467.6 | 169.1 | - | - | 0 | - |
| - | - | 1035 | 169.1 | - | - | 0 | - |
| - | - | 759.4 | 171.1 | - | - | 0 | - |
| - | - | 567.8 | 171.1 | - | - | 0 | - |
| - | - | 639.3 | 173.1 | - | - | 0 | - |
| - | - | 7253 | 173.1 | - | - | 0 | - |
| - | - | 2364 | 175.1 | - | - | 0 | - |
| - | - | 766.9 | 176.1 | - | - | 0 | - |
| - | - | 8346 | 176.1 | - | - | 0 | - |
| - | - | 4917 | 177.1 | - | - | 0 | - |
| - | - | 809.5 | 177.1 | - | - | 0 | - |
| - | - | 504.5 | 178.1 | - | - | 0 | - |
| - | - | 599.4 | 181.1 | - | - | 0 | - |
| - | - | 2.494E+04 | 182.1 | - | - | 0 | - |
| - | - | 2932 | 183.1 | - | - | 0 | - |
| - | - | 539.4 | 183.1 | - | - | 0 | - |
| - | - | 1949 | 185.1 | - | - | 0 | - |
| - | - | 1108 | 186.1 | - | - | 0 | - |
| - | - | 7506 | 188.1 | - | - | 0 | - |
| - | - | 753.7 | 189.1 | - | - | 0 | - |
| - | - | 3242 | 191.1 | - | - | 0 | - |
| - | - | 2108 | 193.1 | - | - | 0 | - |
| - | - | 743.2 | 195.1 | - | - | 0 | - |
| - | - | 486.5 | 195.1 | - | - | 0 | - |
| - | - | 960.7 | 197.1 | - | - | 0 | - |
| - | - | 518.6 | 199.1 | - | - | 0 | - |
| - | - | 535.4 | 199.1 | - | - | 0 | - |
| - | - | 917.1 | 200.1 | - | - | 0 | - |
| - | - | 6189 | 201.1 | - | - | 0 | - |
| - | - | 585.7 | 203.1 | - | - | 0 | - |
| - | - | 1200 | 203.1 | - | - | 0 | - |
| 2 | a | 2170 | 203.1 | 1.513E-05 | 0.07448 | +1 | 2 |
| - | - | 1277 | 204.1 | - | - | 0 | - |
| 9 | y | 4310 | 205.1 | 9.017E-05 | 0.4396 | +1 | 1 |
| - | - | 1491 | 207.1 | - | - | 0 | - |
| - | - | 768 | 209.1 | - | - | 0 | - |
| - | - | 973.4 | 214.1 | - | - | 0 | - |
| - | - | 821.8 | 215.1 | - | - | 0 | - |
| - | - | 565.7 | 217.1 | - | - | 0 | - |
| - | - | 1667 | 217.1 | - | - | 0 | - |
| - | - | 1485 | 219.1 | - | - | 0 | - |
| - | - | 517.7 | 219.7 | - | - | 0 | - |
| - | - | 2345 | 221.1 | - | - | 0 | - |
| - | - | 7170 | 221.1 | - | - | 0 | - |
| 2 | a | 1.123E+05 | 221.1 | 0.0002231 | 1.009 | +1 | 2 |
| - | - | 1.388E+04 | 222.1 | - | - | 0 | - |
| - | - | 1131 | 223.1 | - | - | 0 | - |
| - | - | 940.9 | 223.1 | - | - | 0 | - |
| - | - | 1003 | 225.2 | - | - | 0 | - |
| - | - | 821 | 226.1 | - | - | 0 | - |
| - | - | 771.3 | 226.2 | - | - | 0 | - |
| - | - | 565.6 | 227.1 | - | - | 0 | - |
| - | - | 1092 | 229.1 | - | - | 0 | - |
| - | - | 736.4 | 229.2 | - | - | 0 | - |
| - | - | 533.7 | 229.2 | - | - | 0 | - |
| - | - | 1828 | 231.1 | - | - | 0 | - |
| - | - | 556.6 | 231.1 | - | - | 0 | - |
| 2 | b | 1403 | 231.1 | 0.000355 | 1.536 | +1 | 2 |
| - | - | 915.5 | 233.1 | - | - | 0 | - |
| - | - | 938 | 233.1 | - | - | 0 | - |
| - | - | 627.1 | 233.1 | - | - | 0 | - |
| - | - | 3325 | 235.1 | - | - | 0 | - |
| - | - | 1229 | 239.1 | - | - | 0 | - |
| - | - | 726.7 | 239.1 | - | - | 0 | - |
| - | - | 845.8 | 239.2 | - | - | 0 | - |
| - | - | 717.5 | 240.1 | - | - | 0 | - |
| - | - | 1485 | 241.1 | - | - | 0 | - |
| - | - | 647.4 | 248.1 | - | - | 0 | - |
| 2 | b | 1.777E+04 | 249.1 | 0.0002579 | 1.035 | +1 | 2 |
| - | - | 2485 | 250.1 | - | - | 0 | - |
| - | - | 2040 | 251.1 | - | - | 0 | - |
| - | - | 1859 | 253.1 | - | - | 0 | - |
| - | - | 2294 | 253.2 | - | - | 0 | - |
| - | - | 1221 | 257.1 | - | - | 0 | - |
| - | - | 789.2 | 257.2 | - | - | 0 | - |
| - | - | 3952 | 263.1 | - | - | 0 | - |
| - | - | 510.3 | 263.6 | - | - | 0 | - |
| - | - | 668.6 | 274.1 | - | - | 0 | - |
| - | - | 1406 | 279.1 | - | - | 0 | - |
| - | - | 687.6 | 283 | - | - | 0 | - |
| - | - | 974.3 | 283.1 | - | - | 0 | - |
| - | - | 1456 | 285 | - | - | 0 | - |
| - | - | 2904 | 285.1 | - | - | 0 | - |
| - | - | 1449 | 292.1 | - | - | 0 | - |
| - | - | 2365 | 296.2 | - | - | 0 | - |
| - | - | 746.6 | 297.1 | - | - | 0 | - |
| - | - | 1331 | 302.1 | - | - | 0 | - |
| - | - | 1288 | 312.2 | - | - | 0 | - |
| - | - | 1100 | 314.1 | - | - | 0 | - |
| - | - | 2033 | 319.1 | - | - | 0 | - |
| - | - | 4277 | 324.1 | - | - | 0 | - |
| - | - | 1051 | 325.1 | - | - | 0 | - |
| - | - | 934.9 | 325.1 | - | - | 0 | - |
| - | - | 1.805E+04 | 329.1 | - | - | 0 | - |
| - | - | 3933 | 330.2 | - | - | 0 | - |
| - | - | 839 | 331.2 | - | - | 0 | - |
| - | - | 3365 | 340.2 | - | - | 0 | - |
| 8 | y | 2.259E+04 | 342.2 | 0.0003061 | 0.8947 | +1 | 2 |
| - | - | 3802 | 343.2 | - | - | 0 | - |
| 3 | b | 1361 | 346.1 | 0.0009388 | 2.712 | +1 | 3 |
| - | - | 1565 | 350.1 | - | - | 0 | - |
| - | - | 1709 | 355.1 | - | - | 0 | - |
| - | - | 674.9 | 356.1 | - | - | 0 | - |
| - | - | 1313 | 356.1 | - | - | 0 | - |
| - | - | 4092 | 357.2 | - | - | 0 | - |
| - | - | 881 | 358.2 | - | - | 0 | - |
| - | - | 857.8 | 358.2 | - | - | 0 | - |
| 3 | b | 4417 | 364.2 | 0.0001044 | 0.2866 | +1 | 3 |
| - | - | 1136 | 365.2 | - | - | 0 | - |
| - | - | 620.5 | 366.1 | - | - | 0 | - |
| - | - | 1412 | 368.2 | - | - | 0 | - |
| - | - | 3098 | 378.1 | - | - | 0 | - |
| - | - | 584.6 | 379.1 | - | - | 0 | - |
| - | - | 725.2 | 383.2 | - | - | 0 | - |
| - | - | 868.2 | 392.2 | - | - | 0 | - |
| - | - | 4320 | 394.1 | - | - | 0 | - |
| - | - | 1238 | 395.1 | - | - | 0 | - |
| - | - | 615.9 | 397.2 | - | - | 0 | - |
| - | - | 676.7 | 401.2 | - | - | 0 | - |
| - | - | 659.3 | 413.7 | - | - | 0 | - |
| - | - | 2412 | 425.2 | - | - | 0 | - |
| - | - | 1046 | 425.2 | - | - | 0 | - |
| - | - | 2179 | 427.7 | - | - | 0 | - |
| - | - | 1497 | 428.2 | - | - | 0 | - |
| - | - | 2760 | 436.7 | - | - | 0 | - |
| - | - | 689.7 | 453.3 | - | - | 0 | - |
| 4 | b | 655.1 | 461.2 | 0.0001253 | 0.2717 | +1 | 4 |
| - | - | 2962 | 465.2 | - | - | 0 | - |
| - | - | 751.9 | 466.2 | - | - | 0 | - |
| - | - | 713.6 | 470.3 | - | - | 0 | - |
[truncated: 1,006,577 more chars]
