# Supplementary material for: A Handle on Mass Coincidence Errors in De Novo Sequencing of Antibodies by Bottom-up Proteomics
Source: J Proteome Res. 2024 Jun 27;23(8):3552–9. doi: 10.1021/acs.jproteome.4c00188 (PMC11301774; doi:10.1021/acs.jproteome.4c00188)
Supplement: Supplementary file 1 — pr4c00188_si_001.zip [file pr4c00188_si_001.zip › supplementary data/xln-disambiguation/2023-12-13@14-36-36 f59/report/reads/Combined_010.html]

Details Combined\_010 | Stitch OverviewUndefined

# Read Combined\_010

## Sequence (length=15)

JJSEVSDRPSGVSSR

## Spectrum 4095? Spectrum 4095 The raw spectrum of this peptide as annotated by Hecklib. The fragments are coloured according to ion type (see legend). Any peaks with a star '\*' as text can be hovered over to see the full details, first the ion type second the mass shift type. By hovering over the amino acids in the peptide or ions in the legend the corresponding peaks are highlighted. By toggling the 'Unassigned' label you can turn the background (unassigned) peaks on or off in the plot. By updating the slider in the Ion legend you can update the spectrum to only show the top X% of the peaks with labels. The top X% means any peak that is within X% of the highest intensity. By dragging in the spectrum you can zoom in to a specific part of the spectrum and use 'Zoom Out' to get back to the original zoom level. The annotation of the spectrum is based on the given sequence in the peptides file and is done with different software so inconsistencies are likely. The peaks are annotated based on the given sequence, with 20 ppm tolerance.

Copy Data

### Spectrum 4095 (TSV)

#### Preview

```
Loading example...
```

*Click on the button to copy the data to your clipboard.*

Mz MinMz MaxIntensity Max

WidthHeightPeptide font sizePeptide stroke widthSpectrum font sizeSpectrum stroke widthCompact peptide

Ion legend

wxyz

abcd

OtherUnassignedIonChargePositionShow for top:%

JJSEVSDRPSGVSSR

02.56e+45.11e+47.67e+41.02e+5

Zoom Out

z+11y+11y+11w+12y+12z+12y+12w+13c+13y+13z+13y+13z+28w+14y+28y+14y+14c+28y+313c+14c+314z+29y+29z+314z+15y+314c+29y+15y+210z+210y+210z+16w+16c+15z+211y+211y+16y+16z+16y+16w+212c+212z+212y+212c+16z+213y+213y+213z+213y+213y+17w+214y+214z+214y+214w+18c+17z+18y+18z+18y+18c+18z+19z+19y+19c+19z+110y+110c+110z+111y+111c+111c+112y+112w+113c+113y+113c+114z+114

0621124218642485

Fragment Matches Table

Show background peaks

| Position | Ion type | Intensity | mz Theoretical | mz Error (Th) | mz Error (ppm) | Charge | Series Number |
| --- | --- | --- | --- | --- | --- | --- | --- |
| - | - | 373.7 | 123.9 | - | - | 0 | - |
| - | - | 452.9 | 124.1 | - | - | 0 | - |
| - | - | 376.3 | 124.4 | - | - | 0 | - |
| - | - | 1627 | 125.1 | - | - | 0 | - |
| - | - | 1909 | 126.1 | - | - | 0 | - |
| - | - | 555.4 | 130.1 | - | - | 0 | - |
| - | - | 1465 | 131.1 | - | - | 0 | - |
| - | - | 596.3 | 133.1 | - | - | 0 | - |
| - | - | 472.7 | 138.2 | - | - | 0 | - |
| 15 | z | 413.3 | 142.1 | 5.696E-06 | 0.04009 | +1 | 1 |
| - | - | 667.4 | 148.9 | - | - | 0 | - |
| 15 | y | 836.4 | 158.1 | 0.0002636 | 1.667 | +1 | 1 |
| - | - | 1800 | 163.1 | - | - | 0 | - |
| - | - | 467.3 | 164.2 | - | - | 0 | - |
| - | - | 3568 | 168.1 | - | - | 0 | - |
| - | - | 1393 | 173.5 | - | - | 0 | - |
| 15 | y | 5167 | 175.1 | 0.00028 | 1.599 | +1 | 1 |
| - | - | 538.1 | 183.1 | - | - | 0 | - |
| - | - | 1.326E+04 | 185.1 | - | - | 0 | - |
| - | - | 570.4 | 186.1 | - | - | 0 | - |
| - | - | 542.3 | 189.1 | - | - | 0 | - |
| - | - | 3.039E+04 | 199.2 | - | - | 0 | - |
| - | - | 2407 | 200.1 | - | - | 0 | - |
| - | - | 4112 | 200.2 | - | - | 0 | - |
| - | - | 514.6 | 202.1 | - | - | 0 | - |
| - | - | 677.9 | 212.1 | - | - | 0 | - |
| - | - | 1694 | 217.1 | - | - | 0 | - |
| - | - | 977.7 | 226.1 | - | - | 0 | - |
| - | - | 659 | 226.2 | - | - | 0 | - |
| - | - | 1.607E+04 | 227.2 | - | - | 0 | - |
| - | - | 1894 | 228.2 | - | - | 0 | - |
| 14 | w | 2451 | 229.1 | 0.0004728 | 2.063 | +1 | 2 |
| - | - | 793.1 | 233.2 | - | - | 0 | - |
| - | - | 592.2 | 234.1 | - | - | 0 | - |
| - | - | 641.9 | 244.1 | - | - | 0 | - |
| 14 | y | 2.489E+04 | 245.1 | 0.0003091 | 1.261 | +1 | 2 |
| 14 | z | 4112 | 246.1 | 0.0009394 | 3.817 | +1 | 2 |
| - | - | 1112 | 255.1 | - | - | 0 | - |
| - | - | 591.4 | 261.2 | - | - | 0 | - |
| 14 | y | 2447 | 262.2 | 0.0001932 | 0.7371 | +1 | 2 |
| - | - | 3627 | 270.1 | - | - | 0 | - |
| - | - | 616.2 | 272.2 | - | - | 0 | - |
| - | - | 651 | 293.3 | - | - | 0 | - |
| - | - | 929.8 | 294.2 | - | - | 0 | - |
| - | - | 585.1 | 303.2 | - | - | 0 | - |
| - | - | 1807 | 314.2 | - | - | 0 | - |
| 13 | w | 1.205E+04 | 316.2 | 0.0004726 | 1.495 | +1 | 3 |
| - | - | 2448 | 317.2 | - | - | 0 | - |
| - | - | 3226 | 331.1 | - | - | 0 | - |
| 3 | c | 873.4 | 331.2 | 0.002316 | 6.991 | +1 | 3 |
| 13 | y | 4570 | 332.2 | 0.0003089 | 0.93 | +1 | 3 |
| 13 | z | 1.021E+04 | 333.2 | 0.0001438 | 0.4316 | +1 | 3 |
| - | - | 1130 | 334.2 | - | - | 0 | - |
| 13 | y | 2762 | 349.2 | 5.611E-05 | 0.1607 | +1 | 3 |
| - | - | 6593 | 357.2 | - | - | 0 | - |
| - | - | 615.3 | 363.6 | - | - | 0 | - |
| - | - | 544.6 | 378.2 | - | - | 0 | - |
| - | - | 1347 | 391.7 | - | - | 0 | - |
| - | - | 956.8 | 392.2 | - | - | 0 | - |
| 8 | z | 2313 | 415.2 | 0.003227 | 7.772 | +2 | 8 |
| - | - | 1211 | 415.7 | - | - | 0 | - |
| 12 | w | 1.022E+04 | 417.2 | 0.0005236 | 1.255 | +1 | 4 |
| - | - | 1274 | 418.2 | - | - | 0 | - |
| 8 | y | 2110 | 423.2 | 0.0009756 | 2.305 | +2 | 8 |
| - | - | 1058 | 423.7 | - | - | 0 | - |
| - | - | 672.6 | 425.2 | - | - | 0 | - |
| - | - | 791.1 | 429.2 | - | - | 0 | - |
| 12 | y | 683.6 | 431.2 | 0.001506 | 3.491 | +1 | 4 |
| - | - | 1552 | 443.3 | - | - | 0 | - |
| - | - | 1205 | 443.3 | - | - | 0 | - |
| - | - | 721.2 | 444.3 | - | - | 0 | - |
| - | - | 1067 | 446.2 | - | - | 0 | - |
| - | - | 652.2 | 447.2 | - | - | 0 | - |
| 12 | y | 9224 | 448.3 | 0.0006218 | 1.387 | +1 | 4 |
| - | - | 1759 | 449.3 | - | - | 0 | - |
| 8 | c | 717.7 | 450.7 | 0.002218 | 4.92 | +2 | 8 |
| - | - | 803.1 | 451.2 | - | - | 0 | - |
| 3 | y | 1483 | 454.9 | 0.001283 | 2.821 | +3 | 13 |
| - | - | 626.2 | 455.3 | - | - | 0 | - |
| 4 | c | 6835 | 460.3 | 0.0006772 | 1.471 | +1 | 4 |
| - | - | 1945 | 461.3 | - | - | 0 | - |
| - | - | 606.3 | 461.9 | - | - | 0 | - |
| 14 | c | 2395 | 472.2 | 0.007897 | 16.72 | +3 | 14 |
| 7 | z | 2146 | 472.7 | 0.000162 | 0.3427 | +2 | 9 |
| - | - | 2882 | 474.2 | - | - | 0 | - |
| 7 | y | 1336 | 480.7 | 0.0004435 | 0.9226 | +2 | 9 |
| 2 | z | 1432 | 481.2 | 0.005404 | 11.23 | +3 | 14 |
| - | - | 539 | 481.6 | - | - | 0 | - |
| 11 | z | 2194 | 489.3 | 0.0004761 | 0.9731 | +1 | 5 |
| - | - | 1993 | 490.3 | - | - | 0 | - |
| 2 | y | 865.3 | 492.6 | 0.0007175 | 1.457 | +3 | 14 |
| - | - | 569.6 | 495.9 | - | - | 0 | - |
| 9 | c | 647.8 | 499.3 | 0.006098 | 12.21 | +2 | 9 |
| - | - | 2149 | 499.3 | - | - | 0 | - |
| - | - | 594.5 | 504.3 | - | - | 0 | - |
| 11 | y | 3.149E+04 | 505.3 | 0.0004593 | 0.9091 | +1 | 5 |
| - | - | 6753 | 506.3 | - | - | 0 | - |
| - | - | 1182 | 507.3 | - | - | 0 | - |
| - | - | 1145 | 515.3 | - | - | 0 | - |
| 6 | y | 1717 | 515.7 | 0.0005115 | 0.9918 | +2 | 10 |
| 6 | z | 2986 | 516.3 | 0.0004103 | 0.7947 | +2 | 10 |
| - | - | 892.3 | 516.3 | - | - | 0 | - |
| - | - | 4482 | 516.8 | - | - | 0 | - |
| - | - | 911 | 517.3 | - | - | 0 | - |
| 6 | y | 1.035E+04 | 524.3 | 0.004266 | 8.137 | +2 | 10 |
| - | - | 1894 | 524.6 | - | - | 0 | - |
| - | - | 4704 | 524.8 | - | - | 0 | - |
| - | - | 1795 | 524.9 | - | - | 0 | - |
| - | - | 2124 | 525.3 | - | - | 0 | - |
| - | - | 647.5 | 529.3 | - | - | 0 | - |
| - | - | 1381 | 529.3 | - | - | 0 | - |
| - | - | 640.1 | 530.2 | - | - | 0 | - |
| - | - | 3845 | 530.3 | - | - | 0 | - |
| - | - | 3322 | 530.6 | - | - | 0 | - |
| - | - | 1383 | 530.9 | - | - | 0 | - |
| - | - | 1478 | 541.3 | - | - | 0 | - |
| - | - | 3548 | 542.3 | - | - | 0 | - |
| - | - | 1209 | 543.3 | - | - | 0 | - |
| - | - | 748.9 | 551.3 | - | - | 0 | - |
| - | - | 857.3 | 551.8 | - | - | 0 | - |
| 10 | z | 791.2 | 558.3 | 0.0006019 | 1.078 | +1 | 6 |
| - | - | 2112 | 558.3 | - | - | 0 | - |
| 10 | w | 1523 | 559.3 | 0.0001791 | 0.3202 | +1 | 6 |
| 5 | c | 5801 | 559.3 | 0.0005065 | 0.9056 | +1 | 5 |
| - | - | 621.5 | 560.3 | - | - | 0 | - |
| - | - | 1907 | 560.3 | - | - | 0 | - |
| 5 | z | 2.294E+04 | 565.8 | 1.031E-05 | 0.01823 | +2 | 11 |
| - | - | 1.498E+04 | 566.3 | - | - | 0 | - |
| - | - | 5863 | 566.8 | - | - | 0 | - |
| - | - | 1578 | 567.3 | - | - | 0 | - |
| 5 | y | 1.321E+04 | 573.8 | 8.81E-05 | 0.1535 | +2 | 11 |
| 10 | y | 6453 | 574.3 | 0.003684 | 6.415 | +1 | 6 |
| - | - | 2527 | 574.8 | - | - | 0 | - |
| 10 | y | 956.6 | 575.3 | 0.0005647 | 0.9817 | +1 | 6 |
| 10 | z | 5914 | 576.3 | 0.0003633 | 0.6305 | +1 | 6 |
| - | - | 1307 | 577.3 | - | - | 0 | - |
| - | - | 872.1 | 586.3 | - | - | 0 | - |
| - | - | 1614 | 587.3 | - | - | 0 | - |
| 10 | y | 839.5 | 592.3 | 0.0008101 | 1.368 | +1 | 6 |
| - | - | 2579 | 600.3 | - | - | 0 | - |
| - | - | 1320 | 600.3 | - | - | 0 | - |
| 4 | w | 1.021E+04 | 600.8 | 0.0002989 | 0.4975 | +2 | 12 |
| - | - | 7019 | 601.3 | - | - | 0 | - |
| - | - | 3512 | 601.8 | - | - | 0 | - |
| - | - | 1015 | 603.4 | - | - | 0 | - |
| - | - | 789.7 | 615.3 | - | - | 0 | - |
| - | - | 950.3 | 619.3 | - | - | 0 | - |
| 12 | c | 1556 | 629.3 | 0.006528 | 10.37 | +2 | 12 |
| 4 | z | 2.289E+04 | 630.3 | 0.0001775 | 0.2816 | +2 | 12 |
| - | - | 1.744E+04 | 630.8 | - | - | 0 | - |
| - | - | 6587 | 631.3 | - | - | 0 | - |
| - | - | 2579 | 631.8 | - | - | 0 | - |
| - | - | 903 | 633.4 | - | - | 0 | - |
| - | - | 7474 | 637.8 | - | - | 0 | - |
| 4 | y | 2.711E+04 | 638.3 | 0.0003955 | 0.6195 | +2 | 12 |
| - | - | 1.36E+04 | 638.8 | - | - | 0 | - |
| - | - | 4913 | 639.3 | - | - | 0 | - |
| - | - | 1547 | 639.8 | - | - | 0 | - |
| - | - | 752.1 | 642.4 | - | - | 0 | - |
| - | - | 2082 | 644.3 | - | - | 0 | - |
| - | - | 1765 | 644.8 | - | - | 0 | - |
| - | - | 1049 | 645.4 | - | - | 0 | - |
| 6 | c | 2.196E+04 | 646.4 | 0.0004241 | 0.6561 | +1 | 6 |
| - | - | 7372 | 647.4 | - | - | 0 | - |
| - | - | 1134 | 648.4 | - | - | 0 | - |
| - | - | 1387 | 651.8 | - | - | 0 | - |
| 3 | z | 1405 | 665.3 | 0.01038 | 15.6 | +2 | 13 |
| - | - | 1020 | 665.8 | - | - | 0 | - |
| - | - | 891 | 666.3 | - | - | 0 | - |
| 3 | y | 1604 | 672.8 | 0.0008734 | 1.298 | +2 | 13 |
| 3 | y | 865 | 673.3 | 0.006508 | 9.666 | +2 | 13 |
| 3 | z | 1.587E+04 | 673.8 | 0.0002156 | 0.3199 | +2 | 13 |
| - | - | 2.034E+04 | 674.3 | - | - | 0 | - |
| - | - | 1.055E+04 | 674.8 | - | - | 0 | - |
| - | - | 3765 | 675.3 | - | - | 0 | - |
| - | - | 854.3 | 675.8 | - | - | 0 | - |
| - | - | 802.1 | 676.9 | - | - | 0 | - |
| - | - | 1050 | 680.8 | - | - | 0 | - |
| - | - | 2008 | 681.3 | - | - | 0 | - |
| 3 | y | 1.012E+05 | 681.8 | 6.983E-05 | 0.1024 | +2 | 13 |
| - | - | 6.734E+04 | 682.3 | - | - | 0 | - |
| - | - | 1115 | 682.4 | - | - | 0 | - |
| - | - | 2.779E+04 | 682.8 | - | - | 0 | - |
| - | - | 8690 | 683.3 | - | - | 0 | - |
| - | - | 1245 | 683.8 | - | - | 0 | - |
| - | - | 4339 | 684.4 | - | - | 0 | - |
| - | - | 3886 | 684.9 | - | - | 0 | - |
| - | - | 2955 | 685.4 | - | - | 0 | - |
| - | - | 1269 | 685.9 | - | - | 0 | - |
| - | - | 847 | 686.3 | - | - | 0 | - |
| - | - | 1199 | 687.3 | - | - | 0 | - |
| - | - | 722.9 | 688.3 | - | - | 0 | - |
| 9 | y | 2823 | 689.4 | 0.0007195 | 1.044 | +1 | 7 |
| - | - | 1737 | 690.4 | - | - | 0 | - |
| - | - | 1097 | 692.4 | - | - | 0 | - |
| - | - | 819.5 | 699.4 | - | - | 0 | - |
| - | - | 1737 | 700.4 | - | - | 0 | - |
| - | - | 1046 | 700.9 | - | - | 0 | - |
| - | - | 710.1 | 708.4 | - | - | 0 | - |
| 2 | w | 1752 | 715.8 | 0.001807 | 2.524 | +2 | 14 |
| - | - | 1892 | 716.4 | - | - | 0 | - |
| - | - | 962.8 | 716.9 | - | - | 0 | - |
| - | - | 1836 | 717.4 | - | - | 0 | - |
| - | - | 1846 | 718.4 | - | - | 0 | - |
| 2 | y | 765.2 | 729.4 | 0.006284 | 8.616 | +2 | 14 |
| 2 | z | 2570 | 730.4 | 0.0001758 | 0.2406 | +2 | 14 |
| - | - | 872.3 | 730.9 | - | - | 0 | - |
| - | - | 1764 | 736.9 | - | - | 0 | - |
| - | - | 714.3 | 737.4 | - | - | 0 | - |
| - | - | 1134 | 737.9 | - | - | 0 | - |
| 2 | y | 6655 | 738.4 | 0.0001521 | 0.206 | +2 | 14 |
| - | - | 4425 | 738.9 | - | - | 0 | - |
| - | - | 1950 | 739.4 | - | - | 0 | - |
| - | - | 856.4 | 742.4 | - | - | 0 | - |
| 8 | w | 1674 | 743.4 | 0.002484 | 3.341 | +1 | 8 |
| - | - | 1799 | 744.4 | - | - | 0 | - |
| - | - | 927 | 745.4 | - | - | 0 | - |
| - | - | 845.9 | 750.9 | - | - | 0 | - |
| - | - | 1153 | 751.4 | - | - | 0 | - |
| - | - | 836.9 | 751.9 | - | - | 0 | - |
| - | - | 702.3 | 756.4 | - | - | 0 | - |
| - | - | 1592 | 757.4 | - | - | 0 | - |
| - | - | 3770 | 758.4 | - | - | 0 | - |
| - | - | 4226 | 758.9 | - | - | 0 | - |
| - | - | 2829 | 759.4 | - | - | 0 | - |
| - | - | 1201 | 759.9 | - | - | 0 | - |
| - | - | 1186 | 760.4 | - | - | 0 | - |
| 7 | c | 4.115E+04 | 761.4 | 0.0001534 | 0.2015 | +1 | 7 |
| - | - | 1.672E+04 | 762.4 | - | - | 0 | - |
| - | - | 4309 | 763.4 | - | - | 0 | - |
| - | - | 2088 | 764.4 | - | - | 0 | - |
| - | - | 5182 | 764.9 | - | - | 0 | - |
| - | - | 7.38E+04 | 765.4 | - | - | 0 | - |
| - | - | 5.919E+04 | 765.9 | - | - | 0 | - |
| - | - | 2.6E+04 | 766.4 | - | - | 0 | - |
| - | - | 8434 | 766.9 | - | - | 0 | - |
| - | - | 2231 | 767.4 | - | - | 0 | - |
| - | - | 1179 | 771.9 | - | - | 0 | - |
| - | - | 3082 | 772.4 | - | - | 0 | - |
| - | - | 2611 | 772.9 | - | - | 0 | - |
| - | - | 2803 | 773.4 | - | - | 0 | - |
| - | - | 2.003E+04 | 773.9 | - | - | 0 | - |
| - | - | 1.605E+04 | 774.4 | - | - | 0 | - |
| - | - | 1.148E+04 | 774.9 | - | - | 0 | - |
| - | - | 2592 | 775.4 | - | - | 0 | - |
| - | - | 785.4 | 775.9 | - | - | 0 | - |
| - | - | 2211 | 777.9 | - | - | 0 | - |
| - | - | 2065 | 778.4 | - | - | 0 | - |
| - | - | 1036 | 778.9 | - | - | 0 | - |
| - | - | 915.1 | 780.4 | - | - | 0 | - |
| - | - | 911.5 | 780.9 | - | - | 0 | - |
| - | - | 1277 | 782.4 | - | - | 0 | - |
| - | - | 862.1 | 785.9 | - | - | 0 | - |
| - | - | 8484 | 786.4 | - | - | 0 | - |
| - | - | 7.704E+04 | 786.9 | - | - | 0 | - |
| - | - | 6.341E+04 | 787.4 | - | - | 0 | - |
| - | - | 2.913E+04 | 787.9 | - | - | 0 | - |
| - | - | 9191 | 788.4 | - | - | 0 | - |
| - | - | 2024 | 788.9 | - | - | 0 | - |
| - | - | 1253 | 793.9 | - | - | 0 | - |
| - | - | 4581 | 794.4 | - | - | 0 | - |
| - | - | 5.587E+04 | 794.9 | - | - | 0 | - |
| - | - | 6.845E+04 | 795.4 | - | - | 0 | - |
| - | - | 4.104E+04 | 795.9 | - | - | 0 | - |
| - | - | 1.497E+04 | 796.4 | - | - | 0 | - |
| - | - | 4742 | 796.9 | - | - | 0 | - |
| - | - | 699 | 797.4 | - | - | 0 | - |
| - | - | 751.7 | 798.4 | - | - | 0 | - |
| 8 | z | 2712 | 811.4 | 0.002933 | 3.615 | +1 | 8 |
| - | - | 933.4 | 812.4 | - | - | 0 | - |
| 8 | y | 1029 | 828.4 | 0.0007223 | 0.8718 | +1 | 8 |
| 8 | z | 3.213E+04 | 829.4 | 6.341E-05 | 0.07645 | +1 | 8 |
| - | - | 1.833E+04 | 830.4 | - | - | 0 | - |
| - | - | 5897 | 831.4 | - | - | 0 | - |
| - | - | 2344 | 832.5 | - | - | 0 | - |
| - | - | 1037 | 841.5 | - | - | 0 | - |
| - | - | 1543 | 844.4 | - | - | 0 | - |
| 8 | y | 5707 | 845.5 | 0.00066 | 0.7807 | +1 | 8 |
| - | - | 1703 | 846.5 | - | - | 0 | - |
| - | - | 1240 | 847.5 | - | - | 0 | - |
| - | - | 2405 | 873.5 | - | - | 0 | - |
| - | - | 1010 | 874.5 | - | - | 0 | - |
| - | - | 1180 | 876.4 | - | - | 0 | - |
| 8 | c | 4844 | 900.5 | 0.001288 | 1.431 | +1 | 8 |
| - | - | 2318 | 901.5 | - | - | 0 | - |
| - | - | 930.1 | 902.5 | - | - | 0 | - |
| 7 | z | 870.7 | 926.5 | 0.003075 | 3.319 | +1 | 9 |
| - | - | 1079 | 931.5 | - | - | 0 | - |
| - | - | 670.1 | 940.6 | - | - | 0 | - |
| 7 | z | 1.32E+04 | 944.5 | 0.0003951 | 0.4183 | +1 | 9 |
| - | - | 9444 | 945.5 | - | - | 0 | - |
| - | - | 3239 | 946.5 | - | - | 0 | - |
| - | - | 1669 | 947.5 | - | - | 0 | - |
| - | - | 1167 | 956.5 | - | - | 0 | - |
| 7 | y | 1833 | 960.5 | 0.001755 | 1.827 | +1 | 9 |
| - | - | 895.5 | 961.5 | - | - | 0 | - |
| - | - | 3563 | 970.5 | - | - | 0 | - |
| - | - | 1825 | 971.5 | - | - | 0 | - |
| - | - | 1886 | 980.5 | - | - | 0 | - |
| - | - | 1749 | 1014 | - | - | 0 | - |
| 9 | c | 3.133E+04 | 1015 | 0.0003312 | 0.3265 | +1 | 9 |
| - | - | 1.768E+04 | 1016 | - | - | 0 | - |
| - | - | 5310 | 1017 | - | - | 0 | - |
| - | - | 1683 | 1018 | - | - | 0 | - |
| - | - | 676.6 | 1022 | - | - | 0 | - |
| 6 | z | 5529 | 1031 | 0.0003801 | 0.3685 | +1 | 10 |
| - | - | 6831 | 1033 | - | - | 0 | - |
| - | - | 2808 | 1034 | - | - | 0 | - |
| - | - | 1164 | 1035 | - | - | 0 | - |
| - | - | 1602 | 1040 | - | - | 0 | - |
| - | - | 2365 | 1041 | - | - | 0 | - |
| - | - | 859.4 | 1042 | - | - | 0 | - |
| - | - | 1910 | 1043 | - | - | 0 | - |
| - | - | 631.3 | 1043 | - | - | 0 | - |
| - | - | 790.6 | 1044 | - | - | 0 | - |
| - | - | 1237 | 1045 | - | - | 0 | - |
| - | - | 919.3 | 1047 | - | - | 0 | - |
| 6 | y | 2836 | 1048 | 0.0006715 | 0.6411 | +1 | 10 |
| - | - | 2.168E+04 | 1058 | - | - | 0 | - |
| - | - | 1.331E+04 | 1059 | - | - | 0 | - |
| - | - | 5982 | 1060 | - | - | 0 | - |
| - | - | 1972 | 1061 | - | - | 0 | - |
| - | - | 869.1 | 1063 | - | - | 0 | - |
| - | - | 1020 | 1101 | - | - | 0 | - |
| 10 | c | 4.247E+04 | 1102 | 0.0003463 | 0.3144 | +1 | 10 |
| - | - | 2.401E+04 | 1103 | - | - | 0 | - |
| - | - | 9205 | 1104 | - | - | 0 | - |
| - | - | 1521 | 1105 | - | - | 0 | - |
| - | - | 6531 | 1115 | - | - | 0 | - |
| - | - | 4670 | 1116 | - | - | 0 | - |
| - | - | 2013 | 1117 | - | - | 0 | - |
| 5 | z | 6041 | 1131 | 0.001777 | 1.572 | +1 | 11 |
| - | - | 1.547E+04 | 1132 | - | - | 0 | - |
| - | - | 9187 | 1133 | - | - | 0 | - |
| - | - | 3002 | 1134 | - | - | 0 | - |
| - | - | 886.1 | 1138 | - | - | 0 | - |
| - | - | 1550 | 1139 | - | - | 0 | - |
| 5 | y | 1549 | 1147 | 0.001227 | 1.07 | +1 | 11 |
| - | - | 1074 | 1148 | - | - | 0 | - |
| - | - | 967.6 | 1149 | - | - | 0 | - |
| 11 | c | 2.317E+04 | 1159 | 0.001342 | 1.158 | +1 | 11 |
| - | - | 1.562E+04 | 1160 | - | - | 0 | - |
| - | - | 6005 | 1161 | - | - | 0 | - |
| - | - | 934.6 | 1162 | - | - | 0 | - |
| - | - | 713.1 | 1213 | - | - | 0 | - |
| - | - | 4266 | 1214 | - | - | 0 | - |
| - | - | 3322 | 1215 | - | - | 0 | - |
| - | - | 1813 | 1216 | - | - | 0 | - |
| - | - | 2491 | 1217 | - | - | 0 | - |
| - | - | 688.6 | 1257 | - | - | 0 | - |
| 12 | c | 2.441E+04 | 1258 | 0.002251 | 1.79 | +1 | 12 |
| - | - | 1.555E+04 | 1259 | - | - | 0 | - |
| - | - | 5570 | 1260 | - | - | 0 | - |
| - | - | 2.173E+04 | 1261 | - | - | 0 | - |
| - | - | 1.312E+04 | 1262 | - | - | 0 | - |
| - | - | 5351 | 1263 | - | - | 0 | - |
| - | - | 1424 | 1264 | - | - | 0 | - |
| - | - | 723.6 | 1275 | - | - | 0 | - |
| 4 | y | 7084 | 1276 | 0.003524 | 2.763 | +1 | 12 |
| - | - | 5165 | 1277 | - | - | 0 | - |
| - | - | 1948 | 1278 | - | - | 0 | - |
| - | - | 1165 | 1279 | - | - | 0 | - |
| - | - | 782.9 | 1284 | - | - | 0 | - |
| - | - | 3655 | 1301 | - | - | 0 | - |
| - | - | 3739 | 1302 | - | - | 0 | - |
| - | - | 1785 | 1303 | - | - | 0 | - |
| 3 | w | 1500 | 1330 | 0.00471 | 3.542 | +1 | 13 |
| - | - | 2143 | 1331 | - | - | 0 | - |
| - | - | 1164 | 1332 | - | - | 0 | - |
| 13 | c | 4.167E+04 | 1345 | 0.002297 | 1.708 | +1 | 13 |
| - | - | 3.123E+04 | 1346 | - | - | 0 | - |
| - | - | 1.235E+04 | 1347 | - | - | 0 | - |
| - | - | 1.83E+04 | 1348 | - | - | 0 | - |
| - | - | 1.345E+04 | 1349 | - | - | 0 | - |
| - | - | 4670 | 1350 | - | - | 0 | - |
| - | - | 1665 | 1351 | - | - | 0 | - |
| - | - | 937.6 | 1354 | - | - | 0 | - |
| 3 | y | 1065 | 1363 | 0.004425 | 3.247 | +1 | 13 |
| - | - | 1653 | 1364 | - | - | 0 | - |
| - | - | 740.1 | 1365 | - | - | 0 | - |
| - | - | 1032 | 1369 | - | - | 0 | - |
| - | - | 1518 | 1370 | - | - | 0 | - |
| - | - | 911.1 | 1372 | - | - | 0 | - |
| - | - | 3486 | 1388 | - | - | 0 | - |
| - | - | 3399 | 1389 | - | - | 0 | - |
| - | - | 1528 | 1390 | - | - | 0 | - |
| - | - | 678.9 | 1417 | - | - | 0 | - |
| - | - | 976.3 | 1430 | - | - | 0 | - |
| - | - | 1118 | 1431 | - | - | 0 | - |
| 14 | c | 5.615E+04 | 1432 | 0.002709 | 1.892 | +1 | 14 |
| - | - | 4.334E+04 | 1433 | - | - | 0 | - |
| - | - | 1.882E+04 | 1434 | - | - | 0 | - |
| - | - | 4784 | 1435 | - | - | 0 | - |
| - | - | 1341 | 1436 | - | - | 0 | - |
| 2 | z | 890.9 | 1460 | 0.02216 | 15.18 | +1 | 14 |
| - | - | 3034 | 1461 | - | - | 0 | - |
| - | - | 1918 | 1462 | - | - | 0 | - |
| - | - | 910.8 | 1463 | - | - | 0 | - |
| - | - | 825.6 | 1472 | - | - | 0 | - |
| - | - | 910.9 | 1473 | - | - | 0 | - |
| - | - | 1098 | 1474 | - | - | 0 | - |
| - | - | 803.4 | 1475 | - | - | 0 | - |
| - | - | 733.9 | 1488 | - | - | 0 | - |
| - | - | 5535 | 1489 | - | - | 0 | - |
| - | - | 4053 | 1490 | - | - | 0 | - |
| - | - | 2637 | 1491 | - | - | 0 | - |
| - | - | 1007 | 1492 | - | - | 0 | - |
| - | - | 1369 | 1513 | - | - | 0 | - |
| - | - | 2150 | 1514 | - | - | 0 | - |
| - | - | 1348 | 1515 | - | - | 0 | - |
| - | - | 757 | 1516 | - | - | 0 | - |
| - | - | 913.8 | 1517 | - | - | 0 | - |
| - | - | 886.3 | 1529 | - | - | 0 | - |
| - | - | 1690 | 1530 | - | - | 0 | - |
| - | - | 1.071E+04 | 1531 | - | - | 0 | - |
| - | - | 1.366E+04 | 1532 | - | - | 0 | - |
| - | - | 9407 | 1533 | - | - | 0 | - |
| - | - | 4066 | 1534 | - | - | 0 | - |
| - | - | 1781 | 1535 | - | - | 0 | - |
| - | - | 778.2 | 1544 | - | - | 0 | - |
| - | - | 3812 | 1545 | - | - | 0 | - |
| - | - | 4695 | 1546 | - | - | 0 | - |
| - | - | 6526 | 1547 | - | - | 0 | - |
| - | - | 7831 | 1548 | - | - | 0 | - |
| - | - | 3694 | 1549 | - | - | 0 | - |
| - | - | 1357 | 1550 | - | - | 0 | - |
| - | - | 2668 | 1556 | - | - | 0 | - |
| - | - | 6244 | 1557 | - | - | 0 | - |
| - | - | 4463 | 1558 | - | - | 0 | - |
| - | - | 2821 | 1559 | - | - | 0 | - |
| - | - | 697.1 | 1560 | - | - | 0 | - |
| - | - | 2023 | 1562 | - | - | 0 | - |
| - | - | 1.051E+04 | 1563 | - | - | 0 | - |
| - | - | 7630 | 1564 | - | - | 0 | - |
| - | - | 4113 | 1565 | - | - | 0 | - |
| - | - | 860.8 | 1566 | - | - | 0 | - |
| - | - | 2091 | 1572 | - | - | 0 | - |
| - | - | 1.444E+04 | 1573 | - | - | 0 | - |
| - | - | 8.54E+04 | 1574 | - | - | 0 | - |
| - | - | 6.072E+04 | 1575 | - | - | 0 | - |
| - | - | 3.186E+04 | 1576 | - | - | 0 | - |
| - | - | 9223 | 1577 | - | - | 0 | - |
| - | - | 2487 | 1578 | - | - | 0 | - |
| - | - | 1330 | 1588 | - | - | 0 | - |
| - | - | 7328 | 1589 | - | - | 0 | - |
| - | - | 2.483E+04 | 1590 | - | - | 0 | - |
| - | - | 8.176E+04 | 1591 | - | - | 0 | - |
| - | - | 5.976E+04 | 1592 | - | - | 0 | - |
| - | - | 2.801E+04 | 1593 | - | - | 0 | - |
| - | - | 8507 | 1594 | - | - | 0 | - |
| - | - | 1937 | 1595 | - | - | 0 | - |
| - | - | 681.4 | 1884 | - | - | 0 | - |
| - | - | 795.2 | 2460 | - | - | 0 | - |

m/z Charge Intensity FragmentType MassShift Position
123.9146499633789 0 373.69495
124.08241271972656 0 452.87756
124.38890838623047 0 376.27667
125.10768127441406 0 1627.0758
126.09161376953125 0 1908.8469
130.0615692138672 0 555.41156
131.12939453125 0 1465.1594
133.06114196777344 0 596.2612
138.1892852783203 0 472.71954
142.0736846923828 0 413.3337 z Ammonia loss 14
148.94776916503906 0 667.3628
158.09266662597656 0 836.4271 y Ammonia loss 14
163.07156372070312 0 1800.1489
164.21897888183594 0 467.25333
168.1134796142578 0 3567.813
173.4515838623047 0 1392.6841
175.11923217773438 0 5167.2266 y 14
183.11294555664062 0 538.11285
185.13990783691406 0 13257.199
186.14418029785156 0 570.4292
189.0866241455078 0 542.34534
199.18072509765625 0 30388.236
200.12725830078125 0 2406.5293
200.18408203125 0 4111.868
202.142578125 0 514.58215
212.10308837890625 0 677.94867
217.08221435546875 0 1694.0579
226.130615234375 0 977.7367
226.16812133789062 0 658.9799
227.17564392089844 0 16067.281
228.17965698242188 0 1894.3247
229.12998962402344 0 2451.1511 w 13
233.16458129882812 0 793.1449
234.10763549804688 0 592.17163
244.1167449951172 0 641.9075
245.12474060058594 0 24894.625 y Ammonia loss 13
246.13131713867188 0 4111.9873 z 13
255.10952758789062 0 1111.8522
261.1616516113281 0 591.4032
262.1507873535156 0 2447.114 y 13
270.1196594238281 0 3627.384
272.1720275878906 0 616.2013
293.3086853027344 0 650.9608
294.1743469238281 0 929.8043
303.15386962890625 0 585.07886
314.2078552246094 0 1807.2018
316.1620178222656 0 12051.422 w 12
317.1584777832031 0 2448.0625
331.1490173339844 0 3226.2144
331.2362976074219 0 873.37103 c 2
332.1567687988281 0 4569.7217 y Ammonia loss 12
333.1644287109375 0 10206.214 z 12
334.1689758300781 0 1129.965
349.1829528808594 0 2762.3992 y 12
357.1518859863281 0 6592.8564
363.5715637207031 0 615.33
378.2175598144531 0 544.5865
391.72503662109375 0 1347.0233
392.22515869140625 0 956.77185
415.2268981933594 0 2312.696 z 7
415.7243957519531 0 1210.9937
417.2097473144531 0 10224.912 w 11
418.2111511230469 0 1273.7914
423.2340087890625 0 2110.4736 y 7
423.73492431640625 0 1057.8448
425.2400817871094 0 672.629
429.23712158203125 0 791.1326
431.22637939453125 0 683.56683 y Ammonia loss 11
443.25042724609375 0 1552.1731
443.30963134765625 0 1204.6243
444.2560729980469 0 721.2014
446.2343444824219 0 1067.3217
447.2066650390625 0 652.21704
448.2520446777344 0 9223.92 y 11
449.2552185058594 0 1758.7764
450.7451171875 0 717.7113 c Ammonia loss 7
451.2428894042969 0 803.05334
454.89306640625 0 1483.3613 y 2
455.2895202636719 0 626.2378
460.2772521972656 0 6834.937 c 3
461.280517578125 0 1944.5679
461.92315673828125 0 606.285
472.2518310546875 0 2395.3223 c Ammonia loss 13
472.7373046875 0 2146.0176 z 6
474.2319641113281 0 2881.8167
480.7469482421875 0 1335.7167 y 6
481.248779296875 0 1431.5604 z Water loss 1
481.6166687011719 0 539.01416
489.254638671875 0 2193.8687 z 10
490.261474609375 0 1992.8557
492.5871887207031 0 865.30475 y 1
495.91448974609375 0 569.6013
499.26318359375 0 647.75824 c Ammonia loss 8
499.3002014160156 0 2149.4832
504.2626647949219 0 594.5403
505.2733459472656 0 31487.955 y 10
506.2760314941406 0 6753.4917
507.28057861328125 0 1182.1118
515.3294677734375 0 1145.373
515.749755859375 0 1716.7344 y Ammonia loss 5
516.2527465820312 0 2985.6575 z 5
516.336181640625 0 892.2705
516.756103515625 0 4481.73
517.2592163085938 0 910.96716
524.2667846679688 0 10350.26 y 5
524.6122436523438 0 1893.9756
524.7639770507812 0 4703.947
524.9466552734375 0 1795.1599
525.2681274414062 0 2124.1914
529.2516479492188 0 647.5391
529.2988891601562 0 1380.506
530.2221069335938 0 640.1313
530.2831420898438 0 3845.4194
530.616455078125 0 3321.8281
530.9478149414062 0 1383.2135
541.3123168945312 0 1477.6191
542.318115234375 0 3547.986
543.3218994140625 0 1209.0934
551.279052734375 0 748.8777
551.7835693359375 0 857.28235
558.2750244140625 0 791.1644 z Water loss 9
558.3368530273438 0 2112.3315
559.2836303710938 0 1523.0294 w 9
559.344482421875 0 5801.0312 c 4
560.2753295898438 0 621.53687
560.3485107421875 0 1907.487
565.787353515625 0 22942.986 z 4
566.2891845703125 0 14984.074
566.7908935546875 0 5863.3135
567.2918090820312 0 1577.5524
573.7968139648438 0 13210.699 y 4
574.2980346679688 0 6452.819 y Water loss 9
574.7979125976562 0 2526.7695
575.2789306640625 0 956.6116 y Ammonia loss 9
576.2858276367188 0 5914.4204 z 9
577.2920532226562 0 1307.3074
586.3297729492188 0 872.1142
587.3382568359375 0 1613.7103
592.3057250976562 0 839.54535 y 9
600.2742309570312 0 2579.0208
600.3306884765625 0 1320.3838
600.8023071289062 0 10209.269 w 3
601.3033447265625 0 7019.2285
601.8056640625 0 3512.4556
603.3711547851562 0 1015.32043
615.2735595703125 0 789.66815
619.342041015625 0 950.3298
629.3500366210938 0 1555.897 c 11
630.308837890625 0 22893.26 z 3
630.8106079101562 0 17444.156
631.3110961914062 0 6587.098
631.8125 0 2578.8877
633.3833618164062 0 902.97925
637.8145141601562 0 7474.4736
638.317626953125 0 27112.693 y 3
638.8193359375 0 13604.181
639.3204345703125 0 4913.406
639.8214111328125 0 1546.9144
642.356689453125 0 752.0977
644.3191528320312 0 2081.601
644.8194580078125 0 1765.4248
645.371337890625 0 1049.0807
646.37744140625 0 21955.805 c 5
647.3807373046875 0 7371.598
648.383056640625 0 1133.8412
651.82958984375 0 1386.5931
665.32177734375 0 1404.8502 z Ammonia loss 2
665.81884765625 0 1019.89655
666.318115234375 0 891.00476
672.827880859375 0 1603.7612 y Water loss 2
673.3272705078125 0 864.9809 y Ammonia loss 2
673.8248901367188 0 15871.821 z 2
674.3276977539062 0 20342.3
674.8292236328125 0 10553.859
675.3309326171875 0 3764.7607
675.83447265625 0 854.2764
676.8917846679688 0 802.11383
680.8264770507812 0 1050.422
681.3304443359375 0 2007.7329
681.8341064453125 0 101206.38 y 2
682.33544921875 0 67341.41
682.4046020507812 0 1114.5905
682.837158203125 0 27787.857
683.3380737304688 0 8690.272
683.836181640625 0 1245.1052
684.398193359375 0 4339.052
684.8995361328125 0 3885.6394
685.3994140625 0 2954.6897
685.9010009765625 0 1268.5127
686.3206176757812 0 846.9897
687.3333740234375 0 1198.8513
688.3494262695312 0 722.9357
689.3583984375 0 2823.2751 y 8
690.3611450195312 0 1736.6294
692.3788452148438 0 1096.933
699.3795776367188 0 819.5107
700.3841552734375 0 1737.1661
700.8936157226562 0 1046.3151
708.3642578125 0 710.13385
715.8453369140625 0 1751.6437 w 1
716.3518676757812 0 1891.7708
716.8515014648438 0 962.7937
717.389404296875 0 1836.1942
718.395751953125 0 1846.3954
729.364501953125 0 765.15076 y Water loss 1
730.3668823242188 0 2570.4756 z 1
730.8698120117188 0 872.332
736.9091186523438 0 1764.0702
737.411865234375 0 714.30286
737.9146118164062 0 1133.7942
738.376220703125 0 6654.5645 y 1
738.8776245117188 0 4425.191
739.3780517578125 0 1949.6361
742.3912963867188 0 856.42035
743.3707275390625 0 1674.3552 w 7
744.3736572265625 0 1799.4917
745.3739624023438 0 926.9663
750.897216796875 0 845.9343
751.4026489257812 0 1153.18
751.9058837890625 0 836.89984
756.4208374023438 0 702.3459
757.4000244140625 0 1592.012
758.3882446289062 0 3770.181
758.8837890625 0 4226.28
759.3865966796875 0 2829.369
759.8834838867188 0 1201.0005
760.3934936523438 0 1185.641
761.4041137695312 0 41154.08 c 6
762.4070434570312 0 16722.51
763.4093017578125 0 4308.705
764.4083862304688 0 2087.5222
764.9124145507812 0 5182.33
765.3822631835938 0 73799.42
765.8834838867188 0 59185.086
766.3848266601562 0 25995.455
766.8860473632812 0 8433.698
767.3867797851562 0 2231.0173
771.8961791992188 0 1179.0546
772.4133911132812 0 3081.9727
772.913330078125 0 2611.273
773.4144287109375 0 2802.5103
773.9080200195312 0 20034.3
774.4085693359375 0 16047.194
774.9093627929688 0 11479.693
775.4141845703125 0 2592.4507
775.9081420898438 0 785.4045
777.904052734375 0 2211.0806
778.4083251953125 0 2065.1611
778.8987426757812 0 1036.4651
780.4121704101562 0 915.0847
780.9205932617188 0 911.4715
782.4441528320312 0 1277.1957
785.9046020507812 0 862.1413
786.4136962890625 0 8483.63
786.9094848632812 0 77037.305
787.4108276367188 0 63407.176
787.91162109375 0 29127.43
788.4126586914062 0 9190.784
788.9156494140625 0 2023.964
793.9077758789062 0 1253.2428
794.4148559570312 0 4580.956
794.91796875 0 55868.402
795.4200439453125 0 68451.61
795.9219360351562 0 41041.27
796.4227294921875 0 14974.581
796.9215087890625 0 4742.491
797.4281616210938 0 698.9526
798.4326782226562 0 751.70087
811.4324340820312 0 2712.3354 z Water loss 7
812.431640625 0 933.4146
828.4315185546875 0 1028.5703 y Ammonia loss 7
829.4400024414062 0 32132.488 z 7
830.4434204101562 0 18334.385
831.4464111328125 0 5897.2666
832.4581298828125 0 2344.2441
841.5303344726562 0 1037.1104
844.4495849609375 0 1542.7496
845.4581298828125 0 5706.6514 y 7
846.4630737304688 0 1702.8961
847.470458984375 0 1240.0272
873.4913940429688 0 2405.3438
874.4949340820312 0 1009.66376
876.4163818359375 0 1179.5294
900.4772338867188 0 4844.407 c Ammonia loss 7
901.4800415039062 0 2318.3057
902.484375 0 930.1285
926.453369140625 0 870.7116 z Water loss 6
931.5341796875 0 1079.0609
940.55029296875 0 670.06805
944.4666137695312 0 13203.57 z 6
945.4719848632812 0 9444.137
946.4745483398438 0 3239.069
947.480712890625 0 1668.5741
956.5487060546875 0 1166.8062
960.4874877929688 0 1833.4312 y 6
961.4957885742188 0 895.47925
970.5383911132812 0 3562.8933
971.5413818359375 0 1824.6663
980.4663696289062 0 1886.4741
1013.58447265625 0 1749.3303
1014.5581665039062 0 31329.178 c 8
1015.559814453125 0 17680.252
1016.56396484375 0 5310.0654
1017.5687255859375 0 1683.4272
1022.4562377929688 0 676.56647
1031.4986572265625 0 5528.9775 z 5
1032.504638671875 0 6830.866
1033.510009765625 0 2807.9326
1034.5135498046875 0 1164.1989
1039.5672607421875 0 1602.3677
1040.5645751953125 0 2364.902
1041.5740966796875 0 859.39136
1042.5732421875 0 1909.7092
1043.412841796875 0 631.25244
1043.576416015625 0 790.60645
1044.61328125 0 1236.7784
1046.507568359375 0 919.3354
1047.51708984375 0 2835.7302 y 5
1057.576416015625 0 21682.645
1058.57958984375 0 13309.952
1059.585693359375 0 5982.1094
1060.588623046875 0 1972.4124
1062.5322265625 0 869.1199
1100.5841064453125 0 1019.88965
1101.5902099609375 0 42471.29 c 9
1102.593017578125 0 24007.54
1103.595703125 0 9205.329
1104.592041015625 0 1521.2219
1114.5946044921875 0 6531.347
1115.5997314453125 0 4670.3994
1116.603759765625 0 2012.7073
1130.565673828125 0 6041.31 z 4
1131.57373046875 0 15473.064
1132.5775146484375 0 9186.589
1133.5782470703125 0 3002.2349
1137.666259765625 0 886.12317
1138.66455078125 0 1549.8428
1146.58740234375 0 1549.3564 y 4
1147.5889892578125 0 1073.5905
1148.5947265625 0 967.6354
1158.6099853515625 0 23169.795 c 10
1159.613525390625 0 15624.081
1160.6138916015625 0 6004.608
1161.6295166015625 0 934.57556
1212.6563720703125 0 713.08435
1213.6646728515625 0 4265.5024
1214.6663818359375 0 3321.767
1215.6715087890625 0 1813.2141
1216.6668701171875 0 2491.2158
1256.6636962890625 0 688.5753
1257.677490234375 0 24407.6 c 11
1258.6788330078125 0 15547.57
1259.6751708984375 0 5569.9443
1260.6163330078125 0 21732.594
1261.6182861328125 0 13124.749
1262.6187744140625 0 5350.569
1263.6356201171875 0 1423.844
1274.619140625 0 723.6213
1275.625244140625 0 7083.743 y 3
1276.6307373046875 0 5165.0947
1277.634521484375 0 1948.2886
1278.6396484375 0 1164.5331
1283.6815185546875 0 782.88525
1300.6959228515625 0 3655.0496
1301.6986083984375 0 3739.1887
1302.699462890625 0 1785.2048
1329.64404296875 0 1499.6655 w 2
1330.6292724609375 0 2142.7375
1331.6292724609375 0 1164.0333
1344.70947265625 0 41671.46 c 12
1345.71240234375 0 31233.545
1346.70751953125 0 12354.266
1347.6519775390625 0 18303.049
1348.6494140625 0 13445.278
1349.65380859375 0 4669.9023
1350.6575927734375 0 1664.8525
1353.7760009765625 0 937.57556
1362.6563720703125 0 1064.7428 y 2
1363.6644287109375 0 1653.4307
1364.6705322265625 0 740.0645
1368.7911376953125 0 1031.6222
1369.796630859375 0 1518.1887
1371.8177490234375 0 911.078
1387.7318115234375 0 3485.756
1388.7288818359375 0 3398.798
1389.741943359375 0 1527.7659
1416.7314453125 0 678.8511
1429.7215576171875 0 976.2922
1430.734375 0 1118.3718
1431.7410888671875 0 56153.727 c 13
1432.74267578125 0 43339.73
1433.745849609375 0 18820.37
1434.748779296875 0 4784.3237
1435.74560546875 0 1340.6986
1459.7039794921875 0 890.9119 z 1
1460.7294921875 0 3033.6426
1461.73828125 0 1918.0511
1462.7335205078125 0 910.83185
1471.784423828125 0 825.5887
1472.786865234375 0 910.88666
1473.7423095703125 0 1097.6536
1474.78369140625 0 803.4156
1487.784912109375 0 733.87305
1488.7403564453125 0 5535.357
1489.744384765625 0 4053.4597
1490.743896484375 0 2637.33
1491.7545166015625 0 1007.156
1512.791748046875 0 1368.8699
1513.785888671875 0 2149.8848
1514.7843017578125 0 1347.753
1515.7916259765625 0 757.02026
1516.758056640625 0 913.7841
1528.8095703125 0 886.3369
1529.7886962890625 0 1689.905
1530.7896728515625 0 10707.874
1531.7919921875 0 13656.965
1532.79443359375 0 9407.397
1533.7960205078125 0 4066.3694
1534.8035888671875 0 1780.9235
1543.8336181640625 0 778.17975
1544.8299560546875 0 3812.2925
1545.824951171875 0 4694.7075
1546.8218994140625 0 6525.557
1547.818603515625 0 7831.0474
1548.8140869140625 0 3694.165
1549.8197021484375 0 1356.9823
1555.802978515625 0 2667.7373
1556.790283203125 0 6244.286
1557.7943115234375 0 4462.726
1558.794189453125 0 2820.9963
1559.7967529296875 0 697.099
1561.83203125 0 2023.3583
1562.8453369140625 0 10506.443
1563.8504638671875 0 7630.0166
1564.85498046875 0 4113.136
1565.86279296875 0 860.8126
1571.8055419921875 0 2090.9116
1572.8134765625 0 14442.681
1573.814697265625 0 85400.26
1574.8173828125 0 60719.715
1575.8193359375 0 31862.031
1576.8206787109375 0 9223.321
1577.8193359375 0 2486.692
1587.8231201171875 0 1330.4069
1588.8260498046875 0 7327.942
1589.83203125 0 24832.795
1590.8387451171875 0 81763.4
1591.8416748046875 0 59757.316
1592.8448486328125 0 28010.562
1593.846923828125 0 8506.956
1594.8428955078125 0 1937.3308
1884.0838623046875 0 681.36957
2460.2685546875 0 795.17426

Spectrum Details

|  |  |
| --- | --- |
| Matched peaks? Matched peaksThe total absolute number of peaks matched. Additionally in brackets the total fraction of peaks matched and the total number of peaks is shown. | 79 (17.44% of 453) |
| FDR? FDRThe false discovery rate estimated for this peptide. It is calculated by matching all theoretical fragments with a non-integer shift with the raw peaks for this spectrum. This is done with 40 different shifts. The resulting percentage is the average number of annotated peaks over the number of annotated peaks with the correct spectrum. | 1.33% |
| Satellite FDR? Satellite FDRSee the FDR for details on its calculation. This satellite ion specific FDR only contains the satellite ions (d/w) for I/L/J positions. | 0.00% |
| PSM Score? PSM ScoreThe PSM Score as given by Hecklib to this annotated spectrum. It is shown with three significant figures. | 600 |

## Spectrum 3905? Spectrum 3905 The raw spectrum of this peptide as annotated by Hecklib. The fragments are coloured according to ion type (see legend). Any peaks with a star '\*' as text can be hovered over to see the full details, first the ion type second the mass shift type. By hovering over the amino acids in the peptide or ions in the legend the corresponding peaks are highlighted. By toggling the 'Unassigned' label you can turn the background (unassigned) peaks on or off in the plot. By updating the slider in the Ion legend you can update the spectrum to only show the top X% of the peaks with labels. The top X% means any peak that is within X% of the highest intensity. By dragging in the spectrum you can zoom in to a specific part of the spectrum and use 'Zoom Out' to get back to the original zoom level. The annotation of the spectrum is based on the given sequence in the peptides file and is done with different software so inconsistencies are likely. The peaks are annotated based on the given sequence, with 20 ppm tolerance.

Copy Data

### Spectrum 3905 (TSV)

#### Preview

```
Loading example...
```

*Click on the button to copy the data to your clipboard.*

Mz MinMz MaxIntensity Max

WidthHeightPeptide font sizePeptide stroke widthSpectrum font sizeSpectrum stroke widthCompact peptide

Ion legend

wxyz

abcd

OtherUnassignedIonChargePositionShow for top:%

JJSEVSDRPSGVSSR

04.42e+48.85e+41.33e+51.77e+5

Zoom Out

y+11y+11w+12y+12z+12y+12z+38w+13y+13z+13y+27y+13c+312z+28w+14y+28y+14y+14y+14y+313c+28y+313c+14c+314z+29y+29y+314y+314z+15y+314y+15y+210z+210y+210z+16w+16c+15z+211y+211y+16y+16z+16y+16w+212z+212c+212y+212z+212y+212c+16z+213y+213y+213z+213y+213y+17w+214y+214z+214y+214w+18c+17z+18z+18y+18z+18y+18w+19c+18z+19y+19z+19y+19c+19y+110z+110y+110c+110z+111y+111c+111c+112y+112w+113c+113y+113c+114y+114

0804160824123216

Fragment Matches Table

Show background peaks

| Position | Ion type | Intensity | mz Theoretical | mz Error (Th) | mz Error (ppm) | Charge | Series Number |
| --- | --- | --- | --- | --- | --- | --- | --- |
| - | - | 1940 | 125.1 | - | - | 0 | - |
| - | - | 4750 | 126.1 | - | - | 0 | - |
| - | - | 1743 | 131.1 | - | - | 0 | - |
| - | - | 419.1 | 132 | - | - | 0 | - |
| - | - | 471.5 | 136.5 | - | - | 0 | - |
| - | - | 502 | 143.1 | - | - | 0 | - |
| - | - | 414.7 | 143.8 | - | - | 0 | - |
| - | - | 436.9 | 146.1 | - | - | 0 | - |
| - | - | 1094 | 148.9 | - | - | 0 | - |
| 15 | y | 1326 | 158.1 | 0.000172 | 1.088 | +1 | 1 |
| - | - | 2095 | 160 | - | - | 0 | - |
| - | - | 494.3 | 160.3 | - | - | 0 | - |
| - | - | 427 | 160.7 | - | - | 0 | - |
| - | - | 4905 | 168.1 | - | - | 0 | - |
| - | - | 657.7 | 169.1 | - | - | 0 | - |
| - | - | 1189 | 173.5 | - | - | 0 | - |
| 15 | y | 8719 | 175.1 | 0.0003258 | 1.86 | +1 | 1 |
| - | - | 573.1 | 175.9 | - | - | 0 | - |
| - | - | 595.9 | 176.1 | - | - | 0 | - |
| - | - | 5289 | 178.1 | - | - | 0 | - |
| - | - | 2.117E+04 | 185.1 | - | - | 0 | - |
| - | - | 2350 | 186.1 | - | - | 0 | - |
| - | - | 848.8 | 189.1 | - | - | 0 | - |
| - | - | 5.575E+04 | 199.2 | - | - | 0 | - |
| - | - | 3769 | 200.1 | - | - | 0 | - |
| - | - | 7209 | 200.2 | - | - | 0 | - |
| - | - | 1662 | 201.1 | - | - | 0 | - |
| - | - | 886.4 | 201.1 | - | - | 0 | - |
| - | - | 720.4 | 216.1 | - | - | 0 | - |
| - | - | 600 | 216.7 | - | - | 0 | - |
| - | - | 2659 | 217.1 | - | - | 0 | - |
| - | - | 898.1 | 226.1 | - | - | 0 | - |
| - | - | 777.4 | 226.2 | - | - | 0 | - |
| - | - | 2.911E+04 | 227.2 | - | - | 0 | - |
| - | - | 3527 | 228.2 | - | - | 0 | - |
| 14 | w | 4308 | 229.1 | 0.0003354 | 1.464 | +1 | 2 |
| - | - | 586.2 | 229.2 | - | - | 0 | - |
| - | - | 781 | 230.1 | - | - | 0 | - |
| - | - | 3079 | 233.2 | - | - | 0 | - |
| - | - | 1003 | 244.1 | - | - | 0 | - |
| 14 | y | 3.918E+04 | 245.1 | 0.0004312 | 1.759 | +1 | 2 |
| 14 | z | 6224 | 246.1 | 0.001534 | 6.234 | +1 | 2 |
| - | - | 647.9 | 247.1 | - | - | 0 | - |
| - | - | 1579 | 249.2 | - | - | 0 | - |
| - | - | 1182 | 255.1 | - | - | 0 | - |
| - | - | 3244 | 261.2 | - | - | 0 | - |
| 14 | y | 5641 | 262.2 | 0.0002035 | 0.7763 | +1 | 2 |
| - | - | 700.3 | 262.2 | - | - | 0 | - |
| - | - | 3831 | 270.1 | - | - | 0 | - |
| - | - | 1194 | 272.2 | - | - | 0 | - |
| 8 | z | 1927 | 277.2 | 0.003368 | 12.15 | +3 | 8 |
| - | - | 9212 | 279.1 | - | - | 0 | - |
| - | - | 891.5 | 280.1 | - | - | 0 | - |
| - | - | 567.6 | 299.2 | - | - | 0 | - |
| - | - | 1104 | 302.2 | - | - | 0 | - |
| - | - | 1047 | 303.2 | - | - | 0 | - |
| - | - | 695.8 | 304.1 | - | - | 0 | - |
| - | - | 1914 | 314.2 | - | - | 0 | - |
| 13 | w | 2.012E+04 | 316.2 | 0.0005946 | 1.881 | +1 | 3 |
| - | - | 2927 | 317.2 | - | - | 0 | - |
| - | - | 980.5 | 323.2 | - | - | 0 | - |
| - | - | 5404 | 331.1 | - | - | 0 | - |
| 13 | y | 6254 | 332.2 | 0.0001563 | 0.4706 | +1 | 3 |
| 13 | z | 1.338E+04 | 333.2 | 0.0003574 | 1.073 | +1 | 3 |
| - | - | 1783 | 334.2 | - | - | 0 | - |
| 9 | y | 634.4 | 345.2 | 0.000994 | 2.88 | +2 | 7 |
| - | - | 574.3 | 345.7 | - | - | 0 | - |
| - | - | 1759 | 347.2 | - | - | 0 | - |
| - | - | 793.6 | 349.1 | - | - | 0 | - |
| 13 | y | 4751 | 349.2 | 0.0004932 | 1.412 | +1 | 3 |
| - | - | 1050 | 350.2 | - | - | 0 | - |
| - | - | 9138 | 357.2 | - | - | 0 | - |
| - | - | 711.2 | 359.2 | - | - | 0 | - |
| - | - | 667.3 | 373.2 | - | - | 0 | - |
| - | - | 2137 | 389.7 | - | - | 0 | - |
| - | - | 799.2 | 390.2 | - | - | 0 | - |
| - | - | 1517 | 390.7 | - | - | 0 | - |
| - | - | 666.5 | 391.2 | - | - | 0 | - |
| - | - | 7797 | 391.7 | - | - | 0 | - |
| - | - | 4122 | 392.2 | - | - | 0 | - |
| - | - | 2614 | 398.7 | - | - | 0 | - |
| - | - | 1667 | 399.2 | - | - | 0 | - |
| - | - | 1876 | 399.7 | - | - | 0 | - |
| - | - | 1156 | 400.2 | - | - | 0 | - |
| 12 | c | 754.5 | 414.2 | 0.0006884 | 1.662 | +3 | 12 |
| 8 | z | 5719 | 415.2 | 0.00222 | 5.346 | +2 | 8 |
| - | - | 2710 | 415.7 | - | - | 0 | - |
| 12 | w | 1.517E+04 | 417.2 | 0.0007982 | 1.913 | +1 | 4 |
| - | - | 3072 | 418.2 | - | - | 0 | - |
| 8 | y | 3489 | 423.2 | 0.0003042 | 0.7188 | +2 | 8 |
| - | - | 1527 | 423.7 | - | - | 0 | - |
| - | - | 1544 | 425.2 | - | - | 0 | - |
| - | - | 739.4 | 426.2 | - | - | 0 | - |
| - | - | 1484 | 429.2 | - | - | 0 | - |
| 12 | y | 644.6 | 430.2 | 0.00218 | 5.067 | +1 | 4 |
| 12 | y | 1559 | 431.2 | 0.0005901 | 1.368 | +1 | 4 |
| - | - | 1087 | 433.7 | - | - | 0 | - |
| - | - | 2940 | 442.7 | - | - | 0 | - |
| - | - | 4436 | 443.2 | - | - | 0 | - |
| - | - | 1449 | 443.3 | - | - | 0 | - |
| - | - | 588.8 | 444.3 | - | - | 0 | - |
| - | - | 1232 | 446.2 | - | - | 0 | - |
| - | - | 731.2 | 447.2 | - | - | 0 | - |
| - | - | 878.6 | 447.2 | - | - | 0 | - |
| 12 | y | 1.294E+04 | 448.3 | 0.0008049 | 1.796 | +1 | 4 |
| 3 | y | 637.8 | 449.2 | 0.008159 | 18.16 | +3 | 13 |
| - | - | 3073 | 449.3 | - | - | 0 | - |
| 8 | c | 755.4 | 450.7 | 0.0005699 | 1.264 | +2 | 8 |
| 3 | y | 1041 | 454.9 | 0.0002456 | 0.5399 | +3 | 13 |
| - | - | 746.9 | 455.2 | - | - | 0 | - |
| - | - | 884 | 455.3 | - | - | 0 | - |
| - | - | 751.6 | 460.2 | - | - | 0 | - |
| 4 | c | 1.048E+04 | 460.3 | 0.0007992 | 1.736 | +1 | 4 |
| - | - | 3033 | 461.3 | - | - | 0 | - |
| - | - | 1116 | 465.3 | - | - | 0 | - |
| 14 | c | 2574 | 472.2 | 0.00808 | 17.11 | +3 | 14 |
| 7 | z | 3203 | 472.7 | 0.001017 | 2.15 | +2 | 9 |
| - | - | 930.1 | 473.2 | - | - | 0 | - |
| - | - | 4794 | 474.2 | - | - | 0 | - |
| 7 | y | 3630 | 480.7 | 0.0008403 | 1.748 | +2 | 9 |
| 2 | y | 829 | 486.6 | 0.001859 | 3.82 | +3 | 14 |
| 2 | y | 606.1 | 486.9 | 0.007319 | 15.03 | +3 | 14 |
| - | - | 608.4 | 489.1 | - | - | 0 | - |
| 11 | z | 4233 | 489.3 | 0.0005677 | 1.16 | +1 | 5 |
| - | - | 2977 | 490.3 | - | - | 0 | - |
| 2 | y | 1062 | 492.6 | 0.001053 | 2.138 | +3 | 14 |
| - | - | 641.9 | 492.9 | - | - | 0 | - |
| - | - | 811.3 | 493.3 | - | - | 0 | - |
| - | - | 1393 | 495.9 | - | - | 0 | - |
| - | - | 946.6 | 496.3 | - | - | 0 | - |
| - | - | 2433 | 499.3 | - | - | 0 | - |
| - | - | 607.6 | 504.3 | - | - | 0 | - |
| 11 | y | 4.779E+04 | 505.3 | 0.000795 | 1.573 | +1 | 5 |
| - | - | 1.12E+04 | 506.3 | - | - | 0 | - |
| - | - | 2102 | 507.3 | - | - | 0 | - |
| - | - | 784.4 | 514.3 | - | - | 0 | - |
| - | - | 1915 | 515.2 | - | - | 0 | - |
| - | - | 2093 | 515.3 | - | - | 0 | - |
| 6 | y | 3307 | 515.7 | 0.0005261 | 1.02 | +2 | 10 |
| 6 | z | 4504 | 516.3 | 0.0001051 | 0.2036 | +2 | 10 |
| - | - | 1036 | 516.3 | - | - | 0 | - |
| - | - | 5465 | 516.8 | - | - | 0 | - |
| - | - | 3630 | 517.3 | - | - | 0 | - |
| - | - | 701.6 | 517.3 | - | - | 0 | - |
| - | - | 853.2 | 520.3 | - | - | 0 | - |
| - | - | 3638 | 522.3 | - | - | 0 | - |
| - | - | 1173 | 523.8 | - | - | 0 | - |
| 6 | y | 1.884E+04 | 524.3 | 0.002374 | 4.528 | +2 | 10 |
| - | - | 6030 | 524.6 | - | - | 0 | - |
| - | - | 7524 | 524.8 | - | - | 0 | - |
| - | - | 2304 | 524.9 | - | - | 0 | - |
| - | - | 3439 | 525.3 | - | - | 0 | - |
| - | - | 3752 | 528.8 | - | - | 0 | - |
| - | - | 1.495E+04 | 529.3 | - | - | 0 | - |
| - | - | 4225 | 529.8 | - | - | 0 | - |
| - | - | 5069 | 530.3 | - | - | 0 | - |
| - | - | 4145 | 530.6 | - | - | 0 | - |
| - | - | 1412 | 530.8 | - | - | 0 | - |
| - | - | 3853 | 531 | - | - | 0 | - |
| - | - | 6745 | 531.3 | - | - | 0 | - |
| - | - | 2744 | 531.8 | - | - | 0 | - |
| - | - | 1842 | 541.3 | - | - | 0 | - |
| - | - | 6350 | 542.3 | - | - | 0 | - |
| - | - | 1261 | 543.3 | - | - | 0 | - |
| - | - | 1035 | 546.3 | - | - | 0 | - |
| 10 | z | 651.2 | 558.3 | 0.009896 | 17.73 | +1 | 6 |
| - | - | 2605 | 558.3 | - | - | 0 | - |
| 10 | w | 1837 | 559.3 | 0.001705 | 3.048 | +1 | 6 |
| 5 | c | 8314 | 559.3 | 4.28E-05 | 0.07652 | +1 | 5 |
| - | - | 2134 | 560.3 | - | - | 0 | - |
| - | - | 702.1 | 561.3 | - | - | 0 | - |
| 5 | z | 3.808E+04 | 565.8 | 0.000539 | 0.9527 | +2 | 11 |
| - | - | 2.423E+04 | 566.3 | - | - | 0 | - |
| - | - | 7802 | 566.8 | - | - | 0 | - |
| - | - | 2325 | 567.3 | - | - | 0 | - |
| - | - | 1125 | 571.3 | - | - | 0 | - |
| - | - | 732.7 | 572.3 | - | - | 0 | - |
| - | - | 4794 | 572.4 | - | - | 0 | - |
| - | - | 873.8 | 573.3 | - | - | 0 | - |
| 5 | y | 2.038E+04 | 573.8 | 0.0003322 | 0.579 | +2 | 11 |
| 10 | y | 8961 | 574.3 | 0.003928 | 6.84 | +1 | 6 |
| - | - | 4582 | 574.8 | - | - | 0 | - |
| 10 | y | 2856 | 575.3 | 0.00264 | 4.589 | +1 | 6 |
| 10 | z | 7761 | 576.3 | 0.0003691 | 0.6405 | +1 | 6 |
| - | - | 2543 | 577.3 | - | - | 0 | - |
| - | - | 1034 | 585.3 | - | - | 0 | - |
| - | - | 2421 | 586.3 | - | - | 0 | - |
| - | - | 665.1 | 587.3 | - | - | 0 | - |
| 10 | y | 1529 | 592.3 | 0.0008989 | 1.518 | +1 | 6 |
| - | - | 640.9 | 593.3 | - | - | 0 | - |
| - | - | 3382 | 600.3 | - | - | 0 | - |
| 4 | w | 1.497E+04 | 600.8 | 0.0007872 | 1.31 | +2 | 12 |
| - | - | 8685 | 601.3 | - | - | 0 | - |
| - | - | 4084 | 601.8 | - | - | 0 | - |
| - | - | 655.3 | 602.3 | - | - | 0 | - |
| - | - | 1160 | 603.4 | - | - | 0 | - |
| - | - | 738 | 608.3 | - | - | 0 | - |
| - | - | 780.2 | 611.3 | - | - | 0 | - |
| 4 | z | 668.3 | 621.3 | 0.002536 | 4.081 | +2 | 12 |
| - | - | 974 | 627.4 | - | - | 0 | - |
| 12 | c | 2197 | 629.3 | 0.006833 | 10.86 | +2 | 12 |
| 4 | y | 888.1 | 629.8 | 0.00763 | 12.11 | +2 | 12 |
| 4 | z | 3.574E+04 | 630.3 | 0.0006048 | 0.9595 | +2 | 12 |
| - | - | 2.63E+04 | 630.8 | - | - | 0 | - |
| - | - | 1.159E+04 | 631.3 | - | - | 0 | - |
| - | - | 3210 | 631.8 | - | - | 0 | - |
| - | - | 763.1 | 637.3 | - | - | 0 | - |
| - | - | 1.589E+04 | 637.8 | - | - | 0 | - |
| 4 | y | 3.628E+04 | 638.3 | 0.0002124 | 0.3327 | +2 | 12 |
| - | - | 2.213E+04 | 638.8 | - | - | 0 | - |
| - | - | 8363 | 639.3 | - | - | 0 | - |
| - | - | 1480 | 639.8 | - | - | 0 | - |
| - | - | 3681 | 644.3 | - | - | 0 | - |
| - | - | 2420 | 644.8 | - | - | 0 | - |
| - | - | 894.8 | 645.4 | - | - | 0 | - |
| 6 | c | 3.557E+04 | 646.4 | 0.0006682 | 1.034 | +1 | 6 |
| - | - | 639 | 647.3 | - | - | 0 | - |
| - | - | 1.09E+04 | 647.4 | - | - | 0 | - |
| - | - | 2238 | 648.4 | - | - | 0 | - |
| - | - | 1956 | 651.8 | - | - | 0 | - |
| - | - | 886.8 | 652.3 | - | - | 0 | - |
| 3 | z | 1951 | 665.3 | 0.00995 | 14.96 | +2 | 13 |
| - | - | 1952 | 665.8 | - | - | 0 | - |
| - | - | 1524 | 666.3 | - | - | 0 | - |
| - | - | 743.1 | 670.4 | - | - | 0 | - |
| 3 | y | 1587 | 672.8 | 0.001606 | 2.387 | +2 | 13 |
| 3 | y | 2836 | 673.3 | 0.007485 | 11.12 | +2 | 13 |
| 3 | z | 2.364E+04 | 673.8 | 0.0008259 | 1.226 | +2 | 13 |
| - | - | 3.194E+04 | 674.3 | - | - | 0 | - |
| - | - | 1.723E+04 | 674.8 | - | - | 0 | - |
| - | - | 5956 | 675.3 | - | - | 0 | - |
| - | - | 1045 | 675.8 | - | - | 0 | - |
| - | - | 2403 | 680.8 | - | - | 0 | - |
| - | - | 3202 | 681.3 | - | - | 0 | - |
| 3 | y | 1.752E+05 | 681.8 | 0.0005581 | 0.8185 | +2 | 13 |
| - | - | 1.131E+05 | 682.3 | - | - | 0 | - |
| - | - | 4.947E+04 | 682.8 | - | - | 0 | - |
| - | - | 1.379E+04 | 683.3 | - | - | 0 | - |
| - | - | 3575 | 683.8 | - | - | 0 | - |
| - | - | 1690 | 684.4 | - | - | 0 | - |
| - | - | 1819 | 685.4 | - | - | 0 | - |
| - | - | 832.9 | 686.3 | - | - | 0 | - |
| - | - | 773.1 | 686.4 | - | - | 0 | - |
| - | - | 1988 | 687.3 | - | - | 0 | - |
| - | - | 1002 | 688.3 | - | - | 0 | - |
| 9 | y | 5183 | 689.4 | 0.0009027 | 1.309 | +1 | 7 |
| - | - | 2308 | 690.4 | - | - | 0 | - |
| - | - | 659.4 | 691.4 | - | - | 0 | - |
| - | - | 716.1 | 697.4 | - | - | 0 | - |
| - | - | 1591 | 699.4 | - | - | 0 | - |
| - | - | 763.7 | 700.4 | - | - | 0 | - |
| - | - | 2092 | 701.4 | - | - | 0 | - |
| - | - | 1011 | 711.4 | - | - | 0 | - |
| - | - | 2138 | 712.4 | - | - | 0 | - |
| - | - | 2669 | 713.4 | - | - | 0 | - |
| - | - | 812.8 | 714.4 | - | - | 0 | - |
| 2 | w | 2840 | 715.8 | 0.001367 | 1.909 | +2 | 14 |
| - | - | 2476 | 716.4 | - | - | 0 | - |
| - | - | 939 | 716.8 | - | - | 0 | - |
| - | - | 3517 | 717.4 | - | - | 0 | - |
| - | - | 4852 | 718.4 | - | - | 0 | - |
| - | - | 1667 | 719.4 | - | - | 0 | - |
| 2 | y | 1412 | 729.4 | 0.0007347 | 1.007 | +2 | 14 |
| 2 | z | 3545 | 730.4 | 0.0001905 | 0.2608 | +2 | 14 |
| - | - | 1494 | 730.9 | - | - | 0 | - |
| - | - | 639.4 | 736.4 | - | - | 0 | - |
| 2 | y | 1.07E+04 | 738.4 | 0.0004573 | 0.6193 | +2 | 14 |
| - | - | 7651 | 738.9 | - | - | 0 | - |
| - | - | 4651 | 739.4 | - | - | 0 | - |
| - | - | 1031 | 739.9 | - | - | 0 | - |
| 8 | w | 3028 | 743.4 | 0.002118 | 2.849 | +1 | 8 |
| - | - | 3612 | 744.4 | - | - | 0 | - |
| - | - | 620.2 | 745.4 | - | - | 0 | - |
| - | - | 2928 | 750.9 | - | - | 0 | - |
| - | - | 2080 | 751.4 | - | - | 0 | - |
| - | - | 1250 | 751.9 | - | - | 0 | - |
| - | - | 1085 | 756.9 | - | - | 0 | - |
| - | - | 1005 | 757.4 | - | - | 0 | - |
| - | - | 3708 | 758.4 | - | - | 0 | - |
| - | - | 5751 | 758.9 | - | - | 0 | - |
| - | - | 5764 | 759.4 | - | - | 0 | - |
| - | - | 1158 | 759.9 | - | - | 0 | - |
| - | - | 1896 | 760.4 | - | - | 0 | - |
| 7 | c | 6.599E+04 | 761.4 | 0.0006417 | 0.8427 | +1 | 7 |
| - | - | 2.607E+04 | 762.4 | - | - | 0 | - |
| - | - | 7172 | 763.4 | - | - | 0 | - |
| - | - | 3135 | 764.4 | - | - | 0 | - |
| - | - | 5911 | 764.9 | - | - | 0 | - |
| - | - | 1.092E+05 | 765.4 | - | - | 0 | - |
| - | - | 9.096E+04 | 765.9 | - | - | 0 | - |
| - | - | 4.55E+04 | 766.4 | - | - | 0 | - |
| - | - | 1.311E+04 | 766.9 | - | - | 0 | - |
| - | - | 3429 | 767.4 | - | - | 0 | - |
| - | - | 1087 | 771.4 | - | - | 0 | - |
| - | - | 846.1 | 771.9 | - | - | 0 | - |
| - | - | 3261 | 772.4 | - | - | 0 | - |
| - | - | 2993 | 772.9 | - | - | 0 | - |
| - | - | 3194 | 773.4 | - | - | 0 | - |
| - | - | 3.004E+04 | 773.9 | - | - | 0 | - |
| - | - | 2.544E+04 | 774.4 | - | - | 0 | - |
| - | - | 1.263E+04 | 774.9 | - | - | 0 | - |
| - | - | 3658 | 775.4 | - | - | 0 | - |
| - | - | 1213 | 775.9 | - | - | 0 | - |
| - | - | 1500 | 777.9 | - | - | 0 | - |
| - | - | 4322 | 778.4 | - | - | 0 | - |
| - | - | 2061 | 778.9 | - | - | 0 | - |
| - | - | 1850 | 779.4 | - | - | 0 | - |
| - | - | 1212 | 779.9 | - | - | 0 | - |
| - | - | 2576 | 780.4 | - | - | 0 | - |
| - | - | 643.8 | 780.9 | - | - | 0 | - |
| - | - | 5120 | 782.4 | - | - | 0 | - |
| - | - | 1.359E+04 | 783.4 | - | - | 0 | - |
| - | - | 2531 | 784.4 | - | - | 0 | - |
| - | - | 2402 | 785.9 | - | - | 0 | - |
| - | - | 8007 | 786.4 | - | - | 0 | - |
| - | - | 1.176E+05 | 786.9 | - | - | 0 | - |
| - | - | 9.468E+04 | 787.4 | - | - | 0 | - |
| - | - | 4.777E+04 | 787.9 | - | - | 0 | - |
| - | - | 1.464E+04 | 788.4 | - | - | 0 | - |
| - | - | 3892 | 788.9 | - | - | 0 | - |
| - | - | 1048 | 793.4 | - | - | 0 | - |
| - | - | 2651 | 793.9 | - | - | 0 | - |
| - | - | 6071 | 794.4 | - | - | 0 | - |
| - | - | 7.598E+04 | 794.9 | - | - | 0 | - |
| - | - | 9.814E+04 | 795.4 | - | - | 0 | - |
| - | - | 5.846E+04 | 795.9 | - | - | 0 | - |
| - | - | 2.728E+04 | 796.4 | - | - | 0 | - |
| - | - | 7858 | 796.9 | - | - | 0 | - |
| - | - | 3407 | 797.4 | - | - | 0 | - |
| - | - | 2042 | 798.4 | - | - | 0 | - |
| - | - | 760.4 | 799.4 | - | - | 0 | - |
| - | - | 1584 | 800.5 | - | - | 0 | - |
| 8 | z | 3216 | 811.4 | 0.0001253 | 0.1544 | +1 | 8 |
| 8 | z | 1461 | 812.4 | 0.01245 | 15.32 | +1 | 8 |
| 8 | y | 1011 | 828.4 | 0.001577 | 1.903 | +1 | 8 |
| 8 | z | 5.13E+04 | 829.4 | 0.0004859 | 0.5858 | +1 | 8 |
| - | - | 2.846E+04 | 830.4 | - | - | 0 | - |
| - | - | 7261 | 831.4 | - | - | 0 | - |
| - | - | 1408 | 832.4 | - | - | 0 | - |
| - | - | 1969 | 839.5 | - | - | 0 | - |
| - | - | 1206 | 840.5 | - | - | 0 | - |
| - | - | 4309 | 841.5 | - | - | 0 | - |
| - | - | 2542 | 842.5 | - | - | 0 | - |
| - | - | 2927 | 844.5 | - | - | 0 | - |
| 8 | y | 6725 | 845.5 | 7.239E-05 | 0.08562 | +1 | 8 |
| - | - | 3153 | 846.5 | - | - | 0 | - |
| - | - | 1525 | 847.5 | - | - | 0 | - |
| - | - | 859.5 | 858.5 | - | - | 0 | - |
| - | - | 948.1 | 866.5 | - | - | 0 | - |
| - | - | 1178 | 872.5 | - | - | 0 | - |
| - | - | 3853 | 873.5 | - | - | 0 | - |
| - | - | 1620 | 874.5 | - | - | 0 | - |
| - | - | 7362 | 884.5 | - | - | 0 | - |
| - | - | 2490 | 885.5 | - | - | 0 | - |
| 7 | w | 1192 | 899.5 | 0.002264 | 2.517 | +1 | 9 |
| 8 | c | 7370 | 900.5 | 0.001288 | 1.431 | +1 | 8 |
| - | - | 5614 | 901.5 | - | - | 0 | - |
| - | - | 2418 | 902.5 | - | - | 0 | - |
| 7 | z | 2153 | 926.5 | 0.001061 | 1.145 | +1 | 9 |
| - | - | 1735 | 927.5 | - | - | 0 | - |
| - | - | 2118 | 929.5 | - | - | 0 | - |
| - | - | 3265 | 932.4 | - | - | 0 | - |
| - | - | 1091 | 933.4 | - | - | 0 | - |
| 7 | y | 802.4 | 943.5 | 0.003096 | 3.282 | +1 | 9 |
| 7 | z | 1.945E+04 | 944.5 | 0.0003373 | 0.3571 | +1 | 9 |
| - | - | 1.62E+04 | 945.5 | - | - | 0 | - |
| - | - | 6377 | 946.5 | - | - | 0 | - |
| - | - | 1211 | 947.5 | - | - | 0 | - |
| - | - | 1827 | 954.5 | - | - | 0 | - |
| - | - | 2165 | 955.5 | - | - | 0 | - |
| - | - | 3150 | 956.5 | - | - | 0 | - |
| - | - | 1467 | 957.6 | - | - | 0 | - |
| - | - | 954.1 | 958.6 | - | - | 0 | - |
| - | - | 768.1 | 959.5 | - | - | 0 | - |
| 7 | y | 2352 | 960.5 | 0.000229 | 0.2384 | +1 | 9 |
| - | - | 963.7 | 961.5 | - | - | 0 | - |
| - | - | 5153 | 970.5 | - | - | 0 | - |
| - | - | 3033 | 971.5 | - | - | 0 | - |
| - | - | 714.6 | 972.5 | - | - | 0 | - |
| - | - | 711.7 | 998.6 | - | - | 0 | - |
| - | - | 849.7 | 999.6 | - | - | 0 | - |
| - | - | 1400 | 1003 | - | - | 0 | - |
| - | - | 1.216E+04 | 1004 | - | - | 0 | - |
| - | - | 9880 | 1006 | - | - | 0 | - |
| - | - | 2058 | 1007 | - | - | 0 | - |
| - | - | 3091 | 1014 | - | - | 0 | - |
| 9 | c | 4.694E+04 | 1015 | 0.0009416 | 0.9281 | +1 | 9 |
| - | - | 2.801E+04 | 1016 | - | - | 0 | - |
| - | - | 8279 | 1017 | - | - | 0 | - |
| - | - | 1719 | 1018 | - | - | 0 | - |
| - | - | 933.8 | 1020 | - | - | 0 | - |
| 6 | y | 1319 | 1030 | 0.003663 | 3.555 | +1 | 10 |
| 6 | z | 7088 | 1031 | 0.0002303 | 0.2233 | +1 | 10 |
| - | - | 9851 | 1033 | - | - | 0 | - |
| - | - | 5548 | 1034 | - | - | 0 | - |
| - | - | 1271 | 1035 | - | - | 0 | - |
| - | - | 3548 | 1040 | - | - | 0 | - |
| - | - | 3454 | 1041 | - | - | 0 | - |
| - | - | 2998 | 1042 | - | - | 0 | - |
| - | - | 3635 | 1043 | - | - | 0 | - |
| - | - | 1535 | 1044 | - | - | 0 | - |
| - | - | 1161 | 1045 | - | - | 0 | - |
| - | - | 1.02E+04 | 1046 | - | - | 0 | - |
| - | - | 3886 | 1047 | - | - | 0 | - |
| 6 | y | 3606 | 1048 | 0.0003053 | 0.2915 | +1 | 10 |
| - | - | 1983 | 1049 | - | - | 0 | - |
| - | - | 874.6 | 1050 | - | - | 0 | - |
| - | - | 1696 | 1057 | - | - | 0 | - |
| - | - | 3.731E+04 | 1058 | - | - | 0 | - |
| - | - | 2.451E+04 | 1059 | - | - | 0 | - |
| - | - | 1.57E+04 | 1060 | - | - | 0 | - |
| - | - | 5309 | 1061 | - | - | 0 | - |
| - | - | 3958 | 1062 | - | - | 0 | - |
| - | - | 9767 | 1063 | - | - | 0 | - |
| - | - | 2996 | 1064 | - | - | 0 | - |
| - | - | 798.8 | 1071 | - | - | 0 | - |
| - | - | 791.5 | 1087 | - | - | 0 | - |
| - | - | 2467 | 1101 | - | - | 0 | - |
| 10 | c | 6.482E+04 | 1102 | 0.0002242 | 0.2035 | +1 | 10 |
| - | - | 3.734E+04 | 1103 | - | - | 0 | - |
| - | - | 1.447E+04 | 1104 | - | - | 0 | - |
| - | - | 3147 | 1105 | - | - | 0 | - |
| - | - | 9499 | 1115 | - | - | 0 | - |
| - | - | 5800 | 1116 | - | - | 0 | - |
| - | - | 3432 | 1117 | - | - | 0 | - |
| 5 | z | 9944 | 1131 | 0.0001905 | 0.1685 | +1 | 11 |
| - | - | 2.264E+04 | 1132 | - | - | 0 | - |
| - | - | 9811 | 1133 | - | - | 0 | - |
| - | - | 3694 | 1134 | - | - | 0 | - |
| - | - | 1117 | 1135 | - | - | 0 | - |
| 5 | y | 2932 | 1147 | 0.002801 | 2.443 | +1 | 11 |
| - | - | 2002 | 1148 | - | - | 0 | - |
| 11 | c | 3.665E+04 | 1159 | 0.0003655 | 0.3154 | +1 | 11 |
| - | - | 2.297E+04 | 1160 | - | - | 0 | - |
| - | - | 8294 | 1161 | - | - | 0 | - |
| - | - | 1907 | 1162 | - | - | 0 | - |
| - | - | 7468 | 1214 | - | - | 0 | - |
| - | - | 4488 | 1215 | - | - | 0 | - |
| - | - | 2990 | 1216 | - | - | 0 | - |
| - | - | 861.1 | 1217 | - | - | 0 | - |
| - | - | 837.4 | 1219 | - | - | 0 | - |
| - | - | 740.5 | 1248 | - | - | 0 | - |
| 12 | c | 3.602E+04 | 1258 | 0.001152 | 0.9163 | +1 | 12 |
| - | - | 2.306E+04 | 1259 | - | - | 0 | - |
| - | - | 7669 | 1260 | - | - | 0 | - |
| - | - | 3.114E+04 | 1261 | - | - | 0 | - |
| - | - | 1.822E+04 | 1262 | - | - | 0 | - |
| - | - | 7840 | 1263 | - | - | 0 | - |
| - | - | 1842 | 1264 | - | - | 0 | - |
| 4 | y | 1.019E+04 | 1276 | 0.001693 | 1.327 | +1 | 12 |
| - | - | 8046 | 1277 | - | - | 0 | - |
| - | - | 2559 | 1278 | - | - | 0 | - |
| - | - | 1540 | 1279 | - | - | 0 | - |
| - | - | 841.3 | 1280 | - | - | 0 | - |
| - | - | 1179 | 1284 | - | - | 0 | - |
| - | - | 756 | 1286 | - | - | 0 | - |
| - | - | 5520 | 1301 | - | - | 0 | - |
| - | - | 4731 | 1302 | - | - | 0 | - |
| - | - | 2953 | 1303 | - | - | 0 | - |
| - | - | 1072 | 1304 | - | - | 0 | - |
| - | - | 882.8 | 1305 | - | - | 0 | - |
| - | - | 969.7 | 1306 | - | - | 0 | - |
| 3 | w | 1443 | 1330 | 0.01435 | 10.8 | +1 | 13 |
| - | - | 2665 | 1331 | - | - | 0 | - |
| - | - | 1854 | 1332 | - | - | 0 | - |
| - | - | 1028 | 1333 | - | - | 0 | - |
| - | - | 1255 | 1343 | - | - | 0 | - |
| 13 | c | 6.453E+04 | 1345 | 0.001321 | 0.982 | +1 | 13 |
| - | - | 4.396E+04 | 1346 | - | - | 0 | - |
| - | - | 608.4 | 1346 | - | - | 0 | - |
| - | - | 1.895E+04 | 1347 | - | - | 0 | - |
| - | - | 2.586E+04 | 1348 | - | - | 0 | - |
| - | - | 1.722E+04 | 1349 | - | - | 0 | - |
| - | - | 7363 | 1350 | - | - | 0 | - |
| - | - | 2295 | 1351 | - | - | 0 | - |
| - | - | 762 | 1352 | - | - | 0 | - |
| 3 | y | 1658 | 1363 | 0.003266 | 2.397 | +1 | 13 |
| - | - | 1490 | 1364 | - | - | 0 | - |
| - | - | 727.5 | 1365 | - | - | 0 | - |
| - | - | 4380 | 1388 | - | - | 0 | - |
| - | - | 4696 | 1389 | - | - | 0 | - |
| - | - | 1956 | 1390 | - | - | 0 | - |
| - | - | 995.3 | 1430 | - | - | 0 | - |
| - | - | 2903 | 1431 | - | - | 0 | - |
| 14 | c | 9.159E+04 | 1432 | 0.001855 | 1.295 | +1 | 14 |
| - | - | 6.735E+04 | 1433 | - | - | 0 | - |
| - | - | 3.049E+04 | 1434 | - | - | 0 | - |
| - | - | 9825 | 1435 | - | - | 0 | - |
| - | - | 1940 | 1436 | - | - | 0 | - |
| - | - | 4219 | 1461 | - | - | 0 | - |
| - | - | 3451 | 1462 | - | - | 0 | - |
| - | - | 1551 | 1463 | - | - | 0 | - |
| - | - | 861.7 | 1474 | - | - | 0 | - |
| - | - | 853.6 | 1475 | - | - | 0 | - |
| 2 | y | 724.2 | 1476 | 0.01027 | 6.957 | +1 | 14 |
| - | - | 8030 | 1489 | - | - | 0 | - |
| - | - | 6746 | 1490 | - | - | 0 | - |
| - | - | 2560 | 1491 | - | - | 0 | - |
| - | - | 1646 | 1492 | - | - | 0 | - |
| - | - | 1259 | 1502 | - | - | 0 | - |
| - | - | 2694 | 1513 | - | - | 0 | - |
| - | - | 2135 | 1514 | - | - | 0 | - |
| - | - | 1379 | 1515 | - | - | 0 | - |
| - | - | 1240 | 1516 | - | - | 0 | - |
| - | - | 868.6 | 1519 | - | - | 0 | - |
| - | - | 735.8 | 1529 | - | - | 0 | - |
| - | - | 2117 | 1530 | - | - | 0 | - |
| - | - | 1.722E+04 | 1531 | - | - | 0 | - |
| - | - | 2.237E+04 | 1532 | - | - | 0 | - |
| - | - | 1.55E+04 | 1533 | - | - | 0 | - |
| - | - | 4465 | 1534 | - | - | 0 | - |
| - | - | 2115 | 1535 | - | - | 0 | - |
| - | - | 1361 | 1544 | - | - | 0 | - |
| - | - | 4296 | 1545 | - | - | 0 | - |
| - | - | 6853 | 1546 | - | - | 0 | - |
| - | - | 9193 | 1547 | - | - | 0 | - |
| - | - | 8693 | 1548 | - | - | 0 | - |
| - | - | 6001 | 1549 | - | - | 0 | - |
| - | - | 2486 | 1550 | - | - | 0 | - |
| - | - | 3257 | 1556 | - | - | 0 | - |
| - | - | 7839 | 1557 | - | - | 0 | - |
| - | - | 7289 | 1558 | - | - | 0 | - |
| - | - | 3241 | 1559 | - | - | 0 | - |
| - | - | 912.7 | 1560 | - | - | 0 | - |
| - | - | 3357 | 1562 | - | - | 0 | - |
| - | - | 1.355E+04 | 1563 | - | - | 0 | - |
| - | - | 1.086E+04 | 1564 | - | - | 0 | - |
| - | - | 5495 | 1565 | - | - | 0 | - |
| - | - | 1884 | 1566 | - | - | 0 | - |
| - | - | 3169 | 1572 | - | - | 0 | - |
| - | - | 1.839E+04 | 1573 | - | - | 0 | - |
| - | - | 1.221E+05 | 1574 | - | - | 0 | - |
| - | - | 9.594E+04 | 1575 | - | - | 0 | - |
| - | - | 4.545E+04 | 1576 | - | - | 0 | - |
| - | - | 1.306E+04 | 1577 | - | - | 0 | - |
| - | - | 3825 | 1578 | - | - | 0 | - |
| - | - | 889.4 | 1587 | - | - | 0 | - |
| - | - | 1963 | 1588 | - | - | 0 | - |
| - | - | 9494 | 1589 | - | - | 0 | - |
| - | - | 3.552E+04 | 1590 | - | - | 0 | - |
| - | - | 1.17E+05 | 1591 | - | - | 0 | - |
| - | - | 8.614E+04 | 1592 | - | - | 0 | - |
| - | - | 3.982E+04 | 1593 | - | - | 0 | - |
| - | - | 1.236E+04 | 1594 | - | - | 0 | - |
| - | - | 3095 | 1595 | - | - | 0 | - |
| - | - | 781.5 | 2904 | - | - | 0 | - |
| - | - | 740.8 | 3183 | - | - | 0 | - |
| - | - | 785.7 | 3184 | - | - | 0 | - |

m/z Charge Intensity FragmentType MassShift Position
125.10769653320312 0 1940.1135
126.09169006347656 0 4750.345
131.12945556640625 0 1743.439
132.0272216796875 0 419.08417
136.47999572753906 0 471.53494
143.1185302734375 0 501.95358
143.78578186035156 0 414.71024
146.0915985107422 0 436.94775
148.947265625 0 1093.8223
158.0925750732422 0 1326.3955 y Ammonia loss 14
160.04315185546875 0 2095.1604
160.27426147460938 0 494.25378
160.69541931152344 0 427.0311
168.11349487304688 0 4905.2285
169.11767578125 0 657.6754
173.45188903808594 0 1188.9608
175.11927795410156 0 8718.747 y 14
175.9103240966797 0 573.09235
176.1222686767578 0 595.8944
178.0535430908203 0 5289.161
185.14004516601562 0 21170.227
186.14337158203125 0 2349.8083
189.08738708496094 0 848.8333
199.18080139160156 0 55746.574
200.1269989013672 0 3769.2458
200.1842803955078 0 7209.0625
201.12347412109375 0 1661.5012
201.13449096679688 0 886.4029
216.14573669433594 0 720.3824
216.66163635253906 0 599.98834
217.08209228515625 0 2659.3455
226.13047790527344 0 898.0899
226.16812133789062 0 777.4442
227.1757354736328 0 29106.951
228.17906188964844 0 3526.5894
229.12985229492188 0 4307.907 w 13
229.18310546875 0 586.23846
230.1140899658203 0 781.0001
233.16513061523438 0 3078.902
244.1173553466797 0 1002.5077
245.12486267089844 0 39175.79 y Ammonia loss 13
246.13072204589844 0 6224.14 z 13
247.1354522705078 0 647.9414
249.16012573242188 0 1578.5981
255.10888671875 0 1182.2528
261.16058349609375 0 3244.3574
262.15118408203125 0 5640.817 y 13
262.16357421875 0 700.2847
270.1200866699219 0 3830.7937
272.1722412109375 0 1194.2728
277.1549072265625 0 1927.2301 z 7
279.10137939453125 0 9212.326
280.1047668457031 0 891.4635
299.1968078613281 0 567.608
302.1715393066406 0 1103.5436
303.1546325683594 0 1046.9906
304.14959716796875 0 695.7819
314.2082214355469 0 1914.1309
316.1621398925781 0 20119.203 w 12
317.1613464355469 0 2927.1768
323.2102966308594 0 980.51135
331.1489562988281 0 5404.076
332.1566162109375 0 6254.256 y Ammonia loss 12
333.1646423339844 0 13377.913 z 12
334.1675109863281 0 1782.9717
345.1834716796875 0 634.4274 y 8
345.683349609375 0 574.336
347.1672058105469 0 1759.1085
349.1149597167969 0 793.64404
349.1835021972656 0 4750.889 y 12
350.1867980957031 0 1050.2687
357.1518859863281 0 9137.837
359.1925048828125 0 711.1817
373.2430725097656 0 667.32526
389.70880126953125 0 2136.9668
390.2095642089844 0 799.1793
390.7166748046875 0 1516.5256
391.21881103515625 0 666.4529
391.7240905761719 0 7797.384
392.2254333496094 0 4121.564
398.7137756347656 0 2613.6248
399.21600341796875 0 1666.8585
399.7212219238281 0 1876.4878
400.2213134765625 0 1156.2764
414.2218933105469 0 754.53406 c Ammonia loss 11
415.22589111328125 0 5719.161 z 7
415.7258605957031 0 2710.2537
417.21002197265625 0 15169.885 w 11
418.2131042480469 0 3071.935
423.23333740234375 0 3489.2532 y 7
423.7351989746094 0 1526.5542
425.23883056640625 0 1544.2297
426.24169921875 0 739.3736
429.234619140625 0 1484.2458
430.2386779785156 0 644.5705 y Water loss 11
431.2254638671875 0 1559.0459 y Ammonia loss 11
433.74053955078125 0 1086.9081
442.7467041015625 0 2940.4731
443.2498474121094 0 4436.333
443.3101806640625 0 1448.6738
444.25457763671875 0 588.7874
446.2373962402344 0 1232.293
447.20733642578125 0 731.1601
447.24066162109375 0 878.5988
448.2522277832031 0 12938.1455 y 11
449.22442626953125 0 637.8349 y Ammonia loss 2
449.2554931640625 0 3073.4868
450.74346923828125 0 755.43506 c Ammonia loss 7
454.89202880859375 0 1041.2316 y 2
455.2291259765625 0 746.8678
455.2851257324219 0 883.9812
460.2413635253906 0 751.5774
460.2773742675781 0 10478.803 c 3
461.2803039550781 0 3033.091
465.260009765625 0 1116.1687
472.25201416015625 0 2573.7795 c Ammonia loss 13
472.7381591796875 0 3202.716 z 6
473.24267578125 0 930.069
474.2310791015625 0 4793.9604
480.7473449707031 0 3629.841 y 6
486.5848083496094 0 828.97107 y Water loss 1
486.91827392578125 0 606.05035 y Ammonia loss 1
489.12994384765625 0 608.44244
489.2547302246094 0 4233.3076 z 10
490.2609558105469 0 2976.953
492.5875244140625 0 1062.092 y 1
492.92340087890625 0 641.8604
493.2523193359375 0 811.2945
495.9151306152344 0 1392.7559
496.2511291503906 0 946.6076
499.2995300292969 0 2433.4905
504.26556396484375 0 607.58417
505.273681640625 0 47786 y 10
506.276123046875 0 11197.209
507.2796325683594 0 2102.1672
514.3232421875 0 784.351
515.2465209960938 0 1914.6603
515.3310546875 0 2092.7117
515.7487182617188 0 3306.8032 y Ammonia loss 5
516.2530517578125 0 4504.1367 z 5
516.3388061523438 0 1035.7426
516.7572021484375 0 5464.9736
517.2583618164062 0 3630.0872
517.3399047851562 0 701.62463
520.279541015625 0 853.23376
522.2637329101562 0 3637.9768
523.7589721679688 0 1172.6794
524.264892578125 0 18835.506 y 5
524.6123657226562 0 6029.556
524.7649536132812 0 7524.491
524.9464111328125 0 2303.9893
525.2670288085938 0 3438.894
528.7908325195312 0 3752.1128
529.2973022460938 0 14946.131
529.7977905273438 0 4224.5425
530.2913818359375 0 5069.3955
530.6161499023438 0 4144.9736
530.8006591796875 0 1411.734
530.9502563476562 0 3853.2705
531.2703247070312 0 6744.9224
531.7706909179688 0 2743.89
541.3128051757812 0 1842.0162
542.318115234375 0 6349.9106
543.3217163085938 0 1260.8167
546.2758178710938 0 1035.2803
558.2855224609375 0 651.15076 z Water loss 9
558.3384399414062 0 2604.7776
559.28515625 0 1837.1283 w 9
559.3450317382812 0 8314.072 c 4
560.3488159179688 0 2133.9995
561.2692260742188 0 702.1316
565.7879028320312 0 38080.617 z 4
566.2893676757812 0 24234.164
566.7911987304688 0 7802.1772
567.29248046875 0 2325.2922
571.3458251953125 0 1125.322
572.3102416992188 0 732.67145
572.351806640625 0 4793.8784
573.2877807617188 0 873.7784
573.7970581054688 0 20379.03 y 4
574.2982788085938 0 8960.967 y Water loss 9
574.7992553710938 0 4582.1543
575.281005859375 0 2856.2236 y Ammonia loss 9
576.2865600585938 0 7760.6953 z 9
577.2894287109375 0 2543.3567
585.2640380859375 0 1034.172
586.3312377929688 0 2421.2646
587.3350830078125 0 665.10693
592.3040161132812 0 1529.1438 y 9
593.3071899414062 0 640.90894
600.2733154296875 0 3381.7002
600.8027954101562 0 14969.919 w 3
601.3038330078125 0 8684.763
601.8052368164062 0 4083.925
602.3035278320312 0 655.3067
603.3743286132812 0 1160.128
608.3109741210938 0 738.0333
611.3384399414062 0 780.19525
621.3008422851562 0 668.2695 z Water loss 3
627.35595703125 0 973.9867
629.350341796875 0 2197.1506 c 11
629.8123779296875 0 888.1448 y Ammonia loss 3
630.3092651367188 0 35743.918 z 3
630.81103515625 0 26299.332
631.3118286132812 0 11588.69
631.812744140625 0 3209.7964
637.3075561523438 0 763.13354
637.8147583007812 0 15887.479
638.3178100585938 0 36280.695 y 3
638.8197021484375 0 22134.629
639.3204956054688 0 8363.434
639.8203735351562 0 1480.3673
644.3182983398438 0 3680.8716
644.8203735351562 0 2419.733
645.37548828125 0 894.8106
646.377685546875 0 35567.938 c 5
647.3213500976562 0 638.99817
647.3807983398438 0 10895.604
648.3831787109375 0 2238.2246
651.8292236328125 0 1955.9652
652.3237915039062 0 886.83905
665.3213500976562 0 1950.6807 z Ammonia loss 2
665.8247680664062 0 1951.7518
666.3259887695312 0 1524.4202
670.3534545898438 0 743.0586
672.8271484375 0 1587.4227 y Water loss 2
673.3282470703125 0 2836.4463 y Ammonia loss 2
673.8255004882812 0 23643.385 z 2
674.3279418945312 0 31938.26
674.8301391601562 0 17225.13
675.3312377929688 0 5956.3843
675.834228515625 0 1044.7172
680.8280029296875 0 2402.6804
681.3296508789062 0 3201.7441
681.8345947265625 0 175204.69 y 2
682.3358154296875 0 113144.14
682.8373413085938 0 49465.35
683.33837890625 0 13793.841
683.83984375 0 3575.2861
684.368408203125 0 1689.7577
685.3869018554688 0 1818.9343
686.3238525390625 0 832.934
686.3964233398438 0 773.12195
687.3416137695312 0 1987.9623
688.3439331054688 0 1001.92993
689.3585815429688 0 5182.824 y 8
690.3607177734375 0 2308.265
691.3699340820312 0 659.3511
697.4403686523438 0 716.0807
699.374267578125 0 1590.8431
700.3773193359375 0 763.65546
701.3939208984375 0 2092.095
711.4156494140625 0 1011.17065
712.423828125 0 2138.193
713.4339599609375 0 2668.5571
714.4410400390625 0 812.8057
715.8485107421875 0 2840.0583 w 1
716.3505859375 0 2475.775
716.84716796875 0 938.9903
717.390869140625 0 3516.8337
718.3984985351562 0 4851.6294
719.4006958007812 0 1666.6991
729.3715209960938 0 1412.3995 y Water loss 1
730.3665161132812 0 3545.444 z 1
730.8674926757812 0 1493.7749
736.406494140625 0 639.3622
738.3765258789062 0 10699.634 y 1
738.8778686523438 0 7650.524
739.3787231445312 0 4651.168
739.884521484375 0 1030.5818
743.370361328125 0 3027.9587 w 7
744.3757934570312 0 3612.3064
745.3812255859375 0 620.2006
750.897216796875 0 2927.7776
751.4017944335938 0 2079.5122
751.8945922851562 0 1250.0255
756.9012451171875 0 1084.7865
757.3950805664062 0 1004.8584
758.388427734375 0 3707.8022
758.8841552734375 0 5751.0005
759.385009765625 0 5763.5605
759.884521484375 0 1157.9652
760.3960571289062 0 1895.6384
761.4046020507812 0 65988.58 c 6
762.4071655273438 0 26073.666
763.4095458984375 0 7172.3853
764.4083862304688 0 3134.9778
764.9102783203125 0 5910.6543
765.3825073242188 0 109156.5
765.8837280273438 0 90962.086
766.3854370117188 0 45498.906
766.8876342773438 0 13108.479
767.3886108398438 0 3428.6133
771.4049682617188 0 1087.1943
771.9089965820312 0 846.1458
772.4132080078125 0 3261.2703
772.9090576171875 0 2993.3691
773.4188232421875 0 3193.973
773.9082641601562 0 30043.684
774.4091796875 0 25439.766
774.9104614257812 0 12628.8
775.4124755859375 0 3657.8733
775.9147338867188 0 1213.0315
777.9017333984375 0 1500.0464
778.4066162109375 0 4322.3784
778.9024658203125 0 2061.0303
779.4036254882812 0 1850.3705
779.904296875 0 1212.4973
780.41943359375 0 2575.855
780.91650390625 0 643.78406
782.4403076171875 0 5119.704
783.438720703125 0 13589.396
784.4413452148438 0 2531.3196
785.90283203125 0 2402.0095
786.413818359375 0 8006.7856
786.9096069335938 0 117625.016
787.4108276367188 0 94677.41
787.9120483398438 0 47770.15
788.4132690429688 0 14641.822
788.91552734375 0 3891.546
793.4025268554688 0 1048.1979
793.9072265625 0 2650.7744
794.4127807617188 0 6070.619
794.918212890625 0 75976.79
795.420654296875 0 98142.68
795.92236328125 0 58460.32
796.4235229492188 0 27279.121
796.9202880859375 0 7858.275
797.4187622070312 0 3406.8904
798.4336547851562 0 2041.5024
799.4370727539062 0 760.39374
800.458740234375 0 1583.58
811.4296264648438 0 3215.673 z Water loss 7
812.4259643554688 0 1460.6362 z Ammonia loss 7
828.4306640625 0 1011.4012 y Ammonia loss 7
829.4405517578125 0 51303.977 z 7
830.4442749023438 0 28460.21
831.4486083984375 0 7260.851
832.4485473632812 0 1407.6362
839.51123046875 0 1969.2256
840.5194091796875 0 1205.6414
841.5296020507812 0 4309.4917
842.5335693359375 0 2542.4065
844.4501342773438 0 2927.364
845.4588623046875 0 6725.1133 y 7
846.46240234375 0 3153.242
847.4631958007812 0 1525.3613
858.48486328125 0 859.5432
866.4722290039062 0 948.1236
872.492919921875 0 1178.176
873.4910888671875 0 3853.3074
874.49462890625 0 1619.8875
884.482421875 0 7361.8794
885.4844970703125 0 2490.4048
899.4716186523438 0 1192.0142 w 6
900.4772338867188 0 7370.1816 c Ammonia loss 7
901.4945068359375 0 5614.0923
902.4901123046875 0 2417.8599
926.4553833007812 0 2152.893 z Water loss 6
927.463623046875 0 1735.4949
929.4827270507812 0 2117.9014
932.4269409179688 0 3264.652
933.4264526367188 0 1090.7834
943.4622802734375 0 802.44763 y Ammonia loss 6
944.4673461914062 0 19451.35 z 6
945.4716186523438 0 16202.113
946.475341796875 0 6376.7163
947.4761352539062 0 1211.3102
954.5383911132812 0 1827.292
955.5394897460938 0 2165.4187
956.5482177734375 0 3149.602
957.5582275390625 0 1466.8088
958.5588989257812 0 954.0663
959.4825439453125 0 768.07404
960.4859619140625 0 2351.7854 y 6
961.4644165039062 0 963.68677
970.5435791015625 0 5152.794
971.5453491210938 0 3033.2717
972.5360107421875 0 714.64966
998.5574340820312 0 711.71356
999.5584106445312 0 849.7406
1003.4912109375 0 1399.7078
1004.495849609375 0 12155.253
1005.5013427734375 0 9879.515
1006.5037231445312 0 2058.3577
1013.5834350585938 0 3090.9368
1014.5587768554688 0 46935.918 c 8
1015.5619506835938 0 28006.352
1016.5638427734375 0 8279.247
1017.5696411132812 0 1718.9624
1019.5090942382812 0 933.7869
1030.487548828125 0 1319.0013 y Ammonia loss 5
1031.499267578125 0 7088.4146 z 5
1032.505615234375 0 9850.6045
1033.5079345703125 0 5548.078
1034.51904296875 0 1270.9486
1039.5660400390625 0 3547.543
1040.56396484375 0 3453.7952
1041.56884765625 0 2998.0728
1042.5692138671875 0 3635.1836
1043.572021484375 0 1534.8273
1044.5260009765625 0 1160.9906
1045.51025390625 0 10201.658
1046.51318359375 0 3885.706
1047.5174560546875 0 3606.0654 y 5
1048.522705078125 0 1983.1022
1049.532470703125 0 874.55774
1056.573486328125 0 1696.3857
1057.5772705078125 0 37308.914
1058.580810546875 0 24510.566
1059.587890625 0 15696.049
1060.59375 0 5308.848
1061.529541015625 0 3958.1584
1062.5355224609375 0 9766.88
1063.5391845703125 0 2995.8694
1070.60791015625 0 798.84906
1086.576416015625 0 791.53595
1100.57958984375 0 2466.7825
1101.590087890625 0 64815.496 c 9
1102.5931396484375 0 37340.676
1103.5948486328125 0 14471.045
1104.600341796875 0 3147.1104
1114.596435546875 0 9498.779
1115.6007080078125 0 5800.248
1116.599853515625 0 3432.1143
1130.5672607421875 0 9943.862 z 4
1131.5743408203125 0 22644.459
1132.5775146484375 0 9810.767
1133.580322265625 0 3693.8499
1134.5789794921875 0 1117.2762
1146.5833740234375 0 2931.7903 y 4
1147.5859375 0 2001.7828
1158.6109619140625 0 36651.375 c 10
1159.613525390625 0 22973.824
1160.6168212890625 0 8293.8545
1161.6192626953125 0 1907.3578
1213.6663818359375 0 7468.1313
1214.668701171875 0 4487.9253
1215.672607421875 0 2990.3845
1216.674072265625 0 861.1377
1218.59326171875 0 837.408
1247.5482177734375 0 740.50775
1257.6785888671875 0 36023.164 c 11
1258.6805419921875 0 23060.467
1259.6734619140625 0 7668.7725
1260.6175537109375 0 31141.295
1261.6199951171875 0 18224.156
1262.6197509765625 0 7839.9644
1263.621826171875 0 1842.4973
1275.6270751953125 0 10193.777 y 3
1276.6300048828125 0 8046.3
1277.634765625 0 2558.6362
1278.6383056640625 0 1540.222
1279.638916015625 0 841.30316
1283.6920166015625 0 1179.3457
1285.6708984375 0 755.95844
1300.6956787109375 0 5519.786
1301.700927734375 0 4731.085
1302.7039794921875 0 2953.4448
1303.7044677734375 0 1071.9133
1304.62158203125 0 882.84467
1305.625732421875 0 969.7096
1329.6536865234375 0 1443.4662 w 2
1330.629150390625 0 2664.7217
1331.63037109375 0 1854.2825
1332.63671875 0 1028.0265
1342.697021484375 0 1255.4824
1344.71044921875 0 64525.95 c 12
1345.713134765625 0 43955.64
1345.87060546875 0 608.41626
1346.70947265625 0 18950.582
1347.6524658203125 0 25857.084
1348.651611328125 0 17218.83
1349.654296875 0 7363.0527
1350.6572265625 0 2295.4045
1351.6551513671875 0 761.96356
1362.6640625 0 1658.2474 y 2
1363.66162109375 0 1489.5692
1364.6568603515625 0 727.49426
1387.7315673828125 0 4380.3037
1388.738525390625 0 4696.368
1389.740234375 0 1956.1814
1429.7208251953125 0 995.28674
1430.7296142578125 0 2902.8352
1431.741943359375 0 91590.03 c 13
1432.7447509765625 0 67345.266
1433.7462158203125 0 30492.707
1434.749755859375 0 9825.245
1435.75244140625 0 1940.372
1460.7298583984375 0 4219.036
1461.7347412109375 0 3450.591
1462.7352294921875 0 1550.9662
1473.729248046875 0 861.68225
1474.7177734375 0 853.59863
1475.755126953125 0 724.2234 y 1
1488.739990234375 0 8029.8237
1489.74560546875 0 6746.162
1490.75537109375 0 2560.231
1491.7559814453125 0 1645.7856
1501.7850341796875 0 1258.9233
1512.7923583984375 0 2693.6697
1513.78564453125 0 2134.6514
1514.77978515625 0 1378.8129
1515.783447265625 0 1239.9371
1518.776611328125 0 868.59766
1528.8165283203125 0 735.80536
1529.7908935546875 0 2117.3413
1530.7901611328125 0 17222.871
1531.7923583984375 0 22370.098
1532.79443359375 0 15503.7295
1533.7987060546875 0 4465.1094
1534.806396484375 0 2115.3184
1543.818359375 0 1360.5452
1544.8272705078125 0 4295.5327
1545.8271484375 0 6852.6753
1546.8216552734375 0 9193.334
1547.8187255859375 0 8693.355
1548.817626953125 0 6001.166
1549.8170166015625 0 2485.5227
1555.802001953125 0 3257.3628
1556.7926025390625 0 7839.05
1557.794921875 0 7288.922
1558.794677734375 0 3241.4485
1559.78076171875 0 912.74414
1561.835693359375 0 3357.0647
1562.8458251953125 0 13554.019
1563.846435546875 0 10859.467
1564.8502197265625 0 5494.626
1565.8492431640625 0 1883.6506
1571.803466796875 0 3169.1304
1572.813232421875 0 18388.27
1573.8154296875 0 122078.87
1574.8187255859375 0 95936.86
1575.81982421875 0 45454.547
1576.823486328125 0 13063.005
1577.8258056640625 0 3825.225
1586.8021240234375 0 889.4312
1587.8121337890625 0 1963.3413
1588.824462890625 0 9494.37
1589.8309326171875 0 35521.965
1590.83984375 0 116955.695
1591.8431396484375 0 86137.47
1592.8458251953125 0 39815.63
1593.8463134765625 0 12356.9795
1594.8392333984375 0 3095.081
2903.702392578125 0 781.45874
3183.11279296875 0 740.8437
3183.864013671875 0 785.6884

Spectrum Details

|  |  |
| --- | --- |
| Matched peaks? Matched peaksThe total absolute number of peaks matched. Additionally in brackets the total fraction of peaks matched and the total number of peaks is shown. | 88 (16.06% of 548) |
| FDR? FDRThe false discovery rate estimated for this peptide. It is calculated by matching all theoretical fragments with a non-integer shift with the raw peaks for this spectrum. This is done with 40 different shifts. The resulting percentage is the average number of annotated peaks over the number of annotated peaks with the correct spectrum. | 0.87% |
| Satellite FDR? Satellite FDRSee the FDR for details on its calculation. This satellite ion specific FDR only contains the satellite ions (d/w) for I/L/J positions. | 0.00% |
| PSM Score? PSM ScoreThe PSM Score as given by Hecklib to this annotated spectrum. It is shown with three significant figures. | 656 |

## Spectrum 3840? Spectrum 3840 The raw spectrum of this peptide as annotated by Hecklib. The fragments are coloured according to ion type (see legend). Any peaks with a star '\*' as text can be hovered over to see the full details, first the ion type second the mass shift type. By hovering over the amino acids in the peptide or ions in the legend the corresponding peaks are highlighted. By toggling the 'Unassigned' label you can turn the background (unassigned) peaks on or off in the plot. By updating the slider in the Ion legend you can update the spectrum to only show the top X% of the peaks with labels. The top X% means any peak that is within X% of the highest intensity. By dragging in the spectrum you can zoom in to a specific part of the spectrum and use 'Zoom Out' to get back to the original zoom level. The annotation of the spectrum is based on the given sequence in the peptides file and is done with different software so inconsistencies are likely. The peaks are annotated based on the given sequence, with 20 ppm tolerance.

Copy Data

### Spectrum 3840 (TSV)

#### Preview

```
Loading example...
```

*Click on the button to copy the data to your clipboard.*

Mz MinMz MaxIntensity Max

WidthHeightPeptide font sizePeptide stroke widthSpectrum font sizeSpectrum stroke widthCompact peptide

Ion legend

wxyz

abcd

OtherUnassignedIonChargePositionShow for top:%

JJSEVSDRPSGVSSR

06.20e+41.24e+51.86e+52.48e+5

Zoom Out

y+11y+11w+12y+12z+12y+12z+38w+13c+13y+13z+13y+13c+312z+28w+14y+28y+14z+14y+14y+313y+313c+28y+313c+14z+15c+314z+29y+29z+314y+314y+15z+15y+314y+15y+210y+210z+210y+210z+16w+16c+15y+211z+211y+211y+16y+16z+16y+16w+212z+212y+212c+212z+212y+212c+16z+213z+213y+213y+213z+213y+213y+17w+214y+214y+214z+214y+214w+18c+17z+18y+18z+18y+18w+19c+18z+19y+19z+19y+19c+19y+110z+110y+110c+110z+111y+111c+111w+112c+112y+112w+113c+113y+113c+114y+114

0844168825323376

Fragment Matches Table

Show background peaks

| Position | Ion type | Intensity | mz Theoretical | mz Error (Th) | mz Error (ppm) | Charge | Series Number |
| --- | --- | --- | --- | --- | --- | --- | --- |
| - | - | 804.6 | 120.1 | - | - | 0 | - |
| - | - | 452.2 | 121.3 | - | - | 0 | - |
| - | - | 3033 | 125.1 | - | - | 0 | - |
| - | - | 4272 | 126.1 | - | - | 0 | - |
| - | - | 411.1 | 129.1 | - | - | 0 | - |
| - | - | 477.2 | 130.1 | - | - | 0 | - |
| - | - | 468.6 | 130.1 | - | - | 0 | - |
| - | - | 778 | 131.1 | - | - | 0 | - |
| - | - | 2241 | 131.1 | - | - | 0 | - |
| - | - | 482.2 | 142.2 | - | - | 0 | - |
| - | - | 719.8 | 143.1 | - | - | 0 | - |
| - | - | 458.8 | 147 | - | - | 0 | - |
| - | - | 401.7 | 149.3 | - | - | 0 | - |
| - | - | 507.1 | 151.3 | - | - | 0 | - |
| 15 | y | 1678 | 158.1 | 0.0004314 | 2.729 | +1 | 1 |
| - | - | 6048 | 168.1 | - | - | 0 | - |
| - | - | 458 | 172.4 | - | - | 0 | - |
| - | - | 2643 | 173.5 | - | - | 0 | - |
| 15 | y | 1.175E+04 | 175.1 | 0.0004326 | 2.47 | +1 | 1 |
| - | - | 2.82E+04 | 185.1 | - | - | 0 | - |
| - | - | 2218 | 186.1 | - | - | 0 | - |
| - | - | 873.8 | 189.1 | - | - | 0 | - |
| - | - | 480.3 | 197.8 | - | - | 0 | - |
| - | - | 7.992E+04 | 199.2 | - | - | 0 | - |
| - | - | 4922 | 200.1 | - | - | 0 | - |
| - | - | 9580 | 200.2 | - | - | 0 | - |
| - | - | 909.5 | 201.1 | - | - | 0 | - |
| - | - | 894.6 | 212.1 | - | - | 0 | - |
| - | - | 3967 | 217.1 | - | - | 0 | - |
| - | - | 1626 | 226.1 | - | - | 0 | - |
| - | - | 1166 | 226.2 | - | - | 0 | - |
| - | - | 3.979E+04 | 227.2 | - | - | 0 | - |
| - | - | 5262 | 228.2 | - | - | 0 | - |
| 14 | w | 5051 | 229.1 | 0.0006559 | 2.862 | +1 | 2 |
| - | - | 1117 | 230.1 | - | - | 0 | - |
| - | - | 4601 | 233.2 | - | - | 0 | - |
| - | - | 659.9 | 234.2 | - | - | 0 | - |
| - | - | 1644 | 244.1 | - | - | 0 | - |
| 14 | y | 4.715E+04 | 245.1 | 0.0005685 | 2.319 | +1 | 2 |
| 14 | z | 7136 | 246.1 | 0.001351 | 5.49 | +1 | 2 |
| - | - | 1196 | 248.1 | - | - | 0 | - |
| - | - | 2050 | 249.2 | - | - | 0 | - |
| - | - | 700.9 | 250.2 | - | - | 0 | - |
| - | - | 2076 | 255.1 | - | - | 0 | - |
| - | - | 727.5 | 260.1 | - | - | 0 | - |
| - | - | 947.3 | 261.1 | - | - | 0 | - |
| - | - | 4658 | 261.2 | - | - | 0 | - |
| 14 | y | 7243 | 262.2 | 0.0004782 | 1.824 | +1 | 2 |
| - | - | 521.6 | 262.2 | - | - | 0 | - |
| - | - | 1322 | 263.2 | - | - | 0 | - |
| - | - | 7496 | 270.1 | - | - | 0 | - |
| - | - | 741.9 | 271.1 | - | - | 0 | - |
| - | - | 1903 | 272.2 | - | - | 0 | - |
| 8 | z | 2303 | 277.2 | 0.003581 | 12.92 | +3 | 8 |
| - | - | 511.4 | 286 | - | - | 0 | - |
| - | - | 747.4 | 296.2 | - | - | 0 | - |
| - | - | 1040 | 299.2 | - | - | 0 | - |
| - | - | 995.7 | 303.2 | - | - | 0 | - |
| - | - | 888 | 304.2 | - | - | 0 | - |
| - | - | 828.6 | 312.2 | - | - | 0 | - |
| - | - | 860.6 | 314.1 | - | - | 0 | - |
| - | - | 4316 | 314.2 | - | - | 0 | - |
| 13 | w | 2.463E+04 | 316.2 | 0.0007777 | 2.46 | +1 | 3 |
| - | - | 4488 | 317.2 | - | - | 0 | - |
| - | - | 781.2 | 323.2 | - | - | 0 | - |
| - | - | 5750 | 331.1 | - | - | 0 | - |
| 3 | c | 997.8 | 331.2 | 5.738E-05 | 0.1732 | +1 | 3 |
| 13 | y | 1.039E+04 | 332.2 | 0.0003089 | 0.93 | +1 | 3 |
| 13 | z | 1.997E+04 | 333.2 | 0.00051 | 1.531 | +1 | 3 |
| - | - | 3377 | 334.2 | - | - | 0 | - |
| - | - | 1019 | 347.2 | - | - | 0 | - |
| - | - | 858.4 | 349.1 | - | - | 0 | - |
| 13 | y | 5793 | 349.2 | 0.0006458 | 1.849 | +1 | 3 |
| - | - | 1.289E+04 | 357.2 | - | - | 0 | - |
| - | - | 2149 | 389.7 | - | - | 0 | - |
| - | - | 2164 | 390.2 | - | - | 0 | - |
| - | - | 2778 | 390.7 | - | - | 0 | - |
| - | - | 1038 | 391.2 | - | - | 0 | - |
| - | - | 1.168E+04 | 391.7 | - | - | 0 | - |
| - | - | 5225 | 392.2 | - | - | 0 | - |
| - | - | 965 | 392.7 | - | - | 0 | - |
| - | - | 4311 | 398.7 | - | - | 0 | - |
| - | - | 1567 | 399.2 | - | - | 0 | - |
| - | - | 4756 | 399.7 | - | - | 0 | - |
| - | - | 2482 | 400.2 | - | - | 0 | - |
| 12 | c | 699.6 | 414.2 | 0.001573 | 3.798 | +3 | 12 |
| 8 | z | 6011 | 415.2 | 0.003197 | 7.698 | +2 | 8 |
| - | - | 1821 | 415.7 | - | - | 0 | - |
| - | - | 614.9 | 416.2 | - | - | 0 | - |
| 12 | w | 2.018E+04 | 417.2 | 0.001073 | 2.572 | +1 | 4 |
| - | - | 3636 | 418.2 | - | - | 0 | - |
| 8 | y | 5300 | 423.2 | 0.0007315 | 1.728 | +2 | 8 |
| - | - | 2061 | 423.7 | - | - | 0 | - |
| - | - | 785.3 | 424.2 | - | - | 0 | - |
| - | - | 2452 | 425.2 | - | - | 0 | - |
| - | - | 2088 | 429.2 | - | - | 0 | - |
| 12 | y | 1201 | 431.2 | 0.0001933 | 0.4483 | +1 | 4 |
| 12 | z | 1033 | 432.2 | 0.0008522 | 1.972 | +1 | 4 |
| - | - | 3749 | 443.3 | - | - | 0 | - |
| - | - | 1147 | 443.3 | - | - | 0 | - |
| - | - | 1031 | 444.3 | - | - | 0 | - |
| - | - | 2665 | 446.2 | - | - | 0 | - |
| - | - | 954.3 | 447.2 | - | - | 0 | - |
| 12 | y | 1.785E+04 | 448.3 | 0.001202 | 2.681 | +1 | 4 |
| 3 | y | 1162 | 448.9 | 0.0004712 | 1.05 | +3 | 13 |
| 3 | y | 1215 | 449.2 | 0.007427 | 16.53 | +3 | 13 |
| - | - | 3421 | 449.3 | - | - | 0 | - |
| 8 | c | 1513 | 450.7 | 0.001028 | 2.28 | +2 | 8 |
| 3 | y | 907.3 | 454.9 | 0.0006118 | 1.345 | +3 | 13 |
| - | - | 852.5 | 455.2 | - | - | 0 | - |
| - | - | 1962 | 455.3 | - | - | 0 | - |
| 4 | c | 1.309E+04 | 460.3 | 0.00141 | 3.062 | +1 | 4 |
| - | - | 3805 | 461.3 | - | - | 0 | - |
| - | - | 970.2 | 462.3 | - | - | 0 | - |
| - | - | 863.5 | 465.3 | - | - | 0 | - |
| - | - | 1198 | 465.8 | - | - | 0 | - |
| 11 | z | 934.7 | 471.2 | 5.447E-05 | 0.1156 | +1 | 5 |
| 14 | c | 6009 | 472.2 | 0.008416 | 17.82 | +3 | 14 |
| 7 | z | 3017 | 472.7 | 0.00184 | 3.893 | +2 | 9 |
| - | - | 2267 | 473.2 | - | - | 0 | - |
| - | - | 4729 | 474.2 | - | - | 0 | - |
| - | - | 1380 | 475.2 | - | - | 0 | - |
| 7 | y | 3714 | 480.7 | 0.001908 | 3.97 | +2 | 9 |
| 2 | z | 1112 | 481.2 | 0.006778 | 14.08 | +3 | 14 |
| 2 | y | 820.1 | 486.6 | 0.00195 | 4.008 | +3 | 14 |
| 11 | y | 891.6 | 487.3 | 0.009026 | 18.52 | +1 | 5 |
| 11 | z | 6136 | 489.3 | 0.001178 | 2.408 | +1 | 5 |
| - | - | 3805 | 490.3 | - | - | 0 | - |
| - | - | 1040 | 491.3 | - | - | 0 | - |
| 2 | y | 2202 | 492.6 | 0.001419 | 2.882 | +3 | 14 |
| - | - | 1402 | 492.9 | - | - | 0 | - |
| - | - | 2274 | 495.9 | - | - | 0 | - |
| - | - | 1627 | 496.3 | - | - | 0 | - |
| - | - | 4364 | 499.3 | - | - | 0 | - |
| - | - | 716.4 | 499.8 | - | - | 0 | - |
| - | - | 900.5 | 500.3 | - | - | 0 | - |
| - | - | 906.6 | 504.3 | - | - | 0 | - |
| 11 | y | 6.31E+04 | 505.3 | 0.001222 | 2.419 | +1 | 5 |
| - | - | 1.359E+04 | 506.3 | - | - | 0 | - |
| - | - | 785.2 | 507.2 | - | - | 0 | - |
| - | - | 2642 | 507.3 | - | - | 0 | - |
| - | - | 1265 | 514.3 | - | - | 0 | - |
| 6 | y | 1893 | 515.3 | 0.008396 | 16.3 | +2 | 10 |
| - | - | 2965 | 515.3 | - | - | 0 | - |
| 6 | y | 2072 | 515.7 | 0.0002674 | 0.5184 | +2 | 10 |
| 6 | z | 5868 | 516.3 | 0.001116 | 2.161 | +2 | 10 |
| - | - | 1884 | 516.3 | - | - | 0 | - |
| - | - | 7599 | 516.8 | - | - | 0 | - |
| - | - | 4246 | 517.3 | - | - | 0 | - |
| - | - | 1254 | 517.8 | - | - | 0 | - |
| - | - | 944 | 518.6 | - | - | 0 | - |
| - | - | 1097 | 520.8 | - | - | 0 | - |
| - | - | 1069 | 523.8 | - | - | 0 | - |
| 6 | y | 2.584E+04 | 524.3 | 0.003411 | 6.507 | +2 | 10 |
| - | - | 8581 | 524.6 | - | - | 0 | - |
| - | - | 1.278E+04 | 524.8 | - | - | 0 | - |
| - | - | 4455 | 524.9 | - | - | 0 | - |
| - | - | 4339 | 525.3 | - | - | 0 | - |
| - | - | 821 | 525.8 | - | - | 0 | - |
| - | - | 1412 | 528.3 | - | - | 0 | - |
| - | - | 5251 | 528.8 | - | - | 0 | - |
| - | - | 4116 | 529.3 | - | - | 0 | - |
| - | - | 7387 | 529.8 | - | - | 0 | - |
| - | - | 1.04E+04 | 530.3 | - | - | 0 | - |
| - | - | 1.046E+04 | 530.6 | - | - | 0 | - |
| - | - | 2534 | 530.8 | - | - | 0 | - |
| - | - | 5488 | 531 | - | - | 0 | - |
| - | - | 1086 | 531.3 | - | - | 0 | - |
| - | - | 1868 | 541.3 | - | - | 0 | - |
| - | - | 7510 | 542.3 | - | - | 0 | - |
| - | - | 1570 | 543.3 | - | - | 0 | - |
| - | - | 1406 | 550.8 | - | - | 0 | - |
| 10 | z | 1951 | 558.3 | 0.003121 | 5.591 | +1 | 6 |
| - | - | 4207 | 558.3 | - | - | 0 | - |
| - | - | 1308 | 558.8 | - | - | 0 | - |
| 10 | w | 3400 | 559.3 | 0.001156 | 2.066 | +1 | 6 |
| 5 | c | 1.1E+04 | 559.3 | 0.001019 | 1.822 | +1 | 5 |
| - | - | 681.2 | 559.8 | - | - | 0 | - |
| - | - | 1411 | 560.3 | - | - | 0 | - |
| - | - | 4303 | 560.3 | - | - | 0 | - |
| 5 | y | 738.6 | 565.3 | 0.004765 | 8.429 | +2 | 11 |
| 5 | z | 4.402E+04 | 565.8 | 0.001332 | 2.355 | +2 | 11 |
| - | - | 2.941E+04 | 566.3 | - | - | 0 | - |
| - | - | 1.033E+04 | 566.8 | - | - | 0 | - |
| - | - | 2842 | 567.3 | - | - | 0 | - |
| - | - | 2196 | 572.3 | - | - | 0 | - |
| - | - | 1801 | 573.3 | - | - | 0 | - |
| 5 | y | 2.827E+04 | 573.8 | 0.0008205 | 1.43 | +2 | 11 |
| 10 | y | 1.699E+04 | 574.3 | 0.004478 | 7.797 | +1 | 6 |
| - | - | 7856 | 574.8 | - | - | 0 | - |
| 10 | y | 3331 | 575.3 | 0.003983 | 6.923 | +1 | 6 |
| 10 | z | 9068 | 576.3 | 0.0006743 | 1.17 | +1 | 6 |
| - | - | 2754 | 577.3 | - | - | 0 | - |
| - | - | 1111 | 585.3 | - | - | 0 | - |
| - | - | 2637 | 586.3 | - | - | 0 | - |
| - | - | 885 | 587.3 | - | - | 0 | - |
| 10 | y | 2359 | 592.3 | 0.002275 | 3.841 | +1 | 6 |
| - | - | 5523 | 600.3 | - | - | 0 | - |
| 4 | w | 2.27E+04 | 600.8 | 0.00152 | 2.529 | +2 | 12 |
| - | - | 1.237E+04 | 601.3 | - | - | 0 | - |
| - | - | 513.2 | 601.4 | - | - | 0 | - |
| - | - | 5356 | 601.8 | - | - | 0 | - |
| - | - | 710.8 | 602.3 | - | - | 0 | - |
| - | - | 1720 | 603.4 | - | - | 0 | - |
| - | - | 833.6 | 614.3 | - | - | 0 | - |
| 4 | z | 715.4 | 621.3 | 0.0043 | 6.921 | +2 | 12 |
| 4 | y | 1220 | 629.3 | 0.009823 | 15.61 | +2 | 12 |
| 12 | c | 2771 | 629.3 | 0.009579 | 15.22 | +2 | 12 |
| 4 | z | 5.645E+04 | 630.3 | 0.001154 | 1.831 | +2 | 12 |
| - | - | 3.488E+04 | 630.8 | - | - | 0 | - |
| - | - | 1.635E+04 | 631.3 | - | - | 0 | - |
| - | - | 4784 | 631.8 | - | - | 0 | - |
| - | - | 999.2 | 632.3 | - | - | 0 | - |
| - | - | 1.784E+04 | 637.8 | - | - | 0 | - |
| 4 | y | 5.529E+04 | 638.3 | 0.0007642 | 1.197 | +2 | 12 |
| - | - | 2.83E+04 | 638.8 | - | - | 0 | - |
| - | - | 1.017E+04 | 639.3 | - | - | 0 | - |
| - | - | 3378 | 639.8 | - | - | 0 | - |
| - | - | 3626 | 644.3 | - | - | 0 | - |
| - | - | 2656 | 644.8 | - | - | 0 | - |
| - | - | 2070 | 645.4 | - | - | 0 | - |
| 6 | c | 4.59E+04 | 646.4 | 0.00134 | 2.072 | +1 | 6 |
| - | - | 1.795E+04 | 647.4 | - | - | 0 | - |
| - | - | 3774 | 648.4 | - | - | 0 | - |
| - | - | 1240 | 651.8 | - | - | 0 | - |
| - | - | 656 | 652.3 | - | - | 0 | - |
| - | - | 686.9 | 657.4 | - | - | 0 | - |
| - | - | 723.6 | 660.3 | - | - | 0 | - |
| 3 | z | 1884 | 664.8 | 0.005071 | 7.627 | +2 | 13 |
| 3 | z | 2703 | 665.3 | 0.009767 | 14.68 | +2 | 13 |
| - | - | 1966 | 665.8 | - | - | 0 | - |
| - | - | 1453 | 666.3 | - | - | 0 | - |
| 3 | y | 4436 | 672.8 | 0.001972 | 2.931 | +2 | 13 |
| 3 | y | 3135 | 673.3 | 0.00718 | 10.66 | +2 | 13 |
| - | - | 384.3 | 673.8 | - | - | 0 | - |
| 3 | z | 3.135E+04 | 673.8 | 0.001864 | 2.766 | +2 | 13 |
| - | - | 4.489E+04 | 674.3 | - | - | 0 | - |
| - | - | 2.258E+04 | 674.8 | - | - | 0 | - |
| - | - | 9245 | 675.3 | - | - | 0 | - |
| - | - | 3167 | 675.8 | - | - | 0 | - |
| - | - | 747.4 | 678.3 | - | - | 0 | - |
| - | - | 2660 | 680.8 | - | - | 0 | - |
| - | - | 3962 | 681.3 | - | - | 0 | - |
| 3 | y | 2.454E+05 | 681.8 | 0.001535 | 2.251 | +2 | 13 |
| - | - | 1.724E+05 | 682.3 | - | - | 0 | - |
| - | - | 7.271E+04 | 682.8 | - | - | 0 | - |
| - | - | 1.833E+04 | 683.3 | - | - | 0 | - |
| - | - | 5252 | 683.8 | - | - | 0 | - |
| - | - | 1515 | 684.4 | - | - | 0 | - |
| - | - | 2414 | 685.4 | - | - | 0 | - |
| - | - | 860.3 | 685.8 | - | - | 0 | - |
| - | - | 699.7 | 686.3 | - | - | 0 | - |
| - | - | 836.7 | 686.4 | - | - | 0 | - |
| - | - | 1149 | 686.8 | - | - | 0 | - |
| - | - | 1887 | 687.3 | - | - | 0 | - |
| 9 | y | 7128 | 689.4 | 0.001086 | 1.575 | +1 | 7 |
| - | - | 2526 | 690.4 | - | - | 0 | - |
| - | - | 674.2 | 691.4 | - | - | 0 | - |
| - | - | 784.8 | 697.4 | - | - | 0 | - |
| - | - | 1309 | 699.4 | - | - | 0 | - |
| - | - | 788.6 | 710.4 | - | - | 0 | - |
| - | - | 1262 | 711.4 | - | - | 0 | - |
| - | - | 2361 | 712.4 | - | - | 0 | - |
| - | - | 3796 | 713.4 | - | - | 0 | - |
| - | - | 1576 | 714.4 | - | - | 0 | - |
| 2 | w | 3919 | 715.8 | 0.002771 | 3.87 | +2 | 14 |
| - | - | 4244 | 716.4 | - | - | 0 | - |
| - | - | 1902 | 716.9 | - | - | 0 | - |
| - | - | 2943 | 717.4 | - | - | 0 | - |
| - | - | 6121 | 718.4 | - | - | 0 | - |
| - | - | 2228 | 719.4 | - | - | 0 | - |
| - | - | 847.2 | 723.4 | - | - | 0 | - |
| 2 | y | 1941 | 729.4 | 0.0004249 | 0.5826 | +2 | 14 |
| 2 | y | 744.4 | 729.9 | 0.009093 | 12.46 | +2 | 14 |
| 2 | z | 3127 | 730.4 | 0.001519 | 2.079 | +2 | 14 |
| - | - | 1532 | 730.9 | - | - | 0 | - |
| - | - | 1354 | 731.4 | - | - | 0 | - |
| 2 | y | 1.6E+04 | 738.4 | 0.001495 | 2.025 | +2 | 14 |
| - | - | 1.101E+04 | 738.9 | - | - | 0 | - |
| - | - | 3817 | 739.4 | - | - | 0 | - |
| - | - | 1343 | 739.9 | - | - | 0 | - |
| 8 | w | 4627 | 743.4 | 0.003827 | 5.148 | +1 | 8 |
| - | - | 3145 | 744.4 | - | - | 0 | - |
| - | - | 1731 | 745.4 | - | - | 0 | - |
| - | - | 848.7 | 749.9 | - | - | 0 | - |
| - | - | 3217 | 750.9 | - | - | 0 | - |
| - | - | 2699 | 751.4 | - | - | 0 | - |
| - | - | 934.8 | 751.9 | - | - | 0 | - |
| - | - | 1437 | 756.4 | - | - | 0 | - |
| - | - | 4745 | 758.4 | - | - | 0 | - |
| - | - | 8124 | 758.9 | - | - | 0 | - |
| - | - | 6418 | 759.4 | - | - | 0 | - |
| - | - | 1616 | 759.9 | - | - | 0 | - |
| - | - | 2045 | 760.4 | - | - | 0 | - |
| 7 | c | 8.2E+04 | 761.4 | 0.00174 | 2.286 | +1 | 7 |
| - | - | 3.542E+04 | 762.4 | - | - | 0 | - |
| - | - | 9536 | 763.4 | - | - | 0 | - |
| - | - | 4109 | 764.4 | - | - | 0 | - |
| - | - | 7874 | 764.9 | - | - | 0 | - |
| - | - | 1.48E+05 | 765.4 | - | - | 0 | - |
| - | - | 1.181E+05 | 765.9 | - | - | 0 | - |
| - | - | 5.914E+04 | 766.4 | - | - | 0 | - |
| - | - | 1.683E+04 | 766.9 | - | - | 0 | - |
| - | - | 4832 | 767.4 | - | - | 0 | - |
| - | - | 1095 | 770.9 | - | - | 0 | - |
| - | - | 1286 | 771.4 | - | - | 0 | - |
| - | - | 1700 | 771.9 | - | - | 0 | - |
| - | - | 4693 | 772.4 | - | - | 0 | - |
| - | - | 4803 | 772.9 | - | - | 0 | - |
| - | - | 4880 | 773.4 | - | - | 0 | - |
| - | - | 4.585E+04 | 773.9 | - | - | 0 | - |
| - | - | 3.535E+04 | 774.4 | - | - | 0 | - |
| - | - | 1.672E+04 | 774.9 | - | - | 0 | - |
| - | - | 5517 | 775.4 | - | - | 0 | - |
| - | - | 1682 | 775.9 | - | - | 0 | - |
| - | - | 4093 | 777.9 | - | - | 0 | - |
| - | - | 5143 | 778.4 | - | - | 0 | - |
| - | - | 2210 | 778.9 | - | - | 0 | - |
| - | - | 3064 | 779.4 | - | - | 0 | - |
| - | - | 1758 | 779.9 | - | - | 0 | - |
| - | - | 1828 | 780.4 | - | - | 0 | - |
| - | - | 1240 | 781.4 | - | - | 0 | - |
| - | - | 7989 | 782.4 | - | - | 0 | - |
| - | - | 4762 | 783.4 | - | - | 0 | - |
| - | - | 1551 | 784.4 | - | - | 0 | - |
| - | - | 3280 | 785.9 | - | - | 0 | - |
| - | - | 1.127E+04 | 786.4 | - | - | 0 | - |
| - | - | 1.504E+05 | 786.9 | - | - | 0 | - |
| - | - | 1.345E+05 | 787.4 | - | - | 0 | - |
| - | - | 6.317E+04 | 787.9 | - | - | 0 | - |
| - | - | 2.102E+04 | 788.4 | - | - | 0 | - |
| - | - | 7817 | 788.9 | - | - | 0 | - |
| - | - | 905.8 | 789.5 | - | - | 0 | - |
| - | - | 857.9 | 793.4 | - | - | 0 | - |
| - | - | 2350 | 793.9 | - | - | 0 | - |
| - | - | 7373 | 794.4 | - | - | 0 | - |
| - | - | 1.125E+05 | 794.9 | - | - | 0 | - |
| - | - | 1.288E+05 | 795.4 | - | - | 0 | - |
| - | - | 8.233E+04 | 795.9 | - | - | 0 | - |
| - | - | 3.656E+04 | 796.4 | - | - | 0 | - |
| - | - | 1.152E+04 | 796.9 | - | - | 0 | - |
| - | - | 4163 | 797.4 | - | - | 0 | - |
| - | - | 3005 | 798.4 | - | - | 0 | - |
| - | - | 1995 | 799.4 | - | - | 0 | - |
| - | - | 848.4 | 800.4 | - | - | 0 | - |
| 8 | z | 3799 | 811.4 | 0.0006746 | 0.8314 | +1 | 8 |
| - | - | 2063 | 812.4 | - | - | 0 | - |
| - | - | 795.8 | 814.4 | - | - | 0 | - |
| 8 | y | 1640 | 828.4 | 0.001475 | 1.78 | +1 | 8 |
| 8 | z | 7.194E+04 | 829.4 | 0.001401 | 1.69 | +1 | 8 |
| - | - | 3.399E+04 | 830.4 | - | - | 0 | - |
| - | - | 1.125E+04 | 831.4 | - | - | 0 | - |
| - | - | 2985 | 832.5 | - | - | 0 | - |
| - | - | 2759 | 839.5 | - | - | 0 | - |
| - | - | 3285 | 840.5 | - | - | 0 | - |
| - | - | 6824 | 841.5 | - | - | 0 | - |
| - | - | 3534 | 842.5 | - | - | 0 | - |
| - | - | 3939 | 844.5 | - | - | 0 | - |
| - | - | 1385 | 845.4 | - | - | 0 | - |
| 8 | y | 1.09E+04 | 845.5 | 0.001049 | 1.241 | +1 | 8 |
| - | - | 3384 | 846.5 | - | - | 0 | - |
| - | - | 2026 | 847.5 | - | - | 0 | - |
| - | - | 3772 | 873.5 | - | - | 0 | - |
| - | - | 2863 | 874.5 | - | - | 0 | - |
| - | - | 747.1 | 875.5 | - | - | 0 | - |
| - | - | 834 | 888.4 | - | - | 0 | - |
| 7 | w | 1736 | 899.5 | 0.001043 | 1.16 | +1 | 9 |
| 8 | c | 1.107E+04 | 900.5 | 5.435E-05 | 0.06036 | +1 | 8 |
| - | - | 4470 | 901.5 | - | - | 0 | - |
| - | - | 1779 | 902.5 | - | - | 0 | - |
| 7 | z | 3151 | 926.5 | 0.001014 | 1.095 | +1 | 9 |
| - | - | 2502 | 927.5 | - | - | 0 | - |
| - | - | 1714 | 928.5 | - | - | 0 | - |
| - | - | 2031 | 929.5 | - | - | 0 | - |
| - | - | 1252 | 930.5 | - | - | 0 | - |
| - | - | 810.8 | 943.2 | - | - | 0 | - |
| 7 | y | 1198 | 943.5 | 0.001542 | 1.635 | +1 | 9 |
| 7 | z | 2.976E+04 | 944.5 | 0.001497 | 1.585 | +1 | 9 |
| - | - | 2.337E+04 | 945.5 | - | - | 0 | - |
| - | - | 8223 | 946.5 | - | - | 0 | - |
| - | - | 2332 | 947.5 | - | - | 0 | - |
| - | - | 2607 | 954.5 | - | - | 0 | - |
| - | - | 3710 | 955.5 | - | - | 0 | - |
| - | - | 6525 | 956.6 | - | - | 0 | - |
| - | - | 3385 | 957.6 | - | - | 0 | - |
| - | - | 1093 | 958.6 | - | - | 0 | - |
| 7 | y | 3250 | 960.5 | 0.001633 | 1.7 | +1 | 9 |
| - | - | 1595 | 961.5 | - | - | 0 | - |
| - | - | 6695 | 970.5 | - | - | 0 | - |
| - | - | 2815 | 971.5 | - | - | 0 | - |
| - | - | 1221 | 987.5 | - | - | 0 | - |
| - | - | 1672 | 999.6 | - | - | 0 | - |
| - | - | 914.2 | 1001 | - | - | 0 | - |
| - | - | 3591 | 1014 | - | - | 0 | - |
| 9 | c | 6.195E+04 | 1015 | 0.002345 | 2.312 | +1 | 9 |
| - | - | 3.46E+04 | 1016 | - | - | 0 | - |
| - | - | 1.075E+04 | 1017 | - | - | 0 | - |
| - | - | 2521 | 1018 | - | - | 0 | - |
| - | - | 1094 | 1029 | - | - | 0 | - |
| 6 | y | 933.3 | 1030 | 0.01245 | 12.08 | +1 | 10 |
| 6 | z | 9795 | 1031 | 0.0003524 | 0.3416 | +1 | 10 |
| - | - | 1.427E+04 | 1033 | - | - | 0 | - |
| - | - | 6299 | 1034 | - | - | 0 | - |
| - | - | 2261 | 1035 | - | - | 0 | - |
| - | - | 2409 | 1040 | - | - | 0 | - |
| - | - | 5397 | 1041 | - | - | 0 | - |
| - | - | 3372 | 1042 | - | - | 0 | - |
| - | - | 5311 | 1043 | - | - | 0 | - |
| - | - | 3505 | 1044 | - | - | 0 | - |
| - | - | 925.8 | 1045 | - | - | 0 | - |
| - | - | 1736 | 1047 | - | - | 0 | - |
| 6 | y | 4611 | 1048 | 0.001282 | 1.223 | +1 | 10 |
| - | - | 2565 | 1049 | - | - | 0 | - |
| - | - | 924.5 | 1050 | - | - | 0 | - |
| - | - | 826.6 | 1054 | - | - | 0 | - |
| - | - | 2400 | 1057 | - | - | 0 | - |
| - | - | 4.799E+04 | 1058 | - | - | 0 | - |
| - | - | 3.596E+04 | 1059 | - | - | 0 | - |
| - | - | 2.537E+04 | 1060 | - | - | 0 | - |
| - | - | 9740 | 1061 | - | - | 0 | - |
| - | - | 973.1 | 1061 | - | - | 0 | - |
| - | - | 3161 | 1062 | - | - | 0 | - |
| - | - | 1245 | 1062 | - | - | 0 | - |
| - | - | 2492 | 1062 | - | - | 0 | - |
| - | - | 1548 | 1064 | - | - | 0 | - |
| - | - | 1127 | 1073 | - | - | 0 | - |
| - | - | 823.4 | 1090 | - | - | 0 | - |
| - | - | 1066 | 1100 | - | - | 0 | - |
| - | - | 3191 | 1101 | - | - | 0 | - |
| 10 | c | 8.455E+04 | 1102 | 0.002177 | 1.977 | +1 | 10 |
| - | - | 5.222E+04 | 1103 | - | - | 0 | - |
| - | - | 1.851E+04 | 1104 | - | - | 0 | - |
| - | - | 4204 | 1105 | - | - | 0 | - |
| - | - | 1.315E+04 | 1115 | - | - | 0 | - |
| - | - | 7991 | 1116 | - | - | 0 | - |
| - | - | 3228 | 1117 | - | - | 0 | - |
| 5 | z | 1.392E+04 | 1131 | 0.00103 | 0.9113 | +1 | 11 |
| - | - | 2.653E+04 | 1132 | - | - | 0 | - |
| - | - | 1.502E+04 | 1133 | - | - | 0 | - |
| - | - | 4596 | 1134 | - | - | 0 | - |
| - | - | 1015 | 1135 | - | - | 0 | - |
| 5 | y | 4392 | 1147 | 0.002326 | 2.028 | +1 | 11 |
| - | - | 2246 | 1148 | - | - | 0 | - |
| - | - | 1104 | 1149 | - | - | 0 | - |
| 11 | c | 5.221E+04 | 1159 | 0.001832 | 1.581 | +1 | 11 |
| - | - | 2.978E+04 | 1160 | - | - | 0 | - |
| - | - | 1.043E+04 | 1161 | - | - | 0 | - |
| - | - | 2122 | 1162 | - | - | 0 | - |
| - | - | 838.8 | 1199 | - | - | 0 | - |
| - | - | 694.3 | 1200 | - | - | 0 | - |
| 4 | w | 1184 | 1201 | 0.02399 | 19.98 | +1 | 12 |
| - | - | 1271 | 1213 | - | - | 0 | - |
| - | - | 1.026E+04 | 1214 | - | - | 0 | - |
| - | - | 5890 | 1215 | - | - | 0 | - |
| - | - | 4735 | 1216 | - | - | 0 | - |
| - | - | 1071 | 1217 | - | - | 0 | - |
| - | - | 1082 | 1218 | - | - | 0 | - |
| - | - | 859.9 | 1234 | - | - | 0 | - |
| - | - | 872.6 | 1256 | - | - | 0 | - |
| - | - | 857.4 | 1257 | - | - | 0 | - |
| 12 | c | 4.774E+04 | 1258 | 0.001533 | 1.219 | +1 | 12 |
| - | - | 3.274E+04 | 1259 | - | - | 0 | - |
| - | - | 1.11E+04 | 1260 | - | - | 0 | - |
| - | - | 4.08E+04 | 1261 | - | - | 0 | - |
| - | - | 2.405E+04 | 1262 | - | - | 0 | - |
| - | - | 8414 | 1263 | - | - | 0 | - |
| - | - | 2510 | 1264 | - | - | 0 | - |
| - | - | 847.3 | 1275 | - | - | 0 | - |
| 4 | y | 1.481E+04 | 1276 | 0.000382 | 0.2995 | +1 | 12 |
| - | - | 9422 | 1277 | - | - | 0 | - |
| - | - | 4631 | 1278 | - | - | 0 | - |
| - | - | 2116 | 1279 | - | - | 0 | - |
| - | - | 1621 | 1283 | - | - | 0 | - |
| - | - | 1187 | 1284 | - | - | 0 | - |
| - | - | 933 | 1286 | - | - | 0 | - |
| - | - | 987.4 | 1287 | - | - | 0 | - |
| - | - | 7655 | 1301 | - | - | 0 | - |
| - | - | 5649 | 1302 | - | - | 0 | - |
| - | - | 3302 | 1303 | - | - | 0 | - |
| - | - | 1261 | 1304 | - | - | 0 | - |
| - | - | 1612 | 1305 | - | - | 0 | - |
| - | - | 1377 | 1306 | - | - | 0 | - |
| 3 | w | 2668 | 1330 | 0.008006 | 6.021 | +1 | 13 |
| - | - | 3854 | 1331 | - | - | 0 | - |
| - | - | 2280 | 1332 | - | - | 0 | - |
| 13 | c | 8.206E+04 | 1345 | 0.0005106 | 0.3797 | +1 | 13 |
| - | - | 6.453E+04 | 1346 | - | - | 0 | - |
| - | - | 2.577E+04 | 1347 | - | - | 0 | - |
| - | - | 3.162E+04 | 1348 | - | - | 0 | - |
| - | - | 2.214E+04 | 1349 | - | - | 0 | - |
| - | - | 1.168E+04 | 1350 | - | - | 0 | - |
| - | - | 2868 | 1351 | - | - | 0 | - |
| 3 | y | 1157 | 1363 | 0.002533 | 1.859 | +1 | 13 |
| - | - | 1937 | 1364 | - | - | 0 | - |
| - | - | 974.9 | 1365 | - | - | 0 | - |
| - | - | 6895 | 1388 | - | - | 0 | - |
| - | - | 5528 | 1389 | - | - | 0 | - |
| - | - | 2884 | 1390 | - | - | 0 | - |
| - | - | 1566 | 1391 | - | - | 0 | - |
| - | - | 887.1 | 1417 | - | - | 0 | - |
| - | - | 1780 | 1430 | - | - | 0 | - |
| - | - | 2887 | 1431 | - | - | 0 | - |
| 14 | c | 1.182E+05 | 1432 | 0.0004646 | 0.3245 | +1 | 14 |
| - | - | 9.061E+04 | 1433 | - | - | 0 | - |
| - | - | 4.164E+04 | 1434 | - | - | 0 | - |
| - | - | 1.284E+04 | 1435 | - | - | 0 | - |
| - | - | 2189 | 1436 | - | - | 0 | - |
| - | - | 834.2 | 1459 | - | - | 0 | - |
| - | - | 4706 | 1461 | - | - | 0 | - |
| - | - | 4687 | 1462 | - | - | 0 | - |
| - | - | 1709 | 1463 | - | - | 0 | - |
| - | - | 827.6 | 1473 | - | - | 0 | - |
| - | - | 1245 | 1474 | - | - | 0 | - |
| - | - | 1196 | 1475 | - | - | 0 | - |
| 2 | y | 961.6 | 1476 | 0.02101 | 14.24 | +1 | 14 |
| - | - | 781 | 1477 | - | - | 0 | - |
| - | - | 8779 | 1489 | - | - | 0 | - |
| - | - | 8902 | 1490 | - | - | 0 | - |
| - | - | 4801 | 1491 | - | - | 0 | - |
| - | - | 1903 | 1492 | - | - | 0 | - |
| - | - | 794.1 | 1502 | - | - | 0 | - |
| - | - | 1579 | 1503 | - | - | 0 | - |
| - | - | 1308 | 1504 | - | - | 0 | - |
| - | - | 1020 | 1505 | - | - | 0 | - |
| - | - | 2493 | 1513 | - | - | 0 | - |
| - | - | 3594 | 1514 | - | - | 0 | - |
| - | - | 3081 | 1515 | - | - | 0 | - |
| - | - | 1455 | 1516 | - | - | 0 | - |
| - | - | 815.6 | 1517 | - | - | 0 | - |
| - | - | 1226 | 1520 | - | - | 0 | - |
| - | - | 1448 | 1529 | - | - | 0 | - |
| - | - | 2570 | 1530 | - | - | 0 | - |
| - | - | 2.32E+04 | 1531 | - | - | 0 | - |
| - | - | 3.06E+04 | 1532 | - | - | 0 | - |
| - | - | 1.751E+04 | 1533 | - | - | 0 | - |
| - | - | 9320 | 1534 | - | - | 0 | - |
| - | - | 2096 | 1535 | - | - | 0 | - |
| - | - | 1815 | 1544 | - | - | 0 | - |
| - | - | 4751 | 1545 | - | - | 0 | - |
| - | - | 1.025E+04 | 1546 | - | - | 0 | - |
| - | - | 1.064E+04 | 1547 | - | - | 0 | - |
| - | - | 1.174E+04 | 1548 | - | - | 0 | - |
| - | - | 6875 | 1549 | - | - | 0 | - |
| - | - | 3783 | 1550 | - | - | 0 | - |
| - | - | 4092 | 1556 | - | - | 0 | - |
| - | - | 1.022E+04 | 1557 | - | - | 0 | - |
| - | - | 6617 | 1558 | - | - | 0 | - |
| - | - | 3674 | 1559 | - | - | 0 | - |
| - | - | 1811 | 1560 | - | - | 0 | - |
| - | - | 1648 | 1561 | - | - | 0 | - |
| - | - | 3709 | 1562 | - | - | 0 | - |
| - | - | 1.588E+04 | 1563 | - | - | 0 | - |
| - | - | 1.578E+04 | 1564 | - | - | 0 | - |
| - | - | 6912 | 1565 | - | - | 0 | - |
| - | - | 1661 | 1566 | - | - | 0 | - |
| - | - | 873.4 | 1570 | - | - | 0 | - |
| - | - | 2580 | 1572 | - | - | 0 | - |
| - | - | 2.158E+04 | 1573 | - | - | 0 | - |
| - | - | 1.455E+05 | 1574 | - | - | 0 | - |
| - | - | 1.136E+05 | 1575 | - | - | 0 | - |
| - | - | 5.688E+04 | 1576 | - | - | 0 | - |
| - | - | 1.622E+04 | 1577 | - | - | 0 | - |
| - | - | 3805 | 1578 | - | - | 0 | - |
| - | - | 1229 | 1587 | - | - | 0 | - |
| - | - | 1315 | 1588 | - | - | 0 | - |
| - | - | 1.205E+04 | 1589 | - | - | 0 | - |
| - | - | 1149 | 1590 | - | - | 0 | - |
| - | - | 4.211E+04 | 1590 | - | - | 0 | - |
| - | - | 1.397E+05 | 1591 | - | - | 0 | - |
| - | - | 1.08E+05 | 1592 | - | - | 0 | - |
| - | - | 5.388E+04 | 1593 | - | - | 0 | - |
| - | - | 1.533E+04 | 1594 | - | - | 0 | - |
| - | - | 3869 | 1595 | - | - | 0 | - |
| - | - | 754.6 | 3343 | - | - | 0 | - |

m/z Charge Intensity FragmentType MassShift Position
120.08098602294922 0 804.5564
121.29129791259766 0 452.20657
125.10765075683594 0 3033.1128
126.09169006347656 0 4272.387
129.10244750976562 0 411.11755
130.0614013671875 0 477.17697
130.1219482421875 0 468.643
131.11854553222656 0 777.99054
131.12960815429688 0 2241.0662
142.21131896972656 0 482.1932
143.11875915527344 0 719.76764
146.98863220214844 0 458.75433
149.33848571777344 0 401.72006
151.3488311767578 0 507.1255
158.09283447265625 0 1677.5612 y Ammonia loss 14
168.11358642578125 0 6048.399
172.42527770996094 0 458.00302
173.45205688476562 0 2642.8987
175.119384765625 0 11745.353 y 14
185.14016723632812 0 28195.934
186.1434783935547 0 2217.6562
189.08740234375 0 873.816
197.84255981445312 0 480.32996
199.180908203125 0 79924.64
200.127197265625 0 4922.348
200.1842498779297 0 9579.798
201.12387084960938 0 909.50507
212.10342407226562 0 894.61395
217.0823211669922 0 3966.905
226.13052368164062 0 1626.1378
226.1676483154297 0 1165.7703
227.1758575439453 0 39792.145
228.17919921875 0 5261.504
229.1301727294922 0 5051.0093 w 13
230.11387634277344 0 1117.2434
233.16534423828125 0 4601.149
234.1687469482422 0 659.8661
244.11715698242188 0 1643.6061
245.125 0 47150.367 y Ammonia loss 13
246.1309051513672 0 7135.8496 z 13
248.07107543945312 0 1195.6985
249.1605224609375 0 2049.6887
250.16383361816406 0 700.8823
255.10934448242188 0 2075.9973
260.13482666015625 0 727.4608
261.14520263671875 0 947.2674
261.1603088378906 0 4657.981
262.1514587402344 0 7243.216 y 13
262.1640930175781 0 521.5974
263.1550598144531 0 1321.6498
270.12017822265625 0 7495.9067
271.12335205078125 0 741.9462
272.1718444824219 0 1903.3223
277.1551208496094 0 2303.1672 z 7
286.0365295410156 0 511.38068
296.1976013183594 0 747.36035
299.19549560546875 0 1040.448
303.154052734375 0 995.71075
304.1512145996094 0 887.95337
312.1552734375 0 828.62256
314.1462707519531 0 860.60785
314.20819091796875 0 4315.7954
316.1623229980469 0 24632.482 w 12
317.15960693359375 0 4487.892
323.2076721191406 0 781.20825
331.1494140625 0 5749.8555
331.2340393066406 0 997.78253 c 2
332.1567687988281 0 10387.215 y Ammonia loss 12
333.164794921875 0 19969.064 z 12
334.167724609375 0 3376.5295
347.16778564453125 0 1019.3971
349.1160583496094 0 858.4349
349.18365478515625 0 5792.9385 y 12
357.1523132324219 0 12886.237
389.7098693847656 0 2148.9143
390.2089538574219 0 2163.6494
390.71673583984375 0 2778.2683
391.2171630859375 0 1038.203
391.7247314453125 0 11679.612
392.2259826660156 0 5224.741
392.72845458984375 0 964.9754
398.7142639160156 0 4310.981
399.2156066894531 0 1566.5637
399.7215270996094 0 4756.1875
400.2232666015625 0 2481.9998
414.22100830078125 0 699.5824 c Ammonia loss 11
415.22686767578125 0 6010.9355 z 7
415.7264099121094 0 1821.0066
416.2316589355469 0 614.9242
417.2102966308594 0 20183.432 w 11
418.2136535644531 0 3636.4424
423.2337646484375 0 5299.922 y 7
423.7357177734375 0 2061.0686
424.23486328125 0 785.3483
425.2403564453125 0 2451.8362
429.2360534667969 0 2087.9875
431.2250671386719 0 1201.0973 y Ammonia loss 11
432.2335510253906 0 1032.6534 z 11
443.2505798339844 0 3748.8435
443.3091125488281 0 1147.2865
444.2533874511719 0 1030.5568
446.2373046875 0 2664.949
447.2081298828125 0 954.3333
448.25262451171875 0 17846.195 y 11
448.88873291015625 0 1162.2821 y Water loss 2
449.22369384765625 0 1214.5629 y Ammonia loss 2
449.2561340332031 0 3421.2112
450.7439270019531 0 1513.4515 c Ammonia loss 7
454.89239501953125 0 907.3279 y 2
455.2288818359375 0 852.54175
455.2852783203125 0 1962.4238
460.2779846191406 0 13088.703 c 3
461.28118896484375 0 3804.6672
462.283203125 0 970.1735
465.2590637207031 0 863.4919
465.757568359375 0 1197.6731
471.24365234375 0 934.6804 z Water loss 10
472.2523498535156 0 6009.205 c Ammonia loss 13
472.7389831542969 0 3017.048 z 6
473.24169921875 0 2267.0295
474.23175048828125 0 4729.4917
475.23626708984375 0 1380.0734
480.7484130859375 0 3713.7566 y 6
481.2501525878906 0 1111.9371 z Water loss 1
486.58489990234375 0 820.1314 y Water loss 1
487.2532958984375 0 891.5515 y Water loss 10
489.2553405761719 0 6136.3867 z 10
490.2618408203125 0 3805.2454
491.26409912109375 0 1040.0555
492.587890625 0 2201.6355 y 1
492.9236755371094 0 1401.5907
495.9154052734375 0 2274.1492
496.2523193359375 0 1626.6536
499.30023193359375 0 4364.376
499.7709045410156 0 716.4094
500.2703552246094 0 900.4533
504.265380859375 0 906.5661
505.27410888671875 0 63103.05 y 10
506.27734375 0 13587.479
507.1850280761719 0 785.186
507.2784423828125 0 2641.8374
514.3246459960938 0 1264.5708
515.2488403320312 0 1892.9059 y Water loss 5
515.3318481445312 0 2964.6548
515.74951171875 0 2071.503 y Ammonia loss 5
516.2542724609375 0 5867.708 z 5
516.3383178710938 0 1884.3043
516.7559814453125 0 7599.3555
517.2589721679688 0 4245.6133
517.75830078125 0 1254.0444
518.610107421875 0 943.95496
520.7921752929688 0 1096.6199
523.76025390625 0 1069.3041
524.2659301757812 0 25835.117 y 5
524.61279296875 0 8581.353
524.76513671875 0 12776.282
524.9468383789062 0 4454.7217
525.267333984375 0 4338.718
525.7691040039062 0 821.01587
528.2509155273438 0 1412.0502
528.7915649414062 0 5251.333
529.2919921875 0 4116.4956
529.7978515625 0 7387.182
530.2847290039062 0 10401.361
530.61669921875 0 10456.086
530.8019409179688 0 2533.741
530.9501953125 0 5487.703
531.291015625 0 1085.6632
541.3121337890625 0 1867.6053
542.319091796875 0 7510.1187
543.322998046875 0 1570.0277
550.7825927734375 0 1406.4647
558.2787475585938 0 1951.1029 z Water loss 9
558.3383178710938 0 4206.794
558.776611328125 0 1307.9944
559.2846069335938 0 3399.526 w 9
559.3460083007812 0 10995.46 c 4
559.7760009765625 0 681.24817
560.2908935546875 0 1410.9486
560.3485717773438 0 4303.311
565.2786865234375 0 738.5816 y Ammonia loss 4
565.7886962890625 0 44020.71 z 4
566.2899169921875 0 29411.21
566.7914428710938 0 10333.69
567.292236328125 0 2842.3865
572.3177490234375 0 2196.0354
573.2936401367188 0 1801.1256
573.7975463867188 0 28269.85 y 4
574.298828125 0 16985.793 y Water loss 9
574.801025390625 0 7856.499
575.2823486328125 0 3331.276 y Ammonia loss 9
576.286865234375 0 9067.55 z 9
577.29248046875 0 2754.4373
585.2686767578125 0 1111.1726
586.3323364257812 0 2637.2476
587.3394775390625 0 884.9655
592.3071899414062 0 2359.2024 y 9
600.274169921875 0 5522.6084
600.8035278320312 0 22702.723 w 3
601.30419921875 0 12371.696
601.3590087890625 0 513.2442
601.804931640625 0 5355.9688
602.2947387695312 0 710.78766
603.373291015625 0 1719.7303
614.3324584960938 0 833.59344
621.3076782226562 0 715.3717 z Water loss 3
629.3029174804688 0 1219.7058 y Water loss 3
629.3530883789062 0 2771.3022 c 11
630.309814453125 0 56446.008 z 3
630.8115844726562 0 34875.445
631.3130493164062 0 16349.376
631.8137817382812 0 4783.828
632.3153076171875 0 999.1537
637.8150634765625 0 17843.084
638.3187866210938 0 55289.61 y 3
638.8203735351562 0 28295.986
639.3214111328125 0 10172.317
639.8234252929688 0 3378.3333
644.3203735351562 0 3626.0833
644.8201904296875 0 2656.3762
645.3697509765625 0 2070.4028
646.3783569335938 0 45902.73 c 5
647.381591796875 0 17946.758
648.3849487304688 0 3773.5967
651.8234252929688 0 1240.055
652.3255004882812 0 656.0493
657.3699340820312 0 686.9208
660.3109741210938 0 723.6002
664.824462890625 0 1883.8934 z Water loss 2
665.3211669921875 0 2702.6284 z Ammonia loss 2
665.824951171875 0 1965.5569
666.3222045898438 0 1453.2968
672.8267822265625 0 4435.508 y Water loss 2
673.3279418945312 0 3134.5015 y Ammonia loss 2
673.7771606445312 0 384.25488
673.8265380859375 0 31351.248 z 2
674.328369140625 0 44893.6
674.8302612304688 0 22575.826
675.3321533203125 0 9245.246
675.8331909179688 0 3167.424
678.3192138671875 0 747.3768
680.8270263671875 0 2660.4941
681.3309936523438 0 3962.2192
681.8355712890625 0 245363.8 y 2
682.3367919921875 0 172404.11
682.837890625 0 72714.02
683.3394775390625 0 18334.473
683.8408203125 0 5252.042
684.3734741210938 0 1514.6704
685.3882446289062 0 2413.8486
685.8170166015625 0 860.291
686.3305053710938 0 699.6581
686.3930053710938 0 836.7497
686.8267822265625 0 1148.8728
687.342529296875 0 1887.4314
689.3587646484375 0 7127.5186 y 8
690.3637084960938 0 2525.545
691.3657836914062 0 674.1861
697.4379272460938 0 784.7597
699.3748168945312 0 1309.1924
710.4135131835938 0 788.5571
711.4189453125 0 1261.9795
712.4260864257812 0 2361.1182
713.4340209960938 0 3796.33
714.4379272460938 0 1576.1879
715.8499145507812 0 3919.405 w 1
716.3511352539062 0 4244.1777
716.85205078125 0 1901.544
717.390625 0 2942.7007
718.3980102539062 0 6121.2417
719.4016723632812 0 2228.4102
723.3610229492188 0 847.1645
729.370361328125 0 1940.737 y Water loss 1
729.8718872070312 0 744.3511 y Ammonia loss 1
730.3682250976562 0 3126.563 z 1
730.866943359375 0 1531.7347
731.367431640625 0 1353.7911
738.3775634765625 0 16004.094 y 1
738.879150390625 0 11014.0205
739.37939453125 0 3816.7903
739.8822631835938 0 1343.2225
743.3720703125 0 4626.654 w 7
744.377197265625 0 3144.843
745.3786010742188 0 1731.4733
749.8775634765625 0 848.66846
750.8971557617188 0 3216.7268
751.4006958007812 0 2698.5105
751.8927001953125 0 934.8023
756.4058227539062 0 1437.1097
758.3895874023438 0 4745.475
758.8831176757812 0 8124.1636
759.385009765625 0 6418.165
759.8816528320312 0 1615.9525
760.3927612304688 0 2044.5028
761.4057006835938 0 81996 c 6
762.4083862304688 0 35420.832
763.4103393554688 0 9536.37
764.411376953125 0 4109.084
764.913330078125 0 7874.084
765.3836059570312 0 148045.73
765.8851928710938 0 118088.266
766.386474609375 0 59135.01
766.8872680664062 0 16832.03
767.3901977539062 0 4832.3335
770.9113159179688 0 1095.2405
771.412353515625 0 1285.7786
771.9022216796875 0 1700.3763
772.4156494140625 0 4692.7095
772.916259765625 0 4803.003
773.4127807617188 0 4879.9927
773.909423828125 0 45850.6
774.4103393554688 0 35353.305
774.9110717773438 0 16720.766
775.4132690429688 0 5516.8125
775.91357421875 0 1681.7706
777.9041137695312 0 4093.4983
778.4085693359375 0 5143.149
778.9048461914062 0 2209.5024
779.4077758789062 0 3063.7034
779.8982543945312 0 1758.4346
780.4122924804688 0 1827.7463
781.4232788085938 0 1239.8323
782.4417114257812 0 7988.591
783.444580078125 0 4761.519
784.4468994140625 0 1550.7576
785.90234375 0 3280.2976
786.4147338867188 0 11265.448
786.910888671875 0 150443.67
787.4117431640625 0 134455.25
787.9132080078125 0 63169.48
788.413818359375 0 21019.256
788.9158935546875 0 7816.9165
789.4531860351562 0 905.8211
793.4160766601562 0 857.89374
793.9102172851562 0 2349.5107
794.412353515625 0 7373.4536
794.9192504882812 0 112457.19
795.4218139648438 0 128774.68
795.9236450195312 0 82334.19
796.4246826171875 0 36557.48
796.9234008789062 0 11518.162
797.4219360351562 0 4162.994
798.4349365234375 0 3004.7256
799.4344482421875 0 1995.2301
800.439453125 0 848.4365
811.43017578125 0 3799.2598 z Water loss 7
812.4315795898438 0 2062.6807
814.4243774414062 0 795.8041
828.4337158203125 0 1640.1726 y Ammonia loss 7
829.4414672851562 0 71944.78 z 7
830.4454956054688 0 33991.027
831.4481201171875 0 11252.863
832.4507446289062 0 2984.6145
839.5151977539062 0 2759.014
840.5184326171875 0 3285.299
841.5298461914062 0 6824.255
842.5333251953125 0 3533.9583
844.4522705078125 0 3939.4753
845.3850708007812 0 1385.3019
845.4598388671875 0 10899.128 y 7
846.46337890625 0 3383.8958
847.4675903320312 0 2025.5431
873.4939575195312 0 3772.2144
874.4962768554688 0 2863.421
875.5000610351562 0 747.11676
888.4468383789062 0 833.9541
899.4703979492188 0 1736.2146 w 6
900.4785766601562 0 11069.486 c Ammonia loss 7
901.4825439453125 0 4469.956
902.4852905273438 0 1779.4077
926.4574584960938 0 3150.7185 z Water loss 6
927.4651489257812 0 2501.5742
928.4749145507812 0 1713.6631
929.480712890625 0 2030.585
930.4874267578125 0 1252.3755
943.2314453125 0 810.8142
943.4576416015625 0 1197.7847 y Ammonia loss 6
944.468505859375 0 29757.514 z 6
945.473388671875 0 23368.082
946.4771118164062 0 8223.472
947.4772338867188 0 2332.176
954.54248046875 0 2606.582
955.5397338867188 0 3710.371
956.5527954101562 0 6524.985
957.5595092773438 0 3384.7573
958.5744018554688 0 1092.795
960.4873657226562 0 3250.1052 y 6
961.4906616210938 0 1594.9073
970.5420532226562 0 6694.548
971.5445556640625 0 2815.0647
987.5036010742188 0 1220.5948
999.5656127929688 0 1672.2244
1000.5726928710938 0 914.1652
1013.5866088867188 0 3590.6086
1014.5601806640625 0 61949.527 c 8
1015.56298828125 0 34598.156
1016.5657958984375 0 10750.516
1017.5643310546875 0 2521.1704
1029.477783203125 0 1093.5137
1030.503662109375 0 933.304 y Ammonia loss 5
1031.4993896484375 0 9795.338 z 5
1032.50732421875 0 14265.245
1033.5101318359375 0 6299.0386
1034.51513671875 0 2261.2869
1039.56787109375 0 2408.995
1040.563720703125 0 5397.2607
1041.565673828125 0 3372.437
1042.572509765625 0 5311.116
1043.574462890625 0 3505.3945
1045.4749755859375 0 925.79395
1046.5029296875 0 1735.6719
1047.51904296875 0 4610.934 y 5
1048.5198974609375 0 2564.6123
1049.520263671875 0 924.4747
1053.9962158203125 0 826.6082
1056.577880859375 0 2400.2383
1057.5784912109375 0 47988.95
1058.583251953125 0 35959.793
1059.5911865234375 0 25373.533
1060.595703125 0 9739.775
1061.4813232421875 0 973.05865
1061.6015625 0 3160.8936
1062.383056640625 0 1244.8351
1062.490478515625 0 2491.8506
1063.50048828125 0 1548.3778
1072.5732421875 0 1126.6261
1089.5654296875 0 823.3801
1099.5693359375 0 1065.924
1100.582275390625 0 3191.0208
1101.592041015625 0 84549.11 c 9
1102.5946044921875 0 52217.805
1103.597412109375 0 18514.836
1104.5965576171875 0 4204.124
1114.5977783203125 0 13147.484
1115.60205078125 0 7990.5146
1116.603759765625 0 3228.2563
1130.5684814453125 0 13920.154 z 4
1131.576171875 0 26531.947
1132.5791015625 0 15016.567
1133.5831298828125 0 4596.0425
1134.5828857421875 0 1014.50464
1146.5885009765625 0 4391.5015 y 4
1147.5909423828125 0 2245.7273
1148.5869140625 0 1103.6958
1158.6131591796875 0 52208.473 c 10
1159.6158447265625 0 29779.283
1160.6173095703125 0 10425.407
1161.6226806640625 0 2122.2668
1198.6485595703125 0 838.7712
1199.6556396484375 0 694.30457
1200.6207275390625 0 1184.1465 w 3
1212.6650390625 0 1270.6108
1213.6680908203125 0 10258.928
1214.6697998046875 0 5889.9277
1215.673583984375 0 4735.298
1216.6917724609375 0 1071.3539
1217.58984375 0 1081.9602
1233.61474609375 0 859.9279
1255.672119140625 0 872.6442
1256.6707763671875 0 857.36523
1257.6812744140625 0 47740.406 c 11
1258.682373046875 0 32736.621
1259.677001953125 0 11097.642
1260.6192626953125 0 40800.406
1261.6219482421875 0 24049.053
1262.6236572265625 0 8413.9795
1263.626953125 0 2509.9714
1274.614013671875 0 847.2636
1275.629150390625 0 14805.88 y 3
1276.63232421875 0 9421.521
1277.6357421875 0 4631.204
1278.63720703125 0 2115.643
1282.68896484375 0 1620.6211
1283.6890869140625 0 1186.6112
1285.693603515625 0 932.9826
1286.6783447265625 0 987.37946
1300.6978759765625 0 7655.4014
1301.701416015625 0 5648.509
1302.7069091796875 0 3301.676
1303.7030029296875 0 1261.4808
1304.6241455078125 0 1612.4689
1305.6246337890625 0 1376.9257
1329.6473388671875 0 2667.5579 w 2
1330.636962890625 0 3854.426
1331.639404296875 0 2279.6118
1344.7122802734375 0 82056.25 c 12
1345.71533203125 0 64534.734
1346.7127685546875 0 25773.928
1347.655517578125 0 31624.74
1348.655517578125 0 22144.908
1349.655517578125 0 11679.603
1350.6622314453125 0 2868.0542
1362.663330078125 0 1156.9346 y 2
1363.6632080078125 0 1936.8739
1364.6575927734375 0 974.8833
1387.7308349609375 0 6894.725
1388.7366943359375 0 5527.7314
1389.7364501953125 0 2884.1807
1390.7476806640625 0 1565.8297
1416.7266845703125 0 887.09235
1429.723876953125 0 1780.4368
1430.73095703125 0 2887.092
1431.7442626953125 0 118187.8 c 13
1432.7474365234375 0 90610.56
1433.74951171875 0 41644.137
1434.7520751953125 0 12842.337
1435.7579345703125 0 2188.8396
1458.7674560546875 0 834.19464
1460.7332763671875 0 4705.6724
1461.7342529296875 0 4686.5425
1462.730712890625 0 1709.2305
1473.2293701171875 0 827.6487
1473.74267578125 0 1245.286
1474.759765625 0 1195.7838
1475.765869140625 0 961.5968 y 1
1476.7567138671875 0 781.01624
1488.743408203125 0 8778.565
1489.7471923828125 0 8901.624
1490.753173828125 0 4800.5264
1491.7586669921875 0 1903.0743
1501.751953125 0 794.1254
1502.788818359375 0 1579.1083
1503.77880859375 0 1308.0005
1504.8031005859375 0 1020.02325
1512.7978515625 0 2492.72
1513.7933349609375 0 3594.2253
1514.78271484375 0 3081.167
1515.7813720703125 0 1455.2886
1516.781982421875 0 815.58026
1519.829833984375 0 1225.7372
1528.8070068359375 0 1448.3546
1529.8004150390625 0 2569.5852
1530.793212890625 0 23198.002
1531.795654296875 0 30602.174
1532.7977294921875 0 17507.467
1533.8011474609375 0 9320.447
1534.804931640625 0 2095.7449
1543.823486328125 0 1815.2906
1544.838134765625 0 4750.8564
1545.827880859375 0 10254.613
1546.8245849609375 0 10638.375
1547.8203125 0 11738.355
1548.8204345703125 0 6875.4297
1549.818359375 0 3783.0322
1555.8111572265625 0 4092.2024
1556.796142578125 0 10224.353
1557.797607421875 0 6616.561
1558.80078125 0 3673.6738
1559.811279296875 0 1810.6023
1560.832763671875 0 1648.0309
1561.8370361328125 0 3708.5583
1562.848388671875 0 15880.71
1563.851806640625 0 15775.028
1564.853759765625 0 6912.0815
1565.8577880859375 0 1660.515
1569.78955078125 0 873.41235
1571.809326171875 0 2579.8982
1572.817138671875 0 21579.373
1573.818115234375 0 145503.02
1574.8212890625 0 113562.83
1575.8236083984375 0 56882.555
1576.8255615234375 0 16222.507
1577.8248291015625 0 3804.664
1586.8265380859375 0 1229.3378
1587.8153076171875 0 1315.4268
1588.8255615234375 0 12047.852
1589.5904541015625 0 1149.1786
1589.8349609375 0 42111.984
1590.84326171875 0 139719.22
1591.8460693359375 0 107998.22
1592.848876953125 0 53881.664
1593.850830078125 0 15333.327
1594.8521728515625 0 3868.5867
3342.7490234375 0 754.58124

Spectrum Details

|  |  |
| --- | --- |
| Matched peaks? Matched peaksThe total absolute number of peaks matched. Additionally in brackets the total fraction of peaks matched and the total number of peaks is shown. | 95 (16.55% of 574) |
| FDR? FDRThe false discovery rate estimated for this peptide. It is calculated by matching all theoretical fragments with a non-integer shift with the raw peaks for this spectrum. This is done with 40 different shifts. The resulting percentage is the average number of annotated peaks over the number of annotated peaks with the correct spectrum. | 1.03% |
| Satellite FDR? Satellite FDRSee the FDR for details on its calculation. This satellite ion specific FDR only contains the satellite ions (d/w) for I/L/J positions. | 0.00% |
| PSM Score? PSM ScoreThe PSM Score as given by Hecklib to this annotated spectrum. It is shown with three significant figures. | 694 |

## Spectrum 4284? Spectrum 4284 The raw spectrum of this peptide as annotated by Hecklib. The fragments are coloured according to ion type (see legend). Any peaks with a star '\*' as text can be hovered over to see the full details, first the ion type second the mass shift type. By hovering over the amino acids in the peptide or ions in the legend the corresponding peaks are highlighted. By toggling the 'Unassigned' label you can turn the background (unassigned) peaks on or off in the plot. By updating the slider in the Ion legend you can update the spectrum to only show the top X% of the peaks with labels. The top X% means any peak that is within X% of the highest intensity. By dragging in the spectrum you can zoom in to a specific part of the spectrum and use 'Zoom Out' to get back to the original zoom level. The annotation of the spectrum is based on the given sequence in the peptides file and is done with different software so inconsistencies are likely. The peaks are annotated based on the given sequence, with 20 ppm tolerance.

Copy Data

### Spectrum 4284 (TSV)

#### Preview

```
Loading example...
```

*Click on the button to copy the data to your clipboard.*

Mz MinMz MaxIntensity Max

WidthHeightPeptide font sizePeptide stroke widthSpectrum font sizeSpectrum stroke widthCompact peptide

Ion legend

wxyz

abcd

OtherUnassignedIonChargePositionShow for top:%

JJSEVSDRPSGVSSR

01.93e+43.85e+45.78e+47.71e+4

Zoom Out

y+11y+11w+12y+12z+12y+12w+13y+13z+13y+13z+28w+14y+28y+14y+14y+313c+14c+314z+29y+29z+314z+15y+15y+210z+210y+210w+16c+15z+211y+211y+16y+16z+16y+16w+212c+212z+212y+212w+17c+16z+213y+213z+213y+213y+17w+214y+214y+214z+214y+214c+17z+18z+18y+18w+19c+18z+19y+19c+19z+110y+110c+110z+111y+111c+111c+112y+112c+113c+114

0805161124163221

Fragment Matches Table

Show background peaks

| Position | Ion type | Intensity | mz Theoretical | mz Error (Th) | mz Error (ppm) | Charge | Series Number |
| --- | --- | --- | --- | --- | --- | --- | --- |
| - | - | 1209 | 125.1 | - | - | 0 | - |
| - | - | 2234 | 126.1 | - | - | 0 | - |
| - | - | 950.4 | 131.1 | - | - | 0 | - |
| - | - | 705.7 | 133.1 | - | - | 0 | - |
| - | - | 359.7 | 135.2 | - | - | 0 | - |
| - | - | 497.6 | 137.8 | - | - | 0 | - |
| - | - | 417.8 | 144.5 | - | - | 0 | - |
| - | - | 582 | 147.1 | - | - | 0 | - |
| - | - | 880.7 | 149 | - | - | 0 | - |
| - | - | 526.8 | 149.3 | - | - | 0 | - |
| - | - | 473.9 | 157.2 | - | - | 0 | - |
| 15 | y | 908.8 | 158.1 | 0.000111 | 0.7019 | +1 | 1 |
| - | - | 459.4 | 160.1 | - | - | 0 | - |
| - | - | 3434 | 168.1 | - | - | 0 | - |
| - | - | 2107 | 173.5 | - | - | 0 | - |
| 15 | y | 4085 | 175.1 | 0.0002648 | 1.512 | +1 | 1 |
| - | - | 966.3 | 185.1 | - | - | 0 | - |
| - | - | 1.051E+04 | 185.1 | - | - | 0 | - |
| - | - | 553.8 | 190.1 | - | - | 0 | - |
| - | - | 2.357E+04 | 199.2 | - | - | 0 | - |
| - | - | 1682 | 200.1 | - | - | 0 | - |
| - | - | 2908 | 200.2 | - | - | 0 | - |
| - | - | 548 | 210.9 | - | - | 0 | - |
| - | - | 988.3 | 217.1 | - | - | 0 | - |
| - | - | 1.177E+04 | 227.2 | - | - | 0 | - |
| - | - | 1594 | 228.2 | - | - | 0 | - |
| 14 | w | 1777 | 229.1 | 0.000488 | 2.13 | +1 | 2 |
| - | - | 487.8 | 230.9 | - | - | 0 | - |
| 14 | y | 1.935E+04 | 245.1 | 0.0003549 | 1.448 | +1 | 2 |
| 14 | z | 2618 | 246.1 | 0.001992 | 8.094 | +1 | 2 |
| - | - | 1198 | 255.1 | - | - | 0 | - |
| - | - | 522.2 | 257.9 | - | - | 0 | - |
| - | - | 566.9 | 261.2 | - | - | 0 | - |
| 14 | y | 1864 | 262.2 | 0.0002645 | 1.009 | +1 | 2 |
| - | - | 2648 | 270.1 | - | - | 0 | - |
| - | - | 604.7 | 277.1 | - | - | 0 | - |
| - | - | 1464 | 314.2 | - | - | 0 | - |
| 13 | w | 8810 | 316.2 | 0.0002284 | 0.7225 | +1 | 3 |
| - | - | 1841 | 317.2 | - | - | 0 | - |
| - | - | 521.9 | 318.4 | - | - | 0 | - |
| - | - | 2132 | 331.1 | - | - | 0 | - |
| 13 | y | 3299 | 332.2 | 0.0002784 | 0.8381 | +1 | 3 |
| 13 | z | 6276 | 333.2 | 0.0003574 | 1.073 | +1 | 3 |
| - | - | 890.9 | 334.2 | - | - | 0 | - |
| 13 | y | 2695 | 349.2 | 0.0005542 | 1.587 | +1 | 3 |
| - | - | 4718 | 357.2 | - | - | 0 | - |
| - | - | 1233 | 391.7 | - | - | 0 | - |
| - | - | 1169 | 392.2 | - | - | 0 | - |
| - | - | 664.8 | 395.2 | - | - | 0 | - |
| 8 | z | 2224 | 415.2 | 0.003471 | 8.36 | +2 | 8 |
| - | - | 613.8 | 415.3 | - | - | 0 | - |
| - | - | 682.2 | 415.7 | - | - | 0 | - |
| 12 | w | 7763 | 417.2 | 0.0005846 | 1.401 | +1 | 4 |
| - | - | 1239 | 418.2 | - | - | 0 | - |
| 8 | y | 1699 | 423.2 | 0.0003367 | 0.7954 | +2 | 8 |
| - | - | 1057 | 423.7 | - | - | 0 | - |
| 12 | y | 741.5 | 431.2 | 0.001353 | 3.138 | +1 | 4 |
| - | - | 1763 | 443.3 | - | - | 0 | - |
| - | - | 623.6 | 444.2 | - | - | 0 | - |
| - | - | 928.4 | 446.2 | - | - | 0 | - |
| 12 | y | 6375 | 448.3 | 0.0009269 | 2.068 | +1 | 4 |
| - | - | 848.2 | 449.3 | - | - | 0 | - |
| 3 | y | 880.2 | 454.9 | 0.0008254 | 1.815 | +3 | 13 |
| - | - | 1133 | 455.2 | - | - | 0 | - |
| - | - | 617.4 | 455.6 | - | - | 0 | - |
| 4 | c | 4348 | 460.3 | 0.0001889 | 0.4104 | +1 | 4 |
| - | - | 1416 | 461.3 | - | - | 0 | - |
| 14 | c | 1853 | 472.2 | 0.008416 | 17.82 | +3 | 14 |
| 7 | z | 874.6 | 472.7 | 0.002024 | 4.281 | +2 | 9 |
| - | - | 1050 | 473.2 | - | - | 0 | - |
| - | - | 2500 | 474.2 | - | - | 0 | - |
| - | - | 571.8 | 475.1 | - | - | 0 | - |
| 7 | y | 666.5 | 480.7 | 0.001847 | 3.843 | +2 | 9 |
| 2 | z | 739.4 | 481.2 | 0.005923 | 12.31 | +3 | 14 |
| 11 | z | 1700 | 489.3 | 0.002368 | 4.84 | +1 | 5 |
| - | - | 1498 | 490.3 | - | - | 0 | - |
| - | - | 664.3 | 496.3 | - | - | 0 | - |
| - | - | 1540 | 499.3 | - | - | 0 | - |
| 11 | y | 2.349E+04 | 505.3 | 0.0005204 | 1.03 | +1 | 5 |
| - | - | 5285 | 506.3 | - | - | 0 | - |
| - | - | 1306 | 515.2 | - | - | 0 | - |
| - | - | 918.3 | 515.3 | - | - | 0 | - |
| 6 | y | 1587 | 515.7 | 0.0002209 | 0.4283 | +2 | 10 |
| 6 | z | 1361 | 516.3 | 0.00157 | 3.041 | +2 | 10 |
| - | - | 710.4 | 516.3 | - | - | 0 | - |
| - | - | 3355 | 516.8 | - | - | 0 | - |
| - | - | 1553 | 517.3 | - | - | 0 | - |
| - | - | 680.6 | 518.6 | - | - | 0 | - |
| 6 | y | 7717 | 524.3 | 0.00335 | 6.39 | +2 | 10 |
| - | - | 2051 | 524.6 | - | - | 0 | - |
| - | - | 3769 | 524.8 | - | - | 0 | - |
| - | - | 1307 | 524.9 | - | - | 0 | - |
| - | - | 1472 | 525.3 | - | - | 0 | - |
| - | - | 865.6 | 529.3 | - | - | 0 | - |
| - | - | 1843 | 529.3 | - | - | 0 | - |
| - | - | 2035 | 530.3 | - | - | 0 | - |
| - | - | 9085 | 530.3 | - | - | 0 | - |
| - | - | 2686 | 530.6 | - | - | 0 | - |
| - | - | 1347 | 530.9 | - | - | 0 | - |
| - | - | 2465 | 531.3 | - | - | 0 | - |
| - | - | 580.4 | 537.8 | - | - | 0 | - |
| - | - | 859.1 | 540.3 | - | - | 0 | - |
| - | - | 2508 | 542.3 | - | - | 0 | - |
| - | - | 930.6 | 543.3 | - | - | 0 | - |
| - | - | 1406 | 558.3 | - | - | 0 | - |
| 10 | w | 630.1 | 559.3 | 0.002804 | 5.013 | +1 | 6 |
| 5 | c | 3708 | 559.3 | 0.0001038 | 0.1856 | +1 | 5 |
| - | - | 1524 | 560.3 | - | - | 0 | - |
| 5 | z | 1.448E+04 | 565.8 | 0.0002338 | 0.4133 | +2 | 11 |
| - | - | 1.178E+04 | 566.3 | - | - | 0 | - |
| - | - | 4656 | 566.8 | - | - | 0 | - |
| - | - | 806.4 | 573.3 | - | - | 0 | - |
| 5 | y | 1.125E+04 | 573.8 | 0.0001491 | 0.2599 | +2 | 11 |
| 10 | y | 5746 | 574.3 | 0.00344 | 5.99 | +1 | 6 |
| - | - | 3066 | 574.8 | - | - | 0 | - |
| 10 | y | 1702 | 575.3 | 0.0002898 | 0.5037 | +1 | 6 |
| 10 | z | 3640 | 576.3 | 0.001224 | 2.123 | +1 | 6 |
| - | - | 691.1 | 577.3 | - | - | 0 | - |
| - | - | 846.8 | 586.3 | - | - | 0 | - |
| 10 | y | 926.3 | 592.3 | 0.0005049 | 0.8524 | +1 | 6 |
| - | - | 2219 | 600.3 | - | - | 0 | - |
| 4 | w | 7197 | 600.8 | 0.000482 | 0.8023 | +2 | 12 |
| - | - | 5244 | 601.3 | - | - | 0 | - |
| - | - | 2601 | 601.8 | - | - | 0 | - |
| - | - | 633 | 602.3 | - | - | 0 | - |
| - | - | 886.2 | 628.8 | - | - | 0 | - |
| 12 | c | 748.2 | 629.3 | 0.01123 | 17.84 | +2 | 12 |
| 4 | z | 1.606E+04 | 630.3 | 0.0004217 | 0.669 | +2 | 12 |
| - | - | 1.265E+04 | 630.8 | - | - | 0 | - |
| - | - | 4554 | 631.3 | - | - | 0 | - |
| - | - | 1437 | 631.8 | - | - | 0 | - |
| - | - | 723 | 632.3 | - | - | 0 | - |
| - | - | 6284 | 637.8 | - | - | 0 | - |
| 4 | y | 2.104E+04 | 638.3 | 9.028E-05 | 0.1414 | +2 | 12 |
| - | - | 1.042E+04 | 638.8 | - | - | 0 | - |
| - | - | 4764 | 639.3 | - | - | 0 | - |
| - | - | 1137 | 639.8 | - | - | 0 | - |
| - | - | 1396 | 644.3 | - | - | 0 | - |
| - | - | 1151 | 644.8 | - | - | 0 | - |
| - | - | 666.6 | 645.3 | - | - | 0 | - |
| - | - | 706.4 | 645.4 | - | - | 0 | - |
| 9 | w | 614.1 | 646.3 | 0.002208 | 3.417 | +1 | 7 |
| 6 | c | 1.786E+04 | 646.4 | 0.0006682 | 1.034 | +1 | 6 |
| - | - | 5970 | 647.4 | - | - | 0 | - |
| - | - | 1563 | 648.4 | - | - | 0 | - |
| 3 | z | 1452 | 665.3 | 0.009767 | 14.68 | +2 | 13 |
| - | - | 1560 | 665.8 | - | - | 0 | - |
| 3 | y | 1006 | 673.3 | 0.005898 | 8.76 | +2 | 13 |
| 3 | z | 1.188E+04 | 673.8 | 0.0004597 | 0.6822 | +2 | 13 |
| - | - | 1.6E+04 | 674.3 | - | - | 0 | - |
| - | - | 6871 | 674.8 | - | - | 0 | - |
| - | - | 3034 | 675.3 | - | - | 0 | - |
| - | - | 1340 | 680.8 | - | - | 0 | - |
| - | - | 1564 | 681.3 | - | - | 0 | - |
| 3 | y | 7.629E+04 | 681.8 | 0.000375 | 0.55 | +2 | 13 |
| - | - | 5.27E+04 | 682.3 | - | - | 0 | - |
| - | - | 2.323E+04 | 682.8 | - | - | 0 | - |
| - | - | 6781 | 683.3 | - | - | 0 | - |
| - | - | 1616 | 683.8 | - | - | 0 | - |
| - | - | 1105 | 687.3 | - | - | 0 | - |
| 9 | y | 2655 | 689.4 | 0.0006843 | 0.9926 | +1 | 7 |
| - | - | 956 | 690.4 | - | - | 0 | - |
| - | - | 634.8 | 711.4 | - | - | 0 | - |
| 2 | w | 1406 | 715.8 | 0.0007695 | 1.075 | +2 | 14 |
| - | - | 1805 | 716.3 | - | - | 0 | - |
| - | - | 721.6 | 717.4 | - | - | 0 | - |
| - | - | 2112 | 718.4 | - | - | 0 | - |
| - | - | 973.3 | 719.4 | - | - | 0 | - |
| 2 | y | 1304 | 729.4 | 0.007566 | 10.37 | +2 | 14 |
| 2 | y | 590.7 | 729.9 | 0.01092 | 14.97 | +2 | 14 |
| 2 | z | 1045 | 730.4 | 0.001838 | 2.517 | +2 | 14 |
| 2 | y | 5609 | 738.4 | 0.0001521 | 0.206 | +2 | 14 |
| - | - | 3158 | 738.9 | - | - | 0 | - |
| - | - | 1356 | 739.4 | - | - | 0 | - |
| - | - | 802.2 | 743.9 | - | - | 0 | - |
| - | - | 1369 | 744.4 | - | - | 0 | - |
| - | - | 815.5 | 745.4 | - | - | 0 | - |
| - | - | 1455 | 750.9 | - | - | 0 | - |
| - | - | 924.9 | 757.4 | - | - | 0 | - |
| - | - | 1521 | 758.4 | - | - | 0 | - |
| - | - | 2609 | 758.9 | - | - | 0 | - |
| - | - | 2703 | 759.4 | - | - | 0 | - |
| - | - | 965.7 | 759.9 | - | - | 0 | - |
| - | - | 1147 | 760.4 | - | - | 0 | - |
| 7 | c | 3.366E+04 | 761.4 | 0.0005196 | 0.6824 | +1 | 7 |
| - | - | 1.25E+04 | 762.4 | - | - | 0 | - |
| - | - | 1080 | 762.5 | - | - | 0 | - |
| - | - | 3277 | 763.4 | - | - | 0 | - |
| - | - | 1815 | 764.4 | - | - | 0 | - |
| - | - | 4594 | 764.9 | - | - | 0 | - |
| - | - | 5.792E+04 | 765.4 | - | - | 0 | - |
| - | - | 4.389E+04 | 765.9 | - | - | 0 | - |
| - | - | 2.224E+04 | 766.4 | - | - | 0 | - |
| - | - | 5667 | 766.9 | - | - | 0 | - |
| - | - | 1784 | 767.4 | - | - | 0 | - |
| - | - | 737.3 | 771.9 | - | - | 0 | - |
| - | - | 1456 | 772.4 | - | - | 0 | - |
| - | - | 1221 | 772.9 | - | - | 0 | - |
| - | - | 2264 | 773.4 | - | - | 0 | - |
| - | - | 1.767E+04 | 773.9 | - | - | 0 | - |
| - | - | 1.284E+04 | 774.4 | - | - | 0 | - |
| - | - | 6030 | 774.9 | - | - | 0 | - |
| - | - | 1854 | 775.4 | - | - | 0 | - |
| - | - | 1490 | 778.4 | - | - | 0 | - |
| - | - | 1261 | 779.4 | - | - | 0 | - |
| - | - | 1200 | 785.9 | - | - | 0 | - |
| - | - | 3472 | 786.4 | - | - | 0 | - |
| - | - | 5.514E+04 | 786.9 | - | - | 0 | - |
| - | - | 4.513E+04 | 787.4 | - | - | 0 | - |
| - | - | 2.238E+04 | 787.9 | - | - | 0 | - |
| - | - | 8785 | 788.4 | - | - | 0 | - |
| - | - | 1518 | 788.9 | - | - | 0 | - |
| - | - | 903.8 | 793.4 | - | - | 0 | - |
| - | - | 920.4 | 793.9 | - | - | 0 | - |
| - | - | 3813 | 794.4 | - | - | 0 | - |
| - | - | 3.787E+04 | 794.9 | - | - | 0 | - |
| - | - | 4.801E+04 | 795.4 | - | - | 0 | - |
| - | - | 2.942E+04 | 795.9 | - | - | 0 | - |
| - | - | 1.215E+04 | 796.4 | - | - | 0 | - |
| - | - | 2147 | 796.9 | - | - | 0 | - |
| - | - | 1593 | 797.4 | - | - | 0 | - |
| 8 | z | 1289 | 811.4 | 0.0006746 | 0.8314 | +1 | 8 |
| 8 | z | 2.548E+04 | 829.4 | 0.0003638 | 0.4387 | +1 | 8 |
| - | - | 1.394E+04 | 830.4 | - | - | 0 | - |
| - | - | 5117 | 831.4 | - | - | 0 | - |
| - | - | 727.6 | 832.5 | - | - | 0 | - |
| - | - | 919.6 | 841.5 | - | - | 0 | - |
| - | - | 1440 | 844.5 | - | - | 0 | - |
| 8 | y | 2723 | 845.5 | 4.968E-05 | 0.05877 | +1 | 8 |
| - | - | 1615 | 846.5 | - | - | 0 | - |
| - | - | 830.9 | 847.5 | - | - | 0 | - |
| - | - | 1592 | 873.5 | - | - | 0 | - |
| 7 | w | 667 | 899.5 | 0.0006771 | 0.7528 | +1 | 9 |
| 8 | c | 3971 | 900.5 | 0.001838 | 2.041 | +1 | 8 |
| - | - | 1705 | 901.5 | - | - | 0 | - |
| 7 | z | 9109 | 944.5 | 0.000212 | 0.2245 | +1 | 9 |
| - | - | 7663 | 945.5 | - | - | 0 | - |
| - | - | 2888 | 946.5 | - | - | 0 | - |
| - | - | 870.7 | 955.5 | - | - | 0 | - |
| - | - | 1008 | 956.5 | - | - | 0 | - |
| - | - | 749.4 | 957.4 | - | - | 0 | - |
| 7 | y | 927.1 | 960.5 | 0.005966 | 6.212 | +1 | 9 |
| - | - | 2018 | 970.5 | - | - | 0 | - |
| - | - | 780.5 | 973.5 | - | - | 0 | - |
| - | - | 1556 | 1014 | - | - | 0 | - |
| 9 | c | 2.388E+04 | 1015 | 0.0006364 | 0.6273 | +1 | 9 |
| - | - | 1.137E+04 | 1016 | - | - | 0 | - |
| - | - | 3945 | 1017 | - | - | 0 | - |
| 6 | z | 2982 | 1031 | 0.0008406 | 0.815 | +1 | 10 |
| - | - | 5171 | 1033 | - | - | 0 | - |
| - | - | 2709 | 1034 | - | - | 0 | - |
| - | - | 574.8 | 1035 | - | - | 0 | - |
| - | - | 1194 | 1040 | - | - | 0 | - |
| - | - | 1105 | 1041 | - | - | 0 | - |
| - | - | 713.8 | 1042 | - | - | 0 | - |
| - | - | 729.5 | 1044 | - | - | 0 | - |
| - | - | 2036 | 1045 | - | - | 0 | - |
| - | - | 1136 | 1046 | - | - | 0 | - |
| 6 | y | 2099 | 1048 | 0.001892 | 1.806 | +1 | 10 |
| - | - | 701 | 1057 | - | - | 0 | - |
| - | - | 1.367E+04 | 1058 | - | - | 0 | - |
| - | - | 1.006E+04 | 1059 | - | - | 0 | - |
| - | - | 3814 | 1060 | - | - | 0 | - |
| - | - | 1038 | 1061 | - | - | 0 | - |
| - | - | 635.4 | 1063 | - | - | 0 | - |
| - | - | 1090 | 1101 | - | - | 0 | - |
| 10 | c | 3.024E+04 | 1102 | 0.0003463 | 0.3144 | +1 | 10 |
| - | - | 1.812E+04 | 1103 | - | - | 0 | - |
| - | - | 7137 | 1104 | - | - | 0 | - |
| - | - | 1503 | 1105 | - | - | 0 | - |
| - | - | 5296 | 1115 | - | - | 0 | - |
| - | - | 2931 | 1116 | - | - | 0 | - |
| - | - | 1120 | 1117 | - | - | 0 | - |
| 5 | z | 4831 | 1131 | 0.001045 | 0.9243 | +1 | 11 |
| - | - | 1.167E+04 | 1132 | - | - | 0 | - |
| - | - | 5599 | 1133 | - | - | 0 | - |
| - | - | 2278 | 1134 | - | - | 0 | - |
| 5 | y | 1758 | 1147 | 0.001837 | 1.603 | +1 | 11 |
| - | - | 1086 | 1148 | - | - | 0 | - |
| 11 | c | 1.748E+04 | 1159 | 0.0004876 | 0.4208 | +1 | 11 |
| - | - | 1.144E+04 | 1160 | - | - | 0 | - |
| - | - | 5617 | 1161 | - | - | 0 | - |
| - | - | 1017 | 1162 | - | - | 0 | - |
| - | - | 3274 | 1214 | - | - | 0 | - |
| - | - | 2315 | 1215 | - | - | 0 | - |
| - | - | 1538 | 1216 | - | - | 0 | - |
| 12 | c | 1.682E+04 | 1258 | 0.001397 | 1.11 | +1 | 12 |
| - | - | 1.254E+04 | 1259 | - | - | 0 | - |
| - | - | 4666 | 1260 | - | - | 0 | - |
| - | - | 1.698E+04 | 1261 | - | - | 0 | - |
| - | - | 1.071E+04 | 1262 | - | - | 0 | - |
| - | - | 4453 | 1263 | - | - | 0 | - |
| - | - | 1159 | 1264 | - | - | 0 | - |
| 4 | y | 6279 | 1276 | 0.0008387 | 0.6575 | +1 | 12 |
| - | - | 4405 | 1277 | - | - | 0 | - |
| - | - | 1462 | 1278 | - | - | 0 | - |
| - | - | 807.9 | 1283 | - | - | 0 | - |
| - | - | 710.7 | 1285 | - | - | 0 | - |
| - | - | 2472 | 1301 | - | - | 0 | - |
| - | - | 2174 | 1302 | - | - | 0 | - |
| - | - | 952 | 1303 | - | - | 0 | - |
| - | - | 1077 | 1305 | - | - | 0 | - |
| - | - | 1168 | 1331 | - | - | 0 | - |
| - | - | 1039 | 1332 | - | - | 0 | - |
| 13 | c | 3.35E+04 | 1345 | 0.001443 | 1.073 | +1 | 13 |
| - | - | 2.238E+04 | 1346 | - | - | 0 | - |
| - | - | 6894 | 1347 | - | - | 0 | - |
| - | - | 1.413E+04 | 1348 | - | - | 0 | - |
| - | - | 1.047E+04 | 1349 | - | - | 0 | - |
| - | - | 4809 | 1350 | - | - | 0 | - |
| - | - | 1242 | 1351 | - | - | 0 | - |
| - | - | 664 | 1365 | - | - | 0 | - |
| - | - | 2457 | 1388 | - | - | 0 | - |
| - | - | 2414 | 1389 | - | - | 0 | - |
| - | - | 1327 | 1390 | - | - | 0 | - |
| - | - | 1236 | 1431 | - | - | 0 | - |
| 14 | c | 4.644E+04 | 1432 | 0.001489 | 1.04 | +1 | 14 |
| - | - | 3.534E+04 | 1433 | - | - | 0 | - |
| - | - | 1067 | 1433 | - | - | 0 | - |
| - | - | 1.613E+04 | 1434 | - | - | 0 | - |
| - | - | 3780 | 1435 | - | - | 0 | - |
| - | - | 1347 | 1436 | - | - | 0 | - |
| - | - | 2718 | 1461 | - | - | 0 | - |
| - | - | 1810 | 1462 | - | - | 0 | - |
| - | - | 750.2 | 1474 | - | - | 0 | - |
| - | - | 3833 | 1489 | - | - | 0 | - |
| - | - | 2862 | 1490 | - | - | 0 | - |
| - | - | 2323 | 1491 | - | - | 0 | - |
| - | - | 1110 | 1513 | - | - | 0 | - |
| - | - | 1799 | 1514 | - | - | 0 | - |
| - | - | 884.7 | 1515 | - | - | 0 | - |
| - | - | 706.6 | 1516 | - | - | 0 | - |
| - | - | 1048 | 1517 | - | - | 0 | - |
| - | - | 1741 | 1530 | - | - | 0 | - |
| - | - | 7571 | 1531 | - | - | 0 | - |
| - | - | 1.151E+04 | 1532 | - | - | 0 | - |
| - | - | 7941 | 1533 | - | - | 0 | - |
| - | - | 3424 | 1534 | - | - | 0 | - |
| - | - | 884.6 | 1535 | - | - | 0 | - |
| - | - | 1954 | 1545 | - | - | 0 | - |
| - | - | 4166 | 1546 | - | - | 0 | - |
| - | - | 5643 | 1547 | - | - | 0 | - |
| - | - | 4880 | 1548 | - | - | 0 | - |
| - | - | 3042 | 1549 | - | - | 0 | - |
| - | - | 1564 | 1550 | - | - | 0 | - |
| - | - | 834.4 | 1551 | - | - | 0 | - |
| - | - | 2108 | 1556 | - | - | 0 | - |
| - | - | 4148 | 1557 | - | - | 0 | - |
| - | - | 3870 | 1558 | - | - | 0 | - |
| - | - | 1186 | 1559 | - | - | 0 | - |
| - | - | 1664 | 1562 | - | - | 0 | - |
| - | - | 8447 | 1563 | - | - | 0 | - |
| - | - | 5621 | 1564 | - | - | 0 | - |
| - | - | 3104 | 1565 | - | - | 0 | - |
| - | - | 1201 | 1566 | - | - | 0 | - |
| - | - | 853.1 | 1572 | - | - | 0 | - |
| - | - | 8733 | 1573 | - | - | 0 | - |
| - | - | 6.692E+04 | 1574 | - | - | 0 | - |
| - | - | 4.945E+04 | 1575 | - | - | 0 | - |
| - | - | 2.406E+04 | 1576 | - | - | 0 | - |
| - | - | 7945 | 1577 | - | - | 0 | - |
| - | - | 1563 | 1578 | - | - | 0 | - |
| - | - | 1085 | 1588 | - | - | 0 | - |
| - | - | 5714 | 1589 | - | - | 0 | - |
| - | - | 1.809E+04 | 1590 | - | - | 0 | - |
| - | - | 6.129E+04 | 1591 | - | - | 0 | - |
| - | - | 4.897E+04 | 1592 | - | - | 0 | - |
| - | - | 1070 | 1592 | - | - | 0 | - |
| - | - | 2.095E+04 | 1593 | - | - | 0 | - |
| - | - | 6153 | 1594 | - | - | 0 | - |
| - | - | 1630 | 1595 | - | - | 0 | - |
| - | - | 782.9 | 2244 | - | - | 0 | - |
| - | - | 815.9 | 2355 | - | - | 0 | - |
| - | - | 726 | 2858 | - | - | 0 | - |
| - | - | 719.8 | 3073 | - | - | 0 | - |
| - | - | 794.4 | 3190 | - | - | 0 | - |

m/z Charge Intensity FragmentType MassShift Position
125.1075439453125 0 1208.7502
126.09156799316406 0 2234.131
131.129150390625 0 950.43365
133.0611114501953 0 705.72974
135.20492553710938 0 359.71408
137.75405883789062 0 497.62973
144.4662628173828 0 417.84412
147.0771026611328 0 582.0059
148.9556884765625 0 880.73236
149.3037567138672 0 526.8447
157.15428161621094 0 473.91595
158.09251403808594 0 908.79877 y Ammonia loss 14
160.13731384277344 0 459.44702
168.11329650878906 0 3433.6973
173.45135498046875 0 2106.6895
175.1192169189453 0 4084.5688 y 14
185.1314697265625 0 966.32446
185.13995361328125 0 10509.739
190.08212280273438 0 553.76575
199.1807098388672 0 23574.447
200.12738037109375 0 1681.5221
200.18394470214844 0 2908.4412
210.87632751464844 0 548.0329
217.0816650390625 0 988.3149
227.17567443847656 0 11770.913
228.17898559570312 0 1593.6248
229.1300048828125 0 1777.4714 w 13
230.94395446777344 0 487.82516
245.12478637695312 0 19346.293 y Ammonia loss 13
246.13026428222656 0 2618.3616 z 13
255.10861206054688 0 1197.5603
257.8526611328125 0 522.23535
261.15936279296875 0 566.85175
262.1512451171875 0 1863.7562 y 13
270.11962890625 0 2648.0354
277.115234375 0 604.6759
314.2080383300781 0 1464.3318
316.1617736816406 0 8810.138 w 12
317.1617126464844 0 1841.3384
318.38824462890625 0 521.93634
331.1489562988281 0 2131.627
332.15673828125 0 3299.0671 y Ammonia loss 12
333.1646423339844 0 6275.627 z 12
334.1684265136719 0 890.85297
349.1835632324219 0 2694.609 y 12
357.1518859863281 0 4718.186
391.7236022949219 0 1233.0596
392.2251281738281 0 1168.5665
395.2273864746094 0 664.79364
415.2271423339844 0 2223.5974 z 7
415.25506591796875 0 613.8315
415.72509765625 0 682.18915
417.2098083496094 0 7762.838 w 11
418.2119140625 0 1238.9202
423.2326965332031 0 1699.1382 y 7
423.7351989746094 0 1056.7106
431.2262268066406 0 741.50195 y Ammonia loss 11
443.2504577636719 0 1763.3289
444.208251953125 0 623.5748
446.2359924316406 0 928.35394
448.2523498535156 0 6374.8765 y 11
449.2557678222656 0 848.2246
454.8926086425781 0 880.18097 y 2
455.2262878417969 0 1132.9355
455.5628967285156 0 617.421
460.2767639160156 0 4348.338 c 3
461.2804870605469 0 1416.4136
472.2523498535156 0 1853.3606 c Ammonia loss 13
472.7391662597656 0 874.5705 z 6
473.2378845214844 0 1050.2764
474.2317810058594 0 2500.244
475.1332702636719 0 571.7807
480.74835205078125 0 666.4673 y 6
481.2492980957031 0 739.37823 z Water loss 1
489.25653076171875 0 1699.849 z 10
490.26239013671875 0 1498.1565
496.25067138671875 0 664.31525
499.29937744140625 0 1540.4426
505.2734069824219 0 23493.074 y 10
506.276611328125 0 5285.1807
515.2462768554688 0 1306.1482
515.330810546875 0 918.2982
515.7490234375 0 1587.1732 y Ammonia loss 5
516.2515869140625 0 1361.2996 z 5
516.3406372070312 0 710.36115
516.7561645507812 0 3355.4448
517.258056640625 0 1552.7965
518.6083374023438 0 680.5995
524.265869140625 0 7716.977 y 5
524.6128540039062 0 2051.4246
524.7637329101562 0 3768.9565
524.9462280273438 0 1306.8414
525.2666625976562 0 1472.0934
529.2949829101562 0 865.6065
529.334716796875 0 1842.6171
530.279541015625 0 2035.3099
530.3178100585938 0 9085.429
530.6162109375 0 2686.0269
530.9486083984375 0 1347.4819
531.3193359375 0 2465.2017
537.755126953125 0 580.42676
540.3248901367188 0 859.0818
542.3172607421875 0 2507.5327
543.3209228515625 0 930.63715
558.3374633789062 0 1406.4124
559.2862548828125 0 630.138 w 9
559.3450927734375 0 3708.1736 c 4
560.3475952148438 0 1523.9193
565.78759765625 0 14480.74 z 4
566.2894287109375 0 11784.306
566.7909545898438 0 4656.069
573.2913208007812 0 806.37915
573.796875 0 11245.299 y 4
574.2977905273438 0 5746.3887 y Water loss 9
574.7991943359375 0 3065.8218
575.278076171875 0 1702.0989 y Ammonia loss 9
576.2874145507812 0 3640.1355 z 9
577.2904663085938 0 691.0897
586.3284301757812 0 846.7645
592.305419921875 0 926.34357 y 9
600.2742309570312 0 2219.364
600.802490234375 0 7197.047 w 3
601.3035888671875 0 5244.3057
601.8062133789062 0 2600.9683
602.2981567382812 0 633.03656
628.80859375 0 886.227
629.354736328125 0 748.22925 c 11
630.30908203125 0 16064.871 z 3
630.81103515625 0 12646.188
631.3120727539062 0 4553.8936
631.8134765625 0 1436.6725
632.3130493164062 0 723.00616
637.8146362304688 0 6284.101
638.3179321289062 0 21043.7 y 3
638.8194580078125 0 10420.897
639.3218383789062 0 4764.4575
639.8237915039062 0 1136.8015
644.3168334960938 0 1395.8766
644.820068359375 0 1150.6445
645.3193359375 0 666.57556
645.372314453125 0 706.3712
646.3176879882812 0 614.06445 w 8
646.377685546875 0 17863.07 c 5
647.3807373046875 0 5970.2686
648.3837890625 0 1563.4524
665.3211669921875 0 1451.8143 z Ammonia loss 2
665.824462890625 0 1559.847
673.32666015625 0 1005.8628 y Ammonia loss 2
673.8251342773438 0 11884.975 z 2
674.3278198242188 0 15999.911
674.8287353515625 0 6870.713
675.3296508789062 0 3034.1848
680.830322265625 0 1340.11
681.3291625976562 0 1564.1196
681.8344116210938 0 76294.164 y 2
682.3355712890625 0 52696.984
682.8372192382812 0 23232.498
683.3380737304688 0 6781.1533
683.8417358398438 0 1616.179
687.342529296875 0 1104.6307
689.3569946289062 0 2655.033 y 8
690.3600463867188 0 955.96954
711.4213256835938 0 634.77747
715.8463745117188 0 1406.1765 w 1
716.3484497070312 0 1804.5525
717.385009765625 0 721.56323
718.3954467773438 0 2112.2927
719.4008178710938 0 973.27313
729.3632202148438 0 1303.7963 y Water loss 1
729.8737182617188 0 590.68475 y Ammonia loss 1
730.3648681640625 0 1044.7446 z 1
738.376220703125 0 5609.2515 y 1
738.8779296875 0 3157.7275
739.3786010742188 0 1355.6895
743.8700561523438 0 802.22565
744.3720092773438 0 1369.2833
745.3755493164062 0 815.4612
750.8982543945312 0 1455.1422
757.401611328125 0 924.94104
758.3883666992188 0 1521.3817
758.8839721679688 0 2609.418
759.3816528320312 0 2702.7007
759.88671875 0 965.65314
760.3881225585938 0 1147.2856
761.4044799804688 0 33661.836 c 6
762.4075317382812 0 12499.369
762.4805297851562 0 1079.9631
763.4105834960938 0 3276.6611
764.4103393554688 0 1814.9143
764.9105224609375 0 4593.75
765.382568359375 0 57919.26
765.8837280273438 0 43889.164
766.3856811523438 0 22239.47
766.88671875 0 5666.7817
767.3875732421875 0 1783.9856
771.9075317382812 0 737.31537
772.4025268554688 0 1455.8033
772.9131469726562 0 1220.8687
773.4094848632812 0 2264.3215
773.908203125 0 17673.48
774.4088134765625 0 12838.863
774.91015625 0 6029.891
775.4083862304688 0 1853.5311
778.40771484375 0 1490.1497
779.3978271484375 0 1261.4927
785.9052734375 0 1199.9564
786.4135131835938 0 3472.2004
786.909912109375 0 55139.92
787.4109497070312 0 45128
787.9118041992188 0 22379.988
788.4127807617188 0 8785.422
788.9116821289062 0 1517.6078
793.4105834960938 0 903.84753
793.9136962890625 0 920.4067
794.4136962890625 0 3812.957
794.9180908203125 0 37868.87
795.4203491210938 0 48007.19
795.9220581054688 0 29417.453
796.4229736328125 0 12151.131
796.92431640625 0 2146.9604
797.4182739257812 0 1593.4768
811.43017578125 0 1289.3575 z Water loss 7
829.4404296875 0 25483.062 z 7
830.4437866210938 0 13938.893
831.4473266601562 0 5116.508
832.451904296875 0 727.57196
841.5297241210938 0 919.5935
844.4520874023438 0 1440.416
845.458740234375 0 2722.8303 y 7
846.4630126953125 0 1615.4132
847.4681396484375 0 830.9134
873.4888305664062 0 1591.6232
899.4700317382812 0 667.00146 w 6
900.4766845703125 0 3971.0474 c Ammonia loss 7
901.4816284179688 0 1704.6815
944.466796875 0 9109.083 z 6
945.4717407226562 0 7662.801
946.474365234375 0 2888.1548
955.53466796875 0 870.65076
956.547607421875 0 1007.73486
957.4279174804688 0 749.35974
960.49169921875 0 927.0749 y 6
970.54443359375 0 2018.2913
973.4531860351562 0 780.50275
1013.5839233398438 0 1556.3998
1014.5584716796875 0 23879.06 c 8
1015.5611572265625 0 11366.394
1016.5639038085938 0 3944.824
1031.4998779296875 0 2982.2773 z 5
1032.506591796875 0 5171.182
1033.5084228515625 0 2708.5417
1034.5162353515625 0 574.766
1039.5621337890625 0 1194.4796
1040.566650390625 0 1105.1832
1041.5721435546875 0 713.75397
1044.4808349609375 0 729.54144
1045.4970703125 0 2036.1965
1046.4931640625 0 1136.1064
1047.515869140625 0 2098.6387 y 5
1057.4608154296875 0 701.0066
1057.5772705078125 0 13672.997
1058.580078125 0 10056.594
1059.58544921875 0 3813.7656
1060.59033203125 0 1037.8503
1062.8621826171875 0 635.4369
1100.5782470703125 0 1090.4381
1101.5902099609375 0 30238.643 c 9
1102.5931396484375 0 18117.27
1103.5941162109375 0 7137.156
1104.591064453125 0 1502.7876
1114.59619140625 0 5295.9233
1115.6016845703125 0 2931.023
1116.60107421875 0 1119.8275
1130.56640625 0 4830.842 z 4
1131.57470703125 0 11666.481
1132.5782470703125 0 5598.72
1133.582275390625 0 2277.6387
1146.5880126953125 0 1757.8322 y 4
1147.590576171875 0 1085.5504
1158.61083984375 0 17483.732 c 10
1159.615234375 0 11439.918
1160.6158447265625 0 5616.9565
1161.619140625 0 1017.3306
1213.664306640625 0 3273.5942
1214.6685791015625 0 2314.664
1215.6683349609375 0 1537.779
1257.6783447265625 0 16818.848 c 11
1258.68017578125 0 12540.559
1259.6744384765625 0 4666.13
1260.61767578125 0 16983.709
1261.6197509765625 0 10714.249
1262.6187744140625 0 4452.8057
1263.6265869140625 0 1158.6244
1275.6279296875 0 6279.217 y 3
1276.630859375 0 4404.794
1277.6358642578125 0 1461.9125
1282.6707763671875 0 807.937
1284.6925048828125 0 710.7085
1300.6949462890625 0 2471.635
1301.7003173828125 0 2173.6248
1302.69482421875 0 952.0362
1304.6165771484375 0 1077.0898
1330.640869140625 0 1167.9268
1331.62841796875 0 1038.9122
1344.7103271484375 0 33498.637 c 12
1345.7135009765625 0 22384.77
1346.7066650390625 0 6894.3105
1347.6513671875 0 14131.217
1348.6522216796875 0 10465.155
1349.654541015625 0 4808.6484
1350.6551513671875 0 1242.126
1364.6588134765625 0 664.0042
1387.730224609375 0 2457.2036
1388.7337646484375 0 2413.672
1389.73779296875 0 1326.8109
1430.7247314453125 0 1235.5859
1431.7423095703125 0 46435.84 c 13
1432.7451171875 0 35341.85
1432.95654296875 0 1066.8146
1433.747802734375 0 16127.919
1434.75048828125 0 3779.5508
1435.75390625 0 1346.7804
1460.7274169921875 0 2717.5063
1461.73486328125 0 1810.017
1473.74462890625 0 750.23267
1488.73779296875 0 3833.1555
1489.7423095703125 0 2861.8813
1490.7576904296875 0 2323.2244
1512.791748046875 0 1109.9922
1513.797119140625 0 1799.0153
1514.787109375 0 884.72455
1515.79443359375 0 706.595
1516.7935791015625 0 1048.167
1529.7926025390625 0 1741.2571
1530.794921875 0 7570.6377
1531.7926025390625 0 11509.462
1532.7957763671875 0 7940.677
1533.795166015625 0 3424.4504
1534.815185546875 0 884.56335
1544.833984375 0 1954.2345
1545.8282470703125 0 4165.8047
1546.823486328125 0 5642.6436
1547.820556640625 0 4880.2954
1548.8162841796875 0 3041.8628
1549.824951171875 0 1564.098
1550.8184814453125 0 834.4211
1555.804443359375 0 2108.4392
1556.794677734375 0 4148.341
1557.7958984375 0 3869.8103
1558.7833251953125 0 1186.1329
1561.8397216796875 0 1663.5752
1562.84619140625 0 8446.742
1563.8470458984375 0 5621.026
1564.849609375 0 3104.0215
1565.8414306640625 0 1200.7899
1571.820068359375 0 853.10895
1572.8128662109375 0 8733.421
1573.81591796875 0 66920.59
1574.8187255859375 0 49445.848
1575.8203125 0 24059.422
1576.82080078125 0 7945.389
1577.8304443359375 0 1562.5739
1587.7996826171875 0 1085.4922
1588.8238525390625 0 5714.461
1589.832763671875 0 18086.713
1590.8399658203125 0 61293.316
1591.84375 0 48968.67
1592.0914306640625 0 1069.6271
1592.8465576171875 0 20954.414
1593.8458251953125 0 6152.645
1594.84814453125 0 1630.4152
2244.36474609375 0 782.9357
2355.304931640625 0 815.86743
2858.462890625 0 726.0202
3072.677490234375 0 719.7795
3189.50341796875 0 794.44495

Spectrum Details

|  |  |
| --- | --- |
| Matched peaks? Matched peaksThe total absolute number of peaks matched. Additionally in brackets the total fraction of peaks matched and the total number of peaks is shown. | 69 (18.35% of 376) |
| FDR? FDRThe false discovery rate estimated for this peptide. It is calculated by matching all theoretical fragments with a non-integer shift with the raw peaks for this spectrum. This is done with 40 different shifts. The resulting percentage is the average number of annotated peaks over the number of annotated peaks with the correct spectrum. | 0.79% |
| Satellite FDR? Satellite FDRSee the FDR for details on its calculation. This satellite ion specific FDR only contains the satellite ions (d/w) for I/L/J positions. | 2.38% |
| PSM Score? PSM ScoreThe PSM Score as given by Hecklib to this annotated spectrum. It is shown with three significant figures. | 476 |

## Spectrum 3970? Spectrum 3970 The raw spectrum of this peptide as annotated by Hecklib. The fragments are coloured according to ion type (see legend). Any peaks with a star '\*' as text can be hovered over to see the full details, first the ion type second the mass shift type. By hovering over the amino acids in the peptide or ions in the legend the corresponding peaks are highlighted. By toggling the 'Unassigned' label you can turn the background (unassigned) peaks on or off in the plot. By updating the slider in the Ion legend you can update the spectrum to only show the top X% of the peaks with labels. The top X% means any peak that is within X% of the highest intensity. By dragging in the spectrum you can zoom in to a specific part of the spectrum and use 'Zoom Out' to get back to the original zoom level. The annotation of the spectrum is based on the given sequence in the peptides file and is done with different software so inconsistencies are likely. The peaks are annotated based on the given sequence, with 20 ppm tolerance.

Copy Data

### Spectrum 3970 (TSV)

#### Preview

```
Loading example...
```

*Click on the button to copy the data to your clipboard.*

Mz MinMz MaxIntensity Max

WidthHeightPeptide font sizePeptide stroke widthSpectrum font sizeSpectrum stroke widthCompact peptide

Ion legend

wxyz

abcd

OtherUnassignedIonChargePositionShow for top:%

JJSEVSDRPSGVSSR

03.44e+46.88e+41.03e+51.38e+5

Zoom Out

y+11y+11w+12y+12z+12y+12w+13c+13y+13z+13y+13z+28w+14y+28y+14y+14y+313c+28y+313c+14z+15c+314z+29y+29z+314z+15y+15y+210y+210z+210y+210z+16w+16c+15z+211y+211y+16y+16z+16y+16w+212c+212y+212z+212y+212c+16z+213y+17y+213y+213z+213y+213y+17w+214w+214y+214z+214y+214w+18c+17z+18y+18z+18y+18c+18z+19z+19y+19c+19z+110y+110c+110y+111z+111y+111c+111c+112y+112w+113c+113y+113c+114z+114y+114

0558111616742232

Fragment Matches Table

Show background peaks

| Position | Ion type | Intensity | mz Theoretical | mz Error (Th) | mz Error (ppm) | Charge | Series Number |
| --- | --- | --- | --- | --- | --- | --- | --- |
| - | - | 367.2 | 122.9 | - | - | 0 | - |
| - | - | 378.4 | 124.9 | - | - | 0 | - |
| - | - | 2032 | 125.1 | - | - | 0 | - |
| - | - | 3691 | 126.1 | - | - | 0 | - |
| - | - | 363.9 | 126.1 | - | - | 0 | - |
| - | - | 1113 | 128.1 | - | - | 0 | - |
| - | - | 476.9 | 128.3 | - | - | 0 | - |
| - | - | 543.3 | 130.1 | - | - | 0 | - |
| - | - | 502.4 | 130.1 | - | - | 0 | - |
| - | - | 1418 | 131.1 | - | - | 0 | - |
| - | - | 415 | 137.8 | - | - | 0 | - |
| - | - | 422.6 | 143 | - | - | 0 | - |
| - | - | 489.9 | 145.1 | - | - | 0 | - |
| - | - | 891.5 | 149 | - | - | 0 | - |
| 15 | y | 804.2 | 158.1 | 0.0005077 | 3.211 | +1 | 1 |
| - | - | 441.5 | 162.9 | - | - | 0 | - |
| - | - | 4334 | 168.1 | - | - | 0 | - |
| - | - | 1039 | 173.4 | - | - | 0 | - |
| 15 | y | 6243 | 175.1 | 0.0003258 | 1.86 | +1 | 1 |
| - | - | 802.3 | 176.1 | - | - | 0 | - |
| - | - | 548.5 | 176.2 | - | - | 0 | - |
| - | - | 1.787E+04 | 185.1 | - | - | 0 | - |
| - | - | 1272 | 186.1 | - | - | 0 | - |
| - | - | 4.817E+04 | 199.2 | - | - | 0 | - |
| - | - | 2530 | 200.1 | - | - | 0 | - |
| - | - | 5406 | 200.2 | - | - | 0 | - |
| - | - | 867 | 201.1 | - | - | 0 | - |
| - | - | 4004 | 217.1 | - | - | 0 | - |
| - | - | 625.8 | 226.1 | - | - | 0 | - |
| - | - | 1107 | 226.2 | - | - | 0 | - |
| - | - | 2.238E+04 | 227.2 | - | - | 0 | - |
| - | - | 3167 | 228.2 | - | - | 0 | - |
| 14 | w | 3411 | 229.1 | 0.0004728 | 2.063 | +1 | 2 |
| - | - | 1514 | 233.2 | - | - | 0 | - |
| - | - | 551 | 234.1 | - | - | 0 | - |
| - | - | 1502 | 244.1 | - | - | 0 | - |
| 14 | y | 3.006E+04 | 245.1 | 0.0004617 | 1.884 | +1 | 2 |
| 14 | z | 3997 | 246.1 | 0.001184 | 4.808 | +1 | 2 |
| - | - | 617.3 | 249.2 | - | - | 0 | - |
| - | - | 1252 | 255.1 | - | - | 0 | - |
| - | - | 784.2 | 260.1 | - | - | 0 | - |
| - | - | 1140 | 261.2 | - | - | 0 | - |
| 14 | y | 3743 | 262.2 | 0.0004476 | 1.708 | +1 | 2 |
| - | - | 816.4 | 263.2 | - | - | 0 | - |
| - | - | 5148 | 270.1 | - | - | 0 | - |
| - | - | 1688 | 272.2 | - | - | 0 | - |
| - | - | 2716 | 304.2 | - | - | 0 | - |
| - | - | 3004 | 314.2 | - | - | 0 | - |
| 13 | w | 1.54E+04 | 316.2 | 0.0005641 | 1.784 | +1 | 3 |
| - | - | 2428 | 317.2 | - | - | 0 | - |
| - | - | 837.3 | 318.2 | - | - | 0 | - |
| - | - | 673.2 | 330.2 | - | - | 0 | - |
| - | - | 4203 | 331.1 | - | - | 0 | - |
| 3 | c | 642.6 | 331.2 | 0.0008508 | 2.569 | +1 | 3 |
| 13 | y | 4981 | 332.2 | 0.0004005 | 1.206 | +1 | 3 |
| 13 | z | 1.239E+04 | 333.2 | 0.0006321 | 1.897 | +1 | 3 |
| - | - | 2127 | 334.2 | - | - | 0 | - |
| - | - | 793 | 340.1 | - | - | 0 | - |
| - | - | 1157 | 347.2 | - | - | 0 | - |
| 13 | y | 4087 | 349.2 | 0.0003101 | 0.8881 | +1 | 3 |
| - | - | 639.3 | 350.2 | - | - | 0 | - |
| - | - | 8212 | 357.2 | - | - | 0 | - |
| - | - | 675.3 | 357.2 | - | - | 0 | - |
| - | - | 729.7 | 375.2 | - | - | 0 | - |
| - | - | 886 | 389.7 | - | - | 0 | - |
| - | - | 3538 | 391.7 | - | - | 0 | - |
| - | - | 1615 | 392.2 | - | - | 0 | - |
| - | - | 1238 | 398.7 | - | - | 0 | - |
| - | - | 641.5 | 399.7 | - | - | 0 | - |
| 8 | z | 4726 | 415.2 | 0.003288 | 7.919 | +2 | 8 |
| - | - | 1812 | 415.7 | - | - | 0 | - |
| 12 | w | 1.279E+04 | 417.2 | 0.0008898 | 2.133 | +1 | 4 |
| - | - | 2492 | 418.2 | - | - | 0 | - |
| - | - | 635.1 | 422.7 | - | - | 0 | - |
| - | - | 699.8 | 423.2 | - | - | 0 | - |
| 8 | y | 3445 | 423.2 | 0.001067 | 2.521 | +2 | 8 |
| - | - | 1554 | 423.7 | - | - | 0 | - |
| - | - | 1741 | 425.2 | - | - | 0 | - |
| - | - | 622.4 | 426.2 | - | - | 0 | - |
| 12 | y | 725.8 | 431.2 | 0.0009053 | 2.099 | +1 | 4 |
| - | - | 1627 | 441.2 | - | - | 0 | - |
| - | - | 2703 | 443.3 | - | - | 0 | - |
| - | - | 696.1 | 443.3 | - | - | 0 | - |
| - | - | 780.5 | 444.3 | - | - | 0 | - |
| - | - | 901.6 | 446.2 | - | - | 0 | - |
| - | - | 851.2 | 447.2 | - | - | 0 | - |
| - | - | 1048 | 447.2 | - | - | 0 | - |
| - | - | 414.8 | 448.2 | - | - | 0 | - |
| 12 | y | 1.308E+04 | 448.3 | 0.000988 | 2.204 | +1 | 4 |
| 3 | y | 793.1 | 449.2 | 0.008831 | 19.66 | +3 | 13 |
| - | - | 2602 | 449.3 | - | - | 0 | - |
| 8 | c | 970.9 | 450.7 | 0.00236 | 5.235 | +2 | 8 |
| - | - | 682.8 | 451.2 | - | - | 0 | - |
| 3 | y | 1235 | 454.9 | 0.001588 | 3.492 | +3 | 13 |
| - | - | 750.8 | 455.3 | - | - | 0 | - |
| - | - | 553.3 | 455.6 | - | - | 0 | - |
| 4 | c | 8320 | 460.3 | 0.001104 | 2.399 | +1 | 4 |
| - | - | 1619 | 461.3 | - | - | 0 | - |
| 11 | z | 758.8 | 471.2 | 0.003106 | 6.592 | +1 | 5 |
| 14 | c | 2395 | 472.2 | 0.007744 | 16.4 | +3 | 14 |
| 7 | z | 2182 | 472.7 | 0.003 | 6.346 | +2 | 9 |
| - | - | 1549 | 473.2 | - | - | 0 | - |
| - | - | 589.7 | 473.7 | - | - | 0 | - |
| - | - | 2678 | 474.2 | - | - | 0 | - |
| - | - | 896.2 | 475.2 | - | - | 0 | - |
| 7 | y | 2238 | 480.7 | 0.001786 | 3.716 | +2 | 9 |
| 2 | z | 1070 | 481.2 | 0.006564 | 13.64 | +3 | 14 |
| 11 | z | 2972 | 489.3 | 0.0008118 | 1.659 | +1 | 5 |
| - | - | 2530 | 490.3 | - | - | 0 | - |
| - | - | 1441 | 495.9 | - | - | 0 | - |
| - | - | 571.7 | 498.3 | - | - | 0 | - |
| - | - | 2495 | 499.3 | - | - | 0 | - |
| 11 | y | 3.927E+04 | 505.3 | 0.001009 | 1.996 | +1 | 5 |
| - | - | 1.016E+04 | 506.3 | - | - | 0 | - |
| - | - | 1057 | 507.3 | - | - | 0 | - |
| - | - | 1215 | 512.2 | - | - | 0 | - |
| - | - | 917.7 | 514.3 | - | - | 0 | - |
| 6 | y | 1745 | 515.3 | 0.008762 | 17.01 | +2 | 10 |
| - | - | 2210 | 515.3 | - | - | 0 | - |
| 6 | y | 2374 | 515.7 | 0.001122 | 2.175 | +2 | 10 |
| 6 | z | 3927 | 516.3 | 0.0006273 | 1.215 | +2 | 10 |
| - | - | 1616 | 516.3 | - | - | 0 | - |
| - | - | 5148 | 516.8 | - | - | 0 | - |
| - | - | 2041 | 517.3 | - | - | 0 | - |
| - | - | 767.8 | 517.8 | - | - | 0 | - |
| - | - | 644.2 | 523.8 | - | - | 0 | - |
| 6 | y | 1.347E+04 | 524.3 | 0.00335 | 6.39 | +2 | 10 |
| - | - | 4643 | 524.6 | - | - | 0 | - |
| - | - | 6827 | 524.8 | - | - | 0 | - |
| - | - | 2029 | 524.9 | - | - | 0 | - |
| - | - | 1775 | 525.3 | - | - | 0 | - |
| - | - | 1077 | 525.8 | - | - | 0 | - |
| - | - | 1076 | 529.3 | - | - | 0 | - |
| - | - | 1239 | 529.8 | - | - | 0 | - |
| - | - | 1634 | 530.2 | - | - | 0 | - |
| - | - | 3509 | 530.3 | - | - | 0 | - |
| - | - | 3525 | 530.3 | - | - | 0 | - |
| - | - | 3114 | 530.6 | - | - | 0 | - |
| - | - | 1813 | 530.9 | - | - | 0 | - |
| - | - | 764.3 | 531.3 | - | - | 0 | - |
| - | - | 1696 | 531.3 | - | - | 0 | - |
| - | - | 853.1 | 537.8 | - | - | 0 | - |
| - | - | 1378 | 538.3 | - | - | 0 | - |
| - | - | 711.6 | 538.8 | - | - | 0 | - |
| - | - | 1029 | 541.3 | - | - | 0 | - |
| - | - | 4076 | 542.3 | - | - | 0 | - |
| - | - | 1412 | 543.3 | - | - | 0 | - |
| 10 | z | 1636 | 558.3 | 0.0007409 | 1.327 | +1 | 6 |
| - | - | 1727 | 558.3 | - | - | 0 | - |
| 10 | w | 2004 | 559.3 | 0.0014 | 2.503 | +1 | 6 |
| 5 | c | 7128 | 559.3 | 0.0007142 | 1.277 | +1 | 5 |
| - | - | 624.4 | 560.3 | - | - | 0 | - |
| - | - | 1691 | 560.3 | - | - | 0 | - |
| 5 | z | 2.885E+04 | 565.8 | 0.0009662 | 1.708 | +2 | 11 |
| - | - | 1.905E+04 | 566.3 | - | - | 0 | - |
| - | - | 5939 | 566.8 | - | - | 0 | - |
| - | - | 2077 | 567.3 | - | - | 0 | - |
| - | - | 755 | 572.8 | - | - | 0 | - |
| 5 | y | 1.496E+04 | 573.8 | 0.0007595 | 1.324 | +2 | 11 |
| 10 | y | 7885 | 574.3 | 0.004417 | 7.691 | +1 | 6 |
| - | - | 3580 | 574.8 | - | - | 0 | - |
| 10 | y | 1698 | 575.3 | 0.004105 | 7.135 | +1 | 6 |
| 10 | z | 6510 | 576.3 | 0.0004301 | 0.7464 | +1 | 6 |
| - | - | 1459 | 577.3 | - | - | 0 | - |
| - | - | 1415 | 586.3 | - | - | 0 | - |
| - | - | 742.5 | 587.8 | - | - | 0 | - |
| 10 | y | 1816 | 592.3 | 0.0002607 | 0.4402 | +1 | 6 |
| - | - | 3079 | 600.3 | - | - | 0 | - |
| 4 | w | 1.433E+04 | 600.8 | 0.0006651 | 1.107 | +2 | 12 |
| - | - | 8022 | 601.3 | - | - | 0 | - |
| - | - | 2464 | 601.8 | - | - | 0 | - |
| - | - | 741.6 | 608.3 | - | - | 0 | - |
| - | - | 961.9 | 629.3 | - | - | 0 | - |
| 12 | c | 2184 | 629.3 | 0.006833 | 10.86 | +2 | 12 |
| 4 | y | 934.1 | 629.8 | 0.0009772 | 1.552 | +2 | 12 |
| 4 | z | 3.162E+04 | 630.3 | 0.001032 | 1.637 | +2 | 12 |
| - | - | 1.986E+04 | 630.8 | - | - | 0 | - |
| - | - | 8923 | 631.3 | - | - | 0 | - |
| - | - | 664.6 | 631.4 | - | - | 0 | - |
| - | - | 1717 | 631.8 | - | - | 0 | - |
| - | - | 9923 | 637.8 | - | - | 0 | - |
| - | - | 768 | 637.9 | - | - | 0 | - |
| 4 | y | 3.314E+04 | 638.3 | 0.0002759 | 0.4323 | +2 | 12 |
| - | - | 1.95E+04 | 638.8 | - | - | 0 | - |
| - | - | 7525 | 639.3 | - | - | 0 | - |
| - | - | 1571 | 639.8 | - | - | 0 | - |
| - | - | 3107 | 644.3 | - | - | 0 | - |
| - | - | 1728 | 644.8 | - | - | 0 | - |
| - | - | 1028 | 645.4 | - | - | 0 | - |
| 6 | c | 2.684E+04 | 646.4 | 0.0009734 | 1.506 | +1 | 6 |
| - | - | 8369 | 647.4 | - | - | 0 | - |
| - | - | 1876 | 648.4 | - | - | 0 | - |
| - | - | 1213 | 651.8 | - | - | 0 | - |
| - | - | 778.5 | 652.3 | - | - | 0 | - |
| 3 | z | 1389 | 665.3 | 0.01087 | 16.33 | +2 | 13 |
| - | - | 964.6 | 665.8 | - | - | 0 | - |
| - | - | 1162 | 666.3 | - | - | 0 | - |
| 9 | y | 972.2 | 672.3 | 0.006118 | 9.099 | +1 | 7 |
| 3 | y | 3058 | 672.8 | 0.001972 | 2.931 | +2 | 13 |
| 3 | y | 2641 | 673.3 | 0.006814 | 10.12 | +2 | 13 |
| 3 | z | 2.079E+04 | 673.8 | 0.001375 | 2.041 | +2 | 13 |
| - | - | 2.488E+04 | 674.3 | - | - | 0 | - |
| - | - | 1.607E+04 | 674.8 | - | - | 0 | - |
| - | - | 4424 | 675.3 | - | - | 0 | - |
| - | - | 1461 | 675.8 | - | - | 0 | - |
| - | - | 717.7 | 680.3 | - | - | 0 | - |
| - | - | 3925 | 680.8 | - | - | 0 | - |
| - | - | 4295 | 681.3 | - | - | 0 | - |
| 3 | y | 1.363E+05 | 681.8 | 0.001046 | 1.535 | +2 | 13 |
| - | - | 9.179E+04 | 682.3 | - | - | 0 | - |
| - | - | 3.624E+04 | 682.8 | - | - | 0 | - |
| - | - | 1.115E+04 | 683.3 | - | - | 0 | - |
| - | - | 1711 | 683.8 | - | - | 0 | - |
| - | - | 923.3 | 685.4 | - | - | 0 | - |
| - | - | 660.9 | 685.8 | - | - | 0 | - |
| - | - | 1688 | 687.3 | - | - | 0 | - |
| 9 | y | 3828 | 689.4 | 0.001025 | 1.486 | +1 | 7 |
| - | - | 1122 | 690.4 | - | - | 0 | - |
| - | - | 1161 | 711.4 | - | - | 0 | - |
| - | - | 1359 | 713.4 | - | - | 0 | - |
| - | - | 881.8 | 714.4 | - | - | 0 | - |
| 2 | w | 3572 | 715.8 | 0.001367 | 1.909 | +2 | 14 |
| - | - | 2192 | 716.3 | - | - | 0 | - |
| - | - | 793.7 | 716.9 | - | - | 0 | - |
| - | - | 1904 | 717.4 | - | - | 0 | - |
| - | - | 4221 | 718.4 | - | - | 0 | - |
| 2 | w | 797.8 | 722.9 | 0.002941 | 4.069 | +2 | 14 |
| 2 | y | 1398 | 729.4 | 0.002256 | 3.093 | +2 | 14 |
| 2 | z | 2750 | 730.4 | 0.0002515 | 0.3443 | +2 | 14 |
| - | - | 1359 | 730.9 | - | - | 0 | - |
| - | - | 697 | 737.4 | - | - | 0 | - |
| 2 | y | 6598 | 738.4 | 0.0008845 | 1.198 | +2 | 14 |
| - | - | 5649 | 738.9 | - | - | 0 | - |
| - | - | 2784 | 739.4 | - | - | 0 | - |
| - | - | 699.2 | 742.4 | - | - | 0 | - |
| 8 | w | 2944 | 743.4 | 0.005353 | 7.2 | +1 | 8 |
| - | - | 3189 | 744.4 | - | - | 0 | - |
| - | - | 2088 | 750.9 | - | - | 0 | - |
| - | - | 2005 | 751.4 | - | - | 0 | - |
| - | - | 1479 | 751.9 | - | - | 0 | - |
| - | - | 1211 | 756.4 | - | - | 0 | - |
| - | - | 1581 | 757.9 | - | - | 0 | - |
| - | - | 4718 | 758.4 | - | - | 0 | - |
| - | - | 4239 | 758.9 | - | - | 0 | - |
| - | - | 4205 | 759.4 | - | - | 0 | - |
| - | - | 1207 | 759.9 | - | - | 0 | - |
| - | - | 1430 | 760.4 | - | - | 0 | - |
| 7 | c | 5.417E+04 | 761.4 | 0.001252 | 1.644 | +1 | 7 |
| - | - | 2.068E+04 | 762.4 | - | - | 0 | - |
| - | - | 5320 | 763.4 | - | - | 0 | - |
| - | - | 2988 | 764.4 | - | - | 0 | - |
| - | - | 7001 | 764.9 | - | - | 0 | - |
| - | - | 9.289E+04 | 765.4 | - | - | 0 | - |
| - | - | 7.62E+04 | 765.9 | - | - | 0 | - |
| - | - | 3.547E+04 | 766.4 | - | - | 0 | - |
| - | - | 1.063E+04 | 766.9 | - | - | 0 | - |
| - | - | 1544 | 767.4 | - | - | 0 | - |
| - | - | 887.9 | 771.9 | - | - | 0 | - |
| - | - | 4191 | 772.4 | - | - | 0 | - |
| - | - | 3604 | 772.9 | - | - | 0 | - |
| - | - | 2112 | 773.4 | - | - | 0 | - |
| - | - | 2.572E+04 | 773.9 | - | - | 0 | - |
| - | - | 2.019E+04 | 774.4 | - | - | 0 | - |
| - | - | 9759 | 774.9 | - | - | 0 | - |
| - | - | 2695 | 775.4 | - | - | 0 | - |
| - | - | 1611 | 777.9 | - | - | 0 | - |
| - | - | 3188 | 778.4 | - | - | 0 | - |
| - | - | 1606 | 778.9 | - | - | 0 | - |
| - | - | 1581 | 779.4 | - | - | 0 | - |
| - | - | 1082 | 779.9 | - | - | 0 | - |
| - | - | 1377 | 780.4 | - | - | 0 | - |
| - | - | 717 | 781.4 | - | - | 0 | - |
| - | - | 2636 | 782.4 | - | - | 0 | - |
| - | - | 2494 | 783.4 | - | - | 0 | - |
| - | - | 3359 | 785.4 | - | - | 0 | - |
| - | - | 4521 | 785.9 | - | - | 0 | - |
| - | - | 9958 | 786.4 | - | - | 0 | - |
| - | - | 9.998E+04 | 786.9 | - | - | 0 | - |
| - | - | 8.002E+04 | 787.4 | - | - | 0 | - |
| - | - | 4.19E+04 | 787.9 | - | - | 0 | - |
| - | - | 1.185E+04 | 788.4 | - | - | 0 | - |
| - | - | 3711 | 788.9 | - | - | 0 | - |
| - | - | 949.3 | 792.9 | - | - | 0 | - |
| - | - | 4915 | 793.4 | - | - | 0 | - |
| - | - | 1.882E+04 | 793.9 | - | - | 0 | - |
| - | - | 1.841E+04 | 794.4 | - | - | 0 | - |
| - | - | 7.009E+04 | 794.9 | - | - | 0 | - |
| - | - | 7.765E+04 | 795.4 | - | - | 0 | - |
| - | - | 4.835E+04 | 795.9 | - | - | 0 | - |
| - | - | 1.932E+04 | 796.4 | - | - | 0 | - |
| - | - | 4726 | 796.9 | - | - | 0 | - |
| - | - | 2165 | 797.4 | - | - | 0 | - |
| - | - | 733.5 | 798.4 | - | - | 0 | - |
| - | - | 4708 | 803.4 | - | - | 0 | - |
| - | - | 2351 | 804.4 | - | - | 0 | - |
| 8 | z | 2728 | 811.4 | 0.0007292 | 0.8986 | +1 | 8 |
| 8 | y | 955.7 | 828.4 | 0.0004171 | 0.5035 | +1 | 8 |
| 8 | z | 4.655E+04 | 829.4 | 0.0008521 | 1.027 | +1 | 8 |
| - | - | 2.162E+04 | 830.4 | - | - | 0 | - |
| - | - | 7293 | 831.4 | - | - | 0 | - |
| - | - | 878.4 | 832.5 | - | - | 0 | - |
| - | - | 1216 | 839.5 | - | - | 0 | - |
| - | - | 925 | 840.5 | - | - | 0 | - |
| - | - | 2086 | 841.5 | - | - | 0 | - |
| - | - | 1161 | 842.5 | - | - | 0 | - |
| - | - | 2022 | 844.4 | - | - | 0 | - |
| - | - | 575.7 | 845.4 | - | - | 0 | - |
| 8 | y | 7001 | 845.5 | 0.001171 | 1.385 | +1 | 8 |
| - | - | 1936 | 846.5 | - | - | 0 | - |
| - | - | 1273 | 847.5 | - | - | 0 | - |
| - | - | 772.3 | 857.4 | - | - | 0 | - |
| - | - | 682.7 | 861.4 | - | - | 0 | - |
| - | - | 2251 | 873.5 | - | - | 0 | - |
| - | - | 895.2 | 874.5 | - | - | 0 | - |
| 8 | c | 6682 | 900.5 | 0.0001764 | 0.1959 | +1 | 8 |
| - | - | 2748 | 901.5 | - | - | 0 | - |
| - | - | 1391 | 902.5 | - | - | 0 | - |
| 7 | z | 2229 | 926.5 | 0.0006481 | 0.6995 | +1 | 9 |
| - | - | 1367 | 927.5 | - | - | 0 | - |
| - | - | 1097 | 928.5 | - | - | 0 | - |
| - | - | 1057 | 929.5 | - | - | 0 | - |
| 7 | z | 1.701E+04 | 944.5 | 0.001009 | 1.068 | +1 | 9 |
| - | - | 1.23E+04 | 945.5 | - | - | 0 | - |
| - | - | 4740 | 946.5 | - | - | 0 | - |
| - | - | 1177 | 947.5 | - | - | 0 | - |
| - | - | 1034 | 954.5 | - | - | 0 | - |
| - | - | 1712 | 955.5 | - | - | 0 | - |
| - | - | 2029 | 956.6 | - | - | 0 | - |
| - | - | 791.2 | 957.5 | - | - | 0 | - |
| - | - | 1038 | 959.5 | - | - | 0 | - |
| 7 | y | 2083 | 960.5 | 0.0007783 | 0.8103 | +1 | 9 |
| - | - | 3535 | 970.5 | - | - | 0 | - |
| - | - | 2106 | 971.5 | - | - | 0 | - |
| - | - | 958.8 | 999.6 | - | - | 0 | - |
| - | - | 725.4 | 1004 | - | - | 0 | - |
| - | - | 855.2 | 1006 | - | - | 0 | - |
| - | - | 755.4 | 1013 | - | - | 0 | - |
| - | - | 2527 | 1014 | - | - | 0 | - |
| 9 | c | 4.266E+04 | 1015 | 0.001613 | 1.59 | +1 | 9 |
| - | - | 2.141E+04 | 1016 | - | - | 0 | - |
| - | - | 6479 | 1017 | - | - | 0 | - |
| - | - | 1951 | 1018 | - | - | 0 | - |
| 6 | z | 6787 | 1031 | 0.001207 | 1.17 | +1 | 10 |
| - | - | 9130 | 1033 | - | - | 0 | - |
| - | - | 4314 | 1034 | - | - | 0 | - |
| - | - | 1182 | 1035 | - | - | 0 | - |
| - | - | 2598 | 1040 | - | - | 0 | - |
| - | - | 1587 | 1041 | - | - | 0 | - |
| - | - | 1628 | 1042 | - | - | 0 | - |
| - | - | 2791 | 1043 | - | - | 0 | - |
| - | - | 1008 | 1047 | - | - | 0 | - |
| 6 | y | 2707 | 1048 | 0.000305 | 0.2912 | +1 | 10 |
| - | - | 2150 | 1049 | - | - | 0 | - |
| - | - | 2.819E+04 | 1058 | - | - | 0 | - |
| - | - | 1.984E+04 | 1059 | - | - | 0 | - |
| - | - | 1.024E+04 | 1060 | - | - | 0 | - |
| - | - | 3899 | 1061 | - | - | 0 | - |
| - | - | 712.1 | 1062 | - | - | 0 | - |
| - | - | 2451 | 1101 | - | - | 0 | - |
| 10 | c | 5.598E+04 | 1102 | 0.001567 | 1.422 | +1 | 10 |
| - | - | 3.246E+04 | 1103 | - | - | 0 | - |
| - | - | 1.101E+04 | 1104 | - | - | 0 | - |
| - | - | 2061 | 1105 | - | - | 0 | - |
| - | - | 8608 | 1115 | - | - | 0 | - |
| - | - | 6665 | 1116 | - | - | 0 | - |
| - | - | 2321 | 1117 | - | - | 0 | - |
| - | - | 758.2 | 1118 | - | - | 0 | - |
| 5 | y | 623.6 | 1130 | 0.0007883 | 0.6979 | +1 | 11 |
| 5 | z | 8029 | 1131 | 0.0009082 | 0.8033 | +1 | 11 |
| - | - | 1.734E+04 | 1132 | - | - | 0 | - |
| - | - | 9509 | 1133 | - | - | 0 | - |
| - | - | 3107 | 1134 | - | - | 0 | - |
| 5 | y | 2748 | 1147 | 0.002814 | 2.454 | +1 | 11 |
| - | - | 1096 | 1148 | - | - | 0 | - |
| - | - | 1213 | 1149 | - | - | 0 | - |
| 11 | c | 3.243E+04 | 1159 | 0.0008552 | 0.7381 | +1 | 11 |
| - | - | 1.896E+04 | 1160 | - | - | 0 | - |
| - | - | 8091 | 1161 | - | - | 0 | - |
| - | - | 1494 | 1162 | - | - | 0 | - |
| - | - | 681.8 | 1204 | - | - | 0 | - |
| - | - | 6407 | 1214 | - | - | 0 | - |
| - | - | 4153 | 1215 | - | - | 0 | - |
| - | - | 2910 | 1216 | - | - | 0 | - |
| - | - | 1240 | 1217 | - | - | 0 | - |
| - | - | 775.8 | 1218 | - | - | 0 | - |
| - | - | 731.8 | 1248 | - | - | 0 | - |
| 12 | c | 3.113E+04 | 1258 | 0.0006786 | 0.5396 | +1 | 12 |
| - | - | 2.059E+04 | 1259 | - | - | 0 | - |
| - | - | 7752 | 1260 | - | - | 0 | - |
| - | - | 2.838E+04 | 1261 | - | - | 0 | - |
| - | - | 1.442E+04 | 1262 | - | - | 0 | - |
| - | - | 5681 | 1263 | - | - | 0 | - |
| - | - | 1282 | 1264 | - | - | 0 | - |
| - | - | 918.1 | 1275 | - | - | 0 | - |
| 4 | y | 1.037E+04 | 1276 | 0.0008387 | 0.6575 | +1 | 12 |
| - | - | 6159 | 1277 | - | - | 0 | - |
| - | - | 2458 | 1278 | - | - | 0 | - |
| - | - | 1384 | 1283 | - | - | 0 | - |
| - | - | 837 | 1285 | - | - | 0 | - |
| - | - | 5546 | 1301 | - | - | 0 | - |
| - | - | 3997 | 1302 | - | - | 0 | - |
| - | - | 2752 | 1303 | - | - | 0 | - |
| - | - | 1570 | 1305 | - | - | 0 | - |
| 3 | w | 2095 | 1330 | 0.01228 | 9.234 | +1 | 13 |
| - | - | 2601 | 1331 | - | - | 0 | - |
| - | - | 1594 | 1332 | - | - | 0 | - |
| - | - | 770.9 | 1344 | - | - | 0 | - |
| 13 | c | 5.226E+04 | 1345 | 0.0005881 | 0.4373 | +1 | 13 |
| - | - | 3.646E+04 | 1346 | - | - | 0 | - |
| - | - | 1.746E+04 | 1347 | - | - | 0 | - |
| - | - | 2.382E+04 | 1348 | - | - | 0 | - |
| - | - | 1.427E+04 | 1349 | - | - | 0 | - |
| - | - | 7120 | 1350 | - | - | 0 | - |
| - | - | 2383 | 1351 | - | - | 0 | - |
| 3 | y | 1538 | 1363 | 0.002227 | 1.635 | +1 | 13 |
| - | - | 2393 | 1364 | - | - | 0 | - |
| - | - | 5757 | 1388 | - | - | 0 | - |
| - | - | 4496 | 1389 | - | - | 0 | - |
| - | - | 1243 | 1390 | - | - | 0 | - |
| - | - | 938.4 | 1391 | - | - | 0 | - |
| - | - | 2031 | 1430 | - | - | 0 | - |
| - | - | 1601 | 1431 | - | - | 0 | - |
| 14 | c | 7.458E+04 | 1432 | 0.0006341 | 0.4429 | +1 | 14 |
| - | - | 6.148E+04 | 1433 | - | - | 0 | - |
| - | - | 2.668E+04 | 1434 | - | - | 0 | - |
| - | - | 7206 | 1435 | - | - | 0 | - |
| - | - | 1347 | 1436 | - | - | 0 | - |
| - | - | 755.5 | 1450 | - | - | 0 | - |
| 2 | z | 884.2 | 1460 | 0.005797 | 3.971 | +1 | 14 |
| - | - | 3801 | 1461 | - | - | 0 | - |
| - | - | 2944 | 1462 | - | - | 0 | - |
| - | - | 1371 | 1463 | - | - | 0 | - |
| - | - | 689.6 | 1474 | - | - | 0 | - |
| - | - | 695.2 | 1475 | - | - | 0 | - |
| 2 | y | 658.2 | 1476 | 0.0001344 | 0.09104 | +1 | 14 |
| - | - | 943.6 | 1488 | - | - | 0 | - |
| - | - | 6952 | 1489 | - | - | 0 | - |
| - | - | 6572 | 1490 | - | - | 0 | - |
| - | - | 3868 | 1491 | - | - | 0 | - |
| - | - | 1771 | 1492 | - | - | 0 | - |
| - | - | 1483 | 1513 | - | - | 0 | - |
| - | - | 1674 | 1514 | - | - | 0 | - |
| - | - | 2139 | 1515 | - | - | 0 | - |
| - | - | 987.1 | 1516 | - | - | 0 | - |
| - | - | 969.5 | 1517 | - | - | 0 | - |
| - | - | 1209 | 1529 | - | - | 0 | - |
| - | - | 2108 | 1530 | - | - | 0 | - |
| - | - | 1.614E+04 | 1531 | - | - | 0 | - |
| - | - | 2.058E+04 | 1532 | - | - | 0 | - |
| - | - | 1.327E+04 | 1533 | - | - | 0 | - |
| - | - | 4383 | 1534 | - | - | 0 | - |
| - | - | 971.8 | 1535 | - | - | 0 | - |
| - | - | 1651 | 1544 | - | - | 0 | - |
| - | - | 3727 | 1545 | - | - | 0 | - |
| - | - | 7159 | 1546 | - | - | 0 | - |
| - | - | 7140 | 1547 | - | - | 0 | - |
| - | - | 8737 | 1548 | - | - | 0 | - |
| - | - | 4784 | 1549 | - | - | 0 | - |
| - | - | 2463 | 1550 | - | - | 0 | - |
| - | - | 2076 | 1556 | - | - | 0 | - |
| - | - | 8358 | 1557 | - | - | 0 | - |
| - | - | 5227 | 1558 | - | - | 0 | - |
| - | - | 2767 | 1559 | - | - | 0 | - |
| - | - | 1793 | 1560 | - | - | 0 | - |
| - | - | 861.1 | 1561 | - | - | 0 | - |
| - | - | 3418 | 1562 | - | - | 0 | - |
| - | - | 1.182E+04 | 1563 | - | - | 0 | - |
| - | - | 8559 | 1564 | - | - | 0 | - |
| - | - | 5706 | 1565 | - | - | 0 | - |
| - | - | 1085 | 1566 | - | - | 0 | - |
| - | - | 1216 | 1570 | - | - | 0 | - |
| - | - | 2034 | 1571 | - | - | 0 | - |
| - | - | 4716 | 1572 | - | - | 0 | - |
| - | - | 1.767E+04 | 1573 | - | - | 0 | - |
| - | - | 1.023E+05 | 1574 | - | - | 0 | - |
| - | - | 7.936E+04 | 1575 | - | - | 0 | - |
| - | - | 3.934E+04 | 1576 | - | - | 0 | - |
| - | - | 1.322E+04 | 1577 | - | - | 0 | - |
| - | - | 3583 | 1578 | - | - | 0 | - |
| - | - | 4284 | 1587 | - | - | 0 | - |
| - | - | 7711 | 1588 | - | - | 0 | - |
| - | - | 1.396E+04 | 1589 | - | - | 0 | - |
| - | - | 1093 | 1590 | - | - | 0 | - |
| - | - | 3.473E+04 | 1590 | - | - | 0 | - |
| - | - | 1.022E+05 | 1591 | - | - | 0 | - |
| - | - | 7.681E+04 | 1592 | - | - | 0 | - |
| - | - | 3.865E+04 | 1593 | - | - | 0 | - |
| - | - | 1.042E+04 | 1594 | - | - | 0 | - |
| - | - | 2902 | 1595 | - | - | 0 | - |
| - | - | 635.5 | 2210 | - | - | 0 | - |

m/z Charge Intensity FragmentType MassShift Position
122.85774993896484 0 367.15152
124.91972351074219 0 378.3758
125.10771942138672 0 2031.951
126.09163665771484 0 3690.8892
126.10456848144531 0 363.92114
128.0821075439453 0 1113.4352
128.31515502929688 0 476.9033
130.06167602539062 0 543.29114
130.1215362548828 0 502.3937
131.12950134277344 0 1418.2523
137.79541015625 0 414.9647
143.02391052246094 0 422.63754
145.10855102539062 0 489.85794
148.95480346679688 0 891.5044
158.09291076660156 0 804.18274 y Ammonia loss 14
162.8594207763672 0 441.4897
168.1134796142578 0 4333.814
173.4387664794922 0 1039.0602
175.11927795410156 0 6243.0835 y 14
176.12295532226562 0 802.32227
176.15106201171875 0 548.5143
185.14007568359375 0 17871.49
186.1433868408203 0 1271.658
199.18084716796875 0 48174.547
200.12704467773438 0 2530.0261
200.18414306640625 0 5406.3604
201.1228790283203 0 866.9515
217.08221435546875 0 4003.7688
226.1300506591797 0 625.7795
226.16798400878906 0 1107.3647
227.17576599121094 0 22377.137
228.17919921875 0 3167.3428
229.12998962402344 0 3410.667 w 13
233.1651611328125 0 1514.1793
234.10911560058594 0 550.99786
244.11729431152344 0 1501.7122
245.12489318847656 0 30058.629 y Ammonia loss 13
246.13107299804688 0 3997.1902 z 13
249.16075134277344 0 617.28625
255.10948181152344 0 1251.8398
260.1359558105469 0 784.2109
261.1601867675781 0 1139.5377
262.15142822265625 0 3742.6897 y 13
263.15386962890625 0 816.36096
270.1201171875 0 5148.4893
272.1716003417969 0 1687.6649
304.1509704589844 0 2715.7546
314.2083435058594 0 3004.1484
316.162109375 0 15404.202 w 12
317.1602783203125 0 2427.8782
318.166015625 0 837.29156
330.1653137207031 0 673.17615
331.1491394042969 0 4202.7363
331.2348327636719 0 642.62256 c 2
332.1568603515625 0 4981.279 y Ammonia loss 12
333.1649169921875 0 12385.206 z 12
334.1679992675781 0 2127.4597
340.115966796875 0 793.0369
347.1678161621094 0 1157.0209
349.1833190917969 0 4087.451 y 12
350.18695068359375 0 639.264
357.1520690917969 0 8211.971
357.2486267089844 0 675.256
375.1615295410156 0 729.70135
389.7081604003906 0 885.95105
391.724365234375 0 3537.903
392.22509765625 0 1615.231
398.7146911621094 0 1237.9014
399.7210693359375 0 641.49744
415.2269592285156 0 4725.876 z 7
415.7247009277344 0 1812.499
417.2101135253906 0 12786.362 w 11
418.2123107910156 0 2491.5938
422.7301025390625 0 635.1457
423.1503601074219 0 699.8488
423.2341003417969 0 3444.8062 y 7
423.7347717285156 0 1553.7158
425.24066162109375 0 1740.8545
426.2421569824219 0 622.3697
431.2239685058594 0 725.8272 y Ammonia loss 11
441.1630859375 0 1627.3165
443.25067138671875 0 2702.9504
443.309814453125 0 696.13855
444.2547912597656 0 780.49774
446.2361755371094 0 901.59357
447.2068786621094 0 851.17566
447.2406005859375 0 1047.6027
448.2166442871094 0 414.80945
448.2524108886719 0 13077.065 y 11
449.22509765625 0 793.0998 y Ammonia loss 2
449.2557678222656 0 2601.5718
450.74053955078125 0 970.9486 c Ammonia loss 7
451.2463073730469 0 682.826
454.89337158203125 0 1234.6893 y 2
455.2864685058594 0 750.8223
455.5610046386719 0 553.30133
460.2776794433594 0 8319.849 c 3
461.2798156738281 0 1619.1385
471.2467041015625 0 758.7558 z Water loss 10
472.2516784667969 0 2395.2249 c Ammonia loss 13
472.7401428222656 0 2182.3645 z 6
473.2444152832031 0 1548.7688
473.7429504394531 0 589.68536
474.2312316894531 0 2677.747
475.2341613769531 0 896.1511
480.748291015625 0 2238.1672 y 6
481.24993896484375 0 1070.2123 z Water loss 1
489.2549743652344 0 2972.2136 z 10
490.2613525390625 0 2529.6375
495.91522216796875 0 1440.7943
498.2901611328125 0 571.66626
499.3000183105469 0 2494.8555
505.2738952636719 0 39274.094 y 10
506.2765808105469 0 10161.34
507.2798767089844 0 1057.2538
512.200439453125 0 1214.5823
514.3242797851562 0 917.74304
515.2484741210938 0 1744.5902 y Water loss 5
515.3316040039062 0 2209.7483
515.7503662109375 0 2374.467 y Ammonia loss 5
516.2537841796875 0 3927.2368 z 5
516.3366088867188 0 1616.4813
516.7567138671875 0 5148.427
517.2593383789062 0 2041.2905
517.756591796875 0 767.8077
523.7548217773438 0 644.2255
524.265869140625 0 13469.617 y 5
524.613037109375 0 4643.2744
524.7643432617188 0 6827.4805
524.9465942382812 0 2029.4315
525.2688598632812 0 1775.4342
525.7657470703125 0 1076.8999
529.2719116210938 0 1075.829
529.7965087890625 0 1238.5315
530.2081298828125 0 1633.7461
530.2842407226562 0 3509.124
530.3191528320312 0 3524.912
530.6168823242188 0 3114.4902
530.9494018554688 0 1813.1613
531.28515625 0 764.3199
531.3228759765625 0 1695.8995
537.7596435546875 0 853.1097
538.2594604492188 0 1378.1735
538.7628784179688 0 711.57324
541.3109741210938 0 1028.8967
542.3189697265625 0 4075.5781
543.32177734375 0 1412.1174
558.2763671875 0 1636.118 z Water loss 9
558.338134765625 0 1727.4645
559.2848510742188 0 2003.7513 w 9
559.345703125 0 7128.0444 c 4
560.2888793945312 0 624.36285
560.3482055664062 0 1690.6149
565.788330078125 0 28854.232 z 4
566.2899780273438 0 19051.938
566.791748046875 0 5939.2017
567.2921752929688 0 2077.4365
572.7882080078125 0 755.00507
573.7974853515625 0 14958.626 y 4
574.2987670898438 0 7885.2393 y Water loss 9
574.8010864257812 0 3580.0698
575.282470703125 0 1697.573 y Ammonia loss 9
576.28662109375 0 6510.0195 z 9
577.29052734375 0 1458.7576
586.3316650390625 0 1414.5369
587.793701171875 0 742.5308
592.30517578125 0 1815.7312 y 9
600.2748413085938 0 3079.3074
600.8026733398438 0 14327.516 w 3
601.303955078125 0 8021.9316
601.8070678710938 0 2463.729
608.3133544921875 0 741.58044
629.2994384765625 0 961.86633
629.350341796875 0 2184.1038 c 11
629.8057250976562 0 934.0562 y Ammonia loss 3
630.3096923828125 0 31615.66 z 3
630.81103515625 0 19861.963
631.3123779296875 0 8923.478
631.3684692382812 0 664.58594
631.8123168945312 0 1717.3873
637.8153076171875 0 9923.305
637.8670043945312 0 768.0165
638.3182983398438 0 33138.438 y 3
638.8199462890625 0 19504.727
639.322021484375 0 7524.7607
639.8218383789062 0 1571.3425
644.3193969726562 0 3106.8523
644.818359375 0 1727.6913
645.369873046875 0 1028.1595
646.3779907226562 0 26836.088 c 5
647.38134765625 0 8368.885
648.38525390625 0 1876.3004
651.8267822265625 0 1213.2518
652.3141479492188 0 778.50934
665.322265625 0 1388.5981 z Ammonia loss 2
665.8247680664062 0 964.6102
666.3236083984375 0 1162.2927
672.3250122070312 0 972.22186 y Ammonia loss 8
672.8267822265625 0 3057.65 y Water loss 2
673.3275756835938 0 2641.4495 y Ammonia loss 2
673.8260498046875 0 20792.557 z 2
674.3280029296875 0 24878.396
674.829833984375 0 16070.822
675.329345703125 0 4423.838
675.8308715820312 0 1461.334
680.3257446289062 0 717.67053
680.8252563476562 0 3924.552
681.3297729492188 0 4295.1943
681.8350830078125 0 136276.78 y 2
682.33642578125 0 91787.93
682.8379516601562 0 36244.363
683.339111328125 0 11154.784
683.8386840820312 0 1711.2802
685.3868408203125 0 923.31525
685.8206176757812 0 660.9096
687.3382568359375 0 1688.0173
689.3587036132812 0 3828.163 y 8
690.3639526367188 0 1122.2258
711.4237060546875 0 1160.7336
713.4305419921875 0 1359.0817
714.380859375 0 881.7509
715.8485107421875 0 3571.9329 w 1
716.3495483398438 0 2192.0984
716.8504638671875 0 793.6867
717.3887329101562 0 1904.2256
718.3975219726562 0 4221.2725
722.85791015625 0 797.75604 w 1
729.3685302734375 0 1397.8749 y Water loss 1
730.366455078125 0 2749.9712 z 1
730.8650512695312 0 1358.874
737.3715209960938 0 696.9746
738.376953125 0 6598.093 y 1
738.878662109375 0 5649.1304
739.3783569335938 0 2784.482
742.3970336914062 0 699.1597
743.3735961914062 0 2943.8086 w 7
744.3701171875 0 3188.5242
750.8948974609375 0 2087.6794
751.3970947265625 0 2004.5077
751.9047241210938 0 1478.8124
756.4065551757812 0 1210.5801
757.8973388671875 0 1580.8806
758.3885498046875 0 4718.0264
758.8844604492188 0 4239.252
759.38330078125 0 4205.444
759.8883056640625 0 1207.4473
760.392333984375 0 1429.739
761.4052124023438 0 54172.805 c 6
762.4077758789062 0 20678.463
763.4093017578125 0 5320.2437
764.4133911132812 0 2987.7888
764.910400390625 0 7000.7896
765.38330078125 0 92888.75
765.8843994140625 0 76198.336
766.3859252929688 0 35470.31
766.8867797851562 0 10627.407
767.3856201171875 0 1544.4504
771.908447265625 0 887.89154
772.4126586914062 0 4190.816
772.91064453125 0 3603.8372
773.4111938476562 0 2112.4062
773.9085693359375 0 25717.592
774.4097290039062 0 20192.312
774.9110107421875 0 9758.549
775.4129028320312 0 2694.971
777.9055786132812 0 1611.3452
778.4060668945312 0 3188.4575
778.905029296875 0 1605.7146
779.406494140625 0 1580.9348
779.9059448242188 0 1082.4738
780.4161376953125 0 1376.5923
781.4204711914062 0 716.95953
782.441650390625 0 2636.3662
783.44140625 0 2494.272
785.3975830078125 0 3358.625
785.9012451171875 0 4520.5996
786.4113159179688 0 9957.923
786.9102783203125 0 99984.75
787.4114990234375 0 80018.95
787.9124755859375 0 41895.477
788.4124145507812 0 11845.088
788.91455078125 0 3711.4387
792.8963623046875 0 949.2896
793.4061889648438 0 4915.146
793.9105224609375 0 18818.096
794.4114379882812 0 18407.34
794.918212890625 0 70091.625
795.4208374023438 0 77646.48
795.92236328125 0 48351.73
796.42333984375 0 19323.266
796.9234619140625 0 4726.0923
797.4193725585938 0 2165.0173
798.4315185546875 0 733.4808
803.4262084960938 0 4708.214
804.4280395507812 0 2351.0925
811.4287719726562 0 2728.3315 z Water loss 7
828.4318237304688 0 955.67377 y Ammonia loss 7
829.44091796875 0 46547.1 z 7
830.4447631835938 0 21617.982
831.44873046875 0 7293.2554
832.451904296875 0 878.3961
839.5147705078125 0 1215.5819
840.5286254882812 0 925.04315
841.5309448242188 0 2086.4973
842.5321655273438 0 1160.7238
844.449951171875 0 2022.0812
845.3846435546875 0 575.6875
845.4599609375 0 7001.317 y 7
846.4642333984375 0 1936.0444
847.4664916992188 0 1273.3292
857.4308471679688 0 772.33673
861.4312133789062 0 682.7247
873.490234375 0 2250.7517
874.4951171875 0 895.15643
900.4786987304688 0 6681.5635 c Ammonia loss 7
901.47998046875 0 2747.9106
902.4844970703125 0 1390.5739
926.4570922851562 0 2228.706 z Water loss 6
927.4598388671875 0 1367.017
928.4693603515625 0 1097.1993
929.4895629882812 0 1056.7506
944.468017578125 0 17006.07 z 6
945.472412109375 0 12296.853
946.4782104492188 0 4739.6045
947.4777221679688 0 1177.3715
954.5410766601562 0 1034.2607
955.5360107421875 0 1711.8217
956.553466796875 0 2028.9009
957.5497436523438 0 791.1722
959.47705078125 0 1037.6477
960.4865112304688 0 2082.6118 y 6
970.5432739257812 0 3534.8398
971.5457153320312 0 2106.3967
999.5662231445312 0 958.8327
1004.49462890625 0 725.3558
1005.5076293945312 0 855.2119
1012.5466918945312 0 755.3883
1013.5849609375 0 2527.4128
1014.5594482421875 0 42660.496 c 8
1015.5628051757812 0 21408.316
1016.5661010742188 0 6479
1017.5750122070312 0 1950.7916
1031.500244140625 0 6787.0566 z 5
1032.5064697265625 0 9130.205
1033.5106201171875 0 4314.1807
1034.51171875 0 1182.243
1039.5667724609375 0 2597.503
1040.565673828125 0 1586.5322
1041.5660400390625 0 1627.8433
1042.5733642578125 0 2790.6118
1046.5111083984375 0 1008.30646
1047.51806640625 0 2706.7078 y 5
1048.5216064453125 0 2149.7986
1057.5777587890625 0 28191.646
1058.580810546875 0 19840.363
1059.588134765625 0 10238.146
1060.592529296875 0 3899.037
1061.6146240234375 0 712.073
1100.580078125 0 2451.425
1101.5914306640625 0 55980.004 c 9
1102.593994140625 0 32463.625
1103.5968017578125 0 11012.446
1104.5968017578125 0 2060.751
1114.5980224609375 0 8607.899
1115.60107421875 0 6665.244
1116.6005859375 0 2320.702
1117.60107421875 0 758.2415
1129.558837890625 0 623.6449 y Ammonia loss 4
1130.568359375 0 8029.242 z 4
1131.5753173828125 0 17340.045
1132.578125 0 9508.834
1133.5811767578125 0 3106.8
1146.5889892578125 0 2747.8435 y 4
1147.59228515625 0 1096.177
1148.58935546875 0 1212.8643
1158.6121826171875 0 32425.36 c 10
1159.61474609375 0 18963.227
1160.6181640625 0 8091.1016
1161.62109375 0 1493.565
1203.6134033203125 0 681.75696
1213.666259765625 0 6407.138
1214.6676025390625 0 4152.664
1215.6746826171875 0 2910.0881
1216.674072265625 0 1240.4282
1217.58251953125 0 775.82434
1247.5672607421875 0 731.7912
1257.680419921875 0 31125.318 c 11
1258.681396484375 0 20594.117
1259.6783447265625 0 7752.3013
1260.6187744140625 0 28383.598
1261.6204833984375 0 14419.094
1262.6199951171875 0 5681.112
1263.634033203125 0 1282.0784
1274.61669921875 0 918.12616
1275.6279296875 0 10368.731 y 3
1276.6319580078125 0 6159.3867
1277.6324462890625 0 2458.447
1282.6854248046875 0 1383.6978
1284.6934814453125 0 836.96027
1300.6982421875 0 5545.627
1301.700439453125 0 3997.1663
1302.700927734375 0 2751.9766
1304.631103515625 0 1569.8967
1329.651611328125 0 2095.0342 w 2
1330.631591796875 0 2601.3975
1331.6368408203125 0 1594.2432
1343.699462890625 0 770.9416
1344.711181640625 0 52261.48 c 12
1345.714599609375 0 36461.89
1346.7095947265625 0 17459.38
1347.6533203125 0 23817.617
1348.6553955078125 0 14269.196
1349.654541015625 0 7120.448
1350.6563720703125 0 2382.6567
1362.6585693359375 0 1538.0071 y 2
1363.6671142578125 0 2393.3975
1387.7305908203125 0 5757.3633
1388.7381591796875 0 4495.9653
1389.735107421875 0 1243.0885
1390.74365234375 0 938.4046
1429.72021484375 0 2031.2435
1430.72900390625 0 1600.6558
1431.7431640625 0 74579.87 c 13
1432.74609375 0 61479.523
1433.7476806640625 0 26684.25
1434.750244140625 0 7205.5986
1435.76123046875 0 1346.9144
1449.732421875 0 755.4655
1459.73193359375 0 884.156 z 1
1460.7296142578125 0 3801.3157
1461.7391357421875 0 2944.081
1462.7491455078125 0 1371.2255
1473.73486328125 0 689.59033
1474.742919921875 0 695.1521
1475.7449951171875 0 658.1723 y 1
1487.74853515625 0 943.5632
1488.74169921875 0 6952.349
1489.745849609375 0 6571.6733
1490.7525634765625 0 3868.2046
1491.7567138671875 0 1770.8824
1512.7860107421875 0 1483.2006
1513.7950439453125 0 1674.4878
1514.7864990234375 0 2139.1523
1515.781982421875 0 987.1038
1516.74365234375 0 969.48987
1528.7781982421875 0 1209.083
1529.78662109375 0 2108.3765
1530.792236328125 0 16138.669
1531.794677734375 0 20578.182
1532.7974853515625 0 13268.669
1533.795166015625 0 4383.442
1534.8060302734375 0 971.8458
1543.8074951171875 0 1650.5387
1544.8258056640625 0 3726.6902
1545.8251953125 0 7158.8154
1546.82373046875 0 7139.7305
1547.8218994140625 0 8736.544
1548.81982421875 0 4783.7476
1549.818359375 0 2462.615
1555.81494140625 0 2075.77
1556.7957763671875 0 8358.168
1557.79736328125 0 5226.5557
1558.7938232421875 0 2766.6653
1559.8062744140625 0 1792.5579
1560.824462890625 0 861.12024
1561.833984375 0 3417.6077
1562.8465576171875 0 11818.269
1563.8492431640625 0 8558.93
1564.8553466796875 0 5706.2104
1565.849853515625 0 1084.5669
1569.7979736328125 0 1216.4431
1570.7950439453125 0 2033.9335
1571.799560546875 0 4715.6353
1572.81494140625 0 17667.723
1573.8167724609375 0 102266.836
1574.81982421875 0 79364.7
1575.821044921875 0 39339.89
1576.82275390625 0 13223.442
1577.8271484375 0 3583.4824
1586.805419921875 0 4284.4326
1587.8126220703125 0 7710.6724
1588.823486328125 0 13958.646
1589.59033203125 0 1092.9174
1589.83203125 0 34727.113
1590.8411865234375 0 102228.79
1591.843994140625 0 76811.69
1592.8470458984375 0 38654.516
1593.849853515625 0 10424.187
1594.8428955078125 0 2902.4844
2209.994384765625 0 635.48724

Spectrum Details

|  |  |
| --- | --- |
| Matched peaks? Matched peaksThe total absolute number of peaks matched. Additionally in brackets the total fraction of peaks matched and the total number of peaks is shown. | 84 (17.14% of 490) |
| FDR? FDRThe false discovery rate estimated for this peptide. It is calculated by matching all theoretical fragments with a non-integer shift with the raw peaks for this spectrum. This is done with 40 different shifts. The resulting percentage is the average number of annotated peaks over the number of annotated peaks with the correct spectrum. | 0.88% |
| Satellite FDR? Satellite FDRSee the FDR for details on its calculation. This satellite ion specific FDR only contains the satellite ions (d/w) for I/L/J positions. | 0.00% |
| PSM Score? PSM ScoreThe PSM Score as given by Hecklib to this annotated spectrum. It is shown with three significant figures. | 656 |

## Spectrum 4542? Spectrum 4542 The raw spectrum of this peptide as annotated by Hecklib. The fragments are coloured according to ion type (see legend). Any peaks with a star '\*' as text can be hovered over to see the full details, first the ion type second the mass shift type. By hovering over the amino acids in the peptide or ions in the legend the corresponding peaks are highlighted. By toggling the 'Unassigned' label you can turn the background (unassigned) peaks on or off in the plot. By updating the slider in the Ion legend you can update the spectrum to only show the top X% of the peaks with labels. The top X% means any peak that is within X% of the highest intensity. By dragging in the spectrum you can zoom in to a specific part of the spectrum and use 'Zoom Out' to get back to the original zoom level. The annotation of the spectrum is based on the given sequence in the peptides file and is done with different software so inconsistencies are likely. The peaks are annotated based on the given sequence, with 20 ppm tolerance.

Copy Data

### Spectrum 4542 (TSV)

#### Preview

```
Loading example...
```

*Click on the button to copy the data to your clipboard.*

Mz MinMz MaxIntensity Max

WidthHeightPeptide font sizePeptide stroke widthSpectrum font sizeSpectrum stroke widthCompact peptide

Ion legend

wxyz

abcd

OtherUnassignedIonChargePositionShow for top:%

JJSEVSDRPSGVSSR

01.36e+42.73e+44.09e+45.45e+4

Zoom Out

y+11w+12y+12z+12y+12w+13y+13z+13y+13z+28w+14y+28z+14y+14c+14c+314z+29z+15y+15y+210z+210y+210c+15z+211y+211y+16y+16z+16w+212z+212y+212c+16w+213y+213y+213z+213y+213y+17z+214y+214c+17z+18z+18y+18c+18z+19z+19c+19z+110y+110c+110z+111y+111c+111c+112z+112y+112z+113c+113c+114

0864172925933458

Fragment Matches Table

Show background peaks

| Position | Ion type | Intensity | mz Theoretical | mz Error (Th) | mz Error (ppm) | Charge | Series Number |
| --- | --- | --- | --- | --- | --- | --- | --- |
| - | - | 1064 | 120.1 | - | - | 0 | - |
| - | - | 785.6 | 125.1 | - | - | 0 | - |
| - | - | 336.2 | 125.8 | - | - | 0 | - |
| - | - | 1807 | 126.1 | - | - | 0 | - |
| - | - | 790.1 | 131.1 | - | - | 0 | - |
| - | - | 395.7 | 132.6 | - | - | 0 | - |
| - | - | 558.1 | 133.1 | - | - | 0 | - |
| - | - | 392.6 | 134.7 | - | - | 0 | - |
| - | - | 682.5 | 136.1 | - | - | 0 | - |
| - | - | 446.5 | 143.1 | - | - | 0 | - |
| - | - | 419.9 | 153.5 | - | - | 0 | - |
| - | - | 1858 | 168.1 | - | - | 0 | - |
| - | - | 469.7 | 168.5 | - | - | 0 | - |
| - | - | 438.3 | 172.5 | - | - | 0 | - |
| - | - | 528.8 | 174.1 | - | - | 0 | - |
| - | - | 484.7 | 174.6 | - | - | 0 | - |
| 15 | y | 3195 | 175.1 | 0.000219 | 1.25 | +1 | 1 |
| - | - | 485.7 | 178.2 | - | - | 0 | - |
| - | - | 7348 | 185.1 | - | - | 0 | - |
| - | - | 679.2 | 186.1 | - | - | 0 | - |
| - | - | 1.795E+04 | 199.2 | - | - | 0 | - |
| - | - | 1262 | 200.1 | - | - | 0 | - |
| - | - | 1363 | 200.2 | - | - | 0 | - |
| - | - | 552.6 | 212.1 | - | - | 0 | - |
| - | - | 626.4 | 215.1 | - | - | 0 | - |
| - | - | 916.9 | 217.1 | - | - | 0 | - |
| - | - | 471.2 | 223 | - | - | 0 | - |
| - | - | 8423 | 227.2 | - | - | 0 | - |
| - | - | 1385 | 228.2 | - | - | 0 | - |
| 14 | w | 1970 | 229.1 | 0.0006101 | 2.663 | +1 | 2 |
| - | - | 640.9 | 234.1 | - | - | 0 | - |
| 14 | y | 1.204E+04 | 245.1 | 0.0003549 | 1.448 | +1 | 2 |
| 14 | z | 1787 | 246.1 | 0.001595 | 6.482 | +1 | 2 |
| - | - | 462.7 | 255.1 | - | - | 0 | - |
| - | - | 677.6 | 261.2 | - | - | 0 | - |
| 14 | y | 2297 | 262.2 | 0.0003561 | 1.358 | +1 | 2 |
| - | - | 2433 | 270.1 | - | - | 0 | - |
| - | - | 816.8 | 272.2 | - | - | 0 | - |
| - | - | 514.4 | 300.1 | - | - | 0 | - |
| - | - | 885.7 | 304.2 | - | - | 0 | - |
| - | - | 1020 | 314.2 | - | - | 0 | - |
| 13 | w | 6985 | 316.2 | 0.00032 | 1.012 | +1 | 3 |
| - | - | 1071 | 317.2 | - | - | 0 | - |
| - | - | 976.5 | 331.1 | - | - | 0 | - |
| 13 | y | 2322 | 332.2 | 0.0004615 | 1.389 | +1 | 3 |
| 13 | z | 4723 | 333.2 | 0.0001743 | 0.5232 | +1 | 3 |
| - | - | 612.9 | 334.2 | - | - | 0 | - |
| 13 | y | 1993 | 349.2 | 0.0004627 | 1.325 | +1 | 3 |
| - | - | 3705 | 357.2 | - | - | 0 | - |
| - | - | 690.7 | 391.7 | - | - | 0 | - |
| 8 | z | 1883 | 415.2 | 0.003502 | 8.433 | +2 | 8 |
| 12 | w | 4558 | 417.2 | 0.0007677 | 1.84 | +1 | 4 |
| - | - | 1096 | 418.2 | - | - | 0 | - |
| 8 | y | 1141 | 423.2 | 0.0002146 | 0.507 | +2 | 8 |
| - | - | 903.5 | 425.2 | - | - | 0 | - |
| 12 | z | 743.8 | 432.2 | 0.002047 | 4.736 | +1 | 4 |
| - | - | 1432 | 443.2 | - | - | 0 | - |
| 12 | y | 4712 | 448.3 | 0.0007438 | 1.659 | +1 | 4 |
| - | - | 1184 | 449.3 | - | - | 0 | - |
| - | - | 553.9 | 453.1 | - | - | 0 | - |
| - | - | 602.7 | 453.3 | - | - | 0 | - |
| 4 | c | 3755 | 460.3 | 0.0009213 | 2.002 | +1 | 4 |
| - | - | 844.9 | 461.3 | - | - | 0 | - |
| 14 | c | 1346 | 472.2 | 0.008233 | 17.43 | +3 | 14 |
| 7 | z | 1034 | 472.7 | 0.000986 | 2.086 | +2 | 9 |
| - | - | 1084 | 474.2 | - | - | 0 | - |
| - | - | 722.2 | 475.2 | - | - | 0 | - |
| 11 | z | 1481 | 489.3 | 0.001117 | 2.283 | +1 | 5 |
| - | - | 1205 | 490.3 | - | - | 0 | - |
| - | - | 1102 | 495.9 | - | - | 0 | - |
| - | - | 684.7 | 499.3 | - | - | 0 | - |
| 11 | y | 1.658E+04 | 505.3 | 0.0005814 | 1.151 | +1 | 5 |
| - | - | 4431 | 506.3 | - | - | 0 | - |
| - | - | 852.9 | 507.3 | - | - | 0 | - |
| - | - | 670 | 515.3 | - | - | 0 | - |
| 6 | y | 1000 | 515.7 | 0.001564 | 3.032 | +2 | 10 |
| 6 | z | 1858 | 516.3 | 0.001665 | 3.225 | +2 | 10 |
| - | - | 736.1 | 516.3 | - | - | 0 | - |
| - | - | 1438 | 516.8 | - | - | 0 | - |
| - | - | 693.4 | 517.3 | - | - | 0 | - |
| - | - | 648.8 | 517.8 | - | - | 0 | - |
| 6 | y | 6563 | 524.3 | 0.002557 | 4.877 | +2 | 10 |
| - | - | 1202 | 524.6 | - | - | 0 | - |
| - | - | 2784 | 524.8 | - | - | 0 | - |
| - | - | 729.6 | 525.3 | - | - | 0 | - |
| - | - | 1023 | 528.7 | - | - | 0 | - |
| - | - | 842 | 529.2 | - | - | 0 | - |
| - | - | 1085 | 529.3 | - | - | 0 | - |
| - | - | 687.9 | 529.7 | - | - | 0 | - |
| - | - | 1063 | 530.3 | - | - | 0 | - |
| - | - | 8802 | 530.3 | - | - | 0 | - |
| - | - | 1776 | 530.6 | - | - | 0 | - |
| - | - | 951 | 530.7 | - | - | 0 | - |
| - | - | 3172 | 531.3 | - | - | 0 | - |
| - | - | 617.2 | 542.3 | - | - | 0 | - |
| - | - | 1334 | 542.3 | - | - | 0 | - |
| - | - | 620.2 | 543.3 | - | - | 0 | - |
| - | - | 1058 | 558.3 | - | - | 0 | - |
| 5 | c | 3184 | 559.3 | 0.0002869 | 0.513 | +1 | 5 |
| - | - | 1143 | 560.4 | - | - | 0 | - |
| 5 | z | 1.167E+04 | 565.8 | 0.0002949 | 0.5212 | +2 | 11 |
| - | - | 7370 | 566.3 | - | - | 0 | - |
| - | - | 2407 | 566.8 | - | - | 0 | - |
| - | - | 732.9 | 567.3 | - | - | 0 | - |
| 5 | y | 6383 | 573.8 | 0.0001491 | 0.2599 | +2 | 11 |
| 10 | y | 2998 | 574.3 | 0.003806 | 6.628 | +1 | 6 |
| - | - | 1678 | 574.8 | - | - | 0 | - |
| 10 | y | 1398 | 575.3 | 7.645E-05 | 0.1329 | +1 | 6 |
| 10 | z | 3538 | 576.3 | 0.0009126 | 1.584 | +1 | 6 |
| - | - | 660.2 | 586.3 | - | - | 0 | - |
| - | - | 1298 | 600.3 | - | - | 0 | - |
| 4 | w | 4981 | 600.8 | 0.0006651 | 1.107 | +2 | 12 |
| - | - | 3207 | 601.3 | - | - | 0 | - |
| - | - | 1407 | 601.8 | - | - | 0 | - |
| 4 | z | 1.14E+04 | 630.3 | 0.0002996 | 0.4753 | +2 | 12 |
| - | - | 9628 | 630.8 | - | - | 0 | - |
| - | - | 4076 | 631.3 | - | - | 0 | - |
| - | - | 1563 | 631.8 | - | - | 0 | - |
| - | - | 697.7 | 632.3 | - | - | 0 | - |
| - | - | 6018 | 637.8 | - | - | 0 | - |
| 4 | y | 1.119E+04 | 638.3 | 0.0005786 | 0.9064 | +2 | 12 |
| - | - | 8144 | 638.8 | - | - | 0 | - |
| - | - | 2597 | 639.3 | - | - | 0 | - |
| - | - | 1095 | 639.8 | - | - | 0 | - |
| - | - | 1284 | 644.3 | - | - | 0 | - |
| 6 | c | 1.167E+04 | 646.4 | 0.0006682 | 1.034 | +1 | 6 |
| - | - | 791.6 | 647.3 | - | - | 0 | - |
| - | - | 4528 | 647.4 | - | - | 0 | - |
| - | - | 661.5 | 648.4 | - | - | 0 | - |
| 3 | w | 1140 | 665.3 | 0.002623 | 3.942 | +2 | 13 |
| 3 | y | 1466 | 672.8 | 0.0006903 | 1.026 | +2 | 13 |
| 3 | y | 1007 | 673.3 | 0.008401 | 12.48 | +2 | 13 |
| 3 | z | 9011 | 673.8 | 0.0003987 | 0.5916 | +2 | 13 |
| - | - | 8275 | 674.3 | - | - | 0 | - |
| - | - | 4739 | 674.8 | - | - | 0 | - |
| - | - | 1683 | 675.3 | - | - | 0 | - |
| - | - | 941.4 | 681.3 | - | - | 0 | - |
| 3 | y | 5.396E+04 | 681.8 | 0.000314 | 0.4605 | +2 | 13 |
| - | - | 3.465E+04 | 682.3 | - | - | 0 | - |
| - | - | 1.692E+04 | 682.8 | - | - | 0 | - |
| - | - | 4081 | 683.3 | - | - | 0 | - |
| - | - | 1128 | 683.8 | - | - | 0 | - |
| 9 | y | 1817 | 689.4 | 0.0001349 | 0.1958 | +1 | 7 |
| - | - | 827 | 701.4 | - | - | 0 | - |
| - | - | 1089 | 716.3 | - | - | 0 | - |
| - | - | 1029 | 717.4 | - | - | 0 | - |
| - | - | 1866 | 718.4 | - | - | 0 | - |
| - | - | 652.3 | 726.3 | - | - | 0 | - |
| 2 | z | 883.7 | 730.4 | 0.001716 | 2.35 | +2 | 14 |
| 2 | y | 3077 | 738.4 | 0.0005193 | 0.7033 | +2 | 14 |
| - | - | 3038 | 738.9 | - | - | 0 | - |
| - | - | 1349 | 739.4 | - | - | 0 | - |
| - | - | 933.3 | 739.9 | - | - | 0 | - |
| - | - | 1044 | 744.4 | - | - | 0 | - |
| - | - | 867.7 | 745.4 | - | - | 0 | - |
| - | - | 1662 | 750.9 | - | - | 0 | - |
| - | - | 1504 | 758.4 | - | - | 0 | - |
| - | - | 1220 | 758.9 | - | - | 0 | - |
| - | - | 1045 | 760.4 | - | - | 0 | - |
| 7 | c | 2.144E+04 | 761.4 | 0.0004586 | 0.6023 | +1 | 7 |
| - | - | 7743 | 762.4 | - | - | 0 | - |
| - | - | 2403 | 763.4 | - | - | 0 | - |
| - | - | 1138 | 764.4 | - | - | 0 | - |
| - | - | 1499 | 764.9 | - | - | 0 | - |
| - | - | 3.673E+04 | 765.4 | - | - | 0 | - |
| - | - | 3.16E+04 | 765.9 | - | - | 0 | - |
| - | - | 1.692E+04 | 766.4 | - | - | 0 | - |
| - | - | 5391 | 766.9 | - | - | 0 | - |
| - | - | 1454 | 767.4 | - | - | 0 | - |
| - | - | 1778 | 772.4 | - | - | 0 | - |
| - | - | 915.9 | 772.9 | - | - | 0 | - |
| - | - | 838.9 | 773.4 | - | - | 0 | - |
| - | - | 1.116E+04 | 773.9 | - | - | 0 | - |
| - | - | 1.016E+04 | 774.4 | - | - | 0 | - |
| - | - | 4187 | 774.9 | - | - | 0 | - |
| - | - | 1383 | 775.4 | - | - | 0 | - |
| - | - | 1167 | 777.9 | - | - | 0 | - |
| - | - | 1177 | 778.4 | - | - | 0 | - |
| - | - | 769.7 | 778.9 | - | - | 0 | - |
| - | - | 686 | 780.4 | - | - | 0 | - |
| - | - | 832.2 | 782.4 | - | - | 0 | - |
| - | - | 776.9 | 785.4 | - | - | 0 | - |
| - | - | 962.3 | 785.9 | - | - | 0 | - |
| - | - | 4458 | 786.4 | - | - | 0 | - |
| - | - | 4.249E+04 | 786.9 | - | - | 0 | - |
| - | - | 3.604E+04 | 787.4 | - | - | 0 | - |
| - | - | 1.609E+04 | 787.9 | - | - | 0 | - |
| - | - | 5013 | 788.4 | - | - | 0 | - |
| - | - | 2260 | 788.9 | - | - | 0 | - |
| - | - | 624.3 | 792.9 | - | - | 0 | - |
| - | - | 1807 | 793.9 | - | - | 0 | - |
| - | - | 2872 | 794.4 | - | - | 0 | - |
| - | - | 2.536E+04 | 794.9 | - | - | 0 | - |
| - | - | 3.255E+04 | 795.4 | - | - | 0 | - |
| - | - | 2.197E+04 | 795.9 | - | - | 0 | - |
| - | - | 8829 | 796.4 | - | - | 0 | - |
| - | - | 2732 | 796.9 | - | - | 0 | - |
| - | - | 806.3 | 797.4 | - | - | 0 | - |
| 8 | z | 994.7 | 811.4 | 0.003299 | 4.066 | +1 | 8 |
| 8 | z | 1.827E+04 | 829.4 | 0.0002465 | 0.2972 | +1 | 8 |
| - | - | 9942 | 830.4 | - | - | 0 | - |
| - | - | 2659 | 831.4 | - | - | 0 | - |
| - | - | 1053 | 844.5 | - | - | 0 | - |
| 8 | y | 2701 | 845.5 | 0.0009042 | 1.069 | +1 | 8 |
| - | - | 1196 | 846.5 | - | - | 0 | - |
| - | - | 1862 | 873.5 | - | - | 0 | - |
| 8 | c | 2365 | 900.5 | 0.0006781 | 0.753 | +1 | 8 |
| - | - | 1539 | 901.5 | - | - | 0 | - |
| 7 | z | 754.7 | 926.5 | 0.006324 | 6.826 | +1 | 9 |
| 7 | z | 6618 | 944.5 | 0.0007003 | 0.7415 | +1 | 9 |
| - | - | 6335 | 945.5 | - | - | 0 | - |
| - | - | 2328 | 946.5 | - | - | 0 | - |
| - | - | 1616 | 970.5 | - | - | 0 | - |
| - | - | 640.8 | 1014 | - | - | 0 | - |
| 9 | c | 1.53E+04 | 1015 | 0.0005754 | 0.5671 | +1 | 9 |
| - | - | 8937 | 1016 | - | - | 0 | - |
| - | - | 901 | 1016 | - | - | 0 | - |
| - | - | 2622 | 1017 | - | - | 0 | - |
| 6 | z | 2238 | 1031 | 0.006728 | 6.522 | +1 | 10 |
| - | - | 3699 | 1033 | - | - | 0 | - |
| - | - | 1954 | 1034 | - | - | 0 | - |
| - | - | 878.6 | 1035 | - | - | 0 | - |
| - | - | 926.5 | 1040 | - | - | 0 | - |
| - | - | 707.1 | 1043 | - | - | 0 | - |
| 6 | y | 1200 | 1048 | 0.001526 | 1.457 | +1 | 10 |
| - | - | 803.3 | 1049 | - | - | 0 | - |
| - | - | 682 | 1057 | - | - | 0 | - |
| - | - | 1.202E+04 | 1058 | - | - | 0 | - |
| - | - | 685 | 1058 | - | - | 0 | - |
| - | - | 6605 | 1059 | - | - | 0 | - |
| - | - | 3226 | 1060 | - | - | 0 | - |
| - | - | 726.8 | 1061 | - | - | 0 | - |
| - | - | 681 | 1062 | - | - | 0 | - |
| - | - | 765.6 | 1088 | - | - | 0 | - |
| - | - | 840.6 | 1101 | - | - | 0 | - |
| 10 | c | 2.127E+04 | 1102 | 0.000142 | 0.1289 | +1 | 10 |
| - | - | 1.361E+04 | 1103 | - | - | 0 | - |
| - | - | 5487 | 1104 | - | - | 0 | - |
| - | - | 1072 | 1105 | - | - | 0 | - |
| - | - | 3892 | 1115 | - | - | 0 | - |
| - | - | 1884 | 1116 | - | - | 0 | - |
| - | - | 1105 | 1117 | - | - | 0 | - |
| 5 | z | 3877 | 1131 | 0.0001757 | 0.1554 | +1 | 11 |
| - | - | 7682 | 1132 | - | - | 0 | - |
| - | - | 3914 | 1133 | - | - | 0 | - |
| - | - | 1003 | 1134 | - | - | 0 | - |
| 5 | y | 1601 | 1147 | 0.004876 | 4.253 | +1 | 11 |
| 11 | c | 1.351E+04 | 1159 | 0.0003669 | 0.3167 | +1 | 11 |
| - | - | 8508 | 1160 | - | - | 0 | - |
| - | - | 2746 | 1161 | - | - | 0 | - |
| - | - | 850.8 | 1162 | - | - | 0 | - |
| - | - | 1895 | 1214 | - | - | 0 | - |
| - | - | 2222 | 1215 | - | - | 0 | - |
| - | - | 1145 | 1216 | - | - | 0 | - |
| 12 | c | 1.348E+04 | 1258 | 0.002617 | 2.081 | +1 | 12 |
| - | - | 7244 | 1259 | - | - | 0 | - |
| 4 | z | 2675 | 1260 | 0.005678 | 4.508 | +1 | 12 |
| - | - | 2596 | 1260 | - | - | 0 | - |
| - | - | 1.155E+04 | 1261 | - | - | 0 | - |
| - | - | 7166 | 1262 | - | - | 0 | - |
| - | - | 3072 | 1263 | - | - | 0 | - |
| - | - | 992.8 | 1264 | - | - | 0 | - |
| 4 | y | 4337 | 1276 | 0.001937 | 1.519 | +1 | 12 |
| - | - | 3055 | 1277 | - | - | 0 | - |
| - | - | 1091 | 1278 | - | - | 0 | - |
| - | - | 1866 | 1301 | - | - | 0 | - |
| - | - | 1663 | 1302 | - | - | 0 | - |
| - | - | 1564 | 1303 | - | - | 0 | - |
| - | - | 698.6 | 1305 | - | - | 0 | - |
| 3 | z | 962.5 | 1330 | 0.01436 | 10.8 | +1 | 13 |
| - | - | 1540 | 1331 | - | - | 0 | - |
| 13 | c | 2.284E+04 | 1345 | 0.001931 | 1.436 | +1 | 13 |
| - | - | 1.719E+04 | 1346 | - | - | 0 | - |
| - | - | 7177 | 1347 | - | - | 0 | - |
| - | - | 1.083E+04 | 1348 | - | - | 0 | - |
| - | - | 7765 | 1349 | - | - | 0 | - |
| - | - | 3446 | 1350 | - | - | 0 | - |
| - | - | 1483 | 1351 | - | - | 0 | - |
| - | - | 921.3 | 1364 | - | - | 0 | - |
| - | - | 631.2 | 1373 | - | - | 0 | - |
| - | - | 1959 | 1388 | - | - | 0 | - |
| - | - | 1404 | 1389 | - | - | 0 | - |
| 14 | c | 3.006E+04 | 1432 | 0.001977 | 1.381 | +1 | 14 |
| - | - | 2.523E+04 | 1433 | - | - | 0 | - |
| - | - | 1.254E+04 | 1434 | - | - | 0 | - |
| - | - | 3596 | 1435 | - | - | 0 | - |
| - | - | 705.5 | 1436 | - | - | 0 | - |
| - | - | 1085 | 1461 | - | - | 0 | - |
| - | - | 1177 | 1462 | - | - | 0 | - |
| - | - | 2936 | 1489 | - | - | 0 | - |
| - | - | 2630 | 1490 | - | - | 0 | - |
| - | - | 775.5 | 1491 | - | - | 0 | - |
| - | - | 892.5 | 1492 | - | - | 0 | - |
| - | - | 661.1 | 1513 | - | - | 0 | - |
| - | - | 1731 | 1514 | - | - | 0 | - |
| - | - | 1033 | 1515 | - | - | 0 | - |
| - | - | 1374 | 1530 | - | - | 0 | - |
| - | - | 6939 | 1531 | - | - | 0 | - |
| - | - | 8404 | 1532 | - | - | 0 | - |
| - | - | 6219 | 1533 | - | - | 0 | - |
| - | - | 2136 | 1534 | - | - | 0 | - |
| - | - | 1500 | 1545 | - | - | 0 | - |
| - | - | 3829 | 1546 | - | - | 0 | - |
| - | - | 3730 | 1547 | - | - | 0 | - |
| - | - | 4690 | 1548 | - | - | 0 | - |
| - | - | 2173 | 1549 | - | - | 0 | - |
| - | - | 1157 | 1550 | - | - | 0 | - |
| - | - | 792 | 1556 | - | - | 0 | - |
| - | - | 2753 | 1557 | - | - | 0 | - |
| - | - | 2460 | 1558 | - | - | 0 | - |
| - | - | 1742 | 1559 | - | - | 0 | - |
| - | - | 736.7 | 1560 | - | - | 0 | - |
| - | - | 973.4 | 1562 | - | - | 0 | - |
| - | - | 5060 | 1563 | - | - | 0 | - |
| - | - | 4883 | 1564 | - | - | 0 | - |
| - | - | 2073 | 1565 | - | - | 0 | - |
| - | - | 857.9 | 1571 | - | - | 0 | - |
| - | - | 2301 | 1572 | - | - | 0 | - |
| - | - | 9472 | 1573 | - | - | 0 | - |
| - | - | 4.499E+04 | 1574 | - | - | 0 | - |
| - | - | 3.597E+04 | 1575 | - | - | 0 | - |
| - | - | 1.849E+04 | 1576 | - | - | 0 | - |
| - | - | 5373 | 1577 | - | - | 0 | - |
| - | - | 1012 | 1578 | - | - | 0 | - |
| - | - | 1160 | 1587 | - | - | 0 | - |
| - | - | 1467 | 1588 | - | - | 0 | - |
| - | - | 5104 | 1589 | - | - | 0 | - |
| - | - | 1.265E+04 | 1590 | - | - | 0 | - |
| - | - | 4.687E+04 | 1591 | - | - | 0 | - |
| - | - | 3.624E+04 | 1592 | - | - | 0 | - |
| - | - | 1.709E+04 | 1593 | - | - | 0 | - |
| - | - | 6880 | 1594 | - | - | 0 | - |
| - | - | 1595 | 1595 | - | - | 0 | - |
| - | - | 912.1 | 2686 | - | - | 0 | - |
| - | - | 679.8 | 2960 | - | - | 0 | - |
| - | - | 644.3 | 3423 | - | - | 0 | - |

m/z Charge Intensity FragmentType MassShift Position
120.08113861083984 0 1063.5834
125.1076889038086 0 785.59265
125.81410217285156 0 336.20172
126.09162139892578 0 1806.8712
131.1295928955078 0 790.1039
132.63450622558594 0 395.67914
133.06106567382812 0 558.0703
134.6832733154297 0 392.56274
136.07565307617188 0 682.51044
143.1181182861328 0 446.5395
153.5372772216797 0 419.8835
168.1134490966797 0 1857.7833
168.52410888671875 0 469.6549
172.45437622070312 0 438.3173
174.05638122558594 0 528.7767
174.59815979003906 0 484.7445
175.11917114257812 0 3194.9915 y 14
178.2040557861328 0 485.73953
185.13990783691406 0 7347.7974
186.14328002929688 0 679.1589
199.18072509765625 0 17952.836
200.12681579589844 0 1261.7703
200.18405151367188 0 1362.9314
212.10348510742188 0 552.5956
215.1394500732422 0 626.44055
217.08204650878906 0 916.9089
223.01121520996094 0 471.206
227.1755828857422 0 8423.145
228.17921447753906 0 1384.8506
229.130126953125 0 1970.0186 w 13
234.11013793945312 0 640.91614
245.12478637695312 0 12036.955 y Ammonia loss 13
246.1306610107422 0 1786.536 z 13
255.11033630371094 0 462.6819
261.1585998535156 0 677.59766
262.1513366699219 0 2296.624 y 13
270.1196594238281 0 2433.3413
272.1726379394531 0 816.8088
300.1042785644531 0 514.3711
304.15106201171875 0 885.6721
314.2077941894531 0 1020.2051
316.161865234375 0 6984.5195 w 12
317.1592712402344 0 1071.4617
331.14923095703125 0 976.48016
332.15692138671875 0 2321.6445 y Ammonia loss 12
333.1644592285156 0 4722.9834 z 12
334.1680908203125 0 612.9287
349.1834716796875 0 1993.1423 y 12
357.1522216796875 0 3704.9634
391.7240295410156 0 690.69354
415.2271728515625 0 1883.4296 z 7
417.2099914550781 0 4557.7505 w 11
418.21087646484375 0 1095.8864
423.2328186035156 0 1140.9114 y 7
425.2386169433594 0 903.46466
432.23065185546875 0 743.8173 z 11
443.25 0 1431.9674
448.2521667480469 0 4711.899 y 11
449.25604248046875 0 1183.6289
453.0549621582031 0 553.92926
453.3111572265625 0 602.65405
460.2774963378906 0 3754.882 c 3
461.2821960449219 0 844.86194
472.2521667480469 0 1346.406 c Ammonia loss 13
472.7381286621094 0 1033.9417 z 6
474.2333984375 0 1083.5828
475.2345886230469 0 722.1969
489.2552795410156 0 1481.169 z 10
490.2593994140625 0 1205.4421
495.9162902832031 0 1101.6194
499.29736328125 0 684.7206
505.2734680175781 0 16579.162 y 10
506.276611328125 0 4430.575
507.2797546386719 0 852.9177
515.3319091796875 0 669.9666
515.7476806640625 0 1000.3957 y Ammonia loss 5
516.2548217773438 0 1858.029 z 5
516.3421020507812 0 736.0524
516.75537109375 0 1437.825
517.2600708007812 0 693.4367
517.758544921875 0 648.76904
524.2650756835938 0 6562.906 y 5
524.6126098632812 0 1202.4779
524.7653198242188 0 2784.0515
525.269775390625 0 729.6492
528.7394409179688 0 1023.0707
529.2383422851562 0 841.9943
529.295166015625 0 1084.7207
529.74072265625 0 687.86035
530.2783813476562 0 1062.9757
530.3176879882812 0 8801.727
530.6156005859375 0 1776.4585
530.7276000976562 0 950.9782
531.3212280273438 0 3172.0764
542.2679443359375 0 617.18933
542.3201293945312 0 1334.1582
543.3198852539062 0 620.1592
558.3333740234375 0 1057.5874
559.3452758789062 0 3184.4658 c 4
560.3504028320312 0 1143.4869
565.7876586914062 0 11666.435 z 4
566.2890625 0 7370.444
566.7897338867188 0 2407.0442
567.2885131835938 0 732.8707
573.796875 0 6383.3384 y 4
574.2981567382812 0 2998.487 y Water loss 9
574.7977294921875 0 1677.5276
575.2784423828125 0 1398.154 y Ammonia loss 9
576.2852783203125 0 3537.6729 z 9
586.32861328125 0 660.1767
600.2760009765625 0 1297.9772
600.8026733398438 0 4980.6665 w 3
601.3030395507812 0 3206.8716
601.8067016601562 0 1406.7598
630.3089599609375 0 11395.949 z 3
630.8107299804688 0 9628.229
631.3115234375 0 4075.8098
631.816162109375 0 1562.9332
632.3164672851562 0 697.7388
637.8145141601562 0 6018.336
638.3174438476562 0 11186.349 y 3
638.8192138671875 0 8143.794
639.3197021484375 0 2596.9512
639.8226318359375 0 1094.5508
644.3170776367188 0 1284.068
646.377685546875 0 11674.01 c 5
647.3226318359375 0 791.6261
647.3810424804688 0 4528.0396
648.384765625 0 661.4692
665.325927734375 0 1139.7994 w 2
672.8280639648438 0 1466.1256 y Water loss 2
673.3291625976562 0 1006.8237 y Ammonia loss 2
673.8250732421875 0 9011.227 z 2
674.3279418945312 0 8274.712
674.8285522460938 0 4739.0215
675.33154296875 0 1683.1346
681.3338623046875 0 941.35767
681.8343505859375 0 53963.434 y 2
682.335693359375 0 34652.66
682.837158203125 0 16922.514
683.3390502929688 0 4080.9492
683.8446655273438 0 1128.337
689.3575439453125 0 1817.388 y 8
701.3571166992188 0 827.0466
716.3495483398438 0 1088.5117
717.3902587890625 0 1028.7279
718.3983764648438 0 1865.7914
726.3145751953125 0 652.31305
730.364990234375 0 883.65643 z 1
738.3755493164062 0 3076.5542 y 1
738.8782348632812 0 3038.0703
739.3807983398438 0 1348.7874
739.8802490234375 0 933.2531
744.368408203125 0 1044.4829
745.3787231445312 0 867.6767
750.8987426757812 0 1661.9097
758.3870849609375 0 1503.7246
758.880615234375 0 1220.3901
760.3862915039062 0 1044.7915
761.4044189453125 0 21440.012 c 6
762.4068603515625 0 7743.231
763.4087524414062 0 2402.7273
764.4144287109375 0 1138.2214
764.9066162109375 0 1499.4388
765.3822021484375 0 36730.77
765.8834838867188 0 31599.809
766.384521484375 0 16916.047
766.8858032226562 0 5390.962
767.3851318359375 0 1454.3445
772.4082641601562 0 1778.4147
772.920654296875 0 915.8799
773.4042358398438 0 838.8854
773.9072875976562 0 11161.767
774.408203125 0 10157.737
774.9095458984375 0 4187.1
775.4092407226562 0 1383.165
777.9007568359375 0 1166.7135
778.4090576171875 0 1177.2521
778.9083862304688 0 769.67584
780.4071655273438 0 685.99274
782.4390869140625 0 832.15186
785.4033203125 0 776.8548
785.9026489257812 0 962.3309
786.4116821289062 0 4457.772
786.9096069335938 0 42486.01
787.4107055664062 0 36040.94
787.9110107421875 0 16086.465
788.4116821289062 0 5013.014
788.91064453125 0 2260.102
792.8966064453125 0 624.3255
793.908203125 0 1807.1106
794.4107055664062 0 2872.2976
794.917724609375 0 25361.164
795.4202270507812 0 32547.658
795.9217529296875 0 21972.367
796.418701171875 0 8828.599
796.905517578125 0 2732.4492
797.4158935546875 0 806.27374
811.4328002929688 0 994.71735 z Water loss 7
829.4398193359375 0 18267.615 z 7
830.4442749023438 0 9941.894
831.4451904296875 0 2658.8215
844.4520874023438 0 1053.383
845.4578857421875 0 2700.8342 y 7
846.4578857421875 0 1196.3347
873.4921264648438 0 1862.1086
900.4778442382812 0 2365.0537 c Ammonia loss 7
901.475830078125 0 1538.7393
926.4627685546875 0 754.7267 z Water loss 6
944.46630859375 0 6617.5835 z 6
945.4724731445312 0 6335.17
946.4762573242188 0 2327.89
970.5435791015625 0 1616.092
1013.5791625976562 0 640.8491
1014.5584106445312 0 15297.14 c 8
1015.5621337890625 0 8936.721
1016.4609985351562 0 900.9564
1016.5651245117188 0 2622.4316
1031.4923095703125 0 2238.095 z 5
1032.5037841796875 0 3698.9363
1033.5096435546875 0 1953.7096
1034.5054931640625 0 878.56824
1039.568359375 0 926.52405
1042.5694580078125 0 707.1015
1047.519287109375 0 1199.9957 y 5
1048.5262451171875 0 803.27454
1057.4683837890625 0 682.02716
1057.5767822265625 0 12017.541
1058.4774169921875 0 684.9558
1058.578369140625 0 6605.4053
1059.5833740234375 0 3225.5881
1060.602783203125 0 726.76807
1062.4951171875 0 680.9609
1087.579833984375 0 765.5924
1100.5760498046875 0 840.5711
1101.5897216796875 0 21267.092 c 9
1102.593505859375 0 13613.798
1103.5947265625 0 5486.956
1104.596923828125 0 1071.9567
1114.596435546875 0 3892.4067
1115.6005859375 0 1883.5349
1116.6043701171875 0 1104.5033
1130.567626953125 0 3877.1511 z 4
1131.574462890625 0 7681.8374
1132.5804443359375 0 3913.52
1133.5911865234375 0 1002.875
1146.581298828125 0 1600.7458 y 4
1158.6116943359375 0 13514.403 c 10
1159.61376953125 0 8508.491
1160.6162109375 0 2745.516
1161.628662109375 0 850.8474
1213.6634521484375 0 1894.9866
1214.66748046875 0 2222.0312
1215.6788330078125 0 1145.1431
1257.6771240234375 0 13483.689 c 11
1258.680419921875 0 7243.986
1259.61572265625 0 2674.9683 z 3
1259.671875 0 2596.1655
1260.6170654296875 0 11554.451
1261.6192626953125 0 7166.0425
1262.6202392578125 0 3071.6606
1263.6240234375 0 992.7738
1275.6268310546875 0 4336.8086 y 3
1276.630126953125 0 3055.3123
1277.631591796875 0 1091.0448
1300.7000732421875 0 1865.6403
1301.70068359375 0 1663.0846
1302.7041015625 0 1563.519
1304.6209716796875 0 698.63873
1329.6298828125 0 962.53864 z Ammonia loss 2
1330.6307373046875 0 1540.2406
1344.7098388671875 0 22836.85 c 12
1345.7135009765625 0 17190.695
1346.7052001953125 0 7176.888
1347.6517333984375 0 10828.547
1348.65234375 0 7764.556
1349.65478515625 0 3446.355
1350.662109375 0 1483.1418
1363.66357421875 0 921.3224
1372.7242431640625 0 631.23083
1387.730224609375 0 1958.8832
1388.7354736328125 0 1404.4846
1431.7418212890625 0 30062.322 c 13
1432.74462890625 0 25232.066
1433.7467041015625 0 12539.822
1434.749755859375 0 3595.6948
1435.7567138671875 0 705.4524
1460.7213134765625 0 1084.6458
1461.7413330078125 0 1177.3954
1488.738525390625 0 2936.2595
1489.7454833984375 0 2630.239
1490.74658203125 0 775.4705
1491.76123046875 0 892.5222
1512.7965087890625 0 661.1061
1513.79052734375 0 1730.5127
1514.7977294921875 0 1033.1779
1529.78955078125 0 1374.4369
1530.7899169921875 0 6938.527
1531.79296875 0 8404.23
1532.7958984375 0 6218.503
1533.7958984375 0 2136.0586
1544.82666015625 0 1499.6625
1545.828369140625 0 3828.6807
1546.826416015625 0 3730.4824
1547.8148193359375 0 4689.924
1548.811767578125 0 2173.4077
1549.8046875 0 1156.839
1555.8077392578125 0 792.0335
1556.7982177734375 0 2752.5308
1557.79443359375 0 2459.541
1558.7978515625 0 1742.3625
1559.79443359375 0 736.6543
1561.83544921875 0 973.3684
1562.8446044921875 0 5060.2197
1563.8507080078125 0 4882.6357
1564.852294921875 0 2072.9792
1570.806396484375 0 857.85284
1571.800048828125 0 2300.6567
1572.8135986328125 0 9471.573
1573.8154296875 0 44985.277
1574.8179931640625 0 35965.062
1575.8194580078125 0 18486.502
1576.822021484375 0 5373.1855
1577.820556640625 0 1011.9313
1586.829833984375 0 1160.3743
1587.8134765625 0 1466.7974
1588.82373046875 0 5104.231
1589.8302001953125 0 12653.743
1590.8397216796875 0 46866.465
1591.84228515625 0 36241.23
1592.842041015625 0 17093.73
1593.83984375 0 6880.4893
1594.828125 0 1595.2063
2685.77294921875 0 912.0725
2960.20556640625 0 679.83636
3423.3251953125 0 644.2529

Spectrum Details

|  |  |
| --- | --- |
| Matched peaks? Matched peaksThe total absolute number of peaks matched. Additionally in brackets the total fraction of peaks matched and the total number of peaks is shown. | 60 (17.86% of 336) |
| FDR? FDRThe false discovery rate estimated for this peptide. It is calculated by matching all theoretical fragments with a non-integer shift with the raw peaks for this spectrum. This is done with 40 different shifts. The resulting percentage is the average number of annotated peaks over the number of annotated peaks with the correct spectrum. | 0.99% |
| Satellite FDR? Satellite FDRSee the FDR for details on its calculation. This satellite ion specific FDR only contains the satellite ions (d/w) for I/L/J positions. | ∞ |
| PSM Score? PSM ScoreThe PSM Score as given by Hecklib to this annotated spectrum. It is shown with three significant figures. | 476 |

## Spectrum 3632? Spectrum 3632 The raw spectrum of this peptide as annotated by Hecklib. The fragments are coloured according to ion type (see legend). Any peaks with a star '\*' as text can be hovered over to see the full details, first the ion type second the mass shift type. By hovering over the amino acids in the peptide or ions in the legend the corresponding peaks are highlighted. By toggling the 'Unassigned' label you can turn the background (unassigned) peaks on or off in the plot. By updating the slider in the Ion legend you can update the spectrum to only show the top X% of the peaks with labels. The top X% means any peak that is within X% of the highest intensity. By dragging in the spectrum you can zoom in to a specific part of the spectrum and use 'Zoom Out' to get back to the original zoom level. The annotation of the spectrum is based on the given sequence in the peptides file and is done with different software so inconsistencies are likely. The peaks are annotated based on the given sequence, with 20 ppm tolerance.

Copy Data

### Spectrum 3632 (TSV)

#### Preview

```
Loading example...
```

*Click on the button to copy the data to your clipboard.*

Mz MinMz MaxIntensity Max

WidthHeightPeptide font sizePeptide stroke widthSpectrum font sizeSpectrum stroke widthCompact peptide

Ion legend

wxyz

abcd

OtherUnassignedIonChargePositionShow for top:%

JJSEVSDRPSGVSSR

04.46e+78.93e+71.34e+81.79e+8

Zoom Out

y+11y+35y+11a+12b+12y+12y+12y+12b+13b+13y+13y+13y+27y+27y+13b+312y+28y+28b+14y+14y+14b+14y+14y+313y+313b+28y+313y+29b+314y+29y+314y+314y+15y+15y+314y+15y+210y+210y+210\*\*b+15b+211y+211y+211y+211y+16y+16y+212y+212y+212y+17y+17y+213y+213y+213y+17b+214y+214y+214y+214b+17\*y+18b+18b+18y+19y+19y+110y+110y+111y+113

034068110211361

Fragment Matches Table

Show background peaks

| Position | Ion type | Intensity | mz Theoretical | mz Error (Th) | mz Error (ppm) | Charge | Series Number |
| --- | --- | --- | --- | --- | --- | --- | --- |
| - | - | 3.613E+04 | 125.1 | - | - | 0 | - |
| - | - | 1.116E+06 | 126.1 | - | - | 0 | - |
| - | - | 7.714E+04 | 127.1 | - | - | 0 | - |
| - | - | 3.79E+04 | 127.1 | - | - | 0 | - |
| - | - | 4.904E+04 | 128.1 | - | - | 0 | - |
| - | - | 1.35E+05 | 129.1 | - | - | 0 | - |
| - | - | 1.941E+05 | 129.1 | - | - | 0 | - |
| - | - | 1.09E+05 | 129.1 | - | - | 0 | - |
| - | - | 7.074E+04 | 130.1 | - | - | 0 | - |
| - | - | 6.153E+05 | 130.1 | - | - | 0 | - |
| - | - | 3.4E+05 | 130.1 | - | - | 0 | - |
| - | - | 4.479E+04 | 131.1 | - | - | 0 | - |
| - | - | 5.32E+04 | 133.1 | - | - | 0 | - |
| - | - | 1.041E+05 | 133.1 | - | - | 0 | - |
| - | - | 4.079E+04 | 136 | - | - | 0 | - |
| - | - | 5.162E+04 | 138.1 | - | - | 0 | - |
| - | - | 7.81E+04 | 139.1 | - | - | 0 | - |
| - | - | 1.282E+05 | 140.1 | - | - | 0 | - |
| - | - | 5.742E+04 | 140.1 | - | - | 0 | - |
| - | - | 5.969E+04 | 141.1 | - | - | 0 | - |
| - | - | 1.387E+05 | 141.1 | - | - | 0 | - |
| - | - | 4.742E+04 | 143.1 | - | - | 0 | - |
| - | - | 3.766E+04 | 143.2 | - | - | 0 | - |
| - | - | 3.453E+05 | 144.1 | - | - | 0 | - |
| - | - | 3.763E+05 | 147.1 | - | - | 0 | - |
| - | - | 4.057E+04 | 150.7 | - | - | 0 | - |
| - | - | 1.033E+05 | 153.1 | - | - | 0 | - |
| - | - | 1.145E+06 | 154.1 | - | - | 0 | - |
| - | - | 8.463E+04 | 155.1 | - | - | 0 | - |
| - | - | 1.204E+05 | 155.1 | - | - | 0 | - |
| - | - | 9.637E+04 | 156.1 | - | - | 0 | - |
| - | - | 4.893E+05 | 157.1 | - | - | 0 | - |
| - | - | 5.35E+05 | 157.1 | - | - | 0 | - |
| - | - | 6.268E+05 | 157.1 | - | - | 0 | - |
| 15 | y | 3.008E+06 | 158.1 | 0.0003856 | 2.439 | +1 | 1 |
| - | - | 7.529E+04 | 158.1 | - | - | 0 | - |
| - | - | 1.92E+05 | 159.1 | - | - | 0 | - |
| - | - | 4.648E+05 | 159.1 | - | - | 0 | - |
| - | - | 4.289E+04 | 165.2 | - | - | 0 | - |
| - | - | 2.004E+05 | 167.1 | - | - | 0 | - |
| - | - | 4.23E+04 | 168.7 | - | - | 0 | - |
| 11 | y | 9.663E+04 | 169.1 | 0.001416 | 8.373 | +3 | 5 |
| - | - | 1.294E+05 | 170 | - | - | 0 | - |
| - | - | 6.831E+04 | 170.5 | - | - | 0 | - |
| - | - | 6.896E+04 | 170.5 | - | - | 0 | - |
| - | - | 1.413E+06 | 171.1 | - | - | 0 | - |
| - | - | 6.247E+04 | 171.2 | - | - | 0 | - |
| - | - | 1.623E+05 | 172.1 | - | - | 0 | - |
| - | - | 1.589E+05 | 172.1 | - | - | 0 | - |
| - | - | 4.51E+05 | 173.1 | - | - | 0 | - |
| - | - | 4.518E+04 | 174.1 | - | - | 0 | - |
| - | - | 8.624E+05 | 175.1 | - | - | 0 | - |
| 15 | y | 5.924E+06 | 175.1 | 0.0004021 | 2.296 | +1 | 1 |
| - | - | 4.323E+04 | 176.1 | - | - | 0 | - |
| - | - | 3.754E+05 | 176.1 | - | - | 0 | - |
| - | - | 1.074E+05 | 182.1 | - | - | 0 | - |
| - | - | 4.842E+04 | 182.2 | - | - | 0 | - |
| - | - | 1.294E+06 | 183.1 | - | - | 0 | - |
| - | - | 1.586E+05 | 184.1 | - | - | 0 | - |
| - | - | 1.209E+05 | 184.1 | - | - | 0 | - |
| - | - | 8.596E+04 | 185.1 | - | - | 0 | - |
| - | - | 6.782E+05 | 185.1 | - | - | 0 | - |
| - | - | 1.168E+05 | 185.1 | - | - | 0 | - |
| - | - | 4.687E+04 | 186.1 | - | - | 0 | - |
| - | - | 6.912E+04 | 187.1 | - | - | 0 | - |
| - | - | 9.052E+05 | 187.1 | - | - | 0 | - |
| - | - | 1.012E+05 | 188.1 | - | - | 0 | - |
| - | - | 7.231E+06 | 189.1 | - | - | 0 | - |
| - | - | 4.881E+05 | 190.1 | - | - | 0 | - |
| - | - | 6.981E+04 | 191.1 | - | - | 0 | - |
| - | - | 7.838E+04 | 192.1 | - | - | 0 | - |
| - | - | 6.509E+04 | 193.1 | - | - | 0 | - |
| - | - | 5.212E+04 | 193.1 | - | - | 0 | - |
| - | - | 1.619E+05 | 194.1 | - | - | 0 | - |
| - | - | 6.994E+04 | 195.1 | - | - | 0 | - |
| - | - | 1.736E+05 | 195.1 | - | - | 0 | - |
| - | - | 5.83E+04 | 197.1 | - | - | 0 | - |
| - | - | 1.431E+05 | 197.2 | - | - | 0 | - |
| - | - | 6.073E+05 | 199.1 | - | - | 0 | - |
| 2 | a | 6.912E+07 | 199.2 | 0.0004032 | 2.024 | +1 | 2 |
| - | - | 6.287E+04 | 200.1 | - | - | 0 | - |
| - | - | 5.321E+04 | 200.1 | - | - | 0 | - |
| - | - | 8.555E+06 | 200.2 | - | - | 0 | - |
| - | - | 2.554E+06 | 201.1 | - | - | 0 | - |
| - | - | 5.889E+04 | 201.1 | - | - | 0 | - |
| - | - | 4.105E+05 | 201.2 | - | - | 0 | - |
| - | - | 3.224E+05 | 202.1 | - | - | 0 | - |
| - | - | 1.955E+05 | 202.1 | - | - | 0 | - |
| - | - | 9.404E+05 | 203.1 | - | - | 0 | - |
| - | - | 1.497E+05 | 203.1 | - | - | 0 | - |
| - | - | 1.001E+05 | 204.1 | - | - | 0 | - |
| - | - | 6.705E+04 | 209.1 | - | - | 0 | - |
| - | - | 1.133E+05 | 209.1 | - | - | 0 | - |
| - | - | 1.46E+05 | 210.1 | - | - | 0 | - |
| - | - | 4.97E+05 | 211.1 | - | - | 0 | - |
| - | - | 4.69E+05 | 212.1 | - | - | 0 | - |
| - | - | 1.101E+05 | 213.2 | - | - | 0 | - |
| - | - | 5.142E+04 | 215.1 | - | - | 0 | - |
| - | - | 1.198E+05 | 216.1 | - | - | 0 | - |
| - | - | 1.236E+07 | 217.1 | - | - | 0 | - |
| - | - | 1.181E+06 | 218.1 | - | - | 0 | - |
| - | - | 1.267E+05 | 219.1 | - | - | 0 | - |
| - | - | 5.199E+04 | 220.1 | - | - | 0 | - |
| - | - | 1.066E+05 | 220.1 | - | - | 0 | - |
| - | - | 1.142E+05 | 220.1 | - | - | 0 | - |
| - | - | 5.816E+05 | 224.1 | - | - | 0 | - |
| - | - | 6.504E+04 | 225.1 | - | - | 0 | - |
| - | - | 1.285E+05 | 226.1 | - | - | 0 | - |
| - | - | 4.149E+05 | 227.1 | - | - | 0 | - |
| 2 | b | 2.08E+07 | 227.2 | 0.0004379 | 1.927 | +1 | 2 |
| - | - | 9.506E+04 | 228.1 | - | - | 0 | - |
| - | - | 2.775E+06 | 228.2 | - | - | 0 | - |
| - | - | 4.905E+05 | 229.1 | - | - | 0 | - |
| - | - | 2.201E+05 | 229.2 | - | - | 0 | - |
| - | - | 3.031E+05 | 230.1 | - | - | 0 | - |
| - | - | 1.287E+05 | 237.1 | - | - | 0 | - |
| - | - | 3.246E+05 | 237.1 | - | - | 0 | - |
| - | - | 7.845E+04 | 241.1 | - | - | 0 | - |
| - | - | 1.18E+05 | 242.1 | - | - | 0 | - |
| - | - | 1.34E+06 | 242.1 | - | - | 0 | - |
| - | - | 1.539E+05 | 243.1 | - | - | 0 | - |
| - | - | 3.186E+05 | 244.1 | - | - | 0 | - |
| 14 | y | 1.633E+05 | 244.1 | 0.0006211 | 2.544 | +1 | 2 |
| - | - | 1.179E+05 | 245.1 | - | - | 0 | - |
| 14 | y | 1.893E+06 | 245.1 | 0.0005075 | 2.07 | +1 | 2 |
| - | - | 6.471E+04 | 245.2 | - | - | 0 | - |
| - | - | 1.58E+05 | 246.1 | - | - | 0 | - |
| - | - | 1.018E+05 | 252.1 | - | - | 0 | - |
| - | - | 1.349E+05 | 253.2 | - | - | 0 | - |
| - | - | 1.649E+05 | 254.1 | - | - | 0 | - |
| - | - | 1.338E+05 | 254.2 | - | - | 0 | - |
| - | - | 8.609E+04 | 255.1 | - | - | 0 | - |
| - | - | 1.374E+06 | 255.1 | - | - | 0 | - |
| - | - | 6.875E+04 | 255.1 | - | - | 0 | - |
| - | - | 1.741E+05 | 256.1 | - | - | 0 | - |
| - | - | 9.325E+04 | 256.1 | - | - | 0 | - |
| - | - | 2.154E+05 | 259.1 | - | - | 0 | - |
| - | - | 6.763E+04 | 260.1 | - | - | 0 | - |
| 14 | y | 1.139E+06 | 262.2 | 0.0003866 | 1.475 | +1 | 2 |
| - | - | 1.311E+05 | 263.2 | - | - | 0 | - |
| - | - | 5.863E+04 | 268.1 | - | - | 0 | - |
| - | - | 2.785E+05 | 270.1 | - | - | 0 | - |
| - | - | 1.171E+05 | 271.1 | - | - | 0 | - |
| - | - | 2.145E+05 | 272.1 | - | - | 0 | - |
| - | - | 1.467E+05 | 279.1 | - | - | 0 | - |
| - | - | 1.67E+05 | 280.1 | - | - | 0 | - |
| - | - | 1.31E+05 | 281.1 | - | - | 0 | - |
| - | - | 2.964E+05 | 284.1 | - | - | 0 | - |
| - | - | 1.6E+05 | 284.2 | - | - | 0 | - |
| - | - | 6.382E+04 | 285.1 | - | - | 0 | - |
| - | - | 2.622E+05 | 288.2 | - | - | 0 | - |
| - | - | 7.31E+04 | 289.2 | - | - | 0 | - |
| - | - | 8.767E+04 | 294.1 | - | - | 0 | - |
| - | - | 9.582E+04 | 295.2 | - | - | 0 | - |
| - | - | 2.462E+05 | 296.1 | - | - | 0 | - |
| - | - | 9.401E+04 | 296.2 | - | - | 0 | - |
| 3 | b | 1.016E+06 | 296.2 | 0.000489 | 1.651 | +1 | 3 |
| - | - | 2.435E+05 | 297.1 | - | - | 0 | - |
| - | - | 1.725E+05 | 297.2 | - | - | 0 | - |
| - | - | 6.366E+05 | 298.1 | - | - | 0 | - |
| - | - | 3.201E+05 | 299.1 | - | - | 0 | - |
| - | - | 7.921E+04 | 301.2 | - | - | 0 | - |
| - | - | 2.411E+05 | 302.1 | - | - | 0 | - |
| - | - | 8.068E+04 | 302.2 | - | - | 0 | - |
| - | - | 6.868E+04 | 306.2 | - | - | 0 | - |
| - | - | 6.131E+04 | 307.2 | - | - | 0 | - |
| - | - | 7.251E+04 | 311.1 | - | - | 0 | - |
| - | - | 1.023E+06 | 312.2 | - | - | 0 | - |
| - | - | 2.46E+05 | 313.2 | - | - | 0 | - |
| - | - | 1.864E+06 | 313.2 | - | - | 0 | - |
| - | - | 6.193E+05 | 314.1 | - | - | 0 | - |
| - | - | 2.375E+05 | 314.2 | - | - | 0 | - |
| 3 | b | 2.402E+06 | 314.2 | 0.0006665 | 2.121 | +1 | 3 |
| - | - | 6.108E+04 | 315.1 | - | - | 0 | - |
| - | - | 2.988E+05 | 315.2 | - | - | 0 | - |
| - | - | 2.532E+06 | 316.2 | - | - | 0 | - |
| - | - | 3.186E+05 | 317.2 | - | - | 0 | - |
| - | - | 5.915E+04 | 323.1 | - | - | 0 | - |
| - | - | 9.339E+04 | 323.2 | - | - | 0 | - |
| - | - | 2.108E+05 | 324.1 | - | - | 0 | - |
| - | - | 8.831E+04 | 324.2 | - | - | 0 | - |
| - | - | 7.762E+04 | 325.1 | - | - | 0 | - |
| - | - | 6.195E+04 | 325.1 | - | - | 0 | - |
| - | - | 7.353E+04 | 326.1 | - | - | 0 | - |
| - | - | 9.097E+04 | 329.1 | - | - | 0 | - |
| - | - | 5.924E+05 | 330.2 | - | - | 0 | - |
| 13 | y | 5.588E+05 | 331.2 | 0.0007829 | 2.364 | +1 | 3 |
| 13 | y | 1.73E+06 | 332.2 | 0.000492 | 1.481 | +1 | 3 |
| - | - | 2.744E+05 | 333.2 | - | - | 0 | - |
| 9 | y | 1.235E+05 | 336.2 | 0.0007222 | 2.148 | +2 | 7 |
| - | - | 1.578E+05 | 340.2 | - | - | 0 | - |
| - | - | 5.219E+04 | 341.2 | - | - | 0 | - |
| - | - | 1.006E+06 | 341.2 | - | - | 0 | - |
| - | - | 1.863E+06 | 342.1 | - | - | 0 | - |
| - | - | 1.633E+05 | 342.2 | - | - | 0 | - |
| - | - | 3.288E+05 | 343.1 | - | - | 0 | - |
| - | - | 7.571E+04 | 344.1 | - | - | 0 | - |
| 9 | y | 9.066E+05 | 345.2 | 0.0006888 | 1.996 | +2 | 7 |
| - | - | 2.792E+05 | 345.7 | - | - | 0 | - |
| 13 | y | 1.551E+06 | 349.2 | 0.0004322 | 1.238 | +1 | 3 |
| - | - | 2.206E+05 | 350.2 | - | - | 0 | - |
| - | - | 7.245E+04 | 354.1 | - | - | 0 | - |
| - | - | 1.501E+05 | 358.2 | - | - | 0 | - |
| - | - | 9.476E+05 | 359.2 | - | - | 0 | - |
| - | - | 2.119E+05 | 360.2 | - | - | 0 | - |
| - | - | 8.859E+04 | 365.2 | - | - | 0 | - |
| - | - | 1.02E+05 | 367.2 | - | - | 0 | - |
| - | - | 6.26E+04 | 369.2 | - | - | 0 | - |
| - | - | 6.472E+04 | 379.2 | - | - | 0 | - |
| - | - | 1.261E+05 | 380.2 | - | - | 0 | - |
| - | - | 9.117E+04 | 381.2 | - | - | 0 | - |
| - | - | 8.521E+04 | 382.2 | - | - | 0 | - |
| - | - | 1.078E+05 | 382.2 | - | - | 0 | - |
| - | - | 8.749E+04 | 383.2 | - | - | 0 | - |
| - | - | 2.353E+05 | 385.2 | - | - | 0 | - |
| - | - | 8.03E+04 | 393.2 | - | - | 0 | - |
| - | - | 2.9E+05 | 393.7 | - | - | 0 | - |
| - | - | 9.561E+04 | 394.2 | - | - | 0 | - |
| - | - | 1.464E+05 | 396.2 | - | - | 0 | - |
| - | - | 3.151E+05 | 397.2 | - | - | 0 | - |
| - | - | 1.038E+05 | 398.2 | - | - | 0 | - |
| - | - | 2.56E+05 | 398.2 | - | - | 0 | - |
| - | - | 5.809E+04 | 398.2 | - | - | 0 | - |
| - | - | 6.813E+04 | 399.2 | - | - | 0 | - |
| - | - | 6.094E+04 | 399.7 | - | - | 0 | - |
| - | - | 2.744E+05 | 400.2 | - | - | 0 | - |
| - | - | 1.968E+05 | 401.2 | - | - | 0 | - |
| - | - | 3.292E+05 | 403.2 | - | - | 0 | - |
| - | - | 1.108E+05 | 405.2 | - | - | 0 | - |
| - | - | 1.231E+05 | 405.7 | - | - | 0 | - |
| - | - | 1.03E+05 | 407.2 | - | - | 0 | - |
| - | - | 2.228E+05 | 408.2 | - | - | 0 | - |
| - | - | 8.786E+04 | 408.7 | - | - | 0 | - |
| - | - | 2.414E+05 | 410.2 | - | - | 0 | - |
| - | - | 1.737E+05 | 411.2 | - | - | 0 | - |
| - | - | 7.153E+04 | 413.2 | - | - | 0 | - |
| - | - | 6.076E+04 | 414.2 | - | - | 0 | - |
| 12 | b | 7.493E+05 | 414.2 | 0.006209 | 14.99 | +3 | 12 |
| 8 | y | 1.053E+06 | 414.7 | 0.002196 | 5.294 | +2 | 8 |
| - | - | 6.149E+05 | 415.2 | - | - | 0 | - |
| - | - | 2.423E+05 | 415.3 | - | - | 0 | - |
| - | - | 1.284E+05 | 415.7 | - | - | 0 | - |
| - | - | 1.039E+05 | 416.2 | - | - | 0 | - |
| - | - | 8.258E+04 | 416.3 | - | - | 0 | - |
| - | - | 8.201E+04 | 418.2 | - | - | 0 | - |
| - | - | 1.3E+05 | 422.2 | - | - | 0 | - |
| 8 | y | 1.522E+07 | 423.2 | 0.000762 | 1.8 | +2 | 8 |
| - | - | 6.572E+06 | 423.7 | - | - | 0 | - |
| - | - | 1.81E+06 | 424.2 | - | - | 0 | - |
| - | - | 2.638E+05 | 424.7 | - | - | 0 | - |
| 4 | b | 1.174E+06 | 425.2 | 0.0006816 | 1.603 | +1 | 4 |
| - | - | 3.324E+05 | 426.2 | - | - | 0 | - |
| - | - | 4.157E+05 | 428.2 | - | - | 0 | - |
| - | - | 7.625E+05 | 429.2 | - | - | 0 | - |
| 12 | y | 4.201E+05 | 430.2 | 0.000868 | 2.017 | +1 | 4 |
| 12 | y | 1.428E+05 | 431.2 | 0.002492 | 5.779 | +1 | 4 |
| - | - | 7.669E+04 | 439.2 | - | - | 0 | - |
| - | - | 1.038E+05 | 440.2 | - | - | 0 | - |
| - | - | 8.424E+05 | 441.2 | - | - | 0 | - |
| - | - | 1.513E+05 | 442.2 | - | - | 0 | - |
| - | - | 1.278E+05 | 443.2 | - | - | 0 | - |
| 4 | b | 1.282E+06 | 443.3 | 0.0008591 | 1.938 | +1 | 4 |
| - | - | 2.857E+05 | 444.3 | - | - | 0 | - |
| 12 | y | 5.383E+05 | 448.3 | 0.0004692 | 1.047 | +1 | 4 |
| 3 | y | 3.256E+05 | 448.9 | 0.0007154 | 1.594 | +3 | 13 |
| 3 | y | 2.604E+05 | 449.2 | 0.005565 | 12.39 | +3 | 13 |
| - | - | 1.366E+05 | 449.3 | - | - | 0 | - |
| - | - | 1.885E+05 | 449.6 | - | - | 0 | - |
| 8 | b | 1.567E+05 | 450.7 | 4.049E-05 | 0.08984 | +2 | 8 |
| - | - | 8.727E+04 | 451.2 | - | - | 0 | - |
| - | - | 1.143E+05 | 452.2 | - | - | 0 | - |
| - | - | 3.677E+05 | 452.3 | - | - | 0 | - |
| - | - | 8.184E+04 | 453.3 | - | - | 0 | - |
| 3 | y | 6.568E+05 | 454.9 | 0.0007339 | 1.613 | +3 | 13 |
| - | - | 4.028E+05 | 455.2 | - | - | 0 | - |
| - | - | 1.839E+05 | 455.6 | - | - | 0 | - |
| - | - | 7.568E+04 | 455.9 | - | - | 0 | - |
| - | - | 7.324E+05 | 458.2 | - | - | 0 | - |
| - | - | 7.909E+04 | 459.2 | - | - | 0 | - |
| - | - | 9.607E+04 | 460.2 | - | - | 0 | - |
| - | - | 8.469E+04 | 463.2 | - | - | 0 | - |
| - | - | 3.306E+05 | 469.3 | - | - | 0 | - |
| - | - | 1.798E+05 | 470.2 | - | - | 0 | - |
| - | - | 9.962E+04 | 470.3 | - | - | 0 | - |
| 7 | y | 1.421E+05 | 471.7 | 0.0004692 | 0.9946 | +2 | 9 |
| - | - | 8.55E+04 | 471.9 | - | - | 0 | - |
| 14 | b | 2.264E+05 | 472.2 | 0.005866 | 12.42 | +3 | 14 |
| - | - | 1.576E+05 | 472.7 | - | - | 0 | - |
| - | - | 9.455E+04 | 478.2 | - | - | 0 | - |
| - | - | 1.328E+05 | 480.3 | - | - | 0 | - |
| 7 | y | 2.851E+06 | 480.7 | 0.0005961 | 1.24 | +2 | 9 |
| - | - | 1.319E+06 | 481.2 | - | - | 0 | - |
| - | - | 3.635E+05 | 481.7 | - | - | 0 | - |
| - | - | 8.389E+04 | 482.2 | - | - | 0 | - |
| - | - | 5.986E+04 | 486.2 | - | - | 0 | - |
| 2 | y | 6.159E+05 | 486.6 | 0.0005159 | 1.06 | +3 | 14 |
| 2 | y | 3.438E+05 | 486.9 | 0.007472 | 15.35 | +3 | 14 |
| 11 | y | 2.283E+05 | 487.3 | 0.007836 | 16.08 | +1 | 5 |
| 11 | y | 1.811E+05 | 488.2 | 0.001587 | 3.251 | +1 | 5 |
| 2 | y | 5.165E+05 | 492.6 | 4.611E-05 | 0.09361 | +3 | 14 |
| - | - | 4.627E+05 | 492.9 | - | - | 0 | - |
| - | - | 2.542E+05 | 493.3 | - | - | 0 | - |
| - | - | 1.2E+05 | 494.7 | - | - | 0 | - |
| - | - | 1.667E+05 | 495.2 | - | - | 0 | - |
| - | - | 8.516E+05 | 495.9 | - | - | 0 | - |
| - | - | 1.224E+05 | 496.2 | - | - | 0 | - |
| - | - | 7.404E+05 | 496.2 | - | - | 0 | - |
| - | - | 9.316E+04 | 496.3 | - | - | 0 | - |
| - | - | 3.506E+05 | 496.6 | - | - | 0 | - |
| - | - | 2.201E+05 | 497.3 | - | - | 0 | - |
| - | - | 1.263E+05 | 498.2 | - | - | 0 | - |
| - | - | 1.932E+05 | 500.2 | - | - | 0 | - |
| 11 | y | 1.752E+06 | 505.3 | 0.0006119 | 1.211 | +1 | 5 |
| - | - | 3.598E+05 | 506.3 | - | - | 0 | - |
| - | - | 8.616E+04 | 507.8 | - | - | 0 | - |
| - | - | 1.636E+05 | 509.3 | - | - | 0 | - |
| - | - | 3.726E+05 | 514.3 | - | - | 0 | - |
| 6 | y | 5.158E+05 | 515.3 | 0.0005837 | 1.133 | +2 | 10 |
| - | - | 8.825E+04 | 515.3 | - | - | 0 | - |
| 6 | y | 3.608E+05 | 515.7 | 0.004357 | 8.447 | +2 | 10 |
| - | - | 3.097E+05 | 516.3 | - | - | 0 | - |
| - | - | 2.306E+05 | 518.2 | - | - | 0 | - |
| - | - | 1.711E+05 | 518.3 | - | - | 0 | - |
| - | - | 2.034E+05 | 518.6 | - | - | 0 | - |
| - | - | 1.189E+05 | 518.9 | - | - | 0 | - |
| - | - | 8.098E+04 | 519.3 | - | - | 0 | - |
| 6 | y | 1.667E+07 | 524.3 | 0.002007 | 3.829 | +2 | 10 |
| 0 | Precursor | 2.881E+06 | 524.6 | 0.006723 | 12.82 | +3 | -1 |
| - | - | 8.372E+06 | 524.8 | - | - | 0 | - |
| - | - | 1.647E+06 | 524.9 | - | - | 0 | - |
| - | - | 2.996E+06 | 525.3 | - | - | 0 | - |
| - | - | 4.895E+05 | 525.8 | - | - | 0 | - |
| - | - | 8.278E+04 | 526.3 | - | - | 0 | - |
| - | - | 1.093E+05 | 529.3 | - | - | 0 | - |
| 0 | Precursor | 1.871E+06 | 530.3 | 0.000457 | 0.8618 | +3 | -1 |
| - | - | 1.897E+06 | 530.6 | - | - | 0 | - |
| - | - | 9.148E+05 | 530.9 | - | - | 0 | - |
| - | - | 2.447E+05 | 531.3 | - | - | 0 | - |
| - | - | 1.496E+05 | 531.6 | - | - | 0 | - |
| - | - | 1.927E+05 | 532.3 | - | - | 0 | - |
| - | - | 1.606E+05 | 533.3 | - | - | 0 | - |
| - | - | 1.107E+05 | 538.3 | - | - | 0 | - |
| - | - | 9.762E+04 | 539.3 | - | - | 0 | - |
| - | - | 6.833E+04 | 542.3 | - | - | 0 | - |
| - | - | 8.146E+04 | 542.3 | - | - | 0 | - |
| 5 | b | 5.076E+05 | 542.3 | 0.001262 | 2.328 | +1 | 5 |
| - | - | 9.922E+04 | 543.3 | - | - | 0 | - |
| - | - | 1.082E+05 | 543.3 | - | - | 0 | - |
| - | - | 1.551E+05 | 552.8 | - | - | 0 | - |
| - | - | 1.433E+05 | 556.3 | - | - | 0 | - |
| - | - | 1.247E+05 | 556.3 | - | - | 0 | - |
| - | - | 1.112E+05 | 556.8 | - | - | 0 | - |
| - | - | 1.123E+05 | 557.3 | - | - | 0 | - |
| - | - | 2.345E+05 | 558.8 | - | - | 0 | - |
| - | - | 2.18E+05 | 559.3 | - | - | 0 | - |
| 11 | b | 7.962E+04 | 562.3 | 0.0004618 | 0.8213 | +2 | 11 |
| 5 | y | 8.68E+05 | 564.8 | 0.0001227 | 0.2173 | +2 | 11 |
| - | - | 1.366E+05 | 565.2 | - | - | 0 | - |
| 5 | y | 9.622E+05 | 565.3 | 0.004757 | 8.415 | +2 | 11 |
| - | - | 4.003E+05 | 565.8 | - | - | 0 | - |
| - | - | 1.799E+05 | 566.3 | - | - | 0 | - |
| - | - | 1.72E+05 | 567.3 | - | - | 0 | - |
| - | - | 1.01E+05 | 569.3 | - | - | 0 | - |
| - | - | 1.076E+05 | 572.3 | - | - | 0 | - |
| - | - | 1.881E+05 | 572.8 | - | - | 0 | - |
| - | - | 1.196E+05 | 573.3 | - | - | 0 | - |
| 5 | y | 2.342E+07 | 573.8 | 0.0003933 | 0.6854 | +2 | 11 |
| 10 | y | 1.389E+07 | 574.3 | 0.003989 | 6.947 | +1 | 6 |
| - | - | 4.48E+06 | 574.8 | - | - | 0 | - |
| - | - | 1.104E+06 | 575.3 | - | - | 0 | - |
| - | - | 1.414E+05 | 575.8 | - | - | 0 | - |
| - | - | 1.802E+05 | 583.2 | - | - | 0 | - |
| - | - | 2.917E+05 | 584.3 | - | - | 0 | - |
| - | - | 2E+05 | 587.3 | - | - | 0 | - |
| 10 | y | 1.859E+06 | 592.3 | 0.0003828 | 0.6463 | +1 | 6 |
| - | - | 4.896E+05 | 593.3 | - | - | 0 | - |
| - | - | 1.626E+05 | 594.3 | - | - | 0 | - |
| - | - | 3.691E+05 | 600.3 | - | - | 0 | - |
| - | - | 1.938E+05 | 602.3 | - | - | 0 | - |
| - | - | 1.062E+05 | 611.3 | - | - | 0 | - |
| - | - | 1.304E+05 | 617.3 | - | - | 0 | - |
| - | - | 1.053E+05 | 621.3 | - | - | 0 | - |
| - | - | 8.632E+04 | 623.3 | - | - | 0 | - |
| - | - | 1.073E+05 | 624.3 | - | - | 0 | - |
| - | - | 1.889E+05 | 626.3 | - | - | 0 | - |
| - | - | 1.081E+05 | 627.3 | - | - | 0 | - |
| 4 | y | 1.535E+06 | 629.3 | 0.001774 | 2.819 | +2 | 12 |
| 4 | y | 1.028E+06 | 629.8 | 0.008301 | 13.18 | +2 | 12 |
| - | - | 6.142E+05 | 630.3 | - | - | 0 | - |
| - | - | 1.194E+05 | 630.8 | - | - | 0 | - |
| - | - | 2.149E+05 | 637.3 | - | - | 0 | - |
| - | - | 1.025E+05 | 637.8 | - | - | 0 | - |
| 4 | y | 1.13E+07 | 638.3 | 0.0002149 | 0.3367 | +2 | 12 |
| - | - | 8.258E+06 | 638.8 | - | - | 0 | - |
| - | - | 3.347E+06 | 639.3 | - | - | 0 | - |
| - | - | 7.39E+05 | 639.8 | - | - | 0 | - |
| - | - | 1.88E+05 | 640.3 | - | - | 0 | - |
| - | - | 3.335E+05 | 641.3 | - | - | 0 | - |
| - | - | 1.177E+05 | 642.3 | - | - | 0 | - |
| - | - | 1.091E+05 | 642.8 | - | - | 0 | - |
| - | - | 8.092E+04 | 643.3 | - | - | 0 | - |
| - | - | 2.271E+05 | 643.4 | - | - | 0 | - |
| - | - | 1.48E+05 | 643.8 | - | - | 0 | - |
| - | - | 7.376E+05 | 646.3 | - | - | 0 | - |
| - | - | 2.319E+05 | 647.3 | - | - | 0 | - |
| - | - | 3.428E+05 | 648.8 | - | - | 0 | - |
| - | - | 2.254E+05 | 649.3 | - | - | 0 | - |
| - | - | 1.001E+05 | 649.8 | - | - | 0 | - |
| - | - | 1.339E+05 | 651.8 | - | - | 0 | - |
| - | - | 2.489E+05 | 652.3 | - | - | 0 | - |
| - | - | 1.242E+05 | 652.8 | - | - | 0 | - |
| - | - | 3.245E+05 | 653.3 | - | - | 0 | - |
| - | - | 3.404E+05 | 654.3 | - | - | 0 | - |
| - | - | 3.579E+05 | 654.8 | - | - | 0 | - |
| - | - | 4.338E+05 | 655.3 | - | - | 0 | - |
| - | - | 1.293E+05 | 655.8 | - | - | 0 | - |
| - | - | 2.019E+05 | 656.3 | - | - | 0 | - |
| - | - | 3.733E+05 | 657.3 | - | - | 0 | - |
| - | - | 4.014E+05 | 657.8 | - | - | 0 | - |
| - | - | 2.274E+05 | 658.3 | - | - | 0 | - |
| - | - | 1.527E+05 | 658.8 | - | - | 0 | - |
| - | - | 4.375E+05 | 659.3 | - | - | 0 | - |
| - | - | 1.806E+05 | 659.8 | - | - | 0 | - |
| - | - | 1.243E+05 | 660.3 | - | - | 0 | - |
| - | - | 1.281E+05 | 660.4 | - | - | 0 | - |
| - | - | 5.62E+05 | 660.8 | - | - | 0 | - |
| - | - | 4.039E+05 | 661.3 | - | - | 0 | - |
| - | - | 1.691E+05 | 661.8 | - | - | 0 | - |
| - | - | 9.916E+05 | 663.8 | - | - | 0 | - |
| - | - | 7.539E+05 | 664.3 | - | - | 0 | - |
| - | - | 3.884E+05 | 664.8 | - | - | 0 | - |
| - | - | 1.672E+05 | 665.3 | - | - | 0 | - |
| - | - | 2.261E+05 | 665.8 | - | - | 0 | - |
| - | - | 4.392E+05 | 666.3 | - | - | 0 | - |
| - | - | 1.656E+06 | 666.8 | - | - | 0 | - |
| - | - | 1.156E+06 | 667.3 | - | - | 0 | - |
| - | - | 4.656E+05 | 667.8 | - | - | 0 | - |
| - | - | 7.411E+04 | 668.3 | - | - | 0 | - |
| - | - | 1.225E+05 | 671.3 | - | - | 0 | - |
| 9 | y | 1.917E+06 | 671.3 | 0.00042 | 0.6256 | +1 | 7 |
| - | - | 1.203E+05 | 671.8 | - | - | 0 | - |
| 9 | y | 1.428E+06 | 672.3 | 0.006089 | 9.057 | +1 | 7 |
| 3 | y | 5.812E+06 | 672.8 | 0.0001642 | 0.244 | +2 | 13 |
| 3 | y | 5.542E+06 | 673.3 | 0.009499 | 14.11 | +2 | 13 |
| - | - | 2.353E+06 | 673.8 | - | - | 0 | - |
| - | - | 2.967E+06 | 674.3 | - | - | 0 | - |
| - | - | 2.624E+05 | 674.8 | - | - | 0 | - |
| - | - | 8.892E+05 | 675.3 | - | - | 0 | - |
| - | - | 2.124E+05 | 676.3 | - | - | 0 | - |
| - | - | 2.104E+05 | 677.8 | - | - | 0 | - |
| - | - | 9.628E+04 | 678.3 | - | - | 0 | - |
| - | - | 1.369E+06 | 680.8 | - | - | 0 | - |
| - | - | 1.251E+06 | 681.3 | - | - | 0 | - |
| 3 | y | 1.768E+08 | 681.8 | 0.0008633 | 1.266 | +2 | 13 |
| - | - | 1.292E+08 | 682.3 | - | - | 0 | - |
| - | - | 5.258E+07 | 682.8 | - | - | 0 | - |
| - | - | 1.367E+07 | 683.3 | - | - | 0 | - |
| - | - | 2.538E+06 | 683.8 | - | - | 0 | - |
| - | - | 8.551E+04 | 684.3 | - | - | 0 | - |
| - | - | 1.495E+06 | 686.8 | - | - | 0 | - |
| - | - | 1.143E+06 | 687.3 | - | - | 0 | - |
| - | - | 3.553E+05 | 687.8 | - | - | 0 | - |
| - | - | 9.999E+04 | 688.3 | - | - | 0 | - |
| 9 | y | 1.22E+07 | 689.4 | 0.0002923 | 0.424 | +1 | 7 |
| - | - | 4.234E+06 | 690.4 | - | - | 0 | - |
| - | - | 1.089E+06 | 691.4 | - | - | 0 | - |
| - | - | 1.599E+05 | 692.4 | - | - | 0 | - |
| 14 | b | 5.976E+05 | 699.3 | 0.007802 | 11.16 | +2 | 14 |
| - | - | 2.692E+05 | 700.3 | - | - | 0 | - |
| - | - | 1.072E+05 | 716.4 | - | - | 0 | - |
| - | - | 1.861E+05 | 717.4 | - | - | 0 | - |
| - | - | 1.504E+05 | 724.4 | - | - | 0 | - |
| - | - | 1.124E+05 | 728.4 | - | - | 0 | - |
| 2 | y | 1.722E+06 | 729.4 | 0.0001244 | 0.1705 | +2 | 14 |
| 2 | y | 1.462E+06 | 729.9 | 0.009215 | 12.63 | +2 | 14 |
| - | - | 6.836E+05 | 730.4 | - | - | 0 | - |
| - | - | 1.607E+05 | 730.9 | - | - | 0 | - |
| - | - | 1.069E+05 | 734.9 | - | - | 0 | - |
| - | - | 2.493E+05 | 737.4 | - | - | 0 | - |
| - | - | 1.717E+05 | 737.9 | - | - | 0 | - |
| 2 | y | 1.445E+07 | 738.4 | 3.002E-05 | 0.04066 | +2 | 14 |
| - | - | 1.218E+07 | 738.9 | - | - | 0 | - |
| - | - | 5.652E+06 | 739.4 | - | - | 0 | - |
| - | - | 1.374E+06 | 739.9 | - | - | 0 | - |
| - | - | 3.106E+05 | 740.4 | - | - | 0 | - |
| - | - | 9.336E+05 | 743.4 | - | - | 0 | - |
| - | - | 4.862E+05 | 743.9 | - | - | 0 | - |
| 7 | b | 3.528E+05 | 744.4 | 0.005341 | 7.175 | +1 | 7 |
| - | - | 1.018E+05 | 744.9 | - | - | 0 | - |
| - | - | 1.695E+05 | 758.4 | - | - | 0 | - |
| - | - | 2.946E+05 | 768.4 | - | - | 0 | - |
| - | - | 5.734E+05 | 770.4 | - | - | 0 | - |
| - | - | 2.295E+05 | 771.4 | - | - | 0 | - |
| - | - | 1.111E+05 | 784.4 | - | - | 0 | - |
| - | - | 2.43E+05 | 785.4 | - | - | 0 | - |
| - | - | 1.065E+05 | 786.4 | - | - | 0 | - |
| 0 | Precursor | 3.069E+05 | 786.4 | 0.01162 | 14.77 | +2 | -1 |
| - | - | 3.799E+05 | 787.4 | - | - | 0 | - |
| - | - | 1.637E+05 | 788.4 | - | - | 0 | - |
| - | - | 4.409E+05 | 798.4 | - | - | 0 | - |
| - | - | 1.996E+05 | 799.4 | - | - | 0 | - |
| - | - | 2.347E+05 | 803.4 | - | - | 0 | - |
| - | - | 1.199E+05 | 804.4 | - | - | 0 | - |
| - | - | 1.363E+05 | 810.4 | - | - | 0 | - |
| - | - | 3.522E+05 | 811.4 | - | - | 0 | - |
| - | - | 1.214E+05 | 812.4 | - | - | 0 | - |
| 8 | y | 1.903E+06 | 828.4 | 0.000295 | 0.3561 | +1 | 8 |
| - | - | 7.959E+05 | 829.4 | - | - | 0 | - |
| - | - | 1.954E+05 | 830.4 | - | - | 0 | - |
| - | - | 1.611E+05 | 855.4 | - | - | 0 | - |
| - | - | 2.336E+05 | 857.4 | - | - | 0 | - |
| - | - | 2.242E+05 | 858.4 | - | - | 0 | - |
| - | - | 1.256E+05 | 859.4 | - | - | 0 | - |
| - | - | 1.648E+05 | 867.4 | - | - | 0 | - |
| - | - | 1.837E+05 | 873.4 | - | - | 0 | - |
| - | - | 1.106E+05 | 881.4 | - | - | 0 | - |
| 8 | b | 7.822E+04 | 883.5 | 0.004448 | 5.034 | +1 | 8 |
| - | - | 2.013E+05 | 885.4 | - | - | 0 | - |
| - | - | 4.509E+05 | 887.4 | - | - | 0 | - |
| - | - | 1.925E+05 | 888.4 | - | - | 0 | - |
| - | - | 1.052E+05 | 889.4 | - | - | 0 | - |
| - | - | 2.549E+05 | 891.4 | - | - | 0 | - |
| - | - | 1.099E+05 | 892.4 | - | - | 0 | - |
| - | - | 1.841E+05 | 897.4 | - | - | 0 | - |
| - | - | 1.586E+05 | 899.5 | - | - | 0 | - |
| 8 | b | 4.72E+05 | 900.5 | 0.0102 | 11.33 | +1 | 8 |
| - | - | 1.069E+05 | 901.5 | - | - | 0 | - |
| - | - | 1.139E+05 | 909.5 | - | - | 0 | - |
| - | - | 9.186E+05 | 915.4 | - | - | 0 | - |
| - | - | 3.08E+05 | 916.4 | - | - | 0 | - |
| - | - | 1.658E+05 | 917.4 | - | - | 0 | - |
| - | - | 8.736E+04 | 918.5 | - | - | 0 | - |
| - | - | 1.568E+05 | 925.5 | - | - | 0 | - |
| - | - | 2.036E+05 | 926.4 | - | - | 0 | - |
| - | - | 1.237E+05 | 927.4 | - | - | 0 | - |
| 7 | y | 1.691E+06 | 943.5 | 0.0004436 | 0.4702 | +1 | 9 |
| - | - | 7.927E+05 | 944.5 | - | - | 0 | - |
| - | - | 4.084E+05 | 945.5 | - | - | 0 | - |
| - | - | 8.488E+04 | 951.5 | - | - | 0 | - |
| - | - | 1.406E+05 | 954.5 | - | - | 0 | - |
| - | - | 1.171E+05 | 957.5 | - | - | 0 | - |
| 7 | y | 7.24E+04 | 960.5 | 0.006607 | 6.879 | +1 | 9 |
| - | - | 1.647E+05 | 968.5 | - | - | 0 | - |
| - | - | 2.127E+05 | 969.5 | - | - | 0 | - |
| - | - | 1.124E+05 | 970.5 | - | - | 0 | - |
| - | - | 2.396E+05 | 972.5 | - | - | 0 | - |
| - | - | 1.422E+05 | 973.5 | - | - | 0 | - |
| - | - | 1.397E+06 | 986.5 | - | - | 0 | - |
| - | - | 1.61E+06 | 987.5 | - | - | 0 | - |
| - | - | 7.058E+05 | 988.5 | - | - | 0 | - |
| - | - | 2.572E+05 | 989.5 | - | - | 0 | - |
| - | - | 2.784E+05 | 990.5 | - | - | 0 | - |
| - | - | 1.326E+05 | 991.5 | - | - | 0 | - |
| - | - | 2.106E+05 | 996.5 | - | - | 0 | - |
| - | - | 1.46E+05 | 997.5 | - | - | 0 | - |
| - | - | 2.025E+05 | 1000 | - | - | 0 | - |
| - | - | 1.944E+05 | 1005 | - | - | 0 | - |
| - | - | 5.926E+05 | 1012 | - | - | 0 | - |
| - | - | 3.69E+05 | 1013 | - | - | 0 | - |
| - | - | 1.382E+06 | 1014 | - | - | 0 | - |
| - | - | 7.891E+05 | 1015 | - | - | 0 | - |
| - | - | 2.525E+05 | 1016 | - | - | 0 | - |
| 6 | y | 4.757E+06 | 1030 | 0.0007337 | 0.712 | +1 | 10 |
| - | - | 2.625E+06 | 1031 | - | - | 0 | - |
| - | - | 1.015E+06 | 1032 | - | - | 0 | - |
| - | - | 1.745E+05 | 1033 | - | - | 0 | - |
| - | - | 1.178E+05 | 1040 | - | - | 0 | - |
| 6 | y | 1.366E+05 | 1048 | 0.003235 | 3.088 | +1 | 10 |
| - | - | 1.243E+05 | 1058 | - | - | 0 | - |
| - | - | 5.169E+05 | 1074 | - | - | 0 | - |
| - | - | 2.348E+05 | 1075 | - | - | 0 | - |
| - | - | 1.309E+05 | 1076 | - | - | 0 | - |
| - | - | 3.779E+05 | 1084 | - | - | 0 | - |
| - | - | 2.388E+05 | 1085 | - | - | 0 | - |
| - | - | 3.202E+05 | 1087 | - | - | 0 | - |
| - | - | 1.809E+05 | 1088 | - | - | 0 | - |
| - | - | 1.057E+05 | 1089 | - | - | 0 | - |
| - | - | 7.431E+05 | 1102 | - | - | 0 | - |
| - | - | 4.703E+05 | 1103 | - | - | 0 | - |
| - | - | 1.937E+05 | 1104 | - | - | 0 | - |
| - | - | 1.521E+05 | 1112 | - | - | 0 | - |
| - | - | 1.813E+05 | 1113 | - | - | 0 | - |
| - | - | 1.097E+05 | 1114 | - | - | 0 | - |
| - | - | 1.839E+05 | 1120 | - | - | 0 | - |
| - | - | 1.169E+05 | 1128 | - | - | 0 | - |
| 5 | y | 1.582E+06 | 1130 | 0.002131 | 1.887 | +1 | 11 |
| - | - | 9.351E+05 | 1131 | - | - | 0 | - |
| - | - | 3.809E+05 | 1132 | - | - | 0 | - |
| - | - | 1.139E+05 | 1133 | - | - | 0 | - |
| - | - | 1.711E+05 | 1143 | - | - | 0 | - |
| - | - | 1.087E+05 | 1144 | - | - | 0 | - |
| - | - | 1.457E+05 | 1145 | - | - | 0 | - |
| - | - | 1.222E+05 | 1159 | - | - | 0 | - |
| - | - | 8.428E+05 | 1161 | - | - | 0 | - |
| - | - | 4.411E+05 | 1162 | - | - | 0 | - |
| - | - | 1.948E+05 | 1163 | - | - | 0 | - |
| - | - | 2.787E+05 | 1171 | - | - | 0 | - |
| - | - | 1.685E+05 | 1172 | - | - | 0 | - |
| - | - | 1.189E+06 | 1189 | - | - | 0 | - |
| - | - | 5.742E+05 | 1190 | - | - | 0 | - |
| - | - | 1.6E+05 | 1191 | - | - | 0 | - |
| - | - | 1.14E+05 | 1206 | - | - | 0 | - |
| - | - | 9.267E+05 | 1207 | - | - | 0 | - |
| - | - | 5.712E+05 | 1208 | - | - | 0 | - |
| - | - | 2.128E+05 | 1209 | - | - | 0 | - |
| - | - | 1.109E+05 | 1216 | - | - | 0 | - |
| - | - | 8.395E+04 | 1241 | - | - | 0 | - |
| - | - | 1.158E+05 | 1274 | - | - | 0 | - |
| - | - | 1.202E+05 | 1303 | - | - | 0 | - |
| 3 | y | 1.735E+05 | 1346 | 0.001617 | 1.201 | +1 | 13 |
| - | - | 1.372E+05 | 1347 | - | - | 0 | - |
| - | - | 9.037E+04 | 1348 | - | - | 0 | - |

m/z Charge Intensity FragmentType MassShift Position
125.10782623291016 0 36132.473
126.05530548095703 0 1116264.1
127.05870056152344 0 77140.46
127.0866928100586 0 37903.957
128.08221435546875 0 49043.953
129.0661163330078 0 135009
129.1027069091797 0 194097.8
129.11386108398438 0 108999.35
130.05003356933594 0 70742.9
130.0614776611328 0 615268.75
130.0978240966797 0 340005
131.08200073242188 0 44790.23
133.0605926513672 0 53204.367
133.09756469726562 0 104138.21
136.03924560546875 0 40786.22
138.0918426513672 0 51622.164
139.08685302734375 0 78098.76
140.08216857910156 0 128198.05
140.14390563964844 0 57418.746
141.0661163330078 0 59693.734
141.102783203125 0 138668.25
143.08187866210938 0 47419.21
143.19224548339844 0 37655.02
144.0658416748047 0 345340.7
147.07672119140625 0 376316.44
150.68080139160156 0 40571.895
153.06622314453125 0 103311.82
154.05023193359375 0 1145199.9
155.05357360839844 0 84633.11
155.11825561523438 0 120434.766
156.1024169921875 0 96374.94
157.0611572265625 0 489328.88
157.09759521484375 0 535018.06
157.1087646484375 0 626794.2
158.09278869628906 0 3007740.5 y Ammonia loss 14
158.11256408691406 0 75285.95
159.09622192382812 0 191963.25
159.1131134033203 0 464811.03
165.22463989257812 0 42886.824
167.0818328857422 0 200443.7
168.658935546875 0 42298.094
169.09722900390625 0 96629.734 y 10
170.0449981689453 0 129432.47
170.51834106445312 0 68306.69
170.52516174316406 0 68960.484
171.07679748535156 0 1412562.1
171.18577575683594 0 62471.31
172.0607147216797 0 162328.08
172.0802459716797 0 158936.72
173.12887573242188 0 450959.44
174.13186645507812 0 45178.59
175.07167053222656 0 862409
175.11935424804688 0 5924248 y 14
176.07516479492188 0 43227.727
176.12277221679688 0 375380.1
182.0928955078125 0 107382.98
182.1548614501953 0 48420.332
183.11312866210938 0 1294315.4
184.108642578125 0 158610.72
184.11695861816406 0 120888.22
185.0561981201172 0 85962.33
185.0924072265625 0 678223.44
185.10256958007812 0 116813.586
186.06121826171875 0 46874.95
187.07139587402344 0 69117.32
187.1080780029297 0 905164
188.11143493652344 0 101247.625
189.08737182617188 0 7230604.5
190.09085083007812 0 488071.62
191.09201049804688 0 69809.305
192.1134490966797 0 78379.54
193.0823974609375 0 65093.254
193.09779357910156 0 52123.027
194.0924835205078 0 161893.66
195.07601928710938 0 69936.87
195.149658203125 0 173639.48
197.0927734375 0 58303.46
197.1652069091797 0 143105.44
199.07177734375 0 607349.8
199.18089294433594 0 69119864 a 1
200.0755157470703 0 62865.99
200.10385131835938 0 53211.184
200.18421936035156 0 8554672
201.12364196777344 0 2554141.8
201.13490295410156 0 58886.75
201.18707275390625 0 410545.47
202.11880493164062 0 322414.75
202.1273651123047 0 195539.88
203.06649780273438 0 940374.6
203.1027069091797 0 149681.53
204.0703582763672 0 100072.234
209.10353088378906 0 67051.57
209.1398468017578 0 113277.54
210.08792114257812 0 145991.06
211.10813903808594 0 496970.03
212.10333251953125 0 468975.4
213.16006469726562 0 110127.6
215.11363220214844 0 51423.83
216.13473510742188 0 119764.55
217.082275390625 0 12361109
218.085693359375 0 1181461.4
219.08712768554688 0 126690.97
220.0723876953125 0 51991.375
220.09291076660156 0 106633.195
220.12918090820312 0 114223.79
224.10325622558594 0 581604.6
225.10702514648438 0 65041.355
226.1192169189453 0 128521.01
227.1141357421875 0 414901.56
227.17584228515625 0 20798856 b 1
228.09783935546875 0 95062.805
228.1791534423828 0 2775234
229.11871337890625 0 490461.1
229.1812744140625 0 220095.06
230.11398315429688 0 303075.97
237.09896850585938 0 128687.89
237.13504028320312 0 324567.2
241.1309051513672 0 78449.16
242.1006317138672 0 117968.43
242.11404418945312 0 1340115.5
243.11720275878906 0 153861.34
244.1294403076172 0 318626.72
244.1410369873047 0 163326.8 y Water loss 13
245.1112823486328 0 117931.57
245.12493896484375 0 1893300.1 y Ammonia loss 13
245.1864776611328 0 64705.23
246.1285400390625 0 157967.98
252.1346893310547 0 101828.3
253.22865295410156 0 134904.25
254.11354064941406 0 164924.3
254.16171264648438 0 133826.64
255.09521484375 0 86091.88
255.1092529296875 0 1373742.2
255.14463806152344 0 68753.805
256.1124572753906 0 174095.06
256.127685546875 0 93254.97
259.1402893066406 0 215426.88
260.1443786621094 0 67627.695
262.1513671875 0 1138628.2 y 13
263.1537780761719 0 131115
268.0921936035156 0 58626.145
270.1451110839844 0 278472.4
271.1416015625 0 117060.8
272.1357421875 0 214518.47
279.10943603515625 0 146708.1
280.12945556640625 0 166989.84
281.1253662109375 0 131017.98
284.1243896484375 0 296379.94
284.1612854003906 0 160001.89
285.1293640136719 0 63822.016
288.1555480957031 0 262247.44
289.15155029296875 0 73096.21
294.145751953125 0 87672.95
295.1759948730469 0 95821.3
296.1357421875 0 246225.45
296.17706298828125 0 94007.76
296.1973571777344 0 1016211.06 b Water loss 2
297.11962890625 0 243513
297.2008972167969 0 172471.19
298.1405334472656 0 636551.06
299.1358642578125 0 320141.6
301.1504821777344 0 79211.914
302.1352233886719 0 241147.6
302.17138671875 0 80682.9
306.1583251953125 0 68676.28
307.1605224609375 0 61307.082
311.1350402832031 0 72511.17
312.1558837890625 0 1022576.3
313.1527099609375 0 246048.78
313.1875915527344 0 1864399.1
314.14666748046875 0 619336.2
314.19049072265625 0 237530.44
314.2080993652344 0 2401857 b 2
315.1497802734375 0 61080.934
315.21112060546875 0 298819.66
316.1508483886719 0 2531817.2
317.15185546875 0 318633.28
323.13775634765625 0 59154.625
323.1707763671875 0 93393.984
324.13055419921875 0 210799.38
324.167724609375 0 88311.07
325.1153564453125 0 77615.81
325.1331481933594 0 61947.277
326.13555908203125 0 73533.61
329.145751953125 0 90969.93
330.16632080078125 0 592410.5
331.1716613769531 0 558814 y Water loss 12
332.1569519042969 0 1729581.8 y Ammonia loss 12
333.1594543457031 0 274371.8
336.17791748046875 0 123517.95 y Water loss 8
340.1507263183594 0 157757.8
341.1588439941406 0 52188.047
341.18255615234375 0 1006043
342.14141845703125 0 1863294
342.1836853027344 0 163258.5
343.14447021484375 0 328769.16
344.1449279785156 0 75712.76
345.18316650390625 0 906574.6 y 8
345.6845703125 0 279241.34
349.1834411621094 0 1551484.5 y 12
350.1859130859375 0 220633.42
354.1427917480469 0 72449.66
358.16046142578125 0 150058.84
359.1675109863281 0 947641.1
360.1693420410156 0 211946.53
365.1806640625 0 88590.625
367.1622619628906 0 102008.19
369.22491455078125 0 62600.75
379.2344055175781 0 64719.914
380.1565246582031 0 126094.49
381.1892395019531 0 91166.42
382.17236328125 0 85207.99
382.2088317871094 0 107845.766
383.1982421875 0 87485.445
385.17218017578125 0 235296.12
393.22015380859375 0 80302.17
393.70941162109375 0 290043.8
394.2100524902344 0 95613.06
396.1870422363281 0 146359.44
397.24554443359375 0 315090.62
398.1702575683594 0 103786.16
398.2133483886719 0 255958.06
398.2413330078125 0 58089.82
399.2238464355469 0 68129.59
399.7130126953125 0 60944.484
400.218505859375 0 274373.12
401.24041748046875 0 196847.27
403.18267822265625 0 329152
405.22381591796875 0 110771.31
405.7159423828125 0 123084.664
407.2304992675781 0 102986.03
408.22845458984375 0 222763.36
408.73223876953125 0 87855.17
410.20361328125 0 241351.53
411.2233581542969 0 173672.9
413.2127685546875 0 71528.41
414.2012939453125 0 60758.035
414.2287902832031 0 749282.4 b 11
414.7219543457031 0 1053319.4 y Ammonia loss 7
415.2227478027344 0 614880.8
415.255615234375 0 242309.88
415.72381591796875 0 128381.55
416.2174987792969 0 103869.68
416.2559509277344 0 82579.29
418.1951599121094 0 82014.02
422.22662353515625 0 129975.62
423.2337951660156 0 15215620 y 7
423.7350769042969 0 6572338.5
424.2361755371094 0 1810305.9
424.7368469238281 0 263773.16
425.2401428222656 0 1173706.9 b Water loss 3
426.2431945800781 0 332393.16
428.2164611816406 0 415687.56
429.2348327636719 0 762522.56
430.239990234375 0 420063.5 y Water loss 11
431.2223815917969 0 142808.56 y Ammonia loss 11
439.19268798828125 0 76692.836
440.2232971191406 0 103786.58
441.210205078125 0 842370.4
442.21307373046875 0 151261.97
443.2181396484375 0 127841.4
443.2508850097656 0 1281621.6 b 3
444.2544250488281 0 285698.4
448.25189208984375 0 538347.1 y 11
448.88897705078125 0 325614.1 y Water loss 2
449.2218322753906 0 260420.33 y Ammonia loss 2
449.25543212890625 0 136615.61
449.556396484375 0 188520.25
450.74285888671875 0 156665.78 b 7
451.2411193847656 0 87273.5
452.2265625 0 114288.35
452.2625427246094 0 367674.62
453.26397705078125 0 81843.6
454.89251708984375 0 656779.94 y 2
455.2262878417969 0 402810.47
455.5613098144531 0 183895.31
455.8984069824219 0 75684.33
458.236083984375 0 732442.56
459.2370300292969 0 79089.41
460.2416687011719 0 96072.54
463.2257995605469 0 84688.016
469.2891845703125 0 330640.06
470.2386474609375 0 179777.22
470.2889099121094 0 99619.18
471.7407531738281 0 142110.67 y Water loss 6
471.908447265625 0 85502.96
472.2380676269531 0 226376.39 b 13
472.7353820800781 0 157647.28
478.20355224609375 0 94547.414
480.2523193359375 0 132848.02
480.7471008300781 0 2850922.8 y 6
481.24853515625 0 1319078.1
481.74945068359375 0 363509.22
482.2479248046875 0 83893.87
486.24371337890625 0 59861.953
486.5834655761719 0 615874.7 y Water loss 1
486.9184265136719 0 343831.56 y Ammonia loss 1
487.2544860839844 0 228349.06 y Water loss 10
488.2479248046875 0 181071.05 y Ammonia loss 10
492.5865173339844 0 516523.28 y 1
492.922119140625 0 462675.66
493.25592041015625 0 254180.75
494.7413635253906 0 120029.16
495.2427673339844 0 166669.56
495.9149475097656 0 851595.8
496.2142028808594 0 122359.99
496.2499084472656 0 740424.44
496.3101501464844 0 93164.08
496.5836181640625 0 350555.44
497.2850341796875 0 220095.39
498.23602294921875 0 126279.83
500.19915771484375 0 193167.83
505.27349853515625 0 1752090.6 y 10
506.27618408203125 0 359804.6
507.7521057128906 0 86161.26
509.2569580078125 0 163609.64
514.3237915039062 0 372559.6
515.2566528320312 0 515759.28 y Water loss 5
515.330810546875 0 88251.055
515.7536010742188 0 360822.25 y Ammonia loss 5
516.257080078125 0 309718.28
518.2094116210938 0 230600.31
518.2734375 0 171127.86
518.609619140625 0 203382.39
518.9439086914062 0 118942.06
519.2738037109375 0 80982.74
524.2645263671875 0 16665974 y 5
524.6123657226562 0 2880941 Precursor Ammonia loss
524.764404296875 0 8371530
524.9464111328125 0 1647311.5
525.2669067382812 0 2995762.2
525.7669067382812 0 489509.16
526.2640380859375 0 82780.2
529.2531127929688 0 109284.24
530.2816162109375 0 1871490.1 Precursor
530.6155395507812 0 1897216.1
530.949951171875 0 914804.25
531.2828369140625 0 244716.97
531.6165161132812 0 149638.19
532.2720947265625 0 192690.58
533.2543334960938 0 160607.58
538.3104858398438 0 110717.586
539.2998657226562 0 97624.67
542.2543334960938 0 68326.13
542.2727661132812 0 81463.05
542.3197021484375 0 507558.4 b 4
543.2512817382812 0 99219.7
543.3235473632812 0 108230.375
552.7857666015625 0 155119.75
556.2814331054688 0 143340.81
556.3207397460938 0 124724.57
556.7730712890625 0 111197.51
557.2742919921875 0 112317.74
558.7901611328125 0 234462.53
559.2886962890625 0 217950.05
562.290283203125 0 79622.54 b Water loss 10
564.7913208007812 0 867999.4 y Water loss 4
565.2384643554688 0 136648.98
565.2882080078125 0 962229.7 y Ammonia loss 4
565.7866821289062 0 400346.97
566.2880859375 0 179914.9
567.2938232421875 0 172008.42
569.2691650390625 0 101024.26
572.2736206054688 0 107587.33
572.7908935546875 0 188130.44
573.2916259765625 0 119551.46
573.797119140625 0 23417830 y 4
574.29833984375 0 13893403 y Water loss 9
574.7998046875 0 4479748
575.3002319335938 0 1103859.9
575.8069458007812 0 141368.02
583.2459106445312 0 180200.52
584.3162841796875 0 291656.4
587.2783813476562 0 200031.98
592.3052978515625 0 1859304.4 y 9
593.3082885742188 0 489566.34
594.3101196289062 0 162617.78
600.2730102539062 0 369065.2
602.2904052734375 0 193810.23
611.3163452148438 0 106154.02
617.2994384765625 0 130415.94
621.3056030273438 0 105301.44
623.3145751953125 0 86318.58
624.3096313476562 0 107310.67
626.325927734375 0 188912.16
627.3301391601562 0 108114.77
629.3145141601562 0 1534811.1 y Water loss 3
629.8130493164062 0 1028207.3 y Ammonia loss 3
630.31591796875 0 614187.25
630.8152465820312 0 119384.37
637.3060913085938 0 214915.77
637.8224487304688 0 102468.19
638.3182373046875 0 11299613 y 3
638.81982421875 0 8258296.5
639.32080078125 0 3346826.2
639.8216552734375 0 739033.94
640.3220825195312 0 188000.12
641.3360595703125 0 333502.88
642.3245849609375 0 117656.02
642.8150634765625 0 109124.21
643.3084106445312 0 80922.66
643.355712890625 0 227068.31
643.8179931640625 0 147998.14
646.3163452148438 0 737593.7
647.3201904296875 0 231878.55
648.8193359375 0 342847.25
649.318359375 0 225403.22
649.8207397460938 0 100107.484
651.8198852539062 0 133891.47
652.3148803710938 0 248880.8
652.818359375 0 124216.54
653.3339233398438 0 324501.75
654.32470703125 0 340440.16
654.8181762695312 0 357858.78
655.3211059570312 0 433834
655.8121948242188 0 129342.17
656.3064575195312 0 201888.86
657.2859497070312 0 373298.12
657.8255004882812 0 401386
658.3260498046875 0 227383.25
658.822998046875 0 152650.53
659.34375 0 437530.56
659.8331909179688 0 180569.5
660.2991943359375 0 124252.1
660.3526000976562 0 128051.29
660.8201293945312 0 562029.7
661.3231811523438 0 403944.34
661.8258666992188 0 169122.45
663.8232421875 0 991611.75
664.320556640625 0 753900.8
664.81689453125 0 388392.12
665.3234252929688 0 167180.28
665.8240966796875 0 226063.89
666.3238525390625 0 439221.16
666.829345703125 0 1656205
667.3302001953125 0 1156372.9
667.832763671875 0 465634.66
668.3333740234375 0 74109.73
671.2847900390625 0 122525.984
671.3475341796875 0 1916769.2 y Water loss 8
671.8168334960938 0 120283.93
672.3372192382812 0 1427679.1 y Ammonia loss 8
672.8289184570312 0 5811809.5 y Water loss 2
673.3302612304688 0 5542163 y Ammonia loss 2
673.82958984375 0 2352663.5
674.314208984375 0 2967246
674.8273315429688 0 262375.8
675.3137817382812 0 889190.44
676.3187255859375 0 212354.02
677.8199462890625 0 210418.27
678.3295288085938 0 96282.445
680.8268432617188 0 1368830
681.3287963867188 0 1250789.4
681.8348999023438 0 176759600 y 2
682.3359375 0 129233640
682.8372192382812 0 52579640
683.3380737304688 0 13668467
683.839111328125 0 2538401.5
684.3281860351562 0 85511.79
686.8263549804688 0 1495138.8
687.3289794921875 0 1143247.5
687.8290405273438 0 355250.7
688.340087890625 0 99988.38
689.3579711914062 0 12197581 y 8
690.3607788085938 0 4233635
691.3626708984375 0 1089073.5
692.3634033203125 0 159863.81
699.3411865234375 0 597591 b Ammonia loss 13
700.3447265625 0 269172.47
716.3635864257812 0 107245.69
717.3528442382812 0 186143.42
724.37353515625 0 150358.84
728.3660888671875 0 112442.055
729.3709106445312 0 1721839.5 y Water loss 1
729.8720092773438 0 1462102.9 y Ammonia loss 1
730.3711547851562 0 683643.06
730.8700561523438 0 160668.39
734.8665771484375 0 106874.766
737.3679809570312 0 249302
737.8715209960938 0 171701.89
738.3760986328125 0 14451505 y 1
738.877685546875 0 12175850
739.3788452148438 0 5652146.5
739.8802490234375 0 1373693.6
740.3790893554688 0 310620.62
743.3687133789062 0 933573.8
743.8696899414062 0 486227.56
744.3720703125 0 352790.16 b 6
744.864013671875 0 101794.234
758.38037109375 0 169460.12
768.3994750976562 0 294608.78
770.4159545898438 0 573437
771.420654296875 0 229498.38
784.388427734375 0 111077.555
785.4268188476562 0 242999.92
786.3612060546875 0 106533.8
786.4164428710938 0 306893.8 Precursor Ammonia loss
787.396240234375 0 379922.6
788.4010009765625 0 163680.02
798.4114990234375 0 440864.06
799.413818359375 0 199582.75
803.4351806640625 0 234690.3
804.4412231445312 0 119916.414
810.4329833984375 0 136328.27
811.4052734375 0 352155.84
812.4009399414062 0 121352.66
828.4319458007812 0 1902642.1 y Ammonia loss 7
829.4345703125 0 795870.7
830.4303588867188 0 195352.61
855.396240234375 0 161107.1
857.4420166015625 0 233554.34
858.397705078125 0 224230.92
859.3887939453125 0 125597.63
867.4349975585938 0 164769.69
873.407470703125 0 183703.52
881.4452514648438 0 110601.74
883.4564208984375 0 78222.664 b Ammonia loss 7
885.4349975585938 0 201282.3
887.4240112304688 0 450940
888.4268188476562 0 192465.61
889.4216918945312 0 105181.39
891.4152221679688 0 254926
892.4271240234375 0 109933.29
897.4036865234375 0 184143.67
899.4666748046875 0 158607.7
900.4683227539062 0 472026.84 b 7
901.4588623046875 0 106882.95
909.4527587890625 0 113896.05
915.4166870117188 0 918571.3
916.418701171875 0 308004.97
917.4175415039062 0 165847.4
918.4646606445312 0 87356.21
925.4501342773438 0 156796.97
926.4468994140625 0 203627.39
927.4476318359375 0 123742.69
943.458740234375 0 1690727.2 y Ammonia loss 6
944.466064453125 0 792650.5
945.4714965820312 0 408436.88
951.4555053710938 0 84881.23
954.46044921875 0 140642.9
957.4699096679688 0 117121.18
960.4791259765625 0 72398.516 y 6
968.4810791015625 0 164666.25
969.4717407226562 0 212707.77
970.4695434570312 0 112352.51
972.470947265625 0 239598.98
973.4830322265625 0 142247.06
986.4896240234375 0 1396708.8
987.4891967773438 0 1609574.2
988.4868774414062 0 705763.1
989.4933471679688 0 257163.75
990.4873657226562 0 278426.34
991.4887084960938 0 132613.33
996.468994140625 0 210550.75
997.4750366210938 0 146019.4
1000.480712890625 0 202501.9
1005.49755859375 0 194447.8
1012.4793701171875 0 592643.1
1013.4859619140625 0 368971.97
1014.4842529296875 0 1382367.2
1015.4869995117188 0 789116.9
1016.49072265625 0 252502.86
1030.490478515625 0 4757102.5 y Ammonia loss 5
1031.4931640625 0 2625424.2
1032.495361328125 0 1015090.5
1033.489501953125 0 174537.08
1040.4833984375 0 117838.414
1047.52099609375 0 136644.78 y 5
1057.510498046875 0 124336.45
1073.5211181640625 0 516908.38
1074.5263671875 0 234810.2
1075.5302734375 0 130917.94
1083.5091552734375 0 377853.56
1084.511474609375 0 238819.92
1086.5516357421875 0 320199.9
1087.551025390625 0 180875.38
1088.5501708984375 0 105681.68
1101.5167236328125 0 743073.3
1102.5205078125 0 470308.22
1103.519775390625 0 193725.03
1111.545654296875 0 152093.4
1112.5443115234375 0 181296.69
1113.5440673828125 0 109696.74
1119.5340576171875 0 183854.48
1127.566650390625 0 116857.24
1129.5574951171875 0 1581567.2 y Ammonia loss 4
1130.560791015625 0 935130.06
1131.5589599609375 0 380854.97
1132.5467529296875 0 113936.25
1142.5374755859375 0 171100.56
1143.5372314453125 0 108687.06
1144.5386962890625 0 145687.08
1158.531494140625 0 122231.57
1160.5531005859375 0 842797.44
1161.556884765625 0 441135.5
1162.5621337890625 0 194839.12
1170.5347900390625 0 278674.62
1171.5421142578125 0 168538.44
1188.5487060546875 0 1188509.9
1189.5494384765625 0 574174.8
1190.5596923828125 0 160014.56
1205.5614013671875 0 114029.76
1206.557861328125 0 926686.8
1207.5596923828125 0 571249.4
1208.5623779296875 0 212755.17
1215.5968017578125 0 110927.07
1240.593994140625 0 83950.23
1273.6466064453125 0 115755.34
1302.6341552734375 0 120233.055
1345.6358642578125 0 173463.73 y Ammonia loss 2
1346.6387939453125 0 137183.14
1347.62744140625 0 90374.69

Spectrum Details

|  |  |
| --- | --- |
| Matched peaks? Matched peaksThe total absolute number of peaks matched. Additionally in brackets the total fraction of peaks matched and the total number of peaks is shown. | 72 (11.76% of 612) |
| FDR? FDRThe false discovery rate estimated for this peptide. It is calculated by matching all theoretical fragments with a non-integer shift with the raw peaks for this spectrum. This is done with 40 different shifts. The resulting percentage is the average number of annotated peaks over the number of annotated peaks with the correct spectrum. | 0.66% |
| Satellite FDR? Satellite FDRSee the FDR for details on its calculation. This satellite ion specific FDR only contains the satellite ions (d/w) for I/L/J positions. | - |
| PSM Score? PSM ScoreThe PSM Score as given by Hecklib to this annotated spectrum. It is shown with three significant figures. | 496 |

## Spectrum 3703? Spectrum 3703 The raw spectrum of this peptide as annotated by Hecklib. The fragments are coloured according to ion type (see legend). Any peaks with a star '\*' as text can be hovered over to see the full details, first the ion type second the mass shift type. By hovering over the amino acids in the peptide or ions in the legend the corresponding peaks are highlighted. By toggling the 'Unassigned' label you can turn the background (unassigned) peaks on or off in the plot. By updating the slider in the Ion legend you can update the spectrum to only show the top X% of the peaks with labels. The top X% means any peak that is within X% of the highest intensity. By dragging in the spectrum you can zoom in to a specific part of the spectrum and use 'Zoom Out' to get back to the original zoom level. The annotation of the spectrum is based on the given sequence in the peptides file and is done with different software so inconsistencies are likely. The peaks are annotated based on the given sequence, with 20 ppm tolerance.

Copy Data

### Spectrum 3703 (TSV)

#### Preview

```
Loading example...
```

*Click on the button to copy the data to your clipboard.*

Mz MinMz MaxIntensity Max

WidthHeightPeptide font sizePeptide stroke widthSpectrum font sizeSpectrum stroke widthCompact peptide

Ion legend

wxyz

abcd

OtherUnassignedIonChargePositionShow for top:%

JJSEVSDRPSGVSSR

01.92e+63.83e+65.75e+67.66e+6

Zoom Out

y+11y+35y+11a+12b+12y+12y+12y+12b+13b+13y+13y+13y+27y+27y+13b+312y+28y+28b+14y+14b+14y+14y+313y+313b+28y+313y+29b+314y+29y+314y+314y+15y+15y+314y+15y+210y+210y+210\*\*b+15y+211y+211y+211y+16y+16b+212y+212y+212y+212y+17y+17y+213y+213y+213y+17b+214y+214y+214y+214b+17y+18b+18b+18y+19y+110y+111

0733146722002933

Fragment Matches Table

Show background peaks

| Position | Ion type | Intensity | mz Theoretical | mz Error (Th) | mz Error (ppm) | Charge | Series Number |
| --- | --- | --- | --- | --- | --- | --- | --- |
| - | - | 2.278E+04 | 120.1 | - | - | 0 | - |
| - | - | 2950 | 120.1 | - | - | 0 | - |
| - | - | 3620 | 121.1 | - | - | 0 | - |
| - | - | 2518 | 122.2 | - | - | 0 | - |
| - | - | 2652 | 122.3 | - | - | 0 | - |
| - | - | 6.575E+04 | 126.1 | - | - | 0 | - |
| - | - | 2522 | 127.7 | - | - | 0 | - |
| - | - | 2567 | 128.6 | - | - | 0 | - |
| - | - | 8369 | 129.1 | - | - | 0 | - |
| - | - | 4.051E+04 | 129.1 | - | - | 0 | - |
| - | - | 4427 | 129.1 | - | - | 0 | - |
| - | - | 5763 | 130.1 | - | - | 0 | - |
| - | - | 3.32E+04 | 130.1 | - | - | 0 | - |
| - | - | 1.72E+04 | 130.1 | - | - | 0 | - |
| - | - | 3852 | 130.1 | - | - | 0 | - |
| - | - | 4713 | 131.1 | - | - | 0 | - |
| - | - | 6859 | 131.1 | - | - | 0 | - |
| - | - | 5103 | 133.1 | - | - | 0 | - |
| - | - | 5425 | 133.1 | - | - | 0 | - |
| - | - | 1.753E+04 | 136.1 | - | - | 0 | - |
| - | - | 5271 | 138.1 | - | - | 0 | - |
| - | - | 6069 | 138.1 | - | - | 0 | - |
| - | - | 7534 | 139.1 | - | - | 0 | - |
| - | - | 1.04E+04 | 140.1 | - | - | 0 | - |
| - | - | 3072 | 140.4 | - | - | 0 | - |
| - | - | 3314 | 141.1 | - | - | 0 | - |
| - | - | 8017 | 141.1 | - | - | 0 | - |
| - | - | 1.48E+04 | 144.1 | - | - | 0 | - |
| - | - | 3460 | 145.1 | - | - | 0 | - |
| - | - | 2.081E+04 | 147.1 | - | - | 0 | - |
| - | - | 4476 | 153.1 | - | - | 0 | - |
| - | - | 6.919E+04 | 154.1 | - | - | 0 | - |
| - | - | 3726 | 154.6 | - | - | 0 | - |
| - | - | 3733 | 155.1 | - | - | 0 | - |
| - | - | 7504 | 155.1 | - | - | 0 | - |
| - | - | 4955 | 156.1 | - | - | 0 | - |
| - | - | 2.788E+04 | 157.1 | - | - | 0 | - |
| - | - | 3.68E+04 | 157.1 | - | - | 0 | - |
| - | - | 3.779E+04 | 157.1 | - | - | 0 | - |
| 15 | y | 1.392E+05 | 158.1 | 0.0005077 | 3.211 | +1 | 1 |
| - | - | 8369 | 159.1 | - | - | 0 | - |
| - | - | 1.958E+04 | 159.1 | - | - | 0 | - |
| - | - | 3028 | 159.1 | - | - | 0 | - |
| - | - | 3443 | 162.5 | - | - | 0 | - |
| - | - | 4121 | 165.1 | - | - | 0 | - |
| - | - | 1.006E+04 | 167.1 | - | - | 0 | - |
| - | - | 3407 | 167.1 | - | - | 0 | - |
| 11 | y | 3103 | 169.1 | 0.00169 | 9.997 | +3 | 5 |
| - | - | 7808 | 170 | - | - | 0 | - |
| - | - | 8.203E+04 | 171.1 | - | - | 0 | - |
| - | - | 1.353E+04 | 172.1 | - | - | 0 | - |
| - | - | 4302 | 172.1 | - | - | 0 | - |
| - | - | 2.748E+04 | 173.1 | - | - | 0 | - |
| - | - | 5.775E+04 | 175.1 | - | - | 0 | - |
| 15 | y | 2.773E+05 | 175.1 | 0.0005699 | 3.255 | +1 | 1 |
| - | - | 2.117E+04 | 176.1 | - | - | 0 | - |
| - | - | 6776 | 182.1 | - | - | 0 | - |
| - | - | 6.385E+04 | 183.1 | - | - | 0 | - |
| - | - | 8707 | 184.1 | - | - | 0 | - |
| - | - | 5316 | 184.1 | - | - | 0 | - |
| - | - | 5679 | 185.1 | - | - | 0 | - |
| - | - | 5.197E+04 | 185.1 | - | - | 0 | - |
| - | - | 7770 | 185.1 | - | - | 0 | - |
| - | - | 5644 | 186.1 | - | - | 0 | - |
| - | - | 4213 | 187.1 | - | - | 0 | - |
| - | - | 5.544E+04 | 187.1 | - | - | 0 | - |
| - | - | 6832 | 188.1 | - | - | 0 | - |
| - | - | 3.797E+05 | 189.1 | - | - | 0 | - |
| - | - | 3.483E+04 | 190.1 | - | - | 0 | - |
| - | - | 6614 | 192.1 | - | - | 0 | - |
| - | - | 4683 | 193.1 | - | - | 0 | - |
| - | - | 9397 | 194.1 | - | - | 0 | - |
| - | - | 6145 | 195.1 | - | - | 0 | - |
| - | - | 5199 | 195.1 | - | - | 0 | - |
| - | - | 1.142E+04 | 195.1 | - | - | 0 | - |
| - | - | 5844 | 197.2 | - | - | 0 | - |
| - | - | 2.883E+04 | 199.1 | - | - | 0 | - |
| 2 | a | 2.957E+06 | 199.2 | 0.0006015 | 3.02 | +1 | 2 |
| - | - | 3.619E+05 | 200.2 | - | - | 0 | - |
| - | - | 1.148E+05 | 201.1 | - | - | 0 | - |
| - | - | 2.349E+04 | 201.2 | - | - | 0 | - |
| - | - | 2.262E+04 | 202.1 | - | - | 0 | - |
| - | - | 1.022E+04 | 202.1 | - | - | 0 | - |
| - | - | 5.57E+04 | 203.1 | - | - | 0 | - |
| - | - | 9457 | 203.1 | - | - | 0 | - |
| - | - | 4690 | 209.1 | - | - | 0 | - |
| - | - | 7124 | 209.1 | - | - | 0 | - |
| - | - | 6537 | 209.1 | - | - | 0 | - |
| - | - | 1.21E+04 | 210.1 | - | - | 0 | - |
| - | - | 1.674E+04 | 211.1 | - | - | 0 | - |
| - | - | 2.823E+04 | 212.1 | - | - | 0 | - |
| - | - | 5759 | 212.1 | - | - | 0 | - |
| - | - | 5805 | 213.1 | - | - | 0 | - |
| - | - | 1.111E+04 | 216.1 | - | - | 0 | - |
| - | - | 6.135E+05 | 217.1 | - | - | 0 | - |
| - | - | 5.002E+04 | 218.1 | - | - | 0 | - |
| - | - | 5746 | 219.1 | - | - | 0 | - |
| - | - | 4148 | 223.1 | - | - | 0 | - |
| - | - | 4.18E+04 | 224.1 | - | - | 0 | - |
| - | - | 6373 | 225.1 | - | - | 0 | - |
| - | - | 1.295E+04 | 226.1 | - | - | 0 | - |
| - | - | 3.225E+04 | 226.2 | - | - | 0 | - |
| - | - | 4713 | 227.1 | - | - | 0 | - |
| - | - | 2.6E+04 | 227.1 | - | - | 0 | - |
| 2 | b | 8.517E+05 | 227.2 | 0.000621 | 2.733 | +1 | 2 |
| - | - | 3984 | 228.1 | - | - | 0 | - |
| - | - | 1.136E+05 | 228.2 | - | - | 0 | - |
| - | - | 2.965E+04 | 229.1 | - | - | 0 | - |
| - | - | 1.363E+04 | 229.2 | - | - | 0 | - |
| - | - | 1.82E+04 | 230.1 | - | - | 0 | - |
| - | - | 3.925E+04 | 233.2 | - | - | 0 | - |
| - | - | 7450 | 234.2 | - | - | 0 | - |
| - | - | 4641 | 237.1 | - | - | 0 | - |
| - | - | 2.118E+04 | 237.1 | - | - | 0 | - |
| - | - | 5017 | 238.1 | - | - | 0 | - |
| - | - | 3988 | 241.2 | - | - | 0 | - |
| - | - | 8.207E+04 | 242.1 | - | - | 0 | - |
| - | - | 7600 | 243.1 | - | - | 0 | - |
| - | - | 1.427E+04 | 244.1 | - | - | 0 | - |
| 14 | y | 9382 | 244.1 | 0.001521 | 6.232 | +1 | 2 |
| 14 | y | 8.766E+04 | 245.1 | 0.0007364 | 3.004 | +1 | 2 |
| - | - | 7778 | 246.1 | - | - | 0 | - |
| - | - | 1.872E+04 | 249.2 | - | - | 0 | - |
| - | - | 4192 | 251.1 | - | - | 0 | - |
| - | - | 5114 | 253.2 | - | - | 0 | - |
| - | - | 8626 | 254.1 | - | - | 0 | - |
| - | - | 7785 | 254.2 | - | - | 0 | - |
| - | - | 7.403E+04 | 255.1 | - | - | 0 | - |
| - | - | 4035 | 255.1 | - | - | 0 | - |
| - | - | 6233 | 256.1 | - | - | 0 | - |
| - | - | 7556 | 259.1 | - | - | 0 | - |
| - | - | 1.388E+04 | 261.2 | - | - | 0 | - |
| 14 | y | 5.931E+04 | 262.2 | 0.0006308 | 2.406 | +1 | 2 |
| - | - | 6763 | 262.2 | - | - | 0 | - |
| - | - | 7543 | 263.2 | - | - | 0 | - |
| - | - | 3990 | 266.1 | - | - | 0 | - |
| - | - | 1.628E+04 | 270.1 | - | - | 0 | - |
| - | - | 8256 | 271.1 | - | - | 0 | - |
| - | - | 7215 | 272.1 | - | - | 0 | - |
| - | - | 7427 | 277.2 | - | - | 0 | - |
| - | - | 5531 | 279.1 | - | - | 0 | - |
| - | - | 4935 | 280.1 | - | - | 0 | - |
| - | - | 6160 | 281.1 | - | - | 0 | - |
| - | - | 1.824E+04 | 284.1 | - | - | 0 | - |
| - | - | 1.034E+04 | 284.2 | - | - | 0 | - |
| - | - | 1.238E+04 | 288.2 | - | - | 0 | - |
| - | - | 6752 | 289.2 | - | - | 0 | - |
| - | - | 5984 | 295.2 | - | - | 0 | - |
| - | - | 1.312E+04 | 296.1 | - | - | 0 | - |
| 3 | b | 4.318E+04 | 296.2 | 0.0008858 | 2.99 | +1 | 3 |
| - | - | 1.378E+04 | 297.1 | - | - | 0 | - |
| - | - | 8692 | 297.2 | - | - | 0 | - |
| - | - | 3.138E+04 | 298.1 | - | - | 0 | - |
| - | - | 1.284E+04 | 299.1 | - | - | 0 | - |
| - | - | 5026 | 301.2 | - | - | 0 | - |
| - | - | 1.362E+04 | 302.1 | - | - | 0 | - |
| - | - | 4147 | 302.2 | - | - | 0 | - |
| - | - | 5068 | 307.2 | - | - | 0 | - |
| - | - | 5673 | 311.1 | - | - | 0 | - |
| - | - | 4.438E+04 | 312.2 | - | - | 0 | - |
| - | - | 1.453E+04 | 313.2 | - | - | 0 | - |
| - | - | 9.794E+04 | 313.2 | - | - | 0 | - |
| - | - | 3.329E+04 | 314.1 | - | - | 0 | - |
| - | - | 1.154E+04 | 314.2 | - | - | 0 | - |
| 3 | b | 9.368E+04 | 314.2 | 0.001002 | 3.19 | +1 | 3 |
| - | - | 1.211E+04 | 315.2 | - | - | 0 | - |
| - | - | 1.202E+05 | 316.2 | - | - | 0 | - |
| - | - | 1.474E+04 | 317.2 | - | - | 0 | - |
| - | - | 3987 | 319.2 | - | - | 0 | - |
| - | - | 7052 | 323.2 | - | - | 0 | - |
| - | - | 1.647E+04 | 324.1 | - | - | 0 | - |
| - | - | 4469 | 325.1 | - | - | 0 | - |
| - | - | 5605 | 329.1 | - | - | 0 | - |
| - | - | 2.297E+04 | 330.2 | - | - | 0 | - |
| 13 | y | 2.231E+04 | 331.2 | 0.0001421 | 0.4289 | +1 | 3 |
| 13 | y | 8.234E+04 | 332.2 | 0.0009803 | 2.951 | +1 | 3 |
| - | - | 1.167E+04 | 333.2 | - | - | 0 | - |
| 9 | y | 6606 | 336.2 | 0.002004 | 5.961 | +2 | 7 |
| - | - | 5392 | 338.1 | - | - | 0 | - |
| - | - | 4717 | 339.2 | - | - | 0 | - |
| - | - | 9383 | 340.1 | - | - | 0 | - |
| - | - | 6.691E+04 | 341.2 | - | - | 0 | - |
| - | - | 9.793E+04 | 342.1 | - | - | 0 | - |
| - | - | 8652 | 342.2 | - | - | 0 | - |
| - | - | 1.493E+04 | 343.1 | - | - | 0 | - |
| - | - | 4372 | 343.2 | - | - | 0 | - |
| - | - | 5837 | 344.1 | - | - | 0 | - |
| 9 | y | 3.283E+04 | 345.2 | 0.0006278 | 1.819 | +2 | 7 |
| - | - | 1.839E+04 | 345.7 | - | - | 0 | - |
| 13 | y | 6.693E+04 | 349.2 | 0.000951 | 2.723 | +1 | 3 |
| - | - | 8275 | 350.2 | - | - | 0 | - |
| - | - | 4304 | 353.2 | - | - | 0 | - |
| - | - | 4.55E+04 | 359.2 | - | - | 0 | - |
| - | - | 6144 | 360.2 | - | - | 0 | - |
| - | - | 4100 | 362.1 | - | - | 0 | - |
| - | - | 7419 | 365.2 | - | - | 0 | - |
| - | - | 5070 | 367.2 | - | - | 0 | - |
| - | - | 4637 | 368.9 | - | - | 0 | - |
| - | - | 5451 | 379.2 | - | - | 0 | - |
| - | - | 1.094E+04 | 380.2 | - | - | 0 | - |
| - | - | 1.44E+04 | 385.2 | - | - | 0 | - |
| - | - | 7138 | 385.7 | - | - | 0 | - |
| - | - | 1.591E+04 | 389.7 | - | - | 0 | - |
| - | - | 1.007E+04 | 390.2 | - | - | 0 | - |
| - | - | 1.417E+04 | 390.7 | - | - | 0 | - |
| - | - | 8654 | 391.2 | - | - | 0 | - |
| - | - | 5.303E+04 | 391.7 | - | - | 0 | - |
| - | - | 1.395E+04 | 392.2 | - | - | 0 | - |
| - | - | 8446 | 392.7 | - | - | 0 | - |
| - | - | 1.31E+04 | 393.7 | - | - | 0 | - |
| - | - | 4888 | 395.2 | - | - | 0 | - |
| - | - | 1.203E+04 | 397.2 | - | - | 0 | - |
| - | - | 6330 | 398.2 | - | - | 0 | - |
| - | - | 1.543E+04 | 398.7 | - | - | 0 | - |
| - | - | 1.188E+04 | 399.2 | - | - | 0 | - |
| - | - | 1.717E+04 | 399.7 | - | - | 0 | - |
| - | - | 2.162E+04 | 400.2 | - | - | 0 | - |
| - | - | 5417 | 401.2 | - | - | 0 | - |
| - | - | 1.807E+04 | 403.2 | - | - | 0 | - |
| - | - | 5224 | 404.2 | - | - | 0 | - |
| - | - | 9892 | 405.2 | - | - | 0 | - |
| - | - | 1.225E+04 | 408.2 | - | - | 0 | - |
| - | - | 1.403E+04 | 410.2 | - | - | 0 | - |
| - | - | 9732 | 411.2 | - | - | 0 | - |
| - | - | 5440 | 413.2 | - | - | 0 | - |
| 12 | b | 3.471E+04 | 414.2 | 0.006422 | 15.5 | +3 | 12 |
| 8 | y | 5.241E+04 | 414.7 | 0.002501 | 6.03 | +2 | 8 |
| - | - | 2.805E+04 | 415.2 | - | - | 0 | - |
| - | - | 1.782E+04 | 415.3 | - | - | 0 | - |
| 8 | y | 6.64E+05 | 423.2 | 0.00122 | 2.882 | +2 | 8 |
| - | - | 2.849E+05 | 423.7 | - | - | 0 | - |
| - | - | 7.399E+04 | 424.2 | - | - | 0 | - |
| - | - | 1.318E+04 | 424.7 | - | - | 0 | - |
| 4 | b | 5.088E+04 | 425.2 | 0.0007731 | 1.818 | +1 | 4 |
| - | - | 1.123E+04 | 426.2 | - | - | 0 | - |
| - | - | 2.496E+04 | 428.2 | - | - | 0 | - |
| - | - | 3.431E+04 | 429.2 | - | - | 0 | - |
| 12 | y | 2.214E+04 | 430.2 | 0.0005018 | 1.166 | +1 | 4 |
| - | - | 9029 | 439.2 | - | - | 0 | - |
| - | - | 5068 | 440.2 | - | - | 0 | - |
| - | - | 4.272E+04 | 441.2 | - | - | 0 | - |
| 4 | b | 4.739E+04 | 443.3 | 0.001408 | 3.177 | +1 | 4 |
| - | - | 1.478E+04 | 444.3 | - | - | 0 | - |
| 12 | y | 2.539E+04 | 448.3 | 0.00111 | 2.476 | +1 | 4 |
| 3 | y | 1.387E+04 | 448.9 | 0.0007459 | 1.662 | +3 | 13 |
| 3 | y | 9504 | 449.2 | 0.0088 | 19.59 | +3 | 13 |
| - | - | 5381 | 449.6 | - | - | 0 | - |
| 8 | b | 1.152E+04 | 450.7 | 0.0009361 | 2.077 | +2 | 8 |
| - | - | 5026 | 451.2 | - | - | 0 | - |
| - | - | 7255 | 452.2 | - | - | 0 | - |
| - | - | 2.126E+04 | 452.3 | - | - | 0 | - |
| 3 | y | 2.469E+04 | 454.9 | 0.0008559 | 1.882 | +3 | 13 |
| - | - | 1.938E+04 | 455.2 | - | - | 0 | - |
| - | - | 1.063E+04 | 455.6 | - | - | 0 | - |
| - | - | 3.824E+04 | 458.2 | - | - | 0 | - |
| - | - | 2.132E+04 | 460.2 | - | - | 0 | - |
| - | - | 6056 | 465.3 | - | - | 0 | - |
| - | - | 1.645E+04 | 469.3 | - | - | 0 | - |
| - | - | 4556 | 470.2 | - | - | 0 | - |
| 7 | y | 7162 | 471.7 | 0.001087 | 2.305 | +2 | 9 |
| 14 | b | 1.681E+04 | 472.2 | 0.008094 | 17.14 | +3 | 14 |
| - | - | 7316 | 472.7 | - | - | 0 | - |
| - | - | 5197 | 478.2 | - | - | 0 | - |
| - | - | 4547 | 479.2 | - | - | 0 | - |
| - | - | 7971 | 480.3 | - | - | 0 | - |
| 7 | y | 1.117E+05 | 480.7 | 0.001298 | 2.7 | +2 | 9 |
| - | - | 4.941E+04 | 481.2 | - | - | 0 | - |
| - | - | 1.62E+04 | 481.8 | - | - | 0 | - |
| 2 | y | 1.059E+04 | 486.6 | 0.0003938 | 0.8094 | +3 | 14 |
| 2 | y | 2.414E+04 | 486.9 | 0.008113 | 16.66 | +3 | 14 |
| 11 | y | 7660 | 487.3 | 0.002678 | 5.497 | +1 | 5 |
| 11 | y | 8311 | 488.2 | 0.002197 | 4.5 | +1 | 5 |
| 2 | y | 2.644E+04 | 492.6 | 0.0008701 | 1.766 | +3 | 14 |
| - | - | 1.634E+04 | 492.9 | - | - | 0 | - |
| - | - | 1.127E+04 | 493.3 | - | - | 0 | - |
| - | - | 3.773E+04 | 495.9 | - | - | 0 | - |
| - | - | 8102 | 496.2 | - | - | 0 | - |
| - | - | 2.077E+04 | 496.3 | - | - | 0 | - |
| - | - | 1.299E+04 | 496.6 | - | - | 0 | - |
| - | - | 8593 | 497.2 | - | - | 0 | - |
| - | - | 1.418E+04 | 497.3 | - | - | 0 | - |
| 11 | y | 8.073E+04 | 505.3 | 0.001131 | 2.238 | +1 | 5 |
| - | - | 1.655E+04 | 506.3 | - | - | 0 | - |
| - | - | 5080 | 513.9 | - | - | 0 | - |
| - | - | 5359 | 514.3 | - | - | 0 | - |
| - | - | 1.909E+04 | 514.3 | - | - | 0 | - |
| 6 | y | 1.799E+04 | 515.3 | 0.001011 | 1.962 | +2 | 10 |
| 6 | y | 1.246E+04 | 515.7 | 0.005455 | 10.58 | +2 | 10 |
| - | - | 1.077E+04 | 516.3 | - | - | 0 | - |
| - | - | 6105 | 516.8 | - | - | 0 | - |
| - | - | 5261 | 517.3 | - | - | 0 | - |
| - | - | 8755 | 518.2 | - | - | 0 | - |
| - | - | 7143 | 518.3 | - | - | 0 | - |
| - | - | 8586 | 518.6 | - | - | 0 | - |
| - | - | 6455 | 518.9 | - | - | 0 | - |
| 6 | y | 7.423E+05 | 524.3 | 0.002618 | 4.993 | +2 | 10 |
| 0 | Precursor | 1.458E+05 | 524.6 | 0.00715 | 13.63 | +3 | -1 |
| - | - | 3.907E+05 | 524.8 | - | - | 0 | - |
| - | - | 6.247E+04 | 524.9 | - | - | 0 | - |
| - | - | 1.202E+05 | 525.3 | - | - | 0 | - |
| - | - | 7617 | 525.6 | - | - | 0 | - |
| - | - | 3.666E+04 | 525.8 | - | - | 0 | - |
| - | - | 1.209E+04 | 528.8 | - | - | 0 | - |
| - | - | 9351 | 529.3 | - | - | 0 | - |
| - | - | 1.972E+04 | 529.8 | - | - | 0 | - |
| 0 | Precursor | 8.976E+04 | 530.3 | 0.001128 | 2.128 | +3 | -1 |
| - | - | 5.192E+04 | 530.6 | - | - | 0 | - |
| - | - | 3.275E+04 | 531 | - | - | 0 | - |
| - | - | 1.355E+04 | 532.3 | - | - | 0 | - |
| - | - | 5724 | 533.3 | - | - | 0 | - |
| - | - | 8106 | 539.3 | - | - | 0 | - |
| 5 | b | 1.414E+04 | 542.3 | 0.0023 | 4.241 | +1 | 5 |
| - | - | 5650 | 543.3 | - | - | 0 | - |
| - | - | 1.047E+04 | 556.3 | - | - | 0 | - |
| - | - | 6432 | 556.8 | - | - | 0 | - |
| - | - | 5506 | 558.8 | - | - | 0 | - |
| - | - | 1.205E+04 | 559.3 | - | - | 0 | - |
| 5 | y | 3.502E+04 | 564.8 | 0.0006707 | 1.188 | +2 | 11 |
| - | - | 7286 | 565.2 | - | - | 0 | - |
| 5 | y | 3.352E+04 | 565.3 | 0.005916 | 10.47 | +2 | 11 |
| - | - | 2.135E+04 | 565.8 | - | - | 0 | - |
| - | - | 8273 | 566.3 | - | - | 0 | - |
| - | - | 7583 | 567.3 | - | - | 0 | - |
| - | - | 8924 | 572.3 | - | - | 0 | - |
| - | - | 6936 | 572.8 | - | - | 0 | - |
| - | - | 1.162E+04 | 573.3 | - | - | 0 | - |
| 5 | y | 9.816E+05 | 573.8 | 0.001187 | 2.068 | +2 | 11 |
| 10 | y | 5.855E+05 | 574.3 | 0.004783 | 8.328 | +1 | 6 |
| - | - | 1.894E+05 | 574.8 | - | - | 0 | - |
| - | - | 3.65E+04 | 575.3 | - | - | 0 | - |
| - | - | 1.352E+04 | 583.2 | - | - | 0 | - |
| - | - | 1.326E+04 | 584.3 | - | - | 0 | - |
| - | - | 5969 | 585.3 | - | - | 0 | - |
| - | - | 1.106E+04 | 587.3 | - | - | 0 | - |
| 10 | y | 1.054E+05 | 592.3 | 0.00142 | 2.398 | +1 | 6 |
| - | - | 3.093E+04 | 593.3 | - | - | 0 | - |
| - | - | 1.011E+04 | 594.3 | - | - | 0 | - |
| - | - | 5844 | 596.3 | - | - | 0 | - |
| - | - | 2.26E+04 | 600.3 | - | - | 0 | - |
| - | - | 7381 | 601.3 | - | - | 0 | - |
| - | - | 7728 | 602.3 | - | - | 0 | - |
| - | - | 9810 | 608.3 | - | - | 0 | - |
| 12 | b | 5777 | 612.3 | 0.0001873 | 0.3059 | +2 | 12 |
| - | - | 5813 | 617.3 | - | - | 0 | - |
| - | - | 5938 | 620.3 | - | - | 0 | - |
| - | - | 5256 | 624.3 | - | - | 0 | - |
| - | - | 1.584E+04 | 626.3 | - | - | 0 | - |
| 4 | y | 8.014E+04 | 629.3 | 0.002812 | 4.468 | +2 | 12 |
| 4 | y | 5E+04 | 629.8 | 0.008729 | 13.86 | +2 | 12 |
| - | - | 2.666E+04 | 630.3 | - | - | 0 | - |
| - | - | 9794 | 636.3 | - | - | 0 | - |
| 4 | y | 4.794E+05 | 638.3 | 0.001191 | 1.867 | +2 | 12 |
| - | - | 3.131E+05 | 638.8 | - | - | 0 | - |
| - | - | 1.055E+05 | 639.3 | - | - | 0 | - |
| - | - | 3.387E+04 | 639.8 | - | - | 0 | - |
| - | - | 1.041E+04 | 640.3 | - | - | 0 | - |
| - | - | 9247 | 641.3 | - | - | 0 | - |
| - | - | 7969 | 642.3 | - | - | 0 | - |
| - | - | 7197 | 642.8 | - | - | 0 | - |
| - | - | 1.353E+04 | 643.4 | - | - | 0 | - |
| - | - | 5963 | 644.4 | - | - | 0 | - |
| - | - | 5287 | 645.4 | - | - | 0 | - |
| - | - | 3.451E+04 | 646.3 | - | - | 0 | - |
| - | - | 8422 | 647.3 | - | - | 0 | - |
| - | - | 1.148E+04 | 648.8 | - | - | 0 | - |
| - | - | 1.034E+04 | 649.3 | - | - | 0 | - |
| - | - | 6021 | 649.8 | - | - | 0 | - |
| - | - | 6877 | 651.3 | - | - | 0 | - |
| - | - | 1.192E+04 | 652.3 | - | - | 0 | - |
| - | - | 1.506E+04 | 653.3 | - | - | 0 | - |
| - | - | 2.037E+04 | 654.3 | - | - | 0 | - |
| - | - | 7703 | 654.8 | - | - | 0 | - |
| - | - | 1.253E+04 | 655.3 | - | - | 0 | - |
| - | - | 1.686E+04 | 657.3 | - | - | 0 | - |
| - | - | 6668 | 657.8 | - | - | 0 | - |
| - | - | 2.59E+04 | 659.3 | - | - | 0 | - |
| - | - | 5606 | 659.8 | - | - | 0 | - |
| - | - | 8112 | 660.3 | - | - | 0 | - |
| - | - | 1.197E+04 | 660.3 | - | - | 0 | - |
| - | - | 2.325E+04 | 660.8 | - | - | 0 | - |
| - | - | 1.587E+04 | 661.3 | - | - | 0 | - |
| - | - | 4.12E+04 | 663.8 | - | - | 0 | - |
| - | - | 3.507E+04 | 664.3 | - | - | 0 | - |
| - | - | 2.125E+04 | 664.8 | - | - | 0 | - |
| - | - | 7348 | 665.3 | - | - | 0 | - |
| - | - | 6834 | 665.8 | - | - | 0 | - |
| - | - | 1.978E+04 | 666.3 | - | - | 0 | - |
| - | - | 8.617E+04 | 666.8 | - | - | 0 | - |
| - | - | 5.136E+04 | 667.3 | - | - | 0 | - |
| - | - | 2.093E+04 | 667.8 | - | - | 0 | - |
| - | - | 6859 | 668.3 | - | - | 0 | - |
| 9 | y | 1.074E+05 | 671.3 | 0.001519 | 2.262 | +1 | 7 |
| 9 | y | 7.573E+04 | 672.3 | 0.00615 | 9.148 | +1 | 7 |
| 3 | y | 2.526E+05 | 672.8 | 0.001263 | 1.877 | +2 | 13 |
| 3 | y | 2.417E+05 | 673.3 | 0.01005 | 14.92 | +2 | 13 |
| - | - | 8.987E+04 | 673.8 | - | - | 0 | - |
| - | - | 1.386E+05 | 674.3 | - | - | 0 | - |
| - | - | 1.324E+04 | 674.8 | - | - | 0 | - |
| - | - | 3.992E+04 | 675.3 | - | - | 0 | - |
| - | - | 6113 | 676.3 | - | - | 0 | - |
| - | - | 5811 | 678.3 | - | - | 0 | - |
| - | - | 7.901E+04 | 680.8 | - | - | 0 | - |
| - | - | 7.155E+04 | 681.3 | - | - | 0 | - |
| 3 | y | 7.584E+06 | 681.8 | 0.00184 | 2.698 | +2 | 13 |
| - | - | 5.303E+06 | 682.3 | - | - | 0 | - |
| - | - | 2.137E+06 | 682.8 | - | - | 0 | - |
| - | - | 5.683E+05 | 683.3 | - | - | 0 | - |
| - | - | 1.273E+05 | 683.8 | - | - | 0 | - |
| - | - | 1.121E+04 | 685.4 | - | - | 0 | - |
| - | - | 5.665E+04 | 686.8 | - | - | 0 | - |
| - | - | 4.305E+04 | 687.3 | - | - | 0 | - |
| - | - | 1.83E+04 | 687.8 | - | - | 0 | - |
| 9 | y | 6.107E+05 | 689.4 | 0.001391 | 2.018 | +1 | 7 |
| - | - | 2.148E+05 | 690.4 | - | - | 0 | - |
| - | - | 4.798E+04 | 691.4 | - | - | 0 | - |
| - | - | 8602 | 692.4 | - | - | 0 | - |
| 14 | b | 3.837E+04 | 699.3 | 0.004384 | 6.268 | +2 | 14 |
| - | - | 1.127E+04 | 700.3 | - | - | 0 | - |
| - | - | 1.056E+04 | 728.4 | - | - | 0 | - |
| 2 | y | 7.571E+04 | 729.4 | 0.001162 | 1.593 | +2 | 14 |
| 2 | y | 3.979E+04 | 729.9 | 0.009765 | 13.38 | +2 | 14 |
| - | - | 1.982E+04 | 730.4 | - | - | 0 | - |
| - | - | 8960 | 730.9 | - | - | 0 | - |
| - | - | 6592 | 737.4 | - | - | 0 | - |
| - | - | 8126 | 737.9 | - | - | 0 | - |
| 2 | y | 6.266E+05 | 738.4 | 0.001007 | 1.363 | +2 | 14 |
| - | - | 4.708E+05 | 738.9 | - | - | 0 | - |
| - | - | 2.006E+05 | 739.4 | - | - | 0 | - |
| - | - | 5.847E+04 | 739.9 | - | - | 0 | - |
| - | - | 1.884E+04 | 740.4 | - | - | 0 | - |
| - | - | 3.438E+04 | 743.4 | - | - | 0 | - |
| - | - | 2.189E+04 | 743.9 | - | - | 0 | - |
| 7 | b | 1.515E+04 | 744.4 | 0.003815 | 5.125 | +1 | 7 |
| - | - | 8331 | 758.4 | - | - | 0 | - |
| - | - | 6033 | 767.4 | - | - | 0 | - |
| - | - | 5793 | 768.4 | - | - | 0 | - |
| - | - | 1.386E+04 | 768.4 | - | - | 0 | - |
| - | - | 7650 | 769.4 | - | - | 0 | - |
| - | - | 2.931E+04 | 770.4 | - | - | 0 | - |
| - | - | 7523 | 771.4 | - | - | 0 | - |
| - | - | 1.103E+04 | 778.4 | - | - | 0 | - |
| - | - | 7168 | 779.4 | - | - | 0 | - |
| - | - | 6856 | 780.4 | - | - | 0 | - |
| - | - | 3.463E+04 | 782.4 | - | - | 0 | - |
| - | - | 1.871E+04 | 783.4 | - | - | 0 | - |
| - | - | 6731 | 784.4 | - | - | 0 | - |
| - | - | 1.482E+04 | 785.4 | - | - | 0 | - |
| - | - | 1.106E+04 | 786.4 | - | - | 0 | - |
| - | - | 7408 | 786.4 | - | - | 0 | - |
| - | - | 7059 | 787.4 | - | - | 0 | - |
| - | - | 1.921E+04 | 796.4 | - | - | 0 | - |
| - | - | 6733 | 797.4 | - | - | 0 | - |
| - | - | 2.328E+04 | 798.4 | - | - | 0 | - |
| - | - | 8375 | 799.4 | - | - | 0 | - |
| - | - | 1.496E+04 | 803.4 | - | - | 0 | - |
| - | - | 6822 | 810.4 | - | - | 0 | - |
| - | - | 2.031E+04 | 811.4 | - | - | 0 | - |
| - | - | 8488 | 812.4 | - | - | 0 | - |
| 8 | y | 8.981E+04 | 828.4 | 0.0009867 | 1.191 | +1 | 8 |
| - | - | 3.497E+04 | 829.4 | - | - | 0 | - |
| - | - | 1.36E+04 | 830.4 | - | - | 0 | - |
| - | - | 6077 | 855.4 | - | - | 0 | - |
| - | - | 1.118E+04 | 857.4 | - | - | 0 | - |
| - | - | 7851 | 858.4 | - | - | 0 | - |
| - | - | 6360 | 868.4 | - | - | 0 | - |
| - | - | 1.147E+04 | 873.4 | - | - | 0 | - |
| - | - | 6149 | 874.4 | - | - | 0 | - |
| 8 | b | 4681 | 883.5 | 0.01044 | 11.82 | +1 | 8 |
| - | - | 1.397E+04 | 885.4 | - | - | 0 | - |
| - | - | 2.149E+04 | 887.4 | - | - | 0 | - |
| - | - | 9808 | 888.4 | - | - | 0 | - |
| - | - | 6285 | 889.4 | - | - | 0 | - |
| - | - | 1.514E+04 | 891.4 | - | - | 0 | - |
| - | - | 6420 | 892.4 | - | - | 0 | - |
| - | - | 7308 | 899.5 | - | - | 0 | - |
| 8 | b | 1.891E+04 | 900.5 | 0.009528 | 10.58 | +1 | 8 |
| - | - | 1.158E+04 | 901.5 | - | - | 0 | - |
| - | - | 1.171E+04 | 909.4 | - | - | 0 | - |
| - | - | 5619 | 914.4 | - | - | 0 | - |
| - | - | 4.478E+04 | 915.4 | - | - | 0 | - |
| - | - | 2.349E+04 | 916.4 | - | - | 0 | - |
| - | - | 6523 | 917.4 | - | - | 0 | - |
| - | - | 5879 | 925.5 | - | - | 0 | - |
| - | - | 7351 | 926.4 | - | - | 0 | - |
| - | - | 1.254E+04 | 927.4 | - | - | 0 | - |
| 7 | y | 6.984E+04 | 943.5 | 0.001326 | 1.406 | +1 | 9 |
| - | - | 3.621E+04 | 944.5 | - | - | 0 | - |
| - | - | 1.288E+04 | 945.5 | - | - | 0 | - |
| - | - | 6055 | 946.5 | - | - | 0 | - |
| - | - | 6155 | 952.5 | - | - | 0 | - |
| - | - | 6720 | 953.5 | - | - | 0 | - |
| - | - | 6229 | 957.5 | - | - | 0 | - |
| - | - | 6503 | 968.5 | - | - | 0 | - |
| - | - | 1.325E+04 | 969.5 | - | - | 0 | - |
| - | - | 7073 | 970.5 | - | - | 0 | - |
| - | - | 1.118E+04 | 972.5 | - | - | 0 | - |
| - | - | 6.476E+04 | 986.5 | - | - | 0 | - |
| - | - | 6.904E+04 | 987.5 | - | - | 0 | - |
| - | - | 3.266E+04 | 988.5 | - | - | 0 | - |
| - | - | 1.163E+04 | 989.5 | - | - | 0 | - |
| - | - | 1.459E+04 | 990.5 | - | - | 0 | - |
| - | - | 6785 | 995.5 | - | - | 0 | - |
| - | - | 1.274E+04 | 996.5 | - | - | 0 | - |
| - | - | 6931 | 997.5 | - | - | 0 | - |
| - | - | 9152 | 1000 | - | - | 0 | - |
| - | - | 7910 | 1006 | - | - | 0 | - |
| - | - | 3.453E+04 | 1012 | - | - | 0 | - |
| - | - | 2.332E+04 | 1013 | - | - | 0 | - |
| - | - | 5.393E+04 | 1014 | - | - | 0 | - |
| - | - | 2.522E+04 | 1015 | - | - | 0 | - |
| 6 | y | 2.344E+05 | 1030 | 0.001097 | 1.065 | +1 | 10 |
| - | - | 1.061E+05 | 1031 | - | - | 0 | - |
| - | - | 4.055E+04 | 1032 | - | - | 0 | - |
| - | - | 8617 | 1033 | - | - | 0 | - |
| - | - | 8959 | 1056 | - | - | 0 | - |
| - | - | 7115 | 1057 | - | - | 0 | - |
| - | - | 1.034E+04 | 1058 | - | - | 0 | - |
| - | - | 7258 | 1059 | - | - | 0 | - |
| - | - | 2.3E+04 | 1074 | - | - | 0 | - |
| - | - | 8624 | 1075 | - | - | 0 | - |
| - | - | 1.147E+04 | 1084 | - | - | 0 | - |
| - | - | 1.028E+04 | 1085 | - | - | 0 | - |
| - | - | 1.557E+04 | 1087 | - | - | 0 | - |
| - | - | 6850 | 1088 | - | - | 0 | - |
| - | - | 3.454E+04 | 1102 | - | - | 0 | - |
| - | - | 2.161E+04 | 1103 | - | - | 0 | - |
| - | - | 1.105E+04 | 1112 | - | - | 0 | - |
| - | - | 6471 | 1113 | - | - | 0 | - |
| - | - | 1.427E+04 | 1120 | - | - | 0 | - |
| 5 | y | 6.364E+04 | 1130 | 0.001409 | 1.247 | +1 | 11 |
| - | - | 3.398E+04 | 1131 | - | - | 0 | - |
| - | - | 7768 | 1132 | - | - | 0 | - |
| - | - | 5647 | 1143 | - | - | 0 | - |
| - | - | 6966 | 1144 | - | - | 0 | - |
| - | - | 7645 | 1145 | - | - | 0 | - |
| - | - | 3.534E+04 | 1161 | - | - | 0 | - |
| - | - | 1.709E+04 | 1162 | - | - | 0 | - |
| - | - | 1.426E+04 | 1171 | - | - | 0 | - |
| - | - | 5810 | 1188 | - | - | 0 | - |
| - | - | 3.782E+04 | 1189 | - | - | 0 | - |
| - | - | 2.909E+04 | 1190 | - | - | 0 | - |
| - | - | 5.023E+04 | 1207 | - | - | 0 | - |
| - | - | 3.006E+04 | 1208 | - | - | 0 | - |
| - | - | 1.111E+04 | 1209 | - | - | 0 | - |
| - | - | 6484 | 1216 | - | - | 0 | - |
| - | - | 6945 | 1302 | - | - | 0 | - |
| - | - | 5654 | 2373 | - | - | 0 | - |
| - | - | 5475 | 2904 | - | - | 0 | - |

m/z Charge Intensity FragmentType MassShift Position
120.08124542236328 0 22778.979
120.08514404296875 0 2949.5374
121.0844955444336 0 3619.571
122.19901275634766 0 2517.7878
122.28245544433594 0 2652.356
126.05543518066406 0 65749.38
127.69400787353516 0 2521.8867
128.62628173828125 0 2567.382
129.06631469726562 0 8369.367
129.10267639160156 0 40513.01
129.11419677734375 0 4427.4243
130.05059814453125 0 5763.468
130.0615692138672 0 33204.645
130.0980224609375 0 17204.357
130.10609436035156 0 3851.5051
131.08192443847656 0 4713.1143
131.118408203125 0 6858.5527
133.0614013671875 0 5102.672
133.09756469726562 0 5424.7046
136.07615661621094 0 17534.412
138.06663513183594 0 5271.1953
138.0915985107422 0 6068.7515
139.08726501464844 0 7533.897
140.08245849609375 0 10400.259
140.39524841308594 0 3071.8674
141.0664520263672 0 3313.963
141.10281372070312 0 8017.4707
144.06590270996094 0 14803.255
145.0609130859375 0 3459.9802
147.0768280029297 0 20808.814
153.06605529785156 0 4475.634
154.05035400390625 0 69191.2
154.6253204345703 0 3726.0967
155.05377197265625 0 3733.0562
155.11822509765625 0 7503.593
156.10198974609375 0 4954.6514
157.06124877929688 0 27877.875
157.09759521484375 0 36801.68
157.10888671875 0 37793.67
158.09291076660156 0 139194.42 y Ammonia loss 14
159.09654235839844 0 8369.1045
159.1132354736328 0 19576.883
159.11927795410156 0 3028.4219
162.53790283203125 0 3442.5337
165.1025848388672 0 4120.6587
167.08212280273438 0 10063.839
167.11903381347656 0 3407.0847
169.09750366210938 0 3103.471 y 10
170.04542541503906 0 7808.184
171.07691955566406 0 82026.305
172.06094360351562 0 13533.684
172.0801544189453 0 4302.467
173.1290283203125 0 27483.836
175.07186889648438 0 57751.332
175.11952209472656 0 277334.56 y 14
176.12301635742188 0 21169.338
182.09307861328125 0 6775.643
183.11341857910156 0 63845.36
184.1087646484375 0 8707.118
184.11685180664062 0 5316.419
185.0558319091797 0 5679.499
185.09263610839844 0 51969.918
185.1031494140625 0 7769.515
186.0956573486328 0 5643.963
187.07147216796875 0 4212.997
187.10826110839844 0 55443.766
188.11190795898438 0 6831.594
189.08758544921875 0 379698.1
190.09091186523438 0 34827.625
192.1138153076172 0 6614.211
193.0980987548828 0 4682.588
194.093017578125 0 9397.38
195.07675170898438 0 6144.8
195.1138153076172 0 5198.7217
195.14967346191406 0 11421.175
197.16464233398438 0 5843.576
199.07183837890625 0 28830.879
199.18109130859375 0 2957183.8 a 1
200.1844482421875 0 361858.7
201.1238250732422 0 114791.125
201.18724060058594 0 23494.38
202.1192169189453 0 22623.15
202.12777709960938 0 10216.11
203.0668182373047 0 55696.766
203.10328674316406 0 9457.247
209.093505859375 0 4689.542
209.10369873046875 0 7124.039
209.13999938964844 0 6536.9766
210.08790588378906 0 12099.921
211.10848999023438 0 16742.234
212.10354614257812 0 28225.266
212.13980102539062 0 5759.4175
213.0878448486328 0 5804.656
216.13525390625 0 11111.215
217.08248901367188 0 613514.75
218.08583068847656 0 50021.11
219.0873565673828 0 5746.0947
223.10821533203125 0 4147.971
224.10360717773438 0 41795.43
225.1071319580078 0 6372.9336
226.1194610595703 0 12947.635
226.15557861328125 0 32245.03
227.0656280517578 0 4712.5103
227.11463928222656 0 26004.96
227.176025390625 0 851699.56 b 1
228.098876953125 0 3984.325
228.17935180664062 0 113587.49
229.11911010742188 0 29647.48
229.1818084716797 0 13630.56
230.1142578125 0 18199.32
233.16543579101562 0 39250.38
234.16900634765625 0 7450.011
237.09930419921875 0 4641.269
237.13523864746094 0 21178.16
238.11915588378906 0 5016.6143
241.19122314453125 0 3987.6929
242.1143035888672 0 82065.98
243.1175994873047 0 7599.986
244.12974548339844 0 14272.454
244.14193725585938 0 9382.261 y Water loss 13
245.1251678466797 0 87661.88 y Ammonia loss 13
246.12908935546875 0 7778.445
249.16030883789062 0 18721.602
251.1146240234375 0 4192.08
253.2286376953125 0 5113.7847
254.11407470703125 0 8626.061
254.16204833984375 0 7785.0107
255.1093292236328 0 74027.73
255.14576721191406 0 4034.8142
256.1121520996094 0 6232.671
259.1413269042969 0 7556.223
261.1605529785156 0 13881.969
262.151611328125 0 59307.24 y 13
262.1656188964844 0 6763.238
263.1544189453125 0 7542.6304
266.1136474609375 0 3990.292
270.1455993652344 0 16280.187
271.1291198730469 0 8256.361
272.1369323730469 0 7215.206
277.1555480957031 0 7426.902
279.1091613769531 0 5530.829
280.13006591796875 0 4935.147
281.1255187988281 0 6160.3286
284.1246643066406 0 18241.158
284.1612548828125 0 10340.446
288.15570068359375 0 12380.867
289.151611328125 0 6752.042
295.17803955078125 0 5983.8765
296.13629150390625 0 13118.611
296.19775390625 0 43179.324 b Water loss 2
297.11993408203125 0 13781.118
297.2012634277344 0 8692.093
298.1405944824219 0 31379.742
299.13690185546875 0 12841.992
301.1518859863281 0 5025.6177
302.1359558105469 0 13615.86
302.17156982421875 0 4147.262
307.1632080078125 0 5068.416
311.13543701171875 0 5673.2085
312.15625 0 44383.906
313.153076171875 0 14527.543
313.1880187988281 0 97937.766
314.1470642089844 0 33289.723
314.1903991699219 0 11543.882
314.20843505859375 0 93682.336 b 2
315.2108154296875 0 12106.86
316.1511535644531 0 120230.41
317.152099609375 0 14741.033
319.1802062988281 0 3987.2683
323.2091064453125 0 7052.2607
324.131591796875 0 16470.209
325.1152038574219 0 4468.577
329.1459655761719 0 5604.75
330.1669921875 0 22967.518
331.17230224609375 0 22307.705 y Water loss 12
332.1574401855469 0 82344.625 y Ammonia loss 12
333.15997314453125 0 11666.993
336.17919921875 0 6605.782 y Water loss 8
338.1361389160156 0 5391.927
339.1659851074219 0 4717.048
340.1496276855469 0 9382.675
341.1830139160156 0 66910.42
342.1418151855469 0 97926.53
342.18310546875 0 8652.018
343.1448059082031 0 14929.862
343.1991271972656 0 4372.287
344.145263671875 0 5837.2183
345.18310546875 0 32830.008 y 8
345.6849060058594 0 18391.85
349.1839599609375 0 66928.55 y 12
350.1858825683594 0 8275.452
353.1949462890625 0 4303.9707
359.16796875 0 45498.316
360.17230224609375 0 6143.5273
362.1482238769531 0 4100.469
365.1826171875 0 7419.3145
367.1622314453125 0 5070.3804
368.89666748046875 0 4636.974
379.2350158691406 0 5451.0557
380.15777587890625 0 10940.195
385.17291259765625 0 14404.069
385.7113952636719 0 7138.2954
389.70928955078125 0 15911.186
390.2098693847656 0 10070.386
390.71490478515625 0 14173.643
391.2178039550781 0 8653.778
391.7249755859375 0 53028.895
392.2270202636719 0 13949.553
392.7288513183594 0 8446.232
393.7102966308594 0 13099.405
395.15478515625 0 4888.214
397.2456359863281 0 12029.836
398.21533203125 0 6329.842
398.71453857421875 0 15432.571
399.2176818847656 0 11880.904
399.7208557128906 0 17170.736
400.22149658203125 0 21624.504
401.24139404296875 0 5417.007
403.1831359863281 0 18065.924
404.18896484375 0 5223.7476
405.22552490234375 0 9892.28
408.2275390625 0 12254.591
410.20526123046875 0 14027.358
411.2250061035156 0 9731.716
413.16986083984375 0 5440.374
414.22900390625 0 34706.008 b 11
414.7222595214844 0 52414.5 y Ammonia loss 7
415.2222595214844 0 28048.668
415.2558898925781 0 17820.955
423.2342529296875 0 663981.3 y 7
423.7355041503906 0 284927.53
424.2366638183594 0 73994.84
424.736328125 0 13178.529
425.240234375 0 50884.195 b Water loss 3
426.2433776855469 0 11226.575
428.21588134765625 0 24957.941
429.2353820800781 0 34310.01
430.2403564453125 0 22143.06 y Water loss 11
439.1935119628906 0 9029.377
440.2255554199219 0 5068.1777
441.2104187011719 0 42718.3
443.2514343261719 0 47385.188 b 3
444.2547912597656 0 14784.42
448.2525329589844 0 25389.156 y 11
448.8890075683594 0 13869.611 y Water loss 2
449.2250671386719 0 9504.065 y Ammonia loss 2
449.55694580078125 0 5380.814
450.74383544921875 0 11521.236 b 7
451.1967468261719 0 5025.7524
452.2269592285156 0 7255.242
452.2626647949219 0 21260.1
454.89263916015625 0 24693.3 y 2
455.2268371582031 0 19380.582
455.5616455078125 0 10631.741
458.2362060546875 0 38241.047
460.2417297363281 0 21316.932
465.2621765136719 0 6056.482
469.2898864746094 0 16448.479
470.23614501953125 0 4556.128
471.7423095703125 0 7161.8804 y Water loss 6
472.23583984375 0 16812.13 b 13
472.7350769042969 0 7316.4663
478.2032775878906 0 5197.185
479.2290954589844 0 4547.364
480.25628662109375 0 7971.1104
480.747802734375 0 111738.14 y 6
481.2495422363281 0 49413.656
481.75030517578125 0 16195.298
486.5833435058594 0 10585.407 y Water loss 1
486.9190673828125 0 24137.254 y Ammonia loss 1
487.2596435546875 0 7660.1704 y Water loss 10
488.244140625 0 8311.065 y Ammonia loss 10
492.58734130859375 0 26437.314 y 1
492.9229431152344 0 16340.905
493.25628662109375 0 11272.453
495.9148864746094 0 37726.156
496.2147216796875 0 8101.7
496.25164794921875 0 20768.057
496.5841369628906 0 12985.791
497.2359924316406 0 8593.204
497.284423828125 0 14175.045
505.2740173339844 0 80728.984 y 10
506.27691650390625 0 16551.76
513.9320068359375 0 5079.7866
514.2599487304688 0 5358.507
514.3251342773438 0 19091.717
515.2562255859375 0 17994.203 y Water loss 5
515.7546997070312 0 12463.135 y Ammonia loss 5
516.2575073242188 0 10769.559
516.757568359375 0 6104.9795
517.27001953125 0 5261.254
518.210205078125 0 8754.914
518.2760009765625 0 7143.241
518.61181640625 0 8585.731
518.9437866210938 0 6455.147
524.26513671875 0 742300.56 y 5
524.61279296875 0 145812.77 Precursor Ammonia loss
524.7650146484375 0 390743.97
524.94677734375 0 62469.406
525.2672729492188 0 120196.664
525.614501953125 0 7616.8223
525.767578125 0 36662.723
528.7910766601562 0 12091.521
529.292236328125 0 9351.173
529.7985229492188 0 19715.842
530.2822875976562 0 89755.26 Precursor
530.6165771484375 0 51924.81
530.950927734375 0 32752.064
532.2727661132812 0 13546.694
533.2543334960938 0 5723.828
539.2965698242188 0 8106.3213
542.3207397460938 0 14143.963 b 4
543.3243408203125 0 5649.568
556.320556640625 0 10465.793
556.7741088867188 0 6432.328
558.7938232421875 0 5506.2153
559.2899780273438 0 12049.292
564.7921142578125 0 35017.195 y Water loss 4
565.2421875 0 7286.4307
565.2893676757812 0 33516.58 y Ammonia loss 4
565.7901611328125 0 21345.027
566.2902221679688 0 8272.973
567.2938842773438 0 7582.9224
572.2828979492188 0 8923.7295
572.7903442382812 0 6935.7036
573.288818359375 0 11619.207
573.7979125976562 0 981592.56 y 4
574.2991333007812 0 585538.44 y Water loss 9
574.8006591796875 0 189378.11
575.3009643554688 0 36498.23
583.2463989257812 0 13523.82
584.3157958984375 0 13255.514
585.3069458007812 0 5969.221
587.2809448242188 0 11060.523
592.3063354492188 0 105402.25 y 9
593.3086547851562 0 30929.096
594.3126831054688 0 10107.252
596.3126831054688 0 5844.309
600.2747192382812 0 22601.629
601.2809448242188 0 7380.661
602.2879638671875 0 7727.8765
608.3098754882812 0 9809.591
612.3167724609375 0 5776.784 b Ammonia loss 11
617.3017578125 0 5813.1035
620.3060302734375 0 5937.514
624.3126831054688 0 5255.955
626.3255004882812 0 15836.668
629.3155517578125 0 80140.83 y Water loss 3
629.8134765625 0 49999.92 y Ammonia loss 3
630.3176879882812 0 26656.443
636.3097534179688 0 9793.5
638.3192138671875 0 479438.97 y 3
638.8206787109375 0 313128.9
639.321533203125 0 105521.445
639.8222045898438 0 33874.67
640.3242797851562 0 10413.212
641.3380126953125 0 9246.601
642.3211669921875 0 7968.9478
642.8142700195312 0 7197.2896
643.35400390625 0 13525.871
644.3514404296875 0 5962.7217
645.3626708984375 0 5287.2734
646.3165283203125 0 34507.43
647.3230590820312 0 8421.869
648.8213500976562 0 11482.218
649.31884765625 0 10341.841
649.8214721679688 0 6021.0747
651.3133544921875 0 6876.94
652.315673828125 0 11917.436
653.3363647460938 0 15060.681
654.3258666992188 0 20372.943
654.82568359375 0 7703.346
655.3178100585938 0 12530.027
657.2882690429688 0 16857.576
657.82373046875 0 6667.764
659.34521484375 0 25903.805
659.8317260742188 0 5605.7944
660.2974243164062 0 8111.7847
660.3488159179688 0 11966.877
660.8189086914062 0 23249.342
661.3262939453125 0 15866.8545
663.82373046875 0 41199.19
664.3217163085938 0 35068.42
664.8220825195312 0 21247.105
665.3212890625 0 7347.994
665.8244018554688 0 6833.821
666.325927734375 0 19778.23
666.8302612304688 0 86174.9
667.3313598632812 0 51362.92
667.8322143554688 0 20928.926
668.3248901367188 0 6859.27
671.3486328125 0 107395.336 y Water loss 8
672.3372802734375 0 75725.9 y Ammonia loss 8
672.8300170898438 0 252550.31 y Water loss 2
673.330810546875 0 241697.47 y Ammonia loss 2
673.8292236328125 0 89874.984
674.3158569335938 0 138551.61
674.82861328125 0 13239.202
675.314453125 0 39916.586
676.3168334960938 0 6113.164
678.3317260742188 0 5811.4824
680.8270263671875 0 79013.57
681.3300170898438 0 71554.89
681.8358764648438 0 7584216.5 y 2
682.3370971679688 0 5302956.5
682.8380126953125 0 2137048.2
683.3389892578125 0 568302.2
683.8400268554688 0 127256.086
685.3885498046875 0 11212.249
686.8283081054688 0 56653.285
687.3309326171875 0 43045.457
687.830322265625 0 18302.533
689.3590698242188 0 610651.3 y 8
690.3616943359375 0 214829.28
691.3643798828125 0 47984.16
692.363525390625 0 8602.467
699.3446044921875 0 38369.97 b Ammonia loss 13
700.34521484375 0 11273.154
728.3687744140625 0 10560.787
729.3719482421875 0 75709.305 y Water loss 1
729.87255859375 0 39785.316 y Ammonia loss 1
730.3736572265625 0 19824.639
730.8716430664062 0 8960.089
737.3690185546875 0 6591.5693
737.8750610351562 0 8126.4023
738.3770751953125 0 626569.44 y 1
738.8784790039062 0 470820.25
739.3798828125 0 200551.88
739.88134765625 0 58467.566
740.381103515625 0 18842.037
743.370361328125 0 34380.902
743.8705444335938 0 21887.828
744.3735961914062 0 15152.827 b 6
758.3833618164062 0 8331.376
767.4130249023438 0 6032.5933
768.3526000976562 0 5792.7847
768.40234375 0 13858.147
769.4072875976562 0 7649.9443
770.4158935546875 0 29305.045
771.4249267578125 0 7522.7197
778.4117431640625 0 11031.782
779.4102783203125 0 7167.5474
780.4329833984375 0 6856.276
782.4419555664062 0 34625.094
783.4435424804688 0 18714.678
784.3884887695312 0 6731.237
785.4259033203125 0 14817.608
786.36962890625 0 11064.375
786.4310302734375 0 7407.6313
787.3911743164062 0 7059.3047
796.4207763671875 0 19211.885
797.423095703125 0 6733.2983
798.4208984375 0 23284.152
799.4190673828125 0 8375.175
803.4395751953125 0 14964.436
810.4336547851562 0 6821.611
811.4072875976562 0 20309.768
812.4107055664062 0 8488.11
828.4332275390625 0 89807.9 y Ammonia loss 7
829.4345092773438 0 34966.31
830.405029296875 0 13595.819
855.3949584960938 0 6077.154
857.4454956054688 0 11184.967
858.3956298828125 0 7850.543
868.4337158203125 0 6359.7114
873.4068603515625 0 11468.358
874.4008178710938 0 6149.1494
883.4415283203125 0 4680.6187 b Ammonia loss 7
885.4415893554688 0 13969.439
887.4223022460938 0 21488.566
888.428466796875 0 9808.367
889.427490234375 0 6284.9355
891.4172973632812 0 15137.476
892.4227294921875 0 6419.7246
899.4700927734375 0 7308.341
900.468994140625 0 18913.396 b 7
901.4523315429688 0 11576.356
909.44189453125 0 11713.26
914.448974609375 0 5619.1865
915.4191284179688 0 44776.668
916.421875 0 23486.05
917.4136962890625 0 6523.053
925.455078125 0 5879.102
926.4258422851562 0 7350.568
927.4495239257812 0 12541.031
943.4605102539062 0 69838.17 y Ammonia loss 6
944.4680786132812 0 36210.316
945.4724731445312 0 12876.82
946.4723510742188 0 6055.473
952.46435546875 0 6154.745
953.4570922851562 0 6719.781
957.4688110351562 0 6229.0396
968.4849853515625 0 6503.3687
969.4754028320312 0 13253.384
970.4752197265625 0 7072.673
972.4776000976562 0 11180.26
986.4920043945312 0 64763.64
987.489013671875 0 69035.03
988.4887084960938 0 32661.857
989.4908447265625 0 11634.13
990.4902954101562 0 14589.525
995.4662475585938 0 6785.2144
996.4734497070312 0 12735.612
997.4696655273438 0 6930.91
1000.4842529296875 0 9152.303
1006.4990844726562 0 7910.098
1012.48046875 0 34532.26
1013.4822998046875 0 23319.502
1014.4856567382812 0 53932.9
1015.4891357421875 0 25224.908
1030.4923095703125 0 234442.14 y Ammonia loss 5
1031.4951171875 0 106127.81
1032.4981689453125 0 40546.29
1033.4959716796875 0 8616.876
1055.511962890625 0 8959.273
1056.5228271484375 0 7115.1694
1057.5184326171875 0 10336.273
1058.53466796875 0 7258.3496
1073.5206298828125 0 23004.482
1074.5301513671875 0 8624.339
1083.5133056640625 0 11474.936
1084.5101318359375 0 10284.317
1086.55224609375 0 15567.071
1087.5660400390625 0 6849.7397
1101.52001953125 0 34538.836
1102.52294921875 0 21606.082
1111.5494384765625 0 11054.265
1112.539794921875 0 6471.069
1119.5316162109375 0 14271.518
1129.56103515625 0 63643.164 y Ammonia loss 4
1130.5615234375 0 33977.24
1131.5616455078125 0 7768.152
1142.546630859375 0 5646.7783
1143.538330078125 0 6966.171
1144.560302734375 0 7644.5825
1160.55322265625 0 35335.89
1161.55615234375 0 17094.883
1170.5418701171875 0 14263.678
1187.55859375 0 5809.861
1188.5511474609375 0 37822.61
1189.552001953125 0 29092.002
1206.560546875 0 50233.39
1207.563232421875 0 30064.393
1208.56591796875 0 11108.366
1215.5977783203125 0 6483.5684
1301.63623046875 0 6945.493
2373.021728515625 0 5653.9414
2904.10595703125 0 5474.5225

Spectrum Details

|  |  |
| --- | --- |
| Matched peaks? Matched peaksThe total absolute number of peaks matched. Additionally in brackets the total fraction of peaks matched and the total number of peaks is shown. | 67 (12.23% of 548) |
| FDR? FDRThe false discovery rate estimated for this peptide. It is calculated by matching all theoretical fragments with a non-integer shift with the raw peaks for this spectrum. This is done with 40 different shifts. The resulting percentage is the average number of annotated peaks over the number of annotated peaks with the correct spectrum. | 0.39% |
| Satellite FDR? Satellite FDRSee the FDR for details on its calculation. This satellite ion specific FDR only contains the satellite ions (d/w) for I/L/J positions. | - |
| PSM Score? PSM ScoreThe PSM Score as given by Hecklib to this annotated spectrum. It is shown with three significant figures. | 421 |

## Spectrum 4798? Spectrum 4798 The raw spectrum of this peptide as annotated by Hecklib. The fragments are coloured according to ion type (see legend). Any peaks with a star '\*' as text can be hovered over to see the full details, first the ion type second the mass shift type. By hovering over the amino acids in the peptide or ions in the legend the corresponding peaks are highlighted. By toggling the 'Unassigned' label you can turn the background (unassigned) peaks on or off in the plot. By updating the slider in the Ion legend you can update the spectrum to only show the top X% of the peaks with labels. The top X% means any peak that is within X% of the highest intensity. By dragging in the spectrum you can zoom in to a specific part of the spectrum and use 'Zoom Out' to get back to the original zoom level. The annotation of the spectrum is based on the given sequence in the peptides file and is done with different software so inconsistencies are likely. The peaks are annotated based on the given sequence, with 20 ppm tolerance.

Copy Data

### Spectrum 4798 (TSV)

#### Preview

```
Loading example...
```

*Click on the button to copy the data to your clipboard.*

Mz MinMz MaxIntensity Max

WidthHeightPeptide font sizePeptide stroke widthSpectrum font sizeSpectrum stroke widthCompact peptide

Ion legend

wxyz

abcd

OtherUnassignedIonChargePositionShow for top:%

JJSEVSDRPSGVSSR

01.07e+42.14e+43.21e+44.28e+4

Zoom Out

y+11w+12y+12z+12y+12w+13y+13z+13y+13z+28w+14y+14c+14z+29y+29c+314z+29y+15z+210y+210w+16c+15z+211y+211y+16y+16z+16w+212c+212z+212y+212c+16y+17y+213z+213y+213y+17w+214z+214y+214w+18c+17z+18c+18y+19z+19y+19c+19z+110y+110c+110z+111c+111c+112y+112c+113c+114

0843168625293373

Fragment Matches Table

Show background peaks

| Position | Ion type | Intensity | mz Theoretical | mz Error (Th) | mz Error (ppm) | Charge | Series Number |
| --- | --- | --- | --- | --- | --- | --- | --- |
| - | - | 570.3 | 120.1 | - | - | 0 | - |
| - | - | 410.6 | 123 | - | - | 0 | - |
| - | - | 899.6 | 125.1 | - | - | 0 | - |
| - | - | 1216 | 126.1 | - | - | 0 | - |
| - | - | 1191 | 133.1 | - | - | 0 | - |
| - | - | 490.6 | 136.1 | - | - | 0 | - |
| - | - | 412.9 | 139.1 | - | - | 0 | - |
| - | - | 824.4 | 149 | - | - | 0 | - |
| - | - | 495.8 | 160.4 | - | - | 0 | - |
| - | - | 511 | 163.1 | - | - | 0 | - |
| - | - | 490.4 | 166.9 | - | - | 0 | - |
| - | - | 1369 | 168.1 | - | - | 0 | - |
| - | - | 1027 | 171.1 | - | - | 0 | - |
| - | - | 1148 | 173.4 | - | - | 0 | - |
| 15 | y | 2615 | 175.1 | 9.907E-06 | 0.05657 | +1 | 1 |
| - | - | 620.3 | 177.1 | - | - | 0 | - |
| - | - | 541.9 | 179.1 | - | - | 0 | - |
| - | - | 439.2 | 179.2 | - | - | 0 | - |
| - | - | 711.3 | 185.1 | - | - | 0 | - |
| - | - | 6141 | 185.1 | - | - | 0 | - |
| - | - | 609.7 | 186.1 | - | - | 0 | - |
| - | - | 1.286E+04 | 187.1 | - | - | 0 | - |
| - | - | 952.5 | 188.1 | - | - | 0 | - |
| - | - | 542.9 | 189.1 | - | - | 0 | - |
| - | - | 739.7 | 199.2 | - | - | 0 | - |
| - | - | 1.145E+04 | 199.2 | - | - | 0 | - |
| - | - | 810.5 | 200.1 | - | - | 0 | - |
| - | - | 1731 | 200.2 | - | - | 0 | - |
| - | - | 483.9 | 201.1 | - | - | 0 | - |
| - | - | 1772 | 214.2 | - | - | 0 | - |
| - | - | 497 | 214.9 | - | - | 0 | - |
| - | - | 679.6 | 215.1 | - | - | 0 | - |
| - | - | 5779 | 227.2 | - | - | 0 | - |
| - | - | 961.8 | 228.2 | - | - | 0 | - |
| 14 | w | 953.6 | 229.1 | 0.0001218 | 0.5317 | +1 | 2 |
| 14 | y | 9895 | 245.1 | 0.0001108 | 0.4519 | +1 | 2 |
| 14 | z | 1802 | 246.1 | 0.001031 | 4.188 | +1 | 2 |
| 14 | y | 931.2 | 262.2 | 0.0003256 | 1.242 | +1 | 2 |
| - | - | 696.2 | 270.1 | - | - | 0 | - |
| - | - | 608.3 | 276.9 | - | - | 0 | - |
| 13 | w | 4339 | 316.2 | 0.00032 | 1.012 | +1 | 3 |
| - | - | 871.3 | 317.2 | - | - | 0 | - |
| - | - | 957.7 | 331.1 | - | - | 0 | - |
| 13 | y | 1445 | 332.2 | 0.0006982 | 2.102 | +1 | 3 |
| - | - | 1085 | 333.1 | - | - | 0 | - |
| 13 | z | 3579 | 333.2 | 0.0001743 | 0.5232 | +1 | 3 |
| - | - | 2117 | 334.2 | - | - | 0 | - |
| - | - | 822.1 | 349.1 | - | - | 0 | - |
| 13 | y | 1227 | 349.2 | 0.0001477 | 0.4229 | +1 | 3 |
| - | - | 2478 | 357.2 | - | - | 0 | - |
| - | - | 665.4 | 399.7 | - | - | 0 | - |
| - | - | 673.1 | 404.2 | - | - | 0 | - |
| 8 | z | 1346 | 415.2 | 0.001243 | 2.995 | +2 | 8 |
| - | - | 2316 | 415.3 | - | - | 0 | - |
| 12 | w | 3123 | 417.2 | 0.0007677 | 1.84 | +1 | 4 |
| - | - | 957.1 | 418.2 | - | - | 0 | - |
| - | - | 769.8 | 430.3 | - | - | 0 | - |
| - | - | 2262 | 439.3 | - | - | 0 | - |
| - | - | 1098 | 440.2 | - | - | 0 | - |
| - | - | 1007 | 443.3 | - | - | 0 | - |
| 12 | y | 3885 | 448.3 | 0.0004387 | 0.9786 | +1 | 4 |
| - | - | 1492 | 457.7 | - | - | 0 | - |
| - | - | 1995 | 458.3 | - | - | 0 | - |
| 4 | c | 2213 | 460.3 | 0.0001889 | 0.4104 | +1 | 4 |
| - | - | 924.3 | 461.3 | - | - | 0 | - |
| - | - | 2611 | 462.7 | - | - | 0 | - |
| - | - | 1295 | 463.2 | - | - | 0 | - |
| 7 | z | 625.4 | 463.7 | 0.002393 | 5.159 | +2 | 9 |
| 7 | y | 2523 | 471.7 | 0.003643 | 7.722 | +2 | 9 |
| 14 | c | 983.2 | 472.2 | 0.004265 | 9.032 | +3 | 14 |
| 7 | z | 980.5 | 472.7 | 0.0006198 | 1.311 | +2 | 9 |
| - | - | 1459 | 474.2 | - | - | 0 | - |
| - | - | 2546 | 475.3 | - | - | 0 | - |
| - | - | 661.4 | 486.3 | - | - | 0 | - |
| - | - | 725.9 | 490.3 | - | - | 0 | - |
| - | - | 761 | 499.3 | - | - | 0 | - |
| - | - | 2104 | 501.3 | - | - | 0 | - |
| - | - | 3440 | 502.3 | - | - | 0 | - |
| - | - | 720.6 | 503.3 | - | - | 0 | - |
| - | - | 701.5 | 504.3 | - | - | 0 | - |
| 11 | y | 1.253E+04 | 505.3 | 0.0004898 | 0.9695 | +1 | 5 |
| - | - | 2844 | 506.3 | - | - | 0 | - |
| - | - | 905.1 | 507.3 | - | - | 0 | - |
| - | - | 648.7 | 513.3 | - | - | 0 | - |
| 6 | z | 1310 | 516.3 | 0.0002272 | 0.4401 | +2 | 10 |
| - | - | 1047 | 516.8 | - | - | 0 | - |
| - | - | 770.4 | 521.3 | - | - | 0 | - |
| 6 | y | 4631 | 524.3 | 0.001214 | 2.316 | +2 | 10 |
| - | - | 1787 | 524.6 | - | - | 0 | - |
| - | - | 2255 | 524.8 | - | - | 0 | - |
| - | - | 987.6 | 524.9 | - | - | 0 | - |
| - | - | 656.8 | 529.8 | - | - | 0 | - |
| - | - | 4.242E+04 | 530.3 | - | - | 0 | - |
| - | - | 1226 | 530.6 | - | - | 0 | - |
| - | - | 1012 | 530.9 | - | - | 0 | - |
| - | - | 1.125E+04 | 531.3 | - | - | 0 | - |
| - | - | 1136 | 542.3 | - | - | 0 | - |
| - | - | 696 | 558.3 | - | - | 0 | - |
| 10 | w | 700.5 | 559.3 | 0.0006673 | 1.193 | +1 | 6 |
| 5 | c | 1845 | 559.3 | 0.001019 | 1.822 | +1 | 5 |
| - | - | 601.7 | 560.3 | - | - | 0 | - |
| 5 | z | 8826 | 565.8 | 5.072E-05 | 0.08965 | +2 | 11 |
| - | - | 3332 | 566.3 | - | - | 0 | - |
| - | - | 2894 | 566.8 | - | - | 0 | - |
| - | - | 730.8 | 567.3 | - | - | 0 | - |
| - | - | 2606 | 572.3 | - | - | 0 | - |
| - | - | 1724 | 573.3 | - | - | 0 | - |
| 5 | y | 5671 | 573.8 | 0.0002102 | 0.3663 | +2 | 11 |
| 10 | y | 3500 | 574.3 | 0.004539 | 7.903 | +1 | 6 |
| - | - | 1072 | 574.8 | - | - | 0 | - |
| 10 | y | 771.3 | 575.3 | 0.001663 | 2.891 | +1 | 6 |
| 10 | z | 1573 | 576.3 | 0.0003633 | 0.6305 | +1 | 6 |
| - | - | 869.3 | 577.3 | - | - | 0 | - |
| - | - | 3719 | 585.3 | - | - | 0 | - |
| - | - | 604.2 | 586.3 | - | - | 0 | - |
| - | - | 993.1 | 600.3 | - | - | 0 | - |
| 4 | w | 2675 | 600.8 | 6.255E-06 | 0.01041 | +2 | 12 |
| - | - | 1899 | 601.3 | - | - | 0 | - |
| - | - | 1355 | 601.8 | - | - | 0 | - |
| 12 | c | 1474 | 629.3 | 0.008664 | 13.77 | +2 | 12 |
| 4 | z | 8524 | 630.3 | 0.0002996 | 0.4753 | +2 | 12 |
| - | - | 7646 | 630.8 | - | - | 0 | - |
| - | - | 3688 | 631.3 | - | - | 0 | - |
| - | - | 834.7 | 631.8 | - | - | 0 | - |
| - | - | 3044 | 637.8 | - | - | 0 | - |
| 4 | y | 8162 | 638.3 | 0.0005786 | 0.9064 | +2 | 12 |
| - | - | 5756 | 638.8 | - | - | 0 | - |
| - | - | 2020 | 639.3 | - | - | 0 | - |
| - | - | 943.6 | 643.4 | - | - | 0 | - |
| - | - | 729.5 | 644.8 | - | - | 0 | - |
| 6 | c | 9591 | 646.4 | 0.000363 | 0.5616 | +1 | 6 |
| - | - | 2796 | 647.4 | - | - | 0 | - |
| - | - | 607.1 | 648.4 | - | - | 0 | - |
| 9 | y | 659.1 | 672.3 | 0.009385 | 13.96 | +1 | 7 |
| 3 | y | 638.5 | 673.3 | 0.01127 | 16.74 | +2 | 13 |
| 3 | z | 5626 | 673.8 | 0.0008869 | 1.316 | +2 | 13 |
| - | - | 8591 | 674.3 | - | - | 0 | - |
| - | - | 4042 | 674.8 | - | - | 0 | - |
| - | - | 1485 | 675.3 | - | - | 0 | - |
| - | - | 1167 | 675.8 | - | - | 0 | - |
| - | - | 959.6 | 681.3 | - | - | 0 | - |
| 3 | y | 3.466E+04 | 681.8 | 6.983E-05 | 0.1024 | +2 | 13 |
| - | - | 2.548E+04 | 682.3 | - | - | 0 | - |
| - | - | 9365 | 682.8 | - | - | 0 | - |
| - | - | 3169 | 683.3 | - | - | 0 | - |
| - | - | 2712 | 686.4 | - | - | 0 | - |
| - | - | 3438 | 687.4 | - | - | 0 | - |
| - | - | 1888 | 688.4 | - | - | 0 | - |
| 9 | y | 1199 | 689.4 | 0.008166 | 11.85 | +1 | 7 |
| - | - | 824 | 702.4 | - | - | 0 | - |
| - | - | 1529 | 711.4 | - | - | 0 | - |
| - | - | 1431 | 711.9 | - | - | 0 | - |
| 2 | w | 1356 | 715.8 | 0.0007084 | 0.9897 | +2 | 14 |
| - | - | 861.5 | 718.4 | - | - | 0 | - |
| - | - | 682.7 | 719.4 | - | - | 0 | - |
| - | - | 1104 | 727.4 | - | - | 0 | - |
| 2 | z | 1084 | 730.4 | 0.001899 | 2.601 | +2 | 14 |
| - | - | 625.4 | 730.9 | - | - | 0 | - |
| 2 | y | 3369 | 738.4 | 0.0006414 | 0.8686 | +2 | 14 |
| - | - | 1356 | 738.9 | - | - | 0 | - |
| 8 | w | 837.6 | 743.4 | 0.003766 | 5.066 | +1 | 8 |
| - | - | 3595 | 744.4 | - | - | 0 | - |
| - | - | 1142 | 745.4 | - | - | 0 | - |
| - | - | 956.5 | 749.9 | - | - | 0 | - |
| - | - | 914.3 | 750.9 | - | - | 0 | - |
| - | - | 1003 | 758.4 | - | - | 0 | - |
| - | - | 1641 | 758.9 | - | - | 0 | - |
| - | - | 1429 | 759.4 | - | - | 0 | - |
| 7 | c | 1.633E+04 | 761.4 | 0.0005196 | 0.6824 | +1 | 7 |
| - | - | 7465 | 762.4 | - | - | 0 | - |
| - | - | 1225 | 763.4 | - | - | 0 | - |
| - | - | 1381 | 764.9 | - | - | 0 | - |
| - | - | 2.637E+04 | 765.4 | - | - | 0 | - |
| - | - | 1.874E+04 | 765.9 | - | - | 0 | - |
| - | - | 1.048E+04 | 766.4 | - | - | 0 | - |
| - | - | 2465 | 766.9 | - | - | 0 | - |
| - | - | 940.6 | 767.4 | - | - | 0 | - |
| - | - | 756.4 | 772.4 | - | - | 0 | - |
| - | - | 724.3 | 772.9 | - | - | 0 | - |
| - | - | 918.5 | 773.4 | - | - | 0 | - |
| - | - | 8012 | 773.9 | - | - | 0 | - |
| - | - | 6226 | 774.4 | - | - | 0 | - |
| - | - | 3231 | 774.9 | - | - | 0 | - |
| - | - | 5134 | 775.4 | - | - | 0 | - |
| - | - | 2059 | 776.4 | - | - | 0 | - |
| - | - | 982.8 | 777.9 | - | - | 0 | - |
| - | - | 843.4 | 779.4 | - | - | 0 | - |
| - | - | 896.2 | 779.9 | - | - | 0 | - |
| - | - | 716.3 | 782.4 | - | - | 0 | - |
| - | - | 691.1 | 785.4 | - | - | 0 | - |
| - | - | 1101 | 785.9 | - | - | 0 | - |
| - | - | 2817 | 786.4 | - | - | 0 | - |
| - | - | 3.057E+04 | 786.9 | - | - | 0 | - |
| - | - | 2.358E+04 | 787.4 | - | - | 0 | - |
| - | - | 1.078E+04 | 787.9 | - | - | 0 | - |
| - | - | 5093 | 788.4 | - | - | 0 | - |
| - | - | 693 | 788.9 | - | - | 0 | - |
| - | - | 700.3 | 793.4 | - | - | 0 | - |
| - | - | 925.7 | 793.9 | - | - | 0 | - |
| - | - | 1122 | 794.4 | - | - | 0 | - |
| - | - | 1.908E+04 | 794.9 | - | - | 0 | - |
| - | - | 2.408E+04 | 795.4 | - | - | 0 | - |
| - | - | 1.477E+04 | 795.9 | - | - | 0 | - |
| - | - | 5781 | 796.4 | - | - | 0 | - |
| - | - | 2390 | 796.9 | - | - | 0 | - |
| - | - | 1182 | 797.4 | - | - | 0 | - |
| - | - | 3067 | 827.4 | - | - | 0 | - |
| - | - | 1663 | 828.4 | - | - | 0 | - |
| 8 | z | 1.244E+04 | 829.4 | 0.0006127 | 0.7387 | +1 | 8 |
| - | - | 6037 | 830.4 | - | - | 0 | - |
| - | - | 3335 | 831.4 | - | - | 0 | - |
| - | - | 1587 | 832.4 | - | - | 0 | - |
| - | - | 2147 | 839.4 | - | - | 0 | - |
| - | - | 875.9 | 844.5 | - | - | 0 | - |
| - | - | 3661 | 845.4 | - | - | 0 | - |
| - | - | 2498 | 846.4 | - | - | 0 | - |
| - | - | 880.8 | 847.4 | - | - | 0 | - |
| - | - | 2405 | 857.4 | - | - | 0 | - |
| - | - | 861 | 858.4 | - | - | 0 | - |
| - | - | 1014 | 871.4 | - | - | 0 | - |
| - | - | 632.5 | 872.4 | - | - | 0 | - |
| - | - | 1203 | 874.4 | - | - | 0 | - |
| - | - | 1026 | 888.5 | - | - | 0 | - |
| 8 | c | 2097 | 900.5 | 0.0005426 | 0.6026 | +1 | 8 |
| - | - | 606.4 | 910.7 | - | - | 0 | - |
| - | - | 650.3 | 914.4 | - | - | 0 | - |
| - | - | 551.7 | 928.4 | - | - | 0 | - |
| - | - | 1077 | 931.4 | - | - | 0 | - |
| - | - | 1009 | 932.4 | - | - | 0 | - |
| 7 | y | 2015 | 943.5 | 0.01695 | 17.97 | +1 | 9 |
| 7 | z | 6032 | 944.5 | 0.002962 | 3.136 | +1 | 9 |
| - | - | 5141 | 945.5 | - | - | 0 | - |
| - | - | 1410 | 946.5 | - | - | 0 | - |
| 7 | y | 1123 | 960.5 | 0.003647 | 3.797 | +1 | 9 |
| - | - | 1217 | 970.5 | - | - | 0 | - |
| - | - | 719.5 | 975.5 | - | - | 0 | - |
| - | - | 3154 | 988.5 | - | - | 0 | - |
| - | - | 2384 | 989.5 | - | - | 0 | - |
| - | - | 1567 | 999.6 | - | - | 0 | - |
| - | - | 1191 | 1001 | - | - | 0 | - |
| 9 | c | 1.454E+04 | 1015 | 0.0002791 | 0.2751 | +1 | 9 |
| - | - | 9584 | 1016 | - | - | 0 | - |
| - | - | 2746 | 1017 | - | - | 0 | - |
| - | - | 1540 | 1018 | - | - | 0 | - |
| - | - | 754.9 | 1019 | - | - | 0 | - |
| 6 | z | 1980 | 1031 | 0.0008406 | 0.815 | +1 | 10 |
| - | - | 2224 | 1033 | - | - | 0 | - |
| - | - | 1212 | 1034 | - | - | 0 | - |
| - | - | 6856 | 1043 | - | - | 0 | - |
| - | - | 4146 | 1044 | - | - | 0 | - |
| - | - | 1.276E+04 | 1045 | - | - | 0 | - |
| - | - | 6451 | 1046 | - | - | 0 | - |
| - | - | 2289 | 1047 | - | - | 0 | - |
| 6 | y | 817.9 | 1048 | 0.001526 | 1.457 | +1 | 10 |
| - | - | 1008 | 1049 | - | - | 0 | - |
| - | - | 7595 | 1058 | - | - | 0 | - |
| - | - | 7685 | 1059 | - | - | 0 | - |
| - | - | 9474 | 1060 | - | - | 0 | - |
| - | - | 5363 | 1061 | - | - | 0 | - |
| - | - | 9861 | 1062 | - | - | 0 | - |
| - | - | 5216 | 1063 | - | - | 0 | - |
| - | - | 681.7 | 1064 | - | - | 0 | - |
| 10 | c | 1.549E+04 | 1102 | 0.0003463 | 0.3144 | +1 | 10 |
| - | - | 8813 | 1103 | - | - | 0 | - |
| - | - | 3456 | 1104 | - | - | 0 | - |
| - | - | 815.3 | 1105 | - | - | 0 | - |
| - | - | 2851 | 1115 | - | - | 0 | - |
| - | - | 2032 | 1116 | - | - | 0 | - |
| - | - | 1181 | 1117 | - | - | 0 | - |
| 5 | z | 2244 | 1131 | 0.001274 | 1.127 | +1 | 11 |
| - | - | 5951 | 1132 | - | - | 0 | - |
| - | - | 3093 | 1133 | - | - | 0 | - |
| - | - | 1105 | 1134 | - | - | 0 | - |
| 11 | c | 9115 | 1159 | 0.0007332 | 0.6328 | +1 | 11 |
| - | - | 7018 | 1160 | - | - | 0 | - |
| - | - | 1368 | 1161 | - | - | 0 | - |
| - | - | 1727 | 1214 | - | - | 0 | - |
| - | - | 1017 | 1215 | - | - | 0 | - |
| 12 | c | 8918 | 1258 | 0.00103 | 0.8193 | +1 | 12 |
| - | - | 5564 | 1259 | - | - | 0 | - |
| - | - | 2051 | 1260 | - | - | 0 | - |
| - | - | 7500 | 1261 | - | - | 0 | - |
| - | - | 3848 | 1262 | - | - | 0 | - |
| - | - | 2077 | 1263 | - | - | 0 | - |
| 4 | y | 2794 | 1276 | 0.0009608 | 0.7532 | +1 | 12 |
| - | - | 1667 | 1277 | - | - | 0 | - |
| - | - | 908.6 | 1278 | - | - | 0 | - |
| - | - | 584.8 | 1281 | - | - | 0 | - |
| - | - | 1189 | 1301 | - | - | 0 | - |
| - | - | 912 | 1302 | - | - | 0 | - |
| - | - | 851.4 | 1303 | - | - | 0 | - |
| - | - | 725.4 | 1304 | - | - | 0 | - |
| - | - | 915.4 | 1331 | - | - | 0 | - |
| 13 | c | 1.447E+04 | 1345 | 0.0008322 | 0.6189 | +1 | 13 |
| - | - | 1.051E+04 | 1346 | - | - | 0 | - |
| - | - | 4684 | 1347 | - | - | 0 | - |
| - | - | 6504 | 1348 | - | - | 0 | - |
| - | - | 5223 | 1349 | - | - | 0 | - |
| - | - | 1902 | 1350 | - | - | 0 | - |
| - | - | 672.3 | 1351 | - | - | 0 | - |
| - | - | 1322 | 1388 | - | - | 0 | - |
| - | - | 1081 | 1389 | - | - | 0 | - |
| - | - | 666.2 | 1416 | - | - | 0 | - |
| 14 | c | 2.29E+04 | 1432 | 0.001 | 0.6986 | +1 | 14 |
| - | - | 1.843E+04 | 1433 | - | - | 0 | - |
| - | - | 8669 | 1434 | - | - | 0 | - |
| - | - | 1418 | 1435 | - | - | 0 | - |
| - | - | 824.8 | 1461 | - | - | 0 | - |
| - | - | 745.3 | 1462 | - | - | 0 | - |
| - | - | 2313 | 1489 | - | - | 0 | - |
| - | - | 1550 | 1490 | - | - | 0 | - |
| - | - | 1010 | 1491 | - | - | 0 | - |
| - | - | 818.8 | 1515 | - | - | 0 | - |
| - | - | 728.1 | 1516 | - | - | 0 | - |
| - | - | 4901 | 1531 | - | - | 0 | - |
| - | - | 6243 | 1532 | - | - | 0 | - |
| - | - | 4129 | 1533 | - | - | 0 | - |
| - | - | 1320 | 1534 | - | - | 0 | - |
| - | - | 710 | 1535 | - | - | 0 | - |
| - | - | 1356 | 1545 | - | - | 0 | - |
| - | - | 3052 | 1546 | - | - | 0 | - |
| - | - | 2127 | 1547 | - | - | 0 | - |
| - | - | 1853 | 1548 | - | - | 0 | - |
| - | - | 1900 | 1549 | - | - | 0 | - |
| - | - | 1006 | 1556 | - | - | 0 | - |
| - | - | 2330 | 1557 | - | - | 0 | - |
| - | - | 2029 | 1558 | - | - | 0 | - |
| - | - | 923.3 | 1559 | - | - | 0 | - |
| - | - | 776.4 | 1562 | - | - | 0 | - |
| - | - | 4330 | 1563 | - | - | 0 | - |
| - | - | 3715 | 1564 | - | - | 0 | - |
| - | - | 1304 | 1565 | - | - | 0 | - |
| - | - | 804 | 1571 | - | - | 0 | - |
| - | - | 1175 | 1572 | - | - | 0 | - |
| - | - | 5299 | 1573 | - | - | 0 | - |
| - | - | 3.279E+04 | 1574 | - | - | 0 | - |
| - | - | 2.597E+04 | 1575 | - | - | 0 | - |
| - | - | 1.308E+04 | 1576 | - | - | 0 | - |
| - | - | 3996 | 1577 | - | - | 0 | - |
| - | - | 1465 | 1578 | - | - | 0 | - |
| - | - | 645.1 | 1587 | - | - | 0 | - |
| - | - | 1878 | 1588 | - | - | 0 | - |
| - | - | 3087 | 1589 | - | - | 0 | - |
| - | - | 9374 | 1590 | - | - | 0 | - |
| - | - | 3.04E+04 | 1591 | - | - | 0 | - |
| - | - | 2.484E+04 | 1592 | - | - | 0 | - |
| - | - | 1.107E+04 | 1593 | - | - | 0 | - |
| - | - | 3091 | 1594 | - | - | 0 | - |
| - | - | 985.9 | 1595 | - | - | 0 | - |
| - | - | 597.2 | 1701 | - | - | 0 | - |
| - | - | 720.3 | 2634 | - | - | 0 | - |
| - | - | 702.5 | 2655 | - | - | 0 | - |
| - | - | 707.8 | 3075 | - | - | 0 | - |
| - | - | 746.9 | 3339 | - | - | 0 | - |

m/z Charge Intensity FragmentType MassShift Position
120.08097076416016 0 570.29694
122.95699310302734 0 410.59894
125.10733795166016 0 899.55853
126.0914077758789 0 1215.9297
133.08596801757812 0 1191.2871
136.0762481689453 0 490.6035
139.1114501953125 0 412.88312
148.95480346679688 0 824.44574
160.3935546875 0 495.81256
163.07086181640625 0 510.97116
166.8554229736328 0 490.3758
168.11349487304688 0 1368.6914
171.1135711669922 0 1027.1707
173.43824768066406 0 1148.1074
175.1189422607422 0 2615.4534 y 14
177.11212158203125 0 620.26764
179.0931854248047 0 541.9393
179.19149780273438 0 439.20718
185.1312255859375 0 711.2796
185.1397705078125 0 6141.113
186.14305114746094 0 609.6917
187.10780334472656 0 12860.603
188.11167907714844 0 952.4884
189.0869598388672 0 542.89703
199.1702423095703 0 739.6529
199.18045043945312 0 11453.598
200.12710571289062 0 810.4628
200.1836395263672 0 1731.063
201.11251831054688 0 483.9115
214.1548614501953 0 1771.572
214.91427612304688 0 497.0028
215.1385498046875 0 679.6459
227.17543029785156 0 5778.7427
228.17892456054688 0 961.79877
229.129638671875 0 953.5686 w 13
245.12454223632812 0 9894.684 y Ammonia loss 13
246.1312255859375 0 1801.9875 z 13
262.15130615234375 0 931.23737 y 13
270.1194152832031 0 696.2015
276.8674011230469 0 608.2751
316.161865234375 0 4338.9243 w 12
317.1597595214844 0 871.25275
331.1484375 0 957.6834
332.15576171875 0 1445.3718 y Ammonia loss 12
333.1192626953125 0 1084.6588
333.1644592285156 0 3579.4824 z 12
334.1756286621094 0 2117.0479
349.11444091796875 0 822.05695
349.182861328125 0 1227.1147 y 12
357.1513366699219 0 2477.7026
399.72418212890625 0 665.3786
404.21392822265625 0 673.0616
415.22491455078125 0 1345.9108 z 7
415.2546691894531 0 2316.3594
417.2099914550781 0 3123.3557 w 11
418.2128601074219 0 957.1009
430.2679138183594 0 769.753
439.2544250488281 0 2261.634
440.2488098144531 0 1098.2413
443.25054931640625 0 1006.95087
448.2518615722656 0 3884.7134 y 11
457.7405700683594 0 1491.9541
458.2613525390625 0 1995.371
460.2767639160156 0 2212.9478 c 3
461.27825927734375 0 924.29474
462.73236083984375 0 2611.4802
463.23583984375 0 1294.5879
463.7342529296875 0 625.37787 z Water loss 6
471.7375793457031 0 2523.4504 y Water loss 6
472.2481994628906 0 983.1984 c Ammonia loss 13
472.7377624511719 0 980.49445 z 6
474.23175048828125 0 1458.863
475.2881774902344 0 2546.441
486.2515869140625 0 661.4372
490.2598876953125 0 725.9089
499.2973327636719 0 761.0159
501.30572509765625 0 2104.17
502.313232421875 0 3440.132
503.318359375 0 720.61914
504.26715087890625 0 701.4971
505.27337646484375 0 12528.872 y 10
506.27587890625 0 2844.0803
507.2770080566406 0 905.13806
513.2749633789062 0 648.651
516.2529296875 0 1310.0428 z 5
516.7553100585938 0 1046.5219
521.2685546875 0 770.44916
524.2637329101562 0 4630.7764 y 5
524.6110229492188 0 1786.7555
524.7634887695312 0 2255.3533
524.945556640625 0 987.5844
529.794189453125 0 656.84875
530.3173828125 0 42424.934
530.6162719726562 0 1226.1466
530.9485473632812 0 1011.9296
531.3206176757812 0 11246.565
542.3186645507812 0 1136.3931
558.331298828125 0 696.00183
559.2841186523438 0 700.5018 w 9
559.3460083007812 0 1845.3231 c 4
560.3464965820312 0 601.6928
565.7874145507812 0 8825.936 z 4
566.289794921875 0 3332.397
566.791748046875 0 2893.6519
567.2958374023438 0 730.77356
572.34326171875 0 2605.7278
573.3477783203125 0 1723.6953
573.7969360351562 0 5671.1323 y 4
574.2988891601562 0 3500.0017 y Water loss 9
574.8010864257812 0 1071.7156
575.280029296875 0 771.32007 y Ammonia loss 9
576.2858276367188 0 1572.9569 z 9
577.2904052734375 0 869.2787
585.2560424804688 0 3718.6997
586.2589111328125 0 604.1807
600.2733154296875 0 993.1254
600.802001953125 0 2675.3047 w 3
601.3034057617188 0 1899.0735
601.80517578125 0 1355.2847
629.3521728515625 0 1473.7281 c 11
630.3089599609375 0 8524.096 z 3
630.8113403320312 0 7646.344
631.3121337890625 0 3687.9058
631.8140869140625 0 834.72327
637.815185546875 0 3043.7842
638.3174438476562 0 8161.524 y 3
638.81982421875 0 5756.029
639.3203735351562 0 2019.8044
643.3525390625 0 943.56006
644.8211669921875 0 729.46216
646.3773803710938 0 9591.306 c 5
647.38037109375 0 2796.2961
648.3827514648438 0 607.10187
672.3405151367188 0 659.1118 y Ammonia loss 8
673.33203125 0 638.50476 y Ammonia loss 2
673.8255615234375 0 5626.1787 z 2
674.3279418945312 0 8591.074
674.829833984375 0 4041.8875
675.3305053710938 0 1485.4235
675.8296508789062 0 1166.9434
681.3318481445312 0 959.63574
681.8341064453125 0 34656.668 y 2
682.3355102539062 0 25480.791
682.8369750976562 0 9364.878
683.3363647460938 0 3168.6943
686.3555297851562 0 2711.8247
687.3622436523438 0 3438.429
688.36767578125 0 1887.9208
689.3658447265625 0 1198.9814 y 8
702.35693359375 0 823.9995
711.3551635742188 0 1528.7252
711.860595703125 0 1430.7427
715.846435546875 0 1355.5934 w 1
718.3950805664062 0 861.4777
719.4039916992188 0 682.67584
727.3736572265625 0 1104.0223
730.3648071289062 0 1083.824 z 1
730.8697509765625 0 625.40137
738.3754272460938 0 3368.6208 y 1
738.8770141601562 0 1356.1555
743.3720092773438 0 837.56226 w 7
744.3936767578125 0 3595.4592
745.3956909179688 0 1141.5018
749.8759155273438 0 956.4564
750.8997802734375 0 914.2503
758.3807373046875 0 1003.43176
758.8807373046875 0 1640.9718
759.3832397460938 0 1428.6844
761.4044799804688 0 16326.344 c 6
762.4073486328125 0 7465.307
763.4078979492188 0 1225.1327
764.9139404296875 0 1380.7214
765.3826293945312 0 26369.625
765.8839111328125 0 18739.586
766.3851318359375 0 10479.434
766.888427734375 0 2465.0566
767.3824462890625 0 940.56506
772.4053344726562 0 756.38074
772.9121704101562 0 724.25024
773.4078979492188 0 918.54205
773.9063720703125 0 8011.7686
774.4089965820312 0 6226.372
774.9102172851562 0 3231.426
775.41064453125 0 5134.4233
776.4102783203125 0 2058.9836
777.8975830078125 0 982.8454
779.4142456054688 0 843.4318
779.9028930664062 0 896.1723
782.4412841796875 0 716.2875
785.3765258789062 0 691.08545
785.88818359375 0 1101.0752
786.412109375 0 2817.0427
786.9092407226562 0 30565.47
787.4105224609375 0 23584.752
787.9119873046875 0 10780.416
788.413330078125 0 5093.1685
788.9115600585938 0 693.02936
793.40478515625 0 700.2901
793.8974609375 0 925.7369
794.4044799804688 0 1122.4556
794.917236328125 0 19075.385
795.420166015625 0 24082.424
795.922119140625 0 14767.473
796.4210205078125 0 5781.448
796.9232788085938 0 2390.2812
797.4185791015625 0 1182.1404
827.405517578125 0 3067.1213
828.4111938476562 0 1663.4845
829.439453125 0 12444.168 z 7
830.442626953125 0 6037.198
831.4028930664062 0 3334.7676
832.4044799804688 0 1586.865
839.4065551757812 0 2147.4958
844.4510498046875 0 875.8668
845.4168090820312 0 3661.36
846.4202880859375 0 2498.3997
847.4164428710938 0 880.75214
857.4173583984375 0 2405.203
858.4231567382812 0 861.027
871.4337158203125 0 1013.6103
872.4412231445312 0 632.50354
874.4423828125 0 1203.3185
888.4589233398438 0 1025.568
900.4790649414062 0 2096.5305 c Ammonia loss 7
910.6825561523438 0 606.4185
914.4113159179688 0 650.27185
928.4395751953125 0 551.7491
931.4464111328125 0 1077.4926
932.4440307617188 0 1008.9127
943.4761352539062 0 2014.6724 y Ammonia loss 6
944.469970703125 0 6032.04 z 6
945.47119140625 0 5140.59
946.4752807617188 0 1410.2231
960.4893798828125 0 1122.9824 y 6
970.5458984375 0 1217.2617
975.4781494140625 0 719.4858
988.5221557617188 0 3154.4841
989.5236206054688 0 2384.2446
999.5519409179688 0 1566.7085
1000.5498046875 0 1190.6522
1014.5575561523438 0 14543.408 c 8
1015.5597534179688 0 9583.815
1016.5615844726562 0 2745.5671
1017.560302734375 0 1539.7158
1018.9257202148438 0 754.8972
1031.4998779296875 0 1980.3494 z 5
1032.5029296875 0 2223.619
1033.51171875 0 1211.528
1042.5460205078125 0 6855.6616
1043.545166015625 0 4146.2754
1044.5263671875 0 12755.511
1045.52734375 0 6450.85
1046.5281982421875 0 2289.1133
1047.5162353515625 0 817.93866 y 5
1048.5333251953125 0 1008.20044
1057.576416015625 0 7595.203
1058.572998046875 0 7684.967
1059.5728759765625 0 9474.304
1060.56396484375 0 5363.3325
1061.550537109375 0 9860.831
1062.552978515625 0 5215.549
1063.5579833984375 0 681.7349
1101.5902099609375 0 15493.942 c 9
1102.5936279296875 0 8813.3545
1103.59375 0 3456.1055
1104.5946044921875 0 815.33386
1114.597900390625 0 2851.0688
1115.599365234375 0 2032.0383
1116.6063232421875 0 1180.5872
1130.5687255859375 0 2243.7546 z 4
1131.5748291015625 0 5950.9844
1132.5762939453125 0 3092.529
1133.56884765625 0 1104.9182
1158.612060546875 0 9114.906 c 10
1159.6136474609375 0 7018.24
1160.6109619140625 0 1368.04
1213.66357421875 0 1727.3137
1214.66748046875 0 1017.1937
1257.6787109375 0 8917.731 c 11
1258.681396484375 0 5563.743
1259.674560546875 0 2051.2942
1260.616943359375 0 7499.8677
1261.6209716796875 0 3848.1038
1262.62548828125 0 2076.6423
1275.6278076171875 0 2794.0105 y 3
1276.62744140625 0 1667.4822
1277.6365966796875 0 908.5828
1281.0447998046875 0 584.75055
1300.6968994140625 0 1188.5579
1301.7042236328125 0 911.9971
1302.6903076171875 0 851.39526
1303.6724853515625 0 725.3962
1330.6336669921875 0 915.4441
1344.7109375 0 14474.144 c 12
1345.7130126953125 0 10506.652
1346.7093505859375 0 4683.826
1347.6575927734375 0 6504.1294
1348.6512451171875 0 5223.0264
1349.6556396484375 0 1901.531
1350.6571044921875 0 672.2959
1387.734130859375 0 1321.8796
1388.7452392578125 0 1080.6503
1415.727294921875 0 666.2204
1431.7427978515625 0 22899.105 c 13
1432.746337890625 0 18425.814
1433.7462158203125 0 8668.805
1434.7474365234375 0 1417.8008
1460.7403564453125 0 824.83234
1461.73095703125 0 745.28156
1488.7457275390625 0 2313.164
1489.74462890625 0 1550.0519
1490.7593994140625 0 1009.5891
1514.7911376953125 0 818.7837
1515.7762451171875 0 728.1112
1530.7904052734375 0 4900.64
1531.7926025390625 0 6243.0674
1532.7967529296875 0 4128.606
1533.7921142578125 0 1320.0979
1534.803955078125 0 710.02997
1544.83056640625 0 1356.3391
1545.8314208984375 0 3051.6177
1546.8172607421875 0 2127.0823
1547.82763671875 0 1852.8575
1548.819580078125 0 1899.6016
1555.8238525390625 0 1006.284
1556.7960205078125 0 2330.0195
1557.7894287109375 0 2029.452
1558.796630859375 0 923.3063
1561.818603515625 0 776.3557
1562.843505859375 0 4330.475
1563.84814453125 0 3714.6328
1564.8585205078125 0 1303.8942
1570.77197265625 0 804.00916
1571.770751953125 0 1174.5438
1572.8094482421875 0 5299.2544
1573.816162109375 0 32792.65
1574.81982421875 0 25969.863
1575.8221435546875 0 13080.85
1576.8238525390625 0 3996.0383
1577.8187255859375 0 1464.669
1586.7666015625 0 645.1139
1587.789306640625 0 1877.7761
1588.80517578125 0 3086.7725
1589.8287353515625 0 9373.645
1590.840576171875 0 30400.135
1591.84423828125 0 24844.762
1592.8480224609375 0 11073.557
1593.8468017578125 0 3090.8674
1594.8485107421875 0 985.91833
1700.57470703125 0 597.1866
2633.66357421875 0 720.31305
2654.6083984375 0 702.54517
3074.62158203125 0 707.8372
3339.248779296875 0 746.85596

Spectrum Details

|  |  |
| --- | --- |
| Matched peaks? Matched peaksThe total absolute number of peaks matched. Additionally in brackets the total fraction of peaks matched and the total number of peaks is shown. | 57 (16.10% of 354) |
| FDR? FDRThe false discovery rate estimated for this peptide. It is calculated by matching all theoretical fragments with a non-integer shift with the raw peaks for this spectrum. This is done with 40 different shifts. The resulting percentage is the average number of annotated peaks over the number of annotated peaks with the correct spectrum. | 0.67% |
| Satellite FDR? Satellite FDRSee the FDR for details on its calculation. This satellite ion specific FDR only contains the satellite ions (d/w) for I/L/J positions. | 0.00% |
| PSM Score? PSM ScoreThe PSM Score as given by Hecklib to this annotated spectrum. It is shown with three significant figures. | 393 |

## Spectrum 4735? Spectrum 4735 The raw spectrum of this peptide as annotated by Hecklib. The fragments are coloured according to ion type (see legend). Any peaks with a star '\*' as text can be hovered over to see the full details, first the ion type second the mass shift type. By hovering over the amino acids in the peptide or ions in the legend the corresponding peaks are highlighted. By toggling the 'Unassigned' label you can turn the background (unassigned) peaks on or off in the plot. By updating the slider in the Ion legend you can update the spectrum to only show the top X% of the peaks with labels. The top X% means any peak that is within X% of the highest intensity. By dragging in the spectrum you can zoom in to a specific part of the spectrum and use 'Zoom Out' to get back to the original zoom level. The annotation of the spectrum is based on the given sequence in the peptides file and is done with different software so inconsistencies are likely. The peaks are annotated based on the given sequence, with 20 ppm tolerance.

Copy Data

### Spectrum 4735 (TSV)

#### Preview

```
Loading example...
```

*Click on the button to copy the data to your clipboard.*

Mz MinMz MaxIntensity Max

WidthHeightPeptide font sizePeptide stroke widthSpectrum font sizeSpectrum stroke widthCompact peptide

Ion legend

wxyz

abcd

OtherUnassignedIonChargePositionShow for top:%

JJSEVSDRPSGVSSR

01.78e+43.57e+45.35e+47.13e+4

Zoom Out

y+11y+35y+11w+12y+12z+12y+12w+13y+13z+13y+13z+28w+14y+28y+14c+14y+29z+15y+15y+210w+16c+15z+211y+211y+16z+16w+212z+212y+212c+16z+17z+213y+213y+17w+214y+214z+214y+214w+18c+17z+18y+18c+18z+19y+19z+19y+19c+19z+110y+110c+110z+111y+111c+111c+112y+112c+113c+114

040380512081611

Fragment Matches Table

Show background peaks

| Position | Ion type | Intensity | mz Theoretical | mz Error (Th) | mz Error (ppm) | Charge | Series Number |
| --- | --- | --- | --- | --- | --- | --- | --- |
| - | - | 468.5 | 121.5 | - | - | 0 | - |
| - | - | 1186 | 126.1 | - | - | 0 | - |
| - | - | 415 | 128.4 | - | - | 0 | - |
| - | - | 563.2 | 131.1 | - | - | 0 | - |
| - | - | 360 | 131.9 | - | - | 0 | - |
| - | - | 1617 | 132.1 | - | - | 0 | - |
| - | - | 2288 | 133.1 | - | - | 0 | - |
| - | - | 1744 | 133.1 | - | - | 0 | - |
| - | - | 456.5 | 143.1 | - | - | 0 | - |
| - | - | 405.6 | 149 | - | - | 0 | - |
| - | - | 2023 | 157.1 | - | - | 0 | - |
| - | - | 516.7 | 157.1 | - | - | 0 | - |
| 15 | y | 516.4 | 158.1 | 3.468E-05 | 0.2194 | +1 | 1 |
| - | - | 447.4 | 158.1 | - | - | 0 | - |
| - | - | 1524 | 168.1 | - | - | 0 | - |
| 11 | y | 855.2 | 169.1 | 0.001355 | 8.012 | +3 | 5 |
| - | - | 439.2 | 171.1 | - | - | 0 | - |
| - | - | 612.1 | 174.1 | - | - | 0 | - |
| 15 | y | 2754 | 175.1 | 0.000132 | 0.7536 | +1 | 1 |
| - | - | 1208 | 177.1 | - | - | 0 | - |
| - | - | 2042 | 185.1 | - | - | 0 | - |
| - | - | 5848 | 185.1 | - | - | 0 | - |
| - | - | 631 | 187.1 | - | - | 0 | - |
| - | - | 777 | 199.2 | - | - | 0 | - |
| - | - | 1.287E+04 | 199.2 | - | - | 0 | - |
| - | - | 1123 | 200.1 | - | - | 0 | - |
| - | - | 1840 | 200.2 | - | - | 0 | - |
| - | - | 1187 | 201.1 | - | - | 0 | - |
| - | - | 1489 | 217.1 | - | - | 0 | - |
| - | - | 546.5 | 226.2 | - | - | 0 | - |
| - | - | 6594 | 227.2 | - | - | 0 | - |
| - | - | 666.3 | 228.2 | - | - | 0 | - |
| 14 | w | 1536 | 229.1 | 0.0004275 | 1.866 | +1 | 2 |
| 14 | y | 9762 | 245.1 | 0.0002707 | 1.104 | +1 | 2 |
| 14 | z | 1171 | 246.1 | 0.0005732 | 2.329 | +1 | 2 |
| - | - | 596.8 | 254.1 | - | - | 0 | - |
| - | - | 678.7 | 255.1 | - | - | 0 | - |
| - | - | 646.5 | 256.1 | - | - | 0 | - |
| - | - | 559.1 | 259.6 | - | - | 0 | - |
| - | - | 501.3 | 261.7 | - | - | 0 | - |
| 14 | y | 1389 | 262.2 | 5.092E-05 | 0.1942 | +1 | 2 |
| - | - | 713.7 | 265.2 | - | - | 0 | - |
| - | - | 1101 | 270.1 | - | - | 0 | - |
| - | - | 626.3 | 272.1 | - | - | 0 | - |
| - | - | 542 | 305.8 | - | - | 0 | - |
| - | - | 1185 | 314.2 | - | - | 0 | - |
| 13 | w | 5409 | 316.2 | 4.531E-05 | 0.1433 | +1 | 3 |
| - | - | 849.5 | 317.2 | - | - | 0 | - |
| - | - | 1134 | 323.1 | - | - | 0 | - |
| - | - | 530.9 | 323.4 | - | - | 0 | - |
| - | - | 1373 | 331.1 | - | - | 0 | - |
| 13 | y | 2041 | 332.2 | 0.0004845 | 1.459 | +1 | 3 |
| - | - | 3953 | 333.1 | - | - | 0 | - |
| 13 | z | 4466 | 333.2 | 0.0005581 | 1.675 | +1 | 3 |
| - | - | 1457 | 349.1 | - | - | 0 | - |
| 13 | y | 1243 | 349.2 | 0.0007275 | 2.083 | +1 | 3 |
| - | - | 692.3 | 353.2 | - | - | 0 | - |
| - | - | 3374 | 357.2 | - | - | 0 | - |
| - | - | 895.9 | 378.2 | - | - | 0 | - |
| - | - | 632.2 | 378.7 | - | - | 0 | - |
| - | - | 675.5 | 395.2 | - | - | 0 | - |
| 8 | z | 1721 | 415.2 | 0.0007246 | 1.745 | +2 | 8 |
| - | - | 7659 | 415.3 | - | - | 0 | - |
| - | - | 544.7 | 416.2 | - | - | 0 | - |
| 12 | w | 3451 | 417.2 | 0.0001783 | 0.4274 | +1 | 4 |
| - | - | 970.4 | 418.2 | - | - | 0 | - |
| 8 | y | 1107 | 423.2 | 0.0005484 | 1.296 | +2 | 8 |
| 12 | y | 3995 | 448.3 | 0.000225 | 0.5021 | +1 | 4 |
| - | - | 1127 | 449.3 | - | - | 0 | - |
| - | - | 1321 | 460.2 | - | - | 0 | - |
| 4 | c | 2925 | 460.3 | 0.0001468 | 0.3189 | +1 | 4 |
| - | - | 686.3 | 460.7 | - | - | 0 | - |
| - | - | 836.3 | 461.2 | - | - | 0 | - |
| - | - | 673.5 | 466.2 | - | - | 0 | - |
| - | - | 1084 | 474.2 | - | - | 0 | - |
| 7 | y | 874.1 | 480.7 | 0.001603 | 3.335 | +2 | 9 |
| - | - | 1112 | 482.3 | - | - | 0 | - |
| 11 | z | 1716 | 489.3 | 0.0003784 | 0.7734 | +1 | 5 |
| - | - | 722.3 | 499.3 | - | - | 0 | - |
| 11 | y | 1.373E+04 | 505.3 | 0.0001815 | 0.3593 | +1 | 5 |
| - | - | 3303 | 506.3 | - | - | 0 | - |
| - | - | 1072 | 512.2 | - | - | 0 | - |
| - | - | 919.7 | 515.3 | - | - | 0 | - |
| - | - | 1572 | 516.8 | - | - | 0 | - |
| 6 | y | 4358 | 524.3 | 0.002374 | 4.528 | +2 | 10 |
| - | - | 973.5 | 524.6 | - | - | 0 | - |
| - | - | 1894 | 524.8 | - | - | 0 | - |
| - | - | 618.8 | 529.2 | - | - | 0 | - |
| - | - | 3422 | 530.2 | - | - | 0 | - |
| - | - | 7.062E+04 | 530.3 | - | - | 0 | - |
| - | - | 1304 | 530.6 | - | - | 0 | - |
| - | - | 779.9 | 531.2 | - | - | 0 | - |
| - | - | 2.061E+04 | 531.3 | - | - | 0 | - |
| - | - | 688.5 | 531.7 | - | - | 0 | - |
| - | - | 1550 | 542.3 | - | - | 0 | - |
| - | - | 948.2 | 558.3 | - | - | 0 | - |
| 10 | w | 565.1 | 559.3 | 0.005611 | 10.03 | +1 | 6 |
| 5 | c | 2860 | 559.3 | 0.001666 | 2.979 | +1 | 5 |
| - | - | 867.4 | 560.3 | - | - | 0 | - |
| 5 | z | 8625 | 565.8 | 0.0005596 | 0.9891 | +2 | 11 |
| - | - | 7163 | 566.3 | - | - | 0 | - |
| - | - | 2160 | 566.8 | - | - | 0 | - |
| - | - | 767.1 | 567.3 | - | - | 0 | - |
| 5 | y | 4675 | 573.8 | 0.001194 | 2.08 | +2 | 11 |
| 10 | y | 2152 | 574.3 | 0.004478 | 7.797 | +1 | 6 |
| - | - | 777.5 | 574.8 | - | - | 0 | - |
| 10 | z | 2089 | 576.3 | 2.879E-06 | 0.004996 | +1 | 6 |
| - | - | 1088 | 586.3 | - | - | 0 | - |
| 4 | w | 3767 | 600.8 | 0.0007387 | 1.229 | +2 | 12 |
| - | - | 2032 | 601.3 | - | - | 0 | - |
| - | - | 924.1 | 601.8 | - | - | 0 | - |
| 4 | z | 1.016E+04 | 630.3 | 0.0004328 | 0.6867 | +2 | 12 |
| - | - | 7534 | 630.8 | - | - | 0 | - |
| - | - | 2174 | 631.3 | - | - | 0 | - |
| - | - | 1007 | 631.8 | - | - | 0 | - |
| - | - | 3053 | 637.8 | - | - | 0 | - |
| 4 | y | 9525 | 638.3 | 0.001311 | 2.054 | +2 | 12 |
| - | - | 7552 | 638.8 | - | - | 0 | - |
| - | - | 3301 | 639.3 | - | - | 0 | - |
| - | - | 653.9 | 644.3 | - | - | 0 | - |
| - | - | 737.7 | 644.8 | - | - | 0 | - |
| 6 | c | 1.042E+04 | 646.4 | 0.0001253 | 0.1938 | +1 | 6 |
| - | - | 3218 | 647.4 | - | - | 0 | - |
| 9 | z | 647.8 | 673.3 | 0.004726 | 7.019 | +1 | 7 |
| 3 | z | 5041 | 673.8 | 0.0002117 | 0.3142 | +2 | 13 |
| - | - | 8282 | 674.3 | - | - | 0 | - |
| - | - | 3652 | 674.8 | - | - | 0 | - |
| - | - | 1861 | 675.3 | - | - | 0 | - |
| 3 | y | 3.879E+04 | 681.8 | 0.0005405 | 0.7928 | +2 | 13 |
| - | - | 2.84E+04 | 682.3 | - | - | 0 | - |
| - | - | 1.081E+04 | 682.8 | - | - | 0 | - |
| - | - | 3729 | 683.3 | - | - | 0 | - |
| 9 | y | 835.5 | 689.4 | 0.00105 | 1.524 | +1 | 7 |
| - | - | 608.3 | 701.4 | - | - | 0 | - |
| - | - | 1275 | 711.9 | - | - | 0 | - |
| 2 | w | 787.8 | 715.8 | 2.397E-05 | 0.03349 | +2 | 14 |
| - | - | 622.2 | 716.9 | - | - | 0 | - |
| - | - | 930.2 | 717.4 | - | - | 0 | - |
| - | - | 861.3 | 718.4 | - | - | 0 | - |
| 2 | y | 747.7 | 729.4 | 0.005491 | 7.528 | +2 | 14 |
| 2 | z | 1205 | 730.4 | 0.002144 | 2.935 | +2 | 14 |
| - | - | 694.1 | 738.3 | - | - | 0 | - |
| 2 | y | 3268 | 738.4 | 0.001496 | 2.026 | +2 | 14 |
| - | - | 1688 | 738.9 | - | - | 0 | - |
| - | - | 1071 | 739.4 | - | - | 0 | - |
| 8 | w | 843.5 | 743.4 | 0.00362 | 4.869 | +1 | 8 |
| - | - | 735 | 744.4 | - | - | 0 | - |
| - | - | 749.2 | 755.4 | - | - | 0 | - |
| - | - | 1149 | 758.4 | - | - | 0 | - |
| - | - | 1557 | 758.9 | - | - | 0 | - |
| - | - | 997.1 | 759.4 | - | - | 0 | - |
| - | - | 795.4 | 760.4 | - | - | 0 | - |
| 7 | c | 1.886E+04 | 761.4 | 0.0008232 | 1.081 | +1 | 7 |
| - | - | 7484 | 762.4 | - | - | 0 | - |
| - | - | 1346 | 763.4 | - | - | 0 | - |
| - | - | 1101 | 764.9 | - | - | 0 | - |
| - | - | 3.182E+04 | 765.4 | - | - | 0 | - |
| - | - | 2.46E+04 | 765.9 | - | - | 0 | - |
| - | - | 1.384E+04 | 766.4 | - | - | 0 | - |
| - | - | 3909 | 766.9 | - | - | 0 | - |
| - | - | 1413 | 767.4 | - | - | 0 | - |
| - | - | 891.6 | 771.9 | - | - | 0 | - |
| - | - | 1076 | 772.4 | - | - | 0 | - |
| - | - | 724.8 | 772.9 | - | - | 0 | - |
| - | - | 1057 | 773.4 | - | - | 0 | - |
| - | - | 9209 | 773.9 | - | - | 0 | - |
| - | - | 6049 | 774.4 | - | - | 0 | - |
| - | - | 4460 | 774.9 | - | - | 0 | - |
| - | - | 999.1 | 775.4 | - | - | 0 | - |
| - | - | 880.5 | 778.9 | - | - | 0 | - |
| - | - | 843.3 | 779.4 | - | - | 0 | - |
| - | - | 1126 | 785.4 | - | - | 0 | - |
| - | - | 1098 | 785.9 | - | - | 0 | - |
| - | - | 3309 | 786.4 | - | - | 0 | - |
| - | - | 2.749E+04 | 786.9 | - | - | 0 | - |
| - | - | 3.026E+04 | 787.4 | - | - | 0 | - |
| - | - | 1.148E+04 | 787.9 | - | - | 0 | - |
| - | - | 3854 | 788.4 | - | - | 0 | - |
| - | - | 1021 | 793.9 | - | - | 0 | - |
| - | - | 1767 | 794.4 | - | - | 0 | - |
| - | - | 2.38E+04 | 794.9 | - | - | 0 | - |
| - | - | 2.829E+04 | 795.4 | - | - | 0 | - |
| - | - | 1.656E+04 | 795.9 | - | - | 0 | - |
| - | - | 6052 | 796.4 | - | - | 0 | - |
| - | - | 2482 | 796.9 | - | - | 0 | - |
| - | - | 949.5 | 801.4 | - | - | 0 | - |
| - | - | 693.1 | 813.4 | - | - | 0 | - |
| - | - | 972 | 828.4 | - | - | 0 | - |
| 8 | z | 1.373E+04 | 829.4 | 0.001528 | 1.843 | +1 | 8 |
| - | - | 7122 | 830.4 | - | - | 0 | - |
| - | - | 2303 | 831.4 | - | - | 0 | - |
| - | - | 675.8 | 839.5 | - | - | 0 | - |
| 8 | y | 1947 | 845.5 | 0.0007821 | 0.9251 | +1 | 8 |
| - | - | 795.3 | 873.5 | - | - | 0 | - |
| 8 | c | 2163 | 900.5 | 0.001777 | 1.973 | +1 | 8 |
| - | - | 1034 | 901.5 | - | - | 0 | - |
| - | - | 1834 | 924.5 | - | - | 0 | - |
| - | - | 1223 | 925.5 | - | - | 0 | - |
| 7 | z | 940.5 | 926.5 | 0.001869 | 2.017 | +1 | 9 |
| - | - | 2166 | 941.5 | - | - | 0 | - |
| 7 | y | 994.3 | 942.5 | 0.01726 | 18.32 | +1 | 9 |
| 7 | z | 5480 | 944.5 | 2.89E-05 | 0.0306 | +1 | 9 |
| - | - | 4434 | 945.5 | - | - | 0 | - |
| - | - | 1737 | 946.5 | - | - | 0 | - |
| - | - | 1057 | 954.5 | - | - | 0 | - |
| - | - | 782.1 | 955.5 | - | - | 0 | - |
| - | - | 911.7 | 956.5 | - | - | 0 | - |
| 7 | y | 669 | 960.5 | 0.005447 | 5.671 | +1 | 9 |
| - | - | 941.5 | 961.5 | - | - | 0 | - |
| - | - | 1735 | 964.5 | - | - | 0 | - |
| - | - | 1965 | 970.5 | - | - | 0 | - |
| - | - | 835.7 | 971.5 | - | - | 0 | - |
| - | - | 649.4 | 972.6 | - | - | 0 | - |
| - | - | 894.3 | 984.5 | - | - | 0 | - |
| - | - | 1208 | 986.5 | - | - | 0 | - |
| - | - | 1669 | 987.5 | - | - | 0 | - |
| - | - | 946.2 | 988.5 | - | - | 0 | - |
| - | - | 821.6 | 999.5 | - | - | 0 | - |
| - | - | 1234 | 1002 | - | - | 0 | - |
| - | - | 746.6 | 1003 | - | - | 0 | - |
| - | - | 2058 | 1004 | - | - | 0 | - |
| - | - | 1142 | 1005 | - | - | 0 | - |
| - | - | 886 | 1014 | - | - | 0 | - |
| 9 | c | 1.384E+04 | 1015 | 0.0005843 | 0.5759 | +1 | 9 |
| - | - | 6909 | 1016 | - | - | 0 | - |
| - | - | 1686 | 1017 | - | - | 0 | - |
| 6 | z | 2380 | 1031 | 0.003038 | 2.945 | +1 | 10 |
| - | - | 3494 | 1033 | - | - | 0 | - |
| - | - | 1439 | 1034 | - | - | 0 | - |
| - | - | 885.3 | 1040 | - | - | 0 | - |
| - | - | 2101 | 1041 | - | - | 0 | - |
| - | - | 2921 | 1042 | - | - | 0 | - |
| - | - | 2155 | 1043 | - | - | 0 | - |
| - | - | 1533 | 1044 | - | - | 0 | - |
| - | - | 1314 | 1045 | - | - | 0 | - |
| - | - | 893.5 | 1046 | - | - | 0 | - |
| 6 | y | 899.2 | 1048 | 0.00763 | 7.283 | +1 | 10 |
| - | - | 7990 | 1058 | - | - | 0 | - |
| - | - | 4909 | 1059 | - | - | 0 | - |
| - | - | 2713 | 1060 | - | - | 0 | - |
| - | - | 2554 | 1061 | - | - | 0 | - |
| - | - | 829.2 | 1101 | - | - | 0 | - |
| 10 | c | 1.815E+04 | 1102 | 0.001241 | 1.126 | +1 | 10 |
| - | - | 1.027E+04 | 1103 | - | - | 0 | - |
| - | - | 4433 | 1104 | - | - | 0 | - |
| - | - | 2774 | 1115 | - | - | 0 | - |
| - | - | 1916 | 1116 | - | - | 0 | - |
| 5 | z | 2734 | 1131 | 0.002022 | 1.788 | +1 | 11 |
| - | - | 7152 | 1132 | - | - | 0 | - |
| - | - | 3066 | 1133 | - | - | 0 | - |
| - | - | 1232 | 1134 | - | - | 0 | - |
| 5 | y | 1641 | 1147 | 0.001214 | 1.059 | +1 | 11 |
| - | - | 650.9 | 1148 | - | - | 0 | - |
| 11 | c | 9443 | 1159 | 0.001952 | 1.685 | +1 | 11 |
| - | - | 6663 | 1160 | - | - | 0 | - |
| - | - | 2452 | 1161 | - | - | 0 | - |
| - | - | 2768 | 1214 | - | - | 0 | - |
| - | - | 1010 | 1215 | - | - | 0 | - |
| 12 | c | 1.063E+04 | 1258 | 0.002251 | 1.79 | +1 | 12 |
| - | - | 7597 | 1259 | - | - | 0 | - |
| - | - | 1997 | 1260 | - | - | 0 | - |
| - | - | 2090 | 1260 | - | - | 0 | - |
| - | - | 8577 | 1261 | - | - | 0 | - |
| - | - | 5617 | 1262 | - | - | 0 | - |
| - | - | 2104 | 1263 | - | - | 0 | - |
| 4 | y | 3455 | 1276 | 0.001815 | 1.423 | +1 | 12 |
| - | - | 2079 | 1277 | - | - | 0 | - |
| - | - | 1383 | 1278 | - | - | 0 | - |
| - | - | 862.5 | 1283 | - | - | 0 | - |
| - | - | 966 | 1301 | - | - | 0 | - |
| - | - | 1156 | 1302 | - | - | 0 | - |
| - | - | 785.5 | 1331 | - | - | 0 | - |
| 13 | c | 1.808E+04 | 1345 | 0.001931 | 1.436 | +1 | 13 |
| - | - | 1.239E+04 | 1346 | - | - | 0 | - |
| - | - | 5973 | 1347 | - | - | 0 | - |
| - | - | 7095 | 1348 | - | - | 0 | - |
| - | - | 5399 | 1349 | - | - | 0 | - |
| - | - | 3311 | 1350 | - | - | 0 | - |
| - | - | 1468 | 1388 | - | - | 0 | - |
| - | - | 774.8 | 1390 | - | - | 0 | - |
| - | - | 805.6 | 1431 | - | - | 0 | - |
| 14 | c | 2.584E+04 | 1432 | 0.002587 | 1.807 | +1 | 14 |
| - | - | 1.71E+04 | 1433 | - | - | 0 | - |
| - | - | 8310 | 1434 | - | - | 0 | - |
| - | - | 2894 | 1435 | - | - | 0 | - |
| - | - | 1074 | 1461 | - | - | 0 | - |
| - | - | 1569 | 1462 | - | - | 0 | - |
| - | - | 2610 | 1489 | - | - | 0 | - |
| - | - | 1688 | 1490 | - | - | 0 | - |
| - | - | 944.9 | 1491 | - | - | 0 | - |
| - | - | 4429 | 1531 | - | - | 0 | - |
| - | - | 6595 | 1532 | - | - | 0 | - |
| - | - | 3682 | 1533 | - | - | 0 | - |
| - | - | 2506 | 1534 | - | - | 0 | - |
| - | - | 1048 | 1535 | - | - | 0 | - |
| - | - | 1466 | 1545 | - | - | 0 | - |
| - | - | 2736 | 1546 | - | - | 0 | - |
| - | - | 3141 | 1547 | - | - | 0 | - |
| - | - | 2985 | 1548 | - | - | 0 | - |
| - | - | 1678 | 1549 | - | - | 0 | - |
| - | - | 1315 | 1556 | - | - | 0 | - |
| - | - | 3213 | 1557 | - | - | 0 | - |
| - | - | 2168 | 1558 | - | - | 0 | - |
| - | - | 1001 | 1562 | - | - | 0 | - |
| - | - | 4421 | 1563 | - | - | 0 | - |
| - | - | 3431 | 1564 | - | - | 0 | - |
| - | - | 1610 | 1565 | - | - | 0 | - |
| - | - | 835 | 1566 | - | - | 0 | - |
| - | - | 1793 | 1572 | - | - | 0 | - |
| - | - | 6039 | 1573 | - | - | 0 | - |
| - | - | 3.654E+04 | 1574 | - | - | 0 | - |
| - | - | 3E+04 | 1575 | - | - | 0 | - |
| - | - | 1.639E+04 | 1576 | - | - | 0 | - |
| - | - | 4297 | 1577 | - | - | 0 | - |
| - | - | 1149 | 1578 | - | - | 0 | - |
| - | - | 922.4 | 1588 | - | - | 0 | - |
| - | - | 4068 | 1589 | - | - | 0 | - |
| - | - | 1.008E+04 | 1590 | - | - | 0 | - |
| - | - | 3.681E+04 | 1591 | - | - | 0 | - |
| - | - | 2.833E+04 | 1592 | - | - | 0 | - |
| - | - | 1.333E+04 | 1593 | - | - | 0 | - |
| - | - | 3974 | 1594 | - | - | 0 | - |
| - | - | 964.9 | 1595 | - | - | 0 | - |

m/z Charge Intensity FragmentType MassShift Position
121.49907684326172 0 468.50027
126.09146881103516 0 1186.2203
128.3719482421875 0 415.03265
131.12918090820312 0 563.21906
131.91085815429688 0 360.0447
132.07669067382812 0 1617.0621
133.06077575683594 0 2288.0083
133.08578491210938 0 1744.4414
143.11793518066406 0 456.53986
149.02354431152344 0 405.58905
157.1332550048828 0 2022.757
157.13963317871094 0 516.6948
158.09243774414062 0 516.41455 y Ammonia loss 14
158.13722229003906 0 447.36053
168.1128692626953 0 1523.7645
169.09716796875 0 855.20154 y 10
171.11265563964844 0 439.1553
174.05477905273438 0 612.0904
175.1188201904297 0 2754.056 y 14
177.11212158203125 0 1208.4832
185.1285400390625 0 2042.3936
185.13954162597656 0 5848.1978
187.1082305908203 0 630.9848
199.17047119140625 0 777.014
199.1802978515625 0 12867.673
200.1265869140625 0 1122.5265
200.183837890625 0 1839.665
201.12335205078125 0 1187.2986
217.1178741455078 0 1488.9839
226.16744995117188 0 546.5213
227.1752166748047 0 6594.4575
228.17945861816406 0 666.27625
229.12908935546875 0 1535.6533 w 13
245.12416076660156 0 9762.242 y Ammonia loss 13
246.13168334960938 0 1171.1702 z 13
254.14942932128906 0 596.7961
255.10853576660156 0 678.6712
256.1285400390625 0 646.4866
259.5525817871094 0 559.12524
261.656005859375 0 501.26086
262.1510314941406 0 1388.8119 y 13
265.1644287109375 0 713.7066
270.1197204589844 0 1101.4056
272.1234130859375 0 626.344
305.7637023925781 0 541.9622
314.2081604003906 0 1185.0417
316.1615905761719 0 5409.357 w 12
317.1603088378906 0 849.4811
323.0976257324219 0 1133.7146
323.38763427734375 0 530.9391
331.1480712890625 0 1372.7013
332.1559753417969 0 2041.2859 y Ammonia loss 12
333.1192321777344 0 3952.9976
333.1637268066406 0 4465.6973 z 12
349.1142578125 0 1457.4509
349.1822814941406 0 1242.6354 y 12
353.2198181152344 0 692.34894
357.1509704589844 0 3374.182
378.2201843261719 0 895.8686
378.7242431640625 0 632.20056
395.22821044921875 0 675.52747
415.2243957519531 0 1721.3162 z 7
415.2538146972656 0 7658.5356
416.22808837890625 0 544.7071
417.20904541015625 0 3451.1265 w 11
418.21148681640625 0 970.4281
423.23358154296875 0 1107.0663 y 7
448.25164794921875 0 3995.1304 y 11
449.2549743652344 0 1127.2499
460.2437744140625 0 1320.7719
460.27642822265625 0 2924.6848 c 3
460.74676513671875 0 686.3126
461.2465515136719 0 836.2583
466.17034912109375 0 673.4921
474.230712890625 0 1084.3002
480.74810791015625 0 874.1299 y 6
482.2611999511719 0 1112.43
489.2537841796875 0 1715.6001 z 10
499.29718017578125 0 722.2564
505.272705078125 0 13728.329 y 10
506.2754211425781 0 3302.5632
512.17578125 0 1072.1273
515.3269653320312 0 919.748
516.7577514648438 0 1571.9385
524.264892578125 0 4358.205 y 5
524.6107788085938 0 973.53265
524.76318359375 0 1893.6753
529.201904296875 0 618.8363
530.1884155273438 0 3422.498
530.3167724609375 0 70615.805
530.6141357421875 0 1304.4666
531.1917724609375 0 779.93256
531.3201293945312 0 20610.814
531.7416381835938 0 688.5072
542.3187866210938 0 1550.0411
558.3382568359375 0 948.15234
559.2890625 0 565.103 w 9
559.3433227539062 0 2860.3826 c 4
560.3465576171875 0 867.38965
565.7868041992188 0 8624.746 z 4
566.2886962890625 0 7162.724
566.7894287109375 0 2159.657
567.2925415039062 0 767.051
573.7955322265625 0 4674.9805 y 4
574.298828125 0 2151.5972 y Water loss 9
574.7976684570312 0 777.45715
576.2861938476562 0 2089.0874 z 9
586.3289794921875 0 1088.4904
600.80126953125 0 3767.027 w 3
601.3067626953125 0 2031.9575
601.8037109375 0 924.05054
630.3082275390625 0 10160.097 z 3
630.8095092773438 0 7533.7935
631.3103637695312 0 2174.1887
631.8136596679688 0 1006.7875
637.8143310546875 0 3052.8328
638.3167114257812 0 9525.067 y 3
638.8189086914062 0 7552.496
639.3189697265625 0 3301.3599
644.3157958984375 0 653.87177
644.81591796875 0 737.72253
646.3768920898438 0 10422.108 c 5
647.3792114257812 0 3218.153
673.334228515625 0 647.7929 z 8
673.824462890625 0 5040.815 z 2
674.3267211914062 0 8281.716
674.8291625976562 0 3652.1445
675.330078125 0 1861.4722
681.83349609375 0 38785.613 y 2
682.3348999023438 0 28399.143
682.836669921875 0 10805.567
683.3369750976562 0 3728.7065
689.3566284179688 0 835.4754 y 8
701.3577270507812 0 608.2849
711.8577880859375 0 1275.4371
715.84716796875 0 787.7558 w 1
716.8512573242188 0 622.22754
717.3927001953125 0 930.2143
718.3966674804688 0 861.27496
729.3652954101562 0 747.6666 y Water loss 1
730.3645629882812 0 1205.2172 z 1
738.2706298828125 0 694.06287
738.3745727539062 0 3268.0356 y 1
738.8748168945312 0 1687.9644
739.3784790039062 0 1070.7971
743.3646240234375 0 843.4556 w 7
744.3762817382812 0 735.033
755.4252319335938 0 749.17926
758.384033203125 0 1149.4729
758.8785400390625 0 1556.7053
759.3848876953125 0 997.10284
760.3896484375 0 795.3941
761.4031372070312 0 18855.574 c 6
762.4064331054688 0 7484.4053
763.408935546875 0 1346.0411
764.9194946289062 0 1100.5216
765.3818359375 0 31820.898
765.883056640625 0 24603.621
766.3839721679688 0 13836.088
766.8851928710938 0 3908.9836
767.3876342773438 0 1413.1821
771.9031372070312 0 891.59985
772.3912963867188 0 1075.8152
772.899658203125 0 724.7581
773.3934936523438 0 1056.5693
773.906982421875 0 9208.749
774.4078979492188 0 6048.9355
774.90966796875 0 4459.6
775.4037475585938 0 999.057
778.8998413085938 0 880.4938
779.4035034179688 0 843.32745
785.4036254882812 0 1126.3273
785.9068603515625 0 1097.6416
786.4097900390625 0 3308.9995
786.9083251953125 0 27490.252
787.4099731445312 0 30256.525
787.9104614257812 0 11478.799
788.41162109375 0 3853.7441
793.9010620117188 0 1021.1248
794.4057006835938 0 1767.2894
794.9169921875 0 23799.662
795.4193725585938 0 28285.285
795.9207153320312 0 16562.352
796.4227294921875 0 6051.8315
796.9209594726562 0 2482.1838
801.4022216796875 0 949.5226
813.3904418945312 0 693.05225
828.4071044921875 0 971.96857
829.4385375976562 0 13732.512 z 7
830.4423828125 0 7121.604
831.4437255859375 0 2302.7722
839.5322875976562 0 675.83105
845.4580078125 0 1946.8817 y 7
873.4851684570312 0 795.29755
900.4767456054688 0 2163.4045 c Ammonia loss 7
901.4754028320312 0 1033.9452
924.4569702148438 0 1833.8599
925.4612426757812 0 1222.9581
926.4583129882812 0 940.5247 z Water loss 6
941.4822998046875 0 2165.9817
942.492431640625 0 994.33813 y Water loss 6
944.4669799804688 0 5480.1206 z 6
945.4714965820312 0 4433.9224
946.4747314453125 0 1736.9512
954.4672241210938 0 1057.4485
955.5408935546875 0 782.13885
956.537841796875 0 911.6592
960.4802856445312 0 669.03534 y 6
961.4835205078125 0 941.5289
964.5159912109375 0 1734.5912
970.5332641601562 0 1964.9053
971.5491943359375 0 835.6826
972.5514526367188 0 649.3501
984.481201171875 0 894.3193
986.51416015625 0 1208.1564
987.526123046875 0 1669.374
988.5131225585938 0 946.1651
999.5147094726562 0 821.61053
1001.5196533203125 0 1234.1587
1002.5166625976562 0 746.5806
1003.5276489257812 0 2057.9937
1004.5305786132812 0 1142.1122
1013.5858764648438 0 885.9775
1014.5572509765625 0 13836.12 c 8
1015.5585327148438 0 6908.96
1016.5604858398438 0 1685.7375
1031.5020751953125 0 2379.785 z 5
1032.507080078125 0 3493.6418
1033.5076904296875 0 1438.8937
1039.564208984375 0 885.2982
1040.505615234375 0 2101.0984
1041.5098876953125 0 2920.747
1042.52685546875 0 2155.1265
1043.526611328125 0 1532.5027
1044.5411376953125 0 1313.5178
1046.4931640625 0 893.4928
1047.5101318359375 0 899.2463 y 5
1057.5689697265625 0 7990.092
1058.5714111328125 0 4909.0557
1059.569580078125 0 2713.4631
1060.53759765625 0 2553.9941
1100.581298828125 0 829.1626
1101.588623046875 0 18152.959 c 9
1102.5908203125 0 10269.73
1103.59375 0 4433.245
1114.593017578125 0 2773.545
1115.6004638671875 0 1915.9564
1130.5654296875 0 2733.9917 z 4
1131.573974609375 0 7152.021
1132.5782470703125 0 3065.6648
1133.5845947265625 0 1232.1232
1146.5849609375 0 1640.503 y 4
1147.5838623046875 0 650.9119
1158.609375 0 9442.898 c 10
1159.61376953125 0 6662.8545
1160.616455078125 0 2451.798
1213.6663818359375 0 2768.16
1214.6676025390625 0 1010.31177
1257.677490234375 0 10634.5205 c 11
1258.679931640625 0 7597.204
1259.6434326171875 0 1997.082
1259.669677734375 0 2090.416
1260.6170654296875 0 8576.994
1261.619140625 0 5617.0664
1262.6197509765625 0 2104.219
1275.626953125 0 3454.9944 y 3
1276.629150390625 0 2079.1526
1277.6339111328125 0 1382.8866
1282.693603515625 0 862.5206
1300.6956787109375 0 966.0135
1301.69384765625 0 1155.5414
1330.636474609375 0 785.486
1344.7098388671875 0 18077.896 c 12
1345.712646484375 0 12386.983
1346.70947265625 0 5973.2485
1347.65185546875 0 7094.924
1348.6531982421875 0 5399.3164
1349.6552734375 0 3310.754
1387.730224609375 0 1467.736
1389.7420654296875 0 774.756
1430.73486328125 0 805.6447
1431.7412109375 0 25841.709 c 13
1432.743896484375 0 17098.129
1433.7474365234375 0 8310.248
1434.75 0 2894.4324
1460.7239990234375 0 1073.7776
1461.733154296875 0 1569.4054
1488.7470703125 0 2609.9954
1489.7376708984375 0 1687.8467
1490.745361328125 0 944.9129
1530.78759765625 0 4429.2686
1531.7935791015625 0 6594.777
1532.796630859375 0 3682.241
1533.794189453125 0 2505.9233
1534.79248046875 0 1048.0806
1544.83251953125 0 1466.0118
1545.8250732421875 0 2735.556
1546.819580078125 0 3141.0515
1547.81689453125 0 2985.4968
1548.81005859375 0 1677.8617
1555.8116455078125 0 1314.6859
1556.7935791015625 0 3213.0505
1557.79150390625 0 2167.839
1561.825439453125 0 1000.9723
1562.8450927734375 0 4420.85
1563.8511962890625 0 3431.0237
1564.8519287109375 0 1609.6819
1565.8438720703125 0 834.95294
1571.798583984375 0 1792.8553
1572.8116455078125 0 6039.0713
1573.8148193359375 0 36543.527
1574.8175048828125 0 30004.28
1575.820068359375 0 16387.385
1576.818115234375 0 4297.0283
1577.8074951171875 0 1149.008
1587.805419921875 0 922.3559
1588.815673828125 0 4068.1877
1589.8310546875 0 10083.111
1590.83984375 0 36807.91
1591.8414306640625 0 28330.822
1592.8443603515625 0 13331.527
1593.840576171875 0 3973.5503
1594.8271484375 0 964.9091

Spectrum Details

|  |  |
| --- | --- |
| Matched peaks? Matched peaksThe total absolute number of peaks matched. Additionally in brackets the total fraction of peaks matched and the total number of peaks is shown. | 58 (17.96% of 323) |
| FDR? FDRThe false discovery rate estimated for this peptide. It is calculated by matching all theoretical fragments with a non-integer shift with the raw peaks for this spectrum. This is done with 40 different shifts. The resulting percentage is the average number of annotated peaks over the number of annotated peaks with the correct spectrum. | 0.90% |
| Satellite FDR? Satellite FDRSee the FDR for details on its calculation. This satellite ion specific FDR only contains the satellite ions (d/w) for I/L/J positions. | 0.00% |
| PSM Score? PSM ScoreThe PSM Score as given by Hecklib to this annotated spectrum. It is shown with three significant figures. | 442 |

## Spectrum 4162? Spectrum 4162 The raw spectrum of this peptide as annotated by Hecklib. The fragments are coloured according to ion type (see legend). Any peaks with a star '\*' as text can be hovered over to see the full details, first the ion type second the mass shift type. By hovering over the amino acids in the peptide or ions in the legend the corresponding peaks are highlighted. By toggling the 'Unassigned' label you can turn the background (unassigned) peaks on or off in the plot. By updating the slider in the Ion legend you can update the spectrum to only show the top X% of the peaks with labels. The top X% means any peak that is within X% of the highest intensity. By dragging in the spectrum you can zoom in to a specific part of the spectrum and use 'Zoom Out' to get back to the original zoom level. The annotation of the spectrum is based on the given sequence in the peptides file and is done with different software so inconsistencies are likely. The peaks are annotated based on the given sequence, with 20 ppm tolerance.

Copy Data

### Spectrum 4162 (TSV)

#### Preview

```
Loading example...
```

*Click on the button to copy the data to your clipboard.*

Mz MinMz MaxIntensity Max

WidthHeightPeptide font sizePeptide stroke widthSpectrum font sizeSpectrum stroke widthCompact peptide

Ion legend

wxyz

abcd

OtherUnassignedIonChargePositionShow for top:%

JJSEVSDRPSGVSSR

01.46e+52.92e+54.38e+55.84e+5

Zoom Out

y+11y+35y+11d+12a+12b+12y+12y+12y+12b+13b+13y+13y+13y+27y+13b+312y+28y+28b+14y+14b+14y+14y+313y+313y+29y+314y+15y+314y+15y+210y+210y+210\*\*b+15b+211y+211y+211y+211y+16y+16y+212y+212y+212y+17y+17y+213y+213y+213y+17b+214y+214y+214y+214y+18y+19y+110y+111

0776155323293106

Fragment Matches Table

Show background peaks

| Position | Ion type | Intensity | mz Theoretical | mz Error (Th) | mz Error (ppm) | Charge | Series Number |
| --- | --- | --- | --- | --- | --- | --- | --- |
| - | - | 3170 | 120.1 | - | - | 0 | - |
| - | - | 402.1 | 121.1 | - | - | 0 | - |
| - | - | 403.3 | 122.1 | - | - | 0 | - |
| - | - | 319 | 122.5 | - | - | 0 | - |
| - | - | 522.7 | 124.1 | - | - | 0 | - |
| - | - | 409.6 | 124.6 | - | - | 0 | - |
| - | - | 461.3 | 125.1 | - | - | 0 | - |
| - | - | 359.2 | 125.6 | - | - | 0 | - |
| - | - | 358.8 | 125.9 | - | - | 0 | - |
| - | - | 5631 | 126.1 | - | - | 0 | - |
| - | - | 525 | 127.1 | - | - | 0 | - |
| - | - | 561.5 | 127.1 | - | - | 0 | - |
| - | - | 1045 | 128.1 | - | - | 0 | - |
| - | - | 617.4 | 128.1 | - | - | 0 | - |
| - | - | 1540 | 129.1 | - | - | 0 | - |
| - | - | 4998 | 129.1 | - | - | 0 | - |
| - | - | 389.7 | 129.1 | - | - | 0 | - |
| - | - | 997.8 | 130.1 | - | - | 0 | - |
| - | - | 2860 | 130.1 | - | - | 0 | - |
| - | - | 1816 | 130.1 | - | - | 0 | - |
| - | - | 2.63E+04 | 133.1 | - | - | 0 | - |
| - | - | 455.9 | 133.1 | - | - | 0 | - |
| - | - | 900 | 134.1 | - | - | 0 | - |
| - | - | 2004 | 136.1 | - | - | 0 | - |
| - | - | 710.8 | 138.1 | - | - | 0 | - |
| - | - | 1410 | 140.1 | - | - | 0 | - |
| - | - | 732.4 | 141.1 | - | - | 0 | - |
| - | - | 895.9 | 141.1 | - | - | 0 | - |
| - | - | 577.9 | 143.1 | - | - | 0 | - |
| - | - | 1215 | 144.1 | - | - | 0 | - |
| - | - | 4599 | 145.1 | - | - | 0 | - |
| - | - | 446.3 | 147.1 | - | - | 0 | - |
| - | - | 2721 | 147.1 | - | - | 0 | - |
| - | - | 488 | 147.1 | - | - | 0 | - |
| - | - | 421 | 152.1 | - | - | 0 | - |
| - | - | 940.8 | 153.1 | - | - | 0 | - |
| - | - | 5893 | 154.1 | - | - | 0 | - |
| - | - | 643.7 | 155.1 | - | - | 0 | - |
| - | - | 1027 | 155.1 | - | - | 0 | - |
| - | - | 721.3 | 156.1 | - | - | 0 | - |
| - | - | 511 | 156.1 | - | - | 0 | - |
| - | - | 2421 | 157.1 | - | - | 0 | - |
| - | - | 3241 | 157.1 | - | - | 0 | - |
| - | - | 2708 | 157.1 | - | - | 0 | - |
| 15 | y | 1.214E+04 | 158.1 | 0.0002941 | 1.86 | +1 | 1 |
| - | - | 521.9 | 159.1 | - | - | 0 | - |
| - | - | 1897 | 159.1 | - | - | 0 | - |
| - | - | 1711 | 163.1 | - | - | 0 | - |
| - | - | 861.6 | 167.1 | - | - | 0 | - |
| 11 | y | 1043 | 169.1 | 0.001767 | 10.45 | +3 | 5 |
| - | - | 531.2 | 171 | - | - | 0 | - |
| - | - | 7000 | 171.1 | - | - | 0 | - |
| - | - | 818.4 | 172.1 | - | - | 0 | - |
| - | - | 549.4 | 173.1 | - | - | 0 | - |
| - | - | 3341 | 173.1 | - | - | 0 | - |
| - | - | 428 | 174.1 | - | - | 0 | - |
| - | - | 3852 | 175.1 | - | - | 0 | - |
| 15 | y | 2.411E+04 | 175.1 | 0.0002953 | 1.686 | +1 | 1 |
| - | - | 1902 | 176.1 | - | - | 0 | - |
| - | - | 2093 | 181.1 | - | - | 0 | - |
| - | - | 5665 | 181.1 | - | - | 0 | - |
| - | - | 557.9 | 181.1 | - | - | 0 | - |
| - | - | 447 | 181.6 | - | - | 0 | - |
| - | - | 567.9 | 182.1 | - | - | 0 | - |
| - | - | 802.4 | 182.1 | - | - | 0 | - |
| - | - | 549.6 | 182.2 | - | - | 0 | - |
| - | - | 5236 | 183.1 | - | - | 0 | - |
| - | - | 574.2 | 184.1 | - | - | 0 | - |
| - | - | 1084 | 184.1 | - | - | 0 | - |
| - | - | 801.5 | 184.1 | - | - | 0 | - |
| - | - | 4115 | 185.1 | - | - | 0 | - |
| - | - | 635.8 | 185.1 | - | - | 0 | - |
| - | - | 608.4 | 185.1 | - | - | 0 | - |
| 2 | d | 1406 | 185.2 | 0.0003214 | 1.736 | +1 | 2 |
| - | - | 896.6 | 187.1 | - | - | 0 | - |
| - | - | 4349 | 187.1 | - | - | 0 | - |
| - | - | 763.6 | 187.1 | - | - | 0 | - |
| - | - | 2.982E+04 | 189.1 | - | - | 0 | - |
| - | - | 7968 | 190.1 | - | - | 0 | - |
| - | - | 1910 | 190.1 | - | - | 0 | - |
| - | - | 856.3 | 194.1 | - | - | 0 | - |
| - | - | 1234 | 195.1 | - | - | 0 | - |
| - | - | 620.2 | 195.1 | - | - | 0 | - |
| - | - | 854.4 | 195.1 | - | - | 0 | - |
| - | - | 1949 | 197.1 | - | - | 0 | - |
| - | - | 1584 | 197.1 | - | - | 0 | - |
| - | - | 746.1 | 197.2 | - | - | 0 | - |
| - | - | 5236 | 199.1 | - | - | 0 | - |
| - | - | 2634 | 199.1 | - | - | 0 | - |
| 2 | a | 2.402E+05 | 199.2 | 0.0003269 | 1.641 | +1 | 2 |
| - | - | 575.5 | 199.4 | - | - | 0 | - |
| - | - | 497.8 | 199.8 | - | - | 0 | - |
| - | - | 2.768E+04 | 200.2 | - | - | 0 | - |
| - | - | 1.141E+04 | 201.1 | - | - | 0 | - |
| - | - | 1119 | 201.2 | - | - | 0 | - |
| - | - | 2256 | 202.1 | - | - | 0 | - |
| - | - | 1938 | 202.1 | - | - | 0 | - |
| - | - | 1049 | 202.1 | - | - | 0 | - |
| - | - | 466.2 | 203 | - | - | 0 | - |
| - | - | 4748 | 203.1 | - | - | 0 | - |
| - | - | 1132 | 203.1 | - | - | 0 | - |
| - | - | 642.5 | 204.1 | - | - | 0 | - |
| - | - | 551.4 | 207.1 | - | - | 0 | - |
| - | - | 8033 | 209.1 | - | - | 0 | - |
| - | - | 1012 | 209.1 | - | - | 0 | - |
| - | - | 525.4 | 209.1 | - | - | 0 | - |
| - | - | 1758 | 210.1 | - | - | 0 | - |
| - | - | 1935 | 211.1 | - | - | 0 | - |
| - | - | 2669 | 212.1 | - | - | 0 | - |
| - | - | 881.3 | 213.1 | - | - | 0 | - |
| - | - | 958.3 | 213.2 | - | - | 0 | - |
| - | - | 978 | 215.1 | - | - | 0 | - |
| - | - | 1674 | 215.1 | - | - | 0 | - |
| - | - | 671.8 | 216.1 | - | - | 0 | - |
| - | - | 4.767E+04 | 217.1 | - | - | 0 | - |
| - | - | 4620 | 218.1 | - | - | 0 | - |
| - | - | 1363 | 221.1 | - | - | 0 | - |
| - | - | 786 | 223.1 | - | - | 0 | - |
| - | - | 730.5 | 223.1 | - | - | 0 | - |
| - | - | 4360 | 224.1 | - | - | 0 | - |
| - | - | 1896 | 225.1 | - | - | 0 | - |
| - | - | 833.1 | 225.1 | - | - | 0 | - |
| - | - | 1271 | 226.1 | - | - | 0 | - |
| - | - | 1714 | 226.2 | - | - | 0 | - |
| - | - | 4061 | 227.1 | - | - | 0 | - |
| - | - | 1616 | 227.1 | - | - | 0 | - |
| 2 | b | 6.796E+04 | 227.2 | 0.00027 | 1.189 | +1 | 2 |
| - | - | 644.3 | 228.1 | - | - | 0 | - |
| - | - | 8593 | 228.2 | - | - | 0 | - |
| - | - | 4641 | 229.1 | - | - | 0 | - |
| - | - | 825.8 | 229.2 | - | - | 0 | - |
| - | - | 1553 | 230.1 | - | - | 0 | - |
| - | - | 1435 | 233.2 | - | - | 0 | - |
| - | - | 719.6 | 237.1 | - | - | 0 | - |
| - | - | 848 | 237.1 | - | - | 0 | - |
| - | - | 807.1 | 239.1 | - | - | 0 | - |
| - | - | 773.4 | 239.1 | - | - | 0 | - |
| - | - | 924.7 | 240.1 | - | - | 0 | - |
| - | - | 5459 | 242.1 | - | - | 0 | - |
| - | - | 771.7 | 243.1 | - | - | 0 | - |
| - | - | 1867 | 243.1 | - | - | 0 | - |
| - | - | 2086 | 244.1 | - | - | 0 | - |
| 14 | y | 776.7 | 244.1 | 0.001323 | 5.419 | +1 | 2 |
| 14 | y | 8371 | 245.1 | 0.0002786 | 1.137 | +1 | 2 |
| - | - | 650.3 | 249.2 | - | - | 0 | - |
| - | - | 573.8 | 252.1 | - | - | 0 | - |
| - | - | 1109 | 254.1 | - | - | 0 | - |
| - | - | 650 | 255.1 | - | - | 0 | - |
| - | - | 4415 | 255.1 | - | - | 0 | - |
| - | - | 800.1 | 256.1 | - | - | 0 | - |
| - | - | 950.1 | 256.1 | - | - | 0 | - |
| - | - | 684.7 | 259.1 | - | - | 0 | - |
| - | - | 828.9 | 259.1 | - | - | 0 | - |
| - | - | 630.9 | 261.2 | - | - | 0 | - |
| 14 | y | 4884 | 262.2 | 0.0002035 | 0.7763 | +1 | 2 |
| - | - | 612.5 | 267.1 | - | - | 0 | - |
| - | - | 1498 | 270.1 | - | - | 0 | - |
| - | - | 606.9 | 271.1 | - | - | 0 | - |
| - | - | 656.7 | 272.1 | - | - | 0 | - |
| - | - | 592.9 | 274.1 | - | - | 0 | - |
| - | - | 7033 | 277.1 | - | - | 0 | - |
| - | - | 578.2 | 278.1 | - | - | 0 | - |
| - | - | 1605 | 284.1 | - | - | 0 | - |
| - | - | 877.7 | 284.2 | - | - | 0 | - |
| - | - | 557.3 | 285.1 | - | - | 0 | - |
| - | - | 1007 | 288.2 | - | - | 0 | - |
| - | - | 746.3 | 289.2 | - | - | 0 | - |
| 3 | b | 3731 | 296.2 | 0.0002144 | 0.7238 | +1 | 3 |
| - | - | 1189 | 297.1 | - | - | 0 | - |
| - | - | 3061 | 298.1 | - | - | 0 | - |
| - | - | 1458 | 299.1 | - | - | 0 | - |
| - | - | 777.1 | 307.2 | - | - | 0 | - |
| - | - | 1847 | 310.1 | - | - | 0 | - |
| - | - | 590 | 312.1 | - | - | 0 | - |
| - | - | 3783 | 312.2 | - | - | 0 | - |
| - | - | 1908 | 313.1 | - | - | 0 | - |
| - | - | 702.2 | 313.2 | - | - | 0 | - |
| - | - | 8799 | 313.2 | - | - | 0 | - |
| - | - | 2277 | 314.1 | - | - | 0 | - |
| - | - | 943.7 | 314.2 | - | - | 0 | - |
| 3 | b | 7829 | 314.2 | 0.000514 | 1.636 | +1 | 3 |
| - | - | 1153 | 315.2 | - | - | 0 | - |
| - | - | 9335 | 316.2 | - | - | 0 | - |
| - | - | 1820 | 317.2 | - | - | 0 | - |
| - | - | 703.8 | 319.2 | - | - | 0 | - |
| - | - | 893.3 | 323.2 | - | - | 0 | - |
| - | - | 606.8 | 324.1 | - | - | 0 | - |
| - | - | 644.6 | 324.2 | - | - | 0 | - |
| - | - | 1390 | 326.1 | - | - | 0 | - |
| - | - | 1782 | 330.2 | - | - | 0 | - |
| 13 | y | 1640 | 331.2 | 0.0001115 | 0.3368 | +1 | 3 |
| 13 | y | 6962 | 332.2 | 0.0003699 | 1.114 | +1 | 3 |
| - | - | 1128 | 333.2 | - | - | 0 | - |
| - | - | 677.3 | 335.1 | - | - | 0 | - |
| - | - | 1.327E+04 | 338.1 | - | - | 0 | - |
| - | - | 1192 | 338.2 | - | - | 0 | - |
| - | - | 2428 | 339.1 | - | - | 0 | - |
| - | - | 2409 | 340.2 | - | - | 0 | - |
| - | - | 5515 | 341.2 | - | - | 0 | - |
| - | - | 7103 | 342.1 | - | - | 0 | - |
| - | - | 672.5 | 342.2 | - | - | 0 | - |
| - | - | 1531 | 343.1 | - | - | 0 | - |
| 9 | y | 3153 | 345.2 | 0.000109 | 0.3158 | +2 | 7 |
| - | - | 1355 | 345.7 | - | - | 0 | - |
| - | - | 722.4 | 347.7 | - | - | 0 | - |
| 13 | y | 6504 | 349.2 | 6.596E-05 | 0.1889 | +1 | 3 |
| - | - | 1335 | 350.2 | - | - | 0 | - |
| - | - | 795.2 | 352.1 | - | - | 0 | - |
| - | - | 628.1 | 353.1 | - | - | 0 | - |
| - | - | 686.5 | 353.2 | - | - | 0 | - |
| - | - | 1910 | 354.1 | - | - | 0 | - |
| - | - | 1128 | 356.1 | - | - | 0 | - |
| - | - | 3641 | 359.2 | - | - | 0 | - |
| - | - | 787.6 | 359.2 | - | - | 0 | - |
| - | - | 1289 | 361.7 | - | - | 0 | - |
| - | - | 984.9 | 370.1 | - | - | 0 | - |
| - | - | 903.7 | 381.1 | - | - | 0 | - |
| - | - | 1040 | 385.2 | - | - | 0 | - |
| - | - | 1772 | 388.1 | - | - | 0 | - |
| - | - | 635.2 | 390.7 | - | - | 0 | - |
| - | - | 2809 | 391.7 | - | - | 0 | - |
| - | - | 637.5 | 393.7 | - | - | 0 | - |
| - | - | 966.6 | 396.2 | - | - | 0 | - |
| - | - | 735.2 | 396.6 | - | - | 0 | - |
| - | - | 753.7 | 397.2 | - | - | 0 | - |
| - | - | 1138 | 398.2 | - | - | 0 | - |
| - | - | 631.8 | 398.7 | - | - | 0 | - |
| - | - | 1250 | 399.2 | - | - | 0 | - |
| - | - | 671.3 | 400.2 | - | - | 0 | - |
| - | - | 944.1 | 400.2 | - | - | 0 | - |
| - | - | 1890 | 403.2 | - | - | 0 | - |
| - | - | 708.7 | 404.2 | - | - | 0 | - |
| - | - | 564.7 | 405.2 | - | - | 0 | - |
| - | - | 3962 | 406.2 | - | - | 0 | - |
| - | - | 744.5 | 407.2 | - | - | 0 | - |
| - | - | 621 | 408.2 | - | - | 0 | - |
| - | - | 734.7 | 410.2 | - | - | 0 | - |
| - | - | 671 | 411.2 | - | - | 0 | - |
| - | - | 2159 | 411.2 | - | - | 0 | - |
| - | - | 1560 | 412.2 | - | - | 0 | - |
| - | - | 621.6 | 412.2 | - | - | 0 | - |
| - | - | 849.3 | 412.7 | - | - | 0 | - |
| 12 | b | 2231 | 414.2 | 0.006087 | 14.69 | +3 | 12 |
| 8 | y | 3843 | 414.7 | 0.001433 | 3.455 | +2 | 8 |
| - | - | 1232 | 415.2 | - | - | 0 | - |
| - | - | 799.8 | 415.3 | - | - | 0 | - |
| - | - | 924.1 | 419.2 | - | - | 0 | - |
| 8 | y | 5.319E+04 | 423.2 | 0.0001841 | 0.4349 | +2 | 8 |
| - | - | 2.01E+04 | 423.7 | - | - | 0 | - |
| - | - | 6011 | 424.2 | - | - | 0 | - |
| - | - | 1250 | 424.7 | - | - | 0 | - |
| 4 | b | 4276 | 425.2 | 8.135E-05 | 0.1913 | +1 | 4 |
| - | - | 1110 | 426.2 | - | - | 0 | - |
| - | - | 1276 | 428.2 | - | - | 0 | - |
| - | - | 2143 | 429.2 | - | - | 0 | - |
| 12 | y | 1682 | 430.2 | 0.001356 | 3.152 | +1 | 4 |
| - | - | 901.2 | 433.2 | - | - | 0 | - |
| - | - | 1161 | 437.2 | - | - | 0 | - |
| - | - | 811 | 440.2 | - | - | 0 | - |
| - | - | 4508 | 441.2 | - | - | 0 | - |
| 4 | b | 3747 | 443.3 | 0.000676 | 1.525 | +1 | 4 |
| - | - | 1193 | 444.3 | - | - | 0 | - |
| - | - | 1175 | 447.7 | - | - | 0 | - |
| 12 | y | 1763 | 448.3 | 0.0001106 | 0.2468 | +1 | 4 |
| 3 | y | 1708 | 448.9 | 0.000105 | 0.234 | +3 | 13 |
| - | - | 6782 | 451.2 | - | - | 0 | - |
| - | - | 1578 | 452.2 | - | - | 0 | - |
| - | - | 1404 | 452.3 | - | - | 0 | - |
| 3 | y | 2401 | 454.9 | 0.0004287 | 0.9424 | +3 | 13 |
| - | - | 1805 | 455.2 | - | - | 0 | - |
| - | - | 1142 | 455.6 | - | - | 0 | - |
| - | - | 3122 | 458.2 | - | - | 0 | - |
| - | - | 1405 | 467.2 | - | - | 0 | - |
| - | - | 2120 | 469.2 | - | - | 0 | - |
| - | - | 1346 | 469.3 | - | - | 0 | - |
| - | - | 1152 | 470.2 | - | - | 0 | - |
| - | - | 584.4 | 479.2 | - | - | 0 | - |
| 7 | y | 9415 | 480.7 | 0.000352 | 0.7322 | +2 | 9 |
| - | - | 4604 | 481.2 | - | - | 0 | - |
| - | - | 1316 | 481.8 | - | - | 0 | - |
| 2 | y | 962.5 | 486.6 | 0.001132 | 2.326 | +3 | 14 |
| - | - | 1017 | 487.2 | - | - | 0 | - |
| 11 | y | 1858 | 487.3 | 0.007531 | 15.46 | +1 | 5 |
| 2 | y | 1713 | 492.6 | 0.00148 | 3.005 | +3 | 14 |
| - | - | 2234 | 492.9 | - | - | 0 | - |
| - | - | 2642 | 495.9 | - | - | 0 | - |
| - | - | 1041 | 496.2 | - | - | 0 | - |
| - | - | 3334 | 496.2 | - | - | 0 | - |
| - | - | 1123 | 496.6 | - | - | 0 | - |
| - | - | 1131 | 500.2 | - | - | 0 | - |
| - | - | 807.2 | 502.2 | - | - | 0 | - |
| - | - | 661.3 | 504.3 | - | - | 0 | - |
| - | - | 1545 | 505.2 | - | - | 0 | - |
| 11 | y | 6199 | 505.3 | 0.0003678 | 0.7279 | +1 | 5 |
| - | - | 1351 | 506.3 | - | - | 0 | - |
| - | - | 861.1 | 512.2 | - | - | 0 | - |
| - | - | 971.8 | 514.3 | - | - | 0 | - |
| 6 | y | 1630 | 515.3 | 0.002049 | 3.976 | +2 | 10 |
| 6 | y | 1758 | 515.7 | 0.004113 | 7.974 | +2 | 10 |
| - | - | 1653 | 516.3 | - | - | 0 | - |
| - | - | 1168 | 518.2 | - | - | 0 | - |
| - | - | 855 | 518.3 | - | - | 0 | - |
| - | - | 3451 | 521.3 | - | - | 0 | - |
| - | - | 1448 | 521.8 | - | - | 0 | - |
| - | - | 753.8 | 522.2 | - | - | 0 | - |
| - | - | 1106 | 522.3 | - | - | 0 | - |
| 6 | y | 5.469E+04 | 524.3 | 0.001458 | 2.781 | +2 | 10 |
| 0 | Precursor | 1.01E+04 | 524.6 | 0.006113 | 11.65 | +3 | -1 |
| - | - | 2.99E+04 | 524.8 | - | - | 0 | - |
| - | - | 4166 | 524.9 | - | - | 0 | - |
| - | - | 1.154E+04 | 525.3 | - | - | 0 | - |
| - | - | 2149 | 525.8 | - | - | 0 | - |
| - | - | 929.2 | 529.3 | - | - | 0 | - |
| - | - | 791.5 | 529.8 | - | - | 0 | - |
| - | - | 892 | 530.2 | - | - | 0 | - |
| 0 | Precursor | 6866 | 530.3 | 0.0001534 | 0.2892 | +3 | -1 |
| - | - | 1875 | 530.3 | - | - | 0 | - |
| - | - | 4729 | 530.6 | - | - | 0 | - |
| - | - | 1919 | 531 | - | - | 0 | - |
| - | - | 1126 | 531.2 | - | - | 0 | - |
| - | - | 822.2 | 532.3 | - | - | 0 | - |
| - | - | 1643 | 540.3 | - | - | 0 | - |
| 5 | b | 1388 | 542.3 | 0.0004467 | 0.8236 | +1 | 5 |
| - | - | 6808 | 550.3 | - | - | 0 | - |
| - | - | 1835 | 551.3 | - | - | 0 | - |
| - | - | 1256 | 556.3 | - | - | 0 | - |
| - | - | 1078 | 558.8 | - | - | 0 | - |
| 11 | b | 697 | 562.3 | 0.00552 | 9.816 | +2 | 11 |
| 5 | y | 3137 | 564.8 | 0.001282 | 2.271 | +2 | 11 |
| 5 | y | 2635 | 565.3 | 0.003902 | 6.903 | +2 | 11 |
| - | - | 1123 | 565.8 | - | - | 0 | - |
| - | - | 1575 | 566.3 | - | - | 0 | - |
| - | - | 804.6 | 567.3 | - | - | 0 | - |
| - | - | 3943 | 568.3 | - | - | 0 | - |
| - | - | 910.3 | 569.3 | - | - | 0 | - |
| - | - | 627 | 572.3 | - | - | 0 | - |
| - | - | 860.5 | 573.3 | - | - | 0 | - |
| 5 | y | 7.6E+04 | 573.8 | 0.0002171 | 0.3783 | +2 | 11 |
| 10 | y | 4.742E+04 | 574.3 | 0.003501 | 6.097 | +1 | 6 |
| - | - | 1.354E+04 | 574.8 | - | - | 0 | - |
| - | - | 4222 | 575.3 | - | - | 0 | - |
| - | - | 1580 | 584.3 | - | - | 0 | - |
| - | - | 766.5 | 585.8 | - | - | 0 | - |
| - | - | 1215 | 587.3 | - | - | 0 | - |
| 10 | y | 8092 | 592.3 | 0.0002886 | 0.4872 | +1 | 6 |
| - | - | 2424 | 593.3 | - | - | 0 | - |
| - | - | 742.8 | 594.3 | - | - | 0 | - |
| - | - | 1799 | 600.3 | - | - | 0 | - |
| - | - | 1239 | 626.3 | - | - | 0 | - |
| - | - | 959.3 | 628.3 | - | - | 0 | - |
| 4 | y | 6407 | 629.3 | 0.00214 | 3.401 | +2 | 12 |
| 4 | y | 3540 | 629.8 | 0.008668 | 13.76 | +2 | 12 |
| - | - | 1633 | 630.3 | - | - | 0 | - |
| - | - | 1224 | 636.3 | - | - | 0 | - |
| 4 | y | 3.671E+04 | 638.3 | 0.0002734 | 0.4283 | +2 | 12 |
| - | - | 2.63E+04 | 638.8 | - | - | 0 | - |
| - | - | 9066 | 639.3 | - | - | 0 | - |
| - | - | 2255 | 639.8 | - | - | 0 | - |
| - | - | 917.8 | 641.3 | - | - | 0 | - |
| - | - | 1372 | 643.4 | - | - | 0 | - |
| - | - | 3004 | 646.3 | - | - | 0 | - |
| - | - | 1057 | 647.3 | - | - | 0 | - |
| - | - | 1561 | 649.3 | - | - | 0 | - |
| - | - | 967 | 652.3 | - | - | 0 | - |
| - | - | 2029 | 653.3 | - | - | 0 | - |
| - | - | 1460 | 654.3 | - | - | 0 | - |
| - | - | 1551 | 654.8 | - | - | 0 | - |
| - | - | 1131 | 655.3 | - | - | 0 | - |
| - | - | 1084 | 656.3 | - | - | 0 | - |
| - | - | 759.3 | 657.3 | - | - | 0 | - |
| - | - | 2322 | 659.3 | - | - | 0 | - |
| - | - | 987.2 | 660.8 | - | - | 0 | - |
| - | - | 855.4 | 661.3 | - | - | 0 | - |
| - | - | 1643 | 663.8 | - | - | 0 | - |
| - | - | 2457 | 664.3 | - | - | 0 | - |
| - | - | 1292 | 664.8 | - | - | 0 | - |
| - | - | 764.6 | 665.8 | - | - | 0 | - |
| - | - | 5630 | 666.8 | - | - | 0 | - |
| - | - | 3262 | 667.3 | - | - | 0 | - |
| - | - | 1827 | 667.8 | - | - | 0 | - |
| 9 | y | 1.047E+04 | 671.3 | 0.0006787 | 1.011 | +1 | 7 |
| 9 | y | 6247 | 672.3 | 0.004319 | 6.425 | +1 | 7 |
| 3 | y | 1.908E+04 | 672.8 | 0.0003241 | 0.4817 | +2 | 13 |
| 3 | y | 1.789E+04 | 673.3 | 0.008645 | 12.84 | +2 | 13 |
| - | - | 7669 | 673.8 | - | - | 0 | - |
| - | - | 1.072E+04 | 674.3 | - | - | 0 | - |
| - | - | 1020 | 674.8 | - | - | 0 | - |
| - | - | 3575 | 675.3 | - | - | 0 | - |
| - | - | 948.8 | 676.3 | - | - | 0 | - |
| - | - | 669.1 | 677.8 | - | - | 0 | - |
| - | - | 941.7 | 678.3 | - | - | 0 | - |
| - | - | 1261 | 679.3 | - | - | 0 | - |
| - | - | 8436 | 680.8 | - | - | 0 | - |
| - | - | 5843 | 681.3 | - | - | 0 | - |
| 3 | y | 5.78E+05 | 681.8 | 0.0001133 | 0.1661 | +2 | 13 |
| - | - | 4.033E+05 | 682.3 | - | - | 0 | - |
| - | - | 1.681E+05 | 682.8 | - | - | 0 | - |
| - | - | 4.741E+04 | 683.3 | - | - | 0 | - |
| - | - | 8894 | 683.8 | - | - | 0 | - |
| - | - | 4231 | 686.8 | - | - | 0 | - |
| - | - | 2768 | 687.3 | - | - | 0 | - |
| - | - | 1196 | 687.8 | - | - | 0 | - |
| 9 | y | 4.602E+04 | 689.4 | 0.000257 | 0.3728 | +1 | 7 |
| - | - | 1.467E+04 | 690.4 | - | - | 0 | - |
| - | - | 3686 | 691.4 | - | - | 0 | - |
| - | - | 2553 | 697.3 | - | - | 0 | - |
| 14 | b | 3424 | 699.3 | 0.007863 | 11.24 | +2 | 14 |
| - | - | 1694 | 700.3 | - | - | 0 | - |
| - | - | 3828 | 722.4 | - | - | 0 | - |
| - | - | 2190 | 723.4 | - | - | 0 | - |
| 2 | y | 6095 | 729.4 | 0.0001808 | 0.2479 | +2 | 14 |
| 2 | y | 3659 | 729.9 | 0.007506 | 10.28 | +2 | 14 |
| - | - | 1417 | 730.4 | - | - | 0 | - |
| - | - | 714.1 | 736.9 | - | - | 0 | - |
| - | - | 692.3 | 737.4 | - | - | 0 | - |
| - | - | 1062 | 737.9 | - | - | 0 | - |
| 2 | y | 4.6E+04 | 738.4 | 0.0007024 | 0.9513 | +2 | 14 |
| - | - | 3.639E+04 | 738.9 | - | - | 0 | - |
| - | - | 1.537E+04 | 739.4 | - | - | 0 | - |
| - | - | 4767 | 739.9 | - | - | 0 | - |
| - | - | 1801 | 743.4 | - | - | 0 | - |
| - | - | 1691 | 743.9 | - | - | 0 | - |
| - | - | 717.6 | 758.4 | - | - | 0 | - |
| - | - | 822.6 | 759.4 | - | - | 0 | - |
| - | - | 1066 | 766.4 | - | - | 0 | - |
| - | - | 1179 | 768.4 | - | - | 0 | - |
| - | - | 2232 | 770.4 | - | - | 0 | - |
| - | - | 751.8 | 779.4 | - | - | 0 | - |
| - | - | 1514 | 782.4 | - | - | 0 | - |
| - | - | 1149 | 783.4 | - | - | 0 | - |
| - | - | 888.9 | 784.4 | - | - | 0 | - |
| - | - | 1185 | 785.4 | - | - | 0 | - |
| - | - | 1040 | 786.4 | - | - | 0 | - |
| - | - | 1048 | 798.4 | - | - | 0 | - |
| - | - | 1028 | 799.4 | - | - | 0 | - |
| - | - | 970.9 | 803.4 | - | - | 0 | - |
| - | - | 798.5 | 805.4 | - | - | 0 | - |
| - | - | 1117 | 811.4 | - | - | 0 | - |
| - | - | 741.3 | 812.4 | - | - | 0 | - |
| - | - | 2109 | 821.5 | - | - | 0 | - |
| - | - | 1344 | 823.4 | - | - | 0 | - |
| - | - | 714.1 | 824.4 | - | - | 0 | - |
| 8 | y | 7201 | 828.4 | 0.0007223 | 0.8718 | +1 | 8 |
| - | - | 2637 | 829.4 | - | - | 0 | - |
| - | - | 851.8 | 830.4 | - | - | 0 | - |
| - | - | 2173 | 841.4 | - | - | 0 | - |
| - | - | 849.4 | 857.4 | - | - | 0 | - |
| - | - | 2137 | 887.4 | - | - | 0 | - |
| - | - | 1490 | 891.4 | - | - | 0 | - |
| - | - | 905.7 | 892.4 | - | - | 0 | - |
| - | - | 1190 | 898.4 | - | - | 0 | - |
| - | - | 924.9 | 899.5 | - | - | 0 | - |
| - | - | 1547 | 900.5 | - | - | 0 | - |
| - | - | 3368 | 915.4 | - | - | 0 | - |
| - | - | 1783 | 916.4 | - | - | 0 | - |
| - | - | 826 | 917.4 | - | - | 0 | - |
| 7 | y | 5153 | 943.5 | 0.0008098 | 0.8584 | +1 | 9 |
| - | - | 3058 | 944.5 | - | - | 0 | - |
| - | - | 983.4 | 945.5 | - | - | 0 | - |
| - | - | 1744 | 969.5 | - | - | 0 | - |
| - | - | 757.6 | 971.5 | - | - | 0 | - |
| - | - | 1257 | 972.5 | - | - | 0 | - |
| - | - | 3553 | 973.4 | - | - | 0 | - |
| - | - | 831.8 | 974.5 | - | - | 0 | - |
| - | - | 5094 | 986.5 | - | - | 0 | - |
| - | - | 5675 | 987.5 | - | - | 0 | - |
| - | - | 3377 | 988.5 | - | - | 0 | - |
| - | - | 1019 | 989.5 | - | - | 0 | - |
| - | - | 1710 | 990.5 | - | - | 0 | - |
| - | - | 988.9 | 991.5 | - | - | 0 | - |
| - | - | 778.5 | 1006 | - | - | 0 | - |
| - | - | 765.5 | 1006 | - | - | 0 | - |
| - | - | 2219 | 1012 | - | - | 0 | - |
| - | - | 1815 | 1013 | - | - | 0 | - |
| - | - | 5473 | 1014 | - | - | 0 | - |
| - | - | 1894 | 1015 | - | - | 0 | - |
| - | - | 1071 | 1016 | - | - | 0 | - |
| 6 | y | 1.667E+04 | 1030 | 0.001344 | 1.304 | +1 | 10 |
| - | - | 8787 | 1031 | - | - | 0 | - |
| - | - | 2698 | 1032 | - | - | 0 | - |
| - | - | 1902 | 1074 | - | - | 0 | - |
[truncated: 382,262 more chars]
