# Supplementary material for: A Handle on Mass Coincidence Errors in De Novo Sequencing of Antibodies by Bottom-up Proteomics
Source: J Proteome Res. 2024 Jun 27;23(8):3552–9. doi: 10.1021/acs.jproteome.4c00188 (PMC11301774; doi:10.1021/acs.jproteome.4c00188)
Supplement: Supplementary file 1 — pr4c00188_si_001.zip [file pr4c00188_si_001.zip › supplementary data/xln-disambiguation/2023-12-13@14-36-36 f59/report/reads/Combined_014.html]

Details Combined\_014 | Stitch OverviewUndefined

# Read Combined\_014

## Sequence (length=14)

SWYQHHPGKAPKJJ

## Spectrum 5528? Spectrum 5528 The raw spectrum of this peptide as annotated by Hecklib. The fragments are coloured according to ion type (see legend). Any peaks with a star '\*' as text can be hovered over to see the full details, first the ion type second the mass shift type. By hovering over the amino acids in the peptide or ions in the legend the corresponding peaks are highlighted. By toggling the 'Unassigned' label you can turn the background (unassigned) peaks on or off in the plot. By updating the slider in the Ion legend you can update the spectrum to only show the top X% of the peaks with labels. The top X% means any peak that is within X% of the highest intensity. By dragging in the spectrum you can zoom in to a specific part of the spectrum and use 'Zoom Out' to get back to the original zoom level. The annotation of the spectrum is based on the given sequence in the peptides file and is done with different software so inconsistencies are likely. The peaks are annotated based on the given sequence, with 20 ppm tolerance.

Copy Data

### Spectrum 5528 (TSV)

#### Preview

```
Loading example...
```

*Click on the button to copy the data to your clipboard.*

Mz MinMz MaxIntensity Max

WidthHeightPeptide font sizePeptide stroke widthSpectrum font sizeSpectrum stroke widthCompact peptide

Ion legend

wxyz

abcd

OtherUnassignedIonChargePositionShow for top:%

SWYQHHPGKAPKJJ

01.06e+52.13e+53.19e+54.26e+5

Zoom Out

y+11y+23b+34a+23y+24a+12b+35y+12a+12b+12b+12y+26y+27y+13b+26y+28b+13b+26b+13y+312y+14b+312y+29b+28b+313b+313b+313y+313y+313y+210y+15\*\*y+210\*b+29b+14b+210b+210b+210y+211y+211b+211y+16b+15b+15y+212y+212b+212b+15b+212y+17b+213b+213b+213y+213b+16b+16y+18b+16y+19y+110b+19b+110b+110b+110

0636127319092546

Fragment Matches Table

Show background peaks

| Position | Ion type | Intensity | mz Theoretical | mz Error (Th) | mz Error (ppm) | Charge | Series Number |
| --- | --- | --- | --- | --- | --- | --- | --- |
| - | - | 450.5 | 120.1 | - | - | 0 | - |
| - | - | 752.7 | 120.1 | - | - | 0 | - |
| - | - | 1250 | 120.1 | - | - | 0 | - |
| - | - | 986.9 | 122.1 | - | - | 0 | - |
| - | - | 1769 | 123.1 | - | - | 0 | - |
| - | - | 358.6 | 125.1 | - | - | 0 | - |
| - | - | 360.1 | 126 | - | - | 0 | - |
| - | - | 2329 | 127.1 | - | - | 0 | - |
| - | - | 3814 | 129.1 | - | - | 0 | - |
| - | - | 9.478E+04 | 129.1 | - | - | 0 | - |
| - | - | 2.505E+04 | 130.1 | - | - | 0 | - |
| - | - | 5536 | 130.1 | - | - | 0 | - |
| - | - | 388.5 | 130.5 | - | - | 0 | - |
| - | - | 2907 | 131.1 | - | - | 0 | - |
| - | - | 8.65E+04 | 132.1 | - | - | 0 | - |
| 14 | y | 1.877E+04 | 132.1 | 0.0004203 | 3.182 | +1 | 1 |
| - | - | 1058 | 133.1 | - | - | 0 | - |
| - | - | 8497 | 133.1 | - | - | 0 | - |
| - | - | 910 | 133.1 | - | - | 0 | - |
| - | - | 439.3 | 134.1 | - | - | 0 | - |
| - | - | 502.9 | 134.1 | - | - | 0 | - |
| - | - | 483.1 | 135.1 | - | - | 0 | - |
| - | - | 488.3 | 135.7 | - | - | 0 | - |
| - | - | 4.997E+04 | 136.1 | - | - | 0 | - |
| - | - | 761.3 | 137.1 | - | - | 0 | - |
| - | - | 4584 | 137.1 | - | - | 0 | - |
| - | - | 2541 | 138.1 | - | - | 0 | - |
| - | - | 1487 | 139.1 | - | - | 0 | - |
| - | - | 5957 | 141.1 | - | - | 0 | - |
| - | - | 645.9 | 142.1 | - | - | 0 | - |
| - | - | 579.3 | 143.1 | - | - | 0 | - |
| - | - | 1587 | 144.1 | - | - | 0 | - |
| - | - | 2479 | 145.1 | - | - | 0 | - |
| - | - | 1590 | 146.1 | - | - | 0 | - |
| - | - | 503.6 | 146.1 | - | - | 0 | - |
| - | - | 569.1 | 146.1 | - | - | 0 | - |
| - | - | 1092 | 147 | - | - | 0 | - |
| - | - | 638.9 | 149 | - | - | 0 | - |
| - | - | 1442 | 151.1 | - | - | 0 | - |
| - | - | 780.6 | 152.1 | - | - | 0 | - |
| - | - | 2306 | 152.1 | - | - | 0 | - |
| - | - | 2102 | 152.1 | - | - | 0 | - |
| - | - | 733.1 | 153.1 | - | - | 0 | - |
| - | - | 747.8 | 153.1 | - | - | 0 | - |
| - | - | 576.5 | 153.1 | - | - | 0 | - |
| - | - | 612.8 | 154.1 | - | - | 0 | - |
| - | - | 545.9 | 154.1 | - | - | 0 | - |
| - | - | 4506 | 155.1 | - | - | 0 | - |
| - | - | 3446 | 155.1 | - | - | 0 | - |
| - | - | 3688 | 155.1 | - | - | 0 | - |
| - | - | 4191 | 156.1 | - | - | 0 | - |
| - | - | 663.2 | 157.1 | - | - | 0 | - |
| - | - | 995.6 | 158.1 | - | - | 0 | - |
| - | - | 9116 | 158.1 | - | - | 0 | - |
| - | - | 4.055E+04 | 159.1 | - | - | 0 | - |
| - | - | 2167 | 160.1 | - | - | 0 | - |
| - | - | 4019 | 160.1 | - | - | 0 | - |
| - | - | 635.4 | 161.1 | - | - | 0 | - |
| - | - | 748.3 | 162.1 | - | - | 0 | - |
| - | - | 2086 | 163.1 | - | - | 0 | - |
| - | - | 545.3 | 164.1 | - | - | 0 | - |
| - | - | 762.2 | 165.1 | - | - | 0 | - |
| - | - | 721.6 | 165.1 | - | - | 0 | - |
| - | - | 3359 | 165.1 | - | - | 0 | - |
| - | - | 2499 | 166.1 | - | - | 0 | - |
| - | - | 700.5 | 167.1 | - | - | 0 | - |
| - | - | 795 | 167.1 | - | - | 0 | - |
| - | - | 865.2 | 168.1 | - | - | 0 | - |
| - | - | 4269 | 169.1 | - | - | 0 | - |
| - | - | 7627 | 169.1 | - | - | 0 | - |
| - | - | 1.476E+04 | 170.1 | - | - | 0 | - |
| - | - | 2150 | 171.1 | - | - | 0 | - |
| - | - | 837.7 | 171.1 | - | - | 0 | - |
| - | - | 1079 | 171.1 | - | - | 0 | - |
| - | - | 1335 | 171.1 | - | - | 0 | - |
| - | - | 5862 | 172.1 | - | - | 0 | - |
| - | - | 739.4 | 175.1 | - | - | 0 | - |
| - | - | 742.5 | 175.1 | - | - | 0 | - |
| - | - | 1413 | 176.1 | - | - | 0 | - |
| - | - | 663.1 | 178.1 | - | - | 0 | - |
| - | - | 521.8 | 179.1 | - | - | 0 | - |
| - | - | 917.1 | 180.1 | - | - | 0 | - |
| - | - | 1107 | 181.1 | - | - | 0 | - |
| - | - | 1.774E+04 | 181.1 | - | - | 0 | - |
| - | - | 2277 | 181.1 | - | - | 0 | - |
| - | - | 1592 | 182.1 | - | - | 0 | - |
| - | - | 3709 | 182.1 | - | - | 0 | - |
| - | - | 595.2 | 182.3 | - | - | 0 | - |
| - | - | 1.316E+04 | 183.1 | - | - | 0 | - |
| - | - | 1749 | 183.1 | - | - | 0 | - |
| - | - | 664.9 | 183.1 | - | - | 0 | - |
| - | - | 739 | 183.1 | - | - | 0 | - |
| - | - | 850.2 | 184.1 | - | - | 0 | - |
| - | - | 1048 | 184.1 | - | - | 0 | - |
| - | - | 2866 | 185.1 | - | - | 0 | - |
| - | - | 639.9 | 185.1 | - | - | 0 | - |
| - | - | 559.8 | 185.1 | - | - | 0 | - |
| - | - | 9998 | 186.1 | - | - | 0 | - |
| - | - | 8.048E+04 | 187.1 | - | - | 0 | - |
| - | - | 1714 | 187.1 | - | - | 0 | - |
| 12 | y | 1451 | 187.1 | 0.000427 | 2.281 | +2 | 3 |
| - | - | 8696 | 188.1 | - | - | 0 | - |
| 4 | b | 1895 | 189.1 | 0.002407 | 12.73 | +3 | 4 |
| - | - | 472.2 | 191.1 | - | - | 0 | - |
| - | - | 1063 | 195.1 | - | - | 0 | - |
| - | - | 1340 | 195.1 | - | - | 0 | - |
| - | - | 601.4 | 197.1 | - | - | 0 | - |
| - | - | 3751 | 197.2 | - | - | 0 | - |
| - | - | 641.2 | 198.1 | - | - | 0 | - |
| - | - | 490.1 | 198.2 | - | - | 0 | - |
| - | - | 2097 | 199.1 | - | - | 0 | - |
| - | - | 1817 | 199.1 | - | - | 0 | - |
| - | - | 919.4 | 199.1 | - | - | 0 | - |
| - | - | 3435 | 199.2 | - | - | 0 | - |
| - | - | 1162 | 200.1 | - | - | 0 | - |
| - | - | 607.1 | 201.1 | - | - | 0 | - |
| - | - | 6622 | 201.1 | - | - | 0 | - |
| - | - | 1196 | 201.1 | - | - | 0 | - |
| - | - | 1188 | 202.1 | - | - | 0 | - |
| - | - | 9106 | 202.1 | - | - | 0 | - |
| - | - | 530.3 | 203.1 | - | - | 0 | - |
| - | - | 695.9 | 203.1 | - | - | 0 | - |
| - | - | 1807 | 203.1 | - | - | 0 | - |
| - | - | 600.9 | 203.1 | - | - | 0 | - |
| - | - | 619.2 | 204.1 | - | - | 0 | - |
| - | - | 635.2 | 205.1 | - | - | 0 | - |
| 3 | a | 1070 | 205.1 | 0.0004716 | 2.3 | +2 | 3 |
| - | - | 1025 | 205.1 | - | - | 0 | - |
| - | - | 6483 | 207.1 | - | - | 0 | - |
| - | - | 559.7 | 207.1 | - | - | 0 | - |
| - | - | 695.7 | 207.1 | - | - | 0 | - |
| - | - | 3479 | 207.1 | - | - | 0 | - |
| - | - | 796.2 | 207.2 | - | - | 0 | - |
| - | - | 970.6 | 208.1 | - | - | 0 | - |
| - | - | 815.6 | 209.1 | - | - | 0 | - |
| - | - | 623.2 | 209.1 | - | - | 0 | - |
| - | - | 814.6 | 209.1 | - | - | 0 | - |
| - | - | 1605 | 211.1 | - | - | 0 | - |
| - | - | 687.8 | 211.1 | - | - | 0 | - |
| - | - | 1291 | 212.1 | - | - | 0 | - |
| - | - | 4326 | 214.1 | - | - | 0 | - |
| - | - | 2103 | 215.1 | - | - | 0 | - |
| - | - | 635.6 | 216.1 | - | - | 0 | - |
| - | - | 543.5 | 216.1 | - | - | 0 | - |
| - | - | 559 | 217 | - | - | 0 | - |
| - | - | 920.8 | 217.1 | - | - | 0 | - |
| - | - | 775.2 | 219.1 | - | - | 0 | - |
| - | - | 7066 | 220.1 | - | - | 0 | - |
| - | - | 9255 | 221.1 | - | - | 0 | - |
| - | - | 764.2 | 221.1 | - | - | 0 | - |
| - | - | 818.9 | 222.1 | - | - | 0 | - |
| - | - | 1089 | 222.1 | - | - | 0 | - |
| - | - | 758.7 | 223.1 | - | - | 0 | - |
| - | - | 1470 | 223.2 | - | - | 0 | - |
| - | - | 2068 | 224.1 | - | - | 0 | - |
| - | - | 4592 | 224.2 | - | - | 0 | - |
| - | - | 533.7 | 225 | - | - | 0 | - |
| - | - | 559.5 | 225.4 | - | - | 0 | - |
| - | - | 750.2 | 226.1 | - | - | 0 | - |
| - | - | 1.097E+05 | 226.2 | - | - | 0 | - |
| - | - | 2130 | 227.1 | - | - | 0 | - |
| - | - | 825.4 | 227.1 | - | - | 0 | - |
| 11 | y | 1.326E+04 | 227.2 | 0.001617 | 7.119 | +2 | 4 |
| - | - | 921.6 | 227.2 | - | - | 0 | - |
| 2 | a | 7124 | 228.1 | 0.0004937 | 2.164 | +1 | 2 |
| - | - | 946.8 | 228.1 | - | - | 0 | - |
| - | - | 1004 | 228.2 | - | - | 0 | - |
| 5 | b | 9273 | 229.1 | 0.001752 | 7.645 | +3 | 5 |
| - | - | 1507 | 229.1 | - | - | 0 | - |
| - | - | 943.9 | 229.2 | - | - | 0 | - |
| - | - | 775.1 | 229.2 | - | - | 0 | - |
| - | - | 2234 | 230.1 | - | - | 0 | - |
| - | - | 3172 | 231.1 | - | - | 0 | - |
| - | - | 1222 | 231.1 | - | - | 0 | - |
| - | - | 923.8 | 232.1 | - | - | 0 | - |
| - | - | 1701 | 233.1 | - | - | 0 | - |
| - | - | 735.7 | 234.1 | - | - | 0 | - |
| - | - | 1608 | 234.1 | - | - | 0 | - |
| - | - | 796.4 | 234.1 | - | - | 0 | - |
| - | - | 1.738E+04 | 235.1 | - | - | 0 | - |
| - | - | 2457 | 236.1 | - | - | 0 | - |
| - | - | 629.7 | 238.1 | - | - | 0 | - |
| - | - | 2023 | 238.1 | - | - | 0 | - |
| - | - | 1235 | 239.1 | - | - | 0 | - |
| - | - | 6210 | 239.2 | - | - | 0 | - |
| - | - | 897.2 | 240.1 | - | - | 0 | - |
| - | - | 637.7 | 240.2 | - | - | 0 | - |
| - | - | 1248 | 241.2 | - | - | 0 | - |
| - | - | 2528 | 242.2 | - | - | 0 | - |
| - | - | 786.9 | 243.2 | - | - | 0 | - |
| - | - | 862.1 | 244.1 | - | - | 0 | - |
| 13 | y | 3.787E+04 | 245.2 | 0.0006001 | 2.448 | +1 | 2 |
| 2 | a | 2.149E+05 | 246.1 | 0.0005644 | 2.293 | +1 | 2 |
| - | - | 4182 | 246.2 | - | - | 0 | - |
| - | - | 4.864E+04 | 247.1 | - | - | 0 | - |
| - | - | 1.112E+04 | 248.1 | - | - | 0 | - |
| - | - | 3513 | 248.1 | - | - | 0 | - |
| - | - | 1.572E+04 | 249.1 | - | - | 0 | - |
| - | - | 853 | 249.1 | - | - | 0 | - |
| - | - | 1060 | 249.1 | - | - | 0 | - |
| - | - | 1664 | 250.1 | - | - | 0 | - |
| - | - | 1281 | 250.1 | - | - | 0 | - |
| - | - | 5098 | 251.2 | - | - | 0 | - |
| - | - | 1486 | 252.2 | - | - | 0 | - |
| 2 | b | 5487 | 256.1 | 0.0005284 | 2.063 | +1 | 2 |
| - | - | 1591 | 257.1 | - | - | 0 | - |
| - | - | 1456 | 257.2 | - | - | 0 | - |
| - | - | 1095 | 258.1 | - | - | 0 | - |
| - | - | 2.556E+04 | 258.1 | - | - | 0 | - |
| - | - | 3956 | 259.1 | - | - | 0 | - |
| - | - | 747.7 | 259.1 | - | - | 0 | - |
| - | - | 1929 | 260.1 | - | - | 0 | - |
| - | - | 1459 | 260.2 | - | - | 0 | - |
| - | - | 972.6 | 263.1 | - | - | 0 | - |
| - | - | 1567 | 264.1 | - | - | 0 | - |
| - | - | 961.8 | 265.1 | - | - | 0 | - |
| - | - | 1241 | 265.2 | - | - | 0 | - |
| - | - | 1.38E+04 | 266.1 | - | - | 0 | - |
| - | - | 1836 | 267.1 | - | - | 0 | - |
| - | - | 608.9 | 267.1 | - | - | 0 | - |
| - | - | 6451 | 270.2 | - | - | 0 | - |
| 2 | b | 1.25E+05 | 274.1 | 0.0004923 | 1.796 | +1 | 2 |
| - | - | 3788 | 275.1 | - | - | 0 | - |
| - | - | 6.159E+04 | 275.1 | - | - | 0 | - |
| - | - | 2053 | 276.1 | - | - | 0 | - |
| - | - | 5810 | 276.1 | - | - | 0 | - |
| - | - | 698.3 | 276.2 | - | - | 0 | - |
| - | - | 1699 | 277.1 | - | - | 0 | - |
| - | - | 1081 | 279.2 | - | - | 0 | - |
| - | - | 662.5 | 280.2 | - | - | 0 | - |
| - | - | 1732 | 283.2 | - | - | 0 | - |
| - | - | 1.937E+04 | 283.2 | - | - | 0 | - |
| - | - | 2088 | 284.2 | - | - | 0 | - |
| - | - | 1989 | 285.1 | - | - | 0 | - |
| - | - | 5678 | 285.2 | - | - | 0 | - |
| - | - | 835.7 | 290.1 | - | - | 0 | - |
| - | - | 6597 | 290.2 | - | - | 0 | - |
| - | - | 1478 | 290.7 | - | - | 0 | - |
| - | - | 968.9 | 291.2 | - | - | 0 | - |
| - | - | 1.236E+04 | 292.1 | - | - | 0 | - |
| - | - | 586.9 | 292.2 | - | - | 0 | - |
| - | - | 1654 | 293.1 | - | - | 0 | - |
| - | - | 5983 | 294.1 | - | - | 0 | - |
| - | - | 736.9 | 295.1 | - | - | 0 | - |
| - | - | 689.1 | 297.2 | - | - | 0 | - |
| - | - | 1.699E+04 | 297.2 | - | - | 0 | - |
| - | - | 2865 | 298.2 | - | - | 0 | - |
| - | - | 916.3 | 299.1 | - | - | 0 | - |
| - | - | 800.3 | 300.1 | - | - | 0 | - |
| - | - | 788.5 | 301.1 | - | - | 0 | - |
| - | - | 3115 | 303.1 | - | - | 0 | - |
| - | - | 787.6 | 303.2 | - | - | 0 | - |
| - | - | 3150 | 305.1 | - | - | 0 | - |
| - | - | 1297 | 305.7 | - | - | 0 | - |
| - | - | 773.4 | 306.1 | - | - | 0 | - |
| - | - | 1012 | 306.1 | - | - | 0 | - |
| - | - | 2663 | 311.2 | - | - | 0 | - |
| - | - | 796.8 | 314.7 | - | - | 0 | - |
| - | - | 907.8 | 318.2 | - | - | 0 | - |
| - | - | 3497 | 320.1 | - | - | 0 | - |
| - | - | 1.653E+04 | 321.2 | - | - | 0 | - |
| - | - | 6158 | 322.2 | - | - | 0 | - |
| - | - | 3364 | 322.2 | - | - | 0 | - |
| - | - | 690.4 | 323.2 | - | - | 0 | - |
| - | - | 913.2 | 324.2 | - | - | 0 | - |
| - | - | 754.3 | 324.7 | - | - | 0 | - |
| - | - | 1048 | 325.2 | - | - | 0 | - |
| - | - | 3652 | 326.2 | - | - | 0 | - |
| - | - | 757.8 | 327.2 | - | - | 0 | - |
| - | - | 3530 | 332.7 | - | - | 0 | - |
| - | - | 1043 | 333.1 | - | - | 0 | - |
| - | - | 1326 | 333.2 | - | - | 0 | - |
| 9 | y | 721.3 | 335.2 | 0.001234 | 3.681 | +2 | 6 |
| - | - | 707.5 | 336.1 | - | - | 0 | - |
| - | - | 2.256E+04 | 336.2 | - | - | 0 | - |
| - | - | 731.1 | 337.2 | - | - | 0 | - |
| - | - | 4124 | 337.2 | - | - | 0 | - |
| - | - | 4647 | 337.7 | - | - | 0 | - |
| - | - | 739.3 | 338.1 | - | - | 0 | - |
| - | - | 887.6 | 339.2 | - | - | 0 | - |
| - | - | 1.675E+04 | 339.2 | - | - | 0 | - |
| - | - | 1444 | 340.2 | - | - | 0 | - |
| - | - | 992.4 | 340.2 | - | - | 0 | - |
| - | - | 3300 | 340.2 | - | - | 0 | - |
| - | - | 1183 | 341.2 | - | - | 0 | - |
| - | - | 1.657E+04 | 346.7 | - | - | 0 | - |
| - | - | 6946 | 347.2 | - | - | 0 | - |
| - | - | 1485 | 347.7 | - | - | 0 | - |
| - | - | 2978 | 350.2 | - | - | 0 | - |
| - | - | 831.1 | 351.2 | - | - | 0 | - |
| - | - | 1867 | 351.2 | - | - | 0 | - |
| - | - | 1062 | 353.2 | - | - | 0 | - |
| - | - | 2.993E+04 | 354.2 | - | - | 0 | - |
| - | - | 1271 | 355.1 | - | - | 0 | - |
| - | - | 5909 | 355.2 | - | - | 0 | - |
| - | - | 789.5 | 355.3 | - | - | 0 | - |
| - | - | 1366 | 355.7 | - | - | 0 | - |
| - | - | 725.8 | 356.2 | - | - | 0 | - |
| - | - | 973.1 | 357.2 | - | - | 0 | - |
| - | - | 3052 | 357.3 | - | - | 0 | - |
| - | - | 4709 | 358.2 | - | - | 0 | - |
| - | - | 2253 | 358.7 | - | - | 0 | - |
| - | - | 879.1 | 359.2 | - | - | 0 | - |
| - | - | 927 | 359.2 | - | - | 0 | - |
| - | - | 725.4 | 360.1 | - | - | 0 | - |
| - | - | 1227 | 360.7 | - | - | 0 | - |
| - | - | 754.1 | 361.7 | - | - | 0 | - |
| - | - | 1205 | 362.7 | - | - | 0 | - |
| 8 | y | 1088 | 363.7 | 0.001035 | 2.844 | +2 | 7 |
| - | - | 830.6 | 365.3 | - | - | 0 | - |
| - | - | 3377 | 366.2 | - | - | 0 | - |
| - | - | 1493 | 367.2 | - | - | 0 | - |
| - | - | 2008 | 368.1 | - | - | 0 | - |
| - | - | 1736 | 368.2 | - | - | 0 | - |
| - | - | 690.7 | 370.2 | - | - | 0 | - |
| 12 | y | 7964 | 373.3 | 0.00044 | 1.179 | +1 | 3 |
| - | - | 672.6 | 374.3 | - | - | 0 | - |
| - | - | 1106 | 374.3 | - | - | 0 | - |
| - | - | 1147 | 379.2 | - | - | 0 | - |
| - | - | 1800 | 380.2 | - | - | 0 | - |
| - | - | 752.9 | 381.2 | - | - | 0 | - |
| - | - | 1414 | 382.3 | - | - | 0 | - |
| - | - | 963.1 | 382.5 | - | - | 0 | - |
| - | - | 1531 | 383.3 | - | - | 0 | - |
| - | - | 4152 | 384.2 | - | - | 0 | - |
| - | - | 1.07E+04 | 385.2 | - | - | 0 | - |
| - | - | 3546 | 385.2 | - | - | 0 | - |
| - | - | 8128 | 386.2 | - | - | 0 | - |
| - | - | 888.4 | 386.2 | - | - | 0 | - |
| - | - | 1923 | 387.2 | - | - | 0 | - |
| - | - | 1081 | 389.8 | - | - | 0 | - |
| - | - | 919 | 392.2 | - | - | 0 | - |
| - | - | 3494 | 392.3 | - | - | 0 | - |
| - | - | 1554 | 393.2 | - | - | 0 | - |
| - | - | 1271 | 393.3 | - | - | 0 | - |
| - | - | 8028 | 394.2 | - | - | 0 | - |
| - | - | 710 | 394.3 | - | - | 0 | - |
| - | - | 2420 | 395.2 | - | - | 0 | - |
| - | - | 1534 | 396.3 | - | - | 0 | - |
| - | - | 906.7 | 397.2 | - | - | 0 | - |
| - | - | 974.4 | 398.2 | - | - | 0 | - |
| - | - | 1439 | 401.2 | - | - | 0 | - |
| - | - | 1666 | 401.3 | - | - | 0 | - |
| - | - | 4771 | 402.2 | - | - | 0 | - |
| - | - | 1.046E+04 | 403.2 | - | - | 0 | - |
| - | - | 780.9 | 403.2 | - | - | 0 | - |
| - | - | 2605 | 403.3 | - | - | 0 | - |
| - | - | 953.4 | 403.8 | - | - | 0 | - |
| - | - | 2300 | 404.2 | - | - | 0 | - |
| - | - | 5113 | 406.2 | - | - | 0 | - |
| - | - | 2709 | 406.3 | - | - | 0 | - |
| - | - | 2339 | 406.7 | - | - | 0 | - |
| - | - | 1638 | 406.8 | - | - | 0 | - |
| - | - | 1.005E+04 | 410.3 | - | - | 0 | - |
| 6 | b | 1.608E+04 | 411.2 | 0.0006014 | 1.463 | +2 | 6 |
| - | - | 1977 | 411.3 | - | - | 0 | - |
| - | - | 1998 | 412.2 | - | - | 0 | - |
| - | - | 2483 | 412.2 | - | - | 0 | - |
| 7 | y | 4.114E+04 | 412.3 | 0.0008672 | 2.103 | +2 | 8 |
| - | - | 2.171E+04 | 412.8 | - | - | 0 | - |
| - | - | 1583 | 413.2 | - | - | 0 | - |
| - | - | 6528 | 413.3 | - | - | 0 | - |
| - | - | 3639 | 415.3 | - | - | 0 | - |
| - | - | 1753 | 415.8 | - | - | 0 | - |
| 3 | b | 921.6 | 419.2 | 0.0002797 | 0.6673 | +1 | 3 |
| - | - | 621.5 | 419.2 | - | - | 0 | - |
| - | - | 1925 | 419.9 | - | - | 0 | - |
| 6 | b | 4673 | 420.2 | 0.000446 | 1.062 | +2 | 6 |
| - | - | 3612 | 420.2 | - | - | 0 | - |
| - | - | 3054 | 420.7 | - | - | 0 | - |
| - | - | 3734 | 422.3 | - | - | 0 | - |
| - | - | 1223 | 422.3 | - | - | 0 | - |
| - | - | 1834 | 423.3 | - | - | 0 | - |
| - | - | 1915 | 425.3 | - | - | 0 | - |
| - | - | 4676 | 427.2 | - | - | 0 | - |
| - | - | 2468 | 427.7 | - | - | 0 | - |
| - | - | 836.3 | 428.2 | - | - | 0 | - |
| - | - | 953.7 | 428.3 | - | - | 0 | - |
| - | - | 5.173E+04 | 429.2 | - | - | 0 | - |
| - | - | 1.287E+04 | 430.2 | - | - | 0 | - |
| - | - | 5557 | 431.2 | - | - | 0 | - |
| - | - | 1270 | 432.2 | - | - | 0 | - |
| - | - | 934.3 | 433.3 | - | - | 0 | - |
| - | - | 964.4 | 436.7 | - | - | 0 | - |
| 3 | b | 9560 | 437.2 | 0.0007014 | 1.604 | +1 | 3 |
| - | - | 3098 | 438.2 | - | - | 0 | - |
| - | - | 1234 | 438.6 | - | - | 0 | - |
| - | - | 1628 | 438.9 | - | - | 0 | - |
| - | - | 810.6 | 439.2 | - | - | 0 | - |
| - | - | 655.4 | 439.2 | - | - | 0 | - |
| - | - | 1276 | 439.3 | - | - | 0 | - |
| - | - | 1242 | 442.7 | - | - | 0 | - |
| - | - | 1417 | 444.2 | - | - | 0 | - |
| - | - | 1851 | 445.3 | - | - | 0 | - |
| - | - | 982.5 | 446.2 | - | - | 0 | - |
| - | - | 635.3 | 448.2 | - | - | 0 | - |
| - | - | 694.9 | 450.1 | - | - | 0 | - |
| - | - | 617.2 | 450.3 | - | - | 0 | - |
| - | - | 1638 | 451.2 | - | - | 0 | - |
| - | - | 3203 | 451.3 | - | - | 0 | - |
| - | - | 2167 | 451.7 | - | - | 0 | - |
| - | - | 922.9 | 451.7 | - | - | 0 | - |
| - | - | 766.7 | 452.2 | - | - | 0 | - |
| - | - | 795 | 452.3 | - | - | 0 | - |
| - | - | 1552 | 452.3 | - | - | 0 | - |
| - | - | 664.6 | 453.3 | - | - | 0 | - |
| - | - | 3288 | 454.2 | - | - | 0 | - |
| - | - | 1107 | 455.2 | - | - | 0 | - |
| - | - | 3624 | 455.3 | - | - | 0 | - |
| - | - | 924.3 | 456.3 | - | - | 0 | - |
| - | - | 2048 | 457.2 | - | - | 0 | - |
| - | - | 867.4 | 458.2 | - | - | 0 | - |
| - | - | 1998 | 460.2 | - | - | 0 | - |
| - | - | 696.4 | 460.7 | - | - | 0 | - |
| - | - | 1140 | 461.2 | - | - | 0 | - |
| 3 | y | 8465 | 463.6 | 0.0006605 | 1.425 | +3 | 12 |
| - | - | 8630 | 463.9 | - | - | 0 | - |
| - | - | 854.7 | 464.2 | - | - | 0 | - |
| - | - | 2649 | 464.3 | - | - | 0 | - |
| - | - | 1213 | 464.6 | - | - | 0 | - |
| - | - | 912.5 | 466.9 | - | - | 0 | - |
| - | - | 817.4 | 467.3 | - | - | 0 | - |
| - | - | 4585 | 467.3 | - | - | 0 | - |
| - | - | 2617 | 468.3 | - | - | 0 | - |
| - | - | 1251 | 469.8 | - | - | 0 | - |
| - | - | 1103 | 470.3 | - | - | 0 | - |
| 11 | y | 3.437E+04 | 470.3 | 0.0007157 | 1.522 | +1 | 4 |
| - | - | 1273 | 470.6 | - | - | 0 | - |
| - | - | 8478 | 471.3 | - | - | 0 | - |
| - | - | 2523 | 471.8 | - | - | 0 | - |
| - | - | 2664 | 472.3 | - | - | 0 | - |
| - | - | 1207 | 472.3 | - | - | 0 | - |
| - | - | 822.1 | 472.6 | - | - | 0 | - |
| 12 | b | 1.579E+04 | 473.2 | 0.001494 | 3.158 | +3 | 12 |
| - | - | 1.473E+04 | 473.6 | - | - | 0 | - |
| - | - | 1430 | 473.7 | - | - | 0 | - |
| - | - | 4820 | 473.9 | - | - | 0 | - |
| - | - | 1775 | 474.2 | - | - | 0 | - |
| - | - | 1566 | 474.3 | - | - | 0 | - |
| - | - | 2454 | 474.8 | - | - | 0 | - |
| - | - | 1906 | 475.3 | - | - | 0 | - |
| - | - | 787.5 | 475.8 | - | - | 0 | - |
| - | - | 5774 | 476.3 | - | - | 0 | - |
| - | - | 4418 | 476.6 | - | - | 0 | - |
| - | - | 2588 | 478.2 | - | - | 0 | - |
| 6 | y | 1.03E+04 | 480.8 | 0.0006166 | 1.282 | +2 | 9 |
| - | - | 5733 | 481.3 | - | - | 0 | - |
| - | - | 2002 | 481.9 | - | - | 0 | - |
| - | - | 3912 | 482.3 | - | - | 0 | - |
| - | - | 3728 | 482.3 | - | - | 0 | - |
| - | - | 2979 | 482.8 | - | - | 0 | - |
| - | - | 927.7 | 483.3 | - | - | 0 | - |
| - | - | 7099 | 483.8 | - | - | 0 | - |
| - | - | 3577 | 484.3 | - | - | 0 | - |
| - | - | 921.6 | 484.8 | - | - | 0 | - |
| - | - | 862.8 | 486.2 | - | - | 0 | - |
| - | - | 884.7 | 488.3 | - | - | 0 | - |
| - | - | 4760 | 491.3 | - | - | 0 | - |
| - | - | 1035 | 492.3 | - | - | 0 | - |
| - | - | 668.1 | 492.8 | - | - | 0 | - |
| - | - | 1495 | 494.2 | - | - | 0 | - |
| - | - | 1137 | 495.9 | - | - | 0 | - |
| - | - | 1625 | 496.3 | - | - | 0 | - |
| - | - | 867.6 | 496.8 | - | - | 0 | - |
| 8 | b | 1883 | 497.2 | 0.000289 | 0.5813 | +2 | 8 |
| - | - | 1948 | 497.7 | - | - | 0 | - |
| - | - | 7271 | 501.6 | - | - | 0 | - |
| - | - | 5848 | 501.9 | - | - | 0 | - |
| - | - | 3944 | 502.3 | - | - | 0 | - |
| - | - | 1583 | 502.6 | - | - | 0 | - |
| - | - | 2690 | 503.2 | - | - | 0 | - |
| 13 | b | 1.215E+04 | 504.9 | 0.0001473 | 0.2916 | +3 | 13 |
| 13 | b | 1.53E+04 | 505.3 | 0.005852 | 11.58 | +3 | 13 |
| - | - | 6882 | 505.6 | - | - | 0 | - |
| - | - | 3828 | 505.9 | - | - | 0 | - |
| - | - | 802.7 | 506.3 | - | - | 0 | - |
| - | - | 1851 | 508.8 | - | - | 0 | - |
| - | - | 1819 | 509.3 | - | - | 0 | - |
| 13 | b | 6.906E+04 | 510.9 | 0.000593 | 1.161 | +3 | 13 |
| - | - | 6.636E+04 | 511.3 | - | - | 0 | - |
| - | - | 2.819E+04 | 511.6 | - | - | 0 | - |
| - | - | 7660 | 511.9 | - | - | 0 | - |
| - | - | 2705 | 512.3 | - | - | 0 | - |
| - | - | 7593 | 516.9 | - | - | 0 | - |
| - | - | 9868 | 517.3 | - | - | 0 | - |
| - | - | 5356 | 517.6 | - | - | 0 | - |
| - | - | 1775 | 517.9 | - | - | 0 | - |
| - | - | 1254 | 517.9 | - | - | 0 | - |
| - | - | 847.9 | 519.6 | - | - | 0 | - |
| 2 | y | 2712 | 519.9 | 0.0001021 | 0.1963 | +3 | 13 |
| - | - | 872.1 | 520.2 | - | - | 0 | - |
| - | - | 2611 | 520.3 | - | - | 0 | - |
| - | - | 1791 | 520.4 | - | - | 0 | - |
| - | - | 1226 | 520.6 | - | - | 0 | - |
| - | - | 2490 | 521.2 | - | - | 0 | - |
| - | - | 930 | 523.4 | - | - | 0 | - |
| 2 | y | 1.171E+04 | 525.6 | 0.0001018 | 0.1937 | +3 | 13 |
| - | - | 9944 | 526 | - | - | 0 | - |
| - | - | 5653 | 526.3 | - | - | 0 | - |
| - | - | 2082 | 526.6 | - | - | 0 | - |
| - | - | 1962 | 528.2 | - | - | 0 | - |
| - | - | 907 | 529.2 | - | - | 0 | - |
| - | - | 1783 | 529.8 | - | - | 0 | - |
| - | - | 1723 | 530.3 | - | - | 0 | - |
| - | - | 1216 | 530.8 | - | - | 0 | - |
| - | - | 2329 | 531.2 | - | - | 0 | - |
| - | - | 1242 | 535.3 | - | - | 0 | - |
| - | - | 735.3 | 535.6 | - | - | 0 | - |
| - | - | 1047 | 537.3 | - | - | 0 | - |
| - | - | 823.7 | 537.6 | - | - | 0 | - |
| - | - | 2.747E+04 | 538.3 | - | - | 0 | - |
| - | - | 2192 | 538.4 | - | - | 0 | - |
| - | - | 4315 | 538.8 | - | - | 0 | - |
| - | - | 7805 | 539.3 | - | - | 0 | - |
| - | - | 7369 | 539.3 | - | - | 0 | - |
| - | - | 2044 | 539.6 | - | - | 0 | - |
| - | - | 2903 | 539.8 | - | - | 0 | - |
| - | - | 1016 | 540 | - | - | 0 | - |
| - | - | 1216 | 540.3 | - | - | 0 | - |
| - | - | 1022 | 540.3 | - | - | 0 | - |
| - | - | 804.8 | 540.6 | - | - | 0 | - |
| 5 | y | 781.6 | 540.8 | 0.006103 | 11.28 | +2 | 10 |
| - | - | 589.9 | 541.3 | - | - | 0 | - |
| 10 | y | 9739 | 541.4 | 0.0008333 | 1.539 | +1 | 5 |
| - | - | 879.1 | 542.3 | - | - | 0 | - |
| - | - | 3326 | 542.4 | - | - | 0 | - |
| - | - | 1852 | 543 | - | - | 0 | - |
| - | - | 1116 | 543.3 | - | - | 0 | - |
| - | - | 724 | 544.3 | - | - | 0 | - |
| - | - | 2640 | 547.8 | - | - | 0 | - |
| - | - | 1009 | 548.2 | - | - | 0 | - |
| - | - | 1948 | 548.3 | - | - | 0 | - |
| 0 | Precursor | 1.953E+04 | 548.6 | 0.0005156 | 0.9398 | +3 | -1 |
| 0 | Precursor | 2.31E+04 | 549 | 0.005579 | 10.16 | +3 | -1 |
| - | - | 1686 | 549.2 | - | - | 0 | - |
| - | - | 9260 | 549.3 | - | - | 0 | - |
| 5 | y | 1.671E+04 | 549.3 | 0.001068 | 1.944 | +2 | 10 |
| - | - | 7357 | 549.6 | - | - | 0 | - |
| - | - | 1.031E+04 | 549.8 | - | - | 0 | - |
| - | - | 1616 | 550 | - | - | 0 | - |
| - | - | 4840 | 550.3 | - | - | 0 | - |
| - | - | 1132 | 550.8 | - | - | 0 | - |
| - | - | 735.7 | 551.4 | - | - | 0 | - |
| - | - | 1456 | 553.3 | - | - | 0 | - |
| - | - | 3571 | 554 | - | - | 0 | - |
| - | - | 825.6 | 554.3 | - | - | 0 | - |
| - | - | 2627 | 554.3 | - | - | 0 | - |
| 0 | Precursor | 3.929E+05 | 554.6 | 0.0007172 | 1.293 | +3 | -1 |
| - | - | 4.214E+05 | 555 | - | - | 0 | - |
| - | - | 2.22E+05 | 555.3 | - | - | 0 | - |
| - | - | 5035 | 555.6 | - | - | 0 | - |
| - | - | 7.324E+04 | 555.6 | - | - | 0 | - |
| - | - | 2756 | 555.9 | - | - | 0 | - |
| - | - | 2.322E+04 | 556 | - | - | 0 | - |
| - | - | 1173 | 557.3 | - | - | 0 | - |
| 9 | b | 2376 | 561.3 | 0.0009032 | 1.609 | +2 | 9 |
| - | - | 919.8 | 561.3 | - | - | 0 | - |
| - | - | 3281 | 561.8 | - | - | 0 | - |
| - | - | 1480 | 562.3 | - | - | 0 | - |
| 4 | b | 2130 | 565.2 | 0.001114 | 1.971 | +1 | 4 |
| - | - | 3.405E+04 | 566.2 | - | - | 0 | - |
| - | - | 1.176E+04 | 567.2 | - | - | 0 | - |
| - | - | 1260 | 567.4 | - | - | 0 | - |
| - | - | 1713 | 568.3 | - | - | 0 | - |
| - | - | 899.3 | 568.4 | - | - | 0 | - |
| - | - | 4600 | 570.2 | - | - | 0 | - |
| - | - | 896.9 | 570.3 | - | - | 0 | - |
| - | - | 2124 | 571.2 | - | - | 0 | - |
| - | - | 1.82E+04 | 572.8 | - | - | 0 | - |
| - | - | 1.049E+04 | 573.3 | - | - | 0 | - |
| - | - | 5567 | 573.8 | - | - | 0 | - |
| - | - | 7046 | 577.4 | - | - | 0 | - |
| - | - | 2731 | 578.4 | - | - | 0 | - |
| - | - | 2.26E+04 | 579.4 | - | - | 0 | - |
| - | - | 6915 | 580.4 | - | - | 0 | - |
| - | - | 1218 | 581.4 | - | - | 0 | - |
| - | - | 1181 | 587.3 | - | - | 0 | - |
| 10 | b | 5180 | 587.8 | 0.0005681 | 0.9666 | +2 | 10 |
| 10 | b | 2873 | 588.3 | 0.009293 | 15.8 | +2 | 10 |
| - | - | 1384 | 588.8 | - | - | 0 | - |
| - | - | 7769 | 595.4 | - | - | 0 | - |
| - | - | 1916 | 595.8 | - | - | 0 | - |
| - | - | 848.1 | 596.3 | - | - | 0 | - |
| - | - | 2365 | 596.4 | - | - | 0 | - |
| 10 | b | 4899 | 596.8 | 7.554E-05 | 0.1266 | +2 | 10 |
| - | - | 1984 | 597.3 | - | - | 0 | - |
| - | - | 1604 | 597.8 | - | - | 0 | - |
| - | - | 2279 | 598.2 | - | - | 0 | - |
| - | - | 1067 | 602.3 | - | - | 0 | - |
| - | - | 9299 | 604.4 | - | - | 0 | - |
| 4 | y | 1.324E+04 | 604.8 | 0.004341 | 7.177 | +2 | 11 |
| - | - | 8385 | 605.4 | - | - | 0 | - |
| - | - | 3710 | 605.9 | - | - | 0 | - |
| - | - | 1442 | 606.8 | - | - | 0 | - |
| - | - | 4596 | 610.3 | - | - | 0 | - |
| - | - | 1201 | 611.3 | - | - | 0 | - |
| - | - | 2025 | 611.8 | - | - | 0 | - |
| - | - | 2697 | 612.3 | - | - | 0 | - |
| 4 | y | 1.291E+04 | 613.4 | 0.0007098 | 1.157 | +2 | 11 |
| - | - | 7769 | 613.9 | - | - | 0 | - |
| - | - | 3362 | 614.4 | - | - | 0 | - |
| - | - | 1765 | 615.3 | - | - | 0 | - |
| - | - | 8760 | 615.3 | - | - | 0 | - |
| - | - | 6706 | 615.9 | - | - | 0 | - |
| - | - | 2286 | 616.4 | - | - | 0 | - |
| - | - | 1.44E+04 | 620.3 | - | - | 0 | - |
| - | - | 1.279E+04 | 620.8 | - | - | 0 | - |
| - | - | 4196 | 621.3 | - | - | 0 | - |
| - | - | 1249 | 623.4 | - | - | 0 | - |
| - | - | 792.2 | 624.4 | - | - | 0 | - |
| - | - | 1021 | 628.3 | - | - | 0 | - |
| - | - | 3.717E+04 | 629.3 | - | - | 0 | - |
| - | - | 2.617E+04 | 629.8 | - | - | 0 | - |
| - | - | 1.053E+04 | 630.3 | - | - | 0 | - |
| - | - | 2519 | 630.8 | - | - | 0 | - |
| - | - | 982 | 632.3 | - | - | 0 | - |
| - | - | 4332 | 638.4 | - | - | 0 | - |
| - | - | 2539 | 638.9 | - | - | 0 | - |
| - | - | 970.8 | 643.3 | - | - | 0 | - |
| - | - | 756.8 | 644.8 | - | - | 0 | - |
| 11 | b | 2226 | 645.3 | 0.0001513 | 0.2345 | +2 | 11 |
| - | - | 1661 | 645.8 | - | - | 0 | - |
| - | - | 1420 | 647.4 | - | - | 0 | - |
| - | - | 2117 | 653.8 | - | - | 0 | - |
| - | - | 1631 | 654.3 | - | - | 0 | - |
| - | - | 1728 | 654.8 | - | - | 0 | - |
| - | - | 1679 | 656.3 | - | - | 0 | - |
| - | - | 2949 | 657.3 | - | - | 0 | - |
| - | - | 2403 | 658.3 | - | - | 0 | - |
| - | - | 943.9 | 659.3 | - | - | 0 | - |
| - | - | 6849 | 664.5 | - | - | 0 | - |
| - | - | 2754 | 665.5 | - | - | 0 | - |
| - | - | 1781 | 665.8 | - | - | 0 | - |
| - | - | 986.3 | 666.3 | - | - | 0 | - |
| - | - | 1004 | 666.5 | - | - | 0 | - |
| 9 | y | 3924 | 669.5 | 0.001207 | 1.803 | +1 | 6 |
| - | - | 1608 | 670.5 | - | - | 0 | - |
| - | - | 993.1 | 672.4 | - | - | 0 | - |
| - | - | 8872 | 674.3 | - | - | 0 | - |
| - | - | 2.604E+04 | 674.4 | - | - | 0 | - |
| - | - | 3178 | 675.3 | - | - | 0 | - |
| - | - | 1.139E+04 | 675.4 | - | - | 0 | - |
| - | - | 2025 | 676.4 | - | - | 0 | - |
| - | - | 4248 | 677.4 | - | - | 0 | - |
| - | - | 2655 | 677.9 | - | - | 0 | - |
| - | - | 2116 | 678.4 | - | - | 0 | - |
| - | - | 851.3 | 680.3 | - | - | 0 | - |
| 5 | b | 2119 | 684.3 | 0.0006797 | 0.9933 | +1 | 5 |
| 5 | b | 1802 | 685.3 | 0.01135 | 16.57 | +1 | 5 |
| - | - | 6949 | 685.9 | - | - | 0 | - |
| 3 | y | 7430 | 686.4 | 0.006734 | 9.811 | +2 | 12 |
| - | - | 3814 | 686.9 | - | - | 0 | - |
| - | - | 1410 | 687.4 | - | - | 0 | - |
| - | - | 2.448E+04 | 692.4 | - | - | 0 | - |
| - | - | 1.049E+04 | 693.4 | - | - | 0 | - |
| - | - | 2393 | 694.5 | - | - | 0 | - |
| 3 | y | 1.213E+05 | 694.9 | 0.0004176 | 0.601 | +2 | 12 |
| - | - | 1.019E+05 | 695.4 | - | - | 0 | - |
| - | - | 4.549E+04 | 695.9 | - | - | 0 | - |
| - | - | 1.422E+04 | 696.4 | - | - | 0 | - |
| - | - | 1941 | 696.9 | - | - | 0 | - |
| - | - | 1772 | 698.4 | - | - | 0 | - |
| 12 | b | 1498 | 700.8 | 0.00385 | 5.493 | +2 | 12 |
| 5 | b | 1.461E+04 | 702.3 | 0.00043 | 0.6123 | +1 | 5 |
| - | - | 6915 | 703.3 | - | - | 0 | - |
| - | - | 1190 | 704.3 | - | - | 0 | - |
| - | - | 3459 | 707.3 | - | - | 0 | - |
| - | - | 832.5 | 707.8 | - | - | 0 | - |
| - | - | 2098 | 708.3 | - | - | 0 | - |
| - | - | 1222 | 708.5 | - | - | 0 | - |
| 12 | b | 2.678E+04 | 709.4 | 0.0003408 | 0.4804 | +2 | 12 |
| - | - | 2.376E+04 | 709.9 | - | - | 0 | - |
| - | - | 666.2 | 710.3 | - | - | 0 | - |
| - | - | 1.045E+04 | 710.4 | - | - | 0 | - |
| - | - | 3730 | 710.5 | - | - | 0 | - |
| - | - | 3005 | 710.9 | - | - | 0 | - |
| - | - | 986.3 | 711.4 | - | - | 0 | - |
| - | - | 1032 | 711.5 | - | - | 0 | - |
| - | - | 1046 | 713.4 | - | - | 0 | - |
| - | - | 1260 | 713.9 | - | - | 0 | - |
| - | - | 1688 | 714.4 | - | - | 0 | - |
| - | - | 5204 | 716.4 | - | - | 0 | - |
| - | - | 1232 | 717.4 | - | - | 0 | - |
| - | - | 1131 | 720.3 | - | - | 0 | - |
| - | - | 972.7 | 720.4 | - | - | 0 | - |
| - | - | 2444 | 721.4 | - | - | 0 | - |
| - | - | 1094 | 722.4 | - | - | 0 | - |
| - | - | 833.5 | 722.9 | - | - | 0 | - |
| - | - | 2629 | 724.3 | - | - | 0 | - |
| - | - | 1090 | 725.3 | - | - | 0 | - |
| 8 | y | 9935 | 726.5 | 0.0004344 | 0.5979 | +1 | 7 |
| - | - | 3799 | 727.5 | - | - | 0 | - |
| - | - | 2666 | 735.3 | - | - | 0 | - |
| - | - | 836.7 | 736.3 | - | - | 0 | - |
| - | - | 1107 | 737.4 | - | - | 0 | - |
| - | - | 987.8 | 738.4 | - | - | 0 | - |
| - | - | 803 | 739.4 | - | - | 0 | - |
| - | - | 1582 | 740.3 | - | - | 0 | - |
| - | - | 2173 | 751.9 | - | - | 0 | - |
| - | - | 3452 | 752.3 | - | - | 0 | - |
| - | - | 2003 | 752.4 | - | - | 0 | - |
| - | - | 1067 | 753.3 | - | - | 0 | - |
| - | - | 949 | 756.4 | - | - | 0 | - |
| 13 | b | 2562 | 756.9 | 0.001741 | 2.3 | +2 | 13 |
| 13 | b | 2254 | 757.4 | 0.006679 | 8.818 | +2 | 13 |
| - | - | 1787 | 757.9 | - | - | 0 | - |
| - | - | 856 | 759 | - | - | 0 | - |
| 13 | b | 9393 | 765.9 | 4.171E-06 | 0.005445 | +2 | 13 |
| - | - | 6640 | 766.4 | - | - | 0 | - |
| - | - | 4914 | 766.9 | - | - | 0 | - |
| - | - | 1812 | 767.4 | - | - | 0 | - |
| - | - | 4087 | 774.9 | - | - | 0 | - |
| - | - | 3287 | 775.4 | - | - | 0 | - |
| - | - | 2111 | 775.9 | - | - | 0 | - |
| 2 | y | 3449 | 787.9 | 0.0001898 | 0.2409 | +2 | 13 |
| - | - | 2456 | 788.4 | - | - | 0 | - |
| - | - | 924.9 | 788.5 | - | - | 0 | - |
| - | - | 1784 | 788.9 | - | - | 0 | - |
| - | - | 2063 | 794.3 | - | - | 0 | - |
| - | - | 873.9 | 797.3 | - | - | 0 | - |
| - | - | 5493 | 805.5 | - | - | 0 | - |
| - | - | 2634 | 806.5 | - | - | 0 | - |
| - | - | 1.515E+04 | 811.4 | - | - | 0 | - |
| - | - | 4907 | 811.5 | - | - | 0 | - |
| - | - | 8035 | 812.4 | - | - | 0 | - |
| - | - | 2329 | 812.5 | - | - | 0 | - |
| - | - | 2195 | 813.4 | - | - | 0 | - |
| 6 | b | 2533 | 821.3 | 0.000676 | 0.823 | +1 | 6 |
| 6 | b | 1971 | 822.3 | 0.006092 | 7.408 | +1 | 6 |
| - | - | 1148 | 823.4 | - | - | 0 | - |
| 7 | y | 5.517E+04 | 823.5 | 3.871E-05 | 0.047 | +1 | 8 |
| - | - | 2.546E+04 | 824.5 | - | - | 0 | - |
| - | - | 1251 | 824.6 | - | - | 0 | - |
| - | - | 6520 | 825.5 | - | - | 0 | - |
| - | - | 1197 | 826.5 | - | - | 0 | - |
| - | - | 4368 | 829.5 | - | - | 0 | - |
| - | - | 2487 | 830.5 | - | - | 0 | - |
| 6 | b | 3.278E+04 | 839.4 | 1.019E-05 | 0.01214 | +1 | 6 |
| - | - | 1.623E+04 | 840.4 | - | - | 0 | - |
| - | - | 5366 | 841.4 | - | - | 0 | - |
| - | - | 4283 | 848.4 | - | - | 0 | - |
| - | - | 2473 | 849.4 | - | - | 0 | - |
| - | - | 3530 | 853.5 | - | - | 0 | - |
| - | - | 1454 | 854.5 | - | - | 0 | - |
| - | - | 795.7 | 855.5 | - | - | 0 | - |
| - | - | 2316 | 891.5 | - | - | 0 | - |
| - | - | 938.7 | 892.5 | - | - | 0 | - |
| - | - | 1.127E+04 | 901.4 | - | - | 0 | - |
| - | - | 6239 | 902.4 | - | - | 0 | - |
| - | - | 2002 | 903.4 | - | - | 0 | - |
| - | - | 6829 | 919.5 | - | - | 0 | - |
| - | - | 3325 | 920.5 | - | - | 0 | - |
| - | - | 4545 | 942.6 | - | - | 0 | - |
| - | - | 2756 | 943.6 | - | - | 0 | - |
| - | - | 1408 | 944.6 | - | - | 0 | - |
| - | - | 1009 | 948.5 | - | - | 0 | - |
| - | - | 743.6 | 949.6 | - | - | 0 | - |
| 6 | y | 1.082E+04 | 960.6 | 0.0002794 | 0.2909 | +1 | 9 |
| - | - | 698.8 | 961.5 | - | - | 0 | - |
| - | - | 6143 | 961.6 | - | - | 0 | - |
| - | - | 2274 | 962.6 | - | - | 0 | - |
| - | - | 2715 | 966.6 | - | - | 0 | - |
| - | - | 2126 | 967.6 | - | - | 0 | - |
| - | - | 750.4 | 1018 | - | - | 0 | - |
| - | - | 2329 | 1033 | - | - | 0 | - |
| - | - | 3303 | 1034 | - | - | 0 | - |
| - | - | 1104 | 1035 | - | - | 0 | - |
| - | - | 1152 | 1089 | - | - | 0 | - |
| 5 | y | 4453 | 1098 | 0.0006232 | 0.5677 | +1 | 10 |
| - | - | 3248 | 1099 | - | - | 0 | - |
| - | - | 1302 | 1100 | - | - | 0 | - |
| 9 | b | 3346 | 1122 | 0.002025 | 1.806 | +1 | 9 |
| - | - | 1560 | 1123 | - | - | 0 | - |
| - | - | 1566 | 1145 | - | - | 0 | - |
| 10 | b | 5477 | 1175 | 0.0009867 | 0.8401 | +1 | 10 |
| 10 | b | 4331 | 1176 | 0.01671 | 14.21 | +1 | 10 |
| - | - | 1446 | 1177 | - | - | 0 | - |
| 10 | b | 2267 | 1193 | 0.00496 | 4.159 | +1 | 10 |
| - | - | 1033 | 1194 | - | - | 0 | - |
| - | - | 856.3 | 1258 | - | - | 0 | - |
| - | - | 812.7 | 2521 | - | - | 0 | - |

m/z Charge Intensity FragmentType MassShift Position
120.0565185546875 0 450.45718
120.06600189208984 0 752.6646
120.08116912841797 0 1249.7837
122.07173919677734 0 986.8992
123.0558090209961 0 1769.2579
125.07100677490234 0 358.6497
126.03711700439453 0 360.0972
127.08700561523438 0 2328.773
129.0662384033203 0 3813.9534
129.1027069091797 0 94775.16
130.06556701660156 0 25048.703
130.10601806640625 0 5535.9824
130.531494140625 0 388.53265
131.06893920898438 0 2907.4116
132.08123779296875 0 86503.76
132.10232543945312 0 18769.807 y 13
133.06097412109375 0 1057.8383
133.08456420898438 0 8497.253
133.1058349609375 0 909.9638
134.07122802734375 0 439.25235
134.0882568359375 0 502.90842
135.0923614501953 0 483.10373
135.70071411132812 0 488.3128
136.07611083984375 0 49966.152
137.07382202148438 0 761.2919
137.07948303222656 0 4584.117
138.06668090820312 0 2540.881
139.0868682861328 0 1487.0582
141.10267639160156 0 5957.357
142.105712890625 0 645.9159
143.0821990966797 0 579.272
144.08108520507812 0 1586.9867
145.06121826171875 0 2478.9104
146.0605010986328 0 1590.0782
146.07168579101562 0 503.57452
146.12928771972656 0 569.11426
147.04473876953125 0 1091.5985
148.95370483398438 0 638.8678
151.08712768554688 0 1442.104
152.07098388671875 0 780.64044
152.082275390625 0 2305.6548
152.1437530517578 0 2101.7642
153.06642150878906 0 733.0885
153.10279846191406 0 747.7747
153.14781188964844 0 576.4647
154.0612335205078 0 612.83014
154.09796142578125 0 545.9254
155.0819091796875 0 4505.817
155.0931854248047 0 3446.2322
155.11830139160156 0 3687.556
156.07711791992188 0 4191.185
157.06167602539062 0 663.2315
158.06040954589844 0 995.56995
158.0843505859375 0 9115.543
159.09213256835938 0 40551.57
160.07614135742188 0 2167.3157
160.09548950195312 0 4019.4514
161.09237670898438 0 635.3711
162.05494689941406 0 748.30023
163.07162475585938 0 2086.318
164.07138061523438 0 545.31573
165.0662078857422 0 762.1528
165.07725524902344 0 721.5985
165.1027069091797 0 3359.4692
166.0614776611328 0 2499.1714
167.08163452148438 0 700.4559
167.1184844970703 0 795.011
168.11351013183594 0 865.23157
169.07647705078125 0 4269.276
169.09767150878906 0 7627.2607
170.0604705810547 0 14758.021
171.06405639648438 0 2149.6123
171.07730102539062 0 837.70807
171.1132354736328 0 1078.5592
171.14993286132812 0 1334.8263
172.1084747314453 0 5861.83
175.08714294433594 0 739.3643
175.09849548339844 0 742.5368
176.0822296142578 0 1412.8184
178.13438415527344 0 663.07294
179.08294677734375 0 521.78296
180.07752990722656 0 917.0762
181.0609893798828 0 1106.7305
181.09762573242188 0 17741.469
181.13392639160156 0 2277.1858
182.10110473632812 0 1591.8011
182.12928771972656 0 3709.4902
182.2926788330078 0 595.1994
183.0922088623047 0 13164.543
183.11294555664062 0 1749.451
183.1326446533203 0 664.9002
183.14898681640625 0 738.9846
184.07583618164062 0 850.21594
184.09580993652344 0 1047.9417
185.07130432128906 0 2865.8206
185.07980346679688 0 639.895
185.09226989746094 0 559.8101
186.12416076660156 0 9997.934
187.0870819091797 0 80476.055
187.10740661621094 0 1714.3689
187.14453125 0 1451.4321 y 11
188.09051513671875 0 8696.262
189.08743286132812 0 1895.2805 b 3
191.0816192626953 0 472.16507
195.11373901367188 0 1062.6682
195.1496124267578 0 1339.7548
197.12945556640625 0 601.3987
197.16529846191406 0 3751.281
198.08810424804688 0 641.22314
198.16851806640625 0 490.08954
199.08705139160156 0 2097.0151
199.10842895507812 0 1817.4072
199.14427185058594 0 919.4278
199.18081665039062 0 3434.918
200.139404296875 0 1161.5834
201.06739807128906 0 607.0745
201.10264587402344 0 6622.0483
201.12387084960938 0 1195.6694
202.083984375 0 1188.4794
202.10891723632812 0 9106.245
203.06607055664062 0 530.29675
203.09291076660156 0 695.871
203.10360717773438 0 1806.8868
203.11326599121094 0 600.9114
204.07777404785156 0 619.18427
205.08267211914062 0 635.21857
205.09762573242188 0 1070.2405 a 2
205.10838317871094 0 1025.3152
207.097900390625 0 6482.698
207.10972595214844 0 559.6531
207.114013671875 0 695.74365
207.14947509765625 0 3478.7979
207.15939331054688 0 796.20734
208.14462280273438 0 970.5968
209.0930633544922 0 815.61725
209.1033172607422 0 623.1864
209.12864685058594 0 814.5904
211.0869598388672 0 1604.5922
211.14419555664062 0 687.84375
212.13967895507812 0 1291.358
214.109130859375 0 4326.1973
215.13934326171875 0 2103.2717
216.09872436523438 0 635.5619
216.11257934570312 0 543.52167
217.04359436035156 0 559.0441
217.10841369628906 0 920.79425
219.076904296875 0 775.1659
220.11972045898438 0 7066.4893
221.10372924804688 0 9255.494
221.12301635742188 0 764.2453
222.10711669921875 0 818.87524
222.12420654296875 0 1088.5536
223.0638427734375 0 758.7145
223.15562438964844 0 1469.5099
224.11871337890625 0 2067.9727
224.1761932373047 0 4592.015
225.04339599609375 0 533.7187
225.42227172851562 0 559.52783
226.08314514160156 0 750.17725
226.15550231933594 0 109736.04
227.1029815673828 0 2129.5088
227.1394500732422 0 825.4359
227.15882873535156 0 13264.392 y Ammonia loss 10
227.17189025878906 0 921.5963
228.11363220214844 0 7123.5024 a Water loss 1
228.13455200195312 0 946.7726
228.16073608398438 0 1004.3101
229.09756469726562 0 9272.51 b Ammonia loss 4
229.1182861328125 0 1506.597
229.1546630859375 0 943.9004
229.166748046875 0 775.0593
230.10227966308594 0 2233.7644
231.08802795410156 0 3171.629
231.1353302001953 0 1222.4081
232.11927795410156 0 923.8188
233.10379028320312 0 1700.7045
234.10321044921875 0 735.73254
234.1241455078125 0 1608.4629
234.13601684570312 0 796.40826
235.11949157714844 0 17381.59
236.1224822998047 0 2457.3801
238.11807250976562 0 629.7453
238.1302490234375 0 2023.3734
239.08187866210938 0 1234.8708
239.15077209472656 0 6210.239
240.134765625 0 897.21954
240.15516662597656 0 637.67
241.155029296875 0 1248.0262
242.18695068359375 0 2528.3953
243.1819305419922 0 786.85236
244.09365844726562 0 862.13837
245.1865692138672 0 37871.996 y 12
246.124267578125 0 214894.94 a 1
246.1895751953125 0 4181.5234
247.12893676757812 0 48641.17
248.11460876464844 0 11121.167
248.13217163085938 0 3512.5535
249.0986785888672 0 15723.195
249.115234375 0 853.01636
249.11807250976562 0 1060.0356
250.1018524169922 0 1663.8105
250.12240600585938 0 1280.6588
251.1507568359375 0 5097.689
252.1708221435547 0 1486.1377
256.10858154296875 0 5487.0093 b Water loss 1
257.1138916015625 0 1590.6858
257.1607360839844 0 1455.9541
258.0760498046875 0 1095.342
258.0989685058594 0 25560.928
259.1024169921875 0 3956.1663
259.1297912597656 0 747.6749
260.10699462890625 0 1928.6362
260.19781494140625 0 1458.5767
263.1146545410156 0 972.5764
264.1343078613281 0 1567.3462
265.1410827636719 0 961.8413
265.166015625 0 1241.337
266.1251220703125 0 13801.409
267.12835693359375 0 1835.8499
267.14495849609375 0 608.87427
270.1816711425781 0 6451.3154
274.1191101074219 0 125047.9 b 1
275.10369873046875 0 3788.0845
275.1245422363281 0 61585.008
276.1091613769531 0 2052.6858
276.12786865234375 0 5809.585
276.1560363769531 0 698.2774
277.13336181640625 0 1698.8215
279.18121337890625 0 1080.6799
280.1665954589844 0 662.5218
283.1512451171875 0 1731.9713
283.177001953125 0 19365.838
284.1798095703125 0 2087.5952
285.1097412109375 0 1988.967
285.1562194824219 0 5677.5586
290.1147766113281 0 835.70026
290.18475341796875 0 6596.676
290.6861877441406 0 1477.8656
291.15814208984375 0 968.8777
292.1297912597656 0 12362.6045
292.1658935546875 0 586.9372
293.1332702636719 0 1653.8002
294.1202392578125 0 5982.77
295.1227111816406 0 736.85187
297.1565856933594 0 689.13086
297.19268798828125 0 16987.46
298.1959228515625 0 2865.3354
299.0631408691406 0 916.2675
300.0627136230469 0 800.315
301.1285095214844 0 788.546
303.12030029296875 0 3115.3623
303.21783447265625 0 787.6113
305.1287536621094 0 3150.493
305.6646728515625 0 1296.6882
306.1090087890625 0 773.35956
306.1325988769531 0 1011.7732
311.24517822265625 0 2662.9111
314.6693115234375 0 796.8298
318.193603515625 0 907.8465
320.1249694824219 0 3497.0728
321.2291564941406 0 16533.078
322.1556396484375 0 6157.9263
322.2322692871094 0 3363.908
323.1580810546875 0 690.4364
324.2168273925781 0 913.2492
324.71783447265625 0 754.32056
325.22406005859375 0 1047.9783
326.2195129394531 0 3651.7188
327.16815185546875 0 757.7804
332.7296142578125 0 3529.7349
333.1236572265625 0 1042.8165
333.231689453125 0 1326.177
335.23529052734375 0 721.2986 y 8
336.14593505859375 0 707.52576
336.2037048339844 0 22555.582
337.1509094238281 0 731.1097
337.2067565917969 0 4124.4404
337.7220153808594 0 4646.5005
338.1369323730469 0 739.2703
339.21636962890625 0 887.6239
339.23968505859375 0 16747.965
340.1516418457031 0 1444.3038
340.1876220703125 0 992.3884
340.2432861328125 0 3300.0684
341.2185363769531 0 1182.9708
346.72698974609375 0 16568.459
347.2283630371094 0 6945.6743
347.7295837402344 0 1484.7604
350.15020751953125 0 2978.3591
351.1520690917969 0 831.1227
351.23974609375 0 1866.5137
353.2190856933594 0 1062.4935
354.214111328125 0 29927.78
355.0696716308594 0 1271.0062
355.2168273925781 0 5908.7256
355.2707214355469 0 789.5367
355.7317199707031 0 1366.2035
356.23541259765625 0 725.8106
357.1786804199219 0 973.1107
357.25006103515625 0 3052.2961
358.1627197265625 0 4709.351
358.7140808105469 0 2253.1377
359.16680908203125 0 879.0555
359.21490478515625 0 926.9605
360.13604736328125 0 725.38165
360.689208984375 0 1227.054
361.6806640625 0 754.05963
362.6697692871094 0 1205.3159
363.748291015625 0 1088.1915 y 7
365.25482177734375 0 830.6
366.15618896484375 0 3376.9653
367.16162109375 0 1493.0847
368.14715576171875 0 2008.1107
368.1707763671875 0 1735.9988
370.2466125488281 0 690.65106
373.2813720703125 0 7963.9077 y 11
374.2576599121094 0 672.5519
374.28424072265625 0 1105.535
379.2080993652344 0 1147.2397
380.2296142578125 0 1799.7755
381.15203857421875 0 752.90533
382.2826232910156 0 1414.4648
382.54022216796875 0 963.14087
383.2657775878906 0 1531.3002
384.16705322265625 0 4151.5596
385.17303466796875 0 10701.049
385.1990966796875 0 3546.4058
386.1573486328125 0 8128.1143
386.2008361816406 0 888.43884
387.16015625 0 1923.3312
389.77520751953125 0 1080.8291
392.1624450683594 0 919.02325
392.26654052734375 0 3493.5576
393.1682434082031 0 1554.0375
393.26959228515625 0 1271.2758
394.15179443359375 0 8028.5
394.2809753417969 0 709.9993
395.15423583984375 0 2420.2312
396.26104736328125 0 1533.8588
397.2447814941406 0 906.74005
398.2430725097656 0 974.36597
401.1941833496094 0 1439.0667
401.259521484375 0 1666.4908
402.2253723144531 0 4771.2773
403.1842956542969 0 10460.554
403.22967529296875 0 780.9205
403.2692565917969 0 2605.071
403.7688293457031 0 953.4277
404.18719482421875 0 2300.4795
406.1861572265625 0 5112.9043
406.25164794921875 0 2709.2998
406.6869201660156 0 2339.2686
406.75201416015625 0 1638.1644
410.2768859863281 0 10048.322
411.1781311035156 0 16075.276 b Water loss 5
411.279541015625 0 1977.1611
412.1611328125 0 1998.166
412.1834716796875 0 2483.4158
412.2745056152344 0 41136.867 y 6
412.77587890625 0 21707.684
413.1687316894531 0 1582.6674
413.2778015136719 0 6527.6406
415.2564392089844 0 3639.4146
415.7587585449219 0 1753.4365
419.1716613769531 0 921.6499 b Water loss 2
419.21099853515625 0 621.4539
419.9006042480469 0 1924.5011
420.1832580566406 0 4673.124 b 5
420.2352294921875 0 3611.802
420.6845397949219 0 3053.528
422.2768249511719 0 3733.758
422.3092041015625 0 1222.7831
423.27410888671875 0 1834.4111
425.28790283203125 0 1915.1537
427.2441711425781 0 4676.213
427.74481201171875 0 2468.3162
428.249755859375 0 836.3319
428.2879638671875 0 953.6835
429.1888122558594 0 51725.746
430.192138671875 0 12865.838
431.1807556152344 0 5557.473
432.18145751953125 0 1270.4559
433.25653076171875 0 934.30054
436.6778869628906 0 964.4308
437.1826477050781 0 9560.015 b 2
438.1863708496094 0 3097.9207
438.5577087402344 0 1234.4707
438.8911437988281 0 1628.2212
439.19091796875 0 810.6022
439.2239074707031 0 655.368
439.30322265625 0 1275.9971
442.6789245605469 0 1242.3911
444.23138427734375 0 1416.8618
445.2689514160156 0 1850.8649
446.22283935546875 0 982.45966
448.2278137207031 0 635.3456
450.1085510253906 0 694.9254
450.2828063964844 0 617.2433
451.225341796875 0 1637.7008
451.26702880859375 0 3203.3616
451.6837158203125 0 2167.2764
451.72509765625 0 922.93616
452.2259826660156 0 766.6798
452.268798828125 0 794.9615
452.3242492675781 0 1552.2063
453.3251647949219 0 664.58563
454.2093811035156 0 3287.5684
455.2152404785156 0 1106.772
455.2525939941406 0 3623.808
456.2540283203125 0 924.3341
457.1828918457031 0 2048.3462
458.2347106933594 0 867.40424
460.2306213378906 0 1998.4507
460.7308044433594 0 696.36316
461.1816101074219 0 1139.5735
463.5987548828125 0 8465.268 y 2
463.9330139160156 0 8630.031
464.1665344238281 0 854.73975
464.26715087890625 0 2648.924
464.60223388671875 0 1212.9559
466.9178771972656 0 912.54333
467.25787353515625 0 817.3627
467.2983703613281 0 4584.6147
468.2968444824219 0 2617.4128
469.7869567871094 0 1250.76
470.2503356933594 0 1103.1603
470.33441162109375 0 34373.773 y 10
470.582275390625 0 1272.924
471.3377990722656 0 8477.502
471.7984924316406 0 2522.5774
472.301025390625 0 2664.2666
472.33721923828125 0 1207.0704
472.5941162109375 0 822.1406
473.2438049316406 0 15790.569 b 11
473.5773620605469 0 14727.41
473.6973876953125 0 1429.9797
473.9112243652344 0 4819.6455
474.2474365234375 0 1775.3962
474.2603454589844 0 1566.0276
474.78021240234375 0 2453.6082
475.28076171875 0 1905.81
475.7839660644531 0 787.49756
476.2527770996094 0 5773.5854
476.58746337890625 0 4417.578
478.21026611328125 0 2587.7336
480.8037109375 0 10304.003 y 5
481.305419921875 0 5732.552
481.9248046875 0 2002.0051
482.2631530761719 0 3911.676
482.3085632324219 0 3728.109
482.7629089355469 0 2979.2676
483.2637939453125 0 927.6903
483.78662109375 0 7098.626
484.2872314453125 0 3577.1616
484.7880554199219 0 921.616
486.1742248535156 0 862.75104
488.26513671875 0 884.6966
491.2731018066406 0 4759.865
492.27484130859375 0 1034.6089
492.79168701171875 0 668.0877
494.2340087890625 0 1494.9688
495.9336242675781 0 1137.216
496.262451171875 0 1624.644
496.75860595703125 0 867.6377
497.22021484375 0 1883.2834 b 7
497.7229919433594 0 1947.9014
501.6062927246094 0 7271.297
501.9404602050781 0 5848.139
502.2747497558594 0 3943.585
502.6073913574219 0 1583.1436
503.2160949707031 0 2690.0789
504.9336242675781 0 12151.53 b Water loss 12
505.267333984375 0 15300.683 b Ammonia loss 12
505.6015930175781 0 6882.415
505.9356689453125 0 3828.3936
506.26495361328125 0 802.65155
508.7563781738281 0 1850.6018
509.2605285644531 0 1818.928
510.9375915527344 0 69064.6 b 12
511.27191162109375 0 66357.37
511.6062927246094 0 28186.422
511.94024658203125 0 7660.295
512.2739868164062 0 2705.3184
516.9408569335938 0 7592.995
517.2748413085938 0 9868.379
517.6101684570312 0 5355.758
517.8829345703125 0 1775.0142
517.943603515625 0 1253.9347
519.6203002929688 0 847.93604
519.9489135742188 0 2712.0295 y Ammonia loss 1
520.246337890625 0 872.11053
520.2864990234375 0 2610.7915
520.3618774414062 0 1790.7996
520.6220092773438 0 1225.6769
521.2285766601562 0 2490.392
523.3628540039062 0 929.95197
525.6246337890625 0 11714.527 y 1
525.9592895507812 0 9943.726
526.2930297851562 0 5653.08
526.62841796875 0 2081.6956
528.2333984375 0 1961.5641
529.22265625 0 907.00397
529.8027954101562 0 1782.7307
530.2993774414062 0 1723.4545
530.7996826171875 0 1215.9053
531.2102661132812 0 2328.9368
535.293701171875 0 1241.5508
535.6129760742188 0 735.30835
537.2865600585938 0 1047.0834
537.62109375 0 823.7027
538.2523803710938 0 27466.426
538.37158203125 0 2192.2815
538.810791015625 0 4315.1675
539.2548217773438 0 7805.032
539.3048706054688 0 7369.3657
539.6322021484375 0 2044.0162
539.8051147460938 0 2902.7834
539.970947265625 0 1016.391
540.2568359375 0 1215.8821
540.2998046875 0 1022.4078
540.6224365234375 0 804.8151
540.8253784179688 0 781.6109 y Ammonia loss 4
541.32568359375 0 589.85803
541.3716430664062 0 9739.43 y 9
542.2510986328125 0 879.0648
542.3749389648438 0 3325.7236
542.9580688476562 0 1851.51
543.2928466796875 0 1116.1948
544.302001953125 0 724.0377
547.81494140625 0 2639.8372
548.2371215820312 0 1009.48566
548.3154907226562 0 1947.8187
548.6322021484375 0 19527.781 Precursor Water loss
548.9652709960938 0 23095.004 Precursor Ammonia loss
549.2238159179688 0 1686.1853
549.2974853515625 0 9259.645
549.3336181640625 0 16709.559 y 4
549.6326904296875 0 7357.077
549.8348388671875 0 10308.453
549.96484375 0 1616.0083
550.3359985351562 0 4840.373
550.840087890625 0 1131.678
551.375244140625 0 735.6548
553.3028564453125 0 1455.5933
553.9645385742188 0 3571.0823
554.2679443359375 0 825.58215
554.2982177734375 0 2626.5967
554.6359252929688 0 392883.84 Precursor
554.9700317382812 0 421384.03
555.3042602539062 0 222008.52
555.5818481445312 0 5035.367
555.6385498046875 0 73239.9
555.9149169921875 0 2755.8318
555.97265625 0 23224.922
557.2975463867188 0 1173.2229
561.268310546875 0 2376.016 b 8
561.349609375 0 919.7639
561.7692260742188 0 3280.9534
562.2703247070312 0 1480.27
565.2416381835938 0 2129.5928 b 3
566.2474975585938 0 34053.715
567.2498779296875 0 11762.273
567.3978881835938 0 1259.6799
568.2533569335938 0 1712.7361
568.4004516601562 0 899.2836
570.2460327148438 0 4600.322
570.3236694335938 0 896.9228
571.2484741210938 0 2124.362
572.8046875 0 18200.645
573.3056640625 0 10494.404
573.8067626953125 0 5567.213
577.3825073242188 0 7046.174
578.3845825195312 0 2730.7812
579.3619384765625 0 22603.66
580.3650512695312 0 6915.422
581.3702392578125 0 1218.2814
587.278564453125 0 1180.7612
587.78125 0 5179.915 b Water loss 9
588.281982421875 0 2872.7722 b Ammonia loss 9
588.7828369140625 0 1384.2516
595.3938598632812 0 7769.1553
595.8466186523438 0 1915.5405
596.3446655273438 0 848.11914
596.3951416015625 0 2364.5503
596.785888671875 0 4898.5225 b 9
597.2855224609375 0 1983.7014
597.7890014648438 0 1603.9176
598.241455078125 0 2279.2983
602.30224609375 0 1066.9891
604.3568115234375 0 9298.617
604.8529052734375 0 13236.628 y Ammonia loss 3
605.3526000976562 0 8385.451
605.8529663085938 0 3710.3745
606.83544921875 0 1441.5663
610.3209228515625 0 4596.133
611.3234252929688 0 1201.0475
611.8295288085938 0 2024.7878
612.3280029296875 0 2697.1567
613.362548828125 0 12907.66 y 3
613.8643188476562 0 7768.634
614.36474609375 0 3361.7236
615.2677001953125 0 1764.5215
615.3494262695312 0 8759.909
615.8505249023438 0 6706.457
616.3530883789062 0 2286.4358
620.3416137695312 0 14402.781
620.841796875 0 12792.505
621.3417358398438 0 4196.325
623.3877563476562 0 1249.2009
624.3877563476562 0 792.2381
628.3309936523438 0 1021.0147
629.3467407226562 0 37169.664
629.8480834960938 0 26170.562
630.349609375 0 10528.462
630.849853515625 0 2518.7559
632.29931640625 0 982.0021
638.3511962890625 0 4332.355
638.8541870117188 0 2538.9211
643.260986328125 0 970.8444
644.8289794921875 0 756.80396
645.3121948242188 0 2226.4192 b 10
645.8165283203125 0 1661.0149
647.4227905273438 0 1420.4349
653.827880859375 0 2117.3206
654.3289794921875 0 1631.3523
654.8256225585938 0 1728.4705
656.2931518554688 0 1679.1273
657.281005859375 0 2949.4275
658.307861328125 0 2403.49
659.3177490234375 0 943.90643
664.451171875 0 6848.9985
665.4529418945312 0 2753.688
665.8451538085938 0 1780.5361
666.3466186523438 0 986.33
666.4595336914062 0 1004.4538
669.4669799804688 0 3924.296 y 8
670.4689331054688 0 1608.2833
672.3914794921875 0 993.0914
674.3042602539062 0 8871.577
674.4351196289062 0 26044.682
675.3077392578125 0 3178.094
675.437255859375 0 11385.012
676.44140625 0 2024.6974
677.375244140625 0 4248.3677
677.8775024414062 0 2655.349
678.374267578125 0 2115.726
680.3147583007812 0 851.26996
684.28955078125 0 2119.2744 b Water loss 4
685.2842407226562 0 1802.1768 b Ammonia loss 4
685.8892211914062 0 6948.924
686.386962890625 0 7430.349 y Ammonia loss 2
686.8867797851562 0 3813.567
687.387939453125 0 1410.379
692.44580078125 0 24476.195
693.44873046875 0 10487.294
694.4510498046875 0 2392.7732
694.8939208984375 0 121263.766 y 2
695.3952026367188 0 101859.46
695.8966674804688 0 45493.645
696.398193359375 0 14216.433
696.8994750976562 0 1940.5979
698.4114990234375 0 1772.2881
700.8504028320312 0 1497.7838 b Ammonia loss 11
702.2998657226562 0 14612.0205 b 4
703.3021850585938 0 6914.571
704.3038330078125 0 1190.4498
707.3059692382812 0 3459.4377
707.794921875 0 832.4534
708.3099365234375 0 2098.1846
708.4794921875 0 1221.6395
709.3601684570312 0 26780.8 b 11
709.8614501953125 0 23764.951
710.297607421875 0 666.15576
710.3638916015625 0 10447.985
710.4548950195312 0 3730.354
710.8636474609375 0 3004.8162
711.3670654296875 0 986.2772
711.458740234375 0 1032.4366
713.378173828125 0 1046.2505
713.8829345703125 0 1259.8485
714.3800659179688 0 1687.5337
716.4205322265625 0 5203.98
717.4254150390625 0 1231.6758
720.317138671875 0 1131.081
720.3778076171875 0 972.731
721.3533325195312 0 2443.6838
722.3786010742188 0 1094.2709
722.8817138671875 0 833.5021
724.3335571289062 0 2628.8992
725.3333129882812 0 1089.5065
726.4876708984375 0 9935.101 y 7
727.4912109375 0 3799.4841
735.3020629882812 0 2665.5854
736.3004760742188 0 836.7209
737.35107421875 0 1106.8632
738.3740844726562 0 987.7514
739.36767578125 0 802.9957
740.2951049804688 0 1581.8499
751.9014282226562 0 2173.2568
752.3265991210938 0 3451.9314
752.4088134765625 0 2003.039
753.3297729492188 0 1067.1272
756.3894653320312 0 948.9949
756.8948364257812 0 2561.927 b Water loss 12
757.395263671875 0 2254.2117 b Ammonia loss 12
757.9032592773438 0 1786.5153
759.030029296875 0 856.0361
765.90185546875 0 9392.516 b 12
766.403076171875 0 6639.9663
766.9057006835938 0 4914.1323
767.4094848632812 0 1812.2142
774.9065551757812 0 4086.9705
775.4080810546875 0 3287.3591
775.909912109375 0 2110.7617
787.933349609375 0 3449.2983 y 1
788.4334106445312 0 2455.74
788.5131225585938 0 924.9032
788.9353637695312 0 1783.9329
794.3417358398438 0 2062.9878
797.3203735351562 0 873.9133
805.5292358398438 0 5493.0586
806.5335693359375 0 2634.1914
811.3635864257812 0 15153.042
811.4940185546875 0 4907.147
812.3656616210938 0 8035.0933
812.4990234375 0 2328.5176
813.3712158203125 0 2194.6675
821.3471069335938 0 2532.8 b Water loss 5
822.337890625 0 1970.8363 b Ammonia loss 5
823.351318359375 0 1148.2535
823.5400390625 0 55167.66 y 6
824.54296875 0 25459.72
824.6331176757812 0 1250.5078
825.544677734375 0 6519.9067
826.5498657226562 0 1197.4398
829.5038452148438 0 4368.289
830.5072021484375 0 2487.1792
839.3583374023438 0 32783.785 b 5
840.3610229492188 0 16234.791
841.3628540039062 0 5365.5024
848.4151611328125 0 4282.972
849.4205322265625 0 2473.4517
853.4778442382812 0 3529.846
854.4789428710938 0 1453.7216
855.4832763671875 0 795.73865
891.45751953125 0 2316.3345
892.4584350585938 0 938.6594
901.442138671875 0 11267.6045
902.4454956054688 0 6238.7056
903.4440307617188 0 2001.7255
919.4525146484375 0 6828.7104
920.4554443359375 0 3324.627
942.5863037109375 0 4544.667
943.592041015625 0 2755.8867
944.5927734375 0 1408.4716
948.5497436523438 0 1009.2304
949.5517578125 0 743.5562
960.5986328125 0 10819.561 y 5
961.5064697265625 0 698.84656
961.6012573242188 0 6143.423
962.605224609375 0 2273.7803
966.562255859375 0 2715.128
967.5645141601562 0 2126.4326
1017.5020141601562 0 750.4103
1032.5386962890625 0 2328.6743
1033.533203125 0 3302.9546
1034.5377197265625 0 1103.6049
1088.521484375 0 1152.0706
1097.658447265625 0 4452.955 y 4
1098.659423828125 0 3247.839
1099.659423828125 0 1301.7804
1121.5255126953125 0 3346.1326 b 8
1122.529052734375 0 1560.2318
1144.600830078125 0 1565.6581
1174.5531005859375 0 5477.4844 b Water loss 9
1175.5548095703125 0 4331.343 b Ammonia loss 9
1176.5599365234375 0 1446.0409
1192.5596923828125 0 2267.3677 b 9
1193.5689697265625 0 1032.934
1257.682861328125 0 856.3484
2520.533935546875 0 812.6525

Spectrum Details

|  |  |
| --- | --- |
| Matched peaks? Matched peaksThe total absolute number of peaks matched. Additionally in brackets the total fraction of peaks matched and the total number of peaks is shown. | 66 (8.45% of 781) |
| FDR? FDRThe false discovery rate estimated for this peptide. It is calculated by matching all theoretical fragments with a non-integer shift with the raw peaks for this spectrum. This is done with 40 different shifts. The resulting percentage is the average number of annotated peaks over the number of annotated peaks with the correct spectrum. | 3.46% |
| Satellite FDR? Satellite FDRSee the FDR for details on its calculation. This satellite ion specific FDR only contains the satellite ions (d/w) for I/L/J positions. | - |
| PSM Score? PSM ScoreThe PSM Score as given by Hecklib to this annotated spectrum. It is shown with three significant figures. | 360 |

## Spectrum 5306? Spectrum 5306 The raw spectrum of this peptide as annotated by Hecklib. The fragments are coloured according to ion type (see legend). Any peaks with a star '\*' as text can be hovered over to see the full details, first the ion type second the mass shift type. By hovering over the amino acids in the peptide or ions in the legend the corresponding peaks are highlighted. By toggling the 'Unassigned' label you can turn the background (unassigned) peaks on or off in the plot. By updating the slider in the Ion legend you can update the spectrum to only show the top X% of the peaks with labels. The top X% means any peak that is within X% of the highest intensity. By dragging in the spectrum you can zoom in to a specific part of the spectrum and use 'Zoom Out' to get back to the original zoom level. The annotation of the spectrum is based on the given sequence in the peptides file and is done with different software so inconsistencies are likely. The peaks are annotated based on the given sequence, with 20 ppm tolerance.

Copy Data

### Spectrum 5306 (TSV)

#### Preview

```
Loading example...
```

*Click on the button to copy the data to your clipboard.*

Mz MinMz MaxIntensity Max

WidthHeightPeptide font sizePeptide stroke widthSpectrum font sizeSpectrum stroke widthCompact peptide

Ion legend

wxyz

abcd

OtherUnassignedIonChargePositionShow for top:%

SWYQHHPGKAPKJJ

01.32e+62.63e+63.95e+65.26e+6

Zoom Out

y+11a+23y+24a+12b+35y+12a+12b+12y+25b+12y+26y+27y+13b+26y+28b+26b+13y+312y+312b+27y+14b+312y+29b+28b+313b+313b+313y+313y+313y+210y+15\*\*y+210\*b+29b+14b+210b+210b+210y+211y+211b+211y+16b+15b+15y+212y+212b+212b+212b+15b+212y+17b+213b+213b+213y+213y+213b+16b+16y+18b+16y+19b+18y+110b+19b+110b+110b+110

0833166624993332

Fragment Matches Table

Show background peaks

| Position | Ion type | Intensity | mz Theoretical | mz Error (Th) | mz Error (ppm) | Charge | Series Number |
| --- | --- | --- | --- | --- | --- | --- | --- |
| - | - | 9336 | 120.1 | - | - | 0 | - |
| - | - | 6660 | 122.1 | - | - | 0 | - |
| - | - | 1.908E+04 | 123.1 | - | - | 0 | - |
| - | - | 3437 | 124.7 | - | - | 0 | - |
| - | - | 3275 | 124.9 | - | - | 0 | - |
| - | - | 3.006E+04 | 127.1 | - | - | 0 | - |
| - | - | 8069 | 128.1 | - | - | 0 | - |
| - | - | 4.235E+04 | 129.1 | - | - | 0 | - |
| - | - | 1.118E+06 | 129.1 | - | - | 0 | - |
| - | - | 2.775E+05 | 130.1 | - | - | 0 | - |
| - | - | 9544 | 130.1 | - | - | 0 | - |
| - | - | 7.48E+04 | 130.1 | - | - | 0 | - |
| - | - | 3.304E+04 | 131.1 | - | - | 0 | - |
| - | - | 3758 | 131.1 | - | - | 0 | - |
| - | - | 1.143E+06 | 132.1 | - | - | 0 | - |
| 14 | y | 2.195E+05 | 132.1 | 0.0005119 | 3.875 | +1 | 1 |
| - | - | 3975 | 132.2 | - | - | 0 | - |
| - | - | 1.173E+05 | 133.1 | - | - | 0 | - |
| - | - | 5137 | 133.1 | - | - | 0 | - |
| - | - | 1.585E+04 | 133.1 | - | - | 0 | - |
| - | - | 8368 | 134.1 | - | - | 0 | - |
| - | - | 6.749E+05 | 136.1 | - | - | 0 | - |
| - | - | 6.088E+04 | 137.1 | - | - | 0 | - |
| - | - | 6767 | 137.1 | - | - | 0 | - |
| - | - | 2.552E+04 | 138.1 | - | - | 0 | - |
| - | - | 1.526E+04 | 139.1 | - | - | 0 | - |
| - | - | 8.456E+04 | 141.1 | - | - | 0 | - |
| - | - | 6308 | 142.1 | - | - | 0 | - |
| - | - | 5892 | 142.1 | - | - | 0 | - |
| - | - | 2.231E+04 | 144.1 | - | - | 0 | - |
| - | - | 6934 | 145.1 | - | - | 0 | - |
| - | - | 3908 | 146.1 | - | - | 0 | - |
| - | - | 1.218E+04 | 147 | - | - | 0 | - |
| - | - | 4032 | 149.1 | - | - | 0 | - |
| - | - | 8066 | 151.1 | - | - | 0 | - |
| - | - | 2.828E+04 | 152.1 | - | - | 0 | - |
| - | - | 2.413E+04 | 152.1 | - | - | 0 | - |
| - | - | 3983 | 152.2 | - | - | 0 | - |
| - | - | 4.693E+04 | 155.1 | - | - | 0 | - |
| - | - | 2.821E+04 | 155.1 | - | - | 0 | - |
| - | - | 4.02E+04 | 155.1 | - | - | 0 | - |
| - | - | 3.709E+04 | 156.1 | - | - | 0 | - |
| - | - | 5916 | 156.1 | - | - | 0 | - |
| - | - | 4379 | 156.6 | - | - | 0 | - |
| - | - | 5223 | 157.1 | - | - | 0 | - |
| - | - | 1.511E+04 | 158.1 | - | - | 0 | - |
| - | - | 1.135E+05 | 158.1 | - | - | 0 | - |
| - | - | 4796 | 158.1 | - | - | 0 | - |
| - | - | 5.233E+05 | 159.1 | - | - | 0 | - |
| - | - | 2.611E+04 | 160.1 | - | - | 0 | - |
| - | - | 9371 | 160.1 | - | - | 0 | - |
| - | - | 5.354E+04 | 160.1 | - | - | 0 | - |
| - | - | 3732 | 163 | - | - | 0 | - |
| - | - | 9646 | 164.1 | - | - | 0 | - |
| - | - | 3.165E+04 | 165.1 | - | - | 0 | - |
| - | - | 1.397E+04 | 166.1 | - | - | 0 | - |
| - | - | 5962 | 166.1 | - | - | 0 | - |
| - | - | 1.578E+04 | 168.1 | - | - | 0 | - |
| - | - | 5.779E+04 | 169.1 | - | - | 0 | - |
| - | - | 7119 | 169.1 | - | - | 0 | - |
| - | - | 1.042E+05 | 169.1 | - | - | 0 | - |
| - | - | 8155 | 169.1 | - | - | 0 | - |
| - | - | 6228 | 169.2 | - | - | 0 | - |
| - | - | 1.707E+05 | 170.1 | - | - | 0 | - |
| - | - | 7699 | 170.1 | - | - | 0 | - |
| - | - | 7505 | 170.1 | - | - | 0 | - |
| - | - | 2.509E+04 | 171.1 | - | - | 0 | - |
| - | - | 4834 | 171.1 | - | - | 0 | - |
| - | - | 7.416E+04 | 172.1 | - | - | 0 | - |
| - | - | 1.361E+04 | 173.5 | - | - | 0 | - |
| - | - | 7370 | 175.1 | - | - | 0 | - |
| - | - | 2.521E+04 | 176.1 | - | - | 0 | - |
| - | - | 5634 | 179.1 | - | - | 0 | - |
| - | - | 1.002E+04 | 180.1 | - | - | 0 | - |
| - | - | 6277 | 181.1 | - | - | 0 | - |
| - | - | 2.155E+05 | 181.1 | - | - | 0 | - |
| - | - | 2.112E+04 | 181.1 | - | - | 0 | - |
| - | - | 1.533E+04 | 182.1 | - | - | 0 | - |
| - | - | 3.763E+04 | 182.1 | - | - | 0 | - |
| - | - | 1.598E+05 | 183.1 | - | - | 0 | - |
| - | - | 7269 | 183.1 | - | - | 0 | - |
| - | - | 1.493E+04 | 184.1 | - | - | 0 | - |
| - | - | 2.081E+04 | 184.1 | - | - | 0 | - |
| - | - | 3.766E+04 | 185.1 | - | - | 0 | - |
| - | - | 5695 | 185.1 | - | - | 0 | - |
| - | - | 5268 | 186.1 | - | - | 0 | - |
| - | - | 1.181E+05 | 186.1 | - | - | 0 | - |
| - | - | 9.878E+05 | 187.1 | - | - | 0 | - |
| - | - | 8069 | 188.1 | - | - | 0 | - |
| - | - | 1.112E+05 | 188.1 | - | - | 0 | - |
| - | - | 5586 | 189.1 | - | - | 0 | - |
| - | - | 4645 | 193.1 | - | - | 0 | - |
| - | - | 1.392E+04 | 195.1 | - | - | 0 | - |
| - | - | 4200 | 195.5 | - | - | 0 | - |
| - | - | 5.755E+04 | 197.2 | - | - | 0 | - |
| - | - | 7548 | 198.1 | - | - | 0 | - |
| - | - | 7763 | 198.2 | - | - | 0 | - |
| - | - | 3.707E+04 | 199.1 | - | - | 0 | - |
| - | - | 7877 | 199.1 | - | - | 0 | - |
| - | - | 3.454E+04 | 199.2 | - | - | 0 | - |
| - | - | 5812 | 200.1 | - | - | 0 | - |
| - | - | 2.655E+04 | 200.1 | - | - | 0 | - |
| - | - | 8.836E+04 | 201.1 | - | - | 0 | - |
| - | - | 2.098E+04 | 202.1 | - | - | 0 | - |
| - | - | 1.122E+05 | 202.1 | - | - | 0 | - |
| - | - | 7746 | 203.1 | - | - | 0 | - |
| - | - | 1.205E+04 | 204.1 | - | - | 0 | - |
| 3 | a | 5666 | 205.1 | 0.0005632 | 2.746 | +2 | 3 |
| - | - | 5.135E+04 | 207.1 | - | - | 0 | - |
| - | - | 5430 | 208.2 | - | - | 0 | - |
| - | - | 8918 | 209.1 | - | - | 0 | - |
| - | - | 2.466E+04 | 211.1 | - | - | 0 | - |
| - | - | 7638 | 213.1 | - | - | 0 | - |
| - | - | 4.234E+04 | 214.1 | - | - | 0 | - |
| - | - | 1.127E+04 | 216.1 | - | - | 0 | - |
| - | - | 1.489E+04 | 217.1 | - | - | 0 | - |
| - | - | 7250 | 219.1 | - | - | 0 | - |
| - | - | 9.047E+04 | 220.1 | - | - | 0 | - |
| - | - | 1.157E+05 | 221.1 | - | - | 0 | - |
| - | - | 6808 | 221.1 | - | - | 0 | - |
| - | - | 9394 | 222.1 | - | - | 0 | - |
| - | - | 7204 | 222.1 | - | - | 0 | - |
| - | - | 2.124E+04 | 224.1 | - | - | 0 | - |
| - | - | 5149 | 224.1 | - | - | 0 | - |
| - | - | 6.215E+04 | 224.2 | - | - | 0 | - |
| - | - | 6525 | 225.1 | - | - | 0 | - |
| - | - | 1.109E+04 | 225.2 | - | - | 0 | - |
| - | - | 8316 | 225.2 | - | - | 0 | - |
| - | - | 1.407E+06 | 226.2 | - | - | 0 | - |
| - | - | 5360 | 227.1 | - | - | 0 | - |
| - | - | 7682 | 227.1 | - | - | 0 | - |
| 11 | y | 1.705E+05 | 227.2 | 0.001739 | 7.656 | +2 | 4 |
| - | - | 1.099E+04 | 227.2 | - | - | 0 | - |
| 2 | a | 1.034E+05 | 228.1 | 0.0006615 | 2.9 | +1 | 2 |
| - | - | 1.102E+04 | 228.1 | - | - | 0 | - |
| - | - | 1.437E+04 | 228.2 | - | - | 0 | - |
| 5 | b | 1.116E+05 | 229.1 | 0.002026 | 8.844 | +3 | 5 |
| - | - | 1.643E+04 | 229.1 | - | - | 0 | - |
| - | - | 3.062E+04 | 230.1 | - | - | 0 | - |
| - | - | 4.859E+04 | 231.1 | - | - | 0 | - |
| - | - | 2.24E+04 | 231.1 | - | - | 0 | - |
| - | - | 6240 | 232.1 | - | - | 0 | - |
| - | - | 1.428E+04 | 233.1 | - | - | 0 | - |
| - | - | 7601 | 234.1 | - | - | 0 | - |
| - | - | 2.017E+05 | 235.1 | - | - | 0 | - |
| - | - | 2.374E+04 | 236.1 | - | - | 0 | - |
| - | - | 9656 | 236.1 | - | - | 0 | - |
| - | - | 2.17E+04 | 238.1 | - | - | 0 | - |
| - | - | 1.083E+04 | 239.1 | - | - | 0 | - |
| - | - | 7.908E+04 | 239.2 | - | - | 0 | - |
| - | - | 1.032E+04 | 240.2 | - | - | 0 | - |
| - | - | 1.205E+04 | 241.2 | - | - | 0 | - |
| - | - | 2.549E+04 | 242.2 | - | - | 0 | - |
| - | - | 1.979E+04 | 243.2 | - | - | 0 | - |
| - | - | 7274 | 244.1 | - | - | 0 | - |
| - | - | 6257 | 244.2 | - | - | 0 | - |
| - | - | 6668 | 245.2 | - | - | 0 | - |
| - | - | 6263 | 245.2 | - | - | 0 | - |
| 13 | y | 4.462E+05 | 245.2 | 0.000768 | 3.132 | +1 | 2 |
| 2 | a | 2.716E+06 | 246.1 | 0.0007933 | 3.223 | +1 | 2 |
| - | - | 6.031E+04 | 246.2 | - | - | 0 | - |
| - | - | 6.202E+05 | 247.1 | - | - | 0 | - |
| - | - | 6480 | 247.2 | - | - | 0 | - |
| - | - | 1.549E+05 | 248.1 | - | - | 0 | - |
| - | - | 5.134E+04 | 248.1 | - | - | 0 | - |
| - | - | 1.972E+05 | 249.1 | - | - | 0 | - |
| - | - | 1.67E+04 | 249.1 | - | - | 0 | - |
| - | - | 2.139E+04 | 250.1 | - | - | 0 | - |
| - | - | 1.319E+04 | 250.1 | - | - | 0 | - |
| - | - | 7558 | 251.1 | - | - | 0 | - |
| - | - | 7001 | 251.2 | - | - | 0 | - |
| - | - | 2.616E+04 | 252.2 | - | - | 0 | - |
| - | - | 4742 | 253.2 | - | - | 0 | - |
| - | - | 6720 | 255.1 | - | - | 0 | - |
| 2 | b | 7.092E+04 | 256.1 | 0.000681 | 2.659 | +1 | 2 |
| - | - | 1.683E+04 | 257.1 | - | - | 0 | - |
| - | - | 2.265E+04 | 257.1 | - | - | 0 | - |
| - | - | 2.678E+04 | 257.2 | - | - | 0 | - |
| - | - | 1.449E+04 | 258.1 | - | - | 0 | - |
| - | - | 3.398E+05 | 258.1 | - | - | 0 | - |
| - | - | 5516 | 259.1 | - | - | 0 | - |
| - | - | 4.929E+04 | 259.1 | - | - | 0 | - |
| - | - | 1.25E+04 | 259.1 | - | - | 0 | - |
| - | - | 2.619E+04 | 260.1 | - | - | 0 | - |
| - | - | 2.04E+04 | 260.2 | - | - | 0 | - |
| - | - | 1.133E+04 | 262.2 | - | - | 0 | - |
| - | - | 1.244E+04 | 263.1 | - | - | 0 | - |
| - | - | 1.002E+04 | 264.1 | - | - | 0 | - |
| - | - | 2.336E+04 | 265.1 | - | - | 0 | - |
| - | - | 1.431E+04 | 265.2 | - | - | 0 | - |
| - | - | 1.774E+05 | 266.1 | - | - | 0 | - |
| - | - | 2.201E+04 | 267.1 | - | - | 0 | - |
| - | - | 7763 | 267.1 | - | - | 0 | - |
| - | - | 6.617E+04 | 270.2 | - | - | 0 | - |
| 10 | y | 1.613E+04 | 271.2 | 0.004351 | 16.04 | +2 | 5 |
| - | - | 1.276E+04 | 273.1 | - | - | 0 | - |
| - | - | 1.12E+04 | 273.1 | - | - | 0 | - |
| 2 | b | 1.647E+06 | 274.1 | 0.0007059 | 2.575 | +1 | 2 |
| - | - | 5.714E+04 | 275.1 | - | - | 0 | - |
| - | - | 7.047E+05 | 275.1 | - | - | 0 | - |
| - | - | 2.67E+04 | 276.1 | - | - | 0 | - |
| - | - | 8.422E+04 | 276.1 | - | - | 0 | - |
| - | - | 1.47E+04 | 277.1 | - | - | 0 | - |
| - | - | 1.956E+04 | 279.2 | - | - | 0 | - |
| - | - | 3.265E+04 | 283.2 | - | - | 0 | - |
| - | - | 2.255E+05 | 283.2 | - | - | 0 | - |
| - | - | 3.193E+04 | 284.2 | - | - | 0 | - |
| - | - | 1.463E+04 | 285.1 | - | - | 0 | - |
| - | - | 6.857E+04 | 285.2 | - | - | 0 | - |
| - | - | 6746 | 285.2 | - | - | 0 | - |
| - | - | 1.48E+04 | 286.2 | - | - | 0 | - |
| - | - | 7177 | 290.1 | - | - | 0 | - |
| - | - | 6.454E+04 | 290.2 | - | - | 0 | - |
| - | - | 2.212E+04 | 290.7 | - | - | 0 | - |
| - | - | 2.065E+04 | 291.2 | - | - | 0 | - |
| - | - | 1.84E+05 | 292.1 | - | - | 0 | - |
| - | - | 2.551E+04 | 293.1 | - | - | 0 | - |
| - | - | 8.47E+04 | 294.1 | - | - | 0 | - |
| - | - | 9712 | 295.1 | - | - | 0 | - |
| - | - | 1.902E+05 | 297.2 | - | - | 0 | - |
| - | - | 3.941E+04 | 298.2 | - | - | 0 | - |
| - | - | 8582 | 300.2 | - | - | 0 | - |
| - | - | 6115 | 301.1 | - | - | 0 | - |
| - | - | 4.453E+04 | 303.1 | - | - | 0 | - |
| - | - | 5624 | 303.2 | - | - | 0 | - |
| - | - | 4.751E+04 | 305.1 | - | - | 0 | - |
| - | - | 1.33E+04 | 305.7 | - | - | 0 | - |
| - | - | 9338 | 306.1 | - | - | 0 | - |
| - | - | 8133 | 308.1 | - | - | 0 | - |
| - | - | 8259 | 309.2 | - | - | 0 | - |
| - | - | 4.236E+04 | 311.2 | - | - | 0 | - |
| - | - | 7757 | 312.2 | - | - | 0 | - |
| - | - | 1.023E+04 | 313.2 | - | - | 0 | - |
| - | - | 6045 | 314.7 | - | - | 0 | - |
| - | - | 1.19E+04 | 318.2 | - | - | 0 | - |
| - | - | 6397 | 319.2 | - | - | 0 | - |
| - | - | 4.922E+04 | 320.1 | - | - | 0 | - |
| - | - | 7929 | 320.2 | - | - | 0 | - |
| - | - | 1.594E+04 | 321.1 | - | - | 0 | - |
| - | - | 2.324E+05 | 321.2 | - | - | 0 | - |
| - | - | 7.142E+04 | 322.2 | - | - | 0 | - |
| - | - | 4.558E+04 | 322.2 | - | - | 0 | - |
| - | - | 1.526E+04 | 323.2 | - | - | 0 | - |
| - | - | 6904 | 323.2 | - | - | 0 | - |
| - | - | 1.923E+04 | 324.2 | - | - | 0 | - |
| - | - | 5589 | 324.7 | - | - | 0 | - |
| - | - | 5249 | 325.2 | - | - | 0 | - |
| - | - | 4.758E+04 | 326.2 | - | - | 0 | - |
| - | - | 2.673E+04 | 332.7 | - | - | 0 | - |
| - | - | 1.443E+04 | 333.1 | - | - | 0 | - |
| - | - | 1.461E+04 | 333.2 | - | - | 0 | - |
| - | - | 7654 | 333.7 | - | - | 0 | - |
| 9 | y | 1.135E+04 | 335.2 | 0.001238 | 3.692 | +2 | 6 |
| - | - | 8532 | 336.1 | - | - | 0 | - |
| - | - | 2.898E+05 | 336.2 | - | - | 0 | - |
| - | - | 5.113E+04 | 337.2 | - | - | 0 | - |
| - | - | 5.685E+04 | 337.7 | - | - | 0 | - |
| - | - | 9334 | 338.2 | - | - | 0 | - |
| - | - | 2.202E+04 | 338.2 | - | - | 0 | - |
| - | - | 2.297E+05 | 339.2 | - | - | 0 | - |
| - | - | 2.1E+04 | 340.2 | - | - | 0 | - |
| - | - | 3.533E+04 | 340.2 | - | - | 0 | - |
| - | - | 6086 | 341.2 | - | - | 0 | - |
| - | - | 9682 | 342.2 | - | - | 0 | - |
| - | - | 6056 | 343.2 | - | - | 0 | - |
| - | - | 2.123E+05 | 346.7 | - | - | 0 | - |
| - | - | 8.97E+04 | 347.2 | - | - | 0 | - |
| - | - | 1.928E+04 | 347.7 | - | - | 0 | - |
| - | - | 7925 | 348.1 | - | - | 0 | - |
| - | - | 1.206E+04 | 349.7 | - | - | 0 | - |
| - | - | 2.411E+04 | 350.2 | - | - | 0 | - |
| - | - | 6491 | 350.2 | - | - | 0 | - |
| - | - | 2.947E+04 | 351.2 | - | - | 0 | - |
| - | - | 1.54E+04 | 353.2 | - | - | 0 | - |
| - | - | 1.228E+04 | 354.2 | - | - | 0 | - |
| - | - | 4.196E+05 | 354.2 | - | - | 0 | - |
| - | - | 8093 | 354.7 | - | - | 0 | - |
| - | - | 6.118E+04 | 355.2 | - | - | 0 | - |
| - | - | 7398 | 355.3 | - | - | 0 | - |
| - | - | 2.78E+04 | 355.7 | - | - | 0 | - |
| - | - | 7458 | 356.2 | - | - | 0 | - |
| - | - | 1.367E+04 | 357.2 | - | - | 0 | - |
| - | - | 4.489E+04 | 357.3 | - | - | 0 | - |
| - | - | 5.278E+04 | 358.2 | - | - | 0 | - |
| - | - | 8792 | 358.3 | - | - | 0 | - |
| - | - | 2.269E+04 | 358.7 | - | - | 0 | - |
| - | - | 9235 | 359.2 | - | - | 0 | - |
| - | - | 9228 | 359.2 | - | - | 0 | - |
| - | - | 9393 | 362.7 | - | - | 0 | - |
| 8 | y | 1.236E+04 | 363.7 | 0.000943 | 2.592 | +2 | 7 |
| - | - | 7155 | 365.3 | - | - | 0 | - |
| - | - | 2.812E+04 | 366.2 | - | - | 0 | - |
| - | - | 2.241E+04 | 367.2 | - | - | 0 | - |
| - | - | 3.413E+04 | 368.1 | - | - | 0 | - |
| - | - | 1.743E+04 | 368.2 | - | - | 0 | - |
| - | - | 9913 | 369.2 | - | - | 0 | - |
| - | - | 8174 | 369.2 | - | - | 0 | - |
| - | - | 1.165E+04 | 370.2 | - | - | 0 | - |
| 12 | y | 9.585E+04 | 373.3 | 0.0008977 | 2.405 | +1 | 3 |
| - | - | 7840 | 374.3 | - | - | 0 | - |
| - | - | 2.076E+04 | 374.3 | - | - | 0 | - |
| - | - | 8558 | 376.7 | - | - | 0 | - |
| - | - | 1.812E+04 | 380.2 | - | - | 0 | - |
| - | - | 1.518E+04 | 382.2 | - | - | 0 | - |
| - | - | 2.174E+04 | 382.3 | - | - | 0 | - |
| - | - | 1.818E+04 | 383.3 | - | - | 0 | - |
| - | - | 5.321E+04 | 384.2 | - | - | 0 | - |
| - | - | 1.52E+05 | 385.2 | - | - | 0 | - |
| - | - | 5.672E+04 | 385.2 | - | - | 0 | - |
| - | - | 8.485E+04 | 386.2 | - | - | 0 | - |
| - | - | 1.535E+04 | 386.2 | - | - | 0 | - |
| - | - | 2.026E+04 | 387.2 | - | - | 0 | - |
| - | - | 7292 | 388.2 | - | - | 0 | - |
| - | - | 6282 | 389.3 | - | - | 0 | - |
| - | - | 1.519E+04 | 392.2 | - | - | 0 | - |
| - | - | 5.973E+04 | 392.3 | - | - | 0 | - |
| - | - | 1.202E+04 | 393.2 | - | - | 0 | - |
| - | - | 1.442E+04 | 393.3 | - | - | 0 | - |
| - | - | 1.06E+05 | 394.2 | - | - | 0 | - |
| - | - | 1.101E+04 | 394.3 | - | - | 0 | - |
| - | - | 2.332E+04 | 395.2 | - | - | 0 | - |
| - | - | 2.531E+04 | 396.3 | - | - | 0 | - |
| - | - | 9456 | 397.2 | - | - | 0 | - |
| - | - | 9755 | 397.7 | - | - | 0 | - |
| - | - | 1.049E+04 | 398.2 | - | - | 0 | - |
| - | - | 1.981E+04 | 401.2 | - | - | 0 | - |
| - | - | 4.483E+04 | 402.2 | - | - | 0 | - |
| - | - | 1.415E+05 | 403.2 | - | - | 0 | - |
| - | - | 1.044E+04 | 403.2 | - | - | 0 | - |
| - | - | 2.803E+04 | 403.3 | - | - | 0 | - |
| - | - | 2.039E+04 | 403.8 | - | - | 0 | - |
| - | - | 3.103E+04 | 404.2 | - | - | 0 | - |
| - | - | 7759 | 404.3 | - | - | 0 | - |
| - | - | 6.808E+04 | 406.2 | - | - | 0 | - |
| - | - | 5.169E+04 | 406.3 | - | - | 0 | - |
| - | - | 3.117E+04 | 406.7 | - | - | 0 | - |
| - | - | 6070 | 406.7 | - | - | 0 | - |
| - | - | 3E+04 | 406.8 | - | - | 0 | - |
| - | - | 1.21E+04 | 407.2 | - | - | 0 | - |
| - | - | 6345 | 407.5 | - | - | 0 | - |
| - | - | 8628 | 408.2 | - | - | 0 | - |
| - | - | 1.223E+05 | 410.3 | - | - | 0 | - |
| 6 | b | 1.928E+05 | 411.2 | 0.00112 | 2.724 | +2 | 6 |
| - | - | 2.106E+04 | 411.3 | - | - | 0 | - |
| - | - | 1.858E+04 | 412.2 | - | - | 0 | - |
| - | - | 3.09E+04 | 412.2 | - | - | 0 | - |
| 7 | y | 5.548E+05 | 412.3 | 0.001203 | 2.918 | +2 | 8 |
| - | - | 2.432E+05 | 412.8 | - | - | 0 | - |
| - | - | 6892 | 413.2 | - | - | 0 | - |
| - | - | 6.579E+04 | 413.3 | - | - | 0 | - |
| - | - | 8732 | 413.8 | - | - | 0 | - |
| - | - | 5.103E+04 | 415.3 | - | - | 0 | - |
| - | - | 2.568E+04 | 415.8 | - | - | 0 | - |
| - | - | 2.143E+04 | 419.9 | - | - | 0 | - |
| 6 | b | 8.219E+04 | 420.2 | 0.0006291 | 1.497 | +2 | 6 |
| - | - | 3.55E+04 | 420.2 | - | - | 0 | - |
| - | - | 3.873E+04 | 420.7 | - | - | 0 | - |
| - | - | 8084 | 421.2 | - | - | 0 | - |
| - | - | 4.737E+04 | 422.3 | - | - | 0 | - |
| - | - | 1.071E+04 | 422.3 | - | - | 0 | - |
| - | - | 2.609E+04 | 423.3 | - | - | 0 | - |
| - | - | 1.455E+04 | 424.3 | - | - | 0 | - |
| - | - | 1.209E+04 | 424.7 | - | - | 0 | - |
| - | - | 1.816E+04 | 425.3 | - | - | 0 | - |
| - | - | 5873 | 425.7 | - | - | 0 | - |
| - | - | 3.198E+04 | 427.2 | - | - | 0 | - |
| - | - | 2.411E+04 | 427.7 | - | - | 0 | - |
| - | - | 6160 | 428.2 | - | - | 0 | - |
| - | - | 9592 | 428.3 | - | - | 0 | - |
| - | - | 6.662E+05 | 429.2 | - | - | 0 | - |
| - | - | 1.729E+05 | 430.2 | - | - | 0 | - |
| - | - | 8.7E+04 | 431.2 | - | - | 0 | - |
| - | - | 2.671E+04 | 432.2 | - | - | 0 | - |
| - | - | 1.162E+04 | 433.3 | - | - | 0 | - |
| 3 | b | 1.287E+05 | 437.2 | 0.00119 | 2.721 | +1 | 3 |
| - | - | 3.151E+04 | 438.2 | - | - | 0 | - |
| - | - | 1.801E+04 | 438.6 | - | - | 0 | - |
| - | - | 1.813E+04 | 438.9 | - | - | 0 | - |
| - | - | 1.785E+04 | 439.3 | - | - | 0 | - |
| - | - | 1.147E+04 | 444.2 | - | - | 0 | - |
| - | - | 1.073E+04 | 444.6 | - | - | 0 | - |
| - | - | 1.351E+04 | 445.3 | - | - | 0 | - |
| - | - | 1.148E+04 | 446.2 | - | - | 0 | - |
| - | - | 1.047E+04 | 450.3 | - | - | 0 | - |
| - | - | 2.665E+04 | 451.2 | - | - | 0 | - |
| - | - | 3.94E+04 | 451.3 | - | - | 0 | - |
| - | - | 1.424E+04 | 451.7 | - | - | 0 | - |
| - | - | 1.224E+04 | 451.9 | - | - | 0 | - |
| - | - | 1.514E+04 | 452.3 | - | - | 0 | - |
| - | - | 3.054E+04 | 454.2 | - | - | 0 | - |
| - | - | 1.067E+04 | 455.2 | - | - | 0 | - |
| - | - | 4.271E+04 | 455.3 | - | - | 0 | - |
| - | - | 1.38E+04 | 456.3 | - | - | 0 | - |
| - | - | 2.163E+04 | 457.2 | - | - | 0 | - |
| - | - | 1.208E+04 | 457.6 | - | - | 0 | - |
| 3 | y | 8695 | 457.9 | 0.005126 | 11.19 | +3 | 12 |
| - | - | 2.574E+04 | 460.2 | - | - | 0 | - |
| - | - | 1.212E+04 | 461.2 | - | - | 0 | - |
| - | - | 7658 | 461.2 | - | - | 0 | - |
| - | - | 1.281E+04 | 463.3 | - | - | 0 | - |
| 3 | y | 1.404E+05 | 463.6 | 0.001149 | 2.478 | +3 | 12 |
| - | - | 9.144E+04 | 463.9 | - | - | 0 | - |
| - | - | 3.14E+04 | 464.3 | - | - | 0 | - |
| - | - | 1.264E+04 | 464.6 | - | - | 0 | - |
| - | - | 8287 | 466.9 | - | - | 0 | - |
| - | - | 9579 | 467.3 | - | - | 0 | - |
| - | - | 6.135E+04 | 467.3 | - | - | 0 | - |
| - | - | 3.125E+04 | 468.3 | - | - | 0 | - |
| 7 | b | 8548 | 468.7 | 0.003147 | 6.715 | +2 | 7 |
| - | - | 1.12E+04 | 469.8 | - | - | 0 | - |
| - | - | 1.708E+04 | 470.3 | - | - | 0 | - |
| 11 | y | 4.137E+05 | 470.3 | 0.001234 | 2.625 | +1 | 4 |
| - | - | 2.259E+04 | 470.6 | - | - | 0 | - |
| - | - | 6507 | 470.9 | - | - | 0 | - |
| - | - | 1.137E+05 | 471.3 | - | - | 0 | - |
| - | - | 4.03E+04 | 471.8 | - | - | 0 | - |
| - | - | 2.037E+04 | 472.3 | - | - | 0 | - |
| - | - | 1.913E+04 | 472.3 | - | - | 0 | - |
| - | - | 7167 | 472.8 | - | - | 0 | - |
| 12 | b | 1.389E+05 | 473.2 | 0.001677 | 3.545 | +3 | 12 |
| - | - | 1.808E+05 | 473.6 | - | - | 0 | - |
| - | - | 8.836E+04 | 473.9 | - | - | 0 | - |
| - | - | 1.725E+04 | 474.3 | - | - | 0 | - |
| - | - | 1.136E+04 | 474.6 | - | - | 0 | - |
| - | - | 3.363E+04 | 474.8 | - | - | 0 | - |
| - | - | 1.478E+04 | 475.3 | - | - | 0 | - |
| - | - | 1.165E+04 | 475.8 | - | - | 0 | - |
| - | - | 1.809E+04 | 475.9 | - | - | 0 | - |
| - | - | 4.417E+04 | 476.3 | - | - | 0 | - |
| - | - | 4.409E+04 | 476.6 | - | - | 0 | - |
| - | - | 2.189E+04 | 476.9 | - | - | 0 | - |
| - | - | 3.157E+04 | 478.2 | - | - | 0 | - |
| - | - | 1.112E+04 | 479.2 | - | - | 0 | - |
| 6 | y | 1.333E+05 | 480.8 | 0.001196 | 2.488 | +2 | 9 |
| - | - | 6.539E+04 | 481.3 | - | - | 0 | - |
| - | - | 2.005E+04 | 481.8 | - | - | 0 | - |
| - | - | 3.377E+04 | 481.9 | - | - | 0 | - |
| - | - | 5.634E+04 | 482.3 | - | - | 0 | - |
| - | - | 4.285E+04 | 482.3 | - | - | 0 | - |
| - | - | 1.746E+04 | 482.6 | - | - | 0 | - |
| - | - | 3.077E+04 | 482.8 | - | - | 0 | - |
| - | - | 6826 | 482.9 | - | - | 0 | - |
| - | - | 1.298E+04 | 483.3 | - | - | 0 | - |
| - | - | 9.344E+04 | 483.8 | - | - | 0 | - |
| - | - | 3.604E+04 | 484.3 | - | - | 0 | - |
| - | - | 1.456E+04 | 484.8 | - | - | 0 | - |
| - | - | 5.651E+04 | 491.3 | - | - | 0 | - |
| - | - | 8424 | 491.8 | - | - | 0 | - |
| - | - | 1.111E+04 | 492.3 | - | - | 0 | - |
| - | - | 9358 | 492.8 | - | - | 0 | - |
| - | - | 6608 | 494.3 | - | - | 0 | - |
| - | - | 8744 | 495.9 | - | - | 0 | - |
| - | - | 1.354E+04 | 496.3 | - | - | 0 | - |
| 8 | b | 2.509E+04 | 497.2 | 0.0008994 | 1.809 | +2 | 8 |
| - | - | 1.132E+04 | 497.7 | - | - | 0 | - |
| - | - | 1.455E+04 | 499.3 | - | - | 0 | - |
| - | - | 9.883E+04 | 501.6 | - | - | 0 | - |
| - | - | 1.193E+05 | 501.9 | - | - | 0 | - |
| - | - | 4.923E+04 | 502.3 | - | - | 0 | - |
| - | - | 1.058E+04 | 502.6 | - | - | 0 | - |
| - | - | 7919 | 502.9 | - | - | 0 | - |
| - | - | 4.019E+04 | 503.2 | - | - | 0 | - |
| 13 | b | 1.55E+05 | 504.9 | 0.001337 | 2.649 | +3 | 13 |
| 13 | b | 1.536E+05 | 505.3 | 0.00631 | 12.49 | +3 | 13 |
| - | - | 8.892E+04 | 505.6 | - | - | 0 | - |
| - | - | 8554 | 505.7 | - | - | 0 | - |
| - | - | 3.782E+04 | 505.9 | - | - | 0 | - |
| - | - | 1.21E+04 | 506.3 | - | - | 0 | - |
| - | - | 2.267E+04 | 508.8 | - | - | 0 | - |
| - | - | 1.325E+04 | 509.3 | - | - | 0 | - |
| - | - | 7015 | 510.3 | - | - | 0 | - |
| - | - | 1.422E+04 | 510.4 | - | - | 0 | - |
| 13 | b | 7.69E+05 | 510.9 | 0.001203 | 2.355 | +3 | 13 |
| - | - | 7.766E+05 | 511.3 | - | - | 0 | - |
| - | - | 3.714E+05 | 511.6 | - | - | 0 | - |
| - | - | 1.262E+05 | 511.9 | - | - | 0 | - |
| - | - | 4.024E+04 | 512.3 | - | - | 0 | - |
| - | - | 6983 | 513.9 | - | - | 0 | - |
| - | - | 1.281E+04 | 514.3 | - | - | 0 | - |
| - | - | 1.082E+04 | 514.6 | - | - | 0 | - |
| - | - | 1.164E+05 | 516.9 | - | - | 0 | - |
| - | - | 1.506E+05 | 517.3 | - | - | 0 | - |
| - | - | 5.355E+04 | 517.6 | - | - | 0 | - |
| - | - | 1.88E+04 | 517.9 | - | - | 0 | - |
| - | - | 8721 | 519.6 | - | - | 0 | - |
| 2 | y | 3.27E+04 | 519.9 | 0.002706 | 5.204 | +3 | 13 |
| - | - | 1.353E+04 | 520.2 | - | - | 0 | - |
| - | - | 4.587E+04 | 520.3 | - | - | 0 | - |
| - | - | 1.961E+04 | 520.4 | - | - | 0 | - |
| - | - | 1.203E+04 | 520.6 | - | - | 0 | - |
| - | - | 1.169E+04 | 521 | - | - | 0 | - |
| - | - | 3.802E+04 | 521.2 | - | - | 0 | - |
| - | - | 9948 | 522.3 | - | - | 0 | - |
| - | - | 1.667E+04 | 523.4 | - | - | 0 | - |
| - | - | 8607 | 524.8 | - | - | 0 | - |
| - | - | 1.384E+04 | 525.3 | - | - | 0 | - |
| 2 | y | 1.524E+05 | 525.6 | 0.001139 | 2.168 | +3 | 13 |
| - | - | 1.231E+04 | 525.8 | - | - | 0 | - |
| - | - | 1.382E+05 | 526 | - | - | 0 | - |
| - | - | 6.464E+04 | 526.3 | - | - | 0 | - |
| - | - | 1.238E+04 | 526.6 | - | - | 0 | - |
| - | - | 2.247E+04 | 528.2 | - | - | 0 | - |
| - | - | 2.18E+04 | 529.2 | - | - | 0 | - |
| - | - | 2.009E+04 | 529.8 | - | - | 0 | - |
| - | - | 2.944E+04 | 530.3 | - | - | 0 | - |
| - | - | 1.88E+04 | 530.8 | - | - | 0 | - |
| - | - | 2.527E+04 | 531.2 | - | - | 0 | - |
| - | - | 9472 | 531.3 | - | - | 0 | - |
| - | - | 8071 | 535.3 | - | - | 0 | - |
| - | - | 8055 | 535.6 | - | - | 0 | - |
| - | - | 8404 | 536.3 | - | - | 0 | - |
| - | - | 8499 | 537.3 | - | - | 0 | - |
| - | - | 3.788E+05 | 538.3 | - | - | 0 | - |
| - | - | 2.689E+04 | 538.4 | - | - | 0 | - |
| - | - | 4.996E+04 | 538.8 | - | - | 0 | - |
| - | - | 8473 | 539 | - | - | 0 | - |
| - | - | 1.027E+05 | 539.3 | - | - | 0 | - |
| - | - | 1.067E+05 | 539.3 | - | - | 0 | - |
| - | - | 7952 | 539.4 | - | - | 0 | - |
| - | - | 2.794E+04 | 539.6 | - | - | 0 | - |
| - | - | 4.315E+04 | 539.8 | - | - | 0 | - |
| - | - | 2.265E+04 | 540 | - | - | 0 | - |
| - | - | 1.785E+04 | 540.3 | - | - | 0 | - |
| - | - | 1.591E+04 | 540.3 | - | - | 0 | - |
| - | - | 1.458E+04 | 540.3 | - | - | 0 | - |
| 5 | y | 1.041E+04 | 540.8 | 0.005736 | 10.61 | +2 | 10 |
| - | - | 9069 | 541.3 | - | - | 0 | - |
| 10 | y | 1.431E+05 | 541.4 | 0.001688 | 3.118 | +1 | 5 |
| - | - | 2.057E+04 | 542.3 | - | - | 0 | - |
| - | - | 4.282E+04 | 542.4 | - | - | 0 | - |
| - | - | 2.122E+04 | 543 | - | - | 0 | - |
| - | - | 8641 | 543.3 | - | - | 0 | - |
| - | - | 1.409E+04 | 544.6 | - | - | 0 | - |
| - | - | 1.065E+04 | 544.8 | - | - | 0 | - |
| - | - | 9553 | 545.3 | - | - | 0 | - |
| - | - | 5.767E+04 | 547.8 | - | - | 0 | - |
| - | - | 1.603E+04 | 548.2 | - | - | 0 | - |
| - | - | 3.28E+04 | 548.3 | - | - | 0 | - |
| 0 | Precursor | 2.247E+05 | 548.6 | 0.001431 | 2.608 | +3 | -1 |
| - | - | 1.343E+04 | 548.8 | - | - | 0 | - |
| 0 | Precursor | 2.798E+05 | 549 | 0.00619 | 11.28 | +3 | -1 |
| - | - | 2.167E+04 | 549.2 | - | - | 0 | - |
| - | - | 9.997E+04 | 549.3 | - | - | 0 | - |
| 5 | y | 2.152E+05 | 549.3 | 0.001556 | 2.833 | +2 | 10 |
| - | - | 1.019E+05 | 549.6 | - | - | 0 | - |
| - | - | 1.463E+05 | 549.8 | - | - | 0 | - |
| - | - | 3.011E+04 | 550 | - | - | 0 | - |
| - | - | 8883 | 550.2 | - | - | 0 | - |
| - | - | 1.027E+04 | 550.3 | - | - | 0 | - |
| - | - | 5.712E+04 | 550.3 | - | - | 0 | - |
| - | - | 9268 | 550.8 | - | - | 0 | - |
| - | - | 1.316E+04 | 553.2 | - | - | 0 | - |
| - | - | 1.012E+04 | 553.3 | - | - | 0 | - |
| - | - | 1.745E+04 | 554 | - | - | 0 | - |
| - | - | 5.19E+04 | 554.3 | - | - | 0 | - |
| 0 | Precursor | 5.189E+06 | 554.6 | 0.001266 | 2.283 | +3 | -1 |
| - | - | 5.211E+06 | 555 | - | - | 0 | - |
| - | - | 2.845E+06 | 555.3 | - | - | 0 | - |
| - | - | 1.007E+06 | 555.6 | - | - | 0 | - |
| - | - | 1.146E+04 | 555.8 | - | - | 0 | - |
| - | - | 2.984E+05 | 556 | - | - | 0 | - |
| - | - | 1.696E+04 | 557.3 | - | - | 0 | - |
| 9 | b | 4.451E+04 | 561.3 | 0.0007201 | 1.283 | +2 | 9 |
| - | - | 3.164E+04 | 561.8 | - | - | 0 | - |
| - | - | 1.741E+04 | 562.3 | - | - | 0 | - |
| 4 | b | 4.073E+04 | 565.2 | 0.0008091 | 1.431 | +1 | 4 |
| - | - | 7880 | 565.3 | - | - | 0 | - |
| - | - | 4.603E+05 | 566.2 | - | - | 0 | - |
| - | - | 1.386E+05 | 567.3 | - | - | 0 | - |
| - | - | 1.747E+04 | 567.4 | - | - | 0 | - |
| - | - | 2.916E+04 | 568.3 | - | - | 0 | - |
| - | - | 9577 | 568.4 | - | - | 0 | - |
| - | - | 8.017E+04 | 570.2 | - | - | 0 | - |
| - | - | 2.26E+04 | 571.2 | - | - | 0 | - |
| - | - | 2.05E+05 | 572.8 | - | - | 0 | - |
| - | - | 1.663E+05 | 573.3 | - | - | 0 | - |
| - | - | 6.107E+04 | 573.8 | - | - | 0 | - |
| - | - | 8.962E+04 | 577.4 | - | - | 0 | - |
| - | - | 3.04E+04 | 578.4 | - | - | 0 | - |
| - | - | 2.89E+05 | 579.4 | - | - | 0 | - |
| - | - | 7.716E+04 | 580.4 | - | - | 0 | - |
| - | - | 2.468E+04 | 581.4 | - | - | 0 | - |
| - | - | 1.214E+04 | 587.3 | - | - | 0 | - |
| 10 | b | 4.613E+04 | 587.8 | 0.001423 | 2.42 | +2 | 10 |
| 10 | b | 3.324E+04 | 588.3 | 0.009232 | 15.69 | +2 | 10 |
| - | - | 7819 | 588.3 | - | - | 0 | - |
| - | - | 1.163E+04 | 588.8 | - | - | 0 | - |
| - | - | 7333 | 589.3 | - | - | 0 | - |
| - | - | 6850 | 595.4 | - | - | 0 | - |
| - | - | 8.609E+04 | 595.4 | - | - | 0 | - |
| - | - | 1.836E+04 | 595.8 | - | - | 0 | - |
| - | - | 1.012E+04 | 596.3 | - | - | 0 | - |
| - | - | 2.911E+04 | 596.4 | - | - | 0 | - |
| 10 | b | 5.225E+04 | 596.8 | 0.0004738 | 0.7939 | +2 | 10 |
| - | - | 8374 | 596.8 | - | - | 0 | - |
| - | - | 1.735E+04 | 597.3 | - | - | 0 | - |
| - | - | 1.433E+04 | 597.8 | - | - | 0 | - |
| - | - | 3.298E+04 | 598.2 | - | - | 0 | - |
| - | - | 1.491E+04 | 599.2 | - | - | 0 | - |
| - | - | 2.174E+04 | 602.8 | - | - | 0 | - |
| - | - | 9925 | 603.3 | - | - | 0 | - |
| - | - | 1.137E+05 | 604.4 | - | - | 0 | - |
| 4 | y | 1.696E+05 | 604.8 | 0.005562 | 9.195 | +2 | 11 |
| - | - | 9.958E+04 | 605.4 | - | - | 0 | - |
| - | - | 3.652E+04 | 605.9 | - | - | 0 | - |
| - | - | 1.083E+04 | 606.4 | - | - | 0 | - |
| - | - | 1.084E+04 | 606.8 | - | - | 0 | - |
| - | - | 1.355E+04 | 607.3 | - | - | 0 | - |
| - | - | 4.753E+04 | 610.3 | - | - | 0 | - |
| - | - | 2.598E+04 | 611.3 | - | - | 0 | - |
| - | - | 2.778E+04 | 611.8 | - | - | 0 | - |
| - | - | 2.241E+04 | 612.3 | - | - | 0 | - |
| - | - | 1.084E+04 | 612.8 | - | - | 0 | - |
| 4 | y | 1.459E+05 | 613.4 | 0.001076 | 1.754 | +2 | 11 |
| - | - | 1.208E+05 | 613.9 | - | - | 0 | - |
| - | - | 4.962E+04 | 614.4 | - | - | 0 | - |
| - | - | 1.752E+04 | 615.3 | - | - | 0 | - |
| - | - | 9.504E+04 | 615.4 | - | - | 0 | - |
| - | - | 5.95E+04 | 615.9 | - | - | 0 | - |
| - | - | 2.524E+04 | 616.4 | - | - | 0 | - |
| - | - | 9211 | 616.9 | - | - | 0 | - |
| - | - | 8061 | 618.9 | - | - | 0 | - |
| - | - | 1.942E+05 | 620.3 | - | - | 0 | - |
| - | - | 1.117E+05 | 620.8 | - | - | 0 | - |
| - | - | 7.196E+04 | 621.3 | - | - | 0 | - |
| - | - | 1.15E+04 | 621.4 | - | - | 0 | - |
| - | - | 1.81E+04 | 621.8 | - | - | 0 | - |
| - | - | 2.157E+04 | 623.4 | - | - | 0 | - |
| - | - | 1.85E+04 | 628.3 | - | - | 0 | - |
| - | - | 4.447E+05 | 629.3 | - | - | 0 | - |
| - | - | 3.211E+05 | 629.8 | - | - | 0 | - |
| - | - | 1.228E+05 | 630.3 | - | - | 0 | - |
| - | - | 2.439E+04 | 630.9 | - | - | 0 | - |
| - | - | 1.425E+04 | 632.3 | - | - | 0 | - |
| - | - | 4.701E+04 | 638.4 | - | - | 0 | - |
| - | - | 2.743E+04 | 638.9 | - | - | 0 | - |
| - | - | 1.36E+04 | 639.4 | - | - | 0 | - |
| - | - | 8453 | 643.3 | - | - | 0 | - |
| 11 | b | 2.988E+04 | 645.3 | 3.179E-05 | 0.04926 | +2 | 11 |
| - | - | 1.783E+04 | 645.8 | - | - | 0 | - |
| - | - | 1.878E+04 | 647.4 | - | - | 0 | - |
| - | - | 2.811E+04 | 653.8 | - | - | 0 | - |
| - | - | 1.676E+04 | 654.3 | - | - | 0 | - |
| - | - | 1.552E+04 | 654.8 | - | - | 0 | - |
| - | - | 1.212E+04 | 656.3 | - | - | 0 | - |
| - | - | 3.188E+04 | 657.3 | - | - | 0 | - |
| - | - | 7241 | 657.3 | - | - | 0 | - |
| - | - | 9215 | 657.8 | - | - | 0 | - |
| - | - | 2.118E+04 | 658.3 | - | - | 0 | - |
| - | - | 1.614E+04 | 659.3 | - | - | 0 | - |
| - | - | 9.366E+04 | 664.5 | - | - | 0 | - |
| - | - | 3.479E+04 | 665.5 | - | - | 0 | - |
| - | - | 1.391E+04 | 665.8 | - | - | 0 | - |
| - | - | 8562 | 666.5 | - | - | 0 | - |
| - | - | 8398 | 667.4 | - | - | 0 | - |
| - | - | 1.468E+04 | 668.3 | - | - | 0 | - |
| 9 | y | 4.997E+04 | 669.5 | 0.001512 | 2.259 | +1 | 6 |
| - | - | 2.53E+04 | 670.5 | - | - | 0 | - |
| - | - | 1.354E+04 | 671.9 | - | - | 0 | - |
| - | - | 1.824E+04 | 672.4 | - | - | 0 | - |
| - | - | 7015 | 672.9 | - | - | 0 | - |
| - | - | 1.024E+05 | 674.3 | - | - | 0 | - |
| - | - | 3.096E+05 | 674.4 | - | - | 0 | - |
| - | - | 4.485E+04 | 675.3 | - | - | 0 | - |
| - | - | 1.346E+05 | 675.4 | - | - | 0 | - |
| - | - | 2.866E+04 | 676.4 | - | - | 0 | - |
| - | - | 5.597E+04 | 677.4 | - | - | 0 | - |
| - | - | 4.26E+04 | 677.9 | - | - | 0 | - |
| - | - | 2.535E+04 | 678.4 | - | - | 0 | - |
| 5 | b | 3.237E+04 | 684.3 | 0.00245 | 3.58 | +1 | 5 |
| 5 | b | 1.113E+04 | 685.3 | 0.01306 | 19.06 | +1 | 5 |
| - | - | 9.557E+04 | 685.9 | - | - | 0 | - |
| 3 | y | 8.985E+04 | 686.4 | 0.008138 | 11.86 | +2 | 12 |
| - | - | 4.306E+04 | 686.9 | - | - | 0 | - |
| - | - | 1.816E+04 | 687.4 | - | - | 0 | - |
| - | - | 3.284E+05 | 692.4 | - | - | 0 | - |
| - | - | 1.275E+05 | 693.4 | - | - | 0 | - |
| - | - | 8755 | 694.4 | - | - | 0 | - |
| - | - | 3.006E+04 | 694.5 | - | - | 0 | - |
| 3 | y | 1.608E+06 | 694.9 | 0.001333 | 1.918 | +2 | 12 |
| - | - | 1.235E+06 | 695.4 | - | - | 0 | - |
| - | - | 5.923E+05 | 695.9 | - | - | 0 | - |
| - | - | 1.617E+05 | 696.4 | - | - | 0 | - |
| - | - | 3.498E+04 | 696.9 | - | - | 0 | - |
| - | - | 3.149E+04 | 698.4 | - | - | 0 | - |
| 12 | b | 1.464E+04 | 700.4 | 0.01258 | 17.96 | +2 | 12 |
| 12 | b | 1.764E+04 | 700.8 | 0.005925 | 8.454 | +2 | 12 |
| - | - | 7202 | 701.4 | - | - | 0 | - |
| - | - | 8516 | 701.9 | - | - | 0 | - |
| 5 | b | 1.803E+05 | 702.3 | 0.001529 | 2.177 | +1 | 5 |
| - | - | 8.219E+04 | 703.3 | - | - | 0 | - |
| - | - | 2.179E+04 | 704.3 | - | - | 0 | - |
| - | - | 4.757E+04 | 707.3 | - | - | 0 | - |
| - | - | 2.438E+04 | 708.3 | - | - | 0 | - |
| - | - | 1.097E+04 | 708.5 | - | - | 0 | - |
| 12 | b | 3.542E+05 | 709.4 | 0.001562 | 2.201 | +2 | 12 |
| - | - | 2.816E+05 | 709.9 | - | - | 0 | - |
| - | - | 1.215E+05 | 710.4 | - | - | 0 | - |
| - | - | 4.644E+04 | 710.5 | - | - | 0 | - |
| - | - | 3.384E+04 | 710.9 | - | - | 0 | - |
| - | - | 1.672E+04 | 711.4 | - | - | 0 | - |
| - | - | 1.613E+04 | 711.5 | - | - | 0 | - |
| - | - | 8200 | 712.5 | - | - | 0 | - |
| - | - | 7646 | 713.4 | - | - | 0 | - |
| - | - | 1.313E+04 | 714.4 | - | - | 0 | - |
| - | - | 5.608E+04 | 716.4 | - | - | 0 | - |
| - | - | 2.424E+04 | 717.4 | - | - | 0 | - |
| - | - | 6861 | 718.4 | - | - | 0 | - |
| - | - | 1.107E+04 | 720.3 | - | - | 0 | - |
| - | - | 1.607E+04 | 720.4 | - | - | 0 | - |
| - | - | 2.52E+04 | 721.4 | - | - | 0 | - |
| - | - | 8852 | 722.4 | - | - | 0 | - |
| - | - | 1.607E+04 | 722.9 | - | - | 0 | - |
| - | - | 1.317E+04 | 723.4 | - | - | 0 | - |
| - | - | 2.841E+04 | 724.3 | - | - | 0 | - |
| - | - | 1.219E+04 | 725.3 | - | - | 0 | - |
| 8 | y | 1.241E+05 | 726.5 | 0.0009837 | 1.354 | +1 | 7 |
| - | - | 4.533E+04 | 727.5 | - | - | 0 | - |
| - | - | 4.587E+04 | 735.3 | - | - | 0 | - |
| - | - | 1.335E+04 | 736.3 | - | - | 0 | - |
| - | - | 1.352E+04 | 737.4 | - | - | 0 | - |
| - | - | 1.479E+04 | 738.4 | - | - | 0 | - |
| - | - | 1.147E+04 | 739.4 | - | - | 0 | - |
| - | - | 8379 | 740.4 | - | - | 0 | - |
| - | - | 1.574E+04 | 751.9 | - | - | 0 | - |
| - | - | 3.741E+04 | 752.3 | - | - | 0 | - |
| - | - | 2.184E+04 | 752.4 | - | - | 0 | - |
| - | - | 2.122E+04 | 753.3 | - | - | 0 | - |
| 13 | b | 3.511E+04 | 756.9 | 0.002104 | 2.78 | +2 | 13 |
| 13 | b | 4.248E+04 | 757.4 | 0.01077 | 14.22 | +2 | 13 |
| - | - | 3.792E+04 | 757.9 | - | - | 0 | - |
| - | - | 1.457E+04 | 758.4 | - | - | 0 | - |
| 13 | b | 1.155E+05 | 765.9 | 0.001949 | 2.545 | +2 | 13 |
| - | - | 9326 | 766.3 | - | - | 0 | - |
| - | - | 1.253E+05 | 766.4 | - | - | 0 | - |
| - | - | 5.288E+04 | 766.9 | - | - | 0 | - |
| - | - | 1.945E+04 | 767.4 | - | - | 0 | - |
| - | - | 5.696E+04 | 774.9 | - | - | 0 | - |
| - | - | 5.467E+04 | 775.4 | - | - | 0 | - |
| - | - | 1.802E+04 | 775.9 | - | - | 0 | - |
| - | - | 1.027E+04 | 776.4 | - | - | 0 | - |
| - | - | 1.147E+04 | 777.5 | - | - | 0 | - |
| 2 | y | 7881 | 779.4 | 0.006812 | 8.739 | +2 | 13 |
| - | - | 7160 | 780.3 | - | - | 0 | - |
| - | - | 9813 | 787.5 | - | - | 0 | - |
| 2 | y | 4.136E+04 | 787.9 | 0.002204 | 2.797 | +2 | 13 |
| - | - | 4.225E+04 | 788.4 | - | - | 0 | - |
| - | - | 1.707E+04 | 788.9 | - | - | 0 | - |
| - | - | 7528 | 789.4 | - | - | 0 | - |
| - | - | 9889 | 792.9 | - | - | 0 | - |
| - | - | 1.476E+04 | 793.4 | - | - | 0 | - |
| - | - | 2.456E+04 | 794.3 | - | - | 0 | - |
| - | - | 1.537E+04 | 795.4 | - | - | 0 | - |
| - | - | 1.869E+04 | 801.5 | - | - | 0 | - |
| - | - | 6.207E+04 | 805.5 | - | - | 0 | - |
| - | - | 3.124E+04 | 806.5 | - | - | 0 | - |
| - | - | 1.428E+04 | 807.5 | - | - | 0 | - |
| - | - | 1.94E+05 | 811.4 | - | - | 0 | - |
| - | - | 5.781E+04 | 811.5 | - | - | 0 | - |
| - | - | 8.429E+04 | 812.4 | - | - | 0 | - |
| - | - | 2.823E+04 | 812.5 | - | - | 0 | - |
| - | - | 2.217E+04 | 813.4 | - | - | 0 | - |
| - | - | 8896 | 814.4 | - | - | 0 | - |
| 6 | b | 2.843E+04 | 821.3 | 0.0003616 | 0.4403 | +1 | 6 |
| 6 | b | 1.934E+04 | 822.3 | 0.01268 | 15.42 | +1 | 6 |
| - | - | 8178 | 822.5 | - | - | 0 | - |
| - | - | 1.612E+04 | 823.3 | - | - | 0 | - |
| 7 | y | 7.55E+05 | 823.5 | 0.001259 | 1.529 | +1 | 8 |
| - | - | 3.434E+05 | 824.5 | - | - | 0 | - |
| - | - | 9.12E+04 | 825.5 | - | - | 0 | - |
| - | - | 1.205E+04 | 826.5 | - | - | 0 | - |
| - | - | 5.635E+04 | 829.5 | - | - | 0 | - |
| - | - | 2.715E+04 | 830.5 | - | - | 0 | - |
| - | - | 1.081E+04 | 831.5 | - | - | 0 | - |
| 6 | b | 4.082E+05 | 839.4 | 0.001027 | 1.224 | +1 | 6 |
| - | - | 2.089E+05 | 840.4 | - | - | 0 | - |
| - | - | 6.039E+04 | 841.4 | - | - | 0 | - |
| - | - | 8708 | 842.4 | - | - | 0 | - |
| - | - | 1.126E+04 | 847.5 | - | - | 0 | - |
| - | - | 6.824E+04 | 848.4 | - | - | 0 | - |
| - | - | 3.741E+04 | 849.4 | - | - | 0 | - |
| - | - | 7036 | 850.4 | - | - | 0 | - |
| - | - | 3.522E+04 | 853.5 | - | - | 0 | - |
| - | - | 2.209E+04 | 854.5 | - | - | 0 | - |
| - | - | 9240 | 866.4 | - | - | 0 | - |
| - | - | 2.363E+04 | 891.5 | - | - | 0 | - |
| - | - | 1.069E+04 | 892.5 | - | - | 0 | - |
| - | - | 1.39E+05 | 901.4 | - | - | 0 | - |
| - | - | 8.528E+04 | 902.4 | - | - | 0 | - |
| - | - | 1.874E+04 | 903.4 | - | - | 0 | - |
| - | - | 7.072E+04 | 919.5 | - | - | 0 | - |
| - | - | 5.287E+04 | 920.5 | - | - | 0 | - |
| - | - | 9001 | 921.5 | - | - | 0 | - |
| - | - | 4.106E+04 | 942.6 | - | - | 0 | - |
| - | - | 3.6E+04 | 943.6 | - | - | 0 | - |
| - | - | 7015 | 944.6 | - | - | 0 | - |
| - | - | 1.496E+04 | 948.6 | - | - | 0 | - |
| 6 | y | 1.381E+05 | 960.6 | 0.0007582 | 0.7893 | +1 | 9 |
| - | - | 9498 | 961.5 | - | - | 0 | - |
| - | - | 7.036E+04 | 961.6 | - | - | 0 | - |
| - | - | 1.848E+04 | 962.6 | - | - | 0 | - |
| - | - | 7141 | 963.6 | - | - | 0 | - |
| - | - | 1.157E+04 | 964.5 | - | - | 0 | - |
| - | - | 3.366E+04 | 966.6 | - | - | 0 | - |
| - | - | 1.855E+04 | 967.6 | - | - | 0 | - |
| 8 | b | 1.223E+04 | 993.4 | 0.00246 | 2.477 | +1 | 8 |
| - | - | 8758 | 994.4 | - | - | 0 | - |
| - | - | 1.618E+04 | 1010 | - | - | 0 | - |
| - | - | 1.013E+04 | 1011 | - | - | 0 | - |
| - | - | 7233 | 1017 | - | - | 0 | - |
| - | - | 2.248E+04 | 1033 | - | - | 0 | - |
| - | - | 3.18E+04 | 1034 | - | - | 0 | - |
| - | - | 1.132E+04 | 1035 | - | - | 0 | - |
| - | - | 1.346E+04 | 1088 | - | - | 0 | - |
| 5 | y | 4.842E+04 | 1098 | 0.0008673 | 0.7902 | +1 | 10 |
| - | - | 2.957E+04 | 1099 | - | - | 0 | - |
| - | - | 1.661E+04 | 1100 | - | - | 0 | - |
| 9 | b | 3.371E+04 | 1122 | 0.0004159 | 0.3708 | +1 | 9 |
| - | - | 2.669E+04 | 1123 | - | - | 0 | - |
| - | - | 1.856E+04 | 1145 | - | - | 0 | - |
| - | - | 1.174E+04 | 1146 | - | - | 0 | - |
| - | - | 1.094E+04 | 1147 | - | - | 0 | - |
| - | - | 8888 | 1165 | - | - | 0 | - |
| 10 | b | 7.48E+04 | 1175 | 0.0001322 | 0.1126 | +1 | 10 |
| 10 | b | 5.982E+04 | 1176 | 0.01781 | 15.15 | +1 | 10 |
| - | - | 1.398E+04 | 1177 | - | - | 0 | - |
| 10 | b | 2.849E+04 | 1193 | 0.0001674 | 0.1403 | +1 | 10 |
| - | - | 2.378E+04 | 1194 | - | - | 0 | - |
| - | - | 8384 | 1195 | - | - | 0 | - |
| - | - | 7175 | 2015 | - | - | 0 | - |
| - | - | 5725 | 2188 | - | - | 0 | - |
| - | - | 7062 | 2709 | - | - | 0 | - |
| - | - | 6881 | 2710 | - | - | 0 | - |
| - | - | 6924 | 2926 | - | - | 0 | - |
| - | - | 6994 | 3255 | - | - | 0 | - |
| - | - | 6799 | 3299 | - | - | 0 | - |

m/z Charge Intensity FragmentType MassShift Position
120.05616760253906 0 9335.62
122.07197570800781 0 6659.9927
123.0558090209961 0 19080.555
124.70914459228516 0 3436.7227
124.9344711303711 0 3274.5254
127.08711242675781 0 30055.32
128.07115173339844 0 8069.1245
129.0662841796875 0 42348.914
129.102783203125 0 1118315
130.06564331054688 0 277525.47
130.10052490234375 0 9543.871
130.1061248779297 0 74802.46
131.06900024414062 0 33037.586
131.07334899902344 0 3757.7832
132.08131408691406 0 1143495
132.1024169921875 0 219479.03 y 13
132.19078063964844 0 3974.8774
133.0846405029297 0 117340.555
133.100341796875 0 5136.6074
133.1058807373047 0 15852.439
134.08807373046875 0 8367.782
136.07620239257812 0 674938.4
137.0795135498047 0 60880.883
137.1079559326172 0 6766.6504
138.06661987304688 0 25519.441
139.08705139160156 0 15255.027
141.10279846191406 0 84560.4
142.06573486328125 0 6308.2915
142.10617065429688 0 5891.849
144.08135986328125 0 22313.717
145.06134033203125 0 6934.351
146.12893676757812 0 3907.8625
147.04469299316406 0 12179.828
149.07159423828125 0 4032.319
151.08688354492188 0 8066.132
152.08226013183594 0 28280.902
152.1438446044922 0 24125.053
152.1763916015625 0 3983.2795
155.0819549560547 0 46926.58
155.09329223632812 0 28209.715
155.11846923828125 0 40196.23
156.077392578125 0 37093.418
156.12193298339844 0 5915.679
156.56243896484375 0 4379.274
157.0765838623047 0 5222.6543
158.0603485107422 0 15114.788
158.0843963623047 0 113487.586
158.09616088867188 0 4796.414
159.0922088623047 0 523318.3
160.0760955810547 0 26112.61
160.08892822265625 0 9370.707
160.0955810546875 0 53544.457
163.0257568359375 0 3731.6477
164.07093811035156 0 9646.402
165.10275268554688 0 31646.193
166.0616455078125 0 13972.028
166.1056671142578 0 5961.577
168.11373901367188 0 15778.89
169.0766143798828 0 57790.215
169.08938598632812 0 7118.894
169.0977020263672 0 104202.33
169.1054229736328 0 8155.405
169.17025756835938 0 6227.7715
170.06057739257812 0 170683.12
170.07925415039062 0 7699.3335
170.10159301757812 0 7504.8706
171.06385803222656 0 25093.46
171.09307861328125 0 4834.1284
172.10861206054688 0 74161.164
173.45156860351562 0 13610.314
175.0980224609375 0 7369.801
176.0823974609375 0 25213.129
179.0823211669922 0 5633.8
180.0773162841797 0 10024.161
181.06118774414062 0 6276.806
181.09776306152344 0 215507.12
181.13426208496094 0 21115.016
182.10107421875 0 15332.58
182.1294403076172 0 37633.22
183.09225463867188 0 159801.31
183.1014404296875 0 7268.872
184.07595825195312 0 14925.828
184.09555053710938 0 20810.176
185.07151794433594 0 37661.85
185.10772705078125 0 5694.668
186.07937622070312 0 5268.1377
186.1243133544922 0 118114.24
187.0872039794922 0 987835.2
188.08184814453125 0 8069.2593
188.0905303955078 0 111190.48
189.09371948242188 0 5585.9883
193.1328887939453 0 4645.0835
195.14976501464844 0 13921.081
195.46792602539062 0 4199.9097
197.1653289794922 0 57549.9
198.10250854492188 0 7548.4243
198.16844177246094 0 7762.555
199.08706665039062 0 37072.043
199.10906982421875 0 7877.309
199.18084716796875 0 34543.895
200.08987426757812 0 5812.009
200.1400604248047 0 26552.207
201.10287475585938 0 88360.74
202.08676147460938 0 20983.51
202.10894775390625 0 112193.125
203.11375427246094 0 7745.506
204.07696533203125 0 12048.505
205.09771728515625 0 5666.3667 a 2
207.14979553222656 0 51353.83
208.1546630859375 0 5429.759
209.1288299560547 0 8918.301
211.08697509765625 0 24664.346
213.0769805908203 0 7637.709
214.109375 0 42344.957
216.11386108398438 0 11271.906
217.1086883544922 0 14891.007
219.0883026123047 0 7250.48
220.11990356445312 0 90473.81
221.10403442382812 0 115732.06
221.12278747558594 0 6808.0225
222.10667419433594 0 9394.17
222.1239471435547 0 7204.1943
224.11892700195312 0 21244.07
224.139892578125 0 5148.806
224.17640686035156 0 62148.953
225.0978546142578 0 6525.155
225.160400390625 0 11087.711
225.18038940429688 0 8315.584
226.1557159423828 0 1406759.4
227.08370971679688 0 5359.7646
227.1405029296875 0 7681.973
227.15895080566406 0 170510.66 y Ammonia loss 10
227.17684936523438 0 10991.877
228.11380004882812 0 103440.34 a Water loss 1
228.13424682617188 0 11024.911
228.16183471679688 0 14365.5205
229.09783935546875 0 111556.36 b Ammonia loss 4
229.11837768554688 0 16425.297
230.10284423828125 0 30623.05
231.08851623535156 0 48588.3
231.13583374023438 0 22396.572
232.11984252929688 0 6239.7407
233.1042022705078 0 14283.884
234.13497924804688 0 7601.397
235.11964416503906 0 201742.66
236.12283325195312 0 23739.8
236.13963317871094 0 9655.861
238.1304168701172 0 21697.193
239.08250427246094 0 10833.235
239.15101623535156 0 79080.57
240.15496826171875 0 10321.354
241.15573120117188 0 12052.871
242.1869659423828 0 25493.877
243.1824188232422 0 19785.414
244.10858154296875 0 7273.6934
244.1664276123047 0 6257.1113
245.17144775390625 0 6668.2812
245.1754608154297 0 6263.3955
245.18673706054688 0 446228.75 y 12
246.12449645996094 0 2715933.8 a 1
246.19009399414062 0 60310.336
247.1292266845703 0 620182.06
247.1925506591797 0 6479.733
248.11473083496094 0 154880.08
248.13165283203125 0 51341.92
249.0989227294922 0 197203.03
249.116943359375 0 16704.982
250.1021728515625 0 21389.295
250.12240600585938 0 13191.739
251.1294403076172 0 7558.378
251.1505126953125 0 7001.0874
252.1714630126953 0 26156.098
253.17433166503906 0 4741.88
255.07656860351562 0 6719.5337
256.1087341308594 0 70921.73 b Water loss 1
257.0926208496094 0 16829.86
257.1147155761719 0 22649.584
257.1615295410156 0 26784.984
258.07586669921875 0 14491.419
258.0992431640625 0 339805.75
259.0798645019531 0 5516.078
259.1025695800781 0 49287.285
259.13018798828125 0 12496.638
260.107177734375 0 26190.941
260.1977233886719 0 20399.387
262.15533447265625 0 11328.981
263.11383056640625 0 12441.438
264.1356506347656 0 10019.675
265.1410217285156 0 23355.547
265.16656494140625 0 14311.818
266.1253662109375 0 177414
267.12872314453125 0 22008.059
267.1448059082031 0 7762.5034
270.1818542480469 0 66167.695
271.1846923828125 0 16128.07 y 9
273.1098327636719 0 12760.724
273.1356201171875 0 11203.482
274.11932373046875 0 1647415.8 b 1
275.1036682128906 0 57140.742
275.12481689453125 0 704707.1
276.10833740234375 0 26697.016
276.1277160644531 0 84217.54
277.1334533691406 0 14701.645
279.1817626953125 0 19559.48
283.15191650390625 0 32652.26
283.1772766113281 0 225473.25
284.1805114746094 0 31925.945
285.1106872558594 0 14630.189
285.1564025878906 0 68566.94
285.1817321777344 0 6745.541
286.1598815917969 0 14801.721
290.11474609375 0 7176.827
290.1850280761719 0 64541.832
290.6862487792969 0 22121.176
291.15740966796875 0 20649.988
292.1299743652344 0 183955.62
293.133544921875 0 25510.998
294.1204833984375 0 84699.06
295.1250305175781 0 9712.438
297.1929931640625 0 190233.25
298.1966247558594 0 39406.207
300.2039794921875 0 8582.431
301.1283874511719 0 6115.1646
303.12103271484375 0 44529.656
303.21820068359375 0 5623.856
305.12921142578125 0 47512.19
305.6658630371094 0 13295.827
306.13250732421875 0 9337.615
308.1256408691406 0 8132.6724
309.1671142578125 0 8258.72
311.24505615234375 0 42363.547
312.24871826171875 0 7756.7427
313.2240295410156 0 10226.582
314.67071533203125 0 6045.4463
318.194091796875 0 11904.788
319.17791748046875 0 6397.0493
320.1252746582031 0 49220.637
320.1519470214844 0 7928.6553
321.1293640136719 0 15936.065
321.22943115234375 0 232441.69
322.1560363769531 0 71418.16
322.2326965332031 0 45583.86
323.15924072265625 0 15261.236
323.2347717285156 0 6903.809
324.2165222167969 0 19233.986
324.71795654296875 0 5589.138
325.2220458984375 0 5249.0967
326.2196350097656 0 47582.566
332.7294616699219 0 26728.617
333.1235656738281 0 14432.355
333.2313232421875 0 14610.018
333.7332763671875 0 7653.9946
335.2377624511719 0 11353.572 y 8
336.14605712890625 0 8532.301
336.2039794921875 0 289833.1
337.2067565917969 0 51131.3
337.72216796875 0 56847.055
338.1629943847656 0 9333.754
338.223388671875 0 22023.395
339.2400207519531 0 229689.67
340.15289306640625 0 21002.762
340.2432861328125 0 35333.688
341.2192687988281 0 6086.233
342.1678466796875 0 9682.417
343.21343994140625 0 6056.0127
346.7272033691406 0 212291.3
347.22845458984375 0 89695.195
347.7292175292969 0 19279.113
348.1461486816406 0 7925.216
349.7102966308594 0 12056.701
350.1512756347656 0 24106.982
350.2115173339844 0 6491.488
351.2400817871094 0 29466.773
353.2188720703125 0 15396.864
354.171875 0 12280.731
354.2144775390625 0 419646.88
354.6564636230469 0 8092.513
355.2177734375 0 61179.363
355.2704162597656 0 7398.4497
355.73291015625 0 27803.031
356.17333984375 0 7457.705
357.1798095703125 0 13665.792
357.25079345703125 0 44893.76
358.16326904296875 0 52778.547
358.2531433105469 0 8792.142
358.71514892578125 0 22694.41
359.16632080078125 0 9234.92
359.2159423828125 0 9228.004
362.6706237792969 0 9392.98
363.7481994628906 0 12359.7295 y 7
365.2541809082031 0 7155.263
366.1571350097656 0 28124.268
367.1623840332031 0 22410.143
368.1481628417969 0 34125.93
368.1725158691406 0 17430.443
369.1521911621094 0 9912.5205
369.1775817871094 0 8173.884
370.2459716796875 0 11646.16
373.2818298339844 0 95847.75 y 11
374.2586975097656 0 7839.5117
374.2848205566406 0 20762.229
376.66680908203125 0 8558.081
380.2291259765625 0 18118.654
382.2076416015625 0 15183.041
382.2816467285156 0 21739.969
383.2662658691406 0 18179.682
384.1678466796875 0 53207.24
385.17364501953125 0 151975.75
385.1997985839844 0 56716.3
386.1578063964844 0 84845.125
386.2010498046875 0 15352.845
387.16082763671875 0 20263.428
388.16351318359375 0 7292.1743
389.2701416015625 0 6281.5317
392.162109375 0 15188.744
392.2666015625 0 59727.902
393.1685485839844 0 12016.374
393.271728515625 0 14417.631
394.1519775390625 0 105970.32
394.2812194824219 0 11009.546
395.15460205078125 0 23317.27
396.2617492675781 0 25314.746
397.2431945800781 0 9455.714
397.6722412109375 0 9755.078
398.2414245605469 0 10493.849
401.1943664550781 0 19814.297
402.2259521484375 0 44825.773
403.18475341796875 0 141546.62
403.22723388671875 0 10435.997
403.2695617675781 0 28029.62
403.76861572265625 0 20392.943
404.1878356933594 0 31029.691
404.2680358886719 0 7758.9824
406.1861877441406 0 68078.18
406.2511901855469 0 51693.54
406.6879577636719 0 31167.176
406.72357177734375 0 6069.5327
406.7532958984375 0 29998.45
407.1920471191406 0 12102.561
407.51885986328125 0 6344.759
408.2220764160156 0 8628.369
410.2774658203125 0 122318.6
411.17864990234375 0 192810.92 b Water loss 5
411.2803039550781 0 21063.527
412.1603698730469 0 18577.014
412.1824035644531 0 30896.164
412.27484130859375 0 554760.8 y 6
412.77618408203125 0 243164.78
413.1649475097656 0 6892.3257
413.278076171875 0 65786.125
413.7796936035156 0 8732.272
415.2572021484375 0 51032.348
415.75921630859375 0 25679.229
419.8998718261719 0 21427.469
420.1834411621094 0 82191.53 b 5
420.2355041503906 0 35500.918
420.68560791015625 0 38731.918
421.18548583984375 0 8083.7734
422.2774353027344 0 47370.312
422.31011962890625 0 10708.876
423.27685546875 0 26093.96
424.3294372558594 0 14548.67
424.712890625 0 12093.402
425.2878723144531 0 18164.248
425.70782470703125 0 5872.761
427.2444152832031 0 31980.307
427.7464294433594 0 24108.012
428.2488098144531 0 6159.7964
428.2876281738281 0 9592.296
429.18939208984375 0 666157.9
430.1921081542969 0 172867.64
431.1805419921875 0 86999.875
432.1836853027344 0 26710.416
433.25567626953125 0 11618.637
437.1831359863281 0 128733.88 b 2
438.1865234375 0 31510.328
438.55767822265625 0 18013.38
438.8912658691406 0 18129.271
439.3043212890625 0 17853.748
444.23321533203125 0 11466.675
444.5667724609375 0 10729.029
445.2688903808594 0 13513.377
446.2198791503906 0 11475.445
450.2823181152344 0 10465.284
451.2268981933594 0 26646.562
451.26800537109375 0 39401.75
451.7270812988281 0 14244.078
451.9196472167969 0 12242.415
452.324951171875 0 15136.305
454.2099914550781 0 30542.754
455.2157897949219 0 10674.726
455.253173828125 0 42711.8
456.2566223144531 0 13795.997
457.183837890625 0 21628.625
457.5955810546875 0 12081.68
457.9277038574219 0 8694.713 y Ammonia loss 2
460.23193359375 0 25735.11
461.1826171875 0 12118.809
461.2418518066406 0 7658.0166
463.27801513671875 0 12810.578
463.5992431640625 0 140439.72 y 2
463.9337463378906 0 91437.46
464.2683410644531 0 31400.72
464.6015625 0 12638.36
466.9214782714844 0 8286.568
467.26031494140625 0 9578.594
467.2991027832031 0 61346.848
468.29754638671875 0 31254.605
468.71234130859375 0 8547.56 b 6
469.7890625 0 11200.057
470.25067138671875 0 17082.436
470.3349304199219 0 413731.84 y 10
470.58099365234375 0 22590.375
470.9086608886719 0 6506.874
471.3381042480469 0 113650.83
471.79949951171875 0 40300.55
472.2992858886719 0 20373.889
472.3403625488281 0 19130.426
472.80352783203125 0 7167.2866
473.2439880371094 0 138893.08 b 11
473.5778503417969 0 180771.47
473.9122009277344 0 88357.17
474.2660827636719 0 17248.443
474.5819396972656 0 11361.611
474.7809143066406 0 33626.98
475.280029296875 0 14776.197
475.7847900390625 0 11649.241
475.9239807128906 0 18085.26
476.2543640136719 0 44168.652
476.5879821777344 0 44088.97
476.9215087890625 0 21886.346
478.2090759277344 0 31570.867
479.2145080566406 0 11119.31
480.8042907714844 0 133343.08 y 5
481.30621337890625 0 65388.355
481.8053894042969 0 20047.326
481.9276428222656 0 33772.074
482.2629089355469 0 56340.824
482.3094482421875 0 42847.953
482.5958251953125 0 17464.348
482.7625732421875 0 30772.877
482.9305114746094 0 6825.8857
483.2604675292969 0 12977.503
483.7864074707031 0 93440.96
484.2870788574219 0 36042.438
484.7891540527344 0 14560.746
491.2732238769531 0 56511.215
491.77899169921875 0 8424.475
492.2769470214844 0 11108.826
492.7900695800781 0 9358.016
494.26556396484375 0 6607.859
495.93280029296875 0 8743.5205
496.265869140625 0 13540.742
497.2208251953125 0 25086.238 b 7
497.7239074707031 0 11323.206
499.25970458984375 0 14553.08
501.6067199707031 0 98825.87
501.94085693359375 0 119302.44
502.2747497558594 0 49225.74
502.6106872558594 0 10582.813
502.94207763671875 0 7919.054
503.2158508300781 0 40188.66
504.934814453125 0 154978.38 b Water loss 12
505.2677917480469 0 153604.84 b Ammonia loss 12
505.6026916503906 0 88921.43
505.7338562011719 0 8554.471
505.93609619140625 0 37820.62
506.26727294921875 0 12097.192
508.7579345703125 0 22672.393
509.2625427246094 0 13246.445
510.3001708984375 0 7014.767
510.37762451171875 0 14220.928
510.9382019042969 0 769010.06 b 12
511.27239990234375 0 776591.44
511.6067810058594 0 371375.94
511.9410095214844 0 126231.94
512.2753295898438 0 40242.703
513.9496459960938 0 6983.4536
514.2776489257812 0 12814.1875
514.6115112304688 0 10821.777
516.9414672851562 0 116424.086
517.2758178710938 0 150579.73
517.6099243164062 0 53545.152
517.9426879882812 0 18803.57
519.6194458007812 0 8721.36
519.9517211914062 0 32703.05 y Ammonia loss 1
520.2421264648438 0 13528.229
520.2855834960938 0 45870.504
520.3615112304688 0 19612.55
520.62060546875 0 12034.408
520.9528198242188 0 11685.277
521.2267456054688 0 38020.742
522.2622680664062 0 9948.439
523.3624877929688 0 16668.904
524.8131103515625 0 8606.761
525.3089599609375 0 13844.662
525.6256713867188 0 152426.69 y 1
525.8045043945312 0 12311.734
525.9596557617188 0 138174.84
526.2936401367188 0 64638.957
526.6278686523438 0 12384.66
528.2333374023438 0 22472.568
529.2237548828125 0 21795.92
529.8057861328125 0 20094.707
530.2991943359375 0 29436.701
530.7991943359375 0 18802.219
531.2116088867188 0 25269.398
531.3013305664062 0 9471.802
535.2919921875 0 8071.2036
535.613037109375 0 8054.752
536.2520141601562 0 8404.228
537.2905883789062 0 8499.489
538.2532958984375 0 378765.97
538.3727416992188 0 26886.879
538.8099365234375 0 49957.125
538.9566650390625 0 8472.692
539.2554321289062 0 102735.94
539.3036499023438 0 106694.94
539.3701171875 0 7952.215
539.633056640625 0 27936.771
539.8056640625 0 43154.414
539.9684448242188 0 22648.457
540.2586669921875 0 17846.023
540.2996215820312 0 15912.753
540.3289794921875 0 14582.265
540.8250122070312 0 10412.946 y Ammonia loss 4
541.3292236328125 0 9069.079
541.3724975585938 0 143052.06 y 9
542.2521362304688 0 20565.963
542.3756103515625 0 42819.65
542.9629516601562 0 21222.459
543.2950439453125 0 8641.029
544.6318359375 0 14093.142
544.75634765625 0 10647.947
545.30126953125 0 9552.956
547.816162109375 0 57665.32
548.2356567382812 0 16030.48
548.3155517578125 0 32796.484
548.6331176757812 0 224686.25 Precursor Water loss
548.8162231445312 0 13427.819
548.9658813476562 0 279822.84 Precursor Ammonia loss
549.2232055664062 0 21666.045
549.2965698242188 0 99974.76
549.3341064453125 0 215202.64 y 4
549.6321411132812 0 101863.12
549.8352661132812 0 146274.16
549.9641723632812 0 30106.924
550.2232666015625 0 8883.243
550.29443359375 0 10273.729
550.337158203125 0 57124.098
550.8380737304688 0 9267.578
553.2198486328125 0 13156.506
553.2649536132812 0 10118.671
553.9628295898438 0 17452.389
554.2999267578125 0 51903.438
554.636474609375 0 5188832.5 Precursor
554.970703125 0 5211137.5
555.3048706054688 0 2844816.5
555.6390991210938 0 1007323.75
555.7867431640625 0 11455.983
555.9730224609375 0 298427.53
557.296142578125 0 16956.984
561.2681274414062 0 44510.52 b 8
561.7697143554688 0 31639.406
562.2706298828125 0 17406.537
565.2413330078125 0 40734.516 b 3
565.3013305664062 0 7880.2285
566.2481079101562 0 460341.06
567.2508544921875 0 138557.02
567.3972778320312 0 17466.105
568.2539672851562 0 29160.809
568.4046630859375 0 9577.159
570.2470092773438 0 80165.59
571.2493286132812 0 22603.822
572.8052978515625 0 205006.2
573.3062744140625 0 166278.88
573.8082885742188 0 61065.27
577.3834228515625 0 89621.13
578.3846435546875 0 30400.09
579.362548828125 0 289011.2
580.3658447265625 0 77155.484
581.3677978515625 0 24679.145
587.2733154296875 0 12143.533
587.7821044921875 0 46128.69 b Water loss 9
588.2819213867188 0 33240.18 b Ammonia loss 9
588.3284301757812 0 7819.261
588.78466796875 0 11627.706
589.3261108398438 0 7333.1973
595.3538818359375 0 6849.5547
595.3941040039062 0 86090.54
595.8485107421875 0 18355.012
596.3461303710938 0 10115.279
596.3975219726562 0 29105.18
596.7864379882812 0 52245.383 b 9
596.8406982421875 0 8374.338
597.28857421875 0 17350.695
597.7889404296875 0 14333.792
598.2420654296875 0 32982.086
599.2451171875 0 14913.101
602.8228149414062 0 21735.152
603.3212280273438 0 9924.536
604.3582763671875 0 113661.35
604.8541259765625 0 169612.17 y Ammonia loss 3
605.3541259765625 0 99580.64
605.8549194335938 0 36515.004
606.350341796875 0 10831.089
606.8350830078125 0 10843.041
607.3406372070312 0 13552.047
610.3213500976562 0 47531.27
611.324951171875 0 25984.693
611.829833984375 0 27778.275
612.3312377929688 0 22414.871
612.8302001953125 0 10839.595
613.3629150390625 0 145948.61 y 3
613.8646240234375 0 120789.516
614.3652954101562 0 49616.383
615.2667236328125 0 17518.787
615.3501586914062 0 95035.2
615.8517456054688 0 59501.105
616.3523559570312 0 25244.889
616.85107421875 0 9211.406
618.8534545898438 0 8060.6797
620.3421630859375 0 194201.88
620.843017578125 0 111714.875
621.3432006835938 0 71957.35
621.4059448242188 0 11503.633
621.8419189453125 0 18097.955
623.3876953125 0 21567.426
628.3321533203125 0 18498.436
629.3472900390625 0 444729.9
629.8487548828125 0 321128.66
630.3499755859375 0 122822.67
630.8521728515625 0 24392.05
632.294189453125 0 14251.509
638.3528442382812 0 47010.41
638.8550415039062 0 27426.623
639.3544311523438 0 13602.242
643.2633056640625 0 8452.839
645.3123779296875 0 29877.223 b 10
645.8168334960938 0 17834.59
647.4287719726562 0 18779.059
653.827392578125 0 28112.904
654.3301391601562 0 16764.715
654.829345703125 0 15516.123
656.2946166992188 0 12120.752
657.2787475585938 0 31875.518
657.3306884765625 0 7241.3374
657.8353881835938 0 9215.283
658.309326171875 0 21175.812
659.3138427734375 0 16143.782
664.4518432617188 0 93656.8
665.4552612304688 0 34791.574
665.8462524414062 0 13914.127
666.4573974609375 0 8561.942
667.3516845703125 0 8397.533
668.3270263671875 0 14675.916
669.46728515625 0 49972.86 y 8
670.4694213867188 0 25304.402
671.8933715820312 0 13538.086
672.3947143554688 0 18244.207
672.8926391601562 0 7015.3823
674.3052368164062 0 102427.914
674.4364013671875 0 309593.94
675.30810546875 0 44854.688
675.4385986328125 0 134592.75
676.4420166015625 0 28661.389
677.3761596679688 0 55965.723
677.8779296875 0 42599.02
678.3755493164062 0 25352.832
684.2913208007812 0 32372.115 b Water loss 4
685.2859497070312 0 11130.45 b Ammonia loss 4
685.888916015625 0 95571.67
686.3883666992188 0 89847.12 y Ammonia loss 2
686.8873291015625 0 43059.76
687.3886108398438 0 18164.447
692.4465942382812 0 328385.94
693.4489135742188 0 127548.305
694.3912963867188 0 8754.574
694.4518432617188 0 30055.244
694.8948364257812 0 1607548 y 2
695.3963623046875 0 1235247.9
695.8973388671875 0 592262.6
696.3988647460938 0 161713.06
696.8988647460938 0 34979.93
698.4110107421875 0 31493.535
700.3671264648438 0 14635.796 b Water loss 11
700.8524780273438 0 17641.752 b Ammonia loss 11
701.3568115234375 0 7201.8193
701.8598022460938 0 8516.431
702.3009643554688 0 180251.84 b 4
703.3040771484375 0 82192.77
704.3037109375 0 21785.299
707.3063354492188 0 47573.438
708.3106079101562 0 24378.953
708.48193359375 0 10968.713
709.3613891601562 0 354174.62 b 11
709.8623657226562 0 281645.06
710.3638916015625 0 121548.15
710.458251953125 0 46436.06
710.8651123046875 0 33839.93
711.3672485351562 0 16716.002
711.4602661132812 0 16134.462
712.4742431640625 0 8199.533
713.3792114257812 0 7646.2065
714.382568359375 0 13134.001
716.421630859375 0 56076.598
717.4243774414062 0 24241.879
718.3670043945312 0 6861.422
720.3124389648438 0 11073.811
720.37109375 0 16066.729
721.3539428710938 0 25200.49
722.3877563476562 0 8851.758
722.88671875 0 16066.782
723.3818359375 0 13169.236
724.33251953125 0 28407.998
725.3347778320312 0 12194.5205
726.4882202148438 0 124112.125 y 7
727.4904174804688 0 45331
735.3007202148438 0 45867.145
736.3017578125 0 13354.381
737.3521728515625 0 13515.261
738.3805541992188 0 14785.625
739.371337890625 0 11471.859
740.3837890625 0 8378.789
751.9066162109375 0 15736.191
752.3280639648438 0 37412.61
752.4075927734375 0 21840.018
753.330810546875 0 21216.791
756.898681640625 0 35108.445 b Water loss 12
757.3993530273438 0 42478.215 b Ammonia loss 12
757.8993530273438 0 37916.36
758.408935546875 0 14570.23
765.90380859375 0 115548.91 b 12
766.3302001953125 0 9325.921
766.4047241210938 0 125282.266
766.9063720703125 0 52883.945
767.4124755859375 0 19452.518
774.90771484375 0 56959.582
775.4103393554688 0 54673.97
775.912353515625 0 18018.463
776.4122314453125 0 10272.81
777.5390014648438 0 11471.378
779.4266967773438 0 7881.037 y Ammonia loss 1
780.3218383789062 0 7160.2275
787.519287109375 0 9812.848
787.9353637695312 0 41356.527 y 1
788.4358520507812 0 42246.688
788.9366455078125 0 17071.73
789.4406127929688 0 7528.2925
792.9321899414062 0 9888.913
793.3544921875 0 14764.611
794.34130859375 0 24559.385
795.3526611328125 0 15368.612
801.5091552734375 0 18688.69
805.5320434570312 0 62068.574
806.5297241210938 0 31244.602
807.5337524414062 0 14284.98
811.364013671875 0 194022.9
811.494873046875 0 57813.867
812.3682250976562 0 84286.78
812.4966430664062 0 28232.75
813.3712158203125 0 22167.227
814.3839721679688 0 8896.208
821.34814453125 0 28428.12 b Water loss 5
822.344482421875 0 19339.172 b Ammonia loss 5
822.529296875 0 8178.104
823.3482666015625 0 16115.855
823.541259765625 0 754977.9 y 6
824.544189453125 0 343403.12
825.5470581054688 0 91199.336
826.5492553710938 0 12052.726
829.504150390625 0 56348.055
830.5095825195312 0 27150.928
831.513427734375 0 10805.503
839.359375 0 408180.38 b 5
840.362548828125 0 208921.62
841.3661499023438 0 60394.07
842.3718872070312 0 8707.856
847.5136108398438 0 11255.178
848.4170532226562 0 68237.8
849.4208984375 0 37410.03
850.4215087890625 0 7036.1323
853.4747924804688 0 35218.008
854.481201171875 0 22093.293
866.4033813476562 0 9240.018
891.4581298828125 0 23630.797
892.4611206054688 0 10688.214
901.4439697265625 0 139004.17
902.446044921875 0 85275.766
903.449951171875 0 18739.844
919.4539184570312 0 70718.05
920.4570922851562 0 52871.76
921.4625244140625 0 9000.983
942.59033203125 0 41057.195
943.5923461914062 0 35996.766
944.6041870117188 0 7015.4624
948.55712890625 0 14963.607
960.5996704101562 0 138073.6 y 5
961.4990844726562 0 9497.91
961.6018676757812 0 70358.09
962.6067504882812 0 18479.209
963.6104125976562 0 7141.216
964.5128173828125 0 11568.115
966.5637817382812 0 33662.8
967.5614624023438 0 18545.326
993.4301147460938 0 12225.598 b 7
994.4440307617188 0 8757.917
1010.4661865234375 0 16182.278
1011.4563598632812 0 10128.078
1016.5039672851562 0 7232.8823
1032.5382080078125 0 22480.008
1033.5374755859375 0 31798.658
1034.5482177734375 0 11320.731
1087.5211181640625 0 13459.846
1097.65869140625 0 48423.47 y 4
1098.6607666015625 0 29574.95
1099.6622314453125 0 16611.105
1121.5279541015625 0 33712.953 b 8
1122.5330810546875 0 26687.848
1144.6029052734375 0 18559.797
1145.60888671875 0 11743.084
1146.6087646484375 0 10935.851
1164.5703125 0 8887.645
1174.553955078125 0 74800.13 b Water loss 9
1175.555908203125 0 59817.574 b Ammonia loss 9
1176.5562744140625 0 13975.772
1192.5648193359375 0 28494.064 b 9
1193.566650390625 0 23783.994
1194.57861328125 0 8383.508
2015.07666015625 0 7175.4644
2187.9150390625 0 5724.7954
2709.309814453125 0 7062.3228
2709.870849609375 0 6881.481
2925.899169921875 0 6923.8784
3255.452880859375 0 6993.9653
3299.4013671875 0 6799.4385

Spectrum Details

|  |  |
| --- | --- |
| Matched peaks? Matched peaksThe total absolute number of peaks matched. Additionally in brackets the total fraction of peaks matched and the total number of peaks is shown. | 69 (8.25% of 836) |
| FDR? FDRThe false discovery rate estimated for this peptide. It is calculated by matching all theoretical fragments with a non-integer shift with the raw peaks for this spectrum. This is done with 40 different shifts. The resulting percentage is the average number of annotated peaks over the number of annotated peaks with the correct spectrum. | 3.73% |
| Satellite FDR? Satellite FDRSee the FDR for details on its calculation. This satellite ion specific FDR only contains the satellite ions (d/w) for I/L/J positions. | - |
| PSM Score? PSM ScoreThe PSM Score as given by Hecklib to this annotated spectrum. It is shown with three significant figures. | 360 |

## Spectrum 5459? Spectrum 5459 The raw spectrum of this peptide as annotated by Hecklib. The fragments are coloured according to ion type (see legend). Any peaks with a star '\*' as text can be hovered over to see the full details, first the ion type second the mass shift type. By hovering over the amino acids in the peptide or ions in the legend the corresponding peaks are highlighted. By toggling the 'Unassigned' label you can turn the background (unassigned) peaks on or off in the plot. By updating the slider in the Ion legend you can update the spectrum to only show the top X% of the peaks with labels. The top X% means any peak that is within X% of the highest intensity. By dragging in the spectrum you can zoom in to a specific part of the spectrum and use 'Zoom Out' to get back to the original zoom level. The annotation of the spectrum is based on the given sequence in the peptides file and is done with different software so inconsistencies are likely. The peaks are annotated based on the given sequence, with 20 ppm tolerance.

Copy Data

### Spectrum 5459 (TSV)

#### Preview

```
Loading example...
```

*Click on the button to copy the data to your clipboard.*

Mz MinMz MaxIntensity Max

WidthHeightPeptide font sizePeptide stroke widthSpectrum font sizeSpectrum stroke widthCompact peptide

Ion legend

wxyz

abcd

OtherUnassignedIonChargePositionShow for top:%

SWYQHHPGKAPKJJ

01.40e+52.80e+54.19e+55.59e+5

Zoom Out

y+11y+12z+27z+13y+13y+28c+26w+14c+13y+312y+14c+312y+29c+28c+313z+313z+15y+313z+210y+15y+210c+14c+14w+211c+14w+16c+210y+211z+211y+211z+16c+211y+16y+212z+212y+212c+15c+212z+17c+212c+15y+17c+213c+213y+213z+213w+18y+213y+18c+16z+19c+17y+19c+18y+110z+110y+110c+19c+19y+111z+111y+111c+111z+112y+112c+112c+113z+113

047194214131885

Fragment Matches Table

Show background peaks

| Position | Ion type | Intensity | mz Theoretical | mz Error (Th) | mz Error (ppm) | Charge | Series Number |
| --- | --- | --- | --- | --- | --- | --- | --- |
| - | - | 640.6 | 124.7 | - | - | 0 | - |
| - | - | 528.8 | 124.8 | - | - | 0 | - |
| - | - | 515.1 | 125.7 | - | - | 0 | - |
| - | - | 463.2 | 127.5 | - | - | 0 | - |
| - | - | 510.7 | 128.8 | - | - | 0 | - |
| - | - | 1918 | 129.1 | - | - | 0 | - |
| - | - | 1052 | 130.1 | - | - | 0 | - |
| - | - | 1845 | 132.1 | - | - | 0 | - |
| 14 | y | 3627 | 132.1 | 0.0002372 | 1.796 | +1 | 1 |
| - | - | 1253 | 136.1 | - | - | 0 | - |
| - | - | 527.4 | 141.5 | - | - | 0 | - |
| - | - | 1107 | 146.1 | - | - | 0 | - |
| - | - | 4361 | 153.1 | - | - | 0 | - |
| - | - | 622.3 | 164 | - | - | 0 | - |
| - | - | 633.2 | 168.1 | - | - | 0 | - |
| - | - | 616 | 172.3 | - | - | 0 | - |
| - | - | 1928 | 173.4 | - | - | 0 | - |
| - | - | 605.8 | 175.8 | - | - | 0 | - |
| - | - | 698.8 | 181.7 | - | - | 0 | - |
| - | - | 1639 | 186.1 | - | - | 0 | - |
| - | - | 3355 | 187.1 | - | - | 0 | - |
| - | - | 704.7 | 199.2 | - | - | 0 | - |
| - | - | 674.4 | 209.5 | - | - | 0 | - |
| - | - | 692.1 | 209.9 | - | - | 0 | - |
| - | - | 821.8 | 210.1 | - | - | 0 | - |
| - | - | 1073 | 214.2 | - | - | 0 | - |
| - | - | 1001 | 215.1 | - | - | 0 | - |
| - | - | 2237 | 223.1 | - | - | 0 | - |
| - | - | 3215 | 226.2 | - | - | 0 | - |
| - | - | 731.1 | 227.2 | - | - | 0 | - |
| - | - | 975 | 228.1 | - | - | 0 | - |
| - | - | 622.1 | 243.2 | - | - | 0 | - |
| - | - | 5270 | 245.1 | - | - | 0 | - |
| 13 | y | 6944 | 245.2 | 0.0002186 | 0.8917 | +1 | 2 |
| - | - | 2.101E+04 | 246.1 | - | - | 0 | - |
| - | - | 899.1 | 246.2 | - | - | 0 | - |
| - | - | 2814 | 247.1 | - | - | 0 | - |
| - | - | 1651 | 255.1 | - | - | 0 | - |
| - | - | 1729 | 258.1 | - | - | 0 | - |
| - | - | 830.5 | 267.1 | - | - | 0 | - |
| - | - | 1629 | 273.1 | - | - | 0 | - |
| - | - | 2.566E+04 | 274.1 | - | - | 0 | - |
| - | - | 4196 | 275.1 | - | - | 0 | - |
| - | - | 3090 | 281.2 | - | - | 0 | - |
| - | - | 849.2 | 282.2 | - | - | 0 | - |
| - | - | 3038 | 292.1 | - | - | 0 | - |
| - | - | 1332 | 297.2 | - | - | 0 | - |
| - | - | 1776 | 301.2 | - | - | 0 | - |
| - | - | 3342 | 309.2 | - | - | 0 | - |
| - | - | 2337 | 314.2 | - | - | 0 | - |
| - | - | 1719 | 338.1 | - | - | 0 | - |
| - | - | 902.6 | 338.2 | - | - | 0 | - |
| - | - | 3068 | 346.7 | - | - | 0 | - |
| 8 | z | 780 | 347.2 | 0.00475 | 13.68 | +2 | 7 |
| - | - | 2323 | 349.1 | - | - | 0 | - |
| - | - | 1026 | 350.3 | - | - | 0 | - |
| - | - | 788.1 | 355.1 | - | - | 0 | - |
| 12 | z | 1E+04 | 357.3 | 0.0001821 | 0.5097 | +1 | 3 |
| - | - | 3981 | 358.3 | - | - | 0 | - |
| - | - | 1257 | 371.1 | - | - | 0 | - |
| 12 | y | 2882 | 373.3 | 0.0001399 | 0.3747 | +1 | 3 |
| - | - | 1709 | 382.3 | - | - | 0 | - |
| - | - | 1783 | 394.3 | - | - | 0 | - |
| - | - | 1619 | 399.3 | - | - | 0 | - |
| 7 | y | 1.266E+04 | 412.3 | 0.000501 | 1.215 | +2 | 8 |
| - | - | 6346 | 412.8 | - | - | 0 | - |
| - | - | 3109 | 413.2 | - | - | 0 | - |
| - | - | 1280 | 413.3 | - | - | 0 | - |
| - | - | 980.6 | 414.2 | - | - | 0 | - |
| 6 | c | 1801 | 420.2 | 0.0008122 | 1.933 | +2 | 6 |
| - | - | 939.2 | 420.7 | - | - | 0 | - |
| - | - | 1569 | 423.2 | - | - | 0 | - |
| 11 | w | 5672 | 427.3 | 0.0004649 | 1.088 | +1 | 4 |
| - | - | 901.1 | 428.3 | - | - | 0 | - |
| - | - | 2249 | 428.3 | - | - | 0 | - |
| - | - | 1215 | 429.2 | - | - | 0 | - |
| - | - | 834.4 | 434.2 | - | - | 0 | - |
| - | - | 1591 | 435.2 | - | - | 0 | - |
| - | - | 1242 | 436.2 | - | - | 0 | - |
| - | - | 4531 | 437.2 | - | - | 0 | - |
| - | - | 1053 | 438.2 | - | - | 0 | - |
| - | - | 1240 | 439.2 | - | - | 0 | - |
| - | - | 1006 | 451.2 | - | - | 0 | - |
| - | - | 4933 | 453.2 | - | - | 0 | - |
| 3 | c | 9969 | 454.2 | 0.0001825 | 0.4017 | +1 | 3 |
| - | - | 2747 | 455.2 | - | - | 0 | - |
| 3 | y | 3674 | 463.6 | 0.0009047 | 1.951 | +3 | 12 |
| - | - | 2910 | 463.9 | - | - | 0 | - |
| - | - | 3579 | 465.2 | - | - | 0 | - |
| - | - | 1.055E+04 | 468.3 | - | - | 0 | - |
| - | - | 3329 | 469.3 | - | - | 0 | - |
| - | - | 2378 | 469.3 | - | - | 0 | - |
| - | - | 1628 | 470.3 | - | - | 0 | - |
| 11 | y | 4662 | 470.3 | 0.0001969 | 0.4185 | +1 | 4 |
| - | - | 1043 | 472.3 | - | - | 0 | - |
| 12 | c | 4621 | 473.2 | 0.0004568 | 0.9652 | +3 | 12 |
| - | - | 4526 | 473.6 | - | - | 0 | - |
| - | - | 1991 | 473.9 | - | - | 0 | - |
| - | - | 4072 | 477.2 | - | - | 0 | - |
| - | - | 1607 | 478.2 | - | - | 0 | - |
| - | - | 2054 | 480.2 | - | - | 0 | - |
| 6 | y | 1961 | 480.8 | 0.001624 | 3.377 | +2 | 9 |
| - | - | 1844 | 481.3 | - | - | 0 | - |
| - | - | 3145 | 482.3 | - | - | 0 | - |
| - | - | 1156 | 483.3 | - | - | 0 | - |
| 8 | c | 764.8 | 497.2 | 0.0007163 | 1.441 | +2 | 8 |
| - | - | 857.7 | 498.3 | - | - | 0 | - |
| - | - | 1269 | 501.6 | - | - | 0 | - |
| - | - | 999.7 | 501.9 | - | - | 0 | - |
| - | - | 6023 | 504.9 | - | - | 0 | - |
| - | - | 6193 | 505.3 | - | - | 0 | - |
| - | - | 1706 | 505.6 | - | - | 0 | - |
| - | - | 873.6 | 507.3 | - | - | 0 | - |
| 13 | c | 2.302E+04 | 510.9 | 0.0005319 | 1.041 | +3 | 13 |
| - | - | 1.822E+04 | 511.3 | - | - | 0 | - |
| - | - | 1.003E+04 | 511.6 | - | - | 0 | - |
| - | - | 3877 | 511.9 | - | - | 0 | - |
| - | - | 1122 | 512.3 | - | - | 0 | - |
| - | - | 5297 | 516.9 | - | - | 0 | - |
| - | - | 2998 | 517.3 | - | - | 0 | - |
| - | - | 1006 | 517.6 | - | - | 0 | - |
| - | - | 1169 | 517.9 | - | - | 0 | - |
| 2 | z | 1617 | 520.3 | 0.003768 | 7.243 | +3 | 13 |
| - | - | 2203 | 523.2 | - | - | 0 | - |
| 10 | z | 4.114E+04 | 525.4 | 0.0005144 | 0.9792 | +1 | 5 |
| 2 | y | 3434 | 525.6 | 0.0001423 | 0.2708 | +3 | 13 |
| - | - | 4485 | 526 | - | - | 0 | - |
| - | - | 1919 | 526.3 | - | - | 0 | - |
| - | - | 1.298E+04 | 526.4 | - | - | 0 | - |
| - | - | 2059 | 527.4 | - | - | 0 | - |
| - | - | 1008 | 536.2 | - | - | 0 | - |
| - | - | 1568 | 538.2 | - | - | 0 | - |
| - | - | 1770 | 538.3 | - | - | 0 | - |
| - | - | 2830 | 539.3 | - | - | 0 | - |
| - | - | 1172 | 540 | - | - | 0 | - |
| 5 | z | 1990 | 541.3 | 0.0007395 | 1.366 | +2 | 10 |
| 10 | y | 5638 | 541.4 | 0.001016 | 1.877 | +1 | 5 |
| - | - | 1166 | 541.8 | - | - | 0 | - |
| - | - | 1542 | 542.4 | - | - | 0 | - |
| - | - | 1988 | 546.2 | - | - | 0 | - |
| - | - | 765.8 | 547.2 | - | - | 0 | - |
| - | - | 5912 | 548.6 | - | - | 0 | - |
| - | - | 8449 | 549 | - | - | 0 | - |
| - | - | 4633 | 549.3 | - | - | 0 | - |
| 5 | y | 3692 | 549.3 | 0.002533 | 4.611 | +2 | 10 |
| - | - | 1520 | 549.6 | - | - | 0 | - |
| - | - | 3765 | 549.8 | - | - | 0 | - |
| - | - | 1084 | 550 | - | - | 0 | - |
| - | - | 690.9 | 553.3 | - | - | 0 | - |
| - | - | 1617 | 554 | - | - | 0 | - |
| - | - | 1944 | 554.3 | - | - | 0 | - |
| - | - | 1.936E+05 | 554.6 | - | - | 0 | - |
| - | - | 1.825E+05 | 555 | - | - | 0 | - |
| - | - | 9.881E+04 | 555.3 | - | - | 0 | - |
| - | - | 3.098E+04 | 555.6 | - | - | 0 | - |
| - | - | 1018 | 555.7 | - | - | 0 | - |
| - | - | 8261 | 556 | - | - | 0 | - |
| - | - | 5608 | 556.3 | - | - | 0 | - |
| - | - | 1116 | 561.8 | - | - | 0 | - |
| - | - | 1.199E+04 | 563.2 | - | - | 0 | - |
| 4 | c | 4267 | 564.3 | 0.006508 | 11.53 | +1 | 4 |
| 4 | c | 5120 | 565.2 | 0.001358 | 2.403 | +1 | 4 |
| - | - | 3924 | 566.2 | - | - | 0 | - |
| - | - | 3081 | 566.4 | - | - | 0 | - |
| - | - | 1334 | 567.3 | - | - | 0 | - |
| - | - | 979.9 | 569.8 | - | - | 0 | - |
| - | - | 2092 | 572.8 | - | - | 0 | - |
| - | - | 1394 | 573.3 | - | - | 0 | - |
| 4 | w | 1.136E+04 | 576.3 | 0.0007904 | 1.371 | +2 | 11 |
| - | - | 6942 | 576.8 | - | - | 0 | - |
| - | - | 2342 | 577.3 | - | - | 0 | - |
| - | - | 2020 | 577.8 | - | - | 0 | - |
| - | - | 1174 | 579.4 | - | - | 0 | - |
| - | - | 8264 | 581.3 | - | - | 0 | - |
| 4 | c | 4.522E+04 | 582.3 | 0.0005052 | 0.8676 | +1 | 4 |
| - | - | 3670 | 582.4 | - | - | 0 | - |
| - | - | 1.405E+04 | 583.3 | - | - | 0 | - |
| - | - | 2154 | 584.3 | - | - | 0 | - |
| 9 | w | 6817 | 595.4 | 0.0005836 | 0.9801 | +1 | 6 |
| - | - | 3418 | 596.4 | - | - | 0 | - |
| 10 | c | 1036 | 596.8 | 0.0003517 | 0.5893 | +2 | 10 |
| - | - | 1739 | 597.4 | - | - | 0 | - |
| - | - | 1523 | 604.4 | - | - | 0 | - |
| 4 | y | 3130 | 604.8 | 0.006355 | 10.51 | +2 | 11 |
| 4 | z | 3.266E+04 | 605.4 | 0.0005504 | 0.9092 | +2 | 11 |
| - | - | 3.432E+04 | 605.9 | - | - | 0 | - |
| - | - | 1.944E+04 | 606.4 | - | - | 0 | - |
| - | - | 6671 | 606.9 | - | - | 0 | - |
| - | - | 1037 | 607.4 | - | - | 0 | - |
| - | - | 1827 | 609.5 | - | - | 0 | - |
| - | - | 6650 | 610.4 | - | - | 0 | - |
| - | - | 2302 | 611.4 | - | - | 0 | - |
| - | - | 1206 | 612.9 | - | - | 0 | - |
| 4 | y | 1.095E+04 | 613.4 | 0.0002215 | 0.3612 | +2 | 11 |
| - | - | 6707 | 613.9 | - | - | 0 | - |
| - | - | 2146 | 614.4 | - | - | 0 | - |
| - | - | 949.5 | 617.3 | - | - | 0 | - |
| - | - | 1096 | 620.3 | - | - | 0 | - |
| - | - | 982.8 | 620.8 | - | - | 0 | - |
| - | - | 787 | 622.3 | - | - | 0 | - |
| - | - | 2093 | 624.4 | - | - | 0 | - |
| - | - | 5679 | 629.3 | - | - | 0 | - |
| - | - | 3597 | 629.8 | - | - | 0 | - |
| - | - | 1776 | 630.3 | - | - | 0 | - |
| - | - | 1200 | 637.4 | - | - | 0 | - |
| - | - | 1502 | 639.4 | - | - | 0 | - |
| - | - | 2508 | 645.8 | - | - | 0 | - |
| - | - | 1484 | 646.3 | - | - | 0 | - |
| - | - | 2502 | 651.4 | - | - | 0 | - |
| - | - | 2107 | 651.9 | - | - | 0 | - |
| 9 | z | 3.303E+04 | 653.4 | 0.0007052 | 1.079 | +1 | 6 |
| 11 | c | 2899 | 653.8 | 0.001955 | 2.99 | +2 | 11 |
| - | - | 1999 | 654.3 | - | - | 0 | - |
| - | - | 1.716E+04 | 654.5 | - | - | 0 | - |
| - | - | 3862 | 655.5 | - | - | 0 | - |
| - | - | 1241 | 657.3 | - | - | 0 | - |
| - | - | 4419 | 657.9 | - | - | 0 | - |
| - | - | 1303 | 658.4 | - | - | 0 | - |
| - | - | 1935 | 658.9 | - | - | 0 | - |
| - | - | 4740 | 659.3 | - | - | 0 | - |
| - | - | 1320 | 659.4 | - | - | 0 | - |
| - | - | 1444 | 660.3 | - | - | 0 | - |
| - | - | 1734 | 665.4 | - | - | 0 | - |
| - | - | 1956 | 665.9 | - | - | 0 | - |
| 9 | y | 1305 | 669.5 | 0.004591 | 6.858 | +1 | 6 |
| - | - | 3304 | 674.3 | - | - | 0 | - |
| - | - | 2349 | 675.3 | - | - | 0 | - |
| - | - | 1383 | 685.9 | - | - | 0 | - |
| 3 | y | 3514 | 686.4 | 0.005025 | 7.321 | +2 | 12 |
| 3 | z | 4.186E+04 | 686.9 | 0.0003802 | 0.5536 | +2 | 12 |
| - | - | 3.235E+04 | 687.4 | - | - | 0 | - |
| - | - | 1.629E+04 | 687.9 | - | - | 0 | - |
| - | - | 7667 | 688.4 | - | - | 0 | - |
| - | - | 1377 | 688.9 | - | - | 0 | - |
| - | - | 1410 | 692.4 | - | - | 0 | - |
| 3 | y | 4.183E+04 | 694.9 | 0.0006617 | 0.9523 | +2 | 12 |
| - | - | 3.373E+04 | 695.4 | - | - | 0 | - |
| - | - | 1.733E+04 | 695.9 | - | - | 0 | - |
| - | - | 4576 | 696.4 | - | - | 0 | - |
| - | - | 1567 | 696.9 | - | - | 0 | - |
| 5 | c | 3132 | 702.3 | 5.829E-05 | 0.083 | +1 | 5 |
| - | - | 1586 | 703.3 | - | - | 0 | - |
| - | - | 1510 | 708.4 | - | - | 0 | - |
| 12 | c | 8527 | 709.4 | 0.001134 | 1.599 | +2 | 12 |
| - | - | 6631 | 709.9 | - | - | 0 | - |
| - | - | 2875 | 710.4 | - | - | 0 | - |
| 8 | z | 5418 | 710.5 | 0.0001765 | 0.2485 | +1 | 7 |
| - | - | 4069 | 711.5 | - | - | 0 | - |
| - | - | 1211 | 712.5 | - | - | 0 | - |
| - | - | 2483 | 717.4 | - | - | 0 | - |
| 12 | c | 1.055E+05 | 717.9 | 0.0003719 | 0.5181 | +2 | 12 |
| - | - | 8.537E+04 | 718.4 | - | - | 0 | - |
| - | - | 4.025E+04 | 718.9 | - | - | 0 | - |
| 5 | c | 8.906E+04 | 719.3 | 0.0003701 | 0.5146 | +1 | 5 |
| - | - | 3165 | 719.9 | - | - | 0 | - |
| - | - | 4.106E+04 | 720.3 | - | - | 0 | - |
| - | - | 9045 | 721.3 | - | - | 0 | - |
| - | - | 1131 | 722.3 | - | - | 0 | - |
| 8 | y | 3321 | 726.5 | 6.819E-05 | 0.09386 | +1 | 7 |
| - | - | 1279 | 727.5 | - | - | 0 | - |
| - | - | 1561 | 730.9 | - | - | 0 | - |
| - | - | 1981 | 731.4 | - | - | 0 | - |
| - | - | 1029 | 731.9 | - | - | 0 | - |
| - | - | 1125 | 736.4 | - | - | 0 | - |
| - | - | 1159 | 744.9 | - | - | 0 | - |
| - | - | 1386 | 750.9 | - | - | 0 | - |
| - | - | 1964 | 751.4 | - | - | 0 | - |
| - | - | 3153 | 752.4 | - | - | 0 | - |
| - | - | 2263 | 752.9 | - | - | 0 | - |
| - | - | 1696 | 753.4 | - | - | 0 | - |
| - | - | 1334 | 756.9 | - | - | 0 | - |
| - | - | 1208 | 757.9 | - | - | 0 | - |
| - | - | 1544 | 758.4 | - | - | 0 | - |
| - | - | 4150 | 758.9 | - | - | 0 | - |
| - | - | 4225 | 759.4 | - | - | 0 | - |
| - | - | 1072 | 759.9 | - | - | 0 | - |
| 13 | c | 2915 | 765.9 | 0.002742 | 3.581 | +2 | 13 |
| - | - | 5658 | 766.4 | - | - | 0 | - |
| - | - | 4298 | 766.9 | - | - | 0 | - |
| - | - | 4999 | 767.4 | - | - | 0 | - |
| - | - | 1382 | 767.9 | - | - | 0 | - |
| - | - | 1150 | 768.4 | - | - | 0 | - |
| - | - | 1807 | 773.4 | - | - | 0 | - |
| - | - | 1369 | 773.9 | - | - | 0 | - |
| 13 | c | 6.673E+04 | 774.4 | 0.0007594 | 0.9806 | +2 | 13 |
| - | - | 6.683E+04 | 774.9 | - | - | 0 | - |
| - | - | 3.311E+04 | 775.4 | - | - | 0 | - |
| - | - | 9630 | 775.9 | - | - | 0 | - |
| - | - | 3204 | 776.4 | - | - | 0 | - |
| - | - | 2006 | 777.9 | - | - | 0 | - |
| - | - | 1575 | 778.4 | - | - | 0 | - |
| - | - | 2756 | 778.9 | - | - | 0 | - |
| 2 | y | 2298 | 779.4 | 0.002478 | 3.179 | +2 | 13 |
| 2 | z | 9584 | 779.9 | 0.0009459 | 1.213 | +2 | 13 |
| - | - | 1.337E+04 | 780.4 | - | - | 0 | - |
| 7 | w | 1541 | 780.5 | 0.0004898 | 0.6276 | +1 | 8 |
| - | - | 7264 | 780.9 | - | - | 0 | - |
| - | - | 2689 | 781.4 | - | - | 0 | - |
| - | - | 1417 | 781.5 | - | - | 0 | - |
| - | - | 1574 | 781.9 | - | - | 0 | - |
| - | - | 1716 | 782.4 | - | - | 0 | - |
| 2 | y | 6076 | 787.9 | 0.01043 | 13.24 | +2 | 13 |
| - | - | 5909 | 788.4 | - | - | 0 | - |
| - | - | 3718 | 788.9 | - | - | 0 | - |
| - | - | 1179 | 789.4 | - | - | 0 | - |
| - | - | 8495 | 790.9 | - | - | 0 | - |
| - | - | 7937 | 791.4 | - | - | 0 | - |
| - | - | 3493 | 791.9 | - | - | 0 | - |
| - | - | 3052 | 792.4 | - | - | 0 | - |
| - | - | 1518 | 792.9 | - | - | 0 | - |
| - | - | 1237 | 794.4 | - | - | 0 | - |
| - | - | 1443 | 794.4 | - | - | 0 | - |
| - | - | 1074 | 794.9 | - | - | 0 | - |
| - | - | 5683 | 795.4 | - | - | 0 | - |
| - | - | 7483 | 795.9 | - | - | 0 | - |
| - | - | 2398 | 796.4 | - | - | 0 | - |
| - | - | 6555 | 796.4 | - | - | 0 | - |
| - | - | 4874 | 796.9 | - | - | 0 | - |
| - | - | 2071 | 797.4 | - | - | 0 | - |
| - | - | 3217 | 797.4 | - | - | 0 | - |
| - | - | 1515 | 797.9 | - | - | 0 | - |
| - | - | 5869 | 800.4 | - | - | 0 | - |
| - | - | 7038 | 800.9 | - | - | 0 | - |
| - | - | 6868 | 801.4 | - | - | 0 | - |
| - | - | 4097 | 801.9 | - | - | 0 | - |
| - | - | 4390 | 802.4 | - | - | 0 | - |
| - | - | 4709 | 802.9 | - | - | 0 | - |
| - | - | 2643 | 803.4 | - | - | 0 | - |
| - | - | 3334 | 803.9 | - | - | 0 | - |
| - | - | 5210 | 804.4 | - | - | 0 | - |
| - | - | 2449 | 804.9 | - | - | 0 | - |
| - | - | 1863 | 809 | - | - | 0 | - |
| - | - | 1.382E+04 | 809.4 | - | - | 0 | - |
| - | - | 1.413E+04 | 809.9 | - | - | 0 | - |
| - | - | 8430 | 810.4 | - | - | 0 | - |
| - | - | 4828 | 810.9 | - | - | 0 | - |
| - | - | 1.061E+04 | 811.4 | - | - | 0 | - |
| - | - | 9401 | 812.4 | - | - | 0 | - |
| - | - | 2377 | 813.4 | - | - | 0 | - |
| - | - | 1066 | 814.4 | - | - | 0 | - |
| - | - | 3346 | 814.9 | - | - | 0 | - |
| - | - | 2039 | 815.4 | - | - | 0 | - |
| - | - | 1024 | 815.9 | - | - | 0 | - |
| - | - | 1837 | 822.4 | - | - | 0 | - |
| - | - | 2009 | 822.5 | - | - | 0 | - |
| - | - | 9069 | 822.9 | - | - | 0 | - |
| - | - | 5.319E+04 | 823.4 | - | - | 0 | - |
| 7 | y | 1.157E+04 | 823.5 | 3.871E-05 | 0.047 | +1 | 8 |
| - | - | 5.347E+04 | 823.9 | - | - | 0 | - |
| - | - | 2.698E+04 | 824.4 | - | - | 0 | - |
| - | - | 5990 | 824.5 | - | - | 0 | - |
| - | - | 9305 | 824.9 | - | - | 0 | - |
| - | - | 2745 | 825.4 | - | - | 0 | - |
| - | - | 1818 | 825.5 | - | - | 0 | - |
| - | - | 1297 | 830.4 | - | - | 0 | - |
| - | - | 7968 | 830.9 | - | - | 0 | - |
| - | - | 1.23E+05 | 831.5 | - | - | 0 | - |
| - | - | 5.537E+05 | 832 | - | - | 0 | - |
| - | - | 4.946E+05 | 832.5 | - | - | 0 | - |
| - | - | 2.61E+05 | 833 | - | - | 0 | - |
| - | - | 8.964E+04 | 833.5 | - | - | 0 | - |
| - | - | 2.426E+04 | 834 | - | - | 0 | - |
| 6 | c | 6989 | 839.4 | 0.0005391 | 0.6423 | +1 | 6 |
| - | - | 4187 | 840.4 | - | - | 0 | - |
| - | - | 1277 | 841.4 | - | - | 0 | - |
| - | - | 6522 | 850.5 | - | - | 0 | - |
| - | - | 3678 | 851.5 | - | - | 0 | - |
| - | - | 5924 | 852.5 | - | - | 0 | - |
| - | - | 2177 | 853.5 | - | - | 0 | - |
| - | - | 1793 | 854.5 | - | - | 0 | - |
| - | - | 2722 | 855.5 | - | - | 0 | - |
| - | - | 1419 | 856.5 | - | - | 0 | - |
| - | - | 1360 | 873.5 | - | - | 0 | - |
| - | - | 880.3 | 874.5 | - | - | 0 | - |
| - | - | 1868 | 883.4 | - | - | 0 | - |
| - | - | 984.8 | 884.4 | - | - | 0 | - |
| - | - | 1956 | 909.4 | - | - | 0 | - |
| - | - | 1366 | 910.4 | - | - | 0 | - |
| 6 | z | 8.995E+04 | 944.6 | 0.0003783 | 0.4005 | +1 | 9 |
| - | - | 5.354E+04 | 945.6 | - | - | 0 | - |
| - | - | 1.634E+04 | 946.6 | - | - | 0 | - |
| - | - | 4849 | 947.6 | - | - | 0 | - |
| 7 | c | 9581 | 953.4 | 0.001609 | 1.688 | +1 | 7 |
| - | - | 5432 | 954.4 | - | - | 0 | - |
| - | - | 1829 | 955.4 | - | - | 0 | - |
| 6 | y | 8268 | 960.6 | 0.0002089 | 0.2174 | +1 | 9 |
| - | - | 4787 | 961.6 | - | - | 0 | - |
| - | - | 1659 | 962.6 | - | - | 0 | - |
| - | - | 1854 | 968.6 | - | - | 0 | - |
| - | - | 4246 | 982.5 | - | - | 0 | - |
| - | - | 4269 | 983.6 | - | - | 0 | - |
| - | - | 1449 | 984.6 | - | - | 0 | - |
| - | - | 1329 | 988.6 | - | - | 0 | - |
| - | - | 3960 | 1009 | - | - | 0 | - |
| 8 | c | 7.73E+04 | 1010 | 0.0003484 | 0.3448 | +1 | 8 |
| - | - | 4.92E+04 | 1011 | - | - | 0 | - |
| - | - | 1.452E+04 | 1012 | - | - | 0 | - |
| - | - | 2970 | 1013 | - | - | 0 | - |
| 5 | y | 1584 | 1081 | 0.0001947 | 0.1802 | +1 | 10 |
| 5 | z | 4.574E+04 | 1082 | 0.0005484 | 0.507 | +1 | 10 |
| - | - | 1.004E+05 | 1083 | - | - | 0 | - |
| - | - | 5.747E+04 | 1084 | - | - | 0 | - |
| - | - | 1.989E+04 | 1085 | - | - | 0 | - |
| - | - | 3376 | 1086 | - | - | 0 | - |
| - | - | 3187 | 1095 | - | - | 0 | - |
| - | - | 2595 | 1096 | - | - | 0 | - |
| - | - | 2420 | 1096 | - | - | 0 | - |
| - | - | 3821 | 1097 | - | - | 0 | - |
| - | - | 759.4 | 1098 | - | - | 0 | - |
| 5 | y | 1.049E+04 | 1098 | 0.002062 | 1.879 | +1 | 10 |
| - | - | 4917 | 1099 | - | - | 0 | - |
| - | - | 1419 | 1100 | - | - | 0 | - |
| 9 | c | 1200 | 1122 | 0.0006601 | 0.5885 | +1 | 9 |
| - | - | 1295 | 1138 | - | - | 0 | - |
| 9 | c | 1.027E+05 | 1139 | 0.0007223 | 0.6344 | +1 | 9 |
| - | - | 6.936E+04 | 1140 | - | - | 0 | - |
| - | - | 2.694E+04 | 1141 | - | - | 0 | - |
| - | - | 5855 | 1142 | - | - | 0 | - |
| - | - | 2235 | 1146 | - | - | 0 | - |
| - | - | 2157 | 1147 | - | - | 0 | - |
| - | - | 989.1 | 1154 | - | - | 0 | - |
| - | - | 1845 | 1166 | - | - | 0 | - |
| - | - | 3700 | 1167 | - | - | 0 | - |
| - | - | 956.1 | 1168 | - | - | 0 | - |
| 4 | y | 926.3 | 1209 | 0.00162 | 1.34 | +1 | 11 |
| 4 | z | 1.155E+04 | 1210 | 0.0005647 | 0.4668 | +1 | 11 |
| - | - | 6.072E+04 | 1211 | - | - | 0 | - |
| - | - | 3.874E+04 | 1212 | - | - | 0 | - |
| - | - | 1.537E+04 | 1213 | - | - | 0 | - |
| - | - | 4427 | 1214 | - | - | 0 | - |
| 4 | y | 3461 | 1226 | 0.0007033 | 0.5738 | +1 | 11 |
| - | - | 2745 | 1227 | - | - | 0 | - |
| - | - | 941.7 | 1237 | - | - | 0 | - |
| - | - | 1817 | 1259 | - | - | 0 | - |
| - | - | 1684 | 1260 | - | - | 0 | - |
| - | - | 1229 | 1263 | - | - | 0 | - |
| - | - | 1954 | 1264 | - | - | 0 | - |
| - | - | 2793 | 1292 | - | - | 0 | - |
| - | - | 1493 | 1293 | - | - | 0 | - |
| - | - | 2650 | 1306 | - | - | 0 | - |
| 11 | c | 4.785E+04 | 1307 | 4.403E-05 | 0.0337 | +1 | 11 |
| - | - | 3.452E+04 | 1308 | - | - | 0 | - |
| - | - | 1.676E+04 | 1309 | - | - | 0 | - |
| - | - | 4606 | 1310 | - | - | 0 | - |
| 3 | z | 5787 | 1373 | 0.001363 | 0.9925 | +1 | 12 |
| - | - | 2.798E+04 | 1374 | - | - | 0 | - |
| - | - | 2.292E+04 | 1375 | - | - | 0 | - |
| - | - | 9859 | 1376 | - | - | 0 | - |
| - | - | 2838 | 1377 | - | - | 0 | - |
| 3 | y | 2116 | 1389 | 0.00506 | 3.643 | +1 | 12 |
| - | - | 2517 | 1390 | - | - | 0 | - |
| - | - | 1245 | 1402 | - | - | 0 | - |
| - | - | 2752 | 1419 | - | - | 0 | - |
| - | - | 4169 | 1420 | - | - | 0 | - |
| - | - | 1878 | 1421 | - | - | 0 | - |
| 12 | c | 1.57E+04 | 1435 | 0.001623 | 1.131 | +1 | 12 |
| - | - | 2.177E+04 | 1436 | - | - | 0 | - |
| - | - | 1.519E+04 | 1437 | - | - | 0 | - |
| - | - | 5321 | 1438 | - | - | 0 | - |
| - | - | 2061 | 1439 | - | - | 0 | - |
| - | - | 2642 | 1505 | - | - | 0 | - |
| - | - | 1330 | 1506 | - | - | 0 | - |
| - | - | 1985 | 1532 | - | - | 0 | - |
| - | - | 2783 | 1533 | - | - | 0 | - |
| - | - | 1497 | 1534 | - | - | 0 | - |
| 13 | c | 7720 | 1548 | 0.001093 | 0.7058 | +1 | 13 |
| - | - | 1.643E+04 | 1549 | - | - | 0 | - |
| - | - | 1.026E+04 | 1550 | - | - | 0 | - |
| - | - | 4307 | 1551 | - | - | 0 | - |
| - | - | 2011 | 1552 | - | - | 0 | - |
| 2 | z | 2992 | 1559 | 0.001574 | 1.01 | +1 | 13 |
| - | - | 1.026E+04 | 1560 | - | - | 0 | - |
| - | - | 7715 | 1561 | - | - | 0 | - |
| - | - | 5269 | 1562 | - | - | 0 | - |
| - | - | 1698 | 1563 | - | - | 0 | - |
| - | - | 3750 | 1581 | - | - | 0 | - |
| - | - | 2965 | 1582 | - | - | 0 | - |
| - | - | 1424 | 1583 | - | - | 0 | - |
| - | - | 1385 | 1584 | - | - | 0 | - |
| - | - | 1010 | 1591 | - | - | 0 | - |
| - | - | 1154 | 1601 | - | - | 0 | - |
| - | - | 1500 | 1602 | - | - | 0 | - |
| - | - | 1176 | 1603 | - | - | 0 | - |
| - | - | 4848 | 1618 | - | - | 0 | - |
| - | - | 1.271E+04 | 1619 | - | - | 0 | - |
| - | - | 9909 | 1620 | - | - | 0 | - |
| - | - | 5727 | 1621 | - | - | 0 | - |
| - | - | 1539 | 1622 | - | - | 0 | - |
| - | - | 6540 | 1629 | - | - | 0 | - |
| - | - | 5662 | 1630 | - | - | 0 | - |
| - | - | 2300 | 1631 | - | - | 0 | - |
| - | - | 5154 | 1636 | - | - | 0 | - |
| - | - | 3928 | 1637 | - | - | 0 | - |
| - | - | 2928 | 1638 | - | - | 0 | - |
| - | - | 1577 | 1645 | - | - | 0 | - |
| - | - | 1.229E+04 | 1646 | - | - | 0 | - |
| - | - | 5.273E+04 | 1647 | - | - | 0 | - |
| - | - | 4.824E+04 | 1648 | - | - | 0 | - |
| - | - | 2.278E+04 | 1649 | - | - | 0 | - |
| - | - | 7304 | 1650 | - | - | 0 | - |
| - | - | 1899 | 1651 | - | - | 0 | - |
| - | - | 1.117E+04 | 1662 | - | - | 0 | - |
| - | - | 6.117E+04 | 1663 | - | - | 0 | - |
| - | - | 2.693E+05 | 1664 | - | - | 0 | - |
| - | - | 2.393E+05 | 1665 | - | - | 0 | - |
| - | - | 1.207E+05 | 1666 | - | - | 0 | - |
| - | - | 3.684E+04 | 1667 | - | - | 0 | - |
| - | - | 1.213E+04 | 1668 | - | - | 0 | - |
| - | - | 1115 | 1866 | - | - | 0 | - |

m/z Charge Intensity FragmentType MassShift Position
124.71467590332031 0 640.5883
124.82473754882812 0 528.7971
125.71238708496094 0 515.0658
127.50818634033203 0 463.22693
128.7911834716797 0 510.7017
129.10238647460938 0 1917.6086
130.06536865234375 0 1051.7655
132.0810089111328 0 1844.5024
132.10214233398438 0 3627.4836 y 13
136.07589721679688 0 1253.3531
141.5152130126953 0 527.389
146.09278869628906 0 1106.6724
153.07858276367188 0 4361.2275
164.02366638183594 0 622.2674
168.10133361816406 0 633.17084
172.31005859375 0 616.0124
173.43862915039062 0 1928.4373
175.75599670410156 0 605.81714
181.7494659423828 0 698.8034
186.07847595214844 0 1638.8966
187.08673095703125 0 3355.1218
199.1806640625 0 704.7217
209.54022216796875 0 674.40967
209.9342803955078 0 692.0898
210.1494140625 0 821.80054
214.1551055908203 0 1073.3193
215.1389923095703 0 1001.3379
223.1191864013672 0 2236.5178
226.1551971435547 0 3214.6147
227.1754913330078 0 731.1262
228.11048889160156 0 974.9728
243.2021484375 0 622.0833
245.137451171875 0 5269.7373
245.18618774414062 0 6943.5303 y 12
246.1239471435547 0 21008.033
246.18959045410156 0 899.0804
247.12786865234375 0 2813.54
255.10096740722656 0 1650.6254
258.14532470703125 0 1729.3082
267.1064758300781 0 830.5342
273.1104736328125 0 1629.2363
274.1188049316406 0 25663.096
275.1229248046875 0 4195.8853
281.17352294921875 0 3089.8755
282.17950439453125 0 849.15686
292.12939453125 0 3038.0364
297.19146728515625 0 1332.4003
301.1998291015625 0 1775.5814
309.1558837890625 0 3342.1816
314.20770263671875 0 2336.9055
338.1348571777344 0 1719.4017
338.196533203125 0 902.5911
346.726806640625 0 3067.8975
347.2293701171875 0 779.9588 z Ammonia loss 7
349.1420593261719 0 2323.166
350.26812744140625 0 1026.183
355.06817626953125 0 788.13324
357.26239013671875 0 10004.924 z 11
358.267822265625 0 3981.185
371.09979248046875 0 1256.6587
373.2807922363281 0 2881.6611 y 11
382.28118896484375 0 1709.3297
394.2575988769531 0 1783.179
399.2613525390625 0 1618.704
412.2741394042969 0 12664.859 y 6
412.7757568359375 0 6345.9146
413.2281799316406 0 3109.486
413.27557373046875 0 1280.1488
414.23150634765625 0 980.6197
420.1836242675781 0 1801.2797 c Ammonia loss 5
420.6856994628906 0 939.19684
423.2243347167969 0 1568.8778
427.2919616699219 0 5671.5073 w 10
428.2640380859375 0 901.08734
428.2978210449219 0 2248.972
429.18597412109375 0 1214.9406
434.1932373046875 0 834.42505
435.19122314453125 0 1590.5074
436.17388916015625 0 1241.8254
437.18218994140625 0 4531.081
438.1857604980469 0 1052.8661
439.2200012207031 0 1239.7295
451.220947265625 0 1006.24615
453.2011413574219 0 4932.8667
454.20831298828125 0 9969.141 c 2
455.2120056152344 0 2747.4756
463.5989990234375 0 3673.713 y 2
463.9330749511719 0 2909.5579
465.2091064453125 0 3578.6475
468.3187561035156 0 10550.509
469.2897644042969 0 3329.2979
469.3233642578125 0 2377.7542
470.29486083984375 0 1627.9968
470.3338928222656 0 4662.2266 y 10
472.3034973144531 0 1042.5742
473.2427673339844 0 4620.9985 c Ammonia loss 11
473.5777587890625 0 4525.875
473.90997314453125 0 1990.9249
477.20166015625 0 4071.8867
478.20465087890625 0 1607.3646
480.22259521484375 0 2054.243
480.8047180175781 0 1960.7092 y 5
481.3049621582031 0 1844.0962
482.2983703613281 0 3145.0664
483.3006591796875 0 1156.1655
497.22064208984375 0 764.84235 c Ammonia loss 7
498.32867431640625 0 857.7322
501.60595703125 0 1268.5271
501.9413146972656 0 999.7493
504.93450927734375 0 6022.9766
505.26806640625 0 6192.5938
505.6025695800781 0 1706.4663
507.3424987792969 0 873.5628
510.9375305175781 0 23016.238 c Ammonia loss 12
511.2717590332031 0 18222.303
511.6063232421875 0 10032.104
511.9397277832031 0 3876.8906
512.2709350585938 0 1121.8223
516.941162109375 0 5297.3345
517.2754516601562 0 2998.0703
517.61328125 0 1005.6124
517.94873046875 0 1169.2219
520.2811889648438 0 1617.0875 z 1
523.2420043945312 0 2203.037
525.3526000976562 0 41141.473 z 9
525.6243896484375 0 3434.0613 y 1
525.9595947265625 0 4484.61
526.2941284179688 0 1919.1425
526.3563232421875 0 12976.219
527.3578491210938 0 2058.7314
536.1929321289062 0 1008.1378
538.2496337890625 0 1567.907
538.2889404296875 0 1770.2505
539.2613525390625 0 2830.309
539.9710693359375 0 1172.2559
541.3224487304688 0 1989.505 z 4
541.371826171875 0 5638.14 y 9
541.8252563476562 0 1166.1793
542.375 0 1541.6904
546.2218627929688 0 1988.3452
547.2241821289062 0 765.804
548.6322021484375 0 5912.004
548.96533203125 0 8449.321
549.2978515625 0 4633.356
549.3350830078125 0 3691.7559 y 4
549.6317138671875 0 1520.2092
549.8357543945312 0 3765.225
549.9630126953125 0 1084.3091
553.2877807617188 0 690.9115
553.9620361328125 0 1616.52
554.2923583984375 0 1944.189
554.6358032226562 0 193556.16
554.969970703125 0 182502.4
555.3041381835938 0 98810.46
555.638427734375 0 30978.799
555.7113037109375 0 1017.8056
555.9728393554688 0 8261.002
556.2979736328125 0 5608.114
561.7667236328125 0 1115.6196
563.24951171875 0 11990.408
564.25 0 4266.8013 c Water loss 3
565.2418823242188 0 5119.525 c Ammonia loss 3
566.2462158203125 0 3924.1584
566.4026489257812 0 3081.4392
567.2518920898438 0 1334.1322
569.8168334960938 0 979.8566
572.8050537109375 0 2091.673
573.3053588867188 0 1393.8931
576.338623046875 0 11361.1455 w 3
576.8399658203125 0 6941.5938
577.332275390625 0 2342.005
577.8230590820312 0 2019.9979
579.3635864257812 0 1174.4122
581.2601928710938 0 8263.537
582.267578125 0 45220.406 c 3
582.3737182617188 0 3669.6084
583.2706298828125 0 14049.167
584.2734985351562 0 2154.251
595.3819580078125 0 6816.912 w 8
596.38525390625 0 3417.5178
596.7863159179688 0 1035.6354 c Ammonia loss 9
597.3870849609375 0 1738.7267
604.3568725585938 0 1523.0048
604.8549194335938 0 3130.0952 y Ammonia loss 3
605.35302734375 0 32657.342 z 3
605.8553466796875 0 34320.61
606.357421875 0 19441.234
606.8590698242188 0 6671.055
607.3615112304688 0 1036.863
609.458251953125 0 1826.9169
610.39697265625 0 6649.7017
611.3995361328125 0 2302.1418
612.856689453125 0 1206.0596
613.362060546875 0 10945.606 y 3
613.8640747070312 0 6706.807
614.3648071289062 0 2145.7905
617.2804565429688 0 949.45404
620.3395385742188 0 1095.8892
620.8427734375 0 982.77496
622.32177734375 0 786.9802
624.4217529296875 0 2093.0818
629.3465576171875 0 5678.515
629.8486328125 0 3596.5986
630.3467407226562 0 1776.418
637.4302368164062 0 1199.8722
639.3961181640625 0 1501.7412
645.828125 0 2508.134
646.3255615234375 0 1484.0093
651.3571166992188 0 2502.3213
651.85888671875 0 2107.0422
653.44775390625 0 33025.42 z 8
653.8275756835938 0 2899.445 c 10
654.3236694335938 0 1998.8636
654.4515380859375 0 17159.04
655.455078125 0 3862.3586
657.304443359375 0 1241.3108
657.8662109375 0 4418.873
658.3728637695312 0 1302.8555
658.8568115234375 0 1935.4049
659.2955322265625 0 4739.7344
659.355224609375 0 1320.4569
660.2976684570312 0 1444.4414
665.3592529296875 0 1734.0854
665.8577880859375 0 1955.7449
669.461181640625 0 1305.4053 y 8
674.3057861328125 0 3304.3677
675.311279296875 0 2348.9329
685.8893432617188 0 1382.7634
686.38525390625 0 3513.8813 y Ammonia loss 2
686.884521484375 0 41860.863 z 2
687.38623046875 0 32351.145
687.8882446289062 0 16289.523
688.3895263671875 0 7667.447
688.893310546875 0 1376.7888
692.4469604492188 0 1409.6012
694.8941650390625 0 41826.516 y 2
695.3954467773438 0 33730.594
695.8965454101562 0 17334.504
696.3965454101562 0 4576.1685
696.900634765625 0 1566.6594
702.2993774414062 0 3131.7207 c Ammonia loss 4
703.2991333007812 0 1586.198
708.39013671875 0 1510.1346
709.3609619140625 0 8526.535 c Ammonia loss 11
709.8619995117188 0 6630.5557
710.3610229492188 0 2875.4712
710.4686889648438 0 5417.5044 z 7
711.4737548828125 0 4068.9429
712.4736328125 0 1211.0609
717.367431640625 0 2482.6812
717.8734741210938 0 105518.81 c 11
718.375 0 85365.34
718.876220703125 0 40247.367
719.3263549804688 0 89059.44 c 4
719.876708984375 0 3164.8096
720.32958984375 0 41064.285
721.3323974609375 0 9044.845
722.3372802734375 0 1130.9594
726.4873046875 0 3321.083 y 7
727.492431640625 0 1279.0773
730.8819580078125 0 1560.6257
731.3827514648438 0 1981.3398
731.8798828125 0 1029.3767
736.4064331054688 0 1125.0232
744.9028930664062 0 1159.3475
750.908935546875 0 1386.0026
751.411865234375 0 1963.6335
752.4059448242188 0 3152.9465
752.9094848632812 0 2262.5232
753.4118041992188 0 1696.3818
756.9041137695312 0 1333.6693
757.9078979492188 0 1208.2655
758.4102783203125 0 1544.3579
758.9132080078125 0 4150.0757
759.4137573242188 0 4225.165
759.9071044921875 0 1072.1198
765.9046020507812 0 2915.0227 c Ammonia loss 12
766.4111328125 0 5657.884
766.9154663085938 0 4298.481
767.421142578125 0 4998.5127
767.9329223632812 0 1381.6165
768.3718872070312 0 1149.7325
773.418212890625 0 1806.7052
773.9252319335938 0 1368.973
774.4158935546875 0 66730.83 c 12
774.9170532226562 0 66826.43
775.4183959960938 0 33113.945
775.9191284179688 0 9630.129
776.420166015625 0 3203.5244
777.9222412109375 0 2005.5104
778.4381713867188 0 1574.5787
778.9246826171875 0 2755.5198
779.42236328125 0 2297.7278 y Ammonia loss 1
779.9247436523438 0 9583.777 z 1
780.4248046875 0 13374.599
780.498291015625 0 1540.5652 w 6
780.92724609375 0 7263.8213
781.4232788085938 0 2689.172
781.50439453125 0 1416.8474
781.9232177734375 0 1574.296
782.4221801757812 0 1716.2446
787.9227294921875 0 6076.2764 y 1
788.424072265625 0 5908.6216
788.9288330078125 0 3717.7498
789.42626953125 0 1179.1816
790.9268798828125 0 8495.291
791.4290161132812 0 7936.637
791.9278564453125 0 3493.1284
792.4307861328125 0 3052.1934
792.9358520507812 0 1518.0884
794.3526000976562 0 1237.0023
794.4268798828125 0 1442.6404
794.8994140625 0 1073.598
795.4134521484375 0 5682.9985
795.9147338867188 0 7483.432
796.3530883789062 0 2398.446
796.4229125976562 0 6555.336
796.9218139648438 0 4874.3623
797.3594970703125 0 2070.9614
797.4285278320312 0 3217.4758
797.92919921875 0 1514.7122
800.4384765625 0 5869.3096
800.9418334960938 0 7038.347
801.444091796875 0 6868.261
801.9346923828125 0 4096.6416
802.4236450195312 0 4390.1685
802.9277954101562 0 4709.2383
803.426025390625 0 2642.6497
803.9207153320312 0 3333.6719
804.4236450195312 0 5210.117
804.9261474609375 0 2448.8801
808.9525146484375 0 1862.9606
809.44384765625 0 13819.534
809.9464111328125 0 14125.884
810.443359375 0 8429.679
810.944580078125 0 4828.013
811.3645629882812 0 10613.625
812.3690185546875 0 9401.038
813.3740234375 0 2377.235
814.4315185546875 0 1065.6099
814.9376220703125 0 3345.7517
815.4370727539062 0 2039.3654
815.9368896484375 0 1024.0663
822.4335327148438 0 1836.9218
822.5317993164062 0 2008.9421
822.9456176757812 0 9069.463
823.4418334960938 0 53191.547
823.5400390625 0 11571.972 y 6
823.9426879882812 0 53466.996
824.4434814453125 0 26978.473
824.5435180664062 0 5989.8687
824.9443969726562 0 9304.961
825.4453735351562 0 2744.556
825.5475463867188 0 1818.0363
830.4423217773438 0 1296.7317
830.9461059570312 0 7967.8267
831.4500732421875 0 122997.04
831.9535522460938 0 553663
832.4552001953125 0 494620.2
832.9564208984375 0 261011.86
833.4574584960938 0 89644.945
833.9593505859375 0 24255.748
839.35888671875 0 6988.7334 c Ammonia loss 5
840.3589477539062 0 4186.5435
841.3529052734375 0 1276.9333
850.5288696289062 0 6521.9526
851.5313110351562 0 3677.9563
852.5416870117188 0 5924.1606
853.542236328125 0 2177.089
854.4819946289062 0 1792.5436
855.4939575195312 0 2721.8552
856.4969482421875 0 1419.2634
873.5062866210938 0 1360.4526
874.5005493164062 0 880.30084
883.4102172851562 0 1867.9824
884.4158935546875 0 984.7586
909.4244384765625 0 1956.4403
910.424072265625 0 1365.6493
944.58056640625 0 89948.81 z 5
945.5844116210938 0 53543.56
946.5872192382812 0 16343.783
947.5929565429688 0 4848.56
953.4392700195312 0 9581.059 c 6
954.44189453125 0 5431.9395
955.44287109375 0 1829.0002
960.59912109375 0 8267.895 y 5
961.6024780273438 0 4786.882
962.6046142578125 0 1659.0905
968.5777587890625 0 1854.0248
982.546142578125 0 4246.3633
983.5531005859375 0 4268.5723
984.5596923828125 0 1449.1932
988.5908813476562 0 1329.1292
1009.451416015625 0 3959.734
1010.45947265625 0 77295.23 c 7
1011.4625244140625 0 49199.617
1012.4651489257812 0 14523.187
1013.47021484375 0 2970.2605
1080.6314697265625 0 1584.3158 y Ammonia loss 4
1081.6396484375 0 45737.47 z 4
1082.646240234375 0 100420.766
1083.649169921875 0 57474.36
1084.6533203125 0 19893.488
1085.6572265625 0 3376.4873
1094.5364990234375 0 3187.423
1095.5362548828125 0 2595.0818
1095.634033203125 0 2419.946
1096.6417236328125 0 3821.452
1097.5367431640625 0 759.35095
1097.65576171875 0 10493.479 y 4
1098.6605224609375 0 4917.364
1099.6640625 0 1418.7094
1121.5281982421875 0 1200.2357 c Ammonia loss 8
1137.538330078125 0 1294.9506
1138.5548095703125 0 102667.305 c 8
1139.55712890625 0 69357.35
1140.56103515625 0 26937.652
1141.5633544921875 0 5854.8057
1145.6103515625 0 2235.456
1146.61279296875 0 2157.3345
1153.646728515625 0 989.10223
1165.57763671875 0 1845.4886
1166.5838623046875 0 3700.1133
1167.5849609375 0 956.12384
1208.688232421875 0 926.34625 y Ammonia loss 3
1209.6982421875 0 11547.139 z 3
1210.7052001953125 0 60715.06
1211.708251953125 0 38744.727
1212.71142578125 0 15374.669
1213.7132568359375 0 4427.2495
1225.7156982421875 0 3460.9128 y 3
1226.72021484375 0 2744.947
1236.62060546875 0 941.6799
1258.6990966796875 0 1816.5231
1259.706298828125 0 1683.8561
1262.626708984375 0 1228.9135
1263.6390380859375 0 1954.2739
1291.6295166015625 0 2792.708
1292.6400146484375 0 1493.0145
1305.634033203125 0 2649.5027
1306.6439208984375 0 47845.742 c 10
1307.646484375 0 34521.58
1308.6492919921875 0 16759.58
1309.6522216796875 0 4605.6772
1372.7596435546875 0 5787.1904 z 2
1373.7679443359375 0 27975.28
1374.77099609375 0 22920.309
1375.7724609375 0 9858.637
1376.775634765625 0 2837.7085
1388.7847900390625 0 2116.0913 y 2
1389.781982421875 0 2516.7437
1401.7186279296875 0 1244.9264
1418.7225341796875 0 2752.1406
1419.722412109375 0 4169.163
1420.7225341796875 0 1877.9014
1434.7373046875 0 15703.218 c 11
1435.7430419921875 0 21765.49
1436.74658203125 0 15194.621
1437.752197265625 0 5320.9893
1438.750732421875 0 2061.3438
1504.8070068359375 0 2641.6748
1505.81103515625 0 1330.0642
1531.8040771484375 0 1984.5261
1532.8046875 0 2783.287
1533.80322265625 0 1496.86
1547.8218994140625 0 7719.8247 c 12
1548.8271484375 0 16434.426
1549.8299560546875 0 10264.35
1550.83203125 0 4306.884
1551.8388671875 0 2010.7697
1558.8387451171875 0 2991.902 z 1
1559.8463134765625 0 10262.709
1560.8492431640625 0 7715.373
1561.8497314453125 0 5268.817
1562.842041015625 0 1698.324
1580.8414306640625 0 3749.5283
1581.8509521484375 0 2965.4001
1582.8397216796875 0 1424.1637
1583.863037109375 0 1385.3141
1590.832275390625 0 1009.6437
1600.883056640625 0 1153.9957
1601.8773193359375 0 1499.7136
1602.862060546875 0 1176.0404
1617.882568359375 0 4848.3374
1618.8837890625 0 12711.949
1619.887451171875 0 9909.154
1620.88818359375 0 5726.889
1621.88671875 0 1538.9191
1628.8701171875 0 6540.3535
1629.869873046875 0 5661.98
1630.8795166015625 0 2300.3452
1635.9083251953125 0 5153.807
1636.9088134765625 0 3928.2778
1637.9189453125 0 2927.8975
1644.8753662109375 0 1576.5172
1645.8836669921875 0 12289.307
1646.8802490234375 0 52725.766
1647.8828125 0 48242.008
1648.8861083984375 0 22779.123
1649.888671875 0 7304.311
1650.8856201171875 0 1899.4266
1661.8876953125 0 11171.236
1662.8978271484375 0 61171
1663.90576171875 0 269288.3
1664.9083251953125 0 239314.78
1665.911865234375 0 120657.17
1666.91357421875 0 36843.89
1667.9161376953125 0 12128.67
1865.9827880859375 0 1115.4767

Spectrum Details

|  |  |
| --- | --- |
| Matched peaks? Matched peaksThe total absolute number of peaks matched. Additionally in brackets the total fraction of peaks matched and the total number of peaks is shown. | 68 (13.36% of 509) |
| FDR? FDRThe false discovery rate estimated for this peptide. It is calculated by matching all theoretical fragments with a non-integer shift with the raw peaks for this spectrum. This is done with 40 different shifts. The resulting percentage is the average number of annotated peaks over the number of annotated peaks with the correct spectrum. | 3.12% |
| Satellite FDR? Satellite FDRSee the FDR for details on its calculation. This satellite ion specific FDR only contains the satellite ions (d/w) for I/L/J positions. | - |
| PSM Score? PSM ScoreThe PSM Score as given by Hecklib to this annotated spectrum. It is shown with three significant figures. | 507 |

## Spectrum 5375? Spectrum 5375 The raw spectrum of this peptide as annotated by Hecklib. The fragments are coloured according to ion type (see legend). Any peaks with a star '\*' as text can be hovered over to see the full details, first the ion type second the mass shift type. By hovering over the amino acids in the peptide or ions in the legend the corresponding peaks are highlighted. By toggling the 'Unassigned' label you can turn the background (unassigned) peaks on or off in the plot. By updating the slider in the Ion legend you can update the spectrum to only show the top X% of the peaks with labels. The top X% means any peak that is within X% of the highest intensity. By dragging in the spectrum you can zoom in to a specific part of the spectrum and use 'Zoom Out' to get back to the original zoom level. The annotation of the spectrum is based on the given sequence in the peptides file and is done with different software so inconsistencies are likely. The peaks are annotated based on the given sequence, with 20 ppm tolerance.

Copy Data

### Spectrum 5375 (TSV)

#### Preview

```
Loading example...
```

*Click on the button to copy the data to your clipboard.*

Mz MinMz MaxIntensity Max

WidthHeightPeptide font sizePeptide stroke widthSpectrum font sizeSpectrum stroke widthCompact peptide

Ion legend

wxyz

abcd

OtherUnassignedIonChargePositionShow for top:%

SWYQHHPGKAPKJJ

04.15e+58.30e+51.25e+61.66e+6

Zoom Out

y+11y+12z+13y+13y+28c+26w+14c+13y+312y+14c+312y+29c+313z+15y+313z+210y+15y+210c+29c+14c+14w+211c+14w+16c+210y+211z+211y+211z+16c+211y+16z+212y+212z+212z+17y+212c+15c+212z+17c+212c+15y+17c+213c+213y+213z+213w+18y+213y+18c+16z+19c+17y+19c+18y+110z+110y+110c+19w+111y+111z+111y+111c+111z+112y+112c+112c+113z+113y+113

042184212631685

Fragment Matches Table

Show background peaks

| Position | Ion type | Intensity | mz Theoretical | mz Error (Th) | mz Error (ppm) | Charge | Series Number |
| --- | --- | --- | --- | --- | --- | --- | --- |
| - | - | 1249 | 122.4 | - | - | 0 | - |
| - | - | 4828 | 129.1 | - | - | 0 | - |
| - | - | 2985 | 130.1 | - | - | 0 | - |
| - | - | 6440 | 132.1 | - | - | 0 | - |
| 14 | y | 8245 | 132.1 | 0.0001914 | 1.449 | +1 | 1 |
| - | - | 3833 | 136.1 | - | - | 0 | - |
| - | - | 1382 | 143.8 | - | - | 0 | - |
| - | - | 1590 | 146.1 | - | - | 0 | - |
| - | - | 1.035E+04 | 153.1 | - | - | 0 | - |
| - | - | 1469 | 168.1 | - | - | 0 | - |
| - | - | 1729 | 170.2 | - | - | 0 | - |
| - | - | 4463 | 186.1 | - | - | 0 | - |
| - | - | 8611 | 187.1 | - | - | 0 | - |
| - | - | 3241 | 199.2 | - | - | 0 | - |
| - | - | 2362 | 214.2 | - | - | 0 | - |
| - | - | 1758 | 216.8 | - | - | 0 | - |
| - | - | 4504 | 223.1 | - | - | 0 | - |
| - | - | 6103 | 226.2 | - | - | 0 | - |
| - | - | 2204 | 228.1 | - | - | 0 | - |
| - | - | 1.353E+04 | 245.1 | - | - | 0 | - |
| 13 | y | 1.764E+04 | 245.2 | 0.0001271 | 0.5183 | +1 | 2 |
| - | - | 5.268E+04 | 246.1 | - | - | 0 | - |
| - | - | 2943 | 246.1 | - | - | 0 | - |
| - | - | 2382 | 246.2 | - | - | 0 | - |
| - | - | 6505 | 247.1 | - | - | 0 | - |
| - | - | 3900 | 255.1 | - | - | 0 | - |
| - | - | 2743 | 255.2 | - | - | 0 | - |
| - | - | 1734 | 256.2 | - | - | 0 | - |
| - | - | 7359 | 258.1 | - | - | 0 | - |
| - | - | 1749 | 269 | - | - | 0 | - |
| - | - | 4372 | 273.1 | - | - | 0 | - |
| - | - | 6.865E+04 | 274.1 | - | - | 0 | - |
| - | - | 1.283E+04 | 275.1 | - | - | 0 | - |
| - | - | 8975 | 281.2 | - | - | 0 | - |
| - | - | 2602 | 282.2 | - | - | 0 | - |
| - | - | 9472 | 292.1 | - | - | 0 | - |
| - | - | 2497 | 299.2 | - | - | 0 | - |
| - | - | 5392 | 301.2 | - | - | 0 | - |
| - | - | 9426 | 309.2 | - | - | 0 | - |
| - | - | 2361 | 310.2 | - | - | 0 | - |
| - | - | 3925 | 314.2 | - | - | 0 | - |
| - | - | 2076 | 320.1 | - | - | 0 | - |
| - | - | 2684 | 323.2 | - | - | 0 | - |
| - | - | 5780 | 346.7 | - | - | 0 | - |
| - | - | 6037 | 349.1 | - | - | 0 | - |
| - | - | 2351 | 353.5 | - | - | 0 | - |
| 12 | z | 2.772E+04 | 357.3 | 0.0003652 | 1.022 | +1 | 3 |
| - | - | 1.217E+04 | 358.3 | - | - | 0 | - |
| - | - | 2795 | 359.3 | - | - | 0 | - |
| 12 | y | 6599 | 373.3 | 0.0002263 | 0.6063 | +1 | 3 |
| - | - | 5157 | 382.3 | - | - | 0 | - |
| - | - | 9072 | 394.3 | - | - | 0 | - |
| - | - | 1898 | 395.3 | - | - | 0 | - |
| - | - | 2172 | 399.3 | - | - | 0 | - |
| - | - | 2909 | 401.1 | - | - | 0 | - |
| - | - | 3468 | 406.2 | - | - | 0 | - |
| 7 | y | 3.741E+04 | 412.3 | 0.0007757 | 1.881 | +2 | 8 |
| - | - | 1.766E+04 | 412.8 | - | - | 0 | - |
| - | - | 6387 | 413.2 | - | - | 0 | - |
| - | - | 4602 | 413.3 | - | - | 0 | - |
| 6 | c | 2995 | 420.2 | 0.001972 | 4.693 | +2 | 6 |
| - | - | 3037 | 420.7 | - | - | 0 | - |
| - | - | 2305 | 426.2 | - | - | 0 | - |
| 11 | w | 1.511E+04 | 427.3 | 0.0007395 | 1.731 | +1 | 4 |
| - | - | 5877 | 428.3 | - | - | 0 | - |
| - | - | 2470 | 429.2 | - | - | 0 | - |
| - | - | 8335 | 435.2 | - | - | 0 | - |
| - | - | 2004 | 436.2 | - | - | 0 | - |
| - | - | 1.058E+04 | 437.2 | - | - | 0 | - |
| - | - | 2549 | 438.2 | - | - | 0 | - |
| - | - | 2385 | 439.2 | - | - | 0 | - |
| - | - | 1.49E+04 | 453.2 | - | - | 0 | - |
| 3 | c | 2.723E+04 | 454.2 | 0.0001532 | 0.3373 | +1 | 3 |
| - | - | 7865 | 455.2 | - | - | 0 | - |
| 3 | y | 1.167E+04 | 463.6 | 0.0008131 | 1.754 | +3 | 12 |
| - | - | 7311 | 463.9 | - | - | 0 | - |
| - | - | 9456 | 465.2 | - | - | 0 | - |
| - | - | 3.439E+04 | 468.3 | - | - | 0 | - |
| - | - | 8016 | 469.3 | - | - | 0 | - |
| - | - | 8695 | 469.3 | - | - | 0 | - |
| - | - | 2844 | 470.3 | - | - | 0 | - |
| 11 | y | 1.826E+04 | 470.3 | 0.0001694 | 0.3601 | +1 | 4 |
| - | - | 3744 | 471.3 | - | - | 0 | - |
| 12 | c | 1.852E+04 | 473.2 | 0.00122 | 2.577 | +3 | 12 |
| - | - | 1.008E+04 | 473.6 | - | - | 0 | - |
| - | - | 5249 | 473.9 | - | - | 0 | - |
| - | - | 2974 | 474.2 | - | - | 0 | - |
| - | - | 1.658E+04 | 477.2 | - | - | 0 | - |
| - | - | 4649 | 478.2 | - | - | 0 | - |
| - | - | 2261 | 479.2 | - | - | 0 | - |
| - | - | 3984 | 480.2 | - | - | 0 | - |
| 6 | y | 3070 | 480.8 | 0.000464 | 0.9651 | +2 | 9 |
| - | - | 4860 | 481.3 | - | - | 0 | - |
| - | - | 2435 | 482.3 | - | - | 0 | - |
| - | - | 9366 | 482.3 | - | - | 0 | - |
| - | - | 2620 | 483.8 | - | - | 0 | - |
| - | - | 3963 | 495.4 | - | - | 0 | - |
| - | - | 4099 | 498.3 | - | - | 0 | - |
| - | - | 4511 | 501.6 | - | - | 0 | - |
| - | - | 2694 | 501.9 | - | - | 0 | - |
| - | - | 1.635E+04 | 504.9 | - | - | 0 | - |
| - | - | 1.239E+04 | 505.3 | - | - | 0 | - |
| - | - | 6922 | 505.6 | - | - | 0 | - |
| - | - | 3207 | 507.3 | - | - | 0 | - |
| - | - | 2489 | 510.2 | - | - | 0 | - |
| 13 | c | 7.345E+04 | 510.9 | 0.0006845 | 1.34 | +3 | 13 |
| - | - | 7.02E+04 | 511.3 | - | - | 0 | - |
| - | - | 2.706E+04 | 511.6 | - | - | 0 | - |
| - | - | 9986 | 511.9 | - | - | 0 | - |
| - | - | 4112 | 512.3 | - | - | 0 | - |
| - | - | 8433 | 516.9 | - | - | 0 | - |
| - | - | 8331 | 517.3 | - | - | 0 | - |
| - | - | 3647 | 517.6 | - | - | 0 | - |
| - | - | 9106 | 523.2 | - | - | 0 | - |
| 10 | z | 1.1E+05 | 525.4 | 0.0006975 | 1.328 | +1 | 5 |
| 2 | y | 9103 | 525.6 | 0.000529 | 1.006 | +3 | 13 |
| - | - | 1.26E+04 | 526 | - | - | 0 | - |
| - | - | 8257 | 526.3 | - | - | 0 | - |
| - | - | 3.795E+04 | 526.4 | - | - | 0 | - |
| - | - | 7466 | 527.4 | - | - | 0 | - |
| - | - | 5996 | 538.3 | - | - | 0 | - |
| - | - | 4758 | 539.3 | - | - | 0 | - |
| - | - | 3610 | 539.3 | - | - | 0 | - |
| - | - | 2314 | 540.6 | - | - | 0 | - |
| 5 | z | 5943 | 541.3 | 0.002082 | 3.847 | +2 | 10 |
| 10 | y | 1.218E+04 | 541.4 | 0.001077 | 1.99 | +1 | 5 |
| - | - | 5613 | 541.8 | - | - | 0 | - |
| - | - | 4172 | 542.4 | - | - | 0 | - |
| - | - | 6143 | 546.2 | - | - | 0 | - |
| - | - | 2589 | 547.8 | - | - | 0 | - |
| - | - | 3592 | 548.6 | - | - | 0 | - |
| - | - | 1.901E+04 | 548.6 | - | - | 0 | - |
| - | - | 1.458E+04 | 549 | - | - | 0 | - |
| - | - | 6586 | 549.3 | - | - | 0 | - |
| 5 | y | 1.016E+04 | 549.3 | 0.00119 | 2.166 | +2 | 10 |
| - | - | 7886 | 549.6 | - | - | 0 | - |
| - | - | 1.06E+04 | 549.8 | - | - | 0 | - |
| - | - | 2519 | 550 | - | - | 0 | - |
| - | - | 2785 | 550.3 | - | - | 0 | - |
| - | - | 4489 | 553.4 | - | - | 0 | - |
| - | - | 6580 | 554 | - | - | 0 | - |
| - | - | 3949 | 554.3 | - | - | 0 | - |
| - | - | 5.548E+05 | 554.6 | - | - | 0 | - |
| - | - | 5.668E+05 | 555 | - | - | 0 | - |
| - | - | 3.046E+05 | 555.3 | - | - | 0 | - |
| - | - | 1.013E+05 | 555.6 | - | - | 0 | - |
| - | - | 2587 | 555.7 | - | - | 0 | - |
| - | - | 2.561E+04 | 556 | - | - | 0 | - |
| 9 | c | 3147 | 561.3 | 0.0009643 | 1.718 | +2 | 9 |
| - | - | 3.956E+04 | 563.2 | - | - | 0 | - |
| 4 | c | 1.178E+04 | 564.3 | 0.006874 | 12.18 | +1 | 4 |
| 4 | c | 1.678E+04 | 565.2 | 0.001908 | 3.375 | +1 | 4 |
| - | - | 1.289E+04 | 566.2 | - | - | 0 | - |
| - | - | 9462 | 566.4 | - | - | 0 | - |
| - | - | 3485 | 567.2 | - | - | 0 | - |
| - | - | 3096 | 567.4 | - | - | 0 | - |
| - | - | 2607 | 569.8 | - | - | 0 | - |
| - | - | 2682 | 572.3 | - | - | 0 | - |
| - | - | 5956 | 572.8 | - | - | 0 | - |
| - | - | 3077 | 573.3 | - | - | 0 | - |
| 4 | w | 3.223E+04 | 576.3 | 0.0007294 | 1.266 | +2 | 11 |
| - | - | 2.133E+04 | 576.8 | - | - | 0 | - |
| - | - | 9654 | 577.3 | - | - | 0 | - |
| - | - | 2429 | 579.4 | - | - | 0 | - |
| - | - | 2.385E+04 | 581.3 | - | - | 0 | - |
| 4 | c | 1.269E+05 | 582.3 | 0.0006272 | 1.077 | +1 | 4 |
| - | - | 1.168E+04 | 582.4 | - | - | 0 | - |
| - | - | 3.751E+04 | 583.3 | - | - | 0 | - |
| - | - | 3386 | 583.4 | - | - | 0 | - |
| - | - | 6991 | 584.3 | - | - | 0 | - |
| - | - | 2625 | 587.8 | - | - | 0 | - |
| 9 | w | 2.614E+04 | 595.4 | 0.001133 | 1.903 | +1 | 6 |
| - | - | 9695 | 596.4 | - | - | 0 | - |
| 10 | c | 2776 | 596.8 | 0.005601 | 9.385 | +2 | 10 |
| - | - | 4064 | 597.4 | - | - | 0 | - |
| - | - | 4992 | 604.4 | - | - | 0 | - |
| 4 | y | 7542 | 604.8 | 0.002083 | 3.443 | +2 | 11 |
| 4 | z | 9.228E+04 | 605.4 | 0.0006724 | 1.111 | +2 | 11 |
| - | - | 9.916E+04 | 605.9 | - | - | 0 | - |
| - | - | 4.995E+04 | 606.4 | - | - | 0 | - |
| - | - | 1.233E+04 | 606.9 | - | - | 0 | - |
| - | - | 4969 | 607.4 | - | - | 0 | - |
| - | - | 2355 | 609.4 | - | - | 0 | - |
| - | - | 6484 | 609.5 | - | - | 0 | - |
| - | - | 2.415E+04 | 610.4 | - | - | 0 | - |
| - | - | 7196 | 611.4 | - | - | 0 | - |
| 4 | y | 3.618E+04 | 613.4 | 0.0005877 | 0.9582 | +2 | 11 |
| - | - | 2.087E+04 | 613.9 | - | - | 0 | - |
| - | - | 6332 | 614.4 | - | - | 0 | - |
| - | - | 3601 | 620.3 | - | - | 0 | - |
| - | - | 3208 | 620.8 | - | - | 0 | - |
| - | - | 7468 | 624.4 | - | - | 0 | - |
| - | - | 1.484E+04 | 629.3 | - | - | 0 | - |
| - | - | 1.653E+04 | 629.8 | - | - | 0 | - |
| - | - | 5397 | 630.4 | - | - | 0 | - |
| - | - | 4372 | 637.4 | - | - | 0 | - |
| - | - | 2520 | 638.4 | - | - | 0 | - |
| - | - | 3475 | 645.8 | - | - | 0 | - |
| - | - | 1.041E+04 | 651.4 | - | - | 0 | - |
| - | - | 9722 | 651.9 | - | - | 0 | - |
| - | - | 2757 | 652.4 | - | - | 0 | - |
| 9 | z | 8.353E+04 | 653.4 | 0.001193 | 1.826 | +1 | 6 |
| 11 | c | 7844 | 653.8 | 0.000307 | 0.4696 | +2 | 11 |
| - | - | 8741 | 654.3 | - | - | 0 | - |
| - | - | 4.477E+04 | 654.5 | - | - | 0 | - |
| - | - | 3808 | 654.8 | - | - | 0 | - |
| - | - | 1.314E+04 | 655.5 | - | - | 0 | - |
| - | - | 2993 | 657.3 | - | - | 0 | - |
| - | - | 1.233E+04 | 657.9 | - | - | 0 | - |
| - | - | 8619 | 658.4 | - | - | 0 | - |
| - | - | 9792 | 658.9 | - | - | 0 | - |
| - | - | 1.41E+04 | 659.3 | - | - | 0 | - |
| - | - | 4580 | 659.4 | - | - | 0 | - |
| - | - | 3251 | 659.9 | - | - | 0 | - |
| - | - | 4465 | 660.3 | - | - | 0 | - |
| - | - | 2159 | 664.9 | - | - | 0 | - |
| - | - | 5175 | 665.4 | - | - | 0 | - |
| - | - | 3004 | 666.4 | - | - | 0 | - |
| 9 | y | 3469 | 669.5 | 0.00255 | 3.809 | +1 | 6 |
| - | - | 8097 | 674.3 | - | - | 0 | - |
| - | - | 9700 | 675.3 | - | - | 0 | - |
| - | - | 2795 | 676.3 | - | - | 0 | - |
| - | - | 3191 | 677.4 | - | - | 0 | - |
| - | - | 2707 | 677.9 | - | - | 0 | - |
| 3 | z | 2762 | 678.4 | 0.006331 | 9.332 | +2 | 12 |
| - | - | 7240 | 685.9 | - | - | 0 | - |
| 3 | y | 4684 | 686.4 | 0.008626 | 12.57 | +2 | 12 |
| 3 | z | 1.018E+05 | 686.9 | 0.0005633 | 0.8201 | +2 | 12 |
| - | - | 1.003E+05 | 687.4 | - | - | 0 | - |
| - | - | 5.311E+04 | 687.9 | - | - | 0 | - |
| - | - | 1.759E+04 | 688.4 | - | - | 0 | - |
| - | - | 4878 | 688.9 | - | - | 0 | - |
| - | - | 3507 | 692.4 | - | - | 0 | - |
| 8 | z | 2508 | 693.4 | 0.01025 | 14.78 | +1 | 7 |
| 3 | y | 1.17E+05 | 694.9 | 0.0009059 | 1.304 | +2 | 12 |
| - | - | 8.962E+04 | 695.4 | - | - | 0 | - |
| - | - | 4.765E+04 | 695.9 | - | - | 0 | - |
| - | - | 9127 | 696.4 | - | - | 0 | - |
| 5 | c | 6493 | 702.3 | 0.0009793 | 1.394 | +1 | 5 |
| - | - | 3506 | 703.3 | - | - | 0 | - |
| - | - | 4031 | 708.4 | - | - | 0 | - |
| 12 | c | 1.947E+04 | 709.4 | 0.002599 | 3.664 | +2 | 12 |
| - | - | 2.336E+04 | 709.9 | - | - | 0 | - |
| - | - | 9706 | 710.4 | - | - | 0 | - |
| 8 | z | 1.817E+04 | 710.5 | 0.001397 | 1.967 | +1 | 7 |
| - | - | 9380 | 711.5 | - | - | 0 | - |
| - | - | 2586 | 712.5 | - | - | 0 | - |
| - | - | 4059 | 714.4 | - | - | 0 | - |
| - | - | 4893 | 716.9 | - | - | 0 | - |
| - | - | 5156 | 717.4 | - | - | 0 | - |
| 12 | c | 3.194E+05 | 717.9 | 0.0006161 | 0.8582 | +2 | 12 |
| - | - | 2.664E+05 | 718.4 | - | - | 0 | - |
| - | - | 1.147E+05 | 718.9 | - | - | 0 | - |
| 5 | c | 2.62E+05 | 719.3 | 0.0006753 | 0.9388 | +1 | 5 |
| - | - | 7401 | 719.9 | - | - | 0 | - |
| - | - | 1.13E+05 | 720.3 | - | - | 0 | - |
| - | - | 2.791E+04 | 721.3 | - | - | 0 | - |
| - | - | 5149 | 722.3 | - | - | 0 | - |
| - | - | 2764 | 722.9 | - | - | 0 | - |
| 8 | y | 1.113E+04 | 726.5 | 0.0009084 | 1.25 | +1 | 7 |
| - | - | 2785 | 730.4 | - | - | 0 | - |
| - | - | 2792 | 731.4 | - | - | 0 | - |
| - | - | 3832 | 732.4 | - | - | 0 | - |
| - | - | 2628 | 735.9 | - | - | 0 | - |
| - | - | 3094 | 736.4 | - | - | 0 | - |
| - | - | 2262 | 737.4 | - | - | 0 | - |
| - | - | 3874 | 750.9 | - | - | 0 | - |
| - | - | 2409 | 751.3 | - | - | 0 | - |
| - | - | 4398 | 751.4 | - | - | 0 | - |
| - | - | 4790 | 751.9 | - | - | 0 | - |
| - | - | 8218 | 752.4 | - | - | 0 | - |
| - | - | 3824 | 752.9 | - | - | 0 | - |
| - | - | 2853 | 756.9 | - | - | 0 | - |
| - | - | 3667 | 757.4 | - | - | 0 | - |
| - | - | 4524 | 757.9 | - | - | 0 | - |
| - | - | 7851 | 758.4 | - | - | 0 | - |
| - | - | 1.457E+04 | 758.9 | - | - | 0 | - |
| - | - | 1.079E+04 | 759.4 | - | - | 0 | - |
| - | - | 6706 | 759.9 | - | - | 0 | - |
| 13 | c | 8685 | 765.9 | 0.003109 | 4.059 | +2 | 13 |
| - | - | 1.558E+04 | 766.4 | - | - | 0 | - |
| - | - | 1.209E+04 | 766.9 | - | - | 0 | - |
| - | - | 8321 | 767.4 | - | - | 0 | - |
| - | - | 7123 | 767.9 | - | - | 0 | - |
| - | - | 7253 | 773.4 | - | - | 0 | - |
| - | - | 7029 | 773.9 | - | - | 0 | - |
| 13 | c | 1.851E+05 | 774.4 | 0.001309 | 1.69 | +2 | 13 |
| - | - | 1.837E+05 | 774.9 | - | - | 0 | - |
| - | - | 9.109E+04 | 775.4 | - | - | 0 | - |
| - | - | 3.293E+04 | 775.9 | - | - | 0 | - |
| - | - | 7372 | 776.4 | - | - | 0 | - |
| - | - | 3470 | 778.4 | - | - | 0 | - |
| - | - | 4510 | 778.9 | - | - | 0 | - |
| 2 | y | 7150 | 779.4 | 0.00437 | 5.607 | +2 | 13 |
| 2 | z | 4.303E+04 | 779.9 | 9.143E-05 | 0.1172 | +2 | 13 |
| - | - | 3.411E+04 | 780.4 | - | - | 0 | - |
| 7 | w | 3828 | 780.5 | 0.001466 | 1.879 | +1 | 8 |
| - | - | 1.76E+04 | 780.9 | - | - | 0 | - |
| - | - | 8035 | 781.4 | - | - | 0 | - |
| - | - | 7379 | 781.9 | - | - | 0 | - |
| - | - | 4343 | 782.4 | - | - | 0 | - |
| - | - | 3368 | 787.4 | - | - | 0 | - |
| 2 | y | 2.019E+04 | 787.9 | 0.01202 | 15.25 | +2 | 13 |
| - | - | 1.098E+04 | 788.4 | - | - | 0 | - |
| - | - | 7157 | 788.9 | - | - | 0 | - |
| - | - | 2.513E+04 | 790.9 | - | - | 0 | - |
| - | - | 2.818E+04 | 791.4 | - | - | 0 | - |
| - | - | 1.728E+04 | 791.9 | - | - | 0 | - |
| - | - | 5290 | 792.4 | - | - | 0 | - |
| - | - | 4171 | 792.9 | - | - | 0 | - |
| - | - | 7542 | 794.9 | - | - | 0 | - |
| - | - | 1.793E+04 | 795.4 | - | - | 0 | - |
| - | - | 1.718E+04 | 795.9 | - | - | 0 | - |
| - | - | 9512 | 796.4 | - | - | 0 | - |
| - | - | 1.462E+04 | 796.4 | - | - | 0 | - |
| - | - | 1.63E+04 | 796.9 | - | - | 0 | - |
| - | - | 4595 | 797.4 | - | - | 0 | - |
| - | - | 6112 | 797.4 | - | - | 0 | - |
| - | - | 2878 | 797.9 | - | - | 0 | - |
| - | - | 1.819E+04 | 800.4 | - | - | 0 | - |
| - | - | 2.194E+04 | 800.9 | - | - | 0 | - |
| - | - | 2.34E+04 | 801.4 | - | - | 0 | - |
| - | - | 1.845E+04 | 801.9 | - | - | 0 | - |
| - | - | 9504 | 802.4 | - | - | 0 | - |
| - | - | 1.165E+04 | 802.9 | - | - | 0 | - |
| - | - | 9432 | 803.4 | - | - | 0 | - |
| - | - | 1.385E+04 | 803.9 | - | - | 0 | - |
| - | - | 8333 | 804.4 | - | - | 0 | - |
| - | - | 4140 | 804.9 | - | - | 0 | - |
| - | - | 5029 | 808.9 | - | - | 0 | - |
| - | - | 3.292E+04 | 809.4 | - | - | 0 | - |
| - | - | 3.498E+04 | 809.9 | - | - | 0 | - |
| - | - | 3.001E+04 | 810.4 | - | - | 0 | - |
| - | - | 1.276E+04 | 810.9 | - | - | 0 | - |
| - | - | 2.815E+04 | 811.4 | - | - | 0 | - |
| - | - | 5258 | 811.4 | - | - | 0 | - |
| - | - | 1.986E+04 | 812.4 | - | - | 0 | - |
| - | - | 7402 | 813.4 | - | - | 0 | - |
| - | - | 2980 | 813.9 | - | - | 0 | - |
| - | - | 4563 | 814.4 | - | - | 0 | - |
| - | - | 6001 | 814.9 | - | - | 0 | - |
| - | - | 2805 | 815.4 | - | - | 0 | - |
| - | - | 5109 | 822.5 | - | - | 0 | - |
| - | - | 2.273E+04 | 822.9 | - | - | 0 | - |
| - | - | 1.611E+05 | 823.4 | - | - | 0 | - |
| 7 | y | 3.394E+04 | 823.5 | 0.0002218 | 0.2693 | +1 | 8 |
| - | - | 1.522E+05 | 823.9 | - | - | 0 | - |
| - | - | 8.266E+04 | 824.4 | - | - | 0 | - |
| - | - | 1.584E+04 | 824.5 | - | - | 0 | - |
| - | - | 2.953E+04 | 824.9 | - | - | 0 | - |
| - | - | 9197 | 825.4 | - | - | 0 | - |
| - | - | 6227 | 825.5 | - | - | 0 | - |
| - | - | 4197 | 830.4 | - | - | 0 | - |
| - | - | 1.54E+04 | 830.9 | - | - | 0 | - |
| - | - | 3.654E+05 | 831.5 | - | - | 0 | - |
| - | - | 1.644E+06 | 832 | - | - | 0 | - |
| - | - | 1.475E+06 | 832.5 | - | - | 0 | - |
| - | - | 7.994E+05 | 833 | - | - | 0 | - |
| - | - | 2.498E+05 | 833.5 | - | - | 0 | - |
| - | - | 6.362E+04 | 834 | - | - | 0 | - |
| 6 | c | 2.511E+04 | 839.4 | 0.0006002 | 0.715 | +1 | 6 |
| - | - | 9449 | 840.4 | - | - | 0 | - |
| - | - | 2964 | 841.4 | - | - | 0 | - |
| - | - | 2.039E+04 | 850.5 | - | - | 0 | - |
| - | - | 1.119E+04 | 851.5 | - | - | 0 | - |
| - | - | 2.32E+04 | 852.5 | - | - | 0 | - |
| - | - | 8899 | 853.5 | - | - | 0 | - |
| - | - | 5336 | 854.5 | - | - | 0 | - |
| - | - | 7014 | 855.5 | - | - | 0 | - |
| - | - | 3241 | 856.5 | - | - | 0 | - |
| - | - | 2622 | 873.5 | - | - | 0 | - |
| - | - | 3539 | 883.4 | - | - | 0 | - |
| 6 | z | 2.508E+05 | 944.6 | 0.0009886 | 1.047 | +1 | 9 |
| - | - | 1.489E+05 | 945.6 | - | - | 0 | - |
| - | - | 5.26E+04 | 946.6 | - | - | 0 | - |
| - | - | 9259 | 947.6 | - | - | 0 | - |
| 7 | c | 2.879E+04 | 953.4 | 0.001182 | 1.24 | +1 | 7 |
| - | - | 1.777E+04 | 954.4 | - | - | 0 | - |
| - | - | 4772 | 955.4 | - | - | 0 | - |
| 6 | y | 2.599E+04 | 960.6 | 8.681E-05 | 0.09037 | +1 | 9 |
| - | - | 1.378E+04 | 961.6 | - | - | 0 | - |
| - | - | 3690 | 962.6 | - | - | 0 | - |
| - | - | 5023 | 968.6 | - | - | 0 | - |
| - | - | 3520 | 969.6 | - | - | 0 | - |
| - | - | 1.071E+04 | 982.5 | - | - | 0 | - |
| - | - | 1.401E+04 | 983.6 | - | - | 0 | - |
| - | - | 4762 | 984.6 | - | - | 0 | - |
| - | - | 3175 | 987.6 | - | - | 0 | - |
| - | - | 1.16E+04 | 1009 | - | - | 0 | - |
| 8 | c | 2.32E+05 | 1010 | 0.0008977 | 0.8884 | +1 | 8 |
| - | - | 1.301E+05 | 1011 | - | - | 0 | - |
| - | - | 4.566E+04 | 1012 | - | - | 0 | - |
| - | - | 1.303E+04 | 1013 | - | - | 0 | - |
| - | - | 3453 | 1026 | - | - | 0 | - |
| 5 | y | 4395 | 1081 | 0.003979 | 3.682 | +1 | 10 |
| 5 | z | 1.276E+05 | 1082 | 0.0007926 | 0.7327 | +1 | 10 |
| - | - | 2.848E+05 | 1083 | - | - | 0 | - |
| - | - | 1.662E+05 | 1084 | - | - | 0 | - |
| - | - | 5.727E+04 | 1085 | - | - | 0 | - |
| - | - | 1.151E+04 | 1086 | - | - | 0 | - |
| - | - | 3116 | 1087 | - | - | 0 | - |
| - | - | 7496 | 1095 | - | - | 0 | - |
| - | - | 7220 | 1096 | - | - | 0 | - |
| - | - | 5076 | 1096 | - | - | 0 | - |
| - | - | 1.189E+04 | 1097 | - | - | 0 | - |
| 5 | y | 2.552E+04 | 1098 | 0.0001092 | 0.09952 | +1 | 10 |
| - | - | 1.896E+04 | 1099 | - | - | 0 | - |
| - | - | 4664 | 1100 | - | - | 0 | - |
| - | - | 2815 | 1107 | - | - | 0 | - |
| - | - | 2295 | 1109 | - | - | 0 | - |
| - | - | 2414 | 1124 | - | - | 0 | - |
| - | - | 5503 | 1138 | - | - | 0 | - |
| 9 | c | 3.052E+05 | 1139 | 0.001455 | 1.278 | +1 | 9 |
| - | - | 2.06E+05 | 1140 | - | - | 0 | - |
| - | - | 7.398E+04 | 1141 | - | - | 0 | - |
| - | - | 1.8E+04 | 1142 | - | - | 0 | - |
| - | - | 4030 | 1143 | - | - | 0 | - |
| - | - | 6629 | 1146 | - | - | 0 | - |
| - | - | 4685 | 1147 | - | - | 0 | - |
| 4 | w | 2828 | 1152 | 0.0005421 | 0.4707 | +1 | 11 |
| - | - | 8262 | 1166 | - | - | 0 | - |
| - | - | 7551 | 1167 | - | - | 0 | - |
| - | - | 2714 | 1195 | - | - | 0 | - |
| 4 | y | 4195 | 1209 | 0.002841 | 2.35 | +1 | 11 |
| 4 | z | 3.829E+04 | 1210 | 0.001297 | 1.072 | +1 | 11 |
| - | - | 1.744E+05 | 1211 | - | - | 0 | - |
| - | - | 1.136E+05 | 1212 | - | - | 0 | - |
| - | - | 3.716E+04 | 1213 | - | - | 0 | - |
| - | - | 1.079E+04 | 1214 | - | - | 0 | - |
| 4 | y | 1.151E+04 | 1226 | 0.0001511 | 0.1233 | +1 | 11 |
| - | - | 6449 | 1227 | - | - | 0 | - |
| - | - | 4567 | 1237 | - | - | 0 | - |
| - | - | 5958 | 1259 | - | - | 0 | - |
| - | - | 3925 | 1260 | - | - | 0 | - |
| - | - | 3514 | 1263 | - | - | 0 | - |
| - | - | 6011 | 1264 | - | - | 0 | - |
| - | - | 4419 | 1265 | - | - | 0 | - |
| - | - | 8305 | 1306 | - | - | 0 | - |
| 11 | c | 1.344E+05 | 1307 | 0.0002001 | 0.1531 | +1 | 11 |
| - | - | 1.112E+05 | 1308 | - | - | 0 | - |
| - | - | 4.589E+04 | 1309 | - | - | 0 | - |
| - | - | 1.233E+04 | 1310 | - | - | 0 | - |
| - | - | 4934 | 1311 | - | - | 0 | - |
| 3 | z | 2.201E+04 | 1373 | 0.0002244 | 0.1635 | +1 | 12 |
| - | - | 9.256E+04 | 1374 | - | - | 0 | - |
| - | - | 6.026E+04 | 1375 | - | - | 0 | - |
| - | - | 2.752E+04 | 1376 | - | - | 0 | - |
| - | - | 8069 | 1377 | - | - | 0 | - |
| 3 | y | 4675 | 1389 | 0.001398 | 1.006 | +1 | 12 |
| - | - | 4336 | 1390 | - | - | 0 | - |
| - | - | 1.403E+04 | 1419 | - | - | 0 | - |
| - | - | 1.212E+04 | 1420 | - | - | 0 | - |
| - | - | 5970 | 1421 | - | - | 0 | - |
| 12 | c | 4.778E+04 | 1435 | 0.001379 | 0.9612 | +1 | 12 |
| - | - | 7.595E+04 | 1436 | - | - | 0 | - |
| - | - | 4.219E+04 | 1437 | - | - | 0 | - |
| - | - | 1.775E+04 | 1438 | - | - | 0 | - |
| - | - | 4968 | 1439 | - | - | 0 | - |
| - | - | 2618 | 1504 | - | - | 0 | - |
| - | - | 5266 | 1505 | - | - | 0 | - |
| - | - | 5355 | 1506 | - | - | 0 | - |
| - | - | 2471 | 1507 | - | - | 0 | - |
| - | - | 7264 | 1532 | - | - | 0 | - |
| - | - | 9160 | 1533 | - | - | 0 | - |
| - | - | 3635 | 1534 | - | - | 0 | - |
| 13 | c | 1.975E+04 | 1548 | 0.0001282 | 0.08282 | +1 | 13 |
| - | - | 4.461E+04 | 1549 | - | - | 0 | - |
| - | - | 3.223E+04 | 1550 | - | - | 0 | - |
| - | - | 1.156E+04 | 1551 | - | - | 0 | - |
| - | - | 4992 | 1552 | - | - | 0 | - |
| 2 | z | 5542 | 1559 | 0.00221 | 1.418 | +1 | 13 |
| - | - | 3.509E+04 | 1560 | - | - | 0 | - |
| - | - | 2.91E+04 | 1561 | - | - | 0 | - |
| - | - | 1.24E+04 | 1562 | - | - | 0 | - |
| - | - | 5820 | 1563 | - | - | 0 | - |
| 2 | y | 4192 | 1575 | 0.01078 | 6.843 | +1 | 13 |
| - | - | 2539 | 1576 | - | - | 0 | - |
| - | - | 5794 | 1581 | - | - | 0 | - |
| - | - | 7936 | 1582 | - | - | 0 | - |
| - | - | 3671 | 1583 | - | - | 0 | - |
| - | - | 3729 | 1591 | - | - | 0 | - |
| - | - | 5029 | 1601 | - | - | 0 | - |
| - | - | 4100 | 1602 | - | - | 0 | - |
| - | - | 5889 | 1618 | - | - | 0 | - |
| - | - | 3.77E+04 | 1619 | - | - | 0 | - |
| - | - | 3.174E+04 | 1620 | - | - | 0 | - |
| - | - | 1.64E+04 | 1621 | - | - | 0 | - |
| - | - | 4909 | 1622 | - | - | 0 | - |
| - | - | 2767 | 1628 | - | - | 0 | - |
| - | - | 2.053E+04 | 1629 | - | - | 0 | - |
| - | - | 1.675E+04 | 1630 | - | - | 0 | - |
| - | - | 8669 | 1631 | - | - | 0 | - |
| - | - | 3874 | 1635 | - | - | 0 | - |
| - | - | 1.521E+04 | 1636 | - | - | 0 | - |
| - | - | 1.077E+04 | 1637 | - | - | 0 | - |
| - | - | 8231 | 1638 | - | - | 0 | - |
| - | - | 2370 | 1639 | - | - | 0 | - |
| - | - | 4963 | 1645 | - | - | 0 | - |
| - | - | 4.153E+04 | 1646 | - | - | 0 | - |
| - | - | 1.533E+05 | 1647 | - | - | 0 | - |
| - | - | 1.35E+05 | 1648 | - | - | 0 | - |
| - | - | 7.493E+04 | 1649 | - | - | 0 | - |
| - | - | 2.317E+04 | 1650 | - | - | 0 | - |
| - | - | 5322 | 1651 | - | - | 0 | - |
| - | - | 2.987E+04 | 1662 | - | - | 0 | - |
| - | - | 1.733E+05 | 1663 | - | - | 0 | - |
| - | - | 7.792E+05 | 1664 | - | - | 0 | - |
| - | - | 6.916E+05 | 1665 | - | - | 0 | - |
| - | - | 3.6E+05 | 1666 | - | - | 0 | - |
| - | - | 1.173E+05 | 1667 | - | - | 0 | - |
| - | - | 3.336E+04 | 1668 | - | - | 0 | - |

m/z Charge Intensity FragmentType MassShift Position
122.43207550048828 0 1249.4977
129.10243225097656 0 4827.515
130.0655517578125 0 2984.52
132.0809783935547 0 6440.299
132.1020965576172 0 8245.447 y 13
136.0759735107422 0 3833.3726
143.7855682373047 0 1381.6019
146.09263610839844 0 1589.8337
153.07876586914062 0 10353.659
168.10198974609375 0 1468.5378
170.1539306640625 0 1729.2396
186.0789337158203 0 4462.808
187.0868682861328 0 8611.292
199.1807403564453 0 3240.7812
214.1556854248047 0 2362.4048
216.7711944580078 0 1757.8867
223.11924743652344 0 4504.3296
226.15525817871094 0 6103.2275
228.11184692382812 0 2204.3735
245.13729858398438 0 13528.748
245.18609619140625 0 17638.553 y 12
246.12396240234375 0 52684.34
246.13909912109375 0 2942.548
246.18991088867188 0 2381.674
247.12753295898438 0 6505.2275
255.10011291503906 0 3900.085
255.19451904296875 0 2743.0789
256.20074462890625 0 1733.9437
258.14520263671875 0 7358.9927
268.98162841796875 0 1749.1257
273.1115417480469 0 4372.2554
274.118896484375 0 68646.836
275.12255859375 0 12829.372
281.1737365722656 0 8975.436
282.1793212890625 0 2602.1294
292.12982177734375 0 9472.466
299.2088928222656 0 2496.9287
301.2001953125 0 5392.093
309.1564025878906 0 9425.667
310.1610107421875 0 2360.8784
314.2080078125 0 3924.8013
320.122802734375 0 2075.6526
323.185791015625 0 2684.3416
346.7266845703125 0 5779.834
349.1418762207031 0 6037.259
353.461669921875 0 2351.4072
357.2625732421875 0 27722.951 z 11
358.26800537109375 0 12173.412
359.27099609375 0 2794.5352
373.2811584472656 0 6598.901 y 11
382.2812194824219 0 5157.302
394.2582092285156 0 9072.2
395.2625732421875 0 1897.9485
399.26153564453125 0 2171.8816
401.13690185546875 0 2908.9495
406.18701171875 0 3467.818
412.2744140625 0 37411.62 y 6
412.7757568359375 0 17656.74
413.2276611328125 0 6386.9907
413.2772521972656 0 4601.618
420.1847839355469 0 2995.3442 c Ammonia loss 5
420.6858825683594 0 3037.3406
426.23944091796875 0 2304.6094
427.292236328125 0 15112.284 w 10
428.2980041503906 0 5876.708
429.1904296875 0 2470.3042
435.1915283203125 0 8335.249
436.17352294921875 0 2004.3734
437.1825256347656 0 10581.447
438.186279296875 0 2549.1116
439.1885070800781 0 2384.62
453.2014465332031 0 14896.41
454.2086486816406 0 27230.797 c 2
455.21209716796875 0 7865.043
463.5989074707031 0 11674.078 y 2
463.93365478515625 0 7310.754
465.2096252441406 0 9456.321
468.3187561035156 0 34387.594
469.2891845703125 0 8015.6416
469.3227233886719 0 8694.701
470.2937927246094 0 2843.5151
470.3335266113281 0 18261.09 y 10
471.3385925292969 0 3744.4612
473.2435302734375 0 18516.182 c Ammonia loss 11
473.5762939453125 0 10078.687
473.9121398925781 0 5248.5244
474.24493408203125 0 2973.537
477.2014465332031 0 16580.486
478.2055358886719 0 4649.397
479.2056884765625 0 2261.2122
480.2247009277344 0 3984.2976
480.8035583496094 0 3069.8635 y 5
481.3060607910156 0 4859.965
482.2643127441406 0 2435.062
482.2989501953125 0 9366.098
483.7891845703125 0 2619.8914
495.36529541015625 0 3962.539
498.327392578125 0 4099.111
501.6080017089844 0 4510.89
501.9422302246094 0 2694.0293
504.9329833984375 0 16352.533
505.26666259765625 0 12390.018
505.599365234375 0 6922.4326
507.3428039550781 0 3206.8286
510.2225646972656 0 2489.4775
510.93768310546875 0 73448.41 c Ammonia loss 12
511.27191162109375 0 70198.23
511.6062316894531 0 27057.674
511.940673828125 0 9985.823
512.2704467773438 0 4111.5083
516.940673828125 0 8432.708
517.2760620117188 0 8331.486
517.6087036132812 0 3646.966
523.2413330078125 0 9106.14
525.352783203125 0 110037.99 z 9
525.6250610351562 0 9102.734 y 1
525.9602661132812 0 12600.251
526.2945556640625 0 8257.48
526.3560180664062 0 37947.977
527.3585205078125 0 7465.8726
538.2537841796875 0 5996.472
539.258544921875 0 4757.7285
539.2984619140625 0 3609.6858
540.6119384765625 0 2314.163
541.3211059570312 0 5942.6772 z 4
541.3718872070312 0 12180.767 y 9
541.8262939453125 0 5612.5225
542.3726806640625 0 4172.027
546.2212524414062 0 6143.254
547.8137817382812 0 2588.605
548.5853881835938 0 3591.9333
548.6328735351562 0 19009.516
548.9658813476562 0 14577.756
549.298583984375 0 6586.121
549.333740234375 0 10164.823 y 4
549.6318969726562 0 7886.1772
549.834228515625 0 10603.67
549.9657592773438 0 2519.2056
550.3348388671875 0 2785.414
553.396484375 0 4489.0986
553.9655151367188 0 6579.732
554.3017578125 0 3949.4402
554.635986328125 0 554773.44
554.9700927734375 0 566830.06
555.304443359375 0 304554.66
555.6384887695312 0 101346.82
555.6881103515625 0 2586.7554
555.97265625 0 25607.203
561.2683715820312 0 3146.5222 c Ammonia loss 8
563.249267578125 0 39564.215
564.2496337890625 0 11779.334 c Water loss 3
565.242431640625 0 16780.031 c Ammonia loss 3
566.2451782226562 0 12894.201
566.4039916992188 0 9461.967
567.24951171875 0 3484.94
567.4049682617188 0 3096.2393
569.8164672851562 0 2606.749
572.2647705078125 0 2681.7905
572.8050537109375 0 5955.6055
573.3060302734375 0 3076.727
576.3385620117188 0 32233.705 w 3
576.8399047851562 0 21329.084
577.3355712890625 0 9653.8
579.3603515625 0 2429.169
581.26025390625 0 23847.096
582.2677001953125 0 126947.18 c 3
582.3748779296875 0 11682.403
583.270751953125 0 37512.598
583.37646484375 0 3385.572
584.2728881835938 0 6991.4956
587.7830200195312 0 2624.6555
595.3825073242188 0 26139.186 w 8
596.3860473632812 0 9695.353
596.7915649414062 0 2776.0513 c Ammonia loss 9
597.38916015625 0 4064.1514
604.3501586914062 0 4991.621
604.8506469726562 0 7541.8843 y Ammonia loss 3
605.3531494140625 0 92284.625 z 3
605.8556518554688 0 99156.89
606.357177734375 0 49953.1
606.8600463867188 0 12333.085
607.3580932617188 0 4968.6714
609.4007568359375 0 2354.8403
609.4574584960938 0 6483.727
610.3965454101562 0 24152.943
611.4015502929688 0 7195.6543
613.3624267578125 0 36184.02 y 3
613.8638305664062 0 20874.54
614.3641357421875 0 6331.802
620.3408813476562 0 3600.5435
620.8448486328125 0 3208.1514
624.4209594726562 0 7468.072
629.3464965820312 0 14841.712
629.8486938476562 0 16526.246
630.3504028320312 0 5396.7534
637.4267578125 0 4371.9453
638.4371337890625 0 2519.6284
645.8291625976562 0 3475.2295
651.3583374023438 0 10411.158
651.860107421875 0 9721.769
652.3580322265625 0 2756.8235
653.4482421875 0 83527.98 z 8
653.825927734375 0 7844.2534 c 10
654.3277587890625 0 8741.461
654.4520874023438 0 44767.184
654.8291625976562 0 3808.0662
655.455078125 0 13137.22
657.29931640625 0 2992.5125
657.8674926757812 0 12327.369
658.3692016601562 0 8619.499
658.8593139648438 0 9792.226
659.2944946289062 0 14103.604
659.3567504882812 0 4580.037
659.8544311523438 0 3250.7866
660.2973022460938 0 4464.8784
664.8843383789062 0 2158.6016
665.362060546875 0 5174.9683
666.3588256835938 0 3003.7903
669.4683227539062 0 3469.3293 y 8
674.3056640625 0 8096.9663
675.3126220703125 0 9700.456
676.3134155273438 0 2794.998
677.3758544921875 0 3190.5745
677.880859375 0 2707.0557
678.377197265625 0 2761.9783 z Ammonia loss 2
685.884765625 0 7239.8237
686.3888549804688 0 4684.2456 y Ammonia loss 2
686.8847045898438 0 101847.09 z 2
687.38623046875 0 100310.91
687.8881225585938 0 53112.43
688.3890991210938 0 17591.95
688.8906860351562 0 4877.922
692.4486083984375 0 3506.8618
693.4522094726562 0 2508.173 z Ammonia loss 7
694.8944091796875 0 116988.32 y 2
695.3955078125 0 89618.92
695.8966674804688 0 47646.195
696.396728515625 0 9126.745
702.3004150390625 0 6492.898 c Ammonia loss 4
703.3065795898438 0 3506.1636
708.3875122070312 0 4031.4937
709.3624267578125 0 19465.895 c Ammonia loss 11
709.8630981445312 0 23358.47
710.3619384765625 0 9705.576
710.4699096679688 0 18171.996 z 7
711.4718017578125 0 9379.784
712.4732666015625 0 2586.3738
714.3894653320312 0 4059.0593
716.86767578125 0 4892.984
717.3693237304688 0 5155.542
717.8737182617188 0 319442.4 c 11
718.375244140625 0 266358.12
718.8765258789062 0 114695.766
719.32666015625 0 262028.7 c 4
719.8799438476562 0 7400.528
720.329833984375 0 113008.37
721.33251953125 0 27910.354
722.3348999023438 0 5148.6406
722.8973388671875 0 2764.2874
726.486328125 0 11130.672 y 7
730.3890380859375 0 2785.0298
731.3817749023438 0 2792.3364
732.3899536132812 0 3831.7493
735.9143676757812 0 2627.8943
736.4043579101562 0 3094.3105
737.4027099609375 0 2262.3965
750.9096069335938 0 3874.0217
751.334228515625 0 2409.1802
751.410888671875 0 4398.0283
751.9071044921875 0 4790.3076
752.4119873046875 0 8217.637
752.9086303710938 0 3823.5127
756.9005737304688 0 2853.237
757.3974609375 0 3667.494
757.9223022460938 0 4523.5923
758.4152221679688 0 7850.538
758.9136352539062 0 14570.992
759.4136962890625 0 10786.553
759.9130859375 0 6706.4556
765.9049682617188 0 8685.055 c Ammonia loss 12
766.4124755859375 0 15578.088
766.9114379882812 0 12094.798
767.4194946289062 0 8320.646
767.9259643554688 0 7122.7974
773.4132690429688 0 7253.2085
773.918701171875 0 7029.288
774.4164428710938 0 185085.8 c 12
774.917236328125 0 183652.2
775.41845703125 0 91094.23
775.9198608398438 0 32930.73
776.419677734375 0 7371.9497
778.424072265625 0 3470.3025
778.9280395507812 0 4510.46
779.4242553710938 0 7149.87 y Ammonia loss 1
779.9238891601562 0 43033.152 z 1
780.4257202148438 0 34105.12
780.499267578125 0 3828.3943 w 6
780.9273071289062 0 17603.562
781.4276733398438 0 8034.596
781.919677734375 0 7378.8877
782.425537109375 0 4342.7407
787.4287719726562 0 3367.9263
787.921142578125 0 20193.412 y 1
788.4266967773438 0 10977.444
788.9268188476562 0 7157.32
790.9281005859375 0 25125.602
791.4285278320312 0 28183.865
791.92919921875 0 17279.477
792.4364013671875 0 5289.5073
792.9324340820312 0 4170.6094
794.9161987304688 0 7541.971
795.413818359375 0 17925.086
795.9125366210938 0 17178.54
796.3525390625 0 9511.785
796.4193725585938 0 14617.448
796.92431640625 0 16299.17
797.3541259765625 0 4594.599
797.4251708984375 0 6112.12
797.9280395507812 0 2877.8953
800.439208984375 0 18190.645
800.940185546875 0 21942.777
801.4439697265625 0 23403.87
801.9368286132812 0 18450.744
802.43017578125 0 9504.484
802.92529296875 0 11650.422
803.4262084960938 0 9432.384
803.9253540039062 0 13846.205
804.4251098632812 0 8332.692
804.9302978515625 0 4139.7485
808.9425659179688 0 5029.3765
809.4439697265625 0 32917.3
809.9463500976562 0 34981.668
810.4427490234375 0 30014.107
810.9385375976562 0 12758.009
811.3641967773438 0 28149.703
811.4386596679688 0 5257.708
812.3688354492188 0 19861.482
813.3704223632812 0 7401.8584
813.9224243164062 0 2979.6206
814.437255859375 0 4563.0103
814.9365844726562 0 6000.914
815.442626953125 0 2805.3691
822.5325317382812 0 5108.5083
822.9478149414062 0 22727.148
823.4422607421875 0 161117.67
823.5402221679688 0 33937.1 y 6
823.9425659179688 0 152160.86
824.4436645507812 0 82664.64
824.5433959960938 0 15843.895
824.9447021484375 0 29534.941
825.4465942382812 0 9197.242
825.5464477539062 0 6226.5293
830.4422607421875 0 4196.8496
830.9454956054688 0 15402.183
831.4505615234375 0 365386.9
831.9539794921875 0 1644090.1
832.4556274414062 0 1474952.5
832.9568481445312 0 799412.1
833.4578247070312 0 249797.75
833.9591674804688 0 63618.188
839.3589477539062 0 25106.707 c Ammonia loss 5
840.3640747070312 0 9448.764
841.3637084960938 0 2963.9226
850.52880859375 0 20392.004
851.53076171875 0 11186.624
852.5416870117188 0 23201.023
853.5464477539062 0 8899.493
854.485595703125 0 5336.4834
855.4956665039062 0 7014.3384
856.4948120117188 0 3241.3267
873.504638671875 0 2621.7358
883.40576171875 0 3539.0737
944.5811767578125 0 250821.14 z 5
945.5849609375 0 148921.44
946.5877075195312 0 52604.355
947.5928344726562 0 9258.889
953.4388427734375 0 28790.496 c 6
954.4424438476562 0 17773.154
955.4453125 0 4772.0977
960.5989990234375 0 25991.523 y 5
961.6041259765625 0 13779.491
962.6013793945312 0 3689.5737
968.5746459960938 0 5022.6655
969.5782470703125 0 3519.8535
982.5472412109375 0 10707.265
983.5532836914062 0 14011.256
984.5570068359375 0 4761.5737
987.5806274414062 0 3175.387
1009.4522705078125 0 11600.007
1010.4600219726562 0 231966.8 c 7
1011.462646484375 0 130094.375
1012.4652099609375 0 45662.875
1013.467041015625 0 13034.8
1025.582763671875 0 3453.3818
1080.63525390625 0 4394.6646 y Ammonia loss 4
1081.639892578125 0 127579.88 z 4
1082.6468505859375 0 284774.25
1083.6500244140625 0 166179
1084.6533203125 0 57273.33
1085.655517578125 0 11511.2
1086.668701171875 0 3115.5662
1094.5404052734375 0 7495.835
1095.5413818359375 0 7219.92
1095.6417236328125 0 5075.673
1096.640625 0 11889.191
1097.65771484375 0 25522.69 y 4
1098.6607666015625 0 18956.52
1099.6634521484375 0 4664.2773
1106.53369140625 0 2814.7363
1108.541748046875 0 2295.2212
1123.548583984375 0 2413.94
1137.544189453125 0 5502.5283
1138.5555419921875 0 305162.6 c 8
1139.557861328125 0 205968.55
1140.560546875 0 73977.39
1141.5634765625 0 18002.578
1142.5648193359375 0 4030.4153
1145.616455078125 0 6629.0327
1146.60986328125 0 4684.797
1151.6678466796875 0 2827.5918 w 3
1165.57666015625 0 8262.349
1166.5853271484375 0 7550.897
1194.575927734375 0 2714.1018
1208.68701171875 0 4195.0464 y Ammonia loss 3
1209.698974609375 0 38290.31 z 3
1210.7056884765625 0 174427.62
1211.7081298828125 0 113594.01
1212.7117919921875 0 37160.633
1213.71923828125 0 10792.3955
1225.716552734375 0 11512.668 y 3
1226.7181396484375 0 6448.596
1236.6258544921875 0 4567.0234
1258.694580078125 0 5957.973
1259.6986083984375 0 3925.2598
1262.6214599609375 0 3513.7856
1263.6351318359375 0 6010.517
1264.6427001953125 0 4418.5537
1305.638671875 0 8305.31
1306.6441650390625 0 134373.67 c 10
1307.64697265625 0 111209.63
1308.6502685546875 0 45892.223
1309.6536865234375 0 12329.886
1310.658447265625 0 4934.2026
1372.76123046875 0 22014.326 z 2
1373.7681884765625 0 92562.71
1374.771484375 0 60257.87
1375.7735595703125 0 27521.32
1376.7840576171875 0 8068.9795
1388.7811279296875 0 4675.0547 y 2
1389.7891845703125 0 4336.0845
1418.7196044921875 0 14030.027
1419.7205810546875 0 12124.85
1420.7183837890625 0 5969.6626
1434.737548828125 0 47779.24 c 11
1435.7432861328125 0 75950.86
1436.7469482421875 0 42189.215
1437.7510986328125 0 17750.857
1438.758544921875 0 4967.6494
1503.823486328125 0 2618.4565
1504.81298828125 0 5266.3257
1505.819091796875 0 5354.589
1506.818115234375 0 2470.6636
1531.80517578125 0 7263.957
1532.8077392578125 0 9159.73
1533.8079833984375 0 3635.3076
1547.8231201171875 0 19745.023 c 12
1548.8291015625 0 44614.293
1549.8321533203125 0 32228.443
1550.838623046875 0 11561.792
1551.844970703125 0 4991.51
1558.842529296875 0 5541.572 z 1
1559.8468017578125 0 35094.395
1560.8494873046875 0 29102.139
1561.853759765625 0 12398.014
1562.8453369140625 0 5820.2734
1574.8482666015625 0 4192.242 y 1
1575.8575439453125 0 2539.094
1580.8480224609375 0 5793.51
1581.8406982421875 0 7936.345
1582.8533935546875 0 3670.589
1590.8175048828125 0 3729.3223
1600.8714599609375 0 5028.568
1601.878173828125 0 4100.348
1617.877197265625 0 5889.2803
1618.886474609375 0 37698.508
1619.8870849609375 0 31739.402
1620.887451171875 0 16397.113
1621.8797607421875 0 4908.7705
1627.871826171875 0 2767.3618
1628.87255859375 0 20534.809
1629.8721923828125 0 16745.285
1630.8779296875 0 8668.644
1634.901123046875 0 3874.4192
1635.9107666015625 0 15207.048
1636.9107666015625 0 10771.299
1637.91796875 0 8231.446
1638.91796875 0 2369.6763
1644.870849609375 0 4962.8877
1645.87841796875 0 41531.76
1646.8809814453125 0 153254.12
1647.8839111328125 0 135033.16
1648.8870849609375 0 74929.75
1649.890380859375 0 23172.424
1650.8724365234375 0 5321.509
1661.891845703125 0 29873.715
1662.89892578125 0 173328.03
1663.9068603515625 0 779183.7
1664.9093017578125 0 691568.44
1665.912841796875 0 360012.38
1666.9149169921875 0 117335.94
1667.9158935546875 0 33357.41

Spectrum Details

|  |  |
| --- | --- |
| Matched peaks? Matched peaksThe total absolute number of peaks matched. Additionally in brackets the total fraction of peaks matched and the total number of peaks is shown. | 69 (13.50% of 511) |
| FDR? FDRThe false discovery rate estimated for this peptide. It is calculated by matching all theoretical fragments with a non-integer shift with the raw peaks for this spectrum. This is done with 40 different shifts. The resulting percentage is the average number of annotated peaks over the number of annotated peaks with the correct spectrum. | 3.07% |
| Satellite FDR? Satellite FDRSee the FDR for details on its calculation. This satellite ion specific FDR only contains the satellite ions (d/w) for I/L/J positions. | - |
| PSM Score? PSM ScoreThe PSM Score as given by Hecklib to this annotated spectrum. It is shown with three significant figures. | 527 |

## Spectrum 4749? Spectrum 4749 The raw spectrum of this peptide as annotated by Hecklib. The fragments are coloured according to ion type (see legend). Any peaks with a star '\*' as text can be hovered over to see the full details, first the ion type second the mass shift type. By hovering over the amino acids in the peptide or ions in the legend the corresponding peaks are highlighted. By toggling the 'Unassigned' label you can turn the background (unassigned) peaks on or off in the plot. By updating the slider in the Ion legend you can update the spectrum to only show the top X% of the peaks with labels. The top X% means any peak that is within X% of the highest intensity. By dragging in the spectrum you can zoom in to a specific part of the spectrum and use 'Zoom Out' to get back to the original zoom level. The annotation of the spectrum is based on the given sequence in the peptides file and is done with different software so inconsistencies are likely. The peaks are annotated based on the given sequence, with 20 ppm tolerance.

Copy Data

### Spectrum 4749 (TSV)

#### Preview

```
Loading example...
```

*Click on the button to copy the data to your clipboard.*

Mz MinMz MaxIntensity Max

WidthHeightPeptide font sizePeptide stroke widthSpectrum font sizeSpectrum stroke widthCompact peptide

Ion legend

wxyz

abcd

OtherUnassignedIonChargePositionShow for top:%

SWYQHHPGKAPKJJ

02.39e+44.78e+47.16e+49.55e+4

Zoom Out

y+11y+12c+24z+13y+28w+14c+26c+13y+14c+312c+313z+15y+15c+14w+16c+14z+211y+211z+16z+212y+212z+17c+212c+212y+17c+15c+213w+18c+213y+213z+213y+18c+16z+19y+19c+17c+18z+110y+110c+19z+111c+111z+112y+112c+112c+113

0769153723063074

Fragment Matches Table

Show background peaks

| Position | Ion type | Intensity | mz Theoretical | mz Error (Th) | mz Error (ppm) | Charge | Series Number |
| --- | --- | --- | --- | --- | --- | --- | --- |
| - | - | 346.8 | 127.6 | - | - | 0 | - |
| - | - | 1352 | 129.1 | - | - | 0 | - |
| - | - | 619.1 | 130.1 | - | - | 0 | - |
| 14 | y | 836.7 | 132.1 | 6.937E-05 | 0.5251 | +1 | 1 |
| - | - | 603.3 | 136.1 | - | - | 0 | - |
| - | - | 472.7 | 139.1 | - | - | 0 | - |
| - | - | 493.9 | 142.1 | - | - | 0 | - |
| - | - | 379.2 | 144.1 | - | - | 0 | - |
| - | - | 483.3 | 147.8 | - | - | 0 | - |
| - | - | 476.8 | 153.1 | - | - | 0 | - |
| - | - | 436.6 | 154.3 | - | - | 0 | - |
| - | - | 510.5 | 172.1 | - | - | 0 | - |
| - | - | 1339 | 173.5 | - | - | 0 | - |
| - | - | 471.3 | 198.2 | - | - | 0 | - |
| - | - | 465.2 | 211 | - | - | 0 | - |
| - | - | 589 | 226.2 | - | - | 0 | - |
| - | - | 543.5 | 236.8 | - | - | 0 | - |
| - | - | 600.3 | 241.2 | - | - | 0 | - |
| - | - | 487.2 | 244.1 | - | - | 0 | - |
| - | - | 621.6 | 245.1 | - | - | 0 | - |
| 13 | y | 1699 | 245.2 | 0.0002086 | 0.8508 | +1 | 2 |
| - | - | 684.4 | 255.1 | - | - | 0 | - |
| - | - | 1057 | 262.1 | - | - | 0 | - |
| - | - | 709.8 | 273.1 | - | - | 0 | - |
| - | - | 3735 | 290.1 | - | - | 0 | - |
| 4 | c | 945.7 | 291.1 | 0.001713 | 5.883 | +2 | 4 |
| - | - | 607.7 | 301.2 | - | - | 0 | - |
| - | - | 713.6 | 309.2 | - | - | 0 | - |
| - | - | 703.5 | 314.2 | - | - | 0 | - |
| 12 | z | 1383 | 357.3 | 0.0002757 | 0.7716 | +1 | 3 |
| - | - | 1000 | 358.3 | - | - | 0 | - |
| - | - | 830.8 | 369.2 | - | - | 0 | - |
| 7 | y | 2976 | 412.3 | 0.0002874 | 0.6971 | +2 | 8 |
| - | - | 805.3 | 412.8 | - | - | 0 | - |
| - | - | 616.2 | 413.3 | - | - | 0 | - |
| 11 | w | 1250 | 427.3 | 0.0008921 | 2.088 | +1 | 4 |
| 6 | c | 778 | 428.2 | 0.004073 | 9.512 | +2 | 6 |
| - | - | 565.9 | 432.4 | - | - | 0 | - |
| - | - | 959 | 451.2 | - | - | 0 | - |
| - | - | 661.2 | 453.2 | - | - | 0 | - |
| - | - | 2129 | 468.3 | - | - | 0 | - |
| - | - | 1195 | 469.2 | - | - | 0 | - |
| 3 | c | 3508 | 470.2 | 0.004416 | 9.392 | +1 | 3 |
| 11 | y | 1084 | 470.3 | 0.001817 | 3.864 | +1 | 4 |
| - | - | 810.4 | 471.2 | - | - | 0 | - |
| 12 | c | 789.7 | 478.6 | 0.001725 | 3.605 | +3 | 12 |
| - | - | 1557 | 478.9 | - | - | 0 | - |
| - | - | 594.6 | 479.2 | - | - | 0 | - |
| - | - | 665.9 | 506.9 | - | - | 0 | - |
| - | - | 679 | 507.3 | - | - | 0 | - |
| - | - | 950.6 | 510.3 | - | - | 0 | - |
| - | - | 824.3 | 510.6 | - | - | 0 | - |
| - | - | 764.6 | 510.9 | - | - | 0 | - |
| 13 | c | 5328 | 516.3 | 0.001861 | 3.605 | +3 | 13 |
| - | - | 4995 | 516.6 | - | - | 0 | - |
| - | - | 2692 | 516.9 | - | - | 0 | - |
| - | - | 1184 | 522.3 | - | - | 0 | - |
| 10 | z | 7730 | 525.4 | 0.0003313 | 0.6306 | +1 | 5 |
| - | - | 2330 | 526.4 | - | - | 0 | - |
| - | - | 755.6 | 531.3 | - | - | 0 | - |
| 10 | y | 835.4 | 541.4 | 0.001261 | 2.328 | +1 | 5 |
| - | - | 760.4 | 543.3 | - | - | 0 | - |
| - | - | 591.1 | 544.6 | - | - | 0 | - |
| - | - | 1464 | 554 | - | - | 0 | - |
| - | - | 1226 | 554.3 | - | - | 0 | - |
| - | - | 2065 | 554.6 | - | - | 0 | - |
| - | - | 1021 | 555 | - | - | 0 | - |
| - | - | 1535 | 559.3 | - | - | 0 | - |
| - | - | 3.21E+04 | 560 | - | - | 0 | - |
| - | - | 3.301E+04 | 560.3 | - | - | 0 | - |
| - | - | 1.617E+04 | 560.6 | - | - | 0 | - |
| - | - | 5472 | 561 | - | - | 0 | - |
| - | - | 3889 | 561.3 | - | - | 0 | - |
| - | - | 1960 | 579.2 | - | - | 0 | - |
| 4 | c | 1224 | 581.2 | 0.004217 | 7.256 | +1 | 4 |
| - | - | 891.2 | 582.4 | - | - | 0 | - |
| 9 | w | 2182 | 595.4 | 0.0008813 | 1.48 | +1 | 6 |
| - | - | 885.3 | 596.4 | - | - | 0 | - |
| - | - | 3853 | 597.3 | - | - | 0 | - |
| 4 | c | 8443 | 598.3 | 0.004829 | 8.072 | +1 | 4 |
| - | - | 3702 | 599.3 | - | - | 0 | - |
| 4 | z | 2567 | 605.4 | 0.0009166 | 1.514 | +2 | 11 |
| - | - | 3791 | 605.9 | - | - | 0 | - |
| - | - | 1893 | 606.4 | - | - | 0 | - |
| - | - | 1465 | 610.4 | - | - | 0 | - |
| 4 | y | 1452 | 613.4 | 0.001121 | 1.828 | +2 | 11 |
| - | - | 1301 | 613.9 | - | - | 0 | - |
| - | - | 724.6 | 629.3 | - | - | 0 | - |
| - | - | 737.1 | 629.8 | - | - | 0 | - |
| 9 | z | 4844 | 653.4 | 0.0002169 | 0.332 | +1 | 6 |
| - | - | 3203 | 654.5 | - | - | 0 | - |
| - | - | 947 | 655.5 | - | - | 0 | - |
| - | - | 929.8 | 658.4 | - | - | 0 | - |
| - | - | 694.2 | 667.4 | - | - | 0 | - |
| 3 | z | 5601 | 686.9 | 0.0005353 | 0.7793 | +2 | 12 |
| - | - | 8290 | 687.4 | - | - | 0 | - |
| - | - | 4021 | 687.9 | - | - | 0 | - |
| - | - | 1631 | 688.4 | - | - | 0 | - |
| - | - | 700.9 | 690.3 | - | - | 0 | - |
| 3 | y | 1.063E+04 | 694.9 | 0.000559 | 0.8044 | +2 | 12 |
| - | - | 8986 | 695.4 | - | - | 0 | - |
| - | - | 3673 | 695.9 | - | - | 0 | - |
| - | - | 651.6 | 696 | - | - | 0 | - |
| 8 | z | 2614 | 710.5 | 0.0001155 | 0.1626 | +1 | 7 |
| - | - | 1481 | 711.5 | - | - | 0 | - |
| - | - | 582.4 | 712.5 | - | - | 0 | - |
| 12 | c | 1531 | 717.4 | 0.004425 | 6.169 | +2 | 12 |
| - | - | 1.027E+04 | 721.9 | - | - | 0 | - |
| - | - | 8426 | 722.4 | - | - | 0 | - |
| - | - | 4923 | 722.9 | - | - | 0 | - |
| - | - | 1898 | 723.4 | - | - | 0 | - |
| - | - | 592.7 | 723.9 | - | - | 0 | - |
| 12 | c | 2.148E+04 | 725.9 | 0.00232 | 3.196 | +2 | 12 |
| - | - | 1.343E+04 | 726.4 | - | - | 0 | - |
| 8 | y | 667.8 | 726.5 | 0.002556 | 3.519 | +1 | 7 |
| - | - | 6351 | 726.9 | - | - | 0 | - |
| - | - | 2380 | 727.4 | - | - | 0 | - |
| - | - | 802.7 | 727.9 | - | - | 0 | - |
| 5 | c | 1.501E+04 | 735.3 | 0.00506 | 6.882 | +1 | 5 |
| - | - | 5465 | 736.3 | - | - | 0 | - |
| - | - | 1373 | 737.3 | - | - | 0 | - |
| - | - | 2250 | 758.9 | - | - | 0 | - |
| - | - | 2294 | 759.4 | - | - | 0 | - |
| - | - | 1683 | 765.4 | - | - | 0 | - |
| - | - | 1190 | 765.9 | - | - | 0 | - |
| - | - | 6799 | 766.4 | - | - | 0 | - |
| - | - | 4140 | 766.9 | - | - | 0 | - |
| - | - | 4876 | 767.4 | - | - | 0 | - |
| - | - | 2689 | 767.9 | - | - | 0 | - |
| - | - | 1534 | 768.4 | - | - | 0 | - |
| - | - | 1442 | 772.9 | - | - | 0 | - |
| 13 | c | 2462 | 773.4 | 0.006769 | 8.753 | +2 | 13 |
| - | - | 3145 | 773.9 | - | - | 0 | - |
| - | - | 4180 | 774.4 | - | - | 0 | - |
| 7 | w | 721.1 | 780.5 | 0.0008529 | 1.093 | +1 | 8 |
| 13 | c | 1.353E+04 | 782.4 | 0.002708 | 3.461 | +2 | 13 |
| - | - | 1.293E+04 | 782.9 | - | - | 0 | - |
| - | - | 6485 | 783.4 | - | - | 0 | - |
| - | - | 2246 | 783.9 | - | - | 0 | - |
| - | - | 1406 | 784.4 | - | - | 0 | - |
| 2 | y | 821.6 | 787.4 | 0.01071 | 13.61 | +2 | 13 |
| 2 | z | 900.4 | 787.9 | 0.006871 | 8.721 | +2 | 13 |
| - | - | 662.5 | 795.4 | - | - | 0 | - |
| - | - | 1300 | 798.9 | - | - | 0 | - |
| - | - | 1256 | 799.4 | - | - | 0 | - |
| - | - | 1177 | 799.9 | - | - | 0 | - |
| - | - | 1612 | 804.4 | - | - | 0 | - |
| - | - | 1108 | 804.9 | - | - | 0 | - |
| - | - | 1110 | 808.4 | - | - | 0 | - |
| - | - | 1023 | 808.9 | - | - | 0 | - |
| - | - | 931 | 809.4 | - | - | 0 | - |
| - | - | 915.3 | 809.9 | - | - | 0 | - |
| - | - | 1006 | 810.9 | - | - | 0 | - |
| - | - | 798.7 | 811.9 | - | - | 0 | - |
| - | - | 656.1 | 812.3 | - | - | 0 | - |
| - | - | 906 | 812.4 | - | - | 0 | - |
| - | - | 2080 | 817.4 | - | - | 0 | - |
| - | - | 3004 | 817.9 | - | - | 0 | - |
| - | - | 1466 | 818.4 | - | - | 0 | - |
| - | - | 1165 | 818.9 | - | - | 0 | - |
| - | - | 1330 | 821.9 | - | - | 0 | - |
| - | - | 3758 | 822.4 | - | - | 0 | - |
| - | - | 3377 | 822.9 | - | - | 0 | - |
| - | - | 1540 | 823.4 | - | - | 0 | - |
| 7 | y | 1909 | 823.5 | 0.0004049 | 0.4917 | +1 | 8 |
| - | - | 1076 | 824.5 | - | - | 0 | - |
| - | - | 1603 | 827.4 | - | - | 0 | - |
| - | - | 1422 | 828.4 | - | - | 0 | - |
| - | - | 809.7 | 829.4 | - | - | 0 | - |
| - | - | 823.8 | 829.9 | - | - | 0 | - |
| - | - | 2572 | 830.9 | - | - | 0 | - |
| - | - | 1.55E+04 | 831.4 | - | - | 0 | - |
| - | - | 1.344E+04 | 831.9 | - | - | 0 | - |
| - | - | 6336 | 832.4 | - | - | 0 | - |
| - | - | 3551 | 832.9 | - | - | 0 | - |
| - | - | 794.3 | 833.4 | - | - | 0 | - |
| - | - | 747.2 | 838.4 | - | - | 0 | - |
| - | - | 1139 | 838.9 | - | - | 0 | - |
| - | - | 2.186E+04 | 839.4 | - | - | 0 | - |
| - | - | 9.457E+04 | 840 | - | - | 0 | - |
| - | - | 8.02E+04 | 840.5 | - | - | 0 | - |
| - | - | 5.354E+04 | 841 | - | - | 0 | - |
| - | - | 2.213E+04 | 841.5 | - | - | 0 | - |
| - | - | 1.162E+04 | 841.9 | - | - | 0 | - |
| - | - | 724.5 | 850.5 | - | - | 0 | - |
| - | - | 891.2 | 852.5 | - | - | 0 | - |
| 6 | c | 1101 | 855.3 | 0.005168 | 6.042 | +1 | 6 |
| - | - | 677.1 | 899.4 | - | - | 0 | - |
| 6 | z | 1.369E+04 | 944.6 | 4.899E-05 | 0.05186 | +1 | 9 |
| - | - | 8730 | 945.6 | - | - | 0 | - |
| - | - | 2075 | 946.6 | - | - | 0 | - |
| - | - | 804 | 947.6 | - | - | 0 | - |
| 6 | y | 1030 | 960.6 | 0.001134 | 1.18 | +1 | 9 |
| - | - | 892.3 | 961.6 | - | - | 0 | - |
| 7 | c | 2829 | 969.4 | 0.007032 | 7.254 | +1 | 7 |
| - | - | 1171 | 970.4 | - | - | 0 | - |
| - | - | 898.7 | 983.6 | - | - | 0 | - |
| - | - | 1338 | 1025 | - | - | 0 | - |
| 8 | c | 1.505E+04 | 1026 | 0.004611 | 4.492 | +1 | 8 |
| - | - | 7575 | 1027 | - | - | 0 | - |
| - | - | 2505 | 1028 | - | - | 0 | - |
| 5 | z | 9212 | 1082 | 0.0001822 | 0.1685 | +1 | 10 |
| - | - | 2.149E+04 | 1083 | - | - | 0 | - |
| - | - | 1.259E+04 | 1084 | - | - | 0 | - |
| - | - | 4218 | 1085 | - | - | 0 | - |
| - | - | 580.5 | 1086 | - | - | 0 | - |
| - | - | 1287 | 1086 | - | - | 0 | - |
| 5 | y | 2111 | 1098 | 0.003161 | 2.88 | +1 | 10 |
| - | - | 1448 | 1099 | - | - | 0 | - |
| - | - | 765 | 1105 | - | - | 0 | - |
| - | - | 918.4 | 1109 | - | - | 0 | - |
| - | - | 923.4 | 1112 | - | - | 0 | - |
| - | - | 1576 | 1113 | - | - | 0 | - |
| - | - | 828.5 | 1119 | - | - | 0 | - |
| - | - | 835.1 | 1119 | - | - | 0 | - |
| - | - | 689.6 | 1120 | - | - | 0 | - |
| - | - | 914.5 | 1122 | - | - | 0 | - |
| - | - | 1111 | 1123 | - | - | 0 | - |
| 9 | c | 1.973E+04 | 1155 | 0.004863 | 4.212 | +1 | 9 |
| - | - | 1.195E+04 | 1156 | - | - | 0 | - |
| - | - | 4844 | 1157 | - | - | 0 | - |
| - | - | 1746 | 1158 | - | - | 0 | - |
| - | - | 1095 | 1159 | - | - | 0 | - |
| 4 | z | 3775 | 1210 | 0.002396 | 1.98 | +1 | 11 |
| - | - | 1.309E+04 | 1211 | - | - | 0 | - |
| - | - | 6818 | 1212 | - | - | 0 | - |
| - | - | 3162 | 1213 | - | - | 0 | - |
| - | - | 793.7 | 1214 | - | - | 0 | - |
| - | - | 1033 | 1322 | - | - | 0 | - |
| 11 | c | 8148 | 1323 | 0.003486 | 2.636 | +1 | 11 |
| - | - | 5172 | 1324 | - | - | 0 | - |
| - | - | 3087 | 1325 | - | - | 0 | - |
| - | - | 1063 | 1326 | - | - | 0 | - |
| 3 | z | 1077 | 1373 | 0.006978 | 5.083 | +1 | 12 |
| - | - | 4593 | 1374 | - | - | 0 | - |
| - | - | 5195 | 1375 | - | - | 0 | - |
| - | - | 1934 | 1376 | - | - | 0 | - |
| 3 | y | 2667 | 1389 | 0.003607 | 2.597 | +1 | 12 |
| - | - | 2354 | 1390 | - | - | 0 | - |
| - | - | 1193 | 1391 | - | - | 0 | - |
| - | - | 766.1 | 1443 | - | - | 0 | - |
| - | - | 2955 | 1444 | - | - | 0 | - |
| - | - | 2694 | 1445 | - | - | 0 | - |
| 12 | c | 3232 | 1451 | 0.001297 | 0.8938 | +1 | 12 |
| - | - | 5814 | 1452 | - | - | 0 | - |
| - | - | 4322 | 1453 | - | - | 0 | - |
| - | - | 1193 | 1454 | - | - | 0 | - |
| 13 | c | 1261 | 1564 | 0.004635 | 2.964 | +1 | 13 |
| - | - | 3700 | 1565 | - | - | 0 | - |
| - | - | 2595 | 1566 | - | - | 0 | - |
| - | - | 1510 | 1567 | - | - | 0 | - |
| - | - | 763.2 | 1568 | - | - | 0 | - |
| - | - | 720.5 | 1593 | - | - | 0 | - |
| - | - | 640.3 | 1599 | - | - | 0 | - |
| - | - | 1313 | 1634 | - | - | 0 | - |
| - | - | 2260 | 1635 | - | - | 0 | - |
| - | - | 1795 | 1636 | - | - | 0 | - |
| - | - | 1147 | 1637 | - | - | 0 | - |
| - | - | 1832 | 1645 | - | - | 0 | - |
| - | - | 2103 | 1646 | - | - | 0 | - |
| - | - | 1077 | 1647 | - | - | 0 | - |
| - | - | 1496 | 1652 | - | - | 0 | - |
| - | - | 791.9 | 1653 | - | - | 0 | - |
| - | - | 836.2 | 1654 | - | - | 0 | - |
| - | - | 5468 | 1662 | - | - | 0 | - |
| - | - | 1.408E+04 | 1663 | - | - | 0 | - |
| - | - | 1.219E+04 | 1664 | - | - | 0 | - |
| - | - | 6724 | 1665 | - | - | 0 | - |
| - | - | 3492 | 1666 | - | - | 0 | - |
| - | - | 1528 | 1667 | - | - | 0 | - |
| - | - | 2421 | 1678 | - | - | 0 | - |
| - | - | 1.183E+04 | 1679 | - | - | 0 | - |
| - | - | 5.063E+04 | 1680 | - | - | 0 | - |
| - | - | 4.71E+04 | 1681 | - | - | 0 | - |
| - | - | 3.06E+04 | 1682 | - | - | 0 | - |
| - | - | 1.228E+04 | 1683 | - | - | 0 | - |
| - | - | 9789 | 1684 | - | - | 0 | - |
| - | - | 690 | 2825 | - | - | 0 | - |
| - | - | 695 | 2952 | - | - | 0 | - |
| - | - | 812.9 | 3044 | - | - | 0 | - |

m/z Charge Intensity FragmentType MassShift Position
127.57176971435547 0 346.8379
129.10238647460938 0 1352.2083
130.06556701660156 0 619.09216
132.1019744873047 0 836.70984 y 13
136.0760955810547 0 603.3332
139.05718994140625 0 472.65103
142.09725952148438 0 493.91324
144.07852172851562 0 379.18918
147.820556640625 0 483.30408
153.07875061035156 0 476.84436
154.26792907714844 0 436.63046
172.0714569091797 0 510.46527
173.45132446289062 0 1339.2544
198.23947143554688 0 471.3028
210.9504852294922 0 465.19922
226.15423583984375 0 589.00543
236.8016815185547 0 543.48346
241.1549530029297 0 600.2743
244.09048461914062 0 487.22543
245.1365509033203 0 621.5664
245.18576049804688 0 1698.7666 y 12
255.0760955810547 0 684.3964
262.1186218261719 0 1056.7114
273.08697509765625 0 709.8057
290.113525390625 0 3735.358
291.1171875 0 945.71466 c Ammonia loss 3
301.19903564453125 0 607.7421
309.1553649902344 0 713.6311
314.2076721191406 0 703.4754
357.2619323730469 0 1382.5582 z 11
358.2684631347656 0 999.99164
369.2131652832031 0 830.7919
412.27392578125 0 2975.9104 y 6
412.77587890625 0 805.2595
413.27606201171875 0 616.1884
427.2923889160156 0 1250.33 w 10
428.181884765625 0 778.0131 c Ammonia loss 5
432.3693542480469 0 565.8935
451.1854553222656 0 958.99066
453.1808166503906 0 661.20526
468.3184509277344 0 2129.4463
469.19647216796875 0 1195.417
470.2029113769531 0 3508.345 c 2
470.3318786621094 0 1084.0232 y 10
471.20745849609375 0 810.4072
478.57403564453125 0 789.6961 c Ammonia loss 11
478.9090576171875 0 1556.6664
479.24737548828125 0 594.5946
506.93914794921875 0 665.9008
507.2711486816406 0 678.9867
510.26580810546875 0 950.63873
510.5977478027344 0 824.3108
510.93548583984375 0 764.5705
516.2688598632812 0 5327.8115 c Ammonia loss 12
516.6029663085938 0 4995.349
516.9367065429688 0 2692.0283
522.2732543945312 0 1183.8175
525.3524169921875 0 7729.6343 z 9
526.3560791015625 0 2329.6897
531.2904663085938 0 755.56116
541.3720703125 0 835.3821 y 9
543.2936401367188 0 760.3881
544.628173828125 0 591.0912
553.9638671875 0 1463.8802
554.2943115234375 0 1226.2606
554.6319580078125 0 2065.3418
554.9641723632812 0 1021.26605
559.3101196289062 0 1535.011
559.967041015625 0 32100.814
560.30126953125 0 33006.43
560.63525390625 0 16167.138
560.9680786132812 0 5472.402
561.3007202148438 0 3889.4165
579.2435302734375 0 1960.4489
581.2347412109375 0 1224.0504 c Ammonia loss 3
582.3731079101562 0 891.1929
595.3804931640625 0 2181.9197 w 8
596.3868408203125 0 885.27856
597.2545166015625 0 3852.8105
598.2619018554688 0 8442.835 c 3
599.2649536132812 0 3702.008
605.3533935546875 0 2566.6443 z 3
605.8562622070312 0 3790.6145
606.3573608398438 0 1893.3584
610.3958129882812 0 1465.3628
613.3607177734375 0 1452.3324 y 3
613.8623046875 0 1300.6127
629.3482055664062 0 724.5717
629.8484497070312 0 737.1432
653.447265625 0 4843.9683 z 8
654.4517211914062 0 3203.1345
655.4544677734375 0 947.0421
658.36083984375 0 929.78284
667.4063720703125 0 694.1824
686.8836059570312 0 5600.7563 z 2
687.3864135742188 0 8289.928
687.8881225585938 0 4021.333
688.3869018554688 0 1631.1047
690.2996215820312 0 700.92566
694.8929443359375 0 10630.335 y 2
695.3942260742188 0 8985.835
695.8967895507812 0 3673.2053
695.95751953125 0 651.61755
710.4686279296875 0 2613.86 z 7
711.47265625 0 1480.6473
712.4797973632812 0 582.4335
717.3592529296875 0 1531.2426 c Ammonia loss 11
721.8991088867188 0 10269.116
722.4000854492188 0 8425.558
722.9013671875 0 4922.9897
723.4033813476562 0 1898.2815
723.9154663085938 0 592.7497
725.8704223632812 0 21475.72 c 11
726.3717651367188 0 13431.157
726.4846801757812 0 667.82635 y 7
726.8734741210938 0 6350.967
727.3744506835938 0 2380.1702
727.8705444335938 0 802.6779
735.321044921875 0 15013.273 c 4
736.32373046875 0 5465.0933
737.3253173828125 0 1373.3882
758.9110107421875 0 2249.9253
759.4118041992188 0 2294.0654
765.4104614257812 0 1682.8535
765.9197998046875 0 1189.7692
766.41650390625 0 6798.6655
766.9188232421875 0 4139.888
767.4216918945312 0 4875.8076
767.9259033203125 0 2688.5981
768.4264526367188 0 1533.8558
772.9193115234375 0 1442.1062
773.41162109375 0 2461.781 c Water loss 12
773.9171752929688 0 3145.2776
774.4141845703125 0 4180.3506
780.4969482421875 0 721.08966 w 6
782.412841796875 0 13527.31 c 12
782.9143676757812 0 12933.002
783.415283203125 0 6484.5806
783.9147338867188 0 2245.6504
784.4132080078125 0 1405.5154
787.4255981445312 0 821.6098 y Ammonia loss 1
787.9119262695312 0 900.4297 z 1
795.4194946289062 0 662.4965
798.9220581054688 0 1300.2117
799.4247436523438 0 1256.3032
799.9212036132812 0 1177.0189
804.413330078125 0 1611.8716
804.9207763671875 0 1108.2281
808.4346313476562 0 1110.1937
808.9383544921875 0 1022.9709
809.4420776367188 0 930.9651
809.9083251953125 0 915.288
810.9203491210938 0 1006.27795
811.9129028320312 0 798.705
812.3430786132812 0 656.14703
812.4258422851562 0 905.9866
817.441162109375 0 2079.652
817.9429931640625 0 3004.3057
818.4349365234375 0 1466.1025
818.927734375 0 1165.1451
821.9409790039062 0 1329.6725
822.4338989257812 0 3757.5796
822.9334716796875 0 3376.9492
823.435546875 0 1539.8164
823.5404052734375 0 1908.5253 y 6
824.541748046875 0 1075.8765
827.356201171875 0 1602.9799
828.3583984375 0 1422.156
829.3750610351562 0 809.7358
829.9248657226562 0 823.77216
830.9434814453125 0 2572.0776
831.43994140625 0 15497.669
831.9404296875 0 13443.599
832.44091796875 0 6336.3228
832.9415893554688 0 3551.0928
833.4376831054688 0 794.31836
838.4263916015625 0 747.239
838.9466552734375 0 1138.8392
839.4472045898438 0 21855.508
839.9503784179688 0 94565.74
840.4512939453125 0 80197.23
840.9515380859375 0 53541.008
841.4511108398438 0 22125.557
841.9485473632812 0 11619.775
850.52978515625 0 724.5343
852.5421142578125 0 891.2276
855.353515625 0 1100.7804 c Ammonia loss 5
899.4166259765625 0 677.07275
944.5801391601562 0 13686.892 z 5
945.5833129882812 0 8729.501
946.5870361328125 0 2074.593
947.5894165039062 0 803.98883
960.5977783203125 0 1029.7671 y 5
961.6019287109375 0 892.3455
969.4346923828125 0 2828.5142 c 6
970.4349365234375 0 1170.6194
983.5519409179688 0 898.7015
1025.44921875 0 1338.0111
1026.4537353515625 0 15046.563 c 7
1027.457763671875 0 7575.234
1028.4619140625 0 2504.615
1081.6392822265625 0 9212.288 z 4
1082.6455078125 0 21492.045
1083.648681640625 0 12591.135
1084.65283203125 0 4217.855
1085.5235595703125 0 580.4688
1085.6510009765625 0 1287.1753
1097.6546630859375 0 2111.1882 y 4
1098.65625 0 1447.9132
1104.5162353515625 0 765.0269
1108.501953125 0 918.37177
1111.5404052734375 0 923.448
1112.5322265625 0 1576.3356
1118.531005859375 0 828.4741
1119.4541015625 0 835.07025
1120.0323486328125 0 689.6092
1121.5126953125 0 914.45233
1122.5157470703125 0 1111.0988
1154.5489501953125 0 19727.004 c 8
1155.5526123046875 0 11949.504
1156.5546875 0 4843.9443
1157.5638427734375 0 1745.9454
1158.54931640625 0 1094.9442
1209.7000732421875 0 3775.0312 z 3
1210.7042236328125 0 13085.309
1211.7076416015625 0 6817.5728
1212.7103271484375 0 3162.4285
1213.7196044921875 0 793.73975
1321.6192626953125 0 1033.3911
1322.637451171875 0 8148.4805 c 10
1323.6407470703125 0 5172.2734
1324.63916015625 0 3087.0044
1325.6473388671875 0 1063.2028
1372.7540283203125 0 1077.0286 z 2
1373.765625 0 4592.7944
1374.7708740234375 0 5194.9023
1375.769287109375 0 1934.2852
1388.776123046875 0 2667.0444 y 2
1389.78076171875 0 2353.669
1390.775634765625 0 1193.1385
1442.793701171875 0 766.09326
1443.798095703125 0 2955.1633
1444.797119140625 0 2693.8257
1450.730224609375 0 3231.7808 c 11
1451.7353515625 0 5813.884
1452.7425537109375 0 4322.1367
1453.734375 0 1193.2949
1563.817626953125 0 1261.4724 c 12
1564.8238525390625 0 3700.331
1565.83056640625 0 2594.8164
1566.8275146484375 0 1510.221
1567.8341064453125 0 763.21136
1592.84912109375 0 720.46155
1599.0814208984375 0 640.28217
1633.8807373046875 0 1313.0918
1634.87744140625 0 2260.012
1635.882568359375 0 1795.0111
1636.8974609375 0 1146.977
1644.8594970703125 0 1832.0201
1645.8629150390625 0 2102.7065
1646.879638671875 0 1076.7905
1651.906005859375 0 1496.149
1652.8994140625 0 791.8644
1653.893798828125 0 836.22614
1661.8824462890625 0 5467.752
1662.8787841796875 0 14080.976
1663.87939453125 0 12194.94
1664.8779296875 0 6723.572
1665.88037109375 0 3492.029
1666.861083984375 0 1527.507
1677.8790283203125 0 2420.5066
1678.8902587890625 0 11833.952
1679.900390625 0 50627.977
1680.9029541015625 0 47103
1681.9014892578125 0 30597.396
1682.898193359375 0 12278.841
1683.8916015625 0 9788.541
2825.337158203125 0 689.96
2951.9404296875 0 694.9821
3043.62890625 0 812.8615

Spectrum Details

|  |  |
| --- | --- |
| Matched peaks? Matched peaksThe total absolute number of peaks matched. Additionally in brackets the total fraction of peaks matched and the total number of peaks is shown. | 46 (16.43% of 280) |
| FDR? FDRThe false discovery rate estimated for this peptide. It is calculated by matching all theoretical fragments with a non-integer shift with the raw peaks for this spectrum. This is done with 40 different shifts. The resulting percentage is the average number of annotated peaks over the number of annotated peaks with the correct spectrum. | 2.90% |
| Satellite FDR? Satellite FDRSee the FDR for details on its calculation. This satellite ion specific FDR only contains the satellite ions (d/w) for I/L/J positions. | - |
| PSM Score? PSM ScoreThe PSM Score as given by Hecklib to this annotated spectrum. It is shown with three significant figures. | 344 |

## Spectrum 5672? Spectrum 5672 The raw spectrum of this peptide as annotated by Hecklib. The fragments are coloured according to ion type (see legend). Any peaks with a star '\*' as text can be hovered over to see the full details, first the ion type second the mass shift type. By hovering over the amino acids in the peptide or ions in the legend the corresponding peaks are highlighted. By toggling the 'Unassigned' label you can turn the background (unassigned) peaks on or off in the plot. By updating the slider in the Ion legend you can update the spectrum to only show the top X% of the peaks with labels. The top X% means any peak that is within X% of the highest intensity. By dragging in the spectrum you can zoom in to a specific part of the spectrum and use 'Zoom Out' to get back to the original zoom level. The annotation of the spectrum is based on the given sequence in the peptides file and is done with different software so inconsistencies are likely. The peaks are annotated based on the given sequence, with 20 ppm tolerance.

Copy Data

### Spectrum 5672 (TSV)

#### Preview

```
Loading example...
```

*Click on the button to copy the data to your clipboard.*

Mz MinMz MaxIntensity Max

WidthHeightPeptide font sizePeptide stroke widthSpectrum font sizeSpectrum stroke widthCompact peptide

Ion legend

wxyz

abcd

OtherUnassignedIonChargePositionShow for top:%

SWYQHHPGKAPKJJ

04.67e+49.35e+41.40e+51.87e+5

Zoom Out

y+11y+12z+13y+13y+28w+14c+13y+312y+14c+312c+313z+15y+313y+15y+210c+14c+14w+211c+14w+16y+211z+211y+211z+16c+211y+212z+212z+17y+212c+15c+212z+17c+212c+15y+17c+213z+213y+213y+18c+16z+19c+17y+19c+18z+110y+110c+19y+111z+111y+111c+111z+112y+112c+112c+113z+113

0689137920682757

Fragment Matches Table

Show background peaks

| Position | Ion type | Intensity | mz Theoretical | mz Error (Th) | mz Error (ppm) | Charge | Series Number |
| --- | --- | --- | --- | --- | --- | --- | --- |
| - | - | 363.9 | 120.1 | - | - | 0 | - |
| - | - | 1762 | 129.1 | - | - | 0 | - |
| - | - | 522.5 | 132.1 | - | - | 0 | - |
| 14 | y | 809.3 | 132.1 | 0.0001762 | 1.334 | +1 | 1 |
| - | - | 609.2 | 136.1 | - | - | 0 | - |
| - | - | 447.7 | 139.1 | - | - | 0 | - |
| - | - | 2199 | 153.1 | - | - | 0 | - |
| - | - | 4482 | 173.5 | - | - | 0 | - |
| - | - | 478.3 | 174.1 | - | - | 0 | - |
| - | - | 500.5 | 186.1 | - | - | 0 | - |
| - | - | 774.7 | 187.1 | - | - | 0 | - |
| - | - | 487.3 | 189.4 | - | - | 0 | - |
| - | - | 480.9 | 196.6 | - | - | 0 | - |
| - | - | 531.6 | 201.1 | - | - | 0 | - |
| - | - | 1147 | 211.1 | - | - | 0 | - |
| - | - | 2692 | 215.1 | - | - | 0 | - |
| - | - | 491.1 | 226.2 | - | - | 0 | - |
| - | - | 589.3 | 227.1 | - | - | 0 | - |
| - | - | 729.7 | 229.1 | - | - | 0 | - |
| - | - | 1.465E+04 | 229.2 | - | - | 0 | - |
| - | - | 1527 | 230.2 | - | - | 0 | - |
| - | - | 840.3 | 241.1 | - | - | 0 | - |
| - | - | 1440 | 245.1 | - | - | 0 | - |
| 13 | y | 2386 | 245.2 | 0.0002696 | 1.1 | +1 | 2 |
| - | - | 5670 | 246.1 | - | - | 0 | - |
| - | - | 836.6 | 246.1 | - | - | 0 | - |
| - | - | 518.9 | 246.2 | - | - | 0 | - |
| - | - | 983.2 | 247.1 | - | - | 0 | - |
| - | - | 570.5 | 247.3 | - | - | 0 | - |
| - | - | 642.4 | 258.1 | - | - | 0 | - |
| - | - | 469.8 | 265.1 | - | - | 0 | - |
| - | - | 7452 | 274.1 | - | - | 0 | - |
| - | - | 895.3 | 275.1 | - | - | 0 | - |
| - | - | 1154 | 281.2 | - | - | 0 | - |
| - | - | 1659 | 282.2 | - | - | 0 | - |
| - | - | 1511 | 292.1 | - | - | 0 | - |
| - | - | 934.1 | 299.1 | - | - | 0 | - |
| - | - | 556.3 | 299.2 | - | - | 0 | - |
| - | - | 573.5 | 300.1 | - | - | 0 | - |
| - | - | 1621 | 300.2 | - | - | 0 | - |
| - | - | 1155 | 301.2 | - | - | 0 | - |
| - | - | 1550 | 309.2 | - | - | 0 | - |
| - | - | 2736 | 314.2 | - | - | 0 | - |
| - | - | 646.5 | 315.2 | - | - | 0 | - |
| - | - | 624.3 | 322.3 | - | - | 0 | - |
| - | - | 560 | 331.2 | - | - | 0 | - |
| - | - | 4649 | 340.1 | - | - | 0 | - |
| - | - | 1023 | 341.1 | - | - | 0 | - |
| - | - | 594.2 | 343.2 | - | - | 0 | - |
| - | - | 797.4 | 355.1 | - | - | 0 | - |
| 12 | z | 2077 | 357.3 | 0.0002146 | 0.6008 | +1 | 3 |
| - | - | 1509 | 358.3 | - | - | 0 | - |
| - | - | 915.7 | 371.1 | - | - | 0 | - |
| 12 | y | 1245 | 373.3 | 0.0002314 | 0.62 | +1 | 3 |
| - | - | 760.8 | 382.3 | - | - | 0 | - |
| - | - | 917.6 | 394.3 | - | - | 0 | - |
| - | - | 2172 | 397.2 | - | - | 0 | - |
| - | - | 874 | 398.2 | - | - | 0 | - |
| - | - | 511.2 | 398.6 | - | - | 0 | - |
| - | - | 752.8 | 399.3 | - | - | 0 | - |
| 7 | y | 4300 | 412.3 | 0.0002925 | 0.7094 | +2 | 8 |
| - | - | 2205 | 412.8 | - | - | 0 | - |
| - | - | 567 | 418.2 | - | - | 0 | - |
| 11 | w | 1626 | 427.3 | 0.0002981 | 0.6976 | +1 | 4 |
| - | - | 1782 | 437.2 | - | - | 0 | - |
| - | - | 1176 | 453.2 | - | - | 0 | - |
| 3 | c | 3310 | 454.2 | 0.0006707 | 1.477 | +1 | 3 |
| - | - | 2074 | 455.8 | - | - | 0 | - |
| 3 | y | 1418 | 463.6 | 0.0004469 | 0.964 | +3 | 12 |
| - | - | 977.2 | 463.9 | - | - | 0 | - |
| - | - | 895.4 | 465.2 | - | - | 0 | - |
| - | - | 1760 | 467.8 | - | - | 0 | - |
| - | - | 923.4 | 468.3 | - | - | 0 | - |
| - | - | 3664 | 468.3 | - | - | 0 | - |
| - | - | 659.5 | 469.3 | - | - | 0 | - |
| - | - | 1182 | 469.3 | - | - | 0 | - |
| - | - | 811 | 470.3 | - | - | 0 | - |
| 11 | y | 1089 | 470.3 | 0.001756 | 3.734 | +1 | 4 |
| 12 | c | 1877 | 473.2 | 0.0005198 | 1.098 | +3 | 12 |
| - | - | 916.3 | 474.2 | - | - | 0 | - |
| - | - | 1903 | 477.2 | - | - | 0 | - |
| - | - | 1486 | 480.2 | - | - | 0 | - |
| - | - | 863.1 | 481.3 | - | - | 0 | - |
| - | - | 1250 | 482.3 | - | - | 0 | - |
| - | - | 818.5 | 495.4 | - | - | 0 | - |
| - | - | 990.9 | 501.6 | - | - | 0 | - |
| - | - | 665.4 | 501.9 | - | - | 0 | - |
| - | - | 658 | 504.9 | - | - | 0 | - |
| - | - | 1052 | 505.3 | - | - | 0 | - |
| - | - | 2.083E+04 | 510.3 | - | - | 0 | - |
| 13 | c | 7632 | 510.9 | 0.0005667 | 1.109 | +3 | 13 |
| - | - | 5795 | 511.3 | - | - | 0 | - |
| - | - | 5864 | 511.3 | - | - | 0 | - |
| - | - | 2178 | 511.6 | - | - | 0 | - |
| - | - | 775.9 | 511.9 | - | - | 0 | - |
| - | - | 645.5 | 513.7 | - | - | 0 | - |
| - | - | 2038 | 514.3 | - | - | 0 | - |
| - | - | 776.9 | 517.3 | - | - | 0 | - |
| - | - | 1264 | 523.2 | - | - | 0 | - |
| 10 | z | 1.313E+04 | 525.4 | 0.000279 | 0.5312 | +1 | 5 |
| 2 | y | 1695 | 525.6 | 0.0007121 | 1.355 | +3 | 13 |
| - | - | 1242 | 526 | - | - | 0 | - |
| - | - | 4523 | 526.4 | - | - | 0 | - |
| - | - | 648.3 | 528.2 | - | - | 0 | - |
| - | - | 952.1 | 539.3 | - | - | 0 | - |
| - | - | 659.7 | 539.3 | - | - | 0 | - |
| 10 | y | 1443 | 541.4 | 0.001322 | 2.441 | +1 | 5 |
| - | - | 653.4 | 541.8 | - | - | 0 | - |
| - | - | 2433 | 548.6 | - | - | 0 | - |
| - | - | 731.6 | 549 | - | - | 0 | - |
| - | - | 1359 | 549.3 | - | - | 0 | - |
| 5 | y | 957.5 | 549.3 | 0.005096 | 9.277 | +2 | 10 |
| - | - | 1271 | 549.6 | - | - | 0 | - |
| - | - | 876.9 | 550.3 | - | - | 0 | - |
| - | - | 2198 | 553.3 | - | - | 0 | - |
| - | - | 705.5 | 553.8 | - | - | 0 | - |
| - | - | 5611 | 554.2 | - | - | 0 | - |
| - | - | 734.9 | 554.3 | - | - | 0 | - |
| - | - | 5.642E+04 | 554.6 | - | - | 0 | - |
| - | - | 5.389E+04 | 555 | - | - | 0 | - |
| - | - | 1060 | 555.2 | - | - | 0 | - |
| - | - | 3.363E+04 | 555.3 | - | - | 0 | - |
| - | - | 9232 | 555.6 | - | - | 0 | - |
| - | - | 2535 | 556 | - | - | 0 | - |
| - | - | 3992 | 563.2 | - | - | 0 | - |
| 4 | c | 773.9 | 564.3 | 0.01072 | 19 | +1 | 4 |
| 4 | c | 2610 | 565.2 | 0.0001064 | 0.1882 | +1 | 4 |
| - | - | 912.9 | 566.2 | - | - | 0 | - |
| - | - | 1039 | 566.4 | - | - | 0 | - |
| - | - | 594.2 | 569.8 | - | - | 0 | - |
| - | - | 747.4 | 572.8 | - | - | 0 | - |
| 4 | w | 3152 | 576.3 | 0.0007965 | 1.382 | +2 | 11 |
| - | - | 2123 | 576.8 | - | - | 0 | - |
| - | - | 654 | 577.3 | - | - | 0 | - |
| - | - | 2055 | 581.3 | - | - | 0 | - |
| 4 | c | 1.167E+04 | 582.3 | 0.0007155 | 1.229 | +1 | 4 |
| - | - | 1912 | 582.4 | - | - | 0 | - |
| - | - | 4520 | 583.3 | - | - | 0 | - |
| - | - | 1211 | 584.3 | - | - | 0 | - |
| 9 | w | 3098 | 595.4 | 0.00198 | 3.325 | +1 | 6 |
| - | - | 1076 | 596.4 | - | - | 0 | - |
| - | - | 785.9 | 597.4 | - | - | 0 | - |
| 4 | y | 1178 | 604.8 | 0.001106 | 1.828 | +2 | 11 |
| 4 | z | 7713 | 605.4 | 0.0004262 | 0.7041 | +2 | 11 |
| - | - | 1.152E+04 | 605.9 | - | - | 0 | - |
| - | - | 5204 | 606.4 | - | - | 0 | - |
| - | - | 2111 | 606.9 | - | - | 0 | - |
| - | - | 949 | 609.5 | - | - | 0 | - |
| - | - | 1914 | 610.4 | - | - | 0 | - |
| - | - | 1105 | 611.4 | - | - | 0 | - |
| - | - | 883.8 | 612.9 | - | - | 0 | - |
| 4 | y | 4004 | 613.4 | 0.0002826 | 0.4607 | +2 | 11 |
| - | - | 2719 | 613.9 | - | - | 0 | - |
| - | - | 1195 | 614.4 | - | - | 0 | - |
| - | - | 925 | 620.3 | - | - | 0 | - |
| - | - | 962.1 | 624.4 | - | - | 0 | - |
| - | - | 956.7 | 628.4 | - | - | 0 | - |
| - | - | 1772 | 629.3 | - | - | 0 | - |
| - | - | 1103 | 629.8 | - | - | 0 | - |
| - | - | 764.7 | 637.4 | - | - | 0 | - |
| - | - | 921.2 | 645.8 | - | - | 0 | - |
| - | - | 789.8 | 651.4 | - | - | 0 | - |
| - | - | 931.6 | 651.9 | - | - | 0 | - |
| 9 | z | 9994 | 653.4 | 0.0003934 | 0.6021 | +1 | 6 |
| 11 | c | 1656 | 653.8 | 0.0006695 | 1.024 | +2 | 11 |
| - | - | 5514 | 654.5 | - | - | 0 | - |
| - | - | 1198 | 655.5 | - | - | 0 | - |
| - | - | 825.4 | 657.9 | - | - | 0 | - |
| - | - | 860.1 | 658.9 | - | - | 0 | - |
| - | - | 996.9 | 659.3 | - | - | 0 | - |
| - | - | 1032 | 660.3 | - | - | 0 | - |
| - | - | 667.4 | 660.4 | - | - | 0 | - |
| - | - | 1218 | 675.3 | - | - | 0 | - |
| - | - | 3289 | 682.4 | - | - | 0 | - |
| - | - | 1048 | 683.4 | - | - | 0 | - |
| 3 | y | 807.3 | 686.4 | 0.001261 | 1.838 | +2 | 12 |
| 3 | z | 1.012E+04 | 686.9 | 0.0009625 | 1.401 | +2 | 12 |
| - | - | 1.398E+04 | 687.4 | - | - | 0 | - |
| - | - | 6628 | 687.9 | - | - | 0 | - |
| - | - | 1072 | 688.4 | - | - | 0 | - |
| 8 | z | 1240 | 693.4 | 0.000435 | 0.6273 | +1 | 7 |
| 3 | y | 1.272E+04 | 694.9 | 0.0008031 | 1.156 | +2 | 12 |
| - | - | 8037 | 695.4 | - | - | 0 | - |
| - | - | 4023 | 695.9 | - | - | 0 | - |
| 5 | c | 1246 | 702.3 | 0.002133 | 3.038 | +1 | 5 |
| 12 | c | 1435 | 709.4 | 0.0009409 | 1.326 | +2 | 12 |
| - | - | 2310 | 709.9 | - | - | 0 | - |
| - | - | 915.2 | 710.4 | - | - | 0 | - |
| 8 | z | 1631 | 710.5 | 0.002936 | 4.133 | +1 | 7 |
| - | - | 1243 | 711.5 | - | - | 0 | - |
| 12 | c | 3.407E+04 | 717.9 | 0.0007877 | 1.097 | +2 | 12 |
| - | - | 2.797E+04 | 718.4 | - | - | 0 | - |
| - | - | 1.41E+04 | 718.9 | - | - | 0 | - |
| 5 | c | 2.91E+04 | 719.3 | 0.0006674 | 0.9279 | +1 | 5 |
| - | - | 1.407E+04 | 720.3 | - | - | 0 | - |
| - | - | 3353 | 721.3 | - | - | 0 | - |
| 8 | y | 1030 | 726.5 | 0.00219 | 3.015 | +1 | 7 |
| - | - | 655.1 | 740 | - | - | 0 | - |
| - | - | 1400 | 752.9 | - | - | 0 | - |
| - | - | 996.3 | 758.4 | - | - | 0 | - |
| - | - | 1475 | 758.9 | - | - | 0 | - |
| - | - | 927.8 | 759.4 | - | - | 0 | - |
| - | - | 1794 | 766.4 | - | - | 0 | - |
| - | - | 1063 | 766.9 | - | - | 0 | - |
| - | - | 1567 | 767.4 | - | - | 0 | - |
| - | - | 985.8 | 773.9 | - | - | 0 | - |
| 13 | c | 2.29E+04 | 774.4 | 0.0004613 | 0.5957 | +2 | 13 |
| - | - | 1.896E+04 | 774.9 | - | - | 0 | - |
| - | - | 1.116E+04 | 775.4 | - | - | 0 | - |
| - | - | 3212 | 775.9 | - | - | 0 | - |
| - | - | 1423 | 776.4 | - | - | 0 | - |
| 2 | z | 4735 | 779.9 | 0.001495 | 1.917 | +2 | 13 |
| - | - | 3441 | 780.4 | - | - | 0 | - |
| - | - | 2579 | 780.9 | - | - | 0 | - |
| - | - | 795 | 781.9 | - | - | 0 | - |
| 2 | y | 2003 | 787.9 | 0.01464 | 18.58 | +2 | 13 |
| - | - | 1037 | 788.4 | - | - | 0 | - |
| - | - | 1203 | 788.9 | - | - | 0 | - |
| - | - | 2929 | 790.9 | - | - | 0 | - |
| - | - | 3592 | 791.4 | - | - | 0 | - |
| - | - | 2530 | 791.9 | - | - | 0 | - |
| - | - | 827.7 | 792.4 | - | - | 0 | - |
| - | - | 1043 | 795.4 | - | - | 0 | - |
| - | - | 1485 | 795.9 | - | - | 0 | - |
| - | - | 1094 | 796.4 | - | - | 0 | - |
| - | - | 1805 | 796.9 | - | - | 0 | - |
| - | - | 1711 | 800.4 | - | - | 0 | - |
| - | - | 2111 | 800.9 | - | - | 0 | - |
| - | - | 2545 | 801.4 | - | - | 0 | - |
| - | - | 751.2 | 801.9 | - | - | 0 | - |
| - | - | 756.9 | 802.4 | - | - | 0 | - |
| - | - | 1396 | 802.9 | - | - | 0 | - |
| - | - | 1036 | 804.4 | - | - | 0 | - |
| - | - | 832.8 | 808.9 | - | - | 0 | - |
| - | - | 3304 | 809.4 | - | - | 0 | - |
| - | - | 5885 | 809.9 | - | - | 0 | - |
| - | - | 2578 | 810.4 | - | - | 0 | - |
| - | - | 1945 | 810.9 | - | - | 0 | - |
| - | - | 4468 | 811.4 | - | - | 0 | - |
| - | - | 807.5 | 811.4 | - | - | 0 | - |
| - | - | 3132 | 812.4 | - | - | 0 | - |
| - | - | 858.5 | 813.9 | - | - | 0 | - |
| - | - | 969.6 | 814.4 | - | - | 0 | - |
| - | - | 707.8 | 814.9 | - | - | 0 | - |
| - | - | 783.5 | 815.4 | - | - | 0 | - |
| - | - | 2499 | 822.9 | - | - | 0 | - |
| - | - | 1.716E+04 | 823.4 | - | - | 0 | - |
| 7 | y | 3639 | 823.5 | 0.005393 | 6.549 | +1 | 8 |
| - | - | 1.654E+04 | 823.9 | - | - | 0 | - |
| - | - | 6583 | 824.4 | - | - | 0 | - |
| - | - | 2086 | 824.5 | - | - | 0 | - |
| - | - | 4007 | 824.9 | - | - | 0 | - |
| - | - | 1250 | 825.4 | - | - | 0 | - |
| - | - | 789.2 | 830.4 | - | - | 0 | - |
| - | - | 2311 | 830.9 | - | - | 0 | - |
| - | - | 3.939E+04 | 831.4 | - | - | 0 | - |
| - | - | 1.851E+05 | 832 | - | - | 0 | - |
| - | - | 1.641E+05 | 832.5 | - | - | 0 | - |
| - | - | 8.84E+04 | 833 | - | - | 0 | - |
| - | - | 795.3 | 833.1 | - | - | 0 | - |
| - | - | 2.833E+04 | 833.5 | - | - | 0 | - |
| - | - | 7505 | 834 | - | - | 0 | - |
| - | - | 1501 | 838.5 | - | - | 0 | - |
| 6 | c | 2403 | 839.4 | 0.002146 | 2.557 | +1 | 6 |
| - | - | 856.7 | 839.4 | - | - | 0 | - |
| - | - | 1239 | 840.4 | - | - | 0 | - |
| - | - | 2132 | 850.5 | - | - | 0 | - |
| - | - | 989.6 | 851.5 | - | - | 0 | - |
| - | - | 2162 | 852.5 | - | - | 0 | - |
| - | - | 2101 | 855.5 | - | - | 0 | - |
| - | - | 739 | 855.9 | - | - | 0 | - |
| - | - | 1006 | 856.5 | - | - | 0 | - |
| - | - | 727.7 | 878.4 | - | - | 0 | - |
| - | - | 933.1 | 883.4 | - | - | 0 | - |
| - | - | 1550 | 894.5 | - | - | 0 | - |
| - | - | 762.2 | 903.3 | - | - | 0 | - |
| - | - | 7266 | 910.5 | - | - | 0 | - |
| - | - | 5956 | 911.5 | - | - | 0 | - |
| - | - | 2726 | 912.5 | - | - | 0 | - |
| - | - | 874 | 934.5 | - | - | 0 | - |
| - | - | 864.7 | 935.5 | - | - | 0 | - |
| 6 | z | 3.004E+04 | 944.6 | 0.0007814 | 0.8273 | +1 | 9 |
| - | - | 1.959E+04 | 945.6 | - | - | 0 | - |
| - | - | 5638 | 946.6 | - | - | 0 | - |
| - | - | 999.5 | 947.6 | - | - | 0 | - |
| 7 | c | 3540 | 953.4 | 0.001747 | 1.833 | +1 | 7 |
| - | - | 1792 | 954.4 | - | - | 0 | - |
| 6 | y | 3165 | 960.6 | 0.001439 | 1.498 | +1 | 9 |
| - | - | 997.2 | 961.6 | - | - | 0 | - |
| - | - | 769.9 | 964.4 | - | - | 0 | - |
| - | - | 701 | 968.6 | - | - | 0 | - |
| - | - | 1563 | 982.5 | - | - | 0 | - |
| - | - | 1391 | 983.6 | - | - | 0 | - |
| - | - | 1684 | 1009 | - | - | 0 | - |
| 8 | c | 2.722E+04 | 1010 | 0.0007502 | 0.7425 | +1 | 8 |
| - | - | 1.671E+04 | 1011 | - | - | 0 | - |
| - | - | 6403 | 1012 | - | - | 0 | - |
| - | - | 1157 | 1013 | - | - | 0 | - |
| - | - | 813 | 1081 | - | - | 0 | - |
| 5 | z | 1.37E+04 | 1082 | 0.001283 | 1.186 | +1 | 10 |
| - | - | 3.456E+04 | 1083 | - | - | 0 | - |
| - | - | 2.004E+04 | 1084 | - | - | 0 | - |
| - | - | 6493 | 1085 | - | - | 0 | - |
| - | - | 1994 | 1086 | - | - | 0 | - |
| - | - | 1094 | 1093 | - | - | 0 | - |
| - | - | 1024 | 1094 | - | - | 0 | - |
| - | - | 1827 | 1095 | - | - | 0 | - |
| - | - | 1044 | 1096 | - | - | 0 | - |
| - | - | 1577 | 1097 | - | - | 0 | - |
| 5 | y | 3750 | 1098 | 0.002917 | 2.657 | +1 | 10 |
| - | - | 1899 | 1099 | - | - | 0 | - |
| - | - | 909.4 | 1101 | - | - | 0 | - |
| - | - | 684.5 | 1102 | - | - | 0 | - |
| - | - | 891.2 | 1103 | - | - | 0 | - |
| - | - | 1144 | 1109 | - | - | 0 | - |
| - | - | 840.5 | 1109 | - | - | 0 | - |
| - | - | 1081 | 1110 | - | - | 0 | - |
| - | - | 1644 | 1111 | - | - | 0 | - |
| - | - | 1218 | 1112 | - | - | 0 | - |
| - | - | 661.3 | 1138 | - | - | 0 | - |
| 9 | c | 3.4E+04 | 1139 | 0.0006205 | 0.545 | +1 | 9 |
| - | - | 2.676E+04 | 1140 | - | - | 0 | - |
| - | - | 8013 | 1141 | - | - | 0 | - |
| - | - | 2227 | 1142 | - | - | 0 | - |
| - | - | 738.3 | 1166 | - | - | 0 | - |
| - | - | 728.6 | 1184 | - | - | 0 | - |
| 4 | y | 1056 | 1209 | 0.01016 | 8.41 | +1 | 11 |
| 4 | z | 4323 | 1210 | 0.0006867 | 0.5677 | +1 | 11 |
| - | - | 2.102E+04 | 1211 | - | - | 0 | - |
| - | - | 1.441E+04 | 1212 | - | - | 0 | - |
| - | - | 5128 | 1213 | - | - | 0 | - |
| - | - | 1217 | 1214 | - | - | 0 | - |
| 4 | y | 1605 | 1226 | 0.005464 | 4.458 | +1 | 11 |
| - | - | 903.3 | 1227 | - | - | 0 | - |
| - | - | 1270 | 1306 | - | - | 0 | - |
| 11 | c | 1.758E+04 | 1307 | 0.002119 | 1.622 | +1 | 11 |
| - | - | 1.259E+04 | 1308 | - | - | 0 | - |
| - | - | 5193 | 1309 | - | - | 0 | - |
| - | - | 1085 | 1310 | - | - | 0 | - |
| 3 | z | 2657 | 1373 | 0.002583 | 1.882 | +1 | 12 |
| - | - | 9237 | 1374 | - | - | 0 | - |
| - | - | 9321 | 1375 | - | - | 0 | - |
| - | - | 4108 | 1376 | - | - | 0 | - |
| - | - | 1736 | 1377 | - | - | 0 | - |
| 3 | y | 801.1 | 1389 | 0.003351 | 2.413 | +1 | 12 |
| - | - | 1689 | 1419 | - | - | 0 | - |
| - | - | 1375 | 1420 | - | - | 0 | - |
| 12 | c | 6075 | 1435 | 0.004919 | 3.429 | +1 | 12 |
| - | - | 8103 | 1436 | - | - | 0 | - |
| - | - | 7210 | 1437 | - | - | 0 | - |
| - | - | 1781 | 1438 | - | - | 0 | - |
| - | - | 997.5 | 1505 | - | - | 0 | - |
| - | - | 858.8 | 1532 | - | - | 0 | - |
| - | - | 1067 | 1533 | - | - | 0 | - |
| 13 | c | 1345 | 1548 | 0.005133 | 3.316 | +1 | 13 |
| - | - | 5293 | 1549 | - | - | 0 | - |
| - | - | 4899 | 1550 | - | - | 0 | - |
| - | - | 2406 | 1551 | - | - | 0 | - |
| - | - | 876.3 | 1552 | - | - | 0 | - |
| 2 | z | 845.7 | 1559 | 0.005384 | 3.454 | +1 | 13 |
| - | - | 3410 | 1560 | - | - | 0 | - |
| - | - | 3958 | 1561 | - | - | 0 | - |
| - | - | 1802 | 1562 | - | - | 0 | - |
| - | - | 1227 | 1581 | - | - | 0 | - |
| - | - | 1480 | 1582 | - | - | 0 | - |
| - | - | 911.3 | 1591 | - | - | 0 | - |
| - | - | 837.2 | 1602 | - | - | 0 | - |
| - | - | 1445 | 1618 | - | - | 0 | - |
| - | - | 3678 | 1619 | - | - | 0 | - |
| - | - | 4349 | 1620 | - | - | 0 | - |
| - | - | 1959 | 1621 | - | - | 0 | - |
| - | - | 1708 | 1629 | - | - | 0 | - |
| - | - | 2588 | 1630 | - | - | 0 | - |
| - | - | 1156 | 1631 | - | - | 0 | - |
| - | - | 1793 | 1636 | - | - | 0 | - |
| - | - | 1652 | 1637 | - | - | 0 | - |
| - | - | 783.4 | 1638 | - | - | 0 | - |
| - | - | 4887 | 1646 | - | - | 0 | - |
| - | - | 1.843E+04 | 1647 | - | - | 0 | - |
| - | - | 1.86E+04 | 1648 | - | - | 0 | - |
| - | - | 8040 | 1649 | - | - | 0 | - |
| - | - | 3663 | 1650 | - | - | 0 | - |
| - | - | 948.4 | 1651 | - | - | 0 | - |
| - | - | 4311 | 1662 | - | - | 0 | - |
| - | - | 2.453E+04 | 1663 | - | - | 0 | - |
| - | - | 9.213E+04 | 1664 | - | - | 0 | - |
| - | - | 8.881E+04 | 1665 | - | - | 0 | - |
| - | - | 1109 | 1665 | - | - | 0 | - |
| - | - | 4.865E+04 | 1666 | - | - | 0 | - |
| - | - | 1.658E+04 | 1667 | - | - | 0 | - |
| - | - | 3634 | 1668 | - | - | 0 | - |
| - | - | 622.6 | 1950 | - | - | 0 | - |
| - | - | 698.1 | 2730 | - | - | 0 | - |

m/z Charge Intensity FragmentType MassShift Position
120.06549072265625 0 363.87332
129.1020965576172 0 1762.232
132.0807647705078 0 522.45056
132.10208129882812 0 809.329 y 13
136.07559204101562 0 609.19574
139.07310485839844 0 447.66293
153.0783233642578 0 2198.8035
173.45123291015625 0 4482.144
174.0889434814453 0 478.2546
186.078369140625 0 500.45
187.0861358642578 0 774.6605
189.3968048095703 0 487.26755
196.61134338378906 0 480.9423
201.1235809326172 0 531.5845
211.14358520507812 0 1146.5282
215.13865661621094 0 2692.4426
226.15451049804688 0 491.0938
227.1390838623047 0 589.25494
229.1425018310547 0 729.742
229.15438842773438 0 14650.399
230.15774536132812 0 1526.6915
241.09178161621094 0 840.2639
245.13653564453125 0 1440.2024
245.18569946289062 0 2385.997 y 12
246.12339782714844 0 5670.117
246.1377410888672 0 836.64014
246.18792724609375 0 518.8598
247.1272430419922 0 983.18774
247.3211212158203 0 570.47626
258.1449279785156 0 642.36566
265.1280212402344 0 469.78247
274.11810302734375 0 7452.4097
275.1228942871094 0 895.2923
281.17352294921875 0 1154.2513
282.1807556152344 0 1659.1597
292.1290283203125 0 1510.5563
299.06158447265625 0 934.0572
299.207763671875 0 556.3352
300.06268310546875 0 573.47906
300.1914978027344 0 1621.3755
301.1985778808594 0 1154.9877
309.15545654296875 0 1550.3037
314.2072448730469 0 2736.0847
315.21014404296875 0 646.51825
322.3221130371094 0 624.33624
331.2091369628906 0 560.0445
340.0992431640625 0 4648.984
341.1037902832031 0 1022.8511
343.2439270019531 0 594.2177
355.0677490234375 0 797.36145
357.2619934082031 0 2076.654 z 11
358.26605224609375 0 1508.902
371.09991455078125 0 915.74286
373.28070068359375 0 1244.7853 y 11
382.2804870605469 0 760.7563
394.2567443847656 0 917.60144
397.24407958984375 0 2172.2095
398.2478332519531 0 873.9964
398.5660400390625 0 511.18787
399.25982666015625 0 752.7798
412.2733459472656 0 4300.058 y 6
412.7744445800781 0 2204.651
418.2406005859375 0 567.0105
427.29119873046875 0 1626.1702 w 10
437.1816711425781 0 1781.5381
453.20172119140625 0 1176.1031
454.20782470703125 0 3310.24 c 2
455.7709045410156 0 2073.5957
463.5985412597656 0 1417.7297 y 2
463.929931640625 0 977.1818
465.2079162597656 0 895.44037
467.7506103515625 0 1760.1935
468.2522888183594 0 923.44275
468.318115234375 0 3664.0088
469.28924560546875 0 659.45996
469.3222961425781 0 1181.9421
470.29437255859375 0 810.9928
470.3319396972656 0 1088.513 y 10
473.2417907714844 0 1876.5585 c Ammonia loss 11
474.2459716796875 0 916.2895
477.20013427734375 0 1902.7511
480.2259826660156 0 1485.6371
481.3087158203125 0 863.07355
482.29718017578125 0 1250.0802
495.3658142089844 0 818.4581
501.6058654785156 0 990.94147
501.937255859375 0 665.4354
504.9353942871094 0 657.9839
505.2698974609375 0 1052.3677
510.3283386230469 0 20829.705
510.9364318847656 0 7631.8057 c Ammonia loss 12
511.2706298828125 0 5794.758
511.33154296875 0 5863.9756
511.6044921875 0 2178.1929
511.93853759765625 0 775.85315
513.6951293945312 0 645.5405
514.2971801757812 0 2038.3545
517.2764282226562 0 776.8948
523.2399291992188 0 1263.8296
525.351806640625 0 13134.692 z 9
525.625244140625 0 1695.0101 y 1
525.9586791992188 0 1242.3995
526.354736328125 0 4523.4487
528.2423095703125 0 648.34753
539.2608032226562 0 952.093
539.3040771484375 0 659.7011
541.3721313476562 0 1442.7115 y 9
541.828857421875 0 653.4326
548.6319580078125 0 2432.8728
548.9625244140625 0 731.5532
549.297119140625 0 1359.3331
549.337646484375 0 957.4726 y 4
549.63037109375 0 1270.9879
550.33447265625 0 876.8975
553.3115844726562 0 2197.5164
553.7874145507812 0 705.52625
554.2304077148438 0 5610.514
554.2756958007812 0 734.94464
554.6347045898438 0 56415.91
554.9688720703125 0 53889.184
555.2297973632812 0 1060.2786
555.3031005859375 0 33629.4
555.6373291015625 0 9231.82
555.970703125 0 2535.4253
563.247802734375 0 3991.8752
564.2457885742188 0 773.92896 c Water loss 3
565.2404174804688 0 2609.692 c Ammonia loss 3
566.2440185546875 0 912.9472
566.4030151367188 0 1038.5848
569.8146362304688 0 594.2155
572.8004760742188 0 747.3925
576.3370361328125 0 3152.2668 w 3
576.8386840820312 0 2122.5752
577.3283081054688 0 653.95105
581.2581176757812 0 2054.8682
582.266357421875 0 11672.27 c 3
582.3737182617188 0 1912.1365
583.268310546875 0 4520.4106
584.2716064453125 0 1211.1141
595.37939453125 0 3098.1729 w 8
596.3837280273438 0 1076.0927
597.3859252929688 0 785.8905
604.8496704101562 0 1178.4614 y Ammonia loss 3
605.35205078125 0 7713.101 z 3
605.8536987304688 0 11517.347
606.3555297851562 0 5204.267
606.8572387695312 0 2111.3433
609.4559936523438 0 949.02234
610.3975219726562 0 1913.9965
611.3980102539062 0 1105.3522
612.8558349609375 0 883.7881
613.3621215820312 0 4003.6973 y 3
613.8622436523438 0 2718.8455
614.36279296875 0 1195.4514
620.3412475585938 0 924.9838
624.41748046875 0 962.1223
628.3750610351562 0 956.699
629.3455200195312 0 1771.8315
629.8445434570312 0 1102.7794
637.430419921875 0 764.70154
645.820556640625 0 921.1712
651.3624267578125 0 789.8407
651.8621826171875 0 931.59064
653.4466552734375 0 9994.468 z 8
653.824951171875 0 1656.249 c 10
654.4501342773438 0 5513.8965
655.4545288085938 0 1198.489
657.8656005859375 0 825.3696
658.8535766601562 0 860.106
659.2965698242188 0 996.8597
660.295166015625 0 1031.6653
660.3534545898438 0 667.38995
675.310546875 0 1218.2137
682.3873901367188 0 3289.0623
683.3897094726562 0 1048.0762
686.3789672851562 0 807.3461 y Ammonia loss 2
686.8831787109375 0 10117.445 z 2
687.385009765625 0 13983.05
687.886474609375 0 6628.4004
688.3900756835938 0 1071.5951
693.4415283203125 0 1239.9006 z Ammonia loss 7
694.8927001953125 0 12718.66 y 2
695.3932495117188 0 8037.382
695.8938598632812 0 4022.891
702.2973022460938 0 1245.5541 c Ammonia loss 4
709.35888671875 0 1435.306 c Ammonia loss 11
709.8618774414062 0 2309.5916
710.3637084960938 0 915.1768
710.465576171875 0 1630.7473 z 7
711.4700927734375 0 1243.1718
717.872314453125 0 34067.375 c 11
718.37353515625 0 27965.732
718.8748779296875 0 14101.073
719.3253173828125 0 29099.236 c 4
720.326904296875 0 14066.612
721.3302001953125 0 3352.8196
726.4850463867188 0 1029.8177 y 7
740.0234375 0 655.09216
752.91064453125 0 1399.7161
758.4188842773438 0 996.2716
758.9119262695312 0 1474.5627
759.4085693359375 0 927.79
766.4098510742188 0 1793.7478
766.9158325195312 0 1063.056
767.4080200195312 0 1567.4512
773.924560546875 0 985.8333
774.4146728515625 0 22898.043 c 12
774.91552734375 0 18959.396
775.4170532226562 0 11157.75
775.9174194335938 0 3212.2864
776.4186401367188 0 1422.7009
779.9223022460938 0 4734.697 z 1
780.4265747070312 0 3441.0425
780.9232177734375 0 2578.791
781.9112548828125 0 795.0204
787.9185180664062 0 2003.0654 y 1
788.4190673828125 0 1037.4958
788.9235229492188 0 1202.5133
790.9266967773438 0 2928.851
791.4260864257812 0 3591.9937
791.9273071289062 0 2530.3335
792.4403686523438 0 827.68915
795.4180908203125 0 1042.5347
795.90966796875 0 1485.4514
796.412353515625 0 1093.7395
796.9210815429688 0 1805.2917
800.4368286132812 0 1710.7722
800.9359130859375 0 2110.9114
801.4430541992188 0 2545.0107
801.94287109375 0 751.215
802.4134521484375 0 756.9184
802.9302368164062 0 1396.2778
804.4257202148438 0 1036.0736
808.9423828125 0 832.8243
809.4423828125 0 3304.087
809.9454956054688 0 5885.2896
810.4349365234375 0 2577.5518
810.9301147460938 0 1945.4702
811.3623657226562 0 4467.8057
811.4375610351562 0 807.5201
812.3684692382812 0 3132.3125
813.9107055664062 0 858.4595
814.4320068359375 0 969.6061
814.9439697265625 0 707.8284
815.4364624023438 0 783.51013
822.9458618164062 0 2499.1262
823.4400024414062 0 17157.762
823.5346069335938 0 3639.052 y 6
823.9407348632812 0 16536.777
824.4423828125 0 6582.8066
824.5391235351562 0 2085.736
824.94189453125 0 4007.465
825.4369506835938 0 1249.9875
830.4431762695312 0 789.16766
830.9432373046875 0 2311.2925
831.4488525390625 0 39386.375
831.9521484375 0 185105.62
832.4535522460938 0 164065.66
832.9542846679688 0 88403.305
833.142333984375 0 795.2832
833.4555053710938 0 28325.947
833.9560546875 0 7505.102
838.4521484375 0 1501.0068
839.356201171875 0 2402.626 c Ammonia loss 5
839.44970703125 0 856.65424
840.3558959960938 0 1238.8351
850.5271606445312 0 2132.3384
851.5308837890625 0 989.60834
852.5375366210938 0 2162.1736
855.4911499023438 0 2100.9805
855.9109497070312 0 738.9692
856.488525390625 0 1006.1152
878.4111938476562 0 727.68567
883.4086303710938 0 933.12976
894.51708984375 0 1549.6552
903.3319091796875 0 762.15137
910.5359497070312 0 7266.2725
911.5394897460938 0 5956.305
912.5433349609375 0 2726.1553
934.4880981445312 0 873.98065
935.5009155273438 0 864.7192
944.5794067382812 0 30040.398 z 5
945.5820922851562 0 19594.201
946.58544921875 0 5637.643
947.5807495117188 0 999.45087
953.4359130859375 0 3539.7764 c 6
954.4401245117188 0 1792.1396
960.5974731445312 0 3164.5273 y 5
961.6055297851562 0 997.1809
964.4495849609375 0 769.91986
968.5711669921875 0 701.0212
982.5469970703125 0 1563.1887
983.5552978515625 0 1391.2919
1009.4530639648438 0 1684.4597
1010.4583740234375 0 27218.014 c 7
1011.460693359375 0 16712.307
1012.4627685546875 0 6403.327
1013.4637451171875 0 1156.987
1081.0235595703125 0 812.9552
1081.6378173828125 0 13695.361 z 4
1082.64501953125 0 34555.266
1083.6474609375 0 20043.906
1084.6519775390625 0 6492.947
1085.654052734375 0 1993.5073
1092.5103759765625 0 1094.4537
1093.502197265625 0 1023.96265
1094.5362548828125 0 1827.185
1095.6395263671875 0 1044.3604
1096.639892578125 0 1577.317
1097.6549072265625 0 3749.7195 y 4
1098.6607666015625 0 1898.6729
1100.51318359375 0 909.37335
1102.028564453125 0 684.45435
1102.52294921875 0 891.15576
1108.5311279296875 0 1143.9673
1109.0433349609375 0 840.4865
1109.5189208984375 0 1081.1998
1110.5489501953125 0 1644.13
1111.57421875 0 1218.2007
1137.5472412109375 0 661.2727
1138.553466796875 0 33996.805 c 8
1139.5548095703125 0 26758.955
1140.5582275390625 0 8012.832
1141.5552978515625 0 2226.8276
1165.573486328125 0 738.2821
1183.678955078125 0 728.58075
1208.6796875 0 1056.0076 y Ammonia loss 3
1209.6983642578125 0 4322.9526 z 3
1210.7030029296875 0 21017.56
1211.706298828125 0 14412.792
1212.709716796875 0 5128.142
1213.7154541015625 0 1217.263
1225.7109375 0 1604.8579 y 3
1226.7091064453125 0 903.3484
1305.6387939453125 0 1269.8005
1306.641845703125 0 17582.14 c 10
1307.64453125 0 12586.857
1308.6446533203125 0 5193.0776
1309.647216796875 0 1085.4495
1372.7584228515625 0 2656.7366 z 2
1373.7642822265625 0 9236.557
1374.7696533203125 0 9320.732
1375.765625 0 4107.54
1376.771240234375 0 1735.8967
1388.7830810546875 0 801.1487 y 2
1418.71728515625 0 1688.7263
1419.7119140625 0 1374.7593
1434.7340087890625 0 6075.351 c 11
1435.740966796875 0 8102.6875
1436.74462890625 0 7209.7905
1437.74755859375 0 1781.1185
1504.810791015625 0 997.5412
1531.79541015625 0 858.8159
1532.80615234375 0 1067.458
1547.828125 0 1345.417 c 12
1548.8251953125 0 5293.0303
1549.82958984375 0 4899.1616
1550.8271484375 0 2405.9185
1551.8255615234375 0 876.29535
1558.845703125 0 845.7472 z 1
1559.8443603515625 0 3409.8872
1560.84716796875 0 3958.4316
1561.8485107421875 0 1802.4395
1580.8497314453125 0 1226.8027
1581.84521484375 0 1480.0914
1590.79931640625 0 911.3208
1601.885986328125 0 837.2047
1617.8760986328125 0 1444.5002
1618.880859375 0 3677.772
1619.87841796875 0 4349.316
1620.8856201171875 0 1958.7174
1628.8636474609375 0 1708.2147
1629.8603515625 0 2588.1675
1630.8623046875 0 1155.831
1635.9129638671875 0 1793.3148
1636.91357421875 0 1652.1682
1637.919921875 0 783.4158
1645.875732421875 0 4886.5776
1646.878173828125 0 18433.408
1647.8798828125 0 18596.125
1648.88232421875 0 8040.106
1649.8790283203125 0 3663.4185
1650.8779296875 0 948.4182
1661.88232421875 0 4311.471
1662.89599609375 0 24531.783
1663.904052734375 0 92133.69
1664.9063720703125 0 88806.85
1665.17724609375 0 1108.8507
1665.90966796875 0 48649.504
1666.9105224609375 0 16584.475
1667.9072265625 0 3633.7563
1950.453125 0 622.57025
2729.891357421875 0 698.065

Spectrum Details

|  |  |
| --- | --- |
| Matched peaks? Matched peaksThe total absolute number of peaks matched. Additionally in brackets the total fraction of peaks matched and the total number of peaks is shown. | 56 (14.25% of 393) |
| FDR? FDRThe false discovery rate estimated for this peptide. It is calculated by matching all theoretical fragments with a non-integer shift with the raw peaks for this spectrum. This is done with 40 different shifts. The resulting percentage is the average number of annotated peaks over the number of annotated peaks with the correct spectrum. | 2.68% |
| Satellite FDR? Satellite FDRSee the FDR for details on its calculation. This satellite ion specific FDR only contains the satellite ions (d/w) for I/L/J positions. | - |
| PSM Score? PSM ScoreThe PSM Score as given by Hecklib to this annotated spectrum. It is shown with three significant figures. | 469 |

## Spectrum 5553? Spectrum 5553 The raw spectrum of this peptide as annotated by Hecklib. The fragments are coloured according to ion type (see legend). Any peaks with a star '\*' as text can be hovered over to see the full details, first the ion type second the mass shift type. By hovering over the amino acids in the peptide or ions in the legend the corresponding peaks are highlighted. By toggling the 'Unassigned' label you can turn the background (unassigned) peaks on or off in the plot. By updating the slider in the Ion legend you can update the spectrum to only show the top X% of the peaks with labels. The top X% means any peak that is within X% of the highest intensity. By dragging in the spectrum you can zoom in to a specific part of the spectrum and use 'Zoom Out' to get back to the original zoom level. The annotation of the spectrum is based on the given sequence in the peptides file and is done with different software so inconsistencies are likely. The peaks are annotated based on the given sequence, with 20 ppm tolerance.

Copy Data

### Spectrum 5553 (TSV)

#### Preview

```
Loading example...
```

*Click on the button to copy the data to your clipboard.*

Mz MinMz MaxIntensity Max

WidthHeightPeptide font sizePeptide stroke widthSpectrum font sizeSpectrum stroke widthCompact peptide

Ion legend

wxyz

abcd

OtherUnassignedIonChargePositionShow for top:%

SWYQHHPGKAPKJJ

05.31e+41.06e+51.59e+52.12e+5

Zoom Out

y+33y+11b+44b+45y+23a+23y+36y+24a+12b+35y+24y+37y+12a+12b+48b+12y+25b+12y+38y+39b+411y+26y+27y+310y+13b+39y+413b+310b+310y+413b+310y+311y+311b+26\*\*y+28\*b+26b+13y+312y+312y+14b+312y+29b+28b+313b+313y+313y+313y+210y+15y+210b+29b+29b+14b+210b+210b+210y+211y+211y+16b+15y+212b+15y+17y+18b+16y+19y+19

0757151422713027

Fragment Matches Table

Show background peaks

| Position | Ion type | Intensity | mz Theoretical | mz Error (Th) | mz Error (ppm) | Charge | Series Number |
| --- | --- | --- | --- | --- | --- | --- | --- |
| - | - | 478.3 | 120.1 | - | - | 0 | - |
| - | - | 728.8 | 120.1 | - | - | 0 | - |
| - | - | 1011 | 122.1 | - | - | 0 | - |
| - | - | 3355 | 123.1 | - | - | 0 | - |
| - | - | 748.1 | 123.1 | - | - | 0 | - |
| - | - | 435.6 | 125.1 | - | - | 0 | - |
| 12 | y | 424.1 | 125.1 | 0.002449 | 19.57 | +3 | 3 |
| - | - | 724.5 | 127.1 | - | - | 0 | - |
| - | - | 357.6 | 127.1 | - | - | 0 | - |
| - | - | 3831 | 127.1 | - | - | 0 | - |
| - | - | 490.5 | 128 | - | - | 0 | - |
| - | - | 491.8 | 128.1 | - | - | 0 | - |
| - | - | 716.9 | 128.1 | - | - | 0 | - |
| - | - | 5337 | 129.1 | - | - | 0 | - |
| - | - | 1.374E+05 | 129.1 | - | - | 0 | - |
| - | - | 3.143E+04 | 130.1 | - | - | 0 | - |
| - | - | 1205 | 130.1 | - | - | 0 | - |
| - | - | 8244 | 130.1 | - | - | 0 | - |
| - | - | 3917 | 131 | - | - | 0 | - |
| - | - | 3473 | 131.1 | - | - | 0 | - |
| - | - | 1124 | 131.1 | - | - | 0 | - |
| - | - | 1.193E+05 | 132.1 | - | - | 0 | - |
| 14 | y | 3.437E+04 | 132.1 | 0.0004966 | 3.759 | +1 | 1 |
| - | - | 435 | 133.1 | - | - | 0 | - |
| - | - | 1.09E+04 | 133.1 | - | - | 0 | - |
| - | - | 2277 | 133.1 | - | - | 0 | - |
| - | - | 720.6 | 134.1 | - | - | 0 | - |
| - | - | 804.8 | 134.1 | - | - | 0 | - |
| - | - | 1.609E+05 | 136.1 | - | - | 0 | - |
| - | - | 1203 | 137.1 | - | - | 0 | - |
| - | - | 1.434E+04 | 137.1 | - | - | 0 | - |
| - | - | 542.8 | 137.1 | - | - | 0 | - |
| - | - | 2503 | 138.1 | - | - | 0 | - |
| - | - | 466.1 | 138.1 | - | - | 0 | - |
| - | - | 2962 | 139.1 | - | - | 0 | - |
| - | - | 607.9 | 140.1 | - | - | 0 | - |
| - | - | 7518 | 141.1 | - | - | 0 | - |
| 4 | b | 1811 | 142.1 | 2.449E-05 | 0.1724 | +4 | 4 |
| - | - | 542.8 | 142.1 | - | - | 0 | - |
| - | - | 650.9 | 143.1 | - | - | 0 | - |
| - | - | 408.5 | 144.1 | - | - | 0 | - |
| - | - | 4700 | 144.1 | - | - | 0 | - |
| - | - | 1096 | 145.1 | - | - | 0 | - |
| - | - | 2038 | 146.1 | - | - | 0 | - |
| - | - | 558 | 146.1 | - | - | 0 | - |
| - | - | 6882 | 147 | - | - | 0 | - |
| - | - | 584.1 | 148 | - | - | 0 | - |
| - | - | 811.7 | 148.1 | - | - | 0 | - |
| - | - | 611.6 | 149 | - | - | 0 | - |
| - | - | 545.3 | 149 | - | - | 0 | - |
| - | - | 472.1 | 150.1 | - | - | 0 | - |
| - | - | 1986 | 151.1 | - | - | 0 | - |
| - | - | 1273 | 152.1 | - | - | 0 | - |
| - | - | 2979 | 152.1 | - | - | 0 | - |
| - | - | 1351 | 153.1 | - | - | 0 | - |
| - | - | 925.2 | 154.1 | - | - | 0 | - |
| - | - | 6837 | 155.1 | - | - | 0 | - |
| - | - | 2581 | 155.1 | - | - | 0 | - |
| - | - | 4810 | 155.1 | - | - | 0 | - |
| - | - | 2492 | 156.1 | - | - | 0 | - |
| - | - | 2944 | 156.1 | - | - | 0 | - |
| - | - | 680.8 | 156.1 | - | - | 0 | - |
| - | - | 1145 | 157.1 | - | - | 0 | - |
| - | - | 905.4 | 157.1 | - | - | 0 | - |
| - | - | 757.2 | 157.1 | - | - | 0 | - |
| - | - | 1880 | 158.1 | - | - | 0 | - |
| - | - | 1.504E+04 | 158.1 | - | - | 0 | - |
| - | - | 8.365E+04 | 159.1 | - | - | 0 | - |
| - | - | 1814 | 159.1 | - | - | 0 | - |
| - | - | 3841 | 160.1 | - | - | 0 | - |
| - | - | 9087 | 160.1 | - | - | 0 | - |
| - | - | 777 | 161.1 | - | - | 0 | - |
| - | - | 668.1 | 161.1 | - | - | 0 | - |
| - | - | 635.7 | 163.6 | - | - | 0 | - |
| - | - | 799.8 | 164 | - | - | 0 | - |
| - | - | 2112 | 164.1 | - | - | 0 | - |
| - | - | 1024 | 165.1 | - | - | 0 | - |
| - | - | 5462 | 165.1 | - | - | 0 | - |
| - | - | 2527 | 166.1 | - | - | 0 | - |
| - | - | 1196 | 168.1 | - | - | 0 | - |
| - | - | 8639 | 169.1 | - | - | 0 | - |
| - | - | 9766 | 169.1 | - | - | 0 | - |
| - | - | 839.1 | 169.2 | - | - | 0 | - |
| - | - | 3.958E+04 | 170.1 | - | - | 0 | - |
| - | - | 897.6 | 170.1 | - | - | 0 | - |
| - | - | 4350 | 171.1 | - | - | 0 | - |
| - | - | 1465 | 171.1 | - | - | 0 | - |
| 5 | b | 639.5 | 172.1 | 0.002432 | 14.13 | +4 | 5 |
| - | - | 533.1 | 172.1 | - | - | 0 | - |
| - | - | 8443 | 172.1 | - | - | 0 | - |
| - | - | 947.4 | 173.1 | - | - | 0 | - |
| - | - | 889 | 173.5 | - | - | 0 | - |
| - | - | 577.4 | 174.1 | - | - | 0 | - |
| - | - | 507.7 | 175.1 | - | - | 0 | - |
| - | - | 2129 | 175.1 | - | - | 0 | - |
| - | - | 3758 | 176.1 | - | - | 0 | - |
| - | - | 692.8 | 177.1 | - | - | 0 | - |
| - | - | 760.1 | 178.1 | - | - | 0 | - |
| - | - | 648.3 | 179.1 | - | - | 0 | - |
| - | - | 678.5 | 179.1 | - | - | 0 | - |
| - | - | 634.7 | 179.2 | - | - | 0 | - |
| - | - | 3188 | 181.1 | - | - | 0 | - |
| - | - | 4.097E+04 | 181.1 | - | - | 0 | - |
| - | - | 2413 | 181.1 | - | - | 0 | - |
| - | - | 595.8 | 182.1 | - | - | 0 | - |
| - | - | 537.5 | 182.1 | - | - | 0 | - |
| - | - | 3476 | 182.1 | - | - | 0 | - |
| - | - | 4789 | 182.1 | - | - | 0 | - |
| - | - | 1.688E+04 | 183.1 | - | - | 0 | - |
| - | - | 806.4 | 183.1 | - | - | 0 | - |
| - | - | 1652 | 183.1 | - | - | 0 | - |
| - | - | 601.9 | 183.1 | - | - | 0 | - |
| - | - | 1528 | 183.1 | - | - | 0 | - |
| - | - | 2050 | 184.1 | - | - | 0 | - |
| - | - | 2623 | 184.1 | - | - | 0 | - |
| - | - | 714.8 | 185.1 | - | - | 0 | - |
| - | - | 5237 | 185.1 | - | - | 0 | - |
| - | - | 665.5 | 185.1 | - | - | 0 | - |
| - | - | 1669 | 185.2 | - | - | 0 | - |
| - | - | 1.635E+04 | 186.1 | - | - | 0 | - |
| - | - | 8.867E+04 | 187.1 | - | - | 0 | - |
| - | - | 4179 | 187.1 | - | - | 0 | - |
| - | - | 1045 | 187.1 | - | - | 0 | - |
| 12 | y | 1313 | 187.1 | 0.0004575 | 2.445 | +2 | 3 |
| - | - | 709.8 | 188.1 | - | - | 0 | - |
| - | - | 9746 | 188.1 | - | - | 0 | - |
| - | - | 748.3 | 189.1 | - | - | 0 | - |
| - | - | 461.4 | 191.1 | - | - | 0 | - |
| - | - | 832.5 | 193.1 | - | - | 0 | - |
| - | - | 463.1 | 195.1 | - | - | 0 | - |
| - | - | 856.7 | 195.1 | - | - | 0 | - |
| - | - | 1205 | 195.1 | - | - | 0 | - |
| - | - | 1796 | 196.1 | - | - | 0 | - |
| - | - | 6359 | 197.2 | - | - | 0 | - |
| - | - | 1137 | 198.2 | - | - | 0 | - |
| - | - | 3374 | 199.1 | - | - | 0 | - |
| - | - | 1524 | 199.1 | - | - | 0 | - |
| - | - | 1984 | 199.2 | - | - | 0 | - |
| - | - | 4037 | 199.2 | - | - | 0 | - |
| - | - | 604.9 | 200.1 | - | - | 0 | - |
| - | - | 2307 | 200.1 | - | - | 0 | - |
| - | - | 703 | 201.1 | - | - | 0 | - |
| - | - | 8590 | 201.1 | - | - | 0 | - |
| - | - | 813.3 | 201.1 | - | - | 0 | - |
| - | - | 4651 | 202.1 | - | - | 0 | - |
| - | - | 1.433E+04 | 202.1 | - | - | 0 | - |
| - | - | 1259 | 203.1 | - | - | 0 | - |
| - | - | 1577 | 203.1 | - | - | 0 | - |
| - | - | 605.4 | 203.1 | - | - | 0 | - |
| - | - | 2148 | 204.1 | - | - | 0 | - |
| - | - | 2406 | 204.1 | - | - | 0 | - |
| 3 | a | 762.4 | 205.1 | 0.0005785 | 2.82 | +2 | 3 |
| - | - | 713.1 | 205.1 | - | - | 0 | - |
| - | - | 554.1 | 207.1 | - | - | 0 | - |
| - | - | 3403 | 207.1 | - | - | 0 | - |
| - | - | 897.3 | 208.1 | - | - | 0 | - |
| - | - | 567.8 | 209.1 | - | - | 0 | - |
| - | - | 1700 | 209.1 | - | - | 0 | - |
| - | - | 663.6 | 210.1 | - | - | 0 | - |
| - | - | 2679 | 211.1 | - | - | 0 | - |
| - | - | 803.9 | 212.1 | - | - | 0 | - |
| - | - | 2075 | 212.7 | - | - | 0 | - |
| - | - | 634.4 | 213.1 | - | - | 0 | - |
| - | - | 900.6 | 213.2 | - | - | 0 | - |
| - | - | 3961 | 214.1 | - | - | 0 | - |
| - | - | 1074 | 214.2 | - | - | 0 | - |
| - | - | 1136 | 215.1 | - | - | 0 | - |
| - | - | 1091 | 216.1 | - | - | 0 | - |
| - | - | 1234 | 216.1 | - | - | 0 | - |
| - | - | 585.2 | 216.1 | - | - | 0 | - |
| - | - | 1392 | 217.1 | - | - | 0 | - |
| - | - | 679.3 | 217.2 | - | - | 0 | - |
| 9 | y | 755 | 218.2 | 0.0009903 | 4.54 | +3 | 6 |
| - | - | 1.02E+04 | 220.1 | - | - | 0 | - |
| - | - | 1.469E+04 | 221.1 | - | - | 0 | - |
| - | - | 741.3 | 221.1 | - | - | 0 | - |
| - | - | 1678 | 222.1 | - | - | 0 | - |
| - | - | 1376 | 222.1 | - | - | 0 | - |
| - | - | 763.5 | 223.1 | - | - | 0 | - |
| - | - | 526.3 | 223.2 | - | - | 0 | - |
| - | - | 2369 | 224.1 | - | - | 0 | - |
| - | - | 6212 | 224.2 | - | - | 0 | - |
| - | - | 658.5 | 225.1 | - | - | 0 | - |
| - | - | 2100 | 225.2 | - | - | 0 | - |
| - | - | 822.2 | 225.2 | - | - | 0 | - |
| - | - | 1.134E+05 | 226.2 | - | - | 0 | - |
| - | - | 1049 | 227.1 | - | - | 0 | - |
| - | - | 818.7 | 227.1 | - | - | 0 | - |
| 11 | y | 1.407E+04 | 227.2 | 0.001709 | 7.522 | +2 | 4 |
| - | - | 755.9 | 227.2 | - | - | 0 | - |
| 2 | a | 7557 | 228.1 | 0.0006768 | 2.967 | +1 | 2 |
| - | - | 1466 | 228.1 | - | - | 0 | - |
| - | - | 910.4 | 228.2 | - | - | 0 | - |
| 5 | b | 9180 | 229.1 | 0.00195 | 8.511 | +3 | 5 |
| - | - | 1784 | 229.1 | - | - | 0 | - |
| - | - | 1197 | 229.2 | - | - | 0 | - |
| - | - | 1912 | 229.2 | - | - | 0 | - |
| - | - | 2505 | 230.1 | - | - | 0 | - |
| - | - | 4456 | 231.1 | - | - | 0 | - |
| - | - | 1403 | 231.1 | - | - | 0 | - |
| - | - | 2466 | 231.2 | - | - | 0 | - |
| - | - | 674.4 | 232.1 | - | - | 0 | - |
| - | - | 2636 | 233.1 | - | - | 0 | - |
| - | - | 897.9 | 234.1 | - | - | 0 | - |
| - | - | 992.5 | 234.1 | - | - | 0 | - |
| - | - | 1146 | 234.1 | - | - | 0 | - |
| - | - | 1.785E+04 | 235.1 | - | - | 0 | - |
| 11 | y | 1.691E+04 | 235.7 | 0.0005953 | 2.526 | +2 | 4 |
| - | - | 2586 | 236.1 | - | - | 0 | - |
| - | - | 789.7 | 236.1 | - | - | 0 | - |
| - | - | 5124 | 236.2 | - | - | 0 | - |
| - | - | 805.5 | 237.1 | - | - | 0 | - |
| 8 | y | 817.8 | 237.2 | 0.002094 | 8.829 | +3 | 7 |
| - | - | 1550 | 238.1 | - | - | 0 | - |
| - | - | 6777 | 239.2 | - | - | 0 | - |
| - | - | 1799 | 240.1 | - | - | 0 | - |
| - | - | 1071 | 240.2 | - | - | 0 | - |
| - | - | 1201 | 241.2 | - | - | 0 | - |
| - | - | 3921 | 242.2 | - | - | 0 | - |
| - | - | 1414 | 243.1 | - | - | 0 | - |
| - | - | 1334 | 243.2 | - | - | 0 | - |
| - | - | 644.3 | 244.1 | - | - | 0 | - |
| - | - | 619 | 244.2 | - | - | 0 | - |
| 13 | y | 2.714E+04 | 245.2 | 0.0006459 | 2.634 | +1 | 2 |
| 2 | a | 2.103E+05 | 246.1 | 0.0006559 | 2.665 | +1 | 2 |
| - | - | 3479 | 246.2 | - | - | 0 | - |
| - | - | 762.1 | 246.6 | - | - | 0 | - |
| - | - | 4.976E+04 | 247.1 | - | - | 0 | - |
| - | - | 1.594E+04 | 248.1 | - | - | 0 | - |
| - | - | 3511 | 248.1 | - | - | 0 | - |
| - | - | 2.228E+04 | 249.1 | - | - | 0 | - |
| 8 | b | 1435 | 249.1 | 0.0008093 | 3.249 | +4 | 8 |
| - | - | 914.3 | 249.1 | - | - | 0 | - |
| - | - | 2613 | 250.1 | - | - | 0 | - |
| - | - | 2946 | 250.1 | - | - | 0 | - |
| - | - | 1436 | 251.1 | - | - | 0 | - |
| - | - | 3270 | 251.2 | - | - | 0 | - |
| - | - | 516.4 | 252.2 | - | - | 0 | - |
| - | - | 2248 | 252.2 | - | - | 0 | - |
| - | - | 679.8 | 253.1 | - | - | 0 | - |
| - | - | 839.7 | 253.2 | - | - | 0 | - |
| - | - | 1243 | 255.1 | - | - | 0 | - |
| - | - | 1016 | 255.1 | - | - | 0 | - |
| 2 | b | 5655 | 256.1 | 0.0006505 | 2.54 | +1 | 2 |
| - | - | 1028 | 257.1 | - | - | 0 | - |
| - | - | 2574 | 257.1 | - | - | 0 | - |
| - | - | 2798 | 257.2 | - | - | 0 | - |
| - | - | 3555 | 258.1 | - | - | 0 | - |
| - | - | 3.817E+04 | 258.1 | - | - | 0 | - |
| - | - | 844.1 | 259.1 | - | - | 0 | - |
| - | - | 6307 | 259.1 | - | - | 0 | - |
| - | - | 1391 | 259.1 | - | - | 0 | - |
| - | - | 944.3 | 259.9 | - | - | 0 | - |
| - | - | 5128 | 260.1 | - | - | 0 | - |
| - | - | 3439 | 260.2 | - | - | 0 | - |
| - | - | 1028 | 261.1 | - | - | 0 | - |
| - | - | 1872 | 263.1 | - | - | 0 | - |
| - | - | 2095 | 264.1 | - | - | 0 | - |
| - | - | 1627 | 265.1 | - | - | 0 | - |
| - | - | 1615 | 265.2 | - | - | 0 | - |
| - | - | 928.1 | 266.1 | - | - | 0 | - |
| - | - | 1.391E+04 | 266.1 | - | - | 0 | - |
| - | - | 731.1 | 266.2 | - | - | 0 | - |
| - | - | 1665 | 267.1 | - | - | 0 | - |
| - | - | 1184 | 267.1 | - | - | 0 | - |
| - | - | 2789 | 268.2 | - | - | 0 | - |
| - | - | 2091 | 269.6 | - | - | 0 | - |
| - | - | 741.1 | 270.1 | - | - | 0 | - |
| - | - | 9391 | 270.2 | - | - | 0 | - |
| 10 | y | 5525 | 271.2 | 0.000414 | 1.526 | +2 | 5 |
| - | - | 1343 | 271.7 | - | - | 0 | - |
| - | - | 817.7 | 272.1 | - | - | 0 | - |
| - | - | 687.1 | 273.1 | - | - | 0 | - |
| 2 | b | 8.232E+04 | 274.1 | 0.0005228 | 1.907 | +1 | 2 |
| - | - | 1.055E+04 | 275.1 | - | - | 0 | - |
| - | - | 6.405E+04 | 275.1 | - | - | 0 | - |
| 7 | y | 2822 | 275.2 | 0.0005432 | 1.974 | +3 | 8 |
| - | - | 1423 | 275.5 | - | - | 0 | - |
| - | - | 2893 | 276.1 | - | - | 0 | - |
| - | - | 8850 | 276.1 | - | - | 0 | - |
| - | - | 884.1 | 277.1 | - | - | 0 | - |
| - | - | 553.9 | 277.1 | - | - | 0 | - |
| - | - | 3106 | 277.1 | - | - | 0 | - |
| - | - | 773.3 | 278.1 | - | - | 0 | - |
| - | - | 831.8 | 279.1 | - | - | 0 | - |
| - | - | 1979 | 279.2 | - | - | 0 | - |
| - | - | 1785 | 283.2 | - | - | 0 | - |
| - | - | 2.576E+04 | 283.2 | - | - | 0 | - |
| - | - | 2695 | 283.6 | - | - | 0 | - |
| - | - | 1554 | 284.1 | - | - | 0 | - |
| - | - | 3082 | 284.2 | - | - | 0 | - |
| - | - | 1563 | 285.1 | - | - | 0 | - |
| - | - | 5393 | 285.2 | - | - | 0 | - |
| - | - | 780.2 | 286.2 | - | - | 0 | - |
| - | - | 877 | 288.1 | - | - | 0 | - |
| - | - | 1029 | 290.1 | - | - | 0 | - |
| - | - | 9781 | 290.2 | - | - | 0 | - |
| - | - | 2332 | 290.7 | - | - | 0 | - |
| - | - | 1439 | 291.2 | - | - | 0 | - |
| - | - | 2.04E+04 | 292.1 | - | - | 0 | - |
| - | - | 744.9 | 292.2 | - | - | 0 | - |
| - | - | 3206 | 293.1 | - | - | 0 | - |
| - | - | 5434 | 294.1 | - | - | 0 | - |
| - | - | 2340 | 294.2 | - | - | 0 | - |
| - | - | 722.6 | 295.1 | - | - | 0 | - |
| - | - | 769.2 | 297.2 | - | - | 0 | - |
| - | - | 1.421E+04 | 297.2 | - | - | 0 | - |
| - | - | 2476 | 298.2 | - | - | 0 | - |
| - | - | 1884 | 300.1 | - | - | 0 | - |
| - | - | 1127 | 300.7 | - | - | 0 | - |
| - | - | 4107 | 303.1 | - | - | 0 | - |
| - | - | 677.1 | 303.2 | - | - | 0 | - |
| - | - | 7776 | 305.1 | - | - | 0 | - |
| - | - | 2389 | 305.7 | - | - | 0 | - |
| - | - | 1100 | 306.1 | - | - | 0 | - |
| - | - | 777.3 | 306.2 | - | - | 0 | - |
| - | - | 782.7 | 307.2 | - | - | 0 | - |
| - | - | 1171 | 309.2 | - | - | 0 | - |
| - | - | 746.1 | 309.2 | - | - | 0 | - |
| - | - | 711.2 | 311.2 | - | - | 0 | - |
| - | - | 3467 | 311.2 | - | - | 0 | - |
| - | - | 702.8 | 312.2 | - | - | 0 | - |
| - | - | 785.7 | 313.2 | - | - | 0 | - |
| - | - | 3692 | 314.7 | - | - | 0 | - |
| - | - | 1431 | 315.2 | - | - | 0 | - |
| - | - | 827.5 | 318.2 | - | - | 0 | - |
| - | - | 1018 | 318.2 | - | - | 0 | - |
| - | - | 885.1 | 320.1 | - | - | 0 | - |
| 6 | y | 3224 | 320.9 | 0.0007322 | 2.282 | +3 | 9 |
| - | - | 1118 | 321.2 | - | - | 0 | - |
| - | - | 1.628E+04 | 321.2 | - | - | 0 | - |
| - | - | 1.065E+04 | 322.2 | - | - | 0 | - |
| - | - | 543.9 | 322.2 | - | - | 0 | - |
| - | - | 3750 | 322.2 | - | - | 0 | - |
| - | - | 765 | 323 | - | - | 0 | - |
| 11 | b | 2668 | 323.2 | 0.00115 | 3.56 | +4 | 11 |
| - | - | 3625 | 324.2 | - | - | 0 | - |
| - | - | 1498 | 324.7 | - | - | 0 | - |
| - | - | 744.6 | 325.2 | - | - | 0 | - |
| - | - | 5052 | 326.2 | - | - | 0 | - |
| - | - | 711.1 | 326.7 | - | - | 0 | - |
| - | - | 770.3 | 327.2 | - | - | 0 | - |
| - | - | 1405 | 327.2 | - | - | 0 | - |
| - | - | 1493 | 327.3 | - | - | 0 | - |
| - | - | 5636 | 332.7 | - | - | 0 | - |
| - | - | 3233 | 333.1 | - | - | 0 | - |
| - | - | 2881 | 333.2 | - | - | 0 | - |
| - | - | 1013 | 334.2 | - | - | 0 | - |
| 9 | y | 2187 | 335.2 | 0.0009632 | 2.873 | +2 | 6 |
| - | - | 2.59E+04 | 336.2 | - | - | 0 | - |
| - | - | 4001 | 337.2 | - | - | 0 | - |
| - | - | 7119 | 337.7 | - | - | 0 | - |
| - | - | 627.8 | 338.2 | - | - | 0 | - |
| - | - | 3131 | 338.2 | - | - | 0 | - |
| - | - | 943.9 | 338.2 | - | - | 0 | - |
| - | - | 662 | 338.7 | - | - | 0 | - |
| - | - | 2.713E+04 | 339.2 | - | - | 0 | - |
| - | - | 674.6 | 339.8 | - | - | 0 | - |
| - | - | 2426 | 340.2 | - | - | 0 | - |
| - | - | 5354 | 340.2 | - | - | 0 | - |
| - | - | 718.9 | 341.2 | - | - | 0 | - |
| - | - | 770.6 | 341.2 | - | - | 0 | - |
| - | - | 1173 | 342.2 | - | - | 0 | - |
| - | - | 1235 | 345.6 | - | - | 0 | - |
| - | - | 931 | 346.1 | - | - | 0 | - |
| - | - | 1328 | 346.7 | - | - | 0 | - |
| - | - | 2.351E+04 | 346.7 | - | - | 0 | - |
| - | - | 8306 | 347.2 | - | - | 0 | - |
| - | - | 2170 | 347.7 | - | - | 0 | - |
| - | - | 2197 | 349.7 | - | - | 0 | - |
| - | - | 4128 | 350.2 | - | - | 0 | - |
| - | - | 2327 | 351.2 | - | - | 0 | - |
| - | - | 1429 | 353.2 | - | - | 0 | - |
| - | - | 2170 | 354.2 | - | - | 0 | - |
| - | - | 2376 | 354.2 | - | - | 0 | - |
| - | - | 3.361E+04 | 354.2 | - | - | 0 | - |
| - | - | 1888 | 354.5 | - | - | 0 | - |
| - | - | 1473 | 354.7 | - | - | 0 | - |
| - | - | 6204 | 355.2 | - | - | 0 | - |
| - | - | 5658 | 355.7 | - | - | 0 | - |
| - | - | 794.4 | 356.2 | - | - | 0 | - |
| - | - | 1513 | 356.2 | - | - | 0 | - |
| - | - | 664.8 | 356.7 | - | - | 0 | - |
| - | - | 901 | 357.2 | - | - | 0 | - |
| - | - | 7435 | 357.3 | - | - | 0 | - |
| - | - | 8458 | 358.2 | - | - | 0 | - |
| - | - | 1548 | 358.3 | - | - | 0 | - |
| - | - | 2055 | 358.7 | - | - | 0 | - |
| - | - | 1641 | 359.2 | - | - | 0 | - |
| - | - | 816.3 | 359.2 | - | - | 0 | - |
| - | - | 718.6 | 359.5 | - | - | 0 | - |
| - | - | 1607 | 359.9 | - | - | 0 | - |
| - | - | 911.6 | 360.1 | - | - | 0 | - |
| - | - | 1675 | 360.7 | - | - | 0 | - |
| - | - | 1274 | 360.7 | - | - | 0 | - |
| - | - | 858.1 | 361.2 | - | - | 0 | - |
| - | - | 903.6 | 362.2 | - | - | 0 | - |
| - | - | 2056 | 362.7 | - | - | 0 | - |
| - | - | 728.9 | 362.7 | - | - | 0 | - |
| 8 | y | 1670 | 363.7 | 0.0009735 | 2.676 | +2 | 7 |
| - | - | 724.4 | 365.3 | - | - | 0 | - |
| - | - | 3601 | 366.2 | - | - | 0 | - |
| 5 | y | 3312 | 366.6 | 0.0007991 | 2.18 | +3 | 10 |
| - | - | 2390 | 366.9 | - | - | 0 | - |
| - | - | 1030 | 367.1 | - | - | 0 | - |
| - | - | 2986 | 367.2 | - | - | 0 | - |
| - | - | 4760 | 368.1 | - | - | 0 | - |
| - | - | 892.9 | 368.2 | - | - | 0 | - |
| - | - | 2213 | 369.2 | - | - | 0 | - |
| - | - | 598.9 | 369.7 | - | - | 0 | - |
| - | - | 1071 | 370.5 | - | - | 0 | - |
| - | - | 686.2 | 370.7 | - | - | 0 | - |
| - | - | 1548 | 371.2 | - | - | 0 | - |
| 12 | y | 1.516E+04 | 373.3 | 0.0007146 | 1.914 | +1 | 3 |
| - | - | 785.5 | 374.3 | - | - | 0 | - |
| - | - | 2738 | 374.3 | - | - | 0 | - |
| 9 | b | 1003 | 374.5 | 0.0002519 | 0.6725 | +3 | 9 |
| - | - | 831.1 | 375.2 | - | - | 0 | - |
| - | - | 1542 | 376.7 | - | - | 0 | - |
| - | - | 832.6 | 377 | - | - | 0 | - |
| - | - | 1107 | 377.2 | - | - | 0 | - |
| - | - | 1358 | 378.7 | - | - | 0 | - |
| - | - | 2024 | 379 | - | - | 0 | - |
| - | - | 1142 | 379.2 | - | - | 0 | - |
| - | - | 1373 | 380.2 | - | - | 0 | - |
| - | - | 701.1 | 381.2 | - | - | 0 | - |
| - | - | 6741 | 382.2 | - | - | 0 | - |
| - | - | 1759 | 382.3 | - | - | 0 | - |
| - | - | 3337 | 382.5 | - | - | 0 | - |
| - | - | 754.5 | 382.7 | - | - | 0 | - |
| - | - | 1166 | 382.9 | - | - | 0 | - |
| - | - | 1826 | 383.3 | - | - | 0 | - |
| - | - | 3974 | 384.2 | - | - | 0 | - |
| - | - | 1.276E+04 | 385.2 | - | - | 0 | - |
| - | - | 3323 | 385.2 | - | - | 0 | - |
| - | - | 1.364E+04 | 386.2 | - | - | 0 | - |
| - | - | 1160 | 386.2 | - | - | 0 | - |
| - | - | 721.7 | 386.5 | - | - | 0 | - |
| - | - | 3122 | 387.2 | - | - | 0 | - |
| - | - | 958.6 | 388.2 | - | - | 0 | - |
| - | - | 2613 | 388.9 | - | - | 0 | - |
| - | - | 1486 | 389.2 | - | - | 0 | - |
| - | - | 1759 | 389.3 | - | - | 0 | - |
| - | - | 916.1 | 389.5 | - | - | 0 | - |
| - | - | 975.1 | 389.8 | - | - | 0 | - |
| 2 | y | 7197 | 390.2 | 0.001049 | 2.689 | +4 | 13 |
| - | - | 3916 | 390.5 | - | - | 0 | - |
| - | - | 4582 | 390.7 | - | - | 0 | - |
| 10 | b | 1765 | 392.2 | 0.0004862 | 1.24 | +3 | 10 |
| - | - | 5466 | 392.3 | - | - | 0 | - |
| 10 | b | 2016 | 392.5 | 0.005062 | 12.9 | +3 | 10 |
| - | - | 899.5 | 392.7 | - | - | 0 | - |
| - | - | 691.9 | 393 | - | - | 0 | - |
| - | - | 1237 | 393.2 | - | - | 0 | - |
| - | - | 1932 | 393.3 | - | - | 0 | - |
| - | - | 9941 | 394.2 | - | - | 0 | - |
| 2 | y | 3615 | 394.5 | 0.0003629 | 0.9201 | +4 | 13 |
| - | - | 1186 | 394.7 | - | - | 0 | - |
| - | - | 1399 | 395 | - | - | 0 | - |
| - | - | 2013 | 395.2 | - | - | 0 | - |
| - | - | 1457 | 396.3 | - | - | 0 | - |
| - | - | 655.4 | 397.2 | - | - | 0 | - |
| - | - | 1777 | 397.2 | - | - | 0 | - |
| - | - | 903.9 | 397.6 | - | - | 0 | - |
| 10 | b | 1676 | 398.2 | 0.0008099 | 2.034 | +3 | 10 |
| - | - | 986.9 | 398.2 | - | - | 0 | - |
| - | - | 1482 | 398.5 | - | - | 0 | - |
| - | - | 928.9 | 398.9 | - | - | 0 | - |
| - | - | 1609 | 399.2 | - | - | 0 | - |
| - | - | 762.5 | 400.2 | - | - | 0 | - |
| - | - | 981.1 | 400.2 | - | - | 0 | - |
| - | - | 1294 | 400.5 | - | - | 0 | - |
| - | - | 1095 | 401.2 | - | - | 0 | - |
| - | - | 809 | 401.3 | - | - | 0 | - |
| - | - | 4466 | 402.2 | - | - | 0 | - |
| - | - | 2216 | 402.5 | - | - | 0 | - |
| - | - | 1.174E+04 | 403.2 | - | - | 0 | - |
| - | - | 6311 | 403.2 | - | - | 0 | - |
| - | - | 3561 | 403.3 | - | - | 0 | - |
| 4 | y | 8966 | 403.6 | 0.00291 | 7.21 | +3 | 11 |
| - | - | 1141 | 403.8 | - | - | 0 | - |
| - | - | 5484 | 403.9 | - | - | 0 | - |
| - | - | 2111 | 404.2 | - | - | 0 | - |
| - | - | 2817 | 404.2 | - | - | 0 | - |
| - | - | 928.3 | 404.3 | - | - | 0 | - |
| - | - | 3432 | 404.5 | - | - | 0 | - |
| - | - | 3112 | 404.7 | - | - | 0 | - |
| - | - | 827.8 | 404.9 | - | - | 0 | - |
| - | - | 4729 | 405 | - | - | 0 | - |
| - | - | 3545 | 405.2 | - | - | 0 | - |
| - | - | 6124 | 406.2 | - | - | 0 | - |
| - | - | 3700 | 406.3 | - | - | 0 | - |
| - | - | 2607 | 406.7 | - | - | 0 | - |
| - | - | 2190 | 406.8 | - | - | 0 | - |
| - | - | 1537 | 407.2 | - | - | 0 | - |
| - | - | 2396 | 408.2 | - | - | 0 | - |
| - | - | 2292 | 408.6 | - | - | 0 | - |
| - | - | 1651 | 408.9 | - | - | 0 | - |
| 4 | y | 2885 | 409.2 | 0.001252 | 3.059 | +3 | 11 |
| - | - | 2891 | 409.6 | - | - | 0 | - |
| - | - | 1724 | 409.9 | - | - | 0 | - |
| - | - | 742.4 | 410.2 | - | - | 0 | - |
| - | - | 1.167E+04 | 410.3 | - | - | 0 | - |
| - | - | 4865 | 410.6 | - | - | 0 | - |
| - | - | 5026 | 410.9 | - | - | 0 | - |
| 6 | b | 1.132E+04 | 411.2 | 0.0007845 | 1.908 | +2 | 6 |
| - | - | 829.5 | 411.2 | - | - | 0 | - |
| - | - | 1596 | 411.2 | - | - | 0 | - |
| - | - | 2389 | 411.3 | - | - | 0 | - |
| 0 | Precursor | 1772 | 411.7 | 8.966E-05 | 0.2178 | +4 | -1 |
| 0 | Precursor | 2909 | 412 | 0.003113 | 7.556 | +4 | -1 |
| - | - | 2795 | 412.2 | - | - | 0 | - |
| - | - | 1998 | 412.2 | - | - | 0 | - |
| - | - | 2639 | 412.2 | - | - | 0 | - |
| 7 | y | 4.535E+04 | 412.3 | 0.0009893 | 2.4 | +2 | 8 |
| - | - | 864.5 | 412.5 | - | - | 0 | - |
| - | - | 2.158E+04 | 412.8 | - | - | 0 | - |
| - | - | 1728 | 413.2 | - | - | 0 | - |
| - | - | 6313 | 413.3 | - | - | 0 | - |
| - | - | 812.1 | 413.8 | - | - | 0 | - |
| - | - | 6678 | 413.9 | - | - | 0 | - |
| - | - | 753.5 | 414.2 | - | - | 0 | - |
| - | - | 6908 | 414.2 | - | - | 0 | - |
| - | - | 2821 | 414.6 | - | - | 0 | - |
| - | - | 701.3 | 414.9 | - | - | 0 | - |
| - | - | 837.3 | 415 | - | - | 0 | - |
| - | - | 5047 | 415.3 | - | - | 0 | - |
| - | - | 1672 | 415.8 | - | - | 0 | - |
| 0 | Precursor | 2.609E+04 | 416.2 | 0.0007482 | 1.798 | +4 | -1 |
| - | - | 3237 | 416.3 | - | - | 0 | - |
| - | - | 2.544E+04 | 416.5 | - | - | 0 | - |
| - | - | 1.297E+04 | 416.7 | - | - | 0 | - |
| - | - | 4009 | 417 | - | - | 0 | - |
| - | - | 2399 | 417.2 | - | - | 0 | - |
| - | - | 993.8 | 417.3 | - | - | 0 | - |
| - | - | 779.2 | 417.5 | - | - | 0 | - |
| - | - | 2.976E+04 | 419.9 | - | - | 0 | - |
| 6 | b | 2838 | 420.2 | 0.0006291 | 1.497 | +2 | 6 |
| - | - | 2.281E+04 | 420.2 | - | - | 0 | - |
| - | - | 8707 | 420.6 | - | - | 0 | - |
| - | - | 2460 | 420.7 | - | - | 0 | - |
| - | - | 2213 | 420.9 | - | - | 0 | - |
| - | - | 1277 | 421.2 | - | - | 0 | - |
| - | - | 3111 | 422.3 | - | - | 0 | - |
| - | - | 1645 | 423.3 | - | - | 0 | - |
| - | - | 3100 | 424.3 | - | - | 0 | - |
| - | - | 6618 | 424.7 | - | - | 0 | - |
| - | - | 3245 | 425.2 | - | - | 0 | - |
| - | - | 1772 | 425.3 | - | - | 0 | - |
| - | - | 845.2 | 425.3 | - | - | 0 | - |
| - | - | 6406 | 425.9 | - | - | 0 | - |
| - | - | 2399 | 426.2 | - | - | 0 | - |
| - | - | 930 | 426.6 | - | - | 0 | - |
| - | - | 5085 | 427.2 | - | - | 0 | - |
| - | - | 2075 | 427.7 | - | - | 0 | - |
| - | - | 1215 | 428.3 | - | - | 0 | - |
| - | - | 4.307E+04 | 429.2 | - | - | 0 | - |
| - | - | 1.171E+04 | 430.2 | - | - | 0 | - |
| - | - | 5478 | 431.2 | - | - | 0 | - |
| - | - | 1500 | 432.2 | - | - | 0 | - |
| - | - | 1906 | 433.7 | - | - | 0 | - |
| - | - | 682.8 | 434.2 | - | - | 0 | - |
| - | - | 913.5 | 436.6 | - | - | 0 | - |
| 3 | b | 2434 | 437.2 | 0.0009455 | 2.163 | +1 | 3 |
| - | - | 1630 | 438.2 | - | - | 0 | - |
| - | - | 778.4 | 438.2 | - | - | 0 | - |
| - | - | 2521 | 438.6 | - | - | 0 | - |
| - | - | 3287 | 438.9 | - | - | 0 | - |
| - | - | 2310 | 439.2 | - | - | 0 | - |
| - | - | 886.6 | 439.3 | - | - | 0 | - |
| - | - | 1972 | 442.7 | - | - | 0 | - |
| - | - | 1039 | 444.2 | - | - | 0 | - |
| - | - | 977.5 | 445.2 | - | - | 0 | - |
| - | - | 1719 | 445.3 | - | - | 0 | - |
| - | - | 765.7 | 445.9 | - | - | 0 | - |
| - | - | 4303 | 446.2 | - | - | 0 | - |
| - | - | 1864 | 446.7 | - | - | 0 | - |
| - | - | 760.8 | 447.2 | - | - | 0 | - |
| - | - | 7790 | 448.3 | - | - | 0 | - |
| - | - | 6623 | 448.6 | - | - | 0 | - |
| - | - | 2238 | 448.9 | - | - | 0 | - |
| - | - | 888.9 | 449.3 | - | - | 0 | - |
| - | - | 6348 | 451.2 | - | - | 0 | - |
| - | - | 3508 | 451.3 | - | - | 0 | - |
| - | - | 5089 | 451.7 | - | - | 0 | - |
| - | - | 1.055E+04 | 451.9 | - | - | 0 | - |
| - | - | 1082 | 452.2 | - | - | 0 | - |
| - | - | 8779 | 452.3 | - | - | 0 | - |
| - | - | 1537 | 452.3 | - | - | 0 | - |
| - | - | 5359 | 452.6 | - | - | 0 | - |
| - | - | 712.3 | 452.7 | - | - | 0 | - |
| - | - | 1356 | 452.9 | - | - | 0 | - |
| - | - | 1598 | 453.7 | - | - | 0 | - |
| - | - | 1909 | 454.2 | - | - | 0 | - |
| - | - | 4947 | 455.3 | - | - | 0 | - |
| - | - | 832.8 | 456.3 | - | - | 0 | - |
| - | - | 1807 | 457.2 | - | - | 0 | - |
| - | - | 6147 | 457.6 | - | - | 0 | - |
| 3 | y | 7096 | 457.9 | 0.004851 | 10.59 | +3 | 12 |
| - | - | 6550 | 458.3 | - | - | 0 | - |
| - | - | 1330 | 458.6 | - | - | 0 | - |
| - | - | 792.9 | 458.9 | - | - | 0 | - |
| - | - | 1019 | 459.2 | - | - | 0 | - |
| - | - | 8007 | 460.2 | - | - | 0 | - |
| - | - | 4922 | 460.7 | - | - | 0 | - |
| - | - | 1687 | 461.2 | - | - | 0 | - |
| - | - | 1523 | 462.9 | - | - | 0 | - |
| - | - | 1589 | 463.3 | - | - | 0 | - |
| 3 | y | 1.262E+05 | 463.6 | 0.0009657 | 2.083 | +3 | 12 |
| - | - | 1.025E+05 | 463.9 | - | - | 0 | - |
| - | - | 4.828E+04 | 464.3 | - | - | 0 | - |
| - | - | 1.089E+04 | 464.6 | - | - | 0 | - |
| - | - | 3937 | 464.9 | - | - | 0 | - |
| - | - | 801.4 | 465.3 | - | - | 0 | - |
| - | - | 1616 | 466.9 | - | - | 0 | - |
| - | - | 1670 | 467.3 | - | - | 0 | - |
| - | - | 3843 | 467.3 | - | - | 0 | - |
| - | - | 2370 | 468.3 | - | - | 0 | - |
| - | - | 2389 | 469.8 | - | - | 0 | - |
| - | - | 1825 | 470.2 | - | - | 0 | - |
| 11 | y | 5.232E+04 | 470.3 | 0.0008988 | 1.911 | +1 | 4 |
| - | - | 2882 | 470.6 | - | - | 0 | - |
| - | - | 1247 | 470.9 | - | - | 0 | - |
| - | - | 1.318E+04 | 471.3 | - | - | 0 | - |
| - | - | 4204 | 471.8 | - | - | 0 | - |
| - | - | 1962 | 472.3 | - | - | 0 | - |
| - | - | 2266 | 472.3 | - | - | 0 | - |
| - | - | 1726 | 472.6 | - | - | 0 | - |
| 12 | b | 873.6 | 473.2 | 0.007814 | 16.51 | +3 | 12 |
| - | - | 6296 | 473.3 | - | - | 0 | - |
| - | - | 2509 | 473.6 | - | - | 0 | - |
| - | - | 1535 | 473.9 | - | - | 0 | - |
| - | - | 2218 | 474.3 | - | - | 0 | - |
| - | - | 3174 | 474.8 | - | - | 0 | - |
| - | - | 2441 | 475.3 | - | - | 0 | - |
| - | - | 754.7 | 475.9 | - | - | 0 | - |
| - | - | 8384 | 476.3 | - | - | 0 | - |
| - | - | 1.049E+04 | 476.6 | - | - | 0 | - |
| - | - | 4240 | 476.9 | - | - | 0 | - |
| - | - | 1058 | 477.6 | - | - | 0 | - |
| - | - | 2638 | 478.2 | - | - | 0 | - |
| 6 | y | 1.127E+04 | 480.8 | 0.0009828 | 2.044 | +2 | 9 |
| - | - | 4694 | 481.3 | - | - | 0 | - |
| - | - | 1720 | 481.8 | - | - | 0 | - |
| - | - | 4797 | 481.9 | - | - | 0 | - |
| - | - | 4835 | 482.3 | - | - | 0 | - |
| - | - | 4129 | 482.3 | - | - | 0 | - |
| - | - | 3987 | 482.6 | - | - | 0 | - |
| - | - | 3151 | 482.8 | - | - | 0 | - |
| - | - | 2156 | 482.9 | - | - | 0 | - |
| - | - | 1518 | 483.3 | - | - | 0 | - |
| - | - | 7484 | 483.8 | - | - | 0 | - |
| - | - | 5355 | 484.3 | - | - | 0 | - |
| - | - | 1122 | 484.8 | - | - | 0 | - |
| - | - | 1178 | 487.9 | - | - | 0 | - |
| - | - | 1348 | 488.3 | - | - | 0 | - |
| - | - | 1143 | 488.6 | - | - | 0 | - |
| - | - | 1489 | 490.2 | - | - | 0 | - |
| - | - | 4946 | 491.3 | - | - | 0 | - |
| - | - | 885.2 | 492.3 | - | - | 0 | - |
| - | - | 1878 | 492.8 | - | - | 0 | - |
| - | - | 1764 | 493.3 | - | - | 0 | - |
| - | - | 4015 | 493.8 | - | - | 0 | - |
| - | - | 1571 | 494.2 | - | - | 0 | - |
| - | - | 1197 | 494.3 | - | - | 0 | - |
| 8 | b | 1349 | 497.2 | 0.0003213 | 0.6462 | +2 | 8 |
| - | - | 968.9 | 497.7 | - | - | 0 | - |
| - | - | 834.1 | 501.3 | - | - | 0 | - |
| - | - | 1520 | 501.6 | - | - | 0 | - |
| - | - | 925.1 | 501.9 | - | - | 0 | - |
| - | - | 879.6 | 502.3 | - | - | 0 | - |
| - | - | 3581 | 503.2 | - | - | 0 | - |
| - | - | 1095 | 504.2 | - | - | 0 | - |
| - | - | 1576 | 504.9 | - | - | 0 | - |
| 13 | b | 1660 | 505.3 | 0.008385 | 16.59 | +3 | 13 |
| - | - | 1116 | 505.6 | - | - | 0 | - |
| - | - | 1195 | 506.2 | - | - | 0 | - |
| - | - | 1069 | 508.8 | - | - | 0 | - |
| - | - | 3127 | 509.2 | - | - | 0 | - |
| - | - | 2073 | 509.7 | - | - | 0 | - |
| - | - | 1695 | 510.3 | - | - | 0 | - |
| - | - | 843.6 | 510.6 | - | - | 0 | - |
| 13 | b | 4153 | 510.9 | 0.0005319 | 1.041 | +3 | 13 |
| - | - | 4882 | 511.3 | - | - | 0 | - |
| - | - | 3945 | 511.6 | - | - | 0 | - |
| - | - | 1710 | 511.9 | - | - | 0 | - |
| - | - | 791.8 | 513.9 | - | - | 0 | - |
| - | - | 997 | 514.3 | - | - | 0 | - |
| - | - | 708.7 | 514.6 | - | - | 0 | - |
| - | - | 9579 | 516.9 | - | - | 0 | - |
| - | - | 1.286E+04 | 517.3 | - | - | 0 | - |
| - | - | 5414 | 517.6 | - | - | 0 | - |
| - | - | 1525 | 517.8 | - | - | 0 | - |
| - | - | 1232 | 517.9 | - | - | 0 | - |
| - | - | 1807 | 518.3 | - | - | 0 | - |
| 2 | y | 1.415E+04 | 519.9 | 0.001302 | 2.504 | +3 | 13 |
| - | - | 1170 | 520.2 | - | - | 0 | - |
| - | - | 1.03E+04 | 520.3 | - | - | 0 | - |
| - | - | 2347 | 520.4 | - | - | 0 | - |
| - | - | 5087 | 520.6 | - | - | 0 | - |
| - | - | 1422 | 521 | - | - | 0 | - |
| - | - | 4226 | 521.2 | - | - | 0 | - |
| - | - | 787.5 | 521.3 | - | - | 0 | - |
| - | - | 829.1 | 521.4 | - | - | 0 | - |
| - | - | 1179 | 522.2 | - | - | 0 | - |
| - | - | 1647 | 524.8 | - | - | 0 | - |
| - | - | 1956 | 525.3 | - | - | 0 | - |
| 2 | y | 1.52E+04 | 525.6 | 0.0002239 | 0.4259 | +3 | 13 |
| - | - | 1.808E+04 | 526 | - | - | 0 | - |
| - | - | 9693 | 526.3 | - | - | 0 | - |
| - | - | 916.4 | 526.3 | - | - | 0 | - |
| - | - | 2844 | 526.6 | - | - | 0 | - |
| - | - | 1618 | 527 | - | - | 0 | - |
| - | - | 762.8 | 529.2 | - | - | 0 | - |
| - | - | 1825 | 529.8 | - | - | 0 | - |
| - | - | 3483 | 530.3 | - | - | 0 | - |
| - | - | 1826 | 530.8 | - | - | 0 | - |
| - | - | 3265 | 531.2 | - | - | 0 | - |
| - | - | 1140 | 532.2 | - | - | 0 | - |
| - | - | 2651 | 535.8 | - | - | 0 | - |
| - | - | 1400 | 536.3 | - | - | 0 | - |
| - | - | 1160 | 536.8 | - | - | 0 | - |
| - | - | 819.9 | 537.8 | - | - | 0 | - |
| - | - | 2.62E+04 | 538.3 | - | - | 0 | - |
| - | - | 2585 | 538.4 | - | - | 0 | - |
| - | - | 861.7 | 538.8 | - | - | 0 | - |
| - | - | 4127 | 538.8 | - | - | 0 | - |
| - | - | 8008 | 539.3 | - | - | 0 | - |
| - | - | 8259 | 539.3 | - | - | 0 | - |
| - | - | 3114 | 539.8 | - | - | 0 | - |
| - | - | 1859 | 540.3 | - | - | 0 | - |
| - | - | 971.2 | 540.3 | - | - | 0 | - |
| 5 | y | 803.7 | 540.8 | 0.006103 | 11.28 | +2 | 10 |
| - | - | 864.8 | 541.3 | - | - | 0 | - |
| 10 | y | 1.112E+04 | 541.4 | 0.0007723 | 1.427 | +1 | 5 |
| - | - | 4817 | 542.3 | - | - | 0 | - |
| - | - | 3916 | 542.4 | - | - | 0 | - |
| - | - | 1532 | 543.3 | - | - | 0 | - |
| - | - | 1095 | 544.3 | - | - | 0 | - |
| - | - | 2548 | 544.8 | - | - | 0 | - |
| - | - | 1184 | 545.3 | - | - | 0 | - |
| - | - | 874.8 | 545.8 | - | - | 0 | - |
| - | - | 2851 | 547.8 | - | - | 0 | - |
| - | - | 1163 | 548.2 | - | - | 0 | - |
| - | - | 2766 | 548.3 | - | - | 0 | - |
| - | - | 1796 | 548.8 | - | - | 0 | - |
| - | - | 3587 | 549.2 | - | - | 0 | - |
| 5 | y | 2.908E+04 | 549.3 | 0.0008848 | 1.611 | +2 | 10 |
| - | - | 1.895E+04 | 549.8 | - | - | 0 | - |
| - | - | 7231 | 550.3 | - | - | 0 | - |
| - | - | 797.6 | 550.4 | - | - | 0 | - |
| - | - | 1516 | 550.8 | - | - | 0 | - |
| 9 | b | 718.8 | 552.8 | 0.007098 | 12.84 | +2 | 9 |
| - | - | 1526 | 553.2 | - | - | 0 | - |
| - | - | 1215 | 553.3 | - | - | 0 | - |
| - | - | 897.3 | 553.8 | - | - | 0 | - |
| - | - | 1659 | 555.3 | - | - | 0 | - |
| - | - | 1560 | 556.8 | - | - | 0 | - |
| - | - | 2803 | 557.3 | - | - | 0 | - |
| 9 | b | 7771 | 561.3 | 0.001025 | 1.827 | +2 | 9 |
| - | - | 4972 | 561.8 | - | - | 0 | - |
| - | - | 1420 | 562.3 | - | - | 0 | - |
| 4 | b | 752.2 | 565.2 | 0.001603 | 2.835 | +1 | 4 |
| - | - | 3.314E+04 | 566.2 | - | - | 0 | - |
| - | - | 8918 | 567.2 | - | - | 0 | - |
| - | - | 1453 | 567.4 | - | - | 0 | - |
| - | - | 1870 | 568.3 | - | - | 0 | - |
| - | - | 1.405E+04 | 570.2 | - | - | 0 | - |
| - | - | 4720 | 571.2 | - | - | 0 | - |
| - | - | 1088 | 572.3 | - | - | 0 | - |
| - | - | 1.651E+04 | 572.8 | - | - | 0 | - |
| - | - | 1.024E+04 | 573.3 | - | - | 0 | - |
| - | - | 3129 | 573.8 | - | - | 0 | - |
| - | - | 911.6 | 576.2 | - | - | 0 | - |
| - | - | 8804 | 577.4 | - | - | 0 | - |
| - | - | 1015 | 577.8 | - | - | 0 | - |
| - | - | 3323 | 578.4 | - | - | 0 | - |
| - | - | 1.89E+04 | 579.4 | - | - | 0 | - |
| - | - | 5078 | 580.4 | - | - | 0 | - |
| - | - | 1449 | 581.4 | - | - | 0 | - |
| - | - | 857.7 | 582.8 | - | - | 0 | - |
| - | - | 1516 | 587.3 | - | - | 0 | - |
| 10 | b | 1776 | 587.8 | 0.000385 | 0.655 | +2 | 10 |
| 10 | b | 2844 | 588.3 | 0.007279 | 12.37 | +2 | 10 |
| - | - | 761 | 594.2 | - | - | 0 | - |
| - | - | 787.5 | 595.3 | - | - | 0 | - |
| - | - | 6734 | 595.4 | - | - | 0 | - |
| - | - | 1618 | 595.8 | - | - | 0 | - |
| - | - | 1234 | 596.3 | - | - | 0 | - |
| - | - | 2489 | 596.4 | - | - | 0 | - |
| 10 | b | 1052 | 596.8 | 0.001206 | 2.021 | +2 | 10 |
| - | - | 2068 | 597.3 | - | - | 0 | - |
| - | - | 860.4 | 597.8 | - | - | 0 | - |
| - | - | 7317 | 598.2 | - | - | 0 | - |
| - | - | 2767 | 599.2 | - | - | 0 | - |
| - | - | 899.3 | 600.2 | - | - | 0 | - |
| - | - | 1007 | 600.3 | - | - | 0 | - |
| - | - | 5931 | 604.4 | - | - | 0 | - |
| 4 | y | 1.125E+04 | 604.8 | 0.00373 | 6.168 | +2 | 11 |
| - | - | 7554 | 605.4 | - | - | 0 | - |
| - | - | 2068 | 605.9 | - | - | 0 | - |
| - | - | 6006 | 610.3 | - | - | 0 | - |
| - | - | 2122 | 611.3 | - | - | 0 | - |
| - | - | 1256 | 611.8 | - | - | 0 | - |
| - | - | 1139 | 612.3 | - | - | 0 | - |
| 4 | y | 8720 | 613.4 | 0.0009539 | 1.555 | +2 | 11 |
| - | - | 698.3 | 613.4 | - | - | 0 | - |
| - | - | 4587 | 613.9 | - | - | 0 | - |
| - | - | 2241 | 614.4 | - | - | 0 | - |
| - | - | 743.1 | 614.9 | - | - | 0 | - |
| - | - | 2394 | 615.3 | - | - | 0 | - |
| - | - | 3788 | 615.3 | - | - | 0 | - |
| - | - | 2445 | 615.9 | - | - | 0 | - |
| - | - | 1072 | 616.4 | - | - | 0 | - |
| - | - | 805.7 | 618.9 | - | - | 0 | - |
| - | - | 5543 | 620.3 | - | - | 0 | - |
| - | - | 4312 | 620.8 | - | - | 0 | - |
| - | - | 2033 | 621.3 | - | - | 0 | - |
| - | - | 1164 | 623.4 | - | - | 0 | - |
| - | - | 957.8 | 628.3 | - | - | 0 | - |
| - | - | 1715 | 628.3 | - | - | 0 | - |
| - | - | 1.499E+04 | 629.3 | - | - | 0 | - |
| - | - | 9426 | 629.8 | - | - | 0 | - |
| - | - | 4687 | 630.3 | - | - | 0 | - |
| - | - | 933 | 630.8 | - | - | 0 | - |
| - | - | 8814 | 638.4 | - | - | 0 | - |
| - | - | 5145 | 638.9 | - | - | 0 | - |
| - | - | 4269 | 639.4 | - | - | 0 | - |
| - | - | 1245 | 647.4 | - | - | 0 | - |
| - | - | 773.3 | 648.8 | - | - | 0 | - |
| - | - | 1531 | 657.3 | - | - | 0 | - |
| - | - | 1245 | 657.3 | - | - | 0 | - |
| - | - | 2505 | 657.8 | - | - | 0 | - |
| - | - | 704.3 | 663.3 | - | - | 0 | - |
| - | - | 6514 | 664.5 | - | - | 0 | - |
| - | - | 2169 | 665.5 | - | - | 0 | - |
| - | - | 1620 | 665.8 | - | - | 0 | - |
| - | - | 1630 | 666.3 | - | - | 0 | - |
| - | - | 1307 | 667.3 | - | - | 0 | - |
| - | - | 1616 | 668.3 | - | - | 0 | - |
| 9 | y | 4300 | 669.5 | 0.0006579 | 0.9827 | +1 | 6 |
| - | - | 1629 | 670.5 | - | - | 0 | - |
| - | - | 2205 | 674.3 | - | - | 0 | - |
| - | - | 1.848E+04 | 674.4 | - | - | 0 | - |
| - | - | 933 | 675.3 | - | - | 0 | - |
| - | - | 8696 | 675.4 | - | - | 0 | - |
| - | - | 1752 | 676.4 | - | - | 0 | - |
| - | - | 760.4 | 679.3 | - | - | 0 | - |
| - | - | 1101 | 680.3 | - | - | 0 | - |
| 5 | b | 1448 | 684.3 | 0.0009239 | 1.35 | +1 | 5 |
| - | - | 1433 | 685.4 | - | - | 0 | - |
| - | - | 1677 | 690.3 | - | - | 0 | - |
| - | - | 1.91E+04 | 692.4 | - | - | 0 | - |
| - | - | 7840 | 693.4 | - | - | 0 | - |
| - | - | 1461 | 694.5 | - | - | 0 | - |
| 3 | y | 6299 | 694.9 | 0.0007838 | 1.128 | +2 | 12 |
| - | - | 4491 | 695.4 | - | - | 0 | - |
| - | - | 1265 | 695.9 | - | - | 0 | - |
| - | - | 1496 | 698.4 | - | - | 0 | - |
| 5 | b | 3041 | 702.3 | 0.001468 | 2.09 | +1 | 5 |
| - | - | 1234 | 703.3 | - | - | 0 | - |
| - | - | 8398 | 707.3 | - | - | 0 | - |
| - | - | 3840 | 708.3 | - | - | 0 | - |
| - | - | 938.5 | 708.5 | - | - | 0 | - |
| - | - | 6052 | 710.5 | - | - | 0 | - |
| - | - | 1738 | 711.5 | - | - | 0 | - |
| - | - | 1622 | 713.9 | - | - | 0 | - |
| - | - | 1445 | 714.4 | - | - | 0 | - |
| - | - | 902.4 | 714.9 | - | - | 0 | - |
| - | - | 3457 | 716.4 | - | - | 0 | - |
| - | - | 1765 | 717.4 | - | - | 0 | - |
| - | - | 906.5 | 718.4 | - | - | 0 | - |
| - | - | 1837 | 720.3 | - | - | 0 | - |
| - | - | 3049 | 721.4 | - | - | 0 | - |
| - | - | 1767 | 722.4 | - | - | 0 | - |
| - | - | 1792 | 722.9 | - | - | 0 | - |
| - | - | 2976 | 724.3 | - | - | 0 | - |
| - | - | 1046 | 725.3 | - | - | 0 | - |
| 8 | y | 9577 | 726.5 | 0.0003734 | 0.5139 | +1 | 7 |
| - | - | 4318 | 727.5 | - | - | 0 | - |
| - | - | 784 | 728.5 | - | - | 0 | - |
| - | - | 8619 | 735.3 | - | - | 0 | - |
| - | - | 3595 | 736.3 | - | - | 0 | - |
| - | - | 1048 | 737.4 | - | - | 0 | - |
| - | - | 1716 | 738.4 | - | - | 0 | - |
| - | - | 1542 | 739.4 | - | - | 0 | - |
| - | - | 5176 | 752.3 | - | - | 0 | - |
| - | - | 2068 | 753.3 | - | - | 0 | - |
| - | - | 1116 | 777.5 | - | - | 0 | - |
| - | - | 836.9 | 783.5 | - | - | 0 | - |
| - | - | 2405 | 805.5 | - | - | 0 | - |
| - | - | 1045 | 806.5 | - | - | 0 | - |
| - | - | 1994 | 811.4 | - | - | 0 | - |
| - | - | 2785 | 811.5 | - | - | 0 | - |
| - | - | 1398 | 812.4 | - | - | 0 | - |
| - | - | 1293 | 812.5 | - | - | 0 | - |
| 7 | y | 3.543E+04 | 823.5 | 0.000588 | 0.714 | +1 | 8 |
| - | - | 1.704E+04 | 824.5 | - | - | 0 | - |
| - | - | 4809 | 825.5 | - | - | 0 | - |
| - | - | 1229 | 826.6 | - | - | 0 | - |
| - | - | 2951 | 829.5 | - | - | 0 | - |
| - | - | 891.4 | 830.5 | - | - | 0 | - |
| 6 | b | 5466 | 839.4 | 0.0008443 | 1.006 | +1 | 6 |
| - | - | 2476 | 840.4 | - | - | 0 | - |
| - | - | 730.6 | 847.5 | - | - | 0 | - |
| - | - | 4627 | 848.4 | - | - | 0 | - |
| - | - | 2652 | 849.4 | - | - | 0 | - |
| - | - | 2348 | 853.5 | - | - | 0 | - |
| - | - | 1079 | 866.4 | - | - | 0 | - |
| - | - | 6422 | 901.4 | - | - | 0 | - |
| - | - | 3779 | 902.4 | - | - | 0 | - |
| - | - | 1174 | 903.4 | - | - | 0 | - |
| - | - | 787 | 906.4 | - | - | 0 | - |
| - | - | 729.5 | 907.4 | - | - | 0 | - |
| - | - | 3951 | 919.5 | - | - | 0 | - |
| - | - | 1379 | 920.5 | - | - | 0 | - |
| - | - | 1261 | 942.6 | - | - | 0 | - |
| 6 | y | 721.2 | 943.6 | 0.006067 | 6.43 | +1 | 9 |
| 6 | y | 4131 | 960.6 | 0.0003309 | 0.3445 | +1 | 9 |
| - | - | 1902 | 961.6 | - | - | 0 | - |
| - | - | 1214 | 966.6 | - | - | 0 | - |
| - | - | 908.5 | 1034 | - | - | 0 | - |
| - | - | 791.7 | 1035 | - | - | 0 | - |
| - | - | 937.3 | 1070 | - | - | 0 | - |
| - | - | 1316 | 1089 | - | - | 0 | - |
| - | - | 754.8 | 2997 | - | - | 0 | - |

m/z Charge Intensity FragmentType MassShift Position
120.06586456298828 0 478.2805
120.08129119873047 0 728.7647
122.07164764404297 0 1011.4923
123.05577850341797 0 3355.4436
123.11715698242188 0 748.1233
125.07156372070312 0 435.58987
125.0960464477539 0 424.07077 y 11
127.0504379272461 0 724.50726
127.08348846435547 0 357.60635
127.08705139160156 0 3831.4385
128.03472900390625 0 490.50363
128.07118225097656 0 491.77213
128.08230590820312 0 716.9423
129.0663604736328 0 5336.761
129.10276794433594 0 137426.23
130.0656280517578 0 31428.037
130.10037231445312 0 1205.4894
130.1060791015625 0 8244.339
131.04551696777344 0 3917.046
131.06903076171875 0 3473.3335
131.0818634033203 0 1124.3763
132.081298828125 0 119325.336
132.10240173339844 0 34366.676 y 13
133.06089782714844 0 434.9984
133.08482360839844 0 10895.505
133.105712890625 0 2277.4077
134.07192993164062 0 720.64417
134.08822631835938 0 804.81305
136.07620239257812 0 160920.33
137.07350158691406 0 1203.0891
137.07952880859375 0 14343.709
137.1080780029297 0 542.7981
138.06663513183594 0 2503.2593
138.1033477783203 0 466.0799
139.08706665039062 0 2962.1494
140.08236694335938 0 607.86633
141.10272216796875 0 7517.5894
142.06561279296875 0 1810.6866 b 3
142.10638427734375 0 542.83844
143.11856079101562 0 650.88715
144.0770263671875 0 408.54538
144.08120727539062 0 4699.9214
145.06130981445312 0 1096.1586
146.06065368652344 0 2038.2126
146.1292724609375 0 558.0367
147.04457092285156 0 6882.1997
148.0474395751953 0 584.05475
148.07615661621094 0 811.68134
148.9547882080078 0 611.625
149.02406311035156 0 545.32007
150.06614685058594 0 472.05478
151.08717346191406 0 1986.1028
152.08216857910156 0 1273.1146
152.1438446044922 0 2978.6628
153.10292053222656 0 1350.8275
154.098388671875 0 925.1538
155.0819854736328 0 6837.0767
155.09312438964844 0 2581.2231
155.11830139160156 0 4809.928
156.07667541503906 0 2492.1807
156.08164978027344 0 2944.2068
156.11395263671875 0 680.82965
157.07667541503906 0 1144.5494
157.0843505859375 0 905.36664
157.0972900390625 0 757.21246
158.0605010986328 0 1880.2104
158.08433532714844 0 15041.502
159.09217834472656 0 83646.04
159.1132354736328 0 1814.1372
160.07614135742188 0 3840.7437
160.09552001953125 0 9086.727
161.07937622070312 0 777.0305
161.09848022460938 0 668.11865
163.61387634277344 0 635.69025
164.03439331054688 0 799.842
164.0710906982422 0 2111.9167
165.07754516601562 0 1024.4872
165.10275268554688 0 5462.3896
166.06161499023438 0 2526.7275
168.11380004882812 0 1195.7145
169.0764923095703 0 8638.859
169.0976104736328 0 9765.549
169.17013549804688 0 839.1434
170.060546875 0 39581.82
170.10092163085938 0 897.6474
171.0639190673828 0 4349.9844
171.09210205078125 0 1465.2742
172.07611083984375 0 639.5452 b Ammonia loss 4
172.095947265625 0 533.08875
172.10855102539062 0 8443.239
173.07449340820312 0 947.44165
173.4521942138672 0 888.95496
174.05462646484375 0 577.4343
175.08892822265625 0 507.6931
175.09820556640625 0 2128.7153
176.08238220214844 0 3757.8044
177.11253356933594 0 692.76624
178.13424682617188 0 760.05707
179.08177185058594 0 648.3442
179.0933074951172 0 678.50275
179.1549530029297 0 634.7391
181.0613250732422 0 3188.1394
181.09771728515625 0 40970.254
181.13424682617188 0 2412.7297
182.06051635742188 0 595.77
182.0929718017578 0 537.48016
182.10101318359375 0 3476.2395
182.12930297851562 0 4789.354
183.09219360351562 0 16877.404
183.1016082763672 0 806.379
183.1131591796875 0 1651.7421
183.13272094726562 0 601.9376
183.14988708496094 0 1527.651
184.076171875 0 2049.7202
184.09552001953125 0 2623.151
185.0559539794922 0 714.7929
185.07151794433594 0 5237.0938
185.1077117919922 0 665.513
185.1653289794922 0 1669.0121
186.12420654296875 0 16353.415
187.087158203125 0 88669.234
187.10794067382812 0 4178.5454
187.12811279296875 0 1045.341
187.14456176757812 0 1312.5094 y 11
188.081787109375 0 709.75653
188.09056091308594 0 9746.041
189.1243896484375 0 748.31305
191.09298706054688 0 461.36606
193.09788513183594 0 832.49445
195.08816528320312 0 463.09415
195.11270141601562 0 856.7316
195.14964294433594 0 1204.8975
196.1085662841797 0 1796.305
197.1653289794922 0 6358.7803
198.1683349609375 0 1136.7758
199.08702087402344 0 3374.0576
199.10780334472656 0 1523.8497
199.1695556640625 0 1984.4026
199.18101501464844 0 4036.7422
200.09051513671875 0 604.9222
200.1398468017578 0 2306.9346
201.06918334960938 0 703.0004
201.10272216796875 0 8590.455
201.123779296875 0 813.28326
202.08657836914062 0 4651.185
202.10890197753906 0 14331.302
203.093017578125 0 1259.0276
203.1129608154297 0 1577.3965
203.1383819580078 0 605.408
204.07717895507812 0 2147.8696
204.13475036621094 0 2405.5059
205.0977325439453 0 762.4216 a 2
205.1083984375 0 713.0999
207.1238250732422 0 554.07367
207.14979553222656 0 3403.0676
208.14505004882812 0 897.29694
209.0933380126953 0 567.7719
209.12896728515625 0 1700.1293
210.12448120117188 0 663.6037
211.08702087402344 0 2678.6501
212.13958740234375 0 803.90967
212.66830444335938 0 2074.9956
213.12342834472656 0 634.37445
213.16957092285156 0 900.63995
214.1092529296875 0 3960.8052
214.1916046142578 0 1073.5156
215.13980102539062 0 1136.1351
216.11309814453125 0 1090.6078
216.12420654296875 0 1233.9465
216.135498046875 0 585.17816
217.10899353027344 0 1391.917
217.15560913085938 0 679.26587
218.1502685546875 0 754.95233 y Ammonia loss 8
220.1199188232422 0 10197.573
221.10386657714844 0 14689.4
221.12290954589844 0 741.3487
222.1062774658203 0 1678.108
222.1243896484375 0 1375.7515
223.1083984375 0 763.5498
223.1559295654297 0 526.313
224.11883544921875 0 2369.2847
224.1763153076172 0 6212.0166
225.12326049804688 0 658.5401
225.15997314453125 0 2099.884
225.1799774169922 0 822.16656
226.1555938720703 0 113350.5
227.0821990966797 0 1049.2467
227.1405487060547 0 818.74976
227.15892028808594 0 14071.483 y Ammonia loss 10
227.17379760742188 0 755.8508
228.1138153076172 0 7557.3657 a Water loss 1
228.13455200195312 0 1465.6088
228.16188049316406 0 910.4195
229.09776306152344 0 9179.932 b Ammonia loss 4
229.11805725097656 0 1783.6992
229.15447998046875 0 1196.554
229.1664581298828 0 1911.6348
230.10316467285156 0 2504.8315
231.0882568359375 0 4455.7646
231.1356658935547 0 1402.9724
231.17105102539062 0 2466.3193
232.11978149414062 0 674.4467
233.10414123535156 0 2635.737
234.10350036621094 0 897.8523
234.12356567382812 0 992.4653
234.13525390625 0 1145.9799
235.1195831298828 0 17850.361
235.67108154296875 0 16905.639 y 10
236.12277221679688 0 2586.493
236.1403045654297 0 789.66547
236.17279052734375 0 5124.2847
237.1351776123047 0 805.5322
237.16050720214844 0 817.75165 y Ammonia loss 7
238.13011169433594 0 1550.4283
239.15090942382812 0 6776.7227
240.13514709472656 0 1799.1636
240.15380859375 0 1071.2799
241.15621948242188 0 1200.6364
242.1869354248047 0 3921.3105
243.14756774902344 0 1413.6411
243.1814422607422 0 1333.8722
244.1082763671875 0 644.2555
244.16610717773438 0 619.0426
245.18661499023438 0 27141.67 y 12
246.12435913085938 0 210271.56 a 1
246.1896209716797 0 3478.6907
246.64111328125 0 762.1047
247.129150390625 0 49762.484
248.11465454101562 0 15944.805
248.1316680908203 0 3511.0059
249.09873962402344 0 22284.04
249.11441040039062 0 1435.3911 b 7
249.1326904296875 0 914.26276
250.10189819335938 0 2613.089
250.12313842773438 0 2945.816
251.1287841796875 0 1436.344
251.15101623535156 0 3269.8503
252.15621948242188 0 516.43994
252.17124938964844 0 2247.798
253.09658813476562 0 679.8184
253.1545867919922 0 839.73724
255.0765838623047 0 1243.343
255.1167755126953 0 1015.5749
256.10870361328125 0 5654.9995 b Water loss 1
257.09259033203125 0 1028.0612
257.1143798828125 0 2573.599
257.1614074707031 0 2798.4045
258.0765686035156 0 3554.5037
258.0990905761719 0 38165.188
259.080810546875 0 844.09216
259.10235595703125 0 6306.8184
259.1304626464844 0 1391.2186
259.8507385253906 0 944.2899
260.1072692871094 0 5128.2715
260.19744873046875 0 3438.7556
261.111083984375 0 1028.4609
263.1142883300781 0 1871.6348
264.1349792480469 0 2094.9512
265.1407775878906 0 1626.9808
265.16650390625 0 1614.8302
266.10931396484375 0 928.144
266.12530517578125 0 13905.358
266.1512451171875 0 731.0878
267.1287841796875 0 1665.0006
267.14501953125 0 1184.204
268.16632080078125 0 2788.9578
269.63018798828125 0 2091.3555
270.1264343261719 0 741.07086
270.1817626953125 0 9391.11
271.1886291503906 0 5524.9014 y 9
271.68994140625 0 1343.3794
272.1038513183594 0 817.7278
273.13519287109375 0 687.061
274.119140625 0 82315.055 b 1
275.10345458984375 0 10547.381
275.1250915527344 0 64052.395
275.1853942871094 0 2821.7312 y 6
275.519775390625 0 1422.5548
276.10821533203125 0 2893.2979
276.1285400390625 0 8850.289
277.09344482421875 0 884.09204
277.11590576171875 0 553.88306
277.1335144042969 0 3105.6765
278.1366271972656 0 773.3464
279.1472473144531 0 831.7749
279.18182373046875 0 1978.9163
283.15106201171875 0 1785.4048
283.1771240234375 0 25764.934
283.62738037109375 0 2694.5554
284.1289367675781 0 1553.7731
284.1803283691406 0 3081.7864
285.1101989746094 0 1563.1716
285.15625 0 5393.4634
286.1761169433594 0 780.21674
288.0996398925781 0 877.0204
290.1143798828125 0 1029.2722
290.18499755859375 0 9780.76
290.68682861328125 0 2331.5596
291.15740966796875 0 1438.9298
292.1297607421875 0 20400.158
292.18170166015625 0 744.9497
293.133056640625 0 3206.4744
294.120361328125 0 5433.8535
294.2178649902344 0 2339.7654
295.12322998046875 0 722.6099
297.15374755859375 0 769.1671
297.1927795410156 0 14210.68
298.1958923339844 0 2476.254
300.1372375488281 0 1884.0516
300.6728515625 0 1126.5056
303.12030029296875 0 4107.356
303.21826171875 0 677.12305
305.1293029785156 0 7775.5103
305.6649475097656 0 2389.261
306.1328125 0 1099.7083
306.18035888671875 0 777.32
307.1778564453125 0 782.6653
309.1669921875 0 1170.5894
309.19158935546875 0 746.109
311.173583984375 0 711.2378
311.24517822265625 0 3466.6797
312.2471008300781 0 702.79224
313.2237548828125 0 785.7349
314.6702880859375 0 3692.0674
315.1722106933594 0 1430.6539
318.15167236328125 0 827.4636
318.1923522949219 0 1018.18695
320.12384033203125 0 885.111
320.87188720703125 0 3224.3108 y 5
321.20794677734375 0 1118.4387
321.22930908203125 0 16279.199
322.1556396484375 0 10652.511
322.1754150390625 0 543.8933
322.232177734375 0 3750.2317
323.0200500488281 0 765.009
323.1586608886719 0 2667.594 b 10
324.21673583984375 0 3624.9683
324.7185363769531 0 1497.8057
325.2238464355469 0 744.60016
326.2191467285156 0 5052.1763
326.6630859375 0 711.10565
327.2025451660156 0 770.3383
327.2228698730469 0 1404.8394
327.2759704589844 0 1492.785
332.72979736328125 0 5636.1323
333.1241760253906 0 3233.3608
333.2310485839844 0 2880.717
334.17474365234375 0 1013.0246
335.23748779296875 0 2186.5208 y 8
336.2038269042969 0 25901.291
337.2070007324219 0 4000.9246
337.72198486328125 0 7118.721
338.1907958984375 0 627.773
338.2216796875 0 3130.5537
338.2452697753906 0 943.89014
338.71771240234375 0 661.9547
339.2398986816406 0 27130.508
339.8288879394531 0 674.6068
340.1528625488281 0 2426.2642
340.2431335449219 0 5353.5703
341.219970703125 0 718.93695
341.2442626953125 0 770.5983
342.1679992675781 0 1172.9312
345.6441955566406 0 1235.457
346.14434814453125 0 931.02704
346.66790771484375 0 1328.1757
346.72705078125 0 23506.295
347.2282409667969 0 8305.9375
347.7292175292969 0 2170.3662
349.7085876464844 0 2197.051
350.15045166015625 0 4128.4507
351.2403564453125 0 2327.07
353.2188720703125 0 1428.6982
354.1568298339844 0 2170.1746
354.1752014160156 0 2376.2397
354.2142333984375 0 33605.75
354.5103454589844 0 1887.926
354.6582336425781 0 1473.4225
355.21722412109375 0 6203.9414
355.7323913574219 0 5658.331
356.170654296875 0 794.38306
356.23345947265625 0 1512.7117
356.7328186035156 0 664.8174
357.1780700683594 0 900.9605
357.2503967285156 0 7434.975
358.16302490234375 0 8457.825
358.25286865234375 0 1547.948
358.7143249511719 0 2054.6797
359.1661376953125 0 1641.2067
359.2163391113281 0 816.29694
359.5417785644531 0 718.6086
359.87188720703125 0 1606.6519
360.13470458984375 0 911.58374
360.6648254394531 0 1674.9777
360.6888122558594 0 1273.6022
361.1853942871094 0 858.08636
362.15057373046875 0 903.6483
362.67022705078125 0 2056.2927
362.69677734375 0 728.8691
363.74822998046875 0 1669.902 y 7
365.2543029785156 0 724.38043
366.15692138671875 0 3601.2637
366.5582580566406 0 3312.4883 y 4
366.8927307128906 0 2389.61
367.1396789550781 0 1029.9824
367.162841796875 0 2985.9016
368.1484069824219 0 4760.3354
368.1683654785156 0 892.8579
369.1802062988281 0 2212.766
369.69329833984375 0 598.9474
370.527587890625 0 1070.5264
370.6891174316406 0 686.18207
371.22869873046875 0 1547.8363
373.2816467285156 0 15158.776 y 11
374.2597961425781 0 785.5197
374.2849426269531 0 2737.5876
374.5142822265625 0 1002.5985 b 8
375.185791015625 0 831.0862
376.669189453125 0 1542.2122
376.9589538574219 0 832.6275
377.1711120605469 0 1106.8848
378.7127685546875 0 1358.0746
378.96282958984375 0 2023.8695
379.2083740234375 0 1141.7968
380.230224609375 0 1372.9176
381.15008544921875 0 701.0955
382.2063293457031 0 6740.953
382.2825622558594 0 1759.3276
382.5399475097656 0 3336.9524
382.6721496582031 0 754.45105
382.8732604980469 0 1165.9366
383.2662048339844 0 1825.7911
384.1675720214844 0 3973.907
385.1734924316406 0 12755.176
385.19976806640625 0 3322.8909
386.1575927734375 0 13643.794
386.2018737792969 0 1160.1238
386.46160888671875 0 721.74194
387.1602478027344 0 3121.696
388.1634826660156 0 958.57025
388.8624267578125 0 2612.6099
389.19677734375 0 1485.9291
389.27117919921875 0 1758.5817
389.53216552734375 0 916.0874
389.7725830078125 0 975.09
390.2146301269531 0 7197.3667 y Ammonia loss 1
390.465087890625 0 3915.6377
390.71563720703125 0 4581.657
392.1900329589844 0 1765.4896 b Water loss 9
392.26678466796875 0 5466.0835
392.5226135253906 0 2015.5801 b Ammonia loss 9
392.7496337890625 0 899.5188
392.96893310546875 0 691.8534
393.1675109863281 0 1237.329
393.27032470703125 0 1931.6405
394.1518859863281 0 9941.298
394.4705810546875 0 3615.1711 y 1
394.7232666015625 0 1185.6547
394.972412109375 0 1398.8243
395.1556701660156 0 2013.4856
396.2611083984375 0 1457.4738
397.1779479980469 0 655.4429
397.24542236328125 0 1776.5956
397.5655517578125 0 903.9146
398.1938781738281 0 1676.2408 b 9
398.24432373046875 0 986.9216
398.528076171875 0 1481.8793
398.88970947265625 0 928.89905
399.22369384765625 0 1609.0022
400.174072265625 0 762.5311
400.22503662109375 0 981.129
400.4692077636719 0 1294.2036
401.1923828125 0 1094.7366
401.25921630859375 0 808.9601
402.22515869140625 0 4466.34
402.548828125 0 2216.4993
403.1845397949219 0 11738.4375
403.23992919921875 0 6311.0127
403.2693786621094 0 3561.044
403.571044921875 0 8966.269 y Ammonia loss 3
403.768798828125 0 1140.7913
403.904296875 0 5483.5234
404.187744140625 0 2110.7737
404.2383117675781 0 2816.727
404.26873779296875 0 928.2637
404.4703063964844 0 3432.3215
404.7243347167969 0 3112.307
404.89349365234375 0 827.7936
404.97589111328125 0 4729.1035
405.2279968261719 0 3544.7395
406.1860046386719 0 6123.719
406.2508239746094 0 3699.695
406.6873474121094 0 2606.9976
406.7523498535156 0 2190.3064
407.1918029785156 0 1537.247
408.22222900390625 0 2396.3455
408.5555725097656 0 2291.9363
408.88916015625 0 1651.061
409.2449035644531 0 2884.6348 y 3
409.5785217285156 0 2890.8062
409.912353515625 0 1724.4265
410.24554443359375 0 742.4259
410.2770690917969 0 11674.1
410.56939697265625 0 4865.388
410.9034423828125 0 5025.885
411.1783142089844 0 11318.826 b Water loss 5
411.2074890136719 0 829.46045
411.23785400390625 0 1595.739
411.2803649902344 0 2388.7295
411.7254943847656 0 1771.6936 Precursor Water loss
411.9747009277344 0 2909.2314 Precursor Ammonia loss
412.16131591796875 0 2794.7847
412.1844787597656 0 1998.4951
412.2248840332031 0 2639.437
412.2746276855469 0 45354.02 y 6
412.4781494140625 0 864.5461
412.7759704589844 0 21580.701
413.1673889160156 0 1727.5826
413.2774658203125 0 6312.977
413.7759704589844 0 812.0963
413.8973083496094 0 6678.0522
414.19512939453125 0 753.4658
414.2296142578125 0 6908.0894
414.5627746582031 0 2820.6414
414.8943786621094 0 701.26465
415.0377502441406 0 837.2714
415.25701904296875 0 5047.421
415.7579040527344 0 1671.6123
416.2289733886719 0 26092.908 Precursor
416.2868347167969 0 3236.618
416.4796447753906 0 25437.367
416.73040771484375 0 12972.685
416.9813232421875 0 4009.4883
417.23138427734375 0 2398.5518
417.2691650390625 0 993.77313
417.4835510253906 0 779.2108
419.9007263183594 0 29761.264
420.1834411621094 0 2837.738 b 5
420.2351379394531 0 22812.072
420.56890869140625 0 8707.44
420.6849060058594 0 2459.7688
420.9034729003906 0 2212.8784
421.23736572265625 0 1277.2372
422.27703857421875 0 3111.2053
423.2743225097656 0 1645.1565
424.32904052734375 0 3099.9666
424.7121887207031 0 6618.1367
425.2135009765625 0 3245.3901
425.288330078125 0 1771.9794
425.3338623046875 0 845.21576
425.90399169921875 0 6405.7944
426.2381286621094 0 2399.4907
426.5713806152344 0 930.00977
427.2442626953125 0 5084.9897
427.7448425292969 0 2075.1619
428.28802490234375 0 1214.6073
429.1891174316406 0 43071.285
430.1920471191406 0 11712.554
431.1805725097656 0 5478.4795
432.1818542480469 0 1499.6407
433.7040100097656 0 1906.0565
434.20062255859375 0 682.82214
436.5871276855469 0 913.4715
437.1828918457031 0 2433.6567 b 2
438.1845397949219 0 1630.458
438.22882080078125 0 778.41406
438.55645751953125 0 2521.468
438.8916931152344 0 3286.7227
439.2264404296875 0 2310.4797
439.3043212890625 0 886.61176
442.71295166015625 0 1971.7556
444.23150634765625 0 1039.4854
445.19091796875 0 977.48914
445.2688293457031 0 1719.4136
445.91680908203125 0 765.7239
446.2337646484375 0 4303.3887
446.7361145019531 0 1864.3279
447.2370910644531 0 760.7573
448.2638854980469 0 7789.656
448.5982971191406 0 6622.858
448.93304443359375 0 2237.9946
449.2699279785156 0 888.91895
451.2259216308594 0 6348.12
451.2672424316406 0 3508.4333
451.7244873046875 0 5089.432
451.920166015625 0 10553.876
452.2210998535156 0 1082.1035
452.254150390625 0 8778.786
452.3245544433594 0 1536.7867
452.5882873535156 0 5358.9766
452.7225646972656 0 712.34265
452.9203186035156 0 1355.9338
453.7059326171875 0 1598.0569
454.20855712890625 0 1909.2908
455.2523498535156 0 4946.5347
456.2577209472656 0 832.75757
457.1844482421875 0 1807.1676
457.5950927734375 0 6146.696
457.92742919921875 0 7095.8013 y Ammonia loss 2
458.26019287109375 0 6549.533
458.5927429199219 0 1329.5057
458.9273986816406 0 792.8523
459.2135009765625 0 1018.9614
460.2308654785156 0 8006.798
460.7322998046875 0 4921.975
461.242431640625 0 1687.4526
462.9256896972656 0 1523.064
463.27728271484375 0 1588.9844
463.59906005859375 0 126164 y 2
463.933349609375 0 102535.01
464.2675476074219 0 48283.12
464.6016540527344 0 10889.235
464.9361267089844 0 3936.69
465.2701721191406 0 801.4352
466.92041015625 0 1615.9926
467.2548522949219 0 1669.5879
467.2988586425781 0 3842.8286
468.29437255859375 0 2369.8835
469.7882080078125 0 2389.2808
470.2496032714844 0 1825.2098
470.3345947265625 0 52316.38 y 10
470.5814514160156 0 2882.0078
470.9169006347656 0 1247.0298
471.33770751953125 0 13177.758
471.7985534667969 0 4204.4497
472.2999267578125 0 1962.4484
472.3393859863281 0 2265.8508
472.5948486328125 0 1725.9066
473.2344970703125 0 873.5875 b 11
473.2625732421875 0 6295.865
473.57672119140625 0 2508.7253
473.9134521484375 0 1534.8903
474.2647705078125 0 2217.6438
474.7816162109375 0 3173.714
475.2809143066406 0 2441.1057
475.92425537109375 0 754.7083
476.2522277832031 0 8384.361
476.5868225097656 0 10488.152
476.9209289550781 0 4239.82
477.5929870605469 0 1058.1672
478.20947265625 0 2637.9849
480.8040771484375 0 11266.456 y 5
481.30596923828125 0 4693.9805
481.80596923828125 0 1719.6234
481.9271545410156 0 4797.44
482.2619323730469 0 4834.767
482.309326171875 0 4129.259
482.5924377441406 0 3986.9458
482.7619323730469 0 3151.3396
482.9231262207031 0 2155.522
483.2608947753906 0 1517.8431
483.78594970703125 0 7483.873
484.28765869140625 0 5354.8286
484.78875732421875 0 1121.9755
487.9300842285156 0 1177.6654
488.2648620605469 0 1347.9865
488.60003662109375 0 1143.0874
490.2352600097656 0 1489.3911
491.2732238769531 0 4945.7754
492.27435302734375 0 885.1896
492.79180908203125 0 1878.1367
493.2928771972656 0 1764.1328
493.7984924316406 0 4014.6482
494.2369689941406 0 1570.5985
494.2980651855469 0 1197.0868
497.2196044921875 0 1349.3058 b 7
497.7236633300781 0 968.8723
501.262939453125 0 834.1068
501.60528564453125 0 1519.637
501.9376525878906 0 925.1155
502.27313232421875 0 879.6082
503.2156982421875 0 3580.5586
504.21923828125 0 1094.7739
504.9471435546875 0 1575.9702
505.2698669433594 0 1660.2269 b Ammonia loss 12
505.6002502441406 0 1115.9622
506.2324523925781 0 1195.4846
508.75921630859375 0 1068.9619
509.2393798828125 0 3127.1243
509.7417907714844 0 2073.339
510.2907409667969 0 1695.3492
510.62591552734375 0 843.5817
510.9375305175781 0 4152.661 b 12
511.27178955078125 0 4882.4893
511.6067810058594 0 3944.968
511.94256591796875 0 1709.7119
513.9452514648438 0 791.8263
514.2998046875 0 996.9533
514.6096801757812 0 708.6558
516.9414672851562 0 9579.482
517.2750854492188 0 12862.245
517.610107421875 0 5414.326
517.7501831054688 0 1525.0826
517.9437255859375 0 1231.925
518.254638671875 0 1807.1666
519.9503173828125 0 14145.593 y Ammonia loss 1
520.2418212890625 0 1169.8694
520.2842407226562 0 10298.849
520.3602294921875 0 2347.0635
520.6188354492188 0 5086.9497
520.9500122070312 0 1422.1841
521.22705078125 0 4226.335
521.2847900390625 0 787.488
521.3605346679688 0 829.08997
522.224609375 0 1179.4227
524.8117065429688 0 1646.9825
525.3062744140625 0 1956.2665
525.624755859375 0 15200.531 y 1
525.9595336914062 0 18076.443
526.2937622070312 0 9693.33
526.3314819335938 0 916.43164
526.6285400390625 0 2843.677
526.9618530273438 0 1617.9537
529.2202758789062 0 762.82556
529.8038330078125 0 1825.044
530.2987060546875 0 3483.4697
530.7989501953125 0 1825.9385
531.2108764648438 0 3264.9
532.2132568359375 0 1140.3787
535.7518310546875 0 2650.9546
536.2511596679688 0 1400.023
536.7549438476562 0 1160.4177
537.75244140625 0 819.8763
538.2528076171875 0 26196.08
538.3719482421875 0 2585.0996
538.763671875 0 861.6617
538.8094482421875 0 4126.6865
539.2552490234375 0 8008.4565
539.3043823242188 0 8258.905
539.8038940429688 0 3113.559
540.255615234375 0 1859.2124
540.3018188476562 0 971.1688
540.8253784179688 0 803.69727 y Ammonia loss 4
541.32763671875 0 864.7838
541.37158203125 0 11124.784 y 9
542.251953125 0 4816.7783
542.3746948242188 0 3916.461
543.2574462890625 0 1531.8859
544.2687377929688 0 1094.5061
544.760009765625 0 2547.9841
545.2623291015625 0 1183.9728
545.7604370117188 0 874.7934
547.8146362304688 0 2851.1238
548.236328125 0 1163.0662
548.3123168945312 0 2766.2085
548.8109741210938 0 1795.9769
549.222900390625 0 3586.756
549.3334350585938 0 29076.45 y 4
549.8348388671875 0 18951.668
550.3360595703125 0 7230.795
550.3744506835938 0 797.64716
550.8377075195312 0 1516.4941
552.76123046875 0 718.8291 b Ammonia loss 8
553.2186889648438 0 1526.3779
553.2659301757812 0 1214.6996
553.7702026367188 0 897.286
555.28515625 0 1659.07
556.8201904296875 0 1559.5618
557.2958374023438 0 2803.4075
561.2684326171875 0 7771.0015 b 8
561.7693481445312 0 4972.451
562.2715454101562 0 1419.6116
565.2421264648438 0 752.23663 b 3
566.2476806640625 0 33137.67
567.2498168945312 0 8918.311
567.3997192382812 0 1452.8954
568.2526245117188 0 1870.0392
570.246826171875 0 14050.2
571.2499389648438 0 4720.15
572.2513427734375 0 1087.739
572.8050537109375 0 16513.4
573.3064575195312 0 10236.137
573.8074951171875 0 3129.1492
576.2357788085938 0 911.6224
577.3828735351562 0 8804.123
577.8414306640625 0 1014.7757
578.38427734375 0 3322.5059
579.3621826171875 0 18898.746
580.3649291992188 0 5078.2246
581.369140625 0 1448.6578
582.7914428710938 0 857.6677
587.2734985351562 0 1516.0586
587.7810668945312 0 1775.6959 b Water loss 9
588.2799682617188 0 2844.002 b Ammonia loss 9
594.2400512695312 0 761.0207
595.3477783203125 0 787.49066
595.3929443359375 0 6734.494
595.8462524414062 0 1618.0286
596.3486328125 0 1234.0723
596.3944702148438 0 2489.2893
596.7871704101562 0 1051.8776 b 9
597.2863159179688 0 2067.559
597.7880249023438 0 860.44025
598.242431640625 0 7316.959
599.244873046875 0 2767.25
600.2476806640625 0 899.3007
600.3341064453125 0 1006.9696
604.3562622070312 0 5931.2085
604.852294921875 0 11245.706 y Ammonia loss 3
605.3532104492188 0 7554.1064
605.852783203125 0 2067.7488
610.3209838867188 0 6006.117
611.322509765625 0 2121.87
611.8306274414062 0 1256.3807
612.32763671875 0 1139.0076
613.36279296875 0 8720.162 y 3
613.4064331054688 0 698.28186
613.8650512695312 0 4586.9297
614.3650512695312 0 2241.2798
614.865234375 0 743.1482
615.2673950195312 0 2394.1074
615.3483276367188 0 3787.652
615.8513793945312 0 2445.252
616.3521118164062 0 1072.17
618.8530883789062 0 805.65
620.342041015625 0 5542.959
620.8406372070312 0 4312.363
621.342041015625 0 2033.419
623.3883666992188 0 1164.1342
628.2755737304688 0 957.8485
628.3325805664062 0 1715.0889
629.3462524414062 0 14991.179
629.8482055664062 0 9426.403
630.34912109375 0 4686.822
630.8472900390625 0 932.9933
638.3521118164062 0 8813.692
638.8536987304688 0 5144.671
639.3522338867188 0 4269.0757
647.4268188476562 0 1245.0745
648.8363037109375 0 773.33215
657.2749633789062 0 1531.3921
657.3292846679688 0 1244.5532
657.8330688476562 0 2505.352
663.2986450195312 0 704.34753
664.45166015625 0 6514.2427
665.4541625976562 0 2168.5784
665.8457641601562 0 1619.5533
666.3460693359375 0 1629.5427
667.3446044921875 0 1306.553
668.3253173828125 0 1616.3574
669.4664306640625 0 4299.797 y 8
670.4678344726562 0 1629.2341
674.3026733398438 0 2205.268
674.435546875 0 18478.232
675.3076782226562 0 933.0043
675.4376220703125 0 8696.184
676.4384155273438 0 1751.9701
679.3114013671875 0 760.4277
680.3162841796875 0 1100.7562
684.289794921875 0 1448.4441 b Water loss 4
685.3525390625 0 1432.8654
690.2787475585938 0 1676.8901
692.445556640625 0 19102.676
693.4484252929688 0 7839.6016
694.4515380859375 0 1461.2635
694.894287109375 0 6298.5776 y 2
695.3956298828125 0 4490.897
695.8956298828125 0 1264.9873
698.4124145507812 0 1495.9235
702.3009033203125 0 3041.0576 b 4
703.2994384765625 0 1234.3473
707.3052978515625 0 8398.452
708.3084106445312 0 3840.4414
708.4765625 0 938.5104
710.456787109375 0 6051.834
711.4589233398438 0 1737.523
713.8800048828125 0 1622.3926
714.3788452148438 0 1444.6428
714.8810424804688 0 902.4323
716.4203491210938 0 3456.6174
717.4234619140625 0 1764.7249
718.3667602539062 0 906.528
720.3206176757812 0 1837.4054
721.3529052734375 0 3048.6404
722.3788452148438 0 1767.3489
722.8853759765625 0 1792.3607
724.3338012695312 0 2976.2349
725.3323974609375 0 1045.9764
726.4876098632812 0 9576.86 y 7
727.4898681640625 0 4318.469
728.4918823242188 0 784.03125
735.3005981445312 0 8619.387
736.3036499023438 0 3595.436
737.3541259765625 0 1047.949
738.376708984375 0 1716.3678
739.3688354492188 0 1542.3375
752.3287353515625 0 5175.827
753.3279418945312 0 2067.6235
777.53662109375 0 1115.8226
783.5028076171875 0 836.9417
805.53076171875 0 2404.7566
806.5355834960938 0 1044.7343
811.3657836914062 0 1994.3181
811.495849609375 0 2785.482
812.3681030273438 0 1397.8546
812.499267578125 0 1293.0173
823.5405883789062 0 35431.15 y 6
824.543212890625 0 17037.172
825.5465087890625 0 4808.7056
826.5519409179688 0 1229.3363
829.5040283203125 0 2951.1355
830.5070190429688 0 891.3503
839.3591918945312 0 5466.216 b 5
840.3615112304688 0 2476.1694
847.5133666992188 0 730.58014
848.4147338867188 0 4626.7397
849.4196166992188 0 2651.6074
853.4752197265625 0 2348.423
866.3933715820312 0 1078.7661
901.4420776367188 0 6421.8013
902.4450073242188 0 3778.9585
903.4485473632812 0 1173.6716
906.3983764648438 0 787.0142
907.396240234375 0 729.53174
919.4528198242188 0 3951.4846
920.4572143554688 0 1378.7917
942.5875244140625 0 1260.5247
943.5784301757812 0 721.24133 y Ammonia loss 5
960.5992431640625 0 4131.187 y 5
961.6027221679688 0 1901.5254
966.5628662109375 0 1214.0687
1033.5411376953125 0 908.5308
1034.5245361328125 0 791.7438
1070.498779296875 0 937.32623
1088.5179443359375 0 1316.1306
2997.419921875 0 754.8012

Spectrum Details

|  |  |
| --- | --- |
| Matched peaks? Matched peaksThe total absolute number of peaks matched. Additionally in brackets the total fraction of peaks matched and the total number of peaks is shown. | 70 (7.56% of 926) |
| FDR? FDRThe false discovery rate estimated for this peptide. It is calculated by matching all theoretical fragments with a non-integer shift with the raw peaks for this spectrum. This is done with 40 different shifts. The resulting percentage is the average number of annotated peaks over the number of annotated peaks with the correct spectrum. | 3.78% |
| Satellite FDR? Satellite FDRSee the FDR for details on its calculation. This satellite ion specific FDR only contains the satellite ions (d/w) for I/L/J positions. | - |
| PSM Score? PSM ScoreThe PSM Score as given by Hecklib to this annotated spectrum. It is shown with three significant figures. | 221 |

## Spectrum 5359? Spectrum 5359 The raw spectrum of this peptide as annotated by Hecklib. The fragments are coloured according to ion type (see legend). Any peaks with a star '\*' as text can be hovered over to see the full details, first the ion type second the mass shift type. By hovering over the amino acids in the peptide or ions in the legend the corresponding peaks are highlighted. By toggling the 'Unassigned' label you can turn the background (unassigned) peaks on or off in the plot. By updating the slider in the Ion legend you can update the spectrum to only show the top X% of the peaks with labels. The top X% means any peak that is within X% of the highest intensity. By dragging in the spectrum you can zoom in to a specific part of the spectrum and use 'Zoom Out' to get back to the original zoom level. The annotation of the spectrum is based on the given sequence in the peptides file and is done with different software so inconsistencies are likely. The peaks are annotated based on the given sequence, with 20 ppm tolerance.

Copy Data

### Spectrum 5359 (TSV)

#### Preview

```
Loading example...
```

*Click on the button to copy the data to your clipboard.*

Mz MinMz MaxIntensity Max

WidthHeightPeptide font sizePeptide stroke widthSpectrum font sizeSpectrum stroke widthCompact peptide

Ion legend

wxyz

abcd

OtherUnassignedIonChargePositionShow for top:%

SWYQHHPGKAPKJJ

03.56e+57.12e+51.07e+61.42e+6

Zoom Out

y+11b+44a+23y+24a+12b+35y+24y+12a+12b+48b+12y+25b+12y+38y+39b+411y+26y+27y+310b+39y+13b+39y+413b+310b+310y+413b+310y+311y+311b+26\*\*y+28\*b+26b+13y+312y+312y+14y+29b+28b+313b+313b+313y+313y+313y+210y+15y+210b+29b+14b+210b+210b+210y+211y+211y+16b+15y+212b+15b+212y+17y+18b+16y+19

02755498241098

Fragment Matches Table

Show background peaks

| Position | Ion type | Intensity | mz Theoretical | mz Error (Th) | mz Error (ppm) | Charge | Series Number |
| --- | --- | --- | --- | --- | --- | --- | --- |
| - | - | 4440 | 120.1 | - | - | 0 | - |
| - | - | 5189 | 120.1 | - | - | 0 | - |
| - | - | 3722 | 121 | - | - | 0 | - |
| - | - | 2397 | 122.1 | - | - | 0 | - |
| - | - | 5605 | 122.1 | - | - | 0 | - |
| - | - | 1.894E+04 | 123.1 | - | - | 0 | - |
| - | - | 2410 | 125.1 | - | - | 0 | - |
| - | - | 2300 | 125.1 | - | - | 0 | - |
| - | - | 2.429E+04 | 127.1 | - | - | 0 | - |
| - | - | 1910 | 127.1 | - | - | 0 | - |
| - | - | 2802 | 128.1 | - | - | 0 | - |
| - | - | 1894 | 128.1 | - | - | 0 | - |
| - | - | 2000 | 129 | - | - | 0 | - |
| - | - | 2.808E+04 | 129.1 | - | - | 0 | - |
| - | - | 8.622E+05 | 129.1 | - | - | 0 | - |
| - | - | 2.064E+05 | 130.1 | - | - | 0 | - |
| - | - | 3302 | 130.1 | - | - | 0 | - |
| - | - | 7462 | 130.1 | - | - | 0 | - |
| - | - | 5.296E+04 | 130.1 | - | - | 0 | - |
| - | - | 2534 | 131 | - | - | 0 | - |
| - | - | 2.157E+04 | 131.1 | - | - | 0 | - |
| - | - | 2691 | 131.1 | - | - | 0 | - |
| - | - | 8.282E+05 | 132.1 | - | - | 0 | - |
| 14 | y | 2.196E+05 | 132.1 | 0.0005424 | 4.106 | +1 | 1 |
| - | - | 8.572E+04 | 133.1 | - | - | 0 | - |
| - | - | 1.531E+04 | 133.1 | - | - | 0 | - |
| - | - | 3899 | 134.1 | - | - | 0 | - |
| - | - | 5178 | 134.1 | - | - | 0 | - |
| - | - | 1.093E+06 | 136.1 | - | - | 0 | - |
| - | - | 4826 | 137.1 | - | - | 0 | - |
| - | - | 9.543E+04 | 137.1 | - | - | 0 | - |
| - | - | 1.278E+04 | 138.1 | - | - | 0 | - |
| - | - | 2718 | 138.1 | - | - | 0 | - |
| - | - | 1.355E+04 | 139.1 | - | - | 0 | - |
| - | - | 4233 | 141.1 | - | - | 0 | - |
| - | - | 4.569E+04 | 141.1 | - | - | 0 | - |
| 4 | b | 1.951E+04 | 142.1 | 5.5E-05 | 0.3872 | +4 | 4 |
| - | - | 3.803E+04 | 144.1 | - | - | 0 | - |
| - | - | 3634 | 145.1 | - | - | 0 | - |
| - | - | 5377 | 146.1 | - | - | 0 | - |
| - | - | 2575 | 146.1 | - | - | 0 | - |
| - | - | 4.569E+04 | 147 | - | - | 0 | - |
| - | - | 2981 | 147.1 | - | - | 0 | - |
| - | - | 6079 | 148 | - | - | 0 | - |
| - | - | 2947 | 148.1 | - | - | 0 | - |
| - | - | 3756 | 149.1 | - | - | 0 | - |
| - | - | 2818 | 150.1 | - | - | 0 | - |
| - | - | 1.061E+04 | 151.1 | - | - | 0 | - |
| - | - | 4425 | 152.1 | - | - | 0 | - |
| - | - | 2.036E+04 | 152.1 | - | - | 0 | - |
| - | - | 2266 | 153.1 | - | - | 0 | - |
| - | - | 3324 | 153.1 | - | - | 0 | - |
| - | - | 6897 | 154.1 | - | - | 0 | - |
| - | - | 4.71E+04 | 155.1 | - | - | 0 | - |
| - | - | 1.468E+04 | 155.1 | - | - | 0 | - |
| - | - | 2.793E+04 | 155.1 | - | - | 0 | - |
| - | - | 8334 | 156.1 | - | - | 0 | - |
| - | - | 2.553E+04 | 156.1 | - | - | 0 | - |
| - | - | 4708 | 156.1 | - | - | 0 | - |
| - | - | 6541 | 157.1 | - | - | 0 | - |
| - | - | 3483 | 157.1 | - | - | 0 | - |
| - | - | 3014 | 157.1 | - | - | 0 | - |
| - | - | 1.185E+04 | 158.1 | - | - | 0 | - |
| - | - | 9.79E+04 | 158.1 | - | - | 0 | - |
| - | - | 5.728E+05 | 159.1 | - | - | 0 | - |
| - | - | 2.349E+04 | 160.1 | - | - | 0 | - |
| - | - | 4094 | 160.1 | - | - | 0 | - |
| - | - | 5.431E+04 | 160.1 | - | - | 0 | - |
| - | - | 2708 | 161.1 | - | - | 0 | - |
| - | - | 2530 | 162.1 | - | - | 0 | - |
| - | - | 2540 | 162.5 | - | - | 0 | - |
| - | - | 4688 | 163.6 | - | - | 0 | - |
| - | - | 3008 | 164 | - | - | 0 | - |
| - | - | 1.738E+04 | 164.1 | - | - | 0 | - |
| - | - | 2206 | 164.1 | - | - | 0 | - |
| - | - | 5006 | 165.1 | - | - | 0 | - |
| - | - | 3.367E+04 | 165.1 | - | - | 0 | - |
| - | - | 1.026E+04 | 166.1 | - | - | 0 | - |
| - | - | 3386 | 166.1 | - | - | 0 | - |
| - | - | 2505 | 166.2 | - | - | 0 | - |
| - | - | 2680 | 168.1 | - | - | 0 | - |
| - | - | 7618 | 168.1 | - | - | 0 | - |
| - | - | 6.1E+04 | 169.1 | - | - | 0 | - |
| - | - | 5.809E+04 | 169.1 | - | - | 0 | - |
| - | - | 3994 | 169.2 | - | - | 0 | - |
| - | - | 2.551E+05 | 170.1 | - | - | 0 | - |
| - | - | 2925 | 170.1 | - | - | 0 | - |
| - | - | 3.318E+04 | 171.1 | - | - | 0 | - |
| - | - | 1.117E+04 | 171.1 | - | - | 0 | - |
| - | - | 5.556E+04 | 172.1 | - | - | 0 | - |
| - | - | 3470 | 172.1 | - | - | 0 | - |
| - | - | 9142 | 173.4 | - | - | 0 | - |
| - | - | 2716 | 174.1 | - | - | 0 | - |
| - | - | 1.033E+04 | 175.1 | - | - | 0 | - |
| - | - | 2.341E+04 | 176.1 | - | - | 0 | - |
| - | - | 1.374E+04 | 177.1 | - | - | 0 | - |
| - | - | 3910 | 179.1 | - | - | 0 | - |
| - | - | 4346 | 179.1 | - | - | 0 | - |
| - | - | 2375 | 179.6 | - | - | 0 | - |
| - | - | 4788 | 180.1 | - | - | 0 | - |
| - | - | 1.242E+04 | 181.1 | - | - | 0 | - |
| - | - | 2.736E+05 | 181.1 | - | - | 0 | - |
| - | - | 1.501E+04 | 181.1 | - | - | 0 | - |
| - | - | 3400 | 181.2 | - | - | 0 | - |
| - | - | 4727 | 182.1 | - | - | 0 | - |
| - | - | 2527 | 182.1 | - | - | 0 | - |
| - | - | 2.227E+04 | 182.1 | - | - | 0 | - |
| - | - | 3.417E+04 | 182.1 | - | - | 0 | - |
| - | - | 1.232E+05 | 183.1 | - | - | 0 | - |
| - | - | 4434 | 183.1 | - | - | 0 | - |
| - | - | 4646 | 183.1 | - | - | 0 | - |
| - | - | 1.406E+04 | 184.1 | - | - | 0 | - |
| - | - | 1.51E+04 | 184.1 | - | - | 0 | - |
| - | - | 2.787E+04 | 185.1 | - | - | 0 | - |
| - | - | 2971 | 185.1 | - | - | 0 | - |
| - | - | 5858 | 185.1 | - | - | 0 | - |
| - | - | 6165 | 186.1 | - | - | 0 | - |
| - | - | 1.004E+05 | 186.1 | - | - | 0 | - |
| - | - | 5.796E+05 | 187.1 | - | - | 0 | - |
| - | - | 8509 | 187.1 | - | - | 0 | - |
| - | - | 7.166E+04 | 188.1 | - | - | 0 | - |
| - | - | 2486 | 189.1 | - | - | 0 | - |
| - | - | 4019 | 190.6 | - | - | 0 | - |
| - | - | 2918 | 191.1 | - | - | 0 | - |
| - | - | 2630 | 191.1 | - | - | 0 | - |
| - | - | 2313 | 191.1 | - | - | 0 | - |
| - | - | 3077 | 193.1 | - | - | 0 | - |
| - | - | 2295 | 195.1 | - | - | 0 | - |
| - | - | 4936 | 195.1 | - | - | 0 | - |
| - | - | 9612 | 195.1 | - | - | 0 | - |
| - | - | 6866 | 196.1 | - | - | 0 | - |
| - | - | 4.108E+04 | 197.2 | - | - | 0 | - |
| - | - | 3838 | 198.1 | - | - | 0 | - |
| - | - | 6408 | 198.2 | - | - | 0 | - |
| - | - | 2.665E+04 | 199.1 | - | - | 0 | - |
| - | - | 4317 | 199.1 | - | - | 0 | - |
| - | - | 1.999E+04 | 199.2 | - | - | 0 | - |
| - | - | 2824 | 200.1 | - | - | 0 | - |
| - | - | 1.432E+04 | 200.1 | - | - | 0 | - |
| - | - | 4221 | 201.1 | - | - | 0 | - |
| - | - | 6.169E+04 | 201.1 | - | - | 0 | - |
| - | - | 3867 | 201.1 | - | - | 0 | - |
| - | - | 3468 | 201.1 | - | - | 0 | - |
| - | - | 2.777E+04 | 202.1 | - | - | 0 | - |
| - | - | 9.236E+04 | 202.1 | - | - | 0 | - |
| - | - | 6696 | 203.1 | - | - | 0 | - |
| - | - | 6839 | 203.1 | - | - | 0 | - |
| - | - | 1.12E+04 | 204.1 | - | - | 0 | - |
| 3 | a | 4736 | 205.1 | 0.0007616 | 3.713 | +2 | 3 |
| - | - | 1.14E+04 | 205.1 | - | - | 0 | - |
| - | - | 2.985E+04 | 207.1 | - | - | 0 | - |
| - | - | 4523 | 208.1 | - | - | 0 | - |
| - | - | 2750 | 208.2 | - | - | 0 | - |
| - | - | 7569 | 209.1 | - | - | 0 | - |
| - | - | 4037 | 210.1 | - | - | 0 | - |
| - | - | 1.478E+04 | 211.1 | - | - | 0 | - |
| - | - | 3609 | 212.1 | - | - | 0 | - |
| - | - | 1.308E+04 | 212.1 | - | - | 0 | - |
| - | - | 1.873E+04 | 212.7 | - | - | 0 | - |
| - | - | 4526 | 213.1 | - | - | 0 | - |
| - | - | 2341 | 213.1 | - | - | 0 | - |
| - | - | 9780 | 213.2 | - | - | 0 | - |
| - | - | 3.113E+04 | 214.1 | - | - | 0 | - |
| - | - | 3349 | 214.1 | - | - | 0 | - |
| - | - | 7121 | 214.2 | - | - | 0 | - |
| - | - | 2686 | 215.1 | - | - | 0 | - |
| - | - | 3561 | 215.1 | - | - | 0 | - |
| - | - | 8134 | 216.1 | - | - | 0 | - |
| - | - | 7691 | 217.1 | - | - | 0 | - |
| - | - | 2885 | 219.1 | - | - | 0 | - |
| - | - | 7.102E+04 | 220.1 | - | - | 0 | - |
| - | - | 8.949E+04 | 221.1 | - | - | 0 | - |
| - | - | 8170 | 221.1 | - | - | 0 | - |
| - | - | 3619 | 221.1 | - | - | 0 | - |
| - | - | 8081 | 222.1 | - | - | 0 | - |
| - | - | 6837 | 222.1 | - | - | 0 | - |
| - | - | 2496 | 223.2 | - | - | 0 | - |
| - | - | 1.39E+04 | 224.1 | - | - | 0 | - |
| - | - | 4771 | 224.1 | - | - | 0 | - |
| - | - | 4.04E+04 | 224.2 | - | - | 0 | - |
| - | - | 2903 | 225.1 | - | - | 0 | - |
| - | - | 1.509E+04 | 225.2 | - | - | 0 | - |
| - | - | 4689 | 225.2 | - | - | 0 | - |
| - | - | 7.71E+05 | 226.2 | - | - | 0 | - |
| - | - | 2752 | 227.1 | - | - | 0 | - |
| - | - | 4198 | 227.1 | - | - | 0 | - |
| 11 | y | 8.416E+04 | 227.2 | 0.001754 | 7.723 | +2 | 4 |
| 2 | a | 5.011E+04 | 228.1 | 0.0007226 | 3.168 | +1 | 2 |
| - | - | 8199 | 228.1 | - | - | 0 | - |
| - | - | 6141 | 228.2 | - | - | 0 | - |
| 5 | b | 6.91E+04 | 229.1 | 0.001996 | 8.711 | +3 | 5 |
| - | - | 1.118E+04 | 229.1 | - | - | 0 | - |
| - | - | 9659 | 229.2 | - | - | 0 | - |
| - | - | 5933 | 230.1 | - | - | 0 | - |
| - | - | 2.261E+04 | 230.1 | - | - | 0 | - |
| - | - | 2.806E+04 | 231.1 | - | - | 0 | - |
| - | - | 1.172E+04 | 231.1 | - | - | 0 | - |
| - | - | 3870 | 232.1 | - | - | 0 | - |
| - | - | 6263 | 232.1 | - | - | 0 | - |
| - | - | 4712 | 232.1 | - | - | 0 | - |
| - | - | 2355 | 232.6 | - | - | 0 | - |
| - | - | 1.878E+04 | 233.1 | - | - | 0 | - |
| - | - | 2794 | 234.1 | - | - | 0 | - |
| - | - | 3006 | 234.1 | - | - | 0 | - |
| - | - | 1.427E+05 | 235.1 | - | - | 0 | - |
| 11 | y | 1.144E+05 | 235.7 | 0.0006716 | 2.85 | +2 | 4 |
| - | - | 1.156E+04 | 236.1 | - | - | 0 | - |
| - | - | 4559 | 236.1 | - | - | 0 | - |
| - | - | 3.318E+04 | 236.2 | - | - | 0 | - |
| - | - | 4821 | 237.1 | - | - | 0 | - |
| - | - | 8263 | 238.1 | - | - | 0 | - |
| - | - | 6550 | 239.1 | - | - | 0 | - |
| - | - | 5.553E+04 | 239.2 | - | - | 0 | - |
| - | - | 6265 | 240.1 | - | - | 0 | - |
| - | - | 7395 | 240.2 | - | - | 0 | - |
| - | - | 8523 | 241.2 | - | - | 0 | - |
| - | - | 2.606E+04 | 242.2 | - | - | 0 | - |
| - | - | 4226 | 243.2 | - | - | 0 | - |
| - | - | 7365 | 243.2 | - | - | 0 | - |
| - | - | 2792 | 244.1 | - | - | 0 | - |
| - | - | 6274 | 244.2 | - | - | 0 | - |
| - | - | 2666 | 245.2 | - | - | 0 | - |
| 13 | y | 1.807E+05 | 245.2 | 0.0007985 | 3.257 | +1 | 2 |
| 2 | a | 1.41E+06 | 246.1 | 0.0008085 | 3.285 | +1 | 2 |
| - | - | 2.071E+04 | 246.2 | - | - | 0 | - |
| - | - | 3.24E+05 | 247.1 | - | - | 0 | - |
| - | - | 1.055E+05 | 248.1 | - | - | 0 | - |
| - | - | 2.499E+04 | 248.1 | - | - | 0 | - |
| - | - | 1.472E+05 | 249.1 | - | - | 0 | - |
| 8 | b | 4875 | 249.1 | 0.0008398 | 3.371 | +4 | 8 |
| - | - | 4925 | 249.1 | - | - | 0 | - |
| - | - | 5111 | 249.1 | - | - | 0 | - |
| - | - | 1.596E+04 | 250.1 | - | - | 0 | - |
| - | - | 2.046E+04 | 250.1 | - | - | 0 | - |
| - | - | 7844 | 251.1 | - | - | 0 | - |
| - | - | 1.587E+04 | 252.2 | - | - | 0 | - |
| - | - | 5062 | 253.1 | - | - | 0 | - |
| - | - | 2517 | 253.2 | - | - | 0 | - |
| - | - | 2799 | 254.2 | - | - | 0 | - |
| - | - | 6014 | 254.2 | - | - | 0 | - |
| - | - | 2925 | 255.1 | - | - | 0 | - |
| 2 | b | 3.286E+04 | 256.1 | 0.00062 | 2.421 | +1 | 2 |
| - | - | 6094 | 257.1 | - | - | 0 | - |
| - | - | 1.795E+04 | 257.1 | - | - | 0 | - |
| - | - | 2.044E+04 | 257.2 | - | - | 0 | - |
| - | - | 2.587E+04 | 258.1 | - | - | 0 | - |
| - | - | 2.571E+05 | 258.1 | - | - | 0 | - |
| - | - | 4182 | 259.1 | - | - | 0 | - |
| - | - | 3.581E+04 | 259.1 | - | - | 0 | - |
| - | - | 9850 | 259.1 | - | - | 0 | - |
| - | - | 2917 | 259.8 | - | - | 0 | - |
| - | - | 3.262E+04 | 260.1 | - | - | 0 | - |
| - | - | 3084 | 260.1 | - | - | 0 | - |
| - | - | 1.65E+04 | 260.2 | - | - | 0 | - |
| - | - | 3435 | 261.1 | - | - | 0 | - |
| - | - | 3472 | 261.2 | - | - | 0 | - |
| - | - | 4013 | 262.2 | - | - | 0 | - |
| - | - | 7258 | 263.1 | - | - | 0 | - |
| - | - | 3172 | 264.1 | - | - | 0 | - |
| - | - | 1.677E+04 | 264.1 | - | - | 0 | - |
| - | - | 1.331E+04 | 265.1 | - | - | 0 | - |
| - | - | 1.04E+04 | 265.2 | - | - | 0 | - |
| - | - | 2410 | 266.1 | - | - | 0 | - |
| - | - | 5441 | 266.1 | - | - | 0 | - |
| - | - | 8.53E+04 | 266.1 | - | - | 0 | - |
| - | - | 1.344E+04 | 267.1 | - | - | 0 | - |
| - | - | 5656 | 267.1 | - | - | 0 | - |
| - | - | 1.656E+04 | 269.6 | - | - | 0 | - |
| - | - | 2844 | 270.1 | - | - | 0 | - |
| - | - | 5.5E+04 | 270.2 | - | - | 0 | - |
| 10 | y | 3.745E+04 | 271.2 | 1.329E-05 | 0.04901 | +2 | 5 |
| - | - | 8850 | 271.7 | - | - | 0 | - |
| - | - | 3320 | 272.2 | - | - | 0 | - |
| - | - | 4299 | 273.1 | - | - | 0 | - |
| 2 | b | 5.124E+05 | 274.1 | 0.0006754 | 2.464 | +1 | 2 |
| - | - | 7.063E+04 | 275.1 | - | - | 0 | - |
| - | - | 4.302E+05 | 275.1 | - | - | 0 | - |
| 7 | y | 1.696E+04 | 275.2 | 0.0006042 | 2.196 | +3 | 8 |
| - | - | 8046 | 275.5 | - | - | 0 | - |
| - | - | 4267 | 275.9 | - | - | 0 | - |
| - | - | 1.996E+04 | 276.1 | - | - | 0 | - |
| - | - | 5.457E+04 | 276.1 | - | - | 0 | - |
| - | - | 3583 | 277.1 | - | - | 0 | - |
| - | - | 2.347E+04 | 277.1 | - | - | 0 | - |
| - | - | 3757 | 278.1 | - | - | 0 | - |
| - | - | 5425 | 279.2 | - | - | 0 | - |
| - | - | 1.294E+04 | 279.2 | - | - | 0 | - |
| - | - | 3022 | 280.2 | - | - | 0 | - |
| - | - | 4512 | 282.2 | - | - | 0 | - |
| - | - | 1.698E+04 | 283.2 | - | - | 0 | - |
| - | - | 1.969E+05 | 283.2 | - | - | 0 | - |
| - | - | 1.927E+04 | 283.6 | - | - | 0 | - |
| - | - | 5480 | 284.1 | - | - | 0 | - |
| - | - | 2.748E+04 | 284.2 | - | - | 0 | - |
| - | - | 1.28E+04 | 285.1 | - | - | 0 | - |
| - | - | 3.775E+04 | 285.2 | - | - | 0 | - |
| - | - | 2649 | 286.1 | - | - | 0 | - |
| - | - | 6198 | 286.2 | - | - | 0 | - |
| - | - | 3506 | 288.1 | - | - | 0 | - |
| - | - | 2990 | 290.1 | - | - | 0 | - |
| - | - | 5.858E+04 | 290.2 | - | - | 0 | - |
| - | - | 2.349E+04 | 290.7 | - | - | 0 | - |
| - | - | 1.431E+04 | 291.2 | - | - | 0 | - |
| - | - | 3693 | 291.2 | - | - | 0 | - |
| - | - | 1.295E+05 | 292.1 | - | - | 0 | - |
| - | - | 1.882E+04 | 293.1 | - | - | 0 | - |
| - | - | 3439 | 293.2 | - | - | 0 | - |
| - | - | 3.808E+04 | 294.1 | - | - | 0 | - |
| - | - | 3883 | 294.1 | - | - | 0 | - |
| - | - | 1.328E+04 | 294.2 | - | - | 0 | - |
| - | - | 5317 | 295.1 | - | - | 0 | - |
| - | - | 1E+05 | 297.2 | - | - | 0 | - |
| - | - | 1.791E+04 | 298.2 | - | - | 0 | - |
| - | - | 6032 | 300.1 | - | - | 0 | - |
| - | - | 5880 | 300.2 | - | - | 0 | - |
| - | - | 6318 | 300.7 | - | - | 0 | - |
| - | - | 2.922E+04 | 303.1 | - | - | 0 | - |
| - | - | 2974 | 303.2 | - | - | 0 | - |
| - | - | 3029 | 304.1 | - | - | 0 | - |
| - | - | 5.652E+04 | 305.1 | - | - | 0 | - |
| - | - | 1.709E+04 | 305.7 | - | - | 0 | - |
| - | - | 1.786E+04 | 306.1 | - | - | 0 | - |
| - | - | 3072 | 306.2 | - | - | 0 | - |
| - | - | 3532 | 307.2 | - | - | 0 | - |
| - | - | 4523 | 309.2 | - | - | 0 | - |
| - | - | 4229 | 309.2 | - | - | 0 | - |
| - | - | 3970 | 311.2 | - | - | 0 | - |
| - | - | 2.205E+04 | 311.2 | - | - | 0 | - |
| - | - | 5015 | 312.2 | - | - | 0 | - |
| - | - | 3267 | 313.5 | - | - | 0 | - |
| - | - | 4166 | 314.2 | - | - | 0 | - |
| - | - | 2.457E+04 | 314.7 | - | - | 0 | - |
| - | - | 5386 | 315.2 | - | - | 0 | - |
| - | - | 2920 | 316.9 | - | - | 0 | - |
| - | - | 7802 | 318.2 | - | - | 0 | - |
| - | - | 3550 | 319.2 | - | - | 0 | - |
| - | - | 7209 | 320.1 | - | - | 0 | - |
| 6 | y | 1.61E+04 | 320.9 | 0.0007932 | 2.472 | +3 | 9 |
| - | - | 1.331E+04 | 321.2 | - | - | 0 | - |
| - | - | 1.183E+05 | 321.2 | - | - | 0 | - |
| - | - | 7.419E+04 | 322.2 | - | - | 0 | - |
| - | - | 4696 | 322.2 | - | - | 0 | - |
| - | - | 4942 | 322.2 | - | - | 0 | - |
| - | - | 2.395E+04 | 322.2 | - | - | 0 | - |
| - | - | 3913 | 323.1 | - | - | 0 | - |
| 11 | b | 1.167E+04 | 323.2 | 0.0005706 | 1.766 | +4 | 11 |
| - | - | 1.45E+04 | 324.2 | - | - | 0 | - |
| - | - | 5089 | 324.7 | - | - | 0 | - |
| - | - | 3893 | 325.2 | - | - | 0 | - |
| - | - | 4022 | 325.2 | - | - | 0 | - |
| - | - | 4.075E+04 | 326.2 | - | - | 0 | - |
| - | - | 7880 | 327.2 | - | - | 0 | - |
| - | - | 4407 | 327.2 | - | - | 0 | - |
| - | - | 7070 | 327.3 | - | - | 0 | - |
| - | - | 3261 | 327.7 | - | - | 0 | - |
| - | - | 5556 | 330.1 | - | - | 0 | - |
| - | - | 4135 | 330.2 | - | - | 0 | - |
| - | - | 2781 | 332.1 | - | - | 0 | - |
| - | - | 3268 | 332.7 | - | - | 0 | - |
| - | - | 4.339E+04 | 332.7 | - | - | 0 | - |
| - | - | 1.637E+04 | 333.1 | - | - | 0 | - |
| - | - | 1.476E+04 | 333.2 | - | - | 0 | - |
| - | - | 4139 | 334.1 | - | - | 0 | - |
| - | - | 6628 | 334.2 | - | - | 0 | - |
| - | - | 6826 | 334.7 | - | - | 0 | - |
| 9 | y | 8445 | 335.2 | 0.0005054 | 1.508 | +2 | 6 |
| - | - | 5458 | 335.7 | - | - | 0 | - |
| - | - | 1.638E+05 | 336.2 | - | - | 0 | - |
| - | - | 3232 | 337.2 | - | - | 0 | - |
| - | - | 3.314E+04 | 337.2 | - | - | 0 | - |
| - | - | 6.084E+04 | 337.7 | - | - | 0 | - |
| - | - | 3938 | 338.2 | - | - | 0 | - |
| - | - | 2.611E+04 | 338.2 | - | - | 0 | - |
| - | - | 3037 | 338.7 | - | - | 0 | - |
| - | - | 5249 | 339.2 | - | - | 0 | - |
| - | - | 1.726E+05 | 339.2 | - | - | 0 | - |
| - | - | 3386 | 339.5 | - | - | 0 | - |
| - | - | 2.211E+04 | 340.2 | - | - | 0 | - |
| - | - | 3807 | 340.2 | - | - | 0 | - |
| - | - | 3.092E+04 | 340.2 | - | - | 0 | - |
| - | - | 5545 | 341.2 | - | - | 0 | - |
| - | - | 6057 | 341.2 | - | - | 0 | - |
| - | - | 3319 | 341.2 | - | - | 0 | - |
| - | - | 9490 | 342.2 | - | - | 0 | - |
| - | - | 1.174E+04 | 345.6 | - | - | 0 | - |
| - | - | 5087 | 346.1 | - | - | 0 | - |
| - | - | 5402 | 346.7 | - | - | 0 | - |
| - | - | 1.476E+05 | 346.7 | - | - | 0 | - |
| - | - | 4785 | 347.2 | - | - | 0 | - |
| - | - | 5.357E+04 | 347.2 | - | - | 0 | - |
| - | - | 1.325E+04 | 347.7 | - | - | 0 | - |
| - | - | 9454 | 349.7 | - | - | 0 | - |
| - | - | 2.448E+04 | 350.2 | - | - | 0 | - |
| - | - | 7776 | 350.2 | - | - | 0 | - |
| - | - | 4505 | 350.5 | - | - | 0 | - |
| - | - | 4504 | 351.2 | - | - | 0 | - |
| - | - | 1.21E+04 | 351.2 | - | - | 0 | - |
| - | - | 4253 | 352.2 | - | - | 0 | - |
| - | - | 7130 | 353.2 | - | - | 0 | - |
| - | - | 4299 | 353.9 | - | - | 0 | - |
| - | - | 1.546E+04 | 354.2 | - | - | 0 | - |
| - | - | 9093 | 354.2 | - | - | 0 | - |
| - | - | 2.454E+05 | 354.2 | - | - | 0 | - |
| - | - | 8421 | 354.5 | - | - | 0 | - |
| - | - | 7114 | 354.7 | - | - | 0 | - |
| - | - | 4.008E+04 | 355.2 | - | - | 0 | - |
| - | - | 4784 | 355.3 | - | - | 0 | - |
| - | - | 2.22E+04 | 355.7 | - | - | 0 | - |
| - | - | 6092 | 356.2 | - | - | 0 | - |
| - | - | 3588 | 356.2 | - | - | 0 | - |
| - | - | 8331 | 357.2 | - | - | 0 | - |
| - | - | 5.047E+04 | 357.3 | - | - | 0 | - |
| - | - | 5692 | 357.5 | - | - | 0 | - |
| - | - | 3619 | 357.8 | - | - | 0 | - |
| - | - | 5.405E+04 | 358.2 | - | - | 0 | - |
| - | - | 1.104E+04 | 358.3 | - | - | 0 | - |
| - | - | 1.708E+04 | 358.7 | - | - | 0 | - |
| - | - | 7875 | 359.2 | - | - | 0 | - |
| - | - | 5191 | 359.2 | - | - | 0 | - |
| - | - | 7365 | 359.2 | - | - | 0 | - |
| - | - | 1.188E+04 | 359.5 | - | - | 0 | - |
| - | - | 1.525E+04 | 359.9 | - | - | 0 | - |
| - | - | 7304 | 360.2 | - | - | 0 | - |
| - | - | 1.461E+04 | 360.7 | - | - | 0 | - |
| - | - | 1.245E+04 | 360.7 | - | - | 0 | - |
| - | - | 8981 | 361.2 | - | - | 0 | - |
| - | - | 8165 | 361.7 | - | - | 0 | - |
| - | - | 6205 | 362.2 | - | - | 0 | - |
| - | - | 4861 | 362.7 | - | - | 0 | - |
| - | - | 6774 | 363.2 | - | - | 0 | - |
| - | - | 1.229E+04 | 363.5 | - | - | 0 | - |
| 8 | y | 6156 | 363.7 | 0.0006439 | 1.77 | +2 | 7 |
| - | - | 4648 | 364.2 | - | - | 0 | - |
| - | - | 5590 | 364.2 | - | - | 0 | - |
| - | - | 4160 | 365.5 | - | - | 0 | - |
| - | - | 2.911E+04 | 366.2 | - | - | 0 | - |
| 5 | y | 2.123E+04 | 366.6 | 0.0008906 | 2.43 | +3 | 10 |
| - | - | 2.033E+04 | 366.9 | - | - | 0 | - |
| - | - | 1.974E+04 | 367.2 | - | - | 0 | - |
| - | - | 4779 | 367.2 | - | - | 0 | - |
| - | - | 2.9E+04 | 368.1 | - | - | 0 | - |
| - | - | 7011 | 368.2 | - | - | 0 | - |
| - | - | 4208 | 368.7 | - | - | 0 | - |
| 9 | b | 3342 | 368.8 | 0.006579 | 17.84 | +3 | 9 |
| - | - | 3121 | 369.2 | - | - | 0 | - |
| - | - | 6705 | 369.2 | - | - | 0 | - |
| - | - | 3524 | 369.7 | - | - | 0 | - |
| - | - | 1.005E+04 | 370.2 | - | - | 0 | - |
| - | - | 3655 | 370.5 | - | - | 0 | - |
| - | - | 3082 | 370.7 | - | - | 0 | - |
| - | - | 3.485E+04 | 371.2 | - | - | 0 | - |
| - | - | 3521 | 372.2 | - | - | 0 | - |
| 12 | y | 9.197E+04 | 373.3 | 0.0008977 | 2.405 | +1 | 3 |
| - | - | 6150 | 374.3 | - | - | 0 | - |
| - | - | 1.256E+04 | 374.3 | - | - | 0 | - |
| 9 | b | 1.076E+04 | 374.5 | 0.001167 | 3.117 | +3 | 9 |
| - | - | 6172 | 375.2 | - | - | 0 | - |
| - | - | 5634 | 376.5 | - | - | 0 | - |
| - | - | 9322 | 376.7 | - | - | 0 | - |
| - | - | 3110 | 376.9 | - | - | 0 | - |
| - | - | 4002 | 377.2 | - | - | 0 | - |
| - | - | 1.008E+04 | 378.7 | - | - | 0 | - |
| - | - | 1.173E+04 | 379 | - | - | 0 | - |
| - | - | 7902 | 379.2 | - | - | 0 | - |
| - | - | 1.384E+04 | 380.2 | - | - | 0 | - |
| - | - | 3.436E+04 | 382.2 | - | - | 0 | - |
| - | - | 1.105E+04 | 382.3 | - | - | 0 | - |
| - | - | 5368 | 382.5 | - | - | 0 | - |
| - | - | 1.84E+04 | 382.5 | - | - | 0 | - |
| - | - | 4306 | 382.9 | - | - | 0 | - |
| - | - | 3470 | 383.2 | - | - | 0 | - |
| - | - | 4388 | 383.2 | - | - | 0 | - |
| - | - | 8710 | 383.3 | - | - | 0 | - |
| - | - | 3455 | 383.7 | - | - | 0 | - |
| - | - | 2.626E+04 | 384.2 | - | - | 0 | - |
| - | - | 9.717E+04 | 385.2 | - | - | 0 | - |
| - | - | 2.077E+04 | 385.2 | - | - | 0 | - |
| - | - | 9.587E+04 | 386.2 | - | - | 0 | - |
| - | - | 4300 | 386.2 | - | - | 0 | - |
| - | - | 7458 | 386.5 | - | - | 0 | - |
| - | - | 1.355E+04 | 387.2 | - | - | 0 | - |
| - | - | 4196 | 388.2 | - | - | 0 | - |
| - | - | 2.314E+04 | 388.9 | - | - | 0 | - |
| - | - | 1.477E+04 | 389.2 | - | - | 0 | - |
| - | - | 2.233E+04 | 389.3 | - | - | 0 | - |
| - | - | 3766 | 389.5 | - | - | 0 | - |
| - | - | 9065 | 389.8 | - | - | 0 | - |
| 2 | y | 5.712E+04 | 390.2 | 0.0009577 | 2.454 | +4 | 13 |
| - | - | 5.47E+04 | 390.5 | - | - | 0 | - |
| - | - | 1.853E+04 | 390.7 | - | - | 0 | - |
| - | - | 1.035E+04 | 391 | - | - | 0 | - |
| - | - | 3635 | 392.2 | - | - | 0 | - |
| 10 | b | 1.568E+04 | 392.2 | 0.0009745 | 2.485 | +3 | 10 |
| - | - | 3.664E+04 | 392.3 | - | - | 0 | - |
| 10 | b | 1.204E+04 | 392.5 | 0.003871 | 9.863 | +3 | 10 |
| - | - | 8311 | 392.9 | - | - | 0 | - |
| - | - | 5441 | 393.2 | - | - | 0 | - |
| - | - | 7178 | 393.3 | - | - | 0 | - |
| - | - | 5.852E+04 | 394.2 | - | - | 0 | - |
| - | - | 4066 | 394.3 | - | - | 0 | - |
| 2 | y | 3.78E+04 | 394.5 | 0.001156 | 2.932 | +4 | 13 |
| - | - | 2.33E+04 | 394.7 | - | - | 0 | - |
| - | - | 1.329E+04 | 395 | - | - | 0 | - |
| - | - | 1.371E+04 | 395.2 | - | - | 0 | - |
| - | - | 3998 | 395.2 | - | - | 0 | - |
| - | - | 7065 | 396.3 | - | - | 0 | - |
| - | - | 3705 | 397.2 | - | - | 0 | - |
| - | - | 7748 | 397.2 | - | - | 0 | - |
| - | - | 6927 | 397.6 | - | - | 0 | - |
| - | - | 7029 | 397.7 | - | - | 0 | - |
| - | - | 4408 | 397.9 | - | - | 0 | - |
| 10 | b | 8937 | 398.2 | 0.0005352 | 1.344 | +3 | 10 |
| - | - | 5546 | 398.2 | - | - | 0 | - |
| - | - | 1.469E+04 | 398.5 | - | - | 0 | - |
| - | - | 6930 | 398.9 | - | - | 0 | - |
| - | - | 3761 | 398.9 | - | - | 0 | - |
| - | - | 3.138E+04 | 399.2 | - | - | 0 | - |
| - | - | 9209 | 400.2 | - | - | 0 | - |
| - | - | 4219 | 400.5 | - | - | 0 | - |
| - | - | 7931 | 401.2 | - | - | 0 | - |
| - | - | 9517 | 401.3 | - | - | 0 | - |
| - | - | 3.587E+04 | 402.2 | - | - | 0 | - |
| - | - | 4974 | 402.6 | - | - | 0 | - |
| - | - | 8.748E+04 | 403.2 | - | - | 0 | - |
| - | - | 4.12E+04 | 403.2 | - | - | 0 | - |
| - | - | 1.591E+04 | 403.3 | - | - | 0 | - |
| 4 | y | 7.522E+04 | 403.6 | 0.003703 | 9.176 | +3 | 11 |
| - | - | 6235 | 403.8 | - | - | 0 | - |
| - | - | 2.519E+04 | 403.9 | - | - | 0 | - |
| - | - | 1.758E+04 | 404.2 | - | - | 0 | - |
| - | - | 1.339E+04 | 404.2 | - | - | 0 | - |
| - | - | 5089 | 404.3 | - | - | 0 | - |
| - | - | 1.016E+04 | 404.5 | - | - | 0 | - |
| - | - | 2.825E+04 | 404.7 | - | - | 0 | - |
| - | - | 1.039E+04 | 404.9 | - | - | 0 | - |
| - | - | 1.97E+04 | 405 | - | - | 0 | - |
| - | - | 2707 | 405.2 | - | - | 0 | - |
| - | - | 1.657E+04 | 405.2 | - | - | 0 | - |
| - | - | 3073 | 405.5 | - | - | 0 | - |
| - | - | 3.993E+04 | 406.2 | - | - | 0 | - |
| - | - | 3.505E+04 | 406.3 | - | - | 0 | - |
| - | - | 1.329E+04 | 406.7 | - | - | 0 | - |
| - | - | 1.056E+04 | 406.8 | - | - | 0 | - |
| - | - | 8976 | 407.2 | - | - | 0 | - |
| - | - | 5475 | 407.7 | - | - | 0 | - |
| - | - | 2.28E+04 | 408.2 | - | - | 0 | - |
| - | - | 1.477E+04 | 408.6 | - | - | 0 | - |
| - | - | 8647 | 408.9 | - | - | 0 | - |
| 4 | y | 2.053E+04 | 409.2 | 0.001252 | 3.059 | +3 | 11 |
| - | - | 1.172E+04 | 409.6 | - | - | 0 | - |
| - | - | 5256 | 409.9 | - | - | 0 | - |
| - | - | 8.245E+04 | 410.3 | - | - | 0 | - |
| - | - | 4.376E+04 | 410.6 | - | - | 0 | - |
| - | - | 3.219E+04 | 410.9 | - | - | 0 | - |
| 6 | b | 9.109E+04 | 411.2 | 0.00109 | 2.65 | +2 | 6 |
| - | - | 1.191E+04 | 411.2 | - | - | 0 | - |
| - | - | 1.72E+04 | 411.3 | - | - | 0 | - |
| - | - | 3705 | 411.6 | - | - | 0 | - |
| 0 | Precursor | 1.796E+04 | 411.7 | 0.00107 | 2.599 | +4 | -1 |
| 0 | Precursor | 2.1E+04 | 412 | 0.004029 | 9.779 | +4 | -1 |
| - | - | 1.887E+04 | 412.2 | - | - | 0 | - |
| - | - | 1.451E+04 | 412.2 | - | - | 0 | - |
| - | - | 1.228E+04 | 412.2 | - | - | 0 | - |
| 7 | y | 3.016E+05 | 412.3 | 0.001203 | 2.918 | +2 | 8 |
| - | - | 3986 | 412.5 | - | - | 0 | - |
| - | - | 1.407E+05 | 412.8 | - | - | 0 | - |
| - | - | 1.047E+04 | 413.2 | - | - | 0 | - |
| - | - | 4.078E+04 | 413.3 | - | - | 0 | - |
| - | - | 7430 | 413.8 | - | - | 0 | - |
| - | - | 4.685E+04 | 413.9 | - | - | 0 | - |
| - | - | 9733 | 414.2 | - | - | 0 | - |
| - | - | 4.299E+04 | 414.2 | - | - | 0 | - |
| - | - | 1.522E+04 | 414.6 | - | - | 0 | - |
| - | - | 5442 | 414.9 | - | - | 0 | - |
| - | - | 4214 | 415.2 | - | - | 0 | - |
| - | - | 5689 | 415.2 | - | - | 0 | - |
| - | - | 3.661E+04 | 415.3 | - | - | 0 | - |
| - | - | 8445 | 415.8 | - | - | 0 | - |
| 0 | Precursor | 1.645E+05 | 416.2 | 0.0008702 | 2.091 | +4 | -1 |
| - | - | 1.922E+05 | 416.5 | - | - | 0 | - |
| - | - | 6.357E+04 | 416.7 | - | - | 0 | - |
| - | - | 3.753E+04 | 417 | - | - | 0 | - |
| - | - | 1.645E+04 | 417.2 | - | - | 0 | - |
| - | - | 2.013E+05 | 419.9 | - | - | 0 | - |
| 6 | b | 2.269E+04 | 420.2 | 0.000385 | 0.9163 | +2 | 6 |
| - | - | 1.622E+05 | 420.2 | - | - | 0 | - |
| - | - | 5.307E+04 | 420.6 | - | - | 0 | - |
| - | - | 9145 | 420.7 | - | - | 0 | - |
| - | - | 1.406E+04 | 420.9 | - | - | 0 | - |
| - | - | 2.961E+04 | 422.3 | - | - | 0 | - |
| - | - | 1.13E+04 | 423.3 | - | - | 0 | - |
| - | - | 1.299E+04 | 424.3 | - | - | 0 | - |
| - | - | 2.394E+04 | 424.3 | - | - | 0 | - |
| - | - | 4.711E+04 | 424.7 | - | - | 0 | - |
| - | - | 3763 | 424.8 | - | - | 0 | - |
| - | - | 3532 | 424.9 | - | - | 0 | - |
| - | - | 1.421E+04 | 425.2 | - | - | 0 | - |
| - | - | 1.137E+04 | 425.3 | - | - | 0 | - |
| - | - | 4988 | 425.3 | - | - | 0 | - |
| - | - | 8093 | 425.7 | - | - | 0 | - |
| - | - | 2.486E+04 | 425.9 | - | - | 0 | - |
| - | - | 2.34E+04 | 426.2 | - | - | 0 | - |
| - | - | 8564 | 426.6 | - | - | 0 | - |
| - | - | 5790 | 426.9 | - | - | 0 | - |
| - | - | 2.549E+04 | 427.2 | - | - | 0 | - |
| - | - | 1.114E+04 | 427.7 | - | - | 0 | - |
| - | - | 4345 | 428.2 | - | - | 0 | - |
| - | - | 8853 | 428.3 | - | - | 0 | - |
| - | - | 2.88E+05 | 429.2 | - | - | 0 | - |
| - | - | 7.217E+04 | 430.2 | - | - | 0 | - |
| - | - | 3.959E+04 | 431.2 | - | - | 0 | - |
| - | - | 8719 | 432.2 | - | - | 0 | - |
| - | - | 3880 | 433.2 | - | - | 0 | - |
| - | - | 7269 | 433.7 | - | - | 0 | - |
| - | - | 6230 | 436.6 | - | - | 0 | - |
| 3 | b | 1.89E+04 | 437.2 | 0.001251 | 2.861 | +1 | 3 |
| - | - | 4968 | 438.2 | - | - | 0 | - |
| - | - | 4565 | 438.2 | - | - | 0 | - |
| - | - | 3.176E+04 | 438.6 | - | - | 0 | - |
| - | - | 2.106E+04 | 438.9 | - | - | 0 | - |
| - | - | 7413 | 439.2 | - | - | 0 | - |
| - | - | 5897 | 439.3 | - | - | 0 | - |
| - | - | 5436 | 442.2 | - | - | 0 | - |
| - | - | 1.06E+04 | 442.7 | - | - | 0 | - |
| - | - | 5735 | 443.2 | - | - | 0 | - |
| - | - | 7021 | 444.2 | - | - | 0 | - |
| - | - | 9385 | 444.6 | - | - | 0 | - |
| - | - | 8454 | 445.2 | - | - | 0 | - |
| - | - | 6898 | 445.3 | - | - | 0 | - |
| - | - | 6029 | 445.7 | - | - | 0 | - |
| - | - | 7815 | 445.9 | - | - | 0 | - |
| - | - | 3.403E+04 | 446.2 | - | - | 0 | - |
| - | - | 3472 | 446.6 | - | - | 0 | - |
| - | - | 2.073E+04 | 446.7 | - | - | 0 | - |
| - | - | 4638 | 447.2 | - | - | 0 | - |
| - | - | 4917 | 447.7 | - | - | 0 | - |
| - | - | 5.328E+04 | 448.3 | - | - | 0 | - |
| - | - | 3.517E+04 | 448.6 | - | - | 0 | - |
| - | - | 1.298E+04 | 448.9 | - | - | 0 | - |
| - | - | 5186 | 449.3 | - | - | 0 | - |
| - | - | 4.532E+04 | 451.2 | - | - | 0 | - |
| - | - | 1.453E+04 | 451.3 | - | - | 0 | - |
| - | - | 2.076E+04 | 451.7 | - | - | 0 | - |
| - | - | 7.112E+04 | 451.9 | - | - | 0 | - |
| - | - | 8713 | 452.2 | - | - | 0 | - |
| - | - | 5.808E+04 | 452.3 | - | - | 0 | - |
| - | - | 1.275E+04 | 452.3 | - | - | 0 | - |
| - | - | 3.071E+04 | 452.6 | - | - | 0 | - |
| - | - | 1.186E+04 | 452.9 | - | - | 0 | - |
| - | - | 1.001E+04 | 453.7 | - | - | 0 | - |
| - | - | 8861 | 454.2 | - | - | 0 | - |
| - | - | 3.045E+04 | 455.3 | - | - | 0 | - |
| - | - | 9164 | 456.3 | - | - | 0 | - |
| - | - | 8932 | 457.2 | - | - | 0 | - |
| - | - | 3.856E+04 | 457.6 | - | - | 0 | - |
| 3 | y | 5.281E+04 | 457.9 | 0.005004 | 10.93 | +3 | 12 |
| - | - | 3.183E+04 | 458.3 | - | - | 0 | - |
| - | - | 1.515E+04 | 458.6 | - | - | 0 | - |
| - | - | 7160 | 458.9 | - | - | 0 | - |
| - | - | 7175 | 459.2 | - | - | 0 | - |
| - | - | 6.215E+04 | 460.2 | - | - | 0 | - |
| - | - | 2.593E+04 | 460.7 | - | - | 0 | - |
| - | - | 6544 | 461.2 | - | - | 0 | - |
| - | - | 5605 | 461.2 | - | - | 0 | - |
| - | - | 4931 | 461.6 | - | - | 0 | - |
| - | - | 4471 | 461.9 | - | - | 0 | - |
| - | - | 4102 | 462.9 | - | - | 0 | - |
| - | - | 5120 | 463.3 | - | - | 0 | - |
| 3 | y | 8.344E+05 | 463.6 | 0.001179 | 2.544 | +3 | 12 |
| - | - | 6.962E+05 | 463.9 | - | - | 0 | - |
| - | - | 2.961E+05 | 464.3 | - | - | 0 | - |
| - | - | 8.326E+04 | 464.6 | - | - | 0 | - |
| - | - | 2.242E+04 | 464.9 | - | - | 0 | - |
| - | - | 2.05E+04 | 466.9 | - | - | 0 | - |
| - | - | 3447 | 467.2 | - | - | 0 | - |
| - | - | 1.276E+04 | 467.3 | - | - | 0 | - |
| - | - | 2.573E+04 | 467.3 | - | - | 0 | - |
| - | - | 9625 | 467.6 | - | - | 0 | - |
| - | - | 4032 | 468.3 | - | - | 0 | - |
| - | - | 1.118E+04 | 468.3 | - | - | 0 | - |
| - | - | 1.713E+04 | 469.8 | - | - | 0 | - |
| - | - | 1.606E+04 | 470.2 | - | - | 0 | - |
| 11 | y | 3.347E+05 | 470.3 | 0.001112 | 2.365 | +1 | 4 |
| - | - | 2.185E+04 | 470.6 | - | - | 0 | - |
| - | - | 8681 | 470.9 | - | - | 0 | - |
| - | - | 9.174E+04 | 471.3 | - | - | 0 | - |
| - | - | 2.151E+04 | 471.8 | - | - | 0 | - |
| - | - | 2.187E+04 | 472.3 | - | - | 0 | - |
| - | - | 1.476E+04 | 472.3 | - | - | 0 | - |
| - | - | 3821 | 472.6 | - | - | 0 | - |
| - | - | 5369 | 472.8 | - | - | 0 | - |
| - | - | 4430 | 472.9 | - | - | 0 | - |
| - | - | 3.774E+04 | 473.3 | - | - | 0 | - |
| - | - | 5206 | 473.3 | - | - | 0 | - |
| - | - | 1.47E+04 | 473.6 | - | - | 0 | - |
| - | - | 6914 | 473.9 | - | - | 0 | - |
| - | - | 1.404E+04 | 474.3 | - | - | 0 | - |
| - | - | 2.025E+04 | 474.8 | - | - | 0 | - |
| - | - | 1.578E+04 | 475.3 | - | - | 0 | - |
| - | - | 9194 | 475.9 | - | - | 0 | - |
| - | - | 5.497E+04 | 476.3 | - | - | 0 | - |
| - | - | 5.908E+04 | 476.6 | - | - | 0 | - |
| - | - | 1.965E+04 | 476.9 | - | - | 0 | - |
| - | - | 8969 | 477.3 | - | - | 0 | - |
| - | - | 1.101E+04 | 478.2 | - | - | 0 | - |
| - | - | 5671 | 479.2 | - | - | 0 | - |
| - | - | 5307 | 479.2 | - | - | 0 | - |
| - | - | 3533 | 479.6 | - | - | 0 | - |
| 6 | y | 7.48E+04 | 480.8 | 0.001196 | 2.488 | +2 | 9 |
| - | - | 3.993E+04 | 481.3 | - | - | 0 | - |
| - | - | 1.531E+04 | 481.8 | - | - | 0 | - |
| - | - | 2.877E+04 | 481.9 | - | - | 0 | - |
| - | - | 4.674E+04 | 482.3 | - | - | 0 | - |
| - | - | 2.347E+04 | 482.3 | - | - | 0 | - |
| - | - | 3.025E+04 | 482.6 | - | - | 0 | - |
| - | - | 2.951E+04 | 482.8 | - | - | 0 | - |
| - | - | 5810 | 482.9 | - | - | 0 | - |
| - | - | 4385 | 483.2 | - | - | 0 | - |
| - | - | 1.378E+04 | 483.3 | - | - | 0 | - |
| - | - | 5.165E+04 | 483.8 | - | - | 0 | - |
| - | - | 2.672E+04 | 484.3 | - | - | 0 | - |
| - | - | 1.217E+04 | 484.8 | - | - | 0 | - |
| - | - | 3644 | 485.3 | - | - | 0 | - |
| - | - | 3553 | 487.2 | - | - | 0 | - |
| - | - | 1.193E+04 | 487.9 | - | - | 0 | - |
| - | - | 5850 | 488.3 | - | - | 0 | - |
| - | - | 7038 | 488.6 | - | - | 0 | - |
| - | - | 2.916E+04 | 491.3 | - | - | 0 | - |
| - | - | 4485 | 491.8 | - | - | 0 | - |
| - | - | 7622 | 492.3 | - | - | 0 | - |
| - | - | 1.024E+04 | 492.8 | - | - | 0 | - |
| - | - | 5430 | 493.3 | - | - | 0 | - |
| - | - | 4458 | 494.8 | - | - | 0 | - |
| - | - | 7320 | 495.4 | - | - | 0 | - |
| - | - | 3248 | 495.6 | - | - | 0 | - |
| - | - | 4039 | 495.9 | - | - | 0 | - |
| 8 | b | 1.165E+04 | 497.2 | 0.001845 | 3.711 | +2 | 8 |
| - | - | 1.003E+04 | 497.7 | - | - | 0 | - |
| - | - | 3198 | 498.9 | - | - | 0 | - |
| - | - | 3483 | 499.9 | - | - | 0 | - |
| - | - | 1.567E+04 | 501.6 | - | - | 0 | - |
| - | - | 8209 | 501.9 | - | - | 0 | - |
| - | - | 2.709E+04 | 503.2 | - | - | 0 | - |
| - | - | 3784 | 504.6 | - | - | 0 | - |
| 13 | b | 1.24E+04 | 504.9 | 0.004633 | 9.176 | +3 | 13 |
| 13 | b | 1.889E+04 | 505.3 | 0.008965 | 17.74 | +3 | 13 |
| - | - | 7106 | 505.6 | - | - | 0 | - |
| - | - | 5915 | 505.7 | - | - | 0 | - |
| - | - | 4499 | 505.9 | - | - | 0 | - |
| - | - | 4970 | 506.2 | - | - | 0 | - |
| - | - | 1.419E+04 | 508.8 | - | - | 0 | - |
| - | - | 1.662E+04 | 509.2 | - | - | 0 | - |
| - | - | 9125 | 509.7 | - | - | 0 | - |
| - | - | 6192 | 510.2 | - | - | 0 | - |
| - | - | 6491 | 510.6 | - | - | 0 | - |
| 13 | b | 3.168E+04 | 510.9 | 0.0008371 | 1.638 | +3 | 13 |
| - | - | 4.23E+04 | 511.3 | - | - | 0 | - |
| - | - | 1.853E+04 | 511.6 | - | - | 0 | - |
| - | - | 8307 | 511.9 | - | - | 0 | - |
| - | - | 3339 | 512.3 | - | - | 0 | - |
| - | - | 5489 | 513.9 | - | - | 0 | - |
| - | - | 1.488E+04 | 514.3 | - | - | 0 | - |
| - | - | 1.163E+04 | 514.6 | - | - | 0 | - |
| - | - | 6.754E+04 | 516.9 | - | - | 0 | - |
| - | - | 7.828E+04 | 517.3 | - | - | 0 | - |
| - | - | 2.494E+04 | 517.6 | - | - | 0 | - |
| - | - | 1.088E+04 | 517.8 | - | - | 0 | - |
| - | - | 1.083E+04 | 517.9 | - | - | 0 | - |
| - | - | 1.313E+04 | 518.3 | - | - | 0 | - |
| - | - | 8644 | 519.6 | - | - | 0 | - |
| 2 | y | 7.371E+04 | 519.9 | 0.00118 | 2.269 | +3 | 13 |
| - | - | 4605 | 520.2 | - | - | 0 | - |
| - | - | 6.694E+04 | 520.3 | - | - | 0 | - |
| - | - | 2.171E+04 | 520.4 | - | - | 0 | - |
| - | - | 4.164E+04 | 520.6 | - | - | 0 | - |
| - | - | 1.436E+04 | 521 | - | - | 0 | - |
| - | - | 2.883E+04 | 521.2 | - | - | 0 | - |
| - | - | 4409 | 521.4 | - | - | 0 | - |
| - | - | 4254 | 522.3 | - | - | 0 | - |
| - | - | 9712 | 523.4 | - | - | 0 | - |
| - | - | 4523 | 525.2 | - | - | 0 | - |
| - | - | 1.026E+04 | 525.3 | - | - | 0 | - |
| 2 | y | 1.2E+05 | 525.6 | 0.0008342 | 1.587 | +3 | 13 |
| - | - | 8066 | 525.8 | - | - | 0 | - |
| - | - | 1.427E+05 | 526 | - | - | 0 | - |
| - | - | 6.154E+04 | 526.3 | - | - | 0 | - |
| - | - | 6259 | 526.3 | - | - | 0 | - |
| - | - | 2.185E+04 | 526.6 | - | - | 0 | - |
| - | - | 4046 | 527.2 | - | - | 0 | - |
| - | - | 5952 | 528.2 | - | - | 0 | - |
| - | - | 4838 | 529.2 | - | - | 0 | - |
| - | - | 6429 | 529.3 | - | - | 0 | - |
| - | - | 7758 | 529.8 | - | - | 0 | - |
| - | - | 2.065E+04 | 530.3 | - | - | 0 | - |
| - | - | 1.489E+04 | 530.8 | - | - | 0 | - |
| - | - | 2.047E+04 | 531.2 | - | - | 0 | - |
| - | - | 5367 | 532.2 | - | - | 0 | - |
| - | - | 4109 | 533.8 | - | - | 0 | - |
| - | - | 1.724E+04 | 535.8 | - | - | 0 | - |
| - | - | 1.404E+04 | 536.3 | - | - | 0 | - |
| - | - | 5421 | 536.8 | - | - | 0 | - |
| - | - | 1.873E+05 | 538.3 | - | - | 0 | - |
| - | - | 1.321E+04 | 538.4 | - | - | 0 | - |
| - | - | 2.626E+04 | 538.8 | - | - | 0 | - |
| - | - | 5.632E+04 | 539.3 | - | - | 0 | - |
| - | - | 5.513E+04 | 539.3 | - | - | 0 | - |
| - | - | 2.392E+04 | 539.8 | - | - | 0 | - |
| - | - | 5359 | 540.3 | - | - | 0 | - |
| - | - | 1.143E+04 | 540.3 | - | - | 0 | - |
| 5 | y | 9018 | 540.8 | 0.004211 | 7.786 | +2 | 10 |
| - | - | 5765 | 541.3 | - | - | 0 | - |
| 10 | y | 8.493E+04 | 541.4 | 0.001138 | 2.103 | +1 | 5 |
| - | - | 3.586E+04 | 542.3 | - | - | 0 | - |
| - | - | 2.503E+04 | 542.4 | - | - | 0 | - |
| - | - | 8927 | 543.3 | - | - | 0 | - |
| - | - | 4881 | 543.4 | - | - | 0 | - |
| - | - | 1.035E+04 | 544.3 | - | - | 0 | - |
| - | - | 2.238E+04 | 544.8 | - | - | 0 | - |
| - | - | 1.328E+04 | 545.3 | - | - | 0 | - |
| - | - | 4514 | 545.8 | - | - | 0 | - |
| - | - | 2.044E+04 | 547.8 | - | - | 0 | - |
| - | - | 5739 | 548.2 | - | - | 0 | - |
| - | - | 2.01E+04 | 548.3 | - | - | 0 | - |
| - | - | 4443 | 548.8 | - | - | 0 | - |
| - | - | 1.563E+04 | 549.2 | - | - | 0 | - |
| 5 | y | 2.22E+05 | 549.3 | 0.0008848 | 1.611 | +2 | 10 |
| - | - | 1.228E+05 | 549.8 | - | - | 0 | - |
| - | - | 5468 | 550.2 | - | - | 0 | - |
| - | - | 3.948E+04 | 550.3 | - | - | 0 | - |
| - | - | 3357 | 550.4 | - | - | 0 | - |
| - | - | 1.068E+04 | 550.8 | - | - | 0 | - |
| - | - | 1.508E+04 | 553.2 | - | - | 0 | - |
| - | - | 9219 | 553.3 | - | - | 0 | - |
| - | - | 7482 | 553.8 | - | - | 0 | - |
| - | - | 5303 | 555.3 | - | - | 0 | - |
| - | - | 5330 | 555.8 | - | - | 0 | - |
| - | - | 4194 | 556.8 | - | - | 0 | - |
| - | - | 2.079E+04 | 557.3 | - | - | 0 | - |
| - | - | 4497 | 558.3 | - | - | 0 | - |
| 9 | b | 5.098E+04 | 561.3 | 0.0008422 | 1.501 | +2 | 9 |
| - | - | 5302 | 561.3 | - | - | 0 | - |
| - | - | 3.493E+04 | 561.8 | - | - | 0 | - |
| - | - | 1.483E+04 | 562.3 | - | - | 0 | - |
| - | - | 4573 | 562.8 | - | - | 0 | - |
| - | - | 3408 | 564.3 | - | - | 0 | - |
| - | - | 4413 | 564.8 | - | - | 0 | - |
| 4 | b | 5619 | 565.2 | 0.002823 | 4.995 | +1 | 4 |
| - | - | 3998 | 565.3 | - | - | 0 | - |
| - | - | 2.28E+05 | 566.2 | - | - | 0 | - |
| - | - | 6.911E+04 | 567.3 | - | - | 0 | - |
| - | - | 1.708E+04 | 567.4 | - | - | 0 | - |
| - | - | 1.477E+04 | 568.3 | - | - | 0 | - |
| - | - | 5384 | 568.4 | - | - | 0 | - |
| - | - | 1.069E+05 | 570.2 | - | - | 0 | - |
| - | - | 2.807E+04 | 571.2 | - | - | 0 | - |
| - | - | 5228 | 572.3 | - | - | 0 | - |
| - | - | 9.255E+04 | 572.8 | - | - | 0 | - |
| - | - | 6.057E+04 | 573.3 | - | - | 0 | - |
| - | - | 1.981E+04 | 573.8 | - | - | 0 | - |
| - | - | 5832 | 574.3 | - | - | 0 | - |
| - | - | 6.863E+04 | 577.4 | - | - | 0 | - |
| - | - | 2.48E+04 | 578.4 | - | - | 0 | - |
| - | - | 1.28E+05 | 579.4 | - | - | 0 | - |
| - | - | 4.519E+04 | 580.4 | - | - | 0 | - |
| - | - | 1.114E+04 | 581.4 | - | - | 0 | - |
| - | - | 5622 | 581.8 | - | - | 0 | - |
| - | - | 8561 | 587.3 | - | - | 0 | - |
| 10 | b | 1.35E+04 | 587.8 | 0.001301 | 2.213 | +2 | 10 |
| 10 | b | 1.46E+04 | 588.3 | 0.007095 | 12.06 | +2 | 10 |
| - | - | 4030 | 588.3 | - | - | 0 | - |
| - | - | 8895 | 588.8 | - | - | 0 | - |
| - | - | 4049 | 591.4 | - | - | 0 | - |
| - | - | 4.209E+04 | 595.4 | - | - | 0 | - |
| - | - | 6923 | 595.8 | - | - | 0 | - |
| - | - | 6264 | 596.3 | - | - | 0 | - |
| - | - | 1.743E+04 | 596.4 | - | - | 0 | - |
| 10 | b | 1.41E+04 | 596.8 | 0.001084 | 1.817 | +2 | 10 |
| - | - | 4156 | 596.8 | - | - | 0 | - |
| - | - | 4088 | 597.3 | - | - | 0 | - |
| - | - | 4.511E+04 | 598.2 | - | - | 0 | - |
| - | - | 2.313E+04 | 599.2 | - | - | 0 | - |
| - | - | 7292 | 600.3 | - | - | 0 | - |
| - | - | 4.614E+04 | 604.4 | - | - | 0 | - |
| 4 | y | 7.726E+04 | 604.8 | 0.004341 | 7.177 | +2 | 11 |
| - | - | 5.316E+04 | 605.4 | - | - | 0 | - |
| - | - | 1.639E+04 | 605.9 | - | - | 0 | - |
| - | - | 8974 | 606.4 | - | - | 0 | - |
| - | - | 4.321E+04 | 610.3 | - | - | 0 | - |
| - | - | 1.523E+04 | 611.3 | - | - | 0 | - |
| - | - | 1.239E+04 | 611.8 | - | - | 0 | - |
| - | - | 9249 | 612.3 | - | - | 0 | - |
| 4 | y | 4.809E+04 | 613.4 | 0.0004657 | 0.7592 | +2 | 11 |
| - | - | 2907 | 613.4 | - | - | 0 | - |
| - | - | 3.885E+04 | 613.9 | - | - | 0 | - |
| - | - | 8831 | 614.4 | - | - | 0 | - |
| - | - | 1.072E+04 | 615.3 | - | - | 0 | - |
| - | - | 2.277E+04 | 615.4 | - | - | 0 | - |
| - | - | 1.511E+04 | 615.9 | - | - | 0 | - |
| - | - | 6898 | 616.4 | - | - | 0 | - |
| - | - | 4811 | 618.4 | - | - | 0 | - |
| - | - | 8540 | 618.8 | - | - | 0 | - |
| - | - | 4.299E+04 | 620.3 | - | - | 0 | - |
| - | - | 3.47E+04 | 620.8 | - | - | 0 | - |
| - | - | 1.105E+04 | 621.3 | - | - | 0 | - |
| - | - | 4464 | 621.8 | - | - | 0 | - |
| - | - | 7744 | 623.4 | - | - | 0 | - |
| - | - | 5590 | 624.4 | - | - | 0 | - |
| - | - | 1.252E+04 | 628.3 | - | - | 0 | - |
| - | - | 1.007E+05 | 629.3 | - | - | 0 | - |
| - | - | 7.124E+04 | 629.8 | - | - | 0 | - |
| - | - | 2.845E+04 | 630.3 | - | - | 0 | - |
| - | - | 9286 | 630.9 | - | - | 0 | - |
| - | - | 3803 | 631.3 | - | - | 0 | - |
| - | - | 6.093E+04 | 638.4 | - | - | 0 | - |
| - | - | 4.446E+04 | 638.9 | - | - | 0 | - |
| - | - | 1.532E+04 | 639.4 | - | - | 0 | - |
| - | - | 4400 | 639.9 | - | - | 0 | - |
| - | - | 3532 | 645.8 | - | - | 0 | - |
| - | - | 7803 | 647.4 | - | - | 0 | - |
| - | - | 5530 | 648.4 | - | - | 0 | - |
| - | - | 4976 | 653.8 | - | - | 0 | - |
| - | - | 5521 | 657.3 | - | - | 0 | - |
| - | - | 8150 | 657.3 | - | - | 0 | - |
| - | - | 7609 | 657.8 | - | - | 0 | - |
| - | - | 6017 | 663.3 | - | - | 0 | - |
| - | - | 3.928E+04 | 664.5 | - | - | 0 | - |
| - | - | 1.414E+04 | 665.5 | - | - | 0 | - |
| - | - | 1.485E+04 | 665.8 | - | - | 0 | - |
| - | - | 1.693E+04 | 666.3 | - | - | 0 | - |
| - | - | 6778 | 667.3 | - | - | 0 | - |
| - | - | 1.52E+04 | 668.3 | - | - | 0 | - |
| - | - | 6113 | 669.3 | - | - | 0 | - |
| 9 | y | 3.255E+04 | 669.5 | 0.0005358 | 0.8004 | +1 | 6 |
| - | - | 1.288E+04 | 670.5 | - | - | 0 | - |
| - | - | 1.934E+04 | 674.3 | - | - | 0 | - |
| - | - | 1.354E+05 | 674.4 | - | - | 0 | - |
| - | - | 6332 | 675.3 | - | - | 0 | - |
| - | - | 4.953E+04 | 675.4 | - | - | 0 | - |
| - | - | 1.236E+04 | 676.4 | - | - | 0 | - |
| - | - | 4622 | 677.4 | - | - | 0 | - |
| - | - | 6863 | 679.3 | - | - | 0 | - |
| - | - | 3948 | 680.3 | - | - | 0 | - |
| - | - | 3539 | 680.5 | - | - | 0 | - |
| 5 | b | 8264 | 684.3 | 0.001046 | 1.529 | +1 | 5 |
| - | - | 6084 | 685.3 | - | - | 0 | - |
| - | - | 1.197E+04 | 685.4 | - | - | 0 | - |
| - | - | 4602 | 686.4 | - | - | 0 | - |
| - | - | 9549 | 690.3 | - | - | 0 | - |
| - | - | 3719 | 691.3 | - | - | 0 | - |
| - | - | 1.179E+05 | 692.4 | - | - | 0 | - |
| - | - | 5.23E+04 | 693.4 | - | - | 0 | - |
| - | - | 1.133E+04 | 694.5 | - | - | 0 | - |
| 3 | y | 4.153E+04 | 694.9 | 0.001089 | 1.567 | +2 | 12 |
| - | - | 4.119E+04 | 695.4 | - | - | 0 | - |
| - | - | 1.755E+04 | 695.9 | - | - | 0 | - |
| - | - | 4080 | 696.4 | - | - | 0 | - |
| - | - | 1.122E+04 | 698.4 | - | - | 0 | - |
| - | - | 4484 | 699.4 | - | - | 0 | - |
| 5 | b | 2.56E+04 | 702.3 | 0.002444 | 3.48 | +1 | 5 |
| - | - | 9217 | 703.3 | - | - | 0 | - |
| - | - | 6.24E+04 | 707.3 | - | - | 0 | - |
| - | - | 2.51E+04 | 708.3 | - | - | 0 | - |
| - | - | 8614 | 708.5 | - | - | 0 | - |
| - | - | 6099 | 709.3 | - | - | 0 | - |
| 12 | b | 9674 | 709.4 | 0.003209 | 4.524 | +2 | 12 |
| - | - | 5709 | 709.9 | - | - | 0 | - |
| - | - | 4.382E+04 | 710.5 | - | - | 0 | - |
| - | - | 1.652E+04 | 711.5 | - | - | 0 | - |
| - | - | 4625 | 712.5 | - | - | 0 | - |
| - | - | 5835 | 713.4 | - | - | 0 | - |
| - | - | 8888 | 713.9 | - | - | 0 | - |
| - | - | 7947 | 714.4 | - | - | 0 | - |
| - | - | 4484 | 714.9 | - | - | 0 | - |
| - | - | 2.881E+04 | 716.4 | - | - | 0 | - |
| - | - | 1.026E+04 | 717.4 | - | - | 0 | - |
| - | - | 1.246E+04 | 720.3 | - | - | 0 | - |
| - | - | 7752 | 720.4 | - | - | 0 | - |
| - | - | 1.75E+04 | 721.4 | - | - | 0 | - |
| - | - | 4226 | 722.4 | - | - | 0 | - |
| - | - | 1.239E+04 | 722.9 | - | - | 0 | - |
| - | - | 3707 | 723.4 | - | - | 0 | - |
| - | - | 2.213E+04 | 724.3 | - | - | 0 | - |
| - | - | 7610 | 725.3 | - | - | 0 | - |
| 8 | y | 5.862E+04 | 726.5 | 0.0009227 | 1.27 | +1 | 7 |
| - | - | 2.524E+04 | 727.5 | - | - | 0 | - |
| - | - | 4238 | 728.4 | - | - | 0 | - |
| - | - | 4429 | 728.5 | - | - | 0 | - |
| - | - | 5882 | 731.4 | - | - | 0 | - |
| - | - | 4072 | 732.4 | - | - | 0 | - |
| - | - | 6.007E+04 | 735.3 | - | - | 0 | - |
| - | - | 2.72E+04 | 736.3 | - | - | 0 | - |
| - | - | 1.128E+04 | 737.3 | - | - | 0 | - |
| - | - | 7591 | 738.4 | - | - | 0 | - |
| - | - | 1.019E+04 | 739.4 | - | - | 0 | - |
| - | - | 5183 | 740.4 | - | - | 0 | - |
| - | - | 4677 | 749.3 | - | - | 0 | - |
| - | - | 3.085E+04 | 752.3 | - | - | 0 | - |
| - | - | 1.561E+04 | 753.3 | - | - | 0 | - |
| - | - | 3579 | 754.3 | - | - | 0 | - |
| - | - | 7135 | 756.4 | - | - | 0 | - |
| - | - | 3914 | 777.5 | - | - | 0 | - |
| - | - | 6075 | 793.5 | - | - | 0 | - |
| - | - | 4232 | 794.3 | - | - | 0 | - |
| - | - | 5213 | 801.5 | - | - | 0 | - |
| - | - | 2.063E+04 | 805.5 | - | - | 0 | - |
| - | - | 9636 | 806.5 | - | - | 0 | - |
| - | - | 3711 | 807.5 | - | - | 0 | - |
| - | - | 1.445E+04 | 811.4 | - | - | 0 | - |
| - | - | 1.69E+04 | 811.5 | - | - | 0 | - |
| - | - | 7730 | 812.4 | - | - | 0 | - |
| - | - | 7266 | 812.5 | - | - | 0 | - |
| 7 | y | 2.274E+05 | 823.5 | 0.0007711 | 0.9364 | +1 | 8 |
| - | - | 1.133E+05 | 824.5 | - | - | 0 | - |
| - | - | 2.926E+04 | 825.5 | - | - | 0 | - |
| - | - | 4301 | 826.5 | - | - | 0 | - |
| - | - | 1.441E+04 | 829.5 | - | - | 0 | - |
| - | - | 5224 | 830.5 | - | - | 0 | - |
| 6 | b | 3.517E+04 | 839.4 | 5.085E-05 | 0.06058 | +1 | 6 |
| - | - | 2.015E+04 | 840.4 | - | - | 0 | - |
| - | - | 7565 | 847.5 | - | - | 0 | - |
| - | - | 2.714E+04 | 848.4 | - | - | 0 | - |
| - | - | 1.256E+04 | 849.4 | - | - | 0 | - |
| - | - | 4884 | 850.4 | - | - | 0 | - |
| - | - | 5065 | 852.5 | - | - | 0 | - |
| - | - | 1.013E+04 | 853.5 | - | - | 0 | - |
| - | - | 8111 | 854.5 | - | - | 0 | - |
| - | - | 4312 | 866.4 | - | - | 0 | - |
| - | - | 5748 | 884.4 | - | - | 0 | - |
| - | - | 6342 | 891.5 | - | - | 0 | - |
| - | - | 4.381E+04 | 901.4 | - | - | 0 | - |
| - | - | 2.429E+04 | 902.4 | - | - | 0 | - |
| - | - | 4254 | 903.4 | - | - | 0 | - |
| - | - | 2.743E+04 | 919.5 | - | - | 0 | - |
| - | - | 9342 | 920.5 | - | - | 0 | - |
| - | - | 4675 | 921.5 | - | - | 0 | - |
| - | - | 1.125E+04 | 942.6 | - | - | 0 | - |
| 6 | y | 2.776E+04 | 960.6 | 0.0006361 | 0.6622 | +1 | 9 |
| - | - | 1.246E+04 | 961.6 | - | - | 0 | - |
| - | - | 3797 | 962.6 | - | - | 0 | - |
| - | - | 3968 | 964.5 | - | - | 0 | - |
| - | - | 1.054E+04 | 966.6 | - | - | 0 | - |
| - | - | 5170 | 967.6 | - | - | 0 | - |
| - | - | 5734 | 984.6 | - | - | 0 | - |
| - | - | 6840 | 1033 | - | - | 0 | - |
| - | - | 7009 | 1034 | - | - | 0 | - |
| - | - | 5277 | 1070 | - | - | 0 | - |
| - | - | 5556 | 1088 | - | - | 0 | - |

m/z Charge Intensity FragmentType MassShift Position
120.05255889892578 0 4440.105
120.05657196044922 0 5188.892
121.04007720947266 0 3721.8784
122.06037139892578 0 2396.8523
122.07203674316406 0 5605.316
123.05579376220703 0 18939.494
125.07168579101562 0 2410.2708
125.10835266113281 0 2300.2324
127.08711242675781 0 24292.947
127.12356567382812 0 1910.3345
128.0712127685547 0 2802.2808
128.08164978027344 0 1893.8969
129.03939819335938 0 2000.1936
129.0663299560547 0 28075.469
129.10281372070312 0 862213
130.065673828125 0 206395.05
130.08709716796875 0 3302.1257
130.09996032714844 0 7461.519
130.10610961914062 0 52964.54
131.04586791992188 0 2534.0432
131.0690155029297 0 21572.516
131.07369995117188 0 2690.8335
132.08135986328125 0 828188.5
132.10244750976562 0 219600.36 y 13
133.08514404296875 0 85720.766
133.10586547851562 0 15313.748
134.07179260253906 0 3898.5837
134.08824157714844 0 5178.491
136.07627868652344 0 1093121
137.07366943359375 0 4826.036
137.07958984375 0 95431.36
138.06658935546875 0 12780.958
138.08285522460938 0 2717.609
139.0870819091797 0 13549.642
141.09707641601562 0 4232.871
141.102783203125 0 45693.996
142.06564331054688 0 19511.725 b 3
144.08135986328125 0 38034.28
145.08480834960938 0 3634.0938
146.06063842773438 0 5377.473
146.07131958007812 0 2574.558
147.0446014404297 0 45686.53
147.11349487304688 0 2981.154
148.04782104492188 0 6079.395
148.07659912109375 0 2946.8896
149.08299255371094 0 3756.2507
150.06634521484375 0 2818.1309
151.08717346191406 0 10612.329
152.08248901367188 0 4425.1934
152.1439971923828 0 20363.16
153.0771026611328 0 2266.4004
153.10313415527344 0 3323.6282
154.09786987304688 0 6896.8164
155.08206176757812 0 47096.707
155.0933380126953 0 14684.081
155.1184539794922 0 27928.207
156.07627868652344 0 8333.88
156.08143615722656 0 25525.244
156.1217498779297 0 4707.871
157.0764617919922 0 6540.5347
157.08450317382812 0 3482.823
157.09774780273438 0 3014.0864
158.06053161621094 0 11851.107
158.0844268798828 0 97903.06
159.09225463867188 0 572843.9
160.0762939453125 0 23492.336
160.08848571777344 0 4093.8218
160.09564208984375 0 54314.934
161.08033752441406 0 2707.7654
162.10342407226562 0 2530.161
162.5108184814453 0 2539.87
163.61334228515625 0 4688.476
164.03489685058594 0 3008.265
164.0711212158203 0 17384.25
164.0824432373047 0 2205.5452
165.0776824951172 0 5006.3594
165.102783203125 0 33673.844
166.0616455078125 0 10258.404
166.10623168945312 0 3385.59
166.1678009033203 0 2504.9675
168.08143615722656 0 2679.776
168.11375427246094 0 7617.8564
169.07659912109375 0 60998.945
169.0977020263672 0 58086.297
169.1702880859375 0 3993.6602
170.06063842773438 0 255088
170.10137939453125 0 2924.7117
171.06407165527344 0 33183.375
171.09213256835938 0 11166.307
172.10858154296875 0 55564.953
172.14476013183594 0 3470.3748
173.43984985351562 0 9142.107
174.05612182617188 0 2715.628
175.09817504882812 0 10328.166
176.0824737548828 0 23406.066
177.11293029785156 0 13739.846
179.0824432373047 0 3909.775
179.09307861328125 0 4345.729
179.5849609375 0 2375.0298
180.07774353027344 0 4787.5776
181.0614013671875 0 12421.763
181.0977783203125 0 273610.97
181.1340789794922 0 15005.298
181.17034912109375 0 3400.43
182.06101989746094 0 4727.4604
182.0939178466797 0 2527.234
182.10125732421875 0 22271.477
182.12936401367188 0 34169.582
183.09230041503906 0 123221.734
183.11294555664062 0 4433.679
183.13279724121094 0 4646.417
184.0762939453125 0 14064.519
184.0955810546875 0 15099.1455
185.07151794433594 0 27874.586
185.0803680419922 0 2971.0156
185.10792541503906 0 5858.3174
186.07955932617188 0 6164.673
186.12432861328125 0 100377.68
187.0872344970703 0 579627.4
187.1275634765625 0 8508.955
188.09060668945312 0 71662.586
189.0933380126953 0 2485.747
190.61912536621094 0 4018.642
191.0821990966797 0 2917.5024
191.09335327148438 0 2629.7876
191.11949157714844 0 2312.5537
193.09854125976562 0 3077.1316
195.0886688232422 0 2294.8584
195.11314392089844 0 4935.527
195.14964294433594 0 9611.705
196.10873413085938 0 6865.6104
197.16549682617188 0 41080.625
198.10316467285156 0 3837.8457
198.16868591308594 0 6408.219
199.08714294433594 0 26649.037
199.10850524902344 0 4316.7495
199.18106079101562 0 19988.113
200.09088134765625 0 2823.862
200.14007568359375 0 14316.014
201.0928192138672 0 4220.713
201.10284423828125 0 61686.55
201.1239776611328 0 3866.6611
201.1430206298828 0 3467.795
202.0868377685547 0 27773.57
202.10911560058594 0 92364.6
203.09219360351562 0 6696.2056
203.11289978027344 0 6839.428
204.07728576660156 0 11197.305
205.09791564941406 0 4735.6567 a 2
205.10850524902344 0 11398.019
207.1498565673828 0 29852.314
208.1443634033203 0 4522.551
208.1543426513672 0 2749.7068
209.12918090820312 0 7569.264
210.12396240234375 0 4037.3267
211.0874786376953 0 14784.265
212.09153747558594 0 3609.0688
212.14022827148438 0 13084.619
212.668212890625 0 18733.338
213.0776824951172 0 4525.6055
213.1019744873047 0 2341.0552
213.1703643798828 0 9780.446
214.10926818847656 0 31128.725
214.11973571777344 0 3349.1353
214.1918487548828 0 7121.113
215.1119842529297 0 2686.3828
215.14004516601562 0 3561.023
216.11361694335938 0 8133.926
217.10873413085938 0 7691.1523
219.08937072753906 0 2884.9314
220.1199951171875 0 71016.99
221.10400390625 0 89493
221.1238250732422 0 8169.514
221.1380157470703 0 3619.286
222.10670471191406 0 8080.6235
222.12435913085938 0 6836.6006
223.18101501464844 0 2495.5186
224.11866760253906 0 13902.805
224.13992309570312 0 4770.843
224.1763916015625 0 40400.355
225.12258911132812 0 2902.9238
225.16064453125 0 15092.523
225.17994689941406 0 4688.5376
226.15574645996094 0 770983.75
227.0823211669922 0 2751.692
227.140380859375 0 4198.188
227.15896606445312 0 84159.586 y Ammonia loss 10
228.11386108398438 0 50106.715 a Water loss 1
228.1348114013672 0 8198.953
228.1597137451172 0 6141.459
229.09780883789062 0 69099.97 b Ammonia loss 4
229.11862182617188 0 11175.603
229.16656494140625 0 9658.609
230.0819854736328 0 5932.8154
230.1033477783203 0 22608.598
231.0883331298828 0 28057.533
231.13572692871094 0 11722.238
232.09153747558594 0 3870.0676
232.12008666992188 0 6262.8833
232.1431121826172 0 4712.447
232.64352416992188 0 2354.8977
233.10414123535156 0 18776.686
234.10284423828125 0 2793.511
234.13592529296875 0 3006.2407
235.1196746826172 0 142706.69
235.67115783691406 0 114353.17 y 10
236.12278747558594 0 11563.506
236.1393280029297 0 4559.0664
236.17276000976562 0 33181.617
237.1356964111328 0 4821.357
238.13027954101562 0 8263.258
239.0819091796875 0 6549.671
239.1509246826172 0 55534.24
240.13487243652344 0 6265.2656
240.15420532226562 0 7394.7476
241.15524291992188 0 8523.023
242.18714904785156 0 26058.473
243.16983032226562 0 4225.77
243.18243408203125 0 7364.7373
244.10806274414062 0 2792.4634
244.16592407226562 0 6273.807
245.17535400390625 0 2665.9941
245.186767578125 0 180724.48 y 12
246.12451171875 0 1410228.8 a 1
246.1902313232422 0 20712.162
247.12930297851562 0 324000.7
248.11477661132812 0 105523.27
248.1324920654297 0 24993.758
249.09893798828125 0 147234.44
249.11444091796875 0 4874.9717 b 7
249.12046813964844 0 4925.0864
249.13389587402344 0 5110.611
250.1019744873047 0 15960.9375
250.1232147216797 0 20461.943
251.12954711914062 0 7843.904
252.17129516601562 0 15870.509
253.09815979003906 0 5061.636
253.17648315429688 0 2516.6675
254.15078735351562 0 2798.9358
254.18679809570312 0 6013.5757
255.07742309570312 0 2924.8489
256.1086730957031 0 32856.445 b Water loss 1
257.0918884277344 0 6093.5913
257.1150207519531 0 17951.914
257.16162109375 0 20441.707
258.07623291015625 0 25871.865
258.0992736816406 0 257052.88
259.08111572265625 0 4181.862
259.1025695800781 0 35808.46
259.13043212890625 0 9849.7295
259.849365234375 0 2917.463
260.1075134277344 0 32615.264
260.1341247558594 0 3084.4575
260.1975402832031 0 16501.49
261.1119079589844 0 3435.3523
261.200927734375 0 3472.0457
262.15582275390625 0 4013.2866
263.1153869628906 0 7257.803
264.1212158203125 0 3172.04
264.1348876953125 0 16773.215
265.1408996582031 0 13310.365
265.1666564941406 0 10396.433
266.0782775878906 0 2409.8665
266.1091003417969 0 5441.3247
266.1253967285156 0 85296.18
267.12847900390625 0 13435.312
267.1448059082031 0 5656.3677
269.6302490234375 0 16564.697
270.1302795410156 0 2844.4917
270.1818542480469 0 55003.08
271.1890563964844 0 37449.938 y 9
271.6905212402344 0 8850.049
272.18994140625 0 3319.6807
273.13433837890625 0 4299.128
274.1192932128906 0 512369.4 b 1
275.1036071777344 0 70633.16
275.1253356933594 0 430219.7
275.1854553222656 0 16960.7 y 6
275.5197448730469 0 8046.406
275.85400390625 0 4267.207
276.1081848144531 0 19955.357
276.12841796875 0 54572.164
277.0943908691406 0 3582.991
277.1339111328125 0 23466.424
278.1371765136719 0 3756.5156
279.15203857421875 0 5424.627
279.1825256347656 0 12944.416
280.1658630371094 0 3022.4863
282.1833801269531 0 4512.1294
283.15130615234375 0 16981.799
283.1772766113281 0 196929.58
283.6282043457031 0 19268.572
284.1290588378906 0 5479.787
284.18060302734375 0 27481.27
285.1107177734375 0 12798.541
285.15655517578125 0 37748.89
286.115234375 0 2648.95
286.1599426269531 0 6198.2344
288.1075134277344 0 3505.8313
290.1132507324219 0 2990.376
290.1850891113281 0 58580.016
290.6864318847656 0 23492.008
291.1572265625 0 14314.326
291.18939208984375 0 3692.6992
292.1299133300781 0 129514.234
293.1330871582031 0 18819.719
293.1612548828125 0 3439.0264
294.12042236328125 0 38076.098
294.1367492675781 0 3883.3354
294.2182312011719 0 13276.04
295.1229553222656 0 5316.6626
297.1930236816406 0 100016.26
298.1967468261719 0 17909.078
300.13232421875 0 6031.6997
300.2030029296875 0 5880.4575
300.6723937988281 0 6317.667
303.12109375 0 29220.898
303.21923828125 0 2973.7002
304.12420654296875 0 3028.906
305.12933349609375 0 56520.23
305.6648254394531 0 17092.877
306.1328125 0 17857.9
306.1657409667969 0 3071.9514
307.176025390625 0 3532.3137
309.1676940917969 0 4523.224
309.1919250488281 0 4228.763
311.171630859375 0 3969.823
311.24517822265625 0 22045.979
312.2494201660156 0 5014.7563
313.52734375 0 3267.045
314.2192077636719 0 4166.141
314.67034912109375 0 24572.174
315.1703796386719 0 5386.4316
316.8572082519531 0 2919.7925
318.1925354003906 0 7801.6577
319.17596435546875 0 3549.756
320.12469482421875 0 7209.495
320.8719482421875 0 16103.373 y 5
321.2066650390625 0 13310.516
321.2294006347656 0 118319.95
322.1558837890625 0 74192.555
322.17535400390625 0 4696.2686
322.212890625 0 4941.9375
322.23284912109375 0 23953.889
323.12628173828125 0 3912.5696
323.15924072265625 0 11671.737 b 10
324.2164306640625 0 14502.749
324.71832275390625 0 5088.904
325.18804931640625 0 3892.5657
325.2240905761719 0 4022.2183
326.2197265625 0 40745.16
327.2012023925781 0 7880.245
327.221923828125 0 4406.514
327.27593994140625 0 7069.6694
327.6775207519531 0 3261.0642
330.1087951660156 0 5556.3315
330.176513671875 0 4135.281
332.1214294433594 0 2781.1533
332.6553955078125 0 3268.3494
332.7298583984375 0 43393.97
333.12457275390625 0 16370.226
333.2309265136719 0 14757.087
334.1272888183594 0 4139.2437
334.1756591796875 0 6627.5894
334.6671142578125 0 6825.7764
335.2370300292969 0 8444.782 y 8
335.73876953125 0 5458.3774
336.2039794921875 0 163822.45
337.18695068359375 0 3232.219
337.207275390625 0 33136.773
337.7220153808594 0 60838.113
338.16180419921875 0 3937.6018
338.22308349609375 0 26106.174
338.7249755859375 0 3036.7266
339.1673889160156 0 5248.5093
339.2400207519531 0 172560.69
339.5065612792969 0 3385.8406
340.15283203125 0 22108.137
340.22186279296875 0 3807.3975
340.2433776855469 0 30917.367
341.154052734375 0 5545.188
341.2191162109375 0 6056.5947
341.2445373535156 0 3318.5947
342.1684875488281 0 9490.312
345.64337158203125 0 11735.662
346.1469421386719 0 5086.9614
346.6692810058594 0 5401.735
346.72723388671875 0 147639.27
347.17047119140625 0 4785.1836
347.2287292480469 0 53568.438
347.72979736328125 0 13251.159
349.70916748046875 0 9453.849
350.1510314941406 0 24480.812
350.2098388671875 0 7775.87
350.541015625 0 4504.5103
351.15472412109375 0 4503.5303
351.2403869628906 0 12103.482
352.2418518066406 0 4253.209
353.21856689453125 0 7129.588
353.867431640625 0 4298.6606
354.1570739746094 0 15462.869
354.1757507324219 0 9092.839
354.21441650390625 0 245384.67
354.51043701171875 0 8421.296
354.65771484375 0 7113.9
355.2173767089844 0 40075.05
355.2706604003906 0 4784.1187
355.73248291015625 0 22199.268
356.17041015625 0 6091.9253
356.21875 0 3587.7603
357.17803955078125 0 8331.4795
357.25048828125 0 50468.473
357.50408935546875 0 5691.6704
357.832763671875 0 3618.7498
358.1631164550781 0 54054.15
358.25341796875 0 11038.81
358.7143249511719 0 17078.178
359.1660461425781 0 7875.2803
359.19195556640625 0 5190.761
359.2164611816406 0 7365.4927
359.5429382324219 0 11876.029
359.87249755859375 0 15247.13
360.20672607421875 0 7303.9336
360.66497802734375 0 14614.217
360.68865966796875 0 12448.303
361.1828308105469 0 8981.247
361.6836853027344 0 8165.1914
362.15008544921875 0 6205.4053
362.6702575683594 0 4861.272
363.19720458984375 0 6774.4854
363.5076904296875 0 12287.455
363.7466125488281 0 6156.4014 y 7
364.1766052246094 0 4647.6846
364.249755859375 0 5589.5493
365.5450439453125 0 4159.819
366.1572265625 0 29105.465
366.558349609375 0 21227.533 y 4
366.8925476074219 0 20328.498
367.16265869140625 0 19743.58
367.22857666015625 0 4778.777
368.1490478515625 0 28999.441
368.17193603515625 0 7011.416
368.6539611816406 0 4208.4033
368.8450927734375 0 3341.693 b Ammonia loss 8
369.15216064453125 0 3120.8176
369.1790771484375 0 6705.216
369.6960754394531 0 3523.667
370.1872253417969 0 10051.203
370.5270080566406 0 3655.409
370.6858215332031 0 3081.5251
371.228515625 0 34854.152
372.23187255859375 0 3521.4333
373.2818298339844 0 91970.016 y 11
374.2574768066406 0 6149.5405
374.2848815917969 0 12557.728
374.51519775390625 0 10763.262 b 8
375.18994140625 0 6171.514
376.45556640625 0 5634.3403
376.6676330566406 0 9322.1875
376.8702087402344 0 3110.0286
377.1687316894531 0 4002.045
378.7119445800781 0 10077.33
378.9624938964844 0 11733.722
379.212646484375 0 7902.3643
380.2301025390625 0 13839.593
382.2065734863281 0 34362.383
382.2825927734375 0 11046.868
382.5152893066406 0 5367.5684
382.5400390625 0 18404.611
382.87542724609375 0 4305.52
383.1852722167969 0 3470.1663
383.2140808105469 0 4387.864
383.26629638671875 0 8709.754
383.7059020996094 0 3455.4243
384.1676330566406 0 26256.102
385.1737060546875 0 97171.09
385.19976806640625 0 20770.477
386.15802001953125 0 95872.66
386.2012023925781 0 4299.7026
386.5249938964844 0 7457.773
387.1605224609375 0 13552.55
388.2371826171875 0 4196.1753
388.8625183105469 0 23135.832
389.196533203125 0 14772.616
389.2720642089844 0 22331.275
389.5328063964844 0 3765.9727
389.7737121582031 0 9065.46
390.21453857421875 0 57119.617 y Ammonia loss 1
390.4651794433594 0 54696.684
390.7165222167969 0 18526.305
390.967529296875 0 10349.713
392.1642761230469 0 3635.246
392.1905212402344 0 15680.695 b Water loss 9
392.266357421875 0 36641.63
392.52142333984375 0 12035.754 b Ammonia loss 9
392.8558654785156 0 8311.036
393.16558837890625 0 5440.859
393.26959228515625 0 7178.2593
394.15191650390625 0 58524.223
394.28302001953125 0 4066.3716
394.47137451171875 0 37804.883 y 1
394.7217102050781 0 23299.297
394.9726257324219 0 13286.892
395.1544189453125 0 13706.209
395.2235412597656 0 3997.5315
396.2616882324219 0 7064.6787
397.18218994140625 0 3704.713
397.24554443359375 0 7748.1704
397.5686950683594 0 6927.1777
397.7472229003906 0 7028.697
397.8948669433594 0 4407.762
398.193603515625 0 8936.874 b 9
398.2413635253906 0 5545.8765
398.52825927734375 0 14685.532
398.8631286621094 0 6930.0493
398.891357421875 0 3761.49
399.223876953125 0 31376.76
400.2264404296875 0 9208.636
400.4761657714844 0 4218.666
401.19488525390625 0 7931.041
401.2597351074219 0 9516.727
402.2250061035156 0 35869.99
402.55230712890625 0 4973.518
403.1846923828125 0 87480.305
403.240234375 0 41202.85
403.2703857421875 0 15914.011
403.57183837890625 0 75215.14 y Ammonia loss 3
403.7691955566406 0 6235.388
403.905029296875 0 25189.336
404.18701171875 0 17577.213
404.23779296875 0 13392.063
404.26702880859375 0 5089.3833
404.4713134765625 0 10157.092
404.7257385253906 0 28249.824
404.8947448730469 0 10392.89
404.9774169921875 0 19696.688
405.19146728515625 0 2706.563
405.22833251953125 0 16574.44
405.4809265136719 0 3073.207
406.1861267089844 0 39929.023
406.2508850097656 0 35048.97
406.6880798339844 0 13294.385
406.75213623046875 0 10564.352
407.1931457519531 0 8975.621
407.69281005859375 0 5474.7886
408.2225036621094 0 22801.445
408.5553283691406 0 14768.808
408.8872375488281 0 8646.693
409.2449035644531 0 20526.777 y 3
409.5788879394531 0 11723.912
409.9130859375 0 5256.1797
410.27728271484375 0 82454.78
410.5695495605469 0 43760.395
410.90362548828125 0 32188.523
411.1786193847656 0 91086.016 b Water loss 5
411.2384948730469 0 11913.83
411.2797546386719 0 17204.965
411.57330322265625 0 3705.109
411.7266540527344 0 17961.852 Precursor Water loss
411.9756164550781 0 20996.92 Precursor Ammonia loss
412.1612548828125 0 18865.271
412.1841735839844 0 14507.9375
412.2245178222656 0 12275.955
412.27484130859375 0 301598.4 y 6
412.47674560546875 0 3986.0498
412.7760314941406 0 140723
413.1665954589844 0 10474.657
413.27740478515625 0 40783.06
413.77886962890625 0 7430.0625
[truncated: 730,316 more chars]
