# Supplementary material for: A Handle on Mass Coincidence Errors in De Novo Sequencing of Antibodies by Bottom-up Proteomics
Source: J Proteome Res. 2024 Jun 27;23(8):3552–9. doi: 10.1021/acs.jproteome.4c00188 (PMC11301774; doi:10.1021/acs.jproteome.4c00188)
Supplement: Supplementary file 1 — pr4c00188_si_001.zip [file pr4c00188_si_001.zip › supplementary data/xln-disambiguation/2023-12-13@14-36-36 f59/report/reads/Combined_027.html]

Details Combined\_027 | Stitch OverviewUndefined

# Read Combined\_027

## Sequence (length=13)

TVLHQDWLDGKEY

## Spectrum 7243? Spectrum 7243 The raw spectrum of this peptide as annotated by Hecklib. The fragments are coloured according to ion type (see legend). Any peaks with a star '\*' as text can be hovered over to see the full details, first the ion type second the mass shift type. By hovering over the amino acids in the peptide or ions in the legend the corresponding peaks are highlighted. By toggling the 'Unassigned' label you can turn the background (unassigned) peaks on or off in the plot. By updating the slider in the Ion legend you can update the spectrum to only show the top X% of the peaks with labels. The top X% means any peak that is within X% of the highest intensity. By dragging in the spectrum you can zoom in to a specific part of the spectrum and use 'Zoom Out' to get back to the original zoom level. The annotation of the spectrum is based on the given sequence in the peptides file and is done with different software so inconsistencies are likely. The peaks are annotated based on the given sequence, with 20 ppm tolerance.

Copy Data

### Spectrum 7243 (TSV)

#### Preview

```
Loading example...
```

*Click on the button to copy the data to your clipboard.*

Mz MinMz MaxIntensity Max

WidthHeightPeptide font sizePeptide stroke widthSpectrum font sizeSpectrum stroke widthCompact peptide

Ion legend

wxyz

abcd

OtherUnassignedIonChargePositionShow for top:%

TVLHQDWLDGKEY

04.57e+59.15e+51.37e+61.83e+6

Zoom Out

y+12y+12y+13c+27c+14y+14c+15c+15c+15y+15y+210c+211y+211c+16y+211c+16c+212y+16y+212c+17y+17z+17c+17y+17c+18c+18z+18c+18y+18w+19c+19c+19c+19y+19z+19y+19c+110c+110z+110y+110c+111c+111w+111z+111y+111c+112z+112

0778155623343112

Fragment Matches Table

Show background peaks

| Position | Ion type | Intensity | mz Theoretical | mz Error (Th) | mz Error (ppm) | Charge | Series Number |
| --- | --- | --- | --- | --- | --- | --- | --- |
| - | - | 1618 | 122.9 | - | - | 0 | - |
| - | - | 1681 | 126.5 | - | - | 0 | - |
| - | - | 1537 | 127.7 | - | - | 0 | - |
| - | - | 2.987E+04 | 129.1 | - | - | 0 | - |
| - | - | 2464 | 130.1 | - | - | 0 | - |
| - | - | 1800 | 139.3 | - | - | 0 | - |
| - | - | 2134 | 145.6 | - | - | 0 | - |
| - | - | 2206 | 148.9 | - | - | 0 | - |
| - | - | 2565 | 148.9 | - | - | 0 | - |
| - | - | 1796 | 148.9 | - | - | 0 | - |
| - | - | 2697 | 148.9 | - | - | 0 | - |
| - | - | 3279 | 148.9 | - | - | 0 | - |
| - | - | 3579 | 148.9 | - | - | 0 | - |
| - | - | 5176 | 148.9 | - | - | 0 | - |
| - | - | 5307 | 148.9 | - | - | 0 | - |
| - | - | 1.392E+04 | 148.9 | - | - | 0 | - |
| - | - | 2.303E+04 | 148.9 | - | - | 0 | - |
| - | - | 1.712E+04 | 149 | - | - | 0 | - |
| - | - | 8608 | 149 | - | - | 0 | - |
| - | - | 5345 | 149 | - | - | 0 | - |
| - | - | 5102 | 149 | - | - | 0 | - |
| - | - | 4507 | 149 | - | - | 0 | - |
| - | - | 2217 | 149 | - | - | 0 | - |
| - | - | 2383 | 149 | - | - | 0 | - |
| - | - | 1969 | 149.1 | - | - | 0 | - |
| - | - | 1911 | 149.2 | - | - | 0 | - |
| - | - | 1848 | 150.9 | - | - | 0 | - |
| - | - | 2047 | 163.1 | - | - | 0 | - |
| - | - | 1.788E+04 | 173.1 | - | - | 0 | - |
| - | - | 2010 | 186.1 | - | - | 0 | - |
| - | - | 2.732E+04 | 186.1 | - | - | 0 | - |
| - | - | 2130 | 191.4 | - | - | 0 | - |
| - | - | 2497 | 195.1 | - | - | 0 | - |
| - | - | 3.301E+04 | 201.1 | - | - | 0 | - |
| - | - | 2712 | 202.1 | - | - | 0 | - |
| - | - | 2319 | 247.3 | - | - | 0 | - |
| - | - | 4256 | 251.1 | - | - | 0 | - |
| - | - | 2044 | 263.3 | - | - | 0 | - |
| - | - | 2419 | 267.3 | - | - | 0 | - |
| - | - | 1.06E+04 | 283.1 | - | - | 0 | - |
| 12 | y | 3.146E+04 | 293.1 | 0.000283 | 0.9657 | +1 | 2 |
| - | - | 4965 | 294.1 | - | - | 0 | - |
| - | - | 2851 | 297.2 | - | - | 0 | - |
| - | - | 1.843E+04 | 301.2 | - | - | 0 | - |
| 12 | y | 1.047E+04 | 311.1 | 0.0003192 | 1.026 | +1 | 2 |
| - | - | 8473 | 314.2 | - | - | 0 | - |
| - | - | 6823 | 315.2 | - | - | 0 | - |
| - | - | 2760 | 376.9 | - | - | 0 | - |
| - | - | 2715 | 383.9 | - | - | 0 | - |
| - | - | 4220 | 396.2 | - | - | 0 | - |
| - | - | 2415 | 414.2 | - | - | 0 | - |
| - | - | 8067 | 423.3 | - | - | 0 | - |
| - | - | 2984 | 430.2 | - | - | 0 | - |
| 11 | y | 9236 | 439.2 | 0.0009218 | 2.099 | +1 | 3 |
| 7 | c | 2749 | 440.2 | 0.004014 | 9.118 | +2 | 7 |
| - | - | 3.326E+04 | 451.3 | - | - | 0 | - |
| - | - | 8422 | 452.3 | - | - | 0 | - |
| - | - | 2945 | 458.1 | - | - | 0 | - |
| 4 | c | 3275 | 468.3 | 0.0005964 | 1.274 | +1 | 4 |
| 10 | y | 6.557E+04 | 496.2 | 0.000535 | 1.078 | +1 | 4 |
| - | - | 1.439E+04 | 497.2 | - | - | 0 | - |
| - | - | 3641 | 534.3 | - | - | 0 | - |
| - | - | 3156 | 543.3 | - | - | 0 | - |
| - | - | 1.064E+04 | 552.3 | - | - | 0 | - |
| - | - | 6321 | 553.3 | - | - | 0 | - |
| - | - | 3068 | 555.3 | - | - | 0 | - |
| - | - | 8042 | 577.3 | - | - | 0 | - |
| 5 | c | 3018 | 578.3 | 0.007472 | 12.92 | +1 | 5 |
| 5 | c | 2.065E+04 | 579.3 | 0.0002731 | 0.4714 | +1 | 5 |
| - | - | 7619 | 580.3 | - | - | 0 | - |
| - | - | 1.46E+04 | 595.3 | - | - | 0 | - |
| 5 | c | 3.072E+04 | 596.4 | 0.0008243 | 1.382 | +1 | 5 |
| - | - | 8912 | 597.4 | - | - | 0 | - |
| - | - | 2932 | 600.3 | - | - | 0 | - |
| 9 | y | 3.59E+04 | 611.3 | 0.00047 | 0.7688 | +1 | 5 |
| - | - | 1.273E+04 | 611.8 | - | - | 0 | - |
| - | - | 9321 | 612.3 | - | - | 0 | - |
| - | - | 1.163E+04 | 638.3 | - | - | 0 | - |
| - | - | 9659 | 638.8 | - | - | 0 | - |
| 4 | y | 4.55E+04 | 645.8 | 0.0005208 | 0.8064 | +2 | 10 |
| - | - | 2.949E+04 | 646.3 | - | - | 0 | - |
| - | - | 1.233E+04 | 646.8 | - | - | 0 | - |
| 11 | c | 2.48E+04 | 647.3 | 0.0005467 | 0.8446 | +2 | 11 |
| - | - | 1.853E+04 | 647.8 | - | - | 0 | - |
| - | - | 8028 | 648.3 | - | - | 0 | - |
| - | - | 2.345E+04 | 667.4 | - | - | 0 | - |
| - | - | 2.325E+04 | 668.4 | - | - | 0 | - |
| - | - | 7833 | 669.4 | - | - | 0 | - |
| - | - | 2422 | 688.8 | - | - | 0 | - |
| 3 | y | 5513 | 693.8 | 0.01326 | 19.12 | +2 | 11 |
| 6 | c | 3.924E+04 | 694.4 | 0.001401 | 2.018 | +1 | 6 |
| - | - | 1.174E+04 | 695.4 | - | - | 0 | - |
| - | - | 1.082E+04 | 697.9 | - | - | 0 | - |
| - | - | 3571 | 698.9 | - | - | 0 | - |
| - | - | 3263 | 702.3 | - | - | 0 | - |
| 3 | y | 6.709E+04 | 702.3 | 0.0007437 | 1.059 | +2 | 11 |
| - | - | 6.482E+04 | 702.8 | - | - | 0 | - |
| - | - | 3.622E+04 | 703.3 | - | - | 0 | - |
| - | - | 7786 | 703.8 | - | - | 0 | - |
| - | - | 3131 | 704.3 | - | - | 0 | - |
| - | - | 7141 | 710.4 | - | - | 0 | - |
| 6 | c | 5.699E+04 | 711.4 | 0.001644 | 2.311 | +1 | 6 |
| 12 | c | 1.468E+05 | 711.9 | 0.0001148 | 0.1612 | +2 | 12 |
| - | - | 1.104E+05 | 712.4 | - | - | 0 | - |
| - | - | 4.759E+04 | 712.9 | - | - | 0 | - |
| - | - | 3934 | 713.4 | - | - | 0 | - |
| - | - | 5369 | 720.9 | - | - | 0 | - |
| - | - | 3250 | 721.4 | - | - | 0 | - |
| - | - | 2636 | 721.9 | - | - | 0 | - |
| 8 | y | 1.558E+04 | 724.4 | 0.0003665 | 0.5059 | +1 | 6 |
| - | - | 6240 | 725.4 | - | - | 0 | - |
| 2 | y | 4002 | 751.9 | 0.0002666 | 0.3546 | +2 | 12 |
| - | - | 5123 | 752.4 | - | - | 0 | - |
| - | - | 5173 | 779.4 | - | - | 0 | - |
| - | - | 5061 | 779.9 | - | - | 0 | - |
| - | - | 7121 | 780.4 | - | - | 0 | - |
| - | - | 4008 | 780.9 | - | - | 0 | - |
| - | - | 4142 | 781.4 | - | - | 0 | - |
| - | - | 3729 | 784.9 | - | - | 0 | - |
| - | - | 5.025E+04 | 793.4 | - | - | 0 | - |
| - | - | 5.666E+04 | 793.9 | - | - | 0 | - |
| - | - | 3.306E+04 | 794.4 | - | - | 0 | - |
| - | - | 5611 | 794.9 | - | - | 0 | - |
| - | - | 2.204E+04 | 801.9 | - | - | 0 | - |
| - | - | 1.076E+06 | 802.4 | - | - | 0 | - |
| - | - | 9.753E+05 | 802.9 | - | - | 0 | - |
| - | - | 4.227E+05 | 803.4 | - | - | 0 | - |
| - | - | 5.523E+04 | 803.9 | - | - | 0 | - |
| - | - | 2680 | 852.4 | - | - | 0 | - |
| - | - | 2.275E+04 | 853.4 | - | - | 0 | - |
| - | - | 2.714E+04 | 854.4 | - | - | 0 | - |
| - | - | 9311 | 855.5 | - | - | 0 | - |
| - | - | 3279 | 878.4 | - | - | 0 | - |
| 7 | c | 3.268E+04 | 880.4 | 0.001002 | 1.139 | +1 | 7 |
| - | - | 1.441E+04 | 881.4 | - | - | 0 | - |
| - | - | 2995 | 882.4 | - | - | 0 | - |
| 7 | y | 2915 | 893.4 | 0.01206 | 13.49 | +1 | 7 |
| 7 | z | 1.96E+04 | 894.4 | 0.0004085 | 0.4567 | +1 | 7 |
| - | - | 1.28E+04 | 895.4 | - | - | 0 | - |
| - | - | 2.311E+04 | 896.4 | - | - | 0 | - |
| 7 | c | 9.562E+04 | 897.5 | 0.0008181 | 0.9116 | +1 | 7 |
| - | - | 4.789E+04 | 898.5 | - | - | 0 | - |
| - | - | 7918 | 899.5 | - | - | 0 | - |
| - | - | 5192 | 908.4 | - | - | 0 | - |
| - | - | 5449 | 909.4 | - | - | 0 | - |
| 7 | y | 4.586E+04 | 910.4 | 0.0006999 | 0.7688 | +1 | 7 |
| - | - | 1.821E+04 | 911.4 | - | - | 0 | - |
| - | - | 3993 | 912.4 | - | - | 0 | - |
| - | - | 1.12E+04 | 965.4 | - | - | 0 | - |
| - | - | 5334 | 966.4 | - | - | 0 | - |
| - | - | 1.312E+04 | 966.5 | - | - | 0 | - |
| - | - | 1.634E+04 | 967.5 | - | - | 0 | - |
| - | - | 5916 | 968.5 | - | - | 0 | - |
| - | - | 4337 | 975.5 | - | - | 0 | - |
| - | - | 3097 | 980.5 | - | - | 0 | - |
| - | - | 3.606E+04 | 991.5 | - | - | 0 | - |
| 8 | c | 1.607E+04 | 992.5 | 0.005592 | 5.634 | +1 | 8 |
| 8 | c | 6.239E+04 | 993.5 | 0.0009322 | 0.9382 | +1 | 8 |
| - | - | 3.177E+04 | 994.5 | - | - | 0 | - |
| - | - | 8220 | 995.5 | - | - | 0 | - |
| 6 | z | 3.054E+04 | 1009 | 0.002754 | 2.729 | +1 | 8 |
| - | - | 7.786E+04 | 1010 | - | - | 0 | - |
| - | - | 3.337E+04 | 1010 | - | - | 0 | - |
| 8 | c | 1.889E+05 | 1011 | 0.001081 | 1.07 | +1 | 8 |
| - | - | 1.268E+04 | 1011 | - | - | 0 | - |
| - | - | 9.705E+04 | 1012 | - | - | 0 | - |
| - | - | 2.362E+04 | 1013 | - | - | 0 | - |
| - | - | 6363 | 1024 | - | - | 0 | - |
| 6 | y | 4.009E+04 | 1025 | 0.0001891 | 0.1844 | +1 | 8 |
| - | - | 1.975E+04 | 1026 | - | - | 0 | - |
| - | - | 6071 | 1027 | - | - | 0 | - |
| - | - | 6896 | 1064 | - | - | 0 | - |
| - | - | 4056 | 1065 | - | - | 0 | - |
| - | - | 4357 | 1068 | - | - | 0 | - |
| - | - | 3926 | 1069 | - | - | 0 | - |
| 5 | w | 3516 | 1079 | 0.005371 | 4.975 | +1 | 9 |
| - | - | 4.553E+04 | 1082 | - | - | 0 | - |
| - | - | 4.798E+04 | 1083 | - | - | 0 | - |
| - | - | 2.009E+04 | 1084 | - | - | 0 | - |
| - | - | 4894 | 1085 | - | - | 0 | - |
| - | - | 6602 | 1094 | - | - | 0 | - |
| 9 | c | 7007 | 1108 | 0.003848 | 3.475 | +1 | 9 |
| 9 | c | 8.555E+04 | 1109 | 0.0004173 | 0.3765 | +1 | 9 |
| - | - | 5.374E+04 | 1110 | - | - | 0 | - |
| - | - | 1.371E+04 | 1111 | - | - | 0 | - |
| - | - | 3.417E+04 | 1125 | - | - | 0 | - |
| 9 | c | 4.007E+04 | 1126 | 0.003305 | 2.936 | +1 | 9 |
| - | - | 2.117E+04 | 1127 | - | - | 0 | - |
| - | - | 5999 | 1128 | - | - | 0 | - |
| 5 | y | 9394 | 1136 | 0.008932 | 7.859 | +1 | 9 |
| 5 | z | 2.399E+05 | 1137 | 0.000846 | 0.7437 | +1 | 9 |
| - | - | 1.789E+05 | 1139 | - | - | 0 | - |
| - | - | 6.104E+04 | 1140 | - | - | 0 | - |
| - | - | 8898 | 1141 | - | - | 0 | - |
| - | - | 5379 | 1152 | - | - | 0 | - |
| - | - | 3.527E+04 | 1153 | - | - | 0 | - |
| 5 | y | 1.19E+05 | 1154 | 0.001382 | 1.198 | +1 | 9 |
| - | - | 6.064E+04 | 1155 | - | - | 0 | - |
| - | - | 1.466E+04 | 1156 | - | - | 0 | - |
| - | - | 3151 | 1164 | - | - | 0 | - |
| 10 | c | 3.369E+04 | 1166 | 0.001659 | 1.423 | +1 | 10 |
| - | - | 2.123E+04 | 1167 | - | - | 0 | - |
| - | - | 7581 | 1168 | - | - | 0 | - |
| - | - | 4.694E+04 | 1182 | - | - | 0 | - |
| 10 | c | 3.559E+05 | 1183 | 0.0004763 | 0.4028 | +1 | 10 |
| - | - | 2.271E+05 | 1184 | - | - | 0 | - |
| - | - | 6.695E+04 | 1185 | - | - | 0 | - |
| - | - | 6953 | 1186 | - | - | 0 | - |
| - | - | 3814 | 1204 | - | - | 0 | - |
| - | - | 3720 | 1224 | - | - | 0 | - |
| - | - | 3484 | 1232 | - | - | 0 | - |
| - | - | 3304 | 1267 | - | - | 0 | - |
| - | - | 6166 | 1268 | - | - | 0 | - |
| 4 | z | 2.053E+05 | 1275 | 0.001774 | 1.392 | +1 | 10 |
| - | - | 1.432E+05 | 1276 | - | - | 0 | - |
| - | - | 4.923E+04 | 1277 | - | - | 0 | - |
| - | - | 6312 | 1278 | - | - | 0 | - |
| - | - | 9558 | 1290 | - | - | 0 | - |
| 4 | y | 6.255E+04 | 1291 | 0.002066 | 1.601 | +1 | 10 |
| - | - | 4.529E+04 | 1292 | - | - | 0 | - |
| - | - | 1.363E+04 | 1293 | - | - | 0 | - |
| 11 | c | 2.554E+04 | 1294 | 0.002484 | 1.92 | +1 | 11 |
| - | - | 1.616E+04 | 1295 | - | - | 0 | - |
| - | - | 4163 | 1296 | - | - | 0 | - |
| - | - | 1.946E+04 | 1310 | - | - | 0 | - |
| 11 | c | 1.505E+05 | 1311 | 0.001567 | 1.196 | +1 | 11 |
| - | - | 1.184E+05 | 1312 | - | - | 0 | - |
| - | - | 3.579E+04 | 1313 | - | - | 0 | - |
| - | - | 5744 | 1314 | - | - | 0 | - |
| - | - | 1.628E+04 | 1337 | - | - | 0 | - |
| - | - | 9329 | 1338 | - | - | 0 | - |
| - | - | 4826 | 1344 | - | - | 0 | - |
| 3 | w | 1.688E+04 | 1345 | 0.008854 | 6.585 | +1 | 11 |
| - | - | 1.507E+04 | 1346 | - | - | 0 | - |
| - | - | 4086 | 1347 | - | - | 0 | - |
| - | - | 3239 | 1352 | - | - | 0 | - |
| 3 | z | 1.081E+05 | 1388 | 0.001122 | 0.8083 | +1 | 11 |
| - | - | 9.977E+04 | 1389 | - | - | 0 | - |
| - | - | 3.624E+04 | 1390 | - | - | 0 | - |
| - | - | 4986 | 1391 | - | - | 0 | - |
| - | - | 3.214E+04 | 1396 | - | - | 0 | - |
| - | - | 2.823E+04 | 1397 | - | - | 0 | - |
| - | - | 7946 | 1398 | - | - | 0 | - |
| 3 | y | 4.901E+04 | 1404 | 0.002268 | 1.615 | +1 | 11 |
| - | - | 4.676E+04 | 1405 | - | - | 0 | - |
| - | - | 1.839E+04 | 1406 | - | - | 0 | - |
| - | - | 4355 | 1425 | - | - | 0 | - |
| - | - | 4736 | 1426 | - | - | 0 | - |
| - | - | 4650 | 1431 | - | - | 0 | - |
| - | - | 1.465E+04 | 1439 | - | - | 0 | - |
| 12 | c | 5.181E+05 | 1440 | 0.00168 | 1.167 | +1 | 12 |
| - | - | 3.929E+05 | 1441 | - | - | 0 | - |
| - | - | 1.505E+05 | 1442 | - | - | 0 | - |
| - | - | 1.482E+04 | 1443 | - | - | 0 | - |
| - | - | 4210 | 1475 | - | - | 0 | - |
| 2 | z | 2.226E+04 | 1487 | 0.001909 | 1.284 | +1 | 12 |
| - | - | 2.152E+04 | 1488 | - | - | 0 | - |
| - | - | 9579 | 1489 | - | - | 0 | - |
| - | - | 3443 | 1506 | - | - | 0 | - |
| - | - | 6467 | 1516 | - | - | 0 | - |
| - | - | 1.645E+04 | 1517 | - | - | 0 | - |
| - | - | 1.444E+04 | 1518 | - | - | 0 | - |
| - | - | 7972 | 1519 | - | - | 0 | - |
| - | - | 4110 | 1527 | - | - | 0 | - |
| - | - | 6495 | 1528 | - | - | 0 | - |
| - | - | 5255 | 1529 | - | - | 0 | - |
| - | - | 5459 | 1532 | - | - | 0 | - |
| - | - | 9770 | 1533 | - | - | 0 | - |
| - | - | 1.288E+04 | 1534 | - | - | 0 | - |
| - | - | 8161 | 1535 | - | - | 0 | - |
| - | - | 6316 | 1541 | - | - | 0 | - |
| - | - | 1.261E+04 | 1542 | - | - | 0 | - |
| - | - | 4.301E+04 | 1543 | - | - | 0 | - |
| - | - | 5.089E+04 | 1544 | - | - | 0 | - |
| - | - | 1.159E+05 | 1545 | - | - | 0 | - |
| - | - | 9.233E+04 | 1546 | - | - | 0 | - |
| - | - | 3.674E+04 | 1547 | - | - | 0 | - |
| - | - | 4733 | 1548 | - | - | 0 | - |
| - | - | 1.073E+04 | 1549 | - | - | 0 | - |
| - | - | 1.178E+04 | 1550 | - | - | 0 | - |
| - | - | 8308 | 1551 | - | - | 0 | - |
| - | - | 5.658E+04 | 1559 | - | - | 0 | - |
| - | - | 1.267E+05 | 1560 | - | - | 0 | - |
| - | - | 1.147E+05 | 1561 | - | - | 0 | - |
| - | - | 6.387E+04 | 1562 | - | - | 0 | - |
| - | - | 2.173E+04 | 1563 | - | - | 0 | - |
| - | - | 1.221E+04 | 1569 | - | - | 0 | - |
| - | - | 1.694E+04 | 1570 | - | - | 0 | - |
| - | - | 9192 | 1571 | - | - | 0 | - |
| - | - | 3585 | 1572 | - | - | 0 | - |
| - | - | 3176 | 1576 | - | - | 0 | - |
| - | - | 1.76E+05 | 1577 | - | - | 0 | - |
| - | - | 1.544E+05 | 1578 | - | - | 0 | - |
| - | - | 5308 | 1578 | - | - | 0 | - |
| - | - | 7.211E+04 | 1579 | - | - | 0 | - |
| - | - | 9178 | 1580 | - | - | 0 | - |
| - | - | 1.653E+05 | 1587 | - | - | 0 | - |
| - | - | 4.813E+05 | 1588 | - | - | 0 | - |
| - | - | 3.906E+05 | 1589 | - | - | 0 | - |
| - | - | 1.505E+05 | 1590 | - | - | 0 | - |
| - | - | 1.958E+04 | 1591 | - | - | 0 | - |
| - | - | 2.126E+04 | 1603 | - | - | 0 | - |
| - | - | 7.578E+05 | 1604 | - | - | 0 | - |
| - | - | 1.812E+06 | 1605 | - | - | 0 | - |
| - | - | 1.349E+06 | 1606 | - | - | 0 | - |
| - | - | 5.245E+05 | 1607 | - | - | 0 | - |
| - | - | 6.429E+04 | 1608 | - | - | 0 | - |
| - | - | 3907 | 3081 | - | - | 0 | - |

m/z Charge Intensity FragmentType MassShift Position
122.93534851074219 0 1617.7184
126.45089721679688 0 1681.311
127.67321014404297 0 1536.764
129.10211181640625 0 29872.287
130.10585021972656 0 2464.1118
139.26576232910156 0 1799.7001
145.59568786621094 0 2133.5798
148.8660430908203 0 2206.0464
148.87306213378906 0 2564.812
148.88034057617188 0 1796.4637
148.90185546875 0 2696.5718
148.9088897705078 0 3279.0964
148.91624450683594 0 3579.427
148.92347717285156 0 5175.8213
148.93081665039062 0 5307.4507
148.9381866455078 0 13920.432
148.9458770751953 0 23030.695
148.9624786376953 0 17115.715
148.9701385498047 0 8608.251
148.97732543945312 0 5344.6816
148.98414611816406 0 5102.4897
148.99156188964844 0 4506.977
149.01332092285156 0 2217.163
149.0352325439453 0 2383.324
149.1077117919922 0 1969.4014
149.2371368408203 0 1911.3608
150.8721466064453 0 1848.2457
163.0591583251953 0 2046.7974
173.12828063964844 0 17880.564
186.11618041992188 0 2010.2964
186.12339782714844 0 27315.773
191.39801025390625 0 2129.6353
195.11293029785156 0 2497.4941
201.12301635742188 0 33008.285
202.1261444091797 0 2712.0269
247.31971740722656 0 2319.1045
251.14883422851562 0 4256.4927
263.28558349609375 0 2043.8104
267.26800537109375 0 2418.668
283.1395263671875 0 10603.049
293.1129150390625 0 31457.666 y Water loss 11
294.1164245605469 0 4965.1187
297.15631103515625 0 2850.9744
301.15008544921875 0 18434.91
311.1234436035156 0 10467.84 y 11
314.2078857421875 0 8472.839
315.16656494140625 0 6823.3677
376.89044189453125 0 2760.256
383.9182434082031 0 2715.4736
396.2227783203125 0 4220.0063
414.23284912109375 0 2415.3352
423.27130126953125 0 8067.324
430.1943054199219 0 2984.1055
439.2178039550781 0 9236.299 y 10
440.22320556640625 0 2748.8801 c Water loss 6
451.26593017578125 0 33262.715
452.2705993652344 0 8421.94
458.1185302734375 0 2944.615
468.29229736328125 0 3274.5566 c 3
496.2396545410156 0 65572.055 y 9
497.24334716796875 0 14393.621
534.3260498046875 0 3641.2852
543.2762451171875 0 3156.3645
552.3367309570312 0 10637.914
553.340576171875 0 6320.7656
555.252685546875 0 3067.933
577.3338012695312 0 8042.0503
578.3334350585938 0 3018.3625 c Water loss 4
579.3251953125 0 20650.053 c Ammonia loss 4
580.3265380859375 0 7619.489
595.34375 0 14603.237
596.3506469726562 0 30722.908 c 4
597.35400390625 0 8912.423
600.3136596679688 0 2931.7627
611.2666625976562 0 35904.105 y 8
611.7962036132812 0 12727.103
612.2709350585938 0 9320.837
638.3272705078125 0 11625.174
638.8283081054688 0 9659.245
645.7905883789062 0 45501.465 y 3
646.2916870117188 0 29493.467
646.793701171875 0 12334.028
647.3323974609375 0 24802.043 c Ammonia loss 10
647.8341064453125 0 18532.195
648.3353271484375 0 8027.6406
667.36376953125 0 23448.965
668.3707275390625 0 23245.977
669.3734741210938 0 7833.042
688.8466796875 0 2421.844
693.8331298828125 0 5513.3364 y Ammonia loss 2
694.3504638671875 0 39235.55 c Ammonia loss 5
695.3549194335938 0 11744.003
697.8555297851562 0 10817.7295
698.8596801757812 0 3570.5752
702.2626953125 0 3263.3386
702.3323974609375 0 67094.98 y 2
702.8374633789062 0 64815.465
703.3408813476562 0 36224.168
703.8478393554688 0 7785.534
704.3443603515625 0 3130.9092
710.3707275390625 0 7141.384
711.3767700195312 0 56993.637 c 5
711.8541259765625 0 146756.86 c Ammonia loss 11
712.3565063476562 0 110375.11
712.8565673828125 0 47593.117
713.35595703125 0 3934.2324
720.8593139648438 0 5369.2476
721.3650512695312 0 3249.5415
721.86474609375 0 2636.2166
724.350830078125 0 15575.596 y 7
725.353271484375 0 6240.376
751.8676147460938 0 4002.2542 y 1
752.3698120117188 0 5122.772
779.3860473632812 0 5173.303
779.8873901367188 0 5060.5713
780.3797607421875 0 7121.4595
780.8836059570312 0 4008.387
781.378662109375 0 4142.239
784.8828735351562 0 3729.268
793.3858642578125 0 50254.797
793.884521484375 0 56664.332
794.3853149414062 0 33058.023
794.8837280273438 0 5610.837
801.8981323242188 0 22038.965
802.3909301757812 0 1076178.8
802.892333984375 0 975332.9
803.3939819335938 0 422729.9
803.8943481445312 0 55230.555
852.4362182617188 0 2679.8735
853.4425048828125 0 22746.39
854.4491577148438 0 27137.617
855.4530029296875 0 9311.351
878.4386596679688 0 3278.9758
880.43017578125 0 32682.36 c Ammonia loss 6
881.4346313476562 0 14406.033
882.4376831054688 0 2994.9248
893.416015625 0 2915.2031 y Ammonia loss 6
894.411376953125 0 19599.123 z 6
895.4169311523438 0 12804.64
896.4454956054688 0 23106.645
897.4569091796875 0 95616.55 c 6
898.4607543945312 0 47890.914
899.4607543945312 0 7918.2563
908.4241333007812 0 5191.9893
909.4258422851562 0 5448.9053
910.4298095703125 0 45859.957 y 6
911.4327392578125 0 18213.209
912.4351196289062 0 3992.5046
965.447021484375 0 11195.561
966.4396362304688 0 5333.7754
966.5316772460938 0 13119.694
967.53271484375 0 16344.382
968.5369262695312 0 5915.7695
975.501220703125 0 4336.739
980.4652099609375 0 3096.783
991.5232543945312 0 36064.742
992.525634765625 0 16072.156 c Water loss 7
993.5161743164062 0 62386.188 c Ammonia loss 7
994.5180053710938 0 31770.102
995.5182495117188 0 8219.935
1009.4359741210938 0 30542.133 z 5
1009.5350341796875 0 77856.516
1010.43994140625 0 33368.54
1010.5407104492188 0 188921.27 c 7
1011.44580078125 0 12678.417
1011.5441284179688 0 97053.414
1012.5480346679688 0 23618.637
1024.4552001953125 0 6362.5547
1025.4576416015625 0 40088.81 y 5
1026.4610595703125 0 19747.227
1027.463623046875 0 6071.3364
1063.54541015625 0 6895.944
1064.539306640625 0 4056.4875
1067.5343017578125 0 4356.665
1068.52978515625 0 3925.7395
1079.462646484375 0 3516.3955 w 4
1081.55615234375 0 45531.527
1082.5599365234375 0 47978.316
1083.5645751953125 0 20089.027
1084.5740966796875 0 4893.938
1093.5279541015625 0 6602.371
1107.5543212890625 0 7006.6733 c Water loss 8
1108.5426025390625 0 85554.914 c Ammonia loss 8
1109.5435791015625 0 53737.72
1110.5455322265625 0 13706.178
1124.560791015625 0 34172.086
1125.5654296875 0 40065.547 c 8
1126.5692138671875 0 21167.295
1127.5706787109375 0 5999.3135
1136.4984130859375 0 9393.876 y Ammonia loss 4
1137.4964599609375 0 239934.8 z 4
1138.50146484375 0 178903.47
1139.50537109375 0 61041.613
1140.51171875 0 8897.685
1151.5166015625 0 5379.1934
1152.508544921875 0 35274.195
1153.5146484375 0 118954.59 y 4
1154.5185546875 0 60638.004
1155.52001953125 0 14664.137
1163.5704345703125 0 3151.0059
1165.5653076171875 0 33692.37 c Ammonia loss 9
1166.565673828125 0 21226.742
1167.5740966796875 0 7581.078
1181.5845947265625 0 46937.586
1182.5897216796875 0 355920.9 c 9
1183.5926513671875 0 227094.67
1184.594482421875 0 66953.13
1185.602294921875 0 6953.211
1203.5157470703125 0 3814.3433
1223.5782470703125 0 3719.977
1231.5589599609375 0 3484.0742
1266.673583984375 0 3303.6978
1267.6688232421875 0 6165.5522
1274.554443359375 0 205269.75 z 3
1275.557861328125 0 143157.61
1276.560546875 0 49229.883
1277.575927734375 0 6311.801
1289.56591796875 0 9557.687
1290.5728759765625 0 62551.81 y 3
1291.5771484375 0 45292.918
1292.5797119140625 0 13631.681
1293.6561279296875 0 25535.084 c Ammonia loss 10
1294.660400390625 0 16157.729
1295.6507568359375 0 4163.225
1309.6798095703125 0 19457.236
1310.68359375 0 150479.56 c 10
1311.6864013671875 0 118418.59
1312.6888427734375 0 35789.195
1313.6875 0 5743.537
1336.699462890625 0 16284.648
1337.7022705078125 0 9328.998
1343.6358642578125 0 4825.747
1344.5943603515625 0 16880.031 w 2
1345.59326171875 0 15073.348
1346.5889892578125 0 4086.3394
1351.727294921875 0 3239.0422
1387.63916015625 0 108061.664 z 2
1388.642333984375 0 99773.49
1389.6439208984375 0 36238.31
1390.6468505859375 0 4986.1987
1395.7130126953125 0 32141.014
1396.7137451171875 0 28232.5
1397.7203369140625 0 7946.0396
1403.65673828125 0 49005.945 y 2
1404.659912109375 0 46760.516
1405.664306640625 0 18385.178
1424.7122802734375 0 4354.511
1425.709716796875 0 4735.904
1430.6502685546875 0 4650.2646
1438.7381591796875 0 14653.824
1439.72607421875 0 518083.9 c 11
1440.7294921875 0 392887.53
1441.731689453125 0 150502.75
1442.73046875 0 14821.219
1474.7313232421875 0 4209.805
1486.706787109375 0 22255.936 z 1
1487.7119140625 0 21516.031
1488.7161865234375 0 9579.238
1505.7449951171875 0 3442.9268
1515.763916015625 0 6466.788
1516.7261962890625 0 16453.46
1517.7249755859375 0 14442.368
1518.72265625 0 7971.601
1526.74609375 0 4110.3193
1527.7633056640625 0 6494.5454
1528.7579345703125 0 5255.318
1531.7259521484375 0 5459.131
1532.7611083984375 0 9769.686
1533.7491455078125 0 12881.745
1534.742919921875 0 8161.3804
1540.7750244140625 0 6316.281
1541.76123046875 0 12612.843
1542.768310546875 0 43012.08
1543.7706298828125 0 50887.08
1544.7626953125 0 115868.67
1545.7633056640625 0 92334.02
1546.7645263671875 0 36744.906
1547.7603759765625 0 4732.6323
1548.718505859375 0 10728.954
1549.726806640625 0 11777.881
1550.7264404296875 0 8307.716
1558.7757568359375 0 56584.652
1559.767578125 0 126688.73
1560.7744140625 0 114732.73
1561.7764892578125 0 63868.742
1562.78271484375 0 21732.127
1568.759765625 0 12214.863
1569.7587890625 0 16938.764
1570.7669677734375 0 9191.965
1571.7593994140625 0 3585.096
1575.78759765625 0 3176.4517
1576.7862548828125 0 176016.98
1577.7882080078125 0 154411.08
1578.0335693359375 0 5307.5186
1578.7911376953125 0 72111.81
1579.7952880859375 0 9178.407
1586.769775390625 0 165330.97
1587.759033203125 0 481295.75
1588.7607421875 0 390550.88
1589.76123046875 0 150485.88
1590.7633056640625 0 19578.736
1602.7921142578125 0 21260.82
1603.77392578125 0 757821.3
1604.7796630859375 0 1811625.4
1605.78369140625 0 1349095
1606.787109375 0 524460.94
1607.7890625 0 64287.51
3081.027099609375 0 3906.5366

Spectrum Details

|  |  |
| --- | --- |
| Matched peaks? Matched peaksThe total absolute number of peaks matched. Additionally in brackets the total fraction of peaks matched and the total number of peaks is shown. | 47 (15.26% of 308) |
| FDR? FDRThe false discovery rate estimated for this peptide. It is calculated by matching all theoretical fragments with a non-integer shift with the raw peaks for this spectrum. This is done with 40 different shifts. The resulting percentage is the average number of annotated peaks over the number of annotated peaks with the correct spectrum. | 3.19% |
| Satellite FDR? Satellite FDRSee the FDR for details on its calculation. This satellite ion specific FDR only contains the satellite ions (d/w) for I/L/J positions. | 0.00% |
| PSM Score? PSM ScoreThe PSM Score as given by Hecklib to this annotated spectrum. It is shown with three significant figures. | 493 |

## Spectrum 7331? Spectrum 7331 The raw spectrum of this peptide as annotated by Hecklib. The fragments are coloured according to ion type (see legend). Any peaks with a star '\*' as text can be hovered over to see the full details, first the ion type second the mass shift type. By hovering over the amino acids in the peptide or ions in the legend the corresponding peaks are highlighted. By toggling the 'Unassigned' label you can turn the background (unassigned) peaks on or off in the plot. By updating the slider in the Ion legend you can update the spectrum to only show the top X% of the peaks with labels. The top X% means any peak that is within X% of the highest intensity. By dragging in the spectrum you can zoom in to a specific part of the spectrum and use 'Zoom Out' to get back to the original zoom level. The annotation of the spectrum is based on the given sequence in the peptides file and is done with different software so inconsistencies are likely. The peaks are annotated based on the given sequence, with 20 ppm tolerance.

Copy Data

### Spectrum 7331 (TSV)

#### Preview

```
Loading example...
```

*Click on the button to copy the data to your clipboard.*

Mz MinMz MaxIntensity Max

WidthHeightPeptide font sizePeptide stroke widthSpectrum font sizeSpectrum stroke widthCompact peptide

Ion legend

wxyz

abcd

OtherUnassignedIonChargePositionShow for top:%

TVLHQDWLDGKEY

02.15e+54.30e+56.45e+58.60e+5

Zoom Out

a+12y+34a+12y+11b+12b+12y+23y+24b+25y+12b+13y+25y+37y+25y+12b+13b+26b+26b+26y+26y+26y+39b+310y+13y+13b+27b+27b+14y+13b+27y+27b+14y+27y+14b+28b+28y+14\*\*\*b+29b+29b+15y+29y+29b+15y+15y+15y+210y+210y+210b+16b+16y+211y+211b+16y+211b+212b+212y+16b+212y+16y+212y+212y+212\*b+17b+17b+17y+17b+18b+18b+18y+18y+18b+19b+19y+19y+19y+19b+110

0778155723353114

Fragment Matches Table

Show background peaks

| Position | Ion type | Intensity | mz Theoretical | mz Error (Th) | mz Error (ppm) | Charge | Series Number |
| --- | --- | --- | --- | --- | --- | --- | --- |
| - | - | 8650 | 120.1 | - | - | 0 | - |
| - | - | 1005 | 121 | - | - | 0 | - |
| - | - | 1069 | 121.1 | - | - | 0 | - |
| - | - | 4219 | 122.1 | - | - | 0 | - |
| - | - | 4883 | 123 | - | - | 0 | - |
| - | - | 1507 | 123.1 | - | - | 0 | - |
| - | - | 948.9 | 125.1 | - | - | 0 | - |
| - | - | 761.9 | 125.4 | - | - | 0 | - |
| - | - | 1827 | 126.1 | - | - | 0 | - |
| - | - | 7478 | 127.1 | - | - | 0 | - |
| - | - | 1423 | 127.1 | - | - | 0 | - |
| - | - | 2911 | 127.1 | - | - | 0 | - |
| - | - | 1037 | 128 | - | - | 0 | - |
| - | - | 922.6 | 128.1 | - | - | 0 | - |
| - | - | 5.511E+04 | 128.1 | - | - | 0 | - |
| - | - | 3311 | 129.1 | - | - | 0 | - |
| - | - | 5.652E+05 | 129.1 | - | - | 0 | - |
| - | - | 2.382E+05 | 130.1 | - | - | 0 | - |
| - | - | 3574 | 130.1 | - | - | 0 | - |
| - | - | 3.574E+04 | 130.1 | - | - | 0 | - |
| - | - | 2.291E+04 | 131.1 | - | - | 0 | - |
| - | - | 5.45E+04 | 132.1 | - | - | 0 | - |
| - | - | 891.2 | 133.1 | - | - | 0 | - |
| - | - | 3894 | 133.1 | - | - | 0 | - |
| - | - | 1006 | 135.2 | - | - | 0 | - |
| - | - | 6.728E+04 | 136.1 | - | - | 0 | - |
| - | - | 5881 | 137.1 | - | - | 0 | - |
| - | - | 1088 | 137.2 | - | - | 0 | - |
| - | - | 6234 | 138.1 | - | - | 0 | - |
| - | - | 4064 | 138.1 | - | - | 0 | - |
| - | - | 1151 | 139.1 | - | - | 0 | - |
| - | - | 5655 | 139.1 | - | - | 0 | - |
| - | - | 1.004E+04 | 141.1 | - | - | 0 | - |
| - | - | 5138 | 142.1 | - | - | 0 | - |
| - | - | 3458 | 143 | - | - | 0 | - |
| - | - | 2311 | 144.1 | - | - | 0 | - |
| - | - | 4474 | 145.1 | - | - | 0 | - |
| - | - | 1386 | 146.1 | - | - | 0 | - |
| - | - | 3055 | 147 | - | - | 0 | - |
| - | - | 1.375E+04 | 149 | - | - | 0 | - |
| - | - | 3414 | 150.1 | - | - | 0 | - |
| - | - | 1.11E+04 | 151.1 | - | - | 0 | - |
| - | - | 1110 | 152.1 | - | - | 0 | - |
| - | - | 1037 | 154.1 | - | - | 0 | - |
| - | - | 2585 | 155 | - | - | 0 | - |
| - | - | 2058 | 155.1 | - | - | 0 | - |
| - | - | 3.481E+04 | 155.1 | - | - | 0 | - |
| 2 | a | 2.403E+04 | 155.1 | 0.0003966 | 2.557 | +1 | 2 |
| - | - | 1149 | 156 | - | - | 0 | - |
| - | - | 2875 | 156.1 | - | - | 0 | - |
| - | - | 1764 | 156.1 | - | - | 0 | - |
| - | - | 1451 | 156.1 | - | - | 0 | - |
| - | - | 2184 | 157.1 | - | - | 0 | - |
| - | - | 1113 | 157.1 | - | - | 0 | - |
| - | - | 3018 | 158.1 | - | - | 0 | - |
| - | - | 4.239E+05 | 159.1 | - | - | 0 | - |
| - | - | 4522 | 160.1 | - | - | 0 | - |
| - | - | 2756 | 160.1 | - | - | 0 | - |
| - | - | 4.24E+04 | 160.1 | - | - | 0 | - |
| - | - | 1818 | 161.1 | - | - | 0 | - |
| - | - | 1153 | 162.7 | - | - | 0 | - |
| - | - | 3.203E+04 | 165.1 | - | - | 0 | - |
| - | - | 1333 | 165.1 | - | - | 0 | - |
| - | - | 7.089E+04 | 166.1 | - | - | 0 | - |
| 10 | y | 3243 | 166.1 | 0.001969 | 11.86 | +3 | 4 |
| - | - | 5192 | 166.1 | - | - | 0 | - |
| - | - | 1.368E+04 | 167.1 | - | - | 0 | - |
| - | - | 4712 | 167.1 | - | - | 0 | - |
| - | - | 1101 | 167.1 | - | - | 0 | - |
| - | - | 6946 | 168.1 | - | - | 0 | - |
| - | - | 5292 | 168.1 | - | - | 0 | - |
| - | - | 5467 | 168.1 | - | - | 0 | - |
| - | - | 7294 | 169.1 | - | - | 0 | - |
| - | - | 1106 | 169.1 | - | - | 0 | - |
| - | - | 1.594E+04 | 169.1 | - | - | 0 | - |
| - | - | 1.825E+04 | 170.1 | - | - | 0 | - |
| - | - | 2023 | 171.1 | - | - | 0 | - |
| - | - | 2578 | 171.1 | - | - | 0 | - |
| - | - | 2174 | 171.1 | - | - | 0 | - |
| - | - | 6226 | 172.1 | - | - | 0 | - |
| - | - | 1678 | 172.1 | - | - | 0 | - |
| - | - | 8185 | 173.1 | - | - | 0 | - |
| 2 | a | 8.519E+05 | 173.1 | 0.0004978 | 2.875 | +1 | 2 |
| - | - | 2427 | 173.5 | - | - | 0 | - |
| - | - | 7.427E+04 | 174.1 | - | - | 0 | - |
| - | - | 4728 | 175.1 | - | - | 0 | - |
| - | - | 2135 | 177.1 | - | - | 0 | - |
| - | - | 2.656E+04 | 178.1 | - | - | 0 | - |
| - | - | 1488 | 179.1 | - | - | 0 | - |
| - | - | 5853 | 181.1 | - | - | 0 | - |
| 13 | y | 7.922E+04 | 182.1 | 0.0004954 | 2.721 | +1 | 1 |
| - | - | 6802 | 183.1 | - | - | 0 | - |
| 2 | b | 2.202E+04 | 183.1 | 0.0004008 | 2.189 | +1 | 2 |
| - | - | 2599 | 184.1 | - | - | 0 | - |
| - | - | 1201 | 185.1 | - | - | 0 | - |
| - | - | 3392 | 185.2 | - | - | 0 | - |
| - | - | 1.837E+05 | 186.1 | - | - | 0 | - |
| - | - | 3.825E+04 | 187.1 | - | - | 0 | - |
| - | - | 1.599E+04 | 187.1 | - | - | 0 | - |
| - | - | 1456 | 187.1 | - | - | 0 | - |
| - | - | 4542 | 188.1 | - | - | 0 | - |
| - | - | 4317 | 188.1 | - | - | 0 | - |
| - | - | 6685 | 190.1 | - | - | 0 | - |
| - | - | 4997 | 190.1 | - | - | 0 | - |
| - | - | 3914 | 194.1 | - | - | 0 | - |
| - | - | 4.161E+04 | 195.1 | - | - | 0 | - |
| - | - | 2.959E+04 | 196.1 | - | - | 0 | - |
| - | - | 2706 | 196.1 | - | - | 0 | - |
| - | - | 1667 | 197.1 | - | - | 0 | - |
| - | - | 3215 | 197.1 | - | - | 0 | - |
| - | - | 1455 | 197.2 | - | - | 0 | - |
| - | - | 5290 | 198.1 | - | - | 0 | - |
| - | - | 1.525E+04 | 198.1 | - | - | 0 | - |
| - | - | 3526 | 199.1 | - | - | 0 | - |
| - | - | 3673 | 199.1 | - | - | 0 | - |
| - | - | 1548 | 199.1 | - | - | 0 | - |
| - | - | 2508 | 200.1 | - | - | 0 | - |
| 2 | b | 2.27E+05 | 201.1 | 0.0004257 | 2.117 | +1 | 2 |
| - | - | 2121 | 202.1 | - | - | 0 | - |
| - | - | 2.161E+04 | 202.1 | - | - | 0 | - |
| - | - | 2696 | 204.1 | - | - | 0 | - |
| - | - | 1.708E+04 | 205.1 | - | - | 0 | - |
| - | - | 1651 | 205.1 | - | - | 0 | - |
| - | - | 5441 | 205.1 | - | - | 0 | - |
| - | - | 2179 | 206.1 | - | - | 0 | - |
| - | - | 4475 | 206.1 | - | - | 0 | - |
| - | - | 7481 | 207.2 | - | - | 0 | - |
| - | - | 1782 | 209.1 | - | - | 0 | - |
| - | - | 6253 | 210.1 | - | - | 0 | - |
| 11 | y | 6215 | 211.1 | 0.0005118 | 2.424 | +2 | 3 |
| - | - | 5798 | 212.1 | - | - | 0 | - |
| - | - | 1.956E+04 | 213.1 | - | - | 0 | - |
| - | - | 2111 | 213.2 | - | - | 0 | - |
| - | - | 1587 | 214.1 | - | - | 0 | - |
| - | - | 1183 | 215.1 | - | - | 0 | - |
| - | - | 1707 | 215.1 | - | - | 0 | - |
| - | - | 2254 | 215.1 | - | - | 0 | - |
| - | - | 4183 | 216.1 | - | - | 0 | - |
| - | - | 1251 | 221.1 | - | - | 0 | - |
| - | - | 2352 | 221.1 | - | - | 0 | - |
| - | - | 7312 | 221.1 | - | - | 0 | - |
| - | - | 4076 | 221.1 | - | - | 0 | - |
| - | - | 2459 | 221.1 | - | - | 0 | - |
| - | - | 2678 | 221.1 | - | - | 0 | - |
| - | - | 2052 | 222.1 | - | - | 0 | - |
| - | - | 6066 | 222.1 | - | - | 0 | - |
| - | - | 2951 | 223.1 | - | - | 0 | - |
| - | - | 1.169E+04 | 223.1 | - | - | 0 | - |
| - | - | 1994 | 223.1 | - | - | 0 | - |
| - | - | 7.524E+04 | 223.2 | - | - | 0 | - |
| - | - | 3399 | 224.1 | - | - | 0 | - |
| - | - | 7707 | 224.2 | - | - | 0 | - |
| - | - | 8065 | 225 | - | - | 0 | - |
| - | - | 1441 | 226 | - | - | 0 | - |
| - | - | 1.511E+04 | 226.1 | - | - | 0 | - |
| - | - | 1549 | 226.2 | - | - | 0 | - |
| - | - | 1.158E+04 | 227.1 | - | - | 0 | - |
| - | - | 3133 | 227.2 | - | - | 0 | - |
| - | - | 2776 | 228.1 | - | - | 0 | - |
| - | - | 2.048E+04 | 229.1 | - | - | 0 | - |
| - | - | 7156 | 229.2 | - | - | 0 | - |
| - | - | 1550 | 230.1 | - | - | 0 | - |
| - | - | 2885 | 230.1 | - | - | 0 | - |
| - | - | 2467 | 230.2 | - | - | 0 | - |
| - | - | 1.101E+04 | 233.1 | - | - | 0 | - |
| - | - | 8.248E+04 | 234.1 | - | - | 0 | - |
| - | - | 1.154E+04 | 235.1 | - | - | 0 | - |
| - | - | 2957 | 235.2 | - | - | 0 | - |
| - | - | 2417 | 237.1 | - | - | 0 | - |
| - | - | 8837 | 238.1 | - | - | 0 | - |
| - | - | 2876 | 238.1 | - | - | 0 | - |
| - | - | 2065 | 239.1 | - | - | 0 | - |
| - | - | 1.529E+04 | 239.1 | - | - | 0 | - |
| - | - | 1978 | 239.1 | - | - | 0 | - |
| - | - | 4276 | 240.1 | - | - | 0 | - |
| - | - | 2206 | 240.1 | - | - | 0 | - |
| - | - | 3.201E+04 | 240.1 | - | - | 0 | - |
| - | - | 2514 | 241.1 | - | - | 0 | - |
| - | - | 4121 | 241.1 | - | - | 0 | - |
| - | - | 2021 | 241.1 | - | - | 0 | - |
| - | - | 4307 | 241.2 | - | - | 0 | - |
| - | - | 1582 | 242.1 | - | - | 0 | - |
| - | - | 6282 | 243.1 | - | - | 0 | - |
| - | - | 3.015E+04 | 244.1 | - | - | 0 | - |
| - | - | 3647 | 245.1 | - | - | 0 | - |
| - | - | 4.643E+04 | 247.1 | - | - | 0 | - |
| - | - | 2708 | 247.1 | - | - | 0 | - |
| - | - | 1816 | 247.2 | - | - | 0 | - |
| - | - | 3017 | 248.1 | - | - | 0 | - |
| - | - | 2.011E+04 | 248.1 | - | - | 0 | - |
| 10 | y | 1207 | 248.6 | 3.105E-05 | 0.1249 | +2 | 4 |
| - | - | 2.404E+04 | 249.1 | - | - | 0 | - |
| - | - | 1949 | 249.1 | - | - | 0 | - |
| - | - | 6909 | 249.1 | - | - | 0 | - |
| - | - | 3170 | 250.1 | - | - | 0 | - |
| - | - | 1948 | 250.1 | - | - | 0 | - |
| - | - | 1299 | 250.2 | - | - | 0 | - |
| - | - | 2.79E+05 | 251.2 | - | - | 0 | - |
| - | - | 1901 | 252.1 | - | - | 0 | - |
| - | - | 3.599E+04 | 252.2 | - | - | 0 | - |
| - | - | 1622 | 252.6 | - | - | 0 | - |
| - | - | 1830 | 253.2 | - | - | 0 | - |
| - | - | 1408 | 254.2 | - | - | 0 | - |
| - | - | 1.548E+04 | 255.1 | - | - | 0 | - |
| - | - | 3678 | 256.1 | - | - | 0 | - |
| - | - | 1874 | 256.2 | - | - | 0 | - |
| - | - | 8665 | 257.1 | - | - | 0 | - |
| - | - | 1.215E+04 | 258.1 | - | - | 0 | - |
| - | - | 1628 | 259.1 | - | - | 0 | - |
| - | - | 1963 | 259.1 | - | - | 0 | - |
| - | - | 5544 | 261.1 | - | - | 0 | - |
| - | - | 2135 | 261.1 | - | - | 0 | - |
| - | - | 3724 | 262.1 | - | - | 0 | - |
| - | - | 3.06E+04 | 265.1 | - | - | 0 | - |
| - | - | 2313 | 265.1 | - | - | 0 | - |
| - | - | 7.648E+04 | 266.1 | - | - | 0 | - |
| - | - | 8367 | 267.1 | - | - | 0 | - |
| - | - | 1383 | 268.1 | - | - | 0 | - |
| - | - | 4046 | 268.1 | - | - | 0 | - |
| - | - | 6056 | 268.2 | - | - | 0 | - |
| - | - | 2468 | 269.2 | - | - | 0 | - |
| - | - | 3677 | 269.2 | - | - | 0 | - |
| - | - | 1.041E+04 | 270.1 | - | - | 0 | - |
| - | - | 2059 | 270.1 | - | - | 0 | - |
| - | - | 4283 | 270.6 | - | - | 0 | - |
| - | - | 1.87E+04 | 272.2 | - | - | 0 | - |
| - | - | 3510 | 273.2 | - | - | 0 | - |
| - | - | 1.555E+04 | 274.1 | - | - | 0 | - |
| - | - | 5420 | 274.1 | - | - | 0 | - |
| - | - | 4950 | 275.1 | - | - | 0 | - |
| - | - | 3021 | 275.1 | - | - | 0 | - |
| - | - | 2256 | 275.2 | - | - | 0 | - |
| - | - | 4061 | 276.1 | - | - | 0 | - |
| - | - | 2577 | 276.1 | - | - | 0 | - |
| - | - | 3182 | 276.2 | - | - | 0 | - |
| - | - | 2130 | 276.2 | - | - | 0 | - |
| - | - | 6042 | 279.1 | - | - | 0 | - |
| - | - | 7980 | 280.1 | - | - | 0 | - |
| - | - | 1842 | 280.1 | - | - | 0 | - |
| - | - | 1.123E+04 | 281.1 | - | - | 0 | - |
| - | - | 9141 | 282.2 | - | - | 0 | - |
| - | - | 1.995E+05 | 283.1 | - | - | 0 | - |
| - | - | 1358 | 283.4 | - | - | 0 | - |
| - | - | 1787 | 284.1 | - | - | 0 | - |
| - | - | 8713 | 284.1 | - | - | 0 | - |
| - | - | 2541 | 284.1 | - | - | 0 | - |
| - | - | 2.737E+04 | 284.1 | - | - | 0 | - |
| - | - | 2340 | 285.1 | - | - | 0 | - |
| - | - | 1670 | 285.2 | - | - | 0 | - |
| - | - | 1.491E+04 | 286.1 | - | - | 0 | - |
| - | - | 1764 | 286.2 | - | - | 0 | - |
| - | - | 1554 | 287.1 | - | - | 0 | - |
| - | - | 3686 | 287.2 | - | - | 0 | - |
| - | - | 5657 | 288.6 | - | - | 0 | - |
| 5 | b | 2956 | 290.2 | 0.0001753 | 0.604 | +2 | 5 |
| 12 | y | 1.132E+05 | 293.1 | 0.000663 | 2.262 | +1 | 2 |
| - | - | 2.038E+04 | 294.1 | - | - | 0 | - |
| - | - | 1404 | 295.1 | - | - | 0 | - |
| - | - | 2142 | 295.1 | - | - | 0 | - |
| - | - | 1306 | 296.1 | - | - | 0 | - |
| 3 | b | 3650 | 296.2 | 0.001038 | 3.506 | +1 | 3 |
| - | - | 2283 | 297.1 | - | - | 0 | - |
| 9 | y | 4489 | 297.1 | 0.001226 | 4.126 | +2 | 5 |
| - | - | 3.809E+04 | 297.2 | - | - | 0 | - |
| - | - | 1703 | 297.2 | - | - | 0 | - |
| - | - | 1728 | 297.6 | - | - | 0 | - |
| 7 | y | 4574 | 298.1 | 0.005032 | 16.88 | +3 | 7 |
| - | - | 4439 | 298.2 | - | - | 0 | - |
| - | - | 8.948E+04 | 299.1 | - | - | 0 | - |
| - | - | 2486 | 300.1 | - | - | 0 | - |
| - | - | 1.8E+04 | 300.2 | - | - | 0 | - |
| - | - | 1562 | 301.1 | - | - | 0 | - |
| - | - | 1.035E+05 | 301.2 | - | - | 0 | - |
| - | - | 1.993E+04 | 302.1 | - | - | 0 | - |
| - | - | 1.502E+04 | 302.2 | - | - | 0 | - |
| - | - | 2764 | 303.1 | - | - | 0 | - |
| - | - | 2866 | 305.2 | - | - | 0 | - |
| - | - | 2452 | 305.6 | - | - | 0 | - |
| 9 | y | 1.802E+04 | 306.1 | 0.0008875 | 2.899 | +2 | 5 |
| - | - | 1983 | 306.2 | - | - | 0 | - |
| - | - | 6637 | 306.6 | - | - | 0 | - |
| - | - | 3678 | 307.1 | - | - | 0 | - |
| - | - | 1798 | 309.2 | - | - | 0 | - |
| 12 | y | 1.521E+04 | 311.1 | 0.0005353 | 1.721 | +1 | 2 |
| - | - | 2120 | 312.1 | - | - | 0 | - |
| - | - | 3415 | 314.2 | - | - | 0 | - |
| 3 | b | 9154 | 314.2 | 0.0006971 | 2.218 | +1 | 3 |
| - | - | 6.307E+04 | 315.2 | - | - | 0 | - |
| - | - | 1.19E+04 | 316.2 | - | - | 0 | - |
| - | - | 4019 | 319.2 | - | - | 0 | - |
| - | - | 3923 | 325.2 | - | - | 0 | - |
| - | - | 1.396E+04 | 326.7 | - | - | 0 | - |
| - | - | 3753 | 327.2 | - | - | 0 | - |
| - | - | 1461 | 330.2 | - | - | 0 | - |
| - | - | 1637 | 331.7 | - | - | 0 | - |
| - | - | 6730 | 332.2 | - | - | 0 | - |
| - | - | 1.113E+04 | 333.2 | - | - | 0 | - |
| - | - | 2061 | 334.2 | - | - | 0 | - |
| - | - | 1711 | 338.2 | - | - | 0 | - |
| 6 | b | 2100 | 338.7 | 0.0006688 | 1.975 | +2 | 6 |
| 6 | b | 1342 | 339.2 | 0.004144 | 12.22 | +2 | 6 |
| - | - | 2405 | 339.7 | - | - | 0 | - |
| - | - | 3086 | 340.7 | - | - | 0 | - |
| - | - | 1977 | 341.2 | - | - | 0 | - |
| - | - | 5219 | 342.2 | - | - | 0 | - |
| - | - | 4721 | 343.2 | - | - | 0 | - |
| - | - | 1422 | 343.2 | - | - | 0 | - |
| - | - | 5018 | 344.2 | - | - | 0 | - |
| - | - | 1.138E+04 | 345 | - | - | 0 | - |
| - | - | 8565 | 345.1 | - | - | 0 | - |
| - | - | 2.975E+04 | 346.1 | - | - | 0 | - |
| - | - | 1763 | 346.1 | - | - | 0 | - |
| - | - | 5039 | 347.1 | - | - | 0 | - |
| 6 | b | 1.44E+04 | 347.7 | 0.000727 | 2.091 | +2 | 6 |
| - | - | 3745 | 348.2 | - | - | 0 | - |
| - | - | 1588 | 348.7 | - | - | 0 | - |
| - | - | 1739 | 349.2 | - | - | 0 | - |
| - | - | 2.496E+04 | 350.2 | - | - | 0 | - |
| - | - | 4269 | 351.2 | - | - | 0 | - |
| - | - | 2952 | 352.7 | - | - | 0 | - |
| 8 | y | 5242 | 353.7 | 0.0005148 | 1.456 | +2 | 6 |
| - | - | 1760 | 354.2 | - | - | 0 | - |
| - | - | 2821 | 355.1 | - | - | 0 | - |
| - | - | 1.177E+04 | 359 | - | - | 0 | - |
| - | - | 6442 | 360.7 | - | - | 0 | - |
| - | - | 1.072E+04 | 361.2 | - | - | 0 | - |
| - | - | 1686 | 361.7 | - | - | 0 | - |
| - | - | 9645 | 362.2 | - | - | 0 | - |
| 8 | y | 3.992E+04 | 362.7 | 0.0008783 | 2.422 | +2 | 6 |
| - | - | 4.678E+04 | 363.1 | - | - | 0 | - |
| - | - | 1.528E+04 | 363.2 | - | - | 0 | - |
| - | - | 1500 | 363.7 | - | - | 0 | - |
| - | - | 8181 | 364.1 | - | - | 0 | - |
| - | - | 7058 | 364.1 | - | - | 0 | - |
| - | - | 3348 | 366.2 | - | - | 0 | - |
| - | - | 4234 | 367.1 | - | - | 0 | - |
| - | - | 2241 | 367.2 | - | - | 0 | - |
| - | - | 5842 | 367.2 | - | - | 0 | - |
| - | - | 1468 | 367.7 | - | - | 0 | - |
| - | - | 3719 | 373.2 | - | - | 0 | - |
| - | - | 1634 | 373.7 | - | - | 0 | - |
| - | - | 2308 | 376.2 | - | - | 0 | - |
| - | - | 7160 | 376.2 | - | - | 0 | - |
| - | - | 2236 | 376.7 | - | - | 0 | - |
| - | - | 3199 | 377.1 | - | - | 0 | - |
| - | - | 2158 | 377.3 | - | - | 0 | - |
| - | - | 1122 | 378.2 | - | - | 0 | - |
| - | - | 1570 | 378.2 | - | - | 0 | - |
| - | - | 1.659E+04 | 378.2 | - | - | 0 | - |
| - | - | 7.731E+04 | 379.2 | - | - | 0 | - |
| - | - | 3508 | 380.2 | - | - | 0 | - |
| - | - | 1.622E+04 | 380.2 | - | - | 0 | - |
| - | - | 1.315E+05 | 381.2 | - | - | 0 | - |
| - | - | 4751 | 381.7 | - | - | 0 | - |
| - | - | 2.527E+04 | 382.2 | - | - | 0 | - |
| - | - | 4594 | 382.2 | - | - | 0 | - |
| - | - | 3157 | 383.2 | - | - | 0 | - |
| - | - | 6328 | 384.2 | - | - | 0 | - |
| - | - | 4213 | 384.2 | - | - | 0 | - |
| - | - | 9788 | 385.2 | - | - | 0 | - |
| 5 | y | 3743 | 385.2 | 0.00407 | 10.57 | +3 | 9 |
| - | - | 2154 | 386.2 | - | - | 0 | - |
| 10 | b | 1960 | 389.2 | 0.004715 | 12.11 | +3 | 10 |
| - | - | 8780 | 390.2 | - | - | 0 | - |
| - | - | 2611 | 390.7 | - | - | 0 | - |
| - | - | 6477 | 391.1 | - | - | 0 | - |
| - | - | 2622 | 391.2 | - | - | 0 | - |
| - | - | 3197 | 392.2 | - | - | 0 | - |
| - | - | 2012 | 394.1 | - | - | 0 | - |
| - | - | 1.871E+04 | 394.2 | - | - | 0 | - |
| - | - | 7163 | 395.1 | - | - | 0 | - |
| - | - | 5259 | 395.2 | - | - | 0 | - |
| - | - | 2269 | 395.2 | - | - | 0 | - |
| - | - | 3279 | 395.2 | - | - | 0 | - |
| - | - | 1948 | 396.2 | - | - | 0 | - |
| - | - | 2288 | 396.2 | - | - | 0 | - |
| - | - | 2.681E+04 | 396.2 | - | - | 0 | - |
| - | - | 2407 | 397.2 | - | - | 0 | - |
| - | - | 5531 | 397.2 | - | - | 0 | - |
| - | - | 1414 | 397.7 | - | - | 0 | - |
| - | - | 1.22E+04 | 398.2 | - | - | 0 | - |
| - | - | 1921 | 399.2 | - | - | 0 | - |
| - | - | 5400 | 402.2 | - | - | 0 | - |
| - | - | 1.189E+04 | 402.2 | - | - | 0 | - |
| - | - | 4571 | 403.2 | - | - | 0 | - |
| - | - | 3644 | 404.7 | - | - | 0 | - |
| - | - | 4231 | 405.3 | - | - | 0 | - |
| - | - | 2761 | 406.2 | - | - | 0 | - |
| - | - | 2034 | 407.2 | - | - | 0 | - |
| - | - | 1.062E+04 | 409.1 | - | - | 0 | - |
| - | - | 1723 | 409.2 | - | - | 0 | - |
| - | - | 2472 | 409.2 | - | - | 0 | - |
| - | - | 1820 | 409.7 | - | - | 0 | - |
| - | - | 6418 | 411.2 | - | - | 0 | - |
| - | - | 8.469E+04 | 412.2 | - | - | 0 | - |
| - | - | 7933 | 413.1 | - | - | 0 | - |
| - | - | 4099 | 413.2 | - | - | 0 | - |
| - | - | 1.225E+04 | 413.2 | - | - | 0 | - |
| - | - | 2032 | 413.3 | - | - | 0 | - |
| - | - | 1291 | 414.2 | - | - | 0 | - |
| - | - | 1498 | 414.2 | - | - | 0 | - |
| - | - | 2.526E+04 | 414.2 | - | - | 0 | - |
| - | - | 2116 | 414.3 | - | - | 0 | - |
| - | - | 2.243E+04 | 415 | - | - | 0 | - |
| - | - | 1.016E+04 | 415.2 | - | - | 0 | - |
| - | - | 6712 | 415.2 | - | - | 0 | - |
| - | - | 2310 | 416.2 | - | - | 0 | - |
| - | - | 1.702E+04 | 417.7 | - | - | 0 | - |
| - | - | 2.656E+04 | 418.2 | - | - | 0 | - |
| - | - | 1.297E+04 | 418.7 | - | - | 0 | - |
| - | - | 3968 | 419.2 | - | - | 0 | - |
| 11 | y | 2875 | 421.2 | 0.001891 | 4.49 | +1 | 3 |
| 11 | y | 2399 | 422.2 | 0.0003127 | 0.7406 | +1 | 3 |
| - | - | 2339 | 422.7 | - | - | 0 | - |
| - | - | 5735 | 423.2 | - | - | 0 | - |
| - | - | 3.297E+04 | 423.3 | - | - | 0 | - |
| - | - | 2305 | 423.7 | - | - | 0 | - |
| - | - | 1741 | 424.2 | - | - | 0 | - |
| - | - | 8756 | 424.3 | - | - | 0 | - |
| - | - | 2815 | 424.7 | - | - | 0 | - |
| - | - | 2.171E+05 | 426.7 | - | - | 0 | - |
| - | - | 1.086E+05 | 427.2 | - | - | 0 | - |
| - | - | 3.01E+04 | 427.7 | - | - | 0 | - |
| - | - | 3805 | 428.2 | - | - | 0 | - |
| - | - | 2.926E+04 | 429.2 | - | - | 0 | - |
| - | - | 2.143E+05 | 430.2 | - | - | 0 | - |
| - | - | 4.639E+04 | 431.2 | - | - | 0 | - |
| 7 | b | 2.559E+04 | 431.7 | 0.001112 | 2.577 | +2 | 7 |
| 7 | b | 1.963E+04 | 432.2 | 0.006663 | 15.42 | +2 | 7 |
| - | - | 4814 | 432.7 | - | - | 0 | - |
| - | - | 4025 | 433.2 | - | - | 0 | - |
| 4 | b | 6126 | 433.3 | 0.0003235 | 0.7467 | +1 | 4 |
| - | - | 2011 | 434.3 | - | - | 0 | - |
| - | - | 1368 | 437.2 | - | - | 0 | - |
| - | - | 4420 | 438.2 | - | - | 0 | - |
| - | - | 3669 | 438.7 | - | - | 0 | - |
| 11 | y | 5.65E+04 | 439.2 | 0.001001 | 2.279 | +1 | 3 |
| - | - | 2324 | 440.2 | - | - | 0 | - |
| - | - | 1.289E+04 | 440.2 | - | - | 0 | - |
| 7 | b | 1.083E+05 | 440.7 | 0.001079 | 2.448 | +2 | 7 |
| - | - | 6.018E+04 | 441.2 | - | - | 0 | - |
| - | - | 1561 | 441.3 | - | - | 0 | - |
| - | - | 1.744E+04 | 441.7 | - | - | 0 | - |
| - | - | 5005 | 442.2 | - | - | 0 | - |
| - | - | 2948 | 443.2 | - | - | 0 | - |
| 7 | y | 1.008E+04 | 447.2 | 0.001382 | 3.091 | +2 | 7 |
| - | - | 1699 | 447.2 | - | - | 0 | - |
| - | - | 4050 | 447.7 | - | - | 0 | - |
| - | - | 1.1E+04 | 448.2 | - | - | 0 | - |
| - | - | 1561 | 448.8 | - | - | 0 | - |
| - | - | 3518 | 449.2 | - | - | 0 | - |
| - | - | 1807 | 450.2 | - | - | 0 | - |
| - | - | 1538 | 451.2 | - | - | 0 | - |
| - | - | 2080 | 451.2 | - | - | 0 | - |
| 4 | b | 6.004E+04 | 451.3 | 0.001142 | 2.53 | +1 | 4 |
| - | - | 1.629E+04 | 452.3 | - | - | 0 | - |
| - | - | 2743 | 453.3 | - | - | 0 | - |
| - | - | 3443 | 455.3 | - | - | 0 | - |
| 7 | y | 6840 | 455.7 | 0.0004063 | 0.8917 | +2 | 7 |
| - | - | 6091 | 459.2 | - | - | 0 | - |
| - | - | 6750 | 460.8 | - | - | 0 | - |
| - | - | 6201 | 461.2 | - | - | 0 | - |
| - | - | 1.042E+04 | 461.3 | - | - | 0 | - |
| - | - | 2072 | 462.2 | - | - | 0 | - |
| - | - | 1689 | 462.2 | - | - | 0 | - |
| - | - | 1480 | 464.2 | - | - | 0 | - |
| - | - | 1669 | 465.7 | - | - | 0 | - |
| - | - | 2888 | 466.2 | - | - | 0 | - |
| - | - | 1.505E+04 | 468.3 | - | - | 0 | - |
| - | - | 2793 | 469.3 | - | - | 0 | - |
| - | - | 1361 | 470.2 | - | - | 0 | - |
| - | - | 1.402E+04 | 474.3 | - | - | 0 | - |
| - | - | 1.811E+04 | 474.8 | - | - | 0 | - |
| - | - | 9133 | 475.3 | - | - | 0 | - |
| - | - | 3579 | 475.8 | - | - | 0 | - |
| - | - | 4908 | 476.2 | - | - | 0 | - |
| - | - | 1.158E+04 | 477.2 | - | - | 0 | - |
| 10 | y | 1.275E+04 | 478.2 | 0.0004683 | 0.9793 | +1 | 4 |
| - | - | 6480 | 478.3 | - | - | 0 | - |
| - | - | 1547 | 479.2 | - | - | 0 | - |
| - | - | 1688 | 479.3 | - | - | 0 | - |
| - | - | 2078 | 480.2 | - | - | 0 | - |
| - | - | 2634 | 481.7 | - | - | 0 | - |
| - | - | 4.308E+04 | 483.3 | - | - | 0 | - |
| - | - | 1.838E+04 | 483.8 | - | - | 0 | - |
| - | - | 2183 | 484.2 | - | - | 0 | - |
| - | - | 7974 | 484.3 | - | - | 0 | - |
| - | - | 2306 | 484.8 | - | - | 0 | - |
| 8 | b | 9451 | 488.3 | 0.001561 | 3.197 | +2 | 8 |
| 8 | b | 9542 | 488.7 | 0.007905 | 16.17 | +2 | 8 |
| - | - | 3278 | 489.3 | - | - | 0 | - |
| - | - | 1499 | 489.8 | - | - | 0 | - |
| - | - | 1.431E+05 | 494.2 | - | - | 0 | - |
| - | - | 3.568E+04 | 495.2 | - | - | 0 | - |
| 10 | y | 1.761E+05 | 496.2 | 0.001113 | 2.243 | +1 | 4 |
| - | - | 4.415E+04 | 497.2 | - | - | 0 | - |
| - | - | 1.821E+04 | 497.8 | - | - | 0 | - |
| - | - | 5975 | 498.2 | - | - | 0 | - |
| - | - | 1558 | 502.2 | - | - | 0 | - |
| - | - | 2.052E+04 | 503.1 | - | - | 0 | - |
| - | - | 3922 | 504.2 | - | - | 0 | - |
| - | - | 5166 | 506.2 | - | - | 0 | - |
| - | - | 2898 | 506.3 | - | - | 0 | - |
| - | - | 2269 | 507.3 | - | - | 0 | - |
| - | - | 1.366E+04 | 511.3 | - | - | 0 | - |
| - | - | 2039 | 512.3 | - | - | 0 | - |
| - | - | 2398 | 513.2 | - | - | 0 | - |
| - | - | 2497 | 514.2 | - | - | 0 | - |
| - | - | 2125 | 515.3 | - | - | 0 | - |
| - | - | 6572 | 519.1 | - | - | 0 | - |
| - | - | 5058 | 523.3 | - | - | 0 | - |
| - | - | 2.301E+04 | 525.3 | - | - | 0 | - |
| - | - | 3583 | 526.2 | - | - | 0 | - |
| - | - | 6507 | 526.3 | - | - | 0 | - |
| - | - | 1899 | 527.3 | - | - | 0 | - |
| - | - | 2267 | 528.2 | - | - | 0 | - |
| 0 | Precursor | 4731 | 529.3 | 0.001474 | 2.785 | +3 | -1 |
| 0 | Precursor | 1.001E+04 | 529.6 | 0.007453 | 14.07 | +3 | -1 |
| - | - | 3344 | 529.9 | - | - | 0 | - |
| - | - | 2266 | 530.2 | - | - | 0 | - |
| - | - | 2006 | 531.2 | - | - | 0 | - |
| - | - | 5447 | 532.2 | - | - | 0 | - |
| - | - | 1649 | 534.9 | - | - | 0 | - |
| 0 | Precursor | 3522 | 535.3 | 0.00137 | 2.56 | +3 | -1 |
| - | - | 8788 | 535.6 | - | - | 0 | - |
| - | - | 3795 | 536.2 | - | - | 0 | - |
| - | - | 1951 | 537.7 | - | - | 0 | - |
| - | - | 1698 | 538.8 | - | - | 0 | - |
| - | - | 2.712E+04 | 539.2 | - | - | 0 | - |
| - | - | 6487 | 540.2 | - | - | 0 | - |
| - | - | 5746 | 542.3 | - | - | 0 | - |
| - | - | 5.335E+04 | 543.3 | - | - | 0 | - |
| - | - | 1.428E+04 | 544.3 | - | - | 0 | - |
| - | - | 2766 | 545.3 | - | - | 0 | - |
| 9 | b | 1953 | 545.8 | 0.0006931 | 1.27 | +2 | 9 |
| - | - | 6515 | 546.3 | - | - | 0 | - |
| - | - | 2739 | 546.7 | - | - | 0 | - |
| - | - | 1844 | 547.3 | - | - | 0 | - |
| - | - | 1873 | 548.2 | - | - | 0 | - |
| - | - | 1736 | 548.3 | - | - | 0 | - |
| - | - | 7358 | 549.2 | - | - | 0 | - |
| - | - | 2062 | 550.2 | - | - | 0 | - |
| - | - | 6199 | 551.3 | - | - | 0 | - |
| - | - | 3248 | 551.3 | - | - | 0 | - |
| - | - | 2322 | 552.3 | - | - | 0 | - |
| - | - | 1845 | 553.3 | - | - | 0 | - |
| - | - | 1593 | 554.3 | - | - | 0 | - |
| 9 | b | 5723 | 554.8 | 0.003246 | 5.852 | +2 | 9 |
| - | - | 1.584E+04 | 555.3 | - | - | 0 | - |
| - | - | 8004 | 555.8 | - | - | 0 | - |
| - | - | 2606 | 556.3 | - | - | 0 | - |
| 5 | b | 8027 | 561.3 | 0.000706 | 1.258 | +1 | 5 |
| - | - | 2115 | 562.3 | - | - | 0 | - |
| - | - | 5515 | 563.3 | - | - | 0 | - |
| - | - | 1739 | 564.3 | - | - | 0 | - |
| - | - | 3527 | 564.3 | - | - | 0 | - |
| - | - | 1.014E+04 | 565.3 | - | - | 0 | - |
| - | - | 2.887E+04 | 567.2 | - | - | 0 | - |
| - | - | 6324 | 568.2 | - | - | 0 | - |
| 5 | y | 3018 | 568.7 | 0.009922 | 17.45 | +2 | 9 |
| - | - | 1644 | 569.2 | - | - | 0 | - |
| - | - | 6623 | 575.2 | - | - | 0 | - |
| - | - | 1746 | 575.3 | - | - | 0 | - |
| - | - | 4274 | 576.2 | - | - | 0 | - |
| 5 | y | 1778 | 577.3 | 0.007207 | 12.48 | +2 | 9 |
| - | - | 1769 | 577.8 | - | - | 0 | - |
| - | - | 1478 | 578.3 | - | - | 0 | - |
| - | - | 2166 | 579.3 | - | - | 0 | - |
| 5 | b | 4.086E+04 | 579.3 | 0.001067 | 1.841 | +1 | 5 |
| - | - | 1.199E+04 | 580.3 | - | - | 0 | - |
| - | - | 2660 | 581.3 | - | - | 0 | - |
| - | - | 4432 | 592.3 | - | - | 0 | - |
| - | - | 4262 | 592.3 | - | - | 0 | - |
| 9 | y | 4.564E+04 | 593.3 | 0.0008174 | 1.378 | +1 | 5 |
| - | - | 1.838E+04 | 593.3 | - | - | 0 | - |
| - | - | 2710 | 593.8 | - | - | 0 | - |
| - | - | 1.47E+04 | 594.3 | - | - | 0 | - |
| - | - | 3104 | 594.3 | - | - | 0 | - |
| - | - | 6751 | 595.3 | - | - | 0 | - |
| - | - | 1608 | 596.3 | - | - | 0 | - |
| - | - | 1866 | 597.8 | - | - | 0 | - |
| - | - | 2018 | 598.3 | - | - | 0 | - |
| - | - | 4988 | 600.3 | - | - | 0 | - |
| - | - | 7570 | 602.8 | - | - | 0 | - |
| - | - | 1.056E+04 | 603.3 | - | - | 0 | - |
| - | - | 2420 | 603.8 | - | - | 0 | - |
| - | - | 2103 | 604.3 | - | - | 0 | - |
| - | - | 7.167E+04 | 610.3 | - | - | 0 | - |
| - | - | 4506 | 610.3 | - | - | 0 | - |
| 9 | y | 5.474E+05 | 611.3 | 0.001544 | 2.526 | +1 | 5 |
| - | - | 3.522E+04 | 611.8 | - | - | 0 | - |
| - | - | 1.758E+05 | 612.3 | - | - | 0 | - |
| - | - | 1.072E+04 | 612.8 | - | - | 0 | - |
| - | - | 4.362E+04 | 613.3 | - | - | 0 | - |
| - | - | 6275 | 614.3 | - | - | 0 | - |
| - | - | 3202 | 617.3 | - | - | 0 | - |
| - | - | 3776 | 620.8 | - | - | 0 | - |
| - | - | 6132 | 621.3 | - | - | 0 | - |
| - | - | 3799 | 621.3 | - | - | 0 | - |
| - | - | 2144 | 622.3 | - | - | 0 | - |
| - | - | 2858 | 622.4 | - | - | 0 | - |
| - | - | 3068 | 628.3 | - | - | 0 | - |
| - | - | 1725 | 630.3 | - | - | 0 | - |
| - | - | 5533 | 634.3 | - | - | 0 | - |
| - | - | 9762 | 635.3 | - | - | 0 | - |
| - | - | 4842 | 636.3 | - | - | 0 | - |
| 4 | y | 5522 | 636.8 | 0.001344 | 2.11 | +2 | 10 |
| 4 | y | 3254 | 637.3 | 0.009946 | 15.61 | +2 | 10 |
| - | - | 1772 | 637.8 | - | - | 0 | - |
| - | - | 5078 | 640.3 | - | - | 0 | - |
| - | - | 2521 | 641.3 | - | - | 0 | - |
| - | - | 6141 | 645.3 | - | - | 0 | - |
| 4 | y | 3.198E+04 | 645.8 | 0.001982 | 3.069 | +2 | 10 |
| - | - | 2.691E+04 | 646.3 | - | - | 0 | - |
| - | - | 9354 | 646.8 | - | - | 0 | - |
| - | - | 2695 | 648.3 | - | - | 0 | - |
| - | - | 3128 | 650.3 | - | - | 0 | - |
| - | - | 1709 | 651.3 | - | - | 0 | - |
| - | - | 5.6E+04 | 652.3 | - | - | 0 | - |
| - | - | 2.331E+04 | 653.3 | - | - | 0 | - |
| - | - | 3186 | 654.3 | - | - | 0 | - |
| - | - | 4086 | 658.3 | - | - | 0 | - |
| - | - | 1863 | 659.3 | - | - | 0 | - |
| - | - | 2303 | 660.9 | - | - | 0 | - |
| - | - | 2699 | 661.3 | - | - | 0 | - |
| - | - | 2.053E+04 | 662.3 | - | - | 0 | - |
| - | - | 1.191E+04 | 663.3 | - | - | 0 | - |
| - | - | 4139 | 664.3 | - | - | 0 | - |
| - | - | 1690 | 666.4 | - | - | 0 | - |
| - | - | 2105 | 668.3 | - | - | 0 | - |
| - | - | 1696 | 675.4 | - | - | 0 | - |
| 6 | b | 2.637E+04 | 676.3 | 0.001046 | 1.546 | +1 | 6 |
| 6 | b | 1.679E+04 | 677.3 | 0.008912 | 13.16 | +1 | 6 |
| - | - | 9466 | 678.3 | - | - | 0 | - |
| - | - | 5403 | 679.3 | - | - | 0 | - |
| - | - | 1532 | 679.8 | - | - | 0 | - |
| - | - | 9.325E+04 | 680.3 | - | - | 0 | - |
| - | - | 4.103E+04 | 681.3 | - | - | 0 | - |
| - | - | 8329 | 682.3 | - | - | 0 | - |
| - | - | 2548 | 684.3 | - | - | 0 | - |
| - | - | 8893 | 684.8 | - | - | 0 | - |
| - | - | 6568 | 685.3 | - | - | 0 | - |
| - | - | 2675 | 685.8 | - | - | 0 | - |
| - | - | 2815 | 688.3 | - | - | 0 | - |
| - | - | 3311 | 692.8 | - | - | 0 | - |
| 3 | y | 2.729E+04 | 693.3 | 0.001731 | 2.497 | +2 | 11 |
| 3 | y | 2.853E+04 | 693.8 | 0.008014 | 11.55 | +2 | 11 |
| 6 | b | 2.779E+05 | 694.4 | 0.0003687 | 0.5309 | +1 | 6 |
| - | - | 4801 | 694.8 | - | - | 0 | - |
| - | - | 1.048E+05 | 695.4 | - | - | 0 | - |
| - | - | 2.307E+04 | 696.4 | - | - | 0 | - |
| - | - | 1705 | 697.3 | - | - | 0 | - |
| - | - | 5017 | 697.4 | - | - | 0 | - |
| - | - | 1874 | 697.9 | - | - | 0 | - |
| - | - | 2512 | 698.3 | - | - | 0 | - |
| - | - | 2645 | 700.9 | - | - | 0 | - |
| - | - | 2092 | 701.3 | - | - | 0 | - |
| - | - | 2.902E+04 | 701.8 | - | - | 0 | - |
| - | - | 2563 | 701.9 | - | - | 0 | - |
| 3 | y | 2.168E+05 | 702.3 | 0.002186 | 3.112 | +2 | 11 |
| 12 | b | 1.818E+05 | 702.8 | 0.01253 | 17.83 | +2 | 12 |
| 12 | b | 8.905E+04 | 703.3 | 0.00277 | 3.939 | +2 | 12 |
| - | - | 2.177E+04 | 703.8 | - | - | 0 | - |
| - | - | 7010 | 704.3 | - | - | 0 | - |
| 8 | y | 1.928E+04 | 706.3 | 0.002508 | 3.55 | +1 | 6 |
| - | - | 9166 | 707.3 | - | - | 0 | - |
| - | - | 1898 | 708.3 | - | - | 0 | - |
| - | - | 1.095E+04 | 711.4 | - | - | 0 | - |
| 12 | b | 1.82E+04 | 711.9 | 0.001838 | 2.582 | +2 | 12 |
| - | - | 1.44E+04 | 712.4 | - | - | 0 | - |
| - | - | 5423 | 712.9 | - | - | 0 | - |
| - | - | 2530 | 713.4 | - | - | 0 | - |
| - | - | 2106 | 720.4 | - | - | 0 | - |
| - | - | 1.184E+04 | 721.4 | - | - | 0 | - |
| - | - | 4158 | 722.4 | - | - | 0 | - |
| - | - | 2.115E+04 | 723.4 | - | - | 0 | - |
| 8 | y | 1.818E+05 | 724.4 | 0.00177 | 2.443 | +1 | 6 |
| - | - | 6.997E+04 | 725.4 | - | - | 0 | - |
| - | - | 1.492E+04 | 726.4 | - | - | 0 | - |
| - | - | 2106 | 727.4 | - | - | 0 | - |
| - | - | 9043 | 729.4 | - | - | 0 | - |
| - | - | 2128 | 729.9 | - | - | 0 | - |
| - | - | 3913 | 730.4 | - | - | 0 | - |
| - | - | 6889 | 733.4 | - | - | 0 | - |
| - | - | 6259 | 734.4 | - | - | 0 | - |
| - | - | 2610 | 735.4 | - | - | 0 | - |
| - | - | 1933 | 737.9 | - | - | 0 | - |
| - | - | 2256 | 738.4 | - | - | 0 | - |
| 2 | y | 3716 | 742.9 | 0.004023 | 5.416 | +2 | 12 |
| 2 | y | 4902 | 743.4 | 0.01415 | 19.04 | +2 | 12 |
| - | - | 2139 | 743.9 | - | - | 0 | - |
| - | - | 1762 | 745.4 | - | - | 0 | - |
| - | - | 3951 | 747.4 | - | - | 0 | - |
| - | - | 1841 | 748.4 | - | - | 0 | - |
| - | - | 2401 | 749.3 | - | - | 0 | - |
| - | - | 3.184E+04 | 751.4 | - | - | 0 | - |
| 2 | y | 1.643E+04 | 751.9 | 0.001304 | 1.735 | +2 | 12 |
| - | - | 2.82E+04 | 752.4 | - | - | 0 | - |
| - | - | 1.022E+04 | 752.9 | - | - | 0 | - |
| - | - | 6427 | 753.4 | - | - | 0 | - |
| - | - | 2794 | 760.3 | - | - | 0 | - |
| - | - | 2810 | 761.4 | - | - | 0 | - |
| - | - | 4582 | 762.4 | - | - | 0 | - |
| - | - | 1.671E+04 | 763.4 | - | - | 0 | - |
| - | - | 6690 | 764.4 | - | - | 0 | - |
| - | - | 5894 | 765.4 | - | - | 0 | - |
| - | - | 2441 | 766.4 | - | - | 0 | - |
| - | - | 6990 | 767.3 | - | - | 0 | - |
| - | - | 2688 | 768.3 | - | - | 0 | - |
| - | - | 2084 | 769.4 | - | - | 0 | - |
| - | - | 3277 | 775.4 | - | - | 0 | - |
| - | - | 2689 | 776.4 | - | - | 0 | - |
| - | - | 1.742E+04 | 779.4 | - | - | 0 | - |
| - | - | 9306 | 780.4 | - | - | 0 | - |
| - | - | 7570 | 781.4 | - | - | 0 | - |
| - | - | 4247 | 782.4 | - | - | 0 | - |
| 0 | Precursor | 2.87E+04 | 793.4 | 0.01412 | 17.8 | +2 | -1 |
| - | - | 1.113E+04 | 794.4 | - | - | 0 | - |
| - | - | 9594 | 795.3 | - | - | 0 | - |
| - | - | 3802 | 795.4 | - | - | 0 | - |
| - | - | 4022 | 796.3 | - | - | 0 | - |
| - | - | 6342 | 807.4 | - | - | 0 | - |
| - | - | 5187 | 807.4 | - | - | 0 | - |
| - | - | 2141 | 808.4 | - | - | 0 | - |
| - | - | 3574 | 808.4 | - | - | 0 | - |
| - | - | 4972 | 818.4 | - | - | 0 | - |
| - | - | 3608 | 819.4 | - | - | 0 | - |
| - | - | 1929 | 820.4 | - | - | 0 | - |
| - | - | 2212 | 825.4 | - | - | 0 | - |
| - | - | 3103 | 826.4 | - | - | 0 | - |
| - | - | 3432 | 827.4 | - | - | 0 | - |
| - | - | 6313 | 834.4 | - | - | 0 | - |
| - | - | 8224 | 835.4 | - | - | 0 | - |
| - | - | 1.678E+04 | 836.4 | - | - | 0 | - |
| - | - | 1.07E+04 | 837.5 | - | - | 0 | - |
| - | - | 2445 | 838.5 | - | - | 0 | - |
| - | - | 3942 | 843.4 | - | - | 0 | - |
| - | - | 1.232E+04 | 844.4 | - | - | 0 | - |
| - | - | 4614 | 845.4 | - | - | 0 | - |
| - | - | 8912 | 846.4 | - | - | 0 | - |
| - | - | 6247 | 847.4 | - | - | 0 | - |
| - | - | 3293 | 848.4 | - | - | 0 | - |
| - | - | 6.196E+04 | 852.4 | - | - | 0 | - |
| - | - | 2093 | 853.4 | - | - | 0 | - |
| - | - | 3.299E+04 | 853.4 | - | - | 0 | - |
| - | - | 1.07E+04 | 854.4 | - | - | 0 | - |
| 7 | b | 4.201E+04 | 862.4 | 0.001139 | 1.321 | +1 | 7 |
| 7 | b | 3.155E+04 | 863.4 | 0.01175 | 13.61 | +1 | 7 |
| - | - | 5.354E+04 | 864.5 | - | - | 0 | - |
| - | - | 2.27E+04 | 865.5 | - | - | 0 | - |
| - | - | 6345 | 866.5 | - | - | 0 | - |
| - | - | 1829 | 867.5 | - | - | 0 | - |
| - | - | 1966 | 869.4 | - | - | 0 | - |
| - | - | 6088 | 876.4 | - | - | 0 | - |
| - | - | 2427 | 877.4 | - | - | 0 | - |
| 7 | b | 3.256E+05 | 880.4 | 0.0008897 | 1.011 | +1 | 7 |
| - | - | 1.676E+05 | 881.4 | - | - | 0 | - |
| - | - | 4.917E+04 | 882.4 | - | - | 0 | - |
| - | - | 9036 | 883.4 | - | - | 0 | - |
| - | - | 2423 | 890.4 | - | - | 0 | - |
| - | - | 3954 | 891.4 | - | - | 0 | - |
| - | - | 4710 | 892.5 | - | - | 0 | - |
| - | - | 1913 | 893.5 | - | - | 0 | - |
| - | - | 3485 | 894.5 | - | - | 0 | - |
| - | - | 2079 | 895.4 | - | - | 0 | - |
| - | - | 2081 | 907.4 | - | - | 0 | - |
| - | - | 2.799E+04 | 908.4 | - | - | 0 | - |
| - | - | 1.65E+04 | 909.4 | - | - | 0 | - |
| 7 | y | 2.756E+04 | 910.4 | 0.001558 | 1.712 | +1 | 7 |
| - | - | 1.366E+04 | 911.4 | - | - | 0 | - |
| - | - | 3734 | 912.4 | - | - | 0 | - |
| - | - | 3695 | 936.4 | - | - | 0 | - |
| - | - | 4689 | 937.4 | - | - | 0 | - |
| - | - | 2794 | 947.4 | - | - | 0 | - |
| - | - | 4567 | 954.4 | - | - | 0 | - |
| - | - | 1.019E+04 | 955.4 | - | - | 0 | - |
| - | - | 5964 | 956.4 | - | - | 0 | - |
| - | - | 2372 | 962.4 | - | - | 0 | - |
| - | - | 1686 | 964.5 | - | - | 0 | - |
| - | - | 1.088E+04 | 965.5 | - | - | 0 | - |
| - | - | 5189 | 966.5 | - | - | 0 | - |
| - | - | 2403 | 967.5 | - | - | 0 | - |
| - | - | 7750 | 972.4 | - | - | 0 | - |
| - | - | 4237 | 973.4 | - | - | 0 | - |
| 8 | b | 5062 | 975.5 | 0.001504 | 1.541 | +1 | 8 |
| 8 | b | 4363 | 976.5 | 0.0118 | 12.08 | +1 | 8 |
| - | - | 4897 | 980.5 | - | - | 0 | - |
| - | - | 4531 | 982.5 | - | - | 0 | - |
| - | - | 2080 | 983.5 | - | - | 0 | - |
| 8 | b | 1.556E+04 | 993.5 | 0.0005049 | 0.5082 | +1 | 8 |
| - | - | 1.132E+04 | 994.5 | - | - | 0 | - |
| - | - | 3200 | 995.5 | - | - | 0 | - |
| - | - | 2473 | 1007 | - | - | 0 | - |
| 6 | y | 1706 | 1008 | 0.01539 | 15.26 | +1 | 8 |
| - | - | 1615 | 1010 | - | - | 0 | - |
| - | - | 1890 | 1024 | - | - | 0 | - |
| 6 | y | 1.528E+04 | 1025 | 0.001288 | 1.256 | +1 | 8 |
| - | - | 1.046E+04 | 1026 | - | - | 0 | - |
| - | - | 3257 | 1027 | - | - | 0 | - |
| - | - | 2418 | 1076 | - | - | 0 | - |
| - | - | 2734 | 1077 | - | - | 0 | - |
| 9 | b | 1571 | 1092 | 0.02089 | 19.13 | +1 | 9 |
| - | - | 8192 | 1094 | - | - | 0 | - |
| - | - | 6978 | 1095 | - | - | 0 | - |
| - | - | 2103 | 1096 | - | - | 0 | - |
| 9 | b | 4365 | 1109 | 0.002879 | 2.597 | +1 | 9 |
| - | - | 2089 | 1110 | - | - | 0 | - |
| - | - | 2506 | 1118 | - | - | 0 | - |
| 5 | y | 1.034E+04 | 1136 | 0.001249 | 1.1 | +1 | 9 |
| 5 | y | 1.758E+04 | 1136 | 0.00881 | 7.752 | +1 | 9 |
| - | - | 1.017E+04 | 1137 | - | - | 0 | - |
| - | - | 3162 | 1139 | - | - | 0 | - |
| - | - | 4879 | 1153 | - | - | 0 | - |
| 5 | y | 3.103E+04 | 1154 | 0.001304 | 1.13 | +1 | 9 |
| - | - | 2.049E+04 | 1155 | - | - | 0 | - |
| - | - | 5828 | 1156 | - | - | 0 | - |
| - | - | 1594 | 1165 | - | - | 0 | - |
| 10 | b | 3954 | 1166 | 0.003734 | 3.204 | +1 | 10 |
| - | - | 1810 | 1167 | - | - | 0 | - |
| - | - | 1535 | 1495 | - | - | 0 | - |
| - | - | 1735 | 1711 | - | - | 0 | - |
| - | - | 1371 | 1720 | - | - | 0 | - |
| - | - | 1348 | 1952 | - | - | 0 | - |
| - | - | 1585 | 2769 | - | - | 0 | - |
| - | - | 1386 | 3083 | - | - | 0 | - |

m/z Charge Intensity FragmentType MassShift Position
120.0811767578125 0 8649.986
121.04016876220703 0 1004.66547
121.0845718383789 0 1069.1127
122.07170104980469 0 4218.539
123.0444564819336 0 4882.5874
123.05550384521484 0 1507.0856
125.10821533203125 0 948.9379
125.37583923339844 0 761.8791
126.09159851074219 0 1826.6792
127.05060577392578 0 7478.4526
127.08692932128906 0 1422.9551
127.12323760986328 0 2910.801
128.04901123046875 0 1037.3254
128.0541534423828 0 922.6204
128.10740661621094 0 55114.438
129.06625366210938 0 3310.992
129.10269165039062 0 565230.94
130.06553649902344 0 238166.84
130.09991455078125 0 3574.3108
130.1060028076172 0 35743.445
131.06886291503906 0 22908.508
132.08116149902344 0 54500.523
133.06085205078125 0 891.21844
133.0846405029297 0 3893.977
135.1682586669922 0 1006.20526
136.0760955810547 0 67277.75
137.0793914794922 0 5881.4478
137.16400146484375 0 1087.6134
138.0666046142578 0 6233.968
138.091796875 0 4064.2314
139.05067443847656 0 1151.0259
139.0869598388672 0 5654.8076
141.1026611328125 0 10035.268
142.0654754638672 0 5138.098
143.0457763671875 0 3458.1775
144.08151245117188 0 2310.6348
145.06130981445312 0 4474.455
146.06044006347656 0 1385.8187
147.04440307617188 0 3055.1697
149.04527282714844 0 13746.018
150.0667266845703 0 3413.6245
151.08706665039062 0 11096.127
152.09136962890625 0 1110.4309
154.09823608398438 0 1037.4235
155.0452880859375 0 2585.2375
155.08216857910156 0 2058.2966
155.09315490722656 0 34811.6
155.1182861328125 0 24034.566 a Water loss 1
156.02987670898438 0 1148.7776
156.07681274414062 0 2875.1064
156.0963134765625 0 1763.5522
156.12156677246094 0 1450.6041
157.09765625 0 2184.4023
157.13427734375 0 1113.0303
158.0841064453125 0 3018.3223
159.09214782714844 0 423888.97
160.07603454589844 0 4522.1953
160.08941650390625 0 2755.5054
160.09547424316406 0 42395.215
161.09881591796875 0 1817.5886
162.66802978515625 0 1152.6813
165.05503845214844 0 32029.95
165.0778045654297 0 1332.5521
166.06150817871094 0 70886.375
166.08688354492188 0 3243.0261 y 9
166.09788513183594 0 5191.5933
167.055908203125 0 13676.412
167.06472778320312 0 4712.211
167.11862182617188 0 1100.6453
168.08119201660156 0 6945.633
168.1023712158203 0 5291.71
168.11363220214844 0 5466.9023
169.07643127441406 0 7293.8228
169.08340454101562 0 1106.2295
169.0976104736328 0 15942.399
170.06045532226562 0 18247.807
171.0636444091797 0 2022.851
171.092041015625 0 2578.1753
171.1492919921875 0 2174.4216
172.07215881347656 0 6226.1704
172.10867309570312 0 1677.5334
173.05616760253906 0 8184.721
173.1289520263672 0 851863.94 a 1
173.4528350830078 0 2426.961
174.1322784423828 0 74271.516
175.13426208496094 0 4727.738
177.10272216796875 0 2134.5732
178.1343231201172 0 26559.402
179.137939453125 0 1487.5388
181.06117248535156 0 5853.0967
182.0816650390625 0 79219.055 y 12
183.0856475830078 0 6801.941
183.1132049560547 0 22020.197 b Water loss 1
184.11695861816406 0 2599.1616
185.1288604736328 0 1200.8776
185.1651611328125 0 3391.5015
186.12416076660156 0 183741.56
187.0870361328125 0 38245.766
187.12759399414062 0 15985.395
187.14474487304688 0 1456.1244
188.07081604003906 0 4541.703
188.090576171875 0 4316.8716
190.08287048339844 0 6684.8887
190.13430786132812 0 4997.4956
194.0928497314453 0 3913.8901
195.11326599121094 0 41609.277
196.1084747314453 0 29591.084
196.1165313720703 0 2705.6453
197.11181640625 0 1666.761
197.12855529785156 0 3215.187
197.16529846191406 0 1454.5344
198.0878448486328 0 5290.221
198.128173828125 0 15249.846
199.07167053222656 0 3525.7158
199.08712768554688 0 3673.3594
199.1315460205078 0 1547.8448
200.1435089111328 0 2507.6626
201.12379455566406 0 226962.03 b 1
202.08670043945312 0 2120.6191
202.12701416015625 0 21611.203
204.11407470703125 0 2696.1213
205.09767150878906 0 17080.656
205.10748291015625 0 1650.909
205.1451873779297 0 5441.0635
206.10107421875 0 2178.7527
206.1293487548828 0 4474.8203
207.1608123779297 0 7481.2734
209.05662536621094 0 1782.2583
210.12753295898438 0 6253.4873
211.1082305908203 0 6215.3994 y Water loss 10
212.1398468017578 0 5798.4443
213.123779296875 0 19562.82
213.1601104736328 0 2111.4792
214.11834716796875 0 1586.8372
215.10414123535156 0 1183.4669
215.11741638183594 0 1706.5869
215.13931274414062 0 2253.795
216.0981903076172 0 4183.23
221.0594482421875 0 1251.2124
221.07260131835938 0 2352.3157
221.08482360839844 0 7312.3154
221.1042022705078 0 4075.5671
221.12913513183594 0 2458.6863
221.1400604248047 0 2677.8174
222.08535766601562 0 2052.4993
222.12396240234375 0 6066.0693
223.0641326904297 0 2951.1707
223.10809326171875 0 11693.582
223.11891174316406 0 1993.9552
223.15582275390625 0 75239.73
224.1032257080078 0 3398.5684
224.15911865234375 0 7707.326
225.04342651367188 0 8065.1147
226.04339599609375 0 1440.955
226.082763671875 0 15113.314
226.15538024902344 0 1549.0907
227.06666564941406 0 11579.124
227.1546173095703 0 3133.229
228.13442993164062 0 2775.969
229.11883544921875 0 20483.316
229.1553192138672 0 7155.6333
230.1041717529297 0 1550.1643
230.1221923828125 0 2885.342
230.1503143310547 0 2466.5532
233.14028930664062 0 11008.17
234.124267578125 0 82477.46
235.12750244140625 0 11538.525
235.1560821533203 0 2957.132
237.13555908203125 0 2417.435
238.11907958984375 0 8837.379
238.1307830810547 0 2876.1523
239.08241271972656 0 2065.4917
239.0955352783203 0 15294.385
239.11480712890625 0 1978.463
240.09603881835938 0 4275.852
240.1210174560547 0 2205.924
240.13482666015625 0 32013.293
241.09336853027344 0 2514.1797
241.1188507080078 0 4120.8433
241.13812255859375 0 2021.4436
241.1915283203125 0 4306.7925
242.09280395507812 0 1581.9354
243.10975646972656 0 6281.717
244.09341430664062 0 30150.691
245.09686279296875 0 3646.9453
247.1083526611328 0 46431.645
247.14385986328125 0 2708.319
247.1563720703125 0 1815.801
248.10348510742188 0 3017.218
248.11436462402344 0 20112.25
248.62376403808594 0 1207.121 y 9
249.09873962402344 0 24038.305
249.11260986328125 0 1949.1505
249.1352996826172 0 6909.0986
250.1022186279297 0 3170.4368
250.11842346191406 0 1947.713
250.16680908203125 0 1299.0415
251.15087890625 0 279046.75
252.13409423828125 0 1901.0453
252.15399169921875 0 35988.406
252.60317993164062 0 1621.9037
253.1563262939453 0 1829.9108
254.15155029296875 0 1407.8213
255.14840698242188 0 15481.656
256.1082458496094 0 3677.991
256.1520080566406 0 1873.8695
257.1135559082031 0 8664.929
258.14520263671875 0 12146.988
259.12908935546875 0 1627.9259
259.1498107910156 0 1962.7513
261.1195983886719 0 5544.141
261.13421630859375 0 2134.5793
262.1195983886719 0 3724.315
265.1297912597656 0 30596.39
265.14373779296875 0 2312.7688
266.1253662109375 0 76481.12
267.12823486328125 0 8367.188
268.1142578125 0 1383.0647
268.1300964355469 0 4046.3118
268.1774597167969 0 6056.2275
269.1610412597656 0 2468.2922
269.1856994628906 0 3677.2961
270.1221008300781 0 10410.24
270.1453857421875 0 2059.066
270.6234130859375 0 4283.3438
272.1761779785156 0 18702.582
273.1793518066406 0 3510.1172
274.118896484375 0 15549.645
274.1316223144531 0 5419.9956
275.1029357910156 0 4950.032
275.1212158203125 0 3020.952
275.1500244140625 0 2255.501
276.10906982421875 0 4061.492
276.1260986328125 0 2576.5789
276.1554870605469 0 3182.1873
276.1705017089844 0 2130.1523
279.14544677734375 0 6041.971
280.1298828125 0 7980.1646
280.1466369628906 0 1842.2621
281.0517883300781 0 11227.749
282.1565246582031 0 9140.938
283.14068603515625 0 199512.16
283.35296630859375 0 1357.5121
284.0877380371094 0 1787.0176
284.1039733886719 0 8713.042
284.12408447265625 0 2541.2292
284.14385986328125 0 27365.418
285.14422607421875 0 2339.692
285.15924072265625 0 1670.0244
286.1402587890625 0 14908.315
286.15643310546875 0 1763.9647
287.1428527832031 0 1553.6853
287.172119140625 0 3685.7412
288.619384765625 0 5656.748
290.1659240722656 0 2956.2358 b 4
293.1138610839844 0 113156.05 y Water loss 11
294.1179504394531 0 20382.018
295.1033020019531 0 1403.7208
295.12017822265625 0 2142.4712
296.10614013671875 0 1305.502
296.1979064941406 0 3649.6238 b Water loss 2
297.0835876464844 0 2282.9473
297.1331481933594 0 4489.3374 y Water loss 8
297.15643310546875 0 38087.766
297.1993103027344 0 1702.8221
297.63372802734375 0 1728.1488
298.1398010253906 0 4573.9897 y Water loss 6
298.1595153808594 0 4438.879
299.0624084472656 0 89479.42
300.0634765625 0 2486.2405
300.1688537597656 0 17997.21
301.0589904785156 0 1562.3153
301.1513366699219 0 103467.91
302.1142578125 0 19932.973
302.1545715332031 0 15017.131
303.1180419921875 0 2763.718
305.178466796875 0 2865.513
305.6473388671875 0 2451.8813
306.1380920410156 0 18021.04 y 8
306.2295227050781 0 1982.7198
306.6397399902344 0 6636.575
307.14044189453125 0 3677.948
309.1680603027344 0 1798.0121
311.1242980957031 0 15206.751 y 11
312.1273498535156 0 2119.8662
314.18646240234375 0 3415.4834
314.2081298828125 0 9153.521 b 2
315.16705322265625 0 63073.477
316.17022705078125 0 11901.33
319.1524963378906 0 4018.9739
325.1514892578125 0 3922.6035
326.6644287109375 0 13958.244
327.1661071777344 0 3752.976
330.1664733886719 0 1460.5361
331.6578369140625 0 1637.235
332.2088623046875 0 6729.5435
333.1776123046875 0 11126.287
334.184326171875 0 2060.9346
338.1827697753906 0 1711.1094
338.6749572753906 0 2100.459 b Water loss 5
339.1704406738281 0 1341.5834 b Ammonia loss 5
339.6762390136719 0 2405.4028
340.6619873046875 0 3086.1497
341.1626892089844 0 1977.3928
342.1822204589844 0 5219.4043
343.1619873046875 0 4720.6445
343.1838073730469 0 1422.131
344.1715393066406 0 5018.4297
344.9771423339844 0 11378.182
345.13140869140625 0 8564.724
346.11541748046875 0 29748.762
346.1372375488281 0 1763.0566
347.1178894042969 0 5039.068
347.6802978515625 0 14403.483 b 5
348.1817932128906 0 3745.0684
348.6849060058594 0 1587.8402
349.15130615234375 0 1738.9587
350.219482421875 0 24956.393
351.2216491699219 0 4268.987
352.67437744140625 0 2952.4622
353.6744689941406 0 5242.2505 y Water loss 7
354.1766662597656 0 1760.1376
355.0698547363281 0 2820.6858
359.02899169921875 0 11769.362
360.69561767578125 0 6442.378
361.19781494140625 0 10718.569
361.68914794921875 0 1686.2834
362.1844177246094 0 9645.143
362.68011474609375 0 39921.2 y 7
363.1418151855469 0 46782.58
363.1820068359375 0 15276.644
363.6815185546875 0 1499.6288
364.1256103515625 0 8181.0444
364.1457824707031 0 7058.483
366.17852783203125 0 3348.169
367.1404724121094 0 4233.8315
367.1635437011719 0 2240.9568
367.24615478515625 0 5842.261
367.68505859375 0 1467.6685
373.1856994628906 0 3718.9006
373.6866760253906 0 1633.8551
376.1633605957031 0 2308.12
376.1979675292969 0 7160.2573
376.7003479003906 0 2235.7852
377.146240234375 0 3199.419
377.266357421875 0 2158.149
378.1795654296875 0 1121.8375
378.1847839355469 0 1570.3175
378.2142639160156 0 16587.22
379.20977783203125 0 77314.91
380.1641845703125 0 3507.7815
380.2126159667969 0 16216.327
381.1524658203125 0 131502.98
381.71209716796875 0 4751.2236
382.15557861328125 0 25269.773
382.18878173828125 0 4593.7837
383.1564025878906 0 3157.386
384.167236328125 0 6327.8804
384.1883544921875 0 4212.6963
385.15087890625 0 9787.503
385.17279052734375 0 3742.5852 y 4
386.1794738769531 0 2153.924
389.19744873046875 0 1960.0223 b 9
390.1960144042969 0 8780.034
390.6959533691406 0 2611.0918
391.1368408203125 0 6477.4585
391.1849670410156 0 2622.1384
392.2093811035156 0 3197.059
394.1490783691406 0 2011.8701
394.1728820800781 0 18713.766
395.1344909667969 0 7162.824
395.1575012207031 0 5258.711
395.1790771484375 0 2268.8328
395.2406311035156 0 3279.004
396.1616516113281 0 1948.4733
396.1947326660156 0 2287.5498
396.225341796875 0 26812.95
397.2023620605469 0 2406.5483
397.2283630371094 0 5530.597
397.704833984375 0 1414.4988
398.1771545410156 0 12203.812
399.18353271484375 0 1921.2289
402.1763000488281 0 5399.509
402.1997375488281 0 11887.847
403.20159912109375 0 4571.2705
404.7105712890625 0 3644.384
405.26275634765625 0 4230.9814
406.2453308105469 0 2760.8198
407.2033996582031 0 2034.1193
409.1473083496094 0 10621.621
409.17315673828125 0 1722.9512
409.205322265625 0 2471.7822
409.7015686035156 0 1820.1904
411.1998596191406 0 6417.8677
412.18365478515625 0 84689.67
413.145263671875 0 7933.0005
413.1622009277344 0 4099.319
413.18701171875 0 12253.522
413.25177001953125 0 2032.489
414.1934814453125 0 1290.6696
414.2010498046875 0 1498.1451
414.2355651855469 0 25264.059
414.2646484375 0 2115.6106
415.0377197265625 0 22428.598
415.1980285644531 0 10158.18
415.2389831542969 0 6712.1616
416.2018737792969 0 2310.4897
417.71746826171875 0 17020.293
418.21087646484375 0 26556.04
418.71142578125 0 12970.441
419.2143249511719 0 3968.2769
421.2100524902344 0 2874.7705 y Water loss 10
422.1918640136719 0 2399.2249 y Ammonia loss 10
422.70965576171875 0 2338.667
423.20306396484375 0 5734.7783
423.2723693847656 0 32973.973
423.70513916015625 0 2305.0574
424.2252502441406 0 1740.943
424.2748107910156 0 8756.298
424.73284912109375 0 2814.6138
426.72271728515625 0 217115.17
427.2239990234375 0 108594.195
427.7255859375 0 30098.105
428.2259521484375 0 3805.074
429.2101745605469 0 29257.686
430.1944274902344 0 214305.64
431.1972961425781 0 46385.57
431.7150573730469 0 25587.629 b Water loss 6
432.2126159667969 0 19628.977 b Ammonia loss 6
432.71160888671875 0 4813.6313
433.2102966308594 0 4024.502
433.256103515625 0 6125.7495 b Water loss 3
434.25946044921875 0 2010.6709
437.18719482421875 0 1367.5396
438.1732482910156 0 4420.1777
438.7261962890625 0 3668.9233
439.2197265625 0 56500.68 y 10
440.1855163574219 0 2323.5686
440.2230529785156 0 12892.369
440.7203063964844 0 108304.375 b 6
441.22161865234375 0 60179.426
441.2574462890625 0 1560.6243
441.7230224609375 0 17442.6
442.2249450683594 0 5004.7
443.23968505859375 0 2948.145
447.2070007324219 0 10080.205 y Ammonia loss 6
447.23907470703125 0 1698.9529
447.70977783203125 0 4050.404
448.2054748535156 0 10999.819
448.7676086425781 0 1561.1622
449.21148681640625 0 3518.3774
450.2359313964844 0 1807.1024
451.1749572753906 0 1537.7747
451.23681640625 0 2080.1042
451.2674865722656 0 60044.875 b 3
452.2705993652344 0 16288.472
453.27215576171875 0 2742.6873
455.266845703125 0 3442.6829
455.71929931640625 0 6840.4834 y 6
459.19976806640625 0 6091.238
460.75408935546875 0 6749.801
461.2032470703125 0 6200.9224
461.2539367675781 0 10420.655
462.20794677734375 0 2072.2852
462.2468566894531 0 1689.468
464.2168273925781 0 1480.139
465.7471618652344 0 1669.0934
466.244140625 0 2887.9688
468.2938232421875 0 15053.043
469.2971496582031 0 2793.4373
470.2467956542969 0 1360.7737
474.260009765625 0 14020.09
474.75341796875 0 18108.193
475.2549133300781 0 9133.27
475.7550964355469 0 3578.7976
476.22637939453125 0 4908.3804
477.2107238769531 0 11576.515
478.2291564941406 0 12750.043 y Water loss 9
478.2775573730469 0 6479.942
479.24176025390625 0 1546.7664
479.2817077636719 0 1687.97
480.2215881347656 0 2078.0977
481.7289733886719 0 2633.6492
483.2649841308594 0 43083.66
483.7662048339844 0 18378.623
484.2228088378906 0 2182.9941
484.26751708984375 0 7973.8525
484.7705078125 0 2305.6968
488.2575378417969 0 9451.316 b Water loss 7
488.7558898925781 0 9542.388 b Ammonia loss 7
489.25927734375 0 3277.798
489.7533264160156 0 1498.9905
494.23681640625 0 143069.69
495.23968505859375 0 35682.203
496.2413024902344 0 176143.77 y 9
497.2466125488281 0 44146.414
497.76361083984375 0 18206.107
498.2449645996094 0 5975.4697
502.2296447753906 0 1558.3413
503.10821533203125 0 20518.766
504.2016906738281 0 3921.7468
506.2268371582031 0 5166.152
506.2732238769531 0 2898.0493
507.26068115234375 0 2269.4268
511.2633056640625 0 13659.001
512.2660522460938 0 2038.73
513.207763671875 0 2398.098
514.18994140625 0 2497.409
515.2811279296875 0 2124.668
519.1400756835938 0 6572.224
523.259521484375 0 5057.9634
525.2677001953125 0 23011.053
526.230712890625 0 3583.0117
526.271240234375 0 6506.6025
527.27197265625 0 1898.7697
528.2383422851562 0 2267.385
529.2611694335938 0 4730.62 Precursor Water loss
529.5951538085938 0 10014.822 Precursor Ammonia loss
529.9292602539062 0 3344.4995
530.22216796875 0 2265.8655
531.2203979492188 0 2006.1747
532.194580078125 0 5447.453
534.888671875 0 1649.1097
535.2645874023438 0 3522.0254 Precursor
535.5987548828125 0 8787.811
536.16650390625 0 3795.1206
537.7337036132812 0 1950.8851
538.773193359375 0 1698.2753
539.237060546875 0 27119.035
540.2390747070312 0 6487.0093
542.2933349609375 0 5746.4053
543.278076171875 0 53345.82
544.2816162109375 0 14284.284
545.2830810546875 0 2766.277
545.7701416015625 0 1952.8391 b Water loss 8
546.2503051757812 0 6515.443
546.7495727539062 0 2738.611
547.2811889648438 0 1844.4703
548.2396240234375 0 1873.1737
548.2950439453125 0 1736.4001
549.2210083007812 0 7358.329
550.2114868164062 0 2061.967
551.2584838867188 0 6199.086
551.33056640625 0 3247.6309
552.2586669921875 0 2322.1843
553.2628173828125 0 1844.8689
554.2821044921875 0 1592.5065
554.771484375 0 5723.129 b 8
555.2571411132812 0 15843.161
555.7577514648438 0 8004.3203
556.2559204101562 0 2606.4607
561.3150634765625 0 8027.305 b Water loss 4
562.3214111328125 0 2114.7783
563.2582397460938 0 5515.3853
564.2608642578125 0 1739.046
564.33154296875 0 3526.653
565.2639770507812 0 10141.219
567.2319946289062 0 28874.146
568.235595703125 0 6324.458
568.75830078125 0 3018.2346 y Ammonia loss 4
569.2337036132812 0 1643.9319
575.2465209960938 0 6623.1777
575.2946166992188 0 1746.0106
576.2339477539062 0 4274.159
577.2688598632812 0 1777.5295 y 4
577.7615356445312 0 1768.6211
578.2713623046875 0 1478.217
579.274169921875 0 2165.727
579.3259887695312 0 40856.1 b 4
580.328857421875 0 11989.5
581.3303833007812 0 2659.717
592.2732543945312 0 4431.734
592.3251342773438 0 4261.935
593.2573852539062 0 45639.766 y Water loss 8
593.306396484375 0 18382.926
593.7868041992188 0 2709.674
594.2591552734375 0 14698.167
594.3081665039062 0 3104.4133
595.256591796875 0 6751.47
596.2574462890625 0 1608.0154
597.7977294921875 0 1865.5125
598.300537109375 0 2018.1671
600.3157348632812 0 4988.3037
602.7937622070312 0 7569.8804
603.2904663085938 0 10559.814
603.7963256835938 0 2419.9124
604.2900390625 0 2102.99
610.2840576171875 0 71672.586
610.3302001953125 0 4505.896
611.2686767578125 0 547396.4 y 8
611.798095703125 0 35215.242
612.2718505859375 0 175818.47
612.7998046875 0 10719.093
613.2739868164062 0 43619.633
614.2765502929688 0 6274.9634
617.2845458984375 0 3201.6138
620.8034057617188 0 3775.9407
621.251220703125 0 6131.8325
621.301513671875 0 3799.0994
622.2577514648438 0 2143.9795
622.368896484375 0 2857.7158
628.2748413085938 0 3068.0098
630.2864379882812 0 1724.59
634.3055419921875 0 5533.147
635.2973022460938 0 9762.058
636.3291625976562 0 4842.266
636.7871704101562 0 5521.894 y Water loss 3
637.2877807617188 0 3254.3608 y Ammonia loss 3
637.7892456054688 0 1771.6068
640.2745971679688 0 5077.6523
641.2681884765625 0 2520.891
645.296142578125 0 6141.371
645.7930908203125 0 31983.67 y 3
646.29345703125 0 26910.686
646.795166015625 0 9354.105
648.342529296875 0 2695.4946
650.2694091796875 0 3127.6003
651.2765502929688 0 1709.2915
652.3212890625 0 55998.7
653.3236083984375 0 23312.209
654.3256225585938 0 3185.5173
658.2848510742188 0 4086.3586
659.3157958984375 0 1863.4033
660.8705444335938 0 2302.9216
661.3280639648438 0 2698.957
662.3062133789062 0 20530.627
663.29931640625 0 11905.117
664.3419189453125 0 4138.5537
666.3629760742188 0 1690.0663
668.2807006835938 0 2104.9656
675.3798217773438 0 1696.0261
676.3423461914062 0 26367.28 b Water loss 5
677.334228515625 0 16793.549 b Ammonia loss 5
678.3362426757812 0 9465.974
679.3341064453125 0 5402.622
679.83154296875 0 1531.7565
680.3164672851562 0 93251.64
681.3190307617188 0 41032.285
682.3223876953125 0 8328.831
684.322265625 0 2547.7007
684.8179931640625 0 8893.42
685.318603515625 0 6567.509
685.8224487304688 0 2674.5647
688.3336181640625 0 2815.0442
692.8370971679688 0 3310.8303
693.32958984375 0 27293.14 y Water loss 2
693.827880859375 0 28532.209 y Ammonia loss 2
694.3522338867188 0 277917.8 b 5
694.8287353515625 0 4800.8013
695.3555297851562 0 104772.836
696.3583374023438 0 23072.5
697.290283203125 0 1704.6381
697.3563232421875 0 5017.481
697.8602294921875 0 1873.7523
698.345703125 0 2512.36
700.8926391601562 0 2645.2979
701.337890625 0 2091.625
701.8419799804688 0 29015.934
701.9065551757812 0 2563.3547
702.3353271484375 0 216794.2 y 2
702.83642578125 0 181764.25 b Water loss 11
703.3381958007812 0 89051.76 b Ammonia loss 11
703.8388671875 0 21773.62
704.3384399414062 0 7010.0093
706.3431396484375 0 19284.852 y Water loss 7
707.3446655273438 0 9166.4795
708.3386840820312 0 1897.9121
711.3700561523438 0 10949.913
711.8560791015625 0 18199.287 b 11
712.3591918945312 0 14395.347
712.8599853515625 0 5422.782
713.3562622070312 0 2530.008
720.3742065429688 0 2106.4763
721.36376953125 0 11836.194
722.3670654296875 0 4157.708
723.3685913085938 0 21151.77
724.3529663085938 0 181803.19 y 7
725.3557739257812 0 69974.52
726.3580932617188 0 14922.069
727.3623657226562 0 2106.4082
729.3584594726562 0 9043.174
729.8616333007812 0 2128.1914
730.361572265625 0 3912.634
733.3648681640625 0 6889.1206
734.3563232421875 0 6259.3735
735.3562622070312 0 2610.0176
737.8616943359375 0 1932.892
738.3515625 0 2256.451
742.8660888671875 0 3715.6218 y Water loss 1
743.3682250976562 0 4901.969 y Ammonia loss 1
743.8583374023438 0 2139.32
745.35986328125 0 1762.1047
747.3889770507812 0 3951.0981
748.3795776367188 0 1841.3551
749.33154296875 0 2400.9573
751.3770141601562 0 31843.773
751.86865234375 0 16428.988 y 1
752.3748779296875 0 28202.846
752.87158203125 0 10216.986
753.376220703125 0 6426.9263
760.3053588867188 0 2794.0015
761.3783569335938 0 2810.424
762.4161987304688 0 4582.103
763.3646240234375 0 16709.984
764.3662109375 0 6690.2876
765.4020385742188 0 5894.168
766.4075927734375 0 2440.6992
767.347412109375 0 6989.7173
768.3475341796875 0 2687.755
769.405029296875 0 2084.4397
775.3907470703125 0 3276.9587
776.3707275390625 0 2689.2356
779.3841552734375 0 17423.277
780.38671875 0 9305.569
781.3672485351562 0 7570.317
782.3641967773438 0 4247.024
793.4000244140625 0 28699.365 Precursor Water loss
794.4024658203125 0 11128.257
795.3418579101562 0 9594.383
795.4095458984375 0 3802.3755
796.345703125 0 4021.5242
807.37744140625 0 6342.0723
807.4411010742188 0 5187.069
808.3701782226562 0 2141.4255
808.4314575195312 0 3574.1067
818.4392700195312 0 4971.8145
819.4442138671875 0 3607.8813
820.4362182617188 0 1928.887
825.3858642578125 0 2212.4065
826.377197265625 0 3102.7566
827.3712768554688 0 3431.9387
834.4260864257812 0 6313.227
835.4161987304688 0 8223.879
836.4461059570312 0 16777.463
837.4544677734375 0 10704.716
838.4544067382812 0 2445.0408
843.3966064453125 0 3942.0276
844.388671875 0 12317.377
845.3946533203125 0 4613.7637
846.447509765625 0 8912.421
847.443603515625 0 6246.7197
848.4468994140625 0 3293.4072
852.437255859375 0 61956.465
853.3524169921875 0 2093.4395
853.4403076171875 0 32991.41
854.4420776367188 0 10700.521
862.4217529296875 0 42005.684 b Water loss 6
863.4163818359375 0 31545.443 b Ammonia loss 6
864.4559326171875 0 53540.484
865.4605712890625 0 22700.668
866.4632568359375 0 6345.4004
867.4620971679688 0 1828.7144
869.38525390625 0 1966.4895
876.4365844726562 0 6088.231
877.4367065429688 0 2426.9907
880.4320678710938 0 325623.03 b 6
881.4344482421875 0 167566.64
882.43701171875 0 49170.566
883.4407958984375 0 9036.021
890.4137573242188 0 2422.9172
891.4091796875 0 3954.0725
892.4699096679688 0 4709.5234
893.4727783203125 0 1912.9083
894.453125 0 3484.628
895.447265625 0 2079.1685
907.4397583007812 0 2081.148
908.4276733398438 0 27991.398
909.4326171875 0 16497.074
910.4320678710938 0 27564.443 y 6
911.434814453125 0 13664.713
912.436279296875 0 3733.9658
936.4237670898438 0 3695.4983
937.4144897460938 0 4688.876
947.4387817382812 0 2793.8994
954.4354858398438 0 4566.6655
955.4212646484375 0 10191.582
956.421875 0 5963.827
962.4472045898438 0 2372.3694
964.4705810546875 0 1685.7017
965.4500732421875 0 10882.73
966.4517211914062 0 5189.293
967.4525146484375 0 2402.6418
972.4434204101562 0 7749.5557
973.4470825195312 0 4237.0815
975.503173828125 0 5062.142 b Water loss 7
976.50048828125 0 4362.7065 b Ammonia loss 7
980.4603881835938 0 4897.3623
982.472900390625 0 4531.485
983.4744262695312 0 2079.9275
993.5157470703125 0 15555.412 b 7
994.5202026367188 0 11323.308
995.5261840820312 0 3199.995
1007.4842529296875 0 2473.2666
1008.4462890625 0 1706.1749 y Ammonia loss 5
1009.5018310546875 0 1615.0729
1024.472900390625 0 1889.5896
1025.458740234375 0 15281.217 y 5
1026.4620361328125 0 10463.129
1027.4659423828125 0 3256.6985
1075.541748046875 0 2418.23
1076.5286865234375 0 2733.7312
1091.4947509765625 0 1571.1759 b Ammonia loss 8
1093.544189453125 0 8191.6084
1094.5430908203125 0 6978.2144
1095.5458984375 0 2102.9048
1108.539306640625 0 4365.052 b 8
1109.5416259765625 0 2089.4868
1118.48974609375 0 2506.0698
1135.5067138671875 0 10338.336 y Water loss 4
1136.498291015625 0 17579.895 y Ammonia loss 4
1137.4986572265625 0 10174.933
1138.50048828125 0 3162.262
1152.5313720703125 0 4878.8525
1153.517333984375 0 31025.19 y 4
1154.5198974609375 0 20490.166
1155.522216796875 0 5828.452
1164.5938720703125 0 1594.4017
1165.5673828125 0 3953.8123 b 9
1166.559326171875 0 1809.5311
1494.9261474609375 0 1535.155
1710.91162109375 0 1735.2775
1720.029052734375 0 1371.2191
1951.8946533203125 0 1348.0876
2768.503173828125 0 1584.9862
3082.859619140625 0 1385.8217

Spectrum Details

|  |  |
| --- | --- |
| Matched peaks? Matched peaksThe total absolute number of peaks matched. Additionally in brackets the total fraction of peaks matched and the total number of peaks is shown. | 81 (9.82% of 825) |
| FDR? FDRThe false discovery rate estimated for this peptide. It is calculated by matching all theoretical fragments with a non-integer shift with the raw peaks for this spectrum. This is done with 40 different shifts. The resulting percentage is the average number of annotated peaks over the number of annotated peaks with the correct spectrum. | 0.62% |
| Satellite FDR? Satellite FDRSee the FDR for details on its calculation. This satellite ion specific FDR only contains the satellite ions (d/w) for I/L/J positions. | ∞ |
| PSM Score? PSM ScoreThe PSM Score as given by Hecklib to this annotated spectrum. It is shown with three significant figures. | 604 |

## Spectrum 7274? Spectrum 7274 The raw spectrum of this peptide as annotated by Hecklib. The fragments are coloured according to ion type (see legend). Any peaks with a star '\*' as text can be hovered over to see the full details, first the ion type second the mass shift type. By hovering over the amino acids in the peptide or ions in the legend the corresponding peaks are highlighted. By toggling the 'Unassigned' label you can turn the background (unassigned) peaks on or off in the plot. By updating the slider in the Ion legend you can update the spectrum to only show the top X% of the peaks with labels. The top X% means any peak that is within X% of the highest intensity. By dragging in the spectrum you can zoom in to a specific part of the spectrum and use 'Zoom Out' to get back to the original zoom level. The annotation of the spectrum is based on the given sequence in the peptides file and is done with different software so inconsistencies are likely. The peaks are annotated based on the given sequence, with 20 ppm tolerance.

Copy Data

### Spectrum 7274 (TSV)

#### Preview

```
Loading example...
```

*Click on the button to copy the data to your clipboard.*

Mz MinMz MaxIntensity Max

WidthHeightPeptide font sizePeptide stroke widthSpectrum font sizeSpectrum stroke widthCompact peptide

Ion legend

wxyz

abcd

OtherUnassignedIonChargePositionShow for top:%

TVLHQDWLDGKEY

04.87e+59.75e+51.46e+61.95e+6

Zoom Out

a+12a+12y+11b+12b+12y+23y+24b+25y+12b+13y+25y+37y+25y+12b+13b+26b+26y+26y+26y+39b+310y+13y+13b+27b+27b+14y+13b+27y+27b+14y+27y+14b+28b+28y+14\*\*\*b+29b+15b+15y+29b+15b+210y+15y+15y+210y+210b+211y+210b+16b+16y+211y+211b+16y+211b+212b+212y+16b+212y+16y+212y+212y+212\*b+17b+17b+17y+17b+18b+18b+18y+18b+19b+19y+19y+19y+19b+110

0799159823963195

Fragment Matches Table

Show background peaks

| Position | Ion type | Intensity | mz Theoretical | mz Error (Th) | mz Error (ppm) | Charge | Series Number |
| --- | --- | --- | --- | --- | --- | --- | --- |
| - | - | 5636 | 120.1 | - | - | 0 | - |
| - | - | 1.026E+04 | 122.1 | - | - | 0 | - |
| - | - | 1.179E+04 | 123 | - | - | 0 | - |
| - | - | 2414 | 123.1 | - | - | 0 | - |
| - | - | 3293 | 125.1 | - | - | 0 | - |
| - | - | 1535 | 125.6 | - | - | 0 | - |
| - | - | 1601 | 126 | - | - | 0 | - |
| - | - | 3884 | 126.1 | - | - | 0 | - |
| - | - | 1.455E+04 | 127.1 | - | - | 0 | - |
| - | - | 1538 | 127.1 | - | - | 0 | - |
| - | - | 5685 | 127.1 | - | - | 0 | - |
| - | - | 1642 | 128.1 | - | - | 0 | - |
| - | - | 3597 | 128.1 | - | - | 0 | - |
| - | - | 1.205E+05 | 128.1 | - | - | 0 | - |
| - | - | 1.29E+06 | 129.1 | - | - | 0 | - |
| - | - | 5.292E+05 | 130.1 | - | - | 0 | - |
| - | - | 9603 | 130.1 | - | - | 0 | - |
| - | - | 8.435E+04 | 130.1 | - | - | 0 | - |
| - | - | 5.087E+04 | 131.1 | - | - | 0 | - |
| - | - | 1948 | 131.1 | - | - | 0 | - |
| - | - | 1566 | 131.2 | - | - | 0 | - |
| - | - | 1.257E+05 | 132.1 | - | - | 0 | - |
| - | - | 2446 | 133.1 | - | - | 0 | - |
| - | - | 1.384E+04 | 133.1 | - | - | 0 | - |
| - | - | 2131 | 133.1 | - | - | 0 | - |
| - | - | 1.579E+05 | 136.1 | - | - | 0 | - |
| - | - | 1623 | 136.2 | - | - | 0 | - |
| - | - | 1.377E+04 | 137.1 | - | - | 0 | - |
| - | - | 1.542E+04 | 138.1 | - | - | 0 | - |
| - | - | 5696 | 138.1 | - | - | 0 | - |
| - | - | 1768 | 139.1 | - | - | 0 | - |
| - | - | 1.541E+04 | 139.1 | - | - | 0 | - |
| - | - | 1932 | 139.2 | - | - | 0 | - |
| - | - | 1.983E+04 | 141.1 | - | - | 0 | - |
| - | - | 7678 | 142.1 | - | - | 0 | - |
| - | - | 2321 | 142.1 | - | - | 0 | - |
| - | - | 1814 | 142.6 | - | - | 0 | - |
| - | - | 8487 | 143 | - | - | 0 | - |
| - | - | 2102 | 143.1 | - | - | 0 | - |
| - | - | 7782 | 144.1 | - | - | 0 | - |
| - | - | 1483 | 144.1 | - | - | 0 | - |
| - | - | 9071 | 145.1 | - | - | 0 | - |
| - | - | 2211 | 146.1 | - | - | 0 | - |
| - | - | 6829 | 147 | - | - | 0 | - |
| - | - | 2463 | 148.1 | - | - | 0 | - |
| - | - | 2381 | 149 | - | - | 0 | - |
| - | - | 1.757E+04 | 149 | - | - | 0 | - |
| - | - | 6623 | 150.1 | - | - | 0 | - |
| - | - | 2.476E+04 | 151.1 | - | - | 0 | - |
| - | - | 2716 | 153.1 | - | - | 0 | - |
| - | - | 2752 | 154.1 | - | - | 0 | - |
| - | - | 2700 | 154.1 | - | - | 0 | - |
| - | - | 4053 | 155 | - | - | 0 | - |
| - | - | 3701 | 155.1 | - | - | 0 | - |
| - | - | 7.863E+04 | 155.1 | - | - | 0 | - |
| 2 | a | 5.665E+04 | 155.1 | 0.0003966 | 2.557 | +1 | 2 |
| - | - | 4035 | 156.1 | - | - | 0 | - |
| - | - | 5106 | 156.1 | - | - | 0 | - |
| - | - | 2808 | 156.1 | - | - | 0 | - |
| - | - | 3274 | 156.1 | - | - | 0 | - |
| - | - | 1947 | 157.1 | - | - | 0 | - |
| - | - | 7224 | 157.1 | - | - | 0 | - |
| - | - | 4496 | 157.1 | - | - | 0 | - |
| - | - | 6481 | 158.1 | - | - | 0 | - |
| - | - | 9.717E+05 | 159.1 | - | - | 0 | - |
| - | - | 9117 | 160.1 | - | - | 0 | - |
| - | - | 7912 | 160.1 | - | - | 0 | - |
| - | - | 1.054E+05 | 160.1 | - | - | 0 | - |
| - | - | 5466 | 161.1 | - | - | 0 | - |
| - | - | 8.799E+04 | 165.1 | - | - | 0 | - |
| - | - | 4322 | 165.1 | - | - | 0 | - |
| - | - | 1.617E+05 | 166.1 | - | - | 0 | - |
| - | - | 1.445E+04 | 166.1 | - | - | 0 | - |
| - | - | 1.099E+04 | 167.1 | - | - | 0 | - |
| - | - | 1.09E+04 | 167.1 | - | - | 0 | - |
| - | - | 3045 | 167.1 | - | - | 0 | - |
| - | - | 6454 | 167.1 | - | - | 0 | - |
| - | - | 1.635E+04 | 168.1 | - | - | 0 | - |
| - | - | 1.046E+04 | 168.1 | - | - | 0 | - |
| - | - | 1.261E+04 | 168.1 | - | - | 0 | - |
| - | - | 1.406E+04 | 169.1 | - | - | 0 | - |
| - | - | 2866 | 169.1 | - | - | 0 | - |
| - | - | 3.83E+04 | 169.1 | - | - | 0 | - |
| - | - | 4.701E+04 | 170.1 | - | - | 0 | - |
| - | - | 5061 | 171.1 | - | - | 0 | - |
| - | - | 5164 | 171.1 | - | - | 0 | - |
| - | - | 1.532E+04 | 172.1 | - | - | 0 | - |
| - | - | 1.711E+04 | 173.1 | - | - | 0 | - |
| 2 | a | 1.93E+06 | 173.1 | 0.0004825 | 2.787 | +1 | 2 |
| - | - | 3533 | 173.5 | - | - | 0 | - |
| - | - | 1.696E+05 | 174.1 | - | - | 0 | - |
| - | - | 1.106E+04 | 175.1 | - | - | 0 | - |
| - | - | 2726 | 176.1 | - | - | 0 | - |
| - | - | 3156 | 177.1 | - | - | 0 | - |
| - | - | 5.843E+04 | 178.1 | - | - | 0 | - |
| - | - | 4126 | 179.1 | - | - | 0 | - |
| - | - | 5476 | 179.1 | - | - | 0 | - |
| - | - | 8064 | 181.1 | - | - | 0 | - |
| 13 | y | 1.805E+05 | 182.1 | 0.0004954 | 2.721 | +1 | 1 |
| - | - | 1.64E+04 | 183.1 | - | - | 0 | - |
| 2 | b | 5.006E+04 | 183.1 | 0.0003855 | 2.105 | +1 | 2 |
| - | - | 5178 | 184.1 | - | - | 0 | - |
| - | - | 1.517E+04 | 185.2 | - | - | 0 | - |
| - | - | 4.215E+05 | 186.1 | - | - | 0 | - |
| - | - | 8.119E+04 | 187.1 | - | - | 0 | - |
| - | - | 3.519E+04 | 187.1 | - | - | 0 | - |
| - | - | 7923 | 188.1 | - | - | 0 | - |
| - | - | 2154 | 188.1 | - | - | 0 | - |
| - | - | 7398 | 188.1 | - | - | 0 | - |
| - | - | 2152 | 188.1 | - | - | 0 | - |
| - | - | 1.673E+04 | 190.1 | - | - | 0 | - |
| - | - | 1.412E+04 | 190.1 | - | - | 0 | - |
| - | - | 1.024E+04 | 194.1 | - | - | 0 | - |
| - | - | 9.932E+04 | 195.1 | - | - | 0 | - |
| - | - | 6.703E+04 | 196.1 | - | - | 0 | - |
| - | - | 8471 | 196.1 | - | - | 0 | - |
| - | - | 4574 | 197.1 | - | - | 0 | - |
| - | - | 5261 | 197.1 | - | - | 0 | - |
| - | - | 1.016E+04 | 198.1 | - | - | 0 | - |
| - | - | 3.366E+04 | 198.1 | - | - | 0 | - |
| - | - | 8966 | 199.1 | - | - | 0 | - |
| - | - | 7084 | 199.1 | - | - | 0 | - |
| - | - | 5360 | 199.1 | - | - | 0 | - |
| - | - | 5695 | 200.1 | - | - | 0 | - |
| 2 | b | 5.181E+05 | 201.1 | 0.0003952 | 1.965 | +1 | 2 |
| - | - | 4231 | 202.1 | - | - | 0 | - |
| - | - | 5.208E+04 | 202.1 | - | - | 0 | - |
| - | - | 4338 | 203.1 | - | - | 0 | - |
| - | - | 1957 | 204.1 | - | - | 0 | - |
| - | - | 4458 | 204.1 | - | - | 0 | - |
| - | - | 3313 | 204.1 | - | - | 0 | - |
| - | - | 4.105E+04 | 205.1 | - | - | 0 | - |
| - | - | 6818 | 205.1 | - | - | 0 | - |
| - | - | 3837 | 206.1 | - | - | 0 | - |
| - | - | 9102 | 206.1 | - | - | 0 | - |
| - | - | 1.329E+04 | 207.2 | - | - | 0 | - |
| - | - | 1.645E+04 | 210.1 | - | - | 0 | - |
| 11 | y | 8375 | 211.1 | 0.0003897 | 1.846 | +2 | 3 |
| - | - | 1.258E+04 | 212.1 | - | - | 0 | - |
| - | - | 4.528E+04 | 213.1 | - | - | 0 | - |
| - | - | 6471 | 213.2 | - | - | 0 | - |
| - | - | 3882 | 215.1 | - | - | 0 | - |
| - | - | 2803 | 215.1 | - | - | 0 | - |
| - | - | 1.253E+04 | 216.1 | - | - | 0 | - |
| - | - | 2016 | 218 | - | - | 0 | - |
| - | - | 2332 | 219.1 | - | - | 0 | - |
| - | - | 5395 | 221.1 | - | - | 0 | - |
| - | - | 4247 | 221.1 | - | - | 0 | - |
| - | - | 4034 | 221.1 | - | - | 0 | - |
| - | - | 1.033E+04 | 221.1 | - | - | 0 | - |
| - | - | 2526 | 221.1 | - | - | 0 | - |
| - | - | 5488 | 221.1 | - | - | 0 | - |
| - | - | 1.713E+04 | 222.1 | - | - | 0 | - |
| - | - | 3292 | 222.1 | - | - | 0 | - |
| - | - | 3049 | 223.1 | - | - | 0 | - |
| - | - | 2.449E+04 | 223.1 | - | - | 0 | - |
| - | - | 2552 | 223.1 | - | - | 0 | - |
| - | - | 1.736E+05 | 223.2 | - | - | 0 | - |
| - | - | 7502 | 224.1 | - | - | 0 | - |
| - | - | 1.734E+04 | 224.2 | - | - | 0 | - |
| - | - | 5749 | 225 | - | - | 0 | - |
| - | - | 3491 | 225.1 | - | - | 0 | - |
| - | - | 3798 | 225.2 | - | - | 0 | - |
| - | - | 3.87E+04 | 226.1 | - | - | 0 | - |
| - | - | 3.348E+04 | 227.1 | - | - | 0 | - |
| - | - | 5089 | 227.1 | - | - | 0 | - |
| - | - | 9511 | 227.2 | - | - | 0 | - |
| - | - | 4489 | 228.1 | - | - | 0 | - |
| - | - | 3016 | 228.1 | - | - | 0 | - |
| - | - | 5.383E+04 | 229.1 | - | - | 0 | - |
| - | - | 5221 | 230.1 | - | - | 0 | - |
| - | - | 4105 | 230.2 | - | - | 0 | - |
| - | - | 2.977E+04 | 233.1 | - | - | 0 | - |
| - | - | 1.891E+05 | 234.1 | - | - | 0 | - |
| - | - | 3518 | 234.7 | - | - | 0 | - |
| - | - | 2269 | 235.1 | - | - | 0 | - |
| - | - | 2.874E+04 | 235.1 | - | - | 0 | - |
| - | - | 4875 | 235.2 | - | - | 0 | - |
| - | - | 2317 | 235.6 | - | - | 0 | - |
| - | - | 3679 | 237.1 | - | - | 0 | - |
| - | - | 2.329E+04 | 238.1 | - | - | 0 | - |
| - | - | 4019 | 238.1 | - | - | 0 | - |
| - | - | 3394 | 239.1 | - | - | 0 | - |
| - | - | 1.314E+04 | 239.1 | - | - | 0 | - |
| - | - | 3627 | 239.1 | - | - | 0 | - |
| - | - | 5106 | 240.1 | - | - | 0 | - |
| - | - | 7.171E+04 | 240.1 | - | - | 0 | - |
| - | - | 2615 | 241.1 | - | - | 0 | - |
| - | - | 1.385E+04 | 241.1 | - | - | 0 | - |
| - | - | 1.035E+04 | 241.1 | - | - | 0 | - |
| - | - | 9202 | 241.2 | - | - | 0 | - |
| - | - | 2318 | 242.2 | - | - | 0 | - |
| - | - | 1.156E+04 | 243.1 | - | - | 0 | - |
| - | - | 7.729E+04 | 244.1 | - | - | 0 | - |
| - | - | 6824 | 245.1 | - | - | 0 | - |
| - | - | 1.103E+05 | 247.1 | - | - | 0 | - |
| - | - | 4057 | 247.1 | - | - | 0 | - |
| - | - | 4571 | 248.1 | - | - | 0 | - |
| - | - | 4.13E+04 | 248.1 | - | - | 0 | - |
| 10 | y | 2430 | 248.6 | 0.0001531 | 0.6159 | +2 | 4 |
| - | - | 6.302E+04 | 249.1 | - | - | 0 | - |
| - | - | 4165 | 249.1 | - | - | 0 | - |
| - | - | 4141 | 249.1 | - | - | 0 | - |
| - | - | 1.518E+04 | 249.1 | - | - | 0 | - |
| - | - | 9602 | 250.1 | - | - | 0 | - |
| - | - | 6.869E+05 | 251.2 | - | - | 0 | - |
| - | - | 4442 | 252.1 | - | - | 0 | - |
| - | - | 8.252E+04 | 252.2 | - | - | 0 | - |
| - | - | 6010 | 253.2 | - | - | 0 | - |
| - | - | 3006 | 254.1 | - | - | 0 | - |
| - | - | 2.963E+04 | 255.1 | - | - | 0 | - |
| - | - | 8697 | 256.1 | - | - | 0 | - |
| - | - | 5068 | 256.2 | - | - | 0 | - |
| - | - | 2.032E+04 | 257.1 | - | - | 0 | - |
| - | - | 2.651E+04 | 258.1 | - | - | 0 | - |
| - | - | 3534 | 259.1 | - | - | 0 | - |
| - | - | 4281 | 259.1 | - | - | 0 | - |
| - | - | 1.236E+04 | 261.1 | - | - | 0 | - |
| - | - | 7914 | 261.1 | - | - | 0 | - |
| - | - | 8424 | 262.1 | - | - | 0 | - |
| - | - | 2468 | 263.1 | - | - | 0 | - |
| - | - | 2139 | 263.1 | - | - | 0 | - |
| - | - | 6.084E+04 | 265.1 | - | - | 0 | - |
| - | - | 1.741E+05 | 266.1 | - | - | 0 | - |
| - | - | 1.968E+04 | 267.1 | - | - | 0 | - |
| - | - | 3919 | 267.1 | - | - | 0 | - |
| - | - | 3931 | 267.6 | - | - | 0 | - |
| - | - | 8793 | 268.1 | - | - | 0 | - |
| - | - | 1.479E+04 | 268.2 | - | - | 0 | - |
| - | - | 6328 | 269.2 | - | - | 0 | - |
| - | - | 8709 | 269.2 | - | - | 0 | - |
| - | - | 2.793E+04 | 270.1 | - | - | 0 | - |
| - | - | 4513 | 270.1 | - | - | 0 | - |
| - | - | 2434 | 270.2 | - | - | 0 | - |
| - | - | 4278 | 270.6 | - | - | 0 | - |
| - | - | 3.61E+04 | 272.2 | - | - | 0 | - |
| - | - | 7503 | 273.2 | - | - | 0 | - |
| - | - | 3.375E+04 | 274.1 | - | - | 0 | - |
| - | - | 1.338E+04 | 274.1 | - | - | 0 | - |
| - | - | 5815 | 275.1 | - | - | 0 | - |
| - | - | 5663 | 275.1 | - | - | 0 | - |
| - | - | 1.016E+04 | 276.1 | - | - | 0 | - |
| - | - | 7098 | 276.1 | - | - | 0 | - |
| - | - | 7743 | 276.2 | - | - | 0 | - |
| - | - | 2369 | 276.7 | - | - | 0 | - |
| - | - | 1.517E+04 | 279.1 | - | - | 0 | - |
| - | - | 2702 | 279.6 | - | - | 0 | - |
| - | - | 1.817E+04 | 280.1 | - | - | 0 | - |
| - | - | 3048 | 280.1 | - | - | 0 | - |
| - | - | 1.399E+04 | 281.1 | - | - | 0 | - |
| - | - | 3677 | 281.1 | - | - | 0 | - |
| - | - | 1.509E+04 | 282.2 | - | - | 0 | - |
| - | - | 4.291E+05 | 283.1 | - | - | 0 | - |
| - | - | 3052 | 284.1 | - | - | 0 | - |
| - | - | 1.531E+04 | 284.1 | - | - | 0 | - |
| - | - | 3191 | 284.1 | - | - | 0 | - |
| - | - | 6.142E+04 | 284.1 | - | - | 0 | - |
| - | - | 2707 | 285.1 | - | - | 0 | - |
| - | - | 3793 | 285.1 | - | - | 0 | - |
| - | - | 4645 | 285.2 | - | - | 0 | - |
| - | - | 3.106E+04 | 286.1 | - | - | 0 | - |
| - | - | 3900 | 287.1 | - | - | 0 | - |
| - | - | 9098 | 287.2 | - | - | 0 | - |
| - | - | 4592 | 287.2 | - | - | 0 | - |
| - | - | 6310 | 288.6 | - | - | 0 | - |
| 5 | b | 4218 | 290.2 | 0.0004961 | 1.71 | +2 | 5 |
| - | - | 2487 | 290.7 | - | - | 0 | - |
| 12 | y | 2.564E+05 | 293.1 | 0.0006325 | 2.158 | +1 | 2 |
| - | - | 6782 | 293.2 | - | - | 0 | - |
| - | - | 5.494E+04 | 294.1 | - | - | 0 | - |
| - | - | 3451 | 295.1 | - | - | 0 | - |
| 3 | b | 8294 | 296.2 | 0.0007942 | 2.681 | +1 | 3 |
| - | - | 3350 | 296.7 | - | - | 0 | - |
| - | - | 2737 | 297.1 | - | - | 0 | - |
| 9 | y | 9000 | 297.1 | 0.001348 | 4.537 | +2 | 5 |
| - | - | 7.842E+04 | 297.2 | - | - | 0 | - |
| - | - | 3315 | 297.6 | - | - | 0 | - |
| 7 | y | 8725 | 298.1 | 0.004818 | 16.16 | +3 | 7 |
| - | - | 1.336E+04 | 298.2 | - | - | 0 | - |
| - | - | 8.491E+04 | 299.1 | - | - | 0 | - |
| - | - | 2.519E+04 | 300.2 | - | - | 0 | - |
| - | - | 2.583E+05 | 301.2 | - | - | 0 | - |
| - | - | 4.48E+04 | 302.1 | - | - | 0 | - |
| - | - | 3.596E+04 | 302.2 | - | - | 0 | - |
| - | - | 1.116E+04 | 303.1 | - | - | 0 | - |
| - | - | 4091 | 303.2 | - | - | 0 | - |
| - | - | 6414 | 305.2 | - | - | 0 | - |
| - | - | 3335 | 305.7 | - | - | 0 | - |
| 9 | y | 4.319E+04 | 306.1 | 0.0006434 | 2.102 | +2 | 5 |
| - | - | 4091 | 306.2 | - | - | 0 | - |
| - | - | 1.807E+04 | 306.6 | - | - | 0 | - |
| - | - | 8462 | 307.1 | - | - | 0 | - |
| - | - | 4116 | 309.2 | - | - | 0 | - |
| - | - | 3237 | 310.2 | - | - | 0 | - |
| 12 | y | 2.986E+04 | 311.1 | 0.0004743 | 1.524 | +1 | 2 |
| - | - | 1.481E+04 | 311.1 | - | - | 0 | - |
| - | - | 2631 | 311.7 | - | - | 0 | - |
| - | - | 7266 | 312.1 | - | - | 0 | - |
| - | - | 7958 | 314.2 | - | - | 0 | - |
| 3 | b | 2.309E+04 | 314.2 | 0.0007886 | 2.51 | +1 | 3 |
| - | - | 1.388E+05 | 315.2 | - | - | 0 | - |
| - | - | 4641 | 315.2 | - | - | 0 | - |
| - | - | 2.304E+04 | 316.2 | - | - | 0 | - |
| - | - | 3538 | 317.7 | - | - | 0 | - |
| - | - | 9810 | 319.2 | - | - | 0 | - |
| - | - | 2489 | 320.2 | - | - | 0 | - |
| - | - | 3803 | 322.2 | - | - | 0 | - |
| - | - | 1.038E+04 | 325.2 | - | - | 0 | - |
| - | - | 2330 | 325.2 | - | - | 0 | - |
| - | - | 3.168E+04 | 326.7 | - | - | 0 | - |
| - | - | 1.486E+04 | 327.2 | - | - | 0 | - |
| - | - | 4926 | 327.7 | - | - | 0 | - |
| - | - | 3282 | 331.2 | - | - | 0 | - |
| - | - | 4678 | 331.7 | - | - | 0 | - |
| - | - | 1.099E+04 | 332.2 | - | - | 0 | - |
| - | - | 1.976E+04 | 333.2 | - | - | 0 | - |
| - | - | 3847 | 334.2 | - | - | 0 | - |
| - | - | 2917 | 335.2 | - | - | 0 | - |
| - | - | 3775 | 338.2 | - | - | 0 | - |
| 6 | b | 4311 | 338.7 | 0.0008519 | 2.515 | +2 | 6 |
| - | - | 7622 | 339.2 | - | - | 0 | - |
| - | - | 4224 | 339.7 | - | - | 0 | - |
| - | - | 9754 | 340.7 | - | - | 0 | - |
| - | - | 3092 | 341.2 | - | - | 0 | - |
| - | - | 1.118E+04 | 342.2 | - | - | 0 | - |
| - | - | 1.116E+04 | 343.2 | - | - | 0 | - |
| - | - | 2507 | 343.2 | - | - | 0 | - |
| - | - | 1.422E+04 | 344.2 | - | - | 0 | - |
| - | - | 1.158E+04 | 345 | - | - | 0 | - |
| - | - | 1.789E+04 | 345.1 | - | - | 0 | - |
| - | - | 2834 | 345.2 | - | - | 0 | - |
| - | - | 7.541E+04 | 346.1 | - | - | 0 | - |
| - | - | 4612 | 346.1 | - | - | 0 | - |
| - | - | 3322 | 346.7 | - | - | 0 | - |
| - | - | 1.335E+04 | 347.1 | - | - | 0 | - |
| 6 | b | 4.05E+04 | 347.7 | 0.0006965 | 2.003 | +2 | 6 |
| - | - | 1.477E+04 | 348.2 | - | - | 0 | - |
| - | - | 2777 | 348.7 | - | - | 0 | - |
| - | - | 3119 | 349.1 | - | - | 0 | - |
| - | - | 4007 | 349.2 | - | - | 0 | - |
| - | - | 5.894E+04 | 350.2 | - | - | 0 | - |
| - | - | 3676 | 351.1 | - | - | 0 | - |
| - | - | 4270 | 351.2 | - | - | 0 | - |
| - | - | 7500 | 351.2 | - | - | 0 | - |
| - | - | 4809 | 352.2 | - | - | 0 | - |
| - | - | 4731 | 352.7 | - | - | 0 | - |
| 8 | y | 9196 | 353.7 | 0.0005148 | 1.456 | +2 | 6 |
| - | - | 4243 | 354.2 | - | - | 0 | - |
| - | - | 3164 | 357.2 | - | - | 0 | - |
| - | - | 1.082E+04 | 359 | - | - | 0 | - |
| - | - | 3946 | 360.2 | - | - | 0 | - |
| - | - | 1.832E+04 | 360.7 | - | - | 0 | - |
| - | - | 2.324E+04 | 361.2 | - | - | 0 | - |
| - | - | 2.084E+04 | 362.2 | - | - | 0 | - |
| 8 | y | 1.06E+05 | 362.7 | 0.0007257 | 2.001 | +2 | 6 |
| - | - | 9.942E+04 | 363.1 | - | - | 0 | - |
| - | - | 4.002E+04 | 363.2 | - | - | 0 | - |
| - | - | 7706 | 363.7 | - | - | 0 | - |
| - | - | 1.986E+04 | 364.1 | - | - | 0 | - |
| - | - | 1.883E+04 | 364.1 | - | - | 0 | - |
| - | - | 3500 | 365.1 | - | - | 0 | - |
| - | - | 2932 | 366.2 | - | - | 0 | - |
| - | - | 6505 | 366.2 | - | - | 0 | - |
| - | - | 9709 | 367.1 | - | - | 0 | - |
| - | - | 6685 | 367.2 | - | - | 0 | - |
| - | - | 9698 | 367.2 | - | - | 0 | - |
| - | - | 2887 | 368.1 | - | - | 0 | - |
| - | - | 3891 | 368.2 | - | - | 0 | - |
| - | - | 3726 | 369.2 | - | - | 0 | - |
| - | - | 3342 | 370.2 | - | - | 0 | - |
| - | - | 6082 | 373.2 | - | - | 0 | - |
| - | - | 6273 | 376.2 | - | - | 0 | - |
| - | - | 1.438E+04 | 376.2 | - | - | 0 | - |
| - | - | 4765 | 376.7 | - | - | 0 | - |
| - | - | 5831 | 377.1 | - | - | 0 | - |
| - | - | 4.399E+04 | 378.2 | - | - | 0 | - |
| - | - | 1.695E+05 | 379.2 | - | - | 0 | - |
| - | - | 6681 | 380.2 | - | - | 0 | - |
| - | - | 3.238E+04 | 380.2 | - | - | 0 | - |
| - | - | 2.949E+05 | 381.2 | - | - | 0 | - |
| - | - | 9830 | 381.7 | - | - | 0 | - |
| - | - | 4.717E+04 | 382.2 | - | - | 0 | - |
| - | - | 1.146E+04 | 382.2 | - | - | 0 | - |
| - | - | 4313 | 382.2 | - | - | 0 | - |
| - | - | 2794 | 382.7 | - | - | 0 | - |
| - | - | 8787 | 383.2 | - | - | 0 | - |
| - | - | 3680 | 383.2 | - | - | 0 | - |
| - | - | 1.259E+04 | 384.2 | - | - | 0 | - |
| - | - | 7825 | 384.2 | - | - | 0 | - |
| - | - | 2.698E+04 | 385.2 | - | - | 0 | - |
| 5 | y | 4278 | 385.2 | 0.006512 | 16.91 | +3 | 9 |
| - | - | 4076 | 386.2 | - | - | 0 | - |
| - | - | 4369 | 386.2 | - | - | 0 | - |
| 10 | b | 4310 | 389.2 | 0.000778 | 1.999 | +3 | 10 |
| - | - | 6754 | 390.2 | - | - | 0 | - |
| - | - | 5562 | 390.7 | - | - | 0 | - |
| - | - | 1.124E+04 | 391.1 | - | - | 0 | - |
| - | - | 4361 | 392.2 | - | - | 0 | - |
| - | - | 5017 | 393.2 | - | - | 0 | - |
| - | - | 4.029E+04 | 394.2 | - | - | 0 | - |
| - | - | 2.13E+04 | 395.1 | - | - | 0 | - |
| - | - | 8077 | 395.2 | - | - | 0 | - |
| - | - | 4443 | 395.2 | - | - | 0 | - |
| - | - | 3586 | 395.2 | - | - | 0 | - |
| - | - | 5313 | 396.1 | - | - | 0 | - |
| - | - | 4713 | 396.2 | - | - | 0 | - |
| - | - | 6.534E+04 | 396.2 | - | - | 0 | - |
| - | - | 5273 | 397.2 | - | - | 0 | - |
| - | - | 1.282E+04 | 397.2 | - | - | 0 | - |
| - | - | 3028 | 397.7 | - | - | 0 | - |
| - | - | 2.909E+04 | 398.2 | - | - | 0 | - |
| - | - | 3240 | 398.2 | - | - | 0 | - |
| - | - | 6758 | 399.2 | - | - | 0 | - |
| - | - | 1.612E+04 | 402.2 | - | - | 0 | - |
| - | - | 2.609E+04 | 402.2 | - | - | 0 | - |
| - | - | 2964 | 403.1 | - | - | 0 | - |
| - | - | 1.236E+04 | 403.2 | - | - | 0 | - |
| - | - | 3801 | 404.7 | - | - | 0 | - |
| - | - | 1.181E+04 | 405.3 | - | - | 0 | - |
| - | - | 6326 | 406.2 | - | - | 0 | - |
| - | - | 4062 | 407.2 | - | - | 0 | - |
| - | - | 2.557E+04 | 409.1 | - | - | 0 | - |
| - | - | 4845 | 409.2 | - | - | 0 | - |
| - | - | 3408 | 410.1 | - | - | 0 | - |
| - | - | 1.002E+04 | 411.2 | - | - | 0 | - |
| - | - | 1.921E+05 | 412.2 | - | - | 0 | - |
| - | - | 2.818E+04 | 413.1 | - | - | 0 | - |
| - | - | 3.101E+04 | 413.2 | - | - | 0 | - |
| - | - | 4795 | 414.1 | - | - | 0 | - |
| - | - | 6035 | 414.2 | - | - | 0 | - |
| - | - | 6.01E+04 | 414.2 | - | - | 0 | - |
| - | - | 2.108E+04 | 415 | - | - | 0 | - |
| - | - | 2.455E+04 | 415.2 | - | - | 0 | - |
| - | - | 1.176E+04 | 415.2 | - | - | 0 | - |
| - | - | 6564 | 416.2 | - | - | 0 | - |
| - | - | 3.618E+04 | 417.7 | - | - | 0 | - |
| - | - | 5.146E+04 | 418.2 | - | - | 0 | - |
| - | - | 2.536E+04 | 418.7 | - | - | 0 | - |
| - | - | 3797 | 419.2 | - | - | 0 | - |
| 11 | y | 9610 | 421.2 | 0.0018 | 4.273 | +1 | 3 |
| 11 | y | 4548 | 422.2 | 0.0003892 | 0.9219 | +1 | 3 |
| - | - | 2797 | 422.3 | - | - | 0 | - |
| - | - | 3089 | 422.7 | - | - | 0 | - |
| - | - | 1.381E+04 | 423.2 | - | - | 0 | - |
| - | - | 6.982E+04 | 423.3 | - | - | 0 | - |
| - | - | 4828 | 423.7 | - | - | 0 | - |
| - | - | 3320 | 424.2 | - | - | 0 | - |
| - | - | 2.119E+04 | 424.3 | - | - | 0 | - |
| - | - | 8772 | 424.7 | - | - | 0 | - |
| - | - | 3138 | 425.2 | - | - | 0 | - |
| - | - | 5.155E+05 | 426.7 | - | - | 0 | - |
| - | - | 2.639E+05 | 427.2 | - | - | 0 | - |
| - | - | 7.862E+04 | 427.7 | - | - | 0 | - |
| - | - | 1.253E+04 | 428.2 | - | - | 0 | - |
| - | - | 3.217E+04 | 429.2 | - | - | 0 | - |
| - | - | 3443 | 429.7 | - | - | 0 | - |
| - | - | 5.309E+05 | 430.2 | - | - | 0 | - |
| - | - | 1.097E+05 | 431.2 | - | - | 0 | - |
| 7 | b | 6.066E+04 | 431.7 | 0.0007157 | 1.658 | +2 | 7 |
| 7 | b | 3.063E+04 | 432.2 | 0.00706 | 16.33 | +2 | 7 |
| - | - | 1.134E+04 | 432.7 | - | - | 0 | - |
| - | - | 9996 | 433.2 | - | - | 0 | - |
| 4 | b | 2.06E+04 | 433.3 | 0.0005066 | 1.169 | +1 | 4 |
| - | - | 3924 | 434.3 | - | - | 0 | - |
| - | - | 4013 | 437.2 | - | - | 0 | - |
| - | - | 9008 | 438.2 | - | - | 0 | - |
| - | - | 3749 | 438.7 | - | - | 0 | - |
| 11 | y | 1.235E+05 | 439.2 | 0.001092 | 2.487 | +1 | 3 |
| - | - | 6288 | 440.2 | - | - | 0 | - |
| - | - | 2.938E+04 | 440.2 | - | - | 0 | - |
| 7 | b | 2.452E+05 | 440.7 | 0.001079 | 2.448 | +2 | 7 |
| - | - | 1.403E+05 | 441.2 | - | - | 0 | - |
| - | - | 3.924E+04 | 441.7 | - | - | 0 | - |
| - | - | 7294 | 442.2 | - | - | 0 | - |
| - | - | 2088 | 442.3 | - | - | 0 | - |
| - | - | 2652 | 444.2 | - | - | 0 | - |
| 7 | y | 2.622E+04 | 447.2 | 0.0006194 | 1.385 | +2 | 7 |
| - | - | 5147 | 447.2 | - | - | 0 | - |
| - | - | 9110 | 447.7 | - | - | 0 | - |
| - | - | 2.325E+04 | 448.2 | - | - | 0 | - |
| - | - | 1.025E+04 | 449.2 | - | - | 0 | - |
| - | - | 3876 | 450.2 | - | - | 0 | - |
| - | - | 7001 | 450.2 | - | - | 0 | - |
| - | - | 6252 | 450.3 | - | - | 0 | - |
| 4 | b | 1.421E+05 | 451.3 | 0.001142 | 2.53 | +1 | 4 |
| - | - | 3364 | 452.2 | - | - | 0 | - |
| - | - | 3.421E+04 | 452.3 | - | - | 0 | - |
| - | - | 4676 | 453.3 | - | - | 0 | - |
| - | - | 5224 | 455.2 | - | - | 0 | - |
| - | - | 7060 | 455.3 | - | - | 0 | - |
| 7 | y | 1.61E+04 | 455.7 | 0.001505 | 3.302 | +2 | 7 |
| - | - | 3503 | 456.2 | - | - | 0 | - |
| - | - | 1.181E+04 | 459.2 | - | - | 0 | - |
| - | - | 1.94E+04 | 460.8 | - | - | 0 | - |
| - | - | 1.084E+04 | 461.2 | - | - | 0 | - |
| - | - | 1.274E+04 | 461.3 | - | - | 0 | - |
| - | - | 5866 | 461.8 | - | - | 0 | - |
| - | - | 2895 | 462.2 | - | - | 0 | - |
| - | - | 5146 | 464.2 | - | - | 0 | - |
| - | - | 2559 | 464.2 | - | - | 0 | - |
| - | - | 3290 | 465.8 | - | - | 0 | - |
| - | - | 4684 | 466.2 | - | - | 0 | - |
| - | - | 4.144E+04 | 468.3 | - | - | 0 | - |
| - | - | 8606 | 469.3 | - | - | 0 | - |
| - | - | 2.592E+04 | 474.3 | - | - | 0 | - |
| - | - | 4.039E+04 | 474.8 | - | - | 0 | - |
| - | - | 2.083E+04 | 475.3 | - | - | 0 | - |
| - | - | 4349 | 475.8 | - | - | 0 | - |
| - | - | 1.339E+04 | 476.2 | - | - | 0 | - |
| - | - | 2.686E+04 | 477.2 | - | - | 0 | - |
| 10 | y | 2.563E+04 | 478.2 | 0.0002031 | 0.4246 | +1 | 4 |
| - | - | 1.735E+04 | 478.3 | - | - | 0 | - |
| - | - | 4245 | 479.2 | - | - | 0 | - |
| - | - | 3617 | 479.3 | - | - | 0 | - |
| - | - | 4049 | 479.7 | - | - | 0 | - |
| - | - | 3088 | 480.2 | - | - | 0 | - |
| - | - | 4651 | 481.7 | - | - | 0 | - |
| - | - | 8.649E+04 | 483.3 | - | - | 0 | - |
| - | - | 5.931E+04 | 483.8 | - | - | 0 | - |
| - | - | 6228 | 484.2 | - | - | 0 | - |
| - | - | 1.763E+04 | 484.3 | - | - | 0 | - |
| - | - | 2416 | 484.8 | - | - | 0 | - |
| 8 | b | 2.704E+04 | 488.3 | 0.001439 | 2.947 | +2 | 8 |
| 8 | b | 1.711E+04 | 488.7 | 0.009126 | 18.67 | +2 | 8 |
| - | - | 1.128E+04 | 489.3 | - | - | 0 | - |
| - | - | 3.138E+05 | 494.2 | - | - | 0 | - |
| - | - | 8.139E+04 | 495.2 | - | - | 0 | - |
| 10 | y | 4.226E+05 | 496.2 | 0.001052 | 2.12 | +1 | 4 |
| - | - | 1.121E+05 | 497.2 | - | - | 0 | - |
| - | - | 3.427E+04 | 497.8 | - | - | 0 | - |
| - | - | 1.879E+04 | 498.2 | - | - | 0 | - |
| - | - | 3246 | 502.2 | - | - | 0 | - |
| - | - | 2.037E+04 | 503.1 | - | - | 0 | - |
| - | - | 1.439E+04 | 504.2 | - | - | 0 | - |
| - | - | 4209 | 505.2 | - | - | 0 | - |
| - | - | 2549 | 505.2 | - | - | 0 | - |
| - | - | 8247 | 506.2 | - | - | 0 | - |
| - | - | 4287 | 506.3 | - | - | 0 | - |
| - | - | 7221 | 507.3 | - | - | 0 | - |
| - | - | 2.156E+04 | 511.3 | - | - | 0 | - |
| - | - | 4166 | 512.3 | - | - | 0 | - |
| - | - | 4851 | 513.2 | - | - | 0 | - |
| - | - | 6199 | 514.2 | - | - | 0 | - |
| - | - | 9976 | 515.3 | - | - | 0 | - |
| - | - | 6616 | 519.1 | - | - | 0 | - |
| - | - | 4080 | 519.3 | - | - | 0 | - |
| - | - | 5975 | 522.2 | - | - | 0 | - |
| - | - | 1.099E+04 | 523.3 | - | - | 0 | - |
| - | - | 4384 | 523.6 | - | - | 0 | - |
| - | - | 3098 | 524.3 | - | - | 0 | - |
| - | - | 5.616E+04 | 525.3 | - | - | 0 | - |
| - | - | 1.124E+04 | 526.2 | - | - | 0 | - |
| - | - | 1.249E+04 | 526.3 | - | - | 0 | - |
| - | - | 3380 | 527.2 | - | - | 0 | - |
| 0 | Precursor | 1.64E+04 | 529.3 | 0.001901 | 3.592 | +3 | -1 |
| 0 | Precursor | 2.269E+04 | 529.6 | 0.006782 | 12.81 | +3 | -1 |
| - | - | 1.11E+04 | 529.9 | - | - | 0 | - |
| - | - | 4273 | 530.2 | - | - | 0 | - |
| - | - | 3510 | 530.3 | - | - | 0 | - |
| - | - | 4593 | 531.2 | - | - | 0 | - |
| - | - | 1.438E+04 | 532.2 | - | - | 0 | - |
| - | - | 2988 | 533.2 | - | - | 0 | - |
| 0 | Precursor | 1.334E+04 | 535.3 | 0.0003938 | 0.7357 | +3 | -1 |
| - | - | 1.559E+04 | 535.6 | - | - | 0 | - |
| - | - | 8951 | 535.9 | - | - | 0 | - |
| - | - | 3511 | 536.2 | - | - | 0 | - |
| - | - | 6629 | 536.3 | - | - | 0 | - |
| - | - | 3485 | 537.2 | - | - | 0 | - |
| - | - | 1.157E+04 | 537.7 | - | - | 0 | - |
| - | - | 9512 | 538.3 | - | - | 0 | - |
| - | - | 6391 | 538.8 | - | - | 0 | - |
| - | - | 5.873E+04 | 539.2 | - | - | 0 | - |
| - | - | 4749 | 539.3 | - | - | 0 | - |
| - | - | 1.843E+04 | 540.2 | - | - | 0 | - |
| - | - | 7036 | 541.3 | - | - | 0 | - |
| - | - | 4281 | 542.3 | - | - | 0 | - |
| - | - | 1.288E+05 | 543.3 | - | - | 0 | - |
| - | - | 3.753E+04 | 544.3 | - | - | 0 | - |
| - | - | 7692 | 545.3 | - | - | 0 | - |
| - | - | 1.639E+04 | 546.2 | - | - | 0 | - |
| - | - | 1.043E+04 | 546.8 | - | - | 0 | - |
| - | - | 4509 | 547.2 | - | - | 0 | - |
| - | - | 5655 | 547.8 | - | - | 0 | - |
| - | - | 3659 | 548.2 | - | - | 0 | - |
| - | - | 3257 | 548.3 | - | - | 0 | - |
| - | - | 1.298E+04 | 549.2 | - | - | 0 | - |
| - | - | 8457 | 550.2 | - | - | 0 | - |
| - | - | 1.352E+04 | 551.3 | - | - | 0 | - |
| - | - | 7131 | 551.3 | - | - | 0 | - |
| - | - | 2948 | 552.3 | - | - | 0 | - |
| - | - | 3042 | 552.3 | - | - | 0 | - |
| - | - | 3317 | 553.3 | - | - | 0 | - |
| 9 | b | 1.45E+04 | 554.8 | 0.0002557 | 0.461 | +2 | 9 |
| - | - | 2.99E+04 | 555.3 | - | - | 0 | - |
| - | - | 1.687E+04 | 555.8 | - | - | 0 | - |
| - | - | 5581 | 556.3 | - | - | 0 | - |
| 5 | b | 1.456E+04 | 561.3 | 0.000767 | 1.366 | +1 | 5 |
| 5 | b | 7375 | 562.3 | 0.003507 | 6.237 | +1 | 5 |
| - | - | 1.28E+04 | 563.3 | - | - | 0 | - |
| - | - | 5617 | 564.3 | - | - | 0 | - |
| - | - | 1.98E+04 | 565.3 | - | - | 0 | - |
| - | - | 3817 | 566.3 | - | - | 0 | - |
| - | - | 6.678E+04 | 567.2 | - | - | 0 | - |
| - | - | 1.81E+04 | 568.2 | - | - | 0 | - |
| - | - | 8310 | 568.8 | - | - | 0 | - |
| - | - | 7337 | 569.2 | - | - | 0 | - |
| - | - | 1.566E+04 | 575.2 | - | - | 0 | - |
| - | - | 3584 | 576.2 | - | - | 0 | - |
| - | - | 3284 | 576.3 | - | - | 0 | - |
| - | - | 4511 | 577.2 | - | - | 0 | - |
| 5 | y | 3494 | 577.3 | 0.003606 | 6.246 | +2 | 9 |
| - | - | 4351 | 577.8 | - | - | 0 | - |
| - | - | 4410 | 579.3 | - | - | 0 | - |
| 5 | b | 8.837E+04 | 579.3 | 0.0009445 | 1.63 | +1 | 5 |
| - | - | 2.625E+04 | 580.3 | - | - | 0 | - |
| - | - | 3260 | 581.3 | - | - | 0 | - |
| - | - | 4010 | 581.3 | - | - | 0 | - |
| - | - | 4446 | 582.3 | - | - | 0 | - |
| 10 | b | 4457 | 583.3 | 0.001647 | 2.823 | +2 | 10 |
| - | - | 2930 | 587.3 | - | - | 0 | - |
| - | - | 3660 | 588.8 | - | - | 0 | - |
| - | - | 2812 | 589.3 | - | - | 0 | - |
| - | - | 4100 | 592.3 | - | - | 0 | - |
| - | - | 1.147E+04 | 592.3 | - | - | 0 | - |
| 9 | y | 1.005E+05 | 593.3 | 0.0005733 | 0.9663 | +1 | 5 |
| - | - | 4.794E+04 | 593.3 | - | - | 0 | - |
| - | - | 6232 | 593.8 | - | - | 0 | - |
| - | - | 3.029E+04 | 594.3 | - | - | 0 | - |
| - | - | 1.016E+04 | 594.3 | - | - | 0 | - |
| - | - | 3970 | 594.8 | - | - | 0 | - |
| - | - | 6311 | 595.2 | - | - | 0 | - |
| - | - | 2801 | 596.4 | - | - | 0 | - |
| - | - | 3432 | 597.3 | - | - | 0 | - |
| - | - | 1.055E+04 | 597.8 | - | - | 0 | - |
| - | - | 3668 | 598.3 | - | - | 0 | - |
| - | - | 1.006E+04 | 600.3 | - | - | 0 | - |
| - | - | 2821 | 602.3 | - | - | 0 | - |
| - | - | 2.057E+04 | 602.8 | - | - | 0 | - |
| - | - | 1.956E+04 | 603.3 | - | - | 0 | - |
| - | - | 1.001E+04 | 603.8 | - | - | 0 | - |
| - | - | 4709 | 604.3 | - | - | 0 | - |
| - | - | 8.561E+04 | 610.3 | - | - | 0 | - |
| - | - | 7488 | 610.3 | - | - | 0 | - |
| 9 | y | 1.307E+06 | 611.3 | 0.001483 | 2.426 | +1 | 5 |
| - | - | 8.374E+04 | 611.8 | - | - | 0 | - |
| - | - | 4.078E+05 | 612.3 | - | - | 0 | - |
| - | - | 2.465E+04 | 612.8 | - | - | 0 | - |
| - | - | 8.348E+04 | 613.3 | - | - | 0 | - |
| - | - | 1.613E+04 | 614.3 | - | - | 0 | - |
| - | - | 5718 | 616.3 | - | - | 0 | - |
| - | - | 7229 | 617.3 | - | - | 0 | - |
| - | - | 3016 | 619.3 | - | - | 0 | - |
| - | - | 6376 | 620.8 | - | - | 0 | - |
| - | - | 1.414E+04 | 621.3 | - | - | 0 | - |
| - | - | 7976 | 621.3 | - | - | 0 | - |
| - | - | 3180 | 622.3 | - | - | 0 | - |
| - | - | 5587 | 622.4 | - | - | 0 | - |
| - | - | 2583 | 623.2 | - | - | 0 | - |
| - | - | 3566 | 628.3 | - | - | 0 | - |
| - | - | 3665 | 628.8 | - | - | 0 | - |
| - | - | 1.718E+04 | 634.3 | - | - | 0 | - |
| - | - | 2.155E+04 | 635.3 | - | - | 0 | - |
| - | - | 1.097E+04 | 636.3 | - | - | 0 | - |
| 4 | y | 7394 | 636.8 | 0.0001839 | 0.2888 | +2 | 10 |
| 4 | y | 4253 | 637.3 | 0.005002 | 7.849 | +2 | 10 |
| - | - | 4149 | 637.3 | - | - | 0 | - |
| - | - | 7062 | 637.8 | - | - | 0 | - |
| 11 | b | 6576 | 638.3 | 0.001684 | 2.638 | +2 | 11 |
| - | - | 1.644E+04 | 640.3 | - | - | 0 | - |
| - | - | 3773 | 641.3 | - | - | 0 | - |
| - | - | 1.216E+04 | 645.3 | - | - | 0 | - |
| 4 | y | 8.444E+04 | 645.8 | 0.001432 | 2.218 | +2 | 10 |
| - | - | 7.61E+04 | 646.3 | - | - | 0 | - |
| - | - | 2.432E+04 | 646.8 | - | - | 0 | - |
| - | - | 3501 | 647.3 | - | - | 0 | - |
| - | - | 5030 | 648.3 | - | - | 0 | - |
| - | - | 4264 | 649.3 | - | - | 0 | - |
| - | - | 6332 | 650.3 | - | - | 0 | - |
| - | - | 4144 | 650.3 | - | - | 0 | - |
| - | - | 4126 | 651.3 | - | - | 0 | - |
| - | - | 1.309E+05 | 652.3 | - | - | 0 | - |
| - | - | 4.819E+04 | 653.3 | - | - | 0 | - |
| - | - | 8889 | 654.3 | - | - | 0 | - |
| - | - | 1.079E+04 | 658.3 | - | - | 0 | - |
| - | - | 6748 | 659.3 | - | - | 0 | - |
| - | - | 2734 | 660.3 | - | - | 0 | - |
| - | - | 3382 | 660.9 | - | - | 0 | - |
| - | - | 9412 | 661.3 | - | - | 0 | - |
| - | - | 6311 | 661.8 | - | - | 0 | - |
| - | - | 3.784E+04 | 662.3 | - | - | 0 | - |
| - | - | 3.558E+04 | 663.3 | - | - | 0 | - |
| - | - | 3857 | 664.3 | - | - | 0 | - |
| - | - | 3658 | 664.3 | - | - | 0 | - |
| - | - | 2937 | 666.3 | - | - | 0 | - |
| - | - | 3352 | 666.4 | - | - | 0 | - |
| - | - | 5624 | 668.3 | - | - | 0 | - |
| - | - | 2717 | 670.8 | - | - | 0 | - |
| 6 | b | 5.67E+04 | 676.3 | 0.001046 | 1.546 | +1 | 6 |
| 6 | b | 3.798E+04 | 677.3 | 0.008607 | 12.71 | +1 | 6 |
| - | - | 1.567E+04 | 678.3 | - | - | 0 | - |
| - | - | 1.415E+04 | 679.3 | - | - | 0 | - |
| - | - | 5735 | 679.8 | - | - | 0 | - |
| - | - | 2.289E+05 | 680.3 | - | - | 0 | - |
| - | - | 9.301E+04 | 681.3 | - | - | 0 | - |
| - | - | 1.911E+04 | 682.3 | - | - | 0 | - |
| - | - | 3683 | 683.3 | - | - | 0 | - |
| - | - | 6793 | 684.3 | - | - | 0 | - |
| - | - | 2.107E+04 | 684.8 | - | - | 0 | - |
| - | - | 1.202E+04 | 685.3 | - | - | 0 | - |
| - | - | 8515 | 685.8 | - | - | 0 | - |
| - | - | 2944 | 686.3 | - | - | 0 | - |
| - | - | 4925 | 692.8 | - | - | 0 | - |
| 3 | y | 6.773E+04 | 693.3 | 0.00167 | 2.409 | +2 | 11 |
| 3 | y | 6.121E+04 | 693.8 | 0.008503 | 12.25 | +2 | 11 |
| 6 | b | 6.146E+05 | 694.4 | 0.0004297 | 0.6188 | +1 | 6 |
| - | - | 1.433E+04 | 694.8 | - | - | 0 | - |
| - | - | 2.343E+05 | 695.4 | - | - | 0 | - |
| - | - | 5.721E+04 | 696.4 | - | - | 0 | - |
| - | - | 3240 | 697.3 | - | - | 0 | - |
| - | - | 1.02E+04 | 697.4 | - | - | 0 | - |
| - | - | 4213 | 697.9 | - | - | 0 | - |
| - | - | 7129 | 698.4 | - | - | 0 | - |
| - | - | 3.112E+04 | 701.8 | - | - | 0 | - |
| 3 | y | 5.278E+05 | 702.3 | 0.001637 | 2.33 | +2 | 11 |
| 12 | b | 4.413E+05 | 702.8 | 0.01253 | 17.83 | +2 | 12 |
| 12 | b | 2.019E+05 | 703.3 | 0.003198 | 4.546 | +2 | 12 |
| - | - | 6.474E+04 | 703.8 | - | - | 0 | - |
| - | - | 2.12E+04 | 704.3 | - | - | 0 | - |
| - | - | 5078 | 705.4 | - | - | 0 | - |
| 8 | y | 4.332E+04 | 706.3 | 0.002325 | 3.291 | +1 | 6 |
| - | - | 1.557E+04 | 707.3 | - | - | 0 | - |
| - | - | 4217 | 708.3 | - | - | 0 | - |
| - | - | 1.472E+04 | 711.4 | - | - | 0 | - |
| 12 | b | 3.42E+04 | 711.9 | 0.001533 | 2.154 | +2 | 12 |
| - | - | 4.377E+04 | 712.4 | - | - | 0 | - |
| - | - | 1.624E+04 | 712.9 | - | - | 0 | - |
| - | - | 7745 | 713.4 | - | - | 0 | - |
| - | - | 3222 | 715.3 | - | - | 0 | - |
| - | - | 5148 | 720.4 | - | - | 0 | - |
| - | - | 3114 | 720.9 | - | - | 0 | - |
| - | - | 2.862E+04 | 721.4 | - | - | 0 | - |
| - | - | 9457 | 722.4 | - | - | 0 | - |
| - | - | 3.198E+04 | 723.4 | - | - | 0 | - |
| 8 | y | 4.081E+05 | 724.4 | 0.001648 | 2.275 | +1 | 6 |
| - | - | 1.737E+05 | 725.4 | - | - | 0 | - |
| - | - | 4.146E+04 | 726.4 | - | - | 0 | - |
| - | - | 4979 | 727.4 | - | - | 0 | - |
| - | - | 4929 | 728.9 | - | - | 0 | - |
| - | - | 1.898E+04 | 729.4 | - | - | 0 | - |
| - | - | 9587 | 730.4 | - | - | 0 | - |
| - | - | 1.603E+04 | 733.4 | - | - | 0 | - |
| - | - | 1.616E+04 | 734.4 | - | - | 0 | - |
| - | - | 4404 | 735.4 | - | - | 0 | - |
| - | - | 4897 | 737.9 | - | - | 0 | - |
| - | - | 4473 | 738.4 | - | - | 0 | - |
| 2 | y | 3695 | 742.9 | 0.005366 | 7.223 | +2 | 12 |
| 2 | y | 9679 | 743.4 | 0.01177 | 15.84 | +2 | 12 |
| - | - | 4146 | 743.9 | - | - | 0 | - |
| - | - | 3344 | 744.4 | - | - | 0 | - |
| - | - | 2873 | 746.4 | - | - | 0 | - |
| - | - | 9205 | 747.4 | - | - | 0 | - |
| - | - | 1.037E+04 | 748.4 | - | - | 0 | - |
| - | - | 5931 | 749.3 | - | - | 0 | - |
| - | - | 3427 | 750.4 | - | - | 0 | - |
| - | - | 6.021E+04 | 751.4 | - | - | 0 | - |
| 2 | y | 4.737E+04 | 751.9 | 0.001487 | 1.978 | +2 | 12 |
| - | - | 7.281E+04 | 752.4 | - | - | 0 | - |
| - | - | 1.614E+04 | 752.9 | - | - | 0 | - |
| - | - | 1.561E+04 | 753.4 | - | - | 0 | - |
| - | - | 4086 | 760.3 | - | - | 0 | - |
| - | - | 4698 | 761.4 | - | - | 0 | - |
| - | - | 8278 | 762.4 | - | - | 0 | - |
| - | - | 3.815E+04 | 763.4 | - | - | 0 | - |
| - | - | 1.097E+04 | 764.4 | - | - | 0 | - |
| - | - | 1.594E+04 | 765.4 | - | - | 0 | - |
| - | - | 7682 | 766.4 | - | - | 0 | - |
| - | - | 1.365E+04 | 767.3 | - | - | 0 | - |
| - | - | 4085 | 768.3 | - | - | 0 | - |
| - | - | 4253 | 769.4 | - | - | 0 | - |
| - | - | 7724 | 775.4 | - | - | 0 | - |
| - | - | 8410 | 776.4 | - | - | 0 | - |
| - | - | 4.167E+04 | 779.4 | - | - | 0 | - |
| - | - | 1.651E+04 | 780.4 | - | - | 0 | - |
| - | - | 1.425E+04 | 781.4 | - | - | 0 | - |
| - | - | 8373 | 782.4 | - | - | 0 | - |
| - | - | 2794 | 791.4 | - | - | 0 | - |
| - | - | 3242 | 792.4 | - | - | 0 | - |
| 0 | Precursor | 6.793E+04 | 793.4 | 0.0143 | 18.03 | +2 | -1 |
| - | - | 2.713E+04 | 794.4 | - | - | 0 | - |
| - | - | 2.809E+04 | 795.3 | - | - | 0 | - |
| - | - | 4431 | 795.4 | - | - | 0 | - |
| - | - | 1.031E+04 | 796.3 | - | - | 0 | - |
| - | - | 3608 | 797.3 | - | - | 0 | - |
| - | - | 1.043E+04 | 807.4 | - | - | 0 | - |
| - | - | 7560 | 807.4 | - | - | 0 | - |
| - | - | 9907 | 808.4 | - | - | 0 | - |
| - | - | 3678 | 817.4 | - | - | 0 | - |
| - | - | 7909 | 818.4 | - | - | 0 | - |
| - | - | 7650 | 819.4 | - | - | 0 | - |
| - | - | 3712 | 820.4 | - | - | 0 | - |
| - | - | 6137 | 825.4 | - | - | 0 | - |
| - | - | 1.235E+04 | 826.4 | - | - | 0 | - |
| - | - | 3135 | 827.4 | - | - | 0 | - |
| - | - | 1.211E+04 | 834.4 | - | - | 0 | - |
| - | - | 2.526E+04 | 835.4 | - | - | 0 | - |
| - | - | 5.264E+04 | 836.4 | - | - | 0 | - |
| - | - | 2.089E+04 | 837.5 | - | - | 0 | - |
| - | - | 6326 | 838.5 | - | - | 0 | - |
| - | - | 9180 | 843.4 | - | - | 0 | - |
| - | - | 2.044E+04 | 844.4 | - | - | 0 | - |
| - | - | 1.345E+04 | 845.4 | - | - | 0 | - |
| - | - | 2.038E+04 | 846.4 | - | - | 0 | - |
| - | - | 1.345E+04 | 847.4 | - | - | 0 | - |
| - | - | 1.005E+04 | 852.4 | - | - | 0 | - |
| - | - | 1.48E+05 | 852.4 | - | - | 0 | - |
| - | - | 5105 | 853.4 | - | - | 0 | - |
| - | - | 8.043E+04 | 853.4 | - | - | 0 | - |
| - | - | 1.76E+04 | 854.4 | - | - | 0 | - |
| 7 | b | 8.893E+04 | 862.4 | 0.0008343 | 0.9673 | +1 | 7 |
| 7 | b | 7.164E+04 | 863.4 | 0.01224 | 14.18 | +1 | 7 |
| - | - | 1.105E+05 | 864.5 | - | - | 0 | - |
| - | - | 5.575E+04 | 865.5 | - | - | 0 | - |
| - | - | 2.044E+04 | 866.5 | - | - | 0 | - |
| - | - | 2970 | 867.5 | - | - | 0 | - |
| - | - | 5143 | 869.4 | - | - | 0 | - |
| - | - | 9589 | 876.4 | - | - | 0 | - |
| - | - | 4519 | 877.4 | - | - | 0 | - |
| 7 | b | 7.167E+05 | 880.4 | 0.0007676 | 0.8719 | +1 | 7 |
| - | - | 3.809E+05 | 881.4 | - | - | 0 | - |
| - | - | 1.203E+05 | 882.4 | - | - | 0 | - |
| - | - | 2.156E+04 | 883.4 | - | - | 0 | - |
| - | - | 5099 | 890.4 | - | - | 0 | - |
| - | - | 9915 | 891.4 | - | - | 0 | - |
| - | - | 3760 | 892.4 | - | - | 0 | - |
| - | - | 9595 | 892.5 | - | - | 0 | - |
| - | - | 8167 | 893.5 | - | - | 0 | - |
| - | - | 6106 | 894.4 | - | - | 0 | - |
| - | - | 3284 | 907.4 | - | - | 0 | - |
| - | - | 8.022E+04 | 908.4 | - | - | 0 | - |
| - | - | 4.365E+04 | 909.4 | - | - | 0 | - |
| 7 | y | 7.049E+04 | 910.4 | 0.001436 | 1.578 | +1 | 7 |
| - | - | 3.031E+04 | 911.4 | - | - | 0 | - |
| - | - | 1.009E+04 | 912.4 | - | - | 0 | - |
| - | - | 7365 | 936.4 | - | - | 0 | - |
| - | - | 1.093E+04 | 937.4 | - | - | 0 | - |
| - | - | 4667 | 938.4 | - | - | 0 | - |
| - | - | 8082 | 947.4 | - | - | 0 | - |
| - | - | 5153 | 948.4 | - | - | 0 | - |
| - | - | 1.131E+04 | 954.4 | - | - | 0 | - |
| - | - | 2.485E+04 | 955.4 | - | - | 0 | - |
| - | - | 1.066E+04 | 956.4 | - | - | 0 | - |
| - | - | 4174 | 957.4 | - | - | 0 | - |
| - | - | 3496 | 962.4 | - | - | 0 | - |
| - | - | 4068 | 964.5 | - | - | 0 | - |
| - | - | 2.286E+04 | 965.4 | - | - | 0 | - |
| - | - | 1.226E+04 | 966.4 | - | - | 0 | - |
| - | - | 3326 | 967.5 | - | - | 0 | - |
| - | - | 2.027E+04 | 972.4 | - | - | 0 | - |
| - | - | 1.438E+04 | 973.4 | - | - | 0 | - |
| - | - | 6115 | 974.4 | - | - | 0 | - |
| 8 | b | 5650 | 975.5 | 0.003013 | 3.089 | +1 | 8 |
| 8 | b | 6362 | 976.5 | 0.01534 | 15.7 | +1 | 8 |
| - | - | 7625 | 980.5 | - | - | 0 | - |
| - | - | 3524 | 981.5 | - | - | 0 | - |
| - | - | 7102 | 982.5 | - | - | 0 | - |
| - | - | 4027 | 983.5 | - | - | 0 | - |
| 8 | b | 3.803E+04 | 993.5 | 0.0009322 | 0.9382 | +1 | 8 |
| - | - | 2.229E+04 | 994.5 | - | - | 0 | - |
| - | - | 6279 | 995.5 | - | - | 0 | - |
| - | - | 8483 | 1007 | - | - | 0 | - |
| - | - | 4663 | 1008 | - | - | 0 | - |
| 6 | y | 3.478E+04 | 1025 | 0.002386 | 2.327 | +1 | 8 |
| - | - | 2.241E+04 | 1026 | - | - | 0 | - |
| - | - | 5972 | 1027 | - | - | 0 | - |
| - | - | 3866 | 1076 | - | - | 0 | - |
| - | - | 5523 | 1077 | - | - | 0 | - |
| - | - | 3902 | 1078 | - | - | 0 | - |
| 9 | b | 3475 | 1092 | 0.0193 | 17.68 | +1 | 9 |
| - | - | 2.082E+04 | 1094 | - | - | 0 | - |
| - | - | 1.636E+04 | 1095 | - | - | 0 | - |
| - | - | 4285 | 1096 | - | - | 0 | - |
| - | - | 3250 | 1097 | - | - | 0 | - |
| 9 | b | 1.073E+04 | 1109 | 0.001292 | 1.165 | +1 | 9 |
| - | - | 6271 | 1110 | - | - | 0 | - |
| - | - | 4139 | 1111 | - | - | 0 | - |
| - | - | 4198 | 1118 | - | - | 0 | - |
| - | - | 4769 | 1119 | - | - | 0 | - |
| 5 | y | 2.533E+04 | 1136 | 0.002225 | 1.96 | +1 | 9 |
| 5 | y | 4.094E+04 | 1136 | 0.006613 | 5.819 | +1 | 9 |
| - | - | 2.331E+04 | 1137 | - | - | 0 | - |
| - | - | 1.063E+04 | 1138 | - | - | 0 | - |
| 5 | y | 6.758E+04 | 1154 | 0.00106 | 0.9188 | +1 | 9 |
| - | - | 5.009E+04 | 1155 | - | - | 0 | - |
| - | - | 1.817E+04 | 1156 | - | - | 0 | - |
| - | - | 6260 | 1157 | - | - | 0 | - |
| 10 | b | 6440 | 1166 | 0.0008042 | 0.69 | +1 | 10 |
| - | - | 4575 | 1167 | - | - | 0 | - |
| - | - | 3727 | 1223 | - | - | 0 | - |
| - | - | 3064 | 1287 | - | - | 0 | - |
| - | - | 2744 | 2013 | - | - | 0 | - |
| - | - | 2852 | 3163 | - | - | 0 | - |

m/z Charge Intensity FragmentType MassShift Position
120.08126068115234 0 5635.7485
122.07168579101562 0 10261.165
123.04442596435547 0 11790.01
123.05549621582031 0 2414.1987
125.10782623291016 0 3293.2954
125.5638656616211 0 1535.4692
125.96668243408203 0 1600.5402
126.09197235107422 0 3883.5344
127.05060577392578 0 14552.156
127.08658599853516 0 1537.7372
127.12328338623047 0 5684.993
128.05419921875 0 1641.6833
128.0819854736328 0 3596.9895
128.10739135742188 0 120450.555
129.10267639160156 0 1290455.2
130.06552124023438 0 529168.6
130.09983825683594 0 9603.356
130.10598754882812 0 84349.9
131.06884765625 0 50866.246
131.10824584960938 0 1947.5447
131.22203063964844 0 1566.4266
132.08114624023438 0 125666.04
133.06076049804688 0 2445.5981
133.08448791503906 0 13843.133
133.08934020996094 0 2130.6343
136.0760955810547 0 157948.22
136.20199584960938 0 1623.0112
137.0794219970703 0 13769.69
138.06661987304688 0 15416.894
138.09182739257812 0 5695.9185
139.05026245117188 0 1767.9458
139.0869140625 0 15414.989
139.18386840820312 0 1931.665
141.10269165039062 0 19825.426
142.0654754638672 0 7678.003
142.12351989746094 0 2320.5173
142.63534545898438 0 1814.4125
143.04547119140625 0 8487.125
143.11875915527344 0 2101.9663
144.08114624023438 0 7782.0205
144.08689880371094 0 1483.1349
145.06130981445312 0 9071.229
146.05996704101562 0 2210.7305
147.0446319580078 0 6829.418
148.0609130859375 0 2462.9167
148.95428466796875 0 2381.1191
149.04525756835938 0 17574.928
150.06654357910156 0 6622.8164
151.0869903564453 0 24760.453
153.10250854492188 0 2716.3274
154.06149291992188 0 2752.1409
154.09774780273438 0 2699.5234
155.0458221435547 0 4053.4917
155.0822296142578 0 3701.3684
155.09312438964844 0 78626.49
155.1182861328125 0 56645.918 a Water loss 1
156.07681274414062 0 4034.542
156.09634399414062 0 5106.2856
156.10263061523438 0 2807.5193
156.12156677246094 0 3274.0046
157.0599365234375 0 1947.248
157.09759521484375 0 7224.417
157.13357543945312 0 4496.201
158.08438110351562 0 6481.278
159.09213256835938 0 971727.1
160.07608032226562 0 9116.724
160.08917236328125 0 7912.2363
160.09544372558594 0 105384.086
161.09889221191406 0 5465.555
165.05502319335938 0 87992.23
165.0773162841797 0 4322.0225
166.06149291992188 0 161668.1
166.0979461669922 0 14449.401
167.05615234375 0 10987.563
167.0651092529297 0 10900.412
167.0814971923828 0 3045.445
167.1182403564453 0 6454.2397
168.0812530517578 0 16347.59
168.1022491455078 0 10457.823
168.1135711669922 0 12611.257
169.07640075683594 0 14061.676
169.08387756347656 0 2865.6921
169.09759521484375 0 38299.1
170.0604705810547 0 47005.168
171.0636444091797 0 5060.775
171.09205627441406 0 5164.354
172.0721435546875 0 15322.3125
173.0562286376953 0 17105.734
173.12893676757812 0 1929929.4 a 1
173.45223999023438 0 3533.0676
174.1322784423828 0 169557.77
175.13389587402344 0 11064.365
176.08187866210938 0 2726.2346
177.10279846191406 0 3155.6758
178.13426208496094 0 58432.945
179.11831665039062 0 4126.3164
179.1376190185547 0 5476.48
181.06114196777344 0 8063.5103
182.0816650390625 0 180457.19 y 12
183.08526611328125 0 16398.605
183.11318969726562 0 50058.492 b Water loss 1
184.11642456054688 0 5178.111
185.1652374267578 0 15165.821
186.12417602539062 0 421505.4
187.0870361328125 0 81185.28
187.12759399414062 0 35191.32
188.0709991455078 0 7922.571
188.0804443359375 0 2153.617
188.0906219482422 0 7397.6587
188.12921142578125 0 2152.4941
190.08273315429688 0 16729.09
190.1343536376953 0 14121.066
194.0928192138672 0 10237.709
195.1132049560547 0 99320.695
196.10845947265625 0 67025.45
196.11705017089844 0 8470.511
197.1114501953125 0 4573.7573
197.12881469726562 0 5261.1494
198.0879669189453 0 10164.778
198.12820434570312 0 33657.508
199.07159423828125 0 8966.42
199.08692932128906 0 7084.48
199.13150024414062 0 5360.0977
200.14361572265625 0 5695.326
201.12376403808594 0 518060.78 b 1
202.08677673339844 0 4230.729
202.12709045410156 0 52075.04
203.12918090820312 0 4337.8965
204.0772705078125 0 1956.855
204.11375427246094 0 4458.175
204.13442993164062 0 3312.855
205.09764099121094 0 41051.156
205.14520263671875 0 6817.7407
206.1011505126953 0 3836.58
206.12908935546875 0 9102.301
207.16098022460938 0 13288.499
210.1278076171875 0 16447.652
211.1081085205078 0 8374.921 y Water loss 10
212.13992309570312 0 12580.939
213.123779296875 0 45281.63
213.1602020263672 0 6470.721
215.1185760498047 0 3882.4548
215.1393585205078 0 2802.533
216.09829711914062 0 12533.913
218.02247619628906 0 2015.5652
219.1138458251953 0 2332.1785
221.07156372070312 0 5395.4766
221.0833282470703 0 4246.8477
221.09378051757812 0 4034.0933
221.10382080078125 0 10332.378
221.12921142578125 0 2525.7026
221.14051818847656 0 5488.0327
222.1240692138672 0 17133.121
222.13580322265625 0 3292.43
223.06370544433594 0 3049.2546
223.10812377929688 0 24487.049
223.11920166015625 0 2551.6448
223.15582275390625 0 173646.95
224.10386657714844 0 7501.543
224.15907287597656 0 17337.73
225.04391479492188 0 5748.9565
225.12344360351562 0 3491.0605
225.16090393066406 0 3797.9958
226.08273315429688 0 38699.24
227.0667266845703 0 33477.56
227.08592224121094 0 5088.643
227.1548309326172 0 9511.469
228.0696258544922 0 4488.782
228.13381958007812 0 3015.907
229.1188201904297 0 53830.633
230.12205505371094 0 5221.177
230.15098571777344 0 4104.954
233.14012145996094 0 29770.457
234.12425231933594 0 189114.89
234.65045166015625 0 3518.1655
235.1072235107422 0 2268.572
235.12767028808594 0 28738.246
235.15538024902344 0 4875.4834
235.60598754882812 0 2317.1555
237.1356658935547 0 3678.88
238.119140625 0 23290.645
238.1298828125 0 4018.617
239.08267211914062 0 3394.0652
239.0956573486328 0 13139.138
239.11392211914062 0 3626.8047
240.09609985351562 0 5105.962
240.13478088378906 0 71713.7
241.0967254638672 0 2614.785
241.11851501464844 0 13851.9
241.137939453125 0 10352.756
241.19131469726562 0 9202.253
242.19552612304688 0 2318.2256
243.1097412109375 0 11555.414
244.09335327148438 0 77287.2
245.096435546875 0 6824.371
247.10830688476562 0 110333.695
247.1441650390625 0 4057.05
248.10134887695312 0 4571.223
248.114013671875 0 41298.87
248.62388610839844 0 2429.8008 y 9
249.09877014160156 0 63018.91
249.11502075195312 0 4165.265
249.11892700195312 0 4141.416
249.13531494140625 0 15177.06
250.10208129882812 0 9602.359
251.15087890625 0 686858.94
252.13433837890625 0 4442.4727
252.15406799316406 0 82521.9
253.15606689453125 0 6009.633
254.0766143798828 0 3006.3213
255.14840698242188 0 29630.617
256.1083679199219 0 8697.295
256.1529235839844 0 5068.417
257.1135559082031 0 20322.227
258.1452941894531 0 26514.73
259.12884521484375 0 3534.2195
259.1484375 0 4280.7856
261.1195068359375 0 12364.267
261.1354675292969 0 7913.659
262.1192626953125 0 8423.698
263.1048889160156 0 2467.7522
263.1221923828125 0 2139.026
265.1297302246094 0 60842.684
266.1253662109375 0 174147.84
267.12847900390625 0 19679.088
267.1448974609375 0 3919.0825
267.61212158203125 0 3930.6187
268.1299133300781 0 8792.783
268.1771240234375 0 14790.106
269.1611328125 0 6328.4478
269.18560791015625 0 8708.975
270.1222229003906 0 27927.684
270.1455078125 0 4512.725
270.16015625 0 2433.7214
270.6245422363281 0 4277.939
272.1761474609375 0 36098.016
273.17919921875 0 7502.7666
274.1188049316406 0 33748.36
274.1316223144531 0 13381.351
275.10333251953125 0 5815.4126
275.1230163574219 0 5663.355
276.1092834472656 0 10159.993
276.1274719238281 0 7098.041
276.1558837890625 0 7743.24
276.6714172363281 0 2369.0647
279.145751953125 0 15168.433
279.6144104003906 0 2702.1396
280.12969970703125 0 18172.111
280.14593505859375 0 3048.1882
281.05181884765625 0 13986.056
281.1328430175781 0 3677.046
282.1570129394531 0 15092.552
283.140625 0 429112.25
284.0881652832031 0 3052.3381
284.1036682128906 0 15305.342
284.1239013671875 0 3191.1428
284.1438903808594 0 61422.395
285.0899658203125 0 2706.7603
285.1051940917969 0 3792.6094
285.1575622558594 0 4645.435
286.1403503417969 0 31057.785
287.14208984375 0 3899.7354
287.17181396484375 0 9097.788
287.1883239746094 0 4591.5415
288.61907958984375 0 6310.431
290.1665954589844 0 4218.004 b 4
290.6689758300781 0 2486.9949
293.11383056640625 0 256374.94 y Water loss 11
293.161865234375 0 6781.954
294.11773681640625 0 54942.387
295.1201477050781 0 3451.4082
296.1976623535156 0 8293.904 b Water loss 2
296.66583251953125 0 3349.523
297.0832824707031 0 2737.2043
297.1332702636719 0 8999.883 y Water loss 8
297.1564636230469 0 78424.375
297.6349182128906 0 3314.929
298.1400146484375 0 8725.087 y Water loss 6
298.15948486328125 0 13359.997
299.06231689453125 0 84912.61
300.1689147949219 0 25194.729
301.15130615234375 0 258331.05
302.1142883300781 0 44797.54
302.1546630859375 0 35959.746
303.1172180175781 0 11164.901
303.1682434082031 0 4090.868
305.17889404296875 0 6414.1533
305.6824951171875 0 3335.026
306.1378479003906 0 43194.887 y 8
306.22967529296875 0 4090.9243
306.6395568847656 0 18070.684
307.14093017578125 0 8462.19
309.16693115234375 0 4116.219
310.1536560058594 0 3237.0083
311.1242370605469 0 29864.066 y 11
311.1377868652344 0 14810.062
311.6888427734375 0 2630.9495
312.1280517578125 0 7266.1323
314.18731689453125 0 7957.995
314.2082214355469 0 23093.535 b 2
315.1670227050781 0 138819.75
315.2105407714844 0 4640.5845
316.1707763671875 0 23044.826
317.6594543457031 0 3538.065
319.1521301269531 0 9810.484
320.17169189453125 0 2489.0918
322.2238464355469 0 3802.805
325.1510925292969 0 10384.505
325.1717224121094 0 2330.016
326.66448974609375 0 31681.957
327.16558837890625 0 14864.687
327.6676025390625 0 4926.401
331.1623840332031 0 3282.011
331.6566467285156 0 4678.0513
332.20880126953125 0 10985.618
333.1774597167969 0 19761.562
334.1839599609375 0 3846.8342
335.208984375 0 2917.2148
338.1828918457031 0 3775.4783
338.6751403808594 0 4310.9624 b Water loss 5
339.1731262207031 0 7621.762
339.6765441894531 0 4224.0537
340.662109375 0 9753.731
341.1649169921875 0 3092.478
342.1822814941406 0 11180.487
343.1625061035156 0 11163.202
343.185791015625 0 2507.2375
344.1723937988281 0 14217.404
344.97686767578125 0 11578.654
345.13128662109375 0 17890.219
345.1744689941406 0 2833.9897
346.1153259277344 0 75413.98
346.1374206542969 0 4611.877
346.699462890625 0 3322.1191
347.1182861328125 0 13346.6455
347.6802673339844 0 40498.44 b 5
348.18212890625 0 14765.311
348.68231201171875 0 2776.5298
349.1291809082031 0 3119.0386
349.15130615234375 0 4007.3052
350.21942138671875 0 58943.46
351.1452331542969 0 3676.1487
351.1668395996094 0 4269.613
351.2224426269531 0 7500.1953
352.1630554199219 0 4809.4214
352.6722106933594 0 4730.6543
353.6744689941406 0 9195.754 y Water loss 7
354.1766662597656 0 4242.998
357.1751403808594 0 3163.6995
359.0292663574219 0 10815.1875
360.2027893066406 0 3945.862
360.6955871582031 0 18317.617
361.19805908203125 0 23236.613
362.18402099609375 0 20839.16
362.6799621582031 0 106038.85 y 7
363.1417236328125 0 99423.31
363.1819763183594 0 40018.395
363.6824035644531 0 7706.0747
364.12530517578125 0 19864.975
364.1456604003906 0 18834.537
365.12841796875 0 3499.814
366.1556396484375 0 2932.1843
366.1779479980469 0 6504.512
367.1400451660156 0 9709.202
367.1624450683594 0 6684.666
367.24566650390625 0 9697.977
368.1454772949219 0 2887.0962
368.1922607421875 0 3891.4885
369.1966552734375 0 3726.2588
370.175048828125 0 3342.2048
373.1846618652344 0 6082.232
376.16204833984375 0 6273.097
376.1984558105469 0 14378.553
376.70013427734375 0 4765.456
377.1452941894531 0 5831.146
378.2144775390625 0 43994.09
379.2097473144531 0 169471.6
380.1620788574219 0 6680.6724
380.2126770019531 0 32383.613
381.1524658203125 0 294878.28
381.71160888671875 0 9830.046
382.15521240234375 0 47168.453
382.1888427734375 0 11462.275
382.215087890625 0 4313.059
382.6896057128906 0 2793.775
383.15679931640625 0 8787.491
383.2057800292969 0 3679.9387
384.1663818359375 0 12590.22
384.1888427734375 0 7824.8003
385.15093994140625 0 26981.51
385.17034912109375 0 4278.369 y 4
386.154541015625 0 4075.8213
386.1794128417969 0 4368.7666
389.1935119628906 0 4310.46 b 9
390.197021484375 0 6753.8066
390.6968688964844 0 5561.777
391.135986328125 0 11235.425
392.20794677734375 0 4361.247
393.213134765625 0 5017.0996
394.1730651855469 0 40294.145
395.13543701171875 0 21301.438
395.1567687988281 0 8076.653
395.17889404296875 0 4443.3555
395.240966796875 0 3585.8643
396.1390075683594 0 5312.8906
396.1941223144531 0 4712.9043
396.22515869140625 0 65343.926
397.20306396484375 0 5272.861
397.2283020019531 0 12817.49
397.7044677734375 0 3027.711
398.17730712890625 0 29091.137
398.227294921875 0 3240.457
399.18133544921875 0 6757.9688
402.1766662597656 0 16116.7295
402.1998596191406 0 26094.809
403.136474609375 0 2963.67
403.19976806640625 0 12358.374
404.710205078125 0 3800.9768
405.26123046875 0 11810.484
406.2464904785156 0 6325.7534
407.20379638671875 0 4061.763
409.1475524902344 0 25567.438
409.2019348144531 0 4845.1577
410.1497497558594 0 3407.813
411.20037841796875 0 10024.981
412.1837463378906 0 192065.62
413.1454162597656 0 28182.56
413.1869201660156 0 31012.596
414.14764404296875 0 4794.8027
414.19561767578125 0 6034.902
414.2355651855469 0 60095.28
415.0375061035156 0 21076.773
415.198486328125 0 24548.605
415.23883056640625 0 11762.346
416.2010498046875 0 6564.146
417.717529296875 0 36181.824
418.21087646484375 0 51459.816
418.7117004394531 0 25358.217
419.2136535644531 0 3796.585
421.2099609375 0 9609.87 y Water loss 10
422.19256591796875 0 4547.568 y Ammonia loss 10
422.2516784667969 0 2797.3755
422.7088928222656 0 3088.627
423.2015686035156 0 13805.937
423.27252197265625 0 69818.61
423.70745849609375 0 4827.5273
424.2247009277344 0 3319.6208
424.27532958984375 0 21186.068
424.7353820800781 0 8771.639
425.2350769042969 0 3138.1816
426.7227478027344 0 515548.8
427.22406005859375 0 263857.22
427.7254943847656 0 78619.3
428.2261657714844 0 12531.824
429.210205078125 0 32173.291
429.7296447753906 0 3442.5598
430.19439697265625 0 530864
431.1973571777344 0 109708.484
431.71466064453125 0 60661.848 b Water loss 6
432.2130126953125 0 30629.432 b Ammonia loss 6
432.71307373046875 0 11339.6455
433.2092590332031 0 9996.013
433.25628662109375 0 20596.148 b Water loss 3
434.2600402832031 0 3924.3342
437.1905822753906 0 4013.042
438.1742858886719 0 9007.988
438.7291564941406 0 3749.092
439.2198181152344 0 123505.08 y 10
440.1785888671875 0 6287.754
440.22320556640625 0 29376.623
440.7203063964844 0 245160.11 b 6
441.2216796875 0 140318.12
441.7230224609375 0 39240.367
442.22332763671875 0 7293.611
442.2672119140625 0 2088.41
444.1806335449219 0 2652.4485
447.20623779296875 0 26222.412 y Ammonia loss 6
447.23919677734375 0 5146.5493
447.7077941894531 0 9109.514
448.20526123046875 0 23251.32
449.20880126953125 0 10250.422
450.17413330078125 0 3875.6382
450.2373962402344 0 7000.875
450.2831115722656 0 6251.6895
451.2674865722656 0 142051.72 b 3
452.2396545410156 0 3364.2725
452.2706298828125 0 34210.633
453.2709655761719 0 4675.7666
455.1961364746094 0 5223.9966
455.2673034667969 0 7060.1753
455.72039794921875 0 16095.871 y 6
456.2204284667969 0 3503.051
459.1997985839844 0 11810.153
460.754150390625 0 19403.29
461.20428466796875 0 10838.783
461.2535400390625 0 12742.561
461.7567138671875 0 5865.7427
462.2052001953125 0 2894.8384
464.21649169921875 0 5145.529
464.2497863769531 0 2558.8242
465.75042724609375 0 3289.7754
466.24481201171875 0 4684.1587
468.2939147949219 0 41439.047
469.2960205078125 0 8606.003
474.2594299316406 0 25915.234
474.7545166015625 0 40385.156
475.2540283203125 0 20826.824
475.75372314453125 0 4349.4316
476.2259826660156 0 13386.288
477.21051025390625 0 26857.447
478.2298278808594 0 25632.328 y Water loss 9
478.2777099609375 0 17348.74
479.2372741699219 0 4245.247
479.2841796875 0 3616.9712
479.7479553222656 0 4048.8384
480.2205505371094 0 3087.7405
481.7294616699219 0 4650.843
483.2649230957031 0 86487.37
483.766357421875 0 59314.477
484.22088623046875 0 6228.0923
484.26690673828125 0 17625.324
484.76947021484375 0 2416.3933
488.2574157714844 0 27040.18 b Water loss 7
488.7571105957031 0 17111.84 b Ammonia loss 7
489.2532653808594 0 11284.675
494.23675537109375 0 313827.75
495.2396545410156 0 81394.375
496.2412414550781 0 422584.56 y 9
497.246826171875 0 112124
497.763671875 0 34265.164
498.2433166503906 0 18793.229
502.2298583984375 0 3246.0518
503.1089172363281 0 20370.842
504.20111083984375 0 14389.786
505.19970703125 0 4208.9883
505.2481384277344 0 2549.1138
506.2270202636719 0 8246.837
506.2727966308594 0 4287.36
507.25927734375 0 7220.9395
511.2630920410156 0 21564.389
512.2653198242188 0 4166.4175
513.2123413085938 0 4851.15
514.1885986328125 0 6199.2144
515.282470703125 0 9975.669
519.1399536132812 0 6616.168
519.330810546875 0 4079.7878
522.2335205078125 0 5975.112
523.258544921875 0 10985.967
523.5888061523438 0 4383.5063
524.2518310546875 0 3097.711
525.2669677734375 0 56164.645
526.2314453125 0 11239.3
526.2709350585938 0 12489.303
527.2326049804688 0 3379.6194
529.2615966796875 0 16400.682 Precursor Water loss
529.594482421875 0 22692.879 Precursor Ammonia loss
529.9284057617188 0 11095.883
530.2237548828125 0 4273.4556
530.263916015625 0 3510.0522
531.2205810546875 0 4592.5337
532.1942749023438 0 14383.438
533.1966552734375 0 2988.2783
535.2636108398438 0 13341.861 Precursor
535.598876953125 0 15587.111
535.9334716796875 0 8950.504
536.1658325195312 0 3510.7942
536.2661743164062 0 6628.8203
537.2484130859375 0 3485.107
537.7374877929688 0 11571.005
538.2711791992188 0 9512.348
538.7691650390625 0 6391.2725
539.2368774414062 0 58729.617
539.2821044921875 0 4749.2827
540.2395629882812 0 18430.992
541.2525024414062 0 7035.8633
542.295166015625 0 4281.32
543.278076171875 0 128805.92
544.2807006835938 0 37530.05
545.2828979492188 0 7692.119
546.2496948242188 0 16389.424
546.7513427734375 0 10434.099
547.2452392578125 0 4509.1313
547.7770385742188 0 5654.6943
548.2393798828125 0 3659.304
548.2965087890625 0 3257.167
549.2212524414062 0 12977.8545
550.21044921875 0 8457.227
551.2578125 0 13516.654
551.3319702148438 0 7130.9736
552.2584228515625 0 2948.0266
552.3296508789062 0 3041.7454
553.263916015625 0 3316.629
554.7744750976562 0 14501.373 b 8
555.2576293945312 0 29903.322
555.757080078125 0 16866.686
556.2586669921875 0 5581.4272
561.3151245117188 0 14555.29 b Water loss 4
562.3018798828125 0 7374.7046 b Ammonia loss 4
563.258056640625 0 12803.434
564.2596435546875 0 5617.442
565.26318359375 0 19799.424
566.2679443359375 0 3816.7686
567.2316284179688 0 66776.41
568.23583984375 0 18096.318
568.7598876953125 0 8310.392
569.2401733398438 0 7336.838
575.2465209960938 0 15658.9
576.232177734375 0 3583.785
576.2823486328125 0 3283.5496
577.2139892578125 0 4510.639
577.2652587890625 0 3494.3264 y 4
577.7645874023438 0 4350.7246
579.27392578125 0 4409.6655
579.3258666992188 0 88371.56 b 4
580.3284912109375 0 26251.33
581.2703857421875 0 3259.869
581.332275390625 0 4009.6558
582.3084716796875 0 4445.583
583.287109375 0 4456.564 b 9
587.2547607421875 0 2930.266
588.7960205078125 0 3659.9912
589.306884765625 0 2812.2808
592.2769165039062 0 4099.8755
592.32568359375 0 11470.691
593.2571411132812 0 100535.51 y Water loss 8
593.3062133789062 0 47940.727
593.7844848632812 0 6232.2925
594.2567138671875 0 30290.979
594.3099975585938 0 10159.864
594.7808837890625 0 3970.229
595.2486572265625 0 6311.376
596.3529663085938 0 2800.5142
597.3008422851562 0 3431.782
597.8009643554688 0 10552.307
598.3026733398438 0 3668.174
600.3141479492188 0 10055.535
602.2992553710938 0 2820.7854
602.79150390625 0 20571.523
603.2926025390625 0 19561.312
603.7914428710938 0 10006.808
604.2867431640625 0 4709.241
610.283935546875 0 85613.234
610.3313598632812 0 7487.961
611.2686157226562 0 1306835.6 y 8
611.7976684570312 0 83740.805
612.2718505859375 0 407759.47
612.7996215820312 0 24650.21
613.2740478515625 0 83475.13
614.2767333984375 0 16126.709
616.298095703125 0 5718.4834
617.28515625 0 7228.879
619.2752075195312 0 3016.172
620.803466796875 0 6375.7417
621.252685546875 0 14135.559
621.3018798828125 0 7976.106
622.2552490234375 0 3180.356
622.370849609375 0 5586.721
623.2496948242188 0 2582.8657
628.2760009765625 0 3566.0784
628.777099609375 0 3665.2932
634.3076171875 0 17177.867
635.2969970703125 0 21554.17
636.328125 0 10968.377
636.7860107421875 0 7393.8857 y Water loss 3
637.2828369140625 0 4252.8447 y Ammonia loss 3
637.3394165039062 0 4148.892
637.7880249023438 0 7061.742
638.329345703125 0 6576.3813 b Water loss 10
640.2745361328125 0 16438.896
641.26318359375 0 3772.8604
645.2855834960938 0 12163.268
645.7925415039062 0 84437.37 y 3
646.2933349609375 0 76104.43
646.79541015625 0 24324.941
647.2891845703125 0 3500.7017
648.3444213867188 0 5029.559
649.3428955078125 0 4263.5405
650.2684936523438 0 6331.6953
650.3269653320312 0 4143.721
651.274658203125 0 4125.7
652.3212280273438 0 130906.84
653.32421875 0 48192.49
654.3258666992188 0 8888.792
658.2839965820312 0 10791.659
659.3182983398438 0 6748.3203
660.3198852539062 0 2733.906
660.871826171875 0 3382.1353
661.3300170898438 0 9412.403
661.8320922851562 0 6310.615
662.3060913085938 0 37843.754
663.2953491210938 0 35578.387
664.294189453125 0 3857.3938
664.349609375 0 3657.9363
666.2875366210938 0 2937.2402
666.3525390625 0 3352.083
668.2764282226562 0 5623.8643
670.8245849609375 0 2717.0332
676.3423461914062 0 56702.883 b Water loss 5
677.3339233398438 0 37979.24 b Ammonia loss 5
678.3336181640625 0 15670.672
679.3345336914062 0 14151.325
679.829833984375 0 5734.872
680.31640625 0 228895.25
681.3187255859375 0 93009.13
682.3214721679688 0 19112.572
683.3211669921875 0 3683.0325
684.3216552734375 0 6792.7607
684.8175659179688 0 21068.74
685.3175048828125 0 12015.21
685.8193359375 0 8515.44
686.3201904296875 0 2944.1099
692.8341674804688 0 4925.466
693.3295288085938 0 67730.914 y Water loss 2
693.828369140625 0 61213.77 y Ammonia loss 2
694.352294921875 0 614619.75 b 5
694.8289794921875 0 14331.693
695.355224609375 0 234307.67
696.3577880859375 0 57207.89
697.2879638671875 0 3240.1975
697.3519897460938 0 10198.61
697.8592529296875 0 4212.5786
698.355224609375 0 7129.128
701.842041015625 0 31122.012
702.3347778320312 0 527801.7 y 2
702.83642578125 0 441281.25 b Water loss 11
703.3377685546875 0 201892.69 b Ammonia loss 11
703.8394165039062 0 64742.824
704.3394165039062 0 21198.31
705.3553466796875 0 5077.97
706.3429565429688 0 43315.836 y Water loss 7
707.3452758789062 0 15568.916
708.306640625 0 4217.08
711.3749389648438 0 14723.596
711.8557739257812 0 34202.14 b 11
712.35791015625 0 43772.71
712.857666015625 0 16243.754
713.3598022460938 0 7745.3154
715.3053588867188 0 3221.8064
720.3854370117188 0 5147.53
720.86328125 0 3113.6106
721.3642578125 0 28616.525
722.365478515625 0 9457.404
723.3695068359375 0 31984.15
724.3528442382812 0 408124.16 y 7
725.3554077148438 0 173714.66
726.3582763671875 0 41460.58
727.3577270507812 0 4979.2817
728.8580322265625 0 4928.662
729.3577880859375 0 18983.658
730.3630981445312 0 9586.697
733.3641967773438 0 16027.551
734.3573608398438 0 16164.745
735.3563842773438 0 4403.745
737.8675537109375 0 4896.938
738.3605346679688 0 4473.41
742.867431640625 0 3695.4968 y Water loss 1
743.3658447265625 0 9679.032 y Ammonia loss 1
743.8636474609375 0 4145.725
744.3695068359375 0 3344.1436
746.3502807617188 0 2873.1704
747.3907470703125 0 9204.814
748.384033203125 0 10369.035
749.3372192382812 0 5930.8613
750.3944702148438 0 3427.4265
751.3770751953125 0 60211.027
751.8688354492188 0 47369.535 y 1
752.373291015625 0 72810.88
752.872314453125 0 16136.248
753.3775024414062 0 15607.787
760.3052978515625 0 4086.2163
761.3750610351562 0 4698.3647
762.41552734375 0 8277.626
763.3684692382812 0 38147.33
764.3651733398438 0 10966.632
765.4011840820312 0 15943.116
766.408203125 0 7682.3535
767.3482666015625 0 13650.566
768.34521484375 0 4084.8027
769.409912109375 0 4252.879
775.390380859375 0 7723.531
776.3793334960938 0 8410.489
779.3842163085938 0 41668.31
780.3850708007812 0 16514.338
781.369384765625 0 14248.011
782.3671264648438 0 8373.25
791.38623046875 0 2794.2058
792.437744140625 0 3241.8704
793.4002075195312 0 67930.164 Precursor Water loss
794.4036865234375 0 27130.191
795.342529296875 0 28092.502
795.40771484375 0 4430.781
796.3455200195312 0 10306.909
797.3447265625 0 3607.558
807.3767700195312 0 10426.152
807.4410400390625 0 7560.062
808.3824462890625 0 9906.703
817.40380859375 0 3677.615
818.4415893554688 0 7908.789
819.4359741210938 0 7649.7583
820.4398803710938 0 3711.787
825.3868408203125 0 6137.0684
826.3782348632812 0 12348.624
827.374267578125 0 3134.8672
834.4276733398438 0 12108.862
835.4185180664062 0 25260.057
836.4492797851562 0 52639.79
837.4528198242188 0 20889.791
838.4544677734375 0 6326.0386
843.4031372070312 0 9179.664
844.3892822265625 0 20439.16
845.3927612304688 0 13447.59
846.4466552734375 0 20381.885
847.444091796875 0 13448.598
852.363525390625 0 10047.463
852.4373168945312 0 148018.12
853.3524780273438 0 5105.4546
853.4398193359375 0 80431.836
854.4421997070312 0 17598.326
862.4214477539062 0 88932.73 b Water loss 6
863.4168701171875 0 71635.72 b Ammonia loss 6
864.4558715820312 0 110456.49
865.4601440429688 0 55745.574
866.4629516601562 0 20438.451
867.4647827148438 0 2969.681
869.3908081054688 0 5143.2476
876.436279296875 0 9588.575
877.4334106445312 0 4518.525
880.4319458007812 0 716659.94 b 6
881.4342651367188 0 380891.25
882.4370727539062 0 120344.63
883.43798828125 0 21559.885
890.4139404296875 0 5098.5884
891.4078979492188 0 9915.17
892.39794921875 0 3760.1282
892.4683227539062 0 9594.52
893.4723510742188 0 8166.8926
894.436279296875 0 6106.015
907.4415283203125 0 3283.8809
908.4271240234375 0 80218.79
909.4310913085938 0 43653.617
910.4319458007812 0 70490.01 y 6
911.4344482421875 0 30313.715
912.4384765625 0 10087.626
936.421630859375 0 7364.837
937.4119262695312 0 10927.078
938.416259765625 0 4667.098
947.4393310546875 0 8081.665
948.424072265625 0 5152.6895
954.4321899414062 0 11305.188
955.4207153320312 0 24846.883
956.4215698242188 0 10662.717
957.4220581054688 0 4173.8066
962.4488525390625 0 3496.1643
964.4551391601562 0 4068.311
965.448974609375 0 22861.277
966.4498291015625 0 12262.424
967.4500732421875 0 3325.671
972.4429931640625 0 20272.465
973.4448852539062 0 14377.27
974.4473876953125 0 6115.028
975.5076904296875 0 5649.596 b Water loss 7
976.5040283203125 0 6361.9663 b Ammonia loss 7
980.4602661132812 0 7624.5464
981.4566040039062 0 3524.0786
982.4725341796875 0 7102.12
983.4810791015625 0 4026.9482
993.5161743164062 0 38033.676 b 7
994.5180053710938 0 22286.793
995.5189208984375 0 6279.106
1007.4812622070312 0 8482.783
1008.4948120117188 0 4662.7563
1025.4598388671875 0 34778.51 y 5
1026.4610595703125 0 22409.604
1027.4710693359375 0 5972.21
1075.5318603515625 0 3866.3413
1076.5245361328125 0 5522.954
1077.5257568359375 0 3902.0984
1091.496337890625 0 3475.0796 b Ammonia loss 8
1093.54150390625 0 20819.71
1094.545654296875 0 16357.722
1095.5458984375 0 4284.5947
1096.87548828125 0 3250.4465
1108.5408935546875 0 10726.851 b 8
1109.527099609375 0 6270.9814
1110.544189453125 0 4138.584
1118.4857177734375 0 4198.354
1119.484375 0 4769.0093
1135.5076904296875 0 25331.803 y Water loss 4
1136.49609375 0 40938.836 y Ammonia loss 4
1137.4952392578125 0 23308.922
1138.49951171875 0 10627.299
1153.51708984375 0 67581.93 y 4
1154.5201416015625 0 50087.156
1155.520751953125 0 18168.5
1156.527587890625 0 6260.496
1165.564453125 0 6439.9116 b 9
1166.5711669921875 0 4574.7676
1222.57666015625 0 3726.537
1286.6905517578125 0 3063.8826
2012.9296875 0 2743.7783
3163.44189453125 0 2851.9692

Spectrum Details

|  |  |
| --- | --- |
| Matched peaks? Matched peaksThe total absolute number of peaks matched. Additionally in brackets the total fraction of peaks matched and the total number of peaks is shown. | 79 (8.77% of 901) |
| FDR? FDRThe false discovery rate estimated for this peptide. It is calculated by matching all theoretical fragments with a non-integer shift with the raw peaks for this spectrum. This is done with 40 different shifts. The resulting percentage is the average number of annotated peaks over the number of annotated peaks with the correct spectrum. | 0.72% |
| Satellite FDR? Satellite FDRSee the FDR for details on its calculation. This satellite ion specific FDR only contains the satellite ions (d/w) for I/L/J positions. | ∞ |
| PSM Score? PSM ScoreThe PSM Score as given by Hecklib to this annotated spectrum. It is shown with three significant figures. | 604 |

## Spectrum 7394? Spectrum 7394 The raw spectrum of this peptide as annotated by Hecklib. The fragments are coloured according to ion type (see legend). Any peaks with a star '\*' as text can be hovered over to see the full details, first the ion type second the mass shift type. By hovering over the amino acids in the peptide or ions in the legend the corresponding peaks are highlighted. By toggling the 'Unassigned' label you can turn the background (unassigned) peaks on or off in the plot. By updating the slider in the Ion legend you can update the spectrum to only show the top X% of the peaks with labels. The top X% means any peak that is within X% of the highest intensity. By dragging in the spectrum you can zoom in to a specific part of the spectrum and use 'Zoom Out' to get back to the original zoom level. The annotation of the spectrum is based on the given sequence in the peptides file and is done with different software so inconsistencies are likely. The peaks are annotated based on the given sequence, with 20 ppm tolerance.

Copy Data

### Spectrum 7394 (TSV)

#### Preview

```
Loading example...
```

*Click on the button to copy the data to your clipboard.*

Mz MinMz MaxIntensity Max

WidthHeightPeptide font sizePeptide stroke widthSpectrum font sizeSpectrum stroke widthCompact peptide

Ion legend

wxyz

abcd

OtherUnassignedIonChargePositionShow for top:%

TVLHQDWLDGKEY

01.24e+52.47e+53.71e+54.94e+5

Zoom Out

a+12y+34a+12y+11b+12b+12y+23b+25y+12b+13y+25y+37y+25y+12b+13b+26b+26y+26y+26y+39b+310y+13y+13b+27b+27b+14y+13b+27y+27b+14y+27y+14b+28b+28y+14\*\*\*b+29b+29b+15b+15b+15y+15y+15y+210y+210b+211y+210b+16b+16y+211y+211b+16y+211b+212b+212y+16b+212y+16y+212y+212y+212\*b+17b+17b+17y+17b+18b+18b+18y+18b+19y+19y+19y+19b+110y+110

0872174526173489

Fragment Matches Table

Show background peaks

| Position | Ion type | Intensity | mz Theoretical | mz Error (Th) | mz Error (ppm) | Charge | Series Number |
| --- | --- | --- | --- | --- | --- | --- | --- |
| - | - | 8446 | 120.1 | - | - | 0 | - |
| - | - | 569.9 | 121.1 | - | - | 0 | - |
| - | - | 469.6 | 121.9 | - | - | 0 | - |
| - | - | 2263 | 122.1 | - | - | 0 | - |
| - | - | 2485 | 123 | - | - | 0 | - |
| - | - | 769.5 | 123.1 | - | - | 0 | - |
| - | - | 460.7 | 125 | - | - | 0 | - |
| - | - | 902.9 | 125.1 | - | - | 0 | - |
| - | - | 1224 | 126.1 | - | - | 0 | - |
| - | - | 5867 | 127.1 | - | - | 0 | - |
| - | - | 953 | 127.1 | - | - | 0 | - |
| - | - | 1657 | 127.1 | - | - | 0 | - |
| - | - | 2.764E+04 | 128.1 | - | - | 0 | - |
| - | - | 1904 | 129.1 | - | - | 0 | - |
| - | - | 3.227E+05 | 129.1 | - | - | 0 | - |
| - | - | 1.403E+05 | 130.1 | - | - | 0 | - |
| - | - | 1983 | 130.1 | - | - | 0 | - |
| - | - | 1.861E+04 | 130.1 | - | - | 0 | - |
| - | - | 498.9 | 130.8 | - | - | 0 | - |
| - | - | 1.308E+04 | 131.1 | - | - | 0 | - |
| - | - | 704 | 131.1 | - | - | 0 | - |
| - | - | 3.061E+04 | 132.1 | - | - | 0 | - |
| - | - | 834.3 | 133.1 | - | - | 0 | - |
| - | - | 3231 | 133.1 | - | - | 0 | - |
| - | - | 578.8 | 134 | - | - | 0 | - |
| - | - | 4.319E+04 | 136.1 | - | - | 0 | - |
| - | - | 3302 | 137.1 | - | - | 0 | - |
| - | - | 3975 | 138.1 | - | - | 0 | - |
| - | - | 1406 | 138.1 | - | - | 0 | - |
| - | - | 539.8 | 139.1 | - | - | 0 | - |
| - | - | 3874 | 139.1 | - | - | 0 | - |
| - | - | 5368 | 141.1 | - | - | 0 | - |
| - | - | 1878 | 142.1 | - | - | 0 | - |
| - | - | 1971 | 143 | - | - | 0 | - |
| - | - | 636.7 | 143.1 | - | - | 0 | - |
| - | - | 1013 | 144.1 | - | - | 0 | - |
| - | - | 3393 | 145.1 | - | - | 0 | - |
| - | - | 1189 | 146.1 | - | - | 0 | - |
| - | - | 1789 | 147 | - | - | 0 | - |
| - | - | 1.13E+04 | 149 | - | - | 0 | - |
| - | - | 624.1 | 150 | - | - | 0 | - |
| - | - | 1788 | 150.1 | - | - | 0 | - |
| - | - | 7253 | 151.1 | - | - | 0 | - |
| - | - | 683.5 | 153.1 | - | - | 0 | - |
| - | - | 1070 | 154.1 | - | - | 0 | - |
| - | - | 1495 | 155.1 | - | - | 0 | - |
| - | - | 2.102E+04 | 155.1 | - | - | 0 | - |
| 2 | a | 1.529E+04 | 155.1 | 0.0003356 | 2.163 | +1 | 2 |
| - | - | 1288 | 156.1 | - | - | 0 | - |
| - | - | 760 | 156.1 | - | - | 0 | - |
| - | - | 1122 | 156.1 | - | - | 0 | - |
| - | - | 875.4 | 157.1 | - | - | 0 | - |
| - | - | 1900 | 157.1 | - | - | 0 | - |
| - | - | 3165 | 158.1 | - | - | 0 | - |
| - | - | 2.435E+05 | 159.1 | - | - | 0 | - |
| - | - | 2313 | 160.1 | - | - | 0 | - |
| - | - | 1672 | 160.1 | - | - | 0 | - |
| - | - | 2.772E+04 | 160.1 | - | - | 0 | - |
| - | - | 918.5 | 161.1 | - | - | 0 | - |
| - | - | 1.998E+04 | 165.1 | - | - | 0 | - |
| - | - | 1164 | 165.1 | - | - | 0 | - |
| - | - | 3.752E+04 | 166.1 | - | - | 0 | - |
| 10 | y | 3830 | 166.1 | 0.001786 | 10.76 | +3 | 4 |
| - | - | 2409 | 166.1 | - | - | 0 | - |
| - | - | 1.369E+04 | 167.1 | - | - | 0 | - |
| - | - | 2441 | 167.1 | - | - | 0 | - |
| - | - | 608.9 | 167.1 | - | - | 0 | - |
| - | - | 1369 | 167.1 | - | - | 0 | - |
| - | - | 917.7 | 168.1 | - | - | 0 | - |
| - | - | 3933 | 168.1 | - | - | 0 | - |
| - | - | 3618 | 168.1 | - | - | 0 | - |
| - | - | 3512 | 168.1 | - | - | 0 | - |
| - | - | 4239 | 169.1 | - | - | 0 | - |
| - | - | 1339 | 169.1 | - | - | 0 | - |
| - | - | 9594 | 169.1 | - | - | 0 | - |
| - | - | 1.322E+04 | 170.1 | - | - | 0 | - |
| - | - | 1479 | 171.1 | - | - | 0 | - |
| - | - | 2283 | 171.1 | - | - | 0 | - |
| - | - | 1597 | 171.1 | - | - | 0 | - |
| - | - | 4491 | 172.1 | - | - | 0 | - |
| - | - | 3987 | 173.1 | - | - | 0 | - |
| 2 | a | 4.894E+05 | 173.1 | 0.0004673 | 2.699 | +1 | 2 |
| - | - | 2575 | 173.4 | - | - | 0 | - |
| - | - | 4.308E+04 | 174.1 | - | - | 0 | - |
| - | - | 2110 | 175.1 | - | - | 0 | - |
| - | - | 847.9 | 176.1 | - | - | 0 | - |
| - | - | 1563 | 177.1 | - | - | 0 | - |
| - | - | 1.289E+04 | 178.1 | - | - | 0 | - |
| - | - | 1147 | 179.1 | - | - | 0 | - |
| - | - | 984.3 | 179.1 | - | - | 0 | - |
| - | - | 2833 | 181.1 | - | - | 0 | - |
| 13 | y | 4.778E+04 | 182.1 | 0.0004496 | 2.469 | +1 | 1 |
| - | - | 3756 | 183.1 | - | - | 0 | - |
| 2 | b | 1.283E+04 | 183.1 | 0.0004008 | 2.189 | +1 | 2 |
| - | - | 1265 | 183.1 | - | - | 0 | - |
| - | - | 1498 | 184.1 | - | - | 0 | - |
| - | - | 837.2 | 185.1 | - | - | 0 | - |
| - | - | 3145 | 185.2 | - | - | 0 | - |
| - | - | 1.09E+05 | 186.1 | - | - | 0 | - |
| - | - | 1.791E+04 | 187.1 | - | - | 0 | - |
| - | - | 764.4 | 187.1 | - | - | 0 | - |
| - | - | 8458 | 187.1 | - | - | 0 | - |
| - | - | 2327 | 188.1 | - | - | 0 | - |
| - | - | 2725 | 188.1 | - | - | 0 | - |
| - | - | 861 | 188.1 | - | - | 0 | - |
| - | - | 3984 | 190.1 | - | - | 0 | - |
| - | - | 3032 | 190.1 | - | - | 0 | - |
| - | - | 656.8 | 191.1 | - | - | 0 | - |
| - | - | 688.2 | 193.1 | - | - | 0 | - |
| - | - | 596.8 | 193.1 | - | - | 0 | - |
| - | - | 3969 | 194.1 | - | - | 0 | - |
| - | - | 799.5 | 195.1 | - | - | 0 | - |
| - | - | 2.596E+04 | 195.1 | - | - | 0 | - |
| - | - | 1.554E+04 | 196.1 | - | - | 0 | - |
| - | - | 2218 | 196.1 | - | - | 0 | - |
| - | - | 1394 | 197.1 | - | - | 0 | - |
| - | - | 1931 | 197.1 | - | - | 0 | - |
| - | - | 813.4 | 197.2 | - | - | 0 | - |
| - | - | 2814 | 198.1 | - | - | 0 | - |
| - | - | 8202 | 198.1 | - | - | 0 | - |
| - | - | 1574 | 199.1 | - | - | 0 | - |
| - | - | 1973 | 199.1 | - | - | 0 | - |
| - | - | 1838 | 199.1 | - | - | 0 | - |
| - | - | 726.9 | 200.1 | - | - | 0 | - |
| - | - | 1684 | 200.1 | - | - | 0 | - |
| 2 | b | 1.345E+05 | 201.1 | 0.0003647 | 1.813 | +1 | 2 |
| - | - | 1684 | 202.1 | - | - | 0 | - |
| - | - | 1.213E+04 | 202.1 | - | - | 0 | - |
| - | - | 1110 | 203.1 | - | - | 0 | - |
| - | - | 1604 | 204.1 | - | - | 0 | - |
| - | - | 1.122E+04 | 205.1 | - | - | 0 | - |
| - | - | 662.4 | 205.1 | - | - | 0 | - |
| - | - | 1985 | 205.1 | - | - | 0 | - |
| - | - | 839.9 | 206.1 | - | - | 0 | - |
| - | - | 2025 | 206.1 | - | - | 0 | - |
| - | - | 3520 | 207.2 | - | - | 0 | - |
| - | - | 4193 | 210.1 | - | - | 0 | - |
| 11 | y | 3145 | 211.1 | 0.0003745 | 1.774 | +2 | 3 |
| - | - | 836.8 | 211.1 | - | - | 0 | - |
| - | - | 637.1 | 212.1 | - | - | 0 | - |
| - | - | 2962 | 212.1 | - | - | 0 | - |
| - | - | 1.077E+04 | 213.1 | - | - | 0 | - |
| - | - | 1471 | 213.2 | - | - | 0 | - |
| - | - | 3756 | 215.1 | - | - | 0 | - |
| - | - | 2645 | 216.1 | - | - | 0 | - |
| - | - | 2428 | 221.1 | - | - | 0 | - |
| - | - | 1213 | 221.1 | - | - | 0 | - |
| - | - | 4770 | 221.1 | - | - | 0 | - |
| - | - | 2759 | 221.1 | - | - | 0 | - |
| - | - | 735.4 | 221.1 | - | - | 0 | - |
| - | - | 1664 | 221.1 | - | - | 0 | - |
| - | - | 2208 | 222.1 | - | - | 0 | - |
| - | - | 4160 | 222.1 | - | - | 0 | - |
| - | - | 3425 | 223.1 | - | - | 0 | - |
| - | - | 1337 | 223.1 | - | - | 0 | - |
| - | - | 9063 | 223.1 | - | - | 0 | - |
| - | - | 4.402E+04 | 223.2 | - | - | 0 | - |
| - | - | 3080 | 224.1 | - | - | 0 | - |
| - | - | 4885 | 224.2 | - | - | 0 | - |
| - | - | 7328 | 225 | - | - | 0 | - |
| - | - | 1359 | 225.1 | - | - | 0 | - |
| - | - | 668 | 225.2 | - | - | 0 | - |
| - | - | 2008 | 226 | - | - | 0 | - |
| - | - | 8920 | 226.1 | - | - | 0 | - |
| - | - | 785 | 226.2 | - | - | 0 | - |
| - | - | 879.4 | 227 | - | - | 0 | - |
| - | - | 8433 | 227.1 | - | - | 0 | - |
| - | - | 963.9 | 227.1 | - | - | 0 | - |
| - | - | 1788 | 227.2 | - | - | 0 | - |
| - | - | 3911 | 228.1 | - | - | 0 | - |
| - | - | 9730 | 229.1 | - | - | 0 | - |
| - | - | 864.4 | 230.1 | - | - | 0 | - |
| - | - | 1547 | 230.2 | - | - | 0 | - |
| - | - | 1023 | 231.1 | - | - | 0 | - |
| - | - | 6735 | 233.1 | - | - | 0 | - |
| - | - | 4.764E+04 | 234.1 | - | - | 0 | - |
| - | - | 1145 | 234.7 | - | - | 0 | - |
| - | - | 6337 | 235.1 | - | - | 0 | - |
| - | - | 1360 | 235.6 | - | - | 0 | - |
| - | - | 2055 | 237.1 | - | - | 0 | - |
| - | - | 5559 | 238.1 | - | - | 0 | - |
| - | - | 1001 | 238.1 | - | - | 0 | - |
| - | - | 1.427E+04 | 239.1 | - | - | 0 | - |
| - | - | 3787 | 240.1 | - | - | 0 | - |
| - | - | 1.806E+04 | 240.1 | - | - | 0 | - |
| - | - | 2690 | 241.1 | - | - | 0 | - |
| - | - | 2404 | 241.1 | - | - | 0 | - |
| - | - | 2381 | 241.1 | - | - | 0 | - |
| - | - | 2121 | 241.2 | - | - | 0 | - |
| - | - | 4268 | 243.1 | - | - | 0 | - |
| - | - | 1.943E+04 | 244.1 | - | - | 0 | - |
| - | - | 2143 | 245.1 | - | - | 0 | - |
| - | - | 2.611E+04 | 247.1 | - | - | 0 | - |
| - | - | 1660 | 247.1 | - | - | 0 | - |
| - | - | 1263 | 247.2 | - | - | 0 | - |
| - | - | 2670 | 248.1 | - | - | 0 | - |
| - | - | 9236 | 248.1 | - | - | 0 | - |
| - | - | 1.586E+04 | 249.1 | - | - | 0 | - |
| - | - | 4344 | 249.1 | - | - | 0 | - |
| - | - | 1844 | 250.1 | - | - | 0 | - |
| - | - | 1344 | 250.1 | - | - | 0 | - |
| - | - | 1.644E+05 | 251.2 | - | - | 0 | - |
| - | - | 1515 | 252.1 | - | - | 0 | - |
| - | - | 2.13E+04 | 252.2 | - | - | 0 | - |
| - | - | 708.9 | 253.1 | - | - | 0 | - |
| - | - | 1533 | 253.2 | - | - | 0 | - |
| - | - | 9198 | 255.1 | - | - | 0 | - |
| - | - | 1817 | 256.1 | - | - | 0 | - |
| - | - | 775.4 | 256.2 | - | - | 0 | - |
| - | - | 906.1 | 256.6 | - | - | 0 | - |
| - | - | 3975 | 257.1 | - | - | 0 | - |
| - | - | 8156 | 258.1 | - | - | 0 | - |
| - | - | 3983 | 261.1 | - | - | 0 | - |
| - | - | 1322 | 261.1 | - | - | 0 | - |
| - | - | 2218 | 262.1 | - | - | 0 | - |
| - | - | 1.893E+04 | 265.1 | - | - | 0 | - |
| - | - | 1010 | 265.1 | - | - | 0 | - |
| - | - | 888.8 | 265.2 | - | - | 0 | - |
| - | - | 4.535E+04 | 266.1 | - | - | 0 | - |
| - | - | 4571 | 267.1 | - | - | 0 | - |
| - | - | 1880 | 268.1 | - | - | 0 | - |
| - | - | 4038 | 268.2 | - | - | 0 | - |
| - | - | 2123 | 269.2 | - | - | 0 | - |
| - | - | 3331 | 269.2 | - | - | 0 | - |
| - | - | 6709 | 270.1 | - | - | 0 | - |
| - | - | 1044 | 270.1 | - | - | 0 | - |
| - | - | 2151 | 270.6 | - | - | 0 | - |
| - | - | 8455 | 272.2 | - | - | 0 | - |
| - | - | 1124 | 273.2 | - | - | 0 | - |
| - | - | 8395 | 274.1 | - | - | 0 | - |
| - | - | 3343 | 274.1 | - | - | 0 | - |
| - | - | 2075 | 275.1 | - | - | 0 | - |
| - | - | 999 | 275.1 | - | - | 0 | - |
| - | - | 1009 | 275.2 | - | - | 0 | - |
| - | - | 2488 | 276.1 | - | - | 0 | - |
| - | - | 1499 | 276.1 | - | - | 0 | - |
| - | - | 2490 | 276.2 | - | - | 0 | - |
| - | - | 3477 | 279.1 | - | - | 0 | - |
| - | - | 4034 | 280.1 | - | - | 0 | - |
| - | - | 1.201E+04 | 281.1 | - | - | 0 | - |
| - | - | 906.2 | 281.1 | - | - | 0 | - |
| - | - | 7639 | 282.2 | - | - | 0 | - |
| - | - | 1396 | 283 | - | - | 0 | - |
| - | - | 1.167E+05 | 283.1 | - | - | 0 | - |
| - | - | 5510 | 284.1 | - | - | 0 | - |
| - | - | 968.4 | 284.1 | - | - | 0 | - |
| - | - | 1.606E+04 | 284.1 | - | - | 0 | - |
| - | - | 969.3 | 285 | - | - | 0 | - |
| - | - | 944.1 | 285.1 | - | - | 0 | - |
| - | - | 1359 | 285.1 | - | - | 0 | - |
| - | - | 1185 | 285.2 | - | - | 0 | - |
| - | - | 6694 | 286.1 | - | - | 0 | - |
| - | - | 825.6 | 287.1 | - | - | 0 | - |
| - | - | 1743 | 287.2 | - | - | 0 | - |
| - | - | 1577 | 288.6 | - | - | 0 | - |
| - | - | 822 | 289.1 | - | - | 0 | - |
| 5 | b | 1130 | 290.2 | 0.0009844 | 3.393 | +2 | 5 |
| 12 | y | 6.687E+04 | 293.1 | 0.0005409 | 1.845 | +1 | 2 |
| - | - | 1.34E+04 | 294.1 | - | - | 0 | - |
| - | - | 1414 | 295.1 | - | - | 0 | - |
| - | - | 1069 | 295.1 | - | - | 0 | - |
| - | - | 1360 | 296.1 | - | - | 0 | - |
| 3 | b | 2163 | 296.2 | 0.0006416 | 2.166 | +1 | 3 |
| - | - | 1004 | 296.7 | - | - | 0 | - |
| - | - | 2032 | 297.1 | - | - | 0 | - |
| 9 | y | 1408 | 297.1 | 0.003759 | 12.65 | +2 | 5 |
| - | - | 2.108E+04 | 297.2 | - | - | 0 | - |
| 7 | y | 2010 | 298.1 | 0.005337 | 17.9 | +3 | 7 |
| - | - | 3614 | 298.2 | - | - | 0 | - |
| - | - | 8.637E+04 | 299.1 | - | - | 0 | - |
| - | - | 1869 | 300.1 | - | - | 0 | - |
| - | - | 1.225E+04 | 300.2 | - | - | 0 | - |
| - | - | 1284 | 301.1 | - | - | 0 | - |
| - | - | 6.11E+04 | 301.2 | - | - | 0 | - |
| - | - | 1.093E+04 | 302.1 | - | - | 0 | - |
| - | - | 7615 | 302.2 | - | - | 0 | - |
| - | - | 1276 | 303.1 | - | - | 0 | - |
| - | - | 1989 | 305.2 | - | - | 0 | - |
| - | - | 3388 | 305.6 | - | - | 0 | - |
| 9 | y | 1.126E+04 | 306.1 | 0.0006739 | 2.201 | +2 | 5 |
| - | - | 5445 | 306.6 | - | - | 0 | - |
| - | - | 1400 | 307.1 | - | - | 0 | - |
| - | - | 1024 | 309.2 | - | - | 0 | - |
| - | - | 803.1 | 310.2 | - | - | 0 | - |
| 12 | y | 1.089E+04 | 311.1 | 0.0009931 | 3.192 | +1 | 2 |
| - | - | 2230 | 311.1 | - | - | 0 | - |
| - | - | 1759 | 312.1 | - | - | 0 | - |
| - | - | 1982 | 314.2 | - | - | 0 | - |
| 3 | b | 4935 | 314.2 | 0.0008496 | 2.704 | +1 | 3 |
| - | - | 3.599E+04 | 315.2 | - | - | 0 | - |
| - | - | 1076 | 315.2 | - | - | 0 | - |
| - | - | 5772 | 316.2 | - | - | 0 | - |
| - | - | 1237 | 317.7 | - | - | 0 | - |
| - | - | 1551 | 319.2 | - | - | 0 | - |
| - | - | 2747 | 325.2 | - | - | 0 | - |
| - | - | 7661 | 326.7 | - | - | 0 | - |
| - | - | 3389 | 327.2 | - | - | 0 | - |
| - | - | 1314 | 327.7 | - | - | 0 | - |
| - | - | 3638 | 332.2 | - | - | 0 | - |
| - | - | 6514 | 333.2 | - | - | 0 | - |
| - | - | 1134 | 334.2 | - | - | 0 | - |
| - | - | 1343 | 335.2 | - | - | 0 | - |
| - | - | 1133 | 338.2 | - | - | 0 | - |
| 6 | b | 2261 | 338.7 | 8.894E-05 | 0.2626 | +2 | 6 |
| - | - | 2218 | 340.7 | - | - | 0 | - |
| - | - | 1231 | 341 | - | - | 0 | - |
| - | - | 1745 | 341.2 | - | - | 0 | - |
| - | - | 2707 | 342.2 | - | - | 0 | - |
| - | - | 3537 | 343.2 | - | - | 0 | - |
| - | - | 963 | 343.2 | - | - | 0 | - |
| - | - | 2374 | 344.2 | - | - | 0 | - |
| - | - | 1.178E+04 | 345 | - | - | 0 | - |
| - | - | 4775 | 345.1 | - | - | 0 | - |
| - | - | 1.729E+04 | 346.1 | - | - | 0 | - |
| - | - | 1462 | 346.7 | - | - | 0 | - |
| - | - | 2568 | 347.1 | - | - | 0 | - |
| 6 | b | 9952 | 347.7 | 0.0004829 | 1.389 | +2 | 6 |
| - | - | 2247 | 348.2 | - | - | 0 | - |
| - | - | 1.284E+04 | 350.2 | - | - | 0 | - |
| - | - | 945.5 | 351.2 | - | - | 0 | - |
| - | - | 2626 | 351.2 | - | - | 0 | - |
| - | - | 984.5 | 352.7 | - | - | 0 | - |
| 8 | y | 2631 | 353.7 | 0.001247 | 3.527 | +2 | 6 |
| - | - | 1086 | 354.2 | - | - | 0 | - |
| - | - | 1407 | 355.1 | - | - | 0 | - |
| - | - | 1222 | 356.2 | - | - | 0 | - |
| - | - | 917.3 | 357.2 | - | - | 0 | - |
| - | - | 9929 | 359 | - | - | 0 | - |
| - | - | 1070 | 360.2 | - | - | 0 | - |
| - | - | 4415 | 360.7 | - | - | 0 | - |
| - | - | 735.9 | 361.2 | - | - | 0 | - |
| - | - | 4450 | 361.2 | - | - | 0 | - |
| - | - | 5578 | 362.2 | - | - | 0 | - |
| 8 | y | 2.187E+04 | 362.7 | 0.00039 | 1.075 | +2 | 6 |
| - | - | 2.691E+04 | 363.1 | - | - | 0 | - |
| - | - | 8921 | 363.2 | - | - | 0 | - |
| - | - | 3075 | 363.7 | - | - | 0 | - |
| - | - | 5215 | 364.1 | - | - | 0 | - |
| - | - | 5517 | 364.1 | - | - | 0 | - |
| - | - | 2686 | 366.2 | - | - | 0 | - |
| - | - | 1684 | 367.1 | - | - | 0 | - |
| - | - | 1856 | 367.2 | - | - | 0 | - |
| - | - | 2529 | 367.2 | - | - | 0 | - |
| - | - | 1238 | 367.7 | - | - | 0 | - |
| - | - | 1128 | 368.2 | - | - | 0 | - |
| - | - | 1178 | 368.2 | - | - | 0 | - |
| - | - | 1070 | 369.1 | - | - | 0 | - |
| - | - | 1103 | 369.2 | - | - | 0 | - |
| - | - | 965.1 | 370.1 | - | - | 0 | - |
| - | - | 934.1 | 373.2 | - | - | 0 | - |
| - | - | 904.8 | 375.2 | - | - | 0 | - |
| - | - | 1708 | 376.2 | - | - | 0 | - |
| - | - | 4052 | 376.2 | - | - | 0 | - |
| - | - | 1827 | 377.1 | - | - | 0 | - |
| - | - | 1079 | 377.3 | - | - | 0 | - |
| - | - | 9326 | 378.2 | - | - | 0 | - |
| - | - | 4.532E+04 | 379.2 | - | - | 0 | - |
| - | - | 1169 | 380.2 | - | - | 0 | - |
| - | - | 9664 | 380.2 | - | - | 0 | - |
| - | - | 8.108E+04 | 381.2 | - | - | 0 | - |
| - | - | 2166 | 381.7 | - | - | 0 | - |
| - | - | 1.462E+04 | 382.2 | - | - | 0 | - |
| - | - | 2790 | 382.2 | - | - | 0 | - |
| - | - | 1193 | 382.2 | - | - | 0 | - |
| - | - | 1619 | 383.2 | - | - | 0 | - |
| - | - | 2831 | 384.2 | - | - | 0 | - |
| - | - | 3085 | 384.2 | - | - | 0 | - |
| - | - | 5670 | 385.2 | - | - | 0 | - |
| 5 | y | 1932 | 385.2 | 0.003948 | 10.25 | +3 | 9 |
| - | - | 910.4 | 386.2 | - | - | 0 | - |
| 10 | b | 1582 | 389.2 | 0.0005644 | 1.45 | +3 | 10 |
| - | - | 3664 | 390.2 | - | - | 0 | - |
| - | - | 2876 | 391.1 | - | - | 0 | - |
| - | - | 1570 | 391.2 | - | - | 0 | - |
| - | - | 1591 | 392.2 | - | - | 0 | - |
| - | - | 1322 | 394.1 | - | - | 0 | - |
| - | - | 1.032E+04 | 394.2 | - | - | 0 | - |
| - | - | 4185 | 395.1 | - | - | 0 | - |
| - | - | 2507 | 395.2 | - | - | 0 | - |
| - | - | 1308 | 395.2 | - | - | 0 | - |
| - | - | 1739 | 395.2 | - | - | 0 | - |
| - | - | 1.536E+04 | 396.2 | - | - | 0 | - |
| - | - | 2861 | 397.2 | - | - | 0 | - |
| - | - | 5493 | 398.2 | - | - | 0 | - |
| - | - | 1394 | 398.7 | - | - | 0 | - |
| - | - | 914.6 | 399.2 | - | - | 0 | - |
| - | - | 995.5 | 401.2 | - | - | 0 | - |
| - | - | 3713 | 402.2 | - | - | 0 | - |
| - | - | 6443 | 402.2 | - | - | 0 | - |
| - | - | 2341 | 403.2 | - | - | 0 | - |
| - | - | 1033 | 404.2 | - | - | 0 | - |
| - | - | 1273 | 404.7 | - | - | 0 | - |
| - | - | 841 | 405.2 | - | - | 0 | - |
| - | - | 2762 | 405.3 | - | - | 0 | - |
| - | - | 2063 | 406.2 | - | - | 0 | - |
| - | - | 1051 | 407.2 | - | - | 0 | - |
| - | - | 1114 | 407.2 | - | - | 0 | - |
| - | - | 6374 | 409.1 | - | - | 0 | - |
| - | - | 1898 | 409.2 | - | - | 0 | - |
| - | - | 1040 | 409.7 | - | - | 0 | - |
| - | - | 5107 | 411.2 | - | - | 0 | - |
| - | - | 4.745E+04 | 412.2 | - | - | 0 | - |
| - | - | 5193 | 413.1 | - | - | 0 | - |
| - | - | 2922 | 413.2 | - | - | 0 | - |
| - | - | 7484 | 413.2 | - | - | 0 | - |
| - | - | 1568 | 413.2 | - | - | 0 | - |
| - | - | 1218 | 414.1 | - | - | 0 | - |
| - | - | 1.319E+04 | 414.2 | - | - | 0 | - |
| - | - | 2.157E+04 | 415 | - | - | 0 | - |
| - | - | 3616 | 415.2 | - | - | 0 | - |
| - | - | 2877 | 415.2 | - | - | 0 | - |
| - | - | 1204 | 416.2 | - | - | 0 | - |
| - | - | 1.021E+04 | 417.7 | - | - | 0 | - |
| - | - | 1.323E+04 | 418.2 | - | - | 0 | - |
| - | - | 6109 | 418.7 | - | - | 0 | - |
| - | - | 1645 | 419.2 | - | - | 0 | - |
| 11 | y | 2361 | 421.2 | 0.0005791 | 1.375 | +1 | 3 |
| 11 | y | 1262 | 422.2 | 0.0005113 | 1.211 | +1 | 3 |
| - | - | 880.1 | 422.3 | - | - | 0 | - |
| - | - | 1760 | 422.7 | - | - | 0 | - |
| - | - | 2875 | 423.2 | - | - | 0 | - |
| - | - | 1.834E+04 | 423.3 | - | - | 0 | - |
| - | - | 1724 | 423.7 | - | - | 0 | - |
| - | - | 926.5 | 423.7 | - | - | 0 | - |
| - | - | 5227 | 424.3 | - | - | 0 | - |
| - | - | 1470 | 424.7 | - | - | 0 | - |
| - | - | 907.7 | 425.3 | - | - | 0 | - |
| - | - | 1.307E+05 | 426.7 | - | - | 0 | - |
| - | - | 6.524E+04 | 427.2 | - | - | 0 | - |
| - | - | 1.759E+04 | 427.7 | - | - | 0 | - |
| - | - | 3321 | 428.2 | - | - | 0 | - |
| - | - | 1290 | 429.1 | - | - | 0 | - |
| - | - | 2.667E+04 | 429.2 | - | - | 0 | - |
| - | - | 1.213E+05 | 430.2 | - | - | 0 | - |
| - | - | 2.591E+04 | 431.2 | - | - | 0 | - |
| 7 | b | 1.423E+04 | 431.7 | 0.0006241 | 1.446 | +2 | 7 |
| 7 | b | 6780 | 432.2 | 0.007274 | 16.83 | +2 | 7 |
| - | - | 3993 | 432.7 | - | - | 0 | - |
| - | - | 1052 | 433 | - | - | 0 | - |
| - | - | 1510 | 433.2 | - | - | 0 | - |
| 4 | b | 4886 | 433.3 | 0.0006592 | 1.522 | +1 | 4 |
| - | - | 1145 | 434.3 | - | - | 0 | - |
| - | - | 2762 | 438.2 | - | - | 0 | - |
| - | - | 1229 | 438.7 | - | - | 0 | - |
| 11 | y | 3.447E+04 | 439.2 | 0.0008787 | 2.001 | +1 | 3 |
| - | - | 1563 | 440.2 | - | - | 0 | - |
| - | - | 8359 | 440.2 | - | - | 0 | - |
| 7 | b | 6.405E+04 | 440.7 | 0.0006823 | 1.548 | +2 | 7 |
| - | - | 3.228E+04 | 441.2 | - | - | 0 | - |
| - | - | 9595 | 441.7 | - | - | 0 | - |
| - | - | 1640 | 442.2 | - | - | 0 | - |
| - | - | 1246 | 443.2 | - | - | 0 | - |
| - | - | 863.2 | 444.2 | - | - | 0 | - |
| - | - | 1233 | 446.7 | - | - | 0 | - |
| 7 | y | 4452 | 447.2 | 0.00126 | 2.818 | +2 | 7 |
| - | - | 1350 | 447.7 | - | - | 0 | - |
| - | - | 6524 | 448.2 | - | - | 0 | - |
| - | - | 1425 | 449.2 | - | - | 0 | - |
| - | - | 1602 | 450.2 | - | - | 0 | - |
| - | - | 1853 | 450.3 | - | - | 0 | - |
| 4 | b | 3.774E+04 | 451.3 | 0.0006841 | 1.516 | +1 | 4 |
| - | - | 1.011E+04 | 452.3 | - | - | 0 | - |
| - | - | 1430 | 453.3 | - | - | 0 | - |
| - | - | 1463 | 455.3 | - | - | 0 | - |
| 7 | y | 2103 | 455.7 | 0.0002538 | 0.5568 | +2 | 7 |
| - | - | 2102 | 456.2 | - | - | 0 | - |
| - | - | 3321 | 459.2 | - | - | 0 | - |
| - | - | 3149 | 460.8 | - | - | 0 | - |
| - | - | 3319 | 461.2 | - | - | 0 | - |
| - | - | 4018 | 461.3 | - | - | 0 | - |
| - | - | 984.8 | 462.2 | - | - | 0 | - |
| - | - | 1124 | 462.3 | - | - | 0 | - |
| - | - | 1096 | 464.2 | - | - | 0 | - |
| - | - | 949.7 | 465.7 | - | - | 0 | - |
| - | - | 1168 | 466.2 | - | - | 0 | - |
| - | - | 874 | 466.2 | - | - | 0 | - |
| - | - | 1129 | 468.3 | - | - | 0 | - |
| - | - | 9060 | 468.3 | - | - | 0 | - |
| - | - | 2170 | 469.3 | - | - | 0 | - |
| - | - | 1001 | 472.2 | - | - | 0 | - |
| - | - | 8221 | 474.3 | - | - | 0 | - |
| - | - | 9329 | 474.8 | - | - | 0 | - |
| - | - | 4615 | 475.3 | - | - | 0 | - |
| - | - | 1188 | 475.8 | - | - | 0 | - |
| - | - | 3076 | 476.2 | - | - | 0 | - |
| - | - | 7228 | 477.2 | - | - | 0 | - |
| 10 | y | 6179 | 478.2 | 0.0003768 | 0.7879 | +1 | 4 |
| - | - | 4591 | 478.3 | - | - | 0 | - |
| - | - | 1765 | 479.2 | - | - | 0 | - |
| - | - | 1437 | 479.7 | - | - | 0 | - |
| - | - | 1053 | 480.2 | - | - | 0 | - |
| - | - | 2.64E+04 | 483.3 | - | - | 0 | - |
| - | - | 1.671E+04 | 483.8 | - | - | 0 | - |
| - | - | 1375 | 484.2 | - | - | 0 | - |
| - | - | 3977 | 484.3 | - | - | 0 | - |
| - | - | 1507 | 484.8 | - | - | 0 | - |
| 8 | b | 7700 | 488.3 | 0.001378 | 2.822 | +2 | 8 |
| 8 | b | 3988 | 488.7 | 0.007447 | 15.24 | +2 | 8 |
| - | - | 1892 | 489.3 | - | - | 0 | - |
| - | - | 8.271E+04 | 494.2 | - | - | 0 | - |
| - | - | 840.3 | 495.2 | - | - | 0 | - |
| - | - | 2.229E+04 | 495.2 | - | - | 0 | - |
| 10 | y | 9.704E+04 | 496.2 | 0.0007468 | 1.505 | +1 | 4 |
| - | - | 2.52E+04 | 497.2 | - | - | 0 | - |
| - | - | 8318 | 497.8 | - | - | 0 | - |
| - | - | 4965 | 498.2 | - | - | 0 | - |
| - | - | 2.307E+04 | 503.1 | - | - | 0 | - |
| - | - | 3397 | 504.2 | - | - | 0 | - |
| - | - | 1958 | 506.2 | - | - | 0 | - |
| - | - | 1358 | 507.3 | - | - | 0 | - |
| - | - | 799.4 | 510.3 | - | - | 0 | - |
| - | - | 5106 | 511.3 | - | - | 0 | - |
| - | - | 1345 | 512.3 | - | - | 0 | - |
| - | - | 1317 | 515.3 | - | - | 0 | - |
| - | - | 7093 | 519.1 | - | - | 0 | - |
| - | - | 1119 | 522.2 | - | - | 0 | - |
| - | - | 2621 | 523.3 | - | - | 0 | - |
| - | - | 1935 | 523.6 | - | - | 0 | - |
| - | - | 1196 | 524.3 | - | - | 0 | - |
| - | - | 1.348E+04 | 525.3 | - | - | 0 | - |
| - | - | 2990 | 526.2 | - | - | 0 | - |
| - | - | 3089 | 526.3 | - | - | 0 | - |
| 0 | Precursor | 4634 | 529.3 | 0.001291 | 2.439 | +3 | -1 |
| 0 | Precursor | 5376 | 529.6 | 0.007575 | 14.3 | +3 | -1 |
| - | - | 2757 | 529.9 | - | - | 0 | - |
| - | - | 1087 | 531.2 | - | - | 0 | - |
| - | - | 3701 | 532.2 | - | - | 0 | - |
| - | - | 1354 | 533.2 | - | - | 0 | - |
| - | - | 1992 | 534.9 | - | - | 0 | - |
| 0 | Precursor | 5123 | 535.3 | 0.00076 | 1.42 | +3 | -1 |
| - | - | 2912 | 535.6 | - | - | 0 | - |
| - | - | 1830 | 535.9 | - | - | 0 | - |
| - | - | 4730 | 536.2 | - | - | 0 | - |
| - | - | 1805 | 537.2 | - | - | 0 | - |
| - | - | 2018 | 537.7 | - | - | 0 | - |
| - | - | 1091 | 538.3 | - | - | 0 | - |
| - | - | 1337 | 538.8 | - | - | 0 | - |
| - | - | 1.408E+04 | 539.2 | - | - | 0 | - |
| - | - | 4427 | 540.2 | - | - | 0 | - |
| - | - | 1412 | 541.3 | - | - | 0 | - |
| - | - | 4726 | 542.3 | - | - | 0 | - |
| - | - | 2.63E+04 | 543.3 | - | - | 0 | - |
| - | - | 9098 | 544.3 | - | - | 0 | - |
| - | - | 2021 | 545.3 | - | - | 0 | - |
| 9 | b | 947.2 | 545.8 | 0.004983 | 9.131 | +2 | 9 |
| - | - | 4722 | 546.2 | - | - | 0 | - |
| - | - | 1851 | 546.8 | - | - | 0 | - |
| - | - | 1016 | 547.3 | - | - | 0 | - |
| - | - | 956.4 | 547.8 | - | - | 0 | - |
| - | - | 1324 | 548.2 | - | - | 0 | - |
| - | - | 3125 | 549.2 | - | - | 0 | - |
| - | - | 1526 | 550.2 | - | - | 0 | - |
| - | - | 3830 | 551.3 | - | - | 0 | - |
| - | - | 2112 | 551.3 | - | - | 0 | - |
| - | - | 1918 | 552.3 | - | - | 0 | - |
| - | - | 1245 | 553.3 | - | - | 0 | - |
| 9 | b | 3478 | 554.8 | 0.000744 | 1.341 | +2 | 9 |
| - | - | 6402 | 555.3 | - | - | 0 | - |
| - | - | 5645 | 555.8 | - | - | 0 | - |
| - | - | 797.8 | 556.3 | - | - | 0 | - |
| - | - | 1270 | 560.2 | - | - | 0 | - |
| 5 | b | 3542 | 561.3 | 0.0008891 | 1.584 | +1 | 5 |
| 5 | b | 1218 | 562.3 | 0.003324 | 5.911 | +1 | 5 |
| - | - | 3484 | 563.3 | - | - | 0 | - |
| - | - | 2208 | 564.3 | - | - | 0 | - |
| - | - | 780.7 | 564.3 | - | - | 0 | - |
| - | - | 5852 | 565.3 | - | - | 0 | - |
| - | - | 1562 | 566.3 | - | - | 0 | - |
| - | - | 1.61E+04 | 567.2 | - | - | 0 | - |
| - | - | 938.6 | 567.3 | - | - | 0 | - |
| - | - | 5199 | 568.2 | - | - | 0 | - |
| - | - | 852.7 | 569.2 | - | - | 0 | - |
| - | - | 784.9 | 569.8 | - | - | 0 | - |
| - | - | 4020 | 575.2 | - | - | 0 | - |
| - | - | 2935 | 576.2 | - | - | 0 | - |
| 5 | b | 2.551E+04 | 579.3 | 0.0002731 | 0.4714 | +1 | 5 |
| - | - | 7366 | 580.3 | - | - | 0 | - |
| - | - | 1831 | 581.3 | - | - | 0 | - |
| - | - | 813.9 | 587.3 | - | - | 0 | - |
| - | - | 3424 | 592.3 | - | - | 0 | - |
| - | - | 3493 | 592.3 | - | - | 0 | - |
| 9 | y | 3.154E+04 | 593.3 | 0.0002681 | 0.4519 | +1 | 5 |
| - | - | 1.162E+04 | 593.3 | - | - | 0 | - |
| - | - | 8577 | 594.3 | - | - | 0 | - |
| - | - | 3152 | 594.3 | - | - | 0 | - |
| - | - | 1206 | 594.8 | - | - | 0 | - |
| - | - | 2640 | 595.2 | - | - | 0 | - |
| - | - | 1417 | 596.4 | - | - | 0 | - |
| - | - | 1337 | 597.8 | - | - | 0 | - |
| - | - | 1424 | 600.3 | - | - | 0 | - |
| - | - | 4763 | 602.8 | - | - | 0 | - |
| - | - | 5696 | 603.3 | - | - | 0 | - |
| - | - | 813.9 | 603.4 | - | - | 0 | - |
| - | - | 1903 | 603.8 | - | - | 0 | - |
| - | - | 6.16E+04 | 610.3 | - | - | 0 | - |
| - | - | 2222 | 610.3 | - | - | 0 | - |
| 9 | y | 3.189E+05 | 611.3 | 0.001117 | 1.827 | +1 | 5 |
| - | - | 1.607E+04 | 611.8 | - | - | 0 | - |
| - | - | 9.869E+04 | 612.3 | - | - | 0 | - |
| - | - | 4353 | 612.8 | - | - | 0 | - |
| - | - | 2.238E+04 | 613.3 | - | - | 0 | - |
| - | - | 2939 | 614.3 | - | - | 0 | - |
| - | - | 1106 | 615.3 | - | - | 0 | - |
| - | - | 1142 | 616.3 | - | - | 0 | - |
| - | - | 1896 | 617.3 | - | - | 0 | - |
| - | - | 865.5 | 619.3 | - | - | 0 | - |
| - | - | 4079 | 621.3 | - | - | 0 | - |
| - | - | 1365 | 621.3 | - | - | 0 | - |
| - | - | 1109 | 622.3 | - | - | 0 | - |
| - | - | 3181 | 634.3 | - | - | 0 | - |
| - | - | 4829 | 635.3 | - | - | 0 | - |
| - | - | 3169 | 636.3 | - | - | 0 | - |
| 4 | y | 3476 | 636.8 | 0.000306 | 0.4805 | +2 | 10 |
| 4 | y | 4358 | 637.3 | 0.01104 | 17.33 | +2 | 10 |
| - | - | 1573 | 637.8 | - | - | 0 | - |
| 11 | b | 1150 | 638.3 | 0.005773 | 9.044 | +2 | 11 |
| - | - | 2678 | 640.3 | - | - | 0 | - |
| - | - | 1250 | 641.3 | - | - | 0 | - |
| - | - | 3820 | 645.3 | - | - | 0 | - |
| 4 | y | 2.086E+04 | 645.8 | 0.00186 | 2.88 | +2 | 10 |
| - | - | 1.379E+04 | 646.3 | - | - | 0 | - |
| - | - | 7297 | 646.8 | - | - | 0 | - |
| - | - | 1363 | 647.3 | - | - | 0 | - |
| - | - | 1575 | 648.3 | - | - | 0 | - |
| - | - | 1842 | 650.3 | - | - | 0 | - |
| - | - | 1091 | 650.3 | - | - | 0 | - |
| - | - | 1108 | 651.3 | - | - | 0 | - |
| - | - | 3.316E+04 | 652.3 | - | - | 0 | - |
| - | - | 1160 | 652.8 | - | - | 0 | - |
| - | - | 1.283E+04 | 653.3 | - | - | 0 | - |
| - | - | 2188 | 654.3 | - | - | 0 | - |
| - | - | 2022 | 657.4 | - | - | 0 | - |
| - | - | 2050 | 658.3 | - | - | 0 | - |
| - | - | 1009 | 658.3 | - | - | 0 | - |
| - | - | 1241 | 659.3 | - | - | 0 | - |
| - | - | 1839 | 660.9 | - | - | 0 | - |
| - | - | 2211 | 661.3 | - | - | 0 | - |
| - | - | 1320 | 661.8 | - | - | 0 | - |
| - | - | 1.279E+04 | 662.3 | - | - | 0 | - |
| - | - | 929.8 | 662.8 | - | - | 0 | - |
| - | - | 8104 | 663.3 | - | - | 0 | - |
| - | - | 2774 | 664.3 | - | - | 0 | - |
| - | - | 1086 | 665.3 | - | - | 0 | - |
| - | - | 1570 | 666.4 | - | - | 0 | - |
| - | - | 1668 | 668.3 | - | - | 0 | - |
| 6 | b | 1.797E+04 | 676.3 | 0.0004353 | 0.6436 | +1 | 6 |
| 6 | b | 8643 | 677.3 | 0.006593 | 9.734 | +1 | 6 |
| - | - | 3794 | 678.3 | - | - | 0 | - |
| - | - | 3358 | 679.3 | - | - | 0 | - |
| - | - | 1091 | 679.8 | - | - | 0 | - |
| - | - | 5.895E+04 | 680.3 | - | - | 0 | - |
| - | - | 2.434E+04 | 681.3 | - | - | 0 | - |
| - | - | 4592 | 682.3 | - | - | 0 | - |
| - | - | 2155 | 684.3 | - | - | 0 | - |
| - | - | 3242 | 684.8 | - | - | 0 | - |
| - | - | 2685 | 685.3 | - | - | 0 | - |
| - | - | 1386 | 685.8 | - | - | 0 | - |
| - | - | 1269 | 688.3 | - | - | 0 | - |
| - | - | 959.3 | 692.3 | - | - | 0 | - |
| - | - | 2716 | 692.8 | - | - | 0 | - |
| 3 | y | 1.654E+04 | 693.3 | 0.001609 | 2.321 | +2 | 11 |
| 3 | y | 1.89E+04 | 693.8 | 0.007648 | 11.02 | +2 | 11 |
| 6 | b | 1.664E+05 | 694.4 | 0.0001196 | 0.1723 | +1 | 6 |
| - | - | 2845 | 694.8 | - | - | 0 | - |
| - | - | 6.322E+04 | 695.4 | - | - | 0 | - |
| - | - | 1.424E+04 | 696.4 | - | - | 0 | - |
| - | - | 2678 | 697.4 | - | - | 0 | - |
| - | - | 1852 | 698.4 | - | - | 0 | - |
| - | - | 1240 | 700.9 | - | - | 0 | - |
| - | - | 2.262E+04 | 701.8 | - | - | 0 | - |
| 3 | y | 1.151E+05 | 702.3 | 0.001698 | 2.417 | +2 | 11 |
| 12 | b | 9.805E+04 | 702.8 | 0.01302 | 18.53 | +2 | 12 |
| 12 | b | 4.415E+04 | 703.3 | 0.00332 | 4.72 | +2 | 12 |
| - | - | 1.27E+04 | 703.8 | - | - | 0 | - |
| - | - | 5388 | 704.3 | - | - | 0 | - |
| - | - | 2235 | 705.4 | - | - | 0 | - |
| 8 | y | 9844 | 706.3 | 0.001775 | 2.513 | +1 | 6 |
| - | - | 4260 | 707.3 | - | - | 0 | - |
| - | - | 921.7 | 709.3 | - | - | 0 | - |
| - | - | 6664 | 711.4 | - | - | 0 | - |
| 12 | b | 8696 | 711.9 | 0.003059 | 4.297 | +2 | 12 |
| - | - | 7959 | 712.4 | - | - | 0 | - |
| - | - | 3125 | 712.9 | - | - | 0 | - |
| - | - | 1070 | 719.4 | - | - | 0 | - |
| - | - | 1129 | 720.4 | - | - | 0 | - |
| - | - | 1124 | 720.9 | - | - | 0 | - |
| - | - | 7277 | 721.4 | - | - | 0 | - |
| - | - | 2674 | 722.4 | - | - | 0 | - |
| - | - | 1.606E+04 | 723.4 | - | - | 0 | - |
| 8 | y | 9.565E+04 | 724.4 | 0.001159 | 1.601 | +1 | 6 |
| - | - | 3.873E+04 | 725.4 | - | - | 0 | - |
| - | - | 1.116E+04 | 726.4 | - | - | 0 | - |
| - | - | 1614 | 727.4 | - | - | 0 | - |
| - | - | 888.8 | 728.4 | - | - | 0 | - |
| - | - | 4206 | 729.4 | - | - | 0 | - |
| - | - | 2316 | 730.4 | - | - | 0 | - |
| - | - | 4025 | 733.4 | - | - | 0 | - |
| - | - | 3441 | 734.4 | - | - | 0 | - |
| - | - | 1193 | 737.9 | - | - | 0 | - |
| - | - | 1302 | 738.4 | - | - | 0 | - |
| - | - | 1106 | 739.4 | - | - | 0 | - |
| 2 | y | 1084 | 742.9 | 0.003545 | 4.772 | +2 | 12 |
| 2 | y | 2844 | 743.4 | 0.01336 | 17.97 | +2 | 12 |
| - | - | 1108 | 744.4 | - | - | 0 | - |
| - | - | 1104 | 745.4 | - | - | 0 | - |
| - | - | 2106 | 747.4 | - | - | 0 | - |
| - | - | 1982 | 748.4 | - | - | 0 | - |
| - | - | 1392 | 749.3 | - | - | 0 | - |
| - | - | 2.03E+04 | 751.4 | - | - | 0 | - |
| 2 | y | 1.046E+04 | 751.9 | 0.001976 | 2.628 | +2 | 12 |
| - | - | 1.759E+04 | 752.4 | - | - | 0 | - |
| - | - | 3830 | 752.9 | - | - | 0 | - |
| - | - | 3622 | 753.4 | - | - | 0 | - |
| - | - | 1046 | 754.4 | - | - | 0 | - |
| - | - | 1854 | 760.3 | - | - | 0 | - |
| - | - | 1968 | 761.4 | - | - | 0 | - |
| - | - | 2334 | 762.4 | - | - | 0 | - |
| - | - | 9422 | 763.4 | - | - | 0 | - |
| - | - | 5318 | 764.4 | - | - | 0 | - |
| - | - | 4030 | 765.4 | - | - | 0 | - |
| - | - | 1786 | 766.4 | - | - | 0 | - |
| - | - | 4489 | 767.3 | - | - | 0 | - |
| - | - | 1169 | 768.3 | - | - | 0 | - |
| - | - | 1923 | 769.4 | - | - | 0 | - |
| - | - | 2603 | 776.4 | - | - | 0 | - |
| - | - | 9376 | 779.4 | - | - | 0 | - |
| - | - | 4467 | 780.4 | - | - | 0 | - |
| - | - | 4533 | 781.4 | - | - | 0 | - |
| - | - | 1480 | 782.4 | - | - | 0 | - |
| 0 | Precursor | 1.749E+04 | 793.4 | 0.01351 | 17.03 | +2 | -1 |
| - | - | 8704 | 794.4 | - | - | 0 | - |
| - | - | 6227 | 795.3 | - | - | 0 | - |
| - | - | 1408 | 795.4 | - | - | 0 | - |
| - | - | 1518 | 796.3 | - | - | 0 | - |
| - | - | 4651 | 796.5 | - | - | 0 | - |
| - | - | 1501 | 797.5 | - | - | 0 | - |
| - | - | 3880 | 807.4 | - | - | 0 | - |
| - | - | 2194 | 807.4 | - | - | 0 | - |
| - | - | 2493 | 808.4 | - | - | 0 | - |
| - | - | 2661 | 818.4 | - | - | 0 | - |
| - | - | 2051 | 819.4 | - | - | 0 | - |
| - | - | 1624 | 825.4 | - | - | 0 | - |
| - | - | 1909 | 826.4 | - | - | 0 | - |
| - | - | 1121 | 827.4 | - | - | 0 | - |
| - | - | 1340 | 828.4 | - | - | 0 | - |
| - | - | 4722 | 834.4 | - | - | 0 | - |
| - | - | 6119 | 835.4 | - | - | 0 | - |
| - | - | 1.168E+04 | 836.4 | - | - | 0 | - |
| - | - | 4699 | 837.5 | - | - | 0 | - |
| - | - | 2487 | 838.5 | - | - | 0 | - |
| - | - | 2132 | 843.4 | - | - | 0 | - |
| - | - | 4327 | 844.4 | - | - | 0 | - |
| - | - | 3955 | 845.4 | - | - | 0 | - |
| - | - | 6845 | 846.4 | - | - | 0 | - |
| - | - | 5098 | 847.4 | - | - | 0 | - |
| - | - | 1247 | 848.4 | - | - | 0 | - |
| - | - | 4.2E+04 | 852.4 | - | - | 0 | - |
| - | - | 1.993E+04 | 853.4 | - | - | 0 | - |
| - | - | 6018 | 854.4 | - | - | 0 | - |
| - | - | 963.9 | 857.4 | - | - | 0 | - |
| 7 | b | 2.516E+04 | 862.4 | 0.0001018 | 0.1181 | +1 | 7 |
| 7 | b | 1.859E+04 | 863.4 | 0.012 | 13.89 | +1 | 7 |
| - | - | 2.757E+04 | 864.5 | - | - | 0 | - |
| - | - | 1.456E+04 | 865.5 | - | - | 0 | - |
| - | - | 5112 | 866.5 | - | - | 0 | - |
| - | - | 4322 | 876.4 | - | - | 0 | - |
| - | - | 1343 | 877.4 | - | - | 0 | - |
| - | - | 1275 | 878.4 | - | - | 0 | - |
| 7 | b | 1.975E+05 | 880.4 | 0.0003921 | 0.4453 | +1 | 7 |
| - | - | 1.028E+05 | 881.4 | - | - | 0 | - |
| - | - | 2.999E+04 | 882.4 | - | - | 0 | - |
| - | - | 6238 | 883.4 | - | - | 0 | - |
| - | - | 1303 | 884.4 | - | - | 0 | - |
| - | - | 1734 | 890.4 | - | - | 0 | - |
| - | - | 2257 | 891.4 | - | - | 0 | - |
| - | - | 2663 | 892.5 | - | - | 0 | - |
| - | - | 1287 | 893.5 | - | - | 0 | - |
| - | - | 1198 | 894.4 | - | - | 0 | - |
| - | - | 2276 | 907.4 | - | - | 0 | - |
| - | - | 1.441E+04 | 908.4 | - | - | 0 | - |
| - | - | 8984 | 909.4 | - | - | 0 | - |
| 7 | y | 1.721E+04 | 910.4 | 0.001436 | 1.578 | +1 | 7 |
| - | - | 7636 | 911.4 | - | - | 0 | - |
| - | - | 2366 | 912.4 | - | - | 0 | - |
| - | - | 1054 | 936.4 | - | - | 0 | - |
| - | - | 2910 | 937.4 | - | - | 0 | - |
| - | - | 1378 | 947.4 | - | - | 0 | - |
| - | - | 4273 | 954.4 | - | - | 0 | - |
| - | - | 4942 | 955.4 | - | - | 0 | - |
| - | - | 2852 | 956.4 | - | - | 0 | - |
| - | - | 1279 | 964.5 | - | - | 0 | - |
| - | - | 6231 | 965.4 | - | - | 0 | - |
| - | - | 2483 | 966.4 | - | - | 0 | - |
| - | - | 3035 | 972.4 | - | - | 0 | - |
| - | - | 2674 | 973.4 | - | - | 0 | - |
| 8 | b | 3304 | 975.5 | 2.223E-05 | 0.02279 | +1 | 8 |
| 8 | b | 1903 | 976.5 | 0.01418 | 14.52 | +1 | 8 |
| - | - | 1654 | 980.5 | - | - | 0 | - |
| - | - | 1649 | 982.5 | - | - | 0 | - |
| - | - | 1106 | 990.5 | - | - | 0 | - |
| 8 | b | 1.001E+04 | 993.5 | 0.0004106 | 0.4133 | +1 | 8 |
| - | - | 7558 | 994.5 | - | - | 0 | - |
| - | - | 1165 | 995.5 | - | - | 0 | - |
| - | - | 1570 | 1007 | - | - | 0 | - |
| - | - | 1097 | 1009 | - | - | 0 | - |
| - | - | 1813 | 1024 | - | - | 0 | - |
| 6 | y | 8183 | 1025 | 0.0007994 | 0.7796 | +1 | 8 |
| - | - | 4824 | 1026 | - | - | 0 | - |
| - | - | 1428 | 1027 | - | - | 0 | - |
| - | - | 1282 | 1065 | - | - | 0 | - |
| - | - | 1324 | 1076 | - | - | 0 | - |
| - | - | 1232 | 1078 | - | - | 0 | - |
| - | - | 4226 | 1094 | - | - | 0 | - |
| - | - | 2760 | 1095 | - | - | 0 | - |
| - | - | 1123 | 1096 | - | - | 0 | - |
| 9 | b | 2124 | 1109 | 0.001048 | 0.9449 | +1 | 9 |
| - | - | 1485 | 1110 | - | - | 0 | - |
| - | - | 1491 | 1117 | - | - | 0 | - |
| - | - | 2147 | 1118 | - | - | 0 | - |
| 5 | y | 6228 | 1136 | 0.001249 | 1.1 | +1 | 9 |
| 5 | y | 1.054E+04 | 1136 | 0.006491 | 5.711 | +1 | 9 |
| - | - | 5000 | 1137 | - | - | 0 | - |
| - | - | 1586 | 1138 | - | - | 0 | - |
| - | - | 3930 | 1153 | - | - | 0 | - |
| 5 | y | 1.585E+04 | 1154 | 0.00106 | 0.9188 | +1 | 9 |
| - | - | 1.008E+04 | 1155 | - | - | 0 | - |
| - | - | 3795 | 1156 | - | - | 0 | - |
| - | - | 925 | 1157 | - | - | 0 | - |
| 10 | b | 2266 | 1166 | 0.005565 | 4.774 | +1 | 10 |
| - | - | 882.4 | 1205 | - | - | 0 | - |
| 4 | y | 973.7 | 1291 | 0.005118 | 3.965 | +1 | 10 |
| - | - | 963.5 | 3081 | - | - | 0 | - |
| - | - | 1132 | 3455 | - | - | 0 | - |

m/z Charge Intensity FragmentType MassShift Position
120.0811538696289 0 8445.756
121.08434295654297 0 569.93335
121.92815399169922 0 469.64844
122.07157135009766 0 2263.3984
123.04450988769531 0 2485.313
123.05561065673828 0 769.5132
124.99102783203125 0 460.74606
125.10752868652344 0 902.8716
126.09156799316406 0 1223.9608
127.05061340332031 0 5866.7773
127.08692932128906 0 952.99084
127.12342071533203 0 1656.7916
128.1073760986328 0 27643.783
129.0663604736328 0 1903.9429
129.10267639160156 0 322747.84
130.06552124023438 0 140265.5
130.09983825683594 0 1982.5854
130.10598754882812 0 18614.938
130.78900146484375 0 498.8818
131.06886291503906 0 13081.435
131.1181182861328 0 703.99445
132.08114624023438 0 30608.861
133.06103515625 0 834.34314
133.0845489501953 0 3230.5242
134.04495239257812 0 578.76447
136.07608032226562 0 43186.582
137.07949829101562 0 3302.4238
138.06666564941406 0 3975.2585
138.09158325195312 0 1406.3265
139.0819854736328 0 539.8432
139.0869903564453 0 3873.5156
141.1027069091797 0 5367.975
142.06546020507812 0 1878.4285
143.04568481445312 0 1971.0459
143.08164978027344 0 636.7323
144.08131408691406 0 1013.1926
145.06130981445312 0 3393.2761
146.0606231689453 0 1188.7456
147.04461669921875 0 1789.1641
149.04530334472656 0 11298.224
150.04534912109375 0 624.0867
150.066650390625 0 1787.8827
151.08705139160156 0 7253.494
153.1027069091797 0 683.5373
154.09771728515625 0 1070.3788
155.08180236816406 0 1495.269
155.0931396484375 0 21019.492
155.11822509765625 0 15292.282 a Water loss 1
156.07704162597656 0 1287.6495
156.09632873535156 0 759.9911
156.12149047851562 0 1121.7467
157.0609130859375 0 875.35986
157.09750366210938 0 1900.2062
158.08401489257812 0 3165.3108
159.0921173095703 0 243524.56
160.0758819580078 0 2313.475
160.08926391601562 0 1672.236
160.095458984375 0 27718.436
161.098876953125 0 918.5101
165.0550079345703 0 19975.197
165.0778045654297 0 1164.3998
166.06143188476562 0 37516.96
166.08670043945312 0 3829.7402 y 9
166.09786987304688 0 2409.378
167.05587768554688 0 13693.762
167.06471252441406 0 2440.5598
167.0819091796875 0 608.9237
167.11837768554688 0 1369.195
168.05552673339844 0 917.7028
168.081298828125 0 3933.2925
168.10220336914062 0 3618.0635
168.1135711669922 0 3512.3894
169.07640075683594 0 4239.1997
169.08444213867188 0 1339.4023
169.0976104736328 0 9594.41
170.0604705810547 0 13222.829
171.06370544433594 0 1479.4734
171.09225463867188 0 2282.9504
171.149658203125 0 1596.7542
172.07211303710938 0 4490.713
173.05616760253906 0 3986.6094
173.12892150878906 0 489409.5 a 1
173.4400177001953 0 2574.8074
174.13226318359375 0 43076.664
175.13412475585938 0 2110.0862
176.08290100097656 0 847.86835
177.1028594970703 0 1563.474
178.13426208496094 0 12888.948
179.11819458007812 0 1146.6942
179.13783264160156 0 984.31726
181.06124877929688 0 2832.6033
182.0816192626953 0 47776.492 y 12
183.0852508544922 0 3755.7383
183.1132049560547 0 12825.797 b Water loss 1
183.14930725097656 0 1264.5991
184.11643981933594 0 1497.881
185.09243774414062 0 837.2407
185.1652374267578 0 3145.3376
186.12413024902344 0 109009.36
187.08700561523438 0 17909.996
187.1082305908203 0 764.38635
187.12759399414062 0 8458.302
188.07093811035156 0 2327.0388
188.09071350097656 0 2724.6665
188.12892150878906 0 861.0169
190.08267211914062 0 3983.5593
190.13449096679688 0 3032.2502
191.11875915527344 0 656.841
193.0986785888672 0 688.2083
193.1083526611328 0 596.8116
194.0928192138672 0 3969.0408
195.0913543701172 0 799.4898
195.1132049560547 0 25959.953
196.10842895507812 0 15541.586
196.1168975830078 0 2217.5747
197.1081085205078 0 1394.3418
197.12876892089844 0 1930.7744
197.1649932861328 0 813.43866
198.08767700195312 0 2814.158
198.1280975341797 0 8202.475
199.07162475585938 0 1574.1251
199.0872039794922 0 1973.183
199.13131713867188 0 1837.7579
200.10313415527344 0 726.8924
200.1425323486328 0 1684.4058
201.1237335205078 0 134461.06 b 1
202.08682250976562 0 1683.722
202.12696838378906 0 12125.675
203.12832641601562 0 1110.2494
204.11322021484375 0 1603.8445
205.09756469726562 0 11223.787
205.10806274414062 0 662.4132
205.1450653076172 0 1984.778
206.10101318359375 0 839.8539
206.12896728515625 0 2024.712
207.160888671875 0 3520.2493
210.12753295898438 0 4193.018
211.10809326171875 0 3144.9885 y Water loss 10
211.13156127929688 0 836.84186
212.09046936035156 0 637.0537
212.13966369628906 0 2961.5713
213.12374877929688 0 10772.373
213.1595001220703 0 1471.4066
215.13943481445312 0 3756.1868
216.09815979003906 0 2644.5308
221.05938720703125 0 2427.9683
221.07235717773438 0 1212.8611
221.08474731445312 0 4769.8887
221.10389709472656 0 2758.9255
221.12831115722656 0 735.4187
221.13970947265625 0 1663.7867
222.08535766601562 0 2208.1104
222.12393188476562 0 4159.5605
223.0640106201172 0 3425.1833
223.08265686035156 0 1337.0625
223.1081085205078 0 9062.64
223.15579223632812 0 44021.06
224.10316467285156 0 3079.942
224.1592254638672 0 4885.4785
225.04330444335938 0 7328.4575
225.12368774414062 0 1359.2712
225.16123962402344 0 668.0013
226.0437469482422 0 2007.8872
226.082763671875 0 8919.999
226.1561279296875 0 785.03235
227.02304077148438 0 879.42456
227.0666961669922 0 8433.065
227.08575439453125 0 963.8738
227.1546630859375 0 1788.4202
228.13475036621094 0 3911.098
229.1187286376953 0 9729.828
230.12240600585938 0 864.35156
230.15037536621094 0 1547.4296
231.0878448486328 0 1023.01935
233.14010620117188 0 6735.1914
234.12420654296875 0 47640.88
234.65052795410156 0 1144.5433
235.12754821777344 0 6336.848
235.60604858398438 0 1360.4401
237.1348419189453 0 2054.8594
238.11898803710938 0 5559.115
238.13027954101562 0 1000.5619
239.09544372558594 0 14266.147
240.09609985351562 0 3787.2407
240.13473510742188 0 18060.773
241.09292602539062 0 2689.5803
241.1185760498047 0 2403.5874
241.13784790039062 0 2380.5437
241.19126892089844 0 2120.836
243.1094970703125 0 4267.7256
244.09326171875 0 19430.63
245.09669494628906 0 2142.915
247.1082000732422 0 26111.896
247.14422607421875 0 1659.7825
247.1566162109375 0 1263.0387
248.1031494140625 0 2669.9587
248.11415100097656 0 9235.666
249.0987091064453 0 15860.13
249.1351776123047 0 4343.726
250.10191345214844 0 1844.4573
250.11866760253906 0 1344.1747
251.1508026123047 0 164438.03
252.13377380371094 0 1515.141
252.15394592285156 0 21300.004
253.11851501464844 0 708.9018
253.15621948242188 0 1533.0533
255.1480255126953 0 9197.871
256.10870361328125 0 1816.6045
256.1512145996094 0 775.4055
256.6111755371094 0 906.1081
257.1134033203125 0 3975.2473
258.145263671875 0 8156.229
261.1196594238281 0 3983.247
261.1346740722656 0 1322.0095
262.1193542480469 0 2217.6702
265.12969970703125 0 18926.168
265.1436462402344 0 1010.44055
265.1539611816406 0 888.81104
266.1252746582031 0 45354.17
267.1286926269531 0 4570.989
268.1302185058594 0 1880.4202
268.17706298828125 0 4037.9158
269.16156005859375 0 2123.0986
269.1862487792969 0 3331.1082
270.1218566894531 0 6709.377
270.1457214355469 0 1044.4563
270.623779296875 0 2150.7954
272.1760559082031 0 8455.499
273.17919921875 0 1123.8682
274.11883544921875 0 8395.394
274.1316833496094 0 3343.0823
275.1036682128906 0 2075.0007
275.1206970214844 0 999.0261
275.15032958984375 0 1008.74133
276.1089782714844 0 2487.627
276.1263732910156 0 1498.8685
276.1557312011719 0 2489.805
279.1458740234375 0 3477.0867
280.129638671875 0 4034.4302
281.05181884765625 0 12013.122
281.1331787109375 0 906.22516
282.1567687988281 0 7639.4976
283.0310974121094 0 1395.9752
283.1405334472656 0 116698.81
284.1037292480469 0 5510.3975
284.1235046386719 0 968.4081
284.1434631347656 0 16060.44
285.01043701171875 0 969.3222
285.1062927246094 0 944.0579
285.1436462402344 0 1358.8219
285.1584777832031 0 1185.489
286.140380859375 0 6693.6953
287.14306640625 0 825.56793
287.1718444824219 0 1742.5576
288.6177673339844 0 1576.6993
289.1216735839844 0 821.98376
290.1670837402344 0 1129.5226 b 4
293.1137390136719 0 66870.99 y Water loss 11
294.11773681640625 0 13396.2705
295.1031799316406 0 1413.7975
295.1195373535156 0 1068.7191
296.10498046875 0 1360.0527
296.197509765625 0 2162.994 b Water loss 2
296.6667175292969 0 1004.4825
297.08319091796875 0 2031.9806
297.13568115234375 0 1407.5125 y Water loss 8
297.1562805175781 0 21079.316
298.1394958496094 0 2010.4225 y Water loss 6
298.15924072265625 0 3614.2346
299.062255859375 0 86371.48
300.0625915527344 0 1868.514
300.1680908203125 0 12251.259
301.0634460449219 0 1284.4789
301.1512451171875 0 61104.69
302.1141357421875 0 10927.018
302.1544189453125 0 7615.4917
303.1170349121094 0 1275.6858
305.1793212890625 0 1989.2097
305.64569091796875 0 3388.1597
306.13787841796875 0 11259.35 y 8
306.63916015625 0 5445.316
307.1401672363281 0 1400.4714
309.16748046875 0 1024.3795
310.1545715332031 0 803.085
311.124755859375 0 10894.18 y 11
311.137939453125 0 2229.6328
312.1265869140625 0 1758.9198
314.18701171875 0 1982.0403
314.2082824707031 0 4935.0483 b 2
315.16693115234375 0 35991.98
315.2107849121094 0 1075.5618
316.1704406738281 0 5772.2695
317.6587829589844 0 1237.0149
319.1518859863281 0 1551.4375
325.1508483886719 0 2746.7524
326.6643981933594 0 7661.363
327.1650390625 0 3388.7104
327.666748046875 0 1314.2164
332.2088623046875 0 3638.2734
333.17730712890625 0 6513.7266
334.1850280761719 0 1134.2422
335.207275390625 0 1343.4669
338.18267822265625 0 1133.0931
338.67437744140625 0 2261.182 b Water loss 5
340.6615295410156 0 2217.8657
341.0168762207031 0 1231.17
341.1641845703125 0 1745.2687
342.1820373535156 0 2707.425
343.16192626953125 0 3537.4468
343.1835632324219 0 963.03235
344.1714782714844 0 2374.0122
344.9769592285156 0 11782.902
345.1312561035156 0 4774.7314
346.11505126953125 0 17288.062
346.6983947753906 0 1461.5779
347.11822509765625 0 2567.7317
347.6800537109375 0 9952.201 b 5
348.181640625 0 2246.8599
350.21923828125 0 12838.136
351.1657409667969 0 945.5396
351.2209777832031 0 2626.442
352.671142578125 0 984.53723
353.6752014160156 0 2630.8513 y Water loss 7
354.1757507324219 0 1086.0375
355.0705261230469 0 1406.5674
356.1947326660156 0 1221.9929
357.1731262207031 0 917.2555
359.0286865234375 0 9928.546
360.2027282714844 0 1069.5807
360.69586181640625 0 4415.2104
361.1815490722656 0 735.8945
361.1980895996094 0 4450.495
362.1847229003906 0 5578.32
362.67962646484375 0 21874.56 y 7
363.1414489746094 0 26905.936
363.1816101074219 0 8920.823
363.68280029296875 0 3074.9243
364.124755859375 0 5214.5195
364.1455383300781 0 5516.9927
366.1781311035156 0 2685.9248
367.1401062011719 0 1684.144
367.1626281738281 0 1855.5101
367.24578857421875 0 2529.0715
367.6856384277344 0 1238.4989
368.1663513183594 0 1128.1968
368.1915588378906 0 1178.4526
369.12310791015625 0 1070.4965
369.17529296875 0 1103.3706
370.123291015625 0 965.1128
373.18365478515625 0 934.09265
375.2005920410156 0 904.78644
376.162109375 0 1707.9114
376.1983337402344 0 4052.0044
377.14532470703125 0 1826.5375
377.26708984375 0 1079.2366
378.2139587402344 0 9325.7705
379.20953369140625 0 45318.137
380.1651611328125 0 1169.1443
380.21185302734375 0 9663.879
381.1522521972656 0 81084.414
381.71240234375 0 2165.5576
382.15496826171875 0 14624.233
382.18768310546875 0 2790.3833
382.2137145996094 0 1193.1495
383.15704345703125 0 1618.9525
384.1664123535156 0 2831.2922
384.1886291503906 0 3085.418
385.1509704589844 0 5669.5664
385.17291259765625 0 1931.6393 y 4
386.178955078125 0 910.4105
389.19329833984375 0 1582.0292 b 9
390.1965637207031 0 3664.3745
391.13677978515625 0 2876.3362
391.18389892578125 0 1570.0464
392.2082824707031 0 1591.4364
394.1492614746094 0 1322.1206
394.1729431152344 0 10316.981
395.13494873046875 0 4185.4434
395.1572265625 0 2506.6997
395.18035888671875 0 1308.0968
395.24114990234375 0 1739.2191
396.2247314453125 0 15359.546
397.2275390625 0 2860.6497
398.17730712890625 0 5493.336
398.737548828125 0 1393.63
399.2393493652344 0 914.6188
401.2142639160156 0 995.4579
402.17694091796875 0 3712.621
402.1993713378906 0 6442.6714
403.19964599609375 0 2340.5684
404.2058410644531 0 1033.2229
404.7109680175781 0 1272.7458
405.2101745605469 0 841.0456
405.2613830566406 0 2761.5774
406.2452392578125 0 2062.6284
407.2037353515625 0 1051.4425
407.24365234375 0 1114.4965
409.1473083496094 0 6374.093
409.20513916015625 0 1898.2244
409.7008972167969 0 1040.0475
411.1993408203125 0 5106.958
412.1834716796875 0 47446.664
413.1451110839844 0 5192.964
413.16253662109375 0 2921.5027
413.1868896484375 0 7484.2935
413.2496032714844 0 1567.5194
414.14678955078125 0 1218.0779
414.2354736328125 0 13186.924
415.0372314453125 0 21571.25
415.1982421875 0 3616.3218
415.2391662597656 0 2877.4722
416.2008361816406 0 1204.2274
417.7171630859375 0 10209.638
418.21051025390625 0 13229.32
418.71099853515625 0 6109.433
419.21185302734375 0 1645.2509
421.208740234375 0 2360.721 y Water loss 10
422.19268798828125 0 1261.666 y Ammonia loss 10
422.2541198730469 0 880.06683
422.7098083496094 0 1760.3027
423.2012939453125 0 2874.805
423.2720642089844 0 18338.758
423.7037353515625 0 1723.6923
423.7326965332031 0 926.4551
424.27459716796875 0 5226.641
424.7338562011719 0 1470.3387
425.2746887207031 0 907.7457
426.72247314453125 0 130729.01
427.22381591796875 0 65235.03
427.7252197265625 0 17588.488
428.2256774902344 0 3320.667
429.0882568359375 0 1290.3757
429.2099304199219 0 26671.871
430.19415283203125 0 121285.95
431.19720458984375 0 25907.252
431.7145690917969 0 14228.095 b Water loss 6
432.2132263183594 0 6780.317 b Ammonia loss 6
432.7112731933594 0 3993.2583
433.0466613769531 0 1051.6028
433.20697021484375 0 1509.6208
433.2564392089844 0 4886.2446 b Water loss 3
434.2604064941406 0 1144.8733
438.17425537109375 0 2761.733
438.7320556640625 0 1228.6401
439.2196044921875 0 34472.65 y 10
440.18121337890625 0 1563.4625
440.2223815917969 0 8359.271
440.71990966796875 0 64045.047 b 6
441.22149658203125 0 32283.688
441.7225341796875 0 9594.716
442.22406005859375 0 1639.8827
443.23907470703125 0 1246.0997
444.1815490722656 0 863.2126
446.7414855957031 0 1233.3821
447.2068786621094 0 4451.597 y Ammonia loss 6
447.70489501953125 0 1349.7245
448.2049560546875 0 6523.6274
449.2064514160156 0 1424.5099
450.23583984375 0 1602.1708
450.2825927734375 0 1853.475
451.26702880859375 0 37740.516 b 3
452.27044677734375 0 10106.334
453.2734680175781 0 1430.3463
455.2646179199219 0 1463.2045
455.7191467285156 0 2103.2312 y 6
456.2225036621094 0 2102.1582
459.1994934082031 0 3321.2253
460.753662109375 0 3148.861
461.2046203613281 0 3318.8438
461.2525634765625 0 4017.7297
462.2069396972656 0 984.7635
462.2557678222656 0 1123.6077
464.2138977050781 0 1096.1826
465.74560546875 0 949.67377
466.16778564453125 0 1168.3604
466.2436828613281 0 873.9905
468.2539978027344 0 1129.3855
468.2935485839844 0 9060.382
469.2964172363281 0 2169.5173
472.2196350097656 0 1001.39435
474.25897216796875 0 8220.935
474.75433349609375 0 9329.255
475.2548522949219 0 4614.526
475.7545471191406 0 1187.7013
476.2257385253906 0 3075.5103
477.2108459472656 0 7228.1294
478.229248046875 0 6178.8228 y Water loss 9
478.27752685546875 0 4591.065
479.2356872558594 0 1764.7113
479.744140625 0 1437.3752
480.2251281738281 0 1053.3228
483.2644958496094 0 26400.982
483.76605224609375 0 16712.807
484.22161865234375 0 1374.9557
484.26715087890625 0 3977.0781
484.7692565917969 0 1507.4052
488.2573547363281 0 7700.015 b Water loss 7
488.75543212890625 0 3988.0015 b Ammonia loss 7
489.2544250488281 0 1892.1659
494.2364196777344 0 82707.81
495.2015686035156 0 840.32715
495.23944091796875 0 22286.078
496.2409362792969 0 97043.55 y 9
497.2464294433594 0 25201.246
497.763427734375 0 8317.625
498.24395751953125 0 4965.0073
503.1081848144531 0 23065.834
504.20208740234375 0 3396.7366
506.2259826660156 0 1958.0529
507.2611389160156 0 1357.6106
510.2628479003906 0 799.44696
511.2628479003906 0 5106.151
512.2650146484375 0 1344.8857
515.2842407226562 0 1316.8552
519.1394653320312 0 7093.146
522.2293090820312 0 1118.7424
523.2579956054688 0 2620.7979
523.5887451171875 0 1934.5999
524.2821044921875 0 1195.926
525.2667846679688 0 13484.517
526.2301025390625 0 2990.206
526.2686767578125 0 3089.0098
529.260986328125 0 4633.822 Precursor Water loss
529.5952758789062 0 5376.1147 Precursor Ammonia loss
529.9255981445312 0 2756.7024
531.2216186523438 0 1087.2583
532.1944580078125 0 3700.687
533.2447509765625 0 1353.6124
534.8868408203125 0 1992.0455
535.2639770507812 0 5122.7246 Precursor
535.5971069335938 0 2912.1382
535.9346313476562 0 1830.2985
536.166748046875 0 4729.506
537.2438354492188 0 1805.1626
537.7359008789062 0 2017.9132
538.2730712890625 0 1091.0101
538.77880859375 0 1336.6794
539.2366943359375 0 14078.367
540.2396240234375 0 4426.718
541.254638671875 0 1412.3044
542.2936401367188 0 4725.5376
543.277587890625 0 26303.865
544.2808227539062 0 9097.866
545.2809448242188 0 2021.1154
545.7644653320312 0 947.20044 b Water loss 8
546.25 0 4721.6953
546.7520141601562 0 1850.819
547.2516479492188 0 1015.9165
547.7769775390625 0 956.36957
548.2379760742188 0 1323.6646
549.2207641601562 0 3124.749
550.2072143554688 0 1526.2018
551.2581176757812 0 3830.2283
551.3299560546875 0 2111.9424
552.2581787109375 0 1917.5328
553.2633666992188 0 1244.8809
554.7739868164062 0 3477.6118 b 8
555.2564086914062 0 6402.0557
555.7569580078125 0 5645.3374
556.265869140625 0 797.75146
560.2483520507812 0 1270.318
561.3152465820312 0 3542.3923 b Water loss 4
562.3016967773438 0 1218.2703 b Ammonia loss 4
563.2578125 0 3483.8142
564.2601928710938 0 2208.1052
564.33544921875 0 780.72876
565.2634887695312 0 5852.0215
566.2647094726562 0 1562.0532
567.2313842773438 0 16099.674
567.2802124023438 0 938.60046
568.2347412109375 0 5199.4185
569.2415161132812 0 852.69037
569.8236083984375 0 784.9426
575.2471313476562 0 4020.308
576.2332153320312 0 2934.6484
579.3251953125 0 25514.217 b 4
580.3287353515625 0 7366.01
581.3314819335938 0 1830.9584
587.3340454101562 0 813.88873
592.2730712890625 0 3424.3796
592.3252563476562 0 3493.1714
593.2568359375 0 31537.45 y Water loss 8
593.30615234375 0 11618.547
594.2547607421875 0 8577.438
594.3073120117188 0 3151.767
594.7855224609375 0 1206.1965
595.25 0 2639.7988
596.3516845703125 0 1416.9683
597.7979736328125 0 1337.1938
600.3145751953125 0 1424.4491
602.793212890625 0 4763.014
603.2907104492188 0 5696.3457
603.3605346679688 0 813.8512
603.7915649414062 0 1903.4326
610.2835693359375 0 61602.3
610.3287353515625 0 2222.333
611.2682495117188 0 318883.78 y 8
611.7976684570312 0 16069.522
612.271484375 0 98694.65
612.8013916015625 0 4353.409
613.2733764648438 0 22384.09
614.275146484375 0 2938.7925
615.3176879882812 0 1106.2161
616.3006591796875 0 1141.659
617.2811889648438 0 1895.9087
619.2676391601562 0 865.4591
621.251953125 0 4078.5833
621.299560546875 0 1364.7186
622.2566528320312 0 1108.9961
634.3041381835938 0 3181.4485
635.2962036132812 0 4828.7725
636.3238525390625 0 3168.7659
636.7861328125 0 3476.163 y Water loss 3
637.2888793945312 0 4357.5767 y Ammonia loss 3
637.78662109375 0 1572.9272
638.3334350585938 0 1149.8179 b Water loss 10
640.27490234375 0 2677.5186
641.2759399414062 0 1249.7673
645.2973022460938 0 3820.1504
645.79296875 0 20860.723 y 3
646.2940673828125 0 13788.938
646.7947387695312 0 7296.596
647.2901000976562 0 1363.1769
648.3404541015625 0 1574.6715
650.2684326171875 0 1842.4385
650.3253173828125 0 1090.5322
651.2740478515625 0 1107.5215
652.3206787109375 0 33159.58
652.8245849609375 0 1159.6952
653.32373046875 0 12830.847
654.3252563476562 0 2188.0532
657.3602294921875 0 2021.6633
658.2830810546875 0 2050.228
658.33642578125 0 1008.83527
659.3182983398438 0 1241.2316
660.8706665039062 0 1839.0955
661.3261108398438 0 2211.4219
661.8289184570312 0 1320.3104
662.3056030273438 0 12790.13
662.8306274414062 0 929.84106
663.2957763671875 0 8104.335
664.295166015625 0 2774.205
665.3456420898438 0 1086.1282
666.3561401367188 0 1569.8293
668.2811889648438 0 1668.1078
676.3417358398438 0 17966.758 b Water loss 5
677.3319091796875 0 8642.967 b Ammonia loss 5
678.3360595703125 0 3794.3918
679.3301391601562 0 3358.0508
679.8316040039062 0 1091.0181
680.3157348632812 0 58952.23
681.318359375 0 24340.47
682.3204345703125 0 4592.4463
684.3223876953125 0 2155.2354
684.8176879882812 0 3241.8489
685.3153076171875 0 2685.4897
685.8195190429688 0 1386.1031
688.3336791992188 0 1269.2827
692.2831420898438 0 959.31494
692.8359985351562 0 2715.7212
693.3294677734375 0 16537.697 y Water loss 2
693.8275146484375 0 18904.584 y Ammonia loss 2
694.3517456054688 0 166421.4 b 5
694.8271484375 0 2845.1042
695.354736328125 0 63218.1
696.3568725585938 0 14240.719
697.3515014648438 0 2678.1885
698.3575439453125 0 1852.2484
700.89404296875 0 1240.3636
701.8414306640625 0 22617.705
702.3348388671875 0 115138.66 y 2
702.8359375 0 98048.32 b Water loss 11
703.337646484375 0 44145.887 b Ammonia loss 11
703.8394165039062 0 12704.667
704.3385009765625 0 5387.782
705.358154296875 0 2234.7043
706.3424072265625 0 9844.453 y Water loss 7
707.34326171875 0 4259.7266
709.3150024414062 0 921.659
711.3712768554688 0 6664.449
711.8572998046875 0 8695.627 b 11
712.3583984375 0 7959.3164
712.8592529296875 0 3124.7134
719.391357421875 0 1069.9177
720.3846435546875 0 1129.325
720.8611450195312 0 1124.2526
721.3634033203125 0 7277.0835
722.3648681640625 0 2674.2336
723.3679809570312 0 16063.704
724.3523559570312 0 95645.664 y 7
725.3553466796875 0 38731.03
726.3580322265625 0 11159.033
727.3578491210938 0 1614.3843
728.3726196289062 0 888.7997
729.3594360351562 0 4205.9756
730.3587646484375 0 2315.6929
733.3636474609375 0 4024.9958
734.3530883789062 0 3440.9937
737.8638305664062 0 1193.283
738.3634643554688 0 1302.2968
739.355224609375 0 1105.5901
742.8585205078125 0 1083.8541 y Water loss 1
743.367431640625 0 2844.483 y Ammonia loss 1
744.3560180664062 0 1107.7196
745.364013671875 0 1104.2567
747.3890380859375 0 2105.8162
748.3796997070312 0 1981.649
749.3306884765625 0 1391.6677
751.3768310546875 0 20299.156
751.8693237304688 0 10457.73 y 1
752.3735961914062 0 17594.992
752.8712158203125 0 3830.4717
753.3765258789062 0 3621.6233
754.3790893554688 0 1046.049
760.3064575195312 0 1853.8425
761.3726806640625 0 1967.8796
762.4152221679688 0 2333.7732
763.3658447265625 0 9421.984
764.3655395507812 0 5317.7134
765.4000854492188 0 4029.815
766.4072875976562 0 1786.411
767.3479614257812 0 4488.6143
768.3414306640625 0 1168.8715
769.3995361328125 0 1922.5521
776.3787231445312 0 2603.3481
779.3836669921875 0 9375.878
780.385009765625 0 4467.137
781.3643188476562 0 4533.1143
782.3717651367188 0 1479.5645
793.3994140625 0 17487.64 Precursor Water loss
794.4022827148438 0 8703.791
795.3412475585938 0 6226.7075
795.4085083007812 0 1408.1593
796.3405151367188 0 1517.861
796.468505859375 0 4651.0117
797.4722290039062 0 1501.4993
807.3785400390625 0 3880.4717
807.4453735351562 0 2194.2104
808.3826904296875 0 2493.1365
818.44189453125 0 2660.5557
819.4409790039062 0 2051.3367
825.3867797851562 0 1624.0992
826.3762817382812 0 1908.8374
827.3750610351562 0 1120.7592
828.4361572265625 0 1340.2349
834.4263305664062 0 4722.374
835.4141235351562 0 6118.542
836.4487915039062 0 11680.796
837.4532470703125 0 4698.948
838.4530639648438 0 2486.6875
843.4004516601562 0 2132.0056
844.3889770507812 0 4327.1743
845.3938598632812 0 3954.7764
846.4464111328125 0 6845.0312
847.4450073242188 0 5097.8975
848.4458618164062 0 1247.1047
852.436279296875 0 42002.027
853.4389038085938 0 19932.438
854.441650390625 0 6018.204
857.4461669921875 0 963.93274
862.4207153320312 0 25161.646 b Water loss 6
863.4166259765625 0 18585.193 b Ammonia loss 6
864.453857421875 0 27566.133
865.4584350585938 0 14559.622
866.4617919921875 0 5111.808
876.435302734375 0 4322.149
877.4346313476562 0 1343.2958
878.4368896484375 0 1275.2078
880.4307861328125 0 197536.7 b 6
881.433349609375 0 102810.54
882.4365844726562 0 29986.793
883.4387817382812 0 6238.448
884.4345092773438 0 1302.8961
890.4207153320312 0 1734.131
891.4127197265625 0 2257.266
892.46826171875 0 2663.0227
893.4719848632812 0 1287.3727
894.4432373046875 0 1197.8921
907.442138671875 0 2275.8655
908.4265747070312 0 14413.575
909.4324951171875 0 8984.065
910.4319458007812 0 17211.502 y 6
911.4334106445312 0 7636.333
912.4358520507812 0 2365.735
936.4158935546875 0 1054.3966
937.4164428710938 0 2910.0125
947.4361572265625 0 1378.0979
954.431884765625 0 4273.015
955.42138671875 0 4942.1943
956.4210815429688 0 2851.5276
964.4603271484375 0 1278.7819
965.4484252929688 0 6231.0596
966.4486083984375 0 2482.6846
972.4446411132812 0 3035.4854
973.4425048828125 0 2673.7349
975.5046997070312 0 3304.355 b Water loss 7
976.5028686523438 0 1902.8713 b Ammonia loss 7
980.458984375 0 1654.2672
982.4724731445312 0 1648.762
990.4514770507812 0 1106.4436
993.5148315429688 0 10005.013 b 7
994.5172119140625 0 7558.4365
995.5236206054688 0 1164.7736
1007.4766845703125 0 1570.3733
1008.5011596679688 0 1096.7313
1024.4796142578125 0 1813.26
1025.458251953125 0 8183.039 y 5
1026.4598388671875 0 4823.971
1027.4625244140625 0 1428.0602
1064.5174560546875 0 1282.486
1075.531982421875 0 1323.8494
1077.531494140625 0 1231.729
1093.5400390625 0 4226.4985
1094.55029296875 0 2759.5508
1095.5518798828125 0 1122.641
1108.5411376953125 0 2124.0352 b 8
1109.5313720703125 0 1485.422
1117.4962158203125 0 1491.1777
1118.48681640625 0 2147.119
1135.5067138671875 0 6228.0283 y Water loss 4
1136.4959716796875 0 10536.762 y Ammonia loss 4
1137.496337890625 0 4999.9473
1138.49462890625 0 1585.589
1152.5323486328125 0 3930.1401
1153.51708984375 0 15850.836 y 4
1154.5198974609375 0 10084.531
1155.523681640625 0 3795.4812
1156.530029296875 0 925.0428
1165.5692138671875 0 2265.6182 b 9
1204.5731201171875 0 882.375
1290.56982421875 0 973.7044 y 3
3081.43359375 0 963.4725
3454.69873046875 0 1132.4221

Spectrum Details

|  |  |
| --- | --- |
| Matched peaks? Matched peaksThe total absolute number of peaks matched. Additionally in brackets the total fraction of peaks matched and the total number of peaks is shown. | 78 (9.36% of 833) |
| FDR? FDRThe false discovery rate estimated for this peptide. It is calculated by matching all theoretical fragments with a non-integer shift with the raw peaks for this spectrum. This is done with 40 different shifts. The resulting percentage is the average number of annotated peaks over the number of annotated peaks with the correct spectrum. | 0.73% |
| Satellite FDR? Satellite FDRSee the FDR for details on its calculation. This satellite ion specific FDR only contains the satellite ions (d/w) for I/L/J positions. | ∞ |
| PSM Score? PSM ScoreThe PSM Score as given by Hecklib to this annotated spectrum. It is shown with three significant figures. | 604 |

## Spectrum 6887? Spectrum 6887 The raw spectrum of this peptide as annotated by Hecklib. The fragments are coloured according to ion type (see legend). Any peaks with a star '\*' as text can be hovered over to see the full details, first the ion type second the mass shift type. By hovering over the amino acids in the peptide or ions in the legend the corresponding peaks are highlighted. By toggling the 'Unassigned' label you can turn the background (unassigned) peaks on or off in the plot. By updating the slider in the Ion legend you can update the spectrum to only show the top X% of the peaks with labels. The top X% means any peak that is within X% of the highest intensity. By dragging in the spectrum you can zoom in to a specific part of the spectrum and use 'Zoom Out' to get back to the original zoom level. The annotation of the spectrum is based on the given sequence in the peptides file and is done with different software so inconsistencies are likely. The peaks are annotated based on the given sequence, with 20 ppm tolerance.

Copy Data

### Spectrum 6887 (TSV)

#### Preview

```
Loading example...
```

*Click on the button to copy the data to your clipboard.*

Mz MinMz MaxIntensity Max

WidthHeightPeptide font sizePeptide stroke widthSpectrum font sizeSpectrum stroke widthCompact peptide

Ion legend

wxyz

abcd

OtherUnassignedIonChargePositionShow for top:%

TVLHQDWLDGKEY

08.08e+61.62e+72.42e+73.23e+7

Zoom Out

y+11c+12c+12y+12y+25y+25y+12c+13c+26y+26y+26c+310y+13z+13y+13c+27c+27c+14c+14z+14y+14w+15z+15c+15y+15z+15c+15y+15y+210z+210y+210c+211c+211c+211w+16w+211y+211y+211c+16y+211y+16z+16c+16c+212c+212y+16w+212y+212z+212y+212c+17y+17z+17c+17y+17c+18c+18z+18c+18y+18w+19c+19c+19z+19y+19c+110y+110z+110y+110c+111c+111w+111y+111z+111y+111c+112c+112z+112

0881176126423523

Fragment Matches Table

Show background peaks

| Position | Ion type | Intensity | mz Theoretical | mz Error (Th) | mz Error (ppm) | Charge | Series Number |
| --- | --- | --- | --- | --- | --- | --- | --- |
| - | - | 3.91E+04 | 121.1 | - | - | 0 | - |
| - | - | 4.104E+04 | 127.2 | - | - | 0 | - |
| - | - | 9.743E+04 | 128.1 | - | - | 0 | - |
| - | - | 5.584E+05 | 129.1 | - | - | 0 | - |
| - | - | 1.056E+06 | 130.1 | - | - | 0 | - |
| - | - | 9.327E+04 | 131.1 | - | - | 0 | - |
| - | - | 1.54E+05 | 131.1 | - | - | 0 | - |
| - | - | 3.896E+04 | 134 | - | - | 0 | - |
| - | - | 1.295E+05 | 136.1 | - | - | 0 | - |
| - | - | 3.752E+04 | 138.1 | - | - | 0 | - |
| - | - | 4.628E+04 | 138.1 | - | - | 0 | - |
| - | - | 4.575E+04 | 140.6 | - | - | 0 | - |
| - | - | 3.849E+04 | 141.3 | - | - | 0 | - |
| - | - | 3.903E+04 | 148.8 | - | - | 0 | - |
| - | - | 4.968E+04 | 150.1 | - | - | 0 | - |
| - | - | 3.578E+04 | 152 | - | - | 0 | - |
| - | - | 2.303E+06 | 155.1 | - | - | 0 | - |
| - | - | 1.433E+05 | 156.1 | - | - | 0 | - |
| - | - | 3.954E+04 | 156.6 | - | - | 0 | - |
| - | - | 2.377E+05 | 157.1 | - | - | 0 | - |
| - | - | 9.913E+05 | 159.1 | - | - | 0 | - |
| - | - | 3.998E+04 | 161.3 | - | - | 0 | - |
| - | - | 8.433E+04 | 165.1 | - | - | 0 | - |
| - | - | 8.272E+04 | 171.1 | - | - | 0 | - |
| - | - | 3.864E+06 | 173.1 | - | - | 0 | - |
| - | - | 3.661E+05 | 174.1 | - | - | 0 | - |
| 13 | y | 7.621E+05 | 182.1 | 0.0004343 | 2.385 | +1 | 1 |
| - | - | 6.496E+04 | 183.1 | - | - | 0 | - |
| - | - | 6.473E+04 | 183.1 | - | - | 0 | - |
| - | - | 7.273E+04 | 184.1 | - | - | 0 | - |
| - | - | 2.627E+05 | 186.1 | - | - | 0 | - |
| - | - | 4.338E+04 | 186.6 | - | - | 0 | - |
| - | - | 7.685E+04 | 187.1 | - | - | 0 | - |
| - | - | 1.458E+05 | 188.1 | - | - | 0 | - |
| - | - | 4.534E+04 | 194.1 | - | - | 0 | - |
| - | - | 4.863E+04 | 196 | - | - | 0 | - |
| - | - | 9.743E+04 | 198.1 | - | - | 0 | - |
| 2 | c | 6.688E+04 | 200.1 | 0.0002036 | 1.017 | +1 | 2 |
| - | - | 2.723E+06 | 201.1 | - | - | 0 | - |
| - | - | 2.939E+05 | 202.1 | - | - | 0 | - |
| - | - | 4.873E+04 | 205.7 | - | - | 0 | - |
| - | - | 9.101E+04 | 207.1 | - | - | 0 | - |
| - | - | 9.82E+04 | 213.1 | - | - | 0 | - |
| - | - | 1.22E+06 | 215.1 | - | - | 0 | - |
| - | - | 5.699E+04 | 216.1 | - | - | 0 | - |
| 2 | c | 1.025E+06 | 218.1 | 0.000549 | 2.516 | +1 | 2 |
| - | - | 6.61E+04 | 219.2 | - | - | 0 | - |
| - | - | 1.533E+05 | 223.2 | - | - | 0 | - |
| - | - | 6.577E+04 | 225.1 | - | - | 0 | - |
| - | - | 2.345E+05 | 226.1 | - | - | 0 | - |
| - | - | 1.205E+05 | 228.1 | - | - | 0 | - |
| - | - | 5.742E+04 | 234.1 | - | - | 0 | - |
| - | - | 6.633E+04 | 240.1 | - | - | 0 | - |
| - | - | 2.253E+05 | 243.1 | - | - | 0 | - |
| - | - | 4.084E+05 | 245.1 | - | - | 0 | - |
| - | - | 6.473E+04 | 247.1 | - | - | 0 | - |
| - | - | 7.942E+04 | 251.1 | - | - | 0 | - |
| - | - | 6.338E+05 | 251.2 | - | - | 0 | - |
| - | - | 1.577E+06 | 258.1 | - | - | 0 | - |
| - | - | 1.054E+05 | 258.2 | - | - | 0 | - |
| - | - | 1.764E+05 | 259.1 | - | - | 0 | - |
| - | - | 3.233E+05 | 260.2 | - | - | 0 | - |
| - | - | 7.226E+04 | 266.1 | - | - | 0 | - |
| - | - | 6.845E+04 | 267.7 | - | - | 0 | - |
| - | - | 1.503E+06 | 268.2 | - | - | 0 | - |
| - | - | 1.182E+05 | 269.2 | - | - | 0 | - |
| - | - | 7.732E+04 | 272.2 | - | - | 0 | - |
| - | - | 3.688E+05 | 283.1 | - | - | 0 | - |
| - | - | 8.538E+04 | 285.1 | - | - | 0 | - |
| - | - | 9.925E+04 | 287.2 | - | - | 0 | - |
| - | - | 1.106E+05 | 288.2 | - | - | 0 | - |
| - | - | 9.485E+04 | 288.6 | - | - | 0 | - |
| 12 | y | 6.172E+05 | 293.1 | 0.0005104 | 1.741 | +1 | 2 |
| - | - | 1.29E+05 | 294.1 | - | - | 0 | - |
| 9 | y | 5.35E+04 | 297.1 | 0.0005852 | 1.969 | +2 | 5 |
| - | - | 1.013E+05 | 299.1 | - | - | 0 | - |
| - | - | 2.09E+05 | 300.2 | - | - | 0 | - |
| - | - | 3.315E+05 | 301.2 | - | - | 0 | - |
| - | - | 6.717E+04 | 302.2 | - | - | 0 | - |
| - | - | 4.98E+04 | 305.5 | - | - | 0 | - |
| - | - | 2.066E+05 | 305.6 | - | - | 0 | - |
| 9 | y | 1.972E+05 | 306.1 | 0.001162 | 3.796 | +2 | 5 |
| - | - | 5.865E+04 | 306.2 | - | - | 0 | - |
| - | - | 1.128E+05 | 306.6 | - | - | 0 | - |
| - | - | 1.706E+05 | 307.2 | - | - | 0 | - |
| - | - | 6.018E+04 | 308.2 | - | - | 0 | - |
| 12 | y | 3.452E+05 | 311.1 | 0.0006574 | 2.113 | +1 | 2 |
| - | - | 3.825E+05 | 314.2 | - | - | 0 | - |
| - | - | 2.116E+05 | 315.2 | - | - | 0 | - |
| - | - | 8.879E+04 | 315.2 | - | - | 0 | - |
| - | - | 2.76E+06 | 317.2 | - | - | 0 | - |
| - | - | 3.909E+05 | 318.2 | - | - | 0 | - |
| - | - | 5.266E+05 | 320.2 | - | - | 0 | - |
| - | - | 1.012E+05 | 321.2 | - | - | 0 | - |
| 3 | c | 1.498E+06 | 331.2 | 0.0007593 | 2.292 | +1 | 3 |
| - | - | 2.289E+05 | 332.2 | - | - | 0 | - |
| - | - | 7.331E+04 | 342.1 | - | - | 0 | - |
| - | - | 1.399E+05 | 343.1 | - | - | 0 | - |
| 6 | c | 2.468E+05 | 347.7 | 0.0001777 | 0.5111 | +2 | 6 |
| - | - | 5.873E+04 | 348.2 | - | - | 0 | - |
| - | - | 6.06E+04 | 350.2 | - | - | 0 | - |
| 8 | y | 6.826E+04 | 353.7 | 0.001308 | 3.699 | +2 | 6 |
| - | - | 1.172E+05 | 357.2 | - | - | 0 | - |
| - | - | 1.489E+05 | 362.2 | - | - | 0 | - |
| 8 | y | 6.29E+05 | 362.7 | 0.0007257 | 2.001 | +2 | 6 |
| - | - | 2.035E+05 | 363.2 | - | - | 0 | - |
| - | - | 3.824E+05 | 364.2 | - | - | 0 | - |
| - | - | 9.928E+04 | 365.2 | - | - | 0 | - |
| - | - | 3.263E+05 | 374.2 | - | - | 0 | - |
| - | - | 8.546E+04 | 375.2 | - | - | 0 | - |
| - | - | 1.953E+05 | 378.2 | - | - | 0 | - |
| - | - | 1.436E+05 | 379.2 | - | - | 0 | - |
| - | - | 1.013E+05 | 381.2 | - | - | 0 | - |
| 10 | c | 1.174E+05 | 389.2 | 0.002487 | 6.391 | +3 | 10 |
| - | - | 5.301E+04 | 396.2 | - | - | 0 | - |
| - | - | 8.876E+04 | 406.2 | - | - | 0 | - |
| - | - | 7.232E+04 | 408.3 | - | - | 0 | - |
| - | - | 1.19E+05 | 412.2 | - | - | 0 | - |
| - | - | 7.441E+04 | 417.2 | - | - | 0 | - |
| - | - | 1.094E+05 | 417.7 | - | - | 0 | - |
| - | - | 1.516E+05 | 418.2 | - | - | 0 | - |
| - | - | 6.897E+04 | 420.2 | - | - | 0 | - |
| 11 | y | 7.063E+04 | 421.2 | 0.003051 | 7.244 | +1 | 3 |
| 11 | z | 3.629E+05 | 423.2 | 0.00114 | 2.693 | +1 | 3 |
| - | - | 1.343E+06 | 423.3 | - | - | 0 | - |
| - | - | 8.194E+04 | 424.2 | - | - | 0 | - |
| - | - | 3.604E+05 | 424.3 | - | - | 0 | - |
| - | - | 2.283E+06 | 426.7 | - | - | 0 | - |
| - | - | 1.292E+06 | 427.2 | - | - | 0 | - |
| - | - | 4.065E+05 | 427.7 | - | - | 0 | - |
| - | - | 6.747E+04 | 428.2 | - | - | 0 | - |
| - | - | 3.616E+05 | 429.2 | - | - | 0 | - |
| - | - | 5.434E+05 | 430.2 | - | - | 0 | - |
| - | - | 1.212E+05 | 431.2 | - | - | 0 | - |
| - | - | 3.258E+05 | 431.7 | - | - | 0 | - |
| - | - | 2.513E+05 | 432.2 | - | - | 0 | - |
| - | - | 6.764E+04 | 432.7 | - | - | 0 | - |
| - | - | 8.737E+04 | 438.7 | - | - | 0 | - |
| 11 | y | 5.001E+05 | 439.2 | 0.001275 | 2.904 | +1 | 3 |
| 7 | c | 1.044E+05 | 440.2 | 0.004838 | 10.99 | +2 | 7 |
| 7 | c | 1.629E+06 | 440.7 | 0.000957 | 2.171 | +2 | 7 |
| - | - | 9.459E+05 | 441.2 | - | - | 0 | - |
| - | - | 1.912E+05 | 441.7 | - | - | 0 | - |
| - | - | 6.663E+04 | 448.2 | - | - | 0 | - |
| 4 | c | 2.263E+05 | 450.3 | 0.001393 | 3.093 | +1 | 4 |
| - | - | 3.186E+06 | 451.3 | - | - | 0 | - |
| - | - | 6.773E+05 | 452.3 | - | - | 0 | - |
| - | - | 1.011E+05 | 453.3 | - | - | 0 | - |
| 4 | c | 1.182E+07 | 468.3 | 0.0009905 | 2.115 | +1 | 4 |
| - | - | 3.012E+06 | 469.3 | - | - | 0 | - |
| - | - | 4.567E+05 | 470.3 | - | - | 0 | - |
| - | - | 1.967E+05 | 474.3 | - | - | 0 | - |
| - | - | 1.618E+05 | 474.8 | - | - | 0 | - |
| - | - | 7.743E+04 | 475.3 | - | - | 0 | - |
| 10 | z | 1.173E+06 | 480.2 | 0.001099 | 2.289 | +1 | 4 |
| - | - | 3.858E+05 | 481.2 | - | - | 0 | - |
| - | - | 1.12E+05 | 482.2 | - | - | 0 | - |
| - | - | 9.01E+05 | 483.3 | - | - | 0 | - |
| - | - | 4.212E+05 | 483.8 | - | - | 0 | - |
| - | - | 9.045E+04 | 484.3 | - | - | 0 | - |
| - | - | 1.935E+05 | 488.2 | - | - | 0 | - |
| - | - | 1.702E+05 | 488.3 | - | - | 0 | - |
| - | - | 1.081E+05 | 488.8 | - | - | 0 | - |
| - | - | 4.135E+05 | 489.2 | - | - | 0 | - |
| - | - | 1.333E+05 | 490.2 | - | - | 0 | - |
| - | - | 5.737E+04 | 492.2 | - | - | 0 | - |
| - | - | 1.899E+05 | 494.2 | - | - | 0 | - |
| - | - | 8.783E+05 | 494.3 | - | - | 0 | - |
| - | - | 3.223E+05 | 495.2 | - | - | 0 | - |
| - | - | 2.387E+05 | 495.3 | - | - | 0 | - |
| 10 | y | 2.88E+06 | 496.2 | 0.0008994 | 1.812 | +1 | 4 |
| - | - | 6.603E+05 | 497.2 | - | - | 0 | - |
| - | - | 2.697E+05 | 497.8 | - | - | 0 | - |
| - | - | 1.428E+05 | 498.2 | - | - | 0 | - |
| - | - | 5.774E+04 | 511.3 | - | - | 0 | - |
| - | - | 6.758E+04 | 522.2 | - | - | 0 | - |
| - | - | 2.491E+05 | 523.2 | - | - | 0 | - |
| - | - | 1.83E+05 | 524.2 | - | - | 0 | - |
| - | - | 8.114E+04 | 524.2 | - | - | 0 | - |
| - | - | 7.485E+04 | 525.3 | - | - | 0 | - |
| - | - | 9.578E+04 | 528.9 | - | - | 0 | - |
| - | - | 7.054E+04 | 529.3 | - | - | 0 | - |
| - | - | 1.552E+05 | 529.6 | - | - | 0 | - |
| - | - | 8.653E+04 | 529.9 | - | - | 0 | - |
| - | - | 7.218E+04 | 534.9 | - | - | 0 | - |
| - | - | 3.671E+05 | 535.3 | - | - | 0 | - |
| - | - | 1.74E+05 | 535.6 | - | - | 0 | - |
| - | - | 1.704E+05 | 535.9 | - | - | 0 | - |
| - | - | 6.809E+04 | 536.2 | - | - | 0 | - |
| - | - | 1.555E+06 | 538.3 | - | - | 0 | - |
| - | - | 4.393E+05 | 539.3 | - | - | 0 | - |
| - | - | 1.459E+05 | 540.3 | - | - | 0 | - |
| - | - | 8.959E+04 | 543.3 | - | - | 0 | - |
| 9 | w | 1.724E+06 | 550.3 | 0.0008937 | 1.624 | +1 | 5 |
| - | - | 9.424E+04 | 550.3 | - | - | 0 | - |
| - | - | 5.254E+05 | 551.3 | - | - | 0 | - |
| - | - | 1.474E+05 | 551.3 | - | - | 0 | - |
| - | - | 7.709E+04 | 552.3 | - | - | 0 | - |
| - | - | 9.631E+04 | 552.3 | - | - | 0 | - |
| - | - | 1.544E+05 | 558.3 | - | - | 0 | - |
| - | - | 9.966E+04 | 565.3 | - | - | 0 | - |
| - | - | 1.076E+05 | 569.3 | - | - | 0 | - |
| - | - | 8.513E+04 | 569.8 | - | - | 0 | - |
| - | - | 5.978E+04 | 575.2 | - | - | 0 | - |
| 9 | z | 6.199E+04 | 577.2 | 0.002696 | 4.67 | +1 | 5 |
| - | - | 6.837E+04 | 579.1 | - | - | 0 | - |
| 5 | c | 6.492E+05 | 579.3 | 0.0007004 | 1.209 | +1 | 5 |
| - | - | 1.599E+05 | 580.3 | - | - | 0 | - |
| - | - | 7.591E+04 | 590.8 | - | - | 0 | - |
| - | - | 9.982E+04 | 592.3 | - | - | 0 | - |
| 9 | y | 3.919E+05 | 593.3 | 0.003747 | 6.316 | +1 | 5 |
| - | - | 6.642E+05 | 594.3 | - | - | 0 | - |
| 9 | z | 1.666E+06 | 595.2 | 0.00208 | 3.494 | +1 | 5 |
| - | - | 8.206E+05 | 596.3 | - | - | 0 | - |
| 5 | c | 7.433E+06 | 596.4 | 0.0008847 | 1.483 | +1 | 5 |
| - | - | 1.445E+05 | 597.3 | - | - | 0 | - |
| - | - | 2.463E+06 | 597.4 | - | - | 0 | - |
| - | - | 6.843E+04 | 598.3 | - | - | 0 | - |
| - | - | 5.229E+05 | 598.4 | - | - | 0 | - |
| - | - | 1.037E+05 | 599.4 | - | - | 0 | - |
| - | - | 2.188E+05 | 601.3 | - | - | 0 | - |
| - | - | 1.676E+05 | 602.3 | - | - | 0 | - |
| - | - | 2.859E+05 | 608.3 | - | - | 0 | - |
| - | - | 8.948E+04 | 609.3 | - | - | 0 | - |
| - | - | 2.347E+05 | 609.3 | - | - | 0 | - |
| - | - | 2.484E+06 | 610.3 | - | - | 0 | - |
| - | - | 9.054E+04 | 610.3 | - | - | 0 | - |
| 9 | y | 5.388E+06 | 611.3 | 0.002094 | 3.425 | +1 | 5 |
| - | - | 2.518E+05 | 611.8 | - | - | 0 | - |
| - | - | 1.757E+06 | 612.3 | - | - | 0 | - |
| - | - | 3.581E+05 | 613.3 | - | - | 0 | - |
| - | - | 4.363E+05 | 621.3 | - | - | 0 | - |
| - | - | 1.321E+05 | 622.3 | - | - | 0 | - |
| - | - | 1.03E+05 | 629.8 | - | - | 0 | - |
| - | - | 1.302E+05 | 633.3 | - | - | 0 | - |
| - | - | 2.424E+05 | 633.8 | - | - | 0 | - |
| - | - | 1.181E+05 | 634.3 | - | - | 0 | - |
| - | - | 5.875E+04 | 634.8 | - | - | 0 | - |
| - | - | 6.245E+04 | 635.3 | - | - | 0 | - |
| 4 | y | 8.525E+04 | 637.3 | 0.009153 | 14.36 | +2 | 10 |
| 4 | z | 2.805E+05 | 637.8 | 0.004752 | 7.451 | +2 | 10 |
| - | - | 6.202E+04 | 638.3 | - | - | 0 | - |
| - | - | 2.486E+05 | 645.3 | - | - | 0 | - |
| 4 | y | 4.335E+05 | 645.8 | 0.002958 | 4.581 | +2 | 10 |
| - | - | 7.649E+04 | 645.8 | - | - | 0 | - |
| - | - | 2.201E+05 | 646.3 | - | - | 0 | - |
| - | - | 6.466E+04 | 646.4 | - | - | 0 | - |
| - | - | 1.086E+05 | 646.8 | - | - | 0 | - |
| 11 | c | 1.084E+05 | 646.8 | 0.007452 | 11.52 | +2 | 11 |
| 11 | c | 1.813E+05 | 647.3 | 0.00751 | 11.6 | +2 | 11 |
| - | - | 9.128E+04 | 647.8 | - | - | 0 | - |
| - | - | 1.548E+05 | 648.3 | - | - | 0 | - |
| - | - | 5.228E+04 | 649.3 | - | - | 0 | - |
| - | - | 9.806E+04 | 651.3 | - | - | 0 | - |
| - | - | 1.629E+05 | 652.3 | - | - | 0 | - |
| - | - | 5.85E+05 | 654.9 | - | - | 0 | - |
| - | - | 3.82E+06 | 655.4 | - | - | 0 | - |
| 11 | c | 1.299E+07 | 655.8 | 0.002231 | 3.402 | +2 | 11 |
| - | - | 1.011E+07 | 656.3 | - | - | 0 | - |
| - | - | 4.239E+06 | 656.9 | - | - | 0 | - |
| - | - | 8.338E+05 | 657.4 | - | - | 0 | - |
| - | - | 2.415E+05 | 657.9 | - | - | 0 | - |
| - | - | 8.63E+04 | 663.4 | - | - | 0 | - |
| - | - | 2.512E+06 | 664.3 | - | - | 0 | - |
| - | - | 3.471E+05 | 664.3 | - | - | 0 | - |
| 8 | w | 1.375E+06 | 665.3 | 0.008863 | 13.32 | +1 | 6 |
| - | - | 1.003E+05 | 665.4 | - | - | 0 | - |
| - | - | 3.75E+05 | 666.3 | - | - | 0 | - |
| - | - | 1.835E+05 | 666.4 | - | - | 0 | - |
| - | - | 1.458E+05 | 667.3 | - | - | 0 | - |
| - | - | 5.247E+05 | 667.4 | - | - | 0 | - |
| - | - | 1.033E+06 | 668.4 | - | - | 0 | - |
| - | - | 8.999E+05 | 668.9 | - | - | 0 | - |
| - | - | 4.625E+05 | 669.4 | - | - | 0 | - |
| - | - | 1.861E+05 | 669.9 | - | - | 0 | - |
| - | - | 7.521E+04 | 671.8 | - | - | 0 | - |
| - | - | 8.486E+05 | 672.3 | - | - | 0 | - |
| 3 | w | 1.496E+06 | 672.8 | 0.004512 | 6.706 | +2 | 11 |
| - | - | 1.158E+06 | 673.3 | - | - | 0 | - |
| - | - | 4.498E+05 | 673.8 | - | - | 0 | - |
| - | - | 1.622E+05 | 676.3 | - | - | 0 | - |
| - | - | 6.242E+04 | 677.3 | - | - | 0 | - |
| - | - | 1.479E+05 | 679.3 | - | - | 0 | - |
| - | - | 8.432E+04 | 679.8 | - | - | 0 | - |
| - | - | 4.991E+05 | 680.3 | - | - | 0 | - |
| - | - | 1.652E+05 | 681.3 | - | - | 0 | - |
| - | - | 9.925E+04 | 684.3 | - | - | 0 | - |
| - | - | 6.625E+04 | 684.8 | - | - | 0 | - |
| - | - | 9.22E+04 | 689.9 | - | - | 0 | - |
| - | - | 1.028E+05 | 692.8 | - | - | 0 | - |
| 3 | y | 3.611E+05 | 693.3 | 0.00167 | 2.409 | +2 | 11 |
| 3 | y | 8.508E+05 | 693.8 | 0.0118 | 17.01 | +2 | 11 |
| 6 | c | 1.869E+06 | 694.4 | 0.009275 | 13.36 | +1 | 6 |
| - | - | 7.892E+05 | 694.8 | - | - | 0 | - |
| - | - | 5.272E+05 | 695.4 | - | - | 0 | - |
| - | - | 2.991E+05 | 696.4 | - | - | 0 | - |
| - | - | 1.572E+05 | 697.4 | - | - | 0 | - |
| - | - | 3.584E+05 | 697.9 | - | - | 0 | - |
| - | - | 3.723E+05 | 698.4 | - | - | 0 | - |
| - | - | 2.019E+05 | 698.9 | - | - | 0 | - |
| - | - | 6.702E+04 | 699.4 | - | - | 0 | - |
| - | - | 1.485E+06 | 701.8 | - | - | 0 | - |
| 3 | y | 3.037E+06 | 702.3 | 0.004627 | 6.589 | +2 | 11 |
| - | - | 1.904E+06 | 702.8 | - | - | 0 | - |
| - | - | 8.001E+05 | 703.3 | - | - | 0 | - |
| - | - | 1.62E+05 | 703.8 | - | - | 0 | - |
| 8 | y | 1.383E+05 | 706.3 | 0.003301 | 4.674 | +1 | 6 |
| - | - | 1.619E+06 | 707.3 | - | - | 0 | - |
| 8 | z | 1.78E+06 | 708.3 | 0.004991 | 7.046 | +1 | 6 |
| - | - | 1.774E+05 | 708.9 | - | - | 0 | - |
| - | - | 7.485E+05 | 709.3 | - | - | 0 | - |
| - | - | 1.855E+05 | 710.3 | - | - | 0 | - |
| 6 | c | 6.596E+06 | 711.4 | 0.000675 | 0.9489 | +1 | 6 |
| 12 | c | 2.819E+05 | 711.9 | 0.005317 | 7.47 | +2 | 12 |
| - | - | 2.542E+06 | 712.4 | - | - | 0 | - |
| - | - | 1.383E+05 | 712.9 | - | - | 0 | - |
| - | - | 6.494E+05 | 713.4 | - | - | 0 | - |
| - | - | 7.948E+04 | 713.9 | - | - | 0 | - |
| - | - | 1.167E+05 | 714.4 | - | - | 0 | - |
| - | - | 1.155E+05 | 714.9 | - | - | 0 | - |
| - | - | 1.962E+05 | 715.4 | - | - | 0 | - |
| - | - | 2.213E+05 | 715.8 | - | - | 0 | - |
| - | - | 1.368E+05 | 716.3 | - | - | 0 | - |
| - | - | 8.878E+04 | 716.8 | - | - | 0 | - |
| - | - | 5.697E+06 | 719.9 | - | - | 0 | - |
| 12 | c | 8.615E+06 | 720.4 | 0.005593 | 7.764 | +2 | 12 |
| - | - | 5.956E+06 | 720.9 | - | - | 0 | - |
| - | - | 2.259E+06 | 721.4 | - | - | 0 | - |
| - | - | 5.377E+05 | 721.9 | - | - | 0 | - |
| - | - | 1.062E+05 | 722.4 | - | - | 0 | - |
| - | - | 8.486E+04 | 722.8 | - | - | 0 | - |
| - | - | 7.272E+05 | 723.4 | - | - | 0 | - |
| 8 | y | 1.881E+06 | 724.4 | 0.002868 | 3.96 | +1 | 6 |
| - | - | 7.971E+05 | 725.4 | - | - | 0 | - |
| - | - | 1.658E+05 | 726.4 | - | - | 0 | - |
| - | - | 8.06E+04 | 728.4 | - | - | 0 | - |
| - | - | 6.408E+04 | 728.9 | - | - | 0 | - |
| - | - | 4.293E+05 | 729.4 | - | - | 0 | - |
| - | - | 6.041E+05 | 729.9 | - | - | 0 | - |
| - | - | 3.587E+05 | 730.4 | - | - | 0 | - |
| - | - | 1.375E+05 | 730.9 | - | - | 0 | - |
| 2 | w | 2.354E+05 | 736.3 | 0.0033 | 4.481 | +2 | 12 |
| - | - | 5.251E+05 | 736.9 | - | - | 0 | - |
| - | - | 6.276E+05 | 737.4 | - | - | 0 | - |
| - | - | 3.756E+05 | 737.9 | - | - | 0 | - |
| - | - | 1.05E+05 | 738.4 | - | - | 0 | - |
| - | - | 8.337E+04 | 739.4 | - | - | 0 | - |
| - | - | 8.195E+04 | 740.4 | - | - | 0 | - |
| - | - | 1.291E+05 | 740.9 | - | - | 0 | - |
| - | - | 1.692E+05 | 741.4 | - | - | 0 | - |
| - | - | 8.203E+04 | 742.9 | - | - | 0 | - |
| 2 | y | 2.942E+05 | 743.4 | 0.01464 | 19.69 | +2 | 12 |
| 2 | z | 6.605E+05 | 743.9 | 0.007676 | 10.32 | +2 | 12 |
| - | - | 5.883E+05 | 744.4 | - | - | 0 | - |
| - | - | 2.876E+05 | 744.9 | - | - | 0 | - |
| - | - | 8.134E+04 | 745.4 | - | - | 0 | - |
| - | - | 1.987E+05 | 748.4 | - | - | 0 | - |
| - | - | 2.903E+06 | 748.9 | - | - | 0 | - |
| - | - | 2.336E+06 | 749.4 | - | - | 0 | - |
| - | - | 1.064E+06 | 749.9 | - | - | 0 | - |
| - | - | 5.135E+05 | 750.4 | - | - | 0 | - |
| - | - | 1.888E+05 | 750.9 | - | - | 0 | - |
| - | - | 4.423E+05 | 751.4 | - | - | 0 | - |
| 2 | y | 3.943E+05 | 751.9 | 0.002647 | 3.521 | +2 | 12 |
| - | - | 2.996E+05 | 752.4 | - | - | 0 | - |
| - | - | 8.353E+04 | 752.9 | - | - | 0 | - |
| - | - | 3.088E+05 | 757.9 | - | - | 0 | - |
| - | - | 3.979E+05 | 758.4 | - | - | 0 | - |
| - | - | 3.206E+05 | 758.9 | - | - | 0 | - |
| - | - | 1.432E+05 | 759.4 | - | - | 0 | - |
| - | - | 8.091E+04 | 759.9 | - | - | 0 | - |
| - | - | 1.058E+05 | 762.4 | - | - | 0 | - |
| - | - | 1.102E+05 | 762.9 | - | - | 0 | - |
| - | - | 1.027E+05 | 763.4 | - | - | 0 | - |
| - | - | 1.138E+05 | 764.4 | - | - | 0 | - |
| - | - | 2.649E+05 | 764.9 | - | - | 0 | - |
| - | - | 2.954E+05 | 765.4 | - | - | 0 | - |
| - | - | 1.794E+05 | 765.9 | - | - | 0 | - |
| - | - | 1.584E+05 | 766.4 | - | - | 0 | - |
| - | - | 8.016E+04 | 767.4 | - | - | 0 | - |
| - | - | 1.565E+05 | 770.9 | - | - | 0 | - |
| - | - | 2.13E+05 | 771.4 | - | - | 0 | - |
| - | - | 5.473E+05 | 771.9 | - | - | 0 | - |
| - | - | 1.209E+06 | 772.4 | - | - | 0 | - |
| - | - | 1.196E+06 | 772.9 | - | - | 0 | - |
| - | - | 1.004E+06 | 773.4 | - | - | 0 | - |
| - | - | 3.871E+05 | 773.9 | - | - | 0 | - |
| - | - | 6.506E+04 | 774.4 | - | - | 0 | - |
| - | - | 2.789E+05 | 779.4 | - | - | 0 | - |
| - | - | 1.577E+06 | 779.9 | - | - | 0 | - |
| - | - | 1.369E+06 | 780.4 | - | - | 0 | - |
| - | - | 8.321E+05 | 780.9 | - | - | 0 | - |
| - | - | 3.298E+05 | 781.4 | - | - | 0 | - |
| - | - | 7.43E+04 | 781.9 | - | - | 0 | - |
| - | - | 3.367E+05 | 785.4 | - | - | 0 | - |
| - | - | 5.461E+05 | 785.9 | - | - | 0 | - |
| - | - | 7.761E+05 | 786.4 | - | - | 0 | - |
| - | - | 7.533E+05 | 786.9 | - | - | 0 | - |
| - | - | 5.658E+05 | 787.4 | - | - | 0 | - |
| - | - | 1.819E+05 | 787.9 | - | - | 0 | - |
| - | - | 1.282E+05 | 788.4 | - | - | 0 | - |
| - | - | 4.165E+05 | 793.4 | - | - | 0 | - |
| - | - | 3.224E+06 | 793.9 | - | - | 0 | - |
| - | - | 4.584E+06 | 794.4 | - | - | 0 | - |
| - | - | 3.542E+06 | 794.9 | - | - | 0 | - |
| - | - | 1.614E+06 | 795.4 | - | - | 0 | - |
| - | - | 4.063E+05 | 795.9 | - | - | 0 | - |
| - | - | 9.157E+04 | 796.4 | - | - | 0 | - |
| - | - | 4.217E+06 | 801.9 | - | - | 0 | - |
| - | - | 2.522E+07 | 802.4 | - | - | 0 | - |
| - | - | 3.198E+07 | 802.9 | - | - | 0 | - |
| - | - | 2.084E+07 | 803.4 | - | - | 0 | - |
| - | - | 9.184E+06 | 803.9 | - | - | 0 | - |
| - | - | 2.688E+06 | 804.4 | - | - | 0 | - |
| - | - | 5.172E+05 | 804.9 | - | - | 0 | - |
| - | - | 6.236E+04 | 835.4 | - | - | 0 | - |
| - | - | 7.533E+04 | 836.5 | - | - | 0 | - |
| - | - | 4.149E+05 | 837.4 | - | - | 0 | - |
| - | - | 6.191E+04 | 837.4 | - | - | 0 | - |
| - | - | 9.261E+04 | 837.5 | - | - | 0 | - |
| - | - | 9.461E+05 | 838.4 | - | - | 0 | - |
| - | - | 3.385E+05 | 839.4 | - | - | 0 | - |
| - | - | 1.852E+05 | 840.4 | - | - | 0 | - |
| - | - | 1.089E+05 | 844.4 | - | - | 0 | - |
| - | - | 1.486E+05 | 849.4 | - | - | 0 | - |
| - | - | 1.603E+05 | 850.4 | - | - | 0 | - |
| - | - | 8.546E+04 | 851.4 | - | - | 0 | - |
| - | - | 4.226E+05 | 852.4 | - | - | 0 | - |
| - | - | 4.711E+05 | 853.4 | - | - | 0 | - |
| - | - | 1.769E+05 | 854.4 | - | - | 0 | - |
| - | - | 2.813E+05 | 862.4 | - | - | 0 | - |
| - | - | 1.84E+05 | 863.4 | - | - | 0 | - |
| - | - | 6.974E+05 | 864.5 | - | - | 0 | - |
| - | - | 2.6E+05 | 865.5 | - | - | 0 | - |
| - | - | 1.022E+05 | 876.4 | - | - | 0 | - |
| 7 | c | 4.012E+06 | 880.4 | 0.0008287 | 0.9412 | +1 | 7 |
| - | - | 1.991E+06 | 881.4 | - | - | 0 | - |
| - | - | 6.211E+05 | 882.4 | - | - | 0 | - |
| - | - | 1.12E+05 | 883.4 | - | - | 0 | - |
| 7 | y | 1.512E+05 | 892.4 | 0.01058 | 11.86 | +1 | 7 |
| - | - | 1.359E+06 | 893.4 | - | - | 0 | - |
| 7 | z | 2.49E+06 | 894.4 | 0.005939 | 6.64 | +1 | 7 |
| - | - | 1.008E+06 | 895.4 | - | - | 0 | - |
| - | - | 3.507E+05 | 896.4 | - | - | 0 | - |
| 7 | c | 9.577E+06 | 897.5 | 0.0008909 | 0.9927 | +1 | 7 |
| - | - | 4.862E+06 | 898.5 | - | - | 0 | - |
| - | - | 1.478E+06 | 899.5 | - | - | 0 | - |
| - | - | 3.045E+05 | 900.5 | - | - | 0 | - |
| - | - | 1.171E+05 | 902.4 | - | - | 0 | - |
| - | - | 6.366E+04 | 907.4 | - | - | 0 | - |
| - | - | 7.385E+05 | 909.4 | - | - | 0 | - |
| 7 | y | 9.312E+05 | 910.4 | 0.008577 | 9.421 | +1 | 7 |
| - | - | 4.446E+05 | 911.4 | - | - | 0 | - |
| - | - | 1.61E+05 | 912.4 | - | - | 0 | - |
| - | - | 1.251E+05 | 936.4 | - | - | 0 | - |
| - | - | 2.623E+05 | 937.4 | - | - | 0 | - |
| - | - | 2.059E+05 | 938.4 | - | - | 0 | - |
| - | - | 6.437E+05 | 964.5 | - | - | 0 | - |
| - | - | 1.031E+06 | 965.5 | - | - | 0 | - |
| - | - | 4.095E+05 | 966.5 | - | - | 0 | - |
| - | - | 2.283E+05 | 966.5 | - | - | 0 | - |
| - | - | 1.055E+05 | 967.4 | - | - | 0 | - |
| - | - | 1.894E+05 | 967.5 | - | - | 0 | - |
| - | - | 1.351E+05 | 991.5 | - | - | 0 | - |
| 8 | c | 8.394E+04 | 992.5 | 0.009437 | 9.508 | +1 | 8 |
| 8 | c | 5.943E+05 | 993.5 | 0.001543 | 1.553 | +1 | 8 |
| - | - | 2.891E+05 | 994.5 | - | - | 0 | - |
| - | - | 1.148E+05 | 995.5 | - | - | 0 | - |
| - | - | 2.794E+06 | 1008 | - | - | 0 | - |
| 6 | z | 3.41E+06 | 1009 | 0.00811 | 8.034 | +1 | 8 |
| - | - | 1.069E+06 | 1010 | - | - | 0 | - |
| 8 | c | 7.059E+06 | 1011 | 0.001727 | 1.709 | +1 | 8 |
| - | - | 3.833E+05 | 1011 | - | - | 0 | - |
| - | - | 3.968E+06 | 1012 | - | - | 0 | - |
| - | - | 9.913E+04 | 1012 | - | - | 0 | - |
| - | - | 1.204E+06 | 1013 | - | - | 0 | - |
| - | - | 3.14E+05 | 1014 | - | - | 0 | - |
| - | - | 1.063E+06 | 1024 | - | - | 0 | - |
| 6 | y | 8.367E+05 | 1025 | 0.01472 | 14.35 | +1 | 8 |
| - | - | 4.098E+05 | 1026 | - | - | 0 | - |
| - | - | 1.376E+05 | 1027 | - | - | 0 | - |
| - | - | 8.853E+04 | 1038 | - | - | 0 | - |
| - | - | 2.893E+05 | 1063 | - | - | 0 | - |
| - | - | 2.48E+05 | 1064 | - | - | 0 | - |
| - | - | 9.402E+04 | 1065 | - | - | 0 | - |
| - | - | 1.824E+06 | 1068 | - | - | 0 | - |
| - | - | 1.124E+06 | 1069 | - | - | 0 | - |
| - | - | 3.851E+05 | 1070 | - | - | 0 | - |
| - | - | 1.483E+05 | 1078 | - | - | 0 | - |
| 5 | w | 7.957E+05 | 1079 | 0.00354 | 3.28 | +1 | 9 |
| - | - | 4.167E+05 | 1080 | - | - | 0 | - |
| - | - | 6.293E+05 | 1081 | - | - | 0 | - |
| - | - | 1.379E+05 | 1081 | - | - | 0 | - |
| - | - | 7.809E+05 | 1082 | - | - | 0 | - |
| - | - | 3.212E+05 | 1083 | - | - | 0 | - |
| - | - | 1.456E+05 | 1084 | - | - | 0 | - |
| - | - | 6.686E+04 | 1093 | - | - | 0 | - |
| - | - | 3.701E+05 | 1094 | - | - | 0 | - |
| - | - | 2.672E+05 | 1095 | - | - | 0 | - |
| - | - | 1.018E+05 | 1096 | - | - | 0 | - |
| 9 | c | 1.163E+05 | 1109 | 0.003591 | 3.24 | +1 | 9 |
| - | - | 8.627E+04 | 1110 | - | - | 0 | - |
| - | - | 1.711E+05 | 1111 | - | - | 0 | - |
| - | - | 7.833E+04 | 1120 | - | - | 0 | - |
| - | - | 1.413E+05 | 1124 | - | - | 0 | - |
| - | - | 1.164E+06 | 1125 | - | - | 0 | - |
| 9 | c | 1.662E+06 | 1126 | 0.008658 | 7.692 | +1 | 9 |
| - | - | 8.002E+05 | 1127 | - | - | 0 | - |
| - | - | 2.37E+05 | 1128 | - | - | 0 | - |
| - | - | 2.623E+06 | 1137 | - | - | 0 | - |
| 5 | z | 1.776E+07 | 1137 | 0.002572 | 2.261 | +1 | 9 |
| - | - | 1.09E+07 | 1139 | - | - | 0 | - |
| - | - | 4.119E+06 | 1140 | - | - | 0 | - |
| - | - | 9.663E+05 | 1141 | - | - | 0 | - |
| - | - | 1.14E+05 | 1142 | - | - | 0 | - |
| - | - | 3.666E+05 | 1153 | - | - | 0 | - |
| 5 | y | 8.305E+05 | 1154 | 0.005821 | 5.046 | +1 | 9 |
| - | - | 5.054E+05 | 1155 | - | - | 0 | - |
| - | - | 1.691E+05 | 1156 | - | - | 0 | - |
| - | - | 9.114E+04 | 1169 | - | - | 0 | - |
| - | - | 9.382E+04 | 1170 | - | - | 0 | - |
| - | - | 4.556E+06 | 1182 | - | - | 0 | - |
| 10 | c | 1.047E+07 | 1183 | 0.00636 | 5.378 | +1 | 10 |
| - | - | 6.296E+06 | 1184 | - | - | 0 | - |
| - | - | 2.212E+06 | 1185 | - | - | 0 | - |
| - | - | 4.435E+05 | 1186 | - | - | 0 | - |
| - | - | 1.315E+05 | 1187 | - | - | 0 | - |
| - | - | 1.712E+05 | 1231 | - | - | 0 | - |
| - | - | 1.26E+05 | 1232 | - | - | 0 | - |
| - | - | 3.2E+05 | 1267 | - | - | 0 | - |
| - | - | 5.218E+05 | 1268 | - | - | 0 | - |
| - | - | 2.361E+05 | 1269 | - | - | 0 | - |
| 4 | y | 9.196E+05 | 1274 | 0.02338 | 18.36 | +1 | 10 |
| 4 | z | 3.276E+06 | 1275 | 0.005672 | 4.45 | +1 | 10 |
| - | - | 3.137E+06 | 1276 | - | - | 0 | - |
| - | - | 1.53E+06 | 1277 | - | - | 0 | - |
| - | - | 5E+05 | 1278 | - | - | 0 | - |
| - | - | 8.581E+04 | 1279 | - | - | 0 | - |
| - | - | 1.655E+05 | 1290 | - | - | 0 | - |
| 4 | y | 5.033E+05 | 1291 | 0.002695 | 2.088 | +1 | 10 |
| - | - | 2.954E+05 | 1292 | - | - | 0 | - |
| 11 | c | 1.32E+05 | 1294 | 0.008746 | 6.761 | +1 | 11 |
| - | - | 3.016E+05 | 1295 | - | - | 0 | - |
| - | - | 2.488E+05 | 1296 | - | - | 0 | - |
| - | - | 8.983E+04 | 1297 | - | - | 0 | - |
| - | - | 1.671E+06 | 1310 | - | - | 0 | - |
| 11 | c | 4.877E+06 | 1311 | 0.004048 | 3.088 | +1 | 11 |
| - | - | 3.943E+06 | 1312 | - | - | 0 | - |
| - | - | 1.733E+06 | 1313 | - | - | 0 | - |
| - | - | 4.788E+05 | 1314 | - | - | 0 | - |
| - | - | 9.451E+04 | 1315 | - | - | 0 | - |
| - | - | 1.236E+05 | 1344 | - | - | 0 | - |
| 3 | w | 3.262E+05 | 1345 | 0.005192 | 3.861 | +1 | 11 |
| - | - | 9.22E+04 | 1346 | - | - | 0 | - |
| - | - | 8.747E+04 | 1380 | - | - | 0 | - |
| - | - | 1.086E+05 | 1381 | - | - | 0 | - |
| 3 | y | 6.098E+05 | 1387 | 0.02245 | 16.19 | +1 | 11 |
| 3 | z | 2.851E+06 | 1388 | 0.007057 | 5.086 | +1 | 11 |
| - | - | 3.476E+06 | 1389 | - | - | 0 | - |
| - | - | 2.263E+06 | 1390 | - | - | 0 | - |
| - | - | 8.979E+05 | 1391 | - | - | 0 | - |
| - | - | 1.89E+05 | 1392 | - | - | 0 | - |
| - | - | 3.132E+05 | 1395 | - | - | 0 | - |
| - | - | 8.173E+05 | 1396 | - | - | 0 | - |
| - | - | 8.129E+05 | 1397 | - | - | 0 | - |
| - | - | 3.58E+05 | 1398 | - | - | 0 | - |
| - | - | 1.498E+05 | 1399 | - | - | 0 | - |
| 3 | y | 2.969E+05 | 1404 | 0.006888 | 4.907 | +1 | 11 |
| - | - | 2.811E+05 | 1405 | - | - | 0 | - |
| - | - | 2.217E+05 | 1413 | - | - | 0 | - |
| - | - | 9.504E+04 | 1414 | - | - | 0 | - |
| 12 | c | 3.399E+05 | 1423 | 0.0245 | 17.22 | +1 | 12 |
| - | - | 4.395E+05 | 1424 | - | - | 0 | - |
| - | - | 3.848E+05 | 1425 | - | - | 0 | - |
| - | - | 2.011E+05 | 1426 | - | - | 0 | - |
| - | - | 6.077E+05 | 1439 | - | - | 0 | - |
| 12 | c | 2.156E+06 | 1440 | 0.01382 | 9.601 | +1 | 12 |
| - | - | 2.318E+06 | 1441 | - | - | 0 | - |
| - | - | 1.095E+06 | 1442 | - | - | 0 | - |
| - | - | 4.789E+05 | 1443 | - | - | 0 | - |
| - | - | 1.152E+05 | 1444 | - | - | 0 | - |
| 2 | z | 3.048E+05 | 1487 | 0.01726 | 11.61 | +1 | 12 |
| - | - | 7.509E+05 | 1488 | - | - | 0 | - |
| - | - | 6.131E+05 | 1489 | - | - | 0 | - |
| - | - | 1.854E+05 | 1490 | - | - | 0 | - |
| - | - | 8.647E+04 | 1491 | - | - | 0 | - |
| - | - | 7.698E+04 | 1504 | - | - | 0 | - |
| - | - | 1.084E+05 | 1542 | - | - | 0 | - |
| - | - | 2.739E+05 | 1543 | - | - | 0 | - |
| - | - | 3.244E+05 | 1544 | - | - | 0 | - |
| - | - | 6.647E+05 | 1545 | - | - | 0 | - |
| - | - | 7.623E+05 | 1546 | - | - | 0 | - |
| - | - | 3.963E+05 | 1547 | - | - | 0 | - |
| - | - | 1.584E+05 | 1548 | - | - | 0 | - |
| - | - | 4.642E+05 | 1559 | - | - | 0 | - |
| - | - | 1.905E+06 | 1560 | - | - | 0 | - |
| - | - | 1.653E+06 | 1561 | - | - | 0 | - |
| - | - | 1.077E+06 | 1562 | - | - | 0 | - |
| - | - | 3.789E+05 | 1563 | - | - | 0 | - |
| - | - | 8.09E+04 | 1564 | - | - | 0 | - |
| - | - | 2.118E+05 | 1570 | - | - | 0 | - |
| - | - | 3.663E+05 | 1571 | - | - | 0 | - |
| - | - | 3.699E+05 | 1572 | - | - | 0 | - |
| - | - | 1.259E+05 | 1573 | - | - | 0 | - |
| - | - | 1.875E+05 | 1576 | - | - | 0 | - |
| - | - | 1.033E+06 | 1577 | - | - | 0 | - |
| - | - | 1.156E+06 | 1578 | - | - | 0 | - |
| - | - | 6.736E+05 | 1579 | - | - | 0 | - |
| - | - | 2.922E+05 | 1580 | - | - | 0 | - |
| - | - | 7.379E+05 | 1587 | - | - | 0 | - |
| - | - | 4.35E+06 | 1588 | - | - | 0 | - |
| - | - | 7.105E+06 | 1589 | - | - | 0 | - |
| - | - | 4.886E+06 | 1590 | - | - | 0 | - |
| - | - | 2.5E+06 | 1591 | - | - | 0 | - |
| - | - | 7.238E+05 | 1592 | - | - | 0 | - |
| - | - | 9.879E+04 | 1593 | - | - | 0 | - |
| - | - | 2.9E+05 | 1603 | - | - | 0 | - |
| - | - | 2.238E+06 | 1604 | - | - | 0 | - |
| - | - | 8.123E+06 | 1605 | - | - | 0 | - |
| - | - | 1.32E+07 | 1606 | - | - | 0 | - |
| - | - | 9.209E+06 | 1607 | - | - | 0 | - |
| - | - | 4.224E+06 | 1608 | - | - | 0 | - |
| - | - | 1.259E+06 | 1609 | - | - | 0 | - |
| - | - | 2.306E+05 | 1610 | - | - | 0 | - |
| - | - | 1.063E+05 | 2139 | - | - | 0 | - |
| - | - | 6.622E+04 | 3488 | - | - | 0 | - |

m/z Charge Intensity FragmentType MassShift Position
121.06500244140625 0 39100.88
127.2240982055664 0 41036.598
128.10740661621094 0 97431.91
129.1026153564453 0 558405.3
130.06549072265625 0 1055545.8
131.0689697265625 0 93270.414
131.11822509765625 0 154023.95
133.9840850830078 0 38958.605
136.07611083984375 0 129450.81
138.0660858154297 0 37523.742
138.0911865234375 0 46277.605
140.55209350585938 0 45751.605
141.32044982910156 0 38489.855
148.8430633544922 0 39034.465
150.06680297851562 0 49680.348
152.01950073242188 0 35783.805
155.0930938720703 0 2302751.8
156.09649658203125 0 143265.25
156.55613708496094 0 39538.414
157.13397216796875 0 237650.55
159.09205627441406 0 991254.75
161.2538299560547 0 39978.848
165.05491638183594 0 84326.24
171.11312866210938 0 82715.4
173.12884521484375 0 3863877
174.13230895996094 0 366139.44
182.08160400390625 0 762144.2 y 12
183.08462524414062 0 64958.234
183.11349487304688 0 64734.598
184.06138610839844 0 72729.914
186.12432861328125 0 262670.47
186.5674285888672 0 43384.066
187.08738708496094 0 76854.734
188.1396942138672 0 145754.66
194.09320068359375 0 45341.25
195.95751953125 0 48626.562
198.11317443847656 0 97433.52
200.13955688476562 0 66875.56 c Water loss 1
201.1237335205078 0 2723082.5
202.1270294189453 0 293899.62
205.69387817382812 0 48728.523
207.1130828857422 0 91013.04
213.09971618652344 0 98197.664
215.139404296875 0 1220202.6
216.1433563232422 0 56993.16
218.1504669189453 0 1024993.06 c 1
219.15379333496094 0 66100.305
223.15594482421875 0 153261.98
225.12374877929688 0 65773.414
226.10781860351562 0 234497.08
228.1343231201172 0 120530.84
234.12350463867188 0 57420.484
240.13465881347656 0 66326.42
243.13458251953125 0 225299.56
245.1375274658203 0 408446.53
247.10841369628906 0 64728.434
251.10313415527344 0 79420.02
251.15089416503906 0 633823.7
258.145263671875 0 1576712
258.1600036621094 0 105379.7
259.148193359375 0 176404.95
260.16094970703125 0 323312.62
266.127197265625 0 72259.62
267.6654968261719 0 68448.32
268.1771545410156 0 1502597
269.1808776855469 0 118218.22
272.16021728515625 0 77319.09
283.14068603515625 0 368762.6
285.13104248046875 0 85380.91
287.22064208984375 0 99250.53
288.228515625 0 110631.53
288.6190185546875 0 94854.28
293.11370849609375 0 617245.75 y Water loss 11
294.1177673339844 0 129002.625
297.13250732421875 0 53501.582 y Water loss 8
299.1481628417969 0 101338.266
300.1675109863281 0 209041.86
301.1513977050781 0 331494.72
302.1573486328125 0 67166.5
305.5427551269531 0 49797.1
305.64630126953125 0 206579.78
306.13836669921875 0 197201.61 y 8
306.1818542480469 0 58651.418
306.6392517089844 0 112814.69
307.1895751953125 0 170602.97
308.1947326660156 0 60184.117
311.1244201660156 0 345218.38 y 11
314.20819091796875 0 382530.7
315.1668701171875 0 211615.02
315.21124267578125 0 88787.03
317.1589050292969 0 2760183.5
318.1619873046875 0 390855.16
320.1973876953125 0 526635.5
321.2005920410156 0 101206.84
331.2347412109375 0 1498199 c 2
332.23773193359375 0 228853.03
342.1169128417969 0 73305.914
343.1019592285156 0 139895.88
347.67974853515625 0 246777.7 c Ammonia loss 5
348.18170166015625 0 58726.14
350.22076416015625 0 60597.5
353.6752624511719 0 68256.79 y Water loss 7
357.17742919921875 0 117205.8
362.1873474121094 0 148899.42
362.6799621582031 0 629040.9 y 7
363.1811218261719 0 203516.8
364.1866760253906 0 382439.25
365.1907043457031 0 99278.305
374.179931640625 0 326269.47
375.1824645996094 0 85463.69
378.2078857421875 0 195257.4
379.20855712890625 0 143628.56
381.2251281738281 0 101336.68
389.19024658203125 0 117401.48 c Ammonia loss 9
396.23248291015625 0 53008.74
406.2446594238281 0 88761.13
408.2615051269531 0 72321.59
412.1825256347656 0 118952.51
417.18701171875 0 74413.984
417.7169189453125 0 109390.8
418.2123107910156 0 151603.69
420.1774597167969 0 68968.375
421.2112121582031 0 70634.2 y Water loss 10
423.2011413574219 0 362857.84 z 10
423.272216796875 0 1342585.8
424.20361328125 0 81938.94
424.2747802734375 0 360449.97
426.7225646972656 0 2282589.5
427.22412109375 0 1291809.5
427.7250061035156 0 406518.6
428.22442626953125 0 67470.7
429.2100830078125 0 361575.9
430.1944274902344 0 543374.8
431.1968994140625 0 121239.93
431.7144470214844 0 325846.2
432.2134094238281 0 251335
432.7079772949219 0 67638.71
438.7301025390625 0 87374.82
439.2200012207031 0 500109.47 y 10
440.2223815917969 0 104356.45 c Water loss 6
440.7201843261719 0 1628676.5 c Ammonia loss 6
441.22174072265625 0 945865.2
441.7231140136719 0 191175.72
448.2038879394531 0 66633.32
450.2837219238281 0 226257.9 c Water loss 3
451.2674255371094 0 3185540.5
452.2702331542969 0 677310.56
453.272216796875 0 101096.945
468.29388427734375 0 11820204 c 3
469.29669189453125 0 3012379.2
470.29901123046875 0 456659.3
474.2597961425781 0 196743.9
474.7560729980469 0 161762.36
475.2568664550781 0 77426.69
480.2225646972656 0 1172644.5 z 9
481.2281494140625 0 385763.8
482.2311706542969 0 112022.55
483.2648620605469 0 901045.56
483.7662658691406 0 421201.22
484.2664794921875 0 90446.81
488.22357177734375 0 193505.19
488.25665283203125 0 170180.9
488.754150390625 0 108050.64
489.2080078125 0 413465.97
490.2105712890625 0 133322.08
492.2462463378906 0 57366.38
494.2371520996094 0 189873.97
494.30950927734375 0 878264.44
495.2344970703125 0 322328.56
495.3129577636719 0 238708.17
496.2410888671875 0 2880370.8 y 9
497.2458190917969 0 660339.1
497.7632141113281 0 269714.16
498.2447204589844 0 142769.12
511.2617492675781 0 57743.188
522.24267578125 0 67582
523.2271118164062 0 249107.62
524.1744995117188 0 183027.75
524.2264404296875 0 81138.58
525.2677001953125 0 74853.086
528.9324340820312 0 95782.85
529.2597045898438 0 70538.37
529.595947265625 0 155191.98
529.9267578125 0 86526.27
534.9368896484375 0 72182.51
535.265380859375 0 367071.38
535.60107421875 0 174000.75
535.9334106445312 0 170428.86
536.2330322265625 0 68089.56
538.2517700195312 0 1555226.2
539.25439453125 0 439348.7
540.255615234375 0 145899.3
543.2807006835938 0 89591.71
550.2516479492188 0 1724121.4 w 8
550.2994384765625 0 94237.96
551.2555541992188 0 525388.94
551.3306884765625 0 147358.77
552.2592163085938 0 77085.94
552.3374633789062 0 96307.984
558.3007202148438 0 154356.61
565.262939453125 0 99659.305
569.3112182617188 0 107626.195
569.8114013671875 0 85127.44
575.2454833984375 0 59778.39
577.2405395507812 0 61992.793 z Water loss 8
579.0914306640625 0 68370.67
579.3256225585938 0 649213.56 c Ammonia loss 4
580.3283081054688 0 159936.7
590.8158569335938 0 75905.65
592.2714233398438 0 99822.18
593.2603149414062 0 391856.88 y Water loss 8
594.2637329101562 0 664150.44
595.25048828125 0 1665870.8 z 8
596.2557373046875 0 820585.94
596.3523559570312 0 7433140.5 c 4
597.2633056640625 0 144528.66
597.3550415039062 0 2462519.8
598.262451171875 0 68426.67
598.3572998046875 0 522879.5
599.3606567382812 0 103677.3
601.308349609375 0 218795.02
602.2954711914062 0 167568.03
608.280517578125 0 285867.8
609.2843017578125 0 89480.266
609.337890625 0 234722.98
610.2838745117188 0 2484171.5
610.3299560546875 0 90540.055
611.2692260742188 0 5388244.5 y 8
611.80126953125 0 251788.52
612.272216796875 0 1756905
613.2742919921875 0 358080.8
621.2893676757812 0 436269.94
622.2916259765625 0 132122.94
629.8010864257812 0 102950.37
633.3474731445312 0 130221.91
633.842041015625 0 242377.72
634.3428955078125 0 118140.28
634.841796875 0 58748.9
635.3432006835938 0 62447.508
637.2869873046875 0 85252.36 y Ammonia loss 3
637.7864990234375 0 280529.25 z 3
638.283203125 0 62024.64
645.2984619140625 0 248599.38
645.7940673828125 0 433519.1 y 3
645.8453369140625 0 76493.914
646.2957153320312 0 220087.19
646.35009765625 0 64658.508
646.7920532226562 0 108591.41
646.848388671875 0 108420.664 c Water loss 10
647.3404541015625 0 181309.73 c Ammonia loss 10
647.8401489257812 0 91284.61
648.3406372070312 0 154761.8
649.3431396484375 0 52278.684
651.2904663085938 0 98056.83
652.3214111328125 0 162933.45
654.85107421875 0 584971.75
655.3541259765625 0 3819723.8
655.8484497070312 0 12989158 c 10
656.3494262695312 0 10106434
656.8502197265625 0 4238540.5
657.351806640625 0 833757.56
657.8529052734375 0 241526.47
663.3518676757812 0 86301.32
664.2945556640625 0 2512476.5
664.3448486328125 0 347146.6
665.2865600585938 0 1375051.6 w 7
665.3541259765625 0 100301.54
666.2879638671875 0 375033.5
666.3572998046875 0 183458.5
667.2894287109375 0 145840.6
667.365234375 0 524677.7
668.36328125 0 1032756.9
668.8617553710938 0 899892.06
669.3610229492188 0 462520.66
669.8612670898438 0 186098.27
671.8356323242188 0 75205.7
672.3057861328125 0 848572.4
672.8009033203125 0 1495644.1 w 2
673.3016967773438 0 1157840.2
673.8017578125 0 449834
676.3447875976562 0 162162.22
677.3438720703125 0 62421.164
679.3341674804688 0 147946.05
679.83056640625 0 84316.93
680.3168334960938 0 499124.97
681.3221435546875 0 165156.05
684.3314208984375 0 99246.31
684.8219604492188 0 66250.24
689.8621215820312 0 92201.04
692.8367309570312 0 102782.8
693.3295288085938 0 361050.12 y Water loss 2
693.8316650390625 0 850774.5 y Ammonia loss 2
694.3425903320312 0 1868738 c Ammonia loss 5
694.8284912109375 0 789222.7
695.3507690429688 0 527152.4
696.36328125 0 299086.72
697.3702392578125 0 157158.14
697.8692626953125 0 358372.44
698.3673706054688 0 372284.16
698.8682250976562 0 201914.2
699.3666381835938 0 67021.38
701.8416137695312 0 1485007.6
702.3377685546875 0 3036762.2 y 2
702.8381958007812 0 1903842.8
703.3388061523438 0 800099.56
703.8431396484375 0 162035.83
706.3439331054688 0 138269.77 y Water loss 7
707.3495483398438 0 1619427.1
708.3374633789062 0 1779968.2 z 7
708.8579711914062 0 177405.83
709.3391723632812 0 748521
710.3446044921875 0 185454.19
711.3790893554688 0 6596272 c 5
711.8595581054688 0 281878.03 c Ammonia loss 11
712.3811645507812 0 2542248
712.8550415039062 0 138286.45
713.3823852539062 0 649394.1
713.86669921875 0 79478.61
714.3917236328125 0 116662.625
714.8731689453125 0 115503.18
715.3599853515625 0 196234.42
715.8333129882812 0 221290.77
716.3338623046875 0 136825.56
716.8302612304688 0 88776.89
719.876220703125 0 5697334.5
720.3731079101562 0 8615027 c 11
720.8728637695312 0 5955931.5
721.374267578125 0 2259295.5
721.873291015625 0 537654
722.3814697265625 0 106206.76
722.8406372070312 0 84859.945
723.3668823242188 0 727211.5
724.3540649414062 0 1880994.8 y 7
725.355712890625 0 797132.06
726.3575439453125 0 165767.75
728.376220703125 0 80601.59
728.8746948242188 0 64079.65
729.3613891601562 0 429312.25
729.85888671875 0 604106.3
730.3585205078125 0 358686.6
730.86181640625 0 137528.53
736.3495483398438 0 235385.23 w 1
736.8604736328125 0 525083.1
737.3629760742188 0 627625.1
737.864990234375 0 375569.25
738.3619995117188 0 104960.95
739.352783203125 0 83367.086
740.3630981445312 0 81953.21
740.8657836914062 0 129074.12
741.3682250976562 0 169178.94
742.8788452148438 0 82027.63
743.3687133789062 0 294180.34 y Ammonia loss 1
743.8656616210938 0 660506.5 z 1
744.3646850585938 0 588337.9
744.8663330078125 0 287570.44
745.3628540039062 0 81340.04
748.3748168945312 0 198714.1
748.8676147460938 0 2902629
749.369384765625 0 2335566
749.8712768554688 0 1063633.6
750.3746948242188 0 513531.94
750.8779907226562 0 188838.31
751.3779296875 0 442319.56
751.8699951171875 0 394332.25 y 1
752.3675537109375 0 299553.06
752.8729858398438 0 83529.6
757.8914794921875 0 308813.28
758.38330078125 0 397902.7
758.8724975585938 0 320597
759.372802734375 0 143226.45
759.8680419921875 0 80905.58
762.37353515625 0 105765.49
762.8770141601562 0 110161.19
763.3680419921875 0 102744.516
764.3713989257812 0 113763.56
764.8775634765625 0 264931.3
765.37744140625 0 295445.88
765.8649291992188 0 179370.56
766.3622436523438 0 158374.05
767.3646240234375 0 80160.59
770.8861083984375 0 156492.98
771.3865966796875 0 213033.61
771.8912963867188 0 547264.3
772.3912963867188 0 1209031
772.8834838867188 0 1196308.1
773.3870849609375 0 1003902.3
773.8858642578125 0 387104.47
774.39501953125 0 65056.81
779.3955688476562 0 278895.6
779.894287109375 0 1576915.1
780.3937377929688 0 1368977
780.8947143554688 0 832051.75
781.3925170898438 0 329750.8
781.8953857421875 0 74301.97
785.38623046875 0 336709
785.8847045898438 0 546129.6
786.3822631835938 0 776087.9
786.8770751953125 0 753309.8
787.377197265625 0 565771.3
787.8779907226562 0 181867.22
788.3745727539062 0 128165.17
793.399169921875 0 416473.72
793.891357421875 0 3223657.8
794.3883666992188 0 4583616.5
794.8887939453125 0 3541619
795.3889770507812 0 1614260.2
795.889404296875 0 406311.25
796.385498046875 0 91565.22
801.900146484375 0 4216528.5
802.3972778320312 0 25220720
802.8970947265625 0 31984112
803.3982543945312 0 20836000
803.89990234375 0 9184434
804.4006958007812 0 2687734.2
804.901123046875 0 517199.06
835.4188232421875 0 62358.61
836.4511108398438 0 75325.61
837.3663330078125 0 414935.34
837.4392700195312 0 61911.285
837.4541625976562 0 92614.805
838.3543090820312 0 946071.1
839.3544921875 0 338539.4
840.354248046875 0 185178.53
844.4071655273438 0 108875.17
849.43359375 0 148621.4
850.425048828125 0 160251.47
851.43310546875 0 85458.86
852.4368286132812 0 422595.3
853.443115234375 0 471060.4
854.444580078125 0 176879.56
862.421630859375 0 281342.16
863.4168090820312 0 183953.39
864.45751953125 0 697421.25
865.4595336914062 0 259980.56
876.4360961914062 0 102177.13
880.4320068359375 0 4012432.5 c Ammonia loss 6
881.434814453125 0 1991236.5
882.4381103515625 0 621116.44
883.4400024414062 0 112023.39
892.4093627929688 0 151185.64 y Water loss 6
893.4281005859375 0 1359002.1
894.417724609375 0 2489807 z 6
895.4190063476562 0 1007634.2
896.4215698242188 0 350652.9
897.4586181640625 0 9576859 c 6
898.4614868164062 0 4862264.5
899.464111328125 0 1478089.5
900.468017578125 0 304526.3
902.4146728515625 0 117075.8
907.4469604492188 0 63657.305
909.4462280273438 0 738467.56
910.4390869140625 0 931235.44 y 6
911.4390869140625 0 444644.94
912.440185546875 0 161009.06
936.431396484375 0 125085.6
937.420166015625 0 262314.9
938.4219360351562 0 205916.45
964.4645385742188 0 643709.75
965.4561157226562 0 1030674.56
966.4557495117188 0 409546.75
966.5368041992188 0 228299.19
967.4439086914062 0 105478.68
967.5359497070312 0 189436.97
991.5188598632812 0 135071.27
992.5217895507812 0 83944.984 c Water loss 7
993.5167846679688 0 594300.4 c Ammonia loss 7
994.5184936523438 0 289064.4
995.5217895507812 0 114776.914
1008.455810546875 0 2794352.8
1009.4468383789062 0 3410002.5 z 5
1010.443603515625 0 1069031
1010.5435180664062 0 7059225 c 7
1011.4474487304688 0 383282.62
1011.5459594726562 0 3968137.5
1012.4470825195312 0 99126.15
1012.5486450195312 0 1204411.9
1013.5506591796875 0 313955.5
1024.4769287109375 0 1062516.2
1025.47216796875 0 836652.6 y 5
1026.4735107421875 0 409789.44
1027.4703369140625 0 137641.44
1037.557373046875 0 88528.195
1062.5623779296875 0 289322.5
1063.56103515625 0 247965.44
1064.553955078125 0 94018.875
1067.5396728515625 0 1824075.2
1068.543212890625 0 1123956.4
1069.546142578125 0 385101.12
1078.4884033203125 0 148276.39
1079.4715576171875 0 795738.8 w 4
1080.4678955078125 0 416733.97
1080.5733642578125 0 629283
1081.4595947265625 0 137923.58
1081.5714111328125 0 780929.4
1082.5706787109375 0 321169.16
1083.5718994140625 0 145602.77
1092.5228271484375 0 66859.75
1093.5152587890625 0 370101.1
1094.51953125 0 267194.53
1095.5308837890625 0 101828.76
1108.5457763671875 0 116330.2 c Ammonia loss 8
1109.5433349609375 0 86268.96
1110.5394287109375 0 171064.6
1119.5816650390625 0 78331.51
1123.5758056640625 0 141282.73
1124.58447265625 0 1164344.4
1125.577392578125 0 1662195.5 c 8
1126.57666015625 0 800249.3
1127.5772705078125 0 237032.17
1136.513916015625 0 2623059.8
1137.4998779296875 0 17755968 z 4
1138.502197265625 0 10897770
1139.505126953125 0 4118751.8
1140.5076904296875 0 966319.44
1141.50634765625 0 113992.016
1152.53515625 0 366576.3
1153.5218505859375 0 830541.2 y 4
1154.5233154296875 0 505391.06
1155.5235595703125 0 169130.94
1169.4913330078125 0 91141.04
1170.4921875 0 93819.234
1181.607177734375 0 4555861
1182.5965576171875 0 10472164 c 9
1183.59716796875 0 6296342.5
1184.6002197265625 0 2212332.8
1185.603515625 0 443530.94
1186.6024169921875 0 131520.69
1230.567138671875 0 171174.53
1231.5728759765625 0 126047.67
1266.6796875 0 320036.47
1267.6795654296875 0 521756.94
1268.684326171875 0 236147.14
1273.57177734375 0 919554.2 y Ammonia loss 3
1274.5618896484375 0 3276356.5 z 3
1275.563720703125 0 3136555.2
1276.5665283203125 0 1530059.8
1277.5709228515625 0 499994.1
1278.5675048828125 0 85807.42
1289.581298828125 0 165469.53
1290.57763671875 0 503250.34 y 3
1291.577880859375 0 295389.94
1293.6673583984375 0 131989.69 c Ammonia loss 10
1294.67431640625 0 301574.2
1295.6741943359375 0 248838.17
1296.6678466796875 0 89827.25
1309.6988525390625 0 1671023.4
1310.689208984375 0 4877362.5 c 10
1311.6907958984375 0 3943418
1312.69287109375 0 1733434.9
1313.695068359375 0 478765.25
1314.697021484375 0 94506.1
1343.656494140625 0 123569.76
1344.5906982421875 0 326239.34 w 2
1345.6002197265625 0 92195.8
1379.7115478515625 0 87470.57
1380.7203369140625 0 108583.06
1386.6549072265625 0 609816.2 y Ammonia loss 2
1387.6473388671875 0 2851337.8 z 2
1388.6475830078125 0 3475557.8
1389.65185546875 0 2263432
1390.6531982421875 0 897877.6
1391.6573486328125 0 189036.98
1394.727294921875 0 313193.8
1395.732421875 0 817324.25
1396.7305908203125 0 812892.8
1397.732666015625 0 358028.88
1398.731689453125 0 149774.19
1403.6658935546875 0 296931.28 y 2
1404.666015625 0 281114.7
1412.7489013671875 0 221676.02
1413.7589111328125 0 95039.99
1422.7257080078125 0 339885.25 c Ammonia loss 11
1423.7230224609375 0 439504.5
1424.7197265625 0 384792.9
1425.733642578125 0 201103.3
1438.7418212890625 0 607657.5
1439.7415771484375 0 2155713.5 c 11
1440.7388916015625 0 2317566.5
1441.7420654296875 0 1094883.8
1442.740966796875 0 478900.5
1443.7371826171875 0 115167.39
1486.7259521484375 0 304770.62 z 1
1487.722412109375 0 750941.94
1488.7200927734375 0 613101.7
1489.7236328125 0 185407.95
1490.7122802734375 0 86473.44
1503.7183837890625 0 76977.695
1541.771240234375 0 108423.664
1542.7659912109375 0 273874.8
1543.775634765625 0 324434.2
1544.7786865234375 0 664699.1
1545.774658203125 0 762341.1
1546.773681640625 0 396272.44
1547.780029296875 0 158381.75
1558.7821044921875 0 464205.97
1559.7821044921875 0 1904811.8
1560.7808837890625 0 1653270.8
1561.7838134765625 0 1077242.4
1562.7847900390625 0 378890.78
1563.8011474609375 0 80897.55
1569.7593994140625 0 211752.36
1570.763427734375 0 366283.16
1571.76220703125 0 369893.88
1572.7659912109375 0 125863.414
1575.8031005859375 0 187499.75
1576.8018798828125 0 1032573.75
1577.802978515625 0 1155898.4
1578.804443359375 0 673616.25
1579.8087158203125 0 292181.1
1586.777587890625 0 737915.2
1587.774658203125 0 4350252.5
1588.7706298828125 0 7104683.5
1589.77099609375 0 4886042
1590.771728515625 0 2500417.8
1591.775390625 0 723796.2
1592.771484375 0 98792.445
1602.7894287109375 0 290001.28
1603.7874755859375 0 2238439.5
1604.7928466796875 0 8122818.5
1605.792236328125 0 13202066
1606.7943115234375 0 9209164
1607.7969970703125 0 4223514
1608.8009033203125 0 1259183
1609.7999267578125 0 230590.55
2139.271240234375 0 106290.1
3487.907958984375 0 66217.664

Spectrum Details

|  |  |
| --- | --- |
| Matched peaks? Matched peaksThe total absolute number of peaks matched. Additionally in brackets the total fraction of peaks matched and the total number of peaks is shown. | 78 (12.46% of 626) |
| FDR? FDRThe false discovery rate estimated for this peptide. It is calculated by matching all theoretical fragments with a non-integer shift with the raw peaks for this spectrum. This is done with 40 different shifts. The resulting percentage is the average number of annotated peaks over the number of annotated peaks with the correct spectrum. | 3.21% |
| Satellite FDR? Satellite FDRSee the FDR for details on its calculation. This satellite ion specific FDR only contains the satellite ions (d/w) for I/L/J positions. | 0.00% |
| PSM Score? PSM ScoreThe PSM Score as given by Hecklib to this annotated spectrum. It is shown with three significant figures. | 724 |

## Spectrum 7458? Spectrum 7458 The raw spectrum of this peptide as annotated by Hecklib. The fragments are coloured according to ion type (see legend). Any peaks with a star '\*' as text can be hovered over to see the full details, first the ion type second the mass shift type. By hovering over the amino acids in the peptide or ions in the legend the corresponding peaks are highlighted. By toggling the 'Unassigned' label you can turn the background (unassigned) peaks on or off in the plot. By updating the slider in the Ion legend you can update the spectrum to only show the top X% of the peaks with labels. The top X% means any peak that is within X% of the highest intensity. By dragging in the spectrum you can zoom in to a specific part of the spectrum and use 'Zoom Out' to get back to the original zoom level. The annotation of the spectrum is based on the given sequence in the peptides file and is done with different software so inconsistencies are likely. The peaks are annotated based on the given sequence, with 20 ppm tolerance.

Copy Data

### Spectrum 7458 (TSV)

#### Preview

```
Loading example...
```

*Click on the button to copy the data to your clipboard.*

Mz MinMz MaxIntensity Max

WidthHeightPeptide font sizePeptide stroke widthSpectrum font sizeSpectrum stroke widthCompact peptide

Ion legend

wxyz

abcd

OtherUnassignedIonChargePositionShow for top:%

TVLHQDWLDGKEY

09.07e+41.81e+52.72e+53.63e+5

Zoom Out

y+33a+12y+34a+12y+11b+12b+12y+23y+12b+13y+25y+37y+25y+12b+13b+26b+26b+26y+26y+26y+39y+13y+13b+27b+27b+14y+13b+27y+27b+14y+27y+14b+28b+28y+14\*\*\*b+29b+29b+29b+15b+15b+15y+15y+15y+210y+210y+210b+211b+16b+16y+211y+211b+16y+211b+212b+212y+16b+212y+16y+212y+212y+212\*b+17b+17b+17y+17b+18b+18b+18y+18b+19y+19y+19y+19b+110

0778155623343112

Fragment Matches Table

Show background peaks

| Position | Ion type | Intensity | mz Theoretical | mz Error (Th) | mz Error (ppm) | Charge | Series Number |
| --- | --- | --- | --- | --- | --- | --- | --- |
| - | - | 4691 | 120.1 | - | - | 0 | - |
| - | - | 663.5 | 121.1 | - | - | 0 | - |
| - | - | 351 | 121.2 | - | - | 0 | - |
| - | - | 1573 | 122.1 | - | - | 0 | - |
| - | - | 454.6 | 122.1 | - | - | 0 | - |
| - | - | 2166 | 123 | - | - | 0 | - |
| - | - | 634.4 | 123.1 | - | - | 0 | - |
| - | - | 418.8 | 123.4 | - | - | 0 | - |
| - | - | 358.2 | 123.7 | - | - | 0 | - |
| - | - | 891 | 125.1 | - | - | 0 | - |
| - | - | 811.2 | 126.1 | - | - | 0 | - |
| - | - | 362 | 126.1 | - | - | 0 | - |
| - | - | 4845 | 127.1 | - | - | 0 | - |
| - | - | 444.7 | 127.1 | - | - | 0 | - |
| - | - | 905 | 127.1 | - | - | 0 | - |
| - | - | 2.243E+04 | 128.1 | - | - | 0 | - |
| - | - | 2.368E+05 | 129.1 | - | - | 0 | - |
| - | - | 1.054E+05 | 130.1 | - | - | 0 | - |
| - | - | 1434 | 130.1 | - | - | 0 | - |
| - | - | 1.564E+04 | 130.1 | - | - | 0 | - |
| - | - | 1.104E+04 | 131.1 | - | - | 0 | - |
| - | - | 635.9 | 131.1 | - | - | 0 | - |
| - | - | 2.301E+04 | 132.1 | - | - | 0 | - |
| - | - | 518.4 | 132.5 | - | - | 0 | - |
| - | - | 948.1 | 133.1 | - | - | 0 | - |
| - | - | 2257 | 133.1 | - | - | 0 | - |
| - | - | 3.074E+04 | 136.1 | - | - | 0 | - |
| - | - | 2618 | 137.1 | - | - | 0 | - |
| - | - | 3254 | 138.1 | - | - | 0 | - |
| - | - | 1602 | 138.1 | - | - | 0 | - |
| - | - | 501 | 139.1 | - | - | 0 | - |
| - | - | 3123 | 139.1 | - | - | 0 | - |
| - | - | 4555 | 141.1 | - | - | 0 | - |
| - | - | 1390 | 142.1 | - | - | 0 | - |
| - | - | 1758 | 143 | - | - | 0 | - |
| - | - | 1085 | 144.1 | - | - | 0 | - |
| - | - | 2304 | 145.1 | - | - | 0 | - |
| - | - | 501.3 | 145.1 | - | - | 0 | - |
| - | - | 1253 | 146.1 | - | - | 0 | - |
| - | - | 1494 | 147 | - | - | 0 | - |
| 11 | y | 425.5 | 147.1 | 0.0007637 | 5.193 | +3 | 3 |
| - | - | 814.7 | 148.9 | - | - | 0 | - |
| - | - | 1.25E+04 | 149 | - | - | 0 | - |
| - | - | 1614 | 150.1 | - | - | 0 | - |
| - | - | 5012 | 151.1 | - | - | 0 | - |
| - | - | 551.6 | 152.1 | - | - | 0 | - |
| - | - | 688.4 | 153.1 | - | - | 0 | - |
| - | - | 560.2 | 154.1 | - | - | 0 | - |
| - | - | 539.4 | 155.1 | - | - | 0 | - |
| - | - | 1.275E+04 | 155.1 | - | - | 0 | - |
| 2 | a | 1.122E+04 | 155.1 | 0.0003813 | 2.458 | +1 | 2 |
| - | - | 1150 | 156.1 | - | - | 0 | - |
| - | - | 988.5 | 156.1 | - | - | 0 | - |
| - | - | 644.7 | 156.1 | - | - | 0 | - |
| - | - | 788.3 | 157.1 | - | - | 0 | - |
| - | - | 1272 | 157.1 | - | - | 0 | - |
| - | - | 697.4 | 157.1 | - | - | 0 | - |
| - | - | 1282 | 158.1 | - | - | 0 | - |
| - | - | 1.815E+05 | 159.1 | - | - | 0 | - |
| - | - | 1453 | 160.1 | - | - | 0 | - |
| - | - | 1053 | 160.1 | - | - | 0 | - |
| - | - | 1.925E+04 | 160.1 | - | - | 0 | - |
| - | - | 438.1 | 161.1 | - | - | 0 | - |
| - | - | 1.633E+04 | 165.1 | - | - | 0 | - |
| - | - | 2.749E+04 | 166.1 | - | - | 0 | - |
| 10 | y | 2819 | 166.1 | 0.001802 | 10.85 | +3 | 4 |
| - | - | 1744 | 166.1 | - | - | 0 | - |
| - | - | 1.637E+04 | 167.1 | - | - | 0 | - |
| - | - | 2370 | 167.1 | - | - | 0 | - |
| - | - | 742.2 | 167.1 | - | - | 0 | - |
| - | - | 1234 | 167.1 | - | - | 0 | - |
| - | - | 566.6 | 167.3 | - | - | 0 | - |
| - | - | 429.2 | 167.7 | - | - | 0 | - |
| - | - | 971.2 | 168.1 | - | - | 0 | - |
| - | - | 3221 | 168.1 | - | - | 0 | - |
| - | - | 1575 | 168.1 | - | - | 0 | - |
| - | - | 2270 | 168.1 | - | - | 0 | - |
| - | - | 2220 | 169.1 | - | - | 0 | - |
| - | - | 6731 | 169.1 | - | - | 0 | - |
| - | - | 8240 | 170.1 | - | - | 0 | - |
| - | - | 677.3 | 171.1 | - | - | 0 | - |
| - | - | 733.7 | 171.1 | - | - | 0 | - |
| - | - | 1671 | 171.1 | - | - | 0 | - |
| - | - | 1966 | 171.1 | - | - | 0 | - |
| - | - | 3502 | 172.1 | - | - | 0 | - |
| - | - | 3993 | 173.1 | - | - | 0 | - |
| 2 | a | 3.592E+05 | 173.1 | 0.0004825 | 2.787 | +1 | 2 |
| - | - | 1112 | 173.4 | - | - | 0 | - |
| - | - | 627.8 | 174.1 | - | - | 0 | - |
| - | - | 3.082E+04 | 174.1 | - | - | 0 | - |
| - | - | 2470 | 175.1 | - | - | 0 | - |
| - | - | 466.9 | 176 | - | - | 0 | - |
| - | - | 1247 | 177.1 | - | - | 0 | - |
| - | - | 484.7 | 177.1 | - | - | 0 | - |
| - | - | 1.12E+04 | 178.1 | - | - | 0 | - |
| - | - | 886.7 | 179.1 | - | - | 0 | - |
| - | - | 1896 | 181.1 | - | - | 0 | - |
| 13 | y | 3.512E+04 | 182.1 | 0.0004496 | 2.469 | +1 | 1 |
| - | - | 2975 | 183.1 | - | - | 0 | - |
| 2 | b | 1.015E+04 | 183.1 | 0.0004466 | 2.439 | +1 | 2 |
| - | - | 1076 | 184.1 | - | - | 0 | - |
| - | - | 653 | 185.1 | - | - | 0 | - |
| - | - | 2506 | 185.2 | - | - | 0 | - |
| - | - | 7.231E+04 | 186.1 | - | - | 0 | - |
| - | - | 1.56E+04 | 187.1 | - | - | 0 | - |
| - | - | 666.5 | 187.1 | - | - | 0 | - |
| - | - | 6024 | 187.1 | - | - | 0 | - |
| - | - | 524.1 | 187.1 | - | - | 0 | - |
| - | - | 1606 | 188.1 | - | - | 0 | - |
| - | - | 1579 | 188.1 | - | - | 0 | - |
| - | - | 714.9 | 189.1 | - | - | 0 | - |
| - | - | 3424 | 190.1 | - | - | 0 | - |
| - | - | 2314 | 190.1 | - | - | 0 | - |
| - | - | 676.7 | 193.1 | - | - | 0 | - |
| - | - | 2322 | 194.1 | - | - | 0 | - |
| - | - | 1.839E+04 | 195.1 | - | - | 0 | - |
| - | - | 746.3 | 196.1 | - | - | 0 | - |
| - | - | 1.261E+04 | 196.1 | - | - | 0 | - |
| - | - | 1221 | 196.1 | - | - | 0 | - |
| - | - | 661.1 | 197.1 | - | - | 0 | - |
| - | - | 1006 | 197.1 | - | - | 0 | - |
| - | - | 834.6 | 197.1 | - | - | 0 | - |
| - | - | 2459 | 198.1 | - | - | 0 | - |
| - | - | 6403 | 198.1 | - | - | 0 | - |
| - | - | 1241 | 199.1 | - | - | 0 | - |
| - | - | 1438 | 199.1 | - | - | 0 | - |
| - | - | 1593 | 199.1 | - | - | 0 | - |
| - | - | 923.1 | 199.1 | - | - | 0 | - |
| - | - | 1194 | 200.1 | - | - | 0 | - |
| 2 | b | 9.768E+04 | 201.1 | 0.0003952 | 1.965 | +1 | 2 |
| - | - | 1240 | 202.1 | - | - | 0 | - |
| - | - | 9606 | 202.1 | - | - | 0 | - |
| - | - | 1271 | 203.1 | - | - | 0 | - |
| - | - | 1165 | 203.1 | - | - | 0 | - |
| - | - | 973.5 | 204.1 | - | - | 0 | - |
| - | - | 8332 | 205.1 | - | - | 0 | - |
| - | - | 885.1 | 205.1 | - | - | 0 | - |
| - | - | 1357 | 205.1 | - | - | 0 | - |
| - | - | 769.8 | 206.1 | - | - | 0 | - |
| - | - | 2204 | 206.1 | - | - | 0 | - |
| - | - | 3143 | 207.2 | - | - | 0 | - |
| - | - | 609.6 | 208.1 | - | - | 0 | - |
| - | - | 1045 | 209.1 | - | - | 0 | - |
| - | - | 665.1 | 209.1 | - | - | 0 | - |
| - | - | 3519 | 210.1 | - | - | 0 | - |
| 11 | y | 2318 | 211.1 | 0.0006186 | 2.93 | +2 | 3 |
| - | - | 2581 | 212.1 | - | - | 0 | - |
| - | - | 8387 | 213.1 | - | - | 0 | - |
| - | - | 1417 | 213.2 | - | - | 0 | - |
| - | - | 1024 | 214.1 | - | - | 0 | - |
| - | - | 633.9 | 215.1 | - | - | 0 | - |
| - | - | 1703 | 215.1 | - | - | 0 | - |
| - | - | 1773 | 216.1 | - | - | 0 | - |
| - | - | 1946 | 221.1 | - | - | 0 | - |
| - | - | 953.9 | 221.1 | - | - | 0 | - |
| - | - | 5825 | 221.1 | - | - | 0 | - |
| - | - | 1446 | 221.1 | - | - | 0 | - |
| - | - | 1026 | 221.1 | - | - | 0 | - |
| - | - | 2031 | 222.1 | - | - | 0 | - |
| - | - | 3002 | 222.1 | - | - | 0 | - |
| - | - | 3359 | 223.1 | - | - | 0 | - |
| - | - | 973.6 | 223.1 | - | - | 0 | - |
| - | - | 5381 | 223.1 | - | - | 0 | - |
| - | - | 1314 | 223.1 | - | - | 0 | - |
| - | - | 3.202E+04 | 223.2 | - | - | 0 | - |
| - | - | 1935 | 224.1 | - | - | 0 | - |
| - | - | 3743 | 224.2 | - | - | 0 | - |
| - | - | 7653 | 225 | - | - | 0 | - |
| - | - | 685.7 | 225.1 | - | - | 0 | - |
| - | - | 1852 | 226 | - | - | 0 | - |
| - | - | 8040 | 226.1 | - | - | 0 | - |
| - | - | 931.1 | 226.2 | - | - | 0 | - |
| - | - | 799.6 | 227 | - | - | 0 | - |
| - | - | 1447 | 227 | - | - | 0 | - |
| - | - | 6396 | 227.1 | - | - | 0 | - |
| - | - | 1034 | 227.2 | - | - | 0 | - |
| - | - | 704.3 | 228.1 | - | - | 0 | - |
| - | - | 682.5 | 228.1 | - | - | 0 | - |
| - | - | 2169 | 228.1 | - | - | 0 | - |
| - | - | 7332 | 229.1 | - | - | 0 | - |
| - | - | 1043 | 230.2 | - | - | 0 | - |
| - | - | 5059 | 233.1 | - | - | 0 | - |
| - | - | 3.8E+04 | 234.1 | - | - | 0 | - |
| - | - | 748.6 | 234.7 | - | - | 0 | - |
| - | - | 4662 | 235.1 | - | - | 0 | - |
| - | - | 977.1 | 235.6 | - | - | 0 | - |
| - | - | 1863 | 237.1 | - | - | 0 | - |
| - | - | 4252 | 238.1 | - | - | 0 | - |
| - | - | 880.5 | 238.1 | - | - | 0 | - |
| - | - | 563.5 | 239.1 | - | - | 0 | - |
| - | - | 1.415E+04 | 239.1 | - | - | 0 | - |
| - | - | 4865 | 240.1 | - | - | 0 | - |
| - | - | 1.375E+04 | 240.1 | - | - | 0 | - |
| - | - | 2851 | 241.1 | - | - | 0 | - |
| - | - | 1969 | 241.1 | - | - | 0 | - |
| - | - | 2131 | 241.1 | - | - | 0 | - |
| - | - | 1561 | 241.2 | - | - | 0 | - |
| - | - | 2994 | 243.1 | - | - | 0 | - |
| - | - | 1.551E+04 | 244.1 | - | - | 0 | - |
| - | - | 589.3 | 244.6 | - | - | 0 | - |
| - | - | 1066 | 245.1 | - | - | 0 | - |
| - | - | 1.81E+04 | 247.1 | - | - | 0 | - |
| - | - | 1413 | 247.1 | - | - | 0 | - |
| - | - | 2355 | 248.1 | - | - | 0 | - |
| - | - | 8123 | 248.1 | - | - | 0 | - |
| - | - | 1.18E+04 | 249.1 | - | - | 0 | - |
| - | - | 3041 | 249.1 | - | - | 0 | - |
| - | - | 1402 | 250.1 | - | - | 0 | - |
| - | - | 628.5 | 250.1 | - | - | 0 | - |
| - | - | 677.9 | 250.2 | - | - | 0 | - |
| - | - | 553.1 | 250.3 | - | - | 0 | - |
| - | - | 635.9 | 251 | - | - | 0 | - |
| - | - | 1.21E+05 | 251.2 | - | - | 0 | - |
| - | - | 632.5 | 252.1 | - | - | 0 | - |
| - | - | 1.364E+04 | 252.2 | - | - | 0 | - |
| - | - | 712.5 | 253.1 | - | - | 0 | - |
| - | - | 1571 | 253.2 | - | - | 0 | - |
| - | - | 6790 | 255.1 | - | - | 0 | - |
| - | - | 1617 | 256.1 | - | - | 0 | - |
| - | - | 907.4 | 256.2 | - | - | 0 | - |
| - | - | 1329 | 256.6 | - | - | 0 | - |
| - | - | 3205 | 257.1 | - | - | 0 | - |
| - | - | 5190 | 258.1 | - | - | 0 | - |
| - | - | 975.1 | 259.1 | - | - | 0 | - |
| - | - | 2862 | 261.1 | - | - | 0 | - |
| - | - | 919.8 | 261.1 | - | - | 0 | - |
| - | - | 1357 | 262.1 | - | - | 0 | - |
| - | - | 1.45E+04 | 265.1 | - | - | 0 | - |
| - | - | 3613 | 266.1 | - | - | 0 | - |
| - | - | 2.884E+04 | 266.1 | - | - | 0 | - |
| - | - | 759.4 | 267.1 | - | - | 0 | - |
| - | - | 3286 | 267.1 | - | - | 0 | - |
| - | - | 1015 | 267.1 | - | - | 0 | - |
| - | - | 1407 | 268.1 | - | - | 0 | - |
| - | - | 3414 | 268.2 | - | - | 0 | - |
| - | - | 1398 | 269.2 | - | - | 0 | - |
| - | - | 1733 | 269.2 | - | - | 0 | - |
| - | - | 4669 | 270.1 | - | - | 0 | - |
| - | - | 7326 | 272.2 | - | - | 0 | - |
| - | - | 1501 | 273.2 | - | - | 0 | - |
| - | - | 6925 | 274.1 | - | - | 0 | - |
| - | - | 1667 | 274.1 | - | - | 0 | - |
| - | - | 1577 | 275.1 | - | - | 0 | - |
| - | - | 1442 | 275.1 | - | - | 0 | - |
| - | - | 717.5 | 275.1 | - | - | 0 | - |
| - | - | 1424 | 276.1 | - | - | 0 | - |
| - | - | 2120 | 276.1 | - | - | 0 | - |
| - | - | 2139 | 276.2 | - | - | 0 | - |
| - | - | 2883 | 279.1 | - | - | 0 | - |
| - | - | 3105 | 280.1 | - | - | 0 | - |
| - | - | 9481 | 281.1 | - | - | 0 | - |
| - | - | 6666 | 282.2 | - | - | 0 | - |
| - | - | 829.4 | 282.7 | - | - | 0 | - |
| - | - | 1798 | 283 | - | - | 0 | - |
| - | - | 8.79E+04 | 283.1 | - | - | 0 | - |
| - | - | 3215 | 284.1 | - | - | 0 | - |
| - | - | 799.7 | 284.1 | - | - | 0 | - |
| - | - | 1.15E+04 | 284.1 | - | - | 0 | - |
| - | - | 694.2 | 285.1 | - | - | 0 | - |
| - | - | 1018 | 285.1 | - | - | 0 | - |
| - | - | 1153 | 285.1 | - | - | 0 | - |
| - | - | 728.7 | 285.2 | - | - | 0 | - |
| - | - | 5584 | 286.1 | - | - | 0 | - |
| - | - | 825.4 | 287.1 | - | - | 0 | - |
| - | - | 1761 | 287.2 | - | - | 0 | - |
| - | - | 571.4 | 288.1 | - | - | 0 | - |
| - | - | 1713 | 288.6 | - | - | 0 | - |
| 12 | y | 4.661E+04 | 293.1 | 0.000602 | 2.054 | +1 | 2 |
| - | - | 1477 | 293.2 | - | - | 0 | - |
| - | - | 9563 | 294.1 | - | - | 0 | - |
| - | - | 1245 | 295.1 | - | - | 0 | - |
| - | - | 1130 | 295.1 | - | - | 0 | - |
| - | - | 1186 | 296.1 | - | - | 0 | - |
| 3 | b | 1123 | 296.2 | 0.0008553 | 2.887 | +1 | 3 |
| - | - | 1636 | 297.1 | - | - | 0 | - |
| - | - | 834.9 | 297.1 | - | - | 0 | - |
| 9 | y | 1525 | 297.1 | 0.001989 | 6.694 | +2 | 5 |
| - | - | 1.732E+04 | 297.2 | - | - | 0 | - |
| - | - | 747.8 | 297.2 | - | - | 0 | - |
| 7 | y | 1499 | 298.1 | 0.005154 | 17.29 | +3 | 7 |
| - | - | 1603 | 298.2 | - | - | 0 | - |
| - | - | 9.137E+04 | 299.1 | - | - | 0 | - |
| - | - | 2548 | 300.1 | - | - | 0 | - |
| - | - | 1.173E+04 | 300.2 | - | - | 0 | - |
| - | - | 1624 | 301.1 | - | - | 0 | - |
| - | - | 999.4 | 301.1 | - | - | 0 | - |
| - | - | 4.339E+04 | 301.2 | - | - | 0 | - |
| - | - | 1466 | 301.2 | - | - | 0 | - |
| - | - | 8016 | 302.1 | - | - | 0 | - |
| - | - | 5699 | 302.2 | - | - | 0 | - |
| - | - | 1278 | 303.1 | - | - | 0 | - |
| - | - | 604.7 | 303.2 | - | - | 0 | - |
| - | - | 1021 | 305.2 | - | - | 0 | - |
| - | - | 1991 | 305.6 | - | - | 0 | - |
| 9 | y | 6905 | 306.1 | 0.0008265 | 2.7 | +2 | 5 |
| - | - | 642.8 | 306.2 | - | - | 0 | - |
| - | - | 3437 | 306.6 | - | - | 0 | - |
| - | - | 1857 | 307.1 | - | - | 0 | - |
| - | - | 534.8 | 307.2 | - | - | 0 | - |
| - | - | 600 | 309.2 | - | - | 0 | - |
| 12 | y | 6064 | 311.1 | 0.0003217 | 1.034 | +1 | 2 |
| - | - | 2905 | 311.1 | - | - | 0 | - |
| - | - | 608.4 | 311.7 | - | - | 0 | - |
| - | - | 1556 | 312.1 | - | - | 0 | - |
| - | - | 732.5 | 314.1 | - | - | 0 | - |
| - | - | 1880 | 314.2 | - | - | 0 | - |
| 3 | b | 3944 | 314.2 | 0.0009412 | 2.995 | +1 | 3 |
| - | - | 2.454E+04 | 315.2 | - | - | 0 | - |
| - | - | 4352 | 316.2 | - | - | 0 | - |
| - | - | 890.1 | 317.7 | - | - | 0 | - |
| - | - | 605.5 | 318.1 | - | - | 0 | - |
| - | - | 1797 | 319.2 | - | - | 0 | - |
| - | - | 595 | 324.2 | - | - | 0 | - |
| - | - | 1508 | 325.2 | - | - | 0 | - |
| - | - | 579.5 | 326 | - | - | 0 | - |
| - | - | 6704 | 326.7 | - | - | 0 | - |
| - | - | 628 | 327 | - | - | 0 | - |
| - | - | 2557 | 327.2 | - | - | 0 | - |
| - | - | 2564 | 332.2 | - | - | 0 | - |
| - | - | 4962 | 333.2 | - | - | 0 | - |
| - | - | 982.4 | 334.2 | - | - | 0 | - |
| - | - | 575.3 | 336 | - | - | 0 | - |
| 6 | b | 1092 | 338.7 | 0.0006383 | 1.885 | +2 | 6 |
| 6 | b | 1029 | 339.2 | 0.005335 | 15.73 | +2 | 6 |
| - | - | 1158 | 339.7 | - | - | 0 | - |
| - | - | 2796 | 340.7 | - | - | 0 | - |
| - | - | 704.3 | 341 | - | - | 0 | - |
| - | - | 1242 | 341.2 | - | - | 0 | - |
| - | - | 2445 | 342.2 | - | - | 0 | - |
| - | - | 1627 | 343.2 | - | - | 0 | - |
| - | - | 835.4 | 343.2 | - | - | 0 | - |
| - | - | 1965 | 344.2 | - | - | 0 | - |
| - | - | 1.098E+04 | 345 | - | - | 0 | - |
| - | - | 726.6 | 345 | - | - | 0 | - |
| - | - | 3800 | 345.1 | - | - | 0 | - |
| - | - | 1.34E+04 | 346.1 | - | - | 0 | - |
| - | - | 981 | 346.1 | - | - | 0 | - |
| - | - | 2581 | 347.1 | - | - | 0 | - |
| 6 | b | 7019 | 347.7 | 0.000727 | 2.091 | +2 | 6 |
| - | - | 2454 | 348.2 | - | - | 0 | - |
| - | - | 691 | 348.7 | - | - | 0 | - |
| - | - | 887.8 | 349.2 | - | - | 0 | - |
| - | - | 1.187E+04 | 350.2 | - | - | 0 | - |
| - | - | 974.2 | 351.2 | - | - | 0 | - |
| - | - | 2520 | 351.2 | - | - | 0 | - |
| - | - | 786.1 | 352.2 | - | - | 0 | - |
| - | - | 1817 | 352.7 | - | - | 0 | - |
| 8 | y | 2094 | 353.7 | 0.0004233 | 1.197 | +2 | 6 |
| - | - | 1680 | 354.2 | - | - | 0 | - |
| - | - | 1676 | 355.1 | - | - | 0 | - |
| - | - | 9757 | 359 | - | - | 0 | - |
| - | - | 871.6 | 359.1 | - | - | 0 | - |
| - | - | 709.8 | 360.2 | - | - | 0 | - |
| - | - | 2764 | 360.7 | - | - | 0 | - |
| - | - | 4300 | 361.2 | - | - | 0 | - |
| - | - | 915.4 | 361.7 | - | - | 0 | - |
| - | - | 5333 | 362.2 | - | - | 0 | - |
| 8 | y | 1.725E+04 | 362.7 | 0.0009088 | 2.506 | +2 | 6 |
| - | - | 2.059E+04 | 363.1 | - | - | 0 | - |
| - | - | 6536 | 363.2 | - | - | 0 | - |
| - | - | 1398 | 363.7 | - | - | 0 | - |
| - | - | 3516 | 364.1 | - | - | 0 | - |
| - | - | 3275 | 364.1 | - | - | 0 | - |
| - | - | 1885 | 366.2 | - | - | 0 | - |
| - | - | 1425 | 367.1 | - | - | 0 | - |
| - | - | 1516 | 367.2 | - | - | 0 | - |
| - | - | 1456 | 367.2 | - | - | 0 | - |
| - | - | 1085 | 368.2 | - | - | 0 | - |
| - | - | 1218 | 369.1 | - | - | 0 | - |
| - | - | 1141 | 370.1 | - | - | 0 | - |
| - | - | 802.1 | 370.2 | - | - | 0 | - |
| - | - | 1519 | 373.2 | - | - | 0 | - |
| - | - | 1845 | 376.2 | - | - | 0 | - |
| - | - | 4923 | 376.2 | - | - | 0 | - |
| - | - | 1334 | 376.7 | - | - | 0 | - |
| - | - | 1352 | 377.1 | - | - | 0 | - |
| - | - | 7963 | 378.2 | - | - | 0 | - |
| - | - | 3.071E+04 | 379.2 | - | - | 0 | - |
| - | - | 1098 | 380.2 | - | - | 0 | - |
| - | - | 7215 | 380.2 | - | - | 0 | - |
| - | - | 5.88E+04 | 381.2 | - | - | 0 | - |
| - | - | 1989 | 381.7 | - | - | 0 | - |
| - | - | 1.014E+04 | 382.2 | - | - | 0 | - |
| - | - | 2168 | 382.2 | - | - | 0 | - |
| - | - | 1008 | 382.2 | - | - | 0 | - |
| - | - | 1918 | 383.2 | - | - | 0 | - |
| - | - | 945 | 383.2 | - | - | 0 | - |
| - | - | 2008 | 384.2 | - | - | 0 | - |
| - | - | 3193 | 384.2 | - | - | 0 | - |
| - | - | 3608 | 385.2 | - | - | 0 | - |
| 5 | y | 1345 | 385.2 | 0.003735 | 9.696 | +3 | 9 |
| - | - | 1229 | 386.2 | - | - | 0 | - |
| - | - | 2190 | 390.2 | - | - | 0 | - |
| - | - | 807.6 | 390.7 | - | - | 0 | - |
| - | - | 3067 | 391.1 | - | - | 0 | - |
| - | - | 698.8 | 391.2 | - | - | 0 | - |
| - | - | 730.1 | 392.1 | - | - | 0 | - |
| - | - | 8377 | 394.2 | - | - | 0 | - |
| - | - | 2627 | 395.1 | - | - | 0 | - |
| - | - | 3226 | 395.2 | - | - | 0 | - |
| - | - | 621.9 | 395.2 | - | - | 0 | - |
| - | - | 1396 | 395.2 | - | - | 0 | - |
| - | - | 788.4 | 396.1 | - | - | 0 | - |
| - | - | 1.104E+04 | 396.2 | - | - | 0 | - |
| - | - | 742.1 | 397.2 | - | - | 0 | - |
| - | - | 1757 | 397.2 | - | - | 0 | - |
| - | - | 3560 | 398.2 | - | - | 0 | - |
| - | - | 717.8 | 399.2 | - | - | 0 | - |
| - | - | 702.2 | 401.2 | - | - | 0 | - |
| - | - | 690.6 | 401.2 | - | - | 0 | - |
| - | - | 3309 | 402.2 | - | - | 0 | - |
| - | - | 5203 | 402.2 | - | - | 0 | - |
| - | - | 2357 | 403.2 | - | - | 0 | - |
| - | - | 1747 | 404.7 | - | - | 0 | - |
| - | - | 2390 | 405.3 | - | - | 0 | - |
| - | - | 907.3 | 406.2 | - | - | 0 | - |
| - | - | 1159 | 406.2 | - | - | 0 | - |
| - | - | 1153 | 407.2 | - | - | 0 | - |
| - | - | 582.6 | 407.2 | - | - | 0 | - |
| - | - | 715.5 | 408.7 | - | - | 0 | - |
| - | - | 4271 | 409.1 | - | - | 0 | - |
| - | - | 987.7 | 409.2 | - | - | 0 | - |
| - | - | 1333 | 410.1 | - | - | 0 | - |
| - | - | 4728 | 411.2 | - | - | 0 | - |
| - | - | 3.417E+04 | 412.2 | - | - | 0 | - |
| - | - | 4530 | 413.1 | - | - | 0 | - |
| - | - | 1874 | 413.2 | - | - | 0 | - |
| - | - | 4057 | 413.2 | - | - | 0 | - |
| - | - | 921.9 | 413.3 | - | - | 0 | - |
| - | - | 1165 | 414.1 | - | - | 0 | - |
| - | - | 1212 | 414.2 | - | - | 0 | - |
| - | - | 859.1 | 414.2 | - | - | 0 | - |
| - | - | 8655 | 414.2 | - | - | 0 | - |
| - | - | 2.031E+04 | 415 | - | - | 0 | - |
| - | - | 3434 | 415.2 | - | - | 0 | - |
| - | - | 2120 | 415.2 | - | - | 0 | - |
| - | - | 750.9 | 416.2 | - | - | 0 | - |
| - | - | 6457 | 417.7 | - | - | 0 | - |
| - | - | 1.016E+04 | 418.2 | - | - | 0 | - |
| - | - | 4908 | 418.7 | - | - | 0 | - |
| - | - | 1916 | 419.2 | - | - | 0 | - |
| 11 | y | 1398 | 421.2 | 0.001769 | 4.201 | +1 | 3 |
| 11 | y | 938.4 | 422.2 | 0.001457 | 3.452 | +1 | 3 |
| - | - | 759 | 422.3 | - | - | 0 | - |
| - | - | 1044 | 422.7 | - | - | 0 | - |
| - | - | 1694 | 423.2 | - | - | 0 | - |
| - | - | 1.595E+04 | 423.3 | - | - | 0 | - |
| - | - | 747.3 | 423.7 | - | - | 0 | - |
| - | - | 4005 | 424.3 | - | - | 0 | - |
| - | - | 1450 | 424.7 | - | - | 0 | - |
| - | - | 9.478E+04 | 426.7 | - | - | 0 | - |
| - | - | 5.056E+04 | 427.2 | - | - | 0 | - |
| - | - | 707.6 | 427.3 | - | - | 0 | - |
| - | - | 1.299E+04 | 427.7 | - | - | 0 | - |
| - | - | 2996 | 428.2 | - | - | 0 | - |
| - | - | 1.947E+04 | 429.2 | - | - | 0 | - |
| - | - | 8.576E+04 | 430.2 | - | - | 0 | - |
| - | - | 1.915E+04 | 431.2 | - | - | 0 | - |
| 7 | b | 1.079E+04 | 431.7 | 0.0005326 | 1.234 | +2 | 7 |
| 7 | b | 6875 | 432.2 | 0.006968 | 16.12 | +2 | 7 |
| - | - | 2100 | 432.7 | - | - | 0 | - |
| - | - | 1056 | 433 | - | - | 0 | - |
| - | - | 2501 | 433.2 | - | - | 0 | - |
| 4 | b | 3596 | 433.3 | 0.0005677 | 1.31 | +1 | 4 |
| - | - | 1091 | 434.3 | - | - | 0 | - |
| - | - | 1574 | 438.2 | - | - | 0 | - |
| - | - | 1133 | 438.7 | - | - | 0 | - |
| 11 | y | 2.445E+04 | 439.2 | 0.001092 | 2.487 | +1 | 3 |
| - | - | 1003 | 440.2 | - | - | 0 | - |
| - | - | 5145 | 440.2 | - | - | 0 | - |
| 7 | b | 4.604E+04 | 440.7 | 0.001049 | 2.379 | +2 | 7 |
| - | - | 1143 | 440.9 | - | - | 0 | - |
| - | - | 2.612E+04 | 441.2 | - | - | 0 | - |
| - | - | 5873 | 441.7 | - | - | 0 | - |
| - | - | 1476 | 442.2 | - | - | 0 | - |
| - | - | 861.4 | 442.7 | - | - | 0 | - |
| 7 | y | 2672 | 447.2 | 0.002084 | 4.661 | +2 | 7 |
| - | - | 2098 | 447.7 | - | - | 0 | - |
| - | - | 5031 | 448.2 | - | - | 0 | - |
| - | - | 879.1 | 448.3 | - | - | 0 | - |
| - | - | 1775 | 449.2 | - | - | 0 | - |
| - | - | 1464 | 450.2 | - | - | 0 | - |
| - | - | 1159 | 450.3 | - | - | 0 | - |
| 4 | b | 2.392E+04 | 451.3 | 0.001081 | 2.395 | +1 | 4 |
| - | - | 6736 | 452.3 | - | - | 0 | - |
| - | - | 771.2 | 455.2 | - | - | 0 | - |
| - | - | 1865 | 455.3 | - | - | 0 | - |
| 7 | y | 1337 | 455.7 | 0.001719 | 3.771 | +2 | 7 |
| - | - | 912 | 456.2 | - | - | 0 | - |
| - | - | 2957 | 459.2 | - | - | 0 | - |
| - | - | 2053 | 460.8 | - | - | 0 | - |
| - | - | 3553 | 461.2 | - | - | 0 | - |
| - | - | 3268 | 461.3 | - | - | 0 | - |
| - | - | 937.7 | 462.2 | - | - | 0 | - |
| - | - | 841.8 | 464.2 | - | - | 0 | - |
| - | - | 968.3 | 466.2 | - | - | 0 | - |
| - | - | 7032 | 468.3 | - | - | 0 | - |
| - | - | 1655 | 469.3 | - | - | 0 | - |
| - | - | 6641 | 474.3 | - | - | 0 | - |
| - | - | 7534 | 474.8 | - | - | 0 | - |
| - | - | 2981 | 475.3 | - | - | 0 | - |
| - | - | 1682 | 475.8 | - | - | 0 | - |
| - | - | 2179 | 476.2 | - | - | 0 | - |
| - | - | 717.8 | 476.3 | - | - | 0 | - |
| - | - | 4897 | 477.2 | - | - | 0 | - |
| 10 | y | 4793 | 478.2 | 0.0003862 | 0.8075 | +1 | 4 |
| - | - | 3470 | 478.3 | - | - | 0 | - |
| - | - | 1147 | 479.2 | - | - | 0 | - |
| - | - | 1326 | 479.3 | - | - | 0 | - |
| - | - | 1.767E+04 | 483.3 | - | - | 0 | - |
| - | - | 1.282E+04 | 483.8 | - | - | 0 | - |
| - | - | 862.5 | 484.2 | - | - | 0 | - |
| - | - | 3726 | 484.3 | - | - | 0 | - |
| 8 | b | 4598 | 488.3 | 0.001652 | 3.384 | +2 | 8 |
| 8 | b | 2473 | 488.7 | 0.006684 | 13.68 | +2 | 8 |
| - | - | 1302 | 489.3 | - | - | 0 | - |
| - | - | 5.931E+04 | 494.2 | - | - | 0 | - |
| - | - | 1.413E+04 | 495.2 | - | - | 0 | - |
| 10 | y | 7.57E+04 | 496.2 | 0.001021 | 2.058 | +1 | 4 |
| - | - | 1.673E+04 | 497.2 | - | - | 0 | - |
| - | - | 7117 | 497.8 | - | - | 0 | - |
| - | - | 3426 | 498.2 | - | - | 0 | - |
| - | - | 699.5 | 499.9 | - | - | 0 | - |
| - | - | 2.174E+04 | 503.1 | - | - | 0 | - |
| - | - | 2371 | 504.2 | - | - | 0 | - |
| - | - | 2050 | 506.2 | - | - | 0 | - |
| - | - | 2064 | 507.3 | - | - | 0 | - |
| - | - | 3954 | 511.3 | - | - | 0 | - |
| - | - | 1255 | 512.3 | - | - | 0 | - |
| - | - | 759.2 | 513.2 | - | - | 0 | - |
| - | - | 1114 | 515.3 | - | - | 0 | - |
| - | - | 7656 | 519.1 | - | - | 0 | - |
| - | - | 713.1 | 519.3 | - | - | 0 | - |
| - | - | 866.9 | 520.3 | - | - | 0 | - |
| - | - | 786 | 521.2 | - | - | 0 | - |
| - | - | 1482 | 523.3 | - | - | 0 | - |
| - | - | 1451 | 524.3 | - | - | 0 | - |
| - | - | 1.182E+04 | 525.3 | - | - | 0 | - |
| - | - | 1110 | 526.2 | - | - | 0 | - |
| - | - | 2696 | 526.3 | - | - | 0 | - |
| 0 | Precursor | 2959 | 529.3 | 0.002512 | 4.745 | +3 | -1 |
| 0 | Precursor | 2387 | 529.6 | 0.007636 | 14.42 | +3 | -1 |
| - | - | 2141 | 529.9 | - | - | 0 | - |
| - | - | 676.5 | 531.2 | - | - | 0 | - |
| - | - | 2251 | 532.2 | - | - | 0 | - |
| - | - | 807.5 | 533.2 | - | - | 0 | - |
| - | - | 1055 | 534.3 | - | - | 0 | - |
| - | - | 769.5 | 534.6 | - | - | 0 | - |
| - | - | 881.9 | 534.6 | - | - | 0 | - |
| - | - | 1280 | 534.9 | - | - | 0 | - |
| - | - | 889.7 | 535.2 | - | - | 0 | - |
| 0 | Precursor | 3219 | 535.3 | 0.00076 | 1.42 | +3 | -1 |
| - | - | 1161 | 535.6 | - | - | 0 | - |
| - | - | 2575 | 535.6 | - | - | 0 | - |
| - | - | 2151 | 535.9 | - | - | 0 | - |
| - | - | 5160 | 536.2 | - | - | 0 | - |
| - | - | 739.7 | 536.3 | - | - | 0 | - |
| - | - | 993.1 | 537.2 | - | - | 0 | - |
| - | - | 1419 | 537.7 | - | - | 0 | - |
| - | - | 1037 | 538.3 | - | - | 0 | - |
| - | - | 1.143E+04 | 539.2 | - | - | 0 | - |
| - | - | 853 | 539.3 | - | - | 0 | - |
| - | - | 3005 | 540.2 | - | - | 0 | - |
| - | - | 795.9 | 541.8 | - | - | 0 | - |
| - | - | 3757 | 542.3 | - | - | 0 | - |
| - | - | 1.712E+04 | 543.3 | - | - | 0 | - |
| - | - | 5082 | 544.3 | - | - | 0 | - |
| - | - | 1218 | 545.3 | - | - | 0 | - |
| 9 | b | 1119 | 545.8 | 0.002237 | 4.098 | +2 | 9 |
| 9 | b | 2084 | 546.3 | 0.009625 | 17.62 | +2 | 9 |
| - | - | 977.8 | 547.2 | - | - | 0 | - |
| - | - | 2320 | 549.2 | - | - | 0 | - |
| - | - | 1060 | 550.2 | - | - | 0 | - |
| - | - | 2310 | 551.3 | - | - | 0 | - |
| - | - | 1866 | 551.3 | - | - | 0 | - |
| - | - | 1236 | 552.3 | - | - | 0 | - |
| - | - | 1016 | 553.3 | - | - | 0 | - |
| 9 | b | 2114 | 554.8 | 0.004101 | 7.392 | +2 | 9 |
| - | - | 4799 | 555.3 | - | - | 0 | - |
| - | - | 3492 | 555.8 | - | - | 0 | - |
| 5 | b | 2319 | 561.3 | 0.001499 | 2.671 | +1 | 5 |
| 5 | b | 815.8 | 562.3 | 0.006375 | 11.34 | +1 | 5 |
| - | - | 1889 | 563.3 | - | - | 0 | - |
| - | - | 1425 | 564.3 | - | - | 0 | - |
| - | - | 743.7 | 564.3 | - | - | 0 | - |
| - | - | 3841 | 565.3 | - | - | 0 | - |
| - | - | 1157 | 566.3 | - | - | 0 | - |
| - | - | 1.257E+04 | 567.2 | - | - | 0 | - |
| - | - | 3951 | 568.2 | - | - | 0 | - |
| - | - | 3956 | 575.2 | - | - | 0 | - |
| - | - | 760.7 | 575.3 | - | - | 0 | - |
| - | - | 1953 | 576.2 | - | - | 0 | - |
| 5 | b | 1.666E+04 | 579.3 | 0.0008224 | 1.42 | +1 | 5 |
| - | - | 5994 | 580.3 | - | - | 0 | - |
| - | - | 1189 | 581.3 | - | - | 0 | - |
| - | - | 637.5 | 589.3 | - | - | 0 | - |
| - | - | 2396 | 592.3 | - | - | 0 | - |
| - | - | 3403 | 592.3 | - | - | 0 | - |
| 9 | y | 2.122E+04 | 593.3 | 0.0006343 | 1.069 | +1 | 5 |
| - | - | 7055 | 593.3 | - | - | 0 | - |
| - | - | 6648 | 594.3 | - | - | 0 | - |
| - | - | 1601 | 594.3 | - | - | 0 | - |
| - | - | 855.5 | 595.2 | - | - | 0 | - |
| - | - | 802.1 | 595.3 | - | - | 0 | - |
| - | - | 947.1 | 596.4 | - | - | 0 | - |
| - | - | 900.7 | 597.3 | - | - | 0 | - |
| - | - | 1748 | 597.8 | - | - | 0 | - |
| - | - | 1146 | 600.3 | - | - | 0 | - |
| - | - | 1932 | 602.3 | - | - | 0 | - |
| - | - | 3297 | 602.8 | - | - | 0 | - |
| - | - | 4279 | 603.3 | - | - | 0 | - |
| - | - | 1216 | 603.8 | - | - | 0 | - |
| - | - | 4.745E+04 | 610.3 | - | - | 0 | - |
| - | - | 2090 | 610.3 | - | - | 0 | - |
| 9 | y | 2.295E+05 | 611.3 | 0.001727 | 2.826 | +1 | 5 |
| - | - | 1.182E+04 | 611.8 | - | - | 0 | - |
| - | - | 7.038E+04 | 612.3 | - | - | 0 | - |
| - | - | 2978 | 612.8 | - | - | 0 | - |
| - | - | 1.734E+04 | 613.3 | - | - | 0 | - |
| - | - | 2513 | 614.3 | - | - | 0 | - |
| - | - | 846.4 | 617.3 | - | - | 0 | - |
| - | - | 1632 | 620.8 | - | - | 0 | - |
| - | - | 3994 | 621.3 | - | - | 0 | - |
| - | - | 1820 | 621.3 | - | - | 0 | - |
| - | - | 1461 | 622.3 | - | - | 0 | - |
| - | - | 1336 | 622.4 | - | - | 0 | - |
| - | - | 622.8 | 629.3 | - | - | 0 | - |
| - | - | 777.4 | 630.3 | - | - | 0 | - |
| - | - | 2780 | 634.3 | - | - | 0 | - |
| - | - | 2757 | 635.3 | - | - | 0 | - |
| - | - | 1544 | 636.3 | - | - | 0 | - |
| 4 | y | 2660 | 636.8 | 0.001405 | 2.206 | +2 | 10 |
| 4 | y | 2173 | 637.3 | 0.009885 | 15.51 | +2 | 10 |
| - | - | 924.4 | 637.3 | - | - | 0 | - |
| - | - | 2389 | 640.3 | - | - | 0 | - |
| - | - | 746.4 | 644.3 | - | - | 0 | - |
| - | - | 4053 | 645.3 | - | - | 0 | - |
| 4 | y | 1.511E+04 | 645.8 | 0.002226 | 3.447 | +2 | 10 |
| - | - | 9223 | 646.3 | - | - | 0 | - |
| - | - | 2554 | 646.8 | - | - | 0 | - |
| - | - | 765.8 | 647.3 | - | - | 0 | - |
| 11 | b | 817.6 | 647.3 | 0.004275 | 6.604 | +2 | 11 |
| - | - | 913.9 | 648.3 | - | - | 0 | - |
| - | - | 1824 | 650.3 | - | - | 0 | - |
| - | - | 1460 | 650.3 | - | - | 0 | - |
| - | - | 2.435E+04 | 652.3 | - | - | 0 | - |
| - | - | 8705 | 653.3 | - | - | 0 | - |
| - | - | 1534 | 654.3 | - | - | 0 | - |
| - | - | 1023 | 658.3 | - | - | 0 | - |
| - | - | 2221 | 660.9 | - | - | 0 | - |
| - | - | 1166 | 661.3 | - | - | 0 | - |
| - | - | 1145 | 661.4 | - | - | 0 | - |
| - | - | 7492 | 662.3 | - | - | 0 | - |
| - | - | 743 | 662.8 | - | - | 0 | - |
| - | - | 6051 | 663.3 | - | - | 0 | - |
| - | - | 942.5 | 664.3 | - | - | 0 | - |
| - | - | 1136 | 664.3 | - | - | 0 | - |
| - | - | 661.9 | 666.4 | - | - | 0 | - |
| - | - | 834.8 | 668.3 | - | - | 0 | - |
| - | - | 836.8 | 675.8 | - | - | 0 | - |
| 6 | b | 1.202E+04 | 676.3 | 0.001107 | 1.636 | +1 | 6 |
| 6 | b | 7946 | 677.3 | 0.0108 | 15.95 | +1 | 6 |
| - | - | 1010 | 677.8 | - | - | 0 | - |
| - | - | 2240 | 678.3 | - | - | 0 | - |
| - | - | 1821 | 679.3 | - | - | 0 | - |
| - | - | 4.469E+04 | 680.3 | - | - | 0 | - |
| - | - | 1.842E+04 | 681.3 | - | - | 0 | - |
| - | - | 3980 | 682.3 | - | - | 0 | - |
| - | - | 1034 | 684.3 | - | - | 0 | - |
| - | - | 3436 | 684.8 | - | - | 0 | - |
| - | - | 1963 | 685.3 | - | - | 0 | - |
| - | - | 1060 | 685.8 | - | - | 0 | - |
| - | - | 1903 | 688.3 | - | - | 0 | - |
| - | - | 2709 | 692.8 | - | - | 0 | - |
| 3 | y | 1.352E+04 | 693.3 | 0.00228 | 3.289 | +2 | 11 |
| 3 | y | 1.141E+04 | 693.8 | 0.008319 | 11.99 | +2 | 11 |
| 6 | b | 1.194E+05 | 694.4 | 0.0003687 | 0.5309 | +1 | 6 |
| - | - | 2993 | 694.8 | - | - | 0 | - |
| - | - | 4.718E+04 | 695.4 | - | - | 0 | - |
| - | - | 1.076E+04 | 696.4 | - | - | 0 | - |
| - | - | 1612 | 697.4 | - | - | 0 | - |
| - | - | 901.7 | 697.9 | - | - | 0 | - |
| - | - | 1390 | 698.4 | - | - | 0 | - |
| - | - | 800 | 700.9 | - | - | 0 | - |
| - | - | 1489 | 701.3 | - | - | 0 | - |
| - | - | 2.187E+04 | 701.8 | - | - | 0 | - |
| 3 | y | 8.272E+04 | 702.3 | 0.002613 | 3.721 | +2 | 11 |
| 12 | b | 6.662E+04 | 702.8 | 0.01253 | 17.83 | +2 | 12 |
| 12 | b | 3.119E+04 | 703.3 | 0.003137 | 4.46 | +2 | 12 |
| - | - | 8579 | 703.8 | - | - | 0 | - |
| - | - | 4029 | 704.3 | - | - | 0 | - |
| - | - | 1424 | 705.4 | - | - | 0 | - |
| 8 | y | 7753 | 706.3 | 0.002508 | 3.55 | +1 | 6 |
| - | - | 4132 | 707.3 | - | - | 0 | - |
| - | - | 1023 | 708.3 | - | - | 0 | - |
| - | - | 1702 | 710.4 | - | - | 0 | - |
| - | - | 967 | 710.9 | - | - | 0 | - |
| - | - | 4982 | 711.4 | - | - | 0 | - |
| 12 | b | 6996 | 711.9 | 0.00312 | 4.383 | +2 | 12 |
| - | - | 7378 | 712.4 | - | - | 0 | - |
| - | - | 2789 | 712.9 | - | - | 0 | - |
| - | - | 1188 | 713.4 | - | - | 0 | - |
| - | - | 766.8 | 716.3 | - | - | 0 | - |
| - | - | 1009 | 718.4 | - | - | 0 | - |
| - | - | 1029 | 720.4 | - | - | 0 | - |
| - | - | 5552 | 721.4 | - | - | 0 | - |
| - | - | 2411 | 722.4 | - | - | 0 | - |
| - | - | 1.314E+04 | 723.4 | - | - | 0 | - |
| 8 | y | 7.082E+04 | 724.4 | 0.002075 | 2.865 | +1 | 6 |
| - | - | 2.943E+04 | 725.4 | - | - | 0 | - |
| - | - | 7366 | 726.4 | - | - | 0 | - |
| - | - | 808.4 | 727.4 | - | - | 0 | - |
| - | - | 1102 | 728.4 | - | - | 0 | - |
| - | - | 2159 | 729.4 | - | - | 0 | - |
| - | - | 745.2 | 729.5 | - | - | 0 | - |
| - | - | 1576 | 730.4 | - | - | 0 | - |
| - | - | 3761 | 733.4 | - | - | 0 | - |
| - | - | 2821 | 734.4 | - | - | 0 | - |
| 2 | y | 1115 | 742.9 | 0.002619 | 3.526 | +2 | 12 |
| 2 | y | 2380 | 743.4 | 0.01427 | 19.2 | +2 | 12 |
| - | - | 776.5 | 743.9 | - | - | 0 | - |
| - | - | 1003 | 744.4 | - | - | 0 | - |
| - | - | 1858 | 747.4 | - | - | 0 | - |
| - | - | 2462 | 748.4 | - | - | 0 | - |
| - | - | 1764 | 749.3 | - | - | 0 | - |
| - | - | 900.9 | 750.4 | - | - | 0 | - |
| - | - | 1.327E+04 | 751.4 | - | - | 0 | - |
| 2 | y | 6776 | 751.9 | 0.002647 | 3.521 | +2 | 12 |
| - | - | 1.36E+04 | 752.4 | - | - | 0 | - |
| - | - | 2934 | 752.9 | - | - | 0 | - |
| - | - | 2496 | 753.4 | - | - | 0 | - |
| - | - | 902.7 | 755.4 | - | - | 0 | - |
| - | - | 1350 | 761.4 | - | - | 0 | - |
| - | - | 1652 | 762.4 | - | - | 0 | - |
| - | - | 7072 | 763.4 | - | - | 0 | - |
| - | - | 3755 | 764.4 | - | - | 0 | - |
| - | - | 2708 | 765.4 | - | - | 0 | - |
| - | - | 1683 | 766.4 | - | - | 0 | - |
| - | - | 2959 | 767.3 | - | - | 0 | - |
| - | - | 929.2 | 767.4 | - | - | 0 | - |
| - | - | 1015 | 768.3 | - | - | 0 | - |
| - | - | 1466 | 769.4 | - | - | 0 | - |
| - | - | 1150 | 775.4 | - | - | 0 | - |
| - | - | 1097 | 776.4 | - | - | 0 | - |
| - | - | 8026 | 779.4 | - | - | 0 | - |
| - | - | 3507 | 780.4 | - | - | 0 | - |
| - | - | 3501 | 781.4 | - | - | 0 | - |
| - | - | 1898 | 782.4 | - | - | 0 | - |
| 0 | Precursor | 1.326E+04 | 793.4 | 0.01449 | 18.26 | +2 | -1 |
| - | - | 4548 | 794.4 | - | - | 0 | - |
| - | - | 3176 | 795.3 | - | - | 0 | - |
| - | - | 916.3 | 795.4 | - | - | 0 | - |
| - | - | 1247 | 796.3 | - | - | 0 | - |
| - | - | 3852 | 807.4 | - | - | 0 | - |
| - | - | 1715 | 807.4 | - | - | 0 | - |
| - | - | 1090 | 808.4 | - | - | 0 | - |
| - | - | 1765 | 818.4 | - | - | 0 | - |
| - | - | 1612 | 819.4 | - | - | 0 | - |
| - | - | 860.3 | 820.4 | - | - | 0 | - |
| - | - | 1252 | 825.4 | - | - | 0 | - |
| - | - | 1822 | 826.4 | - | - | 0 | - |
| - | - | 740.4 | 827.4 | - | - | 0 | - |
| - | - | 1826 | 834.4 | - | - | 0 | - |
| - | - | 4122 | 835.4 | - | - | 0 | - |
| - | - | 7887 | 836.5 | - | - | 0 | - |
| - | - | 4736 | 837.5 | - | - | 0 | - |
| - | - | 1799 | 838.5 | - | - | 0 | - |
| - | - | 1663 | 843.4 | - | - | 0 | - |
| - | - | 2972 | 844.4 | - | - | 0 | - |
| - | - | 2419 | 845.4 | - | - | 0 | - |
| - | - | 3481 | 846.4 | - | - | 0 | - |
| - | - | 2757 | 847.4 | - | - | 0 | - |
| - | - | 1575 | 848.4 | - | - | 0 | - |
| - | - | 2.837E+04 | 852.4 | - | - | 0 | - |
| - | - | 1.473E+04 | 853.4 | - | - | 0 | - |
| - | - | 4611 | 854.4 | - | - | 0 | - |
| 7 | b | 1.726E+04 | 862.4 | 0.000346 | 0.4012 | +1 | 7 |
| 7 | b | 1.34E+04 | 863.4 | 0.01297 | 15.03 | +1 | 7 |
| - | - | 2.055E+04 | 864.5 | - | - | 0 | - |
| - | - | 1.308E+04 | 865.5 | - | - | 0 | - |
| - | - | 3619 | 866.5 | - | - | 0 | - |
| - | - | 1116 | 869.4 | - | - | 0 | - |
| - | - | 2781 | 876.4 | - | - | 0 | - |
| - | - | 901.7 | 877.4 | - | - | 0 | - |
| 7 | b | 1.428E+05 | 880.4 | 0.0003404 | 0.3866 | +1 | 7 |
| - | - | 7.637E+04 | 881.4 | - | - | 0 | - |
| - | - | 2.448E+04 | 882.4 | - | - | 0 | - |
| - | - | 3727 | 883.4 | - | - | 0 | - |
| - | - | 1029 | 884.4 | - | - | 0 | - |
| - | - | 1129 | 890.4 | - | - | 0 | - |
| - | - | 1692 | 891.4 | - | - | 0 | - |
| - | - | 2775 | 892.5 | - | - | 0 | - |
| - | - | 1186 | 893.5 | - | - | 0 | - |
| - | - | 925.8 | 894.5 | - | - | 0 | - |
| - | - | 1427 | 895.5 | - | - | 0 | - |
| - | - | 1802 | 907.4 | - | - | 0 | - |
| - | - | 9881 | 908.4 | - | - | 0 | - |
| - | - | 7822 | 909.4 | - | - | 0 | - |
| 7 | y | 1.161E+04 | 910.4 | 0.002108 | 2.315 | +1 | 7 |
| - | - | 3272 | 911.4 | - | - | 0 | - |
| - | - | 1857 | 912.4 | - | - | 0 | - |
| - | - | 1104 | 936.4 | - | - | 0 | - |
| - | - | 1130 | 937.4 | - | - | 0 | - |
| - | - | 1162 | 938.4 | - | - | 0 | - |
| - | - | 987.6 | 947.4 | - | - | 0 | - |
| - | - | 2987 | 954.4 | - | - | 0 | - |
| - | - | 3493 | 955.4 | - | - | 0 | - |
| - | - | 1882 | 956.4 | - | - | 0 | - |
| - | - | 891.5 | 962.4 | - | - | 0 | - |
| - | - | 1095 | 964.5 | - | - | 0 | - |
| - | - | 3506 | 965.4 | - | - | 0 | - |
| - | - | 1813 | 966.5 | - | - | 0 | - |
| - | - | 928.3 | 971.5 | - | - | 0 | - |
| - | - | 2465 | 972.4 | - | - | 0 | - |
| - | - | 1356 | 973.4 | - | - | 0 | - |
| - | - | 916.1 | 974.4 | - | - | 0 | - |
| 8 | b | 1773 | 975.5 | 0.001365 | 1.399 | +1 | 8 |
| 8 | b | 1185 | 976.5 | 0.0179 | 18.33 | +1 | 8 |
| - | - | 814.3 | 981.5 | - | - | 0 | - |
| - | - | 1373 | 982.5 | - | - | 0 | - |
| 8 | b | 8568 | 993.5 | 0.0005659 | 0.5696 | +1 | 8 |
| - | - | 4537 | 994.5 | - | - | 0 | - |
| - | - | 2806 | 995.5 | - | - | 0 | - |
| - | - | 778.6 | 1007 | - | - | 0 | - |
| - | - | 1107 | 1024 | - | - | 0 | - |
| 6 | y | 4594 | 1025 | 0.003363 | 3.279 | +1 | 8 |
| - | - | 2996 | 1026 | - | - | 0 | - |
| - | - | 809 | 1027 | - | - | 0 | - |
| - | - | 935.4 | 1065 | - | - | 0 | - |
| - | - | 755.1 | 1076 | - | - | 0 | - |
| - | - | 1117 | 1077 | - | - | 0 | - |
| - | - | 3343 | 1094 | - | - | 0 | - |
| - | - | 2219 | 1095 | - | - | 0 | - |
| - | - | 946.4 | 1096 | - | - | 0 | - |
| 9 | b | 736.5 | 1109 | 0.002737 | 2.469 | +1 | 9 |
| - | - | 1109 | 1117 | - | - | 0 | - |
| - | - | 1611 | 1118 | - | - | 0 | - |
| - | - | 809.4 | 1119 | - | - | 0 | - |
| 5 | y | 5505 | 1136 | 0.001981 | 1.745 | +1 | 9 |
| 5 | y | 7009 | 1136 | 0.008322 | 7.322 | +1 | 9 |
| - | - | 3565 | 1137 | - | - | 0 | - |
| - | - | 1516 | 1139 | - | - | 0 | - |
| - | - | 2642 | 1153 | - | - | 0 | - |
| 5 | y | 1.091E+04 | 1154 | 0.001914 | 1.66 | +1 | 9 |
| - | - | 6003 | 1155 | - | - | 0 | - |
| - | - | 2527 | 1156 | - | - | 0 | - |
| - | - | 1017 | 1157 | - | - | 0 | - |
| 10 | b | 1961 | 1166 | 0.006053 | 5.193 | +1 | 10 |
| - | - | 1064 | 1183 | - | - | 0 | - |
| - | - | 659.2 | 3081 | - | - | 0 | - |

m/z Charge Intensity FragmentType MassShift Position
120.0811767578125 0 4691.317
121.0843505859375 0 663.5456
121.21499633789062 0 350.9592
122.0716323852539 0 1573.3623
122.14251708984375 0 454.56314
123.04443359375 0 2165.8308
123.0555648803711 0 634.40845
123.39301300048828 0 418.7689
123.74199676513672 0 358.2154
125.10765838623047 0 891.017
126.09171295166016 0 811.1962
126.1018295288086 0 362.02988
127.05062866210938 0 4844.75
127.08773040771484 0 444.74646
127.1233901977539 0 905.0133
128.1073760986328 0 22426.666
129.1026611328125 0 236773.33
130.06552124023438 0 105439.76
130.09982299804688 0 1433.76
130.10598754882812 0 15643.422
131.06886291503906 0 11040.29
131.11846923828125 0 635.88495
132.08116149902344 0 23011.816
132.53701782226562 0 518.3584
133.06112670898438 0 948.06683
133.08460998535156 0 2257.2227
136.0760955810547 0 30735.977
137.07948303222656 0 2617.5789
138.06658935546875 0 3254.1182
138.09176635742188 0 1601.6487
139.0508575439453 0 500.9774
139.08700561523438 0 3123.0786
141.10267639160156 0 4554.7734
142.0655059814453 0 1389.7577
143.04547119140625 0 1757.6964
144.0815887451172 0 1085.4393
145.06101989746094 0 2304.1836
145.08518981933594 0 501.33444
146.060302734375 0 1253.2208
147.04449462890625 0 1493.587
147.07699584960938 0 425.4708 y 10
148.94796752929688 0 814.6537
149.04525756835938 0 12498.76
150.066650390625 0 1614.0977
151.0869140625 0 5012.447
152.09080505371094 0 551.58276
153.10250854492188 0 688.4255
154.09750366210938 0 560.19366
155.08203125 0 539.42786
155.09310913085938 0 12753.498
155.11827087402344 0 11221.993 a Water loss 1
156.0769805908203 0 1150.0275
156.09637451171875 0 988.5332
156.12158203125 0 644.7496
157.06100463867188 0 788.26263
157.0975799560547 0 1271.6581
157.13356018066406 0 697.39734
158.08424377441406 0 1281.5643
159.09213256835938 0 181487.88
160.07598876953125 0 1452.7112
160.0890655517578 0 1053.0791
160.095458984375 0 19250.166
161.09881591796875 0 438.09744
165.0550079345703 0 16325.157
166.0614471435547 0 27493.16
166.0867156982422 0 2818.864 y 9
166.09783935546875 0 1744.3608
167.05589294433594 0 16373.978
167.06504821777344 0 2369.7212
167.0816192626953 0 742.1531
167.11842346191406 0 1233.5715
167.28375244140625 0 566.6411
167.6746063232422 0 429.2287
168.0552978515625 0 971.1789
168.08123779296875 0 3221.0488
168.10220336914062 0 1574.7073
168.11361694335938 0 2270.3835
169.07638549804688 0 2220.2173
169.0975799560547 0 6730.7764
170.0605010986328 0 8240.221
171.0640106201172 0 677.3386
171.0767822265625 0 733.72314
171.09205627441406 0 1671.3356
171.1497039794922 0 1966.1989
172.07211303710938 0 3501.8503
173.0560760498047 0 3992.9539
173.12893676757812 0 359225.72 a 1
173.43882751464844 0 1111.7494
174.05477905273438 0 627.7878
174.13226318359375 0 30821.621
175.13381958007812 0 2470.0542
176.0373077392578 0 466.93774
177.1029510498047 0 1247.0677
177.11183166503906 0 484.7291
178.13436889648438 0 11198.525
179.13751220703125 0 886.6557
181.06134033203125 0 1895.6725
182.0816192626953 0 35122.51 y 12
183.08517456054688 0 2975.005
183.11325073242188 0 10152.891 b Water loss 1
184.11679077148438 0 1075.8584
185.0924530029297 0 653.0006
185.1654510498047 0 2506.2925
186.1241455078125 0 72305.27
187.0869903564453 0 15599.751
187.1077117919922 0 666.4602
187.1276092529297 0 6023.566
187.14443969726562 0 524.0586
188.0710906982422 0 1605.9396
188.09046936035156 0 1579.208
189.0990753173828 0 714.8705
190.08267211914062 0 3424.2327
190.13441467285156 0 2313.6067
193.09754943847656 0 676.73895
194.09278869628906 0 2322.398
195.1132354736328 0 18389.865
196.09805297851562 0 746.2537
196.10845947265625 0 12606.412
196.11666870117188 0 1220.7552
197.07086181640625 0 661.1352
197.11158752441406 0 1005.62946
197.12876892089844 0 834.63544
198.08779907226562 0 2459.0017
198.12803649902344 0 6402.5083
199.07154846191406 0 1241.4612
199.0872039794922 0 1437.8384
199.13186645507812 0 1593.247
199.14437866210938 0 923.06024
200.1435546875 0 1193.7812
201.12376403808594 0 97681.805 b 1
202.0870819091797 0 1240.4355
202.12705993652344 0 9605.764
203.10304260253906 0 1271.3114
203.1294403076172 0 1164.9175
204.1129913330078 0 973.5487
205.09759521484375 0 8332.297
205.107666015625 0 885.0795
205.14547729492188 0 1357.0664
206.1015167236328 0 769.77844
206.12892150878906 0 2204.3062
207.160888671875 0 3142.5686
208.1087646484375 0 609.64026
209.05648803710938 0 1045.2213
209.0929718017578 0 665.0885
210.1275177001953 0 3518.5085
211.10833740234375 0 2318.2988 y Water loss 10
212.13980102539062 0 2581.098
213.1238250732422 0 8387.068
213.160400390625 0 1416.8756
214.1277618408203 0 1024.3645
215.11956787109375 0 633.8684
215.1393280029297 0 1703.0394
216.09820556640625 0 1773.4623
221.059326171875 0 1945.638
221.07220458984375 0 953.8733
221.08489990234375 0 5824.856
221.1039581298828 0 1445.8615
221.14002990722656 0 1025.6644
222.08541870117188 0 2031.082
222.12435913085938 0 3001.6365
223.0643310546875 0 3359.3044
223.08148193359375 0 973.5643
223.10806274414062 0 5381.135
223.1194305419922 0 1314.1958
223.1558074951172 0 32021.412
224.1034393310547 0 1935.0234
224.15919494628906 0 3742.5503
225.04344177246094 0 7653.168
225.1237335205078 0 685.74554
226.04345703125 0 1852.1412
226.08282470703125 0 8040.226
226.15602111816406 0 931.13245
227.02264404296875 0 799.58484
227.04010009765625 0 1447.0896
227.06668090820312 0 6396.3174
227.1537628173828 0 1033.8383
228.07080078125 0 704.3121
228.09808349609375 0 682.4842
228.13487243652344 0 2169.1335
229.11878967285156 0 7332.1064
230.15109252929688 0 1043.4242
233.1400909423828 0 5059.4053
234.12425231933594 0 38001.633
234.65093994140625 0 748.6178
235.12765502929688 0 4661.89
235.6050567626953 0 977.08545
237.1348114013672 0 1862.7429
238.11915588378906 0 4251.795
238.1300811767578 0 880.5254
239.08233642578125 0 563.5052
239.09547424316406 0 14149.678
240.09640502929688 0 4865.4995
240.13479614257812 0 13752.279
241.09329223632812 0 2850.6204
241.11862182617188 0 1968.6555
241.137939453125 0 2131.498
241.19125366210938 0 1561.0593
243.1098175048828 0 2993.7915
244.0933074951172 0 15507.599
244.61099243164062 0 589.3363
245.09642028808594 0 1066.0197
247.10841369628906 0 18098
247.14437866210938 0 1413.4443
248.10313415527344 0 2355.2695
248.11407470703125 0 8123.097
249.0987548828125 0 11800.523
249.13504028320312 0 3040.8203
250.1023712158203 0 1402.1151
250.11793518066406 0 628.4947
250.16795349121094 0 677.92346
250.3199005126953 0 553.0735
251.00486755371094 0 635.9418
251.15084838867188 0 121043.49
252.1363067626953 0 632.45715
252.15406799316406 0 13639.22
253.1192626953125 0 712.47284
253.1555938720703 0 1571.1523
255.1480255126953 0 6789.842
256.10833740234375 0 1617.4545
256.15203857421875 0 907.36017
256.6114196777344 0 1329.1244
257.1135559082031 0 3205.1196
258.1453857421875 0 5189.687
259.1480407714844 0 975.0636
261.1197204589844 0 2861.697
261.134521484375 0 919.8282
262.1195068359375 0 1357.1339
265.1297302246094 0 14502.981
266.1139831542969 0 3613.0432
266.1253662109375 0 28842.99
267.0770263671875 0 759.4328
267.12847900390625 0 3286.1665
267.1448059082031 0 1014.5262
268.1295471191406 0 1407.1243
268.1772766113281 0 3413.6892
269.16131591796875 0 1397.9792
269.18560791015625 0 1733.3582
270.1220397949219 0 4668.516
272.17620849609375 0 7325.5835
273.1800231933594 0 1500.5778
274.1188659667969 0 6925.006
274.1312561035156 0 1667.1814
275.10308837890625 0 1577.221
275.121337890625 0 1441.5986
275.1499328613281 0 717.536
276.10919189453125 0 1423.6339
276.1269226074219 0 2119.8604
276.155517578125 0 2138.6382
279.14581298828125 0 2882.5864
280.1298828125 0 3105.274
281.0517883300781 0 9480.836
282.1568908691406 0 6666.2886
282.6683044433594 0 829.44507
283.0312194824219 0 1798.4498
283.140625 0 87899.74
284.1036682128906 0 3215.4917
284.1233215332031 0 799.7218
284.1435852050781 0 11500.552
285.0887145996094 0 694.2304
285.1060485839844 0 1018.408
285.1431579589844 0 1152.7922
285.1585693359375 0 728.65405
286.14031982421875 0 5584.127
287.1418151855469 0 825.42944
287.1716613769531 0 1760.8793
288.1278991699219 0 571.38367
288.6188659667969 0 1712.9069
293.1138000488281 0 46607.74 y Water loss 11
293.1618957519531 0 1476.7555
294.1178283691406 0 9562.57
295.1036682128906 0 1245.078
295.1204833984375 0 1129.6914
296.10394287109375 0 1185.6123
296.1977233886719 0 1122.5758 b Water loss 2
297.0833740234375 0 1635.8042
297.1013488769531 0 834.9014
297.1339111328125 0 1525.2378 y Water loss 8
297.1564025878906 0 17315.883
297.20001220703125 0 747.7951
298.1396789550781 0 1499.0641 y Water loss 6
298.15863037109375 0 1602.9937
299.0623779296875 0 91366.23
300.06292724609375 0 2548.024
300.16815185546875 0 11734.954
301.06072998046875 0 1624.201
301.1170959472656 0 999.35144
301.1512756347656 0 43390.08
301.1683349609375 0 1465.5953
302.1143798828125 0 8016.492
302.1545715332031 0 5699.0234
303.11822509765625 0 1278.4045
303.1685791015625 0 604.6916
305.1799621582031 0 1021.44806
305.64617919921875 0 1991.286
306.1380310058594 0 6905.373 y 8
306.2287902832031 0 642.8047
306.6398010253906 0 3436.5747
307.14031982421875 0 1857.0289
307.2318420410156 0 534.8017
309.1667175292969 0 600.018
311.12408447265625 0 6064.489 y 11
311.1376647949219 0 2904.6445
311.6877746582031 0 608.39
312.1279296875 0 1555.9181
314.1143493652344 0 732.47925
314.18701171875 0 1880.0978
314.2083740234375 0 3944.3877 b 2
315.1669921875 0 24537.814
316.1706237792969 0 4352.4004
317.65948486328125 0 890.06604
318.1197509765625 0 605.4985
319.15191650390625 0 1797.1908
324.1706848144531 0 595.04755
325.15093994140625 0 1508.0294
326.0198974609375 0 579.53156
326.6645812988281 0 6703.885
326.9665222167969 0 628.00934
327.1658630371094 0 2557.387
332.208740234375 0 2564.0469
333.17742919921875 0 4962.449
334.18511962890625 0 982.4087
336.0015563964844 0 575.31586
338.6749267578125 0 1091.885 b Water loss 5
339.171630859375 0 1028.6539 b Ammonia loss 5
339.6761474609375 0 1157.5765
340.6617431640625 0 2795.66
341.0185852050781 0 704.27386
341.16387939453125 0 1242.2761
342.1820068359375 0 2445.038
343.1617736816406 0 1626.6083
343.1847839355469 0 835.3513
344.17218017578125 0 1965.3647
344.9772033691406 0 10977.75
344.9999084472656 0 726.5555
345.1313781738281 0 3800.396
346.1152648925781 0 13403.497
346.1374206542969 0 980.9954
347.1182861328125 0 2581.1921
347.6802978515625 0 7019.259 b 5
348.1818542480469 0 2453.9028
348.68218994140625 0 691.0358
349.1513366699219 0 887.80005
350.2193603515625 0 11865.747
351.1671447753906 0 974.172
351.2214050292969 0 2519.9802
352.1653747558594 0 786.06433
352.6713562011719 0 1816.6654
353.67437744140625 0 2093.7288 y Water loss 7
354.1762390136719 0 1679.5315
355.070068359375 0 1676.48
359.029052734375 0 9757.049
359.134765625 0 871.6104
360.203369140625 0 709.75464
360.6954040527344 0 2763.6042
361.1976013183594 0 4299.9336
361.6866455078125 0 915.4111
362.1856689453125 0 5333.4595
362.6801452636719 0 17248.738 y 7
363.1416931152344 0 20590.715
363.1819763183594 0 6536.4907
363.6824951171875 0 1397.94
364.12530517578125 0 3516.3267
364.1454772949219 0 3274.8118
366.17816162109375 0 1885.3181
367.1398620605469 0 1424.6761
367.1619567871094 0 1515.5074
367.24530029296875 0 1456.4658
368.1932067871094 0 1085.1375
369.12261962890625 0 1217.8967
370.1224365234375 0 1141.3043
370.17620849609375 0 802.12036
373.1854248046875 0 1519.0228
376.1619873046875 0 1845.1149
376.1985778808594 0 4922.664
376.69866943359375 0 1333.5302
377.14556884765625 0 1351.6747
378.2143859863281 0 7963.266
379.2097473144531 0 30706.723
380.1657409667969 0 1098.1349
380.2126159667969 0 7214.5874
381.15240478515625 0 58796.414
381.7120361328125 0 1988.8477
382.1551208496094 0 10143.747
382.18914794921875 0 2168.1733
382.2137145996094 0 1007.77405
383.1581726074219 0 1918.4303
383.20733642578125 0 945.0019
384.16619873046875 0 2007.9121
384.1883850097656 0 3192.758
385.1509704589844 0 3608.1963
385.1731262207031 0 1344.6729 y 4
386.1550598144531 0 1229.1395
390.196044921875 0 2190.3608
390.698486328125 0 807.6151
391.1363220214844 0 3066.5422
391.18670654296875 0 698.768
392.1195373535156 0 730.07526
394.173095703125 0 8377.457
395.13409423828125 0 2627.2866
395.15777587890625 0 3226.1975
395.2136535644531 0 621.8763
395.2415771484375 0 1395.651
396.1380920410156 0 788.3943
396.2250061035156 0 11042.991
397.2034912109375 0 742.1494
397.2283935546875 0 1756.9711
398.1782531738281 0 3560.0366
399.1814270019531 0 717.83093
401.1826477050781 0 702.2011
401.2146301269531 0 690.5999
402.1767883300781 0 3308.7158
402.1996154785156 0 5203.4087
403.201171875 0 2356.7227
404.7115783691406 0 1746.6919
405.2616271972656 0 2390.2268
406.2110595703125 0 907.2981
406.2451477050781 0 1158.6077
407.2043762207031 0 1153.0826
407.2437744140625 0 582.5587
408.71221923828125 0 715.51135
409.1475524902344 0 4271.1953
409.2075500488281 0 987.72003
410.14849853515625 0 1332.5809
411.199462890625 0 4727.58
412.18359375 0 34170.51
413.1454772949219 0 4529.697
413.1638488769531 0 1873.5231
413.18804931640625 0 4057.2334
413.2516174316406 0 921.9242
414.1488342285156 0 1164.9792
414.1942443847656 0 1211.7921
414.199951171875 0 859.0846
414.2354431152344 0 8655.301
415.0375671386719 0 20314.562
415.1988525390625 0 3434.0205
415.23883056640625 0 2119.9578
416.20086669921875 0 750.9443
417.71734619140625 0 6456.7715
418.21112060546875 0 10156.376
418.71240234375 0 4907.6094
419.2132568359375 0 1916.3959
421.2099304199219 0 1398.4022 y Water loss 10
422.1936340332031 0 938.4496 y Ammonia loss 10
422.2537536621094 0 759.0268
422.7073059082031 0 1043.6455
423.2041015625 0 1694.1802
423.2723083496094 0 15953.364
423.7030334472656 0 747.326
424.27557373046875 0 4004.5671
424.7351989746094 0 1449.7885
426.7226867675781 0 94782.89
427.2240295410156 0 50559.74
427.2586364746094 0 707.6397
427.7254638671875 0 12985.723
428.2253723144531 0 2995.5369
429.210205078125 0 19474.377
430.1943664550781 0 85758.8
431.1973876953125 0 19151.477
431.7144775390625 0 10790.941 b Water loss 6
432.2129211425781 0 6875.4185 b Ammonia loss 6
432.7146911621094 0 2100.2686
433.049560546875 0 1056.373
433.2091369628906 0 2500.7761
433.25634765625 0 3595.603 b Water loss 3
434.2597351074219 0 1090.5114
438.1727600097656 0 1574.1504
438.7308044433594 0 1133.4563
439.2198181152344 0 24451.572 y 10
440.18463134765625 0 1003.3677
440.2227478027344 0 5144.994
440.72027587890625 0 46039.99 b 6
440.91778564453125 0 1143.1488
441.2216491699219 0 26122.406
441.7222900390625 0 5873.0024
442.2244567871094 0 1475.8746
442.7210693359375 0 861.374
447.20770263671875 0 2672.4 y Ammonia loss 6
447.7081604003906 0 2098.034
448.20489501953125 0 5030.8037
448.2648010253906 0 879.0675
449.2091064453125 0 1775.352
450.23583984375 0 1464.4849
450.2826232910156 0 1159.0381
451.2674255371094 0 23918.236 b 3
452.2703552246094 0 6736.2397
455.19647216796875 0 771.24835
455.2660217285156 0 1865.2369
455.7206115722656 0 1337.4535 y 6
456.2191467285156 0 911.9896
459.1993103027344 0 2957.083
460.7528991699219 0 2053.1528
461.2043762207031 0 3553.2603
461.2539367675781 0 3268.077
462.20819091796875 0 937.73145
464.2171325683594 0 841.7606
466.24310302734375 0 968.2761
468.2939758300781 0 7032.3467
469.2977600097656 0 1654.5299
474.2599182128906 0 6641.436
474.75537109375 0 7533.6636
475.2546081542969 0 2981.1846
475.756103515625 0 1681.6414
476.22564697265625 0 2179.3557
476.2608642578125 0 717.80164
477.2111511230469 0 4897.418
478.2300109863281 0 4793.1504 y Water loss 9
478.27813720703125 0 3469.6145
479.23822021484375 0 1146.855
479.2834167480469 0 1326.1304
483.2648010253906 0 17674.494
483.7660827636719 0 12816.167
484.21966552734375 0 862.54553
484.26666259765625 0 3726.4702
488.25762939453125 0 4597.6343 b Water loss 7
488.7546691894531 0 2473.006 b Ammonia loss 7
489.25933837890625 0 1302.0481
494.2366638183594 0 59307.77
495.23956298828125 0 14134.481
496.2412109375 0 75698.56 y 9
497.24652099609375 0 16734.9
497.7634582519531 0 7117.119
498.245361328125 0 3425.9822
499.928955078125 0 699.5477
503.1085205078125 0 21744.572
504.2008972167969 0 2371.443
506.22900390625 0 2049.731
507.2580871582031 0 2064.3108
511.2642822265625 0 3954.4175
512.26806640625 0 1255.3501
513.2081298828125 0 759.19025
515.2857666015625 0 1114.2445
519.1397094726562 0 7656.27
519.3277587890625 0 713.14386
520.2661743164062 0 866.8682
521.2232666015625 0 786.0059
523.2603759765625 0 1481.5657
524.2835693359375 0 1451.09
525.267578125 0 11820.495
526.2335815429688 0 1109.7256
526.2694091796875 0 2696.3352
529.26220703125 0 2959.2917 Precursor Water loss
529.5953369140625 0 2387.231 Precursor Ammonia loss
529.929443359375 0 2141.4924
531.219970703125 0 676.522
532.1959838867188 0 2251.2368
533.1971435546875 0 807.4803
534.3058471679688 0 1055.0299
534.55419921875 0 769.5139
534.634765625 0 881.90485
534.8897094726562 0 1280.0117
535.2232055664062 0 889.6899
535.2639770507812 0 3218.688 Precursor
535.5562133789062 0 1160.8201
535.5992431640625 0 2575.3367
535.9354248046875 0 2150.9678
536.1659545898438 0 5159.9727
536.2637329101562 0 739.72095
537.2440795898438 0 993.1087
537.7384643554688 0 1418.7906
538.2724609375 0 1036.7245
539.2366333007812 0 11428.111
539.2820434570312 0 852.967
540.239990234375 0 3004.795
541.7594604492188 0 795.8975
542.2942504882812 0 3757.421
543.2781982421875 0 17117.324
544.2816772460938 0 5081.9434
545.284423828125 0 1218.4238
545.7672119140625 0 1118.983 b Water loss 8
546.2518310546875 0 2083.877 b Ammonia loss 8
547.2470703125 0 977.8203
549.2202758789062 0 2319.8464
550.2101440429688 0 1060.4606
551.2576904296875 0 2310.3457
551.3306884765625 0 1865.9608
552.2604370117188 0 1235.5045
553.2655639648438 0 1015.90967
554.7706298828125 0 2114.007 b 8
555.2568359375 0 4798.9624
555.7571411132812 0 3492.2603
561.3158569335938 0 2318.943 b Water loss 4
562.3047485351562 0 815.7724 b Ammonia loss 4
563.2588500976562 0 1888.8473
564.2637939453125 0 1425.4393
564.32763671875 0 743.67523
565.2644653320312 0 3841.438
566.2665405273438 0 1157.0275
567.2319946289062 0 12566.308
568.2369995117188 0 3951.4775
575.2481079101562 0 3956.084
575.2931518554688 0 760.65466
576.2385864257812 0 1953.4817
579.3257446289062 0 16657.945 b 4
580.3279418945312 0 5994.279
581.3309936523438 0 1188.8849
589.3094482421875 0 637.48267
592.2723388671875 0 2396.142
592.3253173828125 0 3402.5913
593.2572021484375 0 21224.691 y Water loss 8
593.3062133789062 0 7055.1245
594.2551879882812 0 6647.718
594.309814453125 0 1601.4973
595.2272338867188 0 855.45746
595.26611328125 0 802.07715
596.3533325195312 0 947.1164
597.2986450195312 0 900.72485
597.8027954101562 0 1748.4344
600.3159790039062 0 1145.7177
602.2994384765625 0 1932.4064
602.792724609375 0 3296.596
603.2924194335938 0 4279.4707
603.7935791015625 0 1216.1348
610.2838745117188 0 47453.79
610.3276977539062 0 2089.9348
611.2688598632812 0 229492.45 y 8
611.798828125 0 11816.659
612.2720947265625 0 70376.15
612.8002319335938 0 2977.6426
613.2739868164062 0 17337.744
614.2769165039062 0 2513.0286
617.288818359375 0 846.41144
620.804931640625 0 1632.2692
621.2522583007812 0 3994.1323
621.3033447265625 0 1820.4471
622.2559814453125 0 1461.1357
622.3685302734375 0 1336.0852
629.29931640625 0 622.78107
630.2901611328125 0 777.36694
634.3070068359375 0 2780.184
635.2965698242188 0 2756.8171
636.327392578125 0 1543.5651
636.7872314453125 0 2659.9424 y Water loss 3
637.2877197265625 0 2173.3862 y Ammonia loss 3
637.33984375 0 924.43304
640.2750244140625 0 2389.1067
644.29736328125 0 746.41486
645.296630859375 0 4052.7976
645.7933349609375 0 15108.249 y 3
646.2945556640625 0 9223.454
646.7942504882812 0 2554.0754
647.28662109375 0 765.81793
647.3372192382812 0 817.6249 b 10
648.3421630859375 0 913.9256
650.2703857421875 0 1823.9645
650.3272705078125 0 1459.6696
652.3209838867188 0 24350.855
653.323486328125 0 8705.275
654.3219604492188 0 1534.0266
658.2822875976562 0 1022.80396
660.8733520507812 0 2220.8428
661.3259887695312 0 1165.5272
661.38232421875 0 1144.7954
662.3057250976562 0 7491.7417
662.8274536132812 0 742.97266
663.2994384765625 0 6050.9053
664.2913208007812 0 942.4932
664.3472900390625 0 1136.1743
666.36083984375 0 661.9468
668.283447265625 0 834.823
675.8099975585938 0 836.7538
676.3424072265625 0 12016.918 b Water loss 5
677.3361206054688 0 7945.8447 b Ammonia loss 5
677.8064575195312 0 1010
678.3261108398438 0 2240.0547
679.3328857421875 0 1821.0642
680.3162841796875 0 44694.164
681.3184204101562 0 18416.137
682.3201904296875 0 3980.3896
684.3184814453125 0 1034.4318
684.8189697265625 0 3435.8142
685.3173828125 0 1962.661
685.8209228515625 0 1060.1323
688.3345336914062 0 1903.4294
692.8361206054688 0 2708.5203
693.3301391601562 0 13521.668 y Water loss 2
693.8281860351562 0 11414.457 y Ammonia loss 2
694.3522338867188 0 119359.08 b 5
694.8282470703125 0 2992.5771
695.3551025390625 0 47182.996
696.3579711914062 0 10759.138
697.3507690429688 0 1611.699
697.8607177734375 0 901.70197
698.3500366210938 0 1390.4531
700.8980102539062 0 800.01154
701.3405151367188 0 1488.9387
701.841796875 0 21874.967
702.3357543945312 0 82721.26 y 2
702.83642578125 0 66615.55 b Water loss 11
703.3378295898438 0 31187.377 b Ammonia loss 11
703.8396606445312 0 8578.54
704.3378295898438 0 4029.2336
705.3580322265625 0 1424.296
706.3431396484375 0 7752.591 y Water loss 7
707.3451538085938 0 4131.8296
708.307861328125 0 1022.5425
710.4055786132812 0 1701.9766
710.9064331054688 0 966.95294
711.370361328125 0 4981.6694
711.8573608398438 0 6995.5547 b 11
712.3583374023438 0 7378.275
712.8592529296875 0 2788.8806
713.3572387695312 0 1188.0854
716.34228515625 0 766.8417
718.387451171875 0 1009.4202
720.375 0 1028.969
721.3635864257812 0 5551.6294
722.3660888671875 0 2410.637
723.3684692382812 0 13142.235
724.353271484375 0 70819.7 y 7
725.3557739257812 0 29430.49
726.358642578125 0 7365.515
727.3579711914062 0 808.3893
728.3736572265625 0 1102.1869
729.3605346679688 0 2158.5803
729.4525146484375 0 745.2138
730.3617553710938 0 1575.7147
733.3639526367188 0 3761.2778
734.3565063476562 0 2820.932
742.8646850585938 0 1115.1709 y Water loss 1
743.3683471679688 0 2380.2979 y Ammonia loss 1
743.859375 0 776.4891
744.37939453125 0 1003.14154
747.3930053710938 0 1857.9452
748.3771362304688 0 2461.566
749.33740234375 0 1763.9866
750.391357421875 0 900.9304
751.376953125 0 13268.917
751.8699951171875 0 6775.8965 y 1
752.3744506835938 0 13596.129
752.8716430664062 0 2933.6855
753.3758544921875 0 2496.0984
755.4257202148438 0 902.73376
761.3776245117188 0 1350.0875
762.4169921875 0 1651.7123
763.3668212890625 0 7071.6997
764.3679809570312 0 3755.0706
765.4043579101562 0 2708.4912
766.4057006835938 0 1683.3633
767.3486328125 0 2959.1821
767.4187622070312 0 929.1736
768.3474731445312 0 1015.0852
769.3992919921875 0 1465.5759
775.3956909179688 0 1149.6986
776.378662109375 0 1097.1614
779.3840942382812 0 8025.8125
780.3869018554688 0 3507.2378
781.3700561523438 0 3501.1936
782.3683471679688 0 1897.8635
793.400390625 0 13260.637 Precursor Water loss
794.4019165039062 0 4548.2705
795.3434448242188 0 3176.1528
795.4168090820312 0 916.3455
796.3442993164062 0 1246.9287
807.3776245117188 0 3851.5
807.445068359375 0 1714.6438
808.3696899414062 0 1089.8291
818.44189453125 0 1765.0276
819.4417724609375 0 1612.3031
820.438232421875 0 860.274
825.3941650390625 0 1252.3431
826.3789672851562 0 1821.8444
827.3777465820312 0 740.37537
834.4254760742188 0 1826.1935
835.4174194335938 0 4122.381
836.4503173828125 0 7887.2524
837.4575805664062 0 4736.1294
838.4508056640625 0 1798.937
843.4019165039062 0 1663.4962
844.3912353515625 0 2971.6155
845.3955688476562 0 2419.4546
846.4474487304688 0 3480.8442
847.44482421875 0 2756.8767
848.4466552734375 0 1574.5845
852.4368896484375 0 28371.027
853.4391479492188 0 14734.347
854.4423217773438 0 4610.8022
862.4209594726562 0 17261.068 b Water loss 6
863.4176025390625 0 13398.28 b Ammonia loss 6
864.4564819335938 0 20548.17
865.45947265625 0 13079.098
866.4618530273438 0 3618.7058
869.4093017578125 0 1115.741
876.439208984375 0 2780.8013
877.4351196289062 0 901.6569
880.4315185546875 0 142830.61 b 6
881.43408203125 0 76366.06
882.43701171875 0 24475.56
883.437255859375 0 3726.643
884.426513671875 0 1028.5685
890.4122314453125 0 1128.82
891.4107666015625 0 1691.6223
892.4644165039062 0 2774.579
893.4801635742188 0 1185.9662
894.4519653320312 0 925.781
895.524658203125 0 1427.1833
907.4423217773438 0 1802.2798
908.4282836914062 0 9880.947
909.432861328125 0 7822.4004
910.4326171875 0 11609.628 y 6
911.434814453125 0 3272.0603
912.4390258789062 0 1857.3713
936.4203491210938 0 1104.397
937.4345092773438 0 1129.9828
938.4176635742188 0 1161.8003
947.4298095703125 0 987.6484
954.42919921875 0 2986.81
955.4208374023438 0 3493.4436
956.4249877929688 0 1881.5377
962.4432373046875 0 891.5178
964.4635620117188 0 1095.4558
965.4483032226562 0 3505.9055
966.452392578125 0 1813.3383
971.4573974609375 0 928.3464
972.4493408203125 0 2464.9414
973.443359375 0 1356.0236
974.4489135742188 0 916.1074
975.5060424804688 0 1772.7163 b Water loss 7
976.506591796875 0 1185.2943 b Ammonia loss 7
981.46337890625 0 814.34467
982.4736328125 0 1372.8744
993.5158081054688 0 8567.56 b 7
994.517578125 0 4536.682
995.5185546875 0 2805.561
1007.4802856445312 0 778.6046
1024.475341796875 0 1106.7285
1025.4608154296875 0 4594.1597 y 5
1026.4615478515625 0 2995.692
1027.4576416015625 0 808.9994
1064.5115966796875 0 935.4115
1075.532958984375 0 755.09045
1076.5406494140625 0 1117.3785
1093.5450439453125 0 3342.6106
1094.54833984375 0 2219.3535
1095.550048828125 0 946.4336
1108.544921875 0 736.45404 b 8
1117.49755859375 0 1108.547
1118.485107421875 0 1610.9995
1119.4857177734375 0 809.36414
1135.5074462890625 0 5504.5513 y Water loss 4
1136.497802734375 0 7008.837 y Ammonia loss 4
1137.49609375 0 3565.2854
1138.5028076171875 0 1516.157
1152.5362548828125 0 2641.5117
1153.5179443359375 0 10909.179 y 4
1154.5205078125 0 6002.5913
1155.5230712890625 0 2527.2986
1156.51708984375 0 1017.22504
1165.5697021484375 0 1961.4661 b 9
1182.590576171875 0 1063.5142
3081.01513671875 0 659.1839

Spectrum Details

|  |  |
| --- | --- |
| Matched peaks? Matched peaksThe total absolute number of peaks matched. Additionally in brackets the total fraction of peaks matched and the total number of peaks is shown. | 78 (9.18% of 850) |
| FDR? FDRThe false discovery rate estimated for this peptide. It is calculated by matching all theoretical fragments with a non-integer shift with the raw peaks for this spectrum. This is done with 40 different shifts. The resulting percentage is the average number of annotated peaks over the number of annotated peaks with the correct spectrum. | 1.01% |
| Satellite FDR? Satellite FDRSee the FDR for details on its calculation. This satellite ion specific FDR only contains the satellite ions (d/w) for I/L/J positions. | ∞ |
| PSM Score? PSM ScoreThe PSM Score as given by Hecklib to this annotated spectrum. It is shown with three significant figures. | 583 |

## Spectrum 8106? Spectrum 8106 The raw spectrum of this peptide as annotated by Hecklib. The fragments are coloured according to ion type (see legend). Any peaks with a star '\*' as text can be hovered over to see the full details, first the ion type second the mass shift type. By hovering over the amino acids in the peptide or ions in the legend the corresponding peaks are highlighted. By toggling the 'Unassigned' label you can turn the background (unassigned) peaks on or off in the plot. By updating the slider in the Ion legend you can update the spectrum to only show the top X% of the peaks with labels. The top X% means any peak that is within X% of the highest intensity. By dragging in the spectrum you can zoom in to a specific part of the spectrum and use 'Zoom Out' to get back to the original zoom level. The annotation of the spectrum is based on the given sequence in the peptides file and is done with different software so inconsistencies are likely. The peaks are annotated based on the given sequence, with 20 ppm tolerance.

Copy Data

### Spectrum 8106 (TSV)

#### Preview

```
Loading example...
```

*Click on the button to copy the data to your clipboard.*

Mz MinMz MaxIntensity Max

WidthHeightPeptide font sizePeptide stroke widthSpectrum font sizeSpectrum stroke widthCompact peptide

Ion legend

wxyz

abcd

OtherUnassignedIonChargePositionShow for top:%

TVLHQDWLDGKEY

01.90e+43.80e+45.70e+47.60e+4

Zoom Out

y+11c+12y+12z+12y+12c+13y+26z+13y+13c+27c+14z+14y+14w+15c+15y+15z+15c+15y+15y+210c+211w+16w+211y+211y+211c+16y+211z+16c+16c+212c+212y+16y+212z+212y+212c+17z+17c+17y+17z+18c+18y+18w+19c+19y+19z+19y+19c+110y+110z+110y+110c+111y+111z+111c+112c+112z+112

0750150122513001

Fragment Matches Table

Show background peaks

| Position | Ion type | Intensity | mz Theoretical | mz Error (Th) | mz Error (ppm) | Charge | Series Number |
| --- | --- | --- | --- | --- | --- | --- | --- |
| - | - | 621.1 | 120.1 | - | - | 0 | - |
| - | - | 1361 | 129.1 | - | - | 0 | - |
| - | - | 2466 | 130.1 | - | - | 0 | - |
| - | - | 779.8 | 149 | - | - | 0 | - |
| - | - | 557.9 | 149 | - | - | 0 | - |
| - | - | 5279 | 149 | - | - | 0 | - |
| - | - | 476.5 | 151 | - | - | 0 | - |
| - | - | 427.9 | 155 | - | - | 0 | - |
| - | - | 515.6 | 155.1 | - | - | 0 | - |
| - | - | 6502 | 155.1 | - | - | 0 | - |
| - | - | 720.1 | 156.1 | - | - | 0 | - |
| - | - | 501.2 | 157.1 | - | - | 0 | - |
| - | - | 423.1 | 158.3 | - | - | 0 | - |
| - | - | 2299 | 159.1 | - | - | 0 | - |
| - | - | 473.2 | 159.1 | - | - | 0 | - |
| - | - | 478.7 | 160.9 | - | - | 0 | - |
| - | - | 465.5 | 166.5 | - | - | 0 | - |
| - | - | 2668 | 167.1 | - | - | 0 | - |
| - | - | 1.094E+04 | 173.1 | - | - | 0 | - |
| - | - | 606.3 | 174.1 | - | - | 0 | - |
| 13 | y | 2496 | 182.1 | 2.344E-05 | 0.1287 | +1 | 1 |
| - | - | 970.5 | 186.1 | - | - | 0 | - |
| - | - | 8072 | 201.1 | - | - | 0 | - |
| - | - | 895 | 202.1 | - | - | 0 | - |
| - | - | 2333 | 215.1 | - | - | 0 | - |
| 2 | c | 2110 | 218.1 | 3.086E-05 | 0.1415 | +1 | 2 |
| - | - | 9047 | 221.1 | - | - | 0 | - |
| - | - | 2447 | 222.1 | - | - | 0 | - |
| - | - | 2804 | 223.1 | - | - | 0 | - |
| - | - | 1230 | 223.1 | - | - | 0 | - |
| - | - | 2897 | 225 | - | - | 0 | - |
| - | - | 684.8 | 226 | - | - | 0 | - |
| - | - | 944.9 | 229.2 | - | - | 0 | - |
| - | - | 1.108E+04 | 239.1 | - | - | 0 | - |
| - | - | 3580 | 240.1 | - | - | 0 | - |
| - | - | 2634 | 241.1 | - | - | 0 | - |
| - | - | 540.8 | 242.1 | - | - | 0 | - |
| - | - | 1123 | 243.1 | - | - | 0 | - |
| - | - | 955.7 | 245.1 | - | - | 0 | - |
| - | - | 1520 | 251.2 | - | - | 0 | - |
| - | - | 4372 | 258.1 | - | - | 0 | - |
| - | - | 647.5 | 259.1 | - | - | 0 | - |
| - | - | 1056 | 260.2 | - | - | 0 | - |
| - | - | 3203 | 268.2 | - | - | 0 | - |
| - | - | 587.8 | 280.6 | - | - | 0 | - |
| - | - | 1.776E+04 | 281.1 | - | - | 0 | - |
| 12 | y | 1395 | 293.1 | 0.0003273 | 1.117 | +1 | 2 |
| 12 | z | 2709 | 295.1 | 0.001737 | 5.885 | +1 | 2 |
| - | - | 2650 | 296.1 | - | - | 0 | - |
| - | - | 4151 | 297.1 | - | - | 0 | - |
| - | - | 1612 | 297.1 | - | - | 0 | - |
| - | - | 7.526E+04 | 299.1 | - | - | 0 | - |
| - | - | 1223 | 300.1 | - | - | 0 | - |
| - | - | 842.6 | 301.1 | - | - | 0 | - |
| 12 | y | 965.5 | 311.1 | 0.0002912 | 0.9359 | +1 | 2 |
| - | - | 904.1 | 314.2 | - | - | 0 | - |
| - | - | 5816 | 317.2 | - | - | 0 | - |
| - | - | 1176 | 318.2 | - | - | 0 | - |
| - | - | 786.9 | 320.2 | - | - | 0 | - |
| 3 | c | 3292 | 331.2 | 3.654E-06 | 0.01103 | +1 | 3 |
| - | - | 1239 | 341 | - | - | 0 | - |
| - | - | 553.9 | 348.3 | - | - | 0 | - |
| - | - | 2011 | 355.1 | - | - | 0 | - |
| - | - | 708.5 | 356.1 | - | - | 0 | - |
| - | - | 6189 | 359 | - | - | 0 | - |
| 8 | y | 1090 | 362.7 | 0.001136 | 3.132 | +2 | 6 |
| - | - | 519.5 | 367.8 | - | - | 0 | - |
| - | - | 1498 | 369.1 | - | - | 0 | - |
| - | - | 2918 | 370.1 | - | - | 0 | - |
| - | - | 2225 | 371.1 | - | - | 0 | - |
| - | - | 714 | 374.2 | - | - | 0 | - |
| - | - | 626.8 | 378.2 | - | - | 0 | - |
| - | - | 2.534E+04 | 415 | - | - | 0 | - |
| - | - | 566.2 | 417.7 | - | - | 0 | - |
| - | - | 565.3 | 418.2 | - | - | 0 | - |
| 11 | z | 1168 | 423.2 | 0.0002546 | 0.6017 | +1 | 3 |
| - | - | 3568 | 423.3 | - | - | 0 | - |
| - | - | 1143 | 424.3 | - | - | 0 | - |
| - | - | 4451 | 426.7 | - | - | 0 | - |
| - | - | 2232 | 427.2 | - | - | 0 | - |
| - | - | 747.6 | 427.7 | - | - | 0 | - |
| - | - | 1223 | 429.1 | - | - | 0 | - |
| - | - | 825.2 | 429.2 | - | - | 0 | - |
| - | - | 1089 | 430.2 | - | - | 0 | - |
| - | - | 671.9 | 431.7 | - | - | 0 | - |
| - | - | 601.4 | 432.2 | - | - | 0 | - |
| - | - | 656.2 | 433 | - | - | 0 | - |
| - | - | 855.1 | 438.7 | - | - | 0 | - |
| 11 | y | 940.2 | 439.2 | 0.0007387 | 1.682 | +1 | 3 |
| 7 | c | 3611 | 440.7 | 1.955E-05 | 0.04437 | +2 | 7 |
| - | - | 1798 | 441.2 | - | - | 0 | - |
| - | - | 7668 | 451.3 | - | - | 0 | - |
| - | - | 1382 | 452.3 | - | - | 0 | - |
| 4 | c | 2.873E+04 | 468.3 | 0.0001665 | 0.3556 | +1 | 4 |
| - | - | 7414 | 469.3 | - | - | 0 | - |
| - | - | 1316 | 470.3 | - | - | 0 | - |
| - | - | 613.3 | 471.4 | - | - | 0 | - |
| 10 | z | 3123 | 480.2 | 0.0008856 | 1.844 | +1 | 4 |
| - | - | 789.9 | 481.2 | - | - | 0 | - |
| - | - | 950.6 | 483.3 | - | - | 0 | - |
| - | - | 658.2 | 483.8 | - | - | 0 | - |
| - | - | 671.7 | 489.2 | - | - | 0 | - |
| - | - | 3089 | 494.3 | - | - | 0 | - |
| - | - | 705 | 495.2 | - | - | 0 | - |
| - | - | 630.6 | 495.3 | - | - | 0 | - |
| 10 | y | 7996 | 496.2 | 0.0001993 | 0.4016 | +1 | 4 |
| - | - | 2317 | 497.2 | - | - | 0 | - |
| - | - | 707.2 | 497.8 | - | - | 0 | - |
| - | - | 6.45E+04 | 503.1 | - | - | 0 | - |
| - | - | 2.394E+04 | 519.1 | - | - | 0 | - |
| - | - | 1054 | 529.3 | - | - | 0 | - |
| - | - | 738 | 534.3 | - | - | 0 | - |
| - | - | 1.416E+04 | 536.2 | - | - | 0 | - |
| - | - | 2536 | 538.3 | - | - | 0 | - |
| - | - | 1097 | 539.3 | - | - | 0 | - |
| 9 | w | 3646 | 550.3 | 0.0006496 | 1.181 | +1 | 5 |
| - | - | 2602 | 551.3 | - | - | 0 | - |
| - | - | 723.5 | 551.3 | - | - | 0 | - |
| 5 | c | 996 | 579.3 | 0.0002152 | 0.3714 | +1 | 5 |
| 9 | y | 969.1 | 593.3 | 0.002234 | 3.766 | +1 | 5 |
| - | - | 1361 | 594.3 | - | - | 0 | - |
| 9 | z | 3115 | 595.2 | 0.0007981 | 1.341 | +1 | 5 |
| - | - | 1409 | 596.3 | - | - | 0 | - |
| 5 | c | 2.1E+04 | 596.4 | 3.087E-05 | 0.05177 | +1 | 5 |
| - | - | 5465 | 597.4 | - | - | 0 | - |
| - | - | 1152 | 598.4 | - | - | 0 | - |
| - | - | 4627 | 610.3 | - | - | 0 | - |
| 9 | y | 9747 | 611.3 | 0.0009339 | 1.528 | +1 | 5 |
| - | - | 3263 | 612.3 | - | - | 0 | - |
| - | - | 843.2 | 621.3 | - | - | 0 | - |
| - | - | 1221 | 633.8 | - | - | 0 | - |
| - | - | 775.6 | 645.3 | - | - | 0 | - |
| 4 | y | 860.6 | 645.8 | 0.00168 | 2.602 | +2 | 10 |
| - | - | 867.1 | 646.3 | - | - | 0 | - |
| - | - | 775.7 | 648.3 | - | - | 0 | - |
| - | - | 1750 | 654.8 | - | - | 0 | - |
| - | - | 6987 | 655.4 | - | - | 0 | - |
| 11 | c | 3.154E+04 | 655.8 | 0.001132 | 1.727 | +2 | 11 |
| - | - | 2.477E+04 | 656.3 | - | - | 0 | - |
| - | - | 8638 | 656.8 | - | - | 0 | - |
| - | - | 3472 | 657.4 | - | - | 0 | - |
| - | - | 5593 | 664.3 | - | - | 0 | - |
| - | - | 1035 | 664.3 | - | - | 0 | - |
| 8 | w | 5021 | 665.3 | 0.003126 | 4.698 | +1 | 6 |
| - | - | 1132 | 666.3 | - | - | 0 | - |
| - | - | 752.5 | 666.4 | - | - | 0 | - |
| - | - | 1426 | 667.4 | - | - | 0 | - |
| - | - | 981.3 | 668.4 | - | - | 0 | - |
| - | - | 1933 | 668.9 | - | - | 0 | - |
| - | - | 1355 | 669.9 | - | - | 0 | - |
| - | - | 2009 | 672.3 | - | - | 0 | - |
| 3 | w | 2775 | 672.8 | 0.003779 | 5.617 | +2 | 11 |
| - | - | 3246 | 673.3 | - | - | 0 | - |
| - | - | 1023 | 673.8 | - | - | 0 | - |
| - | - | 1224 | 680.3 | - | - | 0 | - |
| - | - | 621.3 | 688.3 | - | - | 0 | - |
| - | - | 671.9 | 690.3 | - | - | 0 | - |
| 3 | y | 830.4 | 693.3 | 0.006675 | 9.627 | +2 | 11 |
| 3 | y | 1448 | 693.8 | 0.005695 | 8.208 | +2 | 11 |
| 6 | c | 2920 | 694.4 | 0.01269 | 18.28 | +1 | 6 |
| - | - | 1503 | 694.8 | - | - | 0 | - |
| - | - | 2106 | 695.4 | - | - | 0 | - |
| - | - | 809.7 | 698.4 | - | - | 0 | - |
| - | - | 3091 | 701.8 | - | - | 0 | - |
| 3 | y | 7023 | 702.3 | 0.000477 | 0.6792 | +2 | 11 |
| - | - | 2980 | 702.8 | - | - | 0 | - |
| - | - | 1996 | 703.3 | - | - | 0 | - |
| - | - | 2797 | 707.3 | - | - | 0 | - |
| 8 | z | 4477 | 708.3 | 0.004991 | 7.046 | +1 | 6 |
| - | - | 1138 | 709.3 | - | - | 0 | - |
| 6 | c | 1.72E+04 | 711.4 | 0.0005457 | 0.7671 | +1 | 6 |
| 12 | c | 672.6 | 711.9 | 0.01203 | 16.9 | +2 | 12 |
| - | - | 5669 | 712.4 | - | - | 0 | - |
| - | - | 1792 | 713.4 | - | - | 0 | - |
| - | - | 2524 | 718.9 | - | - | 0 | - |
| - | - | 1032 | 719.4 | - | - | 0 | - |
| - | - | 457.5 | 719.8 | - | - | 0 | - |
| - | - | 1.143E+04 | 719.9 | - | - | 0 | - |
| - | - | 706.1 | 719.9 | - | - | 0 | - |
| 12 | c | 2.301E+04 | 720.4 | 0.003395 | 4.713 | +2 | 12 |
| - | - | 1.461E+04 | 720.9 | - | - | 0 | - |
| - | - | 7854 | 721.4 | - | - | 0 | - |
| - | - | 1530 | 721.9 | - | - | 0 | - |
| - | - | 1378 | 723.4 | - | - | 0 | - |
| 8 | y | 4299 | 724.4 | 0.00177 | 2.443 | +1 | 6 |
| - | - | 1554 | 725.4 | - | - | 0 | - |
| - | - | 843.3 | 726.4 | - | - | 0 | - |
| - | - | 1317 | 729.9 | - | - | 0 | - |
| - | - | 677.7 | 730.4 | - | - | 0 | - |
| - | - | 1847 | 736.9 | - | - | 0 | - |
| - | - | 5443 | 737.4 | - | - | 0 | - |
| - | - | 983 | 737.9 | - | - | 0 | - |
| - | - | 912.1 | 741.4 | - | - | 0 | - |
| - | - | 796 | 742.9 | - | - | 0 | - |
| 2 | y | 728.2 | 743.4 | 0.006522 | 8.774 | +2 | 12 |
| 2 | z | 1316 | 743.9 | 0.007248 | 9.744 | +2 | 12 |
| - | - | 1481 | 744.4 | - | - | 0 | - |
| - | - | 628.4 | 748.1 | - | - | 0 | - |
| - | - | 5326 | 748.9 | - | - | 0 | - |
| - | - | 5619 | 749.4 | - | - | 0 | - |
| - | - | 3213 | 749.9 | - | - | 0 | - |
| - | - | 596.8 | 750.4 | - | - | 0 | - |
| - | - | 1471 | 751.4 | - | - | 0 | - |
| 2 | y | 1610 | 751.9 | 0.001731 | 2.303 | +2 | 12 |
| - | - | 856.2 | 758.9 | - | - | 0 | - |
| - | - | 773.6 | 764.9 | - | - | 0 | - |
| - | - | 1187 | 765.4 | - | - | 0 | - |
| - | - | 700.6 | 771.4 | - | - | 0 | - |
| - | - | 1608 | 771.9 | - | - | 0 | - |
| - | - | 2100 | 772.4 | - | - | 0 | - |
| - | - | 4317 | 772.9 | - | - | 0 | - |
| - | - | 3109 | 773.4 | - | - | 0 | - |
| - | - | 878 | 773.9 | - | - | 0 | - |
| - | - | 3690 | 779.9 | - | - | 0 | - |
| - | - | 3472 | 780.4 | - | - | 0 | - |
| - | - | 1126 | 780.9 | - | - | 0 | - |
| - | - | 617.1 | 781.4 | - | - | 0 | - |
| - | - | 1422 | 785.4 | - | - | 0 | - |
| - | - | 1328 | 785.9 | - | - | 0 | - |
| - | - | 2248 | 786.4 | - | - | 0 | - |
| - | - | 1918 | 786.9 | - | - | 0 | - |
| - | - | 2256 | 787.4 | - | - | 0 | - |
| - | - | 1172 | 793.4 | - | - | 0 | - |
| - | - | 8276 | 793.9 | - | - | 0 | - |
| - | - | 1.239E+04 | 794.4 | - | - | 0 | - |
| - | - | 8933 | 794.9 | - | - | 0 | - |
| - | - | 4659 | 795.4 | - | - | 0 | - |
| - | - | 1377 | 795.9 | - | - | 0 | - |
| - | - | 1156 | 800.9 | - | - | 0 | - |
| - | - | 845.9 | 801.3 | - | - | 0 | - |
| - | - | 904.7 | 801.4 | - | - | 0 | - |
| - | - | 943.5 | 801.8 | - | - | 0 | - |
| - | - | 8993 | 801.9 | - | - | 0 | - |
| - | - | 5.343E+04 | 802.4 | - | - | 0 | - |
| - | - | 7.314E+04 | 802.9 | - | - | 0 | - |
| - | - | 4.981E+04 | 803.4 | - | - | 0 | - |
| - | - | 2.183E+04 | 803.9 | - | - | 0 | - |
| - | - | 6316 | 804.4 | - | - | 0 | - |
| - | - | 2787 | 804.9 | - | - | 0 | - |
| - | - | 1457 | 837.4 | - | - | 0 | - |
| - | - | 1836 | 838.4 | - | - | 0 | - |
| - | - | 1329 | 839.4 | - | - | 0 | - |
| - | - | 681.6 | 850.4 | - | - | 0 | - |
| - | - | 797.1 | 852.4 | - | - | 0 | - |
| - | - | 836.8 | 853.4 | - | - | 0 | - |
| - | - | 733.2 | 864.5 | - | - | 0 | - |
| 7 | c | 7084 | 880.4 | 0.001063 | 1.208 | +1 | 7 |
| - | - | 3074 | 881.4 | - | - | 0 | - |
| - | - | 880.8 | 882.4 | - | - | 0 | - |
| - | - | 3607 | 893.4 | - | - | 0 | - |
| 7 | z | 6288 | 894.4 | 0.001972 | 2.205 | +1 | 7 |
| - | - | 1816 | 895.4 | - | - | 0 | - |
| 7 | c | 2.158E+04 | 897.5 | 0.0002688 | 0.2995 | +1 | 7 |
| - | - | 1.001E+04 | 898.5 | - | - | 0 | - |
| - | - | 4039 | 899.5 | - | - | 0 | - |
| - | - | 887.5 | 909.4 | - | - | 0 | - |
| 7 | y | 2773 | 910.4 | 0.003756 | 4.125 | +1 | 7 |
| - | - | 1441 | 911.4 | - | - | 0 | - |
| - | - | 1662 | 964.5 | - | - | 0 | - |
| - | - | 2418 | 965.5 | - | - | 0 | - |
| - | - | 776.8 | 966.5 | - | - | 0 | - |
| - | - | 6115 | 1008 | - | - | 0 | - |
| 6 | z | 1.033E+04 | 1009 | 0.004692 | 4.648 | +1 | 8 |
| - | - | 614 | 1010 | - | - | 0 | - |
| - | - | 4143 | 1010 | - | - | 0 | - |
| 8 | c | 1.729E+04 | 1011 | 0.000384 | 0.38 | +1 | 8 |
| - | - | 947.7 | 1011 | - | - | 0 | - |
| - | - | 8613 | 1012 | - | - | 0 | - |
| - | - | 2720 | 1013 | - | - | 0 | - |
| - | - | 1671 | 1024 | - | - | 0 | - |
| 6 | y | 2911 | 1025 | 0.0113 | 11.02 | +1 | 8 |
| - | - | 1169 | 1026 | - | - | 0 | - |
| - | - | 603.6 | 1063 | - | - | 0 | - |
| - | - | 1079 | 1064 | - | - | 0 | - |
| - | - | 2195 | 1068 | - | - | 0 | - |
| - | - | 2241 | 1068 | - | - | 0 | - |
| - | - | 1333 | 1069 | - | - | 0 | - |
| - | - | 967.5 | 1070 | - | - | 0 | - |
| - | - | 902.6 | 1071 | - | - | 0 | - |
| - | - | 916.6 | 1072 | - | - | 0 | - |
| - | - | 1.106E+04 | 1072 | - | - | 0 | - |
| - | - | 5168 | 1073 | - | - | 0 | - |
| 5 | w | 1079 | 1079 | 0.008057 | 7.464 | +1 | 9 |
| - | - | 953.2 | 1080 | - | - | 0 | - |
| - | - | 1820 | 1081 | - | - | 0 | - |
| - | - | 1819 | 1082 | - | - | 0 | - |
| - | - | 1763 | 1083 | - | - | 0 | - |
| - | - | 904.6 | 1095 | - | - | 0 | - |
| - | - | 2530 | 1125 | - | - | 0 | - |
| 9 | c | 4285 | 1126 | 0.00402 | 3.571 | +1 | 9 |
| - | - | 3196 | 1127 | - | - | 0 | - |
| 5 | y | 5940 | 1136 | 0.02273 | 20 | +1 | 9 |
| 5 | z | 4.188E+04 | 1137 | 0.0001306 | 0.1148 | +1 | 9 |
| - | - | 2.368E+04 | 1139 | - | - | 0 | - |
| - | - | 9569 | 1140 | - | - | 0 | - |
| - | - | 2304 | 1141 | - | - | 0 | - |
| - | - | 896.2 | 1153 | - | - | 0 | - |
| 5 | y | 2129 | 1154 | 0.0046 | 3.988 | +1 | 9 |
| - | - | 1387 | 1155 | - | - | 0 | - |
| - | - | 9571 | 1182 | - | - | 0 | - |
| 10 | c | 2.402E+04 | 1183 | 0.003186 | 2.694 | +1 | 10 |
| - | - | 1.486E+04 | 1184 | - | - | 0 | - |
| - | - | 5647 | 1185 | - | - | 0 | - |
| - | - | 1435 | 1186 | - | - | 0 | - |
| - | - | 1023 | 1187 | - | - | 0 | - |
| - | - | 721.4 | 1224 | - | - | 0 | - |
| - | - | 659.9 | 1266 | - | - | 0 | - |
| - | - | 904 | 1267 | - | - | 0 | - |
| - | - | 869.2 | 1268 | - | - | 0 | - |
| 4 | y | 2012 | 1274 | 0.01606 | 12.61 | +1 | 10 |
| 4 | z | 8545 | 1275 | 0.002986 | 2.343 | +1 | 10 |
| - | - | 7168 | 1276 | - | - | 0 | - |
| - | - | 4165 | 1277 | - | - | 0 | - |
| - | - | 1476 | 1278 | - | - | 0 | - |
| 4 | y | 1024 | 1291 | 0.00123 | 0.9531 | +1 | 10 |
| - | - | 859.9 | 1295 | - | - | 0 | - |
| - | - | 860.5 | 1296 | - | - | 0 | - |
| - | - | 3891 | 1310 | - | - | 0 | - |
| 11 | c | 1.376E+04 | 1311 | 0.001851 | 1.412 | +1 | 11 |
| - | - | 1.16E+04 | 1312 | - | - | 0 | - |
| - | - | 4352 | 1313 | - | - | 0 | - |
| - | - | 1184 | 1314 | - | - | 0 | - |
| 3 | y | 1348 | 1387 | 0.007436 | 5.362 | +1 | 11 |
| 3 | z | 8100 | 1388 | 0.005836 | 4.206 | +1 | 11 |
| - | - | 8303 | 1389 | - | - | 0 | - |
| - | - | 5374 | 1390 | - | - | 0 | - |
| - | - | 1407 | 1391 | - | - | 0 | - |
| - | - | 739.6 | 1395 | - | - | 0 | - |
| - | - | 2093 | 1396 | - | - | 0 | - |
| - | - | 2359 | 1397 | - | - | 0 | - |
| - | - | 833.9 | 1398 | - | - | 0 | - |
| 12 | c | 854.3 | 1423 | 0.01217 | 8.557 | +1 | 12 |
| - | - | 1176 | 1424 | - | - | 0 | - |
| 12 | c | 6486 | 1440 | 0.01041 | 7.227 | +1 | 12 |
| - | - | 7676 | 1441 | - | - | 0 | - |
| - | - | 4460 | 1442 | - | - | 0 | - |
| - | - | 1605 | 1443 | - | - | 0 | - |
| 2 | z | 1052 | 1487 | 0.01567 | 10.54 | +1 | 12 |
| - | - | 1775 | 1488 | - | - | 0 | - |
| - | - | 1540 | 1489 | - | - | 0 | - |
| - | - | 908.6 | 1490 | - | - | 0 | - |
| - | - | 1426 | 1543 | - | - | 0 | - |
| - | - | 928.6 | 1544 | - | - | 0 | - |
| - | - | 2261 | 1545 | - | - | 0 | - |
| - | - | 2420 | 1546 | - | - | 0 | - |
| - | - | 3061 | 1547 | - | - | 0 | - |
| - | - | 828 | 1548 | - | - | 0 | - |
| - | - | 934.1 | 1549 | - | - | 0 | - |
| - | - | 1157 | 1559 | - | - | 0 | - |
| - | - | 4194 | 1560 | - | - | 0 | - |
| - | - | 5867 | 1561 | - | - | 0 | - |
| - | - | 3698 | 1562 | - | - | 0 | - |
| - | - | 1394 | 1563 | - | - | 0 | - |
| - | - | 1600 | 1571 | - | - | 0 | - |
| - | - | 936.7 | 1572 | - | - | 0 | - |
| - | - | 3017 | 1577 | - | - | 0 | - |
| - | - | 2705 | 1578 | - | - | 0 | - |
| - | - | 2657 | 1579 | - | - | 0 | - |
| - | - | 691.5 | 1580 | - | - | 0 | - |
| - | - | 1808 | 1587 | - | - | 0 | - |
| - | - | 1.365E+04 | 1588 | - | - | 0 | - |
| - | - | 2.121E+04 | 1589 | - | - | 0 | - |
| - | - | 960.2 | 1589 | - | - | 0 | - |
| - | - | 1.428E+04 | 1590 | - | - | 0 | - |
| - | - | 6910 | 1591 | - | - | 0 | - |
| - | - | 2652 | 1592 | - | - | 0 | - |
| - | - | 666 | 1603 | - | - | 0 | - |
| - | - | 5943 | 1604 | - | - | 0 | - |
| - | - | 2.158E+04 | 1605 | - | - | 0 | - |
| - | - | 3.848E+04 | 1606 | - | - | 0 | - |
| - | - | 2.616E+04 | 1607 | - | - | 0 | - |
| - | - | 1.228E+04 | 1608 | - | - | 0 | - |
| - | - | 5167 | 1609 | - | - | 0 | - |
| - | - | 2640 | 1610 | - | - | 0 | - |
| - | - | 740.9 | 2195 | - | - | 0 | - |
| - | - | 975.2 | 2963 | - | - | 0 | - |
| - | - | 746.8 | 2972 | - | - | 0 | - |

m/z Charge Intensity FragmentType MassShift Position
120.08094024658203 0 621.14087
129.10238647460938 0 1361.422
130.0651092529297 0 2466.352
148.95416259765625 0 779.7532
149.03929138183594 0 557.92035
149.04495239257812 0 5279.3135
151.0419921875 0 476.47693
155.0412139892578 0 427.90918
155.0870819091797 0 515.6322
155.09275817871094 0 6501.899
156.0960693359375 0 720.10846
157.13369750976562 0 501.1744
158.3282012939453 0 423.05515
159.0916290283203 0 2298.798
159.09869384765625 0 473.15762
160.94061279296875 0 478.68274
166.53457641601562 0 465.48373
167.05538940429688 0 2667.787
173.12844848632812 0 10936.795
174.1326904296875 0 606.3256
182.08114624023438 0 2495.5566 y 12
186.12388610839844 0 970.4724
201.12335205078125 0 8071.7925
202.12623596191406 0 895.01117
215.1389617919922 0 2332.7358
218.14988708496094 0 2110.2258 c 1
221.084228515625 0 9047.383
222.08497619628906 0 2447.0696
223.06346130371094 0 2804.4978
223.0817413330078 0 1229.7097
225.04295349121094 0 2896.766
226.042724609375 0 684.7641
229.15501403808594 0 944.8997
239.09490966796875 0 11084.145
240.09596252441406 0 3579.604
241.091796875 0 2634.4438
242.090576171875 0 540.78595
243.13328552246094 0 1122.6516
245.13645935058594 0 955.68506
251.15011596679688 0 1519.8904
258.1449890136719 0 4372.263
259.1487731933594 0 647.47125
260.15966796875 0 1056.098
268.17669677734375 0 3203.413
280.5548400878906 0 587.82654
281.0511169433594 0 17764.264
293.113525390625 0 1395.001 y Water loss 11
295.1033020019531 0 2708.9167 z 11
296.1038818359375 0 2650.0059
297.0824890136719 0 4151.0728
297.1006164550781 0 1612.125
299.06170654296875 0 75255.08
300.0625 0 1223.2744
301.05877685546875 0 842.6148
311.1240539550781 0 965.5139 y 11
314.2081298828125 0 904.0696
317.1579895019531 0 5816.2876
318.161376953125 0 1176.1477
320.1977844238281 0 786.88226
331.2339782714844 0 3292.1797 c 2
341.017333984375 0 1238.6904
348.253173828125 0 553.853
355.0694580078125 0 2010.816
356.0690002441406 0 708.46954
359.028076171875 0 6188.74
362.6781005859375 0 1089.6324 y 7
367.8006286621094 0 519.51324
369.1227111816406 0 1497.9724
370.1221008300781 0 2917.8037
371.119384765625 0 2224.6528
374.1773681640625 0 713.9685
378.20233154296875 0 626.7545
415.0366516113281 0 25338.28
417.7179260253906 0 566.23224
418.2086181640625 0 565.2646
423.20025634765625 0 1167.5164 z 10
423.2713317871094 0 3568.2817
424.27435302734375 0 1143.4944
426.72161865234375 0 4450.8164
427.2237243652344 0 2232.2603
427.7243957519531 0 747.6217
429.0895080566406 0 1222.8638
429.2096252441406 0 825.21814
430.1942443847656 0 1088.7137
431.7164611816406 0 671.92114
432.2105407714844 0 601.4256
433.04736328125 0 656.2428
438.7288818359375 0 855.05115
439.2179870605469 0 940.20056 y 10
440.7192077636719 0 3610.5679 c Ammonia loss 6
441.22076416015625 0 1797.9608
451.2662658691406 0 7668.2173
452.26898193359375 0 1382.0034
468.2930603027344 0 28728.6 c 3
469.29541015625 0 7414.015
470.2975158691406 0 1315.7174
471.38250732421875 0 613.343
480.22235107421875 0 3122.7783 z 9
481.2242736816406 0 789.8677
483.2633361816406 0 950.5949
483.7640686035156 0 658.15155
489.2073669433594 0 671.6925
494.3083801269531 0 3089.4946
495.2303466796875 0 705.0375
495.3094787597656 0 630.60266
496.239990234375 0 7995.584 y 9
497.2450866699219 0 2316.894
497.7626037597656 0 707.18256
503.1074523925781 0 64495.445
519.1387939453125 0 23936.16
529.2584838867188 0 1054.3909
534.3255004882812 0 738.02966
536.1649780273438 0 14162.294
538.2518310546875 0 2536.4624
539.25537109375 0 1097.0978
550.2514038085938 0 3646.4966 w 8
551.2572021484375 0 2602.1475
551.3276977539062 0 723.4972
579.32470703125 0 995.99493 c Ammonia loss 4
593.2543334960938 0 969.0909 y Water loss 8
594.2651977539062 0 1361.2754
595.2492065429688 0 3114.8906 z 8
596.2571411132812 0 1409.4967
596.3514404296875 0 21003.664 c 4
597.3539428710938 0 5465.4307
598.3532104492188 0 1151.7981
610.2828369140625 0 4627.096
611.26806640625 0 9747.26 y 8
612.2720947265625 0 3263.1257
621.28759765625 0 843.2265
633.837646484375 0 1220.6418
645.2957153320312 0 775.57574
645.7894287109375 0 860.60504 y 3
646.2909545898438 0 867.1252
648.33349609375 0 775.6712
654.847900390625 0 1750.1158
655.3519287109375 0 6986.9585
655.8473510742188 0 31537.262 c 10
656.3480834960938 0 24766.227
656.8499755859375 0 8638.34
657.350341796875 0 3472.3906
664.2930908203125 0 5592.8643
664.3468017578125 0 1035.2087
665.2808227539062 0 5020.783 w 7
666.28271484375 0 1131.7782
666.3556518554688 0 752.5094
667.3649291992188 0 1425.7759
668.3639526367188 0 981.3346
668.8593139648438 0 1933.2488
669.8555908203125 0 1354.5898
672.303955078125 0 2008.6418
672.8001708984375 0 2775.3037 w 2
673.2991943359375 0 3246.27
673.802734375 0 1023.16077
680.3168334960938 0 1223.923
688.3136596679688 0 621.2764
690.34130859375 0 671.8738
693.3345336914062 0 830.43243 y Water loss 2
693.8255615234375 0 1448.4445 y Ammonia loss 2
694.3391723632812 0 2919.632 c Ammonia loss 5
694.8270263671875 0 1502.913
695.3506469726562 0 2106.1714
698.3709106445312 0 809.67554
701.83984375 0 3091.015
702.3336181640625 0 7022.8623 y 2
702.8326416015625 0 2980.409
703.3358154296875 0 1996.1936
707.3462524414062 0 2797.2732
708.3374633789062 0 4477.047 z 7
709.3404541015625 0 1138.2189
711.3778686523438 0 17200.914 c 5
711.8662719726562 0 672.56244 c Ammonia loss 11
712.3801879882812 0 5669.4146
713.3807983398438 0 1791.9186
718.9205932617188 0 2523.6082
719.4235229492188 0 1031.773
719.8048095703125 0 457.5097
719.8751220703125 0 11428.415
719.935302734375 0 706.09454
720.3709106445312 0 23012.934 c 11
720.8706665039062 0 14614.185
721.371826171875 0 7853.788
721.8717041015625 0 1530.4551
723.3692626953125 0 1377.5012
724.3529663085938 0 4298.736 y 7
725.3539428710938 0 1553.5317
726.356689453125 0 843.27167
729.854736328125 0 1316.6348
730.3605346679688 0 677.72925
736.8599853515625 0 1847.3295
737.3602905273438 0 5442.7583
737.8658447265625 0 983
741.3591918945312 0 912.12213
742.8859252929688 0 796.03784
743.360595703125 0 728.21655 y Ammonia loss 1
743.865234375 0 1315.603 z 1
744.3629760742188 0 1480.8588
748.053466796875 0 628.40314
748.8662109375 0 5325.807
749.3684692382812 0 5618.922
749.8695678710938 0 3212.643
750.3905029296875 0 596.8154
751.382080078125 0 1471.0975
751.8690795898438 0 1610.3708 y 1
758.866455078125 0 856.1875
764.8727416992188 0 773.63116
765.3770141601562 0 1187.3181
771.3900146484375 0 700.5604
771.893310546875 0 1608.1562
772.386962890625 0 2099.6545
772.88671875 0 4316.6055
773.3819580078125 0 3108.8306
773.8654174804688 0 877.99744
779.892333984375 0 3690.0542
780.39208984375 0 3472.1282
780.895751953125 0 1125.5488
781.38818359375 0 617.0943
785.3864135742188 0 1422.213
785.880615234375 0 1328.1187
786.3844604492188 0 2247.5273
786.8768920898438 0 1918.0778
787.3773193359375 0 2255.6458
793.3960571289062 0 1171.548
793.8897094726562 0 8275.812
794.38623046875 0 12394.693
794.8861694335938 0 8933.475
795.39013671875 0 4658.708
795.8850708007812 0 1376.9095
800.925537109375 0 1156.1454
801.3196411132812 0 845.89075
801.3884887695312 0 904.6553
801.8263549804688 0 943.5378
801.8991088867188 0 8992.685
802.3955078125 0 53430.93
802.8958740234375 0 73140.414
803.3971557617188 0 49807.246
803.8989868164062 0 21832.271
804.3990478515625 0 6315.822
804.8963012695312 0 2787.0447
837.3638305664062 0 1457.4429
838.3560180664062 0 1836.2561
839.3505249023438 0 1329.1887
850.4237670898438 0 681.64325
852.4408569335938 0 797.05347
853.44189453125 0 836.78754
864.4591674804688 0 733.16046
880.4301147460938 0 7083.6187 c Ammonia loss 6
881.4356079101562 0 3073.6733
882.4453735351562 0 880.8276
893.4255981445312 0 3607.1218
894.4137573242188 0 6288.3994 z 6
895.4180908203125 0 1816.0309
897.4574584960938 0 21579.428 c 6
898.4607543945312 0 10007.014
899.4631958007812 0 4038.9104
909.4424438476562 0 887.5296
910.4342651367188 0 2772.8052 y 6
911.4307250976562 0 1441.0653
964.4627685546875 0 1662.3225
965.4541015625 0 2417.6562
966.5380249023438 0 776.8116
1008.4546508789062 0 6115.2207
1009.4434204101562 0 10325.928 z 5
1009.55126953125 0 614.03894
1010.4403686523438 0 4142.941
1010.5421752929688 0 17285.186 c 7
1011.4446411132812 0 947.65186
1011.544677734375 0 8613.1875
1012.5466918945312 0 2719.9316
1024.476318359375 0 1670.634
1025.46875 0 2911.2024 y 5
1026.4698486328125 0 1168.6865
1062.5797119140625 0 603.6489
1063.5614013671875 0 1079.3606
1067.5394287109375 0 2194.8337
1068.4326171875 0 2241.1875
1068.5455322265625 0 1333.0913
1070.4412841796875 0 967.5146
1071.432861328125 0 902.6486
1071.58984375 0 916.596
1072.4287109375 0 11063.785
1073.4300537109375 0 5168.195
1079.47607421875 0 1079.2964 w 4
1080.4598388671875 0 953.1874
1080.5733642578125 0 1819.5829
1081.564208984375 0 1819.123
1082.5606689453125 0 1763.3981
1094.5302734375 0 904.58777
1124.580322265625 0 2529.8892
1125.57275390625 0 4284.5312 c 8
1126.569091796875 0 3196.0232
1136.51220703125 0 5939.649 y Ammonia loss 4
1137.4974365234375 0 41882.676 z 4
1138.5008544921875 0 23684.604
1139.50341796875 0 9568.635
1140.505615234375 0 2303.7747
1152.5233154296875 0 896.18884
1153.5206298828125 0 2128.698 y 4
1154.5203857421875 0 1387.1295
1181.6048583984375 0 9570.851
1182.5933837890625 0 24022.799 c 9
1183.594970703125 0 14856.345
1184.5977783203125 0 5646.571
1185.604248046875 0 1435.182
1186.6124267578125 0 1023.0155
1223.592041015625 0 721.41235
1265.6732177734375 0 659.8577
1266.6739501953125 0 904.00214
1267.676513671875 0 869.1846
1273.564453125 0 2012.4465 y Ammonia loss 3
1274.5592041015625 0 8545.361 z 3
1275.564208984375 0 7168.4443
1276.564697265625 0 4164.689
1277.5653076171875 0 1475.9451
1290.576171875 0 1023.75885 y 3
1294.6759033203125 0 859.8926
1295.66259765625 0 860.49286
1309.69677734375 0 3891.389
1310.68701171875 0 13755.191 c 10
1311.689208984375 0 11599.92
1312.68994140625 0 4351.822
1313.6868896484375 0 1184.3188
1386.639892578125 0 1347.555 y Ammonia loss 2
1387.6461181640625 0 8099.7803 z 2
1388.6468505859375 0 8303.417
1389.6492919921875 0 5373.981
1390.6427001953125 0 1406.6887
1394.7215576171875 0 739.58374
1395.7376708984375 0 2092.8264
1396.7271728515625 0 2358.781
1397.726318359375 0 833.8799
1422.71337890625 0 854.3473 c Ammonia loss 11
1423.7130126953125 0 1175.52
1439.7381591796875 0 6485.7803 c 11
1440.7374267578125 0 7675.7285
1441.7376708984375 0 4460.4478
1442.7376708984375 0 1604.8807
1486.724365234375 0 1051.6776 z 1
1487.7177734375 0 1775.2706
1488.7181396484375 0 1540.1656
1489.73974609375 0 908.5528
1542.7764892578125 0 1426.3422
1543.76708984375 0 928.5679
1544.78125 0 2260.6855
1545.7742919921875 0 2420.3206
1546.7728271484375 0 3061.0564
1547.775634765625 0 828.01654
1548.7767333984375 0 934.06085
1558.79296875 0 1157.2347
1559.7806396484375 0 4194.1196
1560.7796630859375 0 5867.0884
1561.781005859375 0 3697.5208
1562.7861328125 0 1393.8876
1570.758056640625 0 1600.3005
1571.7449951171875 0 936.74023
1576.7940673828125 0 3016.811
1577.807373046875 0 2705.365
1578.7958984375 0 2657.2534
1579.79931640625 0 691.5069
1586.771240234375 0 1808.3694
1587.772216796875 0 13645.452
1588.767333984375 0 21212.773
1589.1182861328125 0 960.20667
1589.76904296875 0 14278.089
1590.773193359375 0 6909.9707
1591.7691650390625 0 2651.9546
1602.8043212890625 0 666.0165
1603.7857666015625 0 5942.504
1604.7900390625 0 21580.559
1605.7901611328125 0 38478.613
1606.7923583984375 0 26157.963
1607.7960205078125 0 12284.25
1608.798095703125 0 5166.955
1609.788330078125 0 2640.3914
2195.46435546875 0 740.8992
2963.473388671875 0 975.1853
2971.61083984375 0 746.79083

Spectrum Details

|  |  |
| --- | --- |
| Matched peaks? Matched peaksThe total absolute number of peaks matched. Additionally in brackets the total fraction of peaks matched and the total number of peaks is shown. | 57 (15.12% of 377) |
| FDR? FDRThe false discovery rate estimated for this peptide. It is calculated by matching all theoretical fragments with a non-integer shift with the raw peaks for this spectrum. This is done with 40 different shifts. The resulting percentage is the average number of annotated peaks over the number of annotated peaks with the correct spectrum. | 3.26% |
| Satellite FDR? Satellite FDRSee the FDR for details on its calculation. This satellite ion specific FDR only contains the satellite ions (d/w) for I/L/J positions. | 1.19% |
| PSM Score? PSM ScoreThe PSM Score as given by Hecklib to this annotated spectrum. It is shown with three significant figures. | 549 |

## Spectrum 7713? Spectrum 7713 The raw spectrum of this peptide as annotated by Hecklib. The fragments are coloured according to ion type (see legend). Any peaks with a star '\*' as text can be hovered over to see the full details, first the ion type second the mass shift type. By hovering over the amino acids in the peptide or ions in the legend the corresponding peaks are highlighted. By toggling the 'Unassigned' label you can turn the background (unassigned) peaks on or off in the plot. By updating the slider in the Ion legend you can update the spectrum to only show the top X% of the peaks with labels. The top X% means any peak that is within X% of the highest intensity. By dragging in the spectrum you can zoom in to a specific part of the spectrum and use 'Zoom Out' to get back to the original zoom level. The annotation of the spectrum is based on the given sequence in the peptides file and is done with different software so inconsistencies are likely. The peaks are annotated based on the given sequence, with 20 ppm tolerance.

Copy Data

### Spectrum 7713 (TSV)

#### Preview

```
Loading example...
```

*Click on the button to copy the data to your clipboard.*

Mz MinMz MaxIntensity Max

WidthHeightPeptide font sizePeptide stroke widthSpectrum font sizeSpectrum stroke widthCompact peptide

Ion legend

wxyz

abcd

OtherUnassignedIonChargePositionShow for top:%

TVLHQDWLDGKEY

03.51e+47.03e+41.05e+51.41e+5

Zoom Out

y+11c+12y+12z+12y+12c+13c+26y+26y+13c+27c+14c+14z+14y+14w+15c+15y+15z+15c+15y+15z+210y+210c+211w+16w+211y+211y+211c+16y+211z+16c+16c+212c+212y+16w+212y+212z+212y+212c+17z+17c+17y+17c+18z+18c+18y+18w+19c+19z+19y+19c+110y+110z+110y+110c+111y+111z+111y+111c+112c+112z+112

040681312191626

Fragment Matches Table

Show background peaks

| Position | Ion type | Intensity | mz Theoretical | mz Error (Th) | mz Error (ppm) | Charge | Series Number |
| --- | --- | --- | --- | --- | --- | --- | --- |
| - | - | 668.8 | 120.1 | - | - | 0 | - |
| - | - | 485.2 | 128.1 | - | - | 0 | - |
| - | - | 3227 | 129.1 | - | - | 0 | - |
| - | - | 3541 | 130.1 | - | - | 0 | - |
| - | - | 436.6 | 131.1 | - | - | 0 | - |
| - | - | 1031 | 131.1 | - | - | 0 | - |
| - | - | 1084 | 136.1 | - | - | 0 | - |
| - | - | 489.6 | 139.5 | - | - | 0 | - |
| - | - | 588.2 | 148.9 | - | - | 0 | - |
| - | - | 5728 | 149 | - | - | 0 | - |
| - | - | 429.6 | 150.9 | - | - | 0 | - |
| - | - | 1.222E+04 | 155.1 | - | - | 0 | - |
| - | - | 495 | 156.1 | - | - | 0 | - |
| - | - | 1038 | 157.1 | - | - | 0 | - |
| - | - | 3875 | 159.1 | - | - | 0 | - |
| - | - | 502.6 | 160 | - | - | 0 | - |
| - | - | 724.3 | 165.1 | - | - | 0 | - |
| - | - | 1577 | 167.1 | - | - | 0 | - |
| - | - | 586.2 | 171.1 | - | - | 0 | - |
| - | - | 1.673E+04 | 173.1 | - | - | 0 | - |
| - | - | 1499 | 174.1 | - | - | 0 | - |
| - | - | 552.7 | 181.1 | - | - | 0 | - |
| 13 | y | 3263 | 182.1 | 6.812E-05 | 0.3741 | +1 | 1 |
| - | - | 792.6 | 184.1 | - | - | 0 | - |
| - | - | 1267 | 186.1 | - | - | 0 | - |
| - | - | 993.2 | 188.1 | - | - | 0 | - |
| - | - | 466 | 198.2 | - | - | 0 | - |
| - | - | 1.213E+04 | 201.1 | - | - | 0 | - |
| - | - | 992.3 | 202.1 | - | - | 0 | - |
| - | - | 1412 | 213.2 | - | - | 0 | - |
| - | - | 6556 | 215.1 | - | - | 0 | - |
| - | - | 809 | 216.1 | - | - | 0 | - |
| - | - | 520.8 | 216.2 | - | - | 0 | - |
| 2 | c | 3331 | 218.1 | 7.595E-05 | 0.3482 | +1 | 2 |
| - | - | 7143 | 221.1 | - | - | 0 | - |
| - | - | 2597 | 222.1 | - | - | 0 | - |
| - | - | 2621 | 223.1 | - | - | 0 | - |
| - | - | 1461 | 223.1 | - | - | 0 | - |
| - | - | 1113 | 223.2 | - | - | 0 | - |
| - | - | 2176 | 225 | - | - | 0 | - |
| - | - | 614.1 | 226 | - | - | 0 | - |
| - | - | 1027 | 226.1 | - | - | 0 | - |
| - | - | 1676 | 229.2 | - | - | 0 | - |
| - | - | 1.018E+04 | 239.1 | - | - | 0 | - |
| - | - | 4339 | 240.1 | - | - | 0 | - |
| - | - | 1588 | 241.1 | - | - | 0 | - |
| - | - | 1262 | 243.1 | - | - | 0 | - |
| - | - | 1511 | 245.1 | - | - | 0 | - |
| - | - | 2877 | 251.2 | - | - | 0 | - |
| - | - | 570.5 | 252.2 | - | - | 0 | - |
| - | - | 5995 | 258.1 | - | - | 0 | - |
| - | - | 1331 | 260.2 | - | - | 0 | - |
| - | - | 6460 | 268.2 | - | - | 0 | - |
| - | - | 598.6 | 269.2 | - | - | 0 | - |
| - | - | 1.709E+04 | 281.1 | - | - | 0 | - |
| - | - | 568.3 | 283 | - | - | 0 | - |
| - | - | 1551 | 283.1 | - | - | 0 | - |
| - | - | 769.2 | 287.2 | - | - | 0 | - |
| - | - | 960.9 | 288.2 | - | - | 0 | - |
| 12 | y | 2251 | 293.1 | 0.0003883 | 1.325 | +1 | 2 |
| - | - | 932.2 | 294.1 | - | - | 0 | - |
| 12 | z | 3189 | 295.1 | 0.001523 | 5.161 | +1 | 2 |
| - | - | 2243 | 296.1 | - | - | 0 | - |
| - | - | 3177 | 297.1 | - | - | 0 | - |
| - | - | 2139 | 297.1 | - | - | 0 | - |
| - | - | 824.9 | 298.1 | - | - | 0 | - |
| - | - | 6.748E+04 | 299.1 | - | - | 0 | - |
| - | - | 1723 | 300.1 | - | - | 0 | - |
| - | - | 1149 | 301.2 | - | - | 0 | - |
| - | - | 657.7 | 302.2 | - | - | 0 | - |
| - | - | 541.3 | 304.1 | - | - | 0 | - |
| - | - | 1293 | 305.1 | - | - | 0 | - |
| - | - | 811 | 306.1 | - | - | 0 | - |
| - | - | 744.4 | 307.2 | - | - | 0 | - |
| - | - | 557.7 | 308.2 | - | - | 0 | - |
| - | - | 507.4 | 309.2 | - | - | 0 | - |
| 12 | y | 2162 | 311.1 | 0.0004133 | 1.328 | +1 | 2 |
| - | - | 716.8 | 314.1 | - | - | 0 | - |
| - | - | 2057 | 314.2 | - | - | 0 | - |
| - | - | 595.9 | 315.2 | - | - | 0 | - |
| - | - | 1.102E+04 | 317.2 | - | - | 0 | - |
| - | - | 1842 | 318.2 | - | - | 0 | - |
| - | - | 1874 | 320.2 | - | - | 0 | - |
| - | - | 8654 | 323.1 | - | - | 0 | - |
| - | - | 773.8 | 324.1 | - | - | 0 | - |
| - | - | 2993 | 327.2 | - | - | 0 | - |
| 3 | c | 5873 | 331.2 | 0.0001795 | 0.5418 | +1 | 3 |
| - | - | 780.1 | 332.2 | - | - | 0 | - |
| - | - | 1139 | 341 | - | - | 0 | - |
| - | - | 643.5 | 343.1 | - | - | 0 | - |
| - | - | 528.2 | 346.3 | - | - | 0 | - |
| 6 | c | 587.4 | 347.7 | 0.0003411 | 0.9811 | +2 | 6 |
| - | - | 1493 | 355.1 | - | - | 0 | - |
| - | - | 680.7 | 356.1 | - | - | 0 | - |
| - | - | 806.5 | 357.1 | - | - | 0 | - |
| - | - | 5828 | 359 | - | - | 0 | - |
| 8 | y | 1705 | 362.7 | 0.0007257 | 2.001 | +2 | 6 |
| - | - | 1498 | 364.2 | - | - | 0 | - |
| - | - | 1751 | 369.1 | - | - | 0 | - |
| - | - | 3148 | 370.1 | - | - | 0 | - |
| - | - | 2771 | 371.1 | - | - | 0 | - |
| - | - | 762.1 | 372.1 | - | - | 0 | - |
| - | - | 2074 | 374.2 | - | - | 0 | - |
| - | - | 965.5 | 378.2 | - | - | 0 | - |
| - | - | 786.4 | 408.3 | - | - | 0 | - |
| - | - | 1188 | 412.2 | - | - | 0 | - |
| - | - | 2.187E+04 | 415 | - | - | 0 | - |
| - | - | 6178 | 423.3 | - | - | 0 | - |
| - | - | 1547 | 424.3 | - | - | 0 | - |
| - | - | 9214 | 426.7 | - | - | 0 | - |
| - | - | 3323 | 427.2 | - | - | 0 | - |
| - | - | 1442 | 427.7 | - | - | 0 | - |
| - | - | 1591 | 429.1 | - | - | 0 | - |
| - | - | 640.3 | 429.2 | - | - | 0 | - |
| - | - | 2352 | 430.2 | - | - | 0 | - |
| - | - | 1238 | 431.7 | - | - | 0 | - |
| - | - | 1006 | 432.2 | - | - | 0 | - |
| 11 | y | 2589 | 439.2 | 0.0006651 | 1.514 | +1 | 3 |
| 7 | c | 5000 | 440.7 | 0.0001416 | 0.3214 | +2 | 7 |
| - | - | 2338 | 441.2 | - | - | 0 | - |
| - | - | 863.6 | 444.2 | - | - | 0 | - |
| - | - | 994 | 445.2 | - | - | 0 | - |
| 4 | c | 670.8 | 450.3 | 0.0004382 | 0.9732 | +1 | 4 |
| - | - | 1.336E+04 | 451.3 | - | - | 0 | - |
| - | - | 3223 | 452.3 | - | - | 0 | - |
| - | - | 675.5 | 453.3 | - | - | 0 | - |
| 4 | c | 5.233E+04 | 468.3 | 0.0004412 | 0.9421 | +1 | 4 |
| - | - | 1.478E+04 | 469.3 | - | - | 0 | - |
| - | - | 2493 | 470.3 | - | - | 0 | - |
| - | - | 640 | 474.3 | - | - | 0 | - |
| - | - | 1035 | 474.8 | - | - | 0 | - |
| 10 | z | 5198 | 480.2 | 0.0005805 | 1.209 | +1 | 4 |
| - | - | 1934 | 481.2 | - | - | 0 | - |
| - | - | 2324 | 483.3 | - | - | 0 | - |
| - | - | 930.7 | 483.8 | - | - | 0 | - |
| - | - | 2264 | 489.2 | - | - | 0 | - |
| - | - | 949.2 | 494.2 | - | - | 0 | - |
| - | - | 3802 | 494.3 | - | - | 0 | - |
| - | - | 1592 | 495.2 | - | - | 0 | - |
| - | - | 1424 | 495.3 | - | - | 0 | - |
| 10 | y | 1.565E+04 | 496.2 | 0.0004721 | 0.9514 | +1 | 4 |
| - | - | 3276 | 497.2 | - | - | 0 | - |
| - | - | 646.6 | 497.8 | - | - | 0 | - |
| - | - | 5.492E+04 | 503.1 | - | - | 0 | - |
| - | - | 978 | 517.3 | - | - | 0 | - |
| - | - | 2.209E+04 | 519.1 | - | - | 0 | - |
| - | - | 1040 | 523.2 | - | - | 0 | - |
| - | - | 686.9 | 528.9 | - | - | 0 | - |
| - | - | 635.2 | 529.3 | - | - | 0 | - |
| - | - | 1138 | 529.6 | - | - | 0 | - |
| - | - | 753.2 | 529.9 | - | - | 0 | - |
| - | - | 673 | 534.3 | - | - | 0 | - |
| - | - | 3572 | 535.3 | - | - | 0 | - |
| - | - | 1.23E+04 | 536.2 | - | - | 0 | - |
| - | - | 1.026E+04 | 536.3 | - | - | 0 | - |
| - | - | 5113 | 538.3 | - | - | 0 | - |
| - | - | 1452 | 539.3 | - | - | 0 | - |
| 9 | w | 4954 | 550.3 | 0.0007717 | 1.402 | +1 | 5 |
| - | - | 4494 | 551.3 | - | - | 0 | - |
| - | - | 884.6 | 552.3 | - | - | 0 | - |
| 5 | c | 1961 | 579.3 | 0.0007034 | 1.214 | +1 | 5 |
| - | - | 808.3 | 580.3 | - | - | 0 | - |
| 9 | y | 902.3 | 593.3 | 0.0006475 | 1.091 | +1 | 5 |
| - | - | 1602 | 594.3 | - | - | 0 | - |
| 9 | z | 5278 | 595.2 | 0.0009201 | 1.546 | +1 | 5 |
| - | - | 637.2 | 595.3 | - | - | 0 | - |
| - | - | 2032 | 596.3 | - | - | 0 | - |
| 5 | c | 3.081E+04 | 596.4 | 0.0001522 | 0.2553 | +1 | 5 |
| - | - | 9995 | 597.4 | - | - | 0 | - |
| - | - | 2131 | 598.4 | - | - | 0 | - |
| - | - | 790.4 | 602.3 | - | - | 0 | - |
| - | - | 844.6 | 608.3 | - | - | 0 | - |
| - | - | 1234 | 609.3 | - | - | 0 | - |
| - | - | 7901 | 610.3 | - | - | 0 | - |
| 9 | y | 1.94E+04 | 611.3 | 0.001178 | 1.927 | +1 | 5 |
| - | - | 766 | 611.8 | - | - | 0 | - |
| - | - | 6165 | 612.3 | - | - | 0 | - |
| - | - | 1012 | 613.3 | - | - | 0 | - |
| - | - | 1438 | 621.3 | - | - | 0 | - |
| - | - | 1599 | 633.8 | - | - | 0 | - |
| - | - | 1109 | 634.3 | - | - | 0 | - |
| - | - | 1187 | 637.3 | - | - | 0 | - |
| 4 | z | 684.4 | 637.8 | 0.003653 | 5.728 | +2 | 10 |
| 4 | y | 1897 | 645.8 | 0.001493 | 2.312 | +2 | 10 |
| - | - | 1005 | 646.3 | - | - | 0 | - |
| - | - | 604.6 | 651.3 | - | - | 0 | - |
| - | - | 2821 | 654.8 | - | - | 0 | - |
| - | - | 1.401E+04 | 655.4 | - | - | 0 | - |
| 11 | c | 5.846E+04 | 655.8 | 0.001254 | 1.913 | +2 | 11 |
| - | - | 4.473E+04 | 656.3 | - | - | 0 | - |
| - | - | 1.837E+04 | 656.8 | - | - | 0 | - |
| - | - | 4226 | 657.4 | - | - | 0 | - |
| - | - | 958.5 | 657.9 | - | - | 0 | - |
| - | - | 7353 | 664.3 | - | - | 0 | - |
| - | - | 1534 | 664.3 | - | - | 0 | - |
| 8 | w | 9190 | 665.3 | 0.003187 | 4.79 | +1 | 6 |
| - | - | 1184 | 665.3 | - | - | 0 | - |
| - | - | 3472 | 666.3 | - | - | 0 | - |
| - | - | 1730 | 666.4 | - | - | 0 | - |
| - | - | 999 | 667.3 | - | - | 0 | - |
| - | - | 2408 | 667.4 | - | - | 0 | - |
| - | - | 3963 | 668.4 | - | - | 0 | - |
| - | - | 4530 | 668.9 | - | - | 0 | - |
| - | - | 2279 | 669.4 | - | - | 0 | - |
| - | - | 1412 | 669.9 | - | - | 0 | - |
| - | - | 3384 | 672.3 | - | - | 0 | - |
| 3 | w | 5621 | 672.8 | 0.003047 | 4.529 | +2 | 11 |
| - | - | 4469 | 673.3 | - | - | 0 | - |
| - | - | 2011 | 673.8 | - | - | 0 | - |
| - | - | 1342 | 680.3 | - | - | 0 | - |
| - | - | 709.5 | 690.8 | - | - | 0 | - |
| 3 | y | 1707 | 693.3 | 0.0006324 | 0.9121 | +2 | 11 |
| 3 | y | 2339 | 693.8 | 0.008747 | 12.61 | +2 | 11 |
| 6 | c | 5594 | 694.4 | 0.01092 | 15.73 | +1 | 6 |
| - | - | 2604 | 694.8 | - | - | 0 | - |
| - | - | 2579 | 695.4 | - | - | 0 | - |
| - | - | 897.4 | 697.9 | - | - | 0 | - |
| - | - | 1758 | 698.4 | - | - | 0 | - |
| - | - | 812.6 | 698.9 | - | - | 0 | - |
| - | - | 3342 | 701.8 | - | - | 0 | - |
| 3 | y | 9799 | 702.3 | 0.002735 | 3.895 | +2 | 11 |
| - | - | 6758 | 702.8 | - | - | 0 | - |
| - | - | 3533 | 703.3 | - | - | 0 | - |
| - | - | 1497 | 703.8 | - | - | 0 | - |
| - | - | 810.4 | 704.3 | - | - | 0 | - |
| - | - | 5842 | 707.3 | - | - | 0 | - |
| 8 | z | 7528 | 708.3 | 0.003465 | 4.892 | +1 | 6 |
| - | - | 2376 | 709.3 | - | - | 0 | - |
| - | - | 768.9 | 710.3 | - | - | 0 | - |
| 6 | c | 2.632E+04 | 711.4 | 0.0003626 | 0.5097 | +1 | 6 |
| 12 | c | 1459 | 711.9 | 0.004402 | 6.184 | +2 | 12 |
| - | - | 1.117E+04 | 712.4 | - | - | 0 | - |
| - | - | 2279 | 713.4 | - | - | 0 | - |
| - | - | 660.4 | 714.3 | - | - | 0 | - |
| - | - | 948.8 | 718.9 | - | - | 0 | - |
| - | - | 932.2 | 718.9 | - | - | 0 | - |
| - | - | 1234 | 719.4 | - | - | 0 | - |
| - | - | 815.5 | 719.8 | - | - | 0 | - |
| - | - | 2.16E+04 | 719.9 | - | - | 0 | - |
| 12 | c | 4.427E+04 | 720.4 | 0.002724 | 3.781 | +2 | 12 |
| - | - | 2.79E+04 | 720.9 | - | - | 0 | - |
| - | - | 1.292E+04 | 721.4 | - | - | 0 | - |
| - | - | 3326 | 721.9 | - | - | 0 | - |
| - | - | 795.8 | 722.4 | - | - | 0 | - |
| - | - | 2886 | 723.4 | - | - | 0 | - |
| 8 | y | 8369 | 724.4 | 0.001343 | 1.853 | +1 | 6 |
| - | - | 3918 | 725.4 | - | - | 0 | - |
| - | - | 2316 | 729.4 | - | - | 0 | - |
| - | - | 1854 | 729.9 | - | - | 0 | - |
| - | - | 1787 | 730.4 | - | - | 0 | - |
| - | - | 799.1 | 735.9 | - | - | 0 | - |
| 2 | w | 1014 | 736.3 | 0.001766 | 2.398 | +2 | 12 |
| - | - | 2364 | 736.9 | - | - | 0 | - |
| - | - | 5637 | 737.4 | - | - | 0 | - |
| - | - | 2514 | 737.9 | - | - | 0 | - |
| - | - | 902 | 738.4 | - | - | 0 | - |
| - | - | 755 | 741.4 | - | - | 0 | - |
| 2 | y | 878.8 | 743.4 | 0.006461 | 8.692 | +2 | 12 |
| 2 | z | 2671 | 743.9 | 0.004868 | 6.544 | +2 | 12 |
| - | - | 2314 | 744.4 | - | - | 0 | - |
| - | - | 1208 | 744.9 | - | - | 0 | - |
| - | - | 1.111E+04 | 748.9 | - | - | 0 | - |
| - | - | 8290 | 749.4 | - | - | 0 | - |
| - | - | 5458 | 749.9 | - | - | 0 | - |
| - | - | 876.1 | 750.4 | - | - | 0 | - |
| - | - | 2068 | 750.9 | - | - | 0 | - |
| - | - | 1843 | 751.4 | - | - | 0 | - |
| 2 | y | 1659 | 751.9 | 0.0003437 | 0.4572 | +2 | 12 |
| - | - | 1107 | 752.4 | - | - | 0 | - |
| - | - | 1205 | 757.9 | - | - | 0 | - |
| - | - | 1136 | 758.4 | - | - | 0 | - |
| - | - | 896.1 | 758.9 | - | - | 0 | - |
| - | - | 844.9 | 759.4 | - | - | 0 | - |
| - | - | 887 | 764.4 | - | - | 0 | - |
| - | - | 1550 | 764.9 | - | - | 0 | - |
| - | - | 1465 | 765.4 | - | - | 0 | - |
| - | - | 1179 | 766.4 | - | - | 0 | - |
| - | - | 913.6 | 771.4 | - | - | 0 | - |
| - | - | 2616 | 771.9 | - | - | 0 | - |
| - | - | 4507 | 772.4 | - | - | 0 | - |
| - | - | 9283 | 772.9 | - | - | 0 | - |
| - | - | 6820 | 773.4 | - | - | 0 | - |
| - | - | 1855 | 773.9 | - | - | 0 | - |
| - | - | 973 | 774.4 | - | - | 0 | - |
| - | - | 5454 | 779.9 | - | - | 0 | - |
| - | - | 6248 | 780.4 | - | - | 0 | - |
| - | - | 3351 | 780.9 | - | - | 0 | - |
| - | - | 2232 | 781.4 | - | - | 0 | - |
| - | - | 913 | 784.9 | - | - | 0 | - |
| - | - | 1687 | 785.4 | - | - | 0 | - |
| - | - | 3428 | 785.9 | - | - | 0 | - |
| - | - | 2582 | 786.4 | - | - | 0 | - |
| - | - | 4693 | 786.9 | - | - | 0 | - |
| - | - | 2910 | 787.4 | - | - | 0 | - |
| - | - | 1303 | 787.9 | - | - | 0 | - |
| - | - | 1305 | 793.4 | - | - | 0 | - |
| - | - | 1.246E+04 | 793.9 | - | - | 0 | - |
| - | - | 2.239E+04 | 794.4 | - | - | 0 | - |
| - | - | 1.771E+04 | 794.9 | - | - | 0 | - |
| - | - | 7543 | 795.4 | - | - | 0 | - |
| - | - | 1995 | 795.9 | - | - | 0 | - |
| - | - | 966.8 | 797.4 | - | - | 0 | - |
| - | - | 1772 | 801.4 | - | - | 0 | - |
| - | - | 938.4 | 801.5 | - | - | 0 | - |
| - | - | 2209 | 801.8 | - | - | 0 | - |
| - | - | 1.82E+04 | 801.9 | - | - | 0 | - |
| - | - | 9.133E+04 | 802.4 | - | - | 0 | - |
| - | - | 1.391E+05 | 802.9 | - | - | 0 | - |
| - | - | 8.988E+04 | 803.4 | - | - | 0 | - |
| - | - | 4.146E+04 | 803.9 | - | - | 0 | - |
| - | - | 1.634E+04 | 804.4 | - | - | 0 | - |
| - | - | 4061 | 804.9 | - | - | 0 | - |
| - | - | 2160 | 837.4 | - | - | 0 | - |
| - | - | 4693 | 838.4 | - | - | 0 | - |
| - | - | 1920 | 839.4 | - | - | 0 | - |
| - | - | 1256 | 850.4 | - | - | 0 | - |
| - | - | 1219 | 851.4 | - | - | 0 | - |
| - | - | 655.8 | 852.2 | - | - | 0 | - |
| - | - | 1825 | 853.4 | - | - | 0 | - |
| - | - | 1213 | 862.4 | - | - | 0 | - |
| - | - | 1772 | 864.5 | - | - | 0 | - |
| - | - | 830.3 | 865.5 | - | - | 0 | - |
| 7 | c | 1.298E+04 | 880.4 | 0.0005752 | 0.6533 | +1 | 7 |
| - | - | 6990 | 881.4 | - | - | 0 | - |
| - | - | 1602 | 882.4 | - | - | 0 | - |
| - | - | 926.9 | 883.4 | - | - | 0 | - |
| - | - | 5193 | 893.4 | - | - | 0 | - |
| 7 | z | 9944 | 894.4 | 0.002887 | 3.228 | +1 | 7 |
| - | - | 4166 | 895.4 | - | - | 0 | - |
| - | - | 837.1 | 896.4 | - | - | 0 | - |
| 7 | c | 3.891E+04 | 897.5 | 0.0002078 | 0.2315 | +1 | 7 |
| - | - | 2.186E+04 | 898.5 | - | - | 0 | - |
| - | - | 5253 | 899.5 | - | - | 0 | - |
| - | - | 1780 | 900.5 | - | - | 0 | - |
| - | - | 3232 | 909.4 | - | - | 0 | - |
| 7 | y | 5403 | 910.4 | 0.003634 | 3.991 | +1 | 7 |
| - | - | 2572 | 911.4 | - | - | 0 | - |
| - | - | 1345 | 937.4 | - | - | 0 | - |
| - | - | 817.9 | 938.4 | - | - | 0 | - |
| - | - | 2308 | 964.5 | - | - | 0 | - |
| - | - | 5066 | 965.5 | - | - | 0 | - |
| - | - | 1849 | 966.5 | - | - | 0 | - |
| - | - | 919 | 967.5 | - | - | 0 | - |
| - | - | 1105 | 968.5 | - | - | 0 | - |
| - | - | 690.3 | 991.5 | - | - | 0 | - |
| 8 | c | 1907 | 993.5 | 0.0003218 | 0.3239 | +1 | 8 |
| - | - | 1447 | 994.5 | - | - | 0 | - |
| - | - | 8978 | 1008 | - | - | 0 | - |
| 6 | z | 1.547E+04 | 1009 | 0.004753 | 4.709 | +1 | 8 |
| - | - | 972.3 | 1010 | - | - | 0 | - |
| - | - | 6244 | 1010 | - | - | 0 | - |
| 8 | c | 2.939E+04 | 1011 | 0.000384 | 0.38 | +1 | 8 |
| - | - | 1672 | 1011 | - | - | 0 | - |
| - | - | 1.647E+04 | 1012 | - | - | 0 | - |
| - | - | 1129 | 1012 | - | - | 0 | - |
| - | - | 6149 | 1013 | - | - | 0 | - |
| - | - | 819.1 | 1014 | - | - | 0 | - |
| - | - | 3587 | 1024 | - | - | 0 | - |
| 6 | y | 4586 | 1025 | 0.008368 | 8.16 | +1 | 8 |
| - | - | 2244 | 1026 | - | - | 0 | - |
| - | - | 728.1 | 1027 | - | - | 0 | - |
| - | - | 1532 | 1064 | - | - | 0 | - |
| - | - | 764.4 | 1065 | - | - | 0 | - |
| - | - | 4186 | 1068 | - | - | 0 | - |
| - | - | 3419 | 1069 | - | - | 0 | - |
| - | - | 689.5 | 1069 | - | - | 0 | - |
| - | - | 1143 | 1070 | - | - | 0 | - |
| - | - | 1162 | 1071 | - | - | 0 | - |
| 5 | w | 2773 | 1079 | 0.002564 | 2.375 | +1 | 9 |
| - | - | 1850 | 1080 | - | - | 0 | - |
| - | - | 2725 | 1081 | - | - | 0 | - |
| - | - | 743.8 | 1081 | - | - | 0 | - |
| - | - | 5722 | 1082 | - | - | 0 | - |
| - | - | 2378 | 1083 | - | - | 0 | - |
| - | - | 1030 | 1084 | - | - | 0 | - |
| - | - | 758.5 | 1093 | - | - | 0 | - |
| - | - | 1642 | 1094 | - | - | 0 | - |
| - | - | 805.7 | 1095 | - | - | 0 | - |
| - | - | 596.9 | 1109 | - | - | 0 | - |
| - | - | 643.1 | 1110 | - | - | 0 | - |
| - | - | 4579 | 1125 | - | - | 0 | - |
| 9 | c | 7672 | 1126 | 0.004508 | 4.005 | +1 | 9 |
| - | - | 4991 | 1127 | - | - | 0 | - |
| - | - | 1485 | 1128 | - | - | 0 | - |
| - | - | 1.04E+04 | 1137 | - | - | 0 | - |
| 5 | z | 7.661E+04 | 1137 | 0.0002526 | 0.2221 | +1 | 9 |
| - | - | 4.827E+04 | 1139 | - | - | 0 | - |
| - | - | 1.83E+04 | 1140 | - | - | 0 | - |
| - | - | 3441 | 1141 | - | - | 0 | - |
| - | - | 702.3 | 1142 | - | - | 0 | - |
| - | - | 1133 | 1153 | - | - | 0 | - |
| 5 | y | 5166 | 1154 | 0.001792 | 1.554 | +1 | 9 |
| - | - | 2468 | 1155 | - | - | 0 | - |
| - | - | 1334 | 1156 | - | - | 0 | - |
| - | - | 1.614E+04 | 1182 | - | - | 0 | - |
| 10 | c | 4.135E+04 | 1183 | 0.003308 | 2.797 | +1 | 10 |
| - | - | 2.375E+04 | 1184 | - | - | 0 | - |
| - | - | 1.094E+04 | 1185 | - | - | 0 | - |
| - | - | 2939 | 1186 | - | - | 0 | - |
| - | - | 1459 | 1187 | - | - | 0 | - |
| - | - | 1098 | 1224 | - | - | 0 | - |
| - | - | 1755 | 1267 | - | - | 0 | - |
| - | - | 1673 | 1268 | - | - | 0 | - |
| - | - | 1084 | 1269 | - | - | 0 | - |
| 4 | y | 3389 | 1274 | 0.02009 | 15.77 | +1 | 10 |
| 4 | z | 1.462E+04 | 1275 | 0.002376 | 1.864 | +1 | 10 |
| - | - | 1.533E+04 | 1276 | - | - | 0 | - |
| - | - | 6048 | 1277 | - | - | 0 | - |
| - | - | 1779 | 1278 | - | - | 0 | - |
| - | - | 675.1 | 1279 | - | - | 0 | - |
| - | - | 695.8 | 1290 | - | - | 0 | - |
| 4 | y | 2480 | 1291 | 0.004892 | 3.791 | +1 | 10 |
| - | - | 1160 | 1292 | - | - | 0 | - |
| - | - | 1422 | 1295 | - | - | 0 | - |
| - | - | 718.4 | 1296 | - | - | 0 | - |
| - | - | 5608 | 1310 | - | - | 0 | - |
| 11 | c | 2.561E+04 | 1311 | 0.001362 | 1.039 | +1 | 11 |
| - | - | 1.984E+04 | 1312 | - | - | 0 | - |
| - | - | 8289 | 1313 | - | - | 0 | - |
| - | - | 2319 | 1314 | - | - | 0 | - |
| - | - | 854.6 | 1344 | - | - | 0 | - |
| - | - | 768.1 | 1345 | - | - | 0 | - |
| 3 | y | 1442 | 1387 | 0.01171 | 8.444 | +1 | 11 |
| 3 | z | 1.36E+04 | 1388 | 0.003761 | 2.71 | +1 | 11 |
| - | - | 1.477E+04 | 1389 | - | - | 0 | - |
| - | - | 9024 | 1390 | - | - | 0 | - |
| - | - | 3719 | 1391 | - | - | 0 | - |
| - | - | 1070 | 1392 | - | - | 0 | - |
| - | - | 1008 | 1395 | - | - | 0 | - |
| - | - | 4601 | 1396 | - | - | 0 | - |
| - | - | 4618 | 1397 | - | - | 0 | - |
| - | - | 3199 | 1398 | - | - | 0 | - |
| - | - | 939.1 | 1399 | - | - | 0 | - |
| 3 | y | 1490 | 1404 | 0.00469 | 3.342 | +1 | 11 |
| - | - | 1592 | 1405 | - | - | 0 | - |
| - | - | 1043 | 1413 | - | - | 0 | - |
| 12 | c | 1514 | 1423 | 0.02389 | 16.79 | +1 | 12 |
| - | - | 2439 | 1424 | - | - | 0 | - |
| - | - | 2436 | 1425 | - | - | 0 | - |
| - | - | 1081 | 1426 | - | - | 0 | - |
| - | - | 3127 | 1439 | - | - | 0 | - |
| 12 | c | 9250 | 1440 | 0.007597 | 5.277 | +1 | 12 |
| - | - | 1.2E+04 | 1441 | - | - | 0 | - |
| - | - | 8151 | 1442 | - | - | 0 | - |
| - | - | 3197 | 1443 | - | - | 0 | - |
| - | - | 907.8 | 1444 | - | - | 0 | - |
| 2 | z | 1610 | 1487 | 0.01286 | 8.651 | +1 | 12 |
| - | - | 3198 | 1488 | - | - | 0 | - |
| - | - | 2072 | 1489 | - | - | 0 | - |
| - | - | 1372 | 1490 | - | - | 0 | - |
| - | - | 677.6 | 1491 | - | - | 0 | - |
| - | - | 880.7 | 1529 | - | - | 0 | - |
| - | - | 1664 | 1543 | - | - | 0 | - |
| - | - | 1437 | 1544 | - | - | 0 | - |
| - | - | 2154 | 1545 | - | - | 0 | - |
| - | - | 6370 | 1546 | - | - | 0 | - |
| - | - | 4642 | 1547 | - | - | 0 | - |
| - | - | 2201 | 1548 | - | - | 0 | - |
| - | - | 958.3 | 1549 | - | - | 0 | - |
| - | - | 2153 | 1559 | - | - | 0 | - |
| - | - | 7428 | 1560 | - | - | 0 | - |
| - | - | 9745 | 1561 | - | - | 0 | - |
| - | - | 5275 | 1562 | - | - | 0 | - |
| - | - | 2397 | 1563 | - | - | 0 | - |
| - | - | 1207 | 1570 | - | - | 0 | - |
| - | - | 2474 | 1571 | - | - | 0 | - |
| - | - | 1239 | 1572 | - | - | 0 | - |
| - | - | 983.8 | 1573 | - | - | 0 | - |
| - | - | 744.5 | 1576 | - | - | 0 | - |
| - | - | 4570 | 1577 | - | - | 0 | - |
| - | - | 6813 | 1578 | - | - | 0 | - |
| - | - | 4526 | 1579 | - | - | 0 | - |
| - | - | 2287 | 1580 | - | - | 0 | - |
| - | - | 3053 | 1587 | - | - | 0 | - |
| - | - | 1.903E+04 | 1588 | - | - | 0 | - |
| - | - | 3.59E+04 | 1589 | - | - | 0 | - |
| - | - | 2.567E+04 | 1590 | - | - | 0 | - |
| - | - | 1.163E+04 | 1591 | - | - | 0 | - |
| - | - | 4642 | 1592 | - | - | 0 | - |
| - | - | 1363 | 1593 | - | - | 0 | - |
| - | - | 999.7 | 1603 | - | - | 0 | - |
| - | - | 9743 | 1604 | - | - | 0 | - |
| - | - | 3.668E+04 | 1605 | - | - | 0 | - |
| - | - | 6.324E+04 | 1606 | - | - | 0 | - |
| - | - | 4.677E+04 | 1607 | - | - | 0 | - |
| - | - | 2.206E+04 | 1608 | - | - | 0 | - |
| - | - | 7884 | 1609 | - | - | 0 | - |
| - | - | 3423 | 1610 | - | - | 0 | - |

m/z Charge Intensity FragmentType MassShift Position
120.06583404541016 0 668.7516
128.1071319580078 0 485.2024
129.10238647460938 0 3226.6194
130.0653076171875 0 3540.651
131.06863403320312 0 436.60083
131.11817932128906 0 1030.6396
136.0758056640625 0 1084.2795
139.5156707763672 0 489.5873
148.94757080078125 0 588.1633
149.04502868652344 0 5728.491
150.85986328125 0 429.63507
155.09288024902344 0 12215.934
156.09629821777344 0 494.98016
157.1334228515625 0 1037.9093
159.09181213378906 0 3875.2993
160.02146911621094 0 502.5916
165.05455017089844 0 724.3431
167.0554962158203 0 1576.5376
171.1129913330078 0 586.1625
173.12860107421875 0 16726.037
174.13186645507812 0 1499.4857
181.10906982421875 0 552.6512
182.08123779296875 0 3262.5613 y 12
184.06016540527344 0 792.55225
186.12368774414062 0 1267.1764
188.13941955566406 0 993.16187
198.21083068847656 0 465.9615
201.12347412109375 0 12132.795
202.12677001953125 0 992.26056
213.1601104736328 0 1411.9236
215.13916015625 0 6556.314
216.1424560546875 0 808.9974
216.2119598388672 0 520.7579
218.14999389648438 0 3331.361 c 1
221.0845947265625 0 7142.9155
222.084716796875 0 2596.5144
223.06419372558594 0 2620.8164
223.08160400390625 0 1461.1029
223.15567016601562 0 1112.8208
225.04324340820312 0 2176.172
226.04380798339844 0 614.06494
226.10801696777344 0 1027.2568
229.15484619140625 0 1675.7449
239.09506225585938 0 10176.396
240.0957794189453 0 4338.822
241.0925750732422 0 1587.7162
243.13375854492188 0 1262.4827
245.13714599609375 0 1510.8966
251.15029907226562 0 2876.757
252.15664672851562 0 570.53326
258.14508056640625 0 5994.511
260.1597900390625 0 1330.8799
268.1767578125 0 6459.696
269.1814880371094 0 598.5638
281.0513000488281 0 17088.297
283.03021240234375 0 568.25397
283.1399841308594 0 1550.5684
287.2201232910156 0 769.18787
288.22723388671875 0 960.91376
293.11358642578125 0 2250.9558 y Water loss 11
294.11639404296875 0 932.18115
295.103515625 0 3189.4233 z 11
296.10394287109375 0 2242.9512
297.0826110839844 0 3176.5144
297.1007385253906 0 2138.5422
298.1009521484375 0 824.9013
299.0618896484375 0 67477.02
300.0632019042969 0 1722.8198
301.1507873535156 0 1149.029
302.1507568359375 0 657.7052
304.10809326171875 0 541.26935
305.124755859375 0 1292.9049
306.1075439453125 0 810.99316
307.189453125 0 744.4339
308.1934814453125 0 557.6687
309.2047424316406 0 507.41296
311.1241760253906 0 2161.824 y 11
314.1149597167969 0 716.82794
314.2080993652344 0 2057.2908
315.1658630371094 0 595.94617
317.15826416015625 0 11023.204
318.1617126464844 0 1841.6107
320.1966857910156 0 1873.8508
323.1352233886719 0 8653.754
324.1386413574219 0 773.77704
327.203125 0 2992.7815
331.2341613769531 0 5873.4863 c 2
332.23663330078125 0 780.14264
341.0180358886719 0 1139.2722
343.1023864746094 0 643.50494
346.2668151855469 0 528.24
347.6792297363281 0 587.4168 c Ammonia loss 5
355.06964111328125 0 1492.6672
356.0703125 0 680.6775
357.06854248046875 0 806.5323
359.0285949707031 0 5827.895
362.6799621582031 0 1704.8057 y 7
364.18621826171875 0 1497.7001
369.12164306640625 0 1750.6353
370.1229248046875 0 3147.9485
371.12054443359375 0 2770.6865
372.12054443359375 0 762.1432
374.1793518066406 0 2073.5586
378.2130432128906 0 965.5494
408.2606201171875 0 786.35455
412.183349609375 0 1187.58
415.03704833984375 0 21873.887
423.271728515625 0 6177.649
424.27471923828125 0 1546.6936
426.72198486328125 0 9214.431
427.2233581542969 0 3323.1094
427.7247009277344 0 1441.8191
429.0885314941406 0 1590.8389
429.21002197265625 0 640.34265
430.1940002441406 0 2351.7966
431.71368408203125 0 1238.4576
432.2138366699219 0 1006.40094
439.2193908691406 0 2589.328 y 10
440.7190856933594 0 4999.625 c Ammonia loss 6
441.2214050292969 0 2337.5513
444.2486572265625 0 863.6425
445.21673583984375 0 993.9998
[truncated: 1,393,583 more chars]
