# Supplementary material for: A Handle on Mass Coincidence Errors in De Novo Sequencing of Antibodies by Bottom-up Proteomics
Source: J Proteome Res. 2024 Jun 27;23(8):3552–9. doi: 10.1021/acs.jproteome.4c00188 (PMC11301774; doi:10.1021/acs.jproteome.4c00188)
Supplement: Supplementary file 1 — pr4c00188_si_001.zip [file pr4c00188_si_001.zip › supplementary data/xln-disambiguation/2023-12-13@14-36-36 f59/report/reads/Combined_030.html]

Details Combined\_030 | Stitch OverviewUndefined

# Read Combined\_030

## Sequence (length=17)

VTJFPPSSEEJQANKAT

## Spectrum 8009? Spectrum 8009 The raw spectrum of this peptide as annotated by Hecklib. The fragments are coloured according to ion type (see legend). Any peaks with a star '\*' as text can be hovered over to see the full details, first the ion type second the mass shift type. By hovering over the amino acids in the peptide or ions in the legend the corresponding peaks are highlighted. By toggling the 'Unassigned' label you can turn the background (unassigned) peaks on or off in the plot. By updating the slider in the Ion legend you can update the spectrum to only show the top X% of the peaks with labels. The top X% means any peak that is within X% of the highest intensity. By dragging in the spectrum you can zoom in to a specific part of the spectrum and use 'Zoom Out' to get back to the original zoom level. The annotation of the spectrum is based on the given sequence in the peptides file and is done with different software so inconsistencies are likely. The peaks are annotated based on the given sequence, with 20 ppm tolerance.

Copy Data

### Spectrum 8009 (TSV)

#### Preview

```
Loading example...
```

*Click on the button to copy the data to your clipboard.*

Mz MinMz MaxIntensity Max

WidthHeightPeptide font sizePeptide stroke widthSpectrum font sizeSpectrum stroke widthCompact peptide

Ion legend

wxyz

abcd

OtherUnassignedIonChargePositionShow for top:%

VTJFPPSSEEJQANKAT

02.05e+44.09e+46.14e+48.19e+4

Zoom Out

y+11y+12y+12c+12c+12y+37z+13y+13c+13y+27y+14z+14y+14y+15c+29y+29y+15z+315w+16y+211y+211y+16z+16y+212y+212y+16y+212c+212c+16y+213y+213z+213y+213z+17z+214y+17y+214y+214z+214c+17y+214w+18z+215y+215y+215y+215c+215z+18z+18c+18z+216z+216c+216y+18y+216y+216z+216c+216y+18w+19c+19z+19y+19z+110z+110y+110z+110y+110c+110y+111y+111z+111y+111c+111c+111y+112c+112c+112y+113z+113y+113c+113y+114z+114c+114y+114c+114z+115c+115z+116y+116z+116c+116

046492813921856

Fragment Matches Table

Show background peaks

| Position | Ion type | Intensity | mz Theoretical | mz Error (Th) | mz Error (ppm) | Charge | Series Number |
| --- | --- | --- | --- | --- | --- | --- | --- |
| - | - | 696.7 | 120.1 | - | - | 0 | - |
| 17 | y | 1.112E+04 | 120.1 | 8.555E-05 | 0.7125 | +1 | 1 |
| - | - | 1810 | 120.1 | - | - | 0 | - |
| - | - | 547.5 | 121.1 | - | - | 0 | - |
| - | - | 371.4 | 124.6 | - | - | 0 | - |
| - | - | 739.2 | 126.1 | - | - | 0 | - |
| - | - | 960.5 | 128.1 | - | - | 0 | - |
| - | - | 408.5 | 128.1 | - | - | 0 | - |
| - | - | 4562 | 129.1 | - | - | 0 | - |
| - | - | 396.3 | 129.2 | - | - | 0 | - |
| - | - | 746.6 | 131.1 | - | - | 0 | - |
| - | - | 474.5 | 140.1 | - | - | 0 | - |
| - | - | 589.4 | 148.9 | - | - | 0 | - |
| - | - | 1105 | 149 | - | - | 0 | - |
| - | - | 454.2 | 151.3 | - | - | 0 | - |
| - | - | 708.5 | 155.1 | - | - | 0 | - |
| - | - | 565.4 | 155.1 | - | - | 0 | - |
| - | - | 534.1 | 158.1 | - | - | 0 | - |
| - | - | 2070 | 169.1 | - | - | 0 | - |
| - | - | 657.1 | 171.1 | - | - | 0 | - |
| 16 | y | 1295 | 173.1 | 7.911E-05 | 0.4571 | +1 | 2 |
| - | - | 2900 | 173.1 | - | - | 0 | - |
| - | - | 495.3 | 181.1 | - | - | 0 | - |
| - | - | 3672 | 183.1 | - | - | 0 | - |
| - | - | 548.6 | 185.1 | - | - | 0 | - |
| 16 | y | 3994 | 191.1 | 0.0001345 | 0.704 | +1 | 2 |
| - | - | 1702 | 197.1 | - | - | 0 | - |
| 2 | c | 626.5 | 200.1 | 0.0002189 | 1.094 | +1 | 2 |
| - | - | 7029 | 201.1 | - | - | 0 | - |
| - | - | 552.6 | 211.1 | - | - | 0 | - |
| - | - | 8754 | 212.1 | - | - | 0 | - |
| - | - | 795.8 | 213.1 | - | - | 0 | - |
| - | - | 1416 | 215.1 | - | - | 0 | - |
| 2 | c | 4587 | 218.1 | 1.492E-05 | 0.06838 | +1 | 2 |
| - | - | 4220 | 221.1 | - | - | 0 | - |
| - | - | 749.8 | 222.1 | - | - | 0 | - |
| - | - | 763.3 | 223.1 | - | - | 0 | - |
| - | - | 478.8 | 225.8 | - | - | 0 | - |
| - | - | 862.4 | 226.1 | - | - | 0 | - |
| - | - | 988.1 | 227.1 | - | - | 0 | - |
| - | - | 1642 | 227.1 | - | - | 0 | - |
| - | - | 706.2 | 227.1 | - | - | 0 | - |
| - | - | 1176 | 233.2 | - | - | 0 | - |
| - | - | 1014 | 235.1 | - | - | 0 | - |
| - | - | 5012 | 239.1 | - | - | 0 | - |
| - | - | 1903 | 240.1 | - | - | 0 | - |
| - | - | 727.1 | 242.2 | - | - | 0 | - |
| 11 | y | 1364 | 243.1 | 0.003444 | 14.17 | +3 | 7 |
| - | - | 658.9 | 251.2 | - | - | 0 | - |
| - | - | 1394 | 254.1 | - | - | 0 | - |
| - | - | 1005 | 261.2 | - | - | 0 | - |
| - | - | 2040 | 263.1 | - | - | 0 | - |
| - | - | 806 | 272.2 | - | - | 0 | - |
| - | - | 698.4 | 280.2 | - | - | 0 | - |
| - | - | 1261 | 281.1 | - | - | 0 | - |
| - | - | 655.5 | 281.1 | - | - | 0 | - |
| - | - | 851 | 282.1 | - | - | 0 | - |
| - | - | 835.7 | 296.2 | - | - | 0 | - |
| - | - | 1.083E+04 | 296.2 | - | - | 0 | - |
| - | - | 631.1 | 297.1 | - | - | 0 | - |
| - | - | 1001 | 297.2 | - | - | 0 | - |
| - | - | 1634 | 297.2 | - | - | 0 | - |
| - | - | 1104 | 298.2 | - | - | 0 | - |
| - | - | 5175 | 299.1 | - | - | 0 | - |
| - | - | 1.318E+04 | 299.2 | - | - | 0 | - |
| - | - | 1785 | 300.1 | - | - | 0 | - |
| - | - | 1104 | 300.2 | - | - | 0 | - |
| - | - | 657.3 | 301.1 | - | - | 0 | - |
| 15 | z | 6849 | 303.2 | 0.0001004 | 0.3311 | +1 | 3 |
| - | - | 1270 | 304.2 | - | - | 0 | - |
| - | - | 982.8 | 314.2 | - | - | 0 | - |
| - | - | 4472 | 314.2 | - | - | 0 | - |
| - | - | 935.4 | 315.2 | - | - | 0 | - |
| - | - | 5855 | 316.2 | - | - | 0 | - |
| - | - | 911.1 | 317.2 | - | - | 0 | - |
| 15 | y | 4222 | 319.2 | 6.593E-05 | 0.2066 | +1 | 3 |
| - | - | 740.8 | 320.2 | - | - | 0 | - |
| - | - | 1443 | 325.2 | - | - | 0 | - |
| 3 | c | 2.554E+04 | 331.2 | 8.79E-05 | 0.2654 | +1 | 3 |
| - | - | 4461 | 332.2 | - | - | 0 | - |
| - | - | 741.9 | 344.2 | - | - | 0 | - |
| - | - | 986.3 | 345.2 | - | - | 0 | - |
| - | - | 607.7 | 346.1 | - | - | 0 | - |
| - | - | 551.8 | 346.2 | - | - | 0 | - |
| - | - | 653.5 | 347.2 | - | - | 0 | - |
| - | - | 540.6 | 348.2 | - | - | 0 | - |
| - | - | 809 | 350.1 | - | - | 0 | - |
| - | - | 3.835E+04 | 355.1 | - | - | 0 | - |
| - | - | 1.684E+04 | 356.1 | - | - | 0 | - |
| - | - | 9743 | 357.1 | - | - | 0 | - |
| - | - | 1037 | 358.1 | - | - | 0 | - |
| - | - | 1414 | 362.2 | - | - | 0 | - |
| - | - | 894.1 | 370.2 | - | - | 0 | - |
| - | - | 1854 | 371.1 | - | - | 0 | - |
| - | - | 610.8 | 371.2 | - | - | 0 | - |
| - | - | 1293 | 372.1 | - | - | 0 | - |
| - | - | 916.8 | 373.1 | - | - | 0 | - |
| 11 | y | 3372 | 373.2 | 0.004092 | 10.96 | +2 | 7 |
| - | - | 763.6 | 373.7 | - | - | 0 | - |
| - | - | 639.8 | 374.1 | - | - | 0 | - |
| - | - | 1406 | 385.2 | - | - | 0 | - |
| - | - | 699.3 | 385.4 | - | - | 0 | - |
| - | - | 1754 | 386.2 | - | - | 0 | - |
| - | - | 880.6 | 415 | - | - | 0 | - |
| 14 | y | 859.5 | 415.2 | 0.0001008 | 0.2427 | +1 | 4 |
| - | - | 1176 | 416 | - | - | 0 | - |
| - | - | 1075 | 417 | - | - | 0 | - |
| 14 | z | 2280 | 417.2 | 0.0006123 | 1.468 | +1 | 4 |
| - | - | 7195 | 418.2 | - | - | 0 | - |
| - | - | 1167 | 419.2 | - | - | 0 | - |
| 14 | y | 2724 | 433.2 | 0.0007481 | 1.727 | +1 | 4 |
| - | - | 8121 | 433.3 | - | - | 0 | - |
| - | - | 4886 | 434.3 | - | - | 0 | - |
| - | - | 1830 | 435.3 | - | - | 0 | - |
| - | - | 729.2 | 442.2 | - | - | 0 | - |
| - | - | 5846 | 444.2 | - | - | 0 | - |
| - | - | 3144 | 445.3 | - | - | 0 | - |
| - | - | 2588 | 446.3 | - | - | 0 | - |
| - | - | 1514 | 460.3 | - | - | 0 | - |
| - | - | 1.34E+04 | 461.3 | - | - | 0 | - |
| - | - | 3151 | 462.3 | - | - | 0 | - |
| - | - | 743.3 | 463.3 | - | - | 0 | - |
| - | - | 799.1 | 480.7 | - | - | 0 | - |
| - | - | 1450 | 480.8 | - | - | 0 | - |
| - | - | 1270 | 481.2 | - | - | 0 | - |
| 13 | y | 689.8 | 486.3 | 0.001268 | 2.608 | +1 | 5 |
| 9 | c | 9198 | 488.3 | 0.001861 | 3.812 | +2 | 9 |
| - | - | 1116 | 489.1 | - | - | 0 | - |
| - | - | 7165 | 489.3 | - | - | 0 | - |
| - | - | 627.2 | 490.1 | - | - | 0 | - |
| - | - | 1420 | 490.3 | - | - | 0 | - |
| - | - | 2480 | 493.7 | - | - | 0 | - |
| - | - | 1086 | 494.2 | - | - | 0 | - |
| - | - | 731.7 | 497.3 | - | - | 0 | - |
| - | - | 696.4 | 498.2 | - | - | 0 | - |
| 9 | y | 1959 | 502.3 | 0.008705 | 17.33 | +2 | 9 |
| - | - | 3364 | 502.7 | - | - | 0 | - |
| - | - | 2320 | 503.2 | - | - | 0 | - |
| - | - | 740.8 | 503.7 | - | - | 0 | - |
| 13 | y | 9221 | 504.3 | 0.0001028 | 0.2039 | +1 | 5 |
| - | - | 2058 | 505.3 | - | - | 0 | - |
| - | - | 789.6 | 515.3 | - | - | 0 | - |
| - | - | 4717 | 538.3 | - | - | 0 | - |
| - | - | 3371 | 538.8 | - | - | 0 | - |
| 3 | z | 769 | 539.3 | 0.001137 | 2.108 | +3 | 15 |
| - | - | 2266 | 543.3 | - | - | 0 | - |
| - | - | 928.3 | 543.8 | - | - | 0 | - |
| - | - | 6683 | 552.3 | - | - | 0 | - |
| - | - | 4851 | 552.8 | - | - | 0 | - |
| - | - | 1449 | 553.3 | - | - | 0 | - |
| - | - | 894.5 | 554.3 | - | - | 0 | - |
| 12 | w | 1.616E+04 | 558.3 | 0.0002995 | 0.5365 | +1 | 6 |
| - | - | 1775 | 558.3 | - | - | 0 | - |
| - | - | 3962 | 559.3 | - | - | 0 | - |
| - | - | 1247 | 559.3 | - | - | 0 | - |
| - | - | 814.3 | 560.3 | - | - | 0 | - |
| - | - | 1.093E+04 | 566.4 | - | - | 0 | - |
| - | - | 4071 | 567.4 | - | - | 0 | - |
| - | - | 767.7 | 572.3 | - | - | 0 | - |
| - | - | 679.6 | 577.1 | - | - | 0 | - |
| - | - | 1336 | 578.1 | - | - | 0 | - |
| - | - | 1188 | 579.1 | - | - | 0 | - |
| 7 | y | 704.8 | 580.3 | 0.002416 | 4.164 | +2 | 11 |
| 7 | y | 1297 | 580.8 | 0.006736 | 11.6 | +2 | 11 |
| - | - | 1306 | 583.3 | - | - | 0 | - |
| - | - | 727.8 | 584.3 | - | - | 0 | - |
| - | - | 998.4 | 591.3 | - | - | 0 | - |
| - | - | 690.6 | 594.4 | - | - | 0 | - |
| - | - | 680 | 599.3 | - | - | 0 | - |
| - | - | 1991 | 599.8 | - | - | 0 | - |
| - | - | 3473 | 602.8 | - | - | 0 | - |
| - | - | 3200 | 603.3 | - | - | 0 | - |
| - | - | 1043 | 603.8 | - | - | 0 | - |
| - | - | 8593 | 610.2 | - | - | 0 | - |
| - | - | 8211 | 611.2 | - | - | 0 | - |
| - | - | 746.6 | 611.3 | - | - | 0 | - |
| - | - | 7656 | 611.8 | - | - | 0 | - |
| - | - | 6954 | 612.2 | - | - | 0 | - |
| - | - | 720.8 | 612.2 | - | - | 0 | - |
| - | - | 731.9 | 612.2 | - | - | 0 | - |
| - | - | 5423 | 612.3 | - | - | 0 | - |
| - | - | 847 | 612.4 | - | - | 0 | - |
| - | - | 1752 | 612.8 | - | - | 0 | - |
| - | - | 767.1 | 613.2 | - | - | 0 | - |
| 12 | y | 7142 | 614.3 | 0.0109 | 17.74 | +1 | 6 |
| - | - | 753.5 | 614.8 | - | - | 0 | - |
| - | - | 9537 | 615.3 | - | - | 0 | - |
| 12 | z | 3974 | 616.3 | 0.0005355 | 0.8689 | +1 | 6 |
| - | - | 1401 | 616.4 | - | - | 0 | - |
| - | - | 1177 | 617.3 | - | - | 0 | - |
| - | - | 1141 | 617.8 | - | - | 0 | - |
| - | - | 630.7 | 618.3 | - | - | 0 | - |
| - | - | 1754 | 626.3 | - | - | 0 | - |
| - | - | 5045 | 626.8 | - | - | 0 | - |
| - | - | 3215 | 627.3 | - | - | 0 | - |
| - | - | 1921 | 627.3 | - | - | 0 | - |
| - | - | 1146 | 627.4 | - | - | 0 | - |
| - | - | 900.1 | 627.8 | - | - | 0 | - |
| - | - | 1171 | 628.3 | - | - | 0 | - |
| - | - | 2938 | 628.4 | - | - | 0 | - |
| 6 | y | 586.8 | 628.8 | 0.001393 | 2.216 | +2 | 12 |
| 6 | y | 905.5 | 629.3 | 0.005744 | 9.128 | +2 | 12 |
| - | - | 1203 | 629.4 | - | - | 0 | - |
| 12 | y | 5575 | 632.3 | 3.353E-05 | 0.05302 | +1 | 6 |
| - | - | 2243 | 633.3 | - | - | 0 | - |
| - | - | 1616 | 635.3 | - | - | 0 | - |
| - | - | 1175 | 635.8 | - | - | 0 | - |
| - | - | 695.5 | 636.3 | - | - | 0 | - |
| 6 | y | 563.9 | 637.8 | 0.00193 | 3.026 | +2 | 12 |
| - | - | 1049 | 638.3 | - | - | 0 | - |
| - | - | 1540 | 655.3 | - | - | 0 | - |
| - | - | 1208 | 655.8 | - | - | 0 | - |
| - | - | 820.9 | 656.3 | - | - | 0 | - |
| - | - | 1419 | 658.4 | - | - | 0 | - |
| 12 | c | 1151 | 664.8 | 0.005246 | 7.89 | +2 | 12 |
| - | - | 781.6 | 665.3 | - | - | 0 | - |
| - | - | 811 | 668.3 | - | - | 0 | - |
| - | - | 636.4 | 668.8 | - | - | 0 | - |
| - | - | 2542 | 671.4 | - | - | 0 | - |
| 6 | c | 3280 | 672.4 | 0.001744 | 2.594 | +1 | 6 |
| - | - | 1999 | 673.4 | - | - | 0 | - |
| - | - | 907.2 | 674.4 | - | - | 0 | - |
| - | - | 1631 | 676.8 | - | - | 0 | - |
| 5 | y | 7903 | 677.3 | 0.0004926 | 0.7273 | +2 | 13 |
| 5 | y | 6494 | 677.8 | 0.007866 | 11.6 | +2 | 13 |
| 5 | z | 3724 | 678.3 | 0.002977 | 4.388 | +2 | 13 |
| - | - | 1824 | 678.8 | - | - | 0 | - |
| - | - | 5147 | 685.8 | - | - | 0 | - |
| 5 | y | 5.207E+04 | 686.3 | 0.00277 | 4.036 | +2 | 13 |
| - | - | 1.948E+04 | 686.8 | - | - | 0 | - |
| - | - | 1.53E+04 | 687.3 | - | - | 0 | - |
| - | - | 2725 | 687.8 | - | - | 0 | - |
| - | - | 2074 | 688.4 | - | - | 0 | - |
| - | - | 1169 | 697.4 | - | - | 0 | - |
| - | - | 1013 | 716.4 | - | - | 0 | - |
| - | - | 1925 | 722.3 | - | - | 0 | - |
| - | - | 786.5 | 723.4 | - | - | 0 | - |
| - | - | 593.8 | 724.9 | - | - | 0 | - |
| 11 | z | 1.17E+04 | 729.4 | 0.0006151 | 0.8433 | +1 | 7 |
| - | - | 5276 | 730.4 | - | - | 0 | - |
| - | - | 1827 | 731.4 | - | - | 0 | - |
| - | - | 1353 | 740.3 | - | - | 0 | - |
| - | - | 1442 | 742.4 | - | - | 0 | - |
| 4 | z | 875.7 | 742.9 | 0.0002362 | 0.3179 | +2 | 14 |
| 11 | y | 7051 | 745.4 | 0.0009245 | 1.24 | +1 | 7 |
| - | - | 2718 | 746.4 | - | - | 0 | - |
| - | - | 835.6 | 747.4 | - | - | 0 | - |
| - | - | 748.8 | 750.4 | - | - | 0 | - |
| 4 | y | 2043 | 750.9 | 0.00125 | 1.665 | +2 | 14 |
| 4 | y | 1714 | 751.4 | 0.009486 | 12.63 | +2 | 14 |
| 4 | z | 763.6 | 751.9 | 0.007161 | 9.524 | +2 | 14 |
| - | - | 3003 | 757.4 | - | - | 0 | - |
| - | - | 928.3 | 758.4 | - | - | 0 | - |
| - | - | 1963 | 759.4 | - | - | 0 | - |
| 7 | c | 3.302E+04 | 759.4 | 0.0001115 | 0.1468 | +1 | 7 |
| 4 | y | 1.367E+04 | 759.9 | 0.000187 | 0.2461 | +2 | 14 |
| - | - | 7818 | 760.4 | - | - | 0 | - |
| - | - | 1.413E+04 | 760.4 | - | - | 0 | - |
| - | - | 4441 | 760.9 | - | - | 0 | - |
| - | - | 1352 | 761.4 | - | - | 0 | - |
| - | - | 4009 | 761.4 | - | - | 0 | - |
| - | - | 1020 | 762.5 | - | - | 0 | - |
| - | - | 6256 | 764.4 | - | - | 0 | - |
| - | - | 3320 | 765.4 | - | - | 0 | - |
| - | - | 1361 | 766.4 | - | - | 0 | - |
| - | - | 1004 | 790.4 | - | - | 0 | - |
| - | - | 791 | 798.4 | - | - | 0 | - |
| 10 | w | 1.203E+04 | 799.4 | 0.0001187 | 0.1485 | +1 | 8 |
| 3 | z | 997.9 | 799.9 | 0.01155 | 14.43 | +2 | 15 |
| - | - | 4880 | 800.4 | - | - | 0 | - |
| - | - | 1148 | 801.4 | - | - | 0 | - |
| - | - | 5037 | 802.4 | - | - | 0 | - |
| - | - | 2009 | 803.4 | - | - | 0 | - |
| 3 | y | 996.3 | 807.4 | 0.006826 | 8.454 | +2 | 15 |
| 3 | y | 1712 | 807.9 | 0.009141 | 11.32 | +2 | 15 |
| - | - | 858.9 | 808.4 | - | - | 0 | - |
| - | - | 1053 | 813.9 | - | - | 0 | - |
| - | - | 1197 | 815.4 | - | - | 0 | - |
| 3 | y | 3014 | 816.4 | 0.003584 | 4.39 | +2 | 15 |
| - | - | 2507 | 816.9 | - | - | 0 | - |
| - | - | 1123 | 817.4 | - | - | 0 | - |
| - | - | 863.2 | 821.9 | - | - | 0 | - |
| - | - | 4166 | 822.4 | - | - | 0 | - |
| - | - | 4511 | 823.4 | - | - | 0 | - |
| - | - | 1905 | 824.4 | - | - | 0 | - |
| - | - | 1086 | 829.4 | - | - | 0 | - |
| 15 | c | 3.334E+04 | 829.9 | 0.0003095 | 0.3729 | +2 | 15 |
| - | - | 2.889E+04 | 830.4 | - | - | 0 | - |
| - | - | 1.476E+04 | 830.9 | - | - | 0 | - |
| - | - | 6720 | 831.4 | - | - | 0 | - |
| - | - | 1716 | 831.9 | - | - | 0 | - |
| - | - | 745.3 | 835.9 | - | - | 0 | - |
| 10 | z | 688.6 | 840.4 | 0.001088 | 1.295 | +1 | 8 |
| - | - | 813.2 | 840.9 | - | - | 0 | - |
| 10 | z | 1099 | 841.4 | 0.0149 | 17.7 | +1 | 8 |
| - | - | 972.4 | 842.4 | - | - | 0 | - |
| - | - | 1518 | 843.4 | - | - | 0 | - |
| - | - | 1932 | 844 | - | - | 0 | - |
| - | - | 1753 | 844.5 | - | - | 0 | - |
| - | - | 792.9 | 845 | - | - | 0 | - |
| - | - | 875.8 | 845.5 | - | - | 0 | - |
| 8 | c | 2.795E+04 | 846.5 | 6.553E-05 | 0.07742 | +1 | 8 |
| - | - | 1.392E+04 | 847.5 | - | - | 0 | - |
| - | - | 4162 | 848.5 | - | - | 0 | - |
| - | - | 1968 | 848.9 | - | - | 0 | - |
| - | - | 1748 | 849.4 | - | - | 0 | - |
| 2 | z | 3225 | 849.9 | 0.0005881 | 0.6919 | +2 | 16 |
| 2 | z | 2805 | 850.4 | 0.009191 | 10.81 | +2 | 16 |
| - | - | 1163 | 850.9 | - | - | 0 | - |
| 16 | c | 3285 | 856.9 | 0.0004039 | 0.4713 | +2 | 16 |
| 10 | y | 3962 | 857.4 | 0.007891 | 9.203 | +1 | 8 |
| 2 | y | 4690 | 857.9 | 0.01265 | 14.74 | +2 | 16 |
| 2 | y | 2.412E+04 | 858.4 | 0.01674 | 19.5 | +2 | 16 |
| 2 | z | 2513 | 858.9 | 0.01667 | 19.41 | +2 | 16 |
| - | - | 1.034E+04 | 859.4 | - | - | 0 | - |
| - | - | 1197 | 859.9 | - | - | 0 | - |
| - | - | 4199 | 860.4 | - | - | 0 | - |
| - | - | 1123 | 864.5 | - | - | 0 | - |
| - | - | 3451 | 865 | - | - | 0 | - |
| 16 | c | 4.452E+04 | 865.5 | 0.0002986 | 0.3451 | +2 | 16 |
| - | - | 3.841E+04 | 866 | - | - | 0 | - |
| - | - | 2.65E+04 | 866.5 | - | - | 0 | - |
| - | - | 1.142E+04 | 867 | - | - | 0 | - |
| - | - | 2790 | 867.5 | - | - | 0 | - |
| - | - | 1523 | 867.9 | - | - | 0 | - |
| - | - | 1870 | 868.4 | - | - | 0 | - |
| - | - | 1014 | 869.4 | - | - | 0 | - |
| - | - | 766.5 | 870.9 | - | - | 0 | - |
| - | - | 1166 | 871.4 | - | - | 0 | - |
| - | - | 851.9 | 872.5 | - | - | 0 | - |
| - | - | 1529 | 872.9 | - | - | 0 | - |
| - | - | 2848 | 873.5 | - | - | 0 | - |
| 10 | y | 5332 | 874.5 | 1.838E-05 | 0.02102 | +1 | 8 |
| - | - | 1478 | 875.5 | - | - | 0 | - |
| - | - | 2462 | 880.4 | - | - | 0 | - |
| - | - | 6164 | 881 | - | - | 0 | - |
| - | - | 7958 | 881.5 | - | - | 0 | - |
| - | - | 2568 | 882 | - | - | 0 | - |
| - | - | 1386 | 882.5 | - | - | 0 | - |
| - | - | 3672 | 885.5 | - | - | 0 | - |
| - | - | 4602 | 886 | - | - | 0 | - |
| - | - | 2885 | 886.5 | - | - | 0 | - |
| - | - | 818.9 | 887 | - | - | 0 | - |
| - | - | 1548 | 887.5 | - | - | 0 | - |
| - | - | 1700 | 888 | - | - | 0 | - |
| - | - | 1118 | 888.5 | - | - | 0 | - |
| - | - | 1502 | 889 | - | - | 0 | - |
| - | - | 1878 | 889.4 | - | - | 0 | - |
| - | - | 893.3 | 894 | - | - | 0 | - |
| - | - | 2.028E+04 | 894.5 | - | - | 0 | - |
| - | - | 2.024E+04 | 895 | - | - | 0 | - |
| - | - | 1.07E+04 | 895.5 | - | - | 0 | - |
| - | - | 5898 | 896 | - | - | 0 | - |
| - | - | 1542 | 896.5 | - | - | 0 | - |
| - | - | 1473 | 899.5 | - | - | 0 | - |
| - | - | 2466 | 900 | - | - | 0 | - |
| - | - | 852.5 | 902.5 | - | - | 0 | - |
| - | - | 7494 | 907.4 | - | - | 0 | - |
| - | - | 4622 | 908 | - | - | 0 | - |
| - | - | 2081 | 908.4 | - | - | 0 | - |
| - | - | 4386 | 908.5 | - | - | 0 | - |
| - | - | 3640 | 909 | - | - | 0 | - |
| - | - | 1088 | 909.5 | - | - | 0 | - |
| - | - | 877.6 | 915.5 | - | - | 0 | - |
| - | - | 1927 | 916 | - | - | 0 | - |
| - | - | 4.978E+04 | 916.5 | - | - | 0 | - |
| - | - | 8.108E+04 | 917 | - | - | 0 | - |
| - | - | 5.426E+04 | 917.5 | - | - | 0 | - |
| - | - | 3.286E+04 | 918 | - | - | 0 | - |
| - | - | 1.032E+04 | 918.5 | - | - | 0 | - |
| - | - | 3389 | 919 | - | - | 0 | - |
| 9 | w | 5379 | 928.5 | 0.0005365 | 0.5779 | +1 | 9 |
| - | - | 5492 | 929.5 | - | - | 0 | - |
| - | - | 2484 | 930.5 | - | - | 0 | - |
| - | - | 1021 | 931.5 | - | - | 0 | - |
| - | - | 1037 | 939.4 | - | - | 0 | - |
| - | - | 689.8 | 940.5 | - | - | 0 | - |
| - | - | 2549 | 944.5 | - | - | 0 | - |
| - | - | 3704 | 945.5 | - | - | 0 | - |
| - | - | 1598 | 946.5 | - | - | 0 | - |
| - | - | 938.7 | 956.5 | - | - | 0 | - |
| - | - | 1237 | 958.5 | - | - | 0 | - |
| - | - | 1692 | 959.5 | - | - | 0 | - |
| - | - | 5403 | 960.5 | - | - | 0 | - |
| - | - | 2708 | 961.5 | - | - | 0 | - |
| - | - | 1.484E+04 | 973.5 | - | - | 0 | - |
| - | - | 8162 | 974.5 | - | - | 0 | - |
| 9 | c | 4.301E+04 | 975.5 | 0.0005354 | 0.5488 | +1 | 9 |
| - | - | 2.24E+04 | 976.5 | - | - | 0 | - |
| - | - | 7890 | 977.5 | - | - | 0 | - |
| - | - | 1447 | 978.5 | - | - | 0 | - |
| 9 | z | 1.922E+04 | 987.5 | 4.694E-05 | 0.04753 | +1 | 9 |
| - | - | 1.065E+04 | 988.5 | - | - | 0 | - |
| - | - | 3756 | 989.5 | - | - | 0 | - |
| - | - | 938.5 | 990.5 | - | - | 0 | - |
| - | - | 1357 | 1002 | - | - | 0 | - |
| - | - | 1081 | 1003 | - | - | 0 | - |
| 9 | y | 4198 | 1004 | 0.00748 | 7.453 | +1 | 9 |
| - | - | 3682 | 1004 | - | - | 0 | - |
| - | - | 866.9 | 1013 | - | - | 0 | - |
| - | - | 1156 | 1014 | - | - | 0 | - |
| - | - | 1192 | 1018 | - | - | 0 | - |
| - | - | 840.7 | 1019 | - | - | 0 | - |
| - | - | 883.9 | 1032 | - | - | 0 | - |
| - | - | 3277 | 1055 | - | - | 0 | - |
| 8 | z | 2735 | 1057 | 0.003444 | 3.26 | +1 | 10 |
| 8 | z | 1721 | 1057 | 0.01828 | 17.28 | +1 | 10 |
| - | - | 1145 | 1059 | - | - | 0 | - |
| - | - | 1618 | 1060 | - | - | 0 | - |
| - | - | 2802 | 1061 | - | - | 0 | - |
| - | - | 1680 | 1062 | - | - | 0 | - |
| - | - | 1438 | 1073 | - | - | 0 | - |
| 8 | y | 1467 | 1074 | 0.001384 | 1.29 | +1 | 10 |
| 8 | z | 2.094E+04 | 1075 | 0.0008253 | 0.7681 | +1 | 10 |
| - | - | 1.502E+04 | 1076 | - | - | 0 | - |
| - | - | 5781 | 1077 | - | - | 0 | - |
| - | - | 2097 | 1078 | - | - | 0 | - |
| - | - | 4240 | 1088 | - | - | 0 | - |
| - | - | 2232 | 1089 | - | - | 0 | - |
| - | - | 1847 | 1090 | - | - | 0 | - |
| 8 | y | 1.169E+04 | 1091 | 0.001727 | 1.584 | +1 | 10 |
| - | - | 8110 | 1092 | - | - | 0 | - |
| - | - | 2525 | 1093 | - | - | 0 | - |
| - | - | 2747 | 1099 | - | - | 0 | - |
| - | - | 1623 | 1100 | - | - | 0 | - |
| - | - | 3134 | 1104 | - | - | 0 | - |
| 10 | c | 4.582E+04 | 1105 | 0.001136 | 1.029 | +1 | 10 |
| - | - | 2.955E+04 | 1106 | - | - | 0 | - |
| - | - | 1.027E+04 | 1107 | - | - | 0 | - |
| - | - | 3052 | 1108 | - | - | 0 | - |
| - | - | 808.1 | 1109 | - | - | 0 | - |
| - | - | 753.6 | 1119 | - | - | 0 | - |
| - | - | 7800 | 1121 | - | - | 0 | - |
| - | - | 4613 | 1122 | - | - | 0 | - |
| - | - | 1806 | 1123 | - | - | 0 | - |
| 7 | y | 1089 | 1160 | 0.01269 | 10.95 | +1 | 11 |
| 7 | y | 937.4 | 1161 | 0.009639 | 8.306 | +1 | 11 |
| 7 | z | 8957 | 1162 | 0.0004714 | 0.4059 | +1 | 11 |
| - | - | 6635 | 1163 | - | - | 0 | - |
| - | - | 3747 | 1164 | - | - | 0 | - |
| - | - | 1572 | 1165 | - | - | 0 | - |
| - | - | 875 | 1166 | - | - | 0 | - |
| 7 | y | 6011 | 1178 | 0.0004304 | 0.3655 | +1 | 11 |
| - | - | 5934 | 1179 | - | - | 0 | - |
| - | - | 2311 | 1180 | - | - | 0 | - |
| - | - | 1133 | 1181 | - | - | 0 | - |
| - | - | 1605 | 1182 | - | - | 0 | - |
| - | - | 1293 | 1199 | - | - | 0 | - |
| 11 | c | 1510 | 1200 | 0.02349 | 19.58 | +1 | 11 |
| - | - | 1907 | 1201 | - | - | 0 | - |
| - | - | 1137 | 1202 | - | - | 0 | - |
| - | - | 1051 | 1206 | - | - | 0 | - |
| - | - | 1.08E+04 | 1207 | - | - | 0 | - |
| - | - | 8034 | 1208 | - | - | 0 | - |
| - | - | 2563 | 1209 | - | - | 0 | - |
| 11 | c | 2.664E+04 | 1218 | 0.001338 | 1.099 | +1 | 11 |
| - | - | 2.093E+04 | 1219 | - | - | 0 | - |
| - | - | 8206 | 1220 | - | - | 0 | - |
| - | - | 1480 | 1221 | - | - | 0 | - |
| - | - | 5851 | 1223 | - | - | 0 | - |
| - | - | 1.051E+04 | 1224 | - | - | 0 | - |
| - | - | 5454 | 1225 | - | - | 0 | - |
| - | - | 2300 | 1226 | - | - | 0 | - |
| - | - | 829.4 | 1245 | - | - | 0 | - |
| - | - | 2230 | 1253 | - | - | 0 | - |
| - | - | 1350 | 1254 | - | - | 0 | - |
| - | - | 1139 | 1270 | - | - | 0 | - |
| - | - | 1025 | 1271 | - | - | 0 | - |
| 6 | y | 2144 | 1275 | 0.003324 | 2.608 | +1 | 12 |
| - | - | 2258 | 1276 | - | - | 0 | - |
| - | - | 1665 | 1302 | - | - | 0 | - |
| - | - | 1697 | 1303 | - | - | 0 | - |
| - | - | 893.4 | 1328 | - | - | 0 | - |
| 12 | c | 2459 | 1329 | 0.01615 | 12.16 | +1 | 12 |
| - | - | 2422 | 1330 | - | - | 0 | - |
| - | - | 1805 | 1331 | - | - | 0 | - |
| - | - | 749.9 | 1332 | - | - | 0 | - |
| - | - | 3324 | 1345 | - | - | 0 | - |
| 12 | c | 3.581E+04 | 1346 | 0.00242 | 1.799 | +1 | 12 |
| - | - | 2.443E+04 | 1347 | - | - | 0 | - |
| - | - | 1.173E+04 | 1348 | - | - | 0 | - |
| - | - | 3958 | 1349 | - | - | 0 | - |
| - | - | 978.2 | 1350 | - | - | 0 | - |
| - | - | 770.2 | 1353 | - | - | 0 | - |
| 5 | y | 910.4 | 1355 | 0.005308 | 3.918 | +1 | 13 |
| 5 | z | 1063 | 1356 | 0.02177 | 16.06 | +1 | 13 |
| - | - | 2399 | 1371 | - | - | 0 | - |
| 5 | y | 9870 | 1372 | 0.003053 | 2.226 | +1 | 13 |
| - | - | 7254 | 1373 | - | - | 0 | - |
| - | - | 3877 | 1374 | - | - | 0 | - |
| - | - | 2027 | 1375 | - | - | 0 | - |
| - | - | 840.6 | 1398 | - | - | 0 | - |
| - | - | 1003 | 1399 | - | - | 0 | - |
| - | - | 2713 | 1400 | - | - | 0 | - |
| - | - | 4327 | 1401 | - | - | 0 | - |
| - | - | 4202 | 1402 | - | - | 0 | - |
| - | - | 3129 | 1403 | - | - | 0 | - |
| - | - | 1270 | 1404 | - | - | 0 | - |
| - | - | 4411 | 1416 | - | - | 0 | - |
| 13 | c | 2.469E+04 | 1417 | 0.003523 | 2.487 | +1 | 13 |
| - | - | 1.68E+04 | 1418 | - | - | 0 | - |
| - | - | 8412 | 1419 | - | - | 0 | - |
| - | - | 1906 | 1420 | - | - | 0 | - |
| - | - | 2403 | 1431 | - | - | 0 | - |
| - | - | 2075 | 1432 | - | - | 0 | - |
| - | - | 1244 | 1433 | - | - | 0 | - |
| - | - | 933.4 | 1476 | - | - | 0 | - |
| - | - | 1953 | 1487 | - | - | 0 | - |
| - | - | 1692 | 1488 | - | - | 0 | - |
| - | - | 1380 | 1489 | - | - | 0 | - |
| 4 | y | 1572 | 1502 | 0.007695 | 5.124 | +1 | 14 |
| 4 | z | 2.037E+04 | 1503 | 0.002083 | 1.386 | +1 | 14 |
| - | - | 3.356E+04 | 1504 | - | - | 0 | - |
| - | - | 2.315E+04 | 1505 | - | - | 0 | - |
| - | - | 1055 | 1505 | - | - | 0 | - |
| - | - | 9786 | 1506 | - | - | 0 | - |
| - | - | 3315 | 1507 | - | - | 0 | - |
| 14 | c | 1009 | 1514 | 0.003902 | 2.578 | +1 | 14 |
| 4 | y | 2859 | 1519 | 0.005793 | 3.814 | +1 | 14 |
| - | - | 2638 | 1520 | - | - | 0 | - |
| - | - | 2011 | 1521 | - | - | 0 | - |
| - | - | 738 | 1522 | - | - | 0 | - |
| 14 | c | 4.032E+04 | 1531 | 0.00275 | 1.796 | +1 | 14 |
| - | - | 3.334E+04 | 1532 | - | - | 0 | - |
| - | - | 1039 | 1532 | - | - | 0 | - |
| - | - | 1.603E+04 | 1533 | - | - | 0 | - |
| - | - | 6444 | 1534 | - | - | 0 | - |
| - | - | 1583 | 1535 | - | - | 0 | - |
| - | - | 885.5 | 1544 | - | - | 0 | - |
| - | - | 1120 | 1545 | - | - | 0 | - |
| - | - | 2752 | 1615 | - | - | 0 | - |
| 3 | z | 5132 | 1616 | 0.01993 | 12.34 | +1 | 15 |
| - | - | 7393 | 1617 | - | - | 0 | - |
| - | - | 5110 | 1618 | - | - | 0 | - |
| - | - | 2889 | 1619 | - | - | 0 | - |
| - | - | 808.9 | 1633 | - | - | 0 | - |
| - | - | 1893 | 1643 | - | - | 0 | - |
| - | - | 1521 | 1644 | - | - | 0 | - |
| - | - | 1338 | 1645 | - | - | 0 | - |
| 15 | c | 3696 | 1659 | 0.000798 | 0.4811 | +1 | 15 |
| - | - | 1.182E+04 | 1660 | - | - | 0 | - |
| - | - | 9544 | 1661 | - | - | 0 | - |
| - | - | 4313 | 1662 | - | - | 0 | - |
| - | - | 1172 | 1663 | - | - | 0 | - |
| - | - | 1016 | 1673 | - | - | 0 | - |
| - | - | 2732 | 1686 | - | - | 0 | - |
| - | - | 3459 | 1687 | - | - | 0 | - |
| - | - | 2763 | 1688 | - | - | 0 | - |
| - | - | 1009 | 1689 | - | - | 0 | - |
| - | - | 1240 | 1690 | - | - | 0 | - |
| 2 | z | 1425 | 1700 | 0.01516 | 8.918 | +1 | 16 |
| - | - | 1222 | 1701 | - | - | 0 | - |
| - | - | 4904 | 1714 | - | - | 0 | - |
| 2 | y | 3557 | 1715 | 0.0305 | 17.79 | +1 | 16 |
| - | - | 3573 | 1716 | - | - | 0 | - |
| 2 | z | 3913 | 1717 | 0.008142 | 4.742 | +1 | 16 |
| - | - | 9331 | 1718 | - | - | 0 | - |
| - | - | 7767 | 1719 | - | - | 0 | - |
| - | - | 3572 | 1720 | - | - | 0 | - |
| - | - | 1377 | 1721 | - | - | 0 | - |
| 16 | c | 5666 | 1730 | 0.00238 | 1.376 | +1 | 16 |
| - | - | 1.259E+04 | 1731 | - | - | 0 | - |
| - | - | 1.254E+04 | 1732 | - | - | 0 | - |
| - | - | 6199 | 1733 | - | - | 0 | - |
| - | - | 2912 | 1734 | - | - | 0 | - |
| - | - | 1147 | 1761 | - | - | 0 | - |
| - | - | 2811 | 1771 | - | - | 0 | - |
| - | - | 4364 | 1772 | - | - | 0 | - |
| - | - | 3849 | 1773 | - | - | 0 | - |
| - | - | 1007 | 1774 | - | - | 0 | - |
| - | - | 1203 | 1775 | - | - | 0 | - |
| - | - | 5462 | 1788 | - | - | 0 | - |
| - | - | 2.204E+04 | 1789 | - | - | 0 | - |
| - | - | 1.98E+04 | 1790 | - | - | 0 | - |
| - | - | 1.111E+04 | 1791 | - | - | 0 | - |
| - | - | 3739 | 1792 | - | - | 0 | - |
| - | - | 1065 | 1793 | - | - | 0 | - |
| - | - | 1150 | 1799 | - | - | 0 | - |
| - | - | 1663 | 1800 | - | - | 0 | - |
| - | - | 1472 | 1805 | - | - | 0 | - |
| - | - | 4754 | 1806 | - | - | 0 | - |
| - | - | 3916 | 1807 | - | - | 0 | - |
| - | - | 2373 | 1808 | - | - | 0 | - |
| - | - | 842.7 | 1809 | - | - | 0 | - |
| - | - | 991.8 | 1815 | - | - | 0 | - |
| - | - | 5588 | 1816 | - | - | 0 | - |
| - | - | 3.342E+04 | 1817 | - | - | 0 | - |
| - | - | 2.996E+04 | 1818 | - | - | 0 | - |
| - | - | 1.845E+04 | 1819 | - | - | 0 | - |
| - | - | 6392 | 1820 | - | - | 0 | - |
| - | - | 1409 | 1821 | - | - | 0 | - |
| - | - | 3907 | 1832 | - | - | 0 | - |
| - | - | 1.949E+04 | 1833 | - | - | 0 | - |
| - | - | 5.763E+04 | 1834 | - | - | 0 | - |
| - | - | 5.279E+04 | 1835 | - | - | 0 | - |
| - | - | 2.649E+04 | 1836 | - | - | 0 | - |
| - | - | 1.061E+04 | 1837 | - | - | 0 | - |
| - | - | 3358 | 1838 | - | - | 0 | - |

m/z Charge Intensity FragmentType MassShift Position
120.06143188476562 0 696.68396
120.06560516357422 0 11118.019 y 16
120.08100128173828 0 1809.5883
121.0692367553711 0 547.46875
124.55908966064453 0 371.43878
126.05525207519531 0 739.15735
128.09442138671875 0 960.52716
128.10696411132812 0 408.47168
129.10231018066406 0 4562.2944
129.1953125 0 396.3229
131.11807250976562 0 746.56866
140.10699462890625 0 474.4931
148.94754028320312 0 589.4428
149.0448455810547 0 1105.2852
151.33648681640625 0 454.15222
155.09420776367188 0 708.4674
155.1181182861328 0 565.4288
158.14134216308594 0 534.0505
169.1336212158203 0 2069.8303
171.0767822265625 0 657.1482
173.09214782714844 0 1294.6191 y Water loss 15
173.12843322753906 0 2899.6445
181.0966033935547 0 495.31393
183.1128387451172 0 3672.1968
185.1281280517578 0 548.62115
191.10276794433594 0 3994.2212 y 15
197.12840270996094 0 1701.5481
200.1395721435547 0 626.5208 c Water loss 1
201.12327575683594 0 7028.981
211.10830688476562 0 552.59735
212.13937377929688 0 8753.775
213.1427001953125 0 795.75684
215.1392822265625 0 1416.1409
218.14993286132812 0 4586.633 c 1
221.08424377441406 0 4220.372
222.08499145507812 0 749.8085
223.08212280273438 0 763.26935
225.80661010742188 0 478.75153
226.1187286376953 0 862.42975
227.09054565429688 0 988.1465
227.10255432128906 0 1642.3906
227.12693786621094 0 706.22107
233.16494750976562 0 1176.1436
235.10739135742188 0 1013.6435
239.09487915039062 0 5011.5386
240.09573364257812 0 1903.0446
242.1501007080078 0 727.1105
243.14486694335938 0 1363.7866 y Water loss 10
251.1764373779297 0 658.9261
254.14907836914062 0 1394.1986
261.15966796875 0 1005.4822
263.10235595703125 0 2039.936
272.1849670410156 0 805.99274
280.1778259277344 0 698.4395
281.05072021484375 0 1260.9365
281.13665771484375 0 655.50183
282.14544677734375 0 850.9801
296.1772766113281 0 835.7221
296.1968688964844 0 10828.605
297.0820617675781 0 631.09845
297.1562805175781 0 1001.0995
297.2003173828125 0 1633.8605
298.16241455078125 0 1104.414
299.06201171875 0 5174.771
299.1715087890625 0 13179.412
300.0614013671875 0 1784.9369
300.1752014160156 0 1103.9227
301.0579528808594 0 657.2571
303.17877197265625 0 6848.7134 z 14
304.1832275390625 0 1270.0298
314.1842346191406 0 982.837
314.207763671875 0 4471.845
315.21075439453125 0 935.43945
316.186767578125 0 5854.699
317.1905212402344 0 911.1123
319.1976623535156 0 4222.1284 y 14
320.2003479003906 0 740.7932
325.1875 0 1443.2091
331.23406982421875 0 25535.438 c 2
332.2375183105469 0 4461.1816
344.172607421875 0 741.8729
345.1795959472656 0 986.3441
346.1246337890625 0 607.65393
346.1844482421875 0 551.76715
347.19598388671875 0 653.45
348.1918029785156 0 540.60626
350.13433837890625 0 808.9845
355.0697021484375 0 38351.93
356.0704345703125 0 16836.96
357.06756591796875 0 9743.368
358.0696105957031 0 1037.3506
362.2078552246094 0 1413.5902
370.2080383300781 0 894.0711
371.1010437011719 0 1853.8386
371.1926574707031 0 610.83875
372.1015625 0 1292.6399
373.0789489746094 0 916.8136
373.2096862792969 0 3371.7341 y 10
373.71478271484375 0 763.63684
374.08074951171875 0 639.8141
385.2192077636719 0 1406.2267
385.41058349609375 0 699.2544
386.2024230957031 0 1753.8217
415.0357971191406 0 880.58704
415.2298583984375 0 859.51666 y Water loss 13
416.03570556640625 0 1175.9215
417.03411865234375 0 1075.0479
417.222412109375 0 2279.971 z 13
418.2294006347656 0 7195.1724
419.2333068847656 0 1166.9637
433.24127197265625 0 2723.7634 y 13
433.2810974121094 0 8120.567
434.2864990234375 0 4885.598
435.2946472167969 0 1829.8246
442.2394104003906 0 729.2164
444.24578857421875 0 5845.6177
445.2520751953125 0 3143.6953
446.2651672363281 0 2588.2551
460.26812744140625 0 1514.0149
461.276123046875 0 13395.945
462.27911376953125 0 3151.3176
463.2819519042969 0 743.3332
480.716552734375 0 799.12177
480.750732421875 0 1449.7213
481.2488098144531 0 1270.2938
486.2683410644531 0 689.80835 y Water loss 12
488.2590637207031 0 9197.543 c 8
489.05584716796875 0 1115.8505
489.26513671875 0 7165.095
490.0542297363281 0 627.1861
490.2680358886719 0 1419.8853
493.72802734375 0 2480.212
494.2323913574219 0 1086.4792
497.2613525390625 0 731.74744
498.2216491699219 0 696.4088
502.26507568359375 0 1958.8936 y 8
502.7331848144531 0 3364.4785
503.2338562011719 0 2320.2095
503.73516845703125 0 740.7586
504.2777404785156 0 9220.98 y 12
505.27984619140625 0 2057.6526
515.2930908203125 0 789.5569
538.2696533203125 0 4717.083
538.7706909179688 0 3370.6687
539.2733154296875 0 768.95526 z 2
543.2604370117188 0 2265.7397
543.760498046875 0 928.30115
552.2672119140625 0 6683.28
552.7693481445312 0 4851.3193
553.2711791992188 0 1448.6636
554.3196411132812 0 894.46124
558.2879028320312 0 16157.418 w 11
558.3289794921875 0 1774.9677
559.290283203125 0 3961.6
559.3318481445312 0 1247.353
560.2948608398438 0 814.3374
566.4273681640625 0 10933.563
567.4308471679688 0 4070.914
572.3045654296875 0 767.74884
577.1273803710938 0 679.64355
578.1268310546875 0 1335.6788
579.1235961914062 0 1188.3715
580.2807006835938 0 704.8489 y Water loss 6
580.7818603515625 0 1297.1383 y Ammonia loss 6
583.3348388671875 0 1306.02
584.3356323242188 0 727.7889
591.2978515625 0 998.39343
594.3878173828125 0 690.6424
599.2669067382812 0 680.00574
599.8055419921875 0 1990.7046
602.7907104492188 0 3473.1714
603.29296875 0 3200.3223
603.7950439453125 0 1043.2185
610.184326171875 0 8593.225
611.1849975585938 0 8211.243
611.32666015625 0 746.64514
611.7965087890625 0 7655.914
612.18310546875 0 6954.3027
612.2317504882812 0 720.8184
612.2421875 0 731.8558
612.2977294921875 0 5423.461
612.3963623046875 0 846.95337
612.8012084960938 0 1751.8846
613.18359375 0 767.1128
614.3365478515625 0 7141.6445 y Water loss 11
614.82177734375 0 753.47974
615.3448486328125 0 9537.43
616.3169555664062 0 3974.1753 z 11
616.3577880859375 0 1401.0656
617.3201293945312 0 1176.6735
617.8060913085938 0 1140.7832
618.3114013671875 0 630.7427
626.3431396484375 0 1753.6548
626.81201171875 0 5044.642
627.261474609375 0 3215.4858
627.3121337890625 0 1920.9808
627.357177734375 0 1146.1622
627.8157958984375 0 900.11487
628.264892578125 0 1171.4436
628.392578125 0 2938.293
628.80810546875 0 586.82605 y Water loss 5
629.3072509765625 0 905.5447 y Ammonia loss 5
629.3973999023438 0 1202.694
632.336181640625 0 5574.67 y 11
633.3370361328125 0 2243.4092
635.3232421875 0 1615.7711
635.8255615234375 0 1175.4073
636.332275390625 0 695.5097
637.8167114257812 0 563.8762 y 5
638.3157958984375 0 1048.5276
655.3425903320312 0 1539.5638
655.8419799804688 0 1207.7747
656.3387451171875 0 820.90436
658.36669921875 0 1418.888
664.8350219726562 0 1151.0624 c Ammonia loss 11
665.3370361328125 0 781.5896
668.3349609375 0 810.96643
668.8260498046875 0 636.41016
671.3739013671875 0 2541.9683
672.40966796875 0 3279.8433 c 5
673.4097900390625 0 1998.8588
674.4227905273438 0 907.19745
676.8320922851562 0 1631.1814
677.3353881835938 0 7903.17 y Water loss 4
677.8357543945312 0 6494.48 y Ammonia loss 4
678.3347778320312 0 3724.4158 z 4
678.8335571289062 0 1823.7065
685.8372802734375 0 5146.709
686.3439331054688 0 52071.87 y 4
686.8425903320312 0 19479.47
687.3485717773438 0 15303.316
687.8472290039062 0 2725.2693
688.3751831054688 0 2074.4546
697.416748046875 0 1169.472
716.4360961914062 0 1013.40045
722.3480834960938 0 1924.701
723.3538818359375 0 786.51807
724.869140625 0 593.78876
729.4009399414062 0 11698.823 z 10
730.4037475585938 0 5275.701
731.406494140625 0 1826.7053
740.3458862304688 0 1352.7936
742.4135131835938 0 1442.0316
742.8609619140625 0 875.74194 z Water loss 3
745.4212036132812 0 7050.812 y 10
746.4226684570312 0 2717.7275
747.4231567382812 0 835.58514
750.3651123046875 0 748.82983
750.871337890625 0 2043.3398 y Water loss 3
751.37158203125 0 1714.0638 y Ammonia loss 3
751.8731689453125 0 763.6055 z 3
757.4198608398438 0 3002.6448
758.4309692382812 0 928.25476
759.3731689453125 0 1963.4966
759.4400634765625 0 33023.85 c 6
759.8751831054688 0 13674.993 y 3
760.3750610351562 0 7818.0005
760.4435424804688 0 14132.394
760.8778686523438 0 4440.882
761.37744140625 0 1352.3866
761.447509765625 0 4008.7842
762.451904296875 0 1020.4848
764.36083984375 0 6255.659
765.3614501953125 0 3320.217
766.3661499023438 0 1361.0808
790.3980712890625 0 1003.5643
798.38916015625 0 790.9712
799.4307250976562 0 12026.912 w 9
799.9063110351562 0 997.94104 z Ammonia loss 2
800.4310302734375 0 4880.4355
801.43212890625 0 1147.6182
802.3812866210938 0 5037.455
803.385009765625 0 2009.324
807.4189453125 0 996.305 y Water loss 2
807.9132690429688 0 1711.7184 y Ammonia loss 2
808.366455078125 0 858.9135
813.9266357421875 0 1053.2239
815.39306640625 0 1197.2548
816.413818359375 0 3013.6267 y 2
816.9186401367188 0 2507.112
817.4231567382812 0 1122.8007
821.9346313476562 0 863.1866
822.3797607421875 0 4165.635
823.3849487304688 0 4511.0283
824.3896484375 0 1905.2839
829.443359375 0 1085.9658
829.9407348632812 0 33341.22 c 14
830.4422607421875 0 28890.94
830.942626953125 0 14755.6045
831.4439086914062 0 6720.021
831.9419555664062 0 1715.8325
835.93994140625 0 745.3289
840.4324951171875 0 688.5819 z Water loss 9
840.9248046875 0 813.20184
841.4324951171875 0 1099.2399 z Ammonia loss 9
842.4461059570312 0 972.4243
843.4486083984375 0 1517.7545
843.9559936523438 0 1931.9935
844.45458984375 0 1752.7014
844.9552612304688 0 792.8647
845.457763671875 0 875.75793
846.4720458984375 0 27949.777 c 7
847.4746704101562 0 13923.5
848.4769287109375 0 4162.043
848.9317016601562 0 1968.0967
849.433349609375 0 1747.7991
849.9271850585938 0 3225.3901 z Water loss 1
850.4277954101562 0 2804.8455 z Ammonia loss 1
850.9312133789062 0 1163.031
856.9459228515625 0 3285.3052 c Ammonia loss 15
857.4442138671875 0 3962.0574 y Ammonia loss 9
857.9486083984375 0 4690.0225 y Water loss 1
858.4447021484375 0 24116.197 y Ammonia loss 1
858.9485473632812 0 2513.4424 z 1
859.4448852539062 0 10344.589
859.9380493164062 0 1197.047
860.44580078125 0 4199.0405
864.4573974609375 0 1122.9949
864.9605712890625 0 3451.3564
865.4598999023438 0 44515.3 c 15
865.9608154296875 0 38407.523
866.4603881835938 0 26497.152
866.959716796875 0 11420.18
867.456787109375 0 2789.9536
867.94970703125 0 1522.54
868.4034423828125 0 1869.667
869.4031372070312 0 1013.86285
870.94677734375 0 766.5229
871.44677734375 0 1165.7356
872.4583129882812 0 851.9393
872.9473266601562 0 1529.0188
873.4569091796875 0 2848.0378
874.462890625 0 5331.9233 y 9
875.4650268554688 0 1477.8989
880.4489135742188 0 2462.1816
880.9639282226562 0 6163.988
881.4638061523438 0 7957.777
881.966796875 0 2567.7585
882.4686279296875 0 1385.6455
885.466796875 0 3671.5188
885.96923828125 0 4602.333
886.4645385742188 0 2885.0732
886.9652099609375 0 818.8678
887.463134765625 0 1547.6096
887.9620971679688 0 1700.3796
888.4641723632812 0 1118.0537
888.9545288085938 0 1502.4143
889.44921875 0 1878.0476
893.9700927734375 0 893.2771
894.4691162109375 0 20283.959
894.9697265625 0 20238.596
895.4710693359375 0 10701.106
895.9690551757812 0 5898.279
896.466064453125 0 1541.7344
899.4642333984375 0 1473.436
899.9608764648438 0 2466.0515
902.4679565429688 0 852.4573
907.404541015625 0 7494.463
907.9733276367188 0 4622.4233
908.396728515625 0 2080.7876
908.472412109375 0 4386.4683
908.9716186523438 0 3640.4739
909.4736328125 0 1087.5881
915.4640502929688 0 877.59265
915.9666748046875 0 1926.8352
916.4752807617188 0 49775.938
916.977294921875 0 81076.89
917.47900390625 0 54260.26
917.981201171875 0 32860.605
918.4822998046875 0 10321.611
918.9830932617188 0 3389.2366
928.472900390625 0 5378.949 w 8
929.4765625 0 5491.97
930.4811401367188 0 2483.9417
931.4921875 0 1020.5977
939.4413452148438 0 1036.9401
940.475830078125 0 689.7747
944.4716186523438 0 2549.3345
945.4774169921875 0 3703.884
946.4844360351562 0 1598.346
956.462158203125 0 938.65234
958.4854125976562 0 1237.0774
959.4853515625 0 1692.3762
960.4896850585938 0 5403.0137
961.4915771484375 0 2708.2512
973.4948120117188 0 14840.024
974.4968872070312 0 8161.5186
975.5140380859375 0 43008.477 c 8
976.5172729492188 0 22401.248
977.5198974609375 0 7890.1167
978.5191040039062 0 1446.509
987.4866943359375 0 19222.06 z 8
988.4886474609375 0 10647.476
989.4916381835938 0 3756.3428
990.501953125 0 938.49133
1001.5269775390625 0 1357.0725
1002.5226440429688 0 1080.7354
1003.4979858398438 0 4197.589 y 8
1004.4979248046875 0 3682.383
1012.5249633789062 0 866.91
1013.5242309570312 0 1155.7948
1018.4523315429688 0 1191.5348
1019.4691772460938 0 840.7484
1031.5076904296875 0 883.9204
1055.497314453125 0 3277.4722
1056.5047607421875 0 2735.1116 z Water loss 7
1057.510498046875 0 1720.977 z Ammonia loss 7
1058.513427734375 0 1144.5432
1059.512939453125 0 1617.8539
1060.532470703125 0 2802.3347
1061.5338134765625 0 1680.3995
1072.504638671875 0 1438.3601
1073.5123291015625 0 1466.718 y Ammonia loss 7
1074.5179443359375 0 20942.018 z 7
1075.5225830078125 0 15017.986
1076.52587890625 0 5780.8394
1077.5289306640625 0 2097.136
1087.5299072265625 0 4240.1157
1088.5308837890625 0 2232.2097
1089.5301513671875 0 1846.8885
1090.5357666015625 0 11692.886 y 7
1091.5394287109375 0 8110.4272
1092.5401611328125 0 2524.7764
1098.50634765625 0 2746.5854
1099.52001953125 0 1623.3922
1103.5262451171875 0 3133.964
1104.5560302734375 0 45822.855 c 9
1105.5587158203125 0 29552.39
1106.561279296875 0 10266.072
1107.564697265625 0 3051.7712
1108.57763671875 0 808.082
1118.5396728515625 0 753.6067
1120.5523681640625 0 7799.969
1121.556640625 0 4612.5356
1122.558349609375 0 1806.272
1159.5462646484375 0 1088.6387 y Water loss 6
1160.5526123046875 0 937.36926 y Ammonia loss 6
1161.55126953125 0 8957.04 z 6
1162.5531005859375 0 6634.722
1163.5631103515625 0 3746.934
1164.5628662109375 0 1571.7963
1165.56884765625 0 874.98553
1177.569091796875 0 6010.8447 y 6
1178.5733642578125 0 5934.189
1179.5728759765625 0 2311.0344
1180.5802001953125 0 1133.1832
1181.5748291015625 0 1604.6415
1198.608154296875 0 1292.9309
1199.607177734375 0 1509.7904 c Water loss 10
1200.6121826171875 0 1906.9375
1201.615234375 0 1136.5193
1205.5826416015625 0 1051.1027
1206.568603515625 0 10797.521
1207.568603515625 0 8033.9336
1208.5714111328125 0 2562.5044
1217.639892578125 0 26644.934 c 10
1218.6422119140625 0 20927.285
1219.6466064453125 0 8206.347
1220.6395263671875 0 1480.136
1222.5826416015625 0 5851.207
1223.5885009765625 0 10505.1045
1224.59326171875 0 5454.478
1225.59326171875 0 2300.1836
1244.65380859375 0 829.366
1252.6126708984375 0 2229.875
1253.6160888671875 0 1349.8323
1269.6409912109375 0 1139.2095
1270.644287109375 0 1025.1066
1274.6256103515625 0 2144.4827 y 5
1275.6240234375 0 2258.1118
1301.6719970703125 0 1664.5652
1302.6787109375 0 1697.4569
1327.65234375 0 893.44
1328.6571044921875 0 2459.2017 c Ammonia loss 11
1329.658203125 0 2422.3674
1330.6552734375 0 1805.2306
1331.654541015625 0 749.8601
1344.686279296875 0 3324.2021
1345.6973876953125 0 35807.234 c 11
1346.70166015625 0 24433.438
1347.70458984375 0 11729.617
1348.7020263671875 0 3957.9968
1349.700927734375 0 978.22815
1352.6556396484375 0 770.2027
1354.65380859375 0 910.3952 y Ammonia loss 4
1355.6781005859375 0 1062.984 z 4
1370.6683349609375 0 2399.26
1371.6719970703125 0 9870.104 y 4
1372.6785888671875 0 7253.833
1373.685546875 0 3876.5378
1374.6888427734375 0 2026.9241
1397.708984375 0 840.5546
1398.673828125 0 1003.2554
1399.6724853515625 0 2713.214
1400.68359375 0 4326.669
1401.6944580078125 0 4201.5317
1402.694580078125 0 3128.67
1403.6993408203125 0 1269.753
1415.726318359375 0 4411.0522
1416.7333984375 0 24688.152 c 12
1417.7371826171875 0 16798.627
1418.7391357421875 0 8412.495
1419.7396240234375 0 1906.093
1430.7005615234375 0 2403.0144
1431.6988525390625 0 2075.4336
1432.702392578125 0 1244.489
1475.72705078125 0 933.3515
1486.7481689453125 0 1952.5719
1487.769287109375 0 1692.3373
1488.7635498046875 0 1379.8442
1501.724609375 0 1572.2651 y Ammonia loss 3
1502.72265625 0 20371.217 z 3
1503.728759765625 0 33563.965
1504.731201171875 0 23153.047
1504.9486083984375 0 1054.8438
1505.734619140625 0 9786.046
1506.734130859375 0 3315.313
1513.7572021484375 0 1008.67316 c Ammonia loss 13
1518.7376708984375 0 2858.8147 y 3
1519.74267578125 0 2638.0732
1520.7567138671875 0 2010.982
1521.7525634765625 0 737.9565
1530.777099609375 0 40316.656 c 13
1531.7796630859375 0 33343.63
1532.015869140625 0 1039.2485
1532.78271484375 0 16027.735
1533.7867431640625 0 6444.3965
1534.789306640625 0 1582.9382
1543.7750244140625 0 885.49817
1544.7880859375 0 1120.215
1614.8431396484375 0 2751.9893
1615.8287353515625 0 5131.703 z 2
1616.8173828125 0 7392.6465
1617.82470703125 0 5110.1772
1618.82421875 0 2889.4268
1632.8323974609375 0 808.9215
1642.8624267578125 0 1892.9862
1643.859375 0 1521.0769
1644.8660888671875 0 1337.8071
1658.8756103515625 0 3695.659 c 14
1659.8798828125 0 11820.969
1660.8829345703125 0 9544.221
1661.883056640625 0 4313.3115
1662.876708984375 0 1172.1746
1672.847412109375 0 1015.721
1685.8939208984375 0 2732.4727
1686.9053955078125 0 3458.5154
1687.902099609375 0 2763.0125
1688.913818359375 0 1008.73773
1689.9083251953125 0 1239.6312
1699.8450927734375 0 1424.7391 z Ammonia loss 1
1700.8638916015625 0 1221.6735
1713.887939453125 0 4904.2285
1714.8951416015625 0 3557.0032 y Water loss 1
1715.8848876953125 0 3573.3254
1716.8646240234375 0 3912.731 z 1
1717.860595703125 0 9331.384
1718.865478515625 0 7767.077
1719.866455078125 0 3572.08
1720.8802490234375 0 1376.9133
1729.9095458984375 0 5666.0176 c 15
1730.9180908203125 0 12594.802
1731.9212646484375 0 12540.045
1732.9212646484375 0 6198.667
1733.9263916015625 0 2911.5894
1760.9420166015625 0 1147.1172
1770.9217529296875 0 2811.0913
1771.920654296875 0 4364.4917
1772.916748046875 0 3848.8948
1773.9117431640625 0 1006.76685
1774.92626953125 0 1202.5145
1787.93505859375 0 5461.5156
1788.936279296875 0 22040.844
1789.93896484375 0 19804.004
1790.9403076171875 0 11111.916
1791.9427490234375 0 3739.0825
1792.9471435546875 0 1064.81
1798.908447265625 0 1149.8037
1799.9197998046875 0 1663.2184
1804.9483642578125 0 1471.9006
1805.9635009765625 0 4753.7266
1806.962158203125 0 3915.7092
1807.9637451171875 0 2373.2976
1808.964111328125 0 842.7259
1814.9329833984375 0 991.80426
1815.9305419921875 0 5588.0615
1816.93115234375 0 33422.438
1817.93408203125 0 29962.709
1818.9368896484375 0 18452.557
1819.9351806640625 0 6391.639
1820.943359375 0 1409.1987
1831.9415283203125 0 3907.4263
1832.9476318359375 0 19489.297
1833.955322265625 0 57630.895
1834.9598388671875 0 52789.152
1835.9615478515625 0 26490.283
1836.9637451171875 0 10608.607
1837.964111328125 0 3357.6519

Spectrum Details

|  |  |
| --- | --- |
| Matched peaks? Matched peaksThe total absolute number of peaks matched. Additionally in brackets the total fraction of peaks matched and the total number of peaks is shown. | 93 (15.55% of 598) |
| FDR? FDRThe false discovery rate estimated for this peptide. It is calculated by matching all theoretical fragments with a non-integer shift with the raw peaks for this spectrum. This is done with 40 different shifts. The resulting percentage is the average number of annotated peaks over the number of annotated peaks with the correct spectrum. | 1.05% |
| Satellite FDR? Satellite FDRSee the FDR for details on its calculation. This satellite ion specific FDR only contains the satellite ions (d/w) for I/L/J positions. | - |
| PSM Score? PSM ScoreThe PSM Score as given by Hecklib to this annotated spectrum. It is shown with three significant figures. | 833 |

## Spectrum 7949? Spectrum 7949 The raw spectrum of this peptide as annotated by Hecklib. The fragments are coloured according to ion type (see legend). Any peaks with a star '\*' as text can be hovered over to see the full details, first the ion type second the mass shift type. By hovering over the amino acids in the peptide or ions in the legend the corresponding peaks are highlighted. By toggling the 'Unassigned' label you can turn the background (unassigned) peaks on or off in the plot. By updating the slider in the Ion legend you can update the spectrum to only show the top X% of the peaks with labels. The top X% means any peak that is within X% of the highest intensity. By dragging in the spectrum you can zoom in to a specific part of the spectrum and use 'Zoom Out' to get back to the original zoom level. The annotation of the spectrum is based on the given sequence in the peptides file and is done with different software so inconsistencies are likely. The peaks are annotated based on the given sequence, with 20 ppm tolerance.

Copy Data

### Spectrum 7949 (TSV)

#### Preview

```
Loading example...
```

*Click on the button to copy the data to your clipboard.*

Mz MinMz MaxIntensity Max

WidthHeightPeptide font sizePeptide stroke widthSpectrum font sizeSpectrum stroke widthCompact peptide

Ion legend

wxyz

abcd

OtherUnassignedIonChargePositionShow for top:%

VTJFPPSSEEJQANKAT

02.35e+44.70e+47.04e+49.39e+4

Zoom Out

y+11y+12y+12c+12c+12y+37z+38z+13y+26y+26y+13c+13y+27y+14z+14y+14y+313y+15c+29y+29y+15w+210z+315w+16y+211c+211y+16z+16y+212y+16y+212c+212c+16c+212y+213y+213z+213y+213c+213z+17z+214y+17y+214y+214z+214c+17y+214w+18z+215y+215y+215z+215y+215c+215z+18z+18c+18z+216z+216c+216y+18y+216y+216z+216c+216y+18w+19z+19c+19z+19y+19z+110z+110y+110y+110z+110y+110c+110z+111y+111y+111z+111y+111c+111c+111y+112c+112c+112y+113y+113c+113y+114z+114y+114c+114z+115c+115z+116y+116z+116c+116

0775155123263102

Fragment Matches Table

Show background peaks

| Position | Ion type | Intensity | mz Theoretical | mz Error (Th) | mz Error (ppm) | Charge | Series Number |
| --- | --- | --- | --- | --- | --- | --- | --- |
| 17 | y | 1.152E+04 | 120.1 | 0.0001008 | 0.8396 | +1 | 1 |
| - | - | 2237 | 120.1 | - | - | 0 | - |
| - | - | 363.4 | 121.1 | - | - | 0 | - |
| - | - | 405.9 | 125.9 | - | - | 0 | - |
| - | - | 1777 | 126.1 | - | - | 0 | - |
| - | - | 338 | 126.6 | - | - | 0 | - |
| - | - | 807.9 | 128.1 | - | - | 0 | - |
| - | - | 956.6 | 128.1 | - | - | 0 | - |
| - | - | 5174 | 129.1 | - | - | 0 | - |
| - | - | 436.6 | 130.3 | - | - | 0 | - |
| - | - | 1084 | 131.1 | - | - | 0 | - |
| - | - | 391 | 132.6 | - | - | 0 | - |
| - | - | 379.5 | 136.6 | - | - | 0 | - |
| - | - | 407.2 | 137.9 | - | - | 0 | - |
| - | - | 1009 | 140.1 | - | - | 0 | - |
| - | - | 417.4 | 143.7 | - | - | 0 | - |
| - | - | 1019 | 149 | - | - | 0 | - |
| - | - | 642.7 | 149 | - | - | 0 | - |
| - | - | 861.7 | 155.1 | - | - | 0 | - |
| - | - | 625 | 157.1 | - | - | 0 | - |
| - | - | 537 | 158.1 | - | - | 0 | - |
| - | - | 2864 | 169.1 | - | - | 0 | - |
| - | - | 1249 | 171.1 | - | - | 0 | - |
| 16 | y | 1365 | 173.1 | 0.000308 | 1.779 | +1 | 2 |
| - | - | 3363 | 173.1 | - | - | 0 | - |
| - | - | 1460 | 173.4 | - | - | 0 | - |
| - | - | 2508 | 183.1 | - | - | 0 | - |
| 16 | y | 2857 | 191.1 | 0.000104 | 0.5443 | +1 | 2 |
| - | - | 2298 | 197.1 | - | - | 0 | - |
| 2 | c | 689.8 | 200.1 | 0.0004372 | 2.185 | +1 | 2 |
| - | - | 7348 | 201.1 | - | - | 0 | - |
| - | - | 630.8 | 211.1 | - | - | 0 | - |
| - | - | 9954 | 212.1 | - | - | 0 | - |
| - | - | 1236 | 213.1 | - | - | 0 | - |
| - | - | 750.9 | 215.1 | - | - | 0 | - |
| 2 | c | 4409 | 218.1 | 3.018E-05 | 0.1383 | +1 | 2 |
| - | - | 3218 | 221.1 | - | - | 0 | - |
| - | - | 1058 | 222.1 | - | - | 0 | - |
| - | - | 573.6 | 223.1 | - | - | 0 | - |
| - | - | 1350 | 227.1 | - | - | 0 | - |
| - | - | 1873 | 227.1 | - | - | 0 | - |
| - | - | 1507 | 233.2 | - | - | 0 | - |
| - | - | 863.4 | 235.1 | - | - | 0 | - |
| - | - | 4216 | 239.1 | - | - | 0 | - |
| - | - | 1336 | 240.1 | - | - | 0 | - |
| - | - | 566.8 | 241.1 | - | - | 0 | - |
| - | - | 808.2 | 242.2 | - | - | 0 | - |
| 11 | y | 1134 | 243.1 | 0.004314 | 17.74 | +3 | 7 |
| - | - | 1105 | 254.1 | - | - | 0 | - |
| - | - | 598.4 | 256.1 | - | - | 0 | - |
| - | - | 1251 | 261.2 | - | - | 0 | - |
| - | - | 3035 | 263.1 | - | - | 0 | - |
| - | - | 754.1 | 264.1 | - | - | 0 | - |
| - | - | 777.8 | 280.2 | - | - | 0 | - |
| - | - | 1243 | 281.1 | - | - | 0 | - |
| 10 | z | 801.8 | 281.1 | 0.005165 | 18.37 | +3 | 8 |
| - | - | 624.4 | 281.2 | - | - | 0 | - |
| - | - | 630 | 282.1 | - | - | 0 | - |
| - | - | 965.1 | 282.1 | - | - | 0 | - |
| - | - | 1.181E+04 | 296.2 | - | - | 0 | - |
| - | - | 1136 | 297.2 | - | - | 0 | - |
| - | - | 2066 | 297.2 | - | - | 0 | - |
| - | - | 585.5 | 298.1 | - | - | 0 | - |
| - | - | 846.4 | 298.2 | - | - | 0 | - |
| - | - | 4584 | 299.1 | - | - | 0 | - |
| - | - | 1.493E+04 | 299.2 | - | - | 0 | - |
| - | - | 2242 | 300.1 | - | - | 0 | - |
| - | - | 562.5 | 300.2 | - | - | 0 | - |
| - | - | 2249 | 300.2 | - | - | 0 | - |
| - | - | 1064 | 301.1 | - | - | 0 | - |
| 15 | z | 7922 | 303.2 | 3.934E-05 | 0.1298 | +1 | 3 |
| - | - | 1647 | 304.2 | - | - | 0 | - |
| - | - | 729.8 | 307.2 | - | - | 0 | - |
| 12 | y | 798.4 | 307.7 | 9.922E-06 | 0.03225 | +2 | 6 |
| - | - | 713.4 | 314.2 | - | - | 0 | - |
| - | - | 4762 | 314.2 | - | - | 0 | - |
| - | - | 871.4 | 315.2 | - | - | 0 | - |
| - | - | 7705 | 316.2 | - | - | 0 | - |
| 12 | y | 1149 | 316.7 | 3.764E-05 | 0.1189 | +2 | 6 |
| - | - | 523.8 | 317.1 | - | - | 0 | - |
| - | - | 871.9 | 317.2 | - | - | 0 | - |
| 15 | y | 5298 | 319.2 | 2.562E-05 | 0.08026 | +1 | 3 |
| - | - | 946.9 | 325.2 | - | - | 0 | - |
| 3 | c | 2.766E+04 | 331.2 | 8.79E-05 | 0.2654 | +1 | 3 |
| - | - | 5090 | 332.2 | - | - | 0 | - |
| - | - | 669 | 333.2 | - | - | 0 | - |
| - | - | 593.2 | 344.2 | - | - | 0 | - |
| - | - | 867.7 | 345.2 | - | - | 0 | - |
| - | - | 658.5 | 346.2 | - | - | 0 | - |
| - | - | 1185 | 347.2 | - | - | 0 | - |
| - | - | 895 | 350.1 | - | - | 0 | - |
| - | - | 3.708E+04 | 355.1 | - | - | 0 | - |
| - | - | 1.617E+04 | 356.1 | - | - | 0 | - |
| - | - | 8737 | 357.1 | - | - | 0 | - |
| - | - | 882.5 | 358.1 | - | - | 0 | - |
| - | - | 1692 | 362.2 | - | - | 0 | - |
| - | - | 811.2 | 369.2 | - | - | 0 | - |
| - | - | 1106 | 371.1 | - | - | 0 | - |
| - | - | 989.6 | 372.1 | - | - | 0 | - |
| - | - | 1045 | 373.1 | - | - | 0 | - |
| 11 | y | 4325 | 373.2 | 0.004732 | 12.68 | +2 | 7 |
| - | - | 1329 | 378.2 | - | - | 0 | - |
| - | - | 781.5 | 385.2 | - | - | 0 | - |
| - | - | 1325 | 386.2 | - | - | 0 | - |
| - | - | 1792 | 415 | - | - | 0 | - |
| 14 | y | 905.4 | 415.2 | 0.002249 | 5.416 | +1 | 4 |
| - | - | 1112 | 416 | - | - | 0 | - |
| - | - | 911.9 | 417 | - | - | 0 | - |
| 14 | z | 2614 | 417.2 | 0.00107 | 2.565 | +1 | 4 |
| - | - | 7721 | 418.2 | - | - | 0 | - |
| - | - | 2174 | 419.2 | - | - | 0 | - |
| - | - | 813.1 | 426.2 | - | - | 0 | - |
| 14 | y | 2721 | 433.2 | 0.001755 | 4.051 | +1 | 4 |
| - | - | 8883 | 433.3 | - | - | 0 | - |
| - | - | 598.7 | 434.3 | - | - | 0 | - |
| - | - | 4960 | 434.3 | - | - | 0 | - |
| - | - | 678.7 | 434.7 | - | - | 0 | - |
| - | - | 2119 | 435.3 | - | - | 0 | - |
| - | - | 6883 | 444.2 | - | - | 0 | - |
| - | - | 611 | 445.2 | - | - | 0 | - |
| - | - | 2406 | 445.3 | - | - | 0 | - |
| - | - | 875.6 | 446.2 | - | - | 0 | - |
| - | - | 1780 | 446.3 | - | - | 0 | - |
| 5 | y | 861.6 | 451.9 | 0.001335 | 2.955 | +3 | 13 |
| - | - | 723.3 | 453.2 | - | - | 0 | - |
| - | - | 1503 | 460.3 | - | - | 0 | - |
| - | - | 1.288E+04 | 461.3 | - | - | 0 | - |
| - | - | 3667 | 462.3 | - | - | 0 | - |
| - | - | 605.1 | 472.2 | - | - | 0 | - |
| - | - | 698 | 473.2 | - | - | 0 | - |
| 13 | y | 760.3 | 486.3 | 0.0005052 | 1.039 | +1 | 5 |
| 9 | c | 1.127E+04 | 488.3 | 0.001739 | 3.562 | +2 | 9 |
| - | - | 1125 | 488.7 | - | - | 0 | - |
| - | - | 861.6 | 489.1 | - | - | 0 | - |
| - | - | 7632 | 489.3 | - | - | 0 | - |
| - | - | 1230 | 490.1 | - | - | 0 | - |
| - | - | 1492 | 490.3 | - | - | 0 | - |
| - | - | 1757 | 493.7 | - | - | 0 | - |
| - | - | 1422 | 494.2 | - | - | 0 | - |
| - | - | 713.9 | 496.2 | - | - | 0 | - |
| - | - | 979 | 497.3 | - | - | 0 | - |
| - | - | 1175 | 498.2 | - | - | 0 | - |
| 9 | y | 1891 | 502.3 | 0.008674 | 17.27 | +2 | 9 |
| - | - | 2414 | 502.7 | - | - | 0 | - |
| - | - | 1187 | 503.2 | - | - | 0 | - |
| - | - | 1033 | 503.3 | - | - | 0 | - |
| - | - | 589.4 | 503.7 | - | - | 0 | - |
| 13 | y | 8432 | 504.3 | 0.0002859 | 0.567 | +1 | 5 |
| - | - | 1866 | 505.3 | - | - | 0 | - |
| - | - | 998.7 | 515.3 | - | - | 0 | - |
| 8 | w | 795.7 | 529.3 | 0.002202 | 4.16 | +2 | 10 |
| - | - | 765.3 | 529.8 | - | - | 0 | - |
| - | - | 7050 | 538.3 | - | - | 0 | - |
| - | - | 2664 | 538.8 | - | - | 0 | - |
| 3 | z | 2239 | 539.3 | 0.001625 | 3.013 | +3 | 15 |
| - | - | 725.5 | 540.3 | - | - | 0 | - |
| - | - | 1732 | 543.3 | - | - | 0 | - |
| - | - | 1348 | 543.8 | - | - | 0 | - |
| - | - | 752.2 | 544.3 | - | - | 0 | - |
| - | - | 921.5 | 544.8 | - | - | 0 | - |
| - | - | 5422 | 552.3 | - | - | 0 | - |
| - | - | 3865 | 552.8 | - | - | 0 | - |
| - | - | 693.1 | 554.3 | - | - | 0 | - |
| - | - | 1009 | 557.3 | - | - | 0 | - |
| 12 | w | 1.564E+04 | 558.3 | 0.0001774 | 0.3178 | +1 | 6 |
| - | - | 2311 | 558.3 | - | - | 0 | - |
| - | - | 3847 | 559.3 | - | - | 0 | - |
| - | - | 1542 | 559.3 | - | - | 0 | - |
| - | - | 737.7 | 560.3 | - | - | 0 | - |
| - | - | 849.8 | 572.3 | - | - | 0 | - |
| - | - | 1069 | 577.1 | - | - | 0 | - |
| - | - | 701.2 | 578.1 | - | - | 0 | - |
| - | - | 649 | 579.1 | - | - | 0 | - |
| 7 | y | 594.3 | 580.8 | 0.002829 | 4.872 | +2 | 11 |
| - | - | 865.1 | 582.3 | - | - | 0 | - |
| - | - | 876.6 | 582.8 | - | - | 0 | - |
| - | - | 1557 | 583.3 | - | - | 0 | - |
| - | - | 708.4 | 599.3 | - | - | 0 | - |
| - | - | 2208 | 599.8 | - | - | 0 | - |
| 11 | c | 1204 | 600.3 | 0.01013 | 16.88 | +2 | 11 |
| - | - | 747.9 | 600.8 | - | - | 0 | - |
| - | - | 3201 | 602.8 | - | - | 0 | - |
| - | - | 2786 | 603.3 | - | - | 0 | - |
| - | - | 8862 | 610.2 | - | - | 0 | - |
| - | - | 551 | 610.4 | - | - | 0 | - |
| - | - | 9045 | 611.2 | - | - | 0 | - |
| - | - | 9002 | 611.8 | - | - | 0 | - |
| - | - | 6785 | 612.2 | - | - | 0 | - |
| - | - | 1050 | 612.2 | - | - | 0 | - |
| - | - | 5931 | 612.3 | - | - | 0 | - |
| - | - | 1640 | 612.8 | - | - | 0 | - |
| - | - | 998 | 613.2 | - | - | 0 | - |
| 12 | y | 8225 | 614.3 | 0.01169 | 19.03 | +1 | 6 |
| - | - | 1.036E+04 | 615.3 | - | - | 0 | - |
| 12 | z | 3458 | 616.3 | 0.001878 | 3.048 | +1 | 6 |
| - | - | 1696 | 616.4 | - | - | 0 | - |
| - | - | 898.5 | 617.3 | - | - | 0 | - |
| - | - | 1093 | 617.8 | - | - | 0 | - |
| - | - | 1605 | 618.3 | - | - | 0 | - |
| - | - | 929.3 | 618.8 | - | - | 0 | - |
| - | - | 3144 | 626.3 | - | - | 0 | - |
| - | - | 5457 | 626.8 | - | - | 0 | - |
| - | - | 5097 | 627.3 | - | - | 0 | - |
| - | - | 2242 | 627.3 | - | - | 0 | - |
| - | - | 1631 | 628.3 | - | - | 0 | - |
| - | - | 3145 | 628.4 | - | - | 0 | - |
| 6 | y | 814.3 | 628.8 | 0.0007831 | 1.245 | +2 | 12 |
| - | - | 1906 | 629.4 | - | - | 0 | - |
| - | - | 705.4 | 630.4 | - | - | 0 | - |
| - | - | 862.4 | 630.4 | - | - | 0 | - |
| 12 | y | 6222 | 632.3 | 2.751E-05 | 0.0435 | +1 | 6 |
| - | - | 1917 | 633.3 | - | - | 0 | - |
| - | - | 809.1 | 634.3 | - | - | 0 | - |
| - | - | 974.5 | 635.3 | - | - | 0 | - |
| - | - | 1559 | 635.8 | - | - | 0 | - |
| 6 | y | 924.2 | 637.8 | 0.004845 | 7.596 | +2 | 12 |
| - | - | 2887 | 655.3 | - | - | 0 | - |
| - | - | 2754 | 655.8 | - | - | 0 | - |
| - | - | 763.5 | 656.3 | - | - | 0 | - |
| - | - | 928.7 | 658.4 | - | - | 0 | - |
| 12 | c | 1332 | 664.8 | 0.003842 | 5.779 | +2 | 12 |
| - | - | 1194 | 667.4 | - | - | 0 | - |
| - | - | 921.3 | 668.8 | - | - | 0 | - |
| - | - | 2319 | 671.4 | - | - | 0 | - |
| 6 | c | 3716 | 672.4 | 2.56E-05 | 0.03807 | +1 | 6 |
| 12 | c | 627 | 673.4 | 0.01175 | 17.44 | +2 | 12 |
| - | - | 2140 | 673.4 | - | - | 0 | - |
| - | - | 2184 | 676.8 | - | - | 0 | - |
| 5 | y | 7750 | 677.3 | 5.668E-05 | 0.08369 | +2 | 13 |
| 5 | y | 7599 | 677.8 | 0.007927 | 11.69 | +2 | 13 |
| 5 | z | 2774 | 678.3 | 0.004075 | 6.008 | +2 | 13 |
| - | - | 2158 | 678.8 | - | - | 0 | - |
| - | - | 810.6 | 685.3 | - | - | 0 | - |
| - | - | 4574 | 685.8 | - | - | 0 | - |
| 5 | y | 5.648E+04 | 686.3 | 0.002526 | 3.68 | +2 | 13 |
| - | - | 2.152E+04 | 686.8 | - | - | 0 | - |
| - | - | 1.862E+04 | 687.3 | - | - | 0 | - |
| - | - | 2560 | 687.8 | - | - | 0 | - |
| - | - | 1845 | 688.4 | - | - | 0 | - |
| - | - | 1586 | 697.4 | - | - | 0 | - |
| - | - | 1218 | 698.4 | - | - | 0 | - |
| 13 | c | 1051 | 700.4 | 0.009459 | 13.51 | +2 | 13 |
| - | - | 1451 | 716.4 | - | - | 0 | - |
| - | - | 2016 | 722.3 | - | - | 0 | - |
| - | - | 1046 | 723.3 | - | - | 0 | - |
| 11 | z | 1.258E+04 | 729.4 | 0.0006762 | 0.927 | +1 | 7 |
| - | - | 5008 | 730.4 | - | - | 0 | - |
| - | - | 785.3 | 731.4 | - | - | 0 | - |
| - | - | 702.8 | 738.4 | - | - | 0 | - |
| - | - | 1697 | 740.3 | - | - | 0 | - |
| - | - | 1774 | 742.4 | - | - | 0 | - |
| 4 | z | 784.7 | 742.9 | 0.0009076 | 1.222 | +2 | 14 |
| - | - | 1186 | 743.4 | - | - | 0 | - |
| 11 | y | 6625 | 745.4 | 0.0004362 | 0.5852 | +1 | 7 |
| - | - | 3063 | 746.4 | - | - | 0 | - |
| 4 | y | 3444 | 750.9 | 0.000764 | 1.018 | +2 | 14 |
| 4 | y | 2867 | 751.4 | 0.008144 | 10.84 | +2 | 14 |
| 4 | z | 1473 | 751.9 | 0.000447 | 0.5945 | +2 | 14 |
| - | - | 846.1 | 752.4 | - | - | 0 | - |
| - | - | 2162 | 757.4 | - | - | 0 | - |
| - | - | 1823 | 758.4 | - | - | 0 | - |
| - | - | 2632 | 759.4 | - | - | 0 | - |
| 7 | c | 3.355E+04 | 759.4 | 5.048E-05 | 0.06648 | +1 | 7 |
| 4 | y | 1.155E+04 | 759.9 | 0.0006143 | 0.8084 | +2 | 14 |
| - | - | 1.039E+04 | 760.4 | - | - | 0 | - |
| - | - | 1.35E+04 | 760.4 | - | - | 0 | - |
| - | - | 3554 | 760.9 | - | - | 0 | - |
| - | - | 1545 | 761.4 | - | - | 0 | - |
| - | - | 3375 | 761.4 | - | - | 0 | - |
| - | - | 5616 | 764.4 | - | - | 0 | - |
| - | - | 3534 | 765.4 | - | - | 0 | - |
| - | - | 1098 | 766.4 | - | - | 0 | - |
| - | - | 1203 | 798.9 | - | - | 0 | - |
| 10 | w | 1.321E+04 | 799.4 | 0.0003018 | 0.3776 | +1 | 8 |
| 3 | z | 1093 | 799.9 | 0.011 | 13.75 | +2 | 15 |
| - | - | 7236 | 800.4 | - | - | 0 | - |
| - | - | 1547 | 801.4 | - | - | 0 | - |
| - | - | 6079 | 802.4 | - | - | 0 | - |
| - | - | 2965 | 803.4 | - | - | 0 | - |
| 3 | y | 1721 | 807.4 | 0.003347 | 4.145 | +2 | 15 |
| 3 | y | 2075 | 807.9 | 0.009447 | 11.69 | +2 | 15 |
| 3 | z | 1211 | 808.4 | 0.01292 | 15.98 | +2 | 15 |
| - | - | 1236 | 808.9 | - | - | 0 | - |
| - | - | 714.8 | 812.9 | - | - | 0 | - |
| - | - | 1634 | 813.4 | - | - | 0 | - |
| - | - | 953.5 | 813.9 | - | - | 0 | - |
| - | - | 843 | 814.4 | - | - | 0 | - |
| - | - | 1232 | 815.4 | - | - | 0 | - |
| 3 | y | 2878 | 816.4 | 0.003584 | 4.39 | +2 | 15 |
| - | - | 2215 | 816.9 | - | - | 0 | - |
| - | - | 965.9 | 817.4 | - | - | 0 | - |
| - | - | 3968 | 822.4 | - | - | 0 | - |
| - | - | 5093 | 823.4 | - | - | 0 | - |
| - | - | 1920 | 824.4 | - | - | 0 | - |
| - | - | 1434 | 829.4 | - | - | 0 | - |
| 15 | c | 3.702E+04 | 829.9 | 0.0001264 | 0.1523 | +2 | 15 |
| - | - | 3.4E+04 | 830.4 | - | - | 0 | - |
| - | - | 1.616E+04 | 830.9 | - | - | 0 | - |
| - | - | 5920 | 831.4 | - | - | 0 | - |
| - | - | 1006 | 832 | - | - | 0 | - |
| - | - | 896 | 834.9 | - | - | 0 | - |
| - | - | 1318 | 836.4 | - | - | 0 | - |
| - | - | 670 | 836.9 | - | - | 0 | - |
| 10 | z | 1227 | 840.4 | 0.0006206 | 0.7384 | +1 | 8 |
| 10 | z | 992.4 | 841.4 | 0.009159 | 10.88 | +1 | 8 |
| - | - | 1332 | 842.4 | - | - | 0 | - |
| - | - | 3204 | 843.5 | - | - | 0 | - |
| - | - | 2530 | 844 | - | - | 0 | - |
| - | - | 2207 | 844.5 | - | - | 0 | - |
| - | - | 919.4 | 845 | - | - | 0 | - |
| 8 | c | 2.926E+04 | 846.5 | 0.0001876 | 0.2216 | +1 | 8 |
| - | - | 1.419E+04 | 847.5 | - | - | 0 | - |
| - | - | 4388 | 848.5 | - | - | 0 | - |
| - | - | 1783 | 848.9 | - | - | 0 | - |
| - | - | 2082 | 849.4 | - | - | 0 | - |
| 2 | z | 3479 | 849.9 | 0.001809 | 2.128 | +2 | 16 |
| 2 | z | 2209 | 850.4 | 0.004003 | 4.707 | +2 | 16 |
| - | - | 1968 | 850.9 | - | - | 0 | - |
| - | - | 877.1 | 852.4 | - | - | 0 | - |
| 16 | c | 2652 | 856.9 | 3.766E-05 | 0.04395 | +2 | 16 |
| 10 | y | 4822 | 857.4 | 0.007891 | 9.203 | +1 | 8 |
| 2 | y | 3648 | 857.9 | 0.01051 | 12.25 | +2 | 16 |
| 2 | y | 2.368E+04 | 858.4 | 0.01643 | 19.14 | +2 | 16 |
| 2 | z | 4011 | 858.9 | 0.008367 | 9.741 | +2 | 16 |
| - | - | 1.081E+04 | 859.4 | - | - | 0 | - |
| - | - | 1346 | 859.9 | - | - | 0 | - |
| - | - | 2935 | 860.4 | - | - | 0 | - |
| - | - | 1139 | 864 | - | - | 0 | - |
| - | - | 4760 | 865 | - | - | 0 | - |
| 16 | c | 4.939E+04 | 865.5 | 0.0002376 | 0.2745 | +2 | 16 |
| - | - | 5.132E+04 | 866 | - | - | 0 | - |
| - | - | 2.704E+04 | 866.5 | - | - | 0 | - |
| - | - | 1.186E+04 | 867 | - | - | 0 | - |
| - | - | 2998 | 867.5 | - | - | 0 | - |
| - | - | 2023 | 868 | - | - | 0 | - |
| - | - | 2352 | 868.4 | - | - | 0 | - |
| - | - | 1270 | 869.4 | - | - | 0 | - |
| - | - | 967.7 | 871.4 | - | - | 0 | - |
| - | - | 1093 | 872.5 | - | - | 0 | - |
| - | - | 1601 | 872.9 | - | - | 0 | - |
| - | - | 3510 | 873.5 | - | - | 0 | - |
| 10 | y | 4794 | 874.5 | 0.00108 | 1.235 | +1 | 8 |
| - | - | 1862 | 875.5 | - | - | 0 | - |
| - | - | 818.5 | 878.5 | - | - | 0 | - |
| - | - | 4399 | 880.5 | - | - | 0 | - |
| - | - | 7555 | 881 | - | - | 0 | - |
| - | - | 7463 | 881.5 | - | - | 0 | - |
| - | - | 3712 | 882 | - | - | 0 | - |
| - | - | 2176 | 882.5 | - | - | 0 | - |
| - | - | 758.3 | 883 | - | - | 0 | - |
| - | - | 3354 | 885.5 | - | - | 0 | - |
| - | - | 4561 | 886 | - | - | 0 | - |
| - | - | 2867 | 886.5 | - | - | 0 | - |
| - | - | 1097 | 887 | - | - | 0 | - |
| - | - | 2454 | 887.5 | - | - | 0 | - |
| - | - | 1464 | 888 | - | - | 0 | - |
| - | - | 1029 | 888.5 | - | - | 0 | - |
| - | - | 2218 | 888.9 | - | - | 0 | - |
| - | - | 1512 | 889.5 | - | - | 0 | - |
| - | - | 1186 | 894 | - | - | 0 | - |
| - | - | 2.239E+04 | 894.5 | - | - | 0 | - |
| - | - | 2.012E+04 | 895 | - | - | 0 | - |
| - | - | 1.249E+04 | 895.5 | - | - | 0 | - |
| - | - | 6082 | 896 | - | - | 0 | - |
| - | - | 1266 | 896.5 | - | - | 0 | - |
| - | - | 2114 | 899.5 | - | - | 0 | - |
| - | - | 2253 | 900 | - | - | 0 | - |
| - | - | 1569 | 900.5 | - | - | 0 | - |
| - | - | 892 | 902.5 | - | - | 0 | - |
| - | - | 9515 | 907.4 | - | - | 0 | - |
| - | - | 4270 | 908 | - | - | 0 | - |
| - | - | 2894 | 908.4 | - | - | 0 | - |
| - | - | 3897 | 908.5 | - | - | 0 | - |
| - | - | 5410 | 909 | - | - | 0 | - |
| - | - | 832.3 | 910 | - | - | 0 | - |
| - | - | 1453 | 915.5 | - | - | 0 | - |
| - | - | 5.516E+04 | 916.5 | - | - | 0 | - |
| - | - | 9.299E+04 | 917 | - | - | 0 | - |
| - | - | 6.96E+04 | 917.5 | - | - | 0 | - |
| - | - | 3.16E+04 | 918 | - | - | 0 | - |
| - | - | 1.217E+04 | 918.5 | - | - | 0 | - |
| - | - | 3528 | 919 | - | - | 0 | - |
| 9 | w | 6013 | 928.5 | 0.0006586 | 0.7093 | +1 | 9 |
| - | - | 6385 | 929.5 | - | - | 0 | - |
| - | - | 2366 | 930.5 | - | - | 0 | - |
| - | - | 1007 | 931.5 | - | - | 0 | - |
| - | - | 3096 | 944.5 | - | - | 0 | - |
| - | - | 3597 | 945.5 | - | - | 0 | - |
| - | - | 1772 | 946.5 | - | - | 0 | - |
| - | - | 905.9 | 957.5 | - | - | 0 | - |
| - | - | 1474 | 958.5 | - | - | 0 | - |
| - | - | 1468 | 959.5 | - | - | 0 | - |
| - | - | 5485 | 960.5 | - | - | 0 | - |
| - | - | 3101 | 961.5 | - | - | 0 | - |
| 9 | z | 1148 | 969.5 | 0.0131 | 13.52 | +1 | 9 |
| - | - | 1.963E+04 | 973.5 | - | - | 0 | - |
| - | - | 9534 | 974.5 | - | - | 0 | - |
| 9 | c | 4.414E+04 | 975.5 | 0.0007795 | 0.7991 | +1 | 9 |
| - | - | 2.4E+04 | 976.5 | - | - | 0 | - |
| - | - | 7543 | 977.5 | - | - | 0 | - |
| - | - | 1539 | 978.5 | - | - | 0 | - |
| 9 | z | 2.04E+04 | 987.5 | 0.0005352 | 0.542 | +1 | 9 |
| - | - | 1.169E+04 | 988.5 | - | - | 0 | - |
| - | - | 3876 | 989.5 | - | - | 0 | - |
| - | - | 1154 | 990.5 | - | - | 0 | - |
| - | - | 1036 | 1002 | - | - | 0 | - |
| - | - | 909.6 | 1003 | - | - | 0 | - |
| 9 | y | 5558 | 1004 | 0.00986 | 9.825 | +1 | 9 |
| - | - | 3150 | 1005 | - | - | 0 | - |
| - | - | 1237 | 1006 | - | - | 0 | - |
| - | - | 1505 | 1013 | - | - | 0 | - |
| - | - | 923.4 | 1015 | - | - | 0 | - |
| - | - | 1107 | 1017 | - | - | 0 | - |
| - | - | 1346 | 1018 | - | - | 0 | - |
| - | - | 758.9 | 1019 | - | - | 0 | - |
| - | - | 973.3 | 1031 | - | - | 0 | - |
| - | - | 1001 | 1032 | - | - | 0 | - |
| - | - | 826.3 | 1053 | - | - | 0 | - |
| - | - | 4543 | 1056 | - | - | 0 | - |
| 8 | z | 3704 | 1057 | 2.628E-05 | 0.02488 | +1 | 10 |
| 8 | z | 1698 | 1057 | 0.01828 | 17.28 | +1 | 10 |
| - | - | 1169 | 1059 | - | - | 0 | - |
| - | - | 2867 | 1061 | - | - | 0 | - |
| - | - | 1971 | 1062 | - | - | 0 | - |
| - | - | 860 | 1071 | - | - | 0 | - |
| - | - | 1249 | 1072 | - | - | 0 | - |
| 8 | y | 1593 | 1073 | 0.02107 | 19.64 | +1 | 10 |
| 8 | y | 1730 | 1074 | 0.000652 | 0.6074 | +1 | 10 |
| 8 | z | 2.043E+04 | 1075 | 0.0005812 | 0.5409 | +1 | 10 |
| - | - | 1.568E+04 | 1076 | - | - | 0 | - |
| - | - | 6975 | 1077 | - | - | 0 | - |
| - | - | 2245 | 1078 | - | - | 0 | - |
| - | - | 4040 | 1088 | - | - | 0 | - |
| - | - | 2561 | 1089 | - | - | 0 | - |
| - | - | 1506 | 1090 | - | - | 0 | - |
| 8 | y | 1.37E+04 | 1091 | 0.001361 | 1.248 | +1 | 10 |
| - | - | 7676 | 1092 | - | - | 0 | - |
| - | - | 2734 | 1093 | - | - | 0 | - |
| - | - | 817.7 | 1094 | - | - | 0 | - |
| - | - | 1543 | 1099 | - | - | 0 | - |
| - | - | 1488 | 1100 | - | - | 0 | - |
| - | - | 2791 | 1104 | - | - | 0 | - |
| 10 | c | 4.665E+04 | 1105 | 0.00138 | 1.25 | +1 | 10 |
| - | - | 3.146E+04 | 1106 | - | - | 0 | - |
| - | - | 1.143E+04 | 1107 | - | - | 0 | - |
| - | - | 2952 | 1108 | - | - | 0 | - |
| - | - | 882.1 | 1118 | - | - | 0 | - |
| - | - | 904.2 | 1119 | - | - | 0 | - |
| - | - | 8278 | 1121 | - | - | 0 | - |
| - | - | 5892 | 1122 | - | - | 0 | - |
| - | - | 1952 | 1123 | - | - | 0 | - |
| - | - | 1138 | 1134 | - | - | 0 | - |
| 7 | z | 1069 | 1144 | 4.98E-05 | 0.04355 | +1 | 11 |
| 7 | y | 1214 | 1160 | 0.01806 | 15.58 | +1 | 11 |
| 7 | y | 887.4 | 1161 | 0.0008588 | 0.74 | +1 | 11 |
| 7 | z | 1.002E+04 | 1162 | 0.0006272 | 0.54 | +1 | 11 |
| - | - | 6896 | 1163 | - | - | 0 | - |
| - | - | 3804 | 1164 | - | - | 0 | - |
| - | - | 2255 | 1165 | - | - | 0 | - |
| 7 | y | 6402 | 1178 | 0.0003021 | 0.2565 | +1 | 11 |
| - | - | 4414 | 1179 | - | - | 0 | - |
| - | - | 1446 | 1180 | - | - | 0 | - |
| - | - | 1420 | 1181 | - | - | 0 | - |
| - | - | 1905 | 1182 | - | - | 0 | - |
| - | - | 1200 | 1183 | - | - | 0 | - |
| - | - | 918.5 | 1186 | - | - | 0 | - |
| - | - | 2232 | 1199 | - | - | 0 | - |
| 11 | c | 1634 | 1200 | 0.02251 | 18.77 | +1 | 11 |
| - | - | 1632 | 1201 | - | - | 0 | - |
| - | - | 1248 | 1202 | - | - | 0 | - |
| - | - | 1167 | 1206 | - | - | 0 | - |
| - | - | 1.164E+04 | 1207 | - | - | 0 | - |
| - | - | 8976 | 1208 | - | - | 0 | - |
| - | - | 2427 | 1209 | - | - | 0 | - |
| 11 | c | 3.038E+04 | 1218 | 0.001704 | 1.4 | +1 | 11 |
| - | - | 1.967E+04 | 1219 | - | - | 0 | - |
| - | - | 8611 | 1220 | - | - | 0 | - |
| - | - | 2767 | 1221 | - | - | 0 | - |
| - | - | 7141 | 1223 | - | - | 0 | - |
| - | - | 1.14E+04 | 1224 | - | - | 0 | - |
| - | - | 7047 | 1225 | - | - | 0 | - |
| - | - | 1483 | 1226 | - | - | 0 | - |
| - | - | 971.4 | 1236 | - | - | 0 | - |
| - | - | 2736 | 1253 | - | - | 0 | - |
| - | - | 1475 | 1254 | - | - | 0 | - |
| - | - | 1415 | 1270 | - | - | 0 | - |
| - | - | 1191 | 1271 | - | - | 0 | - |
| 6 | y | 3085 | 1275 | 0.0005819 | 0.4565 | +1 | 12 |
| - | - | 2079 | 1276 | - | - | 0 | - |
| - | - | 1063 | 1277 | - | - | 0 | - |
| - | - | 1142 | 1302 | - | - | 0 | - |
| - | - | 1521 | 1303 | - | - | 0 | - |
| - | - | 781.3 | 1304 | - | - | 0 | - |
| 12 | c | 2720 | 1329 | 0.02079 | 15.65 | +1 | 12 |
| - | - | 2908 | 1330 | - | - | 0 | - |
| - | - | 1692 | 1331 | - | - | 0 | - |
| - | - | 4216 | 1345 | - | - | 0 | - |
| 12 | c | 3.969E+04 | 1346 | 0.002176 | 1.617 | +1 | 12 |
| - | - | 3.08E+04 | 1347 | - | - | 0 | - |
| - | - | 1.279E+04 | 1348 | - | - | 0 | - |
| - | - | 3850 | 1349 | - | - | 0 | - |
| 5 | y | 1143 | 1355 | 0.01495 | 11.04 | +1 | 13 |
| - | - | 753.5 | 1358 | - | - | 0 | - |
| - | - | 2117 | 1371 | - | - | 0 | - |
| 5 | y | 1.181E+04 | 1372 | 0.003053 | 2.226 | +1 | 13 |
| - | - | 8972 | 1373 | - | - | 0 | - |
| - | - | 4721 | 1374 | - | - | 0 | - |
| - | - | 1428 | 1375 | - | - | 0 | - |
| - | - | 1555 | 1399 | - | - | 0 | - |
| - | - | 3684 | 1400 | - | - | 0 | - |
| - | - | 5318 | 1401 | - | - | 0 | - |
| - | - | 5105 | 1402 | - | - | 0 | - |
| - | - | 2702 | 1403 | - | - | 0 | - |
| - | - | 953 | 1404 | - | - | 0 | - |
| - | - | 5353 | 1416 | - | - | 0 | - |
| 13 | c | 2.715E+04 | 1417 | 0.003401 | 2.401 | +1 | 13 |
| - | - | 2.122E+04 | 1418 | - | - | 0 | - |
| - | - | 7748 | 1419 | - | - | 0 | - |
| - | - | 3134 | 1420 | - | - | 0 | - |
| - | - | 2321 | 1431 | - | - | 0 | - |
| - | - | 2906 | 1432 | - | - | 0 | - |
| - | - | 1320 | 1433 | - | - | 0 | - |
| - | - | 818.9 | 1444 | - | - | 0 | - |
| - | - | 930.4 | 1486 | - | - | 0 | - |
| - | - | 2174 | 1487 | - | - | 0 | - |
| - | - | 2048 | 1488 | - | - | 0 | - |
| - | - | 1035 | 1489 | - | - | 0 | - |
| 4 | y | 898.6 | 1502 | 0.007206 | 4.799 | +1 | 14 |
| 4 | z | 2.391E+04 | 1503 | 0.002938 | 1.955 | +1 | 14 |
| - | - | 3.587E+04 | 1504 | - | - | 0 | - |
| - | - | 2.491E+04 | 1505 | - | - | 0 | - |
| - | - | 1.077E+04 | 1506 | - | - | 0 | - |
| - | - | 3230 | 1507 | - | - | 0 | - |
| - | - | 772 | 1508 | - | - | 0 | - |
| 4 | y | 3203 | 1519 | 0.0004327 | 0.2849 | +1 | 14 |
| - | - | 2574 | 1520 | - | - | 0 | - |
| - | - | 2078 | 1521 | - | - | 0 | - |
| - | - | 881 | 1522 | - | - | 0 | - |
| - | - | 949.3 | 1529 | - | - | 0 | - |
| - | - | 1214 | 1530 | - | - | 0 | - |
| 14 | c | 4.52E+04 | 1531 | 0.002628 | 1.717 | +1 | 14 |
| - | - | 4.18E+04 | 1532 | - | - | 0 | - |
| - | - | 1.909E+04 | 1533 | - | - | 0 | - |
| - | - | 7935 | 1534 | - | - | 0 | - |
| - | - | 1906 | 1535 | - | - | 0 | - |
| - | - | 1137 | 1544 | - | - | 0 | - |
| - | - | 815.6 | 1545 | - | - | 0 | - |
| - | - | 3222 | 1615 | - | - | 0 | - |
| 3 | z | 5212 | 1616 | 0.01554 | 9.616 | +1 | 15 |
| - | - | 9227 | 1617 | - | - | 0 | - |
| - | - | 6891 | 1618 | - | - | 0 | - |
| - | - | 2141 | 1619 | - | - | 0 | - |
| - | - | 698.2 | 1633 | - | - | 0 | - |
| - | - | 2100 | 1643 | - | - | 0 | - |
| - | - | 1970 | 1644 | - | - | 0 | - |
| - | - | 918 | 1645 | - | - | 0 | - |
| 15 | c | 4788 | 1659 | 0.0009109 | 0.5491 | +1 | 15 |
| - | - | 1.283E+04 | 1660 | - | - | 0 | - |
| - | - | 9232 | 1661 | - | - | 0 | - |
| - | - | 4350 | 1662 | - | - | 0 | - |
| - | - | 2094 | 1663 | - | - | 0 | - |
| - | - | 1501 | 1673 | - | - | 0 | - |
| - | - | 3300 | 1686 | - | - | 0 | - |
| - | - | 3624 | 1687 | - | - | 0 | - |
| - | - | 3130 | 1688 | - | - | 0 | - |
| - | - | 1906 | 1689 | - | - | 0 | - |
| 2 | z | 1599 | 1700 | 0.02261 | 13.3 | +1 | 16 |
| - | - | 1618 | 1701 | - | - | 0 | - |
| - | - | 3876 | 1714 | - | - | 0 | - |
| 2 | y | 4095 | 1715 | 0.03258 | 19 | +1 | 16 |
| - | - | 3250 | 1716 | - | - | 0 | - |
| 2 | z | 3708 | 1717 | 0.00924 | 5.382 | +1 | 16 |
| - | - | 1.082E+04 | 1718 | - | - | 0 | - |
| - | - | 8672 | 1719 | - | - | 0 | - |
| - | - | 4608 | 1720 | - | - | 0 | - |
| - | - | 1881 | 1721 | - | - | 0 | - |
| - | - | 1020 | 1722 | - | - | 0 | - |
| 16 | c | 4579 | 1730 | 0.004211 | 2.434 | +1 | 16 |
| - | - | 1.49E+04 | 1731 | - | - | 0 | - |
| - | - | 1.428E+04 | 1732 | - | - | 0 | - |
| - | - | 7508 | 1733 | - | - | 0 | - |
| - | - | 2937 | 1734 | - | - | 0 | - |
| - | - | 1190 | 1761 | - | - | 0 | - |
| - | - | 1572 | 1762 | - | - | 0 | - |
| - | - | 1033 | 1763 | - | - | 0 | - |
| - | - | 3069 | 1771 | - | - | 0 | - |
| - | - | 3556 | 1772 | - | - | 0 | - |
| - | - | 4200 | 1773 | - | - | 0 | - |
| - | - | 1348 | 1774 | - | - | 0 | - |
| - | - | 1567 | 1775 | - | - | 0 | - |
| - | - | 845.1 | 1777 | - | - | 0 | - |
| - | - | 863.4 | 1787 | - | - | 0 | - |
| - | - | 6278 | 1788 | - | - | 0 | - |
| - | - | 2.573E+04 | 1789 | - | - | 0 | - |
| - | - | 2.071E+04 | 1790 | - | - | 0 | - |
| - | - | 1.233E+04 | 1791 | - | - | 0 | - |
| - | - | 4196 | 1792 | - | - | 0 | - |
| - | - | 1705 | 1793 | - | - | 0 | - |
| - | - | 907.4 | 1800 | - | - | 0 | - |
| - | - | 883.7 | 1801 | - | - | 0 | - |
| - | - | 1207 | 1805 | - | - | 0 | - |
| - | - | 5033 | 1806 | - | - | 0 | - |
| - | - | 4494 | 1807 | - | - | 0 | - |
| - | - | 2967 | 1808 | - | - | 0 | - |
| - | - | 1335 | 1815 | - | - | 0 | - |
| - | - | 8253 | 1816 | - | - | 0 | - |
| - | - | 3.579E+04 | 1817 | - | - | 0 | - |
| - | - | 3.484E+04 | 1818 | - | - | 0 | - |
| - | - | 1.816E+04 | 1819 | - | - | 0 | - |
| - | - | 6248 | 1820 | - | - | 0 | - |
| - | - | 2377 | 1821 | - | - | 0 | - |
| - | - | 4353 | 1832 | - | - | 0 | - |
| - | - | 2.103E+04 | 1833 | - | - | 0 | - |
| - | - | 6.362E+04 | 1834 | - | - | 0 | - |
| - | - | 5.726E+04 | 1835 | - | - | 0 | - |
| - | - | 2.771E+04 | 1836 | - | - | 0 | - |
| - | - | 1.162E+04 | 1837 | - | - | 0 | - |
| - | - | 3427 | 1838 | - | - | 0 | - |
| - | - | 874 | 1839 | - | - | 0 | - |
| - | - | 675.7 | 2325 | - | - | 0 | - |
| - | - | 659 | 3071 | - | - | 0 | - |

m/z Charge Intensity FragmentType MassShift Position
120.06562042236328 0 11523.408 y 16
120.080810546875 0 2237.279
121.06889343261719 0 363.37018
125.88548278808594 0 405.85184
126.0550765991211 0 1776.8569
126.6019515991211 0 338.03076
128.0944061279297 0 807.917
128.10702514648438 0 956.6497
129.10226440429688 0 5174.2876
130.34983825683594 0 436.60165
131.1181182861328 0 1084.4545
132.62591552734375 0 390.97568
136.6066131591797 0 379.48633
137.87278747558594 0 407.2008
140.10711669921875 0 1009.37067
143.68194580078125 0 417.38028
148.95382690429688 0 1018.8542
149.04478454589844 0 642.68207
155.11798095703125 0 861.6861
157.13330078125 0 624.953
158.141845703125 0 537.0474
169.1337890625 0 2863.6948
171.07652282714844 0 1249.0331
173.09237670898438 0 1365.4236 y Water loss 15
173.12843322753906 0 3363.037
173.43881225585938 0 1460.0138
183.11280822753906 0 2507.8835
191.1027374267578 0 2856.581 y 15
197.12855529785156 0 2298.1797
200.138916015625 0 689.7674 c Water loss 1
201.12332153320312 0 7347.5913
211.10816955566406 0 630.7899
212.13931274414062 0 9954.045
213.14273071289062 0 1235.5834
215.1396026611328 0 750.93536
218.1499481201172 0 4408.948 c 1
221.0843505859375 0 3218.033
222.08448791503906 0 1058.3654
223.08203125 0 573.56616
227.09002685546875 0 1349.775
227.1023712158203 0 1873.1945
233.16490173339844 0 1506.709
235.10745239257812 0 863.4167
239.09519958496094 0 4216.048
240.09664916992188 0 1336.2607
241.09242248535156 0 566.75635
242.1501007080078 0 808.23206
243.14573669433594 0 1133.5236 y Water loss 10
254.14984130859375 0 1105.4111
256.1419677734375 0 598.3834
261.1598205566406 0 1251.4275
263.1023864746094 0 3034.7847
264.1073913574219 0 754.0732
280.1782531738281 0 777.8479
281.051025390625 0 1243.0227
281.1388854980469 0 801.837 z Ammonia loss 9
281.18505859375 0 624.41376
282.0514221191406 0 630.04944
282.1445007324219 0 965.1153
296.1969299316406 0 11807.428
297.1551513671875 0 1136.3287
297.2002868652344 0 2066.1892
298.1270751953125 0 585.5158
298.1617736816406 0 846.431
299.0617370605469 0 4584.023
299.1715087890625 0 14933.883
300.0617980957031 0 2241.6523
300.1559753417969 0 562.4711
300.1745300292969 0 2248.5486
301.0603332519531 0 1064.2556
303.1788330078125 0 7922.1494 z 14
304.1830749511719 0 1647.3577
307.1756286621094 0 729.79504
307.6664733886719 0 798.41296 y Water loss 11
314.1866149902344 0 713.3759
314.2074279785156 0 4762.06
315.21142578125 0 871.3882
316.1867370605469 0 7705.005
316.6717834472656 0 1148.611 y 11
317.0541687011719 0 523.75
317.1902160644531 0 871.9004
319.19757080078125 0 5297.6177 y 14
325.18701171875 0 946.92645
331.23406982421875 0 27655.201 c 2
332.2374267578125 0 5089.832
333.2393798828125 0 668.9756
344.17291259765625 0 593.22656
345.1802062988281 0 867.72754
346.18890380859375 0 658.54846
347.1947326660156 0 1185.4178
350.1361389160156 0 895.0099
355.06976318359375 0 37080.09
356.0704040527344 0 16167.544
357.0673828125 0 8737.248
358.0686950683594 0 882.52167
362.2068176269531 0 1692.3315
369.2002868652344 0 811.18304
371.1004638671875 0 1106.4883
372.10076904296875 0 989.6184
373.07928466796875 0 1044.5762
373.20904541015625 0 4324.663 y 10
378.2265930175781 0 1329.1509
385.21844482421875 0 781.4554
386.2028503417969 0 1324.592
415.03704833984375 0 1792.2623
415.2322082519531 0 905.4295 y Water loss 13
416.0376892089844 0 1111.9762
417.0358581542969 0 911.86395
417.2228698730469 0 2613.7942 z 13
418.22955322265625 0 7720.6016
419.23272705078125 0 2173.7317
426.23394775390625 0 813.13916
433.2422790527344 0 2721.0276 y 13
433.28118896484375 0 8883.417
434.2555236816406 0 598.7478
434.28662109375 0 4960.3496
434.7042541503906 0 678.73206
435.29443359375 0 2118.8057
444.2456970214844 0 6882.824
445.20306396484375 0 610.995
445.2531433105469 0 2406.219
446.2352600097656 0 875.6187
446.2648010253906 0 1780.2716
451.89434814453125 0 861.5997 y Water loss 4
453.218505859375 0 723.3415
460.2674560546875 0 1502.754
461.2761535644531 0 12883.7
462.2792663574219 0 3666.8882
472.24554443359375 0 605.0841
473.23382568359375 0 698.0319
486.267578125 0 760.345 y Water loss 12
488.2591857910156 0 11267.053 c 8
488.7345275878906 0 1124.6962
489.05596923828125 0 861.6082
489.26513671875 0 7632.4414
490.0565185546875 0 1229.5596
490.26849365234375 0 1491.8195
493.7278137207031 0 1757.4233
494.22955322265625 0 1421.8601
496.24822998046875 0 713.855
497.2576904296875 0 978.95886
498.21923828125 0 1174.9822
502.2650451660156 0 1891.0902 y 8
502.7322998046875 0 2413.551
503.2325744628906 0 1186.6223
503.2698974609375 0 1032.6888
503.73724365234375 0 589.4461
504.2779235839844 0 8432.016 y 12
505.28033447265625 0 1866.4769
515.2942504882812 0 998.72125
529.2638549804688 0 795.68384 w 7
529.7650756835938 0 765.30383
538.2694091796875 0 7049.682
538.7708740234375 0 2663.786
539.2728271484375 0 2239.2231 z 2
540.31787109375 0 725.5495
543.2610473632812 0 1732.4749
543.76513671875 0 1347.7756
544.2660522460938 0 752.1627
544.7706298828125 0 921.4524
552.267578125 0 5421.577
552.7693481445312 0 3865.3125
554.32080078125 0 693.0858
557.3220825195312 0 1008.8082
558.2880249023438 0 15641.981 w 11
558.329833984375 0 2310.9812
559.2900390625 0 3846.6501
559.3341674804688 0 1542.4291
560.2945556640625 0 737.6785
572.3045654296875 0 849.83124
577.1263427734375 0 1068.8077
578.1263427734375 0 701.15686
579.1215209960938 0 648.95526
580.7779541015625 0 594.289 y Ammonia loss 6
582.281982421875 0 865.1007
582.7792358398438 0 876.6496
583.3366088867188 0 1556.7338
599.2660522460938 0 708.4492
599.8078002929688 0 2207.7295
600.308837890625 0 1204.3954 c Water loss 10
600.8093872070312 0 747.9042
602.7908325195312 0 3201.3562
603.2919921875 0 2785.5063
610.1842651367188 0 8862.088
610.3616333007812 0 551.00494
611.1846313476562 0 9045.362
611.7958984375 0 9002.332
612.182373046875 0 6784.7837
612.2408447265625 0 1049.5511
612.2982177734375 0 5931.0073
612.8023681640625 0 1640.2288
613.1807250976562 0 998.01013
614.3373413085938 0 8225.104 y Water loss 11
615.3448486328125 0 10362.112
616.3156127929688 0 3458.1807 z 11
616.353759765625 0 1696.2216
617.3182983398438 0 898.5086
617.8045043945312 0 1092.6915
618.3072509765625 0 1604.5782
618.805908203125 0 929.3029
626.3419189453125 0 3144.013
626.8125 0 5456.916
627.2615356445312 0 5096.9204
627.31494140625 0 2241.8936
628.2669677734375 0 1630.8917
628.3923950195312 0 3144.5437
628.8087158203125 0 814.2768 y Water loss 5
629.396728515625 0 1905.7325
630.3577270507812 0 705.43646
630.4095458984375 0 862.42334
632.3362426757812 0 6222.092 y 11
633.3388061523438 0 1916.7163
634.341064453125 0 809.11035
635.3252563476562 0 974.53534
635.8245849609375 0 1558.878
637.8099365234375 0 924.1999 y 5
655.3401489257812 0 2886.7563
655.8421630859375 0 2754.1414
656.3426513671875 0 763.52515
658.3619384765625 0 928.6791
664.83642578125 0 1332.0271 c Ammonia loss 11
667.3981323242188 0 1193.5032
668.8251953125 0 921.2698
671.3714599609375 0 2318.6438
672.4078979492188 0 3715.9263 c 5
673.341796875 0 626.982 c 11
673.410400390625 0 2140.017
676.8301391601562 0 2184.2188
677.3359375 0 7749.844 y Water loss 4
677.8358154296875 0 7598.947 y Ammonia loss 4
678.3358764648438 0 2773.6182 z 4
678.832763671875 0 2157.886
685.3242797851562 0 810.6029
685.837890625 0 4573.6377
686.3436889648438 0 56477.61 y 4
686.8426513671875 0 21515.86
687.3477172851562 0 18624.803
687.8453979492188 0 2559.7788
688.3534545898438 0 1844.7242
697.4151611328125 0 1586.0878
698.421630859375 0 1217.9404
700.349365234375 0 1050.9651 c Ammonia loss 12
716.4329833984375 0 1451.1783
722.3475952148438 0 2015.6172
723.3497924804688 0 1045.8584
729.40087890625 0 12579.541 z 10
730.4031372070312 0 5007.9883
731.4085083007812 0 785.31866
738.3751220703125 0 702.83875
740.3443603515625 0 1697.271
742.4156494140625 0 1774.3164
742.8616333007812 0 784.715 z Water loss 3
743.421142578125 0 1186.0101
745.4207153320312 0 6624.703 y 10
746.4223022460938 0 3062.6396
750.8693237304688 0 3443.5618 y Water loss 3
751.3702392578125 0 2867.119 y Ammonia loss 3
751.866455078125 0 1472.9985 z 3
752.3658447265625 0 846.10504
757.4197998046875 0 2161.5515
758.42578125 0 1822.9799
759.3712768554688 0 2632.3425
759.4400024414062 0 33546.32 c 6
759.874755859375 0 11548.593 y 3
760.3744506835938 0 10394.089
760.4436645507812 0 13499.963
760.8782958984375 0 3554.2664
761.3779296875 0 1544.8152
761.4463500976562 0 3375.3774
764.3607177734375 0 5615.999
765.3632202148438 0 3534.355
766.3618774414062 0 1098.3237
798.9033203125 0 1203.4395
799.4305419921875 0 13205.909 w 9
799.90576171875 0 1092.538 z Ammonia loss 2
800.4306640625 0 7235.6655
801.4319458007812 0 1546.5696
802.3821411132812 0 6078.992
803.3837280273438 0 2964.581
807.4154663085938 0 1720.8522 y Water loss 2
807.91357421875 0 2075.2683 y Ammonia loss 2
808.4209594726562 0 1210.6318 z 2
808.9296875 0 1236.193
812.9239501953125 0 714.7635
813.4188842773438 0 1634.1877
813.9207153320312 0 953.46594
814.4282836914062 0 842.9552
815.3845825195312 0 1231.6317
816.413818359375 0 2878.0974 y 2
816.9196166992188 0 2215.4087
817.4208984375 0 965.9111
822.3797607421875 0 3968.2307
823.3820190429688 0 5093.151
824.3878784179688 0 1920.4755
829.443603515625 0 1434.453
829.94091796875 0 37016.63 c 14
830.4420776367188 0 34002.74
830.943359375 0 16164.45
831.44384765625 0 5920.4033
831.9521484375 0 1006.16986
834.9363403320312 0 896.02423
836.4320678710938 0 1318.1403
836.9276733398438 0 670.0089
840.4342041015625 0 1227.3944 z Water loss 9
841.4267578125 0 992.4445 z Ammonia loss 9
842.4421997070312 0 1332.4336
843.451416015625 0 3203.742
843.9549560546875 0 2530.0217
844.4555053710938 0 2207.134
844.9580078125 0 919.3941
846.47216796875 0 29257.805 c 7
847.4744262695312 0 14192.109
848.4765014648438 0 4388.31
848.93115234375 0 1783.3513
849.4306640625 0 2082.488
849.9284057617188 0 3478.9963 z Water loss 1
850.422607421875 0 2208.9363 z Ammonia loss 1
850.9298095703125 0 1967.7782
852.4224853515625 0 877.09314
856.9462890625 0 2651.5728 c Ammonia loss 15
857.4442138671875 0 4821.707 y Ammonia loss 9
857.9464721679688 0 3648.39 y Water loss 1
858.4443969726562 0 23678.434 y Ammonia loss 1
858.9402465820312 0 4011.3735 z 1
859.4447021484375 0 10812.551
859.936767578125 0 1345.8552
860.44580078125 0 2935.4243
863.96435546875 0 1138.5984
864.9608154296875 0 4760.3003
865.4598388671875 0 49390.516 c 15
865.9607543945312 0 51316.996
866.461181640625 0 27036.473
866.961669921875 0 11859.071
867.4600219726562 0 2998.058
867.9603881835938 0 2022.9735
868.4032592773438 0 2351.87
869.4083862304688 0 1270.123
871.4444580078125 0 967.6648
872.4586791992188 0 1093.4417
872.9473266601562 0 1601.1686
873.4536743164062 0 3510.402
874.4617919921875 0 4793.542 y 9
875.462890625 0 1862.271
878.4534912109375 0 818.4717
880.4520874023438 0 4399.358
880.9620971679688 0 7555.026
881.4641723632812 0 7463.0024
881.9686889648438 0 3712.4148
882.4647827148438 0 2175.6177
882.972900390625 0 758.31415
885.462158203125 0 3354.3264
885.9660034179688 0 4560.551
886.46435546875 0 2866.6133
886.9654541015625 0 1097.4773
887.4654541015625 0 2454.1943
887.9666137695312 0 1463.7759
888.4718017578125 0 1029.3972
888.94775390625 0 2217.847
889.4530639648438 0 1512.43
893.9710083007812 0 1185.5878
894.4685668945312 0 22392.785
894.9700317382812 0 20123.775
895.4705200195312 0 12490.665
895.969482421875 0 6082.2744
896.47265625 0 1265.5068
899.4635620117188 0 2114.4744
899.96435546875 0 2253.222
900.4577026367188 0 1569.2872
902.45458984375 0 891.9958
907.4059448242188 0 9515.151
907.9733276367188 0 4270.273
908.4024658203125 0 2893.7507
908.4746704101562 0 3897.1272
908.9707641601562 0 5410.3696
909.9712524414062 0 832.3214
915.472412109375 0 1452.5981
916.4754638671875 0 55157.98
916.9774169921875 0 92994.125
917.4793701171875 0 69599.24
917.98095703125 0 31602.498
918.4822387695312 0 12169.22
918.9822387695312 0 3528.0488
928.4727783203125 0 6013.4453 w 8
929.4794311523438 0 6385.1343
930.4796752929688 0 2365.88
931.4974975585938 0 1006.52295
944.4664916992188 0 3095.8027
945.4762573242188 0 3597.2817
946.4796142578125 0 1772.306
957.4613037109375 0 905.9197
958.4868774414062 0 1474.3646
959.4840087890625 0 1467.6338
960.4892578125 0 5484.9146
961.48974609375 0 3100.5352
969.4630737304688 0 1148.4338 z Water loss 8
973.4945068359375 0 19626.125
974.4976196289062 0 9534.05
975.5137939453125 0 44142.13 c 8
976.5170288085938 0 24004.225
977.5202026367188 0 7542.5317
978.5270385742188 0 1539.4749
987.4862060546875 0 20395.17 z 8
988.489990234375 0 11689.588
989.4929809570312 0 3875.7446
990.4891357421875 0 1154.264
1001.5220947265625 0 1035.969
1002.5254516601562 0 909.61084
1003.49560546875 0 5557.9043 y 8
1004.5005493164062 0 3150.1077
1005.5020141601562 0 1236.9374
1012.5198364257812 0 1505.0969
1015.4895629882812 0 923.38135
1016.5089721679688 0 1106.5411
1018.4569091796875 0 1345.87
1019.4678344726562 0 758.9433
1030.5068359375 0 973.25195
1031.5079345703125 0 1000.68744
1053.4810791015625 0 826.33984
1055.5015869140625 0 4542.736
1056.5081787109375 0 3703.5208 z Water loss 7
1057.510498046875 0 1697.5778 z Ammonia loss 7
1058.519775390625 0 1169.076
1060.532470703125 0 2866.6348
1061.54052734375 0 1971.232
1070.5201416015625 0 859.9943
1071.51171875 0 1249.2789
1072.505859375 0 1593.173 y Water loss 7
1073.5115966796875 0 1729.5599 y Ammonia loss 7
1074.5181884765625 0 20431.914 z 7
1075.523193359375 0 15682.32
1076.5255126953125 0 6975.3276
1077.528564453125 0 2244.6626
1087.529541015625 0 4039.704
1088.532470703125 0 2561.0234
1089.5321044921875 0 1506.44
1090.5361328125 0 13696.504 y 7
1091.538818359375 0 7676.056
1092.541015625 0 2734.1785
1093.5494384765625 0 817.72455
1098.508056640625 0 1542.9812
1099.5133056640625 0 1487.8507
1103.5263671875 0 2790.9102
1104.5557861328125 0 46645.46 c 9
1105.5584716796875 0 31457.764
1106.562744140625 0 11427.803
1107.5635986328125 0 2952.472
1117.5260009765625 0 882.0583
1118.5283203125 0 904.16705
1120.552734375 0 8278.067
1121.5562744140625 0 5891.99
1122.5552978515625 0 1951.6302
1133.5570068359375 0 1138.4916
1143.540283203125 0 1069.4191 z Water loss 6
1159.5408935546875 0 1213.8992 y Water loss 6
1160.5421142578125 0 887.44116 y Ammonia loss 6
1161.5501708984375 0 10021.674 z 6
1162.5538330078125 0 6895.5156
1163.56103515625 0 3804.4587
1164.5628662109375 0 2254.7014
1177.56982421875 0 6402.3066 y 6
1178.57177734375 0 4414.309
1179.5823974609375 0 1446.3154
1180.575439453125 0 1419.6041
1181.5767822265625 0 1905.399
1182.5809326171875 0 1200.1257
1185.53173828125 0 918.5385
1198.610595703125 0 2231.5142
1199.608154296875 0 1633.9797 c Water loss 10
1200.609375 0 1632.4164
1201.6162109375 0 1248.3048
1205.5819091796875 0 1167.0376
1206.565185546875 0 11642.349
1207.56884765625 0 8976.186
1208.5673828125 0 2426.5508
1217.6395263671875 0 30378.148 c 10
1218.6424560546875 0 19673.451
1219.64599609375 0 8611.385
1220.6385498046875 0 2767.2847
1222.582275390625 0 7141.3633
1223.58984375 0 11398.304
1224.5916748046875 0 7047.205
1225.59375 0 1483.0035
1235.609375 0 971.40686
1252.6114501953125 0 2736.1016
1253.6141357421875 0 1474.9187
1269.6395263671875 0 1415.4154
1270.6470947265625 0 1190.9015
1274.6217041015625 0 3084.9724 y 5
1275.626708984375 0 2079.446
1276.62646484375 0 1062.6677
1301.662841796875 0 1142.426
1302.6744384765625 0 1521.414
1303.6400146484375 0 781.3091
1328.6524658203125 0 2719.521 c Ammonia loss 11
1329.6575927734375 0 2908.3247
1330.6539306640625 0 1691.9569
1344.6895751953125 0 4216.0312
1345.6976318359375 0 39688.523 c 11
1346.7001953125 0 30795.797
1347.70361328125 0 12794.064
1348.7078857421875 0 3850.1997
1354.6634521484375 0 1142.5585 y Ammonia loss 4
1357.676025390625 0 753.45404
1370.663330078125 0 2116.5564
1371.6719970703125 0 11806.766 y 4
1372.6776123046875 0 8971.951
1373.6871337890625 0 4721.2715
1374.6961669921875 0 1428.3541
1398.6732177734375 0 1554.8483
1399.6759033203125 0 3683.6719
1400.6783447265625 0 5317.9385
1401.6944580078125 0 5105.491
1402.69775390625 0 2702.3235
1403.6971435546875 0 952.9784
1415.7275390625 0 5353.2373
1416.7335205078125 0 27152.072 c 12
1417.7364501953125 0 21223.031
1418.7392578125 0 7748.3037
1419.7420654296875 0 3133.6511
1430.699951171875 0 2320.6714
1431.6932373046875 0 2905.8303
1432.697998046875 0 1319.7758
1443.736083984375 0 818.9274
1485.7332763671875 0 930.3671
1486.7537841796875 0 2173.7893
1487.7637939453125 0 2047.9148
1488.77490234375 0 1035.4661
1501.72412109375 0 898.5785 y Ammonia loss 3
1502.7218017578125 0 23913.855 z 3
1503.7275390625 0 35872.285
1504.7315673828125 0 24905.24
1505.735595703125 0 10769.822
1506.7332763671875 0 3229.8206
1507.7464599609375 0 772.0301
1518.743896484375 0 3202.8005 y 3
1519.74560546875 0 2574.0269
1520.7454833984375 0 2078.0186
1521.7520751953125 0 880.9992
1528.7642822265625 0 949.3073
1529.775634765625 0 1213.791
1530.7772216796875 0 45204.883 c 13
1531.7801513671875 0 41804.582
1532.7822265625 0 19092.973
1533.7867431640625 0 7935.082
1534.7813720703125 0 1905.6893
1543.781005859375 0 1136.5703
1544.792236328125 0 815.5941
1614.8466796875 0 3221.9397
1615.8243408203125 0 5211.7275 z 2
1616.8206787109375 0 9226.758
1617.8236083984375 0 6891.4707
1618.81884765625 0 2140.8213
1632.870361328125 0 698.18506
1642.8538818359375 0 2100.326
1643.85302734375 0 1969.8948
1644.850341796875 0 917.9799
1658.8739013671875 0 4787.645 c 14
1659.877685546875 0 12830.791
1660.88134765625 0 9231.601
1661.8779296875 0 4350.0376
1662.891357421875 0 2093.7134
1672.8533935546875 0 1501.407
1685.8961181640625 0 3300.199
1686.9000244140625 0 3624.305
1687.8992919921875 0 3129.6309
1688.911865234375 0 1905.5542
1699.8525390625 0 1599.0616 z Ammonia loss 1
1700.8460693359375 0 1617.583
1713.889404296875 0 3876.4385
1714.897216796875 0 4095.3025 y Water loss 1
1715.885009765625 0 3249.5652
1716.86572265625 0 3708.4534 z 1
1717.862548828125 0 10819.652
1718.8665771484375 0 8671.636
1719.8675537109375 0 4608.0854
1720.867919921875 0 1881.1091
1721.8583984375 0 1020.4126
1729.90771484375 0 4578.978 c 15
1730.9154052734375 0 14896.259
1731.9190673828125 0 14280.0205
1732.920166015625 0 7507.999
1733.9140625 0 2936.9385
1760.9495849609375 0 1189.5121
1761.9261474609375 0 1572.0165
1762.923583984375 0 1032.6564
1770.9326171875 0 3068.6792
1771.919189453125 0 3555.8066
1772.918212890625 0 4200.1704
1773.9183349609375 0 1348.2195
1774.914306640625 0 1567.3446
1776.89892578125 0 845.0797
1786.929443359375 0 863.41016
1787.937744140625 0 6277.7017
1788.9373779296875 0 25733.305
1789.9395751953125 0 20706.906
1790.9412841796875 0 12326.269
1791.943603515625 0 4195.6934
1792.93310546875 0 1704.5919
1799.92041015625 0 907.44574
1800.9229736328125 0 883.6949
1804.955810546875 0 1206.7693
1805.95947265625 0 5032.9946
1806.9649658203125 0 4493.5044
1807.9727783203125 0 2967.212
1814.9337158203125 0 1335.353
1815.9302978515625 0 8252.96
1816.9310302734375 0 35794.023
1817.9342041015625 0 34841.6
1818.9361572265625 0 18155.686
1819.9371337890625 0 6248.153
1820.9454345703125 0 2376.5925
1831.942626953125 0 4352.6245
1832.94775390625 0 21026.969
1833.9552001953125 0 63615.38
1834.9593505859375 0 57260.24
1835.96142578125 0 27710.47
1836.9639892578125 0 11615.544
1837.962890625 0 3426.842
1838.958984375 0 873.98895
2325.19921875 0 675.73737
3070.9375 0 658.969

Spectrum Details

|  |  |
| --- | --- |
| Matched peaks? Matched peaksThe total absolute number of peaks matched. Additionally in brackets the total fraction of peaks matched and the total number of peaks is shown. | 101 (16.26% of 621) |
| FDR? FDRThe false discovery rate estimated for this peptide. It is calculated by matching all theoretical fragments with a non-integer shift with the raw peaks for this spectrum. This is done with 40 different shifts. The resulting percentage is the average number of annotated peaks over the number of annotated peaks with the correct spectrum. | 0.78% |
| Satellite FDR? Satellite FDRSee the FDR for details on its calculation. This satellite ion specific FDR only contains the satellite ions (d/w) for I/L/J positions. | - |
| PSM Score? PSM ScoreThe PSM Score as given by Hecklib to this annotated spectrum. It is shown with three significant figures. | 852 |

## Spectrum 7896? Spectrum 7896 The raw spectrum of this peptide as annotated by Hecklib. The fragments are coloured according to ion type (see legend). Any peaks with a star '\*' as text can be hovered over to see the full details, first the ion type second the mass shift type. By hovering over the amino acids in the peptide or ions in the legend the corresponding peaks are highlighted. By toggling the 'Unassigned' label you can turn the background (unassigned) peaks on or off in the plot. By updating the slider in the Ion legend you can update the spectrum to only show the top X% of the peaks with labels. The top X% means any peak that is within X% of the highest intensity. By dragging in the spectrum you can zoom in to a specific part of the spectrum and use 'Zoom Out' to get back to the original zoom level. The annotation of the spectrum is based on the given sequence in the peptides file and is done with different software so inconsistencies are likely. The peaks are annotated based on the given sequence, with 20 ppm tolerance.

Copy Data

### Spectrum 7896 (TSV)

#### Preview

```
Loading example...
```

*Click on the button to copy the data to your clipboard.*

Mz MinMz MaxIntensity Max

WidthHeightPeptide font sizePeptide stroke widthSpectrum font sizeSpectrum stroke widthCompact peptide

Ion legend

wxyz

abcd

OtherUnassignedIonChargePositionShow for top:%

VTJFPPSSEEJQANKAT

01.75e+43.50e+45.25e+47.00e+4

Zoom Out

y+11y+12y+12c+12y+37z+13y+26y+26y+13c+13y+27y+14z+14y+14c+29y+29y+15w+210z+315w+16y+316y+211c+211y+16z+16y+16y+212c+16c+212c+16y+213y+213z+213y+213z+17y+17y+214y+214z+214c+17y+214w+18y+215y+215c+215z+18c+18z+216z+216c+216y+18y+216y+216z+216c+216y+18w+19c+19z+19y+19z+110z+110y+110y+110z+110c+110y+110c+110y+111z+111y+111c+111y+112y+112c+112c+112y+113c+113y+114z+114c+114y+114c+114y+115z+115y+115c+115y+116y+116z+116c+116

0775155023253100

Fragment Matches Table

Show background peaks

| Position | Ion type | Intensity | mz Theoretical | mz Error (Th) | mz Error (ppm) | Charge | Series Number |
| --- | --- | --- | --- | --- | --- | --- | --- |
| - | - | 695.7 | 120.1 | - | - | 0 | - |
| 17 | y | 1.064E+04 | 120.1 | 7.029E-05 | 0.5854 | +1 | 1 |
| - | - | 2015 | 120.1 | - | - | 0 | - |
| - | - | 1158 | 126.1 | - | - | 0 | - |
| - | - | 830.4 | 128.1 | - | - | 0 | - |
| - | - | 4639 | 129.1 | - | - | 0 | - |
| - | - | 394.2 | 129.2 | - | - | 0 | - |
| - | - | 929.7 | 131.1 | - | - | 0 | - |
| - | - | 1187 | 140.1 | - | - | 0 | - |
| - | - | 450.4 | 146.7 | - | - | 0 | - |
| - | - | 1075 | 149 | - | - | 0 | - |
| - | - | 439 | 153.8 | - | - | 0 | - |
| - | - | 575.5 | 155.1 | - | - | 0 | - |
| - | - | 491.9 | 160.6 | - | - | 0 | - |
| - | - | 2654 | 169.1 | - | - | 0 | - |
| - | - | 727.5 | 171.1 | - | - | 0 | - |
| 16 | y | 1382 | 173.1 | 0.0002927 | 1.691 | +1 | 2 |
| - | - | 2950 | 173.1 | - | - | 0 | - |
| - | - | 544.9 | 177.7 | - | - | 0 | - |
| - | - | 516.9 | 181.1 | - | - | 0 | - |
| - | - | 2398 | 183.1 | - | - | 0 | - |
| - | - | 628.4 | 187.1 | - | - | 0 | - |
| 16 | y | 2414 | 191.1 | 6.382E-05 | 0.334 | +1 | 2 |
| - | - | 2236 | 197.1 | - | - | 0 | - |
| - | - | 5983 | 201.1 | - | - | 0 | - |
| - | - | 502.8 | 207.4 | - | - | 0 | - |
| - | - | 527.6 | 211.1 | - | - | 0 | - |
| - | - | 8244 | 212.1 | - | - | 0 | - |
| - | - | 815 | 213.1 | - | - | 0 | - |
| - | - | 871 | 215.1 | - | - | 0 | - |
| - | - | 618.9 | 217.1 | - | - | 0 | - |
| 2 | c | 3148 | 218.1 | 0.000214 | 0.9808 | +1 | 2 |
| - | - | 3301 | 221.1 | - | - | 0 | - |
| - | - | 717.2 | 226.1 | - | - | 0 | - |
| - | - | 1348 | 227.1 | - | - | 0 | - |
| - | - | 1744 | 227.1 | - | - | 0 | - |
| - | - | 1259 | 233.2 | - | - | 0 | - |
| - | - | 1139 | 235.1 | - | - | 0 | - |
| - | - | 600 | 238.2 | - | - | 0 | - |
| - | - | 5430 | 239.1 | - | - | 0 | - |
| - | - | 1594 | 240.1 | - | - | 0 | - |
| - | - | 729.4 | 241.1 | - | - | 0 | - |
| 11 | y | 898.3 | 243.1 | 0.003368 | 13.85 | +3 | 7 |
| - | - | 1014 | 254.2 | - | - | 0 | - |
| - | - | 2808 | 263.1 | - | - | 0 | - |
| - | - | 1051 | 281.1 | - | - | 0 | - |
| - | - | 1198 | 282.1 | - | - | 0 | - |
| - | - | 738.6 | 295.1 | - | - | 0 | - |
| - | - | 1.136E+04 | 296.2 | - | - | 0 | - |
| - | - | 698.4 | 297.1 | - | - | 0 | - |
| - | - | 863.4 | 297.2 | - | - | 0 | - |
| - | - | 1572 | 297.2 | - | - | 0 | - |
| - | - | 785.2 | 298.2 | - | - | 0 | - |
| - | - | 6149 | 299.1 | - | - | 0 | - |
| - | - | 1.431E+04 | 299.2 | - | - | 0 | - |
| - | - | 1232 | 300.1 | - | - | 0 | - |
| - | - | 2019 | 300.2 | - | - | 0 | - |
| - | - | 773 | 301.1 | - | - | 0 | - |
| 15 | z | 6998 | 303.2 | 2.169E-05 | 0.07155 | +1 | 3 |
| - | - | 1125 | 304.2 | - | - | 0 | - |
| 12 | y | 710.8 | 307.7 | 0.0006813 | 2.214 | +2 | 6 |
| - | - | 1105 | 314.2 | - | - | 0 | - |
| - | - | 4226 | 314.2 | - | - | 0 | - |
| - | - | 620 | 315.2 | - | - | 0 | - |
| - | - | 7792 | 316.2 | - | - | 0 | - |
| 12 | y | 1256 | 316.7 | 0.0004344 | 1.372 | +2 | 6 |
| - | - | 664.1 | 317.2 | - | - | 0 | - |
| 15 | y | 3264 | 319.2 | 0.0003711 | 1.163 | +1 | 3 |
| 3 | c | 2.425E+04 | 331.2 | 8.79E-05 | 0.2654 | +1 | 3 |
| - | - | 4363 | 332.2 | - | - | 0 | - |
| - | - | 735.1 | 343.4 | - | - | 0 | - |
| - | - | 783.3 | 345.2 | - | - | 0 | - |
| - | - | 583.5 | 347.2 | - | - | 0 | - |
| - | - | 713.9 | 350.1 | - | - | 0 | - |
| - | - | 3.817E+04 | 355.1 | - | - | 0 | - |
| - | - | 1.663E+04 | 356.1 | - | - | 0 | - |
| - | - | 1.009E+04 | 357.1 | - | - | 0 | - |
| - | - | 1091 | 358.1 | - | - | 0 | - |
| - | - | 1013 | 362.2 | - | - | 0 | - |
| - | - | 615.8 | 369.2 | - | - | 0 | - |
| - | - | 1687 | 371.1 | - | - | 0 | - |
| - | - | 1024 | 372.1 | - | - | 0 | - |
| - | - | 840.7 | 373.1 | - | - | 0 | - |
| 11 | y | 3453 | 373.2 | 0.004763 | 12.76 | +2 | 7 |
| - | - | 855.9 | 378.2 | - | - | 0 | - |
| - | - | 924.3 | 385.2 | - | - | 0 | - |
| - | - | 1711 | 386.2 | - | - | 0 | - |
| - | - | 667.3 | 407.2 | - | - | 0 | - |
| - | - | 2670 | 415 | - | - | 0 | - |
| 14 | y | 752.5 | 415.2 | 0.0001008 | 0.2427 | +1 | 4 |
| 14 | z | 2014 | 417.2 | 0.00165 | 3.955 | +1 | 4 |
| - | - | 5839 | 418.2 | - | - | 0 | - |
| - | - | 578.8 | 419 | - | - | 0 | - |
| - | - | 1533 | 419.2 | - | - | 0 | - |
| 14 | y | 2337 | 433.2 | 0.000626 | 1.445 | +1 | 4 |
| - | - | 7895 | 433.3 | - | - | 0 | - |
| - | - | 845 | 434.3 | - | - | 0 | - |
| - | - | 4359 | 434.3 | - | - | 0 | - |
| - | - | 2693 | 435.3 | - | - | 0 | - |
| - | - | 816 | 442.2 | - | - | 0 | - |
| - | - | 5423 | 444.2 | - | - | 0 | - |
| - | - | 576 | 445.2 | - | - | 0 | - |
| - | - | 2234 | 445.3 | - | - | 0 | - |
| - | - | 1639 | 446.3 | - | - | 0 | - |
| - | - | 1249 | 460.3 | - | - | 0 | - |
| - | - | 1.117E+04 | 461.3 | - | - | 0 | - |
| - | - | 2550 | 462.3 | - | - | 0 | - |
| - | - | 785.6 | 463.3 | - | - | 0 | - |
| - | - | 653.6 | 480.2 | - | - | 0 | - |
| - | - | 1476 | 480.7 | - | - | 0 | - |
| 9 | c | 9223 | 488.3 | 0.001922 | 3.937 | +2 | 9 |
| - | - | 1449 | 489.1 | - | - | 0 | - |
| - | - | 6158 | 489.3 | - | - | 0 | - |
| - | - | 912.8 | 490.1 | - | - | 0 | - |
| - | - | 1844 | 490.3 | - | - | 0 | - |
| - | - | 1904 | 493.7 | - | - | 0 | - |
| - | - | 1163 | 494.2 | - | - | 0 | - |
| - | - | 1044 | 498.2 | - | - | 0 | - |
| 9 | y | 2048 | 502.3 | 0.008278 | 16.48 | +2 | 9 |
| - | - | 3179 | 502.7 | - | - | 0 | - |
| - | - | 2229 | 503.2 | - | - | 0 | - |
| - | - | 828.6 | 503.3 | - | - | 0 | - |
| 13 | y | 8076 | 504.3 | 0.0001333 | 0.2644 | +1 | 5 |
| - | - | 1532 | 505.3 | - | - | 0 | - |
| - | - | 740.2 | 518.2 | - | - | 0 | - |
| 8 | w | 623.6 | 529.3 | 0.004643 | 8.773 | +2 | 10 |
| - | - | 638.2 | 536.3 | - | - | 0 | - |
| - | - | 6252 | 538.3 | - | - | 0 | - |
| - | - | 3655 | 538.8 | - | - | 0 | - |
| 3 | z | 973.2 | 539.3 | 0.002174 | 4.032 | +3 | 15 |
| - | - | 2101 | 543.3 | - | - | 0 | - |
| - | - | 636 | 543.3 | - | - | 0 | - |
| - | - | 1123 | 543.8 | - | - | 0 | - |
| - | - | 1444 | 544.3 | - | - | 0 | - |
| - | - | 6640 | 552.3 | - | - | 0 | - |
| - | - | 812.2 | 552.3 | - | - | 0 | - |
| - | - | 5595 | 552.8 | - | - | 0 | - |
| - | - | 1613 | 553.3 | - | - | 0 | - |
| - | - | 1272 | 554.3 | - | - | 0 | - |
| 12 | w | 1.511E+04 | 558.3 | 0.0004826 | 0.8645 | +1 | 6 |
| - | - | 2710 | 558.3 | - | - | 0 | - |
| - | - | 4718 | 559.3 | - | - | 0 | - |
| - | - | 595.3 | 559.3 | - | - | 0 | - |
| - | - | 1420 | 560.3 | - | - | 0 | - |
| 2 | y | 1470 | 572.3 | 0.01022 | 17.86 | +3 | 16 |
| - | - | 1179 | 577.1 | - | - | 0 | - |
| - | - | 702.9 | 578.1 | - | - | 0 | - |
| - | - | 775.5 | 579.1 | - | - | 0 | - |
| 7 | y | 942.3 | 580.3 | 0.003138 | 5.408 | +2 | 11 |
| - | - | 842.5 | 583.3 | - | - | 0 | - |
| - | - | 800.6 | 591.3 | - | - | 0 | - |
| - | - | 1842 | 599.8 | - | - | 0 | - |
| 11 | c | 1266 | 600.3 | 0.01135 | 18.91 | +2 | 11 |
| - | - | 2963 | 602.8 | - | - | 0 | - |
| - | - | 1736 | 603.3 | - | - | 0 | - |
| - | - | 8401 | 610.2 | - | - | 0 | - |
| - | - | 8783 | 611.2 | - | - | 0 | - |
| - | - | 7301 | 611.8 | - | - | 0 | - |
| - | - | 6641 | 612.2 | - | - | 0 | - |
| - | - | 4486 | 612.3 | - | - | 0 | - |
| - | - | 2311 | 612.8 | - | - | 0 | - |
| - | - | 712.8 | 613.2 | - | - | 0 | - |
| 12 | y | 8255 | 614.3 | 0.01114 | 18.14 | +1 | 6 |
| - | - | 1.106E+04 | 615.3 | - | - | 0 | - |
| 12 | z | 4304 | 616.3 | 0.001512 | 2.453 | +1 | 6 |
| - | - | 1540 | 616.4 | - | - | 0 | - |
| - | - | 1237 | 617.3 | - | - | 0 | - |
| - | - | 1531 | 617.8 | - | - | 0 | - |
| - | - | 2541 | 626.3 | - | - | 0 | - |
| - | - | 3252 | 626.8 | - | - | 0 | - |
| - | - | 3599 | 627.3 | - | - | 0 | - |
| - | - | 1729 | 627.3 | - | - | 0 | - |
| - | - | 1494 | 627.4 | - | - | 0 | - |
| - | - | 910 | 627.8 | - | - | 0 | - |
| - | - | 1423 | 628.3 | - | - | 0 | - |
| - | - | 3443 | 628.4 | - | - | 0 | - |
| - | - | 1330 | 629.4 | - | - | 0 | - |
| - | - | 1251 | 630.4 | - | - | 0 | - |
| 12 | y | 5802 | 632.3 | 0.0001496 | 0.2366 | +1 | 6 |
| - | - | 1226 | 633.3 | - | - | 0 | - |
| - | - | 1192 | 635.3 | - | - | 0 | - |
| 6 | y | 553.3 | 637.8 | 0.00132 | 2.069 | +2 | 12 |
| 6 | c | 789.2 | 654.4 | 0.01176 | 17.97 | +1 | 6 |
| - | - | 2042 | 655.3 | - | - | 0 | - |
| - | - | 1808 | 655.8 | - | - | 0 | - |
| - | - | 937.2 | 656.3 | - | - | 0 | - |
| - | - | 675.8 | 658.2 | - | - | 0 | - |
| - | - | 1023 | 658.4 | - | - | 0 | - |
| 12 | c | 767.3 | 664.8 | 0.005734 | 8.625 | +2 | 12 |
| - | - | 990.7 | 668.3 | - | - | 0 | - |
| - | - | 2738 | 671.4 | - | - | 0 | - |
| 6 | c | 3850 | 672.4 | 0.001002 | 1.49 | +1 | 6 |
| - | - | 1208 | 673.4 | - | - | 0 | - |
| - | - | 1848 | 676.8 | - | - | 0 | - |
| 5 | y | 7562 | 677.3 | 0.0005537 | 0.8174 | +2 | 13 |
| 5 | y | 5782 | 677.8 | 0.008659 | 12.77 | +2 | 13 |
| 5 | z | 3493 | 678.3 | 0.002305 | 3.399 | +2 | 13 |
| - | - | 4529 | 685.8 | - | - | 0 | - |
| 5 | y | 4.995E+04 | 686.3 | 0.002465 | 3.591 | +2 | 13 |
| - | - | 2.066E+04 | 686.8 | - | - | 0 | - |
| - | - | 1.375E+04 | 687.3 | - | - | 0 | - |
| - | - | 3332 | 687.8 | - | - | 0 | - |
| - | - | 3031 | 688.4 | - | - | 0 | - |
| - | - | 1399 | 697.4 | - | - | 0 | - |
| - | - | 1021 | 698.4 | - | - | 0 | - |
| - | - | 852.2 | 713.4 | - | - | 0 | - |
| - | - | 870.1 | 716.4 | - | - | 0 | - |
| - | - | 1796 | 722.3 | - | - | 0 | - |
| - | - | 988.8 | 723.4 | - | - | 0 | - |
| 11 | z | 1.134E+04 | 729.4 | 0.0008593 | 1.178 | +1 | 7 |
| - | - | 3920 | 730.4 | - | - | 0 | - |
| - | - | 1428 | 731.4 | - | - | 0 | - |
| - | - | 1470 | 740.3 | - | - | 0 | - |
| - | - | 2223 | 742.4 | - | - | 0 | - |
| 11 | y | 6890 | 745.4 | 0.0005583 | 0.7489 | +1 | 7 |
| - | - | 2029 | 746.4 | - | - | 0 | - |
| 4 | y | 1039 | 750.9 | 0.002961 | 3.944 | +2 | 14 |
| 4 | y | 1786 | 751.4 | 0.005092 | 6.777 | +2 | 14 |
| 4 | z | 939 | 751.9 | 0.001201 | 1.597 | +2 | 14 |
| - | - | 2292 | 757.4 | - | - | 0 | - |
| - | - | 2065 | 759.4 | - | - | 0 | - |
| 7 | c | 2.977E+04 | 759.4 | 0.0001937 | 0.255 | +1 | 7 |
| 4 | y | 8736 | 759.9 | 0.0004312 | 0.5674 | +2 | 14 |
| - | - | 6643 | 760.4 | - | - | 0 | - |
| - | - | 1.332E+04 | 760.4 | - | - | 0 | - |
| - | - | 2805 | 760.9 | - | - | 0 | - |
| - | - | 2071 | 761.4 | - | - | 0 | - |
| - | - | 4425 | 761.4 | - | - | 0 | - |
| - | - | 5880 | 764.4 | - | - | 0 | - |
| - | - | 3327 | 765.4 | - | - | 0 | - |
| - | - | 1260 | 766.4 | - | - | 0 | - |
| - | - | 988.7 | 798.4 | - | - | 0 | - |
| - | - | 1183 | 798.9 | - | - | 0 | - |
| 10 | w | 1.098E+04 | 799.4 | 0.001645 | 2.057 | +1 | 8 |
| - | - | 5272 | 800.4 | - | - | 0 | - |
| - | - | 1614 | 801.4 | - | - | 0 | - |
| - | - | 4429 | 802.4 | - | - | 0 | - |
| - | - | 2203 | 803.4 | - | - | 0 | - |
| - | - | 848.2 | 803.5 | - | - | 0 | - |
| 3 | y | 1862 | 807.4 | 0.009328 | 11.55 | +2 | 15 |
| - | - | 2240 | 807.9 | - | - | 0 | - |
| - | - | 1050 | 808.4 | - | - | 0 | - |
| - | - | 1195 | 813.9 | - | - | 0 | - |
| - | - | 1897 | 814.4 | - | - | 0 | - |
| - | - | 1228 | 815.4 | - | - | 0 | - |
| 3 | y | 2861 | 816.4 | 0.0001047 | 0.1283 | +2 | 15 |
| - | - | 1605 | 816.9 | - | - | 0 | - |
| - | - | 1026 | 817.4 | - | - | 0 | - |
| - | - | 750.8 | 817.9 | - | - | 0 | - |
| - | - | 3665 | 822.4 | - | - | 0 | - |
| - | - | 5501 | 823.4 | - | - | 0 | - |
| - | - | 2258 | 824.4 | - | - | 0 | - |
| - | - | 858.5 | 829.4 | - | - | 0 | - |
| 15 | c | 2.653E+04 | 829.9 | 0.0005537 | 0.6671 | +2 | 15 |
| - | - | 3.067E+04 | 830.4 | - | - | 0 | - |
| - | - | 1.598E+04 | 830.9 | - | - | 0 | - |
| - | - | 5393 | 831.4 | - | - | 0 | - |
| - | - | 935.7 | 832 | - | - | 0 | - |
| - | - | 1016 | 835.9 | - | - | 0 | - |
| 10 | z | 1660 | 841.4 | 0.01319 | 15.67 | +1 | 8 |
| - | - | 1031 | 842.4 | - | - | 0 | - |
| - | - | 2444 | 843.5 | - | - | 0 | - |
| - | - | 2800 | 844 | - | - | 0 | - |
| - | - | 2321 | 844.5 | - | - | 0 | - |
| 8 | c | 2.461E+04 | 846.5 | 0.0003007 | 0.3552 | +1 | 8 |
| - | - | 1.135E+04 | 847.5 | - | - | 0 | - |
| - | - | 897.8 | 847.9 | - | - | 0 | - |
| - | - | 3519 | 848.5 | - | - | 0 | - |
| - | - | 2773 | 848.9 | - | - | 0 | - |
| - | - | 848.4 | 849.4 | - | - | 0 | - |
| 2 | z | 2621 | 849.9 | 0.002785 | 3.277 | +2 | 16 |
| 2 | z | 3091 | 850.4 | 0.01102 | 12.96 | +2 | 16 |
| - | - | 859.6 | 852.9 | - | - | 0 | - |
| 16 | c | 3473 | 856.9 | 0.002906 | 3.391 | +2 | 16 |
| 10 | y | 4373 | 857.4 | 0.008928 | 10.41 | +1 | 8 |
| 2 | y | 3210 | 857.9 | 0.008316 | 9.693 | +2 | 16 |
| 2 | y | 2.361E+04 | 858.4 | 0.0171 | 19.92 | +2 | 16 |
| 2 | z | 2705 | 858.9 | 0.01063 | 12.37 | +2 | 16 |
| - | - | 1.027E+04 | 859.4 | - | - | 0 | - |
| - | - | 1188 | 859.9 | - | - | 0 | - |
| - | - | 2007 | 860.4 | - | - | 0 | - |
| - | - | 2383 | 865 | - | - | 0 | - |
| 16 | c | 4.247E+04 | 865.5 | 6.547E-06 | 0.007565 | +2 | 16 |
| - | - | 3.878E+04 | 866 | - | - | 0 | - |
| - | - | 2.631E+04 | 866.5 | - | - | 0 | - |
| - | - | 1.146E+04 | 867 | - | - | 0 | - |
| - | - | 5007 | 867.5 | - | - | 0 | - |
| - | - | 2010 | 868.4 | - | - | 0 | - |
| - | - | 776 | 869.4 | - | - | 0 | - |
| - | - | 917 | 871 | - | - | 0 | - |
| - | - | 1184 | 871.5 | - | - | 0 | - |
| - | - | 867.8 | 872 | - | - | 0 | - |
| - | - | 1094 | 872.9 | - | - | 0 | - |
| - | - | 2686 | 873.5 | - | - | 0 | - |
| - | - | 848.3 | 873.9 | - | - | 0 | - |
| 10 | y | 4696 | 874.5 | 0.001385 | 1.584 | +1 | 8 |
| - | - | 1751 | 875.5 | - | - | 0 | - |
| - | - | 2413 | 880.5 | - | - | 0 | - |
| - | - | 6732 | 881 | - | - | 0 | - |
| - | - | 6680 | 881.5 | - | - | 0 | - |
| - | - | 3973 | 882 | - | - | 0 | - |
| - | - | 1883 | 882.5 | - | - | 0 | - |
| - | - | 2007 | 885.5 | - | - | 0 | - |
| - | - | 3629 | 886 | - | - | 0 | - |
| - | - | 3384 | 886.5 | - | - | 0 | - |
| - | - | 1484 | 887 | - | - | 0 | - |
| - | - | 1409 | 888 | - | - | 0 | - |
| - | - | 927.9 | 888.5 | - | - | 0 | - |
| - | - | 1055 | 889 | - | - | 0 | - |
| - | - | 2614 | 889.4 | - | - | 0 | - |
| - | - | 2.154E+04 | 894.5 | - | - | 0 | - |
| - | - | 1.899E+04 | 895 | - | - | 0 | - |
| - | - | 1.038E+04 | 895.5 | - | - | 0 | - |
| - | - | 3521 | 896 | - | - | 0 | - |
| - | - | 1143 | 896.5 | - | - | 0 | - |
| - | - | 980.2 | 899.5 | - | - | 0 | - |
| - | - | 1733 | 900 | - | - | 0 | - |
| - | - | 1390 | 900.5 | - | - | 0 | - |
| - | - | 831.5 | 902 | - | - | 0 | - |
| - | - | 8636 | 907.4 | - | - | 0 | - |
| - | - | 3925 | 908 | - | - | 0 | - |
| - | - | 2581 | 908.4 | - | - | 0 | - |
| - | - | 5058 | 908.5 | - | - | 0 | - |
| - | - | 3538 | 909 | - | - | 0 | - |
| - | - | 1927 | 909.5 | - | - | 0 | - |
| - | - | 1223 | 910 | - | - | 0 | - |
| - | - | 1531 | 916 | - | - | 0 | - |
| - | - | 4.853E+04 | 916.5 | - | - | 0 | - |
| - | - | 6.93E+04 | 917 | - | - | 0 | - |
| - | - | 5.297E+04 | 917.5 | - | - | 0 | - |
| - | - | 2.917E+04 | 918 | - | - | 0 | - |
| - | - | 1.058E+04 | 918.5 | - | - | 0 | - |
| - | - | 2778 | 919 | - | - | 0 | - |
| 9 | w | 5386 | 928.5 | 0.0003534 | 0.3807 | +1 | 9 |
| - | - | 5279 | 929.5 | - | - | 0 | - |
| - | - | 2299 | 930.5 | - | - | 0 | - |
| - | - | 3039 | 944.5 | - | - | 0 | - |
| - | - | 5080 | 945.5 | - | - | 0 | - |
| - | - | 1803 | 946.5 | - | - | 0 | - |
| - | - | 1781 | 959.5 | - | - | 0 | - |
| - | - | 6777 | 960.5 | - | - | 0 | - |
| - | - | 2337 | 961.5 | - | - | 0 | - |
| - | - | 828.8 | 962.5 | - | - | 0 | - |
| - | - | 1.225E+04 | 973.5 | - | - | 0 | - |
| - | - | 7510 | 974.5 | - | - | 0 | - |
| 9 | c | 4.003E+04 | 975.5 | 0.0009016 | 0.9242 | +1 | 9 |
| - | - | 2.006E+04 | 976.5 | - | - | 0 | - |
| - | - | 7939 | 977.5 | - | - | 0 | - |
| - | - | 1994 | 978.5 | - | - | 0 | - |
| 9 | z | 1.888E+04 | 987.5 | 0.0004742 | 0.4802 | +1 | 9 |
| - | - | 9858 | 988.5 | - | - | 0 | - |
| - | - | 3963 | 989.5 | - | - | 0 | - |
| - | - | 887.9 | 990.5 | - | - | 0 | - |
| - | - | 1526 | 1002 | - | - | 0 | - |
| 9 | y | 4518 | 1004 | 0.005404 | 5.385 | +1 | 9 |
| - | - | 2223 | 1005 | - | - | 0 | - |
| - | - | 1303 | 1005 | - | - | 0 | - |
| - | - | 1499 | 1013 | - | - | 0 | - |
| - | - | 970.9 | 1014 | - | - | 0 | - |
| - | - | 984.1 | 1018 | - | - | 0 | - |
| - | - | 840.8 | 1019 | - | - | 0 | - |
| - | - | 823.2 | 1021 | - | - | 0 | - |
| - | - | 772.7 | 1032 | - | - | 0 | - |
| - | - | 4089 | 1055 | - | - | 0 | - |
| 8 | z | 3380 | 1057 | 0.003688 | 3.491 | +1 | 10 |
| 8 | z | 1507 | 1057 | 0.01962 | 18.55 | +1 | 10 |
| - | - | 892.4 | 1059 | - | - | 0 | - |
| - | - | 1062 | 1060 | - | - | 0 | - |
| - | - | 2570 | 1061 | - | - | 0 | - |
| - | - | 1510 | 1062 | - | - | 0 | - |
| 8 | y | 1878 | 1073 | 0.01301 | 12.13 | +1 | 10 |
| 8 | y | 1552 | 1074 | 0.001751 | 1.631 | +1 | 10 |
| 8 | z | 1.997E+04 | 1075 | 0.0005812 | 0.5409 | +1 | 10 |
| - | - | 1.246E+04 | 1076 | - | - | 0 | - |
| - | - | 6937 | 1077 | - | - | 0 | - |
| - | - | 1467 | 1078 | - | - | 0 | - |
| 10 | c | 729.6 | 1087 | 0.01804 | 16.6 | +1 | 10 |
| - | - | 3653 | 1088 | - | - | 0 | - |
| - | - | 1864 | 1089 | - | - | 0 | - |
| - | - | 1822 | 1090 | - | - | 0 | - |
| 8 | y | 1.324E+04 | 1091 | 0.0009947 | 0.9121 | +1 | 10 |
| - | - | 6197 | 1092 | - | - | 0 | - |
| - | - | 2398 | 1093 | - | - | 0 | - |
| - | - | 1137 | 1094 | - | - | 0 | - |
| - | - | 1739 | 1099 | - | - | 0 | - |
| - | - | 815.3 | 1100 | - | - | 0 | - |
| - | - | 842.9 | 1103 | - | - | 0 | - |
| - | - | 2621 | 1104 | - | - | 0 | - |
| 10 | c | 4.416E+04 | 1105 | 0.001747 | 1.581 | +1 | 10 |
| - | - | 2.622E+04 | 1106 | - | - | 0 | - |
| - | - | 8579 | 1107 | - | - | 0 | - |
| - | - | 3009 | 1108 | - | - | 0 | - |
| - | - | 8517 | 1121 | - | - | 0 | - |
| - | - | 5003 | 1122 | - | - | 0 | - |
| - | - | 2068 | 1123 | - | - | 0 | - |
| - | - | 904.8 | 1134 | - | - | 0 | - |
| - | - | 1111 | 1143 | - | - | 0 | - |
| 7 | y | 1016 | 1161 | 0.008419 | 7.254 | +1 | 11 |
| 7 | z | 9591 | 1162 | 1.684E-05 | 0.0145 | +1 | 11 |
| - | - | 6247 | 1163 | - | - | 0 | - |
| - | - | 3986 | 1164 | - | - | 0 | - |
| - | - | 2241 | 1165 | - | - | 0 | - |
| 7 | y | 5162 | 1178 | 0.0006745 | 0.5728 | +1 | 11 |
| - | - | 4960 | 1179 | - | - | 0 | - |
| - | - | 2029 | 1180 | - | - | 0 | - |
| - | - | 1447 | 1181 | - | - | 0 | - |
| - | - | 1192 | 1182 | - | - | 0 | - |
| - | - | 1404 | 1183 | - | - | 0 | - |
| - | - | 3277 | 1199 | - | - | 0 | - |
| - | - | 1497 | 1200 | - | - | 0 | - |
| - | - | 1821 | 1201 | - | - | 0 | - |
| - | - | 1.292E+04 | 1207 | - | - | 0 | - |
| - | - | 9053 | 1208 | - | - | 0 | - |
| - | - | 2310 | 1209 | - | - | 0 | - |
| 11 | c | 2.551E+04 | 1218 | 0.001826 | 1.5 | +1 | 11 |
| - | - | 1.846E+04 | 1219 | - | - | 0 | - |
| - | - | 7426 | 1220 | - | - | 0 | - |
| - | - | 1853 | 1221 | - | - | 0 | - |
| - | - | 7799 | 1223 | - | - | 0 | - |
| - | - | 1.211E+04 | 1224 | - | - | 0 | - |
| - | - | 7131 | 1225 | - | - | 0 | - |
| - | - | 1458 | 1226 | - | - | 0 | - |
| - | - | 2334 | 1253 | - | - | 0 | - |
| - | - | 1507 | 1254 | - | - | 0 | - |
| 6 | y | 901.4 | 1258 | 0.01742 | 13.85 | +1 | 12 |
| - | - | 1486 | 1270 | - | - | 0 | - |
| - | - | 816.7 | 1271 | - | - | 0 | - |
| 6 | y | 2348 | 1275 | 0.0003947 | 0.3096 | +1 | 12 |
| - | - | 1855 | 1276 | - | - | 0 | - |
| - | - | 1010 | 1277 | - | - | 0 | - |
| - | - | 1548 | 1302 | - | - | 0 | - |
| - | - | 784.5 | 1303 | - | - | 0 | - |
| - | - | 852.7 | 1328 | - | - | 0 | - |
| 12 | c | 1449 | 1329 | 0.02067 | 15.56 | +1 | 12 |
| - | - | 2507 | 1330 | - | - | 0 | - |
| - | - | 2399 | 1331 | - | - | 0 | - |
| - | - | 678.6 | 1332 | - | - | 0 | - |
| - | - | 789.8 | 1344 | - | - | 0 | - |
| - | - | 3240 | 1345 | - | - | 0 | - |
| 12 | c | 3.56E+04 | 1346 | 0.002664 | 1.98 | +1 | 12 |
| - | - | 2.999E+04 | 1347 | - | - | 0 | - |
| - | - | 1.009E+04 | 1348 | - | - | 0 | - |
| - | - | 3497 | 1349 | - | - | 0 | - |
| - | - | 2708 | 1371 | - | - | 0 | - |
| 5 | y | 9397 | 1372 | 0.002809 | 2.048 | +1 | 13 |
| - | - | 7692 | 1373 | - | - | 0 | - |
| - | - | 3862 | 1374 | - | - | 0 | - |
| - | - | 1576 | 1375 | - | - | 0 | - |
| - | - | 1375 | 1399 | - | - | 0 | - |
| - | - | 2788 | 1400 | - | - | 0 | - |
| - | - | 4796 | 1401 | - | - | 0 | - |
| - | - | 4672 | 1402 | - | - | 0 | - |
| - | - | 2363 | 1403 | - | - | 0 | - |
| - | - | 1096 | 1404 | - | - | 0 | - |
| - | - | 4567 | 1416 | - | - | 0 | - |
| 13 | c | 2.155E+04 | 1417 | 0.004134 | 2.918 | +1 | 13 |
| - | - | 1.558E+04 | 1418 | - | - | 0 | - |
| - | - | 7139 | 1419 | - | - | 0 | - |
| - | - | 1796 | 1420 | - | - | 0 | - |
| - | - | 2341 | 1431 | - | - | 0 | - |
| - | - | 1728 | 1432 | - | - | 0 | - |
| - | - | 924.7 | 1433 | - | - | 0 | - |
| - | - | 1001 | 1443 | - | - | 0 | - |
| - | - | 966.2 | 1459 | - | - | 0 | - |
| - | - | 1231 | 1487 | - | - | 0 | - |
| - | - | 1595 | 1488 | - | - | 0 | - |
| 4 | y | 1535 | 1502 | 0.005253 | 3.498 | +1 | 14 |
| 4 | z | 2.007E+04 | 1503 | 0.002816 | 1.874 | +1 | 14 |
| - | - | 3.141E+04 | 1504 | - | - | 0 | - |
| - | - | 1.952E+04 | 1505 | - | - | 0 | - |
| - | - | 1.012E+04 | 1506 | - | - | 0 | - |
| - | - | 3259 | 1507 | - | - | 0 | - |
| 14 | c | 1056 | 1514 | 0.01147 | 7.577 | +1 | 14 |
| 4 | y | 3262 | 1519 | 0.0001886 | 0.1242 | +1 | 14 |
| - | - | 2611 | 1520 | - | - | 0 | - |
| - | - | 1667 | 1521 | - | - | 0 | - |
| - | - | 1096 | 1522 | - | - | 0 | - |
| - | - | 914.5 | 1530 | - | - | 0 | - |
| 14 | c | 3.828E+04 | 1531 | 0.002872 | 1.876 | +1 | 14 |
| - | - | 3.434E+04 | 1532 | - | - | 0 | - |
| - | - | 1.606E+04 | 1533 | - | - | 0 | - |
| - | - | 5950 | 1534 | - | - | 0 | - |
| - | - | 1395 | 1535 | - | - | 0 | - |
| 3 | y | 1605 | 1615 | 0.03044 | 18.85 | +1 | 15 |
| 3 | z | 4508 | 1616 | 0.02066 | 12.79 | +1 | 15 |
| - | - | 6757 | 1617 | - | - | 0 | - |
| - | - | 5261 | 1618 | - | - | 0 | - |
| - | - | 1529 | 1619 | - | - | 0 | - |
| 3 | y | 912.7 | 1632 | 0.03038 | 18.62 | +1 | 15 |
| - | - | 902.2 | 1643 | - | - | 0 | - |
| - | - | 1514 | 1644 | - | - | 0 | - |
| - | - | 1115 | 1645 | - | - | 0 | - |
| 15 | c | 4158 | 1659 | 0.00262 | 1.579 | +1 | 15 |
| - | - | 1.043E+04 | 1660 | - | - | 0 | - |
| - | - | 8741 | 1661 | - | - | 0 | - |
| - | - | 5457 | 1662 | - | - | 0 | - |
| - | - | 832.1 | 1663 | - | - | 0 | - |
| - | - | 1571 | 1673 | - | - | 0 | - |
| - | - | 802.9 | 1674 | - | - | 0 | - |
| - | - | 2429 | 1686 | - | - | 0 | - |
| - | - | 3207 | 1687 | - | - | 0 | - |
| - | - | 2108 | 1688 | - | - | 0 | - |
| - | - | 915.1 | 1689 | - | - | 0 | - |
| - | - | 1782 | 1701 | - | - | 0 | - |
| - | - | 2932 | 1714 | - | - | 0 | - |
| 2 | y | 3103 | 1715 | 0.0272 | 15.86 | +1 | 16 |
| 2 | y | 2509 | 1716 | 0.02805 | 16.35 | +1 | 16 |
| 2 | z | 2689 | 1717 | 0.01339 | 7.8 | +1 | 16 |
| - | - | 9204 | 1718 | - | - | 0 | - |
| - | - | 6645 | 1719 | - | - | 0 | - |
| - | - | 3192 | 1720 | - | - | 0 | - |
| 16 | c | 4992 | 1730 | 0.005676 | 3.281 | +1 | 16 |
| - | - | 1.4E+04 | 1731 | - | - | 0 | - |
| - | - | 1.247E+04 | 1732 | - | - | 0 | - |
| - | - | 6436 | 1733 | - | - | 0 | - |
| - | - | 2313 | 1734 | - | - | 0 | - |
| - | - | 970.5 | 1735 | - | - | 0 | - |
| - | - | 1083 | 1762 | - | - | 0 | - |
| - | - | 2466 | 1771 | - | - | 0 | - |
| - | - | 4948 | 1772 | - | - | 0 | - |
| - | - | 3158 | 1773 | - | - | 0 | - |
| - | - | 2541 | 1774 | - | - | 0 | - |
| - | - | 1120 | 1775 | - | - | 0 | - |
| - | - | 6549 | 1788 | - | - | 0 | - |
| - | - | 1.934E+04 | 1789 | - | - | 0 | - |
| - | - | 1.951E+04 | 1790 | - | - | 0 | - |
| - | - | 9028 | 1791 | - | - | 0 | - |
| - | - | 3622 | 1792 | - | - | 0 | - |
| - | - | 1389 | 1800 | - | - | 0 | - |
| - | - | 1263 | 1805 | - | - | 0 | - |
| - | - | 5799 | 1806 | - | - | 0 | - |
| - | - | 4170 | 1807 | - | - | 0 | - |
| - | - | 2658 | 1808 | - | - | 0 | - |
| - | - | 983.6 | 1815 | - | - | 0 | - |
| - | - | 7257 | 1816 | - | - | 0 | - |
| - | - | 3.144E+04 | 1817 | - | - | 0 | - |
| - | - | 2.7E+04 | 1818 | - | - | 0 | - |
| - | - | 1.578E+04 | 1819 | - | - | 0 | - |
| - | - | 5965 | 1820 | - | - | 0 | - |
| - | - | 1534 | 1821 | - | - | 0 | - |
| - | - | 3793 | 1832 | - | - | 0 | - |
| - | - | 2.102E+04 | 1833 | - | - | 0 | - |
| - | - | 5.354E+04 | 1834 | - | - | 0 | - |
| - | - | 4.809E+04 | 1835 | - | - | 0 | - |
| - | - | 2.588E+04 | 1836 | - | - | 0 | - |
| - | - | 9787 | 1837 | - | - | 0 | - |
| - | - | 2609 | 1838 | - | - | 0 | - |
| - | - | 779.6 | 1839 | - | - | 0 | - |
| - | - | 825.3 | 3069 | - | - | 0 | - |

m/z Charge Intensity FragmentType MassShift Position
120.06145477294922 0 695.6927
120.06558990478516 0 10644.592 y 16
120.0809326171875 0 2014.8103
126.0550308227539 0 1157.7925
128.0946502685547 0 830.38763
129.10227966308594 0 4638.9907
129.22695922851562 0 394.20547
131.11801147460938 0 929.6794
140.10690307617188 0 1186.6373
146.6717987060547 0 450.35645
149.0447998046875 0 1075.2426
153.8125762939453 0 439.04633
155.11795043945312 0 575.4869
160.5535888671875 0 491.85083
169.1337432861328 0 2654.1638
171.07699584960938 0 727.4811
173.0923614501953 0 1382.1315 y Water loss 15
173.1284942626953 0 2949.7917
177.67529296875 0 544.94916
181.0967559814453 0 516.9248
183.1127471923828 0 2397.856
187.14402770996094 0 628.4204
191.10256958007812 0 2413.902 y 15
197.1284637451172 0 2236.384
201.12330627441406 0 5982.756
207.37342834472656 0 502.82608
211.10739135742188 0 527.6333
212.13934326171875 0 8243.809
213.14260864257812 0 815.0349
215.1387176513672 0 871.0363
217.13307189941406 0 618.8862
218.1497039794922 0 3147.7217 c 1
221.08433532714844 0 3300.861
226.1187744140625 0 717.24963
227.08999633789062 0 1347.6384
227.10296630859375 0 1744.1807
233.16497802734375 0 1258.8066
235.10702514648438 0 1139.1215
238.15493774414062 0 599.9901
239.09495544433594 0 5430.1455
240.09571838378906 0 1594.1486
241.0916290283203 0 729.372
243.14479064941406 0 898.2774 y Water loss 10
254.15069580078125 0 1013.51337
263.1021423339844 0 2807.7314
281.05145263671875 0 1051.4863
282.1448059082031 0 1197.9187
295.1037902832031 0 738.5546
296.19683837890625 0 11361.847
297.08270263671875 0 698.36523
297.155517578125 0 863.44366
297.2002258300781 0 1572.0522
298.1618347167969 0 785.1759
299.0616760253906 0 6149.4663
299.1715087890625 0 14314.773
300.06329345703125 0 1231.8602
300.1744079589844 0 2018.9429
301.0594787597656 0 772.9765
303.17889404296875 0 6997.7744 z 14
304.1827087402344 0 1124.5336
307.6671447753906 0 710.82446 y Water loss 11
314.18426513671875 0 1104.9292
314.20745849609375 0 4225.806
315.20953369140625 0 620.0324
316.18670654296875 0 7792.422
316.67218017578125 0 1256.1443 y 11
317.1889343261719 0 664.0662
319.1979675292969 0 3263.6194 y 14
331.23406982421875 0 24248.996 c 2
332.2375183105469 0 4362.5054
343.3712463378906 0 735.0915
345.1793518066406 0 783.26654
347.1959228515625 0 583.472
350.13427734375 0 713.8748
355.06976318359375 0 38171.684
356.0702819824219 0 16629.2
357.067626953125 0 10089.685
358.0687561035156 0 1090.9176
362.2061462402344 0 1012.63245
369.2012023925781 0 615.7883
371.1013488769531 0 1687.2555
372.0997314453125 0 1024.017
373.07965087890625 0 840.66833
373.2090148925781 0 3453.2139 y 10
378.2270812988281 0 855.87897
385.2192687988281 0 924.34467
386.2035217285156 0 1711.2217
407.2237548828125 0 667.3139
415.0370178222656 0 2670.3718
415.2298583984375 0 752.4796 y Water loss 13
417.22344970703125 0 2013.876 z 13
418.2296447753906 0 5839.215
418.99517822265625 0 578.83484
419.23309326171875 0 1533.1431
433.24114990234375 0 2337.379 y 13
433.2809753417969 0 7895.476
434.25091552734375 0 844.97076
434.28619384765625 0 4358.6665
435.29486083984375 0 2692.9788
442.23785400390625 0 815.9972
444.2458801269531 0 5423.2163
445.2001647949219 0 576.0258
445.252685546875 0 2233.5051
446.2649841308594 0 1638.6981
460.2676086425781 0 1249.2542
461.27587890625 0 11167.735
462.2790832519531 0 2550.2788
463.2818298339844 0 785.558
480.208984375 0 653.5853
480.7205505371094 0 1476.2212
488.2590026855469 0 9222.817 c 8
489.057373046875 0 1449.2042
489.26470947265625 0 6157.7393
490.05889892578125 0 912.81165
490.26788330078125 0 1843.8018
493.7291564941406 0 1904.1252
494.2294616699219 0 1163.0884
498.2203369140625 0 1044.1583
502.2646484375 0 2047.9257 y 8
502.73211669921875 0 3178.7947
503.2337646484375 0 2228.5188
503.2702941894531 0 828.61273
504.27777099609375 0 8076.141 y 12
505.28106689453125 0 1531.6573
518.2000122070312 0 740.2337
529.2662963867188 0 623.57684 w 7
536.2800903320312 0 638.18286
538.2693481445312 0 6252.1255
538.7711791992188 0 3654.597
539.2722778320312 0 973.1751 z 2
543.2611694335938 0 2100.7974
543.3170776367188 0 635.98914
543.7638549804688 0 1123.4258
544.26513671875 0 1443.6423
552.2669067382812 0 6640.4624
552.3169555664062 0 812.1667
552.7689208984375 0 5595.1978
553.271728515625 0 1613.1914
554.3172607421875 0 1272.0215
558.2877197265625 0 15105.327 w 11
558.3301391601562 0 2709.75
559.2915649414062 0 4718.3794
559.3310546875 0 595.2604
560.29345703125 0 1420.2976
572.3032836914062 0 1470.4111 y Water loss 1
577.1273193359375 0 1179.459
578.1296997070312 0 702.93634
579.1221313476562 0 775.5068
580.2862548828125 0 942.2793 y Water loss 6
583.3380737304688 0 842.5252
591.2913818359375 0 800.5753
599.8067626953125 0 1841.8367
600.3076171875 0 1265.642 c Water loss 10
602.7908325195312 0 2962.5632
603.2918701171875 0 1736.3708
610.1841430664062 0 8400.849
611.1854248046875 0 8783.417
611.7964477539062 0 7300.8433
612.1828002929688 0 6640.5674
612.2966918945312 0 4486.1753
612.8007202148438 0 2310.901
613.184326171875 0 712.81976
614.3367919921875 0 8255.054 y Water loss 11
615.3448486328125 0 11058.466
616.3159790039062 0 4304.299 z 11
616.3578491210938 0 1539.6042
617.3225708007812 0 1236.7311
617.805908203125 0 1530.8658
626.3426513671875 0 2540.573
626.8116455078125 0 3251.9316
627.260498046875 0 3599.2756
627.310791015625 0 1729.4713
627.3552856445312 0 1494.3419
627.8158569335938 0 909.9957
628.2631225585938 0 1423.1256
628.3927612304688 0 3443.3093
629.3950805664062 0 1330.3497
630.3549194335938 0 1251.0865
632.3363647460938 0 5802.429 y 11
633.33642578125 0 1225.5581
635.3238525390625 0 1191.7262
637.8161010742188 0 553.30304 y 5
654.4091186523438 0 789.1812 c Water loss 5
655.341064453125 0 2042.1375
655.842529296875 0 1808.119
656.3364868164062 0 937.24316
658.2335205078125 0 675.798
658.3690795898438 0 1022.5538
664.8345336914062 0 767.34467 c Ammonia loss 11
668.33056640625 0 990.7204
671.3721923828125 0 2737.9312
672.4069213867188 0 3850.367 c 5
673.4155883789062 0 1208.2761
676.8328247070312 0 1847.9686
677.3353271484375 0 7561.794 y Water loss 4
677.8365478515625 0 5781.937 y Ammonia loss 4
678.3341064453125 0 3492.9517 z 4
685.8370361328125 0 4528.8184
686.3436279296875 0 49952.11 y 4
686.8423461914062 0 20659.107
687.3482666015625 0 13747.774
687.8458251953125 0 3332.3396
688.3546142578125 0 3031.3533
697.415283203125 0 1399.1997
698.4212646484375 0 1020.93036
713.4056396484375 0 852.2203
716.44287109375 0 870.06335
722.3484497070312 0 1796.1183
723.3519287109375 0 988.77814
729.4006958007812 0 11339.072 z 10
730.4041748046875 0 3920.1584
731.40966796875 0 1427.9559
740.344970703125 0 1470.0236
742.4138793945312 0 2223.1965
745.4208374023438 0 6890.0083 y 10
746.423095703125 0 2029.4661
750.8671264648438 0 1039.1274 y Water loss 3
751.3671875 0 1786.1438 y Ammonia loss 3
751.8648071289062 0 939.04803 z 3
757.42138671875 0 2292.425
759.3709106445312 0 2065.1646
759.4397583007812 0 29769.883 c 6
759.8749389648438 0 8735.98 y 3
760.3749389648438 0 6642.873
760.4432983398438 0 13319.851
760.87646484375 0 2805.4617
761.376953125 0 2071.1401
761.4464721679688 0 4424.555
764.3612060546875 0 5879.807
765.3626098632812 0 3327.3193
766.3600463867188 0 1259.9114
798.3911743164062 0 988.6802
798.890869140625 0 1183.2502
799.42919921875 0 10977.121 w 9
800.4308471679688 0 5272.498
801.4359741210938 0 1613.5872
802.3816528320312 0 4428.97
803.3822021484375 0 2202.7886
803.461181640625 0 848.22003
807.4214477539062 0 1861.9136 y Water loss 2
807.92578125 0 2239.5984
808.433349609375 0 1049.9572
813.9231567382812 0 1195.0376
814.4254150390625 0 1896.7983
815.384521484375 0 1228.0444
816.4172973632812 0 2861.342 y 2
816.91650390625 0 1604.8901
817.4193115234375 0 1026.1016
817.923095703125 0 750.84766
822.3775024414062 0 3665.0728
823.3850708007812 0 5500.9487
824.3883666992188 0 2258.1655
829.4432373046875 0 858.46484
829.9404907226562 0 26525.72 c 14
830.4417724609375 0 30668.963
830.943115234375 0 15978.563
831.4432373046875 0 5393.1875
831.950439453125 0 935.6799
835.9376220703125 0 1015.61127
841.4307861328125 0 1660.489 z Ammonia loss 9
842.4362182617188 0 1031.3224
843.4506225585938 0 2443.66
843.9523315429688 0 2799.858
844.45654296875 0 2321.3325
846.4716796875 0 24607.709 c 7
847.4745483398438 0 11346.335
847.9415893554688 0 897.775
848.4763793945312 0 3519.4766
848.9320068359375 0 2773.2358
849.4285278320312 0 848.44055
849.9293823242188 0 2621.1333 z Water loss 1
850.4296264648438 0 3091.1287 z Ammonia loss 1
852.928466796875 0 859.5739
856.9434204101562 0 3473.1838 c Ammonia loss 15
857.4452514648438 0 4372.979 y Ammonia loss 9
857.9442749023438 0 3209.5483 y Water loss 1
858.445068359375 0 23614.49 y Ammonia loss 1
858.9425048828125 0 2704.9177 z 1
859.4454345703125 0 10269.244
859.938720703125 0 1188.1891
860.4494018554688 0 2006.6498
864.9580688476562 0 2383.023
865.4595947265625 0 42467.555 c 15
865.9606323242188 0 38777.11
866.4609375 0 26310.896
866.9593505859375 0 11463.912
867.460693359375 0 5006.5366
868.4017333984375 0 2009.5785
869.4105224609375 0 775.96216
870.9500122070312 0 916.99115
871.4534301757812 0 1184.1
871.956298828125 0 867.83636
872.94287109375 0 1094.4783
873.456298828125 0 2685.9307
873.94091796875 0 848.34955
874.4614868164062 0 4695.9556 y 9
875.46533203125 0 1751.0615
880.45361328125 0 2413.1506
880.963134765625 0 6732.333
881.4605712890625 0 6679.5854
881.9657592773438 0 3972.614
882.4666748046875 0 1883.0986
885.4651489257812 0 2006.6549
885.9649658203125 0 3628.7217
886.4657592773438 0 3383.6677
886.9609375 0 1484.265
887.971435546875 0 1408.6293
888.4717407226562 0 927.8738
888.955078125 0 1055.4893
889.4464111328125 0 2613.8345
894.4688720703125 0 21540.191
894.9697875976562 0 18987.896
895.470947265625 0 10381.562
895.9699096679688 0 3520.964
896.4710693359375 0 1142.889
899.4659423828125 0 980.2487
899.959228515625 0 1732.556
900.4571533203125 0 1389.9124
901.9580078125 0 831.5362
907.4049072265625 0 8635.783
907.9724731445312 0 3924.8188
908.3978881835938 0 2581.178
908.4724731445312 0 5058.3726
908.9710083007812 0 3537.5378
909.4739990234375 0 1927.1152
909.9667358398438 0 1222.6113
915.97265625 0 1531.4542
916.4747314453125 0 48533.098
916.9771118164062 0 69304.984
917.4788208007812 0 52971.855
917.980712890625 0 29169.494
918.4818115234375 0 10576.805
918.9826049804688 0 2777.7056
928.4730834960938 0 5386.094 w 8
929.4779663085938 0 5278.8154
930.4819946289062 0 2299.1604
944.4738159179688 0 3039.131
945.4771728515625 0 5080
946.479248046875 0 1802.9259
959.4896240234375 0 1781.0271
960.4893798828125 0 6777.312
961.4915161132812 0 2337.4211
962.4867553710938 0 828.75287
973.49462890625 0 12248.538
974.4985961914062 0 7509.5337
975.513671875 0 40033.016 c 8
976.5166625976562 0 20061.965
977.5196533203125 0 7939.252
978.52197265625 0 1993.9608
987.4862670898438 0 18876.648 z 8
988.4871215820312 0 9857.757
989.49267578125 0 3963.1887
990.4965209960938 0 887.86176
1001.5252685546875 0 1526.3319
1003.5000610351562 0 4517.7 y 8
1004.5022583007812 0 2222.6445
1005.4910278320312 0 1302.6964
1012.5177001953125 0 1498.6368
1013.519287109375 0 970.93353
1018.4541625976562 0 984.142
1019.4592895507812 0 840.7939
1021.4791259765625 0 823.24963
1031.5018310546875 0 772.7209
1055.49951171875 0 4088.525
1056.5045166015625 0 3379.5837 z Water loss 7
1057.5118408203125 0 1507.2471 z Ammonia loss 7
1058.5040283203125 0 892.44244
1059.5118408203125 0 1061.8564
1060.532470703125 0 2570.3528
1061.534912109375 0 1509.8735
1072.513916015625 0 1878.4806 y Water loss 7
1073.5126953125 0 1551.5817 y Ammonia loss 7
1074.5181884765625 0 19974.027 z 7
1075.5228271484375 0 12458.48
1076.5269775390625 0 6936.5015
1077.5355224609375 0 1467.3867
1086.528564453125 0 729.6356 c Water loss 9
1087.529296875 0 3653.1301
1088.5284423828125 0 1864.1333
1089.53173828125 0 1822.419
1090.5364990234375 0 13238.733 y 7
1091.5384521484375 0 6196.623
1092.5411376953125 0 2397.9597
1093.5419921875 0 1137.0056
1098.5018310546875 0 1738.7721
1099.516845703125 0 815.25934
1102.5452880859375 0 842.86835
1103.5277099609375 0 2621.138
1104.555419921875 0 44162.41 c 9
1105.557861328125 0 26218.74
1106.5606689453125 0 8579.054
1107.564208984375 0 3009.4385
1120.5528564453125 0 8517.204
1121.55615234375 0 5003.027
1122.5589599609375 0 2067.6487
1133.5570068359375 0 904.75854
1142.5301513671875 0 1111.1588
1160.5513916015625 0 1015.9002 y Ammonia loss 6
1161.55078125 0 9591.195 z 6
1162.5533447265625 0 6247.12
1163.55908203125 0 3986.3525
1164.556884765625 0 2241.2505
1177.56884765625 0 5162.0425 y 6
1178.5733642578125 0 4959.6704
1179.5740966796875 0 2028.7639
1180.5709228515625 0 1447.3413
1181.572998046875 0 1192.0359
1182.58984375 0 1404.4271
1198.6094970703125 0 3277.1147
1199.60498046875 0 1496.8423
1200.608642578125 0 1821.3893
1206.5665283203125 0 12917.076
1207.5677490234375 0 9053.001
1208.5709228515625 0 2309.643
1217.639404296875 0 25514.26 c 10
1218.64208984375 0 18463.926
1219.6441650390625 0 7426.428
1220.6474609375 0 1853.3901
1222.5828857421875 0 7799.4
1223.587646484375 0 12111.51
1224.5927734375 0 7131.4937
1225.598388671875 0 1458.4199
1252.6146240234375 0 2333.598
1253.619873046875 0 1506.7709
1257.6131591796875 0 901.3734 y Ammonia loss 5
1269.6400146484375 0 1486.2504
1270.640625 0 816.66724
1274.6226806640625 0 2348.034 y 5
1275.6251220703125 0 1855.0304
1276.628662109375 0 1010.21844
1301.67431640625 0 1547.97
1302.6533203125 0 784.4756
1327.65869140625 0 852.7251
1328.652587890625 0 1449.1246 c Ammonia loss 11
1329.6424560546875 0 2507.054
1330.659423828125 0 2398.5693
1331.6517333984375 0 678.5872
1343.6756591796875 0 789.7853
1344.685302734375 0 3239.734
1345.6971435546875 0 35599.953 c 11
1346.700439453125 0 29992.482
1347.70458984375 0 10088.227
1348.70361328125 0 3496.8345
1370.666015625 0 2708.1414
1371.6722412109375 0 9396.957 y 4
1372.6800537109375 0 7691.503
1373.682861328125 0 3862.1091
1374.69970703125 0 1575.542
1398.6973876953125 0 1375.1616
1399.674072265625 0 2788.1433
1400.6781005859375 0 4795.9062
1401.68994140625 0 4671.9565
1402.6976318359375 0 2363.1792
1403.7003173828125 0 1096.2229
1415.72705078125 0 4566.756
1416.7327880859375 0 21547.156 c 12
1417.73583984375 0 15577.481
1418.73876953125 0 7138.7915
1419.7445068359375 0 1795.6353
1430.697509765625 0 2340.9055
1431.69970703125 0 1728.4412
1432.709228515625 0 924.6582
1442.7374267578125 0 1000.9458
1458.7154541015625 0 966.1849
1486.7545166015625 0 1230.7655
1487.759765625 0 1595.2644
1501.72216796875 0 1535.0818 y Ammonia loss 3
1502.721923828125 0 20070.727 z 3
1503.7276611328125 0 31413.826
1504.7314453125 0 19516.582
1505.7335205078125 0 10116.03
1506.7381591796875 0 3258.882
1513.7647705078125 0 1056.1193 c Ammonia loss 13
1518.74365234375 0 3262.3882 y 3
1519.7447509765625 0 2611.2903
1520.749267578125 0 1667.2034
1521.751708984375 0 1096.3734
1529.76318359375 0 914.5304
1530.7769775390625 0 38280.09 c 13
1531.7794189453125 0 34342.3
1532.78271484375 0 16056.64
1533.78564453125 0 5950.0737
1534.783447265625 0 1395.2397
1614.8314208984375 0 1605.29 y Ammonia loss 2
1615.8294677734375 0 4507.908 z 2
1616.818115234375 0 6756.9834
1617.8228759765625 0 5261.3286
1618.8328857421875 0 1529.0969
1631.85791015625 0 912.69934 y 2
1642.8414306640625 0 902.1647
1643.862060546875 0 1514.3328
1644.85107421875 0 1114.548
1658.8721923828125 0 4157.9463 c 14
1659.879150390625 0 10433.043
1660.882568359375 0 8741.136
1661.88232421875 0 5457.1143
1662.8839111328125 0 832.13055
1672.8487548828125 0 1570.5674
1673.8651123046875 0 802.856
1685.8909912109375 0 2428.805
1686.895751953125 0 3207.414
1687.9046630859375 0 2108.1106
1688.912109375 0 915.139
1700.8529052734375 0 1782.1567
1713.8873291015625 0 2931.8977
1714.891845703125 0 3103.1633 y Water loss 1
1715.876708984375 0 2508.976 y Ammonia loss 1
1716.869873046875 0 2689.307 z 1
1717.86376953125 0 9204.25
1718.8656005859375 0 6644.9937
1719.87451171875 0 3192.129
1729.90625 0 4992.152 c 15
1730.91552734375 0 14000.845
1731.91748046875 0 12474.106
1732.9180908203125 0 6435.847
1733.9212646484375 0 2313.411
1734.894287109375 0 970.45404
1761.92529296875 0 1083.1332
1770.9200439453125 0 2466.3347
1771.9180908203125 0 4948.4077
1772.914794921875 0 3158.4998
1773.9161376953125 0 2540.7158
1774.908447265625 0 1120.3378
1787.9344482421875 0 6549.036
1788.935791015625 0 19339.375
1789.9393310546875 0 19505.484
1790.943115234375 0 9027.509
1791.943115234375 0 3622.0925
1799.9193115234375 0 1389.2721
1804.9547119140625 0 1262.9215
1805.9573974609375 0 5799.4316
1806.9599609375 0 4170.1353
1807.9625244140625 0 2658.253
1814.932373046875 0 983.5895
1815.9287109375 0 7257.4736
1816.9296875 0 31439.072
1817.9322509765625 0 27004.832
1818.9354248046875 0 15775.033
1819.937255859375 0 5965.0664
1820.9412841796875 0 1533.6272
1831.9393310546875 0 3793.2544
1832.946533203125 0 21015.86
1833.95458984375 0 53544.67
1834.95849609375 0 48093.08
1835.9605712890625 0 25883.123
1836.96435546875 0 9786.894
1837.9627685546875 0 2609.494
1838.9517822265625 0 779.57544
3069.306396484375 0 825.2526

Spectrum Details

|  |  |
| --- | --- |
| Matched peaks? Matched peaksThe total absolute number of peaks matched. Additionally in brackets the total fraction of peaks matched and the total number of peaks is shown. | 91 (16.58% of 549) |
| FDR? FDRThe false discovery rate estimated for this peptide. It is calculated by matching all theoretical fragments with a non-integer shift with the raw peaks for this spectrum. This is done with 40 different shifts. The resulting percentage is the average number of annotated peaks over the number of annotated peaks with the correct spectrum. | 0.84% |
| Satellite FDR? Satellite FDRSee the FDR for details on its calculation. This satellite ion specific FDR only contains the satellite ions (d/w) for I/L/J positions. | - |
| PSM Score? PSM ScoreThe PSM Score as given by Hecklib to this annotated spectrum. It is shown with three significant figures. | 813 |

## Spectrum 7881? Spectrum 7881 The raw spectrum of this peptide as annotated by Hecklib. The fragments are coloured according to ion type (see legend). Any peaks with a star '\*' as text can be hovered over to see the full details, first the ion type second the mass shift type. By hovering over the amino acids in the peptide or ions in the legend the corresponding peaks are highlighted. By toggling the 'Unassigned' label you can turn the background (unassigned) peaks on or off in the plot. By updating the slider in the Ion legend you can update the spectrum to only show the top X% of the peaks with labels. The top X% means any peak that is within X% of the highest intensity. By dragging in the spectrum you can zoom in to a specific part of the spectrum and use 'Zoom Out' to get back to the original zoom level. The annotation of the spectrum is based on the given sequence in the peptides file and is done with different software so inconsistencies are likely. The peaks are annotated based on the given sequence, with 20 ppm tolerance.

Copy Data

### Spectrum 7881 (TSV)

#### Preview

```
Loading example...
```

*Click on the button to copy the data to your clipboard.*

Mz MinMz MaxIntensity Max

WidthHeightPeptide font sizePeptide stroke widthSpectrum font sizeSpectrum stroke widthCompact peptide

Ion legend

wxyz

abcd

OtherUnassignedIonChargePositionShow for top:%

VTJFPPSSEEJQANKAT

04.32e+58.63e+51.30e+61.73e+6

Zoom Out

y+13y+14y+15y+15y+16z+16y+16y+212z+213y+213y+213z+213y+213c+213z+17y+17y+214y+214z+214y+214y+215y+215c+216y+18y+216y+216y+216y+18c+19z+19y+19y+110y+110z+110y+110c+110y+111z+111y+111c+111y+112y+112y+112c+112c+112c+112y+113y+113z+113y+113c+113z+114c+114y+114c+114y+115z+115y+115c+115z+116z+116y+116y+116z+116c+116

0797159323903187

Fragment Matches Table

Show background peaks

| Position | Ion type | Intensity | mz Theoretical | mz Error (Th) | mz Error (ppm) | Charge | Series Number |
| --- | --- | --- | --- | --- | --- | --- | --- |
| - | - | 4521 | 120.1 | - | - | 0 | - |
| - | - | 8931 | 129.1 | - | - | 0 | - |
| - | - | 2352 | 148.9 | - | - | 0 | - |
| - | - | 2033 | 169.1 | - | - | 0 | - |
| - | - | 7042 | 173.1 | - | - | 0 | - |
| - | - | 1554 | 173.5 | - | - | 0 | - |
| - | - | 1376 | 177 | - | - | 0 | - |
| - | - | 9279 | 183.1 | - | - | 0 | - |
| - | - | 1282 | 187.1 | - | - | 0 | - |
| - | - | 2.486E+04 | 201.1 | - | - | 0 | - |
| - | - | 3916 | 215.1 | - | - | 0 | - |
| - | - | 2912 | 217.1 | - | - | 0 | - |
| - | - | 2001 | 226.1 | - | - | 0 | - |
| - | - | 1.373E+04 | 233.2 | - | - | 0 | - |
| - | - | 4665 | 243.1 | - | - | 0 | - |
| - | - | 5172 | 245.1 | - | - | 0 | - |
| - | - | 1306 | 246 | - | - | 0 | - |
| - | - | 3684 | 247.1 | - | - | 0 | - |
| - | - | 5845 | 251.2 | - | - | 0 | - |
| - | - | 1.207E+04 | 261.2 | - | - | 0 | - |
| - | - | 3550 | 269.2 | - | - | 0 | - |
| - | - | 1200 | 271.2 | - | - | 0 | - |
| - | - | 2766 | 280.2 | - | - | 0 | - |
| - | - | 2254 | 282.1 | - | - | 0 | - |
| - | - | 2.521E+05 | 296.2 | - | - | 0 | - |
| - | - | 1746 | 297.2 | - | - | 0 | - |
| - | - | 3.973E+04 | 297.2 | - | - | 0 | - |
| - | - | 2902 | 298.2 | - | - | 0 | - |
| - | - | 1827 | 298.2 | - | - | 0 | - |
| - | - | 4659 | 303.2 | - | - | 0 | - |
| - | - | 1395 | 304.2 | - | - | 0 | - |
| - | - | 1145 | 307.4 | - | - | 0 | - |
| - | - | 4302 | 314.2 | - | - | 0 | - |
| - | - | 1.27E+05 | 314.2 | - | - | 0 | - |
| - | - | 1.887E+04 | 315.2 | - | - | 0 | - |
| - | - | 1534 | 317.2 | - | - | 0 | - |
| 15 | y | 1.665E+04 | 319.2 | 0.000188 | 0.589 | +1 | 3 |
| - | - | 4733 | 330.2 | - | - | 0 | - |
| - | - | 1816 | 344.2 | - | - | 0 | - |
| - | - | 1.156E+04 | 346.2 | - | - | 0 | - |
| - | - | 1658 | 347.2 | - | - | 0 | - |
| - | - | 1.099E+04 | 348.2 | - | - | 0 | - |
| - | - | 2136 | 349.2 | - | - | 0 | - |
| - | - | 1655 | 360.2 | - | - | 0 | - |
| - | - | 3.821E+04 | 362.2 | - | - | 0 | - |
| - | - | 6922 | 363.2 | - | - | 0 | - |
| - | - | 1663 | 385.2 | - | - | 0 | - |
| - | - | 1991 | 390.2 | - | - | 0 | - |
| - | - | 2070 | 393.3 | - | - | 0 | - |
| - | - | 2836 | 398.2 | - | - | 0 | - |
| - | - | 7586 | 411.3 | - | - | 0 | - |
| - | - | 3026 | 415.3 | - | - | 0 | - |
| - | - | 1.419E+04 | 416.3 | - | - | 0 | - |
| - | - | 4922 | 417.3 | - | - | 0 | - |
| - | - | 1540 | 425.2 | - | - | 0 | - |
| - | - | 1758 | 431.3 | - | - | 0 | - |
| 14 | y | 1.759E+04 | 433.2 | 0.002366 | 5.46 | +1 | 4 |
| - | - | 1.859E+05 | 433.3 | - | - | 0 | - |
| - | - | 3267 | 434.2 | - | - | 0 | - |
| - | - | 4.336E+04 | 434.3 | - | - | 0 | - |
| - | - | 5216 | 435.3 | - | - | 0 | - |
| - | - | 1465 | 441.9 | - | - | 0 | - |
| - | - | 1564 | 442.2 | - | - | 0 | - |
| - | - | 2520 | 443.2 | - | - | 0 | - |
| - | - | 1.91E+04 | 443.3 | - | - | 0 | - |
| - | - | 4826 | 444.3 | - | - | 0 | - |
| - | - | 2229 | 459.3 | - | - | 0 | - |
| - | - | 1.878E+05 | 461.3 | - | - | 0 | - |
| - | - | 4.525E+04 | 462.3 | - | - | 0 | - |
| - | - | 5589 | 463.3 | - | - | 0 | - |
| 13 | y | 1858 | 487.3 | 0.001994 | 4.092 | +1 | 5 |
| - | - | 2617 | 498.2 | - | - | 0 | - |
| 13 | y | 3.087E+04 | 504.3 | 0.0005301 | 1.051 | +1 | 5 |
| - | - | 5227 | 505.3 | - | - | 0 | - |
| - | - | 1672 | 513.3 | - | - | 0 | - |
| - | - | 7905 | 530.3 | - | - | 0 | - |
| - | - | 2362 | 531.3 | - | - | 0 | - |
| - | - | 6979 | 540.3 | - | - | 0 | - |
| - | - | 3356 | 541.3 | - | - | 0 | - |
| - | - | 2.808E+04 | 558.3 | - | - | 0 | - |
| - | - | 6734 | 559.3 | - | - | 0 | - |
| - | - | 2009 | 582.8 | - | - | 0 | - |
| - | - | 4308 | 591.3 | - | - | 0 | - |
| - | - | 1772 | 591.8 | - | - | 0 | - |
| 12 | y | 4238 | 615.3 | 0.0007587 | 1.233 | +1 | 6 |
| 12 | z | 7537 | 616.3 | 0.0009628 | 1.562 | +1 | 6 |
| - | - | 5329 | 617.3 | - | - | 0 | - |
| - | - | 8180 | 617.8 | - | - | 0 | - |
| - | - | 3988 | 618.3 | - | - | 0 | - |
| - | - | 1873 | 618.8 | - | - | 0 | - |
| - | - | 2.712E+04 | 626.8 | - | - | 0 | - |
| - | - | 3235 | 627.3 | - | - | 0 | - |
| - | - | 1.99E+04 | 627.3 | - | - | 0 | - |
| - | - | 4508 | 627.8 | - | - | 0 | - |
| 12 | y | 1.943E+04 | 632.3 | 0.0006379 | 1.009 | +1 | 6 |
| - | - | 5796 | 633.3 | - | - | 0 | - |
| 6 | y | 2440 | 637.8 | 0.001198 | 1.878 | +2 | 12 |
| - | - | 3052 | 638.3 | - | - | 0 | - |
| - | - | 1471 | 654.4 | - | - | 0 | - |
| - | - | 2013 | 655.3 | - | - | 0 | - |
| - | - | 2372 | 663.3 | - | - | 0 | - |
| - | - | 1480 | 663.8 | - | - | 0 | - |
| 5 | z | 1625 | 669.3 | 0.001972 | 2.947 | +2 | 13 |
| 5 | y | 1.531E+04 | 677.3 | 0.000606 | 0.8947 | +2 | 13 |
| 5 | y | 2.05E+04 | 677.8 | 0.004875 | 7.192 | +2 | 13 |
| 5 | z | 1.182E+04 | 678.3 | 0.002244 | 3.309 | +2 | 13 |
| - | - | 3833 | 678.8 | - | - | 0 | - |
| - | - | 4433 | 685.3 | - | - | 0 | - |
| - | - | 1726 | 685.8 | - | - | 0 | - |
| 5 | y | 6.872E+05 | 686.3 | 0.0006948 | 1.012 | +2 | 13 |
| - | - | 4.947E+05 | 686.8 | - | - | 0 | - |
| - | - | 1.558E+05 | 687.3 | - | - | 0 | - |
| - | - | 1.749E+04 | 687.8 | - | - | 0 | - |
| - | - | 1338 | 692.6 | - | - | 0 | - |
| 13 | c | 1.131E+04 | 700.4 | 0.01086 | 15.51 | +2 | 13 |
| - | - | 6617 | 700.9 | - | - | 0 | - |
| - | - | 2264 | 724.4 | - | - | 0 | - |
| 11 | z | 1.119E+04 | 729.4 | 0.0001269 | 0.1739 | +1 | 7 |
| - | - | 9680 | 730.4 | - | - | 0 | - |
| - | - | 2839 | 731.4 | - | - | 0 | - |
| - | - | 4708 | 740.3 | - | - | 0 | - |
| - | - | 1732 | 741.3 | - | - | 0 | - |
| - | - | 5859 | 742.4 | - | - | 0 | - |
| - | - | 3328 | 743.4 | - | - | 0 | - |
| 11 | y | 9730 | 745.4 | 0.001474 | 1.977 | +1 | 7 |
| - | - | 3719 | 746.4 | - | - | 0 | - |
| 4 | y | 4013 | 750.9 | 0.002898 | 3.86 | +2 | 14 |
| 4 | y | 5507 | 751.4 | 0.009181 | 12.22 | +2 | 14 |
| 4 | z | 2886 | 751.9 | 0.002095 | 2.786 | +2 | 14 |
| - | - | 2140 | 757.4 | - | - | 0 | - |
| 4 | y | 9.491E+04 | 759.9 | 0.0006064 | 0.7981 | +2 | 14 |
| - | - | 8.77E+04 | 760.4 | - | - | 0 | - |
| - | - | 3.444E+04 | 760.9 | - | - | 0 | - |
| - | - | 5063 | 761.4 | - | - | 0 | - |
| - | - | 1367 | 774.3 | - | - | 0 | - |
| 3 | y | 2925 | 807.9 | 0.00731 | 9.049 | +2 | 15 |
| - | - | 2572 | 813.4 | - | - | 0 | - |
| 3 | y | 2.107E+04 | 816.4 | 0.0008718 | 1.068 | +2 | 15 |
| - | - | 2.125E+04 | 816.9 | - | - | 0 | - |
| - | - | 1.023E+04 | 817.4 | - | - | 0 | - |
| - | - | 1571 | 821.9 | - | - | 0 | - |
| - | - | 5307 | 829.4 | - | - | 0 | - |
| - | - | 1758 | 830.5 | - | - | 0 | - |
| - | - | 2186 | 839.4 | - | - | 0 | - |
| - | - | 4221 | 847.9 | - | - | 0 | - |
| - | - | 7244 | 848.4 | - | - | 0 | - |
| - | - | 4647 | 848.9 | - | - | 0 | - |
| - | - | 2267 | 849.4 | - | - | 0 | - |
| 16 | c | 2.04E+04 | 856.9 | 0.001305 | 1.523 | +2 | 16 |
| 10 | y | 2.491E+04 | 857.4 | 0.01192 | 13.9 | +1 | 8 |
| 2 | y | 1.052E+04 | 857.9 | 0.01326 | 15.46 | +2 | 16 |
| 2 | y | 3.044E+04 | 858.4 | 0.0171 | 19.92 | +2 | 16 |
| - | - | 2.616E+04 | 859.5 | - | - | 0 | - |
| - | - | 7610 | 860.5 | - | - | 0 | - |
| 2 | y | 5315 | 866.9 | 0.0003844 | 0.4434 | +2 | 16 |
| - | - | 6973 | 867.4 | - | - | 0 | - |
| - | - | 3325 | 867.9 | - | - | 0 | - |
| - | - | 1.023E+04 | 868.4 | - | - | 0 | - |
| - | - | 4152 | 869.4 | - | - | 0 | - |
| 10 | y | 9591 | 874.5 | 0.002155 | 2.464 | +1 | 8 |
| - | - | 2712 | 875.5 | - | - | 0 | - |
| - | - | 3175 | 886.5 | - | - | 0 | - |
| - | - | 2382 | 898.5 | - | - | 0 | - |
| - | - | 4284 | 899 | - | - | 0 | - |
| - | - | 1.209E+04 | 907.5 | - | - | 0 | - |
| - | - | 2.152E+04 | 908 | - | - | 0 | - |
| - | - | 9939 | 908.5 | - | - | 0 | - |
| - | - | 2767 | 909 | - | - | 0 | - |
| - | - | 2229 | 916 | - | - | 0 | - |
| - | - | 3070 | 916.4 | - | - | 0 | - |
| - | - | 1.863E+05 | 916.5 | - | - | 0 | - |
| - | - | 1.877E+05 | 917 | - | - | 0 | - |
| - | - | 9.062E+04 | 917.5 | - | - | 0 | - |
| - | - | 1.416E+04 | 918 | - | - | 0 | - |
| - | - | 6878 | 939.4 | - | - | 0 | - |
| - | - | 2883 | 940.5 | - | - | 0 | - |
| - | - | 3873 | 958.5 | - | - | 0 | - |
| - | - | 1530 | 971.5 | - | - | 0 | - |
| - | - | 6295 | 973.5 | - | - | 0 | - |
| - | - | 3359 | 974.5 | - | - | 0 | - |
| 9 | c | 2313 | 975.5 | 0.01177 | 12.06 | +1 | 9 |
| 9 | z | 4.973E+04 | 987.5 | 0.0005634 | 0.5706 | +1 | 9 |
| - | - | 6.914E+04 | 988.5 | - | - | 0 | - |
| - | - | 2.883E+04 | 989.5 | - | - | 0 | - |
| - | - | 6204 | 990.5 | - | - | 0 | - |
| - | - | 6275 | 1002 | - | - | 0 | - |
| 9 | y | 1.392E+04 | 1004 | 0.002475 | 2.466 | +1 | 9 |
| - | - | 5719 | 1005 | - | - | 0 | - |
| - | - | 1844 | 1015 | - | - | 0 | - |
| - | - | 1921 | 1032 | - | - | 0 | - |
| - | - | 6962 | 1053 | - | - | 0 | - |
| - | - | 3356 | 1054 | - | - | 0 | - |
| 8 | y | 3315 | 1073 | 0.01655 | 15.43 | +1 | 10 |
| 8 | y | 5772 | 1074 | 0.001911 | 1.781 | +1 | 10 |
| 8 | z | 5.076E+04 | 1075 | 0.0002733 | 0.2543 | +1 | 10 |
| - | - | 1.393E+05 | 1076 | - | - | 0 | - |
| - | - | 6.388E+04 | 1077 | - | - | 0 | - |
| - | - | 1.031E+04 | 1078 | - | - | 0 | - |
| - | - | 4834 | 1088 | - | - | 0 | - |
| - | - | 4233 | 1089 | - | - | 0 | - |
| - | - | 1.774E+04 | 1090 | - | - | 0 | - |
| 8 | y | 6.52E+04 | 1091 | 0.0001402 | 0.1286 | +1 | 10 |
| - | - | 2.908E+04 | 1092 | - | - | 0 | - |
| - | - | 9096 | 1093 | - | - | 0 | - |
| - | - | 2806 | 1104 | - | - | 0 | - |
| 10 | c | 1.737E+04 | 1105 | 0.0007701 | 0.6972 | +1 | 10 |
| - | - | 9354 | 1106 | - | - | 0 | - |
| - | - | 2277 | 1107 | - | - | 0 | - |
| - | - | 4661 | 1117 | - | - | 0 | - |
| - | - | 8254 | 1118 | - | - | 0 | - |
| - | - | 4559 | 1119 | - | - | 0 | - |
| - | - | 1852 | 1156 | - | - | 0 | - |
| - | - | 2897 | 1157 | - | - | 0 | - |
| - | - | 3116 | 1160 | - | - | 0 | - |
| 7 | y | 4910 | 1161 | 0.00317 | 2.731 | +1 | 11 |
| 7 | z | 2.607E+04 | 1162 | 0.0008377 | 0.7211 | +1 | 11 |
| - | - | 5.288E+04 | 1163 | - | - | 0 | - |
| - | - | 2.58E+04 | 1164 | - | - | 0 | - |
| - | - | 9787 | 1165 | - | - | 0 | - |
| - | - | 3077 | 1166 | - | - | 0 | - |
| - | - | 2228 | 1175 | - | - | 0 | - |
| - | - | 6291 | 1177 | - | - | 0 | - |
| 7 | y | 3.982E+04 | 1178 | 0.0009124 | 0.7748 | +1 | 11 |
| - | - | 1.799E+04 | 1179 | - | - | 0 | - |
| - | - | 5431 | 1180 | - | - | 0 | - |
| - | - | 1.802E+04 | 1182 | - | - | 0 | - |
| - | - | 1.296E+04 | 1183 | - | - | 0 | - |
| - | - | 3989 | 1184 | - | - | 0 | - |
| - | - | 1.097E+04 | 1201 | - | - | 0 | - |
| - | - | 6864 | 1202 | - | - | 0 | - |
| - | - | 2765 | 1203 | - | - | 0 | - |
| - | - | 2.015E+04 | 1217 | - | - | 0 | - |
| 11 | c | 4.307E+04 | 1218 | 0.001826 | 1.5 | +1 | 11 |
| - | - | 2.472E+04 | 1219 | - | - | 0 | - |
| - | - | 4282 | 1220 | - | - | 0 | - |
| - | - | 3513 | 1235 | - | - | 0 | - |
| - | - | 4607 | 1236 | - | - | 0 | - |
| - | - | 2.059E+04 | 1253 | - | - | 0 | - |
| - | - | 1.481E+04 | 1254 | - | - | 0 | - |
| - | - | 4125 | 1255 | - | - | 0 | - |
| 6 | y | 2391 | 1257 | 0.009671 | 7.696 | +1 | 12 |
| 6 | y | 2776 | 1258 | 0.007358 | 5.851 | +1 | 12 |
| 6 | y | 8.717E+04 | 1275 | 0.0001505 | 0.1181 | +1 | 12 |
| - | - | 5.54E+04 | 1276 | - | - | 0 | - |
| - | - | 1.524E+04 | 1277 | - | - | 0 | - |
| - | - | 1952 | 1278 | - | - | 0 | - |
| - | - | 3746 | 1300 | - | - | 0 | - |
| - | - | 2372 | 1302 | - | - | 0 | - |
| - | - | 4726 | 1303 | - | - | 0 | - |
| - | - | 3905 | 1311 | - | - | 0 | - |
| - | - | 2539 | 1312 | - | - | 0 | - |
| - | - | 3407 | 1326 | - | - | 0 | - |
| - | - | 4864 | 1327 | - | - | 0 | - |
| 12 | c | 2352 | 1328 | 0.003941 | 2.968 | +1 | 12 |
| 12 | c | 1.915E+04 | 1329 | 0.002361 | 1.777 | +1 | 12 |
| - | - | 1.288E+04 | 1330 | - | - | 0 | - |
| - | - | 5167 | 1331 | - | - | 0 | - |
| - | - | 2304 | 1344 | - | - | 0 | - |
| - | - | 2.602E+04 | 1345 | - | - | 0 | - |
| 12 | c | 1.115E+05 | 1346 | 0.0007114 | 0.5286 | +1 | 12 |
| - | - | 7.612E+04 | 1347 | - | - | 0 | - |
| - | - | 2.593E+04 | 1348 | - | - | 0 | - |
| - | - | 2681 | 1349 | - | - | 0 | - |
| 5 | y | 1.119E+04 | 1354 | 0.002995 | 2.213 | +1 | 13 |
| 5 | y | 2.059E+04 | 1355 | 0.01153 | 8.514 | +1 | 13 |
| 5 | z | 1.211E+04 | 1356 | 0.0005346 | 0.3943 | +1 | 13 |
| - | - | 2523 | 1357 | - | - | 0 | - |
| - | - | 3982 | 1358 | - | - | 0 | - |
| - | - | 1892 | 1359 | - | - | 0 | - |
| - | - | 5234 | 1360 | - | - | 0 | - |
| - | - | 4422 | 1370 | - | - | 0 | - |
| - | - | 3.934E+04 | 1371 | - | - | 0 | - |
| 5 | y | 7.925E+05 | 1372 | 1.037E-06 | 0.0007563 | +1 | 13 |
| - | - | 5.216E+05 | 1373 | - | - | 0 | - |
| - | - | 1.835E+05 | 1374 | - | - | 0 | - |
| - | - | 1.95E+04 | 1375 | - | - | 0 | - |
| - | - | 1807 | 1382 | - | - | 0 | - |
| - | - | 4152 | 1398 | - | - | 0 | - |
| - | - | 1.636E+04 | 1399 | - | - | 0 | - |
| - | - | 3.218E+04 | 1400 | - | - | 0 | - |
| - | - | 2.156E+04 | 1401 | - | - | 0 | - |
| - | - | 1.518E+05 | 1402 | - | - | 0 | - |
| - | - | 1.238E+05 | 1403 | - | - | 0 | - |
| - | - | 4.211E+04 | 1404 | - | - | 0 | - |
| - | - | 4416 | 1405 | - | - | 0 | - |
| - | - | 8.79E+04 | 1416 | - | - | 0 | - |
| 13 | c | 1.27E+05 | 1417 | 0.003645 | 2.573 | +1 | 13 |
| - | - | 6.505E+04 | 1418 | - | - | 0 | - |
| - | - | 1.729E+04 | 1419 | - | - | 0 | - |
| - | - | 6414 | 1431 | - | - | 0 | - |
| - | - | 6756 | 1432 | - | - | 0 | - |
| - | - | 2726 | 1433 | - | - | 0 | - |
| - | - | 9455 | 1487 | - | - | 0 | - |
| - | - | 9353 | 1488 | - | - | 0 | - |
| - | - | 3524 | 1489 | - | - | 0 | - |
| 4 | z | 6.625E+04 | 1503 | 0.0002524 | 0.168 | +1 | 14 |
| - | - | 6.285E+04 | 1504 | - | - | 0 | - |
| - | - | 2.533E+04 | 1505 | - | - | 0 | - |
| - | - | 2895 | 1506 | - | - | 0 | - |
| 14 | c | 6277 | 1514 | 0.0006061 | 0.4004 | +1 | 14 |
| - | - | 6035 | 1515 | - | - | 0 | - |
| - | - | 2504 | 1516 | - | - | 0 | - |
| - | - | 2647 | 1518 | - | - | 0 | - |
| 4 | y | 9.016E+04 | 1519 | 0.0004327 | 0.2849 | +1 | 14 |
| - | - | 6.945E+04 | 1520 | - | - | 0 | - |
| - | - | 2.739E+04 | 1521 | - | - | 0 | - |
| - | - | 2944 | 1522 | - | - | 0 | - |
| - | - | 2355 | 1529 | - | - | 0 | - |
| - | - | 1.239E+04 | 1530 | - | - | 0 | - |
| 14 | c | 2.109E+05 | 1531 | 0.0007966 | 0.5204 | +1 | 14 |
| - | - | 1.687E+05 | 1532 | - | - | 0 | - |
| - | - | 6.864E+04 | 1533 | - | - | 0 | - |
| - | - | 7169 | 1534 | - | - | 0 | - |
| - | - | 2821 | 1544 | - | - | 0 | - |
| - | - | 3236 | 1545 | - | - | 0 | - |
| - | - | 3504 | 1571 | - | - | 0 | - |
| - | - | 5417 | 1572 | - | - | 0 | - |
| - | - | 3183 | 1573 | - | - | 0 | - |
| - | - | 2277 | 1574 | - | - | 0 | - |
| 3 | y | 3807 | 1614 | 0.008232 | 5.101 | +1 | 15 |
| - | - | 2.929E+04 | 1615 | - | - | 0 | - |
| 3 | z | 4.57E+04 | 1616 | 0.02872 | 17.77 | +1 | 15 |
| - | - | 3.433E+04 | 1617 | - | - | 0 | - |
| - | - | 1.195E+04 | 1618 | - | - | 0 | - |
| 3 | y | 3.287E+04 | 1632 | 0.001356 | 0.8309 | +1 | 15 |
| - | - | 2.845E+04 | 1633 | - | - | 0 | - |
| - | - | 1.173E+04 | 1634 | - | - | 0 | - |
| - | - | 2112 | 1645 | - | - | 0 | - |
| - | - | 3649 | 1646 | - | - | 0 | - |
| - | - | 2492 | 1647 | - | - | 0 | - |
| - | - | 3692 | 1655 | - | - | 0 | - |
| - | - | 5370 | 1656 | - | - | 0 | - |
| - | - | 3435 | 1657 | - | - | 0 | - |
| - | - | 2550 | 1658 | - | - | 0 | - |
| 15 | c | 8.572E+04 | 1659 | 0.001888 | 1.138 | +1 | 15 |
| - | - | 9.41E+04 | 1660 | - | - | 0 | - |
| - | - | 4.289E+04 | 1661 | - | - | 0 | - |
| - | - | 1.205E+04 | 1662 | - | - | 0 | - |
| - | - | 5918 | 1663 | - | - | 0 | - |
| - | - | 1.391E+04 | 1672 | - | - | 0 | - |
| - | - | 1.642E+04 | 1673 | - | - | 0 | - |
| - | - | 8620 | 1674 | - | - | 0 | - |
| - | - | 2828 | 1675 | - | - | 0 | - |
| - | - | 2.489E+04 | 1686 | - | - | 0 | - |
| - | - | 2.998E+04 | 1687 | - | - | 0 | - |
| - | - | 1.807E+04 | 1688 | - | - | 0 | - |
| - | - | 7545 | 1689 | - | - | 0 | - |
| - | - | 2963 | 1690 | - | - | 0 | - |
| 2 | z | 7243 | 1699 | 0.006011 | 3.538 | +1 | 16 |
| 2 | z | 7523 | 1700 | 0.02139 | 12.58 | +1 | 16 |
| 2 | y | 3.91E+04 | 1715 | 1.753E-05 | 0.01022 | +1 | 16 |
| 2 | y | 6.367E+04 | 1716 | 0.01426 | 8.31 | +1 | 16 |
| 2 | z | 2.014E+05 | 1717 | 0.00155 | 0.9028 | +1 | 16 |
| - | - | 2.018E+05 | 1718 | - | - | 0 | - |
| - | - | 9.803E+04 | 1719 | - | - | 0 | - |
| - | - | 1.75E+04 | 1720 | - | - | 0 | - |
| 16 | c | 1.468E+05 | 1730 | 0.001038 | 0.5999 | +1 | 16 |
| - | - | 1.395E+05 | 1731 | - | - | 0 | - |
| - | - | 9.673E+04 | 1732 | - | - | 0 | - |
| - | - | 3.859E+04 | 1733 | - | - | 0 | - |
| - | - | 1.492E+04 | 1734 | - | - | 0 | - |
| - | - | 2555 | 1735 | - | - | 0 | - |
| - | - | 8178 | 1744 | - | - | 0 | - |
| - | - | 1.862E+04 | 1745 | - | - | 0 | - |
| - | - | 1.416E+04 | 1746 | - | - | 0 | - |
| - | - | 5745 | 1747 | - | - | 0 | - |
| - | - | 1.027E+04 | 1760 | - | - | 0 | - |
| - | - | 2.242E+04 | 1761 | - | - | 0 | - |
| - | - | 3.437E+04 | 1762 | - | - | 0 | - |
| - | - | 2.015E+04 | 1763 | - | - | 0 | - |
| - | - | 7662 | 1764 | - | - | 0 | - |
| - | - | 1.435E+04 | 1769 | - | - | 0 | - |
| - | - | 1.936E+04 | 1770 | - | - | 0 | - |
| - | - | 1.358E+04 | 1771 | - | - | 0 | - |
| - | - | 7285 | 1772 | - | - | 0 | - |
| - | - | 4858 | 1773 | - | - | 0 | - |
| - | - | 1.603E+04 | 1774 | - | - | 0 | - |
| - | - | 1.701E+04 | 1775 | - | - | 0 | - |
| - | - | 1.052E+04 | 1776 | - | - | 0 | - |
| - | - | 2.297E+04 | 1777 | - | - | 0 | - |
| - | - | 2.014E+04 | 1778 | - | - | 0 | - |
| - | - | 9161 | 1779 | - | - | 0 | - |
| - | - | 3.884E+04 | 1787 | - | - | 0 | - |
| - | - | 2.331E+05 | 1788 | - | - | 0 | - |
| - | - | 2.275E+05 | 1789 | - | - | 0 | - |
| - | - | 1.234E+05 | 1790 | - | - | 0 | - |
| - | - | 2.125E+04 | 1791 | - | - | 0 | - |
| - | - | 2616 | 1792 | - | - | 0 | - |
| - | - | 2407 | 1797 | - | - | 0 | - |
| - | - | 6806 | 1798 | - | - | 0 | - |
| - | - | 4505 | 1799 | - | - | 0 | - |
| - | - | 3483 | 1800 | - | - | 0 | - |
| - | - | 3397 | 1801 | - | - | 0 | - |
| - | - | 3525 | 1802 | - | - | 0 | - |
| - | - | 1.817E+04 | 1805 | - | - | 0 | - |
| - | - | 1.775E+04 | 1806 | - | - | 0 | - |
| - | - | 1.107E+04 | 1807 | - | - | 0 | - |
| - | - | 5210 | 1814 | - | - | 0 | - |
| - | - | 5.442E+04 | 1815 | - | - | 0 | - |
| - | - | 3.547E+05 | 1816 | - | - | 0 | - |
| - | - | 3.416E+05 | 1817 | - | - | 0 | - |
| - | - | 1.735E+05 | 1818 | - | - | 0 | - |
| - | - | 2.587E+04 | 1819 | - | - | 0 | - |
| - | - | 7577 | 1830 | - | - | 0 | - |
| - | - | 2.674E+04 | 1831 | - | - | 0 | - |
| - | - | 6.528E+05 | 1832 | - | - | 0 | - |
| - | - | 1.71E+06 | 1833 | - | - | 0 | - |
| - | - | 1.457E+06 | 1834 | - | - | 0 | - |
| - | - | 6.161E+05 | 1835 | - | - | 0 | - |
| - | - | 8.24E+04 | 1836 | - | - | 0 | - |
| - | - | 2967 | 1865 | - | - | 0 | - |
| - | - | 3525 | 1866 | - | - | 0 | - |
| - | - | 1852 | 3155 | - | - | 0 | - |

m/z Charge Intensity FragmentType MassShift Position
120.0809555053711 0 4521.0728
129.10238647460938 0 8930.654
148.9471435546875 0 2352.3416
169.1337432861328 0 2032.6084
173.12863159179688 0 7041.5557
173.4530792236328 0 1554.0836
177.02857971191406 0 1376.1522
183.11297607421875 0 9278.826
187.14515686035156 0 1281.551
201.12347412109375 0 24855.508
215.13897705078125 0 3916.376
217.13412475585938 0 2911.8364
226.11837768554688 0 2001.41
233.16477966308594 0 13727.597
243.14512634277344 0 4665.0806
245.1287841796875 0 5172.43
246.04254150390625 0 1305.6047
247.14410400390625 0 3684.3115
251.17523193359375 0 5844.555
261.15985107421875 0 12073.432
269.1859436035156 0 3550.1055
271.204833984375 0 1199.5994
280.16607666015625 0 2765.5823
282.14483642578125 0 2254.1284
296.19720458984375 0 252138.9
297.1566162109375 0 1746.291
297.2004699707031 0 39730.277
298.1769104003906 0 2901.9785
298.2037048339844 0 1827.4575
303.17010498046875 0 4659.2666
304.1934814453125 0 1394.5188
307.3913879394531 0 1145.0864
314.1861877441406 0 4301.7783
314.20782470703125 0 127033.984
315.2109069824219 0 18866.723
317.1874084472656 0 1533.735
319.1977844238281 0 16654.951 y 14
330.182373046875 0 4732.505
344.1961364746094 0 1815.9423
346.1766357421875 0 11564.764
347.17779541015625 0 1657.7942
348.1917419433594 0 10992.1
349.1940002441406 0 2136.123
360.22808837890625 0 1654.7795
362.20758056640625 0 38214.723
363.210693359375 0 6921.7236
385.21929931640625 0 1663.1241
390.2022399902344 0 1990.8085
393.251220703125 0 2069.7576
398.2447204589844 0 2835.9546
411.26043701171875 0 7586.256
415.2715759277344 0 3025.586
416.2548522949219 0 14191.055
417.25604248046875 0 4921.5107
425.21502685546875 0 1540.4767
431.2659606933594 0 1757.7015
433.2428894042969 0 17588.057 y 13
433.2816467285156 0 185900.31
434.24884033203125 0 3266.685
434.2848205566406 0 43361.875
435.287353515625 0 5216.461
441.93597412109375 0 1464.8583
442.2415771484375 0 1563.6412
443.23187255859375 0 2519.9963
443.2658996582031 0 19097.549
444.26947021484375 0 4825.856
459.2609558105469 0 2229.0024
461.27655029296875 0 187778.06
462.2796325683594 0 45251.438
463.281494140625 0 5588.5913
487.2530822753906 0 1857.7228 y Ammonia loss 12
498.21893310546875 0 2617.465
504.2781677246094 0 30866.035 y 12
505.281982421875 0 5226.5337
513.2783203125 0 1671.6414
530.3349609375 0 7905.4434
531.3358764648438 0 2362.1445
540.31884765625 0 6978.7637
541.3230590820312 0 3355.773
558.3292236328125 0 28075.98
559.332275390625 0 6734.278
582.778076171875 0 2009.0297
591.2940063476562 0 4308.0728
591.7947387695312 0 1771.8381
615.3104248046875 0 4238.458 y Ammonia loss 11
616.3165283203125 0 7537.1577 z 11
617.3250732421875 0 5329.3765
617.8079223632812 0 8179.696
618.3102416992188 0 3988.2717
618.8040771484375 0 1873.0693
626.8123779296875 0 27115.418
627.2610473632812 0 3234.6353
627.314208984375 0 19896.994
627.81298828125 0 4507.6494
632.3368530273438 0 19434.568 y 11
633.3370971679688 0 5795.671
637.8159790039062 0 2439.8516 y 5
638.3167724609375 0 3051.6138
654.3616333007812 0 1471.1613
655.33837890625 0 2013.3085
663.3391723632812 0 2371.932
663.8378295898438 0 1480.4413
669.3284912109375 0 1624.656 z Water loss 4
677.3364868164062 0 15307.108 y Water loss 4
677.832763671875 0 20496.156 y Ammonia loss 4
678.3340454101562 0 11815.452 z 4
678.8355102539062 0 3832.5068
685.3328247070312 0 4432.6533
685.8308715820312 0 1725.9215
686.3418579101562 0 687205.5 y 4
686.8433227539062 0 494723.6
687.3442993164062 0 155783.77
687.8458251953125 0 17485.75
692.5582885742188 0 1337.7174
700.3479614257812 0 11305.038 c Ammonia loss 12
700.8502807617188 0 6616.8193
724.3993530273438 0 2264.2495
729.4014282226562 0 11194.838 z 10
730.4088134765625 0 9680.48
731.41162109375 0 2839.1853
740.34716796875 0 4707.815
741.3493041992188 0 1731.7667
742.414306640625 0 5858.8203
743.4151000976562 0 3328.1672
745.4217529296875 0 9729.959 y 10
746.4227905273438 0 3718.5244
750.8729858398438 0 4013.1536 y Water loss 3
751.3712768554688 0 5506.684 y Ammonia loss 3
751.8681030273438 0 2886.366 z 3
757.4202270507812 0 2140.403
759.8759765625 0 94906.87 y 3
760.3775024414062 0 87696.45
760.8786010742188 0 34441.133
761.3806762695312 0 5063.499
774.342041015625 0 1366.9186
807.9114379882812 0 2925.0254 y Ammonia loss 2
813.4141845703125 0 2572.2822
816.4182739257812 0 21072.674 y 2
816.9194946289062 0 21253.072
817.4222412109375 0 10225.719
821.9262084960938 0 1570.5948
829.4481201171875 0 5307.363
830.454833984375 0 1757.9484
839.4212646484375 0 2186.329
847.9406127929688 0 4221.2065
848.439697265625 0 7243.6104
848.9395751953125 0 4646.617
849.4320678710938 0 2266.5518
856.9476318359375 0 20398.125 c Ammonia loss 15
857.4482421875 0 24905.104 y Ammonia loss 9
857.94921875 0 10515.634 y Water loss 1
858.445068359375 0 30437.396 y Ammonia loss 1
859.4505004882812 0 26164.613
860.455810546875 0 7609.5503
866.9408569335938 0 5314.703 y 1
867.442138671875 0 6973.193
867.9468994140625 0 3325.4756
868.4055786132812 0 10225.428
869.406494140625 0 4152.428
874.4650268554688 0 9591.054 y 9
875.4696655273438 0 2712.2456
886.46728515625 0 3174.9482
898.4616088867188 0 2381.5754
898.96435546875 0 4284.064
907.469482421875 0 12089.281
907.9705810546875 0 21519.57
908.4717407226562 0 9939.203
908.9624633789062 0 2766.8938
915.9724731445312 0 2229.055
916.36572265625 0 3070.292
916.4762573242188 0 186298.53
916.9773559570312 0 187718.8
917.4786376953125 0 90623.414
917.979736328125 0 14161.729
939.4418334960938 0 6878.314
940.4547729492188 0 2882.987
958.4911499023438 0 3872.7043
971.4800415039062 0 1529.796
973.49560546875 0 6294.8486
974.498046875 0 3359.3958
975.5028076171875 0 2312.6025 c 8
987.4873046875 0 49725.3 z 8
988.4942626953125 0 69136.62
989.4979248046875 0 28831.062
990.50341796875 0 6203.684
1002.4972534179688 0 6274.769
1003.5029907226562 0 13920.838 y 8
1004.5073852539062 0 5719.241
1015.4851684570312 0 1843.6805
1031.5045166015625 0 1921.2036
1053.487548828125 0 6961.6704
1054.4864501953125 0 3355.9893
1072.5103759765625 0 3314.9685 y Water loss 7
1073.509033203125 0 5771.5137 y Ammonia loss 7
1074.51904296875 0 50763.598 z 7
1075.52685546875 0 139261.5
1076.5306396484375 0 63882.47
1077.533935546875 0 10305.188
1087.5306396484375 0 4834.336
1088.5382080078125 0 4233.024
1089.5313720703125 0 17738.613
1090.537353515625 0 65199.34 y 7
1091.5404052734375 0 29080.541
1092.5440673828125 0 9096.323
1103.549560546875 0 2805.9492
1104.556396484375 0 17369.12 c 9
1105.5606689453125 0 9354.114
1106.560546875 0 2277.4636
1116.532958984375 0 4660.8213
1117.534423828125 0 8254.296
1118.538818359375 0 4558.564
1155.581787109375 0 1851.6211
1156.57568359375 0 2896.758
1159.5286865234375 0 3115.8455
1160.546142578125 0 4910.367 y Ammonia loss 6
1161.5516357421875 0 26072.975 z 6
1162.5582275390625 0 52878.92
1163.5621337890625 0 25799.889
1164.5592041015625 0 9787.424
1165.552734375 0 3077.3965
1174.6292724609375 0 2227.96
1176.560302734375 0 6290.8413
1177.5704345703125 0 39815.53 y 6
1178.5712890625 0 17991.793
1179.5789794921875 0 5430.7734
1181.582275390625 0 18021.717
1182.587890625 0 12962.516
1183.59130859375 0 3988.583
1200.615234375 0 10967.332
1201.6151123046875 0 6863.697
1202.617431640625 0 2765.462
1216.6337890625 0 20145.125
1217.639404296875 0 43074.59 c 10
1218.6436767578125 0 24724.17
1219.645751953125 0 4282.2065
1234.6041259765625 0 3513.1726
1235.6134033203125 0 4607.245
1252.616455078125 0 20593.076
1253.6217041015625 0 14811.981
1254.6163330078125 0 4124.522
1256.60205078125 0 2390.884 y Water loss 5
1257.58837890625 0 2775.6333 y Ammonia loss 5
1274.6224365234375 0 87166.5 y 5
1275.6253662109375 0 55395.246
1276.629638671875 0 15239.666
1277.6246337890625 0 1951.7666
1299.6392822265625 0 3746.2246
1301.6807861328125 0 2372.1482
1302.6915283203125 0 4726.089
1310.6724853515625 0 3905.0693
1311.6431884765625 0 2538.9907
1325.6695556640625 0 3406.5884
1326.6728515625 0 4864.01
1327.685302734375 0 2352.3567 c Water loss 11
1328.6708984375 0 19145.646 c Ammonia loss 11
1329.6719970703125 0 12880.017
1330.679443359375 0 5166.589
1343.6959228515625 0 2304.409
1344.692138671875 0 26017.412
1345.6990966796875 0 111498.96 c 11
1346.7021484375 0 76118.13
1347.7056884765625 0 25929.174
1348.7161865234375 0 2680.8796
1353.66748046875 0 11186.8545 y Water loss 4
1354.6600341796875 0 20594.023 y Ammonia loss 4
1355.6568603515625 0 12109.579 z 4
1356.650146484375 0 2522.7883
1357.6827392578125 0 3981.6194
1358.6885986328125 0 1892.449
1359.669921875 0 5233.636
1369.6541748046875 0 4421.801
1370.6680908203125 0 39335.617
1371.675048828125 0 792488.44 y 4
1372.6785888671875 0 521642.6
1373.6812744140625 0 183480.69
1374.6900634765625 0 19503.385
1381.6981201171875 0 1807.335
1397.7120361328125 0 4151.609
1398.6690673828125 0 16360.39
1399.682373046875 0 32176.908
1400.689208984375 0 21559.787
1401.701416015625 0 151772.61
1402.704345703125 0 123787.48
1403.707275390625 0 42106.32
1404.70849609375 0 4416.175
1415.7286376953125 0 87895.12
1416.7332763671875 0 126970.69 c 12
1417.7373046875 0 65051.355
1418.743408203125 0 17290.557
1430.69580078125 0 6413.6675
1431.6939697265625 0 6756.3887
1432.686767578125 0 2726.2449
1486.7659912109375 0 9455.032
1487.7684326171875 0 9352.516
1488.773193359375 0 3523.6736
1502.7244873046875 0 66251.92 z 3
1503.727294921875 0 62848.71
1504.7314453125 0 25328.979
1505.7392578125 0 2894.6287
1513.75390625 0 6276.6343 c Ammonia loss 13
1514.7557373046875 0 6034.613
1515.7730712890625 0 2503.8567
1517.757080078125 0 2646.8333
1518.743896484375 0 90163.586 y 3
1519.7457275390625 0 69454.6
1520.7481689453125 0 27394.021
1521.742919921875 0 2943.898
1528.7757568359375 0 2354.6445
1529.7724609375 0 12389.563
1530.779052734375 0 210914.77 c 13
1531.7818603515625 0 168665
1532.7855224609375 0 68640.87
1533.7821044921875 0 7168.8633
1543.7926025390625 0 2821.0178
1544.7916259765625 0 3236.107
1570.8172607421875 0 3503.7107
1571.8221435546875 0 5416.9136
1572.8094482421875 0 3182.9263
1573.773681640625 0 2276.593
1613.8251953125 0 3806.7258 y Water loss 2
1614.8599853515625 0 29290.441
1615.8375244140625 0 45699.31 z 2
1616.831298828125 0 34325.707
1617.8216552734375 0 11951.854
1631.826171875 0 32873.316 y 2
1632.8302001953125 0 28454.637
1633.8309326171875 0 11728.193
1644.8455810546875 0 2112.199
1645.8382568359375 0 3649.0437
1646.8421630859375 0 2491.804
1654.853759765625 0 3692.252
1655.8509521484375 0 5370.3857
1656.85498046875 0 3435.427
1657.8641357421875 0 2550.246
1658.8729248046875 0 85719.49 c 14
1659.8765869140625 0 94098.14
1660.8701171875 0 42887.387
1661.818359375 0 12052.823
1662.8074951171875 0 5918.354
1671.8455810546875 0 13911.451
1672.84521484375 0 16423.443
1673.8460693359375 0 8619.829
1674.845703125 0 2827.7703
1685.8961181640625 0 24891.115
1686.9022216796875 0 29979.057
1687.9085693359375 0 18073.662
1688.912109375 0 7544.5815
1689.899169921875 0 2962.7666
1698.8519287109375 0 7242.7725 z Water loss 1
1699.851318359375 0 7522.8496 z Ammonia loss 1
1714.8646240234375 0 39099.707 y Water loss 1
1715.8629150390625 0 63672.543 y Ammonia loss 1
1716.8580322265625 0 201426.42 z 1
1717.8597412109375 0 201770.14
1718.86474609375 0 98033.93
1719.8670654296875 0 17503.924
1729.9129638671875 0 146759.72 c 15
1730.9154052734375 0 139530.42
1731.9189453125 0 96732.266
1732.9251708984375 0 38589.926
1733.9248046875 0 14920.5
1734.92822265625 0 2555.4546
1743.8956298828125 0 8177.897
1744.8953857421875 0 18623.283
1745.89892578125 0 14159.807
1746.89453125 0 5745.484
1759.8895263671875 0 10272.98
1760.912109375 0 22415.404
1761.9168701171875 0 34369.594
1762.914306640625 0 20147.44
1763.9122314453125 0 7662.5
1768.936279296875 0 14346.72
1769.9349365234375 0 19356.934
1770.9375 0 13581.811
1771.9354248046875 0 7284.5356
1772.9276123046875 0 4858.0747
1773.9189453125 0 16031.204
1774.919677734375 0 17005.598
1775.9207763671875 0 10524.383
1776.89501953125 0 22970.797
1777.8916015625 0 20141.676
1778.8951416015625 0 9160.739
1786.9449462890625 0 38838.715
1787.93310546875 0 233107.05
1788.935302734375 0 227548.77
1789.9359130859375 0 123419.97
1790.92724609375 0 21249.531
1791.8896484375 0 2616.0054
1796.91064453125 0 2406.615
1797.91259765625 0 6805.858
1798.9132080078125 0 4504.6577
1799.9136962890625 0 3483.4395
1800.8892822265625 0 3396.8643
1801.9063720703125 0 3525.023
1804.957763671875 0 18171.258
1805.9578857421875 0 17753.908
1806.9576416015625 0 11072.519
1813.904052734375 0 5210.277
1814.9381103515625 0 54424.277
1815.9288330078125 0 354702.38
1816.9296875 0 341577.62
1817.931396484375 0 173522.69
1818.9327392578125 0 25865.889
1829.9263916015625 0 7576.755
1830.93603515625 0 26744.457
1831.9447021484375 0 652777.3
1832.95166015625 0 1709681.2
1833.95458984375 0 1457438.8
1834.9580078125 0 616064.7
1835.9588623046875 0 82403.195
1864.9334716796875 0 2966.5625
1865.931640625 0 3524.9658
3155.2294921875 0 1852.0929

Spectrum Details

|  |  |
| --- | --- |
| Matched peaks? Matched peaksThe total absolute number of peaks matched. Additionally in brackets the total fraction of peaks matched and the total number of peaks is shown. | 65 (15.74% of 413) |
| FDR? FDRThe false discovery rate estimated for this peptide. It is calculated by matching all theoretical fragments with a non-integer shift with the raw peaks for this spectrum. This is done with 40 different shifts. The resulting percentage is the average number of annotated peaks over the number of annotated peaks with the correct spectrum. | 0.48% |
| Satellite FDR? Satellite FDRSee the FDR for details on its calculation. This satellite ion specific FDR only contains the satellite ions (d/w) for I/L/J positions. | - |
| PSM Score? PSM ScoreThe PSM Score as given by Hecklib to this annotated spectrum. It is shown with three significant figures. | 627 |

## Spectrum 8046? Spectrum 8046 The raw spectrum of this peptide as annotated by Hecklib. The fragments are coloured according to ion type (see legend). Any peaks with a star '\*' as text can be hovered over to see the full details, first the ion type second the mass shift type. By hovering over the amino acids in the peptide or ions in the legend the corresponding peaks are highlighted. By toggling the 'Unassigned' label you can turn the background (unassigned) peaks on or off in the plot. By updating the slider in the Ion legend you can update the spectrum to only show the top X% of the peaks with labels. The top X% means any peak that is within X% of the highest intensity. By dragging in the spectrum you can zoom in to a specific part of the spectrum and use 'Zoom Out' to get back to the original zoom level. The annotation of the spectrum is based on the given sequence in the peptides file and is done with different software so inconsistencies are likely. The peaks are annotated based on the given sequence, with 20 ppm tolerance.

Copy Data

### Spectrum 8046 (TSV)

#### Preview

```
Loading example...
```

*Click on the button to copy the data to your clipboard.*

Mz MinMz MaxIntensity Max

WidthHeightPeptide font sizePeptide stroke widthSpectrum font sizeSpectrum stroke widthCompact peptide

Ion legend

wxyz

abcd

OtherUnassignedIonChargePositionShow for top:%

VTJFPPSSEEJQANKAT

04.60e+59.19e+51.38e+61.84e+6

Zoom Out

y+13y+14y+15y+16z+16y+16y+212c+212y+213y+213z+213y+213c+213z+17y+17y+214y+214z+214y+214y+215y+215z+215y+215c+216y+18y+216y+216y+216y+18w+19z+19y+19y+110z+110y+110c+110y+111y+111z+111y+111c+111y+112c+112c+112c+112y+113y+113z+113y+113c+113y+114z+114c+114y+114c+114y+115z+115y+115c+115z+116z+116y+116y+116z+116c+116

047194214131885

Fragment Matches Table

Show background peaks

| Position | Ion type | Intensity | mz Theoretical | mz Error (Th) | mz Error (ppm) | Charge | Series Number |
| --- | --- | --- | --- | --- | --- | --- | --- |
| - | - | 5537 | 120.1 | - | - | 0 | - |
| - | - | 1068 | 122.3 | - | - | 0 | - |
| - | - | 8410 | 129.1 | - | - | 0 | - |
| - | - | 1048 | 143.1 | - | - | 0 | - |
| - | - | 1250 | 165.4 | - | - | 0 | - |
| - | - | 4929 | 173.1 | - | - | 0 | - |
| - | - | 1.65E+04 | 173.5 | - | - | 0 | - |
| - | - | 1660 | 183.1 | - | - | 0 | - |
| - | - | 1.061E+04 | 183.1 | - | - | 0 | - |
| - | - | 2.006E+04 | 201.1 | - | - | 0 | - |
| - | - | 5638 | 215.1 | - | - | 0 | - |
| - | - | 2527 | 217.1 | - | - | 0 | - |
| - | - | 2107 | 224.6 | - | - | 0 | - |
| - | - | 2588 | 226.1 | - | - | 0 | - |
| - | - | 1441 | 229 | - | - | 0 | - |
| - | - | 1.351E+04 | 233.2 | - | - | 0 | - |
| - | - | 1744 | 233.6 | - | - | 0 | - |
| - | - | 2007 | 234.2 | - | - | 0 | - |
| - | - | 5060 | 243.1 | - | - | 0 | - |
| - | - | 4520 | 245.1 | - | - | 0 | - |
| - | - | 4506 | 251.2 | - | - | 0 | - |
| - | - | 1614 | 253.4 | - | - | 0 | - |
| - | - | 1.194E+04 | 261.2 | - | - | 0 | - |
| - | - | 2340 | 280.2 | - | - | 0 | - |
| - | - | 2024 | 282.1 | - | - | 0 | - |
| - | - | 1745 | 294.2 | - | - | 0 | - |
| - | - | 2.647E+05 | 296.2 | - | - | 0 | - |
| - | - | 3542 | 297.2 | - | - | 0 | - |
| - | - | 3.919E+04 | 297.2 | - | - | 0 | - |
| - | - | 4765 | 298.2 | - | - | 0 | - |
| - | - | 2775 | 298.2 | - | - | 0 | - |
| - | - | 1561 | 301.1 | - | - | 0 | - |
| - | - | 4420 | 303.2 | - | - | 0 | - |
| - | - | 4855 | 314.2 | - | - | 0 | - |
| - | - | 1.284E+05 | 314.2 | - | - | 0 | - |
| - | - | 1.925E+04 | 315.2 | - | - | 0 | - |
| - | - | 2291 | 317.8 | - | - | 0 | - |
| 15 | y | 1.245E+04 | 319.2 | 0.0003711 | 1.163 | +1 | 3 |
| - | - | 6308 | 330.2 | - | - | 0 | - |
| - | - | 2453 | 331.2 | - | - | 0 | - |
| - | - | 1645 | 345.7 | - | - | 0 | - |
| - | - | 7839 | 346.2 | - | - | 0 | - |
| - | - | 2697 | 347.2 | - | - | 0 | - |
| - | - | 1.203E+04 | 348.2 | - | - | 0 | - |
| - | - | 2460 | 349.2 | - | - | 0 | - |
| - | - | 1645 | 360 | - | - | 0 | - |
| - | - | 2407 | 360.2 | - | - | 0 | - |
| - | - | 3.487E+04 | 362.2 | - | - | 0 | - |
| - | - | 4390 | 363.2 | - | - | 0 | - |
| - | - | 2223 | 385.2 | - | - | 0 | - |
| - | - | 2225 | 390.2 | - | - | 0 | - |
| - | - | 3350 | 398.2 | - | - | 0 | - |
| - | - | 1.144E+04 | 411.3 | - | - | 0 | - |
| - | - | 2282 | 412.3 | - | - | 0 | - |
| - | - | 3695 | 415.3 | - | - | 0 | - |
| - | - | 1.564E+04 | 416.3 | - | - | 0 | - |
| - | - | 5305 | 417.3 | - | - | 0 | - |
| 14 | y | 1.708E+04 | 433.2 | 0.002121 | 4.897 | +1 | 4 |
| - | - | 1.797E+05 | 433.3 | - | - | 0 | - |
| - | - | 3698 | 434.2 | - | - | 0 | - |
| - | - | 4.254E+04 | 434.3 | - | - | 0 | - |
| - | - | 5550 | 435.3 | - | - | 0 | - |
| - | - | 2166 | 442.2 | - | - | 0 | - |
| - | - | 3552 | 443.2 | - | - | 0 | - |
| - | - | 1.823E+04 | 443.3 | - | - | 0 | - |
| - | - | 4458 | 444.3 | - | - | 0 | - |
| - | - | 1935 | 459.3 | - | - | 0 | - |
| - | - | 1.812E+05 | 461.3 | - | - | 0 | - |
| - | - | 4.869E+04 | 462.3 | - | - | 0 | - |
| - | - | 4852 | 463.3 | - | - | 0 | - |
| - | - | 1701 | 469.2 | - | - | 0 | - |
| - | - | 1868 | 498.2 | - | - | 0 | - |
| 13 | y | 3.106E+04 | 504.3 | 0.0001944 | 0.3855 | +1 | 5 |
| - | - | 7204 | 505.3 | - | - | 0 | - |
| - | - | 7468 | 530.3 | - | - | 0 | - |
| - | - | 6836 | 540.3 | - | - | 0 | - |
| - | - | 2.487E+04 | 558.3 | - | - | 0 | - |
| - | - | 9412 | 559.3 | - | - | 0 | - |
| - | - | 1591 | 582.3 | - | - | 0 | - |
| - | - | 2760 | 582.8 | - | - | 0 | - |
| - | - | 2000 | 583.3 | - | - | 0 | - |
| - | - | 3902 | 591.3 | - | - | 0 | - |
| 12 | y | 2108 | 615.3 | 0.003139 | 5.102 | +1 | 6 |
| 12 | z | 5255 | 616.3 | 4.725E-05 | 0.07666 | +1 | 6 |
| - | - | 6963 | 617.3 | - | - | 0 | - |
| - | - | 4181 | 617.8 | - | - | 0 | - |
| - | - | 6266 | 618.3 | - | - | 0 | - |
| - | - | 3.028E+04 | 626.8 | - | - | 0 | - |
| - | - | 4171 | 627.3 | - | - | 0 | - |
| - | - | 2.259E+04 | 627.3 | - | - | 0 | - |
| - | - | 4166 | 627.8 | - | - | 0 | - |
| - | - | 1960 | 628.7 | - | - | 0 | - |
| 12 | y | 1.84E+04 | 632.3 | 0.0003327 | 0.5261 | +1 | 6 |
| - | - | 6667 | 633.3 | - | - | 0 | - |
| 6 | y | 2085 | 637.8 | 0.0007705 | 1.208 | +2 | 12 |
| - | - | 2982 | 663.3 | - | - | 0 | - |
| - | - | 3230 | 663.8 | - | - | 0 | - |
| 12 | c | 2057 | 664.3 | 0.007989 | 12.03 | +2 | 12 |
| - | - | 2221 | 668.8 | - | - | 0 | - |
| 5 | y | 1.924E+04 | 677.3 | 0.0004229 | 0.6244 | +2 | 13 |
| 5 | y | 2.098E+04 | 677.8 | 0.004753 | 7.012 | +2 | 13 |
| 5 | z | 1.317E+04 | 678.3 | 0.0004743 | 0.6992 | +2 | 13 |
| - | - | 2220 | 685.3 | - | - | 0 | - |
| 5 | y | 6.538E+05 | 686.3 | 0.0004506 | 0.6565 | +2 | 13 |
| - | - | 4.895E+05 | 686.8 | - | - | 0 | - |
| - | - | 1.551E+05 | 687.3 | - | - | 0 | - |
| - | - | 1.427E+04 | 687.8 | - | - | 0 | - |
| 13 | c | 8270 | 700.4 | 0.01086 | 15.51 | +2 | 13 |
| - | - | 4666 | 700.8 | - | - | 0 | - |
| - | - | 3194 | 701.3 | - | - | 0 | - |
| 11 | z | 1.439E+04 | 729.4 | 4.785E-06 | 0.00656 | +1 | 7 |
| - | - | 1.085E+04 | 730.4 | - | - | 0 | - |
| - | - | 5728 | 740.3 | - | - | 0 | - |
| - | - | 2134 | 741.3 | - | - | 0 | - |
| - | - | 5405 | 742.4 | - | - | 0 | - |
| - | - | 2691 | 743.4 | - | - | 0 | - |
| 11 | y | 1.254E+04 | 745.4 | 0.0005583 | 0.7489 | +1 | 7 |
| - | - | 3826 | 746.4 | - | - | 0 | - |
| 4 | y | 4940 | 750.9 | 0.001983 | 2.64 | +2 | 14 |
| 4 | y | 6558 | 751.4 | 0.007289 | 9.701 | +2 | 14 |
| 4 | z | 3353 | 751.9 | 0.0001418 | 0.1886 | +2 | 14 |
| 4 | y | 1.009E+05 | 759.9 | 0.0002402 | 0.3161 | +2 | 14 |
| - | - | 7.373E+04 | 760.4 | - | - | 0 | - |
| - | - | 3.581E+04 | 760.9 | - | - | 0 | - |
| 3 | y | 1971 | 807.4 | 0.001393 | 1.726 | +2 | 15 |
| 3 | y | 1954 | 807.9 | 0.005601 | 6.933 | +2 | 15 |
| 3 | z | 1827 | 808.4 | 0.003499 | 4.328 | +2 | 15 |
| - | - | 1899 | 810.3 | - | - | 0 | - |
| - | - | 2535 | 812.9 | - | - | 0 | - |
| 3 | y | 2.165E+04 | 816.4 | 0.00136 | 1.666 | +2 | 15 |
| - | - | 1.867E+04 | 816.9 | - | - | 0 | - |
| - | - | 8542 | 817.4 | - | - | 0 | - |
| - | - | 3711 | 829.4 | - | - | 0 | - |
| - | - | 2802 | 842.9 | - | - | 0 | - |
| - | - | 5139 | 847.9 | - | - | 0 | - |
| - | - | 4900 | 848.4 | - | - | 0 | - |
| - | - | 4405 | 848.9 | - | - | 0 | - |
| 16 | c | 1.753E+04 | 856.9 | 3.766E-05 | 0.04395 | +2 | 16 |
| 10 | y | 2.071E+04 | 857.4 | 0.01003 | 11.69 | +1 | 8 |
| 2 | y | 1.467E+04 | 857.9 | 0.01369 | 15.95 | +2 | 16 |
| 2 | y | 3.351E+04 | 858.4 | 0.016 | 18.64 | +2 | 16 |
| - | - | 2.622E+04 | 859.5 | - | - | 0 | - |
| - | - | 6364 | 860.5 | - | - | 0 | - |
| 2 | y | 3059 | 866.9 | 0.002911 | 3.358 | +2 | 16 |
| - | - | 6831 | 867.4 | - | - | 0 | - |
| - | - | 4290 | 867.9 | - | - | 0 | - |
| - | - | 8631 | 868.4 | - | - | 0 | - |
| - | - | 3468 | 869.4 | - | - | 0 | - |
| 10 | y | 7934 | 874.5 | 0.001117 | 1.277 | +1 | 8 |
| - | - | 3487 | 875.5 | - | - | 0 | - |
| - | - | 3282 | 886.5 | - | - | 0 | - |
| - | - | 2324 | 899.5 | - | - | 0 | - |
| - | - | 1.463E+04 | 907.5 | - | - | 0 | - |
| - | - | 1.536E+04 | 908 | - | - | 0 | - |
| - | - | 1.347E+04 | 908.5 | - | - | 0 | - |
| - | - | 4001 | 909 | - | - | 0 | - |
| - | - | 3187 | 916 | - | - | 0 | - |
| - | - | 1.63E+05 | 916.5 | - | - | 0 | - |
| - | - | 1.8E+05 | 917 | - | - | 0 | - |
| - | - | 1.043E+05 | 917.5 | - | - | 0 | - |
| - | - | 1.348E+04 | 918 | - | - | 0 | - |
| 9 | w | 2043 | 928.5 | 0.0009638 | 1.038 | +1 | 9 |
| - | - | 2079 | 929.5 | - | - | 0 | - |
| - | - | 5151 | 939.4 | - | - | 0 | - |
| - | - | 1861 | 940.4 | - | - | 0 | - |
| - | - | 4717 | 958.5 | - | - | 0 | - |
| - | - | 2791 | 959.5 | - | - | 0 | - |
| - | - | 5883 | 973.5 | - | - | 0 | - |
| - | - | 3665 | 974.5 | - | - | 0 | - |
| - | - | 1933 | 975.3 | - | - | 0 | - |
| 9 | z | 5.159E+04 | 987.5 | 0.0007465 | 0.756 | +1 | 9 |
| - | - | 8.349E+04 | 988.5 | - | - | 0 | - |
| - | - | 2.552E+04 | 989.5 | - | - | 0 | - |
| - | - | 6829 | 990.5 | - | - | 0 | - |
| - | - | 3507 | 1003 | - | - | 0 | - |
| 9 | y | 1.632E+04 | 1004 | 0.004184 | 4.169 | +1 | 9 |
| - | - | 6460 | 1005 | - | - | 0 | - |
| - | - | 2090 | 1032 | - | - | 0 | - |
| - | - | 7153 | 1053 | - | - | 0 | - |
| - | - | 3817 | 1054 | - | - | 0 | - |
| - | - | 2342 | 1073 | - | - | 0 | - |
| 8 | y | 7487 | 1074 | 0.00114 | 1.062 | +1 | 10 |
| 8 | z | 5.046E+04 | 1075 | 2.915E-05 | 0.02713 | +1 | 10 |
| - | - | 1.313E+05 | 1076 | - | - | 0 | - |
| - | - | 7.014E+04 | 1077 | - | - | 0 | - |
| - | - | 1.544E+04 | 1078 | - | - | 0 | - |
| - | - | 5526 | 1088 | - | - | 0 | - |
| - | - | 4483 | 1089 | - | - | 0 | - |
| - | - | 2.183E+04 | 1090 | - | - | 0 | - |
| 8 | y | 6.465E+04 | 1091 | 0.0009947 | 0.9121 | +1 | 10 |
| - | - | 3.215E+04 | 1092 | - | - | 0 | - |
| - | - | 6585 | 1093 | - | - | 0 | - |
| - | - | 4236 | 1104 | - | - | 0 | - |
| 10 | c | 1.468E+04 | 1105 | 0.002235 | 2.023 | +1 | 10 |
| - | - | 9404 | 1106 | - | - | 0 | - |
| - | - | 4537 | 1117 | - | - | 0 | - |
| - | - | 1.001E+04 | 1118 | - | - | 0 | - |
| - | - | 4499 | 1119 | - | - | 0 | - |
| - | - | 2147 | 1157 | - | - | 0 | - |
| 7 | y | 2728 | 1160 | 0.02014 | 17.37 | +1 | 11 |
| 7 | y | 3545 | 1161 | 0.002324 | 2.002 | +1 | 11 |
| 7 | z | 2.392E+04 | 1162 | 0.0009934 | 0.8552 | +1 | 11 |
| - | - | 5.402E+04 | 1163 | - | - | 0 | - |
| - | - | 2.807E+04 | 1164 | - | - | 0 | - |
| - | - | 9814 | 1165 | - | - | 0 | - |
| - | - | 3097 | 1166 | - | - | 0 | - |
| - | - | 4557 | 1177 | - | - | 0 | - |
| 7 | y | 3.42E+04 | 1178 | 5.792E-05 | 0.04918 | +1 | 11 |
| - | - | 2.018E+04 | 1179 | - | - | 0 | - |
| - | - | 2646 | 1180 | - | - | 0 | - |
| - | - | 1.437E+04 | 1182 | - | - | 0 | - |
| - | - | 1.119E+04 | 1183 | - | - | 0 | - |
| - | - | 3624 | 1184 | - | - | 0 | - |
| - | - | 1.055E+04 | 1201 | - | - | 0 | - |
| - | - | 5260 | 1202 | - | - | 0 | - |
| - | - | 2.243E+04 | 1217 | - | - | 0 | - |
| 11 | c | 4.545E+04 | 1218 | 0.002681 | 2.202 | +1 | 11 |
| - | - | 2.666E+04 | 1219 | - | - | 0 | - |
| - | - | 7102 | 1220 | - | - | 0 | - |
| - | - | 2598 | 1231 | - | - | 0 | - |
| - | - | 5242 | 1235 | - | - | 0 | - |
| - | - | 3480 | 1236 | - | - | 0 | - |
| - | - | 2.131E+04 | 1253 | - | - | 0 | - |
| - | - | 1.432E+04 | 1254 | - | - | 0 | - |
| - | - | 3624 | 1255 | - | - | 0 | - |
| 6 | y | 8.972E+04 | 1275 | 0.0002157 | 0.1692 | +1 | 12 |
| - | - | 5.602E+04 | 1276 | - | - | 0 | - |
| - | - | 1.59E+04 | 1277 | - | - | 0 | - |
| - | - | 3030 | 1278 | - | - | 0 | - |
| - | - | 2926 | 1300 | - | - | 0 | - |
| - | - | 3192 | 1301 | - | - | 0 | - |
| - | - | 2165 | 1302 | - | - | 0 | - |
| - | - | 4716 | 1303 | - | - | 0 | - |
| - | - | 3418 | 1311 | - | - | 0 | - |
| - | - | 2935 | 1312 | - | - | 0 | - |
| - | - | 2834 | 1326 | - | - | 0 | - |
| - | - | 4189 | 1327 | - | - | 0 | - |
| 12 | c | 3171 | 1328 | 0.008579 | 6.462 | +1 | 12 |
| 12 | c | 1.437E+04 | 1329 | 0.004436 | 3.338 | +1 | 12 |
| - | - | 1.232E+04 | 1330 | - | - | 0 | - |
| - | - | 4027 | 1331 | - | - | 0 | - |
| - | - | 3.195E+04 | 1345 | - | - | 0 | - |
| 12 | c | 1.199E+05 | 1346 | 0.001444 | 1.073 | +1 | 12 |
| - | - | 7.993E+04 | 1347 | - | - | 0 | - |
| - | - | 2.162E+04 | 1348 | - | - | 0 | - |
| - | - | 2714 | 1349 | - | - | 0 | - |
| 5 | y | 1.145E+04 | 1354 | 0.001033 | 0.7631 | +1 | 13 |
| 5 | y | 1.918E+04 | 1355 | 0.01056 | 7.793 | +1 | 13 |
| 5 | z | 8028 | 1356 | 0.004959 | 3.658 | +1 | 13 |
| - | - | 3535 | 1357 | - | - | 0 | - |
| - | - | 3659 | 1358 | - | - | 0 | - |
| - | - | 2790 | 1359 | - | - | 0 | - |
| - | - | 2891 | 1360 | - | - | 0 | - |
| - | - | 2797 | 1361 | - | - | 0 | - |
| - | - | 2608 | 1370 | - | - | 0 | - |
| - | - | 4.479E+04 | 1371 | - | - | 0 | - |
| 5 | y | 8.064E+05 | 1372 | 0.0008555 | 0.6237 | +1 | 13 |
| - | - | 5.66E+05 | 1373 | - | - | 0 | - |
| - | - | 1.82E+05 | 1374 | - | - | 0 | - |
| - | - | 1.58E+04 | 1375 | - | - | 0 | - |
| - | - | 3257 | 1398 | - | - | 0 | - |
| - | - | 1.565E+04 | 1399 | - | - | 0 | - |
| - | - | 3.146E+04 | 1400 | - | - | 0 | - |
| - | - | 1.707E+04 | 1401 | - | - | 0 | - |
| - | - | 1.622E+05 | 1402 | - | - | 0 | - |
| - | - | 1.219E+05 | 1403 | - | - | 0 | - |
| - | - | 3.865E+04 | 1404 | - | - | 0 | - |
| - | - | 5126 | 1405 | - | - | 0 | - |
| - | - | 9.526E+04 | 1416 | - | - | 0 | - |
| 13 | c | 1.269E+05 | 1417 | 0.0045 | 3.176 | +1 | 13 |
| - | - | 6.594E+04 | 1418 | - | - | 0 | - |
| - | - | 2.026E+04 | 1419 | - | - | 0 | - |
| - | - | 5027 | 1431 | - | - | 0 | - |
| - | - | 5862 | 1432 | - | - | 0 | - |
| - | - | 2978 | 1433 | - | - | 0 | - |
| - | - | 9555 | 1487 | - | - | 0 | - |
| - | - | 1.221E+04 | 1488 | - | - | 0 | - |
| - | - | 4011 | 1489 | - | - | 0 | - |
| 4 | y | 2366 | 1501 | 0.01329 | 8.859 | +1 | 14 |
| 4 | z | 6.658E+04 | 1503 | 0.001839 | 1.224 | +1 | 14 |
| - | - | 6.522E+04 | 1504 | - | - | 0 | - |
| - | - | 3.008E+04 | 1505 | - | - | 0 | - |
| 14 | c | 5692 | 1514 | 0.0006061 | 0.4004 | +1 | 14 |
| - | - | 7555 | 1515 | - | - | 0 | - |
| - | - | 4258 | 1516 | - | - | 0 | - |
| - | - | 3080 | 1518 | - | - | 0 | - |
| 4 | y | 8.841E+04 | 1519 | 5.558E-05 | 0.0366 | +1 | 14 |
| - | - | 7.745E+04 | 1520 | - | - | 0 | - |
| - | - | 2.952E+04 | 1521 | - | - | 0 | - |
| - | - | 1.071E+04 | 1530 | - | - | 0 | - |
| 14 | c | 2.044E+05 | 1531 | 0.001651 | 1.079 | +1 | 14 |
| - | - | 1.828E+05 | 1532 | - | - | 0 | - |
| - | - | 2819 | 1532 | - | - | 0 | - |
| - | - | 6.517E+04 | 1533 | - | - | 0 | - |
| - | - | 1.009E+04 | 1534 | - | - | 0 | - |
| - | - | 2630 | 1544 | - | - | 0 | - |
| - | - | 4976 | 1545 | - | - | 0 | - |
| - | - | 3111 | 1571 | - | - | 0 | - |
| - | - | 4898 | 1572 | - | - | 0 | - |
| - | - | 3440 | 1573 | - | - | 0 | - |
| 3 | y | 3109 | 1614 | 0.00312 | 1.933 | +1 | 15 |
| - | - | 3.021E+04 | 1615 | - | - | 0 | - |
| 3 | z | 4.268E+04 | 1616 | 0.02604 | 16.11 | +1 | 15 |
| - | - | 2.851E+04 | 1617 | - | - | 0 | - |
| - | - | 1.005E+04 | 1618 | - | - | 0 | - |
| - | - | 2774 | 1619 | - | - | 0 | - |
| 3 | y | 2.628E+04 | 1632 | 0.0003793 | 0.2325 | +1 | 15 |
| - | - | 2.791E+04 | 1633 | - | - | 0 | - |
| - | - | 1.334E+04 | 1634 | - | - | 0 | - |
| - | - | 2361 | 1643 | - | - | 0 | - |
| - | - | 2627 | 1644 | - | - | 0 | - |
| - | - | 2981 | 1645 | - | - | 0 | - |
| - | - | 4929 | 1646 | - | - | 0 | - |
| - | - | 3310 | 1647 | - | - | 0 | - |
| - | - | 3990 | 1655 | - | - | 0 | - |
| - | - | 5662 | 1656 | - | - | 0 | - |
| - | - | 3639 | 1657 | - | - | 0 | - |
| 15 | c | 1.033E+05 | 1659 | 0.001888 | 1.138 | +1 | 15 |
| - | - | 9.189E+04 | 1660 | - | - | 0 | - |
| - | - | 4.667E+04 | 1661 | - | - | 0 | - |
| - | - | 1.276E+04 | 1662 | - | - | 0 | - |
| - | - | 4887 | 1663 | - | - | 0 | - |
| - | - | 1.173E+04 | 1672 | - | - | 0 | - |
| - | - | 1.71E+04 | 1673 | - | - | 0 | - |
| - | - | 7474 | 1674 | - | - | 0 | - |
| - | - | 3891 | 1675 | - | - | 0 | - |
| - | - | 3.173E+04 | 1686 | - | - | 0 | - |
| - | - | 3.204E+04 | 1687 | - | - | 0 | - |
| - | - | 2.229E+04 | 1688 | - | - | 0 | - |
| - | - | 9580 | 1689 | - | - | 0 | - |
| 2 | z | 5507 | 1699 | 0.01504 | 8.856 | +1 | 16 |
| 2 | z | 5471 | 1700 | 0.01662 | 9.78 | +1 | 16 |
| - | - | 3159 | 1701 | - | - | 0 | - |
| 2 | y | 3.802E+04 | 1715 | 0.001691 | 0.9864 | +1 | 16 |
| 2 | y | 6.46E+04 | 1716 | 0.01121 | 6.531 | +1 | 16 |
| 2 | z | 2.25E+05 | 1717 | 0.0002073 | 0.1207 | +1 | 16 |
| - | - | 2.246E+05 | 1718 | - | - | 0 | - |
| - | - | 2885 | 1719 | - | - | 0 | - |
| - | - | 9.441E+04 | 1719 | - | - | 0 | - |
| - | - | 1.899E+04 | 1720 | - | - | 0 | - |
| 16 | c | 1.572E+05 | 1730 | 0.0001829 | 0.1057 | +1 | 16 |
| - | - | 1.575E+05 | 1731 | - | - | 0 | - |
| - | - | 1.072E+05 | 1732 | - | - | 0 | - |
| - | - | 4.416E+04 | 1733 | - | - | 0 | - |
| - | - | 2.103E+04 | 1734 | - | - | 0 | - |
| - | - | 3103 | 1743 | - | - | 0 | - |
| - | - | 1.181E+04 | 1744 | - | - | 0 | - |
| - | - | 2.299E+04 | 1745 | - | - | 0 | - |
| - | - | 1.565E+04 | 1746 | - | - | 0 | - |
| - | - | 7300 | 1747 | - | - | 0 | - |
| - | - | 2821 | 1759 | - | - | 0 | - |
| - | - | 1.28E+04 | 1760 | - | - | 0 | - |
| - | - | 2.678E+04 | 1761 | - | - | 0 | - |
| - | - | 3.123E+04 | 1762 | - | - | 0 | - |
| - | - | 2.201E+04 | 1763 | - | - | 0 | - |
| - | - | 9400 | 1764 | - | - | 0 | - |
| - | - | 1.92E+04 | 1769 | - | - | 0 | - |
| - | - | 2.483E+04 | 1770 | - | - | 0 | - |
| - | - | 1.601E+04 | 1771 | - | - | 0 | - |
| - | - | 9402 | 1772 | - | - | 0 | - |
| - | - | 4448 | 1773 | - | - | 0 | - |
| - | - | 1.548E+04 | 1774 | - | - | 0 | - |
| - | - | 1.98E+04 | 1775 | - | - | 0 | - |
| - | - | 9388 | 1776 | - | - | 0 | - |
| - | - | 2.083E+04 | 1777 | - | - | 0 | - |
| - | - | 2.455E+04 | 1778 | - | - | 0 | - |
| - | - | 8891 | 1779 | - | - | 0 | - |
| - | - | 3177 | 1786 | - | - | 0 | - |
| - | - | 4.218E+04 | 1787 | - | - | 0 | - |
| - | - | 2.663E+05 | 1788 | - | - | 0 | - |
| - | - | 2.554E+05 | 1789 | - | - | 0 | - |
| - | - | 1.253E+05 | 1790 | - | - | 0 | - |
| - | - | 2.222E+04 | 1791 | - | - | 0 | - |
| - | - | 5387 | 1797 | - | - | 0 | - |
| - | - | 8289 | 1798 | - | - | 0 | - |
| - | - | 6913 | 1799 | - | - | 0 | - |
| - | - | 4382 | 1800 | - | - | 0 | - |
| - | - | 3956 | 1801 | - | - | 0 | - |
| - | - | 2550 | 1803 | - | - | 0 | - |
| - | - | 1.872E+04 | 1805 | - | - | 0 | - |
| - | - | 2.315E+04 | 1806 | - | - | 0 | - |
| - | - | 1.127E+04 | 1807 | - | - | 0 | - |
| - | - | 4164 | 1814 | - | - | 0 | - |
| - | - | 6.072E+04 | 1815 | - | - | 0 | - |
| - | - | 4.009E+05 | 1816 | - | - | 0 | - |
| - | - | 3.731E+05 | 1817 | - | - | 0 | - |
| - | - | 1.965E+05 | 1818 | - | - | 0 | - |
| - | - | 2963 | 1818 | - | - | 0 | - |
| - | - | 3.043E+04 | 1819 | - | - | 0 | - |
| - | - | 3451 | 1820 | - | - | 0 | - |
| - | - | 6246 | 1830 | - | - | 0 | - |
| - | - | 2.609E+04 | 1831 | - | - | 0 | - |
| - | - | 6.786E+05 | 1832 | - | - | 0 | - |
| - | - | 1.82E+06 | 1833 | - | - | 0 | - |
| - | - | 1.572E+06 | 1834 | - | - | 0 | - |
| - | - | 6.62E+05 | 1835 | - | - | 0 | - |
| - | - | 9.563E+04 | 1836 | - | - | 0 | - |
| - | - | 5579 | 1866 | - | - | 0 | - |

m/z Charge Intensity FragmentType MassShift Position
120.08097839355469 0 5536.8774
122.33557891845703 0 1067.5835
129.1023712158203 0 8410.003
143.12428283691406 0 1047.7318
165.4038848876953 0 1249.9261
173.1286163330078 0 4929.395
173.4501953125 0 16499.486
183.10523986816406 0 1659.5571
183.11297607421875 0 10610.647
201.12335205078125 0 20055.432
215.13912963867188 0 5637.737
217.1337890625 0 2526.752
224.60670471191406 0 2107.0093
226.11875915527344 0 2587.7578
229.0153045654297 0 1440.8817
233.16502380371094 0 13512.206
233.60089111328125 0 1743.7103
234.1679229736328 0 2007.4443
243.1453094482422 0 5060.2075
245.12835693359375 0 4519.5933
251.1758270263672 0 4506.033
253.39984130859375 0 1613.523
261.1598815917969 0 11938.391
280.164306640625 0 2339.7998
282.1460266113281 0 2023.6187
294.1814880371094 0 1745.4854
296.1971435546875 0 264691.56
297.1565246582031 0 3542.2383
297.2004089355469 0 39190.51
298.1767883300781 0 4764.852
298.201904296875 0 2775.2996
301.08428955078125 0 1560.9652
303.1708679199219 0 4419.997
314.1861572265625 0 4854.794
314.2077331542969 0 128420.52
315.2109680175781 0 19251.762
317.8280944824219 0 2290.988
319.1979675292969 0 12454.946 y 14
330.1822814941406 0 6308.478
331.1854553222656 0 2453.3079
345.7443542480469 0 1644.9579
346.1758728027344 0 7838.8784
347.1797790527344 0 2696.6267
348.1916198730469 0 12026.337
349.1960754394531 0 2460.0376
360.0343933105469 0 1644.9562
360.22802734375 0 2406.9622
362.2074279785156 0 34867.734
363.2109375 0 4390.421
385.2208557128906 0 2223.406
390.20355224609375 0 2225.4539
398.245849609375 0 3350.4573
411.2607727050781 0 11438.161
412.26470947265625 0 2282.254
415.2695617675781 0 3695.1416
416.25469970703125 0 15644.145
417.255859375 0 5304.749
433.2426452636719 0 17077.066 y 13
433.2814636230469 0 179701.23
434.24853515625 0 3698.133
434.2843933105469 0 42538.52
435.2874755859375 0 5550.2935
442.24700927734375 0 2166.1216
443.2301330566406 0 3551.7283
443.26568603515625 0 18225.738
444.2690734863281 0 4457.9077
459.2615966796875 0 1935.1113
461.2764587402344 0 181210.62
462.2794189453125 0 48685.023
463.2828674316406 0 4852.3306
469.24432373046875 0 1700.544
498.21826171875 0 1868.3374
504.27783203125 0 31060.414 y 12
505.2801818847656 0 7204.231
530.3342895507812 0 7468.375
540.3175048828125 0 6836.3438
558.3291625976562 0 24867.979
559.3319702148438 0 9411.829
582.2835693359375 0 1590.9889
582.7810668945312 0 2760.1133
583.2803955078125 0 2000.0458
591.2950439453125 0 3901.7893
615.3128051757812 0 2107.588 y Ammonia loss 11
616.3174438476562 0 5255.0747 z 11
617.3250122070312 0 6962.742
617.810302734375 0 4180.994
618.3080444335938 0 6266.0054
626.8126220703125 0 30283.225
627.26025390625 0 4170.8555
627.3140869140625 0 22589.74
627.8171997070312 0 4166.0483
628.731689453125 0 1960.114
632.3365478515625 0 18402.014 y 11
633.33984375 0 6666.5674
637.8155517578125 0 2085.3152 y 5
663.3402099609375 0 2982.3125
663.8370971679688 0 3230.0044
664.3402709960938 0 2056.7202 c Water loss 11
668.826171875 0 2221.3833
677.3363037109375 0 19241.467 y Water loss 4
677.8326416015625 0 20979.865 y Ammonia loss 4
678.332275390625 0 13174.197 z 4
685.3318481445312 0 2219.5732
686.3416137695312 0 653821 y 4
686.8429565429688 0 489535.12
687.3438720703125 0 155066.64
687.8455810546875 0 14270.105
700.3479614257812 0 8269.562 c Ammonia loss 12
700.849853515625 0 4666.366
701.346923828125 0 3194.143
729.4015502929688 0 14393.622 z 10
730.4068603515625 0 10846.628
740.3464965820312 0 5728.129
741.3467407226562 0 2133.552
742.4148559570312 0 5405.313
743.4163208007812 0 2691.0315
745.4208374023438 0 12537.878 y 10
746.4171142578125 0 3825.8337
750.8720703125 0 4940.125 y Water loss 3
751.369384765625 0 6557.617 y Ammonia loss 3
751.8661499023438 0 3352.603 z 3
759.8756103515625 0 100913.83 y 3
760.376953125 0 73725.25
760.8781127929688 0 35814.91
807.4135131835938 0 1971.0668 y Water loss 2
807.9097290039062 0 1954.0134 y Ammonia loss 2
808.404541015625 0 1827.1187 z 2
810.2609252929688 0 1899.1115
812.9097900390625 0 2534.9695
816.4187622070312 0 21654.78 y 2
816.9195556640625 0 18672.877
817.4204711914062 0 8541.786
829.4388427734375 0 3711.2524
842.9432373046875 0 2801.9956
847.9402465820312 0 5138.891
848.4385986328125 0 4900.131
848.9385986328125 0 4404.714
856.9462890625 0 17525.508 c Ammonia loss 15
857.4463500976562 0 20712.533 y Ammonia loss 9
857.9496459960938 0 14665.226 y Water loss 1
858.4439697265625 0 33509.32 y Ammonia loss 1
859.4500122070312 0 26221.09
860.4528198242188 0 6364.25
866.9441528320312 0 3059.0068 y 1
867.4407958984375 0 6831.2095
867.9445190429688 0 4289.812
868.4036865234375 0 8631.29
869.4034423828125 0 3468.4727
874.4639892578125 0 7934.3276 y 9
875.4623413085938 0 3487.19
886.4605102539062 0 3281.9734
899.4602661132812 0 2324.2551
907.470703125 0 14627.937
907.96875 0 15356.42
908.4700927734375 0 13469.71
908.9693603515625 0 4000.7883
915.964599609375 0 3187.1604
916.4758911132812 0 163024.83
916.9766845703125 0 180019.62
917.4783935546875 0 104312.414
917.9784545898438 0 13477.065
928.4724731445312 0 2042.9987 w 8
929.48291015625 0 2078.891
939.440185546875 0 5150.5967
940.4459228515625 0 1860.6488
958.4898071289062 0 4717.454
959.48974609375 0 2791.3784
973.489501953125 0 5883.363
974.5005493164062 0 3665.4197
975.3219604492188 0 1932.7743
987.4874877929688 0 51586.19 z 8
988.4937744140625 0 83489.09
989.4971313476562 0 25523.646
990.5 0 6829.382
1002.5005493164062 0 3507.1113
1003.5012817382812 0 16321.071 y 8
1004.5103149414062 0 6460.4727
1031.502685546875 0 2090.256
1053.4857177734375 0 7153.248
1054.49560546875 0 3817.373
1072.5006103515625 0 2342.2874
1073.5120849609375 0 7487.3926 y Ammonia loss 7
1074.518798828125 0 50463.562 z 7
1075.526123046875 0 131348.77
1076.5294189453125 0 70142.84
1077.53271484375 0 15438.128
1087.53173828125 0 5526.456
1088.5333251953125 0 4483.3677
1089.530517578125 0 21834.543
1090.5364990234375 0 64645.13 y 7
1091.5396728515625 0 32152.96
1092.5418701171875 0 6585.0205
1103.5462646484375 0 4236.412
1104.554931640625 0 14680.751 c 9
1105.5596923828125 0 9403.877
1116.53369140625 0 4537.4
1117.5369873046875 0 10010.215
1118.5421142578125 0 4499.273
1156.567626953125 0 2147.2432
1159.538818359375 0 2727.5093 y Water loss 6
1160.5406494140625 0 3545.22 y Ammonia loss 6
1161.5498046875 0 23924.31 z 6
1162.5567626953125 0 54017.977
1163.5615234375 0 28073.36
1164.557373046875 0 9814.338
1165.556884765625 0 3097.3416
1176.5604248046875 0 4557.0337
1177.569580078125 0 34204.51 y 6
1178.5697021484375 0 20183.148
1179.574951171875 0 2646.2405
1181.5819091796875 0 14374.038
1182.5845947265625 0 11191.569
1183.5972900390625 0 3624.0278
1200.6119384765625 0 10553.452
1201.613037109375 0 5260.1924
1216.634521484375 0 22433.596
1217.6385498046875 0 45448.07 c 10
1218.6431884765625 0 26660.518
1219.6456298828125 0 7102.267
1230.603759765625 0 2598.314
1234.603759765625 0 5242.294
1235.602783203125 0 3480.3853
1252.6170654296875 0 21311.316
1253.6195068359375 0 14321.442
1254.609130859375 0 3623.6912
1274.6220703125 0 89716.33 y 5
1275.6251220703125 0 56021.344
1276.6268310546875 0 15903.209
1277.6268310546875 0 3029.5789
1299.6357421875 0 2925.771
1300.638427734375 0 3191.5168
1301.6680908203125 0 2165.337
1302.675048828125 0 4716.083
1310.661376953125 0 3417.986
1311.6632080078125 0 2934.5562
1325.669921875 0 2833.9263
1326.6729736328125 0 4189.0225
1327.6806640625 0 3170.6213 c Water loss 11
1328.6688232421875 0 14366.873 c Ammonia loss 11
1329.6685791015625 0 12317.791
1330.6798095703125 0 4027.1685
1344.69091796875 0 31945.322
1345.6983642578125 0 119948.336 c 11
1346.70166015625 0 79928.56
1347.703857421875 0 21624
1348.7127685546875 0 2714.156
1353.6634521484375 0 11447.957 y Water loss 4
1354.6590576171875 0 19181.959 y Ammonia loss 4
1355.6513671875 0 8028.2817 z 4
1356.6497802734375 0 3535.056
1357.6739501953125 0 3658.57
1358.675537109375 0 2789.7905
1359.6712646484375 0 2891.1575
1360.6749267578125 0 2797.3806
1369.671630859375 0 2607.8833
1370.6666259765625 0 44785.31
1371.6741943359375 0 806432.56 y 4
1372.6773681640625 0 565997.4
1373.680419921875 0 182015.62
1374.6876220703125 0 15803.857
1397.720458984375 0 3257.4458
1398.6676025390625 0 15654.261
1399.680419921875 0 31464.113
1400.6837158203125 0 17069.873
1401.7005615234375 0 162160.36
1402.7032470703125 0 121921.67
1403.7069091796875 0 38646.973
1404.7081298828125 0 5126.1953
1415.728271484375 0 95257.18
1416.732421875 0 126938.2 c 12
1417.7364501953125 0 65936.35
1418.7415771484375 0 20257.81
1430.7010498046875 0 5026.6763
1431.697021484375 0 5862.034
1432.7001953125 0 2978.2393
1486.7635498046875 0 9554.889
1487.767822265625 0 12211.611
1488.7733154296875 0 4010.6938
1500.7196044921875 0 2366.185 y Water loss 3
1502.722900390625 0 66576.52 z 3
1503.7252197265625 0 65218.027
1504.7283935546875 0 30075.615
1513.75390625 0 5692.182 c Ammonia loss 13
1514.7525634765625 0 7555.3833
1515.7642822265625 0 4258.0444
1517.7508544921875 0 3079.7163
1518.743408203125 0 88405.164 y 3
1519.744140625 0 77454.76
1520.7457275390625 0 29520.568
1529.767578125 0 10706.896
1530.7781982421875 0 204373.77 c 13
1531.78076171875 0 182753.36
1532.0167236328125 0 2819.47
1532.7845458984375 0 65170.977
1533.7862548828125 0 10086.607
1543.7803955078125 0 2630.0925
1544.7916259765625 0 4975.6694
1570.8238525390625 0 3110.973
1571.80712890625 0 4898.4004
1572.8153076171875 0 3440.3464
1613.8138427734375 0 3109.1565 y Water loss 2
1614.85498046875 0 30206.645
1615.8348388671875 0 42677.32 z 2
1616.828125 0 28511.348
1617.8173828125 0 10051.303
1618.8277587890625 0 2773.7917
1631.8271484375 0 26284.635 y 2
1632.828125 0 27911.762
1633.8331298828125 0 13339.054
1642.86865234375 0 2360.5398
1643.82421875 0 2626.737
1644.84765625 0 2980.6592
1645.825927734375 0 4929.323
1646.8275146484375 0 3310.289
1654.849609375 0 3990.1565
1655.8505859375 0 5661.6343
1656.8489990234375 0 3638.7288
1658.8729248046875 0 103346.37 c 14
1659.8743896484375 0 91894.44
1660.865234375 0 46668.824
1661.8138427734375 0 12758.486
1662.804443359375 0 4887.4204
1671.8453369140625 0 11725.871
1672.8443603515625 0 17100.652
1673.83642578125 0 7473.798
1674.8214111328125 0 3891.3938
1685.896484375 0 31733.93
1686.900634765625 0 32039.438
1687.906982421875 0 22294.658
1688.9053955078125 0 9580.019
1698.8609619140625 0 5506.699 z Water loss 1
1699.8465576171875 0 5470.661 z Ammonia loss 1
1700.8468017578125 0 3159.2952
1714.8663330078125 0 38021.418 y Water loss 1
1715.85986328125 0 64597.883 y Ammonia loss 1
1716.856689453125 0 225034.52 z 1
1717.85888671875 0 224608.12
1718.585693359375 0 2885.127
1718.8631591796875 0 94405.82
1719.8660888671875 0 18990.238
1729.9117431640625 0 157214.23 c 15
1730.9132080078125 0 157543.39
1731.9176025390625 0 107227.38
1732.924072265625 0 44155.117
1733.9267578125 0 21034.023
1742.8968505859375 0 3103.0981
1743.90185546875 0 11807.759
1744.8896484375 0 22991.28
1745.886962890625 0 15653.821
1746.8975830078125 0 7300.183
1758.9049072265625 0 2821.0696
1759.88525390625 0 12797.362
1760.912841796875 0 26779.547
1761.9154052734375 0 31231.264
1762.91162109375 0 22008.764
1763.9093017578125 0 9400.474
1768.9371337890625 0 19196.611
1769.9346923828125 0 24830.637
1770.9368896484375 0 16014.409
1771.9403076171875 0 9402.392
1772.9058837890625 0 4448.4316
1773.9180908203125 0 15476.642
1774.91748046875 0 19797.668
1775.9180908203125 0 9388.227
1776.8917236328125 0 20830.314
1777.889892578125 0 24554.459
1778.8961181640625 0 8890.531
1785.9105224609375 0 3176.739
1786.9425048828125 0 42180.8
1787.9312744140625 0 266268.78
1788.9334716796875 0 255437.61
1789.9342041015625 0 125341.12
1790.9271240234375 0 22217.496
1796.938232421875 0 5387.2563
1797.9207763671875 0 8289.117
1798.918212890625 0 6912.9253
1799.930908203125 0 4381.516
1800.911376953125 0 3956.168
1802.93603515625 0 2549.7405
1804.9549560546875 0 18716.443
1805.9560546875 0 23150.736
1806.9554443359375 0 11272.854
1813.91943359375 0 4163.7183
1814.9375 0 60724.74
1815.927001953125 0 400854.12
1816.92822265625 0 373142
1817.9293212890625 0 196481.84
1818.233154296875 0 2963.345
1818.9307861328125 0 30427.498
1819.92822265625 0 3451.0256
1829.912353515625 0 6246.2114
1830.9337158203125 0 26093.506
1831.943359375 0 678603.1
1832.9503173828125 0 1820112.1
1833.9530029296875 0 1572178.1
1834.9560546875 0 662022.5
1835.9564208984375 0 95631.8
1865.942626953125 0 5578.991

Spectrum Details

|  |  |
| --- | --- |
| Matched peaks? Matched peaksThe total absolute number of peaks matched. Additionally in brackets the total fraction of peaks matched and the total number of peaks is shown. | 65 (16.33% of 398) |
| FDR? FDRThe false discovery rate estimated for this peptide. It is calculated by matching all theoretical fragments with a non-integer shift with the raw peaks for this spectrum. This is done with 40 different shifts. The resulting percentage is the average number of annotated peaks over the number of annotated peaks with the correct spectrum. | 0.33% |
| Satellite FDR? Satellite FDRSee the FDR for details on its calculation. This satellite ion specific FDR only contains the satellite ions (d/w) for I/L/J positions. | - |
| PSM Score? PSM ScoreThe PSM Score as given by Hecklib to this annotated spectrum. It is shown with three significant figures. | 574 |

## Spectrum 8281? Spectrum 8281 The raw spectrum of this peptide as annotated by Hecklib. The fragments are coloured according to ion type (see legend). Any peaks with a star '\*' as text can be hovered over to see the full details, first the ion type second the mass shift type. By hovering over the amino acids in the peptide or ions in the legend the corresponding peaks are highlighted. By toggling the 'Unassigned' label you can turn the background (unassigned) peaks on or off in the plot. By updating the slider in the Ion legend you can update the spectrum to only show the top X% of the peaks with labels. The top X% means any peak that is within X% of the highest intensity. By dragging in the spectrum you can zoom in to a specific part of the spectrum and use 'Zoom Out' to get back to the original zoom level. The annotation of the spectrum is based on the given sequence in the peptides file and is done with different software so inconsistencies are likely. The peaks are annotated based on the given sequence, with 20 ppm tolerance.

Copy Data

### Spectrum 8281 (TSV)

#### Preview

```
Loading example...
```

*Click on the button to copy the data to your clipboard.*

Mz MinMz MaxIntensity Max

WidthHeightPeptide font sizePeptide stroke widthSpectrum font sizeSpectrum stroke widthCompact peptide

Ion legend

wxyz

abcd

OtherUnassignedIonChargePositionShow for top:%

VTJFPPSSEEJQANKAT

01.33e+52.66e+53.98e+55.31e+5

Zoom Out

y+13y+14y+15y+15y+16z+16y+16y+213y+213z+213y+213z+17y+17y+214c+214y+214y+215y+215y+215c+216y+18y+216z+18y+216y+18z+19y+19y+110y+110z+110y+110c+110y+111z+111y+111c+111y+112c+112c+112y+113y+113z+113y+113c+113c+113z+114c+114y+114c+114y+115z+115y+115c+115z+116z+116y+116y+116z+116c+116

046492713911854

Fragment Matches Table

Show background peaks

| Position | Ion type | Intensity | mz Theoretical | mz Error (Th) | mz Error (ppm) | Charge | Series Number |
| --- | --- | --- | --- | --- | --- | --- | --- |
| - | - | 1173 | 120.1 | - | - | 0 | - |
| - | - | 348.4 | 128.5 | - | - | 0 | - |
| - | - | 2004 | 129.1 | - | - | 0 | - |
| - | - | 399.5 | 133.2 | - | - | 0 | - |
| - | - | 415.6 | 137.5 | - | - | 0 | - |
| - | - | 407.7 | 144.3 | - | - | 0 | - |
| - | - | 505.7 | 148.8 | - | - | 0 | - |
| - | - | 445.5 | 148.9 | - | - | 0 | - |
| - | - | 821 | 148.9 | - | - | 0 | - |
| - | - | 615 | 148.9 | - | - | 0 | - |
| - | - | 704.6 | 148.9 | - | - | 0 | - |
| - | - | 761.4 | 148.9 | - | - | 0 | - |
| - | - | 946.6 | 148.9 | - | - | 0 | - |
| - | - | 1224 | 148.9 | - | - | 0 | - |
| - | - | 1321 | 148.9 | - | - | 0 | - |
| - | - | 3119 | 148.9 | - | - | 0 | - |
| - | - | 5299 | 148.9 | - | - | 0 | - |
| - | - | 3960 | 149 | - | - | 0 | - |
| - | - | 1749 | 149 | - | - | 0 | - |
| - | - | 1401 | 149 | - | - | 0 | - |
| - | - | 932.9 | 149 | - | - | 0 | - |
| - | - | 629 | 149 | - | - | 0 | - |
| - | - | 508.8 | 149 | - | - | 0 | - |
| - | - | 392.8 | 149 | - | - | 0 | - |
| - | - | 430.3 | 149 | - | - | 0 | - |
| - | - | 562 | 149 | - | - | 0 | - |
| - | - | 446.4 | 151.7 | - | - | 0 | - |
| - | - | 552.3 | 160.6 | - | - | 0 | - |
| - | - | 1461 | 173.1 | - | - | 0 | - |
| - | - | 435.1 | 174.3 | - | - | 0 | - |
| - | - | 2884 | 183.1 | - | - | 0 | - |
| - | - | 6664 | 201.1 | - | - | 0 | - |
| - | - | 469 | 201.1 | - | - | 0 | - |
| - | - | 1448 | 215.1 | - | - | 0 | - |
| - | - | 765.4 | 217.1 | - | - | 0 | - |
| - | - | 4182 | 233.2 | - | - | 0 | - |
| - | - | 544.4 | 234.2 | - | - | 0 | - |
| - | - | 1470 | 243.1 | - | - | 0 | - |
| - | - | 988.2 | 245.1 | - | - | 0 | - |
| - | - | 1169 | 251.2 | - | - | 0 | - |
| - | - | 2939 | 261.2 | - | - | 0 | - |
| - | - | 811.6 | 262.2 | - | - | 0 | - |
| - | - | 713.8 | 280.2 | - | - | 0 | - |
| - | - | 869.1 | 282.1 | - | - | 0 | - |
| - | - | 6.28E+04 | 296.2 | - | - | 0 | - |
| - | - | 1.007E+04 | 297.2 | - | - | 0 | - |
| - | - | 667.6 | 298.2 | - | - | 0 | - |
| - | - | 1047 | 298.2 | - | - | 0 | - |
| - | - | 1306 | 303.2 | - | - | 0 | - |
| - | - | 3.567E+04 | 314.2 | - | - | 0 | - |
| - | - | 4601 | 315.2 | - | - | 0 | - |
| 15 | y | 3562 | 319.2 | 5.614E-05 | 0.1759 | +1 | 3 |
| - | - | 1146 | 330.2 | - | - | 0 | - |
| - | - | 2946 | 346.2 | - | - | 0 | - |
| - | - | 2538 | 348.2 | - | - | 0 | - |
| - | - | 1005 | 349.2 | - | - | 0 | - |
| - | - | 783.3 | 360.2 | - | - | 0 | - |
| - | - | 9908 | 362.2 | - | - | 0 | - |
| - | - | 2031 | 363.2 | - | - | 0 | - |
| - | - | 622.9 | 389.3 | - | - | 0 | - |
| - | - | 726.3 | 390.2 | - | - | 0 | - |
| - | - | 2427 | 411.3 | - | - | 0 | - |
| - | - | 1328 | 415.3 | - | - | 0 | - |
| - | - | 4651 | 416.3 | - | - | 0 | - |
| - | - | 976.4 | 417.3 | - | - | 0 | - |
| 14 | y | 4710 | 433.2 | 0.002549 | 5.883 | +1 | 4 |
| - | - | 4.867E+04 | 433.3 | - | - | 0 | - |
| - | - | 925.5 | 434.2 | - | - | 0 | - |
| - | - | 1.275E+04 | 434.3 | - | - | 0 | - |
| - | - | 813.4 | 435.3 | - | - | 0 | - |
| - | - | 687.9 | 441.2 | - | - | 0 | - |
| - | - | 976.7 | 443.2 | - | - | 0 | - |
| - | - | 5882 | 443.3 | - | - | 0 | - |
| - | - | 1358 | 444.3 | - | - | 0 | - |
| - | - | 5.003E+04 | 461.3 | - | - | 0 | - |
| - | - | 1.313E+04 | 462.3 | - | - | 0 | - |
| - | - | 1438 | 463.3 | - | - | 0 | - |
| 13 | y | 648.7 | 487.3 | 0.003581 | 7.349 | +1 | 5 |
| - | - | 846.3 | 498.2 | - | - | 0 | - |
| 13 | y | 8042 | 504.3 | 0.0002554 | 0.5065 | +1 | 5 |
| - | - | 1474 | 505.3 | - | - | 0 | - |
| - | - | 1965 | 530.3 | - | - | 0 | - |
| - | - | 673.4 | 531.3 | - | - | 0 | - |
| - | - | 1534 | 540.3 | - | - | 0 | - |
| - | - | 671 | 541.3 | - | - | 0 | - |
| - | - | 6040 | 558.3 | - | - | 0 | - |
| - | - | 2768 | 559.3 | - | - | 0 | - |
| - | - | 1332 | 582.8 | - | - | 0 | - |
| - | - | 1182 | 583.3 | - | - | 0 | - |
| - | - | 904.1 | 591.3 | - | - | 0 | - |
| 12 | y | 1204 | 615.3 | 0.003444 | 5.598 | +1 | 6 |
| 12 | z | 1204 | 616.3 | 0.001234 | 2.003 | +1 | 6 |
| - | - | 1569 | 617.3 | - | - | 0 | - |
| - | - | 683.6 | 617.8 | - | - | 0 | - |
| - | - | 1790 | 618.3 | - | - | 0 | - |
| - | - | 860.2 | 618.8 | - | - | 0 | - |
| - | - | 647.1 | 624.2 | - | - | 0 | - |
| - | - | 7896 | 626.8 | - | - | 0 | - |
| - | - | 732.6 | 627.3 | - | - | 0 | - |
| - | - | 4137 | 627.3 | - | - | 0 | - |
| - | - | 1098 | 627.8 | - | - | 0 | - |
| 12 | y | 4779 | 632.3 | 0.0007599 | 1.202 | +1 | 6 |
| - | - | 1195 | 633.3 | - | - | 0 | - |
| - | - | 682.8 | 638.3 | - | - | 0 | - |
| 5 | y | 4932 | 677.3 | 0.001583 | 2.336 | +2 | 13 |
| 5 | y | 6842 | 677.8 | 0.004204 | 6.202 | +2 | 13 |
| 5 | z | 2278 | 678.3 | 0.0001971 | 0.2906 | +2 | 13 |
| - | - | 843.3 | 678.8 | - | - | 0 | - |
| - | - | 818.1 | 679.3 | - | - | 0 | - |
| - | - | 1336 | 685.8 | - | - | 0 | - |
| 5 | y | 1.749E+05 | 686.3 | 0.0003896 | 0.5676 | +2 | 13 |
| - | - | 1.321E+05 | 686.8 | - | - | 0 | - |
| - | - | 3.905E+04 | 687.3 | - | - | 0 | - |
| - | - | 4504 | 687.8 | - | - | 0 | - |
| - | - | 917.5 | 700.3 | - | - | 0 | - |
| - | - | 2316 | 700.8 | - | - | 0 | - |
| - | - | 631.8 | 726.2 | - | - | 0 | - |
| 11 | z | 2690 | 729.4 | 0.001216 | 1.667 | +1 | 7 |
| - | - | 2322 | 730.4 | - | - | 0 | - |
| - | - | 1222 | 740.3 | - | - | 0 | - |
| - | - | 1839 | 742.4 | - | - | 0 | - |
| - | - | 614.2 | 743.4 | - | - | 0 | - |
| 11 | y | 3160 | 745.4 | 0.000192 | 0.2576 | +1 | 7 |
| 4 | y | 746.4 | 751.4 | 0.001125 | 1.497 | +2 | 14 |
| 14 | c | 680.5 | 756.9 | 0.00564 | 7.452 | +2 | 14 |
| 4 | y | 2.482E+04 | 759.9 | 0.0003623 | 0.4768 | +2 | 14 |
| - | - | 2.203E+04 | 760.4 | - | - | 0 | - |
| - | - | 1.05E+04 | 760.9 | - | - | 0 | - |
| - | - | 1327 | 761.4 | - | - | 0 | - |
| 3 | y | 857 | 807.4 | 0.001271 | 1.575 | +2 | 15 |
| 3 | y | 924.2 | 807.9 | 0.002245 | 2.778 | +2 | 15 |
| - | - | 963.6 | 812.9 | - | - | 0 | - |
| - | - | 780.6 | 813.4 | - | - | 0 | - |
| 3 | y | 5467 | 816.4 | 0.0008718 | 1.068 | +2 | 15 |
| - | - | 5920 | 816.9 | - | - | 0 | - |
| - | - | 1422 | 817.4 | - | - | 0 | - |
| - | - | 769.6 | 829.4 | - | - | 0 | - |
| - | - | 637.8 | 842.4 | - | - | 0 | - |
| - | - | 670.5 | 843.5 | - | - | 0 | - |
| - | - | 1383 | 847.9 | - | - | 0 | - |
| - | - | 884.8 | 848.4 | - | - | 0 | - |
| 16 | c | 5287 | 856.9 | 0.0004039 | 0.4713 | +2 | 16 |
| 10 | y | 5636 | 857.4 | 0.01143 | 13.33 | +1 | 8 |
| 2 | y | 3811 | 857.9 | 0.01198 | 13.96 | +2 | 16 |
| 10 | z | 8815 | 858.4 | 0.001286 | 1.499 | +1 | 8 |
| - | - | 6692 | 859.4 | - | - | 0 | - |
| - | - | 1921 | 860.4 | - | - | 0 | - |
| 2 | y | 966 | 866.9 | 0.002179 | 2.514 | +2 | 16 |
| - | - | 1483 | 867.4 | - | - | 0 | - |
| - | - | 2662 | 868.4 | - | - | 0 | - |
| - | - | 954.1 | 869.4 | - | - | 0 | - |
| 10 | y | 2507 | 874.5 | 0.0002868 | 0.328 | +1 | 8 |
| - | - | 1064 | 875.5 | - | - | 0 | - |
| - | - | 672.6 | 886.5 | - | - | 0 | - |
| - | - | 4173 | 907.5 | - | - | 0 | - |
| - | - | 4392 | 908 | - | - | 0 | - |
| - | - | 3810 | 908.5 | - | - | 0 | - |
| - | - | 924.2 | 909 | - | - | 0 | - |
| - | - | 1188 | 916 | - | - | 0 | - |
| - | - | 4.186E+04 | 916.5 | - | - | 0 | - |
| - | - | 5.352E+04 | 917 | - | - | 0 | - |
| - | - | 2.806E+04 | 917.5 | - | - | 0 | - |
| - | - | 3801 | 918 | - | - | 0 | - |
| - | - | 2307 | 939.4 | - | - | 0 | - |
| - | - | 752.3 | 958.5 | - | - | 0 | - |
| - | - | 1368 | 973.5 | - | - | 0 | - |
| - | - | 690.8 | 974.5 | - | - | 0 | - |
| 9 | z | 1.339E+04 | 987.5 | 0.0002582 | 0.2615 | +1 | 9 |
| - | - | 1.801E+04 | 988.5 | - | - | 0 | - |
| - | - | 7555 | 989.5 | - | - | 0 | - |
| - | - | 1447 | 990.5 | - | - | 0 | - |
| - | - | 975.9 | 1002 | - | - | 0 | - |
| 9 | y | 4120 | 1004 | 0.003634 | 3.622 | +1 | 9 |
| - | - | 2668 | 1005 | - | - | 0 | - |
| - | - | 1004 | 1053 | - | - | 0 | - |
| - | - | 1226 | 1054 | - | - | 0 | - |
| 8 | y | 1521 | 1073 | 0.01509 | 14.07 | +1 | 10 |
| 8 | y | 2222 | 1074 | 0.00114 | 1.062 | +1 | 10 |
| 8 | z | 1.369E+04 | 1075 | 0.000215 | 0.2001 | +1 | 10 |
| - | - | 3.778E+04 | 1076 | - | - | 0 | - |
| - | - | 1.793E+04 | 1077 | - | - | 0 | - |
| - | - | 4737 | 1078 | - | - | 0 | - |
| - | - | 1672 | 1088 | - | - | 0 | - |
| - | - | 1122 | 1089 | - | - | 0 | - |
| - | - | 4260 | 1090 | - | - | 0 | - |
| 8 | y | 1.53E+04 | 1091 | 0.001239 | 1.136 | +1 | 10 |
| - | - | 7008 | 1092 | - | - | 0 | - |
| - | - | 1821 | 1093 | - | - | 0 | - |
| - | - | 1058 | 1104 | - | - | 0 | - |
| 10 | c | 4143 | 1105 | 0.003456 | 3.129 | +1 | 10 |
| - | - | 2175 | 1106 | - | - | 0 | - |
| - | - | 1373 | 1117 | - | - | 0 | - |
| - | - | 2212 | 1118 | - | - | 0 | - |
| - | - | 1666 | 1119 | - | - | 0 | - |
| 7 | y | 1191 | 1160 | 0.01379 | 11.89 | +1 | 11 |
| 7 | z | 6702 | 1162 | 0.0004714 | 0.4059 | +1 | 11 |
| - | - | 1.535E+04 | 1163 | - | - | 0 | - |
| - | - | 7402 | 1164 | - | - | 0 | - |
| - | - | 2945 | 1165 | - | - | 0 | - |
| - | - | 938.9 | 1175 | - | - | 0 | - |
| - | - | 1617 | 1177 | - | - | 0 | - |
| 7 | y | 9041 | 1178 | 0.0006745 | 0.5728 | +1 | 11 |
| - | - | 4829 | 1179 | - | - | 0 | - |
| - | - | 919.3 | 1180 | - | - | 0 | - |
| - | - | 3573 | 1182 | - | - | 0 | - |
| - | - | 2744 | 1183 | - | - | 0 | - |
| - | - | 3317 | 1201 | - | - | 0 | - |
| - | - | 2126 | 1202 | - | - | 0 | - |
| - | - | 5502 | 1217 | - | - | 0 | - |
| 11 | c | 1.26E+04 | 1218 | 0.002315 | 1.901 | +1 | 11 |
| - | - | 6445 | 1219 | - | - | 0 | - |
| - | - | 1428 | 1220 | - | - | 0 | - |
| - | - | 868.7 | 1235 | - | - | 0 | - |
| - | - | 5646 | 1253 | - | - | 0 | - |
| - | - | 3935 | 1254 | - | - | 0 | - |
| - | - | 1351 | 1255 | - | - | 0 | - |
| 6 | y | 2.08E+04 | 1275 | 0.0002157 | 0.1692 | +1 | 12 |
| - | - | 1.467E+04 | 1276 | - | - | 0 | - |
| - | - | 4553 | 1277 | - | - | 0 | - |
| - | - | 1177 | 1301 | - | - | 0 | - |
| - | - | 1059 | 1302 | - | - | 0 | - |
| - | - | 1491 | 1311 | - | - | 0 | - |
| - | - | 1313 | 1327 | - | - | 0 | - |
| 12 | c | 5886 | 1329 | 0.006755 | 5.084 | +1 | 12 |
| - | - | 3858 | 1330 | - | - | 0 | - |
| - | - | 8531 | 1345 | - | - | 0 | - |
| 12 | c | 3.131E+04 | 1346 | 0.001688 | 1.254 | +1 | 12 |
| - | - | 2.025E+04 | 1347 | - | - | 0 | - |
| - | - | 6913 | 1348 | - | - | 0 | - |
| - | - | 1168 | 1349 | - | - | 0 | - |
| 5 | y | 2871 | 1354 | 0.002864 | 2.116 | +1 | 13 |
| 5 | y | 4070 | 1355 | 0.006162 | 4.549 | +1 | 13 |
| 5 | z | 2809 | 1356 | 0.001633 | 1.205 | +1 | 13 |
| - | - | 1224 | 1357 | - | - | 0 | - |
| - | - | 1100 | 1358 | - | - | 0 | - |
| - | - | 1622 | 1360 | - | - | 0 | - |
| - | - | 1158 | 1361 | - | - | 0 | - |
| - | - | 1073 | 1370 | - | - | 0 | - |
| - | - | 1.253E+04 | 1371 | - | - | 0 | - |
| 5 | y | 2.132E+05 | 1372 | 0.0011 | 0.8017 | +1 | 13 |
| - | - | 1.546E+05 | 1373 | - | - | 0 | - |
| - | - | 5.562E+04 | 1374 | - | - | 0 | - |
| - | - | 5589 | 1375 | - | - | 0 | - |
| - | - | 990.3 | 1398 | - | - | 0 | - |
| - | - | 4628 | 1399 | - | - | 0 | - |
| 13 | c | 7172 | 1400 | 0.02727 | 19.48 | +1 | 13 |
| - | - | 5254 | 1401 | - | - | 0 | - |
| - | - | 3.884E+04 | 1402 | - | - | 0 | - |
| - | - | 2.91E+04 | 1403 | - | - | 0 | - |
| - | - | 8795 | 1404 | - | - | 0 | - |
| - | - | 1226 | 1405 | - | - | 0 | - |
| - | - | 2.526E+04 | 1416 | - | - | 0 | - |
| 13 | c | 3.631E+04 | 1417 | 0.00389 | 2.745 | +1 | 13 |
| - | - | 1.923E+04 | 1418 | - | - | 0 | - |
| - | - | 5443 | 1419 | - | - | 0 | - |
| - | - | 1606 | 1431 | - | - | 0 | - |
| - | - | 1212 | 1432 | - | - | 0 | - |
| - | - | 946.9 | 1474 | - | - | 0 | - |
| - | - | 772 | 1475 | - | - | 0 | - |
| - | - | 3454 | 1487 | - | - | 0 | - |
| - | - | 3080 | 1488 | - | - | 0 | - |
| - | - | 1226 | 1489 | - | - | 0 | - |
| 4 | z | 1.618E+04 | 1503 | 0.0009848 | 0.6554 | +1 | 14 |
| - | - | 1.6E+04 | 1504 | - | - | 0 | - |
| - | - | 7453 | 1505 | - | - | 0 | - |
| - | - | 813 | 1506 | - | - | 0 | - |
| 14 | c | 1694 | 1514 | 0.007206 | 4.761 | +1 | 14 |
| - | - | 1996 | 1515 | - | - | 0 | - |
| - | - | 1292 | 1516 | - | - | 0 | - |
| 4 | y | 2.495E+04 | 1519 | 0.0006659 | 0.4385 | +1 | 14 |
| - | - | 1.943E+04 | 1520 | - | - | 0 | - |
| - | - | 8755 | 1521 | - | - | 0 | - |
| - | - | 903.9 | 1522 | - | - | 0 | - |
| - | - | 2481 | 1530 | - | - | 0 | - |
| 14 | c | 6.02E+04 | 1531 | 0.001895 | 1.238 | +1 | 14 |
| - | - | 5.318E+04 | 1532 | - | - | 0 | - |
| - | - | 1180 | 1532 | - | - | 0 | - |
| - | - | 2.049E+04 | 1533 | - | - | 0 | - |
| - | - | 2454 | 1534 | - | - | 0 | - |
| - | - | 1241 | 1544 | - | - | 0 | - |
| - | - | 1266 | 1545 | - | - | 0 | - |
| - | - | 1624 | 1572 | - | - | 0 | - |
| - | - | 1713 | 1573 | - | - | 0 | - |
| - | - | 828.1 | 1574 | - | - | 0 | - |
| 3 | y | 1584 | 1614 | 0.002266 | 1.404 | +1 | 15 |
| - | - | 5838 | 1615 | - | - | 0 | - |
| 3 | z | 1.276E+04 | 1616 | 0.02567 | 15.89 | +1 | 15 |
| - | - | 8493 | 1617 | - | - | 0 | - |
| - | - | 3485 | 1618 | - | - | 0 | - |
| 3 | y | 7577 | 1632 | 0.003919 | 2.402 | +1 | 15 |
| - | - | 6340 | 1633 | - | - | 0 | - |
| - | - | 2994 | 1634 | - | - | 0 | - |
| - | - | 911.7 | 1645 | - | - | 0 | - |
| - | - | 1520 | 1646 | - | - | 0 | - |
| - | - | 1375 | 1647 | - | - | 0 | - |
| - | - | 1500 | 1655 | - | - | 0 | - |
| - | - | 1590 | 1656 | - | - | 0 | - |
| 15 | c | 2.865E+04 | 1659 | 0.002986 | 1.8 | +1 | 15 |
| - | - | 2.877E+04 | 1660 | - | - | 0 | - |
| - | - | 1.344E+04 | 1661 | - | - | 0 | - |
| - | - | 3736 | 1662 | - | - | 0 | - |
| - | - | 2237 | 1663 | - | - | 0 | - |
| - | - | 911.3 | 1671 | - | - | 0 | - |
| - | - | 3313 | 1672 | - | - | 0 | - |
| - | - | 4342 | 1673 | - | - | 0 | - |
| - | - | 2034 | 1674 | - | - | 0 | - |
| - | - | 8619 | 1686 | - | - | 0 | - |
| - | - | 1.052E+04 | 1687 | - | - | 0 | - |
| - | - | 6526 | 1688 | - | - | 0 | - |
| - | - | 2134 | 1689 | - | - | 0 | - |
| - | - | 1120 | 1690 | - | - | 0 | - |
| - | - | 899.9 | 1698 | - | - | 0 | - |
| 2 | z | 1519 | 1699 | 0.009551 | 5.622 | +1 | 16 |
| 2 | z | 1538 | 1700 | 0.02651 | 15.6 | +1 | 16 |
| 2 | y | 1.051E+04 | 1715 | 0.002459 | 1.434 | +1 | 16 |
| 2 | y | 1.708E+04 | 1716 | 0.01536 | 8.95 | +1 | 16 |
| 2 | z | 6.763E+04 | 1717 | 0.0004514 | 0.2629 | +1 | 16 |
| - | - | 6.178E+04 | 1718 | - | - | 0 | - |
| - | - | 3.052E+04 | 1719 | - | - | 0 | - |
| - | - | 5840 | 1720 | - | - | 0 | - |
| - | - | 1118 | 1727 | - | - | 0 | - |
| - | - | 1185 | 1728 | - | - | 0 | - |
| - | - | 1052 | 1729 | - | - | 0 | - |
| 16 | c | 4.429E+04 | 1730 | 0.0006712 | 0.388 | +1 | 16 |
| - | - | 4.433E+04 | 1731 | - | - | 0 | - |
| - | - | 3.094E+04 | 1732 | - | - | 0 | - |
| - | - | 1.232E+04 | 1733 | - | - | 0 | - |
| - | - | 5110 | 1734 | - | - | 0 | - |
| - | - | 1444 | 1735 | - | - | 0 | - |
| - | - | 1058 | 1742 | - | - | 0 | - |
| - | - | 1176 | 1743 | - | - | 0 | - |
| - | - | 2857 | 1744 | - | - | 0 | - |
| - | - | 5191 | 1745 | - | - | 0 | - |
| - | - | 5771 | 1746 | - | - | 0 | - |
| - | - | 1763 | 1747 | - | - | 0 | - |
| - | - | 877.4 | 1754 | - | - | 0 | - |
| - | - | 1267 | 1759 | - | - | 0 | - |
| - | - | 4049 | 1760 | - | - | 0 | - |
| - | - | 7082 | 1761 | - | - | 0 | - |
| - | - | 9982 | 1762 | - | - | 0 | - |
| - | - | 6237 | 1763 | - | - | 0 | - |
| - | - | 2010 | 1764 | - | - | 0 | - |
| - | - | 5767 | 1769 | - | - | 0 | - |
| - | - | 7566 | 1770 | - | - | 0 | - |
| - | - | 5678 | 1771 | - | - | 0 | - |
| - | - | 2592 | 1772 | - | - | 0 | - |
| - | - | 1734 | 1773 | - | - | 0 | - |
| - | - | 4366 | 1774 | - | - | 0 | - |
| - | - | 5018 | 1775 | - | - | 0 | - |
| - | - | 2013 | 1776 | - | - | 0 | - |
| - | - | 7521 | 1777 | - | - | 0 | - |
| - | - | 5835 | 1778 | - | - | 0 | - |
| - | - | 2941 | 1779 | - | - | 0 | - |
| - | - | 1631 | 1786 | - | - | 0 | - |
| - | - | 1.314E+04 | 1787 | - | - | 0 | - |
| - | - | 8.317E+04 | 1788 | - | - | 0 | - |
| - | - | 7.241E+04 | 1789 | - | - | 0 | - |
| - | - | 3.9E+04 | 1790 | - | - | 0 | - |
| - | - | 7288 | 1791 | - | - | 0 | - |
| - | - | 973 | 1792 | - | - | 0 | - |
| - | - | 2736 | 1798 | - | - | 0 | - |
| - | - | 2590 | 1799 | - | - | 0 | - |
| - | - | 1049 | 1800 | - | - | 0 | - |
| - | - | 995.1 | 1803 | - | - | 0 | - |
| - | - | 6010 | 1805 | - | - | 0 | - |
| - | - | 5569 | 1806 | - | - | 0 | - |
| - | - | 3837 | 1807 | - | - | 0 | - |
| - | - | 2041 | 1814 | - | - | 0 | - |
| - | - | 1.556E+04 | 1815 | - | - | 0 | - |
| - | - | 1.133E+05 | 1816 | - | - | 0 | - |
| - | - | 1.161E+05 | 1817 | - | - | 0 | - |
| - | - | 5.682E+04 | 1818 | - | - | 0 | - |
| - | - | 9360 | 1819 | - | - | 0 | - |
| - | - | 2044 | 1830 | - | - | 0 | - |
| - | - | 7494 | 1831 | - | - | 0 | - |
| - | - | 1.916E+05 | 1832 | - | - | 0 | - |
| - | - | 5.258E+05 | 1833 | - | - | 0 | - |
| - | - | 4.417E+05 | 1834 | - | - | 0 | - |
| - | - | 2.031E+05 | 1835 | - | - | 0 | - |
| - | - | 2.835E+04 | 1836 | - | - | 0 | - |

m/z Charge Intensity FragmentType MassShift Position
120.08103942871094 0 1173.1749
128.50204467773438 0 348.41776
129.1024627685547 0 2003.5388
133.2472381591797 0 399.48773
137.53005981445312 0 415.55002
144.31802368164062 0 407.74838
148.8440399169922 0 505.65167
148.8804473876953 0 445.4587
148.88751220703125 0 820.9501
148.89492797851562 0 615.0221
148.9020538330078 0 704.6363
148.90882873535156 0 761.4099
148.91639709472656 0 946.62415
148.9235076904297 0 1224.1974
148.9306640625 0 1320.6179
148.93804931640625 0 3118.6348
148.94580078125 0 5299.3794
148.9622802734375 0 3960.2458
148.97000122070312 0 1749.0684
148.97702026367188 0 1400.8641
148.98428344726562 0 932.9145
148.99163818359375 0 628.9628
148.99876403808594 0 508.8083
149.00621032714844 0 392.7972
149.02740478515625 0 430.28592
149.0486602783203 0 562.0084
151.73284912109375 0 446.39694
160.6261749267578 0 552.3387
173.12855529785156 0 1460.713
174.2852325439453 0 435.06256
183.1129913330078 0 2883.8618
201.1233367919922 0 6664.064
201.1490936279297 0 468.962
215.13938903808594 0 1447.6464
217.13414001464844 0 765.38885
233.164794921875 0 4181.551
234.16940307617188 0 544.3715
243.14529418945312 0 1469.9634
245.1285400390625 0 988.2372
251.17498779296875 0 1169.3917
261.1595458984375 0 2939.069
262.1641540527344 0 811.64075
280.1654968261719 0 713.757
282.1439514160156 0 869.0917
296.1971130371094 0 62800.32
297.2003479003906 0 10070.6
298.1754150390625 0 667.5708
298.203369140625 0 1047.1204
303.1702880859375 0 1305.828
314.20770263671875 0 35669.72
315.210693359375 0 4601.3657
319.1975402832031 0 3561.9395 y 14
330.1809387207031 0 1146.1915
346.17620849609375 0 2946.4802
348.1919860839844 0 2537.94
349.1971435546875 0 1005.376
360.2276306152344 0 783.30396
362.2074890136719 0 9908.15
363.2110595703125 0 2031.1508
389.2547912597656 0 622.9059
390.2016906738281 0 726.3138
411.2601318359375 0 2427.3608
415.2701110839844 0 1328.0017
416.2552795410156 0 4651.096
417.2539367675781 0 976.44617
433.2430725097656 0 4709.792 y 13
433.281494140625 0 48670.93
434.2484436035156 0 925.49786
434.28436279296875 0 12751.508
435.2884826660156 0 813.4176
441.2475891113281 0 687.88477
443.23199462890625 0 976.7081
443.2660217285156 0 5882.1396
444.2674560546875 0 1358.2944
461.2764587402344 0 50025.82
462.27978515625 0 13130.8955
463.28216552734375 0 1437.5559
487.2546691894531 0 648.7444 y Ammonia loss 12
498.2213439941406 0 846.2757
504.27789306640625 0 8042.2534 y 12
505.2795715332031 0 1473.7656
530.3340454101562 0 1965.2592
531.3367919921875 0 673.3984
540.318359375 0 1533.7211
541.3240356445312 0 670.9956
558.3289184570312 0 6039.702
559.3325805664062 0 2767.8003
582.7825927734375 0 1331.927
583.2838745117188 0 1182.3583
591.2950439453125 0 904.1444
615.3131103515625 0 1203.9851 y Ammonia loss 11
616.3187255859375 0 1203.8351 z 11
617.3209838867188 0 1569.0746
617.8088989257812 0 683.6445
618.3034057617188 0 1789.8231
618.8060913085938 0 860.16327
624.1806640625 0 647.0506
626.8126220703125 0 7896.4365
627.2627563476562 0 732.5745
627.3131713867188 0 4136.828
627.8189086914062 0 1098.3597
632.3369750976562 0 4778.5547 y 11
633.3360595703125 0 1194.9957
638.3176879882812 0 682.7536
677.3374633789062 0 4931.5083 y Water loss 4
677.8320922851562 0 6841.8096 y Ammonia loss 4
678.3316040039062 0 2278.269 z 4
678.8262939453125 0 843.31824
679.3326416015625 0 818.0688
685.8348388671875 0 1336.4338
686.341552734375 0 174873.19 y 4
686.8427124023438 0 132054.31
687.3436889648438 0 39047.2
687.8453979492188 0 4503.552
700.341796875 0 917.4844
700.847900390625 0 2315.9858
726.2018432617188 0 631.82825
729.4027709960938 0 2690.3403 z 10
730.4055786132812 0 2321.7134
740.345947265625 0 1221.7162
742.4141845703125 0 1839.4009
743.4266967773438 0 614.17706
745.4204711914062 0 3160.1433 y 10
751.3632202148438 0 746.408 y Ammonia loss 3
756.8939208984375 0 680.5047 c Water loss 13
759.875732421875 0 24824.135 y 3
760.376708984375 0 22025.72
760.8779907226562 0 10496.774
761.3809204101562 0 1327.0935
807.4133911132812 0 856.9885 y Water loss 2
807.9063720703125 0 924.212 y Ammonia loss 2
812.9066162109375 0 963.6238
813.4092407226562 0 780.6206
816.4182739257812 0 5467.206 y 2
816.9180908203125 0 5920.475
817.4180297851562 0 1422.4723
829.4469604492188 0 769.5786
842.3838500976562 0 637.7583
843.4528198242188 0 670.47876
847.938720703125 0 1382.6917
848.438720703125 0 884.77167
856.9459228515625 0 5286.6094 c Ammonia loss 15
857.44775390625 0 5635.787 y Ammonia loss 9
857.9479370117188 0 3810.7117 y Water loss 1
858.4454345703125 0 8814.888 z 9
859.4493408203125 0 6691.59
860.448974609375 0 1920.9443
866.9434204101562 0 966.00085 y 1
867.4412231445312 0 1482.547
868.4036254882812 0 2661.527
869.4061889648438 0 954.099
874.4625854492188 0 2507.1235 y 9
875.4637451171875 0 1064.1387
886.4584350585938 0 672.598
907.4702758789062 0 4172.7046
907.9693603515625 0 4391.8555
908.4714965820312 0 3809.541
908.9714965820312 0 924.1878
915.967529296875 0 1188.3463
916.4755249023438 0 41858.062
916.9766235351562 0 53523.72
917.4774169921875 0 28063.314
917.977783203125 0 3800.969
939.4418334960938 0 2307.116
958.4860229492188 0 752.26117
973.49267578125 0 1367.7882
974.4971923828125 0 690.7649
987.4869995117188 0 13385.599 z 8
988.4931640625 0 18010.994
989.496337890625 0 7555.472
990.5030517578125 0 1446.5286
1002.4930419921875 0 975.89813
1003.5018310546875 0 4119.6265 y 8
1004.5029296875 0 2667.9983
1053.48193359375 0 1004.46265
1054.48193359375 0 1225.9421
1072.5118408203125 0 1520.8933 y Water loss 7
1073.5120849609375 0 2221.7114 y Ammonia loss 7
1074.5185546875 0 13690.953 z 7
1075.5260009765625 0 37781.08
1076.5286865234375 0 17931.67
1077.531982421875 0 4736.974
1087.5247802734375 0 1672.1361
1088.5380859375 0 1121.7858
1089.5318603515625 0 4260.4126
1090.5362548828125 0 15301.0625 y 7
1091.5391845703125 0 7007.585
1092.543701171875 0 1821.4319
1103.5570068359375 0 1057.8566
1104.5537109375 0 4142.931 c 9
1105.5645751953125 0 2174.6602
1116.534912109375 0 1372.6525
1117.5345458984375 0 2211.5322
1118.5360107421875 0 1666.4504
1159.545166015625 0 1191.3713 y Water loss 6
1161.55126953125 0 6702.196 z 6
1162.5567626953125 0 15345.081
1163.56005859375 0 7402.302
1164.558837890625 0 2944.9253
1174.64404296875 0 938.8995
1176.5616455078125 0 1616.9663
1177.56884765625 0 9041.163 y 6
1178.5728759765625 0 4828.9893
1179.5733642578125 0 919.3293
1181.5811767578125 0 3573.4473
1182.5797119140625 0 2744.0847
1200.612548828125 0 3316.7402
1201.62158203125 0 2126.2007
1216.63232421875 0 5502.4966
1217.638916015625 0 12598.07 c 10
1218.6441650390625 0 6444.8784
1219.6387939453125 0 1427.9846
1234.5989990234375 0 868.683
1252.6143798828125 0 5645.713
1253.619140625 0 3935.1467
1254.6142578125 0 1350.757
1274.6220703125 0 20804.207 y 5
1275.62353515625 0 14672.432
1276.6275634765625 0 4553.432
1300.645263671875 0 1177.4052
1301.6815185546875 0 1059.333
1310.657958984375 0 1491.153
1326.660888671875 0 1312.6725
1328.66650390625 0 5886.0933 c Ammonia loss 11
1329.6702880859375 0 3857.7227
1344.6910400390625 0 8531.476
1345.6981201171875 0 31309.768 c 11
1346.700927734375 0 20249.21
1347.7052001953125 0 6913.3926
1348.7027587890625 0 1168.1542
1353.66162109375 0 2871.2773 y Water loss 4
1354.6546630859375 0 4070.2036 y Ammonia loss 4
1355.657958984375 0 2809.0422 z 4
1356.65234375 0 1223.9213
1357.678466796875 0 1099.7977
1359.67431640625 0 1622.151
1360.677001953125 0 1157.6929
1369.6683349609375 0 1072.871
1370.6658935546875 0 12532.973
1371.6739501953125 0 213213.8 y 4
1372.6767578125 0 154566.06
1373.6793212890625 0 55618.25
1374.683349609375 0 5588.761
1397.7078857421875 0 990.2998
1398.6673583984375 0 4628.3438
1399.68310546875 0 7172.42 c Ammonia loss 12
1400.685546875 0 5253.66
1401.7003173828125 0 38836.04
1402.702880859375 0 29103.426
1403.7061767578125 0 8794.851
1404.7015380859375 0 1225.7474
1415.7279052734375 0 25258.438
1416.7330322265625 0 36311.883 c 12
1417.736572265625 0 19233.176
1418.7412109375 0 5442.641
1430.6954345703125 0 1606.4103
1431.697998046875 0 1212.4272
1473.7379150390625 0 946.9007
1474.7286376953125 0 772.0498
1486.7640380859375 0 3453.7827
1487.7672119140625 0 3079.959
1488.7608642578125 0 1226.2584
1502.7237548828125 0 16184.89 z 3
1503.7249755859375 0 16000.2295
1504.72509765625 0 7453.118
1505.7403564453125 0 812.98627
1513.74609375 0 1694.0989 c Ammonia loss 13
1514.74853515625 0 1995.9083
1515.762451171875 0 1291.8132
1518.7427978515625 0 24947.582 y 3
1519.7454833984375 0 19434.965
1520.7452392578125 0 8754.95
1521.740478515625 0 903.9313
1529.7703857421875 0 2480.9707
1530.7779541015625 0 60198.03 c 13
1531.77978515625 0 53178.383
1532.0155029296875 0 1180.245
1532.78369140625 0 20487.48
1533.7822265625 0 2453.5273
1543.7818603515625 0 1241.1144
1544.7894287109375 0 1266.1455
1571.816162109375 0 1624.1198
1572.8082275390625 0 1712.5027
1573.7852783203125 0 828.0782
1613.814697265625 0 1584.1075 y Water loss 2
1614.850830078125 0 5837.7026
1615.83447265625 0 12755.254 z 2
1616.82666015625 0 8493.114
1617.8255615234375 0 3484.6228
1631.8236083984375 0 7577.071 y 2
1632.828857421875 0 6339.6094
1633.8388671875 0 2994.3726
1644.832275390625 0 911.72205
1645.845703125 0 1520.1056
1646.83056640625 0 1374.8208
1654.834228515625 0 1500.4138
1655.8636474609375 0 1590.2681
1658.871826171875 0 28646.652 c 14
1659.8746337890625 0 28767.848
1660.8629150390625 0 13436.354
1661.810546875 0 3735.6677
1662.79296875 0 2237.3308
1670.878662109375 0 911.3019
1671.8465576171875 0 3313.01
1672.840576171875 0 4341.784
1673.84130859375 0 2034.2279
1685.8978271484375 0 8618.526
1686.899658203125 0 10519.215
1687.90380859375 0 6525.84
1688.91162109375 0 2134.176
1689.9161376953125 0 1119.9773
1697.8375244140625 0 899.93964
1698.85546875 0 1518.81 z Water loss 1
1699.8564453125 0 1537.5518 z Ammonia loss 1
1714.8621826171875 0 10508.114 y Water loss 1
1715.864013671875 0 17075.617 y Ammonia loss 1
1716.85693359375 0 67633.914 z 1
1717.8582763671875 0 61780
1718.863037109375 0 30522.33
1719.865966796875 0 5839.7466
1726.9068603515625 0 1117.8395
1727.937255859375 0 1185.3096
1728.9039306640625 0 1051.6825
1729.9112548828125 0 44291.12 c 15
1730.912841796875 0 44331.734
1731.9180908203125 0 30935.285
1732.92236328125 0 12317.654
1733.928955078125 0 5110.4
1734.922607421875 0 1444.3655
1741.8746337890625 0 1057.804
1742.875244140625 0 1176.2382
1743.9053955078125 0 2857.0342
1744.8968505859375 0 5190.8633
1745.8897705078125 0 5771.256
1746.899658203125 0 1763.1917
1753.937744140625 0 877.3529
1758.9190673828125 0 1266.7766
1759.8890380859375 0 4048.8425
1760.9150390625 0 7082.457
1761.91552734375 0 9982.237
1762.916748046875 0 6237.128
1763.9007568359375 0 2009.6387
1768.9342041015625 0 5767.029
1769.9356689453125 0 7565.561
1770.93359375 0 5678.398
1771.9354248046875 0 2591.5461
1772.9190673828125 0 1734.3997
1773.9112548828125 0 4366.428
1774.9195556640625 0 5018.023
1775.90625 0 2013.3594
1776.888427734375 0 7521.255
1777.8890380859375 0 5834.645
1778.8941650390625 0 2941.468
1785.9158935546875 0 1631.3887
1786.9423828125 0 13137.733
1787.931640625 0 83167.414
1788.933837890625 0 72408.84
1789.9337158203125 0 38995.566
1790.9276123046875 0 7288.181
1791.9075927734375 0 973.0036
1797.914794921875 0 2736.1782
1798.9149169921875 0 2590.01
1799.8936767578125 0 1048.9102
1802.947998046875 0 995.0648
1804.955322265625 0 6010.151
1805.95654296875 0 5568.5166
1806.9586181640625 0 3836.7844
1813.905517578125 0 2040.5425
1814.9344482421875 0 15560.187
1815.9268798828125 0 113342.02
1816.9281005859375 0 116116.59
1817.9283447265625 0 56823.062
1818.929443359375 0 9360.454
1829.9276123046875 0 2043.959
1830.9296875 0 7493.6367
1831.9432373046875 0 191630.16
1832.949462890625 0 525769.4
1833.952392578125 0 441694.66
1834.9552001953125 0 203091.42
1835.9554443359375 0 28350.594

Spectrum Details

|  |  |
| --- | --- |
| Matched peaks? Matched peaksThe total absolute number of peaks matched. Additionally in brackets the total fraction of peaks matched and the total number of peaks is shown. | 59 (15.53% of 380) |
| FDR? FDRThe false discovery rate estimated for this peptide. It is calculated by matching all theoretical fragments with a non-integer shift with the raw peaks for this spectrum. This is done with 40 different shifts. The resulting percentage is the average number of annotated peaks over the number of annotated peaks with the correct spectrum. | 0.56% |
| Satellite FDR? Satellite FDRSee the FDR for details on its calculation. This satellite ion specific FDR only contains the satellite ions (d/w) for I/L/J positions. | - |
| PSM Score? PSM ScoreThe PSM Score as given by Hecklib to this annotated spectrum. It is shown with three significant figures. | 574 |

## Spectrum 7814? Spectrum 7814 The raw spectrum of this peptide as annotated by Hecklib. The fragments are coloured according to ion type (see legend). Any peaks with a star '\*' as text can be hovered over to see the full details, first the ion type second the mass shift type. By hovering over the amino acids in the peptide or ions in the legend the corresponding peaks are highlighted. By toggling the 'Unassigned' label you can turn the background (unassigned) peaks on or off in the plot. By updating the slider in the Ion legend you can update the spectrum to only show the top X% of the peaks with labels. The top X% means any peak that is within X% of the highest intensity. By dragging in the spectrum you can zoom in to a specific part of the spectrum and use 'Zoom Out' to get back to the original zoom level. The annotation of the spectrum is based on the given sequence in the peptides file and is done with different software so inconsistencies are likely. The peaks are annotated based on the given sequence, with 20 ppm tolerance.

Copy Data

### Spectrum 7814 (TSV)

#### Preview

```
Loading example...
```

*Click on the button to copy the data to your clipboard.*

Mz MinMz MaxIntensity Max

WidthHeightPeptide font sizePeptide stroke widthSpectrum font sizeSpectrum stroke widthCompact peptide

Ion legend

wxyz

abcd

OtherUnassignedIonChargePositionShow for top:%

VTJFPPSSEEJQANKAT

02.88e+55.77e+58.65e+51.15e+6

Zoom Out

y+13y+14y+15y+15y+16y+16z+16y+16y+212c+212y+213y+213z+213y+213c+213z+17y+17y+214y+214z+214c+214y+214z+215y+215y+215c+216y+18y+216y+216y+216y+18c+19z+19y+19y+110y+110z+110y+110c+110y+111z+111y+111c+111y+112y+112y+112c+112c+112c+112y+113y+113z+113y+113c+113y+114y+114z+114c+114y+114c+114y+115z+115y+115c+115z+116z+116y+116y+116z+116c+116

047194214131885

Fragment Matches Table

Show background peaks

| Position | Ion type | Intensity | mz Theoretical | mz Error (Th) | mz Error (ppm) | Charge | Series Number |
| --- | --- | --- | --- | --- | --- | --- | --- |
| - | - | 2824 | 120.1 | - | - | 0 | - |
| - | - | 466.3 | 120.1 | - | - | 0 | - |
| - | - | 580.5 | 125.9 | - | - | 0 | - |
| - | - | 5050 | 129.1 | - | - | 0 | - |
| - | - | 542.4 | 129.8 | - | - | 0 | - |
| - | - | 1127 | 148.9 | - | - | 0 | - |
| - | - | 751.4 | 148.9 | - | - | 0 | - |
| - | - | 842.1 | 148.9 | - | - | 0 | - |
| - | - | 1058 | 148.9 | - | - | 0 | - |
| - | - | 1418 | 148.9 | - | - | 0 | - |
| - | - | 1742 | 148.9 | - | - | 0 | - |
| - | - | 3121 | 148.9 | - | - | 0 | - |
| - | - | 5427 | 148.9 | - | - | 0 | - |
| - | - | 7283 | 149 | - | - | 0 | - |
| - | - | 4789 | 149 | - | - | 0 | - |
| - | - | 2203 | 149 | - | - | 0 | - |
| - | - | 1756 | 149 | - | - | 0 | - |
| - | - | 1901 | 149 | - | - | 0 | - |
| - | - | 1221 | 149 | - | - | 0 | - |
| - | - | 1026 | 149 | - | - | 0 | - |
| - | - | 687 | 149 | - | - | 0 | - |
| - | - | 1019 | 149 | - | - | 0 | - |
| - | - | 603.7 | 149 | - | - | 0 | - |
| - | - | 773.3 | 149.1 | - | - | 0 | - |
| - | - | 3117 | 173.1 | - | - | 0 | - |
| - | - | 5571 | 183.1 | - | - | 0 | - |
| - | - | 818.8 | 185.3 | - | - | 0 | - |
| - | - | 651.7 | 193.2 | - | - | 0 | - |
| - | - | 1.481E+04 | 201.1 | - | - | 0 | - |
| - | - | 2880 | 215.1 | - | - | 0 | - |
| - | - | 2189 | 217.1 | - | - | 0 | - |
| - | - | 1084 | 219.1 | - | - | 0 | - |
| - | - | 1239 | 226.1 | - | - | 0 | - |
| - | - | 9342 | 233.2 | - | - | 0 | - |
| - | - | 2739 | 243.1 | - | - | 0 | - |
| - | - | 2543 | 245.1 | - | - | 0 | - |
| - | - | 1903 | 247.1 | - | - | 0 | - |
| - | - | 4450 | 251.2 | - | - | 0 | - |
| - | - | 961.9 | 252.2 | - | - | 0 | - |
| - | - | 863.2 | 253.2 | - | - | 0 | - |
| - | - | 6935 | 261.2 | - | - | 0 | - |
| - | - | 1320 | 262.2 | - | - | 0 | - |
| - | - | 954.1 | 269.2 | - | - | 0 | - |
| - | - | 919.8 | 273.1 | - | - | 0 | - |
| - | - | 1940 | 280.2 | - | - | 0 | - |
| - | - | 2427 | 282.1 | - | - | 0 | - |
| - | - | 1.52E+05 | 296.2 | - | - | 0 | - |
| - | - | 1817 | 297.2 | - | - | 0 | - |
| - | - | 2.459E+04 | 297.2 | - | - | 0 | - |
| - | - | 2541 | 298.2 | - | - | 0 | - |
| - | - | 2127 | 298.2 | - | - | 0 | - |
| - | - | 2575 | 303.2 | - | - | 0 | - |
| - | - | 941.5 | 303.7 | - | - | 0 | - |
| - | - | 2994 | 314.2 | - | - | 0 | - |
| - | - | 7.951E+04 | 314.2 | - | - | 0 | - |
| - | - | 1311 | 315.2 | - | - | 0 | - |
| - | - | 1.339E+04 | 315.2 | - | - | 0 | - |
| - | - | 1461 | 317.2 | - | - | 0 | - |
| 15 | y | 9216 | 319.2 | 0.0004321 | 1.354 | +1 | 3 |
| - | - | 3527 | 330.2 | - | - | 0 | - |
| - | - | 1634 | 344.2 | - | - | 0 | - |
| - | - | 6724 | 346.2 | - | - | 0 | - |
| - | - | 1848 | 347.2 | - | - | 0 | - |
| - | - | 5726 | 348.2 | - | - | 0 | - |
| - | - | 2071 | 349.2 | - | - | 0 | - |
| - | - | 2171 | 360.2 | - | - | 0 | - |
| - | - | 2.289E+04 | 362.2 | - | - | 0 | - |
| - | - | 3721 | 363.2 | - | - | 0 | - |
| - | - | 1391 | 385.2 | - | - | 0 | - |
| - | - | 1388 | 390.2 | - | - | 0 | - |
| - | - | 1288 | 393.3 | - | - | 0 | - |
| - | - | 2124 | 398.2 | - | - | 0 | - |
| - | - | 7002 | 411.3 | - | - | 0 | - |
| - | - | 1157 | 412.3 | - | - | 0 | - |
| - | - | 2935 | 415.3 | - | - | 0 | - |
| - | - | 8979 | 416.3 | - | - | 0 | - |
| - | - | 2223 | 417.3 | - | - | 0 | - |
| - | - | 894.4 | 425.1 | - | - | 0 | - |
| 14 | y | 1.261E+04 | 433.2 | 0.002366 | 5.46 | +1 | 4 |
| - | - | 1.178E+05 | 433.3 | - | - | 0 | - |
| - | - | 1804 | 434.2 | - | - | 0 | - |
| - | - | 3.192E+04 | 434.3 | - | - | 0 | - |
| - | - | 2930 | 435.3 | - | - | 0 | - |
| - | - | 970.1 | 442.2 | - | - | 0 | - |
| - | - | 2358 | 443.2 | - | - | 0 | - |
| - | - | 1.437E+04 | 443.3 | - | - | 0 | - |
| - | - | 3252 | 444.3 | - | - | 0 | - |
| - | - | 1045 | 445.2 | - | - | 0 | - |
| - | - | 1.271E+05 | 461.3 | - | - | 0 | - |
| - | - | 2.892E+04 | 462.3 | - | - | 0 | - |
| - | - | 3014 | 463.3 | - | - | 0 | - |
| 13 | y | 1074 | 487.3 | 0.003733 | 7.662 | +1 | 5 |
| - | - | 1574 | 498.2 | - | - | 0 | - |
| 13 | y | 2.059E+04 | 504.3 | 0.0005301 | 1.051 | +1 | 5 |
| - | - | 3863 | 505.3 | - | - | 0 | - |
| - | - | 5855 | 530.3 | - | - | 0 | - |
| - | - | 2467 | 531.3 | - | - | 0 | - |
| - | - | 4696 | 540.3 | - | - | 0 | - |
| - | - | 992.1 | 541.3 | - | - | 0 | - |
| - | - | 1.787E+04 | 558.3 | - | - | 0 | - |
| - | - | 5223 | 559.3 | - | - | 0 | - |
| - | - | 1266 | 560.3 | - | - | 0 | - |
| - | - | 1524 | 582.8 | - | - | 0 | - |
| - | - | 1972 | 591.3 | - | - | 0 | - |
| - | - | 3335 | 591.8 | - | - | 0 | - |
| 12 | y | 1356 | 614.3 | 0.001681 | 2.736 | +1 | 6 |
| 12 | y | 2948 | 615.3 | 0.001674 | 2.721 | +1 | 6 |
| 12 | z | 3894 | 616.3 | 0.0005631 | 0.9137 | +1 | 6 |
| - | - | 2364 | 617.3 | - | - | 0 | - |
| - | - | 3953 | 617.8 | - | - | 0 | - |
| - | - | 2976 | 618.3 | - | - | 0 | - |
| - | - | 1323 | 618.8 | - | - | 0 | - |
| - | - | 2.158E+04 | 626.8 | - | - | 0 | - |
| - | - | 2196 | 627.3 | - | - | 0 | - |
| - | - | 1.233E+04 | 627.3 | - | - | 0 | - |
| - | - | 2096 | 627.8 | - | - | 0 | - |
| 12 | y | 1.246E+04 | 632.3 | 0.0005768 | 0.9122 | +1 | 6 |
| - | - | 4472 | 633.3 | - | - | 0 | - |
| - | - | 1084 | 635.8 | - | - | 0 | - |
| 6 | y | 3254 | 637.8 | 0.0006485 | 1.017 | +2 | 12 |
| - | - | 2640 | 638.3 | - | - | 0 | - |
| - | - | 1457 | 655.3 | - | - | 0 | - |
| - | - | 1816 | 655.4 | - | - | 0 | - |
| - | - | 876.2 | 656.3 | - | - | 0 | - |
| - | - | 3320 | 663.3 | - | - | 0 | - |
| 12 | c | 1377 | 664.8 | 0.005978 | 8.992 | +2 | 12 |
| 5 | y | 1.333E+04 | 677.3 | 0.000545 | 0.8046 | +2 | 13 |
| 5 | y | 1.196E+04 | 677.8 | 0.00695 | 10.25 | +2 | 13 |
| 5 | z | 9259 | 678.3 | 0.001878 | 2.769 | +2 | 13 |
| - | - | 2779 | 678.8 | - | - | 0 | - |
| - | - | 1446 | 685.3 | - | - | 0 | - |
| - | - | 1715 | 685.8 | - | - | 0 | - |
| 5 | y | 4.473E+05 | 686.3 | 0.0007558 | 1.101 | +2 | 13 |
| - | - | 3.233E+05 | 686.8 | - | - | 0 | - |
| - | - | 1.075E+05 | 687.3 | - | - | 0 | - |
| - | - | 1.012E+04 | 687.8 | - | - | 0 | - |
| 13 | c | 5885 | 700.4 | 0.01056 | 15.08 | +2 | 13 |
| - | - | 3789 | 700.8 | - | - | 0 | - |
| - | - | 1176 | 701.3 | - | - | 0 | - |
| - | - | 1132 | 724.4 | - | - | 0 | - |
| 11 | z | 7129 | 729.4 | 0.0008497 | 1.165 | +1 | 7 |
| - | - | 6115 | 730.4 | - | - | 0 | - |
| - | - | 2402 | 731.4 | - | - | 0 | - |
| - | - | 3667 | 740.3 | - | - | 0 | - |
| - | - | 4095 | 742.4 | - | - | 0 | - |
| - | - | 1798 | 743.4 | - | - | 0 | - |
| 11 | y | 7136 | 745.4 | 0.0003751 | 0.5033 | +1 | 7 |
| - | - | 2687 | 746.4 | - | - | 0 | - |
| 4 | y | 3309 | 750.9 | 0.0001537 | 0.2047 | +2 | 14 |
| 4 | y | 5294 | 751.4 | 0.007106 | 9.458 | +2 | 14 |
| 4 | z | 2732 | 751.9 | 0.002095 | 2.786 | +2 | 14 |
| - | - | 1084 | 752.4 | - | - | 0 | - |
| 14 | c | 1569 | 757.4 | 0.009238 | 12.2 | +2 | 14 |
| 4 | y | 7.123E+04 | 759.9 | 0.0006675 | 0.8784 | +2 | 14 |
| - | - | 5.599E+04 | 760.4 | - | - | 0 | - |
| - | - | 2.423E+04 | 760.9 | - | - | 0 | - |
| - | - | 1608 | 761.4 | - | - | 0 | - |
| 3 | z | 822.3 | 799.4 | 0.004137 | 5.175 | +2 | 15 |
| 3 | y | 2566 | 807.9 | 0.00908 | 11.24 | +2 | 15 |
| - | - | 2357 | 812.9 | - | - | 0 | - |
| - | - | 1926 | 813.4 | - | - | 0 | - |
| 3 | y | 1.56E+04 | 816.4 | 0.001116 | 1.367 | +2 | 15 |
| - | - | 1.269E+04 | 816.9 | - | - | 0 | - |
| - | - | 6413 | 817.4 | - | - | 0 | - |
| - | - | 2686 | 829.4 | - | - | 0 | - |
| - | - | 1519 | 830.4 | - | - | 0 | - |
| - | - | 1448 | 842.9 | - | - | 0 | - |
| - | - | 1334 | 847.9 | - | - | 0 | - |
| - | - | 4496 | 848.4 | - | - | 0 | - |
| - | - | 2628 | 848.9 | - | - | 0 | - |
| 16 | c | 1.34E+04 | 856.9 | 0.0009389 | 1.096 | +2 | 16 |
| 10 | y | 1.321E+04 | 857.4 | 0.01192 | 13.9 | +1 | 8 |
| 2 | y | 6550 | 857.9 | 0.01448 | 16.88 | +2 | 16 |
| 2 | y | 2.074E+04 | 858.4 | 0.01667 | 19.42 | +2 | 16 |
| - | - | 1.792E+04 | 859.5 | - | - | 0 | - |
| - | - | 5360 | 860.5 | - | - | 0 | - |
| 2 | y | 5654 | 866.9 | 0.0008363 | 0.9646 | +2 | 16 |
| - | - | 3894 | 867.4 | - | - | 0 | - |
| - | - | 1445 | 867.9 | - | - | 0 | - |
| - | - | 3858 | 868.4 | - | - | 0 | - |
| - | - | 2513 | 869.4 | - | - | 0 | - |
| 10 | y | 8884 | 874.5 | 0.001117 | 1.277 | +1 | 8 |
| - | - | 2794 | 875.5 | - | - | 0 | - |
| - | - | 1051 | 886 | - | - | 0 | - |
| - | - | 1266 | 886.5 | - | - | 0 | - |
| - | - | 1933 | 899 | - | - | 0 | - |
| - | - | 9803 | 907.5 | - | - | 0 | - |
| - | - | 1.451E+04 | 908 | - | - | 0 | - |
| - | - | 9415 | 908.5 | - | - | 0 | - |
| - | - | 2937 | 909 | - | - | 0 | - |
| - | - | 1.262E+05 | 916.5 | - | - | 0 | - |
| - | - | 1.216E+05 | 917 | - | - | 0 | - |
| - | - | 1729 | 917.1 | - | - | 0 | - |
| - | - | 6.928E+04 | 917.5 | - | - | 0 | - |
| - | - | 9051 | 918 | - | - | 0 | - |
| - | - | 3689 | 939.4 | - | - | 0 | - |
| - | - | 1657 | 940.5 | - | - | 0 | - |
| - | - | 2993 | 958.5 | - | - | 0 | - |
| - | - | 2187 | 959.5 | - | - | 0 | - |
| - | - | 4077 | 973.5 | - | - | 0 | - |
| - | - | 2144 | 974.5 | - | - | 0 | - |
| 9 | c | 1693 | 975.5 | 0.002489 | 2.551 | +1 | 9 |
| 9 | z | 3.453E+04 | 987.5 | 0.001174 | 1.189 | +1 | 9 |
| - | - | 4.439E+04 | 988.5 | - | - | 0 | - |
| - | - | 1.958E+04 | 989.5 | - | - | 0 | - |
| - | - | 4389 | 990.5 | - | - | 0 | - |
| - | - | 3764 | 1002 | - | - | 0 | - |
| 9 | y | 1.153E+04 | 1004 | 0.002719 | 2.709 | +1 | 9 |
| - | - | 4309 | 1005 | - | - | 0 | - |
| - | - | 1226 | 1032 | - | - | 0 | - |
| - | - | 4846 | 1053 | - | - | 0 | - |
| - | - | 2210 | 1054 | - | - | 0 | - |
| - | - | 1424 | 1059 | - | - | 0 | - |
| - | - | 1183 | 1062 | - | - | 0 | - |
| 8 | y | 1583 | 1073 | 0.01057 | 9.857 | +1 | 10 |
| 8 | y | 4622 | 1074 | 4.168E-05 | 0.03882 | +1 | 10 |
| 8 | z | 3.179E+04 | 1075 | 0.000215 | 0.2001 | +1 | 10 |
| - | - | 8.946E+04 | 1076 | - | - | 0 | - |
| - | - | 4.369E+04 | 1077 | - | - | 0 | - |
| - | - | 1498 | 1077 | - | - | 0 | - |
| - | - | 1.005E+04 | 1078 | - | - | 0 | - |
| - | - | 1172 | 1085 | - | - | 0 | - |
| - | - | 1001 | 1087 | - | - | 0 | - |
| - | - | 3612 | 1088 | - | - | 0 | - |
| - | - | 1977 | 1089 | - | - | 0 | - |
| - | - | 1.449E+04 | 1090 | - | - | 0 | - |
| 8 | y | 4.354E+04 | 1091 | 0.0002623 | 0.2405 | +1 | 10 |
| - | - | 1.881E+04 | 1092 | - | - | 0 | - |
| - | - | 4390 | 1093 | - | - | 0 | - |
| - | - | 2904 | 1104 | - | - | 0 | - |
| 10 | c | 1.172E+04 | 1105 | 0.0008168 | 0.7395 | +1 | 10 |
| - | - | 4818 | 1106 | - | - | 0 | - |
| - | - | 1252 | 1107 | - | - | 0 | - |
| - | - | 3398 | 1117 | - | - | 0 | - |
| - | - | 5377 | 1118 | - | - | 0 | - |
| - | - | 3158 | 1119 | - | - | 0 | - |
| - | - | 1977 | 1160 | - | - | 0 | - |
| 7 | y | 3162 | 1161 | 0.00269 | 2.318 | +1 | 11 |
| 7 | z | 1.787E+04 | 1162 | 0.0002273 | 0.1957 | +1 | 11 |
| - | - | 3.823E+04 | 1163 | - | - | 0 | - |
| - | - | 1.995E+04 | 1164 | - | - | 0 | - |
| - | - | 7580 | 1165 | - | - | 0 | - |
| - | - | 2354 | 1166 | - | - | 0 | - |
| - | - | 1347 | 1174 | - | - | 0 | - |
| - | - | 1343 | 1175 | - | - | 0 | - |
| - | - | 3770 | 1177 | - | - | 0 | - |
| 7 | y | 2.386E+04 | 1178 | 0.0004241 | 0.3602 | +1 | 11 |
| - | - | 1.27E+04 | 1179 | - | - | 0 | - |
| - | - | 2645 | 1180 | - | - | 0 | - |
| - | - | 1.125E+04 | 1182 | - | - | 0 | - |
| - | - | 6653 | 1183 | - | - | 0 | - |
| - | - | 2864 | 1184 | - | - | 0 | - |
| - | - | 7776 | 1201 | - | - | 0 | - |
| - | - | 4191 | 1202 | - | - | 0 | - |
| - | - | 1796 | 1203 | - | - | 0 | - |
| - | - | 1.401E+04 | 1217 | - | - | 0 | - |
| 11 | c | 2.556E+04 | 1218 | 0.001826 | 1.5 | +1 | 11 |
| - | - | 1.523E+04 | 1219 | - | - | 0 | - |
| - | - | 2998 | 1220 | - | - | 0 | - |
| - | - | 3109 | 1235 | - | - | 0 | - |
| - | - | 2190 | 1236 | - | - | 0 | - |
| - | - | 1055 | 1237 | - | - | 0 | - |
| - | - | 1.497E+04 | 1253 | - | - | 0 | - |
| - | - | 9127 | 1254 | - | - | 0 | - |
| - | - | 2943 | 1255 | - | - | 0 | - |
| 6 | y | 1406 | 1257 | 0.0002171 | 0.1728 | +1 | 12 |
| 6 | y | 2069 | 1258 | 0.0112 | 8.903 | +1 | 12 |
| - | - | 1387 | 1274 | - | - | 0 | - |
| 6 | y | 5.255E+04 | 1275 | 0.0002726 | 0.2139 | +1 | 12 |
| - | - | 3.839E+04 | 1276 | - | - | 0 | - |
| - | - | 1.287E+04 | 1277 | - | - | 0 | - |
| - | - | 3524 | 1302 | - | - | 0 | - |
| - | - | 2501 | 1303 | - | - | 0 | - |
| - | - | 1682 | 1304 | - | - | 0 | - |
| - | - | 1671 | 1311 | - | - | 0 | - |
| - | - | 1798 | 1326 | - | - | 0 | - |
| - | - | 1415 | 1327 | - | - | 0 | - |
| 12 | c | 2060 | 1328 | 0.01676 | 12.62 | +1 | 12 |
| 12 | c | 1.345E+04 | 1329 | 0.002849 | 2.144 | +1 | 12 |
| - | - | 7150 | 1330 | - | - | 0 | - |
| - | - | 3813 | 1331 | - | - | 0 | - |
| - | - | 2.116E+04 | 1345 | - | - | 0 | - |
| 12 | c | 7.825E+04 | 1346 | 0.0007114 | 0.5286 | +1 | 12 |
| - | - | 4.948E+04 | 1347 | - | - | 0 | - |
| - | - | 1.603E+04 | 1348 | - | - | 0 | - |
| - | - | 2150 | 1349 | - | - | 0 | - |
| 5 | y | 9617 | 1354 | 0.00323 | 2.386 | +1 | 13 |
| 5 | y | 1.187E+04 | 1355 | 0.01092 | 8.063 | +1 | 13 |
| 5 | z | 7630 | 1356 | 0.005173 | 3.816 | +1 | 13 |
| - | - | 2361 | 1357 | - | - | 0 | - |
| - | - | 3642 | 1358 | - | - | 0 | - |
| - | - | 1226 | 1359 | - | - | 0 | - |
| - | - | 2188 | 1360 | - | - | 0 | - |
| - | - | 1780 | 1361 | - | - | 0 | - |
| - | - | 2438 | 1370 | - | - | 0 | - |
| - | - | 2.668E+04 | 1371 | - | - | 0 | - |
| 5 | y | 5.131E+05 | 1372 | 0.0001231 | 0.08975 | +1 | 13 |
| - | - | 3.616E+05 | 1373 | - | - | 0 | - |
| - | - | 1.18E+05 | 1374 | - | - | 0 | - |
| - | - | 1.171E+04 | 1375 | - | - | 0 | - |
| - | - | 2534 | 1398 | - | - | 0 | - |
| - | - | 1.02E+04 | 1399 | - | - | 0 | - |
| - | - | 1.895E+04 | 1400 | - | - | 0 | - |
| - | - | 1.197E+04 | 1401 | - | - | 0 | - |
| - | - | 1.016E+05 | 1402 | - | - | 0 | - |
| - | - | 7.398E+04 | 1403 | - | - | 0 | - |
| - | - | 2.828E+04 | 1404 | - | - | 0 | - |
| - | - | 4379 | 1405 | - | - | 0 | - |
| - | - | 5.609E+04 | 1416 | - | - | 0 | - |
| 13 | c | 8.014E+04 | 1417 | 0.003645 | 2.573 | +1 | 13 |
| - | - | 4.547E+04 | 1418 | - | - | 0 | - |
| - | - | 1.074E+04 | 1419 | - | - | 0 | - |
| - | - | 1230 | 1420 | - | - | 0 | - |
| - | - | 3106 | 1431 | - | - | 0 | - |
| - | - | 3699 | 1432 | - | - | 0 | - |
| - | - | 1063 | 1433 | - | - | 0 | - |
| - | - | 1266 | 1434 | - | - | 0 | - |
| - | - | 5903 | 1487 | - | - | 0 | - |
| - | - | 4868 | 1488 | - | - | 0 | - |
| - | - | 3084 | 1489 | - | - | 0 | - |
| 4 | y | 1497 | 1501 | 0.01759 | 11.72 | +1 | 14 |
| 4 | y | 1076 | 1502 | 0.02222 | 14.8 | +1 | 14 |
| 4 | z | 4.512E+04 | 1503 | 0.0004965 | 0.3304 | +1 | 14 |
| - | - | 4.019E+04 | 1504 | - | - | 0 | - |
| - | - | 1.609E+04 | 1505 | - | - | 0 | - |
| 14 | c | 4932 | 1514 | 0.001094 | 0.7229 | +1 | 14 |
| - | - | 4523 | 1515 | - | - | 0 | - |
| - | - | 2857 | 1516 | - | - | 0 | - |
| - | - | 2753 | 1518 | - | - | 0 | - |
| 4 | y | 5.355E+04 | 1519 | 0.0001776 | 0.117 | +1 | 14 |
| - | - | 4.653E+04 | 1520 | - | - | 0 | - |
| - | - | 1.644E+04 | 1521 | - | - | 0 | - |
| - | - | 1822 | 1522 | - | - | 0 | - |
| - | - | 8451 | 1530 | - | - | 0 | - |
| 14 | c | 1.286E+05 | 1531 | 0.0007966 | 0.5204 | +1 | 14 |
| - | - | 1.131E+05 | 1532 | - | - | 0 | - |
| - | - | 4.349E+04 | 1533 | - | - | 0 | - |
| - | - | 6439 | 1534 | - | - | 0 | - |
| - | - | 3292 | 1544 | - | - | 0 | - |
| - | - | 1969 | 1545 | - | - | 0 | - |
| - | - | 1500 | 1558 | - | - | 0 | - |
| - | - | 1421 | 1571 | - | - | 0 | - |
| - | - | 3036 | 1572 | - | - | 0 | - |
| - | - | 1715 | 1573 | - | - | 0 | - |
| 3 | y | 2287 | 1614 | 0.0004348 | 0.2694 | +1 | 15 |
| - | - | 1.962E+04 | 1615 | - | - | 0 | - |
| 3 | z | 3.084E+04 | 1616 | 0.02787 | 17.25 | +1 | 15 |
| - | - | 2.122E+04 | 1617 | - | - | 0 | - |
| - | - | 7866 | 1618 | - | - | 0 | - |
| 3 | y | 2.091E+04 | 1632 | 0.0005014 | 0.3073 | +1 | 15 |
| - | - | 1.826E+04 | 1633 | - | - | 0 | - |
| - | - | 7786 | 1634 | - | - | 0 | - |
| - | - | 1386 | 1643 | - | - | 0 | - |
| - | - | 1846 | 1645 | - | - | 0 | - |
| - | - | 1688 | 1646 | - | - | 0 | - |
| - | - | 1496 | 1647 | - | - | 0 | - |
| - | - | 3948 | 1655 | - | - | 0 | - |
| - | - | 2733 | 1656 | - | - | 0 | - |
| - | - | 2007 | 1658 | - | - | 0 | - |
| 15 | c | 6.057E+04 | 1659 | 0.002132 | 1.285 | +1 | 15 |
| - | - | 6.187E+04 | 1660 | - | - | 0 | - |
| - | - | 2.724E+04 | 1661 | - | - | 0 | - |
| - | - | 7415 | 1662 | - | - | 0 | - |
| - | - | 3323 | 1663 | - | - | 0 | - |
| - | - | 9519 | 1672 | - | - | 0 | - |
| - | - | 1.275E+04 | 1673 | - | - | 0 | - |
| - | - | 5998 | 1674 | - | - | 0 | - |
| - | - | 1720 | 1675 | - | - | 0 | - |
| - | - | 1.82E+04 | 1686 | - | - | 0 | - |
| - | - | 1.729E+04 | 1687 | - | - | 0 | - |
| - | - | 1.462E+04 | 1688 | - | - | 0 | - |
| - | - | 6096 | 1689 | - | - | 0 | - |
| - | - | 2904 | 1690 | - | - | 0 | - |
| - | - | 1419 | 1698 | - | - | 0 | - |
| 2 | z | 3793 | 1699 | 0.007598 | 4.473 | +1 | 16 |
| 2 | z | 2970 | 1700 | 0.009667 | 5.687 | +1 | 16 |
| - | - | 1729 | 1701 | - | - | 0 | - |
| 2 | y | 2.117E+04 | 1715 | 0.002337 | 1.363 | +1 | 16 |
| 2 | y | 3.879E+04 | 1716 | 0.01414 | 8.238 | +1 | 16 |
| 2 | z | 1.37E+05 | 1717 | 0.002038 | 1.187 | +1 | 16 |
| - | - | 1.27E+05 | 1718 | - | - | 0 | - |
| - | - | 6.402E+04 | 1719 | - | - | 0 | - |
| - | - | 1.133E+04 | 1720 | - | - | 0 | - |
| - | - | 2025 | 1729 | - | - | 0 | - |
| 16 | c | 9.034E+04 | 1730 | 0.0007936 | 0.4588 | +1 | 16 |
| - | - | 9.679E+04 | 1731 | - | - | 0 | - |
| - | - | 6.707E+04 | 1732 | - | - | 0 | - |
| - | - | 2.61E+04 | 1733 | - | - | 0 | - |
| - | - | 1.134E+04 | 1734 | - | - | 0 | - |
| - | - | 2022 | 1735 | - | - | 0 | - |
| - | - | 5688 | 1744 | - | - | 0 | - |
| - | - | 1.028E+04 | 1745 | - | - | 0 | - |
| - | - | 8506 | 1746 | - | - | 0 | - |
| - | - | 4965 | 1747 | - | - | 0 | - |
| - | - | 1304 | 1748 | - | - | 0 | - |
| - | - | 7384 | 1760 | - | - | 0 | - |
| - | - | 1.532E+04 | 1761 | - | - | 0 | - |
| - | - | 1.879E+04 | 1762 | - | - | 0 | - |
| - | - | 1.418E+04 | 1763 | - | - | 0 | - |
| - | - | 4040 | 1764 | - | - | 0 | - |
| - | - | 1.031E+04 | 1769 | - | - | 0 | - |
| - | - | 1.268E+04 | 1770 | - | - | 0 | - |
| - | - | 7629 | 1771 | - | - | 0 | - |
| - | - | 4924 | 1772 | - | - | 0 | - |
| - | - | 3100 | 1773 | - | - | 0 | - |
| - | - | 1.162E+04 | 1774 | - | - | 0 | - |
| - | - | 1.277E+04 | 1775 | - | - | 0 | - |
| - | - | 4886 | 1776 | - | - | 0 | - |
| - | - | 1.39E+04 | 1777 | - | - | 0 | - |
| - | - | 1.563E+04 | 1778 | - | - | 0 | - |
| - | - | 6899 | 1779 | - | - | 0 | - |
| - | - | 1652 | 1780 | - | - | 0 | - |
| - | - | 2577 | 1786 | - | - | 0 | - |
| - | - | 2.449E+04 | 1787 | - | - | 0 | - |
| - | - | 1.567E+05 | 1788 | - | - | 0 | - |
| - | - | 1.516E+05 | 1789 | - | - | 0 | - |
| - | - | 7.489E+04 | 1790 | - | - | 0 | - |
| - | - | 1.332E+04 | 1791 | - | - | 0 | - |
| - | - | 2095 | 1792 | - | - | 0 | - |
| - | - | 1803 | 1797 | - | - | 0 | - |
| - | - | 5167 | 1798 | - | - | 0 | - |
| - | - | 5018 | 1799 | - | - | 0 | - |
| - | - | 1484 | 1802 | - | - | 0 | - |
| - | - | 1213 | 1803 | - | - | 0 | - |
| - | - | 1.402E+04 | 1805 | - | - | 0 | - |
| - | - | 1.349E+04 | 1806 | - | - | 0 | - |
| - | - | 8109 | 1807 | - | - | 0 | - |
| - | - | 1457 | 1813 | - | - | 0 | - |
| - | - | 3502 | 1814 | - | - | 0 | - |
| - | - | 3.696E+04 | 1815 | - | - | 0 | - |
| - | - | 2.303E+05 | 1816 | - | - | 0 | - |
| - | - | 2.24E+05 | 1817 | - | - | 0 | - |
| - | - | 1.171E+05 | 1818 | - | - | 0 | - |
| - | - | 1.875E+04 | 1819 | - | - | 0 | - |
| - | - | 1420 | 1820 | - | - | 0 | - |
| - | - | 4624 | 1830 | - | - | 0 | - |
| - | - | 1.566E+04 | 1831 | - | - | 0 | - |
| - | - | 4.355E+05 | 1832 | - | - | 0 | - |
| - | - | 1.142E+06 | 1833 | - | - | 0 | - |
| - | - | 9.657E+05 | 1834 | - | - | 0 | - |
| - | - | 4.07E+05 | 1835 | - | - | 0 | - |
| - | - | 5.35E+04 | 1836 | - | - | 0 | - |
| - | - | 2023 | 1866 | - | - | 0 | - |

m/z Charge Intensity FragmentType MassShift Position
120.08102416992188 0 2824.1958
120.13362884521484 0 466.28857
125.88520050048828 0 580.45734
129.10240173339844 0 5050.359
129.82728576660156 0 542.41254
148.8828887939453 0 1127.4854
148.89064025878906 0 751.44446
148.905029296875 0 842.12067
148.91200256347656 0 1058.4781
148.9197998046875 0 1418.2476
148.9264678955078 0 1741.9448
148.93357849121094 0 3120.6853
148.94137573242188 0 5426.7075
148.95790100097656 0 7282.9067
148.9657440185547 0 4788.657
148.97299194335938 0 2203.3447
148.98019409179688 0 1755.555
148.98736572265625 0 1901.2825
148.9945068359375 0 1221.3206
149.00173950195312 0 1025.5151
149.0086212158203 0 686.97375
149.0161895751953 0 1018.807
149.02294921875 0 603.7326
149.05264282226562 0 773.2812
173.12876892089844 0 3116.7744
183.1129608154297 0 5571.359
185.32168579101562 0 818.8198
193.15292358398438 0 651.7317
201.12350463867188 0 14812.793
215.13916015625 0 2879.5063
217.13375854492188 0 2189.2698
219.1492462158203 0 1083.5284
226.1190185546875 0 1239.0282
233.1650848388672 0 9341.76
243.14535522460938 0 2739.2952
245.12872314453125 0 2542.6606
247.14474487304688 0 1902.6436
251.17562866210938 0 4449.979
252.1782684326172 0 961.86707
253.2028045654297 0 863.2349
261.16009521484375 0 6935.378
262.1637878417969 0 1320.0171
269.185302734375 0 954.10516
273.07135009765625 0 919.7587
280.165771484375 0 1940.4592
282.14508056640625 0 2426.916
296.1972351074219 0 152024.62
297.1571350097656 0 1816.5426
297.2004699707031 0 24586.02
298.1764831542969 0 2540.805
298.2035827636719 0 2126.6467
303.1701354980469 0 2575.0325
303.67816162109375 0 941.4764
314.18487548828125 0 2994.3657
314.2078857421875 0 79507.29
315.1895446777344 0 1311.497
315.21099853515625 0 13393.435
317.1865234375 0 1460.8081
319.1980285644531 0 9216.291 y 14
330.1814880371094 0 3527.0198
344.1973571777344 0 1634.2585
346.17657470703125 0 6724
347.18035888671875 0 1847.7015
348.1921691894531 0 5726.0586
349.19427490234375 0 2071.3384
360.2284851074219 0 2170.8174
362.2075500488281 0 22890.477
363.2098693847656 0 3720.9377
385.2196350097656 0 1391.212
390.20196533203125 0 1388.0365
393.251708984375 0 1287.8495
398.2431640625 0 2123.6667
411.2608947753906 0 7001.875
412.2650146484375 0 1157.2756
415.271240234375 0 2934.8503
416.2550048828125 0 8978.513
417.256591796875 0 2223.4739
425.11993408203125 0 894.4486
433.2428894042969 0 12606.858 y 13
433.2816467285156 0 117809.195
434.24859619140625 0 1803.6328
434.28472900390625 0 31917.535
435.28814697265625 0 2930.4023
442.2415466308594 0 970.1318
443.2296142578125 0 2357.6987
443.2660827636719 0 14369.436
444.269775390625 0 3252.1978
445.244140625 0 1045.0656
461.2766418457031 0 127114.02
462.27972412109375 0 28915.213
463.28289794921875 0 3013.8284
487.25482177734375 0 1073.6461 y Ammonia loss 12
498.2198181152344 0 1573.5953
504.2781677246094 0 20593.223 y 12
505.28094482421875 0 3863.4607
530.3341674804688 0 5855.18
531.3380126953125 0 2467.1858
540.3189697265625 0 4695.641
541.3184814453125 0 992.1016
558.3294677734375 0 17873.438
559.3319091796875 0 5223.2227
560.33154296875 0 1265.7242
582.7817993164062 0 1524.2084
591.2907104492188 0 1971.7505
591.7942504882812 0 3334.7493
614.3273315429688 0 1355.715 y Water loss 11
615.3113403320312 0 2947.784 y Ammonia loss 11
616.3180541992188 0 3894.1128 z 11
617.322998046875 0 2363.9438
617.807373046875 0 3953.1184
618.3095092773438 0 2976.463
618.8021240234375 0 1322.7424
626.8125610351562 0 21582.662
627.26171875 0 2195.6035
627.3141479492188 0 12326.07
627.8141479492188 0 2096.4648
632.3367919921875 0 12462.841 y 11
633.3394775390625 0 4471.5347
635.8165893554688 0 1083.7593
637.8154296875 0 3254.3713 y 5
638.317138671875 0 2640.0146
655.3348999023438 0 1456.66
655.3876953125 0 1815.5117
656.3273315429688 0 876.17053
663.3366088867188 0 3320
664.8342895507812 0 1377.2267 c Ammonia loss 11
677.33642578125 0 13329.244 y Water loss 4
677.8348388671875 0 11959.391 y Ammonia loss 4
678.3336791992188 0 9258.57 z 4
678.83447265625 0 2778.9595
685.33447265625 0 1446.2798
685.8331909179688 0 1714.8784
686.3419189453125 0 447346 y 4
686.84326171875 0 323330.84
687.344482421875 0 107541.69
687.8460083007812 0 10119.557
700.3482666015625 0 5884.6855 c Ammonia loss 12
700.8491821289062 0 3788.6062
701.3485717773438 0 1176.0186
724.4043579101562 0 1131.9808
729.4024047851562 0 7128.511 z 10
730.4071044921875 0 6115.345
731.4111328125 0 2401.769
740.3457641601562 0 3666.71
742.4132690429688 0 4095.3354
743.4215698242188 0 1797.5161
745.420654296875 0 7136.182 y 10
746.4237060546875 0 2687.1504
750.8699340820312 0 3309.4307 y Water loss 3
751.3692016601562 0 5294.211 y Ammonia loss 3
751.8681030273438 0 2732.0989 z 3
752.3577270507812 0 1083.7555
757.3895263671875 0 1569.0568 c Ammonia loss 13
759.8760375976562 0 71227.69 y 3
760.3772583007812 0 55993.832
760.8790283203125 0 24233.17
761.3807983398438 0 1608.4207
799.3986206054688 0 822.34033 z Water loss 2
807.9132080078125 0 2565.887 y Ammonia loss 2
812.9169311523438 0 2357.0989
813.411376953125 0 1926.3951
816.4185180664062 0 15598.975 y 2
816.9198608398438 0 12687.332
817.4225463867188 0 6412.929
829.4454956054688 0 2686.156
830.44921875 0 1518.9426
842.944580078125 0 1448.1332
847.9427490234375 0 1333.8284
848.4404907226562 0 4496.1753
848.938232421875 0 2628.498
856.947265625 0 13400.695 c Ammonia loss 15
857.4482421875 0 13210.876 y Ammonia loss 9
857.950439453125 0 6549.623 y Water loss 1
858.4446411132812 0 20741.158 y Ammonia loss 1
859.450439453125 0 17917.643
860.4539184570312 0 5360.1226
866.9420776367188 0 5653.697 y 1
867.4436645507812 0 3893.7668
867.9415893554688 0 1444.502
868.4061889648438 0 3858.2793
869.409912109375 0 2512.8025
874.4639892578125 0 8884.034 y 9
875.466064453125 0 2794.0266
885.9661254882812 0 1051.0239
886.473388671875 0 1266.4681
898.9617919921875 0 1932.9434
907.4706420898438 0 9803.241
907.9696655273438 0 14507.36
908.4712524414062 0 9414.541
908.9708251953125 0 2937.076
916.476318359375 0 126247.734
916.9771728515625 0 121617.45
917.083984375 0 1728.993
917.4789428710938 0 69280.695
917.9798583984375 0 9050.557
939.4411010742188 0 3688.8765
940.4511108398438 0 1657.0293
958.48974609375 0 2992.5413
959.4893188476562 0 2186.6592
973.497314453125 0 4077.0083
974.4984130859375 0 2143.784
975.5120849609375 0 1692.7515 c 8
987.4879150390625 0 34529.402 z 8
988.494140625 0 44392.37
989.4990234375 0 19577.283
990.4990844726562 0 4389.1187
1002.49755859375 0 3763.893
1003.5027465820312 0 11526.496 y 8
1004.5054931640625 0 4308.5664
1031.50390625 0 1225.6006
1053.4853515625 0 4846.045
1054.4921875 0 2210.0667
1058.5072021484375 0 1423.6538
1061.5396728515625 0 1182.8221
1072.516357421875 0 1582.6918 y Water loss 7
1073.510986328125 0 4621.8174 y Ammonia loss 7
1074.5185546875 0 31785.553 z 7
1075.5272216796875 0 89463.38
1076.5301513671875 0 43690.863
1076.6619873046875 0 1498.4283
1077.53271484375 0 10048.968
1084.5277099609375 0 1171.6709
1086.5074462890625 0 1000.6635
1087.531494140625 0 3612.0085
1088.5465087890625 0 1977.1328
1089.5316162109375 0 14494.946
1090.5372314453125 0 43543.152 y 7
1091.5404052734375 0 18810.322
1092.54296875 0 4389.9624
1103.5501708984375 0 2904.371
1104.5579833984375 0 11719.268 c 9
1105.56005859375 0 4818.078
1106.56787109375 0 1252.3168
1116.529296875 0 3398.398
1117.535400390625 0 5377.3467
1118.5396728515625 0 3157.7683
1159.5291748046875 0 1977.3054
1160.540283203125 0 3161.554 y Ammonia loss 6
1161.551025390625 0 17871.559 z 6
1162.5576171875 0 38230.93
1163.56298828125 0 19950.5
1164.5601806640625 0 7579.521
1165.558349609375 0 2354.3086
1173.6199951171875 0 1347.1736
1174.63916015625 0 1343.3717
1176.556884765625 0 3770.3123
1177.5699462890625 0 23862.098 y 6
1178.5721435546875 0 12704.943
1179.573486328125 0 2644.5662
1181.581298828125 0 11252.078
1182.585205078125 0 6652.6206
1183.5975341796875 0 2863.5212
1200.61474609375 0 7776.126
1201.6207275390625 0 4190.9224
1202.624755859375 0 1796.1407
1216.63330078125 0 14008.538
1217.639404296875 0 25558.91 c 10
1218.642822265625 0 15225.29
1219.6424560546875 0 2997.879
1234.610107421875 0 3108.6968
1235.60302734375 0 2189.6301
1236.5792236328125 0 1055.4335
1252.6163330078125 0 14970.19
1253.6229248046875 0 9127.081
1254.615966796875 0 2942.6765
1256.6119384765625 0 1405.798 y Water loss 5
1257.60693359375 0 2069.175 y Ammonia loss 5
1273.613037109375 0 1387.2454
1274.62255859375 0 52546.883 y 5
1275.6259765625 0 38389.598
1276.6279296875 0 12874.609
1301.6715087890625 0 3523.5896
1302.69482421875 0 2501.1653
1303.6990966796875 0 1682.2299
1310.6717529296875 0 1671.0018
1325.6785888671875 0 1797.8789
1326.67236328125 0 1415.4788
1327.6724853515625 0 2059.6624 c Water loss 11
1328.67041015625 0 13451.278 c Ammonia loss 11
1329.6722412109375 0 7149.635
1330.6832275390625 0 3813.3674
1344.69140625 0 21158.697
1345.6990966796875 0 78249.65 c 11
1346.703125 0 49483.7
1347.7049560546875 0 16030.146
1348.713134765625 0 2150.2493
1353.6612548828125 0 9617.003 y Water loss 4
1354.659423828125 0 11873.238 y Ammonia loss 4
1355.6614990234375 0 7630.332 z 4
1356.6558837890625 0 2360.8816
1357.6834716796875 0 3642.443
1358.68701171875 0 1226.0819
1359.672607421875 0 2187.5505
1360.6630859375 0 1779.5479
1369.65673828125 0 2437.8035
1370.66650390625 0 26684.098
1371.6749267578125 0 513080.22 y 4
1372.678466796875 0 361641.66
1373.6812744140625 0 118002.69
1374.6895751953125 0 11709.388
1397.7293701171875 0 2534.374
1398.671630859375 0 10197.337
1399.6807861328125 0 18949.623
1400.6878662109375 0 11969.009
1401.7010498046875 0 101606.77
1402.7041015625 0 73983.91
1403.7073974609375 0 28280.082
1404.7027587890625 0 4378.7207
1415.7288818359375 0 56085.367
1416.7332763671875 0 80139.9 c 12
1417.7376708984375 0 45467.21
1418.7420654296875 0 10735.827
1419.751708984375 0 1230.083
1430.6988525390625 0 3105.868
1431.7069091796875 0 3699.0652
1432.694580078125 0 1063.2341
1433.6907958984375 0 1265.9858
1486.76416015625 0 5903.4756
1487.7667236328125 0 4868.113
1488.772216796875 0 3084.262
1500.75048828125 0 1497.0674 y Water loss 3
1501.7391357421875 0 1075.9984 y Ammonia loss 3
1502.7242431640625 0 45117.17 z 3
1503.7281494140625 0 40186.945
1504.732177734375 0 16087.765
1513.75439453125 0 4931.522 c Ammonia loss 13
1514.7550048828125 0 4523.2104
1515.7674560546875 0 2856.7527
1517.7327880859375 0 2753.1064
1518.7432861328125 0 53548.64 y 3
1519.745361328125 0 46531.35
1520.7481689453125 0 16437.354
1521.7490234375 0 1821.5035
1529.770751953125 0 8451.211
1530.779052734375 0 128615.79 c 13
1531.781982421875 0 113101.22
1532.78564453125 0 43492.863
1533.791015625 0 6439.4873
1543.783935546875 0 3292.2905
1544.787109375 0 1969.0074
1557.789794921875 0 1499.9158
1570.8187255859375 0 1421.0421
1571.8182373046875 0 3036.0618
1572.7921142578125 0 1715.2147
1613.8165283203125 0 2286.9946 y Water loss 2
1614.857421875 0 19621.72
1615.836669921875 0 30843.432 z 2
1616.829345703125 0 21218.617
1617.8209228515625 0 7865.7075
1631.8270263671875 0 20914.934 y 2
1632.82763671875 0 18258.299
1633.8345947265625 0 7786.417
1642.8621826171875 0 1386.3269
1644.84423828125 0 1846.0281
1645.8380126953125 0 1687.9381
1646.834716796875 0 1496.1249
1654.8531494140625 0 3947.7527
1655.858154296875 0 2732.9763
1657.849609375 0 2007.0406
1658.8726806640625 0 60566.93 c 14
1659.8763427734375 0 61865.586
1660.8681640625 0 27237.004
1661.815673828125 0 7415.0713
1662.7930908203125 0 3323.0422
1671.8436279296875 0 9519.385
1672.8431396484375 0 12748.447
1673.8443603515625 0 5997.7573
1674.8382568359375 0 1720.0752
1685.8968505859375 0 18196.475
1686.90234375 0 17286.818
1687.9068603515625 0 14615.792
1688.9080810546875 0 6095.78
1689.9078369140625 0 2904.3257
1697.849609375 0 1419.0265
1698.853515625 0 3793.3455 z Water loss 1
1699.839599609375 0 2969.7454 z Ammonia loss 1
1700.85205078125 0 1729.319
1714.8623046875 0 21168.13 y Water loss 1
1715.86279296875 0 38787.38 y Ammonia loss 1
1716.8585205078125 0 136969.72 z 1
1717.85986328125 0 127005.47
1718.8648681640625 0 64019.445
1719.8690185546875 0 11333.577
1728.9056396484375 0 2024.502
1729.9127197265625 0 90344.22 c 15
1730.9156494140625 0 96792.836
1731.9183349609375 0 67066.555
1732.923828125 0 26097.848
1733.924072265625 0 11338.733
1734.932373046875 0 2021.5789
1743.90625 0 5687.526
1744.8939208984375 0 10276.234
1745.8919677734375 0 8506.176
1746.9041748046875 0 4965.3105
1747.8988037109375 0 1304.072
1759.8887939453125 0 7383.626
1760.9141845703125 0 15320.523
1761.91748046875 0 18793.088
1762.9178466796875 0 14176.8545
1763.9110107421875 0 4040.1277
1768.93408203125 0 10310.414
1769.9339599609375 0 12681.885
1770.9395751953125 0 7628.7495
1771.94140625 0 4923.8394
1772.92333984375 0 3100.4636
1773.9146728515625 0 11624.366
1774.91748046875 0 12773.02
1775.9163818359375 0 4886.3584
1776.8941650390625 0 13895.866
1777.89208984375 0 15628.303
1778.896484375 0 6898.879
1779.8880615234375 0 1651.8073
1785.917236328125 0 2577.2512
1786.94677734375 0 24486.578
1787.93310546875 0 156664.69
1788.93505859375 0 151627.62
1789.9359130859375 0 74887.66
1790.9307861328125 0 13318.206
1791.893798828125 0 2094.8184
1796.9412841796875 0 1802.5502
1797.912353515625 0 5167.384
1798.924560546875 0 5017.9434
1801.9068603515625 0 1484.0692
1802.939453125 0 1212.7926
1804.95751953125 0 14022.818
1805.9581298828125 0 13493.773
1806.9595947265625 0 8109.056
1812.900634765625 0 1456.8407
1813.9173583984375 0 3502.0964
1814.937744140625 0 36960.86
1815.9278564453125 0 230267.69
1816.929443359375 0 224044.3
1817.9306640625 0 117149.59
1818.930908203125 0 18748.678
1819.918701171875 0 1420.2955
1829.927978515625 0 4623.6953
1830.9354248046875 0 15661.489
1831.9444580078125 0 435492.3
1832.951416015625 0 1142439.5
1833.954345703125 0 965719.25
1834.9578857421875 0 407006.16
1835.9581298828125 0 53503.03
1865.9400634765625 0 2022.7128

Spectrum Details

|  |  |
| --- | --- |
| Matched peaks? Matched peaksThe total absolute number of peaks matched. Additionally in brackets the total fraction of peaks matched and the total number of peaks is shown. | 70 (15.80% of 443) |
| FDR? FDRThe false discovery rate estimated for this peptide. It is calculated by matching all theoretical fragments with a non-integer shift with the raw peaks for this spectrum. This is done with 40 different shifts. The resulting percentage is the average number of annotated peaks over the number of annotated peaks with the correct spectrum. | 0.51% |
| Satellite FDR? Satellite FDRSee the FDR for details on its calculation. This satellite ion specific FDR only contains the satellite ions (d/w) for I/L/J positions. | - |
| PSM Score? PSM ScoreThe PSM Score as given by Hecklib to this annotated spectrum. It is shown with three significant figures. | 681 |

## Spectrum 8106? Spectrum 8106 The raw spectrum of this peptide as annotated by Hecklib. The fragments are coloured according to ion type (see legend). Any peaks with a star '\*' as text can be hovered over to see the full details, first the ion type second the mass shift type. By hovering over the amino acids in the peptide or ions in the legend the corresponding peaks are highlighted. By toggling the 'Unassigned' label you can turn the background (unassigned) peaks on or off in the plot. By updating the slider in the Ion legend you can update the spectrum to only show the top X% of the peaks with labels. The top X% means any peak that is within X% of the highest intensity. By dragging in the spectrum you can zoom in to a specific part of the spectrum and use 'Zoom Out' to get back to the original zoom level. The annotation of the spectrum is based on the given sequence in the peptides file and is done with different software so inconsistencies are likely. The peaks are annotated based on the given sequence, with 20 ppm tolerance.

Copy Data

### Spectrum 8106 (TSV)

#### Preview

```
Loading example...
```

*Click on the button to copy the data to your clipboard.*

Mz MinMz MaxIntensity Max

WidthHeightPeptide font sizePeptide stroke widthSpectrum font sizeSpectrum stroke widthCompact peptide

Ion legend

wxyz

abcd

OtherUnassignedIonChargePositionShow for top:%

VTJFPPSSEEJQANKAT

03.36e+56.73e+51.01e+61.35e+6

Zoom Out

y+11a+12y+12a+12b+12y+12b+12y+25a+13a+13b+13y+13y+13y+26b+13y+13b+28y+14b+14b+14y+15y+15y+15b+210b+15b+15b+211y+16y+16y+212y+16y+212b+212b+212y+213y+213y+213b+213b+213y+17b+17y+17y+214y+214b+214y+214y+215b+215y+215b+18b+216b+216y+18b+216y+18y+216y+216y+18\*\*\*b+19y+19y+19b+110y+110y+110b+110y+110y+111y+111y+111b+111b+111y+112y+112y+112b+112b+112b+112y+113y+113y+113b+113b+113b+113b+114y+114y+114b+114y+114y+115

0861172125823442

Fragment Matches Table

Show background peaks

| Position | Ion type | Intensity | mz Theoretical | mz Error (Th) | mz Error (ppm) | Charge | Series Number |
| --- | --- | --- | --- | --- | --- | --- | --- |
| 17 | y | 8626 | 120.1 | 0.0003297 | 2.746 | +1 | 1 |
| - | - | 1.181E+05 | 120.1 | - | - | 0 | - |
| - | - | 8964 | 121.1 | - | - | 0 | - |
| - | - | 1051 | 121.2 | - | - | 0 | - |
| - | - | 2.45E+04 | 126.1 | - | - | 0 | - |
| - | - | 2336 | 127.1 | - | - | 0 | - |
| - | - | 3.172E+04 | 128.1 | - | - | 0 | - |
| - | - | 6639 | 129.1 | - | - | 0 | - |
| - | - | 2.114E+05 | 129.1 | - | - | 0 | - |
| - | - | 5069 | 130.1 | - | - | 0 | - |
| - | - | 1.127E+04 | 130.1 | - | - | 0 | - |
| - | - | 8415 | 131.1 | - | - | 0 | - |
| - | - | 2472 | 132.1 | - | - | 0 | - |
| - | - | 1231 | 133.1 | - | - | 0 | - |
| - | - | 5764 | 138.1 | - | - | 0 | - |
| - | - | 1.814E+04 | 138.1 | - | - | 0 | - |
| - | - | 1305 | 139.1 | - | - | 0 | - |
| - | - | 955.1 | 139.1 | - | - | 0 | - |
| - | - | 9755 | 139.1 | - | - | 0 | - |
| - | - | 1391 | 139.1 | - | - | 0 | - |
| - | - | 1121 | 139.1 | - | - | 0 | - |
| - | - | 1868 | 139.1 | - | - | 0 | - |
| - | - | 2225 | 141.1 | - | - | 0 | - |
| - | - | 2175 | 141.1 | - | - | 0 | - |
| - | - | 1514 | 143.1 | - | - | 0 | - |
| - | - | 1234 | 144.1 | - | - | 0 | - |
| - | - | 6400 | 147.1 | - | - | 0 | - |
| - | - | 3667 | 153.1 | - | - | 0 | - |
| - | - | 7826 | 154.1 | - | - | 0 | - |
| - | - | 3104 | 154.1 | - | - | 0 | - |
| - | - | 6652 | 155.1 | - | - | 0 | - |
| 2 | a | 3.283E+04 | 155.1 | 0.000305 | 1.967 | +1 | 2 |
| - | - | 2034 | 156.1 | - | - | 0 | - |
| - | - | 7187 | 157.1 | - | - | 0 | - |
| - | - | 2031 | 157.1 | - | - | 0 | - |
| - | - | 2.787E+04 | 157.1 | - | - | 0 | - |
| - | - | 3400 | 165.1 | - | - | 0 | - |
| - | - | 4188 | 166.1 | - | - | 0 | - |
| - | - | 1.832E+04 | 167.1 | - | - | 0 | - |
| - | - | 1.362E+04 | 167.1 | - | - | 0 | - |
| - | - | 2108 | 168.1 | - | - | 0 | - |
| - | - | 2269 | 168.1 | - | - | 0 | - |
| - | - | 3251 | 169.1 | - | - | 0 | - |
| - | - | 1367 | 169.1 | - | - | 0 | - |
| - | - | 3.189E+04 | 169.1 | - | - | 0 | - |
| - | - | 1693 | 170.1 | - | - | 0 | - |
| - | - | 2779 | 171.1 | - | - | 0 | - |
| - | - | 1584 | 171.1 | - | - | 0 | - |
| - | - | 4295 | 172.1 | - | - | 0 | - |
| 16 | y | 9248 | 173.1 | 0.0003385 | 1.956 | +1 | 2 |
| 2 | a | 1.038E+05 | 173.1 | 0.0003147 | 1.818 | +1 | 2 |
| - | - | 6444 | 174.1 | - | - | 0 | - |
| - | - | 1.074E+04 | 175.1 | - | - | 0 | - |
| - | - | 2334 | 176.1 | - | - | 0 | - |
| - | - | 3233 | 180.1 | - | - | 0 | - |
| - | - | 9416 | 181.1 | - | - | 0 | - |
| - | - | 6175 | 182.1 | - | - | 0 | - |
| - | - | 1.119E+04 | 182.1 | - | - | 0 | - |
| - | - | 1.061E+04 | 183.1 | - | - | 0 | - |
| 2 | b | 3.875E+05 | 183.1 | 0.000355 | 1.939 | +1 | 2 |
| - | - | 1.02E+04 | 184.1 | - | - | 0 | - |
| - | - | 3.245E+04 | 184.1 | - | - | 0 | - |
| - | - | 3.66E+04 | 185.1 | - | - | 0 | - |
| - | - | 3320 | 185.1 | - | - | 0 | - |
| - | - | 7065 | 186.1 | - | - | 0 | - |
| - | - | 2394 | 186.1 | - | - | 0 | - |
| - | - | 5.643E+04 | 187.1 | - | - | 0 | - |
| - | - | 3785 | 188.1 | - | - | 0 | - |
| - | - | 7728 | 189.1 | - | - | 0 | - |
| - | - | 2812 | 191.1 | - | - | 0 | - |
| 16 | y | 1.017E+04 | 191.1 | 0.0003176 | 1.662 | +1 | 2 |
| - | - | 2339 | 195.1 | - | - | 0 | - |
| - | - | 3.148E+04 | 195.1 | - | - | 0 | - |
| - | - | 3026 | 196.1 | - | - | 0 | - |
| - | - | 1225 | 196.1 | - | - | 0 | - |
| - | - | 1111 | 197.1 | - | - | 0 | - |
| - | - | 2924 | 197.1 | - | - | 0 | - |
| - | - | 4.094E+04 | 197.1 | - | - | 0 | - |
| - | - | 6671 | 198.1 | - | - | 0 | - |
| - | - | 4156 | 198.1 | - | - | 0 | - |
| - | - | 1.491E+04 | 199.1 | - | - | 0 | - |
| - | - | 6288 | 199.1 | - | - | 0 | - |
| - | - | 2992 | 199.1 | - | - | 0 | - |
| - | - | 1.106E+04 | 200.1 | - | - | 0 | - |
| - | - | 1.436E+04 | 200.1 | - | - | 0 | - |
| 2 | b | 1.932E+05 | 201.1 | 0.0002731 | 1.358 | +1 | 2 |
| - | - | 1.606E+04 | 202.1 | - | - | 0 | - |
| - | - | 1797 | 203.1 | - | - | 0 | - |
| - | - | 1233 | 207.1 | - | - | 0 | - |
| - | - | 1880 | 208.1 | - | - | 0 | - |
| - | - | 1.965E+04 | 208.1 | - | - | 0 | - |
| - | - | 5.051E+04 | 209.1 | - | - | 0 | - |
| - | - | 3097 | 210.1 | - | - | 0 | - |
| - | - | 2442 | 210.1 | - | - | 0 | - |
| - | - | 2707 | 211.1 | - | - | 0 | - |
| - | - | 5905 | 212.1 | - | - | 0 | - |
| - | - | 4270 | 213.1 | - | - | 0 | - |
| - | - | 3259 | 213.1 | - | - | 0 | - |
| - | - | 1800 | 214.2 | - | - | 0 | - |
| - | - | 7.431E+04 | 215.1 | - | - | 0 | - |
| - | - | 7950 | 216.1 | - | - | 0 | - |
| - | - | 3.669E+04 | 217.1 | - | - | 0 | - |
| - | - | 4.41E+04 | 217.1 | - | - | 0 | - |
| - | - | 2494 | 218.1 | - | - | 0 | - |
| - | - | 1372 | 218.1 | - | - | 0 | - |
| - | - | 4548 | 218.1 | - | - | 0 | - |
| - | - | 3079 | 218.1 | - | - | 0 | - |
| - | - | 1.577E+04 | 219.1 | - | - | 0 | - |
| - | - | 1620 | 220.2 | - | - | 0 | - |
| - | - | 2243 | 224.2 | - | - | 0 | - |
| - | - | 1.702E+04 | 225.1 | - | - | 0 | - |
| - | - | 9485 | 225.1 | - | - | 0 | - |
| - | - | 3752 | 225.2 | - | - | 0 | - |
| - | - | 1.717E+05 | 226.1 | - | - | 0 | - |
| - | - | 4667 | 227.1 | - | - | 0 | - |
| - | - | 1.434E+04 | 227.1 | - | - | 0 | - |
| - | - | 2119 | 228.1 | - | - | 0 | - |
| - | - | 1355 | 230.6 | - | - | 0 | - |
| - | - | 4467 | 231.1 | - | - | 0 | - |
| - | - | 9.625E+04 | 233.2 | - | - | 0 | - |
| - | - | 1.52E+04 | 234.2 | - | - | 0 | - |
| - | - | 1.05E+04 | 236.1 | - | - | 0 | - |
| - | - | 1544 | 236.1 | - | - | 0 | - |
| - | - | 2212 | 236.1 | - | - | 0 | - |
| - | - | 1669 | 237.1 | - | - | 0 | - |
| - | - | 1.016E+04 | 241.1 | - | - | 0 | - |
| - | - | 1398 | 241.2 | - | - | 0 | - |
| - | - | 3153 | 241.2 | - | - | 0 | - |
| - | - | 1337 | 242.1 | - | - | 0 | - |
| - | - | 3.269E+04 | 242.2 | - | - | 0 | - |
| - | - | 5112 | 243.1 | - | - | 0 | - |
| - | - | 5.69E+04 | 243.1 | - | - | 0 | - |
| 13 | y | 7882 | 244.1 | 0.0009445 | 3.869 | +2 | 5 |
| - | - | 5652 | 244.1 | - | - | 0 | - |
| - | - | 2.818E+04 | 245.1 | - | - | 0 | - |
| - | - | 3556 | 246.1 | - | - | 0 | - |
| - | - | 1.738E+04 | 247.1 | - | - | 0 | - |
| - | - | 3083 | 248.1 | - | - | 0 | - |
| - | - | 5.177E+04 | 251.2 | - | - | 0 | - |
| - | - | 3347 | 252.1 | - | - | 0 | - |
| - | - | 5807 | 252.1 | - | - | 0 | - |
| - | - | 1716 | 252.2 | - | - | 0 | - |
| - | - | 4962 | 252.2 | - | - | 0 | - |
| - | - | 2674 | 253.1 | - | - | 0 | - |
| - | - | 4.331E+04 | 254.1 | - | - | 0 | - |
| - | - | 1.18E+04 | 254.2 | - | - | 0 | - |
| - | - | 2773 | 255.1 | - | - | 0 | - |
| - | - | 1737 | 255.2 | - | - | 0 | - |
| - | - | 2047 | 257.2 | - | - | 0 | - |
| - | - | 2418 | 258.1 | - | - | 0 | - |
| - | - | 2.962E+04 | 259.1 | - | - | 0 | - |
| - | - | 2595 | 260.1 | - | - | 0 | - |
| - | - | 3.466E+04 | 261.2 | - | - | 0 | - |
| - | - | 5873 | 262.1 | - | - | 0 | - |
| - | - | 4168 | 262.2 | - | - | 0 | - |
| - | - | 3008 | 263.1 | - | - | 0 | - |
| - | - | 8031 | 264.1 | - | - | 0 | - |
| - | - | 4604 | 268.1 | - | - | 0 | - |
| - | - | 3213 | 268.2 | - | - | 0 | - |
| 3 | a | 1.032E+04 | 268.2 | 0.000378 | 1.409 | +1 | 3 |
| - | - | 1626 | 269.1 | - | - | 0 | - |
| - | - | 1863 | 269.2 | - | - | 0 | - |
| - | - | 1.421E+04 | 269.2 | - | - | 0 | - |
| - | - | 1677 | 270.1 | - | - | 0 | - |
| - | - | 6164 | 270.2 | - | - | 0 | - |
| - | - | 1463 | 271.2 | - | - | 0 | - |
| - | - | 2.142E+04 | 272.1 | - | - | 0 | - |
| - | - | 3181 | 273.1 | - | - | 0 | - |
| - | - | 2.208E+04 | 275.2 | - | - | 0 | - |
| - | - | 3410 | 278.1 | - | - | 0 | - |
| - | - | 1.959E+04 | 279.1 | - | - | 0 | - |
| - | - | 8387 | 280.1 | - | - | 0 | - |
| - | - | 2086 | 280.1 | - | - | 0 | - |
| - | - | 3.311E+04 | 280.2 | - | - | 0 | - |
| - | - | 3239 | 281.2 | - | - | 0 | - |
| - | - | 2488 | 282.1 | - | - | 0 | - |
| - | - | 6.463E+04 | 282.1 | - | - | 0 | - |
| - | - | 7340 | 283.1 | - | - | 0 | - |
| - | - | 2.562E+04 | 285.2 | - | - | 0 | - |
| - | - | 4107 | 285.2 | - | - | 0 | - |
| - | - | 2.021E+04 | 286.1 | - | - | 0 | - |
| - | - | 3763 | 286.2 | - | - | 0 | - |
| 3 | a | 3279 | 286.2 | 6.725E-05 | 0.235 | +1 | 3 |
| - | - | 2327 | 287.1 | - | - | 0 | - |
| - | - | 2019 | 289.2 | - | - | 0 | - |
| - | - | 1606 | 292.1 | - | - | 0 | - |
| - | - | 1779 | 294.1 | - | - | 0 | - |
| - | - | 6469 | 294.2 | - | - | 0 | - |
| - | - | 1481 | 295.2 | - | - | 0 | - |
| 3 | b | 9.893E+05 | 296.2 | 0.0005806 | 1.96 | +1 | 3 |
| - | - | 8488 | 297.1 | - | - | 0 | - |
| - | - | 5.502E+04 | 297.2 | - | - | 0 | - |
| - | - | 1.389E+05 | 297.2 | - | - | 0 | - |
| - | - | 2001 | 298.1 | - | - | 0 | - |
| - | - | 6502 | 298.2 | - | - | 0 | - |
| - | - | 3.402E+04 | 298.2 | - | - | 0 | - |
| - | - | 7704 | 298.2 | - | - | 0 | - |
| - | - | 2533 | 299.1 | - | - | 0 | - |
| - | - | 7492 | 299.2 | - | - | 0 | - |
| - | - | 2527 | 300.1 | - | - | 0 | - |
| - | - | 3041 | 300.2 | - | - | 0 | - |
| - | - | 2829 | 300.2 | - | - | 0 | - |
| - | - | 2487 | 301.2 | - | - | 0 | - |
| 15 | y | 6110 | 301.2 | 0.0004377 | 1.453 | +1 | 3 |
| 15 | y | 3004 | 302.2 | 0.0002173 | 0.7192 | +1 | 3 |
| - | - | 3.975E+04 | 303.2 | - | - | 0 | - |
| - | - | 1.143E+04 | 304.1 | - | - | 0 | - |
| - | - | 6198 | 304.2 | - | - | 0 | - |
| - | - | 4209 | 306.1 | - | - | 0 | - |
| - | - | 1913 | 307.1 | - | - | 0 | - |
| 12 | y | 4582 | 308.2 | 0.002845 | 9.231 | +2 | 6 |
| - | - | 4968 | 310.1 | - | - | 0 | - |
| - | - | 1623 | 311.2 | - | - | 0 | - |
| - | - | 1587 | 312.2 | - | - | 0 | - |
| - | - | 5768 | 313.2 | - | - | 0 | - |
| - | - | 2209 | 314.1 | - | - | 0 | - |
| - | - | 7286 | 314.1 | - | - | 0 | - |
| - | - | 3.916E+04 | 314.2 | - | - | 0 | - |
| 3 | b | 2.457E+05 | 314.2 | 0.0006055 | 1.927 | +1 | 3 |
| - | - | 1989 | 315.2 | - | - | 0 | - |
| - | - | 5743 | 315.2 | - | - | 0 | - |
| - | - | 4.03E+04 | 315.2 | - | - | 0 | - |
| - | - | 2357 | 316.2 | - | - | 0 | - |
| - | - | 8610 | 317.2 | - | - | 0 | - |
| - | - | 1834 | 318.1 | - | - | 0 | - |
| 15 | y | 5.921E+04 | 319.2 | 0.0004627 | 1.449 | +1 | 3 |
| - | - | 9680 | 320.2 | - | - | 0 | - |
| - | - | 1840 | 321.2 | - | - | 0 | - |
| - | - | 2112 | 322.2 | - | - | 0 | - |
| - | - | 4939 | 323.2 | - | - | 0 | - |
| - | - | 1907 | 324.2 | - | - | 0 | - |
| - | - | 2637 | 325.2 | - | - | 0 | - |
| - | - | 3421 | 326.2 | - | - | 0 | - |
| - | - | 1.808E+04 | 328.1 | - | - | 0 | - |
| - | - | 2080 | 328.2 | - | - | 0 | - |
| - | - | 1985 | 329.1 | - | - | 0 | - |
| - | - | 2183 | 329.2 | - | - | 0 | - |
| - | - | 2.545E+04 | 330.2 | - | - | 0 | - |
| - | - | 3040 | 331.2 | - | - | 0 | - |
| - | - | 7575 | 333.2 | - | - | 0 | - |
| - | - | 8452 | 334.2 | - | - | 0 | - |
| - | - | 2139 | 335.2 | - | - | 0 | - |
| - | - | 4307 | 336.2 | - | - | 0 | - |
| - | - | 8318 | 337.2 | - | - | 0 | - |
| - | - | 3715 | 338.1 | - | - | 0 | - |
| - | - | 5844 | 339.2 | - | - | 0 | - |
| - | - | 1756 | 339.2 | - | - | 0 | - |
| - | - | 2455 | 340.2 | - | - | 0 | - |
| - | - | 1.86E+04 | 341.2 | - | - | 0 | - |
| - | - | 6638 | 342.2 | - | - | 0 | - |
| - | - | 9519 | 344.2 | - | - | 0 | - |
| - | - | 4.701E+04 | 346.1 | - | - | 0 | - |
| - | - | 3.709E+04 | 346.2 | - | - | 0 | - |
| - | - | 7445 | 347.1 | - | - | 0 | - |
| - | - | 7004 | 347.2 | - | - | 0 | - |
| - | - | 2.885E+04 | 348.2 | - | - | 0 | - |
| - | - | 1680 | 349.2 | - | - | 0 | - |
| - | - | 5392 | 349.2 | - | - | 0 | - |
| - | - | 6862 | 350.2 | - | - | 0 | - |
| - | - | 2.845E+04 | 351.2 | - | - | 0 | - |
| - | - | 1450 | 351.2 | - | - | 0 | - |
| - | - | 5331 | 352.2 | - | - | 0 | - |
| - | - | 1.55E+04 | 353.2 | - | - | 0 | - |
| - | - | 1.196E+04 | 354.2 | - | - | 0 | - |
| - | - | 2495 | 354.2 | - | - | 0 | - |
| - | - | 9435 | 355.2 | - | - | 0 | - |
| - | - | 2804 | 356.1 | - | - | 0 | - |
| - | - | 1908 | 356.2 | - | - | 0 | - |
| - | - | 6615 | 360.2 | - | - | 0 | - |
| - | - | 8.354E+04 | 362.2 | - | - | 0 | - |
| - | - | 2077 | 363.2 | - | - | 0 | - |
| - | - | 1.251E+04 | 363.2 | - | - | 0 | - |
| - | - | 1.658E+04 | 365.1 | - | - | 0 | - |
| - | - | 6047 | 365.3 | - | - | 0 | - |
| - | - | 3185 | 366.1 | - | - | 0 | - |
| - | - | 1.031E+04 | 367.2 | - | - | 0 | - |
| - | - | 1713 | 368.2 | - | - | 0 | - |
| - | - | 7968 | 368.2 | - | - | 0 | - |
| - | - | 2126 | 369.1 | - | - | 0 | - |
| - | - | 4.43E+04 | 369.2 | - | - | 0 | - |
| - | - | 2181 | 370.1 | - | - | 0 | - |
| - | - | 7227 | 370.2 | - | - | 0 | - |
| - | - | 5714 | 370.7 | - | - | 0 | - |
| - | - | 1772 | 371.2 | - | - | 0 | - |
| - | - | 1.024E+04 | 371.2 | - | - | 0 | - |
| - | - | 1.028E+04 | 372.2 | - | - | 0 | - |
| - | - | 1.491E+04 | 373.2 | - | - | 0 | - |
| - | - | 2493 | 374.2 | - | - | 0 | - |
| - | - | 2138 | 375.2 | - | - | 0 | - |
| - | - | 1965 | 377.2 | - | - | 0 | - |
| - | - | 2194 | 379.2 | - | - | 0 | - |
| - | - | 1736 | 380.2 | - | - | 0 | - |
| - | - | 5171 | 381.2 | - | - | 0 | - |
| - | - | 2.727E+04 | 383.2 | - | - | 0 | - |
| - | - | 7055 | 383.3 | - | - | 0 | - |
| - | - | 4887 | 384.2 | - | - | 0 | - |
| - | - | 2.553E+04 | 385.2 | - | - | 0 | - |
| - | - | 2787 | 386.2 | - | - | 0 | - |
| - | - | 2514 | 386.2 | - | - | 0 | - |
| - | - | 2276 | 387.2 | - | - | 0 | - |
| - | - | 1798 | 388.2 | - | - | 0 | - |
| - | - | 2048 | 390.2 | - | - | 0 | - |
| - | - | 4640 | 390.2 | - | - | 0 | - |
| - | - | 5122 | 393.2 | - | - | 0 | - |
| - | - | 1.27E+04 | 393.3 | - | - | 0 | - |
| - | - | 3914 | 394.3 | - | - | 0 | - |
| - | - | 2202 | 395.2 | - | - | 0 | - |
| - | - | 6861 | 397.1 | - | - | 0 | - |
| - | - | 1895 | 398.1 | - | - | 0 | - |
| - | - | 2706 | 398.2 | - | - | 0 | - |
| - | - | 1.905E+04 | 398.2 | - | - | 0 | - |
| - | - | 5004 | 399.2 | - | - | 0 | - |
| - | - | 4527 | 399.2 | - | - | 0 | - |
| - | - | 4178 | 400.2 | - | - | 0 | - |
| - | - | 5.214E+04 | 401.2 | - | - | 0 | - |
| - | - | 2739 | 401.2 | - | - | 0 | - |
| - | - | 9303 | 402.2 | - | - | 0 | - |
| - | - | 3418 | 405.2 | - | - | 0 | - |
| - | - | 1840 | 406.2 | - | - | 0 | - |
| - | - | 2171 | 407.2 | - | - | 0 | - |
| - | - | 9933 | 407.2 | - | - | 0 | - |
| - | - | 1.804E+04 | 408.2 | - | - | 0 | - |
| - | - | 2208 | 409.2 | - | - | 0 | - |
| - | - | 4356 | 410.2 | - | - | 0 | - |
| - | - | 1763 | 411.2 | - | - | 0 | - |
| - | - | 2.811E+04 | 411.3 | - | - | 0 | - |
| - | - | 4798 | 412.3 | - | - | 0 | - |
| - | - | 2550 | 413.2 | - | - | 0 | - |
| - | - | 1.526E+04 | 415.1 | - | - | 0 | - |
| 8 | b | 5623 | 415.2 | 0.00442 | 10.64 | +2 | 8 |
| - | - | 1.136E+04 | 415.3 | - | - | 0 | - |
| - | - | 3065 | 416.1 | - | - | 0 | - |
| - | - | 2432 | 416.2 | - | - | 0 | - |
| - | - | 3.847E+04 | 416.3 | - | - | 0 | - |
| - | - | 1.084E+04 | 417.3 | - | - | 0 | - |
| - | - | 2636 | 418.3 | - | - | 0 | - |
| - | - | 2153 | 419.2 | - | - | 0 | - |
| - | - | 2576 | 422.2 | - | - | 0 | - |
| - | - | 2979 | 423.2 | - | - | 0 | - |
| - | - | 7521 | 424.2 | - | - | 0 | - |
| - | - | 4835 | 425.1 | - | - | 0 | - |
| - | - | 1.674E+04 | 425.2 | - | - | 0 | - |
| - | - | 2977 | 426.2 | - | - | 0 | - |
| - | - | 9128 | 427.2 | - | - | 0 | - |
| - | - | 2138 | 428.2 | - | - | 0 | - |
| - | - | 1903 | 429.2 | - | - | 0 | - |
| - | - | 2443 | 429.2 | - | - | 0 | - |
| - | - | 2492 | 431.3 | - | - | 0 | - |
| - | - | 2.345E+04 | 433.2 | - | - | 0 | - |
| 14 | y | 4.91E+04 | 433.2 | 0.001908 | 4.403 | +1 | 4 |
| - | - | 3.102E+05 | 433.3 | - | - | 0 | - |
| - | - | 3468 | 434.2 | - | - | 0 | - |
| - | - | 7540 | 434.2 | - | - | 0 | - |
| - | - | 6843 | 434.2 | - | - | 0 | - |
| - | - | 7.942E+04 | 434.3 | - | - | 0 | - |
| - | - | 5921 | 434.7 | - | - | 0 | - |
| - | - | 6153 | 435.2 | - | - | 0 | - |
| - | - | 9966 | 435.3 | - | - | 0 | - |
| - | - | 4768 | 440.2 | - | - | 0 | - |
| - | - | 8551 | 441.2 | - | - | 0 | - |
| - | - | 2917 | 441.3 | - | - | 0 | - |
| - | - | 1.383E+04 | 442.2 | - | - | 0 | - |
| - | - | 6034 | 443.1 | - | - | 0 | - |
| - | - | 3307 | 443.2 | - | - | 0 | - |
| - | - | 1.583E+04 | 443.2 | - | - | 0 | - |
| 4 | b | 3.545E+04 | 443.3 | 0.0009533 | 2.151 | +1 | 4 |
| - | - | 3176 | 444.2 | - | - | 0 | - |
| - | - | 2512 | 444.2 | - | - | 0 | - |
| - | - | 7687 | 444.3 | - | - | 0 | - |
| - | - | 4796 | 445.2 | - | - | 0 | - |
| - | - | 2000 | 446.3 | - | - | 0 | - |
| - | - | 2411 | 448.2 | - | - | 0 | - |
| - | - | 3357 | 451.2 | - | - | 0 | - |
| - | - | 9078 | 452.2 | - | - | 0 | - |
| - | - | 2580 | 453.2 | - | - | 0 | - |
| - | - | 2045 | 459.2 | - | - | 0 | - |
| - | - | 1.272E+04 | 459.2 | - | - | 0 | - |
| - | - | 4978 | 459.3 | - | - | 0 | - |
| - | - | 3708 | 460.2 | - | - | 0 | - |
| - | - | 1922 | 460.3 | - | - | 0 | - |
| 4 | b | 2.756E+05 | 461.3 | 0.0008561 | 1.856 | +1 | 4 |
| - | - | 1.641E+04 | 462.2 | - | - | 0 | - |
| - | - | 6.843E+04 | 462.3 | - | - | 0 | - |
| - | - | 2977 | 463.2 | - | - | 0 | - |
| - | - | 7709 | 463.3 | - | - | 0 | - |
| - | - | 1993 | 464.2 | - | - | 0 | - |
| - | - | 5556 | 466.2 | - | - | 0 | - |
| - | - | 3700 | 468.2 | - | - | 0 | - |
| - | - | 1750 | 468.3 | - | - | 0 | - |
| - | - | 6784 | 469.2 | - | - | 0 | - |
| - | - | 3.746E+04 | 470.2 | - | - | 0 | - |
| - | - | 1.035E+04 | 471.2 | - | - | 0 | - |
| - | - | 4088 | 476.2 | - | - | 0 | - |
| - | - | 3775 | 477.3 | - | - | 0 | - |
| - | - | 8133 | 478.2 | - | - | 0 | - |
| - | - | 4642 | 479.2 | - | - | 0 | - |
| - | - | 2.941E+04 | 480.2 | - | - | 0 | - |
| - | - | 8298 | 481.2 | - | - | 0 | - |
| - | - | 5667 | 482.2 | - | - | 0 | - |
| - | - | 1971 | 483.2 | - | - | 0 | - |
| - | - | 1.264E+04 | 484.2 | - | - | 0 | - |
| 13 | y | 5955 | 486.3 | 0.0004746 | 0.9761 | +1 | 5 |
| - | - | 1950 | 486.3 | - | - | 0 | - |
| 13 | y | 7954 | 487.3 | 0.00178 | 3.653 | +1 | 5 |
| - | - | 2929 | 492.2 | - | - | 0 | - |
| - | - | 1.267E+04 | 494.2 | - | - | 0 | - |
| - | - | 2462 | 495.2 | - | - | 0 | - |
| - | - | 6204 | 495.3 | - | - | 0 | - |
| - | - | 1.819E+04 | 496.3 | - | - | 0 | - |
| - | - | 2897 | 497.3 | - | - | 0 | - |
| - | - | 1.005E+05 | 498.2 | - | - | 0 | - |
| - | - | 2.383E+04 | 499.2 | - | - | 0 | - |
| - | - | 7696 | 500.2 | - | - | 0 | - |
| - | - | 1789 | 501.2 | - | - | 0 | - |
| - | - | 2.251E+04 | 502.2 | - | - | 0 | - |
| - | - | 4368 | 503.2 | - | - | 0 | - |
| - | - | 2059 | 503.3 | - | - | 0 | - |
| 13 | y | 6.937E+04 | 504.3 | 0.0006216 | 1.233 | +1 | 5 |
| - | - | 1.474E+04 | 505.3 | - | - | 0 | - |
| - | - | 1785 | 506.2 | - | - | 0 | - |
| - | - | 1790 | 506.3 | - | - | 0 | - |
| - | - | 5300 | 510.2 | - | - | 0 | - |
| - | - | 2.185E+04 | 512.2 | - | - | 0 | - |
| - | - | 2420 | 512.3 | - | - | 0 | - |
| - | - | 5376 | 513.2 | - | - | 0 | - |
| - | - | 6786 | 513.3 | - | - | 0 | - |
| - | - | 3168 | 515.2 | - | - | 0 | - |
| - | - | 1965 | 516.2 | - | - | 0 | - |
| - | - | 4896 | 518.2 | - | - | 0 | - |
| - | - | 1787 | 521.3 | - | - | 0 | - |
| - | - | 3655 | 524.2 | - | - | 0 | - |
| - | - | 2053 | 527.2 | - | - | 0 | - |
| - | - | 6356 | 528.2 | - | - | 0 | - |
| - | - | 7.272E+04 | 530.2 | - | - | 0 | - |
| - | - | 2.163E+04 | 530.3 | - | - | 0 | - |
| - | - | 1.836E+04 | 531.2 | - | - | 0 | - |
| - | - | 5550 | 531.3 | - | - | 0 | - |
| - | - | 2727 | 532.2 | - | - | 0 | - |
| 10 | b | 1918 | 535.3 | 0.008904 | 16.63 | +2 | 10 |
| - | - | 1867 | 536.2 | - | - | 0 | - |
| - | - | 1685 | 538.3 | - | - | 0 | - |
| - | - | 7403 | 538.3 | - | - | 0 | - |
| - | - | 1669 | 539.3 | - | - | 0 | - |
| 5 | b | 1.456E+04 | 540.3 | 0.0009238 | 1.71 | +1 | 5 |
| - | - | 2461 | 541.3 | - | - | 0 | - |
| - | - | 8307 | 546.2 | - | - | 0 | - |
| - | - | 2467 | 547.2 | - | - | 0 | - |
| - | - | 2652 | 553.3 | - | - | 0 | - |
| - | - | 5869 | 555.3 | - | - | 0 | - |
| - | - | 4098 | 556.2 | - | - | 0 | - |
| - | - | 2182 | 556.3 | - | - | 0 | - |
| 5 | b | 4.109E+04 | 558.3 | 0.000552 | 0.9887 | +1 | 5 |
| - | - | 1.058E+04 | 559.3 | - | - | 0 | - |
| - | - | 3873 | 563.2 | - | - | 0 | - |
| - | - | 2104 | 564.2 | - | - | 0 | - |
| - | - | 9419 | 569.3 | - | - | 0 | - |
| - | - | 9634 | 569.8 | - | - | 0 | - |
| - | - | 3353 | 570.3 | - | - | 0 | - |
| - | - | 4892 | 571.3 | - | - | 0 | - |
| - | - | 2918 | 573.8 | - | - | 0 | - |
| - | - | 3139 | 574.3 | - | - | 0 | - |
| - | - | 1.66E+04 | 578.3 | - | - | 0 | - |
| - | - | 7591 | 578.8 | - | - | 0 | - |
| - | - | 3814 | 579.3 | - | - | 0 | - |
| - | - | 1.622E+04 | 581.3 | - | - | 0 | - |
| - | - | 7752 | 582.3 | - | - | 0 | - |
| - | - | 2.353E+04 | 582.8 | - | - | 0 | - |
| - | - | 1.226E+04 | 583.3 | - | - | 0 | - |
| - | - | 1738 | 583.8 | - | - | 0 | - |
| - | - | 4422 | 587.3 | - | - | 0 | - |
| - | - | 5680 | 591.2 | - | - | 0 | - |
| - | - | 7271 | 591.3 | - | - | 0 | - |
| 11 | b | 5858 | 591.8 | 0.009798 | 16.56 | +2 | 11 |
| - | - | 3000 | 592.3 | - | - | 0 | - |
| - | - | 1.077E+04 | 597.3 | - | - | 0 | - |
| - | - | 4455 | 598.3 | - | - | 0 | - |
| - | - | 7.077E+04 | 599.3 | - | - | 0 | - |
| - | - | 1.876E+04 | 600.3 | - | - | 0 | - |
| - | - | 1952 | 601.3 | - | - | 0 | - |
| - | - | 3653 | 604.3 | - | - | 0 | - |
| - | - | 3469 | 604.8 | - | - | 0 | - |
| - | - | 4337 | 607.3 | - | - | 0 | - |
| - | - | 1637 | 608.3 | - | - | 0 | - |
| - | - | 4448 | 608.4 | - | - | 0 | - |
| - | - | 3856 | 608.8 | - | - | 0 | - |
| - | - | 2.071E+04 | 609.3 | - | - | 0 | - |
| - | - | 4191 | 609.3 | - | - | 0 | - |
| - | - | 2339 | 609.3 | - | - | 0 | - |
| - | - | 6115 | 609.8 | - | - | 0 | - |
| - | - | 5291 | 610.3 | - | - | 0 | - |
| - | - | 1892 | 610.3 | - | - | 0 | - |
| - | - | 1.399E+04 | 612.8 | - | - | 0 | - |
| - | - | 1.22E+04 | 613.3 | - | - | 0 | - |
| - | - | 2904 | 613.8 | - | - | 0 | - |
| 12 | y | 9832 | 614.3 | 8.896E-05 | 0.1448 | +1 | 6 |
| 12 | y | 3.692E+04 | 615.3 | 0.005406 | 8.786 | +1 | 6 |
| - | - | 8894 | 616.3 | - | - | 0 | - |
| - | - | 2586 | 617.3 | - | - | 0 | - |
| - | - | 6.536E+04 | 617.8 | - | - | 0 | - |
| - | - | 5.337E+04 | 618.3 | - | - | 0 | - |
| - | - | 2.043E+04 | 618.8 | - | - | 0 | - |
| - | - | 5241 | 619.3 | - | - | 0 | - |
| - | - | 1802 | 624.3 | - | - | 0 | - |
| - | - | 1.084E+04 | 625.3 | - | - | 0 | - |
| - | - | 5625 | 626.3 | - | - | 0 | - |
| - | - | 9951 | 626.4 | - | - | 0 | - |
| - | - | 1.452E+05 | 626.8 | - | - | 0 | - |
| - | - | 1.6E+05 | 627.3 | - | - | 0 | - |
| - | - | 9.555E+04 | 627.3 | - | - | 0 | - |
| - | - | 3.407E+04 | 627.8 | - | - | 0 | - |
| - | - | 4.19E+04 | 628.3 | - | - | 0 | - |
| - | - | 2351 | 628.3 | - | - | 0 | - |
| 6 | y | 3161 | 628.8 | 0.002431 | 3.866 | +2 | 12 |
| - | - | 6637 | 629.3 | - | - | 0 | - |
| - | - | 2720 | 629.3 | - | - | 0 | - |
| - | - | 3241 | 629.8 | - | - | 0 | - |
| 12 | y | 4.827E+04 | 632.3 | 0.0005768 | 0.9122 | +1 | 6 |
| - | - | 1.285E+04 | 633.3 | - | - | 0 | - |
| - | - | 5841 | 635.8 | - | - | 0 | - |
| - | - | 5005 | 636.3 | - | - | 0 | - |
| - | - | 2448 | 636.8 | - | - | 0 | - |
| 6 | y | 2.046E+04 | 637.8 | 0.0002822 | 0.4425 | +2 | 12 |
| - | - | 1.268E+04 | 638.3 | - | - | 0 | - |
| - | - | 3599 | 638.8 | - | - | 0 | - |
| - | - | 4299 | 640.3 | - | - | 0 | - |
| - | - | 4698 | 642.3 | - | - | 0 | - |
| - | - | 3.263E+04 | 643.3 | - | - | 0 | - |
| - | - | 1.071E+04 | 644.3 | - | - | 0 | - |
| - | - | 6474 | 645.3 | - | - | 0 | - |
| - | - | 1885 | 646.3 | - | - | 0 | - |
| - | - | 3070 | 653.3 | - | - | 0 | - |
| - | - | 9762 | 655.3 | - | - | 0 | - |
| 12 | b | 5200 | 655.8 | 0.006873 | 10.48 | +2 | 12 |
| 12 | b | 2193 | 656.3 | 0.01273 | 19.39 | +2 | 12 |
| - | - | 2672 | 656.8 | - | - | 0 | - |
| - | - | 2553 | 657.3 | - | - | 0 | - |
| - | - | 4915 | 658.3 | - | - | 0 | - |
| - | - | 2174 | 659.3 | - | - | 0 | - |
| - | - | 1.202E+04 | 660.3 | - | - | 0 | - |
| - | - | 4005 | 661.3 | - | - | 0 | - |
| - | - | 5314 | 663.3 | - | - | 0 | - |
| - | - | 4059 | 663.8 | - | - | 0 | - |
| - | - | 4239 | 664.3 | - | - | 0 | - |
| - | - | 1906 | 667.3 | - | - | 0 | - |
| - | - | 2643 | 668.3 | - | - | 0 | - |
| - | - | 1.21E+04 | 668.8 | - | - | 0 | - |
| - | - | 7449 | 669.3 | - | - | 0 | - |
| - | - | 4442 | 674.3 | - | - | 0 | - |
| 5 | y | 4.854E+04 | 677.3 | 0.000545 | 0.8046 | +2 | 13 |
| 5 | y | 6.635E+04 | 677.8 | 0.004448 | 6.562 | +2 | 13 |
| - | - | 3.479E+04 | 678.3 | - | - | 0 | - |
| - | - | 1.212E+04 | 678.8 | - | - | 0 | - |
| - | - | 2352 | 679.3 | - | - | 0 | - |
| - | - | 2086 | 684.4 | - | - | 0 | - |
| - | - | 4874 | 685.3 | - | - | 0 | - |
| - | - | 3352 | 685.8 | - | - | 0 | - |
| 5 | y | 1.124E+06 | 686.3 | 0.0007558 | 1.101 | +2 | 13 |
| - | - | 8.527E+05 | 686.8 | - | - | 0 | - |
| - | - | 2.748E+05 | 687.3 | - | - | 0 | - |
| - | - | 2.351E+04 | 687.8 | - | - | 0 | - |
| 13 | b | 2545 | 691.8 | 0.005218 | 7.542 | +2 | 13 |
| - | - | 1.094E+04 | 694.3 | - | - | 0 | - |
| - | - | 3817 | 695.3 | - | - | 0 | - |
| - | - | 2697 | 698.3 | - | - | 0 | - |
| 13 | b | 1.357E+04 | 700.4 | 0.01214 | 17.34 | +2 | 13 |
| - | - | 1.261E+04 | 700.8 | - | - | 0 | - |
| - | - | 3348 | 701.3 | - | - | 0 | - |
| - | - | 4635 | 704.3 | - | - | 0 | - |
| - | - | 2384 | 705.3 | - | - | 0 | - |
| - | - | 2086 | 709.3 | - | - | 0 | - |
| - | - | 8.951E+04 | 712.4 | - | - | 0 | - |
| - | - | 2.921E+04 | 713.4 | - | - | 0 | - |
| - | - | 4367 | 714.4 | - | - | 0 | - |
| - | - | 2357 | 721.4 | - | - | 0 | - |
| - | - | 2180 | 721.7 | - | - | 0 | - |
| - | - | 1.208E+04 | 722.3 | - | - | 0 | - |
| - | - | 4703 | 723.3 | - | - | 0 | - |
| - | - | 2001 | 725.3 | - | - | 0 | - |
| - | - | 2577 | 726.3 | - | - | 0 | - |
| - | - | 2979 | 727.3 | - | - | 0 | - |
| - | - | 2184 | 728.3 | - | - | 0 | - |
| 11 | y | 2700 | 728.4 | 0.002205 | 3.027 | +1 | 7 |
| - | - | 1814 | 731.4 | - | - | 0 | - |
| - | - | 2391 | 735.3 | - | - | 0 | - |
| - | - | 2042 | 737.4 | - | - | 0 | - |
| - | - | 4529 | 738.3 | - | - | 0 | - |
| - | - | 5057 | 738.4 | - | - | 0 | - |
| - | - | 6849 | 739.4 | - | - | 0 | - |
| - | - | 1.055E+05 | 740.3 | - | - | 0 | - |
| - | - | 3.995E+04 | 741.3 | - | - | 0 | - |
| - | - | 6995 | 742.4 | - | - | 0 | - |
| 7 | b | 7146 | 742.4 | 0.001941 | 2.615 | +1 | 7 |
| - | - | 2124 | 743.4 | - | - | 0 | - |
| - | - | 4228 | 743.4 | - | - | 0 | - |
| - | - | 5850 | 745.3 | - | - | 0 | - |
| 11 | y | 3.776E+04 | 745.4 | 0.0006803 | 0.9127 | +1 | 7 |
| - | - | 2245 | 746.3 | - | - | 0 | - |
| - | - | 1.389E+04 | 746.4 | - | - | 0 | - |
| 4 | y | 6999 | 750.9 | 9.045E-05 | 0.1205 | +2 | 14 |
| 4 | y | 4795 | 751.4 | 0.005824 | 7.752 | +2 | 14 |
| - | - | 4826 | 751.9 | - | - | 0 | - |
| - | - | 7475 | 753.3 | - | - | 0 | - |
| - | - | 6073 | 754.3 | - | - | 0 | - |
| - | - | 4768 | 755.3 | - | - | 0 | - |
| - | - | 6563 | 755.4 | - | - | 0 | - |
| - | - | 2690 | 756.4 | - | - | 0 | - |
| - | - | 2953 | 756.9 | - | - | 0 | - |
| 14 | b | 5.045E+04 | 757.4 | 0.006143 | 8.111 | +2 | 14 |
| - | - | 1.735E+04 | 758.4 | - | - | 0 | - |
| - | - | 4129 | 759.4 | - | - | 0 | - |
| 4 | y | 1.495E+05 | 759.9 | 0.0005454 | 0.7178 | +2 | 14 |
| - | - | 1.35E+05 | 760.4 | - | - | 0 | - |
| - | - | 5.439E+04 | 760.9 | - | - | 0 | - |
| - | - | 8869 | 761.4 | - | - | 0 | - |
| - | - | 1760 | 766.4 | - | - | 0 | - |
| - | - | 2.677E+04 | 771.4 | - | - | 0 | - |
| - | - | 1.18E+04 | 772.4 | - | - | 0 | - |
| - | - | 4857 | 774.3 | - | - | 0 | - |
| - | - | 1993 | 775.3 | - | - | 0 | - |
| - | - | 2181 | 797.4 | - | - | 0 | - |
| 3 | y | 2795 | 807.9 | 0.01268 | 15.7 | +2 | 15 |
| - | - | 2769 | 809.4 | - | - | 0 | - |
| - | - | 3373 | 809.5 | - | - | 0 | - |
| - | - | 2874 | 811.4 | - | - | 0 | - |
| 15 | b | 4266 | 812.9 | 0.0001886 | 0.2321 | +2 | 15 |
| - | - | 4253 | 813.4 | - | - | 0 | - |
| - | - | 3749 | 814.4 | - | - | 0 | - |
| 3 | y | 3.647E+04 | 816.4 | 0.0008718 | 1.068 | +2 | 15 |
| - | - | 2.88E+04 | 816.9 | - | - | 0 | - |
| - | - | 1.673E+04 | 817.4 | - | - | 0 | - |
| - | - | 2597 | 821.9 | - | - | 0 | - |
| - | - | 3001 | 822.4 | - | - | 0 | - |
| - | - | 4081 | 823.4 | - | - | 0 | - |
| - | - | 7777 | 824.4 | - | - | 0 | - |
| - | - | 3443 | 825.4 | - | - | 0 | - |
| - | - | 3918 | 828.4 | - | - | 0 | - |
| 8 | b | 8079 | 829.4 | 0.004208 | 5.073 | +1 | 8 |
| - | - | 2701 | 830.4 | - | - | 0 | - |
| - | - | 3701 | 832.4 | - | - | 0 | - |
| - | - | 2130 | 839.4 | - | - | 0 | - |
| - | - | 1.554E+04 | 840.4 | - | - | 0 | - |
| - | - | 7776 | 841.4 | - | - | 0 | - |
| - | - | 2.12E+04 | 842.4 | - | - | 0 | - |
| - | - | 2268 | 843 | - | - | 0 | - |
| - | - | 8021 | 843.4 | - | - | 0 | - |
| 16 | b | 2923 | 847.9 | 0.001591 | 1.877 | +2 | 16 |
| 16 | b | 8985 | 848.4 | 0.004509 | 5.314 | +2 | 16 |
| - | - | 6480 | 848.9 | - | - | 0 | - |
| - | - | 3862 | 849.4 | - | - | 0 | - |
| - | - | 1.708E+04 | 850.4 | - | - | 0 | - |
| - | - | 1.024E+04 | 851.4 | - | - | 0 | - |
| - | - | 4545 | 852.4 | - | - | 0 | - |
| 10 | y | 3318 | 856.5 | 0.005578 | 6.513 | +1 | 8 |
| 16 | b | 2.801E+04 | 856.9 | 0.0002675 | 0.3122 | +2 | 16 |
| 10 | y | 3.907E+04 | 857.4 | 0.011 | 12.83 | +1 | 8 |
| 2 | y | 1.406E+04 | 857.9 | 0.01106 | 12.89 | +2 | 16 |
| - | - | 6036 | 858.4 | - | - | 0 | - |
| - | - | 1898 | 859.4 | - | - | 0 | - |
| - | - | 1996 | 860.4 | - | - | 0 | - |
| - | - | 2743 | 866.4 | - | - | 0 | - |
| 2 | y | 1.069E+04 | 866.9 | 0.00108 | 1.246 | +2 | 16 |
| - | - | 1.587E+04 | 867.4 | - | - | 0 | - |
| - | - | 3818 | 867.9 | - | - | 0 | - |
| - | - | 1.341E+05 | 868.4 | - | - | 0 | - |
| - | - | 5.69E+04 | 869.4 | - | - | 0 | - |
| - | - | 1.067E+04 | 870.4 | - | - | 0 | - |
| 10 | y | 2.575E+04 | 874.5 | 0.0001405 | 0.1606 | +1 | 8 |
| - | - | 1.036E+04 | 875.5 | - | - | 0 | - |
| - | - | 4525 | 884.4 | - | - | 0 | - |
| - | - | 7001 | 885.4 | - | - | 0 | - |
| - | - | 1991 | 886 | - | - | 0 | - |
| - | - | 2140 | 886.4 | - | - | 0 | - |
| - | - | 8797 | 887.4 | - | - | 0 | - |
| - | - | 2985 | 888.4 | - | - | 0 | - |
| - | - | 3342 | 893.4 | - | - | 0 | - |
| - | - | 8823 | 894.4 | - | - | 0 | - |
| - | - | 5427 | 895.4 | - | - | 0 | - |
| - | - | 2978 | 899 | - | - | 0 | - |
| - | - | 3121 | 899.5 | - | - | 0 | - |
| - | - | 3755 | 900.4 | - | - | 0 | - |
| - | - | 2048 | 904.4 | - | - | 0 | - |
| 0 | Precursor | 2.251E+04 | 907.5 | 0.0009644 | 1.063 | +2 | -1 |
| 0 | Precursor | 4.151E+04 | 908 | 0.008346 | 9.192 | +2 | -1 |
| - | - | 2.078E+04 | 908.5 | - | - | 0 | - |
| - | - | 4853 | 909 | - | - | 0 | - |
| - | - | 1996 | 909.5 | - | - | 0 | - |
| - | - | 2.261E+04 | 911.4 | - | - | 0 | - |
| - | - | 9881 | 912.4 | - | - | 0 | - |
| - | - | 2147 | 913.4 | - | - | 0 | - |
| - | - | 2362 | 915.5 | - | - | 0 | - |
| - | - | 2577 | 916 | - | - | 0 | - |
| - | - | 3430 | 916.4 | - | - | 0 | - |
| 0 | Precursor | 2.463E+05 | 916.5 | 0.0005038 | 0.5498 | +2 | -1 |
| - | - | 2.816E+05 | 917 | - | - | 0 | - |
| - | - | 1.337E+05 | 917.5 | - | - | 0 | - |
| - | - | 2.014E+04 | 918 | - | - | 0 | - |
| - | - | 1.225E+04 | 921.4 | - | - | 0 | - |
| - | - | 1.307E+04 | 922.4 | - | - | 0 | - |
| - | - | 6385 | 923.4 | - | - | 0 | - |
| - | - | 2468 | 936.4 | - | - | 0 | - |
| - | - | 2091 | 938.4 | - | - | 0 | - |
| - | - | 1.011E+05 | 939.4 | - | - | 0 | - |
| - | - | 5.118E+04 | 940.4 | - | - | 0 | - |
| - | - | 1.042E+04 | 941.4 | - | - | 0 | - |
| - | - | 4823 | 953.5 | - | - | 0 | - |
| - | - | 5237 | 954.5 | - | - | 0 | - |
| - | - | 1.562E+04 | 956.5 | - | - | 0 | - |
| - | - | 8013 | 957.4 | - | - | 0 | - |
| 9 | b | 5926 | 958.5 | 0.005541 | 5.781 | +1 | 9 |
| - | - | 3435 | 970.4 | - | - | 0 | - |
| - | - | 9609 | 971.5 | - | - | 0 | - |
| - | - | 2580 | 972.5 | - | - | 0 | - |
| - | - | 1934 | 974.4 | - | - | 0 | - |
| 9 | y | 2648 | 986.5 | 0.0008811 | 0.8932 | +1 | 9 |
| - | - | 3176 | 987.5 | - | - | 0 | - |
| - | - | 3403 | 988.5 | - | - | 0 | - |
| 9 | y | 1.705E+04 | 1004 | 0.0007046 | 0.7021 | +1 | 9 |
| - | - | 9698 | 1005 | - | - | 0 | - |
| - | - | 2659 | 1006 | - | - | 0 | - |
| - | - | 1.06E+04 | 1008 | - | - | 0 | - |
| - | - | 3200 | 1009 | - | - | 0 | - |
| - | - | 8395 | 1015 | - | - | 0 | - |
| - | - | 3126 | 1016 | - | - | 0 | - |
| - | - | 3274 | 1018 | - | - | 0 | - |
| - | - | 2685 | 1019 | - | - | 0 | - |
| - | - | 8724 | 1025 | - | - | 0 | - |
| - | - | 3603 | 1026 | - | - | 0 | - |
| - | - | 5816 | 1035 | - | - | 0 | - |
| - | - | 1.828E+04 | 1036 | - | - | 0 | - |
| - | - | 8170 | 1037 | - | - | 0 | - |
| - | - | 2399 | 1038 | - | - | 0 | - |
| - | - | 8076 | 1041 | - | - | 0 | - |
| - | - | 9084 | 1041 | - | - | 0 | - |
| - | - | 3082 | 1042 | - | - | 0 | - |
| - | - | 7.659E+04 | 1053 | - | - | 0 | - |
| - | - | 4.527E+04 | 1054 | - | - | 0 | - |
| - | - | 1.211E+04 | 1055 | - | - | 0 | - |
| - | - | 9914 | 1059 | - | - | 0 | - |
| - | - | 9891 | 1060 | - | - | 0 | - |
| - | - | 1889 | 1067 | - | - | 0 | - |
| - | - | 3012 | 1068 | - | - | 0 | - |
| - | - | 4893 | 1069 | - | - | 0 | - |
| 10 | b | 3227 | 1070 | 0.01688 | 15.78 | +1 | 10 |
| - | - | 2.629E+04 | 1071 | - | - | 0 | - |
| - | - | 1.469E+04 | 1072 | - | - | 0 | - |
| 8 | y | 6185 | 1073 | 0.006421 | 5.987 | +1 | 10 |
| 8 | y | 5048 | 1074 | 0.009319 | 8.681 | +1 | 10 |
| - | - | 1.728E+04 | 1085 | - | - | 0 | - |
| - | - | 1.039E+04 | 1086 | - | - | 0 | - |
| - | - | 1.063E+04 | 1087 | - | - | 0 | - |
| 10 | b | 1.037E+04 | 1088 | 0.001321 | 1.214 | +1 | 10 |
| - | - | 5134 | 1089 | - | - | 0 | - |
| 8 | y | 3.072E+04 | 1091 | 0.0004701 | 0.4311 | +1 | 10 |
| - | - | 1.544E+04 | 1092 | - | - | 0 | - |
| - | - | 6038 | 1093 | - | - | 0 | - |
| - | - | 4104 | 1121 | - | - | 0 | - |
| - | - | 2474 | 1122 | - | - | 0 | - |
| - | - | 2527 | 1128 | - | - | 0 | - |
| - | - | 4318 | 1129 | - | - | 0 | - |
| - | - | 1.235E+04 | 1138 | - | - | 0 | - |
| - | - | 1.461E+04 | 1139 | - | - | 0 | - |
| - | - | 7500 | 1140 | - | - | 0 | - |
| - | - | 1990 | 1141 | - | - | 0 | - |
| - | - | 2338 | 1147 | - | - | 0 | - |
| - | - | 2.607E+04 | 1156 | - | - | 0 | - |
| - | - | 2.012E+04 | 1157 | - | - | 0 | - |
| - | - | 3470 | 1158 | - | - | 0 | - |
| 7 | y | 6764 | 1160 | 0.001951 | 1.682 | +1 | 11 |
| 7 | y | 7081 | 1161 | 0.007198 | 6.202 | +1 | 11 |
| - | - | 2706 | 1162 | - | - | 0 | - |
| - | - | 5187 | 1164 | - | - | 0 | - |
| - | - | 1.797E+04 | 1165 | - | - | 0 | - |
| - | - | 1.028E+04 | 1166 | - | - | 0 | - |
| - | - | 2875 | 1167 | - | - | 0 | - |
| 7 | y | 4.019E+04 | 1178 | 6.415E-05 | 0.05448 | +1 | 11 |
| - | - | 2.203E+04 | 1179 | - | - | 0 | - |
| - | - | 7730 | 1180 | - | - | 0 | - |
| - | - | 8.936E+04 | 1182 | - | - | 0 | - |
| 11 | b | 6.131E+04 | 1183 | 0.02123 | 17.95 | +1 | 11 |
| - | - | 1.844E+04 | 1184 | - | - | 0 | - |
| - | - | 1908 | 1185 | - | - | 0 | - |
| 11 | b | 1.631E+04 | 1201 | 0.006893 | 5.742 | +1 | 11 |
| - | - | 1.186E+04 | 1202 | - | - | 0 | - |
| - | - | 3405 | 1203 | - | - | 0 | - |
| - | - | 2618 | 1208 | - | - | 0 | - |
| - | - | 2574 | 1209 | - | - | 0 | - |
| - | - | 5607 | 1218 | - | - | 0 | - |
| - | - | 3384 | 1219 | - | - | 0 | - |
| - | - | 6287 | 1225 | - | - | 0 | - |
| - | - | 4017 | 1226 | - | - | 0 | - |
| - | - | 4.132E+04 | 1235 | - | - | 0 | - |
| - | - | 3.526E+04 | 1236 | - | - | 0 | - |
| - | - | 1.489E+04 | 1237 | - | - | 0 | - |
| - | - | 4184 | 1238 | - | - | 0 | - |
| - | - | 2799 | 1240 | - | - | 0 | - |
| - | - | 8.661E+04 | 1253 | - | - | 0 | - |
| - | - | 6.199E+04 | 1254 | - | - | 0 | - |
| - | - | 1.574E+04 | 1255 | - | - | 0 | - |
| - | - | 2289 | 1256 | - | - | 0 | - |
| 6 | y | 9117 | 1257 | 0.001004 | 0.7986 | +1 | 12 |
| 6 | y | 2.044E+04 | 1258 | 0.002041 | 1.623 | +1 | 12 |
| - | - | 1.218E+04 | 1259 | - | - | 0 | - |
| - | - | 5498 | 1271 | - | - | 0 | - |
| - | - | 2806 | 1272 | - | - | 0 | - |
| - | - | 2250 | 1273 | - | - | 0 | - |
| 6 | y | 2.253E+05 | 1275 | 0.0009481 | 0.7438 | +1 | 12 |
| - | - | 1.474E+05 | 1276 | - | - | 0 | - |
| - | - | 5.086E+04 | 1277 | - | - | 0 | - |
| - | - | 5490 | 1278 | - | - | 0 | - |
| - | - | 2792 | 1293 | - | - | 0 | - |
| - | - | 2051 | 1294 | - | - | 0 | - |
| - | - | 3427 | 1301 | - | - | 0 | - |
| - | - | 1.274E+04 | 1310 | - | - | 0 | - |
| 12 | b | 1.433E+04 | 1311 | 0.006007 | 4.583 | +1 | 12 |
| 12 | b | 7320 | 1312 | 0.01747 | 13.32 | +1 | 12 |
| - | - | 4008 | 1313 | - | - | 0 | - |
| - | - | 6407 | 1326 | - | - | 0 | - |
| - | - | 6234 | 1327 | - | - | 0 | - |
| - | - | 6828 | 1328 | - | - | 0 | - |
| 12 | b | 3.332E+04 | 1329 | 0.006023 | 4.533 | +1 | 12 |
| - | - | 2.367E+04 | 1330 | - | - | 0 | - |
| - | - | 7327 | 1331 | - | - | 0 | - |
| - | - | 3857 | 1336 | - | - | 0 | - |
| - | - | 7988 | 1337 | - | - | 0 | - |
| - | - | 7289 | 1338 | - | - | 0 | - |
| - | - | 3422 | 1339 | - | - | 0 | - |
| 5 | y | 4.504E+04 | 1354 | 0.001888 | 1.394 | +1 | 13 |
| 5 | y | 7.577E+04 | 1355 | 0.004942 | 3.648 | +1 | 13 |
| - | - | 4.027E+04 | 1356 | - | - | 0 | - |
| - | - | 1.412E+04 | 1357 | - | - | 0 | - |
| - | - | 5875 | 1370 | - | - | 0 | - |
| 5 | y | 1.333E+06 | 1372 | 0.001588 | 1.158 | +1 | 13 |
| - | - | 9.439E+05 | 1373 | - | - | 0 | - |
| - | - | 3.273E+05 | 1374 | - | - | 0 | - |
| - | - | 3.305E+04 | 1375 | - | - | 0 | - |
| 13 | b | 5031 | 1382 | 0.005716 | 4.137 | +1 | 13 |
| 13 | b | 5869 | 1383 | 0.003648 | 2.638 | +1 | 13 |
| 13 | b | 2.184E+04 | 1400 | 0.006515 | 4.655 | +1 | 13 |
| - | - | 1.655E+04 | 1401 | - | - | 0 | - |
| - | - | 5776 | 1402 | - | - | 0 | - |
| 14 | b | 2599 | 1497 | 0.003851 | 2.573 | +1 | 14 |
| 4 | y | 2877 | 1501 | 0.005482 | 3.653 | +1 | 14 |
| 4 | y | 3800 | 1502 | 0.0002399 | 0.1597 | +1 | 14 |
| 14 | b | 1.106E+04 | 1514 | 0.0009723 | 0.6423 | +1 | 14 |
| - | - | 9070 | 1515 | - | - | 0 | - |
| - | - | 4307 | 1516 | - | - | 0 | - |
| 4 | y | 7.147E+04 | 1519 | 0.002253 | 1.483 | +1 | 14 |
| - | - | 6.024E+04 | 1520 | - | - | 0 | - |
| - | - | 1305 | 1520 | - | - | 0 | - |
| - | - | 2.393E+04 | 1521 | - | - | 0 | - |
| 3 | y | 1.336E+04 | 1632 | 0.005628 | 3.449 | +1 | 15 |
| - | - | 1.146E+04 | 1633 | - | - | 0 | - |
| - | - | 4863 | 1634 | - | - | 0 | - |
| - | - | 2063 | 2235 | - | - | 0 | - |
| - | - | 1998 | 3072 | - | - | 0 | - |
| - | - | 1937 | 3408 | - | - | 0 | - |

m/z Charge Intensity FragmentType MassShift Position
120.06584930419922 0 8626.144 y 16
120.08109283447266 0 118124.445
121.08441162109375 0 8963.634
121.23117065429688 0 1050.6869
126.0552749633789 0 24496.156
127.05858612060547 0 2336.182
128.10728454589844 0 31716.725
129.06619262695312 0 6638.7295
129.10255432128906 0 211360.02
130.0501708984375 0 5069.45
130.1058807373047 0 11270.714
131.1181182861328 0 8415.057
132.07693481445312 0 2472.2598
133.06121826171875 0 1230.605
138.0916748046875 0 5763.8164
138.12802124023438 0 18142.87
139.05038452148438 0 1304.9515
139.08274841308594 0 955.1086
139.08688354492188 0 9754.693
139.0927734375 0 1391.031
139.09490966796875 0 1120.9705
139.13148498535156 0 1868.1005
141.06637573242188 0 2225.004
141.10264587402344 0 2174.9111
143.1175537109375 0 1513.724
144.06578063964844 0 1233.5667
147.07667541503906 0 6399.501
153.10244750976562 0 3666.7576
154.0865936279297 0 7825.639
154.09791564941406 0 3104.1646
155.0818634033203 0 6651.9834
155.11819458007812 0 32832.457 a Water loss 1
156.1218719482422 0 2034.1859
157.06101989746094 0 7186.785
157.0908203125 0 2030.6165
157.09747314453125 0 27866.828
165.10238647460938 0 3400.4878
166.0867919921875 0 4188.2773
167.08180236816406 0 18322.11
167.11822509765625 0 13616.513
168.0849151611328 0 2107.9373
168.12123107910156 0 2268.733
169.0611114501953 0 3251.415
169.09756469726562 0 1367.1417
169.13385009765625 0 31893.9
170.1378936767578 0 1693.0033
171.07679748535156 0 2778.5447
171.11402893066406 0 1584.0377
172.10816955566406 0 4294.937
173.0924072265625 0 9248.095 y Water loss 15
173.12876892089844 0 103846.266 a 1
174.12818908691406 0 6443.8335
175.0716094970703 0 10736.217
176.10757446289062 0 2333.8296
180.11349487304688 0 3233.2334
181.0974578857422 0 9416.167
182.0927734375 0 6175.156
182.12916564941406 0 11193.107
183.07691955566406 0 10606.1875
183.1131591796875 0 387544.12 b Water loss 1
184.10826110839844 0 10196.731
184.11663818359375 0 32452.516
185.0923614501953 0 36601.523
185.12852478027344 0 3320.177
186.08758544921875 0 7064.692
186.09552001953125 0 2394.2969
187.1444549560547 0 56433.305
188.1481475830078 0 3785.1357
189.08731079101562 0 7728.4883
191.08154296875 0 2812.1848
191.1029510498047 0 10168.666 y 15
195.0770263671875 0 2339.3062
195.11309814453125 0 31481.123
196.11672973632812 0 3026.3022
196.134033203125 0 1225.2286
197.0919647216797 0 1111.4817
197.1188201904297 0 2923.8691
197.12875366210938 0 40942.305
198.1239013671875 0 6670.578
198.13241577148438 0 4155.672
199.07162475585938 0 14906.437
199.10789489746094 0 6287.8174
199.11839294433594 0 2992.3918
200.10336303710938 0 11064.118
200.1395263671875 0 14364.264
201.12364196777344 0 193235.61 b 1
202.12713623046875 0 16064.59
203.103271484375 0 1796.6782
207.11228942871094 0 1232.5061
208.09751892089844 0 1879.6228
208.1083526611328 0 19651.648
209.09239196777344 0 50512.66
210.0962677001953 0 3096.5981
210.12388610839844 0 2441.7947
211.14442443847656 0 2706.9373
212.13954162597656 0 5905.236
213.0873565673828 0 4270.3896
213.12356567382812 0 3258.847
214.1544952392578 0 1799.6261
215.13934326171875 0 74308.41
216.1427459716797 0 7949.672
217.0822296142578 0 36685.6
217.13381958007812 0 44096.305
218.08627319335938 0 2493.8384
218.11544799804688 0 1371.7942
218.13720703125 0 4547.5117
218.14952087402344 0 3079.279
219.14968872070312 0 15773.753
220.15286254882812 0 1620.4172
224.17616271972656 0 2242.7053
225.12351989746094 0 17017.68
225.13514709472656 0 9485.072
225.16012573242188 0 3752.257
226.11895751953125 0 171668.3
227.1029052734375 0 4666.767
227.12246704101562 0 14342.865
228.13482666015625 0 2119.2244
230.5900421142578 0 1355.1584
231.09779357910156 0 4466.7534
233.1652374267578 0 96254.04
234.16859436035156 0 15196.113
236.10345458984375 0 10495.324
236.1204071044922 0 1543.6854
236.13999938964844 0 2211.6536
237.08743286132812 0 1668.5786
241.082275390625 0 10162.575
241.17849731445312 0 1398.004
241.19100952148438 0 3153.3801
242.11260986328125 0 1336.7883
242.15032958984375 0 32687.648
243.1338348388672 0 5112.4683
243.14572143554688 0 56897.598
244.130126953125 0 7882.0024 y Ammonia loss 12
244.1486053466797 0 5652.2544
245.12892150878906 0 28178.38
246.13223266601562 0 3556.0564
247.14451599121094 0 17381.338
248.14842224121094 0 3083.2627
251.17576599121094 0 51773.832
252.0985107421875 0 3347.211
252.13482666015625 0 5806.948
252.16796875 0 1716.3625
252.17938232421875 0 4961.8325
253.1306915283203 0 2674.4458
254.11387634277344 0 43305.27
254.150390625 0 11796.678
255.11819458007812 0 2772.735
255.15419006347656 0 1737.2098
257.1647033691406 0 2047.386
258.1094665527344 0 2418.2324
259.0927429199219 0 29622.15
260.0964660644531 0 2594.958
261.1600341796875 0 34660.79
262.1189880371094 0 5873.3564
262.1640319824219 0 4168.4927
263.1387634277344 0 3008.2217
264.13458251953125 0 8030.9863
268.0933532714844 0 4603.7495
268.16619873046875 0 3212.6152
268.20233154296875 0 10322.043 a Water loss 2
269.075927734375 0 1626.0404
269.160400390625 0 1862.6008
269.186279296875 0 14206.202
270.1445007324219 0 1676.7155
270.18194580078125 0 6163.895
271.18170166015625 0 1463.0936
272.1242370605469 0 21423.148
273.12786865234375 0 3180.7085
275.1756591796875 0 22075.727
278.1497802734375 0 3410.0374
279.14556884765625 0 19589.848
280.1294250488281 0 8386.634
280.1496887207031 0 2085.9204
280.1659240722656 0 33106.45
281.1701354980469 0 3239.3748
282.10821533203125 0 2487.6797
282.14520263671875 0 64633.79
283.1484069824219 0 7339.6094
285.16021728515625 0 25617.691
285.1974182128906 0 4106.7334
286.1037902832031 0 20208.082
286.16302490234375 0 3762.6294
286.21258544921875 0 3278.5315 a 2
287.1075439453125 0 2327.3088
289.1512145996094 0 2018.8519
292.13006591796875 0 1605.7728
294.1476135253906 0 1778.8508
294.1816711425781 0 6469.211
295.1839904785156 0 1480.6649
296.19744873046875 0 989319.3 b Water loss 2
297.1199951171875 0 8488.037
297.1563415527344 0 55021.63
297.2005310058594 0 138876.08
298.1037902832031 0 2000.5469
298.1588439941406 0 6502.187
298.1766357421875 0 34023.49
298.2033996582031 0 7704.078
299.08795166015625 0 2533.0933
299.176513671875 0 7492.2876
300.1194763183594 0 2526.925
300.1551818847656 0 3040.784
300.170166015625 0 2828.6963
301.1553039550781 0 2486.997
301.1874694824219 0 6110.131 y Water loss 14
302.1712646484375 0 3004.4934 y Ammonia loss 14
303.1708679199219 0 39754.926
304.1147155761719 0 11429.408
304.1722412109375 0 6197.9287
306.1452941894531 0 4209.1
307.1405944824219 0 1913.482
308.16131591796875 0 4581.6865 y Ammonia loss 11
310.1038818359375 0 4968.069
311.171875 0 1623.2433
312.1911926269531 0 1586.9425
313.1884460449219 0 5768.1216
314.0992126464844 0 2209.4624
314.1466979980469 0 7285.6333
314.1835021972656 0 39163.31
314.2080383300781 0 245683.06 b 2
315.1676330566406 0 1988.931
315.1863098144531 0 5743.0654
315.211181640625 0 40301.465
316.2146911621094 0 2357.4146
317.1864013671875 0 8610.215
318.130615234375 0 1834.0238
319.19805908203125 0 59205.24 y 14
320.201171875 0 9679.948
321.1553955078125 0 1840.2998
322.1770324707031 0 2111.7397
323.1718444824219 0 4939.184
324.1542663574219 0 1906.7379
325.1512756347656 0 2637.0178
326.17041015625 0 3421.3801
328.11444091796875 0 18084.035
328.1647644042969 0 2079.569
329.1163024902344 0 1984.7103
329.1828918457031 0 2183.3264
330.1817321777344 0 25453.871
331.185302734375 0 3040.4785
333.1564025878906 0 7574.9937
334.2129211425781 0 8451.5205
335.2156677246094 0 2138.7815
336.1557922363281 0 4307.154
337.1512145996094 0 8317.984
338.1349792480469 0 3714.9102
339.1667785644531 0 5844.1055
339.20355224609375 0 1756.0839
340.1985778808594 0 2455.4307
341.1841735839844 0 18597.096
342.181396484375 0 6637.8687
344.1974792480469 0 9518.771
346.1250915527344 0 47012.484
346.17657470703125 0 37087.336
347.1298522949219 0 7445.345
347.1791687011719 0 7003.904
348.1921081542969 0 28849.496
349.1529541015625 0 1679.6124
349.19561767578125 0 5391.683
350.1829833984375 0 6861.623
351.1667175292969 0 28448.428
351.18511962890625 0 1450.0144
352.1700439453125 0 5330.975
353.182373046875 0 15499.556
354.1659240722656 0 11959.628
354.18377685546875 0 2494.5042
355.1625061035156 0 9434.701
356.14404296875 0 2803.7407
356.16552734375 0 1908.3995
360.2284240722656 0 6614.969
362.2078552246094 0 83542.45
363.1669921875 0 2076.8718
363.21051025390625 0 12509.826
365.1458740234375 0 16579.041
365.2549133300781 0 6046.785
366.14947509765625 0 3184.9963
367.20843505859375 0 10311.696
368.1659851074219 0 1713.4437
368.19305419921875 0 7967.931
369.1387939453125 0 2125.9197
369.17724609375 0 44300.11
370.12384033203125 0 2180.9185
370.17987060546875 0 7226.79
370.67718505859375 0 5714.0044
371.1544189453125 0 1772.1981
371.19256591796875 0 10236.915
372.1767272949219 0 10281.331
373.17266845703125 0 14908.181
374.17462158203125 0 2493.1543
375.1676940917969 0 2138.2617
377.21893310546875 0 1964.569
379.1959533691406 0 2193.602
380.1948547363281 0 1736.1238
381.177001953125 0 5171.4463
383.1567077636719 0 27272.91
383.2659606933594 0 7055.0137
384.1594543457031 0 4887.4116
385.219970703125 0 25525.709
386.2018737792969 0 2787.4941
386.2259826660156 0 2514.2239
387.1509094238281 0 2275.849
388.22406005859375 0 1798.279
390.1763610839844 0 2048.1873
390.2030029296875 0 4639.824
393.17742919921875 0 5122.233
393.25067138671875 0 12701.738
394.25262451171875 0 3914.3308
395.22991943359375 0 2202.4172
397.1356201171875 0 6860.6323
398.1178894042969 0 1895.4402
398.2041320800781 0 2706.0012
398.2442932128906 0 19050.209
399.1882629394531 0 5004.2925
399.2465515136719 0 4527.1235
400.2237548828125 0 4177.7188
401.1673583984375 0 52143.27
401.2169189453125 0 2739.0278
402.1701354980469 0 9303.209
405.1617126464844 0 3418.1484
406.1630859375 0 1840.0952
407.15838623046875 0 2171.2542
407.2036437988281 0 9932.809
408.18853759765625 0 18036.434
409.1936340332031 0 2208.4827
410.2031555175781 0 4355.969
411.1539611816406 0 1763.3027
411.2608947753906 0 28108.79
412.2633361816406 0 4798.1875
413.2064514160156 0 2549.647
415.14666748046875 0 15264.728
415.23077392578125 0 5623.003 b 7
415.2711486816406 0 11360.645
416.14862060546875 0 3065.2852
416.18963623046875 0 2431.7358
416.25518798828125 0 38472.96
417.2561340332031 0 10840.436
418.2572021484375 0 2635.7969
419.1770324707031 0 2153.262
422.2049865722656 0 2576.0732
423.18695068359375 0 2978.5488
424.2290344238281 0 7521.263
425.1306457519531 0 4835.248
425.2150573730469 0 16742.162
426.21636962890625 0 2977.0764
427.232666015625 0 9128.095
428.23529052734375 0 2138.1777
429.16790771484375 0 1903.4174
429.2126159667969 0 2443.4285
431.2646484375 0 2492.1436
433.15740966796875 0 23453
433.242431640625 0 49100.4 y 13
433.2817687988281 0 310156.78
434.16082763671875 0 3467.9106
434.20428466796875 0 7539.8213
434.2455139160156 0 6843.3647
434.2848815917969 0 79418.87
434.7059020996094 0 5920.7373
435.2080993652344 0 6153.233
435.2872009277344 0 9965.899
440.2152404785156 0 4768.444
441.19903564453125 0 8550.544
441.2515563964844 0 2917.415
442.2403259277344 0 13831.38
443.14056396484375 0 6034.167
443.1751403808594 0 3306.8076
443.22991943359375 0 15830.36
443.2662353515625 0 35446.523 b Water loss 3
444.1873474121094 0 3176.142
444.23602294921875 0 2512.2998
444.2690734863281 0 7686.866
445.2444763183594 0 4795.627
446.2500305175781 0 2000.4272
448.18377685546875 0 2410.6682
451.23138427734375 0 3356.6233
452.2147521972656 0 9077.618
453.21881103515625 0 2579.5881
459.1733703613281 0 2045.3134
459.2090759277344 0 12723.18
459.26104736328125 0 4978.4194
460.21343994140625 0 3708.3572
460.2642822265625 0 1921.6537
461.2767028808594 0 275617.97 b 3
462.1993103027344 0 16410.338
462.27972412109375 0 68434.94
463.2033996582031 0 2977.1582
463.2834167480469 0 7708.5044
464.2144775390625 0 1992.9375
466.19366455078125 0 5556.0356
468.2096862792969 0 3699.9497
468.2564392089844 0 1750.3538
469.2447204589844 0 6784.205
470.2254638671875 0 37459.164
471.22833251953125 0 10353.129
476.1792297363281 0 4087.7205
477.25750732421875 0 3774.9917
478.2408142089844 0 8132.657
479.2262878417969 0 4642.2046
480.20977783203125 0 29410.629
481.21270751953125 0 8298.273
482.2243347167969 0 5666.542
483.2117614746094 0 1971.079
484.20452880859375 0 12639.59
486.2675476074219 0 5955.088 y Water loss 12
486.3069152832031 0 1950.013
487.25286865234375 0 7954.264 y Ammonia loss 12
492.2086181640625 0 2929.1946
494.1888122558594 0 12671.087
495.19378662109375 0 2462.2578
495.2677307128906 0 6203.8765
496.25164794921875 0 18190.43
497.25244140625 0 2896.5264
498.2203063964844 0 100533.555
499.22314453125 0 23827.797
500.2352600097656 0 7696.3896
501.21673583984375 0 1788.5823
502.2151794433594 0 22511.166
503.21771240234375 0 4367.6934
503.2608337402344 0 2058.758
504.27825927734375 0 69367.414 y 12
505.28076171875 0 14737.171
506.2354431152344 0 1785.2914
506.2845153808594 0 1789.8573
510.2218322753906 0 5300.176
512.199462890625 0 21848.8
512.3237915039062 0 2419.7478
513.2033081054688 0 5375.984
513.2783203125 0 6786.351
515.2487182617188 0 3168.1257
516.2418212890625 0 1964.5072
518.2457885742188 0 4895.758
521.2727661132812 0 1787.3345
524.236083984375 0 3654.529
527.2464599609375 0 2052.9023
528.2301025390625 0 6355.999
530.2098388671875 0 72721.55
530.334228515625 0 21632.814
531.213134765625 0 18356.416
531.3375244140625 0 5549.819
532.216064453125 0 2726.6492
535.2547607421875 0 1917.846 b Water loss 9
536.2356567382812 0 1866.9993
538.2564086914062 0 1684.8048
538.298828125 0 7403.0234
539.3037719726562 0 1669.4674
540.3189697265625 0 14556.581 b Water loss 4
541.3235473632812 0 2461.3513
546.2407836914062 0 8307.216
547.2396240234375 0 2467.4458
553.262939453125 0 2652.0645
555.3247680664062 0 5869.4126
556.224365234375 0 4097.7925
556.2688598632812 0 2182.337
558.3291625976562 0 41094.24 b 4
559.3333129882812 0 10583.441
563.246337890625 0 3872.8496
564.24072265625 0 2103.7744
569.2797241210938 0 9419.177
569.7793579101562 0 9633.659
570.2738037109375 0 3352.5168
571.2731323242188 0 4892.003
573.776611328125 0 2917.9858
574.2700805664062 0 3139.089
578.2860717773438 0 16596.342
578.7870483398438 0 7590.6807
579.28466796875 0 3814.4407
581.2576904296875 0 16220.452
582.2891235351562 0 7751.8135
582.7826538085938 0 23532.875
583.2823486328125 0 12256.09
583.7877197265625 0 1737.8044
587.267578125 0 4422.173
591.2427368164062 0 5680.2607
591.2950439453125 0 7271.377
591.7958984375 0 5857.936 b Water loss 10
592.2997436523438 0 3000.2239
597.2916259765625 0 10767.249
598.28466796875 0 4454.9126
599.2677612304688 0 70773.62
600.2719116210938 0 18762.637
601.279052734375 0 1951.8745
604.3062744140625 0 3653.207
604.8043212890625 0 3468.5413
607.272216796875 0 4337.1377
608.2716674804688 0 1637.2823
608.3511352539062 0 4447.665
608.8012084960938 0 3855.7378
609.2518310546875 0 20706.393
609.2952270507812 0 4190.83
609.341064453125 0 2339.3228
609.7969360351562 0 6114.6025
610.2540283203125 0 5291.423
610.3499755859375 0 1892.056
612.8150024414062 0 13991.744
613.31640625 0 12198.701
613.8180541992188 0 2903.989
614.3255615234375 0 9832.129 y Water loss 11
615.3042602539062 0 36923.023 y Ammonia loss 11
616.3067626953125 0 8894.123
617.3024291992188 0 2586.4495
617.8071899414062 0 65356.863
618.3062744140625 0 53368.94
618.8047485351562 0 20427.145
619.3031616210938 0 5241.3564
624.2979736328125 0 1802.1177
625.2846069335938 0 10836.838
626.2847900390625 0 5624.9414
626.3624267578125 0 9951.194
626.8126220703125 0 145229.33
627.26220703125 0 160031.05
627.314453125 0 95546.94
627.8153076171875 0 34065.34
628.2658081054688 0 41898.184
628.3231811523438 0 2350.638
628.8070678710938 0 3160.607 y Water loss 5
629.267333984375 0 6636.915
629.3157348632812 0 2719.7468
629.8067626953125 0 3240.7893
632.3367919921875 0 48274.516 y 11
633.339599609375 0 12845.93
635.8190307617188 0 5840.6626
636.3184814453125 0 5004.5117
636.8209228515625 0 2448.419
637.8150634765625 0 20462.4 y 5
638.3162231445312 0 12683.514
638.82080078125 0 3598.552
640.2935180664062 0 4299.2964
642.3110961914062 0 4697.672
643.294189453125 0 32628.736
644.2937622070312 0 10706.72
645.2908935546875 0 6473.8247
646.2871704101562 0 1884.8015
653.277099609375 0 3070.0493
655.3411865234375 0 9762.205
655.8418579101562 0 5199.7505 b Water loss 11
656.3397216796875 0 2193.4348 b Ammonia loss 11
656.815673828125 0 2671.572
657.3218383789062 0 2553.0886
658.3045043945312 0 4915.0254
659.3135375976562 0 2174.0427
660.3204345703125 0 12023.236
661.3235473632812 0 4005.032
663.3380126953125 0 5313.748
663.8383178710938 0 4059.3445
664.3359375 0 4239.378
667.3380737304688 0 1905.6729
668.3311157226562 0 2643.415
668.8257446289062 0 12104.124
669.3225708007812 0 7449.15
674.3002319335938 0 4441.5034
677.33642578125 0 48535.62 y Water loss 4
677.8323364257812 0 66347.375 y Ammonia loss 4
678.3327026367188 0 34786.21
678.8319091796875 0 12124.107
679.2590942382812 0 2351.625
684.3719482421875 0 2085.815
685.3330078125 0 4874.258
685.8375244140625 0 3351.778
686.3419189453125 0 1124319 y 4
686.8432006835938 0 852732.1
687.3440551757812 0 274775.34
687.8448486328125 0 23507.22
691.84033203125 0 2545.0474 b Ammonia loss 12
694.341796875 0 10943.746
695.34521484375 0 3817.014
698.298583984375 0 2697.4536
700.3466796875 0 13568.349 b 12
700.8492431640625 0 12610.087
701.3482055664062 0 3347.947
704.3263549804688 0 4634.9834
705.3344116210938 0 2384.227
709.321533203125 0 2085.62
712.3517456054688 0 89509.47
713.35498046875 0 29214.309
714.3526611328125 0 4367.0757
721.3633422851562 0 2357.224
721.6515502929688 0 2179.5127
722.3372192382812 0 12078.133
723.3394165039062 0 4702.762
725.3451538085938 0 2000.6805
726.3342895507812 0 2576.6736
727.3269653320312 0 2979.0027
728.3284301757812 0 2183.6833
728.3959350585938 0 2700.002 y Ammonia loss 10
731.3630981445312 0 1814.0385
735.3328247070312 0 2391.0396
737.3926391601562 0 2042.4672
738.2987060546875 0 4529.196
738.3778076171875 0 5056.597
739.3622436523438 0 6848.911
740.346923828125 0 105487.53
741.3499145507812 0 39949.77
742.351806640625 0 6994.9414
742.4153442382812 0 7145.6646 b 6
743.3502197265625 0 2123.6306
743.4179077148438 0 4227.676
745.337890625 0 5849.5586
745.4209594726562 0 37761.32 y 10
746.3496704101562 0 2245.4436
746.4238891601562 0 13885.515
750.8701782226562 0 6999.312 y Water loss 3
751.367919921875 0 4795.0503 y Ammonia loss 3
751.8687744140625 0 4825.8994
753.3407592773438 0 7474.643
754.3383178710938 0 6072.8213
755.3237915039062 0 4767.8745
755.4052734375 0 6563.3896
756.3980712890625 0 2689.7922
756.89013671875 0 2953.1135
757.3741455078125 0 50449.043 b 13
758.3761596679688 0 17348.41
759.371337890625 0 4128.589
759.8759155273438 0 149463.44 y 3
760.3771362304688 0 135003.28
760.8784790039062 0 54390.125
761.3787231445312 0 8868.644
766.36328125 0 1759.7998
771.3521728515625 0 26769.78
772.3541259765625 0 11802.027
774.3338623046875 0 4857.446
775.335205078125 0 1993.1874
797.3700561523438 0 2181.3816
807.9168090820312 0 2794.7437 y Ammonia loss 2
809.3740844726562 0 2769.053
809.4627075195312 0 3372.9158
811.3795776367188 0 2874.3523
812.914306640625 0 4266.4688 b Ammonia loss 14
813.4175415039062 0 4253.1836
814.3965454101562 0 3748.7988
816.4182739257812 0 36472.656 y 2
816.9197387695312 0 28800.158
817.4197998046875 0 16726.809
821.93017578125 0 2596.553
822.4271850585938 0 3000.7246
823.394287109375 0 4081.3213
824.380859375 0 7776.8613
825.3695678710938 0 3443.2314
828.4038696289062 0 3918.4714
829.4412231445312 0 8079.2085 b 7
830.4497680664062 0 2700.8542
832.3834838867188 0 3700.8293
839.42431640625 0 2129.7363
840.4109497070312 0 15535.653
841.4107055664062 0 7775.6587
842.390625 0 21200.443
842.9562377929688 0 2268.023
843.3906860351562 0 8021.1523
847.939453125 0 2923.3389 b Water loss 15
848.4375610351562 0 8985.161 b Ammonia loss 15
848.9415283203125 0 6479.5356
849.4304809570312 0 3862.0454
850.3952026367188 0 17075.293
851.3858032226562 0 10242.379
852.3934936523438 0 4545.4
856.4578857421875 0 3317.567 y Water loss 9
856.9465942382812 0 28005.422 b 15
857.4473266601562 0 39069.277 y Ammonia loss 9
857.947021484375 0 14063.858 y Water loss 1
858.4453125 0 6035.6826
859.4236450195312 0 1897.6755
860.42236328125 0 1996.3501
866.4338989257812 0 2742.7378
866.9423217773438 0 10690.912 y 1
867.4390869140625 0 15865.673
867.939208984375 0 3817.5417
868.4056396484375 0 134062.4
869.408447265625 0 56903.63
870.410400390625 0 10665.203
874.4630126953125 0 25754.29 y 9
875.4636840820312 0 10355.289
884.4420166015625 0 4525.351
885.4462280273438 0 7001.2026
885.9690551757812 0 1990.5734
886.4366455078125 0 2139.695
887.413818359375 0 8796.595
888.4193725585938 0 2985.4026
893.4375610351562 0 3342.243
894.42333984375 0 8822.953
895.4296875 0 5427.4224
898.955810546875 0 2978.0588
899.4645385742188 0 3120.9556
900.4463500976562 0 3754.5237
904.4218139648438 0 2047.5786
907.4711303710938 0 22505.41 Precursor Water loss
907.9705200195312 0 41509.035 Precursor Ammonia loss
908.469970703125 0 20778.008
908.9682006835938 0 4852.5044
909.4558715820312 0 1996.1438
911.4454956054688 0 22608.076
912.4489135742188 0 9881.138
913.4483642578125 0 2147.0586
915.46728515625 0 2362.2944
915.9724731445312 0 2577.418
916.366455078125 0 3430.2131
916.4759521484375 0 246325.31 Precursor
916.9768676757812 0 281562.1
917.4782104492188 0 133667.66
917.9785766601562 0 20140.908
921.4300537109375 0 12246.98
922.4199829101562 0 13072.41
923.4210205078125 0 6384.6206
936.44677734375 0 2467.9404
938.4476928710938 0 2091.3213
939.44140625 0 101115.05
940.444580078125 0 51179.26
941.446044921875 0 10422.039
953.4644775390625 0 4823.212
954.4606323242188 0 5237.467
956.452392578125 0 15624.93
957.4472045898438 0 8013.084
958.4824829101562 0 5925.8394 b 8
970.4459838867188 0 3434.9546
971.478271484375 0 9608.877
972.4808349609375 0 2579.6516
974.44287109375 0 1934.3472
986.4797973632812 0 2647.8452 y Ammonia loss 8
987.4758911132812 0 3176.2046
988.47705078125 0 3403.4148
1003.5047607421875 0 17053.963 y 8
1004.5078735351562 0 9698.284
1005.5025634765625 0 2659.3416
1008.462646484375 0 10601.975
1009.4703979492188 0 3199.962
1015.4736328125 0 8394.882
1016.4750366210938 0 3125.501
1018.4525146484375 0 3274.037
1019.4398193359375 0 2685.1726
1025.4888916015625 0 8723.592
1026.495361328125 0 3603.3938
1035.471923828125 0 5816
1036.4608154296875 0 18278.104
1037.465576171875 0 8170.0596
1038.485595703125 0 2399.1667
1040.50048828125 0 8076.134
1041.4945068359375 0 9084.471
1042.49462890625 0 3081.6167
1053.4853515625 0 76593.94
1054.4859619140625 0 45269.582
1055.4886474609375 0 12114.125
1058.5115966796875 0 9913.576
1059.5145263671875 0 9890.925
1066.5174560546875 0 1889.2747
1067.508056640625 0 3011.581
1068.502197265625 0 4892.584
1069.503173828125 0 3227.2297 b Water loss 9
1070.5115966796875 0 26288.236
1071.5113525390625 0 14687.971
1072.5205078125 0 6185.2466 y Water loss 7
1073.520263671875 0 5048.025 y Ammonia loss 7
1084.52734375 0 17278.502
1085.5284423828125 0 10385.88
1086.51708984375 0 10629.359
1087.529296875 0 10367.901 b 9
1088.5302734375 0 5134.1284
1090.5379638671875 0 30718.344 y 7
1091.5401611328125 0 15437.539
1092.5411376953125 0 6037.898
1120.53564453125 0 4104.287
1121.531005859375 0 2473.8674
1127.5673828125 0 2527.2888
1128.55712890625 0 4318.1045
1137.5533447265625 0 12354.592
1138.544677734375 0 14606.573
1139.5447998046875 0 7500.4575
1140.546142578125 0 1989.6139
1146.5350341796875 0 2338.0938
1155.56396484375 0 26071.902
1156.5657958984375 0 20116.78
1157.5675048828125 0 3470.3164
1159.5570068359375 0 6764.216 y Water loss 6
1160.5501708984375 0 7080.9473 y Ammonia loss 6
1161.552978515625 0 2706.389
1163.571533203125 0 5187.2153
1164.5557861328125 0 17973.115
1165.55810546875 0 10278.785
1166.5587158203125 0 2874.7734
1177.5694580078125 0 40188.996 y 6
1178.5704345703125 0 22026.803
1179.57177734375 0 7729.5093
1181.5791015625 0 89359.2
1182.5828857421875 0 61306.543 b Water loss 10
1183.583251953125 0 18442.945
1184.566162109375 0 1908.2509
1200.6077880859375 0 16309.157 b 10
1201.6134033203125 0 11861.729
1202.6165771484375 0 3404.8528
1207.6064453125 0 2618.395
1208.603271484375 0 2574.4915
1217.591552734375 0 5606.536
1218.5877685546875 0 3383.9702
1224.6190185546875 0 6287.327
1225.6220703125 0 4017.1985
1234.604736328125 0 41324.266
1235.6005859375 0 35261.645
1236.5977783203125 0 14893.377
1237.592529296875 0 4183.775
1239.589111328125 0 2798.8926
1252.615478515625 0 86611.055
1253.6182861328125 0 61985.504
1254.618896484375 0 15735.148
1255.617919921875 0 2288.5632
1256.6107177734375 0 9117.122 y Water loss 5
1257.5977783203125 0 20435.758 y Ammonia loss 5
1258.5994873046875 0 12177.842
1270.628173828125 0 5498.3955
1271.62939453125 0 2806.4443
1272.603759765625 0 2250.0586
1274.621337890625 0 225298.92 y 5
1275.6236572265625 0 147396.22
1276.6263427734375 0 50856.71
1277.6246337890625 0 5490.3296
1292.6527099609375 0 2792.0637
1293.6461181640625 0 2051.048
1300.6492919921875 0 3427.1682
1309.66748046875 0 12743.6455
1310.668701171875 0 14331.545 b Water loss 11
1311.6641845703125 0 7319.684 b Ammonia loss 11
1312.6463623046875 0 4008.2944
1325.664794921875 0 6406.5493
1326.66552734375 0 6234.392
1327.65966796875 0 6827.74
1328.667236328125 0 33319.53 b 11
1329.6702880859375 0 23669.96
1330.67724609375 0 7326.559
1335.6463623046875 0 3856.747
1336.6422119140625 0 7988.3657
1337.63916015625 0 7289.0146
1338.637451171875 0 3421.9011
1353.66259765625 0 45036.074 y Water loss 4
1354.6534423828125 0 75766.875 y Ammonia loss 4
1355.6546630859375 0 40267.215
1356.6556396484375 0 14121.418
1369.6510009765625 0 5875.027
1371.6734619140625 0 1332500.4 y 4
1372.6763916015625 0 943923.6
1373.678466796875 0 327258.16
1374.6795654296875 0 33046.45
1381.694091796875 0 5031.142 b Water loss 12
1382.68017578125 0 5869.4517 b Ammonia loss 12
1399.703857421875 0 21839.44 b 12
1400.7060546875 0 16546.97
1401.707763671875 0 5775.707
1496.722900390625 0 2598.615 b Ammonia loss 13
1500.7274169921875 0 2877.4373 y Water loss 3
1501.7166748046875 0 3800.4397 y Ammonia loss 3
1513.7542724609375 0 11062.202 b 13
1514.751220703125 0 9070.497
1515.746337890625 0 4307.4634
1518.7412109375 0 71473.02 y 3
1519.7432861328125 0 60237.37
1519.959228515625 0 1305.1681
1520.746826171875 0 23926.393
1631.8218994140625 0 13362.671 y 2
1632.8271484375 0 11458.376
1633.8189697265625 0 4863.4487
2234.514404296875 0 2063.2102
3072.246337890625 0 1997.6465
3408.21044921875 0 1937.1259

Spectrum Details

|  |  |
| --- | --- |
| Matched peaks? Matched peaksThe total absolute number of peaks matched. Additionally in brackets the total fraction of peaks matched and the total number of peaks is shown. | 92 (10.75% of 856) |
| FDR? FDRThe false discovery rate estimated for this peptide. It is calculated by matching all theoretical fragments with a non-integer shift with the raw peaks for this spectrum. This is done with 40 different shifts. The resulting percentage is the average number of annotated peaks over the number of annotated peaks with the correct spectrum. | 0.65% |
| Satellite FDR? Satellite FDRSee the FDR for details on its calculation. This satellite ion specific FDR only contains the satellite ions (d/w) for I/L/J positions. | - |
| PSM Score? PSM ScoreThe PSM Score as given by Hecklib to this annotated spectrum. It is shown with three significant figures. | 1.07E+03 |

## Spectrum 8067? Spectrum 8067 The raw spectrum of this peptide as annotated by Hecklib. The fragments are coloured according to ion type (see legend). Any peaks with a star '\*' as text can be hovered over to see the full details, first the ion type second the mass shift type. By hovering over the amino acids in the peptide or ions in the legend the corresponding peaks are highlighted. By toggling the 'Unassigned' label you can turn the background (unassigned) peaks on or off in the plot. By updating the slider in the Ion legend you can update the spectrum to only show the top X% of the peaks with labels. The top X% means any peak that is within X% of the highest intensity. By dragging in the spectrum you can zoom in to a specific part of the spectrum and use 'Zoom Out' to get back to the original zoom level. The annotation of the spectrum is based on the given sequence in the peptides file and is done with different software so inconsistencies are likely. The peaks are annotated based on the given sequence, with 20 ppm tolerance.

Copy Data

### Spectrum 8067 (TSV)

#### Preview

```
Loading example...
```

*Click on the button to copy the data to your clipboard.*

Mz MinMz MaxIntensity Max

WidthHeightPeptide font sizePeptide stroke widthSpectrum font sizeSpectrum stroke widthCompact peptide

Ion legend

wxyz

abcd

OtherUnassignedIonChargePositionShow for top:%

VTJFPPSSEEJQANKAT

02.79e+45.57e+48.36e+41.11e+5

Zoom Out

y+11a+12y+12a+12b+12y+12b+12b+36y+37y+25a+13b+25b+13y+13y+26b+13y+26y+13b+26y+27b+28b+28y+14y+28y+14b+14y+313y+313b+14b+29y+15y+15y+15y+210y+315b+15b+210y+210b+315b+15y+211y+211b+211b+211\*y+16y+16y+212y+212y+16y+212b+212b+212b+212y+213y+213y+213b+213b+213b+17y+17y+17b+17y+17y+214y+214b+214y+214y+215y+215b+18b+215y+215b+18b+216y+18b+216y+18y+216y+216y+216y+18b+19y+19y+19b+110y+110b+110y+110y+111y+111y+111b+111b+111y+112y+112y+112b+112y+113y+113b+113b+114

0857171425713428

Fragment Matches Table

Show background peaks

| Position | Ion type | Intensity | mz Theoretical | mz Error (Th) | mz Error (ppm) | Charge | Series Number |
| --- | --- | --- | --- | --- | --- | --- | --- |
| 17 | y | 4.802E+04 | 120.1 | 0.0002763 | 2.301 | +1 | 1 |
| - | - | 3.921E+04 | 120.1 | - | - | 0 | - |
| - | - | 278.4 | 121.1 | - | - | 0 | - |
| - | - | 277.8 | 121.1 | - | - | 0 | - |
| - | - | 1833 | 121.1 | - | - | 0 | - |
| - | - | 3676 | 121.1 | - | - | 0 | - |
| - | - | 4205 | 126.1 | - | - | 0 | - |
| - | - | 756.4 | 127.1 | - | - | 0 | - |
| - | - | 1.393E+04 | 128.1 | - | - | 0 | - |
| - | - | 2757 | 129.1 | - | - | 0 | - |
| - | - | 477.7 | 129.1 | - | - | 0 | - |
| - | - | 6.79E+04 | 129.1 | - | - | 0 | - |
| - | - | 3850 | 130.1 | - | - | 0 | - |
| - | - | 1208 | 130.1 | - | - | 0 | - |
| - | - | 399.3 | 130.1 | - | - | 0 | - |
| - | - | 629.3 | 130.1 | - | - | 0 | - |
| - | - | 3581 | 130.1 | - | - | 0 | - |
| - | - | 3105 | 131.1 | - | - | 0 | - |
| - | - | 747 | 132.1 | - | - | 0 | - |
| - | - | 517 | 133.1 | - | - | 0 | - |
| - | - | 375 | 134 | - | - | 0 | - |
| - | - | 1649 | 136.1 | - | - | 0 | - |
| - | - | 2852 | 138.1 | - | - | 0 | - |
| - | - | 1482 | 138.1 | - | - | 0 | - |
| - | - | 1910 | 139.1 | - | - | 0 | - |
| - | - | 1543 | 141.1 | - | - | 0 | - |
| - | - | 519.7 | 142.1 | - | - | 0 | - |
| - | - | 1670 | 147.1 | - | - | 0 | - |
| - | - | 668.2 | 148.1 | - | - | 0 | - |
| - | - | 1077 | 149 | - | - | 0 | - |
| - | - | 2672 | 149 | - | - | 0 | - |
| - | - | 485.2 | 152.1 | - | - | 0 | - |
| - | - | 1705 | 154.1 | - | - | 0 | - |
| - | - | 2753 | 154.1 | - | - | 0 | - |
| - | - | 4131 | 155.1 | - | - | 0 | - |
| 2 | a | 9715 | 155.1 | 0.0002288 | 1.475 | +1 | 2 |
| - | - | 1630 | 157.1 | - | - | 0 | - |
| - | - | 6237 | 157.1 | - | - | 0 | - |
| - | - | 5027 | 159.1 | - | - | 0 | - |
| - | - | 1543 | 165.1 | - | - | 0 | - |
| - | - | 888.9 | 166.1 | - | - | 0 | - |
| - | - | 3173 | 167.1 | - | - | 0 | - |
| - | - | 3347 | 167.1 | - | - | 0 | - |
| - | - | 2537 | 167.1 | - | - | 0 | - |
| - | - | 512.6 | 168.1 | - | - | 0 | - |
| - | - | 711.5 | 169.1 | - | - | 0 | - |
| - | - | 1.012E+04 | 169.1 | - | - | 0 | - |
| - | - | 468.6 | 170.1 | - | - | 0 | - |
| - | - | 753.4 | 170.1 | - | - | 0 | - |
| - | - | 1848 | 171.1 | - | - | 0 | - |
| - | - | 2891 | 172.1 | - | - | 0 | - |
| - | - | 476.5 | 172.1 | - | - | 0 | - |
| 16 | y | 7662 | 173.1 | 0.0001707 | 0.986 | +1 | 2 |
| 2 | a | 3.486E+04 | 173.1 | 0.0002384 | 1.377 | +1 | 2 |
| - | - | 2169 | 173.4 | - | - | 0 | - |
| - | - | 2637 | 174.1 | - | - | 0 | - |
| - | - | 1811 | 175.1 | - | - | 0 | - |
| - | - | 885.3 | 180.1 | - | - | 0 | - |
| - | - | 1629 | 181.1 | - | - | 0 | - |
| - | - | 3456 | 182.1 | - | - | 0 | - |
| - | - | 3276 | 182.1 | - | - | 0 | - |
| - | - | 3726 | 183.1 | - | - | 0 | - |
| 2 | b | 4.431E+04 | 183.1 | 0.0002024 | 1.106 | +1 | 2 |
| - | - | 547.1 | 184.1 | - | - | 0 | - |
| - | - | 2218 | 184.1 | - | - | 0 | - |
| - | - | 4256 | 184.1 | - | - | 0 | - |
| - | - | 6874 | 185.1 | - | - | 0 | - |
| - | - | 658.4 | 185.1 | - | - | 0 | - |
| - | - | 2740 | 186.1 | - | - | 0 | - |
| - | - | 1.464E+04 | 187.1 | - | - | 0 | - |
| - | - | 637.2 | 188.1 | - | - | 0 | - |
| - | - | 3537 | 189.1 | - | - | 0 | - |
| - | - | 860.2 | 191.1 | - | - | 0 | - |
| 16 | y | 1.092E+04 | 191.1 | 0.0002566 | 1.343 | +1 | 2 |
| - | - | 856.8 | 191.1 | - | - | 0 | - |
| - | - | 1144 | 192.1 | - | - | 0 | - |
| - | - | 585 | 195.1 | - | - | 0 | - |
| - | - | 6813 | 195.1 | - | - | 0 | - |
| - | - | 508.9 | 197.1 | - | - | 0 | - |
| - | - | 1.426E+04 | 197.1 | - | - | 0 | - |
| - | - | 2720 | 198.1 | - | - | 0 | - |
| - | - | 680.9 | 198.1 | - | - | 0 | - |
| - | - | 4523 | 199.1 | - | - | 0 | - |
| - | - | 693.5 | 199.1 | - | - | 0 | - |
| - | - | 1674 | 199.1 | - | - | 0 | - |
| - | - | 1067 | 199.1 | - | - | 0 | - |
| - | - | 4561 | 200.1 | - | - | 0 | - |
| - | - | 3384 | 200.1 | - | - | 0 | - |
| - | - | 979.2 | 201.1 | - | - | 0 | - |
| 2 | b | 4.332E+04 | 201.1 | 0.0001663 | 0.8269 | +1 | 2 |
| - | - | 4231 | 202.1 | - | - | 0 | - |
| - | - | 464.5 | 203.1 | - | - | 0 | - |
| - | - | 735.4 | 208.1 | - | - | 0 | - |
| - | - | 5199 | 208.1 | - | - | 0 | - |
| - | - | 1.175E+04 | 209.1 | - | - | 0 | - |
| - | - | 1503 | 210.1 | - | - | 0 | - |
| - | - | 564.6 | 210.1 | - | - | 0 | - |
| - | - | 728.3 | 211.1 | - | - | 0 | - |
| - | - | 1710 | 212.1 | - | - | 0 | - |
| - | - | 1031 | 213.1 | - | - | 0 | - |
| - | - | 708.6 | 213.1 | - | - | 0 | - |
| - | - | 648.7 | 214.1 | - | - | 0 | - |
| - | - | 650 | 214.2 | - | - | 0 | - |
| - | - | 1.647E+04 | 215.1 | - | - | 0 | - |
| - | - | 1812 | 216.1 | - | - | 0 | - |
| - | - | 8437 | 217.1 | - | - | 0 | - |
| - | - | 4937 | 217.1 | - | - | 0 | - |
| - | - | 1.154E+04 | 217.1 | - | - | 0 | - |
| - | - | 832.3 | 218.1 | - | - | 0 | - |
| - | - | 1544 | 218.1 | - | - | 0 | - |
| - | - | 1040 | 218.1 | - | - | 0 | - |
| 6 | b | 802.7 | 219.1 | 0.001645 | 7.509 | +3 | 6 |
| - | - | 1240 | 219.1 | - | - | 0 | - |
| - | - | 4525 | 221.1 | - | - | 0 | - |
| - | - | 718.4 | 222.1 | - | - | 0 | - |
| - | - | 754.5 | 223.1 | - | - | 0 | - |
| - | - | 791.8 | 223.1 | - | - | 0 | - |
| - | - | 570.8 | 224.1 | - | - | 0 | - |
| - | - | 1852 | 225 | - | - | 0 | - |
| - | - | 5349 | 225.1 | - | - | 0 | - |
| - | - | 2810 | 225.1 | - | - | 0 | - |
| - | - | 4.843E+04 | 226.1 | - | - | 0 | - |
| - | - | 753.4 | 227 | - | - | 0 | - |
| - | - | 1341 | 227.1 | - | - | 0 | - |
| - | - | 4925 | 227.1 | - | - | 0 | - |
| - | - | 525.3 | 228.1 | - | - | 0 | - |
| - | - | 747.3 | 228.1 | - | - | 0 | - |
| - | - | 1729 | 231.1 | - | - | 0 | - |
| - | - | 1.931E+04 | 233.2 | - | - | 0 | - |
| - | - | 652.9 | 234.1 | - | - | 0 | - |
| - | - | 2401 | 234.1 | - | - | 0 | - |
| - | - | 518 | 234.1 | - | - | 0 | - |
| - | - | 2178 | 234.2 | - | - | 0 | - |
| - | - | 5248 | 235.1 | - | - | 0 | - |
| - | - | 2684 | 236.1 | - | - | 0 | - |
| - | - | 722.4 | 236.1 | - | - | 0 | - |
| - | - | 888.9 | 238.1 | - | - | 0 | - |
| - | - | 1.08E+04 | 239.1 | - | - | 0 | - |
| - | - | 545.9 | 239.6 | - | - | 0 | - |
| - | - | 3468 | 240.1 | - | - | 0 | - |
| - | - | 3139 | 241.1 | - | - | 0 | - |
| - | - | 1.001E+04 | 242.2 | - | - | 0 | - |
| - | - | 1356 | 243.1 | - | - | 0 | - |
| 11 | y | 1.824E+04 | 243.1 | 0.004177 | 17.18 | +3 | 7 |
| 13 | y | 1865 | 244.1 | 0.0004562 | 1.869 | +2 | 5 |
| - | - | 1959 | 244.1 | - | - | 0 | - |
| - | - | 6678 | 245.1 | - | - | 0 | - |
| - | - | 1648 | 246.1 | - | - | 0 | - |
| - | - | 1758 | 247.1 | - | - | 0 | - |
| - | - | 691.3 | 248.1 | - | - | 0 | - |
| - | - | 530.7 | 251.2 | - | - | 0 | - |
| - | - | 6753 | 251.2 | - | - | 0 | - |
| - | - | 1068 | 252.1 | - | - | 0 | - |
| - | - | 1144 | 252.1 | - | - | 0 | - |
| - | - | 737.7 | 252.2 | - | - | 0 | - |
| - | - | 1120 | 253.1 | - | - | 0 | - |
| - | - | 6874 | 254.1 | - | - | 0 | - |
| - | - | 1588 | 254.2 | - | - | 0 | - |
| - | - | 564.6 | 254.3 | - | - | 0 | - |
| - | - | 1263 | 257.2 | - | - | 0 | - |
| - | - | 554.5 | 258.1 | - | - | 0 | - |
| - | - | 6276 | 259.1 | - | - | 0 | - |
| - | - | 8844 | 261.2 | - | - | 0 | - |
| - | - | 1979 | 262.1 | - | - | 0 | - |
| - | - | 791.5 | 262.2 | - | - | 0 | - |
| - | - | 8688 | 263.1 | - | - | 0 | - |
| - | - | 529.7 | 264.1 | - | - | 0 | - |
| - | - | 872.3 | 264.1 | - | - | 0 | - |
| - | - | 7435 | 264.1 | - | - | 0 | - |
| - | - | 1265 | 265.1 | - | - | 0 | - |
| - | - | 639.8 | 266.1 | - | - | 0 | - |
| - | - | 1160 | 268.1 | - | - | 0 | - |
| 3 | a | 1261 | 268.2 | 0.0002323 | 0.8662 | +1 | 3 |
| - | - | 1550 | 269.2 | - | - | 0 | - |
| - | - | 632.2 | 270.2 | - | - | 0 | - |
| - | - | 2677 | 272.1 | - | - | 0 | - |
| - | - | 1885 | 274.1 | - | - | 0 | - |
| - | - | 1706 | 275.2 | - | - | 0 | - |
| - | - | 624.9 | 276.1 | - | - | 0 | - |
| - | - | 548.2 | 277.1 | - | - | 0 | - |
| - | - | 4945 | 279.1 | - | - | 0 | - |
| 5 | b | 752.4 | 279.7 | 0.0001168 | 0.4176 | +2 | 5 |
| - | - | 937 | 280.1 | - | - | 0 | - |
| - | - | 2286 | 280.1 | - | - | 0 | - |
| - | - | 1003 | 280.1 | - | - | 0 | - |
| - | - | 4050 | 280.2 | - | - | 0 | - |
| - | - | 1615 | 281.1 | - | - | 0 | - |
| - | - | 1.722E+04 | 282.1 | - | - | 0 | - |
| - | - | 1909 | 283.1 | - | - | 0 | - |
| - | - | 1.195E+04 | 285 | - | - | 0 | - |
| - | - | 3221 | 285.2 | - | - | 0 | - |
| - | - | 3914 | 286 | - | - | 0 | - |
| - | - | 3742 | 286.1 | - | - | 0 | - |
| - | - | 936.8 | 287 | - | - | 0 | - |
| - | - | 613.7 | 294.2 | - | - | 0 | - |
| - | - | 3272 | 296.1 | - | - | 0 | - |
| - | - | 5090 | 296.2 | - | - | 0 | - |
| 3 | b | 1.104E+05 | 296.2 | 0.0002449 | 0.8268 | +1 | 3 |
| - | - | 2781 | 297.1 | - | - | 0 | - |
| - | - | 1.705E+04 | 297.2 | - | - | 0 | - |
| - | - | 1253 | 297.2 | - | - | 0 | - |
| - | - | 2.075E+04 | 297.2 | - | - | 0 | - |
| - | - | 2530 | 298.2 | - | - | 0 | - |
| - | - | 4656 | 298.2 | - | - | 0 | - |
| - | - | 1629 | 298.2 | - | - | 0 | - |
| - | - | 1.423E+04 | 299.1 | - | - | 0 | - |
| - | - | 915.7 | 299.2 | - | - | 0 | - |
| - | - | 763 | 299.2 | - | - | 0 | - |
| - | - | 4156 | 300.1 | - | - | 0 | - |
| - | - | 1507 | 300.2 | - | - | 0 | - |
| - | - | 1720 | 301.1 | - | - | 0 | - |
| 15 | y | 2295 | 301.2 | 0.0003767 | 1.251 | +1 | 3 |
| - | - | 4124 | 303.2 | - | - | 0 | - |
| - | - | 1742 | 304.1 | - | - | 0 | - |
| - | - | 1057 | 306.1 | - | - | 0 | - |
| - | - | 748.3 | 307.1 | - | - | 0 | - |
| 12 | y | 7216 | 307.7 | 0.0002541 | 0.8258 | +2 | 6 |
| - | - | 1938 | 308.2 | - | - | 0 | - |
| - | - | 1397 | 310.1 | - | - | 0 | - |
| - | - | 671.7 | 313.2 | - | - | 0 | - |
| - | - | 2321 | 313.2 | - | - | 0 | - |
| - | - | 2857 | 314.1 | - | - | 0 | - |
| - | - | 1.621E+04 | 314.2 | - | - | 0 | - |
| 3 | b | 2.24E+04 | 314.2 | 0.0003003 | 0.9558 | +1 | 3 |
| - | - | 2312 | 315.2 | - | - | 0 | - |
| - | - | 4505 | 315.2 | - | - | 0 | - |
| - | - | 9103 | 316.2 | - | - | 0 | - |
| 12 | y | 3040 | 316.7 | 8.443E-05 | 0.2666 | +2 | 6 |
| - | - | 2781 | 317.2 | - | - | 0 | - |
| 15 | y | 1.559E+04 | 319.2 | 0.0003101 | 0.9714 | +1 | 3 |
| - | - | 1569 | 320.2 | - | - | 0 | - |
| - | - | 968.1 | 323.2 | - | - | 0 | - |
| - | - | 749.1 | 324.2 | - | - | 0 | - |
| - | - | 1127 | 325.1 | - | - | 0 | - |
| - | - | 2918 | 328.1 | - | - | 0 | - |
| 6 | b | 775.4 | 328.2 | 0.0005598 | 1.706 | +2 | 6 |
| - | - | 1145 | 329.2 | - | - | 0 | - |
| - | - | 3285 | 330.2 | - | - | 0 | - |
| - | - | 536.1 | 331.1 | - | - | 0 | - |
| - | - | 617.3 | 332.9 | - | - | 0 | - |
| - | - | 3746 | 333.2 | - | - | 0 | - |
| - | - | 952.9 | 334.2 | - | - | 0 | - |
| - | - | 1035 | 337.2 | - | - | 0 | - |
| - | - | 1456 | 339.2 | - | - | 0 | - |
| - | - | 3383 | 341.2 | - | - | 0 | - |
| - | - | 2428 | 342.2 | - | - | 0 | - |
| - | - | 1809 | 344.2 | - | - | 0 | - |
| - | - | 1405 | 345 | - | - | 0 | - |
| - | - | 7252 | 346.1 | - | - | 0 | - |
| - | - | 4813 | 346.2 | - | - | 0 | - |
| - | - | 946.9 | 347.1 | - | - | 0 | - |
| - | - | 3056 | 348.2 | - | - | 0 | - |
| - | - | 1029 | 349.2 | - | - | 0 | - |
| - | - | 1780 | 350.1 | - | - | 0 | - |
| - | - | 3123 | 350.2 | - | - | 0 | - |
| - | - | 1.033E+04 | 351.2 | - | - | 0 | - |
| - | - | 954.6 | 351.2 | - | - | 0 | - |
| - | - | 2317 | 352.2 | - | - | 0 | - |
| - | - | 5311 | 353.2 | - | - | 0 | - |
| - | - | 1440 | 354.2 | - | - | 0 | - |
| - | - | 1125 | 354.2 | - | - | 0 | - |
| - | - | 4.117E+04 | 355.1 | - | - | 0 | - |
| - | - | 1795 | 355.2 | - | - | 0 | - |
| - | - | 1.698E+04 | 356.1 | - | - | 0 | - |
| - | - | 9382 | 357.1 | - | - | 0 | - |
| - | - | 998 | 359 | - | - | 0 | - |
| - | - | 798.7 | 360.2 | - | - | 0 | - |
| - | - | 9193 | 362.2 | - | - | 0 | - |
| - | - | 835.3 | 363.2 | - | - | 0 | - |
| - | - | 2602 | 363.2 | - | - | 0 | - |
| - | - | 2304 | 365.1 | - | - | 0 | - |
| - | - | 4409 | 367.2 | - | - | 0 | - |
| - | - | 3023 | 368.2 | - | - | 0 | - |
| - | - | 1.131E+04 | 369.2 | - | - | 0 | - |
| - | - | 896.9 | 369.2 | - | - | 0 | - |
| - | - | 2029 | 370.2 | - | - | 0 | - |
| - | - | 1343 | 371.1 | - | - | 0 | - |
| - | - | 2629 | 371.2 | - | - | 0 | - |
| - | - | 706 | 371.2 | - | - | 0 | - |
| - | - | 688 | 372.2 | - | - | 0 | - |
| - | - | 804.5 | 372.2 | - | - | 0 | - |
| - | - | 3776 | 373.1 | - | - | 0 | - |
| - | - | 3185 | 373.2 | - | - | 0 | - |
| 11 | y | 4457 | 373.2 | 0.0003946 | 1.057 | +2 | 7 |
| - | - | 1926 | 373.7 | - | - | 0 | - |
| - | - | 2113 | 374.1 | - | - | 0 | - |
| - | - | 1018 | 374.2 | - | - | 0 | - |
| - | - | 713.3 | 381.2 | - | - | 0 | - |
| - | - | 971.8 | 382.2 | - | - | 0 | - |
| - | - | 3638 | 383.2 | - | - | 0 | - |
| - | - | 1031 | 383.3 | - | - | 0 | - |
| - | - | 983.6 | 384.2 | - | - | 0 | - |
| - | - | 725 | 384.3 | - | - | 0 | - |
| - | - | 505.9 | 385.2 | - | - | 0 | - |
| - | - | 1.489E+04 | 385.2 | - | - | 0 | - |
| - | - | 893.7 | 386.2 | - | - | 0 | - |
| - | - | 2650 | 386.2 | - | - | 0 | - |
| - | - | 1447 | 393.2 | - | - | 0 | - |
| - | - | 1164 | 397.1 | - | - | 0 | - |
| - | - | 1371 | 397.2 | - | - | 0 | - |
| - | - | 824.5 | 397.7 | - | - | 0 | - |
| - | - | 794.9 | 398.2 | - | - | 0 | - |
| - | - | 1454 | 398.2 | - | - | 0 | - |
| - | - | 794.2 | 399.2 | - | - | 0 | - |
| - | - | 1236 | 400.2 | - | - | 0 | - |
| - | - | 948.2 | 401 | - | - | 0 | - |
| - | - | 8401 | 401.2 | - | - | 0 | - |
| - | - | 2163 | 402.2 | - | - | 0 | - |
| 8 | b | 1456 | 406.2 | 6.321E-05 | 0.1556 | +2 | 8 |
| - | - | 798.6 | 406.7 | - | - | 0 | - |
| - | - | 2259 | 407.2 | - | - | 0 | - |
| - | - | 758.7 | 407.7 | - | - | 0 | - |
| - | - | 7576 | 408.2 | - | - | 0 | - |
| - | - | 918.9 | 409.2 | - | - | 0 | - |
| - | - | 719.4 | 410.2 | - | - | 0 | - |
| - | - | 747.1 | 411.2 | - | - | 0 | - |
| - | - | 3362 | 411.3 | - | - | 0 | - |
| - | - | 1318 | 412.3 | - | - | 0 | - |
| - | - | 644.8 | 413.2 | - | - | 0 | - |
| - | - | 1968 | 415 | - | - | 0 | - |
| - | - | 1615 | 415.1 | - | - | 0 | - |
| 8 | b | 1716 | 415.2 | 0.00442 | 10.64 | +2 | 8 |
| - | - | 1380 | 415.3 | - | - | 0 | - |
| - | - | 1461 | 416 | - | - | 0 | - |
| 14 | y | 1166 | 416.2 | 0.004287 | 10.3 | +1 | 4 |
| - | - | 4071 | 416.3 | - | - | 0 | - |
| - | - | 1298 | 417.3 | - | - | 0 | - |
| - | - | 4142 | 419 | - | - | 0 | - |
| - | - | 1852 | 420 | - | - | 0 | - |
| - | - | 852.8 | 421 | - | - | 0 | - |
| - | - | 4349 | 424.2 | - | - | 0 | - |
| - | - | 774.4 | 425.1 | - | - | 0 | - |
| - | - | 6617 | 425.2 | - | - | 0 | - |
| - | - | 864.9 | 426.2 | - | - | 0 | - |
| - | - | 1698 | 427.2 | - | - | 0 | - |
| 10 | y | 755.1 | 429.2 | 0.006315 | 14.71 | +2 | 8 |
| - | - | 2619 | 433.2 | - | - | 0 | - |
| 14 | y | 1.549E+04 | 433.2 | 0.000626 | 1.445 | +1 | 4 |
| - | - | 3.319E+04 | 433.3 | - | - | 0 | - |
| - | - | 1020 | 434.2 | - | - | 0 | - |
| - | - | 2873 | 434.2 | - | - | 0 | - |
| - | - | 7944 | 434.3 | - | - | 0 | - |
| - | - | 994.1 | 434.7 | - | - | 0 | - |
| - | - | 1857 | 435.2 | - | - | 0 | - |
| - | - | 1280 | 435.3 | - | - | 0 | - |
| - | - | 727.3 | 438.2 | - | - | 0 | - |
| - | - | 1687 | 440.2 | - | - | 0 | - |
| - | - | 919.5 | 441.2 | - | - | 0 | - |
| - | - | 934.5 | 441.3 | - | - | 0 | - |
| - | - | 5349 | 442.2 | - | - | 0 | - |
| - | - | 1001 | 443.1 | - | - | 0 | - |
| - | - | 2173 | 443.2 | - | - | 0 | - |
| 4 | b | 3110 | 443.3 | 0.0007397 | 1.669 | +1 | 4 |
| - | - | 895 | 444.3 | - | - | 0 | - |
| - | - | 1136 | 445.2 | - | - | 0 | - |
| - | - | 667.4 | 445.2 | - | - | 0 | - |
| - | - | 1680 | 451.2 | - | - | 0 | - |
| 5 | y | 2745 | 451.9 | 0.001519 | 3.36 | +3 | 13 |
| 5 | y | 4094 | 452.2 | 0.003824 | 8.457 | +3 | 13 |
| - | - | 1328 | 452.6 | - | - | 0 | - |
| - | - | 1007 | 453.2 | - | - | 0 | - |
| - | - | 905.5 | 454.2 | - | - | 0 | - |
| - | - | 588.4 | 458.3 | - | - | 0 | - |
| - | - | 1717 | 459.2 | - | - | 0 | - |
| - | - | 1455 | 459.3 | - | - | 0 | - |
| - | - | 964.6 | 461.2 | - | - | 0 | - |
| 4 | b | 3.222E+04 | 461.3 | 0.0001848 | 0.4005 | +1 | 4 |
| - | - | 3147 | 462.2 | - | - | 0 | - |
| - | - | 9370 | 462.3 | - | - | 0 | - |
| - | - | 1013 | 463.3 | - | - | 0 | - |
| - | - | 768.1 | 464.2 | - | - | 0 | - |
| - | - | 718.3 | 464.3 | - | - | 0 | - |
| - | - | 602.2 | 465.2 | - | - | 0 | - |
| - | - | 620.3 | 466.2 | - | - | 0 | - |
| - | - | 1245 | 468.2 | - | - | 0 | - |
| - | - | 1994 | 468.3 | - | - | 0 | - |
| - | - | 2268 | 469.2 | - | - | 0 | - |
| - | - | 9865 | 470.2 | - | - | 0 | - |
| - | - | 1077 | 470.7 | - | - | 0 | - |
| - | - | 2384 | 471.2 | - | - | 0 | - |
| - | - | 1045 | 472.2 | - | - | 0 | - |
| - | - | 737.3 | 476.2 | - | - | 0 | - |
| - | - | 2684 | 477.3 | - | - | 0 | - |
| - | - | 5616 | 478.2 | - | - | 0 | - |
| - | - | 3983 | 479.2 | - | - | 0 | - |
| 9 | b | 961.8 | 479.7 | 0.0009763 | 2.035 | +2 | 9 |
| - | - | 1.024E+04 | 480.2 | - | - | 0 | - |
| - | - | 1273 | 480.2 | - | - | 0 | - |
| - | - | 1026 | 480.7 | - | - | 0 | - |
| - | - | 1541 | 480.8 | - | - | 0 | - |
| - | - | 3482 | 481.2 | - | - | 0 | - |
| - | - | 891.5 | 482.2 | - | - | 0 | - |
| - | - | 1567 | 484.2 | - | - | 0 | - |
| 13 | y | 5002 | 486.3 | 0.0004136 | 0.8506 | +1 | 5 |
| 13 | y | 2840 | 487.3 | 0.001211 | 2.485 | +1 | 5 |
| - | - | 1087 | 488.3 | - | - | 0 | - |
| - | - | 799.2 | 488.7 | - | - | 0 | - |
| - | - | 833.3 | 489.1 | - | - | 0 | - |
| - | - | 684.4 | 490.1 | - | - | 0 | - |
| - | - | 889.1 | 490.2 | - | - | 0 | - |
| - | - | 4217 | 493.7 | - | - | 0 | - |
| - | - | 1083 | 494.2 | - | - | 0 | - |
| - | - | 1494 | 494.2 | - | - | 0 | - |
| - | - | 626.7 | 494.7 | - | - | 0 | - |
| - | - | 3404 | 495.3 | - | - | 0 | - |
| - | - | 1.31E+04 | 496.3 | - | - | 0 | - |
| - | - | 2812 | 497.3 | - | - | 0 | - |
| - | - | 3.079E+04 | 498.2 | - | - | 0 | - |
| - | - | 7211 | 499.2 | - | - | 0 | - |
| - | - | 1628 | 500.2 | - | - | 0 | - |
| - | - | 3199 | 502.2 | - | - | 0 | - |
| - | - | 4602 | 502.7 | - | - | 0 | - |
| - | - | 1677 | 503.2 | - | - | 0 | - |
| 13 | y | 3.604E+04 | 504.3 | 1.127E-05 | 0.02235 | +1 | 5 |
| - | - | 8484 | 505.3 | - | - | 0 | - |
| - | - | 1455 | 506.3 | - | - | 0 | - |
| - | - | 1028 | 508.2 | - | - | 0 | - |
| - | - | 817.8 | 510.2 | - | - | 0 | - |
| - | - | 2292 | 512.2 | - | - | 0 | - |
| - | - | 4770 | 513.3 | - | - | 0 | - |
| - | - | 741.4 | 514.3 | - | - | 0 | - |
| - | - | 1059 | 515.2 | - | - | 0 | - |
| - | - | 870.3 | 518.2 | - | - | 0 | - |
| - | - | 718.6 | 526.3 | - | - | 0 | - |
| - | - | 703.3 | 528.2 | - | - | 0 | - |
| - | - | 937.8 | 529.3 | - | - | 0 | - |
| - | - | 1746 | 529.8 | - | - | 0 | - |
| - | - | 1.062E+04 | 530.2 | - | - | 0 | - |
| - | - | 2686 | 530.3 | - | - | 0 | - |
| - | - | 2685 | 531.2 | - | - | 0 | - |
| - | - | 1274 | 531.3 | - | - | 0 | - |
| - | - | 638.4 | 534.8 | - | - | 0 | - |
| - | - | 693.3 | 535.9 | - | - | 0 | - |
| - | - | 748.1 | 536.3 | - | - | 0 | - |
| 8 | y | 800.5 | 536.8 | 0.002088 | 3.89 | +2 | 10 |
| - | - | 5403 | 538.3 | - | - | 0 | - |
| - | - | 3367 | 538.8 | - | - | 0 | - |
| 3 | y | 609.5 | 538.9 | 0.003567 | 6.619 | +3 | 15 |
| 5 | b | 3375 | 540.3 | 0.0006186 | 1.145 | +1 | 5 |
| - | - | 665 | 541.3 | - | - | 0 | - |
| - | - | 716.8 | 542.8 | - | - | 0 | - |
| - | - | 2542 | 543.3 | - | - | 0 | - |
| - | - | 1113 | 543.8 | - | - | 0 | - |
| 10 | b | 2517 | 544.3 | 0.001857 | 3.412 | +2 | 10 |
| - | - | 878.7 | 544.8 | - | - | 0 | - |
| 8 | y | 707.9 | 545.8 | 0.00438 | 8.025 | +2 | 10 |
| - | - | 829 | 546.2 | - | - | 0 | - |
| 15 | b | 899.4 | 548 | 0.001294 | 2.362 | +3 | 15 |
| - | - | 6234 | 552.3 | - | - | 0 | - |
| - | - | 4305 | 552.8 | - | - | 0 | - |
| - | - | 1577 | 553.3 | - | - | 0 | - |
| - | - | 774.5 | 553.3 | - | - | 0 | - |
| - | - | 2184 | 555.3 | - | - | 0 | - |
| - | - | 1369 | 556.3 | - | - | 0 | - |
| - | - | 1103 | 556.3 | - | - | 0 | - |
| 5 | b | 7845 | 558.3 | 0.0004246 | 0.7604 | +1 | 5 |
| - | - | 2323 | 559.3 | - | - | 0 | - |
| - | - | 988.7 | 560.8 | - | - | 0 | - |
| - | - | 800.8 | 561.3 | - | - | 0 | - |
| - | - | 800.8 | 563.2 | - | - | 0 | - |
| - | - | 757.4 | 566.4 | - | - | 0 | - |
| - | - | 1177 | 569.3 | - | - | 0 | - |
| - | - | 2289 | 569.8 | - | - | 0 | - |
| - | - | 707.1 | 570.3 | - | - | 0 | - |
| - | - | 602.6 | 571.3 | - | - | 0 | - |
| - | - | 737 | 573.8 | - | - | 0 | - |
| - | - | 1023 | 574.3 | - | - | 0 | - |
| - | - | 833.7 | 578.1 | - | - | 0 | - |
| - | - | 1984 | 578.3 | - | - | 0 | - |
| - | - | 1190 | 578.8 | - | - | 0 | - |
| - | - | 1905 | 579.3 | - | - | 0 | - |
| 7 | y | 1698 | 580.3 | 0.005102 | 8.792 | +2 | 11 |
| 7 | y | 1198 | 580.8 | 0.008078 | 13.91 | +2 | 11 |
| - | - | 2587 | 581.3 | - | - | 0 | - |
| - | - | 1961 | 582.3 | - | - | 0 | - |
| - | - | 3904 | 582.8 | - | - | 0 | - |
| - | - | 2398 | 583.3 | - | - | 0 | - |
| - | - | 1464 | 583.8 | - | - | 0 | - |
| - | - | 660.6 | 586.3 | - | - | 0 | - |
| - | - | 2202 | 591.2 | - | - | 0 | - |
| - | - | 2285 | 591.3 | - | - | 0 | - |
| 11 | b | 1801 | 591.8 | 0.007479 | 12.64 | +2 | 11 |
| - | - | 1033 | 592.2 | - | - | 0 | - |
| - | - | 3515 | 597.3 | - | - | 0 | - |
| - | - | 2188 | 598.3 | - | - | 0 | - |
| - | - | 1.375E+04 | 599.3 | - | - | 0 | - |
| - | - | 1541 | 599.6 | - | - | 0 | - |
| - | - | 904.4 | 600 | - | - | 0 | - |
| - | - | 4538 | 600.3 | - | - | 0 | - |
| 11 | b | 844.7 | 600.8 | 0.001775 | 2.954 | +2 | 11 |
| - | - | 829.9 | 601.3 | - | - | 0 | - |
| - | - | 719.2 | 602.1 | - | - | 0 | - |
| - | - | 2850 | 602.8 | - | - | 0 | - |
| - | - | 3076 | 603.3 | - | - | 0 | - |
| - | - | 970.5 | 604.3 | - | - | 0 | - |
| - | - | 826.1 | 606.8 | - | - | 0 | - |
| - | - | 1224 | 608.4 | - | - | 0 | - |
| - | - | 1516 | 608.8 | - | - | 0 | - |
| - | - | 6847 | 609.3 | - | - | 0 | - |
| - | - | 877.6 | 609.3 | - | - | 0 | - |
| - | - | 1005 | 609.3 | - | - | 0 | - |
| - | - | 1438 | 609.8 | - | - | 0 | - |
| - | - | 4307 | 610.2 | - | - | 0 | - |
| - | - | 1634 | 610.3 | - | - | 0 | - |
| - | - | 4694 | 611.2 | - | - | 0 | - |
| 0 | Precursor | 882.8 | 611.3 | 0.002386 | 3.904 | +3 | -1 |
| - | - | 5312 | 611.8 | - | - | 0 | - |
| - | - | 4354 | 612.2 | - | - | 0 | - |
| - | - | 866.9 | 612.2 | - | - | 0 | - |
| - | - | 3582 | 612.3 | - | - | 0 | - |
| - | - | 3833 | 612.8 | - | - | 0 | - |
| - | - | 2349 | 613.3 | - | - | 0 | - |
| - | - | 743.4 | 613.8 | - | - | 0 | - |
| 12 | y | 1.019E+04 | 614.3 | 0.001188 | 1.933 | +1 | 6 |
| 12 | y | 2.077E+04 | 615.3 | 3.472E-05 | 0.05642 | +1 | 6 |
| - | - | 6552 | 616.3 | - | - | 0 | - |
| - | - | 3649 | 617.3 | - | - | 0 | - |
| - | - | 1.501E+04 | 617.8 | - | - | 0 | - |
| - | - | 1.181E+04 | 618.3 | - | - | 0 | - |
| - | - | 7848 | 618.8 | - | - | 0 | - |
| - | - | 2186 | 619.3 | - | - | 0 | - |
| - | - | 938.6 | 620.3 | - | - | 0 | - |
| - | - | 977.4 | 620.8 | - | - | 0 | - |
| - | - | 1096 | 625.3 | - | - | 0 | - |
| - | - | 4540 | 626.4 | - | - | 0 | - |
| - | - | 3.176E+04 | 626.8 | - | - | 0 | - |
| - | - | 6.148E+04 | 627.3 | - | - | 0 | - |
| - | - | 2.039E+04 | 627.3 | - | - | 0 | - |
| - | - | 9294 | 627.8 | - | - | 0 | - |
| - | - | 1.939E+04 | 628.3 | - | - | 0 | - |
| - | - | 1681 | 628.3 | - | - | 0 | - |
| 6 | y | 3519 | 628.8 | 0.0009259 | 1.472 | +2 | 12 |
| - | - | 2623 | 629.3 | - | - | 0 | - |
| 6 | y | 1728 | 629.3 | 0.009834 | 15.63 | +2 | 12 |
| - | - | 1972 | 629.8 | - | - | 0 | - |
| 12 | y | 2.983E+04 | 632.3 | 0.0003387 | 0.5356 | +1 | 6 |
| - | - | 9630 | 633.3 | - | - | 0 | - |
| - | - | 1577 | 634.3 | - | - | 0 | - |
| - | - | 683.8 | 635.8 | - | - | 0 | - |
| 6 | y | 5294 | 637.8 | 0.0003891 | 0.6101 | +2 | 12 |
| - | - | 3257 | 638.3 | - | - | 0 | - |
| - | - | 1646 | 638.8 | - | - | 0 | - |
| - | - | 4005 | 643.3 | - | - | 0 | - |
| - | - | 1946 | 644.3 | - | - | 0 | - |
| - | - | 2912 | 645.3 | - | - | 0 | - |
| - | - | 825.9 | 646.3 | - | - | 0 | - |
| - | - | 958 | 646.8 | - | - | 0 | - |
| - | - | 610.3 | 647.8 | - | - | 0 | - |
| - | - | 1.101E+04 | 655.3 | - | - | 0 | - |
| 12 | b | 8796 | 655.8 | 0.006323 | 9.642 | +2 | 12 |
| 12 | b | 4352 | 656.3 | 0.01181 | 18 | +2 | 12 |
| - | - | 1097 | 656.8 | - | - | 0 | - |
| - | - | 828.6 | 658.3 | - | - | 0 | - |
| - | - | 794.2 | 659.8 | - | - | 0 | - |
| - | - | 1601 | 660.3 | - | - | 0 | - |
| 12 | b | 746 | 664.8 | 0.01104 | 16.61 | +2 | 12 |
| - | - | 1086 | 667.3 | - | - | 0 | - |
| - | - | 3291 | 668.3 | - | - | 0 | - |
| - | - | 4606 | 668.8 | - | - | 0 | - |
| - | - | 2371 | 669.3 | - | - | 0 | - |
| - | - | 954.5 | 669.8 | - | - | 0 | - |
| - | - | 1017 | 676.3 | - | - | 0 | - |
| 5 | y | 3.591E+04 | 677.3 | 0.0006757 | 0.9976 | +2 | 13 |
| 5 | y | 3.172E+04 | 677.8 | 0.00634 | 9.353 | +2 | 13 |
| - | - | 1.662E+04 | 678.3 | - | - | 0 | - |
| - | - | 5757 | 678.8 | - | - | 0 | - |
| - | - | 1522 | 679.3 | - | - | 0 | - |
| - | - | 1395 | 684.4 | - | - | 0 | - |
| 5 | y | 7.457E+04 | 686.3 | 0.000587 | 0.8552 | +2 | 13 |
| - | - | 5.669E+04 | 686.8 | - | - | 0 | - |
| - | - | 522.3 | 686.9 | - | - | 0 | - |
| - | - | 2.207E+04 | 687.3 | - | - | 0 | - |
| - | - | 6778 | 687.8 | - | - | 0 | - |
| - | - | 894.7 | 688.3 | - | - | 0 | - |
| 13 | b | 886.4 | 691.4 | 0.009365 | 13.55 | +2 | 13 |
| - | - | 2292 | 694.3 | - | - | 0 | - |
| - | - | 1223 | 695.3 | - | - | 0 | - |
| - | - | 972.4 | 698.3 | - | - | 0 | - |
| 13 | b | 3987 | 700.4 | 0.01257 | 17.95 | +2 | 13 |
| - | - | 3897 | 700.8 | - | - | 0 | - |
| - | - | 1404 | 701.4 | - | - | 0 | - |
| - | - | 1644 | 704.3 | - | - | 0 | - |
| - | - | 1342 | 710.4 | - | - | 0 | - |
| - | - | 921.6 | 711.4 | - | - | 0 | - |
| - | - | 9541 | 712.4 | - | - | 0 | - |
| - | - | 3694 | 713.4 | - | - | 0 | - |
| - | - | 826.2 | 714.4 | - | - | 0 | - |
| - | - | 2976 | 722.3 | - | - | 0 | - |
| - | - | 927.4 | 723.3 | - | - | 0 | - |
| 7 | b | 1891 | 724.4 | 0.0007996 | 1.104 | +1 | 7 |
| - | - | 1289 | 726.3 | - | - | 0 | - |
| 11 | y | 1469 | 727.4 | 4.649E-05 | 0.06392 | +1 | 7 |
| 11 | y | 2206 | 728.4 | 0.001839 | 2.524 | +1 | 7 |
| - | - | 2326 | 728.9 | - | - | 0 | - |
| - | - | 1888 | 729.4 | - | - | 0 | - |
| - | - | 1132 | 730.4 | - | - | 0 | - |
| - | - | 797.8 | 731.4 | - | - | 0 | - |
| - | - | 1666 | 737.4 | - | - | 0 | - |
| - | - | 1394 | 738.4 | - | - | 0 | - |
| - | - | 1075 | 739.4 | - | - | 0 | - |
| - | - | 2.227E+04 | 740.3 | - | - | 0 | - |
| - | - | 8870 | 741.3 | - | - | 0 | - |
| - | - | 2958 | 742.3 | - | - | 0 | - |
| 7 | b | 3886 | 742.4 | 0.0008427 | 1.135 | +1 | 7 |
| - | - | 737.8 | 742.9 | - | - | 0 | - |
| - | - | 1429 | 743.4 | - | - | 0 | - |
| - | - | 860.1 | 745.3 | - | - | 0 | - |
| 11 | y | 1.332E+04 | 745.4 | 0.0007235 | 0.9706 | +1 | 7 |
| - | - | 5299 | 746.4 | - | - | 0 | - |
| - | - | 1272 | 747.4 | - | - | 0 | - |
| - | - | 1088 | 749.3 | - | - | 0 | - |
| 4 | y | 9339 | 750.9 | 0.0008861 | 1.18 | +2 | 14 |
| 4 | y | 8337 | 751.4 | 0.007228 | 9.62 | +2 | 14 |
| - | - | 4192 | 751.9 | - | - | 0 | - |
| - | - | 1785 | 752.4 | - | - | 0 | - |
| - | - | 813.9 | 752.9 | - | - | 0 | - |
| - | - | 1540 | 753.3 | - | - | 0 | - |
| - | - | 2770 | 755.4 | - | - | 0 | - |
| - | - | 1295 | 756.4 | - | - | 0 | - |
| 14 | b | 7694 | 757.4 | 0.006753 | 8.916 | +2 | 14 |
| - | - | 3624 | 758.4 | - | - | 0 | - |
| - | - | 1659 | 759.4 | - | - | 0 | - |
| 4 | y | 8639 | 759.9 | 0.0006143 | 0.8084 | +2 | 14 |
| - | - | 9520 | 760.4 | - | - | 0 | - |
| - | - | 4001 | 760.9 | - | - | 0 | - |
| - | - | 920.7 | 761.4 | - | - | 0 | - |
| - | - | 762.5 | 761.9 | - | - | 0 | - |
| - | - | 4536 | 771.4 | - | - | 0 | - |
| - | - | 2217 | 772.4 | - | - | 0 | - |
| - | - | 775.3 | 773.4 | - | - | 0 | - |
| - | - | 4241 | 774.3 | - | - | 0 | - |
| - | - | 2222 | 775.3 | - | - | 0 | - |
| - | - | 1475 | 793.4 | - | - | 0 | - |
| - | - | 773 | 797.4 | - | - | 0 | - |
| - | - | 1114 | 806.4 | - | - | 0 | - |
| 3 | y | 1925 | 807.4 | 0.00233 | 2.885 | +2 | 15 |
| 3 | y | 3446 | 807.9 | 0.006761 | 8.369 | +2 | 15 |
| - | - | 2065 | 808.4 | - | - | 0 | - |
| - | - | 1359 | 808.9 | - | - | 0 | - |
| 8 | b | 3428 | 811.4 | 0.001578 | 1.945 | +1 | 8 |
| 15 | b | 2031 | 812.4 | 0.01239 | 15.25 | +2 | 15 |
| - | - | 2229 | 813.4 | - | - | 0 | - |
| 3 | y | 1837 | 816.4 | 0.001081 | 1.324 | +2 | 15 |
| - | - | 1380 | 816.9 | - | - | 0 | - |
| - | - | 681.5 | 822.4 | - | - | 0 | - |
| 8 | b | 3261 | 829.4 | 0.001767 | 2.13 | +1 | 8 |
| - | - | 1628 | 830.4 | - | - | 0 | - |
| - | - | 1009 | 832.4 | - | - | 0 | - |
| - | - | 1800 | 839.4 | - | - | 0 | - |
| - | - | 2734 | 840.4 | - | - | 0 | - |
| - | - | 2407 | 841.4 | - | - | 0 | - |
| - | - | 1752 | 842.4 | - | - | 0 | - |
| - | - | 1178 | 842.9 | - | - | 0 | - |
| - | - | 897.9 | 843.4 | - | - | 0 | - |
| - | - | 811.7 | 845.4 | - | - | 0 | - |
| 16 | b | 1834 | 848.4 | 0.002022 | 2.383 | +2 | 16 |
| - | - | 829.9 | 848.9 | - | - | 0 | - |
| - | - | 3014 | 850.4 | - | - | 0 | - |
| - | - | 1982 | 851.4 | - | - | 0 | - |
| - | - | 884.9 | 852.4 | - | - | 0 | - |
| 10 | y | 1744 | 856.5 | 0.004798 | 5.602 | +1 | 8 |
| 16 | b | 2151 | 856.9 | 0.002967 | 3.463 | +2 | 16 |
| 10 | y | 3994 | 857.4 | 0.006914 | 8.064 | +1 | 8 |
| 2 | y | 2505 | 857.9 | 0.004166 | 4.855 | +2 | 16 |
| 2 | y | 2116 | 858.4 | 0.008679 | 10.11 | +2 | 16 |
| - | - | 1991 | 859.4 | - | - | 0 | - |
| - | - | 1056 | 861.4 | - | - | 0 | - |
| - | - | 2693 | 863.4 | - | - | 0 | - |
| 2 | y | 899.7 | 866.9 | 7.924E-05 | 0.0914 | +2 | 16 |
| - | - | 1470 | 867.4 | - | - | 0 | - |
| - | - | 2.585E+04 | 868.4 | - | - | 0 | - |
| - | - | 1.377E+04 | 869.4 | - | - | 0 | - |
| - | - | 3235 | 870.4 | - | - | 0 | - |
| - | - | 977.4 | 871.4 | - | - | 0 | - |
| - | - | 3671 | 873.5 | - | - | 0 | - |
| 10 | y | 6906 | 874.5 | 0.001263 | 1.445 | +1 | 8 |
| - | - | 1561 | 875.5 | - | - | 0 | - |
| - | - | 4443 | 879.4 | - | - | 0 | - |
| - | - | 1921 | 880.4 | - | - | 0 | - |
| - | - | 1103 | 885.4 | - | - | 0 | - |
| - | - | 4135 | 887.4 | - | - | 0 | - |
| - | - | 1908 | 888.4 | - | - | 0 | - |
| - | - | 3934 | 889.4 | - | - | 0 | - |
| - | - | 1487 | 890.4 | - | - | 0 | - |
| - | - | 803.3 | 895.4 | - | - | 0 | - |
| - | - | 1.627E+04 | 907.4 | - | - | 0 | - |
| - | - | 8028 | 908.4 | - | - | 0 | - |
| - | - | 979.3 | 909.4 | - | - | 0 | - |
| - | - | 2673 | 911.4 | - | - | 0 | - |
| - | - | 1136 | 912.4 | - | - | 0 | - |
| - | - | 1564 | 921.4 | - | - | 0 | - |
| - | - | 2676 | 922.4 | - | - | 0 | - |
| - | - | 1.279E+04 | 939.4 | - | - | 0 | - |
| - | - | 7953 | 940.4 | - | - | 0 | - |
| - | - | 3566 | 941.5 | - | - | 0 | - |
| - | - | 837 | 942.5 | - | - | 0 | - |
| - | - | 867 | 953.5 | - | - | 0 | - |
| - | - | 855.5 | 954.5 | - | - | 0 | - |
| - | - | 3698 | 956.4 | - | - | 0 | - |
| - | - | 1987 | 957.5 | - | - | 0 | - |
| 9 | b | 8360 | 958.5 | 0.001696 | 1.77 | +1 | 9 |
| - | - | 7277 | 959.5 | - | - | 0 | - |
| - | - | 1.098E+04 | 960.5 | - | - | 0 | - |
| - | - | 4553 | 961.5 | - | - | 0 | - |
| - | - | 2590 | 971.5 | - | - | 0 | - |
| 9 | y | 1055 | 986.5 | 0.0008201 | 0.8313 | +1 | 9 |
| - | - | 1039 | 987.5 | - | - | 0 | - |
| - | - | 2197 | 988.5 | - | - | 0 | - |
| - | - | 1567 | 989.5 | - | - | 0 | - |
| - | - | 1619 | 1000 | - | - | 0 | - |
| 9 | y | 1847 | 1004 | 8.886E-05 | 0.08855 | +1 | 9 |
| - | - | 1007 | 1005 | - | - | 0 | - |
| - | - | 841.9 | 1008 | - | - | 0 | - |
| - | - | 1749 | 1015 | - | - | 0 | - |
| - | - | 1614 | 1016 | - | - | 0 | - |
| - | - | 1268 | 1025 | - | - | 0 | - |
| - | - | 2653 | 1036 | - | - | 0 | - |
| - | - | 939.8 | 1037 | - | - | 0 | - |
| - | - | 917.9 | 1041 | - | - | 0 | - |
| - | - | 1640 | 1041 | - | - | 0 | - |
| - | - | 924.3 | 1053 | - | - | 0 | - |
| - | - | 1.024E+04 | 1053 | - | - | 0 | - |
| - | - | 6235 | 1054 | - | - | 0 | - |
| - | - | 2258 | 1055 | - | - | 0 | - |
| - | - | 873.8 | 1056 | - | - | 0 | - |
| - | - | 3160 | 1059 | - | - | 0 | - |
| - | - | 2583 | 1060 | - | - | 0 | - |
| - | - | 878 | 1061 | - | - | 0 | - |
| - | - | 1099 | 1067 | - | - | 0 | - |
| 10 | b | 3246 | 1070 | 0.005893 | 5.51 | +1 | 10 |
| - | - | 5571 | 1071 | - | - | 0 | - |
| - | - | 3675 | 1072 | - | - | 0 | - |
| 8 | y | 1644 | 1073 | 0.01265 | 11.79 | +1 | 10 |
| - | - | 4120 | 1076 | - | - | 0 | - |
| - | - | 2229 | 1077 | - | - | 0 | - |
| - | - | 1621 | 1085 | - | - | 0 | - |
| - | - | 1831 | 1086 | - | - | 0 | - |
| - | - | 2497 | 1087 | - | - | 0 | - |
| 10 | b | 2.434E+04 | 1088 | 0.003518 | 3.235 | +1 | 10 |
| - | - | 1.325E+04 | 1089 | - | - | 0 | - |
| - | - | 5818 | 1090 | - | - | 0 | - |
| 8 | y | 2884 | 1091 | 0.003314 | 3.039 | +1 | 10 |
| - | - | 1541 | 1102 | - | - | 0 | - |
| - | - | 5292 | 1104 | - | - | 0 | - |
| - | - | 2729 | 1105 | - | - | 0 | - |
| - | - | 814 | 1106 | - | - | 0 | - |
| - | - | 751.1 | 1128 | - | - | 0 | - |
| - | - | 1059 | 1130 | - | - | 0 | - |
| - | - | 1593 | 1138 | - | - | 0 | - |
| - | - | 2492 | 1139 | - | - | 0 | - |
| - | - | 1058 | 1140 | - | - | 0 | - |
| - | - | 4346 | 1156 | - | - | 0 | - |
| - | - | 2540 | 1157 | - | - | 0 | - |
| - | - | 1124 | 1158 | - | - | 0 | - |
| 7 | y | 921.9 | 1160 | 0.004031 | 3.476 | +1 | 11 |
| 7 | y | 801.9 | 1161 | 0.007442 | 6.412 | +1 | 11 |
| - | - | 3166 | 1165 | - | - | 0 | - |
| - | - | 2011 | 1166 | - | - | 0 | - |
| 7 | y | 1680 | 1178 | 0.0003021 | 0.2565 | +1 | 11 |
| - | - | 1068 | 1179 | - | - | 0 | - |
| - | - | 9552 | 1182 | - | - | 0 | - |
| 11 | b | 5708 | 1183 | 0.02331 | 19.71 | +1 | 11 |
| - | - | 2834 | 1184 | - | - | 0 | - |
| 11 | b | 3910 | 1201 | 0.009457 | 7.877 | +1 | 11 |
| - | - | 2025 | 1202 | - | - | 0 | - |
| - | - | 783.3 | 1203 | - | - | 0 | - |
| - | - | 1382 | 1213 | - | - | 0 | - |
| - | - | 1362 | 1218 | - | - | 0 | - |
| - | - | 5886 | 1235 | - | - | 0 | - |
| - | - | 4675 | 1236 | - | - | 0 | - |
| - | - | 2053 | 1237 | - | - | 0 | - |
| - | - | 1.461E+04 | 1253 | - | - | 0 | - |
| - | - | 1.009E+04 | 1254 | - | - | 0 | - |
| - | - | 4775 | 1255 | - | - | 0 | - |
| - | - | 1009 | 1256 | - | - | 0 | - |
| 6 | y | 2513 | 1257 | 0.0007054 | 0.5614 | +1 | 12 |
| 6 | y | 1985 | 1258 | 0.002408 | 1.914 | +1 | 12 |
| - | - | 1492 | 1259 | - | - | 0 | - |
| 6 | y | 8009 | 1275 | 0.004366 | 3.425 | +1 | 12 |
| - | - | 4616 | 1276 | - | - | 0 | - |
| - | - | 1086 | 1277 | - | - | 0 | - |
| 12 | b | 1733 | 1329 | 0.01005 | 7.565 | +1 | 12 |
| - | - | 1101 | 1330 | - | - | 0 | - |
| 5 | y | 904.4 | 1354 | 0.01031 | 7.617 | +1 | 13 |
| 5 | y | 2684 | 1372 | 0.005006 | 3.649 | +1 | 13 |
| - | - | 2421 | 1373 | - | - | 0 | - |
| - | - | 894.1 | 1374 | - | - | 0 | - |
| 13 | b | 1835 | 1400 | 0.01225 | 8.754 | +1 | 13 |
| - | - | 1716 | 1401 | - | - | 0 | - |
| 14 | b | 1231 | 1514 | 0.001347 | 0.8899 | +1 | 14 |
| - | - | 1032 | 1515 | - | - | 0 | - |
| - | - | 612.8 | 2226 | - | - | 0 | - |
| - | - | 839.7 | 3070 | - | - | 0 | - |
| - | - | 676.3 | 3394 | - | - | 0 | - |

m/z Charge Intensity FragmentType MassShift Position
120.0657958984375 0 48024.516 y 16
120.08103942871094 0 39209.15
121.06279754638672 0 278.37137
121.06446075439453 0 277.82547
121.0692138671875 0 1832.9965
121.08441162109375 0 3675.7322
126.05525970458984 0 4204.773
127.08673095703125 0 756.4404
128.1072540283203 0 13933.934
129.06607055664062 0 2756.5444
129.07064819335938 0 477.6919
129.10250854492188 0 67897.15
130.05014038085938 0 3850.486
130.0655517578125 0 1208.2766
130.0820770263672 0 399.25488
130.10073852539062 0 629.2786
130.10586547851562 0 3581.4673
131.1180877685547 0 3105.4004
132.0769500732422 0 747.02795
133.06101989746094 0 516.9579
133.9902801513672 0 375.0168
136.07583618164062 0 1649.4329
138.0916290283203 0 2852.0662
138.12811279296875 0 1482.4999
139.08706665039062 0 1909.7589
141.066162109375 0 1542.5334
142.06101989746094 0 519.6732
147.07664489746094 0 1669.8123
148.0606231689453 0 668.16327
148.95445251464844 0 1077.4479
149.0449676513672 0 2672.1077
152.07052612304688 0 485.18164
154.08653259277344 0 1705.263
154.0977325439453 0 2753.2502
155.0817413330078 0 4131.2
155.1181182861328 0 9715.455 a Water loss 1
157.06112670898438 0 1630.2992
157.09739685058594 0 6236.781
159.09188842773438 0 5026.732
165.10247802734375 0 1542.8506
166.08644104003906 0 888.87976
167.0556640625 0 3172.5674
167.08177185058594 0 3346.9631
167.1180419921875 0 2536.587
168.05577087402344 0 512.6298
169.06048583984375 0 711.49133
169.13375854492188 0 10117.613
170.1185302734375 0 468.57315
170.1376190185547 0 753.4204
171.07687377929688 0 1848.4126
172.10850524902344 0 2890.6692
172.14340209960938 0 476.50848
173.0922393798828 0 7661.835 y Water loss 15
173.12869262695312 0 34856.57 a 1
173.4391632080078 0 2169.2195
174.1318359375 0 2637.0679
175.07174682617188 0 1811.0322
180.1133270263672 0 885.31195
181.09716796875 0 1628.6167
182.0926055908203 0 3455.7783
182.12905883789062 0 3275.5166
183.07666015625 0 3726.3354
183.11300659179688 0 44311.043 b Water loss 1
184.0797882080078 0 547.13043
184.10818481445312 0 2218.2515
184.11643981933594 0 4255.614
185.09230041503906 0 6874.01
185.12814331054688 0 658.3997
186.0874481201172 0 2739.7876
187.14439392089844 0 14636.527
188.1485595703125 0 637.2087
189.0872344970703 0 3537.4778
191.0820770263672 0 860.21484
191.10289001464844 0 10920.467 y 15
191.1179962158203 0 856.82806
192.1060028076172 0 1144.0942
195.07614135742188 0 584.9567
195.1129913330078 0 6812.7935
197.10386657714844 0 508.88766
197.12864685058594 0 14263.762
198.123779296875 0 2719.6038
198.13259887695312 0 680.8679
199.07156372070312 0 4522.5425
199.0811004638672 0 693.4666
199.10780334472656 0 1674.445
199.1186065673828 0 1066.716
200.10308837890625 0 4561.3193
200.1395263671875 0 3383.7651
201.0868682861328 0 979.1575
201.12353515625 0 43321.914 b 1
202.12692260742188 0 4230.5034
203.11343383789062 0 464.53824
208.09825134277344 0 735.3702
208.1083221435547 0 5198.933
209.09228515625 0 11752.357
210.0955810546875 0 1502.7673
210.12368774414062 0 564.60016
211.14486694335938 0 728.3157
212.13975524902344 0 1709.7249
213.08750915527344 0 1030.5618
213.1234130859375 0 708.618
214.1298065185547 0 648.6729
214.1558837890625 0 650.0023
215.13922119140625 0 16468.547
216.14231872558594 0 1811.7446
217.0821075439453 0 8436.835
217.09710693359375 0 4937.434
217.1336669921875 0 11536.149
218.0848388671875 0 832.3103
218.13723754882812 0 1544.0406
218.14930725097656 0 1040.3608
219.1336212158203 0 802.67975 b 5
219.1498565673828 0 1240.1613
221.08448791503906 0 4525.036
222.08677673339844 0 718.4202
223.063232421875 0 754.53186
223.08163452148438 0 791.7627
224.13987731933594 0 570.7931
225.0431671142578 0 1852.469
225.12342834472656 0 5349.114
225.1351776123047 0 2810.1516
226.1188201904297 0 48425.16
227.0400390625 0 753.356
227.10281372070312 0 1341.156
227.12222290039062 0 4924.818
228.0972137451172 0 525.2783
228.1342315673828 0 747.26074
231.09805297851562 0 1729.4808
233.16500854492188 0 19312.95
234.0880126953125 0 652.9052
234.12387084960938 0 2401.2246
234.1391143798828 0 517.9609
234.16868591308594 0 2177.9102
235.10775756835938 0 5247.9575
236.10301208496094 0 2683.7197
236.1400604248047 0 722.4394
238.1182861328125 0 888.8644
239.09518432617188 0 10796.418
239.625244140625 0 545.8529
240.0963592529297 0 3467.903
241.0820770263672 0 3139.4492
242.15011596679688 0 10013.415
243.13375854492188 0 1355.8356
243.14559936523438 0 18236.588 y Water loss 10
244.129638671875 0 1864.8458 y Ammonia loss 12
244.14862060546875 0 1959.1309
245.1287078857422 0 6678.214
246.12379455566406 0 1648.4781
247.14422607421875 0 1758.0353
248.13754272460938 0 691.2504
251.15170288085938 0 530.6731
251.1756134033203 0 6753.405
252.09762573242188 0 1067.6371
252.1343231201172 0 1143.6858
252.18051147460938 0 737.74664
253.1296844482422 0 1120.0886
254.11349487304688 0 6873.5645
254.1503143310547 0 1588.1411
254.25306701660156 0 564.57886
257.19769287109375 0 1262.916
258.1089172363281 0 554.48474
259.0925598144531 0 6276.25
261.15972900390625 0 8844.467
262.1188049316406 0 1979.4249
262.16339111328125 0 791.49713
263.1027526855469 0 8687.501
264.0671081542969 0 529.6774
264.1059265136719 0 872.259
264.1344299316406 0 7434.5737
265.1369323730469 0 1265.3438
266.1489562988281 0 639.82
268.0927734375 0 1159.8506
268.20172119140625 0 1260.9089 a Water loss 2
269.1861877441406 0 1549.7689
270.18023681640625 0 632.17145
272.1241455078125 0 2677.1987
274.11907958984375 0 1884.9441
275.1753234863281 0 1705.7295
276.1201171875 0 624.9443
277.11944580078125 0 548.2325
279.1454162597656 0 4945.0015
279.6680603027344 0 752.4024 b 4
280.0930480957031 0 936.97925
280.12957763671875 0 2286.1365
280.1495361328125 0 1002.5733
280.1663818359375 0 4049.563
281.0510559082031 0 1614.9362
282.14501953125 0 17219.732
283.1478576660156 0 1909.0294
285.00982666015625 0 11950.774
285.1599426269531 0 3220.8884
286.01019287109375 0 3914.0513
286.1034851074219 0 3742.0667
287.00750732421875 0 936.7593
294.1813659667969 0 613.74554
296.13555908203125 0 3272.0227
296.1731262207031 0 5089.798
296.1971130371094 0 110384.4 b Water loss 2
297.1195983886719 0 2780.8098
297.156005859375 0 17051.398
297.17572021484375 0 1253.3801
297.2002868652344 0 20748.316
298.1584777832031 0 2530.4927
298.176513671875 0 4655.5693
298.2025451660156 0 1628.731
299.0619201660156 0 14234.566
299.1537780761719 0 915.66394
299.1730651855469 0 762.9898
300.06231689453125 0 4155.792
300.15582275390625 0 1507.3674
301.0592346191406 0 1719.8116
301.1874084472656 0 2295.487 y Water loss 14
303.17041015625 0 4123.571
304.1142883300781 0 1741.771
306.1455993652344 0 1056.8099
307.1404113769531 0 748.2577
307.6667175292969 0 7216.367 y Water loss 11
308.1668395996094 0 1937.7676
310.1033935546875 0 1396.587
313.16436767578125 0 671.6675
313.1881103515625 0 2321.3916
314.1466979980469 0 2857.1343
314.1827392578125 0 16209.371
314.2077331542969 0 22396.014 b 2
315.1856689453125 0 2311.8992
315.2106628417969 0 4504.966
316.1868591308594 0 9103.295
316.6716613769531 0 3040.3496 y 11
317.1883850097656 0 2781.42
319.1979064941406 0 15591.029 y 14
320.200927734375 0 1568.5281
323.1712646484375 0 968.0824
324.15350341796875 0 749.092
325.1499938964844 0 1126.6285
328.1141357421875 0 2917.6194
328.19488525390625 0 775.4498 b 5
329.18231201171875 0 1145.4987
330.18194580078125 0 3284.8704
331.13873291015625 0 536.0911
332.85784912109375 0 617.28815
333.1556396484375 0 3745.763
334.2125244140625 0 952.87225
337.1531066894531 0 1035.4984
339.1661071777344 0 1456.4655
341.1835021972656 0 3383.0525
342.1788635253906 0 2427.659
344.1989440917969 0 1809.109
344.9773254394531 0 1404.741
346.1245422363281 0 7251.6694
346.1763916015625 0 4812.752
347.12933349609375 0 946.8884
348.19207763671875 0 3055.7178
349.1957702636719 0 1028.5277
350.1346130371094 0 1780.1908
350.1822814941406 0 3123.23
351.16632080078125 0 10330.47
351.18731689453125 0 954.6348
352.16998291015625 0 2317.421
353.18182373046875 0 5311.0156
354.16595458984375 0 1440.2141
354.1866455078125 0 1124.9917
355.0699462890625 0 41173.51
355.1628723144531 0 1794.6003
356.07073974609375 0 16978.295
357.06817626953125 0 9382.257
359.02752685546875 0 998.02185
360.2293701171875 0 798.7301
362.2074890136719 0 9193.145
363.1665344238281 0 835.2689
363.2092590332031 0 2601.868
365.145751953125 0 2303.5796
367.2083740234375 0 4408.8726
368.1926574707031 0 3023.3103
369.1768798828125 0 11314.1
369.20050048828125 0 896.8711
370.1803283691406 0 2029.0508
371.10137939453125 0 1343.2532
371.19171142578125 0 2629.4004
371.2425537109375 0 706.0242
372.1747741699219 0 687.9941
372.1993103027344 0 804.5128
373.08056640625 0 3775.5398
373.1717834472656 0 3184.8691
373.21417236328125 0 4456.8325 y 10
373.7151184082031 0 1926.2533
374.080078125 0 2113.0566
374.21673583984375 0 1017.62604
381.1759033203125 0 713.29175
382.20849609375 0 971.75726
383.15631103515625 0 3637.9573
383.2655944824219 0 1031.2002
384.15802001953125 0 983.59863
384.2664489746094 0 725.00464
385.19488525390625 0 505.88504
385.2194519042969 0 14894.95
386.198974609375 0 893.68274
386.2230529785156 0 2650.3987
393.25 0 1446.8503
397.1355285644531 0 1164.3792
397.2165832519531 0 1370.8147
397.71795654296875 0 824.5032
398.20257568359375 0 794.9391
398.2447204589844 0 1454.2769
399.1867980957031 0 794.24255
400.22296142578125 0 1236.0046
400.9850158691406 0 948.19214
401.166748046875 0 8400.959
402.1705017089844 0 2162.6257
406.22100830078125 0 1455.6565 b Water loss 7
406.7228698730469 0 798.6044
407.2039794921875 0 2259.3157
407.7218322753906 0 758.6749
408.1877746582031 0 7575.907
409.1900634765625 0 918.92487
410.20123291015625 0 719.4287
411.20623779296875 0 747.1253
411.2598876953125 0 3361.9727
412.2641906738281 0 1317.9573
413.21136474609375 0 644.8197
415.036376953125 0 1968.1492
415.1452941894531 0 1615.4526
415.23077392578125 0 1716.2404 b 7
415.26959228515625 0 1379.8008
416.0377197265625 0 1461.3948
416.21826171875 0 1166.3794 y Ammonia loss 13
416.25445556640625 0 4071.2783
417.2555236816406 0 1298.1886
418.9956359863281 0 4142.048
419.9960021972656 0 1851.7594
420.99371337890625 0 852.8156
424.2290954589844 0 4348.9565
425.1341552734375 0 774.35175
425.2143249511719 0 6616.8667
426.2189025878906 0 864.92676
427.2303771972656 0 1698.2725
429.2154846191406 0 755.11896 y Ammonia loss 9
433.1560363769531 0 2618.775
433.24114990234375 0 15494.303 y 13
433.28118896484375 0 33186.72
434.2056884765625 0 1019.94525
434.2440490722656 0 2872.9849
434.283935546875 0 7943.6724
434.7067565917969 0 994.072
435.207275390625 0 1856.7645
435.2862854003906 0 1280.1307
438.2362365722656 0 727.2543
440.2110900878906 0 1687.3329
441.1971130371094 0 919.5009
441.2513427734375 0 934.5265
442.24053955078125 0 5348.5093
443.1422119140625 0 1001.27203
443.2316589355469 0 2173.1138
443.2660217285156 0 3110.0984 b Water loss 3
444.26739501953125 0 895.04047
445.2021484375 0 1135.9623
445.2417907714844 0 667.41174
451.2305603027344 0 1679.8165
451.89453125 0 2744.78 y Water loss 4
452.2171936035156 0 4094.2852 y Ammonia loss 4
452.56060791015625 0 1327.7206
453.2145690917969 0 1006.7681
454.2044372558594 0 905.4873
458.2717590332031 0 588.3773
459.2099304199219 0 1716.6965
459.26055908203125 0 1455.4662
461.2178039550781 0 964.59485
461.2760314941406 0 32221.896 b 3
462.1990661621094 0 3146.7324
462.2790832519531 0 9369.82
463.28204345703125 0 1012.72864
464.21856689453125 0 768.0994
464.25341796875 0 718.349
465.2444152832031 0 602.21344
466.194091796875 0 620.27234
468.20733642578125 0 1245.3232
468.2559814453125 0 1993.593
469.2444763183594 0 2267.5142
470.2250061035156 0 9864.717
470.7320556640625 0 1076.9933
471.2281494140625 0 2383.7603
472.2433166503906 0 1045.104
476.17779541015625 0 737.33594
477.25634765625 0 2684.137
478.24151611328125 0 5615.8506
479.2272644042969 0 3982.6465
479.7486267089844 0 961.8219 b 8
480.20916748046875 0 10242.91
480.25 0 1272.7227
480.71734619140625 0 1026.2009
480.7504577636719 0 1540.6195
481.21380615234375 0 3481.743
482.2216491699219 0 891.5295
484.2033996582031 0 1567.2208
486.2674865722656 0 5002.3774 y Water loss 12
487.2498779296875 0 2840.1108 y Ammonia loss 12
488.2512512207031 0 1087.4738
488.73614501953125 0 799.19025
489.0557556152344 0 833.33124
490.05438232421875 0 684.4365
490.20758056640625 0 889.1347
493.7272644042969 0 4216.7607
494.1895446777344 0 1083.3036
494.2291564941406 0 1493.8466
494.7300720214844 0 626.6862
495.2669677734375 0 3403.9011
496.25146484375 0 13102.471
497.2531433105469 0 2812.2258
498.2197265625 0 30793.086
499.2225036621094 0 7211.073
500.2315368652344 0 1628.0963
502.2145690917969 0 3199.3298
502.7328186035156 0 4601.5713
503.2301025390625 0 1676.9421
504.27764892578125 0 36039.914 y 12
505.2799377441406 0 8484.002
506.2847595214844 0 1454.712
508.2190856933594 0 1027.8705
510.2195739746094 0 817.7798
512.1988525390625 0 2291.7227
513.2774047851562 0 4769.533
514.2630004882812 0 741.42865
515.2481689453125 0 1059.2914
518.2052612304688 0 870.2771
526.25537109375 0 718.5944
528.232177734375 0 703.2901
529.2637939453125 0 937.78345
529.7642211914062 0 1745.6252
530.208984375 0 10623.659
530.3327026367188 0 2685.9814
531.2120361328125 0 2684.9763
531.3370971679688 0 1274.4637
534.7532958984375 0 638.4446
535.9453735351562 0 693.2618
536.2757568359375 0 748.132
536.7650146484375 0 800.50116 y Water loss 7
538.2691650390625 0 5402.889
538.7705688476562 0 3367.4
538.9420776367188 0 609.48615 y Ammonia loss 2
540.3186645507812 0 3375.0837 b Water loss 4
541.3221435546875 0 665.0074
542.7689208984375 0 716.8098
543.2625732421875 0 2541.6145
543.766357421875 0 1112.7965
544.26708984375 0 2516.9465 b 9
544.771728515625 0 878.6639
545.7680053710938 0 707.88684 y 7
546.2387084960938 0 828.9821
547.95556640625 0 899.43866 b 14
552.2669677734375 0 6233.998
552.7674560546875 0 4304.7217
553.265380859375 0 1576.6024
553.3099365234375 0 774.45074
555.324951171875 0 2183.5366
556.276123046875 0 1369.417
556.3275146484375 0 1102.9636
558.3281860351562 0 7845.359 b 4
559.3309326171875 0 2323.2783
560.7757568359375 0 988.7459
561.2837524414062 0 800.8181
563.2450561523438 0 800.75073
566.42919921875 0 757.3908
569.2914428710938 0 1176.8818
569.7769165039062 0 2288.5146
570.2781372070312 0 707.07074
571.2710571289062 0 602.6495
573.7738037109375 0 736.97864
574.2744140625 0 1022.736
578.1233520507812 0 833.69934
578.2847900390625 0 1984.4685
578.7872924804688 0 1189.8284
579.2868041992188 0 1905.0498
580.2780151367188 0 1697.8768 y Water loss 6
580.783203125 0 1197.8932 y Ammonia loss 6
581.257568359375 0 2586.5408
582.28662109375 0 1961.1456
582.7826538085938 0 3904.232
583.2823486328125 0 2397.6748
583.7860717773438 0 1464.1161
586.3348388671875 0 660.5515
591.2413330078125 0 2202.2104
591.2936401367188 0 2285.0962
591.7982177734375 0 1801.0977 b Water loss 10
592.2401123046875 0 1032.7224
597.2951049804688 0 3514.8975
598.2892456054688 0 2187.627
599.2672119140625 0 13746.321
599.6446533203125 0 1540.9509
599.979736328125 0 904.39154
600.27001953125 0 4537.7944
600.8092041015625 0 844.6509 b 10
601.2734375 0 829.89667
602.0714111328125 0 719.1532
602.7910766601562 0 2850.2983
603.29296875 0 3075.852
604.2990112304688 0 970.53595
606.8123168945312 0 826.1294
608.3538818359375 0 1224.444
608.8018798828125 0 1516.3348
609.2509155273438 0 6846.5537
609.2940673828125 0 877.6154
609.3405151367188 0 1005.2865
609.8005981445312 0 1438.1439
610.1839599609375 0 4306.665
610.2571411132812 0 1633.9833
611.1843872070312 0 4693.7446
611.32177734375 0 882.79614 Precursor
611.796875 0 5311.7266
612.182373046875 0 4354.1743
612.244140625 0 866.8823
612.2994384765625 0 3581.6997
612.8114624023438 0 3832.9268
613.3143920898438 0 2349.3398
613.8119506835938 0 743.395
614.324462890625 0 10192.999 y Water loss 11
615.3096313476562 0 20767.424 y Ammonia loss 11
616.31103515625 0 6551.596
617.3130493164062 0 3648.6265
617.8065795898438 0 15006.078
618.3063354492188 0 11813.079
618.8051147460938 0 7848.389
619.3040161132812 0 2185.8755
620.301025390625 0 938.64215
620.798095703125 0 977.41895
625.2828369140625 0 1095.6151
626.3626708984375 0 4539.564
626.8116455078125 0 31761.191
627.261474609375 0 61479.883
627.3135375976562 0 20385.654
627.813232421875 0 9293.98
628.2647705078125 0 19391.66
628.3153686523438 0 1681.3538
628.8104248046875 0 3518.767 y Water loss 5
629.2657470703125 0 2622.642
629.3113403320312 0 1727.8452 y Ammonia loss 5
629.8050537109375 0 1972.025
632.3358764648438 0 29830.764 y 11
633.337158203125 0 9629.734
634.341064453125 0 1577.0768
635.814453125 0 683.8328
637.8143920898438 0 5294.2964 y 5
638.3168334960938 0 3257.4058
638.814697265625 0 1645.5092
643.2942504882812 0 4004.7725
644.29248046875 0 1945.9271
645.300048828125 0 2912.4028
646.2863159179688 0 825.88464
646.8289794921875 0 958.0437
647.8173217773438 0 610.30316
655.3394165039062 0 11007.565
655.84130859375 0 8795.566 b Water loss 11
656.3388061523438 0 4352.294 b Ammonia loss 11
656.8427124023438 0 1097.4958
658.3032836914062 0 828.55005
659.8196411132812 0 794.1873
660.3192138671875 0 1600.8224
664.8292236328125 0 745.98206 b 11
667.33837890625 0 1085.5024
668.3313598632812 0 3291.2178
668.8245849609375 0 4606.0835
669.32421875 0 2370.5317
669.825439453125 0 954.5444
676.3274536132812 0 1016.71277
677.335205078125 0 35908.875 y Water loss 4
677.834228515625 0 31719.002 y Ammonia loss 4
678.3342895507812 0 16624.52
678.835693359375 0 5757.2495
679.3306884765625 0 1521.5404
684.3668823242188 0 1395.2474
686.340576171875 0 74572.82 y 4
686.8416748046875 0 56692.395
686.895751953125 0 522.27997
687.343505859375 0 22071.639
687.844482421875 0 6777.715
688.3380737304688 0 894.6647
691.3441772460938 0 886.43933 b Water loss 12
694.3387451171875 0 2292.0352
695.34375 0 1223.2955
698.3385009765625 0 972.38196
700.3462524414062 0 3986.628 b 12
700.8489990234375 0 3897.2266
701.3502807617188 0 1404.4237
704.3276977539062 0 1644.1556
710.3814086914062 0 1342.0621
711.3844604492188 0 921.5645
712.3504638671875 0 9540.654
713.3530883789062 0 3694.257
714.35888671875 0 826.2331
722.3343505859375 0 2976.2773
723.3384399414062 0 927.43317
724.4020385742188 0 1891.0887 b Water loss 6
726.338134765625 0 1288.6146
727.40966796875 0 1469.1597 y Water loss 10
728.3955688476562 0 2206.2935 y Ammonia loss 10
728.8744506835938 0 2325.5933
729.3818359375 0 1888.2736
730.3862915039062 0 1132.0793
731.3528442382812 0 797.8408
737.3929443359375 0 1666.2661
738.3797607421875 0 1394.1151
739.3697509765625 0 1074.6405
740.3458862304688 0 22266.139
741.3492431640625 0 8870.434
742.34814453125 0 2957.8733
742.4142456054688 0 3885.9424 b 6
742.8621826171875 0 737.76886
743.4171142578125 0 1428.8707
745.3451538085938 0 860.11395
745.4195556640625 0 13315.387 y 10
746.4220581054688 0 5298.935
747.4239501953125 0 1271.5322
749.3342895507812 0 1088.3918
750.8692016601562 0 9338.551 y Water loss 3
751.3693237304688 0 8336.686 y Ammonia loss 3
751.8697509765625 0 4191.669
752.3745727539062 0 1784.6344
752.8701782226562 0 813.9464
753.34033203125 0 1540.4843
755.4030151367188 0 2769.6023
756.406982421875 0 1295.2798
757.37353515625 0 7693.6846 b 13
758.3738403320312 0 3623.9238
759.3801879882812 0 1659.3208
759.874755859375 0 8638.542 y 3
760.37548828125 0 9519.977
760.8768920898438 0 4001.4004
761.379638671875 0 920.6645
761.8939819335938 0 762.49243
771.3508911132812 0 4535.797
772.3507080078125 0 2217.1257
773.3548583984375 0 775.2922
774.329833984375 0 4240.932
775.333740234375 0 2222.0415
793.4176025390625 0 1475.1763
797.376220703125 0 772.9552
806.3594970703125 0 1114.0935
807.4097900390625 0 1925.3583 y Water loss 2
807.910888671875 0 3445.5427 y Ammonia loss 2
808.4117431640625 0 2065.3193
808.9130859375 0 1359.1165
811.4332885742188 0 3427.6306 b Water loss 7
812.4348754882812 0 2031.3916 b Water loss 14
813.4318237304688 0 2228.835
816.4163208007812 0 1837.1328 y 2
816.9192504882812 0 1379.7336
822.4022216796875 0 681.4703
829.4436645507812 0 3261.34 b 7
830.444580078125 0 1628.4991
832.3862915039062 0 1008.55743
839.423583984375 0 1800.3898
840.4061279296875 0 2734.216
841.418212890625 0 2407.454
842.400146484375 0 1751.7473
842.9468994140625 0 1177.7698
843.38623046875 0 897.89233
845.396240234375 0 811.6579
848.4310302734375 0 1834.4622 b Ammonia loss 15
848.9434204101562 0 829.9213
850.39599609375 0 3013.8293
851.3904418945312 0 1982.0801
852.382080078125 0 884.8851
856.447509765625 0 1743.9052 y Water loss 9
856.943359375 0 2150.809 b 15
857.4432373046875 0 3994.4768 y Ammonia loss 9
857.9401245117188 0 2504.602 y Water loss 1
858.4366455078125 0 2116.2546 y Ammonia loss 1
859.4262084960938 0 1990.7994
861.396728515625 0 1056.3738
[truncated: 390,249 more chars]
