# Supplementary material for: A Handle on Mass Coincidence Errors in De Novo Sequencing of Antibodies by Bottom-up Proteomics
Source: J Proteome Res. 2024 Jun 27;23(8):3552–9. doi: 10.1021/acs.jproteome.4c00188 (PMC11301774; doi:10.1021/acs.jproteome.4c00188)
Supplement: Supplementary file 1 — pr4c00188_si_001.zip [file pr4c00188_si_001.zip › supplementary data/xln-disambiguation/2023-12-13@14-36-36 f59/report/reads/Combined_056.html]

Details Combined\_056 | Stitch OverviewUndefined

# Read Combined\_056

## Sequence (length=15)

JHQDWLDGKEYKCKK

## Spectrum 5173? Spectrum 5173 The raw spectrum of this peptide as annotated by Hecklib. The fragments are coloured according to ion type (see legend). Any peaks with a star '\*' as text can be hovered over to see the full details, first the ion type second the mass shift type. By hovering over the amino acids in the peptide or ions in the legend the corresponding peaks are highlighted. By toggling the 'Unassigned' label you can turn the background (unassigned) peaks on or off in the plot. By updating the slider in the Ion legend you can update the spectrum to only show the top X% of the peaks with labels. The top X% means any peak that is within X% of the highest intensity. By dragging in the spectrum you can zoom in to a specific part of the spectrum and use 'Zoom Out' to get back to the original zoom level. The annotation of the spectrum is based on the given sequence in the peptides file and is done with different software so inconsistencies are likely. The peaks are annotated based on the given sequence, with 20 ppm tolerance.

Copy Data

### Spectrum 5173 (TSV)

#### Preview

```
Loading example...
```

*Click on the button to copy the data to your clipboard.*

Mz MinMz MaxIntensity Max

WidthHeightPeptide font sizePeptide stroke widthSpectrum font sizeSpectrum stroke widthCompact peptide

Ion legend

wxyz

abcd

OtherUnassignedIonChargePositionShow for top:%

JHQDWLDGKEYKCKK

04.72e+49.44e+41.42e+51.89e+5

Zoom Out

c+12z+12y+12c+13c+13c+14c+14y+27y+27w+14y+28c+314z+14c+314y+29c+210y+14y+314y+314z+314y+314c+15c+15c+212z+15y+15c+16c+16y+212w+16w+213c+213z+213y+213y+213z+213y+213z+16z+16c+214c+17c+214y+16c+17z+214y+214c+18w+17c+18y+17z+17y+17z+18c+19y+18c+19z+19y+19c+110y+110z+110y+110c+111y+111y+111z+111c+112c+112c+112z+112y+112z+112y+112w+113c+113z+113c+114

0769153823073075

Fragment Matches Table

Show background peaks

| Position | Ion type | Intensity | mz Theoretical | mz Error (Th) | mz Error (ppm) | Charge | Series Number |
| --- | --- | --- | --- | --- | --- | --- | --- |
| - | - | 424 | 122.8 | - | - | 0 | - |
| - | - | 2126 | 129.1 | - | - | 0 | - |
| - | - | 387.4 | 136.1 | - | - | 0 | - |
| - | - | 594.8 | 149 | - | - | 0 | - |
| - | - | 1108 | 155.1 | - | - | 0 | - |
| - | - | 3939 | 166.1 | - | - | 0 | - |
| - | - | 515.2 | 170.8 | - | - | 0 | - |
| - | - | 485.4 | 171.1 | - | - | 0 | - |
| - | - | 1802 | 173.1 | - | - | 0 | - |
| - | - | 586.7 | 173.1 | - | - | 0 | - |
| - | - | 577.4 | 178.1 | - | - | 0 | - |
| - | - | 3705 | 185.2 | - | - | 0 | - |
| - | - | 470.3 | 190 | - | - | 0 | - |
| - | - | 686.2 | 201.1 | - | - | 0 | - |
| - | - | 568.5 | 204.1 | - | - | 0 | - |
| - | - | 1664 | 205.1 | - | - | 0 | - |
| - | - | 907.2 | 207.2 | - | - | 0 | - |
| - | - | 481 | 214 | - | - | 0 | - |
| - | - | 619.4 | 217.1 | - | - | 0 | - |
| - | - | 3739 | 223.2 | - | - | 0 | - |
| - | - | 665.5 | 224.2 | - | - | 0 | - |
| - | - | 607.2 | 230.2 | - | - | 0 | - |
| - | - | 5915 | 234.1 | - | - | 0 | - |
| - | - | 735.3 | 239.1 | - | - | 0 | - |
| - | - | 592.9 | 242.1 | - | - | 0 | - |
| - | - | 527.7 | 248.1 | - | - | 0 | - |
| - | - | 1411 | 250.1 | - | - | 0 | - |
| - | - | 2.763E+04 | 251.2 | - | - | 0 | - |
| - | - | 3512 | 252.2 | - | - | 0 | - |
| - | - | 1089 | 260.1 | - | - | 0 | - |
| - | - | 575.6 | 263.6 | - | - | 0 | - |
| - | - | 551.3 | 264.1 | - | - | 0 | - |
| - | - | 1025 | 266.1 | - | - | 0 | - |
| 2 | c | 1.651E+04 | 268.2 | 0.0001351 | 0.5039 | +1 | 2 |
| - | - | 1834 | 269.2 | - | - | 0 | - |
| - | - | 495.9 | 277.2 | - | - | 0 | - |
| - | - | 1499 | 283.2 | - | - | 0 | - |
| - | - | 1577 | 294.2 | - | - | 0 | - |
| - | - | 665.3 | 299.2 | - | - | 0 | - |
| - | - | 520.7 | 311.1 | - | - | 0 | - |
| 14 | z | 2.981E+04 | 317.2 | 0.004402 | 13.88 | +1 | 2 |
| - | - | 6433 | 318.2 | - | - | 0 | - |
| - | - | 991 | 319.2 | - | - | 0 | - |
| 14 | y | 2974 | 333.2 | 0.004724 | 14.18 | +1 | 2 |
| - | - | 856.6 | 342 | - | - | 0 | - |
| - | - | 610.5 | 343 | - | - | 0 | - |
| - | - | 2867 | 352.2 | - | - | 0 | - |
| - | - | 2373 | 353.2 | - | - | 0 | - |
| - | - | 2632 | 355.1 | - | - | 0 | - |
| - | - | 2021 | 356.1 | - | - | 0 | - |
| - | - | 1629 | 357.1 | - | - | 0 | - |
| - | - | 2512 | 359 | - | - | 0 | - |
| - | - | 2329 | 360 | - | - | 0 | - |
| - | - | 1254 | 361 | - | - | 0 | - |
| - | - | 562.8 | 370.1 | - | - | 0 | - |
| 3 | c | 4384 | 379.2 | 0.0001201 | 0.3167 | +1 | 3 |
| - | - | 868.2 | 380.2 | - | - | 0 | - |
| - | - | 646.6 | 383.2 | - | - | 0 | - |
| - | - | 2.438E+04 | 387.2 | - | - | 0 | - |
| - | - | 5002 | 388.2 | - | - | 0 | - |
| - | - | 642.7 | 393.1 | - | - | 0 | - |
| - | - | 4863 | 395.2 | - | - | 0 | - |
| 3 | c | 3.982E+04 | 396.2 | 2.735E-05 | 0.06902 | +1 | 3 |
| - | - | 7042 | 397.2 | - | - | 0 | - |
| - | - | 831.5 | 398.2 | - | - | 0 | - |
| - | - | 1308 | 401.2 | - | - | 0 | - |
| - | - | 3115 | 429.1 | - | - | 0 | - |
| - | - | 5854 | 430.1 | - | - | 0 | - |
| - | - | 5026 | 431.1 | - | - | 0 | - |
| - | - | 1435 | 432.1 | - | - | 0 | - |
| - | - | 802.7 | 451.2 | - | - | 0 | - |
| - | - | 7199 | 467.2 | - | - | 0 | - |
| - | - | 3286 | 468.3 | - | - | 0 | - |
| - | - | 834.1 | 469.3 | - | - | 0 | - |
| - | - | 585.9 | 474.3 | - | - | 0 | - |
| 4 | c | 4946 | 494.2 | 0.00445 | 9.003 | +1 | 4 |
| - | - | 1422 | 495.2 | - | - | 0 | - |
| - | - | 979.1 | 496.3 | - | - | 0 | - |
| - | - | 792.8 | 497.8 | - | - | 0 | - |
| 4 | c | 3.284E+04 | 511.3 | 3.768E-05 | 0.0737 | +1 | 4 |
| - | - | 8310 | 512.3 | - | - | 0 | - |
| 9 | y | 1029 | 513.3 | 0.00935 | 18.22 | +2 | 7 |
| - | - | 5747 | 515.3 | - | - | 0 | - |
| - | - | 2397 | 516.3 | - | - | 0 | - |
| 9 | y | 729.4 | 521.8 | 0.0008728 | 1.673 | +2 | 7 |
| - | - | 2301 | 547.3 | - | - | 0 | - |
| 12 | w | 1009 | 548.2 | 0.006916 | 12.61 | +1 | 4 |
| 8 | y | 5308 | 550.3 | 0.004769 | 8.666 | +2 | 8 |
| - | - | 3400 | 550.8 | - | - | 0 | - |
| - | - | 1425 | 551.3 | - | - | 0 | - |
| - | - | 926.9 | 551.8 | - | - | 0 | - |
| - | - | 829.2 | 555.3 | - | - | 0 | - |
| - | - | 683.9 | 590.3 | - | - | 0 | - |
| - | - | 1119 | 597 | - | - | 0 | - |
| - | - | 2036 | 597.3 | - | - | 0 | - |
| 14 | c | 3672 | 601.6 | 0.0006828 | 1.135 | +3 | 14 |
| - | - | 3126 | 602 | - | - | 0 | - |
| - | - | 1962 | 602.3 | - | - | 0 | - |
| - | - | 733.8 | 602.6 | - | - | 0 | - |
| 12 | z | 2.787E+04 | 606.3 | 0.008869 | 14.63 | +1 | 4 |
| 14 | c | 1.214E+04 | 607.3 | 0.009165 | 15.09 | +3 | 14 |
| 7 | y | 2663 | 607.8 | 0.003287 | 5.408 | +2 | 9 |
| - | - | 3989 | 608.3 | - | - | 0 | - |
| - | - | 641.8 | 608.8 | - | - | 0 | - |
| 10 | c | 1205 | 611.8 | 0.000174 | 0.2845 | +2 | 10 |
| - | - | 1057 | 612.3 | - | - | 0 | - |
| - | - | 3050 | 619.2 | - | - | 0 | - |
| - | - | 5379 | 622.2 | - | - | 0 | - |
| 12 | y | 1.084E+04 | 622.3 | 0.008978 | 14.43 | +1 | 4 |
| - | - | 4622 | 623.2 | - | - | 0 | - |
| - | - | 2920 | 623.3 | - | - | 0 | - |
| - | - | 794.3 | 624.2 | - | - | 0 | - |
| - | - | 1023 | 624.3 | - | - | 0 | - |
| - | - | 968 | 625.3 | - | - | 0 | - |
| - | - | 859.6 | 625.7 | - | - | 0 | - |
| 2 | y | 1609 | 626 | 2.092E-05 | 0.03342 | +3 | 14 |
| 2 | y | 779.4 | 626.3 | 0.01186 | 18.94 | +3 | 14 |
| 2 | z | 921.8 | 626.6 | 0.0006569 | 1.048 | +3 | 14 |
| - | - | 1355 | 628.6 | - | - | 0 | - |
| - | - | 2770 | 629 | - | - | 0 | - |
| - | - | 1948 | 629.3 | - | - | 0 | - |
| - | - | 1660 | 629.6 | - | - | 0 | - |
| - | - | 806.5 | 631.6 | - | - | 0 | - |
| 2 | y | 1.194E+04 | 632 | 0.003073 | 4.863 | +3 | 14 |
| - | - | 9726 | 632.3 | - | - | 0 | - |
| - | - | 5275 | 632.6 | - | - | 0 | - |
| - | - | 3434 | 633 | - | - | 0 | - |
| - | - | 843 | 633.3 | - | - | 0 | - |
| - | - | 981.6 | 634.3 | - | - | 0 | - |
| - | - | 1.541E+04 | 634.6 | - | - | 0 | - |
| - | - | 1.806E+04 | 635 | - | - | 0 | - |
| - | - | 8994 | 635.3 | - | - | 0 | - |
| - | - | 4242 | 635.6 | - | - | 0 | - |
| - | - | 1659 | 636 | - | - | 0 | - |
| - | - | 1817 | 637.2 | - | - | 0 | - |
| - | - | 845.3 | 637.3 | - | - | 0 | - |
| - | - | 945.7 | 637.4 | - | - | 0 | - |
| - | - | 891.8 | 638.3 | - | - | 0 | - |
| - | - | 1089 | 640.6 | - | - | 0 | - |
| - | - | 871.4 | 641.3 | - | - | 0 | - |
| - | - | 847.5 | 650.3 | - | - | 0 | - |
| - | - | 739.5 | 650.6 | - | - | 0 | - |
| - | - | 618.8 | 651.3 | - | - | 0 | - |
| - | - | 1271 | 653.3 | - | - | 0 | - |
| - | - | 2074 | 654.3 | - | - | 0 | - |
| - | - | 2723 | 658 | - | - | 0 | - |
| - | - | 3787 | 658.3 | - | - | 0 | - |
| - | - | 2126 | 658.6 | - | - | 0 | - |
| - | - | 1400 | 659 | - | - | 0 | - |
| - | - | 1.215E+04 | 663.7 | - | - | 0 | - |
| - | - | 1.373E+04 | 664 | - | - | 0 | - |
| - | - | 8834 | 664.3 | - | - | 0 | - |
| - | - | 7277 | 664.7 | - | - | 0 | - |
| - | - | 3268 | 665 | - | - | 0 | - |
| - | - | 1054 | 668.3 | - | - | 0 | - |
| - | - | 867.4 | 668.3 | - | - | 0 | - |
| - | - | 1958 | 669 | - | - | 0 | - |
| - | - | 1.136E+04 | 669.3 | - | - | 0 | - |
| - | - | 1.575E+05 | 669.7 | - | - | 0 | - |
| - | - | 1.869E+05 | 670 | - | - | 0 | - |
| - | - | 1.233E+05 | 670.3 | - | - | 0 | - |
| - | - | 4.748E+04 | 670.7 | - | - | 0 | - |
| - | - | 1.69E+04 | 671 | - | - | 0 | - |
| - | - | 1320 | 678.4 | - | - | 0 | - |
| 5 | c | 2559 | 680.3 | 0.001259 | 1.851 | +1 | 5 |
| - | - | 893.9 | 681.3 | - | - | 0 | - |
| 5 | c | 2.657E+04 | 697.3 | 0.0002042 | 0.2929 | +1 | 5 |
| - | - | 9485 | 698.3 | - | - | 0 | - |
| - | - | 1958 | 699.3 | - | - | 0 | - |
| - | - | 922.9 | 711.3 | - | - | 0 | - |
| - | - | 980.7 | 712.3 | - | - | 0 | - |
| 12 | c | 3358 | 757.4 | 0.004063 | 5.365 | +2 | 12 |
| - | - | 2390 | 757.9 | - | - | 0 | - |
| - | - | 1098 | 758.4 | - | - | 0 | - |
| - | - | 2394 | 766.4 | - | - | 0 | - |
| - | - | 1887 | 767.4 | - | - | 0 | - |
| - | - | 954 | 768.4 | - | - | 0 | - |
| 11 | z | 2.488E+04 | 769.4 | 0.008721 | 11.34 | +1 | 5 |
| - | - | 1.293E+04 | 770.4 | - | - | 0 | - |
| - | - | 4175 | 771.4 | - | - | 0 | - |
| - | - | 994.9 | 772.4 | - | - | 0 | - |
| - | - | 792.2 | 777.4 | - | - | 0 | - |
| 11 | y | 5697 | 785.4 | 0.008769 | 11.16 | +1 | 5 |
| - | - | 2847 | 786.4 | - | - | 0 | - |
| - | - | 902.7 | 787.4 | - | - | 0 | - |
| 6 | c | 3392 | 793.4 | 0.001241 | 1.564 | +1 | 6 |
| - | - | 1293 | 794.4 | - | - | 0 | - |
| - | - | 1194 | 807.4 | - | - | 0 | - |
| - | - | 720.9 | 808.4 | - | - | 0 | - |
| - | - | 2000 | 809.4 | - | - | 0 | - |
| 6 | c | 3.371E+04 | 810.4 | 0.0002228 | 0.2749 | +1 | 6 |
| - | - | 1.418E+04 | 811.4 | - | - | 0 | - |
| - | - | 4071 | 812.4 | - | - | 0 | - |
| 4 | y | 4163 | 814.9 | 0.005124 | 6.288 | +2 | 12 |
| - | - | 3539 | 815.4 | - | - | 0 | - |
| - | - | 1845 | 815.9 | - | - | 0 | - |
| - | - | 641.9 | 816.4 | - | - | 0 | - |
| - | - | 1032 | 817.4 | - | - | 0 | - |
| - | - | 1546 | 818.4 | - | - | 0 | - |
| - | - | 720.2 | 818.9 | - | - | 0 | - |
| - | - | 1788 | 826.4 | - | - | 0 | - |
| - | - | 1334 | 826.9 | - | - | 0 | - |
| 10 | w | 3988 | 839.4 | 0.009446 | 11.25 | +1 | 6 |
| - | - | 1229 | 840.4 | - | - | 0 | - |
| 3 | w | 1129 | 841.9 | 0.000519 | 0.6165 | +2 | 13 |
| - | - | 1514 | 842.9 | - | - | 0 | - |
| - | - | 1633 | 843.4 | - | - | 0 | - |
| - | - | 685.9 | 843.9 | - | - | 0 | - |
| 13 | c | 2880 | 846.4 | 0.000502 | 0.5931 | +2 | 13 |
| - | - | 1635 | 846.9 | - | - | 0 | - |
| - | - | 757.5 | 847.4 | - | - | 0 | - |
| - | - | 867.3 | 860.4 | - | - | 0 | - |
| - | - | 886.7 | 861.4 | - | - | 0 | - |
| 3 | z | 983.8 | 861.9 | 0.004204 | 4.878 | +2 | 13 |
| - | - | 9481 | 867.4 | - | - | 0 | - |
| - | - | 4171 | 868.4 | - | - | 0 | - |
| 3 | y | 2901 | 869.9 | 0.005937 | 6.825 | +2 | 13 |
| 3 | y | 9273 | 870.4 | 0.0001021 | 0.1173 | +2 | 13 |
| 3 | z | 8889 | 870.9 | 0.005764 | 6.618 | +2 | 13 |
| - | - | 5556 | 871.4 | - | - | 0 | - |
| - | - | 2706 | 871.9 | - | - | 0 | - |
| - | - | 875.8 | 872.4 | - | - | 0 | - |
| - | - | 726.7 | 873.9 | - | - | 0 | - |
| - | - | 635 | 874.4 | - | - | 0 | - |
| 3 | y | 1.396E+04 | 878.9 | 0.004139 | 4.709 | +2 | 13 |
| - | - | 1.561E+04 | 879.4 | - | - | 0 | - |
| - | - | 9617 | 879.9 | - | - | 0 | - |
| 10 | z | 3814 | 880.4 | 0.01589 | 18.05 | +1 | 6 |
| - | - | 1631 | 880.9 | - | - | 0 | - |
| - | - | 4117 | 881.4 | - | - | 0 | - |
| - | - | 4103 | 882.4 | - | - | 0 | - |
| - | - | 1849 | 883.4 | - | - | 0 | - |
| - | - | 1894 | 888.4 | - | - | 0 | - |
| - | - | 3621 | 888.9 | - | - | 0 | - |
| - | - | 1875 | 889.4 | - | - | 0 | - |
| - | - | 974.6 | 889.9 | - | - | 0 | - |
| - | - | 969.3 | 892.5 | - | - | 0 | - |
| - | - | 1043 | 893.5 | - | - | 0 | - |
| - | - | 988.5 | 893.9 | - | - | 0 | - |
| - | - | 2169 | 894.4 | - | - | 0 | - |
| - | - | 823.3 | 894.9 | - | - | 0 | - |
| - | - | 732.6 | 896 | - | - | 0 | - |
| 10 | z | 2.528E+04 | 898.4 | 0.008956 | 9.969 | +1 | 6 |
| - | - | 1.272E+04 | 899.4 | - | - | 0 | - |
| - | - | 5294 | 900.4 | - | - | 0 | - |
| - | - | 1195 | 901.4 | - | - | 0 | - |
| 14 | c | 2837 | 901.9 | 0.0006304 | 0.6989 | +2 | 14 |
| - | - | 2720 | 902.4 | - | - | 0 | - |
| - | - | 1204 | 902.9 | - | - | 0 | - |
| - | - | 955.2 | 903.4 | - | - | 0 | - |
| - | - | 977.4 | 903.9 | - | - | 0 | - |
| 7 | c | 4860 | 908.4 | 0.003351 | 3.688 | +1 | 7 |
| - | - | 1905 | 909 | - | - | 0 | - |
| - | - | 3152 | 909.4 | - | - | 0 | - |
| - | - | 2143 | 909.9 | - | - | 0 | - |
| 14 | c | 2.352E+04 | 910.4 | 0.002085 | 2.29 | +2 | 14 |
| - | - | 2.353E+04 | 910.9 | - | - | 0 | - |
| - | - | 1.29E+04 | 911.4 | - | - | 0 | - |
| - | - | 5613 | 911.9 | - | - | 0 | - |
| - | - | 1762 | 912.4 | - | - | 0 | - |
| 10 | y | 4717 | 914.4 | 0.008149 | 8.911 | +1 | 6 |
| - | - | 1601 | 915.4 | - | - | 0 | - |
| - | - | 1416 | 916 | - | - | 0 | - |
| - | - | 2416 | 917.4 | - | - | 0 | - |
| - | - | 2567 | 917.9 | - | - | 0 | - |
| - | - | 788.6 | 918.4 | - | - | 0 | - |
| - | - | 691.4 | 920.5 | - | - | 0 | - |
| - | - | 1008 | 923 | - | - | 0 | - |
| - | - | 728.1 | 923.5 | - | - | 0 | - |
| - | - | 1043 | 924 | - | - | 0 | - |
| - | - | 5737 | 924.4 | - | - | 0 | - |
| 7 | c | 1.903E+04 | 925.5 | 0.001043 | 1.127 | +1 | 7 |
| - | - | 7854 | 926.5 | - | - | 0 | - |
| - | - | 1901 | 927.5 | - | - | 0 | - |
| - | - | 1720 | 929 | - | - | 0 | - |
| - | - | 1054 | 930 | - | - | 0 | - |
| - | - | 867.8 | 930.5 | - | - | 0 | - |
| - | - | 795.3 | 931 | - | - | 0 | - |
| - | - | 843.1 | 931.4 | - | - | 0 | - |
| - | - | 1136 | 932 | - | - | 0 | - |
| - | - | 2327 | 935.5 | - | - | 0 | - |
| - | - | 974.9 | 936 | - | - | 0 | - |
| - | - | 1562 | 936.5 | - | - | 0 | - |
| - | - | 1885 | 937 | - | - | 0 | - |
| - | - | 1136 | 937.5 | - | - | 0 | - |
| - | - | 7352 | 938 | - | - | 0 | - |
| - | - | 1.107E+04 | 938.5 | - | - | 0 | - |
| - | - | 8170 | 939 | - | - | 0 | - |
| 2 | z | 1.449E+04 | 939.4 | 0.006956 | 7.404 | +2 | 14 |
| - | - | 1.101E+04 | 939.9 | - | - | 0 | - |
| - | - | 9550 | 940.4 | - | - | 0 | - |
| - | - | 2581 | 940.9 | - | - | 0 | - |
| - | - | 745 | 943 | - | - | 0 | - |
| - | - | 1040 | 943.5 | - | - | 0 | - |
| - | - | 902.1 | 944 | - | - | 0 | - |
| - | - | 873.5 | 944.5 | - | - | 0 | - |
| - | - | 1090 | 945.5 | - | - | 0 | - |
| - | - | 790.5 | 946 | - | - | 0 | - |
| - | - | 3036 | 946.5 | - | - | 0 | - |
| - | - | 1291 | 947 | - | - | 0 | - |
| 2 | y | 1257 | 947.4 | 0.002965 | 3.129 | +2 | 14 |
| - | - | 823.8 | 948.4 | - | - | 0 | - |
| - | - | 3400 | 950.5 | - | - | 0 | - |
| - | - | 4661 | 951 | - | - | 0 | - |
| - | - | 3571 | 951.5 | - | - | 0 | - |
| - | - | 3320 | 952 | - | - | 0 | - |
| - | - | 5247 | 952.5 | - | - | 0 | - |
| - | - | 3404 | 953 | - | - | 0 | - |
| - | - | 1788 | 953.5 | - | - | 0 | - |
| - | - | 2012 | 954 | - | - | 0 | - |
| - | - | 884.9 | 954.5 | - | - | 0 | - |
| - | - | 833.8 | 959 | - | - | 0 | - |
| - | - | 4122 | 959.5 | - | - | 0 | - |
| - | - | 4.319E+04 | 960 | - | - | 0 | - |
| - | - | 4.57E+04 | 960.5 | - | - | 0 | - |
| - | - | 3.114E+04 | 961 | - | - | 0 | - |
| - | - | 1.265E+04 | 961.5 | - | - | 0 | - |
| - | - | 3641 | 962 | - | - | 0 | - |
| - | - | 3101 | 965 | - | - | 0 | - |
| 8 | c | 2470 | 965.4 | 0.01662 | 17.21 | +1 | 8 |
| - | - | 3991 | 966 | - | - | 0 | - |
| - | - | 4448 | 966.5 | - | - | 0 | - |
| - | - | 2217 | 967 | - | - | 0 | - |
| - | - | 2615 | 967.5 | - | - | 0 | - |
| - | - | 6088 | 968 | - | - | 0 | - |
| 9 | w | 8419 | 968.4 | 0.01449 | 14.96 | +1 | 7 |
| - | - | 4002 | 969 | - | - | 0 | - |
| - | - | 2854 | 969.5 | - | - | 0 | - |
| - | - | 1280 | 972.5 | - | - | 0 | - |
| - | - | 2076 | 973 | - | - | 0 | - |
| - | - | 3087 | 973.5 | - | - | 0 | - |
| - | - | 1.11E+04 | 974 | - | - | 0 | - |
| - | - | 3.573E+04 | 974.5 | - | - | 0 | - |
| - | - | 8.612E+04 | 975 | - | - | 0 | - |
| - | - | 7.68E+04 | 975.5 | - | - | 0 | - |
| - | - | 4.981E+04 | 976 | - | - | 0 | - |
| - | - | 1.843E+04 | 976.5 | - | - | 0 | - |
| - | - | 6147 | 977 | - | - | 0 | - |
| - | - | 772.8 | 978.5 | - | - | 0 | - |
| - | - | 750.3 | 980.5 | - | - | 0 | - |
| - | - | 2.129E+04 | 981.5 | - | - | 0 | - |
| - | - | 1.739E+04 | 982 | - | - | 0 | - |
| 8 | c | 4.102E+04 | 982.5 | 0.0038 | 3.867 | +1 | 8 |
| - | - | 7679 | 983 | - | - | 0 | - |
| - | - | 1.772E+04 | 983.5 | - | - | 0 | - |
| - | - | 4100 | 984.5 | - | - | 0 | - |
| - | - | 1036 | 985.5 | - | - | 0 | - |
| - | - | 2724 | 986.5 | - | - | 0 | - |
| - | - | 2450 | 987 | - | - | 0 | - |
| - | - | 2839 | 987.5 | - | - | 0 | - |
| - | - | 811 | 988 | - | - | 0 | - |
| - | - | 890.2 | 991 | - | - | 0 | - |
| - | - | 1066 | 994 | - | - | 0 | - |
| - | - | 1641 | 995 | - | - | 0 | - |
| - | - | 7507 | 995.5 | - | - | 0 | - |
| - | - | 2.829E+04 | 996 | - | - | 0 | - |
| - | - | 2.89E+04 | 996.5 | - | - | 0 | - |
| - | - | 1.556E+04 | 997 | - | - | 0 | - |
| - | - | 9575 | 997.5 | - | - | 0 | - |
| - | - | 2428 | 998 | - | - | 0 | - |
| - | - | 905.4 | 1002 | - | - | 0 | - |
| - | - | 1660 | 1003 | - | - | 0 | - |
| - | - | 7935 | 1003 | - | - | 0 | - |
| - | - | 7.35E+04 | 1004 | - | - | 0 | - |
| - | - | 1.422E+05 | 1004 | - | - | 0 | - |
| - | - | 1.058E+05 | 1005 | - | - | 0 | - |
| - | - | 6.134E+04 | 1005 | - | - | 0 | - |
| - | - | 2.296E+04 | 1006 | - | - | 0 | - |
| - | - | 6239 | 1006 | - | - | 0 | - |
| - | - | 852.3 | 1012 | - | - | 0 | - |
| 9 | y | 1179 | 1025 | 0.002948 | 2.877 | +1 | 7 |
| 9 | z | 2.16E+04 | 1027 | 0.009315 | 9.074 | +1 | 7 |
| - | - | 2.182E+04 | 1028 | - | - | 0 | - |
| - | - | 1.027E+04 | 1029 | - | - | 0 | - |
| - | - | 2937 | 1030 | - | - | 0 | - |
| - | - | 1140 | 1040 | - | - | 0 | - |
| - | - | 1723 | 1042 | - | - | 0 | - |
| 9 | y | 7419 | 1043 | 0.007775 | 7.458 | +1 | 7 |
| - | - | 4392 | 1044 | - | - | 0 | - |
| - | - | 1760 | 1045 | - | - | 0 | - |
| - | - | 1947 | 1067 | - | - | 0 | - |
| - | - | 1686 | 1068 | - | - | 0 | - |
| 8 | z | 1.105E+04 | 1084 | 0.00905 | 8.352 | +1 | 8 |
| - | - | 1.29E+04 | 1085 | - | - | 0 | - |
| - | - | 6293 | 1086 | - | - | 0 | - |
| - | - | 2058 | 1087 | - | - | 0 | - |
| - | - | 846.6 | 1088 | - | - | 0 | - |
| 9 | c | 877.2 | 1093 | 0.0008868 | 0.8117 | +1 | 9 |
| - | - | 2744 | 1099 | - | - | 0 | - |
| 8 | y | 7225 | 1100 | 0.01129 | 10.27 | +1 | 8 |
| - | - | 3691 | 1101 | - | - | 0 | - |
| - | - | 1183 | 1102 | - | - | 0 | - |
| - | - | 1619 | 1108 | - | - | 0 | - |
| - | - | 1030 | 1109 | - | - | 0 | - |
| - | - | 2560 | 1110 | - | - | 0 | - |
| 9 | c | 3.112E+04 | 1111 | 0.0005872 | 0.5287 | +1 | 9 |
| - | - | 2.142E+04 | 1112 | - | - | 0 | - |
| - | - | 6874 | 1113 | - | - | 0 | - |
| - | - | 944.8 | 1114 | - | - | 0 | - |
| - | - | 2442 | 1137 | - | - | 0 | - |
| - | - | 9623 | 1142 | - | - | 0 | - |
| - | - | 6243 | 1143 | - | - | 0 | - |
| - | - | 3006 | 1144 | - | - | 0 | - |
| - | - | 1812 | 1155 | - | - | 0 | - |
| - | - | 1064 | 1156 | - | - | 0 | - |
| - | - | 2790 | 1196 | - | - | 0 | - |
| - | - | 2462 | 1197 | - | - | 0 | - |
| - | - | 819.7 | 1198 | - | - | 0 | - |
| 7 | z | 2.598E+04 | 1199 | 0.008649 | 7.216 | +1 | 9 |
| - | - | 1.721E+04 | 1200 | - | - | 0 | - |
| - | - | 8906 | 1201 | - | - | 0 | - |
| - | - | 2573 | 1202 | - | - | 0 | - |
| - | - | 1149 | 1214 | - | - | 0 | - |
| 7 | y | 6214 | 1215 | 0.008208 | 6.758 | +1 | 9 |
| - | - | 3711 | 1216 | - | - | 0 | - |
| - | - | 1279 | 1217 | - | - | 0 | - |
| - | - | 2675 | 1239 | - | - | 0 | - |
| 10 | c | 2.993E+04 | 1240 | 0.0005778 | 0.4661 | +1 | 10 |
| - | - | 1.904E+04 | 1241 | - | - | 0 | - |
| - | - | 6990 | 1242 | - | - | 0 | - |
| - | - | 2272 | 1243 | - | - | 0 | - |
| - | - | 3219 | 1268 | - | - | 0 | - |
| - | - | 2050 | 1269 | - | - | 0 | - |
| - | - | 1928 | 1270 | - | - | 0 | - |
| 6 | y | 1266 | 1311 | 0.01521 | 11.6 | +1 | 10 |
| 6 | z | 1.334E+04 | 1312 | 0.00824 | 6.282 | +1 | 10 |
| - | - | 1.123E+04 | 1313 | - | - | 0 | - |
| - | - | 5777 | 1314 | - | - | 0 | - |
| - | - | 2124 | 1315 | - | - | 0 | - |
| - | - | 727.3 | 1323 | - | - | 0 | - |
| - | - | 859 | 1327 | - | - | 0 | - |
| 6 | y | 2765 | 1328 | 0.01207 | 9.093 | +1 | 10 |
| - | - | 1804 | 1329 | - | - | 0 | - |
| - | - | 1121 | 1330 | - | - | 0 | - |
| - | - | 824.3 | 1337 | - | - | 0 | - |
| - | - | 800.5 | 1339 | - | - | 0 | - |
| - | - | 818.4 | 1342 | - | - | 0 | - |
| - | - | 5810 | 1359 | - | - | 0 | - |
| - | - | 3695 | 1360 | - | - | 0 | - |
| - | - | 1013 | 1361 | - | - | 0 | - |
| - | - | 741.3 | 1390 | - | - | 0 | - |
| - | - | 4383 | 1402 | - | - | 0 | - |
| 11 | c | 3.294E+04 | 1403 | 0.001162 | 0.8285 | +1 | 11 |
| - | - | 2.511E+04 | 1404 | - | - | 0 | - |
| - | - | 1.135E+04 | 1405 | - | - | 0 | - |
| - | - | 3485 | 1406 | - | - | 0 | - |
| - | - | 1383 | 1407 | - | - | 0 | - |
| - | - | 1296 | 1442 | - | - | 0 | - |
| - | - | 1029 | 1482 | - | - | 0 | - |
| - | - | 2982 | 1487 | - | - | 0 | - |
| - | - | 2278 | 1488 | - | - | 0 | - |
| 5 | y | 873.8 | 1496 | 0.02833 | 18.94 | +1 | 11 |
| 5 | y | 2594 | 1497 | 0.007674 | 5.127 | +1 | 11 |
| 5 | z | 1.312E+04 | 1498 | 0.007719 | 5.154 | +1 | 11 |
| - | - | 1.545E+04 | 1499 | - | - | 0 | - |
| - | - | 6951 | 1500 | - | - | 0 | - |
| - | - | 2659 | 1501 | - | - | 0 | - |
| - | - | 1287 | 1502 | - | - | 0 | - |
| 12 | c | 1954 | 1513 | 0.02819 | 18.63 | +1 | 12 |
| 12 | c | 7014 | 1514 | 0.006832 | 4.513 | +1 | 12 |
| - | - | 5491 | 1515 | - | - | 0 | - |
| - | - | 3294 | 1516 | - | - | 0 | - |
| - | - | 1211 | 1517 | - | - | 0 | - |
| - | - | 811.4 | 1522 | - | - | 0 | - |
| - | - | 825.2 | 1523 | - | - | 0 | - |
| - | - | 1638 | 1525 | - | - | 0 | - |
| - | - | 1621 | 1526 | - | - | 0 | - |
| - | - | 1014 | 1527 | - | - | 0 | - |
| - | - | 880.3 | 1529 | - | - | 0 | - |
| - | - | 3980 | 1530 | - | - | 0 | - |
| 12 | c | 2.069E+04 | 1531 | 0.001521 | 0.9934 | +1 | 12 |
| - | - | 1.922E+04 | 1532 | - | - | 0 | - |
| - | - | 8509 | 1533 | - | - | 0 | - |
| - | - | 3013 | 1534 | - | - | 0 | - |
| - | - | 831.8 | 1540 | - | - | 0 | - |
| - | - | 969.5 | 1551 | - | - | 0 | - |
| - | - | 1535 | 1552 | - | - | 0 | - |
| - | - | 1033 | 1553 | - | - | 0 | - |
| - | - | 1599 | 1554 | - | - | 0 | - |
| - | - | 1324 | 1555 | - | - | 0 | - |
| - | - | 1452 | 1556 | - | - | 0 | - |
| - | - | 2978 | 1557 | - | - | 0 | - |
| - | - | 2654 | 1558 | - | - | 0 | - |
| - | - | 905.7 | 1559 | - | - | 0 | - |
| - | - | 7392 | 1569 | - | - | 0 | - |
| - | - | 4604 | 1570 | - | - | 0 | - |
| - | - | 3525 | 1571 | - | - | 0 | - |
| - | - | 1688 | 1572 | - | - | 0 | - |
| 4 | z | 724.1 | 1596 | 0.001155 | 0.7239 | +1 | 12 |
| - | - | 947.3 | 1600 | - | - | 0 | - |
| 4 | y | 1767 | 1612 | 0.008527 | 5.29 | +1 | 12 |
| 4 | z | 2.524E+04 | 1613 | 0.01049 | 6.506 | +1 | 12 |
| - | - | 3.224E+04 | 1614 | - | - | 0 | - |
| - | - | 2.037E+04 | 1615 | - | - | 0 | - |
| - | - | 8610 | 1616 | - | - | 0 | - |
| - | - | 3514 | 1617 | - | - | 0 | - |
| - | - | 1147 | 1618 | - | - | 0 | - |
| - | - | 1780 | 1628 | - | - | 0 | - |
| 4 | y | 6935 | 1629 | 0.01152 | 7.071 | +1 | 12 |
| - | - | 5633 | 1630 | - | - | 0 | - |
| - | - | 3577 | 1631 | - | - | 0 | - |
| - | - | 1602 | 1632 | - | - | 0 | - |
| - | - | 1911 | 1633 | - | - | 0 | - |
| - | - | 1005 | 1634 | - | - | 0 | - |
| - | - | 1323 | 1654 | - | - | 0 | - |
| - | - | 977.3 | 1667 | - | - | 0 | - |
| 3 | w | 1019 | 1683 | 0.002184 | 1.298 | +1 | 13 |
| - | - | 1174 | 1684 | - | - | 0 | - |
| - | - | 1282 | 1685 | - | - | 0 | - |
| - | - | 4083 | 1691 | - | - | 0 | - |
| 13 | c | 3.733E+04 | 1692 | 0.006301 | 3.724 | +1 | 13 |
| - | - | 3.193E+04 | 1693 | - | - | 0 | - |
| - | - | 1.945E+04 | 1694 | - | - | 0 | - |
| - | - | 6820 | 1695 | - | - | 0 | - |
| - | - | 2019 | 1696 | - | - | 0 | - |
| - | - | 1140 | 1697 | - | - | 0 | - |
| 3 | z | 1.35E+04 | 1741 | 0.01145 | 6.579 | +1 | 13 |
| - | - | 2.146E+04 | 1742 | - | - | 0 | - |
| - | - | 1.444E+04 | 1743 | - | - | 0 | - |
| - | - | 7331 | 1744 | - | - | 0 | - |
| - | - | 2442 | 1745 | - | - | 0 | - |
| - | - | 1378 | 1758 | - | - | 0 | - |
| - | - | 1023 | 1759 | - | - | 0 | - |
| - | - | 2641 | 1777 | - | - | 0 | - |
| - | - | 1900 | 1778 | - | - | 0 | - |
| - | - | 1407 | 1779 | - | - | 0 | - |
| 14 | c | 3375 | 1820 | 0.008246 | 4.531 | +1 | 14 |
| - | - | 6777 | 1821 | - | - | 0 | - |
| - | - | 4432 | 1822 | - | - | 0 | - |
| - | - | 3361 | 1823 | - | - | 0 | - |
| - | - | 897.5 | 1848 | - | - | 0 | - |
| - | - | 2296 | 1861 | - | - | 0 | - |
| - | - | 1708 | 1862 | - | - | 0 | - |
| - | - | 1245 | 1875 | - | - | 0 | - |
| - | - | 3413 | 1876 | - | - | 0 | - |
| - | - | 4206 | 1877 | - | - | 0 | - |
| - | - | 3726 | 1878 | - | - | 0 | - |
| - | - | 8006 | 1879 | - | - | 0 | - |
| - | - | 8425 | 1880 | - | - | 0 | - |
| - | - | 4936 | 1881 | - | - | 0 | - |
| - | - | 2476 | 1882 | - | - | 0 | - |
| - | - | 1296 | 1890 | - | - | 0 | - |
| - | - | 1705 | 1891 | - | - | 0 | - |
| - | - | 2104 | 1892 | - | - | 0 | - |
| - | - | 2055 | 1893 | - | - | 0 | - |
| - | - | 1355 | 1901 | - | - | 0 | - |
| - | - | 1001 | 1903 | - | - | 0 | - |
| - | - | 1431 | 1904 | - | - | 0 | - |
| - | - | 1766 | 1905 | - | - | 0 | - |
| - | - | 3184 | 1917 | - | - | 0 | - |
| - | - | 7426 | 1918 | - | - | 0 | - |
| - | - | 9848 | 1919 | - | - | 0 | - |
| - | - | 1.204E+04 | 1920 | - | - | 0 | - |
| - | - | 9471 | 1921 | - | - | 0 | - |
| - | - | 7585 | 1922 | - | - | 0 | - |
| - | - | 4443 | 1923 | - | - | 0 | - |
| - | - | 1628 | 1924 | - | - | 0 | - |
| - | - | 2553 | 1932 | - | - | 0 | - |
| - | - | 5597 | 1933 | - | - | 0 | - |
| - | - | 3925 | 1934 | - | - | 0 | - |
| - | - | 1867 | 1935 | - | - | 0 | - |
| - | - | 2184 | 1936 | - | - | 0 | - |
| - | - | 1140 | 1937 | - | - | 0 | - |
| - | - | 1569 | 1947 | - | - | 0 | - |
| - | - | 4147 | 1948 | - | - | 0 | - |
| - | - | 1.948E+04 | 1949 | - | - | 0 | - |
| - | - | 2.906E+04 | 1950 | - | - | 0 | - |
| - | - | 2.256E+04 | 1951 | - | - | 0 | - |
| - | - | 1.198E+04 | 1952 | - | - | 0 | - |
| - | - | 4806 | 1953 | - | - | 0 | - |
| - | - | 1630 | 1954 | - | - | 0 | - |
| - | - | 1662 | 1962 | - | - | 0 | - |
| - | - | 7266 | 1963 | - | - | 0 | - |
| - | - | 1.581E+04 | 1964 | - | - | 0 | - |
| - | - | 1.311E+04 | 1965 | - | - | 0 | - |
| - | - | 9092 | 1966 | - | - | 0 | - |
| - | - | 2580 | 1967 | - | - | 0 | - |
| - | - | 1499 | 1974 | - | - | 0 | - |
| - | - | 1610 | 1975 | - | - | 0 | - |
| - | - | 6842 | 1980 | - | - | 0 | - |
| - | - | 1.791E+04 | 1981 | - | - | 0 | - |
| - | - | 1.744E+04 | 1982 | - | - | 0 | - |
| - | - | 9248 | 1983 | - | - | 0 | - |
| - | - | 4092 | 1984 | - | - | 0 | - |
| - | - | 993.3 | 1985 | - | - | 0 | - |
| - | - | 1292 | 1990 | - | - | 0 | - |
| - | - | 1.214E+04 | 1991 | - | - | 0 | - |
| - | - | 3.549E+04 | 1992 | - | - | 0 | - |
| - | - | 3.254E+04 | 1993 | - | - | 0 | - |
| - | - | 1.922E+04 | 1994 | - | - | 0 | - |
| - | - | 9448 | 1995 | - | - | 0 | - |
| - | - | 2835 | 1996 | - | - | 0 | - |
| - | - | 1687 | 2006 | - | - | 0 | - |
| - | - | 9165 | 2007 | - | - | 0 | - |
| - | - | 4.207E+04 | 2008 | - | - | 0 | - |
| - | - | 1.411E+05 | 2009 | - | - | 0 | - |
| - | - | 1.291E+05 | 2010 | - | - | 0 | - |
| - | - | 8.324E+04 | 2011 | - | - | 0 | - |
| - | - | 3.561E+04 | 2012 | - | - | 0 | - |
| - | - | 1.178E+04 | 2013 | - | - | 0 | - |
| - | - | 1488 | 2211 | - | - | 0 | - |
| - | - | 1029 | 2213 | - | - | 0 | - |
| - | - | 923.1 | 3044 | - | - | 0 | - |
| - | - | 1077 | 3045 | - | - | 0 | - |

m/z Charge Intensity FragmentType MassShift Position
122.76618194580078 0 423.99423
129.102294921875 0 2126.071
136.07603454589844 0 387.3844
149.0448760986328 0 594.8163
155.0929718017578 0 1107.9855
166.06112670898438 0 3939.419
170.77484130859375 0 515.234
171.07635498046875 0 485.35712
173.0919189453125 0 1802.0122
173.12779235839844 0 586.73975
178.13429260253906 0 577.3931
185.16488647460938 0 3705.107
190.02943420410156 0 470.33463
201.1239776611328 0 686.19055
204.13470458984375 0 568.52716
205.1182098388672 0 1664.3701
207.1607208251953 0 907.1769
214.0253143310547 0 480.99585
217.1182403564453 0 619.44257
223.1552734375 0 3738.9543
224.15939331054688 0 665.4793
230.1864013671875 0 607.2183
234.12376403808594 0 5914.6167
239.09437561035156 0 735.268
242.1494903564453 0 592.9291
248.11276245117188 0 527.71783
250.14215087890625 0 1410.5587
251.15023803710938 0 27634.842
252.15382385253906 0 3512.3865
260.1228332519531 0 1089.0496
263.5672607421875 0 575.5914
264.1180419921875 0 551.34235
266.1243591308594 0 1025.2748
268.1766662597656 0 16508.783 c 1
269.17999267578125 0 1833.6509
277.1537780761719 0 495.85602
283.152099609375 0 1498.6022
294.1919860839844 0 1577.0101
299.18408203125 0 665.2928
311.14166259765625 0 520.7455
317.19464111328125 0 29813.514 z 13
318.19976806640625 0 6433.157
319.2019958496094 0 990.95544
333.2130432128906 0 2973.5125 y 13
342.0182189941406 0 856.56964
343.0179748535156 0 610.51685
352.2212829589844 0 2867.2983
353.2283020019531 0 2373.0251
355.0698547363281 0 2632.2512
356.0709533691406 0 2021.1926
357.06805419921875 0 1628.5546
359.0284118652344 0 2511.8384
360.0293884277344 0 2329.1008
361.0254821777344 0 1253.533
370.1250915527344 0 562.8267
379.2087097167969 0 4384.2686 c Ammonia loss 2
380.2112731933594 0 868.2495
383.2275695800781 0 646.6435
387.2238464355469 0 24375.611
388.2268371582031 0 5002.4146
393.1458740234375 0 642.69073
395.2277526855469 0 4862.6157
396.2353515625 0 39818.453 c 2
397.23846435546875 0 7041.8125
398.2407531738281 0 831.46466
401.240478515625 0 1307.5391
429.0892028808594 0 3115.1367
430.0894470214844 0 5853.604
431.08740234375 0 5025.6626
432.08782958984375 0 1435.4006
451.230712890625 0 802.6582
467.248779296875 0 7199.08
468.2549743652344 0 3286.0562
469.2578125 0 834.078
474.2735595703125 0 585.9008
494.2313232421875 0 4946.4307 c Ammonia loss 3
495.234619140625 0 1422.2124
496.2511291503906 0 979.07104
497.7558288574219 0 792.84344
511.2623596191406 0 32841.684 c 3
512.265380859375 0 8310.122
513.26611328125 0 1028.8099 y Ammonia loss 8
515.3192749023438 0 5747.052
516.3229370117188 0 2396.8462
521.7691650390625 0 729.402 y 8
547.2908935546875 0 2301.0474
548.2406005859375 0 1008.54376 w 11
550.2760009765625 0 5307.753 y 7
550.7781982421875 0 3400.017
551.2811279296875 0 1424.6464
551.7782592773438 0 926.90216
555.2701416015625 0 829.18097
590.284423828125 0 683.8962
596.9518432617188 0 1118.914
597.282470703125 0 2036.1782
601.6233520507812 0 3672.0017 c Ammonia loss 13
601.9556274414062 0 3126.3867
602.2905883789062 0 1962.282
602.6263427734375 0 733.8331
606.3043212890625 0 27867.096 z 11
607.3087158203125 0 12139.064 c 13
607.7909545898438 0 2662.7788 y 6
608.30322265625 0 3988.7507
608.7948608398438 0 641.8215
611.7960205078125 0 1205.3334 c Ammonia loss 9
612.297607421875 0 1057.029
619.2296752929688 0 3050.1536
622.2415771484375 0 5379.491
622.3229370117188 0 10840.356 y 11
623.2433471679688 0 4622.054
623.32568359375 0 2920.099
624.2467041015625 0 794.25867
624.32275390625 0 1023.4453
625.3131103515625 0 967.9753
625.6502685546875 0 859.6421
625.96435546875 0 1608.7615 y Water loss 1
626.30419921875 0 779.44104 y Ammonia loss 1
626.6276245117188 0 921.8051 z 1
628.6441040039062 0 1354.5278
628.9744262695312 0 2769.9583
629.3078002929688 0 1947.991
629.6435546875 0 1659.7249
631.6380615234375 0 806.4592
631.9647827148438 0 11940.189 y 1
632.2998046875 0 9726.242
632.6331176757812 0 5275.059
632.9686279296875 0 3433.7556
633.3059692382812 0 842.9735
634.3169555664062 0 981.61804
634.6453857421875 0 15406.377
634.9796142578125 0 18058.074
635.3125610351562 0 8994.094
635.6467895507812 0 4242.236
635.9807739257812 0 1659.3127
637.2396850585938 0 1816.9883
637.3026733398438 0 845.33716
637.3554077148438 0 945.6924
638.3048706054688 0 891.7906
640.6455688476562 0 1089.0941
641.3202514648438 0 871.3853
650.3245849609375 0 847.5256
650.6493530273438 0 739.52435
651.3021850585938 0 618.78284
653.326171875 0 1271.1093
654.3336791992188 0 2074.068
657.9799194335938 0 2723.0288
658.3140869140625 0 3787.3445
658.6471557617188 0 2126.415
658.9857788085938 0 1400.3765
663.655029296875 0 12147.366
663.9884033203125 0 13734.93
664.3229370117188 0 8834.236
664.6560668945312 0 7276.5913
664.98828125 0 3268.1343
668.2843627929688 0 1053.8186
668.3469848632812 0 867.35754
668.9871826171875 0 1957.7803
669.3308715820312 0 11362.984
669.659912109375 0 157469.94
669.9940185546875 0 186891.03
670.328125 0 123309.44
670.6616821289062 0 47481.277
670.9952392578125 0 16895.25
678.378662109375 0 1320.227
680.3163452148438 0 2558.9736 c Ammonia loss 4
681.3169555664062 0 893.8614
697.3414306640625 0 26567.95 c 4
698.3447265625 0 9485.096
699.3482055664062 0 1958.1746
711.30029296875 0 922.9253
712.3036499023438 0 980.70703
757.3712768554688 0 3357.8857 c Ammonia loss 11
757.8740234375 0 2389.534
758.3753662109375 0 1098.4266
766.409912109375 0 2393.6218
767.4157104492188 0 1886.8905
768.4198608398438 0 953.966
769.3677978515625 0 24881.215 z 10
770.3712768554688 0 12929.934
771.370361328125 0 4174.9717
772.3712158203125 0 994.8836
777.3624877929688 0 792.19696
785.386474609375 0 5696.6094 y 10
786.3892822265625 0 2846.9856
787.3839721679688 0 902.654
793.400390625 0 3391.951 c Ammonia loss 5
794.4000854492188 0 1293.1921
807.4266967773438 0 1194.2538
808.4262084960938 0 720.9373
809.4136962890625 0 2000.3248
810.4254760742188 0 33712.594 c 5
811.4281616210938 0 14181.062
812.4301147460938 0 4070.7708
814.88427734375 0 4163.486 y 3
815.3869018554688 0 3538.8408
815.8873901367188 0 1844.9285
816.3872680664062 0 641.9005
817.39208984375 0 1031.5643
818.385986328125 0 1546.1444
818.8851928710938 0 720.15717
826.395263671875 0 1788.0463
826.8920288085938 0 1334.432
839.3963623046875 0 3988.2703 w 9
840.3998413085938 0 1229.3833
841.8952026367188 0 1129.0387 w 2
842.9412841796875 0 1514.2433
843.4435424804688 0 1633.2933
843.9495239257812 0 685.86145
846.397705078125 0 2880.3767 c 12
846.897216796875 0 1634.5084
847.39892578125 0 757.49066
860.4367065429688 0 867.28546
861.4005737304688 0 886.73816
861.8998413085938 0 983.814 z Water loss 2
867.4247436523438 0 9480.773
868.4263305664062 0 4170.8286
869.907470703125 0 2900.6536 y Water loss 2
870.405517578125 0 9273.37 y Ammonia loss 2
870.903564453125 0 8888.56 z 2
871.4049682617188 0 5556.245
871.9085083007812 0 2705.871
872.4112548828125 0 875.78204
873.9170532226562 0 726.73566
874.4358520507812 0 634.95917
878.91455078125 0 13964.713 y 2
879.4158935546875 0 15610.455
879.9171752929688 0 9617.305
880.4244384765625 0 3813.862 z Water loss 9
880.9182739257812 0 1630.8234
881.4408569335938 0 4116.8623
882.4446411132812 0 4103.245
883.4481811523438 0 1849.4363
888.4356079101562 0 1894.1714
888.9391479492188 0 3620.9412
889.4365844726562 0 1874.6069
889.9376220703125 0 974.64764
892.485595703125 0 969.2635
893.4807739257812 0 1042.7921
893.9472045898438 0 988.53186
894.44873046875 0 2168.754
894.9476928710938 0 823.27875
895.9532470703125 0 732.5949
898.41015625 0 25281.072 z 9
899.412841796875 0 12719.776
900.4126586914062 0 5294.3237
901.4156494140625 0 1194.6886
901.9330444335938 0 2836.541 c Ammonia loss 13
902.435546875 0 2720.116
902.9322509765625 0 1203.9412
903.432861328125 0 955.1959
903.92529296875 0 977.36945
908.429443359375 0 4860.3604 c Ammonia loss 6
908.9539184570312 0 1904.8368
909.4338989257812 0 3152.1775
909.946533203125 0 2142.9211
910.443603515625 0 23517.05 c 13
910.9445190429688 0 23526.977
911.4444580078125 0 12902.458
911.9437255859375 0 5613.272
912.4491577148438 0 1762.2828
914.4296875 0 4716.952 y 9
915.4283447265625 0 1600.9352
915.9678955078125 0 1416.0117
917.4417114257812 0 2416.484
917.9407348632812 0 2566.821
918.4443969726562 0 788.56604
920.4680786132812 0 691.42535
922.9754028320312 0 1008.3321
923.4608764648438 0 728.0542
923.9530639648438 0 1043.364
924.448974609375 0 5736.801
925.4515991210938 0 19029.682 c 6
926.4551391601562 0 7853.626
927.4609985351562 0 1900.6895
928.9739379882812 0 1720.2865
929.9788208007812 0 1054.4998
930.4737548828125 0 867.79663
930.9653930664062 0 795.3013
931.4436645507812 0 843.1094
931.958740234375 0 1136.1527
935.5169067382812 0 2327.4226
935.98876953125 0 974.85706
936.502685546875 0 1562.2958
936.9857177734375 0 1884.7472
937.49365234375 0 1136.3523
937.9736328125 0 7352.038
938.466064453125 0 11068.025
938.9599609375 0 8170.482
939.4457397460938 0 14490.324 z 1
939.939208984375 0 11011.317
940.4426879882812 0 9549.814
940.9422607421875 0 2581.061
942.978759765625 0 744.96246
943.4757690429688 0 1039.5342
943.9747314453125 0 902.0936
944.4785766601562 0 873.52356
945.4619140625 0 1089.7997
945.9619750976562 0 790.52423
946.46337890625 0 3035.9956
946.9513549804688 0 1290.9855
947.4511108398438 0 1256.7351 y 1
948.4462890625 0 823.7588
950.4779052734375 0 3400.0256
950.9788818359375 0 4660.551
951.4758911132812 0 3571.2488
951.9837036132812 0 3320.2732
952.4736328125 0 5246.5176
952.9722900390625 0 3403.8005
953.4675903320312 0 1788.2543
953.95361328125 0 2011.9932
954.4539794921875 0 884.89734
958.9896240234375 0 833.75385
959.4874267578125 0 4121.851
959.9779663085938 0 43191.188
960.4797973632812 0 45703.613
960.9807739257812 0 31137.865
961.4800415039062 0 12653.576
961.980712890625 0 3641.3445
964.9758911132812 0 3100.9814
965.4641723632812 0 2469.8274 c Ammonia loss 7
965.9761352539062 0 3991.3442
966.4743041992188 0 4447.7627
966.9730224609375 0 2216.8608
967.4739990234375 0 2615.3623
967.9611206054688 0 6087.6987
968.462890625 0 8419 w 8
968.9624633789062 0 4002.171
969.4639282226562 0 2853.8833
972.4777221679688 0 1280.3923
972.9784545898438 0 2076.1606
973.478759765625 0 3087.1025
973.9784545898438 0 11098.476
974.469970703125 0 35731.56
974.9784545898438 0 86121.75
975.4808349609375 0 76796.47
975.9832153320312 0 49805.23
976.4835205078125 0 18429.148
976.9859619140625 0 6146.6616
978.4810180664062 0 772.8034
980.4773559570312 0 750.25494
981.4783325195312 0 21285.1
981.9837036132812 0 17392.049
982.4779052734375 0 41015.793 c 7
982.9847412109375 0 7678.829
983.4793090820312 0 17723.553
984.4816284179688 0 4100.239
985.48388671875 0 1036.4368
986.4808349609375 0 2723.8906
986.9761352539062 0 2450.1733
987.4771118164062 0 2839.2285
987.9761962890625 0 810.97144
990.973876953125 0 890.16943
993.9552001953125 0 1065.8005
994.97021484375 0 1641.2235
995.4844360351562 0 7506.8105
995.9773559570312 0 28286.908
996.4790649414062 0 28895.33
996.97802734375 0 15563.8
997.4801025390625 0 9574.566
997.9784545898438 0 2428.2666
1002.4596557617188 0 905.4144
1002.9759521484375 0 1659.7518
1003.4894409179688 0 7934.847
1003.9866943359375 0 73502.59
1004.4883422851562 0 142202.08
1004.9898071289062 0 105813.73
1005.4905395507812 0 61341.082
1005.9903564453125 0 22955.166
1006.4913330078125 0 6239.398
1012.4583740234375 0 852.27734
1024.519287109375 0 1178.8713 y Water loss 8
1026.5047607421875 0 21603.453 z 8
1027.5101318359375 0 21817.83
1028.510986328125 0 10272.609
1029.5167236328125 0 2936.6917
1039.5294189453125 0 1139.7137
1041.517822265625 0 1723.2808
1042.5250244140625 0 7419.2964 y 8
1043.526611328125 0 4392.1597
1044.5283203125 0 1760.4395
1066.552734375 0 1947.2838
1067.552490234375 0 1686.1287
1083.5264892578125 0 11048.243 z 7
1084.531005859375 0 12896.072
1085.534912109375 0 6292.5435
1086.5350341796875 0 2058.3943
1088.197265625 0 846.5899
1092.5576171875 0 877.15576 c Water loss 8
1098.53662109375 0 2744.1978
1099.54296875 0 7225.1772 y 7
1100.547119140625 0 3690.6433
1101.5465087890625 0 1182.8202
1107.567626953125 0 1619.0458
1108.54931640625 0 1029.5406
1109.57568359375 0 2560.1343
1110.5684814453125 0 31117.082 c 8
1111.5712890625 0 21416.746
1112.5721435546875 0 6874.0044
1113.5733642578125 0 944.77496
1136.58447265625 0 2442.319
1141.5545654296875 0 9623.061
1142.55859375 0 6242.7935
1143.5587158203125 0 3006.4656
1154.565673828125 0 1812.2106
1155.567138671875 0 1064.3531
1195.600341796875 0 2789.856
1196.60595703125 0 2461.5337
1197.5826416015625 0 819.71985
1198.5538330078125 0 25979.625 z 6
1199.5574951171875 0 17210.295
1200.55859375 0 8905.585
1201.55908203125 0 2573.0964
1213.5703125 0 1149.4595
1214.572998046875 0 6213.574 y 6
1215.5762939453125 0 3710.729
1216.5728759765625 0 1278.5952
1238.6116943359375 0 2674.6204
1239.611083984375 0 29928.354 c 9
1240.6134033203125 0 19039.625
1241.6158447265625 0 6990.077
1242.617919921875 0 2271.8577
1267.6412353515625 0 3219.0066
1268.6407470703125 0 2049.6216
1269.592529296875 0 1927.6873
1310.6539306640625 0 1265.9402 y Ammonia loss 5
1311.6383056640625 0 13342.307 z 5
1312.639892578125 0 11233.751
1313.6427001953125 0 5777.123
1314.6475830078125 0 2123.6968
1322.64892578125 0 727.2813
1326.647705078125 0 859.00494
1327.6531982421875 0 2764.7634 y 5
1328.6519775390625 0 1803.9578
1329.64990234375 0 1121.3956
1336.6236572265625 0 824.2615
1338.70751953125 0 800.4547
1341.60693359375 0 818.44574
1358.660400390625 0 5810.319
1359.6632080078125 0 3695.4136
1360.6702880859375 0 1012.5561
1390.1793212890625 0 741.3357
1401.6776123046875 0 4383.2627
1402.673828125 0 32940.23 c 10
1403.677490234375 0 25113.094
1404.67919921875 0 11346.031
1405.681640625 0 3485.0918
1406.70556640625 0 1383.4741
1441.6478271484375 0 1296.1715
1481.71923828125 0 1028.5215
1486.754150390625 0 2982.3074
1487.7635498046875 0 2278.1748
1495.7056884765625 0 873.8254 y Water loss 4
1496.7257080078125 0 2594.1802 y Ammonia loss 4
1497.7181396484375 0 13115.314 z 4
1498.7183837890625 0 15454.323
1499.720703125 0 6951.296
1500.7215576171875 0 2659.0715
1501.730712890625 0 1286.7971
1512.731201171875 0 1953.9166 c Water loss 11
1513.736572265625 0 7013.906 c Ammonia loss 11
1514.7391357421875 0 5491.0415
1515.745849609375 0 3294.23
1516.74853515625 0 1210.8739
1521.748291015625 0 811.4396
1522.744873046875 0 825.1593
1524.7486572265625 0 1637.807
1525.73681640625 0 1621.1167
1526.737060546875 0 1013.95685
1528.767333984375 0 880.26263
1529.77197265625 0 3980.0225
1530.7684326171875 0 20692.514 c 11
1531.77197265625 0 19224.902
1532.772705078125 0 8509.472
1533.772216796875 0 3013.3853
1539.6695556640625 0 831.8042
1550.7520751953125 0 969.4571
1551.7496337890625 0 1535.434
1552.7257080078125 0 1033.036
1553.72705078125 0 1599.4424
1554.73291015625 0 1324.489
1555.7464599609375 0 1451.8602
1556.759765625 0 2977.9482
1557.7867431640625 0 2653.6687
1558.791259765625 0 905.71246
1568.75146484375 0 7392.486
1569.7520751953125 0 4603.584
1570.757080078125 0 3525.223
1571.75634765625 0 1687.9061
1595.72509765625 0 724.0804 z Ammonia loss 3
1599.74609375 0 947.3101
1611.7364501953125 0 1766.7395 y Ammonia loss 3
1612.7423095703125 0 25236.002 z 3
1613.7457275390625 0 32244.58
1614.7486572265625 0 20372.797
1615.7530517578125 0 8609.598
1616.7552490234375 0 3514.1023
1617.7626953125 0 1147.3966
1627.7479248046875 0 1780.3811
1628.760009765625 0 6935.0034 y 3
1629.7615966796875 0 5632.756
1630.7608642578125 0 3577.3977
1631.7723388671875 0 1602.0258
1632.77294921875 0 1911.4229
1633.77587890625 0 1005.28094
1653.7923583984375 0 1323.359
1666.8494873046875 0 977.3175
1682.7799072265625 0 1019.16846 w 2
1683.7742919921875 0 1173.69
1684.794189453125 0 1281.6742
1690.7880859375 0 4083.0813
1691.7828369140625 0 37333.438 c 12
1692.7861328125 0 31929.469
1693.787109375 0 19453.352
1694.7916259765625 0 6819.7397
1695.7900390625 0 2018.635
1696.804443359375 0 1139.6847
1740.7999267578125 0 13499.243 z 2
1741.80419921875 0 21456.018
1742.809326171875 0 14444.899
1743.811279296875 0 7330.5396
1744.809326171875 0 2442.3374
1757.8212890625 0 1377.791
1758.846435546875 0 1022.7373
1776.871826171875 0 2640.5154
1777.8779296875 0 1899.9705
1778.88134765625 0 1407.2341
1819.8758544921875 0 3375.4314 c 13
1820.8792724609375 0 6776.872
1821.8857421875 0 4432.2593
1822.8870849609375 0 3360.7366
1847.9375 0 897.547
1860.93603515625 0 2295.7295
1861.94091796875 0 1708.2078
1874.9447021484375 0 1244.8004
1875.944091796875 0 3412.86
1876.94287109375 0 4206.281
1877.9178466796875 0 3726.1277
1878.8701171875 0 8005.501
1879.8699951171875 0 8424.566
1880.87255859375 0 4936.0605
1881.872314453125 0 2476.0535
1889.9569091796875 0 1296.4124
1890.9683837890625 0 1704.9482
1891.96240234375 0 2104.4622
1892.950927734375 0 2054.7083
1900.9666748046875 0 1354.731
1902.9105224609375 0 1000.7037
1903.9454345703125 0 1431.4912
1904.9439697265625 0 1766.3926
1916.9847412109375 0 3183.9382
1917.989013671875 0 7426.4717
1918.9742431640625 0 9848.474
1919.9599609375 0 12039.142
1920.9608154296875 0 9471.269
1921.9591064453125 0 7585.158
1922.9674072265625 0 4443.2837
1923.9647216796875 0 1628.0518
1931.94091796875 0 2553.3718
1932.9461669921875 0 5597.026
1933.9405517578125 0 3925.4526
1934.952392578125 0 1867.4907
1935.9384765625 0 2183.6829
1936.9393310546875 0 1140.0077
1946.947265625 0 1569.1318
1947.9517822265625 0 4147.1807
1948.95458984375 0 19478.08
1949.959716796875 0 29060.783
1950.9610595703125 0 22558.926
1951.9658203125 0 11976.93
1952.9635009765625 0 4806.2495
1953.9505615234375 0 1630.28
1961.9759521484375 0 1661.665
1962.964599609375 0 7266.2314
1963.9627685546875 0 15811.809
1964.9615478515625 0 13105.0205
1965.9619140625 0 9092.351
1966.962646484375 0 2580.085
1973.9588623046875 0 1498.8123
1974.9251708984375 0 1610.2881
1979.9727783203125 0 6842.105
1980.978759765625 0 17910.455
1981.982421875 0 17436.277
1982.9844970703125 0 9247.919
1983.986572265625 0 4092.2415
1984.99755859375 0 993.33026
1989.954833984375 0 1292.2416
1990.9583740234375 0 12135.193
1991.95166015625 0 35486.83
1992.9542236328125 0 32536.643
1993.95458984375 0 19220.084
1994.9544677734375 0 9448.27
1995.95751953125 0 2834.9082
2005.9737548828125 0 1686.6721
2006.9619140625 0 9165.265
2007.9713134765625 0 42073.68
2008.975341796875 0 141078.03
2009.9766845703125 0 129065.58
2010.97998046875 0 83236.57
2011.9808349609375 0 35614.043
2012.9786376953125 0 11784.555
2211.047607421875 0 1487.5735
2213.0556640625 0 1029.1849
3044.3212890625 0 923.08813
3044.94873046875 0 1076.677

Spectrum Details

|  |  |
| --- | --- |
| Matched peaks? Matched peaksThe total absolute number of peaks matched. Additionally in brackets the total fraction of peaks matched and the total number of peaks is shown. | 77 (12.75% of 604) |
| FDR? FDRThe false discovery rate estimated for this peptide. It is calculated by matching all theoretical fragments with a non-integer shift with the raw peaks for this spectrum. This is done with 40 different shifts. The resulting percentage is the average number of annotated peaks over the number of annotated peaks with the correct spectrum. | 0.99% |
| Satellite FDR? Satellite FDRSee the FDR for details on its calculation. This satellite ion specific FDR only contains the satellite ions (d/w) for I/L/J positions. | - |
| PSM Score? PSM ScoreThe PSM Score as given by Hecklib to this annotated spectrum. It is shown with three significant figures. | 671 |

## Spectrum 5138? Spectrum 5138 The raw spectrum of this peptide as annotated by Hecklib. The fragments are coloured according to ion type (see legend). Any peaks with a star '\*' as text can be hovered over to see the full details, first the ion type second the mass shift type. By hovering over the amino acids in the peptide or ions in the legend the corresponding peaks are highlighted. By toggling the 'Unassigned' label you can turn the background (unassigned) peaks on or off in the plot. By updating the slider in the Ion legend you can update the spectrum to only show the top X% of the peaks with labels. The top X% means any peak that is within X% of the highest intensity. By dragging in the spectrum you can zoom in to a specific part of the spectrum and use 'Zoom Out' to get back to the original zoom level. The annotation of the spectrum is based on the given sequence in the peptides file and is done with different software so inconsistencies are likely. The peaks are annotated based on the given sequence, with 20 ppm tolerance.

Copy Data

### Spectrum 5138 (TSV)

#### Preview

```
Loading example...
```

*Click on the button to copy the data to your clipboard.*

Mz MinMz MaxIntensity Max

WidthHeightPeptide font sizePeptide stroke widthSpectrum font sizeSpectrum stroke widthCompact peptide

Ion legend

wxyz

abcd

OtherUnassignedIonChargePositionShow for top:%

JHQDWLDGKEYKCKK

01.94e+43.87e+45.81e+47.75e+4

Zoom Out

y+45z+23c+12y+24z+12y+12c+25y+38c+13y+25c+13c+26y+39w+26z+310y+310y+26z+26y+414c+14y+311c+312c+312c+14y+27z+27y+27z+28w+14y+28c+29w+29y+313y+313z+313y+313z+29y+29y+29z+29c+314c+314z+14c+314y+29c+210c+210y+14y+314z+314w+210y+210y+210z+210y+210c+15c+15c+15c+211y+211y+211z+211c+212c+212c+212z+15w+212y+15c+16y+212z+212c+16y+212w+16w+213c+213y+213z+213y+16z+16c+214c+17c+214y+16c+17z+214y+214c+18c+18z+17z+18y+18c+19z+19c+110y+110z+110c+111z+111c+112c+112z+112w+113c+113z+113z+114

0780156123413122

Fragment Matches Table

Show background peaks

| Position | Ion type | Intensity | mz Theoretical | mz Error (Th) | mz Error (ppm) | Charge | Series Number |
| --- | --- | --- | --- | --- | --- | --- | --- |
| - | - | 406.6 | 126.1 | - | - | 0 | - |
| - | - | 463.6 | 129.1 | - | - | 0 | - |
| - | - | 1951 | 129.1 | - | - | 0 | - |
| - | - | 2203 | 130.1 | - | - | 0 | - |
| - | - | 530.5 | 131.1 | - | - | 0 | - |
| - | - | 793.3 | 131.1 | - | - | 0 | - |
| - | - | 514.1 | 133.1 | - | - | 0 | - |
| - | - | 654.9 | 136.1 | - | - | 0 | - |
| - | - | 465.7 | 140.1 | - | - | 0 | - |
| - | - | 497.9 | 141.2 | - | - | 0 | - |
| - | - | 456.8 | 143.1 | - | - | 0 | - |
| - | - | 420.7 | 146.1 | - | - | 0 | - |
| - | - | 430.2 | 148.5 | - | - | 0 | - |
| - | - | 1068 | 149 | - | - | 0 | - |
| - | - | 382.8 | 152 | - | - | 0 | - |
| - | - | 3626 | 155.1 | - | - | 0 | - |
| - | - | 1585 | 159.1 | - | - | 0 | - |
| - | - | 3579 | 166.1 | - | - | 0 | - |
| - | - | 686.2 | 168.1 | - | - | 0 | - |
| - | - | 4204 | 171.1 | - | - | 0 | - |
| - | - | 1.586E+04 | 173.1 | - | - | 0 | - |
| - | - | 578.3 | 173.1 | - | - | 0 | - |
| - | - | 755 | 173.5 | - | - | 0 | - |
| - | - | 643.3 | 174.1 | - | - | 0 | - |
| - | - | 1415 | 174.1 | - | - | 0 | - |
| - | - | 573.8 | 175.1 | - | - | 0 | - |
| - | - | 1685 | 183.1 | - | - | 0 | - |
| - | - | 429.2 | 183.5 | - | - | 0 | - |
| - | - | 3935 | 185.2 | - | - | 0 | - |
| - | - | 2232 | 189.1 | - | - | 0 | - |
| - | - | 2572 | 191.1 | - | - | 0 | - |
| - | - | 513.9 | 191.1 | - | - | 0 | - |
| 11 | y | 484.2 | 197.1 | 0.001262 | 6.401 | +4 | 5 |
| - | - | 711.9 | 201.1 | - | - | 0 | - |
| - | - | 5627 | 201.1 | - | - | 0 | - |
| - | - | 1958 | 202.1 | - | - | 0 | - |
| - | - | 1291 | 202.1 | - | - | 0 | - |
| - | - | 3951 | 203.1 | - | - | 0 | - |
| - | - | 1586 | 205.1 | - | - | 0 | - |
| - | - | 4437 | 215.1 | - | - | 0 | - |
| - | - | 1027 | 217.1 | - | - | 0 | - |
| - | - | 634.5 | 218.1 | - | - | 0 | - |
| - | - | 500.9 | 220.9 | - | - | 0 | - |
| - | - | 3939 | 223.2 | - | - | 0 | - |
| 13 | z | 1.564E+04 | 231.1 | 0.001974 | 8.541 | +2 | 3 |
| - | - | 805.3 | 231.1 | - | - | 0 | - |
| - | - | 827.2 | 233.1 | - | - | 0 | - |
| - | - | 3852 | 234.1 | - | - | 0 | - |
| - | - | 620.3 | 239.1 | - | - | 0 | - |
| - | - | 867.3 | 250.1 | - | - | 0 | - |
| - | - | 627 | 250.2 | - | - | 0 | - |
| - | - | 1.682E+04 | 251.2 | - | - | 0 | - |
| - | - | 2441 | 252.2 | - | - | 0 | - |
| - | - | 1581 | 260.1 | - | - | 0 | - |
| - | - | 1044 | 261.1 | - | - | 0 | - |
| 2 | c | 2066 | 268.2 | 0.0002572 | 0.9591 | +1 | 2 |
| - | - | 695 | 270.1 | - | - | 0 | - |
| - | - | 1907 | 274.1 | - | - | 0 | - |
| - | - | 1563 | 281.1 | - | - | 0 | - |
| - | - | 609.5 | 282 | - | - | 0 | - |
| - | - | 1223 | 282.2 | - | - | 0 | - |
| - | - | 1659 | 283.2 | - | - | 0 | - |
| - | - | 4086 | 284.2 | - | - | 0 | - |
| - | - | 1864 | 286.2 | - | - | 0 | - |
| - | - | 976.3 | 288.2 | - | - | 0 | - |
| - | - | 3300 | 294.2 | - | - | 0 | - |
| - | - | 1.361E+04 | 296.2 | - | - | 0 | - |
| - | - | 2727 | 297.2 | - | - | 0 | - |
| - | - | 7772 | 299.1 | - | - | 0 | - |
| - | - | 1106 | 299.2 | - | - | 0 | - |
| - | - | 845.4 | 300.1 | - | - | 0 | - |
| - | - | 473.9 | 300.1 | - | - | 0 | - |
| - | - | 6857 | 302.2 | - | - | 0 | - |
| - | - | 1046 | 303.2 | - | - | 0 | - |
| 12 | y | 587.5 | 311.7 | 0.00477 | 15.31 | +2 | 4 |
| - | - | 2211 | 312.2 | - | - | 0 | - |
| - | - | 5362 | 314.2 | - | - | 0 | - |
| 14 | z | 1042 | 317.2 | 0.003761 | 11.86 | +1 | 2 |
| - | - | 1268 | 318.2 | - | - | 0 | - |
| - | - | 3626 | 326.7 | - | - | 0 | - |
| - | - | 2203 | 327.2 | - | - | 0 | - |
| - | - | 596.1 | 327.7 | - | - | 0 | - |
| - | - | 6966 | 330.2 | - | - | 0 | - |
| - | - | 1729 | 331.2 | - | - | 0 | - |
| - | - | 694.9 | 331.7 | - | - | 0 | - |
| 14 | y | 595.8 | 333.2 | 0.004693 | 14.09 | +1 | 2 |
| 5 | c | 2409 | 340.7 | 0.0004145 | 1.217 | +2 | 5 |
| - | - | 1154 | 341.2 | - | - | 0 | - |
| - | - | 537.2 | 351.2 | - | - | 0 | - |
| - | - | 1086 | 352.2 | - | - | 0 | - |
| - | - | 3536 | 355.2 | - | - | 0 | - |
| - | - | 1123 | 359 | - | - | 0 | - |
| 8 | y | 1260 | 367.2 | 0.002563 | 6.98 | +3 | 8 |
| - | - | 634.2 | 367.5 | - | - | 0 | - |
| - | - | 1009 | 367.7 | - | - | 0 | - |
| - | - | 584.4 | 369.4 | - | - | 0 | - |
| - | - | 555 | 371.2 | - | - | 0 | - |
| - | - | 4248 | 373.2 | - | - | 0 | - |
| - | - | 1138 | 373.2 | - | - | 0 | - |
| - | - | 774.4 | 378.2 | - | - | 0 | - |
| 3 | c | 7307 | 379.2 | 0.0004253 | 1.121 | +1 | 3 |
| - | - | 676.7 | 380.2 | - | - | 0 | - |
| - | - | 1175 | 383.2 | - | - | 0 | - |
| - | - | 2277 | 383.2 | - | - | 0 | - |
| - | - | 1299 | 384.2 | - | - | 0 | - |
| - | - | 3196 | 387.2 | - | - | 0 | - |
| - | - | 869.3 | 388.2 | - | - | 0 | - |
| - | - | 728.5 | 389.2 | - | - | 0 | - |
| - | - | 567.1 | 390 | - | - | 0 | - |
| 11 | y | 1982 | 393.2 | 0.004727 | 12.02 | +2 | 5 |
| 3 | c | 1.035E+04 | 396.2 | 3.171E-06 | 0.008002 | +1 | 3 |
| 6 | c | 634.9 | 397.2 | 0.003147 | 7.922 | +2 | 6 |
| - | - | 1649 | 397.2 | - | - | 0 | - |
| - | - | 743.1 | 399.2 | - | - | 0 | - |
| - | - | 515.2 | 401.2 | - | - | 0 | - |
| - | - | 1.148E+04 | 401.2 | - | - | 0 | - |
| - | - | 3312 | 402.2 | - | - | 0 | - |
| 7 | y | 1093 | 405.5 | 0.001249 | 3.081 | +3 | 9 |
| - | - | 1192 | 407.7 | - | - | 0 | - |
| - | - | 754.1 | 409.2 | - | - | 0 | - |
| - | - | 2850 | 415 | - | - | 0 | - |
| - | - | 695.8 | 415.2 | - | - | 0 | - |
| - | - | 813.2 | 416 | - | - | 0 | - |
| - | - | 931.6 | 417.2 | - | - | 0 | - |
| 10 | w | 2.396E+04 | 420.2 | 0.004394 | 10.46 | +2 | 6 |
| - | - | 9205 | 420.7 | - | - | 0 | - |
| - | - | 4103 | 421.2 | - | - | 0 | - |
| - | - | 1228 | 421.7 | - | - | 0 | - |
| - | - | 675.3 | 430.1 | - | - | 0 | - |
| - | - | 1180 | 431.2 | - | - | 0 | - |
| 6 | z | 719.5 | 432.2 | 0.007323 | 16.94 | +3 | 10 |
| - | - | 1153 | 435.2 | - | - | 0 | - |
| - | - | 919.1 | 435.7 | - | - | 0 | - |
| - | - | 969.1 | 436.2 | - | - | 0 | - |
| - | - | 698.1 | 442.2 | - | - | 0 | - |
| 6 | y | 1536 | 443.2 | 0.003127 | 7.056 | +3 | 10 |
| - | - | 1513 | 444.2 | - | - | 0 | - |
| - | - | 1168 | 445.7 | - | - | 0 | - |
| - | - | 1304 | 448.3 | - | - | 0 | - |
| 10 | y | 1276 | 448.7 | 0.003071 | 6.844 | +2 | 6 |
| - | - | 1312 | 449.2 | - | - | 0 | - |
| 10 | z | 2270 | 449.7 | 0.004152 | 9.232 | +2 | 6 |
| - | - | 1076 | 450.2 | - | - | 0 | - |
| - | - | 602.7 | 454.3 | - | - | 0 | - |
| - | - | 5573 | 458.2 | - | - | 0 | - |
| - | - | 2997 | 458.7 | - | - | 0 | - |
| - | - | 1689 | 459.2 | - | - | 0 | - |
| - | - | 626.1 | 459.7 | - | - | 0 | - |
| - | - | 1204 | 460.2 | - | - | 0 | - |
| - | - | 4031 | 460.7 | - | - | 0 | - |
| - | - | 2062 | 461.2 | - | - | 0 | - |
| - | - | 1487 | 462.2 | - | - | 0 | - |
| - | - | 900.6 | 466.2 | - | - | 0 | - |
| - | - | 4299 | 466.3 | - | - | 0 | - |
| - | - | 2069 | 467.2 | - | - | 0 | - |
| - | - | 1129 | 468.3 | - | - | 0 | - |
| - | - | 1230 | 468.8 | - | - | 0 | - |
| - | - | 885.5 | 474 | - | - | 0 | - |
| 2 | y | 1055 | 474.2 | 0.002788 | 5.879 | +4 | 14 |
| - | - | 1001 | 474.5 | - | - | 0 | - |
| - | - | 678.1 | 475.3 | - | - | 0 | - |
| - | - | 797.3 | 476.2 | - | - | 0 | - |
| - | - | 733.8 | 476.8 | - | - | 0 | - |
| - | - | 978.4 | 483.3 | - | - | 0 | - |
| - | - | 1921 | 484.2 | - | - | 0 | - |
| - | - | 2.567E+04 | 484.3 | - | - | 0 | - |
| - | - | 1713 | 484.7 | - | - | 0 | - |
| - | - | 1603 | 485.2 | - | - | 0 | - |
| - | - | 7584 | 485.3 | - | - | 0 | - |
| - | - | 912.2 | 485.7 | - | - | 0 | - |
| - | - | 807.3 | 486.2 | - | - | 0 | - |
| - | - | 1707 | 486.3 | - | - | 0 | - |
| - | - | 888.5 | 487.3 | - | - | 0 | - |
| - | - | 3053 | 488.3 | - | - | 0 | - |
| - | - | 639.6 | 489.8 | - | - | 0 | - |
| - | - | 4379 | 493.7 | - | - | 0 | - |
| 4 | c | 7441 | 494.2 | 0.0003111 | 0.6295 | +1 | 4 |
| - | - | 1751 | 494.7 | - | - | 0 | - |
| - | - | 1594 | 495.2 | - | - | 0 | - |
| - | - | 1029 | 496.2 | - | - | 0 | - |
| - | - | 1843 | 496.8 | - | - | 0 | - |
| - | - | 813.9 | 497.3 | - | - | 0 | - |
| - | - | 1037 | 498 | - | - | 0 | - |
| - | - | 999 | 498.2 | - | - | 0 | - |
| - | - | 1111 | 498.5 | - | - | 0 | - |
| 5 | y | 985.5 | 499.2 | 0.006299 | 12.62 | +3 | 11 |
| - | - | 4138 | 501.3 | - | - | 0 | - |
| - | - | 7077 | 502.2 | - | - | 0 | - |
| - | - | 3.49E+04 | 502.3 | - | - | 0 | - |
| - | - | 1.508E+04 | 502.5 | - | - | 0 | - |
| - | - | 1.916E+04 | 502.8 | - | - | 0 | - |
| - | - | 8044 | 503 | - | - | 0 | - |
| - | - | 3399 | 503.1 | - | - | 0 | - |
| - | - | 7966 | 503.2 | - | - | 0 | - |
| - | - | 8230 | 503.3 | - | - | 0 | - |
| - | - | 697.1 | 503.5 | - | - | 0 | - |
| - | - | 1939 | 503.8 | - | - | 0 | - |
| - | - | 3422 | 503.8 | - | - | 0 | - |
| - | - | 1271 | 504.1 | - | - | 0 | - |
| - | - | 2080 | 504.2 | - | - | 0 | - |
| - | - | 1718 | 504.3 | - | - | 0 | - |
| 12 | c | 748.8 | 504.9 | 0.002925 | 5.792 | +3 | 12 |
| 12 | c | 742.1 | 505.3 | 0.004178 | 8.27 | +3 | 12 |
| - | - | 636.6 | 506.7 | - | - | 0 | - |
| 4 | c | 1.87E+04 | 511.3 | 0.0001149 | 0.2248 | +1 | 4 |
| - | - | 4726 | 512.3 | - | - | 0 | - |
| 9 | y | 918.3 | 512.8 | 0.006577 | 12.83 | +2 | 7 |
| - | - | 1145 | 513.3 | - | - | 0 | - |
| 9 | z | 1079 | 513.8 | 0.00384 | 7.474 | +2 | 7 |
| - | - | 852.8 | 514.3 | - | - | 0 | - |
| - | - | 4649 | 515.3 | - | - | 0 | - |
| - | - | 1877 | 516.3 | - | - | 0 | - |
| 9 | y | 5932 | 521.8 | 0.004474 | 8.575 | +2 | 7 |
| - | - | 2206 | 522.3 | - | - | 0 | - |
| - | - | 981.7 | 522.8 | - | - | 0 | - |
| - | - | 661.4 | 533.8 | - | - | 0 | - |
| - | - | 1212 | 538.3 | - | - | 0 | - |
| 8 | z | 2711 | 542.3 | 0.005539 | 10.21 | +2 | 8 |
| - | - | 1593 | 542.8 | - | - | 0 | - |
| - | - | 1423 | 543.3 | - | - | 0 | - |
| - | - | 1151 | 546.3 | - | - | 0 | - |
| - | - | 691.9 | 546.8 | - | - | 0 | - |
| - | - | 2204 | 547.3 | - | - | 0 | - |
| 12 | w | 1051 | 548.2 | 0.009479 | 17.29 | +1 | 4 |
| - | - | 921.3 | 548.3 | - | - | 0 | - |
| - | - | 2271 | 549.3 | - | - | 0 | - |
| 8 | y | 5480 | 550.3 | 0.006173 | 11.22 | +2 | 8 |
| - | - | 3953 | 550.8 | - | - | 0 | - |
| - | - | 804.7 | 553.8 | - | - | 0 | - |
| - | - | 1742 | 554.3 | - | - | 0 | - |
| - | - | 1575 | 554.8 | - | - | 0 | - |
| - | - | 7619 | 555.3 | - | - | 0 | - |
| 9 | c | 2.339E+04 | 555.8 | 0.001622 | 2.919 | +2 | 9 |
| - | - | 1.249E+04 | 556.3 | - | - | 0 | - |
| - | - | 5139 | 556.8 | - | - | 0 | - |
| - | - | 1475 | 557.3 | - | - | 0 | - |
| - | - | 755.2 | 563.8 | - | - | 0 | - |
| - | - | 1884 | 564.2 | - | - | 0 | - |
| - | - | 3267 | 568.8 | - | - | 0 | - |
| - | - | 2277 | 569.3 | - | - | 0 | - |
| - | - | 2255 | 570.3 | - | - | 0 | - |
| - | - | 2119 | 570.7 | - | - | 0 | - |
| - | - | 1.459E+04 | 571.3 | - | - | 0 | - |
| - | - | 8541 | 571.8 | - | - | 0 | - |
| - | - | 3535 | 572.3 | - | - | 0 | - |
| 7 | w | 5806 | 577.3 | 0.005473 | 9.481 | +2 | 9 |
| - | - | 3285 | 577.8 | - | - | 0 | - |
| - | - | 2011 | 578.3 | - | - | 0 | - |
| - | - | 648.3 | 578.8 | - | - | 0 | - |
| 3 | y | 974.1 | 580.3 | 0.003433 | 5.917 | +3 | 13 |
| 3 | y | 929.8 | 580.6 | 0.005964 | 10.27 | +3 | 13 |
| 3 | z | 821.8 | 580.9 | 0.003457 | 5.951 | +3 | 13 |
| 3 | y | 991.1 | 586.3 | 0.002994 | 5.106 | +3 | 13 |
| - | - | 914.7 | 586.6 | - | - | 0 | - |
| 7 | z | 671.2 | 590.8 | 0.005742 | 9.72 | +2 | 9 |
| - | - | 700.7 | 593.3 | - | - | 0 | - |
| - | - | 666.9 | 598.3 | - | - | 0 | - |
| 7 | y | 1610 | 598.8 | 0.003986 | 6.656 | +2 | 9 |
| 7 | y | 5038 | 599.3 | 0.006692 | 11.17 | +2 | 9 |
| 7 | z | 1.326E+04 | 599.8 | 0.002531 | 4.219 | +2 | 9 |
| - | - | 8192 | 600.3 | - | - | 0 | - |
| - | - | 988.8 | 600.4 | - | - | 0 | - |
| - | - | 3729 | 600.8 | - | - | 0 | - |
| 14 | c | 1034 | 601.3 | 0.0006195 | 1.03 | +3 | 14 |
| 14 | c | 1289 | 601.6 | 0.002186 | 3.633 | +3 | 14 |
| - | - | 915.1 | 602 | - | - | 0 | - |
| - | - | 861 | 602.3 | - | - | 0 | - |
| - | - | 755.8 | 603 | - | - | 0 | - |
| - | - | 1101 | 603.3 | - | - | 0 | - |
| 12 | z | 1.497E+04 | 606.3 | 0.009846 | 16.24 | +1 | 4 |
| - | - | 1101 | 606.6 | - | - | 0 | - |
| - | - | 5523 | 607 | - | - | 0 | - |
| 14 | c | 1.909E+04 | 607.3 | 0.0009248 | 1.523 | +3 | 14 |
| - | - | 1.167E+04 | 607.6 | - | - | 0 | - |
| 7 | y | 1.809E+04 | 607.8 | 0.003592 | 5.91 | +2 | 9 |
| - | - | 4592 | 608 | - | - | 0 | - |
| - | - | 1.235E+04 | 608.3 | - | - | 0 | - |
| - | - | 4667 | 608.8 | - | - | 0 | - |
| - | - | 1668 | 609.3 | - | - | 0 | - |
| - | - | 1092 | 611.6 | - | - | 0 | - |
| 10 | c | 2799 | 611.8 | 0.005319 | 8.694 | +2 | 10 |
| - | - | 768.1 | 612 | - | - | 0 | - |
| - | - | 1788 | 612.3 | - | - | 0 | - |
| - | - | 2641 | 612.8 | - | - | 0 | - |
| - | - | 2228 | 613.3 | - | - | 0 | - |
| - | - | 888.7 | 613.8 | - | - | 0 | - |
| - | - | 660.2 | 615.3 | - | - | 0 | - |
| - | - | 8432 | 619.8 | - | - | 0 | - |
| 10 | c | 2.336E+04 | 620.3 | 0.002298 | 3.705 | +2 | 10 |
| - | - | 1.166E+04 | 620.8 | - | - | 0 | - |
| - | - | 7690 | 621.3 | - | - | 0 | - |
| - | - | 680.9 | 621.6 | - | - | 0 | - |
| - | - | 1154 | 621.8 | - | - | 0 | - |
| 12 | y | 8840 | 622.3 | 0.009832 | 15.8 | +1 | 4 |
| - | - | 2525 | 623.3 | - | - | 0 | - |
| - | - | 1200 | 624.3 | - | - | 0 | - |
| - | - | 1021 | 624.7 | - | - | 0 | - |
| - | - | 839.2 | 625.3 | - | - | 0 | - |
| - | - | 1168 | 625.7 | - | - | 0 | - |
| 2 | y | 2024 | 626 | 0.01247 | 19.92 | +3 | 14 |
| - | - | 5367 | 626.3 | - | - | 0 | - |
| 2 | z | 5054 | 626.6 | 0.01076 | 17.17 | +3 | 14 |
| - | - | 2419 | 627 | - | - | 0 | - |
| - | - | 708.3 | 627.3 | - | - | 0 | - |
| - | - | 670.6 | 628.1 | - | - | 0 | - |
| - | - | 882.8 | 630.6 | - | - | 0 | - |
| - | - | 1036 | 631.3 | - | - | 0 | - |
| - | - | 1493 | 631.6 | - | - | 0 | - |
| - | - | 928.5 | 632.6 | - | - | 0 | - |
| - | - | 5650 | 634.3 | - | - | 0 | - |
| - | - | 2172 | 634.6 | - | - | 0 | - |
| 6 | w | 1.019E+04 | 634.8 | 0.0004513 | 0.711 | +2 | 10 |
| - | - | 734.8 | 635 | - | - | 0 | - |
| - | - | 6621 | 635.3 | - | - | 0 | - |
| - | - | 2497 | 635.6 | - | - | 0 | - |
| - | - | 2164 | 635.8 | - | - | 0 | - |
| - | - | 1343 | 636 | - | - | 0 | - |
| - | - | 767.1 | 636.3 | - | - | 0 | - |
| - | - | 7008 | 640 | - | - | 0 | - |
| - | - | 2.834E+04 | 640.3 | - | - | 0 | - |
| - | - | 2.44E+04 | 640.7 | - | - | 0 | - |
| - | - | 1.411E+04 | 641 | - | - | 0 | - |
| - | - | 7867 | 641.3 | - | - | 0 | - |
| - | - | 2316 | 641.7 | - | - | 0 | - |
| - | - | 966.1 | 642.3 | - | - | 0 | - |
| - | - | 713 | 643.3 | - | - | 0 | - |
| - | - | 759.7 | 644.3 | - | - | 0 | - |
| - | - | 1609 | 644.7 | - | - | 0 | - |
| - | - | 706.1 | 645.3 | - | - | 0 | - |
| - | - | 1756 | 645.6 | - | - | 0 | - |
| - | - | 2098 | 646 | - | - | 0 | - |
| - | - | 894.3 | 646.3 | - | - | 0 | - |
| - | - | 1824 | 649.3 | - | - | 0 | - |
| - | - | 6673 | 649.6 | - | - | 0 | - |
| - | - | 2.09E+04 | 650 | - | - | 0 | - |
| - | - | 2.952E+04 | 650.3 | - | - | 0 | - |
| - | - | 2.439E+04 | 650.7 | - | - | 0 | - |
| - | - | 1.62E+04 | 651 | - | - | 0 | - |
| - | - | 4421 | 651.3 | - | - | 0 | - |
| - | - | 1792 | 651.7 | - | - | 0 | - |
| - | - | 2426 | 652.3 | - | - | 0 | - |
| - | - | 1374 | 653.3 | - | - | 0 | - |
| - | - | 1866 | 654.3 | - | - | 0 | - |
| - | - | 3627 | 654.7 | - | - | 0 | - |
| - | - | 4958 | 655 | - | - | 0 | - |
| 6 | y | 2864 | 655.3 | 0.00372 | 5.677 | +2 | 10 |
| - | - | 1686 | 655.7 | - | - | 0 | - |
| 6 | y | 2382 | 655.8 | 0.007629 | 11.63 | +2 | 10 |
| 6 | z | 5638 | 656.3 | 0.002998 | 4.567 | +2 | 10 |
| - | - | 2519 | 656.8 | - | - | 0 | - |
| - | - | 1929 | 657.3 | - | - | 0 | - |
| - | - | 1488 | 657.8 | - | - | 0 | - |
| - | - | 834.9 | 658 | - | - | 0 | - |
| - | - | 1558 | 658.3 | - | - | 0 | - |
| - | - | 817.6 | 663.3 | - | - | 0 | - |
| - | - | 1465 | 663.7 | - | - | 0 | - |
| - | - | 3118 | 663.8 | - | - | 0 | - |
| - | - | 3231 | 664 | - | - | 0 | - |
| 6 | y | 1.071E+04 | 664.3 | 0.005096 | 7.672 | +2 | 10 |
| - | - | 3385 | 664.7 | - | - | 0 | - |
| - | - | 4897 | 664.8 | - | - | 0 | - |
| - | - | 2209 | 665 | - | - | 0 | - |
| - | - | 3904 | 665.3 | - | - | 0 | - |
| - | - | 1360 | 669 | - | - | 0 | - |
| - | - | 1.609E+04 | 669.3 | - | - | 0 | - |
| - | - | 6.254E+04 | 669.7 | - | - | 0 | - |
| - | - | 7.673E+04 | 670 | - | - | 0 | - |
| - | - | 5.05E+04 | 670.3 | - | - | 0 | - |
| - | - | 3.024E+04 | 670.7 | - | - | 0 | - |
| - | - | 1.205E+04 | 671 | - | - | 0 | - |
| - | - | 5316 | 671.3 | - | - | 0 | - |
| - | - | 1768 | 671.7 | - | - | 0 | - |
| - | - | 701.1 | 672 | - | - | 0 | - |
| - | - | 849.3 | 672.3 | - | - | 0 | - |
| - | - | 2069 | 674.3 | - | - | 0 | - |
| - | - | 740.3 | 675.3 | - | - | 0 | - |
| - | - | 933.9 | 678.3 | - | - | 0 | - |
| 5 | c | 964.4 | 679.3 | 0.008835 | 13 | +1 | 5 |
| - | - | 2640 | 679.8 | - | - | 0 | - |
| 5 | c | 8441 | 680.3 | 0.0007711 | 1.134 | +1 | 5 |
| - | - | 2719 | 681.3 | - | - | 0 | - |
| - | - | 2183 | 690.3 | - | - | 0 | - |
| 5 | c | 2.85E+04 | 697.3 | 0.0002653 | 0.3804 | +1 | 5 |
| - | - | 1.066E+04 | 698.3 | - | - | 0 | - |
| - | - | 1736 | 699.4 | - | - | 0 | - |
| - | - | 1209 | 700.4 | - | - | 0 | - |
| - | - | 8415 | 701.3 | - | - | 0 | - |
| 11 | c | 2.036E+04 | 701.8 | 0.001884 | 2.685 | +2 | 11 |
| - | - | 1.291E+04 | 702.3 | - | - | 0 | - |
| - | - | 3233 | 702.8 | - | - | 0 | - |
| - | - | 1852 | 703.4 | - | - | 0 | - |
| - | - | 1050 | 704.4 | - | - | 0 | - |
| - | - | 760 | 706.3 | - | - | 0 | - |
| - | - | 5924 | 716.4 | - | - | 0 | - |
| - | - | 2194 | 717.4 | - | - | 0 | - |
| - | - | 1059 | 721.3 | - | - | 0 | - |
| - | - | 1093 | 722.3 | - | - | 0 | - |
| - | - | 1301 | 727.4 | - | - | 0 | - |
| 5 | y | 904.3 | 748.4 | 0.01213 | 16.2 | +2 | 11 |
| 5 | y | 3519 | 748.9 | 0.00496 | 6.623 | +2 | 11 |
| 5 | z | 8071 | 749.4 | 0.002981 | 3.978 | +2 | 11 |
| - | - | 4358 | 749.9 | - | - | 0 | - |
| - | - | 2006 | 750.4 | - | - | 0 | - |
| - | - | 1084 | 750.9 | - | - | 0 | - |
| - | - | 1676 | 754.9 | - | - | 0 | - |
| - | - | 2325 | 755.9 | - | - | 0 | - |
| 12 | c | 1348 | 756.9 | 0.009858 | 13.03 | +2 | 12 |
| 12 | c | 5292 | 757.4 | 2.588E-05 | 0.03418 | +2 | 12 |
| - | - | 2892 | 757.9 | - | - | 0 | - |
| - | - | 1068 | 758.4 | - | - | 0 | - |
| - | - | 748.2 | 758.9 | - | - | 0 | - |
| - | - | 735.7 | 761.4 | - | - | 0 | - |
| - | - | 769.6 | 763.4 | - | - | 0 | - |
| - | - | 1755 | 765.4 | - | - | 0 | - |
| 12 | c | 4645 | 765.9 | 0.001827 | 2.385 | +2 | 12 |
| - | - | 3887 | 766.4 | - | - | 0 | - |
| - | - | 764 | 767.3 | - | - | 0 | - |
| - | - | 933.2 | 767.4 | - | - | 0 | - |
| 11 | z | 2.388E+04 | 769.4 | 0.009271 | 12.05 | +1 | 5 |
| - | - | 1.196E+04 | 770.4 | - | - | 0 | - |
| - | - | 889.3 | 770.9 | - | - | 0 | - |
| - | - | 5452 | 771.4 | - | - | 0 | - |
| - | - | 1425 | 772.4 | - | - | 0 | - |
| - | - | 886.1 | 777.4 | - | - | 0 | - |
| - | - | 829.6 | 777.9 | - | - | 0 | - |
| - | - | 783.1 | 778.9 | - | - | 0 | - |
| 4 | w | 1034 | 784.4 | 0.00569 | 7.254 | +2 | 12 |
| - | - | 4343 | 784.9 | - | - | 0 | - |
| 11 | y | 1.028E+04 | 785.4 | 0.009989 | 12.72 | +1 | 5 |
| - | - | 975.4 | 785.9 | - | - | 0 | - |
| - | - | 3748 | 786.4 | - | - | 0 | - |
| - | - | 2050 | 787.4 | - | - | 0 | - |
| 6 | c | 1808 | 793.4 | 0.002665 | 3.359 | +1 | 6 |
| - | - | 3666 | 803.4 | - | - | 0 | - |
| 4 | y | 5819 | 806.4 | 0.005892 | 7.307 | +2 | 12 |
| 4 | z | 1.197E+04 | 806.9 | 0.002354 | 2.917 | +2 | 12 |
| - | - | 1.186E+04 | 807.4 | - | - | 0 | - |
| - | - | 5199 | 807.9 | - | - | 0 | - |
| - | - | 1745 | 808.4 | - | - | 0 | - |
| - | - | 906.3 | 809.4 | - | - | 0 | - |
| 6 | c | 4.76E+04 | 810.4 | 0.0009552 | 1.179 | +1 | 6 |
| - | - | 2.184E+04 | 811.4 | - | - | 0 | - |
| - | - | 4911 | 812.4 | - | - | 0 | - |
| - | - | 4271 | 814.4 | - | - | 0 | - |
| 4 | y | 1903 | 814.9 | 0.005856 | 7.187 | +2 | 12 |
| - | - | 3100 | 815.4 | - | - | 0 | - |
| - | - | 951.4 | 816.4 | - | - | 0 | - |
| - | - | 1180 | 819.4 | - | - | 0 | - |
| - | - | 1538 | 831.4 | - | - | 0 | - |
| - | - | 1098 | 832.4 | - | - | 0 | - |
| 10 | w | 2635 | 839.4 | 0.008347 | 9.944 | +1 | 6 |
| - | - | 2266 | 840.4 | - | - | 0 | - |
| - | - | 1186 | 841.4 | - | - | 0 | - |
| 3 | w | 1729 | 841.9 | 0.0009463 | 1.124 | +2 | 13 |
| - | - | 1237 | 842.4 | - | - | 0 | - |
| - | - | 960.4 | 843 | - | - | 0 | - |
| - | - | 1203 | 843.4 | - | - | 0 | - |
| - | - | 4032 | 845.9 | - | - | 0 | - |
| 13 | c | 1.477E+04 | 846.4 | 0.0002304 | 0.2722 | +2 | 13 |
| - | - | 9149 | 846.9 | - | - | 0 | - |
| - | - | 6204 | 847.4 | - | - | 0 | - |
| - | - | 2336 | 847.9 | - | - | 0 | - |
| - | - | 1388 | 848.4 | - | - | 0 | - |
| - | - | 1253 | 848.9 | - | - | 0 | - |
| - | - | 835.4 | 849.4 | - | - | 0 | - |
| - | - | 1.052E+04 | 867.4 | - | - | 0 | - |
| - | - | 5409 | 868.4 | - | - | 0 | - |
| - | - | 1507 | 869.4 | - | - | 0 | - |
| 3 | y | 7624 | 870.4 | 0.006938 | 7.971 | +2 | 13 |
| 3 | z | 1.334E+04 | 870.9 | 0.00143 | 1.642 | +2 | 13 |
| - | - | 8507 | 871.4 | - | - | 0 | - |
| - | - | 5729 | 871.9 | - | - | 0 | - |
| - | - | 2252 | 872.4 | - | - | 0 | - |
| - | - | 801.1 | 872.9 | - | - | 0 | - |
| - | - | 781.2 | 875.4 | - | - | 0 | - |
| - | - | 818.6 | 879.4 | - | - | 0 | - |
| - | - | 1238 | 880.5 | - | - | 0 | - |
| - | - | 3384 | 881.4 | - | - | 0 | - |
| - | - | 1611 | 881.9 | - | - | 0 | - |
| - | - | 1926 | 882.4 | - | - | 0 | - |
| - | - | 4210 | 888.4 | - | - | 0 | - |
| - | - | 1953 | 888.9 | - | - | 0 | - |
| - | - | 1469 | 889.4 | - | - | 0 | - |
| - | - | 1179 | 890.5 | - | - | 0 | - |
| 10 | y | 722.2 | 896.4 | 0.01383 | 15.43 | +1 | 6 |
| 10 | z | 2.84E+04 | 898.4 | 0.009017 | 10.04 | +1 | 6 |
| - | - | 1.864E+04 | 899.4 | - | - | 0 | - |
| - | - | 9737 | 900.4 | - | - | 0 | - |
| - | - | 2422 | 901.4 | - | - | 0 | - |
| 14 | c | 788.4 | 901.9 | 0.009236 | 10.24 | +2 | 14 |
| 7 | c | 1397 | 908.4 | 0.004815 | 5.301 | +1 | 7 |
| - | - | 1740 | 910 | - | - | 0 | - |
| 14 | c | 9963 | 910.4 | 0.002085 | 2.29 | +2 | 14 |
| - | - | 6390 | 910.9 | - | - | 0 | - |
| - | - | 4144 | 911.4 | - | - | 0 | - |
| - | - | 2369 | 911.9 | - | - | 0 | - |
| 10 | y | 2770 | 914.4 | 0.00998 | 10.91 | +1 | 6 |
| - | - | 3597 | 915.4 | - | - | 0 | - |
| - | - | 2853 | 916.4 | - | - | 0 | - |
| - | - | 1009 | 917.4 | - | - | 0 | - |
| - | - | 1778 | 924.5 | - | - | 0 | - |
| 7 | c | 8234 | 925.5 | 0.001399 | 1.511 | +1 | 7 |
| - | - | 4253 | 926.5 | - | - | 0 | - |
| - | - | 968.5 | 927.5 | - | - | 0 | - |
| - | - | 898.2 | 930 | - | - | 0 | - |
| - | - | 2891 | 930.5 | - | - | 0 | - |
| - | - | 2344 | 931 | - | - | 0 | - |
| - | - | 1652 | 931.5 | - | - | 0 | - |
| - | - | 900.9 | 932 | - | - | 0 | - |
| - | - | 3923 | 932.5 | - | - | 0 | - |
| - | - | 2071 | 933.5 | - | - | 0 | - |
| - | - | 952.1 | 935.5 | - | - | 0 | - |
| - | - | 1952 | 937.5 | - | - | 0 | - |
| - | - | 1031 | 938 | - | - | 0 | - |
| - | - | 7715 | 938.5 | - | - | 0 | - |
| - | - | 2065 | 939 | - | - | 0 | - |
| 2 | z | 8550 | 939.4 | 0.003111 | 3.311 | +2 | 14 |
| - | - | 1.045E+04 | 939.9 | - | - | 0 | - |
| - | - | 6112 | 940.4 | - | - | 0 | - |
| - | - | 2276 | 940.9 | - | - | 0 | - |
| - | - | 3390 | 945.5 | - | - | 0 | - |
| - | - | 2375 | 946.5 | - | - | 0 | - |
| 2 | y | 826.8 | 947.4 | 0.009496 | 10.02 | +2 | 14 |
| - | - | 1811 | 952 | - | - | 0 | - |
| - | - | 2717 | 952.5 | - | - | 0 | - |
| - | - | 1830 | 953 | - | - | 0 | - |
| - | - | 1587 | 953.5 | - | - | 0 | - |
| - | - | 841.4 | 958 | - | - | 0 | - |
| - | - | 1134 | 959.5 | - | - | 0 | - |
| - | - | 4091 | 960 | - | - | 0 | - |
| - | - | 5960 | 960.5 | - | - | 0 | - |
| - | - | 3313 | 961 | - | - | 0 | - |
| - | - | 1620 | 961.5 | - | - | 0 | - |
| - | - | 1497 | 962.5 | - | - | 0 | - |
| - | - | 974 | 963.5 | - | - | 0 | - |
| 8 | c | 904.1 | 965.4 | 0.01906 | 19.74 | +1 | 8 |
| - | - | 4705 | 966.5 | - | - | 0 | - |
| - | - | 4274 | 967 | - | - | 0 | - |
| - | - | 3330 | 967.5 | - | - | 0 | - |
| - | - | 1828 | 968 | - | - | 0 | - |
| - | - | 1757 | 968.5 | - | - | 0 | - |
| - | - | 1914 | 973.5 | - | - | 0 | - |
| - | - | 975.8 | 974 | - | - | 0 | - |
| - | - | 6982 | 974.5 | - | - | 0 | - |
| - | - | 1.726E+04 | 975 | - | - | 0 | - |
| - | - | 1.517E+04 | 975.5 | - | - | 0 | - |
| - | - | 8880 | 976 | - | - | 0 | - |
| - | - | 4619 | 976.5 | - | - | 0 | - |
| - | - | 1836 | 977 | - | - | 0 | - |
| - | - | 1522 | 977.5 | - | - | 0 | - |
| - | - | 8964 | 981.5 | - | - | 0 | - |
| - | - | 8443 | 982 | - | - | 0 | - |
| 8 | c | 3.215E+04 | 982.5 | 0.00618 | 6.29 | +1 | 8 |
| - | - | 6647 | 983 | - | - | 0 | - |
| - | - | 1.284E+04 | 983.5 | - | - | 0 | - |
| - | - | 1278 | 984 | - | - | 0 | - |
| - | - | 3547 | 984.5 | - | - | 0 | - |
| - | - | 1211 | 987 | - | - | 0 | - |
| - | - | 1552 | 987.5 | - | - | 0 | - |
| - | - | 961.7 | 988 | - | - | 0 | - |
| - | - | 5624 | 988.5 | - | - | 0 | - |
| - | - | 3906 | 989.5 | - | - | 0 | - |
| - | - | 2128 | 990.5 | - | - | 0 | - |
| - | - | 2727 | 995.5 | - | - | 0 | - |
| - | - | 1.338E+04 | 996 | - | - | 0 | - |
| - | - | 2.655E+04 | 996.5 | - | - | 0 | - |
| - | - | 2.29E+04 | 997 | - | - | 0 | - |
| - | - | 1.314E+04 | 997.5 | - | - | 0 | - |
| - | - | 6466 | 998 | - | - | 0 | - |
| - | - | 2448 | 998.5 | - | - | 0 | - |
| - | - | 1049 | 999 | - | - | 0 | - |
| - | - | 1640 | 1003 | - | - | 0 | - |
| - | - | 5454 | 1004 | - | - | 0 | - |
| - | - | 1.85E+04 | 1004 | - | - | 0 | - |
| - | - | 2.731E+04 | 1005 | - | - | 0 | - |
| - | - | 2.754E+04 | 1005 | - | - | 0 | - |
| - | - | 1.49E+04 | 1006 | - | - | 0 | - |
| - | - | 9964 | 1006 | - | - | 0 | - |
| - | - | 3120 | 1007 | - | - | 0 | - |
| - | - | 3533 | 1007 | - | - | 0 | - |
| - | - | 2075 | 1008 | - | - | 0 | - |
| - | - | 983.8 | 1008 | - | - | 0 | - |
| 9 | z | 3663 | 1027 | 0.00785 | 7.647 | +1 | 7 |
| - | - | 8202 | 1028 | - | - | 0 | - |
| - | - | 3737 | 1029 | - | - | 0 | - |
| - | - | 1906 | 1030 | - | - | 0 | - |
| - | - | 1664 | 1067 | - | - | 0 | - |
| - | - | 1171 | 1068 | - | - | 0 | - |
| 8 | z | 1243 | 1084 | 0.009904 | 9.141 | +1 | 8 |
| - | - | 3486 | 1085 | - | - | 0 | - |
| - | - | 1329 | 1086 | - | - | 0 | - |
| 8 | y | 1740 | 1100 | 0.01422 | 12.94 | +1 | 8 |
| - | - | 1463 | 1101 | - | - | 0 | - |
| - | - | 8483 | 1110 | - | - | 0 | - |
| 9 | c | 2.855E+04 | 1111 | 0.001122 | 1.01 | +1 | 9 |
| - | - | 1.843E+04 | 1112 | - | - | 0 | - |
| - | - | 6195 | 1113 | - | - | 0 | - |
| - | - | 1395 | 1114 | - | - | 0 | - |
| - | - | 1271 | 1141 | - | - | 0 | - |
| - | - | 1150 | 1142 | - | - | 0 | - |
| - | - | 1373 | 1143 | - | - | 0 | - |
| - | - | 1723 | 1196 | - | - | 0 | - |
| - | - | 3321 | 1197 | - | - | 0 | - |
| - | - | 1653 | 1198 | - | - | 0 | - |
| 7 | z | 6401 | 1199 | 0.008563 | 7.144 | +1 | 9 |
| - | - | 1.329E+04 | 1200 | - | - | 0 | - |
| - | - | 6478 | 1201 | - | - | 0 | - |
| - | - | 2967 | 1202 | - | - | 0 | - |
| - | - | 1556 | 1203 | - | - | 0 | - |
| - | - | 2333 | 1239 | - | - | 0 | - |
| 10 | c | 6808 | 1240 | 0.004793 | 3.867 | +1 | 10 |
| - | - | 5907 | 1241 | - | - | 0 | - |
| - | - | 3319 | 1242 | - | - | 0 | - |
| - | - | 1052 | 1243 | - | - | 0 | - |
| 6 | y | 1005 | 1311 | 0.01692 | 12.91 | +1 | 10 |
| 6 | z | 3968 | 1312 | 0.006286 | 4.792 | +1 | 10 |
| - | - | 6573 | 1313 | - | - | 0 | - |
| - | - | 2972 | 1314 | - | - | 0 | - |
| - | - | 1666 | 1315 | - | - | 0 | - |
| - | - | 893.2 | 1317 | - | - | 0 | - |
| - | - | 1588 | 1359 | - | - | 0 | - |
| - | - | 2018 | 1360 | - | - | 0 | - |
| - | - | 1220 | 1361 | - | - | 0 | - |
| - | - | 984.8 | 1387 | - | - | 0 | - |
| - | - | 1656 | 1402 | - | - | 0 | - |
| 11 | c | 4077 | 1403 | 0.005063 | 3.61 | +1 | 11 |
| - | - | 3712 | 1404 | - | - | 0 | - |
| - | - | 1906 | 1405 | - | - | 0 | - |
| - | - | 1125 | 1440 | - | - | 0 | - |
| 5 | z | 2758 | 1498 | 0.001192 | 0.7958 | +1 | 11 |
| - | - | 3232 | 1499 | - | - | 0 | - |
| - | - | 2459 | 1500 | - | - | 0 | - |
| - | - | 989.5 | 1501 | - | - | 0 | - |
| 12 | c | 1013 | 1514 | 0.008671 | 5.728 | +1 | 12 |
| - | - | 1246 | 1515 | - | - | 0 | - |
| - | - | 756.6 | 1530 | - | - | 0 | - |
| 12 | c | 2094 | 1531 | 0.002863 | 1.871 | +1 | 12 |
| - | - | 2226 | 1532 | - | - | 0 | - |
| - | - | 922.1 | 1533 | - | - | 0 | - |
| - | - | 1473 | 1555 | - | - | 0 | - |
| - | - | 1158 | 1569 | - | - | 0 | - |
| 4 | z | 3301 | 1613 | 0.008783 | 5.446 | +1 | 12 |
| - | - | 6950 | 1614 | - | - | 0 | - |
| - | - | 4052 | 1615 | - | - | 0 | - |
| - | - | 1707 | 1616 | - | - | 0 | - |
| 3 | w | 890 | 1683 | 0.01869 | 11.11 | +1 | 13 |
| - | - | 874.9 | 1691 | - | - | 0 | - |
| 13 | c | 2986 | 1692 | 0.002639 | 1.56 | +1 | 13 |
| - | - | 3526 | 1693 | - | - | 0 | - |
| - | - | 2046 | 1694 | - | - | 0 | - |
| - | - | 959.4 | 1695 | - | - | 0 | - |
| 3 | z | 3041 | 1741 | 0.007102 | 4.08 | +1 | 13 |
| - | - | 4417 | 1742 | - | - | 0 | - |
| - | - | 3009 | 1743 | - | - | 0 | - |
| - | - | 897.4 | 1744 | - | - | 0 | - |
| - | - | 818.7 | 1745 | - | - | 0 | - |
| - | - | 967.1 | 1777 | - | - | 0 | - |
| - | - | 1286 | 1778 | - | - | 0 | - |
| - | - | 1001 | 1822 | - | - | 0 | - |
| 2 | z | 1149 | 1878 | 0.03132 | 16.68 | +1 | 14 |
| - | - | 2000 | 1879 | - | - | 0 | - |
| - | - | 1325 | 1880 | - | - | 0 | - |
| - | - | 1160 | 1904 | - | - | 0 | - |
| - | - | 865.5 | 1919 | - | - | 0 | - |
| - | - | 803.1 | 1920 | - | - | 0 | - |
| - | - | 1345 | 1921 | - | - | 0 | - |
| - | - | 812.3 | 1922 | - | - | 0 | - |
| - | - | 948.7 | 1923 | - | - | 0 | - |
| - | - | 1044 | 1933 | - | - | 0 | - |
| - | - | 950.4 | 1949 | - | - | 0 | - |
| - | - | 1724 | 1950 | - | - | 0 | - |
| - | - | 2414 | 1951 | - | - | 0 | - |
| - | - | 1805 | 1952 | - | - | 0 | - |
| - | - | 931.2 | 1964 | - | - | 0 | - |
| - | - | 1467 | 1965 | - | - | 0 | - |
| - | - | 804.4 | 1981 | - | - | 0 | - |
| - | - | 2160 | 1992 | - | - | 0 | - |
| - | - | 2774 | 1993 | - | - | 0 | - |
| - | - | 1822 | 1994 | - | - | 0 | - |
| - | - | 1219 | 1995 | - | - | 0 | - |
| - | - | 825.9 | 1996 | - | - | 0 | - |
| - | - | 2235 | 2008 | - | - | 0 | - |
| - | - | 3396 | 2009 | - | - | 0 | - |
| - | - | 3285 | 2010 | - | - | 0 | - |
| - | - | 2457 | 2011 | - | - | 0 | - |
| - | - | 853.7 | 2012 | - | - | 0 | - |
| - | - | 697.7 | 3091 | - | - | 0 | - |

m/z Charge Intensity FragmentType MassShift Position
126.09109497070312 0 406.60056
129.06573486328125 0 463.58682
129.10226440429688 0 1951.3761
130.06517028808594 0 2203.0537
131.06895446777344 0 530.546
131.11814880371094 0 793.326
133.0612030029297 0 514.0798
136.0756072998047 0 654.92224
140.08212280273438 0 465.74033
141.2064208984375 0 497.89572
143.0821533203125 0 456.81866
146.09268188476562 0 420.6979
148.54071044921875 0 430.17416
149.04489135742188 0 1067.6699
152.0164337158203 0 382.76074
155.0928497314453 0 3626.2412
159.09152221679688 0 1584.9064
166.0611572265625 0 3578.6772
168.13818359375 0 686.2072
171.07644653320312 0 4204.3706
173.092041015625 0 15859.181
173.1285858154297 0 578.33356
173.4540557861328 0 755.00104
174.08750915527344 0 643.2625
174.09542846679688 0 1414.5194
175.07130432128906 0 573.8351
183.1126708984375 0 1685.4214
183.5270233154297 0 429.17285
185.16488647460938 0 3934.9998
189.08709716796875 0 2232.3694
191.10250854492188 0 2572.4185
191.11111450195312 0 513.9003
197.10552978515625 0 484.2049 y 10
201.08702087402344 0 711.9261
201.1233367919922 0 5627.0166
202.08212280273438 0 1957.6812
202.12689208984375 0 1291.4786
203.1024169921875 0 3950.5818
205.11842346191406 0 1586.2957
215.13888549804688 0 4436.963
217.11842346191406 0 1026.722
218.1496124267578 0 634.5107
220.87364196777344 0 500.91187
223.15538024902344 0 3939.3638
231.09750366210938 0 15644.382 z Ammonia loss 12
231.110595703125 0 805.2627
233.13995361328125 0 827.1591
234.1238250732422 0 3852.4
239.14002990722656 0 620.3447
250.1429901123047 0 867.31866
250.16632080078125 0 627.02673
251.15013122558594 0 16818.988
252.15354919433594 0 2441.244
260.1239013671875 0 1580.6086
261.1184387207031 0 1044.2118
268.1765441894531 0 2065.5508 c 1
270.1444091796875 0 695.01245
274.1185302734375 0 1907.3405
281.05096435546875 0 1563.3718
282.0486145019531 0 609.54785
282.1816101074219 0 1223.229
283.1501159667969 0 1659.3202
284.1605529785156 0 4086.1587
286.1761474609375 0 1863.6233
288.1549987792969 0 976.3171
294.1810607910156 0 3300.2449
296.1967468261719 0 13613.618
297.2001953125 0 2726.6255
299.0617370605469 0 7772.309
299.2074279785156 0 1106.1381
300.06268310546875 0 845.3826
300.118896484375 0 473.85773
302.17095947265625 0 6856.5703
303.1751403808594 0 1045.7972
311.6648254394531 0 587.4724 y 11
312.1915588378906 0 2211.2175
314.20758056640625 0 5361.6743
317.1952819824219 0 1041.9816 z 13
318.2024841308594 0 1267.8016
326.6636047363281 0 3626.4004
327.166015625 0 2203.3997
327.6670227050781 0 596.0953
330.2024230957031 0 6966.277
331.2055969238281 0 1728.6462
331.6559143066406 0 694.91644
333.21307373046875 0 595.75195 y 13
340.6607666015625 0 2408.5168 c Ammonia loss 4
341.16131591796875 0 1153.7565
351.2135314941406 0 537.1776
352.2214660644531 0 1085.9371
355.19720458984375 0 3536.123
359.02825927734375 0 1122.7637
367.1870422363281 0 1259.9733 y 7
367.5184020996094 0 634.21704
367.68035888671875 0 1008.62384
369.37359619140625 0 584.43396
371.1936340332031 0 554.99084
373.20819091796875 0 4248.169
373.2428283691406 0 1137.8456
378.20196533203125 0 774.3588
379.2084045410156 0 7307.4106 c Ammonia loss 2
380.2112121582031 0 676.72864
383.2055969238281 0 1174.9147
383.2288513183594 0 2276.859
384.2306823730469 0 1299.2808
387.2236633300781 0 3196.4302
388.2260437011719 0 869.25684
389.2029113769531 0 728.49884
390.03411865234375 0 567.13916
393.196533203125 0 1982.1477 y 10
396.2353820800781 0 10353.725 c 2
397.20635986328125 0 634.93823 c Ammonia loss 5
397.23858642578125 0 1649.4327
399.22418212890625 0 743.0514
401.20849609375 0 515.21893
401.23944091796875 0 11481.88
402.24249267578125 0 3311.851
405.5306701660156 0 1092.9863 y 6
407.7058410644531 0 1192.483
409.2156066894531 0 754.08545
415.03643798828125 0 2850.399
415.2424621582031 0 695.8071
416.03631591796875 0 813.2059
417.2331848144531 0 931.6232
420.2021484375 0 23961.518 w 9
420.70330810546875 0 9204.778
421.203857421875 0 4102.8306
421.7036437988281 0 1227.9888
430.09027099609375 0 675.2597
431.2271728515625 0 1179.8835
432.2041931152344 0 719.5401 z Ammonia loss 5
435.2267761230469 0 1152.9131
435.7237854003906 0 919.0593
436.221435546875 0 969.10754
442.2274475097656 0 698.1174
443.2234802246094 0 1535.5515 y 5
444.2307434082031 0 1512.8284
445.7308654785156 0 1167.6
448.255615234375 0 1303.7225
448.7142028808594 0 1275.841 y Water loss 9
449.2242736816406 0 1311.9338
449.71734619140625 0 2270.4573 z 9
450.2206115722656 0 1076.2683
454.25933837890625 0 602.7008
458.2297058105469 0 5573.0034
458.72943115234375 0 2996.7705
459.227783203125 0 1689.1328
459.7287292480469 0 626.10864
460.23809814453125 0 1203.6758
460.73492431640625 0 4030.8677
461.2368469238281 0 2062.3599
462.2016296386719 0 1486.8613
466.2340087890625 0 900.5933
466.2661437988281 0 4299.166
467.24658203125 0 2068.8906
468.2593688964844 0 1128.556
468.76531982421875 0 1230.1718
473.9807434082031 0 885.4684
474.2304992675781 0 1054.6458 y 1
474.4765930175781 0 1001.3098
475.2972717285156 0 678.1365
476.22857666015625 0 797.279
476.7522277832031 0 733.83356
483.2613830566406 0 978.3782
484.1984558105469 0 1921.09
484.27679443359375 0 25668.379
484.74859619140625 0 1713.1445
485.2344665527344 0 1602.6631
485.28009033203125 0 7583.812
485.72796630859375 0 912.1904
486.2210998535156 0 807.2859
486.29144287109375 0 1707.1985
487.2878723144531 0 888.5239
488.2931213378906 0 3053.2195
489.80914306640625 0 639.58124
493.747314453125 0 4379.2695
494.236083984375 0 7441.264 c Ammonia loss 3
494.7445068359375 0 1750.6686
495.2376403808594 0 1594.1589
496.2489318847656 0 1029.1119
496.7749938964844 0 1842.8354
497.27685546875 0 813.8697
497.9958190917969 0 1037.0665
498.243408203125 0 998.99744
498.4973449707031 0 1111.14
499.24322509765625 0 985.50916 y Water loss 4
501.2723693847656 0 4137.658
502.2496337890625 0 7076.8726
502.2875061035156 0 34904.96
502.4990234375 0 15076.093
502.75042724609375 0 19155.72
502.9995422363281 0 8044.343
503.1077575683594 0 3399.0496
503.24993896484375 0 7965.7007
503.2911071777344 0 8229.559
503.4997253417969 0 697.14935
503.751220703125 0 1939.0771
503.8067626953125 0 3421.5068
504.10760498046875 0 1270.8776
504.2425537109375 0 2079.9019
504.303466796875 0 1717.6727
504.9217224121094 0 748.7796 c Water loss 11
505.24847412109375 0 742.09467 c Ammonia loss 11
506.7303771972656 0 636.6272
511.26220703125 0 18695.564 c 3
512.2649536132812 0 4726.203
512.7581787109375 0 918.266 y Water loss 8
513.2673950195312 0 1144.6506
513.7568359375 0 1079.3527 z 8
514.2578735351562 0 852.84796
515.31884765625 0 4649.348
516.32275390625 0 1876.7639
521.7655639648438 0 5932.47 y 8
522.2679443359375 0 2205.954
522.7664184570312 0 981.66626
533.7794189453125 0 661.44836
538.2713012695312 0 1212.4546
542.265869140625 0 2710.8582 z 7
542.7684326171875 0 1592.971
543.2647094726562 0 1422.7286
546.2620239257812 0 1150.6692
546.7691650390625 0 691.92487
547.2862548828125 0 2204.0303
548.238037109375 0 1051.1112 w 11
548.2908325195312 0 921.28485
549.2685546875 0 2271.4038
550.2745971679688 0 5480.231 y 7
550.7772216796875 0 3953.1921
553.7962646484375 0 804.72534
554.2919921875 0 1742.0668
554.7862548828125 0 1575.1877
555.2957153320312 0 7619.166
555.789794921875 0 23387.992 c 8
556.2904052734375 0 12489.27
556.7913208007812 0 5139.4365
557.2898559570312 0 1474.6005
563.7567749023438 0 755.2449
564.2449951171875 0 1883.6986
568.7962036132812 0 3267.4978
569.2960815429688 0 2277.0571
570.274658203125 0 2254.6184
570.7489013671875 0 2118.599
571.281005859375 0 14588.708
571.7822265625 0 8540.623
572.2837524414062 0 3534.521
577.2805786132812 0 5805.5767 w 6
577.7835693359375 0 3284.6099
578.2813110351562 0 2010.7898
578.7865600585938 0 648.2949
580.2745971679688 0 974.08545 y Water loss 2
580.6119995117188 0 929.75055 y Ammonia loss 2
580.9454345703125 0 821.82806 z 2
586.2845458984375 0 991.07367 y 2
586.6137084960938 0 914.7279
590.7853393554688 0 671.2487 z Water loss 6
593.28173828125 0 700.6694
598.29931640625 0 666.9434
598.7849731445312 0 1610.386 y Water loss 6
599.2876586914062 0 5038.171 y Ammonia loss 6
599.7823486328125 0 13261.001 z 6
600.282958984375 0 8192.083
600.370361328125 0 988.7656
600.7842407226562 0 3729.0383
601.29541015625 0 1033.6278 c Water loss 13
601.626220703125 0 1288.6227 c Ammonia loss 13
601.95947265625 0 915.0582
602.2894287109375 0 860.954
602.96044921875 0 755.7553
603.2838134765625 0 1101.3872
606.3033447265625 0 14967.534 z 11
606.6341552734375 0 1101.4635
606.9686279296875 0 5523.1196
607.3004760742188 0 19089.695 c 13
607.6325073242188 0 11672.876
607.7906494140625 0 18092.312 y 6
607.9671630859375 0 4592.1577
608.2943115234375 0 12346.413
608.7918701171875 0 4667.387
609.2977294921875 0 1667.92
611.6329956054688 0 1091.8156
611.801513671875 0 2799.2915 c Ammonia loss 9
611.9664916992188 0 768.0686
612.300537109375 0 1787.7391
612.7994995117188 0 2640.9863
613.301513671875 0 2227.8943
613.8025512695312 0 888.6641
615.304931640625 0 660.1508
619.8172607421875 0 8431.539
620.311767578125 0 23362.94 c 9
620.8118286132812 0 11655.192
621.3124389648438 0 7690.0513
621.640625 0 680.8887
621.8137817382812 0 1153.757
622.3220825195312 0 8840.026 y 11
623.324951171875 0 2524.7576
624.3241577148438 0 1200.476
624.6661376953125 0 1021.0726
625.3153686523438 0 839.19885
625.6508178710938 0 1167.5996
625.976806640625 0 2023.5334 y Water loss 1
626.3064575195312 0 5367.1147
626.6390380859375 0 5053.955 z 1
626.9714965820312 0 2418.8877
627.3056030273438 0 708.2503
628.0895385742188 0 670.6019
630.6359252929688 0 882.84564
631.3121337890625 0 1036.4504
631.6375122070312 0 1493.37
632.6373901367188 0 928.5009
634.3048095703125 0 5649.9004
634.6488647460938 0 2172.082
634.799072265625 0 10185.909 w 5
634.9905395507812 0 734.80475
635.3024291992188 0 6620.857
635.6449584960938 0 2496.5889
635.7982788085938 0 2163.5623
635.9805908203125 0 1342.8943
636.3198852539062 0 767.09204
639.9918212890625 0 7007.9546
640.32275390625 0 28343.3
640.6560668945312 0 24396.576
640.990234375 0 14105.006
641.3238525390625 0 7867.265
641.6569213867188 0 2315.7952
642.3109741210938 0 966.11615
643.327880859375 0 712.9662
644.3153686523438 0 759.74414
644.6568603515625 0 1608.9702
645.30322265625 0 706.11145
645.6459350585938 0 1756.1012
645.9788818359375 0 2098.46
646.3189697265625 0 894.3345
649.3250122070312 0 1823.778
649.6488037109375 0 6672.84
649.9769897460938 0 20899.318
650.3191528320312 0 29516.717
650.6533203125 0 24392.229
650.9912719726562 0 16197.562
651.3253173828125 0 4420.571
651.6528930664062 0 1791.8711
652.3187866210938 0 2425.8816
653.3248291015625 0 1374.3262
654.3283081054688 0 1865.7709
654.66064453125 0 3627.1406
654.9945678710938 0 4957.919
655.3272705078125 0 2863.6858 y Water loss 5
655.6552124023438 0 1686.036
655.8306274414062 0 2382.2112 y Ammonia loss 5
656.3239135742188 0 5637.837 z 5
656.8257446289062 0 2518.7346
657.3257446289062 0 1929.1234
657.83251953125 0 1488.1135
657.9901123046875 0 834.85803
658.3199462890625 0 1557.9224
663.3306884765625 0 817.644
663.6561889648438 0 1465.0227
663.8387451171875 0 3118.291
663.9917602539062 0 3230.6968
664.3311767578125 0 10712.327 y 5
664.6570434570312 0 3385.0178
664.8342895507812 0 4897.402
664.9864501953125 0 2209.0867
665.330322265625 0 3903.9724
668.9910888671875 0 1359.5153
669.3309936523438 0 16090.358
669.6622314453125 0 62539.586
669.99609375 0 76726.45
670.3297119140625 0 50499.133
670.6634521484375 0 30237.807
670.9970092773438 0 12054.1875
671.331298828125 0 5315.6284
671.6610717773438 0 1768.1077
671.993408203125 0 701.14795
672.3253784179688 0 849.2835
674.324462890625 0 2069.0542
675.3224487304688 0 740.3002
678.329833984375 0 933.90686
679.3399047851562 0 964.3546 c Water loss 4
679.83544921875 0 2639.7065
680.3158569335938 0 8441.075 c Ammonia loss 4
681.3197631835938 0 2719.3623
690.338623046875 0 2182.8926
697.3413696289062 0 28497.326 c 4
698.3436279296875 0 10658.44
699.3501586914062 0 1736.1047
700.3590698242188 0 1208.9698
701.3485717773438 0 8415.495
701.843017578125 0 20356.639 c 10
702.3432006835938 0 12910.455
702.8462524414062 0 3232.6348
703.3516235351562 0 1852.125
704.367431640625 0 1049.954
706.330078125 0 760.04565
716.4047241210938 0 5923.7656
717.4019165039062 0 2193.6807
721.3306884765625 0 1058.5259
722.3336791992188 0 1093.0537
727.3593139648438 0 1300.9418
748.3585205078125 0 904.3232 y Water loss 4
748.8676147460938 0 3519.061 y Ammonia loss 4
749.3635864257812 0 8070.5513 z 4
749.8650512695312 0 4358.1533
750.364013671875 0 2005.994
750.8634643554688 0 1083.662
754.8832397460938 0 1675.8846
755.8861694335938 0 2324.8896
756.8734741210938 0 1347.8329 c Water loss 11
757.3753662109375 0 5291.9326 c Ammonia loss 11
757.8734130859375 0 2892.3184
758.3768920898438 0 1067.6951
758.8883666992188 0 748.22107
761.3841552734375 0 735.69183
763.3626098632812 0 769.6261
765.3980102539062 0 1754.6587
765.8904418945312 0 4645.457 c 11
766.3952026367188 0 3887.2798
767.3423461914062 0 764.04614
767.4146118164062 0 933.1793
769.3672485351562 0 23878.006 z 10
770.3703002929688 0 11958.124
770.8643188476562 0 889.25586
771.3717651367188 0 5452.316
772.3726806640625 0 1425.2323
777.3729858398438 0 886.1436
777.8656005859375 0 829.604
778.8929443359375 0 783.05585
784.3869018554688 0 1034.4523 w 3
784.88134765625 0 4342.7583
785.38525390625 0 10279.752 y 10
785.8768310546875 0 975.3527
786.3875732421875 0 3747.806
787.3958129882812 0 2050.474
793.396484375 0 1808.3899 c Ammonia loss 5
803.4234008789062 0 3665.8972
806.3820190429688 0 5818.9106 y Ammonia loss 3
806.877685546875 0 11969.599 z 3
807.37890625 0 11855.051
807.8798828125 0 5199.001
808.3784790039062 0 1745.4343
809.40380859375 0 906.2855
810.4247436523438 0 47600.383 c 5
811.427734375 0 21836.562
812.4301147460938 0 4910.82
814.4033813476562 0 4271.3076
814.883544921875 0 1903.4044 y 3
815.3975219726562 0 3099.7747
816.4005737304688 0 951.35834
819.3981323242188 0 1179.9558
831.4324340820312 0 1538.4344
832.4246826171875 0 1098.4548
839.3974609375 0 2635.15 w 9
840.4039916992188 0 2266.439
841.3939208984375 0 1185.6873
841.8956298828125 0 1728.8823 w 2
842.3995361328125 0 1236.9426
842.954833984375 0 960.38275
843.4442138671875 0 1202.8392
845.9014282226562 0 4032.41
846.3984375 0 14765.792 c 12
846.8978881835938 0 9149.43
847.3997192382812 0 6203.5913
847.9015502929688 0 2335.6528
848.4028930664062 0 1388.2792
848.91455078125 0 1253.4238
849.4083251953125 0 835.35406
867.423095703125 0 10515.282
868.425537109375 0 5409.3784
869.4261474609375 0 1506.6763
870.412353515625 0 7624.36 y Ammonia loss 2
870.9078979492188 0 13336.855 z 2
871.4105224609375 0 8507.328
871.9104614257812 0 5729.0195
872.41015625 0 2251.852
872.9049072265625 0 801.0729
875.4428100585938 0 781.1559
879.414306640625 0 818.5918
880.4534912109375 0 1237.7417
881.4429931640625 0 3384.45
881.9441528320312 0 1611.142
882.4420776367188 0 1926.458
888.4415893554688 0 4209.9023
888.9380493164062 0 1953.0066
889.4410400390625 0 1468.7488
890.45166015625 0 1179.4901
896.4411010742188 0 722.18365 y Water loss 9
898.4100952148438 0 28403.158 z 9
899.4144897460938 0 18640.547
900.4168090820312 0 9737.048
901.4212036132812 0 2421.8123
901.941650390625 0 788.3524 c Ammonia loss 13
908.430908203125 0 1397.3812 c Ammonia loss 6
909.953369140625 0 1739.7374
910.443603515625 0 9962.977 c 13
910.9439697265625 0 6389.5024
911.4450073242188 0 4144.224
911.9490356445312 0 2368.541
914.4278564453125 0 2770.2883 y 9
915.4424438476562 0 3597.3777
916.44580078125 0 2853.2334
917.4330444335938 0 1009.07526
924.4550170898438 0 1777.6388
925.4540405273438 0 8233.557 c 6
926.4559326171875 0 4253.4756
927.4502563476562 0 968.5489
929.9647216796875 0 898.1852
930.4733276367188 0 2890.748
930.9712524414062 0 2343.9697
931.47314453125 0 1652.426
931.9607543945312 0 900.89874
932.478515625 0 3922.9075
933.4721069335938 0 2071.3567
935.4592895507812 0 952.1073
937.4793701171875 0 1951.7544
937.9774169921875 0 1030.8329
938.47119140625 0 7714.7134
938.9649047851562 0 2065.0542
939.44189453125 0 8549.872 z 1
939.9395751953125 0 10450.536
940.4415283203125 0 6112.045
940.9424438476562 0 2276.4563
945.4832153320312 0 3389.62
946.4864501953125 0 2374.51
947.4576416015625 0 826.8025 y 1
951.9696044921875 0 1811.0608
952.47998046875 0 2717.1155
952.9769287109375 0 1829.7842
953.4738159179688 0 1586.9427
957.9816284179688 0 841.43945
959.4812622070312 0 1134.2035
959.9818725585938 0 4091.2085
960.482177734375 0 5960.2397
960.9830932617188 0 3313.3955
961.4840698242188 0 1619.8655
962.4752197265625 0 1496.5023
963.458984375 0 974.0482
965.4666137695312 0 904.1274 c Ammonia loss 7
966.4774169921875 0 4704.795
966.9745483398438 0 4274.449
967.4760131835938 0 3330.3425
967.9749145507812 0 1828.044
968.4774169921875 0 1757.201
973.4767456054688 0 1913.6522
973.9791259765625 0 975.77563
974.4864501953125 0 6981.9165
974.9841918945312 0 17255.467
975.486083984375 0 15174.228
975.9874877929688 0 8879.531
976.4889526367188 0 4618.543
976.9871215820312 0 1835.547
977.4835815429688 0 1521.6512
981.4878540039062 0 8964.123
981.9888916015625 0 8442.982
982.4802856445312 0 32154.723 c 7
982.98828125 0 6647.166
983.4786987304688 0 12835.899
983.9878540039062 0 1278.3842
984.4855346679688 0 3546.686
986.9781494140625 0 1210.9789
987.4667358398438 0 1552.3253
987.985107421875 0 961.70905
988.4779663085938 0 5623.9224
989.4763793945312 0 3906.0483
990.4743041992188 0 2127.5896
995.4892578125 0 2726.6675
995.9857788085938 0 13379.845
996.4830322265625 0 26551.271
996.9833984375 0 22903.252
997.484130859375 0 13138.199
997.9850463867188 0 6465.7705
998.483642578125 0 2448.2366
998.9881591796875 0 1049.316
1003.4879150390625 0 1639.9623
1003.9901123046875 0 5454.01
1004.494873046875 0 18501.664
1004.995361328125 0 27311.098
1005.4966430664062 0 27536.016
1005.9960327148438 0 14898.782
1006.4965209960938 0 9964.143
1006.9957885742188 0 3119.6719
1007.4849853515625 0 3532.9849
1007.6144409179688 0 2075.4697
1008.4760131835938 0 983.79114
1026.5062255859375 0 3662.784 z 8
1027.5113525390625 0 8202.398
1028.5155029296875 0 3736.5862
1029.514892578125 0 1905.8313
1066.55224609375 0 1664.2449
1067.5614013671875 0 1171.409
1083.525634765625 0 1243.4727 z 7
1084.5382080078125 0 3486.2969
1085.5384521484375 0 1329.02
1099.5400390625 0 1740.4167 y 7
1100.5499267578125 0 1463.4869
1109.583984375 0 8483.255
1110.5701904296875 0 28548.607 c 8
1111.5733642578125 0 18431.355
1112.5755615234375 0 6194.756
1113.574462890625 0 1394.6581
1140.56103515625 0 1270.6984
1141.55712890625 0 1150.1807
1142.5623779296875 0 1372.5121
1195.6082763671875 0 1722.7136
1196.6031494140625 0 3320.9185
1197.5936279296875 0 1653.064
1198.571044921875 0 6401.2065 z 6
1199.56298828125 0 13285.48
1200.56396484375 0 6478.359
1201.5667724609375 0 2967.4514
1202.561279296875 0 1555.7585
1238.6214599609375 0 2333.4385
1239.616455078125 0 6808.4346 c 9
1240.6185302734375 0 5907.3247
1241.6180419921875 0 3319.1719
1242.6185302734375 0 1051.6244
1310.6556396484375 0 1005.36786 y Ammonia loss 5
1311.65283203125 0 3968.4895 z 5
1312.64697265625 0 6573.119
1313.652099609375 0 2971.5286
1314.6583251953125 0 1666.0679
1316.6678466796875 0 893.23346
1358.6710205078125 0 1588.4241
1359.670166015625 0 2018.0115
1360.6689453125 0 1219.799
1386.6517333984375 0 984.765
1401.683349609375 0 1656.2604
1402.6800537109375 0 4077.0845 c 10
1403.68115234375 0 3712.3481
1404.6827392578125 0 1906.3762
1439.71875 0 1125.291
1497.72705078125 0 2758.1213 z 4
1498.725341796875 0 3231.6016
1499.725830078125 0 2459.3635
1500.72509765625 0 989.4506
1513.7520751953125 0 1013.43005 c Ammonia loss 11
1514.7598876953125 0 1246.4716
1529.7802734375 0 756.56396
1530.76708984375 0 2094.0623 c 11
1531.7763671875 0 2226.4058
1532.78125 0 922.06995
1554.740966796875 0 1472.5391
1568.7464599609375 0 1158.0262
1612.7440185546875 0 3300.6406 z 3
1613.7509765625 0 6949.603
1614.75390625 0 4051.6787
1615.754150390625 0 1707.4375
1682.80078125 0 890.0386 w 2
1690.7962646484375 0 874.948
1691.7864990234375 0 2985.9976 c 12
1692.791748046875 0 3525.6965
1693.7921142578125 0 2045.9524
1694.7978515625 0 959.3737
1740.8184814453125 0 3040.8467 z 2
1741.8121337890625 0 4417.0693
1742.81494140625 0 3009.3704
1743.8128662109375 0 897.40485
1744.7899169921875 0 818.6576
1776.87255859375 0 967.054
1777.8831787109375 0 1285.8857
1821.892578125 0 1000.75726
1877.901611328125 0 1148.8789 z 1
1878.8719482421875 0 2000.3616
1879.865966796875 0 1325.2571
1903.9453125 0 1160.2526
1918.98583984375 0 865.5171
1919.98388671875 0 803.13306
1920.9752197265625 0 1344.5906
1921.9522705078125 0 812.256
1922.9705810546875 0 948.6618
1932.9193115234375 0 1044.3871
1948.968505859375 0 950.3836
1949.9599609375 0 1723.5684
1950.975341796875 0 2413.8071
1951.977783203125 0 1805.3833
1963.9873046875 0 931.1571
1964.972412109375 0 1466.941
1980.9669189453125 0 804.3612
1991.9580078125 0 2160.3396
1992.9609375 0 2773.883
1993.9599609375 0 1822.3881
1994.9532470703125 0 1219.3656
1995.9664306640625 0 825.9374
2007.96435546875 0 2235.0837
2008.9788818359375 0 3396.1099
2009.9876708984375 0 3284.9233
2010.9765625 0 2457.1812
2011.9796142578125 0 853.71954
3090.9775390625 0 697.70825

Spectrum Details

|  |  |
| --- | --- |
| Matched peaks? Matched peaksThe total absolute number of peaks matched. Additionally in brackets the total fraction of peaks matched and the total number of peaks is shown. | 106 (15.43% of 687) |
| FDR? FDRThe false discovery rate estimated for this peptide. It is calculated by matching all theoretical fragments with a non-integer shift with the raw peaks for this spectrum. This is done with 40 different shifts. The resulting percentage is the average number of annotated peaks over the number of annotated peaks with the correct spectrum. | 0.40% |
| Satellite FDR? Satellite FDRSee the FDR for details on its calculation. This satellite ion specific FDR only contains the satellite ions (d/w) for I/L/J positions. | 0.00% |
| PSM Score? PSM ScoreThe PSM Score as given by Hecklib to this annotated spectrum. It is shown with three significant figures. | 487 |

## Spectrum 4177? Spectrum 4177 The raw spectrum of this peptide as annotated by Hecklib. The fragments are coloured according to ion type (see legend). Any peaks with a star '\*' as text can be hovered over to see the full details, first the ion type second the mass shift type. By hovering over the amino acids in the peptide or ions in the legend the corresponding peaks are highlighted. By toggling the 'Unassigned' label you can turn the background (unassigned) peaks on or off in the plot. By updating the slider in the Ion legend you can update the spectrum to only show the top X% of the peaks with labels. The top X% means any peak that is within X% of the highest intensity. By dragging in the spectrum you can zoom in to a specific part of the spectrum and use 'Zoom Out' to get back to the original zoom level. The annotation of the spectrum is based on the given sequence in the peptides file and is done with different software so inconsistencies are likely. The peaks are annotated based on the given sequence, with 20 ppm tolerance.

Copy Data

### Spectrum 4177 (TSV)

#### Preview

```
Loading example...
```

*Click on the button to copy the data to your clipboard.*

Mz MinMz MaxIntensity Max

WidthHeightPeptide font sizePeptide stroke widthSpectrum font sizeSpectrum stroke widthCompact peptide

Ion legend

wxyz

abcd

OtherUnassignedIonChargePositionShow for top:%

JHQDWLDGKEYKCKK

01.69e+43.37e+45.06e+46.75e+4

Zoom Out

c+46c+12z+12y+12c+25y+38c+13y+25c+13y+39w+26w+27c+14y+311y+311z+27c+14y+27y+27z+28y+28c+29c+29c+29w+29y+313y+313z+14y+29y+29z+29z+14c+314y+29c+314y+14c+210y+314w+210y+314y+210z+210y+210c+15c+211c+15z+211y+211y+211z+211c+212y+15z+15c+212y+15c+16z+212y+212c+16w+16w+213c+213y+213z+213z+16y+16z+16c+214y+16c+214c+17c+17z+214c+18z+17z+18c+19z+19y+19c+110z+110c+111z+111c+112c+112z+112c+113z+113

0512102515372049

Fragment Matches Table

Show background peaks

| Position | Ion type | Intensity | mz Theoretical | mz Error (Th) | mz Error (ppm) | Charge | Series Number |
| --- | --- | --- | --- | --- | --- | --- | --- |
| - | - | 361.3 | 126.1 | - | - | 0 | - |
| - | - | 1534 | 129.1 | - | - | 0 | - |
| - | - | 571.6 | 136.1 | - | - | 0 | - |
| - | - | 443.4 | 137.1 | - | - | 0 | - |
| - | - | 612.2 | 146.1 | - | - | 0 | - |
| - | - | 532.6 | 149 | - | - | 0 | - |
| - | - | 474.5 | 149.1 | - | - | 0 | - |
| - | - | 2880 | 155.1 | - | - | 0 | - |
| - | - | 429.4 | 163.1 | - | - | 0 | - |
| - | - | 3185 | 166.1 | - | - | 0 | - |
| - | - | 607.9 | 171.9 | - | - | 0 | - |
| - | - | 558.4 | 173.1 | - | - | 0 | - |
| - | - | 1177 | 173.4 | - | - | 0 | - |
| - | - | 1280 | 175.1 | - | - | 0 | - |
| - | - | 889.9 | 181.2 | - | - | 0 | - |
| - | - | 1029 | 183.1 | - | - | 0 | - |
| - | - | 3050 | 185.2 | - | - | 0 | - |
| - | - | 513.5 | 187.1 | - | - | 0 | - |
| 6 | c | 2840 | 203.1 | 0.000282 | 1.389 | +4 | 6 |
| - | - | 1409 | 205.1 | - | - | 0 | - |
| - | - | 4627 | 209.2 | - | - | 0 | - |
| - | - | 533.3 | 210.2 | - | - | 0 | - |
| - | - | 1106 | 213.1 | - | - | 0 | - |
| - | - | 1825 | 215.1 | - | - | 0 | - |
| - | - | 665.9 | 216.1 | - | - | 0 | - |
| - | - | 2056 | 223.2 | - | - | 0 | - |
| - | - | 800.6 | 230.1 | - | - | 0 | - |
| - | - | 3199 | 234.1 | - | - | 0 | - |
| - | - | 633.8 | 235.1 | - | - | 0 | - |
| - | - | 1.089E+04 | 237.2 | - | - | 0 | - |
| - | - | 2111 | 238.2 | - | - | 0 | - |
| - | - | 1.232E+04 | 251.2 | - | - | 0 | - |
| - | - | 1633 | 252.2 | - | - | 0 | - |
| 2 | c | 937.8 | 268.2 | 0.0004752 | 1.772 | +1 | 2 |
| - | - | 631.3 | 278.1 | - | - | 0 | - |
| - | - | 662.3 | 282.2 | - | - | 0 | - |
| - | - | 536.7 | 290.8 | - | - | 0 | - |
| - | - | 1412 | 299.1 | - | - | 0 | - |
| - | - | 693.2 | 300.1 | - | - | 0 | - |
| - | - | 895.3 | 301.1 | - | - | 0 | - |
| - | - | 772 | 309.2 | - | - | 0 | - |
| 14 | z | 813.3 | 317.2 | 0.004188 | 13.2 | +1 | 2 |
| - | - | 767.5 | 318.2 | - | - | 0 | - |
| 14 | y | 598.3 | 333.2 | 0.005975 | 17.93 | +1 | 2 |
| - | - | 2270 | 334.7 | - | - | 0 | - |
| - | - | 1042 | 335.2 | - | - | 0 | - |
| 5 | c | 1484 | 348.7 | 0.002449 | 7.025 | +2 | 5 |
| 8 | y | 1213 | 367.2 | 0.003387 | 9.224 | +3 | 8 |
| - | - | 751.9 | 376.1 | - | - | 0 | - |
| 3 | c | 3321 | 379.2 | 0.0001811 | 0.4776 | +1 | 3 |
| - | - | 595.1 | 385.2 | - | - | 0 | - |
| - | - | 2838 | 387.2 | - | - | 0 | - |
| - | - | 661.9 | 389.5 | - | - | 0 | - |
| 11 | y | 673.3 | 393.2 | 0.004757 | 12.1 | +2 | 5 |
| - | - | 672.6 | 394.2 | - | - | 0 | - |
| - | - | 589.1 | 396.2 | - | - | 0 | - |
| 3 | c | 3705 | 396.2 | 0.0002473 | 0.6242 | +1 | 3 |
| - | - | 3197 | 401 | - | - | 0 | - |
| - | - | 2370 | 402 | - | - | 0 | - |
| - | - | 1681 | 403 | - | - | 0 | - |
| 7 | y | 1035 | 405.5 | 0.001829 | 4.511 | +3 | 9 |
| - | - | 3930 | 419 | - | - | 0 | - |
| - | - | 2045 | 420 | - | - | 0 | - |
| 10 | w | 1.024E+04 | 420.2 | 0.004119 | 9.803 | +2 | 6 |
| - | - | 5774 | 420.7 | - | - | 0 | - |
| - | - | 1580 | 421 | - | - | 0 | - |
| - | - | 2341 | 421.2 | - | - | 0 | - |
| - | - | 733.3 | 421.7 | - | - | 0 | - |
| - | - | 843 | 431.2 | - | - | 0 | - |
| - | - | 580.4 | 449.1 | - | - | 0 | - |
| - | - | 1422 | 460.7 | - | - | 0 | - |
| - | - | 1095 | 461.2 | - | - | 0 | - |
| - | - | 689.3 | 462.2 | - | - | 0 | - |
| - | - | 794.8 | 468.3 | - | - | 0 | - |
| - | - | 1522 | 468.8 | - | - | 0 | - |
| - | - | 647.3 | 472.2 | - | - | 0 | - |
| - | - | 599.6 | 474.3 | - | - | 0 | - |
| - | - | 843.1 | 479.2 | - | - | 0 | - |
| - | - | 1671 | 484.3 | - | - | 0 | - |
| 9 | w | 1240 | 484.7 | 0.006708 | 13.84 | +2 | 7 |
| - | - | 1041 | 484.8 | - | - | 0 | - |
| - | - | 6045 | 489.1 | - | - | 0 | - |
| - | - | 3694 | 490.1 | - | - | 0 | - |
| - | - | 2643 | 491.1 | - | - | 0 | - |
| 4 | c | 1.689E+04 | 494.2 | 0.0003297 | 0.6671 | +1 | 4 |
| - | - | 4674 | 495.2 | - | - | 0 | - |
| - | - | 677.8 | 495.8 | - | - | 0 | - |
| - | - | 1511 | 496.2 | - | - | 0 | - |
| - | - | 2232 | 496.8 | - | - | 0 | - |
| - | - | 974.2 | 497.3 | - | - | 0 | - |
| - | - | 732.1 | 502 | - | - | 0 | - |
| - | - | 2588 | 504.2 | - | - | 0 | - |
| 5 | y | 2977 | 504.6 | 0.0008189 | 1.623 | +3 | 11 |
| 5 | y | 2284 | 504.9 | 0.004977 | 9.858 | +3 | 11 |
| 9 | z | 885.6 | 505.2 | 0.007716 | 15.27 | +2 | 7 |
| - | - | 7482 | 506.2 | - | - | 0 | - |
| - | - | 9711 | 506.3 | - | - | 0 | - |
| - | - | 596.2 | 506.3 | - | - | 0 | - |
| - | - | 985.5 | 506.3 | - | - | 0 | - |
| - | - | 1.014E+04 | 506.5 | - | - | 0 | - |
| - | - | 595.2 | 506.6 | - | - | 0 | - |
| - | - | 7627 | 506.7 | - | - | 0 | - |
| - | - | 5857 | 507 | - | - | 0 | - |
| - | - | 880.5 | 507.2 | - | - | 0 | - |
| - | - | 1897 | 507.3 | - | - | 0 | - |
| - | - | 808.7 | 507.5 | - | - | 0 | - |
| - | - | 1982 | 507.9 | - | - | 0 | - |
| 4 | c | 1.373E+04 | 511.3 | 0.0003429 | 0.6706 | +1 | 4 |
| - | - | 4390 | 512.3 | - | - | 0 | - |
| 9 | y | 751 | 512.8 | 0.002121 | 4.137 | +2 | 7 |
| - | - | 563.1 | 513.7 | - | - | 0 | - |
| - | - | 2611 | 515.3 | - | - | 0 | - |
| - | - | 1128 | 516.3 | - | - | 0 | - |
| - | - | 783.4 | 518.8 | - | - | 0 | - |
| 9 | y | 3394 | 521.8 | 0.004535 | 8.691 | +2 | 7 |
| - | - | 2029 | 522.3 | - | - | 0 | - |
| - | - | 660 | 522.8 | - | - | 0 | - |
| - | - | 794.4 | 533.8 | - | - | 0 | - |
| - | - | 937.3 | 534.3 | - | - | 0 | - |
| - | - | 1305 | 537.3 | - | - | 0 | - |
| 8 | z | 1454 | 542.3 | 0.004501 | 8.3 | +2 | 8 |
| - | - | 1165 | 542.8 | - | - | 0 | - |
| - | - | 1350 | 547.3 | - | - | 0 | - |
| - | - | 788.8 | 548.2 | - | - | 0 | - |
| 8 | y | 3398 | 550.3 | 0.003304 | 6.004 | +2 | 8 |
| - | - | 2953 | 550.8 | - | - | 0 | - |
| - | - | 650.8 | 551.3 | - | - | 0 | - |
| - | - | 2059 | 552.3 | - | - | 0 | - |
| - | - | 1396 | 553.3 | - | - | 0 | - |
| - | - | 1116 | 554.3 | - | - | 0 | - |
| 9 | c | 1758 | 554.8 | 0.006534 | 11.78 | +2 | 9 |
| 9 | c | 1798 | 555.3 | 0.00476 | 8.573 | +2 | 9 |
| - | - | 782.2 | 555.8 | - | - | 0 | - |
| - | - | 3681 | 563.3 | - | - | 0 | - |
| 9 | c | 1.86E+04 | 563.8 | 0.003143 | 5.575 | +2 | 9 |
| - | - | 1003 | 564.2 | - | - | 0 | - |
| - | - | 9200 | 564.3 | - | - | 0 | - |
| - | - | 3128 | 564.8 | - | - | 0 | - |
| - | - | 1180 | 565.3 | - | - | 0 | - |
| - | - | 1651 | 570.3 | - | - | 0 | - |
| - | - | 2588 | 570.3 | - | - | 0 | - |
| - | - | 1800 | 570.7 | - | - | 0 | - |
| - | - | 801 | 571.2 | - | - | 0 | - |
| - | - | 1.12E+04 | 571.3 | - | - | 0 | - |
| - | - | 586.6 | 571.3 | - | - | 0 | - |
| - | - | 6449 | 571.8 | - | - | 0 | - |
| - | - | 1809 | 572.3 | - | - | 0 | - |
| - | - | 1222 | 572.8 | - | - | 0 | - |
| - | - | 702.9 | 576.8 | - | - | 0 | - |
| 7 | w | 2252 | 577.3 | 0.001811 | 3.138 | +2 | 9 |
| - | - | 1451 | 577.8 | - | - | 0 | - |
| - | - | 862.8 | 578.8 | - | - | 0 | - |
| 3 | y | 952.3 | 585.6 | 0.003355 | 5.73 | +3 | 13 |
| 3 | y | 1180 | 585.9 | 0.002868 | 4.895 | +3 | 13 |
| - | - | 888.1 | 586.6 | - | - | 0 | - |
| - | - | 689.1 | 588.3 | - | - | 0 | - |
| 12 | z | 715.1 | 589.3 | 0.00234 | 3.97 | +1 | 4 |
| - | - | 632.1 | 592.3 | - | - | 0 | - |
| 7 | y | 1490 | 598.8 | 0.003375 | 5.637 | +2 | 9 |
| 7 | y | 2556 | 599.3 | 0.005044 | 8.417 | +2 | 9 |
| 7 | z | 1.127E+04 | 599.8 | 0.003812 | 6.356 | +2 | 9 |
| - | - | 7178 | 600.3 | - | - | 0 | - |
| - | - | 3279 | 600.8 | - | - | 0 | - |
| - | - | 656.3 | 600.9 | - | - | 0 | - |
| - | - | 1300 | 601.3 | - | - | 0 | - |
| - | - | 714.1 | 602.3 | - | - | 0 | - |
| 12 | z | 9765 | 606.3 | 0.008869 | 14.63 | +1 | 4 |
| - | - | 718.7 | 606.8 | - | - | 0 | - |
| 14 | c | 1151 | 607 | 0.008917 | 14.69 | +3 | 14 |
| - | - | 5446 | 607.3 | - | - | 0 | - |
| - | - | 1381 | 607.6 | - | - | 0 | - |
| 7 | y | 1.46E+04 | 607.8 | 0.003958 | 6.512 | +2 | 9 |
| - | - | 8632 | 608.3 | - | - | 0 | - |
| - | - | 4167 | 608.8 | - | - | 0 | - |
| - | - | 1223 | 609.3 | - | - | 0 | - |
| - | - | 982.8 | 611.6 | - | - | 0 | - |
| - | - | 749.9 | 612 | - | - | 0 | - |
| - | - | 2455 | 612.3 | - | - | 0 | - |
| 14 | c | 8572 | 612.6 | 0.0004536 | 0.7405 | +3 | 14 |
| - | - | 1992 | 612.8 | - | - | 0 | - |
| - | - | 8124 | 613 | - | - | 0 | - |
| - | - | 6473 | 613.3 | - | - | 0 | - |
| - | - | 1248 | 613.6 | - | - | 0 | - |
| - | - | 1002 | 615.3 | - | - | 0 | - |
| - | - | 1005 | 615.8 | - | - | 0 | - |
| - | - | 1841 | 621 | - | - | 0 | - |
| - | - | 3179 | 621.3 | - | - | 0 | - |
| - | - | 2127 | 621.6 | - | - | 0 | - |
| - | - | 984.9 | 622 | - | - | 0 | - |
| 12 | y | 6535 | 622.3 | 0.009588 | 15.41 | +1 | 4 |
| - | - | 2732 | 623.3 | - | - | 0 | - |
| - | - | 1732 | 624.3 | - | - | 0 | - |
| - | - | 894.3 | 625.3 | - | - | 0 | - |
| - | - | 2631 | 626.3 | - | - | 0 | - |
| - | - | 2928 | 626.6 | - | - | 0 | - |
| - | - | 673.6 | 626.8 | - | - | 0 | - |
| - | - | 2464 | 627 | - | - | 0 | - |
| - | - | 986.4 | 627.3 | - | - | 0 | - |
| - | - | 3792 | 627.8 | - | - | 0 | - |
| 10 | c | 1.451E+04 | 628.3 | 0.00327 | 5.205 | +2 | 10 |
| - | - | 1015 | 628.4 | - | - | 0 | - |
| - | - | 1.079E+04 | 628.8 | - | - | 0 | - |
| - | - | 4327 | 629.3 | - | - | 0 | - |
| - | - | 1492 | 629.8 | - | - | 0 | - |
| - | - | 715.2 | 630.6 | - | - | 0 | - |
| - | - | 762 | 631 | - | - | 0 | - |
| 2 | y | 1599 | 631.3 | 0.0106 | 16.79 | +3 | 14 |
| - | - | 2141 | 631.6 | - | - | 0 | - |
| - | - | 1007 | 632 | - | - | 0 | - |
| - | - | 1974 | 634.3 | - | - | 0 | - |
| 6 | w | 3659 | 634.8 | 0.002893 | 4.557 | +2 | 10 |
| - | - | 3228 | 635.3 | - | - | 0 | - |
| - | - | 1150 | 635.8 | - | - | 0 | - |
| - | - | 773.5 | 636.3 | - | - | 0 | - |
| 2 | y | 1224 | 637.3 | 0.0008499 | 1.334 | +3 | 14 |
| - | - | 713.9 | 637.4 | - | - | 0 | - |
| - | - | 1191 | 640 | - | - | 0 | - |
| - | - | 1236 | 640.3 | - | - | 0 | - |
| - | - | 1166 | 640.6 | - | - | 0 | - |
| - | - | 729.4 | 641 | - | - | 0 | - |
| - | - | 1059 | 641.3 | - | - | 0 | - |
| - | - | 4435 | 645.3 | - | - | 0 | - |
| - | - | 1.891E+04 | 645.7 | - | - | 0 | - |
| - | - | 2.259E+04 | 646 | - | - | 0 | - |
| - | - | 1.029E+04 | 646.3 | - | - | 0 | - |
| - | - | 3436 | 646.7 | - | - | 0 | - |
| - | - | 2603 | 647 | - | - | 0 | - |
| - | - | 1489 | 649.7 | - | - | 0 | - |
| - | - | 1210 | 650 | - | - | 0 | - |
| - | - | 771.7 | 650.3 | - | - | 0 | - |
| - | - | 1495 | 651.3 | - | - | 0 | - |
| - | - | 983.5 | 651.6 | - | - | 0 | - |
| - | - | 842.3 | 654.7 | - | - | 0 | - |
| - | - | 1931 | 655 | - | - | 0 | - |
| - | - | 9875 | 655.3 | - | - | 0 | - |
| - | - | 1.525E+04 | 655.6 | - | - | 0 | - |
| 6 | y | 1505 | 655.8 | 0.007079 | 10.79 | +2 | 10 |
| - | - | 1.519E+04 | 656 | - | - | 0 | - |
| 6 | z | 1.201E+04 | 656.3 | 0.005195 | 7.915 | +2 | 10 |
| - | - | 5419 | 656.7 | - | - | 0 | - |
| - | - | 1789 | 656.8 | - | - | 0 | - |
| - | - | 1256 | 657 | - | - | 0 | - |
| - | - | 1467 | 657.3 | - | - | 0 | - |
| - | - | 1448 | 660 | - | - | 0 | - |
| - | - | 2595 | 660.3 | - | - | 0 | - |
| - | - | 849.4 | 663.8 | - | - | 0 | - |
| 6 | y | 8496 | 664.3 | 0.003693 | 5.558 | +2 | 10 |
| - | - | 4599 | 664.8 | - | - | 0 | - |
| - | - | 1773 | 665.3 | - | - | 0 | - |
| - | - | 863.1 | 665.8 | - | - | 0 | - |
| - | - | 971.1 | 669 | - | - | 0 | - |
| - | - | 3353 | 669.3 | - | - | 0 | - |
| - | - | 4973 | 669.7 | - | - | 0 | - |
| - | - | 754.5 | 669.8 | - | - | 0 | - |
| - | - | 3573 | 670 | - | - | 0 | - |
| - | - | 2554 | 670.3 | - | - | 0 | - |
| - | - | 898.1 | 673.7 | - | - | 0 | - |
| - | - | 1173 | 674 | - | - | 0 | - |
| - | - | 831.6 | 674.3 | - | - | 0 | - |
| - | - | 1476 | 674.4 | - | - | 0 | - |
| - | - | 6917 | 674.7 | - | - | 0 | - |
| - | - | 4.251E+04 | 675 | - | - | 0 | - |
| - | - | 6.679E+04 | 675.3 | - | - | 0 | - |
| - | - | 4.98E+04 | 675.7 | - | - | 0 | - |
| - | - | 3.003E+04 | 676 | - | - | 0 | - |
| - | - | 1.206E+04 | 676.3 | - | - | 0 | - |
| - | - | 4959 | 676.7 | - | - | 0 | - |
| - | - | 896 | 677 | - | - | 0 | - |
| - | - | 1295 | 682.4 | - | - | 0 | - |
| - | - | 1165 | 682.4 | - | - | 0 | - |
| - | - | 1631 | 687.8 | - | - | 0 | - |
| - | - | 853.7 | 688.3 | - | - | 0 | - |
| - | - | 768.1 | 688.8 | - | - | 0 | - |
| - | - | 4619 | 690.8 | - | - | 0 | - |
| - | - | 1.456E+04 | 691.3 | - | - | 0 | - |
| - | - | 9123 | 691.8 | - | - | 0 | - |
| - | - | 7999 | 692.3 | - | - | 0 | - |
| - | - | 2335 | 692.8 | - | - | 0 | - |
| - | - | 970.3 | 693.3 | - | - | 0 | - |
| 5 | c | 4850 | 696.3 | 0.00479 | 6.879 | +1 | 5 |
| - | - | 1564 | 697.3 | - | - | 0 | - |
| - | - | 3287 | 709.3 | - | - | 0 | - |
| 11 | c | 1.378E+04 | 709.8 | 0.003588 | 5.055 | +2 | 11 |
| - | - | 1.017E+04 | 710.3 | - | - | 0 | - |
| - | - | 3932 | 710.8 | - | - | 0 | - |
| - | - | 1737 | 711.3 | - | - | 0 | - |
| - | - | 850.3 | 711.8 | - | - | 0 | - |
| 5 | c | 1.304E+04 | 713.3 | 0.00473 | 6.631 | +1 | 5 |
| - | - | 4868 | 714.3 | - | - | 0 | - |
| - | - | 1489 | 715.3 | - | - | 0 | - |
| - | - | 688.7 | 726.4 | - | - | 0 | - |
| - | - | 983.5 | 726.9 | - | - | 0 | - |
| - | - | 1137 | 727.4 | - | - | 0 | - |
| - | - | 1463 | 733.4 | - | - | 0 | - |
| - | - | 839.6 | 733.9 | - | - | 0 | - |
| - | - | 953.9 | 734.4 | - | - | 0 | - |
| - | - | 811.6 | 742.4 | - | - | 0 | - |
| 5 | z | 1218 | 748.8 | 0.004612 | 6.159 | +2 | 11 |
| - | - | 705.5 | 753.4 | - | - | 0 | - |
| - | - | 1.188E+04 | 755.4 | - | - | 0 | - |
| - | - | 7987 | 755.9 | - | - | 0 | - |
| 5 | y | 7905 | 756.4 | 0.002915 | 3.855 | +2 | 11 |
| - | - | 3694 | 756.4 | - | - | 0 | - |
| 5 | y | 6782 | 756.9 | 0.00764 | 10.09 | +2 | 11 |
| 5 | z | 2619 | 757.4 | 0.002812 | 3.713 | +2 | 11 |
| - | - | 1231 | 757.9 | - | - | 0 | - |
| 12 | c | 751.6 | 765.4 | 0.01052 | 13.74 | +2 | 12 |
| 11 | y | 796.2 | 768.4 | 0.01133 | 14.75 | +1 | 5 |
| 11 | z | 1.625E+04 | 769.4 | 0.009515 | 12.37 | +1 | 5 |
| - | - | 7016 | 770.4 | - | - | 0 | - |
| - | - | 2698 | 771.4 | - | - | 0 | - |
| - | - | 1041 | 772.4 | - | - | 0 | - |
| 12 | c | 4039 | 773.9 | 0.003836 | 4.957 | +2 | 12 |
| - | - | 1930 | 774.4 | - | - | 0 | - |
| - | - | 1164 | 774.9 | - | - | 0 | - |
| - | - | 1212 | 782.4 | - | - | 0 | - |
| - | - | 732.7 | 783.4 | - | - | 0 | - |
| 11 | y | 4838 | 785.4 | 0.01011 | 12.87 | +1 | 5 |
| - | - | 1606 | 786.4 | - | - | 0 | - |
| - | - | 811.9 | 787.4 | - | - | 0 | - |
| - | - | 1905 | 789.5 | - | - | 0 | - |
| - | - | 1052 | 790.5 | - | - | 0 | - |
| - | - | 1148 | 792.9 | - | - | 0 | - |
| 6 | c | 1010 | 809.4 | 0.007457 | 9.213 | +1 | 6 |
| - | - | 1369 | 810.4 | - | - | 0 | - |
| - | - | 1065 | 812.9 | - | - | 0 | - |
| 4 | z | 7962 | 814.9 | 0.001626 | 1.996 | +2 | 12 |
| - | - | 6514 | 815.4 | - | - | 0 | - |
| - | - | 3768 | 815.9 | - | - | 0 | - |
| - | - | 2534 | 816.4 | - | - | 0 | - |
| 4 | y | 1219 | 822.9 | 0.0006732 | 0.8181 | +2 | 12 |
| - | - | 942.8 | 823.4 | - | - | 0 | - |
| - | - | 712.7 | 823.9 | - | - | 0 | - |
| - | - | 1236 | 825.4 | - | - | 0 | - |
| 6 | c | 3.338E+04 | 826.4 | 0.005016 | 6.07 | +1 | 6 |
| - | - | 859.6 | 827.3 | - | - | 0 | - |
| - | - | 1.372E+04 | 827.4 | - | - | 0 | - |
| - | - | 3843 | 828.4 | - | - | 0 | - |
| - | - | 804.7 | 829.4 | - | - | 0 | - |
| 10 | w | 1705 | 839.4 | 0.007492 | 8.926 | +1 | 6 |
| - | - | 1171 | 840.4 | - | - | 0 | - |
| 3 | w | 1308 | 849.9 | 0.001927 | 2.268 | +2 | 13 |
| - | - | 2425 | 853.9 | - | - | 0 | - |
| 13 | c | 8981 | 854.4 | 0.001385 | 1.621 | +2 | 13 |
| - | - | 8580 | 854.9 | - | - | 0 | - |
| - | - | 4717 | 855.4 | - | - | 0 | - |
| - | - | 1156 | 855.9 | - | - | 0 | - |
| - | - | 783.6 | 876.4 | - | - | 0 | - |
| 3 | y | 1251 | 878.4 | 0.007238 | 8.24 | +2 | 13 |
| 3 | z | 6207 | 878.9 | 0.0007025 | 0.7993 | +2 | 13 |
| - | - | 4751 | 879.4 | - | - | 0 | - |
| - | - | 2745 | 879.9 | - | - | 0 | - |
| 10 | z | 1254 | 880.4 | 0.003518 | 3.996 | +1 | 6 |
| - | - | 8672 | 883.4 | - | - | 0 | - |
| - | - | 4152 | 884.4 | - | - | 0 | - |
| - | - | 1490 | 885.4 | - | - | 0 | - |
| - | - | 969 | 887.4 | - | - | 0 | - |
| - | - | 1623 | 889.4 | - | - | 0 | - |
| 10 | y | 3947 | 896.4 | 0.00858 | 9.572 | +1 | 6 |
| - | - | 2845 | 896.9 | - | - | 0 | - |
| - | - | 2590 | 897.4 | - | - | 0 | - |
| 10 | z | 2.096E+04 | 898.4 | 0.008101 | 9.018 | +1 | 6 |
| - | - | 1.333E+04 | 899.4 | - | - | 0 | - |
| - | - | 4513 | 900.4 | - | - | 0 | - |
| - | - | 1775 | 901.4 | - | - | 0 | - |
| - | - | 1132 | 902 | - | - | 0 | - |
| - | - | 868.5 | 903.4 | - | - | 0 | - |
| 14 | c | 823.1 | 909.9 | 0.01057 | 11.62 | +2 | 14 |
| - | - | 962.3 | 910.4 | - | - | 0 | - |
| 10 | y | 2243 | 914.4 | 0.01047 | 11.45 | +1 | 6 |
| - | - | 1153 | 915.4 | - | - | 0 | - |
| - | - | 1688 | 917.9 | - | - | 0 | - |
| 14 | c | 3966 | 918.4 | 0.002854 | 3.107 | +2 | 14 |
| - | - | 4819 | 918.9 | - | - | 0 | - |
| - | - | 3249 | 919.4 | - | - | 0 | - |
| - | - | 1033 | 919.9 | - | - | 0 | - |
| 7 | c | 662.4 | 924.4 | 0.009933 | 10.74 | +1 | 7 |
| - | - | 3098 | 939 | - | - | 0 | - |
| - | - | 1824 | 939.5 | - | - | 0 | - |
| - | - | 1052 | 940 | - | - | 0 | - |
| - | - | 1245 | 940.4 | - | - | 0 | - |
| 7 | c | 5555 | 941.4 | 0.004624 | 4.911 | +1 | 7 |
| - | - | 2709 | 942.5 | - | - | 0 | - |
| - | - | 740.8 | 945.5 | - | - | 0 | - |
| - | - | 2220 | 946 | - | - | 0 | - |
| - | - | 2682 | 946.5 | - | - | 0 | - |
| - | - | 2028 | 947 | - | - | 0 | - |
| 2 | z | 3147 | 947.4 | 0.00451 | 4.76 | +2 | 14 |
| - | - | 4548 | 947.9 | - | - | 0 | - |
| - | - | 3372 | 948.4 | - | - | 0 | - |
| - | - | 1155 | 948.9 | - | - | 0 | - |
| - | - | 2321 | 954.5 | - | - | 0 | - |
| - | - | 1084 | 959.5 | - | - | 0 | - |
| - | - | 1614 | 960 | - | - | 0 | - |
| - | - | 1135 | 960.5 | - | - | 0 | - |
| - | - | 939.7 | 961 | - | - | 0 | - |
| - | - | 1188 | 961.5 | - | - | 0 | - |
| - | - | 3357 | 968 | - | - | 0 | - |
| - | - | 4740 | 968.5 | - | - | 0 | - |
| - | - | 2171 | 969 | - | - | 0 | - |
| - | - | 1234 | 969.5 | - | - | 0 | - |
| - | - | 1003 | 973.5 | - | - | 0 | - |
| - | - | 774.3 | 974 | - | - | 0 | - |
| - | - | 3794 | 974.5 | - | - | 0 | - |
| - | - | 3586 | 975 | - | - | 0 | - |
| - | - | 3001 | 975.5 | - | - | 0 | - |
| - | - | 1552 | 976 | - | - | 0 | - |
| - | - | 699.2 | 980.5 | - | - | 0 | - |
| - | - | 1432 | 981.5 | - | - | 0 | - |
| - | - | 2125 | 982 | - | - | 0 | - |
| - | - | 4204 | 982.5 | - | - | 0 | - |
| - | - | 1.064E+04 | 983 | - | - | 0 | - |
| - | - | 1.356E+04 | 983.5 | - | - | 0 | - |
| - | - | 7797 | 984 | - | - | 0 | - |
| - | - | 4613 | 984.5 | - | - | 0 | - |
| - | - | 1877 | 985 | - | - | 0 | - |
| - | - | 1548 | 989.5 | - | - | 0 | - |
| - | - | 4694 | 990 | - | - | 0 | - |
| - | - | 4746 | 990.5 | - | - | 0 | - |
| - | - | 3459 | 991 | - | - | 0 | - |
| - | - | 1595 | 991.5 | - | - | 0 | - |
| - | - | 904.7 | 992 | - | - | 0 | - |
| - | - | 4296 | 992.6 | - | - | 0 | - |
| - | - | 3584 | 993.6 | - | - | 0 | - |
| - | - | 1175 | 994.6 | - | - | 0 | - |
| - | - | 2182 | 995.5 | - | - | 0 | - |
| - | - | 2722 | 996 | - | - | 0 | - |
| - | - | 1648 | 996.5 | - | - | 0 | - |
| - | - | 3819 | 997.5 | - | - | 0 | - |
| 8 | c | 1.577E+04 | 998.5 | 0.006597 | 6.608 | +1 | 8 |
| - | - | 8562 | 999.5 | - | - | 0 | - |
| - | - | 2784 | 1000 | - | - | 0 | - |
| - | - | 3805 | 1003 | - | - | 0 | - |
| - | - | 1.548E+04 | 1004 | - | - | 0 | - |
| - | - | 2.249E+04 | 1004 | - | - | 0 | - |
| - | - | 1.674E+04 | 1005 | - | - | 0 | - |
| - | - | 1.045E+04 | 1005 | - | - | 0 | - |
| - | - | 3402 | 1006 | - | - | 0 | - |
| - | - | 3087 | 1006 | - | - | 0 | - |
| - | - | 1755 | 1011 | - | - | 0 | - |
| - | - | 1864 | 1011 | - | - | 0 | - |
| - | - | 1949 | 1011 | - | - | 0 | - |
| - | - | 1092 | 1012 | - | - | 0 | - |
| - | - | 3570 | 1012 | - | - | 0 | - |
| - | - | 1.321E+04 | 1012 | - | - | 0 | - |
| - | - | 2.093E+04 | 1013 | - | - | 0 | - |
| - | - | 2.246E+04 | 1013 | - | - | 0 | - |
| - | - | 1.346E+04 | 1014 | - | - | 0 | - |
| - | - | 6763 | 1014 | - | - | 0 | - |
| - | - | 3779 | 1015 | - | - | 0 | - |
| - | - | 1262 | 1015 | - | - | 0 | - |
| 9 | z | 1280 | 1027 | 0.01005 | 9.787 | +1 | 7 |
| - | - | 5247 | 1028 | - | - | 0 | - |
| - | - | 3849 | 1029 | - | - | 0 | - |
| 8 | z | 1854 | 1084 | 0.003401 | 3.139 | +1 | 8 |
| - | - | 3200 | 1085 | - | - | 0 | - |
| - | - | 1837 | 1086 | - | - | 0 | - |
| - | - | 1537 | 1111 | - | - | 0 | - |
| - | - | 3270 | 1126 | - | - | 0 | - |
| 9 | c | 1.445E+04 | 1127 | 0.006849 | 6.08 | +1 | 9 |
| - | - | 1.05E+04 | 1128 | - | - | 0 | - |
| - | - | 3670 | 1129 | - | - | 0 | - |
| - | - | 1089 | 1130 | - | - | 0 | - |
| - | - | 959.3 | 1141 | - | - | 0 | - |
| - | - | 1846 | 1142 | - | - | 0 | - |
| - | - | 1091 | 1143 | - | - | 0 | - |
| 7 | z | 3097 | 1199 | 0.004046 | 3.376 | +1 | 9 |
| - | - | 9020 | 1200 | - | - | 0 | - |
| - | - | 4972 | 1201 | - | - | 0 | - |
| - | - | 2564 | 1202 | - | - | 0 | - |
| - | - | 1212 | 1213 | - | - | 0 | - |
| - | - | 1056 | 1214 | - | - | 0 | - |
| 7 | y | 1222 | 1215 | 0.004058 | 3.341 | +1 | 9 |
| - | - | 913.6 | 1240 | - | - | 0 | - |
| - | - | 1152 | 1255 | - | - | 0 | - |
| 10 | c | 4225 | 1256 | 0.009666 | 7.699 | +1 | 10 |
| - | - | 3715 | 1257 | - | - | 0 | - |
| - | - | 1577 | 1258 | - | - | 0 | - |
| 6 | z | 1227 | 1312 | 0.01215 | 9.26 | +1 | 10 |
| - | - | 4128 | 1313 | - | - | 0 | - |
| - | - | 1897 | 1314 | - | - | 0 | - |
| - | - | 1017 | 1375 | - | - | 0 | - |
| - | - | 1958 | 1376 | - | - | 0 | - |
| - | - | 948 | 1377 | - | - | 0 | - |
| - | - | 2476 | 1382 | - | - | 0 | - |
| - | - | 3206 | 1383 | - | - | 0 | - |
| - | - | 1845 | 1384 | - | - | 0 | - |
| 11 | c | 1794 | 1419 | 0.0103 | 7.262 | +1 | 11 |
| - | - | 3395 | 1420 | - | - | 0 | - |
| - | - | 1981 | 1421 | - | - | 0 | - |
| - | - | 1140 | 1513 | - | - | 0 | - |
| 5 | z | 1767 | 1514 | 0.01217 | 8.039 | +1 | 11 |
| - | - | 1391 | 1515 | - | - | 0 | - |
| 12 | c | 867.3 | 1530 | 0.001471 | 0.9613 | +1 | 12 |
| - | - | 766.7 | 1545 | - | - | 0 | - |
| 12 | c | 1860 | 1547 | 0.0004227 | 0.2733 | +1 | 12 |
| - | - | 1447 | 1548 | - | - | 0 | - |
| 4 | z | 1013 | 1629 | 0.002559 | 1.571 | +1 | 12 |
| - | - | 3926 | 1630 | - | - | 0 | - |
| - | - | 2761 | 1631 | - | - | 0 | - |
| - | - | 1436 | 1632 | - | - | 0 | - |
| - | - | 1004 | 1707 | - | - | 0 | - |
| 13 | c | 2118 | 1708 | 0.006751 | 3.953 | +1 | 13 |
| - | - | 2201 | 1709 | - | - | 0 | - |
| - | - | 1238 | 1710 | - | - | 0 | - |
| - | - | 930.2 | 1711 | - | - | 0 | - |
| 3 | z | 2167 | 1757 | 0.0007203 | 0.41 | +1 | 13 |
| - | - | 2608 | 1758 | - | - | 0 | - |
| - | - | 2738 | 1759 | - | - | 0 | - |
| - | - | 1864 | 1760 | - | - | 0 | - |
| - | - | 969.6 | 1837 | - | - | 0 | - |
| - | - | 807.8 | 1895 | - | - | 0 | - |
| - | - | 787.1 | 1896 | - | - | 0 | - |
| - | - | 726.1 | 1936 | - | - | 0 | - |
| - | - | 790.4 | 1937 | - | - | 0 | - |
| - | - | 1260 | 1938 | - | - | 0 | - |
| - | - | 1156 | 1949 | - | - | 0 | - |
| - | - | 1771 | 1966 | - | - | 0 | - |
| - | - | 1373 | 1967 | - | - | 0 | - |
| - | - | 1398 | 1968 | - | - | 0 | - |
| - | - | 743.3 | 1982 | - | - | 0 | - |
| - | - | 699.3 | 2007 | - | - | 0 | - |
| - | - | 1930 | 2008 | - | - | 0 | - |
| - | - | 2956 | 2009 | - | - | 0 | - |
| - | - | 2028 | 2010 | - | - | 0 | - |
| - | - | 1213 | 2011 | - | - | 0 | - |
| - | - | 922.2 | 2024 | - | - | 0 | - |
| - | - | 2965 | 2025 | - | - | 0 | - |
| - | - | 2828 | 2026 | - | - | 0 | - |
| - | - | 2114 | 2027 | - | - | 0 | - |
| - | - | 1354 | 2028 | - | - | 0 | - |
| - | - | 946.5 | 2029 | - | - | 0 | - |

m/z Charge Intensity FragmentType MassShift Position
126.07347869873047 0 361.33362
129.10214233398438 0 1534.0074
136.07545471191406 0 571.6359
137.1147003173828 0 443.3905
146.092529296875 0 612.2398
149.04483032226562 0 532.648
149.0934600830078 0 474.54825
155.09280395507812 0 2879.6836
163.0718994140625 0 429.39572
166.06117248535156 0 3184.621
171.87277221679688 0 607.88055
173.09234619140625 0 558.37115
173.43861389160156 0 1176.7358
175.0865936279297 0 1279.7109
181.169677734375 0 889.9325
183.11288452148438 0 1029.3505
185.16490173339844 0 3050.3398
187.1077880859375 0 513.4907
203.1024627685547 0 2839.5005 c Ammonia loss 5
205.11849975585938 0 1408.587
209.16481018066406 0 4627.392
210.1687469482422 0 533.33795
213.08731079101562 0 1105.8872
215.1385498046875 0 1825.1399
216.1432342529297 0 665.9248
223.1552734375 0 2055.5264
230.11399841308594 0 800.6032
234.12376403808594 0 3199.4778
235.12701416015625 0 633.8166
237.15988159179688 0 10893.929
238.1632843017578 0 2110.7124
251.15029907226562 0 12318.047
252.15403747558594 0 1633.105
268.1772766113281 0 937.7697 c 1
278.1492004394531 0 631.2514
282.18133544921875 0 662.2719
290.8166198730469 0 536.6838
299.0619201660156 0 1411.5126
300.0614013671875 0 693.2294
301.0591125488281 0 895.3038
309.2038269042969 0 772.046
317.1948547363281 0 813.3437 z 13
318.2027893066406 0 767.54724
333.2117919921875 0 598.2901 y 13
334.6611328125 0 2270.4863
335.1625061035156 0 1041.744
348.65863037109375 0 1483.5472 c Ammonia loss 4
367.18621826171875 0 1212.8185 y 7
376.1369934082031 0 751.93036
379.2086486816406 0 3321.3286 c Ammonia loss 2
385.2293701171875 0 595.0829
387.2236633300781 0 2838.387
389.4936218261719 0 661.85034
393.1965026855469 0 673.26294 y 10
394.1986999511719 0 672.56366
396.1923828125 0 589.05457
396.2356262207031 0 3705.1987 c 2
400.9852600097656 0 3196.5232
401.9850158691406 0 2369.6672
402.9835205078125 0 1680.6588
405.53009033203125 0 1034.5402 y 6
418.9955139160156 0 3929.7483
419.9953308105469 0 2045.0903
420.2024230957031 0 10239.581 w 9
420.7036437988281 0 5774.3457
420.9939270019531 0 1579.7695
421.2043151855469 0 2340.777
421.7023620605469 0 733.30365
431.2286376953125 0 842.9678
449.1242370605469 0 580.42896
460.7371826171875 0 1422.2771
461.23956298828125 0 1094.852
462.20294189453125 0 689.2884
468.2659912109375 0 794.7841
468.76519775390625 0 1522.0383
472.2498779296875 0 647.3441
474.2786865234375 0 599.5666
479.22552490234375 0 843.05206
484.2503967285156 0 1670.731
484.72113037109375 0 1240.3713 w 8
484.7557373046875 0 1041.2365
489.0555419921875 0 6045.3433
490.05670166015625 0 3694.287
491.05596923828125 0 2643.0022
494.2354431152344 0 16891.998 c Ammonia loss 3
495.2379150390625 0 4674.4185
495.765625 0 677.81934
496.2491455078125 0 1511.2825
496.7753601074219 0 2232.046
497.2807922363281 0 974.18726
501.9952697753906 0 732.1358
504.2444763183594 0 2587.8833
504.5787048339844 0 2976.6877 y Water loss 4
504.9125061035156 0 2283.8064 y Ammonia loss 4
505.23968505859375 0 885.5709 z Ammonia loss 8
506.22576904296875 0 7482.3516
506.2599792480469 0 9711.444
506.2978210449219 0 596.18524
506.3053283691406 0 985.50433
506.4967956542969 0 10137.353
506.58203125 0 595.2167
506.7455749511719 0 7627.084
506.99749755859375 0 5856.5723
507.2283935546875 0 880.50604
507.26544189453125 0 1897.3162
507.4990539550781 0 808.73956
507.9436950683594 0 1981.5173
511.2626647949219 0 13726.505 c 3
512.2650756835938 0 4390.3594
512.7626342773438 0 750.98254 y Water loss 8
513.7391967773438 0 563.1052
515.3191528320312 0 2611.0618
516.3240356445312 0 1127.8441
518.7636108398438 0 783.36426
521.7655029296875 0 3393.754 y 8
522.2679443359375 0 2029.2672
522.7625122070312 0 659.98737
533.7755126953125 0 794.4218
534.2723388671875 0 937.2899
537.2789306640625 0 1305.2755
542.2669067382812 0 1453.8483 z 7
542.7686157226562 0 1164.5635
547.2913818359375 0 1350.3455
548.2365112304688 0 788.7583
550.2774658203125 0 3398.2031 y 7
550.7772827148438 0 2953.436
551.2784423828125 0 650.7871
552.2642822265625 0 2058.9446
553.3092041015625 0 1396.3977
554.2925415039062 0 1115.6663
554.784423828125 0 1757.7463 c Water loss 8
555.274658203125 0 1798.4146 c Ammonia loss 8
555.7786254882812 0 782.1653
563.2936401367188 0 3681.0056
563.7863159179688 0 18598.623 c 8
564.2423095703125 0 1002.94525
564.28662109375 0 9199.603
564.7899780273438 0 3127.5933
565.2909545898438 0 1179.6255
570.271240234375 0 1651.1904
570.3251953125 0 2587.8726
570.7474975585938 0 1800.2463
571.2378540039062 0 801.0018
571.2811889648438 0 11199.263
571.3282470703125 0 586.59534
571.7828369140625 0 6449.241
572.2833862304688 0 1809.1696
572.7882080078125 0 1221.8738
576.7948608398438 0 702.9299
577.2842407226562 0 2252.3354 w 6
577.7861938476562 0 1451.083
578.7888793945312 0 862.7991
585.6046752929688 0 952.3397 y Water loss 2
585.9389038085938 0 1179.9572 y Ammonia loss 2
586.605224609375 0 888.13995
588.2811279296875 0 689.1335
589.2843017578125 0 715.082 z Ammonia loss 11
592.2789306640625 0 632.1427
598.7855834960938 0 1489.8608 y Water loss 6
599.2860107421875 0 2555.5564 y Ammonia loss 6
599.7810668945312 0 11268.865 z 6
600.2817993164062 0 7178.2783
600.7836303710938 0 3279.364
600.946044921875 0 656.3029
601.2881469726562 0 1300.3518
602.281494140625 0 714.11676
606.3043212890625 0 9764.931 z 11
606.802001953125 0 718.6946
606.9629516601562 0 1150.678 c Ammonia loss 13
607.3043823242188 0 5446.4004
607.6373901367188 0 1380.8583
607.790283203125 0 14603.6455 y 6
608.2924194335938 0 8631.625
608.7926025390625 0 4166.534
609.2933349609375 0 1223.0465
611.6434936523438 0 982.7674
611.9729614257812 0 749.8975
612.3006591796875 0 2455.0432
612.6300048828125 0 8572.437 c 13
612.8018798828125 0 1992.269
612.96435546875 0 8124.035
613.2996826171875 0 6473.0713
613.626953125 0 1248.0867
615.3087768554688 0 1002.30585
615.8062744140625 0 1005.0374
620.970458984375 0 1841.2736
621.30517578125 0 3178.9482
621.6390991210938 0 2127.1218
621.9733276367188 0 984.85596
622.3223266601562 0 6535.2354 y 11
623.3262939453125 0 2732.0452
624.3273315429688 0 1732.3273
625.323486328125 0 894.2535
626.3063354492188 0 2631.4236
626.6386108398438 0 2928.0762
626.8162231445312 0 673.61676
626.9722290039062 0 2464.2708
627.3096923828125 0 986.3698
627.8145141601562 0 3792.3318
628.3077392578125 0 14507.603 c 9
628.373291015625 0 1014.53754
628.8087158203125 0 10792.915
629.3106689453125 0 4326.8193
629.8115234375 0 1492.4473
630.644287109375 0 715.2203
630.9874877929688 0 761.9954
631.304931640625 0 1599.4928 y Water loss 1
631.6432495117188 0 2141.4548
631.9744262695312 0 1006.83374
634.3140258789062 0 1974.2659
634.796630859375 0 3659.0564 w 5
635.2984619140625 0 3227.6328
635.7980346679688 0 1150.266
636.298828125 0 773.52405
637.2987060546875 0 1223.9413 y 1
637.3576049804688 0 713.87695
639.9813232421875 0 1191.266
640.3174438476562 0 1235.8137
640.6446533203125 0 1166.1428
640.9784545898438 0 729.3959
641.2938842773438 0 1058.9891
645.3257446289062 0 4434.6445
645.654052734375 0 18905.797
645.9876098632812 0 22589.617
646.3215942382812 0 10291.691
646.654296875 0 3436.0627
646.9912109375 0 2602.7
649.6516723632812 0 1488.8146
649.9853515625 0 1210.4845
650.313232421875 0 771.74817
651.3091430664062 0 1495.3027
651.645263671875 0 983.5415
654.6575927734375 0 842.29034
654.9815063476562 0 1930.8018
655.3101806640625 0 9875.088
655.6488647460938 0 15246.446
655.830078125 0 1504.8109 y Ammonia loss 5
655.9871826171875 0 15185.532
656.3217163085938 0 12007.328 z 5
656.6578979492188 0 5418.756
656.825927734375 0 1789.4877
656.9901123046875 0 1256.4634
657.3241577148438 0 1467.095
659.9940185546875 0 1448.3074
660.3260498046875 0 2594.8672
663.8386840820312 0 849.4388
664.3325805664062 0 8496.269 y 5
664.8330078125 0 4598.7285
665.3375854492188 0 1772.5422
665.8331909179688 0 863.0801
668.9827880859375 0 971.1036
669.3245849609375 0 3353.2954
669.6557006835938 0 4973.2783
669.8477783203125 0 754.45374
669.9908447265625 0 3573.2415
670.3192749023438 0 2553.956
673.700439453125 0 898.1199
674.0374755859375 0 1172.7463
674.2920532226562 0 831.5517
674.3643798828125 0 1475.5791
674.6617431640625 0 6917.4985
674.9931640625 0 42507.55
675.3270874023438 0 66786.79
675.6610717773438 0 49798.965
675.9952392578125 0 30032.396
676.3294677734375 0 12059.622
676.6632080078125 0 4959.0293
676.999755859375 0 896.04706
682.36376953125 0 1295.0171
682.4263916015625 0 1165.4188
687.8298950195312 0 1630.563
688.3367919921875 0 853.7323
688.8382568359375 0 768.0746
690.8436279296875 0 4618.9033
691.3394165039062 0 14561.068
691.8406372070312 0 9122.79
692.3402099609375 0 7998.6357
692.842041015625 0 2334.9446
693.3421630859375 0 970.2751
696.3098754882812 0 4849.9116 c Ammonia loss 4
697.3126831054688 0 1563.9563
709.3474731445312 0 3287.1492
709.8397216796875 0 13778.02 c 10
710.3407592773438 0 10173.368
710.84228515625 0 3931.846
711.3432006835938 0 1736.836
711.8474731445312 0 850.26715
713.3363647460938 0 13035.009 c 4
714.3394775390625 0 4868.3057
715.3411865234375 0 1488.8157
726.3594970703125 0 688.7266
726.8617553710938 0 983.5173
727.3611450195312 0 1137.0452
733.3516845703125 0 1462.8151
733.8599853515625 0 839.6316
734.3587036132812 0 953.9037
742.3543701171875 0 811.6426
748.8529052734375 0 1217.8374 z Ammonia loss 4
753.3980102539062 0 705.5011
755.4022216796875 0 11876.299
755.86474609375 0 7987.2695
756.3627319335938 0 7904.709 y Water loss 4
756.415283203125 0 3693.6873
756.8652954101562 0 6782.216 y Ammonia loss 4
757.3643798828125 0 2619.1924 z 4
757.8595581054688 0 1230.9998
765.380859375 0 751.6413 c Ammonia loss 11
768.3573608398438 0 796.16974 y Ammonia loss 10
769.3670043945312 0 16252.907 z 10
770.3711547851562 0 7016.0244
771.3729248046875 0 2698.3608
772.3688354492188 0 1041.4989
773.887451171875 0 4038.7205 c 11
774.3881225585938 0 1929.8727
774.8970947265625 0 1164.2384
782.4039916992188 0 1211.5598
783.4095458984375 0 732.7007
785.3851318359375 0 4838.368 y 10
786.386962890625 0 1606.3389
787.3860473632812 0 811.8828
789.4595947265625 0 1905.3884
790.466796875 0 1051.6417
792.8762817382812 0 1147.7583
809.3966064453125 0 1009.908 c Ammonia loss 5
810.39404296875 0 1369.1033
812.8853759765625 0 1065.3116
814.8734130859375 0 7962.0703 z 3
815.3740234375 0 6513.5273
815.87548828125 0 3767.5354
816.3753662109375 0 2534.1436
822.8837280273438 0 1218.613 y 3
823.3761596679688 0 942.8231
823.8871459960938 0 712.7417
825.4093017578125 0 1235.8445
826.4207153320312 0 33384.395 c 5
827.34130859375 0 859.56433
827.4229125976562 0 13717.731
828.4269409179688 0 3843.4116
829.4334106445312 0 804.65027
839.3983154296875 0 1705.1974 w 9
840.4005737304688 0 1170.9424
849.8877563476562 0 1307.964 w 2
853.8999633789062 0 2424.9277
854.3945922851562 0 8980.778 c 12
854.8956909179688 0 8580.182
855.3961181640625 0 4716.793
855.8938598632812 0 1155.5985
876.4189453125 0 783.63513
878.4076538085938 0 1250.8497 y Ammonia loss 2
878.9036254882812 0 6207.2144 z 2
879.40625 0 4750.64
879.90380859375 0 2744.9778
880.405029296875 0 1253.922 z Water loss 9
883.417236328125 0 8671.946
884.4209594726562 0 4152.271
885.4261474609375 0 1489.6852
887.4132690429688 0 968.99567
889.4341430664062 0 1622.7915
896.4358520507812 0 3946.5586 y Water loss 9
896.9369506835938 0 2844.5525
897.4348754882812 0 2590.1296
898.4110107421875 0 20957.549 z 9
899.4146118164062 0 13328.726
900.4160766601562 0 4513.236
901.4130249023438 0 1775.3582
901.9846801757812 0 1131.9624
903.42529296875 0 868.5265
909.93798828125 0 823.0556 c Ammonia loss 13
910.4364013671875 0 962.27527
914.4273681640625 0 2242.6953 y 9
915.4304809570312 0 1152.6223
917.944091796875 0 1687.9244
918.4435424804688 0 3965.5186 c 13
918.9435424804688 0 4818.6235
919.4415893554688 0 3249.194
919.9465942382812 0 1032.5801
924.426025390625 0 662.432 c Ammonia loss 6
938.9675903320312 0 3098.1062
939.4700317382812 0 1824.1417
939.9639282226562 0 1052.4963
940.44482421875 0 1244.9447
941.447265625 0 5555.4727 c 6
942.4514770507812 0 2709.2375
945.4609375 0 740.79443
945.9711303710938 0 2220.1562
946.475341796875 0 2682.0664
946.9683227539062 0 2027.8883
947.4382934570312 0 3147.4216 z 1
947.9351196289062 0 4547.6523
948.4361572265625 0 3371.668
948.9319458007812 0 1154.768
954.45849609375 0 2320.8127
959.472412109375 0 1083.8323
959.9718627929688 0 1614.2465
960.4683227539062 0 1134.6123
960.9744873046875 0 939.67487
961.4735107421875 0 1188.3992
967.9772338867188 0 3356.909
968.4786376953125 0 4740.1045
968.97998046875 0 2170.9194
969.4862670898438 0 1233.9498
973.4655151367188 0 1002.6925
973.9801025390625 0 774.3317
974.4773559570312 0 3793.5432
974.9742431640625 0 3585.921
975.4752197265625 0 3000.7705
975.9776000976562 0 1551.5634
980.4928588867188 0 699.20667
981.47998046875 0 1431.6791
981.9884033203125 0 2125.2292
982.482421875 0 4203.5605
982.9816284179688 0 10637.963
983.4819946289062 0 13559.841
983.9830322265625 0 7796.932
984.483154296875 0 4612.828
984.9857788085938 0 1877.1224
989.4873657226562 0 1548.012
989.9887084960938 0 4694.0903
990.4835205078125 0 4746.345
990.988525390625 0 3458.6284
991.48193359375 0 1594.6696
991.9861450195312 0 904.7362
992.5643310546875 0 4295.839
993.5658569335938 0 3584.3564
994.5736694335938 0 1174.8525
995.4774780273438 0 2181.5842
995.9781494140625 0 2722.1677
996.4769287109375 0 1648.332
997.4804077148438 0 3819.141
998.470703125 0 15771.828 c 7
999.4744262695312 0 8562.172
1000.4789428710938 0 2783.6538
1003.4888916015625 0 3805.321
1003.9846801757812 0 15476.277
1004.4822998046875 0 22490.393
1004.9818115234375 0 16744.781
1005.483154296875 0 10448.41
1005.9795532226562 0 3402.006
1006.4893798828125 0 3087.3286
1010.5706787109375 0 1754.5947
1011.0532836914062 0 1863.7634
1011.4767456054688 0 1948.6614
1011.58203125 0 1091.8951
1011.985107421875 0 3570.4446
1012.4856567382812 0 13213.426
1012.990966796875 0 20929.822
1013.49072265625 0 22464.145
1013.9935913085938 0 13455.653
1014.490234375 0 6763.201
1014.9993896484375 0 3778.921
1015.4834594726562 0 1261.6014
1026.5040283203125 0 1279.7551 z 8
1027.511962890625 0 5246.6284
1028.513427734375 0 3848.523
1083.5389404296875 0 1853.5491 z 7
1084.5367431640625 0 3200.3484
1085.5413818359375 0 1836.7595
1110.53857421875 0 1536.5007
1125.5771484375 0 3269.5542
1126.56591796875 0 14448.34 c 8
1127.5679931640625 0 10498.724
1128.5682373046875 0 3669.6147
1129.5760498046875 0 1089.2297
1140.550537109375 0 959.3487
1141.5548095703125 0 1846.1753
1142.553466796875 0 1091.1606
1198.5665283203125 0 3096.7104 z 6
1199.563720703125 0 9020.075
1200.564208984375 0 4972.333
1201.5623779296875 0 2564.2737
1212.5894775390625 0 1212.3579
1213.5977783203125 0 1056.0087
1214.5771484375 0 1221.5645 y 6
1239.6031494140625 0 913.5753
1254.625244140625 0 1151.6998
1255.611328125 0 4225.439 c 9
1256.612060546875 0 3714.5557
1257.6136474609375 0 1577.207
1311.65869140625 0 1226.5057 z 5
1312.6451416015625 0 4127.8794
1313.6451416015625 0 1896.9061
1374.666015625 0 1016.67236
1375.669189453125 0 1958.1177
1376.6636962890625 0 947.9532
1381.67578125 0 2475.7695
1382.674072265625 0 3206.098
1383.681640625 0 1845.3151
1418.67529296875 0 1793.5513 c 10
1419.674560546875 0 3395.119
1420.674560546875 0 1980.5077
1512.7421875 0 1139.5676
1513.72802734375 0 1766.8716 z 4
1514.7337646484375 0 1390.5342
1529.73193359375 0 867.2545 c Ammonia loss 11
1545.095947265625 0 766.70544
1546.7603759765625 0 1860.0028 c 11
1547.7703857421875 0 1446.7576
1628.745361328125 0 1013.06366 z 3
1629.74169921875 0 3925.8264
1630.7470703125 0 2761.2576
1631.7470703125 0 1435.9146
1706.7894287109375 0 1004.3336
1707.785888671875 0 2117.9414 c 12
1708.7825927734375 0 2201.1758
1709.7845458984375 0 1237.9071
1710.7999267578125 0 930.2334
1756.8006591796875 0 2166.546 z 2
1757.801513671875 0 2607.6096
1758.8067626953125 0 2738.3364
1759.8157958984375 0 1863.7861
1836.8988037109375 0 969.63135
1894.8624267578125 0 807.8451
1895.852783203125 0 787.0602
1935.96875 0 726.14703
1936.9710693359375 0 790.39667
1937.966064453125 0 1260.2993
1948.9515380859375 0 1155.7363
1965.9525146484375 0 1770.6362
1966.9622802734375 0 1372.6354
1967.9449462890625 0 1397.6184
1981.9610595703125 0 743.3261
2006.9613037109375 0 699.3178
2007.9554443359375 0 1929.6282
2008.9576416015625 0 2956.3486
2009.9490966796875 0 2028.4061
2010.97021484375 0 1212.9578
2023.9696044921875 0 922.1865
2024.97705078125 0 2965.3381
2025.974609375 0 2828.1785
2026.9786376953125 0 2113.796
2027.96826171875 0 1354.24
2029.014892578125 0 946.53107

Spectrum Details

|  |  |
| --- | --- |
| Matched peaks? Matched peaksThe total absolute number of peaks matched. Additionally in brackets the total fraction of peaks matched and the total number of peaks is shown. | 88 (16.54% of 532) |
| FDR? FDRThe false discovery rate estimated for this peptide. It is calculated by matching all theoretical fragments with a non-integer shift with the raw peaks for this spectrum. This is done with 40 different shifts. The resulting percentage is the average number of annotated peaks over the number of annotated peaks with the correct spectrum. | 0.62% |
| Satellite FDR? Satellite FDRSee the FDR for details on its calculation. This satellite ion specific FDR only contains the satellite ions (d/w) for I/L/J positions. | 0.00% |
| PSM Score? PSM ScoreThe PSM Score as given by Hecklib to this annotated spectrum. It is shown with three significant figures. | 470 |

## Spectrum 5273? Spectrum 5273 The raw spectrum of this peptide as annotated by Hecklib. The fragments are coloured according to ion type (see legend). Any peaks with a star '\*' as text can be hovered over to see the full details, first the ion type second the mass shift type. By hovering over the amino acids in the peptide or ions in the legend the corresponding peaks are highlighted. By toggling the 'Unassigned' label you can turn the background (unassigned) peaks on or off in the plot. By updating the slider in the Ion legend you can update the spectrum to only show the top X% of the peaks with labels. The top X% means any peak that is within X% of the highest intensity. By dragging in the spectrum you can zoom in to a specific part of the spectrum and use 'Zoom Out' to get back to the original zoom level. The annotation of the spectrum is based on the given sequence in the peptides file and is done with different software so inconsistencies are likely. The peaks are annotated based on the given sequence, with 20 ppm tolerance.

Copy Data

### Spectrum 5273 (TSV)

#### Preview

```
Loading example...
```

*Click on the button to copy the data to your clipboard.*

Mz MinMz MaxIntensity Max

WidthHeightPeptide font sizePeptide stroke widthSpectrum font sizeSpectrum stroke widthCompact peptide

Ion legend

wxyz

abcd

OtherUnassignedIonChargePositionShow for top:%

JHQDWLDGKEYKCKK

04.36e+48.72e+41.31e+51.74e+5

Zoom Out

y+43c+24c+12z+36z+12c+37z+24y+24z+12y+12c+25y+37y+37c+514y+38z+38y+38y+411y+411z+411c+13y+25z+25y+25c+13c+26z+412y+39y+39z+39y+39w+26w+310z+310y+310y+310y+413y+310y+26y+26z+26c+414c+27c+414y+26c+27z+414z+414y+414y+414z+414y+414z+13c+28c+28w+27c+28c+14z+311c+14y+311y+311z+311c+312c+312c+14y+27z+27y+27w+312y+312y+28y+28z+28y+312w+14y+28c+29w+313c+313w+29y+313y+313z+313y+313z+14z+29z+29y+29y+29z+29c+314c+314z+14c+314y+29c+210c+210c+210y+14z+314y+314w+210z+210y+210y+210z+210y+210c+15c+15c+211c+211c+15c+211y+211z+211c+212c+212y+15z+15w+212y+15c+16y+212z+212c+16y+212w+16w+213c+213z+213z+213y+213y+16z+16c+17c+214y+16c+17z+214c+18c+18z+17z+18y+18c+19z+19c+110z+110c+111z+111

047594914241899

Fragment Matches Table

Show background peaks

| Position | Ion type | Intensity | mz Theoretical | mz Error (Th) | mz Error (ppm) | Charge | Series Number |
| --- | --- | --- | --- | --- | --- | --- | --- |
| 13 | y | 636 | 120.1 | 0.0004713 | 3.926 | +4 | 3 |
| - | - | 501.8 | 120.1 | - | - | 0 | - |
| - | - | 355.1 | 122 | - | - | 0 | - |
| - | - | 627 | 127.1 | - | - | 0 | - |
| - | - | 455.9 | 127.1 | - | - | 0 | - |
| - | - | 472.1 | 128.1 | - | - | 0 | - |
| - | - | 359.1 | 129 | - | - | 0 | - |
| - | - | 5536 | 129 | - | - | 0 | - |
| - | - | 531.8 | 129.1 | - | - | 0 | - |
| - | - | 1.455E+04 | 129.1 | - | - | 0 | - |
| - | - | 4.229E+04 | 130.1 | - | - | 0 | - |
| - | - | 482.6 | 130.1 | - | - | 0 | - |
| - | - | 3544 | 131.1 | - | - | 0 | - |
| - | - | 1571 | 131.1 | - | - | 0 | - |
| - | - | 656 | 132.1 | - | - | 0 | - |
| - | - | 3576 | 132.1 | - | - | 0 | - |
| - | - | 687.4 | 132.1 | - | - | 0 | - |
| - | - | 3729 | 133.1 | - | - | 0 | - |
| - | - | 2493 | 133.1 | - | - | 0 | - |
| - | - | 3862 | 136.1 | - | - | 0 | - |
| - | - | 397.8 | 139 | - | - | 0 | - |
| - | - | 3374 | 139 | - | - | 0 | - |
| - | - | 548.4 | 144.1 | - | - | 0 | - |
| - | - | 468.1 | 146.1 | - | - | 0 | - |
| - | - | 1402 | 146.1 | - | - | 0 | - |
| - | - | 666.4 | 147 | - | - | 0 | - |
| - | - | 962 | 147.1 | - | - | 0 | - |
| - | - | 1.559E+04 | 155.1 | - | - | 0 | - |
| - | - | 700.7 | 156.1 | - | - | 0 | - |
| - | - | 1214 | 156.1 | - | - | 0 | - |
| - | - | 815.5 | 156.1 | - | - | 0 | - |
| - | - | 1.329E+04 | 157 | - | - | 0 | - |
| - | - | 742.4 | 157.1 | - | - | 0 | - |
| - | - | 611.4 | 158.1 | - | - | 0 | - |
| - | - | 1.756E+04 | 159.1 | - | - | 0 | - |
| - | - | 554.6 | 159.1 | - | - | 0 | - |
| - | - | 1739 | 160.1 | - | - | 0 | - |
| - | - | 525 | 164.1 | - | - | 0 | - |
| - | - | 1853 | 164.1 | - | - | 0 | - |
| - | - | 1.298E+04 | 166.1 | - | - | 0 | - |
| - | - | 747.8 | 167.1 | - | - | 0 | - |
| - | - | 1217 | 168.1 | - | - | 0 | - |
| - | - | 9040 | 170.1 | - | - | 0 | - |
| - | - | 940.1 | 171.1 | - | - | 0 | - |
| - | - | 548.1 | 171.1 | - | - | 0 | - |
| - | - | 2998 | 172.1 | - | - | 0 | - |
| - | - | 2582 | 173.1 | - | - | 0 | - |
| - | - | 599.2 | 173.1 | - | - | 0 | - |
| - | - | 981.8 | 173.4 | - | - | 0 | - |
| - | - | 1722 | 174.1 | - | - | 0 | - |
| - | - | 1894 | 177.1 | - | - | 0 | - |
| - | - | 1233 | 178.1 | - | - | 0 | - |
| - | - | 5444 | 183.1 | - | - | 0 | - |
| - | - | 660.5 | 183.1 | - | - | 0 | - |
| - | - | 783.3 | 183.1 | - | - | 0 | - |
| - | - | 718.5 | 183.1 | - | - | 0 | - |
| - | - | 953.4 | 185.1 | - | - | 0 | - |
| - | - | 1.605E+04 | 185.1 | - | - | 0 | - |
| - | - | 936.8 | 185.1 | - | - | 0 | - |
| - | - | 1.228E+04 | 185.2 | - | - | 0 | - |
| - | - | 505.1 | 186.1 | - | - | 0 | - |
| - | - | 473.3 | 186.1 | - | - | 0 | - |
| - | - | 3167 | 186.1 | - | - | 0 | - |
| - | - | 1177 | 186.2 | - | - | 0 | - |
| - | - | 3813 | 187.1 | - | - | 0 | - |
| - | - | 1278 | 187.1 | - | - | 0 | - |
| - | - | 499.7 | 189.1 | - | - | 0 | - |
| - | - | 7604 | 190.1 | - | - | 0 | - |
| - | - | 916.7 | 192.1 | - | - | 0 | - |
| - | - | 2467 | 198.1 | - | - | 0 | - |
| - | - | 2882 | 199.2 | - | - | 0 | - |
| - | - | 2179 | 201.1 | - | - | 0 | - |
| - | - | 570.7 | 202.1 | - | - | 0 | - |
| - | - | 1537 | 203.1 | - | - | 0 | - |
| - | - | 958.7 | 203.2 | - | - | 0 | - |
| - | - | 7068 | 205.1 | - | - | 0 | - |
| - | - | 1074 | 207.2 | - | - | 0 | - |
| - | - | 574.7 | 209.2 | - | - | 0 | - |
| - | - | 1606 | 210.1 | - | - | 0 | - |
| - | - | 1078 | 211.6 | - | - | 0 | - |
| - | - | 8734 | 213.1 | - | - | 0 | - |
| - | - | 659.5 | 215.1 | - | - | 0 | - |
| - | - | 1.219E+04 | 217 | - | - | 0 | - |
| - | - | 600.1 | 221.1 | - | - | 0 | - |
| - | - | 1605 | 223.1 | - | - | 0 | - |
| - | - | 1.216E+04 | 223.2 | - | - | 0 | - |
| - | - | 1367 | 224.2 | - | - | 0 | - |
| - | - | 499.7 | 225.1 | - | - | 0 | - |
| - | - | 722.6 | 226.2 | - | - | 0 | - |
| - | - | 882.7 | 227.1 | - | - | 0 | - |
| - | - | 543.3 | 227.1 | - | - | 0 | - |
| - | - | 551.9 | 229.1 | - | - | 0 | - |
| - | - | 5765 | 229.1 | - | - | 0 | - |
| - | - | 676.7 | 229.2 | - | - | 0 | - |
| - | - | 690 | 230.1 | - | - | 0 | - |
| - | - | 3107 | 233.1 | - | - | 0 | - |
| - | - | 1.258E+04 | 234.1 | - | - | 0 | - |
| - | - | 1902 | 235.1 | - | - | 0 | - |
| - | - | 1609 | 235.2 | - | - | 0 | - |
| - | - | 620.4 | 236.1 | - | - | 0 | - |
| - | - | 2017 | 236.6 | - | - | 0 | - |
| - | - | 703 | 241.1 | - | - | 0 | - |
| - | - | 990.3 | 244.1 | - | - | 0 | - |
| - | - | 733.2 | 245.1 | - | - | 0 | - |
| - | - | 833.9 | 246.1 | - | - | 0 | - |
| - | - | 1313 | 246.1 | - | - | 0 | - |
| - | - | 731.1 | 247.1 | - | - | 0 | - |
| 4 | c | 2906 | 247.6 | 0.0003546 | 1.432 | +2 | 4 |
| - | - | 1990 | 250.1 | - | - | 0 | - |
| - | - | 1533 | 250.1 | - | - | 0 | - |
| - | - | 2145 | 250.2 | - | - | 0 | - |
| - | - | 5.376E+04 | 251.2 | - | - | 0 | - |
| - | - | 7401 | 252.2 | - | - | 0 | - |
| - | - | 651.4 | 252.6 | - | - | 0 | - |
| - | - | 1178 | 255.2 | - | - | 0 | - |
| - | - | 720.2 | 256.6 | - | - | 0 | - |
| - | - | 2.212E+04 | 259.2 | - | - | 0 | - |
| - | - | 1674 | 261.1 | - | - | 0 | - |
| - | - | 1752 | 264.2 | - | - | 0 | - |
| - | - | 1765 | 265.1 | - | - | 0 | - |
| - | - | 767.1 | 265.6 | - | - | 0 | - |
| - | - | 1503 | 266.1 | - | - | 0 | - |
| 2 | c | 1.009E+04 | 268.2 | 0.0004142 | 1.544 | +1 | 2 |
| - | - | 2114 | 269.1 | - | - | 0 | - |
| - | - | 1692 | 269.2 | - | - | 0 | - |
| - | - | 6347 | 270.1 | - | - | 0 | - |
| - | - | 671.4 | 270.2 | - | - | 0 | - |
| - | - | 831.3 | 270.6 | - | - | 0 | - |
| - | - | 1962 | 272.1 | - | - | 0 | - |
| - | - | 1464 | 272.2 | - | - | 0 | - |
| - | - | 7656 | 273.1 | - | - | 0 | - |
| - | - | 1500 | 274.1 | - | - | 0 | - |
| - | - | 1.014E+04 | 274.1 | - | - | 0 | - |
| - | - | 2823 | 274.7 | - | - | 0 | - |
| - | - | 917 | 275.2 | - | - | 0 | - |
| - | - | 895.4 | 279.2 | - | - | 0 | - |
| - | - | 659.8 | 280.2 | - | - | 0 | - |
| - | - | 5185 | 282.2 | - | - | 0 | - |
| - | - | 1219 | 283.1 | - | - | 0 | - |
| - | - | 1099 | 284.1 | - | - | 0 | - |
| - | - | 845 | 285.2 | - | - | 0 | - |
| - | - | 977.4 | 286.1 | - | - | 0 | - |
| - | - | 976 | 288.2 | - | - | 0 | - |
| - | - | 3505 | 290.1 | - | - | 0 | - |
| - | - | 687.5 | 292.2 | - | - | 0 | - |
| 10 | z | 1057 | 294.1 | 0.005451 | 18.53 | +3 | 6 |
| - | - | 1903 | 294.2 | - | - | 0 | - |
| - | - | 1349 | 295.1 | - | - | 0 | - |
| - | - | 918.8 | 296.2 | - | - | 0 | - |
| - | - | 854.7 | 297.1 | - | - | 0 | - |
| - | - | 686.6 | 297.2 | - | - | 0 | - |
| 14 | z | 963.9 | 300.2 | 0.001046 | 3.485 | +1 | 2 |
| - | - | 1243 | 301.2 | - | - | 0 | - |
| - | - | 1380 | 302.1 | - | - | 0 | - |
| 7 | c | 6899 | 303.2 | 7.267E-05 | 0.2397 | +3 | 7 |
| 12 | z | 2399 | 303.7 | 0.005174 | 17.04 | +2 | 4 |
| - | - | 1070 | 304.2 | - | - | 0 | - |
| - | - | 772.8 | 307.1 | - | - | 0 | - |
| - | - | 1475 | 309.1 | - | - | 0 | - |
| - | - | 694.3 | 309.6 | - | - | 0 | - |
| 12 | y | 6768 | 311.7 | 0.003885 | 12.47 | +2 | 4 |
| - | - | 1863 | 312.2 | - | - | 0 | - |
| - | - | 773.2 | 315.2 | - | - | 0 | - |
| 14 | z | 2993 | 317.2 | 0.003731 | 11.76 | +1 | 2 |
| - | - | 2402 | 317.7 | - | - | 0 | - |
| - | - | 7405 | 318.2 | - | - | 0 | - |
| - | - | 1102 | 318.2 | - | - | 0 | - |
| - | - | 2310 | 318.7 | - | - | 0 | - |
| - | - | 666.5 | 319.2 | - | - | 0 | - |
| - | - | 1241 | 323.1 | - | - | 0 | - |
| - | - | 3.289E+04 | 326.7 | - | - | 0 | - |
| - | - | 1.337E+04 | 327.2 | - | - | 0 | - |
| - | - | 3032 | 327.7 | - | - | 0 | - |
| - | - | 3462 | 329.2 | - | - | 0 | - |
| - | - | 815.9 | 329.2 | - | - | 0 | - |
| - | - | 4070 | 331.7 | - | - | 0 | - |
| - | - | 1915 | 332.2 | - | - | 0 | - |
| - | - | 669.1 | 332.2 | - | - | 0 | - |
| 14 | y | 2719 | 333.2 | 0.004053 | 12.16 | +1 | 2 |
| - | - | 1062 | 337.8 | - | - | 0 | - |
| - | - | 625.9 | 338.2 | - | - | 0 | - |
| - | - | 700.1 | 338.7 | - | - | 0 | - |
| - | - | 1544 | 339.7 | - | - | 0 | - |
| - | - | 901.5 | 340.2 | - | - | 0 | - |
| 5 | c | 1.36E+04 | 340.7 | 0.0007146 | 2.098 | +2 | 5 |
| - | - | 6605 | 341.2 | - | - | 0 | - |
| - | - | 1358 | 341.7 | - | - | 0 | - |
| 9 | y | 711.4 | 342.5 | 0.002357 | 6.881 | +3 | 7 |
| 9 | y | 563.2 | 348.2 | 0.002092 | 6.008 | +3 | 7 |
| - | - | 2159 | 349.2 | - | - | 0 | - |
| - | - | 1523 | 350.2 | - | - | 0 | - |
| - | - | 634 | 350.5 | - | - | 0 | - |
| - | - | 678.5 | 350.8 | - | - | 0 | - |
| - | - | 1341 | 351.2 | - | - | 0 | - |
| - | - | 1181 | 353.7 | - | - | 0 | - |
| - | - | 649.9 | 359.2 | - | - | 0 | - |
| - | - | 1034 | 360.7 | - | - | 0 | - |
| 14 | c | 1211 | 361.2 | 0.001756 | 4.863 | +5 | 14 |
| - | - | 662.5 | 361.2 | - | - | 0 | - |
| - | - | 3926 | 361.2 | - | - | 0 | - |
| 8 | y | 1285 | 361.5 | 0.003184 | 8.807 | +3 | 8 |
| 8 | z | 1098 | 361.9 | 0.001532 | 4.233 | +3 | 8 |
| - | - | 1077 | 362.2 | - | - | 0 | - |
| - | - | 2072 | 363.2 | - | - | 0 | - |
| - | - | 1228 | 364.2 | - | - | 0 | - |
| - | - | 680.7 | 364.7 | - | - | 0 | - |
| - | - | 1350 | 365.9 | - | - | 0 | - |
| - | - | 628.8 | 366.2 | - | - | 0 | - |
| 8 | y | 2.619E+04 | 367.2 | 0.002838 | 7.728 | +3 | 8 |
| - | - | 1.618E+04 | 367.5 | - | - | 0 | - |
| - | - | 1479 | 367.7 | - | - | 0 | - |
| - | - | 4920 | 367.9 | - | - | 0 | - |
| - | - | 545.8 | 368.1 | - | - | 0 | - |
| - | - | 2514 | 368.2 | - | - | 0 | - |
| - | - | 1081 | 369.2 | - | - | 0 | - |
| - | - | 1126 | 371.2 | - | - | 0 | - |
| - | - | 1805 | 374.2 | - | - | 0 | - |
| 5 | y | 5628 | 374.7 | 0.00452 | 12.06 | +4 | 11 |
| 5 | y | 3242 | 374.9 | 0.00125 | 3.334 | +4 | 11 |
| 5 | z | 2789 | 375.2 | 0.006956 | 18.54 | +4 | 11 |
| - | - | 724.4 | 375.4 | - | - | 0 | - |
| - | - | 699.7 | 375.7 | - | - | 0 | - |
| - | - | 1398 | 376.2 | - | - | 0 | - |
| - | - | 2584 | 378.2 | - | - | 0 | - |
| - | - | 859.5 | 378.2 | - | - | 0 | - |
| 3 | c | 1.503E+04 | 379.2 | 0.0003072 | 0.81 | +1 | 3 |
| - | - | 512.3 | 379.2 | - | - | 0 | - |
| - | - | 833.6 | 379.5 | - | - | 0 | - |
| - | - | 1545 | 379.7 | - | - | 0 | - |
| - | - | 2595 | 380.2 | - | - | 0 | - |
| - | - | 646 | 380.2 | - | - | 0 | - |
| - | - | 2048 | 381.2 | - | - | 0 | - |
| - | - | 758.8 | 381.5 | - | - | 0 | - |
| - | - | 1.131E+04 | 383.2 | - | - | 0 | - |
| - | - | 1033 | 383.2 | - | - | 0 | - |
| - | - | 6575 | 383.7 | - | - | 0 | - |
| - | - | 1997 | 384.2 | - | - | 0 | - |
| - | - | 1283 | 384.2 | - | - | 0 | - |
| 11 | y | 1108 | 384.7 | 0.004575 | 11.89 | +2 | 5 |
| 11 | z | 6479 | 385.2 | 0.002353 | 6.109 | +2 | 5 |
| - | - | 2.012E+04 | 385.2 | - | - | 0 | - |
| - | - | 8743 | 385.5 | - | - | 0 | - |
| - | - | 4603 | 385.9 | - | - | 0 | - |
| - | - | 1683 | 386.2 | - | - | 0 | - |
| - | - | 2622 | 386.2 | - | - | 0 | - |
| - | - | 768.6 | 386.5 | - | - | 0 | - |
| - | - | 3.096E+04 | 387.2 | - | - | 0 | - |
| - | - | 901.1 | 388.2 | - | - | 0 | - |
| - | - | 6585 | 388.2 | - | - | 0 | - |
| - | - | 962.7 | 388.7 | - | - | 0 | - |
| - | - | 3506 | 389.2 | - | - | 0 | - |
| - | - | 988.9 | 389.2 | - | - | 0 | - |
| - | - | 826.3 | 390.2 | - | - | 0 | - |
| 11 | y | 2.18E+04 | 393.2 | 0.004055 | 10.31 | +2 | 5 |
| - | - | 1.012E+04 | 393.7 | - | - | 0 | - |
| - | - | 2922 | 394.2 | - | - | 0 | - |
| - | - | 1084 | 394.7 | - | - | 0 | - |
| - | - | 1043 | 395.2 | - | - | 0 | - |
| - | - | 1648 | 395.4 | - | - | 0 | - |
| - | - | 1228 | 395.6 | - | - | 0 | - |
| 3 | c | 2.445E+04 | 396.2 | 0.0006746 | 1.702 | +1 | 3 |
| 6 | c | 1.015E+04 | 397.2 | 0.0006748 | 1.699 | +2 | 6 |
| - | - | 5810 | 397.2 | - | - | 0 | - |
| - | - | 5158 | 397.7 | - | - | 0 | - |
| - | - | 2461 | 398.2 | - | - | 0 | - |
| - | - | 1028 | 398.2 | - | - | 0 | - |
| - | - | 2612 | 398.6 | - | - | 0 | - |
| - | - | 3290 | 398.8 | - | - | 0 | - |
| - | - | 5541 | 399 | - | - | 0 | - |
| - | - | 2785 | 399.2 | - | - | 0 | - |
| - | - | 713 | 399.4 | - | - | 0 | - |
| 4 | z | 691.5 | 399.4 | 0.002723 | 6.818 | +4 | 12 |
| 7 | y | 1492 | 399.5 | 0.003496 | 8.75 | +3 | 9 |
| 7 | y | 709.4 | 399.9 | 0.001209 | 3.024 | +3 | 9 |
| 7 | z | 1706 | 400.2 | 0.0005725 | 1.431 | +3 | 9 |
| - | - | 1269 | 401.2 | - | - | 0 | - |
| - | - | 2225 | 401.2 | - | - | 0 | - |
| - | - | 2857 | 401.3 | - | - | 0 | - |
| - | - | 678.6 | 402 | - | - | 0 | - |
| - | - | 4105 | 402.2 | - | - | 0 | - |
| - | - | 832.4 | 402.2 | - | - | 0 | - |
| - | - | 2537 | 402.3 | - | - | 0 | - |
| - | - | 6052 | 402.4 | - | - | 0 | - |
| - | - | 2149 | 402.6 | - | - | 0 | - |
| - | - | 1347 | 402.8 | - | - | 0 | - |
| - | - | 962.5 | 403.2 | - | - | 0 | - |
| - | - | 942.9 | 403.2 | - | - | 0 | - |
| - | - | 824.1 | 403.3 | - | - | 0 | - |
| - | - | 714.2 | 403.6 | - | - | 0 | - |
| - | - | 3067 | 404.2 | - | - | 0 | - |
| - | - | 1853 | 404.7 | - | - | 0 | - |
| 7 | y | 8987 | 405.5 | 0.002196 | 5.414 | +3 | 9 |
| - | - | 8297 | 405.9 | - | - | 0 | - |
| - | - | 1858 | 406.2 | - | - | 0 | - |
| - | - | 754.7 | 409.1 | - | - | 0 | - |
| - | - | 671 | 409.2 | - | - | 0 | - |
| - | - | 734.9 | 413.9 | - | - | 0 | - |
| - | - | 1376 | 418.2 | - | - | 0 | - |
| 10 | w | 1.443E+05 | 420.2 | 0.003722 | 8.858 | +2 | 6 |
| - | - | 6.483E+04 | 420.7 | - | - | 0 | - |
| - | - | 2.045E+04 | 421.2 | - | - | 0 | - |
| - | - | 4679 | 421.7 | - | - | 0 | - |
| - | - | 935.8 | 422.2 | - | - | 0 | - |
| - | - | 819.4 | 423.2 | - | - | 0 | - |
| 6 | w | 1.302E+04 | 423.5 | 0.002147 | 5.068 | +3 | 10 |
| - | - | 7600 | 423.9 | - | - | 0 | - |
| - | - | 3177 | 424.2 | - | - | 0 | - |
| - | - | 796.6 | 424.5 | - | - | 0 | - |
| - | - | 1213 | 430.2 | - | - | 0 | - |
| - | - | 1022 | 431.2 | - | - | 0 | - |
| 6 | z | 895.5 | 432.2 | 0.004943 | 11.44 | +3 | 10 |
| - | - | 830.6 | 434.2 | - | - | 0 | - |
| - | - | 1032 | 435.2 | - | - | 0 | - |
| - | - | 1294 | 435.2 | - | - | 0 | - |
| - | - | 1107 | 436.2 | - | - | 0 | - |
| 6 | y | 1543 | 437.2 | 0.002047 | 4.682 | +3 | 10 |
| 6 | y | 2186 | 437.6 | 0.003291 | 7.521 | +3 | 10 |
| - | - | 932.7 | 438.7 | - | - | 0 | - |
| - | - | 747.3 | 439.2 | - | - | 0 | - |
| - | - | 706.5 | 439.7 | - | - | 0 | - |
| 3 | y | 776.5 | 440 | 0.002532 | 5.755 | +4 | 13 |
| - | - | 1504 | 440.7 | - | - | 0 | - |
| - | - | 806.9 | 441.2 | - | - | 0 | - |
| - | - | 1140 | 442.9 | - | - | 0 | - |
| - | - | 1229 | 443.2 | - | - | 0 | - |
| 6 | y | 1.963E+04 | 443.2 | 0.001968 | 4.44 | +3 | 10 |
| - | - | 1.424E+04 | 443.6 | - | - | 0 | - |
| - | - | 3615 | 443.9 | - | - | 0 | - |
| - | - | 1794 | 444.2 | - | - | 0 | - |
| - | - | 1194 | 445.7 | - | - | 0 | - |
| - | - | 1688 | 446.2 | - | - | 0 | - |
| - | - | 713.4 | 446.7 | - | - | 0 | - |
| - | - | 2288 | 447.2 | - | - | 0 | - |
| - | - | 1941 | 448.2 | - | - | 0 | - |
| 10 | y | 2642 | 448.7 | 0.003498 | 7.796 | +2 | 6 |
| 10 | y | 1084 | 449.2 | 0.005379 | 11.97 | +2 | 6 |
| 10 | z | 2738 | 449.7 | 0.002226 | 4.951 | +2 | 6 |
| - | - | 1175 | 450.2 | - | - | 0 | - |
| 14 | c | 805.5 | 451.2 | 0.003148 | 6.977 | +4 | 14 |
| - | - | 871.2 | 451.9 | - | - | 0 | - |
| - | - | 865.1 | 452.2 | - | - | 0 | - |
| - | - | 1728 | 452.2 | - | - | 0 | - |
| - | - | 1034 | 452.5 | - | - | 0 | - |
| - | - | 865.6 | 452.7 | - | - | 0 | - |
| - | - | 685.9 | 453 | - | - | 0 | - |
| - | - | 885.9 | 453.6 | - | - | 0 | - |
| - | - | 853.1 | 454.6 | - | - | 0 | - |
| 7 | c | 1.269E+04 | 454.7 | 0.001089 | 2.394 | +2 | 7 |
| - | - | 1214 | 455 | - | - | 0 | - |
| - | - | 6545 | 455.2 | - | - | 0 | - |
| 14 | c | 3692 | 455.7 | 0.004986 | 10.94 | +4 | 14 |
| - | - | 1563 | 456 | - | - | 0 | - |
| - | - | 2314 | 456.2 | - | - | 0 | - |
| - | - | 639.4 | 456.7 | - | - | 0 | - |
| 10 | y | 3438 | 457.7 | 0.003379 | 7.382 | +2 | 6 |
| - | - | 1949 | 458.2 | - | - | 0 | - |
| - | - | 625.8 | 459 | - | - | 0 | - |
| - | - | 830.7 | 459.2 | - | - | 0 | - |
| - | - | 796.7 | 459.5 | - | - | 0 | - |
| - | - | 1588 | 459.7 | - | - | 0 | - |
| - | - | 1185 | 460 | - | - | 0 | - |
| - | - | 841.4 | 460.2 | - | - | 0 | - |
| - | - | 1.929E+04 | 460.7 | - | - | 0 | - |
| - | - | 9533 | 461.2 | - | - | 0 | - |
| - | - | 1027 | 461.6 | - | - | 0 | - |
| - | - | 3615 | 461.7 | - | - | 0 | - |
| - | - | 2820 | 462.2 | - | - | 0 | - |
| - | - | 1301 | 462.2 | - | - | 0 | - |
| - | - | 1954 | 462.7 | - | - | 0 | - |
| 7 | c | 1102 | 463.2 | 0.000528 | 1.14 | +2 | 7 |
| - | - | 2438 | 465.5 | - | - | 0 | - |
| 2 | z | 1861 | 465.7 | 0.00109 | 2.34 | +4 | 14 |
| 2 | z | 1348 | 466 | 0.008949 | 19.21 | +4 | 14 |
| - | - | 3374 | 466.2 | - | - | 0 | - |
| - | - | 2672 | 467.3 | - | - | 0 | - |
| - | - | 8385 | 468.3 | - | - | 0 | - |
| - | - | 930 | 468.3 | - | - | 0 | - |
| - | - | 5177 | 468.8 | - | - | 0 | - |
| - | - | 2619 | 469.2 | - | - | 0 | - |
| - | - | 899.8 | 469.5 | - | - | 0 | - |
| 2 | y | 2185 | 469.7 | 0.002292 | 4.879 | +4 | 14 |
| 2 | y | 7205 | 470 | 0.008235 | 17.52 | +4 | 14 |
| 2 | z | 7842 | 470.2 | 0.007103 | 15.11 | +4 | 14 |
| - | - | 1164 | 470.3 | - | - | 0 | - |
| - | - | 4129 | 470.5 | - | - | 0 | - |
| - | - | 1053 | 470.7 | - | - | 0 | - |
| - | - | 732.3 | 473 | - | - | 0 | - |
| - | - | 1672 | 473.2 | - | - | 0 | - |
| - | - | 779.8 | 473.5 | - | - | 0 | - |
| - | - | 2470 | 473.7 | - | - | 0 | - |
| - | - | 901 | 473.9 | - | - | 0 | - |
| - | - | 2410 | 474 | - | - | 0 | - |
| 2 | y | 7819 | 474.2 | 0.0005994 | 1.264 | +4 | 14 |
| - | - | 7247 | 474.5 | - | - | 0 | - |
| - | - | 4101 | 474.7 | - | - | 0 | - |
| - | - | 2260 | 476 | - | - | 0 | - |
| - | - | 854.7 | 476.2 | - | - | 0 | - |
| - | - | 1827 | 476.5 | - | - | 0 | - |
| - | - | 2227 | 476.7 | - | - | 0 | - |
| - | - | 1184 | 477 | - | - | 0 | - |
| - | - | 3250 | 477.2 | - | - | 0 | - |
| - | - | 993.6 | 477.5 | - | - | 0 | - |
| 13 | z | 1903 | 478.2 | 0.0009191 | 1.922 | +1 | 3 |
| - | - | 923.6 | 479.2 | - | - | 0 | - |
| - | - | 986.5 | 479.6 | - | - | 0 | - |
| - | - | 1353 | 479.7 | - | - | 0 | - |
| - | - | 1033 | 479.9 | - | - | 0 | - |
| - | - | 1672 | 480.2 | - | - | 0 | - |
| - | - | 3.646E+04 | 480.5 | - | - | 0 | - |
| - | - | 3.744E+04 | 480.7 | - | - | 0 | - |
| - | - | 1.958E+04 | 481 | - | - | 0 | - |
| - | - | 1.072E+04 | 481.2 | - | - | 0 | - |
| - | - | 3256 | 481.5 | - | - | 0 | - |
| - | - | 1163 | 481.7 | - | - | 0 | - |
| 8 | c | 4991 | 482.7 | 0.0005532 | 1.146 | +2 | 8 |
| 8 | c | 7675 | 483.2 | 0.002564 | 5.306 | +2 | 8 |
| - | - | 1602 | 483.5 | - | - | 0 | - |
| - | - | 2469 | 483.7 | - | - | 0 | - |
| - | - | 1665 | 484 | - | - | 0 | - |
| - | - | 1.108E+04 | 484.2 | - | - | 0 | - |
| - | - | 3764 | 484.5 | - | - | 0 | - |
| - | - | 759.4 | 484.6 | - | - | 0 | - |
| 9 | w | 5579 | 484.7 | 0.002955 | 6.096 | +2 | 7 |
| - | - | 2852 | 484.8 | - | - | 0 | - |
| - | - | 1561 | 485 | - | - | 0 | - |
| - | - | 3037 | 485.2 | - | - | 0 | - |
| - | - | 1607 | 485.7 | - | - | 0 | - |
| - | - | 969.2 | 487.3 | - | - | 0 | - |
| - | - | 3299 | 487.5 | - | - | 0 | - |
| - | - | 7.966E+04 | 487.7 | - | - | 0 | - |
| - | - | 7.516E+04 | 488 | - | - | 0 | - |
| - | - | 4.501E+04 | 488.2 | - | - | 0 | - |
| - | - | 2.186E+04 | 488.5 | - | - | 0 | - |
| - | - | 1.183E+04 | 488.7 | - | - | 0 | - |
| - | - | 4145 | 489 | - | - | 0 | - |
| - | - | 2522 | 489.2 | - | - | 0 | - |
| - | - | 1282 | 491.2 | - | - | 0 | - |
| 8 | c | 7289 | 491.7 | 0.001008 | 2.05 | +2 | 8 |
| - | - | 770.6 | 492 | - | - | 0 | - |
| - | - | 4909 | 492.2 | - | - | 0 | - |
| - | - | 2925 | 492.7 | - | - | 0 | - |
| 4 | c | 791.8 | 493.3 | 0.002393 | 4.852 | +1 | 4 |
| - | - | 2403 | 493.6 | - | - | 0 | - |
| 5 | z | 1650 | 493.9 | 0.00907 | 18.36 | +3 | 11 |
| 4 | c | 3.412E+04 | 494.2 | 0.003076 | 6.224 | +1 | 4 |
| - | - | 812.9 | 494.5 | - | - | 0 | - |
| - | - | 8559 | 495.2 | - | - | 0 | - |
| - | - | 784.5 | 495.8 | - | - | 0 | - |
| - | - | 887.9 | 496.2 | - | - | 0 | - |
| - | - | 1178 | 496.3 | - | - | 0 | - |
| - | - | 4140 | 496.8 | - | - | 0 | - |
| - | - | 3515 | 497.3 | - | - | 0 | - |
| - | - | 2706 | 497.8 | - | - | 0 | - |
| - | - | 4346 | 498.2 | - | - | 0 | - |
| - | - | 3653 | 498.5 | - | - | 0 | - |
| - | - | 3112 | 498.7 | - | - | 0 | - |
| - | - | 970 | 499 | - | - | 0 | - |
| 5 | y | 8597 | 499.2 | 0.008465 | 16.96 | +3 | 11 |
| - | - | 884.7 | 499.5 | - | - | 0 | - |
| 5 | y | 9583 | 499.6 | 0.002578 | 5.16 | +3 | 11 |
| - | - | 1748 | 499.7 | - | - | 0 | - |
| 5 | z | 7693 | 499.9 | 0.003131 | 6.264 | +3 | 11 |
| - | - | 5981 | 500.2 | - | - | 0 | - |
| - | - | 1947 | 500.6 | - | - | 0 | - |
| - | - | 1013 | 500.9 | - | - | 0 | - |
| - | - | 1548 | 502 | - | - | 0 | - |
| - | - | 4072 | 502.2 | - | - | 0 | - |
| - | - | 1.209E+05 | 502.5 | - | - | 0 | - |
| - | - | 1.728E+05 | 502.7 | - | - | 0 | - |
| - | - | 1.27E+05 | 503 | - | - | 0 | - |
| - | - | 5.97E+04 | 503.2 | - | - | 0 | - |
| - | - | 2.197E+04 | 503.5 | - | - | 0 | - |
| - | - | 8123 | 503.7 | - | - | 0 | - |
| - | - | 2801 | 504 | - | - | 0 | - |
| - | - | 1586 | 504.2 | - | - | 0 | - |
| 12 | c | 6470 | 505.3 | 0.002347 | 4.646 | +3 | 12 |
| - | - | 6724 | 505.6 | - | - | 0 | - |
| - | - | 3024 | 505.7 | - | - | 0 | - |
| - | - | 2631 | 505.9 | - | - | 0 | - |
| - | - | 5658 | 506.3 | - | - | 0 | - |
| - | - | 806.7 | 506.6 | - | - | 0 | - |
| - | - | 1726 | 506.7 | - | - | 0 | - |
| - | - | 899.9 | 506.9 | - | - | 0 | - |
| - | - | 1773 | 507.3 | - | - | 0 | - |
| - | - | 948.2 | 510.3 | - | - | 0 | - |
| - | - | 938.4 | 510.6 | - | - | 0 | - |
| 12 | c | 4.44E+04 | 510.9 | 0.0009084 | 1.778 | +3 | 12 |
| 4 | c | 7.929E+04 | 511.3 | 0.0008006 | 1.566 | +1 | 4 |
| - | - | 1.618E+04 | 511.6 | - | - | 0 | - |
| - | - | 6261 | 511.9 | - | - | 0 | - |
| - | - | 1.278E+04 | 512.3 | - | - | 0 | - |
| 9 | y | 1839 | 512.8 | 0.005051 | 9.85 | +2 | 7 |
| - | - | 1522 | 513.2 | - | - | 0 | - |
| - | - | 2072 | 513.3 | - | - | 0 | - |
| 9 | z | 1.995E+04 | 513.8 | 0.004023 | 7.831 | +2 | 7 |
| - | - | 1.7E+04 | 514.3 | - | - | 0 | - |
| - | - | 1565 | 514.6 | - | - | 0 | - |
| - | - | 7038 | 514.8 | - | - | 0 | - |
| - | - | 1273 | 514.9 | - | - | 0 | - |
| - | - | 1162 | 515.3 | - | - | 0 | - |
| - | - | 7144 | 515.3 | - | - | 0 | - |
| - | - | 877.4 | 515.8 | - | - | 0 | - |
| - | - | 2026 | 516.3 | - | - | 0 | - |
| - | - | 992.6 | 517.3 | - | - | 0 | - |
| - | - | 1064 | 517.6 | - | - | 0 | - |
| - | - | 1211 | 518.3 | - | - | 0 | - |
| - | - | 1592 | 519.9 | - | - | 0 | - |
| - | - | 1694 | 520.3 | - | - | 0 | - |
| - | - | 1760 | 521.3 | - | - | 0 | - |
| 9 | y | 1.592E+04 | 521.8 | 0.003558 | 6.82 | +2 | 7 |
| - | - | 9707 | 522.3 | - | - | 0 | - |
| - | - | 3606 | 522.8 | - | - | 0 | - |
| 4 | w | 1257 | 523.3 | 0.009058 | 17.31 | +3 | 12 |
| - | - | 2117 | 523.6 | - | - | 0 | - |
| - | - | 1852 | 523.9 | - | - | 0 | - |
| - | - | 1203 | 525.3 | - | - | 0 | - |
| - | - | 1152 | 525.4 | - | - | 0 | - |
| - | - | 3171 | 526.3 | - | - | 0 | - |
| - | - | 1434 | 526.8 | - | - | 0 | - |
| - | - | 761.9 | 528.3 | - | - | 0 | - |
| - | - | 1010 | 529.3 | - | - | 0 | - |
| - | - | 848 | 529.6 | - | - | 0 | - |
| - | - | 1343 | 530.3 | - | - | 0 | - |
| - | - | 1168 | 530.6 | - | - | 0 | - |
| - | - | 991.2 | 533.8 | - | - | 0 | - |
| - | - | 2177 | 534.3 | - | - | 0 | - |
| - | - | 1114 | 534.3 | - | - | 0 | - |
| - | - | 2383 | 535 | - | - | 0 | - |
| - | - | 3548 | 535.3 | - | - | 0 | - |
| - | - | 5358 | 535.6 | - | - | 0 | - |
| - | - | 1.285E+04 | 535.9 | - | - | 0 | - |
| - | - | 1.259E+04 | 536.3 | - | - | 0 | - |
| - | - | 6707 | 536.6 | - | - | 0 | - |
| - | - | 4310 | 536.9 | - | - | 0 | - |
| - | - | 3288 | 537.3 | - | - | 0 | - |
| 4 | y | 795.3 | 537.9 | 0.002703 | 5.025 | +3 | 12 |
| - | - | 948.1 | 538.3 | - | - | 0 | - |
| - | - | 844.8 | 538.6 | - | - | 0 | - |
| - | - | 833.7 | 539.2 | - | - | 0 | - |
| 8 | y | 2655 | 541.3 | 0.005834 | 10.78 | +2 | 8 |
| 8 | y | 1612 | 541.8 | 0.006919 | 12.77 | +2 | 8 |
| 8 | z | 1.358E+04 | 542.3 | 0.004257 | 7.85 | +2 | 8 |
| - | - | 6972 | 542.8 | - | - | 0 | - |
| - | - | 3493 | 543.3 | - | - | 0 | - |
| 4 | y | 3348 | 543.6 | 0.002098 | 3.859 | +3 | 12 |
| - | - | 1364 | 543.8 | - | - | 0 | - |
| - | - | 4077 | 543.9 | - | - | 0 | - |
| - | - | 1696 | 544.3 | - | - | 0 | - |
| - | - | 2392 | 546.3 | - | - | 0 | - |
| - | - | 2391 | 546.8 | - | - | 0 | - |
| - | - | 4438 | 547.3 | - | - | 0 | - |
| - | - | 2043 | 547.8 | - | - | 0 | - |
| 12 | w | 1992 | 548.2 | 0.00832 | 15.17 | +1 | 4 |
| - | - | 1793 | 548.3 | - | - | 0 | - |
| - | - | 1103 | 548.8 | - | - | 0 | - |
| - | - | 2714 | 549.6 | - | - | 0 | - |
| - | - | 1197 | 549.8 | - | - | 0 | - |
| - | - | 1987 | 549.9 | - | - | 0 | - |
| 8 | y | 3.442E+04 | 550.3 | 0.004158 | 7.557 | +2 | 8 |
| - | - | 1193 | 550.6 | - | - | 0 | - |
| - | - | 2.095E+04 | 550.8 | - | - | 0 | - |
| - | - | 5788 | 551.3 | - | - | 0 | - |
| - | - | 1118 | 551.6 | - | - | 0 | - |
| - | - | 1502 | 551.8 | - | - | 0 | - |
| - | - | 2384 | 552.3 | - | - | 0 | - |
| - | - | 1252 | 554.3 | - | - | 0 | - |
| - | - | 1957 | 554.8 | - | - | 0 | - |
| - | - | 6201 | 555.3 | - | - | 0 | - |
| 9 | c | 1.4E+05 | 555.8 | 0.0002796 | 0.5031 | +2 | 9 |
| - | - | 9.141E+04 | 556.3 | - | - | 0 | - |
| - | - | 2.892E+04 | 556.8 | - | - | 0 | - |
| - | - | 1099 | 556.9 | - | - | 0 | - |
| - | - | 7323 | 557.3 | - | - | 0 | - |
| - | - | 1001 | 557.6 | - | - | 0 | - |
| - | - | 1769 | 557.8 | - | - | 0 | - |
| - | - | 783.3 | 560.9 | - | - | 0 | - |
| - | - | 1687 | 561.3 | - | - | 0 | - |
| 3 | w | 5.271E+04 | 561.6 | 0.002263 | 4.029 | +3 | 13 |
| - | - | 4.797E+04 | 561.9 | - | - | 0 | - |
| - | - | 2.494E+04 | 562.3 | - | - | 0 | - |
| - | - | 7428 | 562.6 | - | - | 0 | - |
| - | - | 1918 | 562.6 | - | - | 0 | - |
| - | - | 3769 | 562.9 | - | - | 0 | - |
| - | - | 2219 | 563.3 | - | - | 0 | - |
| - | - | 1165 | 563.3 | - | - | 0 | - |
| - | - | 7296 | 563.8 | - | - | 0 | - |
| - | - | 999.1 | 563.9 | - | - | 0 | - |
| - | - | 4383 | 564.3 | - | - | 0 | - |
| - | - | 1069 | 564.3 | - | - | 0 | - |
| 13 | c | 2.725E+04 | 564.6 | 0.0009495 | 1.682 | +3 | 13 |
| - | - | 1673 | 564.8 | - | - | 0 | - |
| - | - | 2.445E+04 | 564.9 | - | - | 0 | - |
| - | - | 1.358E+04 | 565.3 | - | - | 0 | - |
| - | - | 7145 | 565.6 | - | - | 0 | - |
| - | - | 3279 | 565.9 | - | - | 0 | - |
| - | - | 4654 | 566.3 | - | - | 0 | - |
| - | - | 4019 | 566.6 | - | - | 0 | - |
| - | - | 2604 | 566.9 | - | - | 0 | - |
| - | - | 2706 | 567.2 | - | - | 0 | - |
| - | - | 2579 | 567.3 | - | - | 0 | - |
| - | - | 908.8 | 568.2 | - | - | 0 | - |
| - | - | 1658 | 568.3 | - | - | 0 | - |
| - | - | 5113 | 568.8 | - | - | 0 | - |
| - | - | 2168 | 569.3 | - | - | 0 | - |
| - | - | 1014 | 569.8 | - | - | 0 | - |
| - | - | 796.5 | 569.9 | - | - | 0 | - |
| - | - | 3557 | 570.3 | - | - | 0 | - |
| - | - | 4351 | 570.8 | - | - | 0 | - |
| - | - | 2096 | 571.3 | - | - | 0 | - |
| - | - | 2566 | 571.8 | - | - | 0 | - |
| - | - | 972.2 | 572.3 | - | - | 0 | - |
| - | - | 1864 | 573 | - | - | 0 | - |
| - | - | 1807 | 573.3 | - | - | 0 | - |
| - | - | 805.1 | 574 | - | - | 0 | - |
| - | - | 1109 | 574.3 | - | - | 0 | - |
| 7 | w | 2406 | 577.3 | 0.005351 | 9.27 | +2 | 9 |
| - | - | 918.4 | 577.3 | - | - | 0 | - |
| - | - | 1.286E+04 | 577.8 | - | - | 0 | - |
| - | - | 1630 | 578 | - | - | 0 | - |
| - | - | 8608 | 578.3 | - | - | 0 | - |
| - | - | 9505 | 578.8 | - | - | 0 | - |
| - | - | 3878 | 579.3 | - | - | 0 | - |
| - | - | 1466 | 579.8 | - | - | 0 | - |
| 3 | y | 1175 | 580.3 | 0.003739 | 6.443 | +3 | 13 |
| 3 | y | 2344 | 580.6 | 0.002119 | 3.649 | +3 | 13 |
| 3 | z | 1.349E+04 | 580.9 | 0.002768 | 4.766 | +3 | 13 |
| - | - | 1.197E+04 | 581.3 | - | - | 0 | - |
| - | - | 5006 | 581.6 | - | - | 0 | - |
| - | - | 2090 | 581.9 | - | - | 0 | - |
| - | - | 4503 | 582.3 | - | - | 0 | - |
| - | - | 1570 | 583.3 | - | - | 0 | - |
| - | - | 962.8 | 585.8 | - | - | 0 | - |
| 3 | y | 7628 | 586.3 | 0.001218 | 2.077 | +3 | 13 |
| - | - | 5568 | 586.6 | - | - | 0 | - |
| - | - | 2449 | 586.9 | - | - | 0 | - |
| - | - | 3020 | 587.3 | - | - | 0 | - |
| - | - | 1965 | 587.6 | - | - | 0 | - |
| - | - | 1016 | 588 | - | - | 0 | - |
| - | - | 7575 | 588.3 | - | - | 0 | - |
| - | - | 6053 | 588.6 | - | - | 0 | - |
| - | - | 2639 | 589 | - | - | 0 | - |
| 12 | z | 841.5 | 589.3 | 0.00234 | 3.97 | +1 | 4 |
| 7 | z | 2303 | 590.8 | 0.004095 | 6.931 | +2 | 9 |
| 7 | z | 2913 | 591.3 | 0.009279 | 15.69 | +2 | 9 |
| - | - | 1087 | 592 | - | - | 0 | - |
| - | - | 1645 | 592.3 | - | - | 0 | - |
| - | - | 8990 | 592.6 | - | - | 0 | - |
| - | - | 8127 | 593 | - | - | 0 | - |
| - | - | 3515 | 593.3 | - | - | 0 | - |
| - | - | 1217 | 593.6 | - | - | 0 | - |
| - | - | 1078 | 597 | - | - | 0 | - |
| - | - | 1758 | 597.3 | - | - | 0 | - |
| - | - | 2756 | 598.3 | - | - | 0 | - |
| 7 | y | 2716 | 598.8 | 0.007001 | 11.69 | +2 | 9 |
| 7 | y | 4095 | 599.3 | 0.003579 | 5.972 | +2 | 9 |
| 7 | z | 4.241E+04 | 599.8 | 0.003324 | 5.542 | +2 | 9 |
| - | - | 4.448E+04 | 600.3 | - | - | 0 | - |
| - | - | 2.494E+04 | 600.8 | - | - | 0 | - |
| 14 | c | 8928 | 601.3 | 0.009225 | 15.34 | +3 | 14 |
| 14 | c | 2762 | 601.6 | 0.00481 | 7.996 | +3 | 14 |
| - | - | 2152 | 601.8 | - | - | 0 | - |
| - | - | 2339 | 602 | - | - | 0 | - |
| - | - | 1759 | 602.3 | - | - | 0 | - |
| - | - | 2667 | 602.6 | - | - | 0 | - |
| - | - | 1160 | 602.8 | - | - | 0 | - |
| - | - | 7676 | 602.9 | - | - | 0 | - |
| - | - | 9119 | 603.3 | - | - | 0 | - |
| - | - | 2875 | 603.6 | - | - | 0 | - |
| - | - | 1306 | 604 | - | - | 0 | - |
| - | - | 1021 | 604.3 | - | - | 0 | - |
| 12 | z | 3.034E+04 | 606.3 | 0.007771 | 12.82 | +1 | 4 |
| - | - | 2084 | 606.6 | - | - | 0 | - |
| - | - | 9579 | 607 | - | - | 0 | - |
| 14 | c | 5.026E+04 | 607.3 | 0.0006807 | 1.121 | +3 | 14 |
| - | - | 3.887E+04 | 607.6 | - | - | 0 | - |
| 7 | y | 4.638E+04 | 607.8 | 0.003714 | 6.11 | +2 | 9 |
| - | - | 2.202E+04 | 608 | - | - | 0 | - |
| - | - | 3.874E+04 | 608.3 | - | - | 0 | - |
| - | - | 2172 | 608.6 | - | - | 0 | - |
| - | - | 1.189E+04 | 608.8 | - | - | 0 | - |
| - | - | 4466 | 609.3 | - | - | 0 | - |
| - | - | 5381 | 610.3 | - | - | 0 | - |
| 10 | c | 2703 | 611.3 | 0.007312 | 11.96 | +2 | 10 |
| 10 | c | 1.061E+04 | 611.8 | 0.002756 | 4.504 | +2 | 10 |
| - | - | 6237 | 612 | - | - | 0 | - |
| - | - | 1.739E+04 | 612.3 | - | - | 0 | - |
| - | - | 3212 | 612.6 | - | - | 0 | - |
| - | - | 3909 | 612.8 | - | - | 0 | - |
| - | - | 2048 | 613 | - | - | 0 | - |
| - | - | 2753 | 613.3 | - | - | 0 | - |
| - | - | 1824 | 615.3 | - | - | 0 | - |
| - | - | 888.6 | 616.3 | - | - | 0 | - |
| - | - | 968 | 618.3 | - | - | 0 | - |
| - | - | 1079 | 619.8 | - | - | 0 | - |
| - | - | 897.4 | 620 | - | - | 0 | - |
| 10 | c | 5.594E+04 | 620.3 | 0.001139 | 1.836 | +2 | 10 |
| - | - | 5639 | 620.6 | - | - | 0 | - |
| - | - | 3.649E+04 | 620.8 | - | - | 0 | - |
| - | - | 1.051E+04 | 621 | - | - | 0 | - |
| - | - | 1334 | 621.2 | - | - | 0 | - |
| - | - | 2.262E+04 | 621.3 | - | - | 0 | - |
| - | - | 5646 | 621.7 | - | - | 0 | - |
| - | - | 4684 | 621.8 | - | - | 0 | - |
| - | - | 1821 | 622 | - | - | 0 | - |
| - | - | 1034 | 622.2 | - | - | 0 | - |
| 12 | y | 1.668E+04 | 622.3 | 0.009039 | 14.52 | +1 | 4 |
| - | - | 4676 | 623.3 | - | - | 0 | - |
| - | - | 3694 | 625.7 | - | - | 0 | - |
| - | - | 5818 | 626 | - | - | 0 | - |
| - | - | 7835 | 626.3 | - | - | 0 | - |
| 2 | z | 1.543E+04 | 626.6 | 0.003188 | 5.088 | +3 | 14 |
| - | - | 1155 | 626.8 | - | - | 0 | - |
| - | - | 1.549E+04 | 627 | - | - | 0 | - |
| - | - | 9644 | 627.3 | - | - | 0 | - |
| - | - | 4956 | 627.6 | - | - | 0 | - |
| - | - | 863 | 627.8 | - | - | 0 | - |
| - | - | 3237 | 628 | - | - | 0 | - |
| - | - | 1139 | 628.3 | - | - | 0 | - |
| - | - | 955.4 | 630 | - | - | 0 | - |
| - | - | 1641 | 630.3 | - | - | 0 | - |
| - | - | 6128 | 630.6 | - | - | 0 | - |
| - | - | 7368 | 631 | - | - | 0 | - |
| - | - | 4260 | 631.3 | - | - | 0 | - |
| - | - | 1334 | 631.6 | - | - | 0 | - |
| 2 | y | 3100 | 632 | 8.267E-05 | 0.1308 | +3 | 14 |
| - | - | 4079 | 632.3 | - | - | 0 | - |
| - | - | 1044 | 632.6 | - | - | 0 | - |
| - | - | 1021 | 633.3 | - | - | 0 | - |
| - | - | 2497 | 634.3 | - | - | 0 | - |
| - | - | 2981 | 634.6 | - | - | 0 | - |
| 6 | w | 2.604E+04 | 634.8 | 0.003625 | 5.711 | +2 | 10 |
| - | - | 3992 | 635 | - | - | 0 | - |
| - | - | 1.855E+04 | 635.3 | - | - | 0 | - |
| - | - | 2633 | 635.7 | - | - | 0 | - |
| - | - | 6731 | 635.8 | - | - | 0 | - |
| - | - | 2759 | 636 | - | - | 0 | - |
| - | - | 2278 | 636.3 | - | - | 0 | - |
| - | - | 1246 | 636.8 | - | - | 0 | - |
| - | - | 1047 | 637.4 | - | - | 0 | - |
| - | - | 1054 | 638.3 | - | - | 0 | - |
| - | - | 1532 | 639 | - | - | 0 | - |
| - | - | 1568 | 639.3 | - | - | 0 | - |
| - | - | 1054 | 639.7 | - | - | 0 | - |
| - | - | 1.868E+04 | 640.3 | - | - | 0 | - |
| - | - | 2.423E+04 | 640.7 | - | - | 0 | - |
| - | - | 1099 | 640.8 | - | - | 0 | - |
| - | - | 1.709E+04 | 641 | - | - | 0 | - |
| - | - | 1.068E+04 | 641.3 | - | - | 0 | - |
| - | - | 3836 | 641.7 | - | - | 0 | - |
| - | - | 1325 | 642 | - | - | 0 | - |
| - | - | 5385 | 643.8 | - | - | 0 | - |
| - | - | 5581 | 644.3 | - | - | 0 | - |
| - | - | 6271 | 644.7 | - | - | 0 | - |
| - | - | 1714 | 644.8 | - | - | 0 | - |
| - | - | 1.084E+04 | 645 | - | - | 0 | - |
| - | - | 6128 | 645.3 | - | - | 0 | - |
| - | - | 6917 | 645.7 | - | - | 0 | - |
| - | - | 4754 | 646 | - | - | 0 | - |
| - | - | 4065 | 646.3 | - | - | 0 | - |
| - | - | 2243 | 646.7 | - | - | 0 | - |
| 6 | z | 1302 | 647.3 | 0.004124 | 6.371 | +2 | 10 |
| - | - | 2599 | 649.3 | - | - | 0 | - |
| - | - | 1370 | 649.7 | - | - | 0 | - |
| - | - | 1.619E+04 | 650 | - | - | 0 | - |
| - | - | 7.619E+04 | 650.3 | - | - | 0 | - |
| - | - | 8.224E+04 | 650.7 | - | - | 0 | - |
| - | - | 1791 | 650.8 | - | - | 0 | - |
| - | - | 4.694E+04 | 651 | - | - | 0 | - |
| - | - | 2.535E+04 | 651.3 | - | - | 0 | - |
| - | - | 1.036E+04 | 651.7 | - | - | 0 | - |
| - | - | 1182 | 651.8 | - | - | 0 | - |
| - | - | 2109 | 652 | - | - | 0 | - |
| - | - | 7035 | 652.3 | - | - | 0 | - |
| - | - | 3286 | 653.3 | - | - | 0 | - |
| - | - | 2117 | 654.3 | - | - | 0 | - |
| - | - | 1373 | 654.7 | - | - | 0 | - |
| - | - | 3967 | 655 | - | - | 0 | - |
| 6 | y | 7242 | 655.3 | 0.005796 | 8.844 | +2 | 10 |
| - | - | 4128 | 655.7 | - | - | 0 | - |
| 6 | y | 1998 | 655.8 | 0.0002435 | 0.3713 | +2 | 10 |
| - | - | 3882 | 656 | - | - | 0 | - |
| 6 | z | 2.319E+04 | 656.3 | 0.003669 | 5.59 | +2 | 10 |
| - | - | 1.927E+04 | 656.8 | - | - | 0 | - |
| - | - | 1015 | 657 | - | - | 0 | - |
| - | - | 1.224E+04 | 657.3 | - | - | 0 | - |
| - | - | 4874 | 657.8 | - | - | 0 | - |
| - | - | 2162 | 658.3 | - | - | 0 | - |
| - | - | 2432 | 658.7 | - | - | 0 | - |
| - | - | 3655 | 659 | - | - | 0 | - |
| - | - | 3923 | 659.3 | - | - | 0 | - |
| - | - | 2472 | 659.7 | - | - | 0 | - |
| - | - | 2356 | 663.3 | - | - | 0 | - |
| - | - | 2691 | 663.8 | - | - | 0 | - |
| - | - | 2393 | 664 | - | - | 0 | - |
| 6 | y | 3.523E+04 | 664.3 | 0.005341 | 8.039 | +2 | 10 |
| - | - | 9.192E+04 | 664.7 | - | - | 0 | - |
| - | - | 1.278E+04 | 664.8 | - | - | 0 | - |
| - | - | 1.001E+05 | 665 | - | - | 0 | - |
| - | - | 5.363E+04 | 665.3 | - | - | 0 | - |
| - | - | 2.294E+04 | 665.7 | - | - | 0 | - |
| - | - | 3387 | 665.8 | - | - | 0 | - |
| - | - | 1.04E+04 | 666 | - | - | 0 | - |
| - | - | 6255 | 666.3 | - | - | 0 | - |
| - | - | 1.926E+04 | 669.7 | - | - | 0 | - |
| - | - | 4.211E+04 | 670 | - | - | 0 | - |
| - | - | 6.876E+04 | 670.3 | - | - | 0 | - |
| - | - | 5.274E+04 | 670.7 | - | - | 0 | - |
| - | - | 2.945E+04 | 671 | - | - | 0 | - |
| - | - | 1.388E+04 | 671.3 | - | - | 0 | - |
| - | - | 6006 | 671.7 | - | - | 0 | - |
| - | - | 2775 | 671.8 | - | - | 0 | - |
| - | - | 1794 | 672 | - | - | 0 | - |
| - | - | 2921 | 672.3 | - | - | 0 | - |
| - | - | 1705 | 672.8 | - | - | 0 | - |
| 5 | c | 2108 | 679.3 | 0.01024 | 15.07 | +1 | 5 |
| - | - | 3.043E+04 | 679.8 | - | - | 0 | - |
| 5 | c | 4.95E+04 | 680.3 | 0.006081 | 8.939 | +1 | 5 |
| - | - | 1.012E+04 | 680.8 | - | - | 0 | - |
| - | - | 2.222E+04 | 681.3 | - | - | 0 | - |
| - | - | 5177 | 682.3 | - | - | 0 | - |
| - | - | 1984 | 683.3 | - | - | 0 | - |
| - | - | 1092 | 683.8 | - | - | 0 | - |
| - | - | 1149 | 685.3 | - | - | 0 | - |
| - | - | 1.364E+04 | 691.3 | - | - | 0 | - |
| - | - | 1.053E+04 | 691.8 | - | - | 0 | - |
| - | - | 5534 | 692.3 | - | - | 0 | - |
| 11 | c | 2242 | 692.8 | 0.003321 | 4.794 | +2 | 11 |
| 11 | c | 1112 | 693.3 | 0.005759 | 8.307 | +2 | 11 |
| - | - | 2997 | 693.8 | - | - | 0 | - |
| - | - | 2134 | 694.3 | - | - | 0 | - |
| 5 | c | 2.735E+04 | 697.3 | 0.0008334 | 1.195 | +1 | 5 |
| - | - | 1.355E+04 | 698.3 | - | - | 0 | - |
| - | - | 2830 | 699.3 | - | - | 0 | - |
| - | - | 1717 | 701.3 | - | - | 0 | - |
| 11 | c | 4.351E+04 | 701.8 | 0.0006635 | 0.9454 | +2 | 11 |
| - | - | 3.617E+04 | 702.3 | - | - | 0 | - |
| - | - | 1.484E+04 | 702.8 | - | - | 0 | - |
| - | - | 4933 | 703.3 | - | - | 0 | - |
| - | - | 2052 | 703.9 | - | - | 0 | - |
| - | - | 3419 | 705.4 | - | - | 0 | - |
| - | - | 2391 | 705.9 | - | - | 0 | - |
| - | - | 2079 | 711.3 | - | - | 0 | - |
| - | - | 1103 | 711.4 | - | - | 0 | - |
| - | - | 2097 | 714.4 | - | - | 0 | - |
| - | - | 1089 | 719.3 | - | - | 0 | - |
| - | - | 980.4 | 719.9 | - | - | 0 | - |
| - | - | 4275 | 720.4 | - | - | 0 | - |
| - | - | 2811 | 720.9 | - | - | 0 | - |
| - | - | 3718 | 721.3 | - | - | 0 | - |
| - | - | 2447 | 722.4 | - | - | 0 | - |
| - | - | 1335 | 723.4 | - | - | 0 | - |
| - | - | 2331 | 726.9 | - | - | 0 | - |
| - | - | 1023 | 727.4 | - | - | 0 | - |
| - | - | 1470 | 728.4 | - | - | 0 | - |
| - | - | 962.1 | 741.4 | - | - | 0 | - |
| - | - | 3035 | 742.3 | - | - | 0 | - |
| - | - | 2461 | 742.9 | - | - | 0 | - |
| - | - | 1091 | 743.4 | - | - | 0 | - |
| - | - | 3794 | 744.4 | - | - | 0 | - |
| - | - | 2593 | 744.9 | - | - | 0 | - |
| - | - | 1056 | 745.4 | - | - | 0 | - |
| 5 | y | 2430 | 748.9 | 0.006393 | 8.537 | +2 | 11 |
| 5 | z | 1.229E+04 | 749.4 | 0.003775 | 5.037 | +2 | 11 |
| - | - | 1.559E+04 | 749.9 | - | - | 0 | - |
| - | - | 1.106E+04 | 750.4 | - | - | 0 | - |
| - | - | 5235 | 750.9 | - | - | 0 | - |
| - | - | 1919 | 751.4 | - | - | 0 | - |
| - | - | 1262 | 752.4 | - | - | 0 | - |
| 12 | c | 3574 | 757.4 | 0.002538 | 3.351 | +2 | 12 |
| - | - | 4411 | 757.9 | - | - | 0 | - |
| - | - | 3207 | 758.4 | - | - | 0 | - |
| - | - | 1142 | 758.9 | - | - | 0 | - |
| - | - | 2278 | 762.9 | - | - | 0 | - |
| - | - | 1202 | 763.4 | - | - | 0 | - |
| - | - | 1262 | 763.9 | - | - | 0 | - |
| - | - | 2428 | 765.4 | - | - | 0 | - |
| 12 | c | 4211 | 765.9 | 0.001095 | 1.429 | +2 | 12 |
| - | - | 9597 | 766.4 | - | - | 0 | - |
| - | - | 5364 | 766.9 | - | - | 0 | - |
| - | - | 3087 | 767.4 | - | - | 0 | - |
| 11 | y | 2884 | 768.4 | 0.009929 | 12.92 | +1 | 5 |
| 11 | z | 1.691E+04 | 769.4 | 0.009026 | 11.73 | +1 | 5 |
| - | - | 1.24E+04 | 770.4 | - | - | 0 | - |
| - | - | 1183 | 770.9 | - | - | 0 | - |
| - | - | 4932 | 771.4 | - | - | 0 | - |
| - | - | 1789 | 776.4 | - | - | 0 | - |
| - | - | 1883 | 777.4 | - | - | 0 | - |
| - | - | 3591 | 777.9 | - | - | 0 | - |
| - | - | 2588 | 778.4 | - | - | 0 | - |
| - | - | 1218 | 778.9 | - | - | 0 | - |
| - | - | 1228 | 779.4 | - | - | 0 | - |
| 4 | w | 2276 | 784.4 | 0.001295 | 1.651 | +2 | 12 |
| - | - | 1721 | 784.9 | - | - | 0 | - |
| 11 | y | 8142 | 785.4 | 0.009196 | 11.71 | +1 | 5 |
| - | - | 2858 | 785.9 | - | - | 0 | - |
| - | - | 3146 | 786.4 | - | - | 0 | - |
| - | - | 1987 | 787.4 | - | - | 0 | - |
| 6 | c | 2.049E+04 | 793.4 | 0.0006305 | 0.7947 | +1 | 6 |
| - | - | 1.275E+04 | 794.4 | - | - | 0 | - |
| - | - | 3965 | 795.4 | - | - | 0 | - |
| - | - | 1385 | 796.4 | - | - | 0 | - |
| - | - | 1269 | 802.4 | - | - | 0 | - |
| - | - | 3071 | 802.9 | - | - | 0 | - |
| - | - | 2808 | 803.4 | - | - | 0 | - |
| - | - | 3211 | 803.9 | - | - | 0 | - |
| - | - | 3374 | 804.4 | - | - | 0 | - |
| - | - | 1693 | 804.9 | - | - | 0 | - |
| - | - | 1736 | 805.4 | - | - | 0 | - |
| 4 | y | 2541 | 806.4 | 0.001559 | 1.933 | +2 | 12 |
| 4 | z | 9965 | 806.9 | 0.003391 | 4.203 | +2 | 12 |
| - | - | 2.272E+04 | 807.4 | - | - | 0 | - |
| - | - | 1.721E+04 | 807.9 | - | - | 0 | - |
| - | - | 7468 | 808.4 | - | - | 0 | - |
| - | - | 3355 | 808.9 | - | - | 0 | - |
| - | - | 1915 | 809.4 | - | - | 0 | - |
| 6 | c | 8.342E+04 | 810.4 | 0.0003265 | 0.4029 | +1 | 6 |
| - | - | 4.455E+04 | 811.4 | - | - | 0 | - |
| - | - | 1.236E+04 | 812.4 | - | - | 0 | - |
| - | - | 1150 | 813.4 | - | - | 0 | - |
| 4 | y | 2344 | 814.9 | 0.003964 | 4.865 | +2 | 12 |
| - | - | 1550 | 815.4 | - | - | 0 | - |
| - | - | 2241 | 816.9 | - | - | 0 | - |
| - | - | 4239 | 817.4 | - | - | 0 | - |
| - | - | 2822 | 817.9 | - | - | 0 | - |
| - | - | 1719 | 818.4 | - | - | 0 | - |
| - | - | 1094 | 823.9 | - | - | 0 | - |
| - | - | 2192 | 824.4 | - | - | 0 | - |
| - | - | 1111 | 824.9 | - | - | 0 | - |
| - | - | 2867 | 826.9 | - | - | 0 | - |
| - | - | 2683 | 827.4 | - | - | 0 | - |
| - | - | 1924 | 828.4 | - | - | 0 | - |
| - | - | 1808 | 838.4 | - | - | 0 | - |
| - | - | 1347 | 838.9 | - | - | 0 | - |
| 10 | w | 2793 | 839.4 | 0.003647 | 4.345 | +1 | 6 |
| - | - | 3225 | 840.4 | - | - | 0 | - |
| - | - | 3361 | 841.4 | - | - | 0 | - |
| 3 | w | 6986 | 841.9 | 0.005158 | 6.126 | +2 | 13 |
| - | - | 5695 | 842.4 | - | - | 0 | - |
| - | - | 1346 | 842.9 | - | - | 0 | - |
| - | - | 1075 | 843.4 | - | - | 0 | - |
| - | - | 1201 | 845.4 | - | - | 0 | - |
| 13 | c | 8232 | 846.4 | 0.001662 | 1.963 | +2 | 13 |
| - | - | 9975 | 846.9 | - | - | 0 | - |
| - | - | 6671 | 847.4 | - | - | 0 | - |
| - | - | 3965 | 847.9 | - | - | 0 | - |
| - | - | 1850 | 848.4 | - | - | 0 | - |
| - | - | 1028 | 848.9 | - | - | 0 | - |
| - | - | 1641 | 859.4 | - | - | 0 | - |
| - | - | 1664 | 859.9 | - | - | 0 | - |
| - | - | 2241 | 860.4 | - | - | 0 | - |
| - | - | 1144 | 861.4 | - | - | 0 | - |
| 3 | z | 1271 | 862.4 | 0.005802 | 6.728 | +2 | 13 |
| - | - | 1040 | 865.4 | - | - | 0 | - |
| - | - | 1180 | 866.4 | - | - | 0 | - |
| - | - | 2325 | 867.4 | - | - | 0 | - |
| 3 | z | 1.078E+04 | 870.9 | 0.003993 | 4.585 | +2 | 13 |
| - | - | 3.708E+04 | 871.4 | - | - | 0 | - |
| - | - | 3.461E+04 | 871.9 | - | - | 0 | - |
| - | - | 1.341E+04 | 872.4 | - | - | 0 | - |
| - | - | 5685 | 872.9 | - | - | 0 | - |
| - | - | 2964 | 873.4 | - | - | 0 | - |
| 3 | y | 2349 | 878.9 | 0.003041 | 3.459 | +2 | 13 |
| - | - | 2832 | 879.4 | - | - | 0 | - |
| - | - | 945 | 879.9 | - | - | 0 | - |
| - | - | 2653 | 880.4 | - | - | 0 | - |
| - | - | 2141 | 880.9 | - | - | 0 | - |
| - | - | 6825 | 881.4 | - | - | 0 | - |
| - | - | 2195 | 881.9 | - | - | 0 | - |
| - | - | 4560 | 882.4 | - | - | 0 | - |
| - | - | 1253 | 883.5 | - | - | 0 | - |
| - | - | 2042 | 888.4 | - | - | 0 | - |
| - | - | 9715 | 888.9 | - | - | 0 | - |
| - | - | 8702 | 889.4 | - | - | 0 | - |
| - | - | 5176 | 889.9 | - | - | 0 | - |
| - | - | 2022 | 890.4 | - | - | 0 | - |
| - | - | 1008 | 895.9 | - | - | 0 | - |
| 10 | y | 1243 | 896.4 | 0.002904 | 3.24 | +1 | 6 |
| 10 | z | 1.311E+04 | 898.4 | 0.00859 | 9.561 | +1 | 6 |
| - | - | 1.855E+04 | 899.4 | - | - | 0 | - |
| - | - | 7782 | 900.4 | - | - | 0 | - |
| - | - | 2307 | 901.4 | - | - | 0 | - |
| - | - | 1718 | 902.4 | - | - | 0 | - |
| - | - | 980.5 | 902.9 | - | - | 0 | - |
| - | - | 1111 | 903.4 | - | - | 0 | - |
| 7 | c | 9180 | 908.4 | 0.0007871 | 0.8664 | +1 | 7 |
| - | - | 5298 | 909.4 | - | - | 0 | - |
| 14 | c | 6018 | 910.4 | 0.007273 | 7.989 | +2 | 14 |
| - | - | 4684 | 910.9 | - | - | 0 | - |
| - | - | 3236 | 911.4 | - | - | 0 | - |
| - | - | 1004 | 911.9 | - | - | 0 | - |
| 10 | y | 2892 | 914.4 | 0.009492 | 10.38 | +1 | 6 |
| - | - | 988 | 915.4 | - | - | 0 | - |
| - | - | 1553 | 923.5 | - | - | 0 | - |
| 7 | c | 4974 | 925.5 | 0.0001883 | 0.2035 | +1 | 7 |
| - | - | 4154 | 926.5 | - | - | 0 | - |
| - | - | 1227 | 927.5 | - | - | 0 | - |
| - | - | 2274 | 931 | - | - | 0 | - |
| - | - | 2174 | 931.5 | - | - | 0 | - |
| - | - | 2805 | 932 | - | - | 0 | - |
| - | - | 5138 | 938.5 | - | - | 0 | - |
| - | - | 1262 | 939 | - | - | 0 | - |
| 2 | z | 4412 | 939.4 | 0.01825 | 19.42 | +2 | 14 |
| - | - | 1.427E+04 | 939.9 | - | - | 0 | - |
| - | - | 1.415E+04 | 940.4 | - | - | 0 | - |
| - | - | 6609 | 940.9 | - | - | 0 | - |
| - | - | 3882 | 941.4 | - | - | 0 | - |
| - | - | 989.7 | 945.5 | - | - | 0 | - |
| - | - | 995.3 | 946 | - | - | 0 | - |
| - | - | 1473 | 946.5 | - | - | 0 | - |
| - | - | 3384 | 952.5 | - | - | 0 | - |
| - | - | 2660 | 953 | - | - | 0 | - |
| - | - | 2626 | 953.5 | - | - | 0 | - |
| - | - | 1449 | 954 | - | - | 0 | - |
| - | - | 1182 | 954.5 | - | - | 0 | - |
| - | - | 1332 | 958.5 | - | - | 0 | - |
| - | - | 1259 | 960.5 | - | - | 0 | - |
| - | - | 2443 | 961 | - | - | 0 | - |
| - | - | 1763 | 961.5 | - | - | 0 | - |
| - | - | 1292 | 962 | - | - | 0 | - |
| - | - | 1038 | 964 | - | - | 0 | - |
| 8 | c | 1054 | 964.5 | 0.007895 | 8.185 | +1 | 8 |
| - | - | 3518 | 966.5 | - | - | 0 | - |
| - | - | 2405 | 967 | - | - | 0 | - |
| - | - | 6714 | 967.5 | - | - | 0 | - |
| - | - | 4940 | 968 | - | - | 0 | - |
| - | - | 3764 | 968.5 | - | - | 0 | - |
| - | - | 973.6 | 969.5 | - | - | 0 | - |
| - | - | 2116 | 974.5 | - | - | 0 | - |
| - | - | 4590 | 975 | - | - | 0 | - |
| - | - | 7399 | 975.5 | - | - | 0 | - |
| - | - | 5638 | 976 | - | - | 0 | - |
| - | - | 3127 | 976.5 | - | - | 0 | - |
| - | - | 1956 | 977 | - | - | 0 | - |
| - | - | 933.8 | 981.5 | - | - | 0 | - |
| 8 | c | 1.928E+04 | 982.5 | 0.0007479 | 0.7612 | +1 | 8 |
| - | - | 1701 | 983 | - | - | 0 | - |
| - | - | 1.406E+04 | 983.5 | - | - | 0 | - |
| - | - | 5623 | 984.5 | - | - | 0 | - |
| - | - | 1553 | 985.5 | - | - | 0 | - |
| - | - | 1286 | 991 | - | - | 0 | - |
| - | - | 9439 | 996.5 | - | - | 0 | - |
| - | - | 8346 | 997 | - | - | 0 | - |
| - | - | 7048 | 997.5 | - | - | 0 | - |
| - | - | 3092 | 998 | - | - | 0 | - |
| - | - | 5237 | 1005 | - | - | 0 | - |
| - | - | 7956 | 1005 | - | - | 0 | - |
| - | - | 5393 | 1006 | - | - | 0 | - |
| - | - | 1409 | 1010 | - | - | 0 | - |
| 9 | z | 2920 | 1027 | 0.009315 | 9.074 | +1 | 7 |
| - | - | 1.076E+04 | 1028 | - | - | 0 | - |
| - | - | 5126 | 1029 | - | - | 0 | - |
| - | - | 2507 | 1030 | - | - | 0 | - |
| - | - | 1699 | 1052 | - | - | 0 | - |
| - | - | 1811 | 1067 | - | - | 0 | - |
| 8 | z | 1479 | 1084 | 0.009294 | 8.577 | +1 | 8 |
| - | - | 2844 | 1085 | - | - | 0 | - |
| - | - | 1849 | 1086 | - | - | 0 | - |
| - | - | 4014 | 1095 | - | - | 0 | - |
| - | - | 2915 | 1096 | - | - | 0 | - |
| 8 | y | 1354 | 1100 | 0.007632 | 6.941 | +1 | 8 |
| 9 | c | 1.784E+04 | 1111 | 0.0008313 | 0.7486 | +1 | 9 |
| - | - | 1.635E+04 | 1112 | - | - | 0 | - |
| - | - | 7988 | 1113 | - | - | 0 | - |
| - | - | 2423 | 1114 | - | - | 0 | - |
| - | - | 1384 | 1137 | - | - | 0 | - |
| - | - | 1155 | 1141 | - | - | 0 | - |
| - | - | 881.6 | 1142 | - | - | 0 | - |
| - | - | 1113 | 1182 | - | - | 0 | - |
| - | - | 1153 | 1197 | - | - | 0 | - |
| 7 | z | 1093 | 1199 | 0.002668 | 2.226 | +1 | 9 |
| - | - | 7305 | 1200 | - | - | 0 | - |
| - | - | 4866 | 1201 | - | - | 0 | - |
| - | - | 1190 | 1202 | - | - | 0 | - |
| - | - | 1158 | 1224 | - | - | 0 | - |
| - | - | 1373 | 1225 | - | - | 0 | - |
| - | - | 930.5 | 1226 | - | - | 0 | - |
| 10 | c | 4532 | 1240 | 0.002165 | 1.746 | +1 | 10 |
| - | - | 3291 | 1241 | - | - | 0 | - |
| - | - | 2733 | 1242 | - | - | 0 | - |
| - | - | 786 | 1243 | - | - | 0 | - |
| - | - | 1122 | 1265 | - | - | 0 | - |
| - | - | 1209 | 1266 | - | - | 0 | - |
| - | - | 1187 | 1267 | - | - | 0 | - |
| 6 | z | 1620 | 1312 | 0.01581 | 12.05 | +1 | 10 |
| - | - | 5098 | 1313 | - | - | 0 | - |
| - | - | 4850 | 1314 | - | - | 0 | - |
| - | - | 2175 | 1315 | - | - | 0 | - |
| - | - | 985.8 | 1345 | - | - | 0 | - |
| - | - | 1174 | 1359 | - | - | 0 | - |
| - | - | 4171 | 1360 | - | - | 0 | - |
| - | - | 2740 | 1361 | - | - | 0 | - |
| - | - | 1344 | 1362 | - | - | 0 | - |
| - | - | 1022 | 1388 | - | - | 0 | - |
| 11 | c | 2576 | 1403 | 0.003481 | 2.482 | +1 | 11 |
| - | - | 3499 | 1404 | - | - | 0 | - |
| - | - | 2251 | 1405 | - | - | 0 | - |
| 5 | z | 1562 | 1498 | 0.002959 | 1.975 | +1 | 11 |
| - | - | 1236 | 1499 | - | - | 0 | - |
| - | - | 1014 | 1516 | - | - | 0 | - |
| - | - | 880.5 | 1533 | - | - | 0 | - |
| - | - | 1211 | 1614 | - | - | 0 | - |
| - | - | 1034 | 1615 | - | - | 0 | - |
| - | - | 930.9 | 1693 | - | - | 0 | - |
| - | - | 852.6 | 1694 | - | - | 0 | - |
| - | - | 1664 | 1743 | - | - | 0 | - |
| - | - | 1040 | 1744 | - | - | 0 | - |
| - | - | 799.5 | 1880 | - | - | 0 | - |

m/z Charge Intensity FragmentType MassShift Position
120.05758666992188 0 636.0194 y Ammonia loss 12
120.06596374511719 0 501.75964
121.96086120605469 0 355.08963
127.08695983886719 0 626.9649
127.1121826171875 0 455.94553
128.09487915039062 0 472.08057
129.0144500732422 0 359.1314
129.01858520507812 0 5535.9834
129.06642150878906 0 531.76855
129.1025848388672 0 14550.411
130.06546020507812 0 42292.766
130.10609436035156 0 482.55603
131.06883239746094 0 3543.5276
131.11822509765625 0 1570.7872
132.0660858154297 0 656.04895
132.0811767578125 0 3575.8425
132.10231018066406 0 687.44775
133.0611114501953 0 3728.846
133.08615112304688 0 2492.8354
136.07606506347656 0 3861.9011
138.99818420410156 0 397.80753
139.00291442871094 0 3374.3057
144.08131408691406 0 548.39606
146.06063842773438 0 468.09042
146.0928192138672 0 1402.0101
147.04483032226562 0 666.4442
147.0764617919922 0 961.9766
155.09307861328125 0 15594.27
156.06613159179688 0 700.7218
156.09625244140625 0 1214.465
156.1383514404297 0 815.4525
157.01348876953125 0 13292.084
157.13394165039062 0 742.4111
158.06088256835938 0 611.43005
159.09202575683594 0 17559.293
159.11363220214844 0 554.5662
160.09536743164062 0 1738.9287
164.0712890625 0 524.99304
164.082275390625 0 1853.0112
166.0614471435547 0 12981.335
167.0650634765625 0 747.80115
168.1384735107422 0 1216.9459
170.0604248046875 0 9039.727
171.06869506835938 0 940.1232
171.13845825195312 0 548.1371
172.0719451904297 0 2998.266
173.05613708496094 0 2581.7998
173.09251403808594 0 599.24445
173.43910217285156 0 981.842
174.07667541503906 0 1721.5656
177.1124267578125 0 1893.8234
178.1343231201172 0 1232.5046
183.1130828857422 0 5444.428
183.12246704101562 0 660.5209
183.1259765625 0 783.34406
183.1498565673828 0 718.5292
185.07179260253906 0 953.447
185.0812225341797 0 16045.137
185.09141540527344 0 936.83246
185.1652069091797 0 12283.735
186.05227661132812 0 505.081
186.07858276367188 0 473.3156
186.1239471435547 0 3166.6375
186.17298889160156 0 1177.2518
187.08702087402344 0 3813.125
187.10818481445312 0 1277.8904
189.09901428222656 0 499.65738
190.08267211914062 0 7604.463
192.0765838623047 0 916.7029
198.12803649902344 0 2466.6177
199.1697235107422 0 2881.6167
201.12342834472656 0 2179.2686
202.11874389648438 0 570.7031
203.10264587402344 0 1536.6478
203.15078735351562 0 958.7173
205.11868286132812 0 7067.971
207.16055297851562 0 1073.6611
209.17083740234375 0 574.6763
210.1279754638672 0 1606.3247
211.6295928955078 0 1078.028
213.07615661621094 0 8733.914
215.13966369628906 0 659.5292
217.03468322753906 0 12189.625
221.13865661621094 0 600.06635
223.1192626953125 0 1605.0193
223.1557159423828 0 12161.679
224.15875244140625 0 1366.7909
225.12457275390625 0 499.71808
226.15524291992188 0 722.6314
227.0669403076172 0 882.70807
227.1378936767578 0 543.28217
229.0825653076172 0 551.8717
229.11874389648438 0 5765.1006
229.1550750732422 0 676.6632
230.1225128173828 0 690.0164
233.13987731933594 0 3106.5542
234.1241455078125 0 12581.605
235.1276092529297 0 1901.8564
235.1542510986328 0 1608.7094
236.13827514648438 0 620.38477
236.63352966308594 0 2016.9585
241.1432647705078 0 702.9943
244.09266662597656 0 990.34674
245.14755249023438 0 733.22504
246.1099395751953 0 833.87335
246.12350463867188 0 1313.4419
247.0756072998047 0 731.0777
247.6211700439453 0 2905.741 c Ammonia loss 3
250.10800170898438 0 1989.7836
250.14273071289062 0 1533.4504
250.166748046875 0 2144.6934
251.15072631835938 0 53764.938
252.15399169921875 0 7401.4395
252.60440063476562 0 651.40405
255.15020751953125 0 1178.3503
256.6317443847656 0 720.21246
259.1542663574219 0 22118.025
261.1195983886719 0 1673.6459
264.17059326171875 0 1751.6957
265.1436767578125 0 1764.6904
265.6354064941406 0 767.1106
266.1250915527344 0 1503.4799
268.1772155761719 0 10086.178 c 1
269.1383972167969 0 2113.928
269.1806945800781 0 1691.9806
270.1219787597656 0 6347.0513
270.1811828613281 0 671.3652
270.623291015625 0 831.28705
272.1065979003906 0 1962.1548
272.1763000488281 0 1463.6418
273.09686279296875 0 7656.1294
274.1194763183594 0 1500.0686
274.1495361328125 0 10138.159
274.65142822265625 0 2823.1765
275.1517639160156 0 916.9837
279.18084716796875 0 895.4426
280.1504821777344 0 659.7584
282.18170166015625 0 5184.8184
283.1390380859375 0 1218.699
284.119873046875 0 1099.3882
285.15716552734375 0 845.0375
286.1405334472656 0 977.4091
288.1556091308594 0 976.0321
290.11724853515625 0 3504.5881
292.1651916503906 0 687.459
294.146484375 0 1057.3167 z Water loss 9
294.193115234375 0 1902.7219
295.1041259765625 0 1348.8508
296.1972351074219 0 918.7582
297.1280212402344 0 854.6516
297.19390869140625 0 686.55237
300.17144775390625 0 963.91315 z Ammonia loss 13
301.151611328125 0 1243.2802
302.14404296875 0 1379.8049
303.15228271484375 0 6899.4287 c Water loss 6
303.6550598144531 0 2398.6738 z 11
304.155029296875 0 1070.1533
307.14324951171875 0 772.82
309.1455078125 0 1475.3777
309.6473693847656 0 694.3095
311.66571044921875 0 6768.3784 y 11
312.1667785644531 0 1863.0514
315.1663818359375 0 773.24
317.1953125 0 2992.9146 z 13
317.65869140625 0 2401.6658
318.15130615234375 0 7404.714
318.201416015625 0 1101.6602
318.65301513671875 0 2309.5857
319.15521240234375 0 666.5207
323.14190673828125 0 1241.1482
326.6643981933594 0 32886.605
327.16595458984375 0 13368.976
327.66741943359375 0 3032.3403
329.1606140136719 0 3461.509
329.1913146972656 0 815.9119
331.6564025878906 0 4070.0513
332.1532897949219 0 1914.6992
332.1934814453125 0 669.0614
333.2137145996094 0 2719.4565 y 13
337.84857177734375 0 1061.8289
338.1824645996094 0 625.94366
338.672607421875 0 700.0501
339.6950988769531 0 1543.969
340.19891357421875 0 901.5344
340.6618957519531 0 13602.115 c Ammonia loss 4
341.16339111328125 0 6605.0874
341.6640319824219 0 1358.2141
342.50457763671875 0 711.35785 y Ammonia loss 8
348.18035888671875 0 563.22925 y 8
349.18914794921875 0 2159.2126
350.1796875 0 1523.431
350.51239013671875 0 633.96704
350.8472900390625 0 678.4986
351.2151794433594 0 1340.737
353.66839599609375 0 1180.5498
359.1502685546875 0 649.86017
360.6944885253906 0 1033.5314
361.17877197265625 0 1210.801 c Water loss 13
361.2015380859375 0 662.46106
361.2225646972656 0 3925.7515
361.51727294921875 0 1285.2737 y Ammonia loss 7
361.8515625 0 1097.8098 z 7
362.1825866699219 0 1076.878
363.2021179199219 0 2072.4675
364.19647216796875 0 1228.4958
364.69598388671875 0 680.6779
365.8548889160156 0 1350.4161
366.1907653808594 0 628.82324
367.186767578125 0 26193.21 y 7
367.5210266113281 0 16179.134
367.6825866699219 0 1478.6715
367.85491943359375 0 4919.9644
368.1105651855469 0 545.78864
368.1873779296875 0 2514.2244
369.2126159667969 0 1081.1816
371.1920471191406 0 1125.6875
374.20123291015625 0 1805.4581
374.6934814453125 0 5627.943 y Water loss 4
374.9337158203125 0 3241.5735 y Ammonia loss 4
375.1938781738281 0 2788.7478 z 4
375.43511962890625 0 724.44055
375.696533203125 0 699.66626
376.19281005859375 0 1398.1438
378.2030029296875 0 2583.5337
378.2268371582031 0 859.49774
379.2091369628906 0 15032.437 c Ammonia loss 2
379.2356262207031 0 512.2632
379.521484375 0 833.59064
379.742431640625 0 1545.2107
380.2118835449219 0 2595.2048
380.2388000488281 0 646.041
381.1516418457031 0 2047.6696
381.5211486816406 0 758.7638
383.2060546875 0 11307.13
383.2305603027344 0 1032.9191
383.7076110839844 0 6574.551
384.2079162597656 0 1997.2726
384.23333740234375 0 1282.7334
384.68341064453125 0 1107.8982 y Ammonia loss 10
385.1895446777344 0 6479.289 z 10
385.24371337890625 0 20119.582
385.5257873535156 0 8742.589
385.8596496582031 0 4603.3647
386.1949157714844 0 1682.7983
386.24688720703125 0 2621.9312
386.52581787109375 0 768.6065
387.2243347167969 0 30955.139
388.1991271972656 0 901.1135
388.2266540527344 0 6584.9546
388.6913757324219 0 962.7128
389.18701171875 0 3506.2427
389.22857666015625 0 988.92346
390.19085693359375 0 826.33075
393.19720458984375 0 21797.791 y 10
393.6990966796875 0 10117.23
394.1983947753906 0 2922.2712
394.7002258300781 0 1083.8578
395.19366455078125 0 1042.8258
395.39300537109375 0 1648.0558
395.5929870605469 0 1227.8346
396.2360534667969 0 24454.24 c 2
397.2038879394531 0 10145.3955 c Ammonia loss 5
397.2395324707031 0 5810.442
397.7057800292969 0 5158.1514
398.2063903808594 0 2460.5923
398.2364807128906 0 1028.0302
398.5968322753906 0 2612.0642
398.7958984375 0 3289.568
398.9953308105469 0 5541.1978
399.1959228515625 0 2784.9531
399.3965759277344 0 712.96564
399.43829345703125 0 691.49866 z Water loss 3
399.52490234375 0 1492.42 y Water loss 6
399.8551940917969 0 709.4186 y Ammonia loss 6
400.1917724609375 0 1706.2328 z 6
401.1882019042969 0 1268.5728
401.23895263671875 0 2225.481
401.2854919433594 0 2856.506
401.958251953125 0 678.5705
402.21246337890625 0 4104.653
402.2405090332031 0 832.4026
402.27081298828125 0 2537.4922
402.4000244140625 0 6052.0967
402.59967041015625 0 2148.6594
402.8015441894531 0 1346.5549
403.188720703125 0 962.5288
403.2159729003906 0 942.9414
403.27996826171875 0 824.14667
403.56878662109375 0 714.1652
404.21673583984375 0 3066.7966
404.71875 0 1852.8208
405.52972412109375 0 8986.564 y 6
405.8640441894531 0 8297.216
406.19781494140625 0 1858.0844
409.1461486816406 0 754.73846
409.22039794921875 0 670.98755
413.8849792480469 0 734.90625
418.2144470214844 0 1375.7266
420.20281982421875 0 144316.3 w 9
420.7043151855469 0 64826.344
421.20458984375 0 20449.195
421.7051696777344 0 4679.376
422.20550537109375 0 935.8279
423.2247314453125 0 819.44586
423.5332946777344 0 13016.1 w 5
423.8679504394531 0 7600.375
424.2012634277344 0 3177.1929
424.5339050292969 0 796.5669
430.19696044921875 0 1213.3815
431.22943115234375 0 1021.5161
432.2065734863281 0 895.52374 z Ammonia loss 5
434.20684814453125 0 830.6285
435.1655578613281 0 1031.6415
435.22412109375 0 1293.8329
436.2272033691406 0 1107.1873
437.2210388183594 0 1542.7264 y Water loss 5
437.55438232421875 0 2185.5586 y Ammonia loss 5
438.7422790527344 0 932.69934
439.24359130859375 0 747.30743
439.74176025390625 0 706.4689
439.96551513671875 0 776.5101 y 2
440.71746826171875 0 1504.3539
441.2207946777344 0 806.9237
442.8941345214844 0 1139.8531
443.192138671875 0 1228.5214
443.2246398925781 0 19626.184 y 5
443.558837890625 0 14236.358
443.8928527832031 0 3614.9143
444.2270202636719 0 1793.7074
445.71136474609375 0 1194.0708
446.2121887207031 0 1687.731
446.74420166015625 0 713.3842
447.2204895019531 0 2287.5017
448.22198486328125 0 1941.2892
448.7137756347656 0 2641.6873 y Water loss 9
449.21466064453125 0 1084.0813 y Ammonia loss 9
449.7109680175781 0 2738.0037 z 9
450.20709228515625 0 1174.8706
451.22698974609375 0 805.4702 c Water loss 13
451.8853454589844 0 871.2036
452.19036865234375 0 865.09064
452.2250671386719 0 1727.7583
452.4727478027344 0 1034.4181
452.7202453613281 0 865.56323
452.97369384765625 0 685.89307
453.5728454589844 0 885.9088
454.570556640625 0 853.08984
454.7177734375 0 12688.802 c Ammonia loss 6
454.98870849609375 0 1214.4412
455.2188720703125 0 6545.3657
455.72149658203125 0 3691.7673 c 13
455.96612548828125 0 1563.4349
456.21832275390625 0 2313.8308
456.7312927246094 0 639.38995
457.71917724609375 0 3438.4207 y 9
458.22137451171875 0 1949.4143
458.9821472167969 0 625.8471
459.2175598144531 0 830.7413
459.47076416015625 0 796.6809
459.724365234375 0 1588.249
459.9728088378906 0 1184.6799
460.2254333496094 0 841.4088
460.7359619140625 0 19293.74
461.23681640625 0 9533.181
461.5516662597656 0 1027.4572
461.7366027832031 0 3614.9236
462.20147705078125 0 2820.1682
462.2358703613281 0 1301.2511
462.7345275878906 0 1953.6655
463.22943115234375 0 1102.3883 c 6
465.4668884277344 0 2438.2937
465.71929931640625 0 1860.8789 z Water loss 1
465.975341796875 0 1347.9421 z Ammonia loss 1
466.2409362792969 0 3373.9033
467.2508239746094 0 2671.5293
468.2636413574219 0 8384.915
468.3190612792969 0 929.9776
468.7655944824219 0 5177.2607
469.2364501953125 0 2619.3926
469.4820861816406 0 899.79224
469.7227783203125 0 2185.11 y Water loss 1
469.97930908203125 0 7204.913 y Ammonia loss 1
470.2301330566406 0 7842.4434 z 1
470.3324890136719 0 1163.8291
470.47998046875 0 4129.2856
470.7267150878906 0 1053.3877
472.9721374511719 0 732.31946
473.2349548339844 0 1672.1667
473.48272705078125 0 779.7624
473.7339782714844 0 2470.2686
473.90350341796875 0 901.04114
473.98046875 0 2410.242
474.22711181640625 0 7819.3096 y 1
474.47735595703125 0 7246.5547
474.7272033691406 0 4101.339
475.9867248535156 0 2260.4019
476.2461853027344 0 854.70593
476.4885559082031 0 1826.8402
476.7376403808594 0 2226.6562
476.9823303222656 0 1183.792
477.2289733886719 0 3249.8726
477.4798583984375 0 993.6256
478.2191467285156 0 1902.6409 z 12
479.22674560546875 0 923.6093
479.5701599121094 0 986.53314
479.74957275390625 0 1352.6852
479.90301513671875 0 1033.1985
480.2466735839844 0 1671.9574
480.4934387207031 0 36458.555
480.74407958984375 0 37438.625
480.9941101074219 0 19583.895
481.2449035644531 0 10722.224
481.4949035644531 0 3256.0786
481.7441101074219 0 1163.482
482.7359619140625 0 4991.3623 c Water loss 7
483.22998046875 0 7675.138 c Ammonia loss 7
483.4842834472656 0 1602.36
483.7318420410156 0 2468.9807
483.98883056640625 0 1664.5211
484.24969482421875 0 11076.222
484.48773193359375 0 3763.5923
484.5745849609375 0 759.44055
484.7248840332031 0 5578.508 w 8
484.75433349609375 0 2851.996
484.9862365722656 0 1561.364
485.2286682128906 0 3036.5825
485.7259826660156 0 1607.4164
487.29144287109375 0 969.2484
487.47918701171875 0 3299.2278
487.7295227050781 0 79661.19
487.9828186035156 0 75161.69
488.23419189453125 0 45014.445
488.4842224121094 0 21857.377
488.7453308105469 0 11834.211
488.9949951171875 0 4144.575
489.2475891113281 0 2521.7239
491.2456970214844 0 1282.0369
491.74169921875 0 7289.324 c 7
491.9961242675781 0 770.6002
492.2417907714844 0 4909.4434
492.7423400878906 0 2925.1404
493.254150390625 0 791.8311 c Water loss 3
493.56512451171875 0 2402.561
493.90087890625 0 1650.4556 z Water loss 4
494.2326965332031 0 34121.6 c Ammonia loss 3
494.4875793457031 0 812.87415
495.2371520996094 0 8558.74
495.76666259765625 0 784.4785
496.2309875488281 0 887.9125
496.2620544433594 0 1177.6381
496.7750244140625 0 4140.287
497.2764892578125 0 3515.41
497.7552795410156 0 2706.0884
498.24658203125 0 4345.5083
498.49371337890625 0 3652.6956
498.7457580566406 0 3111.8604
498.9961242675781 0 969.9971
499.2410583496094 0 8596.66 y Water loss 4
499.4936828613281 0 884.68915
499.574951171875 0 9583.404 y Ammonia loss 4
499.74468994140625 0 1747.6268
499.91033935546875 0 7692.622 z 4
500.2451171875 0 5981.0107
500.5769348144531 0 1946.754
500.91064453125 0 1013.2421
501.9929504394531 0 1548.3102
502.2493591308594 0 4072.0273
502.4974365234375 0 120858.12
502.7483215332031 0 172756.2
502.99896240234375 0 126976.625
503.2494812011719 0 59700.01
503.5001220703125 0 21970.03
503.7497253417969 0 8123.051
504.0008239746094 0 2801.104
504.2481994628906 0 1585.7329
505.25030517578125 0 6469.969 c Ammonia loss 11
505.5850524902344 0 6724.3
505.74053955078125 0 3023.8843
505.9179992675781 0 2631.0613
506.2596130371094 0 5657.548
506.5916442871094 0 806.6595
506.7334289550781 0 1726.162
506.9320068359375 0 899.8937
507.26544189453125 0 1772.9486
510.26068115234375 0 948.1784
510.5951843261719 0 938.3962
510.9290771484375 0 44399.336 c 11
511.26312255859375 0 79290.92 c 3
511.5971984863281 0 16178.947
511.9314880371094 0 6261.291
512.2659301757812 0 12781.556
512.7597045898438 0 1839.0457 y Water loss 8
513.2328491210938 0 1521.5796
513.2683715820312 0 2072.1975
513.7566528320312 0 19952.924 z 8
514.2575073242188 0 17000.133
514.5803833007812 0 1565.1874
514.7603149414062 0 7038.226
514.9116821289062 0 1272.7313
515.2577514648438 0 1161.9734
515.3197021484375 0 7144.465
515.7651977539062 0 877.3606
516.323486328125 0 2025.9633
517.2808227539062 0 992.62146
517.5963134765625 0 1063.6268
518.2881469726562 0 1211.2523
519.93603515625 0 1592.4443
520.266845703125 0 1694.4055
521.2770385742188 0 1760.335
521.7664794921875 0 15915.946 y 8
522.2677001953125 0 9707.311
522.76806640625 0 3605.5713
523.265625 0 1257.3237 w 3
523.591064453125 0 2116.5928
523.9259643554688 0 1852.2637
525.2731323242188 0 1203.31
525.35107421875 0 1151.6924
526.2553100585938 0 3171.1646
526.75341796875 0 1434.4385
528.2548828125 0 761.92535
529.28173828125 0 1009.61224
529.618896484375 0 848.00055
530.27880859375 0 1342.535
530.6096801757812 0 1167.6279
533.7716674804688 0 991.16187
534.2691650390625 0 2176.524
534.3111572265625 0 1114.2476
534.95166015625 0 2383.2139
535.2835083007812 0 3548.4783
535.6165771484375 0 5358.49
535.9474487304688 0 12853.473
536.2827758789062 0 12591.944
536.617919921875 0 6706.8926
536.9493408203125 0 4309.517
537.2789306640625 0 3287.8896
537.9225463867188 0 795.2517 y Ammonia loss 3
538.2755737304688 0 948.0903
538.58984375 0 844.79083
539.2377319335938 0 833.715
541.2696533203125 0 2654.999 y Water loss 7
541.7744140625 0 1612.043 y Ammonia loss 7
542.2671508789062 0 13577.449 z 7
542.7684936523438 0 6971.616
543.271728515625 0 3493.2083
543.59326171875 0 3347.757 y 3
543.7683715820312 0 1364.1069
543.927734375 0 4077.49
544.262939453125 0 1695.8259
546.2619018554688 0 2391.562
546.764404296875 0 2390.733
547.2864379882812 0 4437.969
547.7753295898438 0 2043.2793
548.2391967773438 0 1992.3945 w 11
548.2900390625 0 1792.9792
548.7501220703125 0 1102.9207
549.608154296875 0 2713.9604
549.7605590820312 0 1197.3949
549.93994140625 0 1987.3206
550.276611328125 0 34418.785 y 7
550.59814453125 0 1192.7112
550.7784423828125 0 20948.047
551.2774658203125 0 5787.7617
551.5958862304688 0 1118.341
551.7786865234375 0 1501.6724
552.2619018554688 0 2384.268
554.2930297851562 0 1252.0198
554.7877197265625 0 1957.2595
555.2700805664062 0 6201.128
555.7884521484375 0 140021.27 c 8
556.2901000976562 0 91405.91
556.791015625 0 28922.146
556.9360961914062 0 1098.7402
557.291259765625 0 7323.2524
557.61376953125 0 1001.3587
557.7904663085938 0 1768.8838
560.9307250976562 0 783.3074
561.2692260742188 0 1687.0513
561.5966186523438 0 52709.48 w 2
561.9309692382812 0 47974.664
562.2648315429688 0 24935.314
562.5980224609375 0 7427.66
562.635009765625 0 1917.5764
562.9320678710938 0 3768.8442
563.2664184570312 0 2219.027
563.3126831054688 0 1165.1864
563.7816162109375 0 7296.3857
563.9264526367188 0 999.1099
564.279296875 0 4383.0566
564.3247680664062 0 1068.8641
564.6002807617188 0 27246.66 c 12
564.781982421875 0 1673.0232
564.934326171875 0 24452.035
565.2693481445312 0 13584.148
565.6027221679688 0 7144.8706
565.9356079101562 0 3278.5068
566.2752075195312 0 4653.927
566.6077270507812 0 4019.1604
566.9425659179688 0 2604.458
567.2302856445312 0 2706.2693
567.2798461914062 0 2578.9397
568.2335205078125 0 908.83606
568.2830810546875 0 1658.2361
568.7957763671875 0 5112.6406
569.2950439453125 0 2168.1296
569.7769775390625 0 1014.2404
569.9351806640625 0 796.5186
570.2761840820312 0 3557.2812
570.7772216796875 0 4351.181
571.2794799804688 0 2096.3872
571.7813110351562 0 2566.468
572.2809448242188 0 972.22784
572.9553833007812 0 1863.6174
573.2881469726562 0 1806.925
573.9573974609375 0 805.11035
574.287841796875 0 1108.8834
577.2807006835938 0 2405.7678 w 6
577.3304443359375 0 918.4301
577.7857055664062 0 12856.072
577.9584350585938 0 1629.8088
578.2861328125 0 8608.285
578.78759765625 0 9505.242
579.2874145507812 0 3878.1409
579.7852783203125 0 1466.0671
580.2742919921875 0 1175.2875 y Water loss 2
580.608154296875 0 2344.0874 y Ammonia loss 2
580.939208984375 0 13485.001 z 2
581.2738647460938 0 11969.251
581.6078491210938 0 5006.3936
581.943359375 0 2089.9004
582.2742309570312 0 4503.0264
583.2810668945312 0 1570.4255
585.7825927734375 0 962.8078
586.2803344726562 0 7628.4775 y 2
586.6142578125 0 5568.45
586.9484252929688 0 2449.1184
587.2837524414062 0 3019.8838
587.6240234375 0 1965.4609
587.9584350585938 0 1015.586
588.2838134765625 0 7575.0996
588.6168212890625 0 6052.798
588.9503784179688 0 2638.8884
589.2843017578125 0 841.5392 z Ammonia loss 11
590.78369140625 0 2303.1274 z Water loss 6
591.2808837890625 0 2913.3987 z Ammonia loss 6
591.9570922851562 0 1086.8082
592.290283203125 0 1644.7747
592.6272583007812 0 8990.195
592.9602661132812 0 8127.323
593.2913818359375 0 3515.02
593.6300048828125 0 1217.2185
596.9635009765625 0 1077.769
597.2886962890625 0 1758.235
598.302734375 0 2756.0342
598.7959594726562 0 2715.9834 y Water loss 6
599.2845458984375 0 4094.632 y Ammonia loss 6
599.7815551757812 0 42414.773 z 6
600.2835693359375 0 44482.273
600.7850341796875 0 24936.926
601.2868041992188 0 8928.211 c Water loss 13
601.6288452148438 0 2761.7017 c Ammonia loss 13
601.7861938476562 0 2152.3513
601.9573974609375 0 2339.1013
602.290283203125 0 1758.5911
602.619873046875 0 2667.324
602.794677734375 0 1159.8376
602.94775390625 0 7676.2637
603.2828369140625 0 9119.121
603.6170043945312 0 2875.2195
603.9521484375 0 1306.2698
604.282470703125 0 1020.8088
606.305419921875 0 30344.152 z 11
606.6343383789062 0 2084.1772
606.9683837890625 0 9579.167
607.3002319335938 0 50255.734 c 13
607.6328735351562 0 38871.41
607.79052734375 0 46384.145 y 6
607.9661254882812 0 22020.025
608.2947387695312 0 38738.543
608.6328735351562 0 2172.195
608.7926635742188 0 11893.814
609.2975463867188 0 4466.42
610.2854614257812 0 5381.4346
611.296875 0 2702.9702 c Water loss 9
611.7989501953125 0 10606.145 c Ammonia loss 9
611.9610595703125 0 6236.532
612.2997436523438 0 17392.428
612.63134765625 0 3211.8345
612.8046875 0 3908.802
612.9602661132812 0 2047.8882
613.3021240234375 0 2752.7202
615.303955078125 0 1823.9857
616.30224609375 0 888.5668
618.3338012695312 0 967.96356
619.8154296875 0 1078.9512
619.9900512695312 0 897.3969
620.3106079101562 0 55941.645 c 9
620.6387939453125 0 5639.427
620.812255859375 0 36494.9
620.9789428710938 0 10509.714
621.24169921875 0 1334.3423
621.3137817382812 0 22624.424
621.6505126953125 0 5646.419
621.814453125 0 4683.7266
621.9745483398438 0 1821.1934
622.242431640625 0 1034.0645
622.3228759765625 0 16681.861 y 11
623.3262939453125 0 4676.039
625.6504516601562 0 3694.0818
625.9833984375 0 5818.0547
626.3092041015625 0 7835.419
626.6314697265625 0 15433.0205 z 1
626.8190307617188 0 1154.9362
626.9631958007812 0 15489.257
627.2982788085938 0 9643.708
627.6300048828125 0 4956.0146
627.8202514648438 0 862.983
627.9640502929688 0 3237.1973
628.3048706054688 0 1138.8868
629.9913330078125 0 955.4099
630.3109130859375 0 1641.1028
630.6437377929688 0 6128.075
630.9796752929688 0 7367.8965
631.3060302734375 0 4259.502
631.6474609375 0 1333.922
631.9677734375 0 3100.0657 y 1
632.3016357421875 0 4079.1472
632.6358642578125 0 1043.9908
633.3131713867188 0 1020.9955
634.3215942382812 0 2497.4636
634.6484985351562 0 2981.186
634.7958984375 0 26038.627 w 5
634.98193359375 0 3991.5276
635.2999267578125 0 18554.025
635.654296875 0 2633.1636
635.7977905273438 0 6730.92
635.988525390625 0 2759.184
636.33740234375 0 2278.2817
636.8042602539062 0 1245.5125
637.3553466796875 0 1046.561
638.2701416015625 0 1054.1337
638.9849853515625 0 1531.7965
639.3214721679688 0 1568.1465
639.6607666015625 0 1053.6548
640.322265625 0 18684.191
640.6561889648438 0 24232.91
640.816650390625 0 1099.2549
640.990234375 0 17090.816
641.3241577148438 0 10684.982
641.6563720703125 0 3836.1497
641.9948120117188 0 1324.6866
643.8240966796875 0 5385.0176
644.3229370117188 0 5581.282
644.652587890625 0 6271.3574
644.8248291015625 0 1713.9276
644.98583984375 0 10835.033
645.3202514648438 0 6128.2295
645.650146484375 0 6916.503
645.9824829101562 0 4754.3574
646.3167114257812 0 4064.5422
646.6500854492188 0 2242.812
647.3175048828125 0 1301.5648 z Water loss 5
649.3215942382812 0 2599.487
649.6534423828125 0 1369.691
649.9813232421875 0 16186.448
650.3230590820312 0 76188.26
650.6586303710938 0 82243.33
650.82470703125 0 1790.7579
650.9927978515625 0 46941.5
651.326416015625 0 25347.416
651.66162109375 0 10361.13
651.8107299804688 0 1181.9093
651.9955444335938 0 2108.7446
652.3206787109375 0 7034.6094
653.323974609375 0 3286.1936
654.3294067382812 0 2117.2485
654.6580810546875 0 1373.2484
654.9932250976562 0 3967.4985
655.3251953125 0 7242.326 y Water loss 5
655.6581420898438 0 4127.7305
655.8232421875 0 1997.9546 y Ammonia loss 5
655.9929809570312 0 3881.8518
656.3232421875 0 23193.262 z 5
656.8246459960938 0 19268.996
656.994140625 0 1014.524
657.3264770507812 0 12236.996
657.831787109375 0 4873.8755
658.332763671875 0 2162.094
658.6502075195312 0 2431.7473
658.9864501953125 0 3654.8984
659.3192138671875 0 3922.5168
659.6527709960938 0 2472.0647
663.2909545898438 0 2355.6174
663.82666015625 0 2690.5852
663.9911499023438 0 2393.1694
664.3309326171875 0 35233.184 y 5
664.6568603515625 0 91918.5
664.8333740234375 0 12783.948
664.990966796875 0 100141.58
665.3264770507812 0 53634.023
665.6585693359375 0 22941.39
665.8351440429688 0 3387.1367
665.9925537109375 0 10398.263
666.327392578125 0 6255.231
669.6602172851562 0 19257.414
669.9954833984375 0 42111.98
670.3306884765625 0 68758.125
670.6654663085938 0 52743.555
670.999755859375 0 29454.63
671.3325805664062 0 13881.6455
671.6676635742188 0 6005.785
671.8247680664062 0 2774.8833
672.0036010742188 0 1793.6306
672.31494140625 0 2920.8894
672.8106689453125 0 1704.9093
679.34130859375 0 2107.8276 c Water loss 4
679.8351440429688 0 30431.402
680.3211669921875 0 49501.324 c Ammonia loss 4
680.8384399414062 0 10124.786
681.3214721679688 0 22223.547
682.3275756835938 0 5177.4824
683.3295288085938 0 1984.3473
683.8358764648438 0 1092.2682
685.3243408203125 0 1148.5508
691.3373413085938 0 13642.617
691.8385620117188 0 10528.337
692.3389282226562 0 5534.2153
692.8391723632812 0 2242.2092 c Water loss 10
693.3336181640625 0 1112.3965 c Ammonia loss 10
693.8339233398438 0 2996.8413
694.337158203125 0 2133.9724
697.3424682617188 0 27354.271 c 4
698.3468627929688 0 13549.24
699.3455810546875 0 2830.1172
701.3442993164062 0 1717.2139
701.841796875 0 43510.457 c 10
702.3434448242188 0 36170.88
702.8458251953125 0 14840.766
703.3477172851562 0 4932.589
703.8529663085938 0 2051.6794
705.3558959960938 0 3418.653
705.8533325195312 0 2391.178
711.307861328125 0 2078.9126
711.3677368164062 0 1102.6022
714.377197265625 0 2096.9014
719.330810546875 0 1088.9775
719.8541259765625 0 980.4417
720.3560791015625 0 4275.292
720.8605346679688 0 2811.3577
721.3480224609375 0 3717.783
722.3579711914062 0 2447.0352
723.3654174804688 0 1334.7301
726.8587036132812 0 2331.3845
727.3576049804688 0 1023.082
728.3655395507812 0 1470.0737
741.3665771484375 0 962.1426
742.3475952148438 0 3034.5957
742.850341796875 0 2461.2217
743.3502807617188 0 1091.2218
744.3857421875 0 3794.3179
744.8867797851562 0 2593.4087
745.39111328125 0 1055.512
748.8562622070312 0 2429.8345 y Ammonia loss 4
749.36279296875 0 12293.692 z 4
749.8650512695312 0 15591.488
750.367431640625 0 11061.844
750.8692626953125 0 5234.7285
751.3658447265625 0 1918.6252
752.3788452148438 0 1262.1588
757.372802734375 0 3574.1558 c Ammonia loss 11
757.874755859375 0 4411.344
758.378173828125 0 3207.0757
758.884033203125 0 1142.4734
762.864501953125 0 2278.0896
763.3673095703125 0 1202.1371
763.8621826171875 0 1262.3126
765.4025268554688 0 2427.918
765.8897094726562 0 4211.079 c 11
766.3985595703125 0 9597.262
766.89306640625 0 5364.419
767.4059448242188 0 3086.8599
768.3587646484375 0 2884.1777 y Ammonia loss 10
769.3674926757812 0 16910.809 z 10
770.3724975585938 0 12399.53
770.8724365234375 0 1182.6306
771.3723754882812 0 4931.8394
776.3776245117188 0 1788.623
777.3712158203125 0 1883.1284
777.8704833984375 0 3591.2227
778.37060546875 0 2587.8755
778.87841796875 0 1217.778
779.4012451171875 0 1227.683
784.3825073242188 0 2276.0283 w 3
784.8782958984375 0 1720.627
785.3860473632812 0 8141.744 y 10
785.8827514648438 0 2857.793
786.3870849609375 0 3145.679
787.3978881835938 0 1987.0693
793.3997802734375 0 20491.764 c Ammonia loss 5
794.4033203125 0 12749.156
795.408203125 0 3965.0205
796.415283203125 0 1384.6147
802.4306640625 0 1268.7397
802.9241943359375 0 3070.6755
803.4207763671875 0 2808.3926
803.9170532226562 0 3210.9468
804.423828125 0 3374.2317
804.92724609375 0 1692.5034
805.4188232421875 0 1736.408
806.377685546875 0 2540.6797 y Ammonia loss 3
806.8766479492188 0 9965.494 z 3
807.3793334960938 0 22723.957
807.8812866210938 0 17208.229
808.3825073242188 0 7468.149
808.8839111328125 0 3354.8682
809.388427734375 0 1914.657
810.426025390625 0 83415.65 c 5
811.4291381835938 0 44547.5
812.4324951171875 0 12357.347
813.4405517578125 0 1150.382
814.8854370117188 0 2344.183 y 3
815.3840942382812 0 1550.114
816.8905029296875 0 2240.6538
817.3916625976562 0 4239.1934
817.8920288085938 0 2822.3894
818.394775390625 0 1718.9397
823.9066162109375 0 1094.1908
824.4011840820312 0 2191.999
824.8953247070312 0 1110.8109
826.8995971679688 0 2866.684
827.401123046875 0 2683.1973
828.40283203125 0 1924.4172
838.39013671875 0 1808.4094
838.8877563476562 0 1347.0714
839.4021606445312 0 2792.7925 w 9
840.4041137695312 0 3224.7332
841.4026489257812 0 3361.3796
841.8998413085938 0 6985.6016 w 2
842.4015502929688 0 5694.899
842.904052734375 0 1345.5706
843.40478515625 0 1075.493
845.4014282226562 0 1201.3958
846.3965454101562 0 8232.214 c 12
846.8987426757812 0 9975.086
847.4008178710938 0 6670.925
847.9011840820312 0 3965.2761
848.4021606445312 0 1849.8213
848.9033203125 0 1027.854
859.43408203125 0 1641.3131
859.9343872070312 0 1663.842
860.4293823242188 0 2241.0833
861.431640625 0 1143.6014
862.40185546875 0 1271.3329 z Ammonia loss 2
865.42626953125 0 1040.1251
866.4297485351562 0 1179.9349
867.4313354492188 0 2325.397
870.9053344726562 0 10779.23 z 2
871.4088745117188 0 37081.895
871.9102172851562 0 34612.38
872.4110107421875 0 13414.424
872.9127807617188 0 5685.1465
873.4158325195312 0 2964.152
878.9156494140625 0 2349.0579 y 2
879.417724609375 0 2831.5227
879.9281616210938 0 945.00146
880.4266967773438 0 2652.822
880.9268798828125 0 2140.693
881.4390869140625 0 6824.541
881.9403686523438 0 2194.7368
882.4436645507812 0 4560.0747
883.4527587890625 0 1252.9178
888.43896484375 0 2041.8623
888.93994140625 0 9715.381
889.4429931640625 0 8702.417
889.9412841796875 0 5175.885
890.440673828125 0 2021.6831
895.9317626953125 0 1008.4755
896.43017578125 0 1243.3213 y Water loss 9
898.4105224609375 0 13114.591 z 9
899.4166870117188 0 18554.852
900.4188842773438 0 7782.1694
901.4213256835938 0 2307.0144
902.4287109375 0 1718.2924
902.9319458007812 0 980.5149
903.4357299804688 0 1110.7648
908.4268798828125 0 9179.587 c Ammonia loss 6
909.4314575195312 0 5297.6514
910.4384155273438 0 6018.475 c 13
910.9391479492188 0 4683.9463
911.4449462890625 0 3236.1716
911.9487915039062 0 1003.53534
914.4283447265625 0 2891.9504 y 9
915.4353637695312 0 987.99084
923.45751953125 0 1552.9186
925.4524536132812 0 4973.632 c 6
926.4581298828125 0 4154.1914
927.4589233398438 0 1227.3495
930.9683227539062 0 2273.8354
931.4697265625 0 2174.2988
931.97314453125 0 2805.4006
938.4661865234375 0 5138.204
938.9701538085938 0 1262.3203
939.45703125 0 4411.716 z 1
939.9392700195312 0 14274.62
940.4407958984375 0 14149.3955
940.94091796875 0 6608.9897
941.4423828125 0 3881.7332
945.48388671875 0 989.7325
945.9796752929688 0 995.336
946.4853515625 0 1472.6549
952.4735107421875 0 3383.8274
952.9771728515625 0 2660.365
953.4769287109375 0 2626.02
953.9772338867188 0 1449.2927
954.477294921875 0 1181.8936
958.4784545898438 0 1331.5752
960.4813232421875 0 1258.8881
960.9813232421875 0 2443.2212
961.4818115234375 0 1762.5104
961.9840698242188 0 1292.4161
963.9682006835938 0 1038.1694
964.471435546875 0 1054.104 c Water loss 7
966.4595947265625 0 3518.4858
966.9776000976562 0 2404.899
967.47216796875 0 6714.213
967.9798583984375 0 4939.7124
968.4828491210938 0 3763.5774
969.5014038085938 0 973.602
974.4776000976562 0 2115.7056
974.984130859375 0 4590.31
975.4845581054688 0 7398.686
975.9871215820312 0 5638.419
976.4886474609375 0 3126.763
976.9937133789062 0 1955.925
981.4724731445312 0 933.8108
982.474853515625 0 19284.729 c 7
982.9824829101562 0 1701.0848
983.4802856445312 0 14056.991
984.4832763671875 0 5623.1235
985.4885864257812 0 1552.6821
990.9825439453125 0 1286.4524
996.48193359375 0 9439.403
996.9832763671875 0 8345.689
997.4843139648438 0 7048.2744
997.9853515625 0 3091.9583
1004.9917602539062 0 5236.772
1005.4951171875 0 7956.2563
1005.9972534179688 0 5392.916
1010.4658813476562 0 1408.5436
1026.5047607421875 0 2919.747 z 8
1027.512451171875 0 10763.558
1028.5157470703125 0 5126.0483
1029.5162353515625 0 2507.1375
1051.5546875 0 1699.234
1066.553466796875 0 1811.1691
1083.5262451171875 0 1478.6597 z 7
1084.536376953125 0 2844.02
1085.532958984375 0 1849.4253
1094.5469970703125 0 4013.5251
1095.552734375 0 2914.5833
1099.546630859375 0 1354.493 y 7
1110.5682373046875 0 17838.56 c 8
1111.5723876953125 0 16347.527
1112.578125 0 7987.928
1113.5838623046875 0 2422.5833
1136.52685546875 0 1384.2124
1140.54736328125 0 1155.1368
1141.557861328125 0 881.5934
1181.6036376953125 0 1113.3319
1196.6021728515625 0 1152.7286
1198.559814453125 0 1092.6464 z 6
1199.5609130859375 0 7304.872
1200.5615234375 0 4866.3457
1201.5758056640625 0 1190.005
1223.586669921875 0 1157.9645
1224.61083984375 0 1373.1641
1225.602783203125 0 930.53284
1239.6094970703125 0 4532.145 c 9
1240.615478515625 0 3290.5183
1241.6226806640625 0 2732.8494
1242.6221923828125 0 785.95685
1264.6092529296875 0 1122.0659
1265.6163330078125 0 1208.7954
1266.6142578125 0 1186.6646
1311.6307373046875 0 1620.393 z 5
1312.641845703125 0 5097.7134
1313.6458740234375 0 4849.952
1314.642333984375 0 2175.2727
1344.6502685546875 0 985.7899
1358.656494140625 0 1174.2015
1359.6658935546875 0 4171.3267
1360.6689453125 0 2740.3586
1361.6771240234375 0 1343.7861
1387.6583251953125 0 1022.34216
1402.6715087890625 0 2576.0215 c 10
1403.6766357421875 0 3498.895
1404.6873779296875 0 2251.2412
1497.722900390625 0 1561.7681 z 4
1498.72119140625 0 1236.3064
1515.7564697265625 0 1014.3819
1532.789306640625 0 880.4684
1613.74658203125 0 1210.7501
1614.734375 0 1034.0631
1692.7918701171875 0 930.90796
1693.7952880859375 0 852.61707
1742.812744140625 0 1664.011
1743.8006591796875 0 1040.21
1879.8873291015625 0 799.4868

Spectrum Details

|  |  |
| --- | --- |
| Matched peaks? Matched peaksThe total absolute number of peaks matched. Additionally in brackets the total fraction of peaks matched and the total number of peaks is shown. | 151 (13.64% of 1107) |
| FDR? FDRThe false discovery rate estimated for this peptide. It is calculated by matching all theoretical fragments with a non-integer shift with the raw peaks for this spectrum. This is done with 40 different shifts. The resulting percentage is the average number of annotated peaks over the number of annotated peaks with the correct spectrum. | 1.12% |
| Satellite FDR? Satellite FDRSee the FDR for details on its calculation. This satellite ion specific FDR only contains the satellite ions (d/w) for I/L/J positions. | 2.38% |
| PSM Score? PSM ScoreThe PSM Score as given by Hecklib to this annotated spectrum. It is shown with three significant figures. | 452 |

## Spectrum 5486? Spectrum 5486 The raw spectrum of this peptide as annotated by Hecklib. The fragments are coloured according to ion type (see legend). Any peaks with a star '\*' as text can be hovered over to see the full details, first the ion type second the mass shift type. By hovering over the amino acids in the peptide or ions in the legend the corresponding peaks are highlighted. By toggling the 'Unassigned' label you can turn the background (unassigned) peaks on or off in the plot. By updating the slider in the Ion legend you can update the spectrum to only show the top X% of the peaks with labels. The top X% means any peak that is within X% of the highest intensity. By dragging in the spectrum you can zoom in to a specific part of the spectrum and use 'Zoom Out' to get back to the original zoom level. The annotation of the spectrum is based on the given sequence in the peptides file and is done with different software so inconsistencies are likely. The peaks are annotated based on the given sequence, with 20 ppm tolerance.

Copy Data

### Spectrum 5486 (TSV)

#### Preview

```
Loading example...
```

*Click on the button to copy the data to your clipboard.*

Mz MinMz MaxIntensity Max

WidthHeightPeptide font sizePeptide stroke widthSpectrum font sizeSpectrum stroke widthCompact peptide

Ion legend

wxyz

abcd

OtherUnassignedIonChargePositionShow for top:%

JHQDWLDGKEYKCKK

08.74e+31.75e+42.62e+43.50e+4

Zoom Out

w+22c+12c+25y+38c+13y+25c+13c+310w+26y+310z+26y+414c+14c+14y+27w+14y+28c+29w+29y+313y+29z+29z+14c+314y+29c+210c+210c+210z+314y+14y+314y+314y+314w+210z+210y+210z+210y+210c+15c+15c+15c+211y+211z+211c+212c+212y+15z+15y+15c+16y+212z+212c+16y+212w+213c+213y+213z+213y+213z+16c+17c+214c+17z+214y+214c+18c+18w+17c+18z+17c+19z+19c+110z+110c+111z+111c+112c+112z+112c+113z+113

0508101615242032

Fragment Matches Table

Show background peaks

| Position | Ion type | Intensity | mz Theoretical | mz Error (Th) | mz Error (ppm) | Charge | Series Number |
| --- | --- | --- | --- | --- | --- | --- | --- |
| - | - | 391.3 | 120.1 | - | - | 0 | - |
| - | - | 355.7 | 120.9 | - | - | 0 | - |
| - | - | 391.5 | 129.1 | - | - | 0 | - |
| - | - | 2291 | 129.1 | - | - | 0 | - |
| - | - | 1969 | 130.1 | - | - | 0 | - |
| 14 | w | 431 | 130.1 | 0.0004984 | 3.832 | +2 | 2 |
| - | - | 506.5 | 133.1 | - | - | 0 | - |
| - | - | 1297 | 133.1 | - | - | 0 | - |
| - | - | 679.8 | 136.1 | - | - | 0 | - |
| - | - | 411 | 138.8 | - | - | 0 | - |
| - | - | 540.1 | 140.1 | - | - | 0 | - |
| - | - | 559.3 | 147.1 | - | - | 0 | - |
| - | - | 866.4 | 149 | - | - | 0 | - |
| - | - | 1114 | 149 | - | - | 0 | - |
| - | - | 2091 | 155.1 | - | - | 0 | - |
| - | - | 459.6 | 156.1 | - | - | 0 | - |
| - | - | 464.2 | 156.6 | - | - | 0 | - |
| - | - | 900.4 | 159.1 | - | - | 0 | - |
| - | - | 452.2 | 165.1 | - | - | 0 | - |
| - | - | 1509 | 166.1 | - | - | 0 | - |
| - | - | 596.5 | 167.1 | - | - | 0 | - |
| - | - | 480.7 | 168.1 | - | - | 0 | - |
| - | - | 1054 | 171.1 | - | - | 0 | - |
| - | - | 998.5 | 173.1 | - | - | 0 | - |
| - | - | 3785 | 173.5 | - | - | 0 | - |
| - | - | 638.8 | 174.1 | - | - | 0 | - |
| - | - | 508.7 | 175.1 | - | - | 0 | - |
| - | - | 628.7 | 177.1 | - | - | 0 | - |
| - | - | 945.1 | 183.1 | - | - | 0 | - |
| - | - | 2631 | 185.2 | - | - | 0 | - |
| - | - | 973.1 | 187.1 | - | - | 0 | - |
| - | - | 1372 | 191.1 | - | - | 0 | - |
| - | - | 1248 | 199.1 | - | - | 0 | - |
| - | - | 645.8 | 201.1 | - | - | 0 | - |
| - | - | 1361 | 202.1 | - | - | 0 | - |
| - | - | 2466 | 203.1 | - | - | 0 | - |
| - | - | 561.9 | 205.1 | - | - | 0 | - |
| - | - | 1085 | 207.1 | - | - | 0 | - |
| - | - | 1825 | 209.1 | - | - | 0 | - |
| - | - | 725.5 | 213.1 | - | - | 0 | - |
| - | - | 7861 | 215.1 | - | - | 0 | - |
| - | - | 667.9 | 216.1 | - | - | 0 | - |
| - | - | 638.4 | 217.1 | - | - | 0 | - |
| - | - | 1172 | 217.2 | - | - | 0 | - |
| - | - | 1738 | 218.1 | - | - | 0 | - |
| - | - | 1785 | 223.2 | - | - | 0 | - |
| - | - | 1670 | 227.1 | - | - | 0 | - |
| - | - | 639.8 | 229.1 | - | - | 0 | - |
| - | - | 762.2 | 229.2 | - | - | 0 | - |
| - | - | 640.8 | 230.1 | - | - | 0 | - |
| - | - | 924.4 | 231.2 | - | - | 0 | - |
| - | - | 2669 | 234.1 | - | - | 0 | - |
| - | - | 816.2 | 239.1 | - | - | 0 | - |
| - | - | 808.9 | 242.2 | - | - | 0 | - |
| - | - | 9716 | 251.1 | - | - | 0 | - |
| - | - | 1745 | 252.2 | - | - | 0 | - |
| - | - | 730.7 | 261.1 | - | - | 0 | - |
| 2 | c | 1200 | 268.2 | 0.0001351 | 0.5039 | +1 | 2 |
| - | - | 550.9 | 275.1 | - | - | 0 | - |
| - | - | 1952 | 281.1 | - | - | 0 | - |
| - | - | 495.2 | 282.1 | - | - | 0 | - |
| - | - | 868.7 | 282.2 | - | - | 0 | - |
| - | - | 1184 | 283.2 | - | - | 0 | - |
| - | - | 536.4 | 293.6 | - | - | 0 | - |
| - | - | 8037 | 299.1 | - | - | 0 | - |
| - | - | 903.7 | 299.1 | - | - | 0 | - |
| - | - | 673.7 | 299.2 | - | - | 0 | - |
| - | - | 1576 | 300.1 | - | - | 0 | - |
| - | - | 682.7 | 300.1 | - | - | 0 | - |
| - | - | 1201 | 302.2 | - | - | 0 | - |
| - | - | 562.6 | 310.1 | - | - | 0 | - |
| - | - | 1910 | 326.7 | - | - | 0 | - |
| - | - | 508.9 | 329.1 | - | - | 0 | - |
| - | - | 928.1 | 332.2 | - | - | 0 | - |
| - | - | 1379 | 338.1 | - | - | 0 | - |
| - | - | 1497 | 340.2 | - | - | 0 | - |
| 5 | c | 1088 | 340.7 | 0.0002619 | 0.7689 | +2 | 5 |
| - | - | 767.4 | 354.1 | - | - | 0 | - |
| - | - | 1596 | 359 | - | - | 0 | - |
| - | - | 1201 | 367.2 | - | - | 0 | - |
| 8 | y | 606.3 | 367.2 | 0.005462 | 14.88 | +3 | 8 |
| - | - | 556.5 | 367.7 | - | - | 0 | - |
| 3 | c | 4688 | 379.2 | 0.0005779 | 1.524 | +1 | 3 |
| - | - | 1006 | 380.2 | - | - | 0 | - |
| - | - | 2073 | 387.2 | - | - | 0 | - |
| 11 | y | 1340 | 393.2 | 0.004269 | 10.86 | +2 | 5 |
| - | - | 1250 | 393.7 | - | - | 0 | - |
| 3 | c | 4479 | 396.2 | 0.0006377 | 1.609 | +1 | 3 |
| - | - | 1157 | 397.2 | - | - | 0 | - |
| - | - | 664.8 | 407.7 | - | - | 0 | - |
| 10 | c | 645.6 | 408.2 | 0.001677 | 4.108 | +3 | 10 |
| - | - | 2058 | 415 | - | - | 0 | - |
| - | - | 1314 | 416 | - | - | 0 | - |
| 10 | w | 9720 | 420.2 | 0.004516 | 10.75 | +2 | 6 |
| - | - | 5197 | 420.7 | - | - | 0 | - |
| - | - | 1450 | 421.2 | - | - | 0 | - |
| - | - | 1036 | 421.2 | - | - | 0 | - |
| - | - | 732.3 | 431.2 | - | - | 0 | - |
| - | - | 703.2 | 435.7 | - | - | 0 | - |
| 6 | y | 614.1 | 443.2 | 0.008773 | 19.79 | +3 | 10 |
| - | - | 1282 | 444.2 | - | - | 0 | - |
| - | - | 805.8 | 444.3 | - | - | 0 | - |
| - | - | 761.4 | 449.2 | - | - | 0 | - |
| 10 | z | 1663 | 449.7 | 0.00641 | 14.25 | +2 | 6 |
| - | - | 7528 | 457.3 | - | - | 0 | - |
| - | - | 604.8 | 457.7 | - | - | 0 | - |
| - | - | 3160 | 458.2 | - | - | 0 | - |
| - | - | 2861 | 458.3 | - | - | 0 | - |
| - | - | 1958 | 458.7 | - | - | 0 | - |
| - | - | 4610 | 459.3 | - | - | 0 | - |
| - | - | 1147 | 460.3 | - | - | 0 | - |
| - | - | 1755 | 460.7 | - | - | 0 | - |
| - | - | 1010 | 461.2 | - | - | 0 | - |
| - | - | 989.4 | 466.2 | - | - | 0 | - |
| - | - | 791.7 | 467.2 | - | - | 0 | - |
| - | - | 1381 | 467.2 | - | - | 0 | - |
| - | - | 1195 | 468.3 | - | - | 0 | - |
| - | - | 690.5 | 468.8 | - | - | 0 | - |
| - | - | 1711 | 469.2 | - | - | 0 | - |
| - | - | 704.8 | 470.2 | - | - | 0 | - |
| - | - | 809 | 471.3 | - | - | 0 | - |
| - | - | 1007 | 472.3 | - | - | 0 | - |
| 2 | y | 1161 | 474.2 | 0.0005078 | 1.071 | +4 | 14 |
| - | - | 1132 | 474.5 | - | - | 0 | - |
| - | - | 712.9 | 476.5 | - | - | 0 | - |
| - | - | 1415 | 483.2 | - | - | 0 | - |
| - | - | 1647 | 484.2 | - | - | 0 | - |
| - | - | 903 | 484.7 | - | - | 0 | - |
| - | - | 3054 | 485.2 | - | - | 0 | - |
| - | - | 4256 | 486.3 | - | - | 0 | - |
| - | - | 789.4 | 487.2 | - | - | 0 | - |
| - | - | 712.1 | 487.3 | - | - | 0 | - |
| - | - | 2156 | 488.3 | - | - | 0 | - |
| - | - | 3913 | 493.7 | - | - | 0 | - |
| 4 | c | 4393 | 494.2 | 0.003271 | 6.619 | +1 | 4 |
| - | - | 1439 | 494.7 | - | - | 0 | - |
| - | - | 676.9 | 495.2 | - | - | 0 | - |
| - | - | 729.6 | 496.3 | - | - | 0 | - |
| - | - | 766.6 | 498.2 | - | - | 0 | - |
| - | - | 2490 | 501.2 | - | - | 0 | - |
| - | - | 1683 | 501.3 | - | - | 0 | - |
| - | - | 1428 | 501.7 | - | - | 0 | - |
| - | - | 5016 | 502.3 | - | - | 0 | - |
| - | - | 5088 | 502.5 | - | - | 0 | - |
| - | - | 1.213E+04 | 502.8 | - | - | 0 | - |
| - | - | 4070 | 503 | - | - | 0 | - |
| - | - | 4363 | 503.1 | - | - | 0 | - |
| - | - | 4859 | 503.3 | - | - | 0 | - |
| - | - | 643.4 | 503.5 | - | - | 0 | - |
| - | - | 897.9 | 503.8 | - | - | 0 | - |
| - | - | 1573 | 504.1 | - | - | 0 | - |
| - | - | 2052 | 504.3 | - | - | 0 | - |
| - | - | 2527 | 504.3 | - | - | 0 | - |
| - | - | 606.1 | 504.3 | - | - | 0 | - |
| - | - | 849.2 | 506.3 | - | - | 0 | - |
| 4 | c | 1.302E+04 | 511.3 | 0.000298 | 0.5829 | +1 | 4 |
| - | - | 2716 | 512.3 | - | - | 0 | - |
| - | - | 1754 | 515.3 | - | - | 0 | - |
| - | - | 651.9 | 516.3 | - | - | 0 | - |
| - | - | 1789 | 519.3 | - | - | 0 | - |
| 9 | y | 2304 | 521.8 | 0.005267 | 10.1 | +2 | 7 |
| - | - | 1059 | 522.3 | - | - | 0 | - |
| - | - | 988.4 | 522.8 | - | - | 0 | - |
| - | - | 764.5 | 528.3 | - | - | 0 | - |
| - | - | 1111 | 530.8 | - | - | 0 | - |
| - | - | 644.1 | 535.3 | - | - | 0 | - |
| - | - | 1175 | 547.3 | - | - | 0 | - |
| 12 | w | 730.8 | 548.2 | 0.008991 | 16.4 | +1 | 4 |
| 8 | y | 2865 | 550.3 | 0.006905 | 12.55 | +2 | 8 |
| - | - | 1998 | 550.8 | - | - | 0 | - |
| - | - | 811.3 | 553.8 | - | - | 0 | - |
| - | - | 895.4 | 554.8 | - | - | 0 | - |
| - | - | 3113 | 555.3 | - | - | 0 | - |
| 9 | c | 9628 | 555.8 | 0.0006458 | 1.162 | +2 | 9 |
| - | - | 4587 | 556.3 | - | - | 0 | - |
| - | - | 1883 | 556.8 | - | - | 0 | - |
| - | - | 866.4 | 563.8 | - | - | 0 | - |
| - | - | 1700 | 564.3 | - | - | 0 | - |
| - | - | 1024 | 568.8 | - | - | 0 | - |
| - | - | 683.7 | 569.3 | - | - | 0 | - |
| - | - | 833.8 | 570 | - | - | 0 | - |
| - | - | 1551 | 570.3 | - | - | 0 | - |
| - | - | 3371 | 571.3 | - | - | 0 | - |
| - | - | 2179 | 571.8 | - | - | 0 | - |
| - | - | 1194 | 572.3 | - | - | 0 | - |
| 7 | w | 1847 | 577.3 | 0.006511 | 11.28 | +2 | 9 |
| - | - | 1467 | 577.8 | - | - | 0 | - |
| - | - | 1604 | 578.3 | - | - | 0 | - |
| 3 | y | 999.6 | 580.3 | 0.003006 | 5.181 | +3 | 13 |
| - | - | 646 | 581.3 | - | - | 0 | - |
| - | - | 646.3 | 584.3 | - | - | 0 | - |
| - | - | 679.8 | 586.6 | - | - | 0 | - |
| - | - | 734.4 | 588 | - | - | 0 | - |
| - | - | 723.3 | 593 | - | - | 0 | - |
| - | - | 1896 | 597.3 | - | - | 0 | - |
| - | - | 738.2 | 597.6 | - | - | 0 | - |
| - | - | 906.4 | 597.8 | - | - | 0 | - |
| - | - | 841.4 | 598 | - | - | 0 | - |
| - | - | 575.9 | 598.8 | - | - | 0 | - |
| 7 | y | 2036 | 599.3 | 0.007241 | 12.08 | +2 | 9 |
| 7 | z | 5694 | 599.8 | 0.003263 | 5.44 | +2 | 9 |
| - | - | 3838 | 600.3 | - | - | 0 | - |
| - | - | 1193 | 600.8 | - | - | 0 | - |
| - | - | 751.6 | 601.4 | - | - | 0 | - |
| - | - | 1150 | 602 | - | - | 0 | - |
| - | - | 1184 | 603 | - | - | 0 | - |
| - | - | 882 | 603.3 | - | - | 0 | - |
| - | - | 668.6 | 604.8 | - | - | 0 | - |
| - | - | 759.3 | 605 | - | - | 0 | - |
| 12 | z | 7670 | 606.3 | 0.009602 | 15.84 | +1 | 4 |
| - | - | 2470 | 607 | - | - | 0 | - |
| 14 | c | 1.11E+04 | 607.3 | 0.0004366 | 0.7189 | +3 | 14 |
| - | - | 6795 | 607.6 | - | - | 0 | - |
| 7 | y | 8386 | 607.8 | 0.003714 | 6.11 | +2 | 9 |
| - | - | 2550 | 608 | - | - | 0 | - |
| - | - | 6072 | 608.3 | - | - | 0 | - |
| - | - | 2608 | 608.8 | - | - | 0 | - |
| - | - | 1246 | 609.3 | - | - | 0 | - |
| 10 | c | 818.9 | 611.3 | 0.002027 | 3.315 | +2 | 10 |
| - | - | 798 | 611.6 | - | - | 0 | - |
| 10 | c | 926.6 | 611.8 | 0.005929 | 9.692 | +2 | 10 |
| - | - | 981.3 | 612 | - | - | 0 | - |
| - | - | 1635 | 612.3 | - | - | 0 | - |
| - | - | 1069 | 612.8 | - | - | 0 | - |
| - | - | 1145 | 613.3 | - | - | 0 | - |
| - | - | 808.8 | 614.6 | - | - | 0 | - |
| - | - | 803.6 | 616.3 | - | - | 0 | - |
| - | - | 790.2 | 617.6 | - | - | 0 | - |
| - | - | 3518 | 619.8 | - | - | 0 | - |
| 10 | c | 1.018E+04 | 620.3 | 0.001383 | 2.229 | +2 | 10 |
| - | - | 5449 | 620.8 | - | - | 0 | - |
| 2 | z | 859.6 | 621 | 0.008905 | 14.34 | +3 | 14 |
| - | - | 2840 | 621.3 | - | - | 0 | - |
| - | - | 705.9 | 621.6 | - | - | 0 | - |
| - | - | 1385 | 621.8 | - | - | 0 | - |
| 12 | y | 3757 | 622.3 | 0.008734 | 14.03 | +1 | 4 |
| - | - | 735 | 623.3 | - | - | 0 | - |
| - | - | 1079 | 625.6 | - | - | 0 | - |
| 2 | y | 990.8 | 626 | 0.00112 | 1.789 | +3 | 14 |
| 2 | y | 3034 | 626.3 | 0.01039 | 16.6 | +3 | 14 |
| - | - | 2115 | 626.6 | - | - | 0 | - |
| - | - | 1490 | 627 | - | - | 0 | - |
| - | - | 990.4 | 628 | - | - | 0 | - |
| - | - | 831.8 | 631.3 | - | - | 0 | - |
| - | - | 945 | 631.6 | - | - | 0 | - |
| 2 | y | 1575 | 632 | 0.002646 | 4.187 | +3 | 14 |
| - | - | 1009 | 632.3 | - | - | 0 | - |
| - | - | 677.5 | 633 | - | - | 0 | - |
| - | - | 2805 | 634.3 | - | - | 0 | - |
| - | - | 1475 | 634.6 | - | - | 0 | - |
| 6 | w | 6588 | 634.8 | 0.001489 | 2.346 | +2 | 10 |
| - | - | 1232 | 635 | - | - | 0 | - |
| - | - | 4930 | 635.3 | - | - | 0 | - |
| - | - | 1038 | 635.6 | - | - | 0 | - |
| - | - | 2676 | 635.8 | - | - | 0 | - |
| - | - | 2722 | 636.3 | - | - | 0 | - |
| - | - | 824 | 636.6 | - | - | 0 | - |
| - | - | 956.3 | 637 | - | - | 0 | - |
| - | - | 1474 | 637.3 | - | - | 0 | - |
| - | - | 793.2 | 639.3 | - | - | 0 | - |
| - | - | 3154 | 640 | - | - | 0 | - |
| - | - | 1.211E+04 | 640.3 | - | - | 0 | - |
| - | - | 8351 | 640.7 | - | - | 0 | - |
| - | - | 7869 | 641 | - | - | 0 | - |
| - | - | 2038 | 641.3 | - | - | 0 | - |
| - | - | 1097 | 641.7 | - | - | 0 | - |
| - | - | 806.9 | 642.6 | - | - | 0 | - |
| - | - | 891.7 | 644.3 | - | - | 0 | - |
| - | - | 1251 | 644.6 | - | - | 0 | - |
| - | - | 1685 | 645 | - | - | 0 | - |
| - | - | 1629 | 645.3 | - | - | 0 | - |
| - | - | 2355 | 645.6 | - | - | 0 | - |
| - | - | 4051 | 646 | - | - | 0 | - |
| - | - | 3580 | 646.3 | - | - | 0 | - |
| - | - | 1105 | 646.6 | - | - | 0 | - |
| - | - | 985.5 | 647 | - | - | 0 | - |
| 6 | z | 1096 | 647.3 | 0.01011 | 15.61 | +2 | 10 |
| - | - | 918.2 | 647.6 | - | - | 0 | - |
| - | - | 1575 | 648.3 | - | - | 0 | - |
| - | - | 941.9 | 648.6 | - | - | 0 | - |
| - | - | 753.4 | 649 | - | - | 0 | - |
| - | - | 1000 | 649.3 | - | - | 0 | - |
| - | - | 2996 | 649.6 | - | - | 0 | - |
| - | - | 9374 | 650 | - | - | 0 | - |
| - | - | 1.599E+04 | 650.3 | - | - | 0 | - |
| - | - | 9455 | 650.6 | - | - | 0 | - |
| - | - | 6115 | 651 | - | - | 0 | - |
| - | - | 4743 | 651.3 | - | - | 0 | - |
| - | - | 3146 | 651.6 | - | - | 0 | - |
| - | - | 1771 | 652 | - | - | 0 | - |
| - | - | 857.9 | 652.3 | - | - | 0 | - |
| - | - | 746.7 | 652.6 | - | - | 0 | - |
| - | - | 833.3 | 653 | - | - | 0 | - |
| - | - | 950.7 | 653.3 | - | - | 0 | - |
| - | - | 910.4 | 653.6 | - | - | 0 | - |
| - | - | 1165 | 654.3 | - | - | 0 | - |
| - | - | 1761 | 655 | - | - | 0 | - |
| - | - | 818.2 | 655.3 | - | - | 0 | - |
| - | - | 861.6 | 655.6 | - | - | 0 | - |
| 6 | y | 863.8 | 655.8 | 0.002624 | 4.001 | +2 | 10 |
| 6 | z | 3395 | 656.3 | 0.00666 | 10.15 | +2 | 10 |
| - | - | 2876 | 656.6 | - | - | 0 | - |
| - | - | 1036 | 656.8 | - | - | 0 | - |
| - | - | 3497 | 657 | - | - | 0 | - |
| - | - | 3032 | 657.3 | - | - | 0 | - |
| - | - | 1142 | 657.6 | - | - | 0 | - |
| - | - | 1131 | 658 | - | - | 0 | - |
| - | - | 969.1 | 658.3 | - | - | 0 | - |
| - | - | 1845 | 659 | - | - | 0 | - |
| - | - | 903.6 | 660 | - | - | 0 | - |
| - | - | 1579 | 662.3 | - | - | 0 | - |
| - | - | 2997 | 662.6 | - | - | 0 | - |
| - | - | 898.1 | 663 | - | - | 0 | - |
| - | - | 1238 | 664 | - | - | 0 | - |
| 6 | y | 6172 | 664.3 | 0.008576 | 12.91 | +2 | 10 |
| - | - | 1658 | 664.6 | - | - | 0 | - |
| - | - | 1999 | 664.8 | - | - | 0 | - |
| - | - | 1842 | 665 | - | - | 0 | - |
| - | - | 3099 | 665.3 | - | - | 0 | - |
| - | - | 1151 | 665.7 | - | - | 0 | - |
| - | - | 924.5 | 668.3 | - | - | 0 | - |
| - | - | 1672 | 668.7 | - | - | 0 | - |
| - | - | 985.5 | 669 | - | - | 0 | - |
| - | - | 4484 | 669.3 | - | - | 0 | - |
| - | - | 2.478E+04 | 669.7 | - | - | 0 | - |
| - | - | 3.46E+04 | 670 | - | - | 0 | - |
| - | - | 2.594E+04 | 670.3 | - | - | 0 | - |
| - | - | 1.249E+04 | 670.7 | - | - | 0 | - |
| - | - | 6833 | 671 | - | - | 0 | - |
| - | - | 3326 | 671.3 | - | - | 0 | - |
| - | - | 1863 | 671.7 | - | - | 0 | - |
| - | - | 861.2 | 675.3 | - | - | 0 | - |
| 5 | c | 742.9 | 679.3 | 0.008346 | 12.29 | +1 | 5 |
| 5 | c | 4231 | 680.3 | 0.0001444 | 0.2122 | +1 | 5 |
| - | - | 1201 | 681.3 | - | - | 0 | - |
| - | - | 1062 | 682.3 | - | - | 0 | - |
| - | - | 767.9 | 683.3 | - | - | 0 | - |
| - | - | 1958 | 690.3 | - | - | 0 | - |
| - | - | 1686 | 691.3 | - | - | 0 | - |
| - | - | 1052 | 691.8 | - | - | 0 | - |
| - | - | 831.4 | 695.9 | - | - | 0 | - |
| 5 | c | 1.247E+04 | 697.3 | 0.0005094 | 0.7305 | +1 | 5 |
| - | - | 5679 | 698.3 | - | - | 0 | - |
| - | - | 1835 | 699.3 | - | - | 0 | - |
| - | - | 1314 | 699.8 | - | - | 0 | - |
| - | - | 1119 | 700.3 | - | - | 0 | - |
| - | - | 2255 | 701.3 | - | - | 0 | - |
| 11 | c | 9892 | 701.8 | 0.002311 | 3.293 | +2 | 11 |
| - | - | 6106 | 702.3 | - | - | 0 | - |
| - | - | 2242 | 702.8 | - | - | 0 | - |
| - | - | 851.4 | 703.3 | - | - | 0 | - |
| - | - | 838.5 | 704.4 | - | - | 0 | - |
| - | - | 752.1 | 711.3 | - | - | 0 | - |
| - | - | 906.1 | 713.3 | - | - | 0 | - |
| - | - | 673.7 | 714.3 | - | - | 0 | - |
| - | - | 4366 | 716.4 | - | - | 0 | - |
| - | - | 1434 | 717.4 | - | - | 0 | - |
| - | - | 773.8 | 726.4 | - | - | 0 | - |
| 5 | y | 1667 | 748.9 | 0.005875 | 7.845 | +2 | 11 |
| 5 | z | 4065 | 749.4 | 0.00292 | 3.897 | +2 | 11 |
| - | - | 4253 | 749.9 | - | - | 0 | - |
| - | - | 1937 | 750.4 | - | - | 0 | - |
| - | - | 876.1 | 750.9 | - | - | 0 | - |
| - | - | 1076 | 754.4 | - | - | 0 | - |
| - | - | 2931 | 754.9 | - | - | 0 | - |
| - | - | 1584 | 755.4 | - | - | 0 | - |
| 12 | c | 2604 | 757.4 | 2.588E-05 | 0.03418 | +2 | 12 |
| - | - | 1428 | 757.9 | - | - | 0 | - |
| - | - | 955.8 | 758.4 | - | - | 0 | - |
| - | - | 780.9 | 761.4 | - | - | 0 | - |
| 12 | c | 1450 | 765.9 | 0.002559 | 3.342 | +2 | 12 |
| - | - | 1421 | 766.4 | - | - | 0 | - |
| 11 | y | 955.8 | 768.4 | 0.01078 | 14.03 | +1 | 5 |
| 11 | z | 9780 | 769.4 | 0.009332 | 12.13 | +1 | 5 |
| - | - | 5536 | 770.4 | - | - | 0 | - |
| - | - | 1916 | 771.4 | - | - | 0 | - |
| - | - | 1675 | 784.9 | - | - | 0 | - |
| 11 | y | 4172 | 785.4 | 0.01054 | 13.42 | +1 | 5 |
| - | - | 1748 | 786.4 | - | - | 0 | - |
| - | - | 976.6 | 787.4 | - | - | 0 | - |
| - | - | 746 | 789.4 | - | - | 0 | - |
| 6 | c | 1370 | 793.4 | 0.004191 | 5.283 | +1 | 6 |
| - | - | 805.3 | 798.4 | - | - | 0 | - |
| - | - | 2110 | 803.4 | - | - | 0 | - |
| - | - | 1032 | 804.4 | - | - | 0 | - |
| 4 | y | 1853 | 806.4 | 0.01047 | 12.98 | +2 | 12 |
| 4 | z | 5686 | 806.9 | 0.001499 | 1.858 | +2 | 12 |
| - | - | 6753 | 807.4 | - | - | 0 | - |
| - | - | 3698 | 807.9 | - | - | 0 | - |
| 6 | c | 2.253E+04 | 810.4 | 0.0002838 | 0.3502 | +1 | 6 |
| - | - | 9656 | 811.4 | - | - | 0 | - |
| - | - | 2380 | 812.4 | - | - | 0 | - |
| - | - | 2641 | 814.4 | - | - | 0 | - |
| 4 | y | 1569 | 814.9 | 0.005368 | 6.587 | +2 | 12 |
| - | - | 2064 | 815.4 | - | - | 0 | - |
| - | - | 1813 | 831.4 | - | - | 0 | - |
| - | - | 1375 | 839.4 | - | - | 0 | - |
| - | - | 1234 | 841.4 | - | - | 0 | - |
| 3 | w | 1356 | 841.9 | 0.0008853 | 1.052 | +2 | 13 |
| - | - | 1776 | 842.4 | - | - | 0 | - |
| - | - | 816.7 | 843.4 | - | - | 0 | - |
| - | - | 1234 | 845.9 | - | - | 0 | - |
| 13 | c | 5188 | 846.4 | 0.0007462 | 0.8816 | +2 | 13 |
| - | - | 4776 | 846.9 | - | - | 0 | - |
| - | - | 3087 | 847.4 | - | - | 0 | - |
| - | - | 923.7 | 849.4 | - | - | 0 | - |
| - | - | 738.3 | 862.9 | - | - | 0 | - |
| - | - | 3302 | 867.4 | - | - | 0 | - |
| - | - | 923.1 | 868.4 | - | - | 0 | - |
| 3 | y | 3101 | 870.4 | 0.006816 | 7.831 | +2 | 13 |
| 3 | z | 6127 | 870.9 | 0.001003 | 1.151 | +2 | 13 |
| - | - | 5658 | 871.4 | - | - | 0 | - |
| - | - | 2264 | 871.9 | - | - | 0 | - |
| - | - | 807 | 872.4 | - | - | 0 | - |
| - | - | 854.2 | 874.4 | - | - | 0 | - |
| - | - | 877.4 | 878.4 | - | - | 0 | - |
| 3 | y | 714.6 | 878.9 | 0.001271 | 1.446 | +2 | 13 |
| - | - | 1141 | 879.4 | - | - | 0 | - |
| - | - | 1777 | 881.4 | - | - | 0 | - |
| - | - | 794.2 | 882.4 | - | - | 0 | - |
| - | - | 1703 | 888.4 | - | - | 0 | - |
| - | - | 1044 | 888.9 | - | - | 0 | - |
| 10 | z | 1.175E+04 | 898.4 | 0.008346 | 9.289 | +1 | 6 |
| - | - | 9452 | 899.4 | - | - | 0 | - |
| - | - | 4287 | 900.4 | - | - | 0 | - |
| - | - | 1465 | 901.4 | - | - | 0 | - |
| - | - | 856.5 | 902.4 | - | - | 0 | - |
| - | - | 710.9 | 904.9 | - | - | 0 | - |
| 7 | c | 1220 | 908.4 | 0.0005557 | 0.6117 | +1 | 7 |
| 14 | c | 3642 | 910.4 | 0.003855 | 4.234 | +2 | 14 |
| - | - | 3465 | 910.9 | - | - | 0 | - |
| - | - | 2841 | 911.4 | - | - | 0 | - |
| - | - | 1060 | 911.9 | - | - | 0 | - |
| - | - | 803.7 | 912.4 | - | - | 0 | - |
| - | - | 954 | 914.4 | - | - | 0 | - |
| - | - | 1518 | 915.4 | - | - | 0 | - |
| - | - | 934.6 | 916.4 | - | - | 0 | - |
| - | - | 697.2 | 918.9 | - | - | 0 | - |
| 7 | c | 2968 | 925.5 | 0.001226 | 1.325 | +1 | 7 |
| - | - | 2025 | 926.5 | - | - | 0 | - |
| - | - | 819.2 | 926.9 | - | - | 0 | - |
| - | - | 841.1 | 930.5 | - | - | 0 | - |
| - | - | 1645 | 931 | - | - | 0 | - |
| - | - | 1658 | 931.5 | - | - | 0 | - |
| - | - | 2419 | 932.5 | - | - | 0 | - |
| - | - | 969.6 | 932.9 | - | - | 0 | - |
| - | - | 949.9 | 933.5 | - | - | 0 | - |
| - | - | 847.3 | 933.9 | - | - | 0 | - |
| - | - | 968.5 | 937.5 | - | - | 0 | - |
| - | - | 3962 | 938.5 | - | - | 0 | - |
| - | - | 1869 | 939 | - | - | 0 | - |
| 2 | z | 3594 | 939.4 | 0.01257 | 13.38 | +2 | 14 |
| - | - | 3935 | 939.9 | - | - | 0 | - |
| - | - | 2616 | 940.4 | - | - | 0 | - |
| - | - | 1695 | 940.9 | - | - | 0 | - |
| - | - | 1171 | 941.5 | - | - | 0 | - |
| - | - | 828.5 | 942.4 | - | - | 0 | - |
| - | - | 841 | 944.5 | - | - | 0 | - |
| - | - | 2261 | 945.5 | - | - | 0 | - |
| - | - | 750 | 946 | - | - | 0 | - |
| - | - | 1783 | 946.5 | - | - | 0 | - |
| - | - | 891.8 | 947 | - | - | 0 | - |
| 2 | y | 1060 | 947.4 | 0.01603 | 16.92 | +2 | 14 |
| - | - | 869.3 | 951.4 | - | - | 0 | - |
| - | - | 1182 | 952 | - | - | 0 | - |
| - | - | 998.5 | 953 | - | - | 0 | - |
| - | - | 1052 | 953.5 | - | - | 0 | - |
| - | - | 885.1 | 954 | - | - | 0 | - |
| - | - | 1805 | 955.5 | - | - | 0 | - |
| - | - | 1675 | 960 | - | - | 0 | - |
| - | - | 4008 | 960.5 | - | - | 0 | - |
| - | - | 1090 | 961 | - | - | 0 | - |
| - | - | 1263 | 961.5 | - | - | 0 | - |
| - | - | 1615 | 962.5 | - | - | 0 | - |
| - | - | 1038 | 963 | - | - | 0 | - |
| - | - | 1552 | 963.5 | - | - | 0 | - |
| 8 | c | 2009 | 964.5 | 0.004862 | 5.041 | +1 | 8 |
| 8 | c | 744.9 | 965.4 | 0.006667 | 6.906 | +1 | 8 |
| - | - | 1156 | 966 | - | - | 0 | - |
| - | - | 1138 | 966.5 | - | - | 0 | - |
| - | - | 1473 | 967 | - | - | 0 | - |
| - | - | 3352 | 967.5 | - | - | 0 | - |
| - | - | 1448 | 968 | - | - | 0 | - |
| 9 | w | 3053 | 968.4 | 0.01589 | 16.41 | +1 | 7 |
| - | - | 2077 | 969 | - | - | 0 | - |
| - | - | 1642 | 969.5 | - | - | 0 | - |
| - | - | 1036 | 971 | - | - | 0 | - |
| - | - | 896.3 | 971.5 | - | - | 0 | - |
| - | - | 1512 | 972.5 | - | - | 0 | - |
| - | - | 1046 | 973 | - | - | 0 | - |
| - | - | 811.4 | 973.5 | - | - | 0 | - |
| - | - | 2500 | 974 | - | - | 0 | - |
| - | - | 2051 | 974.5 | - | - | 0 | - |
| - | - | 8173 | 975 | - | - | 0 | - |
| - | - | 7717 | 975.5 | - | - | 0 | - |
| - | - | 4115 | 976 | - | - | 0 | - |
| - | - | 4206 | 976.5 | - | - | 0 | - |
| - | - | 2551 | 977 | - | - | 0 | - |
| - | - | 2529 | 977.5 | - | - | 0 | - |
| - | - | 767.6 | 978 | - | - | 0 | - |
| - | - | 1510 | 978.5 | - | - | 0 | - |
| - | - | 1503 | 980.5 | - | - | 0 | - |
| - | - | 4198 | 981.5 | - | - | 0 | - |
| - | - | 3001 | 982 | - | - | 0 | - |
| 8 | c | 1.341E+04 | 982.5 | 0.003983 | 4.054 | +1 | 8 |
| - | - | 2636 | 983 | - | - | 0 | - |
| - | - | 7118 | 983.5 | - | - | 0 | - |
| - | - | 2864 | 984.5 | - | - | 0 | - |
| - | - | 3005 | 985 | - | - | 0 | - |
| - | - | 5265 | 985.5 | - | - | 0 | - |
| - | - | 2567 | 986 | - | - | 0 | - |
| - | - | 2591 | 986.5 | - | - | 0 | - |
| - | - | 2837 | 987.5 | - | - | 0 | - |
| - | - | 1011 | 988 | - | - | 0 | - |
| - | - | 3378 | 988.5 | - | - | 0 | - |
| - | - | 2806 | 989.5 | - | - | 0 | - |
| - | - | 914.9 | 990 | - | - | 0 | - |
| - | - | 2212 | 993.5 | - | - | 0 | - |
| - | - | 3067 | 994 | - | - | 0 | - |
| - | - | 2432 | 994.5 | - | - | 0 | - |
| - | - | 1650 | 995 | - | - | 0 | - |
| - | - | 2009 | 995.5 | - | - | 0 | - |
| - | - | 4944 | 996 | - | - | 0 | - |
| - | - | 1.3E+04 | 996.5 | - | - | 0 | - |
| - | - | 1.052E+04 | 997 | - | - | 0 | - |
| - | - | 6334 | 997.5 | - | - | 0 | - |
| - | - | 2947 | 998 | - | - | 0 | - |
| - | - | 1506 | 998.5 | - | - | 0 | - |
| - | - | 1278 | 999 | - | - | 0 | - |
| - | - | 1366 | 999.5 | - | - | 0 | - |
| - | - | 2454 | 1002 | - | - | 0 | - |
| - | - | 1224 | 1003 | - | - | 0 | - |
| - | - | 2120 | 1003 | - | - | 0 | - |
| - | - | 3616 | 1004 | - | - | 0 | - |
| - | - | 9742 | 1004 | - | - | 0 | - |
| - | - | 1.481E+04 | 1005 | - | - | 0 | - |
| - | - | 1.084E+04 | 1005 | - | - | 0 | - |
| - | - | 6460 | 1006 | - | - | 0 | - |
| - | - | 4351 | 1006 | - | - | 0 | - |
| - | - | 1552 | 1007 | - | - | 0 | - |
| - | - | 1892 | 1007 | - | - | 0 | - |
| - | - | 1576 | 1008 | - | - | 0 | - |
| 9 | z | 1267 | 1027 | 0.006507 | 6.339 | +1 | 7 |
| - | - | 3915 | 1028 | - | - | 0 | - |
| - | - | 1999 | 1029 | - | - | 0 | - |
| - | - | 999.8 | 1085 | - | - | 0 | - |
| - | - | 2688 | 1110 | - | - | 0 | - |
| 9 | c | 1.308E+04 | 1111 | 0.001244 | 1.12 | +1 | 9 |
| - | - | 7726 | 1112 | - | - | 0 | - |
| - | - | 3216 | 1113 | - | - | 0 | - |
| - | - | 1103 | 1142 | - | - | 0 | - |
| - | - | 864.8 | 1196 | - | - | 0 | - |
| - | - | 1901 | 1197 | - | - | 0 | - |
| - | - | 850.2 | 1198 | - | - | 0 | - |
| 7 | z | 2277 | 1199 | 0.004535 | 3.783 | +1 | 9 |
| - | - | 6041 | 1200 | - | - | 0 | - |
| - | - | 3016 | 1201 | - | - | 0 | - |
| - | - | 1798 | 1202 | - | - | 0 | - |
| 10 | c | 2992 | 1240 | 0.004427 | 3.571 | +1 | 10 |
| - | - | 3190 | 1241 | - | - | 0 | - |
| - | - | 1641 | 1242 | - | - | 0 | - |
| 6 | z | 1377 | 1312 | 0.002503 | 1.908 | +1 | 10 |
| - | - | 3012 | 1313 | - | - | 0 | - |
| - | - | 1572 | 1314 | - | - | 0 | - |
| - | - | 1042 | 1359 | - | - | 0 | - |
| 11 | c | 2059 | 1403 | 0.008237 | 5.873 | +1 | 11 |
| - | - | 1656 | 1404 | - | - | 0 | - |
| - | - | 867.2 | 1405 | - | - | 0 | - |
| 5 | z | 1175 | 1498 | 0.004912 | 3.279 | +1 | 11 |
| - | - | 1949 | 1499 | - | - | 0 | - |
| - | - | 1986 | 1500 | - | - | 0 | - |
| - | - | 847.7 | 1511 | - | - | 0 | - |
| 12 | c | 879.4 | 1514 | 0.002804 | 1.852 | +1 | 12 |
| 12 | c | 1009 | 1531 | 0.01191 | 7.778 | +1 | 12 |
| - | - | 784.2 | 1532 | - | - | 0 | - |
| 4 | z | 1624 | 1613 | 0.0002498 | 0.1549 | +1 | 12 |
| - | - | 3158 | 1614 | - | - | 0 | - |
| - | - | 1932 | 1615 | - | - | 0 | - |
| - | - | 1095 | 1616 | - | - | 0 | - |
| 13 | c | 1581 | 1692 | 0.006517 | 3.852 | +1 | 13 |
| - | - | 1686 | 1693 | - | - | 0 | - |
| - | - | 907.3 | 1694 | - | - | 0 | - |
| 3 | z | 1789 | 1741 | 0.005027 | 2.888 | +1 | 13 |
| - | - | 2365 | 1742 | - | - | 0 | - |
| - | - | 1283 | 1743 | - | - | 0 | - |
| - | - | 1080 | 1744 | - | - | 0 | - |
| - | - | 762.2 | 1761 | - | - | 0 | - |
| - | - | 1203 | 1821 | - | - | 0 | - |
| - | - | 981.8 | 1921 | - | - | 0 | - |
| - | - | 901.9 | 1951 | - | - | 0 | - |
| - | - | 900.2 | 1952 | - | - | 0 | - |
| - | - | 1028 | 1992 | - | - | 0 | - |
| - | - | 1115 | 1993 | - | - | 0 | - |
| - | - | 1223 | 2009 | - | - | 0 | - |
| - | - | 1191 | 2010 | - | - | 0 | - |
| - | - | 1310 | 2011 | - | - | 0 | - |
| - | - | 898 | 2012 | - | - | 0 | - |

m/z Charge Intensity FragmentType MassShift Position
120.06556701660156 0 391.27307
120.87273406982422 0 355.701
129.06585693359375 0 391.53955
129.10218811035156 0 2290.9841
130.06507873535156 0 1968.6411
130.06982421875 0 431.01505 w 13
133.0603485107422 0 506.4843
133.08592224121094 0 1297.4045
136.0753936767578 0 679.8111
138.76266479492188 0 411.01904
140.08218383789062 0 540.14764
147.0767822265625 0 559.3007
148.95376586914062 0 866.3606
149.04478454589844 0 1113.6504
155.0924530029297 0 2090.5374
156.07638549804688 0 459.58148
156.62660217285156 0 464.18396
159.09170532226562 0 900.3568
165.0775146484375 0 452.18518
166.06077575683594 0 1508.6202
167.05508422851562 0 596.4502
168.13803100585938 0 480.67642
171.14898681640625 0 1054.4928
173.0917510986328 0 998.5471
173.45086669921875 0 3785.4575
174.08746337890625 0 638.7856
175.07090759277344 0 508.6932
177.1115264892578 0 628.73224
183.11270141601562 0 945.1224
185.16465759277344 0 2630.9092
187.10745239257812 0 973.11194
191.10252380371094 0 1372.0096
199.1438751220703 0 1247.6875
201.12294006347656 0 645.82965
202.0822296142578 0 1361.1685
203.10231018066406 0 2465.9783
205.11766052246094 0 561.8966
207.0977783203125 0 1084.7858
209.09181213378906 0 1824.969
213.12310791015625 0 725.4658
215.13877868652344 0 7861.232
216.1422576904297 0 667.90753
217.08213806152344 0 638.4095
217.154541015625 0 1171.7046
218.1135711669922 0 1738.4081
223.1551055908203 0 1784.77
227.1026153564453 0 1670.086
229.11810302734375 0 639.76953
229.15419006347656 0 762.1632
230.1127471923828 0 640.82007
231.1695556640625 0 924.433
234.1232452392578 0 2668.581
239.0950927734375 0 816.1555
242.15008544921875 0 808.888
251.14996337890625 0 9715.968
252.15328979492188 0 1744.9205
261.11883544921875 0 730.7437
268.1766662597656 0 1200.2195 c 1
275.1235656738281 0 550.9427
281.0507507324219 0 1951.5717
282.1247253417969 0 495.19824
282.1817626953125 0 868.73175
283.151611328125 0 1183.7795
293.6220397949219 0 536.43976
299.06146240234375 0 8037.0654
299.13421630859375 0 903.67096
299.2097473144531 0 673.729
300.06353759765625 0 1575.8436
300.131103515625 0 682.6548
302.1708984375 0 1201.2701
310.14093017578125 0 562.62146
326.66387939453125 0 1909.7942
329.1457214355469 0 508.91525
332.1820983886719 0 928.1316
338.1349182128906 0 1378.7478
340.1866149902344 0 1497.3679
340.6609191894531 0 1088.4316 c Ammonia loss 4
354.1296081542969 0 767.38354
359.02801513671875 0 1595.9742
367.1612548828125 0 1201.3864
367.18414306640625 0 606.31946 y 7
367.679443359375 0 556.48737
379.208251953125 0 4687.8906 c Ammonia loss 2
380.2107849121094 0 1006.0929
387.22308349609375 0 2072.8242
393.1969909667969 0 1340.2903 y 10
393.6980895996094 0 1249.5319
396.2347412109375 0 4478.558 c 2
397.2387390136719 0 1157.4868
407.7070617675781 0 664.8267
408.1982116699219 0 645.6269 c Ammonia loss 9
415.03662109375 0 2058.436
416.03594970703125 0 1313.8425
420.2020263671875 0 9719.635 w 9
420.7034606933594 0 5197.1895
421.2015380859375 0 1450.4354
421.232421875 0 1035.5292
431.2247009277344 0 732.2945
435.7214050292969 0 703.24243
443.21783447265625 0 614.14685 y 5
444.2330017089844 0 1282.4883
444.2794494628906 0 805.8473
449.221435546875 0 761.4403
449.7196044921875 0 1663.3267 z 9
457.2657775878906 0 7528.402
457.71307373046875 0 604.7822
458.2300720214844 0 3160.1313
458.2692565917969 0 2860.853
458.7288513183594 0 1957.8936
459.2798156738281 0 4610.07
460.2834167480469 0 1147.3802
460.73370361328125 0 1755.2731
461.2373962402344 0 1010.4957
466.2401428222656 0 989.44196
467.21356201171875 0 791.66406
467.2477111816406 0 1380.6913
468.2630310058594 0 1194.6349
468.7653503417969 0 690.45386
469.2274475097656 0 1711.0638
470.2325439453125 0 704.7638
471.2677001953125 0 809.0045
472.30059814453125 0 1007.10864
474.2272033691406 0 1160.6962 y 1
474.47705078125 0 1132.0208
476.4849853515625 0 712.86017
483.2076110839844 0 1415.0824
484.24169921875 0 1647.0364
484.74835205078125 0 903.03577
485.2355651855469 0 3054.1177
486.2919921875 0 4256.223
487.2403259277344 0 789.391
487.28460693359375 0 712.1308
488.2933654785156 0 2155.5469
493.74664306640625 0 3913.3555
494.2390441894531 0 4393.3076 c Ammonia loss 3
494.74774169921875 0 1438.5985
495.2404479980469 0 676.92505
496.2533874511719 0 729.55096
498.2425537109375 0 766.5653
501.2185974121094 0 2489.9185
501.3037109375 0 1682.9049
501.74798583984375 0 1427.8234
502.2681884765625 0 5016.0015
502.4976806640625 0 5087.558
502.75030517578125 0 12128.0205
502.9975891113281 0 4069.7715
503.10723876953125 0 4363.409
503.25140380859375 0 4858.6396
503.505859375 0 643.41077
503.7688903808594 0 897.937
504.1068115234375 0 1572.8892
504.2630615234375 0 2051.7983
504.3019104003906 0 2527.2075
504.3371276855469 0 606.07477
506.26171875 0 849.24915
511.26202392578125 0 13016.724 c 3
512.2645263671875 0 2715.6816
515.317626953125 0 1753.9855
516.3256225585938 0 651.8749
519.2962036132812 0 1788.9868
521.7647705078125 0 2303.9263 y 8
522.2681884765625 0 1059.0822
522.7671508789062 0 988.407
528.2685546875 0 764.4627
530.775634765625 0 1111.4442
535.2879638671875 0 644.1455
547.2901000976562 0 1174.9323
548.238525390625 0 730.77185 w 11
550.2738647460938 0 2865.1501 y 7
550.7781982421875 0 1997.9092
553.7952880859375 0 811.3269
554.7932739257812 0 895.43146
555.2954711914062 0 3113.298
555.788818359375 0 9628.201 c 8
556.2889404296875 0 4586.7563
556.7893676757812 0 1882.8138
563.7691650390625 0 866.3883
564.2723388671875 0 1699.6967
568.7979125976562 0 1024.2936
569.2920532226562 0 683.7429
569.9581909179688 0 833.8179
570.275146484375 0 1551.3372
571.2814331054688 0 3371.3755
571.7810668945312 0 2178.9734
572.2798461914062 0 1194.3928
577.279541015625 0 1846.9847 w 6
577.7847290039062 0 1466.6042
578.2835693359375 0 1603.5209
580.2750244140625 0 999.6131 y Water loss 2
581.2813110351562 0 646.01154
584.3497924804688 0 646.2586
586.6096801757812 0 679.81287
587.955322265625 0 734.4381
592.9505004882812 0 723.28455
597.2898559570312 0 1895.6478
597.6278076171875 0 738.18646
597.79833984375 0 906.394
597.9638671875 0 841.37897
598.8082275390625 0 575.9135
599.2882080078125 0 2036.24 y Ammonia loss 6
599.7816162109375 0 5694.5 z 6
600.2816772460938 0 3837.8364
600.7813720703125 0 1192.7432
601.3746948242188 0 751.5646
601.9583740234375 0 1149.6735
602.9502563476562 0 1183.6194
603.28173828125 0 882.00934
604.7919921875 0 668.59467
604.9688110351562 0 759.3325
606.3035888671875 0 7669.7656 z 11
606.96875 0 2470.2937
607.2999877929688 0 11096.0625 c 13
607.6326293945312 0 6795.0674
607.79052734375 0 8385.553 y 6
607.9652709960938 0 2549.919
608.2944946289062 0 6072.4155
608.7921142578125 0 2608.3604
609.2969360351562 0 1246.4242
611.3062133789062 0 818.949 c Water loss 9
611.6296997070312 0 797.9818
611.8021240234375 0 926.61597 c Ammonia loss 9
611.9601440429688 0 981.32654
612.2973022460938 0 1634.6154
612.80126953125 0 1068.539
613.3017578125 0 1144.8386
614.6343994140625 0 808.79865
616.2883911132812 0 803.5777
617.6256103515625 0 790.248
619.816162109375 0 3517.8677
620.3108520507812 0 10178.277 c 9
620.8113403320312 0 5449.389
620.961669921875 0 859.57855 z Ammonia loss 1
621.30859375 0 2839.7683
621.6270141601562 0 705.8816
621.8135375976562 0 1384.6783
622.3231811523438 0 3756.849 y 11
623.3312377929688 0 734.9612
625.6481323242188 0 1078.5847
625.9654541015625 0 990.7586 y Water loss 1
626.302734375 0 3034.2744 y Ammonia loss 1
626.6409301757812 0 2114.9985
626.9685668945312 0 1489.9799
627.9668579101562 0 990.40234
631.29052734375 0 831.8153
631.6358642578125 0 945.0205
631.9652099609375 0 1574.7574 y 1
632.3060913085938 0 1008.83124
632.9696655273438 0 677.516
634.308837890625 0 2804.9277
634.6473999023438 0 1474.998
634.7980346679688 0 6587.553 w 5
634.9805297851562 0 1232.1327
635.30029296875 0 4929.6685
635.6486206054688 0 1038.3282
635.8005981445312 0 2675.7634
636.2991943359375 0 2722.4224
636.6300659179688 0 823.9996
636.9671630859375 0 956.2529
637.3082885742188 0 1473.8228
639.3140869140625 0 793.17633
639.9901733398438 0 3153.687
640.3213500976562 0 12107.139
640.6549072265625 0 8351.111
640.9876098632812 0 7869.1216
641.3176879882812 0 2038.3225
641.6513671875 0 1096.642
642.634521484375 0 806.8629
644.30712890625 0 891.6533
644.6409301757812 0 1250.6705
644.9698486328125 0 1684.7615
645.3055419921875 0 1628.6504
645.644287109375 0 2354.8164
645.978759765625 0 4050.5303
646.3128051757812 0 3580.0544
646.6461181640625 0 1104.7566
646.97802734375 0 985.48645
647.3115234375 0 1096.4182 z Water loss 5
647.6348876953125 0 918.18146
648.3101806640625 0 1575.2369
648.64453125 0 941.91327
648.9752197265625 0 753.4167
649.319091796875 0 999.99994
649.6446533203125 0 2995.5327
649.9758911132812 0 9374.028
650.317138671875 0 15990.368
650.6478271484375 0 9454.504
650.9865112304688 0 6114.955
651.3169555664062 0 4743.3003
651.6477661132812 0 3146.203
651.9778442382812 0 1771.4348
652.310791015625 0 857.93695
652.6355590820312 0 746.66235
652.973876953125 0 833.3028
653.3170776367188 0 950.6602
653.6326293945312 0 910.3975
654.3209838867188 0 1165.2721
654.9945678710938 0 1761.0667
655.3172607421875 0 818.23895
655.6454467773438 0 861.6461
655.8256225585938 0 863.82074 y Ammonia loss 5
656.3202514648438 0 3395.1143 z 5
656.64208984375 0 2875.5623
656.82275390625 0 1036.077
656.9754028320312 0 3496.6775
657.3103637695312 0 3031.7356
657.6439819335938 0 1141.8193
657.9775390625 0 1130.9186
658.313232421875 0 969.10516
658.9866943359375 0 1845.1344
659.9880981445312 0 903.5524
662.3038330078125 0 1579.3835
662.6438598632812 0 2997.264
662.9737548828125 0 898.12317
663.99169921875 0 1237.9705
664.3276977539062 0 6172.135 y 5
664.6497802734375 0 1658.3889
664.8328857421875 0 1999.1013
664.9837036132812 0 1841.9741
665.3251953125 0 3098.9082
665.6548461914062 0 1151.4531
668.314697265625 0 924.4666
668.6517333984375 0 1671.6886
668.9871215820312 0 985.53076
669.3289794921875 0 4483.5195
669.6609497070312 0 24780.031
669.994873046875 0 34604.324
670.3292846679688 0 25944.35
670.6622314453125 0 12492.585
670.9961547851562 0 6832.668
671.327880859375 0 3325.8672
671.6608276367188 0 1863.4089
675.32763671875 0 861.19293
679.3394165039062 0 742.8631 c Water loss 4
680.31494140625 0 4230.6963 c Ammonia loss 4
681.3152465820312 0 1200.94
682.3278198242188 0 1061.5521
683.3389282226562 0 767.87006
690.3427124023438 0 1957.521
691.3314819335938 0 1685.9758
691.8297729492188 0 1052.247
695.9256591796875 0 831.3684
697.3411254882812 0 12470.636 c 4
698.3448486328125 0 5678.6797
699.344970703125 0 1834.5577
699.8394775390625 0 1314.4036
700.3436889648438 0 1119.0765
701.349365234375 0 2254.544
701.8434448242188 0 9891.948 c 10
702.3429565429688 0 6105.5986
702.8450317382812 0 2242.4092
703.3489379882812 0 851.4459
704.3668212890625 0 838.4674
711.333984375 0 752.12427
713.3421020507812 0 906.07776
714.3309326171875 0 673.6699
716.4039306640625 0 4365.6846
717.4066772460938 0 1433.9082
726.3633422851562 0 773.80695
748.8685302734375 0 1667.4498 y Ammonia loss 4
749.3636474609375 0 4065.3423 z 4
749.8627319335938 0 4252.556
750.3646850585938 0 1937.2479
750.8645629882812 0 876.07336
754.3675537109375 0 1075.762
754.8677978515625 0 2930.7747
755.361572265625 0 1583.5372
757.3753662109375 0 2604.4043 c Ammonia loss 11
757.8726806640625 0 1428.0114
758.3706665039062 0 955.81256
761.3798217773438 0 780.896
765.8911743164062 0 1450.4042 c 11
766.3952026367188 0 1421.4498
768.35791015625 0 955.7861 y Ammonia loss 10
769.3671875 0 9779.986 z 10
770.3671264648438 0 5535.64
771.3682250976562 0 1916.3751
784.8814086914062 0 1674.7032
785.3847045898438 0 4172.457 y 10
786.3798828125 0 1748.016
787.3991088867188 0 976.6186
789.3598022460938 0 746.0398
793.3949584960938 0 1370.1093 c Ammonia loss 5
798.3836059570312 0 805.3499
803.427490234375 0 2110.1746
804.4265747070312 0 1031.5677
806.3865966796875 0 1852.8704 y Ammonia loss 3
806.8785400390625 0 5686.471 z 3
807.3784790039062 0 6752.75
807.8765869140625 0 3697.5144
810.4254150390625 0 22533.541 c 5
811.4277954101562 0 9656.378
812.427490234375 0 2380.1147
814.404296875 0 2641.2605
814.884033203125 0 1569.2904 y 3
815.3960571289062 0 2063.7473
831.4290161132812 0 1813.4193
839.386474609375 0 1374.5149
841.4016723632812 0 1233.6898
841.8955688476562 0 1356.3379 w 2
842.3987426757812 0 1776.2565
843.384521484375 0 816.7225
845.9004516601562 0 1234.0966
846.3974609375 0 5188.095 c 12
846.8978271484375 0 4776.2866
847.3984375 0 3087.023
849.4197998046875 0 923.6654
862.9015502929688 0 738.30963
867.4227294921875 0 3301.6736
868.423583984375 0 923.1416
870.4122314453125 0 3100.6382 y Ammonia loss 2
870.9083251953125 0 6127.353 z 2
871.407958984375 0 5657.823
871.9082641601562 0 2264.0066
872.4139404296875 0 807.00433
874.4221801757812 0 854.208
878.4029541015625 0 877.42316
878.9174194335938 0 714.5733 y 2
879.40673828125 0 1140.688
881.435302734375 0 1777.4713
882.4406127929688 0 794.23566
888.4365844726562 0 1702.7692
888.9429321289062 0 1043.8746
898.4107666015625 0 11747.983 z 9
899.4153442382812 0 9452.157
900.419189453125 0 4287.4956
901.417236328125 0 1465.0975
902.4296875 0 856.4594
904.9269409179688 0 710.9197
908.425537109375 0 1219.8319 c Ammonia loss 6
910.4418334960938 0 3642.0393 c 13
910.9437866210938 0 3464.9717
911.4448852539062 0 2841.4868
911.9464111328125 0 1059.7092
912.4419555664062 0 803.6869
914.4185180664062 0 953.9699
915.4423217773438 0 1518.4584
916.4434814453125 0 934.616
918.94140625 0 697.23724
925.451416015625 0 2968.33 c 6
926.4512939453125 0 2024.772
926.9381713867188 0 819.2112
930.4559326171875 0 841.1073
930.9676513671875 0 1645.4845
931.470458984375 0 1657.798
932.4775390625 0 2418.5923
932.9388427734375 0 969.55084
933.4772338867188 0 949.8515
933.9342041015625 0 847.3048
937.4628295898438 0 968.49884
938.47265625 0 3962.3938
938.9635009765625 0 1868.8748
939.4513549804688 0 3593.8115 z 1
939.93994140625 0 3935.206
940.4467163085938 0 2616.166
940.9415283203125 0 1694.6768
941.45263671875 0 1170.9266
942.4296264648438 0 828.5095
944.4557495117188 0 841.0485
945.4834594726562 0 2260.9404
945.9640502929688 0 749.9685
946.4697265625 0 1783.2595
946.9552612304688 0 891.83026
947.4641723632812 0 1060.3229 y 1
951.4442749023438 0 869.2545
951.9617919921875 0 1181.9166
952.9780883789062 0 998.49335
953.4666137695312 0 1051.7534
953.9714965820312 0 885.11566
955.4583129882812 0 1804.9519
959.9746704101562 0 1675.3127
960.4766235351562 0 4008.3215
960.9741821289062 0 1090.4003
961.4713134765625 0 1263.442
962.4589233398438 0 1615.168
962.9608154296875 0 1038.4152
963.4622802734375 0 1552.4998
964.4586791992188 0 2008.9587 c Water loss 7
965.4542236328125 0 744.8636 c Ammonia loss 7
965.9667358398438 0 1156.3606
966.4737548828125 0 1138.4415
966.9750366210938 0 1473.0988
967.4730224609375 0 3352.0376
967.968994140625 0 1448.1543
968.4642944335938 0 3052.9978 w 8
968.961669921875 0 2076.7993
969.4657592773438 0 1642.4225
970.9640502929688 0 1036.1735
971.4586181640625 0 896.3433
972.45947265625 0 1511.5249
972.968505859375 0 1046.3524
973.47314453125 0 811.3849
973.9794921875 0 2499.8267
974.4777221679688 0 2051.2314
974.98388671875 0 8173.057
975.4830932617188 0 7717.3057
975.982666015625 0 4115.315
976.475830078125 0 4205.5933
976.97314453125 0 2551.4722
977.4712524414062 0 2529.4246
977.9609985351562 0 767.58453
978.4532470703125 0 1509.9487
980.4677124023438 0 1502.7832
981.4835815429688 0 4198.235
981.9861450195312 0 3000.5632
982.4780883789062 0 13411.7705 c 7
982.988525390625 0 2636.3623
983.4786376953125 0 7118.0396
984.4686889648438 0 2863.9805
984.9600219726562 0 3004.9834
985.4721069335938 0 5265.455
985.9658203125 0 2566.7327
986.4701538085938 0 2590.5054
987.4696655273438 0 2836.9856
987.965576171875 0 1011.0646
988.4776611328125 0 3377.9014
989.4801025390625 0 2805.9973
989.955322265625 0 914.8982
993.4598999023438 0 2212.26
993.96826171875 0 3066.9668
994.4660034179688 0 2432.2166
994.9683837890625 0 1649.7999
995.474365234375 0 2008.7794
995.9822998046875 0 4944.09
996.481689453125 0 12997.2705
996.9804077148438 0 10520.452
997.4849853515625 0 6333.703
997.9804077148438 0 2946.981
998.4768676757812 0 1506.4501
998.9577026367188 0 1277.5452
999.4560546875 0 1365.6715
1002.4686279296875 0 2454.0469
1002.9644775390625 0 1223.7083
1003.4862670898438 0 2119.8218
1003.9832763671875 0 3616.404
1004.4949340820312 0 9742.365
1004.9935302734375 0 14808.939
1005.4943237304688 0 10841
1005.99609375 0 6460.3438
1006.4937133789062 0 4351.4053
1006.987060546875 0 1552.3519
1007.4805908203125 0 1891.7803
1008.449462890625 0 1575.742
1026.507568359375 0 1266.9149 z 8
1027.5098876953125 0 3914.654
1028.5087890625 0 1999.4427
1084.529296875 0 999.7715
1109.5849609375 0 2688.3367
1110.5703125 0 13081.935 c 8
1111.5716552734375 0 7726.045
1112.571044921875 0 3216.1174
1141.5494384765625 0 1102.7635
1195.5933837890625 0 864.84247
1196.599609375 0 1900.6547
1197.59716796875 0 850.18024
1198.5670166015625 0 2277.1 z 6
1199.563232421875 0 6041.077
1200.568359375 0 3016.3533
1201.5679931640625 0 1798.198
1239.6160888671875 0 2992.0237 c 9
1240.61669921875 0 3190.0938
1241.6260986328125 0 1640.5123
1311.64404296875 0 1376.5424 z 5
1312.647216796875 0 3011.5647
1313.6517333984375 0 1572.1185
1358.6685791015625 0 1041.703
1402.6832275390625 0 2059.4595 c 10
1403.679931640625 0 1655.6554
1404.68408203125 0 867.2152
1497.720947265625 0 1174.6929 z 4
1498.7197265625 0 1949.2772
1499.7227783203125 0 1985.899
1510.751953125 0 847.711
1513.7406005859375 0 879.35657 c Ammonia loss 11
1530.7818603515625 0 1009.4373 c 11
1531.772705078125 0 784.19543
1612.7530517578125 0 1624.3616 z 3
1613.7548828125 0 3157.7585
1614.7564697265625 0 1932.3634
1615.7750244140625 0 1094.5487
1691.795654296875 0 1581.262 c 12
1692.7950439453125 0 1685.9648
1693.787841796875 0 907.3124
1740.81640625 0 1788.9966 z 2
1741.806396484375 0 2364.6877
1742.8101806640625 0 1283.0938
1743.832275390625 0 1079.98
1760.843017578125 0 762.1617
1820.8837890625 0 1203.2698
1920.959228515625 0 981.82556
1951.004150390625 0 901.8732
1951.955810546875 0 900.1642
1991.953857421875 0 1027.6595
1992.96044921875 0 1114.741
2008.9647216796875 0 1223.2427
2009.974365234375 0 1191.3364
2010.98095703125 0 1310.3871
2011.9896240234375 0 897.9803

Spectrum Details

|  |  |
| --- | --- |
| Matched peaks? Matched peaksThe total absolute number of peaks matched. Additionally in brackets the total fraction of peaks matched and the total number of peaks is shown. | 81 (13.57% of 597) |
| FDR? FDRThe false discovery rate estimated for this peptide. It is calculated by matching all theoretical fragments with a non-integer shift with the raw peaks for this spectrum. This is done with 40 different shifts. The resulting percentage is the average number of annotated peaks over the number of annotated peaks with the correct spectrum. | 0.38% |
| Satellite FDR? Satellite FDRSee the FDR for details on its calculation. This satellite ion specific FDR only contains the satellite ions (d/w) for I/L/J positions. | 0.00% |
| PSM Score? PSM ScoreThe PSM Score as given by Hecklib to this annotated spectrum. It is shown with three significant figures. | 385 |

## Spectrum 5469? Spectrum 5469 The raw spectrum of this peptide as annotated by Hecklib. The fragments are coloured according to ion type (see legend). Any peaks with a star '\*' as text can be hovered over to see the full details, first the ion type second the mass shift type. By hovering over the amino acids in the peptide or ions in the legend the corresponding peaks are highlighted. By toggling the 'Unassigned' label you can turn the background (unassigned) peaks on or off in the plot. By updating the slider in the Ion legend you can update the spectrum to only show the top X% of the peaks with labels. The top X% means any peak that is within X% of the highest intensity. By dragging in the spectrum you can zoom in to a specific part of the spectrum and use 'Zoom Out' to get back to the original zoom level. The annotation of the spectrum is based on the given sequence in the peptides file and is done with different software so inconsistencies are likely. The peaks are annotated based on the given sequence, with 20 ppm tolerance.

Copy Data

### Spectrum 5469 (TSV)

#### Preview

```
Loading example...
```

*Click on the button to copy the data to your clipboard.*

Mz MinMz MaxIntensity Max

WidthHeightPeptide font sizePeptide stroke widthSpectrum font sizeSpectrum stroke widthCompact peptide

Ion legend

wxyz

abcd

OtherUnassignedIonChargePositionShow for top:%

JHQDWLDGKEYKCKK

01.01e+42.01e+43.02e+44.03e+4

Zoom Out

c+46y+11c+12y+12c+25y+411c+13y+25c+13w+26z+26c+14y+311c+14y+27z+28z+28w+14y+28c+29w+29y+313z+29y+29y+29z+29c+314y+14z+14c+314y+29c+210c+210c+210y+14y+314z+314y+314w+210z+210z+210y+210z+210y+210c+15c+15c+211y+211z+211z+15c+212c+212y+15z+15y+15c+16y+212z+212c+16y+212c+213y+213z+213z+16c+214c+17c+214y+16c+17z+214y+214c+18c+18w+17c+18z+17c+19z+19c+110z+110c+111z+111z+112c+113z+113

0770153923093079

Fragment Matches Table

Show background peaks

| Position | Ion type | Intensity | mz Theoretical | mz Error (Th) | mz Error (ppm) | Charge | Series Number |
| --- | --- | --- | --- | --- | --- | --- | --- |
| - | - | 429.1 | 126.1 | - | - | 0 | - |
| - | - | 3547 | 129.1 | - | - | 0 | - |
| - | - | 2247 | 130.1 | - | - | 0 | - |
| - | - | 382.7 | 131.4 | - | - | 0 | - |
| - | - | 429 | 133.1 | - | - | 0 | - |
| - | - | 1276 | 133.1 | - | - | 0 | - |
| - | - | 722.9 | 136.1 | - | - | 0 | - |
| - | - | 436.3 | 140.1 | - | - | 0 | - |
| - | - | 464.6 | 144.5 | - | - | 0 | - |
| - | - | 803.9 | 146.1 | - | - | 0 | - |
| - | - | 1067 | 148.9 | - | - | 0 | - |
| - | - | 488.3 | 149 | - | - | 0 | - |
| - | - | 1245 | 149 | - | - | 0 | - |
| - | - | 3161 | 155.1 | - | - | 0 | - |
| - | - | 454.8 | 156.1 | - | - | 0 | - |
| - | - | 516.7 | 156.9 | - | - | 0 | - |
| - | - | 775.7 | 159.1 | - | - | 0 | - |
| - | - | 733 | 162.1 | - | - | 0 | - |
| - | - | 462.7 | 164.1 | - | - | 0 | - |
| - | - | 605.1 | 165.1 | - | - | 0 | - |
| - | - | 3310 | 166.1 | - | - | 0 | - |
| - | - | 1607 | 168.1 | - | - | 0 | - |
| - | - | 1194 | 173.1 | - | - | 0 | - |
| - | - | 4354 | 173.5 | - | - | 0 | - |
| - | - | 556.3 | 174.1 | - | - | 0 | - |
| - | - | 489.2 | 176.4 | - | - | 0 | - |
| - | - | 1103 | 177.1 | - | - | 0 | - |
| - | - | 1499 | 183.1 | - | - | 0 | - |
| - | - | 455.9 | 184.7 | - | - | 0 | - |
| - | - | 2797 | 185.2 | - | - | 0 | - |
| - | - | 1079 | 187.1 | - | - | 0 | - |
| - | - | 1753 | 191.1 | - | - | 0 | - |
| 6 | c | 1179 | 199.1 | 0.003032 | 15.23 | +4 | 6 |
| - | - | 1624 | 202.1 | - | - | 0 | - |
| - | - | 1548 | 203.1 | - | - | 0 | - |
| - | - | 687.4 | 204.1 | - | - | 0 | - |
| 15 | y | 869.2 | 205.1 | 0.003892 | 18.98 | +1 | 1 |
| - | - | 454.5 | 208 | - | - | 0 | - |
| - | - | 914.6 | 209.1 | - | - | 0 | - |
| - | - | 712.8 | 213.1 | - | - | 0 | - |
| - | - | 9141 | 215.1 | - | - | 0 | - |
| - | - | 974.2 | 217.2 | - | - | 0 | - |
| - | - | 600.6 | 217.3 | - | - | 0 | - |
| - | - | 1131 | 218.1 | - | - | 0 | - |
| - | - | 524 | 218.2 | - | - | 0 | - |
| - | - | 683.4 | 221.1 | - | - | 0 | - |
| - | - | 2524 | 223.2 | - | - | 0 | - |
| - | - | 3345 | 227.1 | - | - | 0 | - |
| - | - | 566.2 | 229.2 | - | - | 0 | - |
| - | - | 597.2 | 230.2 | - | - | 0 | - |
| - | - | 791.6 | 231.2 | - | - | 0 | - |
| - | - | 2981 | 234.1 | - | - | 0 | - |
| - | - | 589.5 | 235.1 | - | - | 0 | - |
| - | - | 668.6 | 242.2 | - | - | 0 | - |
| - | - | 1208 | 246.2 | - | - | 0 | - |
| - | - | 1.497E+04 | 251.2 | - | - | 0 | - |
| - | - | 1632 | 252.2 | - | - | 0 | - |
| - | - | 901.7 | 261.1 | - | - | 0 | - |
| 2 | c | 1510 | 268.2 | 0.0003226 | 1.203 | +1 | 2 |
| - | - | 763.2 | 274.1 | - | - | 0 | - |
| - | - | 944 | 275.1 | - | - | 0 | - |
| - | - | 1052 | 281.1 | - | - | 0 | - |
| - | - | 1120 | 283.2 | - | - | 0 | - |
| - | - | 7005 | 299.1 | - | - | 0 | - |
| - | - | 1118 | 300.1 | - | - | 0 | - |
| - | - | 1712 | 300.2 | - | - | 0 | - |
| - | - | 779.4 | 302.2 | - | - | 0 | - |
| - | - | 797.9 | 317.1 | - | - | 0 | - |
| - | - | 703.7 | 318.2 | - | - | 0 | - |
| - | - | 551.3 | 319.2 | - | - | 0 | - |
| - | - | 2006 | 326.7 | - | - | 0 | - |
| 14 | y | 1108 | 333.2 | 0.002832 | 8.499 | +1 | 2 |
| - | - | 1268 | 338.1 | - | - | 0 | - |
| - | - | 1976 | 340.2 | - | - | 0 | - |
| 5 | c | 674.5 | 340.7 | 0.002485 | 7.294 | +2 | 5 |
| - | - | 499.2 | 341.2 | - | - | 0 | - |
| - | - | 740 | 349.7 | - | - | 0 | - |
| - | - | 803.7 | 359 | - | - | 0 | - |
| 5 | y | 664 | 374.7 | 0.005221 | 13.94 | +4 | 11 |
| - | - | 746.9 | 378.2 | - | - | 0 | - |
| 3 | c | 5220 | 379.2 | 0.0005513 | 1.454 | +1 | 3 |
| - | - | 1452 | 380.2 | - | - | 0 | - |
| - | - | 612.4 | 381.2 | - | - | 0 | - |
| - | - | 735 | 383.2 | - | - | 0 | - |
| - | - | 629 | 386.2 | - | - | 0 | - |
| - | - | 2036 | 387.2 | - | - | 0 | - |
| 11 | y | 1448 | 393.2 | 0.003597 | 9.149 | +2 | 5 |
| 3 | c | 5743 | 396.2 | 0.0005525 | 1.394 | +1 | 3 |
| - | - | 1024 | 397.2 | - | - | 0 | - |
| - | - | 695.4 | 403.5 | - | - | 0 | - |
| - | - | 749.6 | 407.2 | - | - | 0 | - |
| - | - | 3123 | 415 | - | - | 0 | - |
| - | - | 721.6 | 416 | - | - | 0 | - |
| 10 | w | 1.004E+04 | 420.2 | 0.003661 | 8.713 | +2 | 6 |
| - | - | 392.3 | 420.2 | - | - | 0 | - |
| - | - | 4436 | 420.7 | - | - | 0 | - |
| - | - | 1817 | 421.2 | - | - | 0 | - |
| - | - | 782.2 | 428.3 | - | - | 0 | - |
| - | - | 632.2 | 435.7 | - | - | 0 | - |
| - | - | 620.5 | 445.2 | - | - | 0 | - |
| - | - | 768.8 | 447.9 | - | - | 0 | - |
| 10 | z | 2013 | 449.7 | 0.005373 | 11.95 | +2 | 6 |
| - | - | 6557 | 457.3 | - | - | 0 | - |
| - | - | 3101 | 458.2 | - | - | 0 | - |
| - | - | 3064 | 458.3 | - | - | 0 | - |
| - | - | 1983 | 458.7 | - | - | 0 | - |
| - | - | 5585 | 459.3 | - | - | 0 | - |
| - | - | 1322 | 460.3 | - | - | 0 | - |
| - | - | 1556 | 460.7 | - | - | 0 | - |
| - | - | 763.2 | 461.2 | - | - | 0 | - |
| - | - | 1287 | 467.2 | - | - | 0 | - |
| - | - | 880.1 | 468.3 | - | - | 0 | - |
| - | - | 1194 | 469.2 | - | - | 0 | - |
| - | - | 1008 | 472.3 | - | - | 0 | - |
| - | - | 773.3 | 478.3 | - | - | 0 | - |
| - | - | 587.1 | 481.2 | - | - | 0 | - |
| - | - | 1150 | 484.2 | - | - | 0 | - |
| - | - | 2587 | 485.2 | - | - | 0 | - |
| - | - | 785.3 | 485.3 | - | - | 0 | - |
| - | - | 5698 | 486.3 | - | - | 0 | - |
| - | - | 1262 | 487.2 | - | - | 0 | - |
| - | - | 1047 | 488.3 | - | - | 0 | - |
| - | - | 911.3 | 489.2 | - | - | 0 | - |
| - | - | 698.5 | 492.5 | - | - | 0 | - |
| - | - | 3482 | 493.7 | - | - | 0 | - |
| 4 | c | 6577 | 494.2 | 0.001776 | 3.593 | +1 | 4 |
| - | - | 1288 | 494.7 | - | - | 0 | - |
| - | - | 1260 | 495.2 | - | - | 0 | - |
| - | - | 611.7 | 496.3 | - | - | 0 | - |
| - | - | 784.4 | 498.3 | - | - | 0 | - |
| - | - | 1195 | 498.7 | - | - | 0 | - |
| 5 | y | 1408 | 499.2 | 0.004712 | 9.438 | +3 | 11 |
| - | - | 2511 | 501.2 | - | - | 0 | - |
| - | - | 1309 | 501.8 | - | - | 0 | - |
| - | - | 4983 | 502.3 | - | - | 0 | - |
| - | - | 5398 | 502.5 | - | - | 0 | - |
| - | - | 1.223E+04 | 502.8 | - | - | 0 | - |
| - | - | 3321 | 503 | - | - | 0 | - |
| - | - | 3808 | 503.1 | - | - | 0 | - |
| - | - | 7091 | 503.2 | - | - | 0 | - |
| - | - | 832.1 | 503.3 | - | - | 0 | - |
| - | - | 1245 | 503.8 | - | - | 0 | - |
| - | - | 1327 | 504.1 | - | - | 0 | - |
| - | - | 2047 | 504.3 | - | - | 0 | - |
| - | - | 3121 | 504.3 | - | - | 0 | - |
| - | - | 971.3 | 504.3 | - | - | 0 | - |
| - | - | 853.5 | 505.6 | - | - | 0 | - |
| - | - | 986.8 | 506.3 | - | - | 0 | - |
| - | - | 642.2 | 506.7 | - | - | 0 | - |
| - | - | 934.1 | 507.3 | - | - | 0 | - |
| 4 | c | 1.442E+04 | 511.3 | 0.001136 | 2.223 | +1 | 4 |
| - | - | 992.6 | 511.3 | - | - | 0 | - |
| - | - | 3840 | 512.3 | - | - | 0 | - |
| - | - | 928.4 | 513.3 | - | - | 0 | - |
| - | - | 1950 | 515.3 | - | - | 0 | - |
| - | - | 762.4 | 519.3 | - | - | 0 | - |
| - | - | 1505 | 519.3 | - | - | 0 | - |
| 9 | y | 2901 | 521.8 | 0.003314 | 6.352 | +2 | 7 |
| - | - | 792.2 | 522.3 | - | - | 0 | - |
| - | - | 835 | 522.8 | - | - | 0 | - |
| - | - | 826.8 | 527.8 | - | - | 0 | - |
| - | - | 835.9 | 529.3 | - | - | 0 | - |
| 8 | z | 663.7 | 533.3 | 0.009167 | 17.19 | +2 | 8 |
| - | - | 1639 | 535.3 | - | - | 0 | - |
| - | - | 637.2 | 536.3 | - | - | 0 | - |
| 8 | z | 1472 | 542.3 | 0.005294 | 9.763 | +2 | 8 |
| - | - | 825.4 | 543.3 | - | - | 0 | - |
| - | - | 935.6 | 547.3 | - | - | 0 | - |
| - | - | 588.9 | 547.8 | - | - | 0 | - |
| 12 | w | 1124 | 548.2 | 0.009723 | 17.74 | +1 | 4 |
| - | - | 799.4 | 548.8 | - | - | 0 | - |
| - | - | 624.6 | 549.3 | - | - | 0 | - |
| - | - | 1493 | 550.2 | - | - | 0 | - |
| 8 | y | 2180 | 550.3 | 0.002388 | 4.34 | +2 | 8 |
| - | - | 1574 | 550.8 | - | - | 0 | - |
| - | - | 871.4 | 552.3 | - | - | 0 | - |
| - | - | 1190 | 555.3 | - | - | 0 | - |
| - | - | 1977 | 555.3 | - | - | 0 | - |
| 9 | c | 8855 | 555.8 | 0.001378 | 2.48 | +2 | 9 |
| - | - | 4498 | 556.3 | - | - | 0 | - |
| - | - | 1207 | 556.8 | - | - | 0 | - |
| - | - | 634.5 | 563.3 | - | - | 0 | - |
| - | - | 5212 | 564.3 | - | - | 0 | - |
| - | - | 2539 | 564.8 | - | - | 0 | - |
| - | - | 898.6 | 565.3 | - | - | 0 | - |
| - | - | 815.5 | 565.8 | - | - | 0 | - |
| - | - | 868.2 | 568.8 | - | - | 0 | - |
| - | - | 2521 | 571.3 | - | - | 0 | - |
| - | - | 2272 | 571.8 | - | - | 0 | - |
| - | - | 2066 | 572.3 | - | - | 0 | - |
| - | - | 788.1 | 573.3 | - | - | 0 | - |
| 7 | w | 2485 | 577.3 | 0.00407 | 7.05 | +2 | 9 |
| - | - | 1909 | 577.8 | - | - | 0 | - |
| - | - | 1272 | 578.3 | - | - | 0 | - |
| 3 | y | 1262 | 580.3 | 0.003433 | 5.917 | +3 | 13 |
| - | - | 953 | 590.6 | - | - | 0 | - |
| 7 | z | 657.6 | 590.8 | 0.006648 | 11.25 | +2 | 9 |
| - | - | 1541 | 591.3 | - | - | 0 | - |
| - | - | 794.8 | 592.8 | - | - | 0 | - |
| - | - | 1069 | 596.3 | - | - | 0 | - |
| - | - | 2120 | 597.3 | - | - | 0 | - |
| - | - | 937.3 | 597.6 | - | - | 0 | - |
| - | - | 876.3 | 598 | - | - | 0 | - |
| - | - | 763.7 | 598.3 | - | - | 0 | - |
| 7 | y | 1054 | 598.8 | 0.001911 | 3.191 | +2 | 9 |
| - | - | 756.5 | 599 | - | - | 0 | - |
| 7 | y | 2932 | 599.3 | 0.007791 | 13 | +2 | 9 |
| 7 | z | 6086 | 599.8 | 0.002286 | 3.812 | +2 | 9 |
| - | - | 3560 | 600.3 | - | - | 0 | - |
| - | - | 1697 | 600.8 | - | - | 0 | - |
| 14 | c | 848.7 | 601.3 | 0.001657 | 2.756 | +3 | 14 |
| - | - | 750.6 | 601.4 | - | - | 0 | - |
| - | - | 771.5 | 602 | - | - | 0 | - |
| - | - | 903.7 | 602.3 | - | - | 0 | - |
| - | - | 786.2 | 602.9 | - | - | 0 | - |
| - | - | 983.7 | 605 | - | - | 0 | - |
| 12 | y | 1061 | 605.3 | 0.008674 | 14.33 | +1 | 4 |
| - | - | 810.7 | 605.6 | - | - | 0 | - |
| 12 | z | 6026 | 606.3 | 0.009052 | 14.93 | +1 | 4 |
| - | - | 740 | 606.6 | - | - | 0 | - |
| - | - | 2072 | 607 | - | - | 0 | - |
| 14 | c | 9289 | 607.3 | 0.002146 | 3.533 | +3 | 14 |
| - | - | 4762 | 607.6 | - | - | 0 | - |
| 7 | y | 7885 | 607.8 | 0.00347 | 5.709 | +2 | 9 |
| - | - | 3238 | 608 | - | - | 0 | - |
| - | - | 7591 | 608.3 | - | - | 0 | - |
| - | - | 1081 | 608.6 | - | - | 0 | - |
| - | - | 1815 | 608.8 | - | - | 0 | - |
| - | - | 600.2 | 609 | - | - | 0 | - |
| - | - | 994.3 | 609.3 | - | - | 0 | - |
| 10 | c | 1670 | 611.3 | 0.003772 | 6.17 | +2 | 10 |
| 10 | c | 846.7 | 611.8 | 0.01051 | 17.17 | +2 | 10 |
| - | - | 1006 | 612 | - | - | 0 | - |
| - | - | 2691 | 612.3 | - | - | 0 | - |
| - | - | 944.2 | 612.6 | - | - | 0 | - |
| - | - | 1811 | 612.8 | - | - | 0 | - |
| - | - | 1039 | 613.6 | - | - | 0 | - |
| - | - | 924.7 | 615.3 | - | - | 0 | - |
| - | - | 1146 | 615.6 | - | - | 0 | - |
| - | - | 816.3 | 616.6 | - | - | 0 | - |
| - | - | 813 | 617.3 | - | - | 0 | - |
| - | - | 1005 | 619.3 | - | - | 0 | - |
| - | - | 3956 | 619.8 | - | - | 0 | - |
| 10 | c | 1.437E+04 | 620.3 | 0.001749 | 2.82 | +2 | 10 |
| - | - | 809.7 | 620.6 | - | - | 0 | - |
| - | - | 7589 | 620.8 | - | - | 0 | - |
| - | - | 1309 | 621 | - | - | 0 | - |
| - | - | 4003 | 621.3 | - | - | 0 | - |
| - | - | 1316 | 621.8 | - | - | 0 | - |
| - | - | 941.1 | 622 | - | - | 0 | - |
| 12 | y | 3935 | 622.3 | 0.01166 | 18.74 | +1 | 4 |
| - | - | 809.2 | 622.6 | - | - | 0 | - |
| - | - | 1096 | 623.3 | - | - | 0 | - |
| - | - | 1147 | 623.6 | - | - | 0 | - |
| - | - | 1215 | 625.6 | - | - | 0 | - |
| 2 | y | 2688 | 626 | 0.01082 | 17.29 | +3 | 14 |
| - | - | 3055 | 626.3 | - | - | 0 | - |
| 2 | z | 4301 | 626.6 | 0.01143 | 18.24 | +3 | 14 |
| - | - | 705.5 | 626.8 | - | - | 0 | - |
| - | - | 1479 | 627 | - | - | 0 | - |
| - | - | 2992 | 627.3 | - | - | 0 | - |
| - | - | 922.2 | 627.6 | - | - | 0 | - |
| - | - | 1179 | 628 | - | - | 0 | - |
| - | - | 1580 | 629.3 | - | - | 0 | - |
| - | - | 588.3 | 629.6 | - | - | 0 | - |
| - | - | 813.8 | 629.8 | - | - | 0 | - |
| - | - | 666.6 | 630 | - | - | 0 | - |
| - | - | 990.2 | 630.3 | - | - | 0 | - |
| - | - | 1051 | 630.6 | - | - | 0 | - |
| - | - | 1746 | 631 | - | - | 0 | - |
| - | - | 2692 | 631.3 | - | - | 0 | - |
| - | - | 2171 | 631.6 | - | - | 0 | - |
| 2 | y | 2728 | 632 | 0.0002658 | 0.4206 | +3 | 14 |
| - | - | 2480 | 632.3 | - | - | 0 | - |
| - | - | 1563 | 633 | - | - | 0 | - |
| - | - | 959.3 | 633.3 | - | - | 0 | - |
| - | - | 829.9 | 634 | - | - | 0 | - |
| - | - | 2712 | 634.3 | - | - | 0 | - |
| - | - | 1083 | 634.6 | - | - | 0 | - |
| 6 | w | 5770 | 634.8 | 0.001123 | 1.769 | +2 | 10 |
| - | - | 1008 | 635 | - | - | 0 | - |
| - | - | 5546 | 635.3 | - | - | 0 | - |
| - | - | 1953 | 635.6 | - | - | 0 | - |
| - | - | 2351 | 635.8 | - | - | 0 | - |
| - | - | 1960 | 636 | - | - | 0 | - |
| - | - | 2764 | 636.3 | - | - | 0 | - |
| - | - | 2578 | 636.6 | - | - | 0 | - |
| - | - | 2574 | 637 | - | - | 0 | - |
| - | - | 2334 | 637.3 | - | - | 0 | - |
| - | - | 2279 | 637.6 | - | - | 0 | - |
| - | - | 2277 | 638 | - | - | 0 | - |
| - | - | 1672 | 638.3 | - | - | 0 | - |
| - | - | 1156 | 638.6 | - | - | 0 | - |
| - | - | 898.9 | 639.3 | - | - | 0 | - |
| - | - | 2029 | 640 | - | - | 0 | - |
| - | - | 1.432E+04 | 640.3 | - | - | 0 | - |
| - | - | 1.451E+04 | 640.7 | - | - | 0 | - |
| - | - | 8352 | 641 | - | - | 0 | - |
| - | - | 5193 | 641.3 | - | - | 0 | - |
| - | - | 2953 | 641.6 | - | - | 0 | - |
| - | - | 2281 | 642 | - | - | 0 | - |
| - | - | 3022 | 642.3 | - | - | 0 | - |
| - | - | 3390 | 642.6 | - | - | 0 | - |
| - | - | 3334 | 643 | - | - | 0 | - |
| - | - | 1059 | 643.3 | - | - | 0 | - |
| - | - | 1529 | 644 | - | - | 0 | - |
| - | - | 2079 | 644.3 | - | - | 0 | - |
| - | - | 1772 | 644.6 | - | - | 0 | - |
| - | - | 2164 | 645 | - | - | 0 | - |
| - | - | 3764 | 645.3 | - | - | 0 | - |
| - | - | 6958 | 645.6 | - | - | 0 | - |
| - | - | 9487 | 646 | - | - | 0 | - |
| - | - | 6740 | 646.3 | - | - | 0 | - |
| - | - | 3883 | 646.6 | - | - | 0 | - |
| - | - | 2074 | 647 | - | - | 0 | - |
| 6 | z | 1954 | 647.3 | 0.01188 | 18.35 | +2 | 10 |
| - | - | 1204 | 647.6 | - | - | 0 | - |
| 6 | z | 1309 | 647.8 | 0.0009536 | 1.472 | +2 | 10 |
| - | - | 2782 | 648 | - | - | 0 | - |
| - | - | 2601 | 648.3 | - | - | 0 | - |
| - | - | 1663 | 648.6 | - | - | 0 | - |
| - | - | 2039 | 649 | - | - | 0 | - |
| - | - | 1745 | 649.3 | - | - | 0 | - |
| - | - | 4058 | 649.6 | - | - | 0 | - |
| - | - | 9012 | 650 | - | - | 0 | - |
| - | - | 1.679E+04 | 650.3 | - | - | 0 | - |
| - | - | 1.376E+04 | 650.7 | - | - | 0 | - |
| - | - | 1.153E+04 | 651 | - | - | 0 | - |
| - | - | 8593 | 651.3 | - | - | 0 | - |
| - | - | 9160 | 651.6 | - | - | 0 | - |
| - | - | 4219 | 652 | - | - | 0 | - |
| - | - | 4661 | 652.3 | - | - | 0 | - |
| - | - | 2680 | 652.6 | - | - | 0 | - |
| - | - | 915.3 | 653 | - | - | 0 | - |
| - | - | 2366 | 653.3 | - | - | 0 | - |
| - | - | 1578 | 653.6 | - | - | 0 | - |
| - | - | 986.2 | 654 | - | - | 0 | - |
| - | - | 2058 | 654.7 | - | - | 0 | - |
| - | - | 1749 | 655 | - | - | 0 | - |
| 6 | y | 1162 | 655.3 | 0.007383 | 11.27 | +2 | 10 |
| 6 | z | 8038 | 656.3 | 0.01227 | 18.7 | +2 | 10 |
| - | - | 6234 | 656.6 | - | - | 0 | - |
| - | - | 1581 | 656.8 | - | - | 0 | - |
| - | - | 8488 | 657 | - | - | 0 | - |
| - | - | 7984 | 657.3 | - | - | 0 | - |
| - | - | 4384 | 657.6 | - | - | 0 | - |
| - | - | 3268 | 658 | - | - | 0 | - |
| - | - | 782 | 658.3 | - | - | 0 | - |
| - | - | 1270 | 658.6 | - | - | 0 | - |
| - | - | 2306 | 659 | - | - | 0 | - |
| - | - | 2103 | 659.3 | - | - | 0 | - |
| - | - | 1636 | 659.6 | - | - | 0 | - |
| - | - | 1102 | 660 | - | - | 0 | - |
| - | - | 1253 | 660.3 | - | - | 0 | - |
| - | - | 900.3 | 660.6 | - | - | 0 | - |
| - | - | 1033 | 661.3 | - | - | 0 | - |
| - | - | 1913 | 662 | - | - | 0 | - |
| - | - | 4823 | 662.3 | - | - | 0 | - |
| - | - | 7749 | 662.6 | - | - | 0 | - |
| - | - | 5990 | 663 | - | - | 0 | - |
| - | - | 3630 | 663.3 | - | - | 0 | - |
| - | - | 2631 | 663.6 | - | - | 0 | - |
| - | - | 1215 | 663.8 | - | - | 0 | - |
| - | - | 2332 | 664 | - | - | 0 | - |
| 6 | y | 5953 | 664.3 | 0.008453 | 12.72 | +2 | 10 |
| - | - | 3590 | 664.7 | - | - | 0 | - |
| - | - | 3098 | 664.8 | - | - | 0 | - |
| - | - | 3693 | 665 | - | - | 0 | - |
| - | - | 2457 | 665.3 | - | - | 0 | - |
| - | - | 2428 | 665.7 | - | - | 0 | - |
| - | - | 1626 | 666.3 | - | - | 0 | - |
| - | - | 1203 | 668 | - | - | 0 | - |
| - | - | 3456 | 668.3 | - | - | 0 | - |
| - | - | 3250 | 668.6 | - | - | 0 | - |
| - | - | 2975 | 669 | - | - | 0 | - |
| - | - | 7585 | 669.3 | - | - | 0 | - |
| - | - | 2.342E+04 | 669.7 | - | - | 0 | - |
| - | - | 3.987E+04 | 670 | - | - | 0 | - |
| - | - | 2.813E+04 | 670.3 | - | - | 0 | - |
| - | - | 1.309E+04 | 670.7 | - | - | 0 | - |
| - | - | 7452 | 671 | - | - | 0 | - |
| - | - | 4112 | 671.3 | - | - | 0 | - |
| - | - | 1456 | 671.7 | - | - | 0 | - |
| - | - | 1010 | 672 | - | - | 0 | - |
| - | - | 1018 | 673.4 | - | - | 0 | - |
| - | - | 945.5 | 674.3 | - | - | 0 | - |
| 5 | c | 4108 | 680.3 | 0.001443 | 2.12 | +1 | 5 |
| - | - | 2170 | 681.3 | - | - | 0 | - |
| - | - | 3119 | 682.3 | - | - | 0 | - |
| - | - | 1579 | 683.3 | - | - | 0 | - |
| - | - | 1575 | 690.3 | - | - | 0 | - |
| - | - | 1295 | 691.3 | - | - | 0 | - |
| - | - | 938.5 | 691.8 | - | - | 0 | - |
| - | - | 1029 | 694.8 | - | - | 0 | - |
| - | - | 807.8 | 695.9 | - | - | 0 | - |
| 5 | c | 1.609E+04 | 697.3 | 0.0004061 | 0.5824 | +1 | 5 |
| - | - | 8286 | 698.3 | - | - | 0 | - |
| - | - | 2423 | 699.3 | - | - | 0 | - |
| - | - | 1064 | 699.8 | - | - | 0 | - |
| - | - | 1673 | 700.3 | - | - | 0 | - |
| - | - | 2941 | 701.3 | - | - | 0 | - |
| 11 | c | 8676 | 701.8 | 0.00225 | 3.206 | +2 | 11 |
| - | - | 9048 | 702.3 | - | - | 0 | - |
| - | - | 2541 | 702.8 | - | - | 0 | - |
| - | - | 1112 | 703.3 | - | - | 0 | - |
| - | - | 782.5 | 708.8 | - | - | 0 | - |
| - | - | 822.9 | 711.3 | - | - | 0 | - |
| - | - | 1104 | 712.3 | - | - | 0 | - |
| - | - | 1206 | 712.8 | - | - | 0 | - |
| - | - | 1583 | 713.3 | - | - | 0 | - |
| - | - | 1026 | 714.3 | - | - | 0 | - |
| - | - | 3957 | 716.4 | - | - | 0 | - |
| - | - | 1399 | 717.4 | - | - | 0 | - |
| - | - | 1154 | 721.3 | - | - | 0 | - |
| - | - | 879.9 | 721.8 | - | - | 0 | - |
| - | - | 1165 | 722.3 | - | - | 0 | - |
| - | - | 1215 | 723.3 | - | - | 0 | - |
| - | - | 882.1 | 735.3 | - | - | 0 | - |
| - | - | 894.8 | 742.8 | - | - | 0 | - |
| - | - | 950 | 746.3 | - | - | 0 | - |
| 5 | y | 904.7 | 748.9 | 0.005814 | 7.764 | +2 | 11 |
| 5 | z | 6938 | 749.4 | 0.00292 | 3.897 | +2 | 11 |
| - | - | 3182 | 749.9 | - | - | 0 | - |
| - | - | 2309 | 750.4 | - | - | 0 | - |
| 11 | z | 1164 | 752.3 | 0.01472 | 19.56 | +1 | 5 |
| - | - | 1028 | 754.4 | - | - | 0 | - |
| - | - | 2184 | 754.9 | - | - | 0 | - |
| - | - | 1780 | 755.4 | - | - | 0 | - |
| 12 | c | 2002 | 757.4 | 0.002354 | 3.109 | +2 | 12 |
| - | - | 1922 | 757.9 | - | - | 0 | - |
| - | - | 1683 | 758.4 | - | - | 0 | - |
| 12 | c | 1652 | 765.9 | 0.0006673 | 0.8713 | +2 | 12 |
| - | - | 2057 | 766.4 | - | - | 0 | - |
| 11 | y | 885.1 | 768.4 | 0.00938 | 12.21 | +1 | 5 |
| 11 | z | 1.001E+04 | 769.4 | 0.008355 | 10.86 | +1 | 5 |
| - | - | 5913 | 770.4 | - | - | 0 | - |
| - | - | 3041 | 771.4 | - | - | 0 | - |
| - | - | 2278 | 784.9 | - | - | 0 | - |
| 11 | y | 4847 | 785.4 | 0.009867 | 12.56 | +1 | 5 |
| - | - | 1224 | 786.4 | - | - | 0 | - |
| - | - | 815.6 | 788.4 | - | - | 0 | - |
| 6 | c | 1620 | 793.4 | 0.002401 | 3.026 | +1 | 6 |
| - | - | 955.8 | 797.4 | - | - | 0 | - |
| - | - | 2417 | 803.4 | - | - | 0 | - |
| 4 | y | 2732 | 806.4 | 0.009493 | 11.77 | +2 | 12 |
| 4 | z | 7939 | 806.9 | 0.0008888 | 1.102 | +2 | 12 |
| - | - | 6023 | 807.4 | - | - | 0 | - |
| - | - | 3494 | 807.9 | - | - | 0 | - |
| - | - | 1054 | 808.4 | - | - | 0 | - |
| - | - | 813.4 | 809.4 | - | - | 0 | - |
| 6 | c | 2.586E+04 | 810.4 | 0.0005707 | 0.7041 | +1 | 6 |
| - | - | 1475 | 811.4 | - | - | 0 | - |
| - | - | 9387 | 811.4 | - | - | 0 | - |
| - | - | 1206 | 812.4 | - | - | 0 | - |
| - | - | 2539 | 812.4 | - | - | 0 | - |
| - | - | 2330 | 814.4 | - | - | 0 | - |
| 4 | y | 1865 | 814.9 | 0.0004852 | 0.5955 | +2 | 12 |
| - | - | 1410 | 815.4 | - | - | 0 | - |
| - | - | 1050 | 826.4 | - | - | 0 | - |
| - | - | 1028 | 827.4 | - | - | 0 | - |
| - | - | 1841 | 831.4 | - | - | 0 | - |
| - | - | 1778 | 831.8 | - | - | 0 | - |
| - | - | 1065 | 832.4 | - | - | 0 | - |
| - | - | 3705 | 837.5 | - | - | 0 | - |
| - | - | 4048 | 837.8 | - | - | 0 | - |
| - | - | 1292 | 838.1 | - | - | 0 | - |
| - | - | 1076 | 838.5 | - | - | 0 | - |
| - | - | 805 | 839.4 | - | - | 0 | - |
| - | - | 1683 | 840.4 | - | - | 0 | - |
| - | - | 2245 | 842.4 | - | - | 0 | - |
| - | - | 842.6 | 843.4 | - | - | 0 | - |
| - | - | 823.7 | 845.4 | - | - | 0 | - |
| - | - | 2258 | 845.9 | - | - | 0 | - |
| 13 | c | 6433 | 846.4 | 0.001146 | 1.354 | +2 | 13 |
| - | - | 4051 | 846.9 | - | - | 0 | - |
| - | - | 2267 | 847.4 | - | - | 0 | - |
| - | - | 1177 | 847.9 | - | - | 0 | - |
| - | - | 1257 | 848.9 | - | - | 0 | - |
| - | - | 1154 | 851 | - | - | 0 | - |
| - | - | 896.3 | 854.4 | - | - | 0 | - |
| - | - | 742.1 | 860.4 | - | - | 0 | - |
| - | - | 1050 | 862.9 | - | - | 0 | - |
| - | - | 3045 | 867.4 | - | - | 0 | - |
| - | - | 1179 | 868.4 | - | - | 0 | - |
| - | - | 938.6 | 869.4 | - | - | 0 | - |
| 3 | y | 2528 | 870.4 | 0.004497 | 5.166 | +2 | 13 |
| 3 | z | 8373 | 870.9 | 0.004177 | 4.796 | +2 | 13 |
| - | - | 5106 | 871.4 | - | - | 0 | - |
| - | - | 2828 | 871.9 | - | - | 0 | - |
| - | - | 2173 | 872.4 | - | - | 0 | - |
| - | - | 945.5 | 873.4 | - | - | 0 | - |
| - | - | 829.7 | 878.4 | - | - | 0 | - |
| - | - | 883.1 | 878.9 | - | - | 0 | - |
| - | - | 4131 | 881.4 | - | - | 0 | - |
| - | - | 1257 | 883.4 | - | - | 0 | - |
| - | - | 1192 | 888.4 | - | - | 0 | - |
| - | - | 903.4 | 888.9 | - | - | 0 | - |
| - | - | 1075 | 889.9 | - | - | 0 | - |
| 10 | z | 1.061E+04 | 898.4 | 0.006881 | 7.659 | +1 | 6 |
| - | - | 9753 | 899.4 | - | - | 0 | - |
| - | - | 4902 | 900.4 | - | - | 0 | - |
| 14 | c | 1176 | 901.4 | 0.01749 | 19.41 | +2 | 14 |
| - | - | 956.7 | 902.4 | - | - | 0 | - |
| 7 | c | 1195 | 908.4 | 0.004767 | 5.248 | +1 | 7 |
| 14 | c | 4009 | 910.4 | 0.00117 | 1.285 | +2 | 14 |
| - | - | 4575 | 910.9 | - | - | 0 | - |
| - | - | 2201 | 911.4 | - | - | 0 | - |
| - | - | 1265 | 911.9 | - | - | 0 | - |
| - | - | 824.7 | 913.4 | - | - | 0 | - |
| 10 | y | 1476 | 914.4 | 0.004426 | 4.84 | +1 | 6 |
| - | - | 2245 | 915.4 | - | - | 0 | - |
| - | - | 1061 | 917.9 | - | - | 0 | - |
| - | - | 949.1 | 918.9 | - | - | 0 | - |
| - | - | 1174 | 919.4 | - | - | 0 | - |
| - | - | 1069 | 922.4 | - | - | 0 | - |
| - | - | 1311 | 923.4 | - | - | 0 | - |
| - | - | 1870 | 924.5 | - | - | 0 | - |
| 7 | c | 4895 | 925.5 | 0.0002493 | 0.2694 | +1 | 7 |
| - | - | 2575 | 926.5 | - | - | 0 | - |
| - | - | 1177 | 927.5 | - | - | 0 | - |
| - | - | 841.8 | 930.5 | - | - | 0 | - |
| - | - | 1940 | 931 | - | - | 0 | - |
| - | - | 1574 | 931.5 | - | - | 0 | - |
| - | - | 3264 | 932.5 | - | - | 0 | - |
| - | - | 1606 | 938 | - | - | 0 | - |
| - | - | 4692 | 938.5 | - | - | 0 | - |
| - | - | 2204 | 939 | - | - | 0 | - |
| 2 | z | 6187 | 939.4 | 0.01794 | 19.1 | +2 | 14 |
| - | - | 6547 | 939.9 | - | - | 0 | - |
| - | - | 4636 | 940.4 | - | - | 0 | - |
| - | - | 2141 | 940.9 | - | - | 0 | - |
| - | - | 957.2 | 941.5 | - | - | 0 | - |
| - | - | 1651 | 942.5 | - | - | 0 | - |
| - | - | 986.9 | 943.4 | - | - | 0 | - |
| - | - | 1161 | 943.9 | - | - | 0 | - |
| - | - | 1643 | 945.5 | - | - | 0 | - |
| - | - | 1967 | 946.5 | - | - | 0 | - |
| - | - | 1759 | 947 | - | - | 0 | - |
| 2 | y | 3090 | 947.4 | 0.01023 | 10.8 | +2 | 14 |
| - | - | 1093 | 948 | - | - | 0 | - |
| - | - | 1163 | 948.5 | - | - | 0 | - |
| - | - | 1192 | 949.5 | - | - | 0 | - |
| - | - | 910.2 | 950.4 | - | - | 0 | - |
| - | - | 1583 | 953 | - | - | 0 | - |
| - | - | 1635 | 953.5 | - | - | 0 | - |
| - | - | 1640 | 954 | - | - | 0 | - |
| - | - | 2654 | 954.5 | - | - | 0 | - |
| - | - | 2192 | 955 | - | - | 0 | - |
| - | - | 3027 | 955.5 | - | - | 0 | - |
| - | - | 2781 | 956 | - | - | 0 | - |
| - | - | 1680 | 957 | - | - | 0 | - |
| - | - | 2140 | 957.4 | - | - | 0 | - |
| - | - | 1906 | 959.5 | - | - | 0 | - |
| - | - | 3017 | 960 | - | - | 0 | - |
| - | - | 3951 | 960.5 | - | - | 0 | - |
| - | - | 1835 | 961 | - | - | 0 | - |
| - | - | 1256 | 961.5 | - | - | 0 | - |
| - | - | 1848 | 962 | - | - | 0 | - |
| - | - | 2572 | 962.5 | - | - | 0 | - |
| - | - | 2678 | 963 | - | - | 0 | - |
| - | - | 3090 | 963.5 | - | - | 0 | - |
| - | - | 2718 | 964 | - | - | 0 | - |
| 8 | c | 1776 | 964.5 | 0.01078 | 11.18 | +1 | 8 |
| - | - | 2013 | 965 | - | - | 0 | - |
| 8 | c | 1835 | 965.4 | 0.0124 | 12.85 | +1 | 8 |
| - | - | 1288 | 966 | - | - | 0 | - |
| - | - | 3069 | 966.5 | - | - | 0 | - |
| - | - | 2462 | 967 | - | - | 0 | - |
| - | - | 3848 | 967.5 | - | - | 0 | - |
| - | - | 4422 | 968 | - | - | 0 | - |
| 9 | w | 6473 | 968.4 | 0.01718 | 17.73 | +1 | 7 |
| - | - | 3685 | 969 | - | - | 0 | - |
| - | - | 3417 | 969.5 | - | - | 0 | - |
| - | - | 1166 | 970 | - | - | 0 | - |
| - | - | 3053 | 970.5 | - | - | 0 | - |
| - | - | 1791 | 971 | - | - | 0 | - |
| - | - | 2639 | 971.5 | - | - | 0 | - |
| - | - | 1559 | 972 | - | - | 0 | - |
| - | - | 3248 | 972.5 | - | - | 0 | - |
| - | - | 1243 | 973 | - | - | 0 | - |
| - | - | 2396 | 973.5 | - | - | 0 | - |
| - | - | 2450 | 974 | - | - | 0 | - |
| - | - | 2729 | 974.5 | - | - | 0 | - |
| - | - | 7929 | 975 | - | - | 0 | - |
| - | - | 8510 | 975.5 | - | - | 0 | - |
| - | - | 6552 | 976 | - | - | 0 | - |
| - | - | 7535 | 976.5 | - | - | 0 | - |
| - | - | 6614 | 977 | - | - | 0 | - |
| - | - | 5787 | 977.5 | - | - | 0 | - |
| - | - | 3933 | 978 | - | - | 0 | - |
| - | - | 2824 | 978.5 | - | - | 0 | - |
| - | - | 1652 | 979 | - | - | 0 | - |
| - | - | 1891 | 980 | - | - | 0 | - |
| - | - | 1599 | 980.5 | - | - | 0 | - |
| - | - | 1683 | 981 | - | - | 0 | - |
| - | - | 4472 | 981.5 | - | - | 0 | - |
| - | - | 3343 | 982 | - | - | 0 | - |
| 8 | c | 1.362E+04 | 982.5 | 0.005081 | 5.172 | +1 | 8 |
| - | - | 2981 | 983 | - | - | 0 | - |
| - | - | 7362 | 983.5 | - | - | 0 | - |
| - | - | 1241 | 984 | - | - | 0 | - |
| - | - | 5619 | 984.5 | - | - | 0 | - |
| - | - | 7816 | 985 | - | - | 0 | - |
| - | - | 8586 | 985.5 | - | - | 0 | - |
| - | - | 3986 | 986 | - | - | 0 | - |
| - | - | 3708 | 986.5 | - | - | 0 | - |
| - | - | 2497 | 987 | - | - | 0 | - |
| - | - | 2131 | 987.5 | - | - | 0 | - |
| - | - | 2670 | 988 | - | - | 0 | - |
| - | - | 4839 | 988.5 | - | - | 0 | - |
| - | - | 2282 | 989 | - | - | 0 | - |
| - | - | 4611 | 989.5 | - | - | 0 | - |
| - | - | 2040 | 990.5 | - | - | 0 | - |
| - | - | 1218 | 991.5 | - | - | 0 | - |
| - | - | 2293 | 993 | - | - | 0 | - |
| - | - | 7632 | 993.5 | - | - | 0 | - |
| - | - | 7738 | 994 | - | - | 0 | - |
| - | - | 5726 | 994.5 | - | - | 0 | - |
| - | - | 4081 | 995 | - | - | 0 | - |
| - | - | 2559 | 995.5 | - | - | 0 | - |
| - | - | 8644 | 996 | - | - | 0 | - |
| - | - | 1.668E+04 | 996.5 | - | - | 0 | - |
| - | - | 1.247E+04 | 997 | - | - | 0 | - |
| - | - | 1.006E+04 | 997.5 | - | - | 0 | - |
| - | - | 4813 | 998 | - | - | 0 | - |
| - | - | 4273 | 998.5 | - | - | 0 | - |
| - | - | 3169 | 999 | - | - | 0 | - |
| - | - | 2388 | 999.5 | - | - | 0 | - |
| - | - | 3680 | 1002 | - | - | 0 | - |
| - | - | 2904 | 1003 | - | - | 0 | - |
| - | - | 3811 | 1003 | - | - | 0 | - |
| - | - | 4263 | 1004 | - | - | 0 | - |
| - | - | 9629 | 1004 | - | - | 0 | - |
| - | - | 1.549E+04 | 1005 | - | - | 0 | - |
| - | - | 1.279E+04 | 1005 | - | - | 0 | - |
| - | - | 7256 | 1006 | - | - | 0 | - |
| - | - | 4463 | 1006 | - | - | 0 | - |
| - | - | 2404 | 1007 | - | - | 0 | - |
| - | - | 1972 | 1007 | - | - | 0 | - |
| - | - | 1082 | 1008 | - | - | 0 | - |
| 9 | z | 1728 | 1027 | 0.01041 | 10.14 | +1 | 7 |
| - | - | 3422 | 1028 | - | - | 0 | - |
| - | - | 1486 | 1029 | - | - | 0 | - |
| - | - | 1056 | 1030 | - | - | 0 | - |
| - | - | 1099 | 1067 | - | - | 0 | - |
| - | - | 1734 | 1085 | - | - | 0 | - |
| - | - | 3770 | 1110 | - | - | 0 | - |
| 9 | c | 1.597E+04 | 1111 | 0.002465 | 2.219 | +1 | 9 |
| - | - | 9736 | 1112 | - | - | 0 | - |
| - | - | 2894 | 1113 | - | - | 0 | - |
| - | - | 872.2 | 1197 | - | - | 0 | - |
| 7 | z | 2667 | 1199 | 0.008929 | 7.45 | +1 | 9 |
| - | - | 5582 | 1200 | - | - | 0 | - |
| - | - | 3534 | 1201 | - | - | 0 | - |
| - | - | 1157 | 1202 | - | - | 0 | - |
| - | - | 999.6 | 1216 | - | - | 0 | - |
| - | - | 933.4 | 1226 | - | - | 0 | - |
| - | - | 1252 | 1239 | - | - | 0 | - |
| 10 | c | 4533 | 1240 | 0.005404 | 4.359 | +1 | 10 |
| - | - | 3391 | 1241 | - | - | 0 | - |
| - | - | 2213 | 1242 | - | - | 0 | - |
| 6 | z | 1927 | 1312 | 0.007263 | 5.537 | +1 | 10 |
| - | - | 3269 | 1313 | - | - | 0 | - |
| - | - | 1705 | 1314 | - | - | 0 | - |
| - | - | 949 | 1359 | - | - | 0 | - |
| - | - | 1035 | 1402 | - | - | 0 | - |
| 11 | c | 1716 | 1403 | 0.004209 | 3.001 | +1 | 11 |
| - | - | 2215 | 1404 | - | - | 0 | - |
| - | - | 1162 | 1405 | - | - | 0 | - |
| - | - | 833.4 | 1406 | - | - | 0 | - |
| - | - | 873.5 | 1475 | - | - | 0 | - |
| 5 | z | 1300 | 1498 | 0.003877 | 2.589 | +1 | 11 |
| - | - | 2331 | 1499 | - | - | 0 | - |
| - | - | 1366 | 1500 | - | - | 0 | - |
| - | - | 1205 | 1515 | - | - | 0 | - |
| 4 | z | 1060 | 1613 | 0.009272 | 5.749 | +1 | 12 |
| - | - | 3363 | 1614 | - | - | 0 | - |
| - | - | 1959 | 1615 | - | - | 0 | - |
| - | - | 1114 | 1616 | - | - | 0 | - |
| 13 | c | 859 | 1692 | 0.0157 | 9.28 | +1 | 13 |
| - | - | 2296 | 1693 | - | - | 0 | - |
| - | - | 807.1 | 1695 | - | - | 0 | - |
| 3 | z | 1327 | 1741 | 0.001609 | 0.9242 | +1 | 13 |
| - | - | 2392 | 1742 | - | - | 0 | - |
| - | - | 2280 | 1743 | - | - | 0 | - |
| - | - | 1665 | 1744 | - | - | 0 | - |
| - | - | 981.5 | 1821 | - | - | 0 | - |
| - | - | 966.3 | 1879 | - | - | 0 | - |
| - | - | 772.4 | 1904 | - | - | 0 | - |
| - | - | 2050 | 1950 | - | - | 0 | - |
| - | - | 1777 | 1951 | - | - | 0 | - |
| - | - | 1136 | 1953 | - | - | 0 | - |
| - | - | 1186 | 1954 | - | - | 0 | - |
| - | - | 1051 | 1970 | - | - | 0 | - |
| - | - | 1034 | 1971 | - | - | 0 | - |
| - | - | 924.5 | 1972 | - | - | 0 | - |
| - | - | 820.8 | 1989 | - | - | 0 | - |
| - | - | 880.1 | 1992 | - | - | 0 | - |
| - | - | 1064 | 1993 | - | - | 0 | - |
| - | - | 739.5 | 1994 | - | - | 0 | - |
| - | - | 876.2 | 2008 | - | - | 0 | - |
| - | - | 1784 | 2009 | - | - | 0 | - |
| - | - | 1541 | 2010 | - | - | 0 | - |
| - | - | 1622 | 2011 | - | - | 0 | - |
| - | - | 831.7 | 3048 | - | - | 0 | - |
| - | - | 727.3 | 3048 | - | - | 0 | - |

m/z Charge Intensity FragmentType MassShift Position
126.06696319580078 0 429.1314
129.1025390625 0 3546.7751
130.06536865234375 0 2246.5876
131.36260986328125 0 382.7321
133.06076049804688 0 428.97433
133.08616638183594 0 1275.9016
136.0760040283203 0 722.9231
140.0823516845703 0 436.26303
144.4639892578125 0 464.5679
146.09286499023438 0 803.916
148.9470977783203 0 1067.3966
149.02391052246094 0 488.25885
149.04502868652344 0 1245.1785
155.09300231933594 0 3161.189
156.07757568359375 0 454.82562
156.91632080078125 0 516.742
159.0921173095703 0 775.71063
162.05499267578125 0 733.0351
164.08236694335938 0 462.73465
165.10272216796875 0 605.0873
166.0614013671875 0 3310.0044
168.1388397216797 0 1607.4196
173.0924530029297 0 1193.985
173.45025634765625 0 4353.9297
174.08802795410156 0 556.33997
176.35350036621094 0 489.20627
177.1128387451172 0 1103.0203
183.11312866210938 0 1499.1802
184.65748596191406 0 455.9182
185.1650848388672 0 2797.3633
187.10804748535156 0 1078.7114
191.10292053222656 0 1753.4258
199.1082763671875 0 1179.3348 c Ammonia loss 5
202.08273315429688 0 1624.3937
203.1028289794922 0 1548.4617
204.13497924804688 0 687.3815
205.11891174316406 0 869.20746 y 14
207.96127319335938 0 454.48993
209.09259033203125 0 914.5517
213.12332153320312 0 712.76514
215.13938903808594 0 9140.86
217.1551055908203 0 974.1633
217.33831787109375 0 600.599
218.1143341064453 0 1130.7343
218.1586151123047 0 524.0056
221.08445739746094 0 683.43634
223.15562438964844 0 2523.9343
227.10289001464844 0 3345.1282
229.15542602539062 0 566.1804
230.1611328125 0 597.2449
231.17076110839844 0 791.56586
234.12416076660156 0 2980.9216
235.1292724609375 0 589.529
242.1505584716797 0 668.6425
246.1813201904297 0 1208.0283
251.15069580078125 0 14972.269
252.15379333496094 0 1632.2151
261.1196594238281 0 901.73334
268.1771240234375 0 1510.22 c 1
274.1178894042969 0 763.1935
275.12396240234375 0 944.0164
281.0514831542969 0 1051.641
283.1519470214844 0 1120.4614
299.0622863769531 0 7005.028
300.0633850097656 0 1117.5931
300.1923828125 0 1711.5189
302.1716613769531 0 779.3627
317.1464538574219 0 797.9454
318.2040100097656 0 703.69586
319.1643981933594 0 551.3285
326.66461181640625 0 2006.183
333.2149353027344 0 1108.0691 y 13
338.1352233886719 0 1267.5216
340.1874084472656 0 1975.6989
340.6636657714844 0 674.474 c Ammonia loss 4
341.16259765625 0 499.15244
349.6809387207031 0 739.9987
359.0278625488281 0 803.71985
374.6941833496094 0 663.9974 y Water loss 4
378.20184326171875 0 746.9471
379.2093811035156 0 5220.039 c Ammonia loss 2
380.21221923828125 0 1451.7769
381.1521911621094 0 612.4363
383.207275390625 0 735.031
386.15582275390625 0 629.0261
387.2236022949219 0 2035.6477
393.1976623535156 0 1448.1337 y 10
396.2359313964844 0 5742.912 c 2
397.23968505859375 0 1023.83026
403.49945068359375 0 695.3849
407.1970520019531 0 749.55096
415.0380554199219 0 3122.9534
416.0382385253906 0 721.6178
420.202880859375 0 10043.66 w 9
420.2326354980469 0 392.25357
420.7045593261719 0 4435.549
421.20452880859375 0 1816.5947
428.2875061035156 0 782.2116
435.721923828125 0 632.23834
445.1958923339844 0 620.47797
447.92401123046875 0 768.75586
449.71856689453125 0 2012.9232 z 9
457.2664489746094 0 6556.659
458.23065185546875 0 3101.1702
458.27032470703125 0 3063.9128
458.7304992675781 0 1983.1766
459.2804870605469 0 5585.3857
460.28594970703125 0 1322.4258
460.735595703125 0 1555.598
461.2358093261719 0 763.23425
467.2488098144531 0 1287.2363
468.2628479003906 0 880.1143
469.2305603027344 0 1194.4655
472.3017883300781 0 1007.8528
478.2501220703125 0 773.3347
481.2317810058594 0 587.073
484.2444763183594 0 1149.8817
485.23193359375 0 2587.3252
485.3110656738281 0 785.2585
486.29302978515625 0 5697.8833
487.2413330078125 0 1261.6718
488.294189453125 0 1046.6472
489.23419189453125 0 911.25977
492.48846435546875 0 698.49866
493.74993896484375 0 3482.1807
494.237548828125 0 6577.309 c Ammonia loss 3
494.7453918457031 0 1287.922
495.2372741699219 0 1259.8745
496.2513427734375 0 611.6936
498.2567138671875 0 784.4034
498.74700927734375 0 1195.3539
499.24481201171875 0 1407.8893 y Water loss 4
501.21978759765625 0 2510.6284
501.75067138671875 0 1308.9888
502.2720947265625 0 4983.119
502.49853515625 0 5397.505
502.7514953613281 0 12232.711
502.9986267089844 0 3320.7556
503.1084289550781 0 3807.956
503.2498474121094 0 7091.476
503.3219299316406 0 832.0844
503.75738525390625 0 1245.1764
504.1068115234375 0 1326.5104
504.2640380859375 0 2046.7585
504.3029479980469 0 3120.8828
504.339599609375 0 971.32874
505.5876159667969 0 853.4808
506.2618103027344 0 986.8057
506.7278137207031 0 642.2198
507.2644958496094 0 934.0696
511.2634582519531 0 14418.312 c 3
511.3028259277344 0 992.57214
512.2652587890625 0 3839.9854
513.2696533203125 0 928.44794
515.3193969726562 0 1950.4215
519.2750854492188 0 762.4114
519.2984619140625 0 1504.8818
521.7667236328125 0 2901.4937 y 8
522.2697143554688 0 792.2058
522.7700805664062 0 834.95154
527.7677001953125 0 826.83734
529.2651977539062 0 835.9175
533.2569580078125 0 663.6565 z Water loss 7
535.2921142578125 0 1638.8097
536.2870483398438 0 637.23425
542.26611328125 0 1472.1093 z 7
543.2703857421875 0 825.42334
547.2897338867188 0 935.5733
547.7724609375 0 588.9025
548.23779296875 0 1124.3337 w 11
548.75390625 0 799.429
549.2625732421875 0 624.59235
550.2457275390625 0 1492.6919
550.2783813476562 0 2180.268 y 7
550.7778930664062 0 1574.4719
552.2601928710938 0 871.41626
555.26220703125 0 1190.0326
555.3007202148438 0 1976.553
555.78955078125 0 8854.62 c 8
556.2899780273438 0 4497.599
556.79296875 0 1207.255
563.2689819335938 0 634.5386
564.274169921875 0 5211.5405
564.7720336914062 0 2539.4048
565.2757568359375 0 898.5838
565.7708129882812 0 815.45953
568.7974853515625 0 868.16547
571.280517578125 0 2520.8352
571.781982421875 0 2271.7087
572.2784423828125 0 2066.3823
573.2723999023438 0 788.06085
577.281982421875 0 2485.3943 w 6
577.7830810546875 0 1908.8378
578.29052734375 0 1271.9021
580.2745971679688 0 1262.1891 y Water loss 2
590.627685546875 0 952.9637
590.77294921875 0 657.628 z Water loss 6
591.2853393554688 0 1541.4532
592.781005859375 0 794.82855
596.2890625 0 1069.4542
597.29443359375 0 2119.7512
597.6299438476562 0 937.344
597.9612426757812 0 876.32153
598.302001953125 0 763.74866
598.7870483398438 0 1053.9481 y Water loss 6
598.9632568359375 0 756.50085
599.2887573242188 0 2931.6772 y Ammonia loss 6
599.7825927734375 0 6085.6787 z 6
600.2833862304688 0 3560.1963
600.7843627929688 0 1697.3167
601.2943725585938 0 848.71814 c Water loss 13
601.3784790039062 0 750.601
601.9605102539062 0 771.5225
602.28955078125 0 903.6969
602.9476928710938 0 786.19696
604.967041015625 0 983.6616
605.2966918945312 0 1060.7782 y Ammonia loss 11
605.6286010742188 0 810.7398
606.3041381835938 0 6026.014 z 11
606.6345825195312 0 739.9537
606.9677124023438 0 2072.033
607.3016967773438 0 9289.015 c 13
607.63134765625 0 4762.2344
607.790771484375 0 7885.145 y 6
607.96533203125 0 3237.581
608.2941284179688 0 7591.4824
608.6292724609375 0 1080.9915
608.7928466796875 0 1815.4921
608.9572143554688 0 600.1674
609.2903442382812 0 994.3051
611.3004150390625 0 1669.5487 c Water loss 9
611.8067016601562 0 846.67957 c Ammonia loss 9
611.9697265625 0 1006.2181
612.3029174804688 0 2690.9531
612.62939453125 0 944.24023
612.8035888671875 0 1810.6959
613.6179809570312 0 1038.6357
615.3059692382812 0 924.73627
615.6278686523438 0 1146.1222
616.6298217773438 0 816.3006
617.289306640625 0 813.02686
619.2993774414062 0 1005.07074
619.8177490234375 0 3956.427
620.3112182617188 0 14370.472 c 9
620.6378784179688 0 809.7109
620.81298828125 0 7588.552
620.97412109375 0 1308.8417
621.3108520507812 0 4002.89
621.815185546875 0 1315.5679
621.9694213867188 0 941.0856
622.3202514648438 0 3934.5166 y 11
622.632080078125 0 809.2049
623.3282470703125 0 1096.1742
623.6314086914062 0 1147.0582
625.6424560546875 0 1214.828
625.9751586914062 0 2688.05 y Water loss 1
626.3096313476562 0 3055.0623
626.6397094726562 0 4301.158 z 1
626.8064575195312 0 705.5129
626.9635620117188 0 1478.5542
627.2955322265625 0 2992.218
627.6316528320312 0 922.16705
627.968017578125 0 1179.285
629.3013916015625 0 1580.382
629.6351928710938 0 588.26935
629.8084106445312 0 813.756
629.9671630859375 0 666.5975
630.3191528320312 0 990.21533
630.6367797851562 0 1051.0934
630.971923828125 0 1746.2678
631.3033447265625 0 2692.0522
631.63623046875 0 2171.4668
631.9675903320312 0 2728.0698 y 1
632.3018798828125 0 2480.2104
632.9760131835938 0 1563.321
633.2997436523438 0 959.31195
633.9700927734375 0 829.8621
634.3041381835938 0 2712.116
634.6401977539062 0 1083.4717
634.7984008789062 0 5769.729 w 5
634.9862060546875 0 1007.80273
635.30078125 0 5545.6807
635.6396484375 0 1952.9454
635.7984008789062 0 2351.4282
635.9678344726562 0 1959.6447
636.300537109375 0 2763.523
636.6375122070312 0 2577.5225
636.971435546875 0 2573.6738
637.304931640625 0 2334.3796
637.6392822265625 0 2278.809
637.9712524414062 0 2277.095
638.304931640625 0 1672.3558
638.6310424804688 0 1156.042
639.3097534179688 0 898.9095
639.9910278320312 0 2029.0936
640.3219604492188 0 14322.057
640.6553955078125 0 14505.254
640.9892578125 0 8351.79
641.318359375 0 5193.23
641.6472778320312 0 2953.399
641.9767456054688 0 2281.4216
642.3082885742188 0 3021.9956
642.6423950195312 0 3390.2605
642.971923828125 0 3333.673
643.3094482421875 0 1059.3903
643.9677124023438 0 1528.9645
644.3046264648438 0 2078.531
644.6392822265625 0 1771.7131
644.979736328125 0 2164.0454
645.3099975585938 0 3764.2598
645.6437377929688 0 6958.028
645.9791259765625 0 9486.685
646.3139038085938 0 6739.9976
646.64892578125 0 3882.501
646.9811401367188 0 2073.7832
647.3097534179688 0 1953.8578 z Water loss 5
647.641357421875 0 1203.8486
647.8126831054688 0 1308.955 z Ammonia loss 5
647.9752807617188 0 2782.354
648.3069458007812 0 2601.2017
648.6405029296875 0 1663.0297
648.9747314453125 0 2038.6437
649.3184204101562 0 1744.8223
649.6451416015625 0 4057.8186
649.9765014648438 0 9011.854
650.3167724609375 0 16794.932
650.651611328125 0 13759.524
650.98291015625 0 11534.715
651.3116455078125 0 8592.568
651.64697265625 0 9160.456
651.9798583984375 0 4219.214
652.3124389648438 0 4661.412
652.64697265625 0 2679.8662
652.9735717773438 0 915.2756
653.3095703125 0 2365.6794
653.6432495117188 0 1577.7937
653.9761962890625 0 986.1621
654.6559448242188 0 2057.8408
654.98681640625 0 1748.6329
655.3236083984375 0 1161.7655 y Water loss 5
656.3146362304688 0 8037.91 z 5
656.6419677734375 0 6234.261
656.8232421875 0 1580.8119
656.9771728515625 0 8487.53
657.3121337890625 0 7984.1587
657.645751953125 0 4384.451
657.9764404296875 0 3268.342
658.3062744140625 0 782.0361
658.6426391601562 0 1270.3695
658.9785766601562 0 2306.4653
659.3088989257812 0 2102.7583
659.6470336914062 0 1635.7239
659.9761352539062 0 1101.5845
660.313720703125 0 1252.8002
660.642333984375 0 900.3483
661.29931640625 0 1032.9286
661.9743041992188 0 1913.0192
662.3079223632812 0 4822.9824
662.6438598632812 0 7749.092
662.9783325195312 0 5989.6655
663.3121948242188 0 3629.6025
663.64697265625 0 2630.8337
663.8384399414062 0 1215.1914
663.9801025390625 0 2332.2805
664.3278198242188 0 5952.7427 y 5
664.6561889648438 0 3589.965
664.834228515625 0 3097.8691
664.9848022460938 0 3692.7112
665.326171875 0 2457.115
665.656494140625 0 2428.217
666.3010864257812 0 1626.1565
667.9782104492188 0 1202.5867
668.3158569335938 0 3456.2107
668.6488647460938 0 3250.3906
668.9835205078125 0 2974.5598
669.3280639648438 0 7584.7485
669.6621704101562 0 23424.328
669.9962768554688 0 39868.695
670.3289184570312 0 28128.09
670.6632690429688 0 13087.583
670.9964599609375 0 7452.457
671.32666015625 0 4111.7827
671.66162109375 0 1455.9744
671.9865112304688 0 1009.7412
673.354736328125 0 1018.3715
674.3204345703125 0 945.4875
680.3165283203125 0 4108.1133 c Ammonia loss 4
681.3197021484375 0 2169.8054
682.3348388671875 0 3119.351
683.3397216796875 0 1579.4911
690.3402709960938 0 1574.8376
691.3336791992188 0 1295.1154
691.83251953125 0 938.4561
694.83154296875 0 1029.0991
695.925048828125 0 807.7752
697.342041015625 0 16093.482 c 4
698.3452758789062 0 8286.149
699.3430786132812 0 2423.381
699.8427124023438 0 1063.6605
700.3427124023438 0 1672.6176
701.3478393554688 0 2941.0818
701.8433837890625 0 8676.398 c 10
702.3428955078125 0 9047.619
702.8487548828125 0 2541.4167
703.348388671875 0 1111.7992
708.8365478515625 0 782.54596
711.31396484375 0 822.8694
712.3341064453125 0 1104.4948
712.8434448242188 0 1206.343
713.334716796875 0 1582.9999
714.3328247070312 0 1026.1919
716.4048461914062 0 3956.706
717.4093017578125 0 1398.546
721.3370971679688 0 1154.1843
721.8363647460938 0 879.87897
722.345458984375 0 1165.2985
723.3413696289062 0 1215.2375
735.3375244140625 0 882.0807
742.837890625 0 894.79724
746.3402099609375 0 950.03613
748.8684692382812 0 904.6935 y Ammonia loss 4
749.3636474609375 0 6938.42 z 4
749.86328125 0 3182.4172
750.366943359375 0 2309.143
752.3646850585938 0 1163.7163 z Ammonia loss 10
754.3682250976562 0 1028.2517
754.8633422851562 0 2183.566
755.3624877929688 0 1780.1958
757.3729858398438 0 2002.169 c Ammonia loss 11
757.8780517578125 0 1921.5259
758.3751831054688 0 1682.6105
765.8892822265625 0 1651.6523 c 11
766.3958740234375 0 2057.1794
768.3593139648438 0 885.13214 y Ammonia loss 10
769.3681640625 0 10007.624 z 10
770.3693237304688 0 5912.7266
771.364501953125 0 3040.9739
784.8784790039062 0 2277.9104
785.3853759765625 0 4846.577 y 10
786.3881225585938 0 1224.3898
788.3511352539062 0 815.59235
793.4015502929688 0 1619.6975 c Ammonia loss 5
797.3780517578125 0 955.799
803.42529296875 0 2416.8203
806.3856201171875 0 2732.4304 y Ammonia loss 3
806.879150390625 0 7939.0806 z 3
807.3801879882812 0 6023.4126
807.8794555664062 0 3494.0972
808.3761596679688 0 1053.7723
809.4092407226562 0 813.36237
810.42626953125 0 25861.494 c 5
811.3624267578125 0 1474.9111
811.4283447265625 0 9386.82
812.36962890625 0 1206.0066
812.4313354492188 0 2539.0378
814.4069213867188 0 2329.9321
814.888916015625 0 1865.2001 y 3
815.396484375 0 1409.7549
826.4139404296875 0 1050.0525
827.38916015625 0 1027.9353
831.4468994140625 0 1840.5083
831.7852783203125 0 1778.064
832.4435424804688 0 1064.5905
837.455322265625 0 3705.071
837.7884521484375 0 4047.7117
838.12255859375 0 1292.3043
838.4600830078125 0 1076.4196
839.3885498046875 0 804.99615
840.4005126953125 0 1682.7268
842.4051513671875 0 2244.7007
843.3998413085938 0 842.5665
845.3863525390625 0 823.69147
845.9020385742188 0 2258.3088
846.3993530273438 0 6433.274 c 12
846.8984375 0 4051.304
847.3997192382812 0 2267.0212
847.9038696289062 0 1176.5222
848.914306640625 0 1257.2903
850.9686279296875 0 1153.5817
854.3948974609375 0 896.3109
860.41552734375 0 742.12164
862.8931884765625 0 1049.8579
867.4256591796875 0 3044.6519
868.4288330078125 0 1179.3993
869.43359375 0 938.62006
870.409912109375 0 2527.6125 y Ammonia loss 2
870.9051513671875 0 8372.79 z 2
871.4086303710938 0 5106.2627
871.9085693359375 0 2827.9583
872.4104614257812 0 2172.7314
873.4202880859375 0 945.49023
878.4033813476562 0 829.73834
878.8971557617188 0 883.07227
881.4369506835938 0 4130.6436
883.4124145507812 0 1257.4559
888.4348754882812 0 1191.5004
888.9468383789062 0 903.44556
889.90087890625 0 1074.9478
898.4122314453125 0 10613.153 z 9
899.4158325195312 0 9752.856
900.4212646484375 0 4902.4854
901.4229125976562 0 1176.2518 c Water loss 13
902.4386596679688 0 956.7333
908.4213256835938 0 1195.1694 c Ammonia loss 6
910.4445190429688 0 4009.468 c 13
910.9445190429688 0 4575.4053
911.4409790039062 0 2201.172
911.9481201171875 0 1264.5817
913.4296875 0 824.7024
914.4334106445312 0 1476.1993 y 9
915.4432373046875 0 2244.99
917.9276123046875 0 1061.2623
918.9452514648438 0 949.0944
919.44384765625 0 1173.9178
922.4402465820312 0 1068.9565
923.4425659179688 0 1310.9194
924.4527587890625 0 1869.8911
925.452392578125 0 4895.0376 c 6
926.4501342773438 0 2574.511
927.4535522460938 0 1176.9163
930.4576416015625 0 841.84546
930.9761962890625 0 1939.8994
931.4744262695312 0 1574.4056
932.4783325195312 0 3263.888
937.9619750976562 0 1605.8823
938.4710083007812 0 4692.218
938.9580078125 0 2203.8188
939.4567260742188 0 6186.984 z 1
939.94287109375 0 6546.937
940.4462280273438 0 4636.498
940.9430541992188 0 2140.5032
941.4524536132812 0 957.2423
942.4513549804688 0 1651.37
943.431884765625 0 986.931
943.936279296875 0 1161.1434
945.479248046875 0 1642.6774
946.4766845703125 0 1967.1074
946.9603881835938 0 1759.1664
947.4583740234375 0 3090.3188 y 1
947.951171875 0 1092.8984
948.466552734375 0 1162.9661
949.4563598632812 0 1191.76
950.448974609375 0 910.2247
952.9703369140625 0 1582.941
953.4674072265625 0 1634.971
953.9649658203125 0 1639.886
954.4502563476562 0 2654.0732
954.9580078125 0 2192.2996
955.4570922851562 0 3026.988
955.9555053710938 0 2781.3254
956.95556640625 0 1680.3667
957.442138671875 0 2139.9783
959.4534912109375 0 1905.9117
959.974853515625 0 3017.2385
960.4719848632812 0 3950.8303
960.9708251953125 0 1835.3704
961.4734497070312 0 1256.1558
961.9666748046875 0 1847.9072
962.45556640625 0 2571.9277
962.9544677734375 0 2677.9216
963.4569091796875 0 3090.2417
963.9570922851562 0 2717.8325
964.4527587890625 0 1776.1301 c Water loss 7
964.9583740234375 0 2013.4207
965.4599609375 0 1834.6611 c Ammonia loss 7
965.9641723632812 0 1288.3523
966.4705200195312 0 3068.717
966.9686279296875 0 2461.7542
967.47119140625 0 3847.6812
967.9617919921875 0 4421.558
968.465576171875 0 6473.0737 w 8
968.9663696289062 0 3684.5044
969.4641723632812 0 3416.791
969.963623046875 0 1166.4797
970.4536743164062 0 3053.3906
970.953857421875 0 1791.1864
971.4591674804688 0 2639.224
971.9558715820312 0 1559.1453
972.4622192382812 0 3248.051
972.956787109375 0 1243.3829
973.4642333984375 0 2396.3967
973.9701538085938 0 2450.394
974.4829711914062 0 2728.6587
974.981689453125 0 7929.46
975.4811401367188 0 8510.458
975.9788208007812 0 6552.1885
976.4705200195312 0 7534.8516
976.9646606445312 0 6613.8057
977.4656372070312 0 5787.3135
977.9677124023438 0 3933.4348
978.4713134765625 0 2823.6365
978.9625854492188 0 1652.4308
979.9625244140625 0 1890.7487
980.4629516601562 0 1599.1273
980.9637451171875 0 1682.5204
981.4813842773438 0 4471.577
981.9873046875 0 3342.7383
982.4791870117188 0 13616.299 c 7
982.9828491210938 0 2981.248
983.4791870117188 0 7362.4224
983.9710083007812 0 1240.9252
984.4650268554688 0 5619.368
984.9603271484375 0 7816.0723
985.46435546875 0 8586.444
985.9611206054688 0 3985.7659
986.4661865234375 0 3708.1643
986.9666748046875 0 2497.347
987.4678344726562 0 2131.3403
987.9650268554688 0 2670.248
988.476318359375 0 4839.17
988.968505859375 0 2281.771
989.4764404296875 0 4610.793
990.4683837890625 0 2039.6858
991.4663696289062 0 1218.2069
992.962646484375 0 2293.0398
993.46435546875 0 7631.888
993.9653930664062 0 7737.924
994.4656372070312 0 5726.4536
994.9635620117188 0 4081.3914
995.4717407226562 0 2558.6482
995.98095703125 0 8644.338
996.4811401367188 0 16681.256
996.9788818359375 0 12470.8545
997.4786376953125 0 10060.2295
997.9778442382812 0 4812.8
998.4763793945312 0 4273.4937
998.974853515625 0 3169.0027
999.4666748046875 0 2387.6428
1002.4686279296875 0 3680.2273
1002.9747314453125 0 2903.7727
1003.4829711914062 0 3810.8892
1003.9869995117188 0 4263.2275
1004.4913940429688 0 9628.706
1004.9913940429688 0 15493.572
1005.494873046875 0 12786.95
1005.9927368164062 0 7255.55
1006.4944458007812 0 4463.1826
1006.98388671875 0 2403.8071
1007.4776611328125 0 1971.7418
1008.4650268554688 0 1082.2314
1026.503662109375 0 1727.5255 z 8
1027.5107421875 0 3421.8267
1028.514892578125 0 1485.8641
1029.515380859375 0 1055.519
1066.55810546875 0 1099.0559
1084.5380859375 0 1733.9109
1109.58251953125 0 3770.2295
1110.571533203125 0 15973.993 c 8
1111.57421875 0 9735.782
1112.566162109375 0 2893.744
1196.59765625 0 872.2358
1198.5714111328125 0 2666.9797 z 6
1199.5662841796875 0 5581.515
1200.5660400390625 0 3533.778
1201.5640869140625 0 1156.7554
1215.5894775390625 0 999.56085
1225.613525390625 0 933.41296
1238.6182861328125 0 1251.6587
1239.6170654296875 0 4533.187 c 9
1240.61572265625 0 3391.013
1241.6202392578125 0 2213.1663
1311.65380859375 0 1927.1901 z 5
1312.6488037109375 0 3268.853
1313.649169921875 0 1705.3224
1358.6761474609375 0 949.01807
1401.6640625 0 1034.7388
1402.67919921875 0 1716.4648 c 10
1403.6822509765625 0 2214.7695
1404.680908203125 0 1161.7673
1405.6842041015625 0 833.44147
1474.68115234375 0 873.4744
1497.729736328125 0 1299.689 z 4
1498.721923828125 0 2330.9062
1499.7296142578125 0 1366.2976
1514.74853515625 0 1204.7748
1612.7435302734375 0 1059.8646 z 3
1613.7520751953125 0 3362.7239
1614.7550048828125 0 1959.0723
1615.74658203125 0 1113.6248
1691.7734375 0 858.97986 c 12
1692.8023681640625 0 2296.3696
1694.7911376953125 0 807.1461
1740.81298828125 0 1326.5327 z 2
1741.8167724609375 0 2391.7593
1742.8077392578125 0 2279.9697
1743.818115234375 0 1665.075
1820.8814697265625 0 981.527
1878.895751953125 0 966.2675
1903.914794921875 0 772.411
1949.9620361328125 0 2050.3223
1950.940185546875 0 1777.081
1952.9256591796875 0 1135.8945
1953.9234619140625 0 1186.0474
1969.9090576171875 0 1051.486
1970.91552734375 0 1034.0211
1971.9010009765625 0 924.5437
1988.9339599609375 0 820.80334
1991.91455078125 0 880.08954
1992.976318359375 0 1063.9285
1993.9427490234375 0 739.4827
2007.982666015625 0 876.17786
2008.9696044921875 0 1784.3684
2009.97900390625 0 1540.6205
2010.984619140625 0 1621.7006
3047.835205078125 0 831.72375
3048.419921875 0 727.2502

Spectrum Details

|  |  |
| --- | --- |
| Matched peaks? Matched peaksThe total absolute number of peaks matched. Additionally in brackets the total fraction of peaks matched and the total number of peaks is shown. | 85 (12.04% of 706) |
| FDR? FDRThe false discovery rate estimated for this peptide. It is calculated by matching all theoretical fragments with a non-integer shift with the raw peaks for this spectrum. This is done with 40 different shifts. The resulting percentage is the average number of annotated peaks over the number of annotated peaks with the correct spectrum. | 0.59% |
| Satellite FDR? Satellite FDRSee the FDR for details on its calculation. This satellite ion specific FDR only contains the satellite ions (d/w) for I/L/J positions. | 0.00% |
| PSM Score? PSM ScoreThe PSM Score as given by Hecklib to this annotated spectrum. It is shown with three significant figures. | 419 |

## Spectrum 5830? Spectrum 5830 The raw spectrum of this peptide as annotated by Hecklib. The fragments are coloured according to ion type (see legend). Any peaks with a star '\*' as text can be hovered over to see the full details, first the ion type second the mass shift type. By hovering over the amino acids in the peptide or ions in the legend the corresponding peaks are highlighted. By toggling the 'Unassigned' label you can turn the background (unassigned) peaks on or off in the plot. By updating the slider in the Ion legend you can update the spectrum to only show the top X% of the peaks with labels. The top X% means any peak that is within X% of the highest intensity. By dragging in the spectrum you can zoom in to a specific part of the spectrum and use 'Zoom Out' to get back to the original zoom level. The annotation of the spectrum is based on the given sequence in the peptides file and is done with different software so inconsistencies are likely. The peaks are annotated based on the given sequence, with 20 ppm tolerance.

Copy Data

### Spectrum 5830 (TSV)

#### Preview

```
Loading example...
```

*Click on the button to copy the data to your clipboard.*

Mz MinMz MaxIntensity Max

WidthHeightPeptide font sizePeptide stroke widthSpectrum font sizeSpectrum stroke widthCompact peptide

Ion legend

wxyz

abcd

OtherUnassignedIonChargePositionShow for top:%

JHQDWLDGKEYKCKK

04.37e+38.74e+31.31e+41.75e+4

Zoom Out

c+33c+13c+13w+26z+26y+26c+14c+14y+27z+28y+28c+29w+29y+29z+29z+14c+314y+29c+210y+14y+314w+210z+210y+210c+15c+15c+211z+211c+212z+15y+15y+212z+212c+16w+16c+213y+213z+213z+16c+214c+17z+214c+18z+17c+19z+19c+110z+110y+111z+111c+113z+113

0508101515232030

Fragment Matches Table

Show background peaks

| Position | Ion type | Intensity | mz Theoretical | mz Error (Th) | mz Error (ppm) | Charge | Series Number |
| --- | --- | --- | --- | --- | --- | --- | --- |
| - | - | 601.7 | 129.1 | - | - | 0 | - |
| - | - | 989.1 | 129.1 | - | - | 0 | - |
| - | - | 1076 | 130.1 | - | - | 0 | - |
| - | - | 395.4 | 131.1 | - | - | 0 | - |
| - | - | 830 | 132.1 | - | - | 0 | - |
| 3 | c | 475.7 | 132.7 | 0.001274 | 9.599 | +3 | 3 |
| - | - | 1552 | 133.1 | - | - | 0 | - |
| - | - | 422.8 | 133.1 | - | - | 0 | - |
| - | - | 432.8 | 134 | - | - | 0 | - |
| - | - | 458.3 | 140.1 | - | - | 0 | - |
| - | - | 444.5 | 145.3 | - | - | 0 | - |
| - | - | 368.5 | 147.9 | - | - | 0 | - |
| - | - | 390 | 148.5 | - | - | 0 | - |
| - | - | 669.8 | 148.9 | - | - | 0 | - |
| - | - | 1258 | 149 | - | - | 0 | - |
| - | - | 1005 | 155.1 | - | - | 0 | - |
| - | - | 689.6 | 156.1 | - | - | 0 | - |
| - | - | 444.6 | 162.3 | - | - | 0 | - |
| - | - | 953.9 | 166.1 | - | - | 0 | - |
| - | - | 788.8 | 174.1 | - | - | 0 | - |
| - | - | 1359 | 177.1 | - | - | 0 | - |
| - | - | 2059 | 185.2 | - | - | 0 | - |
| - | - | 699.5 | 187.1 | - | - | 0 | - |
| - | - | 1571 | 191.1 | - | - | 0 | - |
| - | - | 538.3 | 194.3 | - | - | 0 | - |
| - | - | 839.3 | 199.2 | - | - | 0 | - |
| - | - | 737.7 | 201.1 | - | - | 0 | - |
| - | - | 1642 | 202.1 | - | - | 0 | - |
| - | - | 3784 | 203.1 | - | - | 0 | - |
| - | - | 1106 | 207.1 | - | - | 0 | - |
| - | - | 8607 | 215.1 | - | - | 0 | - |
| - | - | 710.1 | 219.1 | - | - | 0 | - |
| - | - | 589.8 | 223.2 | - | - | 0 | - |
| - | - | 560.5 | 227.1 | - | - | 0 | - |
| - | - | 499.1 | 228.9 | - | - | 0 | - |
| - | - | 2328 | 229.2 | - | - | 0 | - |
| - | - | 605.6 | 230.2 | - | - | 0 | - |
| - | - | 661.1 | 234.1 | - | - | 0 | - |
| - | - | 1237 | 239.1 | - | - | 0 | - |
| - | - | 4487 | 251.2 | - | - | 0 | - |
| - | - | 481.7 | 269.2 | - | - | 0 | - |
| - | - | 537.4 | 276.3 | - | - | 0 | - |
| - | - | 1927 | 281.1 | - | - | 0 | - |
| - | - | 631.8 | 282 | - | - | 0 | - |
| - | - | 582.4 | 282.2 | - | - | 0 | - |
| - | - | 4265 | 289.1 | - | - | 0 | - |
| - | - | 7562 | 299.1 | - | - | 0 | - |
| - | - | 581.5 | 299.1 | - | - | 0 | - |
| - | - | 1681 | 300.1 | - | - | 0 | - |
| - | - | 1316 | 302.2 | - | - | 0 | - |
| - | - | 3053 | 314.2 | - | - | 0 | - |
| - | - | 579.2 | 318.2 | - | - | 0 | - |
| - | - | 1118 | 326.7 | - | - | 0 | - |
| - | - | 536.4 | 342.2 | - | - | 0 | - |
| - | - | 1902 | 359 | - | - | 0 | - |
| - | - | 839.8 | 360 | - | - | 0 | - |
| 3 | c | 1894 | 379.2 | 0.000761 | 2.007 | +1 | 3 |
| - | - | 1157 | 387.2 | - | - | 0 | - |
| 3 | c | 1925 | 396.2 | 0.0008208 | 2.072 | +1 | 3 |
| - | - | 635.6 | 407.7 | - | - | 0 | - |
| - | - | 654.1 | 414.2 | - | - | 0 | - |
| - | - | 3798 | 415 | - | - | 0 | - |
| - | - | 1257 | 416 | - | - | 0 | - |
| 10 | w | 5510 | 420.2 | 0.004302 | 10.24 | +2 | 6 |
| - | - | 2186 | 420.7 | - | - | 0 | - |
| - | - | 577.5 | 438.2 | - | - | 0 | - |
| - | - | 903.4 | 444.2 | - | - | 0 | - |
| - | - | 841.7 | 444.3 | - | - | 0 | - |
| - | - | 835.1 | 449.2 | - | - | 0 | - |
| 10 | z | 1447 | 449.7 | 0.006197 | 13.78 | +2 | 6 |
| - | - | 602.6 | 450.2 | - | - | 0 | - |
| 10 | y | 620.9 | 457.7 | 0.004234 | 9.249 | +2 | 6 |
| - | - | 3645 | 458.2 | - | - | 0 | - |
| - | - | 1140 | 458.7 | - | - | 0 | - |
| - | - | 1429 | 459.3 | - | - | 0 | - |
| - | - | 716.1 | 460.7 | - | - | 0 | - |
| - | - | 860.1 | 480.7 | - | - | 0 | - |
| - | - | 834.2 | 484.2 | - | - | 0 | - |
| - | - | 876.6 | 485.2 | - | - | 0 | - |
| - | - | 805 | 487.2 | - | - | 0 | - |
| - | - | 1759 | 488.3 | - | - | 0 | - |
| - | - | 2627 | 493.7 | - | - | 0 | - |
| 4 | c | 2206 | 494.2 | 0.007849 | 15.88 | +1 | 4 |
| - | - | 967.8 | 495.3 | - | - | 0 | - |
| - | - | 768.5 | 496.2 | - | - | 0 | - |
| - | - | 2092 | 501.3 | - | - | 0 | - |
| - | - | 805.9 | 501.7 | - | - | 0 | - |
| - | - | 590 | 502.2 | - | - | 0 | - |
| - | - | 2273 | 502.3 | - | - | 0 | - |
| - | - | 2704 | 502.5 | - | - | 0 | - |
| - | - | 5071 | 502.8 | - | - | 0 | - |
| - | - | 2151 | 503 | - | - | 0 | - |
| - | - | 3080 | 503.1 | - | - | 0 | - |
| - | - | 4307 | 503.3 | - | - | 0 | - |
| - | - | 2124 | 503.3 | - | - | 0 | - |
| - | - | 972.5 | 503.8 | - | - | 0 | - |
| - | - | 2030 | 504.1 | - | - | 0 | - |
| - | - | 1252 | 504.3 | - | - | 0 | - |
| 4 | c | 6172 | 511.3 | 0.0006032 | 1.18 | +1 | 4 |
| - | - | 1528 | 512.3 | - | - | 0 | - |
| - | - | 1123 | 515.3 | - | - | 0 | - |
| - | - | 608.8 | 517.3 | - | - | 0 | - |
| 9 | y | 1192 | 521.8 | 0.004779 | 9.159 | +2 | 7 |
| - | - | 888.6 | 522.3 | - | - | 0 | - |
| 8 | z | 875.5 | 542.3 | 0.001693 | 3.123 | +2 | 8 |
| - | - | 664.5 | 545.4 | - | - | 0 | - |
| - | - | 1338 | 547.3 | - | - | 0 | - |
| 8 | y | 1069 | 550.3 | 0.0019 | 3.453 | +2 | 8 |
| - | - | 1514 | 555.3 | - | - | 0 | - |
| 9 | c | 5013 | 555.8 | 0.001012 | 1.821 | +2 | 9 |
| - | - | 1767 | 556.3 | - | - | 0 | - |
| - | - | 906.5 | 556.8 | - | - | 0 | - |
| - | - | 853.9 | 570.3 | - | - | 0 | - |
| - | - | 1726 | 571.3 | - | - | 0 | - |
| 7 | w | 980.9 | 577.3 | 0.002849 | 4.935 | +2 | 9 |
| - | - | 822.2 | 577.8 | - | - | 0 | - |
| - | - | 705.4 | 578.3 | - | - | 0 | - |
| - | - | 784.3 | 596.3 | - | - | 0 | - |
| 7 | y | 2528 | 599.3 | 0.007119 | 11.88 | +2 | 9 |
| 7 | z | 3996 | 599.8 | 0.002592 | 4.321 | +2 | 9 |
| - | - | 864.7 | 600.3 | - | - | 0 | - |
| 12 | z | 4184 | 606.3 | 0.008198 | 13.52 | +1 | 4 |
| 14 | c | 4927 | 607.3 | 0.0008638 | 1.422 | +3 | 14 |
| - | - | 2357 | 607.6 | - | - | 0 | - |
| 7 | y | 4316 | 607.8 | 0.003592 | 5.91 | +2 | 9 |
| - | - | 1790 | 608 | - | - | 0 | - |
| - | - | 2807 | 608.3 | - | - | 0 | - |
| - | - | 1004 | 608.8 | - | - | 0 | - |
| - | - | 1202 | 612.3 | - | - | 0 | - |
| - | - | 641.1 | 612.6 | - | - | 0 | - |
| - | - | 1072 | 613.8 | - | - | 0 | - |
| - | - | 616.5 | 614.1 | - | - | 0 | - |
| - | - | 1748 | 619.8 | - | - | 0 | - |
| 10 | c | 4426 | 620.3 | 0.001139 | 1.836 | +2 | 10 |
| - | - | 3394 | 620.8 | - | - | 0 | - |
| - | - | 1498 | 621.3 | - | - | 0 | - |
| 12 | y | 1882 | 622.3 | 0.008856 | 14.23 | +1 | 4 |
| - | - | 706.4 | 623.3 | - | - | 0 | - |
| 2 | y | 1578 | 626.3 | 0.01186 | 18.94 | +3 | 14 |
| - | - | 865 | 627 | - | - | 0 | - |
| - | - | 1717 | 634.3 | - | - | 0 | - |
| - | - | 922.2 | 634.6 | - | - | 0 | - |
| 6 | w | 2116 | 634.8 | 0.001062 | 1.672 | +2 | 10 |
| - | - | 1184 | 635.3 | - | - | 0 | - |
| - | - | 1641 | 635.8 | - | - | 0 | - |
| - | - | 1167 | 636.7 | - | - | 0 | - |
| - | - | 760.8 | 637.3 | - | - | 0 | - |
| - | - | 2020 | 640 | - | - | 0 | - |
| - | - | 6804 | 640.3 | - | - | 0 | - |
| - | - | 5176 | 640.7 | - | - | 0 | - |
| - | - | 3685 | 641 | - | - | 0 | - |
| - | - | 1493 | 641.3 | - | - | 0 | - |
| - | - | 1181 | 645.6 | - | - | 0 | - |
| - | - | 1026 | 646 | - | - | 0 | - |
| - | - | 1480 | 649.6 | - | - | 0 | - |
| - | - | 4878 | 650 | - | - | 0 | - |
| - | - | 8802 | 650.3 | - | - | 0 | - |
| - | - | 4874 | 650.7 | - | - | 0 | - |
| - | - | 3201 | 651 | - | - | 0 | - |
| - | - | 2207 | 651.3 | - | - | 0 | - |
| - | - | 1161 | 651.6 | - | - | 0 | - |
| - | - | 849.8 | 652 | - | - | 0 | - |
| - | - | 1037 | 653.7 | - | - | 0 | - |
| - | - | 1745 | 654.3 | - | - | 0 | - |
| - | - | 952.3 | 654.7 | - | - | 0 | - |
| - | - | 815.6 | 655 | - | - | 0 | - |
| 6 | z | 1830 | 656.3 | 0.00312 | 4.753 | +2 | 10 |
| - | - | 839.4 | 656.8 | - | - | 0 | - |
| - | - | 1106 | 657 | - | - | 0 | - |
| - | - | 991.6 | 659.7 | - | - | 0 | - |
| - | - | 768.2 | 660 | - | - | 0 | - |
| - | - | 2061 | 664 | - | - | 0 | - |
| 6 | y | 3905 | 664.3 | 0.004425 | 6.661 | +2 | 10 |
| - | - | 2218 | 664.7 | - | - | 0 | - |
| - | - | 1379 | 664.8 | - | - | 0 | - |
| - | - | 798.7 | 665 | - | - | 0 | - |
| - | - | 732.9 | 668 | - | - | 0 | - |
| - | - | 1364 | 669 | - | - | 0 | - |
| - | - | 4908 | 669.3 | - | - | 0 | - |
| - | - | 1.488E+04 | 669.7 | - | - | 0 | - |
| - | - | 1.73E+04 | 670 | - | - | 0 | - |
| - | - | 1.25E+04 | 670.3 | - | - | 0 | - |
| - | - | 5827 | 670.7 | - | - | 0 | - |
| - | - | 4482 | 671 | - | - | 0 | - |
| - | - | 1520 | 672.3 | - | - | 0 | - |
| - | - | 1222 | 674.3 | - | - | 0 | - |
| - | - | 965.4 | 679.8 | - | - | 0 | - |
| 5 | c | 2078 | 680.3 | 0.001487 | 2.186 | +1 | 5 |
| - | - | 839 | 690.3 | - | - | 0 | - |
| 5 | c | 6879 | 697.3 | 0.0006315 | 0.9055 | +1 | 5 |
| - | - | 1930 | 698.3 | - | - | 0 | - |
| - | - | 2583 | 701.3 | - | - | 0 | - |
| 11 | c | 3833 | 701.8 | 0.003349 | 4.772 | +2 | 11 |
| - | - | 2540 | 702.3 | - | - | 0 | - |
| - | - | 1447 | 702.8 | - | - | 0 | - |
| - | - | 3096 | 716.4 | - | - | 0 | - |
| - | - | 1593 | 717.4 | - | - | 0 | - |
| - | - | 785.8 | 726.4 | - | - | 0 | - |
| - | - | 1189 | 744.4 | - | - | 0 | - |
| 5 | z | 1169 | 749.4 | 0.005972 | 7.969 | +2 | 11 |
| - | - | 2057 | 749.9 | - | - | 0 | - |
| - | - | 1232 | 750.4 | - | - | 0 | - |
| - | - | 825.9 | 750.9 | - | - | 0 | - |
| - | - | 1164 | 757.9 | - | - | 0 | - |
| - | - | 918.7 | 765.4 | - | - | 0 | - |
| 12 | c | 850.3 | 765.9 | 0.00439 | 5.733 | +2 | 12 |
| - | - | 1642 | 766.4 | - | - | 0 | - |
| 11 | z | 4944 | 769.4 | 0.01031 | 13.4 | +1 | 5 |
| - | - | 3335 | 770.4 | - | - | 0 | - |
| - | - | 1330 | 771.4 | - | - | 0 | - |
| 11 | y | 2023 | 785.4 | 0.003398 | 4.326 | +1 | 5 |
| - | - | 2043 | 803.4 | - | - | 0 | - |
| 4 | y | 916.3 | 806.4 | 0.003634 | 4.507 | +2 | 12 |
| 4 | z | 2301 | 806.9 | 0.0008278 | 1.026 | +2 | 12 |
| - | - | 1787 | 807.4 | - | - | 0 | - |
| - | - | 987.7 | 807.9 | - | - | 0 | - |
| 6 | c | 1.216E+04 | 810.4 | 0.001016 | 1.254 | +1 | 6 |
| - | - | 5637 | 811.4 | - | - | 0 | - |
| - | - | 1623 | 812.4 | - | - | 0 | - |
| - | - | 2625 | 814.4 | - | - | 0 | - |
| - | - | 1520 | 815.4 | - | - | 0 | - |
| - | - | 748.8 | 817.8 | - | - | 0 | - |
| - | - | 1573 | 831.4 | - | - | 0 | - |
| - | - | 1103 | 832.4 | - | - | 0 | - |
| - | - | 2165 | 837.8 | - | - | 0 | - |
| - | - | 1824 | 838.1 | - | - | 0 | - |
| 10 | w | 1007 | 839.4 | 0.002854 | 3.4 | +1 | 6 |
| 13 | c | 3186 | 846.4 | 0.004198 | 4.959 | +2 | 13 |
| - | - | 2321 | 846.9 | - | - | 0 | - |
| - | - | 1220 | 847.4 | - | - | 0 | - |
| - | - | 910.3 | 847.9 | - | - | 0 | - |
| - | - | 1734 | 867.4 | - | - | 0 | - |
| 3 | y | 1046 | 870.4 | 0.01103 | 12.67 | +2 | 13 |
| 3 | z | 3345 | 870.9 | 0.0007586 | 0.8711 | +2 | 13 |
| - | - | 2102 | 871.4 | - | - | 0 | - |
| - | - | 1331 | 871.9 | - | - | 0 | - |
| - | - | 782.4 | 881.4 | - | - | 0 | - |
| - | - | 840.5 | 887.9 | - | - | 0 | - |
| - | - | 1131 | 888.4 | - | - | 0 | - |
| - | - | 1039 | 889.4 | - | - | 0 | - |
| - | - | 1105 | 890.4 | - | - | 0 | - |
| 10 | z | 6589 | 898.4 | 0.009566 | 10.65 | +1 | 6 |
| - | - | 3668 | 899.4 | - | - | 0 | - |
| - | - | 2344 | 900.4 | - | - | 0 | - |
| 14 | c | 1673 | 910.4 | 0.003164 | 3.475 | +2 | 14 |
| - | - | 2542 | 910.9 | - | - | 0 | - |
| - | - | 814.8 | 915.4 | - | - | 0 | - |
| - | - | 1064 | 916.4 | - | - | 0 | - |
| - | - | 1103 | 924.5 | - | - | 0 | - |
| 7 | c | 1418 | 925.5 | 0.0008493 | 0.9177 | +1 | 7 |
| - | - | 766.5 | 926.5 | - | - | 0 | - |
| - | - | 1496 | 932.5 | - | - | 0 | - |
| - | - | 992.5 | 933.5 | - | - | 0 | - |
| - | - | 1429 | 938.5 | - | - | 0 | - |
| 2 | z | 1902 | 939.4 | 0.009458 | 10.07 | +2 | 14 |
| - | - | 1829 | 939.9 | - | - | 0 | - |
| - | - | 722.4 | 940.3 | - | - | 0 | - |
| - | - | 1691 | 940.4 | - | - | 0 | - |
| - | - | 1017 | 945.5 | - | - | 0 | - |
| - | - | 1334 | 960 | - | - | 0 | - |
| - | - | 2624 | 960.5 | - | - | 0 | - |
| - | - | 1043 | 961 | - | - | 0 | - |
| - | - | 898 | 961.5 | - | - | 0 | - |
| - | - | 688.3 | 965.2 | - | - | 0 | - |
| - | - | 858.7 | 966.5 | - | - | 0 | - |
| - | - | 1018 | 967 | - | - | 0 | - |
| - | - | 863.5 | 967.5 | - | - | 0 | - |
| - | - | 1245 | 968 | - | - | 0 | - |
| - | - | 1620 | 974.5 | - | - | 0 | - |
| - | - | 4281 | 975 | - | - | 0 | - |
| - | - | 3464 | 975.5 | - | - | 0 | - |
| - | - | 2887 | 976 | - | - | 0 | - |
| - | - | 1085 | 976.5 | - | - | 0 | - |
| - | - | 934.5 | 980.5 | - | - | 0 | - |
| - | - | 866.3 | 981 | - | - | 0 | - |
| - | - | 3632 | 981.5 | - | - | 0 | - |
| - | - | 2477 | 982 | - | - | 0 | - |
| 8 | c | 7725 | 982.5 | 0.007279 | 7.408 | +1 | 8 |
| - | - | 1914 | 983 | - | - | 0 | - |
| - | - | 3395 | 983.5 | - | - | 0 | - |
| - | - | 898.6 | 984.5 | - | - | 0 | - |
| - | - | 1019 | 985.5 | - | - | 0 | - |
| - | - | 1769 | 987.5 | - | - | 0 | - |
| - | - | 2786 | 988.5 | - | - | 0 | - |
| - | - | 2671 | 989.5 | - | - | 0 | - |
| - | - | 1070 | 990.5 | - | - | 0 | - |
| - | - | 1108 | 995.5 | - | - | 0 | - |
| - | - | 2746 | 996 | - | - | 0 | - |
| - | - | 7344 | 996.5 | - | - | 0 | - |
| - | - | 6495 | 997 | - | - | 0 | - |
| - | - | 3879 | 997.5 | - | - | 0 | - |
| - | - | 2249 | 998 | - | - | 0 | - |
| - | - | 1663 | 998.5 | - | - | 0 | - |
| - | - | 949.3 | 999.5 | - | - | 0 | - |
| - | - | 2524 | 1004 | - | - | 0 | - |
| - | - | 1728 | 1004 | - | - | 0 | - |
| - | - | 7631 | 1004 | - | - | 0 | - |
| - | - | 7652 | 1005 | - | - | 0 | - |
| - | - | 8400 | 1005 | - | - | 0 | - |
| - | - | 3397 | 1006 | - | - | 0 | - |
| - | - | 3983 | 1006 | - | - | 0 | - |
| - | - | 1026 | 1007 | - | - | 0 | - |
| - | - | 1027 | 1007 | - | - | 0 | - |
| - | - | 1618 | 1008 | - | - | 0 | - |
| 9 | z | 943.7 | 1009 | 0.006194 | 6.142 | +1 | 7 |
| - | - | 2820 | 1028 | - | - | 0 | - |
| - | - | 1120 | 1029 | - | - | 0 | - |
| - | - | 2020 | 1110 | - | - | 0 | - |
| 9 | c | 6697 | 1111 | 0.005516 | 4.967 | +1 | 9 |
| - | - | 3841 | 1112 | - | - | 0 | - |
| - | - | 1725 | 1113 | - | - | 0 | - |
| - | - | 819.2 | 1197 | - | - | 0 | - |
| 7 | z | 827.8 | 1199 | 0.01137 | 9.487 | +1 | 9 |
| - | - | 3061 | 1200 | - | - | 0 | - |
| - | - | 1243 | 1201 | - | - | 0 | - |
| 10 | c | 1829 | 1240 | 0.001986 | 1.602 | +1 | 10 |
| - | - | 1460 | 1241 | - | - | 0 | - |
| - | - | 755.1 | 1242 | - | - | 0 | - |
| - | - | 856.2 | 1257 | - | - | 0 | - |
| 6 | z | 1411 | 1312 | 0.008117 | 6.188 | +1 | 10 |
| - | - | 920.7 | 1313 | - | - | 0 | - |
| - | - | 751.1 | 1326 | - | - | 0 | - |
| - | - | 920.6 | 1404 | - | - | 0 | - |
| 5 | y | 664.8 | 1497 | 0.002669 | 1.783 | +1 | 11 |
| 5 | z | 1133 | 1498 | 0.01682 | 11.23 | +1 | 11 |
| - | - | 954.5 | 1499 | - | - | 0 | - |
| - | - | 1801 | 1614 | - | - | 0 | - |
| 13 | c | 821.4 | 1692 | 0.002 | 1.182 | +1 | 13 |
| 3 | z | 665.2 | 1741 | 0.001931 | 1.109 | +1 | 13 |
| - | - | 833.7 | 1742 | - | - | 0 | - |
| - | - | 881.9 | 1743 | - | - | 0 | - |
| - | - | 1276 | 2009 | - | - | 0 | - |
| - | - | 1277 | 2010 | - | - | 0 | - |

m/z Charge Intensity FragmentType MassShift Position
129.0658721923828 0 601.6983
129.10235595703125 0 989.06775
130.06536865234375 0 1076.3232
131.11788940429688 0 395.4239
132.1020050048828 0 829.98834
132.7487030029297 0 475.7306 c 2
133.0858917236328 0 1551.6216
133.09173583984375 0 422.8467
134.00567626953125 0 432.75745
140.08187866210938 0 458.32172
145.27166748046875 0 444.47092
147.8960723876953 0 368.47137
148.52574157714844 0 390.02893
148.94760131835938 0 669.8233
149.0447540283203 0 1258.3706
155.0928192138672 0 1005.46295
156.07681274414062 0 689.6483
162.34298706054688 0 444.61768
166.06088256835938 0 953.9198
174.0871124267578 0 788.76544
177.1122589111328 0 1358.7135
185.16470336914062 0 2059.2373
187.1077423095703 0 699.452
191.1028289794922 0 1570.7046
194.29266357421875 0 538.3169
199.16888427734375 0 839.2736
201.1233673095703 0 737.69775
202.08200073242188 0 1641.7483
203.1025848388672 0 3784.2676
207.09796142578125 0 1105.8746
215.13894653320312 0 8606.946
219.1337127685547 0 710.14545
223.1553497314453 0 589.7734
227.13919067382812 0 560.46124
228.87734985351562 0 499.13367
229.1545867919922 0 2327.9778
230.15797424316406 0 605.59564
234.12376403808594 0 661.0907
239.09530639648438 0 1237.0802
251.1502685546875 0 4487.046
269.18524169921875 0 481.72452
276.2595520019531 0 537.39014
281.0510559082031 0 1926.8915
282.0493469238281 0 631.82874
282.18023681640625 0 582.37555
289.12164306640625 0 4264.9976
299.0616149902344 0 7561.739
299.13543701171875 0 581.4913
300.0624084472656 0 1680.6085
302.1698913574219 0 1315.538
314.20770263671875 0 3053.2388
318.2001953125 0 579.2011
326.6634216308594 0 1118.092
342.1669921875 0 536.41486
359.02825927734375 0 1901.6151
360.02813720703125 0 839.79047
379.20806884765625 0 1893.7505 c Ammonia loss 2
387.2234802246094 0 1157.1494
396.23455810546875 0 1925.2325 c 2
407.71270751953125 0 635.55676
414.1942138671875 0 654.0914
415.036376953125 0 3798.0483
416.0380859375 0 1256.5502
420.2022399902344 0 5509.6255 w 9
420.7030944824219 0 2185.9722
438.1763916015625 0 577.5285
444.2328796386719 0 903.4463
444.2796936035156 0 841.69476
449.22015380859375 0 835.0916
449.7193908691406 0 1446.9373 z 9
450.2218322753906 0 602.58575
457.71832275390625 0 620.8695 y 9
458.22998046875 0 3644.903
458.72979736328125 0 1140.3862
459.2789306640625 0 1429.1145
460.73486328125 0 716.1023
480.73199462890625 0 860.07404
484.2458801269531 0 834.22943
485.2374572753906 0 876.62665
487.2356872558594 0 805.03937
488.2926940917969 0 1758.8973
493.7494812011719 0 2626.7542
494.2436218261719 0 2206.201 c Ammonia loss 3
495.3029479980469 0 967.8001
496.2464904785156 0 768.4653
501.30474853515625 0 2092.3076
501.7456970214844 0 805.9205
502.2088928222656 0 589.95685
502.2665710449219 0 2272.5166
502.4980773925781 0 2704.03
502.7532043457031 0 5070.8325
502.996826171875 0 2150.5596
503.1086730957031 0 3080.2878
503.2560729980469 0 4306.8145
503.3078918457031 0 2123.8513
503.7582092285156 0 972.49225
504.1095886230469 0 2029.8812
504.2667236328125 0 1251.7391
511.26171875 0 6171.822 c 3
512.2634887695312 0 1528.417
515.3201293945312 0 1123.481
517.2705078125 0 608.78906
521.7652587890625 0 1191.5828 y 8
522.2658081054688 0 888.6042
542.2697143554688 0 875.4735 z 7
545.3758544921875 0 664.45544
547.290283203125 0 1337.9082
550.2788696289062 0 1069.4193 y 7
555.2980346679688 0 1514.1011
555.7891845703125 0 5012.686 c 8
556.2901000976562 0 1767.4902
556.7916259765625 0 906.4842
570.2797241210938 0 853.9468
571.2817993164062 0 1725.7404
577.283203125 0 980.878 w 6
577.784912109375 0 822.15674
578.289306640625 0 705.3684
596.3224487304688 0 784.2806
599.2880859375 0 2527.7595 y Ammonia loss 6
599.7822875976562 0 3996.1396 z 6
600.2783203125 0 864.694
606.3049926757812 0 4184.4165 z 11
607.3004150390625 0 4927.22 c 13
607.6331176757812 0 2357.3499
607.7906494140625 0 4316.3984 y 6
607.9677124023438 0 1789.7391
608.2921752929688 0 2806.9404
608.7905883789062 0 1004.44617
612.3009033203125 0 1202.1016
612.6290283203125 0 641.0784
613.82080078125 0 1071.7653
614.0704345703125 0 616.50507
619.817626953125 0 1747.8398
620.3106079101562 0 4425.799 c 9
620.8113403320312 0 3394.4705
621.3123779296875 0 1498.3953
622.3230590820312 0 1881.592 y 11
623.3264770507812 0 706.4126
626.30419921875 0 1578.341 y Ammonia loss 1
626.9639892578125 0 864.9604
634.3058471679688 0 1716.6261
634.644775390625 0 922.1696
634.7984619140625 0 2116.2698 w 5
635.3035888671875 0 1184.0948
635.7977294921875 0 1641.4829
636.6516723632812 0 1167.2764
637.3008422851562 0 760.82446
639.9921875 0 2020.3215
640.3232421875 0 6803.97
640.6549682617188 0 5175.8857
640.9891967773438 0 3684.5215
641.3226318359375 0 1493.0436
645.6453857421875 0 1181.2451
645.9795532226562 0 1025.957
649.6491088867188 0 1479.6489
649.9775390625 0 4877.92
650.3206787109375 0 8801.629
650.6536865234375 0 4873.947
650.9849853515625 0 3200.5005
651.3153076171875 0 2206.6455
651.6491088867188 0 1161.0944
651.9815063476562 0 849.8252
653.66796875 0 1036.8351
654.341064453125 0 1745.1351
654.6648559570312 0 952.2955
654.9993896484375 0 815.63965
656.3237915039062 0 1829.7134 z 5
656.8250732421875 0 839.3586
656.9736328125 0 1106.4918
659.667724609375 0 991.6012
659.9908447265625 0 768.23517
663.9932250976562 0 2061.065
664.3318481445312 0 3905.1973 y 5
664.6591186523438 0 2217.921
664.8347778320312 0 1378.6979
665.004150390625 0 798.6659
667.9974975585938 0 732.85034
669.0010375976562 0 1363.9745
669.3319702148438 0 4907.5156
669.66259765625 0 14876.789
669.9957275390625 0 17299.746
670.3306884765625 0 12495.343
670.6635131835938 0 5827.401
670.9949340820312 0 4482.3384
672.326904296875 0 1519.9215
674.3214721679688 0 1221.9045
679.8359985351562 0 965.3774
680.3135986328125 0 2077.7593 c Ammonia loss 4
690.3427124023438 0 839.00104
697.3410034179688 0 6878.871 c 4
698.3447875976562 0 1930.4674
701.348876953125 0 2583.0513
701.844482421875 0 3833.1592 c 10
702.34326171875 0 2540.4917
702.8441772460938 0 1446.5967
716.4046630859375 0 3096.1958
717.4059448242188 0 1592.6659
726.3587646484375 0 785.8026
744.3572387695312 0 1189.2992
749.360595703125 0 1169.4916 z 4
749.8656616210938 0 2056.5146
750.3701171875 0 1232.0912
750.872314453125 0 825.88116
757.8758544921875 0 1164.4836
765.3922119140625 0 918.6565
765.8930053710938 0 850.33887 c 11
766.3920288085938 0 1642.2161
769.3662109375 0 4943.8364 z 10
770.3665771484375 0 3335.4048
771.3709716796875 0 1330.0089
785.391845703125 0 2022.974 y 10
803.4292602539062 0 2042.5052
806.3797607421875 0 916.32117 y Ammonia loss 3
806.8792114257812 0 2301.309 z 3
807.3796997070312 0 1786.7345
807.8821411132812 0 987.67786
810.4246826171875 0 12161.127 c 5
811.4276123046875 0 5636.9395
812.4317016601562 0 1622.9559
814.4050903320312 0 2624.7617
815.4013671875 0 1520.382
817.7777709960938 0 748.7588
831.43017578125 0 1572.5427
832.4346313476562 0 1103.4763
837.7918090820312 0 2164.9292
838.1170654296875 0 1824.297
839.4029541015625 0 1006.87244 w 9
846.4024047851562 0 3186.3525 c 12
846.8986206054688 0 2320.7563
847.3995361328125 0 1219.8367
847.8971557617188 0 910.3435
867.4248046875 0 1733.7823
870.4164428710938 0 1046.0765 y Ammonia loss 2
870.9085693359375 0 3344.707 z 2
871.409423828125 0 2102.4312
871.9121704101562 0 1330.5033
881.4457397460938 0 782.3548
887.922119140625 0 840.4966
888.4345703125 0 1130.6271
889.438232421875 0 1039.0474
890.4297485351562 0 1104.8319
898.4095458984375 0 6588.749 z 9
899.415771484375 0 3667.956
900.419677734375 0 2344.4355
910.4488525390625 0 1672.9917 c 13
910.9461669921875 0 2542.13
915.4442138671875 0 814.79706
916.4428100585938 0 1064.2522
924.4569702148438 0 1103.165
925.4534912109375 0 1418.3152 c 6
926.465576171875 0 766.47943
932.4775390625 0 1495.6873
933.4857177734375 0 992.51044
938.467529296875 0 1429.3419
939.4482421875 0 1901.7922 z 1
939.938232421875 0 1828.5067
940.337890625 0 722.36896
940.4485473632812 0 1691.4154
945.48828125 0 1016.6829
959.9816284179688 0 1334.3551
960.4735107421875 0 2624.2446
960.974365234375 0 1043.4235
961.4727783203125 0 897.98694
965.2152709960938 0 688.3231
966.4784545898438 0 858.71466
966.9757080078125 0 1018.042
967.4786987304688 0 863.54803
967.9799194335938 0 1245.4221
974.4862060546875 0 1619.951
974.986083984375 0 4281.159
975.4844970703125 0 3463.8706
975.987548828125 0 2887.4067
976.4862670898438 0 1085.286
980.4979858398438 0 934.457
981.006103515625 0 866.31433
981.4922485351562 0 3631.5723
981.9955444335938 0 2477.2666
982.4813842773438 0 7724.7695 c 7
982.9900512695312 0 1914.4403
983.48046875 0 3394.8027
984.47509765625 0 898.5858
985.4779663085938 0 1018.95074
987.4971313476562 0 1769.1945
988.4865112304688 0 2785.726
989.4803466796875 0 2670.733
990.4723510742188 0 1069.8905
995.4938354492188 0 1107.9344
995.986572265625 0 2746.0813
996.4844360351562 0 7344.463
996.9844360351562 0 6495.1973
997.4871215820312 0 3879.301
997.9815673828125 0 2249.066
998.4794921875 0 1663.4238
999.4832153320312 0 949.27423
1003.5003662109375 0 2523.8398
1003.9873657226562 0 1727.7773
1004.4989624023438 0 7630.8867
1004.9957275390625 0 7651.845
1005.4996337890625 0 8400.192
1005.99755859375 0 3397.4978
1006.4953002929688 0 3982.5354
1006.9963989257812 0 1026.193
1007.4990234375 0 1027.1897
1007.9931640625 0 1617.731
1008.5097045898438 0 943.69604 z Water loss 8
1027.51171875 0 2820.4426
1028.5244140625 0 1119.7917
1109.5802001953125 0 2020.4062
1110.5745849609375 0 6697.132 c 8
1111.5732421875 0 3840.6108
1112.577880859375 0 1724.6877
1196.6019287109375 0 819.15
1198.5738525390625 0 827.7635 z 6
1199.5634765625 0 3060.8123
1200.569091796875 0 1242.6923
1239.6136474609375 0 1829.2101 c 9
1240.6214599609375 0 1459.9824
1241.6275634765625 0 755.0643
1256.697509765625 0 856.244
1311.6546630859375 0 1411.1565 z 5
1312.6455078125 0 920.66876
1325.6558837890625 0 751.1213
1403.6888427734375 0 920.6015
1496.720703125 0 664.7692 y Ammonia loss 4
1497.74267578125 0 1132.8397 z 4
1498.724853515625 0 954.50085
1613.7447509765625 0 1800.5852
1691.7911376953125 0 821.4078 c 12
1740.8094482421875 0 665.2167 z 2
1741.805419921875 0 833.7228
1742.8260498046875 0 881.87555
2008.965576171875 0 1275.627
2009.9595947265625 0 1277.1348

Spectrum Details

|  |  |
| --- | --- |
| Matched peaks? Matched peaksThe total absolute number of peaks matched. Additionally in brackets the total fraction of peaks matched and the total number of peaks is shown. | 52 (15.62% of 333) |
| FDR? FDRThe false discovery rate estimated for this peptide. It is calculated by matching all theoretical fragments with a non-integer shift with the raw peaks for this spectrum. This is done with 40 different shifts. The resulting percentage is the average number of annotated peaks over the number of annotated peaks with the correct spectrum. | 0.73% |
| Satellite FDR? Satellite FDRSee the FDR for details on its calculation. This satellite ion specific FDR only contains the satellite ions (d/w) for I/L/J positions. | 0.00% |
| PSM Score? PSM ScoreThe PSM Score as given by Hecklib to this annotated spectrum. It is shown with three significant figures. | 231 |

## Spectrum 5013? Spectrum 5013 The raw spectrum of this peptide as annotated by Hecklib. The fragments are coloured according to ion type (see legend). Any peaks with a star '\*' as text can be hovered over to see the full details, first the ion type second the mass shift type. By hovering over the amino acids in the peptide or ions in the legend the corresponding peaks are highlighted. By toggling the 'Unassigned' label you can turn the background (unassigned) peaks on or off in the plot. By updating the slider in the Ion legend you can update the spectrum to only show the top X% of the peaks with labels. The top X% means any peak that is within X% of the highest intensity. By dragging in the spectrum you can zoom in to a specific part of the spectrum and use 'Zoom Out' to get back to the original zoom level. The annotation of the spectrum is based on the given sequence in the peptides file and is done with different software so inconsistencies are likely. The peaks are annotated based on the given sequence, with 20 ppm tolerance.

Copy Data

### Spectrum 5013 (TSV)

#### Preview

```
Loading example...
```

*Click on the button to copy the data to your clipboard.*

Mz MinMz MaxIntensity Max

WidthHeightPeptide font sizePeptide stroke widthSpectrum font sizeSpectrum stroke widthCompact peptide

Ion legend

wxyz

abcd

OtherUnassignedIonChargePositionShow for top:%

JHQDWLDGKEYKCKK

07.33e+41.47e+52.20e+52.93e+5

Zoom Out

c+23y+45y+11y+23z+23c+24c+24c+12z+24z+12c+37z+24y+24z+12y+410y+410y+12c+513c+25y+37y+37z+37y+37z+38c+514y+38z+38y+38z+411y+411y+411z+411c+13y+25z+25w+412y+25c+13c+26y+39y+39z+39y+39c+310w+26w+310z+413z+413z+310y+413y+413z+413y+310y+310z+310y+413z+26y+310y+26y+26z+26c+414c+27c+27c+414y+26c+27z+414z+414y+414y+414z+414y+414y+13z+13c+28c+28w+27c+28c+14z+311c+14y+311y+311z+311z+27c+312c+312c+312c+14y+27z+27y+27z+312y+28y+28z+28y+312w+14y+28c+29w+313c+313w+29y+313y+313z+313y+313z+29z+29y+29y+29z+29c+314c+314z+14c+314y+29c+210c+210z+314y+14z+314y+314w+210z+210y+210y+210z+210y+210c+15c+15c+211c+211c+15c+211y+211y+211z+211c+212c+212y+15z+15w+212y+15c+16z+212y+212z+212c+16y+212w+16w+213c+213y+213z+213y+213z+16y+16z+16c+214c+17c+214y+16c+17y+214z+214c+18z+17c+19y+18c+19z+19c+110z+110c+111

0867173326003466

Fragment Matches Table

Show background peaks

| Position | Ion type | Intensity | mz Theoretical | mz Error (Th) | mz Error (ppm) | Charge | Series Number |
| --- | --- | --- | --- | --- | --- | --- | --- |
| - | - | 389.8 | 120.9 | - | - | 0 | - |
| - | - | 728.7 | 126.1 | - | - | 0 | - |
| - | - | 459.7 | 126.1 | - | - | 0 | - |
| - | - | 728.9 | 127.1 | - | - | 0 | - |
| - | - | 864.4 | 128.1 | - | - | 0 | - |
| - | - | 3774 | 129 | - | - | 0 | - |
| - | - | 694 | 129.1 | - | - | 0 | - |
| - | - | 2.629E+04 | 129.1 | - | - | 0 | - |
| - | - | 9.562E+04 | 130.1 | - | - | 0 | - |
| - | - | 1877 | 130.1 | - | - | 0 | - |
| - | - | 604.7 | 131.1 | - | - | 0 | - |
| - | - | 9297 | 131.1 | - | - | 0 | - |
| - | - | 812.3 | 131.1 | - | - | 0 | - |
| - | - | 1700 | 131.1 | - | - | 0 | - |
| - | - | 563.7 | 132.1 | - | - | 0 | - |
| - | - | 534.3 | 132.1 | - | - | 0 | - |
| - | - | 2877 | 132.1 | - | - | 0 | - |
| - | - | 1524 | 132.1 | - | - | 0 | - |
| - | - | 859 | 133.1 | - | - | 0 | - |
| - | - | 1.392E+04 | 133.1 | - | - | 0 | - |
| - | - | 465.2 | 133.1 | - | - | 0 | - |
| - | - | 1033 | 134.1 | - | - | 0 | - |
| - | - | 499.1 | 136.1 | - | - | 0 | - |
| - | - | 5864 | 136.1 | - | - | 0 | - |
| - | - | 401.3 | 137.1 | - | - | 0 | - |
| - | - | 390.6 | 137.9 | - | - | 0 | - |
| - | - | 1328 | 138.1 | - | - | 0 | - |
| - | - | 2820 | 139 | - | - | 0 | - |
| - | - | 434.9 | 140.3 | - | - | 0 | - |
| - | - | 615.6 | 141.1 | - | - | 0 | - |
| - | - | 585.9 | 143 | - | - | 0 | - |
| - | - | 449.5 | 143.1 | - | - | 0 | - |
| - | - | 2815 | 144.1 | - | - | 0 | - |
| - | - | 2173 | 145.1 | - | - | 0 | - |
| - | - | 1795 | 146.1 | - | - | 0 | - |
| - | - | 414.7 | 146.1 | - | - | 0 | - |
| - | - | 722.8 | 147 | - | - | 0 | - |
| - | - | 820.5 | 147.1 | - | - | 0 | - |
| - | - | 457.8 | 154.1 | - | - | 0 | - |
| - | - | 1.751E+04 | 155.1 | - | - | 0 | - |
| - | - | 759.1 | 156.1 | - | - | 0 | - |
| - | - | 902.7 | 156.1 | - | - | 0 | - |
| - | - | 1590 | 156.1 | - | - | 0 | - |
| - | - | 7593 | 157 | - | - | 0 | - |
| - | - | 669.4 | 157.1 | - | - | 0 | - |
| - | - | 1045 | 158.1 | - | - | 0 | - |
| - | - | 4.701E+04 | 159.1 | - | - | 0 | - |
| - | - | 4371 | 160.1 | - | - | 0 | - |
| - | - | 2189 | 164.1 | - | - | 0 | - |
| - | - | 467.6 | 165.1 | - | - | 0 | - |
| - | - | 1.577E+04 | 166.1 | - | - | 0 | - |
| - | - | 1393 | 167.1 | - | - | 0 | - |
| - | - | 1006 | 168.1 | - | - | 0 | - |
| - | - | 537 | 169.1 | - | - | 0 | - |
| - | - | 1.737E+04 | 170.1 | - | - | 0 | - |
| - | - | 1071 | 171.1 | - | - | 0 | - |
| - | - | 824.9 | 171.1 | - | - | 0 | - |
| - | - | 709.4 | 171.1 | - | - | 0 | - |
| - | - | 3952 | 172.1 | - | - | 0 | - |
| - | - | 1791 | 173.1 | - | - | 0 | - |
| - | - | 389.3 | 173.4 | - | - | 0 | - |
| - | - | 2677 | 173.5 | - | - | 0 | - |
| - | - | 2734 | 174.1 | - | - | 0 | - |
| - | - | 1768 | 176.1 | - | - | 0 | - |
| - | - | 568.9 | 176.6 | - | - | 0 | - |
| - | - | 2673 | 178.1 | - | - | 0 | - |
| - | - | 552.3 | 179.1 | - | - | 0 | - |
| - | - | 534.2 | 181.1 | - | - | 0 | - |
| - | - | 6530 | 183.1 | - | - | 0 | - |
| - | - | 831.7 | 183.1 | - | - | 0 | - |
| - | - | 996.3 | 183.1 | - | - | 0 | - |
| - | - | 859.3 | 183.1 | - | - | 0 | - |
| - | - | 1021 | 185.1 | - | - | 0 | - |
| - | - | 1.008E+04 | 185.1 | - | - | 0 | - |
| - | - | 855.3 | 185.1 | - | - | 0 | - |
| - | - | 1.539E+04 | 185.2 | - | - | 0 | - |
| - | - | 701.4 | 186.1 | - | - | 0 | - |
| - | - | 3193 | 186.1 | - | - | 0 | - |
| - | - | 1189 | 186.2 | - | - | 0 | - |
| - | - | 6943 | 187.1 | - | - | 0 | - |
| - | - | 1404 | 187.1 | - | - | 0 | - |
| - | - | 918.4 | 188.1 | - | - | 0 | - |
| - | - | 802.4 | 189.1 | - | - | 0 | - |
| - | - | 2.91E+04 | 190.1 | - | - | 0 | - |
| 3 | c | 1332 | 190.1 | 0.0006047 | 3.181 | +2 | 3 |
| - | - | 500.1 | 190.6 | - | - | 0 | - |
| - | - | 1091 | 191.1 | - | - | 0 | - |
| - | - | 1891 | 192.1 | - | - | 0 | - |
| - | - | 473.4 | 192.5 | - | - | 0 | - |
| - | - | 752.6 | 195.1 | - | - | 0 | - |
| 11 | y | 521.5 | 197.1 | 0.00355 | 18.01 | +4 | 5 |
| - | - | 8004 | 198.1 | - | - | 0 | - |
| - | - | 515.3 | 199.1 | - | - | 0 | - |
| - | - | 2640 | 199.2 | - | - | 0 | - |
| - | - | 785.2 | 200.1 | - | - | 0 | - |
| - | - | 503.2 | 201.1 | - | - | 0 | - |
| - | - | 958.2 | 202.1 | - | - | 0 | - |
| - | - | 793.7 | 203.1 | - | - | 0 | - |
| - | - | 3139 | 203.1 | - | - | 0 | - |
| - | - | 1069 | 203.1 | - | - | 0 | - |
| - | - | 1286 | 203.2 | - | - | 0 | - |
| - | - | 674.6 | 203.6 | - | - | 0 | - |
| 15 | y | 1.213E+04 | 205.1 | 0.003969 | 19.35 | +1 | 1 |
| - | - | 482.2 | 205.1 | - | - | 0 | - |
| - | - | 1312 | 206.1 | - | - | 0 | - |
| - | - | 1833 | 207.2 | - | - | 0 | - |
| - | - | 1010 | 208.1 | - | - | 0 | - |
| - | - | 4287 | 210.1 | - | - | 0 | - |
| - | - | 848 | 211.1 | - | - | 0 | - |
| - | - | 5958 | 211.6 | - | - | 0 | - |
| - | - | 1735 | 212.1 | - | - | 0 | - |
| - | - | 4945 | 213.1 | - | - | 0 | - |
| - | - | 504.9 | 213.2 | - | - | 0 | - |
| - | - | 967.6 | 215.1 | - | - | 0 | - |
| - | - | 8590 | 217 | - | - | 0 | - |
| - | - | 4113 | 223.1 | - | - | 0 | - |
| - | - | 1.691E+04 | 223.2 | - | - | 0 | - |
| - | - | 604.7 | 224.1 | - | - | 0 | - |
| - | - | 2037 | 224.2 | - | - | 0 | - |
| - | - | 580.4 | 226.1 | - | - | 0 | - |
| - | - | 1510 | 227.1 | - | - | 0 | - |
| - | - | 587.6 | 227.1 | - | - | 0 | - |
| - | - | 746.5 | 228.1 | - | - | 0 | - |
| - | - | 1428 | 228.1 | - | - | 0 | - |
| - | - | 606.8 | 229.1 | - | - | 0 | - |
| - | - | 4485 | 229.1 | - | - | 0 | - |
| - | - | 1152 | 230.2 | - | - | 0 | - |
| - | - | 4667 | 233.1 | - | - | 0 | - |
| - | - | 1227 | 233.6 | - | - | 0 | - |
| - | - | 1.958E+04 | 234.1 | - | - | 0 | - |
| - | - | 2920 | 235.1 | - | - | 0 | - |
| - | - | 2377 | 236.6 | - | - | 0 | - |
| 13 | y | 2289 | 239.1 | 1.441E-06 | 0.006025 | +2 | 3 |
| 13 | z | 1193 | 239.6 | 0.002172 | 9.063 | +2 | 3 |
| - | - | 728.4 | 240.2 | - | - | 0 | - |
| - | - | 556 | 243.1 | - | - | 0 | - |
| - | - | 644.4 | 243.1 | - | - | 0 | - |
| - | - | 2737 | 244.1 | - | - | 0 | - |
| - | - | 507.9 | 245.1 | - | - | 0 | - |
| - | - | 1057 | 245.1 | - | - | 0 | - |
| - | - | 1603 | 246.1 | - | - | 0 | - |
| 4 | c | 7578 | 247.6 | 0.0003015 | 1.218 | +2 | 4 |
| - | - | 1764 | 248.1 | - | - | 0 | - |
| - | - | 2190 | 250.1 | - | - | 0 | - |
| - | - | 2006 | 250.1 | - | - | 0 | - |
| - | - | 2239 | 250.2 | - | - | 0 | - |
| - | - | 9.861E+04 | 251.2 | - | - | 0 | - |
| - | - | 1.325E+04 | 252.2 | - | - | 0 | - |
| - | - | 2908 | 252.6 | - | - | 0 | - |
| - | - | 1124 | 253.2 | - | - | 0 | - |
| - | - | 2924 | 255.1 | - | - | 0 | - |
| - | - | 2025 | 255.8 | - | - | 0 | - |
| 4 | c | 723.3 | 256.1 | 0.004147 | 16.19 | +2 | 4 |
| - | - | 810.2 | 256.6 | - | - | 0 | - |
| - | - | 2051 | 258.2 | - | - | 0 | - |
| - | - | 1124 | 258.7 | - | - | 0 | - |
| - | - | 576.4 | 259.1 | - | - | 0 | - |
| - | - | 1.262E+04 | 259.2 | - | - | 0 | - |
| - | - | 3216 | 261.1 | - | - | 0 | - |
| - | - | 2820 | 261.6 | - | - | 0 | - |
| - | - | 644.6 | 262.1 | - | - | 0 | - |
| - | - | 2331 | 264.2 | - | - | 0 | - |
| - | - | 2481 | 265.1 | - | - | 0 | - |
| - | - | 1370 | 265.6 | - | - | 0 | - |
| - | - | 3460 | 266.1 | - | - | 0 | - |
| - | - | 674.1 | 266.6 | - | - | 0 | - |
| - | - | 577.5 | 267.1 | - | - | 0 | - |
| 2 | c | 1.16E+04 | 268.2 | 0.0006888 | 2.569 | +1 | 2 |
| - | - | 1585 | 269.1 | - | - | 0 | - |
| - | - | 1819 | 269.2 | - | - | 0 | - |
| - | - | 2.02E+04 | 270.1 | - | - | 0 | - |
| - | - | 5388 | 270.6 | - | - | 0 | - |
| - | - | 5176 | 272.1 | - | - | 0 | - |
| - | - | 6374 | 272.2 | - | - | 0 | - |
| - | - | 3824 | 273.1 | - | - | 0 | - |
| - | - | 737.6 | 273.1 | - | - | 0 | - |
| - | - | 958.2 | 273.2 | - | - | 0 | - |
| - | - | 3654 | 274.1 | - | - | 0 | - |
| - | - | 1.743E+04 | 274.1 | - | - | 0 | - |
| - | - | 4706 | 274.7 | - | - | 0 | - |
| - | - | 1764 | 275.1 | - | - | 0 | - |
| - | - | 1027 | 275.2 | - | - | 0 | - |
| - | - | 703.1 | 276.1 | - | - | 0 | - |
| - | - | 729.4 | 277.2 | - | - | 0 | - |
| - | - | 1189 | 282.1 | - | - | 0 | - |
| - | - | 7910 | 282.2 | - | - | 0 | - |
| - | - | 3836 | 283.1 | - | - | 0 | - |
| - | - | 1027 | 283.2 | - | - | 0 | - |
| - | - | 1646 | 284.1 | - | - | 0 | - |
| - | - | 3829 | 284.1 | - | - | 0 | - |
| - | - | 649.5 | 284.1 | - | - | 0 | - |
| - | - | 879.6 | 284.6 | - | - | 0 | - |
| - | - | 1437 | 285.2 | - | - | 0 | - |
| - | - | 1423 | 286.1 | - | - | 0 | - |
| - | - | 6972 | 290.1 | - | - | 0 | - |
| - | - | 1250 | 292.2 | - | - | 0 | - |
| - | - | 1430 | 294.1 | - | - | 0 | - |
| - | - | 798.2 | 294.1 | - | - | 0 | - |
| - | - | 945.1 | 294.2 | - | - | 0 | - |
| - | - | 1033 | 295.1 | - | - | 0 | - |
| 12 | z | 905.6 | 295.1 | 0.0008682 | 2.941 | +2 | 4 |
| - | - | 2160 | 297.1 | - | - | 0 | - |
| - | - | 779 | 297.2 | - | - | 0 | - |
| - | - | 677.6 | 297.6 | - | - | 0 | - |
| 14 | z | 2760 | 300.2 | 0.0009852 | 3.282 | +1 | 2 |
| - | - | 1249 | 301.1 | - | - | 0 | - |
| - | - | 3106 | 301.2 | - | - | 0 | - |
| - | - | 3577 | 302.1 | - | - | 0 | - |
| - | - | 2015 | 302.1 | - | - | 0 | - |
| - | - | 718.6 | 302.5 | - | - | 0 | - |
| - | - | 799.1 | 302.7 | - | - | 0 | - |
| - | - | 611.3 | 303.1 | - | - | 0 | - |
| 7 | c | 1.013E+04 | 303.2 | 0.0002253 | 0.7431 | +3 | 7 |
| - | - | 2968 | 303.2 | - | - | 0 | - |
| 12 | z | 3759 | 303.7 | 0.005387 | 17.74 | +2 | 4 |
| - | - | 2134 | 304.2 | - | - | 0 | - |
| - | - | 882.9 | 305.6 | - | - | 0 | - |
| - | - | 620.3 | 306.1 | - | - | 0 | - |
| - | - | 5467 | 309.1 | - | - | 0 | - |
| - | - | 669.8 | 309.2 | - | - | 0 | - |
| - | - | 1129 | 309.6 | - | - | 0 | - |
| - | - | 838.2 | 309.7 | - | - | 0 | - |
| - | - | 2741 | 311.1 | - | - | 0 | - |
| - | - | 887.5 | 311.6 | - | - | 0 | - |
| 12 | y | 9507 | 311.7 | 0.003641 | 11.68 | +2 | 4 |
| - | - | 1743 | 312.2 | - | - | 0 | - |
| - | - | 558.1 | 312.7 | - | - | 0 | - |
| - | - | 1694 | 315.2 | - | - | 0 | - |
| 14 | z | 4186 | 317.2 | 0.003425 | 10.8 | +1 | 2 |
| - | - | 5005 | 317.7 | - | - | 0 | - |
| - | - | 1.999E+04 | 318.2 | - | - | 0 | - |
| - | - | 1268 | 318.2 | - | - | 0 | - |
| - | - | 9064 | 318.7 | - | - | 0 | - |
| - | - | 719.7 | 318.8 | - | - | 0 | - |
| - | - | 1883 | 319.2 | - | - | 0 | - |
| - | - | 844.1 | 320.2 | - | - | 0 | - |
| - | - | 694.1 | 321.2 | - | - | 0 | - |
| - | - | 1000 | 322.8 | - | - | 0 | - |
| - | - | 3105 | 323.1 | - | - | 0 | - |
| - | - | 1768 | 323.6 | - | - | 0 | - |
| - | - | 870.5 | 324.7 | - | - | 0 | - |
| - | - | 4317 | 326.5 | - | - | 0 | - |
| - | - | 8.255E+04 | 326.7 | - | - | 0 | - |
| - | - | 1754 | 326.8 | - | - | 0 | - |
| - | - | 3.331E+04 | 327.2 | - | - | 0 | - |
| - | - | 1388 | 327.2 | - | - | 0 | - |
| - | - | 8306 | 327.7 | - | - | 0 | - |
| 6 | y | 1219 | 328.2 | 0.0003304 | 1.007 | +4 | 10 |
| - | - | 3849 | 329.2 | - | - | 0 | - |
| - | - | 1225 | 331.5 | - | - | 0 | - |
| - | - | 1.245E+04 | 331.7 | - | - | 0 | - |
| - | - | 774.7 | 331.7 | - | - | 0 | - |
| - | - | 1410 | 331.9 | - | - | 0 | - |
| - | - | 4396 | 332.2 | - | - | 0 | - |
| - | - | 865.5 | 332.7 | - | - | 0 | - |
| 6 | y | 1013 | 332.7 | 0.0006495 | 1.952 | +4 | 10 |
| 14 | y | 5780 | 333.2 | 0.003351 | 10.06 | +1 | 2 |
| - | - | 680.6 | 334.2 | - | - | 0 | - |
| - | - | 639.1 | 335.2 | - | - | 0 | - |
| - | - | 1663 | 337.8 | - | - | 0 | - |
| - | - | 960.6 | 338.1 | - | - | 0 | - |
| - | - | 1387 | 338.2 | - | - | 0 | - |
| - | - | 614.9 | 338.5 | - | - | 0 | - |
| - | - | 1640 | 338.7 | - | - | 0 | - |
| 13 | c | 1443 | 339.2 | 0.00316 | 9.318 | +5 | 13 |
| - | - | 629.1 | 339.7 | - | - | 0 | - |
| - | - | 1075 | 339.7 | - | - | 0 | - |
| - | - | 3441 | 339.7 | - | - | 0 | - |
| - | - | 1785 | 340.2 | - | - | 0 | - |
| 5 | c | 3.36E+04 | 340.7 | 0.00105 | 3.083 | +2 | 5 |
| - | - | 1.843E+04 | 341.2 | - | - | 0 | - |
| - | - | 1079 | 341.2 | - | - | 0 | - |
| - | - | 4248 | 341.7 | - | - | 0 | - |
| - | - | 2977 | 341.8 | - | - | 0 | - |
| 9 | y | 1825 | 342.2 | 0.002812 | 8.219 | +3 | 7 |
| 9 | y | 2401 | 342.5 | 0.000251 | 0.7329 | +3 | 7 |
| 9 | z | 2260 | 342.8 | 0.00111 | 3.237 | +3 | 7 |
| - | - | 1446 | 343.2 | - | - | 0 | - |
| - | - | 858.5 | 346.1 | - | - | 0 | - |
| - | - | 1109 | 347.2 | - | - | 0 | - |
| - | - | 1583 | 347.9 | - | - | 0 | - |
| 9 | y | 1800 | 348.2 | 0.001509 | 4.335 | +3 | 7 |
| - | - | 2181 | 349.2 | - | - | 0 | - |
| - | - | 1112 | 349.7 | - | - | 0 | - |
| - | - | 1919 | 351.2 | - | - | 0 | - |
| - | - | 640.3 | 352.5 | - | - | 0 | - |
| - | - | 640.5 | 352.7 | - | - | 0 | - |
| - | - | 2426 | 353.7 | - | - | 0 | - |
| - | - | 1691 | 354.2 | - | - | 0 | - |
| - | - | 1906 | 355.2 | - | - | 0 | - |
| - | - | 2185 | 355.7 | - | - | 0 | - |
| 8 | z | 801.1 | 355.8 | 0.003217 | 9.04 | +3 | 8 |
| - | - | 933.7 | 356.2 | - | - | 0 | - |
| - | - | 3036 | 356.8 | - | - | 0 | - |
| - | - | 2634 | 357.2 | - | - | 0 | - |
| - | - | 868.1 | 357.7 | - | - | 0 | - |
| - | - | 662.1 | 358.2 | - | - | 0 | - |
| - | - | 2811 | 360.7 | - | - | 0 | - |
| 14 | c | 2409 | 361.2 | 0.000624 | 1.728 | +5 | 14 |
| - | - | 1942 | 361.2 | - | - | 0 | - |
| - | - | 2451 | 361.2 | - | - | 0 | - |
| 8 | y | 1771 | 361.5 | 0.0002645 | 0.7316 | +3 | 8 |
| 8 | z | 7961 | 361.9 | 0.002222 | 6.14 | +3 | 8 |
| - | - | 5243 | 362.2 | - | - | 0 | - |
| - | - | 690.5 | 362.5 | - | - | 0 | - |
| - | - | 806.6 | 362.7 | - | - | 0 | - |
| - | - | 1558 | 363.1 | - | - | 0 | - |
| - | - | 955.4 | 363.2 | - | - | 0 | - |
| - | - | 1564 | 364.1 | - | - | 0 | - |
| - | - | 3435 | 364.2 | - | - | 0 | - |
| - | - | 2038 | 364.7 | - | - | 0 | - |
| - | - | 1071 | 365.7 | - | - | 0 | - |
| - | - | 1687 | 366.2 | - | - | 0 | - |
| 8 | y | 4.785E+04 | 367.2 | 0.002136 | 5.817 | +3 | 8 |
| - | - | 2.843E+04 | 367.5 | - | - | 0 | - |
| - | - | 1440 | 367.7 | - | - | 0 | - |
| - | - | 1152 | 367.7 | - | - | 0 | - |
| - | - | 1.282E+04 | 367.9 | - | - | 0 | - |
| - | - | 4686 | 368.2 | - | - | 0 | - |
| - | - | 1028 | 369.2 | - | - | 0 | - |
| - | - | 1023 | 370.4 | - | - | 0 | - |
| - | - | 654.2 | 370.5 | - | - | 0 | - |
| 5 | z | 1279 | 370.7 | 0.002884 | 7.781 | +4 | 11 |
| - | - | 1613 | 371.2 | - | - | 0 | - |
| - | - | 841.7 | 372.4 | - | - | 0 | - |
| - | - | 4844 | 374.2 | - | - | 0 | - |
| 5 | y | 1.177E+04 | 374.7 | 0.004672 | 12.47 | +4 | 11 |
| 5 | y | 1.062E+04 | 374.9 | 0.001189 | 3.171 | +4 | 11 |
| 5 | z | 9303 | 375.2 | 0.0004864 | 1.296 | +4 | 11 |
| - | - | 4300 | 375.4 | - | - | 0 | - |
| - | - | 1268 | 375.7 | - | - | 0 | - |
| - | - | 619.7 | 375.9 | - | - | 0 | - |
| - | - | 3778 | 376.2 | - | - | 0 | - |
| - | - | 1015 | 376.5 | - | - | 0 | - |
| - | - | 6152 | 378.2 | - | - | 0 | - |
| - | - | 864.6 | 378.2 | - | - | 0 | - |
| 3 | c | 3.222E+04 | 379.2 | 0.001009 | 2.661 | +1 | 3 |
| - | - | 1836 | 379.4 | - | - | 0 | - |
| - | - | 5426 | 380.2 | - | - | 0 | - |
| - | - | 7919 | 381.2 | - | - | 0 | - |
| - | - | 2.179E+04 | 381.2 | - | - | 0 | - |
| - | - | 8988 | 381.5 | - | - | 0 | - |
| - | - | 3423 | 381.9 | - | - | 0 | - |
| - | - | 1327 | 382.2 | - | - | 0 | - |
| - | - | 1110 | 382.2 | - | - | 0 | - |
| - | - | 3.111E+04 | 383.2 | - | - | 0 | - |
| - | - | 1.733E+04 | 383.7 | - | - | 0 | - |
| - | - | 4642 | 384.2 | - | - | 0 | - |
| 11 | y | 2333 | 384.7 | 0.002896 | 7.529 | +2 | 5 |
| - | - | 805.9 | 384.7 | - | - | 0 | - |
| - | - | 2429 | 385.2 | - | - | 0 | - |
| 11 | z | 6248 | 385.2 | 0.001804 | 4.683 | +2 | 5 |
| - | - | 3738 | 385.5 | - | - | 0 | - |
| - | - | 787.3 | 385.7 | - | - | 0 | - |
| - | - | 2606 | 385.9 | - | - | 0 | - |
| - | - | 1919 | 386.2 | - | - | 0 | - |
| - | - | 1166 | 386.5 | - | - | 0 | - |
| - | - | 1394 | 387.2 | - | - | 0 | - |
| - | - | 3.987E+04 | 387.2 | - | - | 0 | - |
| - | - | 1598 | 388.2 | - | - | 0 | - |
| - | - | 8852 | 388.2 | - | - | 0 | - |
| - | - | 3663 | 388.7 | - | - | 0 | - |
| - | - | 792.6 | 388.9 | - | - | 0 | - |
| - | - | 8190 | 389.2 | - | - | 0 | - |
| - | - | 791.9 | 389.2 | - | - | 0 | - |
| - | - | 916.2 | 389.2 | - | - | 0 | - |
| - | - | 1094 | 390 | - | - | 0 | - |
| - | - | 5019 | 390.2 | - | - | 0 | - |
| - | - | 1285 | 390.5 | - | - | 0 | - |
| - | - | 737.3 | 391.1 | - | - | 0 | - |
| - | - | 1708 | 391.2 | - | - | 0 | - |
| - | - | 795.9 | 392.2 | - | - | 0 | - |
| 4 | w | 738.8 | 392.7 | 0.00415 | 10.57 | +4 | 12 |
| 11 | y | 5.024E+04 | 393.2 | 0.003475 | 8.839 | +2 | 5 |
| - | - | 762.9 | 393.5 | - | - | 0 | - |
| - | - | 803.2 | 393.6 | - | - | 0 | - |
| - | - | 2.322E+04 | 393.7 | - | - | 0 | - |
| - | - | 8478 | 394.2 | - | - | 0 | - |
| - | - | 1618 | 394.7 | - | - | 0 | - |
| - | - | 2303 | 395 | - | - | 0 | - |
| - | - | 2153 | 395.1 | - | - | 0 | - |
| - | - | 6039 | 395.2 | - | - | 0 | - |
| - | - | 915.6 | 395.2 | - | - | 0 | - |
| - | - | 6770 | 395.4 | - | - | 0 | - |
| - | - | 3729 | 395.6 | - | - | 0 | - |
| - | - | 2667 | 395.8 | - | - | 0 | - |
| 3 | c | 3.763E+04 | 396.2 | 0.001041 | 2.627 | +1 | 3 |
| 6 | c | 2.52E+04 | 397.2 | 0.001102 | 2.775 | +2 | 6 |
| - | - | 7430 | 397.2 | - | - | 0 | - |
| - | - | 1.187E+04 | 397.7 | - | - | 0 | - |
| - | - | 1801 | 398.2 | - | - | 0 | - |
| - | - | 3108 | 398.2 | - | - | 0 | - |
| - | - | 826.4 | 398.2 | - | - | 0 | - |
| - | - | 1.136E+04 | 398.6 | - | - | 0 | - |
| - | - | 2.814E+04 | 398.8 | - | - | 0 | - |
| - | - | 2.609E+04 | 399 | - | - | 0 | - |
| - | - | 1.702E+04 | 399.2 | - | - | 0 | - |
| - | - | 7520 | 399.4 | - | - | 0 | - |
| 7 | y | 3227 | 399.5 | 0.002153 | 5.389 | +3 | 9 |
| - | - | 3072 | 399.6 | - | - | 0 | - |
| - | - | 812.1 | 399.8 | - | - | 0 | - |
| 7 | y | 3998 | 399.9 | 0.0006904 | 1.727 | +3 | 9 |
| 7 | z | 2799 | 400.2 | 0.0008777 | 2.193 | +3 | 9 |
| - | - | 745.7 | 400.5 | - | - | 0 | - |
| - | - | 764.7 | 400.8 | - | - | 0 | - |
| - | - | 1035 | 401.2 | - | - | 0 | - |
| - | - | 1185 | 401.3 | - | - | 0 | - |
| - | - | 2428 | 402 | - | - | 0 | - |
| - | - | 1.938E+04 | 402.2 | - | - | 0 | - |
| - | - | 2.17E+04 | 402.4 | - | - | 0 | - |
| - | - | 1.622E+04 | 402.6 | - | - | 0 | - |
| - | - | 8306 | 402.8 | - | - | 0 | - |
| - | - | 3323 | 403 | - | - | 0 | - |
| - | - | 2982 | 403.2 | - | - | 0 | - |
| - | - | 2287 | 404.2 | - | - | 0 | - |
| - | - | 1751 | 404.7 | - | - | 0 | - |
| - | - | 1164 | 405.2 | - | - | 0 | - |
| 7 | y | 4.299E+04 | 405.5 | 0.001799 | 4.436 | +3 | 9 |
| - | - | 2.5E+04 | 405.9 | - | - | 0 | - |
| - | - | 1.072E+04 | 406.2 | - | - | 0 | - |
| - | - | 3833 | 406.5 | - | - | 0 | - |
| - | - | 732.3 | 407.2 | - | - | 0 | - |
| 10 | c | 1040 | 408.2 | 0.001161 | 2.845 | +3 | 10 |
| - | - | 2967 | 408.9 | - | - | 0 | - |
| - | - | 1303 | 409.1 | - | - | 0 | - |
| - | - | 709.6 | 409.2 | - | - | 0 | - |
| - | - | 886 | 409.5 | - | - | 0 | - |
| - | - | 1223 | 411.2 | - | - | 0 | - |
| - | - | 996.4 | 411.7 | - | - | 0 | - |
| - | - | 1679 | 412.2 | - | - | 0 | - |
| - | - | 704.2 | 413.1 | - | - | 0 | - |
| - | - | 777.8 | 413.2 | - | - | 0 | - |
| - | - | 1092 | 413.9 | - | - | 0 | - |
| - | - | 976 | 414.2 | - | - | 0 | - |
| - | - | 1354 | 414.6 | - | - | 0 | - |
| - | - | 958.2 | 414.9 | - | - | 0 | - |
| - | - | 779.2 | 415.2 | - | - | 0 | - |
| - | - | 1031 | 415.2 | - | - | 0 | - |
| - | - | 1338 | 415.7 | - | - | 0 | - |
| - | - | 1014 | 416.7 | - | - | 0 | - |
| - | - | 2004 | 418.2 | - | - | 0 | - |
| - | - | 818.3 | 419.7 | - | - | 0 | - |
| 10 | w | 2.028E+05 | 420.2 | 0.003234 | 7.696 | +2 | 6 |
| - | - | 9.086E+04 | 420.7 | - | - | 0 | - |
| - | - | 3.326E+04 | 421.2 | - | - | 0 | - |
| - | - | 1.089E+04 | 421.7 | - | - | 0 | - |
| - | - | 1375 | 422.2 | - | - | 0 | - |
| - | - | 916.6 | 422.7 | - | - | 0 | - |
| 6 | w | 1.64E+04 | 423.5 | 0.001658 | 3.915 | +3 | 10 |
| - | - | 1.133E+04 | 423.9 | - | - | 0 | - |
| - | - | 4455 | 424.2 | - | - | 0 | - |
| - | - | 1327 | 424.5 | - | - | 0 | - |
| - | - | 1228 | 424.7 | - | - | 0 | - |
| - | - | 777 | 424.9 | - | - | 0 | - |
| - | - | 1359 | 425.2 | - | - | 0 | - |
| - | - | 893.9 | 427.2 | - | - | 0 | - |
| - | - | 951.3 | 427.7 | - | - | 0 | - |
| - | - | 7998 | 430.2 | - | - | 0 | - |
| - | - | 3098 | 431.2 | - | - | 0 | - |
| - | - | 1110 | 431.2 | - | - | 0 | - |
| 3 | z | 823 | 431.5 | 0.003116 | 7.222 | +4 | 13 |
| 3 | z | 1707 | 431.7 | 0.004085 | 9.462 | +4 | 13 |
| 6 | z | 986.7 | 432.2 | 0.0001533 | 0.3547 | +3 | 10 |
| - | - | 2453 | 434.2 | - | - | 0 | - |
| - | - | 1933 | 435.2 | - | - | 0 | - |
| - | - | 4375 | 435.2 | - | - | 0 | - |
| 3 | y | 2051 | 435.5 | 0.0007473 | 1.716 | +4 | 13 |
| 3 | y | 1906 | 435.7 | 0.001875 | 4.304 | +4 | 13 |
| 3 | z | 1686 | 436 | 0.0008742 | 2.005 | +4 | 13 |
| - | - | 2169 | 436.2 | - | - | 0 | - |
| 6 | y | 4441 | 437.2 | 0.0007655 | 1.751 | +3 | 10 |
| 6 | y | 6633 | 437.6 | 0.004634 | 10.59 | +3 | 10 |
| 6 | z | 1471 | 437.9 | 0.002371 | 5.415 | +3 | 10 |
| - | - | 1112 | 438.2 | - | - | 0 | - |
| - | - | 1326 | 438.2 | - | - | 0 | - |
| - | - | 692.2 | 438.6 | - | - | 0 | - |
| - | - | 1623 | 438.7 | - | - | 0 | - |
| - | - | 1439 | 439.2 | - | - | 0 | - |
| 3 | y | 1315 | 440 | 0.0009166 | 2.083 | +4 | 13 |
| 10 | z | 892.8 | 440.7 | 0.008671 | 19.68 | +2 | 6 |
| - | - | 883.1 | 441.2 | - | - | 0 | - |
| - | - | 763 | 442.7 | - | - | 0 | - |
| - | - | 3211 | 442.9 | - | - | 0 | - |
| 6 | y | 5.811E+04 | 443.2 | 0.001449 | 3.269 | +3 | 10 |
| - | - | 3.547E+04 | 443.6 | - | - | 0 | - |
| - | - | 1.503E+04 | 443.9 | - | - | 0 | - |
| - | - | 4918 | 444.2 | - | - | 0 | - |
| - | - | 1399 | 444.6 | - | - | 0 | - |
| - | - | 2596 | 445.7 | - | - | 0 | - |
| - | - | 1348 | 446.2 | - | - | 0 | - |
| - | - | 977.7 | 446.6 | - | - | 0 | - |
| - | - | 787.9 | 446.7 | - | - | 0 | - |
| - | - | 737.5 | 446.7 | - | - | 0 | - |
| - | - | 6517 | 447.2 | - | - | 0 | - |
| - | - | 931.4 | 447.7 | - | - | 0 | - |
| - | - | 2756 | 448.2 | - | - | 0 | - |
| 10 | y | 5624 | 448.7 | 0.002919 | 6.504 | +2 | 6 |
| 10 | y | 2640 | 449.2 | 0.005531 | 12.31 | +2 | 6 |
| 10 | z | 3754 | 449.7 | 0.002226 | 4.951 | +2 | 6 |
| - | - | 1611 | 450.2 | - | - | 0 | - |
| - | - | 784.5 | 450.7 | - | - | 0 | - |
| 14 | c | 714.2 | 451.2 | 0.008916 | 19.76 | +4 | 14 |
| - | - | 1431 | 452.2 | - | - | 0 | - |
| - | - | 1040 | 452.5 | - | - | 0 | - |
| - | - | 1132 | 452.6 | - | - | 0 | - |
| - | - | 1234 | 452.7 | - | - | 0 | - |
| - | - | 985 | 453.3 | - | - | 0 | - |
| 7 | c | 1112 | 454.2 | 0.0008609 | 1.895 | +2 | 7 |
| - | - | 747.6 | 454.3 | - | - | 0 | - |
| 7 | c | 3558 | 454.7 | 0.00118 | 2.596 | +2 | 7 |
| - | - | 821.9 | 455 | - | - | 0 | - |
| - | - | 1285 | 455.2 | - | - | 0 | - |
| 14 | c | 1959 | 455.7 | 0.0004083 | 0.896 | +4 | 14 |
| - | - | 1471 | 455.9 | - | - | 0 | - |
| - | - | 1974 | 456 | - | - | 0 | - |
| - | - | 2458 | 456.2 | - | - | 0 | - |
| - | - | 1128 | 457.2 | - | - | 0 | - |
| 10 | y | 5552 | 457.7 | 0.002647 | 5.782 | +2 | 6 |
| - | - | 2166 | 458.2 | - | - | 0 | - |
| - | - | 786.8 | 459.2 | - | - | 0 | - |
| - | - | 774.9 | 459.5 | - | - | 0 | - |
| - | - | 2221 | 460 | - | - | 0 | - |
| - | - | 1748 | 460.2 | - | - | 0 | - |
| - | - | 3.26E+04 | 460.7 | - | - | 0 | - |
| - | - | 1757 | 461 | - | - | 0 | - |
| - | - | 1.666E+04 | 461.2 | - | - | 0 | - |
| - | - | 3390 | 461.5 | - | - | 0 | - |
| - | - | 6834 | 461.7 | - | - | 0 | - |
| - | - | 6304 | 462.2 | - | - | 0 | - |
| - | - | 1711 | 462.2 | - | - | 0 | - |
| - | - | 2601 | 462.7 | - | - | 0 | - |
| 7 | c | 2491 | 463.2 | 0.001913 | 4.131 | +2 | 7 |
| - | - | 1248 | 463.7 | - | - | 0 | - |
| - | - | 1947 | 465.2 | - | - | 0 | - |
| - | - | 7000 | 465.5 | - | - | 0 | - |
| 2 | z | 4567 | 465.7 | 0.0004362 | 0.9366 | +4 | 14 |
| - | - | 1135 | 465.9 | - | - | 0 | - |
| 2 | z | 3408 | 466 | 0.004646 | 9.97 | +4 | 14 |
| - | - | 1898 | 466.2 | - | - | 0 | - |
| - | - | 4225 | 466.2 | - | - | 0 | - |
| - | - | 3351 | 466.6 | - | - | 0 | - |
| - | - | 1898 | 466.9 | - | - | 0 | - |
| - | - | 3662 | 467.2 | - | - | 0 | - |
| - | - | 852.7 | 467.8 | - | - | 0 | - |
| - | - | 1.915E+04 | 468.3 | - | - | 0 | - |
| - | - | 1.168E+04 | 468.8 | - | - | 0 | - |
| - | - | 3611 | 469.2 | - | - | 0 | - |
| - | - | 2691 | 469.3 | - | - | 0 | - |
| - | - | 1423 | 469.5 | - | - | 0 | - |
| 2 | y | 6159 | 469.7 | 0.004178 | 8.895 | +4 | 14 |
| 2 | y | 7716 | 470 | 0.004939 | 10.51 | +4 | 14 |
| 2 | z | 9244 | 470.2 | 0.003654 | 7.772 | +4 | 14 |
| - | - | 5760 | 470.5 | - | - | 0 | - |
| - | - | 2295 | 470.7 | - | - | 0 | - |
| - | - | 708.1 | 471.5 | - | - | 0 | - |
| - | - | 1001 | 471.7 | - | - | 0 | - |
| - | - | 2158 | 472 | - | - | 0 | - |
| - | - | 1785 | 473.2 | - | - | 0 | - |
| - | - | 2143 | 473.7 | - | - | 0 | - |
| - | - | 1976 | 474 | - | - | 0 | - |
| 2 | y | 1.549E+04 | 474.2 | 0.0006604 | 1.393 | +4 | 14 |
| - | - | 1.279E+04 | 474.5 | - | - | 0 | - |
| - | - | 7161 | 474.7 | - | - | 0 | - |
| - | - | 3094 | 475 | - | - | 0 | - |
| - | - | 1779 | 475.2 | - | - | 0 | - |
| - | - | 2800 | 475.7 | - | - | 0 | - |
| - | - | 2157 | 476 | - | - | 0 | - |
| - | - | 2588 | 476.2 | - | - | 0 | - |
| - | - | 2834 | 476.2 | - | - | 0 | - |
| - | - | 3992 | 476.5 | - | - | 0 | - |
| - | - | 2230 | 476.7 | - | - | 0 | - |
| - | - | 2038 | 477 | - | - | 0 | - |
| 13 | y | 5068 | 477.2 | 0.004868 | 10.2 | +1 | 3 |
| - | - | 1581 | 477.7 | - | - | 0 | - |
| - | - | 851.6 | 477.8 | - | - | 0 | - |
| 13 | z | 4719 | 478.2 | 0.001957 | 4.092 | +1 | 3 |
| - | - | 1099 | 478.7 | - | - | 0 | - |
| - | - | 1293 | 479.2 | - | - | 0 | - |
| - | - | 1214 | 479.9 | - | - | 0 | - |
| - | - | 909.6 | 480 | - | - | 0 | - |
| - | - | 3744 | 480.2 | - | - | 0 | - |
| - | - | 5.453E+04 | 480.5 | - | - | 0 | - |
| - | - | 5.195E+04 | 480.7 | - | - | 0 | - |
| - | - | 3.673E+04 | 481 | - | - | 0 | - |
| - | - | 1.368E+04 | 481.2 | - | - | 0 | - |
| - | - | 5081 | 481.5 | - | - | 0 | - |
| - | - | 1814 | 481.7 | - | - | 0 | - |
| - | - | 1388 | 482.2 | - | - | 0 | - |
| 8 | c | 5454 | 482.7 | 0.002659 | 5.508 | +2 | 8 |
| 8 | c | 1.137E+04 | 483.2 | 0.003113 | 6.443 | +2 | 8 |
| - | - | 3061 | 483.5 | - | - | 0 | - |
| - | - | 7353 | 483.7 | - | - | 0 | - |
| - | - | 2007 | 484 | - | - | 0 | - |
| - | - | 1.928E+04 | 484.3 | - | - | 0 | - |
| - | - | 7178 | 484.5 | - | - | 0 | - |
| - | - | 5624 | 484.6 | - | - | 0 | - |
| 9 | w | 8547 | 484.7 | 0.002619 | 5.403 | +2 | 7 |
| - | - | 6199 | 484.8 | - | - | 0 | - |
| - | - | 3773 | 484.9 | - | - | 0 | - |
| - | - | 4038 | 485 | - | - | 0 | - |
| - | - | 4604 | 485.2 | - | - | 0 | - |
| - | - | 3015 | 485.2 | - | - | 0 | - |
| - | - | 975.3 | 485.5 | - | - | 0 | - |
| - | - | 1598 | 485.6 | - | - | 0 | - |
| - | - | 1893 | 485.7 | - | - | 0 | - |
| - | - | 1186 | 485.9 | - | - | 0 | - |
| - | - | 1070 | 486.2 | - | - | 0 | - |
| - | - | 940.4 | 487.2 | - | - | 0 | - |
| - | - | 6594 | 487.5 | - | - | 0 | - |
| - | - | 1.34E+05 | 487.7 | - | - | 0 | - |
| - | - | 1.285E+05 | 488 | - | - | 0 | - |
| - | - | 9.235E+04 | 488.2 | - | - | 0 | - |
| - | - | 3.643E+04 | 488.5 | - | - | 0 | - |
| - | - | 1.131E+04 | 488.7 | - | - | 0 | - |
| - | - | 5237 | 489 | - | - | 0 | - |
| - | - | 5572 | 489.2 | - | - | 0 | - |
| - | - | 1254 | 489.7 | - | - | 0 | - |
| - | - | 936.5 | 490.2 | - | - | 0 | - |
| - | - | 1069 | 490.6 | - | - | 0 | - |
| - | - | 2062 | 491.2 | - | - | 0 | - |
| - | - | 1576 | 491.5 | - | - | 0 | - |
| 8 | c | 2.065E+04 | 491.7 | 0.001619 | 3.291 | +2 | 8 |
| - | - | 2074 | 492 | - | - | 0 | - |
| - | - | 1.385E+04 | 492.2 | - | - | 0 | - |
| - | - | 4306 | 492.7 | - | - | 0 | - |
| 4 | c | 2418 | 493.3 | 0.004138 | 8.388 | +1 | 4 |
| - | - | 1582 | 493.6 | - | - | 0 | - |
| - | - | 1139 | 493.7 | - | - | 0 | - |
| 5 | z | 1673 | 493.9 | 0.005957 | 12.06 | +3 | 11 |
| - | - | 1423 | 494 | - | - | 0 | - |
| 4 | c | 7.547E+04 | 494.2 | 0.001581 | 3.199 | +1 | 4 |
| - | - | 1244 | 494.6 | - | - | 0 | - |
| - | - | 2.004E+04 | 495.2 | - | - | 0 | - |
| - | - | 1998 | 495.8 | - | - | 0 | - |
| - | - | 2388 | 496.2 | - | - | 0 | - |
| - | - | 2398 | 496.2 | - | - | 0 | - |
| - | - | 6371 | 496.8 | - | - | 0 | - |
| - | - | 1889 | 497.2 | - | - | 0 | - |
| - | - | 5456 | 497.3 | - | - | 0 | - |
| - | - | 6683 | 497.8 | - | - | 0 | - |
| - | - | 4967 | 498 | - | - | 0 | - |
| - | - | 1.205E+04 | 498.2 | - | - | 0 | - |
| - | - | 1.1E+04 | 498.5 | - | - | 0 | - |
| - | - | 8669 | 498.7 | - | - | 0 | - |
| - | - | 816.7 | 498.9 | - | - | 0 | - |
| - | - | 4184 | 499 | - | - | 0 | - |
| 5 | y | 1.429E+04 | 499.2 | 0.006238 | 12.49 | +3 | 11 |
| 5 | y | 1.467E+04 | 499.6 | 0.001235 | 2.472 | +3 | 11 |
| - | - | 2024 | 499.7 | - | - | 0 | - |
| 5 | z | 1.826E+04 | 499.9 | 0.00246 | 4.921 | +3 | 11 |
| - | - | 1.031E+04 | 500.2 | - | - | 0 | - |
| - | - | 3883 | 500.6 | - | - | 0 | - |
| - | - | 1049 | 500.9 | - | - | 0 | - |
| - | - | 821.5 | 501.3 | - | - | 0 | - |
| - | - | 2804 | 502 | - | - | 0 | - |
| - | - | 1.227E+04 | 502.3 | - | - | 0 | - |
| - | - | 2.129E+05 | 502.5 | - | - | 0 | - |
| - | - | 2.905E+05 | 502.7 | - | - | 0 | - |
| - | - | 2.2E+05 | 503 | - | - | 0 | - |
| - | - | 1.218E+05 | 503.3 | - | - | 0 | - |
| - | - | 4.07E+04 | 503.5 | - | - | 0 | - |
| - | - | 1.702E+04 | 503.8 | - | - | 0 | - |
| - | - | 5834 | 504 | - | - | 0 | - |
| - | - | 807.1 | 504.2 | - | - | 0 | - |
| - | - | 1191 | 504.2 | - | - | 0 | - |
| - | - | 1335 | 504.6 | - | - | 0 | - |
| 9 | z | 813.7 | 504.8 | 0.0005717 | 1.133 | +2 | 7 |
| 12 | c | 2540 | 504.9 | 0.001765 | 3.496 | +3 | 12 |
| 12 | c | 2.019E+04 | 505.3 | 0.000211 | 0.4175 | +3 | 12 |
| - | - | 1.16E+04 | 505.6 | - | - | 0 | - |
| - | - | 1.061E+04 | 505.7 | - | - | 0 | - |
| - | - | 8157 | 505.9 | - | - | 0 | - |
| - | - | 5305 | 506.2 | - | - | 0 | - |
| - | - | 1103 | 506.6 | - | - | 0 | - |
| - | - | 3993 | 506.7 | - | - | 0 | - |
| - | - | 1433 | 507.2 | - | - | 0 | - |
| - | - | 4343 | 508.6 | - | - | 0 | - |
| - | - | 3355 | 508.9 | - | - | 0 | - |
| - | - | 1725 | 509.2 | - | - | 0 | - |
| - | - | 1405 | 509.6 | - | - | 0 | - |
| - | - | 2490 | 510.6 | - | - | 0 | - |
| 12 | c | 6.716E+04 | 510.9 | 0.00158 | 3.092 | +3 | 12 |
| 4 | c | 1.293E+05 | 511.3 | 0.001533 | 2.999 | +1 | 4 |
| - | - | 2.584E+04 | 511.6 | - | - | 0 | - |
| - | - | 9322 | 511.9 | - | - | 0 | - |
| - | - | 2.297E+04 | 512.3 | - | - | 0 | - |
| 9 | y | 5931 | 512.8 | 0.003098 | 6.041 | +2 | 7 |
| - | - | 9956 | 513.2 | - | - | 0 | - |
| - | - | 4196 | 513.3 | - | - | 0 | - |
| 9 | z | 3.168E+04 | 513.8 | 0.003718 | 7.237 | +2 | 7 |
| - | - | 2.27E+04 | 514.3 | - | - | 0 | - |
| - | - | 4400 | 514.6 | - | - | 0 | - |
| - | - | 8632 | 514.8 | - | - | 0 | - |
| - | - | 2081 | 514.9 | - | - | 0 | - |
| - | - | 2768 | 515.3 | - | - | 0 | - |
| - | - | 9436 | 515.3 | - | - | 0 | - |
| - | - | 1380 | 516.2 | - | - | 0 | - |
| - | - | 3023 | 516.3 | - | - | 0 | - |
| - | - | 2882 | 517.3 | - | - | 0 | - |
| - | - | 1733 | 518.3 | - | - | 0 | - |
| - | - | 2349 | 518.8 | - | - | 0 | - |
| - | - | 2596 | 519.3 | - | - | 0 | - |
| - | - | 918.7 | 519.6 | - | - | 0 | - |
| - | - | 3328 | 519.9 | - | - | 0 | - |
| - | - | 2124 | 520.3 | - | - | 0 | - |
| - | - | 1216 | 520.7 | - | - | 0 | - |
| - | - | 2160 | 521.3 | - | - | 0 | - |
| 9 | y | 3.168E+04 | 521.8 | 0.003131 | 6.001 | +2 | 7 |
| - | - | 1.655E+04 | 522.3 | - | - | 0 | - |
| - | - | 5245 | 522.8 | - | - | 0 | - |
| - | - | 2487 | 523.3 | - | - | 0 | - |
| - | - | 1601 | 523.6 | - | - | 0 | - |
| - | - | 1591 | 525.3 | - | - | 0 | - |
| - | - | 2836 | 526.3 | - | - | 0 | - |
| - | - | 1037 | 526.8 | - | - | 0 | - |
| - | - | 2856 | 527.3 | - | - | 0 | - |
| - | - | 2955 | 527.8 | - | - | 0 | - |
| - | - | 2601 | 532.2 | - | - | 0 | - |
| - | - | 1967 | 532.8 | - | - | 0 | - |
| - | - | 1112 | 533.2 | - | - | 0 | - |
| - | - | 969 | 533.2 | - | - | 0 | - |
| - | - | 1745 | 533.3 | - | - | 0 | - |
| - | - | 2755 | 533.8 | - | - | 0 | - |
| - | - | 2460 | 534.3 | - | - | 0 | - |
| - | - | 1293 | 534.3 | - | - | 0 | - |
| - | - | 2546 | 535.2 | - | - | 0 | - |
| - | - | 1027 | 537.3 | - | - | 0 | - |
| 4 | z | 2449 | 538.3 | 0.001967 | 3.653 | +3 | 12 |
| - | - | 1243 | 538.6 | - | - | 0 | - |
| - | - | 891.2 | 538.8 | - | - | 0 | - |
| - | - | 3820 | 539.2 | - | - | 0 | - |
| - | - | 1105 | 540.2 | - | - | 0 | - |
| 8 | y | 8810 | 541.3 | 0.003149 | 5.817 | +2 | 8 |
| 8 | y | 4744 | 541.8 | 0.006736 | 12.43 | +2 | 8 |
| 8 | z | 3.235E+04 | 542.3 | 0.00206 | 3.798 | +2 | 8 |
| - | - | 1.882E+04 | 542.8 | - | - | 0 | - |
| - | - | 1.083E+04 | 543.3 | - | - | 0 | - |
| 4 | y | 3556 | 543.6 | 0.002586 | 4.758 | +3 | 12 |
| - | - | 1902 | 543.8 | - | - | 0 | - |
| - | - | 2262 | 543.9 | - | - | 0 | - |
| - | - | 2360 | 544.3 | - | - | 0 | - |
| - | - | 1049 | 545.3 | - | - | 0 | - |
| - | - | 782.9 | 545.6 | - | - | 0 | - |
| - | - | 8874 | 546.3 | - | - | 0 | - |
| - | - | 4849 | 546.8 | - | - | 0 | - |
| - | - | 4082 | 547.3 | - | - | 0 | - |
| - | - | 1167 | 547.6 | - | - | 0 | - |
| - | - | 2650 | 547.8 | - | - | 0 | - |
| 12 | w | 2909 | 548.2 | 0.006489 | 11.83 | +1 | 4 |
| - | - | 2074 | 548.3 | - | - | 0 | - |
| - | - | 1246 | 549.2 | - | - | 0 | - |
| - | - | 5840 | 549.6 | - | - | 0 | - |
| - | - | 939.4 | 549.8 | - | - | 0 | - |
| - | - | 5899 | 549.9 | - | - | 0 | - |
| 8 | y | 6.514E+04 | 550.3 | 0.003365 | 6.115 | +2 | 8 |
| - | - | 3.659E+04 | 550.8 | - | - | 0 | - |
| - | - | 1082 | 550.9 | - | - | 0 | - |
| - | - | 1.508E+04 | 551.3 | - | - | 0 | - |
| - | - | 1314 | 551.6 | - | - | 0 | - |
| - | - | 5450 | 551.8 | - | - | 0 | - |
| - | - | 3505 | 552.3 | - | - | 0 | - |
| - | - | 2142 | 553.3 | - | - | 0 | - |
| - | - | 1278 | 553.8 | - | - | 0 | - |
| - | - | 3736 | 554.3 | - | - | 0 | - |
| - | - | 3852 | 554.8 | - | - | 0 | - |
| - | - | 3.074E+04 | 555.3 | - | - | 0 | - |
| 9 | c | 2.098E+05 | 555.8 | 0.0007068 | 1.272 | +2 | 9 |
| - | - | 1.399E+05 | 556.3 | - | - | 0 | - |
| - | - | 4.62E+04 | 556.8 | - | - | 0 | - |
| - | - | 2016 | 556.9 | - | - | 0 | - |
| - | - | 1.214E+04 | 557.3 | - | - | 0 | - |
| - | - | 1260 | 557.6 | - | - | 0 | - |
| - | - | 1951 | 557.8 | - | - | 0 | - |
| - | - | 1570 | 559.3 | - | - | 0 | - |
| - | - | 904.4 | 559.6 | - | - | 0 | - |
| - | - | 975.1 | 559.9 | - | - | 0 | - |
| - | - | 991.3 | 560.9 | - | - | 0 | - |
| - | - | 4204 | 561.3 | - | - | 0 | - |
| 3 | w | 7.43E+04 | 561.6 | 0.001591 | 2.833 | +3 | 13 |
| - | - | 6.046E+04 | 561.9 | - | - | 0 | - |
| - | - | 3.646E+04 | 562.3 | - | - | 0 | - |
| - | - | 1.287E+04 | 562.6 | - | - | 0 | - |
| - | - | 3044 | 562.6 | - | - | 0 | - |
| - | - | 2196 | 562.8 | - | - | 0 | - |
| - | - | 3747 | 562.9 | - | - | 0 | - |
| - | - | 1690 | 563 | - | - | 0 | - |
| - | - | 3690 | 563.3 | - | - | 0 | - |
| - | - | 2769 | 563.3 | - | - | 0 | - |
| - | - | 1297 | 563.6 | - | - | 0 | - |
| - | - | 2.517E+04 | 563.8 | - | - | 0 | - |
| - | - | 872.3 | 563.9 | - | - | 0 | - |
| - | - | 2.131E+04 | 564.3 | - | - | 0 | - |
| 13 | c | 5.036E+04 | 564.6 | 2.71E-05 | 0.04799 | +3 | 13 |
| - | - | 7861 | 564.8 | - | - | 0 | - |
| - | - | 4.745E+04 | 564.9 | - | - | 0 | - |
| - | - | 2.928E+04 | 565.3 | - | - | 0 | - |
| - | - | 9979 | 565.6 | - | - | 0 | - |
| - | - | 4268 | 565.9 | - | - | 0 | - |
| - | - | 6581 | 566.3 | - | - | 0 | - |
| - | - | 3896 | 566.6 | - | - | 0 | - |
| - | - | 3389 | 566.9 | - | - | 0 | - |
| - | - | 1.281E+04 | 567.2 | - | - | 0 | - |
| - | - | 2763 | 567.3 | - | - | 0 | - |
| - | - | 4432 | 568.2 | - | - | 0 | - |
| - | - | 2977 | 568.3 | - | - | 0 | - |
| - | - | 1.517E+04 | 568.8 | - | - | 0 | - |
| - | - | 808.5 | 568.9 | - | - | 0 | - |
| - | - | 1.068E+04 | 569.3 | - | - | 0 | - |
| - | - | 4521 | 569.8 | - | - | 0 | - |
| - | - | 1237 | 569.9 | - | - | 0 | - |
| - | - | 2969 | 570.3 | - | - | 0 | - |
| - | - | 2981 | 570.7 | - | - | 0 | - |
| - | - | 4401 | 570.8 | - | - | 0 | - |
| - | - | 5.411E+04 | 571.3 | - | - | 0 | - |
| - | - | 3.224E+04 | 571.8 | - | - | 0 | - |
| - | - | 1.395E+04 | 572.3 | - | - | 0 | - |
| - | - | 4237 | 572.8 | - | - | 0 | - |
| - | - | 2787 | 573 | - | - | 0 | - |
| - | - | 4586 | 573.3 | - | - | 0 | - |
| - | - | 3400 | 573.6 | - | - | 0 | - |
| - | - | 1703 | 574 | - | - | 0 | - |
| - | - | 1810 | 574.6 | - | - | 0 | - |
| - | - | 4579 | 576.3 | - | - | 0 | - |
| - | - | 3216 | 576.8 | - | - | 0 | - |
| 7 | w | 3664 | 577.3 | 0.005901 | 10.22 | +2 | 9 |
| - | - | 4378 | 577.8 | - | - | 0 | - |
| - | - | 3585 | 578 | - | - | 0 | - |
| - | - | 6274 | 578.3 | - | - | 0 | - |
| - | - | 2998 | 578.6 | - | - | 0 | - |
| - | - | 2.126E+04 | 578.8 | - | - | 0 | - |
| - | - | 1.442E+04 | 579.3 | - | - | 0 | - |
| - | - | 6283 | 579.8 | - | - | 0 | - |
| 3 | y | 3852 | 580.3 | 0.003158 | 5.443 | +3 | 13 |
| 3 | y | 6779 | 580.6 | 0.001691 | 2.913 | +3 | 13 |
| 3 | z | 2.095E+04 | 580.9 | 0.001182 | 2.034 | +3 | 13 |
| - | - | 2.126E+04 | 581.3 | - | - | 0 | - |
| - | - | 1193 | 581.3 | - | - | 0 | - |
| - | - | 1.119E+04 | 581.6 | - | - | 0 | - |
| - | - | 3956 | 581.9 | - | - | 0 | - |
| - | - | 3337 | 582.3 | - | - | 0 | - |
| - | - | 1114 | 583 | - | - | 0 | - |
| - | - | 3261 | 583.3 | - | - | 0 | - |
| - | - | 3297 | 583.6 | - | - | 0 | - |
| - | - | 2567 | 583.9 | - | - | 0 | - |
| - | - | 1743 | 584.8 | - | - | 0 | - |
| - | - | 1715 | 585.3 | - | - | 0 | - |
| - | - | 1329 | 585.8 | - | - | 0 | - |
| 3 | y | 1.164E+04 | 586.3 | 3.021E-06 | 0.005153 | +3 | 13 |
| - | - | 9241 | 586.6 | - | - | 0 | - |
| - | - | 6526 | 586.9 | - | - | 0 | - |
| - | - | 2517 | 587.3 | - | - | 0 | - |
| - | - | 2197 | 587.6 | - | - | 0 | - |
| - | - | 1874 | 588 | - | - | 0 | - |
| - | - | 4575 | 588.3 | - | - | 0 | - |
| - | - | 2949 | 588.6 | - | - | 0 | - |
| - | - | 2230 | 589 | - | - | 0 | - |
| - | - | 2702 | 590.3 | - | - | 0 | - |
| 7 | z | 2683 | 590.8 | 0.001409 | 2.385 | +2 | 9 |
| 7 | z | 4025 | 591.3 | 0.01044 | 17.65 | +2 | 9 |
| - | - | 1029 | 591.8 | - | - | 0 | - |
| - | - | 2217 | 592.3 | - | - | 0 | - |
| - | - | 1.126E+04 | 592.6 | - | - | 0 | - |
| - | - | 1.706E+04 | 593 | - | - | 0 | - |
| - | - | 3352 | 593.3 | - | - | 0 | - |
| - | - | 6918 | 593.3 | - | - | 0 | - |
| - | - | 2861 | 593.6 | - | - | 0 | - |
| - | - | 1172 | 593.8 | - | - | 0 | - |
| - | - | 1305 | 594 | - | - | 0 | - |
| - | - | 2521 | 594.3 | - | - | 0 | - |
| - | - | 1230 | 595.2 | - | - | 0 | - |
| - | - | 2614 | 597 | - | - | 0 | - |
| - | - | 2916 | 597.3 | - | - | 0 | - |
| - | - | 2644 | 597.6 | - | - | 0 | - |
| - | - | 2528 | 597.8 | - | - | 0 | - |
| - | - | 1700 | 598 | - | - | 0 | - |
| - | - | 9861 | 598.3 | - | - | 0 | - |
| 7 | y | 1.26E+04 | 598.8 | 0.0002016 | 0.3367 | +2 | 9 |
| 7 | y | 1.524E+04 | 599.3 | 0.005166 | 8.62 | +2 | 9 |
| 7 | z | 6.612E+04 | 599.8 | 0.002286 | 3.812 | +2 | 9 |
| - | - | 6.295E+04 | 600.3 | - | - | 0 | - |
| - | - | 3.27E+04 | 600.8 | - | - | 0 | - |
| 14 | c | 1.071E+04 | 601.3 | 0.00892 | 14.84 | +3 | 14 |
| 14 | c | 3513 | 601.6 | 0.006153 | 10.23 | +3 | 14 |
| - | - | 3428 | 601.8 | - | - | 0 | - |
| - | - | 2967 | 602 | - | - | 0 | - |
| - | - | 3602 | 602.3 | - | - | 0 | - |
| - | - | 3591 | 602.6 | - | - | 0 | - |
| - | - | 3355 | 602.8 | - | - | 0 | - |
| - | - | 1.079E+04 | 602.9 | - | - | 0 | - |
| - | - | 1.421E+04 | 603.3 | - | - | 0 | - |
| - | - | 9433 | 603.6 | - | - | 0 | - |
| - | - | 1269 | 603.8 | - | - | 0 | - |
| - | - | 2300 | 604 | - | - | 0 | - |
| - | - | 1234 | 604.3 | - | - | 0 | - |
| 12 | z | 3.845E+04 | 606.3 | 0.007404 | 12.21 | +1 | 4 |
| - | - | 2480 | 606.6 | - | - | 0 | - |
| - | - | 1224 | 606.8 | - | - | 0 | - |
| - | - | 1.26E+04 | 607 | - | - | 0 | - |
| 14 | c | 7.565E+04 | 607.3 | 0.001291 | 2.126 | +3 | 14 |
| - | - | 5.927E+04 | 607.6 | - | - | 0 | - |
| 7 | y | 2.229E+05 | 607.8 | 0.00292 | 4.805 | +2 | 9 |
| - | - | 3E+04 | 608 | - | - | 0 | - |
| - | - | 1.613E+05 | 608.3 | - | - | 0 | - |
| - | - | 5755 | 608.6 | - | - | 0 | - |
| - | - | 5.989E+04 | 608.8 | - | - | 0 | - |
| - | - | 1.845E+04 | 609.3 | - | - | 0 | - |
| - | - | 4003 | 609.8 | - | - | 0 | - |
| - | - | 1.298E+04 | 610.3 | - | - | 0 | - |
| - | - | 871.1 | 610.8 | - | - | 0 | - |
| - | - | 4274 | 611.3 | - | - | 0 | - |
[truncated: 193,526 more chars]
