# Supplementary material for: A Handle on Mass Coincidence Errors in De Novo Sequencing of Antibodies by Bottom-up Proteomics
Source: J Proteome Res. 2024 Jun 27;23(8):3552–9. doi: 10.1021/acs.jproteome.4c00188 (PMC11301774; doi:10.1021/acs.jproteome.4c00188)
Supplement: Supplementary file 2 — pr4c00188_si_002.pdf [file pr4c00188_si_002.pdf]

Supporting Information to:

A handle on mass coincidence errors in *de novo* sequencing of antibodies by bottom-up proteomics

Douwe Schulte[1], Joost Snijder[1]*

[1] Biomolecular Mass Spectrometry and Proteomics, Bijvoet Center for Biomolecular Research and Utrecht Institute of Pharmaceutical Sciences, Utrecht University, Padualaan 8, 3584, CH, Utrecht, The Netherlands

* corresponding author: j.snijder@uu.nl

Contents:

- Supporting Data S1. All Stitch HTML results related to this study
